# iPhos-PseEn: Identifying phosphorylation sites in proteins by fusing different pseudo components into an ensemble classifier

**DOI:** 10.18632/oncotarget.9987

**Published:** 2016-06-13

**Authors:** Wang-Ren Qiu, Xuan Xiao, Zhao-Chun Xu, Kuo-Chen Chou

**Affiliations:** ^1^ Computer Department, Jingdezhen Ceramic Institute, Jingdezhen, China; ^2^ Department of Computer Science and Bond Life Science Center, University of Missouri, Columbia, MO, USA; ^3^ Gordon Life Science Institute, Boston, MA, USA; ^4^ Center of Excellence in Genomic Medicine Research (CEGMR), King Abdulaziz University, Jeddah, Saudi Arabia; ^5^ Center of Bioinformatics, School of Life Science and Technology, University of Electronic Science and Technology of China, Chengdu, Sichuan, China

**Keywords:** protein phosphorylation, pseudo components, random forests, ensemble classifier

## Abstract

Protein phosphorylation is a posttranslational modification (PTM or PTLM), where a phosphoryl group is added to the residue(s) of a protein molecule. The most commonly phosphorylated amino acids occur at serine (S), threonine (T), and tyrosine (Y). Protein phosphorylation plays a significant role in a wide range of cellular processes; meanwhile its dysregulation is also involved with many diseases. Therefore, from the angles of both basic research and drug development, we are facing a challenging problem: for an uncharacterized protein sequence containing many residues of S, T, or Y, which ones can be phosphorylated, and which ones cannot? To address this problem, we have developed a predictor called iPhos-PseEn by fusing four different pseudo component approaches (amino acids’ disorder scores, nearest neighbor scores, occurrence frequencies, and position weights) into an ensemble classifier via a voting system. Rigorous cross-validations indicated that the proposed predictor remarkably outperformed its existing counterparts. For the convenience of most experimental scientists, a user-friendly web-server for iPhos-PseEn has been established at http://www.jci-bioinfo.cn/iPhos-PseEn, by which users can easily obtain their desired results without the need to go through the complicated mathematical equations involved.

## INTRODUCTION

Cancer and many other major diseases are often caused by varieties of subtle modifications in biological sequences, typically by various types of post-translational modification (PTM or PTLM) in protein [1, 2], post-replication modification (PTRM) in DNA [3] and post-transcription modification (PTCM) in RNA [4]. In order to reveal the pathological mechanisms of these diseases and find new and revolutionary strategies to treat them, many efforts have been made with the aim to identify the possible modified sites in protein (see, e.g., [5–14], DNA [15, 16], and RNA sequences [17, 18]).

Protein phosphorylation is one of the most-studied post-translational modification (PTM or PTLM) that can alter the structural conformation of a protein, causing it to become activated, deactivated, or modifying its function. The most commonly phosphorylated amino acids are serine (S-type), threonine (T-type), and tyrosine (Y-type).

In human cells, phosphorylation also plays a critical role in the transmission of signals controlling a diverse array of cellular functions, such as cell growth, survival differentiation, and metabolism; while its dysregulation is implicated in many diseases. Therefore, information of phosphorylation sites in proteins is significant for both basic research and drug development.

Many efforts have been made to identify the protein phosphorylation. These methods include mass spectroscopy [19, 20], phosphor-specific antibody [21], etc. Unfortunately, these experimental techniques are both time-consuming and expensive. Facing the explosive growth of protein sequences merging in post genomic age, it is highly desired to develop computational methods for effectively identifying the phosphorylation sites in proteins.

Actually, by using computational approaches such as artificial neural networks, hidden Markov models, and support vector machines, some prediction method were developed based on various different features including disorder scores, KNN scores, amino acid frequency [22, 23], and attribute grouping and position weight amino acid composition [24].

In view of its importance and urgency, it is certainly worthwhile to further improve the prediction quality by introducing some novel approaches as elaborated below.

According to the Chou's 5-step rule [25] and demonstrated in a series of recent publications [11, 12, 18, 26–30], to develop a really useful sequence-based predictor for a biological system, we should stick to the following five guidelines and make them crystal clear: (1) how to construct or select a valid benchmark dataset to train and test the predictor; (2) how to formulate the biological sequence samples with an effective mathematical expression that can truly reflect their essential correlation with the target concerned; (3) how to introduce or develop a powerful algorithm (or engine) to run the prediction; (4) how to properly conduct cross-validation tests to objectively evaluate the anticipated accuracy; (5) how to provide a web-server and user guide to make people very easily to get their desired results. Below, we are to address the five procedures one-by-one. However, their order may be changed in order to match the rubric style of Oncotarget.

## RESULTS AND DISCUSSION

### A new ensemble web-server predictor

By fusing four different pseudo component approaches, a new ensemble classifier, named iPhos-PseEn, has been established for predicting phosphorylation sites in proteins.

### Success rates and comparison with the existing methods

The success rates achieved by the iPhos-PseEn predictor via the 5-fold cross validation for S-, T- and Y-type phosphorylation are given in Table 1, where for facilitating comparison the corresponding rates by Musite [22] and PWAAC [24] are also listed. As we can see from the table, compared with its counterparts, iPhos-PseEn is remarkably better than its counterparts in predicting all the three phosphorylation types as measured with all the four metrics, clearly indicating that the proposed predictor not only can achieve higher sensitivity, specificity, and overall accuracy but is also much more stable. As shown from the table, compared with Sp, the improvement in Sn is relatively less significant. This is quite normal because the metrics Sn and Sp are used to measure a predictor from two different angles and hence they are actually constrained with each other [28, 31, 32].

**Table 1:** A comparison of the proposed predictor with the existing methods based on the 5-fold cross-validation on exactly the same benchmark dataset

Prediction method	Metrics	Type of phosphorylation		
		S	T	Y
Musite^a^	Acc (%)^d^	67.22	77.11	71.60
PWAAC ^b^		67.89	66.65	63.04
iPhos-PseEn^c^		79.76	79.88	76.28
Musite^a^	MCC^d^	0.2538	0.2960	0.2472
PWAAC ^b^		0.2342	0.2079	0.1720
iPhos-PseEn^c^		0.3901	0.3444	0.3244
Musite^a^	Sn (%)^d^	76.63	68.26	69.58
PWAAC ^b^		71.74	69.23	67.70
iPhos-PseEn^c^		79.64	71.51	76.18
Musite^a^	Sp (%)^d^	66.28	77.94	71.79
PWAAC ^b^		67.51	66.40	62.61
iPhos-PseEn^c^		79.78	80.68	76.29

a The method developed by Gao et al. [22].

b The method developed by Huang et al. [24].

c The method proposed in this paper.

d See Eq.14 for the definition of metrics.

**Figure 1:**
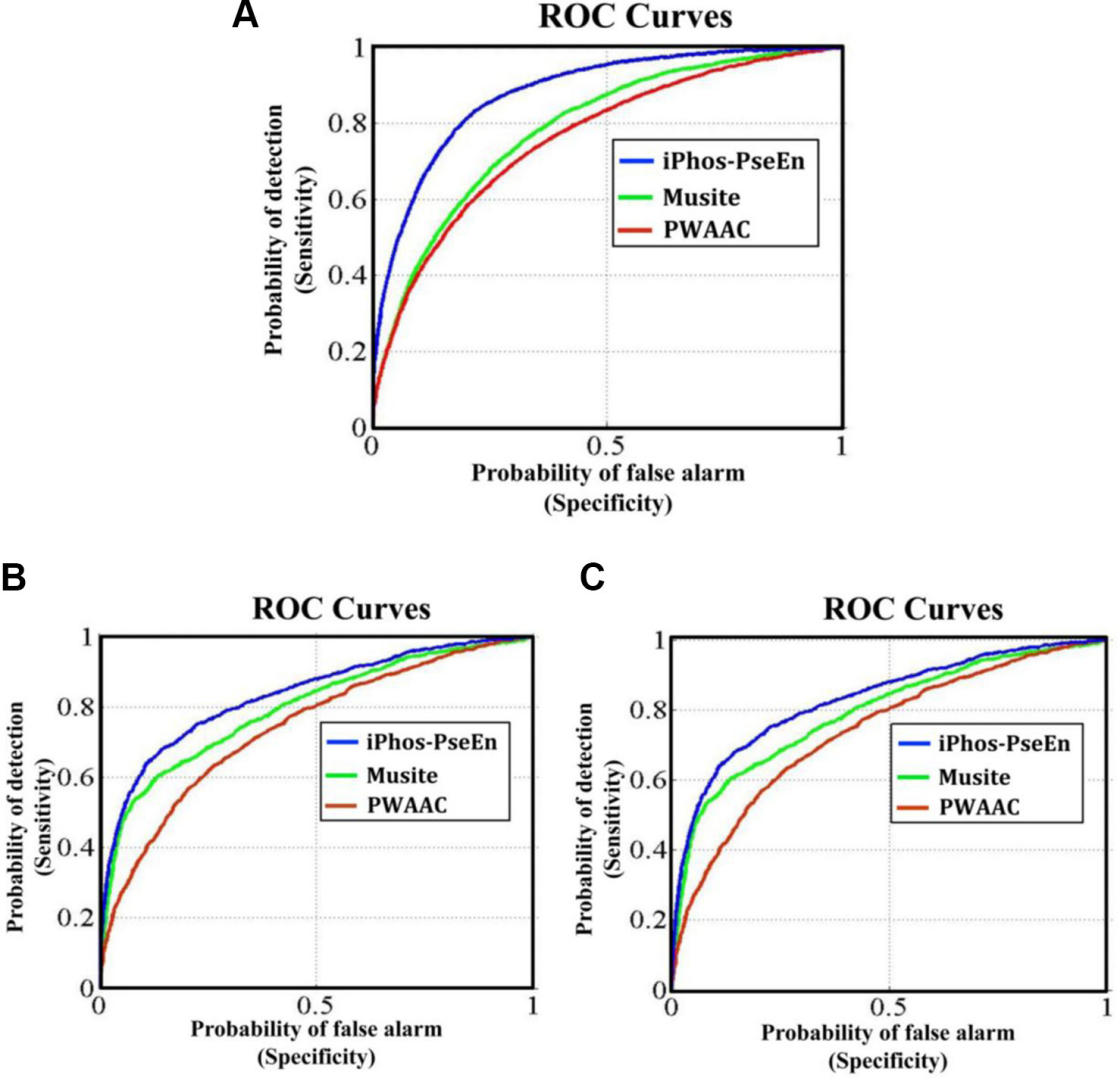
The intuitive graphs of ROC curves to show the performance of Musite, PWAAC, iPhos-PseEn, respectively, for the case of the center residue ⊛ is (A) S, (B) T, and (C) Y See the main text for further explanation.

Graphical approach is a useful vehicle for analyzing complicated biological systems as demonstrated by a series of previous studies (see, e.g., [33–40]). Here, to provide an intuitive comparison, the graph of Receiver Operating Characteristic (ROC) [41, 42] was utilized to show the advantage of iPhos-PseEn over the Musite [22] and PWAAC [24]. In Figure 1 the green and red graphic lines are the ROC curves for the Musite and PWAAC, respectively; while the blue graphic line for the proposed predictor iPhos-PseEn. The area under the ROC curve is called AUC (area under the curve). The greater the AUC value is, the better the predictor will be [41, 42]. As we can see from Figure 1, the area under the blue curve is remarkably greater than that under the red or green line, once again indicating that the proposed predictor is indeed much better than Musite and PWAAC predictors. Therefore, it is anticipated that iPhos-PseEn will become a useful high throughput tool in this important area, or at the very least, play a complementary role to the existing methods.

Why could the proposed method enhance the prediction quality so significantly? The key is the following. Many important features, which have been proved being closely correlated with phosphorylation sites by previous investigators, such as disorder, nearest neighbor scores, amino acid occurrence frequency, and amino acid position weight, are fused into an ensemble classifier via the general PseAAC approach, as will be elaborated in the Materials and Methods section.

### Web server and user guide

As pointed out in two recent review papers [16, 65], a prediction method with its web-server available would practically much more useful. The web-server for iPhos-PseEn has been established. Moreover, to maximize the convenience for users, a step-by-step guide is provided below.

**Figure 2:**
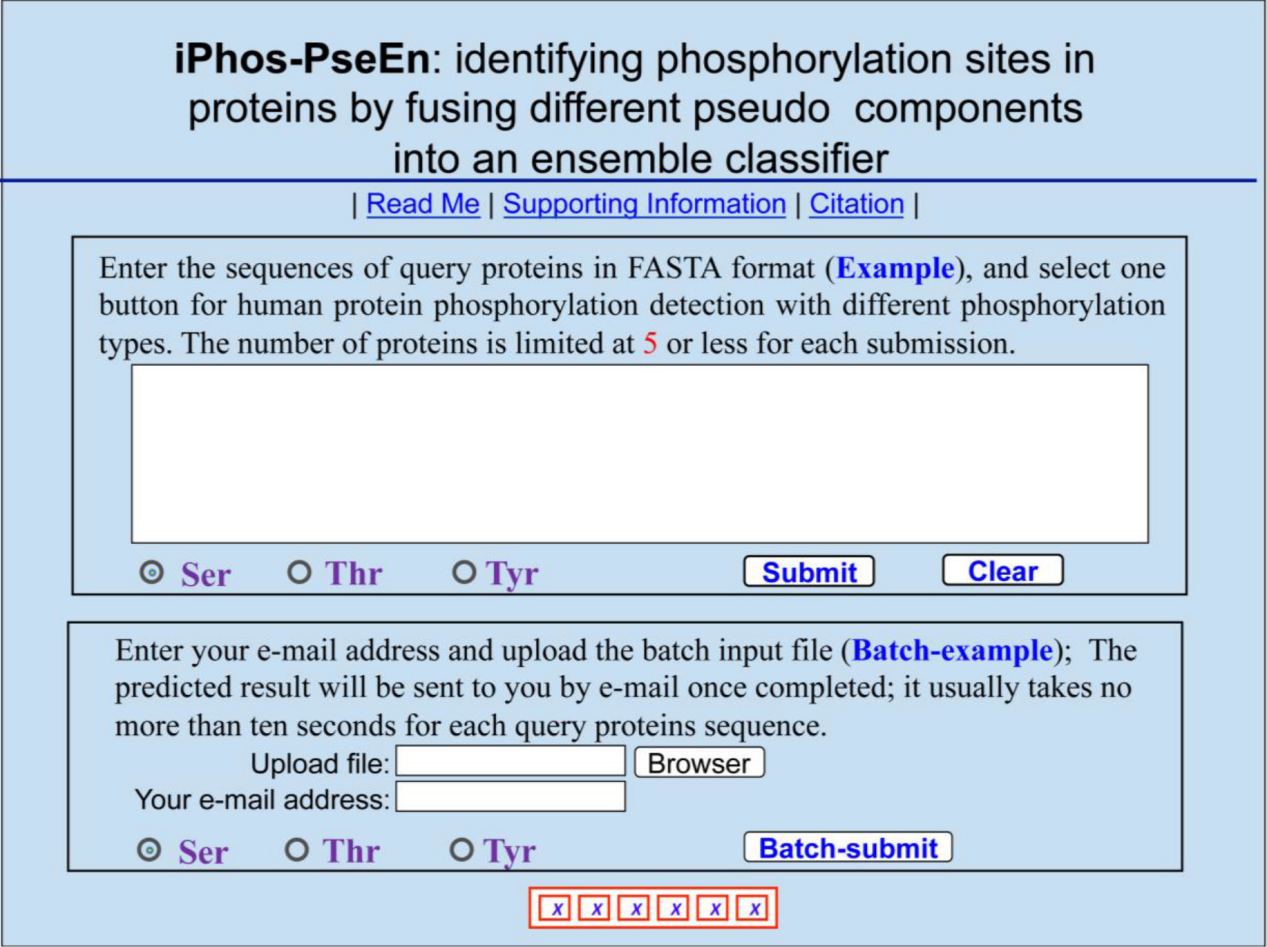
A semi-screenshot to show the top-page of the iPhos-PseEn web-server at http://www.jci-bioinfo.cn/iPhos-PseEn

1. Opening the web-server at http://www.jci-bioinfo.cn/iPhos-PseEn, you will see the top page of iPhos-PseEn on your computer screen, as shown in Figure 2. Click on the Read Me button to see a brief introduction about this predictor.
2. Either type or copy/paste your query protein sequences into the input box at the center of Figure 2. The input sequences should be in the FASTA format. For the examples of sequences in FASTA format, click the Example button right above the input box.
3. Select the phosphorylation type concerned: check on the S, T, or Y button to predict phosphoserine, phosphothreonine, or phosphotyrosine, respectively.
4. Click on the Submit button to see the predicted result. For example, if you use the Sequence_S in the Example window as the input and check on the S button, after 20 seconds or so since your submitting, you will see the following on your screen: Sequence_S contains 11 S residues, of which 2 are predicted to be of phosphorylation site and they are at the sequence positions 2 and 37. If you use the Sequence_T as the input and check on the T button, you will see: Sequence_T contains 11 T residues, of which 5 are of phosphorylation site and at positions 12, 113, 118, 123, and 136. If you use the Sequence_Y as the input and check on the Y button, you will see: Sequence_Y contains 12 Y residues, of which 3 are of phosphorylation site and at the positions 4, 119 and 199. Compared with experimental observations, the above (11 + 11 + 12) = 34 Predicted results contain 1 false negative result $\left(N_{-}^{+}\right)$ that is located at 9th S residues in sequence_S, and 4 false positive results $\left(N_{+}^{-}\right)$ that are located at the 11th, 123th, 136th residues in Sequence_T as well as the 119th Y residue in Sequence_Y. In other words, the total number of phosphorylation sites involved in the above predictions is *N*^+^ = 3 + 2 + 2 = 7, while the total number of non-phosphorylation sites investigated is *N*^−^ = 8 + 9 + 10 = 27. Substituting these data into Eq.14, we have S_n_ = 85.71%, Sp = 85.19%, Acc = 85.29%, and Mcc = 0.6292 quite consistent with the rates in Table 1 obtained by iPhos-PseEn on the benchmark dataset via the 5-fold cross validation test.
5. As shown on the lower panel of Figure 2, you may also choose the batch prediction by entering your e-mail address and your desired batch input file (in FASTA format of course) via the Browse button. To see the sample of batch input file, click on the button Batch-example.
6. Click the Supporting Information button to download the benchmark dataset used in this study.
7. Click the Citation button to find the relevant papers that document the detailed development and algorithm for iPhos-PseEn.

## MATERIALS AND METHODS

### Benchmark dataset

To ensure a high quality, the benchmark dataset used in this study was constructed based on UniProtKB/Swiss-Prot database (released September 2015) at http://www.ebi.ac.uk/uniprot/ according to following the procedures: (1) Open the web site at http://www.uniprot.org/, followed by clicking the button “Advanced”. (2) Select “PTM/Processing” and “Modified residue [FT]” for “Fields”. (3) Select “Any experimental assertion” for “Evidence”. (4) Type “human” for “Term” to do search. (5) Collected were only those proteins that consist of 50 and more amino acid residues to exclude fragments. (6) The proteins thus obtained were subject to a screening operation to remove those sequences that had ≥50% pairwise sequence identity to any other.

After strictly following the aforementioned procedures, we finally obtained 1,770 proteins, of which 638 are non-phosphorylated proteins and 1,132 are phosphorylated proteins. The latter contain 845 phosphoserine proteins, 386 phosphothreonine proteins, and 249 phosphotyrosine proteins. Note that some of phosphorylated proteins may be with multi-label, meaning they may belong to more than one type.

**Figure 3:**
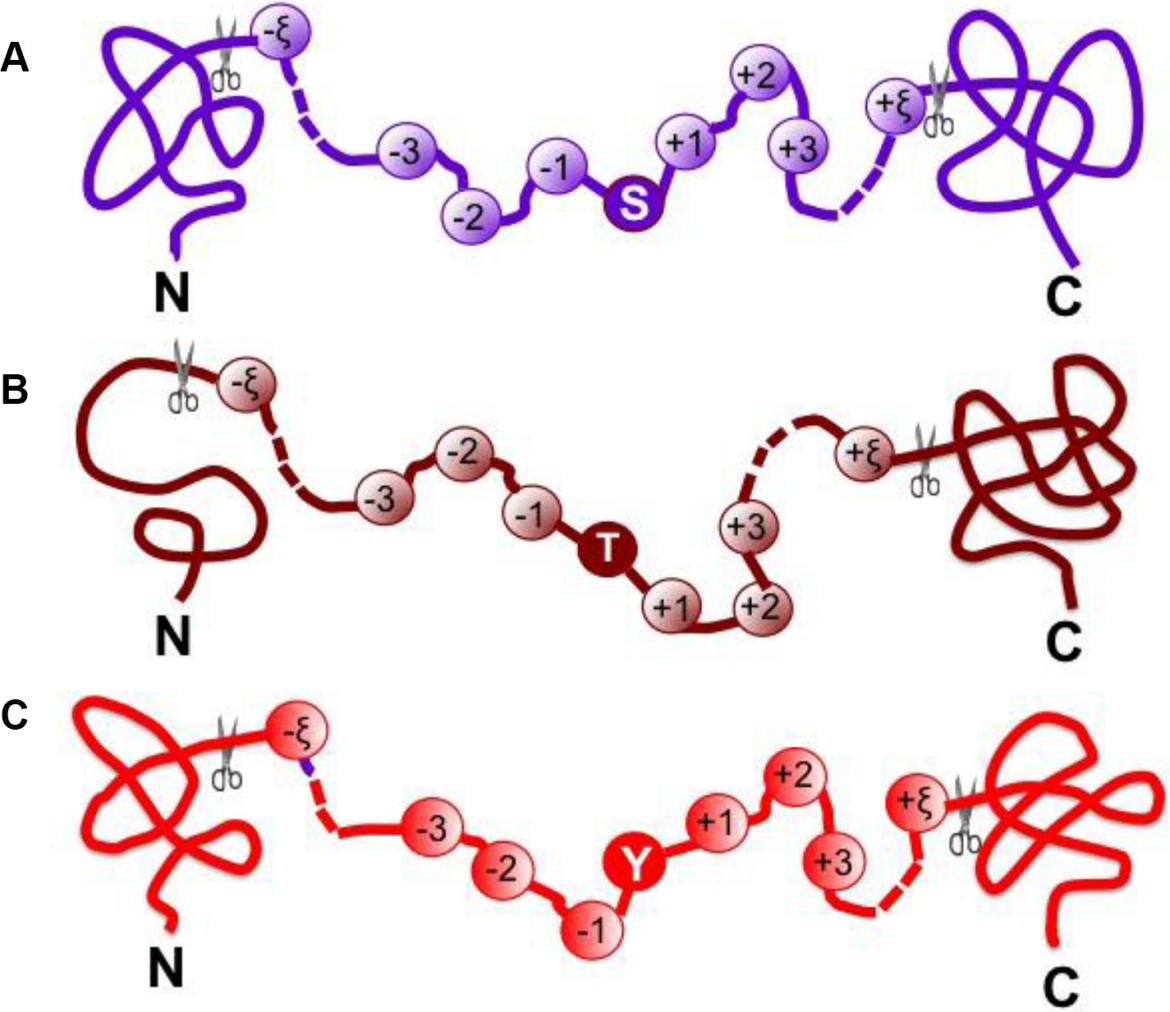
A schematic drawing to show the peptide model **P**_ξ_(⊛) when (A) ⊛ = S, (B) ⊛ = T and (C) ⊛ = Y See Eq.3 as well as the relevant text for further explanation.

For facilitating description later, the Chou's peptide formulation was adopted. The formulation was used to investigate the signal peptide cleavage sites [43], nitrotyrosine sites [9], methylation sites [7], enzyme specificity [44], protein-protein interactions [45], hydroxyproline and hydroxylysine sites [8], and protein-protein binding sites [46]. According to Chou's scheme, a potential phosphorylation site-containing peptide sample can be generally expressed by

$${\text{P}}_{\xi }\left(⊛\right)={\text{R}}_{-\xi }{\text{R}}_{-\left(\xi -1\right)}\cdots {\text{R}}_{-2}{\text{R}}_{-1}⊛{\text{R}}_{+1}{\text{R}}_{+2}\cdots {\text{R}}_{+\left(\xi -1\right)}{\text{R}}_{+\xi }$$ (1)

where the symbol ⊛ denotes the single amino acid code S, T, or Y, the subscript ξ is an integer, R_−ξ_ represents the ξ-th upstream amino acid residue from the center, the R_+ξ_ the ξ-th downstream amino acid residue, and so forth (Figure 3). The (2ξ + 1)-tuple peptide sample ${\text{P}}_{\xi }\left(⊛\right)$ can be further classified into the following two categories:

$${\text{P}}_{\xi }\left(⊛\right)\in \{\begin{array}{cc} {\text{P}}_{\xi }^{+}\left(⊛\right), & \text{if its center is a phosphorylation site}\\ {\text{P}}_{\xi }^{-}\left(⊛\right), & \text{other wise} \end{array}$$ (2)

where ${\text{P}}_{\xi }^{+}\left(⊛\right)$ denotes a true phosphorylation segment with S, T, or Y at its center, ${\text{P}}_{\xi }^{-}\left(⊛\right)$ denotes a corresponding false phosphorylation segment, and the symbol ∈ means “a member of” in the set theory.

In literature the benchmark dataset usually consists of a training dataset and a testing dataset: the former is used for training a model; while the latter, testing the model. But as pointed out in a comprehensive review [47], there is no need to artificially separate a benchmark dataset into the two parts if the prediction model is analyzed with the jackknife test or subsampling (K-fold) cross-validation because the outcome thus obtained is actually from a combination of many different independent dataset tests. Therefore, the benchmark dataset $S_{\xi }\left(⊛\right)$ for the current study can be formulated as

$$\{\begin{array}{ll} S_{\xi }(\text{S})=S_{\xi }^{+}(\text{S})\cup S_{\xi }^{-}(\text{S}), & \text{when}⊛=\text{S}\\ S_{\xi }(\text{T})=S_{\xi }^{+}(\text{T})\cup S_{\xi }^{-}(\text{T}), & \text{when}⊛=\text{T}\\ S_{\xi }(\text{Y})=S_{\xi }^{+}(\text{Y})\cup S_{\xi }^{-}(\text{Y}), & \text{when}⊛=\text{Y} \end{array}$$ (3)

where the positive subset $S_{\xi }^{+}\left(⊛\right)$ only contains the samples of true phosphorylation segments ${\text{P}}_{\xi }^{+}\left(⊛\right)$, and the negative subset $S_{\xi }^{-}\left(⊛\right)$ only contains the samples of false phosphorylation segments ${\text{P}}_{\xi }^{-}\left(⊛\right)$ (see Eq.2); while ∪ represents the symbol for “union” in the set theory.

The detailed procedures to construct the benchmark dataset are as follows. (1) As done in [48], slide the (2ξ + 1)-tuple peptide window along each of the aforementioned 1,770 protein sequences, and collected were only those peptide segments that have S, T, and Y at the center. (2) If the upstream or downstream in a protein sequence was less than ξ or greater than *L*−ξ where *L* is the length of the protein sequence concerned, the lacking residue was filled with the same residue of its closest neighbor. (3) The peptide segment samples thus obtained were put into the positive subset $S_{\xi }^{+}\left(⊛\right)$ if their centers have been experimentally annotated as the phosphorylation sites; otherwise, into the negative subset $S_{\xi }^{-}\left(⊛\right)$. (4) To reduce redundancy, all those peptide samples were removed if they had pairwise sequence identity with any other.

Note that the length of peptide samples and their number thus generated would depend on the ξ value. Many tests by previous investigators [22–24], however, had indicated that it would be most promising when ξ = 6 or the sample's length was 2ξ + 1 = 13 Accordingly, hereafter we only consider the case of ξ = 6; i.e., the samples with 13 amino acid residues. Thus, the benchmark datasets thus obtained for $S_{\xi =6}(\text{S})$, $S_{\xi =6}(\text{T})$, and $S_{\xi =6}(\text{Y})$ are given in Supporting Information S1, S2, and S3, respectively. Listed in Table 2 is a summary of their sizes.

**Table 2:** Summary of phosphorylation site samples in the benchmark dataset^a^

Subset	Phosphorylation type and number of samples		
	⊛ = S	⊛ = T	⊛ = Y
Positive $S_{\xi =6}^{+}\left(⊛\right)$	4,317	923	743
Negative $S_{\xi =6}^{-}\left(⊛\right)$	43,532^b^	9,739^c^	8,061^d^

a See Eqs.1–3 and the relevant text for further explanation.

b Of the negative samples, 21,564 from the 845 phosphoserine proteins and the 21,968 from the 638 non-phosphorylated proteins.

c Of the negative samples, 4,307 from the 386 phosphothreonine proteins and the 5,432 from the 638 non-phosphorylated proteins.

d Of the negative samples, 3,968 from the 249 phosphotyrosine proteins and the 4,362 from the 638 non-phosphorylated proteins.

### Incorporate extracted features into general pseudo amino acid composition

With the avalanche of biological sequence generated in the post-genomic age, one of the most important problems in computational biology is how to formulate a biological sequence with a discrete model or a vector, yet still considerably keep its sequence order information or essential feature. This is because all the existing machine-learning algorithms can only handle vector but not sequence samples, as elaborated in [16].

To address this problem, the pseudo amino acid composition [49, 50] or PseAAC was proposed. Ever since the concept of pseudo amino acid composition or Chou's PseAAC [51–53] was proposed, it has rapidly penetrated into many biomedicine and drug development areas [54–56] and nearly all the areas of computational proteomics (see, e.g., [57–63] as well as a long list of references cited in [64, 65]).

Because it has been widely and increasingly used, recently three powerful open access soft-wares, called ‘PseAAC-Builder’ [51], ‘propy’ [52], and ‘PseAAC-General’ [64], were established: the former two are for generating various modes of Chou's special PseAAC; while the 3rd one for those of Chou's general PseAAC [25], including not only all the special modes of feature vectors for proteins but also the higher level feature vectors such as “Functional Domain” mode (see Eqs.9–10 of [25]), “Gene Ontology” mode (see Eqs.11–12 of [25]), and “Sequential Evolution” or “PSSM” mode (see Eqs.13–14 of [25]). Inspired by the successes of using PseAAC to deal with protein/peptide sequences, three web-servers [66–68] were developed for generating various feature vectors for DNA/RNA sequences. Particularly, recently a powerful web-server called Pse-in-One [69] has been developed that can be used to generate any desired feature vectors for protein/peptide and DNA/RNA sequences according to the need of users’ studies.

According to the general PseAAC [25], the peptide sequence of Eq.1 or Eq.4 can be formulated as

$${\text{P}}_{\xi =6}(⊛)={\left[\Psi_{1}\Psi_{2}\cdots \Psi_{u}\cdots \Psi_{\Omega }\right]}^{\text{T}}$$ (4)

where the components Ψ_*u*_(*u* = 1,2,…,Ω) will be defined by how to extract useful features from the relevant protein/peptide sequence, and **T** is the transpose operator.

### Disorder Score (DS)

Disorder score is a feature to measure the stability of the local structure. Although disordered region does not have fixed three-dimensional structure in proteins, its functional importance has been increasingly recognized [70–73]. It was recently used for identifying protein methylation sites [7]. Particularly, it has been observed that the phosphorylation sites have a strong tendency to be located in disordered regions [74]. Using the VSL2 program [75], the disorder score of each amino acid residues in a protein can be calculated and expressed by

$$\text{DS}=\left[\begin{array}{cccc} d_{1,1} & d_{1,2} & \cdots & d_{1,20}\\ d_{2,1} & d_{2,2} & \cdots & d_{2,20}\\ \vdots & \vdots & \cdots & \vdots \\ d_{k,1} & d_{k,2} & \cdots & d_{k,20}\\ \vdots & \vdots & \vdots & \vdots \\ d_{L,1} & d_{L,2} & \cdots & d_{L,20} \end{array}\right]$$ (5)

where *d_k, j_* is the DS score of the *k*-th amino acid residue (*k* = 1, 2, …, *L*) when its type is (*j* = 1, 2, …, 20). Thus, to reflect the disorder information, the PseAAC of Eq.4 was defined by

$${\text{P}}_{\text{DS}}={\left[{@}_{1}{@}_{2}\cdots {@}_{13}\right]}^{T}$$ (6)

where the components are taken from the disorder score matrix of Eq.5 according to the constituent amino acids in Eq.1 as well as their positions in the relevant protein.

### K Nearest Neighbor Score (KNNS)

Local sequence clusters often exist around phosphorylation sites because the PTM samples in a same family usually share similar patterns. To reflect this kind of patterns, the PseAAC of Eq.4 was defined by

$${\text{P}}_{\text{KNNS}}={\left[\kappa_{1}\kappa_{2}\kappa_{3}\kappa_{4}\kappa_{5}\right]}^{T}$$ (7)

as done in [22, 23] via the BLOSUM62 matrix [76].

### Amino Acid Occurrence Frequency (AAOF)

To reflect the amino acid occurrence frequency, the component in Eq.4 are defined by a 20-D vector; i.e.,

$${\text{P}}_{\text{AAOF}}={\left[f_{1}\;f_{2}\;\cdots \;f_{20}\right]}^{T}$$ (8)

where *f*_1_ is the occurrence frequency of amino acid A in the relevant 13-tuple peptide sample, *f*_2_ is the occurrence frequency of amino acid C, and so forth (according to the alphabetical order of the single-letter codes for 20 native amino acids).

### Position Weight Amino Acid Composition (PWAAC)

Position weight amino acid composition can reveal the sequence-order information around some PTM sites, and it had been used in identifying viral protein phosphorylation sites [24] as well as methylation sites [77]. To reflect this kind of information, the PseAAC of Eq.4 was defined by

$${\text{P}}_{\text{PWAAC}}={\left[{\text{c}}_{1}\;{\text{c}}_{2}\;{\text{c}}_{3}\;{\text{c}}_{4}\;{\text{c}}_{5}\right]}^{T}$$ (9)

where

$$\begin{array}{cc} {\text{c}}_{i}=\frac{1}{\xi \left(\xi +1\right)}\sum_{j=-\xi }^{\xi }\delta_{\text{ij}}\left(j+\frac{\left|j\right|}{\xi }\right) & \left(j=-\xi ,\cdots ,\xi \right) \end{array}$$ (10)

where ξ is the same as in Eq.1, and

$$\delta_{\text{ij}}=\{\begin{array}{ll} 1, & \text{if }i=j\\ 0, & \text{otherwise} \end{array}$$ (11)

### Operation engine

#### Random forests algorithm

Widely used in various areas of computational biology (see, e.g. [11, 12, 27, 45, 46, 78–80]), the random forests (RF) algorithm is a powerful algorithm. Its detailed formulation has been clearly described in [81], and hence there is no need to repeat here.

As shown above, by using DS, KNNS, AAOF, and PWAAC, the sample of Eq.1 can be defined by four different PseAAC vectors, as indicated in Eqs.6, 7, 8, and 9, respectively. Accordingly, we have four different basic RF predictors; i.e.,

$$\{\begin{array}{ll} \text{RF}\left(1\right), & \text{when the sample is based on DS or Eq. 6}\\ \text{RF}\left(2\right), & \text{when the sample is based on KNNS or Eq. 7}\\ \text{RF}\left(3\right), & \text{when the sample is based on AAOF or Eq. 8}\\ \text{RF}\left(4\right), & \text{when the sample is based on PWAAC or Eq. 9} \end{array}$$ (12)

### Ensemble random forests

As demonstrated by a series of previous studies, such as signal peptide prediction [82, 83], membrane protein type classification [84, 85], protein subcellular location prediction [86–88], protein fold pattern recognition [89], enzyme functional classification [90], protein-proteins interaction prediction [45], and protein-protein binding site identification [46], the ensemble predictor formed by fusing an array of individual predictors via a voting system can generate much better prediction quality.

Here, the ensemble predictor is formed by fusing the aforementioned four different individual RF predictor of Eq.12; i.e.,

$${\text{RF}}^{E}=\text{RF}\left(1\right)\forall \text{RF}\left(2\right)\forall \text{RF}\left(3\right)\forall \text{RF}\left(4\right)=\forall_{i=1}^{4}\text{RF}\left(i\right)$$ (13)

where ${\text{RF}}^{E}$ denotes the ensemble predictor, and the symbol ∀ denotes the fusing operator [47]. In the current study, the concrete fusion process can be described as follows. For a query sample of Eq.1, it would be in turn predicted by RF(1), RF(2), RF(3) and RF(4), respectively. If most outcomes indicated that it belonged to phosphorylation segment, its central residue ⊛ was predicted to be phosphorylation site; otherwise, non-phosphorylation site. If there was a tie, the result could be randomly picked between the two. But this kind of tie case rarely happened. For more detailed about this, see a comprehensive review [47] where a crystal clear elucidation with a set of elegant equations are given and hence there is no need to repeat here.

**Figure 4:**
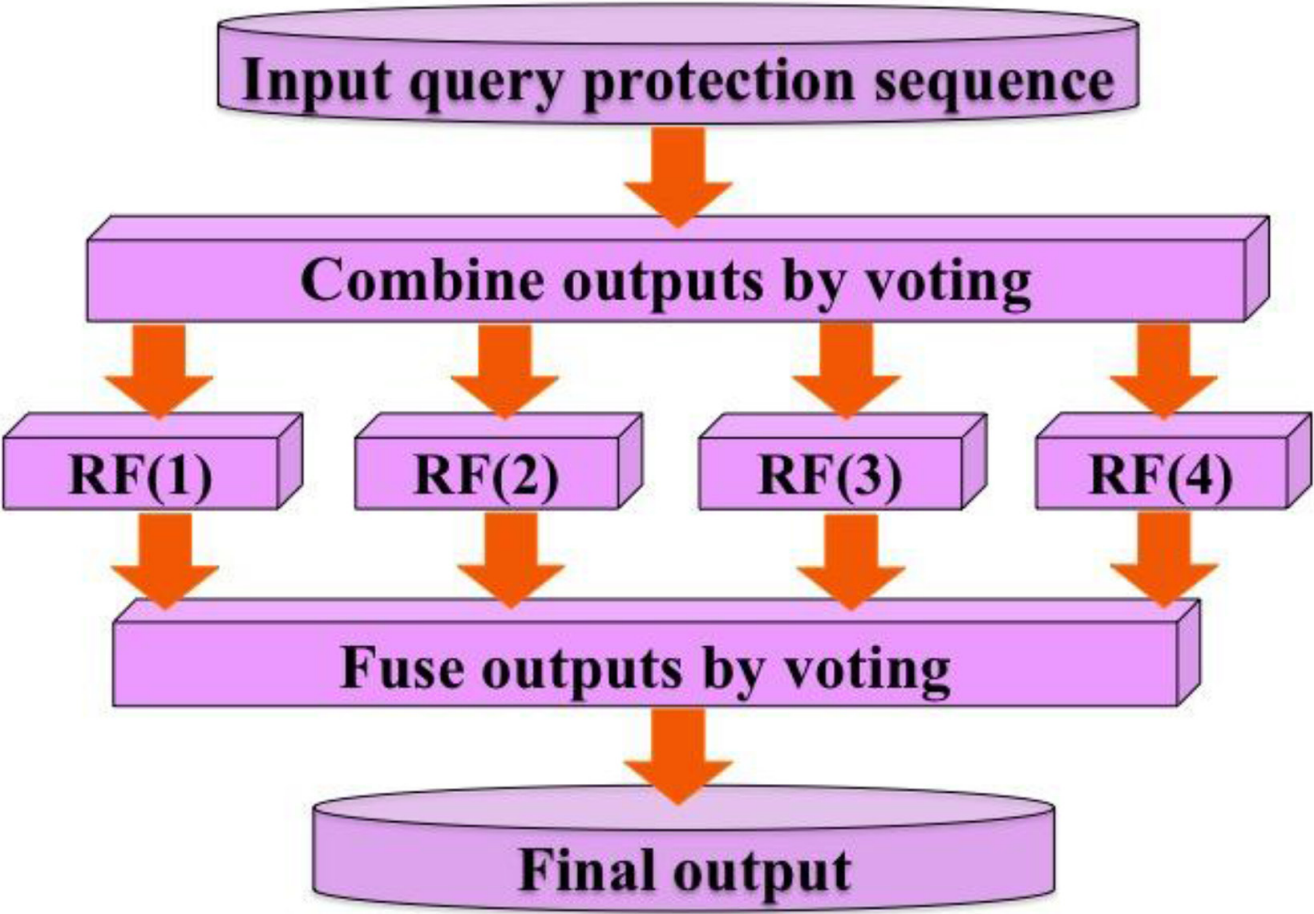
A flow chart to show how the four individual random forest predictors are fused into an ensemble classifier via a voting system See Eqs.12–13 as well as the relevant text for further explanation.

The predictor established via the above procedures is called “iPhos-PseEn”, where “i” stands for identify”, “Phos” for “phosphorylation site”, and “Pse” for “pseudo components”, and “En” for “ensemble”. Depicted in Figure 4 is a flowchart to show how the ensemble predictor is working.

As mentioned in Introduction, among the five guidelines in developing a useful predictor, one of them is how to objectively evaluate its anticipated success rates [25]. To fulfil this, the following two things need to consider: one is what metrics should be used to measure the predictor's quality; the other is what kind of test method should be taken to derive the metrics rates. Below, let us to address such two problems.

### Metrics used to reflect the success rates

A set of four metrics are usually used in literature to measure the quality of a predictor: (1) overall accuracy or Acc; (2) Mathew's correlation coefficient or MCC; (3) sensitivity or Sn; and (4) specificity or Sp [91]. But the conventional formulations for the four metrics are not intuitive, and most experimental scientists feel hard to understand them, particularly for the MCC. Fortunately, if using the symbols introduced by Chou [92] in studying the signal peptides, the set of four metrics can be written as the following forms [5, 93]:

$$\{\begin{array}{ll} \text{sn}=1-\frac{{Ν}_{-}^{+}}{N^{+}} & 0\leq \text{sn}\leq 1\\ \text{sp}=1-\frac{{Ν}_{+}^{-}}{N^{-}} & 0\leq \text{sp}\leq 1\\ \text{Acc}=\Lambda =1-\frac{N_{-}^{+}+N_{+}^{-}}{N^{+}+N^{-}} & 0\leq \text{Acc}\leq 1\\ \text{MCC}=\frac{1-\left(\frac{N_{-}^{+}+N_{+}^{-}}{N^{+}+N^{-}}\right)}{\sqrt{\left(1+\frac{N_{+}^{-}-N_{-}^{+}}{N^{+}}\right)\left(1+\frac{N_{-}^{+}-N_{+}^{-}}{N^{-}}\right)}} & -1\leq \text{MCC}\leq 1 \end{array}$$ (14)

where *N*^+^ represents the total number of true-phosphorylation samples investigated, whereas $N_{-}^{+}$ the number of phosphorylation samples incorrectly predicted to be of false-phosphorylation sample; *N*^−^ the total number of false-phosphorylation samples, whereas $N_{+}^{-}$ the number of false-phosphorylation samples incorrectly predicted to be of true-phosphorylation sample.

According to Eq.14, the following are crystal clear. (1) When $N_{-}^{+}=0$ meaning none of the true-phosphorylation samples is incorrectly predicted to be of false-phosphorylation sample, we have the sensitivity Sn = 1; whereas $N_{-}^{+}=N^{+}$ meaning that all the true-phosphorylation samples are incorrectly predicted to be of false-phosphorylation sample, we have the sensitivity Sn = 0. (2) When $N_{+}^{-}=0$ meaning none of the false-phosphorylation samples is incorrectly predicted to be of true-phosphorylation sample, we have the specificity Sp = 1; whereas $N_{+}^{-}=N^{-}$ meaning that all the false-phosphorylation samples are incorrectly predicted to be of true-phosphorylation sample, we have the specificity Sp = 0. (3) When $N_{-}^{+}=N_{+}^{-}=0$ meaning that none of the true-phosphorylation samples in the positive dataset and none of the false-phosphorylation samples in the negative dataset is incorrectly predicted, we have the overall accuracy Acc = 1 and; Mcc = 1 whereas $N_{-}^{+}=N^{+}$ and $N_{+}^{-}=N^{-}$ meaning that all the true-phosphorylation samples in the positive dataset and all the false-phosphorylation samples in the negative dataset are incorrectly predicted, we have the overall accuracy Acc = 0 and Mcc = −1. (4) When $N_{-}^{+}=N^{+}/2$ and, $N_{+}^{-}=N^{-}/2$ we have Acc = 0.5 and Mcc = 0 meaning no better than random guessing.

As we can see from the above discussion, the set of metrics formulated in Eq.14 has made the meanings of sensitivity, specificity, overall accuracy, and Mathew's correlation coefficient much more intuitive and easier-to-understand, particularly for the meaning of MCC, as unanimously concurred and practically applied by many authors in a series of recent publications (see, e.g., [11, 12, 18, 26, 27, 45, 94–102]).

Note that, of the four metrics in Eq.14, the most important are the Acc and MCC: the former reflects the overall accuracy of a predictor; while the latter, its stability in practical applications. The metrics Sn and Sp are used to measure a predictor from two opposite angles. When, and only when, both Sn and Sp of a tested predictor are higher than those of the other tested predictor, we can say the former predictor is better than the latter one.

Also, it is instructive to point out that the set of equations given in Eq.14 is valid for the single-label systems only. As for the multi-label systems whose emergence has become increasingly often in the system biology [103–105] and system medicine [106], a completely different set of metrics is needed as elucidated in [107].

### Cross-validation

With a set of intuitive evaluation metrics clearly defined, the next step is what kind of validation method should be used to derive the metrics values.

The following three cross-validation methods are often used in literature: (1) independent dataset test, (2) subsampling (or K-fold cross-validation) test, and (3) jackknife test [108]. Of these three, however, the jackknife test is deemed the least arbitrary that can always yield a unique outcome for a given benchmark dataset as elucidated in [25]. Accordingly, the jackknife test has been widely recognized and increasingly used by investigators to examine the quality of various predictors (see, e.g., [57–59, 109–115]).

In this study, however, to reduce the computational time, we adopted the 5-fold cross-validation method, as done by many investigators with SVM as the prediction engine. Given below is a more rigorous description of 5-fold cross-validation on a benchmark dataset S.

First, randomly divided the benchmark dataset S into five groups $S_{1}$, $S_{2}$, $S_{3}$, $S_{4}$, and $S_{5}$, with each having approximately the same number of samples not only for the main-set level but also for all the sub-set levels considered, as can be formulated by

$$S_{1}≜S_{2}≜S_{3}≜S_{4}≜S_{5}$$ (15)

where the symbol ≜ means that the divided datasets are about the same in size, and so are their subsets [27]. Next, each of the five sub-benchmark datasets was singled out one-by-one and tested by the model trained with the remaining four sub-benchmark datasets. The cross-validation process was repeated for five times, with their average as the final outcome. In other words, during the process of 5-fold cross-validation, both the training dataset and testing dataset were actually open, and each sub-benchmark datasets was in turn moved between the two. The 5-fold cross-validation test can exclude the “memory” effect, just like conducting 5 different independent dataset tests.

As we can see from Table 2 or Supporting Information S1, S2, and S3, the negative subset $S_{\xi =6}^{-}\left(⊛\right)$ is much larger than the positive subset $S_{\xi =6}^{+}\left(⊛\right)$. The ratio is about 10:1 for all the three types of phosphorylation. Although this might reflect the real world in which the non-phosphorylation sites are always the majority compared with the phosphorylation ones, a predictor trained by such a highly skewed benchmark dataset would inevitably have the bias consequence that many phosphorylation sites might be mispredicted as non-phosphorylation ones. To deal with this kind of situation, we randomly divide the negative subset into ten groups with each having about the same size. Thus, for each of the three types of phosphorylation, we have ten benchmark datasets in which the positive and negative samples are about the same. It was based on each of such ten datasets that the 5-fold cross-validation was performed, followed by taking an average for the final score.

## CONCLUSIONS

The iPhos-PseEn predictor is a new bioinformatics tool for identifying the phosphorylation sites in proteins. Compared with the existing predictors in this area, its prediction quality is much better, with remarkably higher sensitivity, specificity, overall accuracy, and Mathew's correlation coefficient. For the convenience of most experimental scientists, we have provided its web-server and a step-by-step guide, by which users can easily obtain their desired results without the need to go through the detailed mathematics.

We anticipate that iPhos-PseEn will become a very useful high throughput tool, or at the very least, a complementary tool to the existing methods for predicting the protein phosphorylation sites.

### Online Supporting Information

Please refer to “Supporting information S1, Supporting information S2, Supporting information S3” in Supplementary Materials

## SUPPLEMENTARY MATERIALS FIGURES AND TABLES








