# Supplementary material for: iPhos-PseEn: Identifying phosphorylation sites in proteins by fusing different pseudo components into an ensemble classifier
Source: Oncotarget. 2016 Jun 13;7(32):51270–83. doi: 10.18632/oncotarget.9987 (PMC5239474; doi:10.18632/oncotarget.9987)
Supplement: Supplementary file 1 [file oncotarget-07-51270-s001.pdf]

iPhos-PseEn: Identifying phosphorylation sites in proteins by fusing different pseudo components into an ensemble classifier

Supplementary Materials

Supporting Information S1: The benchmark dataset $\mathbb{S}_{\xi=6}(\mathrm{S})$ used to train and test the model for predicting the possibility of phosphorylation at Ser site. It contains 47,849 samples, of which 4,317 are positive samples and 43,532 are negative samples. All these samples were derived from 1,770 protein sequences in which none has ≥ 50% pairwise sequence identity with any other. See the main text for further explanation. See Supporting_Information_S1

Supporting Information S2: The benchmark dataset $\mathbb{S}_{\xi=6}(\mathrm{T})$ used to train and test the model for predicting the possibility of phosphorylation at Thr site. It contains 10,662 samples, of which 923 are positive samples and 9,739 are negative samples. All these samples were derived from 1,770 protein sequences in which none has ≥ 50% pairwise sequence identity with any other. See the main text for further explanation. See Supporting_Information_S2

Supporting Information S3: The benchmark dataset $\mathbb{S}_{\xi=6}(\mathrm{Y})$ used to train and test the model for predicting the possibility of phosphorylation at Tyr site. It contains 8,804 samples, of which 743 are positive samples and 8,061 are negative samples. All these samples were derived from 1,770 protein sequences in which none has ≥ 50% pairwise sequence identity with any other. See the main text for further explanation. See Supporting_Information_S3
