# Supplementary material for: iPhos-PseEn: Identifying phosphorylation sites in proteins by fusing different pseudo components into an ensemble classifier
Source: Oncotarget. 2016 Jun 13;7(32):51270–83. doi: 10.18632/oncotarget.9987 (PMC5239474; doi:10.18632/oncotarget.9987)
Supplement: Supplementary file 3 [file oncotarget-07-51270-s003.docx]

**Supporting Information S2.** The benchmark dataset $\mathbb{S}_{\xi=6}\left( T \right)$ used to train and test the model for predicting the possibility of phosphorylation at Thr site. It contains 10,662 samples, of which 923 are positive samples and 9,739 are negative samples. All these samples were derived from 1,770 protein sequences in which none has $\geq50\%$ pairwise sequence identity with any other. See the main text for further explanation.

# List of the 923 peptide samples in the positive subset $\mathbb{S}_{\boldsymbol{\xi=6}}^{\mathbf{+}}\boldsymbol{(}\mathbf{T}\boldsymbol{)}$

| Sample # | Protein ID | Site | Sequences |
| --- | --- | --- | --- |
| 1  2  3  4  5  6  7  8  9  10  11  12  13  14  15  16  17  18  19  20  21  22  23  24  25  26  27  28  29  30  31  32  33  34  35  36  37  38  39  40  41  42  43  44  45  46  47  48  49  50  51  52  53  54  55  56  57  58  59  60  61  62  63  64  65  66  67  68  69  70  71  72  73  74  75  76  77  78  79  80  81  82  83  84  85  86  87  88  89  90  91  92  93  94  95  96  97  98  99  100  101  102  103  104  105  106  107  108  109  110  111  112  113  114  115  116  117  118  119  120  121  122  123  124  125  126  127  128  129  130  131  132  133  134  135  136  137  138  139  140  141  142  143  144  145  146  147  148  149  150  151  152  153  154  155  156  157  158  159  160  161  162  163  164  165  166  167  168  169  170  171  172  173  174  175  176  177  178  179  180  181  182  183  184  185  186  187  188  189  190  191  192  193  194  195  196  197  198  199  200  201  202  203  204  205  206  207  208  209  210  211  212  213  214  215  216  217  218  219  220  221  222  223  224  225  226  227  228  229  230  231  232  233  234  235  236  237  238  239  240  241  242  243  244  245  246  247  248  249  250  251  252  253  254  255  256  257  258  259  260  261  262  263  264  265  266  267  268  269  270  271  272  273  274  275  276  277  278  279  280  281  282  283  284  285  286  287  288  289  290  291  292  293  294  295  296  297  298  299  300  301  302  303  304  305  306  307  308  309  310  311  312  313  314  315  316  317  318  319  320  321  322  323  324  325  326  327  328  329  330  331  332  333  334  335  336  337  338  339  340  341  342  343  344  345  346  347  348  349  350  351  352  353  354  355  356  357  358  359  360  361  362  363  364  365  366  367  368  369  370  371  372  373  374  375  376  377  378  379  380  381  382  383  384  385  386  387  388  389  390  391  392  393  394  395  396  397  398  399  400  401  402  403  404  405  406  407  408  409  410  411  412  413  414  415  416  417  418  419  420  421  422  423  424  425  426  427  428  429  430  431  432  433  434  435  436  437  438  439  440  441  442  443  444  445  446  447  448  449  450  451  452  453  454  455  456  457  458  459  460  461  462  463  464  465  466  467  468  469  470  471  472  473  474  475  476  477  478  479  480  481  482  483  484  485  486  487  488  489  490  491  492  493  494  495  496  497  498  499  500  501  502  503  504  505  506  507  508  509  510  511  512  513  514  515  516  517  518  519  520  521  522  523  524  525  526  527  528  529  530  531  532  533  534  535  536  537  538  539  540  541  542  543  544  545  546  547  548  549  550  551  552  553  554  555  556  557  558  559  560  561  562  563  564  565  566  567  568  569  570  571  572  573  574  575  576  577  578  579  580  581  582  583  584  585  586  587  588  589  590  591  592  593  594  595  596  597  598  599  600  601  602  603  604  605  606  607  608  609  610  611  612  613  614  615  616  617  618  619  620  621  622  623  624  625  626  627  628  629  630  631  632  633  634  635  636  637  638  639  640  641  642  643  644  645  646  647  648  649  650  651  652  653  654  655  656  657  658  659  660  661  662  663  664  665  666  667  668  669  670  671  672  673  674  675  676  677  678  679  680  681  682  683  684  685  686  687  688  689  690  691  692  693  694  695  696  697  698  699  700  701  702  703  704  705  706  707  708  709  710  711  712  713  714  715  716  717  718  719  720  721  722  723  724  725  726  727  728  729  730  731  732  733  734  735  736  737  738  739  740  741  742  743  744  745  746  747  748  749  750  751  752  753  754  755  756  757  758  759  760  761  762  763  764  765  766  767  768  769  770  771  772  773  774  775  776  777  778  779  780  781  782  783  784  785  786  787  788  789  790  791  792  793  794  795  796  797  798  799  800  801  802  803  804  805  806  807  808  809  810  811  812  813  814  815  816  817  818  819  820  821  822  823  824  825  826  827  828  829  830  831  832  833  834  835  836  837  838  839  840  841  842  843  844  845  846  847  848  849  850  851  852  853  854  855  856  857  858  859  860  861  862  863  864  865  866  867  868  869  870  871  872  873  874  875  876  877  878  879  880  881  882  883  884  885  886  887  888  889  890  891  892  893  894  895  896  897  898  899  900  901  902  903  904  905  906  907  908  909  910  911  912  913  914  915  916  917  918  919  920  921  922  923 | O00257  O00267  O00267  O00267  O00399  O00418  O00418  O00562  O00562  O14607  O14672  O14713  O14745  O14950  O14965  O14965  O14974  O14974  O15162  O15232  O15240  O15264  O15350  O15357  O15357  O15392  O15392  O15392  O15516  O15516  O15530  O15530  O43164  O43164  O43255  O43255  O43257  O43318  O43318  O43493  O43524  O43524  O43526  O43663  O43663  O43663  O43683  O60285  O60343  O60343  O60566  O60566  O60566  O60610  O75367  O75367  O75449  O75531  O75531  O75533  O75533  O75533  O75533  O75533  O75533  O75533  O75533  O75533  O75533  O75533  O75533  O75533  O75533  O75533  O75533  O75533  O75533  O75533  O75533  O75533  O75533  O75533  O75533  O75581  O75581  O75582  O75582  O75689  O75716  O75821  O75821  O75822  O76024  O76061  O94811  O94811  O94916  O95071  O95071  O95071  O95071  O95071  O95071  O95071  O95251  O95251  O95267  O95551  O95551  O95714  O95714  O95714  O95786  O95835  O95835  O95863  O96017  O96017  O96017  P00740  P01008  P01100  P01100  P01100  P01106  P01106  P02765  P02768  P02768  P02768  P03956  P04004  P04004  P04406  P04406  P04406  P04406  P04406  P04406  P04406  P04637  P04637  P04637  P05067  P05067  P05106  P05107  P05107  P05107  P05412  P05412  P05412  P05412  P05412  P05412  P05412  P05771  P05771  P05771  P05771  P05771  P05771  P05771  P05771  P05814  P06400  P06400  P06400  P06400  P06400  P06400  P06400  P06702  P06748  P06748  P06748  P06748  P06748  P06748  P06748  P07332  P07358  P07900  P07900  P08047  P08047  P08047  P08047  P08047  P08047  P08047  P08833  P08833  P08865  P09603  P0DI83  P10244  P10244  P10244  P10244  P10244  P10244  P10415  P10451  P10451  P10451  P10451  P10636  P10636  P10636  P10636  P10636  P10636  P10636  P11171  P11277  P11309  P11388  P11388  P11388  P11388  P13498  P13569  P13639  P13639  P13639  P13639  P15056  P15056  P15056  P15056  P15056  P15336  P15336  P15336  P15336  P15336  P15927  P15941  P16066  P16066  P16401  P16401  P16401  P16401  P17096  P17096  P17096  P17612  P17612  P17612  P17612  P17661  P17676  P17676  P17676  P18887  P18887  P18887  P18887  P18887  P18887  P18887  P18887  P19021  P19419  P19419  P19419  P19419  P19419  P19429  P19429  P19429  P19429  P19429  P19429  P19525  P19525  P19525  P19525  P19525  P19525  P19525  P20020  P20020  P21127  P21127  P21453  P23246  P23327  P23396  P23396  P23396  P23396  P23443  P23443  P23443  P24385  P24864  P24864  P25963  P25963  P26651  P26678  P27361  P27361  P27361  P27708  P27708  P28749  P28749  P28749  P28749  P29474  P29474  P29474  P29590  P29590  P29590  P30260  P30260  P30260  P30260  P30260  P30260  P30260  P30291  P30291  P30304  P30307  P30307  P30307  P31645  P31645  P31645  P31751  P31751  P31751  P31949  P34947  P35222  P35222  P35236  P35269  P35269  P35269  P35367  P35367  P35367  P35398  P35611  P35611  P35611  P35612  P35612  P35659  P35659  P35659  P35659  P36507  P36507  P36871  P36871  P36871  P38398  P38936  P38936  P41235  P41235  P41235  P41279  P41279  P41743  P41743  P41743  P41743  P42345  P42345  P42345  P42345  P43405  P43405  P43405  P43405  P43405  P43405  P45985  P46060  P46060  P46527  P46527  P46527  P46527  P46695  P46695  P46734  P46937  P46937  P46937  P46937  P46937  P49137  P49137  P49137  P49760  P49760  P49815  P49815  P49815  P50548  P50548  P50548  P50548  P50552  P50552  P50613  P50750  P50750  P50750  P50750  P50750  P50750  P51575  P51587  P51617  P51617  P51617  P51955  P51955  P51955  P52630  P52630  P52732  P52732  P53350  P53350  P53350  P53350  P53667  P53667  P53779  P54198  P54198  P54198  P54646  P54646  P54829  P55211  P57059  P57059  P60484  P60484  P60484  P60484  P62136  P68104  P68400  P68400  P78362  P78527  P78527  P78527  P78536  P80192  P80192  P80192  P98177  Q00534  Q00534  Q00534  Q00534  Q00613  Q00613  Q00987  Q01860  Q02156  Q02156  Q02156  Q02156  Q02156  Q02818  Q04206  Q04206  Q04206  Q04759  Q04759  Q04760  Q05655  Q05655  Q05655  Q05655  Q05655  Q05655  Q06413  Q06413  Q06609  Q06830  Q07820  Q08050  Q08050  Q08050  Q08050  Q08999  Q08999  Q08999  Q09161  Q0VD86  Q12778  Q12834  Q12834  Q12834  Q12888  Q12888  Q12888  Q12888  Q12888  Q12888  Q12888  Q12888  Q12906  Q12906  Q12906  Q12933  Q12933  Q12933  Q13042  Q13042  Q13045  Q13098  Q13153  Q13153  Q13153  Q13153  Q13153  Q13153  Q13163  Q13188  Q13188  Q13188  Q13188  Q13342  Q13522  Q13542  Q13542  Q13542  Q13554  Q13554  Q13554  Q13554  Q13568  Q13627  Q14164  Q14194  Q14194  Q14194  Q14194  Q14207  Q14207  Q14344  Q14457  Q14493  Q14493  Q14493  Q14524  Q14653  Q14653  Q14653  Q14653  Q14653  Q14653  Q14653  Q14654  Q14676  Q14676  Q14676  Q14676  Q14676  Q14676  Q14676  Q14676  Q14676  Q14676  Q14676  Q14676  Q14676  Q14676  Q14676  Q14676  Q14676  Q14676  Q14676  Q14676  Q14676  Q14676  Q14680  Q14680  Q14680  Q14680  Q14680  Q14680  Q14680  Q14680  Q14686  Q14694  Q14694  Q14694  Q14980  Q14980  Q14980  Q14980  Q14980  Q14980  Q14980  Q14980  Q14994  Q15078  Q15080  Q15208  Q15208  Q15291  Q15398  Q15398  Q15398  Q15398  Q15398  Q15398  Q15398  Q15398  Q15554  Q15648  Q15648  Q15648  Q15648  Q15648  Q15648  Q15788  Q15796  Q15796  Q15831  Q15831  Q15831  Q15910  Q15910  Q15910  Q16512  Q16512  Q16512  Q16513  Q16513  Q16513  Q16513  Q16518  Q16518  Q16566  Q16584  Q16584  Q16625  Q16625  Q16637  Q16637  Q16665  Q16695  Q16695  Q16695  Q16695  Q16695  Q2M2Z5  Q2NKX8  Q2NKX8  Q2PPJ7  Q4G163  Q53GL7  Q53HL2  Q53HL2  Q53HL2  Q53HL2  Q53HL2  Q53HL2  Q53HL2  Q5VT25  Q6P5Z2  Q6P5Z2  Q6PKG0  Q6PKG0  Q6PKG0  Q6PKG0  Q6PKG0  Q6Q788  Q6R327  Q71F23  Q71F23  Q7RTN6  Q7RTN6  Q86UC2  Q86W56  Q8IU85  Q8IVT2  Q8IVT2  Q8IVT2  Q8IVT2  Q8IVT2  Q8IVT2  Q8IVT2  Q8IW41  Q8IY84  Q8N122  Q8N163  Q8N163  Q8N163  Q8N264  Q8N4F0  Q8N4N8  Q8NB16  Q8ND76  Q8ND76  Q8ND76  Q8ND76  Q8ND76  Q8NHV4  Q8NHV4  Q8NHW3  Q8NHW3  Q8TAE6  Q8TB45  Q8TB45  Q8TD08  Q8TDC3  Q8WYK2  Q92538  Q92538  Q92597  Q92597  Q92597  Q92597  Q92630  Q92630  Q92794  Q92918  Q92918  Q92918  Q92974  Q92974  Q92997  Q969H0  Q96A00  Q96B36  Q96BR1  Q96EB6  Q96EB6  Q96EB6  Q96EY5  Q96G74  Q96J02  Q96J02  Q96L34  Q96Q89  Q96R06  Q96R06  Q96R06  Q96R06  Q96RG2  Q96RG2  Q99459  Q99459  Q99459  Q99459  Q99459  Q99459  Q99459  Q99459  Q99459  Q99459  Q99459  Q99558  Q99613  Q99640  Q99640  Q99683  Q99683  Q99683  Q99708  Q99958  Q99986  Q9BQ15  Q9BQA1  Q9BUB5  Q9BUB5  Q9BUB5  Q9BWT1  Q9BX84  Q9BXA7  Q9BYG3  Q9BYG3  Q9BYG3  Q9BYG3  Q9GZN2  Q9GZN2  Q9GZX7  Q9H063  Q9H063  Q9H093  Q9H0H5  Q9H0H5  Q9H0H5  Q9H0H5  Q9H0H5  Q9H0H5  Q9H0H5  Q9H0H5  Q9H0K1  Q9H0K1  Q9H1A4  Q9H1A4  Q9H1D0  Q9H211  Q9H2D6  Q9H300  Q9H4X1  Q9H7P6  Q9H7P6  Q9H7P6  Q9H8S9  Q9H8S9  Q9H8S9  Q9H8S9  Q9H8V3  Q9H8V3  Q9H8V3  Q9H8V3  Q9H8Y8  Q9H8Y8  Q9H8Y8  Q9H8Y8  Q9HAW4  Q9HB96  Q9HBH9  Q9HBH9  Q9HBH9  Q9HC16  Q9HC16  Q9HC98  Q9HC98  Q9HCC9  Q9HCX4  Q9NQS7  Q9NQS7  Q9NQS7  Q9NQS7  Q9NQS7  Q9NQS7  Q9NQS7  Q9NQS7  Q9NRD5  Q9NRH2  Q9NRM7  Q9NVI1  Q9NWZ3  Q9NWZ3  Q9NX09  Q9NX09  Q9NYL2  Q9NYL2  Q9NYV4  Q9NYV4  Q9NYV4  Q9NYV4  Q9NYV4  Q9NYV6  Q9NZJ0  Q9NZJ0  Q9NZJ0  Q9P0L2  Q9P0L2  Q9P0L2  Q9P0L2  Q9P1Z0  Q9P1Z0  Q9P1Z0  Q9UBF6  Q9UBF8  Q9UBF8  Q9UBF8  Q9UBR4  Q9UD71  Q9UD71  Q9UJX2  Q9UJX2  Q9UJX2  Q9UJX2  Q9UJY1  Q9UK32  Q9ULC4  Q9UPN6  Q9UPN6  Q9UPZ9  Q9UQ84  Q9UQ84  Q9UQC2  Q9UQC2  Q9UQC2  Q9UQC2  Q9UQC2  Q9UQC2  Q9UQF2  Q9UQF2  Q9UQF2  Q9UQF2  Q9UQL6  Q9Y295  Q9Y2K2  Q9Y2K2  Q9Y2K2  Q9Y371  Q9Y3C5  Q9Y6E0  Q9Y6E0  Q9Y6G9  Q9Y6G9  Q9Y6G9  Q9Y6G9  Q9Y6G9  Q9Y6R1  Q9Y6R1 | 497  775  784  1034  186  348  353  59  287  887  719  38  293  19  287  288  443  696  161  442  424  180  27  165  958  34  48  117  451  461  354  513  246  389  26  119  103  184  187  302  32  179  217  470  481  616  609  211  568  642  792  1008  1042  768  129  178  133  2  3  142  203  207  211  223  227  244  248  257  261  267  273  278  296  299  303  313  326  328  341  350  354  434  436  1479  1493  581  700  276  185  38  41  109  30  254  14  92  135  637  1115  1135  1736  1969  2030  2213  85  88  184  88  92  647  1944  4827  770  246  1079  203  68  383  387  205  63  232  325  331  8  58  319  107  444  446  274  69  76  75  177  182  184  211  229  237  18  55  284  729  743  779  758  759  760  2  8  89  91  93  239  286  17  250  314  324  500  504  635  642  18  252  356  373  821  823  826  841  113  75  95  199  219  234  237  279  421  418  5  7  278  453  640  651  668  681  739  157  193  241  266  69  266  440  444  487  494  520  69  66  185  190  237  470  498  522  529  534  548  744  60  2110  114  282  1205  1343  1470  147  717  54  57  59  435  332  373  396  401  753  52  69  71  73  116  21  1224  532  545  11  39  138  155  39  53  78  49  196  198  202  17  226  235  266  198  202  257  453  457  488  519  523  943  336  353  363  368  417  31  51  78  129  143  181  88  89  90  255  258  446  451  1116  1203  488  595  236  687  76  42  70  221  242  252  412  444  286  77  395  291  299  92  17  198  202  207  456  1884  332  369  385  997  33  495  1175  28  42  409  205  209  244  313  366  430  446  190  239  507  48  67  130  276  613  616  309  449  451  10  485  41  556  66  331  389  446  140  142  279  183  331  445  480  55  611  13  199  289  290  394  396  185  467  507  1394  80  145  166  429  432  80  290  3  9  412  564  1162  2164  2173  2446  256  317  345  384  530  582  261  409  436  157  170  187  198  18  123  222  63  110  119  154  412  25  222  334  127  344  927  1330  1462  3  7  441  526  278  316  170  29  186  350  354  362  363  389  3387  66  209  387  170  175  179  294  800  458  926  6  210  214  498  229  508  221  555  576  586  172  258  255  125  182  322  366  382  383  401  320  432  344  360  492  2609  2638  2647  735  304  305  312  32  49  70  177  325  142  323  419  235  228  309  566  703  710  148  254  435  505  219  538  107  43  50  141  218  451  507  293  300  309  90  163  611  620  627  662  642  974  986  21  182  24  70  106  157  302  543  548  855  922  1214  1372  1609  188  315  592  7  22  117  581  599  818  479  84  185  212  219  230  423  315  117  180  336  384  726  35  37  46  70  287  306  307  401  10  402  501  101  102  509  514  1270  1350  203  119  61  62  171  38  3  75  180  237  244  253  404  341  4  301  331  378  404  449  455  523  1157  1198  1302  1403  1425  1548  1567  1589  1630  1664  1671  1697  1800  1858  56  167  398  409  478  494  518  539  1321  24  42  100  163  211  1776  1804  2000  2015  2055  2106  38  138  154  74  444  252  326  329  338  401  402  639  759  784  230  805  1032  1051  1057  1215  1457  1179  8  220  189  336  363  345  367  487  774  778  914  121  124  628  958  101  105  200  277  708  403  404  25  85  555  4  7  12  81  108  379  813  1063  715  234  101  88  94  106  169  189  204  230  240  718  860  376  526  649  724  845  55  1135  78  110  329  419  286  199  180  164  172  219  224  287  377  577  182  229  706  35  454  484  452  52  125  357  30  37  67  75  331  382  550  53  57  73  241  259  175  189  148  507  1337  328  346  366  375  106  381  369  165  175  355  679  945  346  205  38  246  320  530  544  719  130  507  263  385  214  1644  111  336  937  978  1161  1165  227  377  385  396  404  411  415  424  430  438  442  559  524  17  495  813  838  842  847  247  355  117  5  250  255  385  163  1851  174  223  234  238  279  182  186  27  64  212  208  161  260  342  567  580  588  601  606  25  175  291  537  742  29  2229  69  111  122  204  205  12  35  74  181  359  373  444  846  222  225  415  433  916  346  244  249  379  32  218  202  210  870  15  195  199  213  239  292  406  832  892  82  173  279  952  342  345  23  25  161  628  57  514  692  893  1244  200  464  516  684  5  208  215  613  795  797  983  10  263  438  519  63  34  75  562  565  582  596  63  581  81  6  615  157  581  621  265  278  287  331  385  391  103  205  411  448  292  100  13  163  411  145  135  18  190  213  408  512  513  515  49  254 | LQVKPE**T**PASAAV  PMYGSQ**T**PMYGSG  YGSGSR**T**PMYGSQ  EHLEPI**T**PTKNNK  TMKGSS**T**PVKNNN  LLQSAK**T**ILRGTE  KTILRG**T**EEKCGS  GGSGQY**T**HKVYHV  AASNTG**T**PDGPEA  DKLNPP**T**PSIYLE  PKPLPG**T**LKRRRP  GLSRSS**T**VASLDT  RSASSD**T**SEELNS  KRPQRA**T**SNVFAM  APSSRR**T**TLCGTL  PSSRRT**T**LCGTLD  RLGLRK**T**GSYGAL  RQSRRS**T**QGVTLT  GPSRPF**T**LRIIDN  ARRLVS**T**EDACGC  KRSQEE**T**PGHRRK  HADAEM**T**GYVVTR  SLEPDS**T**YFDLPQ  PLPAPE**T**PTAPAA  PREEPL**T**PRLKPE  LEGCAC**T**PERMAE  GFIHCP**T**ENEPDL  NKIAKE**T**NNKKKE  VSDPSS**T**PTKIPT  IPTDTS**T**PPRQHL  ENLHQQ**T**PPKLTA  EAKNFK**T**FFVHTP  KSSAGD**T**EFVHQN  VITQRE**T**ENNQMT  PPQPQH**T**PSPAAP  TCRGAL**T**PSIRNL  EGPNYL**T**ACAGPP  TACDIQ**T**HMTNNK  DIQTHM**T**NNKGSA  TESGEE**T**DLISPP  SRPRSC**T**WPLQRP  SPDKRL**T**LSQIYE  MDRRGG**T**WKLLGS  YGSAPR**T**PSKRRG  RGLAPN**T**PGKARK  SGILNS**T**NIQSSS  AAQLAS**T**PFHKLP  KDKFLQ**T**FCGSPL  KAKRSL**T**SSLENI  FRRRAH**T**FSHPPS  RNSAEL**T**VIKVSS  NANDEA**T**VSVLGE  WKVGKL**T**SPGALL  VLPFGL**T**PKKLYK  KLEAII**T**PPPAKK  DSTTEG**T**PADGFT  GNRPST**T**VRVHRS  MMMMMM**T**TSQKHR  MMMMMT**T**SQKHRD  FADGGK**T**PDPKMN  KRRWDQ**T**ADQTPG  DQTADQ**T**PGATPK  DQTPGA**T**PKKLSS  SWDQAE**T**PGHTPS  AETPGH**T**PSLRWD  RAKGSE**T**PGATPG  SETPGA**T**PGSKIW  SKIWDP**T**PSHTPA  DPTPSH**T**PAGAAT  TPAGAA**T**PGRGDT  TPGRGD**T**PGHATP  DTPGHA**T**PGHGGA  KNRWDE**T**PKTERD  WDETPK**T**ERDTPG  PKTERD**T**PGHGSG  GSGWAE**T**PRTDRG  GDSIGE**T**PTPGAS  SIGETP**T**PGASKR  KSRWDE**T**PASQMG  SQMGGS**T**PVLTPG  GSTPVL**T**PGKTPI  ARKLTA**T**PTPLGG  KLTATP**T**PLGGMT  SSSTKG**T**YFPAIL  PPPSPA**T**ERSHYT  DNQPLK**T**PCFTLH  SSNPLM**T**PDILGS  FRKRWF**T**MDDRRL  GSRQAL**T**LQDWAA  KGIPLA**T**GDTSPE  PLATGD**T**SPEPEL  EEPKVL**T**PEEQLA  RSRLNA**T**ASLEQE  EPSSRE**T**GRGAKG  AKAANR**T**PPKSPG  IDGRNV**T**VTDVDI  VQQHPS**T**PKRHTV  YKRRRS**T**PAPKEE  KDARGM**T**PFMSAV  AITILE**T**AQKIAK  APSSTS**T**PAASSA  QQTTLD**T**PQLERK  RRSDSM**T**FLGCIP  EPGSIL**T**ELGGFE  RSQQQP**T**PVTPKK  QQPTPV**T**PKKYPL  DWSRKL**T**QRIKSN  KTYVDL**T**NEETTD  DLTNEE**T**TDSTTS  GSDGCK**T**PKLIEK  SAEDSD**T**EDDSEA  SFASDS**T**QDYLTG  SILRLQ**T**WDEAVF  PQVRSV**T**PPPPPR  HAFYEF**T**FRRFFD  GHVRTH**T**GEKPFS  SLETVS**T**QELYSI  ETSLMR**T**LCGTPT  MRTLCG**T**PTYLAP  VDYVNS**T**EAETIL  SPEKKA**T**EDEGSE  GLPEVA**T**PESEEA  ELEPLC**T**PVVTCT  TPVVTC**T**PSCTAY  PLNVSF**T**NRNYDL  KFELLP**T**PPLSPS  HYDLRH**T**FMGVVS  VATLRE**T**YGEMAD  KVPQVS**T**PTLVEV  PQVSTP**T**LVEVSR  QPIGPQ**T**PKACDS  TRGDVF**T**MPEDEY  MPEDEY**T**VYDDGE  INGNPI**T**IFQERD  VEGLMT**T**VHAITA  TTVHAI**T**ATQKTV  VHAITA**T**QKTVDG  NIIPAS**T**GAAKAV  ELNGKL**T**GMAFRV  MAFRVP**T**ANVSVV  PPLSQE**T**FSDLWK  DIEQWF**T**EDPGPD  PGRDRR**T**EEENLR  LKKKQY**T**SIHHGV  EVDAAV**T**PEERHL  YKEATS**T**FTNITY  PLFKSA**T**TTVMNP  LFKSAT**T**TVMNPK  FKSATT**T**VMNPKF  MMMMMM**T**AKMETT  TAKMET**T**FYDDAL  SSNGHI**T**TTPTPT  NGHITT**T**PTPTQF  HITTTP**T**PTQFLC  PEMPGE**T**PPLSPI  LEEKVK**T**LKAQNS  SEGEES**T**VRFARK  IWDWDL**T**SRNDFM  AKISQG**T**KVPEEK  EEKTTN**T**VSKFDN  DGVTTK**T**FCGTPD  TKTFCG**T**PDYIAP  NFDKEF**T**RQPVEL  RQPVEL**T**PTDKLF  LALARE**T**IESLSS  INGSPR**T**PRRGQN  SFETQR**T**PRKSNL  NVIPPH**T**PVRTVM  ISEGLP**T**PTKMTP  EGLPTP**T**KMTPRS  PTPTKM**T**PRSRIL  IGESFG**T**SEKFQK  PGLGEG**T**PPPPPP  GSPIKV**T**LATLKM  LGGFEI**T**PPVVLR  KKSIRD**T**PAKNAQ  DSKPSS**T**PRSKGQ  FKKQEK**T**PKTPKG  QEKTPK**T**PKGPSS  KNCFRM**T**DQEAIQ  EREGGR**T**PTLEIL  DRNKRD**T**MVEDLV  MMMPEE**T**QTQDQP  MPEETQ**T**QDQPME  VSAATL**T**PSSQAV  GPIIIR**T**PTVGPN  GKVYGK**T**SHLRAH  AHLRWH**T**GERPFM  YCGKRF**T**RSDELQ  RHKRTH**T**GEKKFA  EGSGTA**T**PSALIT  LWDAIS**T**YDGSKA  LAKAQE**T**SGEEIS  APEFTA**T**QPEVAD  SFEPPE**T**PVVKDS  PRVIFG**T**PRARVI  DLDAVR**T**PEPLEE  DSCNSL**T**PKSTPV  SLTPKS**T**PVKTLP  QKVVVT**T**PLHRDK  PLHRDK**T**PLHQKH  NTPHTP**T**PFKNAL  RDPVAR**T**SPLQTP  AVSSEE**T**NDFKQE  IQYPDA**T**DEDITS  ATDEDI**T**SHMESE  DDQSAE**T**HSHKQS  GKTKIA**T**PRGAAP  TPPAPK**T**PPSSGE  SPGSPG**T**PGSRSR  PGSRSR**T**PSLPTP  RTPSLP**T**PPTREP  KVAVVR**T**PPKSPS  DSPQLA**T**LADEVS  KASNGD**T**PTHEDL  VSHHAA**T**ERTSPV  CNDLHA**T**KLAPGK  KDKLDE**T**GNSLKV  KAKGKK**T**QMAEVL  SDFDEK**T**DDEDFV  RKRKPS**T**SDDSDS  RPQIGG**T**IKQPPS  FSIVQK**T**PLQMNG  SARAGE**T**RFTDTR  AGETRF**T**DTRKDE  ETRFTD**T**RKDEQE  IMGPNY**T**PGKKED  IGPQIL**T**SPSPSK  PNVHIN**T**IEPVNI  GDGGST**T**GLSATP  TTGLSA**T**PPASLP  ACASPK**T**PIQAGG  KHKHEM**T**LKFGPA  VIVADQ**T**PTPTRF  VADQTP**T**PTRFLK  DQTPTP**T**RFLKNC  DLSPLA**T**PIIRSK  GGAGGY**T**QSPGGF  YVPPSS**T**DRSPYE  SAGSRL**T**LSGRGS  NYGSLL**T**TEGQFQ  APAETA**T**PAPVEK  AAKRKA**T**GPPVSE  KKPAGA**T**PKKAKK  KKAVKK**T**PKKAKK  PPVSPG**T**ALVGSQ  EPSEVP**T**PKRPRG  TRKTTT**T**PGRKPR  QFERIK**T**LGTGSF  KRVKGR**T**WTLCGT  VKGRTW**T**LCGTPE  TWTLCG**T**PEYLAP  VSSYRR**T**FGGAPG  SSGSLS**T**SSSSSP  SSSPPG**T**PSPADA  KSKAKK**T**VDKHSD  FSRINK**T**SPVTAS  NKTSPV**T**ASDPAG  NQEEKK**T**PSKPPA  SPQKPP**T**PEETKA  PPTPEE**T**KAASPV  AEDSGD**T**EDELRR  DPYAGS**T**DENTDS  GSTDEN**T**DSEEHQ  GFDRLS**T**EGSDQE  GPGPER**T**PGSGSG  APGPAL**T**PSLLPT  LPTHTL**T**PVLLTP  LTPVLL**T**PSSLPP  SVDGLS**T**PVVLSP  NYRAYA**T**EPHAKK  RKLQLK**T**LLLQIA  KGRALS**T**RCQPLE  TEIADL**T**QKIFDL  GKFKRP**T**LRRVRI  QVKKED**T**EKENRE  VSPLLL**T**TTNSSE  SPLLLT**T**TNSSEG  PLLLTT**T**NSSEGL  LPDMKE**T**KYTVDK  MKETKY**T**VDKRFG  KNDGKR**T**RSKGTL  RTRSKG**T**LRYMSP  GLNRIQ**T**QMDVVN  IPLIDD**T**DAEDDA  SLREIN**T**ILKAQH  SPLKAY**T**PVVVTL  TRSRRL**T**FRKNIS  RGMGPG**T**PAGYGR  ENKDVS**T**ENGHHF  GVEVRV**T**PTRTEI  RRIREL**T**AVVQKR  DEILPT**T**PISEQK  MPQPVP**T**AAAAAA  DGTVTH**T**FCGTIE  QVFLGF**T**YVAPSV  FIGSPR**T**PVSPVK  EVDLAC**T**PTDVRD  PCSLIP**T**PDKEDD  LPSGLL**T**PPQSGK  DEESYD**T**ESEFTE  SEFTEF**T**EDELPY  ELSPSP**T**SPTATS  AIRRAS**T**IEMPQQ  DPEHDH**T**GFLTEY  DHTGFL**T**EYVATR  LTEYVA**T**RWYRAP  VYFLPI**T**PHYVTQ  EPELMG**T**PDGTCY  AEEEIG**T**PRKFTR  RSFAPS**T**PLTGRR  EKEAVI**T**PVASAT  KNGSGL**T**PRSALL  GKQGPA**T**PAPEPS  GITRKK**T**FKEVAN  VTSRIR**T**QSFSLQ  TMPPPE**T**PSEGRQ  SPSPSP**T**ERAPAS  SPEAAS**T**PRDPID  ETVLTE**T**PQDTIE  TETPQD**T**IELNRL  AVISPD**T**VPLGTG  VIDVPS**T**GAPSKK  PQVLSP**T**ITSPPN  NDSLEI**T**KLDSSI  GKISTI**T**PQIQAF  LFDTPH**T**PKSLLS  VNINPF**T**PDSLLL  FRTKSR**T**WAGEKS  CPDVPR**T**PVGKFL  SILSGG**T**PKRCLD  AQLLCS**T**PNGLDR  IWKGVK**T**SGKVVW  RIIKSI**T**PETPTE  KSITPE**T**PTEIPC  DGATMK**T**FCGTPE  FTAQSI**T**ITPPDR  AQSITI**T**PPDRYD  ISSPTE**T**ERCIES  DIEQFS**T**VKGVNL  IHSGAT**T**TAPSLS  RTSMGG**T**QQQFVE  PICSVN**T**PREVTL  EEKKAP**T**PQEKKR  GNSRPG**T**PSAEGG  PPSGKT**T**PNSGDV  RYLKYR**T**KTRASA  LKYRTK**T**RASATI  LKSPSQ**T**PKEMKS  GEAEPL**T**PTYNIS  CEIQVR**T**LASAGG  KQQREK**T**RWLNSG  KEDGHR**T**STSAVP  EQKKRV**T**MILQSP  PAKEAE**T**KSPLVS  AAEGEG**T**PTQPAS  LPKSKK**T**CSKGSK  KKADSS**T**TKKNQN  KADSST**T**KKNQNS  RLNQPG**T**PTRTAV  NQPGTP**T**RTAVVV  NKFKPF**T**VEIVDS  ANDKVY**T**VEKADN  VFRLSG**T**GSAGAT  QSDILT**T**QQRDTM  PKLYLP**T**GPRRGR  GRKRRQ**T**SMTDFY  VLSRQI**T**SPVSGI  PRGQAA**T**PETPQP  QAATPE**T**PQPSPP  SSVRYG**T**VEDLLA  PKDLRG**T**EIYMSP  MMMMMP**T**QRDSST  TQRDSS**T**MSHTVA  PGDTTS**T**FCGTPN  NEPVQL**T**PDDDDI  IHPIVR**T**LDQSPE  PSLQVI**T**SKQRPR  QRPRKL**T**LMGSNG  KRSRTR**T**DSYSAG  GLLRVL**T**VPCQKI  GNRQES**T**VSFNPY  EALPMD**T**EVYESP  GSGNFG**T**VKKGYY  NYYKAQ**T**HGKWPV  MKGSEV**T**AMLEKG  VDSIAK**T**RDAGCR  QGEKSA**T**PSRKIL  PPADVS**T**FLAFPS  IRKRPA**T**DDSSTQ  NKRANR**T**EENVSD  AGSVEQ**T**PKKPGL  GLRRRQ**T**TTTTTT  TILQAP**T**PAPSTI  AASLAP**T**PVSAVL  VDSVAK**T**MDAGCK  VRGDSE**T**DLEALF  HSRQAS**T**DAGTAG  GTAGAL**T**PQHVRA  VSGPAA**T**PTAQHL  SYSVPR**T**PDDFLN  PPPQPP**T**PALPHP  SHNSLT**T**PCYTPY  STKVPQ**T**PLHTSR  RRRRSR**T**FSRSSS  HSTIVS**T**RHYRAP  LLSFDD**T**PEKDSF  LGMDRR**T**DAYSRS  LRPRGY**T**ISDSAP  MMMMMK**T**PADTGF  MKTPAD**T**GFAFPD  SEEVEV**T**DISDED  EAGGPL**T**PRRVSS  ARRRKA**T**QVGEKT  WEKNST**T**LPRMKS  SPNRAY**T**HQVVTR  AKIGQG**T**FGEVFK  SQPNRY**T**NRVVTL  RKGSQI**T**QQSTNQ  QITQQS**T**NQSRNP  QSRNPA**T**TNQTEF  SRNPAT**T**NQTEFE  LAATSS**T**LGLQEN  RLKRRC**T**TSLIKE  ERSGQR**T**ASVLWP  CEISRG**T**HNFSEE  TQTVRG**T**LAYLPE  RILNHD**T**SFAKTF  DTSFAK**T**FVGTPY  AKTFVG**T**PYYMSP  YQDDPL**T**KGVDLR  DLRHLN**T**EPMEIF  SDLQNK**T**QELETT  DIPTGT**T**PQRKSY  MMSAAV**T**AGKLAR  DGERKK**T**LCGTPN  KKTLCG**T**PNYIAP  KAGANI**T**PREGDE  ILEING**T**PIRNVP  DRKKRY**T**VVGNPY  GTSFMM**T**PYVVTR  SPSVLT**T**PSKIEP  TERSKA**T**PGAPAL  PALTSM**T**PTAVER  DGEFLR**T**SCGSPN  DPLKRA**T**IKDIRE  LTLDMC**T**PGCNEE  EVLRPE**T**PRPVDI  SGEPLS**T**WCGSPP  GVDRQR**T**VESLQN  SSSTSV**T**PDVSDN  HYRYSD**T**TDSDPE  YRYSDT**T**DSDPEN  DQHTQI**T**KVVVVV  PGGRPI**T**PPRNSA  VRDMRQ**T**VAVGVI  SMPGGS**T**PVSSAN  GISSVP**T**PSPLGP  SHDRSR**T**VSASST  TPMFVE**T**QASQGT  AGQIRA**T**QQQHDF  QHDFTL**T**QTADGR  PFPAPQ**T**PGRLQP  AREWHR**T**TKMSAA  REWHRT**T**KMSAAG  KMSAAG**T**YAWMAP  SRPRSC**T**WPLPRP  KRVRVQ**T**GEEGMP  VLRHLE**T**FEHPNV  SFQMAL**T**SVVVTL  NTSELN**T**AAAAAA  SVTKLL**T**DVQLMK  RPSSVD**T**LLSPTA  EFEREE**T**QDKEES  ARKRKR**T**SIENRV  GLKKQE**T**PDQVGS  LADLGV**T**PDKITN  NGVTTT**T**FCGTPD  NFDQDF**T**REEPVL  REEPVL**T**LVDEAI  DPQNQH**T**FEARDL  VAIVFR**T**PPYADP  TQAGEG**T**LSEALL  AITRLV**T**GAQRPP  AINSRE**T**MFHKER  GDAKTN**T**FCGTPD  LTHNWG**T**EDDETQ  STERGK**T**LVQKKP  LVQKKP**T**MYPEWK  DEAKFP**T**MNRRGA  AANSRD**T**IFQKER  FELYRA**T**FYAAEI  GESRAS**T**FCGTPD  SAQSLA**T**PVVSVA  PVVSVA**T**PTLPGQ  RKGRGE**T**RICKIY  HLAWVN**T**PKKQGG  DGSLPS**T**PPPAEE  TLPISS**T**PSKSVL  KSVLPR**T**PESWRL  PESWRL**T**PPAKVG  LMDLST**T**PLQSAP  IAGSPL**T**PRRVTE  VMRSSS**T**LPVPQP  PSSAPP**T**PTRLTG  PHKRRK**T**SDANET  EEDRFL**T**PGRAQL  PRPRSC**T**WPLPRP  SSKVQT**T**PSKPGG  QPENSQ**T**PTKKEH  LYSQKA**T**PGSSRK  EPEVLS**T**QEDLFD  DEDGEN**T**QIEDTE  NTQIED**T**EPMSPV  ELQQPQ**T**QEKTSN  PPLTGA**T**PPLIGH  SAPGDD**T**ESLHSQ  GVSQTG**T**PVCEED  GPYEAV**T**PLTKAA  KVLAGE**T**LSVNDP  QQREDI**T**QSAQHA  EKLFPD**T**PLALDA  MAAASV**T**PPGSLE  QPGFSK**T**LLGTKL  GCTWKG**T**LKEYES  AEETGL**T**PLETSR  RPSLEE**T**FEIEMN  HRPRHA**T**VSRSLE  GSQGEL**T**PANSQS  PSDFEH**T**IHVGFD  DDDDDA**T**PPPVIA  IEPLPV**T**PTRDVA  PTRDVA**T**SPISPT  PTENNT**T**PPDALT  EQSKRS**T**MVGTPY  VNSIAK**T**YVGTNA  IRLRNK**T**LIEDEI  TMAKRN**T**VIGTPF  SVESVG**T**MRATST  TMKRNA**T**SPQVQR  PVEAER**T**PWNCIF  IRRRRP**T**PATLVL  PHDYCT**T**PGGTLF  GTLFST**T**PGGTRI  NSPMAQ**T**PPCHLP  MMHRQE**T**VECLKK  LKGAIL**T**TMLATR  KGAILT**T**MLATRN  SDSANT**T**IEDEDA  SIPVAP**T**PPRRVR  EKLPDG**T**WNLKKT  LRSRLR**T**LAEVLS  AALVGG**T**TMIIDH  ALVGGT**T**MIIDHV  VYEVPA**T**PKYATP  ATPKYA**T**PAPSAK  DLPVPR**T**PGSGAG  SRTTSA**T**PLKDNT  LLARRP**T**KGIHEY  SRRLKV**T**GDLFDI  RRPESF**T**TPEGPK  RPESFT**T**PEGPKP  PGIHPK**T**PNKFKK  QARGST**T**LQESRE  MMMMMG**T**PKPRIL  DKPDLP**T**WKRNFR  PSLDNP**T**PFPNLG  SEVGDR**T**LPGWPV  LPGWPV**T**LPDPGM  DPGMSL**T**DRGVMS  SHPLSL**T**SDQYKA  NTIKVP**T**PLCTAR  MMMMED**T**QAIDWD  PGEDSD**T**DVDDDS  GFIDSD**T**DAEEER  SQAGSD**T**DVEEGK  MVINSD**T**DDEEEV  QRSQTT**T**ERDSDT  TERDSD**T**DVEEEE  TTVDIN**T**QVEKEV  NRSSVK**T**PEPVVP  SRSSVK**T**PETVVP  STDRPV**T**PKPTSR  NRSSGK**T**PETLVP  STDQPV**T**PEPTSQ  STDQPV**T**PEPTSR  NRSSVK**T**PESIVP  SRNQLV**T**PEPTSR  STDQPV**T**PKLTSR  SDLEPF**T**PTDQSV  PTDQSV**T**PEAIAQ  AMPVPT**T**PEFQSP  AGRSRF**T**PELQPK  KEEDVV**T**PKPGKR  DLPRIK**T**EIEALK  KDYHLQ**T**CCGSLA  QFTKYW**T**ESNGVE  VESKSL**T**PALCRT  NQCLKE**T**PIKIPV  GTDKLM**T**GVISPE  QAHMEE**T**PKRKGA  GLDKVI**T**VLTRSK  QSNSGA**T**KRASPS  FNQFFV**T**PRSSVE  GTVLCG**T**QAVDKL  CTASKI**T**PDGITK  PSTCSS**T**FPEELS  MGDILQ**T**PQFQMR  LESLYF**T**PIPARS  LDSGRK**T**RSARRR  PHQGPG**T**PESKKA  CFPRPM**T**PRDRHE  AFSILN**T**PKKLGN  AAAIGA**T**PRAKGK  KGFFRR**T**VSKSIG  AVTSAG**T**PKRVIV  RRLRPR**T**RKVKSV  AHARKE**T**EFLRLK  WVFINY**T**YKRFEG  QDLVNR**T**PWKKCC  LKTYQV**T**PMTPRS  YQVTPM**T**PRSANA  SANAFL**T**PSYTWT  LNKNEA**T**TKNLNG  NKNEAT**T**KNLNGL  PSQRLG**T**PKSVNK  ELNSSI**T**SQDVLM  ILEEGE**T**KISQSE  MSKDPT**T**QKLRND  DCPAIG**T**PLRDSS  SSNRPF**T**PPTSTG  SAGRSQ**T**PPGVAT  TPPGVA**T**PPIPKI  MKPVPG**T**PPSSKA  SKSPAY**T**PQNLDS  YGTNPG**T**PPASTS  SSILPF**T**PPVVKR  SNYIPE**T**PPPGYI  LLTTGG**T**LKISDL  DRWRSM**T**VVPYLE  EDDIIY**T**QDFTVP  TAERIK**T**PPKRPG  NSSRPS**T**PTINVL  PAEDVD**T**PPRKKK  YGDRTS**T**FCGTPE  TSTFCG**T**PEFLAP  FTGEAP**T**LSPPRD  ITDCPR**T**PDTPNN  CPRTPD**T**PNNDPR  SEYKPD**T**PQSGLE  SEAPIL**T**PPREPR  EKRIVI**T**EFGTCA  VITEFG**T**CAFPDP  HQVLMK**T**VCGTPG  AREWHK**T**TQMSAA  TPDSPP**T**PAPLLL  HYETDY**T**TGGESC  YETDYT**T**GGESCD  VLFRRG**T**GQSDDS  KSQKKN**T**AASLQQ  PFSTQD**T**DLDLEM  MMMMAR**T**KQTARK  MARTKQ**T**ARKSTG  QTARKS**T**GGKAPR  IAQDFK**T**DLRFQS  VGLFED**T**NLCVIH  STSSDL**T**ISISED  SADSIA**T**LPKGFG  KQFDAS**T**PKNDIS  MRFRSA**T**TSGAPG  SQQKTS**T**IDDSKD  GLPPGT**T**PQRLEQ  ALEEAA**T**ADLDIT  TADLDI**T**EINKLT  TAEAIQ**T**PLKSAK  RSSRAN**T**VTPAVG  KPTPGL**T**PRFDSR  KTPGLR**T**PAAGER  SKEIFL**T**VPVGGG  SSVAVG**T**PDYISP  FGDRTS**T**FCGTPE  GLPPAL**T**PPAPHS  GVEGPR**T**PKYMNN  GSPRAV**T**PVPTKT  ILIVTQ**T**PHYMRR  EQFDTL**T**PEPPVD  REHRPR**T**ASISSS  MAREPA**T**LKDSLE  RRIRTL**T**EPSVDF  DPPLHS**T**AIYADE  AKRSSD**T**SGNEAS  LSDSLT**T**STPRPS  GIFGLV**T**NLEELE  FLDRPP**T**PLFIPA  NDDHSD**T**DSEENR  KGDVMS**T**ACGTPG  VATLQG**T**PDHGDP  DHGDPR**T**PGPPRS  SSPARG**T**PAGTTP  GTPAGT**T**PGASQA  SVESPG**T**PKETPI  HVGRAS**T**PDWVSE  PEVFSP**T**PDENSD  DQGDLM**T**PQFTPY  KGEMLN**T**FCGSPP  TEGGSL**T**PVRDSP  PPPGLL**T**PPVATE  AEAAPP**T**QEAQGE  SRRNAE**T**PEATTQ  LEKTQT**T**PNGSLQ  QRALQV**T**VPHFLD  IPQKNQ**T**ASGDSL  ELRKTQ**T**SMSLGT  ESYRPD**T**DLSRED  DLSRED**T**GCNLQH  DHPRAS**T**IFLSKS  FLSKSQ**T**DVREKR  ASADNL**T**LPRWSP  RSINTD**T**LSKETD  PINGSS**T**PNPKIA  PGSLSS**T**PLSTPC  SSTPLS**T**PCSSVP  HQQGKV**T**VKYDRK  SPSSQE**T**HDSPFC  HDNRKS**T**SFMSVS  PEDQAV**T**EYVATR  GDSLLE**T**SCGSPH  RTDSVK**T**PESEGN  KLMEII**T**VENPKM  KIHRSA**T**DADVVN  RLMRSR**T**ASGSSV  TRSRSH**T**SEGTRS  TRSRSH**T**SEGAHL  GAHLDI**T**PNSGAA  KRTVLT**T**QPNGLT  EHQRVY**T**YIQSRF  GRKRKI**T**LSSQSA  SAQIGA**T**LARRLS  RLSFIG**T**PYWMAP  TRPPAN**T**ARLQPP  GVELLL**T**PREPAL  DSSDPD**T**GSEEEG  SPRGCF**T**LPRSEP  LVPCSA**T**PTTFGD  KRHARV**T**VKYDRR  PRPRLN**T**SDFQKL  ISDTTT**T**FCGTPE  LSELPP**T**PLHVSE  SSSPER**T**SPPDSS  GGAGFG**T**DGDDQE  LSGKTK**T**VPGYLR  AGADRA**T**SPLVSL  SRPPPP**T**PRRPAS  HFTRTT**T**WQRPTL  LGSKLD**T**FCGSPP  KHPGCT**T**PVTVKI  IPQISS**T**PKTSEE  RILGSD**T**ESWMSP  DEEPES**T**PVPLLG  EMSIMT**T**ELQSLC  RGKLFY**T**FCGTIE  FYTFCG**T**IEYCAP  ALGFYD**T**SEENYQ  ALTNVD**T**PLKGGL  LKGGLN**T**PLHESD  SDFSGV**T**PQRQVV  QRQVVQ**T**PNTVLS  PNTVLS**T**PFRTPS  LSTPFR**T**PSNGAE  NGAEGL**T**PRSGTT  TPRSGT**T**PKPVIN  KPVINS**T**PGRTPL  NSTPGR**T**PLRDKL  GDYIPG**T**ETHMAP  AHQRQL**T**PPEGSS  PMPTEG**T**PPPLSG  LSLFED**T**LDPTTT  DNVLIN**T**YSGVLK  INPCTE**T**FTGTLQ  TETFTG**T**LQYMAP  RYIPPN**T**PENFWE  PRSAAS**T**PAGSPD  LKAKTI**T**KKRKKE  PNPEYS**T**QQAPNK  MMMRKE**T**PPPLVP  SCTPIT**T**PELTTP  TTPELT**T**PCGSAE  PEKGLP**T**PQVLQR  RRPRRR**T**FPGVAS  NQVKPQ**T**IPYTPR  RMALSK**T**FCGSPA  VSGTLD**T**PEKTVD  VDSQGP**T**PVCTPT  GPTPVC**T**PTFLER  EIQETQ**T**PTHSRK  TGGLFN**T**PPPTPP  FNTPPP**T**PPEQDK  AKGRRE**T**YLCYVV  ALSPPQ**T**SGLSPS  ISGSTY**T**PSEAGN  QGKFLQ**T**FCGSPL  DISFDK**T**DESLDW  PWNSDS**T**LNSRQL  IPTLIG**T**PVKIGE  AFSTPQ**T**PDIKVS  LLGPVT**T**PEHQLL  EHQLLK**T**PSSSSL  SQRVRS**T**LTKNTP  STLTKN**T**PRFGSK  FYDIEG**T**LGKGNF  SGELLA**T**WCGSPP  FSEQGG**T**PQNVAT  PLDGVS**T**PKPLSK  ERLRQG**T**LRRDLR  PKLACR**T**PSPARP  AEEREH**T**LRRCQQ  RRSDPG**T**SGEAYK  LLSATV**T**PQKAKL  LIDIKD**T**LPVGFI  HDSSQP**T**TPSQSS  DSSQPT**T**PSQSSA  SSRSSK**T**FKPKKN  LKHAEA**T**LGSGNL  INMLYG**T**ITEFCT  EEAHLN**T**SFKHFI  LYEKAN**T**PELKKS  SMLSLN**T**PNSNRK  KSSKSS**T**PVPSKQ  AFSFSK**T**PKRALR  PGQMAG**T**PITPLK  MAGTPI**T**PLKDGF  SLTVDV**T**PPTAKA  DRVGDS**T**PVSEKP  ELLDLC**T**GKFTSQ  GLLRLC**T**WLLALS  DCSPIS**T**PELLTP  STPELL**T**PCGSAE  PENTLP**T**PMVLQR  ILSRRN**T**VWLCYE  VRGRHE**T**YLCYEV  FFSSET**T**AAHSLV  AHSLVG**T**PYYMSP  KPVRVC**T**HCYMFH  NMQRRH**T**TLREKG  TEPLPR**T**LSPTPA  PRTLSP**T**PASATA  TSQGIP**T**SDEEST  VSSLMA**T**PQDPKG  LPDNFS**T**PTGSRT  SWPHND**T**EIANST  LNSDDS**T**DDEAHP  PRYHKR**T**SSAVWN  RSIKGK**T**KVEVAK  PGKKLT**T**SCGSLA  PSFQSK**T**PPETGG  DADVSV**T**QRTAFQ  SEKFAQ**T**VMTSRI  FAQTVM**T**SRIVGT  PSSLPR**T**PTPDRP  SLPRTP**T**PDRPPR  SRFHNH**T**THMSLV  IKYQQI**T**PVNQSR  KDMGLV**T**PEAASL  LKEEIV**T**PKETET  PEPKAI**T**PPQQPY  EESRPY**T**NKVITL  PQGPRR**T**PTMPQE  ARYVPS**T**PWFLMP  LPLPSN**T**PTFSIK  SSSPPI**T**PPASET  RSPSSQ**T**PNSRRQ  MMMSAR**T**PLPTVN  GFSNEF**T**VGNKLD  VGNKLD**T**FCGSPP  GSSSRS**T**FHGEQL  AERPGG**T**PTPVIA  RPGGTP**T**PVIAYS  APPAPP**T**PPPPTL  VEDGEE**T**CALASH  LSPAPD**T**GLSPSK  LPECGI**T**HEQRAG  QLAHTP**T**AFKRDP  KCSDCH**T**PLAERC  IRRRRP**T**PAMLFR  PNPCAY**T**PPSLKA  LRNQGE**T**PTTEVP  QGETPT**T**EVPAPF  SLSANN**T**PTRRVS  LNLSSV**T**PPPPPP  SSAWPG**T**LRSGMV  ENGLLL**T**PCYTAN  EGPFYP**T**LRLLHK  MMEAVK**T**FNSELY  KETVQT**T**QSPTPV  RSKPPY**T**DYVSTR  DKATVF**T**DEESYS  LGDFSR**T**PSPSPS  TEFRDS**T**YDLPRS  LASHGH**T**KGSLTG  SLTGSE**T**DNEDVY  QIPRTF**T**LDKNHN  SRSVAA**T**IPRRNT  TIPRRN**T**LPAMDN  IDATGD**T**PGAEDD  LKTGEQ**T**PPHEHI  DESDSA**T**VYDNCA  CLSEDS**T**PDEPDV  DGTVIS**T**FKKRAV  YEFTTL**T**TVPGVI  YYEIDR**T**IGKGNF  PGQLLK**T**WCGSPP  LSMRRH**T**VGVADP  SALNFL**T**PLRNFI  WLMRSF**T**CPSCME  LNKRRA**T**LPHPGG  TQIKRN**T**FVGTPF  SPQRRN**T**ASQEDK  PGGSPR**T**PNRSVS  PVTVSP**T**TPTSPT  VTVSPT**T**PTSPTE  VSPTTP**T**SPTEGE  RRHKRK**T**GHKEKK  MTHRNL**T**SSSLND |

# (2) List of the 9,739 peptide samples in the negative subset $\mathbb{S}_{\boldsymbol{\xi=6}}^{\mathbf{-}}\boldsymbol{(}\mathbf{T}\boldsymbol{)}$

| Sample # | Protein ID | Site | Sequences |
| --- | --- | --- | --- |
| 1  2  3  4  5  6  7  8  9  10  11  12  13  14  15  16  17  18  19  20  21  22  23  24  25  26  27  28  29  30  31  32  33  34  35  36  37  38  39  40  41  42  43  44  45  46  47  48  49  50  51  52  53  54  55  56  57  58  59  60  61  62  63  64  65  66  67  68  69  70  71  72  73  74  75  76  77  78  79  80  81  82  83  84  85  86  87  88  89  90  91  92  93  94  95  96  97  98  99  100  101  102  103  104  105  106  107  108  109  110  111  112  113  114  115  116  117  118  119  120  121  122  123  124  125  126  127  128  129  130  131  132  133  134  135  136  137  138  139  140  141  142  143  144  145  146  147  148  149  150  151  152  153  154  155  156  157  158  159  160  161  162  163  164  165  166  167  168  169  170  171  172  173  174  175  176  177  178  179  180  181  182  183  184  185  186  187  188  189  190  191  192  193  194  195  196  197  198  199  200  201  202  203  204  205  206  207  208  209  210  211  212  213  214  215  216  217  218  219  220  221  222  223  224  225  226  227  228  229  230  231  232  233  234  235  236  237  238  239  240  241  242  243  244  245  246  247  248  249  250  251  252  253  254  255  256  257  258  259  260  261  262  263  264  265  266  267  268  269  270  271  272  273  274  275  276  277  278  279  280  281  282  283  284  285  286  287  288  289  290  291  292  293  294  295  296  297  298  299  300  301  302  303  304  305  306  307  308  309  310  311  312  313  314  315  316  317  318  319  320  321  322  323  324  325  326  327  328  329  330  331  332  333  334  335  336  337  338  339  340  341  342  343  344  345  346  347  348  349  350  351  352  353  354  355  356  357  358  359  360  361  362  363  364  365  366  367  368  369  370  371  372  373  374  375  376  377  378  379  380  381  382  383  384  385  386  387  388  389  390  391  392  393  394  395  396  397  398  399  400  401  402  403  404  405  406  407  408  409  410  411  412  413  414  415  416  417  418  419  420  421  422  423  424  425  426  427  428  429  430  431  432  433  434  435  436  437  438  439  440  441  442  443  444  445  446  447  448  449  450  451  452  453  454  455  456  457  458  459  460  461  462  463  464  465  466  467  468  469  470  471  472  473  474  475  476  477  478  479  480  481  482  483  484  485  486  487  488  489  490  491  492  493  494  495  496  497  498  499  500  501  502  503  504  505  506  507  508  509  510  511  512  513  514  515  516  517  518  519  520  521  522  523  524  525  526  527  528  529  530  531  532  533  534  535  536  537  538  539  540  541  542  543  544  545  546  547  548  549  550  551  552  553  554  555  556  557  558  559  560  561  562  563  564  565  566  567  568  569  570  571  572  573  574  575  576  577  578  579  580  581  582  583  584  585  586  587  588  589  590  591  592  593  594  595  596  597  598  599  600  601  602  603  604  605  606  607  608  609  610  611  612  613  614  615  616  617  618  619  620  621  622  623  624  625  626  627  628  629  630  631  632  633  634  635  636  637  638  639  640  641  642  643  644  645  646  647  648  649  650  651  652  653  654  655  656  657  658  659  660  661  662  663  664  665  666  667  668  669  670  671  672  673  674  675  676  677  678  679  680  681  682  683  684  685  686  687  688  689  690  691  692  693  694  695  696  697  698  699  700  701  702  703  704  705  706  707  708  709  710  711  712  713  714  715  716  717  718  719  720  721  722  723  724  725  726  727  728  729  730  731  732  733  734  735  736  737  738  739  740  741  742  743  744  745  746  747  748  749  750  751  752  753  754  755  756  757  758  759  760  761  762  763  764  765  766  767  768  769  770  771  772  773  774  775  776  777  778  779  780  781  782  783  784  785  786  787  788  789  790  791  792  793  794  795  796  797  798  799  800  801  802  803  804  805  806  807  808  809  810  811  812  813  814  815  816  817  818  819  820  821  822  823  824  825  826  827  828  829  830  831  832  833  834  835  836  837  838  839  840  841  842  843  844  845  846  847  848  849  850  851  852  853  854  855  856  857  858  859  860  861  862  863  864  865  866  867  868  869  870  871  872  873  874  875  876  877  878  879  880  881  882  883  884  885  886  887  888  889  890  891  892  893  894  895  896  897  898  899  900  901  902  903  904  905  906  907  908  909  910  911  912  913  914  915  916  917  918  919  920  921  922  923  924  925  926  927  928  929  930  931  932  933  934  935  936  937  938  939  940  941  942  943  944  945  946  947  948  949  950  951  952  953  954  955  956  957  958  959  960  961  962  963  964  965  966  967  968  969  970  971  972  973  974  975  976  977  978  979  980  981  982  983  984  985  986  987  988  989  990  991  992  993  994  995  996  997  998  999  1000  1001  1002  1003  1004  1005  1006  1007  1008  1009  1010  1011  1012  1013  1014  1015  1016  1017  1018  1019  1020  1021  1022  1023  1024  1025  1026  1027  1028  1029  1030  1031  1032  1033  1034  1035  1036  1037  1038  1039  1040  1041  1042  1043  1044  1045  1046  1047  1048  1049  1050  1051  1052  1053  1054  1055  1056  1057  1058  1059  1060  1061  1062  1063  1064  1065  1066  1067  1068  1069  1070  1071  1072  1073  1074  1075  1076  1077  1078  1079  1080  1081  1082  1083  1084  1085  1086  1087  1088  1089  1090  1091  1092  1093  1094  1095  1096  1097  1098  1099  1100  1101  1102  1103  1104  1105  1106  1107  1108  1109  1110  1111  1112  1113  1114  1115  1116  1117  1118  1119  1120  1121  1122  1123  1124  1125  1126  1127  1128  1129  1130  1131  1132  1133  1134  1135  1136  1137  1138  1139  1140  1141  1142  1143  1144  1145  1146  1147  1148  1149  1150  1151  1152  1153  1154  1155  1156  1157  1158  1159  1160  1161  1162  1163  1164  1165  1166  1167  1168  1169  1170  1171  1172  1173  1174  1175  1176  1177  1178  1179  1180  1181  1182  1183  1184  1185  1186  1187  1188  1189  1190  1191  1192  1193  1194  1195  1196  1197  1198  1199  1200  1201  1202  1203  1204  1205  1206  1207  1208  1209  1210  1211  1212  1213  1214  1215  1216  1217  1218  1219  1220  1221  1222  1223  1224  1225  1226  1227  1228  1229  1230  1231  1232  1233  1234  1235  1236  1237  1238  1239  1240  1241  1242  1243  1244  1245  1246  1247  1248  1249  1250  1251  1252  1253  1254  1255  1256  1257  1258  1259  1260  1261  1262  1263  1264  1265  1266  1267  1268  1269  1270  1271  1272  1273  1274  1275  1276  1277  1278  1279  1280  1281  1282  1283  1284  1285  1286  1287  1288  1289  1290  1291  1292  1293  1294  1295  1296  1297  1298  1299  1300  1301  1302  1303  1304  1305  1306  1307  1308  1309  1310  1311  1312  1313  1314  1315  1316  1317  1318  1319  1320  1321  1322  1323  1324  1325  1326  1327  1328  1329  1330  1331  1332  1333  1334  1335  1336  1337  1338  1339  1340  1341  1342  1343  1344  1345  1346  1347  1348  1349  1350  1351  1352  1353  1354  1355  1356  1357  1358  1359  1360  1361  1362  1363  1364  1365  1366  1367  1368  1369  1370  1371  1372  1373  1374  1375  1376  1377  1378  1379  1380  1381  1382  1383  1384  1385  1386  1387  1388  1389  1390  1391  1392  1393  1394  1395  1396  1397  1398  1399  1400  1401  1402  1403  1404  1405  1406  1407  1408  1409  1410  1411  1412  1413  1414  1415  1416  1417  1418  1419  1420  1421  1422  1423  1424  1425  1426  1427  1428  1429  1430  1431  1432  1433  1434  1435  1436  1437  1438  1439  1440  1441  1442  1443  1444  1445  1446  1447  1448  1449  1450  1451  1452  1453  1454  1455  1456  1457  1458  1459  1460  1461  1462  1463  1464  1465  1466  1467  1468  1469  1470  1471  1472  1473  1474  1475  1476  1477  1478  1479  1480  1481  1482  1483  1484  1485  1486  1487  1488  1489  1490  1491  1492  1493  1494  1495  1496  1497  1498  1499  1500  1501  1502  1503  1504  1505  1506  1507  1508  1509  1510  1511  1512  1513  1514  1515  1516  1517  1518  1519  1520  1521  1522  1523  1524  1525  1526  1527  1528  1529  1530  1531  1532  1533  1534  1535  1536  1537  1538  1539  1540  1541  1542  1543  1544  1545  1546  1547  1548  1549  1550  1551  1552  1553  1554  1555  1556  1557  1558  1559  1560  1561  1562  1563  1564  1565  1566  1567  1568  1569  1570  1571  1572  1573  1574  1575  1576  1577  1578  1579  1580  1581  1582  1583  1584  1585  1586  1587  1588  1589  1590  1591  1592  1593  1594  1595  1596  1597  1598  1599  1600  1601  1602  1603  1604  1605  1606  1607  1608  1609  1610  1611  1612  1613  1614  1615  1616  1617  1618  1619  1620  1621  1622  1623  1624  1625  1626  1627  1628  1629  1630  1631  1632  1633  1634  1635  1636  1637  1638  1639  1640  1641  1642  1643  1644  1645  1646  1647  1648  1649  1650  1651  1652  1653  1654  1655  1656  1657  1658  1659  1660  1661  1662  1663  1664  1665  1666  1667  1668  1669  1670  1671  1672  1673  1674  1675  1676  1677  1678  1679  1680  1681  1682  1683  1684  1685  1686  1687  1688  1689  1690  1691  1692  1693  1694  1695  1696  1697  1698  1699  1700  1701  1702  1703  1704  1705  1706  1707  1708  1709  1710  1711  1712  1713  1714  1715  1716  1717  1718  1719  1720  1721  1722  1723  1724  1725  1726  1727  1728  1729  1730  1731  1732  1733  1734  1735  1736  1737  1738  1739  1740  1741  1742  1743  1744  1745  1746  1747  1748  1749  1750  1751  1752  1753  1754  1755  1756  1757  1758  1759  1760  1761  1762  1763  1764  1765  1766  1767  1768  1769  1770  1771  1772  1773  1774  1775  1776  1777  1778  1779  1780  1781  1782  1783  1784  1785  1786  1787  1788  1789  1790  1791  1792  1793  1794  1795  1796  1797  1798  1799  1800  1801  1802  1803  1804  1805  1806  1807  1808  1809  1810  1811  1812  1813  1814  1815  1816  1817  1818  1819  1820  1821  1822  1823  1824  1825  1826  1827  1828  1829  1830  1831  1832  1833  1834  1835  1836  1837  1838  1839  1840  1841  1842  1843  1844  1845  1846  1847  1848  1849  1850  1851  1852  1853  1854  1855  1856  1857  1858  1859  1860  1861  1862  1863  1864  1865  1866  1867  1868  1869  1870  1871  1872  1873  1874  1875  1876  1877  1878  1879  1880  1881  1882  1883  1884  1885  1886  1887  1888  1889  1890  1891  1892  1893  1894  1895  1896  1897  1898  1899  1900  1901  1902  1903  1904  1905  1906  1907  1908  1909  1910  1911  1912  1913  1914  1915  1916  1917  1918  1919  1920  1921  1922  1923  1924  1925  1926  1927  1928  1929  1930  1931  1932  1933  1934  1935  1936  1937  1938  1939  1940  1941  1942  1943  1944  1945  1946  1947  1948  1949  1950  1951  1952  1953  1954  1955  1956  1957  1958  1959  1960  1961  1962  1963  1964  1965  1966  1967  1968  1969  1970  1971  1972  1973  1974  1975  1976  1977  1978  1979  1980  1981  1982  1983  1984  1985  1986  1987  1988  1989  1990  1991  1992  1993  1994  1995  1996  1997  1998  1999  2000  2001  2002  2003  2004  2005  2006  2007  2008  2009  2010  2011  2012  2013  2014  2015  2016  2017  2018  2019  2020  2021  2022  2023  2024  2025  2026  2027  2028  2029  2030  2031  2032  2033  2034  2035  2036  2037  2038  2039  2040  2041  2042  2043  2044  2045  2046  2047  2048  2049  2050  2051  2052  2053  2054  2055  2056  2057  2058  2059  2060  2061  2062  2063  2064  2065  2066  2067  2068  2069  2070  2071  2072  2073  2074  2075  2076  2077  2078  2079  2080  2081  2082  2083  2084  2085  2086  2087  2088  2089  2090  2091  2092  2093  2094  2095  2096  2097  2098  2099  2100  2101  2102  2103  2104  2105  2106  2107  2108  2109  2110  2111  2112  2113  2114  2115  2116  2117  2118  2119  2120  2121  2122  2123  2124  2125  2126  2127  2128  2129  2130  2131  2132  2133  2134  2135  2136  2137  2138  2139  2140  2141  2142  2143  2144  2145  2146  2147  2148  2149  2150  2151  2152  2153  2154  2155  2156  2157  2158  2159  2160  2161  2162  2163  2164  2165  2166  2167  2168  2169  2170  2171  2172  2173  2174  2175  2176  2177  2178  2179  2180  2181  2182  2183  2184  2185  2186  2187  2188  2189  2190  2191  2192  2193  2194  2195  2196  2197  2198  2199  2200  2201  2202  2203  2204  2205  2206  2207  2208  2209  2210  2211  2212  2213  2214  2215  2216  2217  2218  2219  2220  2221  2222  2223  2224  2225  2226  2227  2228  2229  2230  2231  2232  2233  2234  2235  2236  2237  2238  2239  2240  2241  2242  2243  2244  2245  2246  2247  2248  2249  2250  2251  2252  2253  2254  2255  2256  2257  2258  2259  2260  2261  2262  2263  2264  2265  2266  2267  2268  2269  2270  2271  2272  2273  2274  2275  2276  2277  2278  2279  2280  2281  2282  2283  2284  2285  2286  2287  2288  2289  2290  2291  2292  2293  2294  2295  2296  2297  2298  2299  2300  2301  2302  2303  2304  2305  2306  2307  2308  2309  2310  2311  2312  2313  2314  2315  2316  2317  2318  2319  2320  2321  2322  2323  2324  2325  2326  2327  2328  2329  2330  2331  2332  2333  2334  2335  2336  2337  2338  2339  2340  2341  2342  2343  2344  2345  2346  2347  2348  2349  2350  2351  2352  2353  2354  2355  2356  2357  2358  2359  2360  2361  2362  2363  2364  2365  2366  2367  2368  2369  2370  2371  2372  2373  2374  2375  2376  2377  2378  2379  2380  2381  2382  2383  2384  2385  2386  2387  2388  2389  2390  2391  2392  2393  2394  2395  2396  2397  2398  2399  2400  2401  2402  2403  2404  2405  2406  2407  2408  2409  2410  2411  2412  2413  2414  2415  2416  2417  2418  2419  2420  2421  2422  2423  2424  2425  2426  2427  2428  2429  2430  2431  2432  2433  2434  2435  2436  2437  2438  2439  2440  2441  2442  2443  2444  2445  2446  2447  2448  2449  2450  2451  2452  2453  2454  2455  2456  2457  2458  2459  2460  2461  2462  2463  2464  2465  2466  2467  2468  2469  2470  2471  2472  2473  2474  2475  2476  2477  2478  2479  2480  2481  2482  2483  2484  2485  2486  2487  2488  2489  2490  2491  2492  2493  2494  2495  2496  2497  2498  2499  2500  2501  2502  2503  2504  2505  2506  2507  2508  2509  2510  2511  2512  2513  2514  2515  2516  2517  2518  2519  2520  2521  2522  2523  2524  2525  2526  2527  2528  2529  2530  2531  2532  2533  2534  2535  2536  2537  2538  2539  2540  2541  2542  2543  2544  2545  2546  2547  2548  2549  2550  2551  2552  2553  2554  2555  2556  2557  2558  2559  2560  2561  2562  2563  2564  2565  2566  2567  2568  2569  2570  2571  2572  2573  2574  2575  2576  2577  2578  2579  2580  2581  2582  2583  2584  2585  2586  2587  2588  2589  2590  2591  2592  2593  2594  2595  2596  2597  2598  2599  2600  2601  2602  2603  2604  2605  2606  2607  2608  2609  2610  2611  2612  2613  2614  2615  2616  2617  2618  2619  2620  2621  2622  2623  2624  2625  2626  2627  2628  2629  2630  2631  2632  2633  2634  2635  2636  2637  2638  2639  2640  2641  2642  2643  2644  2645  2646  2647  2648  2649  2650  2651  2652  2653  2654  2655  2656  2657  2658  2659  2660  2661  2662  2663  2664  2665  2666  2667  2668  2669  2670  2671  2672  2673  2674  2675  2676  2677  2678  2679  2680  2681  2682  2683  2684  2685  2686  2687  2688  2689  2690  2691  2692  2693  2694  2695  2696  2697  2698  2699  2700  2701  2702  2703  2704  2705  2706  2707  2708  2709  2710  2711  2712  2713  2714  2715  2716  2717  2718  2719  2720  2721  2722  2723  2724  2725  2726  2727  2728  2729  2730  2731  2732  2733  2734  2735  2736  2737  2738  2739  2740  2741  2742  2743  2744  2745  2746  2747  2748  2749  2750  2751  2752  2753  2754  2755  2756  2757  2758  2759  2760  2761  2762  2763  2764  2765  2766  2767  2768  2769  2770  2771  2772  2773  2774  2775  2776  2777  2778  2779  2780  2781  2782  2783  2784  2785  2786  2787  2788  2789  2790  2791  2792  2793  2794  2795  2796  2797  2798  2799  2800  2801  2802  2803  2804  2805  2806  2807  2808  2809  2810  2811  2812  2813  2814  2815  2816  2817  2818  2819  2820  2821  2822  2823  2824  2825  2826  2827  2828  2829  2830  2831  2832  2833  2834  2835  2836  2837  2838  2839  2840  2841  2842  2843  2844  2845  2846  2847  2848  2849  2850  2851  2852  2853  2854  2855  2856  2857  2858  2859  2860  2861  2862  2863  2864  2865  2866  2867  2868  2869  2870  2871  2872  2873  2874  2875  2876  2877  2878  2879  2880  2881  2882  2883  2884  2885  2886  2887  2888  2889  2890  2891  2892  2893  2894  2895  2896  2897  2898  2899  2900  2901  2902  2903  2904  2905  2906  2907  2908  2909  2910  2911  2912  2913  2914  2915  2916  2917  2918  2919  2920  2921  2922  2923  2924  2925  2926  2927  2928  2929  2930  2931  2932  2933  2934  2935  2936  2937  2938  2939  2940  2941  2942  2943  2944  2945  2946  2947  2948  2949  2950  2951  2952  2953  2954  2955  2956  2957  2958  2959  2960  2961  2962  2963  2964  2965  2966  2967  2968  2969  2970  2971  2972  2973  2974  2975  2976  2977  2978  2979  2980  2981  2982  2983  2984  2985  2986  2987  2988  2989  2990  2991  2992  2993  2994  2995  2996  2997  2998  2999  3000  3001  3002  3003  3004  3005  3006  3007  3008  3009  3010  3011  3012  3013  3014  3015  3016  3017  3018  3019  3020  3021  3022  3023  3024  3025  3026  3027  3028  3029  3030  3031  3032  3033  3034  3035  3036  3037  3038  3039  3040  3041  3042  3043  3044  3045  3046  3047  3048  3049  3050  3051  3052  3053  3054  3055  3056  3057  3058  3059  3060  3061  3062  3063  3064  3065  3066  3067  3068  3069  3070  3071  3072  3073  3074  3075  3076  3077  3078  3079  3080  3081  3082  3083  3084  3085  3086  3087  3088  3089  3090  3091  3092  3093  3094  3095  3096  3097  3098  3099  3100  3101  3102  3103  3104  3105  3106  3107  3108  3109  3110  3111  3112  3113  3114  3115  3116  3117  3118  3119  3120  3121  3122  3123  3124  3125  3126  3127  3128  3129  3130  3131  3132  3133  3134  3135  3136  3137  3138  3139  3140  3141  3142  3143  3144  3145  3146  3147  3148  3149  3150  3151  3152  3153  3154  3155  3156  3157  3158  3159  3160  3161  3162  3163  3164  3165  3166  3167  3168  3169  3170  3171  3172  3173  3174  3175  3176  3177  3178  3179  3180  3181  3182  3183  3184  3185  3186  3187  3188  3189  3190  3191  3192  3193  3194  3195  3196  3197  3198  3199  3200  3201  3202  3203  3204  3205  3206  3207  3208  3209  3210  3211  3212  3213  3214  3215  3216  3217  3218  3219  3220  3221  3222  3223  3224  3225  3226  3227  3228  3229  3230  3231  3232  3233  3234  3235  3236  3237  3238  3239  3240  3241  3242  3243  3244  3245  3246  3247  3248  3249  3250  3251  3252  3253  3254  3255  3256  3257  3258  3259  3260  3261  3262  3263  3264  3265  3266  3267  3268  3269  3270  3271  3272  3273  3274  3275  3276  3277  3278  3279  3280  3281  3282  3283  3284  3285  3286  3287  3288  3289  3290  3291  3292  3293  3294  3295  3296  3297  3298  3299  3300  3301  3302  3303  3304  3305  3306  3307  3308  3309  3310  3311  3312  3313  3314  3315  3316  3317  3318  3319  3320  3321  3322  3323  3324  3325  3326  3327  3328  3329  3330  3331  3332  3333  3334  3335  3336  3337  3338  3339  3340  3341  3342  3343  3344  3345  3346  3347  3348  3349  3350  3351  3352  3353  3354  3355  3356  3357  3358  3359  3360  3361  3362  3363  3364  3365  3366  3367  3368  3369  3370  3371  3372  3373  3374  3375  3376  3377  3378  3379  3380  3381  3382  3383  3384  3385  3386  3387  3388  3389  3390  3391  3392  3393  3394  3395  3396  3397  3398  3399  3400  3401  3402  3403  3404  3405  3406  3407  3408  3409  3410  3411  3412  3413  3414  3415  3416  3417  3418  3419  3420  3421  3422  3423  3424  3425  3426  3427  3428  3429  3430  3431  3432  3433  3434  3435  3436  3437  3438  3439  3440  3441  3442  3443  3444  3445  3446  3447  3448  3449  3450  3451  3452  3453  3454  3455  3456  3457  3458  3459  3460  3461  3462  3463  3464  3465  3466  3467  3468  3469  3470  3471  3472  3473  3474  3475  3476  3477  3478  3479  3480  3481  3482  3483  3484  3485  3486  3487  3488  3489  3490  3491  3492  3493  3494  3495  3496  3497  3498  3499  3500  3501  3502  3503  3504  3505  3506  3507  3508  3509  3510  3511  3512  3513  3514  3515  3516  3517  3518  3519  3520  3521  3522  3523  3524  3525  3526  3527  3528  3529  3530  3531  3532  3533  3534  3535  3536  3537  3538  3539  3540  3541  3542  3543  3544  3545  3546  3547  3548  3549  3550  3551  3552  3553  3554  3555  3556  3557  3558  3559  3560  3561  3562  3563  3564  3565  3566  3567  3568  3569  3570  3571  3572  3573  3574  3575  3576  3577  3578  3579  3580  3581  3582  3583  3584  3585  3586  3587  3588  3589  3590  3591  3592  3593  3594  3595  3596  3597  3598  3599  3600  3601  3602  3603  3604  3605  3606  3607  3608  3609  3610  3611  3612  3613  3614  3615  3616  3617  3618  3619  3620  3621  3622  3623  3624  3625  3626  3627  3628  3629  3630  3631  3632  3633  3634  3635  3636  3637  3638  3639  3640  3641  3642  3643  3644  3645  3646  3647  3648  3649  3650  3651  3652  3653  3654  3655  3656  3657  3658  3659  3660  3661  3662  3663  3664  3665  3666  3667  3668  3669  3670  3671  3672  3673  3674  3675  3676  3677  3678  3679  3680  3681  3682  3683  3684  3685  3686  3687  3688  3689  3690  3691  3692  3693  3694  3695  3696  3697  3698  3699  3700  3701  3702  3703  3704  3705  3706  3707  3708  3709  3710  3711  3712  3713  3714  3715  3716  3717  3718  3719  3720  3721  3722  3723  3724  3725  3726  3727  3728  3729  3730  3731  3732  3733  3734  3735  3736  3737  3738  3739  3740  3741  3742  3743  3744  3745  3746  3747  3748  3749  3750  3751  3752  3753  3754  3755  3756  3757  3758  3759  3760  3761  3762  3763  3764  3765  3766  3767  3768  3769  3770  3771  3772  3773  3774  3775  3776  3777  3778  3779  3780  3781  3782  3783  3784  3785  3786  3787  3788  3789  3790  3791  3792  3793  3794  3795  3796  3797  3798  3799  3800  3801  3802  3803  3804  3805  3806  3807  3808  3809  3810  3811  3812  3813  3814  3815  3816  3817  3818  3819  3820  3821  3822  3823  3824  3825  3826  3827  3828  3829  3830  3831  3832  3833  3834  3835  3836  3837  3838  3839  3840  3841  3842  3843  3844  3845  3846  3847  3848  3849  3850  3851  3852  3853  3854  3855  3856  3857  3858  3859  3860  3861  3862  3863  3864  3865  3866  3867  3868  3869  3870  3871  3872  3873  3874  3875  3876  3877  3878  3879  3880  3881  3882  3883  3884  3885  3886  3887  3888  3889  3890  3891  3892  3893  3894  3895  3896  3897  3898  3899  3900  3901  3902  3903  3904  3905  3906  3907  3908  3909  3910  3911  3912  3913  3914  3915  3916  3917  3918  3919  3920  3921  3922  3923  3924  3925  3926  3927  3928  3929  3930  3931  3932  3933  3934  3935  3936  3937  3938  3939  3940  3941  3942  3943  3944  3945  3946  3947  3948  3949  3950  3951  3952  3953  3954  3955  3956  3957  3958  3959  3960  3961  3962  3963  3964  3965  3966  3967  3968  3969  3970  3971  3972  3973  3974  3975  3976  3977  3978  3979  3980  3981  3982  3983  3984  3985  3986  3987  3988  3989  3990  3991  3992  3993  3994  3995  3996  3997  3998  3999  4000  4001  4002  4003  4004  4005  4006  4007  4008  4009  4010  4011  4012  4013  4014  4015  4016  4017  4018  4019  4020  4021  4022  4023  4024  4025  4026  4027  4028  4029  4030  4031  4032  4033  4034  4035  4036  4037  4038  4039  4040  4041  4042  4043  4044  4045  4046  4047  4048  4049  4050  4051  4052  4053  4054  4055  4056  4057  4058  4059  4060  4061  4062  4063  4064  4065  4066  4067  4068  4069  4070  4071  4072  4073  4074  4075  4076  4077  4078  4079  4080  4081  4082  4083  4084  4085  4086  4087  4088  4089  4090  4091  4092  4093  4094  4095  4096  4097  4098  4099  4100  4101  4102  4103  4104  4105  4106  4107  4108  4109  4110  4111  4112  4113  4114  4115  4116  4117  4118  4119  4120  4121  4122  4123  4124  4125  4126  4127  4128  4129  4130  4131  4132  4133  4134  4135  4136  4137  4138  4139  4140  4141  4142  4143  4144  4145  4146  4147  4148  4149  4150  4151  4152  4153  4154  4155  4156  4157  4158  4159  4160  4161  4162  4163  4164  4165  4166  4167  4168  4169  4170  4171  4172  4173  4174  4175  4176  4177  4178  4179  4180  4181  4182  4183  4184  4185  4186  4187  4188  4189  4190  4191  4192  4193  4194  4195  4196  4197  4198  4199  4200  4201  4202  4203  4204  4205  4206  4207  4208  4209  4210  4211  4212  4213  4214  4215  4216  4217  4218  4219  4220  4221  4222  4223  4224  4225  4226  4227  4228  4229  4230  4231  4232  4233  4234  4235  4236  4237  4238  4239  4240  4241  4242  4243  4244  4245  4246  4247  4248  4249  4250  4251  4252  4253  4254  4255  4256  4257  4258  4259  4260  4261  4262  4263  4264  4265  4266  4267  4268  4269  4270  4271  4272  4273  4274  4275  4276  4277  4278  4279  4280  4281  4282  4283  4284  4285  4286  4287  4288  4289  4290  4291  4292  4293  4294  4295  4296  4297  4298  4299  4300  4301  4302  4303  4304  4305  4306  4307  4308  4309  4310  4311  4312  4313  4314  4315  4316  4317  4318  4319  4320  4321  4322  4323  4324  4325  4326  4327  4328  4329  4330  4331  4332  4333  4334  4335  4336  4337  4338  4339  4340  4341  4342  4343  4344  4345  4346  4347  4348  4349  4350  4351  4352  4353  4354  4355  4356  4357  4358  4359  4360  4361  4362  4363  4364  4365  4366  4367  4368  4369  4370  4371  4372  4373  4374  4375  4376  4377  4378  4379  4380  4381  4382  4383  4384  4385  4386  4387  4388  4389  4390  4391  4392  4393  4394  4395  4396  4397  4398  4399  4400  4401  4402  4403  4404  4405  4406  4407  4408  4409  4410  4411  4412  4413  4414  4415  4416  4417  4418  4419  4420  4421  4422  4423  4424  4425  4426  4427  4428  4429  4430  4431  4432  4433  4434  4435  4436  4437  4438  4439  4440  4441  4442  4443  4444  4445  4446  4447  4448  4449  4450  4451  4452  4453  4454  4455  4456  4457  4458  4459  4460  4461  4462  4463  4464  4465  4466  4467  4468  4469  4470  4471  4472  4473  4474  4475  4476  4477  4478  4479  4480  4481  4482  4483  4484  4485  4486  4487  4488  4489  4490  4491  4492  4493  4494  4495  4496  4497  4498  4499  4500  4501  4502  4503  4504  4505  4506  4507  4508  4509  4510  4511  4512  4513  4514  4515  4516  4517  4518  4519  4520  4521  4522  4523  4524  4525  4526  4527  4528  4529  4530  4531  4532  4533  4534  4535  4536  4537  4538  4539  4540  4541  4542  4543  4544  4545  4546  4547  4548  4549  4550  4551  4552  4553  4554  4555  4556  4557  4558  4559  4560  4561  4562  4563  4564  4565  4566  4567  4568  4569  4570  4571  4572  4573  4574  4575  4576  4577  4578  4579  4580  4581  4582  4583  4584  4585  4586  4587  4588  4589  4590  4591  4592  4593  4594  4595  4596  4597  4598  4599  4600  4601  4602  4603  4604  4605  4606  4607  4608  4609  4610  4611  4612  4613  4614  4615  4616  4617  4618  4619  4620  4621  4622  4623  4624  4625  4626  4627  4628  4629  4630  4631  4632  4633  4634  4635  4636  4637  4638  4639  4640  4641  4642  4643  4644  4645  4646  4647  4648  4649  4650  4651  4652  4653  4654  4655  4656  4657  4658  4659  4660  4661  4662  4663  4664  4665  4666  4667  4668  4669  4670  4671  4672  4673  4674  4675  4676  4677  4678  4679  4680  4681  4682  4683  4684  4685  4686  4687  4688  4689  4690  4691  4692  4693  4694  4695  4696  4697  4698  4699  4700  4701  4702  4703  4704  4705  4706  4707  4708  4709  4710  4711  4712  4713  4714  4715  4716  4717  4718  4719  4720  4721  4722  4723  4724  4725  4726  4727  4728  4729  4730  4731  4732  4733  4734  4735  4736  4737  4738  4739  4740  4741  4742  4743  4744  4745  4746  4747  4748  4749  4750  4751  4752  4753  4754  4755  4756  4757  4758  4759  4760  4761  4762  4763  4764  4765  4766  4767  4768  4769  4770  4771  4772  4773  4774  4775  4776  4777  4778  4779  4780  4781  4782  4783  4784  4785  4786  4787  4788  4789  4790  4791  4792  4793  4794  4795  4796  4797  4798  4799  4800  4801  4802  4803  4804  4805  4806  4807  4808  4809  4810  4811  4812  4813  4814  4815  4816  4817  4818  4819  4820  4821  4822  4823  4824  4825  4826  4827  4828  4829  4830  4831  4832  4833  4834  4835  4836  4837  4838  4839  4840  4841  4842  4843  4844  4845  4846  4847  4848  4849  4850  4851  4852  4853  4854  4855  4856  4857  4858  4859  4860  4861  4862  4863  4864  4865  4866  4867  4868  4869  4870  4871  4872  4873  4874  4875  4876  4877  4878  4879  4880  4881  4882  4883  4884  4885  4886  4887  4888  4889  4890  4891  4892  4893  4894  4895  4896  4897  4898  4899  4900  4901  4902  4903  4904  4905  4906  4907  4908  4909  4910  4911  4912  4913  4914  4915  4916  4917  4918  4919  4920  4921  4922  4923  4924  4925  4926  4927  4928  4929  4930  4931  4932  4933  4934  4935  4936  4937  4938  4939  4940  4941  4942  4943  4944  4945  4946  4947  4948  4949  4950  4951  4952  4953  4954  4955  4956  4957  4958  4959  4960  4961  4962  4963  4964  4965  4966  4967  4968  4969  4970  4971  4972  4973  4974  4975  4976  4977  4978  4979  4980  4981  4982  4983  4984  4985  4986  4987  4988  4989  4990  4991  4992  4993  4994  4995  4996  4997  4998  4999  5000  5001  5002  5003  5004  5005  5006  5007  5008  5009  5010  5011  5012  5013  5014  5015  5016  5017  5018  5019  5020  5021  5022  5023  5024  5025  5026  5027  5028  5029  5030  5031  5032  5033  5034  5035  5036  5037  5038  5039  5040  5041  5042  5043  5044  5045  5046  5047  5048  5049  5050  5051  5052  5053  5054  5055  5056  5057  5058  5059  5060  5061  5062  5063  5064  5065  5066  5067  5068  5069  5070  5071  5072  5073  5074  5075  5076  5077  5078  5079  5080  5081  5082  5083  5084  5085  5086  5087  5088  5089  5090  5091  5092  5093  5094  5095  5096  5097  5098  5099  5100  5101  5102  5103  5104  5105  5106  5107  5108  5109  5110  5111  5112  5113  5114  5115  5116  5117  5118  5119  5120  5121  5122  5123  5124  5125  5126  5127  5128  5129  5130  5131  5132  5133  5134  5135  5136  5137  5138  5139  5140  5141  5142  5143  5144  5145  5146  5147  5148  5149  5150  5151  5152  5153  5154  5155  5156  5157  5158  5159  5160  5161  5162  5163  5164  5165  5166  5167  5168  5169  5170  5171  5172  5173  5174  5175  5176  5177  5178  5179  5180  5181  5182  5183  5184  5185  5186  5187  5188  5189  5190  5191  5192  5193  5194  5195  5196  5197  5198  5199  5200  5201  5202  5203  5204  5205  5206  5207  5208  5209  5210  5211  5212  5213  5214  5215  5216  5217  5218  5219  5220  5221  5222  5223  5224  5225  5226  5227  5228  5229  5230  5231  5232  5233  5234  5235  5236  5237  5238  5239  5240  5241  5242  5243  5244  5245  5246  5247  5248  5249  5250  5251  5252  5253  5254  5255  5256  5257  5258  5259  5260  5261  5262  5263  5264  5265  5266  5267  5268  5269  5270  5271  5272  5273  5274  5275  5276  5277  5278  5279  5280  5281  5282  5283  5284  5285  5286  5287  5288  5289  5290  5291  5292  5293  5294  5295  5296  5297  5298  5299  5300  5301  5302  5303  5304  5305  5306  5307  5308  5309  5310  5311  5312  5313  5314  5315  5316  5317  5318  5319  5320  5321  5322  5323  5324  5325  5326  5327  5328  5329  5330  5331  5332  5333  5334  5335  5336  5337  5338  5339  5340  5341  5342  5343  5344  5345  5346  5347  5348  5349  5350  5351  5352  5353  5354  5355  5356  5357  5358  5359  5360  5361  5362  5363  5364  5365  5366  5367  5368  5369  5370  5371  5372  5373  5374  5375  5376  5377  5378  5379  5380  5381  5382  5383  5384  5385  5386  5387  5388  5389  5390  5391  5392  5393  5394  5395  5396  5397  5398  5399  5400  5401  5402  5403  5404  5405  5406  5407  5408  5409  5410  5411  5412  5413  5414  5415  5416  5417  5418  5419  5420  5421  5422  5423  5424  5425  5426  5427  5428  5429  5430  5431  5432  5433  5434  5435  5436  5437  5438  5439  5440  5441  5442  5443  5444  5445  5446  5447  5448  5449  5450  5451  5452  5453  5454  5455  5456  5457  5458  5459  5460  5461  5462  5463  5464  5465  5466  5467  5468  5469  5470  5471  5472  5473  5474  5475  5476  5477  5478  5479  5480  5481  5482  5483  5484  5485  5486  5487  5488  5489  5490  5491  5492  5493  5494  5495  5496  5497  5498  5499  5500  5501  5502  5503  5504  5505  5506  5507  5508  5509  5510  5511  5512  5513  5514  5515  5516  5517  5518  5519  5520  5521  5522  5523  5524  5525  5526  5527  5528  5529  5530  5531  5532  5533  5534  5535  5536  5537  5538  5539  5540  5541  5542  5543  5544  5545  5546  5547  5548  5549  5550  5551  5552  5553  5554  5555  5556  5557  5558  5559  5560  5561  5562  5563  5564  5565  5566  5567  5568  5569  5570  5571  5572  5573  5574  5575  5576  5577  5578  5579  5580  5581  5582  5583  5584  5585  5586  5587  5588  5589  5590  5591  5592  5593  5594  5595  5596  5597  5598  5599  5600  5601  5602  5603  5604  5605  5606  5607  5608  5609  5610  5611  5612  5613  5614  5615  5616  5617  5618  5619  5620  5621  5622  5623  5624  5625  5626  5627  5628  5629  5630  5631  5632  5633  5634  5635  5636  5637  5638  5639  5640  5641  5642  5643  5644  5645  5646  5647  5648  5649  5650  5651  5652  5653  5654  5655  5656  5657  5658  5659  5660  5661  5662  5663  5664  5665  5666  5667  5668  5669  5670  5671  5672  5673  5674  5675  5676  5677  5678  5679  5680  5681  5682  5683  5684  5685  5686  5687  5688  5689  5690  5691  5692  5693  5694  5695  5696  5697  5698  5699  5700  5701  5702  5703  5704  5705  5706  5707  5708  5709  5710  5711  5712  5713  5714  5715  5716  5717  5718  5719  5720  5721  5722  5723  5724  5725  5726  5727  5728  5729  5730  5731  5732  5733  5734  5735  5736  5737  5738  5739  5740  5741  5742  5743  5744  5745  5746  5747  5748  5749  5750  5751  5752  5753  5754  5755  5756  5757  5758  5759  5760  5761  5762  5763  5764  5765  5766  5767  5768  5769  5770  5771  5772  5773  5774  5775  5776  5777  5778  5779  5780  5781  5782  5783  5784  5785  5786  5787  5788  5789  5790  5791  5792  5793  5794  5795  5796  5797  5798  5799  5800  5801  5802  5803  5804  5805  5806  5807  5808  5809  5810  5811  5812  5813  5814  5815  5816  5817  5818  5819  5820  5821  5822  5823  5824  5825  5826  5827  5828  5829  5830  5831  5832  5833  5834  5835  5836  5837  5838  5839  5840  5841  5842  5843  5844  5845  5846  5847  5848  5849  5850  5851  5852  5853  5854  5855  5856  5857  5858  5859  5860  5861  5862  5863  5864  5865  5866  5867  5868  5869  5870  5871  5872  5873  5874  5875  5876  5877  5878  5879  5880  5881  5882  5883  5884  5885  5886  5887  5888  5889  5890  5891  5892  5893  5894  5895  5896  5897  5898  5899  5900  5901  5902  5903  5904  5905  5906  5907  5908  5909  5910  5911  5912  5913  5914  5915  5916  5917  5918  5919  5920  5921  5922  5923  5924  5925  5926  5927  5928  5929  5930  5931  5932  5933  5934  5935  5936  5937  5938  5939  5940  5941  5942  5943  5944  5945  5946  5947  5948  5949  5950  5951  5952  5953  5954  5955  5956  5957  5958  5959  5960  5961  5962  5963  5964  5965  5966  5967  5968  5969  5970  5971  5972  5973  5974  5975  5976  5977  5978  5979  5980  5981  5982  5983  5984  5985  5986  5987  5988  5989  5990  5991  5992  5993  5994  5995  5996  5997  5998  5999  6000  6001  6002  6003  6004  6005  6006  6007  6008  6009  6010  6011  6012  6013  6014  6015  6016  6017  6018  6019  6020  6021  6022  6023  6024  6025  6026  6027  6028  6029  6030  6031  6032  6033  6034  6035  6036  6037  6038  6039  6040  6041  6042  6043  6044  6045  6046  6047  6048  6049  6050  6051  6052  6053  6054  6055  6056  6057  6058  6059  6060  6061  6062  6063  6064  6065  6066  6067  6068  6069  6070  6071  6072  6073  6074  6075  6076  6077  6078  6079  6080  6081  6082  6083  6084  6085  6086  6087  6088  6089  6090  6091  6092  6093  6094  6095  6096  6097  6098  6099  6100  6101  6102  6103  6104  6105  6106  6107  6108  6109  6110  6111  6112  6113  6114  6115  6116  6117  6118  6119  6120  6121  6122  6123  6124  6125  6126  6127  6128  6129  6130  6131  6132  6133  6134  6135  6136  6137  6138  6139  6140  6141  6142  6143  6144  6145  6146  6147  6148  6149  6150  6151  6152  6153  6154  6155  6156  6157  6158  6159  6160  6161  6162  6163  6164  6165  6166  6167  6168  6169  6170  6171  6172  6173  6174  6175  6176  6177  6178  6179  6180  6181  6182  6183  6184  6185  6186  6187  6188  6189  6190  6191  6192  6193  6194  6195  6196  6197  6198  6199  6200  6201  6202  6203  6204  6205  6206  6207  6208  6209  6210  6211  6212  6213  6214  6215  6216  6217  6218  6219  6220  6221  6222  6223  6224  6225  6226  6227  6228  6229  6230  6231  6232  6233  6234  6235  6236  6237  6238  6239  6240  6241  6242  6243  6244  6245  6246  6247  6248  6249  6250  6251  6252  6253  6254  6255  6256  6257  6258  6259  6260  6261  6262  6263  6264  6265  6266  6267  6268  6269  6270  6271  6272  6273  6274  6275  6276  6277  6278  6279  6280  6281  6282  6283  6284  6285  6286  6287  6288  6289  6290  6291  6292  6293  6294  6295  6296  6297  6298  6299  6300  6301  6302  6303  6304  6305  6306  6307  6308  6309  6310  6311  6312  6313  6314  6315  6316  6317  6318  6319  6320  6321  6322  6323  6324  6325  6326  6327  6328  6329  6330  6331  6332  6333  6334  6335  6336  6337  6338  6339  6340  6341  6342  6343  6344  6345  6346  6347  6348  6349  6350  6351  6352  6353  6354  6355  6356  6357  6358  6359  6360  6361  6362  6363  6364  6365  6366  6367  6368  6369  6370  6371  6372  6373  6374  6375  6376  6377  6378  6379  6380  6381  6382  6383  6384  6385  6386  6387  6388  6389  6390  6391  6392  6393  6394  6395  6396  6397  6398  6399  6400  6401  6402  6403  6404  6405  6406  6407  6408  6409  6410  6411  6412  6413  6414  6415  6416  6417  6418  6419  6420  6421  6422  6423  6424  6425  6426  6427  6428  6429  6430  6431  6432  6433  6434  6435  6436  6437  6438  6439  6440  6441  6442  6443  6444  6445  6446  6447  6448  6449  6450  6451  6452  6453  6454  6455  6456  6457  6458  6459  6460  6461  6462  6463  6464  6465  6466  6467  6468  6469  6470  6471  6472  6473  6474  6475  6476  6477  6478  6479  6480  6481  6482  6483  6484  6485  6486  6487  6488  6489  6490  6491  6492  6493  6494  6495  6496  6497  6498  6499  6500  6501  6502  6503  6504  6505  6506  6507  6508  6509  6510  6511  6512  6513  6514  6515  6516  6517  6518  6519  6520  6521  6522  6523  6524  6525  6526  6527  6528  6529  6530  6531  6532  6533  6534  6535  6536  6537  6538  6539  6540  6541  6542  6543  6544  6545  6546  6547  6548  6549  6550  6551  6552  6553  6554  6555  6556  6557  6558  6559  6560  6561  6562  6563  6564  6565  6566  6567  6568  6569  6570  6571  6572  6573  6574  6575  6576  6577  6578  6579  6580  6581  6582  6583  6584  6585  6586  6587  6588  6589  6590  6591  6592  6593  6594  6595  6596  6597  6598  6599  6600  6601  6602  6603  6604  6605  6606  6607  6608  6609  6610  6611  6612  6613  6614  6615  6616  6617  6618  6619  6620  6621  6622  6623  6624  6625  6626  6627  6628  6629  6630  6631  6632  6633  6634  6635  6636  6637  6638  6639  6640  6641  6642  6643  6644  6645  6646  6647  6648  6649  6650  6651  6652  6653  6654  6655  6656  6657  6658  6659  6660  6661  6662  6663  6664  6665  6666  6667  6668  6669  6670  6671  6672  6673  6674  6675  6676  6677  6678  6679  6680  6681  6682  6683  6684  6685  6686  6687  6688  6689  6690  6691  6692  6693  6694  6695  6696  6697  6698  6699  6700  6701  6702  6703  6704  6705  6706  6707  6708  6709  6710  6711  6712  6713  6714  6715  6716  6717  6718  6719  6720  6721  6722  6723  6724  6725  6726  6727  6728  6729  6730  6731  6732  6733  6734  6735  6736  6737  6738  6739  6740  6741  6742  6743  6744  6745  6746  6747  6748  6749  6750  6751  6752  6753  6754  6755  6756  6757  6758  6759  6760  6761  6762  6763  6764  6765  6766  6767  6768  6769  6770  6771  6772  6773  6774  6775  6776  6777  6778  6779  6780  6781  6782  6783  6784  6785  6786  6787  6788  6789  6790  6791  6792  6793  6794  6795  6796  6797  6798  6799  6800  6801  6802  6803  6804  6805  6806  6807  6808  6809  6810  6811  6812  6813  6814  6815  6816  6817  6818  6819  6820  6821  6822  6823  6824  6825  6826  6827  6828  6829  6830  6831  6832  6833  6834  6835  6836  6837  6838  6839  6840  6841  6842  6843  6844  6845  6846  6847  6848  6849  6850  6851  6852  6853  6854  6855  6856  6857  6858  6859  6860  6861  6862  6863  6864  6865  6866  6867  6868  6869  6870  6871  6872  6873  6874  6875  6876  6877  6878  6879  6880  6881  6882  6883  6884  6885  6886  6887  6888  6889  6890  6891  6892  6893  6894  6895  6896  6897  6898  6899  6900  6901  6902  6903  6904  6905  6906  6907  6908  6909  6910  6911  6912  6913  6914  6915  6916  6917  6918  6919  6920  6921  6922  6923  6924  6925  6926  6927  6928  6929  6930  6931  6932  6933  6934  6935  6936  6937  6938  6939  6940  6941  6942  6943  6944  6945  6946  6947  6948  6949  6950  6951  6952  6953  6954  6955  6956  6957  6958  6959  6960  6961  6962  6963  6964  6965  6966  6967  6968  6969  6970  6971  6972  6973  6974  6975  6976  6977  6978  6979  6980  6981  6982  6983  6984  6985  6986  6987  6988  6989  6990  6991  6992  6993  6994  6995  6996  6997  6998  6999  7000  7001  7002  7003  7004  7005  7006  7007  7008  7009  7010  7011  7012  7013  7014  7015  7016  7017  7018  7019  7020  7021  7022  7023  7024  7025  7026  7027  7028  7029  7030  7031  7032  7033  7034  7035  7036  7037  7038  7039  7040  7041  7042  7043  7044  7045  7046  7047  7048  7049  7050  7051  7052  7053  7054  7055  7056  7057  7058  7059  7060  7061  7062  7063  7064  7065  7066  7067  7068  7069  7070  7071  7072  7073  7074  7075  7076  7077  7078  7079  7080  7081  7082  7083  7084  7085  7086  7087  7088  7089  7090  7091  7092  7093  7094  7095  7096  7097  7098  7099  7100  7101  7102  7103  7104  7105  7106  7107  7108  7109  7110  7111  7112  7113  7114  7115  7116  7117  7118  7119  7120  7121  7122  7123  7124  7125  7126  7127  7128  7129  7130  7131  7132  7133  7134  7135  7136  7137  7138  7139  7140  7141  7142  7143  7144  7145  7146  7147  7148  7149  7150  7151  7152  7153  7154  7155  7156  7157  7158  7159  7160  7161  7162  7163  7164  7165  7166  7167  7168  7169  7170  7171  7172  7173  7174  7175  7176  7177  7178  7179  7180  7181  7182  7183  7184  7185  7186  7187  7188  7189  7190  7191  7192  7193  7194  7195  7196  7197  7198  7199  7200  7201  7202  7203  7204  7205  7206  7207  7208  7209  7210  7211  7212  7213  7214  7215  7216  7217  7218  7219  7220  7221  7222  7223  7224  7225  7226  7227  7228  7229  7230  7231  7232  7233  7234  7235  7236  7237  7238  7239  7240  7241  7242  7243  7244  7245  7246  7247  7248  7249  7250  7251  7252  7253  7254  7255  7256  7257  7258  7259  7260  7261  7262  7263  7264  7265  7266  7267  7268  7269  7270  7271  7272  7273  7274  7275  7276  7277  7278  7279  7280  7281  7282  7283  7284  7285  7286  7287  7288  7289  7290  7291  7292  7293  7294  7295  7296  7297  7298  7299  7300  7301  7302  7303  7304  7305  7306  7307  7308  7309  7310  7311  7312  7313  7314  7315  7316  7317  7318  7319  7320  7321  7322  7323  7324  7325  7326  7327  7328  7329  7330  7331  7332  7333  7334  7335  7336  7337  7338  7339  7340  7341  7342  7343  7344  7345  7346  7347  7348  7349  7350  7351  7352  7353  7354  7355  7356  7357  7358  7359  7360  7361  7362  7363  7364  7365  7366  7367  7368  7369  7370  7371  7372  7373  7374  7375  7376  7377  7378  7379  7380  7381  7382  7383  7384  7385  7386  7387  7388  7389  7390  7391  7392  7393  7394  7395  7396  7397  7398  7399  7400  7401  7402  7403  7404  7405  7406  7407  7408  7409  7410  7411  7412  7413  7414  7415  7416  7417  7418  7419  7420  7421  7422  7423  7424  7425  7426  7427  7428  7429  7430  7431  7432  7433  7434  7435  7436  7437  7438  7439  7440  7441  7442  7443  7444  7445  7446  7447  7448  7449  7450  7451  7452  7453  7454  7455  7456  7457  7458  7459  7460  7461  7462  7463  7464  7465  7466  7467  7468  7469  7470  7471  7472  7473  7474  7475  7476  7477  7478  7479  7480  7481  7482  7483  7484  7485  7486  7487  7488  7489  7490  7491  7492  7493  7494  7495  7496  7497  7498  7499  7500  7501  7502  7503  7504  7505  7506  7507  7508  7509  7510  7511  7512  7513  7514  7515  7516  7517  7518  7519  7520  7521  7522  7523  7524  7525  7526  7527  7528  7529  7530  7531  7532  7533  7534  7535  7536  7537  7538  7539  7540  7541  7542  7543  7544  7545  7546  7547  7548  7549  7550  7551  7552  7553  7554  7555  7556  7557  7558  7559  7560  7561  7562  7563  7564  7565  7566  7567  7568  7569  7570  7571  7572  7573  7574  7575  7576  7577  7578  7579  7580  7581  7582  7583  7584  7585  7586  7587  7588  7589  7590  7591  7592  7593  7594  7595  7596  7597  7598  7599  7600  7601  7602  7603  7604  7605  7606  7607  7608  7609  7610  7611  7612  7613  7614  7615  7616  7617  7618  7619  7620  7621  7622  7623  7624  7625  7626  7627  7628  7629  7630  7631  7632  7633  7634  7635  7636  7637  7638  7639  7640  7641  7642  7643  7644  7645  7646  7647  7648  7649  7650  7651  7652  7653  7654  7655  7656  7657  7658  7659  7660  7661  7662  7663  7664  7665  7666  7667  7668  7669  7670  7671  7672  7673  7674  7675  7676  7677  7678  7679  7680  7681  7682  7683  7684  7685  7686  7687  7688  7689  7690  7691  7692  7693  7694  7695  7696  7697  7698  7699  7700  7701  7702  7703  7704  7705  7706  7707  7708  7709  7710  7711  7712  7713  7714  7715  7716  7717  7718  7719  7720  7721  7722  7723  7724  7725  7726  7727  7728  7729  7730  7731  7732  7733  7734  7735  7736  7737  7738  7739  7740  7741  7742  7743  7744  7745  7746  7747  7748  7749  7750  7751  7752  7753  7754  7755  7756  7757  7758  7759  7760  7761  7762  7763  7764  7765  7766  7767  7768  7769  7770  7771  7772  7773  7774  7775  7776  7777  7778  7779  7780  7781  7782  7783  7784  7785  7786  7787  7788  7789  7790  7791  7792  7793  7794  7795  7796  7797  7798  7799  7800  7801  7802  7803  7804  7805  7806  7807  7808  7809  7810  7811  7812  7813  7814  7815  7816  7817  7818  7819  7820  7821  7822  7823  7824  7825  7826  7827  7828  7829  7830  7831  7832  7833  7834  7835  7836  7837  7838  7839  7840  7841  7842  7843  7844  7845  7846  7847  7848  7849  7850  7851  7852  7853  7854  7855  7856  7857  7858  7859  7860  7861  7862  7863  7864  7865  7866  7867  7868  7869  7870  7871  7872  7873  7874  7875  7876  7877  7878  7879  7880  7881  7882  7883  7884  7885  7886  7887  7888  7889  7890  7891  7892  7893  7894  7895  7896  7897  7898  7899  7900  7901  7902  7903  7904  7905  7906  7907  7908  7909  7910  7911  7912  7913  7914  7915  7916  7917  7918  7919  7920  7921  7922  7923  7924  7925  7926  7927  7928  7929  7930  7931  7932  7933  7934  7935  7936  7937  7938  7939  7940  7941  7942  7943  7944  7945  7946  7947  7948  7949  7950  7951  7952  7953  7954  7955  7956  7957  7958  7959  7960  7961  7962  7963  7964  7965  7966  7967  7968  7969  7970  7971  7972  7973  7974  7975  7976  7977  7978  7979  7980  7981  7982  7983  7984  7985  7986  7987  7988  7989  7990  7991  7992  7993  7994  7995  7996  7997  7998  7999  8000  8001  8002  8003  8004  8005  8006  8007  8008  8009  8010  8011  8012  8013  8014  8015  8016  8017  8018  8019  8020  8021  8022  8023  8024  8025  8026  8027  8028  8029  8030  8031  8032  8033  8034  8035  8036  8037  8038  8039  8040  8041  8042  8043  8044  8045  8046  8047  8048  8049  8050  8051  8052  8053  8054  8055  8056  8057  8058  8059  8060  8061  8062  8063  8064  8065  8066  8067  8068  8069  8070  8071  8072  8073  8074  8075  8076  8077  8078  8079  8080  8081  8082  8083  8084  8085  8086  8087  8088  8089  8090  8091  8092  8093  8094  8095  8096  8097  8098  8099  8100  8101  8102  8103  8104  8105  8106  8107  8108  8109  8110  8111  8112  8113  8114  8115  8116  8117  8118  8119  8120  8121  8122  8123  8124  8125  8126  8127  8128  8129  8130  8131  8132  8133  8134  8135  8136  8137  8138  8139  8140  8141  8142  8143  8144  8145  8146  8147  8148  8149  8150  8151  8152  8153  8154  8155  8156  8157  8158  8159  8160  8161  8162  8163  8164  8165  8166  8167  8168  8169  8170  8171  8172  8173  8174  8175  8176  8177  8178  8179  8180  8181  8182  8183  8184  8185  8186  8187  8188  8189  8190  8191  8192  8193  8194  8195  8196  8197  8198  8199  8200  8201  8202  8203  8204  8205  8206  8207  8208  8209  8210  8211  8212  8213  8214  8215  8216  8217  8218  8219  8220  8221  8222  8223  8224  8225  8226  8227  8228  8229  8230  8231  8232  8233  8234  8235  8236  8237  8238  8239  8240  8241  8242  8243  8244  8245  8246  8247  8248  8249  8250  8251  8252  8253  8254  8255  8256  8257  8258  8259  8260  8261  8262  8263  8264  8265  8266  8267  8268  8269  8270  8271  8272  8273  8274  8275  8276  8277  8278  8279  8280  8281  8282  8283  8284  8285  8286  8287  8288  8289  8290  8291  8292  8293  8294  8295  8296  8297  8298  8299  8300  8301  8302  8303  8304  8305  8306  8307  8308  8309  8310  8311  8312  8313  8314  8315  8316  8317  8318  8319  8320  8321  8322  8323  8324  8325  8326  8327  8328  8329  8330  8331  8332  8333  8334  8335  8336  8337  8338  8339  8340  8341  8342  8343  8344  8345  8346  8347  8348  8349  8350  8351  8352  8353  8354  8355  8356  8357  8358  8359  8360  8361  8362  8363  8364  8365  8366  8367  8368  8369  8370  8371  8372  8373  8374  8375  8376  8377  8378  8379  8380  8381  8382  8383  8384  8385  8386  8387  8388  8389  8390  8391  8392  8393  8394  8395  8396  8397  8398  8399  8400  8401  8402  8403  8404  8405  8406  8407  8408  8409  8410  8411  8412  8413  8414  8415  8416  8417  8418  8419  8420  8421  8422  8423  8424  8425  8426  8427  8428  8429  8430  8431  8432  8433  8434  8435  8436  8437  8438  8439  8440  8441  8442  8443  8444  8445  8446  8447  8448  8449  8450  8451  8452  8453  8454  8455  8456  8457  8458  8459  8460  8461  8462  8463  8464  8465  8466  8467  8468  8469  8470  8471  8472  8473  8474  8475  8476  8477  8478  8479  8480  8481  8482  8483  8484  8485  8486  8487  8488  8489  8490  8491  8492  8493  8494  8495  8496  8497  8498  8499  8500  8501  8502  8503  8504  8505  8506  8507  8508  8509  8510  8511  8512  8513  8514  8515  8516  8517  8518  8519  8520  8521  8522  8523  8524  8525  8526  8527  8528  8529  8530  8531  8532  8533  8534  8535  8536  8537  8538  8539  8540  8541  8542  8543  8544  8545  8546  8547  8548  8549  8550  8551  8552  8553  8554  8555  8556  8557  8558  8559  8560  8561  8562  8563  8564  8565  8566  8567  8568  8569  8570  8571  8572  8573  8574  8575  8576  8577  8578  8579  8580  8581  8582  8583  8584  8585  8586  8587  8588  8589  8590  8591  8592  8593  8594  8595  8596  8597  8598  8599  8600  8601  8602  8603  8604  8605  8606  8607  8608  8609  8610  8611  8612  8613  8614  8615  8616  8617  8618  8619  8620  8621  8622  8623  8624  8625  8626  8627  8628  8629  8630  8631  8632  8633  8634  8635  8636  8637  8638  8639  8640  8641  8642  8643  8644  8645  8646  8647  8648  8649  8650  8651  8652  8653  8654  8655  8656  8657  8658  8659  8660  8661  8662  8663  8664  8665  8666  8667  8668  8669  8670  8671  8672  8673  8674  8675  8676  8677  8678  8679  8680  8681  8682  8683  8684  8685  8686  8687  8688  8689  8690  8691  8692  8693  8694  8695  8696  8697  8698  8699  8700  8701  8702  8703  8704  8705  8706  8707  8708  8709  8710  8711  8712  8713  8714  8715  8716  8717  8718  8719  8720  8721  8722  8723  8724  8725  8726  8727  8728  8729  8730  8731  8732  8733  8734  8735  8736  8737  8738  8739  8740  8741  8742  8743  8744  8745  8746  8747  8748  8749  8750  8751  8752  8753  8754  8755  8756  8757  8758  8759  8760  8761  8762  8763  8764  8765  8766  8767  8768  8769  8770  8771  8772  8773  8774  8775  8776  8777  8778  8779  8780  8781  8782  8783  8784  8785  8786  8787  8788  8789  8790  8791  8792  8793  8794  8795  8796  8797  8798  8799  8800  8801  8802  8803  8804  8805  8806  8807  8808  8809  8810  8811  8812  8813  8814  8815  8816  8817  8818  8819  8820  8821  8822  8823  8824  8825  8826  8827  8828  8829  8830  8831  8832  8833  8834  8835  8836  8837  8838  8839  8840  8841  8842  8843  8844  8845  8846  8847  8848  8849  8850  8851  8852  8853  8854  8855  8856  8857  8858  8859  8860  8861  8862  8863  8864  8865  8866  8867  8868  8869  8870  8871  8872  8873  8874  8875  8876  8877  8878  8879  8880  8881  8882  8883  8884  8885  8886  8887  8888  8889  8890  8891  8892  8893  8894  8895  8896  8897  8898  8899  8900  8901  8902  8903  8904  8905  8906  8907  8908  8909  8910  8911  8912  8913  8914  8915  8916  8917  8918  8919  8920  8921  8922  8923  8924  8925  8926  8927  8928  8929  8930  8931  8932  8933  8934  8935  8936  8937  8938  8939  8940  8941  8942  8943  8944  8945  8946  8947  8948  8949  8950  8951  8952  8953  8954  8955  8956  8957  8958  8959  8960  8961  8962  8963  8964  8965  8966  8967  8968  8969  8970  8971  8972  8973  8974  8975  8976  8977  8978  8979  8980  8981  8982  8983  8984  8985  8986  8987  8988  8989  8990  8991  8992  8993  8994  8995  8996  8997  8998  8999  9000  9001  9002  9003  9004  9005  9006  9007  9008  9009  9010  9011  9012  9013  9014  9015  9016  9017  9018  9019  9020  9021  9022  9023  9024  9025  9026  9027  9028  9029  9030  9031  9032  9033  9034  9035  9036  9037  9038  9039  9040  9041  9042  9043  9044  9045  9046  9047  9048  9049  9050  9051  9052  9053  9054  9055  9056  9057  9058  9059  9060  9061  9062  9063  9064  9065  9066  9067  9068  9069  9070  9071  9072  9073  9074  9075  9076  9077  9078  9079  9080  9081  9082  9083  9084  9085  9086  9087  9088  9089  9090  9091  9092  9093  9094  9095  9096  9097  9098  9099  9100  9101  9102  9103  9104  9105  9106  9107  9108  9109  9110  9111  9112  9113  9114  9115  9116  9117  9118  9119  9120  9121  9122  9123  9124  9125  9126  9127  9128  9129  9130  9131  9132  9133  9134  9135  9136  9137  9138  9139  9140  9141  9142  9143  9144  9145  9146  9147  9148  9149  9150  9151  9152  9153  9154  9155  9156  9157  9158  9159  9160  9161  9162  9163  9164  9165  9166  9167  9168  9169  9170  9171  9172  9173  9174  9175  9176  9177  9178  9179  9180  9181  9182  9183  9184  9185  9186  9187  9188  9189  9190  9191  9192  9193  9194  9195  9196  9197  9198  9199  9200  9201  9202  9203  9204  9205  9206  9207  9208  9209  9210  9211  9212  9213  9214  9215  9216  9217  9218  9219  9220  9221  9222  9223  9224  9225  9226  9227  9228  9229  9230  9231  9232  9233  9234  9235  9236  9237  9238  9239  9240  9241  9242  9243  9244  9245  9246  9247  9248  9249  9250  9251  9252  9253  9254  9255  9256  9257  9258  9259  9260  9261  9262  9263  9264  9265  9266  9267  9268  9269  9270  9271  9272  9273  9274  9275  9276  9277  9278  9279  9280  9281  9282  9283  9284  9285  9286  9287  9288  9289  9290  9291  9292  9293  9294  9295  9296  9297  9298  9299  9300  9301  9302  9303  9304  9305  9306  9307  9308  9309  9310  9311  9312  9313  9314  9315  9316  9317  9318  9319  9320  9321  9322  9323  9324  9325  9326  9327  9328  9329  9330  9331  9332  9333  9334  9335  9336  9337  9338  9339  9340  9341  9342  9343  9344  9345  9346  9347  9348  9349  9350  9351  9352  9353  9354  9355  9356  9357  9358  9359  9360  9361  9362  9363  9364  9365  9366  9367  9368  9369  9370  9371  9372  9373  9374  9375  9376  9377  9378  9379  9380  9381  9382  9383  9384  9385  9386  9387  9388  9389  9390  9391  9392  9393  9394  9395  9396  9397  9398  9399  9400  9401  9402  9403  9404  9405  9406  9407  9408  9409  9410  9411  9412  9413  9414  9415  9416  9417  9418  9419  9420  9421  9422  9423  9424  9425  9426  9427  9428  9429  9430  9431  9432  9433  9434  9435  9436  9437  9438  9439  9440  9441  9442  9443  9444  9445  9446  9447  9448  9449  9450  9451  9452  9453  9454  9455  9456  9457  9458  9459  9460  9461  9462  9463  9464  9465  9466  9467  9468  9469  9470  9471  9472  9473  9474  9475  9476  9477  9478  9479  9480  9481  9482  9483  9484  9485  9486  9487  9488  9489  9490  9491  9492  9493  9494  9495  9496  9497  9498  9499  9500  9501  9502  9503  9504  9505  9506  9507  9508  9509  9510  9511  9512  9513  9514  9515  9516  9517  9518  9519  9520  9521  9522  9523  9524  9525  9526  9527  9528  9529  9530  9531  9532  9533  9534  9535  9536  9537  9538  9539  9540  9541  9542  9543  9544  9545  9546  9547  9548  9549  9550  9551  9552  9553  9554  9555  9556  9557  9558  9559  9560  9561  9562  9563  9564  9565  9566  9567  9568  9569  9570  9571  9572  9573  9574  9575  9576  9577  9578  9579  9580  9581  9582  9583  9584  9585  9586  9587  9588  9589  9590  9591  9592  9593  9594  9595  9596  9597  9598  9599  9600  9601  9602  9603  9604  9605  9606  9607  9608  9609  9610  9611  9612  9613  9614  9615  9616  9617  9618  9619  9620  9621  9622  9623  9624  9625  9626  9627  9628  9629  9630  9631  9632  9633  9634  9635  9636  9637  9638  9639  9640  9641  9642  9643  9644  9645  9646  9647  9648  9649  9650  9651  9652  9653  9654  9655  9656  9657  9658  9659  9660  9661  9662  9663  9664  9665  9666  9667  9668  9669  9670  9671  9672  9673  9674  9675  9676  9677  9678  9679  9680  9681  9682  9683  9684  9685  9686  9687  9688  9689  9690  9691  9692  9693  9694  9695  9696  9697  9698  9699  9700  9701  9702  9703  9704  9705  9706  9707  9708  9709  9710  9711  9712  9713  9714  9715  9716  9717  9718  9719  9720  9721  9722  9723  9724  9725  9726  9727  9728  9729  9730  9731  9732  9733  9734  9735  9736  9737  9738  9739 | A6ND36  A6ND36  A6ND36  A6ND36  A6NHR9  A6NHR9  A6NHR9  A6NHR9  A8K4G0  A8K4G0  A8K4G0  A8K4G0  O00151  O00151  O00151  O00151  O00159  O00159  O00159  O00159  O00161  O00161  O00161  O00161  O00165  O00165  O00165  O00165  O00168  O00168  O00168  O00168  O00194  O00194  O00194  O00194  O00204  O00204  O00204  O00204  O00213  O00213  O00213  O00213  O00231  O00231  O00231  O00231  O00232  O00232  O00232  O00232  O00257  O00257  O00257  O00257  O00257  O00257  O00257  O00257  O00257  O00257  O00257  O00257  O00264  O00264  O00264  O00264  O00267  O00267  O00267  O00267  O00267  O00267  O00267  O00267  O00267  O00267  O00267  O00267  O00273  O00273  O00273  O00273  O00303  O00303  O00303  O00303  O00330  O00330  O00330  O00330  O00391  O00391  O00391  O00391  O00399  O00399  O00399  O00399  O00399  O00399  O00399  O00399  O00399  O00399  O00399  O00401  O00401  O00401  O00401  O00410  O00410  O00410  O00410  O00418  O00418  O00418  O00418  O00418  O00418  O00418  O00418  O00418  O00418  O00418  O00418  O00429  O00429  O00429  O00429  O00443  O00443  O00443  O00443  O00459  O00459  O00459  O00459  O00560  O00560  O00560  O00560  O00562  O00562  O00562  O00562  O00562  O00562  O00562  O00562  O00562  O00562  O00562  O00562  O00571  O00571  O00571  O00571  O00622  O00622  O00622  O00622  O00763  O00763  O00763  O00763  O14492  O14492  O14492  O14492  O14493  O14493  O14493  O14493  O14519  O14519  O14519  O14519  O14607  O14607  O14607  O14607  O14607  O14607  O14607  O14607  O14607  O14607  O14607  O14607  O14625  O14625  O14653  O14653  O14653  O14653  O14654  O14654  O14654  O14654  O14672  O14672  O14672  O14672  O14672  O14672  O14672  O14672  O14672  O14672  O14672  O14672  O14713  O14713  O14713  O14713  O14713  O14713  O14713  O14713  O14718  O14718  O14718  O14718  O14744  O14744  O14744  O14744  O14745  O14745  O14745  O14745  O14745  O14745  O14745  O14745  O14746  O14746  O14746  O14746  O14757  O14757  O14757  O14757  O14777  O14777  O14777  O14777  O14791  O14791  O14791  O14791  O14818  O14818  O14818  O14818  O14827  O14827  O14827  O14827  O14920  O14920  O14920  O14920  O14936  O14936  O14936  O14936  O14950  O14950  O14950  O14950  O14950  O14950  O14950  O14950  O14950  O14950  O14950  O14950  O14958  O14958  O14958  O14958  O14965  O14965  O14965  O14965  O14965  O14965  O14965  O14965  O14965  O14965  O14965  O14965  O14974  O14974  O14974  O14974  O14974  O14974  O14974  O14974  O14974  O14974  O14974  O14974  O14979  O14979  O14979  O14979  O14994  O14994  O14994  O14994  O15055  O15055  O15055  O15055  O15084  O15084  O15084  O15084  O15105  O15105  O15105  O15105  O15151  O15151  O15151  O15151  O15160  O15160  O15160  O15160  O15162  O15162  O15162  O15162  O15162  O15162  O15162  O15162  O15162  O15162  O15162  O15162  O15169  O15169  O15169  O15169  O15232  O15232  O15232  O15232  O15232  O15232  O15232  O15232  O15232  O15232  O15232  O15232  O15240  O15240  O15240  O15240  O15240  O15240  O15240  O15240  O15240  O15240  O15240  O15240  O15259  O15259  O15259  O15259  O15264  O15264  O15264  O15264  O15264  O15264  O15264  O15264  O15264  O15264  O15264  O15264  O15269  O15269  O15269  O15269  O15287  O15287  O15287  O15287  O15294  O15294  O15294  O15294  O15304  O15304  O15304  O15304  O15350  O15350  O15350  O15350  O15350  O15350  O15350  O15350  O15350  O15350  O15350  O15350  O15357  O15357  O15357  O15357  O15357  O15357  O15357  O15357  O15357  O15357  O15357  O15357  O15372  O15372  O15372  O15372  O15382  O15382  O15382  O15382  O15392  O15392  O15392  O15400  O15400  O15400  O15400  O15446  O15446  O15446  O15446  O15455  O15455  O15455  O15455  O15492  O15492  O15492  O15492  O15516  O15516  O15516  O15516  O15516  O15516  O15516  O15516  O15516  O15516  O15516  O15516  O15530  O15530  O15530  O15530  O15530  O15530  O15530  O15530  O15530  O15530  O15530  O15530  O15547  O15547  O15547  O15547  O15554  O15554  O15554  O15554  O43156  O43156  O43156  O43156  O43164  O43164  O43164  O43164  O43164  O43164  O43164  O43164  O43164  O43164  O43164  O43164  O43175  O43175  O43175  O43175  O43255  O43255  O43255  O43255  O43255  O43255  O43255  O43255  O43255  O43255  O43255  O43255  O43257  O43257  O43257  O43257  O43257  O43264  O43264  O43264  O43264  O43294  O43294  O43294  O43294  O43318  O43318  O43318  O43318  O43318  O43318  O43318  O43318  O43318  O43318  O43318  O43318  O43324  O43324  O43324  O43324  O43353  O43353  O43353  O43353  O43390  O43390  O43390  O43390  O43432  O43432  O43432  O43432  O43463  O43463  O43463  O43463  O43490  O43490  O43490  O43490  O43491  O43491  O43491  O43491  O43493  O43493  O43493  O43493  O43493  O43493  O43493  O43493  O43493  O43493  O43493  O43493  O43504  O43504  O43504  O43504  O43521  O43521  O43521  O43521  O43524  O43524  O43524  O43524  O43524  O43524  O43524  O43524  O43524  O43524  O43524  O43525  O43525  O43525  O43525  O43525  O43525  O43525  O43525  O43525  O43525  O43525  O43525  O43526  O43526  O43526  O43526  O43526  O43526  O43526  O43526  O43526  O43526  O43526  O43526  O43541  O43541  O43541  O43541  O43548  O43548  O43548  O43548  O43561  O43561  O43561  O43561  O43602  O43602  O43602  O43602  O43633  O43633  O43633  O43633  O43663  O43663  O43663  O43663  O43663  O43663  O43663  O43663  O43663  O43663  O43663  O43663  O43683  O43683  O43683  O43683  O43683  O43683  O43683  O43683  O43683  O43683  O43683  O43683  O43760  O43760  O43760  O43760  O43768  O43768  O43768  O43768  O43809  O43809  O43809  O43809  O60256  O60256  O60256  O60256  O60260  O60260  O60260  O60260  O60285  O60285  O60285  O60285  O60285  O60285  O60285  O60285  O60285  O60285  O60285  O60285  O60331  O60331  O60331  O60331  O60343  O60343  O60343  O60343  O60343  O60343  O60343  O60343  O60343  O60343  O60343  O60343  O60493  O60493  O60493  O60493  O60566  O60566  O60566  O60566  O60566  O60566  O60566  O60566  O60566  O60566  O60566  O60566  O60602  O60602  O60602  O60602  O60610  O60610  O60610  O60610  O60610  O60610  O60610  O60610  O60610  O60610  O60610  O60610  O60658  O60658  O60658  O60658  O60674  O60674  O60674  O60674  O60678  O60678  O60678  O60678  O60711  O60711  O60711  O60711  O60716  O60716  O60716  O60716  O60763  O60763  O60763  O60763  O60832  O60832  O60832  O60832  O60841  O60841  O60841  O60841  O60869  O60869  O60869  O60869  O60870  O60870  O60870  O60870  O60885  O60885  O60885  O60885  O60928  O60928  O60928  O60928  O60934  O60934  O60934  O60934  O75030  O75030  O75030  O75030  O75044  O75044  O75044  O75044  O75056  O75056  O75056  O75056  O75116  O75116  O75116  O75116  O75143  O75143  O75143  O75143  O75151  O75151  O75151  O75151  O75154  O75154  O75154  O75154  O75155  O75155  O75155  O75155  O75179  O75179  O75179  O75179  O75348  O75348  O75348  O75348  O75367  O75367  O75367  O75367  O75367  O75367  O75367  O75367  O75367  O75367  O75367  O75367  O75381  O75381  O75381  O75381  O75410  O75410  O75410  O75410  O75449  O75449  O75449  O75449  O75449  O75449  O75449  O75449  O75449  O75449  O75449  O75449  O75496  O75496  O75496  O75496  O75530  O75530  O75530  O75530  O75531  O75533  O75533  O75533  O75533  O75533  O75533  O75533  O75533  O75533  O75533  O75533  O75533  O75563  O75563  O75563  O75563  O75569  O75569  O75569  O75569  O75581  O75581  O75581  O75581  O75581  O75581  O75581  O75581  O75581  O75581  O75581  O75581  O75582  O75582  O75582  O75582  O75582  O75582  O75582  O75582  O75582  O75582  O75582  O75582  O75607  O75607  O75607  O75607  O75636  O75636  O75636  O75636  O75689  O75689  O75689  O75689  O75689  O75689  O75689  O75689  O75689  O75689  O75689  O75689  O75716  O75716  O75716  O75716  O75716  O75716  O75716  O75807  O75807  O75807  O75807  O75821  O75821  O75821  O75821  O75821  O75821  O75821  O75821  O75821  O75821  O75821  O75821  O75822  O75822  O75822  O75822  O75822  O75822  O75822  O75915  O75915  O75915  O75915  O75925  O75925  O75925  O75925  O75934  O75934  O75934  O75934  O75943  O75943  O75943  O75943  O75995  O75995  O75995  O75995  O76021  O76021  O76021  O76021  O76024  O76024  O76024  O76024  O76024  O76024  O76024  O76024  O76024  O76024  O76024  O76024  O76061  O76061  O76061  O76061  O76061  O76061  O76061  O76061  O76061  O76061  O76061  O76061  O76094  O76094  O76094  O76094  O94760  O94760  O94760  O94760  O94763  O94763  O94763  O94763  O94811  O94811  O94811  O94811  O94811  O94811  O94811  O94811  O94811  O94811  O94811  O94832  O94832  O94832  O94832  O94875  O94875  O94875  O94875  O94915  O94915  O94915  O94915  O94916  O94916  O94916  O94916  O94916  O94916  O94916  O94916  O94916  O94916  O94916  O94916  O95069  O95069  O95069  O95069  O95071  O95071  O95071  O95071  O95071  O95071  O95071  O95071  O95071  O95071  O95071  O95071  O95084  O95084  O95084  O95084  O95235  O95235  O95235  O95235  O95251  O95251  O95251  O95251  O95251  O95251  O95251  O95251  O95251  O95251  O95251  O95251  O95267  O95267  O95267  O95267  O95267  O95267  O95267  O95267  O95267  O95267  O95267  O95267  O95278  O95278  O95278  O95278  O95297  O95297  O95297  O95297  O95373  O95373  O95373  O95373  O95453  O95453  O95453  O95453  O95470  O95470  O95470  O95470  O95477  O95477  O95477  O95477  O95551  O95551  O95551  O95551  O95551  O95551  O95551  O95551  O95551  O95551  O95551  O95551  O95630  O95630  O95630  O95630  O95633  O95633  O95633  O95633  O95644  O95644  O95644  O95644  O95714  O95714  O95714  O95714  O95714  O95714  O95714  O95714  O95714  O95714  O95714  O95714  O95777  O95777  O95777  O95777  O95785  O95785  O95785  O95785  O95786  O95786  O95786  O95786  O95786  O95786  O95786  O95786  O95786  O95786  O95786  O95786  O95810  O95810  O95810  O95810  O95835  O95835  O95835  O95835  O95835  O95835  O95835  O95835  O95835  O95835  O95835  O95835  O95863  O95863  O95863  O95863  O95863  O95863  O95863  O95863  O95863  O95866  O95866  O95866  O95866  O95972  O95972  O95972  O95972  O95997  O95997  O95997  O95997  O96013  O96013  O96013  O96013  O96017  O96017  O96017  O96017  O96017  O96017  O96017  O96017  O96017  O96017  O96017  O96017  O96019  O96019  O96019  O96019  O96033  O96033  O96033  P00167  P00167  P00167  P00167  P00325  P00325  P00325  P00325  P00367  P00367  P00367  P00367  P00439  P00439  P00439  P00439  P00441  P00441  P00441  P00441  P00450  P00450  P00450  P00450  P00451  P00451  P00451  P00451  P00480  P00480  P00480  P00480  P00491  P00491  P00491  P00491  P00492  P00492  P00492  P00492  P00558  P00558  P00558  P00558  P00568  P00568  P00568  P00568  P00734  P00734  P00734  P00734  P00736  P00736  P00736  P00736  P00740  P00740  P00740  P00740  P00740  P00740  P00740  P00740  P00740  P00740  P00740  P00740  P00742  P00742  P00742  P00742  P00747  P00747  P00747  P00747  P00749  P00749  P00749  P00749  P00813  P00813  P00813  P00813  P00915  P00915  P00915  P00915  P01008  P01008  P01008  P01008  P01008  P01008  P01008  P01008  P01008  P01008  P01008  P01008  P01009  P01009  P01009  P01009  P01019  P01019  P01019  P01019  P01024  P01024  P01024  P01024  P01033  P01033  P01033  P01033  P01034  P01034  P01034  P01034  P01042  P01042  P01042  P01042  P01100  P01100  P01100  P01100  P01100  P01100  P01100  P01100  P01100  P01100  P01100  P01100  P01106  P01106  P01106  P01106  P01106  P01106  P01106  P01106  P01106  P01106  P01106  P01106  P01112  P01112  P01112  P01112  P01148  P01148  P01148  P01148  P01185  P01185  P01185  P01189  P01189  P01189  P01189  P01236  P01236  P01236  P01236  P01241  P01241  P01241  P01241  P01258  P01258  P01258  P01258  P01266  P01266  P01266  P01266  P01275  P01275  P01275  P01275  P01282  P01282  P01282  P01282  P01286  P01286  P01286  P01303  P01303  P01303  P01303  P01350  P01579  P01579  P01579  P01579  P01591  P01591  P01591  P01591  P01700  P01700  P01700  P01700  P01744  P01744  P01744  P01744  P01772  P01772  P01772  P01814  P01814  P01814  P01814  P01825  P01825  P01825  P01825  P02008  P02008  P02008  P02008  P02511  P02511  P02511  P02511  P02545  P02545  P02545  P02545  P02647  P02647  P02647  P02647  P02649  P02649  P02649  P02649  P02652  P02652  P02652  P02652  P02671  P02671  P02671  P02671  P02675  P02675  P02675  P02675  P02679  P02679  P02679  P02679  P02686  P02686  P02686  P02686  P02730  P02730  P02730  P02730  P02741  P02741  P02741  P02741  P02745  P02745  P02745  P02745  P02746  P02746  P02746  P02746  P02751  P02751  P02751  P02751  P02763  P02763  P02763  P02763  P02765  P02765  P02765  P02765  P02765  P02765  P02765  P02765  P02765  P02765  P02765  P02765  P02766  P02766  P02766  P02766  P02768  P02768  P02768  P02768  P02768  P02768  P02768  P02768  P02768  P02768  P02768  P02768  P02771  P02771  P02771  P02771  P02778  P02778  P02778  P02778  P02786  P02786  P02786  P02786  P02788  P02788  P02788  P02788  P02792  P02792  P02792  P02792  P02814  P03372  P03372  P03372  P03372  P03950  P03950  P03950  P03950  P03956  P03956  P03956  P03956  P03956  P03956  P03956  P03956  P03956  P03956  P03956  P03956  P03999  P03999  P03999  P03999  P04001  P04001  P04001  P04001  P04004  P04004  P04004  P04004  P04004  P04004  P04004  P04004  P04004  P04004  P04004  P04004  P04070  P04070  P04070  P04070  P04075  P04075  P04075  P04075  P04083  P04083  P04083  P04083  P04090  P04090  P04090  P04090  P04114  P04114  P04114  P04114  P04150  P04150  P04150  P04150  P04179  P04179  P04179  P04179  P04181  P04181  P04181  P04181  P04183  P04183  P04183  P04183  P04198  P04198  P04198  P04198  P04264  P04264  P04264  P04264  P04406  P04406  P04406  P04406  P04406  P04406  P04406  P04406  P04406  P04406  P04406  P04406  P04424  P04424  P04424  P04424  P04626  P04626  P04626  P04626  P04629  P04629  P04629  P04629  P04632  P04632  P04632  P04632  P04637  P04637  P04637  P04637  P04637  P04637  P04637  P04637  P04637  P04637  P04637  P04637  P04745  P04745  P04745  P04745  P04792  P04792  P04792  P04792  P05060  P05060  P05060  P05060  P05067  P05067  P05067  P05067  P05067  P05067  P05067  P05067  P05067  P05067  P05067  P05067  P05090  P05090  P05090  P05090  P05106  P05106  P05106  P05106  P05106  P05106  P05106  P05106  P05106  P05106  P05106  P05106  P05107  P05107  P05107  P05107  P05107  P05107  P05107  P05107  P05107  P05107  P05107  P05107  P05109  P05109  P05114  P05114  P05114  P05114  P05161  P05161  P05161  P05161  P05164  P05164  P05164  P05164  P05231  P05231  P05231  P05231  P05386  P05386  P05386  P05412  P05412  P05412  P05412  P05412  P05412  P05412  P05412  P05412  P05451  P05451  P05451  P05451  P05455  P05455  P05455  P05455  P05546  P05546  P05546  P05546  P05549  P05549  P05549  P05549  P05771  P05771  P05771  P05771  P05771  P05771  P05771  P05771  P05771  P05771  P05771  P05771  P05783  P05783  P05783  P05783  P05813  P05813  P05813  P05813  P05814  P05814  P05814  P05814  P05814  P05814  P05814  P05814  P05997  P05997  P05997  P05997  P06213  P06213  P06213  P06213  P06276  P06276  P06276  P06276  P06307  P06307  P06396  P06396  P06396  P06396  P06400  P06400  P06400  P06400  P06400  P06400  P06400  P06400  P06400  P06400  P06400  P06400  P06401  P06401  P06401  P06401  P06454  P06454  P06454  P06454  P06702  P06702  P06702  P06702  P06702  P06730  P06730  P06730  P06730  P06733  P06733  P06733  P06733  P06737  P06737  P06737  P06737  P06744  P06744  P06744  P06744  P06746  P06746  P06746  P06746  P06748  P06748  P06748  P06850  P06850  P07101  P07101  P07101  P07101  P07108  P07108  P07108  P07108  P07148  P07148  P07148  P07148  P07195  P07195  P07195  P07195  P07199  P07199  P07199  P07199  P07204  P07204  P07204  P07204  P07225  P07225  P07225  P07225  P07305  P07305  P07305  P07305  P07311  P07311  P07311  P07311  P07332  P07332  P07332  P07332  P07332  P07332  P07332  P07332  P07332  P07332  P07332  P07332  P07333  P07333  P07333  P07333  P07358  P07358  P07358  P07358  P07358  P07358  P07358  P07358  P07358  P07358  P07358  P07358  P07359  P07359  P07359  P07359  P07360  P07360  P07360  P07360  P07492  P07492  P07492  P07550  P07550  P07550  P07550  P07737  P07737  P07737  P07737  P07741  P07741  P07741  P07741  P07766  P07766  P07766  P07766  P07814  P07814  P07814  P07814  P07900  P07900  P07900  P07900  P07900  P07900  P07900  P07900  P07900  P07900  P07900  P07900  P07910  P07910  P07910  P07910  P07947  P07947  P07947  P07947  P07949  P07949  P07949  P07949  P08047  P08047  P08047  P08047  P08047  P08047  P08047  P08047  P08047  P08047  P08047  P08047  P08069  P08069  P08069  P08069  P08151  P08151  P08151  P08151  P08195  P08195  P08195  P08195  P08397  P08397  P08397  P08397  P08493  P08493  P08514  P08514  P08514  P08514  P08559  P08559  P08559  P08559  P08567  P08567  P08567  P08567  P08581  P08581  P08581  P08581  P08582  P08582  P08582  P08582  P08697  P08697  P08697  P08697  P08709  P08709  P08709  P08709  P08833  P08833  P08833  P08833  P08833  P08833  P08833  P08833  P08833  P08842  P08842  P08842  P08842  P08865  P08865  P08865  P08865  P08865  P08865  P08865  P08865  P08865  P08865  P08865  P08865  P08949  P08949  P08949  P09012  P09012  P09012  P09012  P09038  P09038  P09038  P09038  P09211  P09211  P09211  P09211  P09382  P09382  P09382  P09382  P09417  P09417  P09417  P09417  P09493  P09493  P09493  P09493  P09603  P09603  P09603  P09603  P09603  P09603  P09603  P09603  P09603  P09603  P09603  P09603  P09619  P09619  P09619  P09619  P09683  P09683  P09683  P09683  P09693  P09693  P09693  P09693  P09871  P09871  P09871  P09871  P09874  P09874  P09874  P09874  P09917  P09917  P09917  P09917  P09958  P09958  P09958  P09958  P09960  P09960  P09960  P09960  P0C0L4  P0C0L4  P0C0L4  P0C0L4  P0CG48  P0CG48  P0CG48  P0CG48  P0DI83  P0DI83  P0DI83  P0DI83  P0DI83  P10070  P10070  P10070  P10070  P10071  P10071  P10071  P10071  P10082  P10082  P10082  P10082  P10145  P10145  P10153  P10153  P10153  P10153  P10155  P10155  P10155  P10155  P10244  P10244  P10244  P10244  P10244  P10244  P10244  P10244  P10244  P10244  P10244  P10244  P10275  P10275  P10275  P10275  P10276  P10276  P10276  P10276  P10415  P10415  P10415  P10415  P10415  P10415  P10415  P10415  P10415  P10415  P10451  P10451  P10451  P10451  P10451  P10451  P10451  P10451  P10451  P10451  P10451  P10515  P10515  P10515  P10515  P10588  P10588  P10588  P10588  P10599  P10599  P10599  P10636  P10636  P10636  P10636  P10636  P10636  P10636  P10636  P10636  P10636  P10636  P10636  P10644  P10644  P10644  P10644  P10645  P10645  P10645  P10645  P10721  P10721  P10721  P10721  P10809  P10809  P10809  P10809  P10914  P10914  P10914  P10914  P10997  P10997  P10997  P10997  P11047  P11047  P11047  P11047  P11142  P11142  P11142  P11142  P11171  P11171  P11171  P11171  P11171  P11171  P11171  P11171  P11171  P11171  P11171  P11171  P11226  P11226  P11226  P11226  P11274  P11274  P11274  P11274  P11277  P11277  P11277  P11277  P11277  P11277  P11277  P11277  P11277  P11277  P11277  P11277  P11309  P11309  P11309  P11309  P11309  P11309  P11309  P11309  P11309  P11362  P11362  P11362  P11362  P11387  P11387  P11387  P11387  P11388  P11388  P11388  P11388  P11388  P11388  P11388  P11388  P11388  P11388  P11388  P11388  P11413  P11413  P11413  P11413  P11474  P11474  P11474  P11474  P11488  P11488  P11488  P11488  P11498  P11498  P11498  P11498  P11831  P11831  P11831  P11831  P11926  P11926  P11926  P11926  P12004  P12004  P12004  P12004  P12111  P12111  P12111  P12111  P12235  P12235  P12235  P12235  P12259  P12259  P12259  P12259  P12270  P12270  P12270  P12270  P12273  P12273  P12273  P12273  P12429  P12429  P12429  P12429  P12644  P12644  P12644  P12644  P12814  P12814  P12814  P12814  P12821  P12821  P12821  P12821  P12830  P12830  P12830  P12830  P12956  P12956  P12956  P12956  P13010  P13010  P13010  P13010  P13224  P13224  P13224  P13224  P13498  P13498  P13498  P13498  P13498  P13498  P13498  P13498  P13498  P13500  P13500  P13500  P13500  P13501  P13501  P13501  P13501  P13521  P13521  P13521  P13521  P13569  P13569  P13569  P13569  P13569  P13569  P13569  P13569  P13569  P13569  P13569  P13569  P13611  P13611  P13611  P13611  P13612  P13612  P13612  P13612  P13639  P13639  P13639  P13639  P13639  P13639  P13639  P13639  P13639  P13639  P13639  P13639  P13646  P13646  P13646  P13646  P13688  P13688  P13688  P13688  P13693  P13693  P13693  P13693  P13796  P13796  P13796  P13796  P13798  P13798  P13798  P13798  P13807  P13807  P13807  P13807  P14210  P14210  P14210  P14210  P14314  P14314  P14314  P14314  P14316  P14316  P14316  P14316  P14317  P14317  P14317  P14317  P14410  P14410  P14410  P14410  P14598  P14598  P14598  P14598  P14618  P14618  P14618  P14618  P14625  P14625  P14625  P14625  P14635  P14635  P14635  P14635  P14672  P14672  P14672  P14672  P14678  P14678  P14678  P14678  P14778  P14778  P14778  P14778  P14859  P14859  P14859  P14859  P14866  P14866  P14866  P14866  P15036  P15036  P15036  P15036  P15056  P15056  P15056  P15056  P15056  P15056  P15056  P15056  P15056  P15056  P15056  P15056  P15121  P15121  P15121  P15121  P15151  P15151  P15151  P15151  P15172  P15172  P15172  P15172  P15289  P15289  P15289  P15289  P15311  P15311  P15311  P15311  P15336  P15336  P15336  P15336  P15336  P15336  P15336  P15336  P15336  P15336  P15336  P15336  P15391  P15391  P15391  P15391  P15502  P15502  P15502  P15502  P15529  P15529  P15529  P15529  P15927  P15927  P15927  P15927  P15927  P15927  P15927  P15927  P15927  P15927  P15927  P15927  P15941  P15941  P15941  P15941  P15941  P15941  P15941  P15941  P15941  P15976  P15976  P15976  P15976  P16066  P16066  P16066  P16066  P16066  P16066  P16066  P16066  P16066  P16066  P16066  P16066  P16070  P16070  P16070  P16070  P16150  P16150  P16150  P16150  P16152  P16152  P16152  P16152  P16157  P16157  P16157  P16157  P16220  P16220  P16220  P16220  P16234  P16234  P16234  P16234  P16284  P16284  P16284  P16284  P16401  P16401  P16401  P16401  P16401  P16401  P16401  P16410  P16410  P16410  P16410  P16435  P16435  P16435  P16435  P16930  P16930  P16930  P16930  P16949  P17096  P17096  P17096  P17096  P17096  P17302  P17302  P17302  P17302  P17612  P17612  P17612  P17612  P17612  P17612  P17612  P17612  P17612  P17612  P17661  P17661  P17661  P17661  P17661  P17661  P17661  P17661  P17661  P17661  P17661  P17661  P17676  P17676  P17676  P17676  P17676  P17676  P17676  P17707  P17707  P17707  P17707  P17735  P17735  P17735  P17735  P17858  P17858  P17858  P17858  P17931  P17931  P17931  P17931  P17936  P17936  P17936  P17936  P17948  P17948  P17948  P17948  P17987  P17987  P17987  P17987  P18031  P18031  P18031  P18031  P18124  P18124  P18124  P18124  P18206  P18206  P18206  P18206  P18509  P18669  P18669  P18669  P18669  P18754  P18754  P18754  P18754  P18887  P18887  P18887  P18887  P18887  P18887  P18887  P18887  P18887  P18887  P18887  P18887  P19021  P19021  P19021  P19021  P19021  P19021  P19021  P19021  P19021  P19021  P19021  P19021  P19022  P19022  P19022  P19022  P19113  P19113  P19113  P19113  P19174  P19174  P19174  P19174  P19367  P19367  P19367  P19367  P19404  P19404  P19404  P19404  P19419  P19419  P19419  P19419  P19419  P19419  P19419  P19419  P19419  P19419  P19419  P19419  P19429  P19429  P19525  P19525  P19525  P19525  P19525  P19525  P19525  P19525  P19525  P19525  P19525  P19525  P19793  P19793  P19793  P19793  P19801  P19801  P19801  P19801  P19823  P19823  P19823  P19823  P19827  P19827  P19827  P19827  P19838  P19838  P19838  P19838  P20020  P20020  P20020  P20020  P20020  P20020  P20020  P20020  P20020  P20020  P20020  P20020  P20138  P20138  P20138  P20138  P20155  P20155  P20155  P20155  P20336  P20336  P20336  P20336  P20338  P20338  P20338  P20338  P20366  P20366  P20393  P20393  P20393  P20393  P20472  P20472  P20591  P20591  P20591  P20591  P20749  P20749  P20749  P20749  P20908  P20908  P20908  P20908  P20933  P20933  P20933  P20933  P21127  P21127  P21127  P21127  P21127  P21127  P21127  P21127  P21127  P21127  P21127  P21127  P21283  P21283  P21283  P21283  P21397  P21397  P21397  P21397  P21453  P21453  P21453  P21453  P21453  P21453  P21453  P21453  P21453  P21453  P21453  P21453  P21549  P21549  P21549  P21549  P21675  P21675  P21675  P21675  P21730  P21730  P21730  P21730  P21754  P21754  P21754  P21754  P21796  P21796  P21796  P21796  P21815  P21815  P21815  P21815  P21980  P21980  P21980  P21980  P22061  P22061  P22061  P22061  P22234  P22234  P22234  P22234  P22314  P22314  P22314  P22314  P22466  P22466  P22466  P22681  P22681  P22681  P22681  P22692  P22692  P22692  P22692  P22735  P22735  P22735  P22735  P22736  P22736  P22736  P22736  P22891  P22891  P22891  P22891  P22914  P22914  P22914  P22914  P23025  P23025  P23025  P23025  P23193  P23193  P23193  P23193  P23229  P23229  P23229  P23229  P23246  P23246  P23246  P23246  P23246  P23246  P23246  P23246  P23246  P23246  P23246  P23246  P23297  P23297  P23297  P23327  P23327  P23327  P23327  P23327  P23327  P23327  P23327  P23327  P23327  P23327  P23327  P23396  P23396  P23396  P23396  P23396  P23396  P23396  P23396  P23396  P23434  P23434  P23434  P23434  P23443  P23443  P23443  P23443  P23443  P23443  P23443  P23443  P23443  P23443  P23443  P23443  P23458  P23458  P23458  P23458  P23508  P23508  P23508  P23508  P23526  P23526  P23526  P23526  P23528  P23528  P23528  P23528  P23588  P23588  P23588  P23588  P23760  P23760  P23760  P23760  P24385  P24385  P24385  P24385  P24385  P24385  P24385  P24385  P24385  P24385  P24385  P24390  P24390  P24390  P24390  P24394  P24394  P24394  P24394  P24593  P24593  P24593  P24593  P24666  P24666  P24666  P24666  P24821  P24821  P24821  P24821  P24864  P24864  P24864  P24864  P24864  P24864  P24864  P24864  P24864  P24864  P24864  P24864  P24928  P24928  P24928  P24928  P25205  P25205  P25205  P25205  P25311  P25311  P25311  P25311  P25398  P25398  P25713  P25713  P25788  P25788  P25788  P25788  P25963  P25963  P25963  P25963  P25963  P25963  P25963  P25963  P25963  P25963  P25963  P25963  P26358  P26358  P26358  P26358  P26368  P26368  P26368  P26368  P26378  P26378  P26378  P26378  P26599  P26599  P26599  P26599  P26640  P26640  P26640  P26640  P26641  P26641  P26641  P26641  P26651  P26651  P26651  P26651  P26651  P26651  P26651  P26651  P26651  P26651  P26651  P26651  P26678  P27105  P27105  P27105  P27105  P27361  P27361  P27361  P27361  P27361  P27361  P27361  P27361  P27361  P27361  P27361  P27361  P27695  P27695  P27695  P27695  P27707  P27707  P27707  P27707  P27708  P27708  P27708  P27708  P27708  P27708  P27708  P27708  P27708  P27708  P27708  P27708  P27815  P27815  P27815  P27815  P27816  P27816  P27816  P27816  P28072  P28072  P28072  P28072  P28223  P28223  P28223  P28223  P28370  P28370  P28370  P28370  P28749  P28749  P28749  P28749  P28749  P28749  P28749  P28749  P28749  P28749  P28749  P28749  P29083  P29083  P29083  P29083  P29144  P29144  P29144  P29144  P29317  P29317  P29317  P29317  P29350  P29350  P29350  P29350  P29474  P29474  P29474  P29474  P29474  P29474  P29474  P29474  P29474  P29474  P29474  P29474  P29508  P29508  P29508  P29508  P29590  P29590  P29590  P29590  P29590  P29590  P29590  P29590  P29590  P29590  P29590  P29590  P29597  P29597  P29597  P29597  P29692  P29692  P29692  P29692  P29966  P29966  P29966  P29966  P30040  P30040  P30040  P30040  P30260  P30260  P30260  P30260  P30260  P30260  P30260  P30260  P30260  P30260  P30260  P30260  P30291  P30291  P30291  P30291  P30291  P30291  P30291  P30291  P30291  P30291  P30291  P30291  P30304  P30304  P30304  P30304  P30304  P30304  P30304  P30304  P30304  P30304  P30304  P30304  P30305  P30305  P30305  P30305  P30307  P30307  P30307  P30307  P30307  P30307  P30307  P30307  P30307  P30307  P30307  P30307  P30411  P30411  P30411  P30411  P30519  P30519  P30519  P30519  P30530  P30530  P30530  P30530  P30566  P30566  P30566  P30566  P30793  P30793  P30793  P30793  P30990  P30990  P30990  P30990  P31040  P31040  P31040  P31040  P31151  P31151  P31151  P31151  P31269  P31269  P31269  P31269  P31327  P31327  P31327  P31327  P31645  P31645  P31645  P31645  P31645  P31645  P31645  P31645  P31645  P31645  P31645  P31645  P31751  P31751  P31751  P31751  P31751  P31751  P31751  P31751  P31751  P31751  P31751  P31751  P31948  P31948  P31948  P31948  P31949  P31949  P31949  P31949  P31949  P31949  P31995  P31995  P31995  P31995  P32004  P32004  P32004  P32004  P33121  P33121  P33121  P33121  P33241  P33241  P33241  P33241  P33316  P33316  P33316  P33316  P33764  P33764  P33764  P33764  P33993  P33993  P33993  P33993  P34059  P34059  P34059  P34059  P34096  P34096  P34096  P34096  P34741  P34741  P34741  P34741  P34896  P34896  P34896  P34896  P34947  P34947  P34947  P34947  P34947  P34947  P34947  P34947  P34947  P34947  P34947  P34947  P34972  P34972  P34972  P34972  P34998  P34998  P34998  P34998  P35221  P35221  P35221  P35221  P35222  P35222  P35222  P35222  P35222  P35222  P35222  P35222  P35222  P35222  P35222  P35222  P35236  P35236  P35236  P35236  P35236  P35236  P35236  P35236  P35236  P35236  P35236  P35236  P35269  P35269  P35269  P35269  P35269  P35269  P35269  P35269  P35269  P35269  P35269  P35269  P35270  P35270  P35270  P35270  P35318  P35318  P35318  P35318  P35367  P35367  P35367  P35367  P35367  P35367  P35367  P35367  P35367  P35367  P35367  P35367  P35398  P35398  P35398  P35398  P35398  P35398  P35398  P35398  P35398  P35398  P35398  P35398  P35408  P35408  P35408  P35408  P35520  P35520  P35520  P35520  P35555  P35555  P35555  P35555  P35558  P35558  P35558  P35558  P35568  P35568  P35568  P35568  P35611  P35611  P35611  P35611  P35611  P35611  P35611  P35611  P35611  P35611  P35611  P35611  P35612  P35612  P35612  P35612  P35612  P35612  P35612  P35612  P35612  P35612  P35612  P35637  P35637  P35637  P35637  P35659  P35659  P35659  P35659  P35659  P35659  P35659  P35659  P35659  P35659  P35659  P35659  P35900  P35900  P35900  P35900  P35916  P35916  P35916  P35916  P35968  P35968  P35968  P35968  P36507  P36507  P36507  P36507  P36507  P36507  P36507  P36507  P36507  P36507  P36507  P36578  P36578  P36578  P36578  P36871  P36871  P36871  P36871  P36871  P36871  P36871  P36871  P36871  P36871  P36871  P36871  P36888  P36888  P36888  P36888  P36896  P36896  P36896  P36896  P36955  P36955  P36955  P36955  P37802  P37802  P37802  P37802  P37840  P37840  P37840  P37840  P38117  P38117  P38117  P38117  P38159  P38159  P38159  P38159  P38398  P38398  P38398  P38398  P38398  P38398  P38398  P38398  P38398  P38398  P38398  P38398  P38432  P38432  P38432  P38432  P38646  P38646  P38646  P38646  P38919  P38919  P38919  P38919  P38935  P38935  P38935  P38935  P38936  P38936  P38936  P38936  P38936  P38936  P39748  P39748  P39748  P39748  P40189  P40189  P40189  P40189  P40429  P40429  P40429  P40429  P40763  P40763  P40763  P40763  P40925  P40925  P40925  P40925  P40926  P40926  P40926  P40926  P41091  P41091  P41091  P41091  P41181  P41181  P41181  P41181  P41182  P41182  P41182  P41182  P41212  P41212  P41212  P41212  P41227  P41227  P41227  P41227  P41235  P41235  P41235  P41235  P41235  P41235  P41235  P41235  P41235  P41235  P41235  P41235  P41240  P41240  P41240  P41240  P41279  P41279  P41279  P41279  P41279  P41279  P41279  P41279  P41279  P41279  P41279  P41279  P41597  P41597  P41597  P41597  P41743  P41743  P41743  P41743  P41743  P41743  P41743  P41743  P41743  P41743  P41743  P41743  P42229  P42229  P42229  P42229  P42285  P42285  P42285  P42285  P42338  P42338  P42338  P42338  P42345  P42345  P42345  P42345  P42345  P42345  P42345  P42345  P42345  P42345  P42345  P42345  P42574  P42574  P42574  P42574  P42680  P42680  P42680  P42680  P42684  P42684  P42684  P42684  P42768  P42768  P42768  P42768  P42771  P42771  P42771  P42771  P42858  P42858  P42858  P42858  P43220  P43220  P43220  P43220  P43243  P43243  P43243  P43243  P43268  P43268  P43268  P43268  P43351  P43351  P43351  P43351  P43403  P43403  P43403  P43403  P43405  P43405  P43405  P43405  P43405  P43405  P43405  P43405  P43405  P43405  P43405  P43405  P43686  P43686  P43686  P43686  P45985  P45985  P45985  P45985  P45985  P45985  P45985  P45985  P45985  P45985  P45985  P45985  P46060  P46060  P46060  P46060  P46060  P46060  P46060  P46060  P46060  P46060  P46060  P46060  P46108  P46108  P46108  P46108  P46527  P46527  P46527  P46527  P46527  P46531  P46531  P46531  P46531  P46695  P46695  P46695  P46695  P46695  P46695  P46695  P46734  P46734  P46734  P46734  P46734  P46734  P46734  P46734  P46734  P46734  P46734  P46734  P46736  P46736  P46736  P46736  P46776  P46776  P46776  P46776  P46779  P46779  P46779  P46779  P46782  P46782  P46782  P46782  P46783  P46783  P46783  P46783  P46821  P46821  P46821  P46821  P46937  P46937  P46937  P46937  P46937  P46937  P46937  P46937  P46937  P46937  P46937  P46937  P46940  P46940  P46940  P46940  P47710  P47710  P47710  P47710  P47712  P47712  P47712  P47712  P47756  P47756  P47756  P47756  P47895  P47895  P47895  P47895  P47897  P47897  P47897  P47897  P47914  P47914  P47914  P47914  P48047  P48047  P48047  P48047  P48048  P48048  P48048  P48048  P48059  P48059  P48059  P48059  P48551  P48551  P48551  P48551  P48552  P48552  P48552  P48552  P48643  P48643  P48643  P48643  P48735  P48735  P48735  P48735  P48736  P48736  P48736  P48736  P49023  P49023  P49023  P49023  P49137  P49137  P49137  P49137  P49137  P49137  P49137  P49137  P49137  P49137  P49137  P49137  P49189  P49189  P49189  P49189  P49327  P49327  P49327  P49327  P49368  P49368  P49368  P49368  P49407  P49407  P49407  P49407  P49450  P49450  P49450  P49450  P49459  P49459  P49459  P49459  P49588  P49588  P49588  P49588  P49675  P49675  P49675  P49675  P49682  P49682  P49682  P49682  P49715  P49715  P49715  P49715  P49720  P49720  P49720  P49720  P49736  P49736  P49736  P49736  P49756  P49756  P49756  P49756  P49760  P49760  P49760  P49760  P49760  P49760  P49760  P49760  P49760  P49760  P49760  P49760  P49768  P49768  P49768  P49768  P49789  P49789  P49789  P49789  P49790  P49790  P49790  P49790  P49795  P49795  P49795  P49795  P49802  P49802  P49802  P49802  P49815  P49815  P49815  P49815  P49815  P49815  P49815  P49815  P49815  P49815  P49815  P49815  P49841  P49841  P49841  P49841  P50150  P50150  P50402  P50402  P50402  P50402  P50502  P50502  P50502  P50502  P50548  P50548  P50548  P50548  P50548  P50548  P50548  P50548  P50548  P50548  P50548  P50548  P50552  P50552  P50552  P50552  P50552  P50552  P50552  P50552  P50552  P50552  P50552  P50552  P50583  P50583  P50583  P50583  P50613  P50613  P50613  P50613  P50613  P50613  P50613  P50613  P50613  P50613  P50613  P50613  P50750  P50750  P50750  P50750  P50750  P50750  P50750  P50750  P50750  P50750  P50750  P50895  P50895  P50895  P50895  P50990  P50990  P50990  P50990  P51114  P51114  P51114  P51114  P51397  P51397  P51397  P51397  P51575  P51575  P51575  P51575  P51575  P51575  P51575  P51575  P51575  P51575  P51575  P51575  P51587  P51587  P51587  P51587  P51587  P51587  P51587  P51587  P51587  P51587  P51587  P51587  P51617  P51617  P51617  P51617  P51617  P51617  P51617  P51617  P51617  P51617  P51617  P51617  P51636  P51636  P51636  P51636  P51654  P51654  P51654  P51654  P51809  P51809  P51809  P51809  P51813  P51813  P51813  P51813  P51884  P51884  P51884  P51884  P51946  P51946  P51946  P51946  P51955  P51955  P51955  P51955  P51955  P51955  P51955  P51955  P51955  P51955  P51955  P51955  P51991  P51991  P51991  P51991  P52272  P52272  P52272  P52272  P52292  P52292  P52292  P52292  P52333  P52333  P52333  P52333  P52434  P52434  P52434  P52434  P52435  P52435  P52435  P52435  P52565  P52565  P52565  P52565  P52630  P52630  P52630  P52630  P52630  P52630  P52630  P52630  P52630  P52630  P52630  P52630  P52655  P52655  P52655  P52655  P52732  P52732  P52732  P52732  P52732  P52732  P52732  P52732  P52732  P52732  P52732  P52732  P52735  P52735  P52735  P52735  P52907  P52907  P52907  P52907  P52926  P52926  P52926  P52926  P52945  P52945  P52945  P52945  P53350  P53350  P53350  P53350  P53350  P53350  P53350  P53350  P53350  P53350  P53350  P53350  P53355  P53355  P53355  P53355  P53396  P53396  P53396  P53396  P53667  P53667  P53667  P53667  P53667  P53667  P53667  P53667  P53667  P53667  P53667  P53667  P53674  P53674  P53674  P53674  P53779  P53779  P53779  P53779  P53779  P53779  P53779  P53779  P53779  P53779  P53779  P53779  P53804  P53804  P53804  P53804  P53999  P53999  P54105  P54105  P54105  P54105  P54198  P54198  P54198  P54198  P54198  P54198  P54198  P54198  P54198  P54198  P54198  P54198  P54253  P54253  P54253  P54253  P54259  P54259  P54259  P54259  P54274  P54274  P54274  P54274  P54577  P54577  P54577  P54577  P54646  P54646  P54646  P54646  P54646  P54646  P54646  P54646  P54646  P54646  P54646  P54646  P54753  P54753  P54753  P54753  P54829  P54829  P54829  P54829  P54829  P54829  P54829  P54829  P54829  P54829  P54829  P54829  P55060  P55060  P55060  P55060  P55072  P55072  P55072  P55072  P55211  P55211  P55211  P55211  P55211  P55211  P55211  P55211  P55211  P55211  P55211  P55211  P55212  P55212  P55212  P55212  P55263  P55263  P55263  P55263  P55268  P55268  P55268  P55268  P55273  P55273  P55273  P55273  P55735  P55735  P55735  P55735  P55769  P55769  P55769  P55769  P55795  P55795  P55795  P55795  P55884  P55884  P55884  P55884  P56537  P56537  P56537  P56537  P56817  P56817  P56817  P56817  P56945  P56945  P56945  P56945  P57059  P57059  P57059  P57059  P57059  P57059  P57059  P57059  P57059  P57059  P57059  P57059  P57081  P57081  P57081  P57081  P58340  P58340  P58340  P58340  P59665  P59998  P59998  P59998  P59998  P60228  P60228  P60228  P60228  P60468  P60468  P60468  P60468  P60484  P60484  P60484  P60484  P60484  P60484  P60484  P60484  P60484  P60484  P60484  P60484  P60866  P60866  P60866  P60866  P60953  P60953  P60953  P60953  P61073  P61073  P61073  P61073  P61081  P61081  P61081  P61081  P61086  P61086  P61086  P61086  P61158  P61158  P61158  P61158  P61244  P61244  P61244  P61244  P61289  P61289  P61289  P61289  P61457  P61457  P61457  P61604  P61604  P61604  P61604  P61769  P61769  P61769  P61769  P61803  P61803  P61803  P61803  P61923  P61923  P61923  P61923  P61978  P61978  P61978  P61978  P62136  P62136  P62136  P62136  P62136  P62136  P62136  P62136  P62253  P62253  P62253  P62253  P62258  P62258  P62258  P62258  P62266  P62266  P62266  P62269  P62269  P62269  P62269  P62280  P62280  P62280  P62280  P62306  P62306  P62316  P62316  P62316  P62316  P62495  P62495  P62495  P62495  P62633  P62633  P62633  P62633  P62745  P62745  P62745  P62745  P62753  P62753  P62753  P62753  P62805  P62805  P62805  P62805  P62820  P62820  P62820  P62820  P62826  P62826  P62826  P62826  P62847  P62847  P62847  P62847  P62873  P62873  P62873  P62873  P62877  P62877  P62877  P62877  P62913  P62913  P62913  P62913  P62917  P62917  P62917  P62917  P62937  P62937  P62937  P62937  P62979  P62979  P62979  P62995  P62995  P62995  P62995  P63010  P63010  P63010  P63010  P63165  P63165  P63165  P63165  P63167  P63167  P63167  P63167  P63218  P63218  P63220  P63220  P63241  P63241  P63241  P63241  P63244  P63244  P63244  P63244  P63252  P63252  P63252  P63252  P63279  P63279  P63279  P63279  P63316  P63316  P63316  P63316  P67775  P67775  P67775  P67775  P67809  P67809  P67809  P67809  P67870  P67870  P67870  P67870  P68032  P68032  P68032  P68032  P68104  P68104  P68104  P68104  P68104  P68104  P68104  P68104  P68104  P68104  P68104  P68104  P68363  P68363  P68363  P68363  P68400  P68400  P68400  P68400  P68400  P68400  P68400  P68400  P68400  P68400  P68400  P68871  P68871  P68871  P68871  P78344  P78344  P78344  P78344  P78346  P78346  P78346  P78346  P78347  P78347  P78347  P78347  P78348  P78348  P78348  P78348  P78362  P78362  P78362  P78362  P78362  P78362  P78362  P78362  P78362  P78362  P78362  P78362  P78371  P78371  P78371  P78371  P78527  P78527  P78527  P78527  P78527  P78527  P78527  P78527  P78527  P78527  P78527  P78527  P78536  P78536  P78536  P78536  P78536  P78536  P78536  P78536  P78536  P78536  P78536  P78536  P78543  P78543  P78543  P78543  P80188  P80188  P80188  P80188  P80192  P80192  P80192  P80192  P80192  P80192  P80192  P80192  P80192  P80192  P80192  P80192  P82979  P82979  P82979  P82979  P84090  P84090  P84090  P84090  P98161  P98161  P98161  P98161  P98170  P98170  P98170  P98170  P98177  P98177  P98177  P98177  P98177  P98177  P98177  P98177  P98177  P98177  P98177  P98177  P98179  P98179  P98179  P98179  P99999  P99999  P99999  P99999  Q00059  Q00059  Q00059  Q00059  Q00341  Q00341  Q00341  Q00341  Q00534  Q00534  Q00534  Q00534  Q00534  Q00534  Q00534  Q00534  Q00534  Q00534  Q00534  Q00534  Q00536  Q00536  Q00536  Q00536  Q00610  Q00610  Q00610  Q00610  Q00613  Q00613  Q00613  Q00613  Q00613  Q00613  Q00613  Q00613  Q00613  Q00613  Q00613  Q00613  Q00653  Q00653  Q00653  Q00653  Q00839  Q00839  Q00839  Q00839  Q00987  Q00987  Q00987  Q00987  Q00987  Q00987  Q00987  Q00987  Q00987  Q00987  Q00987  Q00987  Q01081  Q01081  Q01081  Q01081  Q01094  Q01094  Q01094  Q01094  Q01130  Q01130  Q01130  Q01130  Q01469  Q01469  Q01469  Q01469  Q01518  Q01518  Q01518  Q01518  Q01804  Q01804  Q01804  Q01804  Q01826  Q01826  Q01826  Q01826  Q01844  Q01844  Q01844  Q01844  Q01860  Q01860  Q01860  Q01860  Q01860  Q01860  Q01860  Q01860  Q01860  Q01860  Q01860  Q01860  Q01954  Q01954  Q01954  Q01954  Q01974  Q01974  Q01974  Q01974  Q02156  Q02156  Q02156  Q02156  Q02156  Q02156  Q02156  Q02156  Q02156  Q02156  Q02156  Q02156  Q02388  Q02388  Q02388  Q02388  Q02447  Q02447  Q02447  Q02447  Q02763  Q02763  Q02763  Q02763  Q02790  Q02790  Q02790  Q02790  Q02818  Q02818  Q02818  Q02818  Q02818  Q02818  Q02818  Q02818  Q02818  Q02818  Q02818  Q02818  Q02978  Q02978  Q02978  Q02978  Q03135  Q03135  Q03135  Q03135  Q03393  Q03393  Q03393  Q03393  Q03468  Q03468  Q03468  Q03468  Q03721  Q03721  Q03721  Q03721  Q04206  Q04206  Q04206  Q04206  Q04206  Q04206  Q04206  Q04206  Q04206  Q04206  Q04206  Q04206  Q04323  Q04323  Q04323  Q04323  Q04637  Q04637  Q04637  Q04637  Q04759  Q04759  Q04759  Q04759  Q04759  Q04759  Q04759  Q04759  Q04759  Q04759  Q04759  Q04759  Q04760  Q04760  Q04760  Q04760  Q04760  Q04760  Q04760  Q04760  Q04864  Q04864  Q04864  Q04864  Q04912  Q04912  Q04912  Q04912  Q05066  Q05066  Q05066  Q05066  Q05086  Q05086  Q05086  Q05086  Q05209  Q05209  Q05209  Q05209  Q05315  Q05315  Q05315  Q05315  Q05397  Q05397  Q05397  Q05397  Q05655  Q05655  Q05655  Q05655  Q05655  Q05655  Q05655  Q05655  Q05655  Q05655  Q05655  Q05655  Q05707  Q05707  Q05707  Q05707  Q06187  Q06187  Q06187  Q06187  Q06413  Q06413  Q06413  Q06413  Q06413  Q06413  Q06413  Q06413  Q06413  Q06413  Q06413  Q06413  Q06481  Q06481  Q06481  Q06481  Q06609  Q06609  Q06609  Q06609  Q06609  Q06609  Q06609  Q06609  Q06609  Q06609  Q06609  Q06609  Q06787  Q06787  Q06787  Q06787  Q06828  Q06828  Q06828  Q06828  Q06830  Q06830  Q06830  Q06830  Q06830  Q06830  Q06830  Q06830  Q07065  Q07065  Q07065  Q07065  Q07666  Q07666  Q07666  Q07666  Q07817  Q07817  Q07817  Q07817  Q07820  Q07820  Q07820  Q07820  Q07820  Q07820  Q07820  Q07820  Q07820  Q07820  Q07820  Q07820  Q07866  Q07866  Q07866  Q07866  Q07912  Q07912  Q07912  Q07912  Q07954  Q07954  Q07954  Q07954  Q07955  Q07955  Q07955  Q07955  Q08050  Q08050  Q08050  Q08050  Q08050  Q08050  Q08050  Q08050  Q08050  Q08050  Q08050  Q08050  Q08188  Q08188  Q08188  Q08188  Q08345  Q08345  Q08345  Q08345  Q08426  Q08426  Q08426  Q08426  Q08431  Q08431  Q08431  Q08431  Q08495  Q08495  Q08495  Q08495  Q08722  Q08722  Q08722  Q08722  Q08945  Q08945  Q08945  Q08945  Q08999  Q08999  Q08999  Q08999  Q08999  Q08999  Q08999  Q08999  Q08999  Q08999  Q08999  Q08999  Q08J23  Q08J23  Q08J23  Q08J23  Q09028  Q09028  Q09028  Q09028  Q09161  Q09161  Q09161  Q09161  Q09161  Q09161  Q09161  Q09161  Q09161  Q09161  Q09161  Q09161  Q0VD86  Q0VD86  Q0VD86  Q0VD86  Q0VD86  Q0VD86  Q0VD86  Q0VD86  Q12778  Q12778  Q12778  Q12778  Q12778  Q12778  Q12778  Q12778  Q12778  Q12778  Q12778  Q12778  Q12792  Q12792  Q12792  Q12792  Q12834  Q12834  Q12834  Q12834  Q12834  Q12834  Q12834  Q12834  Q12834  Q12834  Q12834  Q12834  Q12841  Q12841  Q12841  Q12841  Q12866  Q12866  Q12866  Q12866  Q12874  Q12874  Q12874  Q12874  Q12888  Q12888  Q12888  Q12888  Q12888  Q12888  Q12888  Q12888  Q12888  Q12888  Q12888  Q12888  Q12906  Q12906  Q12906  Q12906  Q12906  Q12906  Q12906  Q12906  Q12906  Q12906  Q12906  Q12906  Q12933  Q12933  Q12933  Q12933  Q12933  Q12933  Q12933  Q12933  Q12933  Q12933  Q12933  Q12933  Q12962  Q12962  Q12962  Q12962  Q12968  Q12968  Q12968  Q12968  Q12972  Q12972  Q12972  Q12972  Q13002  Q13002  Q13002  Q13002  Q13042  Q13042  Q13042  Q13042  Q13042  Q13042  Q13042  Q13042  Q13042  Q13042  Q13042  Q13042  Q13045  Q13045  Q13045  Q13045  Q13045  Q13045  Q13045  Q13045  Q13045  Q13045  Q13045  Q13045  Q13049  Q13049  Q13049  Q13049  Q13085  Q13085  Q13085  Q13085  Q13098  Q13098  Q13098  Q13098  Q13098  Q13098  Q13098  Q13098  Q13098  Q13098  Q13098  Q13098  Q13115  Q13115  Q13115  Q13115  Q13153  Q13153  Q13153  Q13153  Q13153  Q13153  Q13153  Q13153  Q13153  Q13153  Q13153  Q13153  Q13158  Q13158  Q13158  Q13158  Q13163  Q13163  Q13163  Q13163  Q13163  Q13163  Q13163  Q13163  Q13163  Q13163  Q13163  Q13163  Q13164  Q13164  Q13164  Q13164  Q13188  Q13188  Q13188  Q13188  Q13188  Q13188  Q13188  Q13188  Q13188  Q13188  Q13188  Q13188  Q13191  Q13191  Q13191  Q13191  Q13217  Q13217  Q13217  Q13217  Q13247  Q13247  Q13247  Q13247  Q13257  Q13257  Q13257  Q13257  Q13263  Q13263  Q13263  Q13263  Q13283  Q13283  Q13283  Q13283  Q13285  Q13285  Q13285  Q13285  Q13303  Q13303  Q13303  Q13303  Q13309  Q13309  Q13309  Q13309  Q13310  Q13310  Q13310  Q13310  Q13315  Q13315  Q13315  Q13315  Q13322  Q13322  Q13322  Q13322  Q13330  Q13330  Q13330  Q13330  Q13342  Q13342  Q13342  Q13342  Q13342  Q13342  Q13342  Q13342  Q13342  Q13342  Q13342  Q13342  Q13363  Q13363  Q13363  Q13363  Q13371  Q13371  Q13371  Q13371  Q13404  Q13404  Q13404  Q13404  Q13421  Q13421  Q13421  Q13421  Q13422  Q13422  Q13422  Q13422  Q13426  Q13426  Q13426  Q13426  Q13444  Q13444  Q13444  Q13444  Q13509  Q13509  Q13509  Q13509  Q13522  Q13522  Q13522  Q13522  Q13522  Q13522  Q13522  Q13522  Q13522  Q13522  Q13522  Q13522  Q13526  Q13526  Q13526  Q13526  Q13535  Q13535  Q13535  Q13535  Q13542  Q13542  Q13542  Q13542  Q13542  Q13542  Q13542  Q13542  Q13546  Q13546  Q13546  Q13546  Q13547  Q13547  Q13547  Q13547  Q13554  Q13554  Q13554  Q13554  Q13554  Q13554  Q13554  Q13554  Q13554  Q13554  Q13554  Q13554  Q13561  Q13561  Q13561  Q13561  Q13568  Q13568  Q13568  Q13568  Q13568  Q13568  Q13568  Q13568  Q13568  Q13568  Q13568  Q13568  Q13572  Q13572  Q13572  Q13572  Q13573  Q13573  Q13573  Q13573  Q13601  Q13601  Q13601  Q13601  Q13614  Q13614  Q13614  Q13614  Q13627  Q13627  Q13627  Q13627  Q13627  Q13627  Q13627  Q13627  Q13627  Q13627  Q13627  Q13627  Q13637  Q13637  Q13637  Q13637  Q13671  Q13671  Q13671  Q13671  Q13765  Q13765  Q13765  Q13765  Q13769  Q13769  Q13769  Q13769  Q13829  Q13829  Q13829  Q13829  Q13882  Q13882  Q13882  Q13882  Q13884  Q13884  Q13884  Q13884  Q13905  Q13905  Q13905  Q13905  Q13950  Q13950  Q13950  Q13950  Q14118  Q14118  Q14118  Q14118  Q14141  Q14141  Q14141  Q14141  Q14152  Q14152  Q14152  Q14152  Q14155  Q14155  Q14155  Q14155  Q14164  Q14164  Q14164  Q14164  Q14164  Q14164  Q14164  Q14164  Q14164  Q14164  Q14164  Q14164  Q14194  Q14194  Q14194  Q14194  Q14194  Q14194  Q14194  Q14194  Q14194  Q14194  Q14194  Q14194  Q14207  Q14207  Q14207  Q14207  Q14207  Q14207  Q14207  Q14207  Q14207  Q14207  Q14207  Q14207  Q14242  Q14242  Q14242  Q14242  Q14247  Q14247  Q14247  Q14247  Q14289  Q14289  Q14289  Q14289  Q14315  Q14315  Q14315  Q14315  Q14344  Q14344  Q14344  Q14344  Q14344  Q14344  Q14344  Q14344  Q14344  Q14344  Q14344  Q14344  Q14393  Q14393  Q14393  Q14393  Q14457  Q14457  Q14457  Q14457  Q14457  Q14457  Q14457  Q14457  Q14457  Q14457  Q14457  Q14457  Q14493  Q14493  Q14493  Q14493  Q14493  Q14493  Q14493  Q14493  Q14498  Q14498  Q14498  Q14498  Q14511  Q14511  Q14511  Q14511  Q14524  Q14524  Q14524  Q14524  Q14524  Q14524  Q14524  Q14524  Q14524  Q14524  Q14524  Q14524  Q14526  Q14526  Q14526  Q14526  Q14527  Q14527  Q14527  Q14527  Q14653  Q14653  Q14653  Q14653  Q14653  Q14653  Q14653  Q14653  Q14653  Q14653  Q14653  Q14653  Q14654  Q14654  Q14654  Q14654  Q14654  Q14654  Q14654  Q14654  Q14654  Q14654  Q14654  Q14654  Q14671  Q14671  Q14671  Q14671  Q14674  Q14674  Q14674  Q14674  Q14676  Q14676  Q14676  Q14676  Q14676  Q14676  Q14676  Q14676  Q14676  Q14676  Q14676  Q14676  Q14678  Q14678  Q14678  Q14678  Q14680  Q14680  Q14680  Q14680  Q14680  Q14680  Q14680  Q14680  Q14680  Q14680  Q14680  Q14680  Q14681  Q14681  Q14681  Q14681  Q14683  Q14683  Q14683  Q14683  Q14686  Q14686  Q14686  Q14686  Q14686  Q14686  Q14686  Q14686  Q14686  Q14686  Q14686  Q14686  Q14690  Q14690  Q14690  Q14690  Q14694  Q14694  Q14694  Q14694  Q14694  Q14694  Q14694  Q14694  Q14694  Q14694  Q14694  Q14694  Q14703  Q14703  Q14703  Q14703  Q14739  Q14739  Q14739  Q14739  Q14764  Q14764  Q14764  Q14764  Q14766  Q14766  Q14766  Q14766  Q14790  Q14790  Q14790  Q14790  Q14814  Q14814  Q14814  Q14814  Q14847  Q14847  Q14847  Q14847  Q14865  Q14865  Q14865  Q14865  Q14934  Q14934  Q14934  Q14934  Q14974  Q14974  Q14974  Q14974  Q14980  Q14980  Q14980  Q14980  Q14980  Q14980  Q14980  Q14980  Q14980  Q14980  Q14980  Q14980  Q14994  Q14994  Q14994  Q14994  Q14994  Q14994  Q14994  Q14994  Q14994  Q14994  Q14994  Q14994  Q15004  Q15004  Q15004  Q15004  Q15005  Q15005  Q15005  Q15005  Q15007  Q15007  Q15007  Q15007  Q15020  Q15020  Q15020  Q15020  Q15024  Q15024  Q15024  Q15024  Q15025  Q15025  Q15025  Q15025  Q15027  Q15027  Q15027  Q15027  Q15046  Q15046  Q15046  Q15046  Q15050  Q15050  Q15050  Q15050  Q15054  Q15054  Q15054  Q15054  Q15056  Q15056  Q15056  Q15056  Q15078  Q15078  Q15078  Q15078  Q15078  Q15078  Q15078  Q15078  Q15078  Q15078  Q15078  Q15078  Q15080  Q15080  Q15080  Q15080  Q15080  Q15080  Q15080  Q15080  Q15080  Q15080  Q15080  Q15080  Q15084  Q15084  Q15084  Q15084  Q15118  Q15118  Q15118  Q15118  Q15139  Q15139  Q15139  Q15139  Q15147  Q15147  Q15147  Q15147  Q15149  Q15149  Q15149  Q15149  Q15170  Q15170  Q15185  Q15185  Q15185  Q15208  Q15208  Q15208  Q15208  Q15208  Q15208  Q15208  Q15208  Q15208  Q15208  Q15208  Q15208  Q15287  Q15287  Q15287  Q15287  Q15291  Q15291  Q15291  Q15291  Q15291  Q15291  Q15291  Q15291  Q15291  Q15291  Q15291  Q15291  Q15293  Q15293  Q15293  Q15293  Q15303  Q15303  Q15303  Q15303  Q15334  Q15334  Q15334  Q15334  Q15370  Q15370  Q15370  Q15370  Q15398  Q15398  Q15398  Q15398  Q15398  Q15398  Q15398  Q15398  Q15398  Q15398  Q15398  Q15398  Q15424  Q15424  Q15424  Q15424  Q15427  Q15427  Q15427  Q15427  Q15428  Q15428  Q15428  Q15428  Q15485  Q15485  Q15485  Q15485  Q15545  Q15545  Q15545  Q15545  Q15554  Q15554  Q15554  Q15554  Q15554  Q15554  Q15554  Q15554  Q15554  Q15554  Q15554  Q15554  Q15637  Q15637  Q15637  Q15637  Q15648  Q15648  Q15648  Q15648  Q15648  Q15648  Q15648  Q15648  Q15648  Q15648  Q15648  Q15648  Q15653  Q15653  Q15653  Q15653  Q15654  Q15654  Q15654  Q15654  Q15691  Q15691  Q15691  Q15691  Q15717  Q15717  Q15717  Q15717  Q15726  Q15726  Q15726  Q15726  Q15746  Q15746  Q15746  Q15746  Q15750  Q15750  Q15750  Q15750  Q15788  Q15788  Q15788  Q15788  Q15788  Q15788  Q15788  Q15788  Q15788  Q15788  Q15788  Q15788  Q15796  Q15796  Q15796  Q15796  Q15796  Q15796  Q15796  Q15796  Q15796  Q15796  Q15796  Q15796  Q15831  Q15831  Q15831  Q15831  Q15831  Q15831  Q15831  Q15831  Q15831  Q15831  Q15831  Q15831  Q15835  Q15835  Q15835  Q15835  Q15848  Q15848  Q15848  Q15848  Q15907  Q15907  Q15907  Q15907  Q15910  Q15910  Q15910  Q15910  Q15910  Q15910  Q15910  Q15910  Q15910  Q15910  Q15910  Q15910  Q15942  Q15942  Q15942  Q15942  Q16236  Q16236  Q16236  Q16236  Q16270  Q16270  Q16270  Q16270  Q16401  Q16401  Q16401  Q16401  Q16512  Q16512  Q16512  Q16512  Q16512  Q16512  Q16512  Q16512  Q16512  Q16512  Q16512  Q16512  Q16513  Q16513  Q16513  Q16513  Q16513  Q16513  Q16513  Q16513  Q16513  Q16513  Q16513  Q16513  Q16518  Q16518  Q16518  Q16518  Q16518  Q16518  Q16518  Q16518  Q16518  Q16518  Q16518  Q16518  Q16566  Q16566  Q16566  Q16566  Q16566  Q16566  Q16566  Q16566  Q16566  Q16566  Q16566  Q16566  Q16581  Q16581  Q16581  Q16581  Q16584  Q16584  Q16584  Q16584  Q16584  Q16584  Q16584  Q16584  Q16584  Q16584  Q16584  Q16584  Q16623  Q16623  Q16623  Q16623  Q16625  Q16625  Q16625  Q16625  Q16625  Q16625  Q16625  Q16625  Q16625  Q16625  Q16625  Q16625  Q16637  Q16637  Q16637  Q16637  Q16637  Q16637  Q16637  Q16637  Q16637  Q16643  Q16643  Q16643  Q16643  Q16655  Q16655  Q16655  Q16655  Q16656  Q16656  Q16656  Q16656  Q16658  Q16658  Q16658  Q16658  Q16659  Q16659  Q16659  Q16659  Q16665  Q16665  Q16665  Q16665  Q16665  Q16665  Q16665  Q16665  Q16665  Q16665  Q16665  Q16665  Q16666  Q16666  Q16666  Q16666  Q16695  Q16695  Q16695  Q16695  Q16695  Q16719  Q16719  Q16719  Q16719  Q16773  Q16773  Q16773  Q16773  Q16790  Q16790  Q16790  Q16790  Q16832  Q16832  Q16832  Q16832  Q16853  Q16853  Q16853  Q16853  Q16875  Q16875  Q16875  Q16875  Q1KMD3  Q1KMD3  Q1KMD3  Q1KMD3  Q24JP5  Q24JP5  Q24JP5  Q24JP5  Q2HXU8  Q2HXU8  Q2HXU8  Q2HXU8  Q2M2Z5  Q2M2Z5  Q2M2Z5  Q2M2Z5  Q2M2Z5  Q2M2Z5  Q2M2Z5  Q2M2Z5  Q2M2Z5  Q2M2Z5  Q2M2Z5  Q2M2Z5  Q2NKX8  Q2NKX8  Q2NKX8  Q2NKX8  Q2NKX8  Q2NKX8  Q2NKX8  Q2NKX8  Q2NKX8  Q2NKX8  Q2NKX8  Q2NKX8  Q2PPJ7  Q2PPJ7  Q2PPJ7  Q2PPJ7  Q2PPJ7  Q2PPJ7  Q2PPJ7  Q2PPJ7  Q2PPJ7  Q2PPJ7  Q2PPJ7  Q2PPJ7  Q2TAY7  Q2TAY7  Q2TAY7  Q2TAY7  Q2V2M9  Q2V2M9  Q2V2M9  Q2V2M9  Q2V2M9  Q2V2M9  Q2V2M9  Q2V2M9  Q2V2M9  Q2V2M9  Q2V2M9  Q2V2M9  Q3MHD2  Q3MHD2  Q3MHD2  Q3MHD2  Q3SXM5  Q3SXM5  Q3SXM5  Q3SXM5  Q3V6T2  Q3V6T2  Q3V6T2  Q3V6T2  Q4G163  Q4G163  Q4G163  Q4G163  Q4G163  Q4G163  Q4G163  Q4G163  Q4G163  Q4G163  Q4G163  Q4G163  Q53EL6  Q53EL6  Q53EL6  Q53EL6  Q53ET0  Q53ET0  Q53ET0  Q53ET0  Q53EZ4  Q53EZ4  Q53EZ4  Q53EZ4  Q53FA7  Q53FA7  Q53FA7  Q53FA7  Q53GL7  Q53GL7  Q53GL7  Q53GL7  Q53GL7  Q53GL7  Q53GL7  Q53GL7  Q53GL7  Q53GL7  Q53GL7  Q53GL7  Q53H47  Q53H47  Q53H47  Q53H47  Q53HL2  Q53HL2  Q53HL2  Q53HL2  Q53HL2  Q53HL2  Q53HL2  Q53HL2  Q5FBB7  Q5FBB7  Q5FBB7  Q5FBB7  Q5FYB1  Q5FYB1  Q5FYB1  Q5FYB1  Q5JRA6  Q5JRA6  Q5JRA6  Q5JRA6  Q5JVF3  Q5JVF3  Q5JVF3  Q5JVF3  Q5KSL6  Q5KSL6  Q5KSL6  Q5KSL6  Q5SQ64  Q5SQ64  Q5SQ64  Q5SQ64  Q5T1C6  Q5T1C6  Q5T1C6  Q5T1C6  Q5TA31  Q5TA31  Q5TAQ9  Q5TAQ9  Q5TAQ9  Q5TAQ9  Q5VT25  Q5VT25  Q5VT25  Q5VT25  Q5VT25  Q5VT25  Q5VT25  Q5VT25  Q5VT25  Q5VT25  Q5VT25  Q5VT25  Q5VTD9  Q5VTD9  Q5VTD9  Q5VTD9  Q5VWQ8  Q5VWQ8  Q5VWQ8  Q5VWQ8  Q5VWZ2  Q5VWZ2  Q5VWZ2  Q5VWZ2  Q5VY43  Q5VY43  Q5VY43  Q5VY43  Q5XUX0  Q5XUX0  Q5XUX0  Q5XUX0  Q684P5  Q684P5  Q684P5  Q684P5  Q69YN4  Q69YN4  Q69YN4  Q69YN4  Q6AI12  Q6AI12  Q6AI12  Q6AI12  Q6B0I6  Q6B0I6  Q6B0I6  Q6B0I6  Q6DN03  Q6DN03  Q6DN03  Q6DN03  Q6DN72  Q6DN72  Q6DN72  Q6DN72  Q6GTX8  Q6GTX8  Q6GTX8  Q6GTX8  Q6JBY9  Q6JBY9  Q6JBY9  Q6JBY9  Q6NXS1  Q6NXS1  Q6NXS1  Q6NXS1  Q6NZI2  Q6NZI2  Q6NZI2  Q6NZI2  Q6P1J9  Q6P1J9  Q6P1J9  Q6P1J9  Q6P1N0  Q6P1N0  Q6P1N0  Q6P1N0  Q6P2Q9  Q6P2Q9  Q6P2Q9  Q6P2Q9  Q6P5Z2  Q6P5Z2  Q6P5Z2  Q6P5Z2  Q6P5Z2  Q6P5Z2  Q6P5Z2  Q6P5Z2  Q6P5Z2  Q6P5Z2  Q6P5Z2  Q6P5Z2  Q6P988  Q6P988  Q6P988  Q6P988  Q6PCB0  Q6PCB0  Q6PCB0  Q6PCB0  Q6PCD5  Q6PCD5  Q6PCD5  Q6PCD5  Q6PHR2  Q6PHR2  Q6PHR2  Q6PHR2  Q6PKG0  Q6PKG0  Q6PKG0  Q6PKG0  Q6PKG0  Q6PKG0  Q6PKG0  Q6PKG0  Q6PKG0  Q6PKG0  Q6PKG0  Q6PKG0  Q6Q788  Q6Q788  Q6Q788  Q6Q788  Q6Q788  Q6Q788  Q6Q788  Q6Q788  Q6Q788  Q6Q788  Q6Q788  Q6Q788  Q6R327  Q6R327  Q6R327  Q6R327  Q6R327  Q6R327  Q6R327  Q6R327  Q6R327  Q6R327  Q6R327  Q6R327  Q6S5L8  Q6S5L8  Q6S5L8  Q6S5L8  Q6TGC4  Q6TGC4  Q6TGC4  Q6TGC4  Q6U736  Q6U736  Q6U736  Q6U736  Q6UUV9  Q6UUV9  Q6UUV9  Q6UUV9  Q6UWZ7  Q6UWZ7  Q6UWZ7  Q6UWZ7  Q6WN34  Q6WN34  Q6WN34  Q6WN34  Q6ZMU5  Q6ZMU5  Q6ZMU5  Q6ZMU5  Q6ZN04  Q6ZN04  Q6ZN04  Q6ZN04  Q6ZSY5  Q6ZSY5  Q6ZSY5  Q6ZSY5  Q6ZUJ8  Q6ZUJ8  Q6ZUJ8  Q6ZUJ8  Q70E73  Q70E73  Q70E73  Q70E73  Q712K3  Q712K3  Q712K3  Q712K3  Q71F23  Q71F23  Q71F23  Q71F23  Q71F23  Q71F23  Q71F23  Q71F23  Q71F23  Q71F23  Q71F23  Q71F23  Q71RC2  Q71RC2  Q71RC2  Q71RC2  Q7KZF4  Q7KZF4  Q7KZF4  Q7KZF4  Q7L2H7  Q7L2H7  Q7L2H7  Q7L2H7  Q7L5D6  Q7L5D6  Q7L5D6  Q7L5D6  Q7L5Y1  Q7L5Y1  Q7L5Y1  Q7L5Y1  Q7L804  Q7L804  Q7L804  Q7L804  Q7RTN6  Q7RTN6  Q7RTN6  Q7RTN6  Q7RTN6  Q7RTN6  Q7RTN6  Q7RTN6  Q7RTN6  Q7RTN6  Q7RTN6  Q7RTN6  Q7Z406  Q7Z406  Q7Z406  Q7Z406  Q7Z417  Q7Z417  Q7Z417  Q7Z417  Q7Z4W1  Q7Z4W1  Q7Z4W1  Q7Z4W1  Q7Z5L9  Q7Z5L9  Q7Z5L9  Q7Z5L9  Q7Z6J0  Q7Z6J0  Q7Z6J0  Q7Z6J0  Q86T82  Q86T82  Q86T82  Q86T82  Q86U42  Q86U42  Q86U42  Q86U42  Q86UC2  Q86UC2  Q86UC2  Q86UC2  Q86UC2  Q86UC2  Q86UC2  Q86UC2  Q86UC2  Q86UC2  Q86UC2  Q86UC2  Q86UP2  Q86UP2  Q86UP2  Q86UP2  Q86UQ8  Q86UQ8  Q86UQ8  Q86UQ8  Q86UR1  Q86UR1  Q86UR1  Q86UR1  Q86V48  Q86V48  Q86V48  Q86V48  Q86V81  Q86V81  Q86V81  Q86V81  Q86VP1  Q86VP1  Q86VP1  Q86VP1  Q86W56  Q86W56  Q86W56  Q86W56  Q86W56  Q86W56  Q86W56  Q86W56  Q86W56  Q86W56  Q86W56  Q86W56  Q86WB0  Q86WB0  Q86WB0  Q86WB0  Q86WR7  Q86WR7  Q86WR7  Q86WR7  Q86WV6  Q86WV6  Q86WV6  Q86WV6  Q86X55  Q86X55  Q86X55  Q86X55  Q86XR7  Q86XR7  Q86XR7  Q86XR7  Q86YS7  Q86YS7  Q86YS7  Q86YS7  Q8IU85  Q8IU85  Q8IU85  Q8IU85  Q8IU85  Q8IU85  Q8IU85  Q8IU85  Q8IU85  Q8IU85  Q8IU85  Q8IU85  Q8IUR7  Q8IUR7  Q8IUR7  Q8IUR7  Q8IVP5  Q8IVP5  Q8IVP5  Q8IVP5  Q8IVT2  Q8IVT2  Q8IVT2  Q8IVT2  Q8IVT2  Q8IVT2  Q8IVT2  Q8IVT2  Q8IVT2  Q8IVT2  Q8IVT2  Q8IVT2  Q8IW19  Q8IW19  Q8IW19  Q8IW19  Q8IW41  Q8IW41  Q8IW41  Q8IW41  Q8IW41  Q8IW41  Q8IW41  Q8IW41  Q8IW41  Q8IW41  Q8IW41  Q8IW41  Q8IWL1  Q8IWL1  Q8IWL1  Q8IWL1  Q8IWT6  Q8IWT6  Q8IWT6  Q8IWT6  Q8IX03  Q8IX03  Q8IX03  Q8IX03  Q8IXJ6  Q8IXJ6  Q8IXJ6  Q8IXJ6  Q8IY84  Q8IY84  Q8IY84  Q8IY84  Q8IY84  Q8IY84  Q8IY84  Q8IY84  Q8IY84  Q8IY84  Q8IY84  Q8IY84  Q8IYA6  Q8IYA6  Q8IYA6  Q8IYA6  Q8IYB3  Q8IYB3  Q8IYB3  Q8IYB3  Q8IZP0  Q8IZP0  Q8IZP0  Q8IZP0  Q8IZS5  Q8IZS5  Q8IZS5  Q8IZS5  Q8N122  Q8N122  Q8N122  Q8N122  Q8N122  Q8N122  Q8N122  Q8N122  Q8N122  Q8N122  Q8N122  Q8N122  Q8N163  Q8N163  Q8N163  Q8N163  Q8N163  Q8N163  Q8N163  Q8N163  Q8N163  Q8N163  Q8N163  Q8N163  Q8N1G4  Q8N1G4  Q8N1G4  Q8N1G4  Q8N264  Q8N264  Q8N264  Q8N264  Q8N264  Q8N264  Q8N264  Q8N264  Q8N264  Q8N264  Q8N264  Q8N264  Q8N2E6  Q8N2E6  Q8N2E6  Q8N2E6  Q8N4F0  Q8N4F0  Q8N4F0  Q8N4F0  Q8N4F0  Q8N4F0  Q8N4F0  Q8N4F0  Q8N4F0  Q8N4F0  Q8N4F0  Q8N4F0  Q8N4N8  Q8N4N8  Q8N4N8  Q8N4N8  Q8N4N8  Q8N4N8  Q8N4N8  Q8N4N8  Q8N4N8  Q8N4N8  Q8N4N8  Q8N4N8  Q8N5C8  Q8N5C8  Q8N5C8  Q8N5C8  Q8N5Z0  Q8N5Z0  Q8N5Z0  Q8N5Z0  Q8NB16  Q8NB16  Q8NB16  Q8NB16  Q8NB16  Q8NB16  Q8NB16  Q8NB16  Q8NB16  Q8NB16  Q8NB16  Q8NB16  Q8NBJ4  Q8NBJ4  Q8NBJ4  Q8NBJ4  Q8NBP7  Q8NBP7  Q8NBP7  Q8NBP7  Q8NBX0  Q8NBX0  Q8NBX0  Q8NBX0  Q8NCD3  Q8NCD3  Q8NCD3  Q8NCD3  Q8ND76  Q8ND76  Q8ND76  Q8ND76  Q8ND76  Q8ND76  Q8ND76  Q8ND76  Q8NDC0  Q8NDC0  Q8NDC0  Q8NDC0  Q8NE71  Q8NE71  Q8NE71  Q8NE71  Q8NFW8  Q8NFW8  Q8NFW8  Q8NFW8  Q8NG66  Q8NG66  Q8NG66  Q8NG66  Q8NHV4  Q8NHV4  Q8NHV4  Q8NHV4  Q8NHV4  Q8NHV4  Q8NHV4  Q8NHV4  Q8NHV4  Q8NHV4  Q8NHV4  Q8NHV4  Q8NHW3  Q8NHW3  Q8NHW3  Q8NHW3  Q8TAE6  Q8TAE6  Q8TAE6  Q8TAP9  Q8TAP9  Q8TAP9  Q8TAP9  Q8TB45  Q8TB45  Q8TB45  Q8TB45  Q8TB45  Q8TB45  Q8TB45  Q8TB45  Q8TB45  Q8TB45  Q8TB45  Q8TB45  Q8TCT0  Q8TCT0  Q8TCT0  Q8TCT0  Q8TD08  Q8TD08  Q8TD08  Q8TD08  Q8TD08  Q8TD08  Q8TD08  Q8TD08  Q8TD08  Q8TD08  Q8TD08  Q8TD08  Q8TD30  Q8TD30  Q8TD30  Q8TD30  Q8TDC3  Q8TDC3  Q8TDC3  Q8TDC3  Q8TDC3  Q8TDC3  Q8TDC3  Q8TDC3  Q8TDC3  Q8TDC3  Q8TDC3  Q8TDC3  Q8TEP8  Q8TEP8  Q8TEP8  Q8TEP8  Q8TEW0  Q8TEW0  Q8TEW0  Q8TEW0  Q8TEX9  Q8TEX9  Q8TEX9  Q8TEX9  Q8TF09  Q8TF09  Q8TF09  Q8TF09  Q8TF76  Q8TF76  Q8TF76  Q8TF76  Q8WTQ7  Q8WTQ7  Q8WTQ7  Q8WTQ7  Q8WU03  Q8WU03  Q8WU03  Q8WU03  Q8WUI4  Q8WUI4  Q8WUI4  Q8WUI4  Q8WUM4  Q8WUM4  Q8WUM4  Q8WUM4  Q8WV28  Q8WV28  Q8WV28  Q8WV28  Q8WVM8  Q8WVM8  Q8WVM8  Q8WVM8  Q8WW12  Q8WW12  Q8WW12  Q8WW12  Q8WWA1  Q8WWA1  Q8WWA1  Q8WWA1  Q8WWM7  Q8WWM7  Q8WWM7  Q8WWM7  Q8WX93  Q8WX93  Q8WX93  Q8WX93  Q8WYJ6  Q8WYJ6  Q8WYJ6  Q8WYJ6  Q8WYK2  Q8WYK2  Q8WYK2  Q8WYK2  Q8WYK2  Q8WYK2  Q8WYK2  Q8WYK2  Q8WYL5  Q8WYL5  Q8WYL5  Q8WYL5  Q92504  Q92504  Q92504  Q92504  Q92522  Q92522  Q92522  Q92522  Q92538  Q92538  Q92538  Q92538  Q92538  Q92538  Q92538  Q92538  Q92538  Q92538  Q92538  Q92538  Q92556  Q92556  Q92556  Q92556  Q92563  Q92563  Q92563  Q92563  Q92574  Q92574  Q92574  Q92574  Q92597  Q92597  Q92597  Q92597  Q92597  Q92597  Q92597  Q92597  Q92597  Q92597  Q92597  Q92597  Q92600  Q92600  Q92600  Q92600  Q92616  Q92616  Q92616  Q92616  Q92620  Q92620  Q92620  Q92620  Q92625  Q92625  Q92625  Q92625  Q92630  Q92630  Q92630  Q92630  Q92630  Q92630  Q92630  Q92630  Q92630  Q92630  Q92630  Q92630  Q92673  Q92673  Q92673  Q92673  Q92731  Q92731  Q92731  Q92731  Q92734  Q92734  Q92734  Q92734  Q92736  Q92736  Q92736  Q92736  Q92783  Q92783  Q92783  Q92783  Q92793  Q92793  Q92793  Q92793  Q92794  Q92794  Q92794  Q92794  Q92794  Q92794  Q92794  Q92794  Q92794  Q92794  Q92794  Q92794  Q92804  Q92804  Q92804  Q92804  Q92888  Q92888  Q92888  Q92888  Q92900  Q92900  Q92900  Q92900  Q92905  Q92905  Q92905  Q92905  Q92918  Q92918  Q92918  Q92918  Q92918  Q92918  Q92918  Q92918  Q92918  Q92918  Q92918  Q92918  Q92922  Q92922  Q92922  Q92922  Q92934  Q92934  Q92934  Q92934  Q92945  Q92945  Q92945  Q92945  Q92974  Q92974  Q92974  Q92974  Q92974  Q92974  Q92974  Q92974  Q92974  Q92974  Q92974  Q92974  Q92979  Q92979  Q92979  Q92979  Q92985  Q92985  Q92985  Q92985  Q92993  Q92993  Q92993  Q92993  Q92997  Q92997  Q92997  Q92997  Q92997  Q92997  Q92997  Q92997  Q92997  Q92997  Q92997  Q92997  Q93009  Q93009  Q93009  Q93009  Q93084  Q93084  Q93084  Q93084  Q969H0  Q969H0  Q969H0  Q969H0  Q969H0  Q969H0  Q969H0  Q969H0  Q969H0  Q969H0  Q969H0  Q969H0  Q969M7  Q969M7  Q969M7  Q969M7  Q969Q0  Q969Q0  Q969Q0  Q969Q0  Q969S2  Q969S2  Q969S2  Q969S2  Q969U6  Q969U6  Q969U6  Q969U6  Q96A65  Q96A65  Q96A65  Q96A65  Q96A72  Q96A72  Q96A72  Q96AD5  Q96AD5  Q96AD5  Q96AD5  Q96AX2  Q96AX2  Q96AX2  Q96AX2  Q96B36  Q96B36  Q96B36  Q96B36  Q96B36  Q96B36  Q96B36  Q96B36  Q96B36  Q96B36  Q96B36  Q96B36  Q96BD6  Q96BD6  Q96BD6  Q96BD6  Q96BR1  Q96BR1  Q96BR1  Q96BR1  Q96BR1  Q96BR1  Q96BR1  Q96BR1  Q96BR1  Q96BR1  Q96BR1  Q96BR1  Q96BT3  Q96BT3  Q96BT3  Q96BT3  Q96C19  Q96C19  Q96C19  Q96C19  Q96C36  Q96C36  Q96C36  Q96C36  Q96C86  Q96C86  Q96C86  Q96C86  Q96CF2  Q96CF2  Q96CF2  Q96CF2  Q96CN5  Q96CN5  Q96CN5  Q96CN5  Q96CT7  Q96CT7  Q96CT7  Q96CT7  Q96CV9  Q96CV9  Q96CV9  Q96CV9  Q96DA6  Q96DA6  Q96DA6  Q96DG6  Q96DG6  Q96DG6  Q96DG6  Q96EB6  Q96EB6  Q96EB6  Q96EB6  Q96EB6  Q96EB6  Q96EB6  Q96EB6  Q96EB6  Q96EB6  Q96EB6  Q96EB6  Q96EG1  Q96EG1  Q96EG1  Q96EG1  Q96EP5  Q96EP5  Q96EP5  Q96EP5  Q96EY1  Q96EY1  Q96EY1  Q96EY1  Q96EY5  Q96EY5  Q96EY5  Q96EY5  Q96EY5  Q96EY5  Q96EY5  Q96EY5  Q96EY5  Q96G03  Q96G03  Q96G03  Q96G03  Q96G74  Q96G74  Q96G74  Q96G74  Q96G74  Q96G74  Q96G74  Q96G74  Q96G74  Q96G74  Q96G74  Q96G74  Q96GA7  Q96GA7  Q96GA7  Q96GA7  Q96H79  Q96H79  Q96H79  Q96H79  Q96HC4  Q96HC4  Q96HC4  Q96HC4  Q96HQ2  Q96HQ2  Q96I15  Q96I15  Q96I15  Q96I15  Q96J02  Q96J02  Q96J02  Q96J02  Q96J02  Q96J02  Q96J02  Q96J02  Q96J02  Q96J02  Q96J02  Q96J02  Q96JB2  Q96JB2  Q96JB2  Q96JB2  Q96KP4  Q96KP4  Q96KP4  Q96KP4  Q96KQ7  Q96KQ7  Q96KQ7  Q96KQ7  Q96L34  Q96L34  Q96L34  Q96L34  Q96L34  Q96L34  Q96L34  Q96L34  Q96L34  Q96L34  Q96L34  Q96L34  Q96LA8  Q96LA8  Q96LA8  Q96LA8  Q96LC7  Q96LC7  Q96LC7  Q96LC7  Q96P11  Q96P11  Q96P11  Q96P11  Q96P16  Q96P16  Q96P16  Q96P16  Q96P70  Q96P70  Q96P70  Q96P70  Q96PD5  Q96PD5  Q96PD5  Q96PD5  Q96PU5  Q96PU5  Q96PU5  Q96PU5  Q96PU8  Q96PU8  Q96PU8  Q96PU8  Q96Q83  Q96Q83  Q96Q83  Q96Q83  Q96Q89  Q96Q89  Q96Q89  Q96Q89  Q96Q89  Q96Q89  Q96Q89  Q96Q89  Q96Q89  Q96Q89  Q96Q89  Q96Q89  Q96R06  Q96R06  Q96R06  Q96R06  Q96R06  Q96R06  Q96R06  Q96R06  Q96R06  Q96R06  Q96R06  Q96R06  Q96RG2  Q96RG2  Q96RG2  Q96RG2  Q96RG2  Q96RG2  Q96RG2  Q96RG2  Q96RG2  Q96RG2  Q96RG2  Q96RG2  Q96RI1  Q96RI1  Q96RI1  Q96RI1  Q96RL1  Q96RL1  Q96RL1  Q96RL1  Q96RU2  Q96RU2  Q96RU2  Q96RU2  Q96S19  Q96S19  Q96S19  Q96S19  Q96SB4  Q96SB4  Q96SB4  Q96SB4  Q96SD1  Q96SD1  Q96SD1  Q96SD1  Q96T51  Q96T51  Q96T51  Q96T51  Q96T88  Q96T88  Q96T88  Q96T88  Q96TA1  Q96TA1  Q96TA1  Q96TA1  Q99259  Q99259  Q99259  Q99259  Q99426  Q99426  Q99426  Q99426  Q99459  Q99459  Q99459  Q99459  Q99459  Q99459  Q99459  Q99459  Q99459  Q99459  Q99459  Q99459  Q99490  Q99490  Q99490  Q99490  Q99497  Q99497  Q99497  Q99497  Q99504  Q99504  Q99504  Q99504  Q99536  Q99536  Q99536  Q99536  Q99558  Q99558  Q99558  Q99558  Q99558  Q99558  Q99558  Q99558  Q99558  Q99558  Q99558  Q99558  Q99572  Q99572  Q99572  Q99572  Q99613  Q99613  Q99613  Q99613  Q99613  Q99613  Q99613  Q99613  Q99613  Q99613  Q99613  Q99613  Q99615  Q99615  Q99615  Q99615  Q99623  Q99623  Q99623  Q99623  Q99627  Q99627  Q99627  Q99627  Q99638  Q99638  Q99638  Q99638  Q99640  Q99640  Q99640  Q99640  Q99640  Q99640  Q99640  Q99640  Q99640  Q99640  Q99640  Q99640  Q99661  Q99661  Q99661  Q99661  Q99665  Q99665  Q99665  Q99665  Q99683  Q99683  Q99683  Q99683  Q99683  Q99683  Q99683  Q99683  Q99683  Q99683  Q99683  Q99683  Q99704  Q99704  Q99704  Q99704  Q99708  Q99708  Q99708  Q99708  Q99708  Q99708  Q99708  Q99708  Q99708  Q99708  Q99708  Q99708  Q99759  Q99759  Q99759  Q99759  Q99828  Q99828  Q99828  Q99828  Q99832  Q99832  Q99832  Q99832  Q99935  Q99935  Q99935  Q99935  Q99958  Q99958  Q99958  Q99958  Q99958  Q99958  Q99958  Q99958  Q99958  Q99958  Q99958  Q99958  Q99983  Q99983  Q99983  Q99983  Q99986  Q99986  Q99986  Q99986  Q99986  Q99986  Q99986  Q99986  Q99986  Q99986  Q99986  Q99986  Q9BPZ7  Q9BPZ7  Q9BPZ7  Q9BPZ7  Q9BQ15  Q9BQ15  Q9BQ15  Q9BQ15  Q9BQ15  Q9BQ15  Q9BQ15  Q9BQ15  Q9BQ15  Q9BQ15  Q9BQ15  Q9BQ15  Q9BQA1  Q9BQA1  Q9BQA1  Q9BQA1  Q9BQA1  Q9BQA1  Q9BQA1  Q9BQA1  Q9BQA1  Q9BQA1  Q9BQA1  Q9BQA1  Q9BR39  Q9BR39  Q9BR39  Q9BR39  Q9BR76  Q9BR76  Q9BR76  Q9BR76  Q9BRK3  Q9BRK3  Q9BRK3  Q9BRK3  Q9BSJ6  Q9BSJ6  Q9BSJ6  Q9BSJ6  Q9BT78  Q9BT78  Q9BT78  Q9BT78  Q9BTC0  Q9BTC0  Q9BTC0  Q9BTC0  Q9BTM9  Q9BTY2  Q9BTY2  Q9BTY2  Q9BTY2  Q9BTY7  Q9BTY7  Q9BTY7  Q9BTY7  Q9BUA3  Q9BUA3  Q9BUA3  Q9BUA3  Q9BUB5  Q9BUB5  Q9BUB5  Q9BUB5  Q9BUB5  Q9BUB5  Q9BUB5  Q9BUB5  Q9BUB5  Q9BUB5  Q9BUB5  Q9BUB5  Q9BV73  Q9BV73  Q9BV73  Q9BV73  Q9BV86  Q9BV86  Q9BV86  Q9BV86  Q9BWF3  Q9BWF3  Q9BWF3  Q9BWF3  Q9BWT1  Q9BWT1  Q9BWT1  Q9BWT1  Q9BWT1  Q9BWT1  Q9BWT1  Q9BWT1  Q9BWT1  Q9BWT1  Q9BWT1  Q9BWT1  Q9BX63  Q9BX63  Q9BX63  Q9BX63  Q9BX84  Q9BX84  Q9BX84  Q9BX84  Q9BX84  Q9BX84  Q9BX84  Q9BX84  Q9BX84  Q9BX84  Q9BX84  Q9BX84  Q9BXA7  Q9BXA7  Q9BXA7  Q9BXA7  Q9BXA7  Q9BXA7  Q9BXA7  Q9BXA7  Q9BXA7  Q9BXA7  Q9BXA7  Q9BXM7  Q9BXM7  Q9BXM7  Q9BXM7  Q9BXS0  Q9BXS0  Q9BXS0  Q9BXS0  Q9BXS5  Q9BXS5  Q9BXS5  Q9BXS5  Q9BXS6  Q9BXS6  Q9BXS6  Q9BXS6  Q9BXW9  Q9BXW9  Q9BXW9  Q9BXW9  Q9BY32  Q9BY32  Q9BY32  Q9BY32  Q9BY41  Q9BY41  Q9BY41  Q9BY41  Q9BY44  Q9BY44  Q9BY44  Q9BY44  Q9BY77  Q9BY77  Q9BY77  Q9BY77  Q9BY84  Q9BY84  Q9BY84  Q9BY84  Q9BYE7  Q9BYE7  Q9BYE7  Q9BYE7  Q9BYG3  Q9BYG3  Q9BYG3  Q9BYG3  Q9BYG3  Q9BYG3  Q9BYG3  Q9BYG3  Q9BYG3  Q9BYG3  Q9BYG3  Q9BYG3  Q9BYV9  Q9BYV9  Q9BYV9  Q9BYV9  Q9BZE4  Q9BZE4  Q9BZE4  Q9BZE4  Q9BZL4  Q9BZL4  Q9BZL4  Q9BZL4  Q9BZL4  Q9BZL4  Q9BZL4  Q9BZL4  Q9BZL4  Q9BZL4  Q9BZL4  Q9BZL4  Q9BZL6  Q9BZL6  Q9BZL6  Q9BZL6  Q9BZS1  Q9BZS1  Q9BZS1  Q9BZS1  Q9BZZ5  Q9BZZ5  Q9BZZ5  Q9BZZ5  Q9GZN2  Q9GZN2  Q9GZN2  Q9GZN2  Q9GZN2  Q9GZN2  Q9GZN2  Q9GZN2  Q9GZN2  Q9GZT4  Q9GZT4  Q9GZT4  Q9GZT4  Q9GZT9  Q9GZT9  Q9GZT9  Q9GZT9  Q9GZV1  Q9GZV1  Q9GZV1  Q9GZV1  Q9GZV5  Q9GZV5  Q9GZV5  Q9GZV5  Q9GZX7  Q9GZX7  Q9GZX7  Q9GZX7  Q9GZX7  Q9GZX7  Q9GZX7  Q9GZY6  Q9GZY6  Q9GZY6  Q9GZY6  Q9H063  Q9H063  Q9H063  Q9H063  Q9H063  Q9H063  Q9H063  Q9H063  Q9H093  Q9H093  Q9H093  Q9H093  Q9H093  Q9H093  Q9H093  Q9H093  Q9H093  Q9H093  Q9H093  Q9H093  Q9H0H5  Q9H0H5  Q9H0H5  Q9H0H5  Q9H0H5  Q9H0H5  Q9H0H5  Q9H0H5  Q9H0H5  Q9H0H5  Q9H0H5  Q9H0H5  Q9H0K1  Q9H0K1  Q9H0K1  Q9H0K1  Q9H0K1  Q9H0K1  Q9H0K1  Q9H0K1  Q9H0K1  Q9H0K1  Q9H0K1  Q9H0K1  Q9H0N0  Q9H0N0  Q9H0N0  Q9H0N0  Q9H0S4  Q9H0S4  Q9H0S4  Q9H0S4  Q9H1A4  Q9H1A4  Q9H1A4  Q9H1A4  Q9H1A4  Q9H1A4  Q9H1A4  Q9H1A4  Q9H1A4  Q9H1A4  Q9H1A4  Q9H1A4  Q9H1D0  Q9H1D0  Q9H1D0  Q9H1D0  Q9H1D0  Q9H1D0  Q9H1D0  Q9H1D0  Q9H1D0  Q9H1D0  Q9H1D0  Q9H1D0  Q9H1Y3  Q9H1Y3  Q9H1Y3  Q9H1Y3  Q9H211  Q9H211  Q9H211  Q9H211  Q9H211  Q9H211  Q9H211  Q9H211  Q9H211  Q9H211  Q9H211  Q9H211  Q9H2D6  Q9H2D6  Q9H2D6  Q9H2D6  Q9H2D6  Q9H2D6  Q9H2D6  Q9H2D6  Q9H2D6  Q9H2D6  Q9H2D6  Q9H2D6  Q9H2G4  Q9H2G4  Q9H2G4  Q9H2G4  Q9H2K2  Q9H2K2  Q9H2K2  Q9H2K2  Q9H2K8  Q9H2K8  Q9H2K8  Q9H2K8  Q9H2W2  Q9H2W2  Q9H2W2  Q9H2W2  Q9H300  Q9H300  Q9H300  Q9H300  Q9H300  Q9H300  Q9H300  Q9H300  Q9H300  Q9H300  Q9H300  Q9H300  Q9H307  Q9H307  Q9H307  Q9H307  Q9H3K6  Q9H3K6  Q9H3K6  Q9H3K6  Q9H3Y6  Q9H3Y6  Q9H3Y6  Q9H3Y6  Q9H3Z4  Q9H3Z4  Q9H3Z4  Q9H3Z4  Q9H400  Q9H400  Q9H400  Q9H400  Q9H425  Q9H425  Q9H425  Q9H425  Q9H479  Q9H479  Q9H479  Q9H479  Q9H492  Q9H492  Q9H492  Q9H4X1  Q9H4X1  Q9H4X1  Q9H4X1  Q9H5V8  Q9H5V8  Q9H5V8  Q9H5V8  Q9H706  Q9H706  Q9H706  Q9H706  Q9H7P6  Q9H7P6  Q9H7P6  Q9H7P6  Q9H7P6  Q9H7P6  Q9H7P6  Q9H7P6  Q9H7P6  Q9H7P6  Q9H7P6  Q9H7P6  Q9H7X0  Q9H7X0  Q9H7X0  Q9H7X0  Q9H7Z6  Q9H7Z6  Q9H7Z6  Q9H7Z6  Q9H8M9  Q9H8M9  Q9H8M9  Q9H8M9  Q9H8S9  Q9H8S9  Q9H8S9  Q9H8S9  Q9H8S9  Q9H8S9  Q9H8S9  Q9H8V3  Q9H8V3  Q9H8V3  Q9H8V3  Q9H8V3  Q9H8V3  Q9H8V3  Q9H8V3  Q9H8V3  Q9H8V3  Q9H8V3  Q9H8V3  Q9H8Y8  Q9H8Y8  Q9H8Y8  Q9H8Y8  Q9H8Y8  Q9H8Y8  Q9H8Y8  Q9H8Y8  Q9H8Y8  Q9H8Y8  Q9H8Y8  Q9H8Y8  Q9H9B4  Q9H9B4  Q9H9B4  Q9H9B4  Q9HAW4  Q9HAW4  Q9HAW4  Q9HAW4  Q9HAW4  Q9HAW4  Q9HAW4  Q9HAW4  Q9HAW4  Q9HAW4  Q9HAW4  Q9HAW4  Q9HB71  Q9HB71  Q9HB71  Q9HB71  Q9HB75  Q9HB75  Q9HB75  Q9HB75  Q9HB96  Q9HB96  Q9HB96  Q9HB96  Q9HB96  Q9HB96  Q9HB96  Q9HB96  Q9HB96  Q9HB96  Q9HB96  Q9HB96  Q9HBH9  Q9HBH9  Q9HBH9  Q9HBH9  Q9HBH9  Q9HBH9  Q9HBL0  Q9HBL0  Q9HBL0  Q9HBL0  Q9HBV2  Q9HBV2  Q9HBV2  Q9HBV2  Q9HC16  Q9HC16  Q9HC16  Q9HC16  Q9HC16  Q9HC16  Q9HC16  Q9HC16  Q9HC16  Q9HC16  Q9HC16  Q9HC16  Q9HC77  Q9HC77  Q9HC77  Q9HC77  Q9HC98  Q9HC98  Q9HC98  Q9HC98  Q9HC98  Q9HC98  Q9HC98  Q9HC98  Q9HCC9  Q9HCC9  Q9HCC9  Q9HCC9  Q9HCC9  Q9HCC9  Q9HCC9  Q9HCC9  Q9HCC9  Q9HCC9  Q9HCC9  Q9HCC9  Q9HCE9  Q9HCE9  Q9HCE9  Q9HCE9  Q9HCN4  Q9HCN4  Q9HCN4  Q9HCN4  Q9HCQ7  Q9HCQ7  Q9HCQ7  Q9HCQ7  Q9HCX4  Q9HCX4  Q9HCX4  Q9HCX4  Q9HCX4  Q9HCX4  Q9HCX4  Q9HCX4  Q9HCX4  Q9HCX4  Q9HCX4  Q9HCX4  Q9HD40  Q9HD40  Q9HD40  Q9HD40  Q9NP72  Q9NP72  Q9NP72  Q9NP72  Q9NP79  Q9NP79  Q9NP79  Q9NP79  Q9NPD3  Q9NPD3  Q9NPD3  Q9NPD3  Q9NQ92  Q9NQ92  Q9NQ92  Q9NQ92  Q9NQC3  Q9NQC3  Q9NQC3  Q9NQC3  Q9NQC7  Q9NQC7  Q9NQC7  Q9NQC7  Q9NQR1  Q9NQR1  Q9NQR1  Q9NQR1  Q9NQS7  Q9NQS7  Q9NQS7  Q9NQS7  Q9NQS7  Q9NQS7  Q9NQS7  Q9NQS7  Q9NQS7  Q9NQS7  Q9NQS7  Q9NQS7  Q9NQT5  Q9NQT5  Q9NQT5  Q9NQT5  Q9NQT8  Q9NQT8  Q9NQT8  Q9NQT8  Q9NQX4  Q9NQX4  Q9NQX4  Q9NQX4  Q9NR20  Q9NR20  Q9NR20  Q9NR20  Q9NR22  Q9NR22  Q9NR22  Q9NR22  Q9NR30  Q9NR30  Q9NR30  Q9NR30  Q9NR50  Q9NR50  Q9NR50  Q9NR50  Q9NRD5  Q9NRD5  Q9NRD5  Q9NRD5  Q9NRD5  Q9NRD5  Q9NRD5  Q9NRD5  Q9NRD5  Q9NRD5  Q9NRD5  Q9NRD5  Q9NRF9  Q9NRF9  Q9NRF9  Q9NRF9  Q9NRH2  Q9NRH2  Q9NRH2  Q9NRH2  Q9NRH2  Q9NRH2  Q9NRH2  Q9NRH2  Q9NRH2  Q9NRH2  Q9NRH2  Q9NRH2  Q9NRM7  Q9NRM7  Q9NRM7  Q9NRM7  Q9NRM7  Q9NRM7  Q9NRM7  Q9NRM7  Q9NRM7  Q9NRM7  Q9NRM7  Q9NRM7  Q9NRY4  Q9NRY4  Q9NRY4  Q9NRY4  Q9NS18  Q9NS18  Q9NS18  Q9NS18  Q9NS28  Q9NS28  Q9NS28  Q9NS28  Q9NS56  Q9NS56  Q9NS56  Q9NS56  Q9NS69  Q9NS69  Q9NS69  Q9NS69  Q9NUB1  Q9NUB1  Q9NUB1  Q9NUB1  Q9NUP9  Q9NUP9  Q9NUP9  Q9NUP9  Q9NV92  Q9NV92  Q9NV92  Q9NV92  Q9NVI1  Q9NVI1  Q9NVI1  Q9NVI1  Q9NVI1  Q9NVI1  Q9NVI1  Q9NVI1  Q9NVI1  Q9NVI1  Q9NVI1  Q9NVI1  Q9NVI7  Q9NVI7  Q9NVI7  Q9NVI7  Q9NVJ2  Q9NVJ2  Q9NVJ2  Q9NVJ2  Q9NVX2  Q9NVX2  Q9NVX2  Q9NVX2  Q9NW13  Q9NW13  Q9NW13  Q9NW13  Q9NWH9  Q9NWH9  Q9NWH9  Q9NWH9  Q9NWQ8  Q9NWQ8  Q9NWQ8  Q9NWQ8  Q9NWZ3  Q9NWZ3  Q9NWZ3  Q9NWZ3  Q9NWZ3  Q9NWZ3  Q9NWZ3  Q9NWZ3  Q9NWZ3  Q9NWZ3  Q9NWZ3  Q9NWZ3  Q9NX09  Q9NX09  Q9NX09  Q9NX09  Q9NX09  Q9NX09  Q9NX09  Q9NX09  Q9NX76  Q9NX76  Q9NX76  Q9NX76  Q9NXV6  Q9NXV6  Q9NXV6  Q9NXV6  Q9NYA1  Q9NYA1  Q9NYA1  Q9NYA1  Q9NYF0  Q9NYF0  Q9NYF0  Q9NYF0  Q9NYF8  Q9NYF8  Q9NYF8  Q9NYF8  Q9NYL2  Q9NYL2  Q9NYL2  Q9NYL2  Q9NYL2  Q9NYL2  Q9NYL2  Q9NYL2  Q9NYL2  Q9NYL2  Q9NYL2  Q9NYL2  Q9NYV4  Q9NYV4  Q9NYV4  Q9NYV4  Q9NYV4  Q9NYV4  Q9NYV4  Q9NYV4  Q9NYV4  Q9NYV4  Q9NYV4  Q9NYV4  Q9NYV6  Q9NYV6  Q9NYV6  Q9NYV6  Q9NYV6  Q9NYV6  Q9NYV6  Q9NYV6  Q9NYV6  Q9NYV6  Q9NYV6  Q9NYV6  Q9NZ53  Q9NZ53  Q9NZ53  Q9NZ53  Q9NZC7  Q9NZC7  Q9NZC7  Q9NZC7  Q9NZJ0  Q9NZJ0  Q9NZJ0  Q9NZJ0  Q9NZJ0  Q9NZJ0  Q9NZJ0  Q9NZJ0  Q9NZJ0  Q9NZJ0  Q9NZJ0  Q9NZJ0  Q9NZJ5  Q9NZJ5  Q9NZJ5  Q9NZJ5  Q9NZN3  Q9NZN3  Q9NZN3  Q9NZN3  Q9NZT1  Q9NZT1  Q9NZT1  Q9NZT1  Q9NZU7  Q9NZU7  Q9NZU7  Q9NZU7  Q9P013  Q9P013  Q9P013  Q9P013  Q9P0L2  Q9P0L2  Q9P0L2  Q9P0L2  Q9P0L2  Q9P0L2  Q9P0L2  Q9P0L2  Q9P0L2  Q9P0L2  Q9P0L2  Q9P0L2  Q9P126  Q9P126  Q9P126  Q9P126  Q9P1Z0  Q9P1Z0  Q9P1Z0  Q9P1Z0  Q9P1Z0  Q9P1Z0  Q9P1Z0  Q9P1Z0  Q9P1Z0  Q9P1Z0  Q9P1Z0  Q9P1Z0  Q9P2R3  Q9P2R3  Q9P2R3  Q9P2R3  Q9P2Y5  Q9P2Y5  Q9P2Y5  Q9P2Y5  Q9UBC9  Q9UBC9  Q9UBC9  Q9UBF6  Q9UBF8  Q9UBF8  Q9UBF8  Q9UBF8  Q9UBF8  Q9UBF8  Q9UBF8  Q9UBF8  Q9UBF8  Q9UBF8  Q9UBF8  Q9UBF8  Q9UBK5  Q9UBK5  Q9UBK5  Q9UBL3  Q9UBL3  Q9UBL3  Q9UBL3  Q9UBN4  Q9UBN4  Q9UBN4  Q9UBN4  Q9UBP6  Q9UBP6  Q9UBP6  Q9UBP6  Q9UBQ5  Q9UBQ5  Q9UBQ5  Q9UBQ5  Q9UBR4  Q9UBR4  Q9UBR4  Q9UBR4  Q9UBR4  Q9UBR4  Q9UBR4  Q9UBR4  Q9UBR4  Q9UBR4  Q9UBR4  Q9UBR4  Q9UBW8  Q9UBW8  Q9UBW8  Q9UBW8  Q9UD71  Q9UD71  Q9UER7  Q9UER7  Q9UER7  Q9UER7  Q9UEW8  Q9UEW8  Q9UEW8  Q9UEW8  Q9UG01  Q9UG01  Q9UG01  Q9UG01  Q9UGC7  Q9UGC7  Q9UGC7  Q9UGC7  Q9UGK3  Q9UGK3  Q9UGK3  Q9UGK3  Q9UH99  Q9UH99  Q9UH99  Q9UH99  Q9UHD1  Q9UHD1  Q9UHD1  Q9UHD1  Q9UHD2  Q9UHD2  Q9UHD2  Q9UHD2  Q9UHM6  Q9UHM6  Q9UHM6  Q9UHM6  Q9UHP3  Q9UHP3  Q9UHP3  Q9UHP3  Q9UIA9  Q9UIA9  Q9UIA9  Q9UIA9  Q9UIB8  Q9UIB8  Q9UIB8  Q9UIB8  Q9UIF9  Q9UIF9  Q9UIF9  Q9UIF9  Q9UJ70  Q9UJ70  Q9UJ70  Q9UJ70  Q9UJQ4  Q9UJQ4  Q9UJQ4  Q9UJQ4  Q9UJX2  Q9UJX2  Q9UJX2  Q9UJX2  Q9UJX2  Q9UJX2  Q9UJX2  Q9UJX2  Q9UJX2  Q9UJX2  Q9UJX2  Q9UJX2  Q9UJX4  Q9UJX4  Q9UJX4  Q9UJX4  Q9UJX5  Q9UJX5  Q9UJX5  Q9UJX5  Q9UJX6  Q9UJX6  Q9UJX6  Q9UJX6  Q9UJY1  Q9UJY1  Q9UJY1  Q9UJY1  Q9UJY1  Q9UJY1  Q9UJY1  Q9UJY1  Q9UJZ1  Q9UJZ1  Q9UJZ1  Q9UJZ1  Q9UK32  Q9UK32  Q9UK32  Q9UK32  Q9UK32  Q9UK32  Q9UK32  Q9UK32  Q9UK32  Q9UK32  Q9UK32  Q9UK32  Q9UK55  Q9UK55  Q9UK55  Q9UK55  Q9UK76  Q9UK76  Q9UK76  Q9UKG1  Q9UKG1  Q9UKG1  Q9UKG1  Q9UKI8  Q9UKI8  Q9UKI8  Q9UKI8  Q9UKM9  Q9UKM9  Q9UKM9  Q9UKM9  Q9UKT5  Q9UKT5  Q9UKT5  Q9UKT5  Q9UKV3  Q9UKV3  Q9UKV3  Q9UKV3  Q9UKV8  Q9UKV8  Q9UKV8  Q9UKV8  Q9UL25  Q9UL25  Q9UL25  Q9UL25  Q9UL42  Q9UL42  Q9UL42  Q9UL42  Q9UL46  Q9UL46  Q9UL46  Q9UL46  Q9UL54  Q9UL54  Q9UL54  Q9UL54  Q9ULC4  Q9ULC4  Q9ULC4  Q9ULC4  Q9ULV5  Q9ULV5  Q9ULV5  Q9ULV5  Q9ULV8  Q9ULV8  Q9ULV8  Q9ULV8  Q9UM07  Q9UM07  Q9UM07  Q9UM07  Q9UM11  Q9UM11  Q9UM11  Q9UM11  Q9UM21  Q9UM21  Q9UM21  Q9UM21  Q9UM73  Q9UM73  Q9UM73  Q9UM73  Q9UMD9  Q9UMD9  Q9UMD9  Q9UMD9  Q9UMS4  Q9UMS4  Q9UMS4  Q9UMS4  Q9UNE7  Q9UNE7  Q9UNE7  Q9UNE7  Q9UNF1  Q9UNF1  Q9UNF1  Q9UNF1  Q9UNL4  Q9UNL4  Q9UNL4  Q9UNL4  Q9UNS2  Q9UNS2  Q9UNS2  Q9UNS2  Q9UP65  Q9UP65  Q9UP65  Q9UP65  Q9UPN6  Q9UPN6  Q9UPN6  Q9UPN6  Q9UPN6  Q9UPN6  Q9UPN6  Q9UPN6  Q9UPN6  Q9UPN6  Q9UPN6  Q9UPN6  Q9UPP1  Q9UPP1  Q9UPP1  Q9UPP1  Q9UPT6  Q9UPT6  Q9UPT6  Q9UPT6  Q9UPV0  Q9UPV0  Q9UPV0  Q9UPV0  Q9UPY6  Q9UPY6  Q9UPY6  Q9UPY6  Q9UPZ9  Q9UPZ9  Q9UPZ9  Q9UPZ9  Q9UPZ9  Q9UPZ9  Q9UPZ9  Q9UPZ9  Q9UPZ9  Q9UPZ9  Q9UPZ9  Q9UPZ9  Q9UQ35  Q9UQ35  Q9UQ35  Q9UQ80  Q9UQ80  Q9UQ80  Q9UQ80  Q9UQ84  Q9UQ84  Q9UQ84  Q9UQ84  Q9UQ84  Q9UQ84  Q9UQ84  Q9UQ84  Q9UQ84  Q9UQ84  Q9UQ84  Q9UQ84  Q9UQC2  Q9UQC2  Q9UQC2  Q9UQC2  Q9UQC2  Q9UQC2  Q9UQC2  Q9UQC2  Q9UQC2  Q9UQC2  Q9UQC2  Q9UQC2  Q9UQE7  Q9UQE7  Q9UQE7  Q9UQE7  Q9UQF2  Q9UQF2  Q9UQF2  Q9UQF2  Q9UQF2  Q9UQF2  Q9UQF2  Q9UQF2  Q9UQF2  Q9UQF2  Q9UQF2  Q9UQF2  Q9UQL6  Q9UQL6  Q9UQL6  Q9UQL6  Q9UQL6  Q9UQL6  Q9UQL6  Q9UQL6  Q9UQL6  Q9UQL6  Q9UQL6  Q9UQL6  Q9Y230  Q9Y230  Q9Y230  Q9Y230  Q9Y232  Q9Y232  Q9Y232  Q9Y232  Q9Y233  Q9Y233  Q9Y233  Q9Y233  Q9Y233  Q9Y233  Q9Y233  Q9Y233  Q9Y233  Q9Y233  Q9Y233  Q9Y233  Q9Y237  Q9Y237  Q9Y237  Q9Y241  Q9Y241  Q9Y259  Q9Y259  Q9Y259  Q9Y259  Q9Y262  Q9Y262  Q9Y262  Q9Y262  Q9Y266  Q9Y266  Q9Y266  Q9Y266  Q9Y272  Q9Y272  Q9Y272  Q9Y272  Q9Y283  Q9Y283  Q9Y283  Q9Y283  Q9Y285  Q9Y285  Q9Y285  Q9Y285  Q9Y294  Q9Y294  Q9Y294  Q9Y294  Q9Y295  Q9Y295  Q9Y295  Q9Y295  Q9Y295  Q9Y295  Q9Y295  Q9Y295  Q9Y295  Q9Y295  Q9Y295  Q9Y295  Q9Y2I7  Q9Y2I7  Q9Y2I7  Q9Y2I7  Q9Y2K2  Q9Y2K2  Q9Y2K2  Q9Y2K2  Q9Y2K2  Q9Y2K2  Q9Y2K2  Q9Y2K2  Q9Y2K2  Q9Y2K2  Q9Y2K2  Q9Y2K2  Q9Y2N7  Q9Y2N7  Q9Y2N7  Q9Y2N7  Q9Y2R2  Q9Y2R2  Q9Y2R2  Q9Y2R2  Q9Y2S0  Q9Y2S0  Q9Y2S0  Q9Y2S0  Q9Y2V2  Q9Y2V2  Q9Y2V2  Q9Y2V2  Q9Y2W1  Q9Y2W1  Q9Y2W1  Q9Y2W1  Q9Y2W7  Q9Y2W7  Q9Y2W7  Q9Y2W7  Q9Y2Z0  Q9Y2Z0  Q9Y2Z0  Q9Y2Z0  Q9Y365  Q9Y365  Q9Y365  Q9Y365  Q9Y371  Q9Y371  Q9Y371  Q9Y371  Q9Y371  Q9Y371  Q9Y371  Q9Y371  Q9Y371  Q9Y371  Q9Y371  Q9Y371  Q9Y383  Q9Y383  Q9Y383  Q9Y383  Q9Y3A5  Q9Y3A5  Q9Y3A5  Q9Y3A5  Q9Y3C5  Q9Y3C5  Q9Y3C5  Q9Y3C5  Q9Y3C5  Q9Y3C5  Q9Y3D6  Q9Y3D6  Q9Y3P8  Q9Y3P8  Q9Y3P8  Q9Y3P8  Q9Y3S1  Q9Y3S1  Q9Y3S1  Q9Y3S1  Q9Y3Z3  Q9Y3Z3  Q9Y3Z3  Q9Y3Z3  Q9Y4E8  Q9Y4E8  Q9Y4E8  Q9Y4E8  Q9Y4P1  Q9Y4P1  Q9Y4P1  Q9Y4P1  Q9Y4R8  Q9Y4R8  Q9Y4R8  Q9Y4R8  Q9Y4X4  Q9Y4X4  Q9Y4X4  Q9Y4X4  Q9Y4Z0  Q9Y4Z0  Q9Y4Z0  Q9Y4Z0  Q9Y572  Q9Y572  Q9Y572  Q9Y572  Q9Y5A9  Q9Y5A9  Q9Y5A9  Q9Y5A9  Q9Y5B9  Q9Y5B9  Q9Y5B9  Q9Y5B9  Q9Y5P4  Q9Y5P4  Q9Y5P4  Q9Y5P4  Q9Y5Q6  Q9Y5Q6  Q9Y5Q6  Q9Y5Q6  Q9Y5Y2  Q9Y5Y2  Q9Y5Y2  Q9Y5Y2  Q9Y608  Q9Y608  Q9Y608  Q9Y608  Q9Y657  Q9Y657  Q9Y657  Q9Y657  Q9Y696  Q9Y696  Q9Y696  Q9Y696  Q9Y6E0  Q9Y6E0  Q9Y6E0  Q9Y6E0  Q9Y6E0  Q9Y6E0  Q9Y6E0  Q9Y6E0  Q9Y6E0  Q9Y6E0  Q9Y6E0  Q9Y6E0  Q9Y6F6  Q9Y6F6  Q9Y6F6  Q9Y6F6  Q9Y6G9  Q9Y6G9  Q9Y6G9  Q9Y6G9  Q9Y6G9  Q9Y6G9  Q9Y6G9  Q9Y6G9  Q9Y6G9  Q9Y6G9  Q9Y6G9  Q9Y6G9  Q9Y6I3  Q9Y6I3  Q9Y6I3  Q9Y6I3  Q9Y6K5  Q9Y6K5  Q9Y6K5  Q9Y6K5  Q9Y6K9  Q9Y6K9  Q9Y6K9  Q9Y6K9  Q9Y6M9  Q9Y6M9  Q9Y6M9  Q9Y6M9  Q9Y6N7  Q9Y6N7  Q9Y6N7  Q9Y6N7  Q9Y6Q9  Q9Y6Q9  Q9Y6Q9  Q9Y6Q9  Q9Y6R1  Q9Y6R1  Q9Y6R1  Q9Y6R1  Q9Y6R1  Q9Y6R1  Q9Y6R1  Q9Y6R1  Q9Y6R1  Q9Y6R1  Q9Y6R1  Q9Y6R1 | 509  561  589  658  493  577  1008  1096  33  115  135  167  34  58  62  291  251  533  599  1038  43  72  101  157  28  124  214  236  16  30  70  79  23  24  41  192  55  258  286  337  265  275  340  381  60  195  284  396  110  253  310  374  101  184  242  364  377  429  435  480  512  514  543  551  74  101  146  152  412  449  507  655  731  751  768  791  806  859  981  991  17  68  103  174  255  303  338  344  67  225  242  250  160  221  281  289  28  33  71  74  86  120  133  141  154  161  180  109  170  199  464  269  514  645  700  64  113  121  149  254  283  291  401  590  595  677  694  17  282  306  347  546  689  1372  1519  187  366  487  582  20  200  219  263  49  179  464  523  775  778  810  917  925  1066  1223  1226  204  231  498  542  73  233  245  299  130  1641  1668  1975  104  262  449  457  42  59  139  189  27  31  50  80  386  469  530  537  565  612  758  872  1028  1297  1300  1312  17  65  82  161  177  200  32  41  259  270  79  130  215  221  228  260  287  364  512  540  559  581  21  44  47  59  123  159  160  189  30  140  203  239  29  80  176  597  71  95  156  226  235  260  309  314  726  839  917  1039  31  323  348  362  39  229  350  571  42  276  336  367  8  97  152  183  188  459  630  1172  202  293  439  506  573  608  727  822  8  10  11  66  84  88  97  115  117  128  129  160  66  198  354  372  28  82  95  204  217  233  235  292  333  337  353  384  34  374  399  406  408  453  571  594  649  700  774  873  107  165  185  402  102  283  402  556  134  182  781  1004  308  483  688  961  96  121  235  354  151  222  299  406  27  147  189  313  15  28  121  125  146  150  174  209  218  264  272  308  230  336  543  815  104  120  160  168  195  314  345  368  380  387  429  467  118  123  142  180  185  191  193  244  256  314  451  460  172  291  373  423  17  24  92  203  221  233  239  244  298  304  318  334  93  427  449  453  487  550  568  578  325  643  679  781  42  76  141  151  108  143  158  167  188  251  482  498  524  556  593  595  210  324  333  357  461  493  527  600  670  763  772  824  8  60  123  267  87  207  275  317  21  97  127  66  78  79  237  182  232  421  435  43  86  123  623  10  11  108  159  139  201  209  254  259  277  353  429  503  613  653  834  33  37  54  128  182  186  222  226  255  359  402  522  27  223  340  343  69  89  247  398  129  288  605  977  50  112  131  136  165  200  331  395  447  460  626  648  60  89  125  329  37  75  113  142  143  148  152  196  197  254  275  279  64  71  127  137  143  127  438  498  499  14  155  259  430  126  169  224  285  341  344  382  466  471  487  489  564  35  42  80  93  411  424  479  494  119  165  429  588  253  269  1019  1504  146  309  354  377  377  530  679  833  23  493  801  812  42  80  104  132  146  160  175  216  280  296  318  391  36  56  60  83  34  38  75  101  114  123  261  307  331  363  383  418  458  595  648  91  326  388  446  492  579  663  733  739  746  783  846  133  194  234  296  298  354  359  368  529  653  730  786  176  297  326  416  210  287  308  590  133  136  174  184  55  95  293  414  10  92  107  176  40  78  95  162  241  327  458  490  509  536  539  593  173  343  392  440  444  479  552  589  601  703  764  968  116  127  150  214  14  93  99  112  36  49  102  126  11  85  323  338  125  222  351  410  65  73  155  245  250  274  339  345  357  535  641  647  69  74  78  543  110  274  432  439  448  650  701  709  1147  1153  1242  1261  8  13  38  47  70  85  237  464  493  600  608  654  695  855  1025  1026  62  398  468  491  14  131  284  307  400  532  673  1010  1113  1196  1238  1259  246  412  594  771  267  530  576  979  58  426  443  504  63  64  146  224  59  177  498  528  71  318  490  625  70  129  294  497  13  84  409  1179  8  18  58  74  133  277  313  330  9  221  573  1186  107  194  220  337  98  144  158  362  121  254  336  410  203  446  452  477  110  241  280  300  451  554  711  806  116  132  135  238  97  200  654  986  405  443  587  690  16  93  301  626  978  1042  1830  2223  71  77  82  86  57  77  99  174  175  184  188  219  232  293  325  341  72  164  200  272  197  294  434  559  46  81  132  175  256  264  268  269  343  422  441  457  59  62  180  193  55  285  351  402  66  125  235  362  371  448  543  798  1077  1096  1113  1170  1294  26  85  147  309  71  76  146  204  155  337  435  649  659  784  848  928  1074  1258  1363  1511  57  74  95  100  406  669  713  753  774  775  779  793  18  33  72  141  23  102  218  232  35  97  154  183  198  205  262  307  316  326  329  340  12  105  121  152  195  216  274  208  232  542  563  27  66  89  119  120  121  129  174  206  240  244  276  33  135  157  168  200  204  235  45  78  100  180  39  207  237  412  40  46  101  198  13  38  115  633  103  112  256  280  312  329  388  454  80  170  337  442  461  487  595  600  681  686  699  747  11  18  25  28  50  87  153  180  190  212  220  232  46  214  602  606  91  165  238  273  350  480  504  519  67  94  110  112  155  162  167  169  170  173  210  96  109  566  631  85  90  289  1086  795  1390  2076  2270  85  140  188  261  674  677  852  875  1027  1275  1349  1460  39  219  378  411  605  1057  1071  1198  1242  1400  1698  1699  1760  1838  2159  2671  158  187  227  301  572  634  686  706  67  74  104  115  130  197  302  349  447  452  477  548  134  171  172  243  293  328  414  466  547  553  602  757  111  187  233  274  57  65  205  223  112  395  705  997  44  316  353  458  148  207  280  380  823  947  1437  1455  83  93  96  97  118  180  202  224  230  263  299  308  281  330  341  351  47  54  141  246  405  646  669  756  333  1074  1842  1990  2036  2251  2550  2707  3293  3294  3648  4666  12  17  26  33  871  1072  1153  1584  145  170  206  266  377  409  441  542  628  630  636  674  57  110  196  276  17  307  367  379  487  490  510  813  851  913  928  967  30  53  89  116  177  184  201  229  244  71  73  127  135  41  93  134  316  43  60  71  94  23  187  207  449  45  133  172  205  225  378  401  441  476  506  517  532  158  166  273  386  19  25  43  38  98  102  111  83  132  259  348  239  436  455  524  193  266  278  418  40  55  59  117  49  139  788  1032  500  834  1548  2105  99  150  247  290  13  97  177  202  111  124  126  145  302  352  363  369  39  113  145  157  164  299  322  488  45  52  303  665  29  85  186  190  218  271  290  332  381  386  417  445  39  264  318  430  118  391  393  778  223  335  389  391  42  176  233  294  36  39  56  200  31  41  92  93  185  226  231  243  299  312  332  433  96  137  174  320  207  259  306  470  77  801  824  991  25  49  120  171  97  98  100  135  65  90  609  629  51  65  69  90  114  116  162  169  266  285  329  344  78  95  103  123  173  244  247  248  304  343  350  400  20  124  144  148  15  58  64  65  69  112  133  58  71  181  248  42  80  151  169  53  93  149  174  41  90  95  105  741  754  1432  2561  26  59  102  157  23  70  87  131  12  26  38  13  60  72  83  36  50  95  124  149  107  120  122  133  13  30  44  71  15  77  90  133  69  78  91  21  31  63  94  35  56  73  90  20  24  27  119  42  63  134  158  10  436  534  623  40  185  221  226  11  85  212  243  12  40  73  81  320  383  412  505  81  228  308  407  80  168  247  264  57  62  151  214  197  422  581  837  11  59  94  191  61  102  154  192  55  119  127  227  365  704  746  1079  40  85  105  127  59  88  95  197  202  206  214  233  248  256  270  341  23  69  95  139  76  92  103  149  190  263  379  436  530  532  539  564  38  59  208  457  15  27  31  65  141  305  367  708  36  150  232  468  30  93  132  150  18  182  371  570  574  19  22  31  35  26  76  103  112  115  241  283  288  373  386  389  451  221  239  240  286  70  201  226  315  55  99  113  141  165  219  325  329  400  429  451  456  310  337  413  436  65  123  125  235  95  132  145  216  51  81  83  177  283  328  392  1380  93  152  174  376  9  79  136  174  189  192  254  369  17  72  118  163  8  142  296  298  32  220  328  383  19  52  59  99  104  153  154  176  187  246  277  294  119  313  320  400  127  233  402  472  264  340  352  360  57  85  143  188  81  102  123  125  140  170  230  253  256  304  329  377  86  269  279  422  110  113  151  174  74  330  492  556  22  59  61  157  163  215  276  278  352  367  603  719  54  100  135  180  162  172  208  223  256  311  337  420  456  588  623  674  52  64  82  104  175  263  329  377  391  485  579  708  40  62  52  70  81  83  70  95  101  103  413  417  553  619  110  147  170  190  22  24  84  39  62  90  95  104  126  131  231  297  27  40  56  141  145  244  302  379  66  147  197  442  21  48  349  377  48  54  108  113  134  153  156  212  226  322  412  644  234  404  408  422  15  24  49  79  30  107  110  122  134  160  207  214  125  901  1271  1281  45  816  928  1215  136  434  516  524  20  50  361  367  402  733  58  142  168  183  197  241  271  502  619  645  664  766  157  674  706  829  8  13  87  102  14  18  31  68  87  11  22  167  205  19  229  376  395  229  376  379  800  109  150  309  536  20  101  121  297  46  78  86  108  164  59  212  269  399  18  36  42  51  51  75  81  110  18  87  249  276  64  150  283  500  61  117  421  440  331  478  520  557  77  78  110  119  27  43  47  68  115  279  311  321  332  409  627  646  659  727  753  769  39  79  587  722  75  112  130  183  208  223  288  342  346  371  475  484  320  375  387  460  8  35  74  130  32  35  55  66  164  195  283  90  98  104  106  21  96  132  135  32  43  48  141  63  76  397  1391  36  65  171  174  305  467  498  594  603  683  708  713  9  109  165  201  21  37  107  467  152  170  369  675  12  49  96  98  119  216  250  265  332  355  531  737  28  748  749  1148  59  101  527  528  99  411  426  442  59  145  190  298  17  42  107  156  207  281  54  57  111  240  56  114  157  319  263  511  579  1355  105  287  431  576  75  128  212  321  143  168  241  298  17  23  52  75  98  127  130  167  219  113  117  196  566  23  28  82  94  97  107  125  135  144  188  223  285  52  71  75  11  89  148  213  13  140  231  254  35  62  76  142  58  71  91  98  57  61  187  211  199  201  237  247  13  14  66  96  137  141  229  257  321  387  479  544  95  192  494  592  32  34  69  95  45  54  66  152  181  472  573  604  258  312  327  821  138  428  484  541  33  657  674  785  36  120  150  588  125  303  1410  1721  14  88  389  539  22  50  101  114  153  286  322  812  1240  27  39  183  219  12  13  72  77  39  64  33  46  114  128  45  203  222  260  65  87  168  250  286  396  424  538  601  611  654  655  168  229  342  497  251  253  285  382  56  74  96  122  125  132  178  187  219  231  14  42  73  114  134  138  143  147  152  155  227  50  68  107  288  72  91  213  384  9  30  76  17  30  76  95  123  248  346  397  486  562  580  731  73  106  192  239  17  30  43  353  84  354  391  594  54  114  428  541  76  147  266  296  23  37  42  63  71  902  1420  1529  177  222  341  495  62  120  155  195  490  627  633  716  734  766  789  801  24  31  127  187  403  654  700  891  59  591  670  902  1132  1296  1368  1372  1699  1830  2073  2098  10  53  175  225  287  290  295  371  390  17  79  319  783  258  269  591  718  108  181  193  377  825  964  996  1272  1278  1279  1360  1460  10  76  91  406  44  124  190  391  85  177  258  336  514  584  750  853  231  387  389  429  63  83  176  390  73  196  206  226  22  2560  2980  3076  84  126  233  254  598  1552  1614  2207  1716  1863  2086  2259  70  87  97  104  46  119  121  289  60  210  239  376  240  293  737  820  331  430  755  1082  66  340  358  646  58  188  449  455  19  122  277  524  35  51  57  63  23  29  34  64  74  84  107  111  132  16  33  55  68  30  31  53  66  227  336  418  559  338  382  501  787  791  816  845  854  910  1019  1064  1115  592  1794  2195  3057  19  100  332  941  36  69  73  139  224  355  404  484  666  687  735  779  34  107  298  319  32  241  311  380  39  62  65  81  60  84  394  614  112  179  426  692  49  207  529  715  239  316  406  476  26  115  242  298  127  268  291  338  16  148  272  456  1368  1406  1563  1734  53  133  150  382  114  121  129  522  288  323  376  709  71  321  330  399  76  174  316  486  30  81  90  166  54  210  311  346  500  543  545  551  175  282  437  513  174  200  385  433  57  121  142  176  233  244  263  313  411  458  521  589  114  136  244  266  59  199  309  385  14  105  261  298  70  106  274  391  98  164  299  519  144  145  156  199  305  320  322  325  336  339  449  485  47  112  287  475  248  300  396  485  76  78  292  303  50  58  75  88  98  110  113  118  124  157  206  258  30  37  93  159  384  731  939  1010  1202  101  176  259  263  373  393  429  603  640  694  756  866  886  893  977  1048  234  260  484  515  79  93  235  238  101  118  179  241  267  981  1262  1850  52  186  208  256  105  393  632  674  54  64  235  335  9  25  48  95  99  102  187  72  115  124  197  146  218  296  369  114  171  257  325  102  21  72  75  76  77  39  154  186  204  38  52  66  89  154  184  279  300  301  325  76  77  79  106  149  170  271  283  341  404  445  453  92  94  96  246  299  307  326  34  81  84  323  82  321  334  342  38  193  337  617  98  133  137  175  85  157  195  208  166  198  612  747  60  244  451  507  91  178  224  230  67  121  170  224  64  614  1088  1123  138  66  96  146  159  252  339  379  411  18  42  90  215  279  281  284  296  304  354  367  586  100  143  328  330  487  493  642  684  792  802  809  870  435  615  647  795  149  248  456  544  61  618  624  1185  275  325  330  503  28  44  120  232  27  53  96  126  161  163  199  269  270  293  359  361  119  123  16  149  170  184  197  336  359  425  487  513  529  535  223  266  328  449  68  503  511  531  335  609  781  849  147  659  706  747  24  303  315  566  244  264  276  440  477  516  525  545  672  831  927  945  125  192  268  303  16  33  58  64  105  122  193  199  9  45  137  155  15  100  239  242  326  502  22  83  242  477  501  651  108  363  368  381  188  304  408  990  86  123  149  310  217  319  337  448  541  565  600  675  705  737  751  773  18  26  35  203  167  276  417  489  48  73  108  109  145  193  207  230  257  286  312  371  9  154  160  322  521  929  1422  1703  29  129  240  274  62  114  260  403  51  65  77  175  122  125  181  217  16  42  295  513  25  35  103  129  84  238  309  354  280  682  735  981  35  61  89  126  129  568  615  58  92  151  233  22  461  647  749  27  48  166  196  296  320  358  386  9  32  111  112  51  71  125  239  154  241  258  286  80  718  803  932  138  152  176  294  309  355  368  411  440  441  476  485  11  40  83  27  44  142  207  384  412  469  509  585  610  637  671  26  44  46  53  55  93  170  195  220  40  54  68  121  77  112  137  140  164  235  248  250  288  390  470  487  147  373  901  1025  103  420  562  713  57  106  261  274  70  88  91  129  18  205  355  506  304  397  423  456  12  37  62  105  116  120  128  156  184  191  230  45  85  146  160  133  178  307  487  58  124  131  172  47  79  85  109  212  1667  1811  1973  15  36  52  91  126  165  186  217  228  245  341  362  850  1660  1772  1933  198  555  617  722  35  100  189  261  16  24  15  28  14  36  124  175  71  146  164  168  169  179  185  219  247  257  263  296  37  382  533  1562  66  208  296  450  17  46  202  206  215  217  407  427  166  540  1011  1068  193  287  335  409  26  51  59  95  97  106  111  144  196  238  246  257  8  110  149  177  182  17  43  66  80  85  127  176  223  312  320  337  347  28  53  97  268  98  150  184  235  313  628  656  723  905  924  1080  1772  2069  2074  2076  2114  82  234  392  551  82  722  900  1120  36  56  94  182  311  381  386  413  476  624  842  1018  184  346  391  443  446  627  652  701  714  766  827  1028  87  149  155  332  676  940  1228  1234  395  526  614  898  92  351  475  555  60  162  424  709  854  860  978  1043  1086  1118  1133  1170  35  49  301  357  75  130  181  184  198  328  471  472  473  482  831  833  573  668  1096  1120  125  129  147  256  120  133  143  150  43  62  133  249  67  68  128  185  289  302  327  383  409  419  753  814  217  257  278  285  287  296  437  468  493  543  620  623  46  48  80  202  208  210  242  249  250  288  325  420  76  265  404  419  37  40  83  85  88  89  261  268  270  309  367  442  37  72  162  269  41  51  180  248  125  488  738  844  204  330  347  382  94  112  209  229  43  57  103  129  96  143  163  634  21  34  52  84  61  149  155  218  54  493  544  1270  66  67  192  221  225  233  284  371  409  421  503  519  21  132  133  174  197  248  292  306  313  372  431  468  143  145  332  405  8  33  37  45  51  69  164  197  230  257  492  507  734  1026  90  144  461  464  196  280  305  323  16  145  156  239  15  44  48  64  168  296  415  582  200  248  286  469  16  87  112  128  69  70  101  143  94  178  321  343  10  48  73  182  185  208  255  307  348  404  504  518  116  208  338  347  80  221  244  249  34  372  506  739  75  150  205  214  297  298  371  418  461  551  637  679  15  23  71  83  168  175  195  210  232  280  304  336  22  23  41  122  147  154  397  400  445  479  482  489  129  151  203  212  54  66  114  116  20  36  41  60  147  172  182  188  340  365  382  453  60  63  106  120  208  248  260  264  267  302  468  485  64  175  176  225  13  318  434  493  468  1460  1572  1950  80  271  339  382  231  335  351  793  11  17  222  242  391  417  494  559  565  568  640  724  181  206  207  233  351  460  552  642  657  679  684  19  45  47  338  15  67  79  103  119  220  259  270  321  328  356  360  23  32  273  397  209  289  618  1132  513  577  967  1238  13  17  25  59  87  230  242  339  364  367  386  23  62  107  228  9  115  267  275  326  337  348  355  357  381  404  495  252  255  325  526  93  325  366  377  76  121  372  383  36  60  84  128  22  54  75  81  27  31  234  242  19  85  216  231  212  278  336  367  557  1246  1256  1289  1619  1620  1681  1700  157  172  265  431  62  111  249  347  39  107  278  398  84  144  493  631  55  57  97  105  118  148  61  177  184  347  18  45  80  555  64  132  153  155  54  133  663  756  10  44  139  225  73  109  204  283  26  78  115  369  126  138  159  244  92  246  476  569  142  188  390  431  73  125  218  219  24  35  40  46  68  233  343  348  400  407  410  455  23  75  101  157  48  94  115  135  202  234  278  306  316  322  324  432  77  155  267  290  13  39  67  79  142  158  215  276  395  416  466  511  158  168  526  684  314  368  817  1028  345  454  744  902  15  49  408  665  1138  1149  1469  1540  1830  1833  1837  1983  62  130  140  237  21  126  306  307  68  461  664  704  111  164  375  447  18  79  93  137  1866  2172  2179  2333  175  355  378  440  239  349  679  819  273  282  355  453  297  318  324  357  68  155  231  380  21  65  73  81  117  135  160  161  188  236  264  371  25  107  223  311  20  56  78  98  136  183  209  212  296  302  318  349  16  60  89  94  109  143  259  262  332  419  461  511  111  113  267  289  42  128  135  142  162  822  1281  1986  2541  10  12  23  37  92  135  147  39  48  51  66  123  125  160  257  302  326  330  333  102  128  163  254  8  72  86  95  24  75  80  89  14  25  73  104  101  118  126  148  140  340  801  1712  41  77  83  114  197  255  256  354  361  390  425  493  435  1383  1403  1564  61  67  72  171  52  60  106  426  170  194  227  236  113  140  155  440  23  520  617  641  8  9  133  151  121  130  153  200  129  241  347  352  9  242  283  286  89  260  326  501  553  738  1013  1080  50  76  106  384  146  169  364  435  46  229  733  746  39  236  543  572  66  86  159  195  206  214  221  277  308  311  317  329  44  283  379  473  196  889  1053  1363  96  265  292  318  116  187  227  275  23  58  79  120  56  60  99  107  31  608  668  941  47  66  167  196  100  157  213  360  98  226  230  310  58  141  196  197  9  158  415  570  187  287  634  747  93  168  173  250  287  321  340  379  383  408  425  443  74  116  147  245  19  61  76  91  348  1032  1361  1460  12  28  176  201  83  136  210  250  147  317  659  667  771  938  1071  1160  1206  1485  1542  1576  38  152  330  395  10  11  13  102  184  202  25  46  55  138  97  103  148  156  193  271  278  282  318  357  456  538  12  28  43  76  126  249  284  315  327  328  331  335  39  59  71  90  34  58  96  223  228  231  233  248  287  302  316  332  41  62  87  122  158  191  224  233  249  330  333  276  286  328  539  261  331  337  415  125  259  340  605  11  33  36  94  18  57  104  109  113  155  158  216  222  245  256  302  568  587  1346  1378  1673  1980  2255  2790  2881  3030  3204  3249  88  100  179  234  250  258  322  417  426  512  551  625  62  63  106  121  71  84  180  217  66  88  96  170  36  144  353  640  129  145  162  209  12  190  215  315  13  16  45  78  80  81  102  118  121  217  249  405  60  82  124  179  205  475  507  665  151  232  255  358  8  21  209  551  63  69  104  110  25  77  88  92  76  91  132  142  102  185  202  226  263  305  341  380  385  611  642  828  157  244  253  331  226  294  330  463  643  663  730  885  923  937  972  1039  134  315  547  555  77  217  253  267  13  40  97  100  11  125  154  186  253  259  288  291  317  341  366  440  464  513  539  551  1018  1053  1065  1271  322  348  582  1014  9  113  123  141  144  198  270  413  420  583  606  631  31  38  42  43  13  57  103  131  202  226  281  308  386  405  444  449  806  886  1306  1479  42  45  207  222  223  225  101  260  378  387  462  554  583  735  783  791  823  893  235  510  665  686  351  606  620  1098  269  293  358  426  44  130  193  219  21  26  38  87  221  303  447  485  506  517  524  536  240  793  920  926  125  140  168  192  230  342  372  425  446  457  509  528  152  341  629  784  56  127  249  403  64  80  91  102  107  179  181  208  253  270  317  366  138  182  250  283  83  211  255  297  113  595  667  1221  54  72  75  84  30  135  230  287  19  43  45  105  193  328  337  384  274  347  542  668  45  91  210  213  47  80  94  437  81  644  756  771  32  231  298  395  473  485  495  496  523  526  549  596  109  188  265  331  67  130  140  175  82  15  52  119  123  101  212  248  263  30  41  48  60  26  78  131  167  210  232  277  286  319  321  348  363  58  60  68  102  17  25  75  102  8  241  287  342  20  46  97  124  37  105  144  192  17  126  175  257  68  80  113  124  73  99  135  207  51  76  79  25  27  45  79  24  88  91  93  20  37  50  102  12  66  156  159  9  70  120  440  31  103  151  153  193  226  265  288  60  76  116  119  38  91  166  232  9  34  82  31  69  136  138  28  54  137  146  15  47  26  33  40  75  76  160  234  358  21  38  133  143  19  37  77  129  26  89  181  241  31  74  81  83  43  75  129  137  42  93  206  207  9  14  69  102  65  178  196  249  9  13  64  69  34  73  102  148  84  111  143  249  32  68  73  157  22  86  147  33  129  196  203  290  401  730  913  10  41  42  76  26  53  67  70  50  56  43  52  48  65  136  142  10  50  86  297  75  130  353  400  35  51  108  135  13  53  124  129  98  176  227  258  25  29  30  57  37  90  145  161  68  150  196  279  22  23  82  88  106  158  176  217  227  279  341  452  41  292  361  382  13  17  60  96  108  119  127  129  251  314  326  13  51  88  124  260  301  418  691  59  130  169  234  260  359  785  984  86  214  289  370  41  94  121  327  338  383  426  455  478  498  506  547  165  379  422  474  137  400  432  516  582  1346  1366  1566  1620  2063  2723  4001  62  246  385  396  500  529  541  549  596  631  761  796  26  47  101  136  24  113  133  161  397  404  452  529  554  598  808  876  902  982  1026  1080  81  100  134  192  11  18  76  78  1418  2322  2582  3080  135  369  429  470  60  123  134  172  227  254  255  313  340  384  395  455  43  53  55  89  20  29  41  79  77  78  144  209  201  271  295  340  84  88  92  95  106  119  121  154  182  198  267  282  24  164  385  424  214  473  1031  1250  24  31  97  120  138  269  346  349  367  423  511  516  79  279  636  651  168  367  515  653  16  49  63  158  216  218  288  306  351  365  399  463  11  41  45  148  171  195  302  344  14  25  29  51  59  63  83  126  203  307  407  443  179  213  636  974  478  481  677  755  101  104  108  193  34  92  116  159  163  182  183  225  256  331  351  352  176  444  497  624  127  143  147  215  21  35  60  71  175  181  205  218  314  453  563  691  225  232  512  949  118  189  494  601  237  349  1017  1043  58  65  106  156  39  42  47  160  162  247  270  313  335  343  372  426  103  128  245  283  15  46  90  93  67  106  107  135  513  575  587  1111  53  138  435  594  54  55  60  67  164  229  352  357  365  429  488  501  65  224  227  229  207  834  912  1119  51  85  86  103  128  163  165  210  250  275  307  402  12  27  35  50  56  98  102  112  41  121  254  425  93  352  1002  1120  48  102  169  172  254  471  591  839  106  238  587  593  16  21  111  133  138  678  929  963  38  170  209  242  295  333  526  564  572  594  605  609  267  497  620  1274  339  474  495  602  9  51  60  70  80  187  205  210  317  402  403  450  168  242  303  582  87  88  90  119  123  134  139  143  165  201  225  266  108  371  414  489  49  129  289  313  18  49  54  111  143  156  166  183  186  313  512  555  33  77  191  324  35  41  109  118  29  38  68  70  90  92  102  191  212  259  280  301  127  215  402  464  262  325  432  886  804  1790  2898  3833  29  95  157  187  69  73  89  127  137  166  252  258  264  319  354  605  97  168  425  512  57  379  538  832  27  366  458  548  56  210  247  304  159  203  267  356  44  120  176  208  91  170  385  386  319  647  664  665  678  720  729  732  736  758  991  1019  362  368  583  755  37  72  155  201  209  277  331  395  408  472  513  550  611  737  764  771  53  55  69  117  160  172  215  217  170  231  323  385  397  412  425  467  478  506  627  647  104  107  148  217  55  69  245  265  304  352  373  377  432  443  450  457  72  182  263  284  45  75  563  681  233  288  314  341  205  338  434  519  591  620  919  1012  1020  1198  1273  1812  38  88  140  160  172  261  356  364  452  469  547  683  26  113  134  188  226  279  281  299  349  383  401  405  114  129  179  194  114  837  869  1032  114  125  167  179  380  535  730  753  147  171  177  218  274  328  332  381  481  504  513  517  43  151  273  282  317  619  665  670  826  847  999  1255  300  312  520  526  888  1196  1480  1970  85  128  134  147  193  242  364  367  395  396  405  434  123  188  289  359  22  167  197  214  225  229  236  239  286  292  359  481  21  124  138  151  32  56  57  58  71  88  171  202  241  293  306  397  219  448  509  596  20  93  125  130  156  172  174  292  329  356  364  471  174  219  398  885  8  105  297  305  96  137  185  323  56  136  140  157  191  228  321  728  162  222  248  268  29  75  327  405  86  164  208  302  21  250  284  375  147  217  347  461  664  1840  2348  2556  76  132  178  210  332  467  479  662  58  92  246  263  304  308  480  491  582  617  626  707  154  264  340  391  57  74  229  252  32  44  46  86  36  300  543  619  235  333  454  510  27  35  46  241  178  239  307  382  221  237  274  399  13  38  42  63  75  108  110  121  132  136  153  158  29  79  81  143  285  947  1374  1663  19  21  36  41  45  50  82  86  38  432  456  583  38  277  304  460  94  254  311  328  333  334  359  371  531  541  566  634  335  355  356  384  51  62  71  75  160  168  173  178  183  314  389  415  61  164  288  325  11  117  261  401  91  103  171  179  104  282  377  496  271  408  495  507  553  575  637  642  667  672  689  733  21  39  73  92  218  236  661  666  44  47  92  161  58  322  374  591  91  141  232  302  72  99  121  422  62  285  417  466  187  237  819  950  198  327  405  449  414  483  734  753  65  241  260  418  189  311  366  435  9  586  631  722  11  42  176  238  262  266  302  343  380  384  453  483  13  57  118  218  253  288  303  312  329  384  419  554  26  71  148  158  220  434  443  479  873  963  1334  1366  67  133  143  173  182  240  344  347  15  751  765  876  1240  1372  1563  2186  43  63  87  127  139  241  262  266  293  348  352  365  104  360  370  504  43  48  62  91  98  150  259  284  343  388  417  426  86  123  130  148  226  238  240  260  175  214  330  522  31  537  764  794  37  143  169  449  550  644  902  976  1094  1197  1709  1858  521  579  677  698  16  346  641  861  32  122  126  135  207  219  291  331  333  359  370  390  15  61  62  106  214  224  294  297  298  302  336  360  179  493  615  671  968  1346  1621  1724  341  358  740  1131  1174  1228  1265  1485  1593  1846  1898  2041  35  544  709  740  16  19  300  348  387  394  446  459  542  564  595  600  40  126  143  178  93  682  1079  1134  537  730  807  883  1164  1176  1402  1533  1618  1627  1860  1978  42  54  191  305  95  105  173  238  362  429  554  592  603  661  674  786  42  50  196  251  38  58  256  311  60  222  256  558  187  357  622  1485  77  207  444  467  145  170  274  316  15  113  166  250  82  337  354  741  124  435  645  711  195  205  427  587  289  295  304  508  509  538  688  714  743  782  963  1952  27  116  122  127  150  166  208  214  225  235  252  280  12  30  36  58  89  149  171  190  221  299  356  375  47  705  813  865  39  68  90  123  272  379  457  619  289  587  678  683  56  337  411  460  56  129  210  285  15  174  277  314  38  43  100  217  15  23  28  79  97  119  134  148  172  197  228  283  36  47  85  88  182  211  216  237  251  264  274  327  100  119  223  337  139  338  383  413  279  325  688  844  49  104  122  1021  536  786  1282  4007  27  66  37  69  90  21  26  111  176  183  196  235  243  326  422  431  452  62  89  234  236  20  168  180  192  198  217  232  293  378  425  455  529  32  100  101  211  412  422  699  716  77  251  277  976  13  16  66  74  75  164  210  230  270  278  322  342  502  595  671  799  188  194  340  456  14  50  280  407  80  178  197  326  14  120  143  245  44  84  98  189  186  188  258  280  295  323  324  434  464  483  484  525  101  208  270  533  428  473  531  561  704  747  751  796  905  1110  1409  1550  60  107  239  338  260  309  335  415  33  135  206  249  32  118  180  281  16  54  61  69  15  937  1132  1599  209  315  376  446  342  405  479  639  792  862  885  922  946  1175  1379  1399  66  89  92  98  172  184  197  252  253  372  430  432  13  24  32  71  185  209  212  244  249  328  367  402  212  259  355  381  22  37  185  235  32  43  67  98  53  72  236  248  283  305  313  386  446  460  462  568  250  315  352  440  221  276  395  439  53  125  155  173  118  177  318  335  64  106  157  165  202  323  339  393  527  557  750  894  304  320  330  352  364  372  503  562  711  729  792  814  27  39  78  86  94  129  178  246  306  317  379  392  18  25  57  88  110  112  122  174  291  301  312  443  218  224  337  391  47  157  285  340  425  503  526  637  676  677  688  702  71  118  122  160  86  95  132  158  168  225  267  305  376  393  424  438  37  62  68  69  112  122  128  167  274  343  440  444  631  22  23  66  71  118  324  342  443  178  190  311  356  92  230  347  448  128  149  163  181  376  383  407  420  498  616  700  796  111  586  603  736  23  33  46  59  119  111  138  152  198  239  241  252  282  257  362  427  443  47  56  335  646  69  458  515  657  129  307  416  471  9  244  299  466  133  307  427  823  9  48  158  164  49  75  175  183  239  249  288  445  449  559  576  607  175  201  235  285  303  397  479  511  679  873  1142  1197  26  281  320  355  654  734  754  856  868  1706  1714  1785  29  72  276  399  60  102  272  782  1036  1100  1131  1276  1335  1348  1351  1374  73  75  93  143  76  163  256  290  93  458  898  1261  20  27  30  63  213  291  330  440  499  518  598  661  33  154  229  375  307  385  458  462  39  220  337  411  116  171  201  205  55  100  162  280  322  546  553  564  579  644  827  840  54  380  494  663  13  100  113  139  152  171  185  199  50  146  181  488  102  172  209  254  277  292  708  1093  156  206  267  321  735  813  938  1149  13  158  205  242  82  84  158  221  173  208  144  270  325  391  138  368  434  687  691  746  959  992  1060  1483  1505  1575  189  293  299  310  353  416  655  808  50  67  84  102  36  64  288  512  28  285  329  515  259  510  541  572  315  435  1223  1761  46  74  150  174  73  265  318  469  20  89  110  122  93  134  377  389  123  132  220  250  94  124  246  255  19  92  177  185  71  244  259  292  38  199  235  495  334  418  470  478  231  418  531  1800  45  128  135  275  315  344  470  501  523  527  716  821  97  157  284  427  84  221  389  404  318  352  531  700  17  27  61  105  138  178  295  439  515  530  540  770  782  865  886  978  9  21  34  133  158  236  264  266  292  300  331  352  166  424  653  967  1152  1186  1266  1472  1558  1605  1646  1695  81  159  448  553  63  256  379  597  125  164  184  251  163  191  299  538  58  189  330  374  116  154  379  420  26  50  235  245  269  297  312  559  267  350  379  671  231  302  610  770  60  184  190  762  31  74  185  194  27  159  168  186  208  231  245  261  280  339  381  396  89  116  430  595  179  633  691  841  122  142  242  328  60  184  253  303  18  57  115  191  18  331  409  428  38  42  84  93  98  136  268  285  311  328  347  401  51  390  669  749  117  199  289  437  67  184  190  231  33  147  394  413  91  185  486  706  75  182  589  762  184  202  256  257  126  151  159  161  179  207  277  454  494  510  523  524  770  893  1304  1331  25  46  67  150  66  111  263  317  249  537  733  1004  104  131  201  219  207  366  478  702  11  80  104  174  322  377  411  430  435  772  919  924  229  301  375  446  17  167  227  377  32  84  229  267  88  217  437  541  51  52  90  225  352  547  788  889  28  31  45  117  123  184  269  283  313  352  375  376  77  261  513  576  24  52  77  132  50  74  157  160  179  223  266  317  338  433  459  658  23  128  160  258  25  44  186  211  214  217  249  260  294  307  368  435  133  183  209  229  28  48  579  716  229  363  660  778  48  63  171  218  31  46  52  66  96  114  205  268  287  332  349  367  149  316  449  610  53  288  378  727  50  121  191  458  14  54  68  217  101  264  268  395  639  680  699  714  745  857  954  1088  21  229  232  299  338  383  431  464  488  493  495  790  322  360  470  574  8  97  202  354  449  491  531  554  576  624  675  678  36  84  97  151  22  129  153  190  212  214  237  256  346  378  388  404  180  228  229  237  283  305  355  412  492  514  533  610  452  458  566  570  21  50  190  275  90  175  246  253  273  275  290  340  355  364  368  374  126  146  178  362  339  459  468  472  166  351  362  400  334  351  380  673  103  110  119  177  185  192  201  245  58  61  78  220  449  479  580  737  121  145  191  396  108  302  517  544  51  81  88  123  149  155  292  303  387  451  505  515  75  107  134  261  10  52  140  10  110  167  169  36  84  123  149  164  181  182  295  315  321  387  394  63  85  170  241  62  150  180  196  223  273  296  352  421  440  456  504  117  184  193  207  39  45  56  472  523  529  531  575  583  693  716  734  212  1693  1855  1874  153  184  895  1283  808  871  907  1056  32  38  39  56  128  410  712  764  14  76  168  389  72  79  100  228  247  411  468  646  176  186  497  674  105  152  223  253  8  182  379  449  66  80  139  141  20  28  193  199  683  839  877  964  70  354  425  450  48  64  251  317  12  13  23  31  97  116  138  143  403  678  802  968  270  283  352  387  72  108  135  140  117  319  340  386  556  583  608  707  1407  1417  1742  1825  78  421  447  473  85  125  259  366  308  380  904  1047  22  39  51  61  71  147  248  279  281  282  321  350  13  106  138  202  418  541  1994  2106  287  492  629  1206  522  585  632  986  16  44  105  117  155  168  292  417  468  469  544  548  310  342  1002  1768  73  123  290  384  133  206  280  364  153  989  2107  2510  166  201  206  317  140  1171  1220  1805  454  1009  1035  1263  1308  1382  1424  1559  1712  1762  1803  1979  42  222  266  287  299  366  744  826  28  446  510  805  103  154  310  311  106  204  254  393  505  559  576  629  690  705  750  805  368  398  937  1068  56  80  137  148  94  234  433  660  22  37  82  246  388  483  497  700  707  761  906  915  54  60  112  234  208  255  292  465  59  79  227  372  106  186  205  332  365  371  383  398  459  485  608  695  190  231  511  659  19  501  645  672  138  163  165  166  385  410  416  541  570  616  639  695  70  102  133  135  10  24  62  63  45  97  219  269  87  115  197  354  30  330  406  944  72  102  103  54  119  183  339  38  73  96  160  24  32  90  97  111  113  136  151  160  177  198  233  11  130  160  187  32  60  143  160  317  318  319  324  341  387  451  457  142  261  264  526  8  24  84  129  59  133  160  296  129  136  162  316  32  80  101  103  120  172  173  398  70  91  96  103  36  204  209  460  11  59  72  59  73  171  211  154  196  200  242  344  511  517  554  607  619  679  738  335  360  434  504  22  86  225  269  70  274  375  450  34  115  117  128  186  196  221  243  263  19  59  290  372  195  263  268  305  313  345  389  433  438  449  480  495  18  91  223  302  11  56  124  240  156  208  292  344  105  106  96  124  187  200  88  155  297  353  383  384  503  504  551  657  805  832  84  104  117  125  131  183  270  460  171  602  787  853  93  127  130  300  394  403  415  495  497  507  624  640  21  39  85  175  140  181  366  653  145  195  325  358  42  58  139  296  297  371  701  769  118  213  470  548  121  367  688  711  60  117  230  271  41  142  169  230  241  484  596  606  662  858  976  987  1471  1707  1723  1754  50  114  306  376  400  541  708  720  761  890  920  1106  34  199  356  374  382  709  720  803  823  872  874  1050  324  392  452  456  62  395  549  707  260  516  966  1048  71  77  88  138  15  347  418  537  65  71  291  641  192  378  491  674  23  93  390  492  225  314  651  735  21  93  229  347  10  21  33  230  61  80  84  162  282  352  407  429  510  602  704  751  252  356  773  925  19  34  110  140  24  57  274  334  12  80  162  243  63  66  193  197  359  374  383  448  685  731  823  854  90  283  308  340  66  157  233  322  345  496  561  626  751  790  794  806  71  326  341  360  21  155  263  288  91  101  158  159  189  195  268  351  14  24  71  91  187  217  260  285  325  412  449  461  242  335  461  545  188  322  363  409  77  288  409  428  552  784  840  1053  1059  1308  1345  1361  129  204  232  354  76  126  192  315  323  333  422  430  671  687  693  859  30  263  470  618  20  23  103  142  38  98  519  531  152  155  165  172  82  147  226  280  340  366  385  411  439  446  465  484  85  107  115  359  30  82  102  153  228  256  305  312  353  373  378  386  24  86  415  421  26  30  32  40  47  71  86  137  170  177  180  187  79  87  113  130  197  216  236  242  259  304  315  329  76  122  287  525  143  311  351  434  136  163  185  319  10  38  133  190  179  292  293  320  26  841  959  1046  28  173  259  326  370  97  208  223  299  282  349  357  362  37  51  97  118  163  187  204  246  249  254  276  399  293  389  923  2405  62  93  102  123  76  90  248  285  30  49  60  81  85  181  213  234  269  277  282  341  121  266  486  1226  40  150  240  404  488  509  723  759  881  899  1181  1375  75  81  104  242  294  299  310  326  331  350  353  22  282  324  545  81  299  392  444  70  126  144  328  67  86  94  263  23  193  294  914  73  117  122  163  72  268  298  338  49  160  314  522  47  280  283  356  66  473  489  494  8  17  129  266  34  43  59  70  82  105  154  156  193  220  240  277  170  377  519  640  139  163  308  518  46  136  209  226  290  425  461  472  517  564  705  719  101  434  651  714  73  344  368  407  256  275  440  461  58  92  122  159  163  165  168  176  227  81  92  263  271  232  236  268  296  31  183  216  327  151  175  226  339  40  79  82  110  140  150  158  53  57  147  175  17  20  92  99  112  210  243  245  13  63  227  293  309  365  391  511  520  608  614  616  47  174  186  220  235  238  270  293  435  449  503  617  359  370  450  484  489  608  696  758  805  816  848  863  27  96  151  200  11  32  101  320  183  416  921  1007  1190  1289  1392  1497  1576  1637  1780  1855  151  190  309  316  351  384  406  519  554  598  640  728  189  240  376  389  82  112  152  215  241  257  358  359  402  439  488  519  211  380  396  502  528  620  762  770  836  866  1271  1952  52  459  464  493  402  821  948  1010  445  640  752  762  91  166  168  206  30  34  86  105  168  170  197  213  267  305  307  347  30  235  513  517  26  27  74  76  43  63  431  435  11  146  169  182  76  188  214  269  114  211  300  319  65  147  149  261  50  76  93  99  102  109  120  414  519  589  821  141  348  553  583  27  45  64  72  136  177  226  230  237  281  298  311  47  126  141  142  155  389  399  437  49  61  91  113  61  76  80  101  120  130  150  8  10  14  39  131  222  309  463  500  513  636  713  76  82  85  141  155  168  278  291  300  400  401  423  40  64  102  155  142  271  272  377  467  550  576  724  802  867  1193  1313  68  94  161  183  352  426  485  596  148  297  376  385  395  412  417  485  498  505  520  521  72  100  106  376  393  430  84  410  1229  1667  33  198  215  257  8  16  89  106  117  201  203  231  280  283  311  327  97  424  798  982  20  35  63  70  148  181  183  201  152  201  275  409  468  508  516  630  687  743  793  831  11  139  655  994  140  157  325  348  13  21  105  134  166  204  241  257  422  433  434  439  525  575  769  778  50  325  366  476  32  72  108  154  45  81  135  256  46  113  168  226  48  59  73  176  216  450  512  690  177  451  514  525  90  294  331  348  46  135  189  204  207  250  434  478  507  509  802  807  22  81  122  243  116  170  1017  1575  595  1148  1560  1659  83  294  299  368  122  282  316  347  41  111  569  589  110  163  272  388  24  56  63  71  98  133  159  183  227  367  368  399  16  49  83  114  38  50  84  247  319  378  386  426  521  619  692  739  9  165  168  283  406  616  776  808  819  931  999  1080  97  1052  1118  1160  81  129  135  137  61  73  190  210  173  204  393  566  52  89  103  125  80  137  193  509  74  78  99  174  144  160  204  257  23  187  317  362  450  886  937  990  1079  1124  1151  1206  221  340  386  488  52  61  66  173  40  107  233  268  51  198  385  437  337  395  488  563  31  147  361  404  9  62  76  111  163  208  222  280  351  365  409  424  13  38  47  70  172  176  209  212  11  110  127  172  223  257  320  429  54  139  193  196  455  600  674  790  42  464  494  661  226  290  328  374  471  491  526  537  552  742  769  785  128  255  518  520  542  715  749  770  1070  1341  1375  1435  8  29  55  57  103  141  221  277  292  296  340  517  43  54  133  249  12  38  67  112  41  158  170  196  229  285  291  301  318  429  466  616  110  202  731  987  73  93  94  463  27  40  71  99  43  69  316  324  46  47  84  214  19  32  99  465  473  526  581  596  602  674  708  709  14  93  122  206  189  195  320  373  411  429  511  534  544  571  776  844  181  218  497  676  73  93  229  545  67  107  139  49  14  34  112  234  280  309  349  371  411  476  491  802  20  21  35  25  121  177  616  400  448  563  640  58  155  198  236  28  35  63  80  21  25  88  127  147  156  162  165  194  280  342  372  74  89  224  240  153  154  78  132  556  611  21  224  352  473  151  231  244  1269  134  166  243  348  72  115  282  388  33  187  193  524  79  155  237  294  96  239  503  634  48  83  162  265  286  364  504  680  363  406  436  666  70  148  265  277  66  224  507  1513  13  35  42  303  125  164  420  944  13  43  196  214  227  239  302  333  369  408  531  542  129  156  394  536  199  296  453  656  26  144  398  459  45  74  76  87  114  140  153  176  9  210  306  342  171  215  236  357  365  447  497  530  620  629  684  710  31  79  278  323  38  54  110  231  250  425  489  192  214  283  536  11  175  192  263  60  92  239  240  486  720  868  902  118  293  368  657  50  51  58  102  42  73  198  249  58  123  155  163  185  471  767  1179  19  63  117  130  33  153  319  471  158  201  243  347  130  198  266  324  134  207  387  479  25  76  180  224  533  697  1026  1457  68  292  303  330  317  396  409  478  54  251  271  276  72  86  117  141  35  89  167  230  87  139  245  313  76  123  191  488  27  166  209  237  477  556  614  706  761  899  995  1203  110  351  571  614  357  433  795  925  1311  1328  1330  1332  152  169  188  388  14  195  208  218  248  294  348  387  463  469  478  552  288  1880  1990  60  124  133  382  309  334  405  528  531  594  601  604  628  719  752  824  101  125  183  225  301  363  446  495  526  632  657  662  369  876  1094  1103  36  65  155  226  241  284  296  303  371  492  595  661  31  328  365  379  381  420  459  650  682  790  979  1080  154  176  222  333  25  177  381  473  15  42  161  183  195  224  337  363  442  578  623  763  86  112  118  54  76  32  232  305  365  44  110  468  482  56  175  283  303  46  54  56  95  102  144  235  408  190  326  329  401  98  118  157  176  18  24  44  62  98  141  192  194  204  220  296  306  133  1069  1179  1351  76  116  156  463  478  612  795  902  1020  1060  1072  1240  12  252  430  628  332  445  460  723  25  41  72  86  45  108  129  139  210  297  487  799  113  162  183  231  101  141  234  338  109  132  169  184  17  63  112  124  138  177  187  219  266  269  322  333  23  52  101  111  13  53  89  146  9  30  52  56  60  63  34  100  17  28  39  128  332  615  1144  1469  25  44  74  387  105  197  445  658  70  147  160  325  269  584  596  713  33  115  144  209  9  46  65  123  174  384  403  471  184  197  217  300  494  690  869  903  209  210  473  494  36  61  100  119  30  133  160  203  170  305  470  646  8  31  164  229  55  86  116  215  37  58  131  135  182  184  194  220  297  340  378  421  80  167  283  606  22  144  231  245  285  342  354  389  439  456  503  508  347  517  527  529  222  466  876  1003  50  147  164  409  11  89  144  151  446  730  1028  1475  103  142  347  910  128  207  346  439  480  584  643  652  663  721  802  845 | PVPKPR**T**VPVADV  QRQLSV**T**QDDPES  DDDDYV**T**LSDQDS  PHITRG**T**FVGPQG  EDFDWC**T**PPKKRG  DKQIKF**T**LFKGVI  NIKKDQ**T**LKARIE  KIKVNW**T**PEINKE  PEQGSL**T**VQCHYK  RGPDLG**T**QVKVIV  TTASSP**T**NSNMAV  ILLILV**T**AILWLK  LAISRV**T**PGSKAA  AIDGEN**T**SNMTHL  ENTSNM**T**HLEAQN  VCTDCG**T**NLKQKG  EGGEEE**T**LRRLGL  KLADQR**T**RKSLGR  DKKRPE**T**VATQFK  DGTIDF**T**PGSELL  AGIKTI**T**MLDEQK  MRETEK**T**LTELNK  SGKAYK**T**TWGDGG  EMEENL**T**QVGSIL  PFFGGM**T**RDEDDD  RLREGQ**T**LRDSML  FKSISV**T**KITKPD  VDSEGR**T**ETTVTR  FCVGLL**T**MAKAES  KEHDPF**T**YDYQSL  FNQQQR**T**GEPDEE  PDEEEG**T**FRSSIR  DSGVGK**T**TFLYRY  SGVGKT**T**FLYRYT  NPKFIT**T**VGIDFR  EQCVEK**T**QIPDTV  ISLAEN**T**QDVRDD  NTMSNY**T**LLPPSL  DWKNHF**T**VAQSEA  PSLEPN**T**SLEREP  WMRVQD**T**SGTYYW  YYWHIP**T**GTTQWE  PEEGTL**T**FPAQSL  LGWVEM**T**EEELAP  GSLLAK**T**GQAAEL  ALTSAR**T**TANAIY  RYAGRQ**T**EALKCV  EPPVDK**T**YEAALE  MVQQCC**T**YVEEIT  YRAIYD**T**PCIQAE  DLLKLF**T**TMELMR  KYYTRI**T**MKRMAQ  GLQDSS**T**DNRAKL  KEAPSP**T**CPDLGA  APKEAV**T**GNGIGG  QPLQLT**T**KPDLLA  WDPARN**T**HPPSHH  PCKKRL**T**ARSIST  TARSIS**T**PTCLGG  DLRCVK**T**RSEAGE  AAAAAP**T**TTAEKP  AAAPTT**T**AEKPPA  FGNIII**T**DVTANC  VTANCL**T**VTFKEY  LKRRDF**T**PAELRR  GKVFDV**T**KGRKFY  DDLSDL**T**AAQQET  TAAQQE**T**LSDWES  EVVTES**T**GKEREH  VDGNKI**T**IMPKHE  ILFSDL**T**MHELKV  SKPRDV**T**NFTVGG  KDATES**T**ARVELH  VDRQRL**T**TVGSRR  TSTYGR**T**PMYGSQ  PMYGSQ**T**PLQDGS  PHYGSQ**T**PLHDGS  GTPNPQ**T**PGYPDP  NSSDWV**T**TDIQVK  QVKVRD**T**YLDTQV  ESGEIR**T**LKPCLL  VLAEDG**T**IVDDDD  NNSDGG**T**AWISQE  VQRLQH**T**LQQVLD  VDLIMK**T**CFSPNR  KVSADN**T**VGRFLM  NDLLMV**T**YLANLT  TYLANL**T**QSQIAL  MPSLSP**T**MEEGNI  LVQLKQ**T**GKITES  APTATP**T**APSPLQ  PSPLQA**T**AGPSYP  LESHHD**T**WPPACP  VRRVLN**T**EANVVR  QTTVAP**T**TANKIA  ANKIAP**T**VWKLAD  EIRGDV**T**IGPRTV  VTIGPR**T**VIHPKA  AYPDNI**T**PDTEDP  DNITPD**T**EDPEPK  KPMIIG**T**NNVFEV  GRNVIL**T**SGCIIG  ACCNLN**T**FEVIPE  EVIPEN**T**VIYGAD  CLRRVQ**T**ERPQPQ  ERPQPQ**T**LQLDFL  YHHLKK**T**MKGSST  PRGYFH**T**FAGDTC  IKNPEI**T**TNRFYG  AKKKRL**T**KADIGT  PPTPAP**T**SGIVGA  LKLCGD**T**SLNNMQ  VLEQVV**T**SIASVA  EVALLD**T**QDMENM  EGFVEY**T**EQVVKL  KYYSNL**T**KSERYS  IATERA**T**RHRYNA  HRYNAV**T**GEWLDD  MRECFR**T**KKLSNF  DDNIRL**T**PQAFSH  GVGDLY**T**DPQIHT  PQIHTE**T**GTDFGD  SSPSSA**T**PHSQKL  DVSLKE**T**EENKTK  ETEENK**T**KGFDYL  EAEMLF**T**GGYGLE  RSGDLY**T**QAAEAA  LQDVFN**T**VGADII  LANRNG**T**KYLART  CLPELK**T**RINVLA  ATEYCN**T**IEGTAK  PCKEAM**T**RHPVEE  TEQLQF**T**IFAAHG  EATIFF**T**RLIESS  SLMNAS**T**DVAECD  LPAPLV**T**PEASAE  KIQGEY**T**LTLRKG  IEAFNE**T**IKIFEE  QYLVWL**T**QKGARQ  KVIQAQ**T**AFSANP  PFERTI**T**MHKDST  FKNGKI**T**SIVKDS  ILSTSG**T**VVTITI  LANRPY**T**DGPGGS  SDDWAR**T**AAQTGP  SAFEAV**T**RIHFPE  ALPLLA**T**SSSRYQ  SLLLAD**T**LQTHSS  LADTLQ**T**HSSLFL  KGSELA**T**DPPAQP  VKIRNV**T**SNHRAS  NHRASD**T**VVCEGR  YLIVYV**T**GRPDMQ  EREGPG**T**PPTTLA  GPGTPP**T**TLARGK  TRYTRP**T**PVQKHA  QTGSGK**T**AAFLLP  SPILVA**T**AVAARG  GNLGLA**T**SFFNER  TQPCDH**T**KGLECN  QKCIVQ**T**TSWSQC  CSKTCG**T**GISTRV  PEPVRF**T**YAGCLS  GLEATD**T**NGLSSS  NIRQTT**T**GSAVPI  SLYKEV**T**DSRSGN  LGREVY**T**SNNQLG  EPELAD**T**SALKAA  IIEVRT**T**MPLEMP  VIRQSE**T**RPGEYV  PGEYVL**T**FNFQGK  IGSNIV**T**SQTIWE  NCVVQS**T**GQMQCK  IVPVSW**T**AHNIIQ  CNCPPR**T**DKPYSA  SVHSPS**T**SMATSS  PSTSMA**T**SSQYRQ  PPSLGY**T**QGTGNS  GKEIRP**T**YAGSKS  RCSNTS**T**LAARIK  EVAQVR**T**TGIHNG  GCIPCG**T**SKILGS  SKILGS**T**DTILLG  LQQNTH**T**LPHNHT  EQPLFS**T**GSAQYH  PAVHTK**T**DHSVAS  PPPRPP**T**SPYPPL  RKGPFK**T**IKFGTN  VTNESN**T**QKTYIV  ESNTQK**T**YIVHCH  HDCARK**T**SKSLEN  AVILCA**T**VVQGFP  KIEVII**T**LKENKG  DVQHLQ**T**ALRNFQ  RLTLKG**T**QKKILD  MLGLSN**T**VMRLIE  IGGMLL**T**CVVMFL  LAAVVT**T**PLLSSG  LSSGTP**T**ALIGTG  VFRLCL**T**DEEVVF  VFVRLN**T**EVASVV  LRMKRD**T**SLFSDE  FEGFIQ**T**RGGTFY  LRKKRT**T**SAEKNT  TSAEKN**T**CQLYIQ  CQLYIQ**T**DHLFFK  DTIYQT**T**DFSGIR  ADEKDP**T**NPFRFP  LNTGII**T**VQNYGS  SQGPCC**T**AQCAFK  GICNGF**T**ALCPAS  TDCNRH**T**QVCING  YGLEEC**T**CASSDG  QSSEIS**T**KSKSVD  TVASLD**T**DSTKSS  SLDTDS**T**KSSGQS  SNNNSD**T**CAEFRI  YGIKVS**T**SDQYDV  SLLALK**T**TDASNE  LLALKT**T**DASNEE  ICKVLS**T**AFDSVL  EHNIVA**T**YLIMAG  RRMTTN**T**YIGLIL  NFIVPL**T**VMFYCY  SDQIDV**T**KMSVIM  VPEIAD**T**LGAVAK  SGRDWN**T**LIVGKL  NAPTTH**T**EEYSGE  TVREGQ**T**ICVRFW  ENVEKE**T**HQQVVS  LVVDPE**T**DEQLQK  LRPRLC**T**MKKGPS  RAGGDE**T**KLLVVD  LVVDRE**T**DEFFKK  PLPVPF**T**NGEIQK  PPKQDS**T**APSSTS  STAPSS**T**SSSDPI  IPQDRL**T**EVIASI  QGSILS**T**LLCSLC  DEALGG**T**AFVQMP  LRVISD**T**ASLCYS  LAVNRV**T**EEAVAV  SQPEPR**T**GLSLWD  ISFSQP**T**CPDHML  NSQLLG**T**PGSSQN  QTKEKP**T**FGKLSI  KLFLDY**T**IKCYES  TIKQEN**T**RLQNII  QLVVQT**T**TEERRK  QNVPSG**T**DTGDPQ  GNTYQL**T**RGIGKD  SRGVKL**T**DVAPVS  EGAKSE**T**AEELKK  SYDRAI**T**VFSPDG  CQSHRL**T**VEDPVT  QTDPSG**T**YHAWKA  TDEAIE**T**DDLTIK  IIALNK**T**KERMRP  LLDTSQ**T**FIRQGS  DICFSK**T**LNSCKV  EGTPNF**T**EEGLVN  QQKYTV**T**VDYWSF  QRGTDP**T**YGPNGC  VWHSIQ**T**LKEDCN  QTEFGI**T**SDKLLL  IVPSYR**T**QSSSCE  SDLPST**T**QPKGRQ  DQLDLV**T**YEEVVK  YGTKLE**T**IRKIHE  SSKKAK**T**KTTKKR  KKAKTK**T**TKKRPQ  KAKTKT**T**KKRPQR  SLGKNP**T**DAYLDA  PGPINF**T**MFLTMF  NFTMFL**T**MFGEKL  GEKLNG**T**DPEDVI  CFDEEA**T**GTIQED  DEEATG**T**IQEDYL  YLRELL**T**TMGDRF  LRELLT**T**MGDRFT  FNYIEF**T**RILKHG  VSSDKV**T**QKQFQL  YIKFFA**T**FDKGVA  DDDDLP**T**AEELED  LSGKIN**T**EDDDED  PKRVLV**T**QQFPCQ  QKQLQA**T**SVPHPV  SRPLNN**T**QKSKQP  GYFHDA**T**RVYLIL  EYAPLG**T**VYRELQ  KFDEQR**T**ATYITE  DEQRTA**T**YITELA  RTTLCG**T**LDYLPP  PPFEAN**T**YQETYK  ANTYQE**T**YKRISR  TFPDFV**T**EGARDL  LEHPWI**T**ANSSKP  VVKRQK**T**KVKFDD  EAETDK**T**KPLASV  VAVTTP**T**VSSGQA  VSSGQA**T**PTSPIK  SGQATP**T**SPIKKF  GALAEI**T**ASKEGQ  SSVPST**T**STPTVT  SSTSTT**T**KITTGS  ASTTTL**T**TTTAGT  RSTQGV**T**LTDLQE  RYRPVS**T**SSSTTP  QEQQSD**T**EEGSNK  AAAATR**T**ARQHPP  TSKKDL**T**EYLSRF  IKTDPV**T**GRSRGF  YSGQQS**T**YGKASR  VIDDAH**T**DWSKYF  AKTYAT**T**EAFIDS  LPMPGG**T**APSPLR  DTSQRG**T**PSEDEA  AKGKAS**T**LATLKY  EEMESV**T**SEHIVK  APGLRN**T**SGIDSP  EAPEGG**T**GAMGTT  KSKDGK**T**PLHMTA  YAATSD**T**DGKCLE  KDKWGR**T**ALHRGA  VDENGY**T**PALACA  ADLKAL**T**HSVLKK  VESRGG**T**RTACLL  VPSSAE**T**GGTNYL  DNPDSR**T**LLVHKV  TSRKRT**T**EDDIPT  GSTDLQ**T**NQDVGT  DTDVEV**T**SEDEWQ  SSESQE**T**ISSMGE  VRNVHT**T**DFPGNY  RLQVRC**T**RNPHAA  DLFPEG**T**IRPVHD  IFSVES**T**GVLPPD  NASHPE**T**NLPVGY  PPQYPP**T**AFQGPP  ELLEVL**T**GFETNN  VLTGFE**T**NNKYEI  YFAAED**T**DCCTRN  EDTDCC**T**RNCCGP  MGQEVI**T**LERPLR  IGYVIQ**T**WHPCLP  PCLPKF**T**IQNEKR  KISKHW**T**GILREA  ILREAF**T**DADNFG  FMFFES**T**GSQEQK  QSSGSG**T**GKGISG  ADTLSL**T**DSSVDG  HHVHHS**T**ARPKEQ  QFKELL**T**KKGSYR  EFTKVK**T**FVSRII  DIGPAD**T**RVAVVN  TPLSTG**T**MSGLAI  SGLAIQ**T**AMDEAF  KVAIIV**T**DGRPQD  DRCALN**T**HGCEHI  LNEDRK**T**CSAQDK  ICVNDR**T**GSHHCE  ECYEGY**T**LNADKK  LNADKK**T**CSVRDK  LNEDKK**T**CSATEE  YLQRLN**T**KLDDIL  EALLTE**T**VRSQTH  ETVRSQ**T**HSLPAP  APPRPQ**T**PENGPE  AKRQQE**T**AAAETE  ETAAAE**T**ETRTHT  TETRTH**T**LTRVNL  TRTHTL**T**RVNLES  SGPLPE**T**HKFGEG  GVSSPK**T**HLGEAL  RRQAEA**T**RQAAAQ  EEMDPQ**T**IDSLIE  SLIELS**T**KLHLPA  QQVGDL**T**FKKGEI  DTNGSE**T**GFRAWN  WDATEG**T**IRSRPS  VLSNIH**T**VRATWQ  KQDVNK**T**AWELPK  AWELPK**T**YVSPTH  GLLDVF**T**PASSLR  WMHYNQ**T**VDIWSV  EMLTGK**T**LFKGKD  DYLDQL**T**QILKVT  TQILKV**T**GVPGTE  VTGVPG**T**EFVQKL  DVDKRL**T**AAQALT  TAAQAL**T**HPFFEP  RDPEEE**T**EAQQPF  LEHEKL**T**VDEWKQ  GPPSHK**T**VVNGKE  NRSIAL**T**QARYLE  SIRVVV**T**VEQTEE  VVTVEQ**T**EEELER  ELLFRA**T**PEEKEQ  CPGNRD**T**YFHLLQ  DRRDEA**T**ALWWRL  WRLEAQ**T**KGSHED  ALRLCP**T**HADSLN  VNMNGY**T**KGARNE  FMDYII**T**DQETSP  PVIPMN**T**IAEAVI  QEVFEK**T**KRLLFL  SPKPGP**T**GAPRAA  CGQCVR**T**CWGCGS  CGSVAC**T**LCGLVD  PSSTFD**T**MSPAPV  AKSATW**T**YSPLLK  YCQIAK**T**CPIQIK  IQIKVS**T**PPPPGT  KKAEHV**T**DVVKRC  VGTEFT**T**ILYNFM  VSGSHC**T**PPPPYH  SLVSFL**T**GLGCPN  YHLQNL**T**IEDLGA  QGHDYS**T**AQQLLR  HFRVRH**T**ITIPNR  RVRHTI**T**IPNRGG  LPHLTR**T**LATSCR  VTLGDL**T**KIGKSQ  GKSQKF**T**LSVDVE  DSQEDW**T**TFTHDR  LDEVTV**T**IPHDIY  GGLKEL**T**DLDYRP  RISHVS**T**SSVKTG  DISLRF**T**HLFWFG  ERGSRD**T**YAWHKQ  VKTASR**T**KFFIEF  FIEFYS**T**CLEEYK  DQHLLL**T**VKSMDG  ASRKEG**T**GSTATS  QEEGQG**T**EVVQGV  VGWYQS**T**YYGSFV  NTYMRN**T**SKQQQQ  QPFQNL**T**LHPASS  FPGGSV**T**PVSLLA  NIFVYW**T**HEDGVL  FRVVER**T**ITMKQL  KDHRIS**T**FKNWPF  KQFEEL**T**LGEFLK  KKEFEE**T**AKKVRR  NQLAKE**T**DKYIKE  EFGSLP**T**TPSEQR  FGSLPT**T**PSEQRQ  QRKSRK**T**LCIIIL  KKEMQV**T**EAPVTQ  AAGPVG**T**EPTVET  QAAPTS**T**KKKKKK  ERGHTV**T**EPIQPL  CSHLKL**T**QVPDDL  LDVGFN**T**ISKLEP  KTFAFC**T**NLTELH  LQKNLI**T**SVEKKV  TLAAFP**T**TCLERA  LAAFPT**T**CLERAK  KKIRSA**T**KLASRA  DAAQGK**T**RTLMEK  YVSESV**T**SLLEHL  CHMLRG**T**IDPKEP  DPKEPS**T**YEYVKF  RVCFVA**T**VRLATP  ATVRLA**T**PQFIKE  EPNEEF**T**SRHSLE  QWIWLQ**T**HYYITY  RFDHSP**T**PSASSR  PVMSQA**T**NLPIPQ  IQSGMN**T**GHIGTT  TSLPSQ**T**QSTLTA  QLSRHR**T**DSLPDP  SMVRTQ**T**ESSTPP  TQTESS**T**PPGIPG  GPAMDG**T**AAEPRP  NKVPYV**T**RERDVM  SFDETC**T**RFYTAE  TCTRFY**T**AEIVSA  DMHIQI**T**DFGTAK  QITDFG**T**AKVLSP  VSPELL**T**EKSACK  QTPPKL**T**AYLPAM  SLSASD**T**GLPQRS  VHTPNR**T**YYLMDP  GLLDYK**T**EKYVMT  LETWDP**T**YFKHCR  LIPTAV**T**LGTGAA  TAVTLG**T**GAAWLG  CTISIS**T**FLLLCL  EVQLFM**T**DNGLRD  LWLIPI**T**FLTIGY  LEKQID**T**LAGKLD  VIQGLS**T**LMHSAY  KTGDKL**T**ILIKKI  EHTCQV**T**SFLAFS  GPVYSH**T**LAFKLQ  SFKPCM**T**RHERSL  GSALNQ**T**TESSQS  SEEGRD**T**LGSSTN  DTLGSS**T**NLHNHS  GIALVH**T**DSYDPD  QESLGN**T**VFELEN  SQVDQE**T**GFNRHE  TENNQM**T**SESGAT  PHRFSG**T**EKDQSS  SDESWE**T**LPGKDE  LVLEDH**T**AIGQEQ  IKDDIA**T**ELPCHH  RSATKV**T**ADVINA  VDLEAA**T**RKGILV  RQIPQA**T**ASMKDG  SAFSPH**T**KPWIGL  APPAAA**T**ISAAGP  PQHHEL**T**SLFECP  KLSCCP**T**CRGALT  FPCKYA**T**TGCSLT  PCKYAT**T**GCSLTL  TTGCSL**T**LHHTEK  SLTLHH**T**EKPEHE  HAHKSI**T**TLQGED  AHKSIT**T**LQGEDI  IVLLIG**T**RKQAEN  GNRRRL**T**WEATPR  RLTWEA**T**PRSIHD  FDDDAD**T**GKKKKK  GKKKKK**T**RGDHFK  GFPSPY**T**CVSCGA  CGARYC**T**VRCLGT  TVRCLG**T**HQETRC  EYNCAL**T**EKKYVT  NVPELP**T**PDEDNK  QTLLEA**T**TSSDQC  TLLEAT**T**SSDQCA  LSDLET**T**TSHMPR  ASATSA**T**LELDRL  VCGGCS**T**ALGGSS  FHPDHF**T**CTFCLR  EPLPYY**T**AAHAMS  LLVAGG**T**VLKICD  ILWEVI**T**RRKPFD  EIVKIM**T**HLMRYF  MEQVPA**T**NDTIKR  VPATND**T**IKRLES  VESLPP**T**SEGKRM  PSVRMI**T**TSGPTS  ITTSGP**T**SEKPTR  WTPDDS**T**DTNGSD  PDDSTD**T**NGSDNS  EKDQQN**T**SRLVQE  QIPVLQ**T**NNGPSL  NNGPSL**T**GLTTIA  WLEYRV**T**QVDGHS  SKNDIH**T**LLKDLN  AFCDHK**T**TPCSSA  IINPLS**T**AGNSER  DYELVS**T**KPTRTS  RQLLDT**T**DIQGEE  SKVQES**T**KGPDEA  VQPGIG**T**EVFVGK  RQASRS**T**AYEDYY  DSKRRQ**T**NNQQNW  EGQTSE**T**TAIVSI  PLPPSP**T**TVSSVA  KVQQLM**T**KEKRRP  KYLDSD**T**EKELQA  SHLGRI**T**VENEVD  YVEDVY**T**VDAAYY  AFFATR**T**IRAGEE  PVDMES**T**RMDSNF  VQRQTT**T**VVAGIK  LFRVLD**T**PYLLNE  DAQTIK**T**IHQQRV  VYDDVE**T**IPMKNM  QLGTDA**T**KEKPKE  VCVEHH**T**FYRLVS  PEASPV**T**QAGASV  SVITVE**T**VIQENV  SAGNVS**T**HPSLSQ  AQTPED**T**PNKSGA  SGAEAK**T**QKGSTS  SHPELQ**T**PKDSTG  SGAEAQ**T**PEDSPN  SGAEAK**T**QKDSPS  GSEAQT**T**KDVPNK  SGAEKQ**T**PKDGSN  ELPKAD**T**NQLADK  SPHAFK**T**ESGEET  VKSSEP**T**EDVEPK  FFAYLV**T**AAILVA  NLGCRG**T**LSDEHA  QQAAKL**T**SDPTDI  KLTSDP**T**DIPVVC  QKHDGI**T**VAVHKM  LRPGAP**T**SLQTEP  APTSLQ**T**EPQGNP  SPGPFA**T**RSPLFI  GYFSFD**T**DRSPAP  PGSGPA**T**AAGGLS  GGLSGG**T**QALLQP  DNSNKY**T**KSRGRA  DELDAW**T**DFRSRT  SPIMAS**T**ELDEVQ  SVSKPC**T**VELPRL  NLNDGL**T**ENLMDD  SSFPYT**T**KGSGLG  QENKPA**T**FSSMSH  GSSLYS**T**SANLPV  FDSLIS**T**QNVVGL  IGLLAK**T**PLSRPV  GDKTPK**T**WEGRLI  AWRYYA**T**NPNRID  TKGKLF**T**PLNVDA  SSEDAG**T**GDPMAE  TPGPPS**T**PKHKKS  YYPTKG**T**SSPAEA  SGKVQA**T**PPSSAT  TPPSSA**T**TYVERP  TYVERP**T**VLPILT  RQRRSI**T**RDSDTP  TDTDPF**T**PSGSMP  YILEIV**T**IVVFGV  QGNVFA**T**SALRSL  HSKELV**T**AWYIGF  GRLLAA**T**FTLIGV  LLAATF**T**LIGVSF  ATNLSR**T**DLHSTW  RTDLHS**T**WQYYER  YYERTV**T**VPMYSS  CPCEFV**T**EDLTPG  RMGIPP**T**ETEAYF  PRQGHG**T**SPVGDH  NLRDSD**T**SISIPS  QELKTV**T**YSLLKR  LDLSDS**T**LSYTET  SMSPDA**T**KPSHWC  IFVNSP**T**LDAPGG  FQTDPA**T**DCALRG  FAAVMC**T**VMRCLG  FDSGHD**T**DGNLII  YSQYLS**T**DKLIRI  VWGPSW**T**RLTPVS  PSWTRL**T**PVSLPP  STPATS**T**AAPSAP  SAPALS**T**PGIRDS  VGMCEE**T**DASFNV  FDERDK**T**SRNMRG  SFEQVL**T**DITEAI  SPISTP**T**SPGSLR  LFGRRK**T**PEELLR  VSLKIQ**T**LKSNNS  QAMKGV**T**KAMGTM  ELGLSL**T**DELSNL  DQRLQR**T**EVVKKH  CQKELN**T**LCSELH  QEEGET**T**ILQLEK  FRQHVT**T**LRETKA  VCEGLR**T**QIRELW  FCAEDY**T**ESLLQL  LKNKKQ**T**ETEMLY  KARKLN**T**TTMSNA  RPIFGG**T**VYHSPV  TCSGKK**T**PRTGRH  GKKTPR**T**GRHGAN  SLSDSS**T**VGLQRE  VLNQMI**T**SKSNPG  APCLPV**T**YQQTPV  VPLKAQ**T**VTDSMF  NTSSFH**T**TPNTSL  FHTTPN**T**SLGMVQ  NMFQAP**T**LPDISD  KPTGAR**T**FGERSV  GIRCNK**T**LAPSPK  KSPGDF**T**SAAQLA  ACRLTD**T**DAAIAE  VSSYPN**T**FEWQCK  FTAKCE**T**SGFQCV  LFSALW**T**FLWFVG  VGFCFL**T**NQWAVT  SVRAAI**T**FSFFSI  FTQNAE**T**TEGYQP  ENPAEE**T**GEEKQD  PDKNLV**T**GDHIPT  TGDHIP**T**PQDLPQ  RKSSLV**T**SKLAGG  PLTLER**T**INLYPL  TNYTFG**T**KEPLYE  LLQLGT**T**FFKLPG  GLKRLM**T**EILGRQ  TPPELE**T**KMNITK  SKDVNT**T**IMELLI  EVVVTN**T**IPHEVQ  QCPKIK**T**VDISMI  LAVILH**T**DSRKDS  PTSDKE**T**SVALHL  PDQRKV**T**CEGGNG  EAASKE**T**IKKTTK  ETLGKG**T**YGKVKR  GKVKRA**T**ERFSGR  RLSERE**T**RHFFRQ  LGVLLY**T**LVYGTM  YTLVYG**T**MPFDGF  GEYREP**T**QPSDAR  DWHHRS**T**GLQADT  TGLQAD**T**EAKMKG  GLAKPT**T**SEVMLE  SKYSAG**T**MDPALV  SSFSLL**T**DMDDVT  TDMDDV**T**QVYKQA  VDASGE**T**TYKKTT  ETTYKK**T**TSSTLK  KKTTSS**T**LKGAIQ  ERSPSE**T**SEQPRY  GTSPSA**T**QPNPAV  PEEADG**T**DTHLGL  VDEVML**T**LKQAFS  LKQAFS**T**AAALQS  ALQSAK**T**QIKLCE  SHPPSS**T**KRKLNL  SLPSLH**T**SFSAPS  FSAPSF**T**APSFLK  VEFLKN**T**LPDMNT  TLPDMN**T**SEMEKI  NLLTRE**T**KMKSLI  KMAYQK**T**VEQLRK  AETVAD**T**RRLITK  DTRRLI**T**KPQNLN  DVSNPQ**T**VGVGRG  VGRGRF**T**TYEIRV  YEIRFY**T**GNDPLD  DRYISW**T**EQNYPQ  KSKGKK**T**ARAPII  TTQQER**T**GDQQEE  QKIPGM**T**LSSSVC  TGFRNV**T**ICPNPE  CPNPED**T**CDFARA  TSEDQQ**T**ACGTIY  SASVAS**T**SSIKCL  QHSEYI**T**HEITVL  MNGVFD**T**TFQSHL  NGVFDT**T**FQSHLN  NYIRTV**T**ASSFPF  RDNALT**T**IHFIPS  SCSGDQ**T**PSENPS  LQLAWE**T**ELCWDV  LGPGRG**T**RDKKKG  VSQYLY**T**SKAGMS  RVLEAM**T**ERAEMD  GLKSGT**T**IALKVG  FQILLN**T**VKDSKA  EKQQIA**T**EKQDLE  SSLPGG**T**AIPPPP  LCKLRD**T**KSTDQK  MHSNME**T**LYKELG  NAEGDE**T**GVMDSL  LLASEL**T**KDDAMA  NSETFP**T**ILEEAK  ANPAFE**T**TMGYQS  SSPMPV**T**EALDRV  AALIAA**T**IHDVDH  PVFDRN**T**CSIPKS  LQSAFY**T**EKFEVK  PTLQRP**T**HMNQMV  YGQLHE**T**EVLLKV  IHRDLA**T**RNILVE  FCNRLF**T**SAEETF  KHIDCH**T**TSISDL  DFTLKI**T**RTSMCT  ALKGKV**T**VHKSKK  PAQLVY**T**TNIQEL  AQLVYT**T**NIQELN  QDLGIA**T**VPKGHC  ILDKVL**T**AMNQTW  PLMANG**T**LTRRHQ  SNNYIQ**T**LGRDFR  HALHAL**T**DEVIIP  EWESVL**T**NTAGCL  LIHVLQ**T**DRSDSE  LLQQLC**T**ILMATG  QGSKIQ**T**RVGLLM  ELEGVI**T**KAIYKS  VRTTHY**T**PLACGS  TLDPKV**T**GCLIVC  PLEKLL**T**SHKRLV  DGDSDT**T**KKKKKK  NKSEDS**T**KDDIDL  TVAVKP**T**ENNEEE  KEGKLL**T**KSQREA  GRHFEA**T**DILVSK  AESDWD**T**VTVLRK  LRKKGP**T**AAQAKS  HSITKN**T**AKLDRE  LHHDRV**T**LEVGKV  LCKVDE**T**PKGWYI  KKRTAR**T**DYWLQP  EVIDKY**T**AVVKMI  KLKLDQ**T**HLETVI  AESGPG**T**RLRNLP  PPPVQA**T**PHPFPA  EPPPKK**T**KKNNSS  QKQEPK**T**PVAPKK  KYITSF**T**AAFSFS  IFQVAN**T**RPSPLT  NGKLYQ**T**SVDFHL  SKSPNR**T**DLDIHI  KSGDGI**T**FGVFGS  FTVNNW**T**EECTHL  MVSVKV**T**IKTICA  APVNTT**T**YVADTE  EVLKVQ**T**HLENPT  ALQMAN**T**LPVSGN  RIKELG**T**LIPKSN  LSLIPS**T**GLCSPD  KQEDRQ**T**PRSPDS  RANQQE**T**EQFYFT  TEQFYF**T**KMKEYL  HDLLQK**T**LGESQR  ALAVST**T**PAVLPT  TAAVLD**T**EAPTPR  STLPLG**T**TAPGPT  PETFLT**T**IRDEPE  IQKKLY**T**LEEHLS  QNSQIS**T**EKVNQL  EAEHKA**T**KARLAD  TQKRCL**T**QNDLKM  EIKVSY**T**VYNRLS  KSLLAI**T**RVTPAY  LAITRV**T**PAYRLS  RTAGED**T**GVIYPS  KELRSR**T**FPSAED  NLEFSD**T**RMSSFV  KALRPP**T**SPGVFG  STTPAS**T**TPASTT  AELSPE**T**LCNGQL  HQSGAL**T**MEALED  DEIESL**T**LRLSEE  LNGQII**T**LSIQGA  SLLEKM**T**SSDKDF  IVDTLC**T**NMRSDK  PHVPNV**T**SLCLQY  RLRNEI**T**RLPAIK  GSIANL**T**ELQGVI  AMSNTP**T**HSIAAS  TTTAAN**T**SLMGIK  SVQLPS**T**LSTQSA  SRGSCS**T**EVEKET  TEVEKE**T**QEKMTI  ETQEKM**T**ILQTYF  KMTILQ**T**YFRQNR  AVLEYL**T**AEILEL  NKKGRV**T**PRHILL  QLLKGV**T**IASGGV  AASADS**T**TEGTPA  ASADST**T**EGTPAD  TPADGF**T**VLSTKS  GFTVLS**T**KSLFLG  EAIINP**T**NADIDL  KDDLGN**T**LEKKGG  EELLEK**T**VKNCLA  NGFPKQ**T**AAQLIL  SSYFVS**T**MSSSIK  AFQQSG**T**AADEPS  SVAQTV**T**QLQTTL  AKATTS**T**NWILES  PVSNES**T**SSSPGK  LQDEAM**T**EGSMGV  PPDLKE**T**PGTLSS  PFKPTT**T**LTSSDF  AGIEKE**T**CQKMEE  LYSVKD**T**YLQQKW  SFKLDS**T**PLKAAQ  HGNRPS**T**TVRVHR  KSPAAV**T**EPETNK  PPGTGK**T**LLAKAV  LAKAVA**T**ECKTTF  VATECK**T**TFFNVS  ATECKT**T**FFNVSS  MDGVGG**T**SENDDP  YSGADI**T**NVCRDA  RRIEGL**T**PEEIRN  EEMHMP**T**TMEDFE  HRNDHL**T**STTSSP  DHLTST**T**SSPGVI  FDSEEE**T**VEDSLV  EDSEIG**T**CAEGTV  NTERPD**T**PTNTPN  DYNPNK**T**NRPFIS  PSESNV**T**ILGRFD  PHKAKC**T**TLTHHK  REWLKD**T**CGANAK  YKKHRR**T**MIISPE  SLRWDE**T**PGRAKG  GKTPIG**T**PAMNMA  MNMATP**T**PGHIMS  TGFHMQ**T**EDRTMK  PLLMSP**T**LEDQER  VKQCCG**T**DGVEAN  KAIRRA**T**VNTFGY  PHDVLA**T**LLNNLK  QNRVCT**T**VAIAIV  DYIYAV**T**PLLEDA  YNDDKN**T**YIRYEL  LLADVE**T**FVADIL  FAGPPD**T**ISLASE  WCALSK**T**VFYYYG  QGLWDC**T**GAFSDE  TFTFRV**T**VGDITC  VTVGDI**T**CTGEGT  WRLPEY**T**LSQEGG  ENHISL**T**NVVGHS  SGFMYW**T**DWGEVP  LLLARR**T**DLRRIS  VTRLNG**T**MRKILI  RRISLE**T**NNNNVA  NVAIPL**T**GVKEAS  MDGSER**T**TLVPNV  QDYIYW**T**DWSRRS  LNADNR**T**CSAPTT  KGYMYF**T**NLQERS  QQFTCF**T**GEIDCI  ELDCYP**T**EEPAPQ  SSNSPS**T**HRSYSY  LLKVLG**T**GAYGKV  KISGHD**T**GKLYAM  VQKAKT**T**EHTRTE  TTEHTR**T**ERQVLE  VERPGV**T**NVARSA  LIQGLL**T**VDPNKR  SGAAVH**T**CVKATF  KKTSTS**T**ETRSSS  SHSHGK**T**TPTKTL  HSHGKT**T**PTKTLQ  KTTPTK**T**LQPSNP  DSNNPE**T**LFQFSD  LSQESR**T**RAGGVG  RVPAPV**T**MDSFFF  LTMLCL**T**EGAKDE  GRHQIV**T**MSNDVS  GPACLK**T**QEHPSC  LLSQGA**T**LSGWYH  GKFSEG**T**AGDSLS  HSGRPF**T**TYDADH  PDWASY**T**LGVFIC  SFYYRP**T**PSDCQL  SRKFVL**T**EREGAL  IEHLNA**T**FQPAKI  PHGLQV**T**YLKDNS  YLKDNS**T**RNIFIY  EGYMEK**T**GPKQTE  SKESGY**T**VLHGFP  HGFPPS**T**QGHHWP  HWPHGI**T**IVTPDR  HGITIV**T**PDRKFL  FLFACE**T**ESDQRE  CVCSRG**T**VIIDNK  PFFKRG**T**LWNEIE  DKGNFL**T**EDQILW  HRDLKP**T**NILLGD  WAAQRC**T**ISYRAP  CVIDER**T**DVWSLG  LLNSMM**T**VDPHQR  EAHRTS**T**SALSPG  EEENQA**T**EDKRTE  KRPETP**T**HDPDPE  RFSEKV**T**VHFLAV  EDDKCV**T**SELLKG  INGNIK**T**VTEYKI  RTFRIE**T**RKASKA  PGPNVA**T**TTVSDD  GPNVAT**T**TVSDDV  PNVATT**T**VSDDVS  SDDVSM**T**FITSKE  RCPYKD**T**LGPMQK  LEPVQA**T**QNKTGK  RADDNA**T**IRVTNL  NATIRV**T**NLSEDT  LAKDKT**T**GQSKGF  KVGGGG**T**AGGDRW  LELAKE**T**FGVNNA  SSRDDF**T**EFGKLL  LLKDKI**T**QYEKSL  DDLKKI**T**NSLTVL  KITNSL**T**VLCSEK  GGGLKA**T**MKDDLA  NLLYYQ**T**NYLVVA  VVVLVF**T**GFVWAA  KKRYPT**T**FVMVVM  EGINRL**T**DYISKV  RKHELL**T**KALHLL  RFCLSE**T**SCPQED  PGYLPP**T**KNGVEP  QFKEDG**T**WAPMRS  ALVEEE**T**RRYRPT  TRRYRP**T**KNYLSY  GQKNDI**T**AWQECV  NYEIER**T**IVQLEN  RPKVSS**T**KVTDWV  VSTITA**T**SLGVNN  KIEEVE**T**WLKAQV  ESLGEP**T**QATVPE  SEEMAD**T**LEEGSA  EGSASP**T**SPDYSL  KRPKPK**T**LHELLE  LLNGYQ**T**LEDFKE  RQQARK**T**ASVLSK  PESGDT**T**VKKPES  EIQKHA**T**GKKSPA  KKDARQ**T**PKKPEA  ADGTGP**T**KGDMEI  RQLSSE**T**DLERAV  FIVSNL**T**IDFFAF  TGFFTV**T**SYLSLS  TRRALA**T**EVTAGL  LKVLGQ**T**FITVPV  FTSLEL**T**KIAVTV  LTKIAV**T**VAVCSV  PRAWKE**T**NMARTQ  ETNMAR**T**QILCSH  LEGHRV**T**WTGRFK  ACSPGN**T**STAEEE  RLGQFM**T**LALVLA  LALVLA**T**FDPARG  FDPARG**T**DATNPP  ARGTDA**T**NPPEGP  RLSLQN**T**AEIQHC  LHGICM**T**FLHNAG  AAAQEN**T**RVIVEM  LVNLLL**T**CGEEVK  EVKEAI**T**HSVQVQ  SILSFC**T**SAIQKP  AIQKPP**T**APPERQ  QPQVDR**T**KLSRAH  INKDDV**T**ALHCKV  RSLSED**T**DGTEED  DQIGKG**T**QGATAG  KGTQGA**T**AGASSE  EETALI**T**RPGAPS  KDYAVS**T**VPVADG  HVLLHR**T**PEEYPE  KVDGLL**T**CCSVLI  KRVRIN**T**GKNTTL  PEAFSG**T**VIEKEF  AHPALP**T**IPERKE  LEASEE**T**GKRVSK  HGDARA**T**GREMHG  GRNVTV**T**DVDIVF  KGKSCR**T**ITFEQF  KSCRTI**T**FEQFQE  PIISGV**T**KAISSP  KAISSP**T**VSRLTD  PTVSRL**T**DTTKFT  VSRLTD**T**TKFTGS  SRLTDT**T**KFTGSH  TDTTKF**T**GSHKER  GYKHAG**T**YDQKVQ  KRRSKD**T**CIVISG  ESGAGK**T**EASKYI  RPLTAA**T**LFKNSM  GFAFRQ**T**YEKFLH  DESGIP**T**AIRTTV  PTAIRT**T**VDRPKD  DLPGSS**T**TLTKSF  VGTSRR**T**KFFGTF  WIFAHV**T**QGQDPW  EVENVW**T**TLADGW  ASTQEM**T**VHLLSK  KTYGGD**T**GSPEIS  GACSSF**T**TSSSPT  STPKRH**T**VLYISP  NFSNMS**T**SSYNDN  LENQKG**T**GVKKSP  KAYNPE**T**LTTIQT  NPETLT**T**IQTQDI  FSSTEP**T**VHTRPD  HPQSEN**T**LSNQQQ  VNLFSS**T**KSMMSV  LFSNQN**T**MATMAS  QSESSQ**T**PLFHSS  NCSQLL**T**SGPATL  PRLSFS**T**KPTVLA  KWNVSQ**T**KIRIIS  NHNQEL**T**PCRRTL  IYLNGL**T**PHCAGE  KQEPVK**T**EMGPPP  TTAATG**T**GQGPST  TIPGPS**T**EPSVVE  DIFECR**T**CGLLES  EKCKCK**T**LIAGQK  DILLLD**T**LLGTLV  DEPLER**T**TNSSHA  EPLERT**T**NSSHAN  RSGTIS**T**SAAAAA  EEKLIP**T**WNWMVS  LAEIGL**T**ESEGPP  NSLEDL**T**AEDFRL  TSVKLS**T**GCTGTL  KTYVKG**T**QKLRVG  WIRVKR**T**HVPKGW  CDVKDE**T**YDLLYQ  VVEAMK**T**LLLKER  ILKESL**T**SFYQEE  LAASAS**T**QQLQEV  KAELNS**T**TEELHK  NLQSFG**T**EEPAYS  EEPAYS**T**RRVTRS  RSSGSE**T**EQVVDF  DFSDRE**T**KNTADH  SPPRTP**T**GNAPSS  NSLGHL**T**GKHERH  PLLENL**T**SEYDLD  GRYELD**T**WYHSPY  FLFYVM**T**EADNTG  MTEADN**T**GCHLIG  NVSCIL**T**MPQYMR  KEISQE**T**AVNPVD  FVRYWI**T**EFWVMF  HCRLID**T**TQINAR  CRLIDT**T**QINARD  CVKENP**T**MERSIA  QLQNFN**T**LMAVIG  KVLGEM**T**ELLSSS  DLVHLL**T**LSLDLY  PKPDPK**T**ISKHVQ  PHNFQE**T**TYLKPT  TTYLKP**T**FCDNCA  KNPVAP**T**ENNTSV  LEQEIN**T**LKADND  HHDRCC**T**YNENNL  KHELGI**T**AVMNFQ  AYIWMP**T**PDMSTE  AGVGRS**T**AAVCGW  GTQGKL**T**CKFKST  KFKSTS**T**TGGLTS  DYTGCS**T**SESLSP  RKSPSD**T**EGLVKS  IRVQLT**T**CIHHII  PTTAAQ**T**LLFTAC  NYVTVD**T**DTLLSD  QLQDIA**T**LADQRR  PSVSAL**T**NGFDTP  EFKEMT**T**CVFPRL  EKRLKE**T**PFNPPK  FPKEWK**T**SDLYQL  EGRASG**T**VYSGEE  DSCGCV**T**SGGTES  RAISRN**T**AMLVCS  YQFFVD**T**DWQGGI  DGFNLT**T**SVSMML  TTMSIL**T**GLFPPT  AGEEEW**T**TAPVPQ  FQNGNW**T**MQNPSP  TISEPK**T**YVDLTN  LTNEET**T**DSTTSK  EETTDS**T**TSKISP  ETTDST**T**SKISPS  SMFSLI**T**WNIDGL  HEEGYF**T**AIMLKK  IIPFPS**T**KMMRNL  NELCLM**T**SHLEST  TSHLES**T**RGHAAE  VIFAGD**T**NLRDRE  CQYTWD**T**QMNSNL  NSNLGI**T**AACKLR  TARGVE**T**CGILCG  DQQGLI**T**LGWIHT  HTHPTQ**T**AFLSSV  SSVDLH**T**HCSYQM  QQGQEA**T**CSLVLQ  CSLVLQ**T**DVTRAE  CGSDGA**T**YRDECE  AGSCAG**T**PEEPPG  PKPLSP**T**SYMSPT  WEMEAK**T**DRDLCK  FRNQRI**T**SPVHVS  PCPQRS**T**LMPAAP  ERSAQG**T**SAPLLP  GESIGQ**T**SDISSP  AQGASA**T**VLEETR  IMQSEA**T**KTLCGL  VRSIAL**T**PQVCGA  QFSDMR**T**CRVCPL  VVTESQ**T**YKKRAD  SIHPGV**T**CDGCQM  GQQGNG**T**TTVNRK  QQGNGT**T**TVNRKP  FDRQCS**T**ERRHDP  PLLSLF**T**GYELET  ENYINR**T**VAVITS  RTVAVI**T**SDGRMI  GRMIVG**T**LKGFDQ  LKGFDQ**T**INLILD  DAQSLT**T**CEVCGA  LRQMGV**T**EWYVNG  PLGHSP**T**ASPPPT  VPRPPQ**T**SLVKFV  ILQICS**T**KGMMAG  KENWPK**T**LKLALE  LEDKME**T**SDIQIF  TIICAP**T**GCGKTF  FDECHN**T**SKQHPY  PQVIGL**T**ASVGVG  DASVIA**T**VKHNLE  KALFLY**T**SHLRKY  YHLNPE**T**ITILFV  LNPETI**T**ILFVKT  TILFVK**T**RALVDA  NQNTGM**T**LPAQKC  SQVNAV**T**VLTLLD  QASTSN**T**VSKLLE  NKSLEE**T**LHTVDL  KIKKSL**T**SNHQKI  RQMRPK**T**FPASNY  PPPPLN**T**SPMNPP  NVVPAG**T**VNRQPP  PPPYPL**T**AANGQS  PSATTV**T**AITPAP  TTVTAI**T**PAPIQQ  LKPELQ**T**ALAPTH  FYIAEL**T**CAVESV  TDFGLC**T**GFRWTH  AHSLVG**T**PNYIAP  LLRTGY**T**QLCDWW  KVINWQ**T**SLHIPP  DSNPEF**T**FQQPYD  PEILNP**T**ASLPML  PRVAEL**T**SLSDED  PSSFSS**T**SVSSLE  MHIRSH**T**LPCVCG  LPCVCG**T**CGKAFS  LQGHVR**T**HTGEKP  LRAHLQ**T**HSDVKK  CQACAR**T**FSRMSL  WASSSG**T**PTVPPL  SSSGTP**T**VPPLQP  HVLGDR**T**YCKAPG  CKAPGP**T**HGSVYP  LLAEAP**T**LPLIEE  NRTIGA**T**MVRLVK  YQLVRA**T**VVYRHH  IAPPFY**T**PNYCKG  GRSQVS**T**PRFGKT  PALPKA**T**RKALGT  GTVNRA**T**EKSVKT  FSAKKM**T**EKTVKA  FEHRVH**T**GFDQHE  SGPDVG**T**PQPAGL  AGRPFN**T**YPRADT  SDSILL**T**HDGRVK  SSSSTS**T**MPNSSQ  EPLLKR**T**DKYRTY  NGTFVN**T**ELVGKG  FVFFDL**T**VDDQSV  EYIMSK**T**LGSGAC  SKILGE**T**SLMRTL  VLVSVG**T**AGYNRA  SLKDQI**T**SGKYNF  DPKARF**T**TEEALR  SEENES**T**ALPQVL  VLAQPS**T**SRKRPR  EAEGAE**T**TKRPAV  CKTAVL**T**AFANGR  FANGRS**T**GLILDS  LQVSDS**T**YDEQVA  LIANNT**T**VERRFS  AKSAEI**T**GVRSET  TGVRSE**T**ISVPQE  LWKEIE**T**RHPGLA  HKVYDL**T**KFLEEH  LNKPPE**T**LITTID  PETLIT**T**IDSSSS  SSSSWW**T**NWVIPA  VGEGVT**T**VKPGDK  DGTRRF**T**CRGKPI  EVLKEM**T**DGGVDF  SLDALI**T**HVLPFE  MSWIAD**T**YASTIG  LNAGGV**T**VSYFEW  VSYGRL**T**FKYERD  ARQIMR**T**AMKYNL  WGTVFK**T**LKSLYK  FRVFHC**T**QYIRHG  GSKPMY**T**PEPDIC  VRYDPY**T**QRIEVL  GSIKGL**T**EGLHGF  HEFGDN**T**AGCTSA  DNTAGC**T**SAGPHF  HCIIGR**T**LVVHEK  KLISVD**T**EHSNIY  AIYPDN**T**TDFQRA  VVYRQY**T**DSTFRV  LEMFPR**T**PGIWLL  IYPHGI**T**DVRPLY  QEAKYE**T**FSDDPS  GSLLQG**T**EGAIKW  IIHGIK**T**QGARQK  TRLSTE**T**GFALLG  KQSDLD**T**LAKEAS  GTKLLL**T**NDPLEA  YQVTMK**T**AKVAAS  YEDYKN**T**AEWLLS  YPLWKV**T**FPVRVF  RQRALS**T**WKQMGE  AGPSFE**T**VAECRV  YCNDQS**T**GDIKVI  GGDDLS**T**LTGKNV  DDLSTL**T**GKNVLI  TGKTMQ**T**LLSLVR  AKTGQA**T**VASGIP  EAFARG**T**KALMDE  DEVVKA**T**SRGCIT  TSRGCI**T**IIGGGD  GYTHLS**T**GDLLRS  RRIGQP**T**LLLYVD  VDDNEE**T**IKKRLE  ETYYKA**T**EPVIAF  RNPDSS**T**TGPWCY  EAVEEE**T**GDGLDE  ATSEYQ**T**FFNPRT  CLPDRE**T**AASLLQ  PNNFET**T**TVITVP  TVITVP**T**GYRVKL  GWKLRY**T**TEIIKC  AVRDPN**T**DRWVAT  LLSAEC**T**VFLDHE  ENTERT**T**EFWKQY  RVSVSQ**T**SKLTRA  SQTSKL**T**RAETVF  DNITQS**T**QSFNDF  AAHCVE**T**GVKITV  IEETEH**T**EQKRNV  VLNSYV**T**PICIAD  PLVDRA**T**CLRSTK  ATCLRS**T**KFTIYN  SGGPHV**T**EVEGTS  GKYGIY**T**KVSRYV  NILARV**T**RANSFL  EGFCGG**T**ILSEFY  NRFTKE**T**YDFDIA  VTRFKD**T**YFVTGI  RGTMSK**T**KNGITC  YRGTSS**T**TTTGKK  GTSSTT**T**TGKKCQ  YILQGV**T**SWGLGC  CWVISA**T**HCFIDY  PEQLKM**T**VVKLIS  SLQGRM**T**LTGIVS  QGRMTL**T**GIVSWG  IALPAN**T**AEGLLN  KKYQQQ**T**VVAIDL  AVDILK**T**ERLGHG  ANYSLN**T**DDPLIF  SPVDIK**T**SETKHD  DIKTSE**T**KHDTSL  VSYNPA**T**AKEIIN  TYPGSL**T**HPPLYE  GFWDCV**T**CHGSPV  SPVDIC**T**AKPRDI  ANSRFA**T**TFYQHL  NSRFAT**T**FYQHLA  FGDKSL**T**FNETYQ  KWVSNK**T**EGRITD  KTEGRI**T**DVIPSE  EAINEL**T**VLVLVN  RRVAEG**T**QVLELP  FKGDDI**T**MVLILP  KVEKEL**T**PEVLQE  LNPNRV**T**FKANRP  LGTKAD**T**HDEILE  DSQLQL**T**TGNGLF  TVNFGD**T**EEAKKQ  KLSITG**T**YDLKSV  AQLLLS**T**VVGVFT  RFMQAV**T**GWKTGC  FWVDNS**T**SVSVPM  VYDQSA**T**ALHFLG  SEKTVL**T**PATNHM  LKDSIT**T**WEILAV  ADPFEV**T**VMQDFF  TPVAQM**T**EDAVDA  APSRAC**T**CVPPHP  RAKFVG**T**PEVNQT  DGLLHI**T**TCSFVA  GTHCLW**T**DQLLQG  DVELGR**T**TCTKTQ  VELGRT**T**CTKTQP  LGRTTC**T**KTQPNL  AVPWQG**T**MTLSKS  ITEATK**T**VGSDTF  PVQSGK**T**WQDCEY  ISDFPD**T**TSPKCP  SEINPT**T**QMKESY  NAQDFC**T**DLAVSS  NFIPTV**T**AISTSP  TVTAIS**T**SPDLQW  SVAPSQ**T**RAPHPF  RAGVVK**T**MTGGRA  GVVKTM**T**GGRAQS  NRRREL**T**DTLQAE  DTLQAE**T**DQLEDE  SSMELK**T**EPFDDF  RPSGSE**T**ARSVPD  LCTPVV**T**CTPSCT  TSSFVF**T**YPEADS  PSYVAV**T**PFSLRG  GGGSFS**T**ADQLEM  DQLEMV**T**ELLGGD  CDPDDE**T**FIKNII  GHSVCS**T**SSLYLQ  LVLHEE**T**PPTTSS  HEETPP**T**TSSDSE  EETPPT**T**SSDSEE  KRCHVS**T**HQHNYA  SNNRKC**T**SPRSSD  SPRSSD**T**EENVKR  VILKKA**T**AYILSV  VGKSAL**T**IQLIQN  CDLAAR**T**VESRQA  GIPYIE**T**SAKTRQ  IETSAK**T**RQGVED  AGLILL**T**WCVEGC  VGQLAE**T**QRFECT  TQRFEC**T**THQPRS  QRFECT**T**HQPRSP  LGCFVG**T**AEALRC  NDESCV**T**EPECRE  SDRSNA**T**QLDGPA  PDLSAE**T**PMFPGN  GDEQPL**T**ENPRKY  EFKREL**T**GQRLRE  TSEKSQ**T**PLVTLF  AARCQV**T**LRDLFD  HGRGFI**T**KAINSC  VEIEEQ**T**KRLLEG  SQVHPE**T**KENEIY  HQLAFD**T**YQEFEE  PSNREE**T**QQKSNL  LEEGIQ**T**LMGRLE  TYSKFD**T**NSHNDD  SPADPA**T**LSEDEA  RCGNLS**T**CMLGTY  STCMLG**T**YTQDFN  DFNKFH**T**FPQTAI  EQAFLR**T**VQALLS  NSSMLP**T**LSDTYI  QGDHFG**T**SPRTWF  RVEAAA**T**WYYSLE  QRSLQD**T**EEKSRS  HSQGTF**T**SDYSKY  ERHAEG**T**FTSDVS  FSDEMN**T**ILDNLA  SVLFSQ**T**SAWPLY  ALAEND**T**PYYDVS  HADGVF**T**SDFSKL  HSDAVF**T**DNYTRL  FFFVIL**T**LSNSSH  SPPPPL**T**LRMRRY  YADAIF**T**NSYRKV  LGLSGL**T**LALSLL  HYINLI**T**RQRYGK  KRSSPE**T**LISDLL  LLMRES**T**ENVPRT  PDAPLG**T**GANRDL  DVADNG**T**LFLGIL  IQKSVE**T**IKEDMN  LTNYSV**T**DLNVQR  LSPAAK**T**GKRKRS  LDNQIV**T**ATQSNI  CDEDSA**T**ETCYTY  EDSATE**T**CYTYDR  DRNKCY**T**AVVPLV  PPSVSG**T**PGQRVT  GGSSNG**T**GNNYVY  YQQLPG**T**APKLLI  SGSKSG**T**SASLAI  FLVAAA**T**RVHSQT  NPNSGG**T**NYAPRF  QGRVTM**T**RDASFS  NFDYSY**T**LDVWGQ  SVKGRF**T**ISRDNS  RDNSKN**T**LFLQMD  SLRPED**T**GVYFCA  KQPLTL**T**CTFSGF  SGFSLS**T**SRMRVS  DKFYWS**T**SLRTRL  NPVDTA**T**YYCARV  FSNDYY**T**WVRQPP  YVFYHG**T**SDDTTP  VTMLVD**T**SKNQFS  SVTAAD**T**AVYYCA  MWAKIS**T**QADTIG  ISTQAD**T**IGTETL  QADTIG**T**ETLERL  RFPADF**T**AEAHAA  DLFPTS**T**SLSPFY  APSWFD**T**GLSEMR  VDPLTI**T**SSLSSD  VSGPER**T**IPITRE  PSQRRA**T**RSGAQA  FSQHAR**T**SGRVAV  TALINS**T**GEEVAM  ASSVTV**T**RSYRSV  RVKDLA**T**VYVDVL  HVDALR**T**HLAPYS  EYHAKA**T**EHLSTL  ATEHLS**T**LSEKAK  WAALLV**T**FLAGCQ  RALMDE**T**MKELKA  GRVRAA**T**VGSLAG  EEMGSR**T**RDRLDE  ATVLLL**T**ICSLEG  VSQYFQ**T**VTDYGK  KSKEQL**T**PLIKKA  LIKKAG**T**ELVNFL  GTGGTA**T**WKPGSS  SSVSGS**T**GQWHSE  NNPDWG**T**FEEVSG  TLSGIG**T**LDGFRH  PAKAAA**T**QKKVER  YCRTPC**T**VSCNIP  GFGNVA**T**NTDGKN  NGMFFS**T**YDRDND  HQVENK**T**SEVKQL  QEPCKD**T**VQIHDI  FGHLSP**T**GTTEFW  KIHLIS**T**QSAIPY  ANQNNG**T**SSQDTA  GTSSQD**T**AVTDSK  GSKYLA**T**ASTMDH  QKSHGR**T**QDENPV  QHSSLE**T**QLFCEQ  ALSPAI**T**FGGLLG  LVLMAG**T**FFFAMM  WRMHLF**T**GIQIIC  LCFLVL**T**SLSHAF  VCLHFY**T**ELSSTR  DIGYSF**T**VGGSEI  SPDEIN**T**IYLGGP  GAPGIR**T**GIQGLK  IPGIKG**T**KGSPGN  SGRFVC**T**VPGYYY  SLGFCD**T**TNKGLF  PDGQPG**T**PGIKGE  SGDYKA**T**QKIAFS  KIAFSA**T**RTINVP  NVFLQA**T**DKNSLL  PCVLPF**T**YNGRTF  TTTSTS**T**PVTSNT  WVSASD**T**VSGFRV  QESPKA**T**GVFTTL  ATLDQI**T**GKWFYI  FLREYQ**T**RQDQCI  VQRENG**T**ISRYVG  ILRDTK**T**YMLAFD  PWGYKH**T**LNQIDE  IDTLET**T**CHVLDP  CHVLDP**T**PVARCS  VPLPPS**T**YVEFTV  STYVEF**T**VSGTDC  EFTVSG**T**DCVAKE  CVAKEA**T**EAAKCN  YGFCKA**T**LSEKLG  EVAVTC**T**VFQTQP  FQTQPV**T**SQPQPE  ANEAVP**T**PVVDPD  HPRKTR**T**VVQPSV  VSEAGP**T**GTGESK  PFASGK**T**SESGEL  YKVEID**T**KSYWKA  PYSYST**T**AVVTNP  VTEFAK**T**CVADES  CDKSLH**T**LFGDKL  KLCTVA**T**LRETYG  EVDVMC**T**AFHDNE  RYKAAF**T**ECCQAA  KLVTDL**T**KVHTEC  LAKTYE**T**TLEKCC  ALLVRY**T**KKVPQV  KEFNAE**T**FTFHAD  FNAETF**T**FHADIC  FHADIC**T**LSEKER  KHKPKA**T**KEQLKA  LDSYQC**T**AEISLA  QFVQEA**T**YKEVSK  FQTKAA**T**VTKELR  TRKMAA**T**AATCCQ  CCLIFL**T**LSGIQG  GVPLSR**T**VRCTCI  SRTVRC**T**CISISN  RVEIIA**T**MKKKGE  LDSTDF**T**GTIKLL  GHAHLG**T**GDPYTP  STCRMV**T**SESKNV  WGSGSH**T**LPALLE  VSQPEA**T**KCFQWQ  WNVPIG**T**LRPFLN  AFIRES**T**VFEDLS  RSDTSL**T**WNSVKG  YLQASY**T**YLSLGF  EDEWGK**T**PDAMKA  LCDFLE**T**HFLDEE  KMGDHL**T**NLHRLG  ALAACF**T**PGESQR  MESAKE**T**RYCAVC  PGFVDL**T**LHDQVH  DQSHLA**T**AGSTSS  LATAGS**T**SSHSLQ  VLGLGL**T**PPTLAQ  LGLTPP**T**LAQDNS  QDNSRY**T**HFLTQH  RYTHFL**T**QHYDAK  FPATLE**T**QEQDVD  FFGLKV**T**GKPDAE  VAQFVL**T**EGNPRW  NPRWEQ**T**HLTYRI  WEQTHL**T**YRIENY  LMYPSY**T**FSGDVQ  ACDSKL**T**FDAITT  LTFDAI**T**TIRGEV  SFGFPR**T**VKHIDA  ALSEEN**T**GKTYFF  EENTGK**T**YFFVAN  YKFDPK**T**KRILTL  LICFSY**T**QLLRAL  QQQESA**T**TQKAER  QQESAT**T**QKAERE  LDLRLV**T**IPSFFS  VIASVF**T**NGLVLA  WPHGLK**T**SCGPDV  MIVLMV**T**CCITPL  FFAKSA**T**IYNPVI  SCCTDY**T**AECKPQ  VGGPSL**T**SDLQAQ  KGNPEQ**T**PVLKPE  IDSRPE**T**LHPGRP  KPFDAF**T**DLKNGS  INCQGK**T**YLFKGS  LLFWGR**T**SAGTRQ  GRTSAG**T**RQPQFI  RRPSRA**T**WLSLFS  DWLVPA**T**CEPIQS  YRVNLR**T**RRVDTV  RTRRVD**T**VDPPYP  HLAQPA**T**LSQTIV  NQAGQE**T**LVTGWG  VASFHG**T**WFLVGL  HNYGVY**T**KVSRYL  YRQLLL**T**ADDRVN  AGTNGE**T**TTQGLD  TNGETT**T**QGLDGL  LKPNMV**T**PGHACT  GKPLDE**T**LKKALT  AMKGLG**T**DEDTLI  EILASR**T**NKEIRD  GERRKG**T**DVNVFN  AICGMS**T**WSKRSL  SFINKD**T**ETINMM  INKDTE**T**INMMSE  CCHVGC**T**KRSLAR  GMVAQV**T**QTLKLE  AEAVLK**T**LQELKK  GQPQCS**T**HILQWL  SGGNTS**T**DHFSLR  GLYMGE**T**ETKVMG  AVSAAP**T**EKEFPK  QHLKGQ**T**GTNGGN  SGDDNL**T**SLGTLN  SRAVCG**T**SRQLAP  LAKGDV**T**AQIALQ  KFKEKL**T**AASVGV  DPLQGT**T**GLIPLL  SAISSS**T**DPTSYD  SSSTDP**T**SYDGFG  GVRELC**T**RHQVLF  LPSDVV**T**AVRGKG  PGSPSK**T**RGQIQV  CTHDRN**T**MEALPA  MANAGK**T**VIVAAL  FREAAY**T**KRLGTE  PSCSTS**T**MPGMIC  HGRGPP**T**AGSTAQ  NTKAVT**T**FTITVR  KAVTTF**T**ITVRPK  NYQRRT**T**SSSTRR  LLQQVD**T**STRTHN  ELSQMQ**T**QISETN  YEELQI**T**AGRHGD  RIGRLV**T**RAAFNS  MFQYDS**T**HGKFHG  HGKFHG**T**VKAENG  EYVVES**T**GVFTTM  STGVFT**T**MEKAGA  ISNASC**T**TNCLAP  SNASCT**T**NCLAPL  IVEGLM**T**TVHAIT  ITATQK**T**VDGPSG  VSVVDL**T**CRLEKP  KGILGY**T**EHQVVS  SDTHSS**T**FDAGAG  RNDQVV**T**DLRLWM  CAGLLM**T**LKGLPS  LKGLPS**T**YNKDLQ  AVFMAE**T**KGVALN  DPLNNT**T**PVTGAS  CKGPLP**T**DCCHEQ  QLQVFE**T**LEEITG  ALIHHN**T**HLCFVH  LNRKNV**T**CWAEND  EPAANE**T**VRHGCL  LRLNQP**T**HVNNGN  VNNGNY**T**LLAANP  GGGGGG**T**AMRILG  EPPPPR**T**HYSNIE  DGFGID**T**CRSMVA  DTDRSG**T**ICSSEL  PAPAAP**T**PAAPAP  SVPSQK**T**YQGSYG  GTAKSV**T**CTYSPA  AKSVTC**T**YSPALN  FCQLAK**T**CPVQLW  KQSQHM**T**EVVRRC  EVGSDC**T**TIHYNY  NRRPIL**T**IITLED  PILTII**T**LEDSSG  ELPPGS**T**KRALPN  LDGEYF**T**LQIRGR  SKKGQS**T**SRHKKL  VSYKLC**T**RSGNED  FGNGRV**T**EFKYGA  YGAKLG**T**VIRKWN  VDGQPF**T**NWYDNG  FAPDEL**T**VKTKDG  DELTVK**T**KDGVVE  PPGVDP**T**QVSSSL  PMPKLA**T**QSNEIT  VKDKET**T**ENENTK  KRDHHS**T**HYRASE  QGDLQD**T**KENREE  EEENEL**T**LNEKNF  RALEVP**T**DGNAGL  DSDPSG**T**KTCIDT  DPSGTK**T**CIDTKE  HTVAKE**T**CSEKST  TCSEKS**T**NLHDYG  WWGGAD**T**DYADGS  TSIATT**T**TTTTES  IATTTT**T**TTESVE  SQSLLK**T**TQEPLA  PVKLPT**T**AASTPD  LTETKT**T**VELLPV  ATVIVI**T**LVMLKK  EIEKIP**T**TFENGR  ATPVNL**T**EPAKLE  ALVYSC**T**CIIQLF  VKKMTV**T**DQVNCP  SIQNLG**T**KLATQM  TQMRKL**T**SNLRIG  PCYDMK**T**TCLPMF  KHVLTL**T**DQVTRF  DAIMQA**T**VCDEKI  NHYSAS**T**TMDYPS  NLIFAV**T**ENVVNL  GLKIGD**T**VSFSIE  SLIVQV**T**FDCDCA  YYCNCT**T**RTDTCM  PGSYGD**T**CEKCPT  VKELKD**T**GKDAVN  CQKLNF**T**GPGDPD  DSIRCD**T**RPQLLM  DDIMDP**T**SLAETQ  LSPQKV**T**LYLRPG  GSFVDK**T**VLPFVN  RLLVFA**T**DDGFHF  TSRMVK**T**YEKLTE  HNALPD**T**LKVTYD  FCSNGV**T**HRNQPR  KNCECQ**T**QGRSSQ  CQCERT**T**EGCLNP  AAIVGG**T**VAGIVL  LKKLLE**T**ECPQYI  KELDIN**T**DGAVNF  SDKKVQ**T**KGKRGA  EVANQE**T**KEDLPA  PAENGE**T**KTEESP  ENGETK**T**EESPAS  GLGPGS**T**VLLVVD  NKGRSS**T**YEVRLT  TYEVRL**T**QTVAHL  EVRLTQ**T**VAHLKQ  SEMPEL**T**SMHTLL  ELTSMH**T**LLLREH  LRGLMA**T**PAKLNR  TVGQLG**T**VLRNLK  SGFNEE**T**CLVKII  RAVQMS**T**KVLIQF  ITTPDP**T**TNASLL  QWLQDM**T**THLILR  LHDDEV**T**VTEDKI  DDEVTV**T**EDKINA  GGPAPS**T**AAAPAE  ILKQSM**T**LNLADP  KNSDLL**T**SPDVGL  SNGHIT**T**TPTPTQ  TTTPTP**T**QFLCPK  LCPKNV**T**DEQEGF  ELHSQN**T**LPSVTS  NTLPSV**T**SAAQPV  LKEEPQ**T**VPEMPG  NSELAS**T**ANMLRE  QGQEAQ**T**ELPQAR  ISCPEG**T**NAYRSY  FNEDRE**T**WVDADL  GYCVSL**T**SSTGFQ  NIQMRR**T**LHKAFK  GDLDDQ**T**CREDLH  LRNKEV**T**WEVLEG  HDENGA**T**GPVKRA  HKENTV**T**NDWIPE  LKDQVN**T**FDNIFI  SKYEIT**T**IHNLFR  LFKHQG**T**ITVNEE  EDRHDG**T**SNGTAR  APPLSH**T**PNADFQ  QICKEF**T**DLLAQD  GIQSCL**T**HFNLIS  RFFKQP**T**FCSHCT  TFCSHC**T**DFIWGF  HKFKIH**T**YSSPTF  HTYSSP**T**FCDHCG  QGMKCD**T**CMMNVH  VPSLCG**T**DHTERR  LCGTDH**T**ERRGRI  SESKQK**T**KTIKCS  NPEWNE**T**FRFQLK  VPEEKT**T**NTVSKF  LHSCFQ**T**MDRLYF  PVELTP**T**DKLFIM  IASSGL**T**VEVDAP  SSNSMQ**T**IQKTTT  MQTIQK**T**TTRRIV  GKVVSE**T**NDTKVL  ELETLP**T**TKMAQT  MAQTNP**T**PGSLGP  GKRMEF**T**SSCPNV  WIGYEH**T**SFCGQQ  SSEESI**T**EYKQKV  VPKAKD**T**VYTKGR  AKDTVY**T**KGRVMP  PVLKSP**T**IPFFDP  PQIPKL**T**DLENLH  PQPIPQ**T**LALPPQ  ELLLNP**T**HQIYPV  HQIYPV**T**QPLAPV  GLVPVV**T**GIRGRP  LKGGRG**T**QGPPGA  DPGVHA**T**LKSLSS  LSSQIE**T**MRSPDG  DIRNNL**T**RLHELE  SGLRHF**T**GYRIEL  SVRIRA**T**SLAGNG  KDGVFT**T**SSDMWS  PKPKNA**T**VLIWIY  CPALEF**T**KKFSEW  ETQNNS**T**SWPVFK  WPVFKS**T**EQKYLT  LAAGAL**T**QPVPPA  LRVSQR**T**DGESRA  RKAALK**T**ASDFIT  TASDFI**T**KMDYPK  WRDPDQ**T**DGLGLS  AKRYIE**T**DPANRD  TEEPDF**T**ALCQKL  KEIDTS**T**KVDNAM  FSKLER**T**CELIYL  PSSSIS**T**EINSAL  LKVSWI**T**FLLAKG  LKEPYK**T**AVIPIN  KQLEND**T**RIIEVL  ATYSRS**T**SQNLDS  PKKKGS**T**TRVNST  QKPLKS**T**SLSLFY  AYLRLN**T**LCERLL  FMQRLK**T**NILQYA  PPAAPA**T**QRVLSP  ALSQRF**T**FSPGQD  YAGHDN**T**KPDTSS  VLLLLN**T**IPLEGL  SDAAVD**T**SSEITT  DTSSEI**T**TKDLKE  EEAESA**T**GKRAAE  EDDDVD**T**KKQKTD  LERNIE**T**IINTFH  IETIIN**T**FHQYSV  KLGHPD**T**LNQGEF  IMEDLD**T**NADKQL  MLMARL**T**WASHEK  EPETTP**T**PNPPTT  TTEEEK**T**ESNQEV  DKIAIW**T**TECENR  SHADTA**T**KSGSTT  DSRGNP**T**VEVDLF  GLELLK**T**AIGKAG  SHRSGE**T**EDTFIA  CTGQIK**T**GAPCRS  LALPYD**T**PVPGYM  QKTFAY**T**NHTVLP  FAYTNH**T**VLPEAL  NPKAWN**T**MVLKNI  LRNRSN**T**PILVDG  YTGKTI**T**DVINIG  MDQHFR**T**TPLEKN  DGSAQV**T**SHDAST  GITDML**T**ELANFE  SSINFL**T**RVSGIG  VDEGIK**T**LEDLRK  FTINEY**T**IRPLGV  HQLSLR**T**VSLGAG  IKVTLA**T**LKMSVQ  KMSVQP**T**VSLGGF  PSPEQA**T**ANFFRV  PISLDL**T**FHLLRE  AASYTP**T**PRSPRF  KCHHLV**T**KFDPDL  WKEVYT**T**LKGLYA  EIEKLS**T**LYWFTV  EVRHLK**T**KPSDEE  GHYKQA**T**VGDINT  TVGDIN**T**ERPGML  PGMLDF**T**GKAKWD  GKHFKF**T**ITAGSK  CELETM**T**GEKVKT  TGEKVK**T**VVQLEG  LNGDII**T**NTMTLG  VAEEEA**T**VPNNKI  DKDYSV**T**ANSKIV  IKLKGY**T**NWAIGL  RIHPVS**T**MVKGMY  KYGVAS**T**CRKTNK  RNAAPR**T**PAAPAS  YLKALD**T**RMAAES  EEAQCP**T**LHFLEG  LRGHLM**T**VRSSVA  VTGDNN**T**SYSRWA  ADCDPN**T**QASCEC  DDGFIC**T**DIDECE  AEFDFR**T**YDSEGV  NKHCLV**T**VEKGSY  IRPSTG**T**GVMLAL  LLSVEN**T**VIYRIQ  SIKRLV**T**TGVLKQ  IKRLVT**T**GVLKQT  SVAFKK**T**KKEIKK  EIKKVA**T**PKKASK  VFFRKH**T**QAEGKK  VGWVQN**T**DRGTVQ  QNTDRG**T**VQGQLQ  MQEWLE**T**RGSPKS  RQQLRK**T**YSEQWQ  DVPPCV**T**FDESLL  SVQHTL**T**SVTDEL  DELAVA**T**EMVFRR  RRQEMV**T**QLQQEL  QDDRHS**T**SSSEQE  RLIGVC**T**QKQPIY  QGGDFL**T**FLRTEG  ARLRVK**T**LLQMVG  QVPVKW**T**APEALN  GILLWE**T**FSLGAS  NLSNQQ**T**REFVEK  KPGATV**T**LRCVGN  NATFQN**T**GTYRCT  NLQFGK**T**LGAGAF  SSQGVD**T**YVEMRP  SWSSWT**T**CDPCQK  EVEDCV**T**NRPCRS  GFVCAQ**T**GRCVNR  SGINLF**T**NSFEGP  PHYILN**T**RFRKPY  ESYTPQ**T**QGKYEF  TKRFSH**T**KSVFLH  LFRDFG**T**HYITEA  FGTHYI**T**EAVLGG  MERGDY**T**LNNVHA  PLYELV**T**ATDFAY  DFAYSS**T**VRQNMK  FPTKAH**T**TPWGLF  SITFSK**T**PKSTTE  EPTPSP**T**TSEPVP  PIPTIA**T**SPTILV  LPPGTA**T**LLTLLL  PASPIS**T**IQPKAN  HRAEAT**T**LHVAPQ  HVVVAE**T**DYQSFA  LPAGGG**T**VLTKMY  GGGTVL**T**KMYPRG  LMGKKS**T**GESSSV  KFERLQ**T**VTNYFI  WIVSGL**T**SFLPIQ  TCCDFF**T**NQAYAI  IIMGTF**T**LCWLPF  FSMDLR**T**KSTGGA  STGGAP**T**FNVTVT  TFNVTV**T**KTDKTL  NVTVTK**T**DKTLVL  SFPDFP**T**PGVVFR  GKLPGP**T**LWASYS  VDDLLA**T**GGTMNA  LLATGG**T**MNAACE  EEMGGI**T**QTPYKV  KVSISG**T**TVILTC  GTTVIL**T**CPQYPG  IVDICI**T**GGLLLL  YLARVA**T**TAGLYG  SNLMEH**T**EIDHWL  THALRT**T**EYHDRD  DTGEKL**T**VAENEA  MSLIIN**T**FYSNKE  IRYESL**T**DPSKLD  SAGGSF**T**VRTDTG  GSFTVR**T**DTGEPM  RNPDDI**T**NEEYGE  ELLRYY**T**SASGDE  YYITGE**T**KDQVAN  VSNRLV**T**SPCCIV  CCIVTS**T**YGWTAN  SLEDPQ**T**HANRIY  DPTADD**T**SAAVTE  DTSAAV**T**EEMPPL  SNVTNK**T**DPRSMN  EMYGSV**T**EHPSPS  QRVSGN**T**SRRGKS  AIKKEL**T**QIKQKV  KYRPEN**T**PEPVST  HYGAEP**T**TVSPCP  DYEART**T**EDLSFK  LQTELV**T**KGRVPY  YFSFFN**T**SFPACS  ELCFPE**T**RPSFRI  SISENR**T**MQLAVL  ISSAEM**T**FRRPAQ  HSMDEM**T**AVVKIE  TGSSSS**T**GGGGQE  TGELDL**T**ATQLSQ  ELDLTA**T**QLSQGA  SSGATP**T**SKEQSG  ANQQII**T**NRGSGG  NVLSGQ**T**QYVTNV  ALNGNI**T**LLPVNS  SYSTTT**T**TSNMGI  TNSQGQ**T**PQRVSG  SMPGLQ**T**INLSAL  GSEGSG**T**ATPSAL  ALSLWP**T**SGEICG  VMQVAN**T**TMSSRS  MQVANT**T**MSSRSR  MVAEDF**T**VKIGDF  YGPARE**T**NSCTEG  LQTVIR**T**SPSSLV  SLSHTG**T**TVSRRV  LSHTGT**T**VSRRVG  SKNAEV**T**GTMSQD  LSDSGS**T**GEHTKS  TQYLNA**T**GNRWCS  SQARLL**T**SFLPAQ  IIAMST**T**GDKILD  EKSVVG**T**SSLRRA  SAIILA**T**AGLQRM  QETMQA**T**IHVPAQ  AALAVV**T**LCYESH  NRRNAN**T**FISPQQ  FDLRDE**T**RNVGSQ  TEEAEK**T**PVGSCF  GFSSVV**T**QAGELV  FDGDLN**T**TEYVVG  EGPPVT**T**VLTRED  PVTTVL**T**REDGLK  EAGINP**T**DHLITA  ERAAAS**T**DYYKRG  LKGSTL**T**SPCQDF  KFARKS**T**RRSIRL  IFNHCF**T**GNCVID  NLFEII**T**ADEVHY  NFIYFL**T**VQRETL  ITGKKI**T**KIPLNG  APLEGG**T**RLTICG  YVHVNA**T**YVNVKC  YDQEVG**T**SYYAVA  VVVRAD**T**DGGLIF  IYTAGK**T**YGLVPA  VAFVRH**T**TVFDNT  VCSRDP**T**PEQTHR  LGAQNH**T**LQRLQQ  QWVKEA**T**EGKIQE  ANLSWD**T**LHPPLV  EGRNCE**T**HKDDQL  CSDHTG**T**KRSCRC  AQLCGG**T**LINTIW  STYVPG**T**TNHDIA  LVLLLL**T**VQVGVT  TVQVGV**T**AGAPWQ  ASCSEV**T**RSAGCG  AACGVA**T**ARCARG  QPLHAL**T**RGQGAC  PESPES**T**EITEEE  PESTEI**T**EEELLD  SKALHV**T**NIKKWK  HSRQCE**T**SMDGEA  SSGGLP**T**DEITFA  LPTDEI**T**FAKLLK  LQIVGV**T**LLTLAA  QLCCPS**T**GLSCQC  KFLAAG**T**HLGGTN  GTHLGG**T**NLDFQM  VISSRN**T**GQRAVL  LKFAAA**T**GATPIA  AAATGA**T**PIAGRF  GRFTPG**T**FTNQIQ  PRLLVV**T**DPRADH  ADHQPL**T**EASYVN  SYVNLP**T**IALCNT  VLRMRG**T**ISREHP  AAEKAV**T**KEEFQG  APTAQA**T**EWVGAT  RGNLWA**T**GHFMGK  SPSPLG**T**APHTSL  LGTAPH**T**SLRDQR  ETRPNH**T**IYINNL  RIQYAK**T**DSDIIA  PGMPPM**T**QAPRIM  NHILFL**T**NLPEET  GDVEDV**T**PRPGGC  MAAGSI**T**TLPALP  LASKCV**T**DECFFF  YRSRKY**T**SWYVAL  WKEEVV**T**VETWQE  FQDGDL**T**LYQSNT  LRHLGR**T**LGLYGK  QNQGGK**T**FIVGDQ  AHGDAN**T**IVCNSK  DGGAWG**T**EQREAV  VAEVCI**T**FDQANL  FDQANL**T**VKLPDG  SIIVKM**T**DSFTEQ  KMTDSF**T**EQADQV  LPVTLD**T**PMNRKS  LEFLVE**T**FHDWIT  LEEELK**T**VTNNLK  EELKTV**T**NNLKSL  KLKEAE**T**RAEFAE  FAERSV**T**KLEKSI  AGRCPP**T**TWLGSL  GRCPPT**T**WLGSLL  ETSCQI**T**FEFVDQ  QDIMED**T**MRFRDN  DKACVR**T**FYETPL  VRTFYE**T**PLQLLE  WEDSEG**T**EGSSLL  QRPPRS**T**CQSFEP  IPVPQG**T**ELSPSR  NHTPQK**T**DHPSAL  SVPLTD**T**GHERQS  PEGSPL**T**QDDRQV  NLTGLD**T**GEYFCT  RSYICK**T**TIGDRE  EFEVVS**T**LRLQHV  QLPYDS**T**WELPRD  RRHSDG**T**FTSELS  HSDGTF**T**SELSRL  ENSMAW**T**RLSAGL  WMPLDG**T**WSPWLP  DGSVLL**T**CDAEAK  AEAKNI**T**WFKDGK  KMIGFL**T**EDKKKW  RASDKQ**T**LLPNDQ  CSGDVF**T**ALIGEI  NEYWVL**T**AAHVVE  ISGWGR**T**EKRDRA  VKVEKP**T**ADAEAY  LKKVCS**T**NDLKEL  SDAYYC**T**GDVTAW  CMVKTQ**T**PNRKEW  RKYVKN**T**HATTHN  RRKELE**T**RQKQYR  FDKANA**T**GGGGHV  VWEAIR**T**FTAEVV  QLSEYL**T**VVIFTA  QKVFTN**T**WAVRIP  CQGPAL**T**DCLSCP  LDPVEQ**T**CSRQSQ  EGRGER**T**AFIKDQ  FTRRTL**T**GTAALT  SALQWL**T**PEQTSG  ILPCQD**T**PSVKLT  HDQAVR**T**YQEHKA  DSLSRT**T**NIQGIN  RGLESQ**T**KLVNGQ  KGHVEY**T**MEANED  ERLCRS**T**RQRAAC  LTGKTI**T**LEVEPS  KTLTGK**T**ITLEVE  IFVKTL**T**GKTITL  MQIFVK**T**LTGKTI  PRVIVG**T**IRPRVI  RPPPDG**T**PRPQLA  ASPRPR**T**PVGSPW  PRVVVG**T**PRPRVI  QDEHSG**T**RAEGSR  ALSPAF**T**FPHPIN  LIQPSP**T**FLAQQP  RDSSTS**T**VSSAYT  CPGMTT**T**MSPHAC  VKCSTR**T**DVSEKA  AVASST**T**SNEDES  SPHRNP**T**AASESP  LSMISA**T**RGLSPT  RPWPAL**T**TVLLAL  PWPALT**T**VLLALL  KRDGPD**T**LLSKTF  DTLLSK**T**FFPDGE  RCQCIK**T**YSKPFH  GPHCAN**T**EIIVKL  VKPPQF**T**WAQWFE  TQHINM**T**SQQCTN  IHCNLT**T**PSPQNI  NCRYAQ**T**PANMFY  FGSEGG**T**YYIKEQ  IVTKYI**T**KGWKEV  KALSVE**T**EKLLKY  VREHLL**T**NHLKSK  SHFPNR**T**DQQCQY  LVKGPW**T**KEEDQK  KMLPGR**T**DNAVKN  ELAAAT**T**SKEQEP  EGSPPE**T**SLPYKW  IPISPS**T**EVGGSG  VALSPV**T**ENSTSL  LKPLPQ**T**PHLEED  VKLMMS**T**LPKSLS  SLSLPT**T**APSNSS  KPRSHF**T**TPAPMS  PRSHFT**T**PAPMSS  LSLLGP**T**FPGLSS  YLGGTS**T**ISDNAK  TLELPS**T**LSLYKS  GQESDF**T**APDVWY  QLPGFT**T**LTIADQ  PGFTTL**T**IADQIT  PEQDTM**T**FSDGLT  KMLMKI**T**DLRSIS  SSQPGH**T**PHPAAS  RTSPLQ**T**PAAPGA  PPVVHL**T**LRQAGD  SSQLHL**T**PFTARG  LHLTPF**T**ARGRFA  ARGRFA**T**VVEELF  NIALWM**T**EYLNRH  LNRHLH**T**WIQDNG  SWLSLK**T**LLSLAL  LVGACI**T**LGAYLG  FCLLGI**T**CAIPVK  YPDAVA**T**WLNPDP  NDFKQE**T**LPSKSN  SDDVDD**T**DDSHQS  ESDELV**T**DFPTDL  LVTDFP**T**DLPATE  PTDLPA**T**EVFTPV  PATEVF**T**PVVPTV  FTPVVP**T**VDTYDG  VVPTVD**T**YDGRGD  GKDSYE**T**SQLDDQ  RRNSVT**T**GYGGVR  TPSSGA**T**PRNRLL  PTMQAG**T**IARWEK  RDVPLG**T**PLCIIV  KHYGVF**T**CEGCKS  RRNLSY**T**CRSNRD  ARLLFS**T**VEWARH  GKTPIE**T**LIRDML  KQIESK**T**AFQEAL  VVDFSA**T**WCGPCK  EVKCMP**T**FQFFKK  MEDHAG**T**YGLGDR  KDQGGY**T**MHQDQE  PTAEDV**T**APLVDE  AAAQPH**T**EIPEGT  EAAGHV**T**QEPESG  DGRPPQ**T**AAREAT  PSLGED**T**KEADLP  KKAKTS**T**RSSAKT  KGQANA**T**RIPAKT  AKSRLQ**T**APVPMP  KSKIGS**T**ENLKHQ  LSNVSS**T**GSIDMV  NLQKAG**T**RTDSRE  ISAEVY**T**EEDAAS  VNNEWA**T**SVGEGG  RILMGS**T**LRKRKM  LCAGQV**T**ALPVNS  PMNKGD**T**EVMKCI  VEVISD**T**LSKPSP  QLAKEL**T**AEKRLE  KQNEWI**T**EKAEAT  WIYMNR**T**FTDKWE  TEGGTY**T**FLVSNS  RLSFGK**T**LGAGAF  ADAVAV**T**MGPKGR  EAGDGT**T**TATVLA  TDALNA**T**RAAVEE  VASLLT**T**AEVVVT  KEPDPK**T**WKANFR  GDSSPD**T**FSDGLS  EPGVQP**T**SVYGDF  SLQRVF**T**DLKNMD  LNHLKA**T**PIESHQ  EKRKCN**T**ATCATQ  NTATCA**T**QRLANF  GAILSS**T**NVGSNT  ATNTCG**T**PPEEYC  SSCNPV**T**GQCECL  SAAADA**T**EAKNKA  QLGQLD**T**VDLNKL  RIINEP**T**AAAIAY  IFEVKS**T**AGDTHL  VLVGGS**T**RIPKIQ  SAVDKS**T**GKENKI  SNGDTP**T**HEDLTK  KEIEFG**T**SLDEEI  SLSSAE**T**QPAQEE  PQSKAE**T**ELKASQ  HTFFRL**T**STDTIP  IEVTVP**T**SNGDQT  TSNGDQ**T**QKLAEK  THSPFR**T**LNINGQ  GPPLVK**T**QTVTIS  TETKTI**T**YEAAQT  VLLTAQ**T**ITSETP  PSSTTT**T**QITKTV  SYSETV**T**CEDAQK  CEDAQK**T**CPAVIA  RIKKWL**T**FSLGKQ  EAFLGI**T**DEKTEG  VVVSEA**T**IVGVRK  DRVTRS**T**LVLHDL  PRRQSM**T**VKKGEH  SCVKLQ**T**VHSIPL  EVVQKK**T**FTKWVN  KAITAA**T**LKFTEG  DYGKDL**T**SVLILQ  EMKTLM**T**QIDGVN  KVIQGQ**T**DPEYLL  INDKLL**T**SQDVSY  DELQAT**T**KEKTQH  ATTKEK**T**QHLSAA  FQLKRE**T**DDLEQW  VGLDAS**T**AESFHR  ALEKPT**T**LELKER  QEEEGE**T**AGEAPV  EPHEPL**T**PPFSAL  SHSRNA**T**RSHSHS  GELPNG**T**RVPMEV  DLFDFI**T**ERGALQ  GALLKD**T**VYTDFD  LKDTVY**T**DFDGTR  YTDFDG**T**RVYSPP  RPSDRP**T**FEEIQN  VLLPQE**T**AEIHLH  AVLVTA**T**LCTARP  LAESNR**T**RITGEE  TAGVNT**T**DKEMEV  SPSFPD**T**RSSTCS  KAEEVA**T**FFAKML  MLDHEY**T**TKEIFR  TAKVFR**T**YNASIT  KQIALG**T**SKLNYL  MSCIRV**T**IDPENN  TKFTVE**T**ASREYK  KKMFKQ**T**WMDNMG  PTFDSQ**T**KENMTL  PPKDDH**T**LKFLYD  REYHTD**T**TVKFVV  KLQTSL**T**CNSMVL  QKREPG**T**KTKKQT  TKTKKQ**T**TLAFKP  KTKKQT**T**LAFKPI  SPPKTK**T**SPKLSN  KPDPAK**T**KNRRKR  QVALSR**T**QVCGIL  YARSRL**T**VADIRK  EPFFKA**T**PEEKLK  VYTKMM**T**KKPGMF  APGPAP**T**RCLPGH  SNECEI**T**KRRRKA  AGGPRK**T**AAPVNA  LPLLRQ**T**AGKVLA  AIVRAM**T**TLNIQY  LRSRVK**T**TGIIET  HRYFAT**T**SIVLFL  FVFDAV**T**DIIIKE  MVNGPT**T**PIPVKA  LATRVR**T**HDLKKI  AGLLKP**T**ACTMLV  AFDCTA**T**MKSGNS  PPRSDP**T**TDQRMS  QTSSSG**T**VTLPAT  SSSGTV**T**LPATIM  LGDGSL**T**VLNAFS  KALPRV**T**PFYAVK  VKTLAA**T**GTGFDC  FGATLR**T**SRLLLE  ENMGAY**T**VAAAST  AMGVNL**T**SMSKIL  KEEEAV**T**IEMNEP  NEPVQL**T**FALRYL  PLSSTV**T**LSMSAD  FLSGFP**T**THAQQQ  ALQINN**T**AVGHAL  AAKPAA**T**KPATTK  YHGSFS**T**KKSQPP  VIRYFP**T**QALNFA  GGAAGA**T**SLCFVY  VSYPFD**T**VRRRMM  DIMYTG**T**VDCWRK  YVPESI**T**TLGFCF  YDDPYK**T**DVRTNI  DIPEDT**T**YKKVVF  IRVIPK**T**WNQSIA  TTTPTA**T**VMPTTQ  KKLKSV**T**PVGTEE  PLPPRL**T**IHAPPQ  TTNETA**T**GDDGDE  ECMVVK**T**YLISSI  AFNYKY**T**ACLCDD  CDDNPK**T**FYWDFY  FYWDFY**T**NRTVQI  MLISIL**T**ERSNAQ  LIEILT**T**RTSRQM  EILTTR**T**SRQMKD  DLLDIR**T**EFKKHY  LRDFEA**T**LLQMFG  LVHHNV**T**RWETFD  GLAIEV**T**HLHQTR  KACCVP**T**ELSAIS  DEKAIM**T**YVSSFY  LEWIRR**T**IPWLEN  VENQIL**T**RDAKGI  DFMSRE**T**ADTDTA  VAEEFF**T**SLELSP  LALSVS**T**PEHLHK  TTYSVA**T**VCHPNG  VFDGSI**T**KENYNQ  CTGRQR**T**AYFSLD  DRESFP**T**YTLVVQ  GEGLST**T**ATAVIT  IQYNDP**T**QESIIL  QSEDEL**T**PFDMSI  KASRAR**T**KAGDLR  KRKMPF**T**EKIMAT  TEKIMA**T**PEQVGK  CMDVGF**T**MSNSIP  DVIQHE**T**IGKKFE  RVKKTW**T**VVDAKT  PPAEVT**T**KSQIPL  PCSCAG**T**LVDCGR  TWASLP**T**AFPVDT  TAFPVD**T**TELVLT  TTELVL**T**GNNLTA  SGLILI**T**GGIVAT  TGGIVA**T**AGRFTQ  ATAGRF**T**QWYFGA  KRKKGS**T**MERWGQ  WGQKYM**T**AVVKLF  KLFGPF**T**RNYYVR  AGFLLA**T**ILGTAC  LATILG**T**ACLAIA  VRGEQW**T**PIEPKP  LLLIAA**T**FIPQGL  AINAPV**T**CCYNFT  ASYRRI**T**SSKCPK  EAVIFK**T**IVAKEI  SPYSSD**T**TPCCFA  PYSSDT**T**PCCFAY  IKEYFY**T**SGKCSN  PAVVFV**T**RKNRQV  EEQKLY**T**DDEDDI  QNGERA**T**RLFEKP  KSGYPK**T**PGRAGT  QGSSQE**T**DKLAPV  ILRKIF**T**TISFCI  QKQEYK**T**LEYNLT  SWIMPG**T**IKENII  QNIHRK**T**TASTRK  RKTTAS**T**RKVSLA  RRLSQE**T**GLEISE  SIPAVT**T**WNTYLR  TYLRYI**T**VHKSLI  AVIITS**T**SSYYVF  PYIFVA**T**VPVIVA  SLKGLW**T**LRAFGR  IFFIAV**T**FISILT  SEDTIH**T**HLEDLE  DSTPVF**T**ETNTLE  GLESYT**T**LPEATE  FNTEVA**T**PPFSLL  RAAVRE**T**VMLLLC  GKNPGQ**T**CEQLQL  GAPMQS**T**IREEGR  KFEIRA**T**GFPEPN  HGKSTL**T**DSLVCK  EQERCI**T**IKSTAI  CITIKS**T**AISLFY  SGVCVQ**T**ETVLRQ  LHGWAF**T**LKQFAE  ALLQMI**T**IHLPSP  ISKMVP**T**SDKGRF  FLVKTG**T**ITTFEH  NILTDI**T**KGVQYL  AGFQWA**T**KEGALC  LYASVL**T**AQPRLM  ESQVAG**T**PMFVVK  GVSTCS**T**RFVSGG  TGNEKI**T**MQNLND  AEEWFH**T**KSAELN  MIQTSK**T**EITELR  TFWNPP**T**TAQLTT  YGPDTP**T**ISPSDT  VTGCNR**T**TVKTII  LSQGNT**T**LSINPV  GKMVSR**T**EGNIDD  GPEGEG**T**ESTVIT  GEGTES**T**VITGVD  NHHLQE**T**SFTKEA  TENLMA**T**GDLDQD  FHGLKS**T**DVAKTF  GALEGE**T**REERTF  NPKMVM**T**VFACLM  LRKAGG**T**GPGEEK  AESFFQ**T**KALDVS  MVAQFS**T**PSLPPT  YYRALK**T**RNVPVR  IYTVLQ**T**KAKVTG  IFTTHA**T**LLGRYL  GIPSIS**T**NLSGFG  NSVDTA**T**SSSLST  QRWDHQ**T**PHRHKF  GEGYRG**T**VNTIWN  MGNLSQ**T**RSGLTC  EGDTTP**T**IVNLDH  PRGVSL**T**NHHFYD  GVICEN**T**CKEKGR  THPELD**T**DGDGAL  PSAPDL**T**EPKEEQ  KGKKPK**T**EKEDKV  YLSNMG**T**RGSYLL  KPDLQV**T**IKEESN  ASVIKK**T**SDITQA  VSVSVE**T**QGDDWD  GEVEKH**T**SQKDYS  QERKAV**T**KRSPEA  DPDDVI**T**DIEMVD  FPDFFR**T**STAEWW  SFVNGT**T**TNQCRN  NHNIAN**T**RRQDPA  DGESID**T**YERDLY  IYEFHK**T**LKEMFP  LPTDNQ**T**KKPETY  DGKSTA**T**DITGPI  NRCSES**T**KRKLAS  VAVALD**T**KGPEIR  KGPEIR**T**GLIKGS  LIKGSG**T**AEVELK  RPGSGF**T**NTMRVV  WSSKTE**T**VEEPME  EEKKPK**T**KKVEKT  MAYIHF**T**AEGEVT  EDEDDK**T**VLDLAV  EAKPSA**T**GKVIDK  VDVEQH**T**LAKYLM  KYLMEL**T**MLDYDM  GLTKHM**T**VKNKYA  SSIPPG**T**LTTLWA  LRGALG**T**LNQLAI  STSIFE**T**AGVGQP  SAAFHR**T**PSLLEQ  GRIFIG**T**FKAFDK  ENLVSM**T**VEGPPP  GPPPKD**T**GIARVP  SIAGAP**T**QYPPGR  PNEHKG**T**ITWYKD  GKQYPI**T**RVIEFI  FYKHPF**T**CFAKNT  GICVTL**T**VIIVCS  VTGTSD**T**TSNNTA  ASSASE**T**STTQTT  SASETS**T**TQTTST  TTQTTS**T**PLSSPL  AFVNYS**T**SQKISR  FKNDQD**T**WDYTNP  AVDRAI**T**HLNNNF  NAPLEV**T**EENFFE  KENQEK**T**EDQYEE  NTLGFG**T**EQAPYG  QSFISW**T**GDGWEF  KNIIHK**T**SGKRYV  KQMIKL**T**QEHIEA  ASMDTV**T**SSSSSS  SVFQNP**T**DVARSN  PARCGV**T**VRDSLK  LENVPL**T**THNFVR  VRKTFF**T**LAFCDF  QGFRCQ**T**CGYKFH  LAETAL**T**SGSSPS  SLPGSL**T**NVKALQ  IPDGQI**T**VGQRIG  LFMGYS**T**KPQLAI  FLHEDL**T**VKIGDF  YLIHWP**T**GFKPGK  NVVPSD**T**NILDTW  AAKHNK**T**TAQVLI  VIPKSV**T**PERIAE  VPNMEV**T**HVSQLT  PGFLSG**T**VTVTSL  DKPINT**T**LICNVT  HLCPSS**T**EHASAS  LRDVDL**T**APDGSL  KACKRK**T**TNADRR  IVERIS**T**ESPAAP  ESSGDP**T**QSPDAA  VPVSLC**T**PSRAAL  LPLEEV**T**VAEVLA  LGLLEE**T**LVIFTA  GVFAVR**T**GKYKAH  ELIQDI**T**QKLFFL  MDQHKL**T**RDQWED  RRRKPD**T**IEVQQM  NEEKRI**T**EAEKNE  LPHPES**T**TSDEKE  PHPEST**T**SDEKEV  EVPLAQ**T**AQPTSA  TSSTVI**T**QAPSSN  GSGLVR**T**QSEESR  SLQQPA**T**STTETP  QQPATS**T**TETPAS  ATSTTE**T**PASPAH  AHTTPQ**T**QSTSGR  TPQTQS**T**SGRRRR  VPSSPH**T**EAIQHS  QMADQS**T**EPALSQ  GTSDGP**T**QQLTWS  AWQPGW**T**VNVEGS  WHWLLR**T**GGWKVS  TQPVAR**T**MDFLSP  AGYPTG**T**GVGPQA  GIAGVG**T**PAAAAA  GVGGIP**T**YGVGAG  GVPGVG**T**PAAAAA  YIPPLA**T**HTICDR  PPLATH**T**ICDRNH  VLPPSS**T**KPPALS  LSHSVS**T**SSTTKS  QHIVPC**T**ISQLLS  SQLLSA**T**LVDEVF  VEISQV**T**IVGIIR  HAEKAP**T**NIVYKI  YKIDDM**T**AAPMDV  VRQWVD**T**DDTSSE  WVDTDD**T**SSENTV  DTSSEN**T**VVPPET  TVVPPE**T**YVKVAG  DMNEFT**T**HILEVI  MPANGL**T**VAQNQV  EGHIYS**T**VDDDHF  SGHASS**T**PGGEKE  PGGEKE**T**SATQRS  ASGSAA**T**WGQDVT  PPAHGV**T**SAPDTR  VTSAPD**T**RPAPGS  RPAPGS**T**APPAHG  PPAHGV**T**SAPDNR  TLASHS**T**KTDASS  IFPARD**T**YHPMSE  GWAYGK**T**GLYPAS  STFFSP**T**GSPLNS  RAGTQC**T**NCQTTT  QCTNCQ**T**TTTTLW  TLAHGG**T**VTDGEN  RSFQGV**T**GYLKID  VLNYNG**T**SQELVA  RFVGAC**T**DPPNIC  MFRYSL**T**NDIVKG  DPEQGH**T**VYAKKL  EIIERV**T**RGEQPP  QLKRGE**T**VQAEAF  SDIVGF**T**ALSAES  ALSAES**T**PMQVVT  LRIGIH**T**GPVCAG  GKGKVR**T**YWLLGE  SATATE**T**ATKRQE  SKNHLH**T**TTQMAG  SITLQP**T**ANPNTG  NSQSFS**T**SHEGLE  EGSPLW**T**SIGAST  SPLPEP**T**TYQEVS  STMMSP**T**TSTNAS  MSPTTS**T**NASTVP  FKVADP**T**PFHIQA  KTNFFG**T**RDVCTE  NKFVED**T**KKGVHQ  MAGPKA**T**KSPEEG  RGAQIE**T**KTKDEL  IIALGP**T**GAQFLS  LRCYCM**T**DDKVDK  QEHEEV**T**VEGPLE  ATSSAP**T**VTLVQL  GQYIAI**T**QGGAIQ  QGLQTL**T**MTNAAA  QTYQIR**T**APTSTI  TCYYNH**T**QTEENE  EDSGHY**T**IVAQNE  VKMLKP**T**ARSSEK  GPIYII**T**EYCFYG  QNGKNL**T**LQCFAD  FADVST**T**SHVKPQ  VTESFS**T**PKFHIS  ELESSF**T**HLDQGE  ETAPAE**T**ATPAPV  PAKKKA**T**KKAAGA  PVSELI**T**KAVAAS  SLVSKG**T**LVQTKG  KGTLVQ**T**KGTGAS  LVQTKG**T**GASGSF  AKPKKA**T**KSPAKP  ATEVRV**T**VLRQAD  GNQVNL**T**IQGLRA  GLRAMD**T**GLYICK  KKRSPL**T**TGVYVK  YGEGDP**T**DNAQDF  LEEDFI**T**WREQFW  RKLNQG**T**ERHLMH  CPTSYR**T**ALTYYL  ISQASA**T**MHLPAT  SVVVSG**T**PIRRPM  LGKSFG**T**TVSPWV  FKYMYW**T**MLQQLT  MAEEKL**T**HKMEAN  KQEKDG**T**EKRGRG  NKGAAK**T**RKTTTT  AAKTRK**T**TTTPGR  AKTRKT**T**TTPGRK  KTRKTT**T**TPGRKP  RILLLG**T**AVESAW  RGGLLR**T**YIISIL  SLSAVY**T**CKRDPC  CFLSRP**T**EKTIFI  ESPAQN**T**AHLDQF  RIKTLG**T**GSFGRV  LVKHKE**T**GNHYAM  LKQIEH**T**LNEKRI  AAQIVL**T**FEYLHS  QGYIQV**T**DFGFAK  LLQVDL**T**KRFGNL  NHKWFA**T**TDWIAI  HKWFAT**T**DWIAIY  FKGPGD**T**SNFDDY  RASRLG**T**TRTPSS  ASRLGT**T**RTPSSY  RLGTTR**T**PSSYGA  EFLTTR**T**NEKVEL  LKGREP**T**RVAELY  RQVEVL**T**NQRARV  MSKPDL**T**AALRDI  IRAQYE**T**IAAKNI  IDALKG**T**NDSLMR  LDVEIA**T**YRKLLE  EVHTKK**T**VMIKTI  MIKTIE**T**RDGEVV  QAPAPA**T**ATDTFE  PAPATA**T**DTFEAA  PATATD**T**FEAAPP  DAKAPP**T**ACYAGA  KMRNLE**T**QHKVLE  HKVLEL**T**AENERL  LSRELS**T**LRNLFK  GSGDLR**T**IPRSEW  RRFILK**T**CGTTLL  ILKTCG**T**TLLLKA  DYNFVF**T**SFAKKQ  LSIGDP**T**VFGNLP  RILGPC**T**IVQGAL  KSILCR**T**PGEFYH  GEFYHN**T**LSFLKS  AAVRAV**T**RMGIYV  EVIDAI**T**TTAQSH  TPACVV**T**LSGNQS  MTEKMK**T**DIQRGL  SGQPSA**T**GAYPAT  VPRMLI**T**ILGTVK  LITILG**T**VKPNAN  RVIVCN**T**KLDNNW  QPCGIY**T**ERCGSG  SPSVSS**T**HRVSDP  DYESQS**T**DTQNFS  SESKRE**T**EYGPCR  VTSPNI**T**VTLKKF  FIISNA**T**YKEIGL  KQKMAI**T**KEHSIT  ESSAYL**T**VQGTSD  ITNDGA**T**ILKLLE  DFSLQK**T**KMKLGV  LLVIPN**T**LAVNAA  AGVFEP**T**IVKVKS  QGPLPN**T**CGHFWE  LHFHYT**T**WPDFGV  GIGRSG**T**FCLADT  TFCLAD**T**CLLLMD  YRQMYR**T**EIRMAR  RQIFNG**T**FVKLNK  KKRIAL**T**DNALIA  GGMKKK**T**THFVEG  GKETVQ**T**TEDQIL  LLAVAA**T**APPDAP  EESEQA**T**EMLVHN  RTDAGF**T**LRWVRK  HSDGIF**T**DSYSRY  QKRAIR**T**LWTVLD  RHYGGL**T**GLNKAE  RRYADL**T**EDQLPS  CESLKD**T**IARALP  FCGAYF**T**FAISHE  EEKSIP**T**LISRLP  TNYQLG**T**GQDEDA  SSGGQH**T**VLLVKD  CSSQDS**T**HCAENL  AKAGEK**T**ISVVLQ  YEVLLV**T**SSFMSP  PSYAAA**T**LQASSA  PKLPAP**T**RTPATA  LPAPTR**T**PATAPV  PTRTPA**T**APVPAR  RAQGAV**T**GKPRGE  KPRGEG**T**EPRRPR  KYRPDW**T**RDSTHL  ICAFAN**T**PKYSQV  RVQFVI**T**AQEWDP  PRASMD**T**VHHMLL  ARNAPP**T**RLPKGV  HAVSFM**T**CTQNVA  VSFMTC**T**QNVAPD  PPPGEG**T**WEPEHT  TWEPEH**T**GDFHME  VAVDPG**T**GAIYVS  PLPGQF**T**VPHSLA  VASEDG**T**VYIGDA  GDAHTN**T**VWKFTL  VWKFTL**T**EKLEHR  VPVVLI**T**TLLVIP  GRFAIQ**T**DPNSND  EAETCE**T**PDPNSI  LPLSPV**T**IKRNWT  QLQQPD**T**VEPDAI  GGVLQS**T**VSESTL  CATLGT**T**GVCAFD  VTSQFT**T**RDDILR  NGLHLE**T**LLDPVD  LETRQI**T**WSRGAD  SRQDAG**T**PKFFLT  TPKFFL**T**DNLVFD  PVKGLK**T**GYRAVP  SLEDIR**T**EFDREI  LFEGRI**T**PELLTR  ITPELL**T**RGKFNT  LGLRKQ**T**HNNAVV  VRNLHK**T**VMQNGA  LFVHRD**T**PENNPD  EVATFY**T**MYNRKP  EPAGGL**T**SLTEPP  GHIISW**T**SRDGGE  GLRKNK**T**NMNYDK  EVAGCS**T**EDCPPQ  HAAPGD**T**VSGKPG  RSGLYS**T**FTIQSL  GLYSTF**T**IQSLQP  PSGSRS**T**SPSPLE  RGFVPE**T**TKAEPE  GFVPET**T**KAEPEV  PAVVMD**T**AGQAGG  TPSLLP**T**HTLTPV  SLLPTH**T**LTPVLL  DIEAKV**T**KNITEI  KVTKNI**T**EIADLT  FMEELN**T**YRQKQG  SIGTGS**T**KQEAKQ  QILSEE**T**SVKSDY  SSGSFA**T**TCESQS  NSLVTS**T**LASESS  FDYDPE**T**SDDSLE  NSSRSK**T**KCLFIQ  NIFLVD**T**KQVKIG  LLHVCD**T**AFETSK  FDKKEK**T**LLQKLL  PEDRPN**T**SEILRT  TSEILR**T**LTVWKK  ENEVES**T**SSANED  SPNDPV**T**NICQAA  DGILLA**T**GLHVHR  GDTPID**T**FLMEML  TTMAKN**T**VFLIEM  HGTRLH**T**HLIGNI  LIGNIH**T**HLVHYR  TKNSFQ**T**LQMKLE  TVEAMK**T**ILDDLR  AAKRRI**T**RSILQM  ISTETI**T**LSHGSS  GIYIPP**T**NKFSPK  RTMEQF**T**IHLTVN  THSSSN**T**QRLPDR  LVQDPN**T**GFSVNG  DFQLEV**T**PQNITL  DPSLTH**T**IFNPEV  FGDFSP**T**DVHRQF  FAIVFK**T**PKYKDI  RDLLEV**T**SGLISD  IDESSL**T**GESDHV  PLLLSG**T**HVMEGS  SGRMVV**T**AVGVNS  GLPLAV**T**ISLAYS  AICSDK**T**GTLTMN  ILSYLV**T**GISVNC  SVNCAY**T**SKILPP  RHVGNK**T**ECALLG  NENDIV**T**GLTCIA  ASDIIL**T**DDNFTS  KPLISR**T**MMKNIL  QLVVVF**T**LLFAGE  RMERGS**T**KYSYKS  SLGPRT**T**HSSVLI  IGGAGV**T**ALLALC  NDTHPT**T**GSASPK  LLLLAV**T**FAASLI  LFSKYR**T**PNCSQY  CGSDMS**T**YANECT  TYANEC**T**LCMKIR  ILMYDI**T**NEESFN  WSTQIK**T**YSWDNA  MSESLD**T**ADPAVT  TADPAV**T**GAKQGP  QTAMSE**T**YDFLFK  KDDSNH**T**IGVEFG  DADREV**T**FLEASR  ELMFLE**T**SALTGE  VFFLVS**T**QLFAEE  SHKRHK**T**DSFVGL  SQCPLE**T**SPTQHP  PLETSP**T**QHPTPG  SGSPPA**T**TPHRWE  MFLSRT**T**YSLQEL  VGAFSA**T**DSFDHK  DLSAKE**T**KMLMAA  DPEGDR**T**IGILTK  DMLHTV**T**DMVRLA  FFNLHR**T**AKSKIE  ERLARL**T**QARRRL  PLVNLP**T**PLYPMM  ILRGKA**T**RPASTS  ATRPAS**T**SQPDPS  PDRSAN**T**SPESSS  DCKKKT**T**KFLDRS  EAAKET**T**EVPEEL  GEFTEE**T**IRNLDE  TGFQGK**T**GPPGPP  DELGET**T**LDAMIM  KVLEHT**T**HTLLVG  NEDLST**T**ASQALH  VICANV**T**GSYGAA  VSAHHR**T**MREDYS  EEEEGS**T**SEESEE  EEEEEE**T**GSNSEE  NRIEEG**T**YGVVYR  LPGEVK**T**LMIQLL  LHRDLK**T**SNLLLS  YTPVVV**T**LWYRAP  PAVKKM**T**FSEHPY  LMNKFL**T**YFPGRR  DPSMFP**T**WPAKSE  QRVKRG**T**SPRPPE  DDDLKE**T**GFHLTT  EKTCQQ**T**WEKLHA  EKLHAA**T**SKNNNL  NNNLAV**T**SKFNIP  WIKQYE**T**LAEMVV  IDKICW**T**KTARRF  INAIPP**T**LTAKIH  FPPGIM**T**QYGRVI  AVEITH**T**FWERNL  ENSIKL**T**SVVFIL  LLTIWK**T**KKFHRP  LLLSGA**T**TYKLTP  LLSGAT**T**YKLTPA  AIERYI**T**MLKMKL  ALSSCS**T**VLPLYH  HYILFC**T**TVFTLL  IYSLVR**T**RSRRLT  SLALLK**T**VIIVLS  VGCKVK**T**CDILFR  TNPIIY**T**LTNKEM  EGDNPE**T**IMSSGN  SHKLLV**T**PPKALL  PVLLFL**T**HGESST  THGESS**T**GVLQPL  LRLPTV**T**TVAVPA  DEKEEA**T**SNSPSK  IDDEVR**T**APWNTT  DYYKII**T**RPMDLQ  ADEEEG**T**VQQPQA  NTPVDK**T**SNTLRV  SILLLA**T**ISADRF  ATRSTK**T**LKVVVA  LEPSSP**T**FLLLKK  SKDLFG**T**GKLIRA  DALVYS**T**FLLHDP  PRPGPD**T**LQFTVD  VVSLTL**T**AVILVL  GSANTE**T**TKVTGS  ETKYRW**T**EYGLTF  FTEKWN**T**DNTLGT  FAVGYK**T**DEFQLH  EDATPG**T**GYTGLA  TPGTGY**T**GLAAIQ  GINGTS**T**NSTEAE  AEDTTE**T**GRQGKG  CDLELE**T**NGRDHH  GQPFWL**T**LHFEGR  RCLGIP**T**RVVTNY  LLLCAR**T**VSYNGI  KNGIIK**T**DKVFEV  FEVMLA**T**DRSHYA  ARMVGC**T**GKVIGI  VRKDDP**T**LLSSGR  TRKCGE**T**AFIAPQ  RDLKEV**T**PEGLQM  HKGPDE**T**LRIKAE  ISCPPL**T**PDWGVQ  TFSICD**T**SNFSDY  TLRLAG**T**QPLEVL  FPPDQL**T**SSGAPF  FLDYFK**T**EHKLEI  KEKRGW**T**LNSAGY  SDKNGL**T**SKRELR  ENNIMR**T**IIEFLS  ENLMKK**T**KQTISL  MKKTKQ**T**ISLFKE  RRPLPC**T**PGDCPS  WTGREL**T**NRHSLP  GCGCCA**T**CALGLG  VEKPLH**T**LMHGQG  KIRDRS**T**SGGKMK  WCVDRK**T**GVKLPG  PLQPPT**T**PSPEPE  WQVVDA**T**PQETSS  GASDRV**T**MPVAYK  DIGGNE**T**VTLRQS  LASDPL**T**PEFIKP  AAPAAP**T**ALPSFS  HFSPSQ**T**YEGLRA  FSFSPP**T**GPSPSL  HLLIPR**T**RGLLSG  LTTRPV**T**LVEGEE  MDGSVV**T**REHRGS  AHMVLV**T**KVSRYS  KTGTKI**T**FYEDKN  DCADFH**T**YLSRCN  SGQMYE**T**TEDCPS  GQMYET**T**EDCPSI  ARPYSA**T**AAAATG  APKIID**T**GGGFIL  NHFDLP**T**CDNCRD  SVWKRE**T**IVHQHE  LAAALR**T**GDDYIA  EMRKNL**T**KEAIRE  KTGGTQ**T**DLFTCG  SADEPM**T**TFVVCN  LQRANR**T**GGLYSC  ALEITV**T**NSPSNP  TEVTFD**T**PDLDIN  INSLNL**T**ESHNSR  PPATPP**T**SGAPPG  GPGPTP**T**PPPAVT  PSSGVP**T**TPPQAG  PGEKTY**T**QRCRLF  NLPADI**T**EDEFKR  KAELDD**T**PMRGRQ  LRVRFA**T**HAAALS  DDRGRS**T**GKGIVE  GVFLLT**T**TPRPVI  VFLLTT**T**PRPVIV  YQKERE**T**PPRFAQ  RFAQHG**T**FEYEYS  LETAME**T**LINVFH  LKELLQ**T**ELSGFL  VLVAAL**T**VACNNF  LLPPAM**T**QQLRGD  RNRNNS**T**GVAGLS  GHGSED**T**EDSAEH  EEEEAS**T**EYGHQA  EEEEEI**T**VQFGHY  FQDEYK**T**EVPHHH  HHPPGH**T**VVKDRS  EQGEKG**T**HHGSRD  EDEPRF**T**IIPNPL  EESGED**T**GPQDAQ  SFCNRC**T**ECESCH  CPLVCE**T**VCAPGS  ELNEFL**T**RELAED  EVRVTP**T**RTEIII  RVTPTR**T**EIIILA  EIIILA**T**RTQNVL  IILATR**T**QNVLGE  YAEKVA**T**RGLCAI  VNYYVD**T**AVRHVL  MLPWDP**T**GKIGPK  KDEILP**T**TPISEQ  GVGAVR**T**LRTGPA  LSVRKF**T**EKHEWV  TENGIG**T**VGISNF  PLSGEV**T**EINEAL  KFEISE**T**SVNRGP  FQVRKV**T**GANTGK  VRNAKD**T**AHTKAE  AKDTAH**T**KAERNI  LIYAFQ**T**GGKLYL  QGHVKL**T**DFGLCK  ESIHDG**T**VTHTFC  IHDGTV**T**HTFCGT  LMYDML**T**GAPPFT  SKFTRQ**T**PVDSPD  STANPQ**T**PVEYPM  IEQMDV**T**MSGEAS  KKIPDA**T**PLLDAS  SYFPEI**T**HIVIKE  DPEGDN**T**GEQVAV  IGDFGL**T**KAIETD  DRLQGT**T**IREEDE  NDLLTI**T**LEECKS  SCDTEF**T**KEDEQR  LAELRT**T**CSENEL  AGCLHM**T**VETAVL  YAWKGE**T**DEEYLW  EGYEVT**T**MDEACQ  EGNIFV**T**TTGCID  VDDPYA**T**FVKMLP  YALYDA**T**YETKES  YDATYE**T**KESKKE  AIKKKL**T**GIKHEL  GKTISL**T**DFLAED  WRARPA**T**DSFDDY  ASTSQS**T**RAASIF  TEQQSP**T**SGGGKV  AMPTLP**T**YQLSET  QVMGLL**T**NHGGVP  GGLEPT**T**TVSASC  PPTYST**T**GYSMDP  LCCEVE**T**IRRAYP  MLKAEE**T**CAPSVS  MRKIVA**T**WMLEVC  LQLLGA**T**CMFVAS  ASKMKE**T**IPLTAE  KETIPL**T**AEKLCI  EKLCIY**T**DNSIRP  WNLAAM**T**PHDFIE  IRKHAQ**T**FVALCA  FVALCA**T**DVKFIS  LSYYRL**T**RFLSRV  LFAVVF**T**ARYLDL  YSKFKA**T**YDGNHD  KTGEAE**T**ITSHYL  ALGVYR**T**LYLFNW  GNLTVH**T**NVSDTL  FRIYNV**T**YLEPSL  HWKNCL**T**KLLPCF  NLTCTE**T**PLVIAG  GCGCCM**T**CALAEG  EHEEPT**T**SEMAEE  SEMAEE**T**YSPKIF  VGGAEN**T**AHPRII  RVDSAA**T**SGYEIG  HVARQI**T**KEDFAT  TKEDFA**T**FDYILC  KSNQVK**T**CKAKIE  ICDDGF**T**GEDCSQ  SEPLEI**T**LLAPER  LVTANI**T**DSEALA  IQTIFT**T**IGLLYP  DAKERD**T**MKEDGG  KRKANV**T**VFLQDP  MAKIDR**T**ARDQCG  RVYPNS**T**CKPRII  MLNKEK**T**YLRDQH  YKLHRE**T**FYLAQD  QENVVK**T**LLQLIG  HQFAYV**T**DGACSG  SGDEIL**T**MELMIM  WRLSPL**T**IVSWLN  AMVIRE**T**GSSKLK  DAHNIQ**T**HRDSLD  REGLID**T**AVKTAE  SPSYSP**T**SPSYSP  SPSYSP**T**SPNYTP  SPTYSP**T**SPKGST  VYKDHQ**T**ITIQEM  DNLLHG**T**KKKKEK  SPVTAR**T**LETLIR  EMPQVH**T**PKTADS  SLTYIY**T**GLSKHV  EDIFME**T**LKDIVE  EEECPA**T**LRKYLK  LHNGNG**T**YQSWVV  GVMDVN**T**ALQEVL  LQEVLK**T**ALIHDG  PSGGSC**T**CADSCK  CEGCKC**T**SCKKSC  YDLSAS**T**FSPDGR  AVENSS**T**AIGIRC  MYVHAY**T**LYSAVR  ARQAAK**T**EIEKLQ  PWKQQL**T**EDGDSF  RDFRGN**T**PLHLAC  ASVGVL**T**QSCTTP  VLTQSC**T**TPHLHS  LTQSCT**T**PHLHSI  HSILKA**T**NYNGHT  TNYNGH**T**CLHLAS  EPCNGR**T**ALHLAV  ADVNRV**T**YQGYSP  YSPYQL**T**WGRPST  TWGRPS**T**RIQQQL  DTESEF**T**EFTEDE  LERDSL**T**EKECVK  DEPQML**T**NEKLSI  LINKIE**T**TVPPSG  SQGFPD**T**YRLFGN  RRSKPL**T**RGAKEE  FRSVDE**T**TQAMAF  LVKDSA**T**GLSKGY  KAMQGL**T**GRKFAN  QVSNGP**T**SNTSNG  TTDDSK**T**NLIVNY  QKPSGA**T**EPITVK  GATEPI**T**VKFANN  TVLKII**T**FTKNNQ  LKIITF**T**KNNQFQ  GKPIRI**T**LSKHQN  QEDQGL**T**KDYGNS  ADLAAV**T**ALLLPF  ATTRIE**T**MLGDVA  YDFPAV**T**TAQYSF  PFMPFV**T**EELFQR  TNRWFL**T**CINQPQ  AHLPKS**T**FVLDEF  PEELTQ**T**FMSCNL  DPGSEE**T**QTLVRE  PSDHGG**T**ESSPGW  SSPSGV**T**SRLPGR  RLPGRS**T**SLVEGR  PSPTSP**T**ATSTTP  PTSPTA**T**STTPSR  TPSRYK**T**ELCRTF  KTELCR**T**FSESGR  RHPKYK**T**ELCHKF  LPSGRR**T**SPPPPG  FSAAPG**T**PLARRD  LARRDP**T**PVCCPS  PSCRRA**T**PISVWG  EKVQYL**T**RSAIRR  PPQEIL**T**KDSVTI  TRLLAQ**T**TLRNVL  AHNMQS**T**LDDATD  STLDDA**T**DAWGIK  GGEPRR**T**EGVGPG  DVGPRY**T**QLQYIG  YDHVRK**T**RVAIKK  SPFEHQ**T**YCQRTL  QTYCQR**T**LREIQI  VQDLME**T**DLYKLL  SNLLIN**T**TCDLKI  LNSKGY**T**KSIDIW  LLDRML**T**FNPNKR  NPNKRI**T**VEEALA  EQYYDP**T**DEPVAE  VAEEPF**T**FAMELD  EAKKSK**T**AAKKND  DPPDQK**T**SPSGKP  ILCLQE**T**KCSENK  YAYTFW**T**YMMNAR  WSFTFQ**T**YACLSR  SECMNE**T**EWTIYQ  LQATPE**T**CLHRIY  QEVPIL**T**LDVNED  DWAPLF**T**NANDGS  DPLGIH**T**GESIVV  QTAIKV**T**QHLGIV  ELRNSV**T**GGTAAF  AVKQID**T**VAAEWP  YLTYWG**T**THDLTF  ARQFCQ**T**VGYPCV  FSKAHW**T**PFEGQK  IREELG**T**VNGMTI  GTVNGM**T**ITMVGD  VNGMTI**T**MVGDLK  SLRMPP**T**VRAFVA  RPGLRT**T**RMSWPS  QQLARE**T**LEELDW  AGGRSL**T**CIMYMI  DMSKHM**T**LLADLK  TPSSKP**T**LLANGG  KAKTQP**T**SLPKQP  MPSRPS**T**TPFIDK  PEAGAP**T**SASGLN  EVSTGT**T**IMAVQF  ADSRTT**T**GSYIAN  AVADAV**T**YQLGFH  TYREGM**T**KEECLQ  SYTGRR**T**MQSISN  VNPLVY**T**LFNKTY  YTLFNK**T**YRSAFS  QLILVN**T**IPALAY  EPGPPY**T**TDEHIV  RVFRLI**T**DNTVEE  DGAEPL**T**PEETEE  FQRRCN**T**LISLIE  WTLFVY**T**KGNFRM  TPLGKL**T**AQANVE  TPVASA**T**QSVSRL  TFCQHY**T**QSTDEQ  QHYTQS**T**DEQPGS  RVKEVR**T**DSGSLR  ERYSSP**T**AGSAKR  SILPGQ**T**LLTMAT  APVTGT**T**GHKVTI  GASPKQ**T**NLTKAQ  WTCFEF**T**LVHCPD  RQGEQR**T**KKRVIA  AADGKT**T**RHNYYF  QLFDPM**T**GTFRCT  TGTFRC**T**FCHTEV  LIHEKK**T**SSAMAG  ATWAEV**T**VCSCSS  KKSSNL**T**LPPKYN  MKLLGW**T**HCASFT  THCASF**T**ENWLPI  PHGLTR**T**SVTVSD  KVHEFQ**T**LSPEGS  IHPSCV**T**RQKVIG  SIRLPS**T**SGSEGV  LQDRDG**T**IIHLKY  RVIVMT**T**REVEKG  LMENIS**T**KGLDCD  HAKASR**T**SSKHKE  PPSSPL**T**QPPEGP  EVAATG**T**YQLRES  VDHHAA**T**ASFMKH  FQAACE**T**FCVGED  PRLPPC**T**LRQALT  TLRQAL**T**FFLDIT  HYGVCS**T**WLSQLK  LQPTPM**T**LVFGCR  EPDNPK**T**YVQDIL  FVCGDV**T**MATNVL  VQRILA**T**EGDMEL  LRTQEV**T**SRIRTQ  YSPISI**T**SALGMV  LGAKDN**T**AQQIKK  LKDTLR**T**MGMVDI  GFGSSP**T**STNEEF  LLPCLH**T**LCSGCL  DAQAVC**T**RCKESA  REFLDG**T**RKTNNI  LDGTRK**T**NNIFCS  PNHRTP**T**LTSIYC  VLQRIR**T**GSALVQ  GEDVSN**T**TTAQKR  EDVSNT**T**TAQKRK  DVSNTT**T**AQKRKC  KRKCSQ**T**QCPRKV  RYLSLQ**T**TTLPPA  LSLQTT**T**LPPAQP  ARASPR**T**LNLSQL  MSQVSH**T**HLAFVH  TLYELL**T**HCDSSQ  IAQGQM**T**VLRLTE  SPGHRA**T**APQTQH  RATAPQ**T**QHVSPM  PAKKPA**T**PAEDDE  EDDKVG**T**DLLEEE  AEPGSP**T**AAEGEA  ASAASS**T**SSPKAE  KAEDGA**T**PSPSNE  PSPSNE**T**PKKKKK  GALPLD**T**VTFYKV  VLVKFD**T**QYPYGE  ENPVPY**T**GAVKVG  KSLNIL**T**AFQKKG  LKGHSC**T**TPQCKY  KGHSCT**T**PQCKYL  GHVYCK**T**DRLAKG  CLPNSC**T**TQVPNH  GILPLE**T**PSPGDG  SYLQNY**T**NTPPVI  VARIGQ**T**GTKSVF  RSSRLF**T**SDSSTT  KIPNRK**T**KSKTNK  TNKGGI**T**QPNIND  YKKLGQ**T**HLALMN  MTDADD**T**QLHAAE  SSLFMD**T**EKSGKR  CRRRKR**T**YWNDSC  YELEDE**T**RPAKRI  RPAKRI**T**ITESNM  AKRITI**T**ESNMKS  NMKSRY**T**TEFHEL  NIFISR**T**SIPNAA  GDLGHV**T**RISSPQ  VLQENY**T**HLPKAD  VLSQEF**T**ELLKVM  FTDRMA**T**RSTTQS  RMATRS**T**TQSNRT  SPVTNL**T**VTMDQL  VTNLTV**T**MDQLQG  MGSSES**T**DSGFCL  NFIPLF**T**PQSPVT  TPQSPV**T**ATLSDE  QSPVTA**T**LSDEDD  CMASLW**T**APLVMR  APLVMR**T**TNLDNR  PLVMRT**T**NLDNRC  ESPPGS**T**KRRKSM  ASSPKG**T**IENILD  KKPIVP**T**DGKRVI  PKSQVG**T**LLFRSR  TPAEGD**T**EEDDGF  KAFLLQ**T**VDGKHQ  KYISPE**T**MVALLT  LLLERD**T**SFTVCP  ERDTSF**T**VCPDVP  LSSGEI**T**ATQLTT  SGEITA**T**QLTTSA  ITATQL**T**TSADLD  TATQLT**T**SADLDE  GLCLKK**T**VSLCDI  VSLCDI**T**ITQMLE  LCDITI**T**QMLEED  KYVNPE**T**VAALLS  PIVPLD**T**QKRIII  HHQDHK**T**ELLRCR  VTLQGP**T**LNGTFA  VLFVLA**T**LENIFV  YLALVK**T**MSMGRM  QTERRA**T**VLVLVV  ELLKEG**T**KEAHDR  HDRAEN**T**QFVKDF  ALKLPS**T**GEGTQF  STLARE**T**LEDGFP  VFLGHQ**T**FVSQPG  GEVFEP**T**VERGEL  VWSFGV**T**MWEIAT  GGADPP**T**QPDPKD  VKGTTG**T**QASFLQ  VQWFER**T**LDDSAN  LAEAFL**T**ADTILN  ELPFMA**T**ENIIMA  RQGLLK**T**PWRAAS  TKGYQE**T**ISDVLN  GVVVEA**T**HMCMVM  SKTVTS**T**MLGVFR  FLTNMH**T**SKISKA  VPSWKM**T**LLNVCS  SLEAML**T**IYQLHK  QEDILD**T**GNDKNG  VTKLFP**T**RSHTVA  DAIHYM**T**EQAPAA  GMPFSR**T**EDGKIY  SYVDVG**T**GKVTLE  DMFHKY**T**RRDDKI  EKPSLL**T**MMKENF  ACDKKG**T**NYLADV  LLGDIA**T**DYHKQS  SFQSKA**T**VFGASW  DCPTLD**T**HTLSLT  THTLSL**T**DYACGS  PYTKHQ**T**LELEKE  IVLEDG**T**KMKGYS  ITPQFV**T**EVIKAE  VESIMA**T**EDRQLF  FPFVSK**T**LGVDFI  RHSIPA**T**TTTLVA  HSIPAT**T**TTLVAE  YLISSF**T**DQLPWT  EDNITW**T**LHSTSP  TWTLHS**T**SPAEEF  PAEEFY**T**RHVLQI  GKVVWV**T**ATFPYI  SVVNCM**T**SFVSGF  PSLLFI**T**YAEAIA  ANMPAS**T**FFAIIF  TGPAVL**T**VALIEA  SWFYGI**T**QFCRDV  RGEYIK**T**WRPRYF  SPSDSS**T**TEEMEV  PSDSST**T**EEMEVA  LVREKA**T**GRYYAM  KDEVAH**T**VTESRV  SRERVF**T**EERARF  DGHIKI**T**DFGLCK  GISDGA**T**MKTFCG  MKTFCG**T**PEYLAP  EIRFPR**T**LSPEAK  PFKPQV**T**SEVDTR  LELDQR**T**HFPQFS  LESDPR**T**RTLLSD  SDPRTR**T**LLSDPT  SLAEHR**T**PDVLKK  NRAACY**T**KLLEFQ  AKISSP**T**ETERCI  KDGYNY**T**LSKTEF  NYTLSK**T**EFLSFM  FLSFMN**T**ELAAFT  TELAAF**T**KNQKDP  MMKKLD**T**NSDGQL  KPLVKV**T**FFQNGK  SGDYHC**T**GNIGYT  IIVAVV**T**GIAVAA  RISANS**T**DPVKAA  DLQAND**T**GRYFCL  NDQNNV**T**IMANLK  TTNMVI**T**WKPLRW  FGNISA**T**AGENYS  YFYDDV**T**TLYEGF  IQKGFK**T**APDQFI  YEGYGQ**T**ECTAGC  YGQTEC**T**AGCCLT  PPLSPT**T**KLIDRT  AQSAAK**T**PSCKDI  QKGGSK**T**SSTIKS  RYKFVA**T**GHGKYE  LCYHFL**T**SLLRSA  YSAYDY**T**IPPMEK  EKAVVK**T**DIQIAL  VQALDD**T**ERGSGG  VAAIVC**T**FQEYAG  LQKELA**T**WTPTEF  LATWTP**T**EFRECD  FMSVLD**T**NKDCEV  TVRGIV**T**RVSEVK  QGLLSE**T**YLEAHR  SSGVGL**T**AAVLRD  SLADYI**T**AAYVEM  FPINLK**T**GEANLT  SKPFLG**T**SQRGRY  NTFVFF**T**SDNGAA  EALSRI**T**SVVQQH  LLLLLL**T**LLGLGL  IRSICS**T**TNIQCK  VTDCRD**T**GSSRAP  YRAIAS**T**RRVVIA  VESPEL**T**TSRPLP  ESPELT**T**SRPLPK  NKIPAQ**T**KSPEET  DSLFKR**T**EVLAAV  FIDELE**T**LCQKRA  YKVNPD**T**GYINYD  ALKQAM**T**LEFKVY  ALSEAL**T**ELGYKI  ENIVAN**T**VLLKAR  CEDLRR**T**IDRDYC  FRQFCE**T**RPGLEC  LERQPV**T**KNTFRQ  QPVTKN**T**FRQYRV  ACQVRA**T**GKMYAC  LAYAYE**T**KDALCL  DLHREN**T**VYRDLK  IRGRVG**T**VGYMAP  DRRVLE**T**EEVYSH  FYSKFS**T**GSVSIP  QNEMIE**T**ECFKEL  IGSVTM**T**FTASVG  FSGIIY**T**YGHVLW  APRSSV**T**ETEADG  EADGKI**T**PWPDSR  GVRYNT**T**NNGYRE  GWCRLV**T**AAYNYF  EGCYLH**T**AIVLTY  HTAIVL**T**YSTDRL  LLEPLV**T**QVTTLV  SAIDKM**T**KKTRDL  DAVDDI**T**SIDDFL  KGPLKN**T**SDVISA  GFSQSF**T**QEQVAD  DDAELA**T**RAIPEL  VRTMQN**T**NDVETA  VETARC**T**AGTLHN  VKFLAI**T**TDCLQI  KFLAIT**T**DCLQIL  ALGLHL**T**DPSQRL  DDINVV**T**CAAGIL  GDREDI**T**EPAICA  QDTQRR**T**SMGGTQ  IEAEGA**T**APLTEL  RLSVEL**T**SSLFRT  SRAQPL**T**LSLGAA  SLGAAM**T**QPPPEK  NTPREV**T**LHFLRT  TAGHPL**T**RWALQR  EKVYIA**T**QGPMPN  QGPMPN**T**VSDFWE  SLIVML**T**QLREGK  CVHYWP**T**EEETYG  KECPEY**T**VRQLTI  VEESPE**T**AAHPGP  TGCFIA**T**RIGCQQ  RGGMIQ**T**AEQYQF  VRVPKN**T**TKKYNI  RVPKNT**T**KKYNIM  DKVNFA**T**WNQARL  GGVTEN**T**SYYIFT  HNWYNF**T**PLARHR  PLARHR**T**LTAEEA  SAEGGS**T**SSTLRA  GGSTSS**T**LRAAAS  QPPSGK**T**TPNSGD  LLKKFQ**T**KKTGLS  KFQTKK**T**GLSSEQ  GLSSEQ**T**VNVLAQ  YWALNL**T**SMLCLT  SPGLNR**T**VVNISS  APGPLD**T**DMQQLA  QQLARE**T**SVDPDM  MSSSYP**T**GLADVK  KAGPAQ**T**LIRPQD  FGCRFG**T**CTVQKL  CRFGTC**T**VQKLAH  MCEGNK**T**TMASPQ  LVVVLS**T**ICLVTV  STICLV**T**VGLNLL  SERKLH**T**VGNLYI  KTRASA**T**ILGAWF  NHFMQQ**T**SVRRED  REDKCE**T**DFYDVT  TDFYDV**T**WFKVMT  DEQGLN**T**HGASEI  SRTDSD**T**TTETAP  LRSGSN**T**GLDYIK  EHLHMF**T**IWLGYI  SRGISV**T**KKTHTS  ISVTKK**T**HTSQIE  SQQSNA**T**YSCPRQ  NCLIDR**T**SRNRCQ  NYIDGH**T**PEGSKA  EPICDY**T**PASGFF  FPYCSF**T**NGETSP  SFTNGE**T**SPTVSM  NGETSP**T**VSMAEL  QQITWQ**T**FLQEEI  REDGIL**T**KLICKV  ALCGRH**T**EKLMAF  VCGLAV**T**DLLGTL  WCFIDW**T**TNVTAH  CFIDWT**T**NVTAHA  RRTSLG**T**EQHHAA  QAEVGP**T**GCPHRS  PTVLDR**T**VVDKWF  TITCGH**T**IEILRE  FKQIRL**T**DTLGRL  NGRCIP**T**PGSCRC  NICVFG**T**CHNLPG  LGKAWG**T**PCEMCP  NGQCIN**T**VGSFQC  NCWLAL**T**DPRDVA  MLILGI**T**NPEGEK  FGVAPG**T**SVKTNP  PLASGV**T**ITSWKN  VGRSAV**T**GPGEFW  SSDGEG**T**MSRPAS  SPVSPS**T**NRTHAH  QHLRLS**T**SSGRLL  SRAAVV**T**SPPPTT  TSPPPT**T**APHKER  VNQAGF**T**LHSAIY  CVVHIH**T**PAGAAV  DNLGYR**T**GYPYRY  EVPASV**T**GYSFAS  LFVPLN**T**NPKEVQ  HVIVST**T**GPNPFT  TGPNPF**T**TLTDRE  NPFTTL**T**DRELEE  AATFKP**T**LPDLSP  KKKKFR**T**PSFLKK  VSCSEV**T**ASSLIK  SCFPVD**T**TGFCLH  CFPVDT**T**GFCLHS  HLHTPA**T**AAVSAM  VQWAGS**T**FGPMQK  GSPRPK**T**TWMKAD  KDDSEE**T**VPNPFS  TTEPET**T**QPEGVV  GREEEQ**T**AEEILS  ADTDVD**T**SKDKTE  DTSKDK**T**ESVTSG  SYGAYP**T**QPGQGY  SGYSQS**T**DTSGYG  YSQSTD**T**SGYGQS  KLKGEA**T**VSFDDP  EGEGTP**T**QPASEK  KKVERL**T**MQVSSL  LQREPF**T**IAQGKG  FLSKKK**T**DELRNL  LYNRPG**T**VSSLKK  ARKAKR**T**KCPEIL  EEPPKK**T**AKREKP  KPKQKA**T**SKSKKS  KLKKPP**T**DEELKE  DEELKE**T**IKKLLA  VYENYP**T**YDLTER  YPTYDL**T**ERKDFI  QAPVVS**T**VGMQRL  MQRLGT**T**PSVYGG  VLQQQV**T**VNTEEL  ERDIKK**T**RKIKTV  YLQCET**T**WGDQDF  RSQQTH**T**ELSSIL  NVHLFA**T**PLAASL  QRLRDG**T**RMRAPE  IEGKNK**T**VSTLVI  CTADRS**T**FENLTW  RRLDSI**T**SSQSSA  RPVSVK**T**FEDIPL  PVLPAL**T**INPTIA  ALTINP**T**IAEGPS  AEGPSP**T**SEGASE  RLEAFL**T**QKAKVG  GNGGVV**T**KVQHRP  ANSFVG**T**RSYMAP  PERLQG**T**HYSVQS  LPNGVF**T**PDFQEF  ADLKML**T**NHTFIK  KMLTNH**T**FIKRSE  AGWLCK**T**LRLNQP  SSGKNV**T**LPAVFK  ELAGHQ**T**SAESWG  MFAPTK**T**WRRWHR  RNIPGI**T**LLNVSK  KIVTVK**T**QAYQDQ  IGGIIL**T**ASHNPG  HPDPNL**T**YAADLV  AADLVE**T**MKSGEH  IPYFQQ**T**GVRGFA  FARSMP**T**SGALDR  DRVASA**T**KIALYE  KIALYE**T**PTGWKF  ALYETP**T**GWKFFG  GEESFG**T**GSDHIR  WLSILA**T**RKQSVE  GLRLIF**T**DGSRIV  TIDLNQ**T**PQTTLP  LNQTPQ**T**TLPQLF  SVARND**T**GYYTCS  LGTSCE**T**ILLNSP  SEDLRN**T**HCCYTD  HMEIVG**T**QGKPGI  HDAVTD**T**IDIAPN  PNQRVG**T**KRYMAP  SSTSPT**T**NVLLSP  SPDIHG**T**YKELLD  EDGAGT**T**PSPGLQ  LQPAHL**T**FPLDYH  LIQWIT**T**QCRKDV  NWLKDG**T**VLCELI  KKIQAS**T**MAFKQM  MACVQR**T**LMNLGG  VAAAEK**T**KQGVAE  VVHGVA**T**VAEKTK  AVVTGV**T**AVAQKT  TAVAQK**T**VEGAGS  RVKPDR**T**GVVTDG  DRTGVV**T**DGVKHS  EDPPQR**T**AGVKVE  GVKVET**T**EDLVAK  GGLNTE**T**NEKALE  IKVEQA**T**KPSFES  RDDGYS**T**KDSYSS  YPSSRD**T**RDYAPP  QITPQG**T**RDEISL  EPCGTN**T**HASSLQ  DRRTPS**T**EKKVDL  SENPRD**T**EDVPWI  SGHENK**T**KGDSIQ  QSTRHS**T**VATECL  ECLSKN**T**EENLLS  HHLSEE**T**KCSASL  SPAAAH**T**TDTAGY  PAAAHT**T**DTAGYN  TLTNLI**T**EETTHV  EFVCER**T**LKYFLG  KAVTDQ**T**VSKKNK  NKATCG**T**VGDDNE  DGPSKV**T**LEARNS  CVVNRS**T**DNQRQQ  VGIDLG**T**TNSCVA  AKRQAV**T**NPNNTF  YDLGGG**T**FDISIL  HLNMKL**T**RAQFEG  EVDVTP**T**FDTMGL  DIQVRE**T**QALILA  YDTLTI**T**QAVIFC  IEQYYS**T**QIDEMP  LPSNSF**T**SGDIVG  KLANDV**T**YRRLKK  PLLLVD**T**AGCGLF  TLVEYF**T**QHGEVR  WNFDFV**T**ETPLEG  FDFVTE**T**PLEGDF  GGRRPG**T**SPALLQ  PALLQG**T**AEEDHV  DLSLSC**T**LVPRSG  RRQTSM**T**DFYHSK  NEEGET**T**SHLMGM  KVYAAA**T**EDMDCL  EDMDCL**T**FGSPVL  DDFFKV**T**GSLSSA  LFIFLT**T**ESTGEL  QLHSNF**T**AVCVLK  IPKEQY**T**IINRTA  GFIRNY**T**IFYRTI  LRKRMN**T**NPSRGP  VVRLKP**T**RKFAYL  WKYQAV**T**ATLEEK  YQAVTA**T**LEEKRK  SKESHA**T**LVFHNL  GQANHP**T**AAVVTE  YKIMDA**T**NILVSP  GQFESL**T**FDMELT  PIRVLV**T**GAAGQI  LVLLDI**T**PMMGVL  ANTNCL**T**ASKSAP  LKGEFV**T**TVQQRG  DLSHIE**T**KAAVKG  PRKPGM**T**RDDLFN  GGHAGK**T**IIPLIS  FVKSQE**T**ECTYFS  LTTLDV**T**KLTPLS  ELERNI**T**IKLGYA  TPDEFP**T**DIPGTK  ALPEIF**T**ELEISY  ALSNST**T**AGQAVT  TVELFL**T**LQLVLC  RGENPG**T**PALSIG  KGLEPD**T**DWEERE  LLDFMY**T**SRLNLR  GEYSRP**T**LEVSPN  MHPPKC**T**SCGSQS  SHKTVH**T**GEKPYR  PGNSIH**T**QPEVIL  SRSPIT**T**NHRPSP  KNRTNM**T**YEKMSR  EIMSGR**T**DRLEHL  VPHGHI**T**SLAVKR  LHLYSN**T**LNFQIS  LSEVSE**T**TESTDV  SEVSET**T**ESTDVK  ALDPAY**T**TLEFEN  ENVQVL**T**MGNDTS  LTMGND**T**SPSEGT  TSPSEG**T**NLNAPN  ICGDRA**T**GKHYGA  HLLLGA**T**KRSMVF  LLLLLP**T**LQSITW  PTLQSI**T**WQMIEQ  MQEHMG**T**NVIVAN  NVIVAN**T**MPTHLS  VANTMP**T**HLSNGQ  LPGAVA**T**IVKPLS  KYNFHG**T**AEQDLP  EGVKAG**T**KLSLMP  LLYPPE**T**GLFLVR  QLVEHY**T**SDADGL  YEPSLM**T**MCQDSN  ANHISN**T**AKHFYG  LLNMVI**T**PQNGRY  LIPWKL**T**YRNIGS  AVLWGE**T**VHLFME  FEIIWV**T**KHVLKG  GLSVQM**T**EDVYFP  LCRGHS**T**KADIYS  IYSLGA**T**LIHMQT  TLIHMQ**T**GTPPWV  IHMQTG**T**PPWVKR  SSCTGS**T**EESEML  KKLKCL**T**DIYLLN  LKARTV**T**FGVVTS  IVILLN**T**FQEFFG  DQATQV**T**ETLGMT  SSTMSH**T**VAGGGS  RGDIMI**T**HFEPSI  DNEQLF**T**MKWIDE  EEGDPC**T**VSSQLE  YCANGH**T**FQAKRF  AHCAIC**T**DRIWGL  HSDHAQ**T**VIPYNP  LVRLKK**T**DRIYAM  EGHIKL**T**DYGMCK  TSTFCG**T**PNYIAP  DNPDQN**T**EDYLFQ  LGCHPQ**T**GFADIQ  RLVTQD**T**ENELKK  LKKLQQ**T**QEYFII  QSNRGL**T**KENLVF  VFSKYY**T**PVLAKA  YTDYRP**T**PLQHYI  KGRKGG**T**KGPSNV  NDPNLE**T**VYTLCE  KFAEGI**T**KIKRDI  KGNKLN**T**EETVKV  FKGQLR**T**GDIILH  GKEAMH**T**CLKQSA  RAIEEF**T**LSCAGY  ATTAAT**T**SSNVSV  ELQHYV**T**MELREM  FRPSAF**T**DTQYLQ  LLVVGI**T**DPDPDI  RKAALE**T**VDRLTE  TESLDF**T**DYASRI  YDKKMD**T**NKDDPE  CMIPRD**T**HDGAFY  NITNAT**T**AATTAA  NATTAA**T**TAATAT  AATTAA**T**ATTTAS  TVASKS**T**TTARHN  HKSTGM**T**SRSGTD  EGIIFG**T**NGPVDL  VDLKKI**T**NFFRGD  EFMHIL**T**RVNRKV  SQQKKK**T**SPLNYK  YHPKFW**T**DGSYQC  YHIKET**T**TSPKKY  HIKETT**T**SPKKYY  GFEGDK**T**GGSSPE  ESLAYN**T**FSIKSD  KKRNAP**T**PPKRSS  ADGFSF**T**PAQQEA  LVYSTP**T**PFFHTF  QLPPPP**T**PANEER  PPPPPA**T**GRSGPL  GIQLNK**T**PGAPES  SADWLA**T**AAARGR  ADPATL**T**RPVHDA  REGFLD**T**LVVLHR  RAAAGG**T**RGSNHA  RHSLSS**T**KLLSPQ  EAAREV**T**LARVSG  LARVSG**T**VQQLPA  LSPERR**T**NTPKAI  FRHLHC**T**RNYIHL  LAKSTL**T**LIPLLG  DEHARG**T**LRFIKL  KPLKCP**T**SSLSSG  DSFFGE**T**SHNYHK  FMLQQS**T**NPAPGI  SSVGDE**T**DLANLG  EEVAKN**T**HCSSLP  AYDSDV**T**GCASMY  ASMYLH**T**EGFSGP  ALLDDP**T**NAHFIA  PVSEED**T**VPLSHL  PPAPPV**T**HSTPVT  DFLAGV**T**QELIKT  TQELIK**T**LEDNSE  PAQTSD**T**LALNNQ  ERQLNG**T**YAIAGG  VEKLIA**T**TAHERM  EGTKFD**T**LWQLVE  GTEKAD**T**EEMMRE  FFFGNI**T**REEAED  RKAHHY**T**IERELN  ERELNG**T**YAIAGG  AIAGGR**T**HASPAD  QGVQPK**T**GPFEDL  REYVKQ**T**WNLQGQ  LEKLIA**T**TAHEKM  EKLIAT**T**AHEKMP  VLIGSK**T**NGKFLI  EGKKFD**T**LWQLVE  PCQKIG**T**QGNVNF  LDRKLL**T**LEDKEL  SVSRPQ**T**GLSFLG  AIVGST**T**GSNYYV  KAVAHH**T**TAAFIR  VKVIMA**T**NRADTL  GGSGSG**T**PGPVGS  NPPFKS**T**ARFTLN  HIERLR**T**HSIESS  EQHWDF**T**AEDLKD  VKRIRS**T**VDEKEQ  CMELMS**T**SFDKFY  EILGKI**T**LATVKA  GKITLA**T**VKALNH  VWSLGI**T**LYELAT  TLYELA**T**GRFPYP  SVFDQL**T**QVVKGD  FVNLCL**T**KDESKR  AETLAK**T**QVAGGQ  LRLEGN**T**VGVEAA  HWSDMF**T**GRLRTE  FTGRLR**T**EIPPAL  LGEGLI**T**AGAQLV  KSSACF**T**LQELKL  AVAMAE**T**LKTLRQ  MAETLK**T**LRQVEV  LDLNGN**T**LGEEGC  KILDPN**T**GEPAPV  VLIAQQ**T**DTSDPE  SSFNSN**T**FLTRLL  KIHYLD**T**TTLIEP  HYLDTT**T**LIEPVS  GELVKV**T**KINVSG  RGHFPF**T**HVRLLD  VDHEEL**T**RDLEKH  PANSED**T**HLVDPK  HLVDPK**T**DPSDSQ  DPSDSQ**T**GLAEQC  ATDDSS**T**QNKRAN  NCLLPY**T**GATCEV  PCDARG**T**QNCVQR  LIRNRA**T**DLDARM  GVSSPP**T**SMQSQI  SRSCHP**T**MTILQA  SCHPTM**T**ILQAPT  PTPAPS**T**IPGPRR  SGPEIF**T**FDPLPE  LLFLLL**T**IVFCQI  LEPFNL**T**SEPSDY  YALDLS**T**FLQQHP  PPAPNP**T**PPRNLD  RNLDSR**T**FITIGD  DSRTFI**T**IGDRNF  EADDLV**T**ISELGR  TVDCFY**T**VTFYGA  DCFYTV**T**FYGALF  VLDKNM**T**IPEDIL  VWSLGI**T**MIEMAI  PEFVDF**T**AQCLRK  MEHPFF**T**LHKTKK  FFTLHK**T**KKTDIA  LHKTKK**T**DIAAFV  QLSAAS**T**EAERLA  HSHPHI**T**VWPSHV  FIEDKN**T**KTGRVL  RRIHSL**T**HLDSVT  PSRLRK**T**RKLRGH  NQSFCP**T**VNLDKL  TLVSEQ**T**RVNAAK  NAAKNK**T**GAAPII  IKRNKQ**T**YSTEPN  GQRKPA**T**SYVRTT  ATSYVR**T**TINKNA  NKNARA**T**LSSIRH  APAVAE**T**PDIKLF  LFGKWS**T**DDVQIN  PIVERL**T**NSMMMH  EIIHLL**T**GENPLQ  RRSRPE**T**GRPRPK  ERPARL**T**RGEADR  GEADRD**T**YRRSAV  AGAGSA**T**EFQFRG  HKLLVL**T**GQCFEN  EQSQGS**T**TNSDWM  VAAAVG**T**GATTAA  MEEPSY**T**QDNDLS  QPAPAA**T**QAAPQA  AVMNPK**T**ANVPQT  TANVPQ**T**VPMRLR  ASTDAG**T**AGALTP  NHIDQT**T**TWQDPR  INHKNK**T**TSWLDP  NHKNKT**T**SWLDPR  LRSQLP**T**LEQDGG  LEQDGG**T**QNPVSS  PFLNSG**T**YHSRDE  SVDEMD**T**GDTINQ  ESVLAA**T**KLDKES  YQKELA**T**LQRQSP  AEMDAR**T**ILLNTK  RFQPGE**T**LTEILE  KISLKY**T**AARLHE  ILREKQ**T**DEIKDT  TDEIKD**T**RNESTQ  DTRNES**T**QNCVVA  SDISNP**T**AHENYE  VELFIS**T**TPDSRK  PDSRKR**T**RHFNND  ETLGTA**T**FTVSSM  ELENIT**T**KHIVSN  TAHYKL**T**STVMLW  NLGGSL**T**RQMEKD  ENKIRS**T**LNEIYF  EIYFGK**T**KDIVNG  VERDRA**T**LAALET  LEGCIR**T**LRYFAG  DKIQGK**T**IPTDDN  STDYGL**T**AAVFTK  EQKARE**T**LKNSAL  PRLFTL**T**ALRRRG  IVFIER**T**DFKEEP  PVGLRH**T**GYVIEL  AKSKNH**T**THNQSR  KSKNHT**T**HNQSRK  AKAKDQ**T**KAQAAA  AQAPKR**T**QAPTKA  NGRLSN**T**QGVVSA  VVSAFS**T**MMSVHR  SPLEEA**T**LSELKT  VDMSVK**T**KIQKLG  ENINGL**T**SAFLFS  VTPEGE**T**IILDQI  FHNFSK**T**VEVETP  KTVEVE**T**PHCAMC  NALASA**T**CERCKG  GLAYCE**T**HYNQLF  ACSTCN**T**KLTLKN  TCNTKL**T**LKNKFV  KNCANT**T**RSFCDL  ALVLTS**T**IVTLKW  DESDSD**T**EAAPRT  PSDQSD**T**SESDVD  RTTPVS**T**PPLLTS  PLRDES**T**QEHSER  KTPVSP**T**FPEHLG  KGGNSV**T**SRETQD  VANTMR**T**SLGPNG  VTNDGA**T**ILSMMD  DEIGDG**T**TGVVVL  KNSRAV**T**IFIRGG  RNILGG**T**VFREPI  GWTKPI**T**IGRHAH  HQKGRP**T**STNPIA  EHFLNT**T**DFLDTI  IEFVLP**T**SQRKCK  VIHRST**T**SQTIKV  AMLHDF**T**QQVQVI  EMLQKV**T**LDIKSL  YPTGNH**T**YQEIAV  SPVPAI**T**VNQGEM  GCQKPI**T**GRCITA  KQLNKG**T**FKEQND  IDDYKV**T**SQVLGL  QIFNKR**T**QEKFAL  RGDQAF**T**EREASE  PENLLY**T**SKRPNA  NAILKL**T**DFGFAK  FGFAKE**T**TSHNSL  TSHNSL**T**TPCYTP  ISPGMK**T**RIRMGQ  IRNLLK**T**EPTQRM  LLKTEP**T**QRMTIT  TQRMTI**T**EFMNHP  PWIMQS**T**KVPQTP  RVIATF**T**CSGEKE  LMANFL**T**QGQVCC  MRPCVL**T**NCRDDM  RENGRV**T**IEYYSQ  VLLKPN**T**SVQFLR  RVLFPA**T**GYLSIV  YLPTRV**T**AIHIDP  VAFLTS**T**EPQYGQ  EVGDGT**T**SVIILA  TREEDF**T**RILQME  KPDVVI**T**EKGISD  IRRVRK**T**DNNRIA  EHAYPF**T**FEIPPN  QPTAET**T**RQFLMS  TNNTNK**T**VKKIKI  CKVYTL**T**PFLANN  SPSPTP**T**PGPSRR  RKLQKS**T**HLLIRK  EICVKF**T**RGVDFN  LHAGRV**T**LFPKDV  DGTFKL**T**IEFTEE  KLTIEF**T**EEYPNK  QNRWSP**T**YDVSSI  DVSSIL**T**SIQSLL  YVHSSA**T**IPLDDP  MSNHTA**T**HILNFA  AAKAVY**T**QDCPLA  DVSAQA**T**GKNVGC  GGPTPS**T**WINQVR  GSRLEE**T**LYSDQE  GKDTFI**T**HELAAE  AKRRGS**T**CVLAGM  HLAVAD**T**LLVLTL  LNIVHA**T**QLYRRG  FPQVGR**T**ALRVLQ  DSSWSE**T**SEASYS  KAAVGP**T**GGGGGG  LQPGHP**T**PPPTPV  HPTPPP**T**PVPSPH  KQRNVE**T**QQKVLE  GLAGLA**T**DVQTVA  DFVVSG**T**CAEQMY  IEKDKI**T**TRTLKA  EKDKIT**T**RTLKAR  ESSESF**T**MASSPA  RQVERA**T**EDGEED  GDEIEL**T**GIYHNN  PVSREW**T**LEAGAL  GNARPE**T**VTNDDE  ISKFRD**T**HKKLEE  SASGNA**T**PNTPGD  LIEKIP**T**AKPELF  GDAYYD**T**DYRHSY  RYEIVS**T**LGEGTF  STLGEG**T**FGRVVQ  ELLGLS**T**FDFLKD  LHDNKL**T**HTDLKP  ERSVKS**T**AVRVVD  DHEHHS**T**IVSTRH  EYYVGF**T**LFQTHD  GFTLFQ**T**HDNREH  SRMIRK**T**RKQKYF  LDWDEN**T**SAGRYV  PLRRYL**T**SEAEEH  EEDEEL**T**LKYGAK  DGQLIY**T**PFTEDT  SVIVVM**T**ILLVVL  KYLPEW**T**AWLILA  SVVFLK**T**ELSFAL  VADLFQ**T**TQRVGT  EKHFHG**T**SLTFSM  GPEAGQ**T**VKHVHV  RSGIDI**T**DFQAKR  TGVINS**T**PAPANT  SQPAPP**T**FGTVSS  VFSSSG**T**SFSGRK  EAEKQI**T**GPEEAD  SMSSHD**T**ASPAAP  QEPSAH**T**FDDAQL  RFLSSP**T**YRALLL  EALHLG**T**LMAAHG  VYLCKR**T**MQNKAR  PGCVNT**T**EVDIKK  HTPTPE**T**KPPTED  EVFKAL**T**DNGRHI  SLRNSP**T**SVLPSF  RGSEKK**T**SGPLSP  GPLSPP**T**GPPGPA  AVVPVL**T**ALISYH  SFRARS**T**SLNERP  KLVTVT**T**SVGTGT  TSPGPR**T**APAAKP  RRPTGN**T**SWLMSL  ALKSRA**T**ASNAEK  QIPSYD**T**HKIAVL  RYTEFL**T**GLGRLI  KDGSKV**T**TVVATP  YSRAKQ**T**LPVIYV  TPTARL**T**PLEACA  STPTNA**T**AASDAN  GMSNNS**T**TSISQA  MSNNST**T**SISQAR  LSDTEL**T**TLLRRY  EESYFT**T**RTYGEP  DLSYYP**T**SSSTSF  SSSSWL**T**RRAIRP  DPSVLH**T**EEMRFL  GKVPPA**T**QKAKSE  AKSEEN**T**KEEKPD  KAIDLF**T**DAIKLN  KRILHK**T**KGKRFT  TKGKRF**T**YKFNFN  FRFPPS**T**PSEVLS  SEVLSP**T**EDPRSP  SDCSDG**T**SELEEP  SPALPM**T**PTHLAY  PTHLAY**T**PSPTLS  AYTPSP**T**LSPMYP  RYLQAH**T**QSVYNY  PPMAPE**T**PPVPSS  DGEVFK**T**PRAPPA  SDLQHA**T**AQLSLE  ICSSRA**T**VMLYDD  RWLPAG**T**GPQAFS  QIYHNP**T**ANSFRV  VKYNQA**T**PNFHQW  PPPALP**T**WSVPNG  EASGGP**T**APKAES  TQVGEK**T**PKDESA  PWEKNS**T**TLPRMK  KSSSSV**T**TSETQP  SSSSVT**T**SETQPC  SVTTSE**T**QPCTPS  SETQPC**T**PSSSDY  DGIHHW**T**PPKGHV  ETALRE**T**QEEAGI  IEAGQL**T**IIEGFK  ARNKPK**T**VIYWLA  KARDKN**T**NQIVAI  KDGINR**T**ALREIK  VFDFME**T**DLEVII  SDLDQL**T**RIFETL  LTRIFE**T**LGTPTE  IFETLG**T**PTEEQW  ETLGTP**T**EEQWPD  SLPDYV**T**FKSFPG  CARITA**T**QALKMK  SNRPGP**T**PGCQLP  PNCPVE**T**LKEQSN  AIKRKR**T**EALEQG  KARHRK**T**GQKVAL  KEGFPI**T**ALREIK  LIEICR**T**KASPYN  NVLVKF**T**LSEIKR  AANVLI**T**RDGVLK  YTNRVV**T**LWYRPP  IMAEMW**T**RSPIMQ  PIMQGN**T**EQHQLA  QLCGSI**T**PEVWPN  LKGMLS**T**HLTSMF  MLSTHL**T**SMFEYL  WVGSPS**T**PAGWVR  WVREGD**T**VQLLCR  LTLEGV**T**RGQSGT  HVFHFG**T**VSPQTS  GTVLIK**T**AEELMN  CKTVGA**T**ALPRLT  TALPRL**T**PPVLEE  VPGGGA**T**EIELAK  KTVKKN**T**FFKCTV  IELDED**T**GTFRIY  PFVFVG**T**KESIGN  GEEKIN**T**LKEENT  PEGKLE**T**KAGHPP  VQKHPH**T**GDTKEE  HPHTGD**T**KEEKDK  KHPSPR**T**QHIQQP  FLFEYD**T**PRMVLV  YEKGYQ**T**SSGLIS  NSFVVM**T**NFIVTP  MTNFIV**T**PKQTQG  IVTPKQ**T**QGYCAE  CVAFND**T**VKTCEI  FNDTVK**T**CEIFGW  NAAHMK**T**CLFHKT  TCLFHK**T**LHPLCP  SGQNFS**T**LAEKGG  GGVVGI**T**IDWHCD  HFVENG**T**NYRHLF  SWPATT**T**QNSVAL  STLKKK**T**NKFIYA  DSSKND**T**VCIHKD  FMKEGN**T**QIKEDL  SALAFY**T**SCSRKT  TCGIFS**T**ASGKSV  ATHSLF**T**CPENEE  RPARWY**T**KLGFFP  EHEENT**T**KPYLPS  NIQLAA**T**KKTQYQ  CTSGPY**T**AQIIPG  PVSAQM**T**SKSCKG  DLVHIL**T**HLQLLR  RARDII**T**AWHPPA  SPAPSS**T**KPGPES  RAVMRN**T**VYAVKR  NADLEW**T**AVKQSF  VKQSFL**T**EVEQLS  LDILLG**T**ARAIQF  GVVVLE**T**LAGQRA  GQRAVK**T**HGARTK  KRRPPM**T**QVYERL  NSYVSS**T**GRAHSG  GCPQGD**T**AGESSW  VIAEPV**T**THSFDK  IAEPVT**T**HSFDKV  AGILFA**T**LSCLHI  LMPFVK**T**CLMVLP  CLPKGP**T**CCSRKM  EEKYQL**T**ARLNME  LFPVIY**T**QLMNPG  FPKLIM**T**QVSKSL  IVYLCI**T**DDDFER  IKKRFQ**T**TYGSRA  YGSRAQ**T**ALPYAM  LVDSSV**T**FKTTSR  LFVLTK**T**NLSYYE  KAAPGC**T**LWEAYA  KHYHVH**T**NAENKL  PHLASD**T**IYQIMY  INHNNL**T**ESVGPL  LEDLQL**T**HNKITK  EGLVNL**T**FIHLQH  LPVSLL**T**LYLDNN  SQKRHW**T**FSSEEQ  PEILRK**T**ADDFLN  PSQIAL**T**AILSSA  HEEEEW**T**DDDLVE  DYEVLY**T**IGTGSY  VLYTIG**T**GSYGRC  LDYGSM**T**EAEKQM  DRIIDR**T**NTTLYI  IIDRTN**T**TLYIVM  IDRTNT**T**LYIVME  SVITKG**T**KERQYL  FVLRVM**T**QLTLAL  RVMTQL**T**LALKEC  ALMPPF**T**AFSQKE  ELNEII**T**RMLNLK  NSESQL**T**SKSKCK  HFEKWG**T**LTDCVV  RGFGFV**T**YSCVEE  KPGAHL**T**VKKIFV  TFDDHD**T**VDKIVV  AGRLGS**T**VFVANL  IERMGQ**T**MERIGS  IDRVGQ**T**IERMGS  NLPFDF**T**WKMLKD  TNIASG**T**SEQTKA  LRNLTW**T**LSNLCR  VEQILP**T**LVRLLH  QKEATW**T**MSNITA  APPSEE**T**PLIPQR  SCSLLS**T**EAGALH  QGLSFV**T**RRRIRR  DGEARK**T**EVLLKV  RLVIAS**T**LYEDGT  TLYEDG**T**LDDGEY  RIEGDE**T**STEAAT  TSTEAA**T**RLSAYV  ITINKD**T**KVPNAC  IIIRVQ**T**TPDYSP  SPQEAF**T**NAITDL  AFTNAI**T**DLISEL  VVVTGL**T**LVCSSA  PLELDL**T**GDLESF  MKYIQH**T**YRKGVK  GVKIDK**T**DYMVGS  PFSQDP**T**QLAEMI  IQAKGK**T**PSLDPH  QKILQE**T**LNELDK  LLGRLT**T**LIELLL  QLETWF**T**AGAKLL  LRNAQV**T**ELLQRL  KTGSKF**T**VRTRLL  RKFNIL**T**SNQKTL  LTSNQK**T**LTPEKG  SSEGGI**T**CSWVEH  LQSLPL**T**EIIRHY  LLAGQN**T**VDEVYV  PVQQIL**T**NSGQLL  TTVAAP**T**PAQAQI  QAQITA**T**GQQQPQ  GQELFD**T**ENVVVC  KRTTAA**T**LMNAYS  INQSLL**T**LGRVIT  LGGRTR**T**SIIATI  KTQELE**T**TQKHLQ  EMILSP**T**VVSILK  IFKTSL**T**VADKIE  KLMNLW**T**ERFCAL  IFLDQM**T**IDEDKL  LKLDIP**T**GTTPQR  SYLYPS**T**LVRTEP  ENNKEE**T**IPDVDV  SKVEET**T**EHLVTK  PFPSEE**T**TENDDD  QKVEEC**T**LKVQDG  KMFLRG**T**FYQGYM  YQGYMC**T**KCGVGA  EDQVLI**T**EHGDLG  DVQDSL**T**VSNEAQ  YQTMSD**T**TFKALR  QLPVTR**T**KIDWNK  GAGQPS**T**SAQGQP  KQQQEP**T**GEPSPK  KPAQEE**T**EETSSQ  QEETEE**T**SSQESA  EQYYAA**T**QLYKDP  FPWMKS**T**KAHAWK  RTRTAY**T**RAQLLE  AVMLNL**T**ERHIKI  GKPPFE**T**SCLKET  TSCLKE**T**YLRIKK  IQKMLQ**T**DPTARP  MLQTDP**T**ARPTIN  PARLPI**T**CLTIPP  SNRKPL**T**VLNKGL  EPVVRE**T**GEVVDC  VLFNDS**T**RLILYN  GTESYL**T**VSSHPN  RLPYLR**T**WFRTRS  NFFQDH**T**KLILCP  PLMAAV**T**YIDEKR  AQQLHS**T**GEINIM  KLLSVE**T**PRALHH  HYRGRY**T**VEDIQR  ETSLTN**T**MGGYKE  TYDYAK**T**ILSLMT  GSIANF**T**NVAATF  RSAYDS**T**METMNY  LEVEKI**T**TSKKPN  LTLLCC**T**WREERM  ECFICL**T**CGTFIG  FIGDGD**T**YTLVEH  GHCYYQ**T**VVTPVI  YYQTVV**T**PVIEQI  GTEHSH**T**VRVQGV  HGLGPE**T**SPLSSP  KRLNFI**T**EYIKGG  EYIKGG**T**LRGIIK  PSFFPI**T**VRCCDL  LEHWLE**T**LRMHLA  DRGFWE**T**YRRGES  GAPPAG**T**SPSPGT  SPSPGT**T**LAPTTV  GTTLAP**T**TVPITS  TTLAPT**T**VPITSA  YYCSEP**T**LDVKIA  VEVGDS**T**FTVLKR  RPFQNQ**T**HAKRAY  SLLNVF**T**PQKTLE  VVKSDC**T**LKILDF  MTPYVV**T**RYYRAP  VIEQLG**T**PCPEFM  PKYAGL**T**FPKLFP  LDEREH**T**IEEWKE  MNSEEK**T**KNGVVK  SMSTDQ**T**LASDTD  QTLASD**T**DSSLEA  NEEQKE**T**VDNVQR  ENNRVK**T**RIFLHV  LEDVKP**T**YWAQSH  IHQEVN**T**EPYNPF  PVKKQK**T**GETSRA  KQKTGE**T**SRALSS  NMAGVR**T**EDSIRD  DGMEVD**T**TPTVAG  GMEVDT**T**PTVAGQ  EVDTTP**T**VAGQFE  MVWKRA**T**YIGPST  EREGWK**T**NMDFVG  SRIHQS**T**YGKSLA  KSLAIM**T**EAQLST  DGRRRI**T**PLCIAQ  LSPSVL**T**TPSKIE  PGAPAL**T**SMTPTA  EWETVL**T**SRILTA  ISTLHC**T**GSYVMA  SYVMAL**T**AAATLS  LAGSDM**T**VSQILL  AIIQGR**T**SNSGRQ  RAPGLI**T**PGSPPP  AAPAIV**T**SSPQFA  SCCPER**T**SQLFDL  DVCISL**T**LKNLKN  GPEKGP**T**LAPSPH  PFPPVP**T**VTTSSA  LSTVIA**T**VASSPA  IPYPAG**T**LPNPLL  VVESKR**T**RTITSQ  TEANLD**T**RKSVSD  KESRRA**T**ESRIPV  LKDRWR**T**MKKLKL  IYWGTA**T**TGKPHV  QLSKEY**T**LDVYRL  DQRKIF**T**FAEKYL  PMVPGL**T**GSKMSS  HYVLGD**T**LGVGTF  DTLGVG**T**FGKVKI  IGEHQL**T**GHKVAV  QVISTP**T**DFFMVM  DDEHVP**T**LFKKIR  CEKFEC**T**ESEVMN  RRKNPV**T**GNYVKM  QRSGSS**T**PQRSCS  SSFDST**T**AESHSL  SLSGSL**T**GSLTGS  GSLTGS**T**LSSVSP  PRLGSH**T**MDFFEM  LVIAPG**T**CIPNAV  PSDPTY**T**SSLGGK  QPLLDR**T**VPDYTT  TVPDYT**T**FTTVGD  LVSSLL**T**LLKQLE  AWLDSG**T**WGVPSL  VWHLLR**T**PPEPPT  SRQPSF**T**YSEWME  KPEADP**T**SLTVKS  HSRVCL**T**SPDPDD  EKVYIA**T**QGPIVS  YDGVEI**T**VQKVIH  ISLKSG**T**EERGLK  LKHYWF**T**SWPDQK  TGCFIA**T**SICCQQ  VDILKT**T**CQLRQD  INGVLR**T**AHSLFK  EDQNTL**T**SICEKV  CLSIRI**T**CKANPA  LQNSKT**T**KFIKSF  QLFRGD**T**VLLKGK  DTVEGI**T**GNLFEV  LYGPPG**T**GKTLIA  EQVANE**T**HGHVGA  LIIDLE**T**RGSQAL  ISCLED**T**GQDMLA  LASFLR**T**NRQAAK  AKLSKP**T**LENLTP  PTLENL**T**PVVLRP  RESGLR**T**RTGSNI  SGLRTR**T**GSNIDC  EVKGDL**T**AKKMVL  PGAVYG**T**DGCPVS  VNIFNG**T**SCPSLG  NPEPDA**T**PFQEGL  GSWYVE**T**LDDIFE  AKIEIQ**T**LTGLFK  DVVDNQ**T**EKLDTN  EFTELL**T**LVNRKV  CFASML**T**KKLHFF  YHAGGS**T**QNSIKV  ENNRIF**T**LNLSAP  REQGFE**T**KDIKEI  ATESEV**T**AFAVLD  RIQNVV**T**SFAPQR  GETPSW**T**GSGFVR  DDRIQG**T**LQPHAR  AQELQQ**T**GVLGAF  VMMFGS**T**AIALEL  SPNVQD**T**SGTSPV  VQDTSG**T**SPVHDA  VHDAAR**T**GFLDTL  YGTRLA**T**CSSDRS  ISLLTY**T**GEGQWE  IGLPTS**T**IASCSQ  GGDNKV**T**LWKESV  LADAHL**T**KKLLDL  KGANEA**T**KTLNRG  ANEATK**T**LNRGIS  VIACSV**T**IKEGSQ  SRAEVR**T**HYDPPR  GPDGRV**T**GEADVE  ADVEFA**T**HEDAVA  ELFLNS**T**AGASGG  DFDKYM**T**ISDEWD  RWSPKG**T**YLATFH  GTQGVV**T**NFEIFR  TGRYVV**T**SVSWWS  EGELSD**T**IPVVHA  RNSLPD**T**VQIRRV  AFCGLD**T**TSTELS  GLDTTS**T**ELSVVE  LRLPRE**T**DEEPEE  GYYVEM**T**VGSPPQ  LNILVD**T**GSSNFA  VEGPFV**T**LDMEDC  GPGPPA**T**PAQPQP  PSPPKF**T**SQDSPD  QCEANL**T**TLTNAV  FFTAVA**T**NQPPKI  FYDIER**T**LGKGNF  DGPNLP**T**LRQRVL  FSAHSY**T**SNLGDY  LCPQPQ**T**LVQSVL  STGRRH**T**LAEVST  TRLSPL**T**APCIVV  IVVSPS**T**TASPAE  VVSPST**T**ASPAEG  PAGLSG**T**PATQGL  LSGTPA**T**QGLLGA  LGSQSA**T**PVLQAQ  LRKTTR**T**KGFLGL  RRCTAL**T**FIASEE  SFCLGH**T**EFVSRI  ALLCDG**T**PVVYIF  QSVPES**T**VLKKVS  EDSLTH**T**DVSSFQ  FQASTQ**T**RRAPGG  PGGIKE**T**RKAMRD  KSKNKK**T**GDEEVN  GERRYG**T**CIYQGR  LSAVRA**T**LQAALC  LLLQPV**T**ISRNEK  DISFLI**T**NFHTEQ  LITNFH**T**EQMYKH  FEDPET**T**RQMQST  QSLQQR**T**WLIHWS  YLNAIQ**T**MCPHIL  LTTAVI**T**NKDVRK  ARAAGS**T**VRQRKN  KNASCG**T**RSAGRT  RSAGRT**T**SAGTGG  GMWRFY**T**EDSPGL  GFDLDL**T**YIYPNI  AERHYD**T**AKFNCR  KAGKGR**T**GVMICA  RDKKGV**T**IPSQRR  PMFSGG**T**CNPQFV  SSNSGP**T**RREDKF  FHFWVN**T**FFIPGP  IPGPEE**T**SEKVEN  KEYLVL**T**LTKNDL  YLVLTL**T**KNDLDK  KVKLYF**T**KTVEEP  PEASSS**T**SVTPDV  GPVRMP**T**KTLRIT  VRMPTK**T**LRITTR  RITTRK**T**PCGEGS  EIVKQI**T**SISIEP  DGAVGK**T**CLLISY  LLISYT**T**NKFPSE  PLSYPQ**T**DVFLVC  KWVPEI**T**HHCPKT  EGISIY**T**SDNYTE  RKALKT**T**VILILA  HKWISI**T**EALAFF  GHSSVS**T**ESESSS  EESAGG**T**KGSSKK  ELNLPK**T**CDISFS  PKVKCE**T**MVYHPN  DWKPVL**T**INSIIY  LVDENF**T**ELRGEI  QWAAAM**T**LRTVLL  PEMFKQ**T**ARLWAH  KSWDVE**T**ATELLL  DCGTGY**T**KLGYAG  PENREY**T**AEIMFE  DSGDGV**T**HVIPVA  KWIKQY**T**GINAIS  QILDKA**T**EYIQYM  MRRKNH**T**HQQDID  SSAQLQ**T**NYPSSD  SDNSLY**T**NAKGST  PDPILL**T**NSHDGL  EEAFQG**T**KVFVMP  LIEKCN**T**VKMWVQ  DYRRTV**T**EIDEKE  RAFGFM**T**RVALQA  YNKVHI**T**LSTHEC  VHITLS**T**HECAGL  ERSAAE**T**VTKGGI  SAAETV**T**KGGIML  GKVLQA**T**VVAVGS  LPEYGG**T**KVVLDD  LEAIQR**T**PKIQVY  FYLLYY**T**EFTPTE  LYYTEF**T**PTEKDE  YTEFTP**T**EKDEYA  EEYLSS**T**PQRLKL  LLYILL**T**GALQFG  YCLLVG**T**FPFNSF  DFLFAS**T**ILHLVV  LEPSLY**T**VKAILI  ALLEGL**T**VVYKSS  GEDVPL**T**EQTVSQ  VPLTEQ**T**VSQVLQ  TEQPEE**T**FPNTET  NIKALR**T**DYNASV  LPSPTA**T**SQLPLE  IITITG**T**QDQIQN  GKNVQL**T**ENEIRG  GKQSLE**T**ICLLLA  NIKLWK**T**FTDCFN  KLWKTF**T**DCFNCL  RRIMRP**T**DVPDQG  DRGVSF**T**FGAEVV  AKRQLV**T**LFSAPN  MMSVDE**T**LMCSFQ  VFKAHL**T**FPKDYP  PKMKFI**T**EIWHPN  RWLPIH**T**VETIMI  PIHTVE**T**IMISVI  GMDVEL**T**VEERNL  YRQMVE**T**ELKLIC  MTELPP**T**HPIRLG  DNLTLW**T**SDMQGD  KCRGLR**T**ARKLRS  KKAHLG**T**ALKANP  KNGKKI**T**AFVPND  KIAFAI**T**AIKGVG  EVERVI**T**IMQNPR  RVRGQH**T**KTTGRR  RGQHTK**T**TGRRGR  RVLLGE**T**GKEKLP  KEAIEG**T**YIDKKC  CRPLSK**T**VRFNVL  FNVLKV**T**KAAGTK  PFLNGL**T**GKPVMV  NMQLAN**T**EEYIDG  EEEEFN**T**GPLSVL  GPLSVL**T**QSVKNN  QSVKNN**T**QVLINC  NVKEMW**T**EVPKSG  SVLGAI**T**SVQQRL  SGALFG**T**LQGNTR  GSADFK**T**ELSQSD  KDKSHF**T**DKETGQ  WARECP**T**GGGRGR  RGRGGF**T**SDRGFQ  HIQKDC**T**KVKCYR  CYRCGE**T**GHVAIN  DGACGK**T**CLLIVF  PEVYVP**T**VFENYV  PLSYPD**T**DVILMC  SDEHVR**T**ELARMK  DERKLR**T**FYEKRM  CYRPRR**T**GERKRK  KIQRLV**T**PRVLQH  SSLRAS**T**SKSESS  DNIQGI**T**KPAIRR  RDAVTY**T**EHAKRK  EHAKRK**T**VTAMDV  AKRKTV**T**AMDVVY  TESYIS**T**IGVDFK  GQERFR**T**ITSSYY  GNKCDL**T**TKKVVD  KKVVDY**T**TAKEFA  EKKYVA**T**LGVEVH  IIMFDV**T**SRVTYK  DLEVAQ**T**TALPDE  LEVAQT**T**ALPDED  DTVTIR**T**RKFMTN  RTRKFM**T**NRLLQR  HFGGGK**T**TGFGMI  LYEKKK**T**SRKQRK  YAMHWG**T**DSRLLV  ETGQQT**T**TFTGHT  LSLAPD**T**RLFVSG  TGSDDA**T**CRLFDL  AAMDVD**T**PSGTNS  VDTPSG**T**NSGAGK  ANQASA**T**SEECTV  ATSEEC**T**VAWGVC  ESGDRL**T**RAAKVL  KIAVHC**T**VRGAKA  KNNFSD**T**GNFGFG  ADKKRR**T**GCIGAK  AAEGIH**T**GQFVYC  GTMPEG**T**IVCCLE  ISHNPE**T**KKTRVK  TGRLRG**T**KTVQEK  ADKVPK**T**AENFRA  CQGGDF**T**RHNGTG  FTRHNG**T**GGKSIY  KTSKKI**T**IADCGQ  EVEPSD**T**IENVKA  RKKKSY**T**TPKKNK  CGKCCL**T**YCFNKP  GKSARH**T**PARSRS  FGLSLY**T**TERDLR  RVDFSI**T**KRPHTP  KRPHTP**T**PGIYMG  LAPPLV**T**LLSGEP  LLDLIQ**T**KVNYVV  EISGTF**T**HRQGHI  PGNPNY**T**LSLKCR  QEAKPS**T**EDLGDK  HFKVKM**T**THLKKL  FKVKMT**T**HLKKLK  RIADNH**T**PKELGM  DSVECA**T**QALEKY  DKKYNP**T**WHCIVG  NFGSYV**T**HETKHF  SYVTHE**T**KHFIYF  QHDPLL**T**GVSSST  TGVSSS**T**NPFRPQ  AEVDKV**T**GRFNGQ  FNGQFK**T**YAICGA  EMSTSK**T**GKHGHA  VGIDIF**T**GKKYED  GEEILI**T**VLSAMT  TVLSAM**T**EEAAVA  QMTLRG**T**LKGHNG  KLTRDE**T**NYGIPQ  SGSWDG**T**LRLWDL  WSADGQ**T**LFAGYT  LADIFT**T**CVDIRW  SEVNSF**T**AAFLFS  TYEVPN**T**PLCSAR  VPESTS**T**DTPPDI  TKNPDG**T**MNLMNW  IPGKKG**T**PWEGGL  DWRPAI**T**IKQILL  AQAEAY**T**IYCQNR  AAVEQL**T**EEQKNE  MLGQNP**T**PEELQE  KIMLQA**T**GETITE  ATGETI**T**EDDIEE  YSVETV**T**LLVALK  LSPSID**T**LDHIRA  GQDISE**T**FNHANG  HDRNVV**T**IFSAPN  ALSAAD**T**KPGTTG  ADTKPG**T**TGSGAG  DTKPGT**T**GSGAGS  DKKVIA**T**KVLGTV  QDKFNL**T**GLNEQV  HARYIL**T**NRGIAQ  KCMDVY**T**PKSSRH  DGAYFG**T**GFPHML  SKRGIL**T**LKYPIE  LYASGR**T**TGIVLD  YLMKIL**T**ERGYSF  SAGIHE**T**TYNSIM  VDSGKS**T**TTGHLI  DSGKST**T**TGHLIY  SLWKFE**T**SKYYVT  TSKYYV**T**IIDAPG  KNMITG**T**SQADCA  VNKMDS**T**EPPYSQ  IVKEVS**T**YIKKIG  FKGWKV**T**RKDGNA  GNASGT**T**LLEALD  KPGMVV**T**FAPVNV  MEAAGF**T**AQVIIL  AGAGKV**T**KSAQKA  QMPSDK**T**IGGGDD  LSVAEI**T**NACFEP  INYQPP**T**VVPGGD  CMLSNT**T**AIAEAW  SRARVY**T**DVNTHR  VYTDVN**T**HRPREY  FEAINI**T**NNEKVV  GGPNII**T**LADIVK  KDPVSR**T**PALVFE  FEHVNN**T**DFKQLY  FKQLYQ**T**LTDYDI  QLYQTL**T**DYDIRF  IAKVLG**T**EDLYDY  DHQSRL**T**AREAME  EHPYFY**T**VVKDQA  EEKSAV**T**ALWGKV  SFGDLS**T**PDAVMG  LKGTFA**T**LSELHC  HFGKEF**T**PPVQAA  LCQIMR**T**VGPRLD  RFLLQD**T**VELREH  GHIMPP**T**QSQFGE  QDREWL**T**ELFQQS  AVSELF**T**TLPIVQ  LVCITV**T**EKLPFY  STMRRY**T**ISSALN  ALLHGE**T**RKTAFG  QAGPSE**T**DDVDEK  KWNARI**T**DLRKQV  PFRRPS**T**FGIPRL  TEEIKE**T**DGSSQI  VAASQL**T**FPAVTL  KTMKGG**T**GNGLEI  LPPPWG**T**CKAVTM  EMPCNL**T**RYGKEL  PPLPDP**T**PPEPEE  GWGHFS**T**VWLCWD  AQHYTE**T**ALDEIK  PEVKLK**T**TGLEEA  EAAEAE**T**AKDNGE  WIESPK**T**NGHIEN  NAESDY**T**YSSSYE  QFPEFS**T**SLFSGS  SEGSPL**T**EQEESS  TVSASS**T**GDLPKA  DLPKAK**T**RAADLL  FTEDIQ**T**RQYRSI  LMNIAG**T**TLSSKL  IVLRGA**T**QQILDE  LMAHAV**T**QLANRT  AHSEGN**T**TAGLDM  LIKLLQ**T**FRSSRL  QMFLTQ**T**DTGDDR  TVPEVY**T**PVLEHL  ASGEVR**T**GKWKVP  VEKLDL**T**LEIQTV  RIMEFT**T**TLLNTS  KKDLCN**T**HLMRVL  YSLFSE**T**INTELL  QGLKLA**T**TILQHW  QDPRPA**T**GRFRRR  KGAAGR**T**DLLRLR  PGLLTN**T**MDVFVK  RKRDLQ**T**STHVET  GRGEES**T**TTNYLI  YLNSGL**T**STKNYG  YGKTIL**T**KEADLV  MYLNND**T**CCNSDC  KNCQFE**T**AQKKCQ  QEAINA**T**CKGVSY  KGVSYC**T**GNSSEC  SCACNE**T**DNSCKV  RKGKPC**T**VGFCDM  DHQRMD**T**IQEDPS  KSFEDL**T**DHPVTR  LSSLLR**T**RGCVSE  ALQEAL**T**EHYKHH  LLPSEL**T**LWVDPY  ASCGLL**T**CKNQVL  AQAQDS**T**SDLIPA  CQPGEF**T**LGNIKS  LVRVVS**T**NYNQHA  ITLYGR**T**KELTSE  HSRPSF**T**NILDQL  NILDQL**T**TIEESG  TWEEEL**T**RAALQQ  DFQHKF**T**VQASPT  SPPASP**T**IIPRLR  PKKKGR**T**WGPGTL  SRPRRS**T**SPPSRK  PPLSPC**T**HNPLVN  LTPTHV**T**LTTPSQ  NTQQDS**T**LERPKT  PANSSS**T**ETPSNL  GQSQDS**T**VPLCRA  EEPPEK**T**VDVAAE  TSEIPQ**T**ERMQKR  GISSVP**T**KGLSSD  SSAGTG**T**TEDTEA  ILLVQP**T**KRPEGR  KRPEGR**T**YADYES  LVYRAD**T**QTYQPY  YRADTQ**T**YQPYNK  YTWDFG**T**EEAAPT  GPRGSS**T**VTIPRE  EPNGSA**T**GLTVWL  NYIVML**T**CAVCLV  LDRPSE**T**HADYLL  TRRIDD**T**IFQNPM  QDESSQ**T**SLQKEI  PCGHLV**T**CKQCAE  LGEKVH**T**EGRSEP  APEKRL**T**LAQIYE  YEWMVR**T**VPYFKD  KVHNEA**T**GKSSWW  APPEGA**T**PTSPVG  EEADMW**T**TFRPRS  EADMWT**T**FRPRSS  LDGLNL**T**SSHSLL  VTGPLH**T**YSSSLF  PADVLM**T**QVDPIL  ILSQAP**T**LLLLGG  LGTPVL**T**PPTEAA  VVKDRE**T**QRSRGF  RGFGFI**T**FTNPEH  FGFITF**T**NPEHAS  GKSARG**T**RGGGFG  KCSQCH**T**VEKGGK  KGGKHK**T**GPNLHG  GLFGRK**T**GQAPGY  KKYIPG**T**KMIFVG  QNPDAK**T**TELIRR  NPDAKT**T**ELIRRI  LKRKAM**T**KKKELT  HAKEDE**T**RYHNEM  QIKITG**T**KEGIEK  RTEIVF**T**GEKEQL  EEKKKK**T**TTIAVE  SDSISE**T**VILRGE  RLFDVC**T**VSRTDR  VCTVSR**T**DRETKL  SRTDRE**T**KLTLVF  DRETKL**T**LVFEHV  HVDQDL**T**TYLDKV  PEPGVP**T**ETIKDM  PGVPTE**T**IKDMMF  PQNILV**T**SSGQIK  LTSVVV**T**LWYRAP  LQSSYA**T**PVDLWS  PIEKFV**T**DIDELG  LLLKCL**T**FNPAKR  GRGIDK**T**NGAPEQ  GFGKLE**T**YIKLDK  GTPTEE**T**WPGILS  DGADLL**T**KLLQFE  GNAEES**T**LFCFAV  VKSVDP**T**LALSVY  QNLLIL**T**AIKADR  ARKANS**T**RTWKEV  FLTKLW**T**LVSDPD  LVSDPD**T**DALICW  KPERDD**T**EFQHPC  NIKRKV**T**SVSTLK  IRQDSV**T**KLLTDV  PIISDI**T**ELAPAS  PAPASV**T**ALTDAR  ASVTAL**T**DARGHT  PPSPPP**T**STPEKC  LFSPSV**T**VPDMSL  GFAEDP**T**ISLLTG  PTISLL**T**GSEPPK  GRKTYP**T**VKICNY  FGDFSP**T**DVHKQY  ERQGGR**T**ALHLAT  EELGLV**T**HLVTKL  QPQPPA**T**QQQQPQ  IGWSLT**T**SGMLLG  GKTTWV**T**KHAAEN  ECFDEI**T**YVELQK  TDGAVT**T**SQIPAS  AQKDTY**T**MKEVLF  LGQYIM**T**KRLYDE  LVSRPS**T**SSRRRA  SSSSES**T**GTPSNP  SSESTG**T**PSNPDL  QAGESD**T**DSFEED  ADYWKC**T**SCNEMN  AKLENS**T**QAEEGF  VPDCKK**T**IVNDSR  DYSQPS**T**SSSIIY  HLMACF**T**CAKKLK  LASIFG**T**EKDKVN  RLHNKP**T**FSQTIA  KPTFSQ**T**IALLNI  AELSPV**T**DFREAC  RRIYDI**T**NVLEGI  QWLGSH**T**TVGVGG  FLCPEE**T**VGGISP  PPSSLT**T**DPSQSL  PDVEGM**T**SLKVDN  DNLTYR**T**SPDTLR  YRTSPD**T**LRRVFE  IPRDRY**T**KESRGF  TIKTES**T**LKTTQF  ESTLKT**T**QFSCTL  TADGRK**T**QTVCNF  CVMNNV**T**CTRIYE  IKEFHT**T**GLAWSK  SAPKPQ**T**SPSPKR  VMGKVP**T**ISINKT  MNVLIP**T**EGGDFN  YEKVFK**T**DVSKIV  DSCKSK**T**AAAAAD  VSQAHL**T**PSPVPV  LNRERE**T**VPVELE  PQVKTA**T**IATERN  KTATIA**T**ERNGKP  DEEAIQ**T**LSAQLD  LSVEGN**T**DINTDL  TGAYDT**T**TATVTT  YDTTTA**T**VTTTQA  TATVTT**T**QASYAA  PPSYPP**T**SYSSTQ  GWVDPR**T**WLSFQG  PQGGLE**T**SQPEGE  ASPEPC**T**VTPGAV  LKQKRI**T**LGYTQA  RITLGY**T**QADVGL  GKVFSQ**T**TICRFE  KVFSQT**T**ICRFEA  EICKAE**T**LVQARK  LQCPKP**T**LQQISH  YGSPHF**T**ALYSSV  FPPVSV**T**TLGSPM  PPVSVT**T**LGSPMH  FLRFGE**T**KSIVEL  YKCPGF**T**VTSPDC  FYRSPA**T**PAEVAN  SGAISQ**T**PEQATH  RIQDLD**T**TDTGYY  ATNGMK**T**ITATGV  MKTITA**T**GVLFVR  AFTMIG**T**STHLSD  AVSLKP**T**AWSLRH  VGPRPQ**T**FLLDPY  TATKQK**T**NSPAWH  WHDEFV**T**DVCNGR  GHKFMA**T**YLRQPT  TYLRQP**T**YCSHCR  YQCQVC**T**CVVHKR  CHELII**T**KCAGLK  VTPDKI**T**NSGQRR  DDDVDC**T**MTEKRI  GILNGV**T**TTTFCG  FKPRIK**T**KRDVNN  AGGVPV**T**RPPDDS  RPPDDS**T**SAPRDL  LPVSPV**T**DLQATE  GPSAEV**T**ARTESP  LSATPT**T**IKDEAG  QVQIGF**T**GSSDNG  TLTPVQ**T**LTLGQV  LRRVAC**T**CPNCKE  GVCHED**T**GECICP  EGIQRM**T**PKIVDL  NYSVYT**T**NSDVWS  TPYCGM**T**CAELYE  RVFVHY**T**GWLLDG  GWLLDG**T**KFDSSL  GEVCHI**T**CKPEYA  IIRRIQ**T**RGEGYA  APNKEE**T**PATESP  KEETPA**T**ESPDTG  ATESPD**T**GLYYHR  LELLIQ**T**ATRDLA  LLIQTA**T**RDLAQY  NRFNPK**T**FFILHD  ELEALF**T**KELEKV  NQDRLV**T**LEEFLA  TGEGWE**T**VEMHPA  EMHPAY**T**EEELRR  QRLSQE**T**EALGRS  LKFHPD**T**DDVPVP  TRLGIY**T**VLFERL  KAVIGM**T**AGATGA  PVDIAK**T**RIQNMR  SLWKGF**T**PYYARL  SEGHLY**T**VPIREQ  QVYDAH**T**KEIDLV  IWKASF**T**TFTVTK  ASFTTF**T**VTKYWF  GEIDPA**T**GMVMNL  FADVVS**T**TENVAV  ADVVST**T**ENVAVY  KVKVYE**T**DNNIVV  LFKYQQ**T**GVRWLW  TVIVCP**T**TVMHQW  WVKEFH**T**WWPPFR  DRLGEE**T**NAVSGP  HETYRS**T**LRTLPG  FWGIDE**T**DVEPCC  WAVVTM**T**TLGYGD  ACFLLS**T**GDYACA  GERSTD**T**TKTHPT  ERSTDT**T**KTHPTI  TTKTHP**T**IKINGY  IKINGY**T**GPGTVR  RLCFQV**T**VRDPSG  DIEVYF**T**GPGWEA  PQPYPF**T**SSLSTI  FTSSLS**T**INYDEF  NYDEFP**T**MVFPSG  PPAPKP**T**QAGEGT  IPVAPH**T**TEPMLM  EYPEAI**T**RLVTGA  ILGREP**T**SSEQGG  VRLPDG**T**SLTQTF  PDGTSL**T**QTFRAR  GTSLTQ**T**FRAREQ  RTASTP**T**PPQTGG  PTTEKP**T**VTVNFR  FKLKML**T**EAIMHD  ARPATS**T**LNRFSA  YIQKKP**T**MYPPWD  VDLISE**T**TVELYS  DLISET**T**VELYSL  RKNNGK**T**EIWLEL  FLEMSD**T**KDMNEF  VKCHEF**T**ATFFPQ  CHEFTA**T**FFPQPT  KVIAKC**T**GSAINS  FCEHCG**T**LLWGLA  VHHRCQ**T**KVANLC  ARCLRD**T**EQIFRE  LAEFKK**T**NQFFAI  PPSGGL**T**DEAALS  SDADPS**T**KDFLLQ  DFLLQQ**T**MLRVKD  KSLDFY**T**RVLGMT  TRVLGM**T**LIQKCD  ALSRKA**T**LELTHN  KATLEL**T**HNWGTE  GTEDDE**T**QSYHNG  IPGEHS**T**DNNRTY  VKEAII**T**RIKAGI  PYCKAI**T**EPVTVK  TPRSGN**T**NPLSSF  SVQSLA**T**GPAGDP  LFGVFV**T**GKDGGP  LASLDQ**T**AGATPL  KSLSRI**T**EMQQVE  SKYQCE**T**GENSKG  YQWKML**T**EAEKWP  LYRDDC**T**KATHSR  DDCTKA**T**HSRMEH  SNEKIE**T**AFLNAL  TFFKVE**T**ENKFSF  PDIGMF**T**YDESTK  DTERLP**T**SHTCFN  PKAYVA**T**QGPLAN  SAGCGR**T**GAICAI  LVDHDN**T**SPLFRT  TSPLFR**T**PLSFTN  EAASLS**T**GSTVTI  STGSTV**T**IKGRPL  NGQSSY**T**FDHRIK  WRDISL**T**KFNVSY  GFLNQF**T**EDKPTL  LKAQLS**T**ILEEEK  KVYENV**T**GLVKAV  VGLALR**T**LLATVD  MKEALS**T**ERGKTL  TFFGQP**T**FCSVCK  KIIGRC**T**GTAANS  HNYMSP**T**FCDHCG  EALNQV**T**QRASRR  MQDNSG**T**YGKIWE  LQGLKY**T**FSVDWW  ESIRVD**T**PHYPRW  HYPRWI**T**KESKDI  TKRLGV**T**GNIKIH  IHPFFK**T**INWTLL  FKTINW**T**LLEKRR  KIGILI**T**DGKSQD  KIGETH**T**DIELSG  PLTGVF**T**TEEVPA  ALVSQP**T**RYLHPE  HYVVCS**T**PQSQYY  RPIFII**T**EYMANG  MRHRFQ**T**QQLLEM  MPYERF**T**NSETAE  RKKIQI**T**RIMDER  LIIFNS**T**NKLFQY  LFQYAS**T**DMDKVL  KVLLKY**T**EYNEPH  EPHESR**T**NSDIVE  SMSPGV**T**HRPPSA  LMGGDL**T**SGAGTS  LTSGAG**T**SAGNGY  YPSAIS**T**TYGTEY  SPPRDR**T**TTPSRY  PPRDRT**T**TPSRYP  HSPIGL**T**RPSPDE  NHQHWH**T**VVKEAC  YKSEFP**T**EADLED  MSDKEI**T**HDVKAV  TASISE**T**PVDVRV  LVPMGF**T**TATEFH  VPMGFT**T**ATEFHQ  MGFTTA**T**EFHQRR  LQGGIE**T**GSITEM  IETGSI**T**EMFGEF  EFRTGK**T**QICHTL  KTQICH**T**LAVTCQ  CHTLAV**T**CQLPID  YIDTEG**T**FRPERL  FNTDHQ**T**QLLYQA  LIVDSA**T**ALYRTD  GVAVVI**T**NQVVAQ  TYNEIV**T**IERLRS  FSQPNS**T**KVQRVL  DSIANA**T**VLLDYH  RRGPGY**T**SGTNSE  DPYPYE**T**YEPYPY  GVFDNA**T**GLLWIA  NGLASN**T**FNSSSL  KIPPVN**T**NLENLY  APNFKA**T**AVMPDG  FYPLDF**T**FVCPTE  FTFVCP**T**EIIAFS  VSDPKR**T**IAQDYG  GILRQI**T**VNDLPV  GRSVDE**T**LRLVQA  VQAFQF**T**DKHGEV  WKPGSD**T**IKPDVQ  QHKQDL**T**EKAVKQ  MEALRS**T**LQTMES  VQEQVH**T**LLSQDQ  DLKMLR**T**AVDSLV  HPSVRQ**T**PSRQPP  ATGPDA**T**VGGPAP  KRLQEE**T**GAKISV  PVRGAI**T**RGATVT  DVEENR**T**EAPEGT  TEAPEG**T**ESEMET  RAFSDL**T**SQLHIT  LHITPG**T**AYQSFE  AGSGGA**T**RPGGRL  GGRLLA**T**EKEASA  GASPPS**T**LTPDSR  SPPSTL**T**PDSRRV  AEVPDV**T**ATPARL  VPDVTA**T**PARLLF  LLFFAP**T**RRAAPL  YLREQA**T**GAKDTK  SRKALE**T**LRRVGD  VFSDGV**T**NWGRIV  VAKHLK**T**INQESC  TDVLVR**T**KRDWLV  RDELAN**T**QQKLQK  IPARLR**T**LHNLVI  KFKQAE**T**LYKEIL  VDSPTV**T**TTLKNL  RNLLLA**T**RDLVKI  TLWEMF**T**YGQEPW  RGQNTR**T**LCVGPF  QSPEEP**T**PLPVPL  QQQQVG**T**NKCRVN  SQLGKA**T**ALAIMG  PKNPHC**T**SQEHKC  ECLRFG**T**CSQLCN  LPPDIR**T**KDIEDV  PRSGRG**T**GRGGGG  DVYRDG**T**GVVEFV  RSHEGE**T**AYIRVK  KIINHP**T**MPNTQV  HPTMPN**T**QVVAIP  NIHSII**T**ALTAKG  LRPQTQ**T**SYDAKR  AKRTEV**T**LETLGP  CEQKRE**T**CADGEA  QFAINS**T**ERKRMT  TERKRM**T**LKDIYT  TLKDIY**T**WIEDHF  SANRYL**T**LDQVFK  ELRRNM**T**IKTELP  KTPIKE**T**LPISST  SNGNTL**T**ISISSP  IIFVGS**T**NRIGMI  IGRYIS**T**KAVGSN  KNLSRD**T**KTVTVN  SSWSDS**T**AARHSR  SPALGG**T**FPPAPW  PAWAKP**T**NTQAYS  VWAFGV**T**LWEVLM  VNAIST**T**LLRDIK  GPKPRL**T**SSVKEL  SPTTIA**T**VMNLSK  PTLLPG**T**PARKRG  FPSYTC**T**CLKGYA  YVRLYP**T**SCHTAC  IPDKQI**T**ASSSYK  EVTGII**T**QGARNF  VGGSPQ**T**KHLIED  SLAVVE**T**EWRKRK  LPIRRK**T**RSLPDR  VTNKGR**T**KLPPGV  VIPCFV**T**NMEAQN  CEVTEL**T**REGETI  GGMDEK**T**IALLVA  YSLKNA**T**GLGLIV  LSDFFK**T**HYRLEL  RFYVPP**T**QEDGVD  FARGTT**T**TRSFDF  ARGTTT**T**RSFDFE  GKEENL**T**GFLEPG  LTPRRV**T**EVRADT  RSITSP**T**TLYDRY  SITSPT**T**LYDRYS  SPPAST**T**RRRLFV  GETVSV**T**PVPGQT  PGQTLV**T**MATATV  TLVTMA**T**ATVTAN  MATATV**T**ANNGQT  NENGGI**T**FFPVQV  PTPTRL**T**GANSDM  YIKQIK**T**FAMKYS  KWMPGI**T**QWKVMT  TQWKVM**T**KDGQWF  KMKVIN**T**GIKVWC  ANSPDV**T**AGCDPA  LYDLVM**T**HALEWP  LVLGTH**T**SDEQNH  VLVFDY**T**KHPSKP  SASDDH**T**ICLWDI  LKRRQK**T**HVPMLQ  FTPPPH**T**EDSVYP  HWKERK**T**CAAQLV  QVLAQA**T**EMLYMR  LDTMNT**T**CVDRFI  LDIVPP**T**FSALCP  AFKSKA**T**NDEIFS  IEVFVQ**T**LLHLAA  VDKMIR**T**QIVDCA  DGTSVL**T**PWYKNC  IQQYMV**T**LENLLF  LENLLF**T**AELDPH  NLNQRP**T**PTWLEE  NQRPTP**T**WLEEQH  PPMLRA**T**GCSQLG  ARVRAV**T**YHLEDL  GCGFPS**T**NEYPDL  LEEERA**T**YPQEED  SLASFS**T**VTARRN  ASFSTV**T**ARRNPL  SYADLI**T**KAIESS  RVQNEG**T**GKSSWW  TSSNAS**T**ISGRLS  NLLSSP**T**SLTVST  TQSSPG**T**MMQQTP  SFAPPN**T**SLNSPS  PNYQKY**T**YGQSSM  LLKELL**T**SDSPPH  PHNDIM**T**PVDPGV  PNSVMS**T**YGSQAS  DLMDGD**T**LDFNFD  FPHSVK**T**TTHSWV  KMLYAA**T**RATLKK  YAATRA**T**LKKEFG  SSPAPL**T**AAEEEL  IIILAN**T**TNTELK  SHSAGR**T**PGRTPG  SSSKVQ**T**TPSKPG  NYLAVG**T**SSAEVQ  KRLRNM**T**SHSARV  AEHHVA**T**LSGHSQ  VPLQTF**T**QHQGAV  QSNVLA**T**GGGTSD  LATGGG**T**SDRHIR  VIWKYP**T**MAKVAE  AELKGH**T**SRVLSL  SRVLSL**T**MSPDGA  MSPDGA**T**VASAAA  CGSNGK**T**YLNHCE  ETAINI**T**TYPDQE  CGNWVC**T**AMTCDG  QTEEEM**T**RYVQEL  FPGSLQ**T**DHTPLL  QPGRPH**T**GNVAIP  RRAIEL**T**LHSLGV  KYGDLH**T**YLLYSR  PGWPKE**T**SSALTH  AQRLFS**T**KGKSLE  KGTKRD**T**ERNKDI  GEQRHL**T**HENVQR  TTNSGY**T**RLSDVD  ASTPAT**T**LHLLQL  VSPQAS**T**PISQST  CKLMLS**T**SEYSQS  KTKGDD**T**DTRDDI  DVCIDL**T**CDSGSQ  DIIPPL**T**GATPPL  NLEKPA**T**GERKNG  ERKNGS**T**AVAESV  QDATVQ**T**ERGSGE  RKVTEE**T**EEPIVE  ADQHCR**T**RKYFLC  QNMVSH**T**ERALKA  TEHMTR**T**LRGVMR  IQLAAV**T**EDKYEI  AIVIKN**T**KEPPLS  SLTIHL**T**SPVVRE  CEKSIG**T**ANRPMG  ENPVDY**T**VQIPPS  QIPPST**T**YAITPM  SGPSKK**T**AKLHVA  QDMGLP**T**GAEGRD  YELISE**T**GGSHDK  YGGNSA**T**AGYSQF  SKTLLG**T**KLEAKY  CPSDGC**T**WKGTLK  RCPLML**T**ECPACK  CPKFPL**T**CDGCGK  AIGCLE**T**VEGEKQ  ESLEKK**T**ATFENI  LEKKTA**T**FENIVC  VERVAM**T**AEACSR  LEMEAS**T**YDGVFI  FSPAFY**T**SRYGYK  YLNGDG**T**GRGTHL  DGTGRG**T**HLSLFF  KPVVSS**T**PLVDFL  LEDYTP**T**IPDAVT  HCKMKG**T**ASGSSR  SKDRKY**T**LTMEDL  CPSIQI**T**SISPNC  LFQQDA**T**LSGLVN  GHLLAH**T**PHSVHT  IGLQDI**T**LDDVNE  QIPIDS**T**VSFGAS  ASTRAY**T**LREKPQ  TELDNL**T**EFNTAH  HNKRIS**T**LTIEEG  RITFNK**T**NGLRTD  FSKPFM**T**LGISIL  GIQRVL**T**SDYAFL  QRNCNL**T**QIGGLI  DALDNR**T**LATYSY  EAFDLL**T**SHHMLT  TSHHML**T**AQEEKE  YNKPSE**T**VIPESV  LPVHIG**T**LVELNK  YLSKAT**T**LEKTYG  ATTLEK**T**YGPAWI  GLEYGL**T**NNSKLA  IPQNAS**T**YSAIGY  AVDYFH**T**ALGLRR  GLRRDD**T**FSVTML  DDTFSV**T**MLGHCI  WLKLNR**T**GLCYLP  QLFINL**T**DLLYLD  QWVHVE**T**LNLSRN  LSRNQL**T**SLPSAI  SGIGKL**T**NLEEFM  EDTHYV**T**RMYRVY  WRGAQA**T**LSSTTK  ATLSST**T**KARLFA  SRSLEG**T**EAQVFK  VLTVDY**T**RNAEAV  SNMGWL**T**FTFSLQ  NEQHAF**T**RCFHAW  AVKKPR**T**VNVEDS  SWAMEA**T**ASAAST  VRPKFV**T**CDAEGT  TCDAEG**T**VYFTQG  VERLMK**T**LRDPSL  NRGNIP**T**LNRMSF  FLNFVP**T**VIMDPS  MQPWAQ**T**VVVGRA  LSFVQR**T**FNVDMY  EPPALD**T**AWVEAT  TAWVEA**T**RKKALL  KLEKLD**T**DLKNYK  RARDYC**T**SAKHVI  GERDSQ**T**QAILTK  LAPHVR**T**LYTQIR  HVRTLY**T**QIRNRA  MAAAFN**T**TVAALE  AAAFNT**T**VAALED  ALEDEL**T**QLILEG  DVDQRS**T**TFEKSL  SLREDS**T**VSLVVQ  GCSSCG**T**PLHDQG  GISRSA**T**ICLAYL  RGKTPA**T**PTSQFV  PMRNTS**T**MIGAGS  VKAVSE**T**PAVPPV  APRPEH**T**KSVYTR  PLPVTP**T**RDVATS  TSPISP**T**ENNTTP  SPTENN**T**TPPDAL  TPPDAL**T**RNTEKQ  DALTRN**T**EKQKKK  ASGTVY**T**AMDVAT  TAMDVA**T**GQEVAI  TDVVTE**T**CMDEGQ  LIATNG**T**PELQNP  LSSSEL**T**ELKFLC  QLKVSD**T**KIDSIE  RYPRNL**T**ERVRES  LRIWKN**T**EKENAT  SGAVDW**T**VHSGPQ  QVLPEA**T**TTAFEY  VLPEAT**T**TAFEYE  LPEATT**T**AFEYED  EDGDRI**T**VRSDEE  LSYYYS**T**VMEQQV  DIRYRD**T**LGHGNG  VILLDI**T**LELQKQ  NRISIC**T**EFMDGG  SNMLVN**T**RGQVKL  CDFGVS**T**QLVNSI  PFVHFI**T**QCMRKQ  EHQYFM**T**EYVATR  PGPAPD**T**IDLTLQ  EPRKPV**T**AQERQR  APAPTP**T**PTPVQP  LSEDSL**T**KQPEEV  GSYFKN**T**DLWIVM  IEDEIA**T**ILKSTL  ATILKS**T**LKGLEY  GNILLN**T**EGHAKL  GVAGQL**T**DTMAKR  AGQLTD**T**MAKRNT  ILRDLI**T**EAMEIK  SHTMVK**T**SVESVG  MIEHNS**T**MLESDL  LESDLG**T**MVINSE  ELRQRY**T**AKRQPI  GDNFRI**T**KADAAE  AMALKS**T**IDLTCN  MCTSCL**T**AWQESD  NVKTNR**T**SQDYDQ  VAPGSV**T**SRLGSV  QLKMDF**T**AARLQR  YESVMK**T**EPSIAE  PSIAEY**T**VRSKER  GYSSRR**T**SGRDKY  RQAGEV**T**YADAHK  IEDKPR**T**SHRRSY  PPPKRA**T**SRSRSR  GLTLLV**T**TDLELI  SVIRQI**T**ATVTFL  QITATV**T**FLPLLE  FDLLIY**T**DKDLVV  KYTKDH**T**VRSTGP  ESCDTL**T**CRDCQL  NDAQKV**T**EGQQER  QLATDS**T**FSLDQP  PEERQQ**T**PEVVPD  EPVLEE**T**APEDAQ  VQEDLR**T**FSWASV  SGAVPV**T**GIPPHV  YHYGLL**T**CESCKG  RFQKCL**T**VGMRLE  GSILLV**T**GQEVEL  AALLDY**T**LCHYPH  GINLFD**T**AEVYAA  NRPDPN**T**PMEETV  ARQFNL**T**PPICEQ  AERLGC**T**LPQLAI  NVATSF**T**WGWDSS  SEFALQ**T**LLSSCS  VAHVSE**T**ITQLNL  GIVPDG**T**LQLLKE  AFVHFE**T**QEAADK  FSQFGK**T**LSVKVM  SSPEEA**T**KAVTEM  QSGPRP**T**LRHLAP  DKMDFL**T**IVRECG  KTDFCQ**T**VLPYLI  GNWLAE**T**CLENPA  MDHPHH**T**LFIILA  CSMQSD**T**VPLLQN  EDQQFR**T**SSLPAI  VFSEDG**T**SKVVEI  VDDNSW**T**LVEHHP  YYYMWK**T**TDRYVQ  PKFAMK**T**RQAFYL  LHTTKL**T**RIARRL  DVFYMA**T**EETRKI  EIASAI**T**RPFPFL  RNLVPV**T**RVMYCV  KLLPYD**T**EVLESN  AARTYS**T**APGEKQ  ETFDLK**T**PQVTNE  LKTPQV**T**NEGEPE  SDQACG**T**MDTVDI  DIANNS**T**LGKPKR  RKHKDE**T**VDFKAP  LVKCIQ**T**EDGKWF  GKWFTP**T**EFEIKG  ELFCCD**T**CSRVFH  QALREG**T**RVQSVE  GAFLVN**T**ARGGLV  EIRRAI**T**GRIPDS  VVGVAP**T**GIPAAV  EGISVN**T**GPKGVI  RFKQLE**T**EQREEQ  GASSQF**T**RNALPA  GNFVRV**T**DQLGDD  KGVGDG**T**VSWGLE  EDDEDM**T**LTRWTG  DEDMTL**T**RWTGMI  PFVRFV**T**KINMNG  WVQPSR**T**LAGETG  RREVEK**T**ACPSGK  DAVLPL**T**VAEVQK  GPGPVL**T**VLALLL  SMGLPG**T**LYPVIK  LRPLVQ**T**PPGGSE  ALRVVS**T**SGEQMK  EFSSHI**T**RGEHRF  QVSWEK**T**LESGFV  ESGFVI**T**LTDGHS  HSAWTG**T**VSESEI  EESENQ**T**DLSGLA  LHLPGH**T**CALSWR  QHLLNR**T**LEVALL  AQLVTG**T**SFSGPT  CIMEAS**T**DFLPGL  LKLATP**T**YGDLNH  ATMSGV**T**TSLRFP  PGFAPL**T**ARGSQQ  AFLHWY**T**GEGMDE  PRKIQF**T**VPLLEP  RRPTPA**T**LVLTSD  PATLVL**T**SDQSSP  NPHLKS**T**LAMSPR  RQRKKM**T**RITPTM  EGAAES**T**ETQESR  AAESTE**T**QESRPP  PPGIPD**T**EVESRL  RLGTSG**T**AKKTAE  SGTAKK**T**AECIPK  GSKEPS**T**KEPSTH  STKEPS**T**HIPPLD  YYFNHI**T**NASQWE  WRQEKI**T**RTKEEA  QEKITR**T**KEEALE  ASFALR**T**GEMSGP  HLVEMD**T**DQLKLY  LHSSQM**T**ALPNTP  RLDFST**T**ETQGKD  HFESFI**T**EKKQNI  QSRAIP**T**RTVAIS  RAIPTR**T**VAISDA  LPHDYC**T**TPGGTL  CTTPGG**T**LFSTTP  GGTLFS**T**TPGGTR  STTPGG**T**RIIYDR  PNIPGV**T**SPGTLI  GVTSPG**T**LIEDSK  SLCFHR**T**QGLMIM  YENFQN**T**EGKGTA  HQPSGL**T**SQPQVL  DNTTSL**T**DKHLDP  PHRIRM**T**HNLLLN  LGCFNL**T**IKGHAK  LGGGGY**T**IRNVAR  EEKKEV**T**EEEKTK  LVFDLV**T**GGELFE  LINQML**T**INPAKR  LTTMLA**T**RNFSVG  APATMS**T**AASGTT  STAASG**T**TMGLVE  TAASGT**T**MGLVEQ  KPQTNS**T**KNSAAA  ATSPKG**T**LPPAAL  IPGPLP**T**PSRKQE  KQEIIK**T**TEQLIE  ICDPGL**T**SFEPEA  GQGRPR**T**SQSEET  LVQRLV**T**IKQLHE  LLTHLD**T**TQQMIA  LTHLDT**T**QQMIAN  MRENLA**T**VEGNFA  IPWRHA**T**RHGPSQ  SQDGDN**T**IFKAWA  KAWAKE**T**GKYTEG  KETGKY**T**EGVDEA  LPSLSL**T**EDVKWP  DVKWPP**T**LQPPTL  PTLQPP**T**LRPPTL  PTLRPP**T**LQPPTL  PTLQPP**T**LQPPVV  DKQRFY**T**NQLLDV  QKGQTN**T**PPPFEI  REKKLI**T**VQVVPV  VIIHKL**T**DVILEA  TRVAHG**T**NSHEMA  IIINNQ**T**GQHAVI  VLQGQS**T**AMAATG  SFLPAP**T**QLSQDQ  VIYSKY**T**DLVPKE  KNAKGY**T**IPLDKR  GVPNPR**T**SNEVQY  EHHVNA**T**LDLIEG  GSMTVC**T**TKKTFD  IGPKGS**T**LKALEL  KALELL**T**NCYIMV  TGAVRG**T**LTVTNY  ESQATI**T**RCSQPM  YPNIEE**T**HWLSNL  MTRQFP**T**AFEFNE  FAQQMC**T**ALLFLA  TWNLKK**T**KDGKRE  TSNSVS**T**SPAMEQ  QSQSSG**T**TSSTSS  QAMDCE**T**HSPQVR  SGTEAP**T**QVTVET  RVYNSP**T**NSSSTQ  PTNSSS**T**QDSMEV  STTSSS**T**SSSSTG  STSSSS**T**GNQGNQ  RPVAAN**T**LDFGQN  GPAHYM**T**EGHLTM  AAPAPE**T**REHLFK  ELGVGK**T**SIIKRY  LNWDSR**T**LVRLQL  ERFGNM**T**RVYYKE  QELDQG**T**GAALCF  PGDLGP**T**KREKFK  LNQLCA**T**KFRVTQ  ATKFRV**T**QPNTFG  LEEQDS**T**QATTQQ  QDSTQA**T**TQQAQL  RQVTGV**T**RVTIRK  ENTQTP**T**VQEESE  YELYKY**T**CQELQR  AEEEQT**T**KRRRPT  MNLNIM**T**VKAKVT  NWKGEK**T**NSNDDN  NYLRDD**T**ITLPQN  CNIPII**T**SLKEEE  TSIVYA**T**EKKQTK  HVKRYS**T**YDDRQL  YLAERE**T**VESEPW  QAEGNA**T**GAFLIR  VLSVRD**T**QAVRHY  VHKLML**T**CWCRDP  AYNGIG**T**ATNGSF  RSKDSA**T**AQAWFS  HLFRAE**T**SRDLSH  ENGFSI**T**TEPQEG  DKEMVT**T**VKGVIK  PAELPL**T**DREVEI  KLLRCA**T**SSQPLA  LEWQKQ**T**SEGLAE  GRGKSF**T**LTITVF  LSSTRG**T**GLPAIT  TYTPPV**T**SGMSLG  PYLYYG**T**SSGSYQ  PGYVEP**T**AVATPP  PTRIRT**T**TSGVPR  PSEAPP**T**EVPDRD  DDVYLH**T**VIPAVV  MDTLFN**T**KFEGEP  FAVIGS**T**EELKIG  RQYPWG**T**VQVENE  QAGGSQ**T**LKRDKE  KFCLQY**T**RKAEFR  EMRKNL**T**QDEMQR  GLQAPP**T**RIGLIN  PQLQNN**T**ILRLLQ  SAEQTV**T**WLITLG  TSVGNP**T**IKPHSV  HPSHHG**T**PHTTIN  VIEAYC**T**SAKTRQ  ANYLWH**T**DDLLGQ  AVKVFN**T**TSYLRP  FVSVYG**T**EEYLHP  EIMYRI**T**TEKPAG  PLEWSY**T**LPITCQ  SYTLPI**T**CQLSLG  DQFFAE**T**SDILQR  EAVHKQ**T**SVAPRH  TASSPL**T**LFSTAI  PLTLFS**T**AIPKGL  MEVLQA**T**CRRTLE  FSSVAG**T**PEIQEL  KSIPHI**T**SDRLLI  VPGGVK**T**IEANGR  PGSSLL**T**SFEKWH  ILEMGI**T**GPEGHA  NCPVYI**T**KVMSKS  SLGTDG**T**HYWSKN  KAAAFV**T**SPPLSP  PLSPDP**T**TPDYLT  CGDLQV**T**GSGHCP  NQFVAV**T**STNAAK  DPDKLK**T**ITAKSH  DNNPRR**T**GHRIVA  LISTCQ**T**FILESS  EYVAMK**T**KETSNN  YLSGQF**T**TPPSTG  STGTQV**T**RPSGQI  ESQRKS**T**TLSGPH  FKTAVP**T**EQKCDI  KCDIDI**T**FESVPN  NQMSTE**T**EMAIGI  LIATCV**T**DPTALG  NGNNFS**T**PPRQVL  VNMAAH**T**LMILSR  RASSRS**T**TKKRKI  EMLRNS**T**DTTPLT  AATEAQ**T**TQPVPT  VPTEAQ**T**TPLAAT  AATEAQ**T**TPPAAT  FDYQGK**T**EKHESQ  GKFGVQ**T**DRQDKC  VPVEAV**T**SKTSNI  EAVTSK**T**SNIRAN  SRVKLG**T**LRRPEG  ASSPTL**T**SPMEYP  PVNSLH**T**PPLHRH  TANLDR**T**DDLVYL  PVPKFP**T**RVHVQP  NKANRF**T**VETRGA  SLPVEF**T**IDARDA  RSSHTY**T**RTERTE  CLSREK**T**YVKRLV  GESGKS**T**FLKQMR  REEFRP**T**IYSNVI  KMMSFD**T**RAPMAA  AQGMVE**T**RVFLQY  ECFDSV**T**SILFLV  LMEDRL**T**NRLTES  RLTNRL**T**ESLNIF  ILFLNK**T**DLLEEK  LYHHFT**T**AINTEN  FTTAIN**T**ENIRLV  FRDVKD**T**ILHDNL  KYGSPY**T**KNSGFA  FKRLQP**T**RLVAEF  AEFDFR**T**FDPEGI  DTTIQE**T**VKVNTR  VTIQEL**T**APLLTT  LTAPLL**T**TAQAKP  ETQEEE**T**NSGEEP  PARMMS**T**ESANSF  ESANSF**T**LIGEAS  LLDQLD**T**QLNVTE  QMRYAQ**T**QLDKLK  HSGQFG**T**INNFRL  SYLESL**T**DKSKEL  EVEKGE**T**RFCLPY  GSYSIK**T**QFNSEE  NSEEQW**T**KALKFM  EEDEMR**T**RVNKEM  SKESMS**T**VPADFE  VPADFE**T**DESVLM  INYGKN**T**IAYDRY  SSSEPQ**T**SSQDDF  FDVYSG**T**PTKVRH  VYSGTP**T**KVRHMD  DLEACL**T**EPLRDF  LEEFFS**T**VGKVRD  PLAIGL**T**GQRVLG  GHVTER**T**DASSAS  PDSMTA**T**QLLVPS  RKGDIL**T**VIEQNT  FVMVAK**T**VPDDAK  FIGDTL**T**RQVTAQ  KTIVMA**T**KMAALH  KQARGS**T**TLQESR  IMCTIL**T**NCVFMA  TFTAIY**T**FESLVK  KEHEAL**T**IRGVDT  ADDENS**T**AGESES  GGPQML**T**SQAPCV  CGEWIE**T**MWDCME  LRFVKR**T**TWDFCC  APPDSR**T**WSQVSA  WWRLRK**T**CYHIVE  CLFQIT**T**SAGWDG  DILFAF**T**KRVLGE  SYKDPA**T**LRQHEK  TRQYRL**T**EHMRIH  AELLAQ**T**THFLHD  YPLAKF**T**AELGLS  VWKYLQ**T**VQYGVH  KLGGNN**T**SEKADG  SLIKNI**T**LRRTKT  LVVSQF**T**TFLSLI  WVNKSR**T**RFRIPW  DFSQPD**T**SPDTNG  PDTSPD**T**NGGGST  GGSTSD**T**QEDILD  EWEFEV**T**AFYRGR  RQVFQQ**T**ISCPEG  RLGHCH**T**YWAVSE  FIVDLI**T**FTEGSG  VDLITF**T**EGSGRS  PQDQPW**T**KRLVMV  MVKVVP**T**CLRALV  ASSLEN**T**VDLHIS  PEEYVL**T**RLAEDP  FLQDVF**T**TLVDLK  LQDVFT**T**LVDLKW  LAPSEG**T**AEPCVT  SMIISA**T**IHMQVV  QVVRKT**T**SPEGEV  EGVVET**T**GITTQA  VETTGI**T**TQARTS  ETTGIT**T**QARTSY  ITTQAR**T**SYLADE  YSKFGN**T**IKVPTP  SLLEAL**T**LASARG  RDSAWG**T**SDHSVS  SANQQT**T**PQAQQG  AGGLAG**T**TNGPFR  SAQPAN**T**SLGFGS  QKAAVE**T**SFLDYG  GRGLPC**T**PKPPDR  ATAFLV**T**ESVSIT  PGTVGN**T**LLLTRL  EERIPA**T**PVVIPM  IFHGVG**T**RGPGAP  HCSFQT**T**GTLDEP  PEPHPS**T**STAQPV  LQPSTS**T**DQPVTS  EPTSQA**T**RGRKNR  PVTSEP**T**YQATRG  DRSSVK**T**PETVVP  LVTPEP**T**SRATRC  KEEEED**T**AEKPGK  APKVLF**T**GVVDAR  PQRVVI**T**CPQDFP  DPYFVE**T**PYGYQL  VGTSVE**T**NSVGIS  LDKQTS**T**QTVETR  RSIGVG**T**LLSGHS  YYELHE**T**IGTGGF  LHETIG**T**GGFAKV  HRNNRQ**T**MEDLIS  ASATPF**T**DIKSNN  LSTGAA**T**PRTSQF  PRTSQF**T**KYWTES  MFPEPK**T**PVNKNQ  HKREIL**T**TPNRYT  KVITVL**T**RSKRKG  KLHYNV**T**TTRLVN  FVQKGY**T**LKCQTQ  YTLKCQ**T**QSDFGK  PRPAGP**T**PRGHGR  LIDRDP**T**YFGPIL  HGKLII**T**KELAEE  RDNENR**T**SQGPVK  EGAEDR**T**FARVIV  EKKERL**T**EELKEQ  CFESVA**T**NIDEIY  LSGGEK**T**VAALAL  GQVPST**T**ATTPGN  NKQQFN**T**QNQSNV  HGDPAT**T**ANNDVS  NKDVTL**T**SPLLVN  QNQLMM**T**GPKPGP  PSPLSA**T**QGATPQ  LNPQNS**T**VSVAAV  KASVIP**T**LQDLSS  TSAALP**T**HLQSAL  QSALMS**T**VVTMPN  GQGLDT**T**APGLMG  SKETST**T**ALQASV  STEEGS**T**KRKKSQ  QKGPAK**T**KKLKIE  KPGMLL**T**GTVSSL  NFLTFF**T**GVVDFM  EFILGC**T**ASKITP  ITPDGI**T**KEASYG  GDDSIS**T**EALVNG  LGSDTR**T**AGQPEG  PVAYVE**T**KYSPPA  WCYINA**T**LQALVA  PSNEKL**T**ISNGPK  RNKTSV**T**RQADFV  FVQTPI**T**GIFGGH  SVQGYT**T**KTKQEV  EISRRV**T**LEKLPP  KPTAER**T**AYLLYY  PGCSHL**T**LKVEFS  KVEFSS**T**VVEYEY  PRQVAQ**T**LQADVL  DGLGHG**T**FVAGVI  STSQLY**T**VKYKDG  NDIKPL**T**SFRQRK  LYELWE**T**RVFGVY  TSAVIG**T**SLFQGV  PPRHYC**T**VANPVS  VDAVIL**T**EKTALH  LVTVQD**T**EAHVPD  RKDPQE**T**AKLFSV  PGSQRC**T**KPSCVP  RIKVVF**T**PSICKV  YKRVNN**T**FCQDIN  DGQCVN**T**EGSYNC  RLDLLI**T**YLNTRK  ELCGVM**T**ISDSPR  DILTIL**T**EVNYEV  KQMPQP**T**FTLRKK  NFAMPV**T**VPVSNQ  VTPSLV**T**SSLTDP  PDLRVI**T**SQAGKG  PVVSVA**T**PSLLSQ  GKIVYP**T**EKVNCL  LQRIKK**T**QDQISN  QPHHIP**T**SAPVYQ  VERTGD**T**GMLPAN  YFLPED**T**PQGRNS  YMKERK**T**PIERIP  KQINLW**T**MFQAAQ  THHGQS**T**DHMAVS  ITSISP**T**PEPPAA  HRAHYE**T**EGSRGA  LQSNEV**T**LTLTVP  SATPFG**T**DMDFSP  NVKLAA**T**NALLNS  LNSLEF**T**KANFDK  SVVVRD**T**AAWTVG  QSLLCA**T**LQNVLR  DKNESL**T**MRLHET  TMRLHE**T**LKQCQD  QCQDLK**T**EKSQMD  SLTSEL**T**TLNATI  LTSELT**T**LNATIQ  QAQLAQ**T**LQQQEQ  SEQQKA**T**EKERVA  KESLKV**T**KGSLEE  SELKAE**T**RSLVEQ  EAHQAE**T**EVLRRE  GRQFCS**T**QAALQA  LSLGTI**T**DEEMKT  YHFNAL**T**CEGCKG  QEELIR**T**LLGAHT  TLLGAH**T**RHMGTM  HTRHMG**T**MFEQFV  HHQPLP**T**LAPVLP  HFADIN**T**FMVLQV  CHIVLN**T**TFCLQT  TTFCLQ**T**QNFLCG  CGPLRY**T**IEDGAR  GARVSP**T**VGFQVE  LFHFHG**T**LRKLQL  PDRPGV**T**QRDEID  ADSVPG**T**YRKVVA  KVLGSS**T**SATNST  TSATNS**T**SVSSRK  PVCVRP**T**PKWQKG  GRLTIC**T**ISCFFA  AHRKDP**T**GMDPDD  RFDDKY**T**LKLTFI  QREAEF**T**KSIAKF  VEGMQS**T**ILVLQQ  HREGNT**T**EDDFPS  THQSND**T**DSSHDP  SGKGNR**T**VGSRHV  RAVAAA**T**YKTMGP  SSKDSI**T**VFVSNL  GLPFSC**T**KEELEE  MKMDGM**T**IKENII  RCVEVE**T**DVVSNT  VKAEMG**T**PKLEKP  DCSASA**T**PEFEGR  SSVDLK**T**LCISPR  SPKMEG**T**GKKAVA  KIEMEE**T**DKEQLT  RKQELV**T**QNELLK  QVMDPP**T**ARPTEP  WSRRWF**T**IQSNQL  HPPSLP**T**MADALA  EGRDPL**T**IAMETA  LTIAME**T**ANADIV  QATAAA**T**NHTTDN  HNPEFT**T**CEFYMA  GMKLPE**T**NLFETE  VTCINP**T**FICDHP  LRCAGP**T**PEAELQ  IRPKKK**T**NLVWDE  AAGLHP**T**GHQSKE  KPQLDV**T**RATNKQ  NIDEFV**T**DQNKIV  ANNELT**T**NGHGPP  TEPKLA**T**PAGLKK  DDETKE**T**ENMRKK  SQKELP**T**EPPYTA  PTEPPY**T**AYVGNL  SLKEAL**T**YDGALL  LQLKPR**T**VATPLN  PSYRKA**T**LFEDGA  FEDGAA**T**VGHYTA  ATVGHY**T**AVQNSK  SYQNNI**T**HLNNEN  SCANLS**T**FAQPPP  QLSGSQ**T**GGSSSV  APHPAV**T**SAGTPK  VIVQAS**T**SELLRC  LKHLSP**T**DPVLWL  QDQGFI**T**PANVVF  LQAVLL**T**CLYLSY  ADPHYF**T**QVFSDL  EEKRGF**T**SHFVFV  FVIEVK**T**KGGSKY  SSALAC**T**LPTLPA  LACTLP**T**LPAKVY  EALFDF**T**GNSKLE  KDWLEG**T**VRGATG  GTVRGA**T**GIFPLS  PEEDDP**T**NWLRCY  YEDTIS**T**IKDIAV  EEDLSS**T**PLLKDL  KDLLEL**T**RREFQR  PWKLHI**T**QKDNYR  GVQGFP**T**IKIFGS  DYQGGR**T**GEAIVD  LAAVDA**T**VNQVLA  MWGWLW**T**EAGAQS  KAIYDF**T**DTVIRI  FNYMYS**T**APRPRV  SLEGYG**T**DAVIYI  AWKHYN**T**NHEADD  FVIHSY**T**RPTVCQ  NCLGEV**T**INGDLL  ITKFLI**T**QILVAL  WLQDYQ**T**WLDLRE  EFGFFL**T**WRSEGK  VCVCSG**T**DLVNIS  AENPEV**T**KQWVEG  EIVAQH**T**KEWSEM  CLQRIV**T**KLQMEA  DWSDRN**T**NMTAKK  AEAQQP**T**FDALRD  CRRDDG**T**GQLLLP  RGLRWS**T**LPKSSP  RCSSRR**T**PSAGLS  FEKSKL**T**FSCLGG  DSKHKR**T**DRSILC  QSWPRL**T**KERAKL  HTKERV**T**MTKVTL  VTMTKV**T**LENFYS  LVQKKD**T**GHVYAM  PGGDMM**T**LLMKKD  LLMKKD**T**LTEEET  QFYIAE**T**VLAIDS  SDFGLC**T**GLKKAH  LKKAHR**T**EFYRNL  PPFCSE**T**PQETYK  SDILKP**T**VATSNH  TSNHPE**T**DYKNKD  KRFEGL**T**ARGAIP  DRGRDK**T**RKRRSA  SSSGSS**T**STGSSS  IDGQEI**T**ATAVLA  GQEITA**T**AVLAPW  PEEADG**T**LDCISM  RGEYIY**T**GNAKGK  KILVLK**T**DSQDLV  VASFRV**T**TGTSNT  TTGTSN**T**TAIKSI  SCFLIN**T**ADRIIR  DGREIL**T**CGRDGE  VKILHG**T**RGELLL  DEEVDV**T**SVDPIA  PPDAVQ**T**SLMDEG  KKKPKT**T**NIELQG  ELSQPL**T**AGGAIS  VLRAKP**T**VRKERV  DGDGFV**T**TEELKT  GDGFVT**T**EELKTW  EIVVLE**T**LEDIDK  SWPPNM**T**DFSVFS  VFSNLV**T**IGGRVL  ELVEPL**T**PSGTAP  LRILKE**T**ELKRVK  LHRDAA**T**VTQMHF  WGRDSS**T**VVSSHS  FPTLQP**T**VATTPY  LSQANG**T**PSILLA  IRRHKT**T**IFTDAK  HKTTIF**T**DAKESS  CGFTSQ**T**ARPQAP  RPQAPA**T**VGLAFR  GLVPEK**T**NVKPRA  KDQMEQ**T**KIDNES  RMTRSA**T**QAAKQV  TARKPV**T**RAANEN  VDSEEN**T**LNSQTN  NSQTNA**T**SGMNPD  PLDGLK**T**YQVTPM  FLTPSY**T**WTPLKT  RGIKET**T**CTDLDG  QEECAE**T**AVSVIP  NAGPQN**T**KSEHVK  DNKSLT**T**ECHLLD  AIEDKE**T**INNLDT  TINNLD**T**SSSDFT  ELAEAP**T**EAPSPE  GFVTMS**T**AEEATK  ERNQDA**T**VYVGGL  MPKDRV**T**GQHQGY  GPPSAG**T**PGAGHP  LPPPRP**T**PRPPVP  QGKKHQ**T**NLARRA  AAEPYE**T**IAFKVP  AEGKFW**T**HWNRET  PGVHPP**T**SGVHPP  GVLGAA**T**LLLSFL  FLSGWH**T**IYLPDC  TDGGGW**T**VFQRRV  NNQSFS**T**KDQDND  NLKDRL**T**IELHPD  KTIDKK**T**FYKTAD  CQMLVS**T**VDGDLY  IIAEDE**T**KEAENQ  MIKTEF**T**LTEAVV  KTEFTL**T**EAVVES  QNFSYE**T**FQQKML  AEPYLL**T**MAKKAL  ESAASS**T**GKEDKQ  QLRNPP**T**TIGMMT  LRNPPT**T**IGMMTL  APPSKP**T**VLNQPL  GVEEKE**T**WVEEDE  APDEDS**T**TNITKK  PDEDST**T**NITKKQ  YPFVNR**T**AVMIKD  NTREFR**T**RKKLEE  PLHALV**T**ANTMEN  PWQSSE**T**RSITNT  QNTTTT**T**TSAGTG  GSCVKR**T**ILKEDS  MDVQDS**T**HVSCKL  LSLIAE**T**VEDMVK  NPMSGT**T**TPTNTF  EKPKHQ**T**EDDFQR  APSQCS**T**PPTTYP  CSTPPT**T**YPQPVP  ASKLPS**T**SDDCPA  ASQALN**T**LGVPML  SSSSAS**T**SGKMKS  SIKAKV**T**LQKPGE  LSMTSN**T**ILSADR  VTEDGD**T**ALHLAV  AAILGE**T**STVEKL  LDKPEP**T**CGRSPL  HSSRSQ**T**RLPPTP  DELDRL**T**KKLVHD  GCFVCS**T**CRAQLR  EGCYVA**T**LEKCAT  EPGQEE**T**VRIVAL  SLQLNL**T**KIEQLC  ARQGQE**T**AVAPSL  VNVLKL**T**VEDLEK  VDILYA**T**DEGFVI  YLPQNM**T**QDELRS  GLPRTM**T**QKDVED  GSSEPI**T**VKFAAN  VIRDFN**T**NKCKGF  LLFLCA**T**HFGEPL  EQSLPC**T**ERKPAA  ERKPAA**T**ARLSRR  RLSRRG**T**SLSPPP  SSHISK**T**SLSVDP  RQVKPK**T**VSEEER  PATIIW**T**LNGKTL  CVNKTG**T**RIKLID  LNVDHT**T**ENEDEL  IAAMID**T**EFAKQT  GEMSQP**T**PSPAPA  TLTLQS**T**NTHTQS  TMLSAH**T**KCKLCY  GVARSS**T**LPPSNS  NSPMEG**T**GISLAQ  LVQLLT**T**TAEQQL  TNPTPM**T**KPTPEE  SATARP**T**SRLNRL  QFGQPG**T**GDQIPW  LLCPPT**T**VEGRND  LSGKDE**T**ELAELD  YPPNYG**T**NPGTPP  STDLLK**T**EADGTQ  FADVQC**T**VNLVGG  ELEKAI**T**TQNCNT  EIWGLS**T**PNTIDQ  GLSTPN**T**IDQWDT  TIDQWD**T**TGLYSF  HYQRVE**T**PVLPPV  VLVPRH**T**EILTEL  PPLDDY**T**HSIPEN  PAELSP**T**TLSPVN  AELSPT**T**LSPVNH  YGWHPA**T**VCKIPP  AEYRRQ**T**VTSTPC  YRRQTV**T**STPCWI  QQLGMF**T**EGELMS  MSVGMD**T**FIHRID  IHRIDS**T**EVIYQP  EVLDSE**T**LCRRAV  PGNLLL**T**TGGTLK  PFAADD**T**CRTSQG  ADDTCR**T**SQGSPA  IWSAGV**T**LYNITT  VTLYNI**T**TGLYPF  IPPSPD**T**KDRWRS  IYTQDF**T**VPGQVP  EAAQLS**T**KSRAEG  ACQMKA**T**GKLYAC  LAYAFE**T**KADLCL  TKGYAG**T**PGFMAP  YFALGV**T**LYEMIA  HDQETT**T**QGPGVL  LPKGAC**T**GWMAGI  DKAMLF**T**YDQYQE  DNDSTF**T**GFLLYH  NLLSRF**T**RNEFNL  NLESKS**T**IGVEFA  KAQIWD**T**AGQERY  DIAKHL**T**YENVER  QKILER**T**EILNQE  QPVHIL**T**SVSSLR  MFPDKG**T**AEELKE  EKYKEL**T**EQQLPG  SLHSFH**T**LFCRRC  FHATPN**T**YKRKNT  KRKNTE**T**ALDNKP  SDREAG**T**ETGGEN  FRVLIG**T**YYDNFC  IARLIG**T**KTCRQV  RLIGTK**T**CRQVYE  CKAQCN**T**KQCPCY  PVSLAN**T**QPRGPP  PQPPSF**T**YAQQRE  GAPGPL**T**LKEVEE  EGCYTD**T**LEKCNT  SPEAKL**T**EVDNYH  SLNSDA**T**VNTDFG  KQNGPK**T**PVHSSG  SPGHRK**T**PFTKDK  GCLLGE**T**RDACGC  VCGSDG**T**TYPSGC  TQVSKG**T**CEQGPS  KDIWNV**T**GAQVYL  ENSDAV**T**EILNNA  LKSVMK**T**NDIVRY  IGVAVD**T**VGILGS  KQVLQK**T**GTRFER  NLRRAT**T**DLGRSL  LPDPAA**T**HDGPQS  TYSNGS**T**KDRKLL  DRKLLL**T**AQQMLQ  QAAPDD**T**QGSPDL  CKPAPL**T**GTLEVR  CRDLPE**T**IPWNPT  NTSEVS**T**VLKLDN  RLIPNA**T**GTGTFS  EKLNLG**T**DSDSSP  DNLLLD**T**EGYVKI  PPPFVP**T**LSGRTD  LVAASP**T**LSPRQS  TQNQYS**T**LSKPAA  PAALTG**T**LEVRLM  PGRSKA**T**SVALPG  GWSPSE**T**RSSFMS  SSFMSR**T**SKSKSG  SKQQGK**T**FLRAPQ  PPASDS**T**VTKLDF  EKRIFE**T**VNSVRH  LFACFQ**T**KEHVCF  DNLLLD**T**EGFVKI  MGYGDR**T**STFCGT  ELSSPL**T**AHVTGR  RIPLWL**T**GSLLRC  FKEGHV**T**YHRRFI  HRRFIR**T**DAYVRA  AYVRAM**T**EKRIVI  FRGVEV**T**DNALVN  VSVNGA**T**AHPHIE  VHSFGL**T**PNYIVF  LNNKYR**T**SPFNLF  LFHHIN**T**YEDNGF  NIDKAD**T**GKNLVT  LPNTTA**T**AILCSD  SSCSSV**T**ASAAPG  ASAAPG**T**ASLVPD  ELGRGA**T**SIVYRC  DKKIVR**T**EIGVLL  LKEIFE**T**PTEISL  EIFETP**T**EISLVL  LVLELV**T**GGELFD  ENLLYA**T**PAPDAP  PKKRLT**T**FQALQH  LQHPWV**T**GKAANF  NFVHMD**T**AQKKLQ  AEEKLK**T**VEEAAA  DPSSFQ**T**NDHPWT  TNDHPW**T**VPTVFQ  TPLVAI**T**ITRLVV  VFLVCW**T**PYHIFG  YANPVW**T**ALFDYE  DEDISV**T**AESVRQ  QMSAAG**T**YAWMAP  VAVNKL**T**LPIPST  SREEEL**T**RAAREQ  DFKHRI**T**VQASPG  GPGDSP**T**FPRFRA  RGSSSG**T**PKLIQR  ERGESP**T**TPPTPT  RGESPT**T**PPTPTP  TPAPCP**T**EPPPSP  ICFSLK**T**PDSPPT  PNPDEK**T**KEELEE  DLRIRK**T**QHSTLS  RKTQHS**T**LSRKFV  EITGRT**T**TSEELE  FACVAS**T**LAWDRG  WDRGYG**T**SLLGGS  YGYGGY**T**DPRAAK  ALVIFV**T**SVIRSE  RSEMSR**T**RRYYLS  FYTPAA**T**GLYVDQ  IFFAVK**T**RRKMDR  KNVSAG**T**QDVPSP  SGGNFE**T**PSKRAP  AGRSKR**T**EQDHYE  REYPPI**T**SDQQRQ  YKRNFD**T**GLQEYK  SDIWDD**T**ALIKAY  NGDICE**T**SGKPKT  TSGKPK**T**TPKRKP  SGKPKT**T**PKRKPA  GCIYPA**T**IASIDF  IDFKRE**T**CVVVYT  TCVVVY**T**GYGNRE  NESQVS**T**DESENS  YMSGYH**T**GYYMGF  SPSDSS**T**ASTPVA  APVEPA**T**ADATEI  PATADA**T**EIHDAA  PPEIDI**T**CWDADP  GHGHSY**T**TAEEAA  HGHSYT**T**AEEAAG  KSLHVG**T**QCALTR  GTQCAL**T**RRCPQE  LRKLRA**T**LDEYTT  SLIQVG**T**GATVAT  ELPTTV**T**VAQVNY  AAVGAL**T**GVQDAN  GVDSLI**T**LAFQDQ  QRYSVQ**T**ADHRFL  CAFRTH**T**GKYWTL  SNGKFV**T**SKKNGQ  PSGSQL**T**DDVGSL  EMLTGK**T**LFAGAH  ILLMDE**T**HSHIYN  HTCNYK**T**RSSSYL  LTQFEL**T**GHSVFD  EMREML**T**HRNGLV  KGKEQN**T**QRSFFL  LTSRGR**T**MNIKSA  KMTQLF**T**KVESED  KVESED**T**SSLFDK  APAAGD**T**IISLDF  GSNDTE**T**DDQQLE  PQIQDQ**T**PSPSDG  PTANAT**T**TTATTD  ALSQRT**T**VPEEEL  SGLPQL**T**SYDCEV  TSPAPS**T**SSTVKT  VMVLNA**T**ESFVYE  KKMFHA**T**VATENE  GDKLKL**T**CFELAP  PRKQLA**T**KVARKS  RKSAPA**T**GGVKKP  HRYRPG**T**VALREI  RRYQKS**T**ELLIRK  IHAKRV**T**IMPKDI  GKRPWI**T**GDESIV  ALMNAL**T**VNLHLL  LSFFKP**T**PKRYKI  PREGEE**T**LRIEDI  PGMWER**T**LTIGSA  MWERTL**T**IGSAGK  GKTFSA**T**GWKVGW  SVFHCP**T**QSQAAV  HVVHLS**T**AFARVD  LHTLSD**T**LWGPGD  GLLFAV**T**SVAFLV  RQHRRG**T**KGGVSY  IPDEDI**T**ASSQWS  SQWSES**T**AAKYGR  PSARFV**T**VPLHHR  LLAVCI**T**DDPLCM  LSREEL**T**AVMRFL  FGGLAE**T**VLVVRS  NQVSEH**T**LGTVHT  NDPWAP**T**VDFSDF  VFDATN**T**TRERRH  LKSTIQ**T**AEALRL  LKCPLH**T**VLKLTP  LASPEP**T**KKPRIN  VKRLKV**T**ELRSEL  DEEEDQ**T**LVNLDT  CFEAKV**T**QNLPMK  LPGSGK**T**QWALKY  SVEAAV**T**PAEPYA  ARPAQP**T**LWTAKL  VPVRLV**T**VDGGGA  EARKEE**T**EAREEE  EEVTYA**T**LTFQDS  AALGLV**T**LCLMLL  NSCYYF**T**TNEEKT  TTNEEK**T**WANSRK  EYNQSD**T**CRVKLK  SEKKAH**T**RNQEYL  SMRDFS**T**EHKSPQ  HKSPQP**T**KNFSIP  GEKMPV**T**ASVLSE  LSEEEQ**T**HCLEIG  LSPENR**T**TDLKCD  SEKESS**T**NAPTRE  SSTNAP**T**REPGQT  AADIPI**T**ETEAYQ  ATLQDN**T**NQTENR  GAFETK**T**ANKIAS  PGMRVK**T**FHGPSK  NGVIIT**T**YQMLIN  EAHKIK**T**SSTKSA  LLGTLK**T**FKMEYE  AREKDA**T**PGEKAL  KELLME**T**RSPLAE  RDEGHQ**T**LVFSQS  RIDGTV**T**HLLERE  DHDLMY**T**CDLSVK  YVLSKS**T**KADIGP  GEASKY**T**EEDPSG  DKAAEA**T**NDYETL  PKKDVL**T**RLKHLR  PKPVYI**T**TTRDNE  KKYLTA**T**QNTKNG  LDGGGP**T**EQDKSH  TAVLAR**T**VYGVEM  IVRQKA**T**EVEECQ  AGSGHL**T**VGQQQQ  DTTLGC**T**NEAELS  SMGPWQ**T**CEEDPE  NGSTGQ**T**APYYAT  PYYATS**T**VEVIFH  MFFIAI**T**KKPEVP  LHRALA**T**LQEETT  LKLPDK**T**LIDLYE  MCFSRD**T**EMLATG  TAGTDI**T**VNSVIL  HKLDDC**T**LQLSHN  HSIILR**T**QLSVRV  MTLVNK**T**LSGLPD  APVQPK**T**ESDYIW  AEQFLL**T**LSSISE  GNFLNG**T**NAKAFE  LLHHVC**T**MVVENF  HRERNK**T**RGKMIT  PALGVR**T**RSRASR  GSTSSW**T**MGTDDS  SSWTMG**T**DDSPNV  RIVKSA**T**QVPSQR  EIINDR**T**ETPPPL  INDRTE**T**PPPLAS  LASKAR**T**EKEEKL  MEEVVI**T**PPYQVE  AVVSGA**T**DGIGKA  PYPQYF**T**QLSEDK  YVATSM**T**APSNFL  GISKRT**T**GYWSHS  KFYYQE**T**LQQLIM  HEVNEL**T**SSRLLK  ELVKRA**T**IDIKTL  SQLLKE**T**EVLQTD  EAYVTL**T**SKSSRF  SKSSRF**T**DETEIL  SRFTDE**T**EILKMS  VNSKYS**T**FRDFCS  VTSTLK**T**EEVTSC  SSVSGT**T**CGTDED  VRGSIS**T**PEDSGF  LKNLSK**T**PALQLV  EKLDIL**T**ELKYRN  MVLESL**T**AESLCS  QREQGS**T**LSPWGE  RGLCSR**T**ACGFDF  DEENAG**T**EEIKNE  ENCVYE**T**VVLPLD  LSDLCG**T**VMSTTD  IMVLES**T**GESTFK  LSGGNS**T**SNLTHT  RHVLPT**T**SLGHPS  PKQFSP**T**MSPTLS  SPTMSP**T**LSSITQ  EIAHLK**T**SVDEIT  HSLPQQ**T**KKPESE  QVQFLY**T**SLLKQQ  ITEPLV**T**FQGETE  PEGLTL**T**QAAAIP  GAIPLV**T**AGSQKK  EDFSEA**T**LKFTKG  EATLKF**T**KGAGVN  GCGGVL**T**FREPAD  QGLPPG**T**TPQRLE  NLGLEG**T**LVSLAR  KHALLR**T**GGLVTA  GRGIMT**T**GSGQEP  FQCVFG**T**ERLATA  ERLATA**T**LDTGLE  LEEVDP**T**EALPVL  NAHTLW**T**PDSTGG  EPVAPS**T**VAPRWL  ERLAEN**T**GEFQEV  VRAFYD**T**LDAARS  PAPFQY**T**PDHVVG  NAFGPG**T**ANERTV  FLDRIV**T**CDEKWI  STDFYA**T**GINQLI  SSRVAK**T**NSLRRR  TEINKL**T**AEAIQT  PLKSAK**T**RKVIQV  ERKNLQ**T**ARVKRC  PPSKKR**T**QSIQGK  SRANTV**T**PAVGRL  VSMVKP**T**PGLTPR  DSRVFK**T**PGLRTP  CQIITN**T**STLLKN  QIPLEE**T**ELPGQG  SGEAKS**T**DNVLPR  VNYKEP**T**LASKLR  SRSQLL**T**GRYQIH  TFLGSL**T**GNVDYY  LSGQYS**T**MLYAQR  YLYRYR**T**MGNVAR  MTLDLK**T**KFGSTA  LVSDDE**T**TRLVTS  VGQTDQ**T**DSTGGP  QRVIGD**T**HASEVS  RVCASD**T**RAGIED  LKDDYS**T**AQRVTY  LLGHMP**T**VELLKK  EKLKII**T**YRNLFK  SAEKSA**T**EYADSS  PEDINQ**T**SPRRRS  IVVLNI**T**SYAGGI  NDAVDV**T**FSLKGL  LLFLCG**T**PQAADN  RRMDSV**T**WQEGKG  IIRCLM**T**HNKGVS  ILMLLL**T**MGQGVV  LPSYKR**T**PTEWIQ  SYKRTP**T**EWIQDF  HGGAIA**T**MIDATV  QSVDEK**T**LYSEAT  RRKKAL**T**DYKKLR  DELADP**T**ERFRSL  DWVSSE**T**SALPRP  VAELSA**T**QCCKNT  ASKLVV**T**KEKEKK  ESKANI**T**CLVYSH  GDNKWI**T**TLHYAF  VSSPTD**T**SNFDVD  DVNVQR**T**LDNNLA  EHQQEI**T**KLKTDL  EITKLK**T**DLEKKS  KDKLEK**T**RRESQS  FLAFLN**T**PTDALD  TPLSVH**T**PTLRKK  CVNKAP**T**TCPVPP  SMEWIQ**T**LPLKKV  NLLGLE**T**IRLIYF  KLISNP**T**NFNHIA  RRSHSG**T**RPFACD  QSSNLI**T**HSRKHT  THSRKH**T**GFKPFS  FSCELC**T**KGFQRK  EFAEHI**T**NHYLGL  LIFREN**T**LATKAI  VHTALS**T**PGSGQL  RAGQTP**T**TPGTSE  VLNQDL**T**FQHIKI  APPRSY**T**PMKGGI  FDRFKI**T**NDCPEH  VMCQVL**T**DLIDEE  SFWESF**T**TTTKES  PWEGPH**T**CPQPTV  GLCDRF**T**GQCRCA  TGACTC**T**PGWHGA  RRGPAE**T**AAADSE  QYDNCL**T**YRRIYL  GRRARG**T**KITGDP  YSRVQA**T**FRNADA  EHEVNN**T**FKFGVI  RSHSME**T**MVGGQK  SMEVTK**T**TFSPPV  LFPRLH**T**GSEGQG  ADLERE**T**FKYPNF  GQLVDW**T**MQALNL  QYTSQT**T**RLLALL  LRPLSS**T**GYRPSP  NEVNGW**T**CLHWAC  ADKEIL**T**TKGEMP  QNGGPS**T**PPASPP  EPPLLG**T**FPRDHT  SEILIA**T**PLQQVA  IPFNRI**T**QEAGEF  CGEARV**T**FSMDAF  QELTLQ**T**PAKRPL  GSKKAV**T**KAQKKD  HYNKRS**T**ITSRRS  PASWPS**T**PCPRAP  PRRSPS**T**PAPSES  VMYIPQ**T**FTQTSE  VTLRCQ**T**KLHPLR  YSVVHR**T**SKRSEA  ARSAEF**T**VGRKDS  ELLVKE**T**SGGPDS  GPDSPD**T**EPGSSA  VDVLER**T**ADKATV  GSSQEV**T**YAQLDH  KLQANL**T**FDPAAL  FHSPPS**T**PSSPGV  RRSPSR**T**EKQEED  QEEDRA**T**EEAKNG  GILKNK**T**STTSSM  CRDTET**T**EAMAPG  DEEMLE**T**ADGESM  DGESMN**T**EESNQG  VDQIQL**T**QAQLEE  KEKMEK**T**KVRTRE  EKTRLK**T**KENLEK  RREKLK**T**SRDKLR  WPKNVK**T**NYVVWG  KRSTIK**T**DLDDDI  RVWRTR**T**TILQST  VQKWDV**T**VLELSY  QPPTPA**T**APSTTE  IQGLEA**T**KPTQQS  KAPPSR**T**PQSGSA  QSGSAP**T**AKAPPK  YQRWQF**T**LPMMST  NLRSGR**T**RRALDI  KPVKTL**T**TKERKK  FEGNLT**T**KPINGA  NLRRVA**T**DRRHLG  QGAENM**T**HTCASG  HTCASG**T**PKERKL  GTPVKP**T**ALTGTL  SEGWLR**T**KAKHQR  NRVVGQ**T**GWGQVA  PCSSPS**T**ISPPKG  NFLPKK**T**PLGEEM  YLPQEP**T**SEETPR  EPTSEE**T**PRTKRP  IGFGDR**T**STFCGT  QPFFRT**T**NWQALL  LHLLLN**T**SVTCND  DWPRTR**T**GTGILS  DCVDTI**T**CAPTEA  SHKASK**T**PLKGCP  VGSRPY**T**EFPFGQ  PQQLHA**T**EITSSG  AGRNCT**T**LQGLAP  AYLVTV**T**AAFRSG  GYRCIS**T**WLKGQV  TLRALD**T**SEQERM  TSLETN**T**VVQTYN  PTCKLL**T**KNAIFQ  LDGFIL**T**ERLGSG  GSGTYA**T**VYKAYA  SVENLL**T**EIEILK  LSRFIH**T**RRILPE  LPPVLT**T**VNGQSP  DAINWP**T**PGEIAH  KGSESA**T**YVPVAP  DGFLPI**T**LIASFH  QHYQKE**T**ESAPGS  AVTPVP**T**KTEEVS  EVSNLK**T**LPKGLS  ARSLPT**T**VPESPN  NYRNTR**T**PRTPRT  VGSYGC**T**PQSLPK  LKENGF**T**QHVYHK  KDFQEE**T**VKDYEA  SMAAVL**T**WALALL  LSAFSA**T**QARKGF  WDYFSQ**T**SGDKGR  QQLKPY**T**MDLMEQ  RVVGED**T**KAQLLG  VLSRKL**T**LKAKAL  SRAFAG**T**GTEEGA  AFAGTG**T**EEGAGP  QAFRQD**T**YLQIAA  LQIAAF**T**RAIDQE  APEFQQ**T**DSGKVL  DLWEDI**T**HSLHDQ  LFPSSV**T**NSLIAV  HISVRA**T**ILLGEL  QNNGLL**T**TLSQHY  VLSIRG**T**CVYVLG  DFTPIS**T**VQKTLQ  ENDLKF**T**KNFGTE  STKTIK**T**SHYLTP  GGLPSG**T**GGLVKN  SDWCEQ**T**IHNPLE  KTIPDD**T**PMCRIL  KEKYPQ**T**FDDICL  AEAVLA**T**PPKQPI  PSQESP**T**PLCTLI  HRATAL**T**PDSCPL  VQSQRD**T**SLLKHT  LVRESA**T**SPGQYV  LIDVAN**T**VISEKE  GNHLKE**T**FYVEAI  TSLILD**T**PQAADL  FCLEKL**T**NIPSDQ  GSLITM**T**AVSLDR  AYASFW**T**TMPLVG  PFGTSC**T**LDWWLA  VLEMKL**T**KVAMLI  QSTMTP**T**QPESFS  VPGMEE**T**TSEADK  AGQGMS**T**PGSSPQ  AAMMGL**T**GSHGSL  DVEVVY**T**IDIQKY  SGSCMS**T**GFSRAV  TLMVEH**T**DIPEAS  KRSKAD**T**GSSNQD  SCQHNG**T**MYQHGE  IYCGLT**T**CPEPGC  KGIFHL**T**QIKKVR  TLELKV**T**ASPDKV  LFDAPV**T**AECGHS  EPAADG**T**VLCPCC  VADKPQ**T**EFLMKY  MKYCLV**T**SRLQKI  SLWSKP**T**PSITPT  SLGSAS**T**DSYFGG  SSSAAA**T**QRLADY  ECPVCH**T**AVTQAI  FVVRYE**T**PEGTFW  KNMDDN**T**FAMAEH  RPWPQM**T**LQVSDV  SLGEAG**T**EAQIEV  KVENEY**T**ISVKAP  TLDKLL**T**ESLKNN  GQRQLI**T**LQEQVK  QVDGTP**T**MSLERP  SPLRQE**T**NMANFS  DTKPLV**T**NQHRRT  TNQHRR**T**ASAGTV  TQVAPP**T**PPPPPP  VEGFRI**T**LVDESD  YPYSPP**T**FRFLTK  DGVKVP**T**TLAEYC  AEYCIK**T**KVPSND  KNTLER**T**HSMKDK  SAEKIS**T**QRHEVI  HEVIRT**T**ASSELS  SVTSKK**T**GPLSAQ  IESQSK**T**QKKGKI  KAIGSD**T**SDIVHI  CPEGMK**T**SDIKEL  LPEFEK**T**HLEHQQ  CKAAIA**T**FYVNVK  QLKQLQ**T**KYDELK  EPNVKE**T**YDSSSL  LLFKAR**T**LLGAES  TGIEES**T**DGMILG  SNSAVS**T**EDLKEC  TYLQKE**T**STLQVE  KPSRAS**T**ASPCNN  IRDLKY**T**IENPRH  LSKVHF**T**AERSSY  VFVTEI**T**DDLHFY  AGCPHV**T**LQFADS  NTPVRY**T**VYCSLI  AIQYIP**T**ELDQVR  LIHDLL**T**IFVSAK  YCKIDQ**T**QRKVVV  MSQSKH**T**EARELM  MLVEYS**T**SRGFRS  VDGGKL**T**VFTVLC  LLGNLL**T**SLMGSS  RDVRFP**T**SLGGHG  GCGITF**T**LGKGTE  GVVHLA**T**AAVLNA  QGYPAY**T**TSCAWL  PTHVQV**T**VLQAKD  FEESSE**T**WDSSMN  ESTNPF**T**AKFRAS  SFHMSP**T**SNEDLR  EQPPGD**T**RRKTND  GDTRRK**T**NDASSE  GFEDLM**T**VNLARY  LARYKP**T**GEYVTV  PTGEYV**T**VRRINL  IVPYRA**T**FIADNE  IYSVGI**T**ACELAN  FKDMPA**T**QMLLEK  IPAEEL**T**MSPSRS  GLSDSL**T**TSTPRP  SHPYHR**T**FSPHFH  RPVTPI**T**NFEGSQ  SPQVEW**T**ARRLVW  RNTDQA**T**MPDNTA  RRGMFR**T**VGQLYK  QRYEIL**T**PNAIPK  LSSDEA**T**NPISRV  NNSGYI**T**NGYMGK  ETGPGG**T**SRGKPA  GGQPLL**T**TAANTL  LGDWEA**T**ERALGS  NPTVVM**T**SMGQAT  TSMGQA**T**WSDPHK  DRSGMT**T**GSTLPV  AMIWDF**T**EPVCRG  SLAQPP**T**PQPPPV  KIPMTP**T**SSFVSP  PHSNRT**T**PPEAAQ  PGGGSG**T**NCTNAL  IHGFFP**T**NFVQII  DGWFKG**T**SMHTSK  CGNSSA**T**KPDKDS  QSRLML**T**LQDNSF  GSGIAR**T**IPSLTS  IIPRYL**T**LSSHCT  ETEKPK**T**ITELDP  NVDYGA**T**AEELEA  GSVNRV**T**ILCDKF  NRPGIS**T**TDRGFP  RPGIST**T**DRGFPR  HNTLAP**T**CFKAKL  SALTDR**T**SRAPST  RAPSTY**T**YTSRPR  PSTYTY**T**SRPRAL  RYRDSL**T**QPDEEP  NTYALQ**T**GPLLGR  VDMECQ**T**DAFLDR  YSMVGR**T**VLDMLI  GGPGAM**T**ESLEAS  EQSMSQ**T**RELLLD  GGYLQR**T**TYDRRS  GYLQRT**T**YDRRSS  ELNAIR**T**ENSSLT  EKDLAN**T**GKWLQD  VKAAGD**T**TVIENS  SVSLNQ**T**VTQLQQ  LSSGAE**T**REGLRS  RPRKQA**T**AAGQRK  CACTSV**T**DLTYWG  SQSNPP**T**RISSAL  AFDQAV**T**KDTCMA  HAAIDY**T**QLGLRF  ADRCTS**T**AYQEQR  EPLVTV**T**VQCAFT  EDGISS**T**LPSKES  SDTSSE**T**VFGKRG  TMELPD**T**NGAGVK  TVSEVL**T**RRNRVG  GGAGVE**T**GGKLLV  LFAEFG**T**LKKAAV  VNRGGM**T**RNRGAG  GGTRRG**T**RGGARG  AEQKGL**T**EVTQSL  EEQVQA**T**RQEVVF  NNNNVF**T**KKTGNQ  EDENVP**T**APDPPS  PGCEPC**T**KRPRWG  FKQKTI**T**SWMDTK  SKENNN**T**RIESMM  LESEPQ**T**VTLVPE  SEADEE**T**SPGFDE  EGGESR**T**GMNDLN  KKDSKI**T**DHFMRL  RKEQWE**T**KHQRTE  ETKHQR**T**ERKIPK  IISRLF**T**EVLDHN  SMHIFL**T**ERKLTV  LTERKL**T**VGDVYK  DELDHR**T**DERKTT  DASFGL**T**SSPIPG  ASPTTR**T**RPVTRS  SRENGG**T**EPDASA  SDMNSD**T**SPSCRL  PPPLPS**T**PDPPRR  RPGEWR**T**PAARGP  GPALPS**T**RARQSF  LSACLV**T**LWGLGE  RGSYWR**T**VRACLG  DKLPQQ**T**GDHAGI  YATPLQ**T**LFAMSQ  CSVSRE**T**ECSRVG  YAVEAS**T**MAQHAE  GDTLSG**T**CLLIAN  LIPLAN**T**GIVNHT  VAEHSN**T**TEGPTG  AEHSNT**T**EGPTGK  LHAEDD**T**DEALRV  QTIWKE**T**RNMVQR  REFPFF**T**LTAFPP  MEYEVH**T**QLMNKL  DELIQV**T**VTAVAI  IPGAKI**T**KYLGII  IFEFKE**T**LGTGAF  FKETLG**T**GAFSEV  LAEEKA**T**GKLFAV  VEKGFY**T**EKDAST  TEKDAS**T**LIRQVL  MSTACG**T**PGYVAP  DPNKRY**T**CEQAAR  PWIAGD**T**ALNKNI  RQAFNA**T**AVVRHM  DCLAPS**T**LCSFIS  ERRPRP**T**TVTAVH  RRPRPT**T**VTAVHS  QQETSS**T**ELKTEC  TSAKCL**T**YMCRAG  ILRSLS**T**EQLFRL  IEVKEQ**T**LCILAN  YEVLDL**T**EYARRH  EKYSVA**T**QIVMGG  VGKLAA**T**AVGGGF  NLIEEA**T**EFIKQN  GQDEPQ**T**WPTDHR  YSVHAY**T**GQPSPR  AVRKSS**T**VATLQG  KSSTVA**T**LQGTPD  PGPPRS**T**PLEENV  RGTPAG**T**TPGASQ  KVRAVP**T**WASVQV  RGLRQA**T**DHQELV  TKLSLI**T**APRRER  PYLSPG**T**PQLEFS  AEAKAA**T**SPKATM  LEAIRV**T**RHKNAM  ALAPGE**T**VIGRGP  DNILNE**T**PKSPVI  KTQMTP**T**NSVSFL  GEECKN**T**DQEEST  EYSINW**T**QKLGAG  VCVKKS**T**QERFAL  LMTPQF**T**PYYVAP  KSGIIP**T**SPTPYT  IIPTSP**T**PYTYNK  TSPTPY**T**YNKSCD  SKHHSR**T**IPKDMR  MRRKIM**T**GSFEFP  KPEERL**T**IEGVLD  HPWLNS**T**EALDNV  KRKLLG**T**KPKDSV  CKLLRD**T**LQSFSW  LQGSIM**T**VGEKVF  FVKKYN**T**YAYVGL  GTPVNY**T**NWYRGE  QCVEMY**T**DGQWND  PWWDVF**T**DYISIV  GGTLQV**T**QDKMIC  SINNEG**T**KLIVLN  LQNLAI**T**ANRIET  AIKKAI**T**CGEKEK  ISPRKW**T**QGEVEQ  SEAVGA**T**RIQIAL  RSGERS**T**RWYNLL  RNLFSQ**T**LSLGSQ  RLLDEL**T**LEGVAR  YTQNID**T**LERIAG  KIFSEV**T**PKCEDC  VESGCQ**T**ESSKEG  GQPRQL**T**PFEKLT  TPFEKL**T**QDMSQD  KVVREI**T**LGKRIG  LGIHSL**T**KEKVAI  TKLDQK**T**QRLLSR  AENVFY**T**SNTCVK  YFMVTG**T**MPFRAE  KSILEG**T**YSVPPH  QGVPYP**T**PLEPFQ  HLSETS**T**LKEEEN  LEHLGI**T**EEHIRN  NIEQLK**T**TKQQLT  YERPNE**T**KIRSYP  VTTVNG**T**QTNPNI  RTTEGI**T**SDSLVA  VIKPWI**T**KRVTEI  SKSRSR**T**RSRSPS  KKPPKR**T**SSPPRK  IRRVSR**T**PEPKKI  RKALEE**T**KAYTTQ  NKNTSR**T**HKIIAP  PMSGRG**T**LGRNTP  VAIYDY**T**KDKDDE  QKALKQ**T**KQKKSK  DLSACQ**T**AEKKVI  HDMPDR**T**LAAHQQ  ILLLKV**T**AVIRSR  PQKALE**T**IGANLQ  LPADLF**T**SCLTTP  LFTSCL**T**TPIKIA  CLSQLP**T**IIEEGT  RTDHST**T**IDHNVA  YESNFC**T**VALQFI  ALPSPA**T**TEGGSL  VRDSPC**T**PRLRSV  LQNLSL**T**EESGGA  LDTSSL**T**QSAPAS  KSFISA**T**VQTGFC  SLLLTA**T**DDGAIR  GRNFSG**T**ASTSLL  PYRVHL**T**PYTVDS  VHLTPY**T**VDSPIC  AGAEPI**T**ADSDPA  MAEPRE**T**PEHPLK  PQVLVR**T**AIRCAQ  YLPDVW**T**IMPTLE  QGETEP**T**EQAPDA  AETPEA**T**TQQETD  ATTQQE**T**DTDLPE  TQQETD**T**DLPEAP  PPPGKS**T**KPGAAP  ENPVPL**T**VRVSPE  RFLTSQ**T**KLHEDL  ITNSEK**T**KVKKTT  SKADLA**T**APPHVT  EENNDS**T**ENPQQG  GDRDRM**T**ANHESY  PSFDSN**T**DVHTVA  EIQKKA**T**MGQLQN  AEGLEK**T**QTTPNG  GILNSD**T**LGNPTN  QHNRLS**T**YDNVHQ  QSIDSA**T**WSTSSC  SCRSST**T**TCPEQD  GRSSRA**T**SSSDNS  LEQRNL**T**LETEMM  RNLTLE**T**EMMSLH  LASLRC**T**LGAFCE  VRDPAP**T**KPLVLS  LHGWTG**T**GKSYVS  WVQGNL**T**ACGRSL  PVVGAS**T**PGTVVR  TQSSIR**T**PVVSIS  FDGSNS**T**SHALLV  VNVHLG**T**LIGLNP  SMVSVP**T**VTSDYI  VSVPTV**T**SDYISL  KPIILP**T**DATPFV  TEGSMA**T**VGLSQQ  FVEVLA**T**ASNSAF  KVKLQG**T**TSVLGD  LGDVQL**T**VASSNV  DTDQVR**T**LMGTVF  RALDVN**T**RNPNYE  PLNQRE**T**TLKDLD  LNQRET**T**LKDLDI  KDLDII**T**VPSDNV  ELVYQF**T**AQPLVE  CFAYGQ**T**GSGKTY  DLKVYG**T**FFEIYG  IGNSCR**T**SRQTPV  GQNKPH**T**PFRASK  IGQNSS**T**CMIATI  CENTLN**T**LRYANR  LLGKDT**T**ISGKGS  RVIPNP**T**TVFKIT  TTVFKI**T**VGRATT  RRVSCT**T**AIPTPE  CTTAIP**T**PEEMTR  NPSPIR**T**MTDILS  NMFPFK**T**AVITVE  KNPQKN**T**PKFLYT  LRIGFL**T**GPKPLI  NICRFL**T**ASQDKI  MKEIKE**T**LRQYLP  IAIVRQ**T**FNKEIK  FNKEIK**T**MKKFES  GICIDE**T**VTPPQF  CIDETV**T**PPQFSI  EYCELG**T**LRELLD  SSNFLV**T**QGYQVK  GFELRK**T**QTSMSL  SMSLGT**T**REKTDR  GTTREK**T**DRVKST  TDRVKS**T**AYLSPQ  LQDQLK**T**LQRNYG  QFQKNQ**T**NLERKF  ERIEEV**T**KKGNEA  NEAESE**T**DKQAAL  VITVGA**T**NAQDQP  WQLFCR**T**VWSAHS  SAHSGP**T**RMATAV  GPTRMA**T**AVARCA  DLGVIY**T**RNKMNG  YSQGTG**T**DKNKPN  PNIKIC**T**QVKGPE  KAGGVF**T**PGAAFS  SEPVKG**T**GALRDC  DVSCRK**T**GLKLEK  WKERKV**T**PSKYSS  AITRDG**T**RDHQFP  KYSSCS**T**IFLDDS  IFLDDS**T**VSQPNL  QPNLKY**T**IKCVAL  IYRFVR**T**LFSAAQ  FSAAQL**T**AECAIV  AECAIV**T**LVYLER  YLERLL**T**YAEIDI  QILKDI**T**VEDMNE  GLPPSA**T**PSTVPF  PSATPS**T**VPFGPA  YPSVPP**T**GPPPGP  TPGLYP**T**PSNPFQ  EMQNRP**T**QKFSGG  LMLDEP**T**NHLDLN  QTKEAL**T**RKQQKC  LESHAH**T**IQICKL  VSKDSS**T**SLDAII  VGNIQA**T**SPCLHP  KGVREV**T**EPLNLN  PADACS**T**AQKAVG  DNFCII**T**EYCEGR  CRYSEM**T**LEDKNL  SQPAYR**T**NQQDSD  TSLDTK**T**ITTMAE  NNNFLV**T**ASSSGD  AEGQKQ**T**CVNLNS  CVNLNS**T**SMYLVS  DHKDQV**T**CVTYNW  IILHSV**T**TNLSST  TTNLSS**T**PFGHGS  TISAHK**T**SVQCIA  IAFQYS**T**VLTKSS  DTLSKE**T**DSGKNQ  PRKNPV**T**SSTSVL  QLAKLV**T**SGAESG  ESGNLN**T**SPSSNQ  CAPSPG**T**GGGGGA  PGAVGG**T**SGKPAL  PEALNL**T**PEDAVE  LKQKRR**T**LKNRGY  ATGSSE**T**AGGASG  DSAPVA**T**AAAAGQ  VDCYKP**T**EEFIKE  QNFRPP**T**PPYPGP  SPGQQQ**T**HPQGSP  SQQYSN**T**QTFTGK  QYSNTQ**T**FTGKKG  MAEVLV**T**GEQLRL  EASDRE**T**AIKLMQ  FRKDDG**T**FPLDNE  LMSPEN**T**LLQPRE  GVKYER**T**FMASEF  VQEGEA**T**TRKEAE  QEGEAT**T**RKEAEQ  YFSSSP**T**LSSSPP  VLKRPV**T**SEELLT  TSEELL**T**PGAPYA  LHVDYR**T**VSNLIL  VSNLIL**T**GPRTIV  IIAVEE**T**DVHGKH  EKPYAF**T**VHCVKR  TTDIIV**T**EHANQA  IIPAGS**T**DCVCYS  RTFREI**T**LLQEFG  LLDANC**T**VKLCDF  VTEYVA**T**RWYRAP  LSSHRY**T**LGVDMW  LFPGTS**T**LHQLEL  ALLPPD**T**SPEALD  DKRLSA**T**QALQHP  CGGSSG**T**SREKGP  SAPLLQ**T**ALLGNG  KEAPPL**T**LSLVKP  GAAPSL**T**SQAAAQ  GRRMFS**T**SALQGA  QVMALC**T**YPNLLD  PDNIYL**T**TGASDG  ASDGIS**T**ILKILV  GGGKSR**T**GVMIPI  PYRLEK**T**LGKGQT  TLGKGQ**T**GLVKLG  LGVHCI**T**GQKVAI  RGGGSP**T**SKTQTL  LHSPLH**T**PRASPT  TPRASP**T**GTPGTT  RASPTG**T**PGTTPP  RKMQVP**T**AEEMSS  EEMSSL**T**PESSPE  IYSVTF**T**LISGPS  QAQLLS**T**HDQPSV  EKNGAQ**T**RPAGAP  RASMSD**T**WDLSLP  TTEPGE**T**SESCLE  VYRFRP**T**SVGESR  RNNSFI**T**HSLKFL  ALIGLS**T**SVSDSN  GFLKQN**T**AGSPKT  SLESLQ**T**AVAEVT  ARVMLE**T**QELLRQ  AVLQRK**T**ACQDTD  KTKQGC**T**VAEKSF  LPVLLS**T**AQEADP  FLAKQH**T**DSFQAA  EGIPIR**T**TLDNST  TTLDNS**T**TVQYAG  TLDNST**T**VQYAGL  TMKAKS**T**VRDIDP  PPQKCS**T**PCGPLR  SIYTTA**T**SLSGSL  MDEDLF**T**GDGDYQ  FKTKCN**T**PAMKQI  DNLIAN**T**AYLQAR  FRDFLA**T**VPTFRK  PFKDFV**T**SAFYDK  EMVAGR**T**PFKDYK  DDQDHY**T**NTYHIF  NTYHIF**T**KAPDKL  VISWEQ**T**LQIQGC  ELRMGY**T**VPKYRH  APFALP**T**VSLLPA  SAEDLE**T**DGGGPG  RGSTGD**T**VLLPLA  WAAGSV**T**DLAFKV  ALSREP**T**VDISPD  SPDTVG**T**LSLIML  EGTNFR**T**VLDKAV  ANLKEG**T**KFYNEL  PPVEQE**T**RPVHPA  KARLTS**T**LPALTA  PASPPG**T**ASGRNS  RAGKKP**T**TPLKTT  AAAAAA**T**AAAAAS  INRPDI**T**DTEMET  SMLSDN**T**AKLTSA  SDPDAG**T**PEDKMR  DLPTKP**T**KISKFG  AIGSQT**T**KKASAI  KNIGRD**T**PTSAGP  IGRDTP**T**SAGPNS  SQVHRE**T**EDVDYG  DVDYGE**T**DFHKQD  LGVGLL**T**FASLET  TFASLE**T**VGIYFG  KSTSTP**T**SPGPRT  AIVSSS**T**PQYPSA  HQPQPA**T**TPTGSQ  TNMAHV**T**QAHVQT  ISQIFS**T**SPASLC  NPSGSD**T**TSAEVF  QQVHSP**T**SYLCRP  TKELQN**T**AVAEGQ  INSLFL**T**NLYEDR  EASARL**T**QTLAIE  RRYSWG**T**VEVENP  LSRQSA**T**EIPLPM  IPDPSV**T**TGSLPG  PDPSVT**T**GSLPGL  PGLGPL**T**GLPSSA  LPSSAL**T**VEELKY  NKKKER**T**EFLQRE  MNAELK**T**QIEELK  LNRHRP**T**CIVRTD  PTCIVR**T**DSVKTP  VSRSAS**T**VIAYAM  NAPAIC**T**QPAFLP  ESEKPT**T**NSYLMQ  LRLAGL**T**VSSPLK  GRQERS**T**KEKQSS  EEEEKE**T**RGVQKR  GLGILT**T**MTVLLH  MRLQLL**T**AVGALA  SLAKIY**T**EAKKVP  LLQVKG**T**GANGSF  GAPAAA**T**APAPTA  ATAPAP**T**AHKAKK  NMADAV**T**HARFVG  GSPGYS**T**ATEPGS  PDLQEG**T**HVEKSQ  PRGVRF**T**QSSQKE  NLFEEL**T**KLLSKN  SLDALL**T**VIDSTE  QQEKKE**T**ARPSCE  KLLITG**T**EQFNQK  RDAAHI**T**PDNFEL  FELCVK**T**LRIFVE  LASAHL**T**SAAGDT  VGVPPM**T**LPIILN  GTILRL**T**TSPAQN  RSSIEL**T**KMLCEI  FHPMFF**T**HDRSFE  WKEMRA**T**SEDFNK  GDEALD**T**TKDPCQ  HRIKQP**T**VKLHGN  MFSKLD**T**SADLFL  QLGLEL**T**GTRTHG  NSYGCA**T**STPYST  SKVFGT**T**AGGKGT  QTQRLD**T**SQKRIL  SSSELS**T**PEKPPH  LVEKGE**T**ITGLLQ  QEQDIE**T**LHGSVH  HVTLCG**T**PKGNRP  NRPVIL**T**YHDIGM  IGMNHK**T**CYNPLF  AGAYIL**T**RFALNN  PMPGTH**T**VTLQCP  NSKLDP**T**KTTLLK  KLDPTK**T**TLLKMA  LDPTKT**T**LLKMAD  MPSASM**T**RLMRSR  SHTSEG**T**RSRSHT  AAPVPT**T**LAQVDR  VASHPE**T**RSAFLA  FEYLRL**T**SLGVIG  LAYICQ**T**YERFSH  LWCNRF**T**MEVPKK  SEDALC**T**VLHLTE  SEIMKS**T**SRDAVL  VAGDAL**T**RHLGVI  RRHLGS**T**PRLSRG  EPDKAV**T**EDGKVD  TLIKYM**T**DGILLR  RSTKIY**T**PGRKEQ  PFQLLC**T**AGQSHP  SHPETL**T**HTASPH  SDSDLL**T**CSPTED  TIILSI**T**YKGVKF  APAAYP**T**GRGGDS  TRSGVG**T**GPPSPI  NKRTVL**T**TQPNGL  LTTVGK**T**GLPVVP  VKATPM**T**PEQAMK  QYMQKL**T**AFEHHE  HMLENF**T**FRNHIC  ILAELL**T**GYPLLP  PRYCTV**T**TLSDGS  RYCTVT**T**LSDGSV  RLPKPP**T**GEKTSV  PPTGEK**T**SVKRIT  DKYMFA**T**KVVHLL  RAAQFV**T**RHPINE  ILANQL**T**GLMDMK  YLSFTL**T**MESDIK  GGPGRQ**T**TSPNVL  LPVNRE**T**LKRKVS  RPSAPF**T**EASMMM  DMLLAT**T**SRFREL  GEPGPS**T**NIPEND  AEDRSG**T**PDSIAS  ASYSQQ**T**GPQQPQ  QPRPGF**T**SLPGST  VGLQED**T**TGEACW  LSFIKL**T**PSQEAM  ALPKTY**T**INGVSV  TAALSA**T**DMALAL  VAKDPG**T**VANKKE  STLYPS**T**SSLLTN  STSSLL**T**NHQHEG  LQMLQS**T**DPSDDQ  PKQAAS**T**SGPTPA  WLYNRK**T**SRVYKF  YGKQLC**T**IPRDAA  KRVVQH**T**KGCKRK  STSDWP**T**DNQDGW  PILTKP**T**LKRKKP  HNSSVV**T**ETISET  SSNSPE**T**ETKEPE  EDAAAE**T**AQNDDH  EQPSHD**T**SVVSEQ  SELDLE**T**VQAVQS  LGSIES**T**TENYEN  SMNNSF**T**PAPMIM  LQQLTN**T**IMDPHA  SHPLAG**T**PQAQAT  HGNMMY**T**GPSHHS  SGYGQT**T**DSSYGQ  RDYGPR**T**DADSES  IKTNKK**T**GKPMIN  KPKGEA**T**VSFDDP  AEKPGA**T**DRKGGV  LAPPES**T**DEGAET  YELVAQ**T**VSERKN  EGQLAA**T**ALRKVL  ELLGAD**T**QGSEFE  TFAVDE**T**SVSGYI  PPGTGK**T**VTSATI  DQIGII**T**PYEGQR  ALPVEG**T**ETRVNA  SGIDVS**T**QMLNQQ  TRDSCK**T**TIEAIH  RDSCKT**T**IEAIHG  QDIYQV**T**GSLSEL  IWSLGI**T**AIELAE  HNFIKV**T**LTKSPK  PTPAED**T**PPPLPP  HSTAAW**T**HPSTKD  MSLSGK**T**PHLYSH  LLERKE**T**RAGNPI  LCGALE**T**SVVLLQ  KSVLFH**T**VRFGAL  WLGEMS**T**EHRGPV  TPEIPM**T**EAVEAV  RLECSG**T**ISPHCN  DEQEDL**T**KDMEDP  KKDSEN**T**PVKGGT  QRQQLL**T**ERQNFH  GPRVPL**T**APNGMY  HQQEQP**T**SSSHHG  SSYPAG**T**EDDEGM  RPKSAG**T**ATQMRQ  RQSSSW**T**RVFQSW  IGGDAA**T**TVNNST  NGGQNG**T**VQEIMI  MTFSIP**T**HKCGLV  KQAQVA**T**GGGPGA  RELASK**T**REKEKM  AKDARY**T**NGHLFT  HNRCKD**T**LANCTK  IYELIQ**T**ELHHVR  ECILLV**T**QRITKY  GCLLWK**T**ATGRFK  VLVLLM**T**DVLVFL  NTSPGV**T**ANGEAR  ANGEAR**T**FNGSIE  AVAQQD**T**LMEARF  PQPSRG**T**DRLDLP  LDLPVT**T**RSVHRN  EGASLE**T**VKVGKT  TVKVGK**T**YELLNC  LQVYIH**T**QKNVLI  LTCAKL**T**TAFEEV  GADPVP**T**KAPGEG  QPAALT**T**GEAAAP  PGALDV**T**IMYKGR  RVHLEG**T**QREGVS  RLDEWV**T**HERLDL  KEAKTP**T**KNGLPG  SHDDIV**T**RMKNIE  NVACIL**T**LPPYQR  PPPMER**T**GGIGDS  MSSELE**T**TSFFDS  SRFSSS**T**EQSSAS  PGPITL**T**VAKCWD  AAWVSH**T**AAMTGT  TAAMTG**T**FPAYGM  MSPSLS**T**ITSTSS  TSSIPD**T**ERLDDF  HNVEGF**T**DRREAR  HTVNKI**T**FSEQCY  GSESDH**T**TRSSLR  SVPPEL**T**ASRQSF  IDDDKV**T**FEVFVQ  MNSLLQ**T**LFFTNQ  LSVRHC**T**NAYMLV  DNENPW**T**IFLETV  LRHFSV**T**AEGGLS  SVYCTP**T**RPHPTG  LGIFGD**T**EDVAGK  QRQACR**T**ARCFAR  SREDEH**T**HTNSVT  LSSPFY**T**KTTKMK  SPFYTK**T**TKMKRK  PFYTKT**T**KMKRKL  HDDHVI**T**CLQFCG  KVWSAV**T**GKCLRT  TGKCLR**T**LVGHTG  HTLQGH**T**NRVYSL  RVWDVE**T**GNCIHT  TGQCLQ**T**LQGPNK  NKNFVI**T**SSDDGT  RNGTEE**T**KLLVLD  CFQLTV**T**PDEGYY  PKVKCL**T**KIWHPN  GTGWAP**T**RTLKDV  GWAPTR**T**LKDVVW  VPKTRR**T**FCKKCG  HQPHKV**T**QYKKGK  FRKKAK**T**TKKIVL  RKKAKT**T**KKIVLR  SLWLQD**T**QVHGKK  EPSGQK**T**LDGSSR  AQPVCY**T**LLDQRY  HVVEFS**T**AWLQGK  PCVEVQ**T**LREHTD  SCSKDC**T**VKIWSN  VFGCWL**T**ETSLIS  SKYLIF**T**TGCLTY  LISVIR**T**LSTSDD  QRGENV**T**VENQPR  YLDMKN**T**RTASEP  GVGELT**T**QNTRLQ  IDDSEI**T**KEDDAL  DEHISF**T**TSKIGS  EHISFT**T**SKIGSL  AGALTA**T**ALVTGV  RLGISL**T**RVSDGE  ELKNTI**T**VSPFSG  AMMVPY**T**LPLESA  VMLLGD**T**GVGKTC  FRNKVV**T**VDGVRV  ERFRSV**T**HAYYRD  RSEDGE**T**LAREYG  ERFRAR**T**GTELVL  TELVLL**T**AAPPPP  PQPPSP**T**PSPPRP  PSPPRP**T**LAREDN  EDEDEP**T**ETETSG  EDEPTE**T**ETSGEQ  VMDEDA**T**LQDLPP  ESDPES**T**DDGSLS  GSLSEE**T**PAGPPT  PASALP**T**QQYAKS  GFKEKR**T**EARSSD  LREAED**T**QVFGDL  VTGGIK**T**VDMRDP  LHSVGY**T**TLVGNN  KNQPSK**T**YPAFLE  LDMDDG**T**LSFIVD  EKKKRF**T**VYKVLV  FDKLYN**T**LKKQFP  SQKLHS**T**SQNINL  NPHAKP**T**DFDFLK  GIAISD**T**TTTFCG  IAISDT**T**TTFCGT  AISDTT**T**TFCGTP  TTTFCG**T**PEYLAP  KQPYDN**T**VDWWCL  RPGVSL**T**AWSILE  DIRNFD**T**AFTEET  TAFTEE**T**VPYSVC  ELEPPT**T**LAPGLL  YHSLPC**T**PHTGAE  LPCTPH**T**GAEDAE  RRQGLV**T**DQVSLH  ATDELA**T**KLSRRL  GEGGGE**T**PEQPGL  RVFNPY**T**EFKEFS  KLGAPQ**T**HLGLKN  TRSNKE**T**VKHSDV  VVQEGA**T**VYATGT  SSVGFC**T**EVEEDL  VKLESP**T**VSTLTP  QLNDVK**T**TVVYPA  TVVYPA**T**EKHLQK  DDYRNI**T**LPHLES  RHYQQR**T**LTFALR  LVRLRE**T**EEMLGK  RFEKQL**T**QIDGTL  ALENSH**T**NTEVLR  ENSHTN**T**EVLRNM  KSIQSL**T**LEWNSL  LALKGN**T**TLQQLD  ALKGNT**T**LQQLDL  QTRQEM**T**SMSAEL  LERKKE**T**QRLLEE  KAPRVA**T**SSKVTR  ATSSKV**T**RAQIED  RAQIED**T**LRRDHQ  PNLDTF**T**PEELLQ  SPGPTR**T**VSTGTA  RTVSTG**T**ALSKYR  QEEDLE**T**MTILRA  VVAVGL**T**IAAAGF  GFEPKM**T**KREAAL  ILGVSP**T**ANKGKI  GWQLPN**T**RYIADM  SGNGYT**T**IVPDFF  YNLKNP**T**LFIFAE  KTFSGQ**T**HGFVHR  FGDEII**T**NGFHSC  QHLMIG**T**DPRTIL  IGTDPR**T**ILKDLL  KRKDIN**T**IEDAVK  KLLRNY**T**QNIDTL  VKLSEI**T**EKPPRT  TEKPPR**T**QKELAY  DSSVIV**T**LLDQAA  KNVGSS**T**GEKNER  RTSVAG**T**VRKCWP  SNSDSG**T**CQSPSL  SVKQEV**T**DMNYPS  GSPAKQ**T**TWEGGH  PVNVTS**T**ALLSVL  YKAFYI**T**GGARAC  ISSADY**T**QDPSVT  GLDWST**T**QETLRS  IDPKPC**T**PRGMQP  AGQPPP**T**WQQGYG  TSYIVS**T**PPGGFP  RPGVSL**T**GTKHNP  PFVMRS**T**CRRCGG  YETINV**T**IPPGTQ  TSSGGS**T**MDSSAG  FSAISC**T**VEGAPA  LLPLGA**T**DTAVFD  PLGATD**T**AVFDVR  VRLSGK**T**KTVPGY  GGLLER**T**ASRLGS  LGSRAS**T**LRRNDS  MDGVPF**T**LHPRFE  SAFGDL**T**IKSLAD  GFVVEK**T**AAARLP  ARLDQE**T**AQWLRW  ARMEFG**T**AGLRAA  PDPEFP**T**VKYPNP  RSALKD**T**YMLSST  EAMDPA**T**VEQQEH  YFSNYV**T**EDFTTY  VTEDFT**T**YINRKR  YQYSTG**T**SAVEPI  AVEPIN**T**FHGIHQ  VNPNKA**T**IGVGLG  EDKKRA**T**DWEATN  PRKASA**T**CSSATA  ATCSSA**T**AAASSG  SGLEEW**T**SRSPRQ  KPPSPG**T**VLALAK  SPCAPG**T**SSQFSA  EPFHVV**T**PLLESW  KLGIPA**T**IVLPES  VTLPDI**T**SVAKSL  CLPPSL**T**SVVVIV  TVCSFL**T**KVLCAH  LLQEVE**T**QEGLGD  NRDCWS**T**CTLSHD  NFQIIS**T**YKHMKL  FTPAHA**T**TSSHAS  NVPRQP**T**VTSVCS  LAQITG**T**EHLKES  PGVTSL**T**AAAAFK  EDLPQF**T**TRSELM  DLPQFT**T**RSELMK  PQDIIF**T**SGGTES  QTSKGH**T**GGHHSP  LAAVRP**T**TRLVTI  MLANNE**T**GIVMPV  RVWSHQ**T**LKSDVL  VTNGET**T**CSENGV  TNTNTN**T**SEGATS  HVEKRT**T**WDRPEP  VDHFTR**T**TTWQRP  DHFTRT**T**TWQRPT  VDHNRR**T**TTYIDP  DHNRRT**T**TYIDPR  PQHIKI**T**VTRKTL  HGKFID**T**GFSLPF  YRHYAR**T**SKQIMW  RLLQFV**T**GTCRLP  SVVPES**T**EDILLK  EEERIE**T**AQQFFS  WFAKLQ**T**QMDQDE  MDQDEG**T**KYRQMR  LYGRGS**T**DDKGPV  IFARKD**T**FFKDVD  EAVAAV**T**EEEHKL  YNYIEG**T**KMLAAY  SEPPPA**T**TSPEGQ  CDPLAD**T**IDSSGP  QDSGGW**T**PIIWAA  VNYHGD**T**PLHIAA  IKIIDK**T**QLNPSS  LFEVIE**T**EKTLYL  VIETEK**T**LYLVME  NPAKRC**T**LEQIMK  RAPSDT**T**NGTSSS  TSSSKG**T**SHSKGQ  QRSSSS**T**YHRQRR  ERRKDS**T**STPNNL  RKDSTS**T**PNNLPP  LPPSMM**T**RRNTYV  KLTSKL**T**RRVADE  IGGPEV**T**SCHLPW  GEGGEG**T**EEEDGA  LERPRR**T**KRERDQ  AALRGK**T**VLDVGA  SVLHAR**T**KWLKEG  GFFLKV**T**ALTQKP  PPSFSW**T**GAALSS  ENLGNG**T**SLPVLE  PEPKSS**T**QAPESQ  RVNTLK**T**CSDDVV  LVFPAQ**T**DLHEHP  EEPGAG**T**PSPVRL  QRLVYS**T**CSLCQE  HSRPIV**T**VWEREL  KPNRKL**T**FLYLAN  KKPRKR**T**YEQIKV  SRLPNV**T**GSHMHL  LPIVWN**T**LTESAA  ILYMQI**T**EEQIKV  PAVAQC**T**LHTDDN  VGRLVS**T**LISKAG  LAPDGS**T**VAVEPL  KAKSPP**T**MVDSLL  HWVGAH**T**LGHNSR  WPHFTA**T**VKPRPA  LPTEDP**T**MERPYT  GRARSS**T**VTGGEE  LFEYSA**T**DNYTLQ  DHLSYF**T**FIGRVA  KDMYND**T**LNGSTE  KQLEAE**T**GCKIMV  AAPRII**T**GPAPVL  NGTPHP**T**AAIVPP  HQKPGQ**T**WKNKEH  YGELPY**T**YSRITM  NRIEEN**T**GHTFNS  EENGDY**T**YVERVK  TLYGSL**T**NSLNIS  KVCVPD**T**LNSSQE  EKKEKL**T**LEFKIR  KIREEV**T**QEFTQY  AKDICA**T**KVETEE  HVSSAI**T**EDQKKS  TATRSI**T**NNVSQI  QIKLMH**T**KIDELR  EKMMLI**T**QAKEAE  RPSSKK**T**YSLRSQ  IGVNLA**T**KKKEGT  AKKIIE**T**MSSSKL  PACSSL**T**PSLCKL  ISSTPK**T**SEEAVD  VEDILS**T**CLTPNL  RDAAIG**T**TPFSTC  QEKSTN**T**SQTGLV  ESRRAE**T**LVCCCF  EECKGQ**T**EQLELE  ENSRLA**T**DLRAQL  ELLCQL**T**QSNEEQ  LTLFLQ**T**KLKEKT  PLPNDR**T**FLGSIL  NCQPMA**T**NWIQEK  GPAAQT**T**AEPSRS  AAVVFG**T**VVDIIS  TLLPDG**T**IHGINH  LFGYGK**T**ELLGKN  LLGKNI**T**FLIPGF  DLCGGC**T**GSSSAC  ACYALA**T**DLPGGL  LLLLTG**T**CVDLGQ  CVGHDP**T**EPLEVC  RLNVQV**T**STPVIV  NVQVTS**T**PVIVMR  PKLGKV**T**LEIAIL  KLPGFQ**T**LDHEDQ  IGELKM**T**QEEYAL  CLLGRL**T**ELRTFN  RLTELR**T**FNHHHA  GLQKTK**T**KQSNRA  LLEEEP**T**TSHGQS  TKSDSG**T**AAQTSL  VQPGSR**T**RTKAGR  QDVSEF**T**HKLLDW  PKSKPL**T**SSRSSM  AASLFE**T**NDDHSV  VLKEPP**T**IRPNSP  DSIAAT**T**QAQGLT  TQAQGL**T**NVKAPL  PLHLDV**T**WGWEHW  PRALLI**T**YGPYAI  QARKKR**T**KAKKDK  TLMERD**T**EGGAAE  DSCTPI**T**SEVSDT  PADIWS**T**ACMAFE  LYCSPV**T**KELLLT  TKELLL**T**SPKYRF  ISIKPS**T**MWFGER  SLLNLS**T**NADSQS  EASDIA**T**SVRNLP  EKSVEI**T**KQDTKV  TSFEGK**T**NQVMSS  GHIFCN**T**CSSNEL  DSLSRL**T**KVEELR  DSELSD**T**DSGCCL  AKMASA**T**SSSQRD  LSGNKR**T**AEQSCD  SKELYG**T**WEMLCG  DMDQII**T**SKEHLA  ASPDGV**T**EIRGLL  HPALHT**T**TEDSAG  AGADPN**T**TNLRPT  RFRRTE**T**DFSNLF  VLMEQI**T**LKKMRE  VNATAG**T**TVYGAF  TGVSAP**T**VTVFIS  ISSSLN**T**FRSEKR  RYSRSL**T**IAEFKC  VKPAVV**T**VGDFPE  DPSIKK**T**EWSREE  LAKLMP**T**QWRTIA  MPTQWR**T**IAPIIG  RARLAN**T**QGKKAK  PSAILQ**T**SGVSEF  NSVALR**T**PRTPAS  VVQTPN**T**VLSTPF  LTPRSG**T**TPKPVI  EREIDD**T**YIEDAA  GNKKGK**T**VGFGTN  INRGHM**T**TEAKRA  AHLELR**T**FEELKK  GGSTAS**T**SGGVGA  GIFTKS**T**GGPPGS  EGLEAT**T**PMPSPS  SKVKLR**T**DSQSEA  GAEEME**T**VIPVDV  RAGIKV**T**VAGLAG  AICAGP**T**ALLAHE  MNGGHY**T**YSENRV  QESGEQ**T**ISQVSN  EEIMTC**T**DYIPRS  TTPSKD**T**DDQSRK  KYGKDP**T**VVIGSG  EVAEAA**T**GEDASS  PGPGQL**T**LRLRAC  LIPEAM**T**FEEAAA  PIDYHT**T**DYVDEI  ILNDVI**T**KGTAKE  DVITKG**T**AKEGSE  APYVRN**T**PQFTKP  RNTPQF**T**KPLKEP  LTSLAK**T**WAARGS  REPSPK**T**EDNEGV  NEGVLL**T**EKLKPV  MACAGL**T**SPRIVP  QANYHQ**T**LHAQPR  NKSPPL**T**LSKEES  SQSSRD**T**LSSGVH  RGRPTD**T**PSYFNG  VHSVFD**T**ADYTFP  RLDDKT**T**NVSLYP  NNVEKR**T**LIKVFG  VVYIGS**T**LSYFGL  LTNLIR**T**IRNAMK  DFESHI**T**SYKQNP  DDEDWD**T**GSTSSD  KMFAKG**T**EITHAV  ARGKKG**T**DRAAQI  LEEKGT**T**EEVCRI  IYAKDR**T**DRIRTC  AFRQGL**T**KDAHNA  KMGDWK**T**CHSFII  QEESLR**T**YLFTYS  LRTYLF**T**YSSVYD  DSISME**T**LSDMFE  YGNRAA**T**LMMLGR  AVKLDD**T**YIKAYL  AQCYMD**T**EQYEEA  VYQTEK**T**KEHKQL  GPRGMG**T**ALKLLL  NASQLI**T**QRAQVS  AQNISK**T**IATSQN  LQDESF**T**RGSDSL  DFPGIY**T**TINAHQ  AHQWSE**T**VQPIME  GWQADS**T**TRMVLP  WQADST**T**RMVLPR  EEEADS**T**AKAMVT  TAKAMV**T**EMCLGE  LATLSD**T**DSHSQD  DEAEPS**T**VPGTPP  LAMPMP**T**EGTPPP  PPPLSG**T**PIPVPA  RLFPPR**T**PGWHQL  RGEASE**T**LQSPGY  GILYLQ**T**ELCGPS  WGYLRD**T**LLALAH  LLVELG**T**AGAGEV  LQGSYG**T**AADVFS  YLPPEF**T**AGLSSE  PLGPPA**T**PPGSPP  EAVLAR**T**VGSTST  TPRSRC**T**PRDALD  IKEFRA**T**LECHPL  ARPLVQ**T**IFEGGK  CRTSGQ**T**FANSNS  FRESKL**T**QVLRDS  DFGINL**T**PESPES  TPEEEP**T**GMLDVW  ILHYQV**T**LQELTG  KGSSLP**T**RINIMN  TSSSSA**T**RGRGSS  KARNLY**T**GKELAA  DSNFTD**T**ESRDHG  AFESEP**T**LQSGIN  LVEATK**T**DVTVVR  QTIGFY**T**KQILEG  PCTETF**T**GTLQYM  DSERRA**T**LHRILT  TLHRIL**T**EDQDKI  NSSGTN**T**EDSELT  DVLYYV**T**RDDLKC  RGGMLC**T**LWKAII  SWTLAP**T**DNPPKL  LLSWPY**T**LLRRYG  SGPGTF**T**FQTAQG  LLKAKL**T**DPKEDP  QKVLHE**T**IKVLED  LMNERN**T**LQEENK  YIEQTH**T**KLEHSV  SDSTSK**T**PPQEEL  PQEELP**T**RVSSPV  SPVFGA**T**SSIKSG  LGEQNR**T**EYGKDS  YGKDSN**T**DKHLEP  QYKMDV**T**VIDTKD  SKLGGE**T**VDMDCT  TVDMDC**T**LVSETV  EVGFPS**T**QTCMER  RMPGYE**T**MKNKDT  EYSDRE**T**QLYDKG  SVTRKY**T**RQILEG  SAEELL**T**HHFAQL  AEYQDL**T**FLTKQE  QDLTFL**T**KQEILL  VFSDTA**T**PDIKSH  TGEGED**T**RLSASE  IAEAVR**T**TLGPRG  DGTTSV**T**LLAAEF  IVSVDE**T**IKNPRS  STVDAP**T**AAGRGR  ITTADT**T**ITTNPP  ADTTIT**T**NPPTTA  TTATAT**T**STSTKP  STSTKP**T**MTISSS  SYIALI**T**MAIQNA  GKGSYW**T**LDPDSY  PALPVI**T**KVETLS  NIMTLR**T**SPPGGE  RAMSLY**T**GAERPA  DHPSGP**T**SPLSAL  EGALAA**T**GHHHQH  APQPQP**T**PQPGAA  NHLPGH**T**FAAQQQ  FAAQQQ**T**FPNVRE  LGIENS**T**LGESQV  QLPYRS**T**PPLYRH  DNRKLK**T**IPNIPM  NEIEAV**T**ANSFIN  NSFINA**T**HLKEIN  CFPHIH**T**IYYGEQ  AVGEII**T**DMAKKE  DNGPLF**T**ELKFYQ  IQKWIR**T**RKLKYL  KRFSRK**T**VLQLSL  DGTIEF**T**SIDAHN  CMIQWL**T**GHLPWE  IAKYME**T**VKLLDY  VKLLDY**T**EKPLYE  GGLKAK**T**ITKKRK  EPGVED**T**EWSNTQ  DTEWSN**T**QTEEAI  TEEAIQ**T**RSRTRK  HVTSDD**T**GMCEMV  IRRRSN**T**AQRLER  VEIDPV**T**NQKAST  TNQKAS**T**KFWIKQ  IFIVLE**T**GRVTKT  LETGRV**T**KTKDGH  TGRVTK**T**KDGHEV  DGHEVR**T**CKVADK  CKVADK**T**GSINIS  GDIIRL**T**KGYASV  GCLTLY**T**GRGGDL  PSASQP**T**TGPSAA  GPHPPH**T**PSHPPS  PSHPPS**T**RITRSQ  PPSTRI**T**RSQPNH  RSQPNH**T**PAGPPG  CSAGVQ**T**EAGVAD  AGVADL**T**WVGERG  ELDENE**T**LIVSKF  HDDIVS**T**VSVLSS  RILLWD**T**RCPKPA  APGYLP**T**SLAWHP  FGDENG**T**VSLVDT  TVSLVD**T**KSTSCV  VHSQCV**T**GLVFSP  DFVRDA**T**WSPLNH  NHSLLT**T**VGWDHQ  VHHVVP**T**EPLPAP  HGLGIE**T**KGRWLY  DGYGTE**T**YADGGT  ADIDAT**T**TETYMG  EGSRSV**T**PSEGAG  IIAWHP**T**ARNVLL  HFLNTF**T**SKEPQR  CEPIVM**T**VPRKSD  APASTT**T**AADATP  TDAGLY**T**CNLHHH  TDGPPA**T**PAYWDG  GAPALL**T**CVNRGH  APGPDP**T**LARGHN  RWQNMG**T**SVRRRS  VVSHQE**T**SVGALG  KGSGSP**T**HSLSQK  RSPYSS**T**EPLCSP  LLQNES**T**NEQLQI  MPHQKA**T**TADGSS  PHQKAT**T**ADGSSI  KLYNNI**T**FEELGA  SKEFRK**T**WGFRRT  SMLKDT**T**SQHRAH  CGSGVV**T**TVTVSG  SPPEGD**T**TLFLSR  IKKHRV**T**LPGQEE  VAIRNR**T**DLRFGL  VRGTVV**T**NDRWGA  VKQLVE**T**VSCGGN  GEAIYE**T**HTWRSQ  DPGLHE**T**LLAADP  QRLLPL**T**QYPDSS  RGGVVG**T**LRNCCF  IMLLTA**T**APGRQQ  LQLFSH**T**QLRGPD  SRHPQG**T**KRVGAG  RVGAGD**T**SDWPTV  DTSDWP**T**VLSESS  KRRGRA**T**DSLPGK  EDMYKL**T**SELLGE  VFREVE**T**LYQCQG  EFFEDD**T**RFYLVF  ALDFLH**T**KDKVSL  GLTAAP**T**SLGSSD  ASQVAG**T**TGIAHR  KLNNSC**T**PITTPE  NSCTPI**T**TPELTT  ITTPEL**T**TPCGSA  VFTDQA**T**FYDKRC  SSTMDL**T**LFAAEA  ELSALL**T**QSQKQN  DADKAL**T**LVRSVL  CQMQLE**T**EKERVS  AQAPEA**T**VLEAET  GPNKTG**T**SCALDC  VDMVDI**T**EDFLVQ  FLVQAK**T**YLGEEG  CGLQDF**T**PEPDSY  SKNKSK**T**STKLHV  NISPTC**T**NKELRA  YNYAEQ**T**LSQLPQ  TSGAAA**T**AAAAAA  KLISME**T**SSSSDD  SDNFAN**T**RLQSVR  VREGCR**T**RSQCRH  KFPARS**T**RGATNK  RSTRGA**T**NKKAES  RRARPL**T**RSRSRI  LVRKRK**T**VDGYMN  RSRSSV**T**LPHIIR  NRSLGS**T**CHQCRQ  HQCRQK**T**IDTKTN  KTIDTK**T**NCRNPD  RDGRCA**T**GVLVYL  PSERNG**T**SSTCQD  TRELRR**T**AYSGVP  MGITTA**T**FPILQG  ELELGK**T**HEIEIK  KNPHRC**T**PVCQVC  GGIQNF**T**MPSKFK  PLSKLT**T**LNSMHS  TALLKG**T**NLSASE  KQGPTN**T**LLHHLV  LSGYRI**T**LIDIGL  RPFVSH**T**CTQMLL  LPPTIL**T**LEFKSK  VSIYIF**T**NAIEVV  SEPGKF**T**QKVKVW  VTSERV**T**EMYFQL  VPDVLA**T**EQDIQT  HCSIIK**T**YEIFET  TYEIFE**T**SHGKVY  LLELIK**T**RGALHE  PRSKHL**T**GECKDL  GESSRG**T**EPLWTP  GTEPLW**T**PEPGSD  SDKKSA**T**KLEPEG  PQAQPE**T**KPEGTA  ETKPEG**T**AMQMSR  FPSKPS**T**METEEG  KPSTME**T**EEGPPQ  ALLLRF**T**GKPGRA  RVLRAF**T**SSVPLL  MKNYPC**T**LRQYLC  LLANRL**T**EKCCVE  IHLLPD**T**LDHLKT  NGPKGD**T**GEKGDP  KQGESG**T**RGPKGS  ALQRIT**T**LTVTGP  NLYLVA**T**SKKNAC  FGYPQT**T**DSKILQ  EGHKLE**T**GAPRPP  SPKFKT**T**VGSVKW  ASSCDE**T**EIQISN  QPLGHV**T**KTRRRC  TRRRCK**T**VRVDPD  GPASQS**T**LGLKGS  TEDASK**T**RKQPLS  VDGKDL**T**TKIMQL  SVTAMD**T**LEVISE  TGKEEK**T**SLLLHN  PVLVED**T**CLCFNA  SAYALC**T**FALSTG  CTFALS**T**GDPSQP  PDGYEQ**T**YAEMPK  EMATFH**T**DAYLQH  LQLGAD**T**IAGDPM  LQWQLA**T**LILGGG  PDHEFF**T**AYGPDY  IFSKDG**T**LFAWGN  ENNNFN**T**IANKLH  PVFDFG**T**GPRNAA  QSTPRN**T**VSQSIS  LSQSTR**T**ATFQQR  LSPLEG**T**KMTVNN  LEGTKM**T**VNNLHP  MNGNVI**T**SDQPIL  QDKVLI**T**ELIQHS  IPKKLQ**T**ARPSDS  RLHSVR**T**SSSGTA  RTSSSG**T**AQRSLL  EGVAVV**T**AGSVGA  SVGAAK**T**EGAAAL  INLSEL**T**PYILCS  IGANEG**T**GHFKPL  QVRKRI**T**QRKKQE  KKQEQL**T**PGVVYV  PNLLDE**T**QIFSYF  YFSQFG**T**VTRFRL  LSRSKR**T**GNSKGY  AKIVAE**T**MNNYLF  RYNRNR**T**LTQKLR  NRNRTL**T**QKLRME  SLILQK**T**ESISKT  KKKVSG**T**LDTPEK  TPVCTP**T**FLERRK  KEEIQE**T**QTPTHS  EDEEEE**T**MDSETA  PFDKGI**T**QGDLKT  LETRTR**T**SSSCSS  IKMHKL**T**SEQLEF  ALGRMC**T**VIKRQK  HLSRLP**T**IDPNTR  DDQKIF**T**DLQSEG  AKKVQR**T**VLEKEM  GERRAR**T**VRFERA  ADNEGW**T**PLHVAA  ELLLHD**T**RCWLNG  EARHPR**T**GASALH  GGMDSL**T**HAGQRP  RFGLLK**T**GSSGAL  WLEGTS**T**QAKELR  LRLARI**T**PTPSPK  AKPNVP**T**ASTAPP  RSTQGV**T**LTDLKE  EALTET**T**LRLAQL  VELERA**T**QRQERF  LFKHDP**T**SANLLQ  LFQNNT**T**NRYYKE  RLPERL**T**KFLITQ  RRSVVG**T**PAYLAP  SQLQLP**T**LPLVMV  PPFTYA**T**LIRWAI  EIYHWF**T**RMFAFF  EKGAVW**T**VDELEF  SKNVHS**T**RFVTYF  NLGTLT**T**PVEGLD  SYKSTV**T**LSWKPV  RASEDT**T**SGSPPK  LSLSGQ**T**NLSVLQ  KDPNQF**T**ISRRGG  VSVPAP**T**NVLSLS  SPKPLV**T**PGSTLT  LVTPGS**T**LTLLTR  TPGSTL**T**LLTRAE  STLTLL**T**RAEAGS  AEAGSP**T**GGLFNT  SLPLLH**T**PIPLVS  KPKAVV**T**HSSGNH  NHGQAL**T**YAAKLE  DDIFTV**T**EDEIKC  DEIKCA**T**QLVWER  VRALHD**T**GKFTDG  HDTGKF**T**DGQLVS  KEPGCE**T**IGLLMS  YKINGR**T**KAMVAC  EAVMDG**T**MEDSEA  CDQFRR**T**ALHRAS  QDRLDC**T**AMHWAC  QLWQAD**T**RHALEH  HIEKIT**T**WQDPRK  HPAVSS**T**PVPQRS  SMPNAL**T**TQQQQQ  NVDEMD**T**GENAGQ  KRRDSA**T**SFSLDF  GRCYRV**T**WFTSWS  YRVTWF**T**SWSPCY  LSLRIF**T**ARLYFC  VQIAIM**T**FKDYFY  YFYCWN**T**FVENHE  VENHER**T**FKAWEG  EDQQSF**T**GSRTYS  SFTGSR**T**YSLVGQ  ICKQKT**T**ETGAQQ  ALKTGP**T**SGLCPS  AINSQL**T**VETGDA  SQLTVE**T**GDAHII  DKCSRK**T**LFYLIA  LFYLIA**T**LNESFR  PDYDFS**T**ARSHEF  RSISGS**T**YTPSEA  GSGAEE**T**STMEED  GAEETS**T**MEEDRV  ARRSGP**T**PSAAEL  ETLGKG**T**YGKVKK  VNGKPY**T**GPEVDS  NPTRRA**T**LEDVAS  VNWGYA**T**RVGEQE  PGGGST**T**PGLERQ  QSLHSD**T**ADDTAH  NGKFSQ**T**ALELAA  ELAAPT**T**FGSLDE  DSCFSL**T**DCQEVT  TDCQEV**T**ATYRQA  CQEVTA**T**YRQALR  RKKWQR**T**DHELGK  DSSLVK**T**FKLKKR  REKRRS**T**SRQFVD  SIVAKT**T**VTVPND  PIEAVS**T**IETVPY  AVSTIE**T**VPYWTR  RQLEPR**T**ETDSVG  HDFVSK**T**VIKPES  LKEPLL**T**FRLNRA  MEAAEI**T**DEDNSI  AKVFGP**T**IVAHAV  SKSKSA**T**NLGRQG  RQRRPS**T**IAEQTV  TVAKAQ**T**VGLPVT  PSNMME**T**SIDEGL  SGQRRH**T**LSEVTN  HTLSEV**T**NQLVVM  LQNLAR**T**KGILEL  LSKAQN**T**CQLYCK  PLPRQE**T**PPPSQQ  LQPLPS**T**SGPRAA  AAPPLP**T**QLQQQQ  LQFSYQ**T**CELPSA  PAPDYP**T**PCQYPV  EQSVAK**T**SLITRF  VVVYDI**T**NVNSFQ  AKGLNV**T**FIETRA  EKPQEQ**T**VSEGGC  EEHDSP**T**EASQPI  FKDLGV**T**DVLCEA  ALVLTP**T**RELAFQ  RSILLA**T**DVASRG  VANVWP**T**KYGLLF  DHLWTE**T**ITNIRE  FSFRHS**T**SVSSLA  SDVPSG**T**ETEEED  HLTKLA**T**LNIHDY  PEMEYC**T**DRESYS  WLRAPD**T**MYLLDF  FAKDFM**T**YLSAPN  GRYSLS**T**SNSSIA  YALLEV**T**YKGTQW  VLYECV**T**QETPEM  KCTIDN**T**LDQWLQ  LVFEPM**T**SELYEG  SARATG**T**AFRRSP  RKHTQW**T**YGPLTS  YGPLTS**T**LYDLTE  RQILDQ**T**PVKELV  LYIICF**T**MCCIYR  TSPRDN**T**LLQQKL  QMLGPF**T**IMIQKM  FYIIFQ**T**EDPEEL  PFMYSI**T**YAAFAI  RAQIVA**T**TVMLER  PSVSRS**T**SRSSAN  VHGLGC**T**VDWKSK  CVEDLQ**T**IQVIKI  SIIFII**T**SDESLS  VDDSDK**T**NGSKVD  EVSSPS**T**PEAPDI  GQPPHL**T**SAQDQD  DAGESC**T**PEAEGR  NRSETP**T**FAKVQR  NVGQIK**T**VYPASY  QERSVP**T**FKDGTR  PATEKL**T**TAQEVL  ATEKLT**T**AQEVLA  RPALPA**T**PPATPP  KQLAQM**T**RCPEQE  MVGSCC**T**IMSPGE  HRIRTD**T**YVKLDK  GQKKED**T**GGGGRS  QRENPR**T**PCVQQD  ASSPNR**T**TQRENS  TRDNPT**T**SCAQRD  TRDNPR**T**SCAQRD  SSPNRA**T**RDNPRT  ASSPNR**T**IQQENL  QQENLR**T**SCTRQD  QQDNPK**T**SCTKRD  QRDNPG**T**SSSQCC  LEREEY**T**VLADLP  VELSPL**T**QASPQR  PRLQEE**T**EAAQVL  SDFMET**T**DYFETT  TTDYFE**T**TDNEIT  VPNNET**T**DNNESA  KTKEFL**T**PLHVAS  VRSPGA**T**ADALSS  PYLTLN**T**SGSGTI  KLWERY**T**HRRKEV  KSASLV**T**RQIHEH  ELNKKR**T**QKEMEH  PKNEHK**T**ILKTLK  TLKDEQ**T**RKLAIL  SQRRKR**T**SFSAEQ  NHCAPG**T**ETKCLK  CAPGTE**T**KCLKPQ  QGQNFE**T**CSPLSE  RSCEEL**T**AVLTPP  ELTAVL**T**PPQLLG  IPPVEE**T**VFYPSP  IKPLFF**T**VGFTGC  LSDGQR**T**VTGIIA  DGQRTV**T**GIIAAN  TMIRYF**T**SNPASK  SPMLLS**T**FSHFSL  YVGKVA**T**GRYGPS  IFLPMF**T**FTAGNA  LPMFTF**T**AGNALK  FGIWYV**T**YGHELI  ENIRKL**T**GRDPND  IIKYIR**T**KTKPHL  LAVLQP**T**PQVTQE  QPTPQV**T**QEQGHL  HVEVED**T**TLNRCS  VEVEDT**T**LNRCSC  HAFEQK**T**LTPDQW  FEQKTL**T**PDQWAR  NTDPVP**T**LPAEPC  LALYDF**T**ARCGGE  CPYEGM**T**NHETLQ  GMTNHE**T**LQQIMR  RQRSLS**T**SGESLY  APEGEE**T**EFYVSP  EREATD**T**PIVIQP  ASATET**T**QLTADS  SLSKSD**T**RLHELH  EPQQGK**T**EVTPAA  RRDPGP**T**TDPLDP  PAGRSS**T**CGAGTP  FGDEDL**T**WQDEHS  AEFQSL**T**PSQIKS  QDDGED**T**LFSEPK  SNVVLK**T**GFDFLD  LEALRS**T**GLVRVP  DKFGFH**T**VTCCGF  FGFHTV**T**CCGFIP  FPRSFF**T**AYHRKI  LPVLDK**T**KFLVPD  RLQLNP**T**QAFFLL  SMVSVS**T**PIADIY  EKLNSP**T**DSTPAL  NSPTDS**T**PALLSA  PALLSA**T**VTPQKA  KAKLGD**T**KELEAF  WMNVEK**T**ISCTDH  ISVTLR**T**FAPSFQ  DQVACL**T**FFKERS  DVSSKD**T**DIPLLN  GECNED**T**EVYNIT  AVRDVK**T**DWNEEC  PSIPPR**T**VKPARQ  NISVTK**T**DTNPSE  PPPQRG**T**DQSTMP  SEALPE**T**SMDPIT  SRNRAP**T**GYDVVA  YDVVAQ**T**ADGVDA  IQETVD**T**QEVAFR  QAPPQY**T**FIGELN  PRHISL**T**LPATFR  SLTLPA**T**FRGRNS  FRGRNS**T**RTDYEY  FDLLGI**T**IKSLAE  YEYSFR**T**EQSAAA  RLPPSP**T**RCQQIP  SWYRDI**T**SNKKFF  LKDHIS**T**TAQDHC  IYLHVL**T**TNNTAI  YLHVLT**T**NNTAIN  INHVQK**T**YAEMDP  KDLSQM**T**SITQND  QNDIIS**T**LQSLNM  YKKPPI**T**VDSVCL  CIGLVL**T**LAALVI  IRISCH**T**DCRRRP  EDGSED**T**VSDLSV  LNKNVF**T**SAEELE  EWIAVN**T**VDFFNQ  MLYGTI**T**EFCTEA  TITEFC**T**EASCPV  YHWADG**T**NIKKPI  YIDYLM**T**WVQDQL  DQLDDE**T**LFPSKI  FMSVAK**T**ILKRLF  AENSVL**T**STTGRT  NSVLTS**T**TGRTSL  TSTTGR**T**SLADSS  NLLIGS**T**SYVEEE  ENVFVV**T**DFQDSV  HLVANC**T**QGEKFR  LGDERC**T**HLVVEE  AKELYQ**T**ESNYVN  APEEIK**T**IFGSIP  DIFDVH**T**KIKDDL  SLKEVM**T**HINEDK  RHKVIG**T**FRSPHG  LIYSSK**T**LELRET  TLELRE**T**SVTPSN  LRETSV**T**PSNLWG  YIIGAD**T**VMNESE  LFSLIE**T**HEAKPL  KLYVYN**T**DTDNCR  LSTGVP**T**VPLLPP  QVNQSL**T**SVPPMN  PPMNPA**T**TLPGLM  PSDPAT**T**TAKADA  SDPATT**T**AKADAA  PTAKAP**T**TVEDRV  PRNILL**T**NEQLES  IVPPGL**T**ENELWR  QVPMNM**T**ITGCMM  TAYVSA**T**TGAVAT  LQSGNS**T**DFTTDR  SELSKG**T**TRKERK  ELSKGT**T**RKERKA  AENEVE**T**NALPVV  PQNPEE**T**DEKVEE  KPRAGQ**T**VNVNVI  ADVVPV**T**LAPKKL  SLSSDS**T**LLLFKD  GTSFFP**T**AGGFRS  FCLEDD**T**QSQLLD  AQQGKI**T**AEEEEE  RGPSFM**T**SPSPKH  AVVAPI**T**TGYTVK  KIYITL**T**GVHQVP  RKKVEN**T**RWDYLT  EKPSYD**T**ETDPSE  PAGATA**T**PITIRY  SWGDLE**T**YLEEEA  IFPPGA**T**EEPRRV  YWLWYT**T**KNCVGG  RDLGVG**T**SMEGAS  LQQLLK**T**LEEGLE  GRILSL**T**SSASRL  ASRLLT**T**ALTSFC  SFCAKY**T**YPVCSA  VLQAPG**T**GPAQTE  GTGPAQ**T**ELLCCL  KGLAAT**T**SMAYAK  LMLTVM**T**KYQANI  KYQANI**T**ETQRLG  MALEPN**T**TFLRKS  ALEPNT**T**FLRKSL  KKRGRA**T**DSFSGR  AHARVQ**T**CINLIT  TCINLI**T**SQEYAV  GCAPEN**T**LPTPMV  SCAKDL**T**SFAAEA  PVLVRA**T**SRCLQL  GWPDLH**T**PALEKI  SDSGNS**T**ASTKTD  TGFHGS**T**VSSPQS  YPLNTV**T**FCDLDP  ARGTNV**T**AAVQDA  VATIKF**T**VYTSSE  RSSLPA**T**DAALIF  WGAKAS**T**PEVQSE  KPHFRN**T**VERMYR  ERMYRD**T**FSYNFY  DQEYEV**T**WYISWS  CTRDMA**T**FLAEDP  DPKVTL**T**IFVARL  HSMDPP**T**FTFNFN  MDPPTF**T**FNFNNE  ERMHND**T**WVLLNQ  DQDYRV**T**CFTSWS  YRVTCF**T**SWSPCF  VSLCIF**T**ARIYDD  CQEGLR**T**LAEAGA  DKSETH**T**AFPCIK  QQLQRK**T**ALKNKE  DFDDER**T**WTDLEE  VFEKYT**T**AARTFP  SNNLCH**T**LGPVHP  PQRHPN**T**LSFRCS  SEVYKA**T**CLLDRK  CLLDRK**T**VALKKV  RLIPER**T**VWKYFV  PANVFI**T**ATGVVK  NVFITA**T**GVVKLG  RFFSSE**T**TAAHSL  ALRDLN**T**YTEKMR  IVLFCE**T**VERALD  DLLQTL**T**EEELHT  MDDVEG**T**AEALAR  NLEAEG**T**DGASLA  EMIAHR**T**GGMKLS  GMKLSA**T**VIFNPK  REPKAP**T**SDKCLP  SGSSSS**T**AGSCSS  VADQLQ**T**NYASDL  CALCQE**T**LSSSEL  ACKAPF**T**VIRRKH  ASGAGG**T**SLEGER  AEFGGG**T**RGFSCE  LEPGVF**T**LAEEDD  FLSSGA**T**SSLAAA  ASGTII**T**EALASS  VIYVMD**T**SRSTNP  PSDLIL**T**RGTLDE  DIDHRV**T**EESHEE  KLFILL**T**LATSSL  ATSSLL**T**SNIFCA  ERSAGA**T**ANLPLR  PQRFGR**T**TTAKSV  RFSHDI**T**PIILAA  CKCNEC**T**EKQRKD  SEDPVL**T**ALELSN  RLANIE**T**EFKNDY  LPNETF**T**DYPKQI  QIFRVK**T**TQFSWT  IFRVKT**T**QFSWTE  TTQFSW**T**EMLIMK  PEVAYF**T**YARDKW  QISLGR**T**VKDIFK  RNSENL**T**ANNTLS  NTLSKP**T**RYQKIM  NGWDES**T**LELFLH  SLDVLI**T**LLSLGS  NERLLH**T**PHNPIS  DDNYDK**T**EDVDIE  LRFTDD**T**FDPELA  GQERFR**T**LTPSYY  WLNELE**T**YCTRND  IEASAK**T**CDGVQC  RLYAMQ**T**GMKIDS  GDNEAI**T**QEIVGC  TVFGEL**T**DENVKH  DPALFN**T**ISQGDV  YIEQGN**T**KALAVV  FEAAIL**T**QLHPRS  AGFVDG**T**ALADLS  AARDVH**T**LLDRVV  SSQERE**T**EKAMDR  DRLARG**T**QSIPND  PARGEG**T**HSEEEG  SKMVFE**T**GQFDDA  KEQPGN**T**ISAGQE  DTSFPS**T**PEGIKD  TEKNTS**T**KTSNPF  NAALQE**T**EAPYIS  GRGQGF**T**DGVYQG  VMEELN**T**APVQES  DECAGC**T**DGTFRG  RGTRYF**T**CALKKA  DGENVF**T**GQSKIY  GDLIEI**T**DAKKRE  VDATRE**T**NRLGRL  KCGNCQ**T**KLHDID  EAERMF**T**REFSKE  AAAAAA**T**MALAAP  VTQLMS**T**EPLPRT  PTPASA**T**APTSQG  ASATAP**T**SQGIPT  KGQGVG**T**GRSASK  GQQEAK**T**DQADGP  QPPRSK**T**PSSPCP  RNQMLM**T**PTSAPR  QMLMTP**T**SAPRSV  VQSPAC**T**SYQMTP  CTSYQM**T**PQGHRA  RARAAR**T**VLGQVV  GDRLLV**T**KCGRLR  HVIGIV**T**AKSGDI  IWVKAK**T**IQQTLI  SYTMMG**T**ADQPGL  PKGSRQ**T**LKVREH  SAKDVP**T**GGIFQL  FSHSVS**T**ATLSDA  FFQENP**T**PPSPFG  EGLKKA**T**RVLESH  QLSYFY**T**TMCQNG  EIYERC**T**SLSAVQ  PEKFSK**T**SFDDEH  WSLGCI**T**AELYTG  ITAELY**T**GYPLFP  LTMVLK**T**YDTSFL  LDVGSG**T**GILSMF  KEVDIY**T**VKTEEL  YFNIEF**T**KCHKKM  YLEDYL**T**VRRGEE  KPKSDK**T**EEIAEE  KVVSSK**T**KKVTKN  IGVPSA**T**EIIKAS  LDSVPP**T**AISHFK  LSCDLI**T**DVALHE  FIGVDS**T**GKRLLF  FIKEAN**T**LNLAPY  KDRVTI**T**NCLLMN  TVPGKV**T**LQKDAQ  VQVFDN**T**PAALDG  PAALDG**T**VAAGDE  AAGDEI**T**GVNGRS  EVKGEV**T**IHYNKL  ENMSSG**T**ADALGL  LEELER**T**AELYKG  FYELSQ**T**HRAFGD  GIRLLK**T**IKPMLT  EVDLAH**T**TLAYGL  VDLAHT**T**LAYGLN  GEPSRD**T**RGAAGP  LPNAVI**T**RIIKEA  VFVLYA**T**SCANNF  EFQRFV**T**PLKEAL  KDKDKK**T**DSEEQD  LARHVF**T**GEKVAV  VKVIDK**T**KLDTLA  LYEVID**T**QTKLYL  ECKDLI**T**RMLQRD  IVEALE**T**NRYNHI  DLEDDL**T**ATPLSH  TPLSHA**T**VPQSPA  AGPALS**T**VPPASL  NIASPG**T**VHKRYH  GGNPTN**T**SGTTRR  KMCISS**T**GNAGQV  LPLCEK**T**ISVNIQ  PKTFPA**T**TYSGNS  GKGLMP**T**PVTRRP  LMPTPV**T**RRPSFE  SKTPPE**T**GGYASL  CPVPSR**T**NSFNSH  VENVIK**T**YQQKVN  FYIAEL**T**LAIESV  DGHIKL**T**DFGLCT  CTGFRW**T**HNSKYY  VINWEN**T**LHIPAQ  ISHPMD**T**SNFDPV  SDLVDQ**T**EGCQPV  MHIVEQ**T**EFIDDQ  PVHFEI**T**KGDLSY  TQGKII**T**IRNINK  KPVLYR**T**RCTRLG  TSCSYC**T**MAKKLF  RIFVNG**T**FIGGAT  TFIGGA**T**DTHRLH  IGGATD**T**HRLHKE  PEFHED**T**RSSRSG  GHLAKE**T**RVSPEE  MEQDSY**T**RFLKSD  GRPQRP**T**NLRRRS  YNGSFV**T**PDRRFR  PVNRRT**T**TPPDSG  SDSSVI**T**ISPDEA  KEEKRS**T**SLSSPR  ERLWGL**T**EMFPER  AALWIG**T**TSFMIL  LPVVFE**T**EKLQME  ILLGPN**T**GLSGGM  GPLARD**T**LVWDTP  ERDEPG**T**EVRITY  RIGAVH**T**VIFAGF  WPGMAR**T**IYGDHQ  EVRANA**T**AKATVA  NATAKA**T**VAAFAA  VVELPK**T**EEGLGF  KLVVRY**T**PKVLEE  SAPALE**T**DSSPPP  ITVEVP**T**TSDTEV  AAAAAE**T**SQRIQE  SFCITN**T**IAGRYG  LQEFLQ**T**LREGDL  FKDVPL**T**AEEVEF  ALLLSV**T**RIQRFQ  HRSYVS**T**MILEVV  VLNRVV**T**RASSPI  VLLWRY**T**SIPTSV  LRALDV**T**DKEGEE  LLVTVL**T**SLSKLL  AIVNLR**T**AAPTVC  EASSQA**T**LPNQPV  FHELVQ**T**ALPSGS  LSGSHL**T**PLCYSF  EAMRRA**T**VEREME  RETSRI**T**VLEALR  AIATRN**T**KKNRSL  NAFLYR**T**GQHSNK  SEDMIP**T**VGFNMR  FNMRKV**T**KGNVTI  VTKGNV**T**IKIWDI  KDNIDI**T**LQWLIQ  DVPVDI**T**PDRLQL  RAVTRC**T**SSLEGH  SVRIWD**T**TAGRCE  SASQDR**T**IKVWRA  RGFGYV**T**FSMLED  KDKYKD**T**QSVSAI  AFAQFM**T**QEAAQK  VKKPTG**T**RNLYLA  GDKEKD**T**LKKGPS  SGLSSN**T**KAADLK  SGDKKN**T**SDRSSK  MVILDQ**T**KGDHCR  AIFFVI**T**FLIFLC  AVDTML**T**ARSVDG  CNDLYA**T**VKDFEK  EKATLG**T**NGHHGL  KPITPS**T**YVRCLN  FEALLQ**T**GKSPTS  LLFDWG**T**TNCTVG  VPKTAN**T**LPSKEA  SLEVSD**T**RFHSFS  KGYVNN**T**TVAVKK  AAMVDI**T**TEELKQ  LSCLDG**T**PPLSWH  TSRIVG**T**TAYMAP  ALRGEI**T**PKSDIY  IEDEEK**T**IEDYID  MNDADS**T**SVEAMY  RFSSSS**T**SSSPSS  SAWGSA**T**REEGFD  EGFDRS**T**SLESSD  FGPEED**T**AYLDGV  DPSLVP**T**FQLTLV  VPTFQL**T**LVLRLD  GFSQSL**T**LSTGFR  QSLTLS**T**GFRVIK  AVYSPT**T**EEDPGP  SSDFYI**T**LGTGCV  SIIFVS**T**HDRTSA  RKPENT**T**RAEALT  SVSSQV**T**TAGSGK  SVNSHM**T**QSTDSR  TASSGL**T**SKTSSE  KFSCKL**T**NEDVKQ  SFTLML**T**ERRNHA  DLLTNC**T**LLLCRR  LGEMRF**T**LGTFLR  MRFTLG**T**FLRLAA  SKHLPK**T**AKPASQ  LENGLP**T**VREKTR  KPKHKR**T**DYRRWK  WSQFVQ**T**LPIQTV  SRSRSR**T**YSRSRS  NYKLKE**T**GYVVER  ITVKKE**T**QSPEQV  IDISPS**T**LRKHTR  EKNERL**T**IPSSCP  RCEIEA**T**LERLKK  LTEQSN**T**PLLPSF  FKENNI**T**GKRLLL  GDEIAI**T**YIKDVT  AEILKM**T**KPPFVM  VRTPKS**T**KHVHSI  SIQWSR**T**KPQDEV  VQLAIQ**T**LFTNSD  DLHQPN**T**IPGMPL  VSEGGW**T**KVEYRK  RPSPAK**T**NKERAR  LLKAKQ**T**EKEKSQ  SPSRSH**T**SSNYDS  IVTPKE**T**ETSEKE  TPKETE**T**SEKETP  PPPLPT**T**TPPPQT  YGERRQ**T**ESDWGK  KAKDKD**T**GELVAL  KEGFPI**T**AIREIK  KTSRKE**T**TSGTST  NRTYGN**T**DGPETG  TLVKNR**T**FSGSLS  SVVHAE**T**KLQNYG  AAPLLH**T**RLPGDA  KLGASR**T**GISNMR  TVRFGG**T**VTEVLL  RFGGTV**T**EVLLKY  SSIMYL**T**KDFEQL  NLVSAQ**T**VFLRPC  VRKSER**T**LECYVH  TQTCGG**T**DSTEGL  MDEDEE**T**EHETKA  EETEHE**T**KAGPER  KVDNGK**T**KDLYRD  NFFAAI**T**NKYQLV  PGPEGL**T**STSLLD  LDLLLP**T**GLEPLD  AGSIED**T**SQAQEL  LLLPSV**T**PTTVTP  YAGLDD**T**DSEDEL  VYYANH**T**EEKTQW  YGWEQE**T**DENGQV  RYDGST**T**AMEILQ  SGNDEH**T**SYGETG  EKAVFC**T**GGRDGN  NIMVWD**T**RCNKKD  NTSDKQ**T**PSKPKK  LFQDEN**T**LVSAGA  LDSTGS**T**LFANCT  TLFANC**T**DDNIYM  IYMFNM**T**GLKTSP  NGHQNS**T**FYVKSS  VTSSQS**T**PAKAPR  LPSNTP**T**FSIKTS  KIEGAG**T**SISEPP  SLVIIS**T**LDGRIA  GGKSLT**T**YGLSAY  GISCDQ**T**SSSESQ  HTGQVG**T**KLYMSP  YSTGKT**T**FIRYLL  RIGPEP**T**TDSFIA  IGPEPT**T**DSFIAV  PVDGKI**T**GANAKK  DTDGNG**T**INAQEL  GAALKA**T**GKNLSE  SFQEFL**T**AAKKAR  DGDGHI**T**VDELRR  GPAPRR**T**APPPPG  AKSESK**T**SLLKAA  AFREFD**T**NGDGEI  GDGEIS**T**SELREA  KIKYRQ**T**TQDAPE  IKYRQT**T**QDAPEE  RPTREH**T**TSSSVS  KRFVND**T**LRSEFH  RDTENH**T**SVDGYT  EPHIQP**T**KSSSRQ  KTQLNP**T**SLQKLF  RKLGST**T**VGSKSE  GSKSEM**T**ASPLVG  GKDSSL**T**EMSVSS  AYRPGT**T**QRVPAA  SAHSIS**T**ATPDRT  TATPDR**T**RFPRGS  GEASGR**T**DTSRST  FTWSMK**T**TSSMDP  TWSMKT**T**SSMDPN  ITLNIK**T**RKPALI  QSELKG**T**FKGHKC  FFRHNL**T**WEESKQ  NGKMHP**T**FCENKH  DAWVPP**T**PAPMAT  TPAPMA**T**SQPEED  CERSYV**T**LSSLKR  CIFCWE**T**FVTYYN  YKPKLN**T**LKLYRL  AKRPYK**T**YSQGAP  ASVITY**T**APPRPP  EPAATP**T**SPATAV  TAVSPA**T**AAGPAM  GRTLTY**T**AKPVGG  CAKVCK**T**AAALSR  AEEASE**T**ASLQDP  EELNAS**T**LMNYCA  KMIKSK**T**EYPLHK  GETPLH**T**ACRHGL  VVDAIC**T**RGADMS  TLHLCS**T**EKIYKE  KNSLNP**T**WRSLDF  KLRLTS**T**SNELKK  ETERKI**T**SLSSSL  VPEPGC**T**KVPEPG  VPEPGY**T**KVPEPG  VPEQGY**T**KVPVPG  WDVECD**T**CAICRV  PAPLKP**T**SEPTSG  SLLSVI**T**EGVGEL  AAVASG**T**AKGARR  QRHSRG**T**KLRKLI  RSKSDA**T**ASISLS  EELSSS**T**ESIDNS  PTKEQK**T**QRLISE  ARVWLP**T**AGFDHH  ECENFD**T**TSVPAR  ELPEVH**T**NSCDNI  FSVDSI**T**SQESKE  SMRSIT**T**KLYDGF  PVAAAQ**T**TPGERS  VAAAQT**T**PGERSS  PAFYPG**T**SGSCSG  GAVANA**T**GAEEGE  LQCGIC**T**KWFTAD  SALANL**T**WQSRTQ  VLYHVE**T**EVDGRR  RQGPPP**T**IVEWMI  NSLYLA**T**ISLKIV  QNNAFS**T**LFETLQ  EWKFAR**T**KLWMSY  EFFAPL**T**QNQSHD  FYKGQL**T**KMFFLF  GLVYTI**T**DVLELH  VVGHLG**T**STEEGK  NPENLA**T**LERYVE  LERYVE**T**QAKENA  NPAFFQ**T**TVTAQI  LTNLPH**T**DFTLCK  GAAAVC**T**LGGTRE  VCTLGG**T**REIPLC  FFKRFG**T**KCAACQ  CKRQLA**T**GDEFYL  CKADYE**T**AKQREA  QREAEA**T**AKRPRT  TAKRPR**T**TITAKQ  RPRTTI**T**AKQLET  EQLSSE**T**GLDMRV  GSLGEP**T**QALGRP  SLVYPD**T**SLGLVP  PSSDLS**T**GSSGGY  TVFAYG**T**YADYLA  RNLPPL**T**EAQKNK  KTIKVT**T**AAAAAA  DPEQHL**T**ELREPA  QSAGQK**T**TCGQGL  SAGQKT**T**CGQGLE  ELCKMQ**T**ADHPEV  YINELC**T**VLKAHS  EQPEEL**T**LEEESP  AGMVSS**T**SFNGGV  QQAAPV**T**AAAAAA  ATGGDV**T**RNKVRK  LIEKLL**T**RTPDIA  DIRFEF**T**PGRDTA  SSTIYG**T**ESYVVS  EGHMLQ**T**FDYSRD  PQEREF**T**TAVSSP  EYEREA**T**KKGARG  DLILEV**T**AGVGGQ  KRWHFE**T**LEYFPS  KDLRID**T**KRASGA  TLHDLE**T**FMQGDY  GAFEKL**T**DEIPWG  WKGFIL**T**VVELRV  PGPAPC**T**GGPKPL  AGLADM**T**AELQKK  VAGSQS**T**LFKDSP  YWWAGT**T**WYRLTT  TWYRLT**T**AASLLD  EGVIGV**T**EEQVHH  VKPEVK**T**TEKKEL  DEIKIG**T**SCKNGG  RHDWHQ**T**GGEVTI  VKRSYV**T**MTATKI  PCGSLY**T**VLEEPS  VMYKII**T**GKPSGA  EISDIH**T**KLLRLS  KQLLSL**T**NQCFDI  LPSISP**T**APGTWA  ILLVGL**T**GMLGNL  SSMITL**T**AIALDR  FRAIRE**T**GRALQT  MKAEEE**T**DEEKPK  GQEHWF**T**ELPPVL  SSELPS**T**SPSSVA  PLVGIE**T**LPPDLR  RLIANF**T**VTSLQH  HMLETY**T**PEVTKA  EDPLED**T**GLVQQQ  TDMRCR**T**TFYTAL  TPGDSE**T**APVVTV  MASVNS**T**CNVTLT  EGSCLN**T**FTKNPY  YAASKK**T**IYTYIM  TVSHTT**T**SGILNS  AAEKEM**T**SVVAEN  PAAAFP**T**ASPANK  KEKTYE**T**DLAVLQ  GVEGGG**T**RSEVLL  EADGLS**T**NHWLIG  NHWLIG**T**DKCVER  NFFSSF**T**LMKLRH  VLSHQP**T**SPGSKD  ETALPP**T**PQDISY  VCGHRF**T**TKGNLK  TMALLG**T**DGKRVS  MVPVAV**T**AAVAPV  LLIAGL**T**RERGLL  DVFVEA**T**HVLPLH  ELCNLI**T**DKEMLK  FLSLPD**T**WMKEFF  FLAHIY**T**ELQLIE  RIENMD**T**FSNLLY  DKYRVE**T**CCVIGN  RYLGAW**T**LMGHEY  WYGLGQ**T**YEILKM  LWDEAS**T**CAQKCC  CCAFND**T**REEGKA  EPEVHK**T**SVVGLF  QVFKLY**T**ALQQYF  RAFAGK**T**ANKLMD  SLVTGI**T**ALNSIE  FKIARV**T**GIAGTC  FVQEKN**T**TTSVQD  FVAEFL**T**EHFNEA  TVVLKD**T**VGREGR  LLVAWN**T**VSTGLV  LGLLMG**T**GAQGLR  NTCDII**T**LYISAI  AVELSK**T**DPASLE  PFPDDL**T**ASWPDW  MVPRGP**T**ATARFG  PRGPTA**T**ARFGVP  VPAEGR**T**PPPFPG  EELMVK**T**KDGYVE  IVSKNF**T**KKIQLP  AEVDPV**T**VFASLS  QVPPYS**T**FGESSF  ARAARG**T**GALLLR  VLESEG**T**RESAIN  SNPGDV**T**SMVAQA  SRDVQG**T**DASLDE  SKEVLF**T**EEDVKF  IGHIKL**T**DFGLSK  AYSFCG**T**VEYMAP  SGKPDD**T**FCFDPE  CFDPEF**T**AKTPKD  KRCIHA**T**TNMEFA  RYVYLV**T**DLMKGG  LYVISK**T**VDYLHC  TMLAGY**T**PFANGP  ANGPND**T**PEEILL  LKHSWI**T**HRDQLP  KGAMVA**T**YSALTH  SPQSPE**T**PAPQNQ  QQLAKE**T**SNFGFS  AGKFAS**T**FDKNFR  TTDLVE**T**WLRNMK  LGFDEP**T**EQPVRK  ASNIFG**T**PEENQA  IHENVD**T**DLPGSL  FLANIG**T**SVQNVR  IETMQQ**T**IEDLEV  RPPTAR**T**SSSGSL  GESGGS**T**KSETED  PNSPSP**T**ALAFGD  SFKIIQ**T**DLTMLK  LLIEKS**T**QEKLSS  DTDTFC**T**VLEYCE  LQASNV**T**NKNDPK  RSTAVT**T**SSAKIK  ELQAIK**T**ELTQIK  APQENT**T**SEAGLP  EAASTL**T**RLPIDV  NHYWNE**T**VRDPIL  ILILYS**T**TRKERD  LILYST**T**RKERDR  LSSPSD**T**KAESPA  SAEKHV**T**QRLQPE  PSISIT**T**ESLKSL  RISEDE**T**ERNGDD  KVELEV**T**LPGEGK  RPASHQ**T**FPLQQE  STMIRA**T**ARSAPD  IQFYKS**T**RFKPTR  FNDKHI**T**TLQASF  NDKHIT**T**LQASFL  LQASFL**T**KKLNIG  ILVYDI**T**DEDSFQ  QEVLQE**T**LKSLGR  LELLED**T**DVSAIP  VKEWPV**T**EAEKKR  SLESRR**T**AQVRYL  LNVADL**T**SLRAPL  KCILVI**T**WIQHLI  RVNAVK**T**KVEAFQ  VEAFQT**T**ISKYFS  ANSFVG**T**PYWMAP  HFATIR**T**ASLVSR  ALGPPN**T**GTPIEQ  PPWAIH**T**LASWGL  NCIQLK**T**SVIKGI  EHIEIL**T**VNGELL  IMCPGL**T**SPGAKL  YPAAVD**T**IVAIMA  LVGDPG**T**DHLIRW  RGVQES**T**EARLRE  ECDFCV**T**APPPLP  GLPGAL**T**IYSTPE  TKAPAH**T**FWRESC  RTTIDL**T**CSGHVS  GYMAFL**T**YDEVQE  YWAMDS**T**FELCKI  TGKVKP**T**RAVKDQ  DFFTNH**T**LVLHVA  DFPGLI**T**LTISLL  DFLKSV**T**TLAMKA  FTYSLS**T**KRSSPD  LSVGLG**T**CVYLWS  CIRFWN**T**LTGQPL  FSKTRS**T**KVKWES  LTLSWY**T**TWQNGK  KRVGAE**T**NGSKDA  VVFIGE**T**DIDYVH  LTNLKE**T**FGDSKE  PASESA**T**VTSATF  SGPHGP**T**QAQCNN  SCIVSP**T**PEPHLP  KAAKKP**T**AAEISV  SGYINS**T**GSTRGH  NLAPSL**T**TLSHGT  GTTTTS**T**AYGVKK  TSAACT**T**SVQSDD  GLSLHA**T**GDYLLS  GHSGPI**T**SIAFSE  NGYYLA**T**AADDSS  TEHSGL**T**TGVAFG  CYGRAI**T**RNPLVA  ITPSGI**T**YDRKDI  GHFDPV**T**RSPLTQ  VTRSPL**T**QEQLIP  ATEVSK**T**PEAREA  ATQASS**T**TQLTDT  ADPQAV**T**MPATET  NTKAQE**T**EAAPSQ  RDLDQR**T**EDLKAE  VQLAMQ**T**YEMVDK  KLKLVR**T**SPEYGM  FACVGL**T**TKPRGK  VQLFIS**T**CNGEHI  MNTNQL**T**SIHADL  QQLPKY**T**SQIVGR  IQRLTK**T**FLTLSL  GLLDAV**T**YLAGVS  AKSLQK**T**IQAARS  EARAPE**T**WFEFTP  WSDTYD**T**FKLADT  ISKAKM**T**QITKAA  TPVTPV**T**PANVVQ  LQQLIQ**T**LQIQQQ  VQLQAL**T**AQLTAA  TLSVCS**T**TLWVGQ  LNKGVK**T**EYKQFW  VKETVQ**T**TQSPTP  PVVPPP**T**IPPVVP  NVFNAP**T**KQAEPE  TPGLLG**T**QPPAGP  QSVDNV**T**NPEKRI  DYFEGA**T**SQRKGD  SSKGHD**T**HKGKPV  QTLFIP**T**GWIHAV  SHQALK**T**GSFQKA  LMSNGS**T**KRVKSL  LDMCPE**T**RLDRTG  NSQLLE**T**KNALNV  DANQPG**T**VVDQFT  LTEHVF**T**DPAPTP  DPKSTP**T**PTYYGS  SALSSA**T**PTSTQW  LSSATP**T**STQWAW  SATPTS**T**QWAWDS  DGLKFY**T**DPSYFF  EKMLQD**T**EDKRKE  QKRIDG**T**TREVKK  PPHPPS**T**GLLVTA  RQLGDG**T**YGSVLL  IMAEVY**T**LRPLFP  GASEID**T**IFKICQ  ICQVLG**T**PKKTDW  VPNNLK**T**LIPNAS  HPLGST**T**QNLQDS  QPPLHL**T**YPYKAE  HPQSKI**T**AGLEHK  PSEPVG**T**GNSAPT  TGNSAP**T**QTSYQR  SYQRRD**T**PTLRSA  TSSSGL**T**GNYVPS  AAAKTH**T**TALAGR  RRSRSR**T**PLISRR  SRTSPV**T**RRRSRS  AMIMEE**T**GKIFKK  IANVAH**T**FVVDVA  VDVAQG**T**QVTGRK  KTAENA**T**SGETLE  DDVDPE**T**LSYAGQ  GNKDIN**T**FEQIDD  VERVIS**T**KGLNLP  IQPLDE**T**AVTDKE  LDETAV**T**DKENNL  FESSKF**T**RTISPP  RTISPP**T**LGTLRS  SPPTLG**T**LRSCFS  PSPSPS**T**ALQQFR  DSQSDQ**T**SKLRLS  SADSLS**T**TKIKPL  RDNIQL**T**PEAEED  FYLVAE**T**EEDMNK  NQAEES**T**DSLRNV  SNHMQP**T**LSTSAP  FLMRSD**T**AVQKLA  FKTPSN**T**LCREFG  KPSQAE**T**PRWGSP  VGRSDS**T**NSEDNY  LGYPST**T**LPVHRG  DRKAKP**T**PLDLRN  PHRKPS**T**SSVTSD  TQALQN**T**MQEWTD  NTMQEW**T**DVRQSS  ARLAQA**T**QERTDL  NKRVKD**T**MARSED  PSVDQF**T**GVGIRV  GIRVSF**T**GKQGEM  PPNFRL**T**HDISLE  SLQCKD**T**LSLRPP  GPGSGD**T**YRPKRP  QSGPAP**T**TDRGTS  SPCRRS**T**ATQMAP  TEEIYL**T**PVQRPP  PDAAEP**T**SAFLPP  SAFLPP**T**ESRMSV  ASLSSD**T**SALSYD  GEEQEQ**T**HRAIFR  AMQKIA**T**TRRLTV  KYFGFI**T**KHPADH  LHSIPV**T**VEVKPV  PNSSHS**T**IAENGF  NQFSLY**T**SPSLPN  SLGLQA**T**VTVTNS  GLQATV**T**VTNSHL  TGKFMS**T**SSIPGC  QARQQS**T**LIAVPL  APLSLA**T**VPHQAL  PVKHLF**T**TGVVYD  IGVDSD**T**VWNEMH  LGGYSV**T**ARCFGH  EAQAGE**T**EEAETV  DRPATG**T**GSKVGK  TIYDLG**T**KMIESL  DAMGSQ**T**KFVQCP  ATNRGI**T**RIRGTS  WQYEGP**T**QKLFLK  QKFRKN**T**APSLSS  VQSALS**T**AAADDS  EKAWFQ**T**PYTTFG  QHLTGL**T**DEKVKA  ESVSAE**T**VEKWLK  VAKSRK**T**LLVEDI  TGLESG**T**RIQSVL  LCLPIV**T**AIGDLI  SHQEVA**T**ANLAWA  AGQVAR**T**GEVLNI  DLYTGY**T**TRNILC  SYHSIC**T**SEEWQG  LAALYS**T**STMEQH  RKAIIA**T**DLALYF  RGEETA**T**WISSPS  GDLGWM**T**RGSMVG  MDKPVF**T**DPPVKT  TDPPVK**T**KFGYHI  LKSRGN**T**KMSIHL  FVVGAM**T**VGMGYS  SKCPDT**T**PKRRRA  RKLLES**T**PSPVVF  RPTDYP**T**QEQQLH  LQASMS**T**IEFGYL  RQYEQQ**T**YQVIPE  ERFFKN**T**PWPEAE  SFLKLY**T**TMPVAK  AGFLDL**T**EQEFRI  MAEKLI**T**QTFSHH  LPNYRW**T**QTLSEL  SDLDSE**T**RSMVEK  KSMGLP**T**SDEQKK  IVSRFL**T**GRFEDA  RFEDAY**T**PTIEDF  EDAYTP**T**IEDFHR  RRLSIL**T**GDVFIL  FMKLLL**T**RRANWM  APGEVD**T**QDKNKQ  VADGNV**T**VVDVLT  HKDVIQ**T**LIKGGA  SISKQE**T**ELSPEM  RKNLLR**T**HTTSAS  LLRTHT**T**SASARA  FTKLGI**T**QLRFKP  VTVVLI**T**CTYRGQ  YVNNEY**T**ETELRE  INWEDN**T**EKLEDA  LQSLLS**T**DALPSA  EAEMAR**T**QKNKAT  TQKNKA**T**AHHLGL  LRRELI**T**PKGGGG  GFDVAK**T**GDARIG  AAYEFT**T**LTTVPG  VIAVAR**T**CNLILI  KGGINL**T**ATCPQS  GINLTA**T**CPQSEL  SELDAE**T**VKSILA  IHNADV**T**LRSDAT  KLVRIY**T**KPKGQL  GQLPDY**T**SPVVLP  QLRSLS**T**VLKRLK  REPFLL**T**EKGMRC  SKDASS**T**SSGQSG  FYGHQY**T**RRANAE  LYQVME**T**ERMIYL  KFKQIV**T**AVYFCH  GFSNLF**T**PGQLLK  MQNLQP**T**GQLEYK  SLLQPP**T**LQLLNG  GQIDER**T**LEKTQQ  RTNLMA**T**LSYGHR  PTPPDY**T**RHQQVP  SQASSP**T**PPHGYA  KSSSTL**T**KGCHDS  SPLLLS**T**GGPGDP  SSCYPS**T**CITDIL  QRARST**T**ELRKEK  SLDMKF**T**YCDDRI  GASVAA**T**PSTPLA  SLQDPS**T**PLLNLN  PNLPKS**T**TKAAKM  NSKVPI**T**RTKSTP  LIQQRE**T**KEVDSK  SIETYS**T**SYPDTM  AEGERK**T**ALEMVQ  TDRHCV**T**FVLHEE  EFCGYT**T**THPSES  INLRIQ**T**RGTLPA  VPSPLP**T**RRTRTF  VEGDEV**T**YKMCSI  AVEVVI**T**HLAPGT  PGTKHE**T**WSGHVI  QTFSGG**T**SQDTKA  AGYQSG**T**HQGQFD  KEKQRK**T**EELEEE  EEYTEE**T**EEREES  GLVDED**T**FKLIYA  SILLRG**T**VHEKLK  NKDGYI**T**KEEMLA  NQDGVV**T**IEEFLE  YAAALE**T**FTEGQK  SQSAGI**T**GADANF  IIPEQS**T**FKVLST  FMESGG**T**VLSTNW  FDIARL**T**VNADVG  KNRDVI**T**LRSWLP  RAVSIQ**T**GYLIQS  PKSCVI**T**YLAQVD  LAADAG**T**FLSRAV  EKIMKQ**T**EVLLQP  TEFGPG**T**AYGNAL  LIKCGE**T**QKRIGT  DRELIQ**T**SALNFL  DLDAAK**T**RLKKAK  KAKAAE**T**RNSSEQ  LEGISS**T**HAHHLR  LSNNNQ**T**SVTPVP  NNQTSV**T**PVPSVL  YDAANS**T**ELSLLA  LADEVI**T**VFSVVG  TSRDGD**T**TRQRIK  HDVLSG**T**RMDLGE  ADCDRR**T**EVAKKR  KKRLAE**T**QEEISA  TNQIRL**T**NVAVVR  LDEVLQ**T**HSVFVN  ICKQIL**T**KGEVQV  IHYSVK**T**NKSTKQ  NCLKSP**T**SDDISL  ASFGEG**T**EPDQEP  VPVYHP**T**PSQTRL  HPTPSQ**T**RLATQL  SQTRLA**T**QLTEEE  RLATQL**T**EEEQIR  GSVSKS**T**QFEYAW  VRGLLQ**T**EPQNNQ  VCLWTL**T**SAAMSR  SRGDNC**T**DLLALG  LGIPSI**T**QAWGLW  EEVMCY**T**SLQLRP  CDNIFI**T**GPTGSV  SLASDS**T**FDSGQG  YGGSDV**T**SGKELS  LTSELE**T**SQPLAE  PRTPSN**T**PSAEAD  LHPDYK**T**WGPEQV  IRENEI**T**GALLPC  IIDTMI**T**DAFLKA  KLVSWY**T**LMEGQE  LNKPDS**T**IQDAGL  HGLFKS**T**LVCPEC  SPSEME**T**DEPDDE  TGPTSD**T**GWGCML  QWYGPN**T**VAQVLK  KLAVFD**T**WSSLAV  VGFFCK**T**EDDFND  AMEAVL**T**GLVEAA  RALVAV**T**VTDPAP  PVADYL**T**SQFYAL  FDRPLV**T**FDLLGE  PAVRVK**T**ELLESE  SPSSTS**T**SSSSSS  SSSTVL**T**PGPLVA  PGNVNN**T**IVVPLL  PLSLLK**T**AQNHPM  LREVIC**T**SRDGDK  CYIRGS**T**IKYLRI  RGGIPG**T**GRGQPE  GGSQSG**T**GSGEPG  QVPQAW**T**AGTSSD  QTPETS**T**FRNQMP  YLTMQQ**T**TALPTW  KAPGMN**T**IDQGMA  ALKLGS**T**EVASNV  VGSGSI**T**SNIVAS  QNIGQP**T**QGSPQP  EAKRRL**T**EQKGEQ  VNGFRF**T**SV
[truncated: 1,716 more chars]
