# Supplementary material for: iPhos-PseEn: Identifying phosphorylation sites in proteins by fusing different pseudo components into an ensemble classifier
Source: Oncotarget. 2016 Jun 13;7(32):51270–83. doi: 10.18632/oncotarget.9987 (PMC5239474; doi:10.18632/oncotarget.9987)
Supplement: Supplementary file 4 [file oncotarget-07-51270-s004.docx]

**Supporting Information S3.** The benchmark dataset $\mathbb{S}_{\xi=6}\left( Y \right)$used to train and test the model for predicting the possibility of phosphorylation at Tyr site. It contains 8,804 samples, of which 743 are positive samples and 8,061 are negative samples. All these samples were derived from 1,770 protein sequences in which none has $\geq50\%$ pairwise sequence identity with any other. See the main text for further explanation.

(1) List of the 743 peptide samples in the positive subset $\mathbb{S}_{\boldsymbol{\xi=6}}^{\mathbf{+}}\boldsymbol{(}\mathbf{Y}\boldsymbol{)}$

| Sample # | Protein ID | Site | Sequences |
| --- | --- | --- | --- |
| 1  2  3  4  5  6  7  8  9  10  11  12  13  14  15  16  17  18  19  20  21  22  23  24  25  26  27  28  29  30  31  32  33  34  35  36  37  38  39  40  41  42  43  44  45  46  47  48  49  50  51  52  53  54  55  56  57  58  59  60  61  62  63  64  65  66  67  68  69  70  71  72  73  74  75  76  77  78  79  80  81  82  83  84  85  86  87  88  89  90  91  92  93  94  95  96  97  98  99  100  101  102  103  104  105  106  107  108  109  110  111  112  113  114  115  116  117  118  119  120  121  122  123  124  125  126  127  128  129  130  131  132  133  134  135  136  137  138  139  140  141  142  143  144  145  146  147  148  149  150  151  152  153  154  155  156  157  158  159  160  161  162  163  164  165  166  167  168  169  170  171  172  173  174  175  176  177  178  179  180  181  182  183  184  185  186  187  188  189  190  191  192  193  194  195  196  197  198  199  200  201  202  203  204  205  206  207  208  209  210  211  212  213  214  215  216  217  218  219  220  221  222  223  224  225  226  227  228  229  230  231  232  233  234  235  236  237  238  239  240  241  242  243  244  245  246  247  248  249  250  251  252  253  254  255  256  257  258  259  260  261  262  263  264  265  266  267  268  269  270  271  272  273  274  275  276  277  278  279  280  281  282  283  284  285  286  287  288  289  290  291  292  293  294  295  296  297  298  299  300  301  302  303  304  305  306  307  308  309  310  311  312  313  314  315  316  317  318  319  320  321  322  323  324  325  326  327  328  329  330  331  332  333  334  335  336  337  338  339  340  341  342  343  344  345  346  347  348  349  350  351  352  353  354  355  356  357  358  359  360  361  362  363  364  365  366  367  368  369  370  371  372  373  374  375  376  377  378  379  380  381  382  383  384  385  386  387  388  389  390  391  392  393  394  395  396  397  398  399  400  401  402  403  404  405  406  407  408  409  410  411  412  413  414  415  416  417  418  419  420  421  422  423  424  425  426  427  428  429  430  431  432  433  434  435  436  437  438  439  440  441  442  443  444  445  446  447  448  449  450  451  452  453  454  455  456  457  458  459  460  461  462  463  464  465  466  467  468  469  470  471  472  473  474  475  476  477  478  479  480  481  482  483  484  485  486  487  488  489  490  491  492  493  494  495  496  497  498  499  500  501  502  503  504  505  506  507  508  509  510  511  512  513  514  515  516  517  518  519  520  521  522  523  524  525  526  527  528  529  530  531  532  533  534  535  536  537  538  539  540  541  542  543  544  545  546  547  548  549  550  551  552  553  554  555  556  557  558  559  560  561  562  563  564  565  566  567  568  569  570  571  572  573  574  575  576  577  578  579  580  581  582  583  584  585  586  587  588  589  590  591  592  593  594  595  596  597  598  599  600  601  602  603  604  605  606  607  608  609  610  611  612  613  614  615  616  617  618  619  620  621  622  623  624  625  626  627  628  629  630  631  632  633  634  635  636  637  638  639  640  641  642  643  644  645  646  647  648  649  650  651  652  653  654  655  656  657  658  659  660  661  662  663  664  665  666  667  668  669  670  671  672  673  674  675  676  677  678  679  680  681  682  683  684  685  686  687  688  689  690  691  692  693  694  695  696  697  698  699  700  701  702  703  704  705  706  707  708  709  710  711  712  713  714  715  716  717  718  719  720  721  722  723  724  725  726  727  728  729  730  731  732  733  734  735  736  737  738  739  740  741  742  743 | A8K4G0  O00213  O00401  O00459  O00459  O00459  O14492  O14493  O14654  O14746  O14818  O15162  O15162  O15259  O15259  O15259  O15264  O15269  O15304  O15350  O15350  O15357  O15357  O15357  O15446  O15455  O15455  O15492  O15492  O15530  O15530  O15530  O15547  O43294  O43294  O43353  O43353  O43561  O43561  O43561  O43561  O43561  O43561  O60331  O60331  O60602  O60674  O60674  O60674  O60674  O60674  O60674  O60674  O60674  O60674  O60711  O60711  O60711  O60716  O60716  O60716  O60716  O60716  O60716  O60716  O60716  O75056  O75056  O75056  O75056  O75116  O75563  O75563  O75563  O75563  O75716  O75807  O75807  O75807  O75807  O95297  O95297  O95866  P01100  P01100  P02730  P02730  P02730  P02730  P02730  P03372  P03956  P04083  P04626  P04626  P04626  P04629  P04629  P04629  P04629  P04629  P05060  P05067  P05067  P05106  P05106  P06213  P06213  P06213  P06213  P06213  P06213  P06396  P06396  P06396  P06396  P06396  P07332  P07332  P07333  P07333  P07333  P07333  P07333  P07333  P07333  P07333  P07550  P07766  P07766  P07947  P07947  P07947  P07947  P07947  P07947  P07949  P07949  P07949  P07949  P07949  P07949  P07949  P07949  P07949  P08069  P08069  P08069  P08069  P08581  P08581  P08581  P08581  P08581  P08581  P08581  P08833  P08865  P09038  P09211  P09211  P09619  P09619  P09619  P09619  P09619  P09619  P09619  P09619  P09619  P09619  P09619  P09619  P09619  P09619  P09619  P10275  P10275  P10275  P10275  P10275  P10275  P10275  P10275  P10275  P10275  P10275  P10451  P10636  P10636  P10721  P10721  P10721  P10721  P10721  P10721  P10721  P10721  P10721  P10721  P11171  P11171  P11274  P11274  P11362  P11362  P11362  P11362  P11362  P11362  P11362  P11488  P12004  P12814  P12830  P12830  P12830  P13639  P13639  P14317  P14317  P14317  P14317  P14317  P14778  P15151  P15311  P15311  P15311  P15311  P15391  P15391  P15391  P15391  P15941  P15941  P15941  P15941  P15941  P16234  P16234  P16234  P16234  P16234  P16234  P16234  P16234  P16234  P16234  P16234  P16284  P16284  P16410  P17948  P17948  P17948  P17948  P17948  P17948  P17948  P17948  P18031  P18031  P18206  P18206  P18206  P18669  P19174  P19174  P19174  P19174  P19174  P19174  P19404  P19429  P19525  P19525  P19525  P20138  P20138  P22681  P22681  P22681  P22681  P23246  P23458  P23458  P23458  P24394  P24394  P24394  P24394  P24666  P24666  P25963  P27361  P28370  P29317  P29317  P29317  P29317  P29317  P29350  P29350  P29350  P29350  P29597  P29597  P29597  P30040  P30040  P30530  P30530  P30530  P30530  P31040  P31645  P31645  P31995  P31995  P35222  P35222  P35222  P35222  P35222  P35222  P35568  P35568  P35568  P35568  P35568  P35568  P35568  P35568  P35568  P35916  P35916  P35916  P35916  P35916  P35916  P35916  P35916  P35916  P35916  P35968  P35968  P35968  P35968  P35968  P35968  P35968  P35968  P35968  P35968  P36888  P36888  P36888  P36888  P36888  P36888  P36888  P36888  P36888  P36888  P36896  P37840  P40763  P41240  P41240  P41240  P41597  P41743  P41743  P41743  P42229  P42680  P42680  P42680  P42684  P42684  P42684  P42684  P42684  P42684  P42684  P42684  P42684  P42684  P42684  P42684  P42684  P42684  P42684  P42684  P42768  P43351  P43403  P43403  P43403  P43403  P43403  P43403  P43405  P43405  P43405  P43405  P43405  P43405  P43405  P43405  P43405  P43405  P43405  P43405  P43405  P43405  P43405  P43405  P46108  P46108  P46527  P46527  P46527  P46937  P48551  P48551  P49023  P49023  P49023  P49023  P49789  P49789  P49841  P51636  P51636  P51813  P51813  P51813  P52333  P52333  P52333  P52333  P52333  P52630  P52735  P52735  P52735  P53779  P54753  P55211  P55263  P56945  P56945  P56945  P56945  P56945  P59665  P60484  P60953  P63010  P63244  P63244  P78347  P78347  P78347  P78347  Q02763  Q02763  Q02763  Q02763  Q03135  Q03135  Q03135  Q04759  Q04912  Q04912  Q04912  Q04912  Q05086  Q05397  Q05397  Q05397  Q05397  Q05397  Q05397  Q05397  Q05397  Q05655  Q05655  Q05655  Q05655  Q05655  Q05655  Q06187  Q06187  Q06187  Q06187  Q06187  Q06187  Q06609  Q07666  Q07666  Q07666  Q07912  Q07912  Q07912  Q07912  Q07912  Q07954  Q08345  Q08345  Q08345  Q08345  Q08345  Q08345  Q08345  Q12866  Q12866  Q12866  Q12866  Q12972  Q12972  Q13153  Q13153  Q13153  Q13153  Q13153  Q13191  Q13191  Q13191  Q13191  Q13322  Q13444  Q13444  Q13627  Q13627  Q13627  Q13627  Q13627  Q13627  Q13627  Q13627  Q13627  Q13671  Q13769  Q13882  Q13882  Q13882  Q13882  Q13882  Q13882  Q13882  Q13905  Q14118  Q14247  Q14247  Q14247  Q14247  Q14247  Q14247  Q14289  Q14289  Q14289  Q14289  Q14289  Q14289  Q14289  Q14289  Q14527  Q14680  Q14680  Q15025  Q15118  Q15118  Q15118  Q15139  Q15139  Q15139  Q15139  Q15303  Q15303  Q15303  Q15303  Q15303  Q15303  Q15303  Q15303  Q15303  Q15303  Q15654  Q15746  Q15746  Q15746  Q15746  Q15746  Q15746  Q15746  Q15746  Q15746  Q15746  Q16625  Q16625  Q16625  Q16790  Q16832  Q16832  Q16832  Q16832  Q2HXU8  Q5KSL6  Q5SQ64  Q5VY43  Q6DN72  Q6GTX8  Q6GTX8  Q6PCB0  Q6S5L8  Q6ZUJ8  Q6ZUJ8  Q6ZUJ8  Q6ZUJ8  Q6ZUJ8  Q6ZUJ8  Q6ZUJ8  Q6ZUJ8  Q6ZUJ8  Q70E73  Q70E73  Q70E73  Q8IVP5  Q8IYA6  Q8IZP0  Q8IZP0  Q8IZP0  Q8TD08  Q8WV28  Q8WV28  Q8WV28  Q8WV28  Q8WV28  Q92556  Q92556  Q92556  Q92556  Q92556  Q92630  Q92783  Q92783  Q92783  Q92888  Q96BD6  Q96EY5  Q96G74  Q96J02  Q96LC7  Q96T51  Q96T51  Q99572  Q99638  Q99665  Q99683  Q99704  Q99704  Q99704  Q99704  Q99704  Q99704  Q99704  Q99704  Q9BZL6  Q9BZL6  Q9GZY6  Q9GZY6  Q9GZY6  Q9GZY6  Q9H1A4  Q9H2W2  Q9H3Y6  Q9H400  Q9H400  Q9H400  Q9H5V8  Q9H706  Q9H706  Q9NR20  Q9NRY4  Q9NRY4  Q9NRY4  Q9NV92  Q9NV92  Q9NV92  Q9NV92  Q9NWQ8  Q9NWQ8  Q9NWQ8  Q9NWQ8  Q9NWQ8  Q9NWQ8  Q9NWQ8  Q9NWQ8  Q9NZC7  Q9NZC7  Q9NZJ5  Q9P126  Q9UBK5  Q9UBN4  Q9UBN4  Q9UBR4  Q9UGK3  Q9UGK3  Q9UGK3  Q9UGK3  Q9UHP3  Q9UIB8  Q9UIB8  Q9UJ70  Q9UJX2  Q9UJX5  Q9ULV8  Q9UM73  Q9UM73  Q9UM73  Q9UM73  Q9UM73  Q9UM73  Q9UM73  Q9UPY6  Q9UPY6  Q9UPY6  Q9UPY6  Q9UPZ9  Q9UQC2  Q9UQC2  Q9UQC2  Q9Y3P8  Q9Y3P8  Q9Y3P8  Q9Y3P8  Q9Y3P8  Q9Y6N7  Q9Y6N7  Q9Y6N7 | 188  547  256  464  605  655  629  208  921  707  153  69  74  46  349  721  182  164  34  28  99  986  1135  1162  80  759  858  168  177  9  373  376  64  38  60  381  474  110  156  161  200  220  255  639  649  798  119  372  373  813  868  966  972  1007  1008  22  62  72  112  217  221  228  257  280  865  904  409  419  431  441  722  75  151  197  261  198  262  391  434  512  241  263  211  10  30  8  21  46  359  904  537  360  21  1139  1196  1248  496  676  680  681  791  401  497  757  773  785  401  992  999  1011  1355  1361  86  409  465  603  651  261  713  546  561  699  708  723  809  923  969  141  188  199  32  336  345  426  446  537  806  809  900  905  981  1015  1062  1090  1096  980  1161  1165  1166  1003  1230  1234  1235  1349  1356  1365  158  139  215  4  199  562  579  686  716  740  751  763  771  775  778  857  934  970  1009  1021  223  267  307  346  357  362  363  393  534  551  915  225  18  514  547  553  568  570  703  721  730  823  900  936  222  660  177  246  463  583  585  653  654  730  766  142  211  12  753  754  755  265  373  140  198  222  378  397  496  398  116  146  354  478  348  378  409  439  1203  1212  1218  1229  1243  572  574  720  731  742  754  762  768  849  988  1018  690  713  201  914  1053  1169  1213  1242  1309  1327  1333  20  66  537  822  1133  26  506  771  775  783  977  1253  193  26  101  162  293  340  358  674  700  731  774  293  3  1034  1035  497  575  603  631  132  133  42  204  954  588  628  735  921  930  64  377  536  564  292  604  1054  64  66  703  779  821  866  215  47  142  294  310  64  86  142  331  333  654  465  612  632  662  896  941  989  1179  1229  830  833  853  1068  1230  1231  1265  1333  1337  1363  801  951  996  1054  1059  1175  1214  1305  1309  1319  572  589  591  599  726  768  793  842  955  969  380  125  705  184  304  416  139  265  280  334  694  206  228  519  116  161  174  185  218  231  261  272  299  303  310  439  515  568  683  718  291  104  248  292  315  319  492  493  28  47  131  296  323  348  352  364  484  507  525  526  546  629  630  631  221  239  74  88  89  407  337  512  31  88  118  181  114  145  216  19  27  216  224  566  785  904  939  980  981  690  142  159  172  223  614  153  77  128  249  362  372  410  85  336  64  737  52  228  248  318  398  503  860  992  1102  1108  6  14  25  90  1238  1239  1353  1360  659  5  397  407  570  576  577  861  925  64  155  313  334  374  567  40  223  344  361  551  617  54  435  440  443  284  518  827  859  872  4507  484  513  520  740  792  796  797  749  753  754  872  264  335  131  142  153  201  285  363  665  709  889  67  715  735  111  140  145  159  177  219  319  321  449  36  225  13  61  66  114  342  351  447  504  892  421  446  453  470  486  489  402  579  580  722  819  834  849  881  195  163  367  552  136  243  244  95  432  463  502  875  1035  1056  1150  1162  1188  1202  1242  1258  1284  55  231  464  471  556  611  792  846  1449  1575  1635  368  398  402  449  471  736  740  741  7  78  281  925  371  251  281  83  424  263  419  444  459  513  553  570  594  694  426  456  1226  18  204  53  213  455  177  72  84  96  178  189  18  216  395  511  720  382  198  381  384  738  31  204  175  420  667  389  400  343  28  800  718  296  337  341  362  377  398  409  449  438  717  58  136  193  233  571  20  380  167  200  254  734  105  453  264  308  1087  1105  151  167  171  177  105  163  181  227  317  359  387  417  33  287  619  7  86  959  972  227  22  250  310  322  740  296  316  205  273  469  341  1078  1092  1096  1131  1278  1507  1604  151  248  337  486  159  266  293  452  90  127  148  169  188  1038  1073  1114 | PGEQPI**Y**MNFSEP  QKFQVY**Y**LGNVPV  ETSKVI**Y**DFIEKT  REYDQL**Y**EEYTRT  NETEDQ**Y**ALMEDE  SSQRGC**Y**ACSVVV  RAVENQ**Y**SFYYYY  SAAASN**Y**VVVVVV  ADSSSD**Y**VNMDFT  DPPPEL**Y**FVKVDV  TDPSGT**Y**HAWKAN  VPNQPV**Y**NQPVYN  VYNQPV**Y**NQPVGA  NKRQHI**Y**QRCIQL  NQFRAN**Y**FLQPEL  DLSEQT**Y**DFLGEM  DAEMTG**Y**VVTRWY  TEEAII**Y**SYGFAT  GVCAER**Y**SQEVFE  LEPDST**Y**FDLPQS  VPTHSP**Y**AQPSST  SFNNPA**Y**YVLEGV  TLSEVD**Y**APAGPA  RGLPSD**Y**GRPLSF  AGKRHR**Y**RVLSSC  QFEYAA**Y**IIHAYK  LEEIPD**Y**KLNHAL  LMEKDS**Y**PRFLKS  FLKSPA**Y**RDLAAQ  RTTSQL**Y**DAVPIQ  EDDEDC**Y**GNYDNL  EDCYGN**Y**DNLLSQ  LLAKKG**Y**QERDLE  LTPPPS**Y**GHQPQT  GDKDHL**Y**STVCKP  RKAQDC**Y**FMKLHH  LIMKED**Y**ELVSTK  ANSVAS**Y**ENEGAS  DEDEDD**Y**HNPGYL  DYHNPG**Y**LVVLPD  MESIDD**Y**VNVPES  LDGSRE**Y**VNVSQE  EEGAPD**Y**ENLQEL  APATDI**Y**FPTDER  DERSWV**Y**SPLHYS  VGSLSQ**Y**QLMKHQ  LYRIRF**Y**FPRWYC  VSLIDG**Y**YRLTAD  SLIDGY**Y**RLTADA  SLFTPD**Y**ELLTEN  SVEMCR**Y**DPLQDN  ICKGME**Y**LGTKRY  YLGTKR**Y**IHRDLA  LPQDKE**Y**YKVKEP  PQDKEY**Y**KVKEPG  LQDSDE**Y**SNPAPL  LPAQLV**Y**TTNIQE  IQELNV**Y**SEAQEP  GQIVET**Y**TEEDPE  DGYSRH**Y**EDGYPG  RHYEDG**Y**PGGSDN  PGGSDN**Y**GSLSRV  PSRQDV**Y**GPQPQV  RFHPEP**Y**GLEDDQ  SQSSHS**Y**DDSTLP  KSLDNN**Y**STPNER  LVTLLI**Y**RMKKKD  KKDEGS**Y**TLEEPK  KQASVT**Y**QKPDKQ  DKQEEF**Y**AAAAAA  ADKNKI**Y**ESIEEA  AEDGEE**Y**DDPFAG  SKTVFY**Y**YGSDKD  APDKRI**Y**QFTAAS  PIDDEI**Y**EELPEE  QRCTIS**Y**RAPELF  DPRSWE**Y**RSGEAS  FLKSWV**Y**QPGEDT  FLKAWV**Y**RPGEDT  PFRVAI**Y**VPGEKP  HQGPVI**Y**AQLDHS  KSESVV**Y**ADIRKN  QEPSLL**Y**ADLDHL  SGFNAD**Y**EASSSR  GDSLSY**Y**HSPADS  EELQDD**Y**EDMMEE  NLEQEE**Y**EDPDIP  EATATD**Y**HTTSHP  KPDSSF**Y**KGLDLN  EEGRDE**Y**DEVAMP  KNVVPL**Y**DLLLEM  QNVLHG**Y**PKDIYS  ENEEQE**Y**VQTVKS  CSPQPE**Y**VNQPDV  AVENPE**Y**LTPQGG  TAENPE**Y**LGLDVP  IIENPQ**Y**FSDACV  GMSRDI**Y**STDYYR  DIYSTD**Y**YRVGGR  IYSTDY**Y**RVGGRT  AQAPPV**Y**LDVLGG  DKMAHG**Y**GEESEE  FNMLKK**Y**VRAEQK  KMQQNG**Y**ENPTYK  TANNPL**Y**KEATST  TFTNIT**Y**RGTTTT  LKIRRS**Y**ALVSLS  GPLGPL**Y**ASSNPE  ASSNPE**Y**LSASDV  VFPCSV**Y**VPDEWE  LGFKRS**Y**EEHIPY  YEEHIP**Y**THMNGG  PVPTNL**Y**GDFFTG  DGLGLS**Y**LSSHIA  PVDPAT**Y**GQFYGG  KTPSAA**Y**LWVGTG  LGGKAA**Y**RTSPRL  IQPEAE**Y**QGFLRQ  EEADGV**Y**AASGGL  YKQKPK**Y**QVRWKI  SYEGNS**Y**TFIDPT  PEGGVD**Y**KNIHLE  IHLEKK**Y**VRRDSG  SQGVDT**Y**VEMRPV  IMNDSN**Y**IVKGNA  DRRERD**Y**TNLPSS  LLQPNN**Y**QFCCCC  ITSPFK**Y**QSLLTK  PVPNPD**Y**EPIRKG  KGQRDL**Y**SGLNQR  STSVSH**Y**GAEPTT  DKLVPL**Y**AVVSEE  VSEEPI**Y**IVTEFM  LIEDNE**Y**TARQGA  APEAAL**Y**GRFTIK  TATEPQ**Y**QPGENL  LLLIVE**Y**AKYGSL  IVEYAK**Y**GSLRGF  GLSRDV**Y**EEDSYV  VYEEDS**Y**VKRSQG  NCSEEM**Y**RLMLQC  MVKRRD**Y**LDLAAS  WIENKL**Y**GMSDPN  NTGFPR**Y**PNDSVY  YPNDSV**Y**ANWMLS  ASVNPE**Y**FSAADV  GMTRDI**Y**ETDYYR  DIYETD**Y**YRKGGK  IYETDY**Y**RKGGKG  SNESVD**Y**RATFPE  GLARDM**Y**DKEYYS  DMYDKE**Y**YSVHNK  MYDKEY**Y**SVHNKT  TFIGEH**Y**VHVNAT  VHVNAT**Y**VNVKCV  VKCVAP**Y**PSLLSS  WDAIST**Y**DGSKAL  PLTEAS**Y**VNLPTI  GVCANR**Y**LAMKED  MMMMPP**Y**TVVYFP  FLASPE**Y**VNLPIN  WQKKPR**Y**EIRWKV  SSDGHE**Y**IYVDPM  ITEYCR**Y**GDLVDY  PPSAEL**Y**SNALPV  GESDGG**Y**MDMSKD  KDESVD**Y**VPMLDM  MKGDVK**Y**ADIESS  DIESSN**Y**MAPYDN  SNYMAP**Y**DNYVPS  MAPYDN**Y**VPSAPE  IMRDSN**Y**ISKGST  HASDEI**Y**EIMQKC  EGYKKK**Y**QQVDEE  DTSSVL**Y**TAVQPN  NEGDND**Y**IIPLPD  TSSKDN**Y**LGGTST  LRGDCM**Y**APLLGV  TEDTAE**Y**SPFKGG  PSTLSL**Y**KSGALD  LDEAAA**Y**QSRDYY  AYQSRD**Y**YNFPLA  YQSRDY**Y**NFPLAL  LENPLD**Y**GSAWAA  DSYSGP**Y**GDMRLE  HVLPID**Y**YFPPQK  GKVKPI**Y**FHTQQQ  SRGKDS**Y**ETSQLD  EDHAGT**Y**GLGDRK  SGDRSG**Y**SSPGSP  MILTYK**Y**LQKPMY  YLQKPM**Y**EVQWKV  EINGNN**Y**VYIDPT  NGNNYV**Y**IDPTQL  HAEAAL**Y**KNLLHS  SDSTNE**Y**MDMKPG  MKPGVS**Y**VVPTKA  IKNDSN**Y**VVKGNA  HAPAEM**Y**DIMKTC  ESTNHI**Y**SNLANC  LLDDTV**Y**ECVVEK  LDGENI**Y**IRHSNL  DAEKPF**Y**VNVEFH  CGVDGD**Y**EDAELN  LAGVSE**Y**ELPEDP  RPPGLE**Y**CYNPSH  PGLEYC**Y**NPSHNP  DIHHID**Y**YKKTTN  IHHIDY**Y**KKTTNG  NCTNEL**Y**MMMRDC  LTSNQE**Y**LDLSMP  FERASE**Y**QLNDSA  LTFALR**Y**LNFFTK  SQQTND**Y**MQPEED  DTRDNV**Y**YYDEEG  TRDNVY**Y**YDEEGG  RDNVYY**Y**DEEGGG  KLWGDR**Y**FDPANG  YRCELL**Y**EGPPDD  SAVGFD**Y**KGEVEK  KGFGGQ**Y**GIQKDR  EAPTTA**Y**KKTTPI  PEPEND**Y**EDVEEM  DEPEGD**Y**EEVLEP  LEKIQD**Y**EKMPES  ANGHVS**Y**SAVSRE  ILSDEI**Y**CPPETA  EVHKSG**Y**LSSERL  MLRLQD**Y**EEKTKK  PPPPPV**Y**EPVSYH  SGPQNQ**Y**GNVLSL  GGTAPS**Y**GNPSSD  EEEGEG**Y**EEPDSE  SQDGSG**Y**ENPEDE  FPARDT**Y**HPMSEY  MSEYPT**Y**HTHGRY  YHTHGR**Y**VPPSST  STDRSP**Y**EKVSAG  GGSSLS**Y**TNPAVA  SPDGHE**Y**IYVDPM  DGHEYI**Y**VDPMQL  DESTRS**Y**VILSFE  FENNGD**Y**MDMKQA  QADTTQ**Y**VPMLER  RKEVSK**Y**SDIQRS  DIQRSL**Y**DRPASY  YDRPAS**Y**KKKSML  IMHDSN**Y**VSKGST  VDSDNA**Y**IGVTYK  LSADSG**Y**IIPLPD  LNSDVQ**Y**TEVQVS  KDTETV**Y**SEVRKA  PLTTGV**Y**VKMPPT  IVEYCK**Y**GNLSNY  IYKNPD**Y**VRKGDT  QQDGKD**Y**IPINAI  SSDDVR**Y**VNAFKF  TSMFDD**Y**QGDSST  GKRRFT**Y**DHAELE  CSPPPD**Y**NSVVLY  YNSVVL**Y**STPPII  GSWAAI**Y**QDIRHE  HQEDND**Y**INASLI  NVMMGP**Y**RQDLLA  SFLDSG**Y**RILGAV  VRKTPW**Y**QQQQQQ  NRFSGW**Y**DADLSP  PVNHEW**Y**PHYFVL  GTAEPD**Y**GALYEG  PDYGAL**Y**EGRNPG  GRNPGF**Y**VEANPM  GTERAC**Y**RDMSSF  GSFESR**Y**QQPFED  QINDNY**Y**EDLTAK  RRRSSN**Y**RAYATE  GLSMGN**Y**IGLINR  LAAKLA**Y**LQILSE  RIDGKT**Y**VIKRVK  MDEELH**Y**ASLNFH  KDTSTE**Y**SEVRTQ  SSANAI**Y**SLAARP  GEEDTE**Y**MTPSSR  QIDSCT**Y**EAMYNI  ENEDDG**Y**DVPKPP  RPGEKT**Y**TQRCRL  MMMMMQ**Y**LNIKED  IETDKE**Y**YTVKDD  ETDKEY**Y**TVKDDR  IAGNPA**Y**RSFSNS  SAPTSG**Y**QEFVHA  PPGEAG**Y**KAFSSL  SSGEEG**Y**KPFQDL  LIIEDP**Y**YGNDSD  IIEDPY**Y**GNDSDF  SMKDEE**Y**EQMVKE  TGFLTE**Y**VATRWY  HQLRIQ**Y**GTSKGK  LKPLKT**Y**VDPHTY  GEFGEV**Y**KGMLKT  YLANMN**Y**VHRDLA  SIKMQQ**Y**TEHFMA  HFMAAG**Y**TAIEKV  GDFYDL**Y**GGEKFA  QRAYGP**Y**SVTNCG  KGQESE**Y**GNITYP  KHKEDV**Y**ENLHTK  AEGEPC**Y**IRDSGV  GTRTNV**Y**EGRLRV  VPEGHE**Y**YRVRED  VKFDTQ**Y**PYGEKQ  FDTQYP**Y**GEKQDE  IYNGDY**Y**RQGRIA  DCLDGL**Y**ALMSRC  EPDEIL**Y**VNMDEG  VHPAGR**Y**VLCPST  LRYDTS**Y**FVEYFA  GQISNG**Y**SAVPSP  ELALGQ**Y**HRNGCI  ETADGG**Y**MTLNPR  DDDKNI**Y**LTLPPN  DTSQVL**Y**EWEQGF  ADIDGQ**Y**AMTRAQ  VVNLIN**Y**QDDAEL  VNIMRT**Y**TYEKLL  IMRTYT**Y**EKLLWT  NEGVAT**Y**AAAVLF  EEELSN**Y**ICMGGK  LHTDDG**Y**MPMSPG  RKGSGD**Y**MPMSPK  RVDPNG**Y**MMMSPS  PKSPGE**Y**VNIEFG  ETGTEE**Y**MKMDLG  PSSRGD**Y**MTMQMS  LENGLN**Y**IDLDLV  SEDLSA**Y**ASISFQ  LEEQCE**Y**LSYDAS  QCEYLS**Y**DASQWE  LGRVLG**Y**GAFGKV  IYKDPD**Y**VRKGSA  HSLAAR**Y**YNWVSF  SLAARY**Y**NWVSFP  PMTPTT**Y**KGSVDN  RGGQVF**Y**NSEYGE  VFYNSE**Y**GELSEP  FFTDNS**Y**YYYYYY  GELKTG**Y**LSIVMD  FRQGKD**Y**VGAIPV  EAPEDL**Y**KDFLTL  GLARDI**Y**KDPDYV  IYKDPD**Y**VRKGDA  QQDGKD**Y**IVLPIS  CDPKFH**Y**DNTAGI  SNQTSG**Y**QSGYHS  SGYQSG**Y**HSDDTD  DTDTTV**Y**SSEEAE  YKKQFR**Y**ESQLQM  GSSDNE**Y**FYVDFR  SDNEYF**Y**VDFREY  DFREYE**Y**DLKWEF  EHNFSF**Y**PTFQSH  SEDEIE**Y**ENQKRL  DLLCFA**Y**QVAKGM  IMSDSN**Y**VVRGNA  DAEEAM**Y**QNVDGR  SECPHT**Y**QNRRPF  RVGTKR**Y**MAPEVL  DPDNEA**Y**EMPSEE  PGSAAP**Y**LKTKFI  AAQDEF**Y**RSGWAL  VCEAME**Y**LEGNNF  GCPPAV**Y**EVMKNC  LLTIDR**Y**LAIVHA  VIGRGS**Y**AKVLLV  KKTDRI**Y**AMKVVK  LFFVIE**Y**VNGGDL  AKAVDG**Y**VKPQIK  LERGQE**Y**LILEKN  KYGNEG**Y**IPSNYV  YVLDDQ**Y**TSSSGA  NLFVAL**Y**DFVASG  GWVPSN**Y**ITPVNS  LEKHSW**Y**HGPVSR  SRSAAE**Y**LLSSLI  RYEGRV**Y**HYRINT  TADGKV**Y**VTAESR  LVTTLH**Y**PAPKCN  CNKPTV**Y**GVSPIH  KLGGGQ**Y**GEVYVG  GQYGEV**Y**VGVWKK  VGVWKK**Y**SLTVAV  LMTGDT**Y**TAHAGA  GCPPKV**Y**ELMRAC  SSSVVP**Y**LPRLPI  NQPHKK**Y**ELTGNF  LVPPKC**Y**GGSFAQ  ETSKLI**Y**DFIEDQ  LNNGKF**Y**VGVCAF  KADGLI**Y**CLKEAC  TLNSDG**Y**TPEPAR  PMDTSV**Y**ESPYSD  SVYESP**Y**SDPEEL  LGADDS**Y**YTARSA  GADDSY**Y**TARSAG  REEAED**Y**LVQGGM  LRQSRN**Y**LGGFAL  ENLIRE**Y**VKQTWN  ISRIKS**Y**SFPKPG  TVSFNP**Y**EPELAP  PMDTEV**Y**ESPYAD  EVYESP**Y**ADPEEI  IRPKEV**Y**LDRKLL  VSMGMK**Y**LEESNF  LLVTQH**Y**AKISDF  LRADEN**Y**YKAQTH  RADENY**Y**KAQTHG  APECIN**Y**YKFSSK  ELRLRN**Y**YYDVVN  LRLRNY**Y**YDVVNN  RLRNYY**Y**DVVNNN  GPEPGP**Y**AQPSVN  LQNGPI**Y**ARVIQK  KPLEGK**Y**EWQEVE  GSLPEF**Y**YRPPRP  SLPEFY**Y**RPPRPP  GLSMSS**Y**SVPRTP  RTSGGG**Y**TMHGLT  VDLGDG**Y**IMRRRR  LSEETP**Y**SYPTGN  QSSSPV**Y**GSSAKT  GEEEHV**Y**SFPNKQ  GALSPL**Y**GVPETN  HRNDSI**Y**EELQKH  AAALRV**Y**FQQQQQ  GEPNVS**Y**ICSRYY  FMDDDS**Y**SHHSGL  HHSGLE**Y**ADPEKF  STSLAQ**Y**DSNSKK  SNSKKI**Y**GSQPNF  YVLDDQ**Y**VSSVGT  SLISSD**Y**ELLSDP  LRLVME**Y**LPSGCL  ICKGME**Y**LGSRRC  LPLDKD**Y**YVVREP  PLDKDY**Y**VVREPG  LQERRK**Y**LKHRLI  ENDDDV**Y**RSLEEL  DLGEDI**Y**DCVPCE  DGGDDI**Y**EDIIKV  SFMMTP**Y**VVTRYY  YIDPFT**Y**EDPNEA  GNADLA**Y**ILSMEP  KKFKVE**Y**HAGGST  KAQQGL**Y**QVPGPS  PGPQDI**Y**DVPPVR  PPAEDV**Y**DVPPPA  PPAPDL**Y**DVPPGL  VVDSGV**Y**AVPPPA  RYGTCI**Y**QGRLWA  KDKANR**Y**FSPNFK  TAGQED**Y**DRLRPL  HRQGHI**Y**MEMNFT  TRDETN**Y**GIPQRA  NEGKHL**Y**TLDGGD  ESEDPD**Y**YQYNIQ  TIEDDD**Y**SPPSKR  SHVEDL**Y**VEGLPE  AHPNDL**Y**VEGLPE  IKRMKE**Y**ASKDDH  SRGQEV**Y**VKKTMG  LEERKT**Y**VNTTLY  YVNTTL**Y**EKFTYA  MMSGGK**Y**VDSEGH  DSEGHL**Y**TVPIRE  REQGNI**Y**KPNNKA  ETTVEL**Y**SLAERC  DILDRE**Y**YSVQQH  ILDREY**Y**SVQQHR  ALLGDH**Y**VQLPAT  VQLPAT**Y**MNLGPS  DSHPVL**Y**QSLKDL  MMMAAA**Y**LDPNLN  VSETDD**Y**AEIIDE  IDEEDT**Y**TMPSTR  DFGLSR**Y**MEDSTY  YMEDST**Y**YKASKG  MEDSTY**Y**KASKGK  IGNQHI**Y**QPVGKP  RSNDKV**Y**ENVTGL  TFDAHI**Y**EGRVIQ  KQAKIH**Y**IKNHEF  SEPVGI**Y**QGFEKK  QDNSGT**Y**GKIWEG  LKGRGE**Y**FAIKAL  RVDTPH**Y**PRWITK  VHKLSY**Y**EYDFER  KKVVAL**Y**DYMPMN  STPQSQ**Y**YLAEKH  IPELIN**Y**HQHNSA  YVLDDE**Y**TSSVGS  AQGLRL**Y**RPHLAS  TVEAVA**Y**APKKEL  RPVKGA**Y**REHPYG  AYREHP**Y**GRYYYY  EHPYGR**Y**YYYYYY  PQNDDH**Y**VMQEHR  GVKKPT**Y**DPVSED  FASDPK**Y**ATPQVI  KVSSTH**Y**YLLPER  PSYLER**Y**QRFLRE  NFTNPV**Y**ATLYMG  PREPPP**Y**QEPRPR  LLSNPA**Y**RLLLAT  RLLLAT**Y**ARPPRG  QGPTIS**Y**PMLLHV  GMSRNL**Y**AGDYYR  NLYAGD**Y**YRVQGR  LYAGDY**Y**RVQGRA  GLSKKI**Y**SGDYYR  KIYSGD**Y**YRQGRI  IYSGDY**Y**RQGRIA  NQADVI**Y**VNTQLL  AFSGGL**Y**GGLPPT  EPKKKK**Y**AKEAWP  LDVLEF**Y**NSKKTS  TSNSQK**Y**MSFTDK  DKSAED**Y**NSSNAL  EHTKSV**Y**TRSVIE  GASGTV**Y**TAMDVA  QEQYEL**Y**CEMGST  HLGSEE**Y**DVPPRL  EEDDDE**Y**KIPSSH  NRTSQD**Y**DQLPSC  ASLESL**Y**SACSMQ  VMLGAS**Y**WYRARL  KGPTCQ**Y**RAAQSG  KHINEV**Y**YAKKKR  KVYNDG**Y**DDDNYD  GYDDDN**Y**DYIVKN  EKWMDR**Y**EIDSLI  GQVVKA**Y**DRVEQE  HDTEMK**Y**YIVHLK  QLGQRI**Y**QYIQSR  GQRIYQ**Y**IQSRFY  GHTVAD**Y**LKFKDL  PAQDPL**Y**DVPNAS  IEVKKE**Y**LSSLQP  AHLGPK**Y**VGLWDF  GAVAQG**Y**VPHNYL  GYVPHN**Y**LAERET  EKPSAD**Y**VLSVRD  LIKEDV**Y**LSHDHN  HDHNIP**Y**KWTAPE  LSSFTS**Y**ENPTTT  PIPSVP**Y**APFAAI  YRSPPP**Y**VPPPPP  LPSSPV**Y**EDAASF  TEPEPV**Y**SMEAAD  SMEAAD**Y**REASSQ  YATEAV**Y**ESAEAP  PAEDST**Y**DEYEND  DSTYDE**Y**ENDLGI  SIESDI**Y**AEIPDE  YIEDED**Y**YKASVT  IEDEDY**Y**KASVTR  KPSRPK**Y**RPPPQT  KQMVED**Y**QWLRQE  SLDPMV**Y**MNDKSP  PEKEVG**Y**LEFTGP  RTDDLV**Y**LNVMEL  GRAGPS**Y**SMPVHA  PKGNKD**Y**HLQTCC  TASDKN**Y**VAGLID  GAYPYA**Y**PPMPAM  EDAKAI**Y**DFTDTV  RRLCDL**Y**YINSPE  RLCDLY**Y**INSPEL  FPECGF**Y**GMYDKI  EGWMVH**Y**TSKDTL  DTGSRY**Y**KEIPLS  TANVVY**Y**VGENVV  EGDEKE**Y**NADGGK  NIPPPI**Y**TSRARI  HSPPPA**Y**TPMSGN  ELDEEG**Y**MTPMRD  DKPKQE**Y**LNPVEE  ALDNPE**Y**HNASNG  PKAEDE**Y**VNEPLY  AFDNPD**Y**WNHSLP  TLQHPD**Y**LQEYST  VAENPE**Y**LSEFSL  LPSEQC**Y**QAPGGP  QDDVGV**Y**TCLVVN  EGSIEV**Y**EDAGSH  EDAGSH**Y**LCLLKA  NGQPIQ**Y**ARSTCE  SSRKSE**Y**LLPVAP  PWHAGQ**Y**EILLKN  GGGSDR**Y**GSLRPG  KEPEVD**Y**RTVTIN  ISEGVE**Y**IHKQGI  APEVIN**Y**EPIGYA  DFRQPR**Y**SSGGNF  RTEQDH**Y**ETDYTT  DHYETD**Y**TTGGES  TKGGVS**Y**RPAEVA  QGSNST**Y**DRIFPL  GMSRNL**Y**SGDYYR  NLYSGD**Y**YRIQGR  LYSGDY**Y**RIQGRA  MSEEVT**Y**ATLTFQ  ESATEL**Y**TEPTPE  KPEIQV**Y**ENIHLA  LSSENP**Y**ATIRDL  KDEGVV**Y**SVVHRT  SSQEVT**Y**AQLDHW  MAESIT**Y**AAVARH  HVGSRP**Y**TEFPFG  SKCSSV**Y**ENCLEQ  ETVISY**Y**TDMEEI  EEADAV**Y**ESMAHL  GCDEDL**Y**ESMAAF  AATEDL**Y**VEMLQA  GQEEDV**Y**HTVDDD  LPDNEP**Y**IFKVFA  ERPGNF**Y**VSSESI  RPQSSI**Y**DPFAGM  KVEFGV**Y**ESGPRK  LRASGI**Y**YVPKGK  VYYGQD**Y**RNKYKA  GSHISG**Y**ATLRRG  ESDDDS**Y**EVLDLT  KPDPKL**Y**TRSKPK  LEETKA**Y**TTQSLA  PTVPND**Y**MTSPAR  EKVVAI**Y**DYTKDK  DQAVTE**Y**VATRWY  DDFDSD**Y**ENPDEH  HSDSEM**Y**VMPAEE  ENADDS**Y**EPPPVE  LEDEAD**Y**VVPVED  EDNDEN**Y**IHPTES  IEWPGA**Y**PKLMEI  LNSHDL**Y**QKVAQE  KHHQDA**Y**IRIVLE  KLQNLS**Y**TEILKI  PKEPSN**Y**DFVYDC  HQRVYT**Y**IQSRFY  TTLSTL**Y**PSTSSL  MNEDPM**Y**SMYAKL  DPMYSM**Y**AKLQNQ  DQEAQI**Y**ELVAQT  ELQGLD**Y**CKPTRL  RRNDSI**Y**EASSLY  EEVGAG**Y**NSEDEY  FNQRFI**Y**GNQDLF  SQEELH**Y**ATLNFP  KVELET**Y**KQTRQG  QGLDEM**Y**SDVWKQ  IGSTLS**Y**FGLAAV  RIGDEL**Y**LEPLED  LPTHDG**Y**LPSNID  PERDSR**Y**SQPLHE  DSPPAL**Y**AEPLDS  QRKKPL**Y**WDLYEH  PLYWDL**Y**EHAQQQ  PKEDPI**Y**DEPEGL  VPPQGL**Y**DLPREP  RVKEEG**Y**ELPYNP  NPATDD**Y**AVPPPR  SHNSAL**Y**SQVQKS  NTTNRY**Y**KEIPLS  VVGTPA**Y**LAPEVL  FTGSRT**Y**SLVGQA  DDDANS**Y**ENVLIC  DEESED**Y**QNSASI  EDGEPD**Y**VNGEVA  KLHDSL**Y**NEDCTF  GAAFPA**Y**RAPHAG  LLKDDI**Y**SPSSSS  SPVVAE**Y**ARVQKR  AQVDVL**Y**SRVCKP  GPLENV**Y**ESIREL  DNDSHV**Y**AVIEDT  IKEPVQ**Y**FNSVEE  GKSELP**Y**EELWLE  HQKVYT**Y**IQSRFY  SPEYQD**Y**VYLEGT  GFDPSD**Y**AEPMDA  NEEENI**Y**SVPHDS  DSSPPP**Y**SSITVE  TSDTEV**Y**GEFYPV  EVYGEF**Y**PVPPPY  YPVPPP**Y**SVATSL  LTCMQH**Y**EEVQTS  LGMEGP**Y**EVLKDS  MVEDCL**Y**ETVKEI  KAEFAE**Y**ASVDRN  EEISAM**Y**SSVNKP  SSCNDL**Y**ATVKDF  EEPEPD**Y**EAIQTL  VPKEND**Y**ESISDL  TKDGWV**Y**YANHTE  SPTKND**Y**WAMLAY  KVDDCN**Y**AIKRIR  MQDEDG**Y**ITLNIK  QEDGKV**Y**INMPGR  EDSSID**Y**DLNLPD  TVTHED**Y**VTTRLL  RQRWGQ**Y**FRNMKR  VLPSHY**Y**ESFLEK  FLLDED**Y**EKVLGY  PNQEEN**Y**VTPIGD  DGPAVD**Y**ENQDVA  AAGDPE**Y**LEQPSR  PAESRI**Y**DEILQS  EPVNTV**Y**SEVQFA  GILTHL**Y**RDFDKC  SQIAVA**Y**HNIRDI  YNRKGK**Y**FNVERV  EEQLQL**Y**WAMDST  ELQSPE**Y**KLSKLR  STIMTD**Y**NPNYCF  TDYNPN**Y**CFAGKT  GAFGEV**Y**EGQVSG  GMARDI**Y**RASYYR  SLWNPT**Y**GSWFTE  APGAGH**Y**EDTILK  KDGLKF**Y**TDPSYF  ASDVTD**Y**SYPATP  PAQIIE**Y**YNPSGP  RRIAVE**Y**SDSDDD  KPPYTD**Y**VSTRWY  EFRDST**Y**DLPRSL  TDNEDV**Y**TFKTPS  TNSEDN**Y**VPMNPG  VEEVPL**Y**GNLHYL  AEEVMC**Y**TSLQLR  PGTPVK**Y**SEVVLD  GPEPEL**Y**ASVCAQ  SFPDQA**Y**ANSQPA  LPESTV**Y**GDVDLS  SGQPTP**Y**ATTQLI  EVAPVQ**Y**NIVEQN |

# (2) List of the 8,061 peptide samples in the negative subset $\mathbb{S}_{\boldsymbol{\xi=6}}^{\mathbf{-}}\boldsymbol{(}\mathbf{Y}\boldsymbol{)}$

| Sample # | Protein ID | Site | Sequences |
| --- | --- | --- | --- |
| 1  2  3  4  5  6  7  8  9  10  11  12  13  14  15  16  17  18  19  20  21  22  23  24  25  26  27  28  29  30  31  32  33  34  35  36  37  38  39  40  41  42  43  44  45  46  47  48  49  50  51  52  53  54  55  56  57  58  59  60  61  62  63  64  65  66  67  68  69  70  71  72  73  74  75  76  77  78  79  80  81  82  83  84  85  86  87  88  89  90  91  92  93  94  95  96  97  98  99  100  101  102  103  104  105  106  107  108  109  110  111  112  113  114  115  116  117  118  119  120  121  122  123  124  125  126  127  128  129  130  131  132  133  134  135  136  137  138  139  140  141  142  143  144  145  146  147  148  149  150  151  152  153  154  155  156  157  158  159  160  161  162  163  164  165  166  167  168  169  170  171  172  173  174  175  176  177  178  179  180  181  182  183  184  185  186  187  188  189  190  191  192  193  194  195  196  197  198  199  200  201  202  203  204  205  206  207  208  209  210  211  212  213  214  215  216  217  218  219  220  221  222  223  224  225  226  227  228  229  230  231  232  233  234  235  236  237  238  239  240  241  242  243  244  245  246  247  248  249  250  251  252  253  254  255  256  257  258  259  260  261  262  263  264  265  266  267  268  269  270  271  272  273  274  275  276  277  278  279  280  281  282  283  284  285  286  287  288  289  290  291  292  293  294  295  296  297  298  299  300  301  302  303  304  305  306  307  308  309  310  311  312  313  314  315  316  317  318  319  320  321  322  323  324  325  326  327  328  329  330  331  332  333  334  335  336  337  338  339  340  341  342  343  344  345  346  347  348  349  350  351  352  353  354  355  356  357  358  359  360  361  362  363  364  365  366  367  368  369  370  371  372  373  374  375  376  377  378  379  380  381  382  383  384  385  386  387  388  389  390  391  392  393  394  395  396  397  398  399  400  401  402  403  404  405  406  407  408  409  410  411  412  413  414  415  416  417  418  419  420  421  422  423  424  425  426  427  428  429  430  431  432  433  434  435  436  437  438  439  440  441  442  443  444  445  446  447  448  449  450  451  452  453  454  455  456  457  458  459  460  461  462  463  464  465  466  467  468  469  470  471  472  473  474  475  476  477  478  479  480  481  482  483  484  485  486  487  488  489  490  491  492  493  494  495  496  497  498  499  500  501  502  503  504  505  506  507  508  509  510  511  512  513  514  515  516  517  518  519  520  521  522  523  524  525  526  527  528  529  530  531  532  533  534  535  536  537  538  539  540  541  542  543  544  545  546  547  548  549  550  551  552  553  554  555  556  557  558  559  560  561  562  563  564  565  566  567  568  569  570  571  572  573  574  575  576  577  578  579  580  581  582  583  584  585  586  587  588  589  590  591  592  593  594  595  596  597  598  599  600  601  602  603  604  605  606  607  608  609  610  611  612  613  614  615  616  617  618  619  620  621  622  623  624  625  626  627  628  629  630  631  632  633  634  635  636  637  638  639  640  641  642  643  644  645  646  647  648  649  650  651  652  653  654  655  656  657  658  659  660  661  662  663  664  665  666  667  668  669  670  671  672  673  674  675  676  677  678  679  680  681  682  683  684  685  686  687  688  689  690  691  692  693  694  695  696  697  698  699  700  701  702  703  704  705  706  707  708  709  710  711  712  713  714  715  716  717  718  719  720  721  722  723  724  725  726  727  728  729  730  731  732  733  734  735  736  737  738  739  740  741  742  743  744  745  746  747  748  749  750  751  752  753  754  755  756  757  758  759  760  761  762  763  764  765  766  767  768  769  770  771  772  773  774  775  776  777  778  779  780  781  782  783  784  785  786  787  788  789  790  791  792  793  794  795  796  797  798  799  800  801  802  803  804  805  806  807  808  809  810  811  812  813  814  815  816  817  818  819  820  821  822  823  824  825  826  827  828  829  830  831  832  833  834  835  836  837  838  839  840  841  842  843  844  845  846  847  848  849  850  851  852  853  854  855  856  857  858  859  860  861  862  863  864  865  866  867  868  869  870  871  872  873  874  875  876  877  878  879  880  881  882  883  884  885  886  887  888  889  890  891  892  893  894  895  896  897  898  899  900  901  902  903  904  905  906  907  908  909  910  911  912  913  914  915  916  917  918  919  920  921  922  923  924  925  926  927  928  929  930  931  932  933  934  935  936  937  938  939  940  941  942  943  944  945  946  947  948  949  950  951  952  953  954  955  956  957  958  959  960  961  962  963  964  965  966  967  968  969  970  971  972  973  974  975  976  977  978  979  980  981  982  983  984  985  986  987  988  989  990  991  992  993  994  995  996  997  998  999  1000  1001  1002  1003  1004  1005  1006  1007  1008  1009  1010  1011  1012  1013  1014  1015  1016  1017  1018  1019  1020  1021  1022  1023  1024  1025  1026  1027  1028  1029  1030  1031  1032  1033  1034  1035  1036  1037  1038  1039  1040  1041  1042  1043  1044  1045  1046  1047  1048  1049  1050  1051  1052  1053  1054  1055  1056  1057  1058  1059  1060  1061  1062  1063  1064  1065  1066  1067  1068  1069  1070  1071  1072  1073  1074  1075  1076  1077  1078  1079  1080  1081  1082  1083  1084  1085  1086  1087  1088  1089  1090  1091  1092  1093  1094  1095  1096  1097  1098  1099  1100  1101  1102  1103  1104  1105  1106  1107  1108  1109  1110  1111  1112  1113  1114  1115  1116  1117  1118  1119  1120  1121  1122  1123  1124  1125  1126  1127  1128  1129  1130  1131  1132  1133  1134  1135  1136  1137  1138  1139  1140  1141  1142  1143  1144  1145  1146  1147  1148  1149  1150  1151  1152  1153  1154  1155  1156  1157  1158  1159  1160  1161  1162  1163  1164  1165  1166  1167  1168  1169  1170  1171  1172  1173  1174  1175  1176  1177  1178  1179  1180  1181  1182  1183  1184  1185  1186  1187  1188  1189  1190  1191  1192  1193  1194  1195  1196  1197  1198  1199  1200  1201  1202  1203  1204  1205  1206  1207  1208  1209  1210  1211  1212  1213  1214  1215  1216  1217  1218  1219  1220  1221  1222  1223  1224  1225  1226  1227  1228  1229  1230  1231  1232  1233  1234  1235  1236  1237  1238  1239  1240  1241  1242  1243  1244  1245  1246  1247  1248  1249  1250  1251  1252  1253  1254  1255  1256  1257  1258  1259  1260  1261  1262  1263  1264  1265  1266  1267  1268  1269  1270  1271  1272  1273  1274  1275  1276  1277  1278  1279  1280  1281  1282  1283  1284  1285  1286  1287  1288  1289  1290  1291  1292  1293  1294  1295  1296  1297  1298  1299  1300  1301  1302  1303  1304  1305  1306  1307  1308  1309  1310  1311  1312  1313  1314  1315  1316  1317  1318  1319  1320  1321  1322  1323  1324  1325  1326  1327  1328  1329  1330  1331  1332  1333  1334  1335  1336  1337  1338  1339  1340  1341  1342  1343  1344  1345  1346  1347  1348  1349  1350  1351  1352  1353  1354  1355  1356  1357  1358  1359  1360  1361  1362  1363  1364  1365  1366  1367  1368  1369  1370  1371  1372  1373  1374  1375  1376  1377  1378  1379  1380  1381  1382  1383  1384  1385  1386  1387  1388  1389  1390  1391  1392  1393  1394  1395  1396  1397  1398  1399  1400  1401  1402  1403  1404  1405  1406  1407  1408  1409  1410  1411  1412  1413  1414  1415  1416  1417  1418  1419  1420  1421  1422  1423  1424  1425  1426  1427  1428  1429  1430  1431  1432  1433  1434  1435  1436  1437  1438  1439  1440  1441  1442  1443  1444  1445  1446  1447  1448  1449  1450  1451  1452  1453  1454  1455  1456  1457  1458  1459  1460  1461  1462  1463  1464  1465  1466  1467  1468  1469  1470  1471  1472  1473  1474  1475  1476  1477  1478  1479  1480  1481  1482  1483  1484  1485  1486  1487  1488  1489  1490  1491  1492  1493  1494  1495  1496  1497  1498  1499  1500  1501  1502  1503  1504  1505  1506  1507  1508  1509  1510  1511  1512  1513  1514  1515  1516  1517  1518  1519  1520  1521  1522  1523  1524  1525  1526  1527  1528  1529  1530  1531  1532  1533  1534  1535  1536  1537  1538  1539  1540  1541  1542  1543  1544  1545  1546  1547  1548  1549  1550  1551  1552  1553  1554  1555  1556  1557  1558  1559  1560  1561  1562  1563  1564  1565  1566  1567  1568  1569  1570  1571  1572  1573  1574  1575  1576  1577  1578  1579  1580  1581  1582  1583  1584  1585  1586  1587  1588  1589  1590  1591  1592  1593  1594  1595  1596  1597  1598  1599  1600  1601  1602  1603  1604  1605  1606  1607  1608  1609  1610  1611  1612  1613  1614  1615  1616  1617  1618  1619  1620  1621  1622  1623  1624  1625  1626  1627  1628  1629  1630  1631  1632  1633  1634  1635  1636  1637  1638  1639  1640  1641  1642  1643  1644  1645  1646  1647  1648  1649  1650  1651  1652  1653  1654  1655  1656  1657  1658  1659  1660  1661  1662  1663  1664  1665  1666  1667  1668  1669  1670  1671  1672  1673  1674  1675  1676  1677  1678  1679  1680  1681  1682  1683  1684  1685  1686  1687  1688  1689  1690  1691  1692  1693  1694  1695  1696  1697  1698  1699  1700  1701  1702  1703  1704  1705  1706  1707  1708  1709  1710  1711  1712  1713  1714  1715  1716  1717  1718  1719  1720  1721  1722  1723  1724  1725  1726  1727  1728  1729  1730  1731  1732  1733  1734  1735  1736  1737  1738  1739  1740  1741  1742  1743  1744  1745  1746  1747  1748  1749  1750  1751  1752  1753  1754  1755  1756  1757  1758  1759  1760  1761  1762  1763  1764  1765  1766  1767  1768  1769  1770  1771  1772  1773  1774  1775  1776  1777  1778  1779  1780  1781  1782  1783  1784  1785  1786  1787  1788  1789  1790  1791  1792  1793  1794  1795  1796  1797  1798  1799  1800  1801  1802  1803  1804  1805  1806  1807  1808  1809  1810  1811  1812  1813  1814  1815  1816  1817  1818  1819  1820  1821  1822  1823  1824  1825  1826  1827  1828  1829  1830  1831  1832  1833  1834  1835  1836  1837  1838  1839  1840  1841  1842  1843  1844  1845  1846  1847  1848  1849  1850  1851  1852  1853  1854  1855  1856  1857  1858  1859  1860  1861  1862  1863  1864  1865  1866  1867  1868  1869  1870  1871  1872  1873  1874  1875  1876  1877  1878  1879  1880  1881  1882  1883  1884  1885  1886  1887  1888  1889  1890  1891  1892  1893  1894  1895  1896  1897  1898  1899  1900  1901  1902  1903  1904  1905  1906  1907  1908  1909  1910  1911  1912  1913  1914  1915  1916  1917  1918  1919  1920  1921  1922  1923  1924  1925  1926  1927  1928  1929  1930  1931  1932  1933  1934  1935  1936  1937  1938  1939  1940  1941  1942  1943  1944  1945  1946  1947  1948  1949  1950  1951  1952  1953  1954  1955  1956  1957  1958  1959  1960  1961  1962  1963  1964  1965  1966  1967  1968  1969  1970  1971  1972  1973  1974  1975  1976  1977  1978  1979  1980  1981  1982  1983  1984  1985  1986  1987  1988  1989  1990  1991  1992  1993  1994  1995  1996  1997  1998  1999  2000  2001  2002  2003  2004  2005  2006  2007  2008  2009  2010  2011  2012  2013  2014  2015  2016  2017  2018  2019  2020  2021  2022  2023  2024  2025  2026  2027  2028  2029  2030  2031  2032  2033  2034  2035  2036  2037  2038  2039  2040  2041  2042  2043  2044  2045  2046  2047  2048  2049  2050  2051  2052  2053  2054  2055  2056  2057  2058  2059  2060  2061  2062  2063  2064  2065  2066  2067  2068  2069  2070  2071  2072  2073  2074  2075  2076  2077  2078  2079  2080  2081  2082  2083  2084  2085  2086  2087  2088  2089  2090  2091  2092  2093  2094  2095  2096  2097  2098  2099  2100  2101  2102  2103  2104  2105  2106  2107  2108  2109  2110  2111  2112  2113  2114  2115  2116  2117  2118  2119  2120  2121  2122  2123  2124  2125  2126  2127  2128  2129  2130  2131  2132  2133  2134  2135  2136  2137  2138  2139  2140  2141  2142  2143  2144  2145  2146  2147  2148  2149  2150  2151  2152  2153  2154  2155  2156  2157  2158  2159  2160  2161  2162  2163  2164  2165  2166  2167  2168  2169  2170  2171  2172  2173  2174  2175  2176  2177  2178  2179  2180  2181  2182  2183  2184  2185  2186  2187  2188  2189  2190  2191  2192  2193  2194  2195  2196  2197  2198  2199  2200  2201  2202  2203  2204  2205  2206  2207  2208  2209  2210  2211  2212  2213  2214  2215  2216  2217  2218  2219  2220  2221  2222  2223  2224  2225  2226  2227  2228  2229  2230  2231  2232  2233  2234  2235  2236  2237  2238  2239  2240  2241  2242  2243  2244  2245  2246  2247  2248  2249  2250  2251  2252  2253  2254  2255  2256  2257  2258  2259  2260  2261  2262  2263  2264  2265  2266  2267  2268  2269  2270  2271  2272  2273  2274  2275  2276  2277  2278  2279  2280  2281  2282  2283  2284  2285  2286  2287  2288  2289  2290  2291  2292  2293  2294  2295  2296  2297  2298  2299  2300  2301  2302  2303  2304  2305  2306  2307  2308  2309  2310  2311  2312  2313  2314  2315  2316  2317  2318  2319  2320  2321  2322  2323  2324  2325  2326  2327  2328  2329  2330  2331  2332  2333  2334  2335  2336  2337  2338  2339  2340  2341  2342  2343  2344  2345  2346  2347  2348  2349  2350  2351  2352  2353  2354  2355  2356  2357  2358  2359  2360  2361  2362  2363  2364  2365  2366  2367  2368  2369  2370  2371  2372  2373  2374  2375  2376  2377  2378  2379  2380  2381  2382  2383  2384  2385  2386  2387  2388  2389  2390  2391  2392  2393  2394  2395  2396  2397  2398  2399  2400  2401  2402  2403  2404  2405  2406  2407  2408  2409  2410  2411  2412  2413  2414  2415  2416  2417  2418  2419  2420  2421  2422  2423  2424  2425  2426  2427  2428  2429  2430  2431  2432  2433  2434  2435  2436  2437  2438  2439  2440  2441  2442  2443  2444  2445  2446  2447  2448  2449  2450  2451  2452  2453  2454  2455  2456  2457  2458  2459  2460  2461  2462  2463  2464  2465  2466  2467  2468  2469  2470  2471  2472  2473  2474  2475  2476  2477  2478  2479  2480  2481  2482  2483  2484  2485  2486  2487  2488  2489  2490  2491  2492  2493  2494  2495  2496  2497  2498  2499  2500  2501  2502  2503  2504  2505  2506  2507  2508  2509  2510  2511  2512  2513  2514  2515  2516  2517  2518  2519  2520  2521  2522  2523  2524  2525  2526  2527  2528  2529  2530  2531  2532  2533  2534  2535  2536  2537  2538  2539  2540  2541  2542  2543  2544  2545  2546  2547  2548  2549  2550  2551  2552  2553  2554  2555  2556  2557  2558  2559  2560  2561  2562  2563  2564  2565  2566  2567  2568  2569  2570  2571  2572  2573  2574  2575  2576  2577  2578  2579  2580  2581  2582  2583  2584  2585  2586  2587  2588  2589  2590  2591  2592  2593  2594  2595  2596  2597  2598  2599  2600  2601  2602  2603  2604  2605  2606  2607  2608  2609  2610  2611  2612  2613  2614  2615  2616  2617  2618  2619  2620  2621  2622  2623  2624  2625  2626  2627  2628  2629  2630  2631  2632  2633  2634  2635  2636  2637  2638  2639  2640  2641  2642  2643  2644  2645  2646  2647  2648  2649  2650  2651  2652  2653  2654  2655  2656  2657  2658  2659  2660  2661  2662  2663  2664  2665  2666  2667  2668  2669  2670  2671  2672  2673  2674  2675  2676  2677  2678  2679  2680  2681  2682  2683  2684  2685  2686  2687  2688  2689  2690  2691  2692  2693  2694  2695  2696  2697  2698  2699  2700  2701  2702  2703  2704  2705  2706  2707  2708  2709  2710  2711  2712  2713  2714  2715  2716  2717  2718  2719  2720  2721  2722  2723  2724  2725  2726  2727  2728  2729  2730  2731  2732  2733  2734  2735  2736  2737  2738  2739  2740  2741  2742  2743  2744  2745  2746  2747  2748  2749  2750  2751  2752  2753  2754  2755  2756  2757  2758  2759  2760  2761  2762  2763  2764  2765  2766  2767  2768  2769  2770  2771  2772  2773  2774  2775  2776  2777  2778  2779  2780  2781  2782  2783  2784  2785  2786  2787  2788  2789  2790  2791  2792  2793  2794  2795  2796  2797  2798  2799  2800  2801  2802  2803  2804  2805  2806  2807  2808  2809  2810  2811  2812  2813  2814  2815  2816  2817  2818  2819  2820  2821  2822  2823  2824  2825  2826  2827  2828  2829  2830  2831  2832  2833  2834  2835  2836  2837  2838  2839  2840  2841  2842  2843  2844  2845  2846  2847  2848  2849  2850  2851  2852  2853  2854  2855  2856  2857  2858  2859  2860  2861  2862  2863  2864  2865  2866  2867  2868  2869  2870  2871  2872  2873  2874  2875  2876  2877  2878  2879  2880  2881  2882  2883  2884  2885  2886  2887  2888  2889  2890  2891  2892  2893  2894  2895  2896  2897  2898  2899  2900  2901  2902  2903  2904  2905  2906  2907  2908  2909  2910  2911  2912  2913  2914  2915  2916  2917  2918  2919  2920  2921  2922  2923  2924  2925  2926  2927  2928  2929  2930  2931  2932  2933  2934  2935  2936  2937  2938  2939  2940  2941  2942  2943  2944  2945  2946  2947  2948  2949  2950  2951  2952  2953  2954  2955  2956  2957  2958  2959  2960  2961  2962  2963  2964  2965  2966  2967  2968  2969  2970  2971  2972  2973  2974  2975  2976  2977  2978  2979  2980  2981  2982  2983  2984  2985  2986  2987  2988  2989  2990  2991  2992  2993  2994  2995  2996  2997  2998  2999  3000  3001  3002  3003  3004  3005  3006  3007  3008  3009  3010  3011  3012  3013  3014  3015  3016  3017  3018  3019  3020  3021  3022  3023  3024  3025  3026  3027  3028  3029  3030  3031  3032  3033  3034  3035  3036  3037  3038  3039  3040  3041  3042  3043  3044  3045  3046  3047  3048  3049  3050  3051  3052  3053  3054  3055  3056  3057  3058  3059  3060  3061  3062  3063  3064  3065  3066  3067  3068  3069  3070  3071  3072  3073  3074  3075  3076  3077  3078  3079  3080  3081  3082  3083  3084  3085  3086  3087  3088  3089  3090  3091  3092  3093  3094  3095  3096  3097  3098  3099  3100  3101  3102  3103  3104  3105  3106  3107  3108  3109  3110  3111  3112  3113  3114  3115  3116  3117  3118  3119  3120  3121  3122  3123  3124  3125  3126  3127  3128  3129  3130  3131  3132  3133  3134  3135  3136  3137  3138  3139  3140  3141  3142  3143  3144  3145  3146  3147  3148  3149  3150  3151  3152  3153  3154  3155  3156  3157  3158  3159  3160  3161  3162  3163  3164  3165  3166  3167  3168  3169  3170  3171  3172  3173  3174  3175  3176  3177  3178  3179  3180  3181  3182  3183  3184  3185  3186  3187  3188  3189  3190  3191  3192  3193  3194  3195  3196  3197  3198  3199  3200  3201  3202  3203  3204  3205  3206  3207  3208  3209  3210  3211  3212  3213  3214  3215  3216  3217  3218  3219  3220  3221  3222  3223  3224  3225  3226  3227  3228  3229  3230  3231  3232  3233  3234  3235  3236  3237  3238  3239  3240  3241  3242  3243  3244  3245  3246  3247  3248  3249  3250  3251  3252  3253  3254  3255  3256  3257  3258  3259  3260  3261  3262  3263  3264  3265  3266  3267  3268  3269  3270  3271  3272  3273  3274  3275  3276  3277  3278  3279  3280  3281  3282  3283  3284  3285  3286  3287  3288  3289  3290  3291  3292  3293  3294  3295  3296  3297  3298  3299  3300  3301  3302  3303  3304  3305  3306  3307  3308  3309  3310  3311  3312  3313  3314  3315  3316  3317  3318  3319  3320  3321  3322  3323  3324  3325  3326  3327  3328  3329  3330  3331  3332  3333  3334  3335  3336  3337  3338  3339  3340  3341  3342  3343  3344  3345  3346  3347  3348  3349  3350  3351  3352  3353  3354  3355  3356  3357  3358  3359  3360  3361  3362  3363  3364  3365  3366  3367  3368  3369  3370  3371  3372  3373  3374  3375  3376  3377  3378  3379  3380  3381  3382  3383  3384  3385  3386  3387  3388  3389  3390  3391  3392  3393  3394  3395  3396  3397  3398  3399  3400  3401  3402  3403  3404  3405  3406  3407  3408  3409  3410  3411  3412  3413  3414  3415  3416  3417  3418  3419  3420  3421  3422  3423  3424  3425  3426  3427  3428  3429  3430  3431  3432  3433  3434  3435  3436  3437  3438  3439  3440  3441  3442  3443  3444  3445  3446  3447  3448  3449  3450  3451  3452  3453  3454  3455  3456  3457  3458  3459  3460  3461  3462  3463  3464  3465  3466  3467  3468  3469  3470  3471  3472  3473  3474  3475  3476  3477  3478  3479  3480  3481  3482  3483  3484  3485  3486  3487  3488  3489  3490  3491  3492  3493  3494  3495  3496  3497  3498  3499  3500  3501  3502  3503  3504  3505  3506  3507  3508  3509  3510  3511  3512  3513  3514  3515  3516  3517  3518  3519  3520  3521  3522  3523  3524  3525  3526  3527  3528  3529  3530  3531  3532  3533  3534  3535  3536  3537  3538  3539  3540  3541  3542  3543  3544  3545  3546  3547  3548  3549  3550  3551  3552  3553  3554  3555  3556  3557  3558  3559  3560  3561  3562  3563  3564  3565  3566  3567  3568  3569  3570  3571  3572  3573  3574  3575  3576  3577  3578  3579  3580  3581  3582  3583  3584  3585  3586  3587  3588  3589  3590  3591  3592  3593  3594  3595  3596  3597  3598  3599  3600  3601  3602  3603  3604  3605  3606  3607  3608  3609  3610  3611  3612  3613  3614  3615  3616  3617  3618  3619  3620  3621  3622  3623  3624  3625  3626  3627  3628  3629  3630  3631  3632  3633  3634  3635  3636  3637  3638  3639  3640  3641  3642  3643  3644  3645  3646  3647  3648  3649  3650  3651  3652  3653  3654  3655  3656  3657  3658  3659  3660  3661  3662  3663  3664  3665  3666  3667  3668  3669  3670  3671  3672  3673  3674  3675  3676  3677  3678  3679  3680  3681  3682  3683  3684  3685  3686  3687  3688  3689  3690  3691  3692  3693  3694  3695  3696  3697  3698  3699  3700  3701  3702  3703  3704  3705  3706  3707  3708  3709  3710  3711  3712  3713  3714  3715  3716  3717  3718  3719  3720  3721  3722  3723  3724  3725  3726  3727  3728  3729  3730  3731  3732  3733  3734  3735  3736  3737  3738  3739  3740  3741  3742  3743  3744  3745  3746  3747  3748  3749  3750  3751  3752  3753  3754  3755  3756  3757  3758  3759  3760  3761  3762  3763  3764  3765  3766  3767  3768  3769  3770  3771  3772  3773  3774  3775  3776  3777  3778  3779  3780  3781  3782  3783  3784  3785  3786  3787  3788  3789  3790  3791  3792  3793  3794  3795  3796  3797  3798  3799  3800  3801  3802  3803  3804  3805  3806  3807  3808  3809  3810  3811  3812  3813  3814  3815  3816  3817  3818  3819  3820  3821  3822  3823  3824  3825  3826  3827  3828  3829  3830  3831  3832  3833  3834  3835  3836  3837  3838  3839  3840  3841  3842  3843  3844  3845  3846  3847  3848  3849  3850  3851  3852  3853  3854  3855  3856  3857  3858  3859  3860  3861  3862  3863  3864  3865  3866  3867  3868  3869  3870  3871  3872  3873  3874  3875  3876  3877  3878  3879  3880  3881  3882  3883  3884  3885  3886  3887  3888  3889  3890  3891  3892  3893  3894  3895  3896  3897  3898  3899  3900  3901  3902  3903  3904  3905  3906  3907  3908  3909  3910  3911  3912  3913  3914  3915  3916  3917  3918  3919  3920  3921  3922  3923  3924  3925  3926  3927  3928  3929  3930  3931  3932  3933  3934  3935  3936  3937  3938  3939  3940  3941  3942  3943  3944  3945  3946  3947  3948  3949  3950  3951  3952  3953  3954  3955  3956  3957  3958  3959  3960  3961  3962  3963  3964  3965  3966  3967  3968  3969  3970  3971  3972  3973  3974  3975  3976  3977  3978  3979  3980  3981  3982  3983  3984  3985  3986  3987  3988  3989  3990  3991  3992  3993  3994  3995  3996  3997  3998  3999  4000  4001  4002  4003  4004  4005  4006  4007  4008  4009  4010  4011  4012  4013  4014  4015  4016  4017  4018  4019  4020  4021  4022  4023  4024  4025  4026  4027  4028  4029  4030  4031  4032  4033  4034  4035  4036  4037  4038  4039  4040  4041  4042  4043  4044  4045  4046  4047  4048  4049  4050  4051  4052  4053  4054  4055  4056  4057  4058  4059  4060  4061  4062  4063  4064  4065  4066  4067  4068  4069  4070  4071  4072  4073  4074  4075  4076  4077  4078  4079  4080  4081  4082  4083  4084  4085  4086  4087  4088  4089  4090  4091  4092  4093  4094  4095  4096  4097  4098  4099  4100  4101  4102  4103  4104  4105  4106  4107  4108  4109  4110  4111  4112  4113  4114  4115  4116  4117  4118  4119  4120  4121  4122  4123  4124  4125  4126  4127  4128  4129  4130  4131  4132  4133  4134  4135  4136  4137  4138  4139  4140  4141  4142  4143  4144  4145  4146  4147  4148  4149  4150  4151  4152  4153  4154  4155  4156  4157  4158  4159  4160  4161  4162  4163  4164  4165  4166  4167  4168  4169  4170  4171  4172  4173  4174  4175  4176  4177  4178  4179  4180  4181  4182  4183  4184  4185  4186  4187  4188  4189  4190  4191  4192  4193  4194  4195  4196  4197  4198  4199  4200  4201  4202  4203  4204  4205  4206  4207  4208  4209  4210  4211  4212  4213  4214  4215  4216  4217  4218  4219  4220  4221  4222  4223  4224  4225  4226  4227  4228  4229  4230  4231  4232  4233  4234  4235  4236  4237  4238  4239  4240  4241  4242  4243  4244  4245  4246  4247  4248  4249  4250  4251  4252  4253  4254  4255  4256  4257  4258  4259  4260  4261  4262  4263  4264  4265  4266  4267  4268  4269  4270  4271  4272  4273  4274  4275  4276  4277  4278  4279  4280  4281  4282  4283  4284  4285  4286  4287  4288  4289  4290  4291  4292  4293  4294  4295  4296  4297  4298  4299  4300  4301  4302  4303  4304  4305  4306  4307  4308  4309  4310  4311  4312  4313  4314  4315  4316  4317  4318  4319  4320  4321  4322  4323  4324  4325  4326  4327  4328  4329  4330  4331  4332  4333  4334  4335  4336  4337  4338  4339  4340  4341  4342  4343  4344  4345  4346  4347  4348  4349  4350  4351  4352  4353  4354  4355  4356  4357  4358  4359  4360  4361  4362  4363  4364  4365  4366  4367  4368  4369  4370  4371  4372  4373  4374  4375  4376  4377  4378  4379  4380  4381  4382  4383  4384  4385  4386  4387  4388  4389  4390  4391  4392  4393  4394  4395  4396  4397  4398  4399  4400  4401  4402  4403  4404  4405  4406  4407  4408  4409  4410  4411  4412  4413  4414  4415  4416  4417  4418  4419  4420  4421  4422  4423  4424  4425  4426  4427  4428  4429  4430  4431  4432  4433  4434  4435  4436  4437  4438  4439  4440  4441  4442  4443  4444  4445  4446  4447  4448  4449  4450  4451  4452  4453  4454  4455  4456  4457  4458  4459  4460  4461  4462  4463  4464  4465  4466  4467  4468  4469  4470  4471  4472  4473  4474  4475  4476  4477  4478  4479  4480  4481  4482  4483  4484  4485  4486  4487  4488  4489  4490  4491  4492  4493  4494  4495  4496  4497  4498  4499  4500  4501  4502  4503  4504  4505  4506  4507  4508  4509  4510  4511  4512  4513  4514  4515  4516  4517  4518  4519  4520  4521  4522  4523  4524  4525  4526  4527  4528  4529  4530  4531  4532  4533  4534  4535  4536  4537  4538  4539  4540  4541  4542  4543  4544  4545  4546  4547  4548  4549  4550  4551  4552  4553  4554  4555  4556  4557  4558  4559  4560  4561  4562  4563  4564  4565  4566  4567  4568  4569  4570  4571  4572  4573  4574  4575  4576  4577  4578  4579  4580  4581  4582  4583  4584  4585  4586  4587  4588  4589  4590  4591  4592  4593  4594  4595  4596  4597  4598  4599  4600  4601  4602  4603  4604  4605  4606  4607  4608  4609  4610  4611  4612  4613  4614  4615  4616  4617  4618  4619  4620  4621  4622  4623  4624  4625  4626  4627  4628  4629  4630  4631  4632  4633  4634  4635  4636  4637  4638  4639  4640  4641  4642  4643  4644  4645  4646  4647  4648  4649  4650  4651  4652  4653  4654  4655  4656  4657  4658  4659  4660  4661  4662  4663  4664  4665  4666  4667  4668  4669  4670  4671  4672  4673  4674  4675  4676  4677  4678  4679  4680  4681  4682  4683  4684  4685  4686  4687  4688  4689  4690  4691  4692  4693  4694  4695  4696  4697  4698  4699  4700  4701  4702  4703  4704  4705  4706  4707  4708  4709  4710  4711  4712  4713  4714  4715  4716  4717  4718  4719  4720  4721  4722  4723  4724  4725  4726  4727  4728  4729  4730  4731  4732  4733  4734  4735  4736  4737  4738  4739  4740  4741  4742  4743  4744  4745  4746  4747  4748  4749  4750  4751  4752  4753  4754  4755  4756  4757  4758  4759  4760  4761  4762  4763  4764  4765  4766  4767  4768  4769  4770  4771  4772  4773  4774  4775  4776  4777  4778  4779  4780  4781  4782  4783  4784  4785  4786  4787  4788  4789  4790  4791  4792  4793  4794  4795  4796  4797  4798  4799  4800  4801  4802  4803  4804  4805  4806  4807  4808  4809  4810  4811  4812  4813  4814  4815  4816  4817  4818  4819  4820  4821  4822  4823  4824  4825  4826  4827  4828  4829  4830  4831  4832  4833  4834  4835  4836  4837  4838  4839  4840  4841  4842  4843  4844  4845  4846  4847  4848  4849  4850  4851  4852  4853  4854  4855  4856  4857  4858  4859  4860  4861  4862  4863  4864  4865  4866  4867  4868  4869  4870  4871  4872  4873  4874  4875  4876  4877  4878  4879  4880  4881  4882  4883  4884  4885  4886  4887  4888  4889  4890  4891  4892  4893  4894  4895  4896  4897  4898  4899  4900  4901  4902  4903  4904  4905  4906  4907  4908  4909  4910  4911  4912  4913  4914  4915  4916  4917  4918  4919  4920  4921  4922  4923  4924  4925  4926  4927  4928  4929  4930  4931  4932  4933  4934  4935  4936  4937  4938  4939  4940  4941  4942  4943  4944  4945  4946  4947  4948  4949  4950  4951  4952  4953  4954  4955  4956  4957  4958  4959  4960  4961  4962  4963  4964  4965  4966  4967  4968  4969  4970  4971  4972  4973  4974  4975  4976  4977  4978  4979  4980  4981  4982  4983  4984  4985  4986  4987  4988  4989  4990  4991  4992  4993  4994  4995  4996  4997  4998  4999  5000  5001  5002  5003  5004  5005  5006  5007  5008  5009  5010  5011  5012  5013  5014  5015  5016  5017  5018  5019  5020  5021  5022  5023  5024  5025  5026  5027  5028  5029  5030  5031  5032  5033  5034  5035  5036  5037  5038  5039  5040  5041  5042  5043  5044  5045  5046  5047  5048  5049  5050  5051  5052  5053  5054  5055  5056  5057  5058  5059  5060  5061  5062  5063  5064  5065  5066  5067  5068  5069  5070  5071  5072  5073  5074  5075  5076  5077  5078  5079  5080  5081  5082  5083  5084  5085  5086  5087  5088  5089  5090  5091  5092  5093  5094  5095  5096  5097  5098  5099  5100  5101  5102  5103  5104  5105  5106  5107  5108  5109  5110  5111  5112  5113  5114  5115  5116  5117  5118  5119  5120  5121  5122  5123  5124  5125  5126  5127  5128  5129  5130  5131  5132  5133  5134  5135  5136  5137  5138  5139  5140  5141  5142  5143  5144  5145  5146  5147  5148  5149  5150  5151  5152  5153  5154  5155  5156  5157  5158  5159  5160  5161  5162  5163  5164  5165  5166  5167  5168  5169  5170  5171  5172  5173  5174  5175  5176  5177  5178  5179  5180  5181  5182  5183  5184  5185  5186  5187  5188  5189  5190  5191  5192  5193  5194  5195  5196  5197  5198  5199  5200  5201  5202  5203  5204  5205  5206  5207  5208  5209  5210  5211  5212  5213  5214  5215  5216  5217  5218  5219  5220  5221  5222  5223  5224  5225  5226  5227  5228  5229  5230  5231  5232  5233  5234  5235  5236  5237  5238  5239  5240  5241  5242  5243  5244  5245  5246  5247  5248  5249  5250  5251  5252  5253  5254  5255  5256  5257  5258  5259  5260  5261  5262  5263  5264  5265  5266  5267  5268  5269  5270  5271  5272  5273  5274  5275  5276  5277  5278  5279  5280  5281  5282  5283  5284  5285  5286  5287  5288  5289  5290  5291  5292  5293  5294  5295  5296  5297  5298  5299  5300  5301  5302  5303  5304  5305  5306  5307  5308  5309  5310  5311  5312  5313  5314  5315  5316  5317  5318  5319  5320  5321  5322  5323  5324  5325  5326  5327  5328  5329  5330  5331  5332  5333  5334  5335  5336  5337  5338  5339  5340  5341  5342  5343  5344  5345  5346  5347  5348  5349  5350  5351  5352  5353  5354  5355  5356  5357  5358  5359  5360  5361  5362  5363  5364  5365  5366  5367  5368  5369  5370  5371  5372  5373  5374  5375  5376  5377  5378  5379  5380  5381  5382  5383  5384  5385  5386  5387  5388  5389  5390  5391  5392  5393  5394  5395  5396  5397  5398  5399  5400  5401  5402  5403  5404  5405  5406  5407  5408  5409  5410  5411  5412  5413  5414  5415  5416  5417  5418  5419  5420  5421  5422  5423  5424  5425  5426  5427  5428  5429  5430  5431  5432  5433  5434  5435  5436  5437  5438  5439  5440  5441  5442  5443  5444  5445  5446  5447  5448  5449  5450  5451  5452  5453  5454  5455  5456  5457  5458  5459  5460  5461  5462  5463  5464  5465  5466  5467  5468  5469  5470  5471  5472  5473  5474  5475  5476  5477  5478  5479  5480  5481  5482  5483  5484  5485  5486  5487  5488  5489  5490  5491  5492  5493  5494  5495  5496  5497  5498  5499  5500  5501  5502  5503  5504  5505  5506  5507  5508  5509  5510  5511  5512  5513  5514  5515  5516  5517  5518  5519  5520  5521  5522  5523  5524  5525  5526  5527  5528  5529  5530  5531  5532  5533  5534  5535  5536  5537  5538  5539  5540  5541  5542  5543  5544  5545  5546  5547  5548  5549  5550  5551  5552  5553  5554  5555  5556  5557  5558  5559  5560  5561  5562  5563  5564  5565  5566  5567  5568  5569  5570  5571  5572  5573  5574  5575  5576  5577  5578  5579  5580  5581  5582  5583  5584  5585  5586  5587  5588  5589  5590  5591  5592  5593  5594  5595  5596  5597  5598  5599  5600  5601  5602  5603  5604  5605  5606  5607  5608  5609  5610  5611  5612  5613  5614  5615  5616  5617  5618  5619  5620  5621  5622  5623  5624  5625  5626  5627  5628  5629  5630  5631  5632  5633  5634  5635  5636  5637  5638  5639  5640  5641  5642  5643  5644  5645  5646  5647  5648  5649  5650  5651  5652  5653  5654  5655  5656  5657  5658  5659  5660  5661  5662  5663  5664  5665  5666  5667  5668  5669  5670  5671  5672  5673  5674  5675  5676  5677  5678  5679  5680  5681  5682  5683  5684  5685  5686  5687  5688  5689  5690  5691  5692  5693  5694  5695  5696  5697  5698  5699  5700  5701  5702  5703  5704  5705  5706  5707  5708  5709  5710  5711  5712  5713  5714  5715  5716  5717  5718  5719  5720  5721  5722  5723  5724  5725  5726  5727  5728  5729  5730  5731  5732  5733  5734  5735  5736  5737  5738  5739  5740  5741  5742  5743  5744  5745  5746  5747  5748  5749  5750  5751  5752  5753  5754  5755  5756  5757  5758  5759  5760  5761  5762  5763  5764  5765  5766  5767  5768  5769  5770  5771  5772  5773  5774  5775  5776  5777  5778  5779  5780  5781  5782  5783  5784  5785  5786  5787  5788  5789  5790  5791  5792  5793  5794  5795  5796  5797  5798  5799  5800  5801  5802  5803  5804  5805  5806  5807  5808  5809  5810  5811  5812  5813  5814  5815  5816  5817  5818  5819  5820  5821  5822  5823  5824  5825  5826  5827  5828  5829  5830  5831  5832  5833  5834  5835  5836  5837  5838  5839  5840  5841  5842  5843  5844  5845  5846  5847  5848  5849  5850  5851  5852  5853  5854  5855  5856  5857  5858  5859  5860  5861  5862  5863  5864  5865  5866  5867  5868  5869  5870  5871  5872  5873  5874  5875  5876  5877  5878  5879  5880  5881  5882  5883  5884  5885  5886  5887  5888  5889  5890  5891  5892  5893  5894  5895  5896  5897  5898  5899  5900  5901  5902  5903  5904  5905  5906  5907  5908  5909  5910  5911  5912  5913  5914  5915  5916  5917  5918  5919  5920  5921  5922  5923  5924  5925  5926  5927  5928  5929  5930  5931  5932  5933  5934  5935  5936  5937  5938  5939  5940  5941  5942  5943  5944  5945  5946  5947  5948  5949  5950  5951  5952  5953  5954  5955  5956  5957  5958  5959  5960  5961  5962  5963  5964  5965  5966  5967  5968  5969  5970  5971  5972  5973  5974  5975  5976  5977  5978  5979  5980  5981  5982  5983  5984  5985  5986  5987  5988  5989  5990  5991  5992  5993  5994  5995  5996  5997  5998  5999  6000  6001  6002  6003  6004  6005  6006  6007  6008  6009  6010  6011  6012  6013  6014  6015  6016  6017  6018  6019  6020  6021  6022  6023  6024  6025  6026  6027  6028  6029  6030  6031  6032  6033  6034  6035  6036  6037  6038  6039  6040  6041  6042  6043  6044  6045  6046  6047  6048  6049  6050  6051  6052  6053  6054  6055  6056  6057  6058  6059  6060  6061  6062  6063  6064  6065  6066  6067  6068  6069  6070  6071  6072  6073  6074  6075  6076  6077  6078  6079  6080  6081  6082  6083  6084  6085  6086  6087  6088  6089  6090  6091  6092  6093  6094  6095  6096  6097  6098  6099  6100  6101  6102  6103  6104  6105  6106  6107  6108  6109  6110  6111  6112  6113  6114  6115  6116  6117  6118  6119  6120  6121  6122  6123  6124  6125  6126  6127  6128  6129  6130  6131  6132  6133  6134  6135  6136  6137  6138  6139  6140  6141  6142  6143  6144  6145  6146  6147  6148  6149  6150  6151  6152  6153  6154  6155  6156  6157  6158  6159  6160  6161  6162  6163  6164  6165  6166  6167  6168  6169  6170  6171  6172  6173  6174  6175  6176  6177  6178  6179  6180  6181  6182  6183  6184  6185  6186  6187  6188  6189  6190  6191  6192  6193  6194  6195  6196  6197  6198  6199  6200  6201  6202  6203  6204  6205  6206  6207  6208  6209  6210  6211  6212  6213  6214  6215  6216  6217  6218  6219  6220  6221  6222  6223  6224  6225  6226  6227  6228  6229  6230  6231  6232  6233  6234  6235  6236  6237  6238  6239  6240  6241  6242  6243  6244  6245  6246  6247  6248  6249  6250  6251  6252  6253  6254  6255  6256  6257  6258  6259  6260  6261  6262  6263  6264  6265  6266  6267  6268  6269  6270  6271  6272  6273  6274  6275  6276  6277  6278  6279  6280  6281  6282  6283  6284  6285  6286  6287  6288  6289  6290  6291  6292  6293  6294  6295  6296  6297  6298  6299  6300  6301  6302  6303  6304  6305  6306  6307  6308  6309  6310  6311  6312  6313  6314  6315  6316  6317  6318  6319  6320  6321  6322  6323  6324  6325  6326  6327  6328  6329  6330  6331  6332  6333  6334  6335  6336  6337  6338  6339  6340  6341  6342  6343  6344  6345  6346  6347  6348  6349  6350  6351  6352  6353  6354  6355  6356  6357  6358  6359  6360  6361  6362  6363  6364  6365  6366  6367  6368  6369  6370  6371  6372  6373  6374  6375  6376  6377  6378  6379  6380  6381  6382  6383  6384  6385  6386  6387  6388  6389  6390  6391  6392  6393  6394  6395  6396  6397  6398  6399  6400  6401  6402  6403  6404  6405  6406  6407  6408  6409  6410  6411  6412  6413  6414  6415  6416  6417  6418  6419  6420  6421  6422  6423  6424  6425  6426  6427  6428  6429  6430  6431  6432  6433  6434  6435  6436  6437  6438  6439  6440  6441  6442  6443  6444  6445  6446  6447  6448  6449  6450  6451  6452  6453  6454  6455  6456  6457  6458  6459  6460  6461  6462  6463  6464  6465  6466  6467  6468  6469  6470  6471  6472  6473  6474  6475  6476  6477  6478  6479  6480  6481  6482  6483  6484  6485  6486  6487  6488  6489  6490  6491  6492  6493  6494  6495  6496  6497  6498  6499  6500  6501  6502  6503  6504  6505  6506  6507  6508  6509  6510  6511  6512  6513  6514  6515  6516  6517  6518  6519  6520  6521  6522  6523  6524  6525  6526  6527  6528  6529  6530  6531  6532  6533  6534  6535  6536  6537  6538  6539  6540  6541  6542  6543  6544  6545  6546  6547  6548  6549  6550  6551  6552  6553  6554  6555  6556  6557  6558  6559  6560  6561  6562  6563  6564  6565  6566  6567  6568  6569  6570  6571  6572  6573  6574  6575  6576  6577  6578  6579  6580  6581  6582  6583  6584  6585  6586  6587  6588  6589  6590  6591  6592  6593  6594  6595  6596  6597  6598  6599  6600  6601  6602  6603  6604  6605  6606  6607  6608  6609  6610  6611  6612  6613  6614  6615  6616  6617  6618  6619  6620  6621  6622  6623  6624  6625  6626  6627  6628  6629  6630  6631  6632  6633  6634  6635  6636  6637  6638  6639  6640  6641  6642  6643  6644  6645  6646  6647  6648  6649  6650  6651  6652  6653  6654  6655  6656  6657  6658  6659  6660  6661  6662  6663  6664  6665  6666  6667  6668  6669  6670  6671  6672  6673  6674  6675  6676  6677  6678  6679  6680  6681  6682  6683  6684  6685  6686  6687  6688  6689  6690  6691  6692  6693  6694  6695  6696  6697  6698  6699  6700  6701  6702  6703  6704  6705  6706  6707  6708  6709  6710  6711  6712  6713  6714  6715  6716  6717  6718  6719  6720  6721  6722  6723  6724  6725  6726  6727  6728  6729  6730  6731  6732  6733  6734  6735  6736  6737  6738  6739  6740  6741  6742  6743  6744  6745  6746  6747  6748  6749  6750  6751  6752  6753  6754  6755  6756  6757  6758  6759  6760  6761  6762  6763  6764  6765  6766  6767  6768  6769  6770  6771  6772  6773  6774  6775  6776  6777  6778  6779  6780  6781  6782  6783  6784  6785  6786  6787  6788  6789  6790  6791  6792  6793  6794  6795  6796  6797  6798  6799  6800  6801  6802  6803  6804  6805  6806  6807  6808  6809  6810  6811  6812  6813  6814  6815  6816  6817  6818  6819  6820  6821  6822  6823  6824  6825  6826  6827  6828  6829  6830  6831  6832  6833  6834  6835  6836  6837  6838  6839  6840  6841  6842  6843  6844  6845  6846  6847  6848  6849  6850  6851  6852  6853  6854  6855  6856  6857  6858  6859  6860  6861  6862  6863  6864  6865  6866  6867  6868  6869  6870  6871  6872  6873  6874  6875  6876  6877  6878  6879  6880  6881  6882  6883  6884  6885  6886  6887  6888  6889  6890  6891  6892  6893  6894  6895  6896  6897  6898  6899  6900  6901  6902  6903  6904  6905  6906  6907  6908  6909  6910  6911  6912  6913  6914  6915  6916  6917  6918  6919  6920  6921  6922  6923  6924  6925  6926  6927  6928  6929  6930  6931  6932  6933  6934  6935  6936  6937  6938  6939  6940  6941  6942  6943  6944  6945  6946  6947  6948  6949  6950  6951  6952  6953  6954  6955  6956  6957  6958  6959  6960  6961  6962  6963  6964  6965  6966  6967  6968  6969  6970  6971  6972  6973  6974  6975  6976  6977  6978  6979  6980  6981  6982  6983  6984  6985  6986  6987  6988  6989  6990  6991  6992  6993  6994  6995  6996  6997  6998  6999  7000  7001  7002  7003  7004  7005  7006  7007  7008  7009  7010  7011  7012  7013  7014  7015  7016  7017  7018  7019  7020  7021  7022  7023  7024  7025  7026  7027  7028  7029  7030  7031  7032  7033  7034  7035  7036  7037  7038  7039  7040  7041  7042  7043  7044  7045  7046  7047  7048  7049  7050  7051  7052  7053  7054  7055  7056  7057  7058  7059  7060  7061  7062  7063  7064  7065  7066  7067  7068  7069  7070  7071  7072  7073  7074  7075  7076  7077  7078  7079  7080  7081  7082  7083  7084  7085  7086  7087  7088  7089  7090  7091  7092  7093  7094  7095  7096  7097  7098  7099  7100  7101  7102  7103  7104  7105  7106  7107  7108  7109  7110  7111  7112  7113  7114  7115  7116  7117  7118  7119  7120  7121  7122  7123  7124  7125  7126  7127  7128  7129  7130  7131  7132  7133  7134  7135  7136  7137  7138  7139  7140  7141  7142  7143  7144  7145  7146  7147  7148  7149  7150  7151  7152  7153  7154  7155  7156  7157  7158  7159  7160  7161  7162  7163  7164  7165  7166  7167  7168  7169  7170  7171  7172  7173  7174  7175  7176  7177  7178  7179  7180  7181  7182  7183  7184  7185  7186  7187  7188  7189  7190  7191  7192  7193  7194  7195  7196  7197  7198  7199  7200  7201  7202  7203  7204  7205  7206  7207  7208  7209  7210  7211  7212  7213  7214  7215  7216  7217  7218  7219  7220  7221  7222  7223  7224  7225  7226  7227  7228  7229  7230  7231  7232  7233  7234  7235  7236  7237  7238  7239  7240  7241  7242  7243  7244  7245  7246  7247  7248  7249  7250  7251  7252  7253  7254  7255  7256  7257  7258  7259  7260  7261  7262  7263  7264  7265  7266  7267  7268  7269  7270  7271  7272  7273  7274  7275  7276  7277  7278  7279  7280  7281  7282  7283  7284  7285  7286  7287  7288  7289  7290  7291  7292  7293  7294  7295  7296  7297  7298  7299  7300  7301  7302  7303  7304  7305  7306  7307  7308  7309  7310  7311  7312  7313  7314  7315  7316  7317  7318  7319  7320  7321  7322  7323  7324  7325  7326  7327  7328  7329  7330  7331  7332  7333  7334  7335  7336  7337  7338  7339  7340  7341  7342  7343  7344  7345  7346  7347  7348  7349  7350  7351  7352  7353  7354  7355  7356  7357  7358  7359  7360  7361  7362  7363  7364  7365  7366  7367  7368  7369  7370  7371  7372  7373  7374  7375  7376  7377  7378  7379  7380  7381  7382  7383  7384  7385  7386  7387  7388  7389  7390  7391  7392  7393  7394  7395  7396  7397  7398  7399  7400  7401  7402  7403  7404  7405  7406  7407  7408  7409  7410  7411  7412  7413  7414  7415  7416  7417  7418  7419  7420  7421  7422  7423  7424  7425  7426  7427  7428  7429  7430  7431  7432  7433  7434  7435  7436  7437  7438  7439  7440  7441  7442  7443  7444  7445  7446  7447  7448  7449  7450  7451  7452  7453  7454  7455  7456  7457  7458  7459  7460  7461  7462  7463  7464  7465  7466  7467  7468  7469  7470  7471  7472  7473  7474  7475  7476  7477  7478  7479  7480  7481  7482  7483  7484  7485  7486  7487  7488  7489  7490  7491  7492  7493  7494  7495  7496  7497  7498  7499  7500  7501  7502  7503  7504  7505  7506  7507  7508  7509  7510  7511  7512  7513  7514  7515  7516  7517  7518  7519  7520  7521  7522  7523  7524  7525  7526  7527  7528  7529  7530  7531  7532  7533  7534  7535  7536  7537  7538  7539  7540  7541  7542  7543  7544  7545  7546  7547  7548  7549  7550  7551  7552  7553  7554  7555  7556  7557  7558  7559  7560  7561  7562  7563  7564  7565  7566  7567  7568  7569  7570  7571  7572  7573  7574  7575  7576  7577  7578  7579  7580  7581  7582  7583  7584  7585  7586  7587  7588  7589  7590  7591  7592  7593  7594  7595  7596  7597  7598  7599  7600  7601  7602  7603  7604  7605  7606  7607  7608  7609  7610  7611  7612  7613  7614  7615  7616  7617  7618  7619  7620  7621  7622  7623  7624  7625  7626  7627  7628  7629  7630  7631  7632  7633  7634  7635  7636  7637  7638  7639  7640  7641  7642  7643  7644  7645  7646  7647  7648  7649  7650  7651  7652  7653  7654  7655  7656  7657  7658  7659  7660  7661  7662  7663  7664  7665  7666  7667  7668  7669  7670  7671  7672  7673  7674  7675  7676  7677  7678  7679  7680  7681  7682  7683  7684  7685  7686  7687  7688  7689  7690  7691  7692  7693  7694  7695  7696  7697  7698  7699  7700  7701  7702  7703  7704  7705  7706  7707  7708  7709  7710  7711  7712  7713  7714  7715  7716  7717  7718  7719  7720  7721  7722  7723  7724  7725  7726  7727  7728  7729  7730  7731  7732  7733  7734  7735  7736  7737  7738  7739  7740  7741  7742  7743  7744  7745  7746  7747  7748  7749  7750  7751  7752  7753  7754  7755  7756  7757  7758  7759  7760  7761  7762  7763  7764  7765  7766  7767  7768  7769  7770  7771  7772  7773  7774  7775  7776  7777  7778  7779  7780  7781  7782  7783  7784  7785  7786  7787  7788  7789  7790  7791  7792  7793  7794  7795  7796  7797  7798  7799  7800  7801  7802  7803  7804  7805  7806  7807  7808  7809  7810  7811  7812  7813  7814  7815  7816  7817  7818  7819  7820  7821  7822  7823  7824  7825  7826  7827  7828  7829  7830  7831  7832  7833  7834  7835  7836  7837  7838  7839  7840  7841  7842  7843  7844  7845  7846  7847  7848  7849  7850  7851  7852  7853  7854  7855  7856  7857  7858  7859  7860  7861  7862  7863  7864  7865  7866  7867  7868  7869  7870  7871  7872  7873  7874  7875  7876  7877  7878  7879  7880  7881  7882  7883  7884  7885  7886  7887  7888  7889  7890  7891  7892  7893  7894  7895  7896  7897  7898  7899  7900  7901  7902  7903  7904  7905  7906  7907  7908  7909  7910  7911  7912  7913  7914  7915  7916  7917  7918  7919  7920  7921  7922  7923  7924  7925  7926  7927  7928  7929  7930  7931  7932  7933  7934  7935  7936  7937  7938  7939  7940  7941  7942  7943  7944  7945  7946  7947  7948  7949  7950  7951  7952  7953  7954  7955  7956  7957  7958  7959  7960  7961  7962  7963  7964  7965  7966  7967  7968  7969  7970  7971  7972  7973  7974  7975  7976  7977  7978  7979  7980  7981  7982  7983  7984  7985  7986  7987  7988  7989  7990  7991  7992  7993  7994  7995  7996  7997  7998  7999  8000  8001  8002  8003  8004  8005  8006  8007  8008  8009  8010  8011  8012  8013  8014  8015  8016  8017  8018  8019  8020  8021  8022  8023  8024  8025  8026  8027  8028  8029  8030  8031  8032  8033  8034  8035  8036  8037  8038  8039  8040  8041  8042  8043  8044  8045  8046  8047  8048  8049  8050  8051  8052  8053  8054  8055  8056  8057  8058  8059  8060  8061 | A6ND36  A6ND36  A6ND36  A6NHR9  A6NHR9  A6NHR9  A8K4G0  A8K4G0  A8K4G0  A8K4G0  O00151  O00151  O00151  O00159  O00159  O00159  O00161  O00161  O00165  O00165  O00168  O00168  O00194  O00194  O00194  O00204  O00204  O00204  O00213  O00213  O00213  O00213  O00213  O00213  O00213  O00213  O00231  O00231  O00231  O00232  O00232  O00232  O00257  O00257  O00257  O00264  O00264  O00264  O00267  O00267  O00267  O00273  O00273  O00273  O00303  O00303  O00303  O00330  O00330  O00330  O00391  O00391  O00391  O00399  O00399  O00399  O00401  O00401  O00401  O00401  O00401  O00401  O00401  O00410  O00410  O00410  O00418  O00418  O00418  O00429  O00429  O00429  O00443  O00443  O00443  O00459  O00459  O00459  O00459  O00459  O00459  O00459  O00459  O00459  O00459  O00459  O00459  O00459  O00459  O00459  O00459  O00459  O00459  O00459  O00459  O00459  O00459  O00560  O00560  O00560  O00562  O00562  O00562  O00571  O00571  O00571  O00622  O00622  O00622  O00763  O00763  O00763  O14492  O14492  O14492  O14492  O14492  O14492  O14492  O14493  O14493  O14493  O14493  O14493  O14519  O14519  O14519  O14607  O14607  O14607  O14625  O14653  O14653  O14654  O14654  O14654  O14654  O14654  O14654  O14654  O14654  O14654  O14654  O14654  O14654  O14654  O14654  O14654  O14654  O14654  O14654  O14654  O14654  O14654  O14654  O14654  O14654  O14672  O14672  O14672  O14713  O14713  O14713  O14718  O14718  O14718  O14744  O14744  O14744  O14745  O14745  O14745  O14746  O14746  O14746  O14746  O14746  O14746  O14746  O14746  O14746  O14746  O14746  O14746  O14746  O14746  O14746  O14746  O14746  O14746  O14746  O14746  O14746  O14746  O14746  O14746  O14757  O14757  O14757  O14777  O14777  O14777  O14791  O14791  O14791  O14818  O14818  O14818  O14818  O14818  O14818  O14818  O14827  O14827  O14827  O14920  O14920  O14920  O14936  O14936  O14936  O14950  O14950  O14950  O14958  O14958  O14958  O14965  O14965  O14965  O14974  O14974  O14974  O14979  O14979  O14979  O14994  O14994  O14994  O15055  O15055  O15055  O15084  O15084  O15084  O15105  O15105  O15105  O15151  O15151  O15151  O15160  O15160  O15160  O15162  O15162  O15162  O15162  O15162  O15162  O15162  O15162  O15162  O15169  O15169  O15169  O15232  O15232  O15232  O15240  O15240  O15240  O15259  O15259  O15259  O15259  O15259  O15259  O15259  O15259  O15259  O15259  O15259  O15264  O15264  O15264  O15264  O15264  O15264  O15264  O15264  O15264  O15264  O15264  O15264  O15264  O15264  O15269  O15269  O15269  O15269  O15269  O15269  O15269  O15269  O15269  O15269  O15269  O15269  O15269  O15269  O15287  O15287  O15287  O15294  O15294  O15294  O15304  O15304  O15350  O15350  O15350  O15350  O15350  O15350  O15350  O15350  O15350  O15350  O15350  O15350  O15350  O15350  O15350  O15350  O15350  O15350  O15357  O15357  O15357  O15357  O15357  O15357  O15357  O15357  O15357  O15357  O15357  O15357  O15357  O15357  O15357  O15357  O15357  O15357  O15357  O15357  O15357  O15357  O15357  O15357  O15372  O15372  O15372  O15382  O15382  O15382  O15400  O15400  O15400  O15455  O15455  O15455  O15455  O15455  O15455  O15455  O15455  O15455  O15455  O15455  O15455  O15455  O15455  O15455  O15455  O15516  O15516  O15516  O15530  O15530  O15530  O15530  O15530  O15530  O15530  O15530  O15530  O15530  O15530  O15530  O15530  O15530  O15530  O15530  O15530  O15530  O15530  O15530  O15547  O15547  O15547  O15547  O15547  O15547  O15547  O15547  O15547  O15547  O15547  O15547  O15554  O15554  O15554  O43156  O43156  O43156  O43164  O43164  O43164  O43175  O43175  O43175  O43255  O43255  O43255  O43257  O43257  O43257  O43264  O43264  O43264  O43294  O43294  O43294  O43294  O43318  O43318  O43318  O43324  O43324  O43324  O43353  O43353  O43353  O43353  O43353  O43353  O43353  O43353  O43353  O43353  O43353  O43353  O43353  O43390  O43390  O43390  O43432  O43432  O43432  O43463  O43463  O43463  O43490  O43490  O43490  O43491  O43491  O43491  O43493  O43493  O43493  O43521  O43521  O43521  O43524  O43524  O43524  O43525  O43525  O43525  O43526  O43526  O43526  O43541  O43541  O43541  O43548  O43548  O43548  O43561  O43561  O43561  O43561  O43602  O43602  O43602  O43633  O43663  O43663  O43663  O43683  O43683  O43683  O43760  O43760  O43760  O43768  O43768  O43768  O43809  O43809  O43809  O60256  O60256  O60256  O60260  O60260  O60260  O60285  O60285  O60285  O60331  O60331  O60331  O60331  O60331  O60331  O60331  O60331  O60331  O60331  O60331  O60331  O60331  O60331  O60331  O60331  O60331  O60331  O60331  O60331  O60331  O60331  O60343  O60343  O60343  O60493  O60493  O60493  O60566  O60566  O60566  O60602  O60602  O60602  O60602  O60602  O60602  O60602  O60602  O60602  O60602  O60602  O60602  O60602  O60602  O60602  O60602  O60602  O60602  O60602  O60602  O60602  O60602  O60602  O60602  O60610  O60610  O60610  O60658  O60658  O60658  O60674  O60674  O60674  O60674  O60674  O60674  O60674  O60674  O60674  O60674  O60674  O60674  O60674  O60674  O60674  O60674  O60674  O60674  O60674  O60674  O60674  O60674  O60674  O60674  O60678  O60678  O60678  O60711  O60711  O60711  O60711  O60711  O60711  O60711  O60711  O60716  O60716  O60716  O60716  O60716  O60716  O60716  O60716  O60716  O60716  O60716  O60716  O60716  O60716  O60716  O60716  O60716  O60716  O60716  O60716  O60716  O60716  O60763  O60763  O60763  O60832  O60832  O60832  O60841  O60841  O60841  O60869  O60870  O60870  O60870  O60885  O60885  O60885  O60928  O60928  O60928  O60934  O60934  O60934  O75030  O75030  O75030  O75044  O75044  O75044  O75056  O75056  O75116  O75116  O75116  O75116  O75116  O75116  O75116  O75116  O75116  O75116  O75116  O75116  O75116  O75116  O75116  O75116  O75116  O75116  O75116  O75116  O75116  O75116  O75116  O75116  O75143  O75143  O75143  O75151  O75151  O75151  O75154  O75154  O75154  O75155  O75155  O75155  O75179  O75179  O75179  O75348  O75348  O75367  O75367  O75367  O75381  O75381  O75381  O75410  O75410  O75410  O75449  O75449  O75449  O75496  O75496  O75496  O75530  O75530  O75530  O75531  O75533  O75533  O75533  O75563  O75563  O75563  O75563  O75563  O75563  O75563  O75563  O75563  O75563  O75563  O75563  O75563  O75563  O75569  O75569  O75569  O75581  O75581  O75581  O75582  O75582  O75582  O75636  O75636  O75636  O75689  O75689  O75689  O75716  O75716  O75716  O75716  O75716  O75716  O75716  O75807  O75807  O75807  O75807  O75821  O75821  O75821  O75822  O75822  O75822  O75915  O75915  O75915  O75925  O75925  O75925  O75934  O75934  O75934  O75943  O75943  O75943  O75995  O75995  O75995  O76021  O76021  O76021  O76024  O76024  O76024  O76061  O76061  O76094  O76094  O76094  O94760  O94760  O94760  O94763  O94763  O94763  O94811  O94811  O94811  O94832  O94832  O94832  O94875  O94875  O94875  O94915  O94915  O94915  O94916  O94916  O94916  O95069  O95069  O95069  O95071  O95071  O95071  O95084  O95084  O95084  O95235  O95235  O95235  O95251  O95251  O95251  O95267  O95267  O95267  O95278  O95278  O95278  O95297  O95297  O95297  O95297  O95297  O95297  O95297  O95297  O95373  O95373  O95373  O95453  O95453  O95453  O95470  O95470  O95470  O95477  O95477  O95477  O95551  O95551  O95551  O95630  O95630  O95630  O95633  O95633  O95633  O95644  O95644  O95644  O95714  O95714  O95714  O95777  O95777  O95785  O95785  O95785  O95786  O95786  O95786  O95810  O95810  O95810  O95835  O95835  O95835  O95863  O95863  O95863  O95866  O95866  O95972  O95972  O95972  O95997  O96013  O96013  O96013  O96017  O96017  O96017  O96019  O96019  O96019  O96033  O96033  P00167  P00167  P00167  P00325  P00325  P00325  P00367  P00367  P00367  P00439  P00439  P00439  P00450  P00450  P00450  P00451  P00451  P00451  P00480  P00480  P00480  P00491  P00491  P00491  P00492  P00492  P00492  P00558  P00558  P00558  P00568  P00568  P00568  P00734  P00734  P00734  P00736  P00736  P00736  P00740  P00740  P00740  P00742  P00742  P00742  P00747  P00747  P00747  P00749  P00749  P00749  P00813  P00813  P00813  P00915  P00915  P00915  P01008  P01008  P01008  P01009  P01009  P01009  P01019  P01019  P01019  P01024  P01024  P01024  P01033  P01033  P01033  P01034  P01034  P01034  P01042  P01042  P01042  P01100  P01100  P01100  P01100  P01100  P01106  P01106  P01106  P01112  P01112  P01112  P01148  P01185  P01185  P01189  P01189  P01189  P01236  P01236  P01236  P01241  P01241  P01241  P01258  P01258  P01266  P01266  P01266  P01275  P01275  P01275  P01282  P01282  P01282  P01286  P01286  P01303  P01303  P01303  P01350  P01579  P01579  P01579  P01591  P01591  P01591  P01700  P01700  P01700  P01744  P01744  P01744  P01772  P01772  P01772  P01814  P01814  P01814  P01825  P01825  P01825  P02008  P02008  P02511  P02511  P02545  P02545  P02545  P02647  P02647  P02647  P02649  P02649  P02649  P02652  P02652  P02652  P02671  P02671  P02671  P02675  P02675  P02675  P02679  P02679  P02679  P02686  P02686  P02686  P02730  P02730  P02730  P02730  P02730  P02730  P02730  P02730  P02730  P02730  P02730  P02730  P02730  P02730  P02730  P02730  P02730  P02730  P02741  P02741  P02741  P02745  P02745  P02745  P02746  P02746  P02746  P02751  P02751  P02751  P02763  P02763  P02763  P02765  P02765  P02765  P02766  P02766  P02766  P02768  P02768  P02768  P02771  P02771  P02771  P02786  P02786  P02786  P02788  P02788  P02788  P02792  P02792  P02792  P02808  P02808  P02808  P02814  P02814  P02814  P02818  P02818  P02818  P03372  P03372  P03372  P03372  P03372  P03372  P03372  P03372  P03372  P03372  P03372  P03372  P03372  P03372  P03372  P03372  P03372  P03372  P03372  P03372  P03372  P03372  P03950  P03950  P03950  P03956  P03956  P03956  P03956  P03956  P03956  P03956  P03956  P03956  P03956  P03956  P03956  P03956  P03956  P03956  P03956  P03956  P03956  P03956  P03956  P03956  P03999  P03999  P03999  P04001  P04001  P04001  P04004  P04004  P04004  P04070  P04070  P04070  P04075  P04075  P04075  P04083  P04083  P04083  P04083  P04083  P04083  P04083  P04083  P04083  P04083  P04090  P04090  P04114  P04114  P04114  P04150  P04150  P04150  P04179  P04179  P04179  P04181  P04181  P04181  P04183  P04183  P04183  P04198  P04198  P04198  P04264  P04264  P04264  P04406  P04406  P04406  P04424  P04424  P04424  P04626  P04626  P04626  P04626  P04626  P04626  P04626  P04626  P04626  P04626  P04626  P04626  P04626  P04626  P04626  P04626  P04626  P04626  P04626  P04626  P04626  P04626  P04626  P04626  P04629  P04629  P04629  P04629  P04629  P04629  P04629  P04629  P04632  P04632  P04632  P04637  P04637  P04637  P04745  P04745  P04745  P04792  P04792  P04792  P05060  P05060  P05060  P05060  P05060  P05060  P05060  P05060  P05060  P05060  P05060  P05060  P05060  P05060  P05060  P05060  P05060  P05060  P05060  P05060  P05067  P05067  P05067  P05067  P05067  P05067  P05067  P05067  P05067  P05067  P05067  P05067  P05067  P05067  P05067  P05067  P05067  P05067  P05090  P05090  P05090  P05106  P05106  P05106  P05106  P05106  P05106  P05106  P05106  P05106  P05106  P05106  P05106  P05106  P05106  P05106  P05106  P05106  P05106  P05106  P05106  P05106  P05106  P05106  P05106  P05107  P05107  P05107  P05109  P05109  P05109  P05161  P05161  P05164  P05164  P05164  P05231  P05231  P05231  P05386  P05412  P05412  P05412  P05451  P05451  P05451  P05455  P05455  P05455  P05546  P05546  P05546  P05549  P05549  P05549  P05771  P05771  P05771  P05783  P05783  P05783  P05813  P05813  P05813  P05814  P05814  P05814  P05997  P05997  P05997  P06213  P06213  P06213  P06213  P06213  P06213  P06213  P06213  P06213  P06213  P06213  P06213  P06213  P06213  P06213  P06213  P06213  P06213  P06213  P06213  P06213  P06213  P06213  P06213  P06276  P06276  P06276  P06307  P06307  P06396  P06396  P06396  P06396  P06396  P06396  P06396  P06396  P06396  P06396  P06396  P06396  P06396  P06396  P06396  P06396  P06396  P06400  P06400  P06400  P06401  P06401  P06401  P06702  P06730  P06730  P06730  P06733  P06733  P06733  P06737  P06737  P06737  P06744  P06744  P06744  P06746  P06746  P06746  P06748  P06748  P06748  P06850  P07101  P07101  P07101  P07108  P07108  P07108  P07195  P07195  P07195  P07199  P07199  P07199  P07204  P07204  P07204  P07225  P07225  P07225  P07305  P07305  P07305  P07311  P07332  P07332  P07332  P07332  P07332  P07332  P07332  P07332  P07332  P07332  P07332  P07332  P07332  P07332  P07332  P07332  P07332  P07332  P07332  P07333  P07333  P07333  P07333  P07333  P07333  P07333  P07333  P07333  P07333  P07333  P07333  P07333  P07333  P07333  P07333  P07333  P07333  P07333  P07333  P07333  P07333  P07333  P07333  P07358  P07358  P07358  P07359  P07359  P07359  P07360  P07360  P07360  P07492  P07492  P07550  P07550  P07550  P07550  P07550  P07550  P07550  P07550  P07550  P07550  P07550  P07550  P07550  P07737  P07737  P07737  P07741  P07741  P07741  P07766  P07766  P07766  P07766  P07766  P07766  P07766  P07766  P07766  P07814  P07814  P07814  P07900  P07900  P07900  P07910  P07910  P07910  P07947  P07947  P07947  P07947  P07947  P07947  P07947  P07947  P07947  P07947  P07947  P07947  P07947  P07947  P07947  P07947  P07947  P07947  P07947  P07949  P07949  P07949  P07949  P07949  P07949  P07949  P07949  P07949  P07949  P07949  P07949  P07949  P07949  P07949  P07949  P07949  P07949  P07949  P07949  P07949  P07949  P07949  P07949  P08047  P08047  P08047  P08069  P08069  P08069  P08069  P08069  P08069  P08069  P08069  P08069  P08069  P08069  P08069  P08069  P08069  P08069  P08069  P08069  P08069  P08069  P08069  P08069  P08069  P08069  P08069  P08151  P08151  P08151  P08195  P08195  P08195  P08397  P08397  P08397  P08493  P08493  P08493  P08514  P08514  P08514  P08559  P08559  P08559  P08567  P08567  P08567  P08581  P08581  P08581  P08581  P08581  P08581  P08581  P08581  P08581  P08581  P08581  P08581  P08581  P08581  P08581  P08581  P08581  P08581  P08581  P08581  P08581  P08581  P08581  P08581  P08582  P08582  P08582  P08697  P08697  P08697  P08709  P08709  P08709  P08833  P08833  P08833  P08833  P08842  P08842  P08842  P08865  P08865  P08865  P08865  P08865  P08865  P08949  P09012  P09012  P09012  P09038  P09038  P09038  P09038  P09038  P09038  P09211  P09211  P09211  P09211  P09211  P09211  P09211  P09211  P09211  P09211  P09382  P09382  P09417  P09417  P09493  P09493  P09493  P09603  P09603  P09603  P09619  P09619  P09619  P09619  P09619  P09619  P09619  P09619  P09619  P09619  P09619  P09619  P09619  P09619  P09619  P09619  P09619  P09619  P09619  P09619  P09619  P09619  P09619  P09619  P09693  P09693  P09693  P09871  P09871  P09871  P09874  P09874  P09874  P09917  P09917  P09917  P09958  P09958  P09958  P09960  P09960  P09960  P0C0L4  P0C0L4  P0C0L4  P0CG48  P10070  P10070  P10070  P10071  P10071  P10071  P10082  P10082  P10082  P10145  P10153  P10153  P10153  P10155  P10155  P10155  P10244  P10244  P10244  P10275  P10275  P10275  P10275  P10275  P10275  P10275  P10275  P10275  P10275  P10275  P10275  P10275  P10275  P10275  P10275  P10275  P10275  P10275  P10275  P10275  P10275  P10276  P10276  P10276  P10415  P10415  P10415  P10451  P10451  P10451  P10451  P10451  P10451  P10451  P10515  P10515  P10515  P10588  P10588  P10588  P10599  P10636  P10636  P10636  P10636  P10644  P10644  P10644  P10645  P10645  P10645  P10721  P10721  P10721  P10721  P10721  P10721  P10721  P10721  P10721  P10721  P10721  P10721  P10721  P10721  P10721  P10721  P10721  P10721  P10721  P10721  P10721  P10721  P10721  P10721  P10809  P10809  P10809  P10914  P10914  P10914  P10997  P11047  P11047  P11047  P11142  P11142  P11142  P11171  P11171  P11171  P11171  P11171  P11171  P11171  P11171  P11171  P11171  P11171  P11171  P11171  P11171  P11171  P11226  P11226  P11274  P11274  P11274  P11274  P11274  P11274  P11274  P11274  P11274  P11274  P11274  P11274  P11274  P11274  P11274  P11274  P11274  P11274  P11274  P11274  P11274  P11274  P11274  P11274  P11277  P11277  P11277  P11309  P11309  P11309  P11362  P11362  P11362  P11362  P11362  P11362  P11362  P11362  P11362  P11362  P11362  P11362  P11362  P11362  P11362  P11362  P11362  P11362  P11362  P11362  P11362  P11362  P11387  P11387  P11387  P11388  P11388  P11388  P11413  P11413  P11413  P11474  P11474  P11474  P11488  P11488  P11488  P11488  P11488  P11488  P11488  P11488  P11488  P11488  P11488  P11488  P11498  P11498  P11498  P11831  P11831  P11831  P11926  P11926  P11926  P12004  P12004  P12004  P12004  P12004  P12004  P12111  P12111  P12111  P12235  P12235  P12235  P12259  P12259  P12259  P12270  P12270  P12270  P12273  P12273  P12273  P12429  P12429  P12429  P12644  P12644  P12644  P12814  P12814  P12814  P12814  P12814  P12814  P12814  P12814  P12814  P12814  P12814  P12814  P12814  P12814  P12814  P12814  P12814  P12814  P12814  P12814  P12814  P12814  P12814  P12821  P12821  P12821  P12830  P12830  P12830  P12830  P12830  P12830  P12830  P12830  P12830  P12830  P12830  P12830  P12830  P12830  P12830  P12830  P12830  P12830  P12830  P12830  P12830  P12830  P12956  P12956  P12956  P13010  P13010  P13010  P13224  P13224  P13498  P13498  P13498  P13500  P13500  P13501  P13501  P13501  P13521  P13521  P13521  P13569  P13569  P13569  P13611  P13611  P13611  P13612  P13612  P13612  P13639  P13639  P13639  P13639  P13639  P13639  P13639  P13639  P13639  P13639  P13639  P13639  P13639  P13639  P13639  P13639  P13639  P13639  P13639  P13646  P13646  P13646  P13688  P13688  P13688  P13693  P13693  P13693  P13796  P13796  P13796  P13798  P13798  P13798  P13807  P13807  P13807  P14210  P14210  P14210  P14314  P14314  P14314  P14316  P14316  P14316  P14317  P14317  P14317  P14317  P14317  P14317  P14317  P14317  P14317  P14317  P14317  P14410  P14410  P14410  P14598  P14598  P14598  P14618  P14618  P14618  P14625  P14625  P14625  P14635  P14635  P14635  P14672  P14672  P14672  P14678  P14678  P14778  P14778  P14778  P14778  P14778  P14778  P14778  P14778  P14778  P14778  P14778  P14778  P14778  P14778  P14778  P14778  P14778  P14778  P14778  P14778  P14778  P14778  P14778  P14778  P14859  P14866  P14866  P14866  P15036  P15036  P15036  P15056  P15056  P15056  P15121  P15121  P15121  P15151  P15151  P15151  P15151  P15151  P15151  P15151  P15151  P15151  P15151  P15172  P15172  P15172  P15289  P15289  P15289  P15311  P15311  P15311  P15311  P15311  P15311  P15311  P15311  P15311  P15311  P15311  P15311  P15311  P15311  P15311  P15336  P15336  P15391  P15391  P15391  P15391  P15391  P15391  P15391  P15391  P15391  P15391  P15502  P15502  P15502  P15515  P15515  P15515  P15529  P15529  P15529  P15529  P15529  P15529  P15529  P15529  P15529  P15529  P15529  P15529  P15529  P15529  P15529  P15529  P15529  P15529  P15529  P15529  P15529  P15529  P15529  P15529  P15927  P15927  P15927  P15941  P15941  P15941  P15941  P15941  P15941  P15941  P15941  P15976  P15976  P15976  P16066  P16066  P16066  P16070  P16070  P16070  P16150  P16150  P16152  P16152  P16152  P16157  P16157  P16157  P16220  P16220  P16220  P16234  P16234  P16234  P16234  P16234  P16234  P16234  P16234  P16234  P16234  P16234  P16234  P16234  P16234  P16234  P16234  P16234  P16234  P16234  P16234  P16234  P16234  P16234  P16234  P16284  P16284  P16284  P16284  P16284  P16284  P16284  P16284  P16284  P16284  P16284  P16284  P16284  P16284  P16284  P16284  P16284  P16284  P16284  P16284  P16401  P16410  P16410  P16410  P16410  P16410  P16410  P16410  P16410  P16435  P16435  P16435  P16930  P16930  P16930  P17302  P17302  P17302  P17612  P17612  P17612  P17661  P17661  P17661  P17676  P17676  P17676  P17707  P17707  P17707  P17735  P17735  P17735  P17858  P17858  P17858  P17931  P17931  P17931  P17936  P17936  P17936  P17948  P17948  P17948  P17948  P17948  P17948  P17948  P17948  P17948  P17948  P17948  P17948  P17948  P17948  P17948  P17948  P17948  P17948  P17948  P17948  P17948  P17948  P17948  P17948  P17987  P17987  P17987  P18031  P18031  P18031  P18031  P18031  P18031  P18031  P18031  P18031  P18031  P18031  P18124  P18124  P18124  P18206  P18206  P18206  P18206  P18206  P18206  P18509  P18509  P18509  P18669  P18669  P18669  P18669  P18669  P18669  P18754  P18754  P18754  P18887  P18887  P18887  P19021  P19021  P19021  P19022  P19022  P19022  P19113  P19113  P19113  P19174  P19174  P19174  P19174  P19174  P19174  P19174  P19174  P19174  P19174  P19174  P19174  P19174  P19174  P19174  P19174  P19174  P19174  P19174  P19174  P19174  P19174  P19174  P19174  P19367  P19367  P19367  P19404  P19404  P19404  P19404  P19404  P19404  P19404  P19419  P19419  P19419  P19429  P19429  P19525  P19525  P19525  P19525  P19525  P19525  P19525  P19525  P19525  P19525  P19525  P19525  P19525  P19525  P19525  P19793  P19793  P19793  P19801  P19801  P19801  P19823  P19823  P19823  P19827  P19827  P19827  P19838  P19838  P19838  P20020  P20020  P20020  P20138  P20138  P20138  P20138  P20138  P20138  P20138  P20155  P20155  P20155  P20336  P20336  P20336  P20338  P20338  P20338  P20366  P20366  P20366  P20393  P20393  P20393  P20591  P20591  P20591  P20749  P20749  P20749  P20908  P20908  P20908  P20933  P20933  P20933  P21127  P21127  P21127  P21283  P21283  P21283  P21397  P21397  P21397  P21453  P21453  P21453  P21549  P21549  P21549  P21675  P21675  P21675  P21730  P21730  P21730  P21754  P21754  P21754  P21796  P21796  P21796  P21815  P21815  P21815  P21980  P21980  P21980  P22061  P22061  P22061  P22234  P22234  P22234  P22314  P22314  P22314  P22466  P22681  P22681  P22681  P22681  P22681  P22681  P22681  P22681  P22681  P22681  P22681  P22681  P22681  P22692  P22692  P22692  P22735  P22735  P22735  P22736  P22736  P22736  P22891  P22891  P22891  P22914  P22914  P22914  P23025  P23025  P23025  P23193  P23193  P23193  P23229  P23229  P23229  P23246  P23246  P23246  P23246  P23246  P23246  P23246  P23246  P23246  P23246  P23246  P23246  P23246  P23297  P23297  P23327  P23327  P23327  P23396  P23396  P23396  P23434  P23434  P23434  P23443  P23443  P23443  P23458  P23458  P23458  P23458  P23458  P23458  P23458  P23458  P23458  P23458  P23458  P23458  P23458  P23458  P23458  P23458  P23458  P23458  P23458  P23458  P23458  P23458  P23458  P23458  P23508  P23508  P23508  P23526  P23526  P23526  P23528  P23528  P23528  P23588  P23588  P23588  P23760  P23760  P23760  P24385  P24385  P24385  P24390  P24390  P24390  P24394  P24394  P24394  P24394  P24394  P24394  P24394  P24394  P24394  P24394  P24394  P24394  P24394  P24394  P24593  P24593  P24593  P24666  P24666  P24666  P24666  P24666  P24821  P24821  P24821  P24864  P24864  P24864  P24928  P24928  P24928  P25205  P25205  P25205  P25311  P25311  P25311  P25398  P25398  P25788  P25788  P25788  P25963  P25963  P25963  P25963  P25963  P25963  P25963  P26358  P26358  P26358  P26368  P26368  P26368  P26378  P26378  P26378  P26599  P26599  P26599  P26640  P26640  P26640  P26641  P26641  P26641  P26651  P26651  P26651  P27105  P27105  P27105  P27361  P27361  P27361  P27361  P27361  P27361  P27361  P27361  P27361  P27361  P27361  P27361  P27361  P27361  P27361  P27361  P27361  P27695  P27695  P27695  P27707  P27707  P27707  P27708  P27708  P27708  P27815  P27815  P27815  P27816  P27816  P27816  P28072  P28072  P28072  P28223  P28223  P28223  P28370  P28370  P28370  P28370  P28370  P28370  P28370  P28370  P28370  P28370  P28370  P28370  P28370  P28370  P28370  P28370  P28370  P28370  P28370  P28370  P28370  P28370  P28370  P28370  P28749  P28749  P28749  P29083  P29083  P29083  P29144  P29144  P29144  P29317  P29317  P29317  P29317  P29317  P29317  P29317  P29317  P29317  P29317  P29317  P29317  P29317  P29317  P29317  P29317  P29317  P29317  P29317  P29317  P29317  P29317  P29317  P29317  P29350  P29350  P29350  P29350  P29350  P29350  P29350  P29350  P29350  P29350  P29350  P29350  P29350  P29350  P29350  P29350  P29350  P29350  P29350  P29350  P29350  P29474  P29474  P29474  P29508  P29508  P29508  P29590  P29590  P29590  P29597  P29597  P29597  P29597  P29597  P29597  P29597  P29597  P29597  P29597  P29597  P29597  P29597  P29597  P29597  P29597  P29597  P29597  P29597  P29597  P29597  P29597  P29597  P29597  P29692  P29692  P29692  P30040  P30040  P30040  P30040  P30040  P30040  P30040  P30040  P30040  P30260  P30260  P30260  P30291  P30291  P30291  P30304  P30304  P30304  P30305  P30305  P30305  P30307  P30307  P30307  P30411  P30411  P30411  P30519  P30519  P30519  P30530  P30530  P30530  P30530  P30530  P30530  P30530  P30530  P30530  P30530  P30530  P30530  P30530  P30530  P30530  P30530  P30530  P30530  P30530  P30530  P30530  P30566  P30566  P30566  P30793  P30793  P30793  P30990  P30990  P30990  P31040  P31040  P31040  P31040  P31040  P31040  P31040  P31040  P31040  P31040  P31040  P31040  P31040  P31040  P31040  P31040  P31040  P31040  P31040  P31040  P31040  P31040  P31040  P31151  P31151  P31151  P31269  P31269  P31269  P31327  P31327  P31327  P31645  P31645  P31645  P31645  P31645  P31645  P31645  P31645  P31645  P31645  P31645  P31645  P31645  P31645  P31645  P31645  P31645  P31645  P31645  P31645  P31645  P31645  P31645  P31645  P31751  P31751  P31751  P31948  P31948  P31948  P31949  P31949  P31949  P31995  P31995  P31995  P31995  P31995  P31995  P31995  P32004  P32004  P32004  P33121  P33121  P33121  P33241  P33241  P33241  P33316  P33316  P33316  P33764  P33764  P33764  P33993  P33993  P33993  P34059  P34059  P34059  P34096  P34096  P34096  P34741  P34741  P34741  P34896  P34896  P34896  P34947  P34947  P34947  P34972  P34972  P34972  P34998  P34998  P34998  P35221  P35221  P35221  P35222  P35222  P35222  P35222  P35222  P35222  P35222  P35222  P35222  P35222  P35222  P35236  P35236  P35236  P35269  P35269  P35269  P35270  P35270  P35270  P35318  P35318  P35318  P35367  P35367  P35367  P35398  P35398  P35398  P35408  P35408  P35408  P35520  P35520  P35520  P35555  P35555  P35555  P35558  P35558  P35558  P35568  P35568  P35568  P35568  P35568  P35568  P35568  P35568  P35568  P35568  P35568  P35568  P35568  P35568  P35568  P35568  P35568  P35568  P35568  P35568  P35568  P35568  P35568  P35611  P35611  P35611  P35612  P35612  P35612  P35637  P35637  P35637  P35659  P35659  P35659  P35900  P35900  P35900  P35916  P35916  P35916  P35916  P35916  P35916  P35916  P35916  P35916  P35916  P35916  P35916  P35916  P35916  P35916  P35916  P35916  P35916  P35916  P35916  P35916  P35916  P35916  P35916  P35968  P35968  P35968  P35968  P35968  P35968  P35968  P35968  P35968  P35968  P35968  P35968  P35968  P35968  P35968  P35968  P35968  P35968  P35968  P35968  P35968  P35968  P35968  P35968  P36507  P36507  P36507  P36578  P36578  P36578  P36871  P36871  P36871  P36888  P36888  P36888  P36888  P36888  P36888  P36888  P36888  P36888  P36888  P36888  P36888  P36888  P36888  P36888  P36888  P36888  P36888  P36888  P36888  P36888  P36888  P36888  P36888  P36896  P36896  P36896  P36896  P36896  P36896  P36896  P36896  P36896  P36896  P36896  P36896  P36896  P36896  P36896  P36896  P36896  P36896  P36955  P36955  P36955  P37802  P37802  P37802  P37840  P38117  P38117  P38159  P38159  P38159  P38398  P38398  P38398  P38432  P38432  P38432  P38646  P38646  P38646  P38919  P38919  P38919  P38935  P38935  P38935  P38936  P38936  P39748  P39748  P39748  P40189  P40189  P40189  P40429  P40429  P40429  P40763  P40763  P40763  P40763  P40763  P40763  P40763  P40763  P40763  P40763  P40763  P40763  P40763  P40763  P40763  P40763  P40763  P40763  P40763  P40763  P40925  P40925  P40925  P40926  P40926  P40926  P41091  P41091  P41091  P41181  P41181  P41181  P41182  P41182  P41182  P41212  P41212  P41212  P41227  P41227  P41227  P41235  P41235  P41235  P41240  P41240  P41240  P41240  P41240  P41240  P41240  P41240  P41240  P41240  P41240  P41240  P41240  P41240  P41240  P41240  P41240  P41279  P41279  P41279  P41597  P41597  P41597  P41597  P41597  P41597  P41597  P41597  P41597  P41597  P41597  P41597  P41743  P41743  P41743  P41743  P41743  P41743  P41743  P41743  P41743  P41743  P41743  P41743  P41743  P41743  P41743  P42229  P42229  P42229  P42229  P42229  P42229  P42229  P42229  P42229  P42229  P42229  P42229  P42229  P42229  P42229  P42229  P42229  P42229  P42285  P42285  P42285  P42338  P42338  P42338  P42345  P42345  P42345  P42574  P42574  P42574  P42680  P42680  P42680  P42680  P42680  P42680  P42680  P42680  P42680  P42680  P42680  P42680  P42680  P42680  P42680  P42680  P42680  P42680  P42680  P42680  P42680  P42680  P42680  P42680  P42684  P42684  P42684  P42684  P42684  P42684  P42684  P42684  P42684  P42684  P42684  P42684  P42684  P42684  P42684  P42684  P42684  P42768  P42768  P42768  P42768  P42768  P42768  P42771  P42771  P42858  P42858  P42858  P43220  P43220  P43220  P43243  P43243  P43243  P43268  P43268  P43268  P43351  P43351  P43351  P43351  P43351  P43351  P43351  P43351  P43351  P43351  P43403  P43403  P43403  P43403  P43403  P43403  P43403  P43403  P43403  P43403  P43403  P43403  P43403  P43403  P43403  P43403  P43403  P43403  P43403  P43403  P43403  P43403  P43403  P43403  P43405  P43405  P43405  P43405  P43405  P43405  P43405  P43405  P43405  P43405  P43405  P43405  P43405  P43405  P43405  P43405  P43405  P43405  P43686  P43686  P43686  P45985  P45985  P45985  P46060  P46060  P46108  P46108  P46108  P46108  P46108  P46108  P46108  P46108  P46108  P46531  P46531  P46531  P46695  P46695  P46734  P46734  P46734  P46736  P46736  P46736  P46776  P46776  P46776  P46779  P46779  P46779  P46782  P46782  P46782  P46783  P46783  P46783  P46821  P46821  P46821  P46937  P46937  P46937  P46937  P46937  P46940  P46940  P46940  P47710  P47710  P47710  P47712  P47712  P47712  P47756  P47756  P47756  P47895  P47895  P47895  P47897  P47897  P47897  P47914  P47914  P48047  P48047  P48047  P48048  P48048  P48048  P48059  P48059  P48059  P48551  P48551  P48551  P48551  P48551  P48551  P48551  P48551  P48551  P48551  P48551  P48551  P48551  P48551  P48552  P48552  P48552  P48643  P48643  P48643  P48735  P48735  P48735  P48736  P48736  P48736  P49023  P49023  P49023  P49023  P49023  P49023  P49023  P49023  P49023  P49023  P49023  P49023  P49137  P49137  P49137  P49189  P49189  P49189  P49327  P49327  P49327  P49368  P49368  P49368  P49407  P49407  P49407  P49450  P49459  P49459  P49459  P49588  P49588  P49588  P49675  P49675  P49675  P49682  P49682  P49682  P49715  P49715  P49715  P49720  P49720  P49720  P49736  P49736  P49736  P49756  P49756  P49756  P49760  P49760  P49760  P49768  P49768  P49768  P49790  P49790  P49790  P49795  P49795  P49795  P49802  P49802  P49802  P49815  P49815  P49815  P49841  P49841  P49841  P49841  P49841  P49841  P49841  P49841  P49841  P49841  P49841  P49841  P49841  P49841  P49841  P49841  P49841  P50150  P50402  P50402  P50402  P50502  P50502  P50502  P50548  P50548  P50548  P50552  P50552  P50552  P50583  P50583  P50583  P50613  P50613  P50613  P50750  P50750  P50750  P50895  P50895  P50895  P50990  P50990  P50990  P51114  P51114  P51114  P51575  P51575  P51575  P51587  P51587  P51587  P51617  P51617  P51617  P51636  P51636  P51654  P51654  P51654  P51809  P51809  P51809  P51813  P51813  P51813  P51813  P51813  P51813  P51813  P51813  P51813  P51813  P51813  P51813  P51813  P51813  P51813  P51813  P51813  P51813  P51813  P51813  P51813  P51813  P51813  P51813  P51884  P51884  P51884  P51946  P51946  P51946  P51955  P51955  P51955  P51991  P51991  P51991  P52272  P52272  P52272  P52292  P52292  P52292  P52333  P52333  P52333  P52333  P52333  P52333  P52333  P52333  P52333  P52333  P52333  P52333  P52333  P52333  P52333  P52333  P52333  P52333  P52333  P52333  P52333  P52333  P52333  P52333  P52434  P52434  P52434  P52435  P52435  P52565  P52565  P52565  P52630  P52630  P52630  P52630  P52630  P52630  P52630  P52630  P52630  P52630  P52630  P52630  P52630  P52630  P52630  P52630  P52630  P52655  P52655  P52655  P52732  P52732  P52732  P52735  P52735  P52735  P52735  P52735  P52735  P52735  P52735  P52735  P52735  P52735  P52735  P52735  P52735  P52735  P52735  P52735  P52735  P52735  P52735  P52735  P52735  P52735  P52735  P52907  P52907  P52907  P52945  P52945  P52945  P53350  P53350  P53350  P53355  P53355  P53355  P53396  P53396  P53396  P53667  P53667  P53667  P53674  P53674  P53674  P53779  P53779  P53779  P53779  P53779  P53779  P53779  P53779  P53779  P53779  P53779  P53779  P53779  P53779  P53779  P53779  P53779  P53779  P53779  P53779  P53804  P53804  P53804  P53999  P53999  P54105  P54105  P54105  P54198  P54198  P54198  P54253  P54253  P54253  P54259  P54259  P54259  P54274  P54274  P54274  P54577  P54577  P54577  P54646  P54646  P54646  P54753  P54753  P54753  P54753  P54753  P54753  P54753  P54753  P54753  P54753  P54753  P54753  P54753  P54753  P54753  P54753  P54753  P54753  P54753  P54753  P54753  P54753  P54753  P54753  P54829  P54829  P54829  P55060  P55060  P55060  P55072  P55072  P55072  P55211  P55211  P55211  P55211  P55212  P55212  P55212  P55263  P55263  P55263  P55263  P55263  P55263  P55263  P55263  P55268  P55268  P55268  P55735  P55735  P55735  P55769  P55769  P55769  P55795  P55795  P55795  P55884  P55884  P55884  P56537  P56537  P56537  P56817  P56817  P56817  P56945  P56945  P56945  P56945  P56945  P56945  P56945  P56945  P56945  P56945  P56945  P56945  P56945  P56945  P56945  P56945  P56945  P56945  P56945  P56945  P56945  P56945  P56945  P57059  P57059  P57059  P57081  P57081  P57081  P58340  P58340  P58340  P59665  P59665  P59998  P59998  P59998  P60228  P60228  P60228  P60468  P60484  P60484  P60484  P60484  P60484  P60484  P60484  P60484  P60484  P60484  P60484  P60484  P60484  P60484  P60484  P60484  P60484  P60484  P60484  P60484  P60484  P60484  P60953  P60953  P60953  P60953  P60953  P60953  P61073  P61073  P61073  P61081  P61081  P61081  P61086  P61086  P61086  P61158  P61158  P61158  P61244  P61244  P61244  P61289  P61289  P61289  P61457  P61604  P61604  P61769  P61769  P61769  P61803  P61803  P61803  P61923  P61923  P61923  P61978  P61978  P61978  P62136  P62136  P62136  P62253  P62253  P62253  P62258  P62258  P62258  P62266  P62266  P62269  P62269  P62269  P62280  P62280  P62280  P62306  P62306  P62306  P62316  P62495  P62495  P62495  P62633  P62633  P62633  P62745  P62745  P62753  P62753  P62753  P62805  P62805  P62805  P62820  P62820  P62820  P62826  P62826  P62826  P62847  P62847  P62847  P62873  P62873  P62873  P62913  P62913  P62913  P62917  P62917  P62917  P62937  P62937  P62979  P62979  P62979  P62995  P62995  P62995  P63010  P63010  P63010  P63010  P63010  P63010  P63010  P63010  P63010  P63010  P63010  P63010  P63010  P63010  P63010  P63010  P63010  P63010  P63010  P63010  P63010  P63010  P63010  P63010  P63165  P63165  P63165  P63167  P63167  P63167  P63220  P63220  P63241  P63241  P63241  P63244  P63244  P63244  P63244  P63252  P63252  P63252  P63279  P63279  P63279  P63316  P63316  P67775  P67775  P67775  P67809  P67809  P67809  P67870  P67870  P67870  P68032  P68032  P68032  P68104  P68104  P68104  P68363  P68363  P68363  P68400  P68400  P68400  P68871  P68871  P78344  P78344  P78344  P78346  P78346  P78346  P78347  P78347  P78347  P78347  P78347  P78347  P78347  P78347  P78347  P78347  P78347  P78347  P78347  P78347  P78347  P78347  P78347  P78347  P78347  P78347  P78348  P78348  P78348  P78362  P78362  P78362  P78371  P78371  P78371  P78527  P78527  P78527  P78536  P78536  P78536  P78543  P78543  P78543  P80188  P80188  P80188  P80192  P80192  P80192  P82979  P84090  P84090  P84090  P98161  P98161  P98161  P98170  P98170  P98170  P98177  P98177  P98177  P98179  P98179  P98179  P99999  P99999  P99999  Q00059  Q00059  Q00059  Q00341  Q00341  Q00341  Q00534  Q00534  Q00534  Q00536  Q00536  Q00536  Q00610  Q00610  Q00610  Q00613  Q00613  Q00613  Q00653  Q00653  Q00653  Q00839  Q00839  Q00839  Q00987  Q00987  Q00987  Q01081  Q01081  Q01081  Q01094  Q01094  Q01094  Q01130  Q01130  Q01130  Q01469  Q01518  Q01518  Q01518  Q01804  Q01804  Q01804  Q01826  Q01826  Q01826  Q01844  Q01844  Q01844  Q01860  Q01860  Q01860  Q01954  Q01954  Q01954  Q01974  Q01974  Q01974  Q02156  Q02156  Q02156  Q02388  Q02388  Q02388  Q02447  Q02447  Q02447  Q02763  Q02763  Q02763  Q02763  Q02763  Q02763  Q02763  Q02763  Q02763  Q02763  Q02763  Q02763  Q02763  Q02763  Q02763  Q02763  Q02763  Q02763  Q02763  Q02763  Q02763  Q02763  Q02763  Q02763  Q02790  Q02790  Q02790  Q02818  Q02818  Q02818  Q02978  Q02978  Q02978  Q03135  Q03135  Q03135  Q03135  Q03135  Q03135  Q03393  Q03393  Q03393  Q03468  Q03468  Q03468  Q03721  Q03721  Q03721  Q04206  Q04206  Q04206  Q04323  Q04323  Q04323  Q04637  Q04637  Q04637  Q04759  Q04759  Q04759  Q04759  Q04759  Q04759  Q04759  Q04759  Q04759  Q04759  Q04759  Q04759  Q04759  Q04759  Q04760  Q04760  Q04760  Q04864  Q04864  Q04864  Q04912  Q04912  Q04912  Q04912  Q04912  Q04912  Q04912  Q04912  Q04912  Q04912  Q04912  Q04912  Q04912  Q04912  Q04912  Q04912  Q04912  Q04912  Q04912  Q04912  Q04912  Q04912  Q04912  Q04912  Q05066  Q05066  Q05066  Q05086  Q05086  Q05086  Q05086  Q05086  Q05086  Q05086  Q05086  Q05086  Q05086  Q05086  Q05086  Q05086  Q05086  Q05086  Q05086  Q05086  Q05086  Q05086  Q05086  Q05086  Q05086  Q05086  Q05086  Q05209  Q05209  Q05209  Q05315  Q05315  Q05315  Q05397  Q05397  Q05397  Q05397  Q05397  Q05397  Q05397  Q05397  Q05397  Q05397  Q05397  Q05397  Q05397  Q05397  Q05397  Q05397  Q05397  Q05397  Q05397  Q05397  Q05397  Q05397  Q05397  Q05397  Q05655  Q05655  Q05655  Q05655  Q05655  Q05655  Q05655  Q05655  Q05655  Q05655  Q05655  Q05655  Q05655  Q05655  Q05707  Q05707  Q05707  Q06187  Q06187  Q06187  Q06187  Q06187  Q06187  Q06187  Q06187  Q06187  Q06187  Q06187  Q06187  Q06187  Q06187  Q06187  Q06187  Q06187  Q06187  Q06187  Q06187  Q06187  Q06187  Q06187  Q06187  Q06413  Q06413  Q06413  Q06481  Q06481  Q06481  Q06609  Q06609  Q06609  Q06609  Q06609  Q06609  Q06609  Q06609  Q06609  Q06787  Q06787  Q06787  Q06828  Q06828  Q06828  Q06830  Q06830  Q06830  Q07065  Q07065  Q07065  Q07666  Q07666  Q07666  Q07666  Q07666  Q07666  Q07666  Q07666  Q07666  Q07666  Q07666  Q07666  Q07666  Q07666  Q07666  Q07666  Q07666  Q07666  Q07666  Q07817  Q07817  Q07817  Q07820  Q07820  Q07820  Q07866  Q07866  Q07866  Q07912  Q07912  Q07912  Q07912  Q07912  Q07912  Q07912  Q07912  Q07912  Q07912  Q07912  Q07912  Q07912  Q07912  Q07954  Q07954  Q07954  Q07954  Q07954  Q07954  Q07954  Q07954  Q07954  Q07954  Q07954  Q07954  Q07954  Q07954  Q07954  Q07954  Q07954  Q07954  Q07954  Q07954  Q07954  Q07954  Q07954  Q07954  Q07955  Q07955  Q07955  Q08050  Q08050  Q08050  Q08188  Q08188  Q08188  Q08345  Q08345  Q08345  Q08345  Q08345  Q08345  Q08345  Q08345  Q08345  Q08345  Q08345  Q08345  Q08345  Q08345  Q08345  Q08345  Q08345  Q08345  Q08345  Q08345  Q08345  Q08345  Q08426  Q08426  Q08426  Q08431  Q08431  Q08431  Q08495  Q08495  Q08495  Q08722  Q08722  Q08722  Q08945  Q08945  Q08945  Q08999  Q08999  Q08999  Q08J23  Q08J23  Q08J23  Q09028  Q09028  Q09028  Q09161  Q09161  Q09161  Q0VD86  Q0VD86  Q0VD86  Q12778  Q12778  Q12778  Q12792  Q12792  Q12792  Q12834  Q12834  Q12834  Q12841  Q12841  Q12841  Q12866  Q12866  Q12866  Q12866  Q12866  Q12866  Q12866  Q12866  Q12866  Q12866  Q12866  Q12866  Q12866  Q12866  Q12866  Q12866  Q12866  Q12866  Q12866  Q12866  Q12866  Q12866  Q12866  Q12866  Q12874  Q12874  Q12874  Q12888  Q12888  Q12888  Q12906  Q12906  Q12906  Q12933  Q12933  Q12933  Q12962  Q12962  Q12962  Q12968  Q12968  Q12968  Q12972  Q12972  Q12972  Q12972  Q12972  Q12972  Q13002  Q13002  Q13002  Q13042  Q13042  Q13042  Q13045  Q13045  Q13045  Q13049  Q13049  Q13049  Q13085  Q13085  Q13085  Q13098  Q13098  Q13098  Q13115  Q13115  Q13115  Q13153  Q13153  Q13153  Q13153  Q13153  Q13153  Q13153  Q13153  Q13153  Q13158  Q13163  Q13163  Q13163  Q13164  Q13164  Q13164  Q13188  Q13188  Q13188  Q13191  Q13191  Q13191  Q13191  Q13191  Q13191  Q13191  Q13191  Q13191  Q13191  Q13191  Q13191  Q13191  Q13191  Q13191  Q13191  Q13191  Q13191  Q13191  Q13217  Q13217  Q13217  Q13247  Q13247  Q13247  Q13257  Q13257  Q13257  Q13263  Q13263  Q13263  Q13283  Q13283  Q13283  Q13285  Q13285  Q13285  Q13303  Q13303  Q13303  Q13309  Q13309  Q13309  Q13310  Q13310  Q13310  Q13315  Q13315  Q13315  Q13322  Q13322  Q13322  Q13322  Q13322  Q13322  Q13322  Q13322  Q13322  Q13322  Q13322  Q13322  Q13330  Q13330  Q13330  Q13342  Q13342  Q13342  Q13363  Q13363  Q13363  Q13371  Q13371  Q13371  Q13404  Q13404  Q13404  Q13421  Q13421  Q13421  Q13422  Q13422  Q13422  Q13426  Q13426  Q13426  Q13444  Q13444  Q13444  Q13444  Q13444  Q13444  Q13444  Q13444  Q13444  Q13509  Q13509  Q13509  Q13526  Q13526  Q13526  Q13535  Q13535  Q13535  Q13542  Q13542  Q13546  Q13546  Q13546  Q13547  Q13547  Q13547  Q13554  Q13554  Q13554  Q13561  Q13561  Q13561  Q13568  Q13568  Q13568  Q13572  Q13572  Q13572  Q13573  Q13573  Q13573  Q13601  Q13601  Q13601  Q13614  Q13614  Q13614  Q13627  Q13627  Q13627  Q13627  Q13627  Q13627  Q13627  Q13627  Q13627  Q13627  Q13627  Q13627  Q13627  Q13627  Q13627  Q13627  Q13627  Q13627  Q13627  Q13627  Q13637  Q13637  Q13637  Q13671  Q13671  Q13671  Q13671  Q13671  Q13671  Q13671  Q13671  Q13671  Q13671  Q13671  Q13671  Q13671  Q13765  Q13765  Q13769  Q13769  Q13769  Q13769  Q13769  Q13769  Q13769  Q13769  Q13769  Q13769  Q13769  Q13769  Q13769  Q13769  Q13769  Q13769  Q13769  Q13769  Q13769  Q13769  Q13769  Q13769  Q13769  Q13769  Q13829  Q13829  Q13829  Q13882  Q13882  Q13882  Q13882  Q13882  Q13882  Q13882  Q13882  Q13882  Q13882  Q13884  Q13884  Q13884  Q13905  Q13905  Q13905  Q13905  Q13905  Q13905  Q13905  Q13905  Q13905  Q13905  Q13905  Q13905  Q13905  Q13905  Q13905  Q13905  Q13905  Q13905  Q13905  Q13905  Q13905  Q13905  Q13905  Q13905  Q13950  Q13950  Q13950  Q14118  Q14118  Q14118  Q14118  Q14118  Q14118  Q14118  Q14118  Q14118  Q14118  Q14118  Q14118  Q14118  Q14118  Q14141  Q14141  Q14141  Q14152  Q14152  Q14152  Q14155  Q14155  Q14155  Q14164  Q14164  Q14164  Q14194  Q14194  Q14194  Q14207  Q14207  Q14207  Q14242  Q14242  Q14242  Q14247  Q14247  Q14247  Q14247  Q14247  Q14247  Q14247  Q14247  Q14247  Q14247  Q14247  Q14247  Q14247  Q14247  Q14247  Q14247  Q14247  Q14247  Q14247  Q14247  Q14289  Q14289  Q14289  Q14289  Q14289  Q14289  Q14289  Q14289  Q14289  Q14289  Q14289  Q14289  Q14289  Q14289  Q14289  Q14289  Q14289  Q14289  Q14289  Q14289  Q14289  Q14289  Q14289  Q14289  Q14315  Q14315  Q14315  Q14344  Q14344  Q14344  Q14393  Q14393  Q14393  Q14457  Q14457  Q14457  Q14493  Q14493  Q14493  Q14498  Q14498  Q14498  Q14511  Q14511  Q14511  Q14524  Q14524  Q14524  Q14526  Q14526  Q14526  Q14527  Q14527  Q14527  Q14527  Q14527  Q14527  Q14527  Q14527  Q14527  Q14527  Q14527  Q14527  Q14527  Q14527  Q14527  Q14527  Q14527  Q14527  Q14527  Q14527  Q14653  Q14653  Q14653  Q14654  Q14654  Q14654  Q14671  Q14671  Q14671  Q14674  Q14674  Q14674  Q14676  Q14676  Q14676  Q14678  Q14678  Q14678  Q14680  Q14680  Q14680  Q14680  Q14680  Q14680  Q14680  Q14680  Q14680  Q14680  Q14680  Q14680  Q14680  Q14680  Q14680  Q14680  Q14680  Q14680  Q14680  Q14680  Q14680  Q14680  Q14680  Q14680  Q14681  Q14681  Q14681  Q14683  Q14683  Q14683  Q14686  Q14686  Q14686  Q14690  Q14690  Q14690  Q14694  Q14694  Q14694  Q14703  Q14703  Q14703  Q14739  Q14739  Q14739  Q14764  Q14764  Q14764  Q14766  Q14766  Q14766  Q14790  Q14790  Q14790  Q14814  Q14814  Q14814  Q14847  Q14847  Q14847  Q14865  Q14865  Q14865  Q14934  Q14934  Q14934  Q14974  Q14974  Q14974  Q14980  Q14980  Q14980  Q14994  Q14994  Q14994  Q15004  Q15004  Q15005  Q15005  Q15005  Q15007  Q15007  Q15007  Q15020  Q15020  Q15020  Q15024  Q15024  Q15024  Q15025  Q15025  Q15025  Q15025  Q15025  Q15025  Q15025  Q15025  Q15025  Q15027  Q15027  Q15027  Q15046  Q15046  Q15046  Q15050  Q15054  Q15054  Q15054  Q15056  Q15056  Q15056  Q15078  Q15078  Q15078  Q15080  Q15080  Q15080  Q15084  Q15084  Q15084  Q15118  Q15118  Q15118  Q15118  Q15118  Q15118  Q15118  Q15118  Q15118  Q15118  Q15118  Q15118  Q15118  Q15118  Q15118  Q15118  Q15118  Q15118  Q15118  Q15139  Q15139  Q15139  Q15139  Q15139  Q15139  Q15139  Q15139  Q15139  Q15139  Q15139  Q15139  Q15139  Q15139  Q15139  Q15139  Q15139  Q15139  Q15139  Q15139  Q15147  Q15147  Q15147  Q15149  Q15149  Q15149  Q15185  Q15185  Q15208  Q15208  Q15208  Q15287  Q15287  Q15287  Q15291  Q15291  Q15291  Q15293  Q15293  Q15293  Q15303  Q15303  Q15303  Q15303  Q15303  Q15303  Q15303  Q15303  Q15303  Q15303  Q15303  Q15303  Q15303  Q15303  Q15303  Q15303  Q15303  Q15303  Q15303  Q15303  Q15303  Q15303  Q15303  Q15303  Q15334  Q15334  Q15334  Q15370  Q15398  Q15398  Q15398  Q15424  Q15424  Q15424  Q15427  Q15427  Q15427  Q15428  Q15428  Q15428  Q15485  Q15485  Q15485  Q15545  Q15545  Q15545  Q15554  Q15554  Q15554  Q15637  Q15637  Q15637  Q15648  Q15648  Q15648  Q15653  Q15653  Q15653  Q15654  Q15654  Q15654  Q15654  Q15654  Q15654  Q15654  Q15654  Q15654  Q15654  Q15654  Q15654  Q15691  Q15691  Q15691  Q15717  Q15717  Q15717  Q15726  Q15746  Q15746  Q15746  Q15746  Q15746  Q15746  Q15746  Q15746  Q15746  Q15746  Q15746  Q15746  Q15746  Q15746  Q15746  Q15746  Q15746  Q15746  Q15746  Q15746  Q15746  Q15746  Q15750  Q15750  Q15750  Q15788  Q15788  Q15788  Q15796  Q15796  Q15796  Q15831  Q15831  Q15831  Q15835  Q15835  Q15835  Q15848  Q15848  Q15848  Q15907  Q15907  Q15907  Q15910  Q15910  Q15910  Q15942  Q15942  Q15942  Q16236  Q16236  Q16236  Q16270  Q16270  Q16270  Q16401  Q16401  Q16401  Q16512  Q16512  Q16512  Q16513  Q16513  Q16513  Q16518  Q16518  Q16518  Q16566  Q16566  Q16566  Q16581  Q16581  Q16581  Q16584  Q16584  Q16623  Q16623  Q16623  Q16625  Q16625  Q16625  Q16625  Q16625  Q16625  Q16625  Q16625  Q16625  Q16625  Q16625  Q16625  Q16625  Q16625  Q16625  Q16625  Q16625  Q16625  Q16625  Q16625  Q16625  Q16625  Q16625  Q16625  Q16637  Q16637  Q16637  Q16643  Q16643  Q16643  Q16655  Q16655  Q16655  Q16656  Q16656  Q16656  Q16658  Q16658  Q16658  Q16659  Q16659  Q16659  Q16665  Q16665  Q16665  Q16666  Q16666  Q16666  Q16695  Q16695  Q16695  Q16719  Q16719  Q16719  Q16773  Q16773  Q16773  Q16790  Q16790  Q16790  Q16790  Q16790  Q16832  Q16832  Q16832  Q16832  Q16832  Q16832  Q16832  Q16832  Q16832  Q16832  Q16832  Q16832  Q16832  Q16832  Q16832  Q16832  Q16832  Q16832  Q16832  Q16832  Q16832  Q16832  Q16832  Q16832  Q16853  Q16853  Q16853  Q16875  Q16875  Q16875  Q1KMD3  Q1KMD3  Q1KMD3  Q24JP5  Q24JP5  Q24JP5  Q2HXU8  Q2HXU8  Q2HXU8  Q2HXU8  Q2HXU8  Q2M2Z5  Q2M2Z5  Q2M2Z5  Q2NKX8  Q2NKX8  Q2NKX8  Q2PPJ7  Q2PPJ7  Q2PPJ7  Q2TAY7  Q2TAY7  Q2TAY7  Q2V2M9  Q2V2M9  Q2V2M9  Q3MHD2  Q3MHD2  Q3MHD2  Q3SXM5  Q3SXM5  Q3SXM5  Q3V6T2  Q3V6T2  Q3V6T2  Q4G163  Q4G163  Q4G163  Q53EL6  Q53EL6  Q53EL6  Q53ET0  Q53ET0  Q53ET0  Q53EZ4  Q53EZ4  Q53EZ4  Q53FA7  Q53FA7  Q53FA7  Q53GL7  Q53GL7  Q53GL7  Q53H47  Q53H47  Q53H47  Q53HL2  Q53HL2  Q53HL2  Q5FBB7  Q5FBB7  Q5FBB7  Q5FYB1  Q5FYB1  Q5FYB1  Q5JRA6  Q5JRA6  Q5JRA6  Q5JVF3  Q5JVF3  Q5JVF3  Q5KSL6  Q5KSL6  Q5KSL6  Q5KSL6  Q5KSL6  Q5KSL6  Q5KSL6  Q5KSL6  Q5KSL6  Q5KSL6  Q5KSL6  Q5KSL6  Q5KSL6  Q5KSL6  Q5KSL6  Q5KSL6  Q5KSL6  Q5KSL6  Q5KSL6  Q5KSL6  Q5KSL6  Q5SQ64  Q5SQ64  Q5SQ64  Q5SQ64  Q5SQ64  Q5T1C6  Q5T1C6  Q5T1C6  Q5TA31  Q5TAQ9  Q5TAQ9  Q5TAQ9  Q5VT25  Q5VT25  Q5VT25  Q5VTD9  Q5VTD9  Q5VTD9  Q5VWQ8  Q5VWQ8  Q5VWQ8  Q5VWZ2  Q5VWZ2  Q5VWZ2  Q5VY43  Q5VY43  Q5VY43  Q5VY43  Q5VY43  Q5VY43  Q5VY43  Q5VY43  Q5VY43  Q5VY43  Q5VY43  Q5VY43  Q5VY43  Q5VY43  Q5VY43  Q5VY43  Q5VY43  Q5VY43  Q5VY43  Q5VY43  Q5VY43  Q5VY43  Q5VY43  Q5VY43  Q5XUX0  Q5XUX0  Q5XUX0  Q684P5  Q684P5  Q684P5  Q69YN4  Q69YN4  Q69YN4  Q6AI12  Q6AI12  Q6AI12  Q6B0I6  Q6B0I6  Q6B0I6  Q6DN03  Q6DN03  Q6DN03  Q6DN72  Q6DN72  Q6DN72  Q6DN72  Q6DN72  Q6DN72  Q6DN72  Q6DN72  Q6DN72  Q6DN72  Q6GTX8  Q6GTX8  Q6GTX8  Q6GTX8  Q6GTX8  Q6GTX8  Q6JBY9  Q6NXS1  Q6NXS1  Q6NXS1  Q6NZI2  Q6NZI2  Q6NZI2  Q6P1J9  Q6P1J9  Q6P1J9  Q6P1N0  Q6P1N0  Q6P1N0  Q6P2Q9  Q6P2Q9  Q6P2Q9  Q6P5Z2  Q6P5Z2  Q6P5Z2  Q6P988  Q6P988  Q6P988  Q6PCB0  Q6PCB0  Q6PCB0  Q6PCB0  Q6PCB0  Q6PCB0  Q6PCB0  Q6PCD5  Q6PCD5  Q6PCD5  Q6PHR2  Q6PHR2  Q6PHR2  Q6PKG0  Q6PKG0  Q6PKG0  Q6Q788  Q6Q788  Q6Q788  Q6R327  Q6R327  Q6R327  Q6S5L8  Q6S5L8  Q6S5L8  Q6S5L8  Q6S5L8  Q6S5L8  Q6S5L8  Q6S5L8  Q6S5L8  Q6S5L8  Q6S5L8  Q6S5L8  Q6S5L8  Q6S5L8  Q6S5L8  Q6TGC4  Q6TGC4  Q6TGC4  Q6U736  Q6U736  Q6U736  Q6UUV9  Q6UUV9  Q6UUV9  Q6UWZ7  Q6UWZ7  Q6UWZ7  Q6WN34  Q6WN34  Q6WN34  Q6ZMU5  Q6ZMU5  Q6ZMU5  Q6ZN04  Q6ZN04  Q6ZN04  Q6ZSY5  Q6ZSY5  Q6ZSY5  Q6ZUJ8  Q6ZUJ8  Q6ZUJ8  Q6ZUJ8  Q6ZUJ8  Q6ZUJ8  Q6ZUJ8  Q6ZUJ8  Q6ZUJ8  Q6ZUJ8  Q6ZUJ8  Q6ZUJ8  Q6ZUJ8  Q70E73  Q70E73  Q70E73  Q70E73  Q70E73  Q70E73  Q70E73  Q70E73  Q70E73  Q70E73  Q70E73  Q70E73  Q70E73  Q70E73  Q70E73  Q70E73  Q70E73  Q70E73  Q70E73  Q70E73  Q70E73  Q70E73  Q70E73  Q712K3  Q712K3  Q712K3  Q71F23  Q71F23  Q71F23  Q71RC2  Q71RC2  Q71RC2  Q7KZF4  Q7KZF4  Q7KZF4  Q7L2H7  Q7L2H7  Q7L2H7  Q7L5D6  Q7L5D6  Q7L5D6  Q7L5Y1  Q7L5Y1  Q7L5Y1  Q7L804  Q7L804  Q7L804  Q7RTN6  Q7RTN6  Q7RTN6  Q7Z406  Q7Z406  Q7Z406  Q7Z417  Q7Z417  Q7Z417  Q7Z4W1  Q7Z5L9  Q7Z5L9  Q7Z5L9  Q7Z6J0  Q7Z6J0  Q7Z6J0  Q86T82  Q86T82  Q86T82  Q86U42  Q86U42  Q86U42  Q86UC2  Q86UC2  Q86UC2  Q86UP2  Q86UP2  Q86UP2  Q86UQ8  Q86UQ8  Q86UR1  Q86UR1  Q86UR1  Q86V48  Q86V48  Q86V48  Q86V81  Q86V81  Q86V81  Q86VP1  Q86VP1  Q86VP1  Q86W56  Q86W56  Q86W56  Q86WB0  Q86WB0  Q86WR7  Q86WV6  Q86WV6  Q86WV6  Q86X55  Q86X55  Q86X55  Q86XR7  Q86XR7  Q86XR7  Q86YS7  Q86YS7  Q86YS7  Q8IU85  Q8IU85  Q8IU85  Q8IUR7  Q8IUR7  Q8IUR7  Q8IVP5  Q8IVP5  Q8IVP5  Q8IVP5  Q8IVT2  Q8IVT2  Q8IVT2  Q8IW19  Q8IW19  Q8IW19  Q8IW41  Q8IW41  Q8IW41  Q8IWL1  Q8IWL1  Q8IWL1  Q8IWT6  Q8IWT6  Q8IWT6  Q8IX03  Q8IX03  Q8IX03  Q8IXJ6  Q8IXJ6  Q8IXJ6  Q8IY84  Q8IY84  Q8IY84  Q8IYA6  Q8IYA6  Q8IYA6  Q8IYA6  Q8IYA6  Q8IYA6  Q8IYA6  Q8IYA6  Q8IYA6  Q8IYA6  Q8IYA6  Q8IYA6  Q8IYB3  Q8IYB3  Q8IYB3  Q8IZP0  Q8IZP0  Q8IZP0  Q8IZP0  Q8IZP0  Q8IZP0  Q8IZP0  Q8IZP0  Q8IZP0  Q8IZP0  Q8IZP0  Q8IZP0  Q8IZP0  Q8IZP0  Q8IZP0  Q8IZP0  Q8IZP0  Q8IZP0  Q8IZS5  Q8IZS5  Q8IZS5  Q8N122  Q8N122  Q8N122  Q8N163  Q8N163  Q8N163  Q8N1G4  Q8N1G4  Q8N1G4  Q8N264  Q8N264  Q8N264  Q8N2E6  Q8N2E6  Q8N2E6  Q8N4F0  Q8N4F0  Q8N4F0  Q8N4N8  Q8N4N8  Q8N4N8  Q8N5C8  Q8N5C8  Q8N5C8  Q8N5Z0  Q8N5Z0  Q8N5Z0  Q8NB16  Q8NB16  Q8NB16  Q8NBJ4  Q8NBJ4  Q8NBJ4  Q8NBP7  Q8NBP7  Q8NBP7  Q8NBX0  Q8NBX0  Q8NBX0  Q8NCD3  Q8NCD3  Q8NCD3  Q8ND76  Q8ND76  Q8ND76  Q8NDC0  Q8NDC0  Q8NDC0  Q8NE71  Q8NE71  Q8NE71  Q8NFW8  Q8NFW8  Q8NFW8  Q8NG66  Q8NG66  Q8NG66  Q8NHV4  Q8NHV4  Q8NHV4  Q8NHW3  Q8NHW3  Q8NHW3  Q8TAE6  Q8TAE6  Q8TAE6  Q8TAP9  Q8TAP9  Q8TAP9  Q8TB45  Q8TB45  Q8TB45  Q8TCT0  Q8TCT0  Q8TCT0  Q8TD08  Q8TD08  Q8TD08  Q8TD08  Q8TD08  Q8TD08  Q8TD08  Q8TD08  Q8TD08  Q8TD08  Q8TD08  Q8TD30  Q8TD30  Q8TD30  Q8TDC3  Q8TDC3  Q8TDC3  Q8TEP8  Q8TEP8  Q8TEP8  Q8TEW0  Q8TEW0  Q8TEW0  Q8TEX9  Q8TEX9  Q8TEX9  Q8TF09  Q8TF09  Q8TF76  Q8TF76  Q8TF76  Q8WTQ7  Q8WTQ7  Q8WTQ7  Q8WU03  Q8WU03  Q8WU03  Q8WUI4  Q8WUI4  Q8WUI4  Q8WUM4  Q8WUM4  Q8WUM4  Q8WV28  Q8WV28  Q8WV28  Q8WV28  Q8WV28  Q8WV28  Q8WV28  Q8WVM8  Q8WVM8  Q8WVM8  Q8WWA1  Q8WWA1  Q8WWA1  Q8WWM7  Q8WWM7  Q8WWM7  Q8WX93  Q8WX93  Q8WX93  Q8WYJ6  Q8WYJ6  Q8WYJ6  Q8WYK2  Q8WYL5  Q8WYL5  Q8WYL5  Q92504  Q92504  Q92504  Q92522  Q92522  Q92522  Q92538  Q92538  Q92538  Q92556  Q92556  Q92556  Q92556  Q92556  Q92556  Q92556  Q92556  Q92556  Q92556  Q92556  Q92556  Q92563  Q92563  Q92563  Q92574  Q92574  Q92574  Q92597  Q92597  Q92597  Q92600  Q92600  Q92600  Q92616  Q92616  Q92616  Q92620  Q92620  Q92620  Q92625  Q92625  Q92625  Q92630  Q92630  Q92630  Q92630  Q92630  Q92630  Q92630  Q92630  Q92630  Q92630  Q92630  Q92630  Q92630  Q92630  Q92630  Q92630  Q92630  Q92673  Q92673  Q92673  Q92731  Q92731  Q92731  Q92734  Q92734  Q92734  Q92736  Q92736  Q92736  Q92783  Q92783  Q92783  Q92783  Q92783  Q92783  Q92783  Q92783  Q92783  Q92783  Q92783  Q92783  Q92793  Q92793  Q92793  Q92794  Q92794  Q92794  Q92804  Q92804  Q92804  Q92888  Q92888  Q92888  Q92888  Q92888  Q92888  Q92888  Q92900  Q92900  Q92900  Q92905  Q92905  Q92905  Q92918  Q92918  Q92918  Q92922  Q92922  Q92922  Q92934  Q92934  Q92945  Q92945  Q92945  Q92974  Q92974  Q92974  Q92979  Q92979  Q92979  Q92985  Q92985  Q92985  Q92993  Q92993  Q92993  Q92997  Q92997  Q92997  Q93009  Q93009  Q93009  Q93084  Q93084  Q93084  Q969H0  Q969H0  Q969H0  Q969M7  Q969M7  Q969M7  Q969Q0  Q969Q0  Q969Q0  Q969S2  Q969S2  Q969S2  Q969U6  Q969U6  Q969U6  Q96A00  Q96A00  Q96A65  Q96A65  Q96A65  Q96A72  Q96A72  Q96A72  Q96AD5  Q96AD5  Q96AD5  Q96AX2  Q96AX2  Q96AX2  Q96B36  Q96B36  Q96BD6  Q96BD6  Q96BD6  Q96BD6  Q96BD6  Q96BD6  Q96BD6  Q96BD6  Q96BD6  Q96BR1  Q96BR1  Q96BR1  Q96BT3  Q96BT3  Q96BT3  Q96C19  Q96C19  Q96C36  Q96C36  Q96C36  Q96C86  Q96C86  Q96C86  Q96CF2  Q96CN5  Q96CN5  Q96CT7  Q96CV9  Q96CV9  Q96CV9  Q96DA6  Q96DA6  Q96DA6  Q96DG6  Q96DG6  Q96DG6  Q96EB6  Q96EB6  Q96EB6  Q96EG1  Q96EG1  Q96EG1  Q96EP5  Q96EP5  Q96EP5  Q96EY1  Q96EY1  Q96EY1  Q96EY5  Q96EY5  Q96EY5  Q96EY5  Q96EY5  Q96G03  Q96G03  Q96G03  Q96G74  Q96G74  Q96G74  Q96G74  Q96G74  Q96G74  Q96G74  Q96G74  Q96G74  Q96G74  Q96G74  Q96G74  Q96G74  Q96G74  Q96GA7  Q96GA7  Q96H79  Q96H79  Q96H79  Q96HC4  Q96HC4  Q96HC4  Q96HQ2  Q96HQ2  Q96HQ2  Q96I15  Q96I15  Q96I15  Q96J02  Q96J02  Q96J02  Q96J02  Q96J02  Q96J02  Q96J02  Q96J02  Q96J02  Q96J02  Q96J02  Q96J02  Q96J02  Q96J02  Q96J02  Q96J02  Q96J02  Q96J02  Q96J02  Q96J02  Q96J02  Q96J02  Q96J02  Q96J02  Q96JB2  Q96JB2  Q96JB2  Q96KP4  Q96KP4  Q96KP4  Q96KQ7  Q96KQ7  Q96KQ7  Q96L34  Q96L34  Q96L34  Q96LA8  Q96LA8  Q96LA8  Q96LC7  Q96LC7  Q96LC7  Q96LC7  Q96LC7  Q96LC7  Q96LC7  Q96LC7  Q96LC7  Q96LC7  Q96LC7  Q96LC7  Q96LC7  Q96LC7  Q96P11  Q96P11  Q96P11  Q96P16  Q96P16  Q96P16  Q96P70  Q96P70  Q96P70  Q96PD5  Q96PD5  Q96PD5  Q96PU5  Q96PU5  Q96PU5  Q96PU8  Q96PU8  Q96PU8  Q96Q83  Q96Q83  Q96Q83  Q96Q89  Q96Q89  Q96Q89  Q96R06  Q96R06  Q96R06  Q96RG2  Q96RG2  Q96RG2  Q96RI1  Q96RI1  Q96RI1  Q96RL1  Q96RL1  Q96RL1  Q96RU2  Q96RU2  Q96RU2  Q96S19  Q96S19  Q96S19  Q96SB4  Q96SB4  Q96SB4  Q96SD1  Q96SD1  Q96SD1  Q96T51  Q96T51  Q96T51  Q96T51  Q96T51  Q96T51  Q96T88  Q96T88  Q96T88  Q96TA1  Q96TA1  Q96TA1  Q99259  Q99259  Q99259  Q99426  Q99426  Q99426  Q99459  Q99459  Q99459  Q99490  Q99490  Q99490  Q99490  Q99490  Q99490  Q99490  Q99490  Q99490  Q99490  Q99490  Q99490  Q99490  Q99490  Q99497  Q99497  Q99497  Q99504  Q99504  Q99504  Q99536  Q99536  Q99536  Q99558  Q99558  Q99558  Q99572  Q99572  Q99572  Q99572  Q99572  Q99572  Q99572  Q99572  Q99572  Q99572  Q99572  Q99572  Q99572  Q99572  Q99572  Q99572  Q99572  Q99572  Q99572  Q99572  Q99572  Q99572  Q99572  Q99572  Q99613  Q99613  Q99613  Q99615  Q99615  Q99615  Q99623  Q99623  Q99623  Q99627  Q99627  Q99627  Q99638  Q99638  Q99638  Q99638  Q99640  Q99640  Q99640  Q99661  Q99661  Q99661  Q99665  Q99665  Q99665  Q99665  Q99665  Q99665  Q99665  Q99665  Q99665  Q99665  Q99665  Q99665  Q99665  Q99665  Q99665  Q99665  Q99665  Q99665  Q99665  Q99665  Q99665  Q99665  Q99665  Q99665  Q99683  Q99683  Q99683  Q99683  Q99683  Q99683  Q99683  Q99683  Q99683  Q99683  Q99683  Q99683  Q99683  Q99683  Q99683  Q99683  Q99683  Q99683  Q99683  Q99683  Q99683  Q99683  Q99683  Q99683  Q99704  Q99704  Q99704  Q99704  Q99704  Q99704  Q99704  Q99708  Q99708  Q99708  Q99759  Q99759  Q99759  Q99828  Q99828  Q99832  Q99832  Q99832  Q99935  Q99935  Q99935  Q99958  Q99958  Q99958  Q99983  Q99983  Q99983  Q99986  Q99986  Q99986  Q9BPZ7  Q9BPZ7  Q9BPZ7  Q9BQ15  Q9BQ15  Q9BQ15  Q9BQA1  Q9BQA1  Q9BQA1  Q9BR39  Q9BR39  Q9BR39  Q9BR76  Q9BR76  Q9BR76  Q9BRK3  Q9BRK3  Q9BRK3  Q9BSJ6  Q9BT78  Q9BT78  Q9BT78  Q9BTC0  Q9BTC0  Q9BTC0  Q9BTM9  Q9BTY2  Q9BTY2  Q9BTY2  Q9BTY7  Q9BTY7  Q9BTY7  Q9BUB5  Q9BUB5  Q9BUB5  Q9BV73  Q9BV73  Q9BV73  Q9BV86  Q9BV86  Q9BV86  Q9BWF3  Q9BWF3  Q9BWF3  Q9BWT1  Q9BWT1  Q9BWT1  Q9BX63  Q9BX63  Q9BX63  Q9BX84  Q9BX84  Q9BX84  Q9BXA7  Q9BXA7  Q9BXA7  Q9BXM7  Q9BXM7  Q9BXM7  Q9BXS0  Q9BXS0  Q9BXS0  Q9BXS5  Q9BXS5  Q9BXS5  Q9BXS6  Q9BXS6  Q9BXS6  Q9BXW9  Q9BXW9  Q9BXW9  Q9BY32  Q9BY32  Q9BY32  Q9BY41  Q9BY41  Q9BY41  Q9BY44  Q9BY44  Q9BY44  Q9BY77  Q9BY77  Q9BY77  Q9BY84  Q9BY84  Q9BY84  Q9BYE7  Q9BYE7  Q9BYE7  Q9BYG3  Q9BYG3  Q9BYG3  Q9BYV9  Q9BYV9  Q9BYV9  Q9BZE4  Q9BZE4  Q9BZE4  Q9BZL4  Q9BZL4  Q9BZL4  Q9BZL6  Q9BZL6  Q9BZL6  Q9BZL6  Q9BZL6  Q9BZL6  Q9BZL6  Q9BZL6  Q9BZL6  Q9BZL6  Q9BZL6  Q9BZL6  Q9BZL6  Q9BZL6  Q9BZL6  Q9BZL6  Q9BZL6  Q9BZL6  Q9BZL6  Q9BZS1  Q9BZS1  Q9BZS1  Q9BZZ5  Q9BZZ5  Q9BZZ5  Q9GZN2  Q9GZN2  Q9GZN2  Q9GZT4  Q9GZT4  Q9GZT4  Q9GZT9  Q9GZT9  Q9GZT9  Q9GZV1  Q9GZV1  Q9GZV5  Q9GZV5  Q9GZV5  Q9GZX7  Q9GZX7  Q9GZX7  Q9GZY6  Q9GZY6  Q9GZY6  Q9GZY6  Q9GZY6  Q9GZY6  Q9H063  Q9H063  Q9H063  Q9H093  Q9H093  Q9H093  Q9H0H5  Q9H0H5  Q9H0H5  Q9H0K1  Q9H0K1  Q9H0K1  Q9H0N0  Q9H0N0  Q9H0N0  Q9H0S4  Q9H0S4  Q9H0S4  Q9H1A4  Q9H1A4  Q9H1A4  Q9H1A4  Q9H1A4  Q9H1A4  Q9H1A4  Q9H1A4  Q9H1A4  Q9H1A4  Q9H1A4  Q9H1A4  Q9H1A4  Q9H1A4  Q9H1A4  Q9H1A4  Q9H1A4  Q9H1A4  Q9H1A4  Q9H1A4  Q9H1A4  Q9H1A4  Q9H1A4  Q9H1A4  Q9H1D0  Q9H1D0  Q9H1D0  Q9H1Y3  Q9H1Y3  Q9H1Y3  Q9H211  Q9H211  Q9H211  Q9H2D6  Q9H2D6  Q9H2D6  Q9H2G4  Q9H2G4  Q9H2G4  Q9H2K2  Q9H2K2  Q9H2K2  Q9H2K8  Q9H2K8  Q9H2K8  Q9H2W2  Q9H300  Q9H300  Q9H300  Q9H307  Q9H307  Q9H3Y6  Q9H3Y6  Q9H3Y6  Q9H3Y6  Q9H3Y6  Q9H3Y6  Q9H3Y6  Q9H3Y6  Q9H3Y6  Q9H3Y6  Q9H3Y6  Q9H3Y6  Q9H3Y6  Q9H3Y6  Q9H3Y6  Q9H3Y6  Q9H3Z4  Q9H3Z4  Q9H3Z4  Q9H400  Q9H400  Q9H425  Q9H425  Q9H425  Q9H479  Q9H479  Q9H479  Q9H492  Q9H492  Q9H492  Q9H4X1  Q9H4X1  Q9H5V8  Q9H5V8  Q9H5V8  Q9H5V8  Q9H5V8  Q9H5V8  Q9H5V8  Q9H5V8  Q9H5V8  Q9H5V8  Q9H5V8  Q9H5V8  Q9H5V8  Q9H5V8  Q9H5V8  Q9H706  Q9H706  Q9H706  Q9H706  Q9H706  Q9H706  Q9H706  Q9H706  Q9H706  Q9H706  Q9H706  Q9H706  Q9H706  Q9H706  Q9H706  Q9H706  Q9H706  Q9H706  Q9H706  Q9H706  Q9H706  Q9H706  Q9H706  Q9H7P6  Q9H7P6  Q9H7P6  Q9H7X0  Q9H7X0  Q9H7X0  Q9H7Z6  Q9H7Z6  Q9H7Z6  Q9H8M9  Q9H8M9  Q9H8S9  Q9H8S9  Q9H8S9  Q9H8V3  Q9H8V3  Q9H8V3  Q9H8Y8  Q9H8Y8  Q9H8Y8  Q9H9B4  Q9H9B4  Q9H9B4  Q9HAW4  Q9HAW4  Q9HAW4  Q9HB71  Q9HB71  Q9HB71  Q9HB75  Q9HB75  Q9HB75  Q9HB96  Q9HB96  Q9HB96  Q9HBH9  Q9HBH9  Q9HBH9  Q9HBL0  Q9HBL0  Q9HBL0  Q9HBV2  Q9HBV2  Q9HBV2  Q9HC16  Q9HC16  Q9HC16  Q9HC77  Q9HC77  Q9HC77  Q9HC98  Q9HC98  Q9HC98  Q9HCC9  Q9HCC9  Q9HCC9  Q9HCE9  Q9HCE9  Q9HCE9  Q9HCN4  Q9HCN4  Q9HCN4  Q9HCQ7  Q9HCQ7  Q9HCQ7  Q9HCX4  Q9HCX4  Q9HCX4  Q9HD40  Q9HD40  Q9HD40  Q9NP72  Q9NP72  Q9NP72  Q9NP79  Q9NP79  Q9NP79  Q9NPD3  Q9NPD3  Q9NPD3  Q9NQ92  Q9NQ92  Q9NQ92  Q9NQC3  Q9NQC3  Q9NQC3  Q9NQC7  Q9NQC7  Q9NQC7  Q9NQR1  Q9NQR1  Q9NQR1  Q9NQS7  Q9NQS7  Q9NQS7  Q9NQT5  Q9NQT5  Q9NQT5  Q9NQT8  Q9NQT8  Q9NQT8  Q9NQX4  Q9NQX4  Q9NQX4  Q9NR20  Q9NR20  Q9NR20  Q9NR20  Q9NR20  Q9NR20  Q9NR20  Q9NR20  Q9NR20  Q9NR20  Q9NR20  Q9NR20  Q9NR20  Q9NR20  Q9NR20  Q9NR20  Q9NR20  Q9NR22  Q9NR22  Q9NR22  Q9NR30  Q9NR30  Q9NR30  Q9NR50  Q9NR50  Q9NR50  Q9NRD5  Q9NRD5  Q9NRD5  Q9NRF9  Q9NRF9  Q9NRH2  Q9NRH2  Q9NRH2  Q9NRM7  Q9NRM7  Q9NRM7  Q9NRY4  Q9NRY4  Q9NRY4  Q9NRY4  Q9NRY4  Q9NRY4  Q9NRY4  Q9NRY4  Q9NRY4  Q9NRY4  Q9NRY4  Q9NRY4  Q9NRY4  Q9NRY4  Q9NRY4  Q9NRY4  Q9NRY4  Q9NRY4  Q9NRY4  Q9NRY4  Q9NRY4  Q9NRY4  Q9NRY4  Q9NRY4  Q9NS18  Q9NS18  Q9NS18  Q9NS28  Q9NS28  Q9NS28  Q9NS56  Q9NS56  Q9NS56  Q9NS69  Q9NUB1  Q9NUB1  Q9NUB1  Q9NUP9  Q9NUP9  Q9NUP9  Q9NV92  Q9NV92  Q9NV92  Q9NV92  Q9NV92  Q9NV92  Q9NV92  Q9NVI1  Q9NVI1  Q9NVI1  Q9NVI7  Q9NVI7  Q9NVI7  Q9NVJ2  Q9NVJ2  Q9NVJ2  Q9NVX2  Q9NVX2  Q9NVX2  Q9NW13  Q9NW13  Q9NW13  Q9NWH9  Q9NWH9  Q9NWH9  Q9NWQ8  Q9NWQ8  Q9NWZ3  Q9NWZ3  Q9NWZ3  Q9NX09  Q9NX09  Q9NX09  Q9NX76  Q9NX76  Q9NX76  Q9NXV6  Q9NXV6  Q9NXV6  Q9NYA1  Q9NYA1  Q9NYA1  Q9NYF0  Q9NYF0  Q9NYF0  Q9NYF8  Q9NYF8  Q9NYF8  Q9NYL2  Q9NYL2  Q9NYL2  Q9NYV4  Q9NYV4  Q9NYV4  Q9NYV6  Q9NYV6  Q9NYV6  Q9NZ53  Q9NZ53  Q9NZ53  Q9NZC7  Q9NZC7  Q9NZC7  Q9NZC7  Q9NZC7  Q9NZC7  Q9NZC7  Q9NZC7  Q9NZC7  Q9NZC7  Q9NZJ0  Q9NZJ0  Q9NZJ0  Q9NZJ5  Q9NZJ5  Q9NZJ5  Q9NZJ5  Q9NZJ5  Q9NZJ5  Q9NZJ5  Q9NZJ5  Q9NZJ5  Q9NZJ5  Q9NZJ5  Q9NZJ5  Q9NZJ5  Q9NZJ5  Q9NZJ5  Q9NZJ5  Q9NZJ5  Q9NZJ5  Q9NZJ5  Q9NZJ5  Q9NZJ5  Q9NZJ5  Q9NZJ5  Q9NZJ5  Q9NZN3  Q9NZN3  Q9NZN3  Q9NZT1  Q9NZT1  Q9NZU7  Q9NZU7  Q9P013  Q9P013  Q9P0L2  Q9P0L2  Q9P0L2  Q9P126  Q9P126  Q9P126  Q9P126  Q9P126  Q9P126  Q9P126  Q9P126  Q9P126  Q9P1Z0  Q9P1Z0  Q9P1Z0  Q9P2R3  Q9P2R3  Q9P2R3  Q9P2Y5  Q9P2Y5  Q9P2Y5  Q9UBC9  Q9UBC9  Q9UBC9  Q9UBF8  Q9UBF8  Q9UBF8  Q9UBK5  Q9UBL3  Q9UBL3  Q9UBL3  Q9UBN4  Q9UBN4  Q9UBN4  Q9UBN4  Q9UBN4  Q9UBN4  Q9UBN4  Q9UBN4  Q9UBN4  Q9UBN4  Q9UBN4  Q9UBN4  Q9UBN4  Q9UBN4  Q9UBN4  Q9UBN4  Q9UBN4  Q9UBN4  Q9UBN4  Q9UBN4  Q9UBN4  Q9UBN4  Q9UBN4  Q9UBN4  Q9UBP6  Q9UBP6  Q9UBP6  Q9UBQ5  Q9UBQ5  Q9UBQ5  Q9UBR4  Q9UBR4  Q9UBR4  Q9UBR4  Q9UBR4  Q9UBR4  Q9UBR4  Q9UBR4  Q9UBR4  Q9UBR4  Q9UBW8  Q9UBW8  Q9UBW8  Q9UD71  Q9UD71  Q9UER7  Q9UER7  Q9UER7  Q9UEW8  Q9UEW8  Q9UEW8  Q9UG01  Q9UG01  Q9UG01  Q9UGC7  Q9UGC7  Q9UGC7  Q9UGK3  Q9UGK3  Q9UGK3  Q9UGK3  Q9UGK3  Q9UGK3  Q9UGK3  Q9UGK3  Q9UGK3  Q9UGK3  Q9UH99  Q9UH99  Q9UH99  Q9UHD1  Q9UHD1  Q9UHD1  Q9UHD2  Q9UHD2  Q9UHD2  Q9UHM6  Q9UHM6  Q9UHM6  Q9UHP3  Q9UHP3  Q9UHP3  Q9UHP3  Q9UHP3  Q9UHP3  Q9UHP3  Q9UHP3  Q9UHP3  Q9UHP3  Q9UHP3  Q9UHP3  Q9UHP3  Q9UHP3  Q9UHP3  Q9UHP3  Q9UHP3  Q9UHP3  Q9UHP3  Q9UHP3  Q9UHP3  Q9UHP3  Q9UHP3  Q9UHP3  Q9UIA9  Q9UIA9  Q9UIA9  Q9UIB8  Q9UIB8  Q9UIB8  Q9UIB8  Q9UIB8  Q9UIB8  Q9UIB8  Q9UIB8  Q9UIB8  Q9UIB8  Q9UIB8  Q9UIB8  Q9UIB8  Q9UIF9  Q9UIF9  Q9UIF9  Q9UJ70  Q9UJ70  Q9UJ70  Q9UJ70  Q9UJ70  Q9UJ70  Q9UJ70  Q9UJQ4  Q9UJQ4  Q9UJQ4  Q9UJX2  Q9UJX2  Q9UJX2  Q9UJX2  Q9UJX2  Q9UJX2  Q9UJX2  Q9UJX2  Q9UJX2  Q9UJX2  Q9UJX2  Q9UJX2  Q9UJX2  Q9UJX2  Q9UJX2  Q9UJX2  Q9UJX2  Q9UJX2  Q9UJX2  Q9UJX2  Q9UJX2  Q9UJX2  Q9UJX2  Q9UJX2  Q9UJX4  Q9UJX4  Q9UJX4  Q9UJX5  Q9UJX5  Q9UJX5  Q9UJX5  Q9UJX5  Q9UJX5  Q9UJX5  Q9UJX5  Q9UJX5  Q9UJX5  Q9UJX5  Q9UJX5  Q9UJX5  Q9UJX5  Q9UJX5  Q9UJX5  Q9UJX5  Q9UJX5  Q9UJX5  Q9UJX5  Q9UJX5  Q9UJX5  Q9UJX5  Q9UJX5  Q9UJX6  Q9UJX6  Q9UJX6  Q9UJY1  Q9UJY1  Q9UJY1  Q9UJZ1  Q9UJZ1  Q9UJZ1  Q9UK32  Q9UK32  Q9UK32  Q9UK55  Q9UK55  Q9UK55  Q9UKG1  Q9UKG1  Q9UKG1  Q9UKI8  Q9UKI8  Q9UKI8  Q9UKM9  Q9UKM9  Q9UKM9  Q9UKT5  Q9UKT5  Q9UKT5  Q9UKV3  Q9UKV3  Q9UKV3  Q9UKV8  Q9UKV8  Q9UKV8  Q9UL25  Q9UL25  Q9UL25  Q9UL42  Q9UL42  Q9UL42  Q9UL46  Q9UL46  Q9UL46  Q9UL54  Q9UL54  Q9UL54  Q9ULC4  Q9ULC4  Q9ULC4  Q9ULV5  Q9ULV5  Q9ULV5  Q9ULV8  Q9ULV8  Q9ULV8  Q9ULV8  Q9ULV8  Q9ULV8  Q9ULV8  Q9ULV8  Q9ULV8  Q9ULV8  Q9UM07  Q9UM07  Q9UM07  Q9UM11  Q9UM11  Q9UM11  Q9UM21  Q9UM21  Q9UM21  Q9UM73  Q9UM73  Q9UM73  Q9UM73  Q9UM73  Q9UM73  Q9UM73  Q9UM73  Q9UM73  Q9UM73  Q9UM73  Q9UM73  Q9UM73  Q9UM73  Q9UM73  Q9UM73  Q9UM73  Q9UM73  Q9UM73  Q9UM73  Q9UM73  Q9UM73  Q9UM73  Q9UM73  Q9UMD9  Q9UMD9  Q9UMD9  Q9UMS4  Q9UMS4  Q9UMS4  Q9UNE7  Q9UNE7  Q9UNE7  Q9UNF1  Q9UNF1  Q9UNF1  Q9UNL4  Q9UNL4  Q9UNL4  Q9UNS2  Q9UNS2  Q9UNS2  Q9UP65  Q9UP65  Q9UP65  Q9UPN6  Q9UPN6  Q9UPN6  Q9UPP1  Q9UPP1  Q9UPP1  Q9UPT6  Q9UPT6  Q9UPT6  Q9UPV0  Q9UPV0  Q9UPV0  Q9UPY6  Q9UPY6  Q9UPY6  Q9UPY6  Q9UPY6  Q9UPY6  Q9UPY6  Q9UPY6  Q9UPY6  Q9UPY6  Q9UPY6  Q9UPZ9  Q9UPZ9  Q9UPZ9  Q9UPZ9  Q9UPZ9  Q9UPZ9  Q9UPZ9  Q9UPZ9  Q9UPZ9  Q9UPZ9  Q9UPZ9  Q9UPZ9  Q9UPZ9  Q9UPZ9  Q9UPZ9  Q9UPZ9  Q9UPZ9  Q9UPZ9  Q9UPZ9  Q9UPZ9  Q9UPZ9  Q9UQ35  Q9UQ35  Q9UQ35  Q9UQ80  Q9UQ80  Q9UQ80  Q9UQ84  Q9UQ84  Q9UQ84  Q9UQC2  Q9UQC2  Q9UQC2  Q9UQC2  Q9UQC2  Q9UQC2  Q9UQC2  Q9UQC2  Q9UQC2  Q9UQC2  Q9UQC2  Q9UQC2  Q9UQC2  Q9UQC2  Q9UQC2  Q9UQC2  Q9UQE7  Q9UQE7  Q9UQE7  Q9UQF2  Q9UQF2  Q9UQF2  Q9UQL6  Q9UQL6  Q9UQL6  Q9Y230  Q9Y230  Q9Y230  Q9Y232  Q9Y232  Q9Y232  Q9Y233  Q9Y233  Q9Y233  Q9Y237  Q9Y237  Q9Y241  Q9Y241  Q9Y241  Q9Y259  Q9Y259  Q9Y259  Q9Y262  Q9Y262  Q9Y262  Q9Y266  Q9Y266  Q9Y266  Q9Y272  Q9Y272  Q9Y272  Q9Y283  Q9Y283  Q9Y283  Q9Y285  Q9Y285  Q9Y285  Q9Y294  Q9Y294  Q9Y294  Q9Y295  Q9Y295  Q9Y295  Q9Y2I7  Q9Y2I7  Q9Y2I7  Q9Y2K2  Q9Y2K2  Q9Y2K2  Q9Y2N7  Q9Y2N7  Q9Y2N7  Q9Y2R2  Q9Y2R2  Q9Y2R2  Q9Y2S0  Q9Y2S0  Q9Y2S0  Q9Y2V2  Q9Y2V2  Q9Y2V2  Q9Y2W1  Q9Y2W1  Q9Y2W1  Q9Y2W7  Q9Y2W7  Q9Y2W7  Q9Y2Z0  Q9Y2Z0  Q9Y2Z0  Q9Y365  Q9Y365  Q9Y365  Q9Y371  Q9Y371  Q9Y371  Q9Y383  Q9Y383  Q9Y383  Q9Y3A5  Q9Y3A5  Q9Y3A5  Q9Y3C5  Q9Y3C5  Q9Y3C5  Q9Y3D6  Q9Y3D6  Q9Y3D6  Q9Y3P8  Q9Y3S1  Q9Y3S1  Q9Y3S1  Q9Y3Z3  Q9Y3Z3  Q9Y3Z3  Q9Y4E8  Q9Y4E8  Q9Y4E8  Q9Y4P1  Q9Y4P1  Q9Y4P1  Q9Y4R8  Q9Y4R8  Q9Y4R8  Q9Y4X4  Q9Y4X4  Q9Y4X4  Q9Y4Z0  Q9Y4Z0  Q9Y4Z0  Q9Y572  Q9Y572  Q9Y572  Q9Y572  Q9Y572  Q9Y5A9  Q9Y5A9  Q9Y5A9  Q9Y5A9  Q9Y5A9  Q9Y5A9  Q9Y5B9  Q9Y5B9  Q9Y5B9  Q9Y5B9  Q9Y5B9  Q9Y5B9  Q9Y5B9  Q9Y5P4  Q9Y5P4  Q9Y5P4  Q9Y5P4  Q9Y5P4  Q9Y5P4  Q9Y5Q6  Q9Y5Q6  Q9Y5Y2  Q9Y5Y2  Q9Y608  Q9Y608  Q9Y608  Q9Y608  Q9Y608  Q9Y608  Q9Y657  Q9Y657  Q9Y657  Q9Y657  Q9Y657  Q9Y657  Q9Y696  Q9Y696  Q9Y696  Q9Y696  Q9Y696  Q9Y6E0  Q9Y6E0  Q9Y6E0  Q9Y6E0  Q9Y6E0  Q9Y6F6  Q9Y6F6  Q9Y6F6  Q9Y6F6  Q9Y6F6  Q9Y6F6  Q9Y6G9  Q9Y6G9  Q9Y6G9  Q9Y6G9  Q9Y6G9  Q9Y6I3  Q9Y6I3  Q9Y6I3  Q9Y6I3  Q9Y6I3  Q9Y6K5  Q9Y6K5  Q9Y6K5  Q9Y6K5  Q9Y6K5  Q9Y6K5  Q9Y6K9  Q9Y6K9  Q9Y6K9  Q9Y6K9  Q9Y6M9  Q9Y6M9  Q9Y6M9  Q9Y6M9  Q9Y6M9  Q9Y6M9  Q9Y6M9  Q9Y6N7  Q9Y6N7  Q9Y6N7  Q9Y6N7  Q9Y6N7  Q9Y6N7  Q9Y6N7  Q9Y6N7  Q9Y6N7  Q9Y6N7  Q9Y6N7  Q9Y6N7  Q9Y6N7  Q9Y6N7  Q9Y6N7  Q9Y6N7  Q9Y6N7  Q9Y6N7  Q9Y6N7  Q9Y6N7  Q9Y6N7  Q9Y6N7  Q9Y6N7  Q9Y6N7  Q9Y6Q9  Q9Y6Q9  Q9Y6Q9  Q9Y6Q9  Q9Y6Q9  Q9Y6Q9  Q9Y6Q9  Q9Y6R1  Q9Y6R1  Q9Y6R1  Q9Y6R1  Q9Y6R1  Q9Y6R1  Q9Y6R1 | 119  587  619  53  218  1179  38  45  102  151  102  151  321  553  648  859  99  139  132  207  31  33  27  122  159  16  68  157  117  234  269  270  403  467  546  658  177  309  327  75  111  247  69  150  169  113  164  180  836  921  992  27  75  195  106  190  289  16  255  410  198  300  340  66  112  144  49  76  95  100  106  175  343  417  714  837  59  69  443  344  368  691  152  987  1320  9  13  74  146  331  365  387  405  413  423  428  449  453  460  467  505  577  623  671  683  686  696  46  56  91  17  528  843  38  283  580  102  221  370  1296  2135  2168  52  112  284  415  454  514  546  67  148  165  193  197  42  49  81  137  265  1148  52  75  191  84  95  111  112  151  164  176  191  231  291  343  487  615  656  700  743  779  828  847  899  959  1016  1038  1046  143  163  257  91  117  136  53  95  166  116  179  389  24  38  164  18  122  176  325  333  386  405  462  552  562  576  638  667  717  739  744  772  825  894  946  1002  1010  1044  1089  20  218  390  228  435  453  273  351  354  21  106  110  118  145  176  228  383  861  1157  98  135  571  121  196  708  69  122  156  115  164  178  197  320  338  446  901  920  252  371  382  169  199  280  294  407  529  50  224  444  277  333  407  75  99  210  95  124  306  21  25  36  39  46  101  129  140  205  346  788  822  218  244  379  335  538  566  78  157  206  210  271  475  508  518  538  584  656  10  25  36  70  101  104  129  132  140  188  200  228  258  343  18  23  44  82  128  139  166  178  248  265  267  293  335  431  249  268  367  282  452  676  53  162  85  121  144  152  181  225  240  254  309  355  356  389  407  487  508  518  537  554  22  62  69  102  185  190  467  497  576  610  661  663  671  697  707  747  770  777  815  831  834  886  987  1213  120  140  326  27  168  352  58  69  230  9  74  283  302  307  326  383  462  465  468  556  675  683  733  756  764  245  299  339  108  126  146  156  161  170  185  195  248  273  288  299  333  361  415  485  486  523  524  547  25  30  52  224  230  237  285  294  317  361  369  374  161  326  379  684  974  1077  146  212  576  174  432  495  140  239  263  101  126  135  175  547  649  279  333  357  451  143  500  584  22  122  152  15  23  77  97  113  134  192  198  213  215  240  257  520  208  474  616  42  952  1536  51  220  297  570  742  846  88  258  495  400  430  437  96  162  189  162  193  348  386  563  832  127  251  670  336  358  434  169  353  444  36  45  64  70  139  145  206  72  140  310  345  802  984  988  44  58  70  41  64  70  40  158  202  110  180  291  149  267  391  143  310  478  71  89  108  136  143  155  157  178  205  206  221  224  259  275  317  354  403  431  489  497  549  654  935  1065  1258  22  71  154  115  162  949  33  57  81  127  140  161  209  321  333  345  352  357  364  442  508  513  609  612  671  691  693  696  774  815  1101  1115  1121  194  239  290  20  42  54  62  114  124  132  201  206  254  266  317  423  481  590  637  766  790  913  918  934  940  956  1021  224  333  359  198  203  213  257  281  321  346  375  96  174  193  208  213  241  248  291  296  302  321  334  335  389  395  588  600  656  668  698  701  832  81  590  655  153  273  376  593  919  951  109  95  298  312  118  390  1054  40  209  311  110  176  679  25  142  471  120  161  830  79  86  75  92  118  164  166  171  184  256  271  285  295  303  415  416  450  515  1084  1174  1185  1232  1255  1268  1318  1319  148  231  285  145  677  875  317  435  737  341  883  1095  798  2038  2536  47  87  48  55  337  109  127  290  533  628  719  22  39  378  98  111  150  80  148  308  43  44  928  1167  11  59  93  122  150  152  169  230  237  298  302  325  331  334  145  159  258  66  706  1584  359  375  523  107  207  267  34  131  258  20  33  46  83  144  226  235  118  179  344  484  70  171  305  170  243  246  43  47  97  144  398  556  49  52  193  189  452  457  116  170  278  54  114  142  110  706  735  140  171  132  436  566  161  224  249  296  392  403  201  205  211  114  385  536  133  426  610  1224  2185  2814  191  511  932  12  281  406  542  1956  2731  35  282  302  157  159  558  305  435  530  131  344  704  86  283  304  41  90  96  100  133  153  191  200  328  416  719  76  381  500  29  164  550  1317  1532  2139  61  143  158  50  114  118  142  163  218  47  189  924  2566  3620  4271  8  60  406  409  705  213  426  548  103  395  399  283  769  927  126  158  236  128  140  137  362  378  111  373  382  480  136  327  445  21  186  416  11  63  11  12  35  35  181  320  429  439  512  138  325  377  260  713  1011  1440  1781  1998  167  176  345  88  166  192  81  105  191  76  161  196  95  154  164  67  359  600  222  684  693  352  371  441  84  108  431  111  273  283  235  350  351  67  97  172  21  52  205  163  198  395  162  184  268  37  349  390  210  243  1480  69  85  143  60  68  88  166  234  298  29  106  299  337  345  24  319  402  137  141  157  28  21  80  160  221  237  124  196  213  68  137  190  58  96  325  1114  2478  62  65  110  29  72  102  32  41  29  48  64  87  22  27  121  85  124  132  36  51  88  46  52  126  32  35  115  96  110  111  33  53  94  43  90  48  122  45  211  646  53  139  216  54  92  180  44  64  89  601  809  851  149  315  375  94  375  380  148  261  268  58  278  299  347  390  392  393  413  486  519  534  553  555  596  628  794  818  824  37  72  91  84  144  227  153  200  202  937  1248  1519  109  128  160  56  129  227  89  125  134  365  377  476  60  151  377  88  168  247  112  417  681  29  31  165  35  37  49  30  54  67  52  54  63  43  52  54  60  73  80  130  131  139  150  184  191  195  197  219  246  328  331  459  526  582  583  30  49  118  37  41  42  116  121  210  237  240  260  302  309  332  348  365  390  397  400  403  411  437  445  35  99  189  116  194  284  75  181  379  344  432  441  59  204  343  39  84  156  207  230  240  243  315  319  335  150  164  740  3074  4509  478  663  693  19  33  193  78  133  323  48  61  162  36  338  429  11  373  566  49  314  320  30  263  375  50  64  83  112  274  289  301  303  343  411  554  590  685  735  772  781  835  877  908  923  952  1005  1112  1127  72  359  591  640  701  723  729  757  178  210  217  103  205  220  82  197  348  54  73  133  142  173  332  341  348  362  370  389  429  471  474  506  525  526  566  568  584  601  613  624  72  77  115  168  217  262  308  321  336  378  476  538  549  572  588  681  728  762  42  66  125  136  141  142  148  190  192  204  216  307  315  344  347  374  504  546  557  582  583  620  660  683  695  696  706  130  293  735  16  30  45  96  140  462  516  634  59  125  128  11  10  26  170  43  121  136  23  104  188  193  216  349  11  62  66  109  368  464  24  36  363  93  176  192  56  186  211  48  1374  1403  30  118  134  154  171  259  273  304  394  519  589  606  614  673  735  818  884  888  941  976  1038  1149  1237  1254  61  174  265  65  97  95  110  114  138  160  167  235  301  371  469  474  478  480  491  547  730  767  529  655  749  329  579  777  22  34  91  145  44  257  407  75  156  768  174  327  344  36  142  266  17  29  67  72  230  401  478  29  32  74  84  173  240  74  490  550  193  355  552  182  263  312  28  53  58  12  44  116  142  156  172  186  230  268  461  491  513  614  633  673  734  737  761  799  811  61  100  154  257  276  300  328  338  360  379  400  424  454  483  538  540  571  665  668  832  841  856  866  873  196  315  538  146  244  294  103  132  181  38  74  70  132  174  185  199  209  219  308  316  326  350  354  366  25  60  129  60  101  105  36  52  95  96  99  111  113  149  150  374  1047  1330  139  381  493  57  105  137  16  85  100  102  141  146  159  194  212  222  223  240  367  386  392  473  489  521  529  30  36  52  73  76  96  122  146  204  237  263  314  440  483  508  606  687  752  791  826  864  928  952  1029  327  608  662  43  73  84  113  131  201  203  418  517  526  538  577  656  718  763  869  885  901  923  930  987  1125  1192  1251  312  373  772  312  453  468  46  213  277  76  86  95  402  676  968  82  183  208  130  189  325  41  69  71  84  125  205  234  245  249  291  390  416  566  649  681  738  830  835  971  1093  1159  1194  1284  1295  318  348  527  167  273  340  128  161  437  181  202  212  232  236  257  286  37  39  47  156  202  204  111  31  78  86  166  245  248  253  257  266  8  50  64  80  104  109  112  119  154  180  105  120  16  150  162  214  261  38  81  139  98  117  188  204  205  207  249  270  289  315  343  377  394  482  589  683  692  800  808  880  904  914  921  966  134  160  171  32  401  671  9  583  889  43  182  514  110  313  560  74  384  430  581  946  1693  591  296  1186  1221  933  1254  1383  29  48  55  40  60  125  134  23  46  331  71  201  371  11  19  105  314  406  445  480  482  503  513  530  552  571  593  620  667  739  763  773  781  834  857  19  24  277  18  108  180  33  36  156  165  181  202  246  86  144  446  68  179  338  49  29  441  627  711  122  246  323  194  262  414  95  108  125  146  179  243  259  269  291  337  350  362  373  390  408  441  503  545  578  609  672  747  855  870  26  227  503  109  210  256  70  312  554  633  107  431  525  250  289  303  304  331  340  349  374  396  423  442  459  470  509  767  19  205  58  70  231  276  279  316  328  360  436  513  554  598  644  751  756  844  852  910  930  940  950  977  981  1131  274  347  1802  289  309  324  99  113  154  206  210  228  236  243  280  307  339  374  380  397  558  563  572  605  613  677  701  776  211  538  619  274  328  757  112  343  428  91  113  161  57  70  91  150  151  163  226  254  286  292  298  316  321  503  718  124  173  302  66  316  323  60  114  133  239  249  250  657  926  1906  112  174  251  1056  1989  2095  444  614  2001  71  103  138  15  295  297  87  181  336  161  193  215  241  246  279  319  378  422  466  467  511  514  582  592  674  681  708  780  842  859  867  879  294  550  656  37  68  101  107  190  228  296  302  341  380  413  450  508  523  561  642  663  797  827  835  859  861  217  369  409  316  397  416  113  131  41  54  121  36  51  26  37  52  61  468  494  380  627  1307  1140  3105  3183  228  250  504  79  175  192  232  367  397  411  434  443  457  579  603  634  639  671  730  745  760  787  65  152  356  68  213  394  95  151  159  28  417  502  43  319  479  332  509  604  219  313  513  82  359  416  158  220  262  83  103  124  153  161  175  177  190  360  437  439  422  1231  1417  48  97  279  83  148  444  355  412  727  262  368  404  56  229  448  15  168  94  95  103  144  161  194  200  202  207  259  278  279  306  329  356  367  372  385  388  391  421  431  475  536  317  163  324  375  178  205  438  198  266  519  40  83  178  50  86  121  241  242  251  256  277  364  366  30  193  231  223  429  439  44  49  85  128  137  191  201  205  228  270  291  424  483  499  565  13  429  95  157  259  260  299  421  461  500  508  531  43  103  228  29  46  49  29  53  54  62  68  70  101  117  121  131  132  140  183  189  209  247  248  261  321  328  331  366  368  378  14  20  256  1065  1066  1081  1124  1132  1177  1191  1209  93  223  231  152  228  395  196  412  560  45  95  81  194  253  327  1062  1468  134  175  224  98  102  118  120  136  172  206  225  249  273  375  392  405  555  613  676  685  800  881  913  926  944  958  962  76  83  94  102  107  123  290  302  384  415  442  474  534  537  546  570  571  623  663  728  74  60  89  127  135  139  140  150  177  84  323  575  128  159  286  137  267  301  180  216  331  155  281  363  31  126  205  158  160  318  250  339  354  578  643  754  36  79  89  186  190  210  139  199  268  309  353  403  533  551  584  602  634  642  662  729  794  815  911  990  1002  1048  1076  1085  1100  1184  121  181  214  46  81  124  152  153  176  271  359  405  424  427  51  61  144  100  107  144  160  692  949  43  92  162  50  92  119  133  142  218  316  323  358  136  211  613  161  227  736  534  571  594  81  322  572  93  162  329  338  353  379  407  481  496  509  519  595  633  702  741  747  754  833  991  1003  1062  1121  1162  1196  417  423  725  60  70  113  119  122  130  192  66  67  83  29  112  17  27  118  133  142  176  257  300  323  332  346  404  454  465  472  169  403  408  253  463  490  73  194  505  123  472  568  177  485  582  11  391  412  49  50  59  116  127  129  233  31  39  59  21  84  123  61  101  194  30  35  88  51  95  365  205  584  626  83  240  307  240  1715  1795  167  178  246  67  594  681  181  201  367  100  124  419  198  221  311  54  176  204  110  903  1601  11  258  300  112  142  230  62  67  247  263  265  308  125  351  548  46  147  153  122  231  316  922  978  998  41  83  92  102  114  141  291  307  337  455  552  735  869  871  70  81  197  257  370  570  112  132  279  65  148  231  60  67  94  148  246  257  158  177  273  276  449  1049  251  320  381  470  488  490  527  597  602  624  642  691  698  27  75  314  410  629  34  107  166  84  139  164  198  259  315  89  112  164  217  220  281  312  313  368  412  422  448  463  568  598  704  788  894  933  967  983  1048  1059  1125  110  459  572  100  257  328  82  85  140  33  228  241  20  354  466  17  84  227  125  151  196  38  62  99  147  152  154  159  175  179  197  208  225  252  713  70  106  247  50  58  88  120  143  837  1775  1917  159  176  253  1797  1839  1895  312  357  653  108  144  193  61  114  105  141  150  181  195  248  251  254  289  305  827  863  1084  269  307  395  89  94  348  193  228  247  151  210  428  24  77  145  104  142  284  123  124  252  42  47  53  60  81  119  130  145  148  156  210  222  250  280  329  333  334  118  262  284  131  135  227  550  679  2212  148  286  396  110  113  1001  124  146  177  28  111  139  144  177  186  198  224  228  290  307  416  427  464  475  521  527  529  757  762  806  807  875  898  943  962  985  274  309  469  20  26  188  33  407  707  48  65  67  87  122  123  197  266  334  385  408  469  473  503  575  594  652  685  694  772  791  813  818  847  61  78  79  98  111  176  208  213  214  276  301  306  321  364  374  390  412  414  505  509  541  163  254  609  29  99  119  305  464  826  108  123  259  376  433  471  481  656  689  851  896  914  916  955  962  980  989  1019  1055  1068  1079  1080  1145  1176  18  182  238  47  96  108  115  119  132  151  161  202  58  604  740  132  325  362  391  444  486  396  444  542  212  326  348  95  215  322  75  134  202  115  132  140  253  305  367  371  409  452  481  498  504  634  643  662  698  702  750  759  761  830  13  34  466  75  109  175  105  146  161  55  124  141  156  169  205  211  219  259  263  301  330  365  408  417  436  543  580  597  604  606  629  641  20  54  86  91  107  237  89  389  582  107  110  121  134  163  171  175  176  185  212  267  289  312  350  358  385  410  495  516  568  570  579  592  595  38  316  456  75  236  431  24  30  32  99  111  194  202  205  246  287  325  648  1151  44  230  577  125  234  328  136  144  167  19  55  75  33  88  580  108  170  191  27  52  122  31  44  131  77  82  128  90  212  291  137  190  209  99  241  338  237  280  568  30  254  306  432  489  604  670  709  716  724  748  125  215  340  14  273  462  123  170  196  10  52  95  50  108  431  311  385  507  94  118  127  233  381  518  66  2639  2853  235  493  557  18  46  47  87  107  151  183  431  483  551  558  582  695  732  750  751  764  765  800  820  908  1001  1012  189  395  419  377  440  489  25  122  479  351  354  357  121  159  384  25  113  115  129  230  268  308  361  369  397  435  448  532  548  583  585  651  669  746  812  932  1017  1091  1115  101  137  165  190  194  214  221  224  354  357  365  396  448  480  528  584  640  735  822  927  938  1008  1130  1223  134  233  324  52  122  331  232  466  476  53  141  152  166  172  303  327  328  359  364  376  401  517  566  597  688  696  702  865  874  889  899  913  919  44  83  97  100  151  156  184  251  284  293  297  404  410  426  430  431  463  478  63  232  321  8  22  70  39  16  192  206  288  327  655  831  1113  128  193  461  118  196  652  155  205  396  92  134  651  77  151  69  173  268  380  683  905  112  149  192  14  22  45  68  79  94  176  230  293  360  446  480  539  575  584  640  657  672  674  686  192  210  278  56  80  161  89  238  375  89  178  219  198  557  602  60  114  401  31  43  138  15  23  94  18  48  64  97  112  116  129  133  145  156  214  263  268  277  380  387  403  99  121  194  26  28  49  80  120  124  188  193  222  252  259  305  31  32  93  125  136  169  219  358  368  376  397  419  430  469  584  22  33  90  98  114  171  176  225  405  548  568  665  668  682  683  720  735  738  173  567  745  503  638  913  1220  1804  2225  83  204  226  26  40  41  49  120  130  188  223  233  245  248  281  286  300  312  329  360  429  439  479  513  539  569  572  80  139  213  220  358  363  366  372  388  399  459  481  486  495  502  1119  1123  54  83  88  102  107  212  44  129  987  2741  3126  101  145  291  213  289  520  9  335  402  31  36  51  65  81  120  126  171  205  391  12  46  69  87  164  178  198  204  209  211  221  238  357  397  451  474  506  525  535  540  569  578  597  598  39  64  74  91  203  216  244  246  389  390  431  459  539  547  568  573  602  611  112  239  403  298  307  380  536  550  14  47  60  104  108  136  186  190  251  550  850  1017  67  141  127  214  316  142  209  247  48  61  109  25  40  77  45  56  188  12  68  78  1034  2025  2357  188  247  248  391  444  103  694  1192  109  147  152  352  502  621  79  107  167  327  337  468  435  440  477  29  98  35  82  193  79  269  373  133  154  232  20  29  34  66  70  106  195  206  211  269  306  316  318  411  279  891  1069  12  420  446  238  285  311  88  110  972  33  40  404  409  418  419  463  468  487  522  527  581  176  228  367  119  372  477  604  1047  2462  126  303  437  208  249  272  110  63  82  137  134  295  667  14  68  134  160  205  235  108  147  181  85  96  104  234  327  821  134  475  832  101  347  430  77  181  195  88  260  283  112  146  185  114  231  417  285  1333  1678  56  71  114  117  127  134  140  146  157  161  163  171  221  222  234  288  323  44  85  95  99  151  213  238  16  87  277  39  72  341  82  93  114  12  117  127  92  206  282  186  342  360  173  242  501  68  92  395  274  363  370  42  1757  2567  395  433  474  82  85  264  357  402  54  90  202  40  41  43  78  84  92  120  194  202  234  330  349  365  376  440  476  486  491  500  518  586  632  642  646  47  70  211  59  207  262  88  182  257  83  149  323  100  213  681  203  277  415  67  100  105  190  238  348  399  425  506  536  581  611  633  637  738  762  809  824  886  891  929  994  1018  1023  90  93  115  61  81  110  149  156  22  34  69  177  288  402  437  439  507  626  631  648  665  677  678  833  841  174  291  365  125  211  932  187  213  214  224  283  286  339  372  426  442  444  477  480  549  552  597  671  695  746  747  765  767  825  846  136  247  277  68  153  170  421  570  582  129  491  572  31  517  993  124  139  415  59  196  206  8  32  37  49  64  82  109  143  168  171  228  229  240  268  297  304  358  363  376  395  203  540  856  71  88  38  168  200  84  297  942  135  573  648  220  583  657  124  243  398  52  97  198  236  275  458  68  78  106  134  190  255  292  373  421  441  486  490  519  531  534  593  608  671  754  792  824  839  855  942  193  353  435  69  282  571  138  495  755  251  345  363  397  26  128  239  48  129  130  161  183  223  233  342  394  689  747  24  79  116  11  32  80  219  240  376  232  272  306  23  35  113  76  260  281  12  67  99  107  115  165  179  192  222  224  234  262  267  287  306  327  387  653  664  666  747  815  837  114  331  348  38  317  361  85  113  206  67  80  8  112  127  152  242  256  59  16  27  29  46  65  68  76  88  138  155  174  176  177  178  180  188  225  240  315  346  377  379  23  32  40  51  72  154  65  76  157  71  100  172  51  152  162  16  109  140  73  115  123  182  196  214  70  76  88  83  86  87  16  30  44  11  31  69  138  280  371  93  137  272  50  65  104  121  122  181  27  134  40  77  95  36  55  95  29  39  50  95  201  290  301  75  120  159  74  183  28  48  209  52  73  89  40  80  136  80  98  155  76  81  97  124  145  264  126  131  170  67  186  189  48  79  85  105  148  86  268  269  70  76  121  136  229  276  277  300  328  333  361  405  421  425  455  524  526  574  707  815  874  888  912  928  21  51  91  65  75  77  12  53  69  98  127  140  194  246  302  68  315  326  87  134  137  111  150  152  248  284  72  138  287  46  101  188  171  339  364  141  162  418  185  224  408  23  26  138  36  131  185  751  839  30  136  156  50  79  119  124  138  165  249  251  373  387  414  460  492  519  597  608  652  770  894  920  122  336  509  118  425  561  297  439  456  2299  3102  3280  250  275  547  65  107  123  98  120  135  56  313  698  41  52  72  92  698  1187  2168  154  290  338  106  292  341  129  135  146  47  49  75  37  162  211  40  288  986  170  185  299  176  250  353  417  430  1634  225  240  253  294  326  509  379  795  802  60  104  276  21  78  93  100  128  245  44  115  144  22  153  186  419  71  743  1064  144  240  719  18  86  240  67  162  334  326  657  937  116  693  696  199  250  434  356  537  1024  33  139  657  100  189  193  277  285  307  470  489  506  513  575  611  674  708  897  899  904  954  1012  1015  1039  1055  1068  1080  113  220  411  54  168  179  81  161  272  42  97  100  118  148  151  53  99  128  274  880  1279  109  110  597  66  152  496  179  211  242  146  596  1550  28  36  45  53  121  188  237  239  460  468  484  502  556  598  49  71  75  287  388  463  34  44  61  196  198  245  249  260  293  318  452  482  485  595  766  829  884  980  1017  1097  1163  1198  1288  1317  44  124  164  37  96  127  134  273  283  331  349  380  464  494  502  511  522  556  624  638  668  694  717  720  748  776  852  42  88  194  35  69  100  83  111  122  128  148  194  251  282  304  347  415  441  516  526  528  661  730  742  747  761  898  950  1007  1016  12  52  120  187  238  437  448  453  471  514  525  539  630  646  21  1105  1118  42  100  106  112  134  142  152  225  241  263  268  279  315  334  392  418  461  476  485  511  545  571  591  598  33  216  311  327  340  475  159  178  191  205  216  228  232  301  315  16  166  461  65  232  302  34  38  44  113  322  563  103  145  167  220  241  273  366  369  371  375  380  383  386  387  396  397  399  408  411  22  120  173  15  129  185  361  362  404  22  40  193  232  242  326  361  423  486  635  670  860  868  992  387  617  1043  1341  1443  1492  1528  1587  1605  1622  1717  1757  2000  2298  2406  2700  3049  3066  3072  3135  3160  3212  3243  3915  72  77  226  241  263  377  241  312  440  33  86  118  122  167  183  194  203  209  215  225  253  255  267  543  547  569  586  703  756  869  881  558  582  682  154  323  333  41  64  70  131  225  288  93  311  614  77  226  341  52  612  646  98  181  284  228  253  294  40  77  118  421  424  507  134  241  257  152  167  186  145  152  240  32  59  123  148  173  190  311  344  355  420  488  520  549  676  682  685  713  777  801  810  812  830  834  929  149  196  397  945  1775  1843  690  853  874  78  382  395  97  137  193  86  150  884  48  49  76  124  294  322  462  542  702  13  309  333  846  1129  1160  188  226  507  693  855  1758  152  191  425  154  265  294  55  270  330  334  346  429  441  464  474  133  86  273  333  267  350  686  85  101  198  73  82  92  106  110  133  227  260  266  276  283  299  329  331  337  360  763  802  944  170  233  284  82  102  269  33  64  77  133  186  208  34  56  174  99  138  409  29  151  255  128  336  346  54  238  382  288  1297  1508  15  16  199  249  252  311  320  354  399  404  407  574  359  402  472  130  138  160  76  206  381  17  188  209  60  75  115  346  360  448  205  237  293  66  129  229  100  119  126  151  229  527  568  674  717  200  208  310  23  24  92  352  1107  2322  34  54  192  212  510  13  336  358  17  223  586  54  81  313  30  118  256  12  119  198  44  433  459  111  182  217  93  302  371  40  58  104  112  136  147  220  243  246  327  338  415  462  471  472  633  680  705  720  731  45  54  96  121  148  295  506  523  561  577  578  632  676  681  698  701  112  120  36  37  52  55  57  126  135  166  205  246  269  278  365  366  405  420  433  441  530  551  552  573  609  673  110  230  300  127  154  201  251  261  302  308  364  388  404  155  448  483  61  106  155  159  341  386  390  478  485  554  561  570  579  715  723  738  740  857  891  929  957  970  984  1030  299  400  454  130  157  292  410  513  524  562  575  750  775  831  850  863  886  115  129  256  32  72  93  113  314  364  88  554  568  145  167  431  142  171  849  48  51  357  84  104  154  162  178  191  199  215  228  265  289  302  310  334  433  464  479  501  503  538  46  90  118  129  153  155  162  166  238  265  351  418  440  444  505  507  513  529  539  573  655  664  683  906  1129  2373  2692  44  89  145  523  531  683  328  394  413  95  144  151  15  293  475  106  189  711  339  774  1426  71  437  576  13  19  30  72  73  93  121  271  272  332  394  507  509  510  531  538  678  692  702  720  64  210  408  26  268  326  469  717  819  383  973  2091  130  1968  2025  39  216  826  10  11  73  88  97  122  128  143  174  185  200  218  226  267  269  310  317  396  427  438  464  561  594  638  82  119  158  168  298  516  973  1114  1221  223  466  742  117  364  659  285  628  994  474  498  575  15  100  324  1060  1573  1704  101  235  380  57  72  337  171  186  207  224  398  1163  665  749  792  76  255  544  1433  1865  1903  21  263  330  13  47  74  103  134  33  193  336  48  110  313  110  187  208  10  331  397  411  535  548  550  572  596  166  297  485  95  227  499  146  23  28  344  12  42  101  164  231  243  59  112  244  146  252  316  49  114  163  179  185  233  268  274  298  334  336  357  365  368  376  381  388  403  411  72  98  154  184  252  278  286  443  462  501  581  598  760  774  800  828  842  859  872  880  312  322  775  795  3779  4393  9  14  192  331  443  184  205  207  165  225  416  127  290  320  46  52  86  96  111  115  148  285  378  429  468  469  636  733  807  921  950  984  1081  1128  1221  1262  1266  1268  261  283  873  45  39  123  323  429  736  801  69  80  117  45  117  138  162  202  219  86  104  156  102  256  519  52  129  450  191  340  417  53  114  326  123  131  149  157  213  275  320  327  332  358  430  449  71  119  217  95  200  295  112  59  104  485  581  693  1168  1308  1353  1357  1366  1400  1410  1464  1498  1565  1640  1652  1739  1810  1835  1859  1880  148  250  488  42  297  991  151  225  279  166  261  292  35  165  480  111  162  225  80  81  99  447  518  520  425  496  500  46  366  576  117  178  201  236  243  490  369  768  834  273  436  635  275  299  431  135  136  218  174  184  318  332  591  134  235  243  22  29  104  106  120  126  128  131  171  172  193  213  228  232  234  236  274  287  315  342  443  474  481  493  43  130  268  32  65  597  10  21  104  32  159  270  23  152  458  110  209  589  119  340  659  43  645  705  42  55  100  86  261  376  15  63  338  143  220  289  324  327  32  60  118  122  148  194  211  217  248  254  256  268  305  350  371  387  481  538  656  684  700  722  789  825  176  270  708  50  74  237  677  687  718  247  373  851  150  155  156  244  251  56  153  328  224  529  678  959  1246  1470  77  125  132  178  683  992  8  34  146  45  133  234  39  332  342  93  503  649  131  152  278  121  130  531  95  187  228  16  98  316  30  914  932  192  214  661  54  73  212  57  91  382  17  248  504  312  350  1155  9  204  369  237  246  313  331  334  422  515  664  711  737  861  885  895  908  940  956  1026  1048  1075  1121  1156  24  89  104  115  121  79  127  184  175  335  406  424  151  259  655  102  115  138  17  290  425  58  158  210  72  76  95  182  183  297  355  429  443  615  778  794  804  813  816  817  890  892  894  902  943  979  1007  1019  286  459  468  85  157  241  325  669  725  133  238  359  131  136  333  38  41  43  21  53  81  89  178  239  242  274  329  356  68  99  103  104  115  165  156  56  63  114  156  308  316  54  315  425  359  576  880  256  312  1178  517  664  847  297  325  363  48  119  242  243  279  364  399  181  196  556  25  88  190  379  406  511  30  194  293  257  970  1321  15  30  192  196  303  306  345  374  375  403  413  465  527  558  596  103  318  472  53  56  174  60  133  552  154  179  219  49  149  298  215  346  416  55  342  474  54  114  492  16  26  133  163  195  230  249  262  292  346  365  374  394  67  72  151  308  357  366  417  427  451  452  460  466  481  484  505  510  513  524  602  619  667  823  1218  39  161  228  341  356  382  425  441  619  365  543  895  139  169  192  46  159  294  34  92  154  80  402  448  69  133  256  148  154  599  37  94  428  149  17  117  548  253  500  840  587  907  948  175  266  281  113  158  174  503  1091  1245  16  71  265  356  410  477  799  1036  77  139  166  26  283  768  357  521  832  105  137  53  107  164  245  72  334  416  29  154  215  222  469  585  88  134  244  162  166  331  11  26  48  94  31  68  638  96  438  462  188  189  218  184  186  200  285  362  653  114  155  209  70  123  191  16  137  204  22  37  113  214  310  321  336  344  479  577  659  700  309  596  604  23  32  37  63  136  143  198  311  333  355  421  431  435  447  457  474  484  499  89  93  121  174  793  1280  425  554  761  54  389  448  49  214  607  101  109  185  36  217  436  311  360  365  58  340  501  195  326  397  218  222  343  32  154  351  38  142  183  165  262  274  389  445  618  26  128  275  72  116  130  529  540  827  237  238  266  20  110  588  127  133  301  122  310  314  76  99  137  45  54  103  140  215  385  283  325  453  13  24  89  119  183  195  303  335  340  521  525  150  321  353  100  196  207  446  1388  1887  199  719  1310  190  242  626  42  86  442  464  736  217  370  394  14  71  243  163  589  726  29  39  513  52  119  347  383  394  407  418  48  111  421  117  177  180  309  729  808  11  342  731  117  199  247  37  376  646  805  117  137  429  48  71  91  547  1305  1323  48  60  140  242  295  352  356  386  576  588  646  662  155  278  342  185  704  1156  144  211  307  23  120  134  565  1190  2611  145  568  925  365  455  1024  14  163  178  182  203  210  220  222  240  309  374  380  388  399  419  461  464  1011  1196  1922  16  43  56  229  247  329  1687  3131  4661  219  371  392  393  405  414  428  460  473  480  498  511  1417  1457  2108  913  1978  1986  218  501  549  100  186  225  487  568  618  726  113  958  970  51  54  261  28  232  637  171  209  240  76  110  149  625  689  54  198  510  61  109  212  122  377  428  246  468  472  42  467  653  468  701  1091  122  294  986  193  509  519  75  76  91  26  41  48  218  225  286  136  140  494  41  64  51  76  150  11  42  53  22  44  338  26  193  209  46  180  19  48  93  129  150  161  196  210  226  34  108  188  255  490  543  83  104  13  135  180  113  116  274  42  8  657  38  214  471  533  21  50  98  18  33  229  301  317  699  155  156  244  29  146  265  94  173  427  52  134  210  254  256  236  506  525  181  234  250  257  261  269  292  299  301  329  335  407  514  553  81  284  172  179  241  530  531  557  30  51  81  65  269  403  42  343  344  375  376  397  455  495  506  524  534  589  614  620  629  637  664  704  737  772  799  802  880  883  407  555  733  190  215  456  736  952  1097  59  278  357  51  70  359  45  57  59  116  125  128  149  236  263  321  342  444  597  641  37  46  203  130  140  265  194  396  889  285  435  486  126  211  756  57  292  328  127  141  181  201  1042  1360  125  870  1169  1107  1179  1218  83  375  383  303  523  575  193  220  323  21  139  142  62  181  365  331  369  391  212  223  238  285  310  685  239  256  458  276  342  482  127  340  442  64  197  213  51  100  511  15  431  442  544  545  551  608  699  710  717  870  1038  1113  1130  67  139  141  96  208  532  177  330  349  391  555  682  13  26  40  51  93  122  155  203  257  273  288  291  298  299  336  358  368  382  383  384  400  529  550  553  106  512  898  64  276  351  77  81  193  55  90  125  48  61  181  306  136  273  319  303  390  581  62  71  154  177  180  245  259  290  301  329  336  360  454  479  492  495  500  556  559  576  594  645  678  767  147  167  184  210  224  271  327  390  435  521  570  574  636  673  677  783  814  845  858  882  961  982  1342  1343  30  146  174  203  209  315  402  228  780  820  377  427  532  16  110  111  222  438  53  102  113  56  59  488  39  58  306  142  198  202  268  457  484  74  85  115  36  122  163  82  94  326  283  364  396  278  372  429  187  26  139  155  928  1024  1860  84  304  386  396  175  272  312  48  99  333  66  169  381  19  34  215  37  201  221  262  305  346  19  461  916  832  1341  1710  30  87  192  171  253  404  51  102  431  267  354  403  202  388  399  131  468  733  87  113  164  75  241  368  17  322  349  182  331  334  88  294  558  183  214  340  48  109  183  234  392  480  176  416  539  146  152  687  87  90  146  176  246  272  378  407  418  437  476  549  566  728  742  768  796  810  827  191  342  364  261  317  497  38  42  45  127  207  231  163  390  403  15  293  118  141  305  64  114  144  40  84  95  110  118  119  32  95  166  241  267  307  54  459  538  93  700  787  42  56  223  170  232  370  142  238  322  642  670  709  715  770  862  880  960  1054  1172  1196  1261  1350  1394  1463  1498  1572  1601  1610  1638  1891  107  318  587  66  137  222  170  190  243  1854  2321  2325  87  306  653  572  1060  1151  57  256  556  110  74  227  249  232  243  60  87  121  156  169  180  196  197  240  299  340  400  422  427  443  456  79  120  149  145  235  32  54  229  31  113  296  38  110  113  53  84  57  240  283  284  345  422  428  482  494  540  558  707  743  762  806  30  52  74  86  121  144  164  260  271  339  374  391  423  433  570  571  596  630  650  652  701  767  781  93  176  249  136  164  180  223  277  361  24  37  26  93  114  172  352  547  17  164  166  54  73  133  81  843  887  71  78  199  651  724  880  394  396  500  156  272  292  559  796  1345  149  200  269  59  219  222  221  223  1096  152  224  238  10  239  595  115  150  897  76  219  265  41  44  162  502  603  791  429  433  473  78  88  109  36  37  285  12  76  135  128  155  168  251  694  1057  376  653  947  98  312  318  322  453  822  109  143  225  154  1450  1758  747  889  1559  52  60  92  102  104  164  174  191  239  256  262  270  281  298  301  356  369  76  310  345  298  361  652  94  124  301  253  275  294  47  92  8  322  329  135  312  1051  185  305  340  416  473  477  504  626  633  665  742  840  855  859  954  993  1058  1246  1271  1283  1335  1374  1421  1437  104  113  154  139  181  189  374  706  761  78  366  543  678  58  118  173  105  186  262  284  288  293  314  469  487  1225  421  486  540  85  94  160  128  299  333  49  441  542  407  427  761  299  341  204  262  413  72  136  222  28  89  94  8  115  549  212  289  358  267  269  749  408  635  839  192  715  773  246  738  968  520  526  578  118  391  481  34  61  85  107  238  293  328  338  361  375  79  252  712  186  208  214  333  361  387  391  396  464  474  476  481  506  565  589  654  795  883  885  978  990  1001  1019  1051  253  426  453  13  136  244  259  30  43  60  627  732  61  83  108  109  114  129  148  197  213  309  351  522  211  553  1111  125  329  516  106  138  146  147  397  728  32  489  544  569  14  36  45  58  95  155  241  271  295  347  373  429  445  459  538  540  541  582  604  624  646  668  706  717  50  115  184  32  42  54  77  111  132  145  176  273  306  315  339  378  75  137  156  74  116  259  264  334  125  152  306  154  461  1670  156  169  327  21  36  49  51  133  169  204  213  232  256  69  687  703  26  178  191  95  198  563  330  350  382  69  159  227  239  300  323  425  436  454  581  591  608  623  650  654  785  802  810  880  893  904  916  927  1031  19  393  454  62  81  82  92  107  118  124  129  166  191  271  279  281  34  89  168  97  102  157  183  191  233  331  170  386  528  87  93  115  118  123  175  238  264  308  318  329  340  364  375  388  403  409  420  460  490  498  515  519  520  161  348  721  129  149  188  190  233  242  264  341  361  376  422  478  498  516  557  585  596  625  636  644  675  683  689  721  622  774  798  12  118  174  113  282  316  102  149  492  185  270  292  36  41  161  501  669  733  109  116  124  88  161  343  1056  1086  1225  21  55  57  99  150  159  162  254  355  33  42  187  309  646  1003  79  88  169  62  78  473  83  144  149  238  244  261  277  307  309  402  97  463  636  130  148  189  242  248  316  46  67  240  262  276  406  479  587  635  705  734  739  772  826  849  966  984  1059  1282  1330  1359  1401  1584  1586  1067  1099  1395  406  449  460  63  230  252  425  428  439  49  72  171  95  176  203  77  169  365  100  1016  1110  383  558  852  383  538  647  73  254  1090  63  125  140  156  210  225  250  267  286  327  338  15  40  76  81  87  107  156  165  177  194  228  281  283  316  349  351  473  489  495  555  583  171  967  996  98  247  309  157  271  742  25  48  49  96  192  194  249  324  409  411  476  491  563  584  614  643  137  225  455  269  431  656  246  364  626  387  401  442  86  339  341  76  207  300  71  122  12  43  46  216  272  316  72  137  539  172  223  292  25  65  72  219  243  295  310  312  488  44  53  101  108  212  240  212  713  1921  8  548  623  89  264  304  68  279  528  57  70  121  63  99  109  80  704  880  130  181  203  95  174  277  148  185  222  238  259  316  71  173  371  32  142  202  40  49  85  38  47  82  95  406  912  1231  146  155  450  479  550  906  143  239  269  600  604  765  328  347  368  25  60  68  45  185  205  453  465  60  68  85  153  456  547  18  46  230  565  600  811  975  181  430  461  492  579  612  33  39  128  151  150  207  225  348  379  398  91  98  170  185  220  254  104  128  154  220  225  97  101  208  303  409  271  522  569  620  757  848  101  278  297  305  309  48  75  83  111  548  87  609  917  953  1005  1016  241  308  374  402  9  20  39  73  91  115  118  229  238  307  328  425  442  514  594  630  640  711  747  824  848  932  949  1138  1142  1202  1413  1476  1529  1587  1636  144  158  221  574  740  1111  1390  30  64  579  610  621  677  911 | PLPSLE**Y**WPQKSD  EEDDDD**Y**VTLSDQ  SSVSEE**Y**FEVREH  VGERSD**Y**AGFRAC  ESDHSG**Y**VRPVPV  IIITDQ**Y**GNQIQA  LTVQCH**Y**KQGWET  KQGWET**Y**IKWWCR  RDDADV**Y**WCGIER  SHKRNH**Y**MLLVFV  EGKRHP**Y**KMNLAS  NNPAGL**Y**SSENIS  VTPPEG**Y**EVVTVF  YAGEVT**Y**SVTGFL  IRHQVK**Y**LGLLEN  KNMVWK**Y**CRSISP  FESGKA**Y**KTTWGD  GAASGG**Y**IKRITN  RDSMLK**Y**PDSHQP  QPQPKS**Y**FKSISV  EHDPFT**Y**DYQSLQ  DPFTYD**Y**QSLQIG  GKTTFL**Y**RYTDNK  QLQANA**Y**CENPDI  DKYGIP**Y**FETSAA  PGLWDT**Y**EDDISE  DIFIIT**Y**PKSGTT  DVVVSL**Y**HYSKIA  KGLIHL**Y**SELELS  SQGSPS**Y**GSPEDT  QDTSGT**Y**YWHIPT  DTSGTY**Y**WHIPTG  CIRQLS**Y**HKNNLH  RERDFA**Y**VARDKL  VQKFQV**Y**YLGNVP  AACMLR**Y**QKCLDA  LLESKT**Y**HALSNL  EKALTD**Y**RAELRD  THLAKL**Y**DNLLEQ  AVVKMC**Y**EAKEWD  VQQCCT**Y**VEEITD  LSICKH**Y**RAIYDT  QEQLMG**Y**RKRGPK  PGKSGK**Y**YYQLNS  QPDPKM**Y**DLQYQG  YGPEGP**Y**GVFAGR  SQFTFK**Y**HHVGKL  GEEPTV**Y**SDEEEP  SRAEEE**Y**EYAFDD  APSPAG**Y**QNTHSP  VKVRDT**Y**LDTQVV  CLLRRN**Y**SREQHG  IVDDDD**Y**FLCLPS  KQLLQL**Y**LQALEK  ASIVDS**Y**ERRNEG  VLIHEY**Y**SREAPN  LSTVLQ**Y**AEDVLS  DPRLLR**Y**LVGFPG  ATAGPS**Y**PRPVIP  KLLPEE**Y**QGGSFS  FEKGGS**Y**LGREVA  ADRSKI**Y**MADLES  VAVLAK**Y**FPGRPL  ALIINA**Y**PDNITP  VIESKA**Y**VGRNVI  PENTVI**Y**GADCLR  SAVVQL**Y**AADRNC  DNPQRS**Y**FLRIFD  LWEQEL**Y**NNFVYN  LYNNFV**Y**NSPRGY  YNSPRG**Y**FHTFAG  ITTNRF**Y**GPQVNN  PPPNRM**Y**PPPPPA  PHPRVR**Y**AACNAV  VPLLKF**Y**FHDGVR  EDDNDV**Y**ILTKVS  NSKVNK**Y**YSNLTK  LTKSER**Y**SSSGSP  NSGDSG**Y**PSEKRG  TKFATE**Y**CNTIEG  GGARIC**Y**IFHETF  ELVGQL**Y**KSSLLD  LPGPST**Y**ALPSIY  ALKYEI**Y**LNSSLV  DLCCQA**Y**NLIRKQ  GPEGFQ**Y**RALYPF  FQYRAL**Y**PFRRER  GDFPGT**Y**VEFLGP  LDSESH**Y**RPELPA  LQDAEW**Y**WGDISR  SKIQGE**Y**TLTLRK  FHRDGH**Y**GFSEPL  VDLINH**Y**RHESLA  HESLAQ**Y**NAKLDT  LDTRLL**Y**PVSKYQ  LYPVSK**Y**QQDQIV  GAQLKV**Y**HQQYQD  KVYHQQ**Y**QDKSRE  QDKSRE**Y**DQLYEE  DQLYEE**Y**TRTSQE  EKCSKE**Y**LERFRR  RKIRDQ**Y**LVWLTQ  HEERTW**Y**VGKINR  TKHCVI**Y**RTATGF  FGFAEP**Y**NLYGSL  AEPYNL**Y**GSLKEL  KELVLH**Y**QHASLV  PHDGNL**Y**PRLYPE  YPELSQ**Y**MGLSLN  RPSSIN**Y**MVAPVT  PMSLDE**Y**QVAQLY  ATSSSR**Y**QGAVAT  RIDYSL**Y**CPEALT  TASKGR**Y**IPPHLR  ELAVQI**Y**EEARKF  MAYEHH**Y**KGSSRG  EGRPCE**Y**NSRIYQ  MEPRIL**Y**NPLQGQ  EAAFPF**Y**RLFNDI  MASLEV**Y**VRRGYI  WFPDSA**Y**KTAQAV  GGMKDM**Y**DQVLKF  LRDNPA**Y**DTPDAG  ALKAAP**Y**GHSRSS  VENGAE**Y**ILETID  ELELSD**Y**PWFHGT  ETRPGE**Y**VLTFNF  DITLRS**Y**VRAQDP  DSPGQH**Y**FSSLAA  QMQCKV**Y**DSLLAL  NIIQDF**Y**NPLVAS  EMGASL**Y**VGWAAS  PRTDKP**Y**SAKYSA  KPYSAK**Y**SAARSA  RQLLSD**Y**GPPSLG  GPPSLG**Y**TQGTGN  KEIRPT**Y**AGSKSA  YGLGLV**Y**FYYNAF  KATKES**Y**AIQYLQ  PNLEDL**Y**EANVPV  EKASIM**Y**PSNNCD  RVDQLK**Y**DVQHLQ  RAFQDK**Y**FMIGGM  EVCKRG**Y**LRKQKH  KHGHRR**Y**FVLKLE  APARLE**Y**YENARK  PARLEY**Y**ENARKF  RRVITL**Y**QCFSVS  QRADAR**Y**RHLIAL  LFTQDE**Y**FAMVAE  SEQESW**Y**LLLSRL  AAEPPF**Y**KDVWQV  CGHSEQ**Y**FFLEVG  RARCRS**Y**SISIGA  EGSGGD**Y**MPMNNW  SLKKRS**Y**FGKLTQ  GGRFRL**Y**FCVDRG  EDEDDP**Y**VPMRPG  FEDSRG**Y**MMMFPR  NDSESD**Y**MFMAPG  QNDNSE**Y**VPMLPG  LDKEVS**Y**NWDPKD  SFITKG**Y**KIKPKP  QSAFSN**Y**VNVEFG  IEEEGD**Y**IEVIFN  ADSAIR**Y**DAETGR  AETGRI**Y**VVDPFS  VEPAER**Y**IKDRTL  HEDDIN**Y**PHKYGP  KAIDTI**Y**QTTDFS  PLDLIN**Y**IDVAQQ  IMGVSK**Y**GIKVST  LHRHAL**Y**LIIRMV  LGIFIK**Y**KELRTP  GSWKFG**Y**AGCQVY  IIGWAS**Y**APDPTG  ELNFGA**Y**LGLPAF  TTHTEE**Y**SGEEKT  DRRIKL**Y**AVEKNP  EKGPNG**Y**GFHLHG  KGKLGQ**Y**IRLVEP  KKGPSG**Y**GFNLHS  SLLRSH**Y**REVLPL  TTSVRS**Y**LPNTVT  VCGPPL**Y**QLGAAT  TPCPPV**Y**AETKHF  ETKHFL**Y**SSGDKE  PRLPQR**Y**WQMRPL  NHAQCP**Y**GVLLKT  SSPWQV**Y**GFVRAC  HWLMSV**Y**VVELLR  LLRSFF**Y**VTETTF  KNRLFF**Y**RKSVWS  PIVNMD**Y**VVGART  LFSVLN**Y**ERARRP  VDVTGA**Y**DTIPQD  IKPQNT**Y**CVRRYA  TYCVRR**Y**AVVQKA  LTDLQP**Y**MRQFVA  RIRGKS**Y**VQCQGI  VRGVPE**Y**GCVVNL  LEVQSD**Y**SSYART  TVCTNI**Y**KILLLQ  ILLLQA**Y**RFHACV  DTASLC**Y**SILKAK  TRHRVT**Y**VPLLGS  TLGEGA**Y**GEVQLA  SDSCQE**Y**SDWKEK  LDADKS**Y**QCLKET  NKLFLD**Y**TIKCYE  AENSKG**Y**DFEIKF  ANCLVK**Y**RAQVYV  SLAGNT**Y**QLTRGI  LVLDVV**Y**LVYESK  DVVYLV**Y**ESKHLH  HLFQVE**Y**AQEAVK  DPVTVE**Y**ITRYIA  VEYITR**Y**IASLKQ  ASLKQR**Y**TQSNGR  DGTPRL**Y**QTDPSG  EFLEKN**Y**TDEAIE  PEEIEK**Y**VAEIEK  LETFLT**Y**PMFQIP  TPRHLR**Y**RQPGGQ  VPYLGM**Y**LTDLAF  PLLAME**Y**CQGGDL  IASALR**Y**LHENRI  EQAREL**Y**RRLREK  EAVASH**Y**MRQILE  VVKREP**Y**GKPVDV  GKKKKQ**Y**KDKYLA  KNPTDA**Y**LDAMMN  GTIQED**Y**LRELLT  KKGNFN**Y**IEFTRI  DEEGSL**Y**ILKGDR  FERIED**Y**IKLIGF  KSEDSE**Y**YKAFEE  PNILRL**Y**GYFHDA  SLGVLC**Y**EFLVGK  NTYQET**Y**KRISRV  LRKTGS**Y**GALAEI  TSAGDR**Y**DSLLGR  LEERKP**Y**SSRLEK  EEQIKE**Y**FGAFGE  YGGDQN**Y**SGYGGY  GYDYTG**Y**NYGNYG  LVRQHA**Y**SMALGE  NSLYSV**Y**NFCSKP  VAMAKT**Y**ATTEAF  PFRMTP**Y**LVKVRD  RARNGE**Y**ITLDTS  TKNRSH**Y**SHESGE  PLHAAA**Y**LGDAEI  MISVVK**Y**LLDLGV  GRSPLH**Y**AAANCN  TRVGRL**Y**CVQEPS  VDGVWV**Y**NRSSYP  KGWGQC**Y**TRQFIS  QEQHMV**Y**CGGDLL  KDPSPL**Y**DMLRKN  GNLRSN**Y**TPRSNG  VEKVLV**Y**NNTSIV  DPRLFE**Y**RNQGDE  ARVRDH**Y**IFSVES  TNLPVG**Y**PPQYPP  VGYPPQ**Y**PPTAFQ  FQGPPG**Y**SGYPGP  PPGYSG**Y**PGPQVS  PGPQVS**Y**PPPPAG  CPPGLE**Y**LSQIDQ  FETNNK**Y**EIKNSF  SFGQRV**Y**FAAEDT  PGVPIG**Y**VIQTWH  VDGIPP**Y**RIRKQH  SIVVAY**Y**FCGEPI  KKGSYR**Y**YFKKVS  ASGIEL**Y**AVGVDR  LEEHVF**Y**VETYGV  CECYEG**Y**TLNADK  SDLLLQ**Y**LLQGGA  REEDEV**Y**PPGPYH  ALRRRH**Y**HHALPP  SAPVAN**Y**NQRKEE  WSTGEE**Y**IAVGDF  GLVPRT**Y**LEPYSE  RTYLEP**Y**SEEEEG  NHVSFC**Y**LIVLMR  FELGIS**Y**IRNSTG  PIPAKT**Y**ELFLNG  LNGGTP**Y**EKGIEV  AHGSVF**Y**QIMTMR  IHLLIF**Y**RQILGD  STFLLV**Y**HDCVLP  IRKKGF**Y**KQDVNK  WELPKT**Y**VSPTHV  HVGSGA**Y**GSVCSA  IFAKRA**Y**RELLLL  SSLRNF**Y**DFYLVM  RNFYDF**Y**LVMPFM  SEEKIQ**Y**LVYQML  KIQYLV**Y**QMLKGL  MLKGLK**Y**IHSAGV  YVVTRW**Y**RAPEVI  ILSWMH**Y**NQTVDI  LFKGKD**Y**LDQLTQ  DKAAKS**Y**IQSLPQ  EWKQHI**Y**KEIVNF  EMVQAL**Y**EAPAYH  LYEAPA**Y**HLILEG  LLFSKT**Y**KLQERS  DHPALN**Y**NIVSGP  LASLKK**Y**GVGTCG  CGPRGF**Y**GTFDVH  EAIIYS**Y**GFATIA  ASAIPA**Y**SKRGDI  IVVEGL**Y**MNTGTI  ELVKLK**Y**KYKARI  VKLKYK**Y**KARIFL  RGVTEH**Y**GINIDD  RLSGQG**Y**CFSASL  ALTQAR**Y**LEKEEK  PVLVQV**Y**TALGSC  PQRALL**Y**LVAALK  GDAAEH**Y**LDLLAL  DLAIDT**Y**RRAIEL  PEAIAS**Y**RTALKL  GALFMD**Y**IITDQE  FLGAQA**Y**LDHVWD  VDCSDM**Y**EKVLCT  AASASP**Y**TPEHAA  IPSNTD**Y**PGPHHF  KSATWT**Y**SPLLKK  PLLKKL**Y**CQIAKT  IRAMPV**Y**KKAEHV  GNNLSQ**Y**VDDPVT  QSVVVP**Y**EPPQVG  EFTTIL**Y**NFMCNS  KADEDH**Y**REQQAL  HGDEDT**Y**YLQVRG  GDEDTY**Y**LQVRGR  QPLVDS**Y**RQQQQL  HLQPPS**Y**GPVLSP  CTPPPP**Y**HADPSL  CPNCIE**Y**FTSQGL  QGLQSI**Y**HLQNLT  LKIPEQ**Y**RMTIWR  LKQGHD**Y**STAQQL  SQAPSW**Y**HRDLSR  FALCVL**Y**QKHVHT  QKHVHT**Y**RILPDG  GELIGL**Y**AQPNQG  STVSHD**Y**LKGSYG  DYLKGS**Y**GLDLEA  TIPHDI**Y**VFGTQE  ELTDLD**Y**RPIAMQ  ARRNQN**Y**LDILRL  WFGDLN**Y**RLDMDI  ISFPPT**Y**RYERGS  FPPTYR**Y**ERGSRD  RGSRDT**Y**AWHKQK  RILWKS**Y**PETHII  HIICNS**Y**GCTDDI  KTSDQA**Y**IEFESI  KFFIEF**Y**STCLEE  STCLEE**Y**KKSFEN  ILADIE**Y**LQDQHL  VKSMDG**Y**ESYGEC  MDGYES**Y**GECVVA  GTRERL**Y**EWISID  FNNPAY**Y**VLEGVP  AIGLER**Y**EEGLVH  HLHVGW**Y**QSTYYG  LDSQFS**Y**QHAIEE  AGQINT**Y**CQNIKE  LCGPRR**Y**ASSSFK  AAGTSL**Y**VRPVLI  PVHRIL**Y**KDRNLH  LQQKQQ**Y**TNQLAK  AKETDK**Y**IKEFGS  LSRAAD**Y**QRKSRK  QTLPCI**Y**FWGGLL  AANFTR**Y**SQLTSL  TMLDLS**Y**NNLNVV  WLPQLE**Y**FFLEYN  EYFFLE**Y**NNIQHL  GLFNVR**Y**LNLKRS  GLINLK**Y**LSLSNS  ENIFEI**Y**LSYNKY  FEIYLS**Y**NKYLQL  YLSYNK**Y**LQLTRN  NPGGPI**Y**FLKGLS  PELSSH**Y**LCNTPP  CNTPPH**Y**HGFPVR  GWRISF**Y**WNVSVH  QTEQFE**Y**AAYIIH  AYIIHA**Y**KDKDWV  RTHRPS**Y**EDRVCF  APPIIG**Y**LPFEVL  KGKSCY**Y**RFLTKG  LATSRE**Y**AIKILE  KENKVP**Y**VTRERD  PFFVKL**Y**FTFQDD  QDDEKL**Y**FGLSYA  LYFGLS**Y**AKNGEL  NGELLK**Y**IRKIGS  ETCTRF**Y**TAEIVS  IVSALE**Y**LHGKGI  FVGTAQ**Y**VSPELL  ALGCII**Y**QLVAGL  FRAGNE**Y**LIFQKI  KIIKLE**Y**DFPEKF  CEEMEG**Y**GPLKAH  PPKLTA**Y**LPAMSE  GSNIEQ**Y**IHDLDS  TEGPHL**Y**YVDPVN  EGPHLY**Y**VDPVNK  HTPNRT**Y**YLMDPS  TPNRTY**Y**LMDPSG  EVWRQR**Y**QSHPDA  GWGLLD**Y**KTEKYV  DYKTEK**Y**VMTRNW  QFGIVV**Y**VVGWAL  ETWDPT**Y**FKHCRY  YFKHCR**Y**EPQFSP  EPQFSP**Y**CPVFRI  SGCWPH**Y**SFQLQE  QLQEKS**Y**NFRTAT  RTLLKL**Y**GIRFDI  CDLLLL**Y**VDREAH  DREAHF**Y**WRTKYE  FYWRTK**Y**EEAKAP  AMLLRL**Y**LVPRAV  QEAWMF**Y**KHTRRK  KMHMIL**Y**DLQQNL  VCRACG**Y**DSLQHL  ARAGPV**Y**SHTLAF  SGQQNP**Y**TTNVLQ  NHSEGE**Y**IPGACS  NREAEA**Y**TGLSPP  DPQFLT**Y**MALEER  GMKTIG**Y**DPIISP  ALAGAP**Y**QAVGLV  GVRLLS**Y**QTSLVS  VLFPCK**Y**ATTGCS  LEKQEK**Y**EGHQQF  QAENFA**Y**RLELNG  VAEGPN**Y**LTACAG  CGFPSP**Y**TCVSCG  VSCGAR**Y**CTVRCL  QKQNIL**Y**HLGEEW  HHNNCM**Y**IAHHLL  VLPVNI**Y**CKAMGT  PFCPEC**Y**FERFSP  EREGRP**Y**CRRDFL  GPILDN**Y**ISALSA  ERAGKP**Y**CQPCFL  CSQGVA**Y**LHSMQP  NSIPMA**Y**LTLDHQ  NKSLST**Y**YQQCKK  LSKGNK**Y**SAQGER  ADILLY**Y**GLHRFI  FCHIQH**Y**PGIRQH  ALPTIP**Y**HKLADL  KLADLR**Y**LSRGAS  HKARFS**Y**ILPILG  LGIVTE**Y**MPNGSL  LHRKTE**Y**PDVAWP  IALGVN**Y**LHNMTP  EGGTII**Y**MPPENY  YMPPEN**Y**EPGQKS  SIKHDI**Y**SYAVIT  KHDIYS**Y**AVITWE  NPLQIM**Y**SVSQGH  NEESLP**Y**DIPHRA  QMGLQP**Y**PEILVV  SGQNRG**Y**AFITFC  EDYYDD**Y**YGYDYH  YSGNYG**Y**NNDNQE  QTPTAV**Y**QANQHI  KPRMDQ**Y**FNQMEK  MFFDCL**Y**DEEVIS  VEYLCD**Y**KKIREQ  RAGLPI**Y**ECNSRC  DRQGAT**Y**LFDLDY  KKNRGT**Y**GTLHLQ  IEETKK**Y**GRTIIG  ENGNNG**Y**HKDHVY  LKKQKS**Y**TLVVAK  NLLEKD**Y**FGLLFQ  VEHHTF**Y**RLVSPE  ILVAVL**Y**IAHHNK  RPKASD**Y**QRLDQK  QRLDQK**Y**VLILNV  SRSSSG**Y**FSFDTD  GDEFNA**Y**YARRVF  ILRLLR**Y**IVRLVW  AWGNLS**Y**ADLITR  MVRCVP**Y**FKDKGD  PLSPML**Y**SSSASL  QAAWRY**Y**ATNPNR  MLSRIK**Y**LQTRID  WMREKR**Y**LAEGET  SSEGAL**Y**ILEIVT  LASFLV**Y**LAEKGE  PEPAPP**Y**HSPEDS  HWCSVA**Y**WEHRTR  QAVSIF**Y**DLPQGS  RKVPPG**Y**SIKVFD  NDYGFI**Y**QGSKNW  KDLPPA**Y**GGWQVL  DDITEN**Y**KYEEGS  HRLPGS**Y**DSTSSD  TSSDSL**Y**PRGIQF  APWPPA**Y**PPVTSY  YPPVTS**Y**PPLSQP  AKKVRF**Y**RNGDRY  YRNGDR**Y**FKGIVY  LEEGES**Y**VCSSDN  LVRTRR**Y**VRKFVL  ILCMPH**Y**DIDSAS  YWDQCF**Y**SQEQRQ  VRLKNY**Y**EVHKEL  GAFAQV**Y**EATQGD  SNKPWN**Y**QIDYFG  WNYQID**Y**FGVAAT  IVFSCI**Y**GEGYSN  HESKQM**Y**CVFNRN  NEDACR**Y**GSAIGV  AKLKAK**Y**PSLGQK  LQKGQK**Y**FDSGDY  YFDSGD**Y**NMAKAK  ERTINL**Y**PLTNYT  NFEPPQ**Y**PYIPAH  APLFEL**Y**DNAPGY  IIGVIP**Y**FPYSKQ  QEEIPD**Y**RNAVIV  LKERGA**Y**KIFVMA  YNSFYV**Y**CKGPCQ  LDCFHL**Y**CVTRLN  GTTTQA**Y**RVDERA  KGELYD**Y**ISERRR  WWVNWG**Y**KSSVCD  QQRESG**Y**YSSPER  ASGETT**Y**KKTTSS  IQLGIG**Y**TVGHLS  VLMQDF**Y**VVESIF  DFRFKT**Y**APVAFR  APVAFR**Y**FRELFG  GIRPDD**Y**LYSLCN  RPDDYL**Y**SLCNEP  ASGSLF**Y**VTSDDE  QKLLPG**Y**YMNLNQ  KLLPGY**Y**MNLNQN  TLLPKF**Y**GLYCVQ  PKFYGL**Y**CVQSGG  DLKGST**Y**KRRASK  EKSFPT**Y**KDLDFM  SFKIMD**Y**SLLLGV  VGQKAL**Y**STAMES  IDILQS**Y**RFIKKL  VHRPSF**Y**AERFFK  EREEAQ**Y**DLRGAR  LRGARS**Y**PTLEDE  TSEQPR**Y**RRRTQS  VYSPLH**Y**SAQAPP  QFLALQ**Y**RLRHRL  SRLLHD**Y**HRDLYN  EQEKMA**Y**QKTVEQ  QNLNDA**Y**GPPSNF  STVRRR**Y**SDFEWL  EIIDKS**Y**TPSKIR  LQGEKR**Y**YSDPRF  ISWAEE**Y**EARENF  ANCSSP**Y**QVDLFG  DGRIAF**Y**RFCNLT  LLLSFN**Y**IRTVTA  LELGSQ**Y**TPLTID  LFELRL**Y**FCGLSD  AVLKDG**Y**FRNLKA  NQIRSL**Y**LHPSFG  LAANSL**Y**SRVSVD  KVLNLA**Y**NKINKI  IADEAF**Y**GLDNLQ  QVLNLS**Y**NLLGEL  NLLGEL**Y**SSNFYG  LYSSNF**Y**GLPKVA  GLPKVA**Y**IDLQKN  ENLDIL**Y**FLLRVP  SHLQVL**Y**LNHNYL  LYLNHN**Y**LNSLPP  GPPADI**Y**CVYPDS  ADIYCV**Y**PDSFSG  GFCFIC**Y**KTAQRL  GTEPDM**Y**KYDAYL  EPDMYK**Y**DAYLCF  MYKYDA**Y**LCFSSK  CLEAFS**Y**AQGRCL  FVQKQQ**Y**LRWPED  KDAQEQ**Y**NKLRMM  SNMETL**Y**KELGEY  YKELGE**Y**FLFDPK  PNIMAC**Y**NELLQL  EDRFIQ**Y**ANPAFE  KEWQGI**Y**YAKKKN  TSTSSI**Y**QNGDIS  DPVLQV**Y**LYHSLG  GKSEAD**Y**LTFPSG  TFPSGE**Y**VAEEIC  TRHNVL**Y**RIRFYF  FYFPRW**Y**CSGSNR  SGSNRA**Y**RHGISR  QTPLAI**Y**NSISYK  IYNSIS**Y**KTFLPK  RNLKLK**Y**LINLET  TLQSAF**Y**TEKFEV  EQDLQL**Y**CDFPNI  GNQTGL**Y**VLRCSP  KDLLNC**Y**QMETVR  DKAHRN**Y**SESFFE  FGSLDT**Y**LKKNKN  QRKLQF**Y**EDRHQL  INNCMD**Y**EPDFRP  HDNIVK**Y**KGVCYS  KYKGVC**Y**SAGRRN  IMEYLP**Y**GSLRDY  YGSLRD**Y**LQKHKE  HIKLLQ**Y**TSQICK  ESPIFW**Y**APESLT  GVYFSS**Y**GHYGIH  ISEWMG**Y**FLLFES  AKGGSV**Y**PDICTI  ERSGLA**Y**CPNDYH  AYCPND**Y**HQLFSP  FSPRCA**Y**CAAPIL  EKDKKP**Y**CRKDFL  RPVLEN**Y**LSAMDT  PFCELH**Y**HHRRGT  CISAMG**Y**KFHPEH  EQNDKT**Y**CQPCFN  DHSHLL**Y**STIPRM  SSVSNN**Y**IQTLGR  NGGPGP**Y**VGQAGT  LPRNFH**Y**PPDGYS  HYPPDG**Y**SRHYED  TRIEER**Y**RPSMEG  RPSMEG**Y**RAPSRQ  DQRSMG**Y**DDLDYG  GYDDLD**Y**GMMSDY  YGMMSD**Y**GTARRT  RRRLRS**Y**EDMIGE  EVPSDQ**Y**YWAPLA  VPSDQY**Y**WAPLAQ  KSNAAA**Y**LQHLCY  YLQHLC**Y**RNDKVK  LLRNLS**Y**QVHREI  IPQAER**Y**QEAAPN  TSPARG**Y**ELLFQP  PEVVRI**Y**ISLLKE  CAGRWT**Y**GRYIRS  RWTYGR**Y**IRSALR  LQTIWG**Y**KELRKP  DSEIIG**Y**ALDTLY  ISKHEL**Y**SRASQK  DNIVTH**Y**KNMIRE  QSAGKE**Y**VGIVRL  LDAQWL**Y**DNHKDE  IMERDT**Y**PRKWGL  MSSDSE**Y**DSDDDR  LRVKNQ**Y**EKHKEV  LPLLVA**Y**KEDEIP  PQVIAD**Y**ESGRAI  VHNNIV**Y**NEYISH  KKLGEK**Y**HKKKAI  KEVIDK**Y**TAVVKM  KRLENN**Y**YWNAQE  ALGLHD**Y**CDIIKH  HHKSDP**Y**STGHLR  AQRGLA**Y**LRDAWG  RVSAVL**Y**QERENG  RGSKGE**Y**QIKMEN  SKFRIE**Y**EPLVAC  PIVKPE**Y**FTEFLK  SGINDD**Y**GQLKNF  EEPKTY**Y**ELKSQP  RQQVKQ**Y**LSTTLA  TEANQA**Y**SVPTKM  TTLSDI**Y**LNNIIP  KVLNEL**Y**SVMKTY  GHVADI**Y**LANINK  DELDDL**Y**SGSGSG  SGSGSG**Y**FEQESG  DNFLNR**Y**EKIVKK  QMKAED**Y**DVVKVI  KASQKV**Y**AMKLLS  AFQDDR**Y**LYMVME  QDDRYL**Y**MVMEYM  LYMVME**Y**MPGGDL  VNLMSN**Y**DVPEKW  AVGTPD**Y**ISPEVL  QGGDGF**Y**GRECDW  SVGVFL**Y**EMLVGD  VGDTPF**Y**ADSLVG  DSLVGT**Y**SKIMDH  PFIGFT**Y**YRENLL  FIGFTY**Y**RENLLL  EIQKKL**Y**TLEEHL  QHKNAE**Y**QRKADH  TQQMIK**Y**QKELNE  FGWVKK**Y**VIVSSK  SKKILF**Y**DSEQDK  RIFQIL**Y**ANEGES  VGEKSN**Y**ICHKGH  EFIPTL**Y**HFPTNC  IAPCKV**Y**YDISTA  APCKVY**Y**DISTAK  RKQGHE**Y**VILYRI  PMHPCN**Y**RTAGED  GSADLA**Y**PVVFAA  VPAPTF**Y**VSDVEN  KPVRDE**Y**EYVSDD  CFKDSD**Y**VYPSLE  MGSEST**Y**SECETF  SKKVAR**Y**LHQSGA  NFRLQD**Y**IDRIIV  QESEDE**Y**SDDDDM  VRAAAS**Y**ALGRVG  AAFECM**Y**SLLESC  SQDVQG**Y**ITNQSP  PTAKEH**Y**PVSSPS  GMPFSV**Y**GNAMIP  QAEIEQ**Y**RLQREK  MTILQT**Y**FRQNRD  GVGAPV**Y**MAAVLE  MAAVLE**Y**LTAEIL  LKAISS**Y**FVSTMS  GSRWRD**Y**GALAII  FGFHQL**Y**KKYLLP  EGSTVT**Y**HLLGPQ  PEEDLE**Y**FECSNV  NEWKKK**Y**EETRQE  KKCAQD**Y**LARVKQ  YALLGN**Y**DSAMVY  LDQMNK**Y**LYSVKD  RLEKRI**Y**IPLPSA  ENPSSQ**Y**WKEVAE  KRRKAL**Y**EALKEN  VAEHVQ**Y**MAELIE  KSKKCK**Y**SFKCVN  DADENF**Y**TCAWTY  RDIHRN**Y**VDCVRW  RGFDKA**Y**VVLGQF  YYDQEI**Y**GGSDSR  GKRVKP**Y**LPQICG  MGKDYI**Y**AVTPLL  SSTSSP**Y**PLPEEI  KDVKSI**Y**LQEFQD  SLASER**Y**DKDDEA  FVLKAG**Y**LEKRRK  LSKTVF**Y**YYGSDK  KTVFYY**Y**GSDKDK  EFAIDG**Y**SVRMNN  DIIPED**Y**DERGEL  DERGEL**Y**DDVDHP  GDKSTD**Y**ANFYQG  TDYANF**Y**QGLWDC  KRGDVI**Y**ILSKEY  YILSKE**Y**NRYGWW  SKEYNR**Y**GWWVGE  GWRLPE**Y**TLSQEG  PAHKRE**Y**TTICRL  QGFNIT**Y**LDIDEL  FSHGLI**Y**WSDVSE  VEFGLD**Y**PEGMAV  LSAEEN**Y**ESCPPS  TEMDPT**Y**SPAALP  EKLFQG**Y**SFVAPS  SETEAS**Y**IMRKLV  ATLSGW**Y**HLCLPE  LGEVDH**Y**QLALGK  SNLNGR**Y**AVSEAA  DPDWAS**Y**TLGVFI  EPYSAG**Y**REGFLW  NYLKEG**Y**MEKTGP  IIDNKR**Y**LFIQKL  GEGGFS**Y**VDLVEG  LHDGHF**Y**ALKRIL  ILRLVA**Y**CLRERG  AIHAKG**Y**AHRDLK  SLGCVL**Y**AMMFGE  MFGEGP**Y**DMVFQK  DDDDGM**Y**GEREAT  GVNKFS**Y**PPSHRE  FLKAWV**Y**WPGEDT  HFRGWG**Y**RPGKET  IKTVTE**Y**KIDEDG  WTTRCP**Y**KDTLGP  GVSGFG**Y**DHLILN  KDKITQ**Y**EKSLYY  KDDLAD**Y**GGYDGG  LADYGG**Y**DGGYVQ  VSNLLY**Y**QTNYLV  LYYQTN**Y**LVVAAM  RRMKKR**Y**PTTFVM  LQKLPF**Y**DLLDEL  FMEILK**Y**CTDCDE  SGDNQH**Y**NTSLLA  YRPTKN**Y**LSYLTA  TKNYLS**Y**LTAPDY  SLVSKN**Y**EIERTI  SFHMFP**Y**QSQIAV  GDLFNL**Y**LHQNYI  LYLHQN**Y**IDFFME  SPTSPD**Y**SLDSPG  EPPAPQ**Y**TGPFCG  TLLLNG**Y**QTLEDF  KSRKNN**Y**GLLLNE  EKTEQF**Y**RKLLNK  KKEYKS**Y**EAKLRL  TEVGKH**Y**LQLAGD  WTGRFK**Y**VRVTDI  DWMRCL**Y**GEAYPA  QLQREC**Y**LKHDLC  LLLHEP**Y**VDLVNL  DECLAV**Y**RDLVRN  TQAIQW**Y**QNHQPK  GKLPKN**Y**DPKVTP  ADTFKD**Y**AVSTVP  IAANCI**Y**LNIPNK  PESAKV**Y**EKLKDH  VNGSSS**Y**HSDDDD  RTPADI**Y**RAFVDV  DVVNGE**Y**VPRKSI  LVDESG**Y**VSGYKH  SGYVSG**Y**KHAGTY  YKHAGT**Y**DQKVQG  KTEASK**Y**IMQYIA  IGVLDI**Y**GFEIFD  DFKRLM**Y**NSSNPV  GLYNPP**Y**SAQSHP  MAWGSP**Y**VPEVRS  VSDHSD**Y**IHLEGS  DSSRSI**Y**EVAMQL  TFNLVT**Y**LAELLE  GAKEDM**Y**RINTDA  NMSTSS**Y**NDNTEV  IVKVPP**Y**HDQHIT  LQQSPV**Y**QQTSHM  SRERPG**Y**RAGVAA  GGSDIE**Y**LDFYKP  LKTESI**Y**LNGLTP  LRNNPL**Y**HAGAVA  VFQALI**Y**WIKAMN  ERQDLV**Y**FWTSSP  KPTWPA**Y**RLPVVL  RIHFSG**Y**DNDRPG  DVKDET**Y**DLLYQQ  GQNWLI**Y**TYGVTN  NWLIYT**Y**GVTNSG  EADISM**Y**GKEELL  ENLTSE**Y**DLDLFR  LDHKTL**Y**YDVEPF  KEVLLR**Y**LHNFQG  ICYFVR**Y**WITEFW  DNYRRA**Y**GECTDF  TAQDTL**Y**VLPSPT  RVDTFW**Y**KFLKRE  VCGWLQ**Y**VMGWNL  AKRPAV**Y**IDEEAL  VSALEV**Y**TPKEIF  TVSFFH**Y**SQGQVY  YSQGQV**Y**LGNYPP  QVYLGN**Y**PPFKDR  FIHNGT**Y**ICDVKN  PGHIRL**Y**VVEKEN  MILAVL**Y**RRKNSK  KNSKRD**Y**TGCSTS  LQQTLN**Y**INQGVS  KTMGFC**Y**QILTEP  KYLEMI**Y**SMCKKV  GLCTFK**Y**DYTDSK  QLHEAG**Y**DAYITG  AVNTSK**Y**AESYRI  STKAKN**Y**VNGHCT  ELLVKA**Y**GDFAWS  VFLDSL**Y**STDTVT  MDGKGS**Y**QVKGWK  WVNEFR**Y**GGFSLG  NRFGDG**Y**TIVVRI  ERALNS**Y**FEPPVE  CSYLAL**Y**SPDVIF  EVIPPY**Y**SYLKKR  IRMASI**Y**SEEGNI  AELLKR**Y**TKEYTE  KRYTKE**Y**TEYNEE  GSDGAT**Y**RDECEL  PDLSVM**Y**RGRCRK  GNNNVT**Y**ISSCHM  EEEHYG**Y**ASSNVS  NSEASS**Y**ESNYSY  EELDQL**Y**LDDVNE  NDDYAV**Y**VRENIQ  VESSHP**Y**TDDTST  TEDGEV**Y**TWGDND  TSALEN**Y**INRTVA  QVVLGL**Y**IVRGDN  EASALL**Y**QPYGAA  ALLYQP**Y**GAAVGL  EERKCP**Y**CPDRFH  SDIQIF**Y**QEDPEC  TDEALD**Y**ICKLCA  TSHLRK**Y**NDALII  LTKLSK**Y**QASTSN  QAQKVR**Y**EGSYAL  VRYEGS**Y**ALTSEE  YSGNME**Y**VISRIS  EWVVRL**Y**YSFQDK  VLLRTG**Y**TQLCDW  SLEAEA**Y**AAFPGL  KAFNCK**Y**CNKEYL  HSDVKK**Y**QCQACA  VLGDRT**Y**CKAPGP  PTHGSV**Y**PQLLIP  VRATVV**Y**RHHLQL  SCVPYK**Y**VPISVL  ANGSIL**Y**KEYEGM  PASDDA**Y**PEIEKF  VVIMRD**Y**QHENVV  ENVVEM**Y**NSYLVG  SLVGTP**Y**WMAPEL  LKRTDK**Y**RTYSKK  EATCKL**Y**FYQMLL  QITSGK**Y**NFIPEV  VFDIGS**Y**TVRAGY  IPVHDG**Y**VLQQGI  WISKQE**Y**EEGGKQ  CQVEVL**Y**FAKSAE  FAVRQE**Y**VELGDQ  SDEAVK**Y**YTLEEI  DEAVKY**Y**TLEEIQ  ILHHKV**Y**DLTKFL  VAPPKA**Y**EVRIKM  CGFSTG**Y**GSAVNV  TWKGAV**Y**GGFKSK  MVIPDL**Y**LNAGGV  GGVTVS**Y**FEWLKN  VHSGLA**Y**TMERSA  ANQILS**Y**GAELDA  EKLATI**Y**WFTVEF  KTAIQN**Y**TVTEFQ  QESNRM**Y**SVNGYT  GGMKQK**Y**TVNQCR  YKHRGV**Y**SSDVFD  HLPAAS**Y**RKKDSG  LGLLGP**Y**IRAEVE  MALYNL**Y**PGVFET  NGLSDL**Y**HPIQIL  IQILAD**Y**LTLQEH  VSLLTD**Y**SPQLQK  QGRFHM**Y**EGYPLW  PAMSDA**Y**DRTMRQ  ELQEGT**Y**VMVAGP  FADLLD**Y**IKALNR  FIRLKS**Y**CNDQST  DKFVVG**Y**ALDYNE  VPMPDK**Y**SLEPVA  SKLGDV**Y**VNDAFG  MKKELN**Y**FAKALE  GFLIDG**Y**PREVQQ  KRLETY**Y**KATEPV  EPVIAF**Y**EKRGIV  VEETCS**Y**EEAFEA  RELLES**Y**IDGRIV  CDRDGK**Y**GFYTHV  PDLRCN**Y**SIRVER  IGCSRG**Y**GFYTKV  YTKVLN**Y**VDWIKK  LKFGSG**Y**VSGWGR  SALVLQ**Y**LRVPLV  CAMKGK**Y**GIYTKV  NEFWNK**Y**KDGDQC  KDGLGE**Y**TCTCLE  TRFKDT**Y**FVTGIV  TGNGKN**Y**RGTMSK  PSSGPT**Y**QCLKGT  KGTGEN**Y**RGNVAV  YPKKED**Y**IVYLGR  ECQQPH**Y**YGSEVT  CQQPHY**Y**GSEVTT  FLAKFD**Y**YMPAIA  AKEGVV**Y**VEVRYS  VELCKK**Y**QQQTVV  EQWSKL**Y**PIANGN  KPISVS**Y**NPATAK  LTHPPL**Y**ESVTWI  KLNCRL**Y**RKANKS  DISELV**Y**GAKLQP  EGRDDL**Y**VSDAFH  EDVKKL**Y**HSEAFT  KKQIND**Y**VEKGTQ  WVLLMK**Y**LGNATA  AAGDRV**Y**IHPFHL  LLIQPH**Y**ASDLDK  LVLQGS**Y**DLQDLL  WKIRAY**Y**ENSPQQ  EPTEKF**Y**YIYNEK  PGAVKV**Y**AYYNLE  IKMTKM**Y**KGFQAL  ADIRFV**Y**TPAMES  RGFTKT**Y**TVGCEE  DFAVGE**Y**NKASND  KASNDM**Y**HSRALQ  IVAGVN**Y**FLDVEL  LRHGIQ**Y**FNNNTQ  ECTDNA**Y**IDIQLR  ENNATF**Y**FKIDNV  AGDSLS**Y**YHSPAD  APSAGA**Y**SRAGVV  DLSGSF**Y**AADWEP  TPSCTA**Y**TSSFVF  SSFVFT**Y**PEADSF  SVQPYF**Y**CDEEEN  PSTRKD**Y**PAAKRV  LKKATA**Y**ILSVQA  QDLARS**Y**GIPYIE  RSYGIP**Y**IETSAK  GVEDAF**Y**TLVREI  SSQHWS**Y**GLRPGG  AFSSAC**Y**FQNCPR  RCQEEN**Y**LPSPCQ  RRPVKV**Y**PNGAED  KKDEGP**Y**RMEHFR  PPKDKR**Y**GGFMTS  SWNEPL**Y**HLVTEV  ESRLSA**Y**YNLLHC  SHKIDN**Y**LKLLKC  IPKEQK**Y**SFLQNP  ASDSNV**Y**DLLKDL  KNYGLL**Y**CFRKDM  AALVQN**Y**VQMKAS  TCMLGT**Y**TQDFNK  CRRNGD**Y**QAVQCQ  CLETGE**Y**ARLQAS  VSGPFH**Y**WGPVID  GTFTSD**Y**SKYLDS  TSDYSK**Y**LDSRRA  TSDVSS**Y**LEGQAA  TSAWPL**Y**RAPSAL  AENDTP**Y**YDVSRN  QLSAKK**Y**LESLMG  TLRMRR**Y**ADAIFT  AIFTNS**Y**RKVLGQ  GALAEA**Y**PSKPDN  AEDMAR**Y**YSALRH  LITRQR**Y**GKRSSP  EEEEEA**Y**GWMDFG  LGSLGC**Y**CQDPYV  CYCQDP**Y**VKEAEN  FEKLTN**Y**SVTDLN  LRTRFV**Y**HLSDLC  SATETC**Y**TYDRNK  YDRNKC**Y**TAVVPL  TGNNYV**Y**WYQQLP  APKLLI**Y**RDDKRP  SEDEAH**Y**HCAAWD  SCKASG**Y**TFIDSY  YTFIDS**Y**IHWIRQ  PFWSDY**Y**NFDYSY  GFIFSS**Y**AMYWVR  FSSYAM**Y**WVRQAP  SCFGPD**Y**WGQGTP  VDTATY**Y**CARVVN  VMAGYY**Y**YYMDVW  MAGYYY**Y**YMDVWG  STFSND**Y**YTWVRQ  WIGYVF**Y**HGTSDD  ADTAVY**Y**CARNLI  HPQTKT**Y**FPHFDL  LSELHA**Y**ILRVDP  TSLSPF**Y**LRPPSF  REFHRK**Y**RIPADV  NDRLAV**Y**IDRVRS  DFQKNI**Y**SEELRE  NLVTRS**Y**LLGNSS  KDSGRD**Y**VSQFEG  QEEMEL**Y**RQKVEP  GARLAE**Y**HAKATE  LGRFWD**Y**LRWVQT  MKELKA**Y**KSELEE  QKRLAV**Y**QAGARE  FQTVTD**Y**GKDLME  QAEAKS**Y**FEKSKE  LVNFLS**Y**FVELGT  TFESKS**Y**KMADEA  YGGGWW**Y**NNCQAA  SFRGAD**Y**SLRAVR  SSFQYM**Y**LLKDLW  NTDGKN**Y**CGLPGE  QISVNK**Y**RGTAGN  KAIQLT**Y**NPDESS  HLNGVY**Y**QGGTYS  YYQGGT**Y**SKASTP  QRHGSK**Y**LATAST  QRPGFG**Y**GGRASD  GGRASD**Y**KSAHKG  PGTHKV**Y**VELQEL  EAPHID**Y**TQLGRA  VFRIDA**Y**MAQSRG  ELLRRR**Y**QSSPAK  RDIRRR**Y**PYYLSD  IRRRYP**Y**YLSDIT  RRRYPY**Y**LSDITD  AAVIFI**Y**FAALSP  ETNGLE**Y**IVGRVW  VRFISR**Y**TQEIFS  ISLIFI**Y**ETFSKL  HPLQKT**Y**NYNVLM  LQKTYN**Y**NVLMVP  KFKNSS**Y**FPGKLR  FFIQDT**Y**TQKLSV  LFGIFL**Y**MGVTSL  LFKPPK**Y**HPDVPY  YHPDVP**Y**VKRVKT  KESDTS**Y**VSLKAP  GYSIFS**Y**ATKRQD  WSKDIG**Y**SFTVGG  NPGKVG**Y**PGPSGP  TNQEEP**Y**QNHSGR  PKKGHI**Y**QGSEAD  TNMNNN**Y**EPRSGK  VVTFCD**Y**AYNTFQ  TFCDYA**Y**NTFQVT  ESAVTG**Y**RVDVIP  EYNVSV**Y**TVKDDK  DYTITV**Y**AVTGRG  NGTISR**Y**VGGQEH  LRDTKT**Y**MLAFDV  EQLGEF**Y**EALDCL  QNLPWG**Y**KHTLNQ  GKFSVV**Y**AKCDSS  LLAEKQ**Y**GFCKAT  EFVEGI**Y**KVEIDT  DSGPRR**Y**TIAALL  AALLSP**Y**SYSTTA  ARRHPD**Y**SVVLLL  LRLAKT**Y**ETTLEK  MPCAED**Y**LSVVLN  FVQEAT**Y**KEVSKM  VTSCEA**Y**EEDRET  LRVAKG**Y**QELLEK  MIGYLG**Y**CKGVEP  DENLAL**Y**VENQFR  KDFEDL**Y**TPVNGS  QPRTHY**Y**AVAVVK  MSLDGG**Y**VYTAGK  KTTYEK**Y**LGPQYV  LYLQAS**Y**TYLSLG  LQASYT**Y**LSLGFY  EAGLGE**Y**LFERLT  RIGRFG**Y**GYGPYQ  GRFGYG**Y**GPYQPV  VPEQPL**Y**PQPYQP  RGPRGP**Y**PPGPLA  PPPPPP**Y**GPGRIP  PPPPAP**Y**GPGIFP  VKRPRR**Y**LYQWLG  RPRRYL**Y**QWLGAP  LGAPVP**Y**PDPLEP  RPLGEV**Y**LDSSKP  SSKPAV**Y**NYPEGA  KPAVYN**Y**PEGAAY  YPEGAA**Y**EFNAAA  AANAQV**Y**GQTGLP  GQTGLP**Y**GPGSEA  HGQQVP**Y**YLENEP  GQQVPY**Y**LENEPS  ENEPSG**Y**TVREAG  AGPPAF**Y**RPNSDN  SAKETR**Y**CAVCND  CAVCND**Y**ASGYHY  NDYASG**Y**HYGVWS  YASGYH**Y**GVWSCE  IQGHND**Y**MCPATN  CRLRKC**Y**EVGMMK  AEPPIL**Y**SEYDPT  PILYSE**Y**DPTRPF  LLNSGV**Y**TFLSST  KGMEHL**Y**SMKCKN  SHSLQK**Y**YITGEA  HSLQKY**Y**ITGEAE  AQDNSR**Y**THFLTQ  QGRDDR**Y**CESIMR  PWPPCQ**Y**RATAGF  VDLVQK**Y**LEKYYN  QKYLEK**Y**YNLKND  KYLEKY**Y**NLKNDG  EQTHLT**Y**RIENYT  TYRIEN**Y**TPDLPR  TNNFRE**Y**NLHRVA  DIGALM**Y**PSYTFS  ALMYPS**Y**TFSGDV  DGIQAI**Y**GRSQNP  FFKDRF**Y**MRTNPF  MRTNPF**Y**PEVELN  NGLEAA**Y**EFADRD  FFKGNK**Y**WAVQGQ  GYPKDI**Y**SSFGFP  ENTGKT**Y**FFVANK  FFVANK**Y**WRYDEY  ANKYWR**Y**DEYKRS  YWRYDE**Y**KRSMDP  RSMDPG**Y**PKMIAH  MKDGFF**Y**FFHGTR  FHGTRQ**Y**KFDPKT  APVWAF**Y**LQAAFM  VASCNG**Y**FVFGRH  SCGPDW**Y**TVGTKY  SVVNQV**Y**GYFVLG  IFGWSR**Y**WPHGLK  CFCWGP**Y**AFFACF  TMPEDE**Y**TVYDDG  FRGQYC**Y**ELDEKA  HRNRKG**Y**RSQRGH  LVTGWG**Y**HSSREK  CGLLHN**Y**GVYTKV  YTKVSR**Y**LDWIHG  EENRRF**Y**RQLLLT  DLKRCQ**Y**VTEKVL  LACQGK**Y**TPSGQA  GSAVSP**Y**PTFNPS  QQIKAA**Y**LQETGK  RDINRV**Y**REELKR  SDARAL**Y**EAGERR  ILTTRS**Y**PQLRRV  RRVFQK**Y**TKYSKH  FQKYTK**Y**SKHDMN  NDIKAF**Y**QKMYGI  AFYQKM**Y**GISLCQ  DETKGD**Y**EKILVA  SPSELK**Y**LGLDTH  RKKRQL**Y**SALANK  LVDHFG**Y**TKDDKH  IDFLNN**Y**ALFLSP  SDQLSD**Y**YEKFIA  NCPACR**Y**RKCLQA  QVSYEE**Y**LCMKTL  DEIRMT**Y**IKELGK  LAPALG**Y**LGSRQK  SLPDLP**Y**DYGALE  HAYYLQ**Y**KNVRPD  DVEGRK**Y**FDFLSS  ITKLFN**Y**HKVLPM  GEHGST**Y**GGNPLG  RFQIAQ**Y**KCLVIK  YAKDTR**Y**SSSFCT  CFREAA**Y**TKRLGT  PDEDDF**Y**FGGPDS  YAAPSP**Y**VESEDA  LKKATE**Y**VHSLQA  FSSRSG**Y**RSGGGF  AEAESL**Y**QSKYEE  GSGGGS**Y**GSGGGG  MVYMFQ**Y**DSTHGK  VKLISW**Y**DNEFGY  YDNEFG**Y**SNRVVD  FNASIA**Y**DRHLWE  AEDLIL**Y**CTKEFS  LATDLA**Y**YLVRKG  DMLRHL**Y**QGCQVV  GNLELT**Y**LPTNAS  IQEVQG**Y**VLIAHN  QLFEDN**Y**ALAVLD  CPALVT**Y**NTDTFE  PNPEGR**Y**TFGASC  CVTACP**Y**NYLSTD  TACPYN**Y**LSTDVG  PCARVC**Y**GLGMEH  EITGYL**Y**ISAWPD  QGLPRE**Y**VNARHC  CVACAH**Y**KDPPFC  QQKIRK**Y**TMRRLL  GAFGTV**Y**KGIWIP  EILDEA**Y**VMAGVG  AGVGSP**Y**VSRLLG  IAKGMS**Y**LEDVRL  DIDETE**Y**HADGGK  QSDVWS**Y**GVTVWE  TFGAKP**Y**DGIPAR  ICTIDV**Y**MIMVKC  PLDSTF**Y**RSLLED  PSPLQR**Y**SEDPTV  PSETDG**Y**VAPLTC  ENLTEL**Y**IENQQH  HVNNGN**Y**TLLAAN  LLMVFE**Y**MRHGDL  VAAGMV**Y**LAGLHF  PPESIL**Y**RKFTTE  LWEIFT**Y**GKQPWY  YGKQPW**Y**QLSNTE  ACPPEV**Y**AIMRGC  KRWQAI**Y**KQFDTD  HLNEHL**Y**NMIIRR  NMIIRR**Y**SDESGN  VPSQKT**Y**QGSYGF  GNLRVE**Y**LDDRNT  HSVVVP**Y**EPPEVG  RYQPVS**Y**KLCTRS  RSKIAE**Y**MNHLID  FMLAHP**Y**GFTRVM  GSSWPG**Y**VRPLPP  AVAAPA**Y**SRALSR  RQDEHG**Y**ISRCFT  EPQWSL**Y**PSDSQV  EEEGEN**Y**QKGERG  DHHSTH**Y**RASEEE  SEEEPE**Y**GEEIKG  GEEIKG**Y**PGVQAP  DLEWER**Y**RGRGSE  GRGSEE**Y**RAPRPQ  EEDKRN**Y**PSLELD  GGEPRA**Y**FMSDTR  KELDRN**Y**LNYGEE  DRNYLN**Y**GEEGAP  RFQDKQ**Y**SSHHTA  GELFNP**Y**YDPLQW  ELFNPY**Y**DPLQWK  KNFFPE**Y**NYDWWE  FFPEYN**Y**DWWEKK  EDVNWG**Y**EKRNLA  LDLKRQ**Y**DRVAQL  LDQLLH**Y**RKKSAE  AEFPDF**Y**DSEEPV  KEGILQ**Y**CQEVYP  QYCQEV**Y**PELQIT  PHFVIP**Y**RCLVGE  STNLHD**Y**GMLLPC  GGADTD**Y**ADGSED  EEAEEP**Y**EEATER  AMISRW**Y**FDVTEG  KCAPFF**Y**GGCGGN  NFDTEE**Y**CMAVCG  PDAVDK**Y**LETPGD  RLALEN**Y**ITALQA  THLRVI**Y**ERMNQS  QSLSLL**Y**NVPAVA  LQKEQN**Y**SDDVLA  SEPRIS**Y**GNDALM  FRHDSG**Y**EVHHQK  MLKKKQ**Y**TSIHHG  GYENPT**Y**KFFEQM  NFDVNK**Y**LGRWYE  RCIQAN**Y**SLMENG  WILATD**Y**ENYALV  VRQVED**Y**PVDIYY  DYPVDI**Y**YLMDLS  YPVDIY**Y**LMDLSY  YLMDLS**Y**SMKDDL  DKPVSP**Y**MYISPP  PVSPYM**Y**ISPPEA  ALENPC**Y**DMKTTC  CLPMFG**Y**KHVLTL  VGSDNH**Y**SASTTM  ASTTMD**Y**PSLGLM  ENVVNL**Y**QNYSEL  VNLYQN**Y**SELIPG  QLIVDA**Y**GKIRSK  ECSEED**Y**RPSQQD  GKITGK**Y**CECDDF  DFSCVR**Y**KGEMCS  DSDWTG**Y**YCNCTT  SDWTGY**Y**CNCTTR  CIQPGS**Y**GDTCEK  ENTCNR**Y**CRDEIE  DAVNCT**Y**KNEDDC  CVVRFQ**Y**YEDSSG  VVRFQY**Y**EDSSGK  SGKSIL**Y**VVEEPE  GYPIDL**Y**YLMDLS  HLEDNL**Y**KRSNEF  LSDLRE**Y**RRFEKE  NSIIDV**Y**HKYSLI  GNFHAV**Y**RDDLKK  ETECPQ**Y**IRKKGA  KGRSST**Y**EVRLTQ  QLPLGE**Y**GLKPLS  IITYRD**Y**LPLVLG  FRLDNR**Y**QPMEPN  RKLMEQ**Y**GTPNNI  IDKQIR**Y**ILDGIS  LLEFEV**Y**LEYLQN  FEVYLE**Y**LQNRFE  SELACI**Y**SALILH  KMETTF**Y**DDALNA  PSESGP**Y**GYSNPK  HSEPPV**Y**ANLSNF  PEGTNA**Y**RSYCYY  SGSLVS**Y**KSWGIG  SSVNPG**Y**CVSLTS  ICHQIE**Y**YFGDFN  PEVTDE**Y**KNDVKN  ILFKDD**Y**FAKKNE  VNASSK**Y**EITTIH  FRRNFG**Y**TLRSVN  DILQLE**Y**VGGISM  LTDNIK**Y**EDCEDR  PYFPPP**Y**QPIYPQ  PPYQPI**Y**PQSQDP  KFKIHT**Y**SSPTFC  KGTDEL**Y**AVKILK  QSKGII**Y**RDLKLD  SVQAPS**Y**GARPVS  SSAASV**Y**AGAGGS  QRQAQE**Y**EALLNI  ILERGE**Y**PRWDAW  CYQYPG**Y**RGYQYI  DHHGGD**Y**KHWREW  EHQDKI**Y**PSFQPQ  PQQVVP**Y**PQRAVP  NPTHQI**Y**PVTQPL  TQNGQM**Y**LNRDIW  RGSQFA**Y**GDHQSP  ASQNIT**Y**ICKNSV  GAAGHL**Y**PGEVCP  SRLFFN**Y**ALVIFE  LKELGL**Y**NLMNIT  KNNELC**Y**LATIDW  DSVEDN**Y**IVLNKD  VACRNF**Y**LDGRCV  TCPPPY**Y**HFQDWR  RQGCHQ**Y**VIHNNK  IEEISG**Y**LKIRRS  LLRWEP**Y**WPPDFR  LKPWTQ**Y**AIFVKT  SDERRT**Y**GAKSDI  AKSDII**Y**VQTDAT  ELFELD**Y**CLKGLK  RKTFED**Y**LHNVVF  LRHFTG**Y**RIELQA  NGLIVL**Y**EVSYRR  VLYEVS**Y**RRYGDE  SWTEPT**Y**FYVTDY  VVIGSI**Y**LFLRKR  GSFGMV**Y**EGNARD  IADGMA**Y**LNAKKF  SLAEQP**Y**QGLSNE  FVMDGG**Y**LDQPDN  AFLGIP**Y**AQPPLG  IVVSMN**Y**RVGALG  WAVTSL**Y**EARNRT  GALLAR**Y**IQQARK  RISDRD**Y**MGWMDF  FFTGDA**Y**VILKTV  RNGNLQ**Y**DLHYWL  LQYDLH**Y**WLGNEC  TVQLDD**Y**LNGRAV  SATFLG**Y**FKSGLK  FKSGLK**Y**KKGGVA  GSNSNR**Y**ERLKAT  RKLAKL**Y**KVSNGA  FITKMD**Y**PKQTQV  ATYGQF**Y**GGDSYI  FYGGDS**Y**IILYNY  DSYIIL**Y**NYRHGG  YIILYN**Y**RHGGRQ  RQGQII**Y**NWQGAQ  GKPMII**Y**KGGTSR  LTSAKR**Y**IETDPA  LGWDDD**Y**WSVDPL  LKAFDF**Y**KVIESF  LFYKKV**Y**RLAYLR  LIKEEE**Y**DSIIVF  LLEDES**Y**DGGAGA  EASGCH**Y**GVLTCG  VSGQML**Y**FAPDLI  INTFHQ**Y**SVKLGH  VANPEH**Y**IKHPLQ  LMPGCD**Y**SLFKDG  GESFDD**Y**SDDVCG  GASTGI**Y**EALELR  FFRSGK**Y**DLDFKS  SERLAK**Y**NQLLRI  IRTQQH**Y**YDKCPK  TLGLAA**Y**GYGIRY  IINMLF**Y**HDRFKV  TEALKP**Y**SSGGPR  ALLGIW**Y**INCFGC  MLPYDQ**Y**LHRFAA  SQAIHK**Y**NAYRKA  QRIGLK**Y**FGDFEK  IPKDQY**Y**CGVLYF  PLRPQN**Y**LFGCEL  LKADKD**Y**HFKVDN  EAEAMN**Y**EGSPIK  LRMGEE**Y**FLRLGN  GFSDQV**Y**RQRRKL  EKLSTL**Y**WFTVEF  KDKLRS**Y**ASRIQR  EEMLFI**Y**GHYKQA  LFIYGH**Y**KQATVG  EDAMKA**Y**INKVEE  IVADKD**Y**SVTANS  DSARFR**Y**LMAEKL  MVVESA**Y**EVIKLK  TNKLSP**Y**DKLEGL  GGSFGA**Y**GAQEEA  FGEAMA**Y**FAMVKR  AAVSIT**Y**GTPFAA  CHCYPN**Y**DLVDGE  ARAKME**Y**KCAAPS  DNTPGS**Y**HCSCKN  VNYPGG**Y**TCYCDG  FAGVVL**Y**LKFRLP  STDHPK**Y**SDMIVA  RQSIQK**Y**IKSHYK  KYIKSH**Y**KVGENA  TLISVD**Y**EIFGKV  VKSDRE**Y**AGLLHH  QQLRKT**Y**SEQWQQ  EKLKSQ**Y**RALARD  AQAKRK**Y**QEASKD  DKAKDK**Y**VRSLWK  FAHHNR**Y**VLGVRA  KEILQE**Y**LEISSL  QGFLRQ**Y**GSAPDV  LHEQLW**Y**HGAIPR  SQGKQE**Y**VLSVLW  QSLDNL**Y**RLEGEG  ARILKQ**Y**SHPNIV  TQKQPI**Y**IVMELV  AAAGME**Y**LESKCC  APEALN**Y**GRYSSE  ALNYGR**Y**SSESDV  SLGASP**Y**PNLSNQ  MEQCWA**Y**EPGQRP  PSFSTI**Y**QELQSI  SPHWTL**Y**SDGSSS  SAAIHL**Y**VKDPAR  LMRHTN**Y**SFSPWH  DFHNNR**Y**QKVLTL  FQHAGN**Y**SCVASN  RVVESA**Y**LNLSSE  KVMVEA**Y**PGLQGF  QGFNWT**Y**LGPFSD  ATTKDT**Y**RHTFTL  PSEAGR**Y**SFLARN  FELTLR**Y**PPEVSV  LCAASG**Y**PQPNVT  QVWDDP**Y**PEVLSQ  LEHNQT**Y**ECRAHN  LLLLLL**Y**KYKQKP  LLLLYK**Y**KQKPKY  DPTQLP**Y**NEKWEF  VLVITE**Y**CCYGDL  ITEYCC**Y**GDLLNF  SIFDCV**Y**TVQSDV  QSDVWS**Y**GILLWE  SLGLNP**Y**PGILVN  LVNSKF**Y**KLVKDG  KLVKDG**Y**QMAQPA  VLDHRY**Y**AGGCSP  RSLMLH**Y**EFLQRV  LACEVS**Y**RKNTPI  GELQEL**Y**LKGNEL  DNAENV**Y**VWKQGV  DTDLYD**Y**YPEEDT  WQVRQL**Y**GDTGVL  VVAETD**Y**QSFAVL  TEDQIF**Y**FPKYGF  TVLTKM**Y**PRGNHW  KQQLRE**Y**IRWEEA  LQTVTN**Y**FITSLA  VIAVDR**Y**FAITSP  PIQMHW**Y**RATHQE  QEAINC**Y**ANETCC  FFTNQA**Y**AIASSI  SSIVSF**Y**VPLVIM  VIMVFV**Y**SRVFQE  LIRKEV**Y**ILLNWI  LLNWIG**Y**VNSGFN  GFNPLI**Y**CRSPDF  RSSLKA**Y**GNGYSS  KAYGNG**Y**SSNGNT  TGEQSG**Y**HVEQEK  DAAIVG**Y**KDSPSV  KDRSSF**Y**VNGLTL  LINKKC**Y**EMASHL  HGGRID**Y**IAGLDS  PTLWAS**Y**SLEYGK  ASYSLE**Y**GKAELE  GITQTP**Y**KVSISG  ILTCPQ**Y**PGSEIL  ELEQSG**Y**YVCYPR  LEQSGY**Y**VCYPRG  SGYYVC**Y**PRGSKP  PEDANF**Y**LYLRAR  DANFYL**Y**LRARVC  GLLLLV**Y**YWSKNR  LLLLVY**Y**WSKNRK  GNKYNV**Y**PTYDFA  HDISGC**Y**ILRPWA  IAKCND**Y**RRRLLS  QFGVGF**Y**SAYLVA  EELIPE**Y**LNFIRG  NQKHIY**Y**ITGETK  GFAFVQ**Y**VNERNA  RSAAEM**Y**GSVTEH  DYYDRM**Y**SYPARV  KSPAIK**Y**RPENTP  SVVPSS**Y**PAGLTG  TIFVAL**Y**DYEART  FVALYD**Y**EARTTE  ATGKNG**Y**IPSNYV  GYIPSN**Y**VAPADS  IQAEEW**Y**FGKMGR  ETTKGA**Y**SLSIRD  GDNVKH**Y**KIRKLD  KLDNGG**Y**YITTRA  LDNGGY**Y**ITTRAQ  QKLVKH**Y**TEHADG  KEGDGK**Y**LKLPQL  IADGMA**Y**IERMNY  YIERMN**Y**IHRDLR  TKGRVP**Y**PGMVNR  EQVERG**Y**RMPCPQ  ERPTFE**Y**IQSFLE  QSFLED**Y**FTATEP  KVALGL**Y**FSRDAY  YFSRDA**Y**WEKLYV  AGTPLL**Y**VHALRD  RLGQHL**Y**GTYRTR  QHLYGT**Y**RTRLHE  EDTGLL**Y**LNRSLD  FPLLTV**Y**LKVFLS  PGCARV**Y**FSFFNT  PNISVA**Y**RLLEGE  REQREK**Y**ELVAVC  PFPVTV**Y**DEDDSA  GELVRR**Y**TSTLLP  SGINVQ**Y**KLHSSG  KCAELH**Y**MVVATD  VTVEGS**Y**VAEEAG  RGIKAG**Y**GTCNCF  QAFPVS**Y**SSSGAR  LKGRAG**Y**TTVAVK  PHVIKL**Y**GACSQD  RKVGPG**Y**LGSGGS  ISQGMQ**Y**LAEMKL  SLFDHI**Y**TTQSDV  TLGGNP**Y**PGIPPE  PSDSLI**Y**DDGLSE  FTNANS**Y**STTTTT  EACTCP**Y**CKDSEG  FMCTWS**Y**CGKRFT  IDIRND**Y**QQLKRL  AEDYRS**Y**RFPKLT  LTVITE**Y**LLLFRV  RGWKLF**Y**NYALVI  LKDIGL**Y**NLRNIT  TTINNE**Y**NYRCWT  INNEYN**Y**RCWTTN  EQLEGN**Y**SFYVLD  RYRPPD**Y**RDLISF  LISFTV**Y**YKEAPF  FKNVTE**Y**DGQDAC  LKPWTQ**Y**AVYVKA  RQPQDG**Y**LYRHNY  EKEEAE**Y**RKVFEN  TTAADT**Y**NITDPE  LMYEIK**Y**GSQVED  CVSRQE**Y**RKYGGA  RLNPGN**Y**TARIQA  TDPVFF**Y**VQAKTG  VQAKTG**Y**ENFIHL  FSAADV**Y**VPDEWE  IADGMA**Y**LNANKF  DGVFTT**Y**SDVWSF  MRMCWQ**Y**NPKMRP  EGCRKS**Y**SRLENL  PGCTKR**Y**TDPSSL  CPQQAS**Y**PDPTQE  LDLTPN**Y**RGENSW  AQLLRL**Y**QLMLFT  GTPVFS**Y**GDEIGL  ATLKAS**Y**PGLQFE  HPEECM**Y**AVGQGA  MKDGQL**Y**LTGGVW  REACDD**Y**RLCERY  ERYAMV**Y**GYNAAY  NAAYNR**Y**FRKRRG  DLDRDG**Y**NDIAVA  NEGEGA**Y**EAELAV  NVSSLP**Y**AVPPLS  LKADQL**Y**KQKIIR  IALACK**Y**NGKDEV  GQIFEA**Y**NMAALW  IDLGAL**Y**LSMKDT  SLLNEG**Y**LQPAGD  TADEVH**Y**FLQAAT  MNVNMK**Y**QLPNFT  FLGATN**Y**IYVLNE  GATNYI**Y**VLNEED  LQKVAE**Y**KTGPVL  ALVVDT**Y**YDDQLI  NTINSS**Y**FPDHPL  FLTDQS**Y**IDVLPE  PEFRDS**Y**PIKYVH  DSYPIK**Y**VHAFES  NSGLHS**Y**MEMPLE  RCLQHF**Y**GPNHEH  EARRDE**Y**RTEFTT  ICLPAI**Y**KVFPNS  GHGTTQ**Y**STFSYV  LTLTGN**Y**LNSGNS  ETSIFS**Y**REDPIV  DGILSK**Y**FDLIYV  KYFDLI**Y**VHNPVF  GSELVR**Y**DARVHT  GHFGCV**Y**HGTLLD  PLVVLP**Y**MKHGDL  VAKGMK**Y**LASKKF  TRGAPP**Y**PDVNTF  TFDITV**Y**LLQGRR  MFSSEA**Y**GQKDLL  AWLGHE**Y**LHAMKG  VNNPKN**Y**PSSLCA  RLAARM**Y**LQKGFP  MMQART**Y**PLRWFL  VRLPKL**Y**LKHQMD  KDQLQS**Y**ICFCLP  NGGCEQ**Y**CSDHTG  VGHFGV**Y**TRVSQY  PCRIEL**Y**RVVESL  EEISKF**Y**LPNCNK  CNKNGF**Y**HSRQCE  GLCWCV**Y**PWNGKR  FLGFLH**Y**FRPLNC  IQQPMS**Y**DNLTQR  FLLVLS**Y**LHVHTA  DFQMEQ**Y**IYKRKS  QMEQYI**Y**KRKSDG  RKSDGI**Y**IINLKR  TDSPLR**Y**VDIAIP  EVMPDL**Y**FYRDPE  MPDLYF**Y**RDPEEI  PAPQIQ**Y**RRLLVQ  ELKKSL**Y**AIFSQF  MQGFPF**Y**DKPMRI  KPMRIQ**Y**AKTDSD  KDPKRL**Y**CKNGGF  RLESNN**Y**NTYRSR  SNNYNT**Y**RSRKYT  TYRSRK**Y**TSWYVA  RKYTSW**Y**VALKRT  LKRTGQ**Y**KLGSKT  PPYTVV**Y**FPVRGR  LKASCL**Y**GQLPKF  DGDLTL**Y**QSNTIL  GRTLGL**Y**GKDQQE  EDLRCK**Y**ISLIYT  KYISLI**Y**TNYEAG  SLIYTN**Y**EAGKDD  EAGKDD**Y**VKALPG  QISFAD**Y**NLLDLL  FPLLSA**Y**VGRLSA  VKLPDG**Y**EFKFPN  NLEAIN**Y**MAADGD  ARRVLV**Y**GGRGAL  TPGMIG**Y**GMAKGA  EDADRK**Y**EEVARK  EAQAEK**Y**SQKEDR  DLEDEL**Y**AQKLKY  TEEVSE**Y**CSHMIG  LKDPVC**Y**LKKAFL  ACVRTF**Y**ETPLQL  GLDTGE**Y**FCTHND  DERKRL**Y**IFVPDP  IFEDRS**Y**ICKTTI  EVDSDA**Y**YVYRLQ  VDSDAY**Y**VYRLQV  SDAYYV**Y**RLQVSS  VNFEWT**Y**PRKESG  FLLDMP**Y**HIRSIL  LEDSGT**Y**TCNVTE  TVVESG**Y**VRLLGE  QVVFEA**Y**PPPTVL  NVSETR**Y**VSELTL  VAEAGH**Y**TMRAFH  LETNVT**Y**WEEEQE  DPMQLP**Y**DSTWEL  IYIITE**Y**CRYGDL  YGDLVD**Y**LHRNKH  ESPVLS**Y**MDLVGF  DLVGFS**Y**QVANGM  SIFNSL**Y**TTLSDV  TLGGTP**Y**PELPMN  PMNEQF**Y**NAIKRG  NAIKRG**Y**RMAQPA  RLLGEG**Y**KKKYQQ  VLAVGV**Y**FIAGQD  LPNDQL**Y**QPLKDR  DREDDQ**Y**SHLQGN  PNYPQA**Y**PSEVEK  NGGGGE**Y**HCAGNG  YTRVKN**Y**VDWIMK  ESSDKL**Y**RVEYAK  DDKENR**Y**WIFRSW  EAPVTG**Y**MFGKGI  LLDKPF**Y**NDFERG  VDFVLN**Y**SKAMEN  DFVNDV**Y**VYGMRG  RTKRDV**Y**QEPTDP  GYTNSI**Y**TLSISS  TSEANN**Y**GTLTKF  NGQEVK**Y**ALGERQ  AYSSVP**Y**EKGFAL  DWKDFL**Y**SYFKDK  VDGAKQ**Y**RNGESV  HREELV**Y**ELNPLD  GLDGAT**Y**DLEGHP  GRTLSD**Y**NIQKES  PINPVA**Y**QQILSQ  FGQYPG**Y**SPQGLQ  GIPRVN**Y**MQQLRQ  YRLKAK**Y**AAATGG  PCKAPQ**Y**GNCLNR  LAVVRG**Y**QPCASF  GALVDA**Y**PIKPEA  PEELNR**Y**YASLRH  YASLRH**Y**LNLVTR  CQCIKT**Y**SKPFHP  MQVINN**Y**QRRCKN  NISNCR**Y**AQTPAN  TPANMF**Y**IVACDN  ANSQDG**Y**VWQVTD  GSEGGT**Y**YIKEQK  LIALET**Y**KTGHGL  TDQQCQ**Y**RWLRVL  DCKPPV**Y**LLLELE  VPSVTE**Y**RLDGHT  LGLGRV**Y**PRPPSK  RPPSKT**Y**RGAFQN  RRGPTG**Y**LVLDEE  SPFKGG**Y**TKGLEG  AAAQCR**Y**GDLASL  AEEGQL**Y**GPCGGG  AGAVAP**Y**GYTRPP  AVAPYG**Y**TRPPQG  TAPDVW**Y**PGGMVS  MVSRVP**Y**PSPTCV  GPWMDS**Y**SGPYGD  VLPIDY**Y**FPPQKT  EASGCH**Y**GALTCG  AEGKQK**Y**LCASRN  CRLRKC**Y**EAGMTL  VSHIEG**Y**ECQPIF  QMAVIQ**Y**SWMGLM  VNSRML**Y**FAPDLV  DLVFNE**Y**RMHKSR  MHKSRM**Y**SQCVRM  DELRMN**Y**IKELDR  SCSRRF**Y**QLTKLL  GGHLNG**Y**PVPPYA  GYPVPP**Y**AFFFPP  LRICTR**Y**TPEQDT  REIVMK**Y**IHYKLS  DDFSRR**Y**RRDFAE  ALWMTE**Y**LNRHLH  SEEKQL**Y**NKYPDA  KQLYNK**Y**PDAVAT  VPTVDT**Y**DGRGDS  RGDSVV**Y**GLRSKS  RRPDIQ**Y**PDATDE  EELNGA**Y**KAIPVA  HKQSRL**Y**KRKAND  SPGRRY**Y**SLPPHQ  ESLEEC**Y**MAKILV  KQTIPH**Y**YLSIDV  KSSGKH**Y**GVFTCE  LLRAEP**Y**PAAAGR  QVALTE**Y**VRAQYP  HSLSEK**Y**SNVIFL  RKDQGG**Y**TMHQDQ  PPSSPK**Y**VSSVTS  GSVQIV**Y**KPVDLS  HGAEIV**Y**KSPVVS  KVIPKD**Y**KTMAAL  LRKRKM**Y**EEFLSK  RLGPSD**Y**FGEIAL  SLPSQK**Y**PGPQAE  PHPSLG**Y**KEIRKG  PLQVRG**Y**PEEKKE  ATNTGK**Y**TCTNKH  GLSNSI**Y**VFVRDP  LVDRSL**Y**GKEDND  DPEVTN**Y**SLKGCQ  KSVKRA**Y**HRLCLH  DVSSSV**Y**STWKRE  TKLQEK**Y**NSWHHG  HHGDFN**Y**ERQATL  SGVFMC**Y**ANNTFG  VDLIVE**Y**EAFPKP  EHQQWI**Y**MNRTFT  TDKWED**Y**PKSENE  NESNIR**Y**VSELHL  GTEGGT**Y**TFLVSN  AIAFNV**Y**VNTKPE  EPTIDW**Y**FCPGTE  VGKTSA**Y**FNFAFK  IVMILT**Y**KYLQKP  DPTQLP**Y**DHKWEF  VVEATA**Y**GLIKSD  TLVITE**Y**CCYGDL  SVRIGS**Y**IERDVT  ESDVWS**Y**GIFLWE  SLGSSP**Y**PGMPVD  PHLTRA**Y**AKDVKF  RGYISP**Y**FINTSK  SSSEVG**Y**DAMAGD  SSAVRV**Y**RMLPPL  DSTSDL**Y**NFQVSP  NVDGKG**Y**LLNEPG  NVGSNT**Y**GKRNAV  NCKHNT**Y**GVDCEK  AVISDS**Y**FPRYFI  LHEATD**Y**PWRPAL  PKVQVE**Y**KGETKS  TQTFTT**Y**SDNQPG  VQEAEK**Y**KAEDEK  NLLEED**Y**FGLAIW  TFNVKF**Y**PPDPAQ  TEDITR**Y**YLCLQL  EDITRY**Y**LCLQLR  LALLGS**Y**TIQSEL  QSELGD**Y**DPELHG  ELHGVD**Y**VSDFKL  MELHKS**Y**RSMTPA  AKKLSM**Y**GVDLHK  SSGLLV**Y**KDKLRI  KVLKIS**Y**KRSSFF  PGEQEQ**Y**ESTIGF  GFKLPS**Y**RAAKKL  LGSKFR**Y**SGRTQA  ETKTIT**Y**EAAQTD  SMVAAS**Y**SETVTC  TGNRLT**Y**TNWNEG  ERFRMI**Y**LQTLLA  AKEKKS**Y**DRQRWG  DASRPP**Y**RGRSSE  PWPPLE**Y**QPYQSI  PLEYQP**Y**QSIYVG  TWPRRS**Y**SPRSFE  EDCGGG**Y**TPDCSS  SPSPTT**Y**RMFRDK  FGTPPG**Y**GCAADR  LASEET**Y**LSHLEA  FKVPEL**Y**EIHKEF  RAFVDN**Y**GVAMEM  SLETLL**Y**KPVDRV  GGKTQQ**Y**DCKWYI  QYDCKW**Y**IPLTDL  SRNGKS**Y**TFLISS  FLISSD**Y**ERAEWR  DESPGL**Y**GFLNVI  KQSSNL**Y**CTLEVD  EVDSFG**Y**FVNKAK  KAKTRV**Y**RDTAEP  TLRILC**Y**EKCYNK  LCYEKC**Y**NKTKIP  AGTLKL**Y**FRELPE  TYVVAF**Y**HYFSKM  LQAFST**Y**RTVEKP  SYDLHR**Y**FYTGAE  LLKDTV**Y**TDFDGT  WIRYHR**Y**HGRSAA  SLGILL**Y**DMVCGD  PADSGL**Y**ACVTSS  SGSDTT**Y**FSVNVS  RMPVAP**Y**WTSPEK  DHRIGG**Y**KVRYAT  GGYKVR**Y**ATWSII  PSDKGN**Y**TCIVEN  CIVENE**Y**GSINHT  GSINHT**Y**QLDVVE  EFMCKV**Y**SDPQPH  GPDNLP**Y**VQILKT  FEDAGE**Y**TCLAGN  VMTSPL**Y**LEIIIY  YLEIII**Y**CTGAFL  VGSVIV**Y**KMKSGT  TQDGPL**Y**VIVEYA  LYVIVE**Y**ASKGNL  KGNLRE**Y**LQARRP  DLVSCA**Y**QVARGM  VARGME**Y**LASKKC  ALFDRI**Y**THQSDV  TLGGSP**Y**PGVPVE  SMPLDQ**Y**SPSFPD  WWEEER**Y**PEGIKW  KDSIRY**Y**NKVPVE  PAKILS**Y**NRANRA  RSYVDM**Y**LKDKLD  GGRHVD**Y**VADQIV  VAEMSS**Y**HHGEMS  SYVAGQ**Y**DDAASY  FAAVVL**Y**VENERW  LTYGNR**Y**KNVKLP  VASGYH**Y**GVASCE  IQGSIE**Y**SCPASN  RGGRQK**Y**KRRPEV  IIHQDG**Y**SLEECL  EFIAII**Y**GNTLQS  TTLNIQ**Y**GDSARQ  LNDSAG**Y**YLSDLE  NDSAGY**Y**LSDLER  RLVTPG**Y**VPTEQD  IAALSA**Y**DMVLVE  SICNHR**Y**FATTSI  SICFPD**Y**DGPNTY  YDGPNT**Y**EDAGNY  YEDAGN**Y**IKVQFL  RDVKEI**Y**SHMTCA  DRHGKH**Y**FIEVNS  AQKLLH**Y**LGHVMV  DPSRTK**Y**SLQYYM  AAATGG**Y**GPVSGA  GIMKKA**Y**ELSTLT  SFPITN**Y**LAPVSA  PRVTPF**Y**AVKCND  SEQTFM**Y**YVNDGV  YVNDGV**Y**GSFNCI  SEGFDT**Y**RCDRNL  QEKVSD**Y**EMKLMD  GIPEQE**Y**SCVVKM  VPLVVE**Y**KIADMG  DMGHLK**Y**YLAPKI  MGHLKY**Y**LAPKIE  GKTNFP**Y**VRDFVM  LDYAQR**Y**IFVKSA  AFDFDE**Y**QPEMLE  HKQFWR**Y**FAGNLA  DGLRGL**Y**QGFNVS  KGADIM**Y**TGTVDC  PLRSEA**Y**NTFSER  HYLKSC**Y**TTEFYV  KSWWGD**Y**WEPFRA  KRQREE**Y**ERAQKA  VRQRDM**Y**RILLSQ  ADGNDG**Y**EADDAE  CMVVKT**Y**LISSIP  TFYWDF**Y**TNRTVQ  IKNNRF**Y**TIEILK  RGTVRD**Y**PDFSPS  TEFKKH**Y**GYSLYS  FKKHYG**Y**SLYSAI  DYMRDL**Y**RLQSGE  FHRINI**Y**EVMKPP  PGYQAF**Y**CHGDCP  QRKTAP**Y**KNVNIQ  RPELID**Y**GKLRKD  FDVAEK**Y**LDIPKM  EKAIMT**Y**VSSFYH  TYVSSF**Y**HAFSGA  EQLMED**Y**EKLASD  LEDFRD**Y**RRLHKP  EQVEKG**Y**EEWLLN  MLRQKD**Y**ETATLS  ELNELD**Y**YDSPSV  LNELDY**Y**DSPSVN  ETIDQL**Y**LEYAKR  DQLYLE**Y**AKRAAP  SKIVQT**Y**HVNMAG  MAGTNP**Y**TTITPQ  LSHLRQ**Y**EKSIVN  EKSIVN**Y**KPKIDQ  DNKHTN**Y**TMEHIR  CLISLG**Y**DIGNDP  LAGDKN**Y**ITMDEL  PPDQAE**Y**CIARMA  IARMAP**Y**TGPDSV  VPGALD**Y**MSFSTA  QSWENI**Y**DMVVPF  LCKEAG**Y**EGPLHQ  SKFVEE**Y**DRTSQV  GFDAES**Y**TFTVPR  GRQRTA**Y**FSLDTR  QIHFLV**Y**AWDSTY  YAWDST**Y**RKFSTK  KEGKVF**Y**SITGQG  RERIAT**Y**TLFSHA  DDDVNT**Y**NAAIAY  YNAAIA**Y**TILSQD  RESFPT**Y**TLVVQA  IFNPTT**Y**KGQVPE  PAWEAV**Y**TILNDD  FEAKQQ**Y**ILHVAV  GQEITS**Y**TAQEPD  MEQKIT**Y**RIWRDT  HVKNST**Y**TALIIA  ANWTIQ**Y**NDPTQE  ALEVGD**Y**KINLKL  LMSVPR**Y**LPRPAN  DPTAPP**Y**DSLLVF  SLLVFD**Y**EGSGSE  SDKDQD**Y**DYLNEW  KDQDYD**Y**LNEWGN  FDISLF**Y**RDIISI  RPSLFV**Y**PEESLV  RRNIPP**Y**FVALVP  IIQGFR**Y**GSDIVP  AIVRYA**Y**DKRANP  PHIKHN**Y**ECLVYV  RPERAP**Y**RDLRCV  RGRLLP**Y**LAEDEL  QWYFGA**Y**SIVAGV  FVCLLE**Y**PRGKRK  AIASGI**Y**LLAAVR  APVTCC**Y**NFTNRK  VQRLAS**Y**RRITSS  PASASP**Y**SSDTTP  TPCCFA**Y**IARPLP  HIKEYF**Y**TSGKCS  MIRALE**Y**IENLRQ  VLPRLP**Y**GAGRSR  ENRQMA**Y**ENLNDK  FLQKQE**Y**KTLEYN  EGSSYF**Y**GTFSEL  RKNLDP**Y**EQWSDQ  PGPEQK**Y**ETEGSS  LNGGTC**Y**PTETSY  QGQCYK**Y**FAHRRT  TGSLFV**Y**NITTNK  QVKFGS**Y**LGYSVG  LTLCFS**Y**KGKEVP  TAISLF**Y**ELSEND  LEPEEL**Y**QTFQRI  NVIIST**Y**GEGESG  KQFAEM**Y**VAKFAA  PVTAQK**Y**RCELLY  KGPLMM**Y**ISKMVP  SDKGRF**Y**AFGRVF  RIMGPN**Y**TPGKKE  GKKEDL**Y**LKPIQR  ILMMGR**Y**VEPIED  SDPVVS**Y**RETVSE  NKHNRL**Y**MKARPF  LKQRAR**Y**LAEKYE  RYLAEK**Y**EWDVAE  ITKGVQ**Y**LNEIKD  TARRCL**Y**ASVLTA  RLMEPI**Y**LVEIQC  QVVGGI**Y**GVLNRK  MFVVKA**Y**LPVNES  SGFGGG**Y**GGGLGG  ASPERD**Y**SPYYKT  AETECR**Y**ALQLQQ  LFGYSW**Y**KGERVD  RNDTGP**Y**ECEIQN  REDAGT**Y**WCEVFN  KKYIKD**Y**MKSIKG  MVALLD**Y**REDGVT  EDGVTP**Y**MIFFKD  DTDGNG**Y**ISFNEL  PRVNHL**Y**SDLSDA  WQLMRR**Y**TLNILE  TQYGGQ**Y**RTVHTE  RIVYLQ**Y**PSLIPH  EQENVQ**Y**AGLDFE  FFIAGR**Y**EFSNKG  GVFPSY**Y**EPWGYT  WKYLGR**Y**YMSARH  TCNGES**Y**RGLMDH  QGQGEG**Y**RGTVNT  WMVSLR**Y**RNKHIC  HCTNTG**Y**KPLYIP  EDKMPP**Y**DEQTQA  YLYSQC**Y**ELTTNE  SDLSPE**Y**AVLTST  VSMSEL**Y**PLQISP  NIEGKQ**Y**LSNMGT  PKASHG**Y**GGRFGV  SAVGHE**Y**VAEVEK  KGFGGK**Y**GVERDR  HTSQKD**Y**SRGFGG  RGFGGR**Y**GVEKDK  DKAALG**Y**DYKGET  AALGYD**Y**KGETEK  HESQRD**Y**AKGFGG  VEEEPV**Y**EAEPEP  ISAVAV**Y**DYQGEG  AVAVYD**Y**QGEGSD  HDHGQK**Y**VIILDP  GFQLCR**Y**GYANTS  RNDELN**Y**PPYFPE  RRFTEI**Y**EFHKTL  QGTLTE**Y**CGTLMS  GYFPSM**Y**LQKSGQ  SHGTHE**Y**HAETIK  ITLDNA**Y**MEKCDE  AHQVAR**Y**RPRAPI  EVEEDE**Y**KAFYKS  SDYIKL**Y**VRRVFI  ATLRSG**Y**LLPDTK  SKYEEM**Y**PPEIGD  TPTLQH**Y**LSYTEE  MTVKNK**Y**ATSKHA  KVIEQS**Y**NETWLG  CPESPR**Y**LYIIQN  AEAMGP**Y**VFLLFA  MLQHID**Y**RMRCIL  AGAPTQ**Y**PPGRGG  VEDSGH**Y**YCVVRN  EDSGHY**Y**CVVRNS  VVRNSS**Y**CLRIKI  GGLVCP**Y**MEFFKN  LPKLQW**Y**KDCKPL  EKHRGN**Y**TCHASY  YTCHAS**Y**TYLGKQ  CHASYT**Y**LGKQYP  TYLGKQ**Y**PITRVI  QLSDIA**Y**WKWNGS  PVLGED**Y**YSVENP  VLGEDY**Y**SVENPA  EIESRF**Y**KHPFTC  AYIQLI**Y**PVTNFQ  VCSVFI**Y**KIFKID  IDIVLW**Y**RDSCYD  WYRDSC**Y**DFLPIK  ASDGKT**Y**DAYILY  GKTYDA**Y**ILYPKT  YDAYIL**Y**PKTVGE  LEKQCG**Y**KLFIYG  IYGRDD**Y**VGEDIV  EEQIAM**Y**NALVQD  FWKNVR**Y**HMPVQR  LAMGKL**Y**GNDFSQ  AADNQI**Y**IAGHPA  GGPHGG**Y**HSHYHD  PPPPPE**Y**GPHADS  EKTEDQ**Y**EENSHL  GTEQAP**Y**GMQTQN  KTSGKR**Y**VYRFVC  PECCAV**Y**RIQDGE  RCQTCG**Y**KFHQRC  ILLFMG**Y**STKPQL  VAIDVG**Y**RHIDCA  SKLWCT**Y**HEKGLV  NKPGLK**Y**KPAVNQ  SVTLPC**Y**LQVPNM  QTQGPS**Y**SESKRL  VEDEGN**Y**TCLFVT  TVNLTV**Y**YPPEVS  VNLTVY**Y**PPEVSI  EVSISG**Y**DNNWYL  GYDNNW**Y**LGQNEA  NPEPTG**Y**NWSTTM  LLGIGI**Y**FYWSKC  GIGIYF**Y**WSKCSR  ATTDDF**Y**DDPCFD  GRGGEH**Y**SGDSDA  CYEGAY**Y**NEAPSE  RPFFLY**Y**ASHHTH  HEPPLL**Y**DLSKDP  KDPGEN**Y**NLLGGV  GLREVW**Y**FGLHYV  WYFGLH**Y**VDNKGF  KFRAKF**Y**PEDVAE  AVLLGS**Y**AVQAKF  QAKFGD**Y**NKEVHK  DNAMLE**Y**LKIAQD  AQDLEM**Y**GINYFE  EMYGIN**Y**FEIKNK  ALGLNI**Y**EKDDKL  APDFVF**Y**APRLRI  MGNHEL**Y**MRRRKP  AAELAE**Y**TAKIAL  VYEPVS**Y**HVQESL  GAEPTG**Y**SAELSS  RQGRDK**Y**KTLRQI  VNSARQ**Y**KDLWNM  MQKKSG**Y**HTADKD  QQMGGF**Y**LCQPGP  LMSPKL**Y**VWAKDR  AQDAGK**Y**YCHRGN  QDAGKY**Y**CHRGNL  SAVTLA**Y**LIFCLC  EEDSEF**Y**ENDSNL  FSNAES**Y**ENEDEE  SLGSQS**Y**EDMRGI  DMRGIL**Y**AAPQLR  EEDADS**Y**ENMDNP  VPGGVF**Y**PGAGLG  ADAAAA**Y**KAAKAG  TTGKLP**Y**GYGPGG  EKRHHG**Y**RRKFHE  HREFPF**Y**GDYGSN  FPFYGD**Y**GSNYLY  AMVLLL**Y**SFSDAC  IGKPKP**Y**YEIGER  GKPKPY**Y**EIGERV  IGERVD**Y**KCKKGY  YKCKKG**Y**FYIPPL  CKKGYF**Y**IPPLAT  YRETCP**Y**IRDPLN  VPANGT**Y**EFGYQM  GTYEFG**Y**QMHFIC  FICNEG**Y**YLIGEE  ICNEGY**Y**LIGEEI  IGEEIL**Y**CELKGS  EVEVFE**Y**LDAVTY  YLDAVT**Y**SCDPAP  IGESTI**Y**CGDNSV  GFGKKF**Y**YKATVM  FGKKFY**Y**KATVMF  ECDKGF**Y**LDGSDT  SGPRPT**Y**KPPVSN  KPPVSN**Y**PGYPKP  VSNYPG**Y**PKPEEG  VICVVP**Y**RYLQRR  CVVPYR**Y**LQRRKK  RKKKGT**Y**LTDETH  SYGSSS**Y**GGAGGY  YGGAGG**Y**TQSPGG  SNEGHI**Y**STVDDD  EDPSTD**Y**YQELQR  DPSTDY**Y**QELQRD  EMFLQI**Y**KQGGFL  ETQFNQ**Y**KTEAAS  TEAASR**Y**NLTISD  VALAIV**Y**LIALAV  QCRRKN**Y**GQLDIF  YHPMSE**Y**PTYHTH  IPGGSP**Y**AGWAYG  RDRTGH**Y**LCNACG  CNACGL**Y**HKMNGQ  FGVKDE**Y**ALTTRA  EDDLSH**Y**TRLLRT  FQGVTG**Y**LKIDSS  SGGYIF**Y**TFSTVH  NRWHEG**Y**RQTPKE  TTLLEG**Y**TSHYPH  PLSSKM**Y**TTSITS  LPEPTT**Y**QEVSIK  DFLRKE**Y**GGLDVL  GWPSSA**Y**GVTKIG  GAETPV**Y**LALLPP  VRLLLQ**Y**DAEIDD  TTDFPL**Y**FVIMSR  ANMENL**Y**TALQSI  LSRRPS**Y**RKILND  TVPTPI**Y**QTSSGQ  GTTILQ**Y**AQTTDG  AAHTGL**Y**TCYYNH  GLYTCY**Y**NHTQTE  LEGRHI**Y**IYVPDP  GRHIYI**Y**VPDPDV  PLGMTD**Y**LVIVED  GVVPAS**Y**DSRQGF  TIPFNV**Y**ALKATS  EALKTV**Y**KSGETI  VDLQWT**Y**PGEVKG  PSIKLV**Y**TLTVPE  KIQEIR**Y**RSKLKL  EEDSGH**Y**TIVAQN  EDAVKS**Y**TFELLT  WKQKPR**Y**EIRWRV  VVEGTA**Y**GLSRSQ  IYIITE**Y**CFYGDL  YGDLVN**Y**LHKNRD  DLLSFT**Y**QVARGM  LSDVWS**Y**GILLWE  NKIKSG**Y**RMAKPD  HATSEV**Y**EIMVKC  EKRPSF**Y**HLSEIV  NLLPGQ**Y**KKSYEK  GQYKKS**Y**EKIHLD  QHQMLF**Y**KDDVLF  KDDVLF**Y**NISSMK  MKSTES**Y**FIPEVR  IPEVRI**Y**DSGTYK  IYDSGT**Y**KCTVIV  EKTTAE**Y**QLLVEG  HGNKAV**Y**SVMAMV  VEHSGN**Y**TCKVES  KSDSGT**Y**ICTAGI  SQPRIS**Y**DAQFEV  GTLPIS**Y**QLLKTS  PTEDVE**Y**QCVADN  GSGPIT**Y**KFYREK  PITYKF**Y**REKEGK  KEGKPF**Y**QMTSNA  KEQEGE**Y**YCTAFN  EQEGEY**Y**CTAFNR  IIAAKC**Y**FLRKAK  MEANSH**Y**GHNDDV  DAVESR**Y**SRTEGS  ALAAGG**Y**DVEKNN  ASFVCE**Y**ASPGKA  EVCAAT**Y**MMGNEL  AMDTGL**Y**ICKVEL  CKVELM**Y**PPPYYL  LMYPPP**Y**YLGIGN  MYPPPY**Y**LGIGNG  GNGTQI**Y**VIDPEP  SSGLFF**Y**SFLLTA  RNIIVF**Y**GSQTGT  GDHVAV**Y**PANDSA  SDEDYL**Y**REELAQ  GDYTDF**Y**SSRQHA  LHLPVG**Y**HGRASS  DPRPLP**Y**LCHDEP  EIKKFK**Y**GIEEHG  GSQKYA**Y**FNGCSS  NSSCRN**Y**NKQASE  LIDQQG**Y**IQVTDF  IILSKG**Y**NKAVDW  TSNFDD**Y**EEEEIR  TRVAEL**Y**EEELRE  RDIRAQ**Y**ETIAAK  ASEASG**Y**QDNIAR  EVANFY**Y**EADCLA  DLFSDD**Y**GGKNCK  MAAGFP**Y**ALRAYL  MNSDCW**Y**LYTLDF  SDCWYL**Y**TLDFPE  SAMFND**Y**NFVFTS  ILADEI**Y**GDMVFS  RTPGEF**Y**HNTLSF  SNADLC**Y**GALAAI  MGGYCG**Y**LATVTG  EFLYNL**Y**SSEGKG  LKMLAQ**Y**RISMAA  PAGAGG**Y**PGASYP  APAPGV**Y**PGPPSG  PSGPGA**Y**PSSGQP  AKDSQR**Y**KVDYES  QRYKVD**Y**ESQSTD  SKRETE**Y**GPCRRE  RPFVEM**Y**SEIPEI  IISNAT**Y**KEIGLL  VQMTWS**Y**PDEKNK  NKDKGL**Y**TCRVRS  VAGKRS**Y**RLSMKV  EEDAGN**Y**TILLSI  SRISGI**Y**ICIASN  GRNISF**Y**ITDVPN  TVNKFL**Y**RDVTWI  NNRTMH**Y**SISKQK  LQDSGT**Y**ACRARN  CRARNV**Y**TGEEIL  RDQEAP**Y**LLRNLS  EEDEGV**Y**HCKATN  SEIKTD**Y**LSIIMD  QCERLP**Y**DASKWE  LMVIVE**Y**CKYGNL  EDSDGF**Y**KEPITM  MEDLIS**Y**SFQVAR  GLARDI**Y**KNPDYV  SIFDKI**Y**STKSDV  KSDVWS**Y**GVLLWE  SLGGSP**Y**PGVQMD  GNSGFT**Y**STPAFS  TSVISG**Y**RLACKE  AVLAIK**Y**TDIRGQ  SMLISG**Y**ALNCVV  NKNRNR**Y**RDVSPF  EEAQRS**Y**ILTQGP  SLKCAQ**Y**WPQKEE  SEDIKS**Y**YTVRQL  EDIKSY**Y**TVRQLE  EILHFH**Y**TTWPDF  DQLRFS**Y**LAVIEG  PLNAAP**Y**GIESMS  EDHALS**Y**WKPFLV  VLTAGA**Y**LCYRFL  AGAYLC**Y**RFLFNS  ARRKLI**Y**EKAKHY  KHYHKE**Y**RQMYRT  PYIAWG**Y**PNLKSV  MLQSDP**Y**SVPARD  SVPARD**Y**LIDGSR  CKGILE**Y**LTVAEV  MEDLVT**Y**TKNLGP  PGNQAA**Y**EHFETM  PDMEDD**Y**EPELLL  RPEEEA**Y**GEDGNP  GILNEA**Y**RKVLDQ  AVLGKR**Y**KQRVKN  ALRDAG**Y**EFDICF  RLNERH**Y**GGLTGL  KIWRRS**Y**DVPPPP  EPDHPF**Y**SNISKD  ISKDRR**Y**ADLTED  TGIPIV**Y**ELDKNL  DSEGKA**Y**SLGRAE  SLGRAE**Y**GRLGLG  CGASVG**Y**AVTKDG  IVCSQP**Y**SKDSPF  DPAGPS**Y**AAATLQ  VRPRWI**Y**SCNEKQ  GETGSK**Y**FVLQVH  SCHYKN**Y**PMHVFA  NVFAIS**Y**IPGLLF  MQQNIR**Y**TKLSDP  NVKNNI**Y**NATFLA  TGTLQI**Y**LLDIND  PHMHAY**Y**PALTSW  FWVKDK**Y**KLQQTF  SSFLFS**Y**LSVQTK  SRDFDR**Y**QEDPAF  WLRKQF**Y**SVDRNR  NNPLSH**Y**WISSSH  SSSHNT**Y**LTGDQF  ESSLEA**Y**ARCLRM  DGMPVI**Y**HGHTLT  AFVASE**Y**PVILSI  VPTSMM**Y**SENDIS  IKNGIL**Y**LEDPVN  HEWYPH**Y**FVLTSS  TSSKIY**Y**SEETSS  ETFVGD**Y**TLSFWR  LVFDSL**Y**DLITHY  RNEPNS**Y**AISFRA  VDLISY**Y**EKHPLY  YEKHPL**Y**RKMKLR  RKMKLR**Y**PINEEA  GWWRGD**Y**GGKKQL  ETKAEK**Y**VNKAKG  GKKFLQ**Y**NRLQLS  TGRHCG**Y**VLQPST  EVAGAE**Y**DSTKQK  FLRFVV**Y**EEDMFS  VPLKNN**Y**SEDLEL  GVDGSL**Y**KTHPQY  YKTHPQ**Y**SRRFHK  DDIRTH**Y**DRLVDE  DFTPEN**Y**KRIEAI  EAIVKN**Y**PEGHKA  VPPMRV**Y**EVATFY  YEVATF**Y**TMYNRK  ATFYTM**Y**NRKPVG  RKPVGK**Y**HIQVCT  VQINDN**Y**YEDLTA  LSRALR**Y**YYDKNI  SRALRY**Y**YDKNII  SGQKFV**Y**KFVSYP  SSNYRA**Y**ATEPHA  KVDEER**Y**DIEAKV  MEELNT**Y**RQKQGV  QGVVLK**Y**QELPNS  KRLTVN**Y**EQCASG  GPEGFH**Y**KCKMGQ  KMGQKE**Y**SIGTGS  TSVKSD**Y**LSSGSF  DMKETK**Y**TVDKRF  VIKRVK**Y**NNEKAE  HVNIVH**Y**NGCWDG  CWDGFD**Y**DPETSD  SLESSD**Y**DPENSK  ITKGVD**Y**IHSKKL  SKGTLR**Y**MSPEQI  QISSQD**Y**GKEVDL  GKEVDL**Y**ALGLIL  VRKDLT**Y**TCRDNK  YASLEA**Y**CKHKYP  AYCKHK**Y**PEQPGR  EELARK**Y**ADGEVD  TVYNYD**Y**IWDFIF  YVHATF**Y**TPEGLR  VDQVTL**Y**SYKVQS  WRKLGS**Y**EHRIYL  RNVQFN**Y**PHTSVT  VTAWKQ**Y**RKAAIS  QQLQGF**Y**SQVAKP  VERLWA**Y**LTIQEL  VHPDLA**Y**LQAEGG  GEVTLT**Y**ATGTKE  NMRNDL**Y**QTPLHL  ANNSVA**Y**SGVKNS  VIILVL**Y**FVIDTF  AECTPI**Y**IQYFVK  FFHPIP**Y**YDKNSP  FHPIPY**Y**DKNSPV  NSPVHG**Y**WFREGA  RRDNGS**Y**FFRMER  ERGSTK**Y**SYKSPQ  GSTKYS**Y**KSPQLS  IQLNVT**Y**VPQNPT  FGLFSK**Y**RTPNCS  TPNCSQ**Y**RLPGCP  GSDMST**Y**ANECTL  SDQNFD**Y**MFKILI  TAGQER**Y**RTITTA  STQIKT**Y**SWDNAQ  INVGGK**Y**VKLQIW  ITSRET**Y**NALTNW  MGSGIQ**Y**GDAALR  ANDDLN**Y**WSDWYD  NYWSDW**Y**DSDQIK  ALLKAL**Y**GHGQIS  TQGCPT**Y**FPPSPT  SSSSSF**Y**NGSPPG  RQAPSS**Y**PPTWPP  KTLIKK**Y**IQRQET  FQHLMA**Y**HQEASK  LQDKDT**Y**SWLLKE  ARPEAL**Y**YPGALL  DLEARN**Y**DGLTAL  NVNAQM**Y**SGSSAL  YDYCEH**Y**SPDCDT  RGKLLS**Y**VDAEGN  CATKKG**Y**QKTVLE  RNCQPN**Y**WRNVIP  IPDPSK**Y**CGPYKP  IPGAGA**Y**ADDTAG  TIRNSP**Y**RREDSM  GSPLKA**Y**TPVVVT  TFSEHP**Y**NNLRKR  FVLDSE**Y**LVTLLV  NDWIKQ**Y**ETLAEM  LSQQEY**Y**PYVYYK  SERLVQ**Y**VKGKTY  PIAYLD**Y**NNLWRT  PGIMTQ**Y**GRVIRQ  STVLPL**Y**HKHYIL  LSIVIL**Y**CRIYSL  GTNPII**Y**TLTNKE  SMSKDM**Y**QIMDEI  GELCHR**Y**KCLLLV  QGIDIL**Y**SGSQKA  TEDAVD**Y**SDINEV  RLKDAG**Y**GEKSFF  EIVNVC**Y**QTLTEY  NYTTPD**Y**GHYDDK  FIFWLP**Y**QVTGIM  CINPII**Y**VVAGQG  TDDALV**Y**STFLLH  IPIECR**Y**PRQGNV  DQNASP**Y**HTIVDF  GSLETK**Y**RWTEYG  KYRWTE**Y**GLTFTE  SLIGLG**Y**TQTLKP  VEYEGE**Y**EYTGAN  YEGEYE**Y**TGANEY  GQGYDG**Y**DGQNYY  LEASTG**Y**QGSSFV  PDLQPG**Y**EGWQAL  VPLCIL**Y**EKYRDC  YAKCNP**Y**MDSPQS  GDGRMG**Y**AEEAPY  YAEEAP**Y**DAIHVG  KEGYKF**Y**PPKVEL  QKDKQS**Y**RDLKEV  LRIKAE**Y**EGDGIP  HRQLDS**Y**KNGFLN  LKQFLD**Y**FKTEHK  QGVSML**Y**SFFMPA  TLNSAG**Y**LLGPHA  LKNSPP**Y**ILDLLP  DLLPDT**Y**QHLRTI  RTILSR**Y**EGKMET  TLGENE**Y**FRVFME  EGKERM**Y**EENSQP  IHKPGS**Y**IFRLSC  GQWAIG**Y**VTADGN  GFREGF**Y**LFPDGR  GAPSPN**Y**DDDDDE  PPPDRP**Y**SVGAES  CTYEAM**Y**NIQSQA  NLMSQG**Y**SYQDIQ  MSQGYS**Y**QDIQKA  GMPCGV**Y**TPRCGS  GSGLRC**Y**PPRGVE  RTHEDL**Y**IIPIPN  CPEDIV**Y**VDHEDW  SYLRTG**Y**SVPYGQ  GSKPNV**Y**ANRGSA  FQVYGC**Y**PGPLSG  SSGSDY**Y**GSPCSA  NASCQH**Y**GVRTCE  YEEICV**Y**EEAREV  LPGQES**Y**TCSCAQ  NITVKT**Y**FNRTSQ  NFAGYM**Y**ILPQGE  ILPQGE**Y**PEYQRW  LPSGGQ**Y**KIQIFE  TEAKQE**Y**LLKDCD  IVHQHE**Y**GPEENL  NLEDDM**Y**RKTCTM  LRTGDD**Y**IAIGAD  QIEEAI**Y**QEIRNT  KKKNCT**Y**TQVQTR  DPDQFV**Y**KTRPPR  LGKVFI**Y**HGSANG  SKTVAQ**Y**SGVPWW  RQHHPP**Y**HQQHHQ  KRLFAK**Y**GEPGEV  VRNLSP**Y**VSNELL  AQKNPM**Y**QKERET  QHGTFE**Y**EYSQRW  GTFEYE**Y**SQRWKS  SEMEDA**Y**HEHQAN  RQREES**Y**SRMGYM  SYSRMG**Y**MDPRER  MNMGDP**Y**GSGGQK  GGGGIG**Y**EANPGV  PGTPAG**Y**GRGREE  GRGREE**Y**EGPNKK  GKEGDK**Y**KLSKKE  EVDFQE**Y**VVLVAA  DDVSTE**Y**GHQAHR  EDFQDE**Y**KTEVPH  PGSLCG**Y**CSFCNR  ELAEDG**Y**SGVEVR  QAESLR**Y**KLLGGL  SGDPVN**Y**YVDTAV  ALGDVV**Y**CSLPEV  LVNKSC**Y**EDGWLI  LMSEEA**Y**EKYIKS  EDTACF**Y**LAEISM  FCGTIE**Y**MAPEIL  KLNLPP**Y**LTQEAR  ENTKLW**Y**APNRTI  HYRMRF**Y**FTNWHG  LFAQGQ**Y**DLVKCL  LPKDIS**Y**KRYIPE  DISYKR**Y**IPETLN  ETLTKH**Y**GAEIFE  DGGNVL**Y**YEVMVT  GGNVLY**Y**EVMVTG  EWNNFS**Y**FPEITH  VSLVDG**Y**FRLTAD  TADAHH**Y**LCTDVA  GPICTE**Y**AINKLR  GSEEGM**Y**VLRWSC  QEWQPV**Y**PMSQLS  GTRTHI**Y**SGTLMD  LASALS**Y**LEDKDL  TLWEIC**Y**NGEIPL  KVELCR**Y**DPEGDN  EILRNL**Y**HENIVK  SGSLKE**Y**LPKNKN  LKQQLK**Y**AVQICK  DSPVFW**Y**APECLM  LMQSKF**Y**IASDVW  NCPDEV**Y**QLMRKC  IREEDE**Y**SELRSE  EQCIEA**Y**ELLLAL  EQRLKD**Y**IQQLKN  KAGIPV**Y**AWKGET  QAAMEG**Y**EVTTMD  KPQVDR**Y**RLKNGR  PDKDCR**Y**ALYDAT  DCRYAL**Y**DATYET  ELQANC**Y**EEVKDR  TGGGST**Y**VSKPVS  DKYRDR**Y**DSDRYR  DGYRDG**Y**RDGPRR  PGPGQN**Y**PRSGFP  PDSSSA**Y**CLPSTR  MDPVTG**Y**QYGQYG  ETIRRA**Y**PDANLL  FPLAMN**Y**LDRFLS  NNFLSY**Y**RLTRFL  LWTFSI**Y**LESVAI  ETITSH**Y**LFALGV  LYCDFF**Y**LYITKV  PTCVSD**Y**MSISTC  TELRLL**Y**QLVFLL  VVSADN**Y**TLDLWA  LTWSNP**Y**PPDNYL  PYPPDN**Y**LYNHLT  PPDNYL**Y**NHLTYA  LYNHLT**Y**AVNIWS  PADFRI**Y**NVTYLE  RIYNVT**Y**LEPSLR  LKSGIS**Y**RARVRA  RAWAQC**Y**NTTWSE  TKWHNS**Y**REPFEQ  AVCLLC**Y**VSITKI  LGSGIV**Y**SALTCH  GQSCGV**Y**TERCAQ  CLNEKS**Y**REQVKI  CWCVDK**Y**GMKLPG  SAATSG**Y**EIGNPP  IGNPPD**Y**RGQSCM  DFATFD**Y**ILCMDE  IELLGS**Y**DPQKQL  SDFETV**Y**QQCVRC  DGIELT**Y**GIKDVP  LIPGVE**Y**LVSIIA  RASVTG**Y**LLVYES  MEVCEV**Y**KLHRET  QDFFDR**Y**MATQEN  VSWLNV**Y**MQVAYL  SPTSPS**Y**SPTSPS  SPASPK**Y**TPTSPS  SPTSPT**Y**SPTSPV  SIHGHD**Y**VKKAIL  KSQLLR**Y**VLCTAP  ELVQYA**Y**FKKVLE  KDIVEY**Y**NDSNGS  YYDGKD**Y**IEFNKE  PATLRK**Y**LKYSKN  NCDEPM**Y**VKLVEA  CVVVKD**Y**GKESQA  FRSNFG**Y**NIPLKH  SFMLGS**Y**SVNDGA  NDGAQL**Y**MIDPSG  ILKATN**Y**NGHTCL  LASIHG**Y**LGIVEL  DVNRVT**Y**QGYSPY  RVTYQG**Y**SPYQLT  YQGYSP**Y**QLTWGR  SEDEES**Y**DTESEF  TEDELP**Y**DDCVFG  EDMQLS**Y**IHSKVK  GDDGKT**Y**FYQLWY  GGPNRF**Y**FLEAYN  IGGLPN**Y**LNDDQV  GYAFCE**Y**VDINVT  LLDDEE**Y**EEIVED  TGQSLG**Y**GFVNYI  GYGFVN**Y**IDPKDA  FVTMTN**Y**DEAAMA  IVENLF**Y**PVTLDV  FQALLQ**Y**ADPVSA  LDGQNI**Y**NACCTL  WLRLHT**Y**LAGEAP  LGEVVL**Y**SGARPL  EKGDRI**Y**HQLKKL  ALIAAQ**Y**SGAQVR  ESNAIA**Y**YVSNEE  LGLLDA**Y**LKTRTF  STTPSR**Y**KTELCR  ANRHPK**Y**KTELCH  GSDPDE**Y**ASSGSS  SVDGVV**Y**YRVQNA  VDGVVY**Y**RVQNAT  AALQLR**Y**LQTLTT  FDVGPR**Y**TQLQYI  RYTQLQ**Y**IGEGAY  YIGEGA**Y**GMVSSA  GMVSSA**Y**DHVRKT  PFEHQT**Y**CQRTLR  EAMRDV**Y**IVQDLM  LMETDL**Y**KLLKSQ  SNDHIC**Y**FLYQIL  HICYFL**Y**QILRGL  ILRGLK**Y**IHSANV  YVATRW**Y**RAPEIM  MLNSKG**Y**TKSIDI  IFPGKH**Y**LDQLNH  NMKARN**Y**LQSLPS  EALAHP**Y**LEQYYD  HPYLEQ**Y**YDPTDE  PYLEQY**Y**DPTDEP  PGLSHQ**Y**WSAPSD  LYPNTP**Y**AYTFWT  VGWRLD**Y**FLLSHS  FFERSV**Y**SDRYIF  SVYSDR**Y**IFASNL  LKTNFD**Y**LQEVPI  AAERLG**Y**PVLVRA  PESEQY**Y**IIEVNA  QAENGM**Y**IRMALL  RRESFL**Y**RSDSDY  GNQVSE**Y**ISTTFL  SLTCIM**Y**MIFQER  LEEKMA**Y**QEYPNS  KMAYQE**Y**PNSQNW  VSKKVS**Y**SHIQSK  KEMCYR**Y**REDLMA  QEGGQV**Y**SVPMGG  GYVDAT**Y**REGMTK  NDDTRL**Y**SNDFNS  LQNATN**Y**FLMSLA  LTILYG**Y**RWPLPS  LISAGD**Y**RHRRTE  FEVSPS**Y**VKGGPL  GGPLRD**Y**QIRGLN  NWLISL**Y**ENGVNG  TIALLG**Y**LKHYRN  LGYLKH**Y**RNIPGP  DVCVTS**Y**EMVIKE  KKFHWR**Y**LVIDEA  KKEIKI**Y**LGLSKM  KMQREW**Y**TKILMK  KCCNHP**Y**LFDGAE  AEPGPP**Y**TTDEHI  LDILED**Y**CMWRGY  YCMWRG**Y**EYCRLD  MWRGYE**Y**CRLDGQ  RERKAN**Y**AVDAYF  NYAVDA**Y**FREALR  LEKEIL**Y**YRKTIG  EKEILY**Y**RKTIGY  IKANEK**Y**GRDDID  PEEVME**Y**SAVFWE  DAKIAR**Y**KAPFHQ  TSKGKN**Y**TEEEDR  FDRENV**Y**EELRQC  EHYFKP**Y**ISKLFD  NKEYEE**Y**VLTVGD  LAEILY**Y**KILETV  LKRLAK**Y**VIRGFY  YVIRGF**Y**GIEHAL  EQIEPI**Y**ALLRET  LCRYPE**Y**DGRGVL  KLPANQ**Y**TWSSRG  YRSHEF**Y**KFCSLP  GWLTHP**Y**GKGWDL  MNDMPI**Y**MYSVCN  DMPIYM**Y**SVCNVM  LRTNWV**Y**RGEAER  KETFNL**Y**YAESDL  ETFNLY**Y**AESDLD  LLSVRV**Y**YKKCPE  CLCQAG**Y**EKVEDA  PPSAPH**Y**LTAVGM  CEASVR**Y**SEPPHG  LEPHMN**Y**TFTVEA  QSRVWK**Y**EVTYRK  WKYEVT**Y**RKKGDS  LAPDTT**Y**LVQVQA  QSPEDV**Y**FSKSEQ  YVDPHT**Y**EDPNQA  KTLKAG**Y**TEKQRV  EGVISK**Y**KPMMII  MMIITE**Y**MENGAL  DDPEAT**Y**TTSGGK  APEAIS**Y**RKFTSA  MWEVMT**Y**GERPYW  TYGERP**Y**WELSNH  DCPSAI**Y**QLMMQC  QNSGDF**Y**DLYGGE  LTELVE**Y**YTQQQG  TELVEY**Y**TQQQGV  TIIHLK**Y**PLNCSD  PTSERW**Y**HGHMSG  MCEGGR**Y**TVGGLE  ASGAFV**Y**LRQPYY  VYLRQP**Y**YATRVN  YLRQPY**Y**ATRVNA  NKGKNR**Y**KNILPF  NIPGSD**Y**INANYI  DYINAN**Y**IKNQLL  DENAKT**Y**IASQGC  RNKCVP**Y**WPEVGM  VGMQRA**Y**GPYSVT  EHDTTE**Y**KLRTLQ  IREIWH**Y**QYLSWP  EIWHYQ**Y**LSWPDH  VQTEAQ**Y**KFIYVA  AQYKFI**Y**VAIAQF  EYGNIT**Y**PPAMKN  VAATGT**Y**QLRESE  LVRYAG**Y**RQQDGS  PEQHKS**Y**KIRFNS  KENNIF**Y**SPISIT  NKSTDA**Y**ELKIAN  YLFLQE**Y**LDAIKK  EAVDAR**Y**QRDYEE  SIKGPS**Y**GEDVSN  LKKYSR**Y**LSLQTT  YFRIRF**Y**FRNWHG  PREPAV**Y**RCGPPG  QMVMVK**Y**LATLER  REPLWA**Y**FCDFRD  TADSSH**Y**LCHEVA  RPEDGL**Y**LIHWST  WSTSHP**Y**RLILTV  DIALAF**Y**ETASLM  NIMVTE**Y**VEHGPL  TSQCLT**Y**EPTQRP  TVFHKR**Y**LKKIRD  FGKVSL**Y**CYDPTN  KVSLYC**Y**DPTNDG  DILRTL**Y**HEHIIK  HEHIIK**Y**KGCCED  LQLVME**Y**VPLGSL  LGSLRD**Y**LPRHSI  YLHAQH**Y**IHRDLA  PEGHEY**Y**RVREDG  DSPVFW**Y**APECLK  LKEYKF**Y**YASDVW  KEYKFY**Y**ASDVWS  KCPCEV**Y**HLMKNC  KTVHEK**Y**QGQAPS  WFDKFK**Y**DDAERR  EERLRQ**Y**AEKKAK  KLVPVG**Y**GIRKLQ  LDTVTF**Y**KVIPKS  EVGISD**Y**GDKLNM  MELSEK**Y**KLDKES  KLDKES**Y**PVFYLF  ESYPVF**Y**LFRDGD  FENPVP**Y**TGAVKV  LKGQGV**Y**LGMPGC  PGCLPV**Y**DALAGE  KKWAEQ**Y**LKIMGK  GKAYKA**Y**RLLKGH  PNYAYA**Y**TLLGHE  PKESLV**Y**FLIGKV  KSPAAP**Y**FLGSSF  RLDGCI**Y**AIKRSK  HSHVVR**Y**FSAWAE  CRYPYE**Y**EGGHIK  GPRMCR**Y**VRERDR  YCEPPS**Y**RPMHHE  RELIGD**Y**SKAFLL  VDCRYP**Y**EYEGGH  FCEPQD**Y**RPMNHE  VSRSGL**Y**RSPSMP  GLIEKF**Y**VIDCRY  QGALNL**Y**SQEELF  CTVAEI**Y**LGNLAA  TACVIS**Y**PSLIWE  IASFMA**Y**SNSCLN  LATTAL**Y**FTYSAL  PKAAQK**Y**VERIHY  QQFKQL**Y**RARMNA  LSDTGQ**Y**QCLVFL  FVSQPG**Y**VGLEGL  GLEGLP**Y**FLEEPE  PGLSGI**Y**PLTHCT  LHPHTP**Y**HIRVAC  QGTLLG**Y**RLAYQG  LGYRLA**Y**QGQDTP  TVCVAA**Y**TAAGDG  FSWPWW**Y**VLLGAV  RKKETR**Y**GEVFEP  GELVVR**Y**RVRKSY  YRVRKS**Y**SRRTTE  LHSFLL**Y**SRLGDQ  LGDQPV**Y**LPTQML  IASGME**Y**LSTKRF  GLSKKI**Y**NGDYYR  KIYNGD**Y**YRQGRI  TRGQTP**Y**PGVENS  VENSEI**Y**DYLRQG  NSEIYD**Y**LRQGNR  MDEGGG**Y**PEPPGA  HGSPDS**Y**RSPLAS  FVFSDR**Y**KFRTWR  FLEEEV**Y**PLLKPY  PNLAAA**Y**SSILSS  QFFTKG**Y**QETISD  ARIVEI**Y**SRRLQV  EAMLTI**Y**QLHKIC  IKRKIP**Y**ILKRQL  NKPRRP**Y**ILKRDS  DSISAQ**Y**PVVDHE  NWRWHF**Y**DTVKGS  DQDAIH**Y**MTEQAP  VVELEN**Y**GMPFSR  TEDGKI**Y**QRAFGG  SLLHTL**Y**GRSLRY  YGRSLR**Y**DTSYFV  TSYFVE**Y**FALDLL  VVATGG**Y**GRTYFS  GGYGRT**Y**FSCTSA  FHPTGI**Y**GAGCLI  ERFMER**Y**APVAKD  PEKDHV**Y**LQLHHL  VLPTVH**Y**NMGGIP  GGIPTN**Y**KGQVLR  QIVPGL**Y**ACGEAA  GKISKL**Y**GDLKHL  CALQTI**Y**GAEARK  AHARED**Y**KVRIDE  KVRIDE**Y**DYSKPI  RIDEYD**Y**SKPIQG  RKHTLS**Y**VDVGTG  GKVTLE**Y**RPVIDK  IDMFHK**Y**TRRDDK  DKKGTN**Y**LADVFE  GDIATD**Y**HKQSHG  HHHHHP**Y**VHPQAP  AAPDGR**Y**MRSWLE  LTRDRR**Y**EVARLL  AITDPA**Y**KGQILT  GPIDTE**Y**LFDSFF  AADTIG**Y**PVMIRS  NVWRFP**Y**ICYQNG  RFPYIC**Y**QNGGGA  GAFLLP**Y**TIMAIF  GGIPLF**Y**MELALG  IFKGIG**Y**AICIIA  ICIIAF**Y**IASYYN  AFYIAS**Y**YNTIMA  FYIASY**Y**NTIMAW  IMAWAL**Y**YLISSF  TGNCTN**Y**FSEDNI  LIFTVI**Y**FSIWKG  VTATFP**Y**IILSVL  WRGVLF**Y**LKPNWQ  LLAFAS**Y**NKFNNN  KFNNNC**Y**QDALVT  IFTVLG**Y**MAEMRN  SLLFIT**Y**AEAIAN  VKLLEE**Y**ATGPAV  VAVSWF**Y**GITQFC  QLRLFQ**Y**NYPYWS  RLFQYN**Y**PYWSII  WSIILG**Y**CIGTSS  FICIPT**Y**IAYRLI  IPTYIA**Y**RLIITP  DGSFIG**Y**KERPEA  FCGTPE**Y**LAPEVL  ITPPDR**Y**DSLGLL  PDWGKG**Y**SRKAAA  ELGNDA**Y**KKKDFD  PTFIKG**Y**TRKAAA  IAVFQK**Y**AGKDGY  YAGKDG**Y**NYTLSK  GKDGYN**Y**TLSKTE  THTQPS**Y**RFKANN  NNDSGE**Y**TCQTGQ  HSHSGD**Y**HCTGNI  CTGNIG**Y**TLYSSK  NIGYTL**Y**SSKPVT  AVVALI**Y**CRKKRI  EETNND**Y**ETADGG  GSARHA**Y**YVTVEA  NAPIEK**Y**DIEFED  RSKGGK**Y**SVKDKE  ALTTFW**Y**ATRPKP  IVVMDA**Y**GSELVE  EKIENI**Y**MRSEPV  RPGLHA**Y**EKEDSD  DQWLEQ**Y**TQAIET  ATGHGK**Y**EKVLVE  SARAAG**Y**DLYSAY  LYSAYD**Y**TIPPME  ALPSGC**Y**GRVAPR  VCTFQE**Y**AGRCGD  EFRECD**Y**NKFMSV  EVDFVE**Y**VRSLAC  GKKQFK**Y**GNQLVR  QELLPQ**Y**KEREVV  PESLAD**Y**ITAAYV  AHARNA**Y**TPQEIV  NCHFGP**Y**DNKARP  EMVGRY**Y**EEFPIN  GLVQPS**Y**GQDGMY  TGGSDR**Y**CNLMMQ  RAPNCR**Y**RAIAST  TSDKDM**Y**LDNSSI  EEASGV**Y**PIDDDD  EEDTNV**Y**TEKHSD  NKYSEG**Y**PGQRYY  GYPGQR**Y**YGGTEF  PANFAV**Y**TALVEP  LDSVAE**Y**EVTPDE  RATGKM**Y**ACKRLE  EERALF**Y**AAEILC  RYLCLR**Y**PPSYKA  PLIPND**Y**LLSWLL  SGIIYT**Y**GHVLWK  WAARVN**Y**SECQEI  MFGEGC**Y**LHTAIV  TSETIQ**Y**RKAVKA  HPDVAA**Y**KANRDL  GGGELA**Y**ALNNFD  NYEPGV**Y**TEKVLE  HWQQQS**Y**LDSGIH  VDSVLF**Y**AITTLH  CLQILA**Y**GNQESK  NLTCNN**Y**KNKMMV  NAVRLH**Y**GLPVVV  LFVQLL**Y**SPIENI  EDKPQD**Y**KKRLSV  QGEPLG**Y**RQDDPS  RQDDPS**Y**RSFHSG  SFHSGG**Y**GQDALG  HHPGAD**Y**PVDGLP  HASKDR**Y**KTILPN  PTEEET**Y**GPFQIR  IQTAEQ**Y**QFLHHT  SQNVTE**Y**VVRVPK  EGQEVD**Y**MSDGSS  EDAVRR**Y**LTRKPM  STQVNN**Y**WALNLT  FKGWAL**Y**CAGKAA  NVRVLN**Y**APGPLD  VSVALM**Y**LGSLAF  LRMSSS**Y**PTGLAD  RIRVKR**Y**RQSMNN  LNLLVL**Y**AVRSER  FWLSMD**Y**VASTAS  ILCWIP**Y**FIFFMV  QEEIEN**Y**QNKQRE  VYFDGK**Y**ASPDVF  LHFPPL**Y**KELFTS  GQPLCE**Y**STFILL  AMSVER**Y**LAINHA  INHAYF**Y**SHYVDK  SNPLAH**Y**DTTADE  PDSVRN**Y**MTKFLS  VHEQIQ**Y**HSTGKS  GSRYNA**Y**CCPGWK  CPAGFQ**Y**EQFSGG  DKYDKD**Y**LSGELG  ISFGSG**Y**GGNSLL  GYNFGK**Y**LAHWLS  KLTPIG**Y**IPKEDA  DVRKVG**Y**LRKPKS  GPARLE**Y**YENEKK  PARLEY**Y**ENEKKW  KHLVAL**Y**TRDEHF  AEQDSW**Y**QALLQL  AGEDLS**Y**GDVPPG  KNLIGI**Y**RLCLTS  FISSDE**Y**GSSPCD  TAPNGH**Y**ILSRGG  VASIEE**Y**TEMMPA  TEMMPA**Y**PPGGGS  FVPTRS**Y**PEEGLE  VPSGTS**Y**GKLWTN  LPCTGD**Y**MNMSPV  SSPSDC**Y**YGPEDP  SPSDCY**Y**GPEDPQ  HKPVLS**Y**YSLPRS  KPVLSY**Y**SLPRSF  SSGRLL**Y**AATADD  DSLGGG**Y**CGARLE  GSDQSG**Y**LSGPVA  SCPRQS**Y**VDTSPA  PAAPVS**Y**ADMRTG  VPFGLL**Y**SEVTAS  YRTGYP**Y**RYPALR  PASVTG**Y**SFASDG  MLDNLG**Y**RTGYTY  LNTPNT**Y**LRVNVA  NQFVPL**Y**TDPQEV  TQPGQG**Y**SQQSSQ  SSQSSS**Y**GQPQSG  RGGRGG**Y**DRGGYR  QICKKV**Y**ENYPTY  KKVYEN**Y**PTYDLT  YENYPT**Y**DLTERK  RDYSAY**Y**RQIEEL  EDFRLK**Y**ETERGI  DVKTTE**Y**QLSTLE  DGLVSG**Y**SMTPPT  GSYVCY**Y**KYIKAR  YVCYYK**Y**IKARIE  TTAASS**Y**VFVRDF  ITGNEL**Y**DIQLLP  VTFDWD**Y**PGKQAE  QHDLGS**Y**VCKANN  PVKLAA**Y**PPPEFQ  PPEFQW**Y**KDGKAL  EASTGT**Y**TLALWN  ASSPSI**Y**SRHSRQ  ALTCTA**Y**GVPLPL  ANVSAM**Y**KCVVSN  QDERLI**Y**FYVTTI  SCQADS**Y**KYEHLR  QADSYK**Y**EHLRWY  PEHEGH**Y**VCEVQD  KHCHKK**Y**LSVQAL  EEDAGR**Y**LCSVCN  ADIKTG**Y**LSIIMD  IVEFCK**Y**GNLSNF  MEDLVC**Y**SFQVAR  SIFDKV**Y**TTQSDV  SLGASP**Y**PGVQIN  GNDTGA**Y**KCFYRE  DQHGVV**Y**ITENKN  VSLCAR**Y**PEKRFV  GFTIPS**Y**MISYAG  PSYMIS**Y**AGMVFC  SYQSIM**Y**IVVVVG  IVVVVG**Y**RIYDVV  VVGYRI**Y**DVVLSP  VRIPAK**Y**LGYPPP  PAKYLG**Y**PPPEIK  PPEIKW**Y**KNGIPL  ERDTGN**Y**TVILTN  TLTCTV**Y**AIPPPH  VSVTNP**Y**PCEEWR  ANVSAL**Y**KCEAVN  FENLTW**Y**KLGPQP  LQDQGD**Y**VCLAQD  KEDEGL**Y**TCQACS  HCERLP**Y**DASKWE  FGNLST**Y**LRSKRN  RNEFVP**Y**KTKGAR  LEHLIC**Y**SFQVAK  RMRAPD**Y**TTPEMY  TAGISQ**Y**LQNSKR  PYIVGF**Y**GAFYSD  FVGTRS**Y**MAPERL  IFELLD**Y**IVNEPP  KNNRQP**Y**AVSELA  NTTQKR**Y**AICSAL  MLKLNP**Y**AKTMRR  HGVVGP**Y**VKKILC  SANDKV**Y**TVEKAD  KADNFE**Y**SDPVDG  VGKSSS**Y**PMVSES  ETQAGE**Y**LLFIQS  QSEATN**Y**TILFTV  IRNTLL**Y**TLRRPY  YTLRRP**Y**FRKMEN  YFEMST**Y**STNRTM  ARNDTG**Y**YTCSSS  RNDTGY**Y**TCSSSK  TNSSED**Y**EIDQYE  DYEIDQ**Y**EEFCFS  SVRFKA**Y**PQIRCT  KGLDNG**Y**SISKFC  LVKCCA**Y**NSLGTS  LLICHK**Y**KKQFRY  YVDFRE**Y**EYDLKW  TLSGPI**Y**LIFEYC  IFEYCC**Y**GDLLNY  YGDLLN**Y**LRSKRE  SLFEGI**Y**TIKSDV  KSDVWS**Y**GILLWE  SLGVNP**Y**PGIPVD  PVDANF**Y**KLIQNG  KMDQPF**Y**ATEEIY  YATEEI**Y**IIMQSC  SCLQAN**Y**TCETDG  PAGKPF**Y**CLSSED  RNTHCC**Y**TDYCNR  HCCYTD**Y**CNRIDL  VFLVIN**Y**HQRVYH  NYHQRV**Y**HNRQRL  TLQDLV**Y**DLSTSG  FREAEI**Y**QTVMLR  LWLVSD**Y**HEHGSL  HGSLFD**Y**LNRYTV  FDYLNR**Y**TVTIEG  FKCADI**Y**ALGLVY  YALGLV**Y**WEIARR  GGVHEE**Y**QLPYYD  EEYQLP**Y**YDLVPS  EYQLPY**Y**DLVPSD  PNWWQS**Y**EALRVM  MMRECW**Y**ANGAAR  AVSNFG**Y**DLYRVR  TSLEDF**Y**LDEERT  PKLKLS**Y**EGEVTK  ANRGPA**Y**GLSREV  QKIEKQ**Y**DADLEQ  ELINAL**Y**PEGQAP  TKEGVL**Y**VGSKTK  VKRVID**Y**AVKIRV  RLNEPR**Y**ATLPNI  PSRRDV**Y**LSPRDD  RDSYES**Y**GNSRSA  GGSRDS**Y**SSSRSD  EIKKKK**Y**NQMPVR  DTEGFK**Y**PLGHEV  EIKKQE**Y**EEVVQT  TEPDCK**Y**SKKHWK  KKEKCE**Y**KKKAKN  ETPKKD**Y**SLLPLL  NPNNTF**Y**ATKRLI  VITVPA**Y**FNDSQR  KLFEMA**Y**KKMASE  DIRKLD**Y**GQHVVA  EQIYDV**Y**RYLPPA  RDIEQY**Y**STQIDE  GDIVGL**Y**DAANEG  LDRENS**Y**RLLKLA  DIVPEN**Y**SHENSQ  LGLPKL**Y**LPTGPR  TSMTDF**Y**HSKRRL  HLMGMF**Y**RTIRMM  VKAGKV**Y**AAATED  RLDPNK**Y**PVPENW  KSHLQN**Y**TVNATK  NSKDQM**Y**SDGNFT  EGMPKS**Y**LPQTVR  DGIPPP**Y**DKKKRM  HEVGWK**Y**QAVTAT  EKKIDK**Y**TEVLKT  QQLDTR**Y**LEQLHQ  EQLHQL**Y**SDSFPM  ESQDWA**Y**AASKES  GEIDQQ**Y**SRFLQE  QESNVL**Y**QHNLRR  QFLQSR**Y**LEKPME  DDFDFN**Y**KTLKSQ  LLSAME**Y**VQKTLT  LQQKVS**Y**KGDPIV  KFPELN**Y**QLKIKV  TFETEV**Y**HQGLKI  WASILW**Y**NMLTNN  LGPGVN**Y**SGCQIT  IDLVKK**Y**ILALWN  ALWNEG**Y**IMGFIS  IQSVEP**Y**TKQQLN  AEIIMG**Y**KIMDAT  LVSPLV**Y**LYPDIP  SPLVYL**Y**PDIPKE  EEAFGK**Y**CRPESQ  NHSSTQ**Y**PDVNHA  GKEVGV**Y**EALKDD  ISDGNS**Y**GVPDDL  VSRLTL**Y**DIAHTP  KAAVKG**Y**LGPEQL  FKKHGV**Y**NPNKIF  YANAKI**Y**KLDDPS  IEVVCE**Y**IVKKIP  TELEIS**Y**FLLRRL  VLRAAF**Y**VAAQLL  HLLGIH**Y**TGCSMN  ILGSLL**Y**NYVLFP  PASYSM**Y**SHLPVS  CQASFR**Y**KGNLAS  HSGEKP**Y**KCETCG  LRLQPI**Y**WSRDDV  HSGDVL**Y**ELLQHI  SRALRH**Y**YKLNII  NYQMKY**Y**FYHGLS  SWPQLS**Y**IAEDEN  EVEPKY**Y**ADGEDA  DMDMAD**Y**SAALDP  AALDPA**Y**TTLEFE  VRKNHM**Y**SCRFSR  TECIAK**Y**NFHGTA  TKDPNW**Y**KAKNKV  GIIPAN**Y**VQKREG  QAERLL**Y**PPETGL  VRESTN**Y**PGDYTL  TNYPGD**Y**TLCVSC  DGKVEH**Y**RIMYHA  EHYRIM**Y**HASKLS  SIDEEV**Y**FENLMQ  MQLVEH**Y**TSDADG  DVMLGD**Y**RGNKVA  EEKGGL**Y**IVTEYM  LYIVTE**Y**MAKGSL  KGSLVD**Y**LRSRGR  ILLWEI**Y**SFGRVP  SFGRVP**Y**PRIPLK  PRVEKG**Y**KMDAPD  NTAKHF**Y**GQRPQE  TPQNGR**Y**QIDSDV  ENIAEL**Y**GAVLWG  VTTFFD**Y**DYGAPC  TFFDYD**Y**GAPCHK  QLLPPL**Y**SLVFIF  KCLTDI**Y**LLNLAI  KLFTGL**Y**HIGYFG  GLYHIG**Y**FGGIFF  QKEDSV**Y**VCGPYF  VYVCGP**Y**FPRGWN  LIMVIC**Y**SGILKT  FTIMIV**Y**FLFWTP  FLFWTP**Y**NIVILL  CINPII**Y**AFVGEK  QVRVKA**Y**YRGDIM  VRVKAY**Y**RGDIMI  EEAFRL**Y**ELNKDS  GEDKSI**Y**RRGARR  RRWRKL**Y**CANGHT  GLGRQG**Y**KCINCK  AQTVIP**Y**NPSSHE  EEHARF**Y**SAEISL  ISLALN**Y**LHERGI  HERGII**Y**RDLKLD  HIKLTD**Y**GMCKEG  FCGTPN**Y**IAPEIL  ILRGED**Y**GFSVDW  DQNTED**Y**LFQVIL  EFEGFE**Y**INPLLM  RQMQVL**Y**GQHFPI  PIEVRH**Y**LAQWIE  KIKLGH**Y**ATQLQK  TQLQKT**Y**DRCPLE  CIRHIL**Y**NEQRLV  LQQTQE**Y**FIIQYQ  EYFIIQ**Y**QESLRI  AQTLQQ**Y**RVELAE  NCCVME**Y**HQATGT  SSHLED**Y**SGLSVS  NLPGWN**Y**TFWQWF  RLGDLS**Y**LIYVFP  DLSYLI**Y**VFPDRP  DEVFSK**Y**YTPVLA  EVFSKY**Y**TPVLAK  GGSSAT**Y**MDQAPS  VCPQAP**Y**NMYPQN  QAPYNM**Y**PQNPDH  KTVCAE**Y**AIALAL  SAFHLT**Y**NMVLNL  ISSVRL**Y**IPKDLR  ENKKQP**Y**YYPPFD  DEELSQ**Y**LLQLVQ  GYCVAS**Y**VLGIGD  CRIVKG**Y**TLADEE  FEAVLH**Y**KHQNQA  NLSIQR**Y**AVIPLS  TFRNLK**Y**EVRNKN  STAPGY**Y**SWRNSK  CAMLKQ**Y**ADKLEF  KTSPLN**Y**KERLFV  TKSMLT**Y**YEGRAE  KSMLTY**Y**EGRAEK  GRAEKK**Y**RKGFID  NNIMIK**Y**HPKFWT  FWTDGS**Y**QCCRQT  EIVVAM**Y**DFQAAE  WRARDK**Y**GNEGYI  GYIPSN**Y**VTGKKS  SNNLDQ**Y**EWYCRN  LDQYEW**Y**CRNMNR  SSQPGL**Y**TVSLYT  LYTVSL**Y**TKFGGE  SSGFRH**Y**HIKETT  TTSPKK**Y**YLAEKH  IPEIIE**Y**HKHNAA  TTAGFS**Y**EKWEIN  PKLVQL**Y**GVCTQQ  TQQKPI**Y**IVTEFM  VCEGME**Y**LERNSF  DFGMAR**Y**VLDDQY  PPEVFN**Y**SRFSSK  RMPFEK**Y**TNYEVV  FEKYTN**Y**EVVTMV  EALHRP**Y**GCDVEP  KLRVLG**Y**NQNGEW  LSISLR**Y**EGRVYH  EGRVYH**Y**RINTTA  TLEPPF**Y**IVTEYM  FYIVTE**Y**MPYGNL  VTEYMP**Y**GNLLDY  YGNLLD**Y**LRECNR  TAVVLL**Y**MATQIS  ISSAME**Y**LEKKNF  APESLA**Y**NTFSIK  LWEIAT**Y**GMSPYP  TYGMSP**Y**PGIDLS  IDLSQV**Y**DLLEKG  DLLEKG**Y**RMEQPE  GHQLLD**Y**CSGYVD  LDYCSG**Y**VDCIPQ  TAVVQL**Y**LALPPG  DNPQKS**Y**FIRLYG  SYFIRL**Y**GLQAGR  LWEQEL**Y**SQLVYS  LYSQLV**Y**STPTPF  DITSSR**Y**RGLPAP  PNAPNS**Y**GRRPIQ  HRDVAR**Y**LRAAAG  TRIYRG**Y**NLLPSI  DEILAQ**Y**LVPATC  AAPGSP**Y**HRLLTC  VPQGHV**Y**RFCTAE  EQLLFL**Y**IIYTVG  GIVKYL**Y**EDEGCW  RSQESG**Y**YDRMDY  GLLPKG**Y**PHLCSI  LKLAEP**Y**GKIKNY  RRMKAG**Y**LDQQVP  FREGPP**Y**QRRGAL  SRSLRY**Y**YEKGIM  CFGQCQ**Y**TAEEYQ  QYTAEE**Y**QAIQKA  QRLGPE**Y**ISSRMA  GGQKVC**Y**IEGHRV  ANEMFG**Y**NGWAHS  QLKDGS**Y**HEDVGY  YHEDVG**Y**GVSEGL  CILDKD**Y**LRSLNK  SVEEAR**Y**NSCRPN  SWDLQT**Y**SADQRT  AHLPFF**Y**GSISRA  LRSLGG**Y**VLSLVH  RQLNGT**Y**AIAGGK  AELCEF**Y**SRDPDG  HERMPW**Y**HSSLTR  EAERKL**Y**SGAQTD  RKEQGT**Y**ALSLIY  YALSLI**Y**GKTVYH  IYGKTV**Y**HYLISQ  GKTVYH**Y**LISQDK  QDKAGK**Y**CIPEGT  LWQLVE**Y**LKLKAD  SVRQGV**Y**RMRKKQ  HQLDNP**Y**IVRLIG  VSMGMK**Y**LEEKNF  LLVNRH**Y**AKISDF  KWPLKW**Y**APECIN  RSDVWS**Y**GVTMWE  MWEALS**Y**GQKPYK  SYGQKP**Y**KKMKGP  ECPPEL**Y**ALMSDC  MSDCWI**Y**KWEDRP  QRMRAC**Y**YSLASK  RMRACY**Y**SLASKV  GMSDGL**Y**LLRQSR  GRKAHH**Y**TIEREL  RELNGT**Y**AIAGGR  PADLCH**Y**HSQESD  RDNNGS**Y**ALCLLH  EGKVLH**Y**RIDKDK  WQLVEH**Y**SYKADG  LVEHYS**Y**KADGLL  GTVKKG**Y**YQMKKV  TVKKGY**Y**QMKKVV  QQLDNP**Y**IVRMIG  LGPLNK**Y**LQQNRH  KWPVKW**Y**APECIN  PECINY**Y**KFSSKS  MWEAFS**Y**GQKPYR  SYGQKP**Y**RGMKGS  GCPREM**Y**DLMNLC  MNLCWT**Y**DVENRP  TTGSNY**Y**VRILST  SEFVQK**Y**LGEGPR  KDFEKA**Y**KTVIKK  SLGITL**Y**ELATGR  ATGRFP**Y**PKWNSV  AVEVAC**Y**VCKILD  KAIANL**Y**GPLMAL  HMVQQD**Y**FPKALA  EERSSW**Y**WGRLSR  STSPGD**Y**VLSVSE  NSRVSH**Y**IINSSG  PALLEF**Y**KIHYLD  EFYKIH**Y**LDTTTL  RQEEAE**Y**VRALFD  GMIPVP**Y**VEKYRP  VPYVEK**Y**RPASAS  KRVPNA**Y**DKTALA  LDGPNT**Y**TCVCTE  CRQSED**Y**ESFSCV  PGFTGS**Y**CQHDVN  RSRRVL**Y**PRVVRR  TSEPSD**Y**ALDLST  FYTVTF**Y**GALFRE  DFGISG**Y**LVDSVA  PAERMS**Y**LELMEH  VRTQAM**Y**QMMDQG  QKEEER**Y**ERIEIP  QEEQDA**Y**RRIHSL  RINFDK**Y**HPGYFG  KVGMKH**Y**HLKRNQ  VVRSGY**Y**KVLGKG  KRNKQT**Y**STEPNN  ARNSFR**Y**NGLIHR  RKPATS**Y**VRTTIN  IAVKEK**Y**AKYLPH  PHSAGR**Y**AAKRFR  KGSSNS**Y**AIKKKD  KNRIAI**Y**ELLFKE  FAWRHF**Y**WYLTNE  TNEGIQ**Y**LRDYLH  DAREEE**Y**EPEKME  ESEGYS**Y**ETSTKT  PPGLPV**Y**LDLCYI  TSSGQR**Y**FLNHID  TQDGEI**Y**YINHKN  QDGEIY**Y**INHKNK  FLNSGT**Y**HSRDES  QNRFPD**Y**LEAIPG  DREQTR**Y**KATGLH  HWVKGG**Y**YYYHNL  IIGNLL**Y**YRYMNP  FCRLNE**Y**NQLQLQ  QLAAYP**Y**AVWYYP  PYAVWY**Y**PQIMQY  WVEFSP**Y**EIGMAK  LNLNTS**Y**PLSPLS  EGLKEC**Y**VFKPKN  SPWSNK**Y**DPPLED  NNAFDQ**Y**RDLYFE  SGRTAH**Y**KLTSTV  FVEEQV**Y**SEFVRR  VRRSVE**Y**AKKRPV  TVWINC**Y**NALYAQ  GDKWCI**Y**PTYDYT  IYPTYD**Y**THCLCD  CNALDV**Y**CPVQWE  KPRSQR**Y**ESLKGV  KLDRLA**Y**IAHPKL  RPPVQV**Y**GIEGRY  ASVLNP**Y**VKRSIK  VRIGEK**Y**VDMSVK  LDLKWR**Y**KMTIFI  ISPLTI**Y**HVIDHN  ARMKRG**Y**DNPNFI  ARGLGK**Y**ICQKCH  IFKNDP**Y**HPDHFN  FLGHRH**Y**ERKGLA  NLVLMV**Y**ISLVFG  LVFGIS**Y**DSPDYT  SYDSPD**Y**TDESCT  SIVPTH**Y**TLLYTI  THYTLL**Y**TIMSKP  RSTHEA**Y**VTVLEG  MSGNFT**Y**IIDKLI  LIPNTN**Y**CVSVYL  NYCVSV**Y**LEHSDE  TLKWIG**Y**ICLRNS  DMVEVI**Y**INRKKK  KKKVWD**Y**NYDDES  KVWDYN**Y**DDESDS  PFPEED**Y**SSTEGS  EAHLQQ**Y**SREHAL  SAPEVL**Y**GSLLNQ  KTNPIL**Y**YMLQKG  TLAFDE**Y**GRPFLI  RDNRVV**Y**GGGAAE  CPTLEQ**Y**AMRAFA  AHSCFQ**Y**AIQKKW  DKNKIW**Y**EHRLID  VWACKN**Y**DGDVQS  SVAADF**Y**HRLGPH  GQWYEI**Y**DKYQVV  GHILGN**Y**KSFLGI  EETPYS**Y**PTGNHT  PTGNHT**Y**QEIAVP  ERDGQP**Y**CEKDYH  PYCEKD**Y**HNLFSP  LFSPRC**Y**YCNGPI  FSPRCY**Y**CNGPIL  EKDGKA**Y**CRKDYF  AYCRKD**Y**FDMFAP  RAILEN**Y**ISALNT  EHDGQP**Y**CEVHYH  PYCEVH**Y**HERRGS  EQNDKP**Y**CQNCFL  IGEAIQ**Y**LHSINI  TPCYTP**Y**YVAPEV  ATMRVD**Y**EQIKIK  SWQCLE**Y**YAGLAA  KLKDGY**Y**MRPCVL  RVTIEY**Y**SQLKTV  EAVLAA**Y**WRGQCI  SAKHGL**Y**LPTRVT  EDLGAD**Y**NLSQVC  TVVISA**Y**RKALDD  SDLAQH**Y**LMRANI  GVEQWP**Y**RAVAQA  SLDKEI**Y**YHGEPI  LFNTAQ**Y**KCPVAM  STFCKV**Y**TLTPFL  HLFEDA**Y**LLTLHA  IEFTEE**Y**PNKPPT  MFHPNV**Y**ADGSIC  QENKRE**Y**EKRVSA  IPIERL**Y**VTYFGG  DGIDMA**Y**RVLADH  EAAKAV**Y**TQDCPL  LCAGSS**Y**RHMRNM  RLEETL**Y**SDQELA  QPMERL**Y**EELVER  VHATQL**Y**RRGPPA  NATHCQ**Y**NFPQVG  LVMAYC**Y**AHILAV  GGGDFD**Y**PGAPAG  GRLEPL**Y**ERVGAP  LAGLFP**Y**QPPPPP  GRQIKP**Y**TLMSMV  MVANLL**Y**EKRFGP  KRFGPY**Y**TEPVIA  ESLVVN**Y**EDLAAR  QLSMVK**Y**NCNKCN  RKTFAR**Y**LSFRRD  AFGFCE**Y**KEPEST  RKKTRE**Y**EKEAER  MWRLLI**Y**ETEAKK  YRHSYE**Y**QRENSS  IVSTRH**Y**RAPEVI  NTSAGR**Y**VRENCK  EELTLK**Y**GAKHVI  FFFSFI**Y**LGEVFK  YNVAVD**Y**ITVALL  KEDHLV**Y**ADEESS  LGDSPF**Y**PGKTTY  KLRNTP**Y**QAPVRR  AFLRTE**Y**SEENML  RLIYED**Y**VSILSP  DAQLQI**Y**TLMHRD  YRFQTP**Y**FWPSNC  KTRKSV**Y**GLQNDI  VKEPGR**Y**TFEDAQ  LMEDRA**Y**MEDAPL  DRRTDA**Y**SRSSSV  IVTPLD**Y**ECNLVS  RPQEVS**Y**TDTKVI  GSFGVV**Y**QAKLCD  NIVRLR**Y**FFYSSG  RLRYFF**Y**SSGEKK  EKKDEV**Y**LNLVLD  LNLVLD**Y**VPETVY  YVPETV**Y**RVARHY  YRVARH**Y**SRAKQT  QTLPVI**Y**VKLYMY  VIYVKL**Y**MYQLFR  YVKLYM**Y**QLFRSL  LFRSLA**Y**IHSFGI  SYICSR**Y**YRAPEL  YICSRY**Y**RAPELI  IFGATD**Y**TSSIDV  REMNPN**Y**TEFKFP  CSRLLE**Y**TPTARL  AADLLA**Y**CEAHVR  KEDALL**Y**QSKGYN  GYNDDY**Y**EESYFT  DYYEES**Y**FTTRTY  PRLAIL**Y**AKRASV  LACKLD**Y**DEDASA  AEHRRK**Y**ERKREE  AFPDWA**Y**KPESSP  LSRALR**Y**YYNKRI  TPTHLA**Y**TPSPTL  FSRVQI**Y**HNPTAN  IVRGVK**Y**NQATPN  TPSSSD**Y**SDLQRV  FKRELN**Y**VARNKP  KPKTVI**Y**WLAEVK  SHEHQA**Y**RWLGLE  KSRAKR**Y**EKLDFL  PSHIKA**Y**MLMTLQ  TLQGLE**Y**LHQHWI  RTKASP**Y**NRCKGS  LLGERD**Y**GPPIDL  KDRLKA**Y**VRDPYA  APKITW**Y**RNGQRL  GCRVED**Y**DAADDV  LELRVA**Y**LDPLEL  SIMSKQ**Y**GNEVFL  DAKIAV**Y**SCPFDG  AGILDT**Y**LGKYWA  GDEVEV**Y**SRANDQ  MMKGEF**Y**VIEYAA  GRRGPN**Y**TSGYGT  RHCRPI**Y**EFHGLY  LPKRHY**Y**KQKKFK  KQKKFK**Y**AEDMGP  SSEAPP**Y**NSEPAE  YHSDEV**Y**NDSGYL  QFHTED**Y**FGKESL  AYLPEE**Y**IKTGRL  HGARTK**Y**LKDLVE  PIAMQI**Y**KKHLDP  LFEISK**Y**VMYKFL  ISKYVM**Y**KFLTVF  RMWYCS**Y**CQGLMM  HSQQRQ**Y**RSAYYP  KSFISF**Y**SALPGY  GNYLFH**Y**ICQDRI  KRFQTT**Y**GSRAQT  VSIVFI**Y**IIVSPL  TKTNLS**Y**YEYDKM  KTNLSY**Y**EYDKMK  NLSYYE**Y**DKMKRG  TPVERQ**Y**PFQIVY  YPFQIV**Y**KDGLLY  DGLLYV**Y**ASNEES  PHLLVK**Y**HSGFFV  STTLAQ**Y**DNESKK  NESKKN**Y**GSQPPS  PNFNMQ**Y**IPREDF  SSQVGM**Y**TVSLFS  KGTVKH**Y**HVHTNA  LYLAEN**Y**CFDSIP  IPKLIH**Y**HQHNSA  GKWKGQ**Y**DVAVKM  PKLVKF**Y**GVCSKE  SKEYPI**Y**IVTEYI  IYIVTE**Y**ISNGCL  NGCLLN**Y**LRSHGK  QLLEMC**Y**DVCEGM  APEVFH**Y**FKYSSK  SQGHRL**Y**RPHLAS  LASDTI**Y**QIMYSC  TIYQIM**Y**SCWHEL  CNCPES**Y**PSAMYC  VPPGIK**Y**LYLRNN  VSLLTL**Y**LDNNKI  EMTLCK**Y**YEKRLL  TDAYLL**Y**TPSQIA  RNLVKK**Y**EPPRSE  LYIVME**Y**CEGGDL  FVGTPY**Y**MSPEQM  MLNLKD**Y**HRPSVE  GFGFVT**Y**SCVEEV  RDYFEK**Y**GKIETI  NFGGGN**Y**GGGGNY  KVGEVT**Y**VELLMD  FVANLD**Y**KVGWKK  ECGHVL**Y**ADIKME  RDLVIK**Y**GAVDPL  TCWAIS**Y**LTDGPN  TVEQIV**Y**LVHCGI  SGILPV**Y**HSLFAL  STQVLL**Y**RIRFYF  LYRIRF**Y**FPNWFG  LLKTVS**Y**KACLPP  HSLMAK**Y**IMDLER  VALVDG**Y**FRLTTD  GSRPGS**Y**VLRRSP  NPLGPD**Y**KGCLIR  VQPQSQ**Y**QLSQMT  GSFTKI**Y**RGCRHE  LMSQVS**Y**RHLVLL  LGAIDM**Y**LRKRGH  VVKQLA**Y**ALNYLE  LAYALN**Y**LEDKGL  AKKLQF**Y**EDRQQL  IQQCMA**Y**EPVQRP  WNGAQL**Y**ACQDPT  EERHLK**Y**ISQLGK  SDFIVK**Y**RGVSYG  KYRGVS**Y**GPGRQS  ASRLLL**Y**SSQICK  QSPIFW**Y**APESLS  SFGVVL**Y**ELFTYC  LYELFT**Y**CDKSCS  RADQFE**Y**VMYGKV  QFEYVM**Y**GKVYRI  ATRLSA**Y**VSYGGL  QVLFAG**Y**KVPHPL  VQTTPD**Y**SPQEAF  LKEGVE**Y**RIKISF  DYMVGS**Y**GPRAEE  GPRAEE**Y**EFLTPV  DQLHQL**Y**SHSLLP  PVDIRQ**Y**LAVWIE  FLDQLN**Y**ECGRCS  DVFCFR**Y**KIQAKG  LSCLVS**Y**QDDPLT  LIWDFG**Y**LTLVEQ  ISFTVK**Y**TYQGLK  FTVKYT**Y**QGLKQE  SWQFSS**Y**VGRGLN  DDKVLI**Y**SVQPYT  IYSVQP**Y**TKEVLQ  TEIIRH**Y**QLLTEE  NPLRFL**Y**PRIPRD  DEAFGC**Y**YQEKVN  EAFGCY**Y**QEKVNL  NTVDEV**Y**VSRPSH  SRPSHF**Y**TDGPLM  AANGAQ**Y**IFQPQQ  EDEEED**Y**DDDEEE  NLNGRD**Y**IFSKAI  RSPNEE**Y**TWEEDP  HNKDEV**Y**QILEKG  TPQRKS**Y**LYPSTL  QQPMIR**Y**MQKMGM  QETEAK**Y**YRTLED  ETEAKY**Y**RTLEDI  EDIEKN**Y**MSPLRL  KERLLI**Y**GEYCSH  LLIYGE**Y**CSHMEH  MQRVLK**Y**HLLLKE  MQDLAM**Y**INEVKR  HTKQDR**Y**LFLFDK  VCKRKG**Y**SYELKE  KRKGYS**Y**ELKEII  HGKMWS**Y**GFYLIH  MWSYGF**Y**LIHLQG  FLRGTF**Y**QGYMCT  GTFYQG**Y**MCTKCG  MVAMQN**Y**HGNPAP  EIDYTA**Y**PWFAGN  SHASGT**Y**LIRERP  LLELVE**Y**YQCHSL  LELVEY**Y**QCHSLK  LDTTLK**Y**PYKSRE  TTLKYP**Y**KSRERS  GTAVAR**Y**NFAARD  GDVVRI**Y**SRIGGD  AYVKDH**Y**SNGFCT  TAISEN**Y**QTMSDT  WNKILS**Y**KIGKEM  PPDISP**Y**EVPPLA  KRTRTA**Y**TRAQLL  EFLFNK**Y**ISRPRR  VDYSDK**Y**GLGYQL  LSLLEE**Y**GCCKEL  LASRLR**Y**ARTMVD  ILNGVY**Y**LHSLQI  AAWHGY**Y**SVAKAL  QGCFVD**Y**QDRHGN  IQNRFK**Y**ARVTPD  GMLDFD**Y**VCSRDE  VQILKD**Y**VRQHFP  IGDGDT**Y**TLVEHS  YCGHCY**Y**QTVVTP  LNFITE**Y**IKGGTL  ELPPGN**Y**RLVVFE  SGTWVG**Y**QYPGYR  GYRGYQ**Y**LLEPGD  SLHFLY**Y**CSEPTL  KQVDVS**Y**IAKHYN  SYIAKH**Y**NMSKSK  KVDNQF**Y**SVEVGD  FTVLKR**Y**QNLKPI  GIVCAA**Y**DAVLDR  THAKRA**Y**RELVLM  EEFQDV**Y**LVMELM  DHERMS**Y**LLYQML  RMSYLL**Y**QMLCGI  PYVVTR**Y**YRAPEV  YVVTRY**Y**RAPEVI  VILGMG**Y**KENVDI  LFPGRD**Y**IDQWNK  QPTVRN**Y**VENRPK  VENRPK**Y**AGLTFP  DALQHP**Y**INVWYD  PYINVW**Y**DPAEVE  APPPQI**Y**DKQLDE  EWKELI**Y**KEVMNS  FFTEYK**Y**HITKIV  NYVLVV**Y**GLAISL  VLSTED**Y**TTCFSS  QIGKMR**Y**VSVRDF  LIDIRE**Y**WMDPEG  LGTGTL**Y**IAESRL  GDIPTF**Y**TYEEGL  QSVSSQ**Y**NMAGVR  WSNSGM**Y**LASGGD  AKPSCP**Y**CCCAVG  RHWLLV**Y**ARYLVN  FIGSSQ**Y**SGTYAS  PPTLPP**Y**FMKGSI  VEVLVE**Y**PFFVFG  PPHPQL**Y**PGGTGG  STSQGS**Y**PCSHPS  TATPPG**Y**KPGSPP  CQLRTI**Y**ICQFLT  MEKIKS**Y**VNYVLS  RSGVRK**Y**GEGNWS  GKPHVA**Y**FVPMSK  ELRVSY**Y**ENVIKA  FTFAEK**Y**LPALGY  VFYIPE**Y**LNRSVA  KQDLPS**Y**LFPEDP  KMSLQL**Y**LVDNRS  WEEVSG**Y**DEAMNP  MNPIRT**Y**QVCNVR  RDVQRV**Y**VELKFT  ETFNLF**Y**YEADSD  LSKAGF**Y**LAFQDQ  SVPLKL**Y**CNGDGE  PCPPGS**Y**KAKQGE  GRDDLL**Y**NVICKK  LLAHTR**Y**TFEVQA  SPLPPR**Y**AAVNIT  NGVILD**Y**EMKYFE  LDYEMK**Y**FEKSEG  LRPDAR**Y**VVQVRA  ARTVAG**Y**GQYSRP  VAGYGQ**Y**SRPAEF  HGSDSE**Y**TEKLQQ  APGMKV**Y**IDPFTY  KTLKVG**Y**TERQRR  YLSEMN**Y**VHRDLA  DPSDPT**Y**TSSLGG  ASDVWS**Y**GIVMWE  SYGERP**Y**WDMSNQ  NAVEQD**Y**RLPPPM  AIKMGR**Y**KESFVS  RQPSFT**Y**SEWMEE  DDPLSS**Y**INANYI  VIHTED**Y**RLRLIS  SVTFKN**Y**IKRNWR  ALYAQK**Y**DEEFQR  GSSENE**Y**IMKAIM  EVYLKP**Y**FLEAYR  LQELVQ**Y**PVEHPD  DNDIRK**Y**EMFAQT  QFPGAV**Y**GTDGCP  SDIFVS**Y**STFPGF  PKSGSW**Y**VETLDD  VSVKGI**Y**KQMPGC  TETDAF**Y**KREMFD  GEGNHI**Y**AYDAKI  CEMLGK**Y**GSSLEF  KDFLDK**Y**SLKPND  AHVDAH**Y**YEQNEQ  HVDAHY**Y**EQNEQP  LAAANC**Y**KKEKHL  EKARVC**Y**IAGFFL  PFISQF**Y**KESLMK  LMKVMP**Y**VDILFG  CIRAGH**Y**AASIII  LCRPFF**Y**RDPTKD  LEPGIS**Y**KLHLKL  QATFER**Y**QCHEEG  DAQMDY**Y**GTRLAT  ILASCS**Y**DRKVII  CWAPHD**Y**GLILAC  DVNPKA**Y**PLADAH  VQQSCN**Y**KQLRKG  EDKNVP**Y**VFVRSK  PGAGRG**Y**NSIGRG  GAYGGG**Y**GGYDDY  GAYDHS**Y**VELFLN  DGKTKG**Y**IFLEYA  FTDFDK**Y**MTISDE  AECRDQ**Y**SVIFES  AKLTNT**Y**CLVAIG  GGSENF**Y**SVFEGE  VTTCND**Y**VALVHP  KSGQGY**Y**VEMTVG  IRREWY**Y**EVIIVR  KMDCKE**Y**NYDKSI  VLAKAL**Y**DNVAES  KILVGM**Y**DKKPAG  APPASQ**Y**TPMLPN  PMLPNT**Y**QPQPDS  PQPDSV**Y**LVPTPS  SPATDL**Y**QVPPGP  GPAQDI**Y**QVPPSA  GMGHDI**Y**QVPPSM  TRVGQG**Y**VYEAAQ  VGQGYV**Y**EAAQPE  QPEQDE**Y**DIPRHL  GLLPSQ**Y**GQEVYD  QYGQEV**Y**DTPPMA  DPLLEV**Y**DVPPSV  SNHHAV**Y**DVPPSV  LLREET**Y**DVPPAF  PGPGTL**Y**DVPRER  DSPDGQ**Y**ENSEGG  GGWMED**Y**DYVHLQ  WMEDYD**Y**VHLQGK  RQLLLF**Y**LEQCEA  RSQVTH**Y**SNLLCD  KAAALQ**Y**PSPSAA  NGEMFD**Y**LTSNGH  SLQNSS**Y**NHFAAI  LERLKE**Y**RNAQCA  DDSLFI**Y**DCSAAE  EAPLVL**Y**RPVGDQ  ASFSSL**Y**KATFDN  VSNMRN**Y**MQKLER  SSSVMT**Y**SKIGDE  QSEVLK**Y**KPGRHN  RKNMAC**Y**CRIPAC  IAGERR**Y**GTCIYQ  TATLRP**Y**LSAVRA  RKPVEG**Y**DISFLI  FHTEQM**Y**KHKLVD  GAAEYL**Y**FFRVLV  FLYQPQ**Y**LNAIQT  CPHILR**Y**LTTAVI  GGMWRF**Y**TEDSPG  SRNKRR**Y**QEDGFD  FDLDLT**Y**IYPNII  LDLTYI**Y**PNIIAM  ERLEGV**Y**RNNIDD  SKHKNH**Y**KIYNLC  KNHYKI**Y**NLCAER  LCAERH**Y**DTAKFN  NCRVAQ**Y**PFEDHN  GVMICA**Y**LLHRGK  QEALDF**Y**GEVRTR  IPSQRR**Y**VYYYSY  SQRRYV**Y**YYSYLL  QRRYVY**Y**YSYLLK  RRYVYY**Y**SYLLKN  YVYYYS**Y**LLKNHL  LKNHLD**Y**RPVALL  QLKVKI**Y**SSNSGP  REDKFM**Y**FEFPQP  ADNDKE**Y**LVLTLT  NFKVKL**Y**FTKTVE  DNEPDH**Y**RYSDTT  EPDHYR**Y**SDTTDS  TCLLIS**Y**TTNKFP  NKFPSE**Y**VPTVFD  PTVFDN**Y**AVTVMI  MIGGEP**Y**TLGLFD  RLRPLS**Y**PQTDVF  DLKAVK**Y**VECSAL  VILVMG**Y**QKKLRS  RSMTDK**Y**RLHLSV  LAEKVV**Y**VGVWIP  CPDEGF**Y**KSGKFV  KCETMV**Y**HPNIDL  RSMRGG**Y**IGSTYF  GPPDTP**Y**EGGRYQ  RLWAHV**Y**AGAPVS  PVSSPE**Y**TKKIEN  VDCGTG**Y**TKLGYA  AEPEDH**Y**FLLTEP  FNVPGL**Y**IAVQAV  ATEYIQ**Y**MRRKNH  AQLQTN**Y**PSSDNS  SSDNSL**Y**TNAKGS  LDQISR**Y**YITRAK  VSKIAK**Y**PHVEDY  EIDEKE**Y**ISLRLI  PEWFNV**Y**NKVHIT  KVLLPE**Y**GGTKVV  VLDDKD**Y**FLFRDG  SKDWSF**Y**LLYYTE  WSFYLL**Y**YTEFTP  SFYLLY**Y**TEFTPT  SRFLEE**Y**LSSTPQ  LKLLDA**Y**LLYILL  GALQFG**Y**CLLVGT  ILEPSL**Y**TVKAIL  RLFAKY**Y**DDTYPS  EGLTVV**Y**KSSIDL  CLNYQH**Y**KGSDFD  PPSRRD**Y**DDMSPR  GGSGYD**Y**SYAGGR  YLFLGD**Y**VDRGKQ  NRIYGF**Y**DECKRR  LFSAPN**Y**CGEFDN  GPPDTL**Y**EGGVFK  LTFPKD**Y**PLRPPK  GEDKYG**Y**EKPEER  GESKVF**Y**YKMKGD  ESKVFY**Y**KMKGDY  LNFSVF**Y**YEILNS  KWHDKQ**Y**KKAHLG  VSLLAL**Y**KGKKER  KGVGRR**Y**AHVVLR  MQNPRQ**Y**KIPDWF  DVKDGK**Y**SQVLAN  KEKLPR**Y**YKNIGL  EAIEGT**Y**IDKKCP  RRDYLH**Y**IRKYNR  LKWGME**Y**KGYLVS  LVSVDG**Y**MNMQLA  LANTEE**Y**IDGALS  PVNKDR**Y**ISKMFL  MEKRHN**Y**VRKVAE  KKLIGR**Y**FDEISQ  SQDTGK**Y**CFGVED  LQEDAC**Y**NCGRGG  ADEQKC**Y**SCGEFG  TSEVNC**Y**RCGESG  RLRPLS**Y**PDTDVI  AALQKR**Y**GSQNGC  RKLRTF**Y**EKRMAT  GEEWKG**Y**VVRISG  KEEAAE**Y**AKLLAK  RISGLI**Y**EETRGV  IRDAVT**Y**TEHAKR  TAMDVV**Y**ALKRQG  DTYTES**Y**ISTIGV  RTITSS**Y**YRGAHG  TKKVVD**Y**TTAKEF  GLRDGY**Y**IQAQCA  VTSRVT**Y**KNVPNW  ISAKSN**Y**NFEKPF  TGFGMI**Y**DSLDYA  IYDSLD**Y**AKKNEP  LARHGL**Y**EKKKTS  DNICSI**Y**NLKTRE  LAGHTG**Y**LSCCRF  DQELMT**Y**SHDNII  DPSIGI**Y**GLDFYV  IYGLDF**Y**VVLGRP  RWFQQK**Y**DGIILP  VVFRDP**Y**RFKKRT  LKAGRA**Y**HKYKAK  GRAYHK**Y**KAKRNC  GEKGFG**Y**KGSCFH  TGGKSI**Y**GEKFED  KRKKKS**Y**TTPKKN  KLAVLK**Y**YKVDEN  GKCCLT**Y**CFNKPE  RSRSRS**Y**SRDYRR  RRSPSP**Y**YSRGGY  RSPSPY**Y**SRGGYR  ELKKLV**Y**LYLMNY  YLYLMN**Y**AKSQPD  VDKITE**Y**LCEPLR  LKDEDP**Y**VRKTAA  LDCLSN**Y**NPKDDR  LPKDSD**Y**YNMLLK  PKDSDY**Y**NMLLKK  GEPEVQ**Y**VALRNI  KVFFVK**Y**NDPIYV  KYNDPI**Y**VKLEKL  LAELKE**Y**ATEVDV  IQTKVN**Y**VVQEAI  RDIFRK**Y**PNKYES  RKYPNK**Y**ESIIAT  IWIVGE**Y**AERIDN  DLRDRG**Y**IYWRLL  RDRGYI**Y**WRLLST  GSLASV**Y**HKPPNA  GMAPGG**Y**VAPKAV  NNIDVF**Y**FSCLIP  LQNNNV**Y**TIAKRN  EGQDML**Y**QSLKLT  QPGNPN**Y**TLSLKC  EVSQYI**Y**QVYDSI  DKKEGE**Y**IKLKVI  KKLKES**Y**CQRQGV  EDVIEV**Y**QEQTGG  GRNFGS**Y**VTHETK  ETKHFI**Y**FYLGQV  KHFIYF**Y**LGQVAI  GEFVDL**Y**VPRKCS  NGQFKT**Y**AICGAI  IFTGKK**Y**EDICPS  IGIQDG**Y**LSLLQD  KEIEQK**Y**DCGEEI  TLGVCK**Y**TVQDES  HIGHTG**Y**LNTVTV  CFSPNR**Y**WLCAAT  QTLFAG**Y**TDNLVR  GEKGQR**Y**LADIFT  TQCRSS**Y**LANEIL  ILWGHR**Y**EPVLFE  LFHPNV**Y**PSGTVC  PAQAEA**Y**TIYCQN  AEAYTI**Y**CQNRVE  DKNADG**Y**IDLDEL  NDGRID**Y**DEFLEF  FTDLFD**Y**LPLTAL  QLVMEG**Y**NWCHDR  LDDTLK**Y**SFLQFD  FNVRNG**Y**GFINRN  PVQGSK**Y**AADRNH  YRRNFN**Y**RRRRPE  NEQVPH**Y**RQALDM  AQMLEK**Y**QQGDFG  QFVPRL**Y**GFKIHP  VPIYEG**Y**ALPHAI  APPERK**Y**SVWIGG  WISKQE**Y**DEAGPS  EHALLA**Y**TLGVKQ  DSTEPP**Y**SQKRYE  VESFSD**Y**PPLGRF  TAVVEP**Y**NSILTT  DIERPT**Y**TNLNRL  RAFVHW**Y**VGEGME  THRPRE**Y**WDYESH  PREYWD**Y**ESHVVE  DIRFYM**Y**EILKAL  GRLLVV**Y**PWTQRF  PPVQAA**Y**QKVVAG  TRNVDV**Y**DKRENP  PSPQTI**Y**KWIKDN  ALQVHC**Y**NSNFPK  TAAHLG**Y**SVVAIN  TEKLPF**Y**FKRPPI  LAFELV**Y**SPAIKD  VACIAV**Y**ETDVFV  QKDFVK**Y**CVEEEE  RKTVED**Y**FCFCYG  DYFCFC**Y**GKALGK  TVVPVP**Y**EKMLRD  FKHPEN**Y**DLATLK  SEDPDY**Y**QYNIQA  DPDYYQ**Y**NIQAGP  ELFERK**Y**AQAIKA  GPVTIP**Y**PLFQSH  FRRPST**Y**GIPRLE  GVKEEW**Y**ARITKL  EAKAVP**Y**QKFEAH  FRSPSW**Y**GIPRLE  EAVKVP**Y**PVFESN  SNPEFL**Y**VEGLPE  PELVIS**Y**LPPGMA  SFPEDF**Y**VEGLPE  FPVKVP**Y**RKITIN  SFKAPS**Y**LEISSM  ALLNNR**Y**EIPDTQ  MPGDAP**Y**CTPEQY  HPAGMT**Y**AANILP  VKSAQH**Y**TETALD  PNAESD**Y**TYSSSY  VLIGAG**Y**STPADI  INRQLI**Y**NYPEQL  AVAMES**Y**AKALRM  IADNAG**Y**DSADLV  GIQSSE**Y**FQALVN  VDRAKY**Y**IQNGIQ  VSWVQS**Y**CRLSHC  ESTTTN**Y**LIELID  NAGFKG**Y**GIQIEQ  TCKGVS**Y**CTGNSS  PSKGSG**Y**RCIRIN  TLWVDP**Y**EVSYRI  GSICVL**Y**EEAPLA  RKKKCD**Y**WIRTFV  LGNIKS**Y**PGLTSY  RVVSTN**Y**NQHAMV  CDAPLP**Y**WTAVFE  MSAAGT**Y**AWMAPE  HSPSQS**Y**LCIPFP  IHRLQA**Y**LEEHAE  NSPSIT**Y**DISQLF  DLSCLV**Y**RADTQT  WIKEKI**Y**VLLRRQ  AGPPAQ**Y**SVTLHG  YASRGT**Y**HVRLEV  RRSQRN**Y**LEAHVD  DISDTI**Y**PRNPAM  LARAGF**Y**ALGEGD  EQKGQE**Y**INNIHL  AWGNQS**Y**AELISQ  PASVSS**Y**AGGVPP  TGPLHT**Y**SSSLFS  GGYGYG**Y**GRSRDY  YGRSRD**Y**NGRNQG  QGGYDR**Y**SGGNYR  TGQAPG**Y**SYTAAN  QAPGYS**Y**TAANKN  LENPKK**Y**IPGTKM  SPFSFV**Y**LPRWFS  KRPRSA**Y**NVYVAE  KEDETR**Y**HNEMKS  ESDPPT**Y**KDAFPP  ARIKKI**Y**EEKKKK  PFDLHR**Y**VIGQKG  FGLARI**Y**SFQMAL  VVVTLW**Y**RAPEVL  SALSHP**Y**FQDLER  KLGEGT**Y**ATVYKG  DKDLKQ**Y**LDDCGN  GVGCIF**Y**EMATGR  TSPLLQ**Y**FGILLD  QGQLNK**Y**ESLELC  ETQPIV**Y**GQPQLM  AHSMPK**Y**SRQFSL  VHGSGP**Y**SAPSPA  YSSSSL**Y**APDAVA  VFRTPP**Y**HKMKIE  SKQFTY**Y**PLVEDK  VIEQIV**Y**VIHHAQ  GEEEFS**Y**GYSLKG  RGNNRG**Y**KNQSQG  KNQSQG**Y**NQWQQG  LFYLGQ**Y**IMTKRL  KIYTMI**Y**RNLVVV  DEDDEV**Y**QVTVYQ  KVNCSF**Y**FKIGAC  VEMQEH**Y**DEFFEE  TEMEEK**Y**GEVEEM  LETDHQ**Y**LAESSG  PGEKSR**Y**ETSLNL  DSQRLA**Y**VTCQDL  GRVGDV**Y**IPRDRY  RYGGGG**Y**GRRSRS  SRSRSR**Y**SRSKSR  SKGFDE**Y**MKELGV  APKPGP**Y**VKEMND  VDWVKA**Y**LSIWTE  TDGCHA**Y**LSKNSL  RMACIH**Y**LRENRE  KVPVPV**Y**PHNPWF  YSGRGG**Y**QHVRSE  NPVPLS**Y**VTDAPD  QEFGRW**Y**KHFKKT  EVDVAE**Y**KEEELL  AAAQQG**Y**SAYTAQ  PTAPQA**Y**SQPVQG  QQPPTS**Y**PPQTGS  PPCPPP**Y**EFCGGM  KRITLG**Y**TQADVG  PHFTAL**Y**SSVPFP  LSDSSS**Y**NIVTKF  DGGHEH**Y**FTPGME  HLCQKT**Y**SNKGTF  IIRKTE**Y**GSRLRI  SYGLQP**Y**CGYSNQ  LQPYCG**Y**SNQDVV  VIGKQG**Y**QCQVCT  KFGIHN**Y**KVPTFC  KGKDEV**Y**AVKVLK  VPGATG**Y**RVTWRV  VPGATQ**Y**RIIVRS  LEPGVS**Y**IFSLTP  GGGHGE**Y**LQQQQQ  ATSSGQ**Y**VLPLQN  FVCNWM**Y**CGKRFT  SKINGA**Y**FCEGRV  PQDAGV**Y**SARYIG  GVYSAR**Y**IGGNLF  QEGCKS**Y**VFCLPD  FCLPDP**Y**GCSCAT  ACHPGF**Y**GPDCKL  NISSEP**Y**FGDGPI  YKPVNH**Y**EAWQHI  EIVTLN**Y**LEPRTE  LEPRTE**Y**ELCVQL  SSEDDF**Y**VEVERR  LHPREQ**Y**VVRARV  SSITIR**Y**KVQGKN  LEPETA**Y**QVDIFA  ACEHRG**Y**LYLAIE  EHRGYL**Y**LAIEYA  LYLAIE**Y**APHGNL  VARGMD**Y**LSQKQF  AIESLN**Y**SVYTTN  SLNYSV**Y**TTNSDV  SLGGTP**Y**CGMTCA  EKLPQG**Y**RLEKPL  NCDDEV**Y**DLMRQC  CWREKP**Y**ERPSFA  CKPEYA**Y**GSAGSP  GEHSIV**Y**LKPSYA  AREKKL**Y**ANMFER  GLYYHR**Y**LQEVID  TRDLAQ**Y**DAAHHE  HEEFKR**Y**EMLKEH  EGLRGI**Y**TGLSAG  ADQRRG**Y**KNVFNA  LFKVVR**Y**EGFFSL  LSEKQV**Y**DAHTKE  TFTVTK**Y**WFYRLL  VTKYWF**Y**RLLSAL  ALIWGI**Y**FAILSF  QCISRV**Y**SIYVHT  SRVYSI**Y**VHTVCD  NGHGHN**Y**KVVVTV  LDMDVP**Y**FADVVS  LPVGVL**Y**KVKVYE  ASGFEK**Y**LADQAK  FLRAQK**Y**TYLKMD  DGASPD**Y**VLVEAE  FAYVLN**Y**YRTGKL  AYVLNY**Y**RTGKLH  LLSTGD**Y**ACADGS  TIKING**Y**TGPGTV  EEQRGD**Y**DLNAVR  EPMLME**Y**PEAITR  AERAKK**Y**GGSVGS  PPTKRE**Y**DQCRIQ  LAAVRL**Y**VELHRG  TYAGAY**Y**PAQGVQ  GEQKYE**Y**KSDQWK  MFFDAL**Y**DEDVVK  GEAVNP**Y**CAVLVK  AVLVKE**Y**VESENG  SENGQM**Y**IQKKPT  QKKPTM**Y**PPWDST  MLMNAR**Y**FLEMSD  GLNKQG**Y**QCRQCN  PHRFKV**Y**NYKSPT  RFKVYN**Y**KSPTFC  LFFVME**Y**LNGGDL  NGGDLM**Y**HIQSCH  LSRATF**Y**AAEIIL  HSKGIV**Y**RDLKLD  ILLGQK**Y**NHSVDW  RMDNPF**Y**PRWLEK  KKSLDF**Y**TRVLGM  IMKFSL**Y**FLAYED  SLYFLA**Y**EDKNDI  PDEKDT**Y**GNKAKK  SQAESY**Y**PSPGPI  ASNACI**Y**NNADDI  QCPRTP**Y**AASRDF  RDFDVK**Y**VVPSFS  VQAMVT**Y**EGDRNE  EQGQAS**Y**FYVASS  GQASYF**Y**VASSLD  PKHLVS**Y**SIEYVH  VSYSIE**Y**VHSFHT  HTGAFV**Y**FLTVQP  EPELGD**Y**RELVLD  PEGGQP**Y**PVLRVA  VQVTAL**Y**VTRLDN  LVRSLN**Y**LLYVSN  SLNYLL**Y**VSNFSL  LCGSNF**Y**LHPSGL  GSWTFQ**Y**REDPVV  LCRLPE**Y**VVRDPQ  HAIKFE**Y**IGLGAV  TALVFS**Y**WWRRKQ  LYSGSD**Y**RSGLAL  GHFGVV**Y**HGEYID  PHVLLP**Y**MCHGDL  VARSME**Y**LAEQKF  TRGAPP**Y**RHIDPF  YCPDSL**Y**QVMQQC  ESCNSK**Y**QCETGE  AMHREK**Y**PNYKYR  QLDNRL**Y**RDDCTK  HLIERY**Y**HQLTEG  KGASSA**Y**LENSKG  DFKDVT**Y**LTEEKV  LTEEKV**Y**EILELC  VECDLT**Y**HNVYSR  YSRDPN**Y**LNLFII  IRLWSK**Y**NADQIR  FQQLIT**Y**KVISNE  CLKMVY**Y**ANVVGG  LEMDKD**Y**TFFKVE  TKNLGL**Y**YDNRIR  DNRIRM**Y**SERRIT  RRITVL**Y**SLVQGQ  GQQLNP**Y**LRLKVR  DLKKQL**Y**VEFEGE  VLGLAI**Y**NNCILD  HFPMVV**Y**RKLMGK  LKDLLE**Y**EGNVED  FGNPMM**Y**DLKENG  KEFVNL**Y**SDYILN  VNLYSD**Y**ILNKSV  NESPLK**Y**LFRPEE  LEETTE**Y**DGGYTR  VLLLPE**Y**SSKEKL  RRLSTK**Y**RTEKIY  PSQDSD**Y**INANFI  RLYQFH**Y**VNWPDH  CFLNEP**Y**LQVDFH  VMNSRE**Y**GAWKQQ  SVLPDK**Y**QVMVNG  VKHVAC**Y**GFRLSH  SSVREK**Y**ELAHPP  PPEEWK**Y**ELRIRY  YELRIR**Y**LPKGFL  PTLNFF**Y**QQVKSD  LEKKSN**Y**EVLEKD  EILSPV**Y**RFDKEC  PEEGIS**Y**LTDKGC  QVQTIQ**Y**SNSEDK  ADLIDG**Y**CRLVNG  MPSTRD**Y**EIQRER  DVHQGI**Y**MSPENP  FLQVRK**Y**SLDLAS  LASLIL**Y**AYQLST  SLILYA**Y**QLSTAL  MTKCWA**Y**DPSRRP  RSSEGF**Y**PSPQHM  MVQTNH**Y**QVSGYP  HYQVSG**Y**PGSHGI  AMAGSI**Y**PGQASL  SSPADS**Y**NEGVKL  PAPPEE**Y**VPMVKE  MKLAQQ**Y**VMTSLQ  TSLQQE**Y**KKQMLT  RIAFNS**Y**ELGSLQ  QKKPTM**Y**PEWKST  VLMSVQ**Y**FLEDVD  GLNKQG**Y**KCRQCN  RFKVHN**Y**MSPTFC  NGGDLM**Y**HIQDKG  KGRFEL**Y**RATFYA  LYRATF**Y**AAEIMC  HSKGII**Y**RDLKLD  FCGTPD**Y**IAPEIL  ILQGLK**Y**TFSVDW  SFGVLL**Y**EMLIGQ  VKSPRD**Y**SNFDQE  EKARLS**Y**SDKNLI  PFLAIV**Y**FCTIVQ  AIKHIS**Y**KGGNTK  TGKAIK**Y**VRDTLF  KLSYYE**Y**DFERGR  IIERFP**Y**PFQVVY  YPFQVV**Y**DEGPLY  YDEGPL**Y**VFSPTE  LKNVIR**Y**NSDLVQ  SDLVQK**Y**HPCFWI  FWIDGQ**Y**LCCSQT  VVALYD**Y**MPMNAN  LRKGDE**Y**FILEES  KNGQEG**Y**IPSNYV  GYIPSN**Y**VTEAED  EDSIEM**Y**EWYSKH  SSKAGK**Y**TVSVFA  QGVIRH**Y**VVCSTP  STAGLG**Y**GSWEID  QFGVVK**Y**GKWRGQ  EKLVQL**Y**GVCTKQ  IFIITE**Y**MANGCL  NGCLLN**Y**LREMRH  VCEAME**Y**LESKQF  DFGLSR**Y**VLDDEY  PPEVLM**Y**SKFSSK  VLMWEI**Y**SLGKMP  SLGKMP**Y**ERFTNS  GLMKKA**Y**ELSVLC  TSAGNG**Y**GNPRNS  GQGMGG**Y**PSAIST  AVMPRW**Y**FDLSKG  KCVRFI**Y**GGCGGN  RMALEN**Y**LAALQS  GEGKAM**Y**IDTEGT  LAVAER**Y**GLSGSD  VLDNVA**Y**ARAFNT  HQTQLL**Y**QASAMM  MMVESR**Y**ALLIVD  DSATAL**Y**RTDYSG  ALYRTD**Y**SGRGEL  ASTTRL**Y**LRKGRG  TRICKI**Y**DSPCLP  GSNGAF**Y**KAFVKD  TYDPEN**Y**QLVILS  TDKEKS**Y**VTDDGQ  EGPAYT**Y**GSPSPP  ILLDLS**Y**NHLRKV  LELDLS**Y**NQLQKI  DISLSD**Y**KGKYVV  SDYKGK**Y**VVFFFY  YVVFFF**Y**PLDFTF  ALNFLF**Y**LALVAA  TMESDI**Y**TEVREL  VDSLVA**Y**SVKIET  MEPENK**Y**LPELMA  KDDEEN**Y**LDLFSH  LIPVKQ**Y**PKFNFV  KGGDPK**Y**AHLNMD  GPPCEA**Y**ALMAHA  QFLELS**Y**LNGVPE  PPAPET**Y**EEYGYD  PETYEE**Y**GYDDTY  TYEEYG**Y**DDTYAE  YGYDDT**Y**AEQSYE  TYAEQS**Y**EGYEGY  EQSYEG**Y**EGYYSQ  YEGYEG**Y**YSQSQG  EGYEGY**Y**SQSQGD  SQGDSE**Y**YDYGHG  QGDSEY**Y**DYGHGE  DSEYYD**Y**GHGEVQ  GEVQDS**Y**EAYGQD  QDSYEA**Y**GQDDWN  KLSQKG**Y**SWSQFS  ITPGTA**Y**QSFEQV  AAWMAT**Y**LNDHLE  VIGLNL**Y**CGGAGL  EEELDG**Y**EPEPLG  LEIISR**Y**LREQAT  YEEVEY**Y**YQRALE  EEVEYY**Y**QRALEI  KQAETL**Y**KEILTR  EVQLQQ**Y**FLRLRD  RLSHFE**Y**VKNEDL  RNLIRL**Y**GVVLTP  LGTLSR**Y**AVQVAE  VAEGMG**Y**LESKRF  LWEMFT**Y**GQEPWI  DCPQDI**Y**NVMVQC  EGRAEN**Y**WWRGQN  DRIDEL**Y**LGNPMD  LPPPPA**Y**DDVAQD  SQGQTN**Y**AFVPEQ  VSSTHY**Y**LLPERP  LPERPS**Y**LERYQR  VQRAAQ**Y**LKVEQL  VYWADA**Y**LDYIEV  WMGDNL**Y**WTDDGP  DNDCGD**Y**SDETHA  FEVVIQ**Y**GLATPE  NGLTVD**Y**LEKRIL  LYGGEV**Y**WTDWRT  PFDLQV**Y**HPSRQP  FKKFLL**Y**ARQMEI  DAPYYN**Y**IISFTV  NVTVLD**Y**DAREQR  PLRGKL**Y**WTDGDN  FPESKL**Y**WISSGN  LNGSFR**Y**VVISQG  RGWDTL**Y**WTSYTT  KIERCE**Y**DGSHRY  PRCPLN**Y**FACPSG  NLDGSN**Y**TLLKQG  VALDFD**Y**REQMIY  YREQMI**Y**WTDVTT  SKLNGA**Y**RTVLVS  VQNGYL**Y**WTDWGD  ADARED**Y**IEFASL  LTLFED**Y**VYWTDW  VKAGRV**Y**WTNWHT  DAEDAV**Y**GRDGYD  VYGRDG**Y**DYDGYR  NSRSRS**Y**SPRRSR  ERPPYS**Y**MAMIQF  MTLKDI**Y**TWIEDH  LPRVSS**Y**LVPIQF  GNWSGT**Y**TGGRDP  NLSVDV**Y**YDPMGN  MDVTDK**Y**KYPEGS  DPAKCR**Y**ALGMQD  FPKEEE**Y**LQVDLQ  KEFSRS**Y**RLRYSR  RSYRLR**Y**SRDGRR  ARLVRF**Y**PRADRV  CLRVEL**Y**GCLWRD  RDGLLS**Y**TAPVGQ  PVGQTM**Y**LSEAVY  YLSEAV**Y**LNDSTY  YLNDST**Y**DGHTVG  TVGGLQ**Y**GGLGQL  LRVWPG**Y**DYVGWS  VWPGYD**Y**VGWSNH  HSFSSG**Y**VEMEFE  PTNTQA**Y**SGDYME  QAYSGD**Y**MEPEKP  QNSVPH**Y**AEADIV  VTGGNT**Y**AVPALP  LCMITD**Y**MENGDL  IASGMR**Y**LATLNF  DQGRQV**Y**LSRPPA  ACPQGL**Y**ELMLRC  KRGNRR**Y**CPIPDV  GKGWYQ**Y**DKPLGR  LEKLQK**Y**YRQNPD  RLASHE**Y**LKAFKV  ASYKVA**Y**SNDSAN  SANWTE**Y**QDPRTG  DNQVLG**Y**KDLAAI  RPDLMI**Y**EPHFTY  YEPHFT**Y**SLLEHV  TIIELK**Y**RVVSWF  ILILLH**Y**YVFSTA  QLLGLV**Y**MKFVAS  DFFKTH**Y**RLELME  NMSGSL**Y**EMVSRV  EKAMKE**Y**EGGRGE  RSMSES**Y**TLEGND  DDLVNS**Y**HLLLCA  NKAYEE**Y**VLSVGN  KLFEHY**Y**QELKIV  EGIYTL**Y**PFINSR  KLSSET**Y**SQAKDL  QFDASH**Y**DSEKGE  GHQKEG**Y**GLSWNP  CLSFNP**Y**SEFILA  PHPQEE**Y**LDCLWA  RHILRP**Y**LAFDSI  IFRMFD**Y**TDDPEG  RPMPQR**Y**GDVFWK  CSQLGL**Y**PPEQLP  RVRAVT**Y**HLEDLR  NSPSPN**Y**QKYTYG  SPNYQK**Y**TYGQSS  NSVMST**Y**GSQASH  DVSLHG**Y**KKYLLS  RYHFFL**Y**KHSHEG  ESIVFI**Y**SMPGYT  NRLKVL**Y**SQKATP  SRKTCR**Y**IPSLPD  EIRNDY**Y**LNLVDW  FSKGSN**Y**SEILDK  SEILDK**Y**FKNFDN  ALEDET**Y**ADGAET  REEAKP**Y**PLFPGP  LPHASG**Y**QPALMF  ISVPNI**Y**QDTTIS  HAITQF**Y**PDDEVT  RSDNGS**Y**ICKMKI  IVSDPI**Y**IEVQGL  VPGFDG**Y**SPFRNC  SALPHL**Y**QIKQLQ  LQALAN**Y**SIGVSC  DGELVG**Y**RISHVW  AHGWVD**Y**APSSTP  LIGLIL**Y**ISLAIR  SELVVN**Y**IAKKSF  ILPFMK**Y**GDLHTY  YGDLHT**Y**LLYSRL  LHTYLL**Y**SRLETG  IALGME**Y**LSNRNF  SLADRV**Y**TSKSDV  TRGMTP**Y**PGVQNH  VQNHEM**Y**DYLLHG  NHEMYD**Y**LLHGHR  DCLDEL**Y**EIMYSC  ELYEIM**Y**SCWRTD  KPHEGR**Y**ILNGGS  HDCYLK**Y**INLKAS  LEYLQD**Y**TDRVKP  PIPYWL**Y**KLHGLN  PIGISN**Y**PESTIA  PPFNKQ**Y**TESQLR  ANQLQN**Y**RNYLLP  AGYSQF**Y**SNGGHS  QGKQGG**Y**SQSNYN  SGPPSS**Y**QSSQGG  CVHEGI**Y**EEGISI  IFSPAF**Y**TSRYGY  KMCLRI**Y**LNGDGT  AISNGV**Y**VLPSAA  PDAVTG**Y**YLNRAG  KSKDRK**Y**TLTMED  LQSHKN**Y**EGTCEI  PSRDHL**Y**LPLEPS  HLQSMG**Y**HCSNTG  IIDEKK**Y**YLFGRN  IDEKKY**Y**LFGRNP  VHAALV**Y**HKHLKR  GASTRA**Y**TLREKP  GGLPMP**Y**PNLAPD  APNPAV**Y**NPEAVN  NDRFEG**Y**CIDLLR  LGISIL**Y**RKPNGT  KSKIST**Y**DKMWAF  RKRVRQ**Y**LDQQQY  FAVGCY**Y**LMVGHK  TTLEKT**Y**GPAWIA  DVLTVD**Y**TRNAEA  TMFDTS**Y**SKQVIN  YDDDAE**Y**MKHTRL  KAVLQE**Y**GHEERR  VVEEQS**Y**LLNIAE  GSGVVK**Y**SCLCSA  RQSPNS**Y**VVIMNG  LHRVFH**Y**VLDNLV  YLTPQD**Y**KRVSAL  DTDLKN**Y**KGNSIK  YSRARD**Y**CTSAKH  SHSKIL**Y**ARDVDQ  ERFSSE**Y**PEFCSK  FMEAIE**Y**IDAVKD  ATICLA**Y**LMMKKR  KKKDRF**Y**RSILPG  GDPKKK**Y**TRFEKI  NPNIVN**Y**LDSYLV  VNYLDS**Y**LVGDEL  LWVVME**Y**LAGGSL  TMVGTP**Y**WMAPEV  VVTRKA**Y**GPKVDI  IEGEPP**Y**LNENPL  NPLRAL**Y**LIATNG  DSIEDR**Y**PRNLTE  AMLSYY**Y**STVMEQ  VVKGLT**Y**LWSLKI  RISGEQ**Y**GIHSDV  LFPGKN**Y**VHQLQL  HPFLAK**Y**HDPDDE  TPGVLP**Y**FPPGLP  SPYVVK**Y**YGSYFK  LWIVME**Y**CGAGSV  VIQEIG**Y**NCVADI  LKNSPP**Y**ILDILP  DILPDT**Y**QHLRLI  RLILSK**Y**DDNQKL  QLSENE**Y**FKIYID  NEYFKI**Y**IDSLMK  EGKERM**Y**EEQSQD  DLTCND**Y**ISVFEF  AVTHPG**Y**MAFLTY  YMAFLT**Y**DEVKAR  KARLQK**Y**STKPGS  STKPGS**Y**IFRLSC  GQWAIG**Y**VTGDGN  GSREGF**Y**LYPDGR  REGFYL**Y**PDGRSY  YPDGRS**Y**NPDLTG  KVTQEQ**Y**ELYCEM  IPDLSI**Y**LKGDVF  NRTPSD**Y**DLLIPP  KLMGEG**Y**AFEEVK  AFGSGD**Y**TAAIAF  KISTLY**Y**QLGDHE  LIRDGR**Y**TDATSK  DRDGYS**Y**GSRSGG  TSGRDK**Y**GPPVRT  SRSKDE**Y**EKSRSR  GINSIL**Y**QRGIYP  DLELIK**Y**LNNVVE  QLKDWL**Y**KCSVQK  KDIVEN**Y**FMRDSG  AHQRVK**Y**TKDHTV  DGERTV**Y**CNVHKH  DMLHRF**Y**GKNSSY  KPADAV**Y**GQKEIH  DDSGTF**Y**DQAVVS  NKFGPM**Y**KRDRAL  PPPAPD**Y**VLPPSL  DYTLCH**Y**PHCGDK  MIYSTR**Y**GSPKRQ  ERLQLE**Y**VDVVFA  GIVSGK**Y**DSGIPP  GVCKRW**Y**RLASDE  EFFQLN**Y**LQHLSL  LSLSRC**Y**DIIPET  TRRSLG**Y**AYVNFQ  GFGFVS**Y**EKHEDA  AHLTNQ**Y**MQRVAG  LFQLQI**Y**IHHPKG  LVNILP**Y**FAYEGT  CQTAVT**Y**CKDALE  SFLHHP**Y**YQDKVE  FLHHPY**Y**QDKVEQ  LCQLLV**Y**KSHCVD  FLFRKN**Y**AKYEFF  RKNYAK**Y**EFFKNP  KSWKKL**Y**VCLRRS  LRRSGL**Y**CSTKGT  IAGRKQ**Y**NAPTDH  AFRLLK**Y**GMLLYQ  KYGMLL**Y**QNYRIP  MLLYQN**Y**RIPQQR  IQLVDF**Y**QLNKGV  QVYIPN**Y**NKPNPN  CYTTQS**Y**QWYSWG  KTRQAF**Y**LHTTKL  PDLNEI**Y**RSFQNV  SFQNVC**Y**EHSPLQ  RPRLLP**Y**GKQENS  AVGALM**Y**HTITLT  VLFYDP**Y**LSDGVE  NGAAYR**Y**PPGVVG  EKLQYY**Y**SSSEDE  VIMVHI**Y**EDGIPG  ICLAAE**Y**PAVKFC  GPPRTI**Y**ENRIYS  IECGPK**Y**PEAPPF  AKWQNS**Y**SIKVVL  NAIPFT**Y**EQLDVL  HKLDEL**Y**PQGYPE  TAFYPG**Y**LCSLSP  KPHKCG**Y**CGRSYK  GLPGTL**Y**PVIKEE  GLSDTP**Y**DSSASY  AMEKGK**Y**VGELRK  IRELIC**Y**CLDTIA  ADRDPV**Y**DESTDE  RPTLVW**Y**QPDGTR  TLENCC**Y**QGRVRG  QGRVRG**Y**AGSWVS  LTPERS**Y**TLEQGP  HSEAQK**Y**RDFQHL  HGRCAS**Y**AQQCQS  RNPSGS**Y**VSCTPR  DSNRHC**Y**CEEGWA  LGASYW**Y**RARLHQ  ENTDET**Y**CIDNEA  IDNEAL**Y**DICFRT  DPRHGR**Y**LTVATV  RSSGRV**Y**YFNHIT  SSGRVY**Y**FNHITN  LELING**Y**IQKIKS  LCHLLQ**Y**FLKFVP  ASSDDP**Y**QGPRDI  GSDGKF**Y**IMMCKP  AQLPHD**Y**CTTPGG  GGTRII**Y**DRKFLL  NGGTLY**Y**MAPEHL  TEKSDV**Y**SFAVVL  PVPETN**Y**LGNTPT  TRRKVC**Y**YYDGDV  YNDYFE**Y**FGPDFK  NQNTNE**Y**LEKIKQ  TDEYQL**Y**EDIGKG  EDQHKL**Y**QQIKAG  MDFHRF**Y**FENLLA  VNPNAA**Y**DKFKDK  KTKRTG**Y**ESGEYE  SKVHQL**Y**ETIQRW  QVNSCQ**Y**PGLQWV  PQPYKI**Y**EVCSNG  LEIKFQ**Y**RGRPPR  KGKRVG**Y**WLSEKK  IRKIEA**Y**MEDDRI  NHNAVL**Y**KVFVVG  RREPPP**Y**GYRKGW  DEIYNV**Y**DQAWRG  NLDKDM**Y**GDDLEA  KKTFDP**Y**IIIRAR  ELLTNC**Y**IMVQGN  KNIHPI**Y**NIKSLM  MAKDVT**Y**ICPFTG  SKEDEK**Y**LQAIMD  KLKEIV**Y**PNIEET  MPHSHQ**Y**SDRRQP  QVSALS**Y**SDQIQQ  VDLIKT**Y**KHINEV  HINEVY**Y**AKKKRR  KKERKV**Y**NDGYDD  DDDNYD**Y**IVKNGE  DTEMKY**Y**IVHLKR  VFEMLS**Y**NLYDLL  MLSYNL**Y**DLLRNT  YIQSRF**Y**RSPEVL  VLLGMP**Y**DLAIDM  KDGKRE**Y**KPPGTR  ILRMLD**Y**DPKTRI  KTRIQP**Y**YALQHS  TRIQPY**Y**ALQHSF  RTRPRV**Y**NSPTNS  NQGNQA**Y**QNRPVA  DVNLTV**Y**SNPRQE  IAGHPT**Y**QFSANT  NTGPAH**Y**MTEGHL  TSIIKR**Y**VHQLFS  QLFSQH**Y**RATIGV  NMTRVY**Y**KEAVGA  SFVSSH**Y**ILESPG  VQLICA**Y**CHTRDI  RESSVG**Y**RVPAGS  LQLLRT**Y**SPSAQV  QACKLL**Y**MALRTQ  LLLEAE**Y**MSELLE  LTGEGG**Y**YLTSLS  TGEGGY**Y**LTSLSA  HLLRVA**Y**QDPSSG  TFGLFL**Y**KEQGYH  LYKEQG**Y**HRLPPG  RLPTTG**Y**LVYRRA  TTGYLV**Y**RRAEWP  ITKPDV**Y**KSPASD  SPASDT**Y**IVFGEA  TEQEGK**Y**YSEEAE  EQEGKY**Y**SEEAEV  RDPGRD**Y**ELYKYT  GRDYEL**Y**KYTCQE  DYELYK**Y**TCQELQ  KQKVDA**Y**HLQLQN  QLQNLL**Y**EVMHLQ  VSLEEF**Y**KEAPPD  KRLAEK**Y**RECLSN  SLPVQE**Y**LFMPFD  HLPPPL**Y**VLFVQA  FVQATA**Y**GQACDK  VLHLTF**Y**YLMNLN  LHLTFY**Y**LMNLNI  SVLSCL**Y**PGDHGK  PNPANQ**Y**QFDKVG  ILTLSD**Y**VLELGH  LELGHP**Y**LWVQKL  TVAHED**Y**MELHFT  AGDTNL**Y**YMALIE  GDTNLY**Y**MALIER  VVLNPG**Y**SSIPPV  GEVNVC**Y**KELCGP  RMKPFK**Y**NHPQGF  LMAEAK**Y**YLIQGL  CCTSIV**Y**ATEKKQ  RIHVKR**Y**STYDDR  TQAVRH**Y**KIWRRA  LPELVN**Y**HRAQSL  RKLGSG**Y**FGEVFE  KHILAL**Y**AVVSVG  SVGDPV**Y**IITELM  VAEGMC**Y**LESQNY  YLESQN**Y**IHRDLA  ALSRGH**Y**STKSDV  SRGQVP**Y**PGMSNH  LRVDAG**Y**RMPCPL  DQTQAL**Y**VGDAIL  ISTACT**Y**KNQECR  TIIQSP**Y**EKLKMS  RFLPEG**Y**PLPLDL  VVSALR**Y**FKTIVD  SALSSC**Y**SRVYQS  SCYSRV**Y**QSLANL  SGGSHS**Y**GGESPR  CETLDH**Y**DPDYEF  DHYDPD**Y**EFLQQD  SGCRVS**Y**ERHPSQ  ERHPSQ**Y**DNISGE  NKHMLA**Y**MQLLED  MQLLED**Y**SEPQPS  PQPSMF**Y**QTPQNE  PQNEHI**Y**QQKNKL  RKDLVL**Y**CEAFLT  EAFLTT**Y**RTFISP  LIKKLQ**Y**RYEKFS  KKLQYR**Y**EKFSPF  LDAELF**Y**KIEIPE  HFNNMS**Y**WVRSII  LNNFNS**Y**LAILSA  SEGLAE**Y**CTLIDS  SSSFRA**Y**RAALSE  EPPCIP**Y**LGLILQ  CFQQAH**Y**DMRRND  SSPPWS**Y**DQSYPS  YPATFT**Y**TPPVTS  GTSSGS**Y**QFPMVP  TDKGVH**Y**ISVSAT  VFSIEV**Y**PEDHSE  MSAQLG**Y**PVVGWH  TMTIPG**Y**VEPTAV  DAWVGT**Y**FEVKIP  IPSDTF**Y**DHEDTT  SNSQLM**Y**GLPDSS  HVGKHE**Y**FMHATD  SSEDDV**Y**LHTVIP  IIAMIC**Y**RKKRKG  PLPPPE**Y**PNQSVP  QDTMGE**Y**TPLRDE  DPNAPP**Y**QPPPPF  PKNMTP**Y**RSPPPY  INKEDS**Y**KPIVEF  DAQFEA**Y**LQEELK  MMRARQ**Y**PWGTVQ  PALDVL**Y**DVMKSK  LAKEGL**Y**QYKNIC  EDVVRA**Y**LKMAEE  FDANDL**Y**QGQNFN  SSANIS**Y**LMGNLE  KTLYLT**Y**CANHPS  KVLVME**Y**CSSGSL  DKMNFI**Y**KQFKKS  MRPGLG**Y**NEEQIH  VDITSW**Y**DGVREE  VNSFQV**Y**MAYKDV  HKSAVE**Y**NIFEGM  ELLTLP**Y**LSGQFT  SDPSRS**Y**FVVVNH  VSKDGG**Y**IQLMPA  QATEYE**Y**LDYDFL  EYEYLD**Y**DFLPET  MYPVRN**Y**SPTEMV  PKASHG**Y**GGKFGV  SAVGHE**Y**QSKLSK  HASQKD**Y**SSGFGG  SGFGGK**Y**GVQADR  SAVGFD**Y**QGKTEK  HESQRD**Y**SKGFGG  KGFGGK**Y**GIDKDK  SAVGFE**Y**QGKTEK  HESQKD**Y**VKGFGG  HESQKD**Y**KTGFGG  AAVGFD**Y**KEKLAK  HESQQD**Y**SKGFGG  KGFGGK**Y**GVQKDR  TQVSSA**Y**QKTVPV  FKAELS**Y**RGPVSG  SQQGLA**Y**ATEAVY  AEAPGH**Y**PAEDST  ITAVAL**Y**DYQAAG  AVALYD**Y**QAAGDD  GVCKGR**Y**GLFPAN  ILKVCF**Y**SNSFNP  IRLAEC**Y**GLRLKH  GEVQDK**Y**ECLHVE  VEAEWR**Y**DLQIRY  DRTTLL**Y**FYQQLR  TTLLYF**Y**QQLRND  QQLRND**Y**MQRYAS  NDYMQR**Y**ASKVSE  QQTFQQ**Y**ASLREE  NIDQET**Y**RCELIQ  ADLIDG**Y**CRLQGE  RPGGPQ**Y**GIARED  GFFGEV**Y**EGVYTN  EVYEGV**Y**TNHKGE  WIIMEL**Y**PYGELG  IMELYP**Y**GELGHY  YGELGH**Y**LERNKN  VLTLVL**Y**SLQICK  ICKAMA**Y**LESINC  DFGLSR**Y**IEDEDY  LCPPVL**Y**TLMTRC  MTRCWD**Y**DPSDRP  CSLSDV**Y**QMEKDI  QLPPEG**Y**VVVVKN  PTEPGE**Y**TINILF  GSCGVS**Y**VVQEPG  HMGNRV**Y**NVTYTV  LSREKT**Y**VKRLVK  EFRPTI**Y**SNVIKG  TRVFLQ**Y**LPAIRA  TERGSF**Y**PGSGFA  GSGFAF**Y**SLDYMR  APVTAF**Y**RGCMTL  GLKFQR**Y**RLVPYG  TRFCLP**Y**RMDVEK  GGSGGS**Y**SIKTQF  NKEMAR**Y**KRKLLI  RQKQIN**Y**GKNTIA  GKNTIA**Y**DRYIKE  AMLEAP**Y**KKDENK  TGRSKG**Y**GFITFS  SAQGNV**Y**VKCPSI  APRDTI**Y**QVPPSY  HTTQGV**Y**DIPPSS  RQLLCF**Y**YDQCET  GTCPEG**Y**RCLKAG  IIALDP**Y**YYFQQG  GWMDIM**Y**AAVDSR  LAASSA**Y**LKSLVV  SFGDNL**Y**VCIPCG  MRFTRQ**Y**RLTEHM  RDPVWK**Y**LQTVQY  YLQTVQ**Y**GVHGNF  NFPRLS**Y**PTFFPR  HVVGLR**Y**YTGVVN  VVGLRY**Y**TGVVNN  RDPNNP**Y**DKNAIK  LAGALA**Y**IMDNKL  EQRNDL**Y**YNTITN  QRNDLY**Y**NTITNF  NLLKKE**Y**NVNDDS  RKTAVQ**Y**IESSDS  DVHLNF**Y**VYYGPD  HLNFYV**Y**YGPDRI  LNFYVY**Y**GPDRIR  DIVLTT**Y**NILTHD  NILTHD**Y**GTKGDS  DEERKI**Y**QSVKNE  RATIGR**Y**FNEGTV  GTVLAH**Y**ADVLGL  QICCHT**Y**LLTNAV  AEATGA**Y**VPGRDK  FEVTAF**Y**RGRQVF  SLTSDQ**Y**KAYLQD  DPAEPR**Y**RARQRR  DANSPL**Y**DLAPSD  AEEDGR**Y**SVDYSK  VTPWGV**Y**PASLFQ  TGSSDL**Y**KRTSSS  SSSRLR**Y**GMSDVM  LRDDGV**Y**GGSSKQ  ETSFLD**Y**GENLVQ  PGAPLL**Y**YVNQAR  ADLLCQ**Y**HRLDVS  FLPPDE**Y**VVTDPE  SCCGGT**Y**LPSMPR  VETPYG**Y**QLDLDF  HQLQNG**Y**QGNGDY  MTGLDH**Y**IERIQK  YDELLK**Y**YELHET  DELLKY**Y**ELHETI  QHICQL**Y**HVLETA  IFMVLE**Y**CPGGEL  GGELFD**Y**IISQDR  IVSAVA**Y**VHSQGY  YVHSQG**Y**AHRDLK  NLLFDE**Y**HKLKLI  CCGSLA**Y**AAPELI  LIQGKS**Y**LGSEAD  SMGILL**Y**VLMCGF  DNVMAL**Y**KKIMRG  KIMRGK**Y**DVPKWL  PWIMQD**Y**NYPVEW  IMQDYN**Y**PVEWQS  LISLWQ**Y**DHLTAT  DHLTAT**Y**LLLLAK  TSQFTK**Y**WTESNG  KNKENV**Y**TPKSAV  AVKNEE**Y**FMFPEP  LTTPNR**Y**TTPSKA  RRLKLH**Y**NVTTTR  DFVQKG**Y**TLKCQT  KGDAWV**Y**KRLVED  LNVGGT**Y**FVTTRQ  KDETGA**Y**LIDRDP  LEEAEF**Y**NIASLV  GELAQE**Y**DKRKKE  NQKRPQ**Y**IKAKEN  LYPGSV**Y**GRLIDL  SNRPPG**Y**PSQPVE  NSRKMV**Y**QESPQN  ASPRPY**Y**PQTPNN  LLKAQE**Y**IRQKNK  VLTIKS**Y**GMLVKV  VKSIKD**Y**GVFIQF  GSIDCQ**Y**PGSALA  AYVETK**Y**SPPAIS  RESVQG**Y**TTKTKQ  TNNQVS**Y**TSWFLD  RVLWDQ**Y**HNLRYP  GIMPGR**Y**NQEVGQ  YSFQAF**Y**LVSHPN  VLKLCG**Y**VIFRGA  PYFYII**Y**FTMLLV  RIPPYH**Y**IHVLDQ  QDPFPL**Y**PGEVLE  QGIQDV**Y**VLSEQQ  SNLEGS**Y**MCSCHK  LKDSDD**Y**AQLCNI  PSDKPN**Y**CTPLNT  RAQISA**Y**RVMLYQ  KSKPRG**Y**CLIINN  DSEEQP**Y**LEMDLS  SNKLFQ**Y**ASTDMD  LLKYTE**Y**NEPHES  TAYNTD**Y**QLTSAE  PTSAPV**Y**QQPQQQ  AQSYGG**Y**KEPAAP  GGGGKR**Y**RAVYDY  RNPQIL**Y**CRDTFD  TCTRRH**Y**ERLILP  LLHNSI**Y**PLAAIN  SRPVQV**Y**FYVSNG  YGMPPL**Y**PQTGPP  PFPSDP**Y**GGRGSS  PDIKAQ**Y**QQRWLA  YQYMET**Y**MGPALF  NSAKDC**Y**PAVQKT  RAEKAS**Y**AEQLSM  RLGSPD**Y**GNSALL  PGRNSF**Y**MGTCQD  GDQATG**Y**HFNALT  QLQEPE**Y**VLLAAM  RSINEA**Y**GYQIQH  DSVPGT**Y**RKVVAA  RKAENK**Y**AGGNPV  LLEKYK**Y**VENFGL  VALIWD**Y**MHPFPE  LTIYTS**Y**KEKSIF  ILRWKQ**Y**EAYVQA  LALQKK**Y**SEELKS  NQLSAG**Y**ESVDSP  AVAAAT**Y**KTMGPA  QLSINV**Y**DYNCHV  APRLAE**Y**QAYIDF  EIANTL**Y**RIFNNK  ELSDDP**Y**DCIRLS  TLCKIG**Y**RHVVDA  RGPYRI**Y**DPGGSV  RSMKQQ**Y**EQKITE  LRQKVK**Y**LQDQLS  LTRQRE**Y**QEKEIQ  KASGER**Y**HVEPHP  EHLCGA**Y**PYAYPP  LCGAYP**Y**AYPPMP  DWSQIR**Y**PPPPMA  LFHLPE**Y**TWRLPC  RTARAG**Y**RGRALD  QSNQLV**Y**QKKYKD  VIINQI**Y**EARVEA  VNGEDP**Y**PHKFHV  KDKETR**Y**RQRYLD  KEICNA**Y**TELNDP  WRRRWG**Y**QRARDD  QNKIVT**Y**KWLSYT  TYKWLS**Y**TLGVHV  PDSPGA**Y**EAESPS  TYDDRA**Y**SSFGGG  LPTEPP**Y**TAYVGN  LKEALT**Y**DGALLG  FLCRRC**Y**RLKHLS  VLLTCL**Y**LSYSYM  MGNEIS**Y**PLKPFL  YLIYRR**Y**RQFHAL  IPALNA**Y**MKSLLS  NWLRCY**Y**YEDTIS  GGRSGG**Y**SSGKQG  GESPVD**Y**DGGRTR  AAGRNS**Y**LEVLLK  PGQVDF**Y**ARFSPS  QLVQSW**Y**IQSLQE  AQGVIE**Y**KESFGV  TSQNVQ**Y**FLDRFY  YFLDRF**Y**MSRISI  EVIKDG**Y**ENARRL  QPIQVV**Y**VPSHLY  YVPSHL**Y**HMVFEL  HANRGV**Y**PPIQVH  IDRLFN**Y**MYSTAP  RLFNYM**Y**STAPRP  PLAGFG**Y**GLPISR  LPISRL**Y**AQYFQG  SRLYAQ**Y**FQGDLK  QGDLKL**Y**SLEGYG  LYSLEG**Y**GTDAVI  GTDAVI**Y**IKALST  IERLPV**Y**NKAAWK  KAAWKH**Y**NTNHEA  QDSSGD**Y**SLAHVR  CGFYGM**Y**DKILLF  ALFVHS**Y**RAPAFC  EGCGLN**Y**HKRCAF  RSNSQS**Y**IGRPIH  TFVIHS**Y**TRPTVC  RPTVCQ**Y**CKKLLK  TLRKRH**Y**WRLDSK  NDTGSR**Y**YKEIPL  TTANVV**Y**YVGENV  VDISTV**Y**QIFPDE  GQFGIV**Y**GGKHRK  VLRNKG**Y**NRSLDM  SVGVII**Y**VSLSGT  QNAAFM**Y**PPNPWK  VKMRKR**Y**SVDKTL  HPWLQD**Y**QTWLDL  CKIGER**Y**ITHESD  DLRWEK**Y**AGEQGL  GEQGLQ**Y**PTHLIN  LDRLEL**Y**QEMDHP  DHPLAH**Y**FISSSH  VLRIAV**Y**DDNNKL  TAKKES**Y**SALMRE  GLASYD**Y**VRRRLT  VGSSSS**Y**PISPAV  PASAKW**Y**DRRDYV  WYDRRD**Y**VFIEFC  EEETQF**Y**IAETVL  ETPQET**Y**KKVMNW  DWVFIN**Y**TYKRFE  MEIFST**Y**GKIKMI  PHLSKG**Y**AYVEFE  LSKGYA**Y**VEFENP  FDRRGE**Y**IYTGNA  DRIIRV**Y**DGREIL  DPEENP**Y**GPPPDA  AKVWKD**Y**DRDKDD  EARHLV**Y**ESDKNK  GSQATN**Y**GEDLTK  SDLEQQ**Y**RALRKY  YRALRK**Y**YENCEV  VREVTG**Y**VLVALN  ALNQFR**Y**LPLENL  IRGTKL**Y**EDRYAL  KLYEDR**Y**ALAIFL  ILNGGV**Y**VDQNKF  FNAKYT**Y**GAFCVK  GIHGDP**Y**NAIEAI  IGGRVL**Y**SGLSLL  DNSNLC**Y**YHTINW  NSNLCY**Y**HTINWT  SHDCIY**Y**PWTGHS  GAFGTV**Y**KGIWVP  HGCLLE**Y**VHEHKD  TFGGKP**Y**DGIPTR  ICTIDV**Y**MVMVKC  ARDPQR**Y**LVIQGD  QGVSVP**Y**RAPTST  DSSTQR**Y**SADPTV  TLGKAE**Y**LKNNIL  PDYLQE**Y**STKYFY  QEYSTK**Y**FYKQNG  YSTKYF**Y**KQNGRI  SHSDGS**Y**AVWSVD  TVATTP**Y**GPFPCK  SVACED**Y**AETCLA  PDEQRL**Y**KDDQLL  ENRHKE**Y**ERNRHF  IFKVGR**Y**RPDMPC  LDGLKT**Y**QVTPMT  KNLFSK**Y**GKVVGA  AALDER**Y**HSDFNR  RDGWGG**Y**GSDKRM  SEEDAD**Y**AIKIMN  MNMIKL**Y**GKPIRV  IDEKLL**Y**DTFSAF  DINKDP**Y**FMKNHL  KIGRPG**Y**KVTKQR  LLFQID**Y**PEIAEG  YRDWAT**Y**KQGFGS  VDFEDN**Y**QFAKYR  ADEAEK**Y**NLVLGA  IDKKTF**Y**KTADIC  TVDGDL**Y**PPVEEP  KTAKKK**Y**IESPDV  RWVLKF**Y**FHEALR  VIQNFS**Y**ETFQQK  AAISKN**Y**PFVNRT  REQERA**Y**IVQLQI  FKPPAD**Y**KPPATR  PPPMDQ**Y**LGSTPV  SKMAIM**Y**WKATNA  PTYAPL**Y**ELITQF  IRHQVA**Y**NTLIGS  APLVFG**Y**VTEDGD  STVEKL**Y**AAGAGL  GDEGDE**Y**DDIVVH  RPDRQA**Y**EPPPPP  PPPPPA**Y**RTGSLK  PLPASP**Y**GGPTPA  GPTPAS**Y**TTASTP  EVWGPG**Y**RSQREP  HPPSGE**Y**FGQCGG  LRGQHF**Y**AVERRA  AVERRA**Y**CEGCYV  AYCEGC**Y**VATLEK  RAMGKA**Y**HPGCFT  SFHIGC**Y**KCEECG  GECQGC**Y**PLDGHI  AKLEHE**Y**IQNFKI  KFFDAN**Y**DGKDYD  EKERDF**Y**FGKLRN  KTIKVS**Y**ARPSSE  ALLSQL**Y**HSPARR  FVTMTN**Y**EEAAMA  EKDLPN**Y**NWNSFG  EGRVRG**Y**PEPQVT  EEDRGK**Y**TCEATN  TRDSGT**Y**SCTASN  PEDHGT**Y**TCLAEN  PEDTGT**Y**TCEAWN  PEDRGL**Y**KCVAKN  QEHCGC**Y**TLLVEN  SLTLSW**Y**GSSYDG  SWYGSS**Y**DGGSAV  GSAVQS**Y**SIEIWD  LLPDHE**Y**KFRVRA  VRAINV**Y**GTSEPS  QKVSDF**Y**DIEERL  GKFFKA**Y**SAKEKE  ERECIK**Y**MRQISE  NYEPIG**Y**ATDMWS  SIGVIC**Y**ILVSGL  KDRMKK**Y**MARRKW  KPHVKP**Y**FSKTIR  DCKIEG**Y**PDPEVV  RHFQID**Y**DEDGNC  GDDDAK**Y**TCKAVN  HQLPPQ**Y**QKILER  GDYKVK**Y**GYTDID  VDFAEF**Y**RLWSVD  REQENK**Y**LEELAE  LVRKCI**Y**AFFQPQ  EERPNL**Y**SQPYSS  AIENCE**Y**AFNLKK  ETPPPG**Y**ISEDGE  FWCSIA**Y**YELNQR  LIDGLE**Y**LHSQGI  FEGDNI**Y**KLFENI  LKGMLE**Y**EPAKRF  PSRDKK**Y**LAKLKL  QAPFQE**Y**LGSLYF  PDSKTV**Y**AKDIQD  GEGAYV**Y**RSAFSV  GLYYFA**Y**HITVYM  GERNGL**Y**ADNDND  RAITSA**Y**YRGAVG  AITSAY**Y**RGAVGA  IAKHLT**Y**ENVERW  RVLIGT**Y**YDNFCA  GSSNHV**Y**NYQPCD  SNHVYN**Y**QPCDHP  LQGQQF**Y**SLEGAP  PHCVPD**Y**HKQYAP  PDYHKQ**Y**APRCSV  SQRRKE**Y**ELEKQK  SVESSS**Y**GDTLLG  DEDGKP**Y**SPSEYS  CVCKSR**Y**PVCGSD  VTGAQV**Y**LSCEVI  KVKRGH**Y**GVQRTE  MVTSLA**Y**THHGRQ  THHGRQ**Y**LAQEGV  YLSEGP**Y**YVKPVS  RPARGL**Y**SRSGSL  CKEGMG**Y**GDRTST  VNDEVR**Y**PRFLSA  KLDLLK**Y**SLEQRL  ELEISV**Y**WRDWRS  PQSGLE**Y**SGIQEL  SLWGAN**Y**MDCFES  DKKRKK**Y**LNNKYR  KYCGKP**Y**TYAYGL  RIVEKG**Y**YSERDA  IVEKGY**Y**SERDAA  ILRGCA**Y**GPEVDM  NHNRCG**Y**KFGLSS  LSSSLD**Y**PDFYGD  QGFQDY**Y**NLGQFT  DCLAVA**Y**GVAVNK  MDEATW**Y**LDSDDS  VEVMSE**Y**NATQSD  MIDRIE**Y**NVEHAV  VEHAVD**Y**VERAVS  EFKPNH**Y**APSNDI  APSNDI**Y**GGEMHV  LGGSVG**Y**PYGGSG  GSVGYP**Y**GGSGFG  YGSGYG**Y**GYGYGY  YGYGYG**Y**GYGGYT  YGYGYG**Y**GGYTDP  GYGYGG**Y**TDPRAA  MSRTRR**Y**YLSVII  SRTRRY**Y**LSVIIV  FIATIV**Y**IMGVNP  LYGSQI**Y**ALCNQF  PAATGL**Y**VDQYLY  GLYVDQ**Y**LYHYCV  YVDQYL**Y**HYCVVD  DQYLYH**Y**CVVDPQ  RRKMDR**Y**DKSNIL  WDKEHI**Y**DEQPPN  PSPPSD**Y**VERVDS  FYPESS**Y**KSTPVP  DTGLQE**Y**KSLQSE  REESEE**Y**MAAADE  MAAADE**Y**NRLKQV  VKGSAD**Y**KSKKNH  TALIKA**Y**DKAVAS  VVVYTG**Y**GNREEQ  SMLISW**Y**MSGYHT  AADWAL**Y**TYEDGS  ENQKVM**Y**GFCSVK  TQASEG**Y**FSQSQE  EDAHFI**Y**GYPKKG  KGHGHS**Y**TTAEEA  PNAPPA**Y**EKLSAE  QVHVAT**Y**TEHSML  ENVVRK**Y**KSMILE  TIVKNC**Y**KQHGRE  INCGNK**Y**LTAEAF  SVTRKR**Y**AHLSAR  FFEFCD**Y**NKVAIK  VYIVQE**Y**METDLA  LLSPNN**Y**TKAIDM  AQLEAL**Y**QSSWDS  SDNVNK**Y**MGLTQF  CIVCVN**Y**VVSGII  SATSSP**Y**RDTQSR  LKMREE**Y**DKIQIA  NGFLEV**Y**PFTLVA  RGEFTY**Y**EIQDNT  VKKPHR**Y**RPGTVA  LREIRR**Y**QKSTEL  QEACES**Y**LVGLFE  PKMVKT**Y**LEEELD  VGNVEL**Y**LHDWGV  YLIKHN**Y**GKDKAA  RLDGID**Y**NPWVEF  DFMLNQ**Y**TKTFGY  IIPQGS**Y**FLITDI  DQSHWR**Y**GGDPPW  LGPGRE**Y**RALQLH  PEENSA**Y**EQLLSR  PSDFSR**Y**FQYEGS  FSRYFQ**Y**EGSLTT  NPAICR**Y**PLGMSG  ESTAAK**Y**GRLDSE  IEFAPM**Y**KINYSR  PMYKIN**Y**SRDGTR  DGNSNP**Y**DIFLKD  LDGLVS**Y**NAPAGQ  PGGSII**Y**LNDSVY  YLNDSV**Y**DGAVGY  FTQTHE**Y**HVWPGY  YHVWPG**Y**DYVGWR  VWPGYD**Y**VGWRNE  ESATNG**Y**IEIMFE  FKEVQC**Y**FRSEAS  SAIKCQ**Y**HFADTW  QSDAAM**Y**NNSEAL  PMAPTT**Y**DPMLKV  FPLRPD**Y**QEPSRL  VTGGNT**Y**SVPAVT  LCMITE**Y**MENGDL  DVRTVS**Y**TNLKFM  IASGMK**Y**LSSLNF  CLVGKN**Y**TIKIAD  FCQEQP**Y**SQLSDE  ICPDSV**Y**KLMLSC  PVLFQE**Y**LDIDQM  VFYQGR**Y**YDSLAQ  GFFLRP**Y**NFFDED  PARGKT**Y**ISKKLT  VFNVGE**Y**RREAVK  IQSRIV**Y**YLMNIH  RAYGQQ**Y**WGQPGN  PGNRGG**Y**RNFYDR  RDYYRQ**Y**NRDWQS  PADPPQ**Y**QEVPLD  VTWQLE**Y**PGQAPE  ELELGM**Y**ALLGVF  PKMWQW**Y**QNSCYY  WYQNSC**Y**YFTTNE  YQNSCY**Y**FTTNEE  GSKGCA**Y**FQKGNI  FQKGNI**Y**ISRCSA  CRVKLK**Y**VKLKNY  AMSRGL**Y**QPATIF  PIPVSE**Y**CESENK  QEFVWD**Y**VILDEA  FQQNKD**Y**SVFLLT  SDHDLM**Y**TCDLSV  RVFCYL**Y**ELWYKL  LLPSAE**Y**SSVETD  RDISGK**Y**SWDGKV  KTLIDL**Y**EQVVLE  NLLARS**Y**FDPREA  FDPREA**Y**PDGSSK  LGQIML**Y**VDGMNG  FMLDML**Y**AHNRKS  ILNFDE**Y**ALNKEG  AAPPGE**Y**FSVGSQ  EVVAFD**Y**QSKMLA  VVITPP**Y**QVENCK  TVICDF**Y**SLIRLH  SSGREI**Y**LPIREA  FSRALQ**Y**EYASKG  GTNLDE**Y**VALVDG  ESEVSR**Y**KERLHD  LHDIEF**Y**KARVEE  DNIDKE**Y**LGKKEK  ILTELK**Y**RNLKHI  CQSPAK**Y**QPYKKR  GTPGQV**Y**DVEEVD  DQENCV**Y**ETVVLP  GILCNT**Y**IDSYKG  VSPLRR**Y**TRHIDS  HIDSSP**Y**SPAYLS  PGRQSH**Y**GTPYPP  DQLKAR**Y**STTTLL  NQQWLV**Y**DQQREV  KPESEG**Y**LQEEKQ  GGPENL**Y**VKEVAK  GGGQAQ**Y**VTVPEG  IQEAHK**Y**MEANKN  DELLTL**Y**FENRRR  GRNATV**Y**GKGVYF  LSVQDR**Y**SPPNAD  TKSDSN**Y**IIAIRE  TFVDPT**Y**IGNIGR  SQSTDF**Y**ATGINQ  KEVDNL**Y**NIEILR  EMNWLD**Y**FALGGN  AAGERI**Y**NISGNG  STLLKN**Y**QDNNKM  LRKECY**Y**LTCQLY  DDSDDL**Y**LPTCKY  SLLSFG**Y**LSWDWA  LQSPRE**Y**LYRYRT  TAIPVR**Y**PAENPR  ELDTEY**Y**AVGKED  KSGVEK**Y**PTDKEQ  IYSFMF**Y**LTKSLV  HITINQ**Y**LQQVYE  SNLKDD**Y**STAQRV  ILANLI**Y**MGHVKG  KRWKLR**Y**FLVQGQ  VQGQKL**Y**FAHHPA  IQQGEI**Y**KIPAAE  VGMHCW**Y**SSYSHR  HCWYSS**Y**SHRTQH  HESCGS**Y**QRLQDF  LRKFKQ**Y**LNPSQV  KILKAK**Y**PTEMII  LKSDLM**Y**DRLSVL  EKSATE**Y**ADSSKA  KCVMNN**Y**FGIGLD  DEHPGQ**Y**NSRLKN  LKNKMW**Y**GLLGTK  ELLQRS**Y**RKLEER  VLNITS**Y**AGGINF  NTATTE**Y**EAPAID  GLIKIR**Y**KNAAQM  SMKMWE**Y**KHTEIQ  SLSDEE**Y**AQMQHL  ILNDIF**Y**GQDSGN  FSLKGL**Y**DDTTAF  DNMQAI**Y**VALGEA  LRLLGN**Y**SLWLEG  EEDAGR**Y**WCAVLG  LGQHHN**Y**QNWRVY  YQNWRV**Y**DVLVLK  WKRLPS**Y**KRTPTE  FEYVMF**Y**NDIEKR  ANLNIN**Y**KRPIPL  KKALTD**Y**KKLRAF  EKKVGL**Y**TIYVNP  TELLAS**Y**NDEDIY  HSDGAQ**Y**VKRYKG  QDDNNL**Y**LVMDYY  EDGKGR**Y**GPECDW  REQSEH**Y**SKQLEN  SDSPPF**Y**KPSFSW  DTLATT**Y**GHSYRQ  EHSVSL**Y**GSPLVP  TGRSSY**Y**YRLLRR  KKERNS**Y**LGLVSL  TKAIEE**Y**LKLVGQ  QHIKII**Y**PTAPPR  NKASAV**Y**QALQKS  HSFPNV**Y**HELSKT  PQPTVV**Y**RTVYRQ  VVYRTV**Y**RQVVKT  QCCHGF**Y**ESRGFC  QPCTPG**Y**YGPACQ  PCTPGY**Y**GPACQF  CRCAPG**Y**TGDRCR  LCPDGF**Y**GLSCQA  CQCAPG**Y**TGPHCA  LCPPDT**Y**GVNCSA  SCQPGR**Y**GKRCVP  VALFIG**Y**RHWQKG  HHLAVA**Y**SSGRLD  RLDGSE**Y**VMPDVP  PDVPPS**Y**SHYYSN  PPSYSH**Y**YSNPSY  PSYSHY**Y**SNPSYH  SRLDRS**Y**SYSYSN  LDRSYS**Y**SYSNGP  RSYSYS**Y**SNGPGP  NGPGPF**Y**NKGLIS  GPRESS**Y**MEMKGP  QRDSGT**Y**EQPSPL  GLPPGH**Y**DSPKNS  SHIPGH**Y**DLPPVR  YDNCLT**Y**RRIYLP  SSRNED**Y**PRTCRM  TCRMCF**Y**GTGLIA  QKNKDD**Y**IPYPSI  KGEARA**Y**RRHFLG  RFNPVL**Y**PKASQM  PNFDVE**Y**TAEDLA  PERDDP**Y**PVLFRY  LFFMSE**Y**EATNLL  PAFPFI**Y**TPTAED  EPQNGT**Y**AGPAPA  LTERPC**Y**NRRASK  YWKNRI**Y**NSPIYG  IYNSPI**Y**GADISG  ILQPER**Y**DLWKRG  RSRKES**Y**SIYVYK  KESYSI**Y**VYKVLK  SYSIYV**Y**KVLKRV  GKTVWL**Y**LQAWPN  LSQVKF**Y**RDGKFL  VQSRGQ**Y**SCSGQV  CSGQVM**Y**IPQTFT  EGDSGL**Y**WCEVAP  GSPPIL**Y**SFYLDE  PILYSF**Y**LDEKIV  EQDAGN**Y**SCEAEN  AAALLV**Y**VRSWRK  GEQCPL**Y**ANVHHQ  RESRST**Y**NDTEDV  EGNAGP**Y**RCIYYK  GPYRCI**Y**YKPPKW  PYRCIY**Y**KPPKWS  WSEQSD**Y**LELLVK  LKAEHL**Y**ILIGVS  GSHLPC**Y**NKVRTR  INILAT**Y**HPADKG  HPADKG**Y**GLMKID  EGLEPK**Y**RIQEQE  NFKVMI**Y**QDEVKL  TPDHVV**Y**ARSKTA  RSKTAV**Y**KVPPFT  EGQPRE**Y**YTLDSI  IDTMGT**Y**HGMTLK  ISVTVP**Y**RVVDQP  EQRMER**Y**QVAAAQ  QEAARR**Y**GELTKL  PEVAQQ**Y**QDIMQR  VDDNYF**Y**LFDLKA  QPIRTE**Y**KIAFPY  NSFVSV**Y**SKDNPN  PKPPRL**Y**LPQEPT  EPQARF**Y**VACVVL  GPADLR**Y**FEGEFT  IRRGIR**Y**WNGVVP  FFGYKV**Y**PTLRCP  QEGLRL**Y**IQNLGR  SASVSH**Y**EFSRVR  TGLALV**Y**AKEQLF  LTADSG**Y**YVLELV  TADSGY**Y**VLELVP  LDPDTD**Y**DVALVP  SAAALG**Y**HVQFGP  LAPGTA**Y**LVTVTA  RAPLDA**Y**FQVSRT  DLPATT**Y**DSETRN  DEANYI**Y**AGLANG  RLGSGT**Y**ATVYKA  WDSDNI**Y**LIMEFC  MVCQRQ**Y**DARVDL  GPRTPK**Y**MNNITY  QELLKD**Y**IKRQIE  FVPRQH**Y**QKETES  RKGFWD**Y**FSQTSG  KELFHP**Y**AESLVS  AFRQDT**Y**LQIAAF  ERILAP**Y**TDFHYR  IRGTCV**Y**VLGLIA  ADCNFS**Y**TSSRDA  LAGLVL**Y**VGLFGH  MLHRAK**Y**SRFRNE  LGMGMN**Y**CVRYMG  MNYCVR**Y**MGCVEV  DPDTTD**Y**VAYVAK  TTDYVA**Y**VAKDPV  ELRFKQ**Y**LKNPSL  EREDHE**Y**YNEIPG  REDHEY**Y**NEIPGK  ATEQMA**Y**CPIQCE  QCEKLC**Y**LPGNSK  LFDDPC**Y**INTQAL  LWSEEC**Y**HGKLSR  ATSPGQ**Y**VLSGLQ  VGHLIR**Y**HMDNSL  DKVSVT**Y**YGPNED  QVPLEV**Y**LCRELQ  QAPVEL**Y**SDWLMT  STFGNG**Y**VLYMSS  GNGYVL**Y**MSSRRK  LVGLGD**Y**VPEPFG  GPSRGQ**Y**YGGSLP  QADSCP**Y**GTMYLS  DSQQLG**Y**ASHSGI  RLEHSL**Y**KPQKGL  MSEQLG**Y**KTVSGS  HKINEM**Y**ASLQEE  GESWHP**Y**LEPQGL  CTEGQI**Y**CGLTTC  VTCPTE**Y**PCRHPE  LGSLNS**Y**LEQLRQ  LSEGEH**Y**WEVDVG  PTRIGL**Y**LSFGDG  DEGASL**Y**DSEPRK  NGNGYT**Y**TAGGEA  GLAYAA**Y**ANGLGA  LAQLRR**Y**RPWGGP  PAGGGF**Y**LVPTFS  QELEQL**Y**LSHLSR  CDILIV**Y**SPDAEE  AEEWCQ**Y**LQTLFL  DDEPET**Y**VAAVKK  DEKVVS**Y**SKQQNL  GAETTV**Y**VIVRCK  AKVENE**Y**TISVKA  NVSLKI**Y**SGDLVV  CETVIS**Y**YTDMEE  AFKIVP**Y**NTETLD  LHFAAK**Y**GLKNLT  PGALQA**Y**SVANKH  ANKHGH**Y**PNTIAE  RQFIDE**Y**VETVDM  NMANFS**Y**RFSIYN  SYRFSI**Y**NLNEAL  KPSHAS**Y**SLDDVT  DKSHCG**Y**SLDWSL  MERIEK**Y**ALFKNP  FKNPQN**Y**LLGKKE  KSWKKR**Y**FLLRAS  RASGIY**Y**VPKGKA  LDHVNV**Y**YGQDYR  DHVNVY**Y**GQDYRN  QDYRNK**Y**KAPTDY  YKAPTD**Y**CLVLKH  IQKKSQ**Y**IKYLCC  KSQYIK**Y**LCCDDV  GIRIAK**Y**GKQLYM  KYGKQL**Y**MNYQEA  KQLYMN**Y**QEALKR  KRTESA**Y**DWTSLS  ESMNRP**Y**TSLVPP  PKIVTP**Y**TASQPS  APMFVK**Y**STITRL  PAFPAS**Y**IPPSPP  DQQKAG**Y**GGSHIS  VDESDL**Y**NWEVAI  KGKDKE**Y**AEIIRK  EEDADC**Y**DDDDSG  KQLQTK**Y**DELKER  SLRNAA**Y**FLSNLK  PNVKET**Y**DSSSLP  GHQEQT**Y**LQKETS  VEQNGD**Y**GRGRRT  EPRKLS**Y**AEVCQK  KLNSGD**Y**KTIHLS  GSRLKL**Y**LPKETC  RLNLWR**Y**GDFRAD  SCGAIQ**Y**IPTELD  TLLRLL**Y**EALVDC  VELLGS**Y**TEDNAS  YEAHQM**Y**RTLFFR  LWKEQN**Y**CESRYH  PKQTSS**Y**GGLLGN  MHTDPD**Y**SAAYVV  GDFRGF**Y**RQLTSD  SCIDFR**Y**ITDVLT  QGSPEK**Y**ILFLIV  RQDYFD**Y**ESTNPF  FDATAG**Y**RSLTYE  LPEGGC**Y**ELLTVI  HPNIVP**Y**RATFIA  QQNLQG**Y**DAKSDI  CVVINP**Y**KQLPIY  YKQLPI**Y**TEAIVE  DFSVLH**Y**AGKVDY  HHHHHY**Y**FYNHSH  PKKKTG**Y**GELNGN  GTDGNV**Y**PPGGQP  VTNHSV**Y**CSTKGA  SRRQSC**Y**LCDLPR  PQALER**Y**PLAAAA  GASGEV**Y**CPSGEK  GIFPIS**Y**VEFNSA  GVFPGN**Y**VAPVTR  HRVVVS**Y**PPQSEA  QVIIPR**Y**LTLSSH  TSSSGH**Y**ISDVYD  SGYIFF**Y**MHKEIF  ADARSI**Y**VGNVDY  GFPRAR**Y**RARTTN  SSRSRF**Y**SGFNSR  NPCPWH**Y**LHLSGS  SRAPST**Y**TYTSRP  PCQRSR**Y**RDSLTQ  WEEVQS**Y**IRKRTA  VPSNLS**Y**GEWLHG  KKLDDS**Y**SEAVRQ  LKSLNG**Y**KNLSSG  SVTDLT**Y**WGPAGH  RCTSTA**Y**QEQRPQ  QLGQLS**Y**LAPGED  VVAQHS**Y**SAQGPE  PSVLSR**Y**PPAAQE  TSSITI**Y**PSDSSS  DCTLSV**Y**RQLHNS  RNRPAP**Y**SRPKQL  KKAAVH**Y**DRSGRS  LKAMKQ**Y**NGVPLD  QNVAKS**Y**LPNAHL  KDEKEL**Y**KVHLKN  EQFPPD**Y**DQQVFE  IEFRKR**Y**STKGGE  CSEQNL**Y**PVEDEN  ALHFRR**Y**LDQFVP  PLVCAK**Y**GWVTVE  AFDFDR**Y**KQRCAE  DDESLK**Y**LTHEEK  LSIYFY**Y**SLPNAV  GLAWSY**Y**IGYLRL  VYSNSI**Y**ELLENG  SAGIAL**Y**SHEDVC  GAAVDE**Y**FRQPVV  EPLTHW**Y**QVRCLF  VDTSPG**Y**HESDSK  WCNFQF**Y**TSLMNS  IFQESV**Y**KTQQTI  GNAVVG**Y**LQCFDL  GFCHIP**Y**DELNMP  ASATGV**Y**LAALPT  VALEDI**Y**ESPNHL  VLDAVY**Y**LHRMGI  YEFDSP**Y**WDDISD  LLSRSR**Y**TQEYIC  SRYTQE**Y**ICQIFS  IAMLAD**Y**FKYPSS  NPPPQD**Y**ESDDDS  VLDLTE**Y**ARRHQW  GPMVEK**Y**SVATQI  IASHSG**Y**VQIDWK  GDTSYT**Y**HLVCMG  QRQGVS**Y**SVHAYT  RKKEQW**Y**AGINPS  SLLVDK**Y**IFRILS  PQHKIE**Y**RHNTLP  VGQPNE**Y**DLNDSF  TPQFTP**Y**YVAPQV  PQFTPY**Y**VAPQVL  SPTPYT**Y**NKSCDL  VKKYNT**Y**AYVGLT  KYNTYA**Y**VGLTEG  SPGDFR**Y**SDGTPV  ICYTVY**Y**VHNIKF  IREESS**Y**SDIPDV  WYNHIA**Y**IPIQIG  SAQKEI**Y**QVKQQR  SSSSSK**Y**DPEILK  SDAQGS**Y**KLDEAQ  LEGVAR**Y**MQSERC  AIFEIS**Y**FKKHPE  EAHGTF**Y**TSHCVS  GLVNPH**Y**ARWDRR  PNIIRL**Y**EVVETL  KAENVF**Y**TSNTCV  QRKLQE**Y**LAAKGK  SQNTKP**Y**LKSKNN  CVSSNP**Y**SKPSSK  KPKTDS**Y**NQTKNS  KVNRSQ**Y**ERPNET  ETKIRS**Y**PVTEQR  HTKPRT**Y**PSLLQG  SLLQGE**Y**NNRHPN  KSKGKT**Y**KRPPME  FDVIGL**Y**EEAIKN  KAEQHN**Y**PGIKLQ  ERAVSR**Y**PEMLQE  RSRSRS**Y**SPRRRP  SPSPRR**Y**SPPIQR  PPIQRR**Y**SPSPPP  RALIES**Y**QNLTRV  LTRVAD**Y**CENNYI  DYCENN**Y**IQATDK  SLASVA**Y**QINALA  MERPVR**Y**IRKPID  IRKPID**Y**TVLDDV  LGRNTP**Y**KTLEPV  SAPGSQ**Y**GTMTRQ  STSSGG**Y**RRTPSV  VNGGPL**Y**SQNSIS  PPPPVD**Y**EDEEAA  EAAVVQ**Y**NDPYAD  VQYNDP**Y**ADGDPA  AWAPKN**Y**IEKVVA  VVAIYD**Y**TKDKDD  MEGAII**Y**VIKKND  KNDDGW**Y**EGVCNR  GLFPGN**Y**VESIMH  EPKGNE**Y**GRNYFD  NEYGRN**Y**FDPLMD  DDDRIF**Y**NRLTKL  NKNYTQ**Y**IPLSIY  MRRASS**Y**SSLNSL  NQFTAI**Y**NSSGEL  LQTVVV**Y**LPDVWT  LQRDFG**Y**RVYKML  QYRSLQ**Y**SRQEGL  TLPLLH**Y**LEVSGC  VKGPLL**Y**CARPPQ  LDGNEN**Y**PCLVDA  LKGDQL**Y**YFKDED  ASLLKL**Y**LRELPE  LSHPRD**Y**ESKSDH  TGTGKS**Y**VSSLLA  SSLLAH**Y**LFQGGL  GSSWVV**Y**GTNYRK  NKAALS**Y**VSEIGK  PTVTSD**Y**ISLEVN  GVVNLH**Y**VAPEIF  TGSGKT**Y**TMGGDF  GTFFEI**Y**GGKVYD  IYGGKV**Y**DLLNWK  KYLYME**Y**HSPDDN  QQGPPS**Y**QKQGSH  EKGSHK**Y**QRSSSS  NTPKFL**Y**TVPNGN  DRVIDF**Y**SNQKDA  SSAPSP**Y**LRASFS  NEVSTL**Y**KGEYHR  TLYKGE**Y**HRAPVA  FLVTQG**Y**QVKLAG  IVLGFN**Y**WIASSR  LERKFS**Y**DLSQCI  LRGEDD**Y**NMDENE  EDEDGD**Y**EELVLA  KLPHVD**Y**IEEDSS  GSLVEV**Y**LLDTSI  ADLGVI**Y**TRNKMN  VRRTQR**Y**LYENLE  EESPVQ**Y**AAYVTV  KYSSLI**Y**FDSSAT  DQLHRE**Y**CLSPRN  ASMEVR**Y**QTEGFL  HSRLES**Y**RPDTDL  CVALAI**Y**YHIKNR  SVYAKY**Y**FDLRSL  PAPTGM**Y**PSVPPT  PGPTGP**Y**PTPNMP  PELPRP**Y**GAPTDP  AQRLHY**Y**RGNYMT  MTFKKM**Y**QQKQKE  DGDFED**Y**KREVLE  QGGKMA**Y**YEMRAE  GGKMAY**Y**EMRAEH  RVLRYG**Y**FGKEKL  STAIST**Y**PKTLIA  FCIITE**Y**CEGRDL  EVFEEV**Y**NYLKRA  QVTCVT**Y**NWNDCY  YNWNDC**Y**IASGSL  QCIAFQ**Y**STVLTK  LYWMSG**Y**QHHLNP  AKERDL**Y**KEKYEK  DLYKEK**Y**EKLAGR  GKVTVK**Y**DRKELR  EQLGQL**Y**GCEEEE  EALVDC**Y**KPTEEF  PSPRDG**Y**GSPHHT  PHHTPP**Y**GPRSRP  SQQQFG**Y**SPGQQQ  MRGQRL**Y**EKLMSP  VDSNLL**Y**QFRMNF  NVLHVD**Y**RTVSNL  NSTLLR**Y**SVSLLG  FLSHHC**Y**EGTVSF  FTFVEV**Y**RVKKFQ  PRIVRR**Y**LLRRQL  QLGQGA**Y**GIVWKA  ENDRDI**Y**LVFEFM  HVRSIF**Y**QLLRAT  YVATRW**Y**RAPEVL  LLSSHR**Y**TLGVDM  QALQHP**Y**VQRFHC  QLSVPE**Y**RSRVYQ  EYRSRV**Y**QMILEC  RALLGG**Y**SQAYGT  GGYSQA**Y**GTVCHS  GNSLGS**Y**SASQGV  SFKKVL**Y**EMGPEY  CGYRGG**Y**MEVINL  LKLHDV**Y**ENKKYL  SCGSPH**Y**ACPEVI  VIKGEK**Y**DGRRAD  GVSRLT**Y**VSEPES  TELSTV**Y**LFGGDE  SPREPF**Y**VKHSKY  RKKDEN**Y**RSLPRD  RISHSL**Y**SGIEGL  SEGPSN**Y**DSYKKV  LTTMAP**Y**LSTEDV  VPVITP**Y**LSEVLT  EGIVPQ**Y**DGSSSF  NSTTVQ**Y**AGLLHH  VAPDKE**Y**LLIVIQ  WHSSSM**Y**LLSPLN  SDAEKV**Y**GECSQK  NNRWGE**Y**HPYSNV  KNTGKM**Y**ACKKLD  EKVSYS**Y**PVDWFA  RTPFKD**Y**KEKVSK  QKLQIL**Y**KSLEKS  KDDQDH**Y**TNTYHI  NMLQIG**Y**HLEKYL  PNLKLR**Y**KPKKSL  ERHVLL**Y**GTNPLS  QDPSVL**Y**ISLHRH  KFIQQT**Y**PSGGEE  GEEQAQ**Y**CRAAEE  GQVKEC**Y**QSHRDT  SVPRRD**Y**ASESPA  PFARGE**Y**IDNRSS  VLCKPW**Y**AGACDR  HDSKQP**Y**TLVVFF  FFNKRV**Y**NIPVRF  IEATKQ**Y**ALGRKK  KKNGEE**Y**FGSVAE  VWKVLI**Y**DRFGQD  DLRNQL**Y**ESYYLN  ARKLDV**Y**FEYEEK  KDELQL**Y**GDAPGE  GALLVC**Y**HYYADW  LVCYHY**Y**ADWFMS  IESSPQ**Y**RLRIAM  VQAPQM**Y**PYPVSN  MQPMAH**Y**PSQPVF  SSHESF**Y**DSLSDM  EDDTGR**Y**TCLATN  SSGSFN**Y**ARPKQF  EEQFEQ**Y**LRDESG  EEEIHI**Y**QFPECD  PVRGRR**Y**SWGTVE  TVEELK**Y**ADIRNL  AHWNEA**Y**HFINKA  NKVKPS**Y**KSCADC  KPTTNS**Y**LMQHQE  EHSHGG**Y**GESGAP  AVTLWA**Y**ALGATV  TAGGFI**Y**VATVSV  KNQPGK**Y**SQLVVE  SSLAKI**Y**TEAKKV  GRTYLK**Y**SIKALV  NYDCDY**Y**CSNLFE  DSELPS**Y**HQNDVS  TSDSEV**Y**TDHGRP  SLANHE**Y**FALQHA  ADSSNF**Y**ITEKNR  ESGTER**Y**QKLQKI  DQEIQT**Y**TIAVIN  EMAHQL**Y**VLQVLT  EKRKSM**Y**TRDYKK  SMYTRD**Y**KKLGFI  ALDNML**Y**FAKHHQ  RQDKFW**Y**CRLSPN  NHKVLH**Y**GDLEES  LAFSIL**Y**DSNCQL  APDKHE**Y**CIWTDG  GSDGHT**Y**SSVCKL  AINLDK**Y**EVCIRP  SCDEDG**Y**YRKMQC  HLHASV**Y**ALFHRL  LHNQLL**Y**ERFKRQ  QLHIMD**Y**NETHHE  GTGAGA**Y**ILTRFA  VEVVHT**Y**RQHIVN  LAEAFK**Y**FVQGMG  VDREKI**Y**QWINEL  HIPLFL**Y**PFLHTV  KTRPFE**Y**LRLTSL  GNKVQQ**Y**HRALVA  SQAVAR**Y**QRQAAE  NTVVRA**Y**SDQAIV  RREHGV**Y**ASSKEE  TTQLTQ**Y**LHEDGY  NMLNSM**Y**QLWILG  GPYEAL**Y**NAISCH  EEDEHP**Y**ELLLTA  DLCTFA**Y**ITKDLQ  AAAPAA**Y**PTGRGG  EQAMKQ**Y**MQKLTA  HHEIFS**Y**PEIYFL  FSYPEI**Y**FLGLNA  GPNNGG**Y**DDDQGS  DDDQGS**Y**VQVPHD  PHDHVA**Y**RYEVLK  DHVAYR**Y**EVLKVI  GQVVKA**Y**DHKVHQ  LLSMNL**Y**ELIKKN  DFGSSC**Y**EHQRVY  YEHQRV**Y**TYIQSR  YIQSRF**Y**RAPEVI  VILGAR**Y**GMPIDM  AELLTG**Y**PLLPGE  FVSSKG**Y**PRYCTV  SKGYPR**Y**CTVTTL  MDMKIF**Y**KGKNTG  ANCTAI**Y**HTCEAS  QGPSSD**Y**VVVKMI  LNSPSS**Y**NCSQSI  VDSHHE**Y**PAMTFY  SPAVMN**Y**SIPSNV  QPQQPP**Y**TGAQTQ  GQMYQQ**Y**QQQAGY  NYPAQT**Y**TAQTSQ  DEPQLL**Y**AIENKY  DVQVSC**Y**RILTSL  DKYGEF**Y**GRDRIS  RKVRAI**Y**DFEAAE  MEALSL**Y**TKLMNE  KLQNQP**Y**YMQSSG  LQNQPY**Y**MQSSGV  VSGSQV**Y**AGPPPS  PPPSGA**Y**LVAGNA  MSHLQS**Y**SLPPEQ  QQTQAA**Y**PNTMVS  SVQGNT**Y**PSQAPV  PSQAPV**Y**SPPPAA  TADVTL**Y**QNAGPN  MPQVPN**Y**NLTSST  GMHVQE**Y**GSDCPP  HEILIG**Y**LEYVKK  KQRTAK**Y**VANQPG  EATQEQ**Y**TESEEQ  PHGNMM**Y**TGPSHH  GPSHHS**Y**MNAAGV  FGGHRD**Y**GPRTDA  GGDRGG**Y**GGDRGG  GDRSGG**Y**GGDRSG  KAFLDF**Y**HSFLEK  GRDRAS**Y**EARERH  VNAIGL**Y**MRHLGV  RRQESG**Y**LIEEIG  MQRLTK**Y**PLLLQS  LLRLKD**Y**QRRLDL  TDHKAF**Y**VLFTWD  EDEEDT**Y**YTKDLP  IIPGSV**Y**DRSSQG  GRPSSM**Y**FQTHDQ  WTKDHH**Y**FKYCKI  DHHYFK**Y**CKISAL  LLTNAD**Y**TTGQVF  RLGGGT**Y**GEVFKA  LMTKSG**Y**QPPRLK  VVLLQW**Y**QPMNKF  LTRPNI**Y**LIPDID  SHHIYP**Y**SSSQDD  GFYPDS**Y**DTWVHS  RSRHSS**Y**PAGTED  LWAAQR**Y**GRELRR  TSMTEE**Y**RVPDGM  TKAWEE**Y**YKKIGQ  RQQAAY**Y**GQTPGP  SGMTMC**Y**ACNKSI  DEAEVI**Y**SELMSD  QEKDQK**Y**IFPTLD  VKVGKT**Y**ELLNCD  AGLLQV**Y**IHTQKN  GKVSVE**Y**TEKMVS  ADPHKV**Y**ALSREL  MGKCKV**Y**WEVGGP  RRGSPR**Y**TIYLGF  HRLKPW**Y**FSPYPQ  LNLINY**Y**KGQYIL  NYYKGQ**Y**ILTLSE  VLQRPS**Y**KFFFKS  RREARK**Y**ASNLLK  HHTHPS**Y**GPPGVP  GGHYVV**Y**LNPKGD  KTRSLN**Y**CGHIYT  APKRSR**Y**TYLEKA  IEALKE**Y**EPEMGK  WLRGAV**Y**YFKIAV  LDEALK**Y**LSRNHM  PCKVSE**Y**TSTTGL  AVRCVQ**Y**DGRRVV  RVVSGA**Y**DFMVKV  VTPDEG**Y**YQGGKF  TPDEGY**Y**QGGKFQ  TEVPDA**Y**NMVPPK  PHKVTQ**Y**KKGKDS  AQGRRR**Y**DRKQSG  DRKQSG**Y**GGQTKP  QAQPVC**Y**TLLDQR  TLLDQR**Y**FSGLGN  PQHTQV**Y**QKEQCP  SADMRP**Y**NWSYTQ  RPYNWS**Y**TQFSQF  AEDRHG**Y**IWDRHY  ARVTVK**Y**DRRELQ  GRLEEL**Y**RGMEAD  GRLEEA**Y**EKCDRD  TTAIRT**Y**QSITER  CMASKH**Y**LSATDM  SDFYLR**Y**YVGHKG  YANNSN**Y**KNDVMI  MIRKEA**Y**VHKSVM  GFLGVY**Y**VGVASC  ANATHI**Y**GASAGA  TAMMVP**Y**TLPLES  PPCSPS**Y**DLTGKV  IAKELK**Y**RAGHQA  SFQIRD**Y**VESQKK  RPGPCA**Y**AAHGRG  ALPTQQ**Y**AKSLPV  DMRDPT**Y**RPLKQE  DMPPVS**Y**DVQLLH  IRGKVG**Y**TRGLHV  PLHSVG**Y**TTLVGN  LGRNRL**Y**HDGKNQ  NQPSKT**Y**PAFLEP  FIVDGQ**Y**MGVAFR  LKGKKL**Y**PVVSAV  CEIRMR**Y**LNGLDP  KKRFTV**Y**KVLVSV  VRYPEL**Y**NHPDVR  KLDGKF**Y**AVKVLQ  VGSPNV**Y**HSLPCT  EKCLDK**Y**FQHLCD  RHLPLE**Y**RQLLIP  RRVFNP**Y**TEFKEF  EKMFKQ**Y**DAGRDG  GAGQLA**Y**ALARGF  QEGATV**Y**ATGTHA  SGSGPA**Y**AFMALD  QFSNDI**Y**STYHLF  NDIYST**Y**HLFPPR  HYLPSY**Y**HLHVHF  LGKKQE**Y**LENRIQ  EEFRRS**Y**SRLCRE  QNAVLA**Y**VQASPV  KELEDA**Y**WKDDDK  GTALSK**Y**RSRSAD  RAQMEV**Y**CSDFHA  DSDQQA**Y**LVQRGA  AGFAGR**Y**VLQAMK  AFSGGY**Y**RGGFEP  DKGGSP**Y**IAAKIN  IGHRLE**Y**GGLGRE  VEHIKA**Y**VTKSPV  SPADKP**Y**IDEARR  AMFDIE**Y**FRKDPR  KFAKEI**Y**PGQFQP  SEIEEF**Y**NGLEDE  NFRGFD**Y**YFGIPY  FRGFDY**Y**FGIPYS  GRPFLL**Y**VALAHM  QETLRS**Y**FSQYGE  TEVVMI**Y**DAEKQR  PPPFTS**Y**IVSTPP  PLAKED**Y**YQILGV  SGSQHS**Y**WKGGPT  QSLILS**Y**AEDETD  FAQKSG**Y**FLCLSS  TKTVPG**Y**LRIGDM  YEASSL**Y**GISAMD  ADIEEE**Y**NYGFVV  IEEEYN**Y**GFVVEK  FEDLKK**Y**CFHRSV  YDGKNN**Y**PKACGK  RDLTTG**Y**DDSQPD  YNSEDE**Y**EAAAAR  AVADQV**Y**GDQDMH  RKHCMD**Y**LMKNAD  LMKNAD**Y**FSNYVT  ADYFSN**Y**VTEDFT  TEDFTT**Y**INRKRK  QAMAEM**Y**NRPVEV  NRPVEV**Y**QYSTGT  PVEVYQ**Y**STGTSA  EPIRVS**Y**HRNIHY  YHRNIH**Y**NSVVNP  QVARES**Y**LQWLRD  SPLVSL**Y**PALECR  AVSQQE**Y**LDSMKK  AGIAAA**Y**AARKLG  AALAAI**Y**SGLLRR  PEVCLL**Y**NKGEAL  NKGEAL**Y**GYCNLK  FQIIST**Y**KHMKLH  PYCETD**Y**YALFGT  YCETDY**Y**ALFGTI  FLEALG**Y**TWHDTC  AEQFRS**Y**SESEKQ  LRHLPD**Y**RDPPDG  LFLGCS**Y**NKDLLD  GNPSSP**Y**SAGRKA  PRIGAL**Y**IRGLGE  SPVLLS**Y**GVPFDV  WFGPSP**Y**VEVTVD  DQHGRV**Y**YVDHVE  QHGRVY**Y**VDHVEK  DNMGRI**Y**YVDHFT  NMGRIY**Y**VDHFTR  LESVRN**Y**EQWQLQ  DSNGRV**Y**FVNHNT  TVDGIP**Y**FVDHNR  NRRTTT**Y**IDPRTG  NGPQIA**Y**VRDFKA  FKAKVQ**Y**FRFWCQ  GEEGLD**Y**GGVARE  MYCLFE**Y**AGKDNY  YAGKDN**Y**CLQINP  QINPAS**Y**INPDHL  NPDHLK**Y**FRFIGR  GFSLPF**Y**KRILNK  ECDLEM**Y**FSVDKE  EENKEE**Y**IRMVAE  PQQYLQ**Y**FDAKEL  WQRHAI**Y**RHYART  HAIYRH**Y**ARTSKQ  RLDLPP**Y**KSYEQL  LPPYKS**Y**EQLKEK  KLCVSL**Y**DVFRPL  DLHGMW**Y**PTVRRT  SQGGPK**Y**TLSQQP  FFKDVD**Y**VCISDN  GLRGIC**Y**FFIEVE  KLNRYN**Y**IEGTKM  HLEVAR**Y**MVQRGG  CPEDYK**Y**ISENCE  NKDGEV**Y**CIDARY  QPHVGN**Y**RLLRTI  KYRVPF**Y**MSTDCE  SLTSQK**Y**NEVTAT  QLYYEC**Y**SDVSVH  RVRTDA**Y**RLGILR  LRVLLR**Y**KVGDQE  VPCSFS**Y**PRQDWT  TGSTPA**Y**GYWFKA  STPAYG**Y**WFKAVT  MQDESQ**Y**FFRVER  RVERGS**Y**VRYNFM  RGSYVR**Y**NFMNDG  TQKPDV**Y**IPETLE  VRLRVA**Y**APRDLV  PQGNVP**Y**LEAQKG  AGDSGR**Y**TCRAEN  LDLSVQ**Y**PPENLR  LSLSVH**Y**SPKLLG  HSTILD**Y**INVVPT  KNQKKQ**Y**QLPSFP  QNVKQL**Y**ALVCET  VCETQR**Y**SAVLDA  LHEHPL**Y**RAGHLI  QLKQAL**Y**GDKKPR  KPRKRT**Y**EQIKVD  EHKLEE**Y**KRKLAR  VILPEM**Y**KIFTMA  DDDTFS**Y**TVRIAA  VKGEEI**Y**SMDEGI  GVILGD**Y**LSRTPE  MRSMQR**Y**HQDTQG  VAIVGN**Y**TAALPT  PTMERP**Y**TFKDFL  NLGRTY**Y**VNHNNR  ESVDSE**Y**YNSLKW  RVRKDM**Y**NDTLNG  TPYEYP**Y**TLAPAT  DMRVHP**Y**QRIVTA  IREDIT**Y**QQPRLT  WYGELP**Y**TYSRIT  SLLCNL**Y**RNEKDS  PHRSRE**Y**LRLSSE  SKQVKE**Y**RIQEPN  EEAIQQ**Y**ERACKD  VDPLGN**Y**MVKTIV  LEKTRQ**Y**SQKLGL  DIVQHI**Y**KTLLSI  DEPLAS**Y**IFRQLV  VLMGNP**Y**RGPELE  AAIHPP**Y**LVSKEL  YSNLGF**Y**PQQPEE  SGISDE**Y**ITPMFS  TPMFSF**Y**KSIGEL  LCQLEV**Y**QKSLKM  IERHAM**Y**CNGLME  LVPFRE**Y**QCHVDS  RRLVLS**Y**SLPQNV  FMQELQ**Y**LFALMM  PLQVNG**Y**RNLDEC  LHVLRQ**Y**LDPAQR  RALLIT**Y**GPYAIN  LITYGP**Y**AINGKI  QEDPND**Y**CKGGYH  WIIKSN**Y**QGLPLP  VIEVIN**Y**TQNSNN  IKDFLS**Y**LCPVNA  QSTEPK**Y**KPLGKL  SEEEDD**Y**LFDDPL  RGRAWL**Y**LALMQK  QKKLAD**Y**LKVLID  HLLSEF**Y**EPEALM  VIDFSL**Y**LKDVQD  VLDQKN**Y**VEELNR  ELALPS**Y**PKPVRV  KERGFW**Y**DAEISR  RTAREL**Y**ANVVLG  RSNDGA**Y**SLVLAG  QISDAV**Y**HMVYEQ  RNHVQP**Y**IPSILE  VLKKYD**Y**DSSSVR  VDILLN**Y**VRKTFD  KGYVPF**Y**VNATAG  FQPDKQ**Y**DVSYDT  GVDDKF**Y**SKLDQE  YWIGVR**Y**DEPLGK  SVNGKR**Y**FECQAK  QCKARW**Y**EWLDPS  AQCLEH**Y**EFLLDK  REIDDT**Y**IEDAAD  QRRTTT**Y**LISLTL  RFLTGS**Y**QVLEKT  KTESEQ**Y**KKEMLV  DMKRCS**Y**YETCAT  MKRCSY**Y**ETCATY  YETCAT**Y**GLNVDR  GGHTSD**Y**SSSLPS  KEWKKK**Y**VTLSSN  SNGFLL**Y**HPSIND  HPSIND**Y**IHSTHG  GSLRNI**Y**KAEENF  SWIRAK**Y**EQLLFL  TQLLLW**Y**GADVAA  GRTALF**Y**ARQAGS  AKKEGP**Y**DVVVLP  MMNGGH**Y**TYSENR  NGGHYT**Y**SENRVE  YPGQTQ**Y**QTLQQT  ILGQNQ**Y**QACYPS  FGKKVT**Y**VVIGDG  VNYITA**Y**MVLFDF  CGFHLG**Y**LDGEVE  ARLLAL**Y**NQGHIK  KLKPVD**Y**EYREEV  SLLTGD**Y**IPGTET  PPNQAN**Y**HQTLHA  CSDVFQ**Y**ETNKVT  RIQSMN**Y**GTIKWF  HVIIFS**Y**VCFALV  LVSDKL**Y**QRKEPV  VFDTAD**Y**TFPLQG  QRLCPE**Y**PTRRTL  TGRCVV**Y**EGNQKT  DFPGHN**Y**TTRNIL  IMGIEI**Y**WDCNLD  HHCRPK**Y**SFRRLD  TTNVSL**Y**PGYNFR  VSLYPG**Y**NFRYAK  NFRYAK**Y**YKENNV  FRYAKY**Y**KENNVE  IIQLVV**Y**IGSTLS  DFLIDT**Y**SSNCCR  CCRSHI**Y**PWCKCC  PCVVNE**Y**YYRKKC  CVVNEY**Y**YRKKCE  VVNEYY**Y**RKKCES  PKPTLK**Y**VSFVDE  LQFLLL**Y**QEPLLA  RLRHCA**Y**RCYATW  HCAYRC**Y**ATWRFG  EGVPRF**Y**IRILAD  RILHTY**Y**KFDYKA  YRKNEG**Y**MRRGGY  CPKNAS**Y**YGNRAA  GNYKLA**Y**ELYTEA  EEAVRD**Y**EKVYQT  RIPWFQ**Y**PIIYDI  FQYPII**Y**DIRARP  LSFSRE**Y**TAAVEA  DMNNAR**Y**LWKRIP  RDFPGI**Y**TTINAH  ALVSQA**Y**TSIIAD  NSSRSA**Y**ACFLFA  PLFFQQ**Y**QAATPG  RVILRS**Y**HEEEAD  NDDIDS**Y**MIAMET  KEDGRL**Y**AVKRSM  QEGDPR**Y**MAPELL  QQLRQG**Y**LPPEFT  KVDLTK**Y**LENQAF  LKNQPC**Y**RKLGLE  TLNTLR**Y**ADRVKE  RQGCFH**Y**SRRNKL  RNKLIL**Y**KFDRRI  GRDTHL**Y**TEYTLQ  KQCKDI**Y**CDYLDF  KDIYCD**Y**LDFGIN  VSRCTL**Y**WRDEGL  LLNRLR**Y**RPSNSR  LKPFTE**Y**EFQISS  SSKLHL**Y**KGSWSD  GMLDVW**Y**MKRHID  MKRHID**Y**SRQQIS  RGKILH**Y**QVTLQE  PSAVQE**Y**VVEWRE  WLRSRP**Y**NVSALI  SENIKS**Y**ICYEIR  IKSYIC**Y**EIRVYA  CYEIRV**Y**ALSGDQ  MGCLLH**Y**RIYWKE  LLHYRI**Y**WKERDS  QLCEIP**Y**RVSQNS  LQPRVT**Y**VLWMTA  GIFSTH**Y**FQQKVF  STCAKK**Y**PIAEEK  RQLVDL**Y**KVLESR  TVLDRF**Y**NADIAV  RQPSLF**Y**HLGVRE  ANNIIL**Y**CDTNSD  TMCTGN**Y**TFVPYM  TPHNKV**Y**CCDSSF  QASSSQ**Y**FRESIL  YRDIQD**Y**DSIVKL  QVASDM**Y**CLVGRI  LQSGIN**Y**AVLLLA  VETILI**Y**KHFVKL  LEPTKI**Y**QPSYLS  KIYQPS**Y**LSINNE  SDDFQI**Y**FCTELH  ESDLLE**Y**DYEYDE  LEYDYE**Y**DENGDR  EQTIGF**Y**TKQILE  NVLINT**Y**SGVLKI  FTGTLQ**Y**MAPEII  DKGPRG**Y**GKAADI  TGKPPF**Y**ELGEPQ  SAGSNE**Y**LRSISL  TSSSSE**Y**GSVSPD  TLLDVL**Y**YVTRDD  LLDVLY**Y**VTRDDL  KTWAVL**Y**PASPHG  MLENSL**Y**SPTWEG  CGLHGS**Y**VLRVEA  PLLSWP**Y**TLLRRY  YTLLRR**Y**GRDKVM  PSQDSL**Y**SDPLDS  EGYELP**Y**NPATDD  ILVADT**Y**DQSQSP  KAFVEP**Y**FKGDER  KECEIY**Y**ADMPAE  GAFGRV**Y**LCYDVD  HERIVQ**Y**YGCLRD  SVTGTP**Y**WMSPEV  KELLAE**Y**QDLTFL  PDIKSH**Y**AFRIFD  LKQVKP**Y**VEEGLH  FKKTFS**Y**AGFEMQ  QLLIGA**Y**AKALEI  PSPPPP**Y**DSRLNS  PRLFPG**Y**PNLHFP  FPLRPY**Y**VGPIRI  AGMGRS**Y**APYHHH  GRSYAP**Y**HHHQPA  RSTPPL**Y**RHAAPY  QEPDDD**Y**QTGFPF  GVPFHQ**Y**TLGCVS  RNLEHL**Y**LQNNEI  SDLQKI**Y**EANAKR  QVYLVD**Y**GLAYRY  VDYGLA**Y**RYCPEG  LALVEK**Y**SSPGLT  AIFKLT**Y**LSNHDY  IVLKVN**Y**ILESRA  IRLTKG**Y**ASVFKG  KGCLTL**Y**TGRGGD  SEPNPE**Y**STQQAP  QLEAAR**Y**RSDGAL  VSKFCK**Y**EHDDIV  QVVLSS**Y**RAHAAQ  TKGRWL**Y**KGEWTH  HGFKGR**Y**GIRQSS  DNLRHG**Y**GCTTLP  PDTSVV**Y**VCGKGD  LFQDDL**Y**PDTAGP  ISLREA**Y**VPSKQR  CHLHHH**Y**CGLHER  RRGGYE**Y**SDQKSG  SPLPAK**Y**IDLDKG  AAFRSP**Y**SSTEPL  KDLAGK**Y**RQILEK  ETGQKQ**Y**NVDYKL  LKIARL**Y**LEDDDP  PNVDRT**Y**FPGPPG  SSPDPR**Y**LSVPPS  EFQDAP**Y**NEVTGA  LLGELD**Y**QLQDQD  DKLSWG**Y**RREAGI  VTPDVW**Y**TSKPKE  PKEKLV**Y**AIFLKW  ARAPLH**Y**LAPLLS  LPVDLQ**Y**LPPDKQ  VRDQGA**Y**LILREL  GKFEDM**Y**KLTSEL  REVETL**Y**QCQGNK  SIQEGK**Y**EFPDKD  QAKVLQ**Y**RSWCQE  QEFFKG**Y**LKGEHG  IFSQFD**Y**QDADKA  YSKAKT**Y**WKQIPP  DGMLGG**Y**GHISSI  NLPDEI**Y**HVYSFA  CDIIKN**Y**GFVHIE  GAVRTP**Y**TMSYGD  YGALDA**Y**YKRCRA  NSREKI**Y**NRSLGS  PCLRNR**Y**GEEVRD  ATGVLV**Y**LAKYHG  VKIYFP**Y**KAYPSQ  YLVERD**Y**ESACKI  EVTSLK**Y**STPPYL  PWTRKV**Y**EFYSAP  RQAHSK**Y**GQFLLV  QEPHHH**Y**SAIERN  AKVKSA**Y**SERLKF  TSHGKV**Y**IVMELA  VLQGIP**Y**QPKVYD  GCSAAV**Y**EATMPT  VALAGE**Y**GAVTYR  LPFSSW**Y**VDRGGN  AVVSCL**Y**LGVKTN  LLAQKS**Y**EHMAKI  ATEIID**Y**NGNLHE  EFELMS**Y**RLNTHV  FPGGKE**Y**LMRAHF  IIEKSG**Y**QALPWV  MESIDQ**Y**IERKKK  QSKENN**Y**LNQHVN  VNRINF**Y**KKTYKQ  ASMGAS**Y**SKSLIK  FKFFDT**Y**CQQEVV  PLCLAP**Y**FRLLRL  GGLPGP**Y**IKWFLE  FEDKSA**Y**ALCTFA  DGYEQT**Y**AEMPKA  TFHTDA**Y**LQHLQK  IQDEKY**Y**QICESV  IQQILN**Y**IKGNLK  RGSEGL**Y**MVNGPP  PRNAAY**Y**SPHGHI  VWDVKN**Y**KLISKP  QAKQNL**Y**DLDEDD  DDAITA**Y**KKYNNR  ITAYKK**Y**NNRCLD  SQKVVV**Y**DQSSQD  LGQLLD**Y**EKKIKN  SLTSSW**Y**FATESS  HQTQPL**Y**NIRLDR  KQMHDF**Y**KERGLE  GLLVLH**Y**GLVVSP  LTPGVV**Y**VRHLPN  AETMNN**Y**LFGERL  AKKGID**Y**DFPSLI  YPRYKK**Y**QLACTK  TPFTGN**Y**GQPHVG  LPSSQA**Y**SHGGLM  TLLLCG**Y**PNVGKS  ILDLQK**Y**WDLMNL  DKDDAH**Y**AVQARR  VAASCG**Y**LDIARY  YLDIAR**Y**LLSHGA  GGFRTL**Y**AELRRE  FPECGF**Y**GLYDKI  CGFYGL**Y**DKILLF  ALTVHS**Y**RAPAFC  DGCGLN**Y**HKRCAF  SSSASS**Y**TGRPIE  TFLIHS**Y**TRPTVC  AQSSLG**Y**IPLMRV  EGWVVH**Y**SNKDTL  TLRKRH**Y**WRLDCK  NNTTNR**Y**YKEIPL  VTANAT**Y**FVGEMP  VDIATV**Y**QIFPDE  GQFGVV**Y**GGKHRK  VLLNQG**Y**NRSLDM  SVGVIM**Y**VSLSGT  QNAAFM**Y**PASPWS  VKMRKR**Y**SVDKSL  HPWLQE**Y**QTWLDL  GKMGER**Y**ITHESD  AVPQSS**Y**PLLANG  MRPPFT**Y**ATLIRW  RTLNEI**Y**HWFTRM  STRFVT**Y**FCEQVL  FDKLLE**Y**MPLPPE  NLGNFN**Y**EQRGAF  ILRDWL**Y**LHRYNA  WLYLHR**Y**NAYPSE  LHRYNA**Y**PSEQEK  YGASIV**Y**CEPSDE  KPSVKV**Y**AAEPSN  KLMPNL**Y**PPETIA  QEKANL**Y**PPSNTP  YAITVW**Y**FDADER  ARAKVK**Y**LTGEKG  GVGALA**Y**KAPEAL  AVRLNR**Y**KIIKLL  HLRQQS**Y**DVTDEL  TATGQR**Y**FLNHIE  FLNGGP**Y**HSREQS  ELLFLR**Y**ISDWDL  IFTARL**Y**FCEDRK  IMTFKD**Y**FYCWNT  KRSEKI**Y**QQRSLR  DKLLQF**Y**PSLEDP  DPASSR**Y**QNFSKG  HGSEEA**Y**IDPIAM  DPIAME**Y**YNWGRF  PIAMEY**Y**NWGRFS  IGRIES**Y**SCKMAG  SRKTLF**Y**LIATLN  LAECDI**Y**SYNPDL  SLGVLL**Y**ILVHGT  QISNGA**Y**REPPKP  WWVNWG**Y**ATRVGE  DHELGK**Y**KDLLMK  NSIAAM**Y**QAVGEL  LSLPLE**Y**WSQFMM  ETKSML**Y**LVTEYA  QNTCQL**Y**CKEPPR  SSEQMQ**Y**SPFLSQ  DSFDNT**Y**QAIIGI  FLSKTM**Y**LEDGTI  LPQKPP**Y**SFIDCS  NLRALK**Y**LVMDEA  CAVSSK**Y**QTVEKL  ITFVTQ**Y**DVELFQ  DKSEKA**Y**SSNEVE  GSSRVQ**Y**VVDHAM  TSPFQN**Y**SSIHSQ  QMLVKW**Y**NVHSAP  LMNMMG**Y**NTDRLA  SDDDWE**Y**LLNSDY  YLLNSD**Y**HQNVES  FVLHLV**Y**EELKLN  GMPPYP**Y**LPGICE  VLSIAL**Y**ILGDES  PIRDAI**Y**HCREQP  RVNVVQ**Y**PELSDH  AELANE**Y**AGFLMA  TLNIHD**Y**LTKGHE  VGIGLV**Y**QGTAHR  KHKSPS**Y**QIKEGD  RAPDTM**Y**LLDFVK  LSQAHV**Y**IIAGAC  AKDFMT**Y**LSAPNA  FLGGGR**Y**SLSTSN  HSTDNR**Y**HLQALR  QALRHL**Y**VLAAEP  ALLEVT**Y**KGTQWY  LACFLV**Y**HSVPAP  LNKLLK**Y**EDCKVH  PLTSTL**Y**DLTEID  IDGPAN**Y**NVDLPF  LLVLVL**Y**YKFQRL  TLTVLA**Y**ERYIRV  GVIAHC**Y**GHILYS  GEKAPA**Y**QRFHAL  LVLPYK**Y**QVLAEM  GQIKTV**Y**PASYRF  YAVQRN**Y**GFQIHT  RFTSGK**Y**QDVYVE  GKYQDV**Y**VELSHI  EGGIRA**Y**FTLGAE  RHISMG**Y**KMKLYF  IYEEGN**Y**EEEGSE  ACSYGH**Y**EVAELL  MFGAGI**Y**FAENSS  EQAYPE**Y**LITYQI  AIKKMS**Y**SGKQTH  FRRFVD**Y**CLQKIP  ESQKKQ**Y**KICKEK  VFRRTR**Y**PDIHLR  GTSGEA**Y**KRSALI  HMAANM**Y**VLWSFS  EQFMAV**Y**LSAGVI  NAKIIK**Y**IRTKTK  TKPHLF**Y**IPGRMC  QLFLAL**Y**DFTARC  LEEGGG**Y**IFARRL  LSDQPW**Y**FSGVSR  ESSLGG**Y**SLSVRA  QAKVCH**Y**RVSMAA  AADGSL**Y**LQKGRL  LEELLT**Y**YKANWK  EELLTY**Y**KANWKL  RKLGEG**Y**FGEVWE  SGGEPV**Y**IVTELM  VAEGMS**Y**LEEQRV  APEAAN**Y**RVFSQK  LHEVFT**Y**GQCPYE  TYGQCP**Y**EGMTNH  QQIMRG**Y**RLPRPA  ACPAEV**Y**VLMLEC  RNIYDK**Y**GSLGLY  GLLTCC**Y**CCCCLC  GEETEF**Y**VSPEDL  AGLEAT**Y**SNVGLA  LAGDLA**Y**QTLPLR  ERKRFT**Y**FSSLSP  EKIREK**Y**GPEWAR  KVLPPC**Y**RQEPAP  ISEGRA**Y**DTDAGP  MADLHL**Y**NQKLRE  NHFGRE**Y**RSPSLG  PVIIER**Y**KGEKQL  DEDGFL**Y**MVYASQ  GFLYMV**Y**ASQETF  HERHFH**Y**EEHLER  ESADSL**Y**RNSFSF  LLAKPC**Y**IVISKR  TLMSAN**Y**PEGFPE  KEERVE**Y**YIPGST  EERVEY**Y**IPGSTT  NESNKI**Y**VVDLSN  SCTDHR**Y**CQRKSY  YCQRKS**Y**SLQVPS  CNTSFS**Y**LVASAI  IPSQDL**Y**FGSFCP  TVSFIP**Y**FKEEGV  DTKSKV**Y**LRTPNW  GPAVGI**Y**NDNINT  IEDTMV**Y**GHLLQD  QPEVDT**Y**RPFQGT  ESESEP**Y**TFSHPN  DLLVST**Y**RLPQIA  GLREND**Y**LLIHSC  SLEEGH**Y**VIGPKI  IEIPVH**Y**AGQFKL  AFPERV**Y**VMEDIT  NEDTEV**Y**NITLCT  GQAEIL**Y**AKTFKE  PPPRNP**Y**DLHFIR  IREGHR**Y**KFVNIQ  QFDIDE**Y**SRAVRD  VPNSLS**Y**ARDELT  RLSVCV**Y**GNNLHG  PHDILP**Y**QDSGDS  GDSGSD**Y**LFPEAS  PSPTLS**Y**YSSGLH  SPTLSY**Y**SSGLHN  STPVSC**Y**PCNRVK  LSWPNH**Y**SGASES  LDPSRS**Y**SYPRQK  PSRSYS**Y**PRQKTP  CPKSAS**Y**SLESTD  DLSEDQ**Y**FVKKGM  DIFSAS**Y**PFSSPL  KSKVTR**Y**LCFTRS  KQAPPQ**Y**TFIGEL  YQHSNL**Y**AISAMD  DHCKAI**Y**LHVLTT  HHYLPY**Y**YSIRGV  GFTYVL**Y**INGGHP  MKYEKS**Y**RFHLGQ  LDHKTL**Y**FDVEPF  DLGKLS**Y**RSYWSW  SNILAA**Y**SFVSEN  PERAAL**Y**FVSGVC  PEGSHQ**Y**ELLKHA  MSAGPR**Y**EYHWAD  KCSAPK**Y**IDYLMT  FSCRPL**Y**CTSMMN  RAGETM**Y**LYEKAN  KDLVKT**Y**PPFVNF  GGGTEG**Y**HVLRVQ  AKPLKL**Y**VYNTDT  PLKLYV**Y**NTDTDN  RKIVHD**Y**RQGIVP  ELWRAK**Y**IYDSAF  FNAVVN**Y**TNRSGD  SPEKTT**Y**DSAEEE  EDSQDL**Y**NASPEP  RNHRNQ**Y**QALKPR  APITTG**Y**TVKISN  TVKISN**Y**GWDQSD  NVLKKI**Y**EDGDDD  RRLLER**Y**RGPEPS  RGQVSF**Y**RGAVPV  ELGRRK**Y**QDSIRR  TSFCAK**Y**TYPVCS  FCAKYT**Y**PVCSAL  LTVMTK**Y**QANITE  EEEDRF**Y**LVFEKM  SEEASI**Y**DKRCDL  YILLSG**Y**PPFVGR  MVNGGG**Y**PYESAS  GRSYSP**Y**DYQPCL  QSSASG**Y**QAPSTP  PLNRFK**Y**MWKLLR  TIKFTV**Y**TSSELQ  EQSSVR**Y**KDSTSL  IFRGQV**Y**SELKYH  RGRHET**Y**LCYEVE  HETYLC**Y**EVERMH  TCCFPS**Y**VYPDPT  CFPSYV**Y**PDPTQE  EKYKKN**Y**LPMQGN  ERTVWK**Y**FVQLCS  RIHENG**Y**NFKSDI  SLGCLL**Y**EMAALQ  RFRKWL**Y**KPKRSD  VCGLVV**Y**ADGPLN  GVIGAS**Y**AAGLAK  FFVTAT**Y**ESLLRG  CEEDFI**Y**ENVESE  RQAQHR**Y**QQQQRR  IRDTVK**Y**KEVMKQ  LNQETT**Y**VSNLTR  TSAAEE**Y**EREYRP  LHSKEN**Y**DKYSEP  KENYDK**Y**SEPRGY  CANDLF**Y**SMTCQH  ATEAQL**Y**VDQHVQ  MFNLYS**Y**YRGAKY  KRLIKR**Y**VLKAQV  MSHTNN**Y**PCAYLN  NNYPCA**Y**LNAASA  KESDDN**Y**DKTEDV  TLTPSY**Y**RGAQGV  QGVILV**Y**DVTRRD  LNELET**Y**CTRNDI  RDPVVA**Y**YCRLYA  DPVVAY**Y**CRLYAM  AGSALQ**Y**EDVSTA  LLSDQG**Y**RVDGRR  ALVNCQ**Y**SSATFS  QADGGT**Y**AACVNA  ADQGNP**Y**DADDIQ  PQGDMI**Y**DPSWHH  PPPLIP**Y**YSKMVF  SFKEHE**Y**LGNLST  QETEAP**Y**ISIACD  TISFRI**Y**KGVIQA  KSKNTW**Y**IDEVAE  PLRIYG**Y**VCATKI  DAYMCM**Y**QSPTMS  QSKIYS**Y**MSPNKC  DPSTGC**Y**MYYFQY  YMYYFQ**Y**LSKTYC  QVLAQK**Y**SLVAKQ  ARRKRS**Y**KQAVSE  KINPDN**Y**GMDLNS  DSQQKR**Y**VPVKGD  EPASLS**Y**LSFEGA  QEVGKL**Y**PLEIVF  VSYMEI**Y**NEKVRD  SNLAAS**Y**LNPVKS  FRCNPG**Y**GLLVRP  RAGQVA**Y**LEKLRL  LNIQLT**Y**RVQRLQ  QQLSYF**Y**TTMCQN  KNQLSP**Y**EQSEIL  QSEILG**Y**AELWFL  DDEHGF**Y**LKVLHD  LHDHIA**Y**RYEVLE  DHIAYR**Y**EVLETI  KDKDNT**Y**NVVHMK  HMKDFF**Y**FRNHFC  LLGINL**Y**ELMKNN  PENIVL**Y**QKGQAS  DFGSSC**Y**EHQKVY  YEHQKV**Y**TYIQSR  YIQSRF**Y**RSPEVI  VILGHP**Y**DVAIDM  CITAEL**Y**TGYPLF  AELYTG**Y**PLFPGE  NRGKKR**Y**PDSKDL  TMVLKT**Y**DTSFLD  MTSRDY**Y**FDSYAH  VHALVT**Y**FNIEFT  VFYLED**Y**LTVRRG  FYGGTP**Y**GGQFER  EILSVA**Y**KKDSED  EMPNIS**Y**AWKELK  DSLRYI**Y**PKLKTD  VDLFRA**Y**DASLAM  RSQVRC**Y**VHIMKE  RLTIKK**Y**LDVKFE  EMDDEE**Y**SCIALG  STGNYE**Y**RLILRC  ASVFVL**Y**ATSCAN  KEALEA**Y**RREQKG  AGFKRG**Y**DGKIAG  ALETNR**Y**NHITAT  NHITAT**Y**FLLAER  IEAALE**Y**ISKMGY  PPAAGL**Y**VPHPHH  FFDDNG**Y**PFRCPK  KFVSNL**Y**NQLAKT  MQASPE**Y**QDYVYL  ERRRKL**Y**LAALPL  VPAEQL**Y**EAHLEK  WLEESV**Y**MDIYGK  SVYMDI**Y**GKHQKQ  EYSELF**Y**ELELDA  CTNDDK**Y**VIDGKM  VIDGKM**Y**ELSLRP  HGCLCL**Y**NSKESL  ASAGIG**Y**GRNINE  ELSVLS**Y**HSSFSI  SRLVHG**Y**IVFYSA  HGYIVF**Y**SAKRKA  IEATHM**Y**DNAAEA  EDIEPS**Y**SLFRED  TKGDLS**Y**LDQGHR  ATWESN**Y**FGVPLT  IERCIE**Y**IEATGL  LSTEGI**Y**RVSGNK  PDPLVP**Y**NMQIDL  NHEVFK**Y**VISHLN  LTATRT**Y**QTIIEL  QCPFFF**Y**NRPITE  ELDLLE**Y**GNQFQD  QFQDAL**Y**KMTGER  PLVHQC**Y**LKKSKR  LKAKAI**Y**EKFIQT  AAQSRV**Y**QLMEQD  LMEQDS**Y**TRFLKS  FDQHAN**Y**DCPAPS  YKWEYT**Y**YSRNKD  NHSERK**Y**YYYERH  FVAQKM**Y**RFSRAA  FESTPV**Y**PNAGRY  RTEGGY**Y**QITGRM  AEILSV**Y**QKCKDK  EVYEHV**Y**ETVDIS  EQNSPI**Y**ISRIIP  VKLVVR**Y**TPKVLE  QPGTGR**Y**QVLLNE  ATSLPT**Y**DEAEKA  NTIAGR**Y**GAICGF  IVRFSD**Y**FTGYFN  SDYFTG**Y**FNGQYW  GYFNGQ**Y**WLWWIF  FRGFVN**Y**LKVRNM  LSNIVM**Y**APLVLQ  VTEAFD**Y**LSFLPL  KSKSLN**Y**TGEKKE  LHSGMD**Y**AIMTGG  TLNAFL**Y**RTGQHS  RMYFDK**Y**VLKPAT  RSMWER**Y**CRGVNA  GVNAIV**Y**MIDAAD  DREICC**Y**SISCKE  FSPTGK**Y**LASGSG  MALSTD**Y**ALRTGA  ERALSR**Y**NLVRGQ  ACRGFG**Y**VTFSML  TGTRNL**Y**LAREGL  KGQSLG**Y**AFAEFQ  KNLFGK**Y**GKVLSA  SPGAKC**Y**GIVTMS  FGHGSD**Y**SRQQNR  RFSSLS**Y**KSREED  TVPEST**Y**TSIQGD  GVVYKG**Y**VNNTTV  DDLCLV**Y**VYMPNG  EKTIED**Y**IDKKMN  PEEDTA**Y**LDGVSL  ELLRLA**Y**SEPCGL  VIKKKL**Y**SSEQLL  RSGLAA**Y**FFMGRL  LLILIV**Y**CTPFYE  VYCTPF**Y**ERVDTT  AQEVSE**Y**LSQNPR  VTDAPT**Y**TTRDEL  KICKRK**Y**RGSEIE  YRGRLA**Y**LPVGRV  GVMHLF**Y**VRAGVS  GQVHPN**Y**FWMVSG  KNGNDV**Y**RYPSPL  GNDVYR**Y**PSPLHA  EDEQSN**Y**TTNCFG  TEETED**Y**RQFRKS  NERFTS**Y**QKATEE  EEWDPE**Y**TPKSKK  TCDTYS**Y**GVVLWE  RYPGKF**Y**RVSQSA  GWTKVE**Y**RKKPHR  ASYGQD**Y**DLSPSR  IIGEGT**Y**GQVYKA  DVIKLP**Y**FNTMKP  AAITNK**Y**QLVFCY  YQLVFC**Y**TIIERN  KFIDPI**Y**QVWEDM  LGPTAD**Y**VFPDLT  NLAGKN**Y**IILNMT  EIGIQN**Y**STTSSC  KDGWVY**Y**ANHTEE  VAGDLP**Y**GWEQET  INKRTT**Y**LDPRLA  PTTRQR**Y**DGSTTA  NHLGHF**Y**LVQLLQ  YWAMLA**Y**NRSKLC  HPGNMM**Y**SNIHRS  HRSWWV**Y**TLLFTL  GAATTV**Y**CAAVPE  EGLGGM**Y**FNNCCR  EGFVRL**Y**NTESQS  RKNYTA**Y**RQEPIA  MRKICT**Y**FHRKSQ  SLLESS**Y**KFGDDV  TYGLSA**Y**SGKVRY  YSGKVR**Y**ICSALG  GHLEWE**Y**QFCTPI  LFDDTS**Y**TSNDDV  ATENSV**Y**LGMYRG  SVYLGM**Y**RGQLYL  MYRGQL**Y**LQSSVR  KFSHEE**Y**SNGALS  ALSILQ**Y**PYDNGY  SILQYP**Y**DNGYYL  PYDNGY**Y**LPYYKR  FLDNPH**Y**NKNIRK  CQTENK**Y**DSVSGE  SGYISR**Y**LTDFEP  HPGIVR**Y**FNAWLE  PSEASP**Y**VRSRER  PSSPKV**Y**LYIQMQ  SPKVYL**Y**IQMQLC  LTPMPA**Y**ARHTGQ  QVGTKL**Y**MSPEQI  QIHGNS**Y**SHKVDI  ILFELL**Y**PFSTQM  QKYPCE**Y**VMVQDM  PEVIRV**Y**IGSFWS  GPFGHG**Y**GEGAGE  MYDEIF**Y**TLSPVD  PEEEAQ**Y**KKAFSA  QDGRVN**Y**EEFARM  DKDKDG**Y**INCRDL  CMRTMG**Y**MPTEME  SQLSKQ**Y**SSRDLP  SHTKIK**Y**RQTTQD  QPHIGN**Y**RLQKTI  ERRSVA**Y**NGPPAS  DANNCD**Y**EQKERF  SVMQRN**Y**LQGENE  AKRFCQ**Y**VVKQSE  CDTNWR**Y**YGDSCY  DTNWRY**Y**GDSCYG  YYGDSC**Y**GFFRHN  WEESKQ**Y**CTDMNA  NRNIVE**Y**IKARTH  GNMNCA**Y**FHNGKM  FCENKH**Y**LMCERK  VGGHVL**Y**VCAACE  VFALAE**Y**RTKHEV  PPKKRE**Y**PPPPPE  MSAQLL**Y**KMIKSK  LHMAIA**Y**NHPDVV  CDGSYC**Y**ECTARF  GGKENI**Y**QLLIEW  SELSYI**Y**PIDLNE  ENERLQ**Y**KTPPPS  KVPEPG**Y**TKVPEP  KVPEQG**Y**TKVPVP  KVPVPG**Y**TKLPEP  ISMAIS**Y**LYNSKE  SKDKAP**Y**LIYVEV  DGDMFN**Y**YKMLML  SSLPAF**Y**PGTSGS  GDVLGF**Y**INLPED  HSEIIF**Y**KNGVNQ  FPAISL**Y**KSCTVS  RNVNAP**Y**RDRIPL  SPSEKA**Y**LNAVEK  AVEKGD**Y**ASVKKS  LEEAEI**Y**FKININ  LLSFNV**Y**VGDALL  AAHTNN**Y**EIIKLL  NEFKSE**Y**EELSRQ  LEIILN**Y**RDDNSL  LKLAIK**Y**RQKEFV  PVFSVC**Y**LIAPKS  ICHTAS**Y**LTFLFL  DGGLQD**Y**IHDWWN  FVMNSL**Y**LATISL  IVAFVK**Y**SALNPR  NGLNQL**Y**FYYEET  LNQLYF**Y**YEETKG  NQLYFY**Y**EETKGL  FGLINL**Y**VTNVKA  ATMFGT**Y**NVISLV  AMMNNS**Y**QLIADH  TKLWMS**Y**FEEGGT  SPKSLW**Y**LIKWIW  LRRHHQ**Y**QEVMRN  RNLVKR**Y**VAAMIR  MDWSEL**Y**PEFFAP  RVKVSD**Y**VQDRIR  PTLLAE**Y**AYVLRV  LATLER**Y**VETQAK  QAKENA**Y**DLEANL  LAVLKL**Y**QFNPAF  SRGESV**Y**CKDDFF  RAQDFV**Y**HLHCFA  ATGDEF**Y**LMEDSR  LVCKAD**Y**ETAKQR  ETLKSA**Y**NTSPKP  GPANGL**Y**GSLGEP  LAGPEQ**Y**RELRPG  LRPGSP**Y**GVPPSP  LLSSLV**Y**PDTSLG  TGSSGG**Y**PDFPAS  VFAYGT**Y**ADYLAE  LVIEAV**Y**ADVLRG  QRLEVD**Y**SIGRDI  RPNPCA**Y**TPPSLK  ELRELG**Y**PREEDE  IEQRIP**Y**RGTRYP  PYRGTR**Y**PEVNRR  RHLDLI**Y**NFGCHL  PNVVTY**Y**TSFVVK  MLDIIK**Y**IVNRGE  KEMMKK**Y**GKSFRK  IYGTES**Y**VVSLTT  DNKKLA**Y**LIDIKT  RDERGA**Y**EASLVA  FDMYQQ**Y**AAFKRW  HFETLE**Y**FPSELG  SEKIRT**Y**NFPQNR  GVLPSH**Y**YESFLE  GPCDRD**Y**KKFWAG  LQGLTI**Y**FYNSNR  GLTIYF**Y**NSNRDF  LLPGHL**Y**MMSEVL  QLLLER**Y**PECGNL  THVVRH**Y**KVKREG  KREGPK**Y**VIDVEQ  LDAVVN**Y**FVSHTK  YEKVLG**Y**VEADKE  SDAHTS**Y**YSESLV  APTMAT**Y**QVVELR  NWGHPE**Y**TCIYRF  SDDACT**Y**HPGVPV  LEEVCV**Y**HSGVPI  FHEGMK**Y**WSCCRR  CPCGSL**Y**TVLEEP  KDHQKK**Y**GATVDL  NCMTEI**Y**YQFKKD  AHVLTP**Y**MSSVPA  IHNPII**Y**AITHPK  RRHSRP**Y**PSYRST  QQEETT**Y**YQTALP  RDSRNP**Y**DRKRQD  FMRELR**Y**LFALLV  VGTKRK**Y**VDPSRA  PMVELF**Y**GRFLAV  TEMFGQ**Y**PLQVNG  IKRLKD**Y**LTVLQQ  QQRLER**Y**LSYGSG  LVDVLQ**Y**ALEFAS  RTIELM**Y**SDKSMI  SMIQVP**Y**RLHAVL  QANAGH**Y**WAYIFD  ESRWMK**Y**NDIAVT  YRNASA**Y**CLMYIN  SAYCLM**Y**INDKAQ  SAIKLE**Y**ARLVKL  TPPETD**Y**RLHHVV  LHHVVV**Y**FIQNQA  FRETTM**Y**LIIGLE  NFQRES**Y**IDSLLF  LFLICA**Y**QNNKEL  LLSKGL**Y**RGHDEE  EELISH**Y**RRECLL  PPKLPS**Y**STHELC  NLCKQL**Y**ETTDTT  LAASVP**Y**VKATEP  TIGRCE**Y**EKTCAL  SKTSVA**Y**VTPGDS  TVTHRN**Y**YERIHA  VTHRNY**Y**ERIHAL  HALGPN**Y**NLVISD  MEDAGD**Y**KADINT  NTQADP**Y**TTTKRY  YTTTKR**Y**NLQIYR  RYNLQI**Y**RRLGKP  EEKNVT**Y**NWSPLG  EDQELT**Y**TCTAQN  TFTKNP**Y**AASKKT  ASKKTI**Y**TYIMAS  KKTIYT**Y**IMASRN  SSGEGL**Y**TNGSPM  HHPSVA**Y**DCLWNY  FDSQEL**Y**DSFPDQ  LRDRFP**Y**LSESYL  PYLSES**Y**LITTDA  GDEGSA**Y**WIAHQA  APHDIG**Y**VKQAMF  KQAMFH**Y**FQVPDR  GDPLSR**Y**IFRKAG  HLLPMD**Y**SANAIA  TPQDIS**Y**LAKGKV  YKHKCK**Y**CSKVFG  PGVGPN**Y**NSPRAG  AQDMDA**Y**TLAKAY  YTLAKA**Y**FDVKEY  CNSKKA**Y**FLYMYS  KKAYFL**Y**MYSRYL  LYMYSR**Y**LSGEKK  GFGLYL**Y**GVVLRK  FFLAHI**Y**TELQLI  GFSKSS**Y**IVSQIA  TFSNLL**Y**VRSMKS  MKSELS**Y**LAHNLC  LCEIDK**Y**RVETCC  CCVIGN**Y**YSLRSQ  LKLNPR**Y**LGAWTL  TLMGHE**Y**MEMKNT  SAAIQA**Y**RHAIEV  RDYRAW**Y**GLGQTY  YGLGQT**Y**EILKMP  MPFYCL**Y**YYRRAH  KCYWRA**Y**AVGDVE  EQAAQC**Y**IKYIQD  KYIQDI**Y**SCGEIV  ESTAFR**Y**LAQYYF  FRYLAQ**Y**YFKCKL  RYLAQY**Y**FKCKLW  YTALQQ**Y**FQNGEK  HCLSWL**Y**VLGQKR  YFQARL**Y**HTLGKT  SVLTSF**Y**NAEDES  PTLPKN**Y**SNTSKI  SGFIEL**Y**AYGMFK  FIELYA**Y**GMFKIA  GASEVS**Y**FQLETN  LETNLL**Y**SFLPEV  ISALLQ**Y**INLSLT  QSIESS**Y**SSIQKL  GSESLL**Y**HLSELK  ASWKQK**Y**EPLGLD  AFFRWL**Y**VAMLRM  VERVGQ**Y**LKDEDD  TEGNQW**Y**DFLQNS  SPLLFP**Y**YPRKSL  AICIPL**Y**RDTRSE  KTSNLH**Y**LLFTIL  ILEDSL**Y**KMCILR  KFGSFT**Y**ATTEKV  KVRRSI**Y**SCLDAQ  CLDAQF**Y**DDETVT  LPLSLV**Y**NSEDSA  SEDSAE**Y**QFTGTY  YQFTGT**Y**STRLDE  ESMKAQ**Y**VAGNGF  RAALEA**Y**CKKYEQ  LSLDRI**Y**NMLRMF  LQELQG**Y**LQKKVR  MPFSCH**Y**PSRLRR  VKTKDG**Y**VEVSGK  APQVPP**Y**STFGES  LRIMDP**Y**KASYGV  LTVAEQ**Y**VSAFSK  AQAMGV**Y**GALTKA  PDAGQL**Y**AMKVLK  QTEGKL**Y**LILDFL  VFDDGR**Y**VYLVTD  FNLSKR**Y**FDTECV  IKVPMM**Y**GAGKFA  HVLKLP**Y**QGNATM  ATAISN**Y**MNQLYQ  NYMNQL**Y**QAMHRI  ENDKVK**Y**EVTEDV  DEKKEN**Y**HKHACR  IFFQCL**Y**GRKPFG  QLANDP**Y**LLPHMR  RAASAI**Y**SGYIFD  SGYIFD**Y**DYYRDD  YYRDDF**Y**DRLFDY  LGSTNH**Y**WNETVR  KSSRPM**Y**GAVTSF  KRMPCF**Y**LAHELH  SHCFVT**Y**STVEEA  KFLCAD**Y**AEQDEL  KAAPCI**Y**WLPLTD  PPPIQG**Y**AFKPPP  IPKIDI**Y**HYELDI  KIDIYH**Y**ELDIKP  NGAILV**Y**DITDED  IQEAES**Y**AESVGA  SVGAKH**Y**HTSAKQ  PLLPMR**Y**RKLRVF  RTAQVR**Y**LKTYQE  PEERDG**Y**GRWNHE  EAEEFL**Y**RFLPQK  LPQKII**Y**LNQLLQ  ETHVMD**Y**RALVHE  ELDNLQ**Y**RKMKKI  ELQCRQ**Y**KRKMLL  VGLGAS**Y**LLLCTA  QREGPF**Y**PTLRLL  LRLLHK**Y**PFILPH  GIENIH**Y**LNDGLW  KEVLPQ**Y**FKHSNM  VRQLNM**Y**GFRKVV  PGALTI**Y**STPESR  GDFLLI**Y**LANLEA  LFPGGK**Y**CGHMYQ  KYCGHM**Y**QLTKAP  AVNHPG**Y**MAFLTY  YMAFLT**Y**DEVQER  RDKPGS**Y**IFRPSC  GQWAIG**Y**VSSDGS  GQKDGF**Y**LYPDGK  KDGFYL**Y**PDGKTH  WEAVSI**Y**QFHGQA  KVQISY**Y**GPKTPP  QAPVKL**Y**SDWLSV  INDFFT**Y**HIRHGE  KKGLFT**Y**SLSTKR  GNDVSP**Y**SLSPVS  ELQDDF**Y**LNLVDW  TKQNLD**Y**CFLMMY  YCFLMM**Y**AQEKGI  EFIFMF**Y**KEKPID  PREPLS**Y**SRLQRK  PSLFRV**Y**ARDLLL  PSPSPD**Y**FTWNLT  LSHRSR**Y**GLECSF  FPCELE**Y**SPPLHD  FRVALE**Y**ISSGNR  KLPVGF**Y**CNFEDG  VWHVAA**Y**EGLSLW  SISLDC**Y**LTISGE  AQCNNA**Y**QNSNLS  VPATDT**Y**SISGYG  TYSISG**Y**GAAGGK  EKDDML**Y**ILVGQQ  GGGGAT**Y**VFKMKD  GGGGRA**Y**GAKTDT  SPLGIL**Y**TPALKV  EVNIKH**Y**LNCSHC  SGIMIV**Y**RRKHQE  DIYRAS**Y**YRKGGC  SLGYMP**Y**PSKSNQ  NCPGPV**Y**RIMTQC  TALPIE**Y**GPLVEE  PCGNVN**Y**GYQQQG  GNVNYG**Y**QQQGLP  ASHVVS**Y**LRTSGY  RDDVRQ**Y**LRQYLM  LLQGMA**Y**TVQGPP  FSENGY**Y**LATAAD  FDQSGT**Y**LALGGT  GTDVQI**Y**ICKQWT  PLVAVY**Y**TNRALC  KRDIPD**Y**LCGKIS  TPSGIT**Y**DRKDIE  EFVKQK**Y**LDYARV  KQKYLD**Y**ARVPNS  NSNPPE**Y**EFFWGL  DKLATE**Y**MSSARS  KQIQEA**Y**GKCKEF  VRTSPE**Y**GMPSVT  NGEHIR**Y**ATDTFA  CKENGA**Y**DAKHFL  NFERAL**Y**FYEQAI  LLDAVT**Y**LAGVSG  EEGTLP**Y**PIFAAI  TTHNFL**Y**KHGGIR  STFQNL**Y**RCPGDD  QEGDRD**Y**RFPPIE  HRVLPV**Y**GGPKGL  EMQLKA**Y**EIEKRL  PAGQLS**Y**NLMDTY  KDAEYI**Y**PSLESD  INTDSL**Y**HELSTA  LMERNQ**Y**KERLME  EQKREQ**Y**RQVREH  DITGDI**Y**YFNFAN  LGGDFE**Y**EESLRT  QSKEDL**Y**LDSLSS  NEANNF**Y**IRANSL  NPVADI**Y**NQSDKP  LNILTP**Y**RDDKKD  FYTDPS**Y**FFDLWK  EWNMMA**Y**DKELRP  RLSQSV**Y**HGASSE  DVTDYS**Y**PATPNH  QPVTPS**Y**AAGDVP  QAAEHE**Y**RPPSAS  PMAPAD**Y**GMLPAQ  AQIIEY**Y**NPSGPP  QLGDGT**Y**GSVLLG  KMKRKF**Y**SWEECM  RENDHL**Y**FIFEYM  LYFIFE**Y**MKENLY  YMKENL**Y**QLIKER  AIRNIM**Y**QILQGL  IRSKPP**Y**TDYVST  YVSTRW**Y**RAPEVL  LLRSTN**Y**SSPIDV  CIMAEV**Y**TLRPLF  TDWPEG**Y**QLSSAM  ASQALR**Y**PYFQVG  QALRYP**Y**FQVGHP  KAGPPP**Y**IKPVPP  PPLHLT**Y**PYKAEV  LHLTYP**Y**KAEVSR  APTQTS**Y**QRRDTP  SAAKQH**Y**LKHSRY  YLKHSR**Y**LPGISI  SGLTGN**Y**VPSFLK  PDPSPG**Y**SSLKAM  PEPPKP**Y**SLVRES  SRLLPR**Y**SHSGSS  SGSISP**Y**PKVKAQ  LKSDQD**Y**ILKEGD  GQRTTI**Y**KRDPSK  QPFNVL**Y**EKEGEF  ADAQLA**Y**LNKAGI  ITVPED**Y**INGFIR  NKVPGL**Y**KSSSAD  EKKLRR**Y**AWKKRW  DPDVLE**Y**YKNDHS  PDVLEY**Y**KNDHSK  TSERTF**Y**LVAETE  TSAPQE**Y**LYLHQC  APQEYL**Y**LHQCIS  GQVHGF**Y**SLPKPS  ATPLSA**Y**QIPRTF  ASSCET**Y**EYPQRG  SCETYE**Y**PQRGGE  DNSQSV**Y**IPMSPG  HFDSLG**Y**PSTTLP  NSSSSQ**Y**CRPIST  GDSEEN**Y**VPMQNP  STGSVD**Y**LALDFQ  SDEKVD**Y**VQVDKE  SRSNPY**Y**IVKQGK  MRRALE**Y**TIYNQE  EELDRK**Y**YEVKNK  HRDRIH**Y**QADVRL  SAIGEE**Y**EEAPRP  HPKNNK**Y**FGFITK  LPLPGP**Y**DSRDDF  PNQFSL**Y**TSPSLP  EEPGAG**Y**KKLFSD  EMSEDA**Y**TVLTRI  LETSLR**Y**AIQLIT  ESRSTQ**Y**MKEYQD  LVRWKG**Y**DSEDDT  RQTESA**Y**RYRDIV  TESAYR**Y**RDIVVR  NMQGVV**Y**ELNSYI  IGILEL**Y**RHWGKE  HKNKEL**Y**SDLFDI  NEVAAQ**Y**SEDKAR  VKTKFG**Y**HIIMVE  GVSLPS**Y**EEDQGS  FAAIVA**Y**GLYKLK  IVAYGL**Y**KLKSRG  MNLLEM**Y**SLKDEM  EYSSYN**Y**RGFDIG  LHFIRH**Y**LAEAKK  LIDQKV**Y**ELQASR  LYKELY**Y**RHIYAK  TKVARR**Y**GDFFIR  GADLPN**Y**RWTQTL  IIDGEL**Y**NEVKVE  MVEKMM**Y**DQRQKS  IPAKNC**Y**RMVILG  DFHRKF**Y**SIRGEV  SIRGEV**Y**QLDILD  LLNWQD**Y**EGRTPL  VDVLTS**Y**ESCNIT  GATPLH**Y**AAQSNF  GYGSQG**Y**KYNWKL  GSQGYK**Y**NWKLDE  VNLQMV**Y**DSPLCR  LEWKII**Y**VGSAES  SAESEE**Y**DQVLDS  VLITCT**Y**RGQEFI  VPGVIR**Y**KGAKIQ  KSILAE**Y**KIHNAD  VEGNRV**Y**IPCIYV  DLRACT**Y**CRKIAL  LKCSIE**Y**LYREET  GVYRIG**Y**KNSQNN  PARIGY**Y**EIDRTI  AVQSST**Y**KDSNTL  QQQHML**Y**QQEQHH  EPLDAC**Y**LKALEG  IAEVAG**Y**SPDDLI  QAVTGQ**Y**RFLARS  DILPYD**Y**SRVELS  VQTQEQ**Y**ELVYNA  ALGVYS**Y**IPLVEN  LGNSLR**Y**MIMKNP  EVEFCG**Y**TTTHPS  EASIKD**Y**KDQKAS  ASQGPV**Y**KGVCKC  SDVEGE**Y**VPVEGD  EGDEVT**Y**KMCSIP  RGHNRG**Y**RRPYYF  SPREPG**Y**KAEGKY  TPKSKK**Y**YLHDDR  QGDATT**Y**AHFLFN  DINKDG**Y**ITKEEM  MMGRHT**Y**PILRED  EYHEKN**Y**AAALET  HQSKIK**Y**DWYQTE  ADVKNL**Y**PSSSPY  DYIIMN**Y**SVKHPK  KSCVIT**Y**LAQVDP  YKACLK**Y**PEWKQK  EAQMTY**Y**AQCYQY  GSFPSN**Y**LSNNNQ  ARVLYD**Y**DAANST  LALRAD**Y**EIASKE  REAEEV**Y**RNSMPA  KDKKRS**Y**ESANGR  RFEIAC**Y**KNKVVG  AMKDIH**Y**SVKTNK  VIESED**Y**GQQLEI  QEPPPP**Y**QEQVPV  QVPVPV**Y**HPTPSQ  HLPKGV**Y**DPGRDG  KSTQFE**Y**AWCLVR  CLVRSK**Y**NDDIRK  YLAVGN**Y**RLKEYE  LYGNLH**Y**LQTGRL  QNAAQI**Y**RKVTCG  LPGQPV**Y**PAAFPQ  PDEIAT**Y**MVEHDF  QFQRLR**Y**IKQLGG  QLGGGY**Y**VFPGAS  ILKQIE**Y**RNLFKY  ERTLEV**Y**LVRMDP  MERDDI**Y**VFEINI  DDGKWY**Y**FDDSSV  KSIGQW**Y**GPNTVA  TDINEA**Y**VETLKH  AHYFIG**Y**VGEELI  YLTSQF**Y**ALNYSL  QFYALN**Y**SLRQRM  RFHIDA**Y**VRQGLL  EGCNKV**Y**TKSSHL  HTGEKP**Y**KCTWEG  DELTRH**Y**RKHTGV  LKNGET**Y**NGHLVS  WRMPEC**Y**IRGSTI  RGSTIK**Y**LRIPDE  QHRKWG**Y**DVAVKI  PGGTLG**Y**LAPELF  STASDV**Y**SFGILM  RPLVNI**Y**NCSGVQ  QVGDNN**Y**LTMQQT  SYLPSY**Y**SPSIGF  PSIGFS**Y**SLGEAA  GDTAMP**Y**LTSYGQ  SGYSSN**Y**AYAPSS  NGKGPV**Y**LLFSVN  LKIIAS**Y**KHTTSI  RRVKRL**Y**SNWRKG  VDEEIV**Y**AKSTAL  AIEEKK**Y**LAGADP  MSVEGD**Y**TYLRIN  FVKEIT**Y**RASNIK  GFNGAP**Y**RSTCLL  EAEESD**Y**SKESLG  VDTLQK**Y**FDACAD  EGEMKV**Y**RREVEE  GHEVCN**Y**FWNVDV  DNAIII**Y**QTHKRV  ILCKIT**Y**VANVNP  LKRFTS**Y**VQEKTA  RLCGLE**Y**IRTVIY  YIRTVI**Y**ICASSR  AWGELD**Y**LVVDTP  IEALRP**Y**QPLGAL  DLLSGL**Y**FDQRNY  ASLASL**Y**NGGLYN  TPSECS**Y**YSSRIS  SELRDI**Y**DLKDQI  SEVEEK**Y**KKAMVS  EKNNLI**Y**QVDTLK  SLYLIK**Y**DGFDCV  DGFDCV**Y**GLELNK  TWFYIT**Y**EKDPVL  YQLLDD**Y**KEGDLR  VGKQVE**Y**AKEDGS  DDDFHI**Y**VYDLVK  VLCPPK**Y**LKLSPK  FAKFSA**Y**IKNSRP  LQKLDE**Y**LNSPLP  MTGIWR**Y**LTNAYS  RYLTNA**Y**SRDEFT  SPYVTK**Y**YGSYLK  TKYYGS**Y**LKDTKL  VIKQSA**Y**DSKADI  TELIDR**Y**KRWKAE  ELRGAI**Y**LAEEAC  NFDPLQ**Y**PETTPK  AFRNDS**Y**TLESRI  CEHRET**Y**QKLLED  ENLKRT**Y**EKDHAE  RMEEEA**Y**SKGFQE  LGLYNS**Y**NSCAEQ  KGRGLE**Y**LYLNVH  RKFCLQ**Y**GAALIY  KNIDLV**Y**KYIVQK  YIVQKL**Y**GFPYKI  KLYGFP**Y**KIPAVV  EIADLT**Y**NVVAFS  KNWRHV**Y**KAMTLM  AMTLME**Y**LIKTGS  TLKDFQ**Y**VDRDGK  PGAPPT**Y**ISPLGG  LDCFKS**Y**VDQRAR  LLVKHW**Y**RQVAAQ  VDLIHS**Y**SNAGEY  RLVKHW**Y**QQCTKI  LELVTQ**Y**RQLCIY  IYWTIN**Y**NAKDKT  HQLFQE**Y**DNHIKS  KAQADI**Y**KADFQA  LPPAPA**Y**LSSPLA  CCPKCQ**Y**QAPDMD  FLASGP**Y**LTHQQK  QKVLRL**Y**KRALRH  QRDKYR**Y**FACLMR  AEEEFW**Y**RQHPQP  SPGGTS**Y**ERYDCY  PSEKAM**Y**PDYFAK  KAMYPD**Y**FAKREQ  GKLMIT**Y**TRKSDA  KSDAGK**Y**VCVGTN  ELPKSR**Y**EIRDDH  AGDMGS**Y**TCVAEN  QRSDVG**Y**YICQTL  SIITKA**Y**LEVTDV  GVLQIR**Y**AKLGDT  GATPTS**Y**IIEAFS  LKPNAI**Y**LFLVRA  VRAANA**Y**GISDPS  QGYKIL**Y**RPSGAN  LRKGVN**Y**EIKARP  LGNETR**Y**HINKTV  LVPGIR**Y**SVEVAA  NGLTST**Y**AGIRKV  FTPTVT**Y**QRGGEA  VPPTIP**Y**NQSYDQ  IPYNQS**Y**DQNTGG  HSNSEE**Y**NISVDE  VAAAAE**Y**AGLKVA  HLRRET**Y**TDDLPP  DRKGSS**Y**KGREVL  QEDILP**Y**CRPTFP  MQVLGG**Y**ERGEDN  SENVTQ**Y**LQYKQE  LVNTSV**Y**NILHEE  PEMRQR**Y**ETMQCF  KSPLGF**Y**CDQNPV  NNALLR**Y**LLDRDD  DQKAGL**Y**GQTYPA  QGNPAV**Y**SMVHMN  EGHHTI**Y**IGVHVP  ERISEN**Y**SDKSDI  ASFLVQ**Y**FTRFTE  MIKLAD**Y**YPINSN  SNFKVG**Y**NTLFSC  KKECSK**Y**GGNLVG  LYGVFL**Y**MGVASL |
