# Supplementary material for: iPhos-PseEn: Identifying phosphorylation sites in proteins by fusing different pseudo components into an ensemble classifier
Source: Oncotarget. 2016 Jun 13;7(32):51270–83. doi: 10.18632/oncotarget.9987 (PMC5239474; doi:10.18632/oncotarget.9987)
Supplement: Supplementary file 2 [file oncotarget-07-51270-s002.docx]

**Supporting Information S1.** The benchmark dataset $\mathbb{S}_{\xi=6}\left( \mathbb{S} \right)$used to train and test the model for predicting the possibility of phosphorylation at Ser site. It contains 47,849 samples, of which 4,317 are positive samples and 43,532 are negative samples. All these samples were derived from 1,770 protein sequences in which none has $\geq50\%$ pairwise sequence identity with any other. See the main text for further explanation.

# (1) List of the 4,317 peptide samples in the positive subset $\mathbb{S}_{\boldsymbol{\xi=6}}^{\boldsymbol{+}}\boldsymbol{(}\mathbb{S}\boldsymbol{)}$

| Sample # | Protein ID | Site | Sequences |
| --- | --- | --- | --- |
| 1  2  3  4  5  6  7  8  9  10  11  12  13  14  15  16  17  18  19  20  21  22  23  24  25  26  27  28  29  30  31  32  33  34  35  36  37  38  39  40  41  42  43  44  45  46  47  48  49  50  51  52  53  54  55  56  57  58  59  60  61  62  63  64  65  66  67  68  69  70  71  72  73  74  75  76  77  78  79  80  81  82  83  84  85  86  87  88  89  90  91  92  93  94  95  96  97  98  99  100  101  102  103  104  105  106  107  108  109  110  111  112  113  114  115  116  117  118  119  120  121  122  123  124  125  126  127  128  129  130  131  132  133  134  135  136  137  138  139  140  141  142  143  144  145  146  147  148  149  150  151  152  153  154  155  156  157  158  159  160  161  162  163  164  165  166  167  168  169  170  171  172  173  174  175  176  177  178  179  180  181  182  183  184  185  186  187  188  189  190  191  192  193  194  195  196  197  198  199  200  201  202  203  204  205  206  207  208  209  210  211  212  213  214  215  216  217  218  219  220  221  222  223  224  225  226  227  228  229  230  231  232  233  234  235  236  237  238  239  240  241  242  243  244  245  246  247  248  249  250  251  252  253  254  255  256  257  258  259  260  261  262  263  264  265  266  267  268  269  270  271  272  273  274  275  276  277  278  279  280  281  282  283  284  285  286  287  288  289  290  291  292  293  294  295  296  297  298  299  300  301  302  303  304  305  306  307  308  309  310  311  312  313  314  315  316  317  318  319  320  321  322  323  324  325  326  327  328  329  330  331  332  333  334  335  336  337  338  339  340  341  342  343  344  345  346  347  348  349  350  351  352  353  354  355  356  357  358  359  360  361  362  363  364  365  366  367  368  369  370  371  372  373  374  375  376  377  378  379  380  381  382  383  384  385  386  387  388  389  390  391  392  393  394  395  396  397  398  399  400  401  402  403  404  405  406  407  408  409  410  411  412  413  414  415  416  417  418  419  420  421  422  423  424  425  426  427  428  429  430  431  432  433  434  435  436  437  438  439  440  441  442  443  444  445  446  447  448  449  450  451  452  453  454  455  456  457  458  459  460  461  462  463  464  465  466  467  468  469  470  471  472  473  474  475  476  477  478  479  480  481  482  483  484  485  486  487  488  489  490  491  492  493  494  495  496  497  498  499  500  501  502  503  504  505  506  507  508  509  510  511  512  513  514  515  516  517  518  519  520  521  522  523  524  525  526  527  528  529  530  531  532  533  534  535  536  537  538  539  540  541  542  543  544  545  546  547  548  549  550  551  552  553  554  555  556  557  558  559  560  561  562  563  564  565  566  567  568  569  570  571  572  573  574  575  576  577  578  579  580  581  582  583  584  585  586  587  588  589  590  591  592  593  594  595  596  597  598  599  600  601  602  603  604  605  606  607  608  609  610  611  612  613  614  615  616  617  618  619  620  621  622  623  624  625  626  627  628  629  630  631  632  633  634  635  636  637  638  639  640  641  642  643  644  645  646  647  648  649  650  651  652  653  654  655  656  657  658  659  660  661  662  663  664  665  666  667  668  669  670  671  672  673  674  675  676  677  678  679  680  681  682  683  684  685  686  687  688  689  690  691  692  693  694  695  696  697  698  699  700  701  702  703  704  705  706  707  708  709  710  711  712  713  714  715  716  717  718  719  720  721  722  723  724  725  726  727  728  729  730  731  732  733  734  735  736  737  738  739  740  741  742  743  744  745  746  747  748  749  750  751  752  753  754  755  756  757  758  759  760  761  762  763  764  765  766  767  768  769  770  771  772  773  774  775  776  777  778  779  780  781  782  783  784  785  786  787  788  789  790  791  792  793  794  795  796  797  798  799  800  801  802  803  804  805  806  807  808  809  810  811  812  813  814  815  816  817  818  819  820  821  822  823  824  825  826  827  828  829  830  831  832  833  834  835  836  837  838  839  840  841  842  843  844  845  846  847  848  849  850  851  852  853  854  855  856  857  858  859  860  861  862  863  864  865  866  867  868  869  870  871  872  873  874  875  876  877  878  879  880  881  882  883  884  885  886  887  888  889  890  891  892  893  894  895  896  897  898  899  900  901  902  903  904  905  906  907  908  909  910  911  912  913  914  915  916  917  918  919  920  921  922  923  924  925  926  927  928  929  930  931  932  933  934  935  936  937  938  939  940  941  942  943  944  945  946  947  948  949  950  951  952  953  954  955  956  957  958  959  960  961  962  963  964  965  966  967  968  969  970  971  972  973  974  975  976  977  978  979  980  981  982  983  984  985  986  987  988  989  990  991  992  993  994  995  996  997  998  999  1000  1001  1002  1003  1004  1005  1006  1007  1008  1009  1010  1011  1012  1013  1014  1015  1016  1017  1018  1019  1020  1021  1022  1023  1024  1025  1026  1027  1028  1029  1030  1031  1032  1033  1034  1035  1036  1037  1038  1039  1040  1041  1042  1043  1044  1045  1046  1047  1048  1049  1050  1051  1052  1053  1054  1055  1056  1057  1058  1059  1060  1061  1062  1063  1064  1065  1066  1067  1068  1069  1070  1071  1072  1073  1074  1075  1076  1077  1078  1079  1080  1081  1082  1083  1084  1085  1086  1087  1088  1089  1090  1091  1092  1093  1094  1095  1096  1097  1098  1099  1100  1101  1102  1103  1104  1105  1106  1107  1108  1109  1110  1111  1112  1113  1114  1115  1116  1117  1118  1119  1120  1121  1122  1123  1124  1125  1126  1127  1128  1129  1130  1131  1132  1133  1134  1135  1136  1137  1138  1139  1140  1141  1142  1143  1144  1145  1146  1147  1148  1149  1150  1151  1152  1153  1154  1155  1156  1157  1158  1159  1160  1161  1162  1163  1164  1165  1166  1167  1168  1169  1170  1171  1172  1173  1174  1175  1176  1177  1178  1179  1180  1181  1182  1183  1184  1185  1186  1187  1188  1189  1190  1191  1192  1193  1194  1195  1196  1197  1198  1199  1200  1201  1202  1203  1204  1205  1206  1207  1208  1209  1210  1211  1212  1213  1214  1215  1216  1217  1218  1219  1220  1221  1222  1223  1224  1225  1226  1227  1228  1229  1230  1231  1232  1233  1234  1235  1236  1237  1238  1239  1240  1241  1242  1243  1244  1245  1246  1247  1248  1249  1250  1251  1252  1253  1254  1255  1256  1257  1258  1259  1260  1261  1262  1263  1264  1265  1266  1267  1268  1269  1270  1271  1272  1273  1274  1275  1276  1277  1278  1279  1280  1281  1282  1283  1284  1285  1286  1287  1288  1289  1290  1291  1292  1293  1294  1295  1296  1297  1298  1299  1300  1301  1302  1303  1304  1305  1306  1307  1308  1309  1310  1311  1312  1313  1314  1315  1316  1317  1318  1319  1320  1321  1322  1323  1324  1325  1326  1327  1328  1329  1330  1331  1332  1333  1334  1335  1336  1337  1338  1339  1340  1341  1342  1343  1344  1345  1346  1347  1348  1349  1350  1351  1352  1353  1354  1355  1356  1357  1358  1359  1360  1361  1362  1363  1364  1365  1366  1367  1368  1369  1370  1371  1372  1373  1374  1375  1376  1377  1378  1379  1380  1381  1382  1383  1384  1385  1386  1387  1388  1389  1390  1391  1392  1393  1394  1395  1396  1397  1398  1399  1400  1401  1402  1403  1404  1405  1406  1407  1408  1409  1410  1411  1412  1413  1414  1415  1416  1417  1418  1419  1420  1421  1422  1423  1424  1425  1426  1427  1428  1429  1430  1431  1432  1433  1434  1435  1436  1437  1438  1439  1440  1441  1442  1443  1444  1445  1446  1447  1448  1449  1450  1451  1452  1453  1454  1455  1456  1457  1458  1459  1460  1461  1462  1463  1464  1465  1466  1467  1468  1469  1470  1471  1472  1473  1474  1475  1476  1477  1478  1479  1480  1481  1482  1483  1484  1485  1486  1487  1488  1489  1490  1491  1492  1493  1494  1495  1496  1497  1498  1499  1500  1501  1502  1503  1504  1505  1506  1507  1508  1509  1510  1511  1512  1513  1514  1515  1516  1517  1518  1519  1520  1521  1522  1523  1524  1525  1526  1527  1528  1529  1530  1531  1532  1533  1534  1535  1536  1537  1538  1539  1540  1541  1542  1543  1544  1545  1546  1547  1548  1549  1550  1551  1552  1553  1554  1555  1556  1557  1558  1559  1560  1561  1562  1563  1564  1565  1566  1567  1568  1569  1570  1571  1572  1573  1574  1575  1576  1577  1578  1579  1580  1581  1582  1583  1584  1585  1586  1587  1588  1589  1590  1591  1592  1593  1594  1595  1596  1597  1598  1599  1600  1601  1602  1603  1604  1605  1606  1607  1608  1609  1610  1611  1612  1613  1614  1615  1616  1617  1618  1619  1620  1621  1622  1623  1624  1625  1626  1627  1628  1629  1630  1631  1632  1633  1634  1635  1636  1637  1638  1639  1640  1641  1642  1643  1644  1645  1646  1647  1648  1649  1650  1651  1652  1653  1654  1655  1656  1657  1658  1659  1660  1661  1662  1663  1664  1665  1666  1667  1668  1669  1670  1671  1672  1673  1674  1675  1676  1677  1678  1679  1680  1681  1682  1683  1684  1685  1686  1687  1688  1689  1690  1691  1692  1693  1694  1695  1696  1697  1698  1699  1700  1701  1702  1703  1704  1705  1706  1707  1708  1709  1710  1711  1712  1713  1714  1715  1716  1717  1718  1719  1720  1721  1722  1723  1724  1725  1726  1727  1728  1729  1730  1731  1732  1733  1734  1735  1736  1737  1738  1739  1740  1741  1742  1743  1744  1745  1746  1747  1748  1749  1750  1751  1752  1753  1754  1755  1756  1757  1758  1759  1760  1761  1762  1763  1764  1765  1766  1767  1768  1769  1770  1771  1772  1773  1774  1775  1776  1777  1778  1779  1780  1781  1782  1783  1784  1785  1786  1787  1788  1789  1790  1791  1792  1793  1794  1795  1796  1797  1798  1799  1800  1801  1802  1803  1804  1805  1806  1807  1808  1809  1810  1811  1812  1813  1814  1815  1816  1817  1818  1819  1820  1821  1822  1823  1824  1825  1826  1827  1828  1829  1830  1831  1832  1833  1834  1835  1836  1837  1838  1839  1840  1841  1842  1843  1844  1845  1846  1847  1848  1849  1850  1851  1852  1853  1854  1855  1856  1857  1858  1859  1860  1861  1862  1863  1864  1865  1866  1867  1868  1869  1870  1871  1872  1873  1874  1875  1876  1877  1878  1879  1880  1881  1882  1883  1884  1885  1886  1887  1888  1889  1890  1891  1892  1893  1894  1895  1896  1897  1898  1899  1900  1901  1902  1903  1904  1905  1906  1907  1908  1909  1910  1911  1912  1913  1914  1915  1916  1917  1918  1919  1920  1921  1922  1923  1924  1925  1926  1927  1928  1929  1930  1931  1932  1933  1934  1935  1936  1937  1938  1939  1940  1941  1942  1943  1944  1945  1946  1947  1948  1949  1950  1951  1952  1953  1954  1955  1956  1957  1958  1959  1960  1961  1962  1963  1964  1965  1966  1967  1968  1969  1970  1971  1972  1973  1974  1975  1976  1977  1978  1979  1980  1981  1982  1983  1984  1985  1986  1987  1988  1989  1990  1991  1992  1993  1994  1995  1996  1997  1998  1999  2000  2001  2002  2003  2004  2005  2006  2007  2008  2009  2010  2011  2012  2013  2014  2015  2016  2017  2018  2019  2020  2021  2022  2023  2024  2025  2026  2027  2028  2029  2030  2031  2032  2033  2034  2035  2036  2037  2038  2039  2040  2041  2042  2043  2044  2045  2046  2047  2048  2049  2050  2051  2052  2053  2054  2055  2056  2057  2058  2059  2060  2061  2062  2063  2064  2065  2066  2067  2068  2069  2070  2071  2072  2073  2074  2075  2076  2077  2078  2079  2080  2081  2082  2083  2084  2085  2086  2087  2088  2089  2090  2091  2092  2093  2094  2095  2096  2097  2098  2099  2100  2101  2102  2103  2104  2105  2106  2107  2108  2109  2110  2111  2112  2113  2114  2115  2116  2117  2118  2119  2120  2121  2122  2123  2124  2125  2126  2127  2128  2129  2130  2131  2132  2133  2134  2135  2136  2137  2138  2139  2140  2141  2142  2143  2144  2145  2146  2147  2148  2149  2150  2151  2152  2153  2154  2155  2156  2157  2158  2159  2160  2161  2162  2163  2164  2165  2166  2167  2168  2169  2170  2171  2172  2173  2174  2175  2176  2177  2178  2179  2180  2181  2182  2183  2184  2185  2186  2187  2188  2189  2190  2191  2192  2193  2194  2195  2196  2197  2198  2199  2200  2201  2202  2203  2204  2205  2206  2207  2208  2209  2210  2211  2212  2213  2214  2215  2216  2217  2218  2219  2220  2221  2222  2223  2224  2225  2226  2227  2228  2229  2230  2231  2232  2233  2234  2235  2236  2237  2238  2239  2240  2241  2242  2243  2244  2245  2246  2247  2248  2249  2250  2251  2252  2253  2254  2255  2256  2257  2258  2259  2260  2261  2262  2263  2264  2265  2266  2267  2268  2269  2270  2271  2272  2273  2274  2275  2276  2277  2278  2279  2280  2281  2282  2283  2284  2285  2286  2287  2288  2289  2290  2291  2292  2293  2294  2295  2296  2297  2298  2299  2300  2301  2302  2303  2304  2305  2306  2307  2308  2309  2310  2311  2312  2313  2314  2315  2316  2317  2318  2319  2320  2321  2322  2323  2324  2325  2326  2327  2328  2329  2330  2331  2332  2333  2334  2335  2336  2337  2338  2339  2340  2341  2342  2343  2344  2345  2346  2347  2348  2349  2350  2351  2352  2353  2354  2355  2356  2357  2358  2359  2360  2361  2362  2363  2364  2365  2366  2367  2368  2369  2370  2371  2372  2373  2374  2375  2376  2377  2378  2379  2380  2381  2382  2383  2384  2385  2386  2387  2388  2389  2390  2391  2392  2393  2394  2395  2396  2397  2398  2399  2400  2401  2402  2403  2404  2405  2406  2407  2408  2409  2410  2411  2412  2413  2414  2415  2416  2417  2418  2419  2420  2421  2422  2423  2424  2425  2426  2427  2428  2429  2430  2431  2432  2433  2434  2435  2436  2437  2438  2439  2440  2441  2442  2443  2444  2445  2446  2447  2448  2449  2450  2451  2452  2453  2454  2455  2456  2457  2458  2459  2460  2461  2462  2463  2464  2465  2466  2467  2468  2469  2470  2471  2472  2473  2474  2475  2476  2477  2478  2479  2480  2481  2482  2483  2484  2485  2486  2487  2488  2489  2490  2491  2492  2493  2494  2495  2496  2497  2498  2499  2500  2501  2502  2503  2504  2505  2506  2507  2508  2509  2510  2511  2512  2513  2514  2515  2516  2517  2518  2519  2520  2521  2522  2523  2524  2525  2526  2527  2528  2529  2530  2531  2532  2533  2534  2535  2536  2537  2538  2539  2540  2541  2542  2543  2544  2545  2546  2547  2548  2549  2550  2551  2552  2553  2554  2555  2556  2557  2558  2559  2560  2561  2562  2563  2564  2565  2566  2567  2568  2569  2570  2571  2572  2573  2574  2575  2576  2577  2578  2579  2580  2581  2582  2583  2584  2585  2586  2587  2588  2589  2590  2591  2592  2593  2594  2595  2596  2597  2598  2599  2600  2601  2602  2603  2604  2605  2606  2607  2608  2609  2610  2611  2612  2613  2614  2615  2616  2617  2618  2619  2620  2621  2622  2623  2624  2625  2626  2627  2628  2629  2630  2631  2632  2633  2634  2635  2636  2637  2638  2639  2640  2641  2642  2643  2644  2645  2646  2647  2648  2649  2650  2651  2652  2653  2654  2655  2656  2657  2658  2659  2660  2661  2662  2663  2664  2665  2666  2667  2668  2669  2670  2671  2672  2673  2674  2675  2676  2677  2678  2679  2680  2681  2682  2683  2684  2685  2686  2687  2688  2689  2690  2691  2692  2693  2694  2695  2696  2697  2698  2699  2700  2701  2702  2703  2704  2705  2706  2707  2708  2709  2710  2711  2712  2713  2714  2715  2716  2717  2718  2719  2720  2721  2722  2723  2724  2725  2726  2727  2728  2729  2730  2731  2732  2733  2734  2735  2736  2737  2738  2739  2740  2741  2742  2743  2744  2745  2746  2747  2748  2749  2750  2751  2752  2753  2754  2755  2756  2757  2758  2759  2760  2761  2762  2763  2764  2765  2766  2767  2768  2769  2770  2771  2772  2773  2774  2775  2776  2777  2778  2779  2780  2781  2782  2783  2784  2785  2786  2787  2788  2789  2790  2791  2792  2793  2794  2795  2796  2797  2798  2799  2800  2801  2802  2803  2804  2805  2806  2807  2808  2809  2810  2811  2812  2813  2814  2815  2816  2817  2818  2819  2820  2821  2822  2823  2824  2825  2826  2827  2828  2829  2830  2831  2832  2833  2834  2835  2836  2837  2838  2839  2840  2841  2842  2843  2844  2845  2846  2847  2848  2849  2850  2851  2852  2853  2854  2855  2856  2857  2858  2859  2860  2861  2862  2863  2864  2865  2866  2867  2868  2869  2870  2871  2872  2873  2874  2875  2876  2877  2878  2879  2880  2881  2882  2883  2884  2885  2886  2887  2888  2889  2890  2891  2892  2893  2894  2895  2896  2897  2898  2899  2900  2901  2902  2903  2904  2905  2906  2907  2908  2909  2910  2911  2912  2913  2914  2915  2916  2917  2918  2919  2920  2921  2922  2923  2924  2925  2926  2927  2928  2929  2930  2931  2932  2933  2934  2935  2936  2937  2938  2939  2940  2941  2942  2943  2944  2945  2946  2947  2948  2949  2950  2951  2952  2953  2954  2955  2956  2957  2958  2959  2960  2961  2962  2963  2964  2965  2966  2967  2968  2969  2970  2971  2972  2973  2974  2975  2976  2977  2978  2979  2980  2981  2982  2983  2984  2985  2986  2987  2988  2989  2990  2991  2992  2993  2994  2995  2996  2997  2998  2999  3000  3001  3002  3003  3004  3005  3006  3007  3008  3009  3010  3011  3012  3013  3014  3015  3016  3017  3018  3019  3020  3021  3022  3023  3024  3025  3026  3027  3028  3029  3030  3031  3032  3033  3034  3035  3036  3037  3038  3039  3040  3041  3042  3043  3044  3045  3046  3047  3048  3049  3050  3051  3052  3053  3054  3055  3056  3057  3058  3059  3060  3061  3062  3063  3064  3065  3066  3067  3068  3069  3070  3071  3072  3073  3074  3075  3076  3077  3078  3079  3080  3081  3082  3083  3084  3085  3086  3087  3088  3089  3090  3091  3092  3093  3094  3095  3096  3097  3098  3099  3100  3101  3102  3103  3104  3105  3106  3107  3108  3109  3110  3111  3112  3113  3114  3115  3116  3117  3118  3119  3120  3121  3122  3123  3124  3125  3126  3127  3128  3129  3130  3131  3132  3133  3134  3135  3136  3137  3138  3139  3140  3141  3142  3143  3144  3145  3146  3147  3148  3149  3150  3151  3152  3153  3154  3155  3156  3157  3158  3159  3160  3161  3162  3163  3164  3165  3166  3167  3168  3169  3170  3171  3172  3173  3174  3175  3176  3177  3178  3179  3180  3181  3182  3183  3184  3185  3186  3187  3188  3189  3190  3191  3192  3193  3194  3195  3196  3197  3198  3199  3200  3201  3202  3203  3204  3205  3206  3207  3208  3209  3210  3211  3212  3213  3214  3215  3216  3217  3218  3219  3220  3221  3222  3223  3224  3225  3226  3227  3228  3229  3230  3231  3232  3233  3234  3235  3236  3237  3238  3239  3240  3241  3242  3243  3244  3245  3246  3247  3248  3249  3250  3251  3252  3253  3254  3255  3256  3257  3258  3259  3260  3261  3262  3263  3264  3265  3266  3267  3268  3269  3270  3271  3272  3273  3274  3275  3276  3277  3278  3279  3280  3281  3282  3283  3284  3285  3286  3287  3288  3289  3290  3291  3292  3293  3294  3295  3296  3297  3298  3299  3300  3301  3302  3303  3304  3305  3306  3307  3308  3309  3310  3311  3312  3313  3314  3315  3316  3317  3318  3319  3320  3321  3322  3323  3324  3325  3326  3327  3328  3329  3330  3331  3332  3333  3334  3335  3336  3337  3338  3339  3340  3341  3342  3343  3344  3345  3346  3347  3348  3349  3350  3351  3352  3353  3354  3355  3356  3357  3358  3359  3360  3361  3362  3363  3364  3365  3366  3367  3368  3369  3370  3371  3372  3373  3374  3375  3376  3377  3378  3379  3380  3381  3382  3383  3384  3385  3386  3387  3388  3389  3390  3391  3392  3393  3394  3395  3396  3397  3398  3399  3400  3401  3402  3403  3404  3405  3406  3407  3408  3409  3410  3411  3412  3413  3414  3415  3416  3417  3418  3419  3420  3421  3422  3423  3424  3425  3426  3427  3428  3429  3430  3431  3432  3433  3434  3435  3436  3437  3438  3439  3440  3441  3442  3443  3444  3445  3446  3447  3448  3449  3450  3451  3452  3453  3454  3455  3456  3457  3458  3459  3460  3461  3462  3463  3464  3465  3466  3467  3468  3469  3470  3471  3472  3473  3474  3475  3476  3477  3478  3479  3480  3481  3482  3483  3484  3485  3486  3487  3488  3489  3490  3491  3492  3493  3494  3495  3496  3497  3498  3499  3500  3501  3502  3503  3504  3505  3506  3507  3508  3509  3510  3511  3512  3513  3514  3515  3516  3517  3518  3519  3520  3521  3522  3523  3524  3525  3526  3527  3528  3529  3530  3531  3532  3533  3534  3535  3536  3537  3538  3539  3540  3541  3542  3543  3544  3545  3546  3547  3548  3549  3550  3551  3552  3553  3554  3555  3556  3557  3558  3559  3560  3561  3562  3563  3564  3565  3566  3567  3568  3569  3570  3571  3572  3573  3574  3575  3576  3577  3578  3579  3580  3581  3582  3583  3584  3585  3586  3587  3588  3589  3590  3591  3592  3593  3594  3595  3596  3597  3598  3599  3600  3601  3602  3603  3604  3605  3606  3607  3608  3609  3610  3611  3612  3613  3614  3615  3616  3617  3618  3619  3620  3621  3622  3623  3624  3625  3626  3627  3628  3629  3630  3631  3632  3633  3634  3635  3636  3637  3638  3639  3640  3641  3642  3643  3644  3645  3646  3647  3648  3649  3650  3651  3652  3653  3654  3655  3656  3657  3658  3659  3660  3661  3662  3663  3664  3665  3666  3667  3668  3669  3670  3671  3672  3673  3674  3675  3676  3677  3678  3679  3680  3681  3682  3683  3684  3685  3686  3687  3688  3689  3690  3691  3692  3693  3694  3695  3696  3697  3698  3699  3700  3701  3702  3703  3704  3705  3706  3707  3708  3709  3710  3711  3712  3713  3714  3715  3716  3717  3718  3719  3720  3721  3722  3723  3724  3725  3726  3727  3728  3729  3730  3731  3732  3733  3734  3735  3736  3737  3738  3739  3740  3741  3742  3743  3744  3745  3746  3747  3748  3749  3750  3751  3752  3753  3754  3755  3756  3757  3758  3759  3760  3761  3762  3763  3764  3765  3766  3767  3768  3769  3770  3771  3772  3773  3774  3775  3776  3777  3778  3779  3780  3781  3782  3783  3784  3785  3786  3787  3788  3789  3790  3791  3792  3793  3794  3795  3796  3797  3798  3799  3800  3801  3802  3803  3804  3805  3806  3807  3808  3809  3810  3811  3812  3813  3814  3815  3816  3817  3818  3819  3820  3821  3822  3823  3824  3825  3826  3827  3828  3829  3830  3831  3832  3833  3834  3835  3836  3837  3838  3839  3840  3841  3842  3843  3844  3845  3846  3847  3848  3849  3850  3851  3852  3853  3854  3855  3856  3857  3858  3859  3860  3861  3862  3863  3864  3865  3866  3867  3868  3869  3870  3871  3872  3873  3874  3875  3876  3877  3878  3879  3880  3881  3882  3883  3884  3885  3886  3887  3888  3889  3890  3891  3892  3893  3894  3895  3896  3897  3898  3899  3900  3901  3902  3903  3904  3905  3906  3907  3908  3909  3910  3911  3912  3913  3914  3915  3916  3917  3918  3919  3920  3921  3922  3923  3924  3925  3926  3927  3928  3929  3930  3931  3932  3933  3934  3935  3936  3937  3938  3939  3940  3941  3942  3943  3944  3945  3946  3947  3948  3949  3950  3951  3952  3953  3954  3955  3956  3957  3958  3959  3960  3961  3962  3963  3964  3965  3966  3967  3968  3969  3970  3971  3972  3973  3974  3975  3976  3977  3978  3979  3980  3981  3982  3983  3984  3985  3986  3987  3988  3989  3990  3991  3992  3993  3994  3995  3996  3997  3998  3999  4000  4001  4002  4003  4004  4005  4006  4007  4008  4009  4010  4011  4012  4013  4014  4015  4016  4017  4018  4019  4020  4021  4022  4023  4024  4025  4026  4027  4028  4029  4030  4031  4032  4033  4034  4035  4036  4037  4038  4039  4040  4041  4042  4043  4044  4045  4046  4047  4048  4049  4050  4051  4052  4053  4054  4055  4056  4057  4058  4059  4060  4061  4062  4063  4064  4065  4066  4067  4068  4069  4070  4071  4072  4073  4074  4075  4076  4077  4078  4079  4080  4081  4082  4083  4084  4085  4086  4087  4088  4089  4090  4091  4092  4093  4094  4095  4096  4097  4098  4099  4100  4101  4102  4103  4104  4105  4106  4107  4108  4109  4110  4111  4112  4113  4114  4115  4116  4117  4118  4119  4120  4121  4122  4123  4124  4125  4126  4127  4128  4129  4130  4131  4132  4133  4134  4135  4136  4137  4138  4139  4140  4141  4142  4143  4144  4145  4146  4147  4148  4149  4150  4151  4152  4153  4154  4155  4156  4157  4158  4159  4160  4161  4162  4163  4164  4165  4166  4167  4168  4169  4170  4171  4172  4173  4174  4175  4176  4177  4178  4179  4180  4181  4182  4183  4184  4185  4186  4187  4188  4189  4190  4191  4192  4193  4194  4195  4196  4197  4198  4199  4200  4201  4202  4203  4204  4205  4206  4207  4208  4209  4210  4211  4212  4213  4214  4215  4216  4217  4218  4219  4220  4221  4222  4223  4224  4225  4226  4227  4228  4229  4230  4231  4232  4233  4234  4235  4236  4237  4238  4239  4240  4241  4242  4243  4244  4245  4246  4247  4248  4249  4250  4251  4252  4253  4254  4255  4256  4257  4258  4259  4260  4261  4262  4263  4264  4265  4266  4267  4268  4269  4270  4271  4272  4273  4274  4275  4276  4277  4278  4279  4280  4281  4282  4283  4284  4285  4286  4287  4288  4289  4290  4291  4292  4293  4294  4295  4296  4297  4298  4299  4300  4301  4302  4303  4304  4305  4306  4307  4308  4309  4310  4311  4312  4313  4314  4315  4316  4317 | P11171  P11171  P11171  P11171  P11171  P11171  P11171  P11171  P11171  P11171  P11171  P11171  P11171  Q13542  Q13542  P28223  P01009  P01009  P05067  P05067  P05067  P05067  O95477  O95477  O95477  Q8NE71  Q8NE71  Q8NE71  Q8NE71  Q8NE71  Q8NE71  Q8NE71  Q8NE71  Q13085  Q13085  Q13085  Q13085  Q13085  Q13085  Q13085  Q13085  Q13085  Q13085  Q13085  Q13085  Q13085  O00763  O00763  O00763  O00763  O00763  O00763  Q15027  P12821  Q9UKV3  Q9UKV3  Q9UKV3  Q9UKV3  Q9UKV3  Q9UKV3  Q9UKV3  Q9UKV3  Q9UKV3  Q9UKV3  Q9UKV3  Q9UKV3  Q9UKV3  Q9UKV3  Q9UKV3  Q9UKV3  Q9UKV3  Q9UKV3  Q9UKV3  Q9UKV3  Q9UKV3  Q9UKV3  Q9UKV3  Q9UKV3  Q9UKV3  Q9UKV3  Q9UKV3  Q9UKV3  Q9UKV3  Q9UKV3  Q9UKV3  Q9UKV3  Q9UKV3  P78536  P78536  O75689  P35611  P35611  P35611  P35611  P35611  P35611  P35611  P35611  P35611  P35611  P35611  P35611  P35611  P35611  P35611  P35611  P35611  P35612  P35612  P35612  P35612  P35612  P35612  P35612  P35612  P35612  P35612  P35612  P35612  P35612  P35612  P35612  P07550  P07550  P07550  P07550  P07550  P07550  P07550  Q9GZX7  P31751  P31751  P31751  P31751  P31751  P31751  P31751  P02768  P02768  P02768  P02768  P02768  P19021  P19021  P19021  Q9UJX6  Q9UJX6  Q9UJX6  Q9UJX6  P10275  P10275  P10275  P10275  Q9GZV1  O60678  O60678  Q9HCE9  P16066  P16066  P16066  P16066  P16066  O75179  O75179  O75179  O75179  O75179  O75179  O75179  O75179  O75179  O75179  O75179  O75179  O75179  O75179  O75179  O15084  O15084  P01008  P04083  P04083  P04083  P04083  P05549  Q9H1A4  Q9H1A4  Q9H1A4  Q9H1A4  Q9H1A4  Q9H1A4  Q9H1A4  Q9H1A4  Q9H1A4  Q9H1A4  Q9H1A4  Q9H1A4  Q9H1A4  Q9UJX5  Q9UJX5  Q9UJX5  Q9UJX5  Q9UJX4  Q8IW19  Q06481  P02652  P02652  P04114  P04114  P02649  O14791  O14791  P41181  Q92974  Q92974  Q92974  Q92974  Q92974  Q92974  Q92974  Q92974  Q92974  Q92974  Q92974  Q92974  Q92974  Q92974  Q92974  Q92974  Q92974  Q92974  Q14155  Q14155  Q14155  Q14155  P49407  Q9Y294  P78348  P15336  P15336  P15336  P15336  P15336  P15336  P15336  P15336  P15336  P15336  P15336  O75143  O75143  O75143  Q13315  Q13315  Q13315  Q13315  Q13315  P54259  P54259  P54259  P54259  P54259  P54259  P54259  P54259  P54259  P54259  Q13535  P54253  P54253  O14965  O14965  O14965  O15169  O15169  O15169  O15169  O15169  O15169  Q07817  Q07817  O43521  Q13829  Q13829  Q9BYV9  O75531  Q92934  Q92934  Q92934  Q92934  Q92934  P50895  P50895  P50895  P50895  P56945  P56945  P56945  P56945  P56945  P56945  P10415  P10415  P20749  P20749  Q9NYF8  Q9NYF8  Q9NYF8  Q9NYF8  Q9NYF8  Q9NYF8  Q9NYF8  Q9NYF8  Q9NYF8  Q9NYF8  Q9NYF8  Q9NYF8  Q9NYF8  Q9NYF8  Q9NYF8  Q9NYF8  Q9NYF8  Q9NYF8  Q9NYF8  Q9NYF8  Q9NYF8  Q9NYF8  Q9NYF8  Q9NYF8  Q9NYF8  Q9NYF8  Q9NYF8  P41182  P41182  Q14457  Q14457  Q14457  P30411  P30411  P30411  O95972  Q01954  Q01954  P12644  Q53HL2  Q53HL2  Q53HL2  Q53HL2  Q53HL2  Q53HL2  Q8N4F0  P15056  P15056  P15056  P15056  P15056  P15056  P15056  P15056  P38398  P38398  P38398  P38398  P38398  P38398  P38398  P38398  P38398  P38398  P38398  P38398  P38398  P38398  P38398  P38398  P38398  P38398  P38398  P38398  P38398  P51587  P51587  O60885  O60885  O60885  O60885  O60885  O60885  O60885  O60885  O60885  O60885  Q8TDC3  Q8TDC3  Q8TDC3  P78543  P78543  Q06187  Q06187  Q06187  Q06187  Q06187  Q06187  O60566  O60566  O60566  O60566  O60566  P00736  Q86YS7  Q86YS7  Q86YS7  Q86YS7  Q86YS7  Q86YS7  Q86YS7  Q86YS7  Q6P1N0  Q6P1N0  Q6P1N0  Q6P1N0  Q9Y5P4  Q9Y5P4  Q9Y5P4  P21730  P21730  P21730  P21730  P21730  P21730  Q9NZU7  P19022  P19022  Q86X55  P47710  P47710  P47710  P47710  P47710  P47710  P47710  P47710  P47710  P05814  P05814  P05814  P05814  Q14511  Q14511  P55212  Q14790  Q14790  Q14790  O14958  O14958  P51636  P51636  Q03135  Q13191  Q13191  Q13191  Q13191  Q13191  Q92793  Q92793  Q92793  Q92793  Q92793  Q92793  Q92793  Q96CT7  P14635  P14635  P14635  P14635  P24864  P24864  P24864  P51946  P51946  P51946  P51946  Q8ND76  Q8ND76  Q8ND76  Q8ND76  Q8ND76  Q8ND76  Q8ND76  Q8ND76  Q8ND76  Q8ND76  Q8ND76  Q8ND76  P21127  P21127  P21127  P09693  P09693  P16070  P16070  P16070  P16070  Q15078  Q13042  Q13042  Q13042  Q13042  Q12834  Q12834  Q12834  Q12834  Q12834  P30260  P30260  P30260  P30260  P30260  P30260  P30260  Q99459  Q99459  Q99459  Q99459  Q00536  Q00536  Q00536  Q00536  Q00536  Q00536  Q00536  Q00536  Q00536  P50613  P50613  P50613  P50750  P50750  P50750  P50750  O14519  P38936  P38936  P38936  P38936  P46527  P42771  P42771  P42771  P42771  Q9UPV0  P49715  P49715  P49450  P49450  P49450  P49450  Q9HC77  Q9HC77  Q9HC77  Q96BT3  Q96BT3  Q96BT3  Q96BT3  Q96BT3  Q53EZ4  Q53EZ4  Q53EZ4  Q8TCT0  Q8TCT0  P00450  P13569  P13569  P13569  P13569  P13569  P13569  P13569  P13569  P13569  P13569  P13569  P13569  P13569  Q9UNE7  Q9UNE7  Q9UNE7  Q9UNE7  O14757  O14757  O14757  O14757  O14757  O14757  O96017  O96017  O96017  O96017  P06276  Q96CF2  Q9Y2V2  Q9Y2V2  Q9Y2V2  Q9Y2V2  Q9Y2V2  Q9Y2V2  Q99828  Q9BUA3  Q9BUA3  Q9BUA3  Q9BUA3  Q07065  Q07065  Q07065  Q07065  Q07065  P49760  P49760  P49760  P10645  P10645  P10645  P10645  P10645  P10645  P10645  P10645  P10645  P34972  P34972  P34972  P01024  P01024  P01024  P01024  P01024  P01024  P01024  P01024  P0C0L4  P23528  P23528  P23528  Q9UMD9  P38432  P38432  P38432  P38432  P38432  P38432  P38432  Q99708  Q99708  Q99708  Q99708  Q99708  Q99708  Q99708  Q9BR76  Q9BV73  Q9BV73  Q9BV73  Q9BV73  Q6JBY9  Q6JBY9  Q6JBY9  Q6JBY9  Q6JBY9  Q6JBY9  Q6JBY9  Q6JBY9  Q6JBY9  Q6JBY9  Q6JBY9  Q6JBY9  Q6JBY9  Q6JBY9  Q6JBY9  P23508  Q6WN34  P16220  P16220  P16220  P34998  Q6UUV9  Q53ET0  Q53ET0  Q53ET0  Q53ET0  Q53ET0  Q53ET0  Q53ET0  Q53ET0  Q53ET0  Q53ET0  Q53ET0  Q53ET0  Q53ET0  Q53ET0  Q53ET0  Q53ET0  P02511  P02511  P02511  Q9Y2W7  P68400  P68400  P67870  P67870  P67870  O14936  O14936  O14936  P41240  Q13098  Q13098  Q13098  Q9UNS2  Q9UNS2  Q99627  P13611  Q13363  Q13363  O60716  O60716  O60716  O60716  O60716  O60716  O60716  O60716  O60716  O60716  O60716  O60716  O60716  O60716  O60716  O60716  O60716  O60716  O60716  O60716  O60716  O60716  O60716  O60716  P35222  P35222  P35222  P35222  P35222  P35222  P35222  P35222  P35222  P17302  P17302  P17302  P17302  P17302  P17302  P17302  P17302  P17302  P17302  P17302  P61073  P61073  P61073  P61073  P61073  P61073  P61073  P61073  Q9NQC7  Q9NQC7  O00622  P01034  Q5VWQ8  Q5VWQ8  Q9NYF0  Q9NYF0  P51397  P51397  P51397  P51397  P53355  P53355  P53355  P53355  Q9UER7  Q9UER7  Q9UER7  Q9UER7  Q9UER7  Q9UER7  Q9UER7  Q9UER7  Q9UER7  Q9UER7  Q9Y6G9  Q9Y6G9  Q9Y6G9  Q9Y6G9  Q9Y6G9  Q9Y6G9  Q9Y6G9  Q9Y6G9  P27707  P27707  P27707  Q96SD1  O43602  O43602  O43602  O43602  O43602  O43602  O43602  O43602  O43602  O43602  O43602  O43602  O43602  O43602  O43602  O43602  Q9NX09  Q9NX09  Q9NR30  Q9NR30  Q9NR30  Q9NR30  Q9NR30  Q9NR30  Q9NR30  O00571  O00571  O00571  O00571  O95786  O95786  P35659  P35659  P35659  P35659  P35659  P35659  P35659  P35659  P35659  P35659  P35659  P35659  P35659  P35659  P35659  P35659  P35659  P35659  P35659  P35659  P35659  Q08495  Q08495  Q08495  Q08495  Q08495  Q08495  Q08495  Q08495  Q08495  Q08495  P17661  P17661  P17661  P17661  Q15398  Q15398  Q15398  Q15398  Q15398  Q15398  Q15398  Q15398  Q15398  Q15398  Q15398  Q15398  Q13217  Q9H3Z4  Q9H3Z4  Q9H3Z4  O00429  O00429  O00429  O00429  P26358  P26358  P26358  P26358  P26358  P26358  P26358  P26358  P26358  Q9UKG1  Q9UKG1  Q8TB45  Q8TB45  Q8TB45  Q8TB45  Q8TB45  Q8TB45  Q8TB45  Q8TB45  Q8TB45  Q8TB45  Q8TB45  Q16643  Q16643  Q16643  Q16643  Q16643  Q9BY84  Q9BY84  Q13115  Q13115  P33316  Q92997  Q92997  Q92997  Q92997  P63167  Q13627  Q13627  Q13627  Q13627  Q92630  Q92630  P19525  P19525  P19525  P68104  P29692  P29692  P29692  O00418  O00418  O00418  O00418  O00418  O00418  O00418  O00418  O00418  O00418  O00418  O00418  O00418  O00418  O00418  P13639  P13639  Q14152  Q14152  Q14152  Q14152  Q14152  Q14152  P55884  P55884  P55884  P55884  P55884  P55884  P55884  P55884  P55884  P55884  Q99613  Q99613  Q99613  Q99613  Q99613  Q99613  Q99613  Q99613  Q99613  O00303  O00303  O75821  O75821  O75821  O15372  O75822  O75822  O75822  O75822  P19419  P19419  P19419  P19419  P50402  P50402  P50402  P50402  P50402  P50402  P50402  Q7L5Y1  P14625  P14625  P14625  O43768  O43768  O43768  P29317  P29317  P29317  P29317  P29317  P29317  O95278  Q9Y6I3  Q9Y6I3  Q9Y6I3  Q9Y6I3  Q9Y6I3  Q9Y6I3  P24390  P50548  P50548  P50548  P50548  P50548  P50548  P50548  P50548  P50548  P11474  P11474  Q14674  Q14674  Q14674  Q14674  P03372  P03372  P03372  P03372  Q92731  Q92731  P15036  P15036  P15036  P43268  P43268  P41212  P41212  P41212  Q9H8M9  Q9UQ84  Q9UQ84  Q9UQ84  Q9UQ84  Q9UQ84  Q9UQ84  Q9UQ84  Q9UQ84  Q9UQ84  Q9UQ84  Q99504  Q99504  Q15910  Q15910  P50502  Q6UWZ7  Q6UWZ7  Q6UWZ7  Q16875  Q16875  P12259  Q9BSJ6  Q9BSJ6  Q9BSJ6  Q9BSJ6  Q9BSJ6  A6ND36  A6ND36  A6ND36  A6ND36  A6ND36  A6ND36  A6ND36  A6ND36  A6ND36  P00740  P00740  Q9BXW9  Q9BXW9  Q9BXW9  Q9BXW9  Q9BXW9  Q9BXW9  Q9BXW9  Q9BXW9  Q9BXW9  Q9BXW9  Q13158  Q05397  Q05397  Q05397  Q05397  Q05397  Q05397  Q9HB96  Q9HB96  Q9NVI1  Q9NVI1  Q9NVI1  Q9NVI1  O15287  Q9BX63  Q9BX63  Q9BX63  Q9BX63  Q9BX63  P35555  Q5XUX0  Q5XUX0  Q9UKT5  Q969U6  Q969H0  P39748  P39748  P02771  P02771  P02771  P02771  P02771  P02771  P02765  P02765  P02765  P02765  P02765  P02765  P02671  P02671  P02671  P02671  P02671  P02671  P02671  P02671  P02671  P02671  P02671  P02671  P02671  P02679  P02751  Q13045  Q13045  Q13045  Q14315  Q14315  Q14315  P36888  P36888  P36888  Q06787  Q06787  Q06787  P01100  P01100  Q99958  Q99958  Q99958  Q99958  Q99958  Q99958  Q99958  Q99958  Q99958  Q08050  Q08050  Q08050  Q08050  Q08050  Q12778  Q12778  Q12778  Q12778  Q12778  Q12778  Q12778  Q12778  Q12778  Q12778  Q12778  P98177  P98177  O43524  O43524  O43524  O43524  O43524  O43524  O43524  O43524  O43524  O43524  O43524  O43524  Q9BZS1  Q9BZS1  Q16658  Q16658  Q12841  O95633  Q92945  Q92945  Q92945  Q92945  Q92945  Q9BTY2  P09958  P09958  Q9Y2I7  Q9Y2I7  Q9Y2I7  Q9Y2I7  Q9Y2I7  Q9Y2I7  Q13283  Q13283  Q13283  Q13283  P04406  P04406  P04406  P04406  P04406  P04406  P06744  Q9UQC2  Q9UQC2  Q9UQC2  Q9UQC2  Q9UQC2  Q9UQC2  Q9UQC2  Q9UQC2  Q9UQC2  Q9UQC2  Q9UQC2  Q9UQC2  Q9UQC2  Q9UQC2  Q9UQC2  Q9UQC2  Q9UQC2  P22466  Q14393  P01350  P15976  P15976  P15976  P15976  P15976  P15976  P30793  P30793  P04150  P04150  P04150  P04150  P04150  P04150  P04150  O75496  O75496  O75496  P10070  P10070  P10070  P10070  P10070  P10070  P10071  P10071  P10071  P10071  P10071  P10071  P10071  P14314  P14314  P14314  P14314  P14314  P14314  Q8NBJ4  Q8NBJ4  P13224  P51654  Q13322  Q13322  Q13322  Q13322  Q13322  Q13322  Q3V6T2  Q3V6T2  Q3V6T2  Q3V6T2  Q13002  Q13002  P34947  Q8WTQ7  P49841  P49841  P78347  P78347  P78347  P78347  P78347  P78347  P78347  P14672  P13807  P13807  P13807  P13807  P13807  P13807  P13807  P13807  P13807  P13807  P16401  P16401  P16401  P16401  P16401  Q6DN03  Q6DN03  Q16695  Q16695  Q16695  Q16695  P62805  P62805  Q8TF76  Q8TF76  Q8TF76  Q8TF76  Q9BY41  Q8WUI4  Q8WUI4  Q8WUI4  Q8WUI4  Q8WUI4  Q8WUI4  Q8WUI4  Q13547  Q13547  Q13547  Q9UQL6  Q9UQL6  Q9UQL6  P42858  P42858  P42858  P42858  P42858  P42858  P05546  Q16665  Q16665  Q16665  Q16665  Q16665  P54198  P54198  P54198  P54198  P54198  P54198  P54198  P54198  P54198  P15515  Q8NCD3  Q8NCD3  Q8NCD3  Q8NCD3  Q8NCD3  Q8NCD3  Q8NCD3  Q8NCD3  P17096  P17096  P17096  P17096  P17096  P17096  P05114  P05114  P05114  P05114  P05114  P05114  Q9UK76  Q9UK76  Q9UK76  Q9UK76  P41235  P41235  P41235  P41235  P07910  P07910  P07910  P07910  P07910  P07910  P07910  P07910  P07910  P07910  P07910  P07910  Q1KMD3  Q1KMD3  P61978  P61978  P61978  P61978  P61978  P61978  P14866  P14866  P14866  P14866  P14866  P14866  P35367  P35367  P07900  P07900  P07900  P07900  Q00613  Q00613  Q00613  Q00613  Q00613  Q00613  Q00613  Q00613  Q00613  Q00613  Q00613  Q9ULV5  P04792  P04792  P04792  P04792  P04792  P04792  P04792  P04792  P04792  Q7Z5L9  Q7Z5L9  Q7Z5L9  Q7Z5L9  Q7Z5L9  Q7Z5L9  Q7Z5L9  Q7Z5L9  P08833  P08833  P08833  P08833  P08833  P08833  P08833  P08833  P08833  P17936  P17936  P22692  P24593  Q16270  P54105  P54105  P46695  P46695  Q16666  Q16666  Q16666  Q16666  Q16666  Q16666  Q16666  O60841  O60841  O60841  O60841  O60841  O60841  O60841  O60841  O60841  O60841  O60841  O60841  O60841  O60841  O60841  O60841  O60841  P23588  P23588  P23588  P23588  P23588  P23588  P23588  P23588  P23588  P23588  P23588  P23588  P23588  P23588  P06730  Q04637  Q04637  Q04637  Q04637  Q04637  Q04637  Q04637  Q04637  Q04637  Q04637  O43432  O43432  O43432  O43432  O43432  P56537  P56537  P56537  P56537  P56537  P56537  Q15653  Q15653  Q15653  Q15653  Q15653  P25963  P25963  P25963  P25963  P25963  O14920  O14920  O14920  O14920  O14920  O14920  O14920  O14920  O14920  O14920  O14920  O14920  O14920  O14920  Q14164  Q14164  Q13422  Q13422  Q13422  Q13422  Q13422  Q13422  Q13422  Q13422  Q13422  Q13422  Q13422  Q13422  Q13422  Q13422  P40189  P40189  P40189  P40189  P05231  Q0VD86  Q0VD86  Q0VD86  Q9NQS7  Q9NQS7  Q9NQS7  Q9NQS7  Q9NQS7  Q9NQS7  Q9NQS7  Q9NQS7  Q9NQS7  Q9NQS7  Q9NQS7  Q9NQS7  Q9NQS7  Q9NQS7  Q9NQS7  Q9NQS7  Q9NQS7  Q9NQS7  Q9NQS7  Q9NQS7  Q9NQS7  Q9NQS7  Q9NQS7  Q9NQS7  Q6NXS1  Q6NXS1  Q6NXS1  P46940  P46940  P46940  P46940  Q9NWZ3  Q14653  Q14653  Q14653  Q14653  Q14653  Q14653  Q14653  Q13568  Q13568  Q13568  Q13568  Q13568  Q13568  Q13568  Q92985  Q92985  P35568  P35568  P35568  P35568  P35568  P35568  P35568  P35568  P35568  P35568  P35568  P35568  P13612  P05107  P05107  Q96J02  Q96J02  Q96J02  P19823  P19823  P19823  Q9UQF2  Q9UQF2  Q9UQF2  Q9UQF2  Q9UQF2  Q9UQF2  Q9UQF2  Q9UQF2  Q9UQF2  Q9UQF2  Q9UQF2  Q9UQF2  Q9UQF2  Q9UQF2  Q9UQF2  Q9UQF2  Q9UQF2  Q9UQF2  Q9UQF2  Q9UQF2  Q9UQF2  Q9UQF2  Q9UQF2  Q9UPT6  Q9UPT6  Q9UPT6  Q9UPT6  Q9BR39  Q9BR39  Q9BR39  Q9BR39  P05412  P05412  P05412  P05412  P05412  P13646  P05783  P05783  P05783  P05783  P05783  P05783  P05783  P05783  P05783  P05783  P05783  P05783  P05783  P05783  P05783  P05783  P05783  P05783  P05783  P35900  Q14678  Q14678  P17612  P17612  P17612  Q92993  Q92993  O95251  O95251  O95251  O95251  O95251  O95251  O95251  O95251  Q13303  Q13303  Q13303  Q13303  Q16566  Q16566  Q16566  Q16566  Q14654  O60928  O60928  Q03721  Q03721  Q03721  Q03721  P48048  O95069  O43526  Q9NQT8  Q9NQT8  Q9NQT8  Q9NQT8  Q9NQT8  Q9NQT8  Q9NQT8  Q9NQT8  Q9NQT8  Q9NQT8  Q9NQT8  O95235  O95235  O95235  O95235  O95235  O95235  O95235  O95235  O95235  Q8IX03  Q8IX03  Q8IX03  Q8IX03  Q8IX03  Q8IX03  P52732  Q8N4N8  Q8N4N8  Q99661  Q99661  Q99661  Q99661  Q99661  Q99661  Q99661  Q99661  Q99661  P04183  P04183  P10721  P10721  P10721  P10721  P10721  Q07866  Q07866  P01042  P05771  P05771  Q15139  Q15139  Q15139  Q15139  Q15139  Q15139  Q15139  Q15139  Q15139  Q15139  Q9BZL6  Q9BZL6  Q9BZL6  Q9BZL6  Q9BZL6  Q9BZL6  Q9BZL6  Q05655  Q05655  Q05655  Q05655  Q05655  Q05655  Q05655  Q05655  Q05655  Q05655  Q05655  Q02156  Q02156  Q02156  Q02156  Q02156  Q02156  Q02156  Q02156  Q04759  Q04759  Q04759  Q04759  O60256  O60256  O60256  O75582  O75582  O75582  O75582  O75582  O75582  O75582  O75582  O75582  O75582  O75582  O75582  Q9UK32  Q9UK32  Q9UK32  P23443  P23443  P23443  P23443  P23443  P43405  P43405  P43405  P43405  P43405  P43405  P43405  P43405  P43405  Q86UP2  Q86UP2  Q86UP2  O75449  O75449  O75449  P32004  P32004  P32004  P32004  Q15334  P55268  P11047  P11047  Q6PKG0  Q6PKG0  Q6PKG0  Q6PKG0  Q6PKG0  Q6PKG0  Q6PKG0  Q6PKG0  Q6PKG0  Q6PKG0  Q6PKG0  Q6PKG0  Q6PKG0  Q6PKG0  Q6PKG0  Q6PKG0  Q6PKG0  Q6PKG0  O95835  O95835  O95835  O95835  O95835  Q9NRM7  P05455  Q14739  Q14739  Q14739  Q14739  Q14739  P09382  P17931  P17931  P16150  P16150  P16150  P16150  Q9UBR4  Q9UBR4  Q9UBR4  P53667  P53667  P53667  P53667  P53667  P53667  P09960  P02545  P02545  P02545  P02545  P02545  P02545  P02545  P02545  P02545  P02545  P02545  P02545  P02545  P02545  P02545  P02545  P02545  P02545  P02545  P02545  P02545  P02545  P02545  P09917  P09917  Q96CN5  O75581  O75581  O75581  Q9Y608  Q9Y608  Q9Y608  Q9Y608  Q9Y608  Q9Y608  Q9Y608  P33241  P33241  P33241  P33241  P33241  P33241  P33241  Q9HCC9  Q14766  Q14766  Q16584  Q16584  Q16584  Q16584  Q16584  Q16584  Q16584  Q16584  Q16584  Q16584  Q16584  Q16584  Q16584  Q16584  Q16584  Q16584  Q16584  Q16584  Q16584  Q16584  Q99683  Q99683  Q99683  Q99683  Q99683  P41279  P41279  P41279  P41279  O43318  O43318  O43318  Q99759  Q99759  Q99759  Q99759  P80192  Q92918  Q92918  Q92918  Q92918  Q92918  Q92918  Q9H063  Q9H063  Q9H063  Q9H063  Q9H063  Q9H063  Q8NHW3  Q8NHW3  Q8NHW3  Q8NHW3  P27816  P27816  P27816  P27816  P27816  P27816  P27816  P27816  P27816  P27816  P27816  P27816  P27816  P27816  P27816  P27816  P27816  P27816  P27816  P27816  P27816  P27816  P49137  P49137  P49137  Q8IW41  Q8IW41  Q16655  P29966  P29966  P29966  P29966  P29966  P29966  P29966  P29966  P29966  P29966  P29966  P29966  P29966  P29966  P29966  P29966  P29966  P29966  O15232  Q96EY5  Q96EY5  Q96EY5  Q96EY5  Q96EY5  Q9H7P6  Q9H7P6  Q9H7P6  P02686  P02686  P02686  P02686  P02686  P02686  Q14703  Q07820  Q07820  Q07820  P49736  P49736  P49736  P49736  P49736  P49736  P49736  P49736  P49736  P49736  P25205  P25205  P25205  P25205  P25205  P25205  Q9ULC4  Q13257  Q13257  Q13257  Q00987  Q00987  Q00987  Q00987  Q00987  Q00987  Q00987  Q00987  Q00987  Q00987  Q00987  O15151  O15151  Q14814  Q14814  Q14814  Q14814  Q14814  Q14814  Q06413  Q06413  Q06413  Q14680  Q14680  Q14680  Q14680  Q14680  Q14680  Q14680  Q14680  Q14680  Q14680  Q14680  Q6ZN04  Q08431  P08493  P08493  P08493  Q9UM21  Q5JRA6  Q5JRA6  Q5JRA6  Q5JRA6  Q5JRA6  Q5JRA6  Q5JRA6  Q5JRA6  Q5JRA6  Q5JRA6  Q5JRA6  Q8IVT2  Q8IVT2  Q8IVT2  Q8IVT2  Q8IVT2  Q8IVT2  Q8IVT2  Q8IVT2  Q8IVT2  Q8IVT2  Q8IVT2  Q8IVT2  Q8IVT2  Q8IVT2  Q8IVT2  Q8IVT2  O75030  O75030  O75030  O75030  O75030  Q16659  Q9BYG3  Q9BYG3  Q9BYG3  O14950  P58340  Q8NB16  Q8NB16  Q8NB16  Q9H492  Q9NYL2  Q9NYL2  Q9NYL2  Q9NYL2  Q9NYL2  Q9NYL2  Q9NYL2  P03956  Q13163  P36507  P36507  P36507  P36507  P36507  P46734  P46734  P46734  P30304  P30304  P30304  P30304  P30304  P30304  P30304  P30304  P30304  P30304  P30305  P30305  P30305  P30305  P30305  P30307  P30307  P30307  P30307  P30307  P30307  P30307  P30307  P30307  P30307  P30307  P30307  Q8TAP9  Q8TAP9  Q8TAP9  Q8TAP9  Q8TAP9  Q8TAP9  Q8TAP9  Q8TAP9  Q5VT25  Q5VT25  Q5VT25  Q5VT25  Q5VT25  Q5VT25  Q5VT25  Q5VT25  Q5VT25  Q9Y6F6  Q9Y6F6  Q9Y6F6  Q9Y6F6  Q13421  Q13614  Q13614  P42345  P42345  P42345  P42345  P42345  P42345  P15941  Q9BRK3  P10244  P10244  P04198  P04198  P01106  P01106  P01106  P01106  P01106  O14974  O14974  O14974  O14974  O14974  O14974  O14974  O14974  O14974  O14974  O14974  O14974  O14974  O14974  O14974  O14974  O14974  P41227  P41227  P41227  P41227  P41227  P41227  Q13765  Q13765  Q13765  Q13765  Q13765  P0DI83  P0DI83  P0DI83  P0DI83  P0DI83  O60934  O60934  O60934  O60934  O60934  Q09161  Q09161  P14598  P14598  P14598  P14598  P14598  P14598  Q15080  Q15788  Q15788  Q15788  Q15788  Q15788  Q15788  Q15788  Q15788  Q9Y6Q9  Q9Y6Q9  Q9Y6Q9  Q9Y6Q9  Q9Y6Q9  Q9Y6Q9  Q9Y6Q9  Q14686  O14777  O14777  Q92597  Q92597  Q92597  Q92597  Q92597  Q92597  Q96PU5  Q96PU5  Q96PU5  Q96PU5  Q96PU5  Q96PU5  Q96PU5  Q96PU5  Q8NHV4  Q8NHV4  Q8NHV4  Q8NHV4  Q8NHV4  P51955  P51955  P51955  P51955  P51955  P51955  P51955  P51955  P51955  P51955  P51955  P51955  Q8NG66  Q9HC98  Q9HC98  Q9Y6K9  Q9Y6K9  Q9Y6K9  Q9Y6K9  Q9Y6K9  Q9Y6K9  Q16236  Q16236  O95644  O95644  O95644  Q14934  Q14934  Q14934  Q14934  Q14934  Q14934  O94916  O94916  O94916  P19838  P19838  P19838  P19838  P19838  P19838  P19838  Q00653  Q00653  Q00653  Q00653  Q00653  O14745  O14745  O14745  O14745  O14745  O14745  O14745  O14745  Q96TA1  Q96TA1  Q96TA1  Q96TA1  Q96TA1  Q96TA1  Q96TA1  Q96TA1  Q96TA1  Q96TA1  Q96TA1  Q96TA1  Q96TA1  Q86WB0  Q86WB0  Q86WB0  Q86WB0  Q86WB0  Q86WB0  Q86WB0  Q86WB0  Q86WB0  Q86WB0  Q86WB0  Q86WB0  Q86WB0  P29474  P29474  P29474  P29474  P29474  Q6P988  Q86UR1  Q86UR1  Q14207  Q14207  Q14207  P06748  P06748  P06748  P06748  P06748  P06748  P06748  P06748  P06748  P06748  P06748  P20393  P20393  P22736  P22736  P10588  P10588  Q16656  Q16656  Q16656  Q16656  Q16656  Q08J23  Q08J23  Q08J23  Q08J23  Q08J23  Q08J23  Q08J23  O60285  O60285  O60285  Q02818  Q02818  Q9Y266  Q9Y266  Q9Y266  Q7Z417  Q7Z417  Q7Z417  Q7Z417  Q7Z417  Q7Z417  Q7Z417  Q7Z417  Q7Z417  Q7Z417  Q7Z417  Q7Z417  Q14980  Q14980  Q14980  Q14980  Q14980  Q14980  Q14980  Q14980  Q14980  Q14980  Q14980  Q14980  Q14980  Q14980  Q14980  Q14980  Q14980  Q14980  Q14980  Q14980  Q14980  Q14980  Q14980  Q14980  Q14980  Q14980  Q14980  Q14980  Q14980  Q14980  Q14980  Q14980  Q9BXS6  Q9BXS6  Q9BXS6  Q9BXS6  Q9BXS6  Q9BXS6  Q9BXS6  Q16625  Q16625  Q16625  Q16625  Q16625  Q16625  Q16625  P08559  P08559  P08559  P08559  Q96CV9  Q96CV9  Q96CV9  P10451  P10451  P10451  P10451  P10451  P10451  P10451  P10451  P10451  P10451  P10451  P10451  P10451  P10451  P10451  P10451  P10451  P10451  P10451  P10451  P10451  P10451  P10451  P10451  P10451  P10451  P10451  P10451  P10451  P10451  P10451  P10451  P10451  P10451  P10451  P10451  P10451  P10451  Q96G74  Q96G74  Q96G74  Q96G74  P51575  P51575  O00443  O00443  O00443  O00443  O00443  P04637  P04637  P04637  P04637  P04637  P04637  P04637  P04637  P04637  P04637  P47712  P47712  P47712  P47712  Q9UQ80  Q9UQ80  Q6TGC4  Q6TGC4  Q15004  Q15004  Q13153  Q13153  Q13153  Q13153  Q13153  Q13153  Q13153  Q13153  Q13153  Q13153  O96013  O96013  O96013  O96013  O96013  O96013  O96013  O96013  O96013  O96013  Q8WX93  Q8WX93  Q8WX93  Q8WX93  Q8WX93  Q8WX93  Q8WX93  Q8WX93  Q8WX93  Q8WX93  Q8WX93  Q8WX93  Q8TEW0  Q8TEW0  Q8TEW0  Q8TEW0  Q8TEW0  Q8TEW0  Q8TEW0  Q8TEW0  Q8TEW0  Q8TEW0  Q86W56  Q86W56  Q86W56  Q86W56  Q86W56  Q86W56  Q86W56  Q86W56  Q9H300  Q9H300  O95453  O95453  O95453  O95453  O95453  P23760  P23760  P23760  P49023  P49023  P49023  P49023  P49023  P49023  P49023  P49023  P49023  P49023  P49023  Q9BYE7  Q9BYE7  Q8NBP7  Q8NBP7  Q9Y365  Q9Y365  Q9Y365  Q53EL6  Q53EL6  Q53EL6  Q53EL6  Q53EL6  Q53EL6  Q53EL6  Q53EL6  Q53EL6  Q53EL6  P27815  P27815  P27815  P27815  O60658  O60658  O60658  Q15084  Q15084  Q15084  Q9BY77  Q9BY77  Q9BY77  Q9BY77  Q9BY77  O15530  O15530  O15530  O15530  O15530  O15530  O15530  O15530  O15530  P52945  P35408  P35408  P35408  P35408  Q5VY43  Q5VY43  P36955  P36955  P36955  O15055  O15055  O15055  O15055  O15055  O15055  O15055  O15055  O15055  O15055  O15055  O15055  O15055  O00264  O00264  O00264  Q96PD5  P00439  O75151  O75151  O75151  O75151  O75151  O75151  O75151  Q9UPP1  Q9UPP1  Q9UPP1  Q9UPP1  Q9UPP1  Q9UPP1  Q9UPP1  Q13371  Q13371  Q13371  Q9UBF8  Q9UBF8  Q9UBF8  Q9UBF8  Q9UBF8  Q9UBF8  O60331  Q9HB75  Q9HB75  P11309  P11309  P11309  Q13526  Q13526  Q9Y237  Q9BXM7  Q9BXM7  O00562  O00562  O00562  O00562  O43164  O43164  O43164  O43164  P42338  P48736  P98161  P19174  P19174  Q15149  Q15149  Q15149  Q15149  Q15149  Q15149  Q15149  Q15149  Q15149  Q15149  Q15149  Q15149  Q15149  Q15149  Q15149  Q15149  Q15149  Q15149  Q15149  Q15149  Q15149  Q15149  Q15149  Q15149  Q15149  Q15149  Q15149  Q15149  Q15149  P08567  P08567  P53350  P53350  P53350  P53350  P53350  P53350  P00747  P00747  O00168  O00168  Q96AD5  Q96AD5  Q96AD5  P13796  P13796  P13796  P13796  P13796  P13796  P29590  P29590  P29590  P29590  P29590  P29590  P29590  P29590  P29590  P29590  P29590  P29590  P29590  Q99640  Q99640  Q99640  Q99640  Q99640  Q99640  P14859  P14859  P14859  Q01860  Q01860  Q01860  Q01860  Q01860  P26678  Q6ZSY5  Q6ZSY5  Q6ZSY5  Q6ZSY5  P02812  P02812  P02812  P02812  O43663  O43663  O43663  O43663  O43663  O43663  O43663  P06401  P06401  P06401  P06401  P06401  P06401  P06401  P06401  P06401  P06401  P06401  P78527  P78527  P78527  P78527  P78527  O75569  O75569  O75569  O60260  P01236  P01236  P01236  P01236  P01236  P04070  P07737  P07737  O95084  Q86WR7  Q86WR7  Q86WR7  Q86WR7  Q86WR7  P25788  P25788  P61289  P61289  P49768  P49768  P49768  P49768  P60484  P60484  P60484  P06454  P06454  P06454  P18031  P18031  P18031  P18031  P18031  P18031  Q9Y2R2  Q9Y2R2  Q9Y2R2  Q9Y2R2  P54829  P54829  P35236  P35236  P35236  Q03393  Q6NZI2  Q6NZI2  Q6NZI2  Q6NZI2  Q6NZI2  Q6NZI2  Q6NZI2  Q6NZI2  Q6NZI2  Q6NZI2  Q6NZI2  Q6NZI2  Q6NZI2  O95997  Q14671  Q14671  Q14671  Q14671  Q14671  Q14671  Q14671  Q14671  Q14671  Q14671  P06737  P06737  P27708  P27708  P27708  P27708  O00391  P20338  O75943  O75943  Q99638  Q99638  Q99638  Q99638  Q99638  Q99638  Q99638  P46060  P46060  P46060  P46060  Q9UKM9  Q9UKM9  Q9UKM9  Q9UKM9  Q9UKM9  P10276  P10276  P10276  P10276  Q15291  Q15291  Q15291  Q15291  Q15291  P28749  P28749  P28749  P28749  P28749  P28749  P28749  P28749  P28749  Q08999  Q08999  Q08999  Q08999  Q08999  Q08999  Q08999  Q08999  Q08999  Q08999  Q08999  Q08999  P49756  P49756  P49756  P49756  Q9BWF3  P06400  P06400  P06400  P06400  P06400  P06400  P06400  P18754  P18754  Q15293  Q15293  Q04864  Q04864  Q92900  Q92900  Q92900  Q92900  Q92900  Q92900  P15927  P15927  P15927  P15927  P15927  Q7L804  O75154  O75154  O75154  O75154  O75154  O75154  O75154  Q6PCD5  Q6PCD5  Q9H0H5  Q9H0H5  Q9H0H5  Q9H0H5  Q9H0H5  Q9H0H5  Q9H0H5  Q9H0H5  Q9H0H5  Q9H0H5  Q9H0H5  Q9H0H5  Q2PPJ7  Q2PPJ7  Q2PPJ7  Q2PPJ7  Q2PPJ7  O14827  O14827  Q9NS28  P49795  P49795  P49795  P49802  Q8N264  Q8N264  Q8N264  Q8N264  Q8N264  Q13671  Q13671  Q13671  Q13671  Q13671  Q13671  Q13671  Q13671  Q13546  Q13546  Q13546  Q13546  Q13546  Q13546  Q13546  Q13546  O43353  O43353  O43353  O43353  O43353  O43353  O43353  O43353  Q9Y572  Q9Y572  Q9Y572  Q9Y572  Q9Y572  Q9Y572  Q15835  Q15835  Q15835  P40429  P05386  P05387  P05387  P05387  P05387  P05387  O94763  Q15287  Q15287  Q15287  Q16518  Q684P5  Q684P5  Q684P5  Q684P5  Q684P5  Q8N122  Q8N122  Q8N122  Q8N122  Q8N122  Q8N122  Q8N122  Q8N122  Q8N122  Q8N122  P62979  P23396  P23396  P23396  P62753  P62753  P62753  P62753  P62753  P62753  Q9NQC3  Q9NQC3  Q9NQC3  Q9NQC3  Q9NQC3  Q9NQC3  Q9NQC3  Q9NQC3  Q13950  P19793  P19793  P19793  P19793  P19793  P19793  Q92736  Q92736  Q92736  Q92504  Q92504  Q9Y6R1  Q9Y6R1  Q9Y6R1  Q9Y6R1  Q9Y6R1  Q9HBV2  Q9UJQ4  Q9UJQ4  Q9UJQ4  Q9UJQ4  Q9UJQ4  Q9UJQ4  Q01826  O75995  O75995  O75995  O75995  O75995  O75995  O75995  O75995  P60468  P60468  P60468  P60468  P31645  P05060  P05060  P05060  P05060  P05060  P05060  P05060  P05060  P05060  P05060  P05060  P05060  P05060  P05060  P05060  P05060  P05060  P05060  P05060  P05060  P05060  Q14524  Q14524  Q14524  Q14524  Q14524  Q14524  Q14524  Q14524  Q14524  Q14524  Q14524  P34741  P34741  Q8WYJ6  Q8WYJ6  Q8WYJ6  Q8WYJ6  Q53H47  Q15637  Q15637  Q15637  Q15637  P23246  P23246  P23246  P23246  P23246  P23246  Q96BR1  Q96BR1  Q96BR1  Q96BR1  Q96BR1  Q96BR1  Q5FBB7  Q5FBB7  Q5FBB7  Q5FBB7  Q9Y2Z0  Q9Y2Z0  Q7Z6J0  O43255  O43255  O43255  P21815  P21815  P21815  P21815  P21815  P21815  P21815  P21815  P57059  P57059  Q96EB6  Q96EB6  Q96EB6  Q96EB6  Q96EB6  Q96EB6  Q96EB6  Q96EB6  Q96EB6  Q96EB6  Q96EB6  Q96EB6  Q96EB6  Q8IXJ6  Q8IXJ6  Q8IXJ6  Q8IXJ6  Q8IXJ6  Q15796  Q15796  Q15796  Q15796  Q15796  Q15796  Q15796  Q15796  Q15796  O43541  Q14683  Q14683  Q14683  Q14683  Q14683  Q16637  Q16637  Q16637  Q16637  Q16637  Q16637  O95863  O95863  O95863  O95863  O95863  O95863  O95863  O95863  O95863  O95863  O95863  P01241  P01241  Q92673  Q92673  P08047  P08047  P08047  P08047  P08047  P08047  P08047  P08047  Q96R06  Q96R06  Q96R06  Q96R06  Q96R06  Q96R06  Q96R06  Q96R06  Q9NYA1  Q9Y657  Q9Y657  Q9Y657  P35270  P11277  P11277  P11277  P11277  P11277  P11277  P23327  P23327  P23327  P23327  P23327  P23327  P23327  P11831  P11831  P11831  P11831  P11831  P11831  P11831  P11831  P11831  Q96SB4  Q96SB4  Q96SB4  Q96SB4  Q8IYB3  Q8IYB3  Q8IYB3  Q8IYB3  Q8IYB3  Q8IYB3  Q8IYB3  Q8IYB3  Q8IYB3  Q8IYB3  Q8IYB3  Q8IYB3  Q8IYB3  Q8IYB3  Q8IYB3  Q8IYB3  Q8IYB3  Q8IYB3  Q8IYB3  Q8IYB3  Q8IYB3  Q8IYB3  Q8IYB3  Q8IYB3  Q8IYB3  Q8IYB3  Q8IYB3  Q8IYB3  Q8IYB3  Q8IYB3  Q8IYB3  Q8IYB3  Q8IYB3  Q8IYB3  Q8IYB3  Q8IYB3  Q8IYB3  Q8IYB3  Q8IYB3  Q8IYB3  Q8IYB3  Q8IYB3  Q8IYB3  Q8IYB3  Q8IYB3  Q8IYB3  Q8IYB3  Q8IYB3  Q8IYB3  Q8IYB3  Q8IYB3  Q8IYB3  Q8IYB3  Q8IYB3  Q8IYB3  Q8IYB3  Q8IYB3  Q9UQ35  Q9UQ35  Q9UQ35  Q9UQ35  Q9UQ35  Q9UQ35  Q9UQ35  Q9UQ35  Q9UQ35  Q9UQ35  Q9UQ35  Q9UQ35  Q9UQ35  Q9UQ35  Q9UQ35  Q9UQ35  Q9UQ35  Q9UQ35  Q9UQ35  Q9UQ35  Q9UQ35  Q9UQ35  Q9UQ35  Q9UQ35  Q9UQ35  Q9UQ35  Q9UQ35  Q9UQ35  Q9UQ35  Q9UQ35  Q9UQ35  Q9UQ35  Q9UQ35  Q9UQ35  Q9UQ35  Q9UQ35  Q9UQ35  Q9UQ35  Q9UQ35  Q9UQ35  Q9UQ35  Q9UQ35  Q9UQ35  Q9UQ35  Q9UQ35  Q9UQ35  Q9UQ35  Q9UQ35  Q9UQ35  Q9UQ35  Q9UQ35  Q9UQ35  Q9UQ35  Q9UQ35  Q9UQ35  Q9UQ35  Q9UQ35  Q9UQ35  Q9UQ35  Q9UQ35  Q9UQ35  Q9UQ35  Q9UQ35  Q9UQ35  Q9UQ35  Q9UQ35  Q9UQ35  Q9UQ35  Q9UQ35  Q9UQ35  Q9UQ35  Q9UQ35  Q9UQ35  Q9UQ35  Q9UQ35  Q9UQ35  Q9UQ35  Q9UQ35  Q9UQ35  Q9UQ35  Q9UQ35  Q9UQ35  Q9UQ35  Q9UQ35  Q9UQ35  Q9UQ35  Q9UQ35  Q9UQ35  Q9UQ35  Q9UQ35  Q9UQ35  Q9UQ35  Q9UQ35  Q9UQ35  Q9UQ35  Q9UQ35  Q9UQ35  Q9UQ35  Q9UQ35  Q9UQ35  Q9UQ35  Q9UQ35  Q9UQ35  Q9UQ35  Q9UQ35  Q9UQ35  Q9UQ35  Q9UQ35  Q9UQ35  Q9UQ35  Q9UQ35  Q9UQ35  Q9UQ35  Q9UQ35  Q9UQ35  Q9UQ35  Q9UQ35  Q9UQ35  Q9UQ35  Q9UQ35  Q9UQ35  Q9UQ35  Q9UQ35  Q9UQ35  Q9UQ35  Q9UQ35  Q9UQ35  Q9UQ35  Q9UQ35  Q9UQ35  Q9UQ35  Q9UQ35  Q9UQ35  Q9UQ35  Q9UQ35  Q9UQ35  Q9UQ35  Q9UQ35  Q9UQ35  Q9UQ35  Q9UQ35  Q9UQ35  Q9UQ35  Q9UQ35  Q9UQ35  Q9UQ35  Q9UQ35  Q9UQ35  Q9UQ35  Q9UQ35  Q9UQ35  Q9UQ35  Q9UQ35  Q9UQ35  Q9UQ35  Q9UQ35  Q9UQ35  Q9UQ35  Q9UQ35  Q9UQ35  Q9UQ35  Q9UQ35  Q9UQ35  Q9UQ35  Q9UQ35  Q9UQ35  Q9UQ35  Q9UQ35  Q9UQ35  Q9UQ35  Q9UQ35  Q9UQ35  Q9UQ35  Q9UQ35  Q9UQ35  Q9UQ35  Q9UQ35  Q9UQ35  Q9UQ35  Q9UQ35  Q9UQ35  Q9UQ35  Q9UQ35  Q9UQ35  Q9UQ35  Q9UQ35  Q9UQ35  Q9UQ35  Q9UQ35  Q9UQ35  Q9UQ35  Q9UQ35  Q9UQ35  Q13247  Q13247  Q13247  Q13247  Q13247  Q13247  Q13247  Q8WYL5  Q8WYL5  Q8WYL5  Q08945  Q08945  Q08945  Q08945  Q08945  Q08945  Q08945  Q08945  Q08945  Q08945  Q08945  O00204  O95630  O95630  O95630  O95630  O95630  P49675  P49675  P52630  P52630  P52630  P40763  P02808  P02808  O76061  O76061  Q13285  Q86WV6  Q15831  Q15831  Q15831  O75716  Q15208  Q15208  Q9UEW8  Q9UEW8  Q9UEW8  Q9UEW8  P16949  P16949  P16949  P16949  P16949  Q9UJZ1  Q9UJZ1  P27105  P27105  P27105  Q16623  Q16623  P14410  P63165  P63165  Q9UH99  Q9UH99  Q9UH99  Q9UH99  P07814  P07814  P07814  P07814  P07814  P07814  O14994  O14994  O14994  P37840  P37840  Q24JP5  P35269  P35269  P35269  P35269  P35269  P35269  P35269  P35269  P35269  P35269  P35269  P35269  P35269  Q8N5C8  Q8N5C8  Q8N5C8  Q8N5C8  O75410  O75410  O75410  O75410  O75410  O75410  Q15750  Q15750  Q15750  P21675  P21675  P21675  Q15545  Q15545  Q15545  Q9UL54  Q9UL54  Q9UL54  Q9UL54  Q9UL54  Q9UL54  Q9H2K8  Q9H2K8  Q9H2K8  P10636  P10636  P10636  P10636  P10636  P10636  P10636  P10636  P10636  P10636  P10636  P10636  P10636  P10636  P10636  P10636  P10636  P10636  P10636  P10636  P10636  P10636  Q86VP1  Q86VP1  Q86VP1  Q86VP1  P68363  P68363  P68363  Q13509  Q99426  Q99426  Q99426  O60343  O60343  O60343  O60343  O60343  O60343  O60343  O60343  O60343  Q9UHD2  Q15170  Q15170  Q15170  Q15170  Q15170  Q15170  Q15170  Q15170  Q86XR7  P53999  P53999  P53999  P53999  P53999  P53999  P53999  P53999  P53999  P13693  P13693  P13693  Q15185  Q15185  Q15185  Q15185  Q15185  Q9Y4R8  Q9Y4R8  Q9Y4R8  Q9Y4R8  Q9Y4R8  Q9Y4R8  P24821  P24821  P24821  Q9HBL0  Q9HBL0  Q9HBL0  Q9HBL0  Q9HBL0  Q9HBL0  Q9HBL0  Q9HBL0  Q9HBL0  Q9HBL0  Q9HBL0  Q9HBL0  Q9HBL0  Q9HBL0  Q9HBL0  Q9HBL0  Q9HBL0  Q9HBL0  Q9HBL0  Q9HBL0  Q9HBL0  Q9HBL0  Q9HBL0  P54274  P54274  O14746  O14746  P52655  P52655  P52655  P52655  Q04206  Q04206  Q04206  Q04206  Q04206  Q04206  Q00059  Q00059  Q00059  Q00059  Q00059  P02786  P22735  P22735  P22735  P22735  P22735  O43493  O43493  O43493  O43493  Q5T1C6  Q5T1C6  Q92563  Q13263  Q13263  Q13263  Q13263  Q13263  Q13263  Q13263  Q13263  Q13263  Q13263  Q13263  Q13263  Q13263  Q13263  Q13263  P01033  Q9UKI8  Q9UKI8  Q9UKI8  O60602  P19429  P19429  P19429  P19429  P19429  P19429  P19429  P19429  P19429  P19429  P11387  P11387  P11387  P11387  P11388  P11388  P11388  P11388  P11388  P11388  P11388  P11388  P11388  P11388  P11388  P11388  P11388  P11388  P11388  P11388  P11388  P11388  P11388  P11388  P11388  P11388  P11388  P11388  P11388  Q9NS56  Q9NS56  Q9NS56  Q9NS56  Q9NS56  Q9NS56  Q9NS56  Q12888  Q12888  Q12888  Q12888  Q12888  Q12888  Q12888  Q12888  Q12888  Q12888  Q12888  Q12888  Q12888  Q12888  Q12888  Q12888  Q12888  Q12888  Q12888  Q12888  Q12888  Q12888  Q12888  Q12888  Q12888  Q12888  Q12888  Q12888  Q12888  Q12888  Q12888  Q12888  Q12888  Q12888  Q12888  Q12888  Q12888  Q12888  Q12888  Q12888  Q12888  Q12888  Q12888  Q12888  Q12888  Q12888  Q12888  Q12888  Q12888  Q12888  Q12888  P09493  P09493  P09493  P09493  O94811  O94811  O94811  O94811  O94811  O94811  Q9Y2W1  Q9Y2W1  Q9Y2W1  Q9Y2W1  Q9Y2W1  Q9Y2W1  Q9Y2W1  Q9Y2W1  Q9Y2W1  Q9Y2W1  Q9Y2W1  Q9Y2W1  Q9Y2W1  Q9Y2W1  Q9Y2W1  Q9Y2W1  Q9Y2W1  Q9Y2W1  Q9Y2W1  Q9Y2W1  Q9Y2W1  Q9Y2W1  Q9Y2W1  Q9Y2W1  Q12933  P08582  Q9UBP6  Q92574  Q92574  Q92574  Q92574  P49815  P49815  P49815  P49815  P49815  P49815  P49815  P49815  P49815  P49815  P49815  P49815  P49815  P49815  P49815  P49815  Q9H2G4  Q9H2G4  P53804  O43156  O43156  P26651  P26651  P26651  P26651  P26651  P26651  P26651  P26651  P26651  P26651  P26651  P26651  P26651  P07101  P07101  P07101  Q712K3  P22314  P22314  P22314  P22314  P22314  P22314  P22314  P63279  P49459  Q14694  Q14694  Q14694  Q14694  Q14694  Q14694  Q14694  Q96RU2  Q96RU2  Q86T82  Q86T82  Q86T82  Q93009  Q93009  O95071  O95071  O95071  O95071  O95071  O95071  O95071  O95071  O95071  O95071  O95071  O95071  O95071  O95071  O95071  O95071  O95071  O95071  O95071  O95071  O95071  O95071  O95071  Q04323  Q04323  Q04323  Q96T88  Q96T88  Q96T88  Q96T88  Q96T88  Q96T88  Q96T88  Q96RL1  Q96RL1  Q96RL1  Q96RL1  Q96RL1  Q96RL1  Q96RL1  Q96RL1  Q96RL1  Q96RL1  Q6PHR2  Q6PHR2  Q6PHR2  Q6PHR2  Q6PHR2  P00749  P00749  O60763  O60763  O60763  Q9P2Y5  Q9P2Y5  Q9P2Y5  Q9P2Y5  Q9P2Y5  Q9P2Y5  Q9P2Y5  Q9P2Y5  P50552  P50552  P50552  P50552  P21796  P21796  O15240  Q99986  P04004  P04004  P04004  P04004  Q6PCB0  Q6PCB0  Q6PCB0  O00401  O00401  O00401  P42768  P42768  P30291  P30291  P30291  P30291  P30291  P30291  P30291  P30291  O76024  Q9Y3S1  Q9Y3S1  Q9Y3S1  Q9Y3S1  Q9Y3S1  Q9Y3S1  Q9Y3S1  Q9Y3S1  Q9Y3S1  Q9Y3S1  Q9Y3S1  Q9Y3S1  Q9Y3S1  Q9Y3S1  Q9GZV5  Q9GZV5  Q9GZV5  P98170  P23025  P18887  P18887  P18887  P18887  P18887  P18887  P18887  P18887  P18887  P18887  P18887  P18887  P18887  P18887  P18887  P18887  P13010  P13010  P13010  Q13426  Q13426  Q13426  Q13426  Q13426  Q13426  P12956  P12956  P12956  P12956  P12956  P46937  P46937  P46937  P46937  P46937  P46937  P46937  P46937  P46937  P46937  P46937  P46937  P46937  P46937  P67809  P67809  P67809  P67809  P67809  P67809  Q9UK55 | 14  84  85  149  151  152  188  191  540  542  555  709  712  65  83  280  38  383  198  206  441  730  1042  1296  2054  22  24  105  109  140  166  228  595  5  23  25  29  48  53  78  80  488  1201  1216  1263  1273  35  91  95  200  222  469  554  1299  166  169  208  210  216  240  243  295  328  365  384  386  388  400  410  434  453  478  490  561  655  657  710  714  729  825  838  895  898  987  990  1004  1180  791  819  87  12  59  64  334  358  408  431  436  465  481  586  678  707  710  714  716  726  60  396  530  532  535  592  596  600  604  613  617  693  697  701  703  246  261  262  345  346  355  356  38  34  126  128  131  447  474  478  29  82  89  443  513  929  942  946  218  314  470  534  81  94  256  650  99  25  27  801  519  529  534  538  542  19  130  134  803  1457  1635  1696  1700  1709  2042  2045  2047  2059  2067  2401  1007  1011  68  5  27  34  37  239  51  60  202  286  341  355  362  373  377  547  555  686  688  757  758  777  779  195  116  590  54  68  3279  4048  147  311  314  256  143  163  172  174  645  648  691  696  782  886  896  932  940  941  947  953  956  960  176  249  518  694  412  192  479  62  90  112  121  328  340  367  442  446  490  498  355  356  361  367  1893  1981  1983  2996  34  77  79  101  103  107  661  739  746  748  428  238  775  41  51  342  75  77  217  469  486  493  49  62  69  278  280  521  4  75  91  99  118  134  596  598  600  621  134  139  292  428  437  639  70  87  402  406  102  104  177  181  196  198  222  259  264  268  285  290  297  385  389  397  472  496  502  512  531  578  648  658  660  690  760  333  343  90  93  96  366  373  375  273  537  541  91  110  165  219  224  238  244  60  151  337  365  399  405  446  447  729  114  308  395  398  423  694  753  840  988  1143  1211  1217  1218  1280  1328  1336  1342  1387  1423  1457  1524  755  3291  470  484  488  492  494  498  499  503  601  1117  447  508  563  147  149  21  55  115  180  623  659  435  543  670  676  1043  206  197  200  293  295  643  659  671  852  208  253  324  455  132  315  377  314  317  327  332  334  338  323  96  135  216  31  33  41  71  86  88  89  90  91  21  23  24  25  296  369  257  188  211  387  385  393  23  36  37  282  521  525  529  634  121  1030  1382  1386  2063  2076  2079  141  126  128  133  147  103  387  399  5  132  304  322  25  33  71  73  83  99  100  102  288  295  324  326  115  482  589  145  148  672  686  697  706  8  112  490  560  595  41  72  92  153  161  291  339  386  426  435  438  821  303  358  417  437  12  42  78  82  95  119  138  153  155  7  164  321  175  347  353  357  46  114  130  146  160  10  7  8  140  152  186  190  234  7  17  19  27  589  595  759  47  373  385  386  397  425  428  436  340  408  722  549  660  686  700  712  737  753  768  790  795  813  1444  1456  19  23  25  273  280  286  296  301  317  345  62  73  379  456  226  210  30  32  41  52  146  147  78  148  248  308  310  3  17  19  232  312  34  98  142  142  203  218  270  300  322  333  398  402  335  336  352  38  70  297  303  672  968  1321  1573  918  3  8  156  544  105  184  271  272  301  489  566  326  327  349  664  679  723  745  2  2229  2252  2417  2421  68  83  105  108  116  120  123  177  179  216  244  267  268  284  298  828  182  133  142  271  330  151  70  86  90  136  171  274  306  368  393  433  456  489  490  492  613  624  19  45  59  63  362  370  2  3  209  151  155  313  364  468  474  483  410  423  175  2116  300  422  4  47  125  225  230  252  268  269  288  320  346  349  352  617  811  847  857  859  864  868  879  899  920  943  23  29  33  37  45  191  246  552  675  255  262  314  325  328  330  344  365  368  369  373  319  321  324  325  330  339  348  351  387  418  188  43  728  978  237  827  3  49  51  91  289  308  333  734  25  178  213  495  668  671  688  702  737  739  207  398  405  419  421  487  510  516  11  15  74  645  109  128  155  171  191  196  346  368  375  378  387  408  415  418  430  436  19  121  13  71  89  121  147  171  567  102  131  183  612  854  855  19  32  51  159  201  204  210  227  230  231  232  243  244  251  287  288  296  301  303  306  307  16  18  96  105  156  226  289  333  372  403  12  28  60  68  67  618  627  642  662  725  757  777  806  812  830  839  274  8  10  15  548  607  616  637  127  133  143  152  154  394  509  714  732  401  410  244  258  263  265  282  283  287  293  297  298  299  141  142  337  339  416  446  501  386  391  88  48  125  192  700  88  310  529  748  758  442  449  83  242  456  300  60  133  162  18  27  61  66  71  78  359  366  398  445  470  474  477  491  500  502  595  492  881  882  1198  1336  1364  78  81  83  85  119  125  152  154  164  239  9  11  13  15  16  18  39  166  909  46  258  11  42  189  183  11  13  20  127  324  383  389  422  8  29  49  54  60  87  171  148  64  306  447  2  67  109  570  579  869  892  897  901  25  382  419  420  435  447  454  209  20  185  190  327  431  435  444  531  548  19  22  1126  1396  1399  1508  104  106  118  167  87  105  220  225  295  101  214  22  213  257  114  376  422  454  598  623  639  660  674  714  746  266  472  21  366  346  386  387  406  461  467  859  16  129  131  199  201  4  124  127  356  610  614  616  650  666  114  204  8  222  592  594  717  1401  1404  1412  1423  1435  194  29  580  722  732  887  910  249  374  407  556  730  1121  7  927  930  990  1032  1237  2702  33  278  12  151  227  187  197  111  115  117  344  444  445  134  135  138  325  328  330  22  45  50  56  281  291  294  364  451  501  524  560  609  68  2384  406  436  856  1161  1338  2233  574  759  993  337  370  500  362  374  215  219  232  235  240  251  281  288  367  331  376  693  730  739  212  218  234  235  249  256  287  319  322  325  329  197  262  215  253  280  284  315  399  413  421  551  555  588  626  19  418  38  39  165  255  181  184  193  274  480  301  773  775  299  318  329  1544  1549  1754  149  231  232  373  83  122  148  151  241  312  185  133  140  141  148  149  159  164  210  218  223  264  281  405  480  543  622  623  117  71  96  26  72  142  178  187  310  60  81  113  134  141  203  211  226  267  63  64  184  149  234  236  242  388  1011  664  849  865  877  907  980  1006  24  89  168  383  390  434  187  309  191  352  104  150  418  428  431  476  1387  1417  1675  1837  846  868  484  36  9  389  103  207  210  412  668  784  823  274  8  11  412  641  645  649  653  657  698  727  2  18  107  173  189  15  37  11  29  58  87  2  48  58  93  143  147  39  109  155  181  283  358  364  486  393  421  423  259  498  661  411  432  1179  1199  1870  1874  37  247  551  576  589  657  549  557  584  610  611  612  614  661  687  21  123  140  185  448  473  486  595  642  36  44  49  99  102  103  8  21  25  86  89  99  80  87  88  131  142  167  313  436  113  115  162  166  233  238  239  241  253  260  299  306  161  228  75  116  214  216  284  379  52  101  185  291  298  544  396  398  231  252  263  399  121  230  292  303  307  314  326  344  363  419  444  298  15  26  65  78  82  83  86  98  199  71  175  240  318  360  455  457  460  45  120  123  126  144  156  194  199  242  148  201  255  116  239  102  144  31  126  95  106  153  168  174  575  780  107  113  135  137  164  171  182  183  186  190  214  547  560  588  589  591  595  93  192  219  283  406  418  422  425  445  459  462  498  504  597  209  314  1028  1092  1145  1147  1185  1187  1209  1231  1596  230  232  267  495  1156  166  174  175  235  239  243  19  23  183  313  315  32  36  283  288  293  177  181  670  672  675  682  689  692  695  697  705  733  740  750  172  664  13  63  101  168  196  258  295  361  364  389  391  393  397  445  661  667  782  829  81  23  191  194  119  143  148  197  214  263  269  275  296  306  312  314  330  400  446  476  510  514  828  831  893  894  899  914  121  122  127  2  330  1441  1443  346  14  97  188  386  396  398  427  158  293  301  435  437  440  446  477  479  99  270  307  312  323  345  348  527  629  636  794  1101  1021  745  756  240  273  450  60  466  886  40  152  181  187  193  195  196  214  311  328  330  340  355  366  369  407  409  444  447  469  471  472  473  314  364  365  676  165  469  484  486  58  63  73  243  249  427  7  10  15  18  30  31  34  42  51  53  60  100  177  305  319  323  398  399  401  13  186  325  11  140  339  86  90  50  57  102  124  158  162  164  506  9  20  31  112  12  13  336  360  385  201  287  8  9  15  21  44  348  52  661  1379  1381  1382  1410  1432  1438  1537  1559  1644  1797  7  14  21  528  532  662  668  825  867  542  899  931  947  975  978  1033  147  204  22  95  109  111  115  166  192  621  633  13  231  741  746  821  891  959  521  524  332  16  661  205  208  345  397  401  448  473  738  742  910  200  203  206  214  244  518  876  130  299  302  304  307  503  506  645  654  658  664  234  316  329  346  350  368  388  729  348  676  685  695  219  227  233  212  360  376  381  647  657  691  695  750  752  758  759  232  372  389  394  434  441  447  452  44  202  295  297  316  319  350  379  579  75  156  1084  42  109  170  1163  1181  1194  1248  663  1532  1149  1493  75  90  143  165  215  220  324  327  517  521  548  627  631  766  774  824  851  1040  278  464  613  674  909  83  366  67  71  86  97  99  30  6  12  291  351  355  368  71  234  238  210  302  307  310  323  337  416  12  18  22  212  277  301  307  390  392  395  403  404  407  414  431  458  463  546  612  628  632  636  652  272  524  661  1420  1430  1490  18  190  202  312  320  324  328  24  111  177  188  189  193  252  586  1414  1597  11  281  394  507  524  548  555  556  654  693  705  724  727  740  748  758  770  789  793  815  83  958  966  1029  1033  62  141  400  443  192  389  439  166  250  337  340  308  171  405  407  413  421  586  60  65  68  70  75  214  14  49  61  65  14  60  99  280  358  507  510  580  624  636  643  696  787  797  825  827  928  941  1000  1073  1145  1151  9  272  328  115  212  108  26  27  29  46  63  77  81  101  118  135  145  147  159  163  167  170  262  314  441  163  170  202  207  232  46  101  309  141  174  190  249  295  299  168  121  159  162  12  13  26  27  40  41  53  108  139  381  160  535  668  672  681  711  118  170  178  195  166  240  242  246  260  262  386  395  407  425  429  342  367  121  180  190  231  251  444  59  396  419  171  253  336  343  356  391  407  431  498  505  529  462  42  22  25  28  474  226  229  727  876  1067  1706  1727  1741  1745  1892  1906  78  214  284  382  394  395  397  400  430  471  541  543  575  582  586  675  180  405  414  491  516  189  218  230  247  20  34  125  358  360  12  165  275  302  599  633  727  733  57  311  23  222  226  293  295  3  15  218  76  79  82  88  124  178  279  293  513  519  169  249  323  353  375  38  57  61  64  122  129  168  191  198  214  216  472  47  72  80  82  93  104  124  133  222  234  1127  1545  1651  1669  1693  1719  1721  110  367  657  670  200  6  58  567  1261  2159  2448  2478  2481  1227  228  241  577  261  263  6  62  71  161  329  299  422  432  445  472  473  477  507  601  668  692  695  852  862  871  910  995  182  186  205  209  213  216  43  166  186  191  203  13  78  87  96  123  278  343  397  432  615  7  22  303  304  320  328  345  348  315  22  372  395  517  569  698  1033  1185  214  551  601  728  857  860  867  884  69  165  326  330  332  333  336  364  342  446  448  449  464  479  483  487  397  411  426  516  637  171  241  296  356  365  387  390  397  402  406  428  438  273  37  206  31  43  68  85  376  387  40  215  245  269  294  168  170  213  217  289  344  120  134  155  337  892  903  907  927  932  937  713  715  717  866  870  46  162  269  280  290  291  294  302  568  574  601  603  609  624  638  641  646  665  681  692  696  24  58  62  321  329  335  338  344  354  359  370  395  407  114  633  638  836  1177  81  172  461  775  779  1100  4  10  70  125  137  139  227  242  243  254  260  55  59  341  351  40  83  39  44  46  47  52  139  456  473  593  724  743  751  22  455  600  86  369  139  274  326  112  113  212  214  304  376  572  629  637  652  655  692  162  169  203  271  388  395  820  1187  1225  1601  1721  1724  1728  1757  1760  1769  1772  1788  1789  1792  1800  1830  1833  1834  1840  1862  1887  1969  1991  2003  2077  2087  124  135  240  247  276  311  352  313  321  340  369  370  408  490  232  293  295  300  177  198  342  24  26  27  62  63  76  78  81  99  102  105  108  117  120  123  126  129  191  195  215  219  224  228  234  239  243  254  258  263  267  270  275  280  291  303  308  310  311  64  165  177  452  387  388  60  108  259  327  338  9  15  20  33  37  46  183  269  315  392  437  505  727  729  2  361  10  446  31  72  21  57  115  144  149  174  199  204  220  223  41  104  148  167  181  195  258  267  291  474  401  684  688  893  979  984  1101  1104  1106  1116  1118  1121  25  383  692  695  728  809  827  852  873  962  133  137  197  261  264  291  298  302  65  70  163  557  619  623  628  201  205  209  83  85  106  119  126  130  137  143  244  322  533  30  115  47  688  253  259  284  67  68  71  76  78  80  94  313  317  457  152  165  686  688  20  359  457  129  156  428  5  127  275  383  385  25  241  393  394  396  398  410  501  529  268  374  377  379  382  953  1029  24  114  227  527  530  533  540  662  696  700  714  766  771  945  977  1124  54  57  181  239  16  474  539  681  705  882  899  1056  69  120  651  804  854  857  880  25  293  296  258  266  277  294  428  511  650  299  305  99  189  352  71  108  19  228  402  382  600  621  896  309  323  342  432  1070  1101  4166  1221  1248  125  149  720  1047  1435  1721  1732  2631  2782  2802  3036  4054  4382  4384  4385  4386  4389  4390  4391  4392  4396  4400  4613  4616  4618  4622  4626  4642  4675  113  117  103  137  269  335  375  450  597  688  83  88  372  404  428  5  7  30  257  323  406  8  36  38  40  117  403  504  505  518  527  530  535  565  94  120  160  426  469  473  283  385  448  111  236  289  290  355  16  14  18  420  421  24  52  113  172  195  265  501  513  571  592  615  20  81  102  130  162  190  213  294  345  400  676  893  2056  2612  3205  4026  18  246  287  65  54  62  118  163  194  347  85  138  109  43  166  179  212  215  243  250  24  247  43  310  346  367  370  380  385  2  9  10  50  242  243  352  378  386  35  635  684  692  245  268  44  93  143  19  36  40  118  167  169  202  203  300  365  366  379  387  389  165  19  75  98  106  124  209  229  709  714  806  15  639  1406  1859  1873  1900  426  204  646  656  272  277  328  341  375  380  387  358  428  435  442  106  135  264  288  295  77  96  219  369  350  388  389  497  525  640  650  749  762  964  975  988  1009  1041  639  662  672  948  952  966  971  972  973  981  982  1035  583  677  683  703  309  37  249  567  612  795  807  811  2  11  55  80  267  503  10  31  1089  1107  1110  1127  4  8  23  29  33  227  102  281  348  488  538  647  648  46  63  149  154  157  164  170  203  206  214  257  387  410  600  486  696  820  821  1593  737  746  49  24  97  151  434  391  402  413  415  437  3  16  210  258  333  337  351  609  6  20  25  161  166  303  320  333  168  176  363  393  527  529  531  539  2  164  199  227  299  389  5  21  491  77  101  17  79  86  102  105  372  53  155  157  117  7  45  544  558  564  696  719  721  722  738  792  855  859  863  877  65  6  209  224  235  236  240  242  244  247  7  15  107  152  181  182  361  511  451  21  27  56  70  259  260  2031  2808  2814  275  276  257  262  1026  1029  1034  256  57  307  776  789  852  1019  185  27  34  42  97  110  113  120  320  7  13  14  17  611  99  100  130  149  183  225  259  263  293  294  311  335  367  377  380  405  533  534  617  626  631  36  457  460  483  484  497  510  571  664  667  1503  115  187  206  248  307  315  508  20  80  82  89  8  33  273  283  379  626  126  129  142  461  465  486  14  256  436  507  281  331  304  16  28  68  31  67  74  75  94  100  149  280  186  575  14  26  27  47  159  162  172  173  535  545  659  661  747  23  25  27  368  372  240  245  250  255  458  460  464  465  467  435  358  360  957  966  970  4  5  8  28  31  187  11  82  92  96  100  104  107  111  115  119  246  132  176  114  2206  2  7  59  101  612  641  670  702  43  62  66  135  334  341  362  974  225  109  124  199  213  2043  2114  2117  2123  2125  2128  119  145  333  358  431  494  567  77  79  83  85  103  224  253  435  446  51  309  311  555  227  234  260  389  391  393  402  414  420  429  431  436  450  452  463  465  478  524  526  528  530  532  549  551  560  562  583  597  605  607  616  626  628  636  638  694  695  696  705  707  713  715  738  740  748  752  754  756  769  773  775  777  781  791  795  874  901  24  220  222  295  297  300  322  323  351  353  357  358  377  387  395  398  404  408  424  435  436  437  440  454  484  486  534  536  778  780  783  846  857  864  871  875  876  908  924  935  950  952  954  957  968  970  972  973  974  994  1010  1014  1028  1032  1064  1069  1072  1073  1083  1099  1101  1102  1103  1112  1122  1124  1129  1132  1152  1179  1188  1214  1219  1257  1311  1318  1320  1326  1329  1336  1348  1368  1382  1383  1387  1401  1403  1404  1415  1421  1423  1424  1444  1451  1458  1460  1462  1463  1482  1483  1497  1499  1501  1502  1517  1519  1521  1522  1537  1539  1541  1542  1552  1577  1579  1581  1582  1598  1600  1601  1616  1620  1621  1648  1658  1691  1693  1694  1727  1729  1731  1732  1762  1764  1818  1822  1854  1857  1876  1878  1884  1890  1893  1916  1919  1923  1925  1946  1948  1958  1970  1972  1975  1984  1987  2020  2030  2032  2044  2046  2067  2071  2090  2100  2102  2118  2121  2123  2132  2272  2335  2343  2382  2394  2398  2407  2412  2415  2426  2429  2449  2453  2581  2664  2675  2677  2684  2688  2690  2692  2694  2702  2706  45  81  84  299  303  314  316  37  57  978  437  444  471  510  657  667  668  671  672  673  688  348  2  48  243  245  247  57  195  283  287  753  727  21  22  250  251  203  358  31  325  428  197  264  281  309  370  371  385  16  25  31  38  63  17  330  10  161  244  14  188  7  2  9  12  38  54  116  882  885  886  891  999  1000  9  470  484  87  129  529  217  218  221  224  377  380  381  385  391  431  433  436  449  60  101  492  506  4  10  228  276  381  591  7  378  438  137  307  1826  171  213  264  9  181  414  777  825  827  324  331  442  214  396  515  516  519  531  552  554  579  602  606  610  622  641  669  673  713  717  721  726  733  739  138  225  593  666  48  232  439  172  65  110  128  262  318  341  344  566  570  588  591  751  172  31  32  36  37  41  42  47  48  16  4  9  10  11  13  15  17  19  118  46  53  64  85  113  118  148  151  456  485  487  491  688  836  65  70  72  338  364  378  433  701  764  769  794  899  907  963  967  1124  1177  1192  1269  1294  1309  1314  1381  1393  1446  1588  11  219  227  457  280  281  316  321  276  281  311  468  529  536  55  56  61  160  193  24  24  82  85  92  95  71  221  298  351  37  38  72  19  50  439  471  473  479  489  501  594  683  689  697  752  757  824  178  80  159  743  805  5  6  23  24  42  44  77  150  166  199  10  57  112  506  4  1106  1213  1247  1295  1297  1299  1302  1332  1337  1351  1354  1374  1377  1387  1392  1393  1449  1469  1471  1474  1476  1495  1504  1525  98  499  585  718  864  866  1028  25  105  124  166  176  178  222  265  294  366  380  395  398  452  500  523  525  552  566  580  630  635  639  640  692  727  809  831  834  970  975  1028  1068  1086  1094  1101  1114  1216  1219  1362  1368  1426  1430  1460  1462  1474  1618  1678  1701  1759  1778  61  174  206  283  18  32  35  45  159  160  232  237  240  243  248  253  257  315  320  339  377  379  406  408  535  560  575  622  672  682  684  698  928  939  11  462  27  505  511  598  1100  540  664  939  981  1132  1155  1337  1338  1346  1387  1411  1418  1420  1448  1452  1798  18  20  378  459  828  60  66  88  90  93  169  186  197  218  228  276  296  323  19  62  71  233  4  13  46  810  816  820  835  71  120  226  321  337  365  370  563  576  67  714  628  650  652  18  963  110  327  352  578  612  808  928  1018  1227  1308  1355  1375  1481  1549  1741  1780  1990  2026  2028  2076  2289  2484  2486  199  200  270  76  91  287  298  639  651  707  44  46  101  140  205  402  419  627  653  677  176  300  350  384  464  158  323  50  942  952  493  498  508  522  549  550  571  582  157  239  322  323  13  193  420  342  130  137  312  397  74  80  93  242  484  485  483  484  53  85  123  127  139  150  312  642  32  45  352  356  560  1150  1262  1588  1685  1736  1817  1818  1862  1889  2067  89  105  311  87  196  140  199  204  226  241  259  371  408  409  410  421  446  447  461  485  518  577  579  580  237  256  260  320  327  328  6  51  477  520  550  61  109  127  128  131  138  164  274  289  367  371  397  400  403  102  165  167  174  176  314  56 | VTEAEN**S**QHQQKE  GLSRLF**S**SFLKRP  LSRLFS**S**FLKRPK  PSLDLH**S**LSSAET  LDLHSL**S**SAETQP  DLHSLS**S**AETQPA  IEVKEE**S**PQSKAE  KEESPQ**S**KAETEL  TASKRA**S**RSLDGA  SKRASR**S**LDGAAA  VDSADR**S**PRPTSA  EWDKRL**S**THSPFR  KRLSTH**S**PFRTLN  LLDRRN**S**PMAQTP  NIPGVT**S**PGTLIE  TRAKLA**S**FSFLPQ  AQKTDT**S**HHDQDH  LEAIPM**S**IPPEVK  ESDNVD**S**ADAEED  DAEEDD**S**DVWWGG  FQEKVE**S**LEQEAA  KKKQYT**S**IHHGVV  GMQRKL**S**VALAFV  AADPND**S**DIDPES  GNKRKL**S**TAMALI  GDGEST**S**PSDKVV  GESTSP**S**DKVVKK  ERLKKL**S**VPTSDE  KLSVPT**S**DEEDEV  ALIQDQ**S**EEEEEE  RINKAV**S**EEQQPA  KKAEQG**S**EEEGEG  KNQDEE**S**QEAPEL  MMMDEP**S**PLAQPL  SRFIIG**S**VSEDNS  FIIGSV**S**EDNSED  SVSEDN**S**EDEISN  LEEKEG**S**LSPASV  GSLSPA**S**VGSDTL  ALHIRS**S**MSGLHL  HIRSSM**S**GLHLVK  VSPWGD**S**PIDFED  PTLNRM**S**FSSNLN  GMTHVA**S**VSDVLL  SDSPPQ**S**PTFPEA  PEAGHT**S**LYDEDK  SKPITK**S**KSEANL  AGRRRN**S**LPPSHQ  RNSLPP**S**HQKPPR  ASLGAL**S**LEAYLT  TMRPSM**S**GLHLVK  GIRKAE**S**AEDFPI  IRPRPG**S**LRSKPE  HGPQFG**S**EVELRH  AELEEA**S**AESEDE  EEASAE**S**EDEMIH  SPRKSS**S**ISEEKG  RKSSSI**S**EEKGDS  SEEKGD**S**DDEKPR  ARAAKL**S**EGSQPA  AKLSEG**S**QPAEEE  DDEGQK**S**REAPIL  ERPKTR**S**QEQEVL  KEMKTT**S**PLEEEE  QGLKEK**S**KSPSPP  LKEKSK**S**PSPPRL  EKSKSP**S**PPRLTE  EDRKKA**S**LVALPE  LPEQTA**S**EEETPP  PHPQLH**S**EEEIEP  AVLIQL**S**PPNTDA  QLVGGL**S**PLSSPS  SDTKAE**S**PAEKVP  KEGRRA**S**HTLLPS  SSNSRK**S**LSPGVS  NSRKSL**S**PGVSRD  VQARRL**S**QPESAE  RLSQPE**S**AEKHVT  LQPERG**S**PKKCEA  SFKRKI**S**VVSATK  GVPAGN**S**DTEGGQ  DLHADD**S**RISEDE  ADDSRI**S**EDETER  SISQQK**S**GVSITI  QQKSGV**S**ITIDDP  RTAQVP**S**PPRGKI  PRSRSR**S**RDRRRK  SSTAAK**S**FEDLTD  RQNRVD**S**KETECC  ARARFE**S**KVPSFY  RAAVVT**S**PPPTTA  EQKKRV**S**MILQSP  VSMILQ**S**PAFCEE  QVRTLA**S**AGGPDN  KSRSPG**S**PVGEGT  EKSKKY**S**DVEVPA  GDSGTC**S**PLRHSF  CSPLRH**S**FQKQQR  EGQNGS**S**PKSKTK  EDGHRT**S**TSAVPN  ERKQKG**S**EENLDE  EAPTEA**S**PEPAPD  AAADPG**S**DGSPGK  DPGSDG**S**PGKSPS  DGSPGK**S**PSKKKK  SPGKSP**S**KKKKKF  KKFRTP**S**FLKKSK  VTMILQ**S**PSFREE  EKTKHK**S**EVEIPA  IAEKSR**S**PSTESQ  EKSRSP**S**TESQLM  RSPSTE**S**QLMSKG  APEEPG**S**PAKSAP  PGSPAK**S**APASPV  AKSAPA**S**PVQSPA  PASPVQ**S**PAKEAE  KEAETK**S**PLVSPS  TKSPLV**S**PSKSLE  VTSGPM**S**PEGSPS  PMSPEG**S**PSKSPS  EGSPSK**S**PSKKKK  SPSKSP**S**KKKKKF  FHVQNL**S**QVEQDG  GHGLRR**S**SKFCLK  HGLRRS**S**KFCLKE  LLCLRR**S**SLKAYG  LCLRRS**S**LKAYGN  AYGNGY**S**SNGNTG  YGNGYS**S**NGNTGE  VVKRRD**S**ATSFSL  LLKSDG**S**FIGYKE  MDYKCG**S**PSDSST  YKCGSP**S**DSSTTE  GSPSDS**S**TTEEME  DEFTAQ**S**ITITPP  THFPQF**S**YSASIR  QFSYSA**S**IREEEE  RRDAHK**S**EVAHRF  TCVADE**S**AENCDK  AENCDK**S**LHTLFG  KKVPQV**S**TPTLVE  NRRPCF**S**ALEVDE  LGNFFA**S**RKGYSR  KGFDRL**S**TEGSDQ  RLSTEG**S**DQEKED  YYRLLQ**S**PLCAGC  GPARPA**S**PEAGNT  LETGQD**S**EDDSGE  LHQFSF**S**PEREIR  QQQQET**S**PRQQQQ  QQGEDG**S**PQAHRR  EALEHL**S**PGEQLR  EASSTT**S**PTEETT  ERVRKT**S**LDLRRE  EDLPEL**S**DSGDEA  LPELSD**S**GDEAAW  FGQRVE**S**IGQWQK  EDVEPS**S**LERHLR  HLRSAG**S**RLTLSG  GSRLTL**S**GRGSNY  TLSGRG**S**NYGSLL  RGSNYG**S**LLTTEG  AAEGEG**S**PPAVAA  DEEEEV**S**EVESFI  EVSEVE**S**FILDQD  GYITNQ**S**PESIVE  EANKNA**S**ILLEEL  SNSSRK**S**DNHSPA  CTKSGP**S**PLSSPN  GPSPLS**S**PNGKLT  GKLTVA**S**PKRGQK  EHYPVS**S**PSSPSP  PVSSPS**S**PSPPAQ  SSPSSP**S**PPAQPG  GGVSRN**S**PLDCGS  LDCGSA**S**PNKVAS  SGVRAP**S**PAPSSV  NRYTNT**S**KTVSFE  NTSKTV**S**FEALPI  ATEDEG**S**EQKIPE  MMMAMV**S**EFLKQA  YVQTVK**S**SKGGPG  SKGGPG**S**AVSPYP  GPGSAV**S**PYPTFN  EVQRRL**S**PPECLN  ASELWS**S**DGAAGL  AAGLVG**S**LQEVTI  HEVPPG**S**PREPLP  NVVLKF**S**EQGGTP  PSLHSR**S**PSISNM  ALSRAH**S**PALGVH  PALGVH**S**FSGVQR  QRFNIS**S**HNQSPK  ISSHNQ**S**PKRHSI  LSKLLG**S**LDEVVL  DEVVLL**S**PVPELR  NFDFEG**S**LSPVIA  DFEGSL**S**PVIAPK  EWELDE**S**SDEEEE  WELDES**S**DEEEEA  IKEEVL**S**ESEAEN  EEVLSE**S**EAENQQ  KEELDV**S**VREEEV  QCTLRN**S**QVLDED  VDVRVS**S**EESEEI  LMEKVK**S**PELQAE  KSYFEK**S**KEQLTP  VFPKAV**S**MPSFSI  ELRVRE**S**DEETQI  QAMLGQ**S**TEELRV  RVTEPI**S**AESGEQ  EPISAE**S**GEQVER  EVRRRQ**S**VELHSP  SRRGRS**S**LSLAKS  GHFNDE**S**PLGLRR  GLRRIL**S**QSTDSL  RRILSQ**S**TDSLNM  GLFRSE**S**LESPRG  RSESLE**S**PRGERL  LPLEPD**S**GGNTSP  DSGGNT**S**PGVTAN  KLCRAN**S**RDGEAG  VDPRRR**S**LPAGDA  GDALYL**S**FNPPQP  ERQELG**S**PEERLQ  EERLQD**S**SDPDTG  ERLQDS**S**DPDTGS  SDPDTG**S**EEEGSS  SEEEGS**S**RLSPPH  EGSSRL**S**PPHSPR  RLSPPH**S**PRDFTR  FQGQYR**S**LDMTDN  ASEKPV**S**PKSGTL  SASPRM**S**GFIYQG  KPERKP**S**DEEFAS  EEDGTG**S**PQLNNR  WSTSEN**S**LNVMLE  KEAKRS**S**ADKGVA  GPARND**S**VIVADQ  LFNELA**S**PFENEF  KMPLDL**S**PLATPI  ATPIIR**S**KIEEPS  TTETPA**S**PAHTTP  PQTQST**S**GRRRRA  RNRAAA**S**RCRQKR  DSSEDI**S**VPSSPH  DISVPS**S**PHTEAI  STEPAL**S**QIVMAP  IVMAPS**S**QSQPSG  TETVSN**S**SEGRAS  ETVSNS**S**EGRASP  SSEGRA**S**PHDVLE  TRSLEI**S**QSYTTT  ANLDSE**S**EHFFRC  LAFEEG**S**QSTTIS  FEEGSQ**S**TTISSL  ECKRNL**S**DIDQSF  RSRGRA**S**PGGVST  GRSEEI**S**ESESEE  SEEISE**S**ESEETN  ELPRPQ**S**PSDLDS  PRPQSP**S**DLDSLD  SPSDLD**S**LDGRSL  PGYKPG**S**PPSFRT  EYETPE**S**PVPPAR  PVPPAR**S**PSPPPK  PPARSP**S**PPPKVV  SNSDGI**S**PKRRRL  GLITPG**S**PPPAQQ  TRKRRW**S**APESRK  NPLPVN**S**GQAQRV  QRVLCP**S**NSSQRV  ETYKRI**S**RVEFTF  GYEPEG**S**ASPTPP  EPEGSA**S**PTPPYL  TRTGSE**S**PKVCSD  HEENPE**S**ILDEHV  RTPGRQ**S**PGPGHR  PGPGHR**S**PDSGHV  SEMETP**S**AINGNP  SWHLAD**S**PAVNGA  PLAPPA**S**PGPFAT  RSRSQA**S**PSEDEE  RSQASP**S**EDEETF  TRTRTS**S**SCSSYS  MMMMTT**S**QKHRDF  IRSRHS**S**YPAGTE  GMGEEP**S**PFRGRS  FRGRSR**S**APPNLW  RELRRM**S**DEFVDS  GLPRPK**S**AGTATQ  PGEPGL**S**HSGSEQ  EPGLSH**S**GSEQPE  GLSHSG**S**EQPEQT  GGARGG**S**GGFGDE  YQVPGP**S**PQFQSP  PSPQFQ**S**PPAKQT  VYDVPP**S**VEKGLP  AEGKRL**S**ASSTGS  STGSTR**S**SQSASS  QSRPLP**S**PPKFTS  DPVART**S**PLQTPA  AAGPAL**S**PVPPVV  SASPSS**S**PSQSPP  SSSPSQ**S**PPRDPP  VWNRRH**S**RSPRRG  NRRHSR**S**PRRGRS  GEPQEE**S**PLKSKS  EESPLK**S**KSQEEP  TFEHDP**S**ESIDEF  EHDPSE**S**IDEFNK  LSAYDN**S**PRSPHS  SAKNTP**S**QHSHSI  PSQHSH**S**IQHSPE  SHSIQH**S**PERSGS  NGSSRY**S**PSQNSP  YSPSQN**S**PIHHIP  PIHHIP**S**RRSPAK  EALDYF**S**DKESGK  YFSDKE**S**GKQKFN  KQKFND**S**EGDDTE  YVVERP**S**TTKDKH  VKKETQ**S**PEQVKS  SPEQVK**S**EKLKDL  KDLFDY**S**PPLHKN  STFREE**S**PLRIKM  ASTLVH**S**VKKEQE  HSTRQK**S**PEIHRR  HRRIDI**S**PSTLRK  RIDISP**S**TLRKHT  KKLRCD**S**ADLRHD  SSASPS**S**PSSREE  GLVSPQ**S**PQKSDC  SDCQPN**S**PTESCS  PPARMM**S**TESANS  RMMSTE**S**ANSFTL  STESAN**S**FTLIGE  PIQMEN**S**MGTLRT  MGTLRT**S**ISVERQ  TLRTSI**S**VERQIH  RQADGI**S**AEVTAS  ALPKKK**S**RKSSMP  KKSRKS**S**MPIKIE  DLYRLQ**S**GEEEEE  IQTPLK**S**AKTRKV  GKGKRS**S**RANTVT  NISGNG**S**PLADSK  GSPLAD**S**KEIFLT  PVGGGE**S**LRLLAS  SLRLLA**S**DLQRHS  PHFLDW**S**GEALQP  ARSNPK**S**PQKPIV  LTSPSP**S**KSIPIP  QRDRSS**S**APNVHI  GSTTGL**S**ATPPAS  SATPPA**S**LPGSLT  TLGRRD**S**SDDWEI  LGRRDS**S**DDWEIP  KIHRSA**S**EPSLNR  AKKENN**S**PEHLKD  AEFCNK**S**KQPGLA  SDELLG**S**DDSHDG  LLGSDD**S**HDGESE  NEVDEY**S**GSSEKI  TSKRHD**S**DTFPEL  PKDLML**S**GERVLQ  GHEVNH**S**RETSIE  PLFPIK**S**FVKTKC  MGSSHA**S**QVCSET  GAKKLE**S**SEENLS  SSEENL**S**SEDEEL  SEENLS**S**EDEELP  VILAKA**S**QEHHLS  KQMRHQ**S**ESQGVG  SQGVGL**S**DKELVS  SDKELV**S**DDEERG  DCSGLS**S**QSDILT  VLEQHG**S**QPSNSY  EKAVLT**S**QKSSEY  QNRNYP**S**QEELIK  SDTDFQ**S**QKSLLY  PICTFV**S**PAAQKA  PVVAVS**S**PAVPPP  KVVAPP**S**SSDSSS  PPSSSD**S**SSDSSS  SDSSSD**S**SSDSDS  SSSDSS**S**DSDSST  SSSDSD**S**STDDSE  SSDSDS**S**TDDSEE  DSSTDD**S**EEERAQ  PPPTYE**S**EEEDKC  KEEKIH**S**PIIRSE  SSSPLS**S**PRSPVF  LPGPPG**S**PRSSGG  RNSFLG**S**PRFHRR  VLLGRS**S**PSKNYV  LGRSSP**S**KNYVMA  QQKKKT**S**PLNFKK  RGSKKG**S**IDVEKI  GPLYVF**S**PTEELR  SLKPGS**S**HRKTKK  YRPHLA**S**EKVYTI  DVMDEE**S**SSSSSS  EAELLT**S**AEKRAE  SEKKNK**S**PPADPP  LSIKKL**S**PIIEDS  SPIIED**S**REATHS  KVGKLT**S**PGALLF  EASGYI**S**SLEYPR  ARQRLI**S**LMSGEL  RLISLM**S**GELQRK  LKNQTY**S**FSPSKS  NQTYSF**S**PSKSYS  SEEIIG**S**PIPEPR  RLLRSQ**S**ESSDEV  VTELDL**S**HGKKDA  MTVEKA**S**PVGDGN  ASTPTY**S**PAPTQP  MPPGPC**S**PGPLAQ  LPPDPP**S**PPSQPP  VPKKQN**S**PVAPTA  SLRRHG**S**MVSLVS  YEEGPN**S**LINEEE  PYSRSS**S**MSSIDL  QGRLRK**S**LPSLLR  LRKSLP**S**LLRNVL  NVLTEE**S**VVRESK  ESVVRE**S**KSFTRS  VVRESK**S**FTRSTV  SKSFTR**S**TVDTMA  NGDGEI**S**TSELRE  VRSFPL**S**SEHAKF  EESVKE**S**AEVEEI  IYAVEA**S**TMAQHA  ERLQNP**S**ESSEPI  LQNPSE**S**SEPIPL  EPIPLE**S**REEYMN  KDTRNE**S**TQNCVV  PEKMES**S**ISSSSE  KMESSI**S**SSSEEM  MESSIS**S**SSEEMS  ESSISS**S**SEEMSL  SSISSS**S**EEMSLS  ARETIE**S**LSSSEE  ETIESL**S**SSEESI  TIESLS**S**SEESIT  IESLSS**S**EESITE  VARRHQ**S**LSPNHP  DGINRL**S**FSSTGS  LVNRKV**S**QRRVDF  SKERSS**S**LEGSPD  VMTISD**S**PREQDS  LEMDLS**S**PQTRYI  DDDDDN**S**DEEDND  EEDNDD**S**DDDDDE  DSYSHH**S**GLEYAD  PEKFAD**S**DQDRDP  AMADEL**S**EKQVYD  YSTKPG**S**YIFRLS  QKGIVR**S**PCGSPT  VRSPCG**S**PTGSPK  CGSPTG**S**PKSSPC  RHSRVG**S**DPVLMR  LSAMGK**S**PLSQGD  EDLQGA**S**QVKEET  KSRFVD**S**GEMSES  VDSGEM**S**ESFPYR  QPPRSI**S**PSALQD  LLRTLK**S**PSSPQQ  TLKSPS**S**PQQQQQ  RVLEEG**S**VEARTI  ILVDTA**S**PSPMET  VDTASP**S**PMETSG  PSPMET**S**GCAPAE  DLCQAF**S**DVILAV  IAPSRG**S**PLPVLS  SEQNRA**S**PLPSGL  LLTPPQ**S**GKKQSS  MMMYHN**S**SQKRHW  VGNLRE**S**PLGQEK  EDDDYV**S**KKSKHE  DDDLVE**S**LLLLLL  AHSRLE**S**YRPDTD  RPDTDL**S**REDTGC  ASTIFL**S**KSQTDV  TIFLSK**S**QTDVRE  VREKRK**S**LFINHH  QIARKY**S**SCSTIF  IARKYS**S**CSTIFL  RKYSSC**S**TIFLDD  AEANNL**S**FPLEPL  FPLEPL**S**RERAHK  RSARKR**S**ASADNL  ARKRSA**S**ADNLTL  RRHRSH**S**AEGGKH  EGFPIT**S**LREINT  LAREYG**S**PLKAYT  QDGVRQ**S**RASDKQ  VRQSRA**S**DKQTLL  VCIAVN**S**RRRCGQ  KKLVIN**S**GNGAVE  VEDRKP**S**GLNGEA  NGEASK**S**QEMVHL  GTVLSL**S**PSYRKA  KYLKDE**S**GFKDPS  AIGYIH**S**LMGNFE  TLKNII**S**PPWDFR  TPDSRP**S**LEETFE  AAGPAP**S**PMRAAN  KVQTTP**S**KPGGDR  AQMEVA**S**FLLSKE  RLKVLY**S**QKATPG  KATPGS**S**RKTCRY  LPLETP**S**PGDGSY  FSQSGN**S**REVTPI  RLFTSD**S**STTKEN  QPNIND**S**LEITKL  ITKLDS**S**IISEGK  LDSSII**S**EGKIST  QLHAAE**S**DEFFFF  LPAPQI**S**DAELQE  TPRTPA**S**QDRILQ  TPFRTP**S**NGAEGL  PKPVIN**S**TPGRTP  KIKRQL**S**MTLRGG  SGGGGG**S**DPGEAP  EDLKMG**S**DGESDQ  MGSDGE**S**DQASAT  SSDEVQ**S**PVRVRM  DINKRL**S**LPADIR  EKLTLN**S**PIFDKP  RRLRRV**S**LSEIGF  LRRVSL**S**EIGFGK  MALDVK**S**RAKRYE  LAKSFG**S**PNRAYT  ETLKEQ**S**NPALAI  GLARAF**S**LAKNSQ  PPRRKG**S**QITQQS  SQITQQ**S**TNQSRN  QQSTNQ**S**RNPATT  SDYGPP**S**LGYTQG  EDHVDL**S**LSCTLV  GEQAEG**S**PGGPGD  RKRRQT**S**MTDFYH  KRRLIF**S**KRKPPP  VRVSNG**S**PSLERM  MEPAAG**S**SMEPSA  EPAAGS**S**MEPSAD  AGGTRG**S**NHARID  DAAEGP**S**DIPDDD  GELMLP**S**QGLKTS  PPPPPP**S**HPHPHP  PPTPVP**S**PHPAPA  MGPRRR**S**RKPEAP  EAPRRR**S**PSPTPT  PRRRSP**S**PTPTPG  TPTPGP**S**RRGPSL  QAADEI**S**FSSNSS  SFSSNS**S**FVLKIL  DVDLDL**S**DKDYSS  ALLETA**S**PRKLSG  VTEAEG**S**QGTAEA  ADGPGA**S**SGDEDA  DGPGAS**S**GDEDAS  ASGRAA**S**PESASS  REKVAA**S**PKSPTA  VAASPK**S**PTAALN  TAALNE**S**LVECPK  AQHTVG**S**PRDRKP  RSPRGL**S**PAAHLG  NQCRRQ**S**EDSTFY  EGGITL**S**GGQRAR  SAERRN**S**ILTETL  TETKKQ**S**FKQTGE  GEKRKN**S**ILNPIN  NSIRKF**S**IVQKTP  PLERRL**S**LVPDSE  AILPRI**S**VISTGP  QARRRQ**S**VLNLMT  HRKTTA**S**TRKVSL  ASTRKV**S**LAPQAN  IYSRRL**S**QETGLE  RQAISP**S**DRVKLF  FPHRNS**S**KCKSKP  LGAGGG**S**PEKSPS  GGSPEK**S**PSAQEL  SPEKSP**S**AQELKE  FDPVTR**S**PLTQEQ  KRPRVT**S**GGVSES  SGGVSE**S**PSGFSK  FSKHIQ**S**NLDFSP  QSNLDF**S**PVNSAS  NVKYSS**S**QPEPRT  VQGISF**S**QPTCPD  SSGTLS**S**LETVST  STQELY**S**IPEDQE  KILGET**S**LMRTLC  EVWAEV**S**EKALDL  VTLFGE**S**AGAASV  SSTARR**S**RAASSQ  PRSRER**S**PSPLRG  SRERSP**S**PLRGNV  RGNVVP**S**PLPTRR  RRTRTF**S**ATVRAS  WSGHVI**S**SSSSSS  SGHVIS**S**SSSSSS  RICRVF**S**TSPAKD  VRAEQP**S**PPNSDS  PDPEPP**S**PDSPTE  GLPRAE**S**PSPAPP  PRAESP**S**PAPPPG  MMMMMP**S**AKQRGS  GGHGAA**S**PSEKGA  HGAASP**S**EKGAHP  RERDFT**S**LENTVE  DMEALR**S**TLQTME  KRRRSR**S**WSSSSD  DTDYRH**S**YEYQRE  SSRRAK**S**VEDDAE  SKEAEK**S**GEATDG  PQAEGD**S**EGLSQG  DREKGL**S**AEPGWQ  EIRKGE**S**RSEALA  KGEQEH**S**QQKEEE  LFRGGK**S**GELEQE  QEEERL**S**KEWEDS  RGWRPS**S**REDSLE  PSSRED**S**LEAGLP  KEEAPR**S**SVTETE  EEAPRS**S**VTETEA  ITPWPD**S**RDLDLS  NILRLE**S**EETMVL  GKKLVL**S**SEKTVL  IPIEDG**S**GEVVLS  SGEVVL**S**RKVLLD  AARRRR**S**VQLTEK  IPPADL**S**DQVPDT  SASLLR**S**EETKEN  QTIKSG**S**DEVQVG  KVVARG**S**FEFPVG  MMMMMA**S**GVAVSD  ASGVAV**S**DGVIKV  AEKLGG**S**AVISLE  IGLHSD**S**QEELWM  NGDINL**S**LRKAKK  EEAKRK**S**PKKKEK  TLEARN**S**SEKLPT  LEARNS**S**EKLPTE  AIKLGF**S**LTPSKG  ELTSSY**S**PDVSDY  PRLIIE**S**PSNTSS  ELPTRV**S**SPVFGA  LPTRVS**S**PVFGAT  NTSLSP**S**LLQPGK  DPGADL**S**QYKMDV  IDTKDG**S**QSKLGG  ERKMND**S**LEDMFD  CLADSF**S**QAADEE  MMMMMM**S**FRKVVR  EKERLH**S**PGATST  VQLGEV**S**GVEAEP  TRRLDE**S**LTQSLT  DESLTQ**S**LTSPGP  QNGEEK**S**PPNASH  KFKVKS**S**PLIEKL  ALLPGA**S**PKSPGL  PGASPK**S**PGLKAM  GLKAMV**S**PFHSPP  MVSPFH**S**PPSTPS  PFHSPP**S**TPSSPG  SRRFRR**S**QSDCGE  RFRRSQ**S**DCGELG  KSKAPG**S**PLSSEG  PLRRSP**S**RTEKQE  GEKARR**S**SEEVDG  EKARRS**S**EEVDGQ  QEEVPE**S**PQTSGP  AENRCG**S**PREEKP  PHTNET**S**LLLLLL  DEASEQ**S**DEEDSV  ILSRRP**S**YRKILN  KILNDL**S**SDAPGV  GVVMAS**S**PALPTQ  MTKLRA**S**TTSETI  SWRRTN**S**DSALHQ  SSHYGG**S**LPNVNQ  GLAEFQ**S**PLHSPL  FQSPLH**S**PLDSSR  YSPAYL**S**PPPESS  ALNRTS**S**DSALHT  AMNTGG**S**LPDLTN  SLSGGN**S**TSNLTH  QLQGSH**S**HPSLPA  LGHPSL**S**APALSS  HRRVPL**S**PLSLLA  QLPKQF**S**PTMSPT  LPPYPY**S**SPSLVL  PPYPYS**S**PSLVLP  YPYSSP**S**LVLPTQ  HCSRHG**S**GPNIIL  ILTGDS**S**PGFSKE  PFFPFH**S**PSRLFD  PTSTSL**S**PFYLRP  SFLRAP**S**WFDTGL  PQGSDS**S**DSELEL  SSVPTP**S**PLGPLA  LGPLAG**S**PVIAAA  MMMMMM**S**SSEEVS  MMMMMS**S**SEEVSW  AASNFK**S**PVKTIR  HCVLLA**S**KENSAP  LASKEN**S**APVKLG  AVLAAV**S**SHKFNS  LREKKF**S**TKSDVW  NQIHVK**S**PPREGS  SPPREG**S**QGELTP  ELTPAN**S**QSRMST  VQKSMG**S**QEDDSG  NKPSSY**S**SSSSSS  AGALDV**S**FNKFIP  IESETT**S**EEQIQE  SEPFSF**S**QGPLKD  HPPHAP**S**PGQTVK  MMMMDD**S**EVESTA  LERVRV**S**PQDANP  GAMSVV**S**VETSDD  DGYPGG**S**DNYGSL  GSDNYG**S**LSRVTR  EGYRAP**S**RQDVYG  QVRVGG**S**SVDLHR  VRVGGS**S**VDLHRF  LEDDQR**S**MGYDDL  PRRRLR**S**YEDMIG  AQHERG**S**LASLDS  ERGSLA**S**LDSLRK  SLASLD**S**LRKGGP  TGPHAA**S**CFGAKK  LVLINK**S**GNRSEK  KEGWKK**S**DFQVNL  VNLNNA**S**RSQSSH  LNNASR**S**QSSHSY  RSQSSH**S**YDDSTL  SHSYDD**S**TLPLID  IDRNQK**S**DKKPDR  MGSNTK**S**LDNNYS  NRTLDR**S**GDLGDM  QDEGQE**S**LEEELD  DRKAAV**S**HWQQQS  SHWQQQ**S**YLDSGI  QQSYLD**S**GIHSGA  LDSGIH**S**GATTTA  ATTTAP**S**LSGKGN  RHAIMR**S**PQMVSA  LVKMLG**S**PVDSVL  DTQRRT**S**MGGTQQ  DYKKRL**S**VELTSS  ATSGAL**S**PAKDCG  PAKDCG**S**QKYAYF  QNWANY**S**AEQNRM  RMGQAG**S**TISNSH  QAGSTI**S**NSHAQP  GSTISN**S**HAQPFD  PDDNQN**S**KKLAAG  VDQRPS**S**RASSRA  RPSSRA**S**SRASSR  PSSRAS**S**RASSRP  ASSRAS**S**RPRPDD  AQHALT**S**VSRGSS  HALTSV**S**RGSSLK  TSVSRG**S**SLKILS  SVSRGS**S**LKILSK  SSLKIL**S**KGKRGG  KRGGHS**S**VSTESE  TESESS**S**FHSSSS  ESSSFH**S**SSSSSS  AEDPAK**S**LTEIST  TENRFH**S**LPFSLT  ELGFDA**S**EVELTR  GGPMDA**S**VEEEGV  SPARSS**S**YSEANE  SSSKGD**S**PELKPR  AVCRSL**S**TPQFNS  KILRFR**S**GSLKLM  MMMMMS**S**PPEGKL  DDQEWE**S**PSPPKP  QEWESP**S**PPKPTV  SMDKHP**S**PRTQHI  ALSRKA**S**AVNMEK  RKKWKQ**S**VRLISL  NMSVAR**S**DDTLDE  SSRFPP**S**PLASKP  AAQPGP**S**HPLPNA  ENTASQ**S**PRTRGS  EKELDL**S**ELDDPD  GKDGDK**S**PMSSLQ  KICTLP**S**PPSPLA  TLPSPP**S**PLASLA  SSTRVD**S**PSHGLV  SSLCIP**S**PARLSQ  EEIIVL**S**DSDDDD  IIVLSD**S**DDDDDD  GEDFPA**S**PQRRNT  GRPVDA**S**PRVPGG  PRVPGG**S**PRTPNR  VSSNVA**S**VSPIPA  SNVASV**S**PIPAGS  PPSTKK**S**GQKPVL  RKPVTV**S**PTTPTS  SPTTPT**S**PTEGEA  PKRSCP**S**FSASSE  CPSFSA**S**SEGTRI  FEELTM**S**QKNGGN  LSTNAD**S**QSSSDF  RMNGLP**S**PTHSAH  RTLQAL**S**NEKKAK  IVYAVS**S**DRFRSF  LADLTR**S**LSDNIN  IYTIDG**S**RKIGSM  GSRKIG**S**MDELEE  YAQDDF**S**LDENEC  TAGPKA**S**PTPQKT  PTPQKT**S**AKSPGP  QKTSAK**S**PGPMRR  PMRRSK**S**PADSAN  TPKSKQ**S**PISTPT  PISTPT**S**PGSLRK  TPTSPG**S**LRKHKD  DLYLPL**S**LDDSDS  SLDDSD**S**LGDSMM  TSSSPS**S**LPRTPT  ARLLMP**S**QLVSQV  SDAGLE**S**DTAMKK  SEVDMN**S**PKSKKA  PSQNDI**S**PKTKSL  TKNEEP**S**EEEIDA  GETREK**S**PKLKNG  EAASEE**S**NSEIEQ  KRIGVP**S**ATEIIK  FDDRGR**S**DYDGIG  SRWCDK**S**DEDDWS  PHIESF**S**DVEMGE  SGASSS**S**FSSSRA  PKPKQF**S**SFEKRA  KPKQFS**S**FEKRAK  TPTQPA**S**EKEPEM  PGPREE**S**EEEEDE  EEEKEK**S**LIVEGK  RNAMLK**S**ICEVLD  KSKKTC**S**KGSKKE  KTCSKG**S**KKERNS  SKKERN**S**SGMARK  KCPEIL**S**DESSSD  EILSDE**S**SSDEDE  ILSDES**S**SDEDEK  LSDESS**S**DEDEKK  KKNKEE**S**SDDEDK  KNKEES**S**DDEDKE  DDEDKE**S**EEEPPK  NVKKAD**S**STTKKN  VKKADS**S**TTKKNQ  TKKNQN**S**SKKESE  NSSKKE**S**ESEDSS  SKKESE**S**EDSSDD  ESESED**S**SDDEPL  SESEDS**S**DDEPLI  TSPGSV**S**PSRDSS  PGSVSP**S**RDSSVP  STSPPP**S**PEVWAD  VWADSR**S**PGIISQ  RESVGG**S**PQTKHL  EEEDDD**S**GEEMKA  GTSKSS**S**LPAYGR  RMDRGN**S**LPCVLE  RLERHL**S**AEDFSR  ELKKKA**S**LFFFFF  SSSQRV**S**SYRRTF  PGFPLG**S**PLSSPV  YQVSRT**S**GGAGGL  GAGGLG**S**LRASRL  VELDET**S**QGLVPE  GFFRVE**S**PVKLFS  KLFSGL**S**VSSEGP  RLGTPK**S**VNKAVS  IPQQTT**S**PENAGP  LSSERM**S**LPLLAG  GMELNS**S**ITSQDV  NTASQN**S**ILEEGE  ECHLLD**S**PGLNCS  SPGLNC**S**NPFTQL  EHARHI**S**FGGNLI  GNLITF**S**PLQPGE  LNKLIE**S**AEELIR  ADQRQR**S**LSTSGE  QRQRSL**S**TSGESL  LSTSGE**S**LYHVLG  PASQEP**S**PAASAE  LLAEEK**S**KPIPIM  IPIMPA**S**PQKGHA  PVARKL**S**AREQRD  GMADAN**S**PPKPLS  SPPKPL**S**KPRTPR  TPRRSK**S**DGEAKP  EAKPEP**S**PSPRIT  KPEPSP**S**PRITRK  IFDANE**S**GFESYE  QEKIYI**S**KIVVEF  NIPEMP**S**PKKMHQ  QNKNRI**S**WVGEAV  LEAVTP**S**PSFQQR  FQQRHE**S**LRPAAG  SQETHD**S**PFCLRK  SHDNRK**S**TSFMSV  KSTSFM**S**VSPSKE  TSFMSV**S**PSKEIK  VRRSSM**S**SCGSSG  RRSSMS**S**CGSSGY  MSSCGS**S**GYFSSS  SGYFSS**S**PTLSSS  SSSPTL**S**SSPPVL  SSPTLS**S**SPPVLC  SPTLSS**S**PPVLCN  NGLARL**S**SPVLHR  GLARLS**S**PVLHRL  TPIPTR**S**PSDSST  IPTRSP**S**DSSTAS  PPRGPG**S**PAEDLM  NKLCQF**S**PVQELS  AQRSLL**S**PLHRSG  SLPYLH**S**PITTSP  HSPITT**S**PSCCCC  LPKAGG**S**PAPGPE  YKFFFK**S**MDDDFG  PHAGGG**S**QENLDN  TTSFFD**S**DEDDST  LTASRQ**S**FRMAMG  AILLFK**S**GGGGGG  KIVDFG**S**SCQLGQ  NSGRAR**S**DPTHQH  GADREE**S**PMTGVC  GVCVQQ**S**PVASSS  ELLGMP**S**QKLLDA  QKLLDA**S**KRAKNF  KEKKAV**S**PLLLTT  QRKAKR**S**LAPRFD  GTLRYM**S**PEQISS  MHHEAL**S**EALPGD  RENIQK**S**LAGSSG  PQTQHV**S**PMRQVE  DIDLFG**S**DNEEED  GVDGGQ**S**PRAGHD  AGHDGD**S**DGDSDD  KVNKYY**S**NLTKSE  YSNLTK**S**ERYSSS  KSERYS**S**SGSPAN  SGSPAN**S**FHFKEA  TEEKCG**S**PQVRTL  PQVRTL**S**GSRPPL  SLPSSP**S**SATPHS  GDSGYP**S**EKRGEL  SNRKYE**S**DEDSLG  YESDED**S**LGSSGR  DEDSLG**S**SGRVCV  KWNLLN**S**SRLHLP  LHLPRA**S**AVALEV  VMKFSV**S**PVVRVA  VLCLSK**S**PNKHNR  HTSRTL**S**FGSDLN  ERRLGD**S**SLSRKD  RRLGDS**S**LSRKDS  PRESRP**S**EEREWD  VPPPAL**S**RDRERD  AEKDRE**S**LRRTKN  AEAEAA**S**GPSESP  EAASGP**S**ESPSPP  ASGPSE**S**PSPPAA  GPSESP**S**PPAAEE  RDERSD**S**RAQAVS  SRAQAV**S**EDAGGN  GDADEP**S**FSDPED  ADEPSF**S**DPEDFV  DFVDDV**S**EEELLG  IFLEYA**S**PAHAVD  RFFTTG**S**DSESES  FTTGSD**S**ESESSL  TGSDSE**S**ESSLSG  SDSESE**S**SLSGEE  DSESES**S**LSGEEL  ESESSL**S**GEELVT  KQPLLL**S**EDEEDT  KQNPEQ**S**ADEDAE  GYRQQQ**S**QTAYYY  AAAPAS**S**SDPAAA  IMKTCF**S**PNRVIG  DFDSKP**S**WADQVE  LATGDT**S**PEPELL  AEQLGL**S**TGEKEK  CKEKDF**S**PEALKK  AAAAGD**S**DSWDAD  AAGDSD**S**WDADAF  WDADAF**S**VEDPVR  KKLQEE**S**DLELAK  DLELPL**S**PSLLGG  HFWSTL**S**PIAPRS  SPIAPR**S**PAKLSF  STPVVL**S**PGPQKP  DNYADL**S**DTELTT  HGPVVG**S**TRRLYE  TQRRRL**S**PPSSSA  LSPPSS**S**AASSYS  SAASSY**S**FSDLNS  DALLYQ**S**KGYNDD  THYRPV**S**ASRSSL  DPRMLV**S**CIDFRY  LDGLNA**S**QIRELR  KEEKEE**S**DDEAAV  DLPLNV**S**RETLQQ  MMMMMM**S**QKQEEE  GQKYFD**S**GDYNMA  LPQRKS**S**LVTSKL  NQRARQ**S**PEDVYF  DVYFSK**S**EQLKPL  KFADIV**S**ILDKLI  DFDPRV**S**IRLPST  VSIRLP**S**TSGSEG  LPSTSG**S**EGVPFR  ELLVVG**S**RPELGR  SDPWGG**S**PAKPST  ALPTSG**S**SAGELE  LPTSGS**S**AGELEL  GEVPAR**S**PGAFDM  MSGVRG**S**LAEAVG  LAEAVG**S**PPPAAT  LKGKKL**S**LPAAAA  WAYKPE**S**SPGSRQ  RRLGRG**S**VSDCSD  GSVSDC**S**DGTSEL  VYNYHL**S**PRAFLH  IKVEPI**S**EGESEE  PISEGE**S**EEVEVT  VEVTDI**S**DEDEED  LTPRRV**S**SDLQHA  SLEHRD**S**SSSSSS  IKAEPA**S**PDSPKG  EPASPD**S**PKGSSE  APSTNS**S**PVLKTK  RLKVNF**S**DDSDLE  VNFSDD**S**DLEDPV  FEILRG**S**DGEDSA  PPLNSV**S**PSPLML  LNSVSP**S**PLMLLH  HPPPQL**S**PFLQPH  GRERLA**S**TNDKGS  PTPGHL**S**PLVVHR  YAEPQK**S**PWCEAR  KGGLLD**S**MCPAST  DSMCPA**S**TPSVLS  SDSLLQ**S**WNSQSS  IKKEPQ**S**PRTDPA  PYQHQL**S**EPCPPY  SYTPPE**S**PVPSYA  NMIRRL**S**PAERAQ  SHPKPS**S**PRQEST  NKNVFT**S**AEELER  IWHRNY**S**PRPESG  IVKRPR**S**AELSED  EGNKSL**S**FSEVFV  KFTRTI**S**PPTLGT  DFSRTP**S**PSPSTA  FRRKSD**S**PTSLPE  LKSEES**S**DDESHP  REEACS**S**QSQESG  NIKLLD**S**QSDQTS  GLYKSS**S**ADSLST  STSPSL**S**QTTPSK  SLLLIQ**S**RKNCVN  WRKRVK**S**EYMRLR  NNSSRP**S**TPTINV  QNPANM**S**KYQSNP  DKASKM**S**SPETDE  KASKMS**S**PETDEE  FGEYSR**S**PTFFFF  PLMRRN**S**VTPLAS  SVTPLA**S**PEPTKK  GAGEFK**S**QEHAKH  TSVRRR**S**LQHQEQ  RGAQKG**S**GSPTHS  AQKGSG**S**PTHSLS  LCSPSE**S**DSDLEP  SPSESD**S**DLEPVG  MMMMAF**S**QVQCLD  EYWPQK**S**DRSIPQ  PQKSDR**S**IPQLDL  ALVKAK**S**VDEIAK  PGPRRP**S**VASSVS  RPSVAS**S**VSEEYF  SVASSV**S**EEYFEV  PPRRQL**S**APHITR  VGPQGG**S**PWAQSR  CKDDIN**S**YECWCP  DVDYVN**S**TEAETI  VSKRRL**S**KSEDKE  PEILGD**S**QHADVG  AADRSE**S**PSLTQE  DRSESP**S**LTQERA  DGGPVT**S**QESGQK  QGEEIK**S**QNSQES  EIKSQN**S**QESTAD  ESTADE**S**EDDMSS  SSQASK**S**KATEDG  GEEDEV**S**AGEKEQ  NRSGAM**S**PMSWNS  GTGMER**S**PGAMER  STYYKA**S**KGKLPI  SRPGYP**S**PRSSEG  SEGFYP**S**PQHMVQ  APGHLG**S**LASLSS  LQPQEI**S**PPPTAN  ADGGSA**S**PIKDQP  FLGRIL**S**LTSSAS  GKTIET**S**PSLSRM  LGSLSS**S**QCSQSL  DKSADF**S**QSTSIG  LSEEAS**S**QATLPN  MSRQTT**S**VGSSCL  YLLEAA**S**HLSPEN  EAASHL**S**PENFVE  VISRST**S**PTFNKQ  SENSAS**S**PPRFKT  IKNFKP**S**PSKNKG  NPEPPV**S**GEMDDN  ETAAAD**S**EPDTDP  MKFIYT**S**QYDNCL  PRSGTN**S**PPPPFS  QFNKDD**S**LLLASG  QRRRIT**S**VQPPTG  DCLTFG**S**PVLMRH  MRHLTA**S**EAKKLP  LEKYGH**S**DCCSQS  GHSDCC**S**QSEEGR  SDCCSQ**S**EEGRHN  DFNQFS**S**GEKNIF  KAPQLT**S**SELMAI  APQLTS**S**ELMAIT  VYAKCD**S**SPDSAE  YAKCDS**S**PDSAED  CDSSPD**S**AEDVRK  TFMGVV**S**LGSPSG  GVVSLG**S**PSGEVS  VSLGSP**S**GEVSHP  TAWTAD**S**GEGDFL  VVERHQ**S**ACKDSD  QSACKD**S**DWPFCS  SDWPFC**S**DEDWNY  TSYGTG**S**ETESPR  SPRNPS**S**AGSWNS  NPSSAG**S**WNSGSS  GTWNPG**S**SERGSA  GKEKVT**S**GSTTTT  MDLGTL**S**GIGTLD  AFFDTA**S**TGKTFP  FTNTKE**S**SSHHPG  MADEAG**S**EADHEG  VDKDLQ**S**LEDILH  QADRED**S**REEEEE  LRLAGA**S**PATVAA  LRRRKD**S**AQDDQA  AEAVLQ**S**PGLSGK  PSKVRA**S**GPGLER  EVAVPK**S**PFRVGV  GRERLG**S**FGSITR  KQFRYE**S**QLQMVQ  SGLHGN**S**FHSEDE  QAQVED**S**SSSSSS  EIMPPN**S**LPSNNS  HFSQPN**S**TKVQRV  SEASNA**S**ETESDH  AAHRKG**S**SSNEPS  SSDSLS**S**PTLLAL  KKVVIK**S**EAASPA  IKSEAA**S**PALPVI  TKVETL**S**PESALQ  ETLSPE**S**ALQGSP  ESALQG**S**PRSAAS  ASTPAG**S**PDGSLP  IMTLRT**S**PPGGEL  PPGGEL**S**PGAGRA  HPSGPT**S**PLSALN  KPLDPG**S**PQLPEH  LLPRVS**S**YLVPIQ  VPFGNS**S**PSDIDV  LDTMND**S**LSKILL  KILLDI**S**FPGLDE  SAGWKN**S**IRHNLS  SIRHNL**S**LHSKFI  NEGTGK**S**SWWMLN  EGTGKS**S**WWMLNP  GGKSGK**S**PRRRAA  PRRRAA**S**MDNNSK  QEGAGD**S**PGSQFS  FRPRTS**S**NASTIS  RTSSNA**S**TISGRL  SNASTI**S**GRLSPI  TISGRL**S**PIMTEQ  PRRRAA**S**MDSSSK  FRPRSS**S**NASSVS  SIRHNL**S**LHSRFM  PRRRAV**S**MDNSNK  LQTAPE**S**ADDSPS  PESADD**S**PSQLSK  FRSRTN**S**NASTVS  NITLPP**S**QPSPTG  LMQRSS**S**FPYTTK  PYTTKG**S**GLGSPT  GGSRAL**S**NSVSNM  ALSNSV**S**NMGLSE  TLSDSL**S**GSSLYS  LECDME**S**IIRSEL  SLALGP**S**PGASPS  EFRKKR**S**QRPSRC  KVNASA**S**SLKKKQ  VNASAS**S**LKKKQI  GDSRLD**S**SEFLKF  EPPGGE**S**AEEEEN  GCKVQI**S**PDSGGL  VQISPD**S**GGLPER  LPERSV**S**LTGAPE  ILIQDG**S**QNTNVD  LFIIRG**S**PQQIDH  MTIDKL**S**WGYRRE  WQEECP**S**DSEEDE  EECPSD**S**EEDEGR  QEDAGK**S**PARNRS  SLDRSG**S**PMVPSY  SYETSV**S**PQANRT  ISAMDA**S**PRNISP  ASPRNI**S**PGLQNG  RGTAGK**S**PDLSSQ  TEPQEE**S**EEEVEE  EDAQKS**S**SPAPAD  DAQKSS**S**PAPADI  VELRIN**S**GGKLPN  FQERDP**S**KIKWGD  AKRVII**S**APSADA  NSLKII**S**NASCTT  KIISNA**S**CTTNCL  VPTANV**S**VVDLTC  HFVKLI**S**WYDNEF  PRVWYV**S**NIDGTH  SLRNVS**S**AGHGPR  AGHGPR**S**SPAELS  GHGPRS**S**PAELSS  PAELSS**S**SQHLLR  AELSSS**S**QHLLRE  LRERKS**S**APSHSS  SSAPSH**S**SQPTLF  ENARSA**S**FSQGTR  SQGTRA**S**FLMRSD  ASFLMR**S**DTAVQK  NTEFRD**S**TYDLPR  HGHTKG**S**LTGSET  RLHRAS**S**CETYEY  SVYIPM**S**PGAHHF  DELPFK**S**PITKSW  ALDFQP**S**SPSPHR  LDFQPS**S**PSPHRK  LPAAAS**S**EDIERS  CVEELC**S**REEARE  MDFGRR**S**AEDENN  DPALVS**S**TPESGV  AEAYRH**S**PVFQVY  LKTERL**S**PDLLTL  FFSPTG**S**PLNSAA  NSAAYS**S**PKLRGT  TRNRKA**S**GKGKKK  KGERPR**S**EEDNEL  YSSILS**S**LGENPQ  QGQISL**S**SGETDL  NLNRST**S**VPENPK  VPENPK**S**SASTAV  LEFSSG**S**PGKETN  GKETNE**S**PWRSDL  DENCLL**S**PLAGED  GDLVLS**S**PSNVTL  HLTSTT**S**SPGVIV  LTSTTS**S**PGVIVP  EETVED**S**LVEDSE  SAARGL**S**PADVAQ  SRKRAL**S**ISPLSD  KRALSI**S**PLSDAS  SPLSDA**S**LDLQRM  EGLRPA**S**PLALTQ  RGLRLQ**S**HPSTDG  SHSQSR**S**PGRPTQ  MLNRRD**S**SASTIS  LSSRRS**S**GISPCF  FSSRRS**S**EASQAE  DASRRS**S**EASQSD  HAPRRC**S**DGGAHG  HGVRRA**S**DPVRTG  KRPRGV**S**LTNHHF  KPLYIP**S**NRVNDG  LQAGKK**S**LEDQVE  FEEAER**S**LKDMEE  LKDMEE**S**IRNLEQ  CPFKLV**S**QKPKLG  GNEAVA**S**RDLSEN  QAALSV**S**QENPEM  RAAARL**S**LTDPLV  GKLCAH**S**QQRQYR  SIQPQV**S**PRQRVQ  ELCGPG**S**PPVLTP  QRKALL**S**PFSTPV  TPVRSV**S**ENSLVA  RSVSEN**S**LVAMDF  NILGSQ**S**PLHPST  YKFYDP**S**PPRRRG  NRERQK**S**LTLTPT  HKIKTG**S**PGSEVV  LPISVD**S**PPAAAD  GEFLYK**S**KKNAQL  VEELRM**S**LKCQRR  LDIEQF**S**TVKGVN  LQRRRR**S**LALPGL  GRPRTT**S**FAESCK  RIQAAA**S**TPTNAT  TQANRM**S**VDAVEI  VTDADR**S**ILSPGG  ADRSIL**S**PGGSCG  IPFRRP**S**TYGIPR  NTKALQ**S**PKRPRS  VPFRRP**S**TFGIPR  ESTSSK**S**PPRKIN  ERERPL**S**LLQLLG  PLNRTL**S**MSSLPG  RTLSMS**S**LPGLED  ESLLVG**S**LPDMNK  RYPRPA**S**VPPSPS  PASVPP**S**PSLSRH  PPSPSL**S**RHSSPH  SLSRHS**S**PHQSED  HSSPHQ**S**EDEEDP  EWPRRA**S**CTSSTS  TPSEPL**S**PTSSLG  MMMMMM**S**ETAPAE  PAPVEK**S**PAKKKA  GTGASG**S**FKLNKK  VKKVAK**S**PKKAKA  PKKATK**S**PAKPKA  PAPKKG**S**KKAVTK  KRSRKE**S**YSIYVY  KQTARK**S**TGGKAP  TKVARK**S**APATGG  IRRYQK**S**TELLIR  TDLRFQ**S**SAVMAL  MMMMMM**S**GRGKGG  GGVKRI**S**GLIYEE  ASIGDP**S**QSDDPD  VPKDRP**S**LTVTPK  PFPSRD**S**GRLSPD  RDSGRL**S**PDLSVC  KIPKRA**S**MVHSLI  RTVHPN**S**PGIPYR  PLRKTV**S**EPNLKL  PLLRKE**S**APPSLR  RGPILG**S**PHTPLF  PLSRTR**S**EPLPPS  SEPLPP**S**ATAPPP  PLSRAQ**S**SPAAPA  DAIPEE**S**GDEDED  ACEEEF**S**DSEEEG  EEEFSD**S**EEEGEG  PLRKTA**S**EPNLKV  PLSRTQ**S**SPLPQS  ALGRTQ**S**SPAAPG  TAAKEE**S**GGRSRS  GGGSSC**S**PVLSRK  TNPPSL**S**PIRRKG  QASVPL**S**PKKGSE  SSTKLL**S**PQMSGE  LLSPQM**S**GEEEDS  GGETAQ**S**ADPQWE  TFLSRH**S**LDMKFS  EAKNPF**S**TQDTDL  DDFQLR**S**FDQLSP  LESSSA**S**PESASP  TTSATS**S**PYRDTQ  STPAAL**S**PSVLTT  SVLTTP**S**KIEPMK  GAPALT**S**MTPTAV  PRDLLE**S**SSDSDE  RDLLES**S**SDSDEK  DLLESS**S**DSDEKV  LESSSD**S**DEKVPL  VSLSVQ**S**PAALTA  LKLPIP**S**PQRAFT  SMISAD**S**HEKRHH  GEVDAT**S**DQEESV  APAVPQ**S**PLKNEL  PLPSLA**S**PAVPAP  HREYCL**S**PRNQPR  YRGGPA**S**PGGLQG  LETRRL**S**LPSSKA  QKYCLK**S**PGQMTV  FQKLPS**S**PLGCRK  RKQPPV**S**PGTALV  GTALVG**S**QKEPSE  GSQKEP**S**EVPTPK  EEEEGI**S**QESSEE  EGISQE**S**SEEEQQ  GISQES**S**EEEQQQ  PKRKVS**S**AEGAAK  EEPKRR**S**ARLSAK  RRSARL**S**AKPPAK  ETKTEE**S**PASDEA  TEESPA**S**DEAGEK  GEKEAK**S**DDDDDD  REDLES**S**GLQRRN  GLQRRN**S**SEASSG  LQRRNS**S**EASSGD  PAAPVP**S**PVAPAP  RISTRR**S**SYEDSS  LSRQIT**S**PVSGIN  KIKRLR**S**QVQVSL  PETPQP**S**PPGGSG  SVTEHP**S**PSPLLS  TEHPSP**S**PLLSSS  SKRQRV**S**GNTSRR  RVSGNT**S**RRGKSG  EMKNDK**S**EEEQSS  KSEEEQ**S**SSSVKK  SEEEQS**S**SSVKKD  EEQSSS**S**VKKDET  TNVKME**S**EGGADD  EGGADD**S**AEEGDL  GEDDRD**S**ANGEDD  ANGEDD**S**SSSSSS  DEPEER**S**GDETPG  YHSRSK**S**PLPPEE  RTDYNA**S**VSVPDS  EGLQLP**S**PTATSQ  IILDLI**S**ESPIKG  LDLISE**S**PIKGRA  RDYDDM**S**PRRGPP  YAGGRG**S**YGDLGG  RYYGGG**S**EGGRAP  PHKTPA**S**PVVHIR  ISRPGD**S**DDSRSV  YTNPNL**S**GQGDPG  GQGDPG**S**NPNKRQ  KSERSS**S**GLLEWE  TWKRLR**S**HSRQYV  KRLRSH**S**RQYVSG  ERDKEV**S**DDEAEE  EKEEKE**S**EDKPEI  EIEDVG**S**DEEEEK  DLPLNI**S**REMLQQ  IKRKVT**S**VSTLKS  KYSRQF**S**LEHVHG  ERPLSS**S**PLVRVK  VKEEPP**S**PPQSPR  PPSPPQ**S**PRVEEA  PRVEEA**S**PGRPSS  SVDTLL**S**PTALID  SEPAPA**S**VTALTD  TEGRPP**S**PPPTST  ALLDLF**S**PSVTVP  SIQELL**S**PQEPPR  LKEEPA**S**PGGDGE  SLLRGP**S**WDPFRD  RDWYPH**S**RLFDQA  PPAAIE**S**PAVAAP  AYSRAL**S**RQLSSG  ALSRQL**S**SGVSEI  LSRQLS**S**GVSEIR  QLSSGV**S**EIRHTA  ADRWRV**S**LDVNHF  GPEAAK**S**DETAAK  CFPEGR**S**PPGAAA  PELNRQ**S**PNPRRG  AHKRPA**S**VSSSAA  LHQHGH**S**GPFESK  ARKRKP**S**PEPEGE  STTRRN**S**NSPPSP  TRRNSN**S**PPSPSS  NSNSPP**S**PSSMNQ  ALCPPV**S**ASCSEV  HAAEAG**S**PESPES  EAGSPE**S**PESTEI  SPESPE**S**TEITEE  FHLMAP**S**EEDHSI  ILWDAI**S**TYDGSK  AKAQET**S**GEEISK  TSGEEI**S**KFYLPN  GKRIPG**S**PEIRGD  EDRSAG**S**VESPSV  TDTQNF**S**SESKRE  CHQLAD**S**FREEEE  VKIERD**S**REHEEP  VLVSPL**S**KEDAGE  DEEEED**S**DDDVEP  DPEDED**S**DDYDGE  PGPRRG**S**GPEIFT  LAPTPV**S**AVLEPF  VKGPAL**S**RKRKKE  KEVDAT**S**PAPSTS  EQTQPP**S**PAGAGM  AMGRSP**S**PKTSLS  SPKTSL**S**APPNSS  IEDSAQ**S**DLKEVM  DSSMET**S**PDFFFF  QKGKKQ**S**FDDNDS  SFDDND**S**EELEDK  PKVEMY**S**GSDDDD  VEMYSG**S**DDDDDF  NKKWDG**S**EEDEDN  EEDEDN**S**KKIKER  ERSRIN**S**SGESGD  RSRINS**S**GESGDE  INSSGE**S**GDESDE  GESGDE**S**DEFLQS  PGPNIE**S**GNEDDD  EEEDEE**S**EEEEEE  EGESEG**S**EGDEED  KPSKEM**S**SDSEYD  PSKEMS**S**DSEYDS  KEMSSD**S**EYDSDD  SDSEYD**S**DDDRTK  RSRLPK**S**PPYTAF  RDRNRD**S**DKTDTD  PRRGDD**S**FGDKYR  GRRAFG**S**GYRRDD  PRERHP**S**WRSEET  TQERER**S**RTGSES  ERSRTG**S**ESSQTG  RTGSES**S**QTGTST  RRESEK**S**LENETL  KEEDCH**S**PTSKPP  DCHSPT**S**KPPKPD  PARSQS**S**DTEQQS  SDTEQQ**S**PTSGGG  SKYAAL**S**VDGEDE  TATKSG**S**TTKNRF  GEPYRL**S**PEPTPL  QLMAKG**S**DKRRGG  APGGRL**S**WGKGSS  RVVQRS**S**LSRERG  VQRSSL**S**RERGEK  TPATKR**S**FSKEVE  ATKRSF**S**KEVEER  GLRKAA**S**LTEDRD  AALPPV**S**PLKAAL  REAEEE**S**DHNNNN  PDPVLK**S**PSPVLR  PVLKSP**S**PVLRLV  ELPLPP**S**PTTVSS  NLNSRR**S**PVPAQI  TFMRGG**S**SKDLLD  LVHPKT**S**IEDQDE  EDQDEL**S**SLLQVP  DQDELS**S**LLQVPL  PSTIAT**S**MRDSLI  ATSMRD**S**LIDSLT  RDSLID**S**LTTTTT  ADEWCD**S**GLGSLG  CDSGLG**S**LGPDAA  VALYPD**S**DLEKEE  KSGPCS**S**SSDSDS  GPCSSS**S**DSDSGD  LDDRHD**S**GLDSMK  HDSGLD**S**MKDEEY  LQMLPE**S**EDEESY  ESEDEE**S**YDTESE  ESYDTE**S**EFTEFT  KELDQG**S**LCTSFV  QGSLCT**S**FVGTLQ  KVRGPV**S**GSPDSM  RGPVSG**S**PDSMNA  VSGSPD**S**MNASRL  MNASRL**S**QPGQLM  QPGQLM**S**QPSTAS  QLMSQP**S**TASNSL  SQPSTA**S**NSLPEP  PSTASN**S**LPEPAK  PEPAKK**S**EELVAE  VREQDQ**S**FTALDW  FTALDW**S**WLQTEE  TEEEEH**S**CLEQAS  DDEKFV**S**VYGTEE  AKGAQA**S**PPPIAP  QDMSQV**S**GKESPP  VKVETQ**S**DEENGR  SHRDQG**S**SALSGV  RHIKLH**S**GEKPFK  GHLRTH**S**VGKPHK  DLCKIG**S**ERSLVL  SDTPYD**S**SASYEK  AEGTPR**S**NHSAQD  TPRSNH**S**AQDSAV  PSEREA**S**PSNSCQ  EREASP**S**NSCQDS  EASPSN**S**CQDSTD  SNSCQD**S**TDTESN  RAASEN**S**QDALRV  VPDPSK**S**HIAQWS  SHIAQW**S**PHTPPR  VFSRSE**S**TQPLLD  ESSPDI**S**HFERSK  SNMCES**S**KEALAE  SRVVSR**S**PPPRLP  RAQLLW**S**PWSPLD  LLWSPW**S**PLDQEE  VVGENG**S**VLRRVT  ALAAPS**S**PTPESP  SSPTPE**S**PTMLTK  PLPRTL**S**PTPASA  SQGIPT**S**DEESTP  LRIAQV**S**PGPRDS  SPGPRD**S**PAFPDS  SPAFPD**S**PWRERV  FSTPTG**S**RTDSQS  SQSVRH**S**PIAPSS  SPIAPS**S**PSPQVL  IAPSSP**S**PQVLAQ  LVAKQE**S**VVRRAS  ENNGNN**S**WPHNDT  PREPPQ**S**ARRKRS  ELQPPR**S**KTPSSP  MLMTPT**S**APRSVM  PTSAPR**S**VMKSFI  YGMDLN**S**DDSTDD  DLNSDD**S**TDDEAH  RYHKRT**S**SAVWNS  YHKRTS**S**AVWNSP  SSAVWN**S**PPLQGA  PSSLAY**S**LKKHHH  RIQEQE**S**SGEEDS  IQEQES**S**GEEDSD  SSGEED**S**DLSPEE  MMMMMM**S**AADEVD  LFRALQ**S**PALGLR  PDKMKK**S**KSVKED  KMKKSK**S**VKEDSN  AQTVMT**S**RIVGTT  ILPWLV**S**QLDLGQ  RLAEDR**S**KDPHDP  FPNLGP**S**ENPLKR  RVGGAS**S**LENTVD  TVDLHI**S**NSHPLS  DLHISN**S**HPLSLT  FQGPGE**S**SSSSSS  RMLPSL**S**LTEDVK  ELFGPI**S**LEQVRF  EQVRFP**S**PEDIPS  MFSGEL**S**WSADSI  SGELSW**S**ADSIRL  LSWSAD**S**IRLQIS  SIRLQI**S**NPDLKD  SSLDSS**S**LSLCLS  LDSSSL**S**LCLSSA  FAIAAD**S**EAEQDS  FRPRSK**S**QSSSNC  RRSRTE**S**ITATSP  ESITAT**S**PASMVG  VGGKPG**S**FRVRAS  PASVDG**S**PVSPST  VDGSPV**S**PSTNRT  FRKRTH**S**AGTSPT  PSGRKG**S**GDYMPM  GDYMPM**S**PKSVSA  HLRLST**S**SGRLLY  CRRRHS**S**ETFSST  EENRRD**S**WSYINS  EKEKLK**S**QWNNDN  DNPLFK**S**ATTTVM  RVSGNN**S**PSLSNG  PASVNG**S**PSATSE  WEKRTD**S**NGRVYF  RSLPGE**S**EEMMEE  DFLKRL**S**NENHGI  PEKPEA**S**MEVKGQ  RLTHDI**S**LEEFED  QSQGPG**S**GDTYRP  DTLNNN**S**LGKKHS  SLGKKH**S**WQDRVS  SWQDRV**S**RSSSPL  QDRVSR**S**SSPLKT  DRVSRS**S**SPLKTG  HEHICL**S**DELPPQ  SRMSVS**S**DPDPAA  AGRPHP**S**ISEEEE  RPHPSI**S**EEEEGF  EGFDCL**S**SPERAE  GGGWRG**S**LGEPPP  PPPPRA**S**LSSDTS  PRASLS**S**DTSALS  GDYSDE**S**DSATVY  YSDESD**S**ATVYDN  QPPACL**S**EDSTPD  ACLSED**S**TPDEPD  VFMSGR**S**RSSSAE  MSGRSR**S**SSAESF  SGRSRS**S**SAESFG  GRSRSS**S**AESFGL  AREKRD**S**RNMEVQ  RLDRTG**S**SPTQGI  LDRTGS**S**PTQGIV  AKYKQL**S**PNGGQE  LRTSLS**S**LRSEHS  PQPPRE**S**PQLHER  PRPEGG**S**PSPAGT  PEGGSP**S**PAGTPP  HLRAKN**S**DLLTSP  NSDLLT**S**PDVGLL  GLLKLA**S**PELERL  GETPPL**S**PIDMES  SPIDME**S**QERIKA  SSAGSV**S**PRSTSV  MSFTTR**S**TFSTNY  TTRSTF**S**TNYRSL  FSTNYR**S**LGSVQA  NYRSLG**S**VQAPSY  YGARPV**S**SAASVY  GARPVS**S**AASVYA  PVSSAA**S**VYAGAG  YAGAGG**S**GSRISV  RISVSR**S**TSFRGG  SVSRST**S**FRGGMG  FRGGMG**S**GGLATG  YLDRVR**S**LETENR  ELAMRQ**S**VENDIH  LRRTVQ**S**LEIDLD  MRNLKA**S**LENSLR  KASLEN**S**LREVEA  LGDALD**S**SNSMQT  GDALDS**S**NSMQTI  ALDSSN**S**MQTIQK  SFHRSL**S**SSLQAP  RRPRLA**S**FGGMGT  VRKRSY**S**AGNASQ  AAAKKG**S**EQESVK  RRIGRF**S**EPHARF  EEEIRV**S**INEKCG  KNGLPG**S**RPGSPE  PGSRPG**S**PEREVP  RSSARL**S**QSSQDS  QSSQDS**S**PVRNLQ  QTRSSG**S**ETEQVV  TADHDE**S**PPRTPT  SIAKDM**S**LKDSGS  DMSLKD**S**GSDLSH  SLKDSG**S**DLSHRP  VEEKVG**S**PERPLS  PESTTG**S**PARLSL  SLRQTG**S**PGMIYS  YSTRYG**S**PKRQLQ  KGWRRS**S**LVITTK  VPSCSA**S**SCSSVT  PSCSAS**S**CSSVTA  VKAVVA**S**SRLGSA  KASRDP**S**PIQDGN  KPKFSI**S**PDSLSS  RPSPLT**S**VRVSAV  ICQRRT**S**YLPSEI  ISSVCV**S**SYRGRK  SSVCVS**S**YRGRKS  SYRGRK**S**GNKPPS  SGNKPP**S**KTCLKE  QRARLV**S**KDGRCN  ETRRRL**S**VEIYDK  EAPKRG**S**ILSKPR  DRFSFH**S**PSAQQR  GKLSRR**S**ISSPNV  LSRRSI**S**SPNVNR  SRRSIS**S**PNVNRL  NKGRWE**S**QQDVSQ  APALSV**S**PQNNHS  SPQNNH**S**PDPGLS  KPAKVP**S**PPPVIA  AQDGPP**S**PLSEAS  EAPAPG**S**PFRVRR  SATLSG**S**ATNLAS  MSQGIL**S**PPAGLL  PPAGLL**S**DDDVVV  DDDVVV**S**PMFEST  SFIKEH**S**LQVSPS  EHSLQV**S**PSLEKG  QEARQQ**S**VAHQQS  SVAHQQ**S**GSELAL  KLQGQV**S**AKKRLG  QSSTDC**S**PYARIL  PRSSLS**S**PSPPCS  TETPAP**S**PTVVRP  IRSKTF**S**PGPQSQ  RLNRSD**S**DSSTLS  RMKRPS**S**VKSLRS  RPSSVK**S**LRSERL  INTLER**S**KVEETT  LMKQKK**S**PCLWEI  LDSSKI**S**VLEPPQ  AIKIQR**S**NGLIHS  QKQKRR**S**VNSKIP  PKESLR**S**RSTRMS  ESLRSR**S**TRMSTV  SRSTRM**S**TVSELR  IPLRMV**S**EEMEEQ  NSVRRK**S**CLVKEV  LIPGNL**S**KEEEEL  LSSQMS**S**FNEAMT  PTVLPG**S**PSKTRG  QQILQC**S**PANNNN  KADKRR**S**VRIGSY  RSVRIG**S**YIERDV  RDIKND**S**NYVVKG  EGFRML**S**PEHAPA  HSVRIN**S**VGSTAS  NMEKRR**S**RESLNV  KRRSRE**S**LNVDVV  TTCSKE**S**NEELTE  PSEGEE**S**TVRFAR  NEFAGF**S**YTNPEF  VRRRRL**S**NVSLTG  RRLSNV**S**LTGVST  VVMEEG**S**DDNDSE  DANRTI**S**PSTSNN  TISPST**S**NNIPLM  HYWRLD**S**KCITLF  PLSEIL**S**LEPVKT  RIIGEK**S**FRRSVV  EKSFRR**S**VVGTPA  ALGERV**S**ILLLLL  RRLSST**S**LASGHS  SSTSLA**S**GHSVRL  SLASGH**S**VRLGTS  RLGTSE**S**LPCTAE  SSSSSA**S**SYTGRP  ILQDAP**S**APGHAP  GLAERI**S**VLLLLL  DVDCKQ**S**MRSEDE  QVTQRA**S**RRSDSA  QRASRR**S**DSASSE  ASRRSD**S**ASSEPV  RSDSAS**S**EPVGIY  ENIFGE**S**RASTFC  FGESRA**S**TFCGTP  NEKARL**S**YSDKNL  DKNLID**S**MDQSAF  IDSMDQ**S**AFAGFS  SAFAGF**S**FVNPKF  TPDQVG**S**QRFSVN  PDKITN**S**GQRRKK  LIAGAE**S**PQPASG  EEDRSK**S**APTSPC  SKSAPT**S**PCDQEI  NIRKAL**S**FDNRGE  PDGQLM**S**PGENGE  EEFKGF**S**YFGEDL  QGISWE**S**PLDEVD  NEKPRL**S**FADRAL  DRALIN**S**MDQNMF  NMFRNF**S**FMNPGM  EAQDAE**S**DLVDGR  LVDGRH**S**PPMVRS  SPPMVR**S**VAAIHP  ETERAY**S**FCGTIE  EMDPTY**S**PAALPQ  KLFQGY**S**FVAPSI  YSFVAP**S**ILFKRN  IKKGDF**S**FEGEAW  EAWKNV**S**QEAKDL  EWLQDG**S**QLSSNP  DGSQLS**S**NPLMTP  RKMKKT**S**TSTETR  MKKTST**S**TETRSS  STETRS**S**SSESSH  TETRSS**S**SESSHS  QEKKAY**S**FCGTVE  AKTPKD**S**PGLPAS  QLFKGF**S**FVATSI  RQTPVD**S**PDDSTL  FEPKIR**S**PRRFIG  PRRFIG**S**PRTPVS  SPRTPV**S**PVKFSP  VSPVKF**S**PGDFWG  LYLLRQ**S**RNYLGG  ARDNNG**S**YALCLL  IISRIK**S**YSFPKP  SRIKSY**S**FPKPGH  QGNRQE**S**TVSFNP  RQESTV**S**FNPYEP  DTEVYE**S**PYADPE  EDKELG**S**GNFGTV  YRGMKG**S**EVTAML  NGNLHE**S**DSESVP  KQPTPP**S**EAAASK  KLFPKV**S**VPSNLS  MNKYLY**S**VKDTYL  PVERRP**S**PGPRKR  REEKNK**S**PAAVTE  EDTQVD**S**EARPMK  EYRSLE**S**DNEEKA  FGSSQP**S**LNGDIK  DSSGAT**S**PINPAV  KKSLRQ**S**FRRIRK  EGADPD**S**IEMVAT  RLIEIA**S**RELEKA  MMAGMA**S**QAAQEA  GQQERE**S**PRPLQL  AEGPAI**S**DGEEGG  TTVNGQ**S**PPEHSA  PKQRKG**S**KVGDFG  QEKGEG**S**DSKESP  GSDSKE**S**PKTKSD  DQDETS**S**VKSDGA  ETSSVK**S**DGAGGA  YQKETE**S**APGSPR  TESAPG**S**PRAVTP  PKGLSA**S**LPDLDS  NTFTAW**S**DEESDY  AWSDEE**S**DYEIDD  PSTIAR**S**LPTTVP  PTTVPE**S**PNYRNT  RKTRHS**S**NPPLES  TASISS**S**PSEGTP  GNHKRH**S**VVAGGG  SQTKRY**S**GNMEYV  IPVRSN**S**FNNPLG  KKQITT**S**PITVRK  MMRVGL**S**QDAQDQ  QRCLAH**S**LVGTPN  LREIRY**S**LLPFAN  KKTKFA**S**DDEHDE  RQRKGG**S**TSSSPS  GGSTSS**S**PSRRRG  SRSRSR**S**PGRPPK  PKSARR**S**ASASHQ  SARRSA**S**ASHQAD  VAPDAK**S**FVLNLG  MMADNF**S**LHDALS  SLHDAL**S**GSGNPN  TGALVL**S**RGGKRN  FFGRRK**S**RQGSLA  RKSRQG**S**LAMEEL  KSGSGP**S**LKGEEE  LAERCF**S**RGESVY  FRNMKR**S**RGGSKS  KRSRGG**S**KSDKDS  VDPGCM**S**PDVKNS  SCSIDR**S**PGAGSL  RSPGAG**S**LGSPAS  GAGSLG**S**PASQRK  DLGRSE**S**LRVVCR  HRIFRP**S**DLIHGE  AYVEKF**S**YKSITT  QRRATR**S**GAQASS  SGAQAS**S**TPLSPT  ASSTPL**S**PTRITR  FQKNIY**S**EELRET  LDNARQ**S**AERNSN  SRIRID**S**LSAQLS  SLSAQL**S**QLQKQL  EERLRL**S**PSPTSQ  RLRLSP**S**PTSQRS  LSPSPT**S**QRSRGR  RSRGRA**S**SHSSQT  SRGRAS**S**HSSQTQ  RASSHS**S**QTQGGG  QTQGGG**S**VTKKRK  ESRSSF**S**QHARTS  VRLRNK**S**NEDQSM  KSNEDQ**S**MGNWQI  MRKLVR**S**VTVVED  QVGGPI**S**SGSSAS  VTRSYR**S**VGGSGG  YRSVGG**S**GGGSFG  GGSGGG**S**FGDNLV  YLLGNS**S**PRTQSP  SLERQL**S**LEQEVQ  MRGRKS**S**GFPKSV  LAYVQA**S**PVRTLS  VVHGPA**S**VPLGYV  GYVPHP**S**SLSGSL  ILNPPP**S**PATERS  PVKDRF**S**AEDEAL  YKSDRA**S**PTANSG  GLLRSA**S**LASLYN  RPSSRN**S**ASATTP  SATTPL**S**GNSSRR  PLSGNS**S**RRGSGD  NSSRRG**S**GDTSSL  GPTAQW**S**VEDEEE  EPPQCR**S**PEGEQE  QQPRTP**S**PLVLEG  EGTIEQ**S**SPPLSP  GTIEQS**S**PPLSPT  QSSPPL**S**PTTKLI  KLARQA**S**IELPSM  RLREKC**S**PGGVIG  VPAGES**S**SEAGGE  DALVDF**S**EQYTPE  KSLFLK**S**PLGSWN  HKTTQM**S**AAGTYA  PRDSFH**S**MQEGWK  RITVQA**S**PGLDRR  EVGPGD**S**PTFPRF  QAWGRQ**S**PRRLED  PRRLED**S**SNGERR  RRLEDS**S**NGERRA  GTALLA**S**LGLGRD  PTEPPP**S**PLICFS  SLKTPD**S**PPTPAP  IPVGQR**S**AKSPRR  GQRSAK**S**PRREEE  PRGGTV**S**PPPGTS  PPGTSR**S**APGTPG  TPGTPR**S**PPLGLI  ISRPRP**S**PLRSRI  SAGPRP**S**PLPSPQ  RPSPLP**S**PQPAPR  SDPFWD**S**PPANPF  TRGRGS**S**VGGGSR  SALSAG**S**NEYLRS  EYLRSI**S**LPVPVL  ENFEDH**S**APPSPE  DHSAPP**S**PEEKDS  NDERSK**S**LLLSGQ  TYRNIG**S**DFIPRG  DQPRCQ**S**LDSALL  MLKRQR**S**LYIDLG  MTNNKG**S**AAWMAP  SEGKRM**S**ADMSEI  GQPRRR**S**IQDLTV  PRSRHL**S**VSSQNP  RMSRAQ**S**FPDNRQ  PRGRLR**S**ADSENA  RLRSAD**S**ENALSV  HRTTKM**S**AAGTYA  TLARRL**S**FIGTPY  PKPKFR**S**PSDEGP  PKFRSP**S**DEGPGS  SDEGPG**S**MGDDGQ  GDDGQL**S**PGVLVR  NPIAHI**S**PHRLLA  HVLEAL**S**PPQTSG  LSPPQT**S**GLSPSR  PQTSGL**S**PSRLSK  TSGLSP**S**RLSKSQ  PSRLSK**S**QGGEEE  GSTYTP**S**EAGNEL  GAELPS**S**PLAIEY  HRLPPG**S**LSSTPL  LSTPCS**S**VPSSPS  CSSVPS**S**PSFCAP  DALTEP**S**PDIEGE  DEKTGN**S**ESKKKP  GSDTTG**S**PTEFLE  LAKDME**S**PTKLDV  KETERA**S**PIKMDL  GLLKDM**S**PLSETE  KDMSPL**S**ETEMAL  KDVPPL**S**ETEATP  APTFMI**S**PETVTG  GTGKKC**S**LPAEED  LPAEED**S**VLEKLG  NKELPP**S**PEKKTK  APEKRA**S**PSKPAS  PASAPA**S**RSGSKS  TGPSSR**S**PSTLLP  PSSRSP**S**TLLPKK  RLATNT**S**APDLKN  VRSKVG**S**TENIKH  IVSKKV**S**YSHIQS  AQAKVG**S**LDNVGH  EAQTLD**S**QIQETS  SQIQET**S**IIIIII  SNSQGQ**S**PPVPFP  NHGLAI**S**PGMKTR  HPWIMQ**S**TKVPQT  ELFHRI**S**QHRHFT  SGIIPT**S**PTPYTY  PAYEKL**S**AEQSPP  GEAAVA**S**SPSKAN  EAAVAS**S**PSKANG  AVASSP**S**KANGQE  KVNGDA**S**PAAAES  ELQANG**S**APAADK  EPAAAG**S**GAASPS  AGSGAA**S**PSAAEK  APEAGA**S**PVEKEA  EAAEPG**S**PTAAEG  AASSTS**S**PKAEDG  EDGATP**S**PSNETP  GATPSP**S**NETPKK  KKKKRF**S**FKKSFK  RFSFKK**S**FKLSGF  KKSFKL**S**GFSFKK  FKLSGF**S**FKKNKK  KAAEEP**S**KVEEKK  SACAAP**S**QEAQPE  EARRLV**S**TEDACG  PKPRGL**S**RDMQGL  RDMQGL**S**LDAASQ  TLRRND**S**IYEASS  DSIYEA**S**SLYGIS  FEGKSC**S**PLAFSA  EALPET**S**MDPITG  CFTRSF**S**KENSHL  AARLPP**S**PTRCQQ  ASQKRP**S**QRHGSK  DTGILD**S**IGRFFG  GAPKRG**S**GKDSHH  LSLSRF**S**WGAEGQ  KLGGRD**S**RSGSPM  RDSRSG**S**PMARRR  LRRASL**S**LGSGFW  AADAIM**S**PEEELD  NTSTDG**S**LPSTPP  TDGSLP**S**TPPPAE  ESFTMA**S**SPAQRR  SFTMAS**S**PAQRRR  GNDPLT**S**SPGRSS  NDPLTS**S**PGRSSR  RTDALT**S**SPGRDL  TDALTS**S**PGRDLP  PPFEDE**S**EGLLGT  VEELTA**S**QREAAE  RGLLYD**S**DEEDEE  RIRIQE**S**PGKVAA  TIERRY**S**DLTTLV  TDDPNF**S**QEDQQD  KKRKKR**S**EDESET  KRSEDE**S**ETEDEE  EDEEEK**S**QEDQEQ  YDPYDF**S**DTEEEM  MCPGLT**S**PGAKLY  PEKWEE**S**GPQFIT  PQFITN**S**EEVRLR  TIHKVN**S**MVAYKI  SRRRAI**S**ETEENS  DWLDQD**S**VSDQFS  LDQDSV**S**DQFSVE  SVSDQF**S**VEFEVE  LDSEDY**S**LSEEGQ  SEDYSL**S**EEGQEL  DKITQA**S**QSQESE  QESEDY**S**QPSTSS  SSIIYS**S**QEDVKE  TQDKEE**S**VESSLP  EESVES**S**LPLNAI  KLTHSL**S**TSDITA  DCRRTI**S**APVVRP  DKYRRA**S**EELDGL  TDPRLL**S**PQQPAL  PALQRN**S**VSPGLP  YVSARA**S**PGLLPV  KVIPAK**S**PPPPTH  IKSEPV**S**PSRERS  KLFQYA**S**TDMDKV  IKSEPV**S**PPRDRT  RHEAGR**S**PVDSLS  LQTCCG**S**LAYAAP  DPKKRI**S**MKNLLN  VRLRLS**S**FSCGQA  FSCGQA**S**ATPFTD  IKSNNW**S**LEDVTA  AATPRT**S**QFTKYW  NGVESK**S**LTPALC  NVYTPK**S**AVKNEE  LMTGVI**S**PERRCR  PERRCR**S**VELDLN  GAKVFG**S**LERGLD  LARRVR**S**DPGGGG  GLCEEI**S**QEVRGD  VTLCYE**S**HESMES  CYESHE**S**MESYEL  SHESME**S**YELNPF  SEGLEI**S**KETKDK  AQGEQA**S**FESFEE  EQASFE**S**FEEMLQ  EEDDYP**S**EELLED  EPEGEL**S**KEDHEN  LHEDNF**S**REKTAE  PRSEFG**S**VDGPLP  SGKPSP**S**DPGSGT  TMMNSS**S**RGSSPT  SSSRGS**S**PTRVLD  DEPPPA**S**QSTSQD  SQALKQ**S**PPPPPP  AYTGQP**S**PRGLHS  KGAPHS**S**PARGTP  SLASVE**S**PGTPKE  STPDWV**S**EGPQPG  GLRRAL**S**SDSILS  LRRALS**S**DSILSP  RALSSD**S**ILSPAP  SSDSIL**S**PAPDAR  AYQPYL**S**PGTPQL  MSPRHL**S**ESSGKP  ALRLQK**S**QSSDLL  RLQKSQ**S**SDLLER  LFPEVF**S**PTPDEN  PTPDEN**S**DQNSRS  ENSDQN**S**RSSSQA  ESRIYA**S**EEDDDD  GSSAPN**S**PMAMLT  ARAHGL**S**LIPSTG  PSTGLC**S**PDLVNR  LMDDTL**S**PVGVTD  TSSRRS**S**MSMEET  SHKGHL**S**EGLVTK  KKKKKV**S**GTLDTP  PEKTVD**S**QGPTPV  FLERRK**S**QVAELN  RPQRAT**S**NVFAMF  QMIRSF**S**EPFGRD  EQRMPV**S**PISQGA  LRKTQT**S**MSLGTT  KTQTSM**S**LGTTRE  PFKQRR**S**FADRCK  NHTTHM**S**LVGTFP  LPDKCN**S**FLHNKA  KLERDL**S**FKEQEL  SNPILG**S**PFFSHF  ITPVNQ**S**RSSSPT  ALNPHQ**S**PDFKRS  SPDFKR**S**PRDLHQ  VEKRRN**S**GPVVEK  STQLVN**S**IAKTYV  TIAEGP**S**PTSEGA  SGQLID**S**MANSFV  IDSMAN**S**FVGTRS  EEGEPH**S**ISPRPR  GEPHSI**S**PRPRPP  MMMMME**S**PASSQP  PASMPQ**S**KGKSKR  SGYLVD**S**VAKTMD  NLQRMG**S**SESTDS  RMGSSE**S**TDSGFC  SSESTD**S**GFCLDS  SGFCLD**S**PGPLDS  ALKRSH**S**DSLDHD  FTQRQN**S**APARML  LKRPER**S**QEESPP  STKRRK**S**MSGASP  TWAGEK**S**KREMYS  SKREMY**S**RLKKLL  LRNITN**S**QAPDGR  MEVEEL**S**PLALGR  RLFRSP**S**MPCSVI  QNKRRR**S**VTPPEE  RVLRSK**S**LCHDEI  LLERDT**S**FTVCPD  GKFLGD**S**ANLSIL  GDSANL**S**ILSGGT  ANLSIL**S**GGTPKR  QHLMKC**S**PAQLLC  PAQLLC**S**TPNGLD  EMKYLG**S**PITTVP  DQAEEI**S**DELMEF  DELMEF**S**LKDQEA  RSGLYR**S**PSMPEN  GLYRSP**S**MPENLN  LLVKDM**S**PPPPPP  PRDGYG**S**PHHTPP  SPRHGG**S**FPGGRF  PGGRFG**S**PSPGGY  GRFGSP**S**PGGYPG  PGSYSR**S**PAGSQQ  QQQFGY**S**PGQQQT  TSTPFG**S**GRVREK  VREKRM**S**NELENY  RLADFG**S**CLKLME  EDGTVQ**S**SVAVGT  GKASQP**S**VVISQV  NNKRRY**S**FRVPEE  ALKREF**S**GGSYSA  PSPSEG**S**LSSGGM  DGEDSD**S**PRHSTA  SPRKTK**S**LSLEST  RKTKSL**S**LESTDR  SPHKRL**S**HRHLKV  PLLRGL**S**WDSGPE  RTARSM**S**LTLGKN  MPRRRV**S**VAVVPK  GRFVAE**S**AEVLLP  MMEKSS**S**CESLGS  TSADNF**S**PDLRVL  LAHQLA**S**PGLTTL  MKKLHV**S**TINLQK  IQSIAP**S**LQVITS  SRTRTD**S**YSAGQS  GTTVPE**S**IHSFIG  VPESIH**S**FIGDGL  PSSTDR**S**PYEKVS  LLDLYA**S**GERRAY  IKEEEN**S**EEELAA  KPGLRR**S**PIKKVR  SGEDTL**S**DSDDED  EDTLSD**S**DDEDDE  MMPLNV**S**FTNRNY  LPTPPL**S**PSRRSG  RRSGLC**S**PSYVAV  ARKDSG**S**PNPARG  KRVKLD**S**VRVLRQ  EKRDKK**S**PLIEST  TTATKI**S**PKEEER  EERKDE**S**PATWRL  GLRKTG**S**YGALAE  GVTRSA**S**SPRLSS  VTRSAS**S**PRLSSS  ASSPRL**S**SSLDNK  IPRRLA**S**TSDIEE  KITTGS**S**SAGTQS  VRERRR**S**YLTPVR  SRQARQ**S**RRSTQG  ARQSRR**S**TQGVTL  PREKRR**S**TGVSFW  SFWTQD**S**DENEQE  NEQEQQ**S**DTEEGS  LLGRSG**S**YSYLEE  ALERRI**S**EMEEEL  IENKVE**S**KGNSPP  VESKGN**S**PPSSGE  GLAAED**S**GGDSKD  EDSGGD**S**KDLSEV  GDSKDL**S**EVSETT  KDLSEV**S**ETTEST  ELEEQD**S**TQATTQ  PTVQEE**S**EEEEVD  DIELVM**S**QANVSR  MSQANV**S**RAKAVR  RALKNN**S**NDIVNA  PRDDVG**S**PRPRVI  ARVILG**S**PRPRVI  PRVIVS**S**PWPAVV  PAVVVA**S**PRPRTP  PRVIVG**S**PRARVA  DTGITN**S**QTLIPD  TPGPSL**S**QGVSVD  QKFRML**S**QDAPTV  IPNYQL**S**PTKLPS  PESSKI**S**QENEIG  MSRRRH**S**DENDGG  HKRRKT**S**DANETE  GAPPRR**S**SIRNAH  APPRRS**S**IRNAHS  RSRKRL**S**QDAYRR  DAYRRN**S**VRFLQQ  ARPGPQ**S**PGSPLE  GPQSPG**S**PLEEER  QARGLP**S**QKRLFP  SHKRKG**S**PCDTLA  REHSGL**S**PQDDTN  SVNPSI**S**PAHGVA  NISTLS**S**PVGMTS  QMSSQN**S**PSRLNI  RLLQEG**S**PSDITT  PPRGAF**S**PGMGMQ  TPPAST**S**PFSQLA  LEDINA**S**PEMRQR  GPKLDN**S**PNMNIT  DKESKE**S**SVEGAE  VKQEQL**S**PKKKEN  PYNRAV**S**LDSPVS  RAVSLD**S**PVSVGS  PVSVGS**S**PPVKNI  KDVTLT**S**PLLVNL  GHGSRN**S**QLGIFS  GYPFAL**S**KSSMYT  MTRLMR**S**RTASGS  MRSRTA**S**GSSVTS  SRTASG**S**SVTSLD  RTASGS**S**VTSLDG  SGSSVT**S**LDGTRS  EGTRSR**S**HTSEGA  SRLRSC**S**VTDAVA  QIRRPR**S**LSSPTV  RRPRSL**S**SPTVTL  RPRSLS**S**PTVTLS  LEGAKD**S**PVRRAV  TLSNPQ**S**PQPSPY  PQSPQP**S**PYNSPK  QPSPYN**S**PKPQHK  KNQDFS**S**FDDTGK  SLGDMF**S**PIRDDA  NKGSDE**S**IGKGDG  SGNLNT**S**PSSNQT  SLLERY**S**VNEGLV  ILNHDT**S**FAKTFV  RIPYRY**S**DELNEI  LGEPEK**S**QDSSPV  NLLKNY**S**LLKERK  KERKFL**S**LASNPE  KKKVHF**S**GESKEN  VHFSGE**S**KENIMR  KENIMR**S**ENSESQ  RSENSE**S**QLTSKS  SESQLT**S**KSKCKD  LRAQAL**S**DIEKNY  KNYQLK**S**RQILGM  MLNKNP**S**LRPSAI  RHPNTL**S**FRCSLA  ETTAAH**S**LVGTPY  DVLGEE**S**PLGKPA  AMLHLP**S**EQGAPE  RDAIRQ**S**NQILRE  LLHFQA**S**QREEKE  PAPAYL**S**SPLALP  LPSQRR**S**PPEEPP  REVFDF**S**QRRKEY  ETTMVP**S**PEAKLT  STSPRA**S**VTEESW  CNKRKY**S**LNGRQP  HGSPRV**S**VTDDSW  GGGAFF**S**PSPGSS  GAFFSP**S**PGSSSL  SRFGLG**S**PLPSPR  LGSPLP**S**PRASPR  ALSRRG**S**LGEEGS  AVALPR**S**EEPASC  VSNRGV**S**EKQLTS  TVQQHP**S**TPKRHT  EDLLDN**S**RMSCQD  VQLRRK**S**DLETSE  EVIQAA**S**SPVKTT  TTSQAH**S**LPLSPA  AHSLPL**S**PASTRQ  SDSVCD**S**GVETSF  DSGVET**S**FRKLSF  TSFRKL**S**FTESLT  SPPTSD**S**DSDSEG  PTSDSD**S**DSEGPE  SDSDSD**S**EGPEKD  AEVKED**S**AYGSQS  EDSAYG**S**QSVEQE  RLVEPG**S**PAEKAG  TMKKGP**S**GYGFNL  EIQKEN**S**REALAE  AEAALE**S**PRPALV  ALVRSA**S**SDTSEE  LVRSAS**S**DTSEEL  SASSDT**S**EELNSQ  ELNSQD**S**PPKQDS  HNLYRD**S**MVMHNS  SMVMHN**S**DPNLHL  NSGGGG**S**PSPSTP  GGGGSP**S**PSTPES  SPSTPE**S**ATLSEK  RAKQVV**S**VVQDEE  GLPFEA**S**PESPPP  FEASPE**S**PPPASP  ESPPPA**S**PDGVTE  QGLRPE**S**PPPAGP  GAPAGE**S**PQPKAA  AAPEAS**S**PPASPL  ASSPPA**S**PLQHLL  WGAVVR**S**PEGTPQ  TSATSQ**S**VNGSPQ  SQSVNG**S**PQAEQP  LPLVPE**S**PRRMMT  RRMMTR**S**QDATFS  SQDATF**S**PGSEQA  ATFSPG**S**EQAEKS  SEQAEK**S**PGPIVS  IVSRTR**S**WDSSSP  RSWDSS**S**PVDRPE  PEPEAA**S**PTTRTR  GLEVPS**S**PLRKAK  KRARLC**S**SSSSDT  KLQGRP**S**PGPPAP  RRKRKE**S**SNTDSA  ESSNTD**S**AGALGT  EQLEKG**S**PGGPPP  SRIRTQ**S**FSLQER  QSLYPC**S**AQQLNE  QVQRRG**S**LPPRQV  PAGPRM**S**GAPGRL  PTIILS**S**PTKSPT  LSSPTK**S**PTKNAE  SFPNLD**S**PNVSST  MMMMED**S**MDMDMS  SMDMDM**S**PLRPQN  AMNYEG**S**PIKVTL  VEEDAE**S**EDEEEE  EDVKLL**S**ISGKRS  VKLLSI**S**GKRSAP  RSKGQE**S**FKKQEK  KTPKGP**S**SVEDIK  TPKGPS**S**VEDIKA  KAKMQA**S**IEKGGS  SIEKGG**S**LPKVEA  PTYFPP**S**PTGSLT  PPSPTG**S**LTQDPA  EVVRTD**S**LKGRRG  RRGRLP**S**KPKQPP  SPPGAA**S**DAEPGD  KSFFKR**S**IRRNLS  TEHSML**S**ADEDSP  LSADED**S**PSSPED  ADEDSP**S**SPEDTS  DEDSPS**S**PEDTSY  SSPEDT**S**YDDSDI  RKILRK**S**PHLEKF  RESTQL**S**PADLTE  DPSKLE**S**PSFTGT  VWCRNN**S**GEEFDC  TNESAA**S**TGQPDN  RAGEPN**S**PDAEEA  DAEEAN**S**PDVTAG  GLGAPG**S**PREAVA  EVPGKL**S**PKQSAT  ARQRIR**S**CVSAEN  IKSGKL**S**RELDFV  AKAQRL**S**QETEAL  KNGSLD**S**PGKQDT  KINPEN**S**KLSDLD  HPEMDF**S**KAKFNN  ISLKNL**S**SDEATN  SLKNLS**S**DEATNP  GADNDG**S**GSESGY  DNDGSG**S**ESGYTT  DMLRKS**S**DSKPGV  IQNSSV**S**PTSSSS  ELEKRT**S**PQVLGS  SQNPLA**S**PTNTLL  TNTLLG**S**AKEQRY  GLERND**S**WGSFDL  RNDSWG**S**FDLRAA  YNEAMD**S**PDQQQQ  VPSTCS**S**TFPEEL  TFPEEL**S**PPSHQA  LSGSPA**S**PMGDIL  NEKQAA**S**PLEPKE  ILQGKL**S**QLEEHL  QLEEHL**S**QLQDNP  RERYED**S**QQEEAQ  SQSALA**S**AQRELA  EAERKN**S**LISSLE  ELQAQL**S**QKEQAA  KPQLDL**S**IDSLDL  LDLSID**S**LDLSCE  IDSLDL**S**CEEGTP  VPGEPA**S**PISQRL  EPASPI**S**QRLPPK  LPPKVE**S**LESLYF  KVESLE**S**LYFTPI  SQAPLE**S**SLDSLG  QAPLES**S**LDSLGD  LESSLD**S**LGDVFL  GDVFLD**S**GRKTRS  DVEEPD**S**ANSSFY  EPDSAN**S**SFYSTR  PDSANS**S**FYSTRS  SFYSTR**S**APASQA  SLARLG**S**PDYGNS  RSSARR**S**QAGVSS  ETLRRA**S**MQPIQI  QQRKRV**S**LEPHQG  GPGTPE**S**KKATSC  KALSKA**S**PNTRSG  RSGTRR**S**PRIATT  NHEKQE**S**QDLRAT  ATAKVP**S**PPDEHQ  PPRGRL**S**VASTPI  VASTPI**S**QRRSQG  LKRSAI**S**AAKTGV  SAHVTV**S**GGTPKG  ATQTPV**S**NKKPVF  DVPSPP**S**DYVERV  YVERVD**S**PMAYSS  KRFYPE**S**SYKSTP  FRQPRY**S**SGGNFE  RQPRYS**S**GGNFET  YTTGGE**S**CDELEE  LKQVKG**S**ADYKSK  RYGMGT**S**VERAAA  YRYHGH**S**MSDPGV  YHGHSM**S**DPGVSY  MSDPGV**S**YRTREE  SGSSED**S**FVEIRM  VKEIKH**S**PGPTRT  ALERKN**S**AIPSEL  PVKQAD**S**GSSEEK  KQADSG**S**SEEKQL  QADSGS**S**EEKQLY  APQNAV**S**SEETND  PQNAVS**S**EETNDF  KQETLP**S**KSNESH  ETLPSK**S**NESHDH  PSKSNE**S**HDHMDD  DDDHVD**S**QDSIDS  HVDSQD**S**IDSNDS  SQDSID**S**NDSDDV  SIDSND**S**DDVDDT  VDDTDD**S**HQSDES  TDDSHQ**S**DESHHS  SHQSDE**S**HHSDES  SDESHH**S**DESDEL  SHHSDE**S**DELVTD  TDEDIT**S**HMESEE  ITSHME**S**EELNGA  QDLNAP**S**DWDSRG  APSDWD**S**RGKDSY  DSRGKD**S**YETSQL  KDSYET**S**QLDDQS  SQLDDQ**S**AETHSH  QSAETH**S**HKQSRL  THSHKQ**S**RLYKRK  RKANDE**S**NEHSDV  DESNEH**S**DVIDSQ  HSDVID**S**QELSKV  IDSQEL**S**KVSREF  QELSKV**S**REFHSH  VSREFH**S**HEFHSH  HSHEFH**S**HEDMLV  LVVDPK**S**KEEDKH  HLKFRI**S**HELDSA  ISHELD**S**ASSEVN  HELDSA**S**SEVNNN  ELDSAS**S**EVNNNN  GARPRA**S**PPPQGP  GAVGGG**S**PEREEV  VGAGYN**S**EDEYEA  EEWTSR**S**PRQRSS  RDLAAT**S**STLGLQ  DLAATS**S**TLGLQE  GFELSS**S**TRKKAQ  KLLLDD**S**FETKKT  VSNLQV**S**PKSEDI  RKVNGK**S**LSVATV  TVTRSQ**S**LNIRTT  EPQSDP**S**VEPPLS  SVEPPL**S**QETFSD  LSQETF**S**DLWKLL  PENNVL**S**PLPSQA  VLSPLP**S**QAMDDL  MDDLML**S**PDDIEQ  PHHERC**S**DSDGLA  NLLGRN**S**FEVRVC  PNNTSS**S**PQPKKK  KTEGPD**S**DDDDDD  SNDSSD**S**DDESHE  NTSYPL**S**PLSDFA  QNPSRC**S**VSLSNV  PSRCSV**S**LSNVEA  MMMMMM**S**GEDEQQ  KALLQS**S**ASRKTQ  VEGRAM**S**FQSIIH  GSSFYP**S**AEGRAM  VLGSST**S**ATNSTS  GEFFRL**S**PKDSEK  PPMRNT**S**TMIGAG  KDRFYR**S**ILPGDK  TSNITK**S**EQKKNP  NSQKYM**S**FTDKSA  MSFTDK**S**AEDYNS  PAVPPV**S**EDEDDD  RPEHTK**S**VYTRSV  KSVYTR**S**VIEPLP  TRDVAT**S**PISPTE  VATSPI**S**PTENNT  LPRQWQ**S**LIEESA  NSLRRD**S**PPPPAR  SEAGGG**S**GDRRRA  KSSREG**S**GGPQES  RDKRPL**S**GPDVGT  QPAGLA**S**GAKLAA  RARGAP**S**PGVLGP  VLGPHA**S**EPQLAP  GPPGPR**S**PQREPQ  EVPRRK**S**LVGTPY  LTIGSS**S**PKTGVT  PPPAPR**S**APPSPP  PRSAPP**S**PPFPPP  RTARIA**S**DEEIQG  EYRLER**S**PVDESG  RSPVDE**S**GDEVQY  VNQRGR**S**PRSPSG  RGRSPR**S**PSGHPH  RSPRSP**S**GHPHVR  HVRRPR**S**RSRDSG  RRPRSR**S**RDSGDE  RSRSRD**S**GDENEP  GHMKVF**S**LIQQAV  YYSSRF**S**PDSQYI  KCNELK**S**PGSPPG  ELKSPG**S**PPGPEL  IEGLDE**S**PSRNAA  SADCSL**S**PDVDPV  EGFGRQ**S**MSEKRT  KTRKSK**S**MDLGIA  DDQKAG**S**PSRDVG  SRSGRE**S**VSTASD  ENVSQL**S**LDKSPT  QLSLDK**S**PTEKST  PQNDDH**S**DTDSEE  IDVVPE**S**PLSDVG  VPESPL**S**DVGSED  ESCLGN**S**PPFEKE  PPFEKE**S**EPESPM  KESEPE**S**PMDVDN  KVEPRR**S**DPGTSG  RSDPGT**S**GEAYKR  GALSYV**S**PNTSKC  HYYRNN**S**FTAPST  RMKKEL**S**PAGSIS  ELSPAG**S**ISKNSP  GSISKN**S**PATLFE  RASAPQ**S**DEGSDI  PQSDEG**S**DIDSEP  EGSDID**S**EPDLPL  IHQQPQ**S**SSPVYG  QQPQSS**S**PVYGSS  PQDSVG**S**PCSRVG  EEEHVY**S**FPNKQK  FPNKQK**S**AEPSPT  QKSAEP**S**PTVMST  PTVMST**S**LGSNLS  SLGSNL**S**ELDRLL  NQGEMS**S**PQRVTS  GKTGSS**S**PPGGPP  YHERRG**S**LCSGCQ  PPPPPV**S**PPALTP  EGGRQD**S**EDEEER  LVLALR**S**EEDGLA  RHLAQA**S**QELQQQ  LALSEL**S**VQHADS  SVQHAD**S**LENIDE  GAGGEG**S**DDDTSL  RRLRKN**S**SRDSGR  RLRKNS**S**RDSGRG  KNSSRD**S**GRGDSV  DSGRGD**S**VSDSGS  GRGDSV**S**DSGSDA  GDSVSD**S**GSDALR  GLTVPT**S**PKGRLL  GGKRKD**S**VWGSGG  KDSVWG**S**GGGQQS  GRKRFV**S**EGDGGR  FLYRSD**S**DYDMSP  KTMSRN**S**SVTSEA  YSAIRQ**S**PSPPPE  AIRQSP**S**PPPEEE  VAEDAP**S**PAAPPL  KDRRKG**S**LDVKAV  DGLRRL**S**GNEYVL  IVDAAL**S**ALRQLV  KQGRSD**S**SSKKDV  EDDIDL**S**DVELDD  MMMADI**S**LDELIR  ISLKRS**S**PAAFIN  AAEPVL**S**PLEGTK  LPRRVN**S**ASSSNP  RRVNSA**S**SSNPPA  VLCSCP**S**PSMVRT  KQARAN**S**FVGTAQ  QVSSSS**S**SHSLSA  VSSSSS**S**HSLSAS  SSSSSH**S**LSASDT  SSSHSL**S**ASDTGL  LPQRSG**S**NIEQYI  KGEIPW**S**QELRPE  YYLMDP**S**GNAHKW  PPGLSA**S**PQPSSV  RTSSAM**S**GHSRSF  SAMSGH**S**RSFISR  MSGHSR**S**FISREL  HSRSFI**S**RELKEI  KGPPSG**S**PPRQPP  PVRHPP**S**PPLRRQ  SCQNPA**S**PPEEGS  LYYDLI**S**SPDIHG  FDSRKT**S**LEDFYL  NKTKNR**S**HYSHES  KNRSHY**S**HESGEQ  SHYSHE**S**GEQKKK  GEQKKK**S**VTEMQT  LPGKAE**S**VASLTS  MVEDAA**S**GPESLD  AASGPE**S**LDCLAG  ALACGL**S**QEKEPF  YYLQER**S**KGQPSE  RSKGQP**S**ERTAPG  SEMASA**S**QPEFPS  SAMGRA**S**PPLFQS  NTGMEE**S**EHFIKC  GDQPAA**S**GDSDDD  PAASGD**S**DDDEPP  EEPTVY**S**DEEEPK  RGSQTQ**S**HPDLGT  GLGRKL**S**DFGQET  EKEEPP**S**PIEATP  KSRESA**S**PTIPNL  DEYEYV**S**DDGELK  APKRDL**S**FLLDKK  VYPSLE**S**DEDNPI  SKKRKG**S**DDAPYS  LTQRRP**S**ASSPNN  QDWFHG**S**CVGVEE  KPVKTG**S**PTFVRE  DEFDLD**S**DDELQI  LGTVSN**S**PASQRT  AEYIYP**S**LESDDD  IYPSLE**S**DDDDPA  SDDAPW**S**PKARVT  SSEDED**S**DHEDKD  NSATCH**S**EDSDLE  TCHSED**S**DLEIDD  RELPSL**S**PAPDTG  APDTGL**S**PSKRTH  THQRSK**S**DATASI  NLKRTA**S**NPKVEN  RIRSTR**S**VENLPE  DIRRRL**S**EQLAHT  ERSWVY**S**PLHYSA  NPLGEA**S**PDAPSS  SPDAPS**S**PVAALI  LLSKIN**S**LAHLRA  LLKKVS**S**GFSGVI  FRQRVS**S**ECQHLI  SQSRRP**S**SWRQEK  DFESLA**S**QFSDCS  GKGGAA**S**GSDSAD  WNISAG**S**SSEAIL  LQLPFS**S**WYVDRG  FIDAFA**S**PVEAEG  MNNELL**S**PEFGPV  EGLGRG**S**PEPSAL  EEPSIY**S**PAFPRE  EKNHGS**S**PEQVVR  KVRKLI**S**SSQVDQ  HEAKQR**S**VQRWRE  EDSSEC**S**DGEWSA  VRKDYR**S**SSSSSS  KQGEKH**S**AAAAAA  PLPSRS**S**RGSKVS  KENGDL**S**PFSGTS  GRAREG**S**FESRYQ  VQGPLG**S**PPKRGP  KELEEV**S**PETPVV  SDEGQL**S**PATRGA  QLIRSW**S**LATFRT  QLEPVA**S**PAKKPK  LQSKRA**S**FAEKTA  TAQLER**S**LQEEHV  TQALQQ**S**FLSEKD  EAEPEH**S**FDGLRR  QEAGIL**S**AEELQR  ELVYTD**S**EARDVF  LREGLT**S**IEEVTK  NAGGFR**S**RSSSVG  GGFRSR**S**SSVGSS  GFRSRS**S**SVGSSS  FRSRSS**S**VGSSSS  RSSSVG**S**SSSYPI  SSSVGS**S**SSYPIS  SSVGSS**S**SYPISP  SVGSSS**S**YPISPA  SSSYPI**S**PAVSRT  PISPAV**S**RTQLAS  STKGYY**S**PYSVSG  GYYSPY**S**VSGSGS  YSPYSV**S**GSGSTA  SVSGSG**S**TAGSRT  SGSTAG**S**RTGSRT  AGSRRG**S**FDATGS  ASGSSA**S**LGGPES  QKFARK**S**TRRSIR  RKSTRR**S**IRLPET  KMSMEI**S**IHRSLA  ELCRRR**S**LLELHK  IKKNEY**S**IPKHIN  PSSLDP**S**NRKPLT  VVDCHL**S**DMLQQL  LYNDGD**S**LQYIER  SWPWQV**S**LRTRFG  IPACLP**S**PNYVVA  EGTFRS**S**IRRLST  SSIRRL**S**TRRRRR  MKEQTG**S**ICQYLV  ELRRVQ**S**LPSVPL  WMRNNL**S**LGDALA  MMMARG**S**VSDEEM  MARGSV**S**DEEMME  DGNGYI**S**FNELND  LLREGE**S**LEDLMK  AVVIDM**S**GLREKD  FRNWMN**S**LGVNPR  EPAPAR**S**PRPQQD  SEGRQP**S**PSPSPT  GRQPSP**S**PSPTER  QPSPSP**S**PTERAP  SLQRRL**S**VYRQIV  AVSKKA**S**PEAAST  EARLAR**S**SPEQPR  ARLARS**S**PEQPRP  STSKAV**S**PPHLDG  HLDGPP**S**PRSPVI  GPPSPR**S**PVIGSE  RSPVIG**S**EVFLPN  ISSSED**S**DAENSS  ASETLQ**S**PGYDPS  SRLGHG**S**YGEVFK  KLAEVG**S**HEKVGQ  SLLLDS**S**LSSNWD  RDALDL**S**DINSEP  DLSDIN**S**EPPRGS  PSLEEP**S**DLEELE  RRKKRT**S**IETNIR  SGGTSS**S**PIKAIF  SNSDGA**S**PEPCTV  RKRKRT**S**IENRVR  QKGKRS**S**SDYAQR  KGKRSS**S**DYAQRE  SVTTLG**S**PMHSNN  SAIRRA**S**TIEMPQ  EPPLRH**S**APPSPA  RHSAPP**S**PAAGEP  AIRIPP**S**SPLCGL  IRIPPS**S**PLCGLG  NLNEDV**S**QEESPS  KPQGPP**S**PPGKPQ  KSRSPR**S**PPGKPQ  GDNKSR**S**SRSPPG  DHTPDT**S**FERDVV  AVATIM**S**GSKAKV  NATANS**S**IRPIFG  GGTVYH**S**PVSRLP  PLQRNF**S**INSVAS  PSLSDS**S**TVGLQR  TSGILN**S**TNIQSS  VAGGPP**S**PEVGSP  QDQQSL**S**DVEGAY  AGGSSS**S**PPEKDS  PGQSQP**S**PPACEV  ATQRVL**S**PLMSRS  VLPRGL**S**PARQLL  GAPVKP**S**PQAAAV  PMAPGR**S**PLATTV  FAPPRS**S**PCASST  AEASAR**S**PRSYLV  SQRFTF**S**PGQDIQ  DREKRL**S**FAVPFR  QSYSYS**S**QDPRPA  FVETQA**S**QGTLQT  PLPEDN**S**MNVDQD  MLKKGG**S**WIQEIN  PLERED**S**GTFSLG  DYIQLL**S**EIAKEQ  ITVCHG**S**GISCGN  CDLDQQ**S**IVHIVQ  DRAVVL**S**HYIHNL  YIHNLS**S**EMFSEF  IVSILR**S**WNEPLY  GMELIV**S**QVHPET  DEESRL**S**AYYNLL  GWGYHS**S**REKEAK  LQDGEF**S**MDLRTK  ASHLRR**S**QYYYYY  IYILSS**S**GDGAQH  SSSRSR**S**FTLDDE  APPPLP**S**TPDPPR  RELRAP**S**PPVEHP  AGNEAL**S**PTSPFR  EALSPT**S**PFREGR  EKYAKE**S**LKEEDE  LKEEDE**S**DDDNMM  FRERIT**S**EAEDLV  KIKRPR**S**SNAETL  EHNDRR**S**LGHPEP  EAQRRV**S**KNSKYN  WEAQRD**S**HLGPHR  VQELSS**S**ILAGED  SVTPDV**S**DNEPDH  PDHYRY**S**DTTDSD  YSDTTD**S**DPENEP  MMMMMM**S**DAAVDT  DAAVDT**S**SEITTK  AAVDTS**S**EITTKD  NRYRDV**S**PFDHSR  DKRKDP**S**SVDIKK  KRKDPS**S**VDIKKV  IKEEKG**S**PLNAAP  SRVVGG**S**LRGAQA  RGAQAA**S**PAKGEP  LKLKRQ**S**TKYKAD  EEAGEF**S**PNVPKS  KLRSPK**S**ELHQDR  LHQDRS**S**PPPPLP  LQERRG**S**NVSLTL  GFGYLM**S**PREESA  LQERRG**S**NVALML  LQRQPP**S**PKQLEE  CLGRAQ**S**QEDGDY  QVSRRI**S**FSASHR  QAAEEP**S**GAGSEE  EPSGAG**S**EELIKS  EKVRKV**S**VNVKTV  KLPAKL**S**ISKSLK  PAKLSI**S**KSLKES  AAALEL**S**SDEAVE  AALELS**S**DEAVEV  RDKLRK**S**FTPDHV  GDLRRG**S**SPDVHA  DLRRGS**S**PDVHAL  LEITEE**S**DAVLVD  AVLVDK**S**DSDDDD  LVDKSD**S**DDDDDD  FQLGPP**S**PVKMPS  LWQDSF**S**PHLKHH  IGVAGR**S**QDDAMV  QLGGGG**S**GGGGYN  GGGYNN**S**KHRWPT  AEHQVR**S**MDELNH  EVNSVL**S**PRSESG  EYVLSS**S**PGDSCL  SGSRRD**S**LTGSSD  DSLTGS**S**DLYKRT  SASSLF**S**PSSTLF  EKRRQI**S**IRGIVG  NDPMVG**S**KLKVIF  AGGRRL**S**SFVTKG  PRIHRA**S**DPGLPA  EPKEKS**S**RKVAEP  PVPRQA**S**PQNLGT  RQNVDH**S**QEAAKA  ALRQLR**S**PRRAQA  TWSLPL**S**QNSASE  ASELPA**S**QPQPFS  SDTDSH**S**QDLGSP  HSQDLG**S**PERHQP  LPSISL**S**PGPQPP  KSPGPH**S**EEEDEA  ILAPVR**S**PQGPSP  RSPQGP**S**PVLAED  PVLAED**S**EGEGGG  KVLASL**S**DDEDEE  PAPVLS**S**PPPADV  PPPADV**S**TFLAFP  TFLAFP**S**PEKLLR  GLKRAA**S**AIYSGY  DYRGRL**S**PVPVPR  PQENTT**S**EAGLPQ  EGLLTH**S**EEELEH  EEELEH**S**QDTDAD  IVPSPP**S**PPPLPR  VCQDKS**S**GYHYGV  SSEQRV**S**LDIDLW  VRKRRP**S**RPHMFP  EYEERE**S**EFDIED  PIAAFC**S**SDEELE  IAAFCS**S**DEELED  KGKEKD**S**PFKPKL  KVQAEL**S**QPLTAG  RDMQPL**S**PISVHE  VHERYS**S**PTAGSA  QESKVK**S**PVSLTA  HSLIGA**S**PKQTNL  MDAPPL**S**PFPHIK  IKQQPG**S**PRRISQ  QHSIYI**S**PHKNGS  LYKFNG**S**PSKSLK  IDSDAE**S**PAKRVC  EICIAG**S**PLTPRR  LGRSIT**S**PTTLYD  LYDRYS**S**PPASTT  GSSDSR**S**HQNSPT  SRSHQN**S**PTELNK  RTSRDS**S**PVMRSS  SSPVMR**S**SSTLPV  SPVMRS**S**STLPVP  PVMRSS**S**TLPVPQ  LPVPQP**S**SAPPTP  PVPQPS**S**APPTPT  MDAPPL**S**PYPFVR  IKQEPE**S**EEEEEE  KLGASN**S**PGQPNS  SPGQPN**S**VKRKKL  KFEDED**S**DDVPRK  YYGRDR**S**PLRRAT  EDPEQD**S**GPEDLP  VIPING**S**PRTPRR  LAWLSD**S**PLFDLI  YLSPVR**S**PKKKGS  PYKFPS**S**PLRIPG  GGNIYI**S**PLKSPY  YISPLK**S**PYKISE  MMMMMM**S**PKRIAK  RIAKRR**S**PPADAI  PPEDNQ**S**FQYDHE  QLTPDE**S**KERLGK  MQLRRP**S**DQEVSE  NMMTTS**S**DSMGET  EAYGPS**S**QTLTFL  GADTQG**S**EFEFTD  LSQPEL**S**QDSYLG  QIDVAL**S**QDSTYQ  VALSQD**S**TYQGER  GGVTGL**S**QYYYYY  MMMMWN**S**GFESYG  WNSGFE**S**YGSSSY  AGGYTQ**S**PGGFGS  SPGGFG**S**PAPSQA  FGSPAP**S**QAEKKS  RLSSAH**S**MSDLSG  PRGQLA**S**PDAPGP  CYGGVA**S**AQDEEP  ADSAGG**S**AVPSEC  ATGEQH**S**RLRQEN  KMEREK**S**IEIENL  QRRGRS**S**SMGLQE  RRGRSS**S**MGLQEY  PADVVS**S**QGVPSI  PAEVIS**S**QATPPL  LSTIDE**S**GSILSD  ESGSIL**S**DISFDK  SILSDI**S**FDKTDE  FDKTDE**S**LDWDSS  SLDWDS**S**LVKTFK  PVKKTR**S**IGSAVD  KTRSIG**S**AVDQGN  VDQGNE**S**IVAKTT  TLQPWN**S**DSTLNS  TGLYRI**S**GCDRTV  KTVPLL**S**KVDDIH  LSQRVR**S**TLTKNT  SWGRTY**S**FTSAMS  TVGRSF**S**LSWRSH  ILVRRS**S**SPAELD  LVRRSS**S**PAELDL  TSQGQP**S**PVEPRG  LAVSRT**S**SPVRAR  VRARKL**S**LTSPLN  EKRNRL**S**LLVQKP  DRPPSM**S**SHDTAS  FDKLMH**S**PAGRSV  DYVSIL**S**PKEVSL  KLMKSD**S**YPRFIR  SSMNNG**S**PTALSG  SGSKTN**S**PKNSVH  VHKLDV**S**RSPPLM  KLDVSR**S**PPLMVK  GIVTNG**S**FSSSNA  MMMMME**S**PGESGA  GSPGAP**S**PSSFTT  PQLKAR**S**PQELDQ  ETSSPL**S**PPAVPP  VPGSRG**S**PATSPH  RGSPAT**S**PHLGRR  PLLRSM**S**AAFCSL  VQELRR**S**LSLWEQ  MMQPDM**S**LNVIKM  SSDFLE**S**AELDSG  ESAELD**S**GGFGKV  ADLGLA**S**FKMWSK  ASFKMW**S**KLNNEE  VEEDVK**S**LKKEYS  VVKRMQ**S**LQLDCV  AVPSSR**S**NSATEQ  IADFGL**S**KWRMMS  WRMMSL**S**QSRSSK  SPETSR**S**LPAPQD  HCPGNH**S**WDSTIS  PEILVV**S**RSPSLN  ILVVSR**S**PSLNLL  VVSRSP**S**LNLLQN  NLLQNK**S**MMMMMM  MMMMMM**S**CVKLWP  LADFGL**S**TFQGGS  NVNRKA**S**TASDVY  ELPTEP**S**LVYEAV  NMNAAV**S**TVKDFL  WTAGTS**S**DSMAQP  MMMDFG**S**LETVVA  FIAARG**S**FDGSSS  QDVGAF**S**TVKGVA  YHFRAP**S**RIFWRT  EAKKEE**S**EESDDD  ALGGNS**S**PSAKDI  VSAAPG**S**AAPAAG  AAPAAG**S**APAAAE  DEKKEE**S**EESDDD  KEESEE**S**DDDMGF  KRKRKN**S**TGSGHS  GATKES**S**EKDRGR  KERKRR**S**PSPKPT  RKRRSP**S**PKPTKV  PCKNIF**S**RFFSYF  MFGRKR**S**VSFGGF  LPDRPL**S**PPLTAP  VTKTTF**S**PPVVAA  VKNQSR**S**PIKRRS  SPIKRR**S**GLFPRL  KNYALP**S**PATTEG  CTPRLR**S**VSSYGN  PRLRSV**S**SYGNIR  RLRSVS**S**YGNIRA  ARSLNK**S**LQNLSL  KMRRAS**S**YSSLNS  RVLDTS**S**LTQSAP  TSSLTQ**S**APASPT  TQSAPA**S**PTNKGV  IHQAGG**S**PPASST  YNIQKE**S**TLHLVL  MMAVQI**S**KKRKFV  PLPDHV**S**IVEPKD  LPTTPI**S**EQKGGK  AKRRRL**S**SLRAST  KRRRLS**S**LRASTS  LSSLRA**S**TSKSES  SLRAST**S**KSESSQ  RASTSK**S**ESSQKK  TSKSES**S**QKKKKK  MEDLDQ**S**PLVSSS  LVSSSD**S**PPRPQP  APERQP**S**WDPSPV  PPPASV**S**PQAEPV  APKRRG**S**SGSVDE  PKRRGS**S**GSVDET  KEDEVV**S**SEKAKD  VTEKNT**S**TKTSNP  LYYGTS**S**GSYQFP  VNSSLT**S**PTGRGS  SPTGRG**S**MAAPSL  PISTLS**S**PINGMG  PFSVIS**S**PMGPHS  NMGLNP**S**SPNDPV  MGLNPS**S**PNDPVT  IRGRLL**S**LVEKVT  NRTRRI**S**QTSQVS  SQTSQV**S**VDAAHG  STKEKQ**S**SEEEEK  TKEKQS**S**EEEEKE  RNLTSS**S**LNDISD  SSLNDI**S**DKPEKD  KKKKKG**S**LDSDND  KKGSLD**S**DNDDSD  DSDNDD**S**DCPYSE  FWGAKA**S**TPEVQS  GNDEVA**S**EDEATV  SATSSL**S**PGLAPF  QEYQSR**S**PDILET  TSFQAL**S**PANSQA  VGPSTL**S**PGMTPL  VSKMDG**S**QSGISA  LPPEQW**S**HTTVRN  SLQRSS**S**FKDFAK  FKDFAK**S**KPSSPV  PSSPVV**S**EKEFNL  MMVKAL**S**EEMADT  LEEGSA**S**PTSPDY  GSASPT**S**PDYSLD  PDYSLD**S**PGPEKM  LDYDTG**S**EEAEEG  MPGPTP**S**GTNVGS  SGTNVG**S**SGRSPS  GTNVGS**S**GRSPSK  VGSSGR**S**PSKAVA  KERIIK**S**ITPETP  SEAHES**S**SRGEAG  EAHESS**S**RGEAGA  AEGGGH**S**RERADE  PSDSQV**S**EEVKTR  ERGEDS**S**EEKHLE  THAAGH**S**QEKTHS  SKGQPR**S**QEESEE  PRSQEE**S**EEGEED  RSRPDR**S**SQGGSL  SRPDRS**S**QGGSLP  GHPQEE**S**EESNVS  STHYRA**S**EEEPEY  RYRGRG**S**EEYRAP  RAPRPQ**S**EESWDE  RPQSEE**S**WDEEDK  HGYGEE**S**EEERGL  DPLQWK**S**SHFERR  PLQWKS**S**HFERRD  LHYRKK**S**AEFPDF  FPDFYD**S**EEPVST  DSEEPV**S**THQEAE  EKQARG**S**TTLQES  RGVDTV**S**RSSLEM  DTVSRS**S**LEMSPL  KRRKRM**S**SGTEEC  RRKRMS**S**GTEECG  EDRLPK**S**DSEDGP  RAMNHL**S**LTRGLS  WPLRRT**S**AQGQPS  ARQRAL**S**AVSVLT  RALSAV**S**VLTSAL  AMKKLG**S**KKPQKP  KEKVHL**S**DSERKM  LGERKP**S**SAAYQK  QFPECD**S**DEDEDF  VRGRRY**S**WGTVEV  GARDRA**S**RSKLSR  SKLSRQ**S**ATEIPL  YDNRRR**S**AQWLDQ  SKKRKR**S**RWNQDT  PNPEDR**S**PSPEPI  PEDRSP**S**PEPIYN  PEPIYN**S**EGKRLN  SRDRFR**S**RGGGGG  GLHDFR**S**PPPGMG  RSEEKI**S**DSEGFK  GFKANL**S**LLRRPG  LSVRNL**S**PYVSNE  MGDPYG**S**GGQKFP  DSPKHQ**S**DPSEDE  KHQSDP**S**EDEDER  SSQKLH**S**TSQNIN  EETVPY**S**VCVSSD  PYSVCV**S**SDYSIV  DAFVGF**S**YAPPSE  KKSFQD**S**LEDIKK  KDQVNL**S**PKLIQP  PPETQQ**S**PHLSLK  DLCFLN**S**PIFKQK  NLYPSS**S**PYTRNW  KRAMNK**S**FMESGG  NTKKRH**S**FTSLTM  SANKPC**S**KQPPPQ  QPQHTP**S**PAAPPA  GGAGPV**S**PQHHEL  RVKIED**S**EENGVF  QGSSDS**S**EENGDD  EENGDD**S**SEEEEE  ENGDDS**S**EEEEEE  GENNEE**S**NEDEDS  SNEDED**S**EAENTT  ATKEKE**S**DEEEEE  GYEIYE**S**ENGEPR  LSTWCG**S**PPYAAP  QEGRRA**S**DTSLTQ  ALQPGG**S**PSAAGA  ADREAA**S**SPAGEP  DREAAS**S**PAGEPL  GPGLER**S**PGEPGG  ITNGFH**S**CESDEE  GFHSCE**S**DEEDRA  DRASHA**S**SSDWTP  RASHAS**S**SDWTPR  PTPLHV**S**EDSSSP  SSPERT**S**PPDSSV  HGAEVY**S**DSEDDV  AEVYSD**S**EDDVLS  NYPSNK**S**SSSSSS  VQEAQD**S**DSDSEG  EAQDSD**S**DSEGGA  QDSDSD**S**EGGAAG  NPSTSA**S**PKKSPP  SASPKK**S**PPPAKD  DQQLNQ**S**MDTGSP  QSMDTG**S**PAELSP  GSPAEL**S**PTTLSP  LSPTTL**S**PVNHSL  VLTQMG**S**PSVRCS  TQMGSP**S**VRCSSM  SPSVRC**S**SMSSSS  PSVRCS**S**MSSSSS  VRCSSM**S**SSSSSS  KVPPGY**S**IKVFDF  ERMEEE**S**QSQGRD  MEEESQ**S**QGRDLT  SQEEGS**S**QGEDSV  EDSVSG**S**QRISSI  SGSQRI**S**SIYARE  MMMMAM**S**SGGSGG  MMMAMS**S**GGSGGG  AMSSGG**S**GGGVPE  RRGTGQ**S**DDSDIW  TGQSDD**S**DIWDDT  DNIKPK**S**APWNSF  FLVRKP**S**DPNRKP  SLRLQE**S**PRVAEL  AELTSL**S**DEDSGK  SLSDED**S**GKGSQP  EDSGKG**S**QPPSPP  KGSQPP**S**PPSPAP  QPPSPP**S**PAPSSF  PPSPAP**S**SFSSTS  APSSFS**S**TSVSSL  FSSTSV**S**SLEAEA  ACARTF**S**RMSLLH  SLVYGA**S**DSNVYD  SKFDTN**S**HNDDAL  HWAGEK**S**NVIVAL  PMITGF**S**DDVPMV  MMMMMM**S**DQDHSM  MSDQDH**S**MDEMTA  GQESQP**S**PLALLA  LTATQL**S**QGANGW  CPYCKD**S**EGRGSG  KVYGKT**S**HLRAHL  GKRFTR**S**DELQRH  MRSDHL**S**KHIKTH  TNSGKR**S**PACSSL  LGLQEG**S**NNSSPV  EGSNNS**S**PVDFVN  TIVLVP**S**PLGQQQ  VGRILG**S**DTESWM  DTESWM**S**PLAWLE  LENLRQ**S**LSLPSM  ESLAEM**S**IMTTEL  GSKTPA**S**PVVVQQ  NKDERV**S**ALEVLP  VATSRI**S**DAHLAD  MPDSND**S**PPAERE  QLARET**S**VDPDMR  FGHTVD**S**VEKLIK  AATERT**S**PVSLWS  ERTSPV**S**LWSRLS  SLWSRL**S**SSWESL  WSRLSS**S**WESLQP  LSSSWE**S**LQPEPS  VGDEGV**S**GEEVFA  SEDTED**S**AEHRHH  EEVEAV**S**GEHHHH  EEDEDV**S**TERWHQ  EEDEEV**S**AELGHQ  KEEDPG**S**HEEDDE  PLSPDH**S**EEEEEE  TAGALY**S**GSEGDS  GALYSG**S**EGDSES  SGSEGD**S**ESGEEE  SEGDSE**S**GEEEEL  GLKRSL**S**EMEIGM  CLNSPD**S**PPRSDP  TYQVSE**S**DSSGET  TVLNAF**S**QAPSTM  TMQVSH**S**QVQEPG  EEEILG**S**DDDEQE  PNKQEE**S**ESPVER  KQEESE**S**PVERPL  YLFEPH**S**GEEYTR  PELPEP**S**VKVKEP  VKVKEP**S**VQEATS  PEPKEP**S**PEKNSK  RKTRRL**S**PSASPP  TRRLSP**S**ASPPRR  RLSPSA**S**PPRRRH  RRRHRP**S**PPATPP  PPKTRH**S**PTPQQS  SPTPQQ**S**NRTRKS  TRKSRV**S**VSPGRT  KSRVSV**S**PGRTSG  VSPGRT**S**GKVTKH  GTEKRE**S**PSPAPK  EKRESP**S**PAPKPR  PRKVEL**S**ESEEDK  KVELSE**S**EEDKGG  KMAAAD**S**VQQRRQ  GRRRRH**S**PSRSAS  RRRHSP**S**RSASPS  RHSPSR**S**ASPSPR  SPSRSA**S**PSPRKR  SRSASP**S**PRKRQK  RGRRRR**S**PSPPPT  RRRRSP**S**PPPTRR  PTRRRR**S**PSPAPP  RRRRSP**S**PAPPPR  PRRRTP**S**PPPRRR  PSPRRY**S**PPIQRR  PIQRRY**S**PSPPPK  QRRYSP**S**PPPKRR  PKRRTA**S**PPPPPK  PPKRRA**S**PSPPPK  KRRASP**S**PPPKRR  PPKRRV**S**HSPPPK  KRRVSH**S**PPPKQR  PRAPQT**S**SSPPPV  RAPQTS**S**SPPPVR  APQTSS**S**PPPVRR  PVRRGA**S**SSPQRR  RRGASS**S**PQRRQS  SPQRRQ**S**PSPSTR  QRRQSP**S**PSTRPI  KIKKAA**S**PSPQSV  KKAASP**S**PQSVRR  QSVRRV**S**SSRSVS  RVSSSR**S**VSGSPE  SSSRSV**S**GSPEPA  SRSVSG**S**PEPAAK  KPPAPP**S**PVQSQS  PPSPVQ**S**QSPSTN  SPVQSQ**S**PSTNWS  VQSQSP**S**TNWSPA  SPSTNW**S**PAVPVK  PVKKAK**S**PTPSPS  AKSPTP**S**PSPPRN  PKKETE**S**EAEDNL  MRKAQV**S**PQSSSS  YVQRNL**S**LVRGRR  KKKKHR**S**ESESKK  KKHRSE**S**ESKKRK  TALAGR**S**PSPASG  LAGRSP**S**PASGRR  RSPSPA**S**GRRGEG  TSTQRP**S**SPETAT  STQRPS**S**PETATK  KSATRP**S**PSPERS  ATRPSP**S**PERSST  SPSPER**S**STGPEP  PSPERS**S**TGPEPP  AERHGG**S**PQPLAT  LATTPL**S**QEPVNP  EPVNPP**S**EASPTR  NPPSEA**S**PTRDRS  SPTRDR**S**PPKSPE  DRSPPK**S**PEKLPQ  SESSPP**S**PQPTKV  KVSRHA**S**SSPESP  VSRHAS**S**SPESPK  SRHASS**S**PESPKP  ASSSPE**S**PKPAPA  GSHREI**S**SSPTSK  SRRMGR**S**RSPATA  RMGRSR**S**PATAKR  PQRRGR**S**RSPQRP  RRGRSR**S**PQRPGW  RLSLRR**S**LSGSSP  SLRRSL**S**GSSPCP  RSLSGS**S**PCPKQK  PHPKVK**S**GTPPRQ  RQGSIT**S**PQANEQ  PQANEQ**S**VTPQRR  VTPQRR**S**CFESSP  RRSCFE**S**SPDPEL  RSCFES**S**PDPELK  STPPRQ**S**PSRSSS  KVKAII**S**PRQRSH  SHSGSS**S**PSPSRV  RTTPRR**S**RSVSPC  TPRRSR**S**VSPCSN  RRSRSV**S**PCSNVE  RSVSPC**S**NVESRL  RLLPRY**S**HSGSSS  LPRYSH**S**GSSSPD  RYSHSG**S**SSPDTK  YSHSGS**S**SPDTKV  SHSGSS**S**PDTKVK  SHSGSI**S**PYPKVK  PPGPSL**S**GSKSPC  SLSGSK**S**PCPQEK  KDSLVQ**S**CPGSLS  VQSCPG**S**LSLCAG  KGQSQT**S**PDHRSD  TSPDHR**S**DTSSPE  DHRSDT**S**SPEVRQ  HRSDTS**S**PEVRQS  RQSHSE**S**PSLQSK  SPKGGR**S**RSSSPV  KGGRSR**S**SSPVTE  GGRSRS**S**SPVTEL  GRSRSS**S**PVTELA  TELASR**S**PIRQDR  QDRGEF**S**ASPMLK  RGEFSA**S**PMLKSG  ASPMLK**S**GMSPEQ  MLKSGM**S**PEQSRF  PTVDSN**S**LLGQSR  QEDATA**S**PPRQKD  RQKDKF**S**PFPVQD  TPPRER**S**GAGSSP  RSGAGS**S**PETKEQ  QILSHL**S**SELKEM  WGGPHF**S**PEHKEL  PEHKEL**S**NSPLRE  HKELSN**S**PLRENS  SPLREN**S**FGSPLE  RENSFG**S**PLEFRN  PLEFRN**S**GPLGTE  EMNTGF**S**SEVKED  QLETDP**S**LDMKEQ  TRSSGH**S**SSELSP  RSSGHS**S**SELSPD  HSSSEL**S**PDAVEK  GMSSNQ**S**ISSPVL  SSNQSI**S**SPVLDA  SNQSIS**S**PVLDAV  AVPRTP**S**RERSSS  SRERSS**S**ASSPEM  ERSSSA**S**SPEMKD  RSSSAS**S**PEMKDG  RSRSGS**S**PGLRDG  PGLRDG**S**GTPSRH  GTPSRH**S**LSGSSP  PSRHSL**S**GSSPGM  RHSLSG**S**SPGMKD  HSLSGS**S**PGMKDI  GRSECD**S**SPEPKA  RSECDS**S**PEPKAL  QTPRPR**S**RSPSSP  PRPRSR**S**PSSPEL  PRSRSP**S**SPELNN  RSRSPS**S**PELNNK  TPQRER**S**GSESSV  QRERSG**S**ESSVDQ  ERSGSE**S**SVDQKT  RSGSES**S**VDQKTV  TPLGQR**S**RSGSSQ  LGQRSR**S**GSSQEL  QRSRSG**S**SQELDV  RSRSGS**S**QELDVK  DVKPSA**S**PQERSE  TPLRQR**S**RSGSSP  LRQRSR**S**GSSPEV  QRSRSG**S**SPEVDS  RSRSGS**S**PEVDSK  SPRRSR**S**GSSPEV  RRSRSG**S**SPEVKD  RSRSGS**S**PEVKDK  AAPRAQ**S**GSDSSP  AQSGSD**S**SPEPKA  QSGSDS**S**PEPKAP  SKGRGP**S**PEGSSS  SSSTES**S**PEHPPK  PPRRRS**S**RSSPEL  RRRSSR**S**SPELTR  RRSSRS**S**PELTRK  PPRHRR**S**PSVSSP  RHRRSP**S**VSSPEP  RRSPSV**S**SPEPAE  RSPSVS**S**PEPAEK  TSRRGR**S**PSPKPR  RRGRSP**S**PKPRGL  RRRRGG**S**GYHSRS  GGSGYH**S**RSPARQ  SRKRSR**S**RTSPAP  RSRSRT**S**PAPWKR  PATHRR**S**RSRTPL  THRRSR**S**RTPLIS  SRTPLI**S**RRRSRS  SRRRSR**S**RTSPVS  RSRSRT**S**PVSRRR  RSRSRA**S**PVSRRR  SRASPV**S**RRRSRS  PVSRRR**S**RSRTPP  SRRRSR**S**RTPPVT  PTTRRR**S**RSRTPP  TRRRSR**S**RTPPVT  PVTRRR**S**RSRTPP  PVTRRR**S**RSRTSP  TRRRSR**S**RTSPIT  RSRSRT**S**PITRRR  TRRRSR**S**RTSPVT  RSRSRT**S**PVTRRR  TRRRSR**S**RTPPAI  PAIRRR**S**RSRTPL  IRRRSR**S**RTPLLP  PRKRSR**S**RSPLAI  KRSRSR**S**PLAIRR  TARGKR**S**LTRSPP  KRSLTR**S**PPAIRR  SSDRSR**S**ATPPAT  PATRNH**S**GSRTPP  TRNHSG**S**RTPPVA  SRMSCF**S**RPSMSP  SCFSRP**S**MSPTPL  FSRPSM**S**PTPLDR  PLDRCR**S**PGMLEP  AAMNLA**S**PRTAVA  TPQAPA**S**ANLVGP  NLVGPR**S**AHATAP  SGANLT**S**PRVPLS  SAYERV**S**GRTSPP  RVSGRT**S**PPLLDR  LLDRAR**S**RTPPSA  RSRTPP**S**APSQSR  TPPSAP**S**QSRMTS  TSERAP**S**PSSRMG  RAPSPS**S**RMGQAP  AQDQPR**S**PVPSAF  PRSPVP**S**AFSDQS  ALKRVP**S**PTPAPK  ALPKPA**S**PKKPPP  PPGERR**S**RSPRKP  GERRSR**S**PRKPID  PRKPID**S**LRDSRS  IDSLRD**S**RSLSYS  SLRDSR**S**LSYSPV  RDSRSL**S**YSPVER  SRSLSY**S**PVERRR  VERRRP**S**PQPSPR  RPSPQP**S**PRDQQS  FVEFED**S**RDADDA  RDRDGY**S**YGSRSG  DGYSYG**S**RSGGGG  SRSRSQ**S**RSNSPL  SQSRSN**S**PLPVPP  PPSKAR**S**VSPPPK  SKARSV**S**PPPKRA  DRKLNL**S**LSESFF  FLQQGS**S**PQGQRS  PLKRSH**S**LAKLGS  KEGMNP**S**YDEYAD  YDEYAD**S**DEDQHD  ENANDS**S**DDSGEE  SSNEGD**S**DRDEKK  SSSRQL**S**ESFKSK  KSKEFV**S**SDESSS  SKEFVS**S**DESSSG  FVSSDE**S**SSGENK  VSSDES**S**SGENKS  SSDESS**S**GENKSK  RRRSED**S**EEEELA  EPRPNS**S**PSPSPG  MMMMMM**S**DHGDVS  EIIRMA**S**IYSEEG  LKPGAL**S**NSESIP  PGALSN**S**ESIPTI  ALSNSE**S**IPTIDG  QVRRRS**S**LLGSRL  CAKRRG**S**TCVLAG  KELKGL**S**CLVSYQ  GLSCLV**S**YQDDPL  LGPELE**S**VLESTL  TIDLPM**S**PRTLDS  SMIGAD**S**SEEKFL  MIGADS**S**EEKFLR  HHLPEP**S**SRETGR  HLPEPS**S**RETGRG  YPEPYA**S**PPQPGL  PSTSTM**S**QEPELL  FIHRID**S**TEVIYQ  PVPIPP**S**PDTKDR  SKIRRL**S**ACKQQQ  AQRCTI**S**YRAPEL  TFQNMN**S**KRKAET  RRQLAF**S**TVGTPD  MKKYGK**S**FRKLLS  VRRVPG**S**SGHLHK  RRVPGS**S**GHLHKT  DGDWEW**S**DDEMDE  ELEKRA**S**GQAFEL  AFELIL**S**PRSKES  SPRSKE**S**VPEFPL  VPEFPL**S**PPKKKD  AEERRK**S**HEAEVL  ALLLRG**S**LLASGR  VPGTPD**S**LSSGSS  KRHTRD**S**EAQRLP  LGTKNL**S**QILSDR  SMVITE**S**PAALQL  LRTAKD**S**DDDDDV  IMDSSI**S**KQALSE  MARKKF**S**GLEISL  MMMMMM**S**DQEAKP  DQEAKP**S**TEDLGD  QRLTRY**S**QGDDDG  STLFKD**S**PLRTLK  SNMKRL**S**PAPQLG  SESSRA**S**GLVGRK  QPPLSQ**S**SDSSPT  LSQSSD**S**SPTRNS  SQSSDS**S**PTRNSE  SSPTRN**S**EPAGLE  QGGGLS**S**SGAGEG  GGGLSS**S**GAGEGQ  FLRRRL**S**DSSFMA  QGQQPL**S**PQSGSP  QQRSPG**S**PQLSRA  TVEGAG**S**IAAATG  EAYEMP**S**EEGYQD  EPAAEA**S**DEAERR  EDDLEM**S**SDASDA  DDLEMS**S**DASDAS  EMSSDA**S**DASGEE  SDASDA**S**GEEGGR  KRERKP**S**GGSSRG  RKPSGG**S**SRGNSR  KPSGGS**S**RGNSRP  GSSRGN**S**RPGTPS  SRPGTP**S**AEGGST  LDTGPQ**S**LSGKST  TGPQSL**S**GKSTPQ  QSLSGK**S**TPQPPS  GKTTPN**S**GDVQVT  LYMEYH**S**PDDNRM  GRTLVH**S**SSDGHI  ISVIPG**S**GGEKGS  KYQRSS**S**SGSDDY  MMMMAF**S**PWQILS  SPWQIL**S**PVQWAK  LVPSRR**S**KLRKPK  ELDENT**S**PLLGDA  GPLSQT**S**SKPDPS  VSCGGE**S**PLDGIC  MAAQRR**S**LLQSEQ  MSQPTP**S**PAPAAG  PTLTNQ**S**PTLTLQ  LQPLCH**S**DYDEDD  PPEQCL**S**DDEITM  IGGYEV**S**EEEEDE  EVKRLL**S**TDAEAV  HRQGHD**S**LEHDEL  QDKLNE**S**DEQHQE  AGGRAG**S**LKDPDV  IMAPAN**S**FVGTPY  TVTSHS**S**IIHRLP  IEQQPC**S**PGQEAV  EESGAP**S**PSPQKH  SGAPSP**S**PQKHGS  NGPLNE**S**QEDEED  QEDEED**S**EHGTSL  ATIKSA**S**LVTRQI  GKERPG**S**KEEVDE  DKKAKT**S**TRSSAK  GDRSGY**S**SPGSPG  DRSGYS**S**PGSPGT  GYSSPG**S**PGTPGS  SRSRTP**S**LPTPPT  VRTPPK**S**PSSAKS  TPPKSP**S**SAKSRL  VKSKIG**S**TENLKH  NKKLDL**S**NVQSKC  DLSNVQ**S**KCGSKD  VQSKCG**S**KDNIKH  HVPGGG**S**VQIVYK  VTSKCG**S**LGNIHH  FKDRVQ**S**KIGSLD  VQSKIG**S**LDNITH  AEIVYK**S**PVVSGD  YKSPVV**S**GDTSPR  VVSGDT**S**PRHLSN  TSPRHL**S**NVSSTG  NVSSTG**S**IDMVDS  SIDMVD**S**PQLATL  MEDEGN**S**DMLVVT  EFKKRF**S**DATSKA  YKELKR**S**LENPAE  PPVRVP**S**WGLEDN  IGGGDD**S**FNTFFS  NLNRLI**S**QIVSSI  EEVGVD**S**VEGEGE  TFSVVP**S**PKVSDT  VDDKFY**S**KLDQED  VEKYTI**S**QEAYDQ  RSFLKR**S**KLGRYN  EVVVPG**S**PGDCLP  FRSRCS**S**VTGVQR  PRRRHA**S**APSHVQ  RHASAP**S**HVQPSD  KNKAKR**S**LTSSLE  KRSLTS**S**LENIFS  MRGRLG**S**VDSFER  RLGSVD**S**FERSNS  GRKRTS**S**TCSNES  DDEQFV**S**LYGTEE  WSTLPK**S**SPPRSS  STLPKS**S**PPRSSL  KSSPPR**S**SLRRSS  SSPPRS**S**LRRSSP  RSSLRR**S**SPRRRS  SSLRRS**S**PRRRSS  SSPRRR**S**SFLRSS  SPRRRS**S**FLRSSC  SCPLSL**S**WGKRHS  MMMMPK**S**KELVSS  KSKELV**S**SSSSGS  SKELVS**S**SSSGSD  KELVSS**S**SSGSDS  LVSSSS**S**GSDSDS  SSSSSG**S**DSDSEV  SSSGSD**S**DSEVDK  SGSDSD**S**EVDKKL  QLKEQI**S**DIDDAV  EGNIDD**S**LIGGNA  LIGGNA**S**AEGPEG  EGEGTE**S**TVITGV  KGESGQ**S**WPRLTK  KDWEDD**S**DEDMSN  DSDEDM**S**NFDRFS  DGADDD**S**QDSDDE  DDDSQD**S**DDEKMP  ASEAGT**S**LVPATA  QAQLAG**S**DSDLDS  QLAGSD**S**DLDSDD  SDSDLD**S**DDEFVP  RQGPAG**S**PSRFNS  RLLPPA**S**PPPPPP  PVGSQC**S**VDLESA  CSVDLE**S**ASGEKD  VDLESA**S**GEKDLA  PLIRWD**S**YDNFSG  QGPLDG**S**LYAKVK  KDSLHG**S**TGAVNA  SATAAL**S**PQEKRE  EFPRAA**S**QQEIEQ  PLTQSR**S**GYIPSG  RSGYIP**S**GHSLGT  PPGRSY**S**PYDYQP  LRRRAA**S**DGQYEN  GQYENQ**S**PEATSP  EGTSPS**S**PPPSGV  PSSPPP**S**GVRSPP  GSLGQP**S**PSAQRN  VHTVPG**S**PQARHR  GTNTPP**S**PGFGWR  VATTPG**S**PSLGRH  HSNAIA**S**PGSPSL  HLGGSG**S**VVPGSP  GSVVPG**S**PCLDRH  AAFRQG**S**PTPALP  PEKRRM**S**VGDRAG  YSMPDN**S**PETRAK  SSGPAN**S**TADLLK  VSSAAP**S**PRGCAD  KLLMII**S**QKDTFH  ARRRGG**S**ASRSLP  LLRQHS**S**PWQVYG  DGTGDT**S**SEEDED  GTGDTS**S**EEDEDE  EEEPLN**S**EDDVSD  NSEDDV**S**DEEGQE  MQLRRP**S**DRELSE  PSDREL**S**EPMEFQ  TYETFK**S**IMKKSP  VFTDLA**S**VDNSEF  LPNGLL**S**GDEDFS  GDEDFS**S**IADMDF  CPKKPV**S**SYLRFS  PKKPVS**S**YLRFSK  SSYLRF**S**KEQLPI  KPKRPR**S**AYNVYV  ENWKNL**S**DSEKEL  LSYTRF**S**LARQVD  QPPTTP**S**PEPEPE  GTRRPG**S**RGSDSR  RPGSRG**S**DSRRPV  DSRRPV**S**RGSGVN  RPVSRG**S**GVNAAG  DSPSKS**S**AEAQTP  QTPKDG**S**NKSGAE  HAFKTE**S**GEETDL  KMSGSA**S**SENREG  PELRSF**S**SEEVIL  ELRSFS**S**EEVILK  EDDYIK**S**WEDNQQ  ASAASG**S**PGPGEG  ASAAAS**S**PAGGGA  SKQGSG**S**SQPMEV  VSGVKR**S**RSGEGE  GVKRSR**S**GEGEVS  SGEGEV**S**GLMRKV  RKVPRV**S**LERLDL  LDLTAD**S**QPPVFK  EGPRLA**S**PSGSTS  EEDGSL**S**LDGADS  SLDGAD**S**TGVVAK  GVVAKL**S**PANQRK  RLQEKL**S**PPYSSP  LSPPYS**S**PQEFAQ  PGAGLS**S**QELSGG  DQLLQG**S**EKGFQS  STGSTG**S**CSVGAK  QGGNGS**S**PVRGIP  HMRRSN**S**SGNLHM  QLMKHQ**S**IRGFVQ  MMMADG**S**SDAARE  MMADGS**S**DAAREP  APIRRR**S**SNYRAY  PIRRRS**S**NYRAYA  KKKSKI**S**ASRKLQ  KSKISA**S**RKLQLK  EKGRAL**S**TRCQPL  LRRVRI**S**ADAMMQ  GARAKE**S**LDLRAH  KNIDAL**S**GMEGRK  DHLHND**S**QIEADF  NSEHKD**S**EKKHKE  KENGFS**S**PPQIKD  DTVGCC**S**LRVEHI  MMMMEV**S**PLQPVN  EEENEE**S**DNEKET  MAEVLP**S**PRGQRV  NENTEG**S**PQEDGV  KKRNPW**S**DSESDR  RNPWSD**S**ESDRSS  PWSDSE**S**DRSSDE  DSESDR**S**SDESNF  FTMDLD**S**DEDFSD  DSDEDF**S**DFDEKT  DEDFVP**S**DASPPK  FVPSDA**S**PPKTKT  ELKPQK**S**VVSDLE  PQKSVV**S**DLEADD  ADDVKG**S**VPLSSS  GSVPLS**S**SPPATH  SVPLSS**S**PPATHF  RDPALN**S**GVSQKP  RRKRKP**S**TSDDSD  KRKPST**S**DDSDSN  PSTSDD**S**DSNFEK  TSDDSD**S**NFEKIV  KKSKGE**S**DDFHMD  FHMDFD**S**AVAPRA  IKYLEE**S**DEDDLF  TVPADA**S**PDSKCP  ELVELS**S**DSEDLG  RGDRVY**S**PYNHRH  DRDGYE**S**SYRRRT  RKRKTR**S**LSVEIV  RKTRSL**S**VEIVYE  PSRQLP**S**PRTSLM  CLIIED**S**QPESQV  DTCGSI**S**QVIEQL  SSVLGM**S**VESAPA  AEDTAS**S**QLGFGV  FGVLEL**S**QSQDVE  VLELSQ**S**QDVEEN  IKHEEQ**S**NEDIPI  SEDMPF**S**PKASVA  GLQIQK**S**PEPEVL  SDLVAP**S**PDAFRS  PFIVPS**S**PTEQEG  DKPMDT**S**VLSEEG  MDTSVL**S**EEGGEP  GSLPIP**S**QPQFSH  EIEPKN**S**PEDLGL  LSTSEY**S**QSPKME  TSEYSQ**S**PKMESL  EDTEPM**S**PVLNSK  VPAEND**S**ILMNPA  DGEVQL**S**QNDDKT  GSQAVP**S**PATRSE  PSPATR**S**EALSSV  TRSEAL**S**SVLDQE  RSEALS**S**VLDQEE  SVPLHL**S**LTETQS  SVISID**S**PQKLAI  DTKEEK**S**VEYEGD  PVEQDS**S**QPSLPL  QDSSQP**S**LPLVRA  HDPILG**S**GKGDSG  GSGKGD**S**GAAPDV  VAESVA**S**PQKTMS  LLHFPS**S**QGEEEK  DHTIRQ**S**QQPMKP  QPMKPI**S**PVKDPV  PVKDPV**S**PASQKM  VIQGPS**S**PQGEAM  PGDDTE**S**LHSQGE  DTESLH**S**QGEEEF  GGPGKL**S**PRKGVS  SPRKGV**S**QTGTPV  LGIEDI**S**PNLSPD  DISPNL**S**PDDKSF  AGALRR**S**DSPEIP  ALRRSD**S**PEIPFQ  QAAAGP**S**DGLDAS  TKAADI**S**LDNLVE  TSEEER**S**PAKRGR  GAGEFV**S**PCESGD  DGPTGS**S**EEEEEF  NKQYTE**S**QLRAGA  DELDKY**S**EALKDA  KLVIIE**S**DLERAE  VTNNLK**S**LEAQAE  ALNDMT**S**IIIIII  NRTPPK**S**PGDPSK  RAAKRL**S**LESEGA  KRLSLE**S**EGAGEG  GEGAAA**S**PELSAL  GVTKAI**S**SPTVSR  VTKAIS**S**PTVSRL  ATYGTG**S**ASRASA  GSASRA**S**AVSELS  SRASAV**S**ELSPRE  SAVSEL**S**PRERSP  LSPRER**S**PALKSP  RSPALK**S**PLQSVV  LKSPLQ**S**VVVRRR  LSPSKK**S**PVGKSP  KSPVGK**S**PPSTGS  KEESAA**S**GGAAYT  KIKEKG**S**FSDTGL  KEKGSF**S**DTGLGD  EKPFRG**S**QSPKRY  PFRGSQ**S**PKRYKL  VQEKSS**S**PPPRKT  FPTGKS**S**FSITRE  VNVRMD**S**FDEDLA  SAQSQR**S**PSELFA  AAKNKK**S**PEIHRR  HRRIDI**S**PSTFRK  RIDISP**S**TFRKHG  AHDEMK**S**PREPGY  WAHDKF**S**GEEGEI  EIEDDE**S**GTENRE  SVTPPG**S**LELLQP  VVRRDS**S**HAFTLD  YRQRAH**S**NPMADH  PRGGFD**S**PFYRDS  SPFYRD**S**LPGSQR  TRVGFG**S**GQPPPY  ELFRNK**S**ESQCDE  VMARSL**S**PPPELE  KTSGPL**S**PPTGPP  FRARST**S**LNERPK  FRCRSI**S**VSEHVV  DRVRSM**S**GGHGLR  FLGSAT**S**PGPRTA  DAYSRS**S**SVSSQE  AYSRSS**S**VSSQEE  SSQEEK**S**LHAEEL  PLSKSS**S**SPELQT  ADVGRL**S**PEVKAR  PEVKAR**S**QSGTLD  VKARSQ**S**GTLDGE  EGPLPS**S**SPRSPS  PSSSPR**S**PSGLRP  QRKRLI**S**SVEDFT  TRRLSS**S**ESPQRD  RLSSSE**S**PQRDPP  YTPRSL**S**APIFTT  SDDLNA**S**PKTSAT  VADGNV**S**DFDNEE  LPGRST**S**LVEGRS  SLVEGR**S**CGWVPP  RLGPEL**S**PSPTSP  GPELSP**S**PTSPTA  LSPSPT**S**PTATST  HFIHNP**S**EDLAAP  VLRQSI**S**FSGLPS  PSGRRT**S**PPPPGL  SFSPSS**S**PPPPGD  PGDLPL**S**PSAFSA  RTPSVQ**S**LGSDPD  SLGGSD**S**PVFEAG  PIFNRI**S**VSEEEE  GFRRAV**S**ELDAKQ  YTPTPR**S**PRFIGR  FIGRRQ**S**LIEDAR  CYDDDD**S**GNEESS  MMMMSS**S**PLSKKR  SKKRRV**S**GPDPKP  GMAKNG**S**EADIDE  GVKIHV**S**DQELQS  SDQELQ**S**ANASVD  LQSANA**S**VDDSRL  LKATLP**S**PDKLPG  KDDYPS**S**PPKCKF  DEPNPN**S**PANSQA  SDIVPD**S**PFPGAL  TKPESA**S**PPADGT  SGTLPV**S**QPKSWA  YVETKY**S**PPAISP  YSPPAI**S**PLVSEK  NGPKNH**S**VNEEEQ  EEQGEG**S**EDEWEQ  ERVKEP**S**QDTVAT  SSTNSS**S**QDYSTS  VNSCIT**S**PSTPSK  LALCLD**S**DSEDEL  LCLDSD**S**EDELKR  AGEQQL**S**EPEDME  ELLECL**S**PATSRT  PGRTSD**S**PWFLSG  RWLDGA**S**FDNERG  KNTPVQ**S**PVSLGE  CRFQLR**S**PESLKN  EMGPPP**S**PASTCS  FTAGQE**S**PIILRD  ILHACV**S**VCFPTS  DHQDPV**S**PPIAPP  CKLKRT**S**PTAYCD  RNRKTA**S**PEDSDM  ENKDPL**S**ASSRIG  VYLNQQ**S**GTIRLD  LCNVAE**S**LIVPVR  PQQRRI**S**QSQPVR  STPAAS**S**AGLIYI  ASSYLT**S**ASSLAR  GIDNED**S**EHENDD  HPFFRR**S**DSMTFL  FFRRSD**S**MTFLGC  PSQGLY**S**SSASSG  DEPGEG**S**GVARSF  RKRHGS**S**RSVVDM  RHGSSR**S**VVDMDL  EPGPVP**S**SPSQEP  PGPVPS**S**PSQEPP  FPRRAF**S**EADMER  QLLVRQ**S**LVLPHS  ERDSEL**S**DTDSGC  ERPGEG**S**PMVDNP  NPMRRK**S**GPSCKH  QEGGFA**S**PRTGKG  GKWKRK**S**AGGGPS  LKDRPA**S**GSPFQL  DAFIVI**S**DSDGEE  FIVISD**S**DGEEPK  EAREVN**S**QEEEEE  LATGPS**S**QSHQEK  GSWDQS**S**QPVFEN  TSHGQS**S**QGIVEE  GNSVPA**S**QSVAAL  SEGRLL**S**FLEQSE  GAFRVP**S**PGMEEA  RRDLNE**S**PVKSFV  KHVLRG**S**PLYMAP  KDQEGD**S**AAALSL  LKAIVS**S**SNQALL  AALEVA**S**AAMAKE  KEGLSE**S**VRSSCT  ADGKKP**S**SPPEEL  GFGKEN**S**TDYLYP  RALKSL**S**KKYRLE  EEDELE**S**GDQEDE  EDEDDE**S**EDPGKD  GADVGF**S**GGIPSP  FSGGIP**S**PDKGHR  GHRKRA**S**SENERL  YKTPPP**S**YNSALA  KITSLS**S**SLDTSL  ITSLSS**S**LDTSLD  GEDLVG**S**LNGGHA  HANVHP**S**QEQGEA  HIERRV**S**NAGGPP  AKLRKV**S**KQEEAS  TLPRMK**S**SSSVTT  LPRMKS**S**SSVTTS  YADLGK**S**ARDVFT  GTEFGG**S**IYQKVN  GAEDKR**S**QEETPG  DGKLDL**S**VVENGG  APEVGA**S**KPEGID  KPEGID**S**RPETLH  AMMQRD**S**WEDIFE  QNSRRP**S**RATWLS  TGALRA**S**LVHVGS  SLVHVG**S**RPYTEF  PFGQHS**S**GEAAQD  FDMCGI**S**EAQLKD  RSKAIH**S**SDEDED  SKAIHS**S**DEDEDE  RSRAIH**S**SDEGED  SRAIHS**S**DEGEDQ  HSTGED**S**AFQEPD  PGPAPG**S**PGELEE  EGFGSS**S**PVKSPA  SSSPVK**S**PAAPYF  FLGSSF**S**PVRCGG  GGPGDA**S**PRGCGA  GSGEFG**S**VFKCVK  KMNRSV**S**LTIYYY  RLNATA**S**LEQERS  QRFLRR**S**VVESDQ  ATLKRA**S**FAKSVI  RASFAK**S**VIGTPE  EQQDVG**S**PDKARG  TSGKEL**S**DSCEGA  KAEDML**S**EDTDAD  VDSTIK**S**LDEKLR  SSMTAE**S**SPRSML  GGEAGE**S**SAEPPP  YLDEAP**S**SPDVKL  LDEAPS**S**PDVKLA  PVQKQA**S**LPVSGS  RPSRAG**S**LGPETP  ICSGLA**S**DGGGAR  QHVRSH**S**SPASLQ  GAGAAG**S**PAQQHA  YHSREQ**S**TDSGLG  GRHRKV**S**PNCRFI  SLEVWG**S**QEALEE  QPYSKD**S**PFGLSF  SRINKT**S**PVTASD  TSPVTA**S**DPAGPS  SAASSA**S**PVSRAI  TSKPQE**S**PKGKRK  EEKKTP**S**KPPAQL  ANTPKY**S**QVLGLG  LMAGPG**S**SSEEDE  MAGPGS**S**SEEDEA  AGPGSS**S**EEDEAS  ASHSGG**S**GDEAPK  TQAAGP**S**SPQKPP  QAAGPS**S**PQKPPT  EETKAA**S**PVLQED  DNGAED**S**GDTEDE  EDPYAG**S**TDENTD  QGGAHF**S**VSSLAE  GAHFSV**S**SLAEGS  AHFSVS**S**LAEGSV  ESTDEE**S**ENQTDL  AVSKDD**S**IISSLD  DDSIIS**S**LDVTDI  ISAENM**S**LETLRN  LETLRN**S**SPEDLF  ETLRNS**S**PEDLFD  MMSGWE**S**YYKTEG  SKAMFE**S**QSEDEL  FTYRSD**S**FENPVL  MNKRLG**S**LVDEFK  KHDNEG**S**GSKRPK  VHVRGD**S**ETDLEA  SHSRQA**S**TDAGTA  QHVRAH**S**SPASLQ  HVRAHS**S**PASLQL  AHSSPA**S**LQLGAV  LQLGAV**S**PGTLTP  QHLRQS**S**FEIPDD  AMNQRI**S**QSAPVK  PPLAPQ**S**PQGGVM  TQNPVS**S**PGMSQE  VSSPGM**S**QELRTM  YHSRDE**S**TDSGLS  RDESTD**S**GLSMSS  STDSGL**S**MSSYSV  PRKYLR**S**VGDGET  QQNYQN**S**ESGEKN  NYQNSE**S**GEKNEG  GEKNEG**S**ESAPEG  KNEGSE**S**APEGQA  PPAENS**S**APEAEQ  EDEQEA**S**EEKASE |

# (2) List of the 43,532 peptide samples in the negative subset $\mathbb{S}_{\boldsymbol{\xi=6}}^{\mathbf{-}}\boldsymbol{(}\mathbb{S}\boldsymbol{)}$

| Sample # | Protein ID | Site | Sequences |
| --- | --- | --- | --- |
| 1  2  3  4  5  6  7  8  9  10  11  12  13  14  15  16  17  18  19  20  21  22  23  24  25  26  27  28  29  30  31  32  33  34  35  36  37  38  39  40  41  42  43  44  45  46  47  48  49  50  51  52  53  54  55  56  57  58  59  60  61  62  63  64  65  66  67  68  69  70  71  72  73  74  75  76  77  78  79  80  81  82  83  84  85  86  87  88  89  90  91  92  93  94  95  96  97  98  99  100  101  102  103  104  105  106  107  108  109  110  111  112  113  114  115  116  117  118  119  120  121  122  123  124  125  126  127  128  129  130  131  132  133  134  135  136  137  138  139  140  141  142  143  144  145  146  147  148  149  150  151  152  153  154  155  156  157  158  159  160  161  162  163  164  165  166  167  168  169  170  171  172  173  174  175  176  177  178  179  180  181  182  183  184  185  186  187  188  189  190  191  192  193  194  195  196  197  198  199  200  201  202  203  204  205  206  207  208  209  210  211  212  213  214  215  216  217  218  219  220  221  222  223  224  225  226  227  228  229  230  231  232  233  234  235  236  237  238  239  240  241  242  243  244  245  246  247  248  249  250  251  252  253  254  255  256  257  258  259  260  261  262  263  264  265  266  267  268  269  270  271  272  273  274  275  276  277  278  279  280  281  282  283  284  285  286  287  288  289  290  291  292  293  294  295  296  297  298  299  300  301  302  303  304  305  306  307  308  309  310  311  312  313  314  315  316  317  318  319  320  321  322  323  324  325  326  327  328  329  330  331  332  333  334  335  336  337  338  339  340  341  342  343  344  345  346  347  348  349  350  351  352  353  354  355  356  357  358  359  360  361  362  363  364  365  366  367  368  369  370  371  372  373  374  375  376  377  378  379  380  381  382  383  384  385  386  387  388  389  390  391  392  393  394  395  396  397  398  399  400  401  402  403  404  405  406  407  408  409  410  411  412  413  414  415  416  417  418  419  420  421  422  423  424  425  426  427  428  429  430  431  432  433  434  435  436  437  438  439  440  441  442  443  444  445  446  447  448  449  450  451  452  453  454  455  456  457  458  459  460  461  462  463  464  465  466  467  468  469  470  471  472  473  474  475  476  477  478  479  480  481  482  483  484  485  486  487  488  489  490  491  492  493  494  495  496  497  498  499  500  501  502  503  504  505  506  507  508  509  510  511  512  513  514  515  516  517  518  519  520  521  522  523  524  525  526  527  528  529  530  531  532  533  534  535  536  537  538  539  540  541  542  543  544  545  546  547  548  549  550  551  552  553  554  555  556  557  558  559  560  561  562  563  564  565  566  567  568  569  570  571  572  573  574  575  576  577  578  579  580  581  582  583  584  585  586  587  588  589  590  591  592  593  594  595  596  597  598  599  600  601  602  603  604  605  606  607  608  609  610  611  612  613  614  615  616  617  618  619  620  621  622  623  624  625  626  627  628  629  630  631  632  633  634  635  636  637  638  639  640  641  642  643  644  645  646  647  648  649  650  651  652  653  654  655  656  657  658  659  660  661  662  663  664  665  666  667  668  669  670  671  672  673  674  675  676  677  678  679  680  681  682  683  684  685  686  687  688  689  690  691  692  693  694  695  696  697  698  699  700  701  702  703  704  705  706  707  708  709  710  711  712  713  714  715  716  717  718  719  720  721  722  723  724  725  726  727  728  729  730  731  732  733  734  735  736  737  738  739  740  741  742  743  744  745  746  747  748  749  750  751  752  753  754  755  756  757  758  759  760  761  762  763  764  765  766  767  768  769  770  771  772  773  774  775  776  777  778  779  780  781  782  783  784  785  786  787  788  789  790  791  792  793  794  795  796  797  798  799  800  801  802  803  804  805  806  807  808  809  810  811  812  813  814  815  816  817  818  819  820  821  822  823  824  825  826  827  828  829  830  831  832  833  834  835  836  837  838  839  840  841  842  843  844  845  846  847  848  849  850  851  852  853  854  855  856  857  858  859  860  861  862  863  864  865  866  867  868  869  870  871  872  873  874  875  876  877  878  879  880  881  882  883  884  885  886  887  888  889  890  891  892  893  894  895  896  897  898  899  900  901  902  903  904  905  906  907  908  909  910  911  912  913  914  915  916  917  918  919  920  921  922  923  924  925  926  927  928  929  930  931  932  933  934  935  936  937  938  939  940  941  942  943  944  945  946  947  948  949  950  951  952  953  954  955  956  957  958  959  960  961  962  963  964  965  966  967  968  969  970  971  972  973  974  975  976  977  978  979  980  981  982  983  984  985  986  987  988  989  990  991  992  993  994  995  996  997  998  999  1000  1001  1002  1003  1004  1005  1006  1007  1008  1009  1010  1011  1012  1013  1014  1015  1016  1017  1018  1019  1020  1021  1022  1023  1024  1025  1026  1027  1028  1029  1030  1031  1032  1033  1034  1035  1036  1037  1038  1039  1040  1041  1042  1043  1044  1045  1046  1047  1048  1049  1050  1051  1052  1053  1054  1055  1056  1057  1058  1059  1060  1061  1062  1063  1064  1065  1066  1067  1068  1069  1070  1071  1072  1073  1074  1075  1076  1077  1078  1079  1080  1081  1082  1083  1084  1085  1086  1087  1088  1089  1090  1091  1092  1093  1094  1095  1096  1097  1098  1099  1100  1101  1102  1103  1104  1105  1106  1107  1108  1109  1110  1111  1112  1113  1114  1115  1116  1117  1118  1119  1120  1121  1122  1123  1124  1125  1126  1127  1128  1129  1130  1131  1132  1133  1134  1135  1136  1137  1138  1139  1140  1141  1142  1143  1144  1145  1146  1147  1148  1149  1150  1151  1152  1153  1154  1155  1156  1157  1158  1159  1160  1161  1162  1163  1164  1165  1166  1167  1168  1169  1170  1171  1172  1173  1174  1175  1176  1177  1178  1179  1180  1181  1182  1183  1184  1185  1186  1187  1188  1189  1190  1191  1192  1193  1194  1195  1196  1197  1198  1199  1200  1201  1202  1203  1204  1205  1206  1207  1208  1209  1210  1211  1212  1213  1214  1215  1216  1217  1218  1219  1220  1221  1222  1223  1224  1225  1226  1227  1228  1229  1230  1231  1232  1233  1234  1235  1236  1237  1238  1239  1240  1241  1242  1243  1244  1245  1246  1247  1248  1249  1250  1251  1252  1253  1254  1255  1256  1257  1258  1259  1260  1261  1262  1263  1264  1265  1266  1267  1268  1269  1270  1271  1272  1273  1274  1275  1276  1277  1278  1279  1280  1281  1282  1283  1284  1285  1286  1287  1288  1289  1290  1291  1292  1293  1294  1295  1296  1297  1298  1299  1300  1301  1302  1303  1304  1305  1306  1307  1308  1309  1310  1311  1312  1313  1314  1315  1316  1317  1318  1319  1320  1321  1322  1323  1324  1325  1326  1327  1328  1329  1330  1331  1332  1333  1334  1335  1336  1337  1338  1339  1340  1341  1342  1343  1344  1345  1346  1347  1348  1349  1350  1351  1352  1353  1354  1355  1356  1357  1358  1359  1360  1361  1362  1363  1364  1365  1366  1367  1368  1369  1370  1371  1372  1373  1374  1375  1376  1377  1378  1379  1380  1381  1382  1383  1384  1385  1386  1387  1388  1389  1390  1391  1392  1393  1394  1395  1396  1397  1398  1399  1400  1401  1402  1403  1404  1405  1406  1407  1408  1409  1410  1411  1412  1413  1414  1415  1416  1417  1418  1419  1420  1421  1422  1423  1424  1425  1426  1427  1428  1429  1430  1431  1432  1433  1434  1435  1436  1437  1438  1439  1440  1441  1442  1443  1444  1445  1446  1447  1448  1449  1450  1451  1452  1453  1454  1455  1456  1457  1458  1459  1460  1461  1462  1463  1464  1465  1466  1467  1468  1469  1470  1471  1472  1473  1474  1475  1476  1477  1478  1479  1480  1481  1482  1483  1484  1485  1486  1487  1488  1489  1490  1491  1492  1493  1494  1495  1496  1497  1498  1499  1500  1501  1502  1503  1504  1505  1506  1507  1508  1509  1510  1511  1512  1513  1514  1515  1516  1517  1518  1519  1520  1521  1522  1523  1524  1525  1526  1527  1528  1529  1530  1531  1532  1533  1534  1535  1536  1537  1538  1539  1540  1541  1542  1543  1544  1545  1546  1547  1548  1549  1550  1551  1552  1553  1554  1555  1556  1557  1558  1559  1560  1561  1562  1563  1564  1565  1566  1567  1568  1569  1570  1571  1572  1573  1574  1575  1576  1577  1578  1579  1580  1581  1582  1583  1584  1585  1586  1587  1588  1589  1590  1591  1592  1593  1594  1595  1596  1597  1598  1599  1600  1601  1602  1603  1604  1605  1606  1607  1608  1609  1610  1611  1612  1613  1614  1615  1616  1617  1618  1619  1620  1621  1622  1623  1624  1625  1626  1627  1628  1629  1630  1631  1632  1633  1634  1635  1636  1637  1638  1639  1640  1641  1642  1643  1644  1645  1646  1647  1648  1649  1650  1651  1652  1653  1654  1655  1656  1657  1658  1659  1660  1661  1662  1663  1664  1665  1666  1667  1668  1669  1670  1671  1672  1673  1674  1675  1676  1677  1678  1679  1680  1681  1682  1683  1684  1685  1686  1687  1688  1689  1690  1691  1692  1693  1694  1695  1696  1697  1698  1699  1700  1701  1702  1703  1704  1705  1706  1707  1708  1709  1710  1711  1712  1713  1714  1715  1716  1717  1718  1719  1720  1721  1722  1723  1724  1725  1726  1727  1728  1729  1730  1731  1732  1733  1734  1735  1736  1737  1738  1739  1740  1741  1742  1743  1744  1745  1746  1747  1748  1749  1750  1751  1752  1753  1754  1755  1756  1757  1758  1759  1760  1761  1762  1763  1764  1765  1766  1767  1768  1769  1770  1771  1772  1773  1774  1775  1776  1777  1778  1779  1780  1781  1782  1783  1784  1785  1786  1787  1788  1789  1790  1791  1792  1793  1794  1795  1796  1797  1798  1799  1800  1801  1802  1803  1804  1805  1806  1807  1808  1809  1810  1811  1812  1813  1814  1815  1816  1817  1818  1819  1820  1821  1822  1823  1824  1825  1826  1827  1828  1829  1830  1831  1832  1833  1834  1835  1836  1837  1838  1839  1840  1841  1842  1843  1844  1845  1846  1847  1848  1849  1850  1851  1852  1853  1854  1855  1856  1857  1858  1859  1860  1861  1862  1863  1864  1865  1866  1867  1868  1869  1870  1871  1872  1873  1874  1875  1876  1877  1878  1879  1880  1881  1882  1883  1884  1885  1886  1887  1888  1889  1890  1891  1892  1893  1894  1895  1896  1897  1898  1899  1900  1901  1902  1903  1904  1905  1906  1907  1908  1909  1910  1911  1912  1913  1914  1915  1916  1917  1918  1919  1920  1921  1922  1923  1924  1925  1926  1927  1928  1929  1930  1931  1932  1933  1934  1935  1936  1937  1938  1939  1940  1941  1942  1943  1944  1945  1946  1947  1948  1949  1950  1951  1952  1953  1954  1955  1956  1957  1958  1959  1960  1961  1962  1963  1964  1965  1966  1967  1968  1969  1970  1971  1972  1973  1974  1975  1976  1977  1978  1979  1980  1981  1982  1983  1984  1985  1986  1987  1988  1989  1990  1991  1992  1993  1994  1995  1996  1997  1998  1999  2000  2001  2002  2003  2004  2005  2006  2007  2008  2009  2010  2011  2012  2013  2014  2015  2016  2017  2018  2019  2020  2021  2022  2023  2024  2025  2026  2027  2028  2029  2030  2031  2032  2033  2034  2035  2036  2037  2038  2039  2040  2041  2042  2043  2044  2045  2046  2047  2048  2049  2050  2051  2052  2053  2054  2055  2056  2057  2058  2059  2060  2061  2062  2063  2064  2065  2066  2067  2068  2069  2070  2071  2072  2073  2074  2075  2076  2077  2078  2079  2080  2081  2082  2083  2084  2085  2086  2087  2088  2089  2090  2091  2092  2093  2094  2095  2096  2097  2098  2099  2100  2101  2102  2103  2104  2105  2106  2107  2108  2109  2110  2111  2112  2113  2114  2115  2116  2117  2118  2119  2120  2121  2122  2123  2124  2125  2126  2127  2128  2129  2130  2131  2132  2133  2134  2135  2136  2137  2138  2139  2140  2141  2142  2143  2144  2145  2146  2147  2148  2149  2150  2151  2152  2153  2154  2155  2156  2157  2158  2159  2160  2161  2162  2163  2164  2165  2166  2167  2168  2169  2170  2171  2172  2173  2174  2175  2176  2177  2178  2179  2180  2181  2182  2183  2184  2185  2186  2187  2188  2189  2190  2191  2192  2193  2194  2195  2196  2197  2198  2199  2200  2201  2202  2203  2204  2205  2206  2207  2208  2209  2210  2211  2212  2213  2214  2215  2216  2217  2218  2219  2220  2221  2222  2223  2224  2225  2226  2227  2228  2229  2230  2231  2232  2233  2234  2235  2236  2237  2238  2239  2240  2241  2242  2243  2244  2245  2246  2247  2248  2249  2250  2251  2252  2253  2254  2255  2256  2257  2258  2259  2260  2261  2262  2263  2264  2265  2266  2267  2268  2269  2270  2271  2272  2273  2274  2275  2276  2277  2278  2279  2280  2281  2282  2283  2284  2285  2286  2287  2288  2289  2290  2291  2292  2293  2294  2295  2296  2297  2298  2299  2300  2301  2302  2303  2304  2305  2306  2307  2308  2309  2310  2311  2312  2313  2314  2315  2316  2317  2318  2319  2320  2321  2322  2323  2324  2325  2326  2327  2328  2329  2330  2331  2332  2333  2334  2335  2336  2337  2338  2339  2340  2341  2342  2343  2344  2345  2346  2347  2348  2349  2350  2351  2352  2353  2354  2355  2356  2357  2358  2359  2360  2361  2362  2363  2364  2365  2366  2367  2368  2369  2370  2371  2372  2373  2374  2375  2376  2377  2378  2379  2380  2381  2382  2383  2384  2385  2386  2387  2388  2389  2390  2391  2392  2393  2394  2395  2396  2397  2398  2399  2400  2401  2402  2403  2404  2405  2406  2407  2408  2409  2410  2411  2412  2413  2414  2415  2416  2417  2418  2419  2420  2421  2422  2423  2424  2425  2426  2427  2428  2429  2430  2431  2432  2433  2434  2435  2436  2437  2438  2439  2440  2441  2442  2443  2444  2445  2446  2447  2448  2449  2450  2451  2452  2453  2454  2455  2456  2457  2458  2459  2460  2461  2462  2463  2464  2465  2466  2467  2468  2469  2470  2471  2472  2473  2474  2475  2476  2477  2478  2479  2480  2481  2482  2483  2484  2485  2486  2487  2488  2489  2490  2491  2492  2493  2494  2495  2496  2497  2498  2499  2500  2501  2502  2503  2504  2505  2506  2507  2508  2509  2510  2511  2512  2513  2514  2515  2516  2517  2518  2519  2520  2521  2522  2523  2524  2525  2526  2527  2528  2529  2530  2531  2532  2533  2534  2535  2536  2537  2538  2539  2540  2541  2542  2543  2544  2545  2546  2547  2548  2549  2550  2551  2552  2553  2554  2555  2556  2557  2558  2559  2560  2561  2562  2563  2564  2565  2566  2567  2568  2569  2570  2571  2572  2573  2574  2575  2576  2577  2578  2579  2580  2581  2582  2583  2584  2585  2586  2587  2588  2589  2590  2591  2592  2593  2594  2595  2596  2597  2598  2599  2600  2601  2602  2603  2604  2605  2606  2607  2608  2609  2610  2611  2612  2613  2614  2615  2616  2617  2618  2619  2620  2621  2622  2623  2624  2625  2626  2627  2628  2629  2630  2631  2632  2633  2634  2635  2636  2637  2638  2639  2640  2641  2642  2643  2644  2645  2646  2647  2648  2649  2650  2651  2652  2653  2654  2655  2656  2657  2658  2659  2660  2661  2662  2663  2664  2665  2666  2667  2668  2669  2670  2671  2672  2673  2674  2675  2676  2677  2678  2679  2680  2681  2682  2683  2684  2685  2686  2687  2688  2689  2690  2691  2692  2693  2694  2695  2696  2697  2698  2699  2700  2701  2702  2703  2704  2705  2706  2707  2708  2709  2710  2711  2712  2713  2714  2715  2716  2717  2718  2719  2720  2721  2722  2723  2724  2725  2726  2727  2728  2729  2730  2731  2732  2733  2734  2735  2736  2737  2738  2739  2740  2741  2742  2743  2744  2745  2746  2747  2748  2749  2750  2751  2752  2753  2754  2755  2756  2757  2758  2759  2760  2761  2762  2763  2764  2765  2766  2767  2768  2769  2770  2771  2772  2773  2774  2775  2776  2777  2778  2779  2780  2781  2782  2783  2784  2785  2786  2787  2788  2789  2790  2791  2792  2793  2794  2795  2796  2797  2798  2799  2800  2801  2802  2803  2804  2805  2806  2807  2808  2809  2810  2811  2812  2813  2814  2815  2816  2817  2818  2819  2820  2821  2822  2823  2824  2825  2826  2827  2828  2829  2830  2831  2832  2833  2834  2835  2836  2837  2838  2839  2840  2841  2842  2843  2844  2845  2846  2847  2848  2849  2850  2851  2852  2853  2854  2855  2856  2857  2858  2859  2860  2861  2862  2863  2864  2865  2866  2867  2868  2869  2870  2871  2872  2873  2874  2875  2876  2877  2878  2879  2880  2881  2882  2883  2884  2885  2886  2887  2888  2889  2890  2891  2892  2893  2894  2895  2896  2897  2898  2899  2900  2901  2902  2903  2904  2905  2906  2907  2908  2909  2910  2911  2912  2913  2914  2915  2916  2917  2918  2919  2920  2921  2922  2923  2924  2925  2926  2927  2928  2929  2930  2931  2932  2933  2934  2935  2936  2937  2938  2939  2940  2941  2942  2943  2944  2945  2946  2947  2948  2949  2950  2951  2952  2953  2954  2955  2956  2957  2958  2959  2960  2961  2962  2963  2964  2965  2966  2967  2968  2969  2970  2971  2972  2973  2974  2975  2976  2977  2978  2979  2980  2981  2982  2983  2984  2985  2986  2987  2988  2989  2990  2991  2992  2993  2994  2995  2996  2997  2998  2999  3000  3001  3002  3003  3004  3005  3006  3007  3008  3009  3010  3011  3012  3013  3014  3015  3016  3017  3018  3019  3020  3021  3022  3023  3024  3025  3026  3027  3028  3029  3030  3031  3032  3033  3034  3035  3036  3037  3038  3039  3040  3041  3042  3043  3044  3045  3046  3047  3048  3049  3050  3051  3052  3053  3054  3055  3056  3057  3058  3059  3060  3061  3062  3063  3064  3065  3066  3067  3068  3069  3070  3071  3072  3073  3074  3075  3076  3077  3078  3079  3080  3081  3082  3083  3084  3085  3086  3087  3088  3089  3090  3091  3092  3093  3094  3095  3096  3097  3098  3099  3100  3101  3102  3103  3104  3105  3106  3107  3108  3109  3110  3111  3112  3113  3114  3115  3116  3117  3118  3119  3120  3121  3122  3123  3124  3125  3126  3127  3128  3129  3130  3131  3132  3133  3134  3135  3136  3137  3138  3139  3140  3141  3142  3143  3144  3145  3146  3147  3148  3149  3150  3151  3152  3153  3154  3155  3156  3157  3158  3159  3160  3161  3162  3163  3164  3165  3166  3167  3168  3169  3170  3171  3172  3173  3174  3175  3176  3177  3178  3179  3180  3181  3182  3183  3184  3185  3186  3187  3188  3189  3190  3191  3192  3193  3194  3195  3196  3197  3198  3199  3200  3201  3202  3203  3204  3205  3206  3207  3208  3209  3210  3211  3212  3213  3214  3215  3216  3217  3218  3219  3220  3221  3222  3223  3224  3225  3226  3227  3228  3229  3230  3231  3232  3233  3234  3235  3236  3237  3238  3239  3240  3241  3242  3243  3244  3245  3246  3247  3248  3249  3250  3251  3252  3253  3254  3255  3256  3257  3258  3259  3260  3261  3262  3263  3264  3265  3266  3267  3268  3269  3270  3271  3272  3273  3274  3275  3276  3277  3278  3279  3280  3281  3282  3283  3284  3285  3286  3287  3288  3289  3290  3291  3292  3293  3294  3295  3296  3297  3298  3299  3300  3301  3302  3303  3304  3305  3306  3307  3308  3309  3310  3311  3312  3313  3314  3315  3316  3317  3318  3319  3320  3321  3322  3323  3324  3325  3326  3327  3328  3329  3330  3331  3332  3333  3334  3335  3336  3337  3338  3339  3340  3341  3342  3343  3344  3345  3346  3347  3348  3349  3350  3351  3352  3353  3354  3355  3356  3357  3358  3359  3360  3361  3362  3363  3364  3365  3366  3367  3368  3369  3370  3371  3372  3373  3374  3375  3376  3377  3378  3379  3380  3381  3382  3383  3384  3385  3386  3387  3388  3389  3390  3391  3392  3393  3394  3395  3396  3397  3398  3399  3400  3401  3402  3403  3404  3405  3406  3407  3408  3409  3410  3411  3412  3413  3414  3415  3416  3417  3418  3419  3420  3421  3422  3423  3424  3425  3426  3427  3428  3429  3430  3431  3432  3433  3434  3435  3436  3437  3438  3439  3440  3441  3442  3443  3444  3445  3446  3447  3448  3449  3450  3451  3452  3453  3454  3455  3456  3457  3458  3459  3460  3461  3462  3463  3464  3465  3466  3467  3468  3469  3470  3471  3472  3473  3474  3475  3476  3477  3478  3479  3480  3481  3482  3483  3484  3485  3486  3487  3488  3489  3490  3491  3492  3493  3494  3495  3496  3497  3498  3499  3500  3501  3502  3503  3504  3505  3506  3507  3508  3509  3510  3511  3512  3513  3514  3515  3516  3517  3518  3519  3520  3521  3522  3523  3524  3525  3526  3527  3528  3529  3530  3531  3532  3533  3534  3535  3536  3537  3538  3539  3540  3541  3542  3543  3544  3545  3546  3547  3548  3549  3550  3551  3552  3553  3554  3555  3556  3557  3558  3559  3560  3561  3562  3563  3564  3565  3566  3567  3568  3569  3570  3571  3572  3573  3574  3575  3576  3577  3578  3579  3580  3581  3582  3583  3584  3585  3586  3587  3588  3589  3590  3591  3592  3593  3594  3595  3596  3597  3598  3599  3600  3601  3602  3603  3604  3605  3606  3607  3608  3609  3610  3611  3612  3613  3614  3615  3616  3617  3618  3619  3620  3621  3622  3623  3624  3625  3626  3627  3628  3629  3630  3631  3632  3633  3634  3635  3636  3637  3638  3639  3640  3641  3642  3643  3644  3645  3646  3647  3648  3649  3650  3651  3652  3653  3654  3655  3656  3657  3658  3659  3660  3661  3662  3663  3664  3665  3666  3667  3668  3669  3670  3671  3672  3673  3674  3675  3676  3677  3678  3679  3680  3681  3682  3683  3684  3685  3686  3687  3688  3689  3690  3691  3692  3693  3694  3695  3696  3697  3698  3699  3700  3701  3702  3703  3704  3705  3706  3707  3708  3709  3710  3711  3712  3713  3714  3715  3716  3717  3718  3719  3720  3721  3722  3723  3724  3725  3726  3727  3728  3729  3730  3731  3732  3733  3734  3735  3736  3737  3738  3739  3740  3741  3742  3743  3744  3745  3746  3747  3748  3749  3750  3751  3752  3753  3754  3755  3756  3757  3758  3759  3760  3761  3762  3763  3764  3765  3766  3767  3768  3769  3770  3771  3772  3773  3774  3775  3776  3777  3778  3779  3780  3781  3782  3783  3784  3785  3786  3787  3788  3789  3790  3791  3792  3793  3794  3795  3796  3797  3798  3799  3800  3801  3802  3803  3804  3805  3806  3807  3808  3809  3810  3811  3812  3813  3814  3815  3816  3817  3818  3819  3820  3821  3822  3823  3824  3825  3826  3827  3828  3829  3830  3831  3832  3833  3834  3835  3836  3837  3838  3839  3840  3841  3842  3843  3844  3845  3846  3847  3848  3849  3850  3851  3852  3853  3854  3855  3856  3857  3858  3859  3860  3861  3862  3863  3864  3865  3866  3867  3868  3869  3870  3871  3872  3873  3874  3875  3876  3877  3878  3879  3880  3881  3882  3883  3884  3885  3886  3887  3888  3889  3890  3891  3892  3893  3894  3895  3896  3897  3898  3899  3900  3901  3902  3903  3904  3905  3906  3907  3908  3909  3910  3911  3912  3913  3914  3915  3916  3917  3918  3919  3920  3921  3922  3923  3924  3925  3926  3927  3928  3929  3930  3931  3932  3933  3934  3935  3936  3937  3938  3939  3940  3941  3942  3943  3944  3945  3946  3947  3948  3949  3950  3951  3952  3953  3954  3955  3956  3957  3958  3959  3960  3961  3962  3963  3964  3965  3966  3967  3968  3969  3970  3971  3972  3973  3974  3975  3976  3977  3978  3979  3980  3981  3982  3983  3984  3985  3986  3987  3988  3989  3990  3991  3992  3993  3994  3995  3996  3997  3998  3999  4000  4001  4002  4003  4004  4005  4006  4007  4008  4009  4010  4011  4012  4013  4014  4015  4016  4017  4018  4019  4020  4021  4022  4023  4024  4025  4026  4027  4028  4029  4030  4031  4032  4033  4034  4035  4036  4037  4038  4039  4040  4041  4042  4043  4044  4045  4046  4047  4048  4049  4050  4051  4052  4053  4054  4055  4056  4057  4058  4059  4060  4061  4062  4063  4064  4065  4066  4067  4068  4069  4070  4071  4072  4073  4074  4075  4076  4077  4078  4079  4080  4081  4082  4083  4084  4085  4086  4087  4088  4089  4090  4091  4092  4093  4094  4095  4096  4097  4098  4099  4100  4101  4102  4103  4104  4105  4106  4107  4108  4109  4110  4111  4112  4113  4114  4115  4116  4117  4118  4119  4120  4121  4122  4123  4124  4125  4126  4127  4128  4129  4130  4131  4132  4133  4134  4135  4136  4137  4138  4139  4140  4141  4142  4143  4144  4145  4146  4147  4148  4149  4150  4151  4152  4153  4154  4155  4156  4157  4158  4159  4160  4161  4162  4163  4164  4165  4166  4167  4168  4169  4170  4171  4172  4173  4174  4175  4176  4177  4178  4179  4180  4181  4182  4183  4184  4185  4186  4187  4188  4189  4190  4191  4192  4193  4194  4195  4196  4197  4198  4199  4200  4201  4202  4203  4204  4205  4206  4207  4208  4209  4210  4211  4212  4213  4214  4215  4216  4217  4218  4219  4220  4221  4222  4223  4224  4225  4226  4227  4228  4229  4230  4231  4232  4233  4234  4235  4236  4237  4238  4239  4240  4241  4242  4243  4244  4245  4246  4247  4248  4249  4250  4251  4252  4253  4254  4255  4256  4257  4258  4259  4260  4261  4262  4263  4264  4265  4266  4267  4268  4269  4270  4271  4272  4273  4274  4275  4276  4277  4278  4279  4280  4281  4282  4283  4284  4285  4286  4287  4288  4289  4290  4291  4292  4293  4294  4295  4296  4297  4298  4299  4300  4301  4302  4303  4304  4305  4306  4307  4308  4309  4310  4311  4312  4313  4314  4315  4316  4317  4318  4319  4320  4321  4322  4323  4324  4325  4326  4327  4328  4329  4330  4331  4332  4333  4334  4335  4336  4337  4338  4339  4340  4341  4342  4343  4344  4345  4346  4347  4348  4349  4350  4351  4352  4353  4354  4355  4356  4357  4358  4359  4360  4361  4362  4363  4364  4365  4366  4367  4368  4369  4370  4371  4372  4373  4374  4375  4376  4377  4378  4379  4380  4381  4382  4383  4384  4385  4386  4387  4388  4389  4390  4391  4392  4393  4394  4395  4396  4397  4398  4399  4400  4401  4402  4403  4404  4405  4406  4407  4408  4409  4410  4411  4412  4413  4414  4415  4416  4417  4418  4419  4420  4421  4422  4423  4424  4425  4426  4427  4428  4429  4430  4431  4432  4433  4434  4435  4436  4437  4438  4439  4440  4441  4442  4443  4444  4445  4446  4447  4448  4449  4450  4451  4452  4453  4454  4455  4456  4457  4458  4459  4460  4461  4462  4463  4464  4465  4466  4467  4468  4469  4470  4471  4472  4473  4474  4475  4476  4477  4478  4479  4480  4481  4482  4483  4484  4485  4486  4487  4488  4489  4490  4491  4492  4493  4494  4495  4496  4497  4498  4499  4500  4501  4502  4503  4504  4505  4506  4507  4508  4509  4510  4511  4512  4513  4514  4515  4516  4517  4518  4519  4520  4521  4522  4523  4524  4525  4526  4527  4528  4529  4530  4531  4532  4533  4534  4535  4536  4537  4538  4539  4540  4541  4542  4543  4544  4545  4546  4547  4548  4549  4550  4551  4552  4553  4554  4555  4556  4557  4558  4559  4560  4561  4562  4563  4564  4565  4566  4567  4568  4569  4570  4571  4572  4573  4574  4575  4576  4577  4578  4579  4580  4581  4582  4583  4584  4585  4586  4587  4588  4589  4590  4591  4592  4593  4594  4595  4596  4597  4598  4599  4600  4601  4602  4603  4604  4605  4606  4607  4608  4609  4610  4611  4612  4613  4614  4615  4616  4617  4618  4619  4620  4621  4622  4623  4624  4625  4626  4627  4628  4629  4630  4631  4632  4633  4634  4635  4636  4637  4638  4639  4640  4641  4642  4643  4644  4645  4646  4647  4648  4649  4650  4651  4652  4653  4654  4655  4656  4657  4658  4659  4660  4661  4662  4663  4664  4665  4666  4667  4668  4669  4670  4671  4672  4673  4674  4675  4676  4677  4678  4679  4680  4681  4682  4683  4684  4685  4686  4687  4688  4689  4690  4691  4692  4693  4694  4695  4696  4697  4698  4699  4700  4701  4702  4703  4704  4705  4706  4707  4708  4709  4710  4711  4712  4713  4714  4715  4716  4717  4718  4719  4720  4721  4722  4723  4724  4725  4726  4727  4728  4729  4730  4731  4732  4733  4734  4735  4736  4737  4738  4739  4740  4741  4742  4743  4744  4745  4746  4747  4748  4749  4750  4751  4752  4753  4754  4755  4756  4757  4758  4759  4760  4761  4762  4763  4764  4765  4766  4767  4768  4769  4770  4771  4772  4773  4774  4775  4776  4777  4778  4779  4780  4781  4782  4783  4784  4785  4786  4787  4788  4789  4790  4791  4792  4793  4794  4795  4796  4797  4798  4799  4800  4801  4802  4803  4804  4805  4806  4807  4808  4809  4810  4811  4812  4813  4814  4815  4816  4817  4818  4819  4820  4821  4822  4823  4824  4825  4826  4827  4828  4829  4830  4831  4832  4833  4834  4835  4836  4837  4838  4839  4840  4841  4842  4843  4844  4845  4846  4847  4848  4849  4850  4851  4852  4853  4854  4855  4856  4857  4858  4859  4860  4861  4862  4863  4864  4865  4866  4867  4868  4869  4870  4871  4872  4873  4874  4875  4876  4877  4878  4879  4880  4881  4882  4883  4884  4885  4886  4887  4888  4889  4890  4891  4892  4893  4894  4895  4896  4897  4898  4899  4900  4901  4902  4903  4904  4905  4906  4907  4908  4909  4910  4911  4912  4913  4914  4915  4916  4917  4918  4919  4920  4921  4922  4923  4924  4925  4926  4927  4928  4929  4930  4931  4932  4933  4934  4935  4936  4937  4938  4939  4940  4941  4942  4943  4944  4945  4946  4947  4948  4949  4950  4951  4952  4953  4954  4955  4956  4957  4958  4959  4960  4961  4962  4963  4964  4965  4966  4967  4968  4969  4970  4971  4972  4973  4974  4975  4976  4977  4978  4979  4980  4981  4982  4983  4984  4985  4986  4987  4988  4989  4990  4991  4992  4993  4994  4995  4996  4997  4998  4999  5000  5001  5002  5003  5004  5005  5006  5007  5008  5009  5010  5011  5012  5013  5014  5015  5016  5017  5018  5019  5020  5021  5022  5023  5024  5025  5026  5027  5028  5029  5030  5031  5032  5033  5034  5035  5036  5037  5038  5039  5040  5041  5042  5043  5044  5045  5046  5047  5048  5049  5050  5051  5052  5053  5054  5055  5056  5057  5058  5059  5060  5061  5062  5063  5064  5065  5066  5067  5068  5069  5070  5071  5072  5073  5074  5075  5076  5077  5078  5079  5080  5081  5082  5083  5084  5085  5086  5087  5088  5089  5090  5091  5092  5093  5094  5095  5096  5097  5098  5099  5100  5101  5102  5103  5104  5105  5106  5107  5108  5109  5110  5111  5112  5113  5114  5115  5116  5117  5118  5119  5120  5121  5122  5123  5124  5125  5126  5127  5128  5129  5130  5131  5132  5133  5134  5135  5136  5137  5138  5139  5140  5141  5142  5143  5144  5145  5146  5147  5148  5149  5150  5151  5152  5153  5154  5155  5156  5157  5158  5159  5160  5161  5162  5163  5164  5165  5166  5167  5168  5169  5170  5171  5172  5173  5174  5175  5176  5177  5178  5179  5180  5181  5182  5183  5184  5185  5186  5187  5188  5189  5190  5191  5192  5193  5194  5195  5196  5197  5198  5199  5200  5201  5202  5203  5204  5205  5206  5207  5208  5209  5210  5211  5212  5213  5214  5215  5216  5217  5218  5219  5220  5221  5222  5223  5224  5225  5226  5227  5228  5229  5230  5231  5232  5233  5234  5235  5236  5237  5238  5239  5240  5241  5242  5243  5244  5245  5246  5247  5248  5249  5250  5251  5252  5253  5254  5255  5256  5257  5258  5259  5260  5261  5262  5263  5264  5265  5266  5267  5268  5269  5270  5271  5272  5273  5274  5275  5276  5277  5278  5279  5280  5281  5282  5283  5284  5285  5286  5287  5288  5289  5290  5291  5292  5293  5294  5295  5296  5297  5298  5299  5300  5301  5302  5303  5304  5305  5306  5307  5308  5309  5310  5311  5312  5313  5314  5315  5316  5317  5318  5319  5320  5321  5322  5323  5324  5325  5326  5327  5328  5329  5330  5331  5332  5333  5334  5335  5336  5337  5338  5339  5340  5341  5342  5343  5344  5345  5346  5347  5348  5349  5350  5351  5352  5353  5354  5355  5356  5357  5358  5359  5360  5361  5362  5363  5364  5365  5366  5367  5368  5369  5370  5371  5372  5373  5374  5375  5376  5377  5378  5379  5380  5381  5382  5383  5384  5385  5386  5387  5388  5389  5390  5391  5392  5393  5394  5395  5396  5397  5398  5399  5400  5401  5402  5403  5404  5405  5406  5407  5408  5409  5410  5411  5412  5413  5414  5415  5416  5417  5418  5419  5420  5421  5422  5423  5424  5425  5426  5427  5428  5429  5430  5431  5432  5433  5434  5435  5436  5437  5438  5439  5440  5441  5442  5443  5444  5445  5446  5447  5448  5449  5450  5451  5452  5453  5454  5455  5456  5457  5458  5459  5460  5461  5462  5463  5464  5465  5466  5467  5468  5469  5470  5471  5472  5473  5474  5475  5476  5477  5478  5479  5480  5481  5482  5483  5484  5485  5486  5487  5488  5489  5490  5491  5492  5493  5494  5495  5496  5497  5498  5499  5500  5501  5502  5503  5504  5505  5506  5507  5508  5509  5510  5511  5512  5513  5514  5515  5516  5517  5518  5519  5520  5521  5522  5523  5524  5525  5526  5527  5528  5529  5530  5531  5532  5533  5534  5535  5536  5537  5538  5539  5540  5541  5542  5543  5544  5545  5546  5547  5548  5549  5550  5551  5552  5553  5554  5555  5556  5557  5558  5559  5560  5561  5562  5563  5564  5565  5566  5567  5568  5569  5570  5571  5572  5573  5574  5575  5576  5577  5578  5579  5580  5581  5582  5583  5584  5585  5586  5587  5588  5589  5590  5591  5592  5593  5594  5595  5596  5597  5598  5599  5600  5601  5602  5603  5604  5605  5606  5607  5608  5609  5610  5611  5612  5613  5614  5615  5616  5617  5618  5619  5620  5621  5622  5623  5624  5625  5626  5627  5628  5629  5630  5631  5632  5633  5634  5635  5636  5637  5638  5639  5640  5641  5642  5643  5644  5645  5646  5647  5648  5649  5650  5651  5652  5653  5654  5655  5656  5657  5658  5659  5660  5661  5662  5663  5664  5665  5666  5667  5668  5669  5670  5671  5672  5673  5674  5675  5676  5677  5678  5679  5680  5681  5682  5683  5684  5685  5686  5687  5688  5689  5690  5691  5692  5693  5694  5695  5696  5697  5698  5699  5700  5701  5702  5703  5704  5705  5706  5707  5708  5709  5710  5711  5712  5713  5714  5715  5716  5717  5718  5719  5720  5721  5722  5723  5724  5725  5726  5727  5728  5729  5730  5731  5732  5733  5734  5735  5736  5737  5738  5739  5740  5741  5742  5743  5744  5745  5746  5747  5748  5749  5750  5751  5752  5753  5754  5755  5756  5757  5758  5759  5760  5761  5762  5763  5764  5765  5766  5767  5768  5769  5770  5771  5772  5773  5774  5775  5776  5777  5778  5779  5780  5781  5782  5783  5784  5785  5786  5787  5788  5789  5790  5791  5792  5793  5794  5795  5796  5797  5798  5799  5800  5801  5802  5803  5804  5805  5806  5807  5808  5809  5810  5811  5812  5813  5814  5815  5816  5817  5818  5819  5820  5821  5822  5823  5824  5825  5826  5827  5828  5829  5830  5831  5832  5833  5834  5835  5836  5837  5838  5839  5840  5841  5842  5843  5844  5845  5846  5847  5848  5849  5850  5851  5852  5853  5854  5855  5856  5857  5858  5859  5860  5861  5862  5863  5864  5865  5866  5867  5868  5869  5870  5871  5872  5873  5874  5875  5876  5877  5878  5879  5880  5881  5882  5883  5884  5885  5886  5887  5888  5889  5890  5891  5892  5893  5894  5895  5896  5897  5898  5899  5900  5901  5902  5903  5904  5905  5906  5907  5908  5909  5910  5911  5912  5913  5914  5915  5916  5917  5918  5919  5920  5921  5922  5923  5924  5925  5926  5927  5928  5929  5930  5931  5932  5933  5934  5935  5936  5937  5938  5939  5940  5941  5942  5943  5944  5945  5946  5947  5948  5949  5950  5951  5952  5953  5954  5955  5956  5957  5958  5959  5960  5961  5962  5963  5964  5965  5966  5967  5968  5969  5970  5971  5972  5973  5974  5975  5976  5977  5978  5979  5980  5981  5982  5983  5984  5985  5986  5987  5988  5989  5990  5991  5992  5993  5994  5995  5996  5997  5998  5999  6000  6001  6002  6003  6004  6005  6006  6007  6008  6009  6010  6011  6012  6013  6014  6015  6016  6017  6018  6019  6020  6021  6022  6023  6024  6025  6026  6027  6028  6029  6030  6031  6032  6033  6034  6035  6036  6037  6038  6039  6040  6041  6042  6043  6044  6045  6046  6047  6048  6049  6050  6051  6052  6053  6054  6055  6056  6057  6058  6059  6060  6061  6062  6063  6064  6065  6066  6067  6068  6069  6070  6071  6072  6073  6074  6075  6076  6077  6078  6079  6080  6081  6082  6083  6084  6085  6086  6087  6088  6089  6090  6091  6092  6093  6094  6095  6096  6097  6098  6099  6100  6101  6102  6103  6104  6105  6106  6107  6108  6109  6110  6111  6112  6113  6114  6115  6116  6117  6118  6119  6120  6121  6122  6123  6124  6125  6126  6127  6128  6129  6130  6131  6132  6133  6134  6135  6136  6137  6138  6139  6140  6141  6142  6143  6144  6145  6146  6147  6148  6149  6150  6151  6152  6153  6154  6155  6156  6157  6158  6159  6160  6161  6162  6163  6164  6165  6166  6167  6168  6169  6170  6171  6172  6173  6174  6175  6176  6177  6178  6179  6180  6181  6182  6183  6184  6185  6186  6187  6188  6189  6190  6191  6192  6193  6194  6195  6196  6197  6198  6199  6200  6201  6202  6203  6204  6205  6206  6207  6208  6209  6210  6211  6212  6213  6214  6215  6216  6217  6218  6219  6220  6221  6222  6223  6224  6225  6226  6227  6228  6229  6230  6231  6232  6233  6234  6235  6236  6237  6238  6239  6240  6241  6242  6243  6244  6245  6246  6247  6248  6249  6250  6251  6252  6253  6254  6255  6256  6257  6258  6259  6260  6261  6262  6263  6264  6265  6266  6267  6268  6269  6270  6271  6272  6273  6274  6275  6276  6277  6278  6279  6280  6281  6282  6283  6284  6285  6286  6287  6288  6289  6290  6291  6292  6293  6294  6295  6296  6297  6298  6299  6300  6301  6302  6303  6304  6305  6306  6307  6308  6309  6310  6311  6312  6313  6314  6315  6316  6317  6318  6319  6320  6321  6322  6323  6324  6325  6326  6327  6328  6329  6330  6331  6332  6333  6334  6335  6336  6337  6338  6339  6340  6341  6342  6343  6344  6345  6346  6347  6348  6349  6350  6351  6352  6353  6354  6355  6356  6357  6358  6359  6360  6361  6362  6363  6364  6365  6366  6367  6368  6369  6370  6371  6372  6373  6374  6375  6376  6377  6378  6379  6380  6381  6382  6383  6384  6385  6386  6387  6388  6389  6390  6391  6392  6393  6394  6395  6396  6397  6398  6399  6400  6401  6402  6403  6404  6405  6406  6407  6408  6409  6410  6411  6412  6413  6414  6415  6416  6417  6418  6419  6420  6421  6422  6423  6424  6425  6426  6427  6428  6429  6430  6431  6432  6433  6434  6435  6436  6437  6438  6439  6440  6441  6442  6443  6444  6445  6446  6447  6448  6449  6450  6451  6452  6453  6454  6455  6456  6457  6458  6459  6460  6461  6462  6463  6464  6465  6466  6467  6468  6469  6470  6471  6472  6473  6474  6475  6476  6477  6478  6479  6480  6481  6482  6483  6484  6485  6486  6487  6488  6489  6490  6491  6492  6493  6494  6495  6496  6497  6498  6499  6500  6501  6502  6503  6504  6505  6506  6507  6508  6509  6510  6511  6512  6513  6514  6515  6516  6517  6518  6519  6520  6521  6522  6523  6524  6525  6526  6527  6528  6529  6530  6531  6532  6533  6534  6535  6536  6537  6538  6539  6540  6541  6542  6543  6544  6545  6546  6547  6548  6549  6550  6551  6552  6553  6554  6555  6556  6557  6558  6559  6560  6561  6562  6563  6564  6565  6566  6567  6568  6569  6570  6571  6572  6573  6574  6575  6576  6577  6578  6579  6580  6581  6582  6583  6584  6585  6586  6587  6588  6589  6590  6591  6592  6593  6594  6595  6596  6597  6598  6599  6600  6601  6602  6603  6604  6605  6606  6607  6608  6609  6610  6611  6612  6613  6614  6615  6616  6617  6618  6619  6620  6621  6622  6623  6624  6625  6626  6627  6628  6629  6630  6631  6632  6633  6634  6635  6636  6637  6638  6639  6640  6641  6642  6643  6644  6645  6646  6647  6648  6649  6650  6651  6652  6653  6654  6655  6656  6657  6658  6659  6660  6661  6662  6663  6664  6665  6666  6667  6668  6669  6670  6671  6672  6673  6674  6675  6676  6677  6678  6679  6680  6681  6682  6683  6684  6685  6686  6687  6688  6689  6690  6691  6692  6693  6694  6695  6696  6697  6698  6699  6700  6701  6702  6703  6704  6705  6706  6707  6708  6709  6710  6711  6712  6713  6714  6715  6716  6717  6718  6719  6720  6721  6722  6723  6724  6725  6726  6727  6728  6729  6730  6731  6732  6733  6734  6735  6736  6737  6738  6739  6740  6741  6742  6743  6744  6745  6746  6747  6748  6749  6750  6751  6752  6753  6754  6755  6756  6757  6758  6759  6760  6761  6762  6763  6764  6765  6766  6767  6768  6769  6770  6771  6772  6773  6774  6775  6776  6777  6778  6779  6780  6781  6782  6783  6784  6785  6786  6787  6788  6789  6790  6791  6792  6793  6794  6795  6796  6797  6798  6799  6800  6801  6802  6803  6804  6805  6806  6807  6808  6809  6810  6811  6812  6813  6814  6815  6816  6817  6818  6819  6820  6821  6822  6823  6824  6825  6826  6827  6828  6829  6830  6831  6832  6833  6834  6835  6836  6837  6838  6839  6840  6841  6842  6843  6844  6845  6846  6847  6848  6849  6850  6851  6852  6853  6854  6855  6856  6857  6858  6859  6860  6861  6862  6863  6864  6865  6866  6867  6868  6869  6870  6871  6872  6873  6874  6875  6876  6877  6878  6879  6880  6881  6882  6883  6884  6885  6886  6887  6888  6889  6890  6891  6892  6893  6894  6895  6896  6897  6898  6899  6900  6901  6902  6903  6904  6905  6906  6907  6908  6909  6910  6911  6912  6913  6914  6915  6916  6917  6918  6919  6920  6921  6922  6923  6924  6925  6926  6927  6928  6929  6930  6931  6932  6933  6934  6935  6936  6937  6938  6939  6940  6941  6942  6943  6944  6945  6946  6947  6948  6949  6950  6951  6952  6953  6954  6955  6956  6957  6958  6959  6960  6961  6962  6963  6964  6965  6966  6967  6968  6969  6970  6971  6972  6973  6974  6975  6976  6977  6978  6979  6980  6981  6982  6983  6984  6985  6986  6987  6988  6989  6990  6991  6992  6993  6994  6995  6996  6997  6998  6999  7000  7001  7002  7003  7004  7005  7006  7007  7008  7009  7010  7011  7012  7013  7014  7015  7016  7017  7018  7019  7020  7021  7022  7023  7024  7025  7026  7027  7028  7029  7030  7031  7032  7033  7034  7035  7036  7037  7038  7039  7040  7041  7042  7043  7044  7045  7046  7047  7048  7049  7050  7051  7052  7053  7054  7055  7056  7057  7058  7059  7060  7061  7062  7063  7064  7065  7066  7067  7068  7069  7070  7071  7072  7073  7074  7075  7076  7077  7078  7079  7080  7081  7082  7083  7084  7085  7086  7087  7088  7089  7090  7091  7092  7093  7094  7095  7096  7097  7098  7099  7100  7101  7102  7103  7104  7105  7106  7107  7108  7109  7110  7111  7112  7113  7114  7115  7116  7117  7118  7119  7120  7121  7122  7123  7124  7125  7126  7127  7128  7129  7130  7131  7132  7133  7134  7135  7136  7137  7138  7139  7140  7141  7142  7143  7144  7145  7146  7147  7148  7149  7150  7151  7152  7153  7154  7155  7156  7157  7158  7159  7160  7161  7162  7163  7164  7165  7166  7167  7168  7169  7170  7171  7172  7173  7174  7175  7176  7177  7178  7179  7180  7181  7182  7183  7184  7185  7186  7187  7188  7189  7190  7191  7192  7193  7194  7195  7196  7197  7198  7199  7200  7201  7202  7203  7204  7205  7206  7207  7208  7209  7210  7211  7212  7213  7214  7215  7216  7217  7218  7219  7220  7221  7222  7223  7224  7225  7226  7227  7228  7229  7230  7231  7232  7233  7234  7235  7236  7237  7238  7239  7240  7241  7242  7243  7244  7245  7246  7247  7248  7249  7250  7251  7252  7253  7254  7255  7256  7257  7258  7259  7260  7261  7262  7263  7264  7265  7266  7267  7268  7269  7270  7271  7272  7273  7274  7275  7276  7277  7278  7279  7280  7281  7282  7283  7284  7285  7286  7287  7288  7289  7290  7291  7292  7293  7294  7295  7296  7297  7298  7299  7300  7301  7302  7303  7304  7305  7306  7307  7308  7309  7310  7311  7312  7313  7314  7315  7316  7317  7318  7319  7320  7321  7322  7323  7324  7325  7326  7327  7328  7329  7330  7331  7332  7333  7334  7335  7336  7337  7338  7339  7340  7341  7342  7343  7344  7345  7346  7347  7348  7349  7350  7351  7352  7353  7354  7355  7356  7357  7358  7359  7360  7361  7362  7363  7364  7365  7366  7367  7368  7369  7370  7371  7372  7373  7374  7375  7376  7377  7378  7379  7380  7381  7382  7383  7384  7385  7386  7387  7388  7389  7390  7391  7392  7393  7394  7395  7396  7397  7398  7399  7400  7401  7402  7403  7404  7405  7406  7407  7408  7409  7410  7411  7412  7413  7414  7415  7416  7417  7418  7419  7420  7421  7422  7423  7424  7425  7426  7427  7428  7429  7430  7431  7432  7433  7434  7435  7436  7437  7438  7439  7440  7441  7442  7443  7444  7445  7446  7447  7448  7449  7450  7451  7452  7453  7454  7455  7456  7457  7458  7459  7460  7461  7462  7463  7464  7465  7466  7467  7468  7469  7470  7471  7472  7473  7474  7475  7476  7477  7478  7479  7480  7481  7482  7483  7484  7485  7486  7487  7488  7489  7490  7491  7492  7493  7494  7495  7496  7497  7498  7499  7500  7501  7502  7503  7504  7505  7506  7507  7508  7509  7510  7511  7512  7513  7514  7515  7516  7517  7518  7519  7520  7521  7522  7523  7524  7525  7526  7527  7528  7529  7530  7531  7532  7533  7534  7535  7536  7537  7538  7539  7540  7541  7542  7543  7544  7545  7546  7547  7548  7549  7550  7551  7552  7553  7554  7555  7556  7557  7558  7559  7560  7561  7562  7563  7564  7565  7566  7567  7568  7569  7570  7571  7572  7573  7574  7575  7576  7577  7578  7579  7580  7581  7582  7583  7584  7585  7586  7587  7588  7589  7590  7591  7592  7593  7594  7595  7596  7597  7598  7599  7600  7601  7602  7603  7604  7605  7606  7607  7608  7609  7610  7611  7612  7613  7614  7615  7616  7617  7618  7619  7620  7621  7622  7623  7624  7625  7626  7627  7628  7629  7630  7631  7632  7633  7634  7635  7636  7637  7638  7639  7640  7641  7642  7643  7644  7645  7646  7647  7648  7649  7650  7651  7652  7653  7654  7655  7656  7657  7658  7659  7660  7661  7662  7663  7664  7665  7666  7667  7668  7669  7670  7671  7672  7673  7674  7675  7676  7677  7678  7679  7680  7681  7682  7683  7684  7685  7686  7687  7688  7689  7690  7691  7692  7693  7694  7695  7696  7697  7698  7699  7700  7701  7702  7703  7704  7705  7706  7707  7708  7709  7710  7711  7712  7713  7714  7715  7716  7717  7718  7719  7720  7721  7722  7723  7724  7725  7726  7727  7728  7729  7730  7731  7732  7733  7734  7735  7736  7737  7738  7739  7740  7741  7742  7743  7744  7745  7746  7747  7748  7749  7750  7751  7752  7753  7754  7755  7756  7757  7758  7759  7760  7761  7762  7763  7764  7765  7766  7767  7768  7769  7770  7771  7772  7773  7774  7775  7776  7777  7778  7779  7780  7781  7782  7783  7784  7785  7786  7787  7788  7789  7790  7791  7792  7793  7794  7795  7796  7797  7798  7799  7800  7801  7802  7803  7804  7805  7806  7807  7808  7809  7810  7811  7812  7813  7814  7815  7816  7817  7818  7819  7820  7821  7822  7823  7824  7825  7826  7827  7828  7829  7830  7831  7832  7833  7834  7835  7836  7837  7838  7839  7840  7841  7842  7843  7844  7845  7846  7847  7848  7849  7850  7851  7852  7853  7854  7855  7856  7857  7858  7859  7860  7861  7862  7863  7864  7865  7866  7867  7868  7869  7870  7871  7872  7873  7874  7875  7876  7877  7878  7879  7880  7881  7882  7883  7884  7885  7886  7887  7888  7889  7890  7891  7892  7893  7894  7895  7896  7897  7898  7899  7900  7901  7902  7903  7904  7905  7906  7907  7908  7909  7910  7911  7912  7913  7914  7915  7916  7917  7918  7919  7920  7921  7922  7923  7924  7925  7926  7927  7928  7929  7930  7931  7932  7933  7934  7935  7936  7937  7938  7939  7940  7941  7942  7943  7944  7945  7946  7947  7948  7949  7950  7951  7952  7953  7954  7955  7956  7957  7958  7959  7960  7961  7962  7963  7964  7965  7966  7967  7968  7969  7970  7971  7972  7973  7974  7975  7976  7977  7978  7979  7980  7981  7982  7983  7984  7985  7986  7987  7988  7989  7990  7991  7992  7993  7994  7995  7996  7997  7998  7999  8000  8001  8002  8003  8004  8005  8006  8007  8008  8009  8010  8011  8012  8013  8014  8015  8016  8017  8018  8019  8020  8021  8022  8023  8024  8025  8026  8027  8028  8029  8030  8031  8032  8033  8034  8035  8036  8037  8038  8039  8040  8041  8042  8043  8044  8045  8046  8047  8048  8049  8050  8051  8052  8053  8054  8055  8056  8057  8058  8059  8060  8061  8062  8063  8064  8065  8066  8067  8068  8069  8070  8071  8072  8073  8074  8075  8076  8077  8078  8079  8080  8081  8082  8083  8084  8085  8086  8087  8088  8089  8090  8091  8092  8093  8094  8095  8096  8097  8098  8099  8100  8101  8102  8103  8104  8105  8106  8107  8108  8109  8110  8111  8112  8113  8114  8115  8116  8117  8118  8119  8120  8121  8122  8123  8124  8125  8126  8127  8128  8129  8130  8131  8132  8133  8134  8135  8136  8137  8138  8139  8140  8141  8142  8143  8144  8145  8146  8147  8148  8149  8150  8151  8152  8153  8154  8155  8156  8157  8158  8159  8160  8161  8162  8163  8164  8165  8166  8167  8168  8169  8170  8171  8172  8173  8174  8175  8176  8177  8178  8179  8180  8181  8182  8183  8184  8185  8186  8187  8188  8189  8190  8191  8192  8193  8194  8195  8196  8197  8198  8199  8200  8201  8202  8203  8204  8205  8206  8207  8208  8209  8210  8211  8212  8213  8214  8215  8216  8217  8218  8219  8220  8221  8222  8223  8224  8225  8226  8227  8228  8229  8230  8231  8232  8233  8234  8235  8236  8237  8238  8239  8240  8241  8242  8243  8244  8245  8246  8247  8248  8249  8250  8251  8252  8253  8254  8255  8256  8257  8258  8259  8260  8261  8262  8263  8264  8265  8266  8267  8268  8269  8270  8271  8272  8273  8274  8275  8276  8277  8278  8279  8280  8281  8282  8283  8284  8285  8286  8287  8288  8289  8290  8291  8292  8293  8294  8295  8296  8297  8298  8299  8300  8301  8302  8303  8304  8305  8306  8307  8308  8309  8310  8311  8312  8313  8314  8315  8316  8317  8318  8319  8320  8321  8322  8323  8324  8325  8326  8327  8328  8329  8330  8331  8332  8333  8334  8335  8336  8337  8338  8339  8340  8341  8342  8343  8344  8345  8346  8347  8348  8349  8350  8351  8352  8353  8354  8355  8356  8357  8358  8359  8360  8361  8362  8363  8364  8365  8366  8367  8368  8369  8370  8371  8372  8373  8374  8375  8376  8377  8378  8379  8380  8381  8382  8383  8384  8385  8386  8387  8388  8389  8390  8391  8392  8393  8394  8395  8396  8397  8398  8399  8400  8401  8402  8403  8404  8405  8406  8407  8408  8409  8410  8411  8412  8413  8414  8415  8416  8417  8418  8419  8420  8421  8422  8423  8424  8425  8426  8427  8428  8429  8430  8431  8432  8433  8434  8435  8436  8437  8438  8439  8440  8441  8442  8443  8444  8445  8446  8447  8448  8449  8450  8451  8452  8453  8454  8455  8456  8457  8458  8459  8460  8461  8462  8463  8464  8465  8466  8467  8468  8469  8470  8471  8472  8473  8474  8475  8476  8477  8478  8479  8480  8481  8482  8483  8484  8485  8486  8487  8488  8489  8490  8491  8492  8493  8494  8495  8496  8497  8498  8499  8500  8501  8502  8503  8504  8505  8506  8507  8508  8509  8510  8511  8512  8513  8514  8515  8516  8517  8518  8519  8520  8521  8522  8523  8524  8525  8526  8527  8528  8529  8530  8531  8532  8533  8534  8535  8536  8537  8538  8539  8540  8541  8542  8543  8544  8545  8546  8547  8548  8549  8550  8551  8552  8553  8554  8555  8556  8557  8558  8559  8560  8561  8562  8563  8564  8565  8566  8567  8568  8569  8570  8571  8572  8573  8574  8575  8576  8577  8578  8579  8580  8581  8582  8583  8584  8585  8586  8587  8588  8589  8590  8591  8592  8593  8594  8595  8596  8597  8598  8599  8600  8601  8602  8603  8604  8605  8606  8607  8608  8609  8610  8611  8612  8613  8614  8615  8616  8617  8618  8619  8620  8621  8622  8623  8624  8625  8626  8627  8628  8629  8630  8631  8632  8633  8634  8635  8636  8637  8638  8639  8640  8641  8642  8643  8644  8645  8646  8647  8648  8649  8650  8651  8652  8653  8654  8655  8656  8657  8658  8659  8660  8661  8662  8663  8664  8665  8666  8667  8668  8669  8670  8671  8672  8673  8674  8675  8676  8677  8678  8679  8680  8681  8682  8683  8684  8685  8686  8687  8688  8689  8690  8691  8692  8693  8694  8695  8696  8697  8698  8699  8700  8701  8702  8703  8704  8705  8706  8707  8708  8709  8710  8711  8712  8713  8714  8715  8716  8717  8718  8719  8720  8721  8722  8723  8724  8725  8726  8727  8728  8729  8730  8731  8732  8733  8734  8735  8736  8737  8738  8739  8740  8741  8742  8743  8744  8745  8746  8747  8748  8749  8750  8751  8752  8753  8754  8755  8756  8757  8758  8759  8760  8761  8762  8763  8764  8765  8766  8767  8768  8769  8770  8771  8772  8773  8774  8775  8776  8777  8778  8779  8780  8781  8782  8783  8784  8785  8786  8787  8788  8789  8790  8791  8792  8793  8794  8795  8796  8797  8798  8799  8800  8801  8802  8803  8804  8805  8806  8807  8808  8809  8810  8811  8812  8813  8814  8815  8816  8817  8818  8819  8820  8821  8822  8823  8824  8825  8826  8827  8828  8829  8830  8831  8832  8833  8834  8835  8836  8837  8838  8839  8840  8841  8842  8843  8844  8845  8846  8847  8848  8849  8850  8851  8852  8853  8854  8855  8856  8857  8858  8859  8860  8861  8862  8863  8864  8865  8866  8867  8868  8869  8870  8871  8872  8873  8874  8875  8876  8877  8878  8879  8880  8881  8882  8883  8884  8885  8886  8887  8888  8889  8890  8891  8892  8893  8894  8895  8896  8897  8898  8899  8900  8901  8902  8903  8904  8905  8906  8907  8908  8909  8910  8911  8912  8913  8914  8915  8916  8917  8918  8919  8920  8921  8922  8923  8924  8925  8926  8927  8928  8929  8930  8931  8932  8933  8934  8935  8936  8937  8938  8939  8940  8941  8942  8943  8944  8945  8946  8947  8948  8949  8950  8951  8952  8953  8954  8955  8956  8957  8958  8959  8960  8961  8962  8963  8964  8965  8966  8967  8968  8969  8970  8971  8972  8973  8974  8975  8976  8977  8978  8979  8980  8981  8982  8983  8984  8985  8986  8987  8988  8989  8990  8991  8992  8993  8994  8995  8996  8997  8998  8999  9000  9001  9002  9003  9004  9005  9006  9007  9008  9009  9010  9011  9012  9013  9014  9015  9016  9017  9018  9019  9020  9021  9022  9023  9024  9025  9026  9027  9028  9029  9030  9031  9032  9033  9034  9035  9036  9037  9038  9039  9040  9041  9042  9043  9044  9045  9046  9047  9048  9049  9050  9051  9052  9053  9054  9055  9056  9057  9058  9059  9060  9061  9062  9063  9064  9065  9066  9067  9068  9069  9070  9071  9072  9073  9074  9075  9076  9077  9078  9079  9080  9081  9082  9083  9084  9085  9086  9087  9088  9089  9090  9091  9092  9093  9094  9095  9096  9097  9098  9099  9100  9101  9102  9103  9104  9105  9106  9107  9108  9109  9110  9111  9112  9113  9114  9115  9116  9117  9118  9119  9120  9121  9122  9123  9124  9125  9126  9127  9128  9129  9130  9131  9132  9133  9134  9135  9136  9137  9138  9139  9140  9141  9142  9143  9144  9145  9146  9147  9148  9149  9150  9151  9152  9153  9154  9155  9156  9157  9158  9159  9160  9161  9162  9163  9164  9165  9166  9167  9168  9169  9170  9171  9172  9173  9174  9175  9176  9177  9178  9179  9180  9181  9182  9183  9184  9185  9186  9187  9188  9189  9190  9191  9192  9193  9194  9195  9196  9197  9198  9199  9200  9201  9202  9203  9204  9205  9206  9207  9208  9209  9210  9211  9212  9213  9214  9215  9216  9217  9218  9219  9220  9221  9222  9223  9224  9225  9226  9227  9228  9229  9230  9231  9232  9233  9234  9235  9236  9237  9238  9239  9240  9241  9242  9243  9244  9245  9246  9247  9248  9249  9250  9251  9252  9253  9254  9255  9256  9257  9258  9259  9260  9261  9262  9263  9264  9265  9266  9267  9268  9269  9270  9271  9272  9273  9274  9275  9276  9277  9278  9279  9280  9281  9282  9283  9284  9285  9286  9287  9288  9289  9290  9291  9292  9293  9294  9295  9296  9297  9298  9299  9300  9301  9302  9303  9304  9305  9306  9307  9308  9309  9310  9311  9312  9313  9314  9315  9316  9317  9318  9319  9320  9321  9322  9323  9324  9325  9326  9327  9328  9329  9330  9331  9332  9333  9334  9335  9336  9337  9338  9339  9340  9341  9342  9343  9344  9345  9346  9347  9348  9349  9350  9351  9352  9353  9354  9355  9356  9357  9358  9359  9360  9361  9362  9363  9364  9365  9366  9367  9368  9369  9370  9371  9372  9373  9374  9375  9376  9377  9378  9379  9380  9381  9382  9383  9384  9385  9386  9387  9388  9389  9390  9391  9392  9393  9394  9395  9396  9397  9398  9399  9400  9401  9402  9403  9404  9405  9406  9407  9408  9409  9410  9411  9412  9413  9414  9415  9416  9417  9418  9419  9420  9421  9422  9423  9424  9425  9426  9427  9428  9429  9430  9431  9432  9433  9434  9435  9436  9437  9438  9439  9440  9441  9442  9443  9444  9445  9446  9447  9448  9449  9450  9451  9452  9453  9454  9455  9456  9457  9458  9459  9460  9461  9462  9463  9464  9465  9466  9467  9468  9469  9470  9471  9472  9473  9474  9475  9476  9477  9478  9479  9480  9481  9482  9483  9484  9485  9486  9487  9488  9489  9490  9491  9492  9493  9494  9495  9496  9497  9498  9499  9500  9501  9502  9503  9504  9505  9506  9507  9508  9509  9510  9511  9512  9513  9514  9515  9516  9517  9518  9519  9520  9521  9522  9523  9524  9525  9526  9527  9528  9529  9530  9531  9532  9533  9534  9535  9536  9537  9538  9539  9540  9541  9542  9543  9544  9545  9546  9547  9548  9549  9550  9551  9552  9553  9554  9555  9556  9557  9558  9559  9560  9561  9562  9563  9564  9565  9566  9567  9568  9569  9570  9571  9572  9573  9574  9575  9576  9577  9578  9579  9580  9581  9582  9583  9584  9585  9586  9587  9588  9589  9590  9591  9592  9593  9594  9595  9596  9597  9598  9599  9600  9601  9602  9603  9604  9605  9606  9607  9608  9609  9610  9611  9612  9613  9614  9615  9616  9617  9618  9619  9620  9621  9622  9623  9624  9625  9626  9627  9628  9629  9630  9631  9632  9633  9634  9635  9636  9637  9638  9639  9640  9641  9642  9643  9644  9645  9646  9647  9648  9649  9650  9651  9652  9653  9654  9655  9656  9657  9658  9659  9660  9661  9662  9663  9664  9665  9666  9667  9668  9669  9670  9671  9672  9673  9674  9675  9676  9677  9678  9679  9680  9681  9682  9683  9684  9685  9686  9687  9688  9689  9690  9691  9692  9693  9694  9695  9696  9697  9698  9699  9700  9701  9702  9703  9704  9705  9706  9707  9708  9709  9710  9711  9712  9713  9714  9715  9716  9717  9718  9719  9720  9721  9722  9723  9724  9725  9726  9727  9728  9729  9730  9731  9732  9733  9734  9735  9736  9737  9738  9739  9740  9741  9742  9743  9744  9745  9746  9747  9748  9749  9750  9751  9752  9753  9754  9755  9756  9757  9758  9759  9760  9761  9762  9763  9764  9765  9766  9767  9768  9769  9770  9771  9772  9773  9774  9775  9776  9777  9778  9779  9780  9781  9782  9783  9784  9785  9786  9787  9788  9789  9790  9791  9792  9793  9794  9795  9796  9797  9798  9799  9800  9801  9802  9803  9804  9805  9806  9807  9808  9809  9810  9811  9812  9813  9814  9815  9816  9817  9818  9819  9820  9821  9822  9823  9824  9825  9826  9827  9828  9829  9830  9831  9832  9833  9834  9835  9836  9837  9838  9839  9840  9841  9842  9843  9844  9845  9846  9847  9848  9849  9850  9851  9852  9853  9854  9855  9856  9857  9858  9859  9860  9861  9862  9863  9864  9865  9866  9867  9868  9869  9870  9871  9872  9873  9874  9875  9876  9877  9878  9879  9880  9881  9882  9883  9884  9885  9886  9887  9888  9889  9890  9891  9892  9893  9894  9895  9896  9897  9898  9899  9900  9901  9902  9903  9904  9905  9906  9907  9908  9909  9910  9911  9912  9913  9914  9915  9916  9917  9918  9919  9920  9921  9922  9923  9924  9925  9926  9927  9928  9929  9930  9931  9932  9933  9934  9935  9936  9937  9938  9939  9940  9941  9942  9943  9944  9945  9946  9947  9948  9949  9950  9951  9952  9953  9954  9955  9956  9957  9958  9959  9960  9961  9962  9963  9964  9965  9966  9967  9968  9969  9970  9971  9972  9973  9974  9975  9976  9977  9978  9979  9980  9981  9982  9983  9984  9985  9986  9987  9988  9989  9990  9991  9992  9993  9994  9995  9996  9997  9998  9999  10000  10001  10002  10003  10004  10005  10006  10007  10008  10009  10010  10011  10012  10013  10014  10015  10016  10017  10018  10019  10020  10021  10022  10023  10024  10025  10026  10027  10028  10029  10030  10031  10032  10033  10034  10035  10036  10037  10038  10039  10040  10041  10042  10043  10044  10045  10046  10047  10048  10049  10050  10051  10052  10053  10054  10055  10056  10057  10058  10059  10060  10061  10062  10063  10064  10065  10066  10067  10068  10069  10070  10071  10072  10073  10074  10075  10076  10077  10078  10079  10080  10081  10082  10083  10084  10085  10086  10087  10088  10089  10090  10091  10092  10093  10094  10095  10096  10097  10098  10099  10100  10101  10102  10103  10104  10105  10106  10107  10108  10109  10110  10111  10112  10113  10114  10115  10116  10117  10118  10119  10120  10121  10122  10123  10124  10125  10126  10127  10128  10129  10130  10131  10132  10133  10134  10135  10136  10137  10138  10139  10140  10141  10142  10143  10144  10145  10146  10147  10148  10149  10150  10151  10152  10153  10154  10155  10156  10157  10158  10159  10160  10161  10162  10163  10164  10165  10166  10167  10168  10169  10170  10171  10172  10173  10174  10175  10176  10177  10178  10179  10180  10181  10182  10183  10184  10185  10186  10187  10188  10189  10190  10191  10192  10193  10194  10195  10196  10197  10198  10199  10200  10201  10202  10203  10204  10205  10206  10207  10208  10209  10210  10211  10212  10213  10214  10215  10216  10217  10218  10219  10220  10221  10222  10223  10224  10225  10226  10227  10228  10229  10230  10231  10232  10233  10234  10235  10236  10237  10238  10239  10240  10241  10242  10243  10244  10245  10246  10247  10248  10249  10250  10251  10252  10253  10254  10255  10256  10257  10258  10259  10260  10261  10262  10263  10264  10265  10266  10267  10268  10269  10270  10271  10272  10273  10274  10275  10276  10277  10278  10279  10280  10281  10282  10283  10284  10285  10286  10287  10288  10289  10290  10291  10292  10293  10294  10295  10296  10297  10298  10299  10300  10301  10302  10303  10304  10305  10306  10307  10308  10309  10310  10311  10312  10313  10314  10315  10316  10317  10318  10319  10320  10321  10322  10323  10324  10325  10326  10327  10328  10329  10330  10331  10332  10333  10334  10335  10336  10337  10338  10339  10340  10341  10342  10343  10344  10345  10346  10347  10348  10349  10350  10351  10352  10353  10354  10355  10356  10357  10358  10359  10360  10361  10362  10363  10364  10365  10366  10367  10368  10369  10370  10371  10372  10373  10374  10375  10376  10377  10378  10379  10380  10381  10382  10383  10384  10385  10386  10387  10388  10389  10390  10391  10392  10393  10394  10395  10396  10397  10398  10399  10400  10401  10402  10403  10404  10405  10406  10407  10408  10409  10410  10411  10412  10413  10414  10415  10416  10417  10418  10419  10420  10421  10422  10423  10424  10425  10426  10427  10428  10429  10430  10431  10432  10433  10434  10435  10436  10437  10438  10439  10440  10441  10442  10443  10444  10445  10446  10447  10448  10449  10450  10451  10452  10453  10454  10455  10456  10457  10458  10459  10460  10461  10462  10463  10464  10465  10466  10467  10468  10469  10470  10471  10472  10473  10474  10475  10476  10477  10478  10479  10480  10481  10482  10483  10484  10485  10486  10487  10488  10489  10490  10491  10492  10493  10494  10495  10496  10497  10498  10499  10500  10501  10502  10503  10504  10505  10506  10507  10508  10509  10510  10511  10512  10513  10514  10515  10516  10517  10518  10519  10520  10521  10522  10523  10524  10525  10526  10527  10528  10529  10530  10531  10532  10533  10534  10535  10536  10537  10538  10539  10540  10541  10542  10543  10544  10545  10546  10547  10548  10549  10550  10551  10552  10553  10554  10555  10556  10557  10558  10559  10560  10561  10562  10563  10564  10565  10566  10567  10568  10569  10570  10571  10572  10573  10574  10575  10576  10577  10578  10579  10580  10581  10582  10583  10584  10585  10586  10587  10588  10589  10590  10591  10592  10593  10594  10595  10596  10597  10598  10599  10600  10601  10602  10603  10604  10605  10606  10607  10608  10609  10610  10611  10612  10613  10614  10615  10616  10617  10618  10619  10620  10621  10622  10623  10624  10625  10626  10627  10628  10629  10630  10631  10632  10633  10634  10635  10636  10637  10638  10639  10640  10641  10642  10643  10644  10645  10646  10647  10648  10649  10650  10651  10652  10653  10654  10655  10656  10657  10658  10659  10660  10661  10662  10663  10664  10665  10666  10667  10668  10669  10670  10671  10672  10673  10674  10675  10676  10677  10678  10679  10680  10681  10682  10683  10684  10685  10686  10687  10688  10689  10690  10691  10692  10693  10694  10695  10696  10697  10698  10699  10700  10701  10702  10703  10704  10705  10706  10707  10708  10709  10710  10711  10712  10713  10714  10715  10716  10717  10718  10719  10720  10721  10722  10723  10724  10725  10726  10727  10728  10729  10730  10731  10732  10733  10734  10735  10736  10737  10738  10739  10740  10741  10742  10743  10744  10745  10746  10747  10748  10749  10750  10751  10752  10753  10754  10755  10756  10757  10758  10759  10760  10761  10762  10763  10764  10765  10766  10767  10768  10769  10770  10771  10772  10773  10774  10775  10776  10777  10778  10779  10780  10781  10782  10783  10784  10785  10786  10787  10788  10789  10790  10791  10792  10793  10794  10795  10796  10797  10798  10799  10800  10801  10802  10803  10804  10805  10806  10807  10808  10809  10810  10811  10812  10813  10814  10815  10816  10817  10818  10819  10820  10821  10822  10823  10824  10825  10826  10827  10828  10829  10830  10831  10832  10833  10834  10835  10836  10837  10838  10839  10840  10841  10842  10843  10844  10845  10846  10847  10848  10849  10850  10851  10852  10853  10854  10855  10856  10857  10858  10859  10860  10861  10862  10863  10864  10865  10866  10867  10868  10869  10870  10871  10872  10873  10874  10875  10876  10877  10878  10879  10880  10881  10882  10883  10884  10885  10886  10887  10888  10889  10890  10891  10892  10893  10894  10895  10896  10897  10898  10899  10900  10901  10902  10903  10904  10905  10906  10907  10908  10909  10910  10911  10912  10913  10914  10915  10916  10917  10918  10919  10920  10921  10922  10923  10924  10925  10926  10927  10928  10929  10930  10931  10932  10933  10934  10935  10936  10937  10938  10939  10940  10941  10942  10943  10944  10945  10946  10947  10948  10949  10950  10951  10952  10953  10954  10955  10956  10957  10958  10959  10960  10961  10962  10963  10964  10965  10966  10967  10968  10969  10970  10971  10972  10973  10974  10975  10976  10977  10978  10979  10980  10981  10982  10983  10984  10985  10986  10987  10988  10989  10990  10991  10992  10993  10994  10995  10996  10997  10998  10999  11000  11001  11002  11003  11004  11005  11006  11007  11008  11009  11010  11011  11012  11013  11014  11015  11016  11017  11018  11019  11020  11021  11022  11023  11024  11025  11026  11027  11028  11029  11030  11031  11032  11033  11034  11035  11036  11037  11038  11039  11040  11041  11042  11043  11044  11045  11046  11047  11048  11049  11050  11051  11052  11053  11054  11055  11056  11057  11058  11059  11060  11061  11062  11063  11064  11065  11066  11067  11068  11069  11070  11071  11072  11073  11074  11075  11076  11077  11078  11079  11080  11081  11082  11083  11084  11085  11086  11087  11088  11089  11090  11091  11092  11093  11094  11095  11096  11097  11098  11099  11100  11101  11102  11103  11104  11105  11106  11107  11108  11109  11110  11111  11112  11113  11114  11115  11116  11117  11118  11119  11120  11121  11122  11123  11124  11125  11126  11127  11128  11129  11130  11131  11132  11133  11134  11135  11136  11137  11138  11139  11140  11141  11142  11143  11144  11145  11146  11147  11148  11149  11150  11151  11152  11153  11154  11155  11156  11157  11158  11159  11160  11161  11162  11163  11164  11165  11166  11167  11168  11169  11170  11171  11172  11173  11174  11175  11176  11177  11178  11179  11180  11181  11182  11183  11184  11185  11186  11187  11188  11189  11190  11191  11192  11193  11194  11195  11196  11197  11198  11199  11200  11201  11202  11203  11204  11205  11206  11207  11208  11209  11210  11211  11212  11213  11214  11215  11216  11217  11218  11219  11220  11221  11222  11223  11224  11225  11226  11227  11228  11229  11230  11231  11232  11233  11234  11235  11236  11237  11238  11239  11240  11241  11242  11243  11244  11245  11246  11247  11248  11249  11250  11251  11252  11253  11254  11255  11256  11257  11258  11259  11260  11261  11262  11263  11264  11265  11266  11267  11268  11269  11270  11271  11272  11273  11274  11275  11276  11277  11278  11279  11280  11281  11282  11283  11284  11285  11286  11287  11288  11289  11290  11291  11292  11293  11294  11295  11296  11297  11298  11299  11300  11301  11302  11303  11304  11305  11306  11307  11308  11309  11310  11311  11312  11313  11314  11315  11316  11317  11318  11319  11320  11321  11322  11323  11324  11325  11326  11327  11328  11329  11330  11331  11332  11333  11334  11335  11336  11337  11338  11339  11340  11341  11342  11343  11344  11345  11346  11347  11348  11349  11350  11351  11352  11353  11354  11355  11356  11357  11358  11359  11360  11361  11362  11363  11364  11365  11366  11367  11368  11369  11370  11371  11372  11373  11374  11375  11376  11377  11378  11379  11380  11381  11382  11383  11384  11385  11386  11387  11388  11389  11390  11391  11392  11393  11394  11395  11396  11397  11398  11399  11400  11401  11402  11403  11404  11405  11406  11407  11408  11409  11410  11411  11412  11413  11414  11415  11416  11417  11418  11419  11420  11421  11422  11423  11424  11425  11426  11427  11428  11429  11430  11431  11432  11433  11434  11435  11436  11437  11438  11439  11440  11441  11442  11443  11444  11445  11446  11447  11448  11449  11450  11451  11452  11453  11454  11455  11456  11457  11458  11459  11460  11461  11462  11463  11464  11465  11466  11467  11468  11469  11470  11471  11472  11473  11474  11475  11476  11477  11478  11479  11480  11481  11482  11483  11484  11485  11486  11487  11488  11489  11490  11491  11492  11493  11494  11495  11496  11497  11498  11499  11500  11501  11502  11503  11504  11505  11506  11507  11508  11509  11510  11511  11512  11513  11514  11515  11516  11517  11518  11519  11520  11521  11522  11523  11524  11525  11526  11527  11528  11529  11530  11531  11532  11533  11534  11535  11536  11537  11538  11539  11540  11541  11542  11543  11544  11545  11546  11547  11548  11549  11550  11551  11552  11553  11554  11555  11556  11557  11558  11559  11560  11561  11562  11563  11564  11565  11566  11567  11568  11569  11570  11571  11572  11573  11574  11575  11576  11577  11578  11579  11580  11581  11582  11583  11584  11585  11586  11587  11588  11589  11590  11591  11592  11593  11594  11595  11596  11597  11598  11599  11600  11601  11602  11603  11604  11605  11606  11607  11608  11609  11610  11611  11612  11613  11614  11615  11616  11617  11618  11619  11620  11621  11622  11623  11624  11625  11626  11627  11628  11629  11630  11631  11632  11633  11634  11635  11636  11637  11638  11639  11640  11641  11642  11643  11644  11645  11646  11647  11648  11649  11650  11651  11652  11653  11654  11655  11656  11657  11658  11659  11660  11661  11662  11663  11664  11665  11666  11667  11668  11669  11670  11671  11672  11673  11674  11675  11676  11677  11678  11679  11680  11681  11682  11683  11684  11685  11686  11687  11688  11689  11690  11691  11692  11693  11694  11695  11696  11697  11698  11699  11700  11701  11702  11703  11704  11705  11706  11707  11708  11709  11710  11711  11712  11713  11714  11715  11716  11717  11718  11719  11720  11721  11722  11723  11724  11725  11726  11727  11728  11729  11730  11731  11732  11733  11734  11735  11736  11737  11738  11739  11740  11741  11742  11743  11744  11745  11746  11747  11748  11749  11750  11751  11752  11753  11754  11755  11756  11757  11758  11759  11760  11761  11762  11763  11764  11765  11766  11767  11768  11769  11770  11771  11772  11773  11774  11775  11776  11777  11778  11779  11780  11781  11782  11783  11784  11785  11786  11787  11788  11789  11790  11791  11792  11793  11794  11795  11796  11797  11798  11799  11800  11801  11802  11803  11804  11805  11806  11807  11808  11809  11810  11811  11812  11813  11814  11815  11816  11817  11818  11819  11820  11821  11822  11823  11824  11825  11826  11827  11828  11829  11830  11831  11832  11833  11834  11835  11836  11837  11838  11839  11840  11841  11842  11843  11844  11845  11846  11847  11848  11849  11850  11851  11852  11853  11854  11855  11856  11857  11858  11859  11860  11861  11862  11863  11864  11865  11866  11867  11868  11869  11870  11871  11872  11873  11874  11875  11876  11877  11878  11879  11880  11881  11882  11883  11884  11885  11886  11887  11888  11889  11890  11891  11892  11893  11894  11895  11896  11897  11898  11899  11900  11901  11902  11903  11904  11905  11906  11907  11908  11909  11910  11911  11912  11913  11914  11915  11916  11917  11918  11919  11920  11921  11922  11923  11924  11925  11926  11927  11928  11929  11930  11931  11932  11933  11934  11935  11936  11937  11938  11939  11940  11941  11942  11943  11944  11945  11946  11947  11948  11949  11950  11951  11952  11953  11954  11955  11956  11957  11958  11959  11960  11961  11962  11963  11964  11965  11966  11967  11968  11969  11970  11971  11972  11973  11974  11975  11976  11977  11978  11979  11980  11981  11982  11983  11984  11985  11986  11987  11988  11989  11990  11991  11992  11993  11994  11995  11996  11997  11998  11999  12000  12001  12002  12003  12004  12005  12006  12007  12008  12009  12010  12011  12012  12013  12014  12015  12016  12017  12018  12019  12020  12021  12022  12023  12024  12025  12026  12027  12028  12029  12030  12031  12032  12033  12034  12035  12036  12037  12038  12039  12040  12041  12042  12043  12044  12045  12046  12047  12048  12049  12050  12051  12052  12053  12054  12055  12056  12057  12058  12059  12060  12061  12062  12063  12064  12065  12066  12067  12068  12069  12070  12071  12072  12073  12074  12075  12076  12077  12078  12079  12080  12081  12082  12083  12084  12085  12086  12087  12088  12089  12090  12091  12092  12093  12094  12095  12096  12097  12098  12099  12100  12101  12102  12103  12104  12105  12106  12107  12108  12109  12110  12111  12112  12113  12114  12115  12116  12117  12118  12119  12120  12121  12122  12123  12124  12125  12126  12127  12128  12129  12130  12131  12132  12133  12134  12135  12136  12137  12138  12139  12140  12141  12142  12143  12144  12145  12146  12147  12148  12149  12150  12151  12152  12153  12154  12155  12156  12157  12158  12159  12160  12161  12162  12163  12164  12165  12166  12167  12168  12169  12170  12171  12172  12173  12174  12175  12176  12177  12178  12179  12180  12181  12182  12183  12184  12185  12186  12187  12188  12189  12190  12191  12192  12193  12194  12195  12196  12197  12198  12199  12200  12201  12202  12203  12204  12205  12206  12207  12208  12209  12210  12211  12212  12213  12214  12215  12216  12217  12218  12219  12220  12221  12222  12223  12224  12225  12226  12227  12228  12229  12230  12231  12232  12233  12234  12235  12236  12237  12238  12239  12240  12241  12242  12243  12244  12245  12246  12247  12248  12249  12250  12251  12252  12253  12254  12255  12256  12257  12258  12259  12260  12261  12262  12263  12264  12265  12266  12267  12268  12269  12270  12271  12272  12273  12274  12275  12276  12277  12278  12279  12280  12281  12282  12283  12284  12285  12286  12287  12288  12289  12290  12291  12292  12293  12294  12295  12296  12297  12298  12299  12300  12301  12302  12303  12304  12305  12306  12307  12308  12309  12310  12311  12312  12313  12314  12315  12316  12317  12318  12319  12320  12321  12322  12323  12324  12325  12326  12327  12328  12329  12330  12331  12332  12333  12334  12335  12336  12337  12338  12339  12340  12341  12342  12343  12344  12345  12346  12347  12348  12349  12350  12351  12352  12353  12354  12355  12356  12357  12358  12359  12360  12361  12362  12363  12364  12365  12366  12367  12368  12369  12370  12371  12372  12373  12374  12375  12376  12377  12378  12379  12380  12381  12382  12383  12384  12385  12386  12387  12388  12389  12390  12391  12392  12393  12394  12395  12396  12397  12398  12399  12400  12401  12402  12403  12404  12405  12406  12407  12408  12409  12410  12411  12412  12413  12414  12415  12416  12417  12418  12419  12420  12421  12422  12423  12424  12425  12426  12427  12428  12429  12430  12431  12432  12433  12434  12435  12436  12437  12438  12439  12440  12441  12442  12443  12444  12445  12446  12447  12448  12449  12450  12451  12452  12453  12454  12455  12456  12457  12458  12459  12460  12461  12462  12463  12464  12465  12466  12467  12468  12469  12470  12471  12472  12473  12474  12475  12476  12477  12478  12479  12480  12481  12482  12483  12484  12485  12486  12487  12488  12489  12490  12491  12492  12493  12494  12495  12496  12497  12498  12499  12500  12501  12502  12503  12504  12505  12506  12507  12508  12509  12510  12511  12512  12513  12514  12515  12516  12517  12518  12519  12520  12521  12522  12523  12524  12525  12526  12527  12528  12529  12530  12531  12532  12533  12534  12535  12536  12537  12538  12539  12540  12541  12542  12543  12544  12545  12546  12547  12548  12549  12550  12551  12552  12553  12554  12555  12556  12557  12558  12559  12560  12561  12562  12563  12564  12565  12566  12567  12568  12569  12570  12571  12572  12573  12574  12575  12576  12577  12578  12579  12580  12581  12582  12583  12584  12585  12586  12587  12588  12589  12590  12591  12592  12593  12594  12595  12596  12597  12598  12599  12600  12601  12602  12603  12604  12605  12606  12607  12608  12609  12610  12611  12612  12613  12614  12615  12616  12617  12618  12619  12620  12621  12622  12623  12624  12625  12626  12627  12628  12629  12630  12631  12632  12633  12634  12635  12636  12637  12638  12639  12640  12641  12642  12643  12644  12645  12646  12647  12648  12649  12650  12651  12652  12653  12654  12655  12656  12657  12658  12659  12660  12661  12662  12663  12664  12665  12666  12667  12668  12669  12670  12671  12672  12673  12674  12675  12676  12677  12678  12679  12680  12681  12682  12683  12684  12685  12686  12687  12688  12689  12690  12691  12692  12693  12694  12695  12696  12697  12698  12699  12700  12701  12702  12703  12704  12705  12706  12707  12708  12709  12710  12711  12712  12713  12714  12715  12716  12717  12718  12719  12720  12721  12722  12723  12724  12725  12726  12727  12728  12729  12730  12731  12732  12733  12734  12735  12736  12737  12738  12739  12740  12741  12742  12743  12744  12745  12746  12747  12748  12749  12750  12751  12752  12753  12754  12755  12756  12757  12758  12759  12760  12761  12762  12763  12764  12765  12766  12767  12768  12769  12770  12771  12772  12773  12774  12775  12776  12777  12778  12779  12780  12781  12782  12783  12784  12785  12786  12787  12788  12789  12790  12791  12792  12793  12794  12795  12796  12797  12798  12799  12800  12801  12802  12803  12804  12805  12806  12807  12808  12809  12810  12811  12812  12813  12814  12815  12816  12817  12818  12819  12820  12821  12822  12823  12824  12825  12826  12827  12828  12829  12830  12831  12832  12833  12834  12835  12836  12837  12838  12839  12840  12841  12842  12843  12844  12845  12846  12847  12848  12849  12850  12851  12852  12853  12854  12855  12856  12857  12858  12859  12860  12861  12862  12863  12864  12865  12866  12867  12868  12869  12870  12871  12872  12873  12874  12875  12876  12877  12878  12879  12880  12881  12882  12883  12884  12885  12886  12887  12888  12889  12890  12891  12892  12893  12894  12895  12896  12897  12898  12899  12900  12901  12902  12903  12904  12905  12906  12907  12908  12909  12910  12911  12912  12913  12914  12915  12916  12917  12918  12919  12920  12921  12922  12923  12924  12925  12926  12927  12928  12929  12930  12931  12932  12933  12934  12935  12936  12937  12938  12939  12940  12941  12942  12943  12944  12945  12946  12947  12948  12949  12950  12951  12952  12953  12954  12955  12956  12957  12958  12959  12960  12961  12962  12963  12964  12965  12966  12967  12968  12969  12970  12971  12972  12973  12974  12975  12976  12977  12978  12979  12980  12981  12982  12983  12984  12985  12986  12987  12988  12989  12990  12991  12992  12993  12994  12995  12996  12997  12998  12999  13000  13001  13002  13003  13004  13005  13006  13007  13008  13009  13010  13011  13012  13013  13014  13015  13016  13017  13018  13019  13020  13021  13022  13023  13024  13025  13026  13027  13028  13029  13030  13031  13032  13033  13034  13035  13036  13037  13038  13039  13040  13041  13042  13043  13044  13045  13046  13047  13048  13049  13050  13051  13052  13053  13054  13055  13056  13057  13058  13059  13060  13061  13062  13063  13064  13065  13066  13067  13068  13069  13070  13071  13072  13073  13074  13075  13076  13077  13078  13079  13080  13081  13082  13083  13084  13085  13086  13087  13088  13089  13090  13091  13092  13093  13094  13095  13096  13097  13098  13099  13100  13101  13102  13103  13104  13105  13106  13107  13108  13109  13110  13111  13112  13113  13114  13115  13116  13117  13118  13119  13120  13121  13122  13123  13124  13125  13126  13127  13128  13129  13130  13131  13132  13133  13134  13135  13136  13137  13138  13139  13140  13141  13142  13143  13144  13145  13146  13147  13148  13149  13150  13151  13152  13153  13154  13155  13156  13157  13158  13159  13160  13161  13162  13163  13164  13165  13166  13167  13168  13169  13170  13171  13172  13173  13174  13175  13176  13177  13178  13179  13180  13181  13182  13183  13184  13185  13186  13187  13188  13189  13190  13191  13192  13193  13194  13195  13196  13197  13198  13199  13200  13201  13202  13203  13204  13205  13206  13207  13208  13209  13210  13211  13212  13213  13214  13215  13216  13217  13218  13219  13220  13221  13222  13223  13224  13225  13226  13227  13228  13229  13230  13231  13232  13233  13234  13235  13236  13237  13238  13239  13240  13241  13242  13243  13244  13245  13246  13247  13248  13249  13250  13251  13252  13253  13254  13255  13256  13257  13258  13259  13260  13261  13262  13263  13264  13265  13266  13267  13268  13269  13270  13271  13272  13273  13274  13275  13276  13277  13278  13279  13280  13281  13282  13283  13284  13285  13286  13287  13288  13289  13290  13291  13292  13293  13294  13295  13296  13297  13298  13299  13300  13301  13302  13303  13304  13305  13306  13307  13308  13309  13310  13311  13312  13313  13314  13315  13316  13317  13318  13319  13320  13321  13322  13323  13324  13325  13326  13327  13328  13329  13330  13331  13332  13333  13334  13335  13336  13337  13338  13339  13340  13341  13342  13343  13344  13345  13346  13347  13348  13349  13350  13351  13352  13353  13354  13355  13356  13357  13358  13359  13360  13361  13362  13363  13364  13365  13366  13367  13368  13369  13370  13371  13372  13373  13374  13375  13376  13377  13378  13379  13380  13381  13382  13383  13384  13385  13386  13387  13388  13389  13390  13391  13392  13393  13394  13395  13396  13397  13398  13399  13400  13401  13402  13403  13404  13405  13406  13407  13408  13409  13410  13411  13412  13413  13414  13415  13416  13417  13418  13419  13420  13421  13422  13423  13424  13425  13426  13427  13428  13429  13430  13431  13432  13433  13434  13435  13436  13437  13438  13439  13440  13441  13442  13443  13444  13445  13446  13447  13448  13449  13450  13451  13452  13453  13454  13455  13456  13457  13458  13459  13460  13461  13462  13463  13464  13465  13466  13467  13468  13469  13470  13471  13472  13473  13474  13475  13476  13477  13478  13479  13480  13481  13482  13483  13484  13485  13486  13487  13488  13489  13490  13491  13492  13493  13494  13495  13496  13497  13498  13499  13500  13501  13502  13503  13504  13505  13506  13507  13508  13509  13510  13511  13512  13513  13514  13515  13516  13517  13518  13519  13520  13521  13522  13523  13524  13525  13526  13527  13528  13529  13530  13531  13532  13533  13534  13535  13536  13537  13538  13539  13540  13541  13542  13543  13544  13545  13546  13547  13548  13549  13550  13551  13552  13553  13554  13555  13556  13557  13558  13559  13560  13561  13562  13563  13564  13565  13566  13567  13568  13569  13570  13571  13572  13573  13574  13575  13576  13577  13578  13579  13580  13581  13582  13583  13584  13585  13586  13587  13588  13589  13590  13591  13592  13593  13594  13595  13596  13597  13598  13599  13600  13601  13602  13603  13604  13605  13606  13607  13608  13609  13610  13611  13612  13613  13614  13615  13616  13617  13618  13619  13620  13621  13622  13623  13624  13625  13626  13627  13628  13629  13630  13631  13632  13633  13634  13635  13636  13637  13638  13639  13640  13641  13642  13643  13644  13645  13646  13647  13648  13649  13650  13651  13652  13653  13654  13655  13656  13657  13658  13659  13660  13661  13662  13663  13664  13665  13666  13667  13668  13669  13670  13671  13672  13673  13674  13675  13676  13677  13678  13679  13680  13681  13682  13683  13684  13685  13686  13687  13688  13689  13690  13691  13692  13693  13694  13695  13696  13697  13698  13699  13700  13701  13702  13703  13704  13705  13706  13707  13708  13709  13710  13711  13712  13713  13714  13715  13716  13717  13718  13719  13720  13721  13722  13723  13724  13725  13726  13727  13728  13729  13730  13731  13732  13733  13734  13735  13736  13737  13738  13739  13740  13741  13742  13743  13744  13745  13746  13747  13748  13749  13750  13751  13752  13753  13754  13755  13756  13757  13758  13759  13760  13761  13762  13763  13764  13765  13766  13767  13768  13769  13770  13771  13772  13773  13774  13775  13776  13777  13778  13779  13780  13781  13782  13783  13784  13785  13786  13787  13788  13789  13790  13791  13792  13793  13794  13795  13796  13797  13798  13799  13800  13801  13802  13803  13804  13805  13806  13807  13808  13809  13810  13811  13812  13813  13814  13815  13816  13817  13818  13819  13820  13821  13822  13823  13824  13825  13826  13827  13828  13829  13830  13831  13832  13833  13834  13835  13836  13837  13838  13839  13840  13841  13842  13843  13844  13845  13846  13847  13848  13849  13850  13851  13852  13853  13854  13855  13856  13857  13858  13859  13860  13861  13862  13863  13864  13865  13866  13867  13868  13869  13870  13871  13872  13873  13874  13875  13876  13877  13878  13879  13880  13881  13882  13883  13884  13885  13886  13887  13888  13889  13890  13891  13892  13893  13894  13895  13896  13897  13898  13899  13900  13901  13902  13903  13904  13905  13906  13907  13908  13909  13910  13911  13912  13913  13914  13915  13916  13917  13918  13919  13920  13921  13922  13923  13924  13925  13926  13927  13928  13929  13930  13931  13932  13933  13934  13935  13936  13937  13938  13939  13940  13941  13942  13943  13944  13945  13946  13947  13948  13949  13950  13951  13952  13953  13954  13955  13956  13957  13958  13959  13960  13961  13962  13963  13964  13965  13966  13967  13968  13969  13970  13971  13972  13973  13974  13975  13976  13977  13978  13979  13980  13981  13982  13983  13984  13985  13986  13987  13988  13989  13990  13991  13992  13993  13994  13995  13996  13997  13998  13999  14000  14001  14002  14003  14004  14005  14006  14007  14008  14009  14010  14011  14012  14013  14014  14015  14016  14017  14018  14019  14020  14021  14022  14023  14024  14025  14026  14027  14028  14029  14030  14031  14032  14033  14034  14035  14036  14037  14038  14039  14040  14041  14042  14043  14044  14045  14046  14047  14048  14049  14050  14051  14052  14053  14054  14055  14056  14057  14058  14059  14060  14061  14062  14063  14064  14065  14066  14067  14068  14069  14070  14071  14072  14073  14074  14075  14076  14077  14078  14079  14080  14081  14082  14083  14084  14085  14086  14087  14088  14089  14090  14091  14092  14093  14094  14095  14096  14097  14098  14099  14100  14101  14102  14103  14104  14105  14106  14107  14108  14109  14110  14111  14112  14113  14114  14115  14116  14117  14118  14119  14120  14121  14122  14123  14124  14125  14126  14127  14128  14129  14130  14131  14132  14133  14134  14135  14136  14137  14138  14139  14140  14141  14142  14143  14144  14145  14146  14147  14148  14149  14150  14151  14152  14153  14154  14155  14156  14157  14158  14159  14160  14161  14162  14163  14164  14165  14166  14167  14168  14169  14170  14171  14172  14173  14174  14175  14176  14177  14178  14179  14180  14181  14182  14183  14184  14185  14186  14187  14188  14189  14190  14191  14192  14193  14194  14195  14196  14197  14198  14199  14200  14201  14202  14203  14204  14205  14206  14207  14208  14209  14210  14211  14212  14213  14214  14215  14216  14217  14218  14219  14220  14221  14222  14223  14224  14225  14226  14227  14228  14229  14230  14231  14232  14233  14234  14235  14236  14237  14238  14239  14240  14241  14242  14243  14244  14245  14246  14247  14248  14249  14250  14251  14252  14253  14254  14255  14256  14257  14258  14259  14260  14261  14262  14263  14264  14265  14266  14267  14268  14269  14270  14271  14272  14273  14274  14275  14276  14277  14278  14279  14280  14281  14282  14283  14284  14285  14286  14287  14288  14289  14290  14291  14292  14293  14294  14295  14296  14297  14298  14299  14300  14301  14302  14303  14304  14305  14306  14307  14308  14309  14310  14311  14312  14313  14314  14315  14316  14317  14318  14319  14320  14321  14322  14323  14324  14325  14326  14327  14328  14329  14330  14331  14332  14333  14334  14335  14336  14337  14338  14339  14340  14341  14342  14343  14344  14345  14346  14347  14348  14349  14350  14351  14352  14353  14354  14355  14356  14357  14358  14359  14360  14361  14362  14363  14364  14365  14366  14367  14368  14369  14370  14371  14372  14373  14374  14375  14376  14377  14378  14379  14380  14381  14382  14383  14384  14385  14386  14387  14388  14389  14390  14391  14392  14393  14394  14395  14396  14397  14398  14399  14400  14401  14402  14403  14404  14405  14406  14407  14408  14409  14410  14411  14412  14413  14414  14415  14416  14417  14418  14419  14420  14421  14422  14423  14424  14425  14426  14427  14428  14429  14430  14431  14432  14433  14434  14435  14436  14437  14438  14439  14440  14441  14442  14443  14444  14445  14446  14447  14448  14449  14450  14451  14452  14453  14454  14455  14456  14457  14458  14459  14460  14461  14462  14463  14464  14465  14466  14467  14468  14469  14470  14471  14472  14473  14474  14475  14476  14477  14478  14479  14480  14481  14482  14483  14484  14485  14486  14487  14488  14489  14490  14491  14492  14493  14494  14495  14496  14497  14498  14499  14500  14501  14502  14503  14504  14505  14506  14507  14508  14509  14510  14511  14512  14513  14514  14515  14516  14517  14518  14519  14520  14521  14522  14523  14524  14525  14526  14527  14528  14529  14530  14531  14532  14533  14534  14535  14536  14537  14538  14539  14540  14541  14542  14543  14544  14545  14546  14547  14548  14549  14550  14551  14552  14553  14554  14555  14556  14557  14558  14559  14560  14561  14562  14563  14564  14565  14566  14567  14568  14569  14570  14571  14572  14573  14574  14575  14576  14577  14578  14579  14580  14581  14582  14583  14584  14585  14586  14587  14588  14589  14590  14591  14592  14593  14594  14595  14596  14597  14598  14599  14600  14601  14602  14603  14604  14605  14606  14607  14608  14609  14610  14611  14612  14613  14614  14615  14616  14617  14618  14619  14620  14621  14622  14623  14624  14625  14626  14627  14628  14629  14630  14631  14632  14633  14634  14635  14636  14637  14638  14639  14640  14641  14642  14643  14644  14645  14646  14647  14648  14649  14650  14651  14652  14653  14654  14655  14656  14657  14658  14659  14660  14661  14662  14663  14664  14665  14666  14667  14668  14669  14670  14671  14672  14673  14674  14675  14676  14677  14678  14679  14680  14681  14682  14683  14684  14685  14686  14687  14688  14689  14690  14691  14692  14693  14694  14695  14696  14697  14698  14699  14700  14701  14702  14703  14704  14705  14706  14707  14708  14709  14710  14711  14712  14713  14714  14715  14716  14717  14718  14719  14720  14721  14722  14723  14724  14725  14726  14727  14728  14729  14730  14731  14732  14733  14734  14735  14736  14737  14738  14739  14740  14741  14742  14743  14744  14745  14746  14747  14748  14749  14750  14751  14752  14753  14754  14755  14756  14757  14758  14759  14760  14761  14762  14763  14764  14765  14766  14767  14768  14769  14770  14771  14772  14773  14774  14775  14776  14777  14778  14779  14780  14781  14782  14783  14784  14785  14786  14787  14788  14789  14790  14791  14792  14793  14794  14795  14796  14797  14798  14799  14800  14801  14802  14803  14804  14805  14806  14807  14808  14809  14810  14811  14812  14813  14814  14815  14816  14817  14818  14819  14820  14821  14822  14823  14824  14825  14826  14827  14828  14829  14830  14831  14832  14833  14834  14835  14836  14837  14838  14839  14840  14841  14842  14843  14844  14845  14846  14847  14848  14849  14850  14851  14852  14853  14854  14855  14856  14857  14858  14859  14860  14861  14862  14863  14864  14865  14866  14867  14868  14869  14870  14871  14872  14873  14874  14875  14876  14877  14878  14879  14880  14881  14882  14883  14884  14885  14886  14887  14888  14889  14890  14891  14892  14893  14894  14895  14896  14897  14898  14899  14900  14901  14902  14903  14904  14905  14906  14907  14908  14909  14910  14911  14912  14913  14914  14915  14916  14917  14918  14919  14920  14921  14922  14923  14924  14925  14926  14927  14928  14929  14930  14931  14932  14933  14934  14935  14936  14937  14938  14939  14940  14941  14942  14943  14944  14945  14946  14947  14948  14949  14950  14951  14952  14953  14954  14955  14956  14957  14958  14959  14960  14961  14962  14963  14964  14965  14966  14967  14968  14969  14970  14971  14972  14973  14974  14975  14976  14977  14978  14979  14980  14981  14982  14983  14984  14985  14986  14987  14988  14989  14990  14991  14992  14993  14994  14995  14996  14997  14998  14999  15000  15001  15002  15003  15004  15005  15006  15007  15008  15009  15010  15011  15012  15013  15014  15015  15016  15017  15018  15019  15020  15021  15022  15023  15024  15025  15026  15027  15028  15029  15030  15031  15032  15033  15034  15035  15036  15037  15038  15039  15040  15041  15042  15043  15044  15045  15046  15047  15048  15049  15050  15051  15052  15053  15054  15055  15056  15057  15058  15059  15060  15061  15062  15063  15064  15065  15066  15067  15068  15069  15070  15071  15072  15073  15074  15075  15076  15077  15078  15079  15080  15081  15082  15083  15084  15085  15086  15087  15088  15089  15090  15091  15092  15093  15094  15095  15096  15097  15098  15099  15100  15101  15102  15103  15104  15105  15106  15107  15108  15109  15110  15111  15112  15113  15114  15115  15116  15117  15118  15119  15120  15121  15122  15123  15124  15125  15126  15127  15128  15129  15130  15131  15132  15133  15134  15135  15136  15137  15138  15139  15140  15141  15142  15143  15144  15145  15146  15147  15148  15149  15150  15151  15152  15153  15154  15155  15156  15157  15158  15159  15160  15161  15162  15163  15164  15165  15166  15167  15168  15169  15170  15171  15172  15173  15174  15175  15176  15177  15178  15179  15180  15181  15182  15183  15184  15185  15186  15187  15188  15189  15190  15191  15192  15193  15194  15195  15196  15197  15198  15199  15200  15201  15202  15203  15204  15205  15206  15207  15208  15209  15210  15211  15212  15213  15214  15215  15216  15217  15218  15219  15220  15221  15222  15223  15224  15225  15226  15227  15228  15229  15230  15231  15232  15233  15234  15235  15236  15237  15238  15239  15240  15241  15242  15243  15244  15245  15246  15247  15248  15249  15250  15251  15252  15253  15254  15255  15256  15257  15258  15259  15260  15261  15262  15263  15264  15265  15266  15267  15268  15269  15270  15271  15272  15273  15274  15275  15276  15277  15278  15279  15280  15281  15282  15283  15284  15285  15286  15287  15288  15289  15290  15291  15292  15293  15294  15295  15296  15297  15298  15299  15300  15301  15302  15303  15304  15305  15306  15307  15308  15309  15310  15311  15312  15313  15314  15315  15316  15317  15318  15319  15320  15321  15322  15323  15324  15325  15326  15327  15328  15329  15330  15331  15332  15333  15334  15335  15336  15337  15338  15339  15340  15341  15342  15343  15344  15345  15346  15347  15348  15349  15350  15351  15352  15353  15354  15355  15356  15357  15358  15359  15360  15361  15362  15363  15364  15365  15366  15367  15368  15369  15370  15371  15372  15373  15374  15375  15376  15377  15378  15379  15380  15381  15382  15383  15384  15385  15386  15387  15388  15389  15390  15391  15392  15393  15394  15395  15396  15397  15398  15399  15400  15401  15402  15403  15404  15405  15406  15407  15408  15409  15410  15411  15412  15413  15414  15415  15416  15417  15418  15419  15420  15421  15422  15423  15424  15425  15426  15427  15428  15429  15430  15431  15432  15433  15434  15435  15436  15437  15438  15439  15440  15441  15442  15443  15444  15445  15446  15447  15448  15449  15450  15451  15452  15453  15454  15455  15456  15457  15458  15459  15460  15461  15462  15463  15464  15465  15466  15467  15468  15469  15470  15471  15472  15473  15474  15475  15476  15477  15478  15479  15480  15481  15482  15483  15484  15485  15486  15487  15488  15489  15490  15491  15492  15493  15494  15495  15496  15497  15498  15499  15500  15501  15502  15503  15504  15505  15506  15507  15508  15509  15510  15511  15512  15513  15514  15515  15516  15517  15518  15519  15520  15521  15522  15523  15524  15525  15526  15527  15528  15529  15530  15531  15532  15533  15534  15535  15536  15537  15538  15539  15540  15541  15542  15543  15544  15545  15546  15547  15548  15549  15550  15551  15552  15553  15554  15555  15556  15557  15558  15559  15560  15561  15562  15563  15564  15565  15566  15567  15568  15569  15570  15571  15572  15573  15574  15575  15576  15577  15578  15579  15580  15581  15582  15583  15584  15585  15586  15587  15588  15589  15590  15591  15592  15593  15594  15595  15596  15597  15598  15599  15600  15601  15602  15603  15604  15605  15606  15607  15608  15609  15610  15611  15612  15613  15614  15615  15616  15617  15618  15619  15620  15621  15622  15623  15624  15625  15626  15627  15628  15629  15630  15631  15632  15633  15634  15635  15636  15637  15638  15639  15640  15641  15642  15643  15644  15645  15646  15647  15648  15649  15650  15651  15652  15653  15654  15655  15656  15657  15658  15659  15660  15661  15662  15663  15664  15665  15666  15667  15668  15669  15670  15671  15672  15673  15674  15675  15676  15677  15678  15679  15680  15681  15682  15683  15684  15685  15686  15687  15688  15689  15690  15691  15692  15693  15694  15695  15696  15697  15698  15699  15700  15701  15702  15703  15704  15705  15706  15707  15708  15709  15710  15711  15712  15713  15714  15715  15716  15717  15718  15719  15720  15721  15722  15723  15724  15725  15726  15727  15728  15729  15730  15731  15732  15733  15734  15735  15736  15737  15738  15739  15740  15741  15742  15743  15744  15745  15746  15747  15748  15749  15750  15751  15752  15753  15754  15755  15756  15757  15758  15759  15760  15761  15762  15763  15764  15765  15766  15767  15768  15769  15770  15771  15772  15773  15774  15775  15776  15777  15778  15779  15780  15781  15782  15783  15784  15785  15786  15787  15788  15789  15790  15791  15792  15793  15794  15795  15796  15797  15798  15799  15800  15801  15802  15803  15804  15805  15806  15807  15808  15809  15810  15811  15812  15813  15814  15815  15816  15817  15818  15819  15820  15821  15822  15823  15824  15825  15826  15827  15828  15829  15830  15831  15832  15833  15834  15835  15836  15837  15838  15839  15840  15841  15842  15843  15844  15845  15846  15847  15848  15849  15850  15851  15852  15853  15854  15855  15856  15857  15858  15859  15860  15861  15862  15863  15864  15865  15866  15867  15868  15869  15870  15871  15872  15873  15874  15875  15876  15877  15878  15879  15880  15881  15882  15883  15884  15885  15886  15887  15888  15889  15890  15891  15892  15893  15894  15895  15896  15897  15898  15899  15900  15901  15902  15903  15904  15905  15906  15907  15908  15909  15910  15911  15912  15913  15914  15915  15916  15917  15918  15919  15920  15921  15922  15923  15924  15925  15926  15927  15928  15929  15930  15931  15932  15933  15934  15935  15936  15937  15938  15939  15940  15941  15942  15943  15944  15945  15946  15947  15948  15949  15950  15951  15952  15953  15954  15955  15956  15957  15958  15959  15960  15961  15962  15963  15964  15965  15966  15967  15968  15969  15970  15971  15972  15973  15974  15975  15976  15977  15978  15979  15980  15981  15982  15983  15984  15985  15986  15987  15988  15989  15990  15991  15992  15993  15994  15995  15996  15997  15998  15999  16000  16001  16002  16003  16004  16005  16006  16007  16008  16009  16010  16011  16012  16013  16014  16015  16016  16017  16018  16019  16020  16021  16022  16023  16024  16025  16026  16027  16028  16029  16030  16031  16032  16033  16034  16035  16036  16037  16038  16039  16040  16041  16042  16043  16044  16045  16046  16047  16048  16049  16050  16051  16052  16053  16054  16055  16056  16057  16058  16059  16060  16061  16062  16063  16064  16065  16066  16067  16068  16069  16070  16071  16072  16073  16074  16075  16076  16077  16078  16079  16080  16081  16082  16083  16084  16085  16086  16087  16088  16089  16090  16091  16092  16093  16094  16095  16096  16097  16098  16099  16100  16101  16102  16103  16104  16105  16106  16107  16108  16109  16110  16111  16112  16113  16114  16115  16116  16117  16118  16119  16120  16121  16122  16123  16124  16125  16126  16127  16128  16129  16130  16131  16132  16133  16134  16135  16136  16137  16138  16139  16140  16141  16142  16143  16144  16145  16146  16147  16148  16149  16150  16151  16152  16153  16154  16155  16156  16157  16158  16159  16160  16161  16162  16163  16164  16165  16166  16167  16168  16169  16170  16171  16172  16173  16174  16175  16176  16177  16178  16179  16180  16181  16182  16183  16184  16185  16186  16187  16188  16189  16190  16191  16192  16193  16194  16195  16196  16197  16198  16199  16200  16201  16202  16203  16204  16205  16206  16207  16208  16209  16210  16211  16212  16213  16214  16215  16216  16217  16218  16219  16220  16221  16222  16223  16224  16225  16226  16227  16228  16229  16230  16231  16232  16233  16234  16235  16236  16237  16238  16239  16240  16241  16242  16243  16244  16245  16246  16247  16248  16249  16250  16251  16252  16253  16254  16255  16256  16257  16258  16259  16260  16261  16262  16263  16264  16265  16266  16267  16268  16269  16270  16271  16272  16273  16274  16275  16276  16277  16278  16279  16280  16281  16282  16283  16284  16285  16286  16287  16288  16289  16290  16291  16292  16293  16294  16295  16296  16297  16298  16299  16300  16301  16302  16303  16304  16305  16306  16307  16308  16309  16310  16311  16312  16313  16314  16315  16316  16317  16318  16319  16320  16321  16322  16323  16324  16325  16326  16327  16328  16329  16330  16331  16332  16333  16334  16335  16336  16337  16338  16339  16340  16341  16342  16343  16344  16345  16346  16347  16348  16349  16350  16351  16352  16353  16354  16355  16356  16357  16358  16359  16360  16361  16362  16363  16364  16365  16366  16367  16368  16369  16370  16371  16372  16373  16374  16375  16376  16377  16378  16379  16380  16381  16382  16383  16384  16385  16386  16387  16388  16389  16390  16391  16392  16393  16394  16395  16396  16397  16398  16399  16400  16401  16402  16403  16404  16405  16406  16407  16408  16409  16410  16411  16412  16413  16414  16415  16416  16417  16418  16419  16420  16421  16422  16423  16424  16425  16426  16427  16428  16429  16430  16431  16432  16433  16434  16435  16436  16437  16438  16439  16440  16441  16442  16443  16444  16445  16446  16447  16448  16449  16450  16451  16452  16453  16454  16455  16456  16457  16458  16459  16460  16461  16462  16463  16464  16465  16466  16467  16468  16469  16470  16471  16472  16473  16474  16475  16476  16477  16478  16479  16480  16481  16482  16483  16484  16485  16486  16487  16488  16489  16490  16491  16492  16493  16494  16495  16496  16497  16498  16499  16500  16501  16502  16503  16504  16505  16506  16507  16508  16509  16510  16511  16512  16513  16514  16515  16516  16517  16518  16519  16520  16521  16522  16523  16524  16525  16526  16527  16528  16529  16530  16531  16532  16533  16534  16535  16536  16537  16538  16539  16540  16541  16542  16543  16544  16545  16546  16547  16548  16549  16550  16551  16552  16553  16554  16555  16556  16557  16558  16559  16560  16561  16562  16563  16564  16565  16566  16567  16568  16569  16570  16571  16572  16573  16574  16575  16576  16577  16578  16579  16580  16581  16582  16583  16584  16585  16586  16587  16588  16589  16590  16591  16592  16593  16594  16595  16596  16597  16598  16599  16600  16601  16602  16603  16604  16605  16606  16607  16608  16609  16610  16611  16612  16613  16614  16615  16616  16617  16618  16619  16620  16621  16622  16623  16624  16625  16626  16627  16628  16629  16630  16631  16632  16633  16634  16635  16636  16637  16638  16639  16640  16641  16642  16643  16644  16645  16646  16647  16648  16649  16650  16651  16652  16653  16654  16655  16656  16657  16658  16659  16660  16661  16662  16663  16664  16665  16666  16667  16668  16669  16670  16671  16672  16673  16674  16675  16676  16677  16678  16679  16680  16681  16682  16683  16684  16685  16686  16687  16688  16689  16690  16691  16692  16693  16694  16695  16696  16697  16698  16699  16700  16701  16702  16703  16704  16705  16706  16707  16708  16709  16710  16711  16712  16713  16714  16715  16716  16717  16718  16719  16720  16721  16722  16723  16724  16725  16726  16727  16728  16729  16730  16731  16732  16733  16734  16735  16736  16737  16738  16739  16740  16741  16742  16743  16744  16745  16746  16747  16748  16749  16750  16751  16752  16753  16754  16755  16756  16757  16758  16759  16760  16761  16762  16763  16764  16765  16766  16767  16768  16769  16770  16771  16772  16773  16774  16775  16776  16777  16778  16779  16780  16781  16782  16783  16784  16785  16786  16787  16788  16789  16790  16791  16792  16793  16794  16795  16796  16797  16798  16799  16800  16801  16802  16803  16804  16805  16806  16807  16808  16809  16810  16811  16812  16813  16814  16815  16816  16817  16818  16819  16820  16821  16822  16823  16824  16825  16826  16827  16828  16829  16830  16831  16832  16833  16834  16835  16836  16837  16838  16839  16840  16841  16842  16843  16844  16845  16846  16847  16848  16849  16850  16851  16852  16853  16854  16855  16856  16857  16858  16859  16860  16861  16862  16863  16864  16865  16866  16867  16868  16869  16870  16871  16872  16873  16874  16875  16876  16877  16878  16879  16880  16881  16882  16883  16884  16885  16886  16887  16888  16889  16890  16891  16892  16893  16894  16895  16896  16897  16898  16899  16900  16901  16902  16903  16904  16905  16906  16907  16908  16909  16910  16911  16912  16913  16914  16915  16916  16917  16918  16919  16920  16921  16922  16923  16924  16925  16926  16927  16928  16929  16930  16931  16932  16933  16934  16935  16936  16937  16938  16939  16940  16941  16942  16943  16944  16945  16946  16947  16948  16949  16950  16951  16952  16953  16954  16955  16956  16957  16958  16959  16960  16961  16962  16963  16964  16965  16966  16967  16968  16969  16970  16971  16972  16973  16974  16975  16976  16977  16978  16979  16980  16981  16982  16983  16984  16985  16986  16987  16988  16989  16990  16991  16992  16993  16994  16995  16996  16997  16998  16999  17000  17001  17002  17003  17004  17005  17006  17007  17008  17009  17010  17011  17012  17013  17014  17015  17016  17017  17018  17019  17020  17021  17022  17023  17024  17025  17026  17027  17028  17029  17030  17031  17032  17033  17034  17035  17036  17037  17038  17039  17040  17041  17042  17043  17044  17045  17046  17047  17048  17049  17050  17051  17052  17053  17054  17055  17056  17057  17058  17059  17060  17061  17062  17063  17064  17065  17066  17067  17068  17069  17070  17071  17072  17073  17074  17075  17076  17077  17078  17079  17080  17081  17082  17083  17084  17085  17086  17087  17088  17089  17090  17091  17092  17093  17094  17095  17096  17097  17098  17099  17100  17101  17102  17103  17104  17105  17106  17107  17108  17109  17110  17111  17112  17113  17114  17115  17116  17117  17118  17119  17120  17121  17122  17123  17124  17125  17126  17127  17128  17129  17130  17131  17132  17133  17134  17135  17136  17137  17138  17139  17140  17141  17142  17143  17144  17145  17146  17147  17148  17149  17150  17151  17152  17153  17154  17155  17156  17157  17158  17159  17160  17161  17162  17163  17164  17165  17166  17167  17168  17169  17170  17171  17172  17173  17174  17175  17176  17177  17178  17179  17180  17181  17182  17183  17184  17185  17186  17187  17188  17189  17190  17191  17192  17193  17194  17195  17196  17197  17198  17199  17200  17201  17202  17203  17204  17205  17206  17207  17208  17209  17210  17211  17212  17213  17214  17215  17216  17217  17218  17219  17220  17221  17222  17223  17224  17225  17226  17227  17228  17229  17230  17231  17232  17233  17234  17235  17236  17237  17238  17239  17240  17241  17242  17243  17244  17245  17246  17247  17248  17249  17250  17251  17252  17253  17254  17255  17256  17257  17258  17259  17260  17261  17262  17263  17264  17265  17266  17267  17268  17269  17270  17271  17272  17273  17274  17275  17276  17277  17278  17279  17280  17281  17282  17283  17284  17285  17286  17287  17288  17289  17290  17291  17292  17293  17294  17295  17296  17297  17298  17299  17300  17301  17302  17303  17304  17305  17306  17307  17308  17309  17310  17311  17312  17313  17314  17315  17316  17317  17318  17319  17320  17321  17322  17323  17324  17325  17326  17327  17328  17329  17330  17331  17332  17333  17334  17335  17336  17337  17338  17339  17340  17341  17342  17343  17344  17345  17346  17347  17348  17349  17350  17351  17352  17353  17354  17355  17356  17357  17358  17359  17360  17361  17362  17363  17364  17365  17366  17367  17368  17369  17370  17371  17372  17373  17374  17375  17376  17377  17378  17379  17380  17381  17382  17383  17384  17385  17386  17387  17388  17389  17390  17391  17392  17393  17394  17395  17396  17397  17398  17399  17400  17401  17402  17403  17404  17405  17406  17407  17408  17409  17410  17411  17412  17413  17414  17415  17416  17417  17418  17419  17420  17421  17422  17423  17424  17425  17426  17427  17428  17429  17430  17431  17432  17433  17434  17435  17436  17437  17438  17439  17440  17441  17442  17443  17444  17445  17446  17447  17448  17449  17450  17451  17452  17453  17454  17455  17456  17457  17458  17459  17460  17461  17462  17463  17464  17465  17466  17467  17468  17469  17470  17471  17472  17473  17474  17475  17476  17477  17478  17479  17480  17481  17482  17483  17484  17485  17486  17487  17488  17489  17490  17491  17492  17493  17494  17495  17496  17497  17498  17499  17500  17501  17502  17503  17504  17505  17506  17507  17508  17509  17510  17511  17512  17513  17514  17515  17516  17517  17518  17519  17520  17521  17522  17523  17524  17525  17526  17527  17528  17529  17530  17531  17532  17533  17534  17535  17536  17537  17538  17539  17540  17541  17542  17543  17544  17545  17546  17547  17548  17549  17550  17551  17552  17553  17554  17555  17556  17557  17558  17559  17560  17561  17562  17563  17564  17565  17566  17567  17568  17569  17570  17571  17572  17573  17574  17575  17576  17577  17578  17579  17580  17581  17582  17583  17584  17585  17586  17587  17588  17589  17590  17591  17592  17593  17594  17595  17596  17597  17598  17599  17600  17601  17602  17603  17604  17605  17606  17607  17608  17609  17610  17611  17612  17613  17614  17615  17616  17617  17618  17619  17620  17621  17622  17623  17624  17625  17626  17627  17628  17629  17630  17631  17632  17633  17634  17635  17636  17637  17638  17639  17640  17641  17642  17643  17644  17645  17646  17647  17648  17649  17650  17651  17652  17653  17654  17655  17656  17657  17658  17659  17660  17661  17662  17663  17664  17665  17666  17667  17668  17669  17670  17671  17672  17673  17674  17675  17676  17677  17678  17679  17680  17681  17682  17683  17684  17685  17686  17687  17688  17689  17690  17691  17692  17693  17694  17695  17696  17697  17698  17699  17700  17701  17702  17703  17704  17705  17706  17707  17708  17709  17710  17711  17712  17713  17714  17715  17716  17717  17718  17719  17720  17721  17722  17723  17724  17725  17726  17727  17728  17729  17730  17731  17732  17733  17734  17735  17736  17737  17738  17739  17740  17741  17742  17743  17744  17745  17746  17747  17748  17749  17750  17751  17752  17753  17754  17755  17756  17757  17758  17759  17760  17761  17762  17763  17764  17765  17766  17767  17768  17769  17770  17771  17772  17773  17774  17775  17776  17777  17778  17779  17780  17781  17782  17783  17784  17785  17786  17787  17788  17789  17790  17791  17792  17793  17794  17795  17796  17797  17798  17799  17800  17801  17802  17803  17804  17805  17806  17807  17808  17809  17810  17811  17812  17813  17814  17815  17816  17817  17818  17819  17820  17821  17822  17823  17824  17825  17826  17827  17828  17829  17830  17831  17832  17833  17834  17835  17836  17837  17838  17839  17840  17841  17842  17843  17844  17845  17846  17847  17848  17849  17850  17851  17852  17853  17854  17855  17856  17857  17858  17859  17860  17861  17862  17863  17864  17865  17866  17867  17868  17869  17870  17871  17872  17873  17874  17875  17876  17877  17878  17879  17880  17881  17882  17883  17884  17885  17886  17887  17888  17889  17890  17891  17892  17893  17894  17895  17896  17897  17898  17899  17900  17901  17902  17903  17904  17905  17906  17907  17908  17909  17910  17911  17912  17913  17914  17915  17916  17917  17918  17919  17920  17921  17922  17923  17924  17925  17926  17927  17928  17929  17930  17931  17932  17933  17934  17935  17936  17937  17938  17939  17940  17941  17942  17943  17944  17945  17946  17947  17948  17949  17950  17951  17952  17953  17954  17955  17956  17957  17958  17959  17960  17961  17962  17963  17964  17965  17966  17967  17968  17969  17970  17971  17972  17973  17974  17975  17976  17977  17978  17979  17980  17981  17982  17983  17984  17985  17986  17987  17988  17989  17990  17991  17992  17993  17994  17995  17996  17997  17998  17999  18000  18001  18002  18003  18004  18005  18006  18007  18008  18009  18010  18011  18012  18013  18014  18015  18016  18017  18018  18019  18020  18021  18022  18023  18024  18025  18026  18027  18028  18029  18030  18031  18032  18033  18034  18035  18036  18037  18038  18039  18040  18041  18042  18043  18044  18045  18046  18047  18048  18049  18050  18051  18052  18053  18054  18055  18056  18057  18058  18059  18060  18061  18062  18063  18064  18065  18066  18067  18068  18069  18070  18071  18072  18073  18074  18075  18076  18077  18078  18079  18080  18081  18082  18083  18084  18085  18086  18087  18088  18089  18090  18091  18092  18093  18094  18095  18096  18097  18098  18099  18100  18101  18102  18103  18104  18105  18106  18107  18108  18109  18110  18111  18112  18113  18114  18115  18116  18117  18118  18119  18120  18121  18122  18123  18124  18125  18126  18127  18128  18129  18130  18131  18132  18133  18134  18135  18136  18137  18138  18139  18140  18141  18142  18143  18144  18145  18146  18147  18148  18149  18150  18151  18152  18153  18154  18155  18156  18157  18158  18159  18160  18161  18162  18163  18164  18165  18166  18167  18168  18169  18170  18171  18172  18173  18174  18175  18176  18177  18178  18179  18180  18181  18182  18183  18184  18185  18186  18187  18188  18189  18190  18191  18192  18193  18194  18195  18196  18197  18198  18199  18200  18201  18202  18203  18204  18205  18206  18207  18208  18209  18210  18211  18212  18213  18214  18215  18216  18217  18218  18219  18220  18221  18222  18223  18224  18225  18226  18227  18228  18229  18230  18231  18232  18233  18234  18235  18236  18237  18238  18239  18240  18241  18242  18243  18244  18245  18246  18247  18248  18249  18250  18251  18252  18253  18254  18255  18256  18257  18258  18259  18260  18261  18262  18263  18264  18265  18266  18267  18268  18269  18270  18271  18272  18273  18274  18275  18276  18277  18278  18279  18280  18281  18282  18283  18284  18285  18286  18287  18288  18289  18290  18291  18292  18293  18294  18295  18296  18297  18298  18299  18300  18301  18302  18303  18304  18305  18306  18307  18308  18309  18310  18311  18312  18313  18314  18315  18316  18317  18318  18319  18320  18321  18322  18323  18324  18325  18326  18327  18328  18329  18330  18331  18332  18333  18334  18335  18336  18337  18338  18339  18340  18341  18342  18343  18344  18345  18346  18347  18348  18349  18350  18351  18352  18353  18354  18355  18356  18357  18358  18359  18360  18361  18362  18363  18364  18365  18366  18367  18368  18369  18370  18371  18372  18373  18374  18375  18376  18377  18378  18379  18380  18381  18382  18383  18384  18385  18386  18387  18388  18389  18390  18391  18392  18393  18394  18395  18396  18397  18398  18399  18400  18401  18402  18403  18404  18405  18406  18407  18408  18409  18410  18411  18412  18413  18414  18415  18416  18417  18418  18419  18420  18421  18422  18423  18424  18425  18426  18427  18428  18429  18430  18431  18432  18433  18434  18435  18436  18437  18438  18439  18440  18441  18442  18443  18444  18445  18446  18447  18448  18449  18450  18451  18452  18453  18454  18455  18456  18457  18458  18459  18460  18461  18462  18463  18464  18465  18466  18467  18468  18469  18470  18471  18472  18473  18474  18475  18476  18477  18478  18479  18480  18481  18482  18483  18484  18485  18486  18487  18488  18489  18490  18491  18492  18493  18494  18495  18496  18497  18498  18499  18500  18501  18502  18503  18504  18505  18506  18507  18508  18509  18510  18511  18512  18513  18514  18515  18516  18517  18518  18519  18520  18521  18522  18523  18524  18525  18526  18527  18528  18529  18530  18531  18532  18533  18534  18535  18536  18537  18538  18539  18540  18541  18542  18543  18544  18545  18546  18547  18548  18549  18550  18551  18552  18553  18554  18555  18556  18557  18558  18559  18560  18561  18562  18563  18564  18565  18566  18567  18568  18569  18570  18571  18572  18573  18574  18575  18576  18577  18578  18579  18580  18581  18582  18583  18584  18585  18586  18587  18588  18589  18590  18591  18592  18593  18594  18595  18596  18597  18598  18599  18600  18601  18602  18603  18604  18605  18606  18607  18608  18609  18610  18611  18612  18613  18614  18615  18616  18617  18618  18619  18620  18621  18622  18623  18624  18625  18626  18627  18628  18629  18630  18631  18632  18633  18634  18635  18636  18637  18638  18639  18640  18641  18642  18643  18644  18645  18646  18647  18648  18649  18650  18651  18652  18653  18654  18655  18656  18657  18658  18659  18660  18661  18662  18663  18664  18665  18666  18667  18668  18669  18670  18671  18672  18673  18674  18675  18676  18677  18678  18679  18680  18681  18682  18683  18684  18685  18686  18687  18688  18689  18690  18691  18692  18693  18694  18695  18696  18697  18698  18699  18700  18701  18702  18703  18704  18705  18706  18707  18708  18709  18710  18711  18712  18713  18714  18715  18716  18717  18718  18719  18720  18721  18722  18723  18724  18725  18726  18727  18728  18729  18730  18731  18732  18733  18734  18735  18736  18737  18738  18739  18740  18741  18742  18743  18744  18745  18746  18747  18748  18749  18750  18751  18752  18753  18754  18755  18756  18757  18758  18759  18760  18761  18762  18763  18764  18765  18766  18767  18768  18769  18770  18771  18772  18773  18774  18775  18776  18777  18778  18779  18780  18781  18782  18783  18784  18785  18786  18787  18788  18789  18790  18791  18792  18793  18794  18795  18796  18797  18798  18799  18800  18801  18802  18803  18804  18805  18806  18807  18808  18809  18810  18811  18812  18813  18814  18815  18816  18817  18818  18819  18820  18821  18822  18823  18824  18825  18826  18827  18828  18829  18830  18831  18832  18833  18834  18835  18836  18837  18838  18839  18840  18841  18842  18843  18844  18845  18846  18847  18848  18849  18850  18851  18852  18853  18854  18855  18856  18857  18858  18859  18860  18861  18862  18863  18864  18865  18866  18867  18868  18869  18870  18871  18872  18873  18874  18875  18876  18877  18878  18879  18880  18881  18882  18883  18884  18885  18886  18887  18888  18889  18890  18891  18892  18893  18894  18895  18896  18897  18898  18899  18900  18901  18902  18903  18904  18905  18906  18907  18908  18909  18910  18911  18912  18913  18914  18915  18916  18917  18918  18919  18920  18921  18922  18923  18924  18925  18926  18927  18928  18929  18930  18931  18932  18933  18934  18935  18936  18937  18938  18939  18940  18941  18942  18943  18944  18945  18946  18947  18948  18949  18950  18951  18952  18953  18954  18955  18956  18957  18958  18959  18960  18961  18962  18963  18964  18965  18966  18967  18968  18969  18970  18971  18972  18973  18974  18975  18976  18977  18978  18979  18980  18981  18982  18983  18984  18985  18986  18987  18988  18989  18990  18991  18992  18993  18994  18995  18996  18997  18998  18999  19000  19001  19002  19003  19004  19005  19006  19007  19008  19009  19010  19011  19012  19013  19014  19015  19016  19017  19018  19019  19020  19021  19022  19023  19024  19025  19026  19027  19028  19029  19030  19031  19032  19033  19034  19035  19036  19037  19038  19039  19040  19041  19042  19043  19044  19045  19046  19047  19048  19049  19050  19051  19052  19053  19054  19055  19056  19057  19058  19059  19060  19061  19062  19063  19064  19065  19066  19067  19068  19069  19070  19071  19072  19073  19074  19075  19076  19077  19078  19079  19080  19081  19082  19083  19084  19085  19086  19087  19088  19089  19090  19091  19092  19093  19094  19095  19096  19097  19098  19099  19100  19101  19102  19103  19104  19105  19106  19107  19108  19109  19110  19111  19112  19113  19114  19115  19116  19117  19118  19119  19120  19121  19122  19123  19124  19125  19126  19127  19128  19129  19130  19131  19132  19133  19134  19135  19136  19137  19138  19139  19140  19141  19142  19143  19144  19145  19146  19147  19148  19149  19150  19151  19152  19153  19154  19155  19156  19157  19158  19159  19160  19161  19162  19163  19164  19165  19166  19167  19168  19169  19170  19171  19172  19173  19174  19175  19176  19177  19178  19179  19180  19181  19182  19183  19184  19185  19186  19187  19188  19189  19190  19191  19192  19193  19194  19195  19196  19197  19198  19199  19200  19201  19202  19203  19204  19205  19206  19207  19208  19209  19210  19211  19212  19213  19214  19215  19216  19217  19218  19219  19220  19221  19222  19223  19224  19225  19226  19227  19228  19229  19230  19231  19232  19233  19234  19235  19236  19237  19238  19239  19240  19241  19242  19243  19244  19245  19246  19247  19248  19249  19250  19251  19252  19253  19254  19255  19256  19257  19258  19259  19260  19261  19262  19263  19264  19265  19266  19267  19268  19269  19270  19271  19272  19273  19274  19275  19276  19277  19278  19279  19280  19281  19282  19283  19284  19285  19286  19287  19288  19289  19290  19291  19292  19293  19294  19295  19296  19297  19298  19299  19300  19301  19302  19303  19304  19305  19306  19307  19308  19309  19310  19311  19312  19313  19314  19315  19316  19317  19318  19319  19320  19321  19322  19323  19324  19325  19326  19327  19328  19329  19330  19331  19332  19333  19334  19335  19336  19337  19338  19339  19340  19341  19342  19343  19344  19345  19346  19347  19348  19349  19350  19351  19352  19353  19354  19355  19356  19357  19358  19359  19360  19361  19362  19363  19364  19365  19366  19367  19368  19369  19370  19371  19372  19373  19374  19375  19376  19377  19378  19379  19380  19381  19382  19383  19384  19385  19386  19387  19388  19389  19390  19391  19392  19393  19394  19395  19396  19397  19398  19399  19400  19401  19402  19403  19404  19405  19406  19407  19408  19409  19410  19411  19412  19413  19414  19415  19416  19417  19418  19419  19420  19421  19422  19423  19424  19425  19426  19427  19428  19429  19430  19431  19432  19433  19434  19435  19436  19437  19438  19439  19440  19441  19442  19443  19444  19445  19446  19447  19448  19449  19450  19451  19452  19453  19454  19455  19456  19457  19458  19459  19460  19461  19462  19463  19464  19465  19466  19467  19468  19469  19470  19471  19472  19473  19474  19475  19476  19477  19478  19479  19480  19481  19482  19483  19484  19485  19486  19487  19488  19489  19490  19491  19492  19493  19494  19495  19496  19497  19498  19499  19500  19501  19502  19503  19504  19505  19506  19507  19508  19509  19510  19511  19512  19513  19514  19515  19516  19517  19518  19519  19520  19521  19522  19523  19524  19525  19526  19527  19528  19529  19530  19531  19532  19533  19534  19535  19536  19537  19538  19539  19540  19541  19542  19543  19544  19545  19546  19547  19548  19549  19550  19551  19552  19553  19554  19555  19556  19557  19558  19559  19560  19561  19562  19563  19564  19565  19566  19567  19568  19569  19570  19571  19572  19573  19574  19575  19576  19577  19578  19579  19580  19581  19582  19583  19584  19585  19586  19587  19588  19589  19590  19591  19592  19593  19594  19595  19596  19597  19598  19599  19600  19601  19602  19603  19604  19605  19606  19607  19608  19609  19610  19611  19612  19613  19614  19615  19616  19617  19618  19619  19620  19621  19622  19623  19624  19625  19626  19627  19628  19629  19630  19631  19632  19633  19634  19635  19636  19637  19638  19639  19640  19641  19642  19643  19644  19645  19646  19647  19648  19649  19650  19651  19652  19653  19654  19655  19656  19657  19658  19659  19660  19661  19662  19663  19664  19665  19666  19667  19668  19669  19670  19671  19672  19673  19674  19675  19676  19677  19678  19679  19680  19681  19682  19683  19684  19685  19686  19687  19688  19689  19690  19691  19692  19693  19694  19695  19696  19697  19698  19699  19700  19701  19702  19703  19704  19705  19706  19707  19708  19709  19710  19711  19712  19713  19714  19715  19716  19717  19718  19719  19720  19721  19722  19723  19724  19725  19726  19727  19728  19729  19730  19731  19732  19733  19734  19735  19736  19737  19738  19739  19740  19741  19742  19743  19744  19745  19746  19747  19748  19749  19750  19751  19752  19753  19754  19755  19756  19757  19758  19759  19760  19761  19762  19763  19764  19765  19766  19767  19768  19769  19770  19771  19772  19773  19774  19775  19776  19777  19778  19779  19780  19781  19782  19783  19784  19785  19786  19787  19788  19789  19790  19791  19792  19793  19794  19795  19796  19797  19798  19799  19800  19801  19802  19803  19804  19805  19806  19807  19808  19809  19810  19811  19812  19813  19814  19815  19816  19817  19818  19819  19820  19821  19822  19823  19824  19825  19826  19827  19828  19829  19830  19831  19832  19833  19834  19835  19836  19837  19838  19839  19840  19841  19842  19843  19844  19845  19846  19847  19848  19849  19850  19851  19852  19853  19854  19855  19856  19857  19858  19859  19860  19861  19862  19863  19864  19865  19866  19867  19868  19869  19870  19871  19872  19873  19874  19875  19876  19877  19878  19879  19880  19881  19882  19883  19884  19885  19886  19887  19888  19889  19890  19891  19892  19893  19894  19895  19896  19897  19898  19899  19900  19901  19902  19903  19904  19905  19906  19907  19908  19909  19910  19911  19912  19913  19914  19915  19916  19917  19918  19919  19920  19921  19922  19923  19924  19925  19926  19927  19928  19929  19930  19931  19932  19933  19934  19935  19936  19937  19938  19939  19940  19941  19942  19943  19944  19945  19946  19947  19948  19949  19950  19951  19952  19953  19954  19955  19956  19957  19958  19959  19960  19961  19962  19963  19964  19965  19966  19967  19968  19969  19970  19971  19972  19973  19974  19975  19976  19977  19978  19979  19980  19981  19982  19983  19984  19985  19986  19987  19988  19989  19990  19991  19992  19993  19994  19995  19996  19997  19998  19999  20000  20001  20002  20003  20004  20005  20006  20007  20008  20009  20010  20011  20012  20013  20014  20015  20016  20017  20018  20019  20020  20021  20022  20023  20024  20025  20026  20027  20028  20029  20030  20031  20032  20033  20034  20035  20036  20037  20038  20039  20040  20041  20042  20043  20044  20045  20046  20047  20048  20049  20050  20051  20052  20053  20054  20055  20056  20057  20058  20059  20060  20061  20062  20063  20064  20065  20066  20067  20068  20069  20070  20071  20072  20073  20074  20075  20076  20077  20078  20079  20080  20081  20082  20083  20084  20085  20086  20087  20088  20089  20090  20091  20092  20093  20094  20095  20096  20097  20098  20099  20100  20101  20102  20103  20104  20105  20106  20107  20108  20109  20110  20111  20112  20113  20114  20115  20116  20117  20118  20119  20120  20121  20122  20123  20124  20125  20126  20127  20128  20129  20130  20131  20132  20133  20134  20135  20136  20137  20138  20139  20140  20141  20142  20143  20144  20145  20146  20147  20148  20149  20150  20151  20152  20153  20154  20155  20156  20157  20158  20159  20160  20161  20162  20163  20164  20165  20166  20167  20168  20169  20170  20171  20172  20173  20174  20175  20176  20177  20178  20179  20180  20181  20182  20183  20184  20185  20186  20187  20188  20189  20190  20191  20192  20193  20194  20195  20196  20197  20198  20199  20200  20201  20202  20203  20204  20205  20206  20207  20208  20209  20210  20211  20212  20213  20214  20215  20216  20217  20218  20219  20220  20221  20222  20223  20224  20225  20226  20227  20228  20229  20230  20231  20232  20233  20234  20235  20236  20237  20238  20239  20240  20241  20242  20243  20244  20245  20246  20247  20248  20249  20250  20251  20252  20253  20254  20255  20256  20257  20258  20259  20260  20261  20262  20263  20264  20265  20266  20267  20268  20269  20270  20271  20272  20273  20274  20275  20276  20277  20278  20279  20280  20281  20282  20283  20284  20285  20286  20287  20288  20289  20290  20291  20292  20293  20294  20295  20296  20297  20298  20299  20300  20301  20302  20303  20304  20305  20306  20307  20308  20309  20310  20311  20312  20313  20314  20315  20316  20317  20318  20319  20320  20321  20322  20323  20324  20325  20326  20327  20328  20329  20330  20331  20332  20333  20334  20335  20336  20337  20338  20339  20340  20341  20342  20343  20344  20345  20346  20347  20348  20349  20350  20351  20352  20353  20354  20355  20356  20357  20358  20359  20360  20361  20362  20363  20364  20365  20366  20367  20368  20369  20370  20371  20372  20373  20374  20375  20376  20377  20378  20379  20380  20381  20382  20383  20384  20385  20386  20387  20388  20389  20390  20391  20392  20393  20394  20395  20396  20397  20398  20399  20400  20401  20402  20403  20404  20405  20406  20407  20408  20409  20410  20411  20412  20413  20414  20415  20416  20417  20418  20419  20420  20421  20422  20423  20424  20425  20426  20427  20428  20429  20430  20431  20432  20433  20434  20435  20436  20437  20438  20439  20440  20441  20442  20443  20444  20445  20446  20447  20448  20449  20450  20451  20452  20453  20454  20455  20456  20457  20458  20459  20460  20461  20462  20463  20464  20465  20466  20467  20468  20469  20470  20471  20472  20473  20474  20475  20476  20477  20478  20479  20480  20481  20482  20483  20484  20485  20486  20487  20488  20489  20490  20491  20492  20493  20494  20495  20496  20497  20498  20499  20500  20501  20502  20503  20504  20505  20506  20507  20508  20509  20510  20511  20512  20513  20514  20515  20516  20517  20518  20519  20520  20521  20522  20523  20524  20525  20526  20527  20528  20529  20530  20531  20532  20533  20534  20535  20536  20537  20538  20539  20540  20541  20542  20543  20544  20545  20546  20547  20548  20549  20550  20551  20552  20553  20554  20555  20556  20557  20558  20559  20560  20561  20562  20563  20564  20565  20566  20567  20568  20569  20570  20571  20572  20573  20574  20575  20576  20577  20578  20579  20580  20581  20582  20583  20584  20585  20586  20587  20588  20589  20590  20591  20592  20593  20594  20595  20596  20597  20598  20599  20600  20601  20602  20603  20604  20605  20606  20607  20608  20609  20610  20611  20612  20613  20614  20615  20616  20617  20618  20619  20620  20621  20622  20623  20624  20625  20626  20627  20628  20629  20630  20631  20632  20633  20634  20635  20636  20637  20638  20639  20640  20641  20642  20643  20644  20645  20646  20647  20648  20649  20650  20651  20652  20653  20654  20655  20656  20657  20658  20659  20660  20661  20662  20663  20664  20665  20666  20667  20668  20669  20670  20671  20672  20673  20674  20675  20676  20677  20678  20679  20680  20681  20682  20683  20684  20685  20686  20687  20688  20689  20690  20691  20692  20693  20694  20695  20696  20697  20698  20699  20700  20701  20702  20703  20704  20705  20706  20707  20708  20709  20710  20711  20712  20713  20714  20715  20716  20717  20718  20719  20720  20721  20722  20723  20724  20725  20726  20727  20728  20729  20730  20731  20732  20733  20734  20735  20736  20737  20738  20739  20740  20741  20742  20743  20744  20745  20746  20747  20748  20749  20750  20751  20752  20753  20754  20755  20756  20757  20758  20759  20760  20761  20762  20763  20764  20765  20766  20767  20768  20769  20770  20771  20772  20773  20774  20775  20776  20777  20778  20779  20780  20781  20782  20783  20784  20785  20786  20787  20788  20789  20790  20791  20792  20793  20794  20795  20796  20797  20798  20799  20800  20801  20802  20803  20804  20805  20806  20807  20808  20809  20810  20811  20812  20813  20814  20815  20816  20817  20818  20819  20820  20821  20822  20823  20824  20825  20826  20827  20828  20829  20830  20831  20832  20833  20834  20835  20836  20837  20838  20839  20840  20841  20842  20843  20844  20845  20846  20847  20848  20849  20850  20851  20852  20853  20854  20855  20856  20857  20858  20859  20860  20861  20862  20863  20864  20865  20866  20867  20868  20869  20870  20871  20872  20873  20874  20875  20876  20877  20878  20879  20880  20881  20882  20883  20884  20885  20886  20887  20888  20889  20890  20891  20892  20893  20894  20895  20896  20897  20898  20899  20900  20901  20902  20903  20904  20905  20906  20907  20908  20909  20910  20911  20912  20913  20914  20915  20916  20917  20918  20919  20920  20921  20922  20923  20924  20925  20926  20927  20928  20929  20930  20931  20932  20933  20934  20935  20936  20937  20938  20939  20940  20941  20942  20943  20944  20945  20946  20947  20948  20949  20950  20951  20952  20953  20954  20955  20956  20957  20958  20959  20960  20961  20962  20963  20964  20965  20966  20967  20968  20969  20970  20971  20972  20973  20974  20975  20976  20977  20978  20979  20980  20981  20982  20983  20984  20985  20986  20987  20988  20989  20990  20991  20992  20993  20994  20995  20996  20997  20998  20999  21000  21001  21002  21003  21004  21005  21006  21007  21008  21009  21010  21011  21012  21013  21014  21015  21016  21017  21018  21019  21020  21021  21022  21023  21024  21025  21026  21027  21028  21029  21030  21031  21032  21033  21034  21035  21036  21037  21038  21039  21040  21041  21042  21043  21044  21045  21046  21047  21048  21049  21050  21051  21052  21053  21054  21055  21056  21057  21058  21059  21060  21061  21062  21063  21064  21065  21066  21067  21068  21069  21070  21071  21072  21073  21074  21075  21076  21077  21078  21079  21080  21081  21082  21083  21084  21085  21086  21087  21088  21089  21090  21091  21092  21093  21094  21095  21096  21097  21098  21099  21100  21101  21102  21103  21104  21105  21106  21107  21108  21109  21110  21111  21112  21113  21114  21115  21116  21117  21118  21119  21120  21121  21122  21123  21124  21125  21126  21127  21128  21129  21130  21131  21132  21133  21134  21135  21136  21137  21138  21139  21140  21141  21142  21143  21144  21145  21146  21147  21148  21149  21150  21151  21152  21153  21154  21155  21156  21157  21158  21159  21160  21161  21162  21163  21164  21165  21166  21167  21168  21169  21170  21171  21172  21173  21174  21175  21176  21177  21178  21179  21180  21181  21182  21183  21184  21185  21186  21187  21188  21189  21190  21191  21192  21193  21194  21195  21196  21197  21198  21199  21200  21201  21202  21203  21204  21205  21206  21207  21208  21209  21210  21211  21212  21213  21214  21215  21216  21217  21218  21219  21220  21221  21222  21223  21224  21225  21226  21227  21228  21229  21230  21231  21232  21233  21234  21235  21236  21237  21238  21239  21240  21241  21242  21243  21244  21245  21246  21247  21248  21249  21250  21251  21252  21253  21254  21255  21256  21257  21258  21259  21260  21261  21262  21263  21264  21265  21266  21267  21268  21269  21270  21271  21272  21273  21274  21275  21276  21277  21278  21279  21280  21281  21282  21283  21284  21285  21286  21287  21288  21289  21290  21291  21292  21293  21294  21295  21296  21297  21298  21299  21300  21301  21302  21303  21304  21305  21306  21307  21308  21309  21310  21311  21312  21313  21314  21315  21316  21317  21318  21319  21320  21321  21322  21323  21324  21325  21326  21327  21328  21329  21330  21331  21332  21333  21334  21335  21336  21337  21338  21339  21340  21341  21342  21343  21344  21345  21346  21347  21348  21349  21350  21351  21352  21353  21354  21355  21356  21357  21358  21359  21360  21361  21362  21363  21364  21365  21366  21367  21368  21369  21370  21371  21372  21373  21374  21375  21376  21377  21378  21379  21380  21381  21382  21383  21384  21385  21386  21387  21388  21389  21390  21391  21392  21393  21394  21395  21396  21397  21398  21399  21400  21401  21402  21403  21404  21405  21406  21407  21408  21409  21410  21411  21412  21413  21414  21415  21416  21417  21418  21419  21420  21421  21422  21423  21424  21425  21426  21427  21428  21429  21430  21431  21432  21433  21434  21435  21436  21437  21438  21439  21440  21441  21442  21443  21444  21445  21446  21447  21448  21449  21450  21451  21452  21453  21454  21455  21456  21457  21458  21459  21460  21461  21462  21463  21464  21465  21466  21467  21468  21469  21470  21471  21472  21473  21474  21475  21476  21477  21478  21479  21480  21481  21482  21483  21484  21485  21486  21487  21488  21489  21490  21491  21492  21493  21494  21495  21496  21497  21498  21499  21500  21501  21502  21503  21504  21505  21506  21507  21508  21509  21510  21511  21512  21513  21514  21515  21516  21517  21518  21519  21520  21521  21522  21523  21524  21525  21526  21527  21528  21529  21530  21531  21532  21533  21534  21535  21536  21537  21538  21539  21540  21541  21542  21543  21544  21545  21546  21547  21548  21549  21550  21551  21552  21553  21554  21555  21556  21557  21558  21559  21560  21561  21562  21563  21564  21565  21566  21567  21568  21569  21570  21571  21572  21573  21574  21575  21576  21577  21578  21579  21580  21581  21582  21583  21584  21585  21586  21587  21588  21589  21590  21591  21592  21593  21594  21595  21596  21597  21598  21599  21600  21601  21602  21603  21604  21605  21606  21607  21608  21609  21610  21611  21612  21613  21614  21615  21616  21617  21618  21619  21620  21621  21622  21623  21624  21625  21626  21627  21628  21629  21630  21631  21632  21633  21634  21635  21636  21637  21638  21639  21640  21641  21642  21643  21644  21645  21646  21647  21648  21649  21650  21651  21652  21653  21654  21655  21656  21657  21658  21659  21660  21661  21662  21663  21664  21665  21666  21667  21668  21669  21670  21671  21672  21673  21674  21675  21676  21677  21678  21679  21680  21681  21682  21683  21684  21685  21686  21687  21688  21689  21690  21691  21692  21693  21694  21695  21696  21697  21698  21699  21700  21701  21702  21703  21704  21705  21706  21707  21708  21709  21710  21711  21712  21713  21714  21715  21716  21717  21718  21719  21720  21721  21722  21723  21724  21725  21726  21727  21728  21729  21730  21731  21732  21733  21734  21735  21736  21737  21738  21739  21740  21741  21742  21743  21744  21745  21746  21747  21748  21749  21750  21751  21752  21753  21754  21755  21756  21757  21758  21759  21760  21761  21762  21763  21764  21765  21766  21767  21768  21769  21770  21771  21772  21773  21774  21775  21776  21777  21778  21779  21780  21781  21782  21783  21784  21785  21786  21787  21788  21789  21790  21791  21792  21793  21794  21795  21796  21797  21798  21799  21800  21801  21802  21803  21804  21805  21806  21807  21808  21809  21810  21811  21812  21813  21814  21815  21816  21817  21818  21819  21820  21821  21822  21823  21824  21825  21826  21827  21828  21829  21830  21831  21832  21833  21834  21835  21836  21837  21838  21839  21840  21841  21842  21843  21844  21845  21846  21847  21848  21849  21850  21851  21852  21853  21854  21855  21856  21857  21858  21859  21860  21861  21862  21863  21864  21865  21866  21867  21868  21869  21870  21871  21872  21873  21874  21875  21876  21877  21878  21879  21880  21881  21882  21883  21884  21885  21886  21887  21888  21889  21890  21891  21892  21893  21894  21895  21896  21897  21898  21899  21900  21901  21902  21903  21904  21905  21906  21907  21908  21909  21910  21911  21912  21913  21914  21915  21916  21917  21918  21919  21920  21921  21922  21923  21924  21925  21926  21927  21928  21929  21930  21931  21932  21933  21934  21935  21936  21937  21938  21939  21940  21941  21942  21943  21944  21945  21946  21947  21948  21949  21950  21951  21952  21953  21954  21955  21956  21957  21958  21959  21960  21961  21962  21963  21964  21965  21966  21967  21968  21969  21970  21971  21972  21973  21974  21975  21976  21977  21978  21979  21980  21981  21982  21983  21984  21985  21986  21987  21988  21989  21990  21991  21992  21993  21994  21995  21996  21997  21998  21999  22000  22001  22002  22003  22004  22005  22006  22007  22008  22009  22010  22011  22012  22013  22014  22015  22016  22017  22018  22019  22020  22021  22022  22023  22024  22025  22026  22027  22028  22029  22030  22031  22032  22033  22034  22035  22036  22037  22038  22039  22040  22041  22042  22043  22044  22045  22046  22047  22048  22049  22050  22051  22052  22053  22054  22055  22056  22057  22058  22059  22060  22061  22062  22063  22064  22065  22066  22067  22068  22069  22070  22071  22072  22073  22074  22075  22076  22077  22078  22079  22080  22081  22082  22083  22084  22085  22086  22087  22088  22089  22090  22091  22092  22093  22094  22095  22096  22097  22098  22099  22100  22101  22102  22103  22104  22105  22106  22107  22108  22109  22110  22111  22112  22113  22114  22115  22116  22117  22118  22119  22120  22121  22122  22123  22124  22125  22126  22127  22128  22129  22130  22131  22132  22133  22134  22135  22136  22137  22138  22139  22140  22141  22142  22143  22144  22145  22146  22147  22148  22149  22150  22151  22152  22153  22154  22155  22156  22157  22158  22159  22160  22161  22162  22163  22164  22165  22166  22167  22168  22169  22170  22171  22172  22173  22174  22175  22176  22177  22178  22179  22180  22181  22182  22183  22184  22185  22186  22187  22188  22189  22190  22191  22192  22193  22194  22195  22196  22197  22198  22199  22200  22201  22202  22203  22204  22205  22206  22207  22208  22209  22210  22211  22212  22213  22214  22215  22216  22217  22218  22219  22220  22221  22222  22223  22224  22225  22226  22227  22228  22229  22230  22231  22232  22233  22234  22235  22236  22237  22238  22239  22240  22241  22242  22243  22244  22245  22246  22247  22248  22249  22250  22251  22252  22253  22254  22255  22256  22257  22258  22259  22260  22261  22262  22263  22264  22265  22266  22267  22268  22269  22270  22271  22272  22273  22274  22275  22276  22277  22278  22279  22280  22281  22282  22283  22284  22285  22286  22287  22288  22289  22290  22291  22292  22293  22294  22295  22296  22297  22298  22299  22300  22301  22302  22303  22304  22305  22306  22307  22308  22309  22310  22311  22312  22313  22314  22315  22316  22317  22318  22319  22320  22321  22322  22323  22324  22325  22326  22327  22328  22329  22330  22331  22332  22333  22334  22335  22336  22337  22338  22339  22340  22341  22342  22343  22344  22345  22346  22347  22348  22349  22350  22351  22352  22353  22354  22355  22356  22357  22358  22359  22360  22361  22362  22363  22364  22365  22366  22367  22368  22369  22370  22371  22372  22373  22374  22375  22376  22377  22378  22379  22380  22381  22382  22383  22384  22385  22386  22387  22388  22389  22390  22391  22392  22393  22394  22395  22396  22397  22398  22399  22400  22401  22402  22403  22404  22405  22406  22407  22408  22409  22410  22411  22412  22413  22414  22415  22416  22417  22418  22419  22420  22421  22422  22423  22424  22425  22426  22427  22428  22429  22430  22431  22432  22433  22434  22435  22436  22437  22438  22439  22440  22441  22442  22443  22444  22445  22446  22447  22448  22449  22450  22451  22452  22453  22454  22455  22456  22457  22458  22459  22460  22461  22462  22463  22464  22465  22466  22467  22468  22469  22470  22471  22472  22473  22474  22475  22476  22477  22478  22479  22480  22481  22482  22483  22484  22485  22486  22487  22488  22489  22490  22491  22492  22493  22494  22495  22496  22497  22498  22499  22500  22501  22502  22503  22504  22505  22506  22507  22508  22509  22510  22511  22512  22513  22514  22515  22516  22517  22518  22519  22520  22521  22522  22523  22524  22525  22526  22527  22528  22529  22530  22531  22532  22533  22534  22535  22536  22537  22538  22539  22540  22541  22542  22543  22544  22545  22546  22547  22548  22549  22550  22551  22552  22553  22554  22555  22556  22557  22558  22559  22560  22561  22562  22563  22564  22565  22566  22567  22568  22569  22570  22571  22572  22573  22574  22575  22576  22577  22578  22579  22580  22581  22582  22583  22584  22585  22586  22587  22588  22589  22590  22591  22592  22593  22594  22595  22596  22597  22598  22599  22600  22601  22602  22603  22604  22605  22606  22607  22608  22609  22610  22611  22612  22613  22614  22615  22616  22617  22618  22619  22620  22621  22622  22623  22624  22625  22626  22627  22628  22629  22630  22631  22632  22633  22634  22635  22636  22637  22638  22639  22640  22641  22642  22643  22644  22645  22646  22647  22648  22649  22650  22651  22652  22653  22654  22655  22656  22657  22658  22659  22660  22661  22662  22663  22664  22665  22666  22667  22668  22669  22670  22671  22672  22673  22674  22675  22676  22677  22678  22679  22680  22681  22682  22683  22684  22685  22686  22687  22688  22689  22690  22691  22692  22693  22694  22695  22696  22697  22698  22699  22700  22701  22702  22703  22704  22705  22706  22707  22708  22709  22710  22711  22712  22713  22714  22715  22716  22717  22718  22719  22720  22721  22722  22723  22724  22725  22726  22727  22728  22729  22730  22731  22732  22733  22734  22735  22736  22737  22738  22739  22740  22741  22742  22743  22744  22745  22746  22747  22748  22749  22750  22751  22752  22753  22754  22755  22756  22757  22758  22759  22760  22761  22762  22763  22764  22765  22766  22767  22768  22769  22770  22771  22772  22773  22774  22775  22776  22777  22778  22779  22780  22781  22782  22783  22784  22785  22786  22787  22788  22789  22790  22791  22792  22793  22794  22795  22796  22797  22798  22799  22800  22801  22802  22803  22804  22805  22806  22807  22808  22809  22810  22811  22812  22813  22814  22815  22816  22817  22818  22819  22820  22821  22822  22823  22824  22825  22826  22827  22828  22829  22830  22831  22832  22833  22834  22835  22836  22837  22838  22839  22840  22841  22842  22843  22844  22845  22846  22847  22848  22849  22850  22851  22852  22853  22854  22855  22856  22857  22858  22859  22860  22861  22862  22863  22864  22865  22866  22867  22868  22869  22870  22871  22872  22873  22874  22875  22876  22877  22878  22879  22880  22881  22882  22883  22884  22885  22886  22887  22888  22889  22890  22891  22892  22893  22894  22895  22896  22897  22898  22899  22900  22901  22902  22903  22904  22905  22906  22907  22908  22909  22910  22911  22912  22913  22914  22915  22916  22917  22918  22919  22920  22921  22922  22923  22924  22925  22926  22927  22928  22929  22930  22931  22932  22933  22934  22935  22936  22937  22938  22939  22940  22941  22942  22943  22944  22945  22946  22947  22948  22949  22950  22951  22952  22953  22954  22955  22956  22957  22958  22959  22960  22961  22962  22963  22964  22965  22966  22967  22968  22969  22970  22971  22972  22973  22974  22975  22976  22977  22978  22979  22980  22981  22982  22983  22984  22985  22986  22987  22988  22989  22990  22991  22992  22993  22994  22995  22996  22997  22998  22999  23000  23001  23002  23003  23004  23005  23006  23007  23008  23009  23010  23011  23012  23013  23014  23015  23016  23017  23018  23019  23020  23021  23022  23023  23024  23025  23026  23027  23028  23029  23030  23031  23032  23033  23034  23035  23036  23037  23038  23039  23040  23041  23042  23043  23044  23045  23046  23047  23048  23049  23050  23051  23052  23053  23054  23055  23056  23057  23058  23059  23060  23061  23062  23063  23064  23065  23066  23067  23068  23069  23070  23071  23072  23073  23074  23075  23076  23077  23078  23079  23080  23081  23082  23083  23084  23085  23086  23087  23088  23089  23090  23091  23092  23093  23094  23095  23096  23097  23098  23099  23100  23101  23102  23103  23104  23105  23106  23107  23108  23109  23110  23111  23112  23113  23114  23115  23116  23117  23118  23119  23120  23121  23122  23123  23124  23125  23126  23127  23128  23129  23130  23131  23132  23133  23134  23135  23136  23137  23138  23139  23140  23141  23142  23143  23144  23145  23146  23147  23148  23149  23150  23151  23152  23153  23154  23155  23156  23157  23158  23159  23160  23161  23162  23163  23164  23165  23166  23167  23168  23169  23170  23171  23172  23173  23174  23175  23176  23177  23178  23179  23180  23181  23182  23183  23184  23185  23186  23187  23188  23189  23190  23191  23192  23193  23194  23195  23196  23197  23198  23199  23200  23201  23202  23203  23204  23205  23206  23207  23208  23209  23210  23211  23212  23213  23214  23215  23216  23217  23218  23219  23220  23221  23222  23223  23224  23225  23226  23227  23228  23229  23230  23231  23232  23233  23234  23235  23236  23237  23238  23239  23240  23241  23242  23243  23244  23245  23246  23247  23248  23249  23250  23251  23252  23253  23254  23255  23256  23257  23258  23259  23260  23261  23262  23263  23264  23265  23266  23267  23268  23269  23270  23271  23272  23273  23274  23275  23276  23277  23278  23279  23280  23281  23282  23283  23284  23285  23286  23287  23288  23289  23290  23291  23292  23293  23294  23295  23296  23297  23298  23299  23300  23301  23302  23303  23304  23305  23306  23307  23308  23309  23310  23311  23312  23313  23314  23315  23316  23317  23318  23319  23320  23321  23322  23323  23324  23325  23326  23327  23328  23329  23330  23331  23332  23333  23334  23335  23336  23337  23338  23339  23340  23341  23342  23343  23344  23345  23346  23347  23348  23349  23350  23351  23352  23353  23354  23355  23356  23357  23358  23359  23360  23361  23362  23363  23364  23365  23366  23367  23368  23369  23370  23371  23372  23373  23374  23375  23376  23377  23378  23379  23380  23381  23382  23383  23384  23385  23386  23387  23388  23389  23390  23391  23392  23393  23394  23395  23396  23397  23398  23399  23400  23401  23402  23403  23404  23405  23406  23407  23408  23409  23410  23411  23412  23413  23414  23415  23416  23417  23418  23419  23420  23421  23422  23423  23424  23425  23426  23427  23428  23429  23430  23431  23432  23433  23434  23435  23436  23437  23438  23439  23440  23441  23442  23443  23444  23445  23446  23447  23448  23449  23450  23451  23452  23453  23454  23455  23456  23457  23458  23459  23460  23461  23462  23463  23464  23465  23466  23467  23468  23469  23470  23471  23472  23473  23474  23475  23476  23477  23478  23479  23480  23481  23482  23483  23484  23485  23486  23487  23488  23489  23490  23491  23492  23493  23494  23495  23496  23497  23498  23499  23500  23501  23502  23503  23504  23505  23506  23507  23508  23509  23510  23511  23512  23513  23514  23515  23516  23517  23518  23519  23520  23521  23522  23523  23524  23525  23526  23527  23528  23529  23530  23531  23532  23533  23534  23535  23536  23537  23538  23539  23540  23541  23542  23543  23544  23545  23546  23547  23548  23549  23550  23551  23552  23553  23554  23555  23556  23557  23558  23559  23560  23561  23562  23563  23564  23565  23566  23567  23568  23569  23570  23571  23572  23573  23574  23575  23576  23577  23578  23579  23580  23581  23582  23583  23584  23585  23586  23587  23588  23589  23590  23591  23592  23593  23594  23595  23596  23597  23598  23599  23600  23601  23602  23603  23604  23605  23606  23607  23608  23609  23610  23611  23612  23613  23614  23615  23616  23617  23618  23619  23620  23621  23622  23623  23624  23625  23626  23627  23628  23629  23630  23631  23632  23633  23634  23635  23636  23637  23638  23639  23640  23641  23642  23643  23644  23645  23646  23647  23648  23649  23650  23651  23652  23653  23654  23655  23656  23657  23658  23659  23660  23661  23662  23663  23664  23665  23666  23667  23668  23669  23670  23671  23672  23673  23674  23675  23676  23677  23678  23679  23680  23681  23682  23683  23684  23685  23686  23687  23688  23689  23690  23691  23692  23693  23694  23695  23696  23697  23698  23699  23700  23701  23702  23703  23704  23705  23706  23707  23708  23709  23710  23711  23712  23713  23714  23715  23716  23717  23718  23719  23720  23721  23722  23723  23724  23725  23726  23727  23728  23729  23730  23731  23732  23733  23734  23735  23736  23737  23738  23739  23740  23741  23742  23743  23744  23745  23746  23747  23748  23749  23750  23751  23752  23753  23754  23755  23756  23757  23758  23759  23760  23761  23762  23763  23764  23765  23766  23767  23768  23769  23770  23771  23772  23773  23774  23775  23776  23777  23778  23779  23780  23781  23782  23783  23784  23785  23786  23787  23788  23789  23790  23791  23792  23793  23794  23795  23796  23797  23798  23799  23800  23801  23802  23803  23804  23805  23806  23807  23808  23809  23810  23811  23812  23813  23814  23815  23816  23817  23818  23819  23820  23821  23822  23823  23824  23825  23826  23827  23828  23829  23830  23831  23832  23833  23834  23835  23836  23837  23838  23839  23840  23841  23842  23843  23844  23845  23846  23847  23848  23849  23850  23851  23852  23853  23854  23855  23856  23857  23858  23859  23860  23861  23862  23863  23864  23865  23866  23867  23868  23869  23870  23871  23872  23873  23874  23875  23876  23877  23878  23879  23880  23881  23882  23883  23884  23885  23886  23887  23888  23889  23890  23891  23892  23893  23894  23895  23896  23897  23898  23899  23900  23901  23902  23903  23904  23905  23906  23907  23908  23909  23910  23911  23912  23913  23914  23915  23916  23917  23918  23919  23920  23921  23922  23923  23924  23925  23926  23927  23928  23929  23930  23931  23932  23933  23934  23935  23936  23937  23938  23939  23940  23941  23942  23943  23944  23945  23946  23947  23948  23949  23950  23951  23952  23953  23954  23955  23956  23957  23958  23959  23960  23961  23962  23963  23964  23965  23966  23967  23968  23969  23970  23971  23972  23973  23974  23975  23976  23977  23978  23979  23980  23981  23982  23983  23984  23985  23986  23987  23988  23989  23990  23991  23992  23993  23994  23995  23996  23997  23998  23999  24000  24001  24002  24003  24004  24005  24006  24007  24008  24009  24010  24011  24012  24013  24014  24015  24016  24017  24018  24019  24020  24021  24022  24023  24024  24025  24026  24027  24028  24029  24030  24031  24032  24033  24034  24035  24036  24037  24038  24039  24040  24041  24042  24043  24044  24045  24046  24047  24048  24049  24050  24051  24052  24053  24054  24055  24056  24057  24058  24059  24060  24061  24062  24063  24064  24065  24066  24067  24068  24069  24070  24071  24072  24073  24074  24075  24076  24077  24078  24079  24080  24081  24082  24083  24084  24085  24086  24087  24088  24089  24090  24091  24092  24093  24094  24095  24096  24097  24098  24099  24100  24101  24102  24103  24104  24105  24106  24107  24108  24109  24110  24111  24112  24113  24114  24115  24116  24117  24118  24119  24120  24121  24122  24123  24124  24125  24126  24127  24128  24129  24130  24131  24132  24133  24134  24135  24136  24137  24138  24139  24140  24141  24142  24143  24144  24145  24146  24147  24148  24149  24150  24151  24152  24153  24154  24155  24156  24157  24158  24159  24160  24161  24162  24163  24164  24165  24166  24167  24168  24169  24170  24171  24172  24173  24174  24175  24176  24177  24178  24179  24180  24181  24182  24183  24184  24185  24186  24187  24188  24189  24190  24191  24192  24193  24194  24195  24196  24197  24198  24199  24200  24201  24202  24203  24204  24205  24206  24207  24208  24209  24210  24211  24212  24213  24214  24215  24216  24217  24218  24219  24220  24221  24222  24223  24224  24225  24226  24227  24228  24229  24230  24231  24232  24233  24234  24235  24236  24237  24238  24239  24240  24241  24242  24243  24244  24245  24246  24247  24248  24249  24250  24251  24252  24253  24254  24255  24256  24257  24258  24259  24260  24261  24262  24263  24264  24265  24266  24267  24268  24269  24270  24271  24272  24273  24274  24275  24276  24277  24278  24279  24280  24281  24282  24283  24284  24285  24286  24287  24288  24289  24290  24291  24292  24293  24294  24295  24296  24297  24298  24299  24300  24301  24302  24303  24304  24305  24306  24307  24308  24309  24310  24311  24312  24313  24314  24315  24316  24317  24318  24319  24320  24321  24322  24323  24324  24325  24326  24327  24328  24329  24330  24331  24332  24333  24334  24335  24336  24337  24338  24339  24340  24341  24342  24343  24344  24345  24346  24347  24348  24349  24350  24351  24352  24353  24354  24355  24356  24357  24358  24359  24360  24361  24362  24363  24364  24365  24366  24367  24368  24369  24370  24371  24372  24373  24374  24375  24376  24377  24378  24379  24380  24381  24382  24383  24384  24385  24386  24387  24388  24389  24390  24391  24392  24393  24394  24395  24396  24397  24398  24399  24400  24401  24402  24403  24404  24405  24406  24407  24408  24409  24410  24411  24412  24413  24414  24415  24416  24417  24418  24419  24420  24421  24422  24423  24424  24425  24426  24427  24428  24429  24430  24431  24432  24433  24434  24435  24436  24437  24438  24439  24440  24441  24442  24443  24444  24445  24446  24447  24448  24449  24450  24451  24452  24453  24454  24455  24456  24457  24458  24459  24460  24461  24462  24463  24464  24465  24466  24467  24468  24469  24470  24471  24472  24473  24474  24475  24476  24477  24478  24479  24480  24481  24482  24483  24484  24485  24486  24487  24488  24489  24490  24491  24492  24493  24494  24495  24496  24497  24498  24499  24500  24501  24502  24503  24504  24505  24506  24507  24508  24509  24510  24511  24512  24513  24514  24515  24516  24517  24518  24519  24520  24521  24522  24523  24524  24525  24526  24527  24528  24529  24530  24531  24532  24533  24534  24535  24536  24537  24538  24539  24540  24541  24542  24543  24544  24545  24546  24547  24548  24549  24550  24551  24552  24553  24554  24555  24556  24557  24558  24559  24560  24561  24562  24563  24564  24565  24566  24567  24568  24569  24570  24571  24572  24573  24574  24575  24576  24577  24578  24579  24580  24581  24582  24583  24584  24585  24586  24587  24588  24589  24590  24591  24592  24593  24594  24595  24596  24597  24598  24599  24600  24601  24602  24603  24604  24605  24606  24607  24608  24609  24610  24611  24612  24613  24614  24615  24616  24617  24618  24619  24620  24621  24622  24623  24624  24625  24626  24627  24628  24629  24630  24631  24632  24633  24634  24635  24636  24637  24638  24639  24640  24641  24642  24643  24644  24645  24646  24647  24648  24649  24650  24651  24652  24653  24654  24655  24656  24657  24658  24659  24660  24661  24662  24663  24664  24665  24666  24667  24668  24669  24670  24671  24672  24673  24674  24675  24676  24677  24678  24679  24680  24681  24682  24683  24684  24685  24686  24687  24688  24689  24690  24691  24692  24693  24694  24695  24696  24697  24698  24699  24700  24701  24702  24703  24704  24705  24706  24707  24708  24709  24710  24711  24712  24713  24714  24715  24716  24717  24718  24719  24720  24721  24722  24723  24724  24725  24726  24727  24728  24729  24730  24731  24732  24733  24734  24735  24736  24737  24738  24739  24740  24741  24742  24743  24744  24745  24746  24747  24748  24749  24750  24751  24752  24753  24754  24755  24756  24757  24758  24759  24760  24761  24762  24763  24764  24765  24766  24767  24768  24769  24770  24771  24772  24773  24774  24775  24776  24777  24778  24779  24780  24781  24782  24783  24784  24785  24786  24787  24788  24789  24790  24791  24792  24793  24794  24795  24796  24797  24798  24799  24800  24801  24802  24803  24804  24805  24806  24807  24808  24809  24810  24811  24812  24813  24814  24815  24816  24817  24818  24819  24820  24821  24822  24823  24824  24825  24826  24827  24828  24829  24830  24831  24832  24833  24834  24835  24836  24837  24838  24839  24840  24841  24842  24843  24844  24845  24846  24847  24848  24849  24850  24851  24852  24853  24854  24855  24856  24857  24858  24859  24860  24861  24862  24863  24864  24865  24866  24867  24868  24869  24870  24871  24872  24873  24874  24875  24876  24877  24878  24879  24880  24881  24882  24883  24884  24885  24886  24887  24888  24889  24890  24891  24892  24893  24894  24895  24896  24897  24898  24899  24900  24901  24902  24903  24904  24905  24906  24907  24908  24909  24910  24911  24912  24913  24914  24915  24916  24917  24918  24919  24920  24921  24922  24923  24924  24925  24926  24927  24928  24929  24930  24931  24932  24933  24934  24935  24936  24937  24938  24939  24940  24941  24942  24943  24944  24945  24946  24947  24948  24949  24950  24951  24952  24953  24954  24955  24956  24957  24958  24959  24960  24961  24962  24963  24964  24965  24966  24967  24968  24969  24970  24971  24972  24973  24974  24975  24976  24977  24978  24979  24980  24981  24982  24983  24984  24985  24986  24987  24988  24989  24990  24991  24992  24993  24994  24995  24996  24997  24998  24999  25000  25001  25002  25003  25004  25005  25006  25007  25008  25009  25010  25011  25012  25013  25014  25015  25016  25017  25018  25019  25020  25021  25022  25023  25024  25025  25026  25027  25028  25029  25030  25031  25032  25033  25034  25035  25036  25037  25038  25039  25040  25041  25042  25043  25044  25045  25046  25047  25048  25049  25050  25051  25052  25053  25054  25055  25056  25057  25058  25059  25060  25061  25062  25063  25064  25065  25066  25067  25068  25069  25070  25071  25072  25073  25074  25075  25076  25077  25078  25079  25080  25081  25082  25083  25084  25085  25086  25087  25088  25089  25090  25091  25092  25093  25094  25095  25096  25097  25098  25099  25100  25101  25102  25103  25104  25105  25106  25107  25108  25109  25110  25111  25112  25113  25114  25115  25116  25117  25118  25119  25120  25121  25122  25123  25124  25125  25126  25127  25128  25129  25130  25131  25132  25133  25134  25135  25136  25137  25138  25139  25140  25141  25142  25143  25144  25145  25146  25147  25148  25149  25150  25151  25152  25153  25154  25155  25156  25157  25158  25159  25160  25161  25162  25163  25164  25165  25166  25167  25168  25169  25170  25171  25172  25173  25174  25175  25176  25177  25178  25179  25180  25181  25182  25183  25184  25185  25186  25187  25188  25189  25190  25191  25192  25193  25194  25195  25196  25197  25198  25199  25200  25201  25202  25203  25204  25205  25206  25207  25208  25209  25210  25211  25212  25213  25214  25215  25216  25217  25218  25219  25220  25221  25222  25223  25224  25225  25226  25227  25228  25229  25230  25231  25232  25233  25234  25235  25236  25237  25238  25239  25240  25241  25242  25243  25244  25245  25246  25247  25248  25249  25250  25251  25252  25253  25254  25255  25256  25257  25258  25259  25260  25261  25262  25263  25264  25265  25266  25267  25268  25269  25270  25271  25272  25273  25274  25275  25276  25277  25278  25279  25280  25281  25282  25283  25284  25285  25286  25287  25288  25289  25290  25291  25292  25293  25294  25295  25296  25297  25298  25299  25300  25301  25302  25303  25304  25305  25306  25307  25308  25309  25310  25311  25312  25313  25314  25315  25316  25317  25318  25319  25320  25321  25322  25323  25324  25325  25326  25327  25328  25329  25330  25331  25332  25333  25334  25335  25336  25337  25338  25339  25340  25341  25342  25343  25344  25345  25346  25347  25348  25349  25350  25351  25352  25353  25354  25355  25356  25357  25358  25359  25360  25361  25362  25363  25364  25365  25366  25367  25368  25369  25370  25371  25372  25373  25374  25375  25376  25377  25378  25379  25380  25381  25382  25383  25384  25385  25386  25387  25388  25389  25390  25391  25392  25393  25394  25395  25396  25397  25398  25399  25400  25401  25402  25403  25404  25405  25406  25407  25408  25409  25410  25411  25412  25413  25414  25415  25416  25417  25418  25419  25420  25421  25422  25423  25424  25425  25426  25427  25428  25429  25430  25431  25432  25433  25434  25435  25436  25437  25438  25439  25440  25441  25442  25443  25444  25445  25446  25447  25448  25449  25450  25451  25452  25453  25454  25455  25456  25457  25458  25459  25460  25461  25462  25463  25464  25465  25466  25467  25468  25469  25470  25471  25472  25473  25474  25475  25476  25477  25478  25479  25480  25481  25482  25483  25484  25485  25486  25487  25488  25489  25490  25491  25492  25493  25494  25495  25496  25497  25498  25499  25500  25501  25502  25503  25504  25505  25506  25507  25508  25509  25510  25511  25512  25513  25514  25515  25516  25517  25518  25519  25520  25521  25522  25523  25524  25525  25526  25527  25528  25529  25530  25531  25532  25533  25534  25535  25536  25537  25538  25539  25540  25541  25542  25543  25544  25545  25546  25547  25548  25549  25550  25551  25552  25553  25554  25555  25556  25557  25558  25559  25560  25561  25562  25563  25564  25565  25566  25567  25568  25569  25570  25571  25572  25573  25574  25575  25576  25577  25578  25579  25580  25581  25582  25583  25584  25585  25586  25587  25588  25589  25590  25591  25592  25593  25594  25595  25596  25597  25598  25599  25600  25601  25602  25603  25604  25605  25606  25607  25608  25609  25610  25611  25612  25613  25614  25615  25616  25617  25618  25619  25620  25621  25622  25623  25624  25625  25626  25627  25628  25629  25630  25631  25632  25633  25634  25635  25636  25637  25638  25639  25640  25641  25642  25643  25644  25645  25646  25647  25648  25649  25650  25651  25652  25653  25654  25655  25656  25657  25658  25659  25660  25661  25662  25663  25664  25665  25666  25667  25668  25669  25670  25671  25672  25673  25674  25675  25676  25677  25678  25679  25680  25681  25682  25683  25684  25685  25686  25687  25688  25689  25690  25691  25692  25693  25694  25695  25696  25697  25698  25699  25700  25701  25702  25703  25704  25705  25706  25707  25708  25709  25710  25711  25712  25713  25714  25715  25716  25717  25718  25719  25720  25721  25722  25723  25724  25725  25726  25727  25728  25729  25730  25731  25732  25733  25734  25735  25736  25737  25738  25739  25740  25741  25742  25743  25744  25745  25746  25747  25748  25749  25750  25751  25752  25753  25754  25755  25756  25757  25758  25759  25760  25761  25762  25763  25764  25765  25766  25767  25768  25769  25770  25771  25772  25773  25774  25775  25776  25777  25778  25779  25780  25781  25782  25783  25784  25785  25786  25787  25788  25789  25790  25791  25792  25793  25794  25795  25796  25797  25798  25799  25800  25801  25802  25803  25804  25805  25806  25807  25808  25809  25810  25811  25812  25813  25814  25815  25816  25817  25818  25819  25820  25821  25822  25823  25824  25825  25826  25827  25828  25829  25830  25831  25832  25833  25834  25835  25836  25837  25838  25839  25840  25841  25842  25843  25844  25845  25846  25847  25848  25849  25850  25851  25852  25853  25854  25855  25856  25857  25858  25859  25860  25861  25862  25863  25864  25865  25866  25867  25868  25869  25870  25871  25872  25873  25874  25875  25876  25877  25878  25879  25880  25881  25882  25883  25884  25885  25886  25887  25888  25889  25890  25891  25892  25893  25894  25895  25896  25897  25898  25899  25900  25901  25902  25903  25904  25905  25906  25907  25908  25909  25910  25911  25912  25913  25914  25915  25916  25917  25918  25919  25920  25921  25922  25923  25924  25925  25926  25927  25928  25929  25930  25931  25932  25933  25934  25935  25936  25937  25938  25939  25940  25941  25942  25943  25944  25945  25946  25947  25948  25949  25950  25951  25952  25953  25954  25955  25956  25957  25958  25959  25960  25961  25962  25963  25964  25965  25966  25967  25968  25969  25970  25971  25972  25973  25974  25975  25976  25977  25978  25979  25980  25981  25982  25983  25984  25985  25986  25987  25988  25989  25990  25991  25992  25993  25994  25995  25996  25997  25998  25999  26000  26001  26002  26003  26004  26005  26006  26007  26008  26009  26010  26011  26012  26013  26014  26015  26016  26017  26018  26019  26020  26021  26022  26023  26024  26025  26026  26027  26028  26029  26030  26031  26032  26033  26034  26035  26036  26037  26038  26039  26040  26041  26042  26043  26044  26045  26046  26047  26048  26049  26050  26051  26052  26053  26054  26055  26056  26057  26058  26059  26060  26061  26062  26063  26064  26065  26066  26067  26068  26069  26070  26071  26072  26073  26074  26075  26076  26077  26078  26079  26080  26081  26082  26083  26084  26085  26086  26087  26088  26089  26090  26091  26092  26093  26094  26095  26096  26097  26098  26099  26100  26101  26102  26103  26104  26105  26106  26107  26108  26109  26110  26111  26112  26113  26114  26115  26116  26117  26118  26119  26120  26121  26122  26123  26124  26125  26126  26127  26128  26129  26130  26131  26132  26133  26134  26135  26136  26137  26138  26139  26140  26141  26142  26143  26144  26145  26146  26147  26148  26149  26150  26151  26152  26153  26154  26155  26156  26157  26158  26159  26160  26161  26162  26163  26164  26165  26166  26167  26168  26169  26170  26171  26172  26173  26174  26175  26176  26177  26178  26179  26180  26181  26182  26183  26184  26185  26186  26187  26188  26189  26190  26191  26192  26193  26194  26195  26196  26197  26198  26199  26200  26201  26202  26203  26204  26205  26206  26207  26208  26209  26210  26211  26212  26213  26214  26215  26216  26217  26218  26219  26220  26221  26222  26223  26224  26225  26226  26227  26228  26229  26230  26231  26232  26233  26234  26235  26236  26237  26238  26239  26240  26241  26242  26243  26244  26245  26246  26247  26248  26249  26250  26251  26252  26253  26254  26255  26256  26257  26258  26259  26260  26261  26262  26263  26264  26265  26266  26267  26268  26269  26270  26271  26272  26273  26274  26275  26276  26277  26278  26279  26280  26281  26282  26283  26284  26285  26286  26287  26288  26289  26290  26291  26292  26293  26294  26295  26296  26297  26298  26299  26300  26301  26302  26303  26304  26305  26306  26307  26308  26309  26310  26311  26312  26313  26314  26315  26316  26317  26318  26319  26320  26321  26322  26323  26324  26325  26326  26327  26328  26329  26330  26331  26332  26333  26334  26335  26336  26337  26338  26339  26340  26341  26342  26343  26344  26345  26346  26347  26348  26349  26350  26351  26352  26353  26354  26355  26356  26357  26358  26359  26360  26361  26362  26363  26364  26365  26366  26367  26368  26369  26370  26371  26372  26373  26374  26375  26376  26377  26378  26379  26380  26381  26382  26383  26384  26385  26386  26387  26388  26389  26390  26391  26392  26393  26394  26395  26396  26397  26398  26399  26400  26401  26402  26403  26404  26405  26406  26407  26408  26409  26410  26411  26412  26413  26414  26415  26416  26417  26418  26419  26420  26421  26422  26423  26424  26425  26426  26427  26428  26429  26430  26431  26432  26433  26434  26435  26436  26437  26438  26439  26440  26441  26442  26443  26444  26445  26446  26447  26448  26449  26450  26451  26452  26453  26454  26455  26456  26457  26458  26459  26460  26461  26462  26463  26464  26465  26466  26467  26468  26469  26470  26471  26472  26473  26474  26475  26476  26477  26478  26479  26480  26481  26482  26483  26484  26485  26486  26487  26488  26489  26490  26491  26492  26493  26494  26495  26496  26497  26498  26499  26500  26501  26502  26503  26504  26505  26506  26507  26508  26509  26510  26511  26512  26513  26514  26515  26516  26517  26518  26519  26520  26521  26522  26523  26524  26525  26526  26527  26528  26529  26530  26531  26532  26533  26534  26535  26536  26537  26538  26539  26540  26541  26542  26543  26544  26545  26546  26547  26548  26549  26550  26551  26552  26553  26554  26555  26556  26557  26558  26559  26560  26561  26562  26563  26564  26565  26566  26567  26568  26569  26570  26571  26572  26573  26574  26575  26576  26577  26578  26579  26580  26581  26582  26583  26584  26585  26586  26587  26588  26589  26590  26591  26592  26593  26594  26595  26596  26597  26598  26599  26600  26601  26602  26603  26604  26605  26606  26607  26608  26609  26610  26611  26612  26613  26614  26615  26616  26617  26618  26619  26620  26621  26622  26623  26624  26625  26626  26627  26628  26629  26630  26631  26632  26633  26634  26635  26636  26637  26638  26639  26640  26641  26642  26643  26644  26645  26646  26647  26648  26649  26650  26651  26652  26653  26654  26655  26656  26657  26658  26659  26660  26661  26662  26663  26664  26665  26666  26667  26668  26669  26670  26671  26672  26673  26674  26675  26676  26677  26678  26679  26680  26681  26682  26683  26684  26685  26686  26687  26688  26689  26690  26691  26692  26693  26694  26695  26696  26697  26698  26699  26700  26701  26702  26703  26704  26705  26706  26707  26708  26709  26710  26711  26712  26713  26714  26715  26716  26717  26718  26719  26720  26721  26722  26723  26724  26725  26726  26727  26728  26729  26730  26731  26732  26733  26734  26735  26736  26737  26738  26739  26740  26741  26742  26743  26744  26745  26746  26747  26748  26749  26750  26751  26752  26753  26754  26755  26756  26757  26758  26759  26760  26761  26762  26763  26764  26765  26766  26767  26768  26769  26770  26771  26772  26773  26774  26775  26776  26777  26778  26779  26780  26781  26782  26783  26784  26785  26786  26787  26788  26789  26790  26791  26792  26793  26794  26795  26796  26797  26798  26799  26800  26801  26802  26803  26804  26805  26806  26807  26808  26809  26810  26811  26812  26813  26814  26815  26816  26817  26818  26819  26820  26821  26822  26823  26824  26825  26826  26827  26828  26829  26830  26831  26832  26833  26834  26835  26836  26837  26838  26839  26840  26841  26842  26843  26844  26845  26846  26847  26848  26849  26850  26851  26852  26853  26854  26855  26856  26857  26858  26859  26860  26861  26862  26863  26864  26865  26866  26867  26868  26869  26870  26871  26872  26873  26874  26875  26876  26877  26878  26879  26880  26881  26882  26883  26884  26885  26886  26887  26888  26889  26890  26891  26892  26893  26894  26895  26896  26897  26898  26899  26900  26901  26902  26903  26904  26905  26906  26907  26908  26909  26910  26911  26912  26913  26914  26915  26916  26917  26918  26919  26920  26921  26922  26923  26924  26925  26926  26927  26928  26929  26930  26931  26932  26933  26934  26935  26936  26937  26938  26939  26940  26941  26942  26943  26944  26945  26946  26947  26948  26949  26950  26951  26952  26953  26954  26955  26956  26957  26958  26959  26960  26961  26962  26963  26964  26965  26966  26967  26968  26969  26970  26971  26972  26973  26974  26975  26976  26977  26978  26979  26980  26981  26982  26983  26984  26985  26986  26987  26988  26989  26990  26991  26992  26993  26994  26995  26996  26997  26998  26999  27000  27001  27002  27003  27004  27005  27006  27007  27008  27009  27010  27011  27012  27013  27014  27015  27016  27017  27018  27019  27020  27021  27022  27023  27024  27025  27026  27027  27028  27029  27030  27031  27032  27033  27034  27035  27036  27037  27038  27039  27040  27041  27042  27043  27044  27045  27046  27047  27048  27049  27050  27051  27052  27053  27054  27055  27056  27057  27058  27059  27060  27061  27062  27063  27064  27065  27066  27067  27068  27069  27070  27071  27072  27073  27074  27075  27076  27077  27078  27079  27080  27081  27082  27083  27084  27085  27086  27087  27088  27089  27090  27091  27092  27093  27094  27095  27096  27097  27098  27099  27100  27101  27102  27103  27104  27105  27106  27107  27108  27109  27110  27111  27112  27113  27114  27115  27116  27117  27118  27119  27120  27121  27122  27123  27124  27125  27126  27127  27128  27129  27130  27131  27132  27133  27134  27135  27136  27137  27138  27139  27140  27141  27142  27143  27144  27145  27146  27147  27148  27149  27150  27151  27152  27153  27154  27155  27156  27157  27158  27159  27160  27161  27162  27163  27164  27165  27166  27167  27168  27169  27170  27171  27172  27173  27174  27175  27176  27177  27178  27179  27180  27181  27182  27183  27184  27185  27186  27187  27188  27189  27190  27191  27192  27193  27194  27195  27196  27197  27198  27199  27200  27201  27202  27203  27204  27205  27206  27207  27208  27209  27210  27211  27212  27213  27214  27215  27216  27217  27218  27219  27220  27221  27222  27223  27224  27225  27226  27227  27228  27229  27230  27231  27232  27233  27234  27235  27236  27237  27238  27239  27240  27241  27242  27243  27244  27245  27246  27247  27248  27249  27250  27251  27252  27253  27254  27255  27256  27257  27258  27259  27260  27261  27262  27263  27264  27265  27266  27267  27268  27269  27270  27271  27272  27273  27274  27275  27276  27277  27278  27279  27280  27281  27282  27283  27284  27285  27286  27287  27288  27289  27290  27291  27292  27293  27294  27295  27296  27297  27298  27299  27300  27301  27302  27303  27304  27305  27306  27307  27308  27309  27310  27311  27312  27313  27314  27315  27316  27317  27318  27319  27320  27321  27322  27323  27324  27325  27326  27327  27328  27329  27330  27331  27332  27333  27334  27335  27336  27337  27338  27339  27340  27341  27342  27343  27344  27345  27346  27347  27348  27349  27350  27351  27352  27353  27354  27355  27356  27357  27358  27359  27360  27361  27362  27363  27364  27365  27366  27367  27368  27369  27370  27371  27372  27373  27374  27375  27376  27377  27378  27379  27380  27381  27382  27383  27384  27385  27386  27387  27388  27389  27390  27391  27392  27393  27394  27395  27396  27397  27398  27399  27400  27401  27402  27403  27404  27405  27406  27407  27408  27409  27410  27411  27412  27413  27414  27415  27416  27417  27418  27419  27420  27421  27422  27423  27424  27425  27426  27427  27428  27429  27430  27431  27432  27433  27434  27435  27436  27437  27438  27439  27440  27441  27442  27443  27444  27445  27446  27447  27448  27449  27450  27451  27452  27453  27454  27455  27456  27457  27458  27459  27460  27461  27462  27463  27464  27465  27466  27467  27468  27469  27470  27471  27472  27473  27474  27475  27476  27477  27478  27479  27480  27481  27482  27483  27484  27485  27486  27487  27488  27489  27490  27491  27492  27493  27494  27495  27496  27497  27498  27499  27500  27501  27502  27503  27504  27505  27506  27507  27508  27509  27510  27511  27512  27513  27514  27515  27516  27517  27518  27519  27520  27521  27522  27523  27524  27525  27526  27527  27528  27529  27530  27531  27532  27533  27534  27535  27536  27537  27538  27539  27540  27541  27542  27543  27544  27545  27546  27547  27548  27549  27550  27551  27552  27553  27554  27555  27556  27557  27558  27559  27560  27561  27562  27563  27564  27565  27566  27567  27568  27569  27570  27571  27572  27573  27574  27575  27576  27577  27578  27579  27580  27581  27582  27583  27584  27585  27586  27587  27588  27589  27590  27591  27592  27593  27594  27595  27596  27597  27598  27599  27600  27601  27602  27603  27604  27605  27606  27607  27608  27609  27610  27611  27612  27613  27614  27615  27616  27617  27618  27619  27620  27621  27622  27623  27624  27625  27626  27627  27628  27629  27630  27631  27632  27633  27634  27635  27636  27637  27638  27639  27640  27641  27642  27643  27644  27645  27646  27647  27648  27649  27650  27651  27652  27653  27654  27655  27656  27657  27658  27659  27660  27661  27662  27663  27664  27665  27666  27667  27668  27669  27670  27671  27672  27673  27674  27675  27676  27677  27678  27679  27680  27681  27682  27683  27684  27685  27686  27687  27688  27689  27690  27691  27692  27693  27694  27695  27696  27697  27698  27699  27700  27701  27702  27703  27704  27705  27706  27707  27708  27709  27710  27711  27712  27713  27714  27715  27716  27717  27718  27719  27720  27721  27722  27723  27724  27725  27726  27727  27728  27729  27730  27731  27732  27733  27734  27735  27736  27737  27738  27739  27740  27741  27742  27743  27744  27745  27746  27747  27748  27749  27750  27751  27752  27753  27754  27755  27756  27757  27758  27759  27760  27761  27762  27763  27764  27765  27766  27767  27768  27769  27770  27771  27772  27773  27774  27775  27776  27777  27778  27779  27780  27781  27782  27783  27784  27785  27786  27787  27788  27789  27790  27791  27792  27793  27794  27795  27796  27797  27798  27799  27800  27801  27802  27803  27804  27805  27806  27807  27808  27809  27810  27811  27812  27813  27814  27815  27816  27817  27818  27819  27820  27821  27822  27823  27824  27825  27826  27827  27828  27829  27830  27831  27832  27833  27834  27835  27836  27837  27838  27839  27840  27841  27842  27843  27844  27845  27846  27847  27848  27849  27850  27851  27852  27853  27854  27855  27856  27857  27858  27859  27860  27861  27862  27863  27864  27865  27866  27867  27868  27869  27870  27871  27872  27873  27874  27875  27876  27877  27878  27879  27880  27881  27882  27883  27884  27885  27886  27887  27888  27889  27890  27891  27892  27893  27894  27895  27896  27897  27898  27899  27900  27901  27902  27903  27904  27905  27906  27907  27908  27909  27910  27911  27912  27913  27914  27915  27916  27917  27918  27919  27920  27921  27922  27923  27924  27925  27926  27927  27928  27929  27930  27931  27932  27933  27934  27935  27936  27937  27938  27939  27940  27941  27942  27943  27944  27945  27946  27947  27948  27949  27950  27951  27952  27953  27954  27955  27956  27957  27958  27959  27960  27961  27962  27963  27964  27965  27966  27967  27968  27969  27970  27971  27972  27973  27974  27975  27976  27977  27978  27979  27980  27981  27982  27983  27984  27985  27986  27987  27988  27989  27990  27991  27992  27993  27994  27995  27996  27997  27998  27999  28000  28001  28002  28003  28004  28005  28006  28007  28008  28009  28010  28011  28012  28013  28014  28015  28016  28017  28018  28019  28020  28021  28022  28023  28024  28025  28026  28027  28028  28029  28030  28031  28032  28033  28034  28035  28036  28037  28038  28039  28040  28041  28042  28043  28044  28045  28046  28047  28048  28049  28050  28051  28052  28053  28054  28055  28056  28057  28058  28059  28060  28061  28062  28063  28064  28065  28066  28067  28068  28069  28070  28071  28072  28073  28074  28075  28076  28077  28078  28079  28080  28081  28082  28083  28084  28085  28086  28087  28088  28089  28090  28091  28092  28093  28094  28095  28096  28097  28098  28099  28100  28101  28102  28103  28104  28105  28106  28107  28108  28109  28110  28111  28112  28113  28114  28115  28116  28117  28118  28119  28120  28121  28122  28123  28124  28125  28126  28127  28128  28129  28130  28131  28132  28133  28134  28135  28136  28137  28138  28139  28140  28141  28142  28143  28144  28145  28146  28147  28148  28149  28150  28151  28152  28153  28154  28155  28156  28157  28158  28159  28160  28161  28162  28163  28164  28165  28166  28167  28168  28169  28170  28171  28172  28173  28174  28175  28176  28177  28178  28179  28180  28181  28182  28183  28184  28185  28186  28187  28188  28189  28190  28191  28192  28193  28194  28195  28196  28197  28198  28199  28200  28201  28202  28203  28204  28205  28206  28207  28208  28209  28210  28211  28212  28213  28214  28215  28216  28217  28218  28219  28220  28221  28222  28223  28224  28225  28226  28227  28228  28229  28230  28231  28232  28233  28234  28235  28236  28237  28238  28239  28240  28241  28242  28243  28244  28245  28246  28247  28248  28249  28250  28251  28252  28253  28254  28255  28256  28257  28258  28259  28260  28261  28262  28263  28264  28265  28266  28267  28268  28269  28270  28271  28272  28273  28274  28275  28276  28277  28278  28279  28280  28281  28282  28283  28284  28285  28286  28287  28288  28289  28290  28291  28292  28293  28294  28295  28296  28297  28298  28299  28300  28301  28302  28303  28304  28305  28306  28307  28308  28309  28310  28311  28312  28313  28314  28315  28316  28317  28318  28319  28320  28321  28322  28323  28324  28325  28326  28327  28328  28329  28330  28331  28332  28333  28334  28335  28336  28337  28338  28339  28340  28341  28342  28343  28344  28345  28346  28347  28348  28349  28350  28351  28352  28353  28354  28355  28356  28357  28358  28359  28360  28361  28362  28363  28364  28365  28366  28367  28368  28369  28370  28371  28372  28373  28374  28375  28376  28377  28378  28379  28380  28381  28382  28383  28384  28385  28386  28387  28388  28389  28390  28391  28392  28393  28394  28395  28396  28397  28398  28399  28400  28401  28402  28403  28404  28405  28406  28407  28408  28409  28410  28411  28412  28413  28414  28415  28416  28417  28418  28419  28420  28421  28422  28423  28424  28425  28426  28427  28428  28429  28430  28431  28432  28433  28434  28435  28436  28437  28438  28439  28440  28441  28442  28443  28444  28445  28446  28447  28448  28449  28450  28451  28452  28453  28454  28455  28456  28457  28458  28459  28460  28461  28462  28463  28464  28465  28466  28467  28468  28469  28470  28471  28472  28473  28474  28475  28476  28477  28478  28479  28480  28481  28482  28483  28484  28485  28486  28487  28488  28489  28490  28491  28492  28493  28494  28495  28496  28497  28498  28499  28500  28501  28502  28503  28504  28505  28506  28507  28508  28509  28510  28511  28512  28513  28514  28515  28516  28517  28518  28519  28520  28521  28522  28523  28524  28525  28526  28527  28528  28529  28530  28531  28532  28533  28534  28535  28536  28537  28538  28539  28540  28541  28542  28543  28544  28545  28546  28547  28548  28549  28550  28551  28552  28553  28554  28555  28556  28557  28558  28559  28560  28561  28562  28563  28564  28565  28566  28567  28568  28569  28570  28571  28572  28573  28574  28575  28576  28577  28578  28579  28580  28581  28582  28583  28584  28585  28586  28587  28588  28589  28590  28591  28592  28593  28594  28595  28596  28597  28598  28599  28600  28601  28602  28603  28604  28605  28606  28607  28608  28609  28610  28611  28612  28613  28614  28615  28616  28617  28618  28619  28620  28621  28622  28623  28624  28625  28626  28627  28628  28629  28630  28631  28632  28633  28634  28635  28636  28637  28638  28639  28640  28641  28642  28643  28644  28645  28646  28647  28648  28649  28650  28651  28652  28653  28654  28655  28656  28657  28658  28659  28660  28661  28662  28663  28664  28665  28666  28667  28668  28669  28670  28671  28672  28673  28674  28675  28676  28677  28678  28679  28680  28681  28682  28683  28684  28685  28686  28687  28688  28689  28690  28691  28692  28693  28694  28695  28696  28697  28698  28699  28700  28701  28702  28703  28704  28705  28706  28707  28708  28709  28710  28711  28712  28713  28714  28715  28716  28717  28718  28719  28720  28721  28722  28723  28724  28725  28726  28727  28728  28729  28730  28731  28732  28733  28734  28735  28736  28737  28738  28739  28740  28741  28742  28743  28744  28745  28746  28747  28748  28749  28750  28751  28752  28753  28754  28755  28756  28757  28758  28759  28760  28761  28762  28763  28764  28765  28766  28767  28768  28769  28770  28771  28772  28773  28774  28775  28776  28777  28778  28779  28780  28781  28782  28783  28784  28785  28786  28787  28788  28789  28790  28791  28792  28793  28794  28795  28796  28797  28798  28799  28800  28801  28802  28803  28804  28805  28806  28807  28808  28809  28810  28811  28812  28813  28814  28815  28816  28817  28818  28819  28820  28821  28822  28823  28824  28825  28826  28827  28828  28829  28830  28831  28832  28833  28834  28835  28836  28837  28838  28839  28840  28841  28842  28843  28844  28845  28846  28847  28848  28849  28850  28851  28852  28853  28854  28855  28856  28857  28858  28859  28860  28861  28862  28863  28864  28865  28866  28867  28868  28869  28870  28871  28872  28873  28874  28875  28876  28877  28878  28879  28880  28881  28882  28883  28884  28885  28886  28887  28888  28889  28890  28891  28892  28893  28894  28895  28896  28897  28898  28899  28900  28901  28902  28903  28904  28905  28906  28907  28908  28909  28910  28911  28912  28913  28914  28915  28916  28917  28918  28919  28920  28921  28922  28923  28924  28925  28926  28927  28928  28929  28930  28931  28932  28933  28934  28935  28936  28937  28938  28939  28940  28941  28942  28943  28944  28945  28946  28947  28948  28949  28950  28951  28952  28953  28954  28955  28956  28957  28958  28959  28960  28961  28962  28963  28964  28965  28966  28967  28968  28969  28970  28971  28972  28973  28974  28975  28976  28977  28978  28979  28980  28981  28982  28983  28984  28985  28986  28987  28988  28989  28990  28991  28992  28993  28994  28995  28996  28997  28998  28999  29000  29001  29002  29003  29004  29005  29006  29007  29008  29009  29010  29011  29012  29013  29014  29015  29016  29017  29018  29019  29020  29021  29022  29023  29024  29025  29026  29027  29028  29029  29030  29031  29032  29033  29034  29035  29036  29037  29038  29039  29040  29041  29042  29043  29044  29045  29046  29047  29048  29049  29050  29051  29052  29053  29054  29055  29056  29057  29058  29059  29060  29061  29062  29063  29064  29065  29066  29067  29068  29069  29070  29071  29072  29073  29074  29075  29076  29077  29078  29079  29080  29081  29082  29083  29084  29085  29086  29087  29088  29089  29090  29091  29092  29093  29094  29095  29096  29097  29098  29099  29100  29101  29102  29103  29104  29105  29106  29107  29108  29109  29110  29111  29112  29113  29114  29115  29116  29117  29118  29119  29120  29121  29122  29123  29124  29125  29126  29127  29128  29129  29130  29131  29132  29133  29134  29135  29136  29137  29138  29139  29140  29141  29142  29143  29144  29145  29146  29147  29148  29149  29150  29151  29152  29153  29154  29155  29156  29157  29158  29159  29160  29161  29162  29163  29164  29165  29166  29167  29168  29169  29170  29171  29172  29173  29174  29175  29176  29177  29178  29179  29180  29181  29182  29183  29184  29185  29186  29187  29188  29189  29190  29191  29192  29193  29194  29195  29196  29197  29198  29199  29200  29201  29202  29203  29204  29205  29206  29207  29208  29209  29210  29211  29212  29213  29214  29215  29216  29217  29218  29219  29220  29221  29222  29223  29224  29225  29226  29227  29228  29229  29230  29231  29232  29233  29234  29235  29236  29237  29238  29239  29240  29241  29242  29243  29244  29245  29246  29247  29248  29249  29250  29251  29252  29253  29254  29255  29256  29257  29258  29259  29260  29261  29262  29263  29264  29265  29266  29267  29268  29269  29270  29271  29272  29273  29274  29275  29276  29277  29278  29279  29280  29281  29282  29283  29284  29285  29286  29287  29288  29289  29290  29291  29292  29293  29294  29295  29296  29297  29298  29299  29300  29301  29302  29303  29304  29305  29306  29307  29308  29309  29310  29311  29312  29313  29314  29315  29316  29317  29318  29319  29320  29321  29322  29323  29324  29325  29326  29327  29328  29329  29330  29331  29332  29333  29334  29335  29336  29337  29338  29339  29340  29341  29342  29343  29344  29345  29346  29347  29348  29349  29350  29351  29352  29353  29354  29355  29356  29357  29358  29359  29360  29361  29362  29363  29364  29365  29366  29367  29368  29369  29370  29371  29372  29373  29374  29375  29376  29377  29378  29379  29380  29381  29382  29383  29384  29385  29386  29387  29388  29389  29390  29391  29392  29393  29394  29395  29396  29397  29398  29399  29400  29401  29402  29403  29404  29405  29406  29407  29408  29409  29410  29411  29412  29413  29414  29415  29416  29417  29418  29419  29420  29421  29422  29423  29424  29425  29426  29427  29428  29429  29430  29431  29432  29433  29434  29435  29436  29437  29438  29439  29440  29441  29442  29443  29444  29445  29446  29447  29448  29449  29450  29451  29452  29453  29454  29455  29456  29457  29458  29459  29460  29461  29462  29463  29464  29465  29466  29467  29468  29469  29470  29471  29472  29473  29474  29475  29476  29477  29478  29479  29480  29481  29482  29483  29484  29485  29486  29487  29488  29489  29490  29491  29492  29493  29494  29495  29496  29497  29498  29499  29500  29501  29502  29503  29504  29505  29506  29507  29508  29509  29510  29511  29512  29513  29514  29515  29516  29517  29518  29519  29520  29521  29522  29523  29524  29525  29526  29527  29528  29529  29530  29531  29532  29533  29534  29535  29536  29537  29538  29539  29540  29541  29542  29543  29544  29545  29546  29547  29548  29549  29550  29551  29552  29553  29554  29555  29556  29557  29558  29559  29560  29561  29562  29563  29564  29565  29566  29567  29568  29569  29570  29571  29572  29573  29574  29575  29576  29577  29578  29579  29580  29581  29582  29583  29584  29585  29586  29587  29588  29589  29590  29591  29592  29593  29594  29595  29596  29597  29598  29599  29600  29601  29602  29603  29604  29605  29606  29607  29608  29609  29610  29611  29612  29613  29614  29615  29616  29617  29618  29619  29620  29621  29622  29623  29624  29625  29626  29627  29628  29629  29630  29631  29632  29633  29634  29635  29636  29637  29638  29639  29640  29641  29642  29643  29644  29645  29646  29647  29648  29649  29650  29651  29652  29653  29654  29655  29656  29657  29658  29659  29660  29661  29662  29663  29664  29665  29666  29667  29668  29669  29670  29671  29672  29673  29674  29675  29676  29677  29678  29679  29680  29681  29682  29683  29684  29685  29686  29687  29688  29689  29690  29691  29692  29693  29694  29695  29696  29697  29698  29699  29700  29701  29702  29703  29704  29705  29706  29707  29708  29709  29710  29711  29712  29713  29714  29715  29716  29717  29718  29719  29720  29721  29722  29723  29724  29725  29726  29727  29728  29729  29730  29731  29732  29733  29734  29735  29736  29737  29738  29739  29740  29741  29742  29743  29744  29745  29746  29747  29748  29749  29750  29751  29752  29753  29754  29755  29756  29757  29758  29759  29760  29761  29762  29763  29764  29765  29766  29767  29768  29769  29770  29771  29772  29773  29774  29775  29776  29777  29778  29779  29780  29781  29782  29783  29784  29785  29786  29787  29788  29789  29790  29791  29792  29793  29794  29795  29796  29797  29798  29799  29800  29801  29802  29803  29804  29805  29806  29807  29808  29809  29810  29811  29812  29813  29814  29815  29816  29817  29818  29819  29820  29821  29822  29823  29824  29825  29826  29827  29828  29829  29830  29831  29832  29833  29834  29835  29836  29837  29838  29839  29840  29841  29842  29843  29844  29845  29846  29847  29848  29849  29850  29851  29852  29853  29854  29855  29856  29857  29858  29859  29860  29861  29862  29863  29864  29865  29866  29867  29868  29869  29870  29871  29872  29873  29874  29875  29876  29877  29878  29879  29880  29881  29882  29883  29884  29885  29886  29887  29888  29889  29890  29891  29892  29893  29894  29895  29896  29897  29898  29899  29900  29901  29902  29903  29904  29905  29906  29907  29908  29909  29910  29911  29912  29913  29914  29915  29916  29917  29918  29919  29920  29921  29922  29923  29924  29925  29926  29927  29928  29929  29930  29931  29932  29933  29934  29935  29936  29937  29938  29939  29940  29941  29942  29943  29944  29945  29946  29947  29948  29949  29950  29951  29952  29953  29954  29955  29956  29957  29958  29959  29960  29961  29962  29963  29964  29965  29966  29967  29968  29969  29970  29971  29972  29973  29974  29975  29976  29977  29978  29979  29980  29981  29982  29983  29984  29985  29986  29987  29988  29989  29990  29991  29992  29993  29994  29995  29996  29997  29998  29999  30000  30001  30002  30003  30004  30005  30006  30007  30008  30009  30010  30011  30012  30013  30014  30015  30016  30017  30018  30019  30020  30021  30022  30023  30024  30025  30026  30027  30028  30029  30030  30031  30032  30033  30034  30035  30036  30037  30038  30039  30040  30041  30042  30043  30044  30045  30046  30047  30048  30049  30050  30051  30052  30053  30054  30055  30056  30057  30058  30059  30060  30061  30062  30063  30064  30065  30066  30067  30068  30069  30070  30071  30072  30073  30074  30075  30076  30077  30078  30079  30080  30081  30082  30083  30084  30085  30086  30087  30088  30089  30090  30091  30092  30093  30094  30095  30096  30097  30098  30099  30100  30101  30102  30103  30104  30105  30106  30107  30108  30109  30110  30111  30112  30113  30114  30115  30116  30117  30118  30119  30120  30121  30122  30123  30124  30125  30126  30127  30128  30129  30130  30131  30132  30133  30134  30135  30136  30137  30138  30139  30140  30141  30142  30143  30144  30145  30146  30147  30148  30149  30150  30151  30152  30153  30154  30155  30156  30157  30158  30159  30160  30161  30162  30163  30164  30165  30166  30167  30168  30169  30170  30171  30172  30173  30174  30175  30176  30177  30178  30179  30180  30181  30182  30183  30184  30185  30186  30187  30188  30189  30190  30191  30192  30193  30194  30195  30196  30197  30198  30199  30200  30201  30202  30203  30204  30205  30206  30207  30208  30209  30210  30211  30212  30213  30214  30215  30216  30217  30218  30219  30220  30221  30222  30223  30224  30225  30226  30227  30228  30229  30230  30231  30232  30233  30234  30235  30236  30237  30238  30239  30240  30241  30242  30243  30244  30245  30246  30247  30248  30249  30250  30251  30252  30253  30254  30255  30256  30257  30258  30259  30260  30261  30262  30263  30264  30265  30266  30267  30268  30269  30270  30271  30272  30273  30274  30275  30276  30277  30278  30279  30280  30281  30282  30283  30284  30285  30286  30287  30288  30289  30290  30291  30292  30293  30294  30295  30296  30297  30298  30299  30300  30301  30302  30303  30304  30305  30306  30307  30308  30309  30310  30311  30312  30313  30314  30315  30316  30317  30318  30319  30320  30321  30322  30323  30324  30325  30326  30327  30328  30329  30330  30331  30332  30333  30334  30335  30336  30337  30338  30339  30340  30341  30342  30343  30344  30345  30346  30347  30348  30349  30350  30351  30352  30353  30354  30355  30356  30357  30358  30359  30360  30361  30362  30363  30364  30365  30366  30367  30368  30369  30370  30371  30372  30373  30374  30375  30376  30377  30378  30379  30380  30381  30382  30383  30384  30385  30386  30387  30388  30389  30390  30391  30392  30393  30394  30395  30396  30397  30398  30399  30400  30401  30402  30403  30404  30405  30406  30407  30408  30409  30410  30411  30412  30413  30414  30415  30416  30417  30418  30419  30420  30421  30422  30423  30424  30425  30426  30427  30428  30429  30430  30431  30432  30433  30434  30435  30436  30437  30438  30439  30440  30441  30442  30443  30444  30445  30446  30447  30448  30449  30450  30451  30452  30453  30454  30455  30456  30457  30458  30459  30460  30461  30462  30463  30464  30465  30466  30467  30468  30469  30470  30471  30472  30473  30474  30475  30476  30477  30478  30479  30480  30481  30482  30483  30484  30485  30486  30487  30488  30489  30490  30491  30492  30493  30494  30495  30496  30497  30498  30499  30500  30501  30502  30503  30504  30505  30506  30507  30508  30509  30510  30511  30512  30513  30514  30515  30516  30517  30518  30519  30520  30521  30522  30523  30524  30525  30526  30527  30528  30529  30530  30531  30532  30533  30534  30535  30536  30537  30538  30539  30540  30541  30542  30543  30544  30545  30546  30547  30548  30549  30550  30551  30552  30553  30554  30555  30556  30557  30558  30559  30560  30561  30562  30563  30564  30565  30566  30567  30568  30569  30570  30571  30572  30573  30574  30575  30576  30577  30578  30579  30580  30581  30582  30583  30584  30585  30586  30587  30588  30589  30590  30591  30592  30593  30594  30595  30596  30597  30598  30599  30600  30601  30602  30603  30604  30605  30606  30607  30608  30609  30610  30611  30612  30613  30614  30615  30616  30617  30618  30619  30620  30621  30622  30623  30624  30625  30626  30627  30628  30629  30630  30631  30632  30633  30634  30635  30636  30637  30638  30639  30640  30641  30642  30643  30644  30645  30646  30647  30648  30649  30650  30651  30652  30653  30654  30655  30656  30657  30658  30659  30660  30661  30662  30663  30664  30665  30666  30667  30668  30669  30670  30671  30672  30673  30674  30675  30676  30677  30678  30679  30680  30681  30682  30683  30684  30685  30686  30687  30688  30689  30690  30691  30692  30693  30694  30695  30696  30697  30698  30699  30700  30701  30702  30703  30704  30705  30706  30707  30708  30709  30710  30711  30712  30713  30714  30715  30716  30717  30718  30719  30720  30721  30722  30723  30724  30725  30726  30727  30728  30729  30730  30731  30732  30733  30734  30735  30736  30737  30738  30739  30740  30741  30742  30743  30744  30745  30746  30747  30748  30749  30750  30751  30752  30753  30754  30755  30756  30757  30758  30759  30760  30761  30762  30763  30764  30765  30766  30767  30768  30769  30770  30771  30772  30773  30774  30775  30776  30777  30778  30779  30780  30781  30782  30783  30784  30785  30786  30787  30788  30789  30790  30791  30792  30793  30794  30795  30796  30797  30798  30799  30800  30801  30802  30803  30804  30805  30806  30807  30808  30809  30810  30811  30812  30813  30814  30815  30816  30817  30818  30819  30820  30821  30822  30823  30824  30825  30826  30827  30828  30829  30830  30831  30832  30833  30834  30835  30836  30837  30838  30839  30840  30841  30842  30843  30844  30845  30846  30847  30848  30849  30850  30851  30852  30853  30854  30855  30856  30857  30858  30859  30860  30861  30862  30863  30864  30865  30866  30867  30868  30869  30870  30871  30872  30873  30874  30875  30876  30877  30878  30879  30880  30881  30882  30883  30884  30885  30886  30887  30888  30889  30890  30891  30892  30893  30894  30895  30896  30897  30898  30899  30900  30901  30902  30903  30904  30905  30906  30907  30908  30909  30910  30911  30912  30913  30914  30915  30916  30917  30918  30919  30920  30921  30922  30923  30924  30925  30926  30927  30928  30929  30930  30931  30932  30933  30934  30935  30936  30937  30938  30939  30940  30941  30942  30943  30944  30945  30946  30947  30948  30949  30950  30951  30952  30953  30954  30955  30956  30957  30958  30959  30960  30961  30962  30963  30964  30965  30966  30967  30968  30969  30970  30971  30972  30973  30974  30975  30976  30977  30978  30979  30980  30981  30982  30983  30984  30985  30986  30987  30988  30989  30990  30991  30992  30993  30994  30995  30996  30997  30998  30999  31000  31001  31002  31003  31004  31005  31006  31007  31008  31009  31010  31011  31012  31013  31014  31015  31016  31017  31018  31019  31020  31021  31022  31023  31024  31025  31026  31027  31028  31029  31030  31031  31032  31033  31034  31035  31036  31037  31038  31039  31040  31041  31042  31043  31044  31045  31046  31047  31048  31049  31050  31051  31052  31053  31054  31055  31056  31057  31058  31059  31060  31061  31062  31063  31064  31065  31066  31067  31068  31069  31070  31071  31072  31073  31074  31075  31076  31077  31078  31079  31080  31081  31082  31083  31084  31085  31086  31087  31088  31089  31090  31091  31092  31093  31094  31095  31096  31097  31098  31099  31100  31101  31102  31103  31104  31105  31106  31107  31108  31109  31110  31111  31112  31113  31114  31115  31116  31117  31118  31119  31120  31121  31122  31123  31124  31125  31126  31127  31128  31129  31130  31131  31132  31133  31134  31135  31136  31137  31138  31139  31140  31141  31142  31143  31144  31145  31146  31147  31148  31149  31150  31151  31152  31153  31154  31155  31156  31157  31158  31159  31160  31161  31162  31163  31164  31165  31166  31167  31168  31169  31170  31171  31172  31173  31174  31175  31176  31177  31178  31179  31180  31181  31182  31183  31184  31185  31186  31187  31188  31189  31190  31191  31192  31193  31194  31195  31196  31197  31198  31199  31200  31201  31202  31203  31204  31205  31206  31207  31208  31209  31210  31211  31212  31213  31214  31215  31216  31217  31218  31219  31220  31221  31222  31223  31224  31225  31226  31227  31228  31229  31230  31231  31232  31233  31234  31235  31236  31237  31238  31239  31240  31241  31242  31243  31244  31245  31246  31247  31248  31249  31250  31251  31252  31253  31254  31255  31256  31257  31258  31259  31260  31261  31262  31263  31264  31265  31266  31267  31268  31269  31270  31271  31272  31273  31274  31275  31276  31277  31278  31279  31280  31281  31282  31283  31284  31285  31286  31287  31288  31289  31290  31291  31292  31293  31294  31295  31296  31297  31298  31299  31300  31301  31302  31303  31304  31305  31306  31307  31308  31309  31310  31311  31312  31313  31314  31315  31316  31317  31318  31319  31320  31321  31322  31323  31324  31325  31326  31327  31328  31329  31330  31331  31332  31333  31334  31335  31336  31337  31338  31339  31340  31341  31342  31343  31344  31345  31346  31347  31348  31349  31350  31351  31352  31353  31354  31355  31356  31357  31358  31359  31360  31361  31362  31363  31364  31365  31366  31367  31368  31369  31370  31371  31372  31373  31374  31375  31376  31377  31378  31379  31380  31381  31382  31383  31384  31385  31386  31387  31388  31389  31390  31391  31392  31393  31394  31395  31396  31397  31398  31399  31400  31401  31402  31403  31404  31405  31406  31407  31408  31409  31410  31411  31412  31413  31414  31415  31416  31417  31418  31419  31420  31421  31422  31423  31424  31425  31426  31427  31428  31429  31430  31431  31432  31433  31434  31435  31436  31437  31438  31439  31440  31441  31442  31443  31444  31445  31446  31447  31448  31449  31450  31451  31452  31453  31454  31455  31456  31457  31458  31459  31460  31461  31462  31463  31464  31465  31466  31467  31468  31469  31470  31471  31472  31473  31474  31475  31476  31477  31478  31479  31480  31481  31482  31483  31484  31485  31486  31487  31488  31489  31490  31491  31492  31493  31494  31495  31496  31497  31498  31499  31500  31501  31502  31503  31504  31505  31506  31507  31508  31509  31510  31511  31512  31513  31514  31515  31516  31517  31518  31519  31520  31521  31522  31523  31524  31525  31526  31527  31528  31529  31530  31531  31532  31533  31534  31535  31536  31537  31538  31539  31540  31541  31542  31543  31544  31545  31546  31547  31548  31549  31550  31551  31552  31553  31554  31555  31556  31557  31558  31559  31560  31561  31562  31563  31564  31565  31566  31567  31568  31569  31570  31571  31572  31573  31574  31575  31576  31577  31578  31579  31580  31581  31582  31583  31584  31585  31586  31587  31588  31589  31590  31591  31592  31593  31594  31595  31596  31597  31598  31599  31600  31601  31602  31603  31604  31605  31606  31607  31608  31609  31610  31611  31612  31613  31614  31615  31616  31617  31618  31619  31620  31621  31622  31623  31624  31625  31626  31627  31628  31629  31630  31631  31632  31633  31634  31635  31636  31637  31638  31639  31640  31641  31642  31643  31644  31645  31646  31647  31648  31649  31650  31651  31652  31653  31654  31655  31656  31657  31658  31659  31660  31661  31662  31663  31664  31665  31666  31667  31668  31669  31670  31671  31672  31673  31674  31675  31676  31677  31678  31679  31680  31681  31682  31683  31684  31685  31686  31687  31688  31689  31690  31691  31692  31693  31694  31695  31696  31697  31698  31699  31700  31701  31702  31703  31704  31705  31706  31707  31708  31709  31710  31711  31712  31713  31714  31715  31716  31717  31718  31719  31720  31721  31722  31723  31724  31725  31726  31727  31728  31729  31730  31731  31732  31733  31734  31735  31736  31737  31738  31739  31740  31741  31742  31743  31744  31745  31746  31747  31748  31749  31750  31751  31752  31753  31754  31755  31756  31757  31758  31759  31760  31761  31762  31763  31764  31765  31766  31767  31768  31769  31770  31771  31772  31773  31774  31775  31776  31777  31778  31779  31780  31781  31782  31783  31784  31785  31786  31787  31788  31789  31790  31791  31792  31793  31794  31795  31796  31797  31798  31799  31800  31801  31802  31803  31804  31805  31806  31807  31808  31809  31810  31811  31812  31813  31814  31815  31816  31817  31818  31819  31820  31821  31822  31823  31824  31825  31826  31827  31828  31829  31830  31831  31832  31833  31834  31835  31836  31837  31838  31839  31840  31841  31842  31843  31844  31845  31846  31847  31848  31849  31850  31851  31852  31853  31854  31855  31856  31857  31858  31859  31860  31861  31862  31863  31864  31865  31866  31867  31868  31869  31870  31871  31872  31873  31874  31875  31876  31877  31878  31879  31880  31881  31882  31883  31884  31885  31886  31887  31888  31889  31890  31891  31892  31893  31894  31895  31896  31897  31898  31899  31900  31901  31902  31903  31904  31905  31906  31907  31908  31909  31910  31911  31912  31913  31914  31915  31916  31917  31918  31919  31920  31921  31922  31923  31924  31925  31926  31927  31928  31929  31930  31931  31932  31933  31934  31935  31936  31937  31938  31939  31940  31941  31942  31943  31944  31945  31946  31947  31948  31949  31950  31951  31952  31953  31954  31955  31956  31957  31958  31959  31960  31961  31962  31963  31964  31965  31966  31967  31968  31969  31970  31971  31972  31973  31974  31975  31976  31977  31978  31979  31980  31981  31982  31983  31984  31985  31986  31987  31988  31989  31990  31991  31992  31993  31994  31995  31996  31997  31998  31999  32000  32001  32002  32003  32004  32005  32006  32007  32008  32009  32010  32011  32012  32013  32014  32015  32016  32017  32018  32019  32020  32021  32022  32023  32024  32025  32026  32027  32028  32029  32030  32031  32032  32033  32034  32035  32036  32037  32038  32039  32040  32041  32042  32043  32044  32045  32046  32047  32048  32049  32050  32051  32052  32053  32054  32055  32056  32057  32058  32059  32060  32061  32062  32063  32064  32065  32066  32067  32068  32069  32070  32071  32072  32073  32074  32075  32076  32077  32078  32079  32080  32081  32082  32083  32084  32085  32086  32087  32088  32089  32090  32091  32092  32093  32094  32095  32096  32097  32098  32099  32100  32101  32102  32103  32104  32105  32106  32107  32108  32109  32110  32111  32112  32113  32114  32115  32116  32117  32118  32119  32120  32121  32122  32123  32124  32125  32126  32127  32128  32129  32130  32131  32132  32133  32134  32135  32136  32137  32138  32139  32140  32141  32142  32143  32144  32145  32146  32147  32148  32149  32150  32151  32152  32153  32154  32155  32156  32157  32158  32159  32160  32161  32162  32163  32164  32165  32166  32167  32168  32169  32170  32171  32172  32173  32174  32175  32176  32177  32178  32179  32180  32181  32182  32183  32184  32185  32186  32187  32188  32189  32190  32191  32192  32193  32194  32195  32196  32197  32198  32199  32200  32201  32202  32203  32204  32205  32206  32207  32208  32209  32210  32211  32212  32213  32214  32215  32216  32217  32218  32219  32220  32221  32222  32223  32224  32225  32226  32227  32228  32229  32230  32231  32232  32233  32234  32235  32236  32237  32238  32239  32240  32241  32242  32243  32244  32245  32246  32247  32248  32249  32250  32251  32252  32253  32254  32255  32256  32257  32258  32259  32260  32261  32262  32263  32264  32265  32266  32267  32268  32269  32270  32271  32272  32273  32274  32275  32276  32277  32278  32279  32280  32281  32282  32283  32284  32285  32286  32287  32288  32289  32290  32291  32292  32293  32294  32295  32296  32297  32298  32299  32300  32301  32302  32303  32304  32305  32306  32307  32308  32309  32310  32311  32312  32313  32314  32315  32316  32317  32318  32319  32320  32321  32322  32323  32324  32325  32326  32327  32328  32329  32330  32331  32332  32333  32334  32335  32336  32337  32338  32339  32340  32341  32342  32343  32344  32345  32346  32347  32348  32349  32350  32351  32352  32353  32354  32355  32356  32357  32358  32359  32360  32361  32362  32363  32364  32365  32366  32367  32368  32369  32370  32371  32372  32373  32374  32375  32376  32377  32378  32379  32380  32381  32382  32383  32384  32385  32386  32387  32388  32389  32390  32391  32392  32393  32394  32395  32396  32397  32398  32399  32400  32401  32402  32403  32404  32405  32406  32407  32408  32409  32410  32411  32412  32413  32414  32415  32416  32417  32418  32419  32420  32421  32422  32423  32424  32425  32426  32427  32428  32429  32430  32431  32432  32433  32434  32435  32436  32437  32438  32439  32440  32441  32442  32443  32444  32445  32446  32447  32448  32449  32450  32451  32452  32453  32454  32455  32456  32457  32458  32459  32460  32461  32462  32463  32464  32465  32466  32467  32468  32469  32470  32471  32472  32473  32474  32475  32476  32477  32478  32479  32480  32481  32482  32483  32484  32485  32486  32487  32488  32489  32490  32491  32492  32493  32494  32495  32496  32497  32498  32499  32500  32501  32502  32503  32504  32505  32506  32507  32508  32509  32510  32511  32512  32513  32514  32515  32516  32517  32518  32519  32520  32521  32522  32523  32524  32525  32526  32527  32528  32529  32530  32531  32532  32533  32534  32535  32536  32537  32538  32539  32540  32541  32542  32543  32544  32545  32546  32547  32548  32549  32550  32551  32552  32553  32554  32555  32556  32557  32558  32559  32560  32561  32562  32563  32564  32565  32566  32567  32568  32569  32570  32571  32572  32573  32574  32575  32576  32577  32578  32579  32580  32581  32582  32583  32584  32585  32586  32587  32588  32589  32590  32591  32592  32593  32594  32595  32596  32597  32598  32599  32600  32601  32602  32603  32604  32605  32606  32607  32608  32609  32610  32611  32612  32613  32614  32615  32616  32617  32618  32619  32620  32621  32622  32623  32624  32625  32626  32627  32628  32629  32630  32631  32632  32633  32634  32635  32636  32637  32638  32639  32640  32641  32642  32643  32644  32645  32646  32647  32648  32649  32650  32651  32652  32653  32654  32655  32656  32657  32658  32659  32660  32661  32662  32663  32664  32665  32666  32667  32668  32669  32670  32671  32672  32673  32674  32675  32676  32677  32678  32679  32680  32681  32682  32683  32684  32685  32686  32687  32688  32689  32690  32691  32692  32693  32694  32695  32696  32697  32698  32699  32700  32701  32702  32703  32704  32705  32706  32707  32708  32709  32710  32711  32712  32713  32714  32715  32716  32717  32718  32719  32720  32721  32722  32723  32724  32725  32726  32727  32728  32729  32730  32731  32732  32733  32734  32735  32736  32737  32738  32739  32740  32741  32742  32743  32744  32745  32746  32747  32748  32749  32750  32751  32752  32753  32754  32755  32756  32757  32758  32759  32760  32761  32762  32763  32764  32765  32766  32767  32768  32769  32770  32771  32772  32773  32774  32775  32776  32777  32778  32779  32780  32781  32782  32783  32784  32785  32786  32787  32788  32789  32790  32791  32792  32793  32794  32795  32796  32797  32798  32799  32800  32801  32802  32803  32804  32805  32806  32807  32808  32809  32810  32811  32812  32813  32814  32815  32816  32817  32818  32819  32820  32821  32822  32823  32824  32825  32826  32827  32828  32829  32830  32831  32832  32833  32834  32835  32836  32837  32838  32839  32840  32841  32842  32843  32844  32845  32846  32847  32848  32849  32850  32851  32852  32853  32854  32855  32856  32857  32858  32859  32860  32861  32862  32863  32864  32865  32866  32867  32868  32869  32870  32871  32872  32873  32874  32875  32876  32877  32878  32879  32880  32881  32882  32883  32884  32885  32886  32887  32888  32889  32890  32891  32892  32893  32894  32895  32896  32897  32898  32899  32900  32901  32902  32903  32904  32905  32906  32907  32908  32909  32910  32911  32912  32913  32914  32915  32916  32917  32918  32919  32920  32921  32922  32923  32924  32925  32926  32927  32928  32929  32930  32931  32932  32933  32934  32935  32936  32937  32938  32939  32940  32941  32942  32943  32944  32945  32946  32947  32948  32949  32950  32951  32952  32953  32954  32955  32956  32957  32958  32959  32960  32961  32962  32963  32964  32965  32966  32967  32968  32969  32970  32971  32972  32973  32974  32975  32976  32977  32978  32979  32980  32981  32982  32983  32984  32985  32986  32987  32988  32989  32990  32991  32992  32993  32994  32995  32996  32997  32998  32999  33000  33001  33002  33003  33004  33005  33006  33007  33008  33009  33010  33011  33012  33013  33014  33015  33016  33017  33018  33019  33020  33021  33022  33023  33024  33025  33026  33027  33028  33029  33030  33031  33032  33033  33034  33035  33036  33037  33038  33039  33040  33041  33042  33043  33044  33045  33046  33047  33048  33049  33050  33051  33052  33053  33054  33055  33056  33057  33058  33059  33060  33061  33062  33063  33064  33065  33066  33067  33068  33069  33070  33071  33072  33073  33074  33075  33076  33077  33078  33079  33080  33081  33082  33083  33084  33085  33086  33087  33088  33089  33090  33091  33092  33093  33094  33095  33096  33097  33098  33099  33100  33101  33102  33103  33104  33105  33106  33107  33108  33109  33110  33111  33112  33113  33114  33115  33116  33117  33118  33119  33120  33121  33122  33123  33124  33125  33126  33127  33128  33129  33130  33131  33132  33133  33134  33135  33136  33137  33138  33139  33140  33141  33142  33143  33144  33145  33146  33147  33148  33149  33150  33151  33152  33153  33154  33155  33156  33157  33158  33159  33160  33161  33162  33163  33164  33165  33166  33167  33168  33169  33170  33171  33172  33173  33174  33175  33176  33177  33178  33179  33180  33181  33182  33183  33184  33185  33186  33187  33188  33189  33190  33191  33192  33193  33194  33195  33196  33197  33198  33199  33200  33201  33202  33203  33204  33205  33206  33207  33208  33209  33210  33211  33212  33213  33214  33215  33216  33217  33218  33219  33220  33221  33222  33223  33224  33225  33226  33227  33228  33229  33230  33231  33232  33233  33234  33235  33236  33237  33238  33239  33240  33241  33242  33243  33244  33245  33246  33247  33248  33249  33250  33251  33252  33253  33254  33255  33256  33257  33258  33259  33260  33261  33262  33263  33264  33265  33266  33267  33268  33269  33270  33271  33272  33273  33274  33275  33276  33277  33278  33279  33280  33281  33282  33283  33284  33285  33286  33287  33288  33289  33290  33291  33292  33293  33294  33295  33296  33297  33298  33299  33300  33301  33302  33303  33304  33305  33306  33307  33308  33309  33310  33311  33312  33313  33314  33315  33316  33317  33318  33319  33320  33321  33322  33323  33324  33325  33326  33327  33328  33329  33330  33331  33332  33333  33334  33335  33336  33337  33338  33339  33340  33341  33342  33343  33344  33345  33346  33347  33348  33349  33350  33351  33352  33353  33354  33355  33356  33357  33358  33359  33360  33361  33362  33363  33364  33365  33366  33367  33368  33369  33370  33371  33372  33373  33374  33375  33376  33377  33378  33379  33380  33381  33382  33383  33384  33385  33386  33387  33388  33389  33390  33391  33392  33393  33394  33395  33396  33397  33398  33399  33400  33401  33402  33403  33404  33405  33406  33407  33408  33409  33410  33411  33412  33413  33414  33415  33416  33417  33418  33419  33420  33421  33422  33423  33424  33425  33426  33427  33428  33429  33430  33431  33432  33433  33434  33435  33436  33437  33438  33439  33440  33441  33442  33443  33444  33445  33446  33447  33448  33449  33450  33451  33452  33453  33454  33455  33456  33457  33458  33459  33460  33461  33462  33463  33464  33465  33466  33467  33468  33469  33470  33471  33472  33473  33474  33475  33476  33477  33478  33479  33480  33481  33482  33483  33484  33485  33486  33487  33488  33489  33490  33491  33492  33493  33494  33495  33496  33497  33498  33499  33500  33501  33502  33503  33504  33505  33506  33507  33508  33509  33510  33511  33512  33513  33514  33515  33516  33517  33518  33519  33520  33521  33522  33523  33524  33525  33526  33527  33528  33529  33530  33531  33532  33533  33534  33535  33536  33537  33538  33539  33540  33541  33542  33543  33544  33545  33546  33547  33548  33549  33550  33551  33552  33553  33554  33555  33556  33557  33558  33559  33560  33561  33562  33563  33564  33565  33566  33567  33568  33569  33570  33571  33572  33573  33574  33575  33576  33577  33578  33579  33580  33581  33582  33583  33584  33585  33586  33587  33588  33589  33590  33591  33592  33593  33594  33595  33596  33597  33598  33599  33600  33601  33602  33603  33604  33605  33606  33607  33608  33609  33610  33611  33612  33613  33614  33615  33616  33617  33618  33619  33620  33621  33622  33623  33624  33625  33626  33627  33628  33629  33630  33631  33632  33633  33634  33635  33636  33637  33638  33639  33640  33641  33642  33643  33644  33645  33646  33647  33648  33649  33650  33651  33652  33653  33654  33655  33656  33657  33658  33659  33660  33661  33662  33663  33664  33665  33666  33667  33668  33669  33670  33671  33672  33673  33674  33675  33676  33677  33678  33679  33680  33681  33682  33683  33684  33685  33686  33687  33688  33689  33690  33691  33692  33693  33694  33695  33696  33697  33698  33699  33700  33701  33702  33703  33704  33705  33706  33707  33708  33709  33710  33711  33712  33713  33714  33715  33716  33717  33718  33719  33720  33721  33722  33723  33724  33725  33726  33727  33728  33729  33730  33731  33732  33733  33734  33735  33736  33737  33738  33739  33740  33741  33742  33743  33744  33745  33746  33747  33748  33749  33750  33751  33752  33753  33754  33755  33756  33757  33758  33759  33760  33761  33762  33763  33764  33765  33766  33767  33768  33769  33770  33771  33772  33773  33774  33775  33776  33777  33778  33779  33780  33781  33782  33783  33784  33785  33786  33787  33788  33789  33790  33791  33792  33793  33794  33795  33796  33797  33798  33799  33800  33801  33802  33803  33804  33805  33806  33807  33808  33809  33810  33811  33812  33813  33814  33815  33816  33817  33818  33819  33820  33821  33822  33823  33824  33825  33826  33827  33828  33829  33830  33831  33832  33833  33834  33835  33836  33837  33838  33839  33840  33841  33842  33843  33844  33845  33846  33847  33848  33849  33850  33851  33852  33853  33854  33855  33856  33857  33858  33859  33860  33861  33862  33863  33864  33865  33866  33867  33868  33869  33870  33871  33872  33873  33874  33875  33876  33877  33878  33879  33880  33881  33882  33883  33884  33885  33886  33887  33888  33889  33890  33891  33892  33893  33894  33895  33896  33897  33898  33899  33900  33901  33902  33903  33904  33905  33906  33907  33908  33909  33910  33911  33912  33913  33914  33915  33916  33917  33918  33919  33920  33921  33922  33923  33924  33925  33926  33927  33928  33929  33930  33931  33932  33933  33934  33935  33936  33937  33938  33939  33940  33941  33942  33943  33944  33945  33946  33947  33948  33949  33950  33951  33952  33953  33954  33955  33956  33957  33958  33959  33960  33961  33962  33963  33964  33965  33966  33967  33968  33969  33970  33971  33972  33973  33974  33975  33976  33977  33978  33979  33980  33981  33982  33983  33984  33985  33986  33987  33988  33989  33990  33991  33992  33993  33994  33995  33996  33997  33998  33999  34000  34001  34002  34003  34004  34005  34006  34007  34008  34009  34010  34011  34012  34013  34014  34015  34016  34017  34018  34019  34020  34021  34022  34023  34024  34025  34026  34027  34028  34029  34030  34031  34032  34033  34034  34035  34036  34037  34038  34039  34040  34041  34042  34043  34044  34045  34046  34047  34048  34049  34050  34051  34052  34053  34054  34055  34056  34057  34058  34059  34060  34061  34062  34063  34064  34065  34066  34067  34068  34069  34070  34071  34072  34073  34074  34075  34076  34077  34078  34079  34080  34081  34082  34083  34084  34085  34086  34087  34088  34089  34090  34091  34092  34093  34094  34095  34096  34097  34098  34099  34100  34101  34102  34103  34104  34105  34106  34107  34108  34109  34110  34111  34112  34113  34114  34115  34116  34117  34118  34119  34120  34121  34122  34123  34124  34125  34126  34127  34128  34129  34130  34131  34132  34133  34134  34135  34136  34137  34138  34139  34140  34141  34142  34143  34144  34145  34146  34147  34148  34149  34150  34151  34152  34153  34154  34155  34156  34157  34158  34159  34160  34161  34162  34163  34164  34165  34166  34167  34168  34169  34170  34171  34172  34173  34174  34175  34176  34177  34178  34179  34180  34181  34182  34183  34184  34185  34186  34187  34188  34189  34190  34191  34192  34193  34194  34195  34196  34197  34198  34199  34200  34201  34202  34203  34204  34205  34206  34207  34208  34209  34210  34211  34212  34213  34214  34215  34216  34217  34218  34219  34220  34221  34222  34223  34224  34225  34226  34227  34228  34229  34230  34231  34232  34233  34234  34235  34236  34237  34238  34239  34240  34241  34242  34243  34244  34245  34246  34247  34248  34249  34250  34251  34252  34253  34254  34255  34256  34257  34258  34259  34260  34261  34262  34263  34264  34265  34266  34267  34268  34269  34270  34271  34272  34273  34274  34275  34276  34277  34278  34279  34280  34281  34282  34283  34284  34285  34286  34287  34288  34289  34290  34291  34292  34293  34294  34295  34296  34297  34298  34299  34300  34301  34302  34303  34304  34305  34306  34307  34308  34309  34310  34311  34312  34313  34314  34315  34316  34317  34318  34319  34320  34321  34322  34323  34324  34325  34326  34327  34328  34329  34330  34331  34332  34333  34334  34335  34336  34337  34338  34339  34340  34341  34342  34343  34344  34345  34346  34347  34348  34349  34350  34351  34352  34353  34354  34355  34356  34357  34358  34359  34360  34361  34362  34363  34364  34365  34366  34367  34368  34369  34370  34371  34372  34373  34374  34375  34376  34377  34378  34379  34380  34381  34382  34383  34384  34385  34386  34387  34388  34389  34390  34391  34392  34393  34394  34395  34396  34397  34398  34399  34400  34401  34402  34403  34404  34405  34406  34407  34408  34409  34410  34411  34412  34413  34414  34415  34416  34417  34418  34419  34420  34421  34422  34423  34424  34425  34426  34427  34428  34429  34430  34431  34432  34433  34434  34435  34436  34437  34438  34439  34440  34441  34442  34443  34444  34445  34446  34447  34448  34449  34450  34451  34452  34453  34454  34455  34456  34457  34458  34459  34460  34461  34462  34463  34464  34465  34466  34467  34468  34469  34470  34471  34472  34473  34474  34475  34476  34477  34478  34479  34480  34481  34482  34483  34484  34485  34486  34487  34488  34489  34490  34491  34492  34493  34494  34495  34496  34497  34498  34499  34500  34501  34502  34503  34504  34505  34506  34507  34508  34509  34510  34511  34512  34513  34514  34515  34516  34517  34518  34519  34520  34521  34522  34523  34524  34525  34526  34527  34528  34529  34530  34531  34532  34533  34534  34535  34536  34537  34538  34539  34540  34541  34542  34543  34544  34545  34546  34547  34548  34549  34550  34551  34552  34553  34554  34555  34556  34557  34558  34559  34560  34561  34562  34563  34564  34565  34566  34567  34568  34569  34570  34571  34572  34573  34574  34575  34576  34577  34578  34579  34580  34581  34582  34583  34584  34585  34586  34587  34588  34589  34590  34591  34592  34593  34594  34595  34596  34597  34598  34599  34600  34601  34602  34603  34604  34605  34606  34607  34608  34609  34610  34611  34612  34613  34614  34615  34616  34617  34618  34619  34620  34621  34622  34623  34624  34625  34626  34627  34628  34629  34630  34631  34632  34633  34634  34635  34636  34637  34638  34639  34640  34641  34642  34643  34644  34645  34646  34647  34648  34649  34650  34651  34652  34653  34654  34655  34656  34657  34658  34659  34660  34661  34662  34663  34664  34665  34666  34667  34668  34669  34670  34671  34672  34673  34674  34675  34676  34677  34678  34679  34680  34681  34682  34683  34684  34685  34686  34687  34688  34689  34690  34691  34692  34693  34694  34695  34696  34697  34698  34699  34700  34701  34702  34703  34704  34705  34706  34707  34708  34709  34710  34711  34712  34713  34714  34715  34716  34717  34718  34719  34720  34721  34722  34723  34724  34725  34726  34727  34728  34729  34730  34731  34732  34733  34734  34735  34736  34737  34738  34739  34740  34741  34742  34743  34744  34745  34746  34747  34748  34749  34750  34751  34752  34753  34754  34755  34756  34757  34758  34759  34760  34761  34762  34763  34764  34765  34766  34767  34768  34769  34770  34771  34772  34773  34774  34775  34776  34777  34778  34779  34780  34781  34782  34783  34784  34785  34786  34787  34788  34789  34790  34791  34792  34793  34794  34795  34796  34797  34798  34799  34800  34801  34802  34803  34804  34805  34806  34807  34808  34809  34810  34811  34812  34813  34814  34815  34816  34817  34818  34819  34820  34821  34822  34823  34824  34825  34826  34827  34828  34829  34830  34831  34832  34833  34834  34835  34836  34837  34838  34839  34840  34841  34842  34843  34844  34845  34846  34847  34848  34849  34850  34851  34852  34853  34854  34855  34856  34857  34858  34859  34860  34861  34862  34863  34864  34865  34866  34867  34868  34869  34870  34871  34872  34873  34874  34875  34876  34877  34878  34879  34880  34881  34882  34883  34884  34885  34886  34887  34888  34889  34890  34891  34892  34893  34894  34895  34896  34897  34898  34899  34900  34901  34902  34903  34904  34905  34906  34907  34908  34909  34910  34911  34912  34913  34914  34915  34916  34917  34918  34919  34920  34921  34922  34923  34924  34925  34926  34927  34928  34929  34930  34931  34932  34933  34934  34935  34936  34937  34938  34939  34940  34941  34942  34943  34944  34945  34946  34947  34948  34949  34950  34951  34952  34953  34954  34955  34956  34957  34958  34959  34960  34961  34962  34963  34964  34965  34966  34967  34968  34969  34970  34971  34972  34973  34974  34975  34976  34977  34978  34979  34980  34981  34982  34983  34984  34985  34986  34987  34988  34989  34990  34991  34992  34993  34994  34995  34996  34997  34998  34999  35000  35001  35002  35003  35004  35005  35006  35007  35008  35009  35010  35011  35012  35013  35014  35015  35016  35017  35018  35019  35020  35021  35022  35023  35024  35025  35026  35027  35028  35029  35030  35031  35032  35033  35034  35035  35036  35037  35038  35039  35040  35041  35042  35043  35044  35045  35046  35047  35048  35049  35050  35051  35052  35053  35054  35055  35056  35057  35058  35059  35060  35061  35062  35063  35064  35065  35066  35067  35068  35069  35070  35071  35072  35073  35074  35075  35076  35077  35078  35079  35080  35081  35082  35083  35084  35085  35086  35087  35088  35089  35090  35091  35092  35093  35094  35095  35096  35097  35098  35099  35100  35101  35102  35103  35104  35105  35106  35107  35108  35109  35110  35111  35112  35113  35114  35115  35116  35117  35118  35119  35120  35121  35122  35123  35124  35125  35126  35127  35128  35129  35130  35131  35132  35133  35134  35135  35136  35137  35138  35139  35140  35141  35142  35143  35144  35145  35146  35147  35148  35149  35150  35151  35152  35153  35154  35155  35156  35157  35158  35159  35160  35161  35162  35163  35164  35165  35166  35167  35168  35169  35170  35171  35172  35173  35174  35175  35176  35177  35178  35179  35180  35181  35182  35183  35184  35185  35186  35187  35188  35189  35190  35191  35192  35193  35194  35195  35196  35197  35198  35199  35200  35201  35202  35203  35204  35205  35206  35207  35208  35209  35210  35211  35212  35213  35214  35215  35216  35217  35218  35219  35220  35221  35222  35223  35224  35225  35226  35227  35228  35229  35230  35231  35232  35233  35234  35235  35236  35237  35238  35239  35240  35241  35242  35243  35244  35245  35246  35247  35248  35249  35250  35251  35252  35253  35254  35255  35256  35257  35258  35259  35260  35261  35262  35263  35264  35265  35266  35267  35268  35269  35270  35271  35272  35273  35274  35275  35276  35277  35278  35279  35280  35281  35282  35283  35284  35285  35286  35287  35288  35289  35290  35291  35292  35293  35294  35295  35296  35297  35298  35299  35300  35301  35302  35303  35304  35305  35306  35307  35308  35309  35310  35311  35312  35313  35314  35315  35316  35317  35318  35319  35320  35321  35322  35323  35324  35325  35326  35327  35328  35329  35330  35331  35332  35333  35334  35335  35336  35337  35338  35339  35340  35341  35342  35343  35344  35345  35346  35347  35348  35349  35350  35351  35352  35353  35354  35355  35356  35357  35358  35359  35360  35361  35362  35363  35364  35365  35366  35367  35368  35369  35370  35371  35372  35373  35374  35375  35376  35377  35378  35379  35380  35381  35382  35383  35384  35385  35386  35387  35388  35389  35390  35391  35392  35393  35394  35395  35396  35397  35398  35399  35400  35401  35402  35403  35404  35405  35406  35407  35408  35409  35410  35411  35412  35413  35414  35415  35416  35417  35418  35419  35420  35421  35422  35423  35424  35425  35426  35427  35428  35429  35430  35431  35432  35433  35434  35435  35436  35437  35438  35439  35440  35441  35442  35443  35444  35445  35446  35447  35448  35449  35450  35451  35452  35453  35454  35455  35456  35457  35458  35459  35460  35461  35462  35463  35464  35465  35466  35467  35468  35469  35470  35471  35472  35473  35474  35475  35476  35477  35478  35479  35480  35481  35482  35483  35484  35485  35486  35487  35488  35489  35490  35491  35492  35493  35494  35495  35496  35497  35498  35499  35500  35501  35502  35503  35504  35505  35506  35507  35508  35509  35510  35511  35512  35513  35514  35515  35516  35517  35518  35519  35520  35521  35522  35523  35524  35525  35526  35527  35528  35529  35530  35531  35532  35533  35534  35535  35536  35537  35538  35539  35540  35541  35542  35543  35544  35545  35546  35547  35548  35549  35550  35551  35552  35553  35554  35555  35556  35557  35558  35559  35560  35561  35562  35563  35564  35565  35566  35567  35568  35569  35570  35571  35572  35573  35574  35575  35576  35577  35578  35579  35580  35581  35582  35583  35584  35585  35586  35587  35588  35589  35590  35591  35592  35593  35594  35595  35596  35597  35598  35599  35600  35601  35602  35603  35604  35605  35606  35607  35608  35609  35610  35611  35612  35613  35614  35615  35616  35617  35618  35619  35620  35621  35622  35623  35624  35625  35626  35627  35628  35629  35630  35631  35632  35633  35634  35635  35636  35637  35638  35639  35640  35641  35642  35643  35644  35645  35646  35647  35648  35649  35650  35651  35652  35653  35654  35655  35656  35657  35658  35659  35660  35661  35662  35663  35664  35665  35666  35667  35668  35669  35670  35671  35672  35673  35674  35675  35676  35677  35678  35679  35680  35681  35682  35683  35684  35685  35686  35687  35688  35689  35690  35691  35692  35693  35694  35695  35696  35697  35698  35699  35700  35701  35702  35703  35704  35705  35706  35707  35708  35709  35710  35711  35712  35713  35714  35715  35716  35717  35718  35719  35720  35721  35722  35723  35724  35725  35726  35727  35728  35729  35730  35731  35732  35733  35734  35735  35736  35737  35738  35739  35740  35741  35742  35743  35744  35745  35746  35747  35748  35749  35750  35751  35752  35753  35754  35755  35756  35757  35758  35759  35760  35761  35762  35763  35764  35765  35766  35767  35768  35769  35770  35771  35772  35773  35774  35775  35776  35777  35778  35779  35780  35781  35782  35783  35784  35785  35786  35787  35788  35789  35790  35791  35792  35793  35794  35795  35796  35797  35798  35799  35800  35801  35802  35803  35804  35805  35806  35807  35808  35809  35810  35811  35812  35813  35814  35815  35816  35817  35818  35819  35820  35821  35822  35823  35824  35825  35826  35827  35828  35829  35830  35831  35832  35833  35834  35835  35836  35837  35838  35839  35840  35841  35842  35843  35844  35845  35846  35847  35848  35849  35850  35851  35852  35853  35854  35855  35856  35857  35858  35859  35860  35861  35862  35863  35864  35865  35866  35867  35868  35869  35870  35871  35872  35873  35874  35875  35876  35877  35878  35879  35880  35881  35882  35883  35884  35885  35886  35887  35888  35889  35890  35891  35892  35893  35894  35895  35896  35897  35898  35899  35900  35901  35902  35903  35904  35905  35906  35907  35908  35909  35910  35911  35912  35913  35914  35915  35916  35917  35918  35919  35920  35921  35922  35923  35924  35925  35926  35927  35928  35929  35930  35931  35932  35933  35934  35935  35936  35937  35938  35939  35940  35941  35942  35943  35944  35945  35946  35947  35948  35949  35950  35951  35952  35953  35954  35955  35956  35957  35958  35959  35960  35961  35962  35963  35964  35965  35966  35967  35968  35969  35970  35971  35972  35973  35974  35975  35976  35977  35978  35979  35980  35981  35982  35983  35984  35985  35986  35987  35988  35989  35990  35991  35992  35993  35994  35995  35996  35997  35998  35999  36000  36001  36002  36003  36004  36005  36006  36007  36008  36009  36010  36011  36012  36013  36014  36015  36016  36017  36018  36019  36020  36021  36022  36023  36024  36025  36026  36027  36028  36029  36030  36031  36032  36033  36034  36035  36036  36037  36038  36039  36040  36041  36042  36043  36044  36045  36046  36047  36048  36049  36050  36051  36052  36053  36054  36055  36056  36057  36058  36059  36060  36061  36062  36063  36064  36065  36066  36067  36068  36069  36070  36071  36072  36073  36074  36075  36076  36077  36078  36079  36080  36081  36082  36083  36084  36085  36086  36087  36088  36089  36090  36091  36092  36093  36094  36095  36096  36097  36098  36099  36100  36101  36102  36103  36104  36105  36106  36107  36108  36109  36110  36111  36112  36113  36114  36115  36116  36117  36118  36119  36120  36121  36122  36123  36124  36125  36126  36127  36128  36129  36130  36131  36132  36133  36134  36135  36136  36137  36138  36139  36140  36141  36142  36143  36144  36145  36146  36147  36148  36149  36150  36151  36152  36153  36154  36155  36156  36157  36158  36159  36160  36161  36162  36163  36164  36165  36166  36167  36168  36169  36170  36171  36172  36173  36174  36175  36176  36177  36178  36179  36180  36181  36182  36183  36184  36185  36186  36187  36188  36189  36190  36191  36192  36193  36194  36195  36196  36197  36198  36199  36200  36201  36202  36203  36204  36205  36206  36207  36208  36209  36210  36211  36212  36213  36214  36215  36216  36217  36218  36219  36220  36221  36222  36223  36224  36225  36226  36227  36228  36229  36230  36231  36232  36233  36234  36235  36236  36237  36238  36239  36240  36241  36242  36243  36244  36245  36246  36247  36248  36249  36250  36251  36252  36253  36254  36255  36256  36257  36258  36259  36260  36261  36262  36263  36264  36265  36266  36267  36268  36269  36270  36271  36272  36273  36274  36275  36276  36277  36278  36279  36280  36281  36282  36283  36284  36285  36286  36287  36288  36289  36290  36291  36292  36293  36294  36295  36296  36297  36298  36299  36300  36301  36302  36303  36304  36305  36306  36307  36308  36309  36310  36311  36312  36313  36314  36315  36316  36317  36318  36319  36320  36321  36322  36323  36324  36325  36326  36327  36328  36329  36330  36331  36332  36333  36334  36335  36336  36337  36338  36339  36340  36341  36342  36343  36344  36345  36346  36347  36348  36349  36350  36351  36352  36353  36354  36355  36356  36357  36358  36359  36360  36361  36362  36363  36364  36365  36366  36367  36368  36369  36370  36371  36372  36373  36374  36375  36376  36377  36378  36379  36380  36381  36382  36383  36384  36385  36386  36387  36388  36389  36390  36391  36392  36393  36394  36395  36396  36397  36398  36399  36400  36401  36402  36403  36404  36405  36406  36407  36408  36409  36410  36411  36412  36413  36414  36415  36416  36417  36418  36419  36420  36421  36422  36423  36424  36425  36426  36427  36428  36429  36430  36431  36432  36433  36434  36435  36436  36437  36438  36439  36440  36441  36442  36443  36444  36445  36446  36447  36448  36449  36450  36451  36452  36453  36454  36455  36456  36457  36458  36459  36460  36461  36462  36463  36464  36465  36466  36467  36468  36469  36470  36471  36472  36473  36474  36475  36476  36477  36478  36479  36480  36481  36482  36483  36484  36485  36486  36487  36488  36489  36490  36491  36492  36493  36494  36495  36496  36497  36498  36499  36500  36501  36502  36503  36504  36505  36506  36507  36508  36509  36510  36511  36512  36513  36514  36515  36516  36517  36518  36519  36520  36521  36522  36523  36524  36525  36526  36527  36528  36529  36530  36531  36532  36533  36534  36535  36536  36537  36538  36539  36540  36541  36542  36543  36544  36545  36546  36547  36548  36549  36550  36551  36552  36553  36554  36555  36556  36557  36558  36559  36560  36561  36562  36563  36564  36565  36566  36567  36568  36569  36570  36571  36572  36573  36574  36575  36576  36577  36578  36579  36580  36581  36582  36583  36584  36585  36586  36587  36588  36589  36590  36591  36592  36593  36594  36595  36596  36597  36598  36599  36600  36601  36602  36603  36604  36605  36606  36607  36608  36609  36610  36611  36612  36613  36614  36615  36616  36617  36618  36619  36620  36621  36622  36623  36624  36625  36626  36627  36628  36629  36630  36631  36632  36633  36634  36635  36636  36637  36638  36639  36640  36641  36642  36643  36644  36645  36646  36647  36648  36649  36650  36651  36652  36653  36654  36655  36656  36657  36658  36659  36660  36661  36662  36663  36664  36665  36666  36667  36668  36669  36670  36671  36672  36673  36674  36675  36676  36677  36678  36679  36680  36681  36682  36683  36684  36685  36686  36687  36688  36689  36690  36691  36692  36693  36694  36695  36696  36697  36698  36699  36700  36701  36702  36703  36704  36705  36706  36707  36708  36709  36710  36711  36712  36713  36714  36715  36716  36717  36718  36719  36720  36721  36722  36723  36724  36725  36726  36727  36728  36729  36730  36731  36732  36733  36734  36735  36736  36737  36738  36739  36740  36741  36742  36743  36744  36745  36746  36747  36748  36749  36750  36751  36752  36753  36754  36755  36756  36757  36758  36759  36760  36761  36762  36763  36764  36765  36766  36767  36768  36769  36770  36771  36772  36773  36774  36775  36776  36777  36778  36779  36780  36781  36782  36783  36784  36785  36786  36787  36788  36789  36790  36791  36792  36793  36794  36795  36796  36797  36798  36799  36800  36801  36802  36803  36804  36805  36806  36807  36808  36809  36810  36811  36812  36813  36814  36815  36816  36817  36818  36819  36820  36821  36822  36823  36824  36825  36826  36827  36828  36829  36830  36831  36832  36833  36834  36835  36836  36837  36838  36839  36840  36841  36842  36843  36844  36845  36846  36847  36848  36849  36850  36851  36852  36853  36854  36855  36856  36857  36858  36859  36860  36861  36862  36863  36864  36865  36866  36867  36868  36869  36870  36871  36872  36873  36874  36875  36876  36877  36878  36879  36880  36881  36882  36883  36884  36885  36886  36887  36888  36889  36890  36891  36892  36893  36894  36895  36896  36897  36898  36899  36900  36901  36902  36903  36904  36905  36906  36907  36908  36909  36910  36911  36912  36913  36914  36915  36916  36917  36918  36919  36920  36921  36922  36923  36924  36925  36926  36927  36928  36929  36930  36931  36932  36933  36934  36935  36936  36937  36938  36939  36940  36941  36942  36943  36944  36945  36946  36947  36948  36949  36950  36951  36952  36953  36954  36955  36956  36957  36958  36959  36960  36961  36962  36963  36964  36965  36966  36967  36968  36969  36970  36971  36972  36973  36974  36975  36976  36977  36978  36979  36980  36981  36982  36983  36984  36985  36986  36987  36988  36989  36990  36991  36992  36993  36994  36995  36996  36997  36998  36999  37000  37001  37002  37003  37004  37005  37006  37007  37008  37009  37010  37011  37012  37013  37014  37015  37016  37017  37018  37019  37020  37021  37022  37023  37024  37025  37026  37027  37028  37029  37030  37031  37032  37033  37034  37035  37036  37037  37038  37039  37040  37041  37042  37043  37044  37045  37046  37047  37048  37049  37050  37051  37052  37053  37054  37055  37056  37057  37058  37059  37060  37061  37062  37063  37064  37065  37066  37067  37068  37069  37070  37071  37072  37073  37074  37075  37076  37077  37078  37079  37080  37081  37082  37083  37084  37085  37086  37087  37088  37089  37090  37091  37092  37093  37094  37095  37096  37097  37098  37099  37100  37101  37102  37103  37104  37105  37106  37107  37108  37109  37110  37111  37112  37113  37114  37115  37116  37117  37118  37119  37120  37121  37122  37123  37124  37125  37126  37127  37128  37129  37130  37131  37132  37133  37134  37135  37136  37137  37138  37139  37140  37141  37142  37143  37144  37145  37146  37147  37148  37149  37150  37151  37152  37153  37154  37155  37156  37157  37158  37159  37160  37161  37162  37163  37164  37165  37166  37167  37168  37169  37170  37171  37172  37173  37174  37175  37176  37177  37178  37179  37180  37181  37182  37183  37184  37185  37186  37187  37188  37189  37190  37191  37192  37193  37194  37195  37196  37197  37198  37199  37200  37201  37202  37203  37204  37205  37206  37207  37208  37209  37210  37211  37212  37213  37214  37215  37216  37217  37218  37219  37220  37221  37222  37223  37224  37225  37226  37227  37228  37229  37230  37231  37232  37233  37234  37235  37236  37237  37238  37239  37240  37241  37242  37243  37244  37245  37246  37247  37248  37249  37250  37251  37252  37253  37254  37255  37256  37257  37258  37259  37260  37261  37262  37263  37264  37265  37266  37267  37268  37269  37270  37271  37272  37273  37274  37275  37276  37277  37278  37279  37280  37281  37282  37283  37284  37285  37286  37287  37288  37289  37290  37291  37292  37293  37294  37295  37296  37297  37298  37299  37300  37301  37302  37303  37304  37305  37306  37307  37308  37309  37310  37311  37312  37313  37314  37315  37316  37317  37318  37319  37320  37321  37322  37323  37324  37325  37326  37327  37328  37329  37330  37331  37332  37333  37334  37335  37336  37337  37338  37339  37340  37341  37342  37343  37344  37345  37346  37347  37348  37349  37350  37351  37352  37353  37354  37355  37356  37357  37358  37359  37360  37361  37362  37363  37364  37365  37366  37367  37368  37369  37370  37371  37372  37373  37374  37375  37376  37377  37378  37379  37380  37381  37382  37383  37384  37385  37386  37387  37388  37389  37390  37391  37392  37393  37394  37395  37396  37397  37398  37399  37400  37401  37402  37403  37404  37405  37406  37407  37408  37409  37410  37411  37412  37413  37414  37415  37416  37417  37418  37419  37420  37421  37422  37423  37424  37425  37426  37427  37428  37429  37430  37431  37432  37433  37434  37435  37436  37437  37438  37439  37440  37441  37442  37443  37444  37445  37446  37447  37448  37449  37450  37451  37452  37453  37454  37455  37456  37457  37458  37459  37460  37461  37462  37463  37464  37465  37466  37467  37468  37469  37470  37471  37472  37473  37474  37475  37476  37477  37478  37479  37480  37481  37482  37483  37484  37485  37486  37487  37488  37489  37490  37491  37492  37493  37494  37495  37496  37497  37498  37499  37500  37501  37502  37503  37504  37505  37506  37507  37508  37509  37510  37511  37512  37513  37514  37515  37516  37517  37518  37519  37520  37521  37522  37523  37524  37525  37526  37527  37528  37529  37530  37531  37532  37533  37534  37535  37536  37537  37538  37539  37540  37541  37542  37543  37544  37545  37546  37547  37548  37549  37550  37551  37552  37553  37554  37555  37556  37557  37558  37559  37560  37561  37562  37563  37564  37565  37566  37567  37568  37569  37570  37571  37572  37573  37574  37575  37576  37577  37578  37579  37580  37581  37582  37583  37584  37585  37586  37587  37588  37589  37590  37591  37592  37593  37594  37595  37596  37597  37598  37599  37600  37601  37602  37603  37604  37605  37606  37607  37608  37609  37610  37611  37612  37613  37614  37615  37616  37617  37618  37619  37620  37621  37622  37623  37624  37625  37626  37627  37628  37629  37630  37631  37632  37633  37634  37635  37636  37637  37638  37639  37640  37641  37642  37643  37644  37645  37646  37647  37648  37649  37650  37651  37652  37653  37654  37655  37656  37657  37658  37659  37660  37661  37662  37663  37664  37665  37666  37667  37668  37669  37670  37671  37672  37673  37674  37675  37676  37677  37678  37679  37680  37681  37682  37683  37684  37685  37686  37687  37688  37689  37690  37691  37692  37693  37694  37695  37696  37697  37698  37699  37700  37701  37702  37703  37704  37705  37706  37707  37708  37709  37710  37711  37712  37713  37714  37715  37716  37717  37718  37719  37720  37721  37722  37723  37724  37725  37726  37727  37728  37729  37730  37731  37732  37733  37734  37735  37736  37737  37738  37739  37740  37741  37742  37743  37744  37745  37746  37747  37748  37749  37750  37751  37752  37753  37754  37755  37756  37757  37758  37759  37760  37761  37762  37763  37764  37765  37766  37767  37768  37769  37770  37771  37772  37773  37774  37775  37776  37777  37778  37779  37780  37781  37782  37783  37784  37785  37786  37787  37788  37789  37790  37791  37792  37793  37794  37795  37796  37797  37798  37799  37800  37801  37802  37803  37804  37805  37806  37807  37808  37809  37810  37811  37812  37813  37814  37815  37816  37817  37818  37819  37820  37821  37822  37823  37824  37825  37826  37827  37828  37829  37830  37831  37832  37833  37834  37835  37836  37837  37838  37839  37840  37841  37842  37843  37844  37845  37846  37847  37848  37849  37850  37851  37852  37853  37854  37855  37856  37857  37858  37859  37860  37861  37862  37863  37864  37865  37866  37867  37868  37869  37870  37871  37872  37873  37874  37875  37876  37877  37878  37879  37880  37881  37882  37883  37884  37885  37886  37887  37888  37889  37890  37891  37892  37893  37894  37895  37896  37897  37898  37899  37900  37901  37902  37903  37904  37905  37906  37907  37908  37909  37910  37911  37912  37913  37914  37915  37916  37917  37918  37919  37920  37921  37922  37923  37924  37925  37926  37927  37928  37929  37930  37931  37932  37933  37934  37935  37936  37937  37938  37939  37940  37941  37942  37943  37944  37945  37946  37947  37948  37949  37950  37951  37952  37953  37954  37955  37956  37957  37958  37959  37960  37961  37962  37963  37964  37965  37966  37967  37968  37969  37970  37971  37972  37973  37974  37975  37976  37977  37978  37979  37980  37981  37982  37983  37984  37985  37986  37987  37988  37989  37990  37991  37992  37993  37994  37995  37996  37997  37998  37999  38000  38001  38002  38003  38004  38005  38006  38007  38008  38009  38010  38011  38012  38013  38014  38015  38016  38017  38018  38019  38020  38021  38022  38023  38024  38025  38026  38027  38028  38029  38030  38031  38032  38033  38034  38035  38036  38037  38038  38039  38040  38041  38042  38043  38044  38045  38046  38047  38048  38049  38050  38051  38052  38053  38054  38055  38056  38057  38058  38059  38060  38061  38062  38063  38064  38065  38066  38067  38068  38069  38070  38071  38072  38073  38074  38075  38076  38077  38078  38079  38080  38081  38082  38083  38084  38085  38086  38087  38088  38089  38090  38091  38092  38093  38094  38095  38096  38097  38098  38099  38100  38101  38102  38103  38104  38105  38106  38107  38108  38109  38110  38111  38112  38113  38114  38115  38116  38117  38118  38119  38120  38121  38122  38123  38124  38125  38126  38127  38128  38129  38130  38131  38132  38133  38134  38135  38136  38137  38138  38139  38140  38141  38142  38143  38144  38145  38146  38147  38148  38149  38150  38151  38152  38153  38154  38155  38156  38157  38158  38159  38160  38161  38162  38163  38164  38165  38166  38167  38168  38169  38170  38171  38172  38173  38174  38175  38176  38177  38178  38179  38180  38181  38182  38183  38184  38185  38186  38187  38188  38189  38190  38191  38192  38193  38194  38195  38196  38197  38198  38199  38200  38201  38202  38203  38204  38205  38206  38207  38208  38209  38210  38211  38212  38213  38214  38215  38216  38217  38218  38219  38220  38221  38222  38223  38224  38225  38226  38227  38228  38229  38230  38231  38232  38233  38234  38235  38236  38237  38238  38239  38240  38241  38242  38243  38244  38245  38246  38247  38248  38249  38250  38251  38252  38253  38254  38255  38256  38257  38258  38259  38260  38261  38262  38263  38264  38265  38266  38267  38268  38269  38270  38271  38272  38273  38274  38275  38276  38277  38278  38279  38280  38281  38282  38283  38284  38285  38286  38287  38288  38289  38290  38291  38292  38293  38294  38295  38296  38297  38298  38299  38300  38301  38302  38303  38304  38305  38306  38307  38308  38309  38310  38311  38312  38313  38314  38315  38316  38317  38318  38319  38320  38321  38322  38323  38324  38325  38326  38327  38328  38329  38330  38331  38332  38333  38334  38335  38336  38337  38338  38339  38340  38341  38342  38343  38344  38345  38346  38347  38348  38349  38350  38351  38352  38353  38354  38355  38356  38357  38358  38359  38360  38361  38362  38363  38364  38365  38366  38367  38368  38369  38370  38371  38372  38373  38374  38375  38376  38377  38378  38379  38380  38381  38382  38383  38384  38385  38386  38387  38388  38389  38390  38391  38392  38393  38394  38395  38396  38397  38398  38399  38400  38401  38402  38403  38404  38405  38406  38407  38408  38409  38410  38411  38412  38413  38414  38415  38416  38417  38418  38419  38420  38421  38422  38423  38424  38425  38426  38427  38428  38429  38430  38431  38432  38433  38434  38435  38436  38437  38438  38439  38440  38441  38442  38443  38444  38445  38446  38447  38448  38449  38450  38451  38452  38453  38454  38455  38456  38457  38458  38459  38460  38461  38462  38463  38464  38465  38466  38467  38468  38469  38470  38471  38472  38473  38474  38475  38476  38477  38478  38479  38480  38481  38482  38483  38484  38485  38486  38487  38488  38489  38490  38491  38492  38493  38494  38495  38496  38497  38498  38499  38500  38501  38502  38503  38504  38505  38506  38507  38508  38509  38510  38511  38512  38513  38514  38515  38516  38517  38518  38519  38520  38521  38522  38523  38524  38525  38526  38527  38528  38529  38530  38531  38532  38533  38534  38535  38536  38537  38538  38539  38540  38541  38542  38543  38544  38545  38546  38547  38548  38549  38550  38551  38552  38553  38554  38555  38556  38557  38558  38559  38560  38561  38562  38563  38564  38565  38566  38567  38568  38569  38570  38571  38572  38573  38574  38575  38576  38577  38578  38579  38580  38581  38582  38583  38584  38585  38586  38587  38588  38589  38590  38591  38592  38593  38594  38595  38596  38597  38598  38599  38600  38601  38602  38603  38604  38605  38606  38607  38608  38609  38610  38611  38612  38613  38614  38615  38616  38617  38618  38619  38620  38621  38622  38623  38624  38625  38626  38627  38628  38629  38630  38631  38632  38633  38634  38635  38636  38637  38638  38639  38640  38641  38642  38643  38644  38645  38646  38647  38648  38649  38650  38651  38652  38653  38654  38655  38656  38657  38658  38659  38660  38661  38662  38663  38664  38665  38666  38667  38668  38669  38670  38671  38672  38673  38674  38675  38676  38677  38678  38679  38680  38681  38682  38683  38684  38685  38686  38687  38688  38689  38690  38691  38692  38693  38694  38695  38696  38697  38698  38699  38700  38701  38702  38703  38704  38705  38706  38707  38708  38709  38710  38711  38712  38713  38714  38715  38716  38717  38718  38719  38720  38721  38722  38723  38724  38725  38726  38727  38728  38729  38730  38731  38732  38733  38734  38735  38736  38737  38738  38739  38740  38741  38742  38743  38744  38745  38746  38747  38748  38749  38750  38751  38752  38753  38754  38755  38756  38757  38758  38759  38760  38761  38762  38763  38764  38765  38766  38767  38768  38769  38770  38771  38772  38773  38774  38775  38776  38777  38778  38779  38780  38781  38782  38783  38784  38785  38786  38787  38788  38789  38790  38791  38792  38793  38794  38795  38796  38797  38798  38799  38800  38801  38802  38803  38804  38805  38806  38807  38808  38809  38810  38811  38812  38813  38814  38815  38816  38817  38818  38819  38820  38821  38822  38823  38824  38825  38826  38827  38828  38829  38830  38831  38832  38833  38834  38835  38836  38837  38838  38839  38840  38841  38842  38843  38844  38845  38846  38847  38848  38849  38850  38851  38852  38853  38854  38855  38856  38857  38858  38859  38860  38861  38862  38863  38864  38865  38866  38867  38868  38869  38870  38871  38872  38873  38874  38875  38876  38877  38878  38879  38880  38881  38882  38883  38884  38885  38886  38887  38888  38889  38890  38891  38892  38893  38894  38895  38896  38897  38898  38899  38900  38901  38902  38903  38904  38905  38906  38907  38908  38909  38910  38911  38912  38913  38914  38915  38916  38917  38918  38919  38920  38921  38922  38923  38924  38925  38926  38927  38928  38929  38930  38931  38932  38933  38934  38935  38936  38937  38938  38939  38940  38941  38942  38943  38944  38945  38946  38947  38948  38949  38950  38951  38952  38953  38954  38955  38956  38957  38958  38959  38960  38961  38962  38963  38964  38965  38966  38967  38968  38969  38970  38971  38972  38973  38974  38975  38976  38977  38978  38979  38980  38981  38982  38983  38984  38985  38986  38987  38988  38989  38990  38991  38992  38993  38994  38995  38996  38997  38998  38999  39000  39001  39002  39003  39004  39005  39006  39007  39008  39009  39010  39011  39012  39013  39014  39015  39016  39017  39018  39019  39020  39021  39022  39023  39024  39025  39026  39027  39028  39029  39030  39031  39032  39033  39034  39035  39036  39037  39038  39039  39040  39041  39042  39043  39044  39045  39046  39047  39048  39049  39050  39051  39052  39053  39054  39055  39056  39057  39058  39059  39060  39061  39062  39063  39064  39065  39066  39067  39068  39069  39070  39071  39072  39073  39074  39075  39076  39077  39078  39079  39080  39081  39082  39083  39084  39085  39086  39087  39088  39089  39090  39091  39092  39093  39094  39095  39096  39097  39098  39099  39100  39101  39102  39103  39104  39105  39106  39107  39108  39109  39110  39111  39112  39113  39114  39115  39116  39117  39118  39119  39120  39121  39122  39123  39124  39125  39126  39127  39128  39129  39130  39131  39132  39133  39134  39135  39136  39137  39138  39139  39140  39141  39142  39143  39144  39145  39146  39147  39148  39149  39150  39151  39152  39153  39154  39155  39156  39157  39158  39159  39160  39161  39162  39163  39164  39165  39166  39167  39168  39169  39170  39171  39172  39173  39174  39175  39176  39177  39178  39179  39180  39181  39182  39183  39184  39185  39186  39187  39188  39189  39190  39191  39192  39193  39194  39195  39196  39197  39198  39199  39200  39201  39202  39203  39204  39205  39206  39207  39208  39209  39210  39211  39212  39213  39214  39215  39216  39217  39218  39219  39220  39221  39222  39223  39224  39225  39226  39227  39228  39229  39230  39231  39232  39233  39234  39235  39236  39237  39238  39239  39240  39241  39242  39243  39244  39245  39246  39247  39248  39249  39250  39251  39252  39253  39254  39255  39256  39257  39258  39259  39260  39261  39262  39263  39264  39265  39266  39267  39268  39269  39270  39271  39272  39273  39274  39275  39276  39277  39278  39279  39280  39281  39282  39283  39284  39285  39286  39287  39288  39289  39290  39291  39292  39293  39294  39295  39296  39297  39298  39299  39300  39301  39302  39303  39304  39305  39306  39307  39308  39309  39310  39311  39312  39313  39314  39315  39316  39317  39318  39319  39320  39321  39322  39323  39324  39325  39326  39327  39328  39329  39330  39331  39332  39333  39334  39335  39336  39337  39338  39339  39340  39341  39342  39343  39344  39345  39346  39347  39348  39349  39350  39351  39352  39353  39354  39355  39356  39357  39358  39359  39360  39361  39362  39363  39364  39365  39366  39367  39368  39369  39370  39371  39372  39373  39374  39375  39376  39377  39378  39379  39380  39381  39382  39383  39384  39385  39386  39387  39388  39389  39390  39391  39392  39393  39394  39395  39396  39397  39398  39399  39400  39401  39402  39403  39404  39405  39406  39407  39408  39409  39410  39411  39412  39413  39414  39415  39416  39417  39418  39419  39420  39421  39422  39423  39424  39425  39426  39427  39428  39429  39430  39431  39432  39433  39434  39435  39436  39437  39438  39439  39440  39441  39442  39443  39444  39445  39446  39447  39448  39449  39450  39451  39452  39453  39454  39455  39456  39457  39458  39459  39460  39461  39462  39463  39464  39465  39466  39467  39468  39469  39470  39471  39472  39473  39474  39475  39476  39477  39478  39479  39480  39481  39482  39483  39484  39485  39486  39487  39488  39489  39490  39491  39492  39493  39494  39495  39496  39497  39498  39499  39500  39501  39502  39503  39504  39505  39506  39507  39508  39509  39510  39511  39512  39513  39514  39515  39516  39517  39518  39519  39520  39521  39522  39523  39524  39525  39526  39527  39528  39529  39530  39531  39532  39533  39534  39535  39536  39537  39538  39539  39540  39541  39542  39543  39544  39545  39546  39547  39548  39549  39550  39551  39552  39553  39554  39555  39556  39557  39558  39559  39560  39561  39562  39563  39564  39565  39566  39567  39568  39569  39570  39571  39572  39573  39574  39575  39576  39577  39578  39579  39580  39581  39582  39583  39584  39585  39586  39587  39588  39589  39590  39591  39592  39593  39594  39595  39596  39597  39598  39599  39600  39601  39602  39603  39604  39605  39606  39607  39608  39609  39610  39611  39612  39613  39614  39615  39616  39617  39618  39619  39620  39621  39622  39623  39624  39625  39626  39627  39628  39629  39630  39631  39632  39633  39634  39635  39636  39637  39638  39639  39640  39641  39642  39643  39644  39645  39646  39647  39648  39649  39650  39651  39652  39653  39654  39655  39656  39657  39658  39659  39660  39661  39662  39663  39664  39665  39666  39667  39668  39669  39670  39671  39672  39673  39674  39675  39676  39677  39678  39679  39680  39681  39682  39683  39684  39685  39686  39687  39688  39689  39690  39691  39692  39693  39694  39695  39696  39697  39698  39699  39700  39701  39702  39703  39704  39705  39706  39707  39708  39709  39710  39711  39712  39713  39714  39715  39716  39717  39718  39719  39720  39721  39722  39723  39724  39725  39726  39727  39728  39729  39730  39731  39732  39733  39734  39735  39736  39737  39738  39739  39740  39741  39742  39743  39744  39745  39746  39747  39748  39749  39750  39751  39752  39753  39754  39755  39756  39757  39758  39759  39760  39761  39762  39763  39764  39765  39766  39767  39768  39769  39770  39771  39772  39773  39774  39775  39776  39777  39778  39779  39780  39781  39782  39783  39784  39785  39786  39787  39788  39789  39790  39791  39792  39793  39794  39795  39796  39797  39798  39799  39800  39801  39802  39803  39804  39805  39806  39807  39808  39809  39810  39811  39812  39813  39814  39815  39816  39817  39818  39819  39820  39821  39822  39823  39824  39825  39826  39827  39828  39829  39830  39831  39832  39833  39834  39835  39836  39837  39838  39839  39840  39841  39842  39843  39844  39845  39846  39847  39848  39849  39850  39851  39852  39853  39854  39855  39856  39857  39858  39859  39860  39861  39862  39863  39864  39865  39866  39867  39868  39869  39870  39871  39872  39873  39874  39875  39876  39877  39878  39879  39880  39881  39882  39883  39884  39885  39886  39887  39888  39889  39890  39891  39892  39893  39894  39895  39896  39897  39898  39899  39900  39901  39902  39903  39904  39905  39906  39907  39908  39909  39910  39911  39912  39913  39914  39915  39916  39917  39918  39919  39920  39921  39922  39923  39924  39925  39926  39927  39928  39929  39930  39931  39932  39933  39934  39935  39936  39937  39938  39939  39940  39941  39942  39943  39944  39945  39946  39947  39948  39949  39950  39951  39952  39953  39954  39955  39956  39957  39958  39959  39960  39961  39962  39963  39964  39965  39966  39967  39968  39969  39970  39971  39972  39973  39974  39975  39976  39977  39978  39979  39980  39981  39982  39983  39984  39985  39986  39987  39988  39989  39990  39991  39992  39993  39994  39995  39996  39997  39998  39999  40000  40001  40002  40003  40004  40005  40006  40007  40008  40009  40010  40011  40012  40013  40014  40015  40016  40017  40018  40019  40020  40021  40022  40023  40024  40025  40026  40027  40028  40029  40030  40031  40032  40033  40034  40035  40036  40037  40038  40039  40040  40041  40042  40043  40044  40045  40046  40047  40048  40049  40050  40051  40052  40053  40054  40055  40056  40057  40058  40059  40060  40061  40062  40063  40064  40065  40066  40067  40068  40069  40070  40071  40072  40073  40074  40075  40076  40077  40078  40079  40080  40081  40082  40083  40084  40085  40086  40087  40088  40089  40090  40091  40092  40093  40094  40095  40096  40097  40098  40099  40100  40101  40102  40103  40104  40105  40106  40107  40108  40109  40110  40111  40112  40113  40114  40115  40116  40117  40118  40119  40120  40121  40122  40123  40124  40125  40126  40127  40128  40129  40130  40131  40132  40133  40134  40135  40136  40137  40138  40139  40140  40141  40142  40143  40144  40145  40146  40147  40148  40149  40150  40151  40152  40153  40154  40155  40156  40157  40158  40159  40160  40161  40162  40163  40164  40165  40166  40167  40168  40169  40170  40171  40172  40173  40174  40175  40176  40177  40178  40179  40180  40181  40182  40183  40184  40185  40186  40187  40188  40189  40190  40191  40192  40193  40194  40195  40196  40197  40198  40199  40200  40201  40202  40203  40204  40205  40206  40207  40208  40209  40210  40211  40212  40213  40214  40215  40216  40217  40218  40219  40220  40221  40222  40223  40224  40225  40226  40227  40228  40229  40230  40231  40232  40233  40234  40235  40236  40237  40238  40239  40240  40241  40242  40243  40244  40245  40246  40247  40248  40249  40250  40251  40252  40253  40254  40255  40256  40257  40258  40259  40260  40261  40262  40263  40264  40265  40266  40267  40268  40269  40270  40271  40272  40273  40274  40275  40276  40277  40278  40279  40280  40281  40282  40283  40284  40285  40286  40287  40288  40289  40290  40291  40292  40293  40294  40295  40296  40297  40298  40299  40300  40301  40302  40303  40304  40305  40306  40307  40308  40309  40310  40311  40312  40313  40314  40315  40316  40317  40318  40319  40320  40321  40322  40323  40324  40325  40326  40327  40328  40329  40330  40331  40332  40333  40334  40335  40336  40337  40338  40339  40340  40341  40342  40343  40344  40345  40346  40347  40348  40349  40350  40351  40352  40353  40354  40355  40356  40357  40358  40359  40360  40361  40362  40363  40364  40365  40366  40367  40368  40369  40370  40371  40372  40373  40374  40375  40376  40377  40378  40379  40380  40381  40382  40383  40384  40385  40386  40387  40388  40389  40390  40391  40392  40393  40394  40395  40396  40397  40398  40399  40400  40401  40402  40403  40404  40405  40406  40407  40408  40409  40410  40411  40412  40413  40414  40415  40416  40417  40418  40419  40420  40421  40422  40423  40424  40425  40426  40427  40428  40429  40430  40431  40432  40433  40434  40435  40436  40437  40438  40439  40440  40441  40442  40443  40444  40445  40446  40447  40448  40449  40450  40451  40452  40453  40454  40455  40456  40457  40458  40459  40460  40461  40462  40463  40464  40465  40466  40467  40468  40469  40470  40471  40472  40473  40474  40475  40476  40477  40478  40479  40480  40481  40482  40483  40484  40485  40486  40487  40488  40489  40490  40491  40492  40493  40494  40495  40496  40497  40498  40499  40500  40501  40502  40503  40504  40505  40506  40507  40508  40509  40510  40511  40512  40513  40514  40515  40516  40517  40518  40519  40520  40521  40522  40523  40524  40525  40526  40527  40528  40529  40530  40531  40532  40533  40534  40535  40536  40537  40538  40539  40540  40541  40542  40543  40544  40545  40546  40547  40548  40549  40550  40551  40552  40553  40554  40555  40556  40557  40558  40559  40560  40561  40562  40563  40564  40565  40566  40567  40568  40569  40570  40571  40572  40573  40574  40575  40576  40577  40578  40579  40580  40581  40582  40583  40584  40585  40586  40587  40588  40589  40590  40591  40592  40593  40594  40595  40596  40597  40598  40599  40600  40601  40602  40603  40604  40605  40606  40607  40608  40609  40610  40611  40612  40613  40614  40615  40616  40617  40618  40619  40620  40621  40622  40623  40624  40625  40626  40627  40628  40629  40630  40631  40632  40633  40634  40635  40636  40637  40638  40639  40640  40641  40642  40643  40644  40645  40646  40647  40648  40649  40650  40651  40652  40653  40654  40655  40656  40657  40658  40659  40660  40661  40662  40663  40664  40665  40666  40667  40668  40669  40670  40671  40672  40673  40674  40675  40676  40677  40678  40679  40680  40681  40682  40683  40684  40685  40686  40687  40688  40689  40690  40691  40692  40693  40694  40695  40696  40697  40698  40699  40700  40701  40702  40703  40704  40705  40706  40707  40708  40709  40710  40711  40712  40713  40714  40715  40716  40717  40718  40719  40720  40721  40722  40723  40724  40725  40726  40727  40728  40729  40730  40731  40732  40733  40734  40735  40736  40737  40738  40739  40740  40741  40742  40743  40744  40745  40746  40747  40748  40749  40750  40751  40752  40753  40754  40755  40756  40757  40758  40759  40760  40761  40762  40763  40764  40765  40766  40767  40768  40769  40770  40771  40772  40773  40774  40775  40776  40777  40778  40779  40780  40781  40782  40783  40784  40785  40786  40787  40788  40789  40790  40791  40792  40793  40794  40795  40796  40797  40798  40799  40800  40801  40802  40803  40804  40805  40806  40807  40808  40809  40810  40811  40812  40813  40814  40815  40816  40817  40818  40819  40820  40821  40822  40823  40824  40825  40826  40827  40828  40829  40830  40831  40832  40833  40834  40835  40836  40837  40838  40839  40840  40841  40842  40843  40844  40845  40846  40847  40848  40849  40850  40851  40852  40853  40854  40855  40856  40857  40858  40859  40860  40861  40862  40863  40864  40865  40866  40867  40868  40869  40870  40871  40872  40873  40874  40875  40876  40877  40878  40879  40880  40881  40882  40883  40884  40885  40886  40887  40888  40889  40890  40891  40892  40893  40894  40895  40896  40897  40898  40899  40900  40901  40902  40903  40904  40905  40906  40907  40908  40909  40910  40911  40912  40913  40914  40915  40916  40917  40918  40919  40920  40921  40922  40923  40924  40925  40926  40927  40928  40929  40930  40931  40932  40933  40934  40935  40936  40937  40938  40939  40940  40941  40942  40943  40944  40945  40946  40947  40948  40949  40950  40951  40952  40953  40954  40955  40956  40957  40958  40959  40960  40961  40962  40963  40964  40965  40966  40967  40968  40969  40970  40971  40972  40973  40974  40975  40976  40977  40978  40979  40980  40981  40982  40983  40984  40985  40986  40987  40988  40989  40990  40991  40992  40993  40994  40995  40996  40997  40998  40999  41000  41001  41002  41003  41004  41005  41006  41007  41008  41009  41010  41011  41012  41013  41014  41015  41016  41017  41018  41019  41020  41021  41022  41023  41024  41025  41026  41027  41028  41029  41030  41031  41032  41033  41034  41035  41036  41037  41038  41039  41040  41041  41042  41043  41044  41045  41046  41047  41048  41049  41050  41051  41052  41053  41054  41055  41056  41057  41058  41059  41060  41061  41062  41063  41064  41065  41066  41067  41068  41069  41070  41071  41072  41073  41074  41075  41076  41077  41078  41079  41080  41081  41082  41083  41084  41085  41086  41087  41088  41089  41090  41091  41092  41093  41094  41095  41096  41097  41098  41099  41100  41101  41102  41103  41104  41105  41106  41107  41108  41109  41110  41111  41112  41113  41114  41115  41116  41117  41118  41119  41120  41121  41122  41123  41124  41125  41126  41127  41128  41129  41130  41131  41132  41133  41134  41135  41136  41137  41138  41139  41140  41141  41142  41143  41144  41145  41146  41147  41148  41149  41150  41151  41152  41153  41154  41155  41156  41157  41158  41159  41160  41161  41162  41163  41164  41165  41166  41167  41168  41169  41170  41171  41172  41173  41174  41175  41176  41177  41178  41179  41180  41181  41182  41183  41184  41185  41186  41187  41188  41189  41190  41191  41192  41193  41194  41195  41196  41197  41198  41199  41200  41201  41202  41203  41204  41205  41206  41207  41208  41209  41210  41211  41212  41213  41214  41215  41216  41217  41218  41219  41220  41221  41222  41223  41224  41225  41226  41227  41228  41229  41230  41231  41232  41233  41234  41235  41236  41237  41238  41239  41240  41241  41242  41243  41244  41245  41246  41247  41248  41249  41250  41251  41252  41253  41254  41255  41256  41257  41258  41259  41260  41261  41262  41263  41264  41265  41266  41267  41268  41269  41270  41271  41272  41273  41274  41275  41276  41277  41278  41279  41280  41281  41282  41283  41284  41285  41286  41287  41288  41289  41290  41291  41292  41293  41294  41295  41296  41297  41298  41299  41300  41301  41302  41303  41304  41305  41306  41307  41308  41309  41310  41311  41312  41313  41314  41315  41316  41317  41318  41319  41320  41321  41322  41323  41324  41325  41326  41327  41328  41329  41330  41331  41332  41333  41334  41335  41336  41337  41338  41339  41340  41341  41342  41343  41344  41345  41346  41347  41348  41349  41350  41351  41352  41353  41354  41355  41356  41357  41358  41359  41360  41361  41362  41363  41364  41365  41366  41367  41368  41369  41370  41371  41372  41373  41374  41375  41376  41377  41378  41379  41380  41381  41382  41383  41384  41385  41386  41387  41388  41389  41390  41391  41392  41393  41394  41395  41396  41397  41398  41399  41400  41401  41402  41403  41404  41405  41406  41407  41408  41409  41410  41411  41412  41413  41414  41415  41416  41417  41418  41419  41420  41421  41422  41423  41424  41425  41426  41427  41428  41429  41430  41431  41432  41433  41434  41435  41436  41437  41438  41439  41440  41441  41442  41443  41444  41445  41446  41447  41448  41449  41450  41451  41452  41453  41454  41455  41456  41457  41458  41459  41460  41461  41462  41463  41464  41465  41466  41467  41468  41469  41470  41471  41472  41473  41474  41475  41476  41477  41478  41479  41480  41481  41482  41483  41484  41485  41486  41487  41488  41489  41490  41491  41492  41493  41494  41495  41496  41497  41498  41499  41500  41501  41502  41503  41504  41505  41506  41507  41508  41509  41510  41511  41512  41513  41514  41515  41516  41517  41518  41519  41520  41521  41522  41523  41524  41525  41526  41527  41528  41529  41530  41531  41532  41533  41534  41535  41536  41537  41538  41539  41540  41541  41542  41543  41544  41545  41546  41547  41548  41549  41550  41551  41552  41553  41554  41555  41556  41557  41558  41559  41560  41561  41562  41563  41564  41565  41566  41567  41568  41569  41570  41571  41572  41573  41574  41575  41576  41577  41578  41579  41580  41581  41582  41583  41584  41585  41586  41587  41588  41589  41590  41591  41592  41593  41594  41595  41596  41597  41598  41599  41600  41601  41602  41603  41604  41605  41606  41607  41608  41609  41610  41611  41612  41613  41614  41615  41616  41617  41618  41619  41620  41621  41622  41623  41624  41625  41626  41627  41628  41629  41630  41631  41632  41633  41634  41635  41636  41637  41638  41639  41640  41641  41642  41643  41644  41645  41646  41647  41648  41649  41650  41651  41652  41653  41654  41655  41656  41657  41658  41659  41660  41661  41662  41663  41664  41665  41666  41667  41668  41669  41670  41671  41672  41673  41674  41675  41676  41677  41678  41679  41680  41681  41682  41683  41684  41685  41686  41687  41688  41689  41690  41691  41692  41693  41694  41695  41696  41697  41698  41699  41700  41701  41702  41703  41704  41705  41706  41707  41708  41709  41710  41711  41712  41713  41714  41715  41716  41717  41718  41719  41720  41721  41722  41723  41724  41725  41726  41727  41728  41729  41730  41731  41732  41733  41734  41735  41736  41737  41738  41739  41740  41741  41742  41743  41744  41745  41746  41747  41748  41749  41750  41751  41752  41753  41754  41755  41756  41757  41758  41759  41760  41761  41762  41763  41764  41765  41766  41767  41768  41769  41770  41771  41772  41773  41774  41775  41776  41777  41778  41779  41780  41781  41782  41783  41784  41785  41786  41787  41788  41789  41790  41791  41792  41793  41794  41795  41796  41797  41798  41799  41800  41801  41802  41803  41804  41805  41806  41807  41808  41809  41810  41811  41812  41813  41814  41815  41816  41817  41818  41819  41820  41821  41822  41823  41824  41825  41826  41827  41828  41829  41830  41831  41832  41833  41834  41835  41836  41837  41838  41839  41840  41841  41842  41843  41844  41845  41846  41847  41848  41849  41850  41851  41852  41853  41854  41855  41856  41857  41858  41859  41860  41861  41862  41863  41864  41865  41866  41867  41868  41869  41870  41871  41872  41873  41874  41875  41876  41877  41878  41879  41880  41881  41882  41883  41884  41885  41886  41887  41888  41889  41890  41891  41892  41893  41894  41895  41896  41897  41898  41899  41900  41901  41902  41903  41904  41905  41906  41907  41908  41909  41910  41911  41912  41913  41914  41915  41916  41917  41918  41919  41920  41921  41922  41923  41924  41925  41926  41927  41928  41929  41930  41931  41932  41933  41934  41935  41936  41937  41938  41939  41940  41941  41942  41943  41944  41945  41946  41947  41948  41949  41950  41951  41952  41953  41954  41955  41956  41957  41958  41959  41960  41961  41962  41963  41964  41965  41966  41967  41968  41969  41970  41971  41972  41973  41974  41975  41976  41977  41978  41979  41980  41981  41982  41983  41984  41985  41986  41987  41988  41989  41990  41991  41992  41993  41994  41995  41996  41997  41998  41999  42000  42001  42002  42003  42004  42005  42006  42007  42008  42009  42010  42011  42012  42013  42014  42015  42016  42017  42018  42019  42020  42021  42022  42023  42024  42025  42026  42027  42028  42029  42030  42031  42032  42033  42034  42035  42036  42037  42038  42039  42040  42041  42042  42043  42044  42045  42046  42047  42048  42049  42050  42051  42052  42053  42054  42055  42056  42057  42058  42059  42060  42061  42062  42063  42064  42065  42066  42067  42068  42069  42070  42071  42072  42073  42074  42075  42076  42077  42078  42079  42080  42081  42082  42083  42084  42085  42086  42087  42088  42089  42090  42091  42092  42093  42094  42095  42096  42097  42098  42099  42100  42101  42102  42103  42104  42105  42106  42107  42108  42109  42110  42111  42112  42113  42114  42115  42116  42117  42118  42119  42120  42121  42122  42123  42124  42125  42126  42127  42128  42129  42130  42131  42132  42133  42134  42135  42136  42137  42138  42139  42140  42141  42142  42143  42144  42145  42146  42147  42148  42149  42150  42151  42152  42153  42154  42155  42156  42157  42158  42159  42160  42161  42162  42163  42164  42165  42166  42167  42168  42169  42170  42171  42172  42173  42174  42175  42176  42177  42178  42179  42180  42181  42182  42183  42184  42185  42186  42187  42188  42189  42190  42191  42192  42193  42194  42195  42196  42197  42198  42199  42200  42201  42202  42203  42204  42205  42206  42207  42208  42209  42210  42211  42212  42213  42214  42215  42216  42217  42218  42219  42220  42221  42222  42223  42224  42225  42226  42227  42228  42229  42230  42231  42232  42233  42234  42235  42236  42237  42238  42239  42240  42241  42242  42243  42244  42245  42246  42247  42248  42249  42250  42251  42252  42253  42254  42255  42256  42257  42258  42259  42260  42261  42262  42263  42264  42265  42266  42267  42268  42269  42270  42271  42272  42273  42274  42275  42276  42277  42278  42279  42280  42281  42282  42283  42284  42285  42286  42287  42288  42289  42290  42291  42292  42293  42294  42295  42296  42297  42298  42299  42300  42301  42302  42303  42304  42305  42306  42307  42308  42309  42310  42311  42312  42313  42314  42315  42316  42317  42318  42319  42320  42321  42322  42323  42324  42325  42326  42327  42328  42329  42330  42331  42332  42333  42334  42335  42336  42337  42338  42339  42340  42341  42342  42343  42344  42345  42346  42347  42348  42349  42350  42351  42352  42353  42354  42355  42356  42357  42358  42359  42360  42361  42362  42363  42364  42365  42366  42367  42368  42369  42370  42371  42372  42373  42374  42375  42376  42377  42378  42379  42380  42381  42382  42383  42384  42385  42386  42387  42388  42389  42390  42391  42392  42393  42394  42395  42396  42397  42398  42399  42400  42401  42402  42403  42404  42405  42406  42407  42408  42409  42410  42411  42412  42413  42414  42415  42416  42417  42418  42419  42420  42421  42422  42423  42424  42425  42426  42427  42428  42429  42430  42431  42432  42433  42434  42435  42436  42437  42438  42439  42440  42441  42442  42443  42444  42445  42446  42447  42448  42449  42450  42451  42452  42453  42454  42455  42456  42457  42458  42459  42460  42461  42462  42463  42464  42465  42466  42467  42468  42469  42470  42471  42472  42473  42474  42475  42476  42477  42478  42479  42480  42481  42482  42483  42484  42485  42486  42487  42488  42489  42490  42491  42492  42493  42494  42495  42496  42497  42498  42499  42500  42501  42502  42503  42504  42505  42506  42507  42508  42509  42510  42511  42512  42513  42514  42515  42516  42517  42518  42519  42520  42521  42522  42523  42524  42525  42526  42527  42528  42529  42530  42531  42532  42533  42534  42535  42536  42537  42538  42539  42540  42541  42542  42543  42544  42545  42546  42547  42548  42549  42550  42551  42552  42553  42554  42555  42556  42557  42558  42559  42560  42561  42562  42563  42564  42565  42566  42567  42568  42569  42570  42571  42572  42573  42574  42575  42576  42577  42578  42579  42580  42581  42582  42583  42584  42585  42586  42587  42588  42589  42590  42591  42592  42593  42594  42595  42596  42597  42598  42599  42600  42601  42602  42603  42604  42605  42606  42607  42608  42609  42610  42611  42612  42613  42614  42615  42616  42617  42618  42619  42620  42621  42622  42623  42624  42625  42626  42627  42628  42629  42630  42631  42632  42633  42634  42635  42636  42637  42638  42639  42640  42641  42642  42643  42644  42645  42646  42647  42648  42649  42650  42651  42652  42653  42654  42655  42656  42657  42658  42659  42660  42661  42662  42663  42664  42665  42666  42667  42668  42669  42670  42671  42672  42673  42674  42675  42676  42677  42678  42679  42680  42681  42682  42683  42684  42685  42686  42687  42688  42689  42690  42691  42692  42693  42694  42695  42696  42697  42698  42699  42700  42701  42702  42703  42704  42705  42706  42707  42708  42709  42710  42711  42712  42713  42714  42715  42716  42717  42718  42719  42720  42721  42722  42723  42724  42725  42726  42727  42728  42729  42730  42731  42732  42733  42734  42735  42736  42737  42738  42739  42740  42741  42742  42743  42744  42745  42746  42747  42748  42749  42750  42751  42752  42753  42754  42755  42756  42757  42758  42759  42760  42761  42762  42763  42764  42765  42766  42767  42768  42769  42770  42771  42772  42773  42774  42775  42776  42777  42778  42779  42780  42781  42782  42783  42784  42785  42786  42787  42788  42789  42790  42791  42792  42793  42794  42795  42796  42797  42798  42799  42800  42801  42802  42803  42804  42805  42806  42807  42808  42809  42810  42811  42812  42813  42814  42815  42816  42817  42818  42819  42820  42821  42822  42823  42824  42825  42826  42827  42828  42829  42830  42831  42832  42833  42834  42835  42836  42837  42838  42839  42840  42841  42842  42843  42844  42845  42846  42847  42848  42849  42850  42851  42852  42853  42854  42855  42856  42857  42858  42859  42860  42861  42862  42863  42864  42865  42866  42867  42868  42869  42870  42871  42872  42873  42874  42875  42876  42877  42878  42879  42880  42881  42882  42883  42884  42885  42886  42887  42888  42889  42890  42891  42892  42893  42894  42895  42896  42897  42898  42899  42900  42901  42902  42903  42904  42905  42906  42907  42908  42909  42910  42911  42912  42913  42914  42915  42916  42917  42918  42919  42920  42921  42922  42923  42924  42925  42926  42927  42928  42929  42930  42931  42932  42933  42934  42935  42936  42937  42938  42939  42940  42941  42942  42943  42944  42945  42946  42947  42948  42949  42950  42951  42952  42953  42954  42955  42956  42957  42958  42959  42960  42961  42962  42963  42964  42965  42966  42967  42968  42969  42970  42971  42972  42973  42974  42975  42976  42977  42978  42979  42980  42981  42982  42983  42984  42985  42986  42987  42988  42989  42990  42991  42992  42993  42994  42995  42996  42997  42998  42999  43000  43001  43002  43003  43004  43005  43006  43007  43008  43009  43010  43011  43012  43013  43014  43015  43016  43017  43018  43019  43020  43021  43022  43023  43024  43025  43026  43027  43028  43029  43030  43031  43032  43033  43034  43035  43036  43037  43038  43039  43040  43041  43042  43043  43044  43045  43046  43047  43048  43049  43050  43051  43052  43053  43054  43055  43056  43057  43058  43059  43060  43061  43062  43063  43064  43065  43066  43067  43068  43069  43070  43071  43072  43073  43074  43075  43076  43077  43078  43079  43080  43081  43082  43083  43084  43085  43086  43087  43088  43089  43090  43091  43092  43093  43094  43095  43096  43097  43098  43099  43100  43101  43102  43103  43104  43105  43106  43107  43108  43109  43110  43111  43112  43113  43114  43115  43116  43117  43118  43119  43120  43121  43122  43123  43124  43125  43126  43127  43128  43129  43130  43131  43132  43133  43134  43135  43136  43137  43138  43139  43140  43141  43142  43143  43144  43145  43146  43147  43148  43149  43150  43151  43152  43153  43154  43155  43156  43157  43158  43159  43160  43161  43162  43163  43164  43165  43166  43167  43168  43169  43170  43171  43172  43173  43174  43175  43176  43177  43178  43179  43180  43181  43182  43183  43184  43185  43186  43187  43188  43189  43190  43191  43192  43193  43194  43195  43196  43197  43198  43199  43200  43201  43202  43203  43204  43205  43206  43207  43208  43209  43210  43211  43212  43213  43214  43215  43216  43217  43218  43219  43220  43221  43222  43223  43224  43225  43226  43227  43228  43229  43230  43231  43232  43233  43234  43235  43236  43237  43238  43239  43240  43241  43242  43243  43244  43245  43246  43247  43248  43249  43250  43251  43252  43253  43254  43255  43256  43257  43258  43259  43260  43261  43262  43263  43264  43265  43266  43267  43268  43269  43270  43271  43272  43273  43274  43275  43276  43277  43278  43279  43280  43281  43282  43283  43284  43285  43286  43287  43288  43289  43290  43291  43292  43293  43294  43295  43296  43297  43298  43299  43300  43301  43302  43303  43304  43305  43306  43307  43308  43309  43310  43311  43312  43313  43314  43315  43316  43317  43318  43319  43320  43321  43322  43323  43324  43325  43326  43327  43328  43329  43330  43331  43332  43333  43334  43335  43336  43337  43338  43339  43340  43341  43342  43343  43344  43345  43346  43347  43348  43349  43350  43351  43352  43353  43354  43355  43356  43357  43358  43359  43360  43361  43362  43363  43364  43365  43366  43367  43368  43369  43370  43371  43372  43373  43374  43375  43376  43377  43378  43379  43380  43381  43382  43383  43384  43385  43386  43387  43388  43389  43390  43391  43392  43393  43394  43395  43396  43397  43398  43399  43400  43401  43402  43403  43404  43405  43406  43407  43408  43409  43410  43411  43412  43413  43414  43415  43416  43417  43418  43419  43420  43421  43422  43423  43424  43425  43426  43427  43428  43429  43430  43431  43432  43433  43434  43435  43436  43437  43438  43439  43440  43441  43442  43443  43444  43445  43446  43447  43448  43449  43450  43451  43452  43453  43454  43455  43456  43457  43458  43459  43460  43461  43462  43463  43464  43465  43466  43467  43468  43469  43470  43471  43472  43473  43474  43475  43476  43477  43478  43479  43480  43481  43482  43483  43484  43485  43486  43487  43488  43489  43490  43491  43492  43493  43494  43495  43496  43497  43498  43499  43500  43501  43502  43503  43504  43505  43506  43507  43508  43509  43510  43511  43512  43513  43514  43515  43516  43517  43518  43519  43520  43521  43522  43523  43524  43525  43526  43527  43528  43529  43530  43531  43532 | A6ND36  A6ND36  A6ND36  A6ND36  A6ND36  A6ND36  A6ND36  A6ND36  A6ND36  A6ND36  A6ND36  A6ND36  A6ND36  A6ND36  A6ND36  A6ND36  A6ND36  A6ND36  A6ND36  A6ND36  A6ND36  A6ND36  A6ND36  A6ND36  A6ND36  A6ND36  A6ND36  A6ND36  A6ND36  A6ND36  A6ND36  A6NHR9  A6NHR9  A6NHR9  A6NHR9  A6NHR9  A6NHR9  A6NHR9  A6NHR9  A6NHR9  A6NHR9  A6NHR9  A6NHR9  A6NHR9  A6NHR9  A6NHR9  A6NHR9  A6NHR9  A6NHR9  A6NHR9  A6NHR9  A6NHR9  A6NHR9  A6NHR9  A6NHR9  A6NHR9  A6NHR9  A6NHR9  A6NHR9  A6NHR9  A6NHR9  A6NHR9  A6NHR9  A6NHR9  A8K4G0  A8K4G0  A8K4G0  A8K4G0  A8K4G0  A8K4G0  A8K4G0  A8K4G0  A8K4G0  A8K4G0  A8K4G0  A8K4G0  A8K4G0  A8K4G0  A8K4G0  O00151  O00151  O00151  O00151  O00151  O00151  O00151  O00151  O00151  O00151  O00151  O00151  O00151  O00151  O00151  O00151  O00151  O00151  O00151  O00151  O00151  O00151  O00151  O00151  O00151  O00151  O00159  O00159  O00159  O00159  O00159  O00159  O00159  O00159  O00159  O00159  O00159  O00159  O00159  O00159  O00159  O00159  O00159  O00159  O00159  O00159  O00159  O00159  O00159  O00159  O00159  O00159  O00159  O00159  O00159  O00159  O00159  O00159  O00159  O00161  O00161  O00161  O00161  O00161  O00161  O00161  O00161  O00165  O00165  O00165  O00165  O00165  O00165  O00165  O00165  O00165  O00165  O00165  O00165  O00165  O00165  O00165  O00165  O00165  O00165  O00165  O00165  O00165  O00165  O00165  O00165  O00165  O00168  O00168  O00168  O00168  O00194  O00194  O00194  O00194  O00194  O00194  O00194  O00194  O00194  O00204  O00204  O00204  O00204  O00204  O00204  O00204  O00204  O00204  O00204  O00204  O00204  O00204  O00204  O00204  O00204  O00204  O00204  O00204  O00204  O00204  O00204  O00204  O00204  O00204  O00204  O00204  O00204  O00204  O00204  O00204  O00213  O00213  O00213  O00213  O00213  O00213  O00213  O00213  O00213  O00213  O00213  O00213  O00213  O00213  O00213  O00213  O00213  O00213  O00213  O00213  O00213  O00213  O00213  O00213  O00213  O00213  O00213  O00213  O00213  O00213  O00213  O00213  O00213  O00231  O00231  O00231  O00231  O00231  O00231  O00231  O00231  O00231  O00231  O00231  O00231  O00231  O00231  O00231  O00231  O00231  O00231  O00231  O00231  O00231  O00231  O00231  O00231  O00231  O00231  O00231  O00231  O00231  O00231  O00231  O00232  O00232  O00232  O00232  O00232  O00232  O00232  O00232  O00232  O00232  O00232  O00232  O00232  O00232  O00232  O00232  O00232  O00232  O00232  O00232  O00232  O00232  O00232  O00232  O00232  O00232  O00257  O00257  O00257  O00257  O00257  O00257  O00257  O00257  O00257  O00257  O00257  O00257  O00257  O00257  O00257  O00257  O00257  O00257  O00257  O00257  O00257  O00257  O00257  O00257  O00257  O00257  O00257  O00257  O00257  O00257  O00257  O00257  O00257  O00264  O00264  O00264  O00264  O00264  O00264  O00264  O00267  O00267  O00267  O00267  O00267  O00267  O00267  O00267  O00267  O00267  O00267  O00267  O00267  O00267  O00267  O00267  O00267  O00267  O00267  O00267  O00267  O00267  O00267  O00267  O00267  O00267  O00267  O00267  O00267  O00267  O00267  O00267  O00267  O00273  O00273  O00273  O00273  O00273  O00273  O00273  O00273  O00273  O00273  O00273  O00273  O00273  O00273  O00273  O00273  O00273  O00273  O00273  O00273  O00273  O00273  O00273  O00273  O00273  O00273  O00273  O00273  O00273  O00273  O00273  O00273  O00273  O00303  O00303  O00303  O00303  O00303  O00303  O00303  O00303  O00303  O00303  O00303  O00303  O00303  O00303  O00303  O00303  O00303  O00303  O00303  O00303  O00303  O00303  O00303  O00303  O00303  O00330  O00330  O00330  O00330  O00330  O00330  O00330  O00330  O00330  O00330  O00330  O00330  O00330  O00330  O00330  O00330  O00330  O00330  O00330  O00330  O00330  O00330  O00330  O00330  O00330  O00330  O00330  O00330  O00330  O00330  O00330  O00330  O00330  O00391  O00391  O00391  O00391  O00391  O00391  O00391  O00391  O00391  O00391  O00391  O00391  O00391  O00391  O00391  O00391  O00391  O00391  O00391  O00391  O00391  O00391  O00391  O00391  O00391  O00391  O00391  O00391  O00391  O00391  O00391  O00399  O00399  O00399  O00399  O00399  O00401  O00401  O00401  O00401  O00401  O00401  O00401  O00401  O00401  O00401  O00401  O00401  O00401  O00401  O00401  O00401  O00401  O00401  O00401  O00401  O00401  O00401  O00401  O00401  O00401  O00401  O00401  O00401  O00401  O00410  O00410  O00410  O00410  O00410  O00410  O00410  O00410  O00410  O00410  O00410  O00410  O00410  O00410  O00410  O00410  O00410  O00410  O00410  O00410  O00410  O00410  O00410  O00410  O00410  O00410  O00410  O00410  O00410  O00410  O00410  O00410  O00410  O00418  O00418  O00418  O00418  O00418  O00418  O00418  O00418  O00418  O00418  O00418  O00418  O00418  O00418  O00418  O00418  O00418  O00418  O00418  O00418  O00418  O00418  O00418  O00418  O00418  O00418  O00418  O00418  O00418  O00418  O00418  O00429  O00429  O00429  O00429  O00429  O00429  O00429  O00429  O00429  O00429  O00429  O00429  O00429  O00429  O00429  O00429  O00429  O00429  O00429  O00429  O00429  O00429  O00429  O00429  O00429  O00429  O00429  O00429  O00429  O00429  O00429  O00443  O00443  O00443  O00443  O00443  O00443  O00443  O00443  O00443  O00443  O00443  O00443  O00443  O00443  O00443  O00443  O00443  O00443  O00443  O00443  O00443  O00443  O00443  O00443  O00443  O00443  O00443  O00443  O00443  O00443  O00443  O00459  O00459  O00459  O00459  O00459  O00459  O00459  O00459  O00459  O00459  O00459  O00459  O00459  O00459  O00459  O00459  O00459  O00459  O00459  O00459  O00459  O00459  O00459  O00459  O00459  O00459  O00459  O00459  O00459  O00459  O00459  O00459  O00459  O00560  O00560  O00560  O00560  O00560  O00560  O00560  O00560  O00560  O00560  O00560  O00560  O00560  O00560  O00560  O00560  O00560  O00560  O00560  O00560  O00560  O00560  O00562  O00562  O00562  O00562  O00562  O00562  O00562  O00562  O00562  O00562  O00562  O00562  O00562  O00562  O00562  O00562  O00562  O00562  O00562  O00562  O00562  O00562  O00562  O00562  O00562  O00562  O00562  O00562  O00562  O00562  O00562  O00571  O00571  O00571  O00571  O00571  O00571  O00571  O00571  O00571  O00571  O00571  O00571  O00571  O00571  O00571  O00571  O00571  O00571  O00571  O00571  O00571  O00571  O00571  O00571  O00571  O00571  O00571  O00571  O00571  O00571  O00571  O00622  O00622  O00622  O00622  O00622  O00622  O00622  O00622  O00622  O00622  O00622  O00622  O00622  O00622  O00622  O00622  O00622  O00622  O00622  O00622  O00622  O00622  O00622  O00763  O00763  O00763  O00763  O00763  O00763  O00763  O00763  O00763  O00763  O00763  O00763  O00763  O00763  O00763  O00763  O00763  O00763  O00763  O00763  O00763  O00763  O00763  O00763  O00763  O00763  O00763  O00763  O00763  O00763  O00763  O14492  O14492  O14492  O14492  O14492  O14492  O14492  O14492  O14492  O14492  O14492  O14492  O14492  O14492  O14492  O14492  O14492  O14492  O14492  O14492  O14492  O14492  O14492  O14492  O14492  O14492  O14492  O14492  O14492  O14492  O14492  O14492  O14492  O14493  O14493  O14493  O14493  O14493  O14493  O14493  O14493  O14493  O14493  O14493  O14493  O14493  O14519  O14519  O14519  O14519  O14519  O14519  O14519  O14519  O14519  O14519  O14519  O14607  O14607  O14607  O14607  O14607  O14607  O14607  O14607  O14607  O14607  O14607  O14607  O14607  O14607  O14607  O14607  O14607  O14607  O14607  O14607  O14607  O14607  O14607  O14607  O14607  O14607  O14607  O14607  O14607  O14607  O14607  O14607  O14607  O14625  O14625  O14625  O14653  O14653  O14653  O14653  O14653  O14653  O14653  O14653  O14653  O14653  O14653  O14653  O14654  O14654  O14654  O14654  O14654  O14654  O14654  O14654  O14654  O14654  O14654  O14654  O14654  O14654  O14654  O14654  O14654  O14654  O14654  O14654  O14654  O14654  O14654  O14654  O14654  O14654  O14654  O14654  O14654  O14654  O14654  O14654  O14654  O14672  O14672  O14672  O14672  O14672  O14672  O14672  O14672  O14672  O14672  O14672  O14672  O14672  O14672  O14672  O14672  O14672  O14672  O14672  O14672  O14672  O14672  O14672  O14672  O14672  O14672  O14672  O14672  O14672  O14672  O14672  O14672  O14672  O14713  O14713  O14713  O14713  O14713  O14713  O14713  O14713  O14713  O14713  O14713  O14713  O14713  O14713  O14713  O14713  O14713  O14713  O14713  O14713  O14713  O14713  O14713  O14713  O14713  O14713  O14713  O14713  O14713  O14713  O14718  O14718  O14718  O14718  O14718  O14718  O14718  O14718  O14718  O14718  O14718  O14718  O14718  O14718  O14718  O14718  O14718  O14718  O14718  O14718  O14718  O14718  O14718  O14718  O14718  O14718  O14718  O14718  O14744  O14744  O14744  O14744  O14744  O14744  O14744  O14744  O14744  O14744  O14744  O14744  O14744  O14744  O14744  O14744  O14744  O14744  O14744  O14744  O14744  O14744  O14744  O14744  O14744  O14744  O14744  O14744  O14744  O14744  O14744  O14744  O14744  O14745  O14745  O14745  O14745  O14745  O14745  O14745  O14745  O14745  O14745  O14745  O14745  O14745  O14745  O14745  O14745  O14745  O14745  O14745  O14745  O14745  O14746  O14746  O14746  O14746  O14746  O14746  O14746  O14746  O14746  O14746  O14746  O14746  O14746  O14746  O14746  O14746  O14746  O14746  O14746  O14746  O14746  O14746  O14746  O14746  O14746  O14746  O14746  O14746  O14746  O14746  O14746  O14757  O14757  O14757  O14757  O14757  O14757  O14757  O14757  O14757  O14757  O14757  O14757  O14757  O14757  O14757  O14757  O14757  O14757  O14757  O14757  O14757  O14757  O14757  O14757  O14757  O14757  O14757  O14757  O14757  O14777  O14777  O14777  O14777  O14777  O14777  O14777  O14777  O14777  O14777  O14777  O14777  O14777  O14777  O14777  O14777  O14777  O14777  O14777  O14777  O14777  O14777  O14777  O14777  O14777  O14777  O14777  O14777  O14777  O14777  O14777  O14791  O14791  O14791  O14791  O14791  O14791  O14791  O14791  O14791  O14791  O14791  O14791  O14791  O14791  O14791  O14791  O14791  O14791  O14791  O14791  O14791  O14791  O14791  O14791  O14791  O14791  O14791  O14818  O14818  O14818  O14818  O14818  O14818  O14818  O14818  O14818  O14818  O14818  O14827  O14827  O14827  O14827  O14827  O14827  O14827  O14827  O14827  O14827  O14827  O14827  O14827  O14827  O14827  O14827  O14827  O14827  O14827  O14827  O14827  O14827  O14827  O14827  O14827  O14827  O14827  O14827  O14827  O14827  O14827  O14920  O14920  O14920  O14920  O14920  O14920  O14920  O14920  O14920  O14920  O14920  O14920  O14920  O14920  O14920  O14920  O14920  O14920  O14920  O14920  O14920  O14920  O14920  O14920  O14920  O14920  O14920  O14920  O14920  O14920  O14920  O14936  O14936  O14936  O14936  O14936  O14936  O14936  O14936  O14936  O14936  O14936  O14936  O14936  O14936  O14936  O14936  O14936  O14936  O14936  O14936  O14936  O14936  O14936  O14936  O14936  O14936  O14936  O14936  O14936  O14936  O14936  O14950  O14950  O14958  O14958  O14958  O14958  O14958  O14958  O14958  O14958  O14958  O14958  O14958  O14958  O14958  O14958  O14958  O14958  O14965  O14965  O14965  O14965  O14965  O14965  O14965  O14965  O14965  O14965  O14965  O14965  O14965  O14965  O14965  O14965  O14965  O14965  O14965  O14965  O14965  O14965  O14965  O14965  O14965  O14965  O14974  O14974  O14974  O14974  O14974  O14974  O14974  O14974  O14974  O14974  O14974  O14974  O14974  O14974  O14974  O14974  O14974  O14974  O14974  O14974  O14974  O14974  O14974  O14974  O14974  O14974  O14974  O14974  O14974  O14974  O14974  O14979  O14979  O14979  O14979  O14979  O14979  O14979  O14979  O14979  O14979  O14979  O14979  O14979  O14979  O14979  O14979  O14979  O14979  O14979  O14979  O14979  O14979  O14979  O14979  O14979  O14979  O14979  O14979  O14979  O14979  O14979  O14979  O14994  O14994  O14994  O14994  O14994  O14994  O14994  O14994  O14994  O14994  O14994  O14994  O14994  O14994  O14994  O14994  O14994  O14994  O14994  O14994  O14994  O14994  O14994  O14994  O14994  O14994  O14994  O14994  O14994  O14994  O14994  O15055  O15055  O15055  O15055  O15055  O15055  O15055  O15055  O15055  O15055  O15055  O15055  O15055  O15055  O15055  O15055  O15055  O15055  O15055  O15055  O15055  O15055  O15055  O15055  O15055  O15055  O15055  O15055  O15055  O15055  O15055  O15084  O15084  O15084  O15084  O15084  O15084  O15084  O15084  O15084  O15084  O15084  O15084  O15084  O15084  O15084  O15084  O15084  O15084  O15084  O15084  O15084  O15084  O15084  O15084  O15084  O15084  O15084  O15084  O15084  O15084  O15084  O15105  O15105  O15105  O15105  O15105  O15105  O15105  O15105  O15105  O15105  O15105  O15105  O15105  O15105  O15105  O15105  O15105  O15105  O15105  O15105  O15105  O15105  O15105  O15105  O15105  O15105  O15105  O15105  O15105  O15105  O15105  O15151  O15151  O15151  O15151  O15151  O15151  O15151  O15151  O15151  O15151  O15151  O15151  O15151  O15151  O15151  O15151  O15151  O15151  O15151  O15151  O15151  O15151  O15151  O15151  O15151  O15151  O15151  O15151  O15151  O15151  O15151  O15160  O15160  O15160  O15160  O15160  O15160  O15160  O15160  O15160  O15160  O15160  O15160  O15160  O15160  O15162  O15162  O15162  O15162  O15162  O15162  O15162  O15162  O15162  O15162  O15162  O15162  O15162  O15162  O15162  O15169  O15169  O15169  O15169  O15169  O15169  O15169  O15169  O15169  O15169  O15169  O15169  O15169  O15169  O15169  O15169  O15169  O15169  O15169  O15169  O15169  O15169  O15169  O15169  O15169  O15169  O15169  O15169  O15169  O15169  O15169  O15232  O15232  O15232  O15232  O15232  O15232  O15232  O15232  O15232  O15232  O15232  O15232  O15232  O15232  O15232  O15232  O15232  O15232  O15232  O15232  O15232  O15232  O15232  O15232  O15232  O15232  O15232  O15232  O15232  O15232  O15232  O15240  O15240  O15240  O15240  O15240  O15240  O15240  O15240  O15240  O15240  O15240  O15240  O15240  O15240  O15240  O15240  O15240  O15240  O15240  O15240  O15240  O15240  O15240  O15240  O15240  O15240  O15240  O15240  O15240  O15240  O15240  O15259  O15259  O15259  O15259  O15259  O15259  O15259  O15259  O15259  O15259  O15259  O15259  O15259  O15259  O15259  O15259  O15259  O15259  O15259  O15259  O15259  O15259  O15259  O15259  O15259  O15259  O15259  O15259  O15259  O15259  O15259  O15259  O15259  O15264  O15264  O15264  O15264  O15264  O15264  O15264  O15264  O15264  O15264  O15264  O15264  O15264  O15264  O15264  O15264  O15264  O15264  O15264  O15269  O15269  O15269  O15269  O15269  O15269  O15269  O15269  O15269  O15269  O15269  O15269  O15269  O15269  O15269  O15269  O15269  O15269  O15269  O15269  O15269  O15269  O15269  O15269  O15269  O15269  O15269  O15287  O15287  O15287  O15287  O15287  O15287  O15287  O15287  O15287  O15287  O15287  O15287  O15287  O15287  O15287  O15287  O15287  O15287  O15287  O15287  O15287  O15287  O15287  O15287  O15287  O15287  O15287  O15287  O15287  O15287  O15287  O15294  O15294  O15294  O15294  O15294  O15294  O15294  O15294  O15294  O15294  O15294  O15294  O15294  O15294  O15294  O15294  O15294  O15294  O15294  O15294  O15294  O15294  O15294  O15294  O15294  O15294  O15294  O15294  O15294  O15294  O15294  O15294  O15294  O15304  O15304  O15304  O15304  O15304  O15304  O15304  O15304  O15304  O15304  O15304  O15304  O15350  O15350  O15350  O15350  O15350  O15350  O15350  O15350  O15350  O15350  O15350  O15350  O15350  O15350  O15350  O15350  O15350  O15350  O15350  O15350  O15350  O15350  O15350  O15350  O15350  O15350  O15350  O15350  O15350  O15350  O15350  O15350  O15350  O15357  O15357  O15357  O15357  O15357  O15357  O15357  O15357  O15357  O15357  O15357  O15357  O15357  O15357  O15357  O15357  O15357  O15357  O15357  O15357  O15357  O15357  O15357  O15357  O15357  O15357  O15357  O15357  O15357  O15357  O15357  O15357  O15357  O15372  O15372  O15372  O15372  O15372  O15372  O15372  O15372  O15372  O15372  O15372  O15372  O15372  O15372  O15372  O15372  O15372  O15372  O15372  O15372  O15372  O15372  O15372  O15372  O15372  O15382  O15382  O15382  O15382  O15382  O15382  O15382  O15382  O15382  O15382  O15382  O15382  O15382  O15382  O15382  O15382  O15382  O15392  O15392  O15392  O15392  O15400  O15400  O15400  O15400  O15400  O15400  O15400  O15400  O15400  O15400  O15400  O15400  O15400  O15400  O15400  O15400  O15400  O15400  O15400  O15400  O15400  O15446  O15446  O15446  O15446  O15446  O15446  O15446  O15446  O15446  O15446  O15446  O15446  O15446  O15446  O15446  O15446  O15446  O15446  O15446  O15446  O15446  O15446  O15446  O15446  O15446  O15446  O15446  O15455  O15455  O15455  O15455  O15455  O15455  O15455  O15455  O15455  O15455  O15455  O15455  O15455  O15455  O15455  O15455  O15455  O15455  O15455  O15455  O15455  O15455  O15455  O15455  O15455  O15455  O15455  O15455  O15455  O15455  O15455  O15455  O15455  O15492  O15492  O15492  O15492  O15492  O15492  O15492  O15492  O15492  O15492  O15492  O15492  O15492  O15492  O15492  O15492  O15492  O15492  O15516  O15516  O15516  O15516  O15516  O15516  O15516  O15516  O15516  O15516  O15516  O15516  O15516  O15516  O15516  O15516  O15516  O15516  O15516  O15516  O15516  O15516  O15516  O15516  O15516  O15516  O15516  O15516  O15516  O15516  O15516  O15516  O15516  O15530  O15530  O15530  O15530  O15530  O15530  O15530  O15530  O15530  O15530  O15530  O15530  O15530  O15530  O15530  O15530  O15530  O15530  O15530  O15530  O15530  O15530  O15530  O15530  O15530  O15530  O15530  O15530  O15530  O15530  O15530  O15547  O15547  O15547  O15547  O15547  O15547  O15547  O15547  O15547  O15547  O15547  O15547  O15547  O15547  O15547  O15547  O15547  O15547  O15547  O15547  O15547  O15547  O15547  O15547  O15554  O15554  O15554  O15554  O15554  O15554  O15554  O15554  O15554  O15554  O15554  O15554  O15554  O15554  O15554  O15554  O15554  O15554  O15554  O43156  O43156  O43156  O43156  O43156  O43156  O43156  O43156  O43156  O43156  O43156  O43156  O43156  O43156  O43156  O43156  O43156  O43156  O43156  O43156  O43156  O43156  O43156  O43156  O43156  O43156  O43156  O43156  O43156  O43156  O43156  O43164  O43164  O43164  O43164  O43164  O43164  O43164  O43164  O43164  O43164  O43164  O43164  O43164  O43164  O43164  O43164  O43164  O43164  O43164  O43164  O43164  O43164  O43164  O43164  O43164  O43164  O43164  O43164  O43164  O43164  O43164  O43175  O43175  O43175  O43175  O43175  O43175  O43175  O43175  O43175  O43175  O43175  O43175  O43175  O43175  O43175  O43175  O43175  O43175  O43175  O43175  O43175  O43175  O43175  O43175  O43175  O43175  O43175  O43175  O43175  O43175  O43175  O43175  O43255  O43255  O43255  O43255  O43255  O43255  O43255  O43255  O43255  O43255  O43255  O43255  O43255  O43255  O43255  O43255  O43255  O43255  O43257  O43257  O43257  O43257  O43257  O43264  O43264  O43264  O43264  O43264  O43264  O43264  O43264  O43264  O43264  O43264  O43264  O43264  O43264  O43264  O43264  O43264  O43264  O43264  O43264  O43264  O43264  O43264  O43264  O43264  O43264  O43264  O43264  O43264  O43264  O43264  O43264  O43264  O43294  O43294  O43294  O43294  O43294  O43294  O43294  O43294  O43294  O43294  O43294  O43294  O43294  O43294  O43294  O43294  O43294  O43294  O43294  O43294  O43294  O43294  O43294  O43294  O43294  O43294  O43294  O43294  O43294  O43294  O43294  O43294  O43294  O43318  O43318  O43318  O43318  O43318  O43318  O43318  O43318  O43318  O43318  O43318  O43318  O43318  O43318  O43318  O43318  O43318  O43318  O43318  O43318  O43318  O43318  O43318  O43318  O43318  O43318  O43318  O43318  O43318  O43318  O43318  O43324  O43324  O43324  O43324  O43324  O43324  O43324  O43324  O43324  O43324  O43324  O43324  O43353  O43353  O43353  O43353  O43353  O43353  O43353  O43353  O43353  O43353  O43353  O43353  O43353  O43353  O43353  O43353  O43353  O43353  O43353  O43353  O43353  O43353  O43353  O43353  O43353  O43353  O43353  O43353  O43353  O43353  O43353  O43390  O43390  O43390  O43390  O43390  O43390  O43390  O43390  O43390  O43390  O43390  O43390  O43390  O43390  O43390  O43390  O43390  O43390  O43390  O43390  O43390  O43390  O43390  O43390  O43390  O43390  O43390  O43390  O43432  O43432  O43432  O43432  O43432  O43432  O43432  O43432  O43432  O43432  O43432  O43432  O43432  O43432  O43432  O43432  O43432  O43432  O43432  O43432  O43432  O43432  O43432  O43432  O43432  O43432  O43432  O43432  O43432  O43432  O43432  O43463  O43463  O43463  O43463  O43463  O43463  O43463  O43463  O43463  O43463  O43463  O43463  O43463  O43463  O43463  O43463  O43463  O43463  O43463  O43490  O43490  O43490  O43490  O43490  O43490  O43490  O43490  O43490  O43490  O43490  O43490  O43490  O43490  O43490  O43490  O43490  O43490  O43490  O43490  O43490  O43490  O43490  O43490  O43490  O43490  O43490  O43490  O43490  O43490  O43490  O43490  O43490  O43491  O43491  O43491  O43491  O43491  O43491  O43491  O43491  O43491  O43491  O43491  O43491  O43491  O43491  O43491  O43491  O43491  O43491  O43491  O43491  O43491  O43491  O43491  O43491  O43491  O43491  O43491  O43491  O43491  O43491  O43491  O43491  O43493  O43493  O43493  O43493  O43493  O43493  O43493  O43493  O43493  O43493  O43493  O43493  O43493  O43493  O43493  O43493  O43493  O43493  O43493  O43493  O43493  O43493  O43493  O43493  O43493  O43493  O43493  O43493  O43493  O43493  O43493  O43504  O43504  O43504  O43504  O43504  O43504  O43521  O43521  O43521  O43521  O43521  O43521  O43521  O43521  O43521  O43521  O43521  O43521  O43521  O43521  O43521  O43521  O43521  O43521  O43521  O43524  O43524  O43524  O43524  O43524  O43524  O43524  O43524  O43524  O43524  O43524  O43524  O43524  O43524  O43524  O43524  O43524  O43524  O43524  O43524  O43524  O43524  O43524  O43524  O43524  O43524  O43524  O43524  O43524  O43524  O43524  O43525  O43525  O43525  O43525  O43525  O43525  O43525  O43525  O43525  O43525  O43525  O43525  O43525  O43525  O43525  O43525  O43525  O43525  O43525  O43525  O43525  O43525  O43525  O43525  O43525  O43525  O43525  O43525  O43525  O43525  O43525  O43525  O43525  O43526  O43526  O43526  O43526  O43526  O43526  O43526  O43526  O43526  O43526  O43526  O43526  O43526  O43526  O43526  O43526  O43526  O43526  O43526  O43526  O43526  O43526  O43526  O43526  O43526  O43526  O43526  O43526  O43526  O43526  O43526  O43541  O43541  O43541  O43541  O43541  O43541  O43541  O43541  O43541  O43541  O43541  O43541  O43541  O43541  O43541  O43541  O43541  O43541  O43541  O43541  O43541  O43541  O43541  O43541  O43541  O43541  O43541  O43541  O43541  O43541  O43541  O43548  O43548  O43548  O43548  O43548  O43548  O43548  O43548  O43548  O43548  O43548  O43548  O43548  O43548  O43548  O43548  O43548  O43548  O43548  O43548  O43548  O43548  O43548  O43548  O43548  O43548  O43548  O43548  O43548  O43548  O43548  O43548  O43548  O43561  O43561  O43561  O43561  O43561  O43561  O43561  O43561  O43561  O43561  O43561  O43561  O43561  O43561  O43561  O43561  O43561  O43561  O43561  O43561  O43561  O43561  O43561  O43561  O43561  O43561  O43561  O43561  O43561  O43561  O43561  O43602  O43602  O43602  O43602  O43602  O43602  O43602  O43602  O43602  O43602  O43602  O43602  O43602  O43602  O43602  O43602  O43602  O43602  O43602  O43602  O43602  O43602  O43602  O43602  O43602  O43602  O43602  O43602  O43602  O43602  O43602  O43633  O43633  O43633  O43633  O43633  O43633  O43633  O43633  O43633  O43633  O43633  O43663  O43663  O43663  O43663  O43663  O43663  O43663  O43663  O43663  O43663  O43663  O43663  O43663  O43663  O43663  O43663  O43663  O43663  O43663  O43663  O43663  O43663  O43663  O43663  O43663  O43663  O43663  O43663  O43663  O43663  O43663  O43683  O43683  O43683  O43683  O43683  O43683  O43683  O43683  O43683  O43683  O43683  O43683  O43683  O43683  O43683  O43683  O43683  O43683  O43683  O43683  O43683  O43683  O43683  O43683  O43683  O43683  O43683  O43683  O43683  O43683  O43683  O43683  O43683  O43760  O43760  O43760  O43760  O43760  O43760  O43760  O43760  O43760  O43760  O43760  O43760  O43760  O43760  O43760  O43768  O43768  O43768  O43768  O43768  O43809  O43809  O43809  O43809  O43809  O60256  O60256  O60256  O60256  O60256  O60256  O60256  O60256  O60256  O60256  O60256  O60256  O60256  O60256  O60256  O60256  O60256  O60256  O60256  O60256  O60256  O60256  O60256  O60256  O60256  O60256  O60260  O60260  O60260  O60260  O60260  O60260  O60260  O60260  O60260  O60260  O60260  O60260  O60260  O60260  O60260  O60260  O60260  O60260  O60260  O60260  O60260  O60260  O60260  O60260  O60260  O60260  O60260  O60260  O60260  O60285  O60285  O60285  O60285  O60285  O60285  O60285  O60285  O60285  O60285  O60285  O60285  O60285  O60285  O60285  O60285  O60285  O60285  O60285  O60285  O60285  O60285  O60285  O60285  O60285  O60285  O60285  O60285  O60285  O60285  O60285  O60331  O60331  O60331  O60331  O60331  O60331  O60331  O60331  O60331  O60331  O60331  O60331  O60331  O60331  O60331  O60331  O60331  O60331  O60331  O60331  O60331  O60331  O60331  O60331  O60331  O60331  O60331  O60331  O60331  O60331  O60331  O60343  O60343  O60343  O60343  O60343  O60343  O60343  O60343  O60343  O60343  O60343  O60343  O60343  O60343  O60343  O60343  O60343  O60343  O60343  O60343  O60343  O60343  O60343  O60343  O60343  O60343  O60343  O60343  O60343  O60343  O60343  O60493  O60493  O60493  O60493  O60493  O60493  O60493  O60566  O60566  O60566  O60566  O60566  O60566  O60566  O60566  O60566  O60566  O60566  O60566  O60566  O60566  O60566  O60566  O60566  O60566  O60566  O60566  O60566  O60566  O60566  O60566  O60566  O60566  O60566  O60566  O60566  O60566  O60566  O60602  O60602  O60602  O60602  O60602  O60602  O60602  O60602  O60602  O60602  O60602  O60602  O60602  O60602  O60602  O60602  O60602  O60602  O60602  O60602  O60602  O60602  O60602  O60602  O60602  O60602  O60602  O60602  O60602  O60602  O60602  O60610  O60610  O60610  O60610  O60610  O60610  O60610  O60610  O60610  O60610  O60610  O60610  O60610  O60610  O60610  O60610  O60610  O60610  O60610  O60610  O60610  O60610  O60610  O60610  O60610  O60610  O60610  O60610  O60610  O60610  O60610  O60610  O60610  O60658  O60658  O60658  O60658  O60658  O60658  O60658  O60658  O60658  O60658  O60658  O60658  O60658  O60658  O60658  O60658  O60658  O60658  O60658  O60658  O60658  O60658  O60658  O60658  O60658  O60658  O60658  O60658  O60658  O60658  O60658  O60674  O60674  O60674  O60674  O60674  O60674  O60674  O60674  O60674  O60674  O60674  O60674  O60674  O60674  O60674  O60674  O60674  O60674  O60674  O60674  O60674  O60674  O60674  O60674  O60674  O60674  O60674  O60674  O60674  O60674  O60674  O60674  O60674  O60678  O60678  O60678  O60678  O60678  O60678  O60678  O60678  O60678  O60678  O60678  O60678  O60678  O60678  O60678  O60678  O60678  O60678  O60678  O60678  O60678  O60678  O60678  O60678  O60678  O60678  O60678  O60678  O60678  O60678  O60678  O60711  O60711  O60711  O60711  O60711  O60711  O60711  O60711  O60711  O60711  O60711  O60711  O60711  O60711  O60711  O60711  O60711  O60711  O60711  O60711  O60711  O60711  O60711  O60711  O60711  O60711  O60711  O60716  O60716  O60716  O60716  O60716  O60716  O60716  O60716  O60716  O60716  O60716  O60716  O60716  O60716  O60716  O60716  O60716  O60716  O60716  O60716  O60716  O60716  O60716  O60716  O60716  O60716  O60716  O60716  O60716  O60716  O60716  O60763  O60763  O60763  O60763  O60763  O60763  O60763  O60763  O60763  O60763  O60763  O60763  O60763  O60763  O60763  O60763  O60763  O60763  O60763  O60763  O60763  O60763  O60763  O60763  O60763  O60763  O60763  O60763  O60763  O60763  O60763  O60832  O60832  O60832  O60832  O60832  O60832  O60832  O60832  O60832  O60832  O60832  O60832  O60832  O60832  O60832  O60832  O60832  O60832  O60832  O60832  O60832  O60832  O60832  O60832  O60832  O60832  O60832  O60832  O60832  O60841  O60841  O60841  O60841  O60841  O60841  O60841  O60841  O60841  O60841  O60841  O60841  O60841  O60841  O60841  O60841  O60841  O60841  O60841  O60841  O60841  O60841  O60841  O60841  O60841  O60841  O60841  O60841  O60841  O60841  O60841  O60869  O60869  O60869  O60869  O60869  O60870  O60870  O60870  O60870  O60870  O60870  O60870  O60870  O60870  O60870  O60870  O60870  O60870  O60870  O60870  O60870  O60870  O60870  O60870  O60870  O60870  O60870  O60870  O60870  O60870  O60870  O60870  O60870  O60885  O60885  O60885  O60885  O60885  O60885  O60885  O60885  O60885  O60885  O60885  O60885  O60885  O60885  O60885  O60885  O60885  O60885  O60885  O60885  O60885  O60885  O60885  O60885  O60885  O60885  O60885  O60885  O60885  O60885  O60885  O60928  O60928  O60928  O60928  O60928  O60928  O60928  O60928  O60928  O60928  O60928  O60928  O60928  O60928  O60928  O60928  O60928  O60928  O60928  O60928  O60928  O60928  O60928  O60928  O60928  O60928  O60934  O60934  O60934  O60934  O60934  O60934  O60934  O60934  O60934  O60934  O60934  O60934  O60934  O60934  O60934  O60934  O60934  O60934  O60934  O60934  O60934  O60934  O60934  O60934  O60934  O60934  O60934  O60934  O60934  O60934  O60934  O75030  O75030  O75030  O75030  O75030  O75030  O75030  O75030  O75030  O75030  O75030  O75030  O75030  O75030  O75030  O75030  O75030  O75030  O75030  O75030  O75030  O75030  O75030  O75030  O75030  O75030  O75030  O75030  O75030  O75030  O75030  O75044  O75044  O75044  O75044  O75044  O75044  O75044  O75044  O75044  O75044  O75044  O75044  O75044  O75044  O75044  O75044  O75044  O75044  O75044  O75044  O75044  O75044  O75044  O75044  O75044  O75044  O75044  O75044  O75044  O75044  O75044  O75044  O75044  O75056  O75056  O75056  O75056  O75056  O75056  O75056  O75056  O75056  O75056  O75056  O75056  O75056  O75056  O75056  O75056  O75056  O75056  O75056  O75056  O75056  O75056  O75056  O75056  O75056  O75056  O75056  O75056  O75056  O75116  O75116  O75116  O75116  O75116  O75116  O75116  O75116  O75116  O75116  O75116  O75116  O75116  O75116  O75116  O75116  O75116  O75116  O75116  O75116  O75116  O75116  O75116  O75116  O75116  O75116  O75116  O75116  O75116  O75116  O75116  O75116  O75116  O75143  O75143  O75143  O75143  O75143  O75143  O75143  O75143  O75143  O75143  O75143  O75143  O75143  O75143  O75143  O75143  O75143  O75143  O75143  O75143  O75143  O75143  O75143  O75143  O75143  O75143  O75143  O75143  O75143  O75143  O75143  O75151  O75151  O75151  O75151  O75151  O75151  O75151  O75151  O75151  O75151  O75151  O75151  O75151  O75151  O75151  O75151  O75151  O75151  O75151  O75151  O75151  O75151  O75151  O75151  O75151  O75151  O75151  O75151  O75151  O75151  O75151  O75154  O75154  O75154  O75154  O75154  O75154  O75154  O75154  O75154  O75154  O75154  O75154  O75154  O75154  O75154  O75154  O75154  O75154  O75154  O75154  O75154  O75154  O75154  O75154  O75154  O75154  O75154  O75154  O75154  O75154  O75154  O75155  O75155  O75155  O75155  O75155  O75155  O75155  O75155  O75155  O75155  O75155  O75155  O75155  O75155  O75155  O75155  O75155  O75155  O75155  O75155  O75155  O75155  O75155  O75155  O75155  O75155  O75155  O75155  O75155  O75155  O75155  O75155  O75155  O75179  O75179  O75179  O75179  O75179  O75179  O75179  O75179  O75179  O75179  O75179  O75179  O75179  O75179  O75179  O75179  O75179  O75179  O75179  O75179  O75179  O75179  O75179  O75179  O75179  O75179  O75179  O75179  O75179  O75179  O75179  O75348  O75348  O75348  O75348  O75367  O75367  O75367  O75367  O75367  O75367  O75367  O75367  O75367  O75367  O75367  O75367  O75367  O75367  O75367  O75367  O75367  O75367  O75367  O75367  O75367  O75367  O75367  O75367  O75367  O75367  O75367  O75367  O75367  O75381  O75381  O75381  O75381  O75381  O75381  O75381  O75381  O75381  O75381  O75381  O75381  O75381  O75381  O75381  O75381  O75381  O75381  O75381  O75381  O75381  O75381  O75381  O75381  O75381  O75381  O75381  O75381  O75381  O75381  O75381  O75381  O75381  O75410  O75410  O75410  O75410  O75410  O75410  O75410  O75410  O75410  O75410  O75410  O75410  O75410  O75410  O75410  O75410  O75410  O75410  O75410  O75410  O75410  O75410  O75410  O75410  O75410  O75410  O75410  O75410  O75410  O75410  O75410  O75449  O75449  O75449  O75449  O75449  O75449  O75449  O75449  O75449  O75449  O75449  O75449  O75449  O75449  O75449  O75449  O75449  O75449  O75449  O75449  O75449  O75449  O75449  O75449  O75449  O75449  O75449  O75449  O75449  O75449  O75449  O75496  O75496  O75496  O75496  O75496  O75496  O75496  O75496  O75496  O75496  O75496  O75496  O75496  O75496  O75496  O75496  O75496  O75496  O75530  O75530  O75530  O75530  O75530  O75530  O75530  O75530  O75530  O75530  O75530  O75530  O75530  O75530  O75530  O75530  O75530  O75530  O75530  O75530  O75530  O75530  O75530  O75530  O75530  O75530  O75530  O75530  O75530  O75530  O75530  O75530  O75530  O75531  O75531  O75533  O75533  O75533  O75533  O75533  O75533  O75533  O75533  O75533  O75533  O75533  O75533  O75533  O75533  O75533  O75533  O75533  O75533  O75533  O75533  O75533  O75533  O75533  O75533  O75533  O75533  O75533  O75533  O75533  O75533  O75533  O75533  O75533  O75563  O75563  O75563  O75563  O75563  O75563  O75563  O75563  O75563  O75563  O75563  O75563  O75563  O75563  O75563  O75563  O75563  O75563  O75563  O75563  O75563  O75563  O75563  O75563  O75563  O75563  O75569  O75569  O75569  O75569  O75569  O75569  O75569  O75569  O75569  O75569  O75569  O75569  O75569  O75569  O75569  O75569  O75569  O75569  O75569  O75569  O75569  O75569  O75581  O75581  O75581  O75581  O75581  O75581  O75581  O75581  O75581  O75581  O75581  O75581  O75581  O75581  O75581  O75581  O75581  O75581  O75581  O75581  O75581  O75581  O75581  O75581  O75581  O75581  O75581  O75581  O75581  O75581  O75581  O75582  O75582  O75582  O75582  O75582  O75582  O75582  O75582  O75582  O75582  O75582  O75582  O75582  O75582  O75582  O75582  O75582  O75582  O75582  O75582  O75582  O75582  O75582  O75582  O75582  O75582  O75582  O75582  O75582  O75582  O75582  O75607  O75607  O75607  O75607  O75607  O75607  O75607  O75607  O75607  O75607  O75607  O75607  O75607  O75636  O75636  O75636  O75636  O75636  O75636  O75636  O75636  O75636  O75636  O75636  O75636  O75636  O75636  O75636  O75636  O75636  O75636  O75636  O75636  O75636  O75636  O75636  O75689  O75689  O75689  O75689  O75689  O75689  O75689  O75689  O75689  O75689  O75689  O75689  O75689  O75689  O75689  O75689  O75716  O75716  O75716  O75716  O75716  O75716  O75716  O75716  O75716  O75716  O75716  O75716  O75716  O75716  O75807  O75807  O75807  O75807  O75807  O75807  O75807  O75807  O75807  O75807  O75807  O75807  O75807  O75807  O75807  O75807  O75807  O75807  O75807  O75807  O75807  O75807  O75807  O75807  O75807  O75807  O75807  O75807  O75807  O75807  O75807  O75807  O75807  O75821  O75821  O75821  O75821  O75821  O75821  O75821  O75821  O75821  O75821  O75821  O75821  O75821  O75821  O75821  O75821  O75821  O75822  O75822  O75822  O75822  O75822  O75822  O75822  O75822  O75822  O75915  O75915  O75915  O75915  O75915  O75915  O75915  O75915  O75925  O75925  O75925  O75925  O75925  O75925  O75925  O75925  O75925  O75925  O75925  O75925  O75925  O75925  O75925  O75925  O75925  O75925  O75925  O75925  O75925  O75925  O75925  O75925  O75925  O75925  O75925  O75925  O75925  O75925  O75925  O75925  O75925  O75934  O75934  O75934  O75934  O75934  O75934  O75934  O75934  O75934  O75934  O75934  O75943  O75943  O75943  O75943  O75943  O75943  O75943  O75943  O75943  O75943  O75943  O75943  O75943  O75943  O75943  O75943  O75943  O75943  O75943  O75943  O75943  O75943  O75943  O75943  O75943  O75943  O75943  O75943  O75943  O75943  O75943  O75995  O75995  O75995  O75995  O75995  O75995  O75995  O75995  O75995  O75995  O75995  O75995  O75995  O75995  O75995  O75995  O75995  O75995  O75995  O75995  O75995  O75995  O75995  O75995  O75995  O75995  O75995  O75995  O75995  O75995  O75995  O76021  O76021  O76021  O76021  O76021  O76021  O76021  O76021  O76021  O76021  O76021  O76021  O76021  O76021  O76021  O76021  O76021  O76021  O76021  O76021  O76021  O76021  O76021  O76021  O76021  O76021  O76021  O76021  O76021  O76021  O76021  O76021  O76021  O76024  O76024  O76024  O76024  O76024  O76024  O76024  O76024  O76024  O76024  O76024  O76024  O76024  O76024  O76024  O76024  O76024  O76024  O76024  O76024  O76024  O76024  O76024  O76024  O76024  O76024  O76024  O76024  O76024  O76024  O76024  O76061  O76061  O76061  O76061  O76061  O76061  O76061  O76061  O76061  O76061  O76061  O76061  O76061  O76061  O76061  O76061  O76061  O76061  O76094  O76094  O76094  O76094  O76094  O76094  O76094  O76094  O76094  O76094  O76094  O76094  O76094  O76094  O76094  O76094  O76094  O76094  O76094  O76094  O76094  O76094  O76094  O76094  O76094  O76094  O76094  O76094  O76094  O76094  O76094  O76094  O76094  O94760  O94760  O94760  O94760  O94760  O94760  O94760  O94760  O94760  O94760  O94760  O94760  O94760  O94760  O94760  O94760  O94760  O94763  O94763  O94763  O94763  O94763  O94763  O94763  O94763  O94763  O94763  O94763  O94763  O94763  O94763  O94763  O94763  O94763  O94763  O94763  O94763  O94763  O94763  O94763  O94763  O94763  O94763  O94763  O94763  O94763  O94763  O94763  O94811  O94811  O94811  O94811  O94811  O94811  O94811  O94811  O94811  O94811  O94811  O94811  O94811  O94832  O94832  O94832  O94832  O94832  O94832  O94832  O94832  O94832  O94832  O94832  O94832  O94832  O94832  O94832  O94832  O94832  O94832  O94832  O94832  O94832  O94832  O94832  O94832  O94832  O94832  O94832  O94832  O94832  O94832  O94832  O94832  O94832  O94875  O94875  O94875  O94875  O94875  O94875  O94875  O94875  O94875  O94875  O94875  O94875  O94875  O94875  O94875  O94875  O94875  O94875  O94875  O94875  O94875  O94875  O94875  O94875  O94875  O94875  O94875  O94875  O94875  O94875  O94875  O94875  O94875  O94915  O94915  O94915  O94915  O94915  O94915  O94915  O94915  O94915  O94915  O94915  O94915  O94915  O94915  O94915  O94915  O94915  O94915  O94915  O94915  O94915  O94915  O94915  O94915  O94915  O94915  O94915  O94915  O94915  O94915  O94915  O94915  O94915  O94916  O94916  O94916  O94916  O94916  O94916  O94916  O94916  O94916  O94916  O94916  O94916  O94916  O94916  O94916  O94916  O94916  O94916  O94916  O94916  O94916  O94916  O94916  O94916  O94916  O94916  O94916  O94916  O94916  O94916  O94916  O95069  O95069  O95069  O95069  O95069  O95069  O95069  O95069  O95069  O95069  O95069  O95069  O95069  O95069  O95069  O95069  O95069  O95069  O95069  O95069  O95069  O95069  O95069  O95069  O95069  O95069  O95069  O95071  O95071  O95071  O95071  O95071  O95071  O95071  O95071  O95071  O95071  O95071  O95071  O95071  O95071  O95071  O95071  O95071  O95071  O95071  O95071  O95071  O95071  O95071  O95071  O95071  O95071  O95071  O95071  O95071  O95071  O95071  O95084  O95084  O95084  O95084  O95084  O95084  O95084  O95084  O95084  O95084  O95084  O95084  O95084  O95084  O95084  O95084  O95084  O95084  O95084  O95084  O95084  O95084  O95084  O95084  O95084  O95084  O95084  O95235  O95235  O95235  O95235  O95235  O95235  O95235  O95235  O95235  O95235  O95235  O95235  O95235  O95235  O95235  O95235  O95235  O95235  O95235  O95235  O95235  O95235  O95235  O95235  O95235  O95235  O95235  O95235  O95235  O95235  O95235  O95251  O95251  O95251  O95251  O95251  O95251  O95251  O95251  O95251  O95251  O95251  O95251  O95251  O95251  O95251  O95251  O95251  O95251  O95251  O95251  O95251  O95251  O95251  O95251  O95251  O95251  O95251  O95251  O95251  O95251  O95251  O95267  O95267  O95267  O95267  O95267  O95267  O95267  O95267  O95267  O95267  O95267  O95267  O95267  O95267  O95267  O95267  O95267  O95267  O95267  O95267  O95267  O95267  O95267  O95267  O95267  O95267  O95267  O95267  O95267  O95267  O95267  O95267  O95267  O95278  O95278  O95278  O95278  O95278  O95278  O95278  O95297  O95297  O95297  O95297  O95297  O95297  O95297  O95297  O95297  O95297  O95297  O95297  O95297  O95297  O95297  O95297  O95297  O95297  O95297  O95297  O95297  O95297  O95297  O95297  O95297  O95297  O95297  O95373  O95373  O95373  O95373  O95373  O95373  O95373  O95373  O95373  O95373  O95373  O95373  O95373  O95373  O95373  O95373  O95373  O95373  O95373  O95373  O95373  O95373  O95373  O95373  O95373  O95373  O95373  O95373  O95373  O95373  O95373  O95373  O95373  O95453  O95453  O95453  O95453  O95453  O95453  O95453  O95453  O95453  O95453  O95453  O95453  O95453  O95453  O95453  O95453  O95453  O95453  O95453  O95453  O95453  O95453  O95453  O95453  O95453  O95453  O95453  O95453  O95453  O95453  O95453  O95470  O95470  O95470  O95470  O95470  O95470  O95470  O95470  O95470  O95470  O95470  O95470  O95470  O95470  O95470  O95470  O95470  O95470  O95470  O95470  O95470  O95470  O95470  O95470  O95470  O95470  O95470  O95470  O95470  O95470  O95470  O95470  O95470  O95477  O95477  O95477  O95477  O95477  O95477  O95477  O95477  O95477  O95477  O95477  O95477  O95477  O95477  O95477  O95477  O95477  O95477  O95477  O95477  O95477  O95477  O95477  O95477  O95477  O95477  O95477  O95477  O95477  O95477  O95477  O95551  O95551  O95551  O95551  O95551  O95551  O95551  O95551  O95551  O95551  O95551  O95551  O95551  O95551  O95551  O95551  O95551  O95551  O95551  O95551  O95551  O95551  O95551  O95551  O95551  O95551  O95551  O95630  O95630  O95630  O95630  O95630  O95630  O95630  O95630  O95630  O95630  O95630  O95630  O95630  O95630  O95630  O95630  O95630  O95630  O95630  O95630  O95630  O95630  O95630  O95630  O95633  O95633  O95633  O95633  O95633  O95633  O95633  O95633  O95633  O95633  O95633  O95633  O95633  O95633  O95633  O95633  O95633  O95633  O95633  O95644  O95644  O95644  O95644  O95644  O95644  O95644  O95644  O95644  O95644  O95644  O95644  O95644  O95644  O95644  O95644  O95644  O95644  O95644  O95644  O95644  O95644  O95644  O95644  O95644  O95644  O95644  O95644  O95644  O95644  O95644  O95714  O95714  O95714  O95714  O95714  O95714  O95714  O95714  O95714  O95714  O95714  O95714  O95714  O95714  O95714  O95714  O95714  O95714  O95714  O95714  O95714  O95714  O95714  O95714  O95714  O95714  O95714  O95714  O95714  O95714  O95714  O95714  O95714  O95777  O95777  O95777  O95777  O95777  O95777  O95785  O95785  O95785  O95785  O95785  O95785  O95785  O95785  O95785  O95785  O95785  O95785  O95785  O95785  O95785  O95785  O95785  O95785  O95785  O95785  O95785  O95785  O95785  O95785  O95785  O95785  O95785  O95785  O95785  O95785  O95785  O95785  O95785  O95786  O95786  O95786  O95786  O95786  O95786  O95786  O95786  O95786  O95786  O95786  O95786  O95786  O95786  O95786  O95786  O95786  O95786  O95786  O95786  O95786  O95786  O95786  O95786  O95786  O95786  O95786  O95786  O95786  O95786  O95786  O95810  O95810  O95810  O95810  O95810  O95810  O95810  O95810  O95810  O95810  O95810  O95810  O95810  O95810  O95810  O95810  O95810  O95810  O95810  O95810  O95810  O95810  O95810  O95810  O95810  O95810  O95810  O95810  O95810  O95810  O95810  O95810  O95810  O95835  O95835  O95835  O95835  O95835  O95835  O95835  O95835  O95835  O95835  O95835  O95835  O95835  O95835  O95835  O95835  O95835  O95835  O95835  O95835  O95835  O95835  O95835  O95835  O95835  O95835  O95835  O95835  O95835  O95835  O95835  O95863  O95863  O95863  O95863  O95863  O95863  O95863  O95863  O95863  O95863  O95863  O95863  O95863  O95863  O95863  O95863  O95863  O95863  O95863  O95866  O95866  O95866  O95866  O95866  O95866  O95866  O95866  O95866  O95866  O95866  O95866  O95866  O95866  O95866  O95866  O95866  O95866  O95972  O95972  O95972  O95972  O95972  O95972  O95972  O95972  O95972  O95972  O95972  O95972  O95972  O95972  O95972  O95972  O95972  O95972  O95972  O95972  O95972  O95972  O95972  O95972  O95972  O95972  O95972  O95972  O95972  O95997  O95997  O95997  O95997  O95997  O95997  O95997  O95997  O95997  O95997  O95997  O95997  O95997  O95997  O95997  O95997  O95997  O95997  O96013  O96013  O96013  O96013  O96013  O96013  O96013  O96013  O96013  O96013  O96013  O96013  O96013  O96013  O96013  O96013  O96013  O96013  O96013  O96013  O96013  O96013  O96013  O96013  O96013  O96013  O96013  O96013  O96013  O96013  O96013  O96017  O96017  O96017  O96017  O96017  O96017  O96017  O96017  O96017  O96017  O96017  O96017  O96017  O96017  O96017  O96017  O96017  O96017  O96017  O96017  O96017  O96017  O96017  O96017  O96017  O96017  O96017  O96017  O96017  O96017  O96017  O96019  O96019  O96019  O96019  O96019  O96019  O96019  O96019  O96019  O96019  O96019  O96019  O96019  O96019  O96019  O96019  O96019  O96019  O96019  O96019  O96019  O96019  O96019  O96019  O96019  O96019  O96019  O96019  O96019  O96033  O96033  O96033  P00167  P00167  P00167  P00167  P00167  P00167  P00167  P00167  P00167  P00325  P00325  P00325  P00325  P00325  P00325  P00325  P00325  P00325  P00325  P00325  P00325  P00325  P00325  P00325  P00325  P00325  P00325  P00325  P00367  P00367  P00367  P00367  P00367  P00367  P00367  P00367  P00367  P00367  P00367  P00367  P00367  P00367  P00367  P00367  P00367  P00367  P00367  P00367  P00367  P00367  P00367  P00367  P00367  P00367  P00367  P00367  P00367  P00367  P00367  P00367  P00367  P00439  P00439  P00439  P00439  P00439  P00439  P00439  P00439  P00439  P00439  P00439  P00439  P00439  P00439  P00439  P00439  P00439  P00439  P00439  P00439  P00439  P00439  P00439  P00439  P00439  P00441  P00441  P00441  P00441  P00441  P00441  P00441  P00441  P00441  P00441  P00450  P00450  P00450  P00450  P00450  P00450  P00450  P00450  P00450  P00450  P00450  P00450  P00450  P00450  P00450  P00450  P00450  P00450  P00450  P00450  P00450  P00450  P00450  P00450  P00450  P00450  P00450  P00450  P00450  P00450  P00450  P00451  P00451  P00451  P00451  P00451  P00451  P00451  P00451  P00451  P00451  P00451  P00451  P00451  P00451  P00451  P00451  P00451  P00451  P00451  P00451  P00451  P00451  P00451  P00451  P00451  P00451  P00451  P00451  P00451  P00451  P00451  P00451  P00451  P00480  P00480  P00480  P00480  P00480  P00480  P00480  P00480  P00480  P00480  P00480  P00480  P00480  P00480  P00480  P00480  P00480  P00480  P00480  P00480  P00480  P00480  P00491  P00491  P00491  P00491  P00491  P00491  P00491  P00491  P00491  P00491  P00491  P00491  P00492  P00492  P00492  P00492  P00492  P00492  P00492  P00492  P00492  P00492  P00558  P00558  P00558  P00558  P00558  P00558  P00558  P00558  P00558  P00558  P00558  P00558  P00558  P00558  P00558  P00558  P00558  P00558  P00558  P00558  P00558  P00568  P00568  P00568  P00568  P00568  P00568  P00568  P00568  P00568  P00568  P00568  P00568  P00734  P00734  P00734  P00734  P00734  P00734  P00734  P00734  P00734  P00734  P00734  P00734  P00734  P00734  P00734  P00734  P00734  P00734  P00734  P00734  P00734  P00734  P00734  P00734  P00734  P00734  P00734  P00734  P00734  P00734  P00734  P00734  P00734  P00736  P00736  P00736  P00736  P00736  P00736  P00736  P00736  P00736  P00736  P00736  P00736  P00736  P00736  P00736  P00736  P00736  P00736  P00736  P00736  P00736  P00736  P00736  P00736  P00736  P00736  P00736  P00736  P00736  P00736  P00736  P00740  P00740  P00740  P00740  P00740  P00740  P00740  P00740  P00740  P00740  P00740  P00740  P00740  P00740  P00740  P00740  P00740  P00740  P00740  P00740  P00740  P00740  P00740  P00740  P00740  P00742  P00742  P00742  P00742  P00742  P00742  P00742  P00742  P00742  P00742  P00742  P00742  P00742  P00742  P00742  P00742  P00742  P00742  P00742  P00742  P00742  P00742  P00742  P00742  P00742  P00742  P00742  P00747  P00747  P00747  P00747  P00747  P00747  P00747  P00747  P00747  P00747  P00747  P00747  P00747  P00747  P00747  P00747  P00747  P00747  P00747  P00747  P00747  P00747  P00747  P00747  P00747  P00747  P00747  P00747  P00747  P00747  P00747  P00749  P00749  P00749  P00749  P00749  P00749  P00749  P00749  P00749  P00749  P00749  P00749  P00749  P00749  P00749  P00749  P00749  P00749  P00749  P00749  P00749  P00749  P00749  P00749  P00749  P00749  P00749  P00749  P00749  P00749  P00813  P00813  P00813  P00813  P00813  P00813  P00813  P00813  P00813  P00813  P00813  P00813  P00813  P00813  P00813  P00915  P00915  P00915  P00915  P00915  P00915  P00915  P00915  P00915  P00915  P00915  P00915  P00915  P00915  P00915  P00915  P00915  P00915  P00915  P00915  P00915  P00915  P00915  P00915  P00915  P00915  P00915  P00915  P01008  P01008  P01008  P01008  P01008  P01008  P01008  P01008  P01008  P01008  P01008  P01008  P01008  P01008  P01008  P01008  P01008  P01008  P01008  P01008  P01008  P01008  P01008  P01008  P01008  P01008  P01008  P01008  P01008  P01008  P01008  P01009  P01009  P01009  P01009  P01009  P01009  P01009  P01009  P01009  P01009  P01009  P01009  P01009  P01009  P01009  P01009  P01009  P01009  P01009  P01009  P01019  P01019  P01019  P01019  P01019  P01019  P01019  P01019  P01019  P01019  P01019  P01019  P01019  P01019  P01019  P01019  P01019  P01019  P01019  P01019  P01019  P01019  P01019  P01019  P01019  P01019  P01019  P01019  P01019  P01019  P01019  P01024  P01024  P01024  P01024  P01024  P01024  P01024  P01024  P01024  P01024  P01024  P01024  P01024  P01024  P01024  P01024  P01024  P01024  P01024  P01024  P01024  P01024  P01024  P01024  P01024  P01024  P01024  P01024  P01024  P01024  P01024  P01033  P01033  P01033  P01033  P01033  P01033  P01033  P01033  P01033  P01033  P01033  P01033  P01034  P01034  P01034  P01034  P01034  P01034  P01042  P01042  P01042  P01042  P01042  P01042  P01042  P01042  P01042  P01042  P01042  P01042  P01042  P01042  P01042  P01042  P01042  P01042  P01042  P01042  P01042  P01042  P01042  P01042  P01042  P01042  P01042  P01042  P01042  P01042  P01042  P01100  P01100  P01100  P01100  P01100  P01100  P01100  P01100  P01100  P01100  P01100  P01100  P01100  P01100  P01100  P01100  P01100  P01100  P01100  P01100  P01100  P01100  P01100  P01100  P01100  P01100  P01100  P01100  P01100  P01100  P01100  P01106  P01106  P01106  P01106  P01106  P01106  P01106  P01106  P01106  P01106  P01106  P01106  P01106  P01106  P01106  P01106  P01106  P01106  P01106  P01106  P01106  P01106  P01106  P01106  P01106  P01106  P01106  P01106  P01106  P01106  P01106  P01112  P01112  P01112  P01112  P01112  P01112  P01112  P01112  P01112  P01148  P01148  P01148  P01148  P01148  P01148  P01185  P01185  P01185  P01185  P01185  P01185  P01185  P01185  P01185  P01185  P01189  P01189  P01189  P01189  P01189  P01189  P01189  P01189  P01189  P01189  P01189  P01189  P01189  P01189  P01189  P01189  P01189  P01189  P01189  P01189  P01189  P01236  P01236  P01236  P01236  P01236  P01236  P01236  P01236  P01236  P01236  P01236  P01236  P01236  P01236  P01236  P01241  P01241  P01241  P01241  P01241  P01241  P01241  P01241  P01241  P01241  P01241  P01241  P01241  P01241  P01241  P01241  P01258  P01258  P01258  P01258  P01258  P01258  P01258  P01258  P01258  P01258  P01258  P01258  P01258  P01258  P01266  P01266  P01266  P01266  P01266  P01266  P01266  P01266  P01266  P01266  P01266  P01266  P01266  P01266  P01266  P01266  P01266  P01266  P01266  P01266  P01266  P01266  P01266  P01266  P01266  P01266  P01266  P01266  P01266  P01266  P01266  P01266  P01266  P01275  P01275  P01275  P01275  P01275  P01275  P01275  P01275  P01275  P01275  P01275  P01275  P01275  P01275  P01275  P01275  P01282  P01282  P01282  P01282  P01282  P01282  P01282  P01282  P01282  P01282  P01282  P01282  P01282  P01282  P01282  P01282  P01282  P01282  P01286  P01286  P01286  P01286  P01286  P01286  P01286  P01286  P01286  P01286  P01303  P01303  P01303  P01303  P01303  P01303  P01303  P01303  P01350  P01350  P01350  P01350  P01350  P01579  P01579  P01579  P01579  P01579  P01579  P01579  P01579  P01579  P01579  P01579  P01591  P01591  P01591  P01591  P01591  P01591  P01591  P01591  P01700  P01700  P01700  P01700  P01700  P01700  P01700  P01700  P01700  P01700  P01700  P01700  P01700  P01700  P01700  P01744  P01744  P01744  P01744  P01744  P01744  P01744  P01744  P01744  P01744  P01744  P01744  P01744  P01744  P01744  P01772  P01772  P01772  P01772  P01772  P01772  P01772  P01772  P01772  P01772  P01772  P01772  P01772  P01772  P01772  P01814  P01814  P01814  P01814  P01814  P01814  P01814  P01814  P01814  P01814  P01814  P01825  P01825  P01825  P01825  P01825  P01825  P01825  P01825  P01825  P01825  P01825  P02008  P02008  P02008  P02008  P02008  P02008  P02008  P02008  P02008  P02008  P02008  P02511  P02511  P02511  P02511  P02511  P02511  P02511  P02511  P02511  P02511  P02511  P02511  P02511  P02511  P02545  P02545  P02545  P02545  P02545  P02545  P02545  P02545  P02545  P02545  P02545  P02545  P02545  P02545  P02545  P02545  P02545  P02545  P02545  P02545  P02545  P02545  P02545  P02545  P02545  P02545  P02545  P02545  P02545  P02545  P02545  P02647  P02647  P02647  P02647  P02647  P02647  P02647  P02647  P02647  P02647  P02647  P02647  P02647  P02647  P02647  P02647  P02649  P02649  P02649  P02649  P02649  P02649  P02649  P02649  P02649  P02649  P02649  P02649  P02652  P02652  P02652  P02652  P02652  P02671  P02671  P02671  P02671  P02671  P02671  P02671  P02671  P02671  P02671  P02671  P02671  P02671  P02671  P02671  P02671  P02671  P02671  P02671  P02671  P02671  P02671  P02671  P02671  P02671  P02671  P02671  P02671  P02671  P02671  P02671  P02675  P02675  P02675  P02675  P02675  P02675  P02675  P02675  P02675  P02675  P02675  P02675  P02675  P02675  P02675  P02675  P02675  P02675  P02675  P02675  P02675  P02675  P02675  P02675  P02675  P02675  P02675  P02675  P02675  P02675  P02675  P02675  P02675  P02679  P02679  P02679  P02679  P02679  P02679  P02679  P02679  P02679  P02679  P02679  P02679  P02679  P02679  P02679  P02679  P02679  P02679  P02679  P02679  P02679  P02679  P02679  P02679  P02679  P02679  P02686  P02686  P02686  P02686  P02686  P02686  P02686  P02686  P02686  P02686  P02686  P02686  P02686  P02686  P02686  P02686  P02686  P02686  P02686  P02686  P02686  P02686  P02686  P02686  P02686  P02686  P02686  P02730  P02730  P02730  P02730  P02730  P02730  P02730  P02730  P02730  P02730  P02730  P02730  P02730  P02730  P02730  P02730  P02730  P02730  P02730  P02730  P02730  P02730  P02730  P02730  P02730  P02730  P02730  P02730  P02730  P02730  P02730  P02730  P02730  P02741  P02741  P02741  P02741  P02741  P02741  P02741  P02741  P02741  P02741  P02741  P02741  P02741  P02741  P02741  P02741  P02741  P02741  P02741  P02741  P02741  P02741  P02741  P02745  P02745  P02745  P02745  P02745  P02745  P02745  P02745  P02745  P02745  P02745  P02745  P02745  P02745  P02745  P02745  P02745  P02746  P02746  P02746  P02746  P02746  P02746  P02746  P02746  P02746  P02746  P02746  P02751  P02751  P02751  P02751  P02751  P02751  P02751  P02751  P02751  P02751  P02751  P02751  P02751  P02751  P02751  P02751  P02751  P02751  P02751  P02751  P02751  P02751  P02751  P02751  P02751  P02751  P02751  P02751  P02751  P02751  P02751  P02763  P02763  P02763  P02763  P02763  P02763  P02765  P02765  P02765  P02765  P02765  P02765  P02765  P02765  P02765  P02765  P02765  P02765  P02765  P02765  P02765  P02766  P02766  P02766  P02766  P02766  P02766  P02766  P02766  P02766  P02766  P02766  P02766  P02768  P02768  P02768  P02768  P02768  P02768  P02768  P02768  P02768  P02768  P02768  P02768  P02768  P02768  P02768  P02768  P02768  P02768  P02768  P02768  P02768  P02768  P02771  P02771  P02771  P02771  P02771  P02771  P02771  P02771  P02771  P02771  P02771  P02771  P02771  P02771  P02771  P02771  P02771  P02771  P02771  P02771  P02771  P02771  P02771  P02771  P02771  P02771  P02771  P02771  P02771  P02771  P02771  P02778  P02778  P02778  P02778  P02778  P02778  P02778  P02778  P02786  P02786  P02786  P02786  P02786  P02786  P02786  P02786  P02786  P02786  P02786  P02786  P02786  P02786  P02786  P02786  P02786  P02786  P02786  P02786  P02786  P02786  P02786  P02786  P02786  P02786  P02786  P02786  P02786  P02786  P02786  P02788  P02788  P02788  P02788  P02788  P02788  P02788  P02788  P02788  P02788  P02788  P02788  P02788  P02788  P02788  P02788  P02788  P02788  P02788  P02788  P02788  P02788  P02788  P02788  P02788  P02788  P02788  P02788  P02788  P02788  P02788  P02792  P02792  P02792  P02792  P02792  P02792  P02808  P02812  P02812  P02812  P02812  P02812  P02812  P02812  P02812  P02812  P02812  P02812  P02812  P02812  P02812  P02812  P02812  P02812  P02812  P02812  P02812  P02812  P02814  P02818  P02818  P02818  P02818  P02818  P03372  P03372  P03372  P03372  P03372  P03372  P03372  P03372  P03372  P03372  P03372  P03372  P03372  P03372  P03372  P03372  P03372  P03372  P03372  P03372  P03372  P03372  P03372  P03372  P03372  P03372  P03372  P03372  P03372  P03372  P03372  P03950  P03950  P03950  P03950  P03950  P03950  P03950  P03950  P03956  P03956  P03956  P03956  P03956  P03956  P03956  P03956  P03956  P03956  P03956  P03956  P03956  P03956  P03956  P03956  P03956  P03956  P03999  P03999  P03999  P03999  P03999  P03999  P03999  P03999  P03999  P03999  P03999  P03999  P03999  P03999  P03999  P03999  P03999  P03999  P03999  P03999  P03999  P03999  P03999  P03999  P03999  P04001  P04001  P04001  P04001  P04001  P04001  P04001  P04001  P04001  P04001  P04001  P04001  P04001  P04001  P04001  P04001  P04001  P04001  P04001  P04001  P04001  P04001  P04001  P04001  P04001  P04001  P04001  P04001  P04001  P04004  P04004  P04004  P04004  P04004  P04004  P04004  P04004  P04004  P04004  P04004  P04004  P04004  P04004  P04004  P04004  P04004  P04004  P04004  P04004  P04004  P04004  P04004  P04004  P04004  P04004  P04004  P04004  P04004  P04004  P04070  P04070  P04070  P04070  P04070  P04070  P04070  P04070  P04070  P04070  P04070  P04070  P04070  P04070  P04070  P04070  P04070  P04070  P04070  P04070  P04070  P04070  P04070  P04070  P04070  P04070  P04070  P04070  P04070  P04070  P04070  P04075  P04075  P04075  P04075  P04075  P04075  P04075  P04075  P04075  P04075  P04075  P04075  P04075  P04075  P04075  P04075  P04075  P04075  P04075  P04083  P04083  P04083  P04083  P04083  P04083  P04083  P04083  P04083  P04083  P04083  P04083  P04083  P04083  P04083  P04090  P04090  P04090  P04090  P04090  P04090  P04090  P04090  P04090  P04090  P04090  P04090  P04090  P04090  P04090  P04090  P04090  P04114  P04114  P04114  P04114  P04114  P04114  P04114  P04114  P04114  P04114  P04114  P04114  P04114  P04114  P04114  P04114  P04114  P04114  P04114  P04114  P04114  P04114  P04114  P04114  P04114  P04114  P04114  P04114  P04114  P04114  P04114  P04150  P04150  P04150  P04150  P04150  P04150  P04150  P04150  P04150  P04150  P04150  P04150  P04150  P04150  P04150  P04150  P04150  P04150  P04150  P04150  P04150  P04150  P04150  P04150  P04150  P04150  P04150  P04150  P04150  P04150  P04150  P04179  P04179  P04179  P04179  P04179  P04179  P04179  P04179  P04179  P04181  P04181  P04181  P04181  P04181  P04181  P04181  P04181  P04181  P04181  P04181  P04181  P04181  P04181  P04181  P04181  P04181  P04181  P04181  P04181  P04181  P04183  P04183  P04183  P04183  P04183  P04183  P04183  P04183  P04183  P04198  P04198  P04198  P04198  P04198  P04198  P04198  P04198  P04198  P04198  P04198  P04198  P04198  P04198  P04198  P04198  P04198  P04198  P04198  P04198  P04198  P04198  P04198  P04198  P04198  P04198  P04198  P04198  P04198  P04198  P04198  P04264  P04264  P04264  P04264  P04264  P04264  P04264  P04264  P04264  P04264  P04264  P04264  P04264  P04264  P04264  P04264  P04264  P04264  P04264  P04264  P04264  P04264  P04264  P04264  P04264  P04264  P04264  P04264  P04264  P04264  P04264  P04264  P04264  P04406  P04406  P04406  P04406  P04406  P04406  P04406  P04406  P04406  P04406  P04406  P04406  P04406  P04406  P04424  P04424  P04424  P04424  P04424  P04424  P04424  P04424  P04424  P04424  P04424  P04424  P04424  P04424  P04424  P04424  P04424  P04424  P04424  P04424  P04424  P04424  P04424  P04424  P04424  P04424  P04424  P04424  P04424  P04424  P04424  P04424  P04424  P04626  P04626  P04626  P04626  P04626  P04626  P04626  P04626  P04626  P04626  P04626  P04626  P04626  P04626  P04626  P04626  P04626  P04626  P04626  P04626  P04626  P04626  P04626  P04626  P04626  P04626  P04626  P04626  P04626  P04626  P04626  P04626  P04626  P04629  P04629  P04629  P04629  P04629  P04629  P04629  P04629  P04629  P04629  P04629  P04629  P04629  P04629  P04629  P04629  P04629  P04629  P04629  P04629  P04629  P04629  P04629  P04629  P04629  P04629  P04629  P04629  P04629  P04629  P04629  P04629  P04629  P04632  P04632  P04632  P04632  P04632  P04632  P04632  P04632  P04632  P04632  P04632  P04632  P04632  P04632  P04632  P04637  P04637  P04637  P04637  P04637  P04637  P04637  P04637  P04637  P04637  P04637  P04637  P04637  P04637  P04637  P04637  P04637  P04637  P04637  P04637  P04637  P04637  P04637  P04637  P04637  P04637  P04637  P04745  P04745  P04745  P04745  P04745  P04745  P04745  P04745  P04745  P04745  P04745  P04745  P04745  P04745  P04745  P04745  P04745  P04745  P04745  P04745  P04745  P04745  P04745  P04745  P04745  P04745  P04745  P04745  P04745  P04745  P04745  P04745  P04792  P04792  P04792  P04792  P04792  P04792  P04792  P04792  P04792  P04792  P04792  P04792  P05060  P05060  P05060  P05060  P05060  P05060  P05060  P05060  P05060  P05060  P05060  P05060  P05060  P05060  P05060  P05060  P05060  P05060  P05060  P05060  P05060  P05060  P05060  P05060  P05060  P05060  P05060  P05060  P05060  P05060  P05060  P05067  P05067  P05067  P05067  P05067  P05067  P05067  P05067  P05067  P05067  P05067  P05067  P05067  P05067  P05067  P05067  P05067  P05067  P05067  P05067  P05067  P05067  P05067  P05067  P05067  P05067  P05067  P05067  P05067  P05067  P05067  P05090  P05090  P05090  P05090  P05090  P05090  P05090  P05106  P05106  P05106  P05106  P05106  P05106  P05106  P05106  P05106  P05106  P05106  P05106  P05106  P05106  P05106  P05106  P05106  P05106  P05106  P05106  P05106  P05106  P05106  P05106  P05106  P05106  P05106  P05106  P05106  P05106  P05106  P05106  P05106  P05107  P05107  P05107  P05107  P05107  P05107  P05107  P05107  P05107  P05107  P05107  P05107  P05107  P05107  P05107  P05107  P05107  P05107  P05107  P05107  P05107  P05107  P05107  P05107  P05107  P05107  P05107  P05107  P05107  P05107  P05107  P05109  P05109  P05114  P05114  P05161  P05161  P05161  P05161  P05161  P05161  P05161  P05161  P05161  P05161  P05161  P05161  P05161  P05161  P05164  P05164  P05164  P05164  P05164  P05164  P05164  P05164  P05164  P05164  P05164  P05164  P05164  P05164  P05164  P05164  P05164  P05164  P05164  P05164  P05164  P05164  P05164  P05164  P05164  P05164  P05164  P05164  P05164  P05164  P05164  P05164  P05164  P05231  P05231  P05231  P05231  P05231  P05231  P05231  P05231  P05231  P05231  P05231  P05231  P05231  P05231  P05386  P05386  P05386  P05387  P05387  P05387  P05387  P05387  P05387  P05412  P05412  P05412  P05412  P05412  P05412  P05412  P05412  P05412  P05412  P05412  P05412  P05412  P05412  P05412  P05412  P05412  P05412  P05412  P05412  P05412  P05412  P05412  P05412  P05412  P05451  P05451  P05451  P05451  P05451  P05451  P05451  P05451  P05451  P05451  P05451  P05451  P05451  P05451  P05451  P05451  P05451  P05451  P05451  P05455  P05455  P05455  P05455  P05455  P05455  P05455  P05455  P05455  P05455  P05455  P05455  P05455  P05455  P05455  P05455  P05546  P05546  P05546  P05546  P05546  P05546  P05546  P05546  P05546  P05546  P05546  P05546  P05546  P05546  P05546  P05546  P05546  P05546  P05546  P05546  P05546  P05546  P05546  P05546  P05546  P05546  P05546  P05549  P05549  P05549  P05549  P05549  P05549  P05549  P05549  P05549  P05549  P05549  P05549  P05549  P05549  P05549  P05549  P05549  P05549  P05549  P05549  P05549  P05549  P05549  P05549  P05549  P05549  P05549  P05549  P05549  P05549  P05549  P05771  P05771  P05771  P05771  P05771  P05771  P05771  P05771  P05771  P05771  P05771  P05771  P05771  P05771  P05771  P05771  P05771  P05771  P05771  P05771  P05771  P05771  P05771  P05771  P05771  P05771  P05771  P05771  P05771  P05771  P05771  P05783  P05783  P05783  P05783  P05783  P05783  P05783  P05783  P05783  P05783  P05783  P05783  P05783  P05783  P05783  P05783  P05783  P05813  P05813  P05813  P05813  P05813  P05813  P05813  P05813  P05813  P05813  P05813  P05813  P05813  P05813  P05813  P05813  P05813  P05813  P05813  P05814  P05814  P05814  P05814  P05997  P05997  P05997  P05997  P05997  P05997  P05997  P05997  P05997  P05997  P05997  P05997  P05997  P05997  P05997  P05997  P05997  P05997  P05997  P05997  P05997  P05997  P05997  P05997  P05997  P05997  P05997  P05997  P05997  P05997  P05997  P05997  P05997  P06213  P06213  P06213  P06213  P06213  P06213  P06213  P06213  P06213  P06213  P06213  P06213  P06213  P06213  P06213  P06213  P06213  P06213  P06213  P06213  P06213  P06213  P06213  P06213  P06213  P06213  P06213  P06213  P06213  P06213  P06213  P06213  P06213  P06276  P06276  P06276  P06276  P06276  P06276  P06276  P06276  P06276  P06276  P06276  P06276  P06276  P06276  P06276  P06276  P06276  P06276  P06276  P06276  P06276  P06276  P06276  P06276  P06276  P06276  P06276  P06276  P06276  P06276  P06276  P06307  P06307  P06307  P06307  P06307  P06307  P06307  P06307  P06396  P06396  P06396  P06396  P06396  P06396  P06396  P06396  P06396  P06396  P06396  P06396  P06396  P06396  P06396  P06396  P06396  P06396  P06396  P06396  P06396  P06396  P06396  P06396  P06396  P06396  P06396  P06396  P06396  P06396  P06396  P06396  P06396  P06400  P06400  P06400  P06400  P06400  P06400  P06400  P06400  P06400  P06400  P06400  P06400  P06400  P06400  P06400  P06400  P06400  P06400  P06400  P06400  P06400  P06400  P06400  P06400  P06400  P06400  P06400  P06400  P06400  P06400  P06400  P06401  P06401  P06401  P06401  P06401  P06401  P06401  P06401  P06401  P06401  P06401  P06401  P06401  P06401  P06401  P06401  P06401  P06401  P06401  P06401  P06401  P06401  P06401  P06401  P06401  P06401  P06401  P06401  P06401  P06401  P06401  P06454  P06702  P06702  P06702  P06730  P06730  P06730  P06730  P06730  P06730  P06730  P06730  P06730  P06730  P06730  P06733  P06733  P06733  P06733  P06733  P06733  P06733  P06733  P06733  P06733  P06733  P06733  P06733  P06733  P06733  P06733  P06733  P06733  P06733  P06733  P06733  P06733  P06733  P06733  P06733  P06737  P06737  P06737  P06737  P06737  P06737  P06737  P06737  P06737  P06737  P06737  P06737  P06737  P06737  P06737  P06737  P06737  P06737  P06737  P06737  P06737  P06737  P06737  P06737  P06737  P06737  P06737  P06737  P06737  P06737  P06737  P06744  P06744  P06744  P06744  P06744  P06744  P06744  P06744  P06744  P06744  P06744  P06744  P06744  P06744  P06744  P06744  P06744  P06744  P06744  P06744  P06744  P06744  P06744  P06744  P06744  P06744  P06744  P06744  P06746  P06746  P06746  P06746  P06746  P06746  P06746  P06746  P06746  P06746  P06746  P06746  P06746  P06746  P06746  P06746  P06746  P06746  P06746  P06748  P06748  P06748  P06748  P06748  P06748  P06748  P06748  P06748  P06748  P06748  P06748  P06748  P06748  P06850  P06850  P06850  P06850  P06850  P06850  P06850  P06850  P06850  P06850  P06850  P06850  P06850  P06850  P06850  P07101  P07101  P07101  P07101  P07101  P07101  P07101  P07101  P07101  P07101  P07101  P07101  P07101  P07101  P07101  P07101  P07101  P07101  P07101  P07101  P07101  P07101  P07101  P07101  P07101  P07101  P07101  P07101  P07101  P07101  P07101  P07108  P07108  P07148  P07148  P07148  P07148  P07195  P07195  P07195  P07195  P07195  P07195  P07195  P07195  P07195  P07195  P07195  P07195  P07195  P07195  P07195  P07195  P07195  P07195  P07195  P07195  P07195  P07195  P07195  P07195  P07195  P07195  P07199  P07199  P07199  P07199  P07199  P07199  P07199  P07199  P07199  P07199  P07199  P07199  P07199  P07199  P07199  P07199  P07199  P07199  P07199  P07199  P07199  P07199  P07199  P07199  P07199  P07199  P07199  P07199  P07199  P07199  P07199  P07199  P07199  P07204  P07204  P07204  P07204  P07204  P07204  P07204  P07204  P07204  P07204  P07204  P07204  P07204  P07204  P07204  P07204  P07204  P07204  P07204  P07204  P07204  P07204  P07204  P07204  P07204  P07204  P07204  P07204  P07204  P07204  P07204  P07204  P07225  P07225  P07225  P07225  P07225  P07225  P07225  P07225  P07225  P07225  P07225  P07225  P07225  P07225  P07225  P07225  P07225  P07225  P07225  P07225  P07225  P07225  P07225  P07225  P07225  P07225  P07225  P07225  P07225  P07225  P07225  P07225  P07225  P07305  P07305  P07305  P07305  P07305  P07305  P07305  P07305  P07305  P07305  P07305  P07305  P07305  P07305  P07305  P07305  P07305  P07305  P07311  P07311  P07311  P07311  P07332  P07332  P07332  P07332  P07332  P07332  P07332  P07332  P07332  P07332  P07332  P07332  P07332  P07332  P07332  P07332  P07332  P07332  P07332  P07332  P07332  P07332  P07332  P07332  P07332  P07332  P07332  P07332  P07332  P07332  P07332  P07332  P07332  P07333  P07333  P07333  P07333  P07333  P07333  P07333  P07333  P07333  P07333  P07333  P07333  P07333  P07333  P07333  P07333  P07333  P07333  P07333  P07333  P07333  P07333  P07333  P07333  P07333  P07333  P07333  P07333  P07333  P07333  P07333  P07333  P07333  P07358  P07358  P07358  P07358  P07358  P07358  P07358  P07358  P07358  P07358  P07358  P07358  P07358  P07358  P07358  P07358  P07358  P07358  P07358  P07358  P07358  P07358  P07358  P07358  P07358  P07358  P07358  P07358  P07358  P07358  P07358  P07358  P07358  P07359  P07359  P07359  P07359  P07359  P07359  P07359  P07359  P07359  P07359  P07359  P07359  P07359  P07359  P07359  P07359  P07359  P07359  P07359  P07359  P07359  P07359  P07359  P07359  P07359  P07359  P07359  P07359  P07359  P07359  P07359  P07359  P07359  P07360  P07360  P07360  P07360  P07360  P07360  P07360  P07360  P07360  P07360  P07360  P07492  P07492  P07492  P07492  P07492  P07492  P07492  P07492  P07492  P07492  P07492  P07492  P07492  P07550  P07550  P07550  P07550  P07550  P07550  P07550  P07550  P07550  P07550  P07550  P07550  P07550  P07550  P07550  P07550  P07550  P07550  P07550  P07550  P07737  P07737  P07737  P07737  P07737  P07737  P07737  P07741  P07741  P07741  P07741  P07741  P07741  P07741  P07741  P07741  P07766  P07766  P07766  P07766  P07766  P07766  P07766  P07766  P07766  P07766  P07766  P07814  P07814  P07814  P07814  P07814  P07814  P07814  P07814  P07814  P07814  P07814  P07814  P07814  P07814  P07814  P07814  P07814  P07814  P07814  P07814  P07814  P07814  P07814  P07814  P07814  P07814  P07814  P07814  P07814  P07814  P07814  P07900  P07900  P07900  P07900  P07900  P07900  P07900  P07900  P07900  P07900  P07900  P07900  P07900  P07900  P07900  P07900  P07900  P07900  P07900  P07900  P07900  P07900  P07900  P07900  P07900  P07900  P07900  P07900  P07900  P07900  P07900  P07910  P07910  P07910  P07910  P07910  P07910  P07910  P07910  P07910  P07910  P07910  P07910  P07910  P07910  P07910  P07910  P07910  P07910  P07910  P07910  P07910  P07947  P07947  P07947  P07947  P07947  P07947  P07947  P07947  P07947  P07947  P07947  P07947  P07947  P07947  P07947  P07947  P07947  P07947  P07947  P07947  P07947  P07947  P07947  P07947  P07947  P07947  P07947  P07947  P07947  P07947  P07947  P07947  P07947  P07949  P07949  P07949  P07949  P07949  P07949  P07949  P07949  P07949  P07949  P07949  P07949  P07949  P07949  P07949  P07949  P07949  P07949  P07949  P07949  P07949  P07949  P07949  P07949  P07949  P07949  P07949  P07949  P07949  P07949  P07949  P07949  P07949  P08047  P08047  P08047  P08047  P08047  P08047  P08047  P08047  P08047  P08047  P08047  P08047  P08047  P08047  P08047  P08047  P08047  P08047  P08047  P08047  P08047  P08047  P08047  P08047  P08047  P08047  P08047  P08047  P08047  P08047  P08047  P08069  P08069  P08069  P08069  P08069  P08069  P08069  P08069  P08069  P08069  P08069  P08069  P08069  P08069  P08069  P08069  P08069  P08069  P08069  P08069  P08069  P08069  P08069  P08069  P08069  P08069  P08069  P08069  P08069  P08069  P08069  P08069  P08069  P08151  P08151  P08151  P08151  P08151  P08151  P08151  P08151  P08151  P08151  P08151  P08151  P08151  P08151  P08151  P08151  P08151  P08151  P08151  P08151  P08151  P08151  P08151  P08151  P08151  P08151  P08151  P08151  P08151  P08151  P08151  P08151  P08151  P08195  P08195  P08195  P08195  P08195  P08195  P08195  P08195  P08195  P08195  P08195  P08195  P08195  P08195  P08195  P08195  P08195  P08195  P08195  P08195  P08195  P08195  P08195  P08195  P08195  P08195  P08195  P08195  P08195  P08195  P08195  P08195  P08195  P08397  P08397  P08397  P08397  P08397  P08397  P08397  P08397  P08397  P08397  P08397  P08397  P08397  P08397  P08397  P08397  P08397  P08397  P08397  P08493  P08493  P08514  P08514  P08514  P08514  P08514  P08514  P08514  P08514  P08514  P08514  P08514  P08514  P08514  P08514  P08514  P08514  P08514  P08514  P08514  P08514  P08514  P08514  P08514  P08514  P08514  P08514  P08514  P08514  P08514  P08514  P08514  P08514  P08514  P08559  P08559  P08559  P08559  P08559  P08559  P08559  P08559  P08559  P08559  P08559  P08559  P08559  P08559  P08559  P08567  P08567  P08567  P08567  P08567  P08567  P08567  P08567  P08567  P08567  P08567  P08567  P08567  P08567  P08567  P08567  P08567  P08567  P08567  P08581  P08581  P08581  P08581  P08581  P08581  P08581  P08581  P08581  P08581  P08581  P08581  P08581  P08581  P08581  P08581  P08581  P08581  P08581  P08581  P08581  P08581  P08581  P08581  P08581  P08581  P08581  P08581  P08581  P08581  P08581  P08581  P08581  P08582  P08582  P08582  P08582  P08582  P08582  P08582  P08582  P08582  P08582  P08582  P08582  P08582  P08582  P08582  P08582  P08582  P08582  P08582  P08582  P08582  P08582  P08582  P08582  P08582  P08582  P08582  P08582  P08582  P08582  P08582  P08697  P08697  P08697  P08697  P08697  P08697  P08697  P08697  P08697  P08697  P08697  P08697  P08697  P08697  P08697  P08697  P08697  P08697  P08697  P08697  P08697  P08697  P08697  P08697  P08697  P08697  P08697  P08697  P08697  P08697  P08697  P08697  P08697  P08709  P08709  P08709  P08709  P08709  P08709  P08709  P08709  P08709  P08709  P08709  P08709  P08709  P08709  P08709  P08709  P08709  P08709  P08709  P08709  P08709  P08709  P08709  P08709  P08709  P08709  P08709  P08709  P08709  P08709  P08833  P08833  P08833  P08833  P08833  P08833  P08833  P08833  P08833  P08833  P08833  P08833  P08842  P08842  P08842  P08842  P08842  P08842  P08842  P08842  P08842  P08842  P08842  P08842  P08842  P08842  P08842  P08842  P08842  P08842  P08842  P08842  P08842  P08842  P08842  P08842  P08842  P08842  P08842  P08842  P08842  P08842  P08842  P08842  P08842  P08865  P08865  P08865  P08865  P08865  P08865  P08865  P08865  P08865  P08865  P08865  P08865  P08949  P08949  P08949  P08949  P08949  P08949  P08949  P08949  P08949  P08949  P08949  P08949  P08949  P09012  P09012  P09012  P09012  P09012  P09012  P09012  P09012  P09012  P09012  P09012  P09038  P09038  P09038  P09038  P09038  P09038  P09038  P09038  P09038  P09038  P09038  P09038  P09038  P09038  P09038  P09038  P09211  P09211  P09211  P09211  P09211  P09211  P09211  P09211  P09211  P09211  P09382  P09382  P09382  P09382  P09417  P09417  P09417  P09417  P09417  P09417  P09417  P09417  P09417  P09417  P09417  P09417  P09417  P09417  P09417  P09417  P09417  P09417  P09417  P09493  P09493  P09493  P09493  P09493  P09493  P09493  P09493  P09493  P09493  P09493  P09603  P09603  P09603  P09603  P09603  P09603  P09603  P09603  P09603  P09603  P09603  P09603  P09603  P09603  P09603  P09603  P09603  P09603  P09603  P09603  P09603  P09603  P09603  P09603  P09603  P09603  P09603  P09603  P09603  P09603  P09603  P09603  P09603  P09619  P09619  P09619  P09619  P09619  P09619  P09619  P09619  P09619  P09619  P09619  P09619  P09619  P09619  P09619  P09619  P09619  P09619  P09619  P09619  P09619  P09619  P09619  P09619  P09619  P09619  P09619  P09619  P09619  P09619  P09619  P09619  P09619  P09683  P09683  P09683  P09683  P09683  P09683  P09683  P09683  P09683  P09683  P09683  P09693  P09693  P09693  P09693  P09693  P09693  P09693  P09693  P09871  P09871  P09871  P09871  P09871  P09871  P09871  P09871  P09871  P09871  P09871  P09871  P09871  P09871  P09871  P09871  P09871  P09871  P09871  P09871  P09871  P09871  P09871  P09871  P09871  P09871  P09871  P09871  P09871  P09871  P09871  P09871  P09871  P09874  P09874  P09874  P09874  P09874  P09874  P09874  P09874  P09874  P09874  P09874  P09874  P09874  P09874  P09874  P09874  P09874  P09874  P09874  P09874  P09874  P09874  P09874  P09874  P09874  P09874  P09874  P09874  P09874  P09874  P09874  P09874  P09874  P09917  P09917  P09917  P09917  P09917  P09917  P09917  P09917  P09917  P09917  P09917  P09917  P09917  P09917  P09917  P09917  P09917  P09917  P09917  P09917  P09917  P09917  P09917  P09917  P09917  P09917  P09917  P09917  P09917  P09958  P09958  P09958  P09958  P09958  P09958  P09958  P09958  P09958  P09958  P09958  P09958  P09958  P09958  P09958  P09958  P09958  P09958  P09958  P09958  P09958  P09958  P09958  P09958  P09958  P09958  P09958  P09958  P09958  P09958  P09958  P09960  P09960  P09960  P09960  P09960  P09960  P09960  P09960  P09960  P09960  P09960  P09960  P09960  P09960  P09960  P09960  P09960  P09960  P09960  P09960  P09960  P09960  P09960  P09960  P09960  P09960  P09960  P09960  P09960  P09960  P09960  P0C0L4  P0C0L4  P0C0L4  P0C0L4  P0C0L4  P0C0L4  P0C0L4  P0C0L4  P0C0L4  P0C0L4  P0C0L4  P0C0L4  P0C0L4  P0C0L4  P0C0L4  P0C0L4  P0C0L4  P0C0L4  P0C0L4  P0C0L4  P0C0L4  P0C0L4  P0C0L4  P0C0L4  P0C0L4  P0C0L4  P0C0L4  P0C0L4  P0C0L4  P0C0L4  P0C0L4  P0DI83  P0DI83  P0DI83  P0DI83  P0DI83  P0DI83  P0DI83  P0DI83  P10070  P10070  P10070  P10070  P10070  P10070  P10070  P10070  P10070  P10070  P10070  P10070  P10070  P10070  P10070  P10070  P10070  P10070  P10070  P10070  P10070  P10070  P10070  P10070  P10070  P10070  P10070  P10070  P10070  P10070  P10070  P10071  P10071  P10071  P10071  P10071  P10071  P10071  P10071  P10071  P10071  P10071  P10071  P10071  P10071  P10071  P10071  P10071  P10071  P10071  P10071  P10071  P10071  P10071  P10071  P10071  P10071  P10071  P10071  P10071  P10071  P10071  P10082  P10082  P10082  P10082  P10145  P10145  P10145  P10145  P10145  P10153  P10153  P10153  P10153  P10153  P10153  P10153  P10153  P10155  P10155  P10155  P10155  P10155  P10155  P10155  P10155  P10155  P10155  P10155  P10155  P10155  P10155  P10155  P10155  P10155  P10155  P10155  P10155  P10155  P10155  P10155  P10155  P10155  P10155  P10155  P10244  P10244  P10244  P10244  P10244  P10244  P10244  P10244  P10244  P10244  P10244  P10244  P10244  P10244  P10244  P10244  P10244  P10244  P10244  P10244  P10244  P10244  P10244  P10244  P10244  P10244  P10244  P10244  P10244  P10244  P10244  P10275  P10275  P10275  P10275  P10275  P10275  P10275  P10275  P10275  P10275  P10275  P10275  P10275  P10275  P10275  P10275  P10275  P10275  P10275  P10275  P10275  P10275  P10275  P10275  P10275  P10275  P10275  P10275  P10275  P10275  P10275  P10276  P10276  P10276  P10276  P10276  P10276  P10276  P10276  P10276  P10276  P10276  P10276  P10276  P10276  P10276  P10276  P10276  P10276  P10276  P10276  P10276  P10276  P10276  P10276  P10276  P10276  P10276  P10276  P10276  P10276  P10276  P10415  P10415  P10415  P10415  P10415  P10415  P10415  P10415  P10415  P10415  P10415  P10415  P10415  P10451  P10451  P10451  P10451  P10515  P10515  P10515  P10515  P10515  P10515  P10515  P10515  P10515  P10515  P10515  P10515  P10515  P10515  P10515  P10515  P10515  P10515  P10515  P10515  P10515  P10515  P10515  P10515  P10515  P10515  P10515  P10515  P10515  P10515  P10515  P10515  P10515  P10588  P10588  P10588  P10588  P10588  P10588  P10588  P10588  P10588  P10588  P10588  P10588  P10588  P10588  P10588  P10588  P10588  P10588  P10588  P10588  P10588  P10588  P10588  P10588  P10588  P10599  P10599  P10599  P10599  P10599  P10599  P10636  P10636  P10636  P10636  P10636  P10636  P10636  P10636  P10636  P10636  P10636  P10636  P10636  P10636  P10636  P10636  P10636  P10636  P10636  P10636  P10636  P10636  P10636  P10636  P10636  P10636  P10636  P10636  P10636  P10636  P10636  P10644  P10644  P10644  P10644  P10644  P10644  P10644  P10644  P10644  P10644  P10644  P10644  P10644  P10644  P10644  P10644  P10644  P10644  P10644  P10644  P10644  P10644  P10645  P10645  P10645  P10645  P10645  P10645  P10645  P10645  P10645  P10645  P10645  P10645  P10645  P10645  P10645  P10645  P10645  P10645  P10645  P10645  P10645  P10645  P10645  P10645  P10645  P10645  P10645  P10645  P10721  P10721  P10721  P10721  P10721  P10721  P10721  P10721  P10721  P10721  P10721  P10721  P10721  P10721  P10721  P10721  P10721  P10721  P10721  P10721  P10721  P10721  P10721  P10721  P10721  P10721  P10721  P10721  P10721  P10721  P10721  P10809  P10809  P10809  P10809  P10809  P10809  P10809  P10809  P10809  P10809  P10809  P10809  P10809  P10809  P10809  P10809  P10809  P10809  P10809  P10809  P10809  P10809  P10809  P10809  P10809  P10914  P10914  P10914  P10914  P10914  P10914  P10914  P10914  P10914  P10914  P10914  P10914  P10914  P10914  P10914  P10914  P10914  P10914  P10914  P10914  P10914  P10914  P10914  P10914  P10914  P10914  P10914  P10914  P10914  P10914  P10914  P10914  P10914  P10997  P10997  P10997  P10997  P10997  P10997  P10997  P11047  P11047  P11047  P11047  P11047  P11047  P11047  P11047  P11047  P11047  P11047  P11047  P11047  P11047  P11047  P11047  P11047  P11047  P11047  P11047  P11047  P11047  P11047  P11047  P11047  P11047  P11047  P11047  P11047  P11047  P11047  P11142  P11142  P11142  P11142  P11142  P11142  P11142  P11142  P11142  P11142  P11142  P11142  P11142  P11142  P11142  P11142  P11142  P11142  P11142  P11142  P11142  P11142  P11142  P11142  P11142  P11142  P11142  P11142  P11142  P11142  P11142  P11142  P11142  P11171  P11171  P11171  P11171  P11171  P11171  P11171  P11171  P11171  P11171  P11171  P11171  P11171  P11171  P11171  P11171  P11171  P11171  P11171  P11171  P11171  P11171  P11171  P11171  P11171  P11171  P11171  P11171  P11171  P11171  P11171  P11226  P11226  P11226  P11226  P11226  P11226  P11226  P11226  P11226  P11226  P11226  P11226  P11226  P11226  P11226  P11226  P11274  P11274  P11274  P11274  P11274  P11274  P11274  P11274  P11274  P11274  P11274  P11274  P11274  P11274  P11274  P11274  P11274  P11274  P11274  P11274  P11274  P11274  P11274  P11274  P11274  P11274  P11274  P11274  P11274  P11274  P11274  P11274  P11274  P11277  P11277  P11277  P11277  P11277  P11277  P11277  P11277  P11277  P11277  P11277  P11277  P11277  P11277  P11277  P11277  P11277  P11277  P11277  P11277  P11277  P11277  P11277  P11277  P11277  P11277  P11277  P11277  P11277  P11277  P11277  P11309  P11309  P11309  P11309  P11309  P11309  P11309  P11309  P11309  P11309  P11309  P11309  P11309  P11309  P11309  P11309  P11309  P11309  P11309  P11309  P11309  P11309  P11309  P11309  P11309  P11309  P11309  P11309  P11309  P11309  P11309  P11362  P11362  P11362  P11362  P11362  P11362  P11362  P11362  P11362  P11362  P11362  P11362  P11362  P11362  P11362  P11362  P11362  P11362  P11362  P11362  P11362  P11362  P11362  P11362  P11362  P11362  P11362  P11362  P11362  P11362  P11362  P11362  P11362  P11387  P11387  P11387  P11387  P11387  P11387  P11387  P11387  P11387  P11387  P11387  P11387  P11387  P11387  P11387  P11387  P11387  P11387  P11387  P11387  P11387  P11387  P11387  P11387  P11387  P11387  P11388  P11388  P11388  P11388  P11388  P11388  P11388  P11388  P11388  P11388  P11388  P11388  P11388  P11388  P11388  P11388  P11388  P11388  P11388  P11388  P11388  P11388  P11388  P11388  P11388  P11388  P11388  P11388  P11388  P11388  P11388  P11413  P11413  P11413  P11413  P11413  P11413  P11413  P11413  P11413  P11413  P11413  P11413  P11413  P11413  P11413  P11413  P11413  P11413  P11413  P11413  P11413  P11413  P11413  P11413  P11474  P11474  P11474  P11474  P11474  P11474  P11474  P11474  P11474  P11474  P11474  P11474  P11474  P11474  P11474  P11474  P11474  P11474  P11474  P11474  P11474  P11488  P11488  P11488  P11488  P11488  P11488  P11488  P11488  P11488  P11488  P11488  P11488  P11488  P11488  P11488  P11488  P11488  P11488  P11488  P11488  P11498  P11498  P11498  P11498  P11498  P11498  P11498  P11498  P11498  P11498  P11498  P11498  P11498  P11498  P11498  P11498  P11498  P11498  P11498  P11498  P11498  P11498  P11498  P11498  P11498  P11498  P11498  P11498  P11498  P11498  P11498  P11498  P11498  P11831  P11831  P11831  P11831  P11831  P11831  P11831  P11831  P11831  P11831  P11831  P11831  P11831  P11831  P11831  P11831  P11831  P11831  P11831  P11831  P11831  P11831  P11831  P11831  P11831  P11831  P11831  P11831  P11831  P11831  P11831  P11926  P11926  P11926  P11926  P11926  P11926  P11926  P11926  P11926  P11926  P11926  P11926  P11926  P11926  P11926  P11926  P11926  P11926  P11926  P11926  P11926  P11926  P11926  P11926  P11926  P11926  P11926  P11926  P11926  P11926  P11926  P12004  P12004  P12004  P12004  P12004  P12004  P12004  P12004  P12004  P12004  P12004  P12004  P12004  P12004  P12004  P12004  P12004  P12004  P12004  P12004  P12004  P12004  P12004  P12004  P12111  P12111  P12111  P12111  P12111  P12111  P12111  P12111  P12111  P12111  P12111  P12111  P12111  P12111  P12111  P12111  P12111  P12111  P12111  P12111  P12111  P12111  P12111  P12111  P12111  P12111  P12111  P12111  P12111  P12111  P12111  P12111  P12111  P12235  P12235  P12235  P12235  P12235  P12235  P12235  P12235  P12235  P12235  P12235  P12235  P12235  P12259  P12259  P12259  P12259  P12259  P12259  P12259  P12259  P12259  P12259  P12259  P12259  P12259  P12259  P12259  P12259  P12259  P12259  P12259  P12259  P12259  P12259  P12259  P12259  P12259  P12259  P12259  P12259  P12259  P12259  P12259  P12270  P12270  P12270  P12270  P12270  P12270  P12270  P12270  P12270  P12270  P12270  P12270  P12270  P12270  P12270  P12270  P12270  P12270  P12270  P12270  P12270  P12270  P12270  P12270  P12270  P12270  P12270  P12270  P12270  P12270  P12270  P12270  P12270  P12273  P12273  P12273  P12273  P12429  P12429  P12429  P12429  P12429  P12429  P12429  P12429  P12429  P12429  P12429  P12429  P12429  P12429  P12429  P12429  P12429  P12429  P12429  P12429  P12429  P12429  P12429  P12644  P12644  P12644  P12644  P12644  P12644  P12644  P12644  P12644  P12644  P12644  P12644  P12644  P12644  P12644  P12644  P12644  P12644  P12644  P12644  P12644  P12644  P12644  P12644  P12644  P12644  P12644  P12644  P12644  P12814  P12814  P12814  P12814  P12814  P12814  P12814  P12814  P12814  P12814  P12814  P12814  P12814  P12814  P12814  P12814  P12814  P12814  P12814  P12814  P12814  P12814  P12814  P12814  P12814  P12814  P12814  P12814  P12814  P12814  P12814  P12814  P12814  P12821  P12821  P12821  P12821  P12821  P12821  P12821  P12821  P12821  P12821  P12821  P12821  P12821  P12821  P12821  P12821  P12821  P12821  P12821  P12821  P12821  P12821  P12821  P12821  P12821  P12821  P12821  P12821  P12821  P12821  P12821  P12830  P12830  P12830  P12830  P12830  P12830  P12830  P12830  P12830  P12830  P12830  P12830  P12830  P12830  P12830  P12830  P12830  P12830  P12830  P12830  P12830  P12830  P12830  P12830  P12830  P12830  P12830  P12830  P12830  P12830  P12830  P12830  P12830  P12956  P12956  P12956  P12956  P12956  P12956  P12956  P12956  P12956  P12956  P12956  P12956  P12956  P12956  P12956  P12956  P12956  P12956  P12956  P12956  P12956  P12956  P12956  P12956  P12956  P12956  P12956  P12956  P12956  P12956  P12956  P13010  P13010  P13010  P13010  P13010  P13010  P13010  P13010  P13010  P13010  P13010  P13010  P13010  P13010  P13010  P13010  P13010  P13010  P13010  P13010  P13010  P13010  P13010  P13010  P13010  P13010  P13010  P13010  P13010  P13010  P13010  P13224  P13224  P13224  P13224  P13498  P13498  P13498  P13498  P13498  P13498  P13498  P13500  P13500  P13500  P13500  P13500  P13501  P13501  P13501  P13501  P13501  P13501  P13521  P13521  P13521  P13521  P13521  P13521  P13521  P13521  P13521  P13521  P13521  P13521  P13521  P13521  P13521  P13521  P13521  P13521  P13521  P13521  P13521  P13521  P13521  P13521  P13521  P13521  P13521  P13521  P13521  P13521  P13521  P13521  P13521  P13569  P13569  P13569  P13569  P13569  P13569  P13569  P13569  P13569  P13569  P13569  P13569  P13569  P13569  P13569  P13569  P13569  P13569  P13569  P13569  P13569  P13569  P13569  P13569  P13569  P13569  P13569  P13569  P13569  P13569  P13569  P13611  P13611  P13611  P13611  P13611  P13611  P13611  P13611  P13611  P13611  P13611  P13611  P13611  P13611  P13611  P13611  P13611  P13611  P13611  P13611  P13611  P13611  P13611  P13611  P13611  P13611  P13611  P13611  P13611  P13611  P13611  P13612  P13612  P13612  P13612  P13612  P13612  P13612  P13612  P13612  P13612  P13612  P13612  P13612  P13612  P13612  P13612  P13612  P13612  P13612  P13612  P13612  P13612  P13612  P13612  P13612  P13612  P13612  P13612  P13612  P13612  P13612  P13639  P13639  P13639  P13639  P13639  P13639  P13639  P13639  P13639  P13639  P13639  P13639  P13639  P13639  P13639  P13639  P13639  P13639  P13639  P13639  P13639  P13639  P13639  P13639  P13639  P13639  P13639  P13639  P13639  P13639  P13639  P13646  P13646  P13646  P13646  P13646  P13646  P13646  P13646  P13646  P13646  P13646  P13646  P13646  P13646  P13646  P13646  P13646  P13646  P13646  P13646  P13646  P13646  P13646  P13646  P13646  P13646  P13646  P13646  P13646  P13646  P13646  P13688  P13688  P13688  P13688  P13688  P13688  P13688  P13688  P13688  P13688  P13688  P13688  P13688  P13688  P13688  P13688  P13688  P13688  P13688  P13688  P13688  P13688  P13688  P13688  P13688  P13688  P13688  P13688  P13688  P13688  P13688  P13688  P13688  P13693  P13693  P13693  P13693  P13693  P13796  P13796  P13796  P13796  P13796  P13796  P13796  P13796  P13796  P13796  P13796  P13796  P13796  P13796  P13796  P13796  P13796  P13796  P13796  P13796  P13796  P13796  P13796  P13796  P13796  P13796  P13798  P13798  P13798  P13798  P13798  P13798  P13798  P13798  P13798  P13798  P13798  P13798  P13798  P13798  P13798  P13798  P13798  P13798  P13798  P13798  P13798  P13798  P13798  P13798  P13798  P13798  P13798  P13798  P13798  P13798  P13798  P13798  P13798  P13807  P13807  P13807  P13807  P13807  P13807  P13807  P13807  P13807  P13807  P13807  P13807  P13807  P13807  P13807  P13807  P13807  P13807  P13807  P13807  P13807  P13807  P13807  P13807  P13807  P13807  P13807  P13807  P13807  P13807  P13807  P14210  P14210  P14210  P14210  P14210  P14210  P14210  P14210  P14210  P14210  P14210  P14210  P14210  P14210  P14210  P14210  P14210  P14210  P14210  P14210  P14210  P14210  P14210  P14210  P14210  P14210  P14210  P14210  P14210  P14210  P14210  P14210  P14210  P14314  P14314  P14314  P14314  P14314  P14314  P14314  P14314  P14314  P14314  P14314  P14314  P14314  P14314  P14314  P14314  P14314  P14314  P14314  P14314  P14314  P14314  P14314  P14314  P14314  P14314  P14316  P14316  P14316  P14316  P14316  P14316  P14316  P14316  P14316  P14316  P14316  P14316  P14316  P14316  P14316  P14316  P14316  P14316  P14316  P14316  P14316  P14316  P14316  P14316  P14316  P14316  P14316  P14316  P14316  P14316  P14316  P14316  P14317  P14317  P14317  P14317  P14317  P14317  P14317  P14317  P14317  P14317  P14317  P14317  P14317  P14317  P14317  P14317  P14317  P14317  P14317  P14317  P14317  P14317  P14317  P14317  P14317  P14317  P14317  P14317  P14317  P14317  P14317  P14317  P14317  P14410  P14410  P14410  P14410  P14410  P14410  P14410  P14410  P14410  P14410  P14410  P14410  P14410  P14410  P14410  P14410  P14410  P14410  P14410  P14410  P14410  P14410  P14410  P14410  P14410  P14410  P14410  P14410  P14410  P14410  P14410  P14598  P14598  P14598  P14598  P14598  P14598  P14598  P14598  P14598  P14598  P14598  P14598  P14598  P14598  P14598  P14598  P14598  P14598  P14598  P14598  P14598  P14598  P14618  P14618  P14618  P14618  P14618  P14618  P14618  P14618  P14618  P14618  P14618  P14618  P14618  P14618  P14618  P14618  P14618  P14618  P14618  P14618  P14618  P14618  P14618  P14618  P14618  P14618  P14625  P14625  P14625  P14625  P14625  P14625  P14625  P14625  P14625  P14625  P14625  P14625  P14625  P14625  P14625  P14625  P14625  P14625  P14625  P14625  P14625  P14625  P14625  P14625  P14625  P14625  P14625  P14625  P14625  P14625  P14625  P14635  P14635  P14635  P14635  P14635  P14635  P14635  P14635  P14635  P14635  P14635  P14635  P14635  P14635  P14635  P14635  P14672  P14672  P14672  P14672  P14672  P14672  P14672  P14672  P14672  P14672  P14672  P14672  P14672  P14672  P14672  P14672  P14672  P14672  P14672  P14672  P14672  P14672  P14672  P14672  P14672  P14672  P14672  P14672  P14672  P14672  P14672  P14678  P14678  P14678  P14678  P14778  P14778  P14778  P14778  P14778  P14778  P14778  P14778  P14778  P14778  P14778  P14778  P14778  P14778  P14778  P14778  P14778  P14778  P14778  P14778  P14778  P14778  P14778  P14778  P14778  P14778  P14778  P14778  P14778  P14778  P14778  P14778  P14778  P14859  P14859  P14859  P14859  P14859  P14859  P14859  P14859  P14859  P14859  P14859  P14859  P14859  P14859  P14859  P14859  P14859  P14859  P14859  P14859  P14859  P14859  P14859  P14859  P14859  P14859  P14859  P14859  P14859  P14859  P14859  P14866  P14866  P14866  P14866  P14866  P14866  P14866  P14866  P14866  P14866  P14866  P14866  P14866  P14866  P14866  P14866  P14866  P14866  P14866  P14866  P14866  P14866  P14866  P14866  P14866  P14866  P14866  P14866  P14866  P14866  P14866  P15036  P15036  P15036  P15036  P15036  P15036  P15036  P15036  P15036  P15036  P15036  P15036  P15036  P15036  P15036  P15036  P15036  P15036  P15036  P15036  P15036  P15036  P15036  P15036  P15036  P15036  P15036  P15036  P15036  P15036  P15036  P15056  P15056  P15056  P15056  P15056  P15056  P15056  P15056  P15056  P15056  P15056  P15056  P15056  P15056  P15056  P15056  P15056  P15056  P15056  P15056  P15056  P15056  P15056  P15056  P15056  P15056  P15056  P15056  P15056  P15056  P15056  P15121  P15121  P15121  P15121  P15121  P15121  P15121  P15121  P15121  P15121  P15121  P15121  P15121  P15121  P15121  P15121  P15151  P15151  P15151  P15151  P15151  P15151  P15151  P15151  P15151  P15151  P15151  P15151  P15151  P15151  P15151  P15151  P15151  P15151  P15151  P15151  P15151  P15151  P15151  P15151  P15151  P15151  P15151  P15151  P15151  P15151  P15151  P15151  P15151  P15172  P15172  P15172  P15172  P15172  P15172  P15172  P15172  P15172  P15172  P15172  P15172  P15172  P15172  P15172  P15172  P15172  P15172  P15172  P15172  P15172  P15172  P15172  P15172  P15172  P15172  P15172  P15172  P15172  P15172  P15289  P15289  P15289  P15289  P15289  P15289  P15289  P15289  P15289  P15289  P15289  P15289  P15289  P15289  P15289  P15289  P15289  P15289  P15289  P15289  P15289  P15289  P15289  P15289  P15289  P15289  P15289  P15289  P15311  P15311  P15311  P15311  P15311  P15311  P15311  P15311  P15311  P15311  P15311  P15311  P15311  P15311  P15311  P15311  P15311  P15311  P15336  P15336  P15336  P15336  P15336  P15336  P15336  P15336  P15336  P15336  P15336  P15336  P15336  P15336  P15336  P15336  P15336  P15336  P15336  P15336  P15336  P15336  P15336  P15336  P15336  P15336  P15336  P15336  P15336  P15336  P15336  P15391  P15391  P15391  P15391  P15391  P15391  P15391  P15391  P15391  P15391  P15391  P15391  P15391  P15391  P15391  P15391  P15391  P15391  P15391  P15391  P15391  P15391  P15391  P15391  P15391  P15391  P15391  P15391  P15391  P15391  P15391  P15391  P15391  P15502  P15502  P15502  P15502  P15502  P15502  P15502  P15502  P15502  P15502  P15502  P15502  P15502  P15502  P15502  P15515  P15515  P15515  P15529  P15529  P15529  P15529  P15529  P15529  P15529  P15529  P15529  P15529  P15529  P15529  P15529  P15529  P15529  P15529  P15529  P15529  P15529  P15529  P15529  P15529  P15529  P15529  P15529  P15529  P15529  P15529  P15529  P15927  P15927  P15927  P15927  P15927  P15927  P15927  P15927  P15927  P15927  P15927  P15927  P15927  P15927  P15927  P15927  P15927  P15927  P15927  P15927  P15927  P15927  P15941  P15941  P15941  P15941  P15941  P15941  P15941  P15941  P15941  P15941  P15941  P15941  P15941  P15941  P15976  P15976  P15976  P15976  P15976  P15976  P15976  P15976  P15976  P15976  P15976  P15976  P15976  P15976  P15976  P15976  P15976  P15976  P15976  P15976  P15976  P15976  P15976  P15976  P15976  P15976  P15976  P15976  P15976  P15976  P15976  P16066  P16066  P16066  P16066  P16066  P16066  P16066  P16066  P16066  P16066  P16066  P16066  P16066  P16066  P16066  P16066  P16066  P16066  P16066  P16066  P16066  P16066  P16066  P16066  P16066  P16066  P16066  P16066  P16066  P16066  P16066  P16070  P16070  P16070  P16070  P16070  P16070  P16070  P16070  P16070  P16070  P16070  P16070  P16070  P16070  P16070  P16070  P16070  P16070  P16070  P16070  P16070  P16070  P16070  P16070  P16070  P16070  P16070  P16070  P16070  P16070  P16070  P16150  P16150  P16150  P16150  P16150  P16150  P16150  P16150  P16150  P16150  P16150  P16150  P16150  P16150  P16150  P16150  P16150  P16150  P16150  P16150  P16150  P16150  P16150  P16150  P16150  P16150  P16150  P16150  P16150  P16150  P16150  P16152  P16152  P16152  P16152  P16152  P16152  P16152  P16152  P16152  P16152  P16152  P16152  P16152  P16152  P16157  P16157  P16157  P16157  P16157  P16157  P16157  P16157  P16157  P16157  P16157  P16157  P16157  P16157  P16157  P16157  P16157  P16157  P16157  P16157  P16157  P16157  P16157  P16157  P16157  P16157  P16157  P16157  P16157  P16157  P16157  P16157  P16157  P16220  P16220  P16220  P16220  P16220  P16220  P16220  P16220  P16220  P16220  P16220  P16220  P16220  P16220  P16220  P16220  P16220  P16220  P16220  P16220  P16220  P16220  P16220  P16220  P16220  P16234  P16234  P16234  P16234  P16234  P16234  P16234  P16234  P16234  P16234  P16234  P16234  P16234  P16234  P16234  P16234  P16234  P16234  P16234  P16234  P16234  P16234  P16234  P16234  P16234  P16234  P16234  P16234  P16234  P16234  P16234  P16234  P16234  P16284  P16284  P16284  P16284  P16284  P16284  P16284  P16284  P16284  P16284  P16284  P16284  P16284  P16284  P16284  P16284  P16284  P16284  P16284  P16284  P16284  P16284  P16284  P16284  P16284  P16284  P16284  P16284  P16284  P16284  P16284  P16284  P16284  P16401  P16401  P16401  P16401  P16401  P16401  P16401  P16401  P16410  P16410  P16410  P16410  P16410  P16410  P16410  P16410  P16410  P16410  P16410  P16410  P16410  P16410  P16410  P16435  P16435  P16435  P16435  P16435  P16435  P16435  P16435  P16435  P16435  P16435  P16435  P16435  P16435  P16435  P16435  P16435  P16435  P16435  P16435  P16435  P16435  P16435  P16435  P16435  P16435  P16435  P16435  P16435  P16435  P16435  P16435  P16435  P16930  P16930  P16930  P16930  P16930  P16930  P16930  P16930  P16930  P16930  P16930  P16930  P16930  P16930  P16930  P16930  P16930  P16930  P16930  P16930  P16930  P16930  P16930  P16930  P16930  P16930  P16930  P16949  P16949  P16949  P16949  P17096  P17096  P17096  P17096  P17302  P17302  P17302  P17302  P17302  P17302  P17302  P17302  P17302  P17302  P17302  P17302  P17302  P17302  P17302  P17302  P17302  P17302  P17302  P17302  P17302  P17302  P17302  P17302  P17302  P17612  P17612  P17612  P17612  P17612  P17612  P17612  P17612  P17612  P17612  P17612  P17612  P17612  P17661  P17661  P17661  P17661  P17661  P17661  P17661  P17661  P17661  P17661  P17661  P17661  P17661  P17661  P17661  P17661  P17661  P17661  P17661  P17661  P17661  P17661  P17661  P17661  P17661  P17661  P17661  P17661  P17661  P17676  P17676  P17676  P17676  P17676  P17676  P17676  P17676  P17676  P17676  P17676  P17676  P17676  P17676  P17676  P17676  P17676  P17676  P17676  P17676  P17676  P17676  P17676  P17676  P17676  P17707  P17707  P17707  P17707  P17707  P17707  P17707  P17707  P17707  P17707  P17707  P17707  P17707  P17707  P17707  P17707  P17707  P17707  P17707  P17707  P17707  P17707  P17707  P17707  P17707  P17707  P17707  P17707  P17707  P17707  P17707  P17735  P17735  P17735  P17735  P17735  P17735  P17735  P17735  P17735  P17735  P17735  P17735  P17735  P17735  P17735  P17735  P17735  P17735  P17735  P17735  P17735  P17735  P17735  P17735  P17735  P17735  P17735  P17735  P17735  P17735  P17735  P17735  P17858  P17858  P17858  P17858  P17858  P17858  P17858  P17858  P17858  P17858  P17858  P17858  P17858  P17858  P17858  P17858  P17858  P17858  P17858  P17858  P17858  P17858  P17858  P17858  P17858  P17858  P17858  P17858  P17858  P17858  P17858  P17858  P17858  P17931  P17931  P17931  P17931  P17931  P17931  P17931  P17931  P17931  P17931  P17936  P17936  P17936  P17936  P17936  P17936  P17936  P17936  P17936  P17936  P17936  P17936  P17936  P17936  P17936  P17936  P17936  P17936  P17936  P17936  P17936  P17936  P17936  P17948  P17948  P17948  P17948  P17948  P17948  P17948  P17948  P17948  P17948  P17948  P17948  P17948  P17948  P17948  P17948  P17948  P17948  P17948  P17948  P17948  P17948  P17948  P17948  P17948  P17948  P17948  P17948  P17948  P17948  P17948  P17948  P17948  P17987  P17987  P17987  P17987  P17987  P17987  P17987  P17987  P17987  P17987  P17987  P17987  P17987  P17987  P17987  P17987  P17987  P17987  P17987  P17987  P17987  P17987  P17987  P17987  P17987  P17987  P17987  P17987  P17987  P17987  P17987  P17987  P17987  P18031  P18031  P18031  P18031  P18031  P18031  P18031  P18031  P18031  P18031  P18031  P18031  P18031  P18031  P18031  P18031  P18031  P18031  P18031  P18031  P18031  P18031  P18031  P18031  P18031  P18031  P18124  P18124  P18124  P18124  P18124  P18124  P18206  P18206  P18206  P18206  P18206  P18206  P18206  P18206  P18206  P18206  P18206  P18206  P18206  P18206  P18206  P18206  P18206  P18206  P18206  P18206  P18206  P18206  P18206  P18206  P18206  P18206  P18206  P18206  P18206  P18206  P18206  P18206  P18206  P18509  P18509  P18509  P18509  P18509  P18509  P18509  P18509  P18509  P18509  P18509  P18509  P18509  P18509  P18669  P18669  P18669  P18669  P18669  P18669  P18669  P18669  P18669  P18669  P18669  P18754  P18754  P18754  P18754  P18754  P18754  P18754  P18754  P18754  P18754  P18754  P18754  P18754  P18754  P18754  P18754  P18754  P18754  P18754  P18754  P18754  P18754  P18754  P18754  P18754  P18754  P18754  P18754  P18754  P18754  P18754  P18887  P18887  P18887  P18887  P18887  P18887  P18887  P18887  P18887  P18887  P18887  P18887  P18887  P18887  P18887  P18887  P18887  P18887  P18887  P18887  P18887  P18887  P18887  P18887  P18887  P18887  P18887  P18887  P18887  P18887  P18887  P19021  P19021  P19021  P19021  P19021  P19021  P19021  P19021  P19021  P19021  P19021  P19021  P19021  P19021  P19021  P19021  P19021  P19021  P19021  P19021  P19021  P19021  P19021  P19021  P19021  P19021  P19021  P19021  P19021  P19021  P19021  P19022  P19022  P19022  P19022  P19022  P19022  P19022  P19022  P19022  P19022  P19022  P19022  P19022  P19022  P19022  P19022  P19022  P19022  P19022  P19022  P19022  P19022  P19022  P19022  P19022  P19022  P19022  P19022  P19022  P19022  P19022  P19113  P19113  P19113  P19113  P19113  P19113  P19113  P19113  P19113  P19113  P19113  P19113  P19113  P19113  P19113  P19113  P19113  P19113  P19113  P19113  P19113  P19113  P19113  P19113  P19113  P19113  P19113  P19113  P19113  P19113  P19113  P19113  P19113  P19174  P19174  P19174  P19174  P19174  P19174  P19174  P19174  P19174  P19174  P19174  P19174  P19174  P19174  P19174  P19174  P19174  P19174  P19174  P19174  P19174  P19174  P19174  P19174  P19174  P19174  P19174  P19174  P19174  P19174  P19174  P19367  P19367  P19367  P19367  P19367  P19367  P19367  P19367  P19367  P19367  P19367  P19367  P19367  P19367  P19367  P19367  P19367  P19367  P19367  P19367  P19367  P19367  P19367  P19367  P19367  P19367  P19367  P19367  P19367  P19367  P19367  P19367  P19367  P19404  P19404  P19404  P19404  P19404  P19404  P19419  P19419  P19419  P19419  P19419  P19419  P19419  P19419  P19419  P19419  P19419  P19419  P19419  P19419  P19419  P19419  P19419  P19419  P19419  P19419  P19419  P19419  P19419  P19419  P19419  P19419  P19419  P19419  P19419  P19419  P19419  P19429  P19525  P19525  P19525  P19525  P19525  P19525  P19525  P19525  P19525  P19525  P19525  P19525  P19525  P19525  P19525  P19525  P19525  P19525  P19525  P19525  P19525  P19525  P19525  P19525  P19525  P19525  P19525  P19525  P19525  P19525  P19525  P19793  P19793  P19793  P19793  P19793  P19793  P19793  P19793  P19793  P19793  P19793  P19793  P19793  P19793  P19793  P19793  P19793  P19793  P19793  P19793  P19793  P19793  P19793  P19793  P19793  P19793  P19793  P19793  P19793  P19793  P19793  P19801  P19801  P19801  P19801  P19801  P19801  P19801  P19801  P19801  P19801  P19801  P19801  P19801  P19801  P19801  P19801  P19801  P19801  P19801  P19801  P19801  P19801  P19801  P19801  P19801  P19801  P19801  P19801  P19801  P19801  P19801  P19801  P19801  P19823  P19823  P19823  P19823  P19823  P19823  P19823  P19823  P19823  P19823  P19823  P19823  P19823  P19823  P19823  P19823  P19823  P19823  P19823  P19823  P19823  P19823  P19823  P19823  P19823  P19823  P19823  P19823  P19823  P19823  P19823  P19827  P19827  P19827  P19827  P19827  P19827  P19827  P19827  P19827  P19827  P19827  P19827  P19827  P19827  P19827  P19827  P19827  P19827  P19827  P19827  P19827  P19827  P19827  P19827  P19827  P19827  P19827  P19827  P19827  P19827  P19827  P19827  P19827  P19838  P19838  P19838  P19838  P19838  P19838  P19838  P19838  P19838  P19838  P19838  P19838  P19838  P19838  P19838  P19838  P19838  P19838  P19838  P19838  P19838  P19838  P19838  P19838  P19838  P19838  P19838  P19838  P19838  P19838  P19838  P20020  P20020  P20020  P20020  P20020  P20020  P20020  P20020  P20020  P20020  P20020  P20020  P20020  P20020  P20020  P20020  P20020  P20020  P20020  P20020  P20020  P20020  P20020  P20020  P20020  P20020  P20020  P20020  P20020  P20020  P20020  P20020  P20020  P20138  P20138  P20138  P20138  P20138  P20138  P20138  P20138  P20138  P20138  P20138  P20138  P20138  P20138  P20138  P20138  P20138  P20138  P20138  P20138  P20138  P20138  P20138  P20138  P20138  P20138  P20138  P20138  P20138  P20138  P20155  P20155  P20155  P20155  P20155  P20336  P20336  P20336  P20336  P20336  P20336  P20336  P20336  P20336  P20336  P20336  P20336  P20336  P20336  P20336  P20336  P20338  P20338  P20338  P20338  P20338  P20338  P20338  P20338  P20338  P20338  P20338  P20366  P20366  P20366  P20366  P20366  P20366  P20366  P20366  P20366  P20393  P20393  P20393  P20393  P20393  P20393  P20393  P20393  P20393  P20393  P20393  P20393  P20393  P20393  P20393  P20393  P20393  P20393  P20393  P20393  P20393  P20393  P20393  P20393  P20393  P20393  P20393  P20393  P20393  P20393  P20393  P20472  P20472  P20472  P20472  P20472  P20472  P20591  P20591  P20591  P20591  P20591  P20591  P20591  P20591  P20591  P20591  P20591  P20591  P20591  P20591  P20591  P20591  P20591  P20591  P20591  P20591  P20591  P20591  P20591  P20591  P20591  P20591  P20591  P20591  P20591  P20591  P20591  P20591  P20591  P20749  P20749  P20749  P20749  P20749  P20749  P20749  P20749  P20749  P20749  P20749  P20749  P20749  P20749  P20749  P20749  P20749  P20749  P20749  P20749  P20749  P20749  P20749  P20749  P20749  P20749  P20749  P20749  P20749  P20749  P20749  P20908  P20908  P20908  P20908  P20908  P20908  P20908  P20908  P20908  P20908  P20908  P20908  P20908  P20908  P20908  P20908  P20908  P20908  P20908  P20908  P20908  P20908  P20908  P20908  P20908  P20908  P20908  P20908  P20908  P20908  P20908  P20908  P20908  P20933  P20933  P20933  P20933  P20933  P20933  P20933  P20933  P20933  P20933  P20933  P20933  P20933  P20933  P20933  P20933  P20933  P20933  P20933  P20933  P20933  P21127  P21127  P21127  P21127  P21127  P21127  P21127  P21127  P21127  P21127  P21127  P21127  P21127  P21127  P21127  P21127  P21127  P21127  P21127  P21127  P21127  P21127  P21127  P21127  P21127  P21127  P21127  P21127  P21127  P21127  P21127  P21283  P21283  P21283  P21283  P21283  P21283  P21283  P21283  P21283  P21283  P21283  P21283  P21283  P21283  P21283  P21283  P21283  P21283  P21283  P21283  P21283  P21283  P21397  P21397  P21397  P21397  P21397  P21397  P21397  P21397  P21397  P21397  P21397  P21397  P21397  P21397  P21397  P21397  P21397  P21397  P21397  P21397  P21397  P21397  P21397  P21453  P21453  P21453  P21453  P21453  P21453  P21453  P21453  P21453  P21453  P21453  P21453  P21453  P21453  P21453  P21453  P21453  P21453  P21453  P21453  P21453  P21453  P21453  P21453  P21453  P21453  P21453  P21453  P21453  P21453  P21453  P21453  P21453  P21549  P21549  P21549  P21549  P21549  P21549  P21549  P21549  P21549  P21549  P21549  P21549  P21549  P21549  P21549  P21549  P21549  P21549  P21549  P21549  P21549  P21549  P21549  P21549  P21675  P21675  P21675  P21675  P21675  P21675  P21675  P21675  P21675  P21675  P21675  P21675  P21675  P21675  P21675  P21675  P21675  P21675  P21675  P21675  P21675  P21675  P21675  P21675  P21675  P21675  P21675  P21675  P21675  P21675  P21675  P21730  P21730  P21730  P21730  P21730  P21730  P21730  P21730  P21730  P21730  P21730  P21730  P21730  P21730  P21730  P21730  P21730  P21754  P21754  P21754  P21754  P21754  P21754  P21754  P21754  P21754  P21754  P21754  P21754  P21754  P21754  P21754  P21754  P21754  P21754  P21754  P21754  P21754  P21754  P21754  P21754  P21754  P21754  P21754  P21754  P21754  P21754  P21754  P21754  P21796  P21796  P21796  P21796  P21796  P21796  P21796  P21796  P21796  P21796  P21796  P21796  P21796  P21796  P21796  P21796  P21815  P21815  P21815  P21815  P21815  P21815  P21815  P21815  P21815  P21815  P21815  P21815  P21815  P21815  P21815  P21815  P21815  P21980  P21980  P21980  P21980  P21980  P21980  P21980  P21980  P21980  P21980  P21980  P21980  P21980  P21980  P21980  P21980  P21980  P21980  P21980  P21980  P21980  P21980  P21980  P21980  P21980  P21980  P21980  P21980  P21980  P21980  P21980  P21980  P21980  P22061  P22061  P22061  P22061  P22061  P22061  P22061  P22061  P22061  P22061  P22061  P22061  P22234  P22234  P22234  P22234  P22234  P22234  P22234  P22234  P22234  P22234  P22234  P22234  P22234  P22234  P22234  P22234  P22234  P22234  P22234  P22234  P22234  P22234  P22234  P22234  P22234  P22234  P22234  P22234  P22234  P22234  P22314  P22314  P22314  P22314  P22314  P22314  P22314  P22314  P22314  P22314  P22314  P22314  P22314  P22314  P22314  P22314  P22314  P22314  P22314  P22314  P22314  P22314  P22314  P22314  P22314  P22314  P22314  P22314  P22314  P22314  P22314  P22466  P22466  P22466  P22466  P22466  P22466  P22466  P22466  P22466  P22466  P22466  P22681  P22681  P22681  P22681  P22681  P22681  P22681  P22681  P22681  P22681  P22681  P22681  P22681  P22681  P22681  P22681  P22681  P22681  P22681  P22681  P22681  P22681  P22681  P22681  P22681  P22681  P22681  P22681  P22681  P22681  P22681  P22681  P22692  P22692  P22692  P22692  P22692  P22692  P22692  P22692  P22692  P22692  P22692  P22692  P22692  P22692  P22735  P22735  P22735  P22735  P22735  P22735  P22735  P22735  P22735  P22735  P22735  P22735  P22735  P22735  P22735  P22735  P22735  P22735  P22735  P22735  P22735  P22735  P22735  P22735  P22735  P22735  P22735  P22735  P22735  P22735  P22735  P22736  P22736  P22736  P22736  P22736  P22736  P22736  P22736  P22736  P22736  P22736  P22736  P22736  P22736  P22736  P22736  P22736  P22736  P22736  P22736  P22736  P22736  P22736  P22736  P22736  P22736  P22736  P22736  P22736  P22736  P22736  P22891  P22891  P22891  P22891  P22891  P22891  P22891  P22891  P22891  P22891  P22891  P22891  P22891  P22891  P22891  P22891  P22891  P22891  P22891  P22891  P22891  P22891  P22891  P22891  P22891  P22914  P22914  P22914  P22914  P22914  P22914  P22914  P22914  P22914  P23025  P23025  P23025  P23025  P23025  P23025  P23025  P23025  P23193  P23193  P23193  P23193  P23193  P23193  P23193  P23193  P23193  P23193  P23193  P23193  P23193  P23193  P23193  P23193  P23193  P23193  P23193  P23193  P23193  P23193  P23193  P23229  P23229  P23229  P23229  P23229  P23229  P23229  P23229  P23229  P23229  P23229  P23229  P23229  P23229  P23229  P23229  P23229  P23229  P23229  P23229  P23229  P23229  P23229  P23229  P23229  P23229  P23229  P23229  P23229  P23229  P23229  P23246  P23246  P23246  P23246  P23246  P23246  P23246  P23246  P23246  P23246  P23246  P23246  P23246  P23246  P23246  P23246  P23246  P23246  P23246  P23246  P23246  P23246  P23246  P23246  P23246  P23246  P23246  P23246  P23246  P23246  P23297  P23297  P23297  P23327  P23327  P23327  P23327  P23327  P23327  P23327  P23327  P23327  P23327  P23327  P23327  P23327  P23327  P23327  P23327  P23327  P23327  P23327  P23327  P23327  P23327  P23327  P23327  P23327  P23327  P23327  P23327  P23327  P23327  P23327  P23396  P23396  P23396  P23396  P23396  P23396  P23396  P23434  P23434  P23434  P23434  P23434  P23434  P23434  P23434  P23434  P23434  P23434  P23434  P23434  P23443  P23443  P23443  P23443  P23443  P23443  P23443  P23443  P23443  P23443  P23443  P23443  P23443  P23443  P23443  P23443  P23443  P23443  P23443  P23443  P23443  P23443  P23443  P23443  P23443  P23443  P23458  P23458  P23458  P23458  P23458  P23458  P23458  P23458  P23458  P23458  P23458  P23458  P23458  P23458  P23458  P23458  P23458  P23458  P23458  P23458  P23458  P23458  P23458  P23458  P23458  P23458  P23458  P23458  P23458  P23458  P23458  P23458  P23458  P23508  P23508  P23508  P23508  P23508  P23508  P23508  P23508  P23508  P23508  P23508  P23508  P23508  P23508  P23508  P23508  P23508  P23508  P23508  P23508  P23508  P23508  P23508  P23508  P23508  P23508  P23508  P23508  P23508  P23508  P23508  P23526  P23526  P23526  P23526  P23526  P23526  P23526  P23526  P23526  P23526  P23526  P23526  P23526  P23528  P23528  P23528  P23528  P23528  P23528  P23528  P23528  P23588  P23588  P23588  P23588  P23588  P23588  P23588  P23588  P23588  P23588  P23588  P23588  P23588  P23588  P23588  P23588  P23588  P23588  P23588  P23588  P23588  P23588  P23588  P23588  P23588  P23588  P23588  P23588  P23588  P23588  P23588  P23760  P23760  P23760  P23760  P23760  P23760  P23760  P23760  P23760  P23760  P23760  P23760  P23760  P23760  P23760  P23760  P23760  P23760  P23760  P23760  P23760  P23760  P23760  P23760  P23760  P23760  P23760  P23760  P23760  P23760  P23760  P24385  P24385  P24385  P24385  P24385  P24385  P24385  P24385  P24385  P24385  P24385  P24385  P24385  P24385  P24385  P24385  P24390  P24390  P24390  P24390  P24390  P24390  P24390  P24390  P24390  P24390  P24390  P24390  P24394  P24394  P24394  P24394  P24394  P24394  P24394  P24394  P24394  P24394  P24394  P24394  P24394  P24394  P24394  P24394  P24394  P24394  P24394  P24394  P24394  P24394  P24394  P24394  P24394  P24394  P24394  P24394  P24394  P24394  P24394  P24394  P24394  P24593  P24593  P24593  P24593  P24593  P24593  P24593  P24593  P24593  P24593  P24593  P24593  P24593  P24593  P24593  P24666  P24666  P24666  P24666  P24666  P24666  P24666  P24666  P24666  P24666  P24666  P24821  P24821  P24821  P24821  P24821  P24821  P24821  P24821  P24821  P24821  P24821  P24821  P24821  P24821  P24821  P24821  P24821  P24821  P24821  P24821  P24821  P24821  P24821  P24821  P24821  P24821  P24821  P24821  P24821  P24821  P24821  P24864  P24864  P24864  P24864  P24864  P24864  P24864  P24864  P24864  P24864  P24864  P24864  P24864  P24864  P24864  P24864  P24864  P24864  P24864  P24864  P24864  P24864  P24928  P24928  P24928  P24928  P24928  P24928  P24928  P24928  P24928  P24928  P24928  P24928  P24928  P24928  P24928  P24928  P24928  P24928  P24928  P24928  P24928  P24928  P24928  P24928  P24928  P24928  P25205  P25205  P25205  P25205  P25205  P25205  P25205  P25205  P25205  P25205  P25205  P25205  P25205  P25205  P25205  P25205  P25205  P25205  P25205  P25205  P25205  P25205  P25205  P25205  P25205  P25205  P25205  P25205  P25205  P25205  P25205  P25311  P25311  P25311  P25311  P25311  P25311  P25311  P25311  P25311  P25311  P25311  P25311  P25311  P25311  P25311  P25311  P25311  P25311  P25398  P25398  P25398  P25713  P25713  P25713  P25713  P25713  P25713  P25788  P25788  P25788  P25788  P25788  P25788  P25788  P25788  P25788  P25788  P25788  P25788  P25788  P25788  P25788  P25788  P25788  P25963  P25963  P25963  P25963  P25963  P25963  P25963  P25963  P25963  P25963  P26358  P26358  P26358  P26358  P26358  P26358  P26358  P26358  P26358  P26358  P26358  P26358  P26358  P26358  P26358  P26358  P26358  P26358  P26358  P26358  P26358  P26358  P26358  P26358  P26358  P26358  P26358  P26358  P26358  P26358  P26358  P26368  P26368  P26368  P26368  P26368  P26368  P26368  P26368  P26368  P26368  P26368  P26368  P26368  P26368  P26368  P26368  P26368  P26368  P26368  P26368  P26368  P26368  P26368  P26368  P26368  P26368  P26368  P26368  P26378  P26378  P26378  P26378  P26378  P26378  P26378  P26378  P26378  P26378  P26378  P26378  P26378  P26378  P26378  P26378  P26378  P26378  P26378  P26378  P26378  P26378  P26378  P26378  P26378  P26378  P26378  P26378  P26378  P26378  P26378  P26378  P26378  P26599  P26599  P26599  P26599  P26599  P26599  P26599  P26599  P26599  P26599  P26599  P26599  P26599  P26599  P26599  P26599  P26599  P26599  P26599  P26599  P26599  P26599  P26599  P26599  P26599  P26599  P26599  P26599  P26599  P26599  P26599  P26599  P26599  P26640  P26640  P26640  P26640  P26640  P26640  P26640  P26640  P26640  P26640  P26640  P26640  P26640  P26640  P26640  P26640  P26640  P26640  P26640  P26640  P26640  P26640  P26640  P26640  P26640  P26640  P26640  P26640  P26640  P26640  P26640  P26640  P26640  P26641  P26641  P26641  P26641  P26641  P26641  P26641  P26641  P26641  P26641  P26641  P26641  P26641  P26641  P26641  P26641  P26641  P26641  P26641  P26641  P26641  P26641  P26641  P26641  P26641  P26651  P26651  P26651  P26651  P26651  P26651  P26651  P26651  P26651  P26651  P26651  P26651  P26651  P26651  P26651  P26651  P26651  P26651  P26651  P26651  P26651  P26651  P26651  P26651  P26651  P26651  P26651  P26651  P26651  P26651  P26651  P26678  P27105  P27105  P27105  P27105  P27105  P27105  P27105  P27105  P27105  P27105  P27105  P27105  P27105  P27105  P27105  P27105  P27361  P27361  P27361  P27361  P27361  P27361  P27361  P27361  P27361  P27361  P27361  P27361  P27361  P27361  P27361  P27361  P27361  P27695  P27695  P27695  P27695  P27695  P27695  P27695  P27695  P27695  P27695  P27695  P27695  P27695  P27695  P27695  P27695  P27695  P27695  P27695  P27695  P27707  P27707  P27707  P27707  P27707  P27707  P27707  P27707  P27707  P27707  P27707  P27707  P27707  P27707  P27707  P27707  P27708  P27708  P27708  P27708  P27708  P27708  P27708  P27708  P27708  P27708  P27708  P27708  P27708  P27708  P27708  P27708  P27708  P27708  P27708  P27708  P27708  P27708  P27708  P27708  P27708  P27708  P27708  P27708  P27708  P27708  P27708  P27815  P27815  P27815  P27815  P27815  P27815  P27815  P27815  P27815  P27815  P27815  P27815  P27815  P27815  P27815  P27815  P27815  P27815  P27815  P27815  P27815  P27815  P27815  P27815  P27815  P27815  P27815  P27815  P27815  P27815  P27815  P27816  P27816  P27816  P27816  P27816  P27816  P27816  P27816  P27816  P27816  P27816  P27816  P27816  P27816  P27816  P27816  P27816  P27816  P27816  P27816  P27816  P27816  P27816  P27816  P27816  P27816  P27816  P27816  P27816  P27816  P27816  P28072  P28072  P28072  P28072  P28072  P28072  P28072  P28072  P28072  P28072  P28072  P28072  P28072  P28072  P28072  P28072  P28223  P28223  P28223  P28223  P28223  P28223  P28223  P28223  P28223  P28223  P28223  P28223  P28223  P28223  P28223  P28223  P28223  P28223  P28223  P28223  P28223  P28223  P28223  P28223  P28223  P28223  P28223  P28223  P28223  P28223  P28223  P28370  P28370  P28370  P28370  P28370  P28370  P28370  P28370  P28370  P28370  P28370  P28370  P28370  P28370  P28370  P28370  P28370  P28370  P28370  P28370  P28370  P28370  P28370  P28370  P28370  P28370  P28370  P28370  P28370  P28370  P28370  P28370  P28370  P28749  P28749  P28749  P28749  P28749  P28749  P28749  P28749  P28749  P28749  P28749  P28749  P28749  P28749  P28749  P28749  P28749  P28749  P28749  P28749  P28749  P28749  P28749  P28749  P28749  P28749  P28749  P28749  P28749  P28749  P28749  P29083  P29083  P29083  P29083  P29083  P29083  P29083  P29083  P29083  P29083  P29083  P29083  P29083  P29083  P29083  P29083  P29083  P29083  P29083  P29083  P29083  P29083  P29083  P29083  P29083  P29083  P29144  P29144  P29144  P29144  P29144  P29144  P29144  P29144  P29144  P29144  P29144  P29144  P29144  P29144  P29144  P29144  P29144  P29144  P29144  P29144  P29144  P29144  P29144  P29144  P29144  P29144  P29144  P29144  P29144  P29144  P29144  P29144  P29144  P29317  P29317  P29317  P29317  P29317  P29317  P29317  P29317  P29317  P29317  P29317  P29317  P29317  P29317  P29317  P29317  P29317  P29317  P29317  P29317  P29317  P29317  P29317  P29317  P29317  P29317  P29317  P29317  P29317  P29317  P29317  P29350  P29350  P29350  P29350  P29350  P29350  P29350  P29350  P29350  P29350  P29350  P29350  P29350  P29350  P29350  P29350  P29350  P29350  P29350  P29350  P29350  P29350  P29350  P29350  P29350  P29350  P29350  P29350  P29350  P29350  P29350  P29350  P29474  P29474  P29474  P29474  P29474  P29474  P29474  P29474  P29474  P29474  P29474  P29474  P29474  P29474  P29474  P29474  P29474  P29474  P29474  P29474  P29474  P29474  P29474  P29474  P29474  P29474  P29474  P29474  P29474  P29474  P29474  P29508  P29508  P29508  P29508  P29508  P29508  P29508  P29508  P29508  P29508  P29508  P29508  P29508  P29508  P29508  P29508  P29508  P29508  P29508  P29508  P29508  P29508  P29508  P29508  P29508  P29590  P29590  P29590  P29590  P29590  P29590  P29590  P29590  P29590  P29590  P29590  P29590  P29590  P29590  P29590  P29590  P29590  P29590  P29590  P29590  P29590  P29590  P29590  P29590  P29590  P29590  P29590  P29590  P29590  P29590  P29590  P29597  P29597  P29597  P29597  P29597  P29597  P29597  P29597  P29597  P29597  P29597  P29597  P29597  P29597  P29597  P29597  P29597  P29597  P29597  P29597  P29597  P29597  P29597  P29597  P29597  P29597  P29597  P29597  P29597  P29597  P29597  P29597  P29597  P29692  P29692  P29692  P29692  P29692  P29692  P29692  P29692  P29692  P29692  P29692  P29692  P29692  P29692  P29692  P29692  P29692  P29966  P29966  P29966  P29966  P29966  P29966  P29966  P29966  P29966  P29966  P29966  P29966  P29966  P30040  P30040  P30040  P30040  P30040  P30040  P30040  P30040  P30040  P30040  P30040  P30040  P30040  P30040  P30040  P30040  P30260  P30260  P30260  P30260  P30260  P30260  P30260  P30260  P30260  P30260  P30260  P30260  P30260  P30260  P30260  P30260  P30260  P30260  P30260  P30260  P30260  P30260  P30260  P30260  P30260  P30260  P30260  P30260  P30260  P30260  P30260  P30291  P30291  P30291  P30291  P30291  P30291  P30291  P30291  P30291  P30291  P30291  P30291  P30291  P30291  P30291  P30291  P30291  P30291  P30291  P30291  P30291  P30291  P30291  P30291  P30291  P30291  P30291  P30291  P30291  P30291  P30291  P30304  P30304  P30304  P30304  P30304  P30304  P30304  P30304  P30304  P30304  P30304  P30304  P30304  P30304  P30304  P30304  P30304  P30304  P30304  P30304  P30304  P30304  P30304  P30304  P30304  P30304  P30304  P30304  P30304  P30304  P30304  P30305  P30305  P30305  P30305  P30305  P30305  P30305  P30305  P30305  P30305  P30305  P30305  P30305  P30305  P30305  P30305  P30305  P30305  P30305  P30305  P30305  P30305  P30305  P30305  P30305  P30305  P30305  P30305  P30305  P30305  P30305  P30307  P30307  P30307  P30307  P30307  P30307  P30307  P30307  P30307  P30307  P30307  P30307  P30307  P30307  P30307  P30307  P30307  P30307  P30307  P30307  P30307  P30307  P30307  P30307  P30307  P30307  P30307  P30307  P30307  P30411  P30411  P30411  P30411  P30411  P30411  P30411  P30411  P30411  P30411  P30411  P30411  P30411  P30411  P30411  P30411  P30411  P30411  P30411  P30411  P30411  P30411  P30411  P30411  P30411  P30411  P30411  P30411  P30411  P30411  P30519  P30519  P30519  P30519  P30519  P30519  P30519  P30519  P30519  P30519  P30519  P30530  P30530  P30530  P30530  P30530  P30530  P30530  P30530  P30530  P30530  P30530  P30530  P30530  P30530  P30530  P30530  P30530  P30530  P30530  P30530  P30530  P30530  P30530  P30530  P30530  P30530  P30530  P30530  P30530  P30530  P30530  P30530  P30530  P30566  P30566  P30566  P30566  P30566  P30566  P30566  P30566  P30566  P30566  P30566  P30566  P30566  P30566  P30566  P30566  P30566  P30566  P30566  P30566  P30566  P30566  P30566  P30566  P30566  P30566  P30566  P30566  P30566  P30566  P30793  P30793  P30793  P30793  P30793  P30793  P30793  P30793  P30793  P30793  P30793  P30793  P30793  P30990  P30990  P30990  P30990  P30990  P30990  P30990  P30990  P30990  P30990  P30990  P30990  P31040  P31040  P31040  P31040  P31040  P31040  P31040  P31040  P31040  P31040  P31040  P31040  P31040  P31040  P31040  P31040  P31040  P31040  P31040  P31040  P31040  P31040  P31040  P31040  P31040  P31040  P31040  P31040  P31040  P31040  P31040  P31040  P31040  P31151  P31151  P31151  P31151  P31151  P31151  P31269  P31269  P31269  P31269  P31269  P31269  P31269  P31269  P31269  P31269  P31269  P31269  P31269  P31269  P31269  P31269  P31269  P31327  P31327  P31327  P31327  P31327  P31327  P31327  P31327  P31327  P31327  P31327  P31327  P31327  P31327  P31327  P31327  P31327  P31327  P31327  P31327  P31327  P31327  P31327  P31327  P31327  P31327  P31327  P31327  P31327  P31327  P31327  P31327  P31327  P31645  P31645  P31645  P31645  P31645  P31645  P31645  P31645  P31645  P31645  P31645  P31645  P31645  P31645  P31645  P31645  P31645  P31645  P31645  P31645  P31645  P31645  P31645  P31645  P31645  P31645  P31645  P31645  P31645  P31645  P31645  P31751  P31751  P31751  P31751  P31751  P31751  P31751  P31751  P31751  P31751  P31751  P31751  P31751  P31751  P31751  P31751  P31751  P31948  P31948  P31948  P31948  P31948  P31948  P31948  P31948  P31948  P31948  P31948  P31948  P31948  P31948  P31948  P31948  P31948  P31948  P31949  P31949  P31949  P31949  P31949  P31949  P31995  P31995  P31995  P31995  P31995  P31995  P31995  P31995  P31995  P31995  P31995  P31995  P31995  P31995  P31995  P31995  P31995  P31995  P31995  P31995  P31995  P31995  P31995  P31995  P31995  P32004  P32004  P32004  P32004  P32004  P32004  P32004  P32004  P32004  P32004  P32004  P32004  P32004  P32004  P32004  P32004  P32004  P32004  P32004  P32004  P32004  P32004  P32004  P32004  P32004  P32004  P32004  P32004  P32004  P32004  P32004  P33121  P33121  P33121  P33121  P33121  P33121  P33121  P33121  P33121  P33121  P33121  P33121  P33121  P33121  P33121  P33121  P33121  P33121  P33121  P33121  P33121  P33121  P33121  P33121  P33121  P33121  P33121  P33121  P33121  P33121  P33121  P33241  P33241  P33241  P33241  P33241  P33241  P33241  P33241  P33241  P33241  P33241  P33241  P33241  P33241  P33241  P33241  P33241  P33241  P33241  P33241  P33241  P33241  P33241  P33241  P33241  P33316  P33316  P33316  P33316  P33316  P33316  P33316  P33316  P33316  P33316  P33316  P33316  P33316  P33316  P33316  P33764  P33764  P33993  P33993  P33993  P33993  P33993  P33993  P33993  P33993  P33993  P33993  P33993  P33993  P33993  P33993  P33993  P33993  P33993  P33993  P33993  P33993  P33993  P33993  P33993  P33993  P33993  P33993  P33993  P33993  P33993  P33993  P33993  P33993  P33993  P34059  P34059  P34059  P34059  P34059  P34059  P34059  P34059  P34059  P34059  P34059  P34059  P34059  P34059  P34059  P34059  P34059  P34059  P34059  P34059  P34059  P34059  P34059  P34059  P34059  P34059  P34096  P34096  P34096  P34096  P34096  P34096  P34096  P34096  P34741  P34741  P34741  P34741  P34741  P34741  P34741  P34741  P34741  P34741  P34741  P34741  P34741  P34741  P34741  P34741  P34741  P34741  P34896  P34896  P34896  P34896  P34896  P34896  P34896  P34896  P34896  P34896  P34896  P34896  P34896  P34896  P34896  P34896  P34896  P34896  P34896  P34896  P34896  P34896  P34896  P34896  P34896  P34896  P34896  P34896  P34896  P34896  P34947  P34947  P34947  P34947  P34947  P34947  P34947  P34947  P34947  P34947  P34947  P34947  P34947  P34947  P34947  P34947  P34947  P34947  P34947  P34947  P34947  P34947  P34947  P34947  P34947  P34947  P34947  P34947  P34972  P34972  P34972  P34972  P34972  P34972  P34972  P34972  P34972  P34972  P34972  P34972  P34972  P34972  P34972  P34972  P34972  P34972  P34972  P34972  P34972  P34972  P34972  P34972  P34972  P34972  P34972  P34972  P34972  P34972  P34998  P34998  P34998  P34998  P34998  P34998  P34998  P34998  P34998  P34998  P34998  P34998  P34998  P34998  P34998  P34998  P34998  P34998  P34998  P34998  P34998  P34998  P34998  P34998  P34998  P34998  P34998  P34998  P34998  P35221  P35221  P35221  P35221  P35221  P35221  P35221  P35221  P35221  P35221  P35221  P35221  P35221  P35221  P35221  P35221  P35221  P35221  P35221  P35221  P35221  P35221  P35221  P35221  P35221  P35221  P35221  P35221  P35221  P35221  P35221  P35221  P35221  P35222  P35222  P35222  P35222  P35222  P35222  P35222  P35222  P35222  P35222  P35222  P35222  P35222  P35222  P35222  P35222  P35222  P35222  P35222  P35222  P35222  P35222  P35222  P35222  P35222  P35222  P35222  P35222  P35222  P35222  P35222  P35236  P35236  P35236  P35236  P35236  P35236  P35236  P35236  P35236  P35236  P35236  P35236  P35236  P35236  P35236  P35269  P35269  P35269  P35269  P35269  P35269  P35269  P35269  P35269  P35269  P35269  P35269  P35269  P35269  P35269  P35269  P35269  P35269  P35269  P35269  P35269  P35269  P35269  P35269  P35269  P35269  P35269  P35269  P35269  P35269  P35269  P35270  P35270  P35270  P35270  P35270  P35270  P35270  P35270  P35270  P35270  P35270  P35270  P35270  P35270  P35270  P35270  P35270  P35270  P35270  P35318  P35318  P35318  P35318  P35318  P35318  P35318  P35318  P35318  P35318  P35318  P35318  P35318  P35318  P35318  P35318  P35318  P35367  P35367  P35367  P35367  P35367  P35367  P35367  P35367  P35367  P35367  P35367  P35367  P35367  P35367  P35367  P35367  P35367  P35367  P35367  P35367  P35367  P35367  P35367  P35367  P35367  P35367  P35367  P35367  P35367  P35367  P35367  P35398  P35398  P35398  P35398  P35398  P35398  P35398  P35398  P35398  P35398  P35398  P35398  P35398  P35398  P35398  P35398  P35398  P35398  P35398  P35398  P35398  P35398  P35398  P35398  P35398  P35398  P35398  P35398  P35398  P35398  P35398  P35398  P35398  P35408  P35408  P35408  P35408  P35408  P35408  P35408  P35408  P35408  P35408  P35408  P35408  P35408  P35408  P35408  P35408  P35408  P35408  P35408  P35408  P35408  P35408  P35408  P35408  P35408  P35408  P35408  P35408  P35408  P35408  P35408  P35520  P35520  P35520  P35520  P35520  P35520  P35520  P35520  P35520  P35520  P35520  P35520  P35520  P35520  P35520  P35520  P35520  P35520  P35520  P35520  P35520  P35520  P35520  P35520  P35520  P35520  P35520  P35520  P35520  P35520  P35520  P35520  P35555  P35555  P35555  P35555  P35555  P35555  P35555  P35555  P35555  P35555  P35555  P35555  P35555  P35555  P35555  P35555  P35555  P35555  P35555  P35555  P35555  P35555  P35555  P35555  P35555  P35555  P35555  P35555  P35555  P35555  P35555  P35558  P35558  P35558  P35558  P35558  P35558  P35558  P35558  P35558  P35558  P35558  P35558  P35558  P35558  P35558  P35558  P35558  P35558  P35558  P35558  P35558  P35558  P35558  P35558  P35558  P35558  P35558  P35558  P35558  P35558  P35558  P35558  P35558  P35568  P35568  P35568  P35568  P35568  P35568  P35568  P35568  P35568  P35568  P35568  P35568  P35568  P35568  P35568  P35568  P35568  P35568  P35568  P35568  P35568  P35568  P35568  P35568  P35568  P35568  P35568  P35568  P35568  P35568  P35568  P35611  P35611  P35611  P35611  P35611  P35611  P35611  P35611  P35611  P35611  P35611  P35611  P35611  P35611  P35611  P35611  P35611  P35611  P35611  P35611  P35611  P35611  P35611  P35611  P35611  P35611  P35611  P35611  P35611  P35611  P35611  P35612  P35612  P35612  P35612  P35612  P35612  P35612  P35612  P35612  P35612  P35612  P35612  P35612  P35612  P35612  P35612  P35612  P35612  P35612  P35612  P35612  P35612  P35612  P35612  P35612  P35612  P35612  P35612  P35612  P35612  P35612  P35637  P35637  P35637  P35637  P35637  P35637  P35637  P35637  P35637  P35637  P35637  P35637  P35637  P35637  P35637  P35637  P35637  P35637  P35637  P35637  P35637  P35637  P35637  P35637  P35637  P35637  P35637  P35637  P35637  P35637  P35637  P35637  P35637  P35659  P35659  P35659  P35659  P35659  P35659  P35659  P35659  P35659  P35659  P35659  P35659  P35659  P35659  P35659  P35659  P35659  P35659  P35900  P35900  P35900  P35900  P35900  P35900  P35900  P35900  P35900  P35900  P35900  P35900  P35900  P35900  P35900  P35900  P35900  P35900  P35900  P35900  P35900  P35900  P35900  P35900  P35900  P35900  P35916  P35916  P35916  P35916  P35916  P35916  P35916  P35916  P35916  P35916  P35916  P35916  P35916  P35916  P35916  P35916  P35916  P35916  P35916  P35916  P35916  P35916  P35916  P35916  P35916  P35916  P35916  P35916  P35916  P35916  P35916  P35916  P35916  P35968  P35968  P35968  P35968  P35968  P35968  P35968  P35968  P35968  P35968  P35968  P35968  P35968  P35968  P35968  P35968  P35968  P35968  P35968  P35968  P35968  P35968  P35968  P35968  P35968  P35968  P35968  P35968  P35968  P35968  P35968  P35968  P35968  P36507  P36507  P36507  P36507  P36507  P36507  P36507  P36507  P36507  P36507  P36507  P36507  P36507  P36507  P36507  P36507  P36507  P36507  P36507  P36507  P36578  P36578  P36578  P36578  P36578  P36578  P36578  P36578  P36578  P36578  P36578  P36578  P36578  P36578  P36578  P36578  P36578  P36578  P36578  P36871  P36871  P36871  P36871  P36871  P36871  P36871  P36871  P36871  P36871  P36871  P36871  P36871  P36871  P36871  P36871  P36871  P36871  P36871  P36871  P36871  P36871  P36871  P36871  P36871  P36871  P36871  P36871  P36871  P36871  P36871  P36871  P36871  P36888  P36888  P36888  P36888  P36888  P36888  P36888  P36888  P36888  P36888  P36888  P36888  P36888  P36888  P36888  P36888  P36888  P36888  P36888  P36888  P36888  P36888  P36888  P36888  P36888  P36888  P36888  P36888  P36888  P36888  P36888  P36896  P36896  P36896  P36896  P36896  P36896  P36896  P36896  P36896  P36896  P36896  P36896  P36896  P36896  P36896  P36896  P36896  P36896  P36896  P36896  P36896  P36896  P36896  P36896  P36896  P36896  P36896  P36896  P36896  P36896  P36955  P36955  P36955  P36955  P36955  P36955  P36955  P36955  P36955  P36955  P36955  P36955  P36955  P36955  P36955  P36955  P36955  P36955  P36955  P36955  P36955  P36955  P36955  P36955  P36955  P36955  P36955  P36955  P36955  P36955  P36955  P37802  P37802  P37802  P37802  P37802  P37802  P37802  P37840  P37840  P38117  P38117  P38117  P38117  P38117  P38117  P38159  P38159  P38159  P38159  P38159  P38159  P38159  P38159  P38159  P38159  P38159  P38159  P38159  P38159  P38159  P38159  P38159  P38159  P38159  P38159  P38159  P38159  P38159  P38159  P38159  P38159  P38159  P38159  P38159  P38159  P38159  P38159  P38159  P38398  P38398  P38398  P38398  P38398  P38398  P38398  P38398  P38398  P38398  P38398  P38398  P38398  P38398  P38398  P38398  P38398  P38398  P38398  P38398  P38398  P38398  P38398  P38398  P38398  P38398  P38398  P38398  P38398  P38398  P38398  P38432  P38432  P38432  P38432  P38432  P38432  P38432  P38432  P38432  P38432  P38432  P38432  P38432  P38432  P38432  P38432  P38432  P38432  P38432  P38432  P38432  P38432  P38432  P38432  P38432  P38432  P38432  P38432  P38432  P38432  P38432  P38646  P38646  P38646  P38646  P38646  P38646  P38646  P38646  P38646  P38646  P38646  P38646  P38646  P38646  P38646  P38646  P38646  P38646  P38646  P38646  P38646  P38646  P38646  P38646  P38646  P38646  P38646  P38646  P38646  P38646  P38646  P38646  P38646  P38919  P38919  P38919  P38919  P38919  P38919  P38919  P38919  P38919  P38919  P38919  P38919  P38919  P38919  P38919  P38919  P38919  P38919  P38919  P38935  P38935  P38935  P38935  P38935  P38935  P38935  P38935  P38935  P38935  P38935  P38935  P38935  P38935  P38935  P38935  P38935  P38935  P38935  P38935  P38935  P38935  P38935  P38935  P38935  P38935  P38935  P38935  P38935  P38935  P38935  P38935  P38935  P38936  P38936  P38936  P38936  P38936  P38936  P38936  P38936  P39748  P39748  P39748  P39748  P39748  P39748  P39748  P39748  P39748  P39748  P39748  P39748  P39748  P39748  P39748  P39748  P39748  P39748  P39748  P39748  P39748  P39748  P39748  P39748  P39748  P40189  P40189  P40189  P40189  P40189  P40189  P40189  P40189  P40189  P40189  P40189  P40189  P40189  P40189  P40189  P40189  P40189  P40189  P40189  P40189  P40189  P40189  P40189  P40189  P40189  P40189  P40189  P40189  P40189  P40189  P40189  P40429  P40429  P40763  P40763  P40763  P40763  P40763  P40763  P40763  P40763  P40763  P40763  P40763  P40763  P40763  P40763  P40763  P40763  P40763  P40763  P40763  P40763  P40763  P40763  P40763  P40763  P40763  P40763  P40763  P40763  P40763  P40763  P40763  P40925  P40925  P40925  P40925  P40925  P40925  P40925  P40925  P40925  P40925  P40925  P40925  P40925  P40925  P40925  P40925  P40925  P40925  P40925  P40925  P40925  P40925  P40926  P40926  P40926  P40926  P40926  P40926  P40926  P40926  P40926  P40926  P40926  P40926  P40926  P40926  P40926  P40926  P40926  P40926  P40926  P40926  P40926  P41091  P41091  P41091  P41091  P41091  P41091  P41091  P41091  P41091  P41091  P41091  P41091  P41091  P41091  P41091  P41091  P41091  P41091  P41091  P41091  P41091  P41091  P41091  P41091  P41091  P41091  P41091  P41181  P41181  P41181  P41181  P41181  P41181  P41181  P41181  P41181  P41181  P41181  P41181  P41181  P41181  P41181  P41181  P41182  P41182  P41182  P41182  P41182  P41182  P41182  P41182  P41182  P41182  P41182  P41182  P41182  P41182  P41182  P41182  P41182  P41182  P41182  P41182  P41182  P41182  P41182  P41182  P41182  P41182  P41182  P41182  P41182  P41182  P41182  P41212  P41212  P41212  P41212  P41212  P41212  P41212  P41212  P41212  P41212  P41212  P41212  P41212  P41212  P41212  P41212  P41212  P41212  P41212  P41212  P41212  P41212  P41212  P41212  P41212  P41212  P41212  P41212  P41212  P41212  P41212  P41227  P41227  P41227  P41227  P41227  P41227  P41227  P41227  P41227  P41227  P41227  P41227  P41227  P41235  P41235  P41235  P41235  P41235  P41235  P41235  P41235  P41235  P41235  P41235  P41235  P41235  P41235  P41235  P41235  P41235  P41235  P41235  P41235  P41235  P41235  P41235  P41235  P41235  P41235  P41235  P41235  P41235  P41235  P41235  P41240  P41240  P41240  P41240  P41240  P41240  P41240  P41240  P41240  P41240  P41240  P41240  P41240  P41240  P41240  P41240  P41240  P41240  P41240  P41240  P41240  P41240  P41279  P41279  P41279  P41279  P41279  P41279  P41279  P41279  P41279  P41279  P41279  P41279  P41279  P41279  P41279  P41279  P41279  P41279  P41279  P41279  P41279  P41279  P41279  P41279  P41279  P41279  P41279  P41279  P41279  P41279  P41279  P41597  P41597  P41597  P41597  P41597  P41597  P41597  P41597  P41597  P41597  P41597  P41597  P41597  P41743  P41743  P41743  P41743  P41743  P41743  P41743  P41743  P41743  P41743  P41743  P41743  P41743  P41743  P41743  P41743  P41743  P41743  P41743  P41743  P41743  P41743  P41743  P41743  P41743  P41743  P41743  P41743  P41743  P41743  P41743  P41743  P41743  P42229  P42229  P42229  P42229  P42229  P42229  P42229  P42229  P42229  P42229  P42229  P42229  P42229  P42229  P42229  P42229  P42229  P42229  P42229  P42229  P42229  P42229  P42229  P42229  P42229  P42229  P42229  P42229  P42229  P42229  P42229  P42229  P42229  P42285  P42285  P42285  P42285  P42285  P42285  P42285  P42285  P42285  P42285  P42285  P42285  P42285  P42285  P42285  P42285  P42285  P42285  P42285  P42285  P42285  P42285  P42285  P42285  P42285  P42285  P42285  P42285  P42285  P42285  P42285  P42285  P42285  P42338  P42338  P42338  P42338  P42338  P42338  P42338  P42338  P42338  P42338  P42338  P42338  P42338  P42338  P42338  P42338  P42338  P42338  P42338  P42338  P42338  P42338  P42338  P42338  P42338  P42338  P42338  P42338  P42338  P42338  P42338  P42345  P42345  P42345  P42345  P42345  P42345  P42345  P42345  P42345  P42345  P42345  P42345  P42345  P42345  P42345  P42345  P42345  P42345  P42345  P42345  P42345  P42345  P42345  P42345  P42345  P42345  P42345  P42345  P42345  P42345  P42345  P42574  P42574  P42574  P42574  P42574  P42574  P42574  P42574  P42574  P42574  P42574  P42574  P42574  P42574  P42574  P42574  P42574  P42574  P42574  P42574  P42574  P42574  P42574  P42574  P42574  P42680  P42680  P42680  P42680  P42680  P42680  P42680  P42680  P42680  P42680  P42680  P42680  P42680  P42680  P42680  P42680  P42680  P42680  P42680  P42680  P42680  P42680  P42680  P42680  P42680  P42680  P42680  P42680  P42680  P42680  P42680  P42680  P42680  P42684  P42684  P42684  P42684  P42684  P42684  P42684  P42684  P42684  P42684  P42684  P42684  P42684  P42684  P42684  P42684  P42684  P42684  P42684  P42684  P42684  P42684  P42684  P42684  P42684  P42684  P42684  P42684  P42684  P42684  P42684  P42684  P42684  P42768  P42768  P42768  P42768  P42768  P42768  P42768  P42768  P42768  P42768  P42768  P42768  P42768  P42768  P42768  P42768  P42768  P42768  P42768  P42768  P42768  P42768  P42768  P42768  P42768  P42768  P42768  P42771  P42771  P42771  P42858  P42858  P42858  P42858  P42858  P42858  P42858  P42858  P42858  P42858  P42858  P42858  P42858  P42858  P42858  P42858  P42858  P42858  P42858  P42858  P42858  P42858  P42858  P42858  P42858  P42858  P42858  P42858  P42858  P42858  P42858  P43220  P43220  P43220  P43220  P43220  P43220  P43220  P43220  P43220  P43220  P43220  P43220  P43220  P43220  P43220  P43220  P43220  P43220  P43220  P43220  P43220  P43220  P43220  P43220  P43220  P43220  P43220  P43220  P43220  P43220  P43220  P43220  P43220  P43243  P43243  P43243  P43243  P43243  P43243  P43243  P43243  P43243  P43243  P43243  P43243  P43243  P43243  P43243  P43243  P43243  P43243  P43243  P43243  P43243  P43243  P43243  P43243  P43243  P43243  P43243  P43243  P43243  P43243  P43243  P43243  P43243  P43268  P43268  P43268  P43268  P43268  P43268  P43268  P43268  P43268  P43268  P43268  P43268  P43268  P43268  P43268  P43268  P43268  P43268  P43268  P43268  P43268  P43268  P43268  P43268  P43268  P43268  P43268  P43268  P43268  P43268  P43268  P43351  P43351  P43351  P43351  P43351  P43351  P43351  P43351  P43351  P43351  P43351  P43351  P43351  P43351  P43351  P43351  P43351  P43351  P43351  P43351  P43351  P43351  P43351  P43351  P43351  P43351  P43351  P43351  P43351  P43351  P43351  P43351  P43351  P43403  P43403  P43403  P43403  P43403  P43403  P43403  P43403  P43403  P43403  P43403  P43403  P43403  P43403  P43403  P43403  P43403  P43403  P43403  P43403  P43403  P43403  P43403  P43403  P43403  P43403  P43403  P43403  P43403  P43403  P43403  P43403  P43403  P43405  P43405  P43405  P43405  P43405  P43405  P43405  P43405  P43405  P43405  P43405  P43405  P43405  P43405  P43405  P43405  P43405  P43405  P43405  P43405  P43405  P43405  P43405  P43405  P43405  P43405  P43405  P43686  P43686  P43686  P43686  P43686  P43686  P43686  P43686  P43686  P43686  P43686  P43686  P43686  P43686  P43686  P43686  P43686  P43686  P43686  P43686  P45985  P45985  P45985  P45985  P45985  P45985  P45985  P45985  P45985  P45985  P45985  P45985  P45985  P45985  P45985  P45985  P45985  P45985  P45985  P45985  P45985  P45985  P45985  P45985  P45985  P45985  P45985  P45985  P45985  P45985  P45985  P45985  P45985  P46060  P46060  P46060  P46060  P46060  P46060  P46060  P46060  P46060  P46060  P46060  P46060  P46060  P46060  P46060  P46060  P46060  P46060  P46060  P46060  P46060  P46060  P46060  P46060  P46060  P46060  P46060  P46060  P46060  P46060  P46060  P46108  P46108  P46108  P46108  P46108  P46108  P46108  P46108  P46108  P46108  P46108  P46108  P46108  P46108  P46108  P46108  P46108  P46108  P46108  P46108  P46108  P46108  P46108  P46108  P46108  P46108  P46527  P46527  P46527  P46527  P46527  P46527  P46527  P46527  P46527  P46527  P46527  P46527  P46527  P46527  P46527  P46531  P46531  P46531  P46531  P46531  P46531  P46531  P46531  P46531  P46531  P46531  P46531  P46531  P46531  P46531  P46531  P46531  P46531  P46531  P46531  P46531  P46531  P46531  P46531  P46531  P46531  P46531  P46531  P46531  P46531  P46531  P46531  P46531  P46695  P46695  P46695  P46695  P46695  P46695  P46695  P46695  P46734  P46734  P46734  P46734  P46734  P46734  P46734  P46734  P46734  P46734  P46734  P46734  P46734  P46734  P46734  P46734  P46734  P46734  P46734  P46734  P46736  P46736  P46736  P46736  P46736  P46736  P46736  P46736  P46736  P46736  P46736  P46736  P46736  P46736  P46736  P46736  P46736  P46736  P46736  P46736  P46736  P46736  P46736  P46736  P46736  P46736  P46736  P46776  P46776  P46776  P46776  P46776  P46776  P46779  P46779  P46779  P46779  P46779  P46779  P46779  P46779  P46779  P46779  P46782  P46782  P46782  P46782  P46782  P46782  P46782  P46782  P46782  P46782  P46783  P46783  P46783  P46783  P46783  P46821  P46821  P46821  P46821  P46821  P46821  P46821  P46821  P46821  P46821  P46821  P46821  P46821  P46821  P46821  P46821  P46821  P46821  P46821  P46821  P46821  P46821  P46821  P46821  P46821  P46821  P46821  P46821  P46821  P46821  P46821  P46821  P46821  P46937  P46937  P46937  P46937  P46937  P46937  P46937  P46937  P46937  P46937  P46937  P46937  P46937  P46937  P46937  P46937  P46937  P46937  P46937  P46937  P46937  P46937  P46937  P46937  P46937  P46937  P46937  P46937  P46937  P46937  P46937  P46940  P46940  P46940  P46940  P46940  P46940  P46940  P46940  P46940  P46940  P46940  P46940  P46940  P46940  P46940  P46940  P46940  P46940  P46940  P46940  P46940  P46940  P46940  P46940  P46940  P46940  P46940  P46940  P46940  P46940  P46940  P47710  P47710  P47710  P47710  P47710  P47710  P47710  P47712  P47712  P47712  P47712  P47712  P47712  P47712  P47712  P47712  P47712  P47712  P47712  P47712  P47712  P47712  P47712  P47712  P47712  P47712  P47712  P47712  P47712  P47712  P47712  P47712  P47712  P47712  P47712  P47712  P47712  P47712  P47756  P47756  P47756  P47756  P47756  P47756  P47756  P47756  P47756  P47756  P47756  P47756  P47756  P47756  P47756  P47756  P47756  P47756  P47756  P47756  P47756  P47756  P47756  P47895  P47895  P47895  P47895  P47895  P47895  P47895  P47895  P47895  P47895  P47895  P47895  P47895  P47895  P47895  P47895  P47895  P47895  P47895  P47895  P47895  P47895  P47897  P47897  P47897  P47897  P47897  P47897  P47897  P47897  P47897  P47897  P47897  P47897  P47897  P47897  P47897  P47897  P47897  P47897  P47897  P47897  P47897  P47897  P47897  P47897  P47897  P47897  P47897  P47897  P47897  P47897  P47897  P47897  P47914  P47914  P47914  P47914  P47914  P47914  P48047  P48047  P48047  P48047  P48047  P48047  P48047  P48047  P48047  P48047  P48047  P48047  P48047  P48047  P48047  P48047  P48047  P48047  P48047  P48047  P48048  P48048  P48048  P48048  P48048  P48048  P48048  P48048  P48048  P48048  P48048  P48048  P48048  P48048  P48048  P48048  P48048  P48048  P48048  P48048  P48048  P48048  P48059  P48059  P48059  P48059  P48551  P48551  P48551  P48551  P48551  P48551  P48551  P48551  P48551  P48551  P48551  P48551  P48551  P48551  P48551  P48551  P48551  P48551  P48551  P48551  P48551  P48551  P48551  P48551  P48551  P48551  P48551  P48551  P48551  P48551  P48551  P48551  P48551  P48552  P48552  P48552  P48552  P48552  P48552  P48552  P48552  P48552  P48552  P48552  P48552  P48552  P48552  P48552  P48552  P48552  P48552  P48552  P48552  P48552  P48552  P48552  P48552  P48552  P48552  P48552  P48552  P48552  P48552  P48552  P48552  P48552  P48643  P48643  P48643  P48643  P48643  P48643  P48643  P48643  P48643  P48643  P48643  P48643  P48643  P48643  P48643  P48643  P48643  P48643  P48643  P48643  P48643  P48735  P48735  P48735  P48735  P48735  P48735  P48735  P48735  P48735  P48735  P48735  P48735  P48735  P48735  P48735  P48735  P48735  P48735  P48735  P48735  P48735  P48735  P48736  P48736  P48736  P48736  P48736  P48736  P48736  P48736  P48736  P48736  P48736  P48736  P48736  P48736  P48736  P48736  P48736  P48736  P48736  P48736  P48736  P48736  P48736  P48736  P48736  P48736  P48736  P48736  P48736  P48736  P48736  P49023  P49023  P49023  P49023  P49023  P49023  P49023  P49023  P49023  P49023  P49023  P49023  P49023  P49023  P49023  P49023  P49023  P49023  P49023  P49023  P49023  P49023  P49023  P49023  P49023  P49023  P49023  P49023  P49023  P49023  P49023  P49137  P49137  P49137  P49137  P49137  P49137  P49137  P49137  P49137  P49137  P49137  P49137  P49137  P49137  P49137  P49137  P49137  P49137  P49189  P49189  P49189  P49189  P49189  P49189  P49189  P49189  P49189  P49189  P49189  P49189  P49189  P49189  P49189  P49189  P49189  P49189  P49189  P49189  P49189  P49189  P49189  P49189  P49327  P49327  P49327  P49327  P49327  P49327  P49327  P49327  P49327  P49327  P49327  P49327  P49327  P49327  P49327  P49327  P49327  P49327  P49327  P49327  P49327  P49327  P49327  P49327  P49327  P49327  P49327  P49327  P49327  P49327  P49327  P49327  P49327  P49368  P49368  P49368  P49368  P49368  P49368  P49368  P49368  P49368  P49368  P49368  P49368  P49368  P49368  P49368  P49368  P49368  P49368  P49368  P49368  P49368  P49368  P49368  P49368  P49368  P49368  P49368  P49368  P49368  P49368  P49368  P49368  P49407  P49407  P49407  P49407  P49407  P49407  P49407  P49407  P49407  P49407  P49407  P49407  P49407  P49407  P49407  P49407  P49450  P49450  P49450  P49450  P49450  P49450  P49459  P49459  P49459  P49459  P49459  P49459  P49459  P49459  P49459  P49459  P49459  P49588  P49588  P49588  P49588  P49588  P49588  P49588  P49588  P49588  P49588  P49588  P49588  P49588  P49588  P49588  P49588  P49588  P49588  P49588  P49588  P49588  P49588  P49588  P49588  P49588  P49588  P49588  P49588  P49588  P49588  P49588  P49588  P49588  P49675  P49675  P49675  P49675  P49675  P49675  P49675  P49675  P49675  P49675  P49675  P49675  P49675  P49675  P49675  P49675  P49675  P49682  P49682  P49682  P49682  P49682  P49682  P49682  P49682  P49682  P49682  P49682  P49682  P49682  P49682  P49682  P49682  P49682  P49682  P49682  P49682  P49682  P49682  P49682  P49682  P49715  P49715  P49715  P49715  P49715  P49715  P49715  P49715  P49715  P49715  P49715  P49715  P49715  P49715  P49715  P49715  P49715  P49715  P49720  P49720  P49720  P49720  P49720  P49720  P49736  P49736  P49736  P49736  P49736  P49736  P49736  P49736  P49736  P49736  P49736  P49736  P49736  P49736  P49736  P49736  P49736  P49736  P49736  P49736  P49736  P49736  P49736  P49736  P49736  P49736  P49736  P49736  P49736  P49736  P49736  P49756  P49756  P49756  P49756  P49756  P49756  P49756  P49756  P49756  P49756  P49756  P49756  P49756  P49756  P49756  P49756  P49756  P49756  P49756  P49756  P49756  P49756  P49756  P49756  P49756  P49756  P49756  P49756  P49756  P49756  P49756  P49760  P49760  P49760  P49760  P49760  P49760  P49760  P49760  P49760  P49760  P49760  P49760  P49760  P49760  P49760  P49760  P49760  P49760  P49760  P49760  P49760  P49760  P49760  P49760  P49760  P49760  P49760  P49760  P49760  P49760  P49760  P49768  P49768  P49768  P49768  P49768  P49768  P49768  P49768  P49768  P49768  P49768  P49768  P49768  P49768  P49768  P49768  P49768  P49768  P49768  P49768  P49768  P49768  P49768  P49768  P49768  P49768  P49768  P49768  P49768  P49768  P49789  P49789  P49789  P49789  P49789  P49789  P49789  P49790  P49790  P49790  P49790  P49790  P49790  P49790  P49790  P49790  P49790  P49790  P49790  P49790  P49790  P49790  P49790  P49790  P49790  P49790  P49790  P49790  P49790  P49790  P49790  P49790  P49790  P49790  P49790  P49790  P49790  P49790  P49790  P49790  P49795  P49795  P49795  P49795  P49795  P49795  P49795  P49795  P49795  P49795  P49795  P49795  P49795  P49795  P49795  P49795  P49795  P49795  P49795  P49795  P49795  P49802  P49802  P49802  P49802  P49802  P49802  P49802  P49802  P49802  P49802  P49802  P49802  P49802  P49802  P49802  P49802  P49802  P49802  P49802  P49802  P49802  P49802  P49802  P49802  P49802  P49802  P49802  P49802  P49802  P49802  P49802  P49815  P49815  P49815  P49815  P49815  P49815  P49815  P49815  P49815  P49815  P49815  P49815  P49815  P49815  P49815  P49815  P49815  P49815  P49815  P49815  P49815  P49815  P49815  P49815  P49815  P49815  P49815  P49815  P49815  P49815  P49815  P49841  P49841  P49841  P49841  P49841  P49841  P49841  P49841  P49841  P49841  P49841  P49841  P49841  P49841  P49841  P49841  P49841  P49841  P49841  P49841  P49841  P49841  P49841  P49841  P49841  P50150  P50150  P50150  P50150  P50150  P50402  P50402  P50402  P50402  P50402  P50402  P50402  P50402  P50402  P50402  P50402  P50402  P50402  P50402  P50402  P50402  P50402  P50402  P50402  P50402  P50402  P50402  P50402  P50402  P50402  P50402  P50402  P50402  P50402  P50402  P50402  P50502  P50502  P50502  P50502  P50502  P50502  P50502  P50502  P50502  P50502  P50502  P50502  P50502  P50502  P50502  P50548  P50548  P50548  P50548  P50548  P50548  P50548  P50548  P50548  P50548  P50548  P50548  P50548  P50548  P50548  P50548  P50548  P50548  P50548  P50548  P50548  P50548  P50548  P50548  P50548  P50548  P50548  P50548  P50548  P50548  P50548  P50552  P50552  P50552  P50552  P50552  P50552  P50552  P50552  P50552  P50552  P50552  P50552  P50552  P50552  P50552  P50552  P50552  P50552  P50552  P50552  P50552  P50552  P50552  P50552  P50583  P50583  P50613  P50613  P50613  P50613  P50613  P50613  P50613  P50613  P50613  P50613  P50613  P50613  P50750  P50750  P50750  P50750  P50750  P50750  P50750  P50750  P50750  P50750  P50750  P50750  P50750  P50750  P50895  P50895  P50895  P50895  P50895  P50895  P50895  P50895  P50895  P50895  P50895  P50895  P50895  P50895  P50895  P50895  P50895  P50895  P50895  P50895  P50895  P50895  P50895  P50895  P50895  P50895  P50895  P50895  P50895  P50895  P50895  P50990  P50990  P50990  P50990  P50990  P50990  P50990  P50990  P50990  P50990  P50990  P50990  P50990  P50990  P50990  P50990  P50990  P50990  P50990  P50990  P50990  P50990  P50990  P50990  P50990  P50990  P50990  P50990  P51114  P51114  P51114  P51114  P51114  P51114  P51114  P51114  P51114  P51114  P51114  P51114  P51114  P51114  P51114  P51114  P51114  P51114  P51114  P51114  P51114  P51114  P51114  P51114  P51114  P51114  P51114  P51114  P51114  P51114  P51114  P51114  P51114  P51397  P51397  P51575  P51575  P51575  P51575  P51575  P51575  P51575  P51575  P51575  P51575  P51575  P51575  P51575  P51587  P51587  P51587  P51587  P51587  P51587  P51587  P51587  P51587  P51587  P51587  P51587  P51587  P51587  P51587  P51587  P51587  P51587  P51587  P51587  P51587  P51587  P51587  P51587  P51587  P51587  P51587  P51587  P51587  P51587  P51587  P51617  P51617  P51617  P51617  P51617  P51617  P51617  P51617  P51617  P51617  P51617  P51617  P51617  P51617  P51617  P51617  P51617  P51617  P51617  P51617  P51617  P51617  P51617  P51617  P51617  P51617  P51617  P51617  P51617  P51617  P51617  P51617  P51617  P51636  P51636  P51636  P51636  P51636  P51636  P51636  P51636  P51636  P51636  P51636  P51636  P51654  P51654  P51654  P51654  P51654  P51654  P51654  P51654  P51654  P51654  P51654  P51654  P51654  P51654  P51654  P51654  P51654  P51654  P51654  P51654  P51654  P51654  P51654  P51654  P51654  P51654  P51654  P51654  P51654  P51654  P51654  P51809  P51809  P51809  P51809  P51809  P51809  P51809  P51809  P51809  P51809  P51809  P51809  P51809  P51813  P51813  P51813  P51813  P51813  P51813  P51813  P51813  P51813  P51813  P51813  P51813  P51813  P51813  P51813  P51813  P51813  P51813  P51813  P51813  P51813  P51813  P51813  P51813  P51813  P51813  P51813  P51813  P51813  P51813  P51813  P51813  P51884  P51884  P51884  P51884  P51884  P51884  P51884  P51884  P51884  P51884  P51884  P51884  P51884  P51884  P51884  P51884  P51884  P51884  P51884  P51884  P51884  P51884  P51884  P51884  P51884  P51884  P51884  P51884  P51884  P51946  P51946  P51946  P51946  P51946  P51946  P51946  P51946  P51946  P51946  P51946  P51946  P51946  P51946  P51946  P51946  P51946  P51946  P51946  P51955  P51955  P51955  P51955  P51955  P51955  P51955  P51955  P51955  P51955  P51955  P51955  P51955  P51955  P51955  P51955  P51955  P51955  P51955  P51955  P51991  P51991  P51991  P51991  P51991  P51991  P51991  P51991  P51991  P51991  P51991  P51991  P51991  P51991  P51991  P51991  P51991  P51991  P51991  P51991  P51991  P51991  P51991  P51991  P52272  P52272  P52272  P52272  P52272  P52272  P52272  P52272  P52272  P52272  P52272  P52272  P52272  P52272  P52272  P52272  P52272  P52272  P52272  P52272  P52272  P52272  P52272  P52272  P52272  P52272  P52272  P52272  P52272  P52272  P52272  P52272  P52292  P52292  P52292  P52292  P52292  P52292  P52292  P52292  P52292  P52292  P52292  P52292  P52292  P52292  P52292  P52292  P52292  P52292  P52292  P52292  P52292  P52292  P52292  P52292  P52292  P52292  P52292  P52292  P52292  P52292  P52292  P52292  P52292  P52333  P52333  P52333  P52333  P52333  P52333  P52333  P52333  P52333  P52333  P52333  P52333  P52333  P52333  P52333  P52333  P52333  P52333  P52333  P52333  P52333  P52333  P52333  P52333  P52333  P52333  P52333  P52333  P52333  P52333  P52333  P52333  P52333  P52434  P52434  P52434  P52434  P52434  P52434  P52434  P52434  P52434  P52435  P52435  P52435  P52435  P52435  P52565  P52565  P52565  P52565  P52565  P52565  P52565  P52565  P52565  P52565  P52565  P52565  P52565  P52565  P52565  P52630  P52630  P52630  P52630  P52630  P52630  P52630  P52630  P52630  P52630  P52630  P52630  P52630  P52630  P52630  P52630  P52630  P52630  P52630  P52630  P52630  P52630  P52630  P52630  P52630  P52630  P52630  P52630  P52630  P52630  P52630  P52655  P52655  P52655  P52655  P52655  P52655  P52655  P52655  P52655  P52655  P52655  P52655  P52732  P52732  P52732  P52732  P52732  P52732  P52732  P52732  P52732  P52732  P52732  P52732  P52732  P52732  P52732  P52732  P52732  P52732  P52732  P52732  P52732  P52732  P52732  P52732  P52732  P52732  P52732  P52732  P52732  P52732  P52732  P52735  P52735  P52735  P52735  P52735  P52735  P52735  P52735  P52735  P52735  P52735  P52735  P52735  P52735  P52735  P52735  P52735  P52735  P52735  P52735  P52735  P52735  P52735  P52735  P52735  P52735  P52735  P52735  P52735  P52735  P52735  P52735  P52735  P52907  P52907  P52907  P52907  P52907  P52907  P52907  P52907  P52907  P52907  P52907  P52907  P52907  P52907  P52907  P52907  P52926  P52926  P52926  P52926  P52926  P52926  P52926  P52945  P52945  P52945  P52945  P52945  P52945  P52945  P52945  P52945  P52945  P53350  P53350  P53350  P53350  P53350  P53350  P53350  P53350  P53350  P53350  P53350  P53350  P53350  P53350  P53350  P53350  P53350  P53350  P53350  P53350  P53350  P53350  P53350  P53350  P53350  P53350  P53350  P53350  P53350  P53350  P53350  P53355  P53355  P53355  P53355  P53355  P53355  P53355  P53355  P53355  P53355  P53355  P53355  P53355  P53355  P53355  P53355  P53355  P53355  P53355  P53355  P53355  P53355  P53355  P53355  P53355  P53355  P53355  P53355  P53355  P53355  P53355  P53396  P53396  P53396  P53396  P53396  P53396  P53396  P53396  P53396  P53396  P53396  P53396  P53396  P53396  P53396  P53396  P53396  P53396  P53396  P53396  P53396  P53396  P53396  P53396  P53396  P53396  P53396  P53396  P53396  P53396  P53396  P53396  P53396  P53667  P53667  P53667  P53667  P53667  P53667  P53667  P53667  P53667  P53667  P53667  P53667  P53667  P53667  P53667  P53667  P53667  P53667  P53667  P53667  P53667  P53667  P53667  P53667  P53667  P53667  P53667  P53667  P53667  P53667  P53667  P53674  P53674  P53674  P53674  P53674  P53674  P53674  P53674  P53674  P53674  P53674  P53674  P53674  P53674  P53674  P53674  P53674  P53674  P53674  P53674  P53674  P53674  P53674  P53779  P53779  P53779  P53779  P53779  P53779  P53779  P53779  P53779  P53779  P53779  P53779  P53779  P53779  P53779  P53779  P53779  P53779  P53779  P53779  P53779  P53779  P53779  P53779  P53779  P53779  P53779  P53779  P53779  P53779  P53779  P53779  P53779  P53804  P53804  P53804  P53804  P53804  P53804  P53804  P53804  P53804  P53804  P53804  P53804  P53804  P53804  P53804  P53804  P53804  P53804  P53804  P53804  P53804  P53804  P53804  P53804  P53804  P53804  P53804  P53804  P53804  P53804  P53804  P53999  P53999  P53999  P53999  P53999  P53999  P53999  P53999  P53999  P53999  P53999  P53999  P54105  P54105  P54105  P54105  P54105  P54105  P54105  P54105  P54105  P54105  P54105  P54105  P54105  P54105  P54105  P54105  P54198  P54198  P54198  P54198  P54198  P54198  P54198  P54198  P54198  P54198  P54198  P54198  P54198  P54198  P54198  P54198  P54198  P54198  P54198  P54198  P54198  P54198  P54198  P54198  P54198  P54198  P54198  P54198  P54198  P54198  P54198  P54253  P54253  P54253  P54253  P54253  P54253  P54253  P54253  P54253  P54253  P54253  P54253  P54253  P54253  P54253  P54253  P54253  P54253  P54253  P54253  P54253  P54253  P54253  P54253  P54253  P54253  P54253  P54253  P54253  P54253  P54253  P54259  P54259  P54259  P54259  P54259  P54259  P54259  P54259  P54259  P54259  P54259  P54259  P54259  P54259  P54259  P54259  P54259  P54259  P54259  P54259  P54259  P54259  P54259  P54259  P54259  P54259  P54259  P54259  P54259  P54259  P54259  P54274  P54274  P54274  P54274  P54274  P54274  P54274  P54274  P54274  P54274  P54274  P54274  P54274  P54274  P54274  P54274  P54274  P54274  P54274  P54274  P54274  P54274  P54274  P54274  P54274  P54274  P54274  P54274  P54274  P54274  P54274  P54577  P54577  P54577  P54577  P54577  P54577  P54577  P54577  P54577  P54577  P54577  P54577  P54577  P54577  P54577  P54577  P54577  P54577  P54577  P54577  P54577  P54577  P54577  P54577  P54577  P54577  P54577  P54577  P54577  P54646  P54646  P54646  P54646  P54646  P54646  P54646  P54646  P54646  P54646  P54646  P54646  P54646  P54646  P54646  P54646  P54646  P54646  P54646  P54646  P54646  P54646  P54646  P54646  P54646  P54646  P54646  P54646  P54646  P54646  P54646  P54646  P54646  P54753  P54753  P54753  P54753  P54753  P54753  P54753  P54753  P54753  P54753  P54753  P54753  P54753  P54753  P54753  P54753  P54753  P54753  P54753  P54753  P54753  P54753  P54753  P54753  P54753  P54753  P54753  P54753  P54753  P54753  P54753  P54753  P54753  P54829  P54829  P54829  P54829  P54829  P54829  P54829  P54829  P54829  P54829  P54829  P54829  P54829  P54829  P54829  P54829  P54829  P54829  P54829  P54829  P54829  P54829  P54829  P54829  P54829  P54829  P54829  P54829  P54829  P54829  P54829  P55060  P55060  P55060  P55060  P55060  P55060  P55060  P55060  P55060  P55060  P55060  P55060  P55060  P55060  P55060  P55060  P55060  P55060  P55060  P55060  P55060  P55060  P55060  P55060  P55060  P55060  P55060  P55060  P55060  P55060  P55060  P55060  P55060  P55072  P55072  P55072  P55072  P55072  P55072  P55072  P55072  P55072  P55072  P55072  P55072  P55072  P55072  P55072  P55072  P55072  P55072  P55072  P55072  P55072  P55072  P55072  P55072  P55072  P55072  P55072  P55072  P55072  P55072  P55072  P55072  P55072  P55211  P55211  P55211  P55211  P55211  P55211  P55211  P55211  P55211  P55211  P55211  P55211  P55211  P55211  P55211  P55211  P55211  P55211  P55211  P55211  P55211  P55211  P55211  P55211  P55211  P55211  P55211  P55211  P55211  P55211  P55211  P55211  P55211  P55212  P55212  P55212  P55212  P55212  P55212  P55212  P55212  P55212  P55212  P55212  P55212  P55212  P55263  P55263  P55263  P55263  P55263  P55263  P55263  P55263  P55263  P55263  P55263  P55263  P55263  P55263  P55263  P55263  P55268  P55268  P55268  P55268  P55268  P55268  P55268  P55268  P55268  P55268  P55268  P55268  P55268  P55268  P55268  P55268  P55268  P55268  P55268  P55268  P55268  P55268  P55268  P55268  P55268  P55268  P55268  P55268  P55268  P55268  P55268  P55273  P55273  P55273  P55273  P55273  P55273  P55273  P55735  P55735  P55735  P55735  P55735  P55735  P55735  P55735  P55735  P55735  P55735  P55735  P55735  P55735  P55735  P55735  P55735  P55735  P55735  P55735  P55735  P55735  P55735  P55735  P55735  P55735  P55735  P55735  P55735  P55735  P55735  P55735  P55735  P55769  P55769  P55769  P55769  P55769  P55769  P55769  P55795  P55795  P55795  P55795  P55795  P55795  P55795  P55795  P55795  P55795  P55795  P55795  P55795  P55795  P55795  P55795  P55795  P55795  P55795  P55795  P55795  P55795  P55795  P55795  P55795  P55795  P55795  P55795  P55795  P55795  P55795  P55795  P55795  P55884  P55884  P55884  P55884  P55884  P55884  P55884  P55884  P55884  P55884  P55884  P55884  P55884  P55884  P55884  P55884  P55884  P55884  P55884  P55884  P55884  P55884  P55884  P55884  P55884  P55884  P55884  P55884  P55884  P55884  P55884  P56537  P56537  P56537  P56537  P56537  P56537  P56537  P56537  P56537  P56537  P56537  P56537  P56537  P56817  P56817  P56817  P56817  P56817  P56817  P56817  P56817  P56817  P56817  P56817  P56817  P56817  P56817  P56817  P56817  P56817  P56817  P56817  P56817  P56817  P56817  P56817  P56817  P56817  P56817  P56817  P56817  P56817  P56817  P56817  P56817  P56945  P56945  P56945  P56945  P56945  P56945  P56945  P56945  P56945  P56945  P56945  P56945  P56945  P56945  P56945  P56945  P56945  P56945  P56945  P56945  P56945  P56945  P56945  P56945  P56945  P56945  P56945  P56945  P56945  P56945  P56945  P57059  P57059  P57059  P57059  P57059  P57059  P57059  P57059  P57059  P57059  P57059  P57059  P57059  P57059  P57059  P57059  P57059  P57059  P57059  P57059  P57059  P57059  P57059  P57059  P57059  P57059  P57059  P57059  P57059  P57059  P57059  P57081  P57081  P57081  P57081  P57081  P57081  P57081  P57081  P57081  P57081  P57081  P57081  P57081  P57081  P57081  P57081  P57081  P57081  P57081  P57081  P57081  P57081  P57081  P57081  P57081  P57081  P57081  P57081  P57081  P57081  P57081  P57081  P57081  P58340  P58340  P58340  P58340  P58340  P58340  P58340  P58340  P58340  P58340  P58340  P58340  P58340  P58340  P58340  P58340  P58340  P58340  P58340  P58340  P58340  P58340  P58340  P58340  P58340  P58340  P59665  P59665  P59665  P59998  P59998  P59998  P59998  P59998  P59998  P59998  P59998  P59998  P59998  P59998  P59998  P60228  P60228  P60228  P60228  P60228  P60228  P60228  P60228  P60228  P60228  P60228  P60228  P60228  P60228  P60228  P60228  P60228  P60228  P60228  P60228  P60228  P60228  P60228  P60468  P60468  P60468  P60468  P60468  P60468  P60468  P60468  P60484  P60484  P60484  P60484  P60484  P60484  P60484  P60484  P60484  P60484  P60484  P60484  P60484  P60484  P60484  P60484  P60484  P60484  P60484  P60866  P60866  P60866  P60866  P60866  P60866  P60866  P60953  P60953  P60953  P60953  P60953  P60953  P60953  P60953  P60953  P61073  P61073  P61073  P61073  P61073  P61073  P61073  P61073  P61073  P61073  P61073  P61073  P61073  P61073  P61073  P61073  P61073  P61073  P61073  P61073  P61073  P61073  P61081  P61081  P61081  P61081  P61081  P61081  P61081  P61081  P61081  P61081  P61081  P61086  P61086  P61086  P61086  P61086  P61086  P61086  P61086  P61086  P61086  P61158  P61158  P61158  P61158  P61158  P61158  P61158  P61158  P61158  P61158  P61158  P61158  P61158  P61158  P61158  P61158  P61158  P61158  P61158  P61158  P61244  P61244  P61244  P61244  P61244  P61244  P61244  P61244  P61244  P61244  P61244  P61244  P61244  P61244  P61244  P61244  P61244  P61244  P61244  P61244  P61289  P61289  P61289  P61289  P61289  P61289  P61289  P61289  P61289  P61289  P61289  P61289  P61457  P61457  P61457  P61457  P61604  P61604  P61604  P61604  P61604  P61769  P61769  P61769  P61769  P61769  P61769  P61769  P61769  P61769  P61769  P61769  P61803  P61803  P61803  P61803  P61803  P61803  P61803  P61803  P61803  P61923  P61923  P61923  P61923  P61923  P61923  P61923  P61923  P61923  P61923  P61923  P61923  P61978  P61978  P61978  P61978  P61978  P61978  P61978  P61978  P61978  P61978  P61978  P61978  P61978  P61978  P61978  P61978  P61978  P61978  P61978  P61978  P61978  P61978  P61978  P61978  P61978  P62136  P62136  P62136  P62136  P62136  P62136  P62136  P62136  P62136  P62136  P62136  P62136  P62136  P62136  P62136  P62253  P62253  P62253  P62253  P62253  P62258  P62258  P62258  P62258  P62258  P62258  P62258  P62258  P62258  P62258  P62258  P62258  P62258  P62258  P62266  P62266  P62266  P62266  P62269  P62280  P62280  P62280  P62280  P62280  P62306  P62306  P62316  P62316  P62316  P62316  P62316  P62316  P62316  P62495  P62495  P62495  P62495  P62495  P62495  P62495  P62495  P62495  P62495  P62495  P62495  P62495  P62495  P62495  P62495  P62495  P62495  P62495  P62495  P62495  P62495  P62495  P62495  P62495  P62495  P62633  P62633  P62633  P62633  P62633  P62633  P62633  P62633  P62633  P62633  P62633  P62745  P62745  P62745  P62745  P62745  P62745  P62745  P62745  P62753  P62753  P62753  P62753  P62753  P62753  P62753  P62820  P62820  P62820  P62820  P62820  P62820  P62820  P62820  P62820  P62820  P62820  P62820  P62820  P62826  P62826  P62826  P62826  P62847  P62847  P62873  P62873  P62873  P62873  P62873  P62873  P62873  P62873  P62873  P62873  P62873  P62873  P62873  P62873  P62873  P62873  P62873  P62873  P62873  P62873  P62873  P62873  P62873  P62873  P62873  P62873  P62877  P62877  P62877  P62877  P62877  P62913  P62913  P62913  P62913  P62913  P62913  P62913  P62917  P62917  P62917  P62917  P62917  P62917  P62917  P62917  P62937  P62937  P62937  P62937  P62937  P62937  P62937  P62937  P62979  P62979  P62979  P62979  P62979  P62979  P62995  P62995  P62995  P62995  P62995  P62995  P62995  P62995  P62995  P62995  P62995  P62995  P62995  P62995  P62995  P62995  P62995  P62995  P62995  P62995  P62995  P62995  P62995  P62995  P62995  P62995  P62995  P62995  P62995  P62995  P62995  P62995  P62995  P63010  P63010  P63010  P63010  P63010  P63010  P63010  P63010  P63010  P63010  P63010  P63010  P63010  P63010  P63010  P63010  P63010  P63010  P63010  P63010  P63010  P63010  P63010  P63010  P63010  P63010  P63010  P63010  P63010  P63010  P63010  P63010  P63010  P63165  P63165  P63165  P63165  P63167  P63167  P63167  P63218  P63218  P63218  P63218  P63220  P63220  P63220  P63220  P63220  P63241  P63241  P63241  P63241  P63241  P63241  P63241  P63241  P63244  P63244  P63244  P63244  P63244  P63244  P63244  P63244  P63244  P63244  P63244  P63244  P63244  P63244  P63244  P63244  P63244  P63244  P63244  P63244  P63244  P63244  P63244  P63244  P63244  P63244  P63244  P63244  P63244  P63244  P63252  P63252  P63252  P63252  P63252  P63252  P63252  P63252  P63252  P63252  P63252  P63252  P63252  P63252  P63252  P63252  P63252  P63252  P63252  P63252  P63252  P63252  P63252  P63252  P63252  P63252  P63252  P63252  P63252  P63252  P63279  P63279  P63279  P63316  P63316  P63316  P63316  P63316  P67775  P67775  P67775  P67775  P67775  P67775  P67775  P67775  P67775  P67775  P67775  P67775  P67775  P67775  P67775  P67809  P67809  P67809  P67809  P67809  P67809  P67809  P67870  P67870  P67870  P67870  P67870  P67870  P67870  P68032  P68032  P68032  P68032  P68032  P68032  P68032  P68032  P68032  P68032  P68032  P68032  P68032  P68032  P68032  P68032  P68032  P68032  P68032  P68032  P68032  P68032  P68032  P68104  P68104  P68104  P68104  P68104  P68104  P68104  P68104  P68104  P68104  P68104  P68104  P68104  P68104  P68104  P68104  P68104  P68104  P68104  P68104  P68104  P68104  P68363  P68363  P68363  P68363  P68363  P68363  P68363  P68363  P68363  P68363  P68363  P68363  P68363  P68363  P68363  P68363  P68363  P68363  P68363  P68400  P68400  P68400  P68400  P68400  P68400  P68400  P68400  P68400  P68400  P68400  P68400  P68400  P68400  P68400  P68400  P68400  P68400  P68400  P68400  P68400  P68871  P68871  P68871  P68871  P68871  P78344  P78344  P78344  P78344  P78344  P78344  P78344  P78344  P78344  P78344  P78344  P78344  P78344  P78344  P78344  P78344  P78344  P78344  P78344  P78344  P78344  P78344  P78344  P78344  P78344  P78344  P78344  P78344  P78344  P78344  P78344  P78344  P78344  P78346  P78346  P78346  P78346  P78346  P78346  P78346  P78346  P78346  P78346  P78346  P78346  P78346  P78346  P78346  P78346  P78346  P78346  P78346  P78347  P78347  P78347  P78347  P78347  P78347  P78347  P78347  P78347  P78347  P78347  P78347  P78347  P78347  P78347  P78347  P78347  P78347  P78347  P78347  P78347  P78347  P78347  P78347  P78347  P78347  P78347  P78347  P78347  P78347  P78347  P78348  P78348  P78348  P78348  P78348  P78348  P78348  P78348  P78348  P78348  P78348  P78348  P78348  P78348  P78348  P78348  P78348  P78348  P78348  P78348  P78348  P78348  P78348  P78348  P78348  P78348  P78348  P78362  P78362  P78362  P78362  P78362  P78362  P78362  P78362  P78362  P78362  P78362  P78362  P78362  P78362  P78362  P78362  P78362  P78362  P78362  P78362  P78362  P78362  P78362  P78362  P78362  P78362  P78362  P78362  P78362  P78362  P78362  P78362  P78371  P78371  P78371  P78371  P78371  P78371  P78371  P78371  P78371  P78371  P78371  P78371  P78371  P78371  P78371  P78371  P78371  P78371  P78371  P78371  P78371  P78371  P78371  P78371  P78371  P78371  P78371  P78371  P78371  P78527  P78527  P78527  P78527  P78527  P78527  P78527  P78527  P78527  P78527  P78527  P78527  P78527  P78527  P78527  P78527  P78527  P78527  P78527  P78527  P78527  P78527  P78527  P78527  P78527  P78527  P78527  P78527  P78527  P78527  P78527  P78536  P78536  P78536  P78536  P78536  P78536  P78536  P78536  P78536  P78536  P78536  P78536  P78536  P78536  P78536  P78536  P78536  P78536  P78536  P78536  P78536  P78536  P78536  P78536  P78536  P78536  P78536  P78536  P78536  P78536  P78536  P78543  P78543  P78543  P78543  P78543  P78543  P78543  P78543  P78543  P78543  P78543  P78543  P78543  P78543  P80188  P80188  P80188  P80188  P80188  P80188  P80188  P80188  P80188  P80188  P80188  P80188  P80192  P80192  P80192  P80192  P80192  P80192  P80192  P80192  P80192  P80192  P80192  P80192  P80192  P80192  P80192  P80192  P80192  P80192  P80192  P80192  P80192  P80192  P80192  P80192  P80192  P80192  P80192  P80192  P80192  P80192  P80192  P82979  P82979  P82979  P82979  P82979  P82979  P82979  P82979  P82979  P82979  P82979  P82979  P82979  P84090  P84090  P84090  P84090  P84090  P98161  P98161  P98161  P98161  P98161  P98161  P98161  P98161  P98161  P98161  P98161  P98161  P98161  P98161  P98161  P98161  P98161  P98161  P98161  P98161  P98161  P98161  P98161  P98161  P98161  P98161  P98161  P98161  P98161  P98161  P98161  P98170  P98170  P98170  P98170  P98170  P98170  P98170  P98170  P98170  P98170  P98170  P98170  P98170  P98170  P98170  P98170  P98170  P98170  P98170  P98170  P98170  P98170  P98170  P98170  P98170  P98170  P98170  P98170  P98170  P98170  P98170  P98177  P98177  P98177  P98177  P98177  P98177  P98177  P98177  P98177  P98177  P98177  P98177  P98177  P98177  P98177  P98177  P98177  P98177  P98177  P98177  P98177  P98177  P98177  P98177  P98177  P98177  P98177  P98177  P98177  P98177  P98177  P98179  P98179  P98179  P98179  P98179  P98179  P98179  P98179  P98179  P98179  P98179  P98179  P98179  P99999  P99999  Q00059  Q00059  Q00059  Q00059  Q00059  Q00059  Q00059  Q00059  Q00059  Q00059  Q00059  Q00059  Q00059  Q00059  Q00059  Q00341  Q00341  Q00341  Q00341  Q00341  Q00341  Q00341  Q00341  Q00341  Q00341  Q00341  Q00341  Q00341  Q00341  Q00341  Q00341  Q00341  Q00341  Q00341  Q00341  Q00341  Q00341  Q00341  Q00341  Q00341  Q00341  Q00341  Q00341  Q00341  Q00341  Q00341  Q00341  Q00341  Q00534  Q00534  Q00534  Q00534  Q00534  Q00534  Q00534  Q00534  Q00534  Q00534  Q00534  Q00534  Q00534  Q00534  Q00534  Q00534  Q00534  Q00534  Q00534  Q00536  Q00536  Q00536  Q00536  Q00536  Q00536  Q00536  Q00536  Q00536  Q00536  Q00536  Q00536  Q00536  Q00536  Q00536  Q00536  Q00536  Q00536  Q00536  Q00536  Q00536  Q00536  Q00536  Q00536  Q00536  Q00536  Q00536  Q00536  Q00610  Q00610  Q00610  Q00610  Q00610  Q00610  Q00610  Q00610  Q00610  Q00610  Q00610  Q00610  Q00610  Q00610  Q00610  Q00610  Q00610  Q00610  Q00610  Q00610  Q00610  Q00610  Q00610  Q00610  Q00610  Q00610  Q00610  Q00610  Q00610  Q00610  Q00610  Q00610  Q00610  Q00613  Q00613  Q00613  Q00613  Q00613  Q00613  Q00613  Q00613  Q00613  Q00613  Q00613  Q00613  Q00613  Q00613  Q00613  Q00613  Q00613  Q00613  Q00613  Q00613  Q00613  Q00613  Q00613  Q00613  Q00613  Q00613  Q00613  Q00613  Q00613  Q00613  Q00613  Q00653  Q00653  Q00653  Q00653  Q00653  Q00653  Q00653  Q00653  Q00653  Q00653  Q00653  Q00653  Q00653  Q00653  Q00653  Q00653  Q00653  Q00653  Q00653  Q00653  Q00653  Q00653  Q00653  Q00653  Q00653  Q00653  Q00653  Q00653  Q00653  Q00653  Q00653  Q00839  Q00839  Q00839  Q00839  Q00839  Q00839  Q00839  Q00839  Q00839  Q00839  Q00839  Q00839  Q00839  Q00839  Q00839  Q00839  Q00839  Q00839  Q00839  Q00839  Q00839  Q00839  Q00839  Q00839  Q00839  Q00839  Q00839  Q00839  Q00839  Q00839  Q00839  Q00839  Q00839  Q00987  Q00987  Q00987  Q00987  Q00987  Q00987  Q00987  Q00987  Q00987  Q00987  Q00987  Q00987  Q00987  Q00987  Q00987  Q00987  Q00987  Q00987  Q00987  Q00987  Q00987  Q00987  Q00987  Q00987  Q00987  Q00987  Q00987  Q00987  Q00987  Q00987  Q00987  Q01081  Q01081  Q01081  Q01081  Q01081  Q01081  Q01081  Q01081  Q01081  Q01081  Q01081  Q01081  Q01081  Q01081  Q01081  Q01094  Q01094  Q01094  Q01094  Q01094  Q01094  Q01094  Q01094  Q01094  Q01094  Q01094  Q01094  Q01094  Q01094  Q01094  Q01094  Q01094  Q01094  Q01094  Q01094  Q01094  Q01094  Q01094  Q01094  Q01094  Q01094  Q01094  Q01094  Q01094  Q01094  Q01094  Q01094  Q01094  Q01130  Q01130  Q01130  Q01130  Q01130  Q01130  Q01130  Q01130  Q01130  Q01130  Q01130  Q01130  Q01130  Q01130  Q01130  Q01130  Q01130  Q01130  Q01130  Q01130  Q01130  Q01130  Q01130  Q01130  Q01130  Q01130  Q01130  Q01130  Q01130  Q01130  Q01130  Q01130  Q01130  Q01469  Q01469  Q01469  Q01469  Q01518  Q01518  Q01518  Q01518  Q01518  Q01518  Q01518  Q01518  Q01518  Q01518  Q01518  Q01518  Q01518  Q01518  Q01518  Q01518  Q01518  Q01518  Q01518  Q01518  Q01518  Q01518  Q01518  Q01518  Q01518  Q01518  Q01518  Q01518  Q01518  Q01518  Q01518  Q01518  Q01518  Q01804  Q01804  Q01804  Q01804  Q01804  Q01804  Q01804  Q01804  Q01804  Q01804  Q01804  Q01804  Q01804  Q01804  Q01804  Q01804  Q01804  Q01804  Q01804  Q01804  Q01804  Q01804  Q01804  Q01804  Q01804  Q01804  Q01804  Q01804  Q01804  Q01804  Q01804  Q01804  Q01804  Q01826  Q01826  Q01826  Q01826  Q01826  Q01826  Q01826  Q01826  Q01826  Q01826  Q01826  Q01826  Q01826  Q01826  Q01826  Q01826  Q01826  Q01826  Q01826  Q01826  Q01826  Q01826  Q01826  Q01826  Q01826  Q01826  Q01826  Q01826  Q01826  Q01826  Q01826  Q01844  Q01844  Q01844  Q01844  Q01844  Q01844  Q01844  Q01844  Q01844  Q01844  Q01844  Q01844  Q01844  Q01844  Q01844  Q01844  Q01844  Q01844  Q01844  Q01844  Q01844  Q01844  Q01844  Q01844  Q01844  Q01844  Q01844  Q01844  Q01844  Q01844  Q01844  Q01844  Q01844  Q01860  Q01860  Q01860  Q01860  Q01860  Q01860  Q01860  Q01860  Q01860  Q01860  Q01860  Q01860  Q01860  Q01860  Q01860  Q01860  Q01860  Q01860  Q01954  Q01954  Q01954  Q01954  Q01954  Q01954  Q01954  Q01954  Q01954  Q01954  Q01954  Q01954  Q01954  Q01954  Q01954  Q01954  Q01954  Q01954  Q01954  Q01954  Q01954  Q01954  Q01954  Q01954  Q01954  Q01954  Q01954  Q01954  Q01954  Q01954  Q01954  Q01974  Q01974  Q01974  Q01974  Q01974  Q01974  Q01974  Q01974  Q01974  Q01974  Q01974  Q01974  Q01974  Q01974  Q01974  Q01974  Q01974  Q01974  Q01974  Q01974  Q01974  Q01974  Q01974  Q01974  Q01974  Q01974  Q01974  Q01974  Q01974  Q01974  Q01974  Q01974  Q01974  Q02156  Q02156  Q02156  Q02156  Q02156  Q02156  Q02156  Q02156  Q02156  Q02156  Q02156  Q02156  Q02156  Q02156  Q02156  Q02156  Q02156  Q02156  Q02156  Q02156  Q02156  Q02156  Q02156  Q02156  Q02156  Q02156  Q02156  Q02388  Q02388  Q02388  Q02388  Q02388  Q02388  Q02388  Q02388  Q02388  Q02388  Q02388  Q02388  Q02388  Q02388  Q02388  Q02388  Q02388  Q02388  Q02388  Q02388  Q02388  Q02388  Q02388  Q02388  Q02388  Q02388  Q02388  Q02388  Q02388  Q02388  Q02388  Q02388  Q02388  Q02447  Q02447  Q02447  Q02447  Q02447  Q02447  Q02447  Q02447  Q02447  Q02447  Q02447  Q02447  Q02447  Q02447  Q02447  Q02447  Q02447  Q02447  Q02447  Q02447  Q02447  Q02447  Q02447  Q02447  Q02447  Q02447  Q02447  Q02447  Q02447  Q02447  Q02447  Q02763  Q02763  Q02763  Q02763  Q02763  Q02763  Q02763  Q02763  Q02763  Q02763  Q02763  Q02763  Q02763  Q02763  Q02763  Q02763  Q02763  Q02763  Q02763  Q02763  Q02763  Q02763  Q02763  Q02763  Q02763  Q02763  Q02763  Q02763  Q02763  Q02763  Q02763  Q02763  Q02763  Q02790  Q02790  Q02790  Q02790  Q02790  Q02790  Q02790  Q02790  Q02790  Q02790  Q02790  Q02790  Q02790  Q02790  Q02790  Q02790  Q02790  Q02790  Q02790  Q02790  Q02790  Q02790  Q02790  Q02790  Q02790  Q02790  Q02790  Q02818  Q02818  Q02818  Q02818  Q02818  Q02818  Q02818  Q02818  Q02818  Q02818  Q02978  Q02978  Q02978  Q02978  Q02978  Q02978  Q02978  Q02978  Q02978  Q02978  Q02978  Q02978  Q02978  Q02978  Q02978  Q03135  Q03135  Q03135  Q03135  Q03135  Q03135  Q03135  Q03135  Q03135  Q03393  Q03393  Q03393  Q03393  Q03393  Q03393  Q03468  Q03468  Q03468  Q03468  Q03468  Q03468  Q03468  Q03468  Q03468  Q03468  Q03468  Q03468  Q03468  Q03468  Q03468  Q03468  Q03468  Q03468  Q03468  Q03468  Q03468  Q03468  Q03468  Q03468  Q03468  Q03468  Q03468  Q03468  Q03468  Q03468  Q03468  Q03468  Q03468  Q03721  Q03721  Q03721  Q03721  Q03721  Q03721  Q03721  Q03721  Q03721  Q03721  Q03721  Q03721  Q03721  Q03721  Q03721  Q03721  Q03721  Q03721  Q03721  Q03721  Q03721  Q03721  Q03721  Q03721  Q03721  Q03721  Q03721  Q03721  Q03721  Q03721  Q03721  Q04206  Q04206  Q04206  Q04206  Q04206  Q04206  Q04206  Q04206  Q04206  Q04206  Q04206  Q04206  Q04206  Q04206  Q04206  Q04206  Q04206  Q04206  Q04206  Q04206  Q04206  Q04206  Q04206  Q04206  Q04206  Q04206  Q04206  Q04206  Q04206  Q04206  Q04323  Q04323  Q04323  Q04323  Q04323  Q04323  Q04323  Q04323  Q04323  Q04323  Q04323  Q04323  Q04323  Q04637  Q04637  Q04637  Q04637  Q04637  Q04637  Q04637  Q04637  Q04637  Q04637  Q04637  Q04637  Q04637  Q04637  Q04637  Q04637  Q04637  Q04637  Q04637  Q04637  Q04637  Q04637  Q04637  Q04637  Q04637  Q04637  Q04637  Q04637  Q04637  Q04637  Q04637  Q04759  Q04759  Q04759  Q04759  Q04759  Q04759  Q04759  Q04759  Q04759  Q04759  Q04759  Q04759  Q04759  Q04759  Q04759  Q04759  Q04759  Q04759  Q04759  Q04759  Q04759  Q04759  Q04759  Q04759  Q04759  Q04759  Q04759  Q04760  Q04760  Q04760  Q04760  Q04760  Q04760  Q04760  Q04760  Q04760  Q04760  Q04864  Q04864  Q04864  Q04864  Q04864  Q04864  Q04864  Q04864  Q04864  Q04864  Q04864  Q04864  Q04864  Q04864  Q04864  Q04864  Q04864  Q04864  Q04864  Q04864  Q04864  Q04864  Q04864  Q04864  Q04864  Q04864  Q04864  Q04864  Q04864  Q04864  Q04864  Q04912  Q04912  Q04912  Q04912  Q04912  Q04912  Q04912  Q04912  Q04912  Q04912  Q04912  Q04912  Q04912  Q04912  Q04912  Q04912  Q04912  Q04912  Q04912  Q04912  Q04912  Q04912  Q04912  Q04912  Q04912  Q04912  Q04912  Q04912  Q04912  Q04912  Q04912  Q04912  Q05066  Q05066  Q05066  Q05066  Q05066  Q05066  Q05066  Q05066  Q05066  Q05066  Q05066  Q05066  Q05066  Q05066  Q05066  Q05066  Q05066  Q05066  Q05086  Q05086  Q05086  Q05086  Q05086  Q05086  Q05086  Q05086  Q05086  Q05086  Q05086  Q05086  Q05086  Q05086  Q05086  Q05086  Q05086  Q05086  Q05086  Q05086  Q05086  Q05086  Q05086  Q05086  Q05086  Q05086  Q05086  Q05086  Q05086  Q05086  Q05086  Q05086  Q05086  Q05209  Q05209  Q05209  Q05209  Q05209  Q05209  Q05209  Q05209  Q05209  Q05209  Q05209  Q05209  Q05209  Q05209  Q05209  Q05209  Q05209  Q05209  Q05209  Q05209  Q05209  Q05209  Q05209  Q05209  Q05209  Q05209  Q05209  Q05209  Q05209  Q05209  Q05209  Q05209  Q05209  Q05315  Q05315  Q05315  Q05315  Q05315  Q05315  Q05315  Q05315  Q05315  Q05315  Q05315  Q05397  Q05397  Q05397  Q05397  Q05397  Q05397  Q05397  Q05397  Q05397  Q05397  Q05397  Q05397  Q05397  Q05397  Q05397  Q05397  Q05397  Q05397  Q05397  Q05397  Q05397  Q05397  Q05397  Q05397  Q05397  Q05397  Q05397  Q05397  Q05397  Q05397  Q05397  Q05655  Q05655  Q05655  Q05655  Q05655  Q05655  Q05655  Q05655  Q05655  Q05655  Q05655  Q05655  Q05655  Q05655  Q05655  Q05655  Q05655  Q05655  Q05655  Q05655  Q05655  Q05655  Q05655  Q05655  Q05655  Q05655  Q05707  Q05707  Q05707  Q05707  Q05707  Q05707  Q05707  Q05707  Q05707  Q05707  Q05707  Q05707  Q05707  Q05707  Q05707  Q05707  Q05707  Q05707  Q05707  Q05707  Q05707  Q05707  Q05707  Q05707  Q05707  Q05707  Q05707  Q05707  Q05707  Q05707  Q05707  Q05707  Q05707  Q06187  Q06187  Q06187  Q06187  Q06187  Q06187  Q06187  Q06187  Q06187  Q06187  Q06187  Q06187  Q06187  Q06187  Q06187  Q06187  Q06187  Q06187  Q06187  Q06187  Q06187  Q06187  Q06187  Q06187  Q06187  Q06187  Q06187  Q06187  Q06187  Q06187  Q06187  Q06413  Q06413  Q06413  Q06413  Q06413  Q06413  Q06413  Q06413  Q06413  Q06413  Q06413  Q06413  Q06413  Q06413  Q06413  Q06413  Q06413  Q06413  Q06413  Q06413  Q06413  Q06413  Q06413  Q06413  Q06413  Q06413  Q06413  Q06413  Q06413  Q06413  Q06413  Q06481  Q06481  Q06481  Q06481  Q06481  Q06481  Q06481  Q06481  Q06481  Q06481  Q06481  Q06481  Q06481  Q06481  Q06481  Q06481  Q06481  Q06481  Q06481  Q06481  Q06481  Q06481  Q06481  Q06481  Q06481  Q06481  Q06481  Q06481  Q06481  Q06481  Q06481  Q06609  Q06609  Q06609  Q06609  Q06609  Q06609  Q06609  Q06609  Q06609  Q06609  Q06609  Q06609  Q06609  Q06609  Q06609  Q06609  Q06787  Q06787  Q06787  Q06787  Q06787  Q06787  Q06787  Q06787  Q06787  Q06787  Q06787  Q06787  Q06787  Q06787  Q06787  Q06787  Q06787  Q06787  Q06787  Q06787  Q06787  Q06787  Q06787  Q06787  Q06787  Q06787  Q06787  Q06787  Q06787  Q06787  Q06787  Q06828  Q06828  Q06828  Q06828  Q06828  Q06828  Q06828  Q06828  Q06828  Q06828  Q06828  Q06828  Q06828  Q06828  Q06828  Q06828  Q06828  Q06828  Q06828  Q06828  Q06828  Q06828  Q06828  Q06828  Q06828  Q06828  Q06828  Q06828  Q06828  Q06828  Q06830  Q06830  Q06830  Q06830  Q06830  Q06830  Q06830  Q06830  Q06830  Q06830  Q07065  Q07065  Q07065  Q07065  Q07065  Q07065  Q07065  Q07065  Q07065  Q07065  Q07065  Q07065  Q07065  Q07065  Q07065  Q07065  Q07065  Q07065  Q07065  Q07065  Q07065  Q07065  Q07065  Q07065  Q07065  Q07065  Q07065  Q07065  Q07065  Q07065  Q07065  Q07666  Q07666  Q07666  Q07666  Q07666  Q07666  Q07666  Q07666  Q07666  Q07666  Q07666  Q07666  Q07666  Q07666  Q07666  Q07666  Q07666  Q07666  Q07666  Q07666  Q07666  Q07666  Q07666  Q07666  Q07666  Q07666  Q07666  Q07666  Q07817  Q07817  Q07817  Q07817  Q07817  Q07817  Q07817  Q07817  Q07817  Q07817  Q07817  Q07817  Q07817  Q07817  Q07817  Q07817  Q07817  Q07820  Q07820  Q07820  Q07820  Q07820  Q07820  Q07820  Q07820  Q07820  Q07820  Q07820  Q07820  Q07820  Q07820  Q07820  Q07820  Q07820  Q07820  Q07866  Q07866  Q07866  Q07866  Q07866  Q07866  Q07866  Q07866  Q07866  Q07866  Q07866  Q07866  Q07866  Q07866  Q07866  Q07866  Q07866  Q07866  Q07866  Q07866  Q07866  Q07866  Q07866  Q07866  Q07866  Q07866  Q07866  Q07866  Q07912  Q07912  Q07912  Q07912  Q07912  Q07912  Q07912  Q07912  Q07912  Q07912  Q07912  Q07912  Q07912  Q07912  Q07912  Q07912  Q07912  Q07912  Q07912  Q07912  Q07912  Q07912  Q07912  Q07912  Q07912  Q07912  Q07912  Q07912  Q07912  Q07912  Q07912  Q07912  Q07912  Q07954  Q07954  Q07954  Q07954  Q07954  Q07954  Q07954  Q07954  Q07954  Q07954  Q07954  Q07954  Q07954  Q07954  Q07954  Q07954  Q07954  Q07954  Q07954  Q07954  Q07954  Q07954  Q07954  Q07954  Q07954  Q07954  Q07954  Q07954  Q07954  Q07954  Q07954  Q07954  Q07954  Q07955  Q07955  Q07955  Q07955  Q07955  Q07955  Q07955  Q07955  Q07955  Q07955  Q07955  Q07955  Q07955  Q07955  Q07955  Q07955  Q07955  Q07955  Q07955  Q07955  Q07955  Q07955  Q08050  Q08050  Q08050  Q08050  Q08050  Q08050  Q08050  Q08050  Q08050  Q08050  Q08050  Q08050  Q08050  Q08050  Q08050  Q08050  Q08050  Q08050  Q08050  Q08050  Q08050  Q08050  Q08050  Q08050  Q08050  Q08050  Q08050  Q08050  Q08050  Q08050  Q08050  Q08188  Q08188  Q08188  Q08188  Q08188  Q08188  Q08188  Q08188  Q08188  Q08188  Q08188  Q08188  Q08188  Q08188  Q08188  Q08188  Q08188  Q08188  Q08188  Q08188  Q08188  Q08188  Q08188  Q08188  Q08188  Q08188  Q08188  Q08188  Q08188  Q08188  Q08188  Q08188  Q08188  Q08345  Q08345  Q08345  Q08345  Q08345  Q08345  Q08345  Q08345  Q08345  Q08345  Q08345  Q08345  Q08345  Q08345  Q08345  Q08345  Q08345  Q08345  Q08345  Q08345  Q08345  Q08345  Q08345  Q08345  Q08345  Q08345  Q08345  Q08345  Q08345  Q08345  Q08345  Q08345  Q08345  Q08426  Q08426  Q08426  Q08426  Q08426  Q08426  Q08426  Q08426  Q08426  Q08426  Q08426  Q08426  Q08426  Q08426  Q08426  Q08426  Q08426  Q08426  Q08426  Q08426  Q08426  Q08426  Q08426  Q08426  Q08426  Q08426  Q08426  Q08426  Q08426  Q08426  Q08426  Q08426  Q08426  Q08431  Q08431  Q08431  Q08431  Q08431  Q08431  Q08431  Q08431  Q08431  Q08431  Q08431  Q08431  Q08431  Q08431  Q08431  Q08431  Q08431  Q08431  Q08431  Q08431  Q08431  Q08431  Q08431  Q08431  Q08431  Q08431  Q08431  Q08431  Q08495  Q08495  Q08495  Q08495  Q08495  Q08495  Q08495  Q08495  Q08495  Q08495  Q08495  Q08495  Q08495  Q08495  Q08495  Q08495  Q08495  Q08495  Q08495  Q08495  Q08495  Q08495  Q08495  Q08495  Q08495  Q08495  Q08495  Q08495  Q08495  Q08495  Q08495  Q08722  Q08722  Q08722  Q08722  Q08722  Q08722  Q08722  Q08722  Q08722  Q08722  Q08722  Q08722  Q08722  Q08722  Q08722  Q08722  Q08722  Q08722  Q08722  Q08722  Q08722  Q08945  Q08945  Q08945  Q08945  Q08945  Q08945  Q08945  Q08945  Q08945  Q08945  Q08945  Q08945  Q08945  Q08945  Q08945  Q08945  Q08945  Q08945  Q08945  Q08945  Q08945  Q08945  Q08945  Q08945  Q08945  Q08945  Q08945  Q08945  Q08945  Q08945  Q08945  Q08999  Q08999  Q08999  Q08999  Q08999  Q08999  Q08999  Q08999  Q08999  Q08999  Q08999  Q08999  Q08999  Q08999  Q08999  Q08999  Q08999  Q08999  Q08999  Q08999  Q08999  Q08999  Q08999  Q08999  Q08999  Q08999  Q08999  Q08999  Q08999  Q08999  Q08999  Q08J23  Q08J23  Q08J23  Q08J23  Q08J23  Q08J23  Q08J23  Q08J23  Q08J23  Q08J23  Q08J23  Q08J23  Q08J23  Q08J23  Q08J23  Q08J23  Q08J23  Q08J23  Q08J23  Q08J23  Q08J23  Q08J23  Q08J23  Q08J23  Q08J23  Q08J23  Q08J23  Q08J23  Q08J23  Q08J23  Q08J23  Q09028  Q09028  Q09028  Q09028  Q09028  Q09028  Q09028  Q09028  Q09028  Q09028  Q09028  Q09028  Q09028  Q09028  Q09028  Q09028  Q09028  Q09028  Q09028  Q09028  Q09028  Q09028  Q09028  Q09028  Q09028  Q09028  Q09028  Q09028  Q09028  Q09028  Q09028  Q09028  Q09028  Q09161  Q09161  Q09161  Q09161  Q09161  Q09161  Q09161  Q09161  Q09161  Q09161  Q09161  Q09161  Q09161  Q09161  Q09161  Q09161  Q09161  Q09161  Q09161  Q09161  Q09161  Q09161  Q09161  Q09161  Q09161  Q09161  Q09161  Q09161  Q09161  Q09161  Q09161  Q0VD86  Q0VD86  Q0VD86  Q0VD86  Q0VD86  Q0VD86  Q0VD86  Q0VD86  Q0VD86  Q0VD86  Q0VD86  Q0VD86  Q0VD86  Q0VD86  Q12778  Q12778  Q12778  Q12778  Q12778  Q12778  Q12778  Q12778  Q12778  Q12778  Q12778  Q12778  Q12778  Q12778  Q12778  Q12778  Q12778  Q12778  Q12778  Q12778  Q12778  Q12778  Q12778  Q12778  Q12778  Q12778  Q12778  Q12778  Q12778  Q12778  Q12778  Q12792  Q12792  Q12792  Q12792  Q12792  Q12792  Q12792  Q12792  Q12792  Q12792  Q12792  Q12792  Q12792  Q12792  Q12792  Q12792  Q12792  Q12792  Q12792  Q12792  Q12792  Q12792  Q12792  Q12792  Q12834  Q12834  Q12834  Q12834  Q12834  Q12834  Q12834  Q12834  Q12834  Q12834  Q12834  Q12834  Q12834  Q12834  Q12834  Q12834  Q12834  Q12834  Q12834  Q12834  Q12834  Q12834  Q12834  Q12834  Q12834  Q12834  Q12834  Q12834  Q12834  Q12834  Q12834  Q12841  Q12841  Q12841  Q12841  Q12841  Q12841  Q12841  Q12841  Q12841  Q12841  Q12841  Q12841  Q12841  Q12841  Q12841  Q12841  Q12841  Q12866  Q12866  Q12866  Q12866  Q12866  Q12866  Q12866  Q12866  Q12866  Q12866  Q12866  Q12866  Q12866  Q12866  Q12866  Q12866  Q12866  Q12866  Q12866  Q12866  Q12866  Q12866  Q12866  Q12866  Q12866  Q12866  Q12866  Q12866  Q12866  Q12866  Q12866  Q12866  Q12866  Q12874  Q12874  Q12874  Q12874  Q12874  Q12874  Q12874  Q12874  Q12874  Q12874  Q12874  Q12874  Q12874  Q12874  Q12874  Q12874  Q12874  Q12874  Q12874  Q12874  Q12874  Q12874  Q12874  Q12874  Q12874  Q12874  Q12874  Q12888  Q12888  Q12888  Q12888  Q12888  Q12888  Q12888  Q12888  Q12888  Q12888  Q12888  Q12888  Q12888  Q12888  Q12888  Q12888  Q12888  Q12888  Q12888  Q12888  Q12888  Q12888  Q12888  Q12888  Q12888  Q12888  Q12888  Q12888  Q12888  Q12888  Q12888  Q12906  Q12906  Q12906  Q12906  Q12906  Q12906  Q12906  Q12906  Q12906  Q12906  Q12906  Q12906  Q12906  Q12906  Q12906  Q12906  Q12906  Q12906  Q12906  Q12906  Q12906  Q12906  Q12906  Q12906  Q12906  Q12906  Q12906  Q12906  Q12906  Q12906  Q12906  Q12906  Q12906  Q12933  Q12933  Q12933  Q12933  Q12933  Q12933  Q12933  Q12933  Q12933  Q12933  Q12933  Q12933  Q12933  Q12933  Q12933  Q12933  Q12933  Q12933  Q12933  Q12933  Q12933  Q12933  Q12933  Q12933  Q12933  Q12933  Q12933  Q12933  Q12933  Q12933  Q12933  Q12962  Q12962  Q12962  Q12962  Q12962  Q12962  Q12962  Q12962  Q12962  Q12962  Q12962  Q12962  Q12962  Q12962  Q12962  Q12962  Q12962  Q12962  Q12962  Q12962  Q12968  Q12968  Q12968  Q12968  Q12968  Q12968  Q12968  Q12968  Q12968  Q12968  Q12968  Q12968  Q12968  Q12968  Q12968  Q12968  Q12968  Q12968  Q12968  Q12968  Q12968  Q12968  Q12968  Q12968  Q12968  Q12968  Q12968  Q12968  Q12968  Q12968  Q12968  Q12968  Q12968  Q12972  Q12972  Q12972  Q12972  Q12972  Q12972  Q12972  Q12972  Q12972  Q12972  Q12972  Q12972  Q12972  Q12972  Q12972  Q12972  Q12972  Q12972  Q12972  Q12972  Q13002  Q13002  Q13002  Q13002  Q13002  Q13002  Q13002  Q13002  Q13002  Q13002  Q13002  Q13002  Q13002  Q13002  Q13002  Q13002  Q13002  Q13002  Q13002  Q13002  Q13002  Q13002  Q13002  Q13002  Q13002  Q13002  Q13002  Q13002  Q13002  Q13002  Q13002  Q13042  Q13042  Q13042  Q13042  Q13042  Q13042  Q13042  Q13042  Q13042  Q13042  Q13042  Q13042  Q13042  Q13042  Q13042  Q13042  Q13042  Q13042  Q13042  Q13042  Q13042  Q13042  Q13042  Q13042  Q13042  Q13042  Q13042  Q13042  Q13042  Q13042  Q13042  Q13045  Q13045  Q13045  Q13045  Q13045  Q13045  Q13045  Q13045  Q13045  Q13045  Q13045  Q13045  Q13045  Q13045  Q13045  Q13045  Q13045  Q13045  Q13045  Q13045  Q13045  Q13045  Q13045  Q13045  Q13045  Q13045  Q13045  Q13045  Q13045  Q13045  Q13045  Q13049  Q13049  Q13049  Q13049  Q13049  Q13049  Q13049  Q13049  Q13049  Q13049  Q13049  Q13049  Q13049  Q13049  Q13049  Q13049  Q13049  Q13049  Q13049  Q13049  Q13049  Q13049  Q13049  Q13049  Q13049  Q13049  Q13049  Q13049  Q13049  Q13049  Q13049  Q13049  Q13049  Q13085  Q13085  Q13085  Q13085  Q13085  Q13085  Q13085  Q13085  Q13085  Q13085  Q13085  Q13085  Q13085  Q13085  Q13085  Q13085  Q13085  Q13085  Q13085  Q13085  Q13085  Q13085  Q13085  Q13085  Q13085  Q13085  Q13085  Q13085  Q13085  Q13085  Q13085  Q13098  Q13098  Q13098  Q13098  Q13098  Q13098  Q13098  Q13098  Q13098  Q13098  Q13098  Q13098  Q13098  Q13098  Q13098  Q13098  Q13098  Q13098  Q13098  Q13098  Q13098  Q13098  Q13098  Q13098  Q13098  Q13098  Q13098  Q13098  Q13098  Q13098  Q13098  Q13115  Q13115  Q13115  Q13115  Q13115  Q13115  Q13115  Q13115  Q13115  Q13115  Q13115  Q13115  Q13115  Q13115  Q13115  Q13115  Q13115  Q13115  Q13115  Q13115  Q13115  Q13115  Q13115  Q13115  Q13115  Q13115  Q13115  Q13115  Q13115  Q13115  Q13115  Q13153  Q13153  Q13153  Q13153  Q13153  Q13153  Q13153  Q13153  Q13153  Q13153  Q13153  Q13153  Q13153  Q13153  Q13153  Q13153  Q13153  Q13153  Q13153  Q13153  Q13153  Q13153  Q13153  Q13153  Q13153  Q13153  Q13153  Q13158  Q13158  Q13158  Q13158  Q13158  Q13158  Q13158  Q13158  Q13158  Q13158  Q13158  Q13158  Q13158  Q13158  Q13158  Q13158  Q13158  Q13163  Q13163  Q13163  Q13163  Q13163  Q13163  Q13163  Q13163  Q13163  Q13163  Q13163  Q13163  Q13163  Q13163  Q13163  Q13163  Q13163  Q13163  Q13163  Q13163  Q13163  Q13163  Q13163  Q13163  Q13163  Q13163  Q13163  Q13163  Q13163  Q13163  Q13163  Q13164  Q13164  Q13164  Q13164  Q13164  Q13164  Q13164  Q13164  Q13164  Q13164  Q13164  Q13164  Q13164  Q13164  Q13164  Q13164  Q13164  Q13164  Q13164  Q13164  Q13164  Q13164  Q13164  Q13164  Q13164  Q13164  Q13164  Q13164  Q13164  Q13164  Q13164  Q13164  Q13164  Q13188  Q13188  Q13188  Q13188  Q13188  Q13188  Q13188  Q13188  Q13188  Q13188  Q13188  Q13188  Q13188  Q13188  Q13188  Q13188  Q13188  Q13188  Q13188  Q13188  Q13188  Q13188  Q13188  Q13188  Q13188  Q13188  Q13188  Q13188  Q13188  Q13188  Q13188  Q13191  Q13191  Q13191  Q13191  Q13191  Q13191  Q13191  Q13191  Q13191  Q13191  Q13191  Q13191  Q13191  Q13191  Q13191  Q13191  Q13191  Q13191  Q13191  Q13191  Q13191  Q13191  Q13191  Q13191  Q13191  Q13191  Q13191  Q13191  Q13191  Q13191  Q13191  Q13217  Q13217  Q13217  Q13217  Q13217  Q13217  Q13217  Q13217  Q13217  Q13217  Q13217  Q13217  Q13217  Q13217  Q13217  Q13217  Q13217  Q13217  Q13217  Q13217  Q13217  Q13217  Q13217  Q13217  Q13217  Q13217  Q13217  Q13217  Q13247  Q13247  Q13247  Q13247  Q13247  Q13247  Q13247  Q13247  Q13247  Q13247  Q13247  Q13247  Q13247  Q13247  Q13247  Q13247  Q13247  Q13247  Q13247  Q13247  Q13247  Q13247  Q13247  Q13247  Q13247  Q13247  Q13247  Q13247  Q13247  Q13247  Q13247  Q13257  Q13257  Q13257  Q13257  Q13257  Q13257  Q13257  Q13257  Q13257  Q13257  Q13257  Q13257  Q13257  Q13263  Q13263  Q13263  Q13263  Q13263  Q13263  Q13263  Q13263  Q13263  Q13263  Q13263  Q13263  Q13263  Q13263  Q13263  Q13263  Q13263  Q13263  Q13263  Q13263  Q13263  Q13263  Q13263  Q13263  Q13263  Q13263  Q13263  Q13263  Q13263  Q13263  Q13263  Q13283  Q13283  Q13283  Q13283  Q13283  Q13283  Q13283  Q13283  Q13283  Q13283  Q13283  Q13283  Q13283  Q13283  Q13283  Q13283  Q13283  Q13283  Q13283  Q13283  Q13283  Q13283  Q13285  Q13285  Q13285  Q13285  Q13285  Q13285  Q13285  Q13285  Q13285  Q13285  Q13285  Q13285  Q13285  Q13285  Q13285  Q13303  Q13303  Q13303  Q13303  Q13303  Q13303  Q13303  Q13303  Q13303  Q13303  Q13303  Q13303  Q13303  Q13303  Q13303  Q13303  Q13303  Q13303  Q13303  Q13303  Q13303  Q13303  Q13303  Q13303  Q13309  Q13309  Q13309  Q13309  Q13309  Q13309  Q13309  Q13309  Q13309  Q13309  Q13309  Q13309  Q13309  Q13309  Q13309  Q13309  Q13309  Q13309  Q13309  Q13309  Q13309  Q13309  Q13309  Q13309  Q13309  Q13309  Q13309  Q13309  Q13309  Q13309  Q13309  Q13309  Q13309  Q13310  Q13310  Q13310  Q13310  Q13310  Q13310  Q13310  Q13310  Q13310  Q13310  Q13310  Q13310  Q13310  Q13310  Q13310  Q13310  Q13310  Q13310  Q13310  Q13310  Q13310  Q13310  Q13310  Q13310  Q13310  Q13310  Q13310  Q13310  Q13310  Q13310  Q13310  Q13310  Q13310  Q13315  Q13315  Q13315  Q13315  Q13315  Q13315  Q13315  Q13315  Q13315  Q13315  Q13315  Q13315  Q13315  Q13315  Q13315  Q13315  Q13315  Q13315  Q13315  Q13315  Q13315  Q13315  Q13315  Q13315  Q13315  Q13315  Q13315  Q13315  Q13315  Q13315  Q13315  Q13322  Q13322  Q13322  Q13322  Q13322  Q13322  Q13322  Q13322  Q13322  Q13322  Q13322  Q13322  Q13322  Q13322  Q13322  Q13322  Q13322  Q13322  Q13322  Q13322  Q13322  Q13322  Q13322  Q13322  Q13322  Q13322  Q13322  Q13322  Q13322  Q13322  Q13322  Q13330  Q13330  Q13330  Q13330  Q13330  Q13330  Q13330  Q13330  Q13330  Q13330  Q13330  Q13330  Q13330  Q13330  Q13330  Q13330  Q13330  Q13330  Q13330  Q13330  Q13330  Q13330  Q13330  Q13330  Q13330  Q13330  Q13330  Q13330  Q13330  Q13330  Q13330  Q13330  Q13330  Q13342  Q13342  Q13342  Q13342  Q13342  Q13342  Q13342  Q13342  Q13342  Q13342  Q13342  Q13342  Q13342  Q13342  Q13342  Q13342  Q13342  Q13342  Q13342  Q13342  Q13342  Q13342  Q13342  Q13342  Q13342  Q13342  Q13342  Q13342  Q13342  Q13342  Q13342  Q13342  Q13363  Q13363  Q13363  Q13363  Q13363  Q13363  Q13363  Q13363  Q13363  Q13363  Q13363  Q13363  Q13363  Q13363  Q13363  Q13363  Q13363  Q13363  Q13363  Q13371  Q13371  Q13371  Q13371  Q13371  Q13371  Q13371  Q13371  Q13371  Q13371  Q13371  Q13371  Q13371  Q13371  Q13371  Q13371  Q13371  Q13371  Q13371  Q13404  Q13404  Q13404  Q13404  Q13404  Q13404  Q13404  Q13404  Q13421  Q13421  Q13421  Q13421  Q13421  Q13421  Q13421  Q13421  Q13421  Q13421  Q13421  Q13421  Q13421  Q13421  Q13421  Q13421  Q13421  Q13421  Q13421  Q13421  Q13421  Q13421  Q13421  Q13421  Q13421  Q13421  Q13421  Q13421  Q13421  Q13421  Q13421  Q13422  Q13422  Q13422  Q13422  Q13422  Q13422  Q13422  Q13422  Q13422  Q13422  Q13422  Q13422  Q13422  Q13422  Q13422  Q13422  Q13422  Q13422  Q13422  Q13422  Q13422  Q13422  Q13422  Q13422  Q13422  Q13422  Q13422  Q13422  Q13422  Q13422  Q13422  Q13426  Q13426  Q13426  Q13426  Q13426  Q13426  Q13426  Q13426  Q13426  Q13426  Q13426  Q13426  Q13426  Q13426  Q13426  Q13426  Q13426  Q13426  Q13426  Q13426  Q13426  Q13426  Q13426  Q13426  Q13426  Q13426  Q13426  Q13444  Q13444  Q13444  Q13444  Q13444  Q13444  Q13444  Q13444  Q13444  Q13444  Q13444  Q13444  Q13444  Q13444  Q13444  Q13444  Q13444  Q13444  Q13444  Q13444  Q13444  Q13444  Q13444  Q13444  Q13444  Q13444  Q13444  Q13444  Q13444  Q13444  Q13444  Q13444  Q13444  Q13509  Q13509  Q13509  Q13509  Q13509  Q13509  Q13509  Q13509  Q13509  Q13509  Q13509  Q13509  Q13509  Q13509  Q13509  Q13509  Q13509  Q13509  Q13509  Q13509  Q13509  Q13509  Q13509  Q13509  Q13509  Q13509  Q13509  Q13509  Q13509  Q13509  Q13522  Q13522  Q13522  Q13522  Q13522  Q13522  Q13522  Q13522  Q13522  Q13522  Q13522  Q13522  Q13526  Q13526  Q13526  Q13526  Q13526  Q13526  Q13526  Q13526  Q13526  Q13526  Q13526  Q13526  Q13526  Q13526  Q13526  Q13526  Q13526  Q13526  Q13526  Q13526  Q13526  Q13535  Q13535  Q13535  Q13535  Q13535  Q13535  Q13535  Q13535  Q13535  Q13535  Q13535  Q13535  Q13535  Q13535  Q13535  Q13535  Q13535  Q13535  Q13535  Q13535  Q13535  Q13535  Q13535  Q13535  Q13535  Q13535  Q13535  Q13535  Q13535  Q13535  Q13535  Q13542  Q13542  Q13542  Q13542  Q13542  Q13546  Q13546  Q13546  Q13546  Q13546  Q13546  Q13546  Q13546  Q13546  Q13546  Q13546  Q13546  Q13546  Q13546  Q13546  Q13546  Q13546  Q13546  Q13546  Q13546  Q13546  Q13546  Q13546  Q13546  Q13546  Q13546  Q13546  Q13546  Q13546  Q13546  Q13546  Q13547  Q13547  Q13547  Q13547  Q13547  Q13547  Q13547  Q13547  Q13547  Q13547  Q13547  Q13547  Q13547  Q13547  Q13547  Q13547  Q13547  Q13547  Q13547  Q13547  Q13547  Q13547  Q13547  Q13547  Q13554  Q13554  Q13554  Q13554  Q13554  Q13554  Q13554  Q13554  Q13554  Q13554  Q13554  Q13554  Q13554  Q13554  Q13554  Q13554  Q13554  Q13554  Q13554  Q13554  Q13554  Q13554  Q13554  Q13554  Q13554  Q13554  Q13554  Q13554  Q13554  Q13554  Q13554  Q13554  Q13561  Q13561  Q13561  Q13561  Q13561  Q13561  Q13561  Q13561  Q13561  Q13561  Q13561  Q13561  Q13561  Q13561  Q13561  Q13561  Q13561  Q13561  Q13561  Q13561  Q13561  Q13561  Q13568  Q13568  Q13568  Q13568  Q13568  Q13568  Q13568  Q13568  Q13568  Q13568  Q13568  Q13568  Q13568  Q13568  Q13568  Q13568  Q13568  Q13568  Q13568  Q13568  Q13572  Q13572  Q13572  Q13572  Q13572  Q13572  Q13572  Q13572  Q13572  Q13572  Q13572  Q13572  Q13572  Q13572  Q13572  Q13572  Q13572  Q13572  Q13572  Q13572  Q13572  Q13572  Q13572  Q13572  Q13572  Q13572  Q13572  Q13572  Q13572  Q13572  Q13572  Q13573  Q13573  Q13573  Q13573  Q13573  Q13573  Q13573  Q13573  Q13573  Q13573  Q13573  Q13573  Q13573  Q13573  Q13573  Q13573  Q13573  Q13573  Q13573  Q13573  Q13573  Q13573  Q13573  Q13573  Q13573  Q13573  Q13573  Q13573  Q13573  Q13573  Q13573  Q13573  Q13601  Q13601  Q13601  Q13601  Q13601  Q13601  Q13601  Q13601  Q13601  Q13601  Q13601  Q13601  Q13601  Q13601  Q13601  Q13601  Q13601  Q13601  Q13601  Q13601  Q13601  Q13614  Q13614  Q13614  Q13614  Q13614  Q13614  Q13614  Q13614  Q13614  Q13614  Q13614  Q13614  Q13614  Q13614  Q13614  Q13614  Q13614  Q13614  Q13614  Q13614  Q13614  Q13614  Q13614  Q13614  Q13614  Q13614  Q13614  Q13614  Q13614  Q13614  Q13614  Q13627  Q13627  Q13627  Q13627  Q13627  Q13627  Q13627  Q13627  Q13627  Q13627  Q13627  Q13627  Q13627  Q13627  Q13627  Q13627  Q13627  Q13627  Q13627  Q13627  Q13627  Q13627  Q13627  Q13627  Q13627  Q13627  Q13627  Q13627  Q13627  Q13627  Q13627  Q13637  Q13637  Q13637  Q13637  Q13637  Q13637  Q13637  Q13637  Q13637  Q13637  Q13637  Q13637  Q13637  Q13637  Q13637  Q13671  Q13671  Q13671  Q13671  Q13671  Q13671  Q13671  Q13671  Q13671  Q13671  Q13671  Q13671  Q13671  Q13671  Q13671  Q13671  Q13671  Q13671  Q13671  Q13671  Q13671  Q13671  Q13671  Q13671  Q13671  Q13671  Q13671  Q13671  Q13671  Q13671  Q13671  Q13765  Q13765  Q13765  Q13765  Q13765  Q13765  Q13765  Q13765  Q13765  Q13765  Q13765  Q13765  Q13765  Q13769  Q13769  Q13769  Q13769  Q13769  Q13769  Q13769  Q13769  Q13769  Q13769  Q13769  Q13769  Q13769  Q13769  Q13769  Q13769  Q13769  Q13769  Q13769  Q13769  Q13769  Q13769  Q13769  Q13769  Q13769  Q13769  Q13769  Q13769  Q13769  Q13769  Q13769  Q13769  Q13769  Q13829  Q13829  Q13829  Q13829  Q13829  Q13829  Q13829  Q13829  Q13829  Q13829  Q13829  Q13829  Q13829  Q13829  Q13829  Q13829  Q13829  Q13829  Q13829  Q13829  Q13829  Q13882  Q13882  Q13882  Q13882  Q13882  Q13882  Q13882  Q13882  Q13882  Q13882  Q13882  Q13882  Q13882  Q13882  Q13882  Q13882  Q13882  Q13882  Q13882  Q13882  Q13882  Q13882  Q13882  Q13882  Q13882  Q13882  Q13882  Q13882  Q13882  Q13882  Q13884  Q13884  Q13884  Q13884  Q13884  Q13884  Q13884  Q13884  Q13884  Q13884  Q13884  Q13884  Q13884  Q13884  Q13884  Q13884  Q13884  Q13884  Q13884  Q13884  Q13884  Q13884  Q13884  Q13884  Q13884  Q13884  Q13884  Q13884  Q13884  Q13884  Q13884  Q13884  Q13884  Q13905  Q13905  Q13905  Q13905  Q13905  Q13905  Q13905  Q13905  Q13905  Q13905  Q13905  Q13905  Q13905  Q13905  Q13905  Q13905  Q13905  Q13905  Q13905  Q13905  Q13905  Q13905  Q13905  Q13905  Q13905  Q13905  Q13905  Q13905  Q13905  Q13905  Q13905  Q13905  Q13905  Q13950  Q13950  Q13950  Q13950  Q13950  Q13950  Q13950  Q13950  Q13950  Q13950  Q13950  Q13950  Q13950  Q13950  Q13950  Q13950  Q13950  Q13950  Q13950  Q13950  Q13950  Q13950  Q13950  Q13950  Q13950  Q13950  Q13950  Q13950  Q13950  Q13950  Q13950  Q14118  Q14118  Q14118  Q14118  Q14118  Q14118  Q14118  Q14118  Q14118  Q14118  Q14118  Q14118  Q14118  Q14118  Q14118  Q14118  Q14118  Q14118  Q14118  Q14118  Q14118  Q14118  Q14118  Q14118  Q14118  Q14118  Q14118  Q14118  Q14118  Q14118  Q14118  Q14118  Q14118  Q14141  Q14141  Q14141  Q14141  Q14141  Q14141  Q14141  Q14141  Q14141  Q14141  Q14141  Q14141  Q14141  Q14141  Q14141  Q14141  Q14141  Q14141  Q14141  Q14141  Q14141  Q14141  Q14141  Q14141  Q14152  Q14152  Q14152  Q14152  Q14152  Q14152  Q14152  Q14152  Q14152  Q14152  Q14152  Q14152  Q14152  Q14152  Q14152  Q14152  Q14152  Q14152  Q14152  Q14152  Q14152  Q14152  Q14152  Q14152  Q14152  Q14152  Q14152  Q14152  Q14152  Q14152  Q14152  Q14155  Q14155  Q14155  Q14155  Q14155  Q14155  Q14155  Q14155  Q14155  Q14155  Q14155  Q14155  Q14155  Q14155  Q14155  Q14155  Q14155  Q14155  Q14155  Q14155  Q14155  Q14155  Q14155  Q14155  Q14155  Q14155  Q14155  Q14155  Q14155  Q14155  Q14155  Q14164  Q14164  Q14164  Q14164  Q14164  Q14164  Q14164  Q14164  Q14164  Q14164  Q14164  Q14164  Q14164  Q14164  Q14164  Q14164  Q14164  Q14164  Q14164  Q14164  Q14164  Q14164  Q14164  Q14164  Q14164  Q14164  Q14164  Q14164  Q14164  Q14164  Q14164  Q14194  Q14194  Q14194  Q14194  Q14194  Q14194  Q14194  Q14194  Q14194  Q14194  Q14194  Q14194  Q14194  Q14194  Q14194  Q14194  Q14194  Q14194  Q14194  Q14194  Q14194  Q14194  Q14194  Q14194  Q14194  Q14194  Q14194  Q14194  Q14194  Q14194  Q14194  Q14194  Q14194  Q14207  Q14207  Q14207  Q14207  Q14207  Q14207  Q14207  Q14207  Q14207  Q14207  Q14207  Q14207  Q14207  Q14207  Q14207  Q14207  Q14207  Q14207  Q14207  Q14207  Q14207  Q14207  Q14207  Q14207  Q14207  Q14207  Q14207  Q14207  Q14207  Q14207  Q14207  Q14242  Q14242  Q14242  Q14242  Q14242  Q14242  Q14242  Q14242  Q14242  Q14242  Q14242  Q14242  Q14242  Q14242  Q14242  Q14242  Q14242  Q14242  Q14242  Q14242  Q14242  Q14242  Q14247  Q14247  Q14247  Q14247  Q14247  Q14247  Q14247  Q14247  Q14247  Q14247  Q14247  Q14247  Q14247  Q14247  Q14247  Q14247  Q14247  Q14247  Q14247  Q14247  Q14247  Q14247  Q14247  Q14247  Q14247  Q14247  Q14247  Q14247  Q14247  Q14247  Q14247  Q14247  Q14247  Q14289  Q14289  Q14289  Q14289  Q14289  Q14289  Q14289  Q14289  Q14289  Q14289  Q14289  Q14289  Q14289  Q14289  Q14289  Q14289  Q14289  Q14289  Q14289  Q14289  Q14289  Q14289  Q14289  Q14289  Q14289  Q14289  Q14289  Q14289  Q14289  Q14289  Q14289  Q14289  Q14289  Q14315  Q14315  Q14315  Q14315  Q14315  Q14315  Q14315  Q14315  Q14315  Q14315  Q14315  Q14315  Q14315  Q14315  Q14315  Q14315  Q14315  Q14315  Q14315  Q14315  Q14315  Q14315  Q14315  Q14315  Q14315  Q14315  Q14315  Q14315  Q14315  Q14315  Q14315  Q14344  Q14344  Q14344  Q14344  Q14344  Q14344  Q14344  Q14344  Q14344  Q14344  Q14344  Q14344  Q14344  Q14344  Q14344  Q14344  Q14344  Q14344  Q14344  Q14344  Q14344  Q14344  Q14344  Q14393  Q14393  Q14393  Q14393  Q14393  Q14393  Q14393  Q14393  Q14393  Q14393  Q14393  Q14393  Q14393  Q14393  Q14393  Q14393  Q14393  Q14393  Q14393  Q14393  Q14393  Q14393  Q14393  Q14393  Q14393  Q14393  Q14393  Q14393  Q14393  Q14393  Q14393  Q14457  Q14457  Q14457  Q14457  Q14457  Q14457  Q14457  Q14457  Q14457  Q14457  Q14457  Q14457  Q14457  Q14457  Q14457  Q14457  Q14457  Q14457  Q14457  Q14457  Q14457  Q14457  Q14493  Q14493  Q14493  Q14493  Q14493  Q14493  Q14493  Q14493  Q14493  Q14493  Q14493  Q14493  Q14493  Q14493  Q14493  Q14493  Q14493  Q14493  Q14493  Q14493  Q14493  Q14493  Q14493  Q14493  Q14493  Q14493  Q14493  Q14498  Q14498  Q14498  Q14498  Q14498  Q14498  Q14498  Q14498  Q14498  Q14498  Q14498  Q14498  Q14498  Q14498  Q14498  Q14498  Q14498  Q14498  Q14498  Q14498  Q14498  Q14498  Q14498  Q14498  Q14498  Q14498  Q14498  Q14498  Q14498  Q14498  Q14498  Q14498  Q14498  Q14511  Q14511  Q14511  Q14511  Q14511  Q14511  Q14511  Q14511  Q14511  Q14511  Q14511  Q14511  Q14511  Q14511  Q14511  Q14511  Q14511  Q14511  Q14511  Q14511  Q14511  Q14511  Q14511  Q14511  Q14511  Q14511  Q14511  Q14511  Q14511  Q14511  Q14511  Q14524  Q14524  Q14524  Q14524  Q14524  Q14524  Q14524  Q14524  Q14524  Q14524  Q14524  Q14524  Q14524  Q14524  Q14524  Q14524  Q14524  Q14524  Q14524  Q14524  Q14524  Q14524  Q14524  Q14524  Q14524  Q14524  Q14524  Q14524  Q14524  Q14524  Q14524  Q14526  Q14526  Q14526  Q14526  Q14526  Q14526  Q14526  Q14526  Q14526  Q14526  Q14526  Q14526  Q14526  Q14526  Q14526  Q14526  Q14526  Q14526  Q14526  Q14526  Q14526  Q14526  Q14526  Q14526  Q14526  Q14526  Q14526  Q14526  Q14526  Q14526  Q14526  Q14526  Q14526  Q14527  Q14527  Q14527  Q14527  Q14527  Q14527  Q14527  Q14527  Q14527  Q14527  Q14527  Q14527  Q14527  Q14527  Q14527  Q14527  Q14527  Q14527  Q14527  Q14527  Q14527  Q14527  Q14527  Q14527  Q14527  Q14527  Q14527  Q14527  Q14527  Q14527  Q14527  Q14527  Q14527  Q14653  Q14653  Q14653  Q14653  Q14653  Q14653  Q14653  Q14653  Q14653  Q14653  Q14653  Q14653  Q14653  Q14653  Q14653  Q14653  Q14653  Q14653  Q14653  Q14653  Q14653  Q14653  Q14653  Q14654  Q14654  Q14654  Q14654  Q14654  Q14654  Q14654  Q14654  Q14654  Q14654  Q14654  Q14654  Q14654  Q14654  Q14654  Q14654  Q14654  Q14654  Q14654  Q14654  Q14654  Q14654  Q14671  Q14671  Q14671  Q14671  Q14671  Q14671  Q14671  Q14671  Q14671  Q14671  Q14671  Q14671  Q14671  Q14671  Q14671  Q14671  Q14671  Q14671  Q14671  Q14671  Q14671  Q14671  Q14671  Q14671  Q14671  Q14671  Q14671  Q14671  Q14671  Q14671  Q14671  Q14674  Q14674  Q14674  Q14674  Q14674  Q14674  Q14674  Q14674  Q14674  Q14674  Q14674  Q14674  Q14674  Q14674  Q14674  Q14674  Q14674  Q14674  Q14674  Q14674  Q14674  Q14674  Q14674  Q14674  Q14674  Q14674  Q14674  Q14674  Q14674  Q14674  Q14674  Q14676  Q14676  Q14676  Q14676  Q14676  Q14676  Q14676  Q14676  Q14676  Q14676  Q14676  Q14676  Q14676  Q14676  Q14676  Q14676  Q14676  Q14676  Q14676  Q14676  Q14676  Q14676  Q14676  Q14676  Q14676  Q14676  Q14676  Q14676  Q14676  Q14676  Q14676  Q14676  Q14676  Q14678  Q14678  Q14678  Q14678  Q14678  Q14678  Q14678  Q14678  Q14678  Q14678  Q14678  Q14678  Q14678  Q14678  Q14678  Q14678  Q14678  Q14678  Q14678  Q14678  Q14678  Q14678  Q14678  Q14678  Q14678  Q14678  Q14678  Q14678  Q14678  Q14678  Q14678  Q14680  Q14680  Q14680  Q14680  Q14680  Q14680  Q14680  Q14680  Q14680  Q14680  Q14680  Q14680  Q14680  Q14680  Q14680  Q14680  Q14680  Q14680  Q14680  Q14680  Q14680  Q14680  Q14680  Q14680  Q14680  Q14680  Q14680  Q14681  Q14681  Q14681  Q14681  Q14681  Q14681  Q14681  Q14681  Q14681  Q14681  Q14681  Q14681  Q14681  Q14681  Q14683  Q14683  Q14683  Q14683  Q14683  Q14683  Q14683  Q14683  Q14683  Q14683  Q14683  Q14683  Q14683  Q14683  Q14683  Q14683  Q14683  Q14683  Q14683  Q14683  Q14683  Q14683  Q14683  Q14683  Q14683  Q14683  Q14683  Q14683  Q14683  Q14683  Q14683  Q14686  Q14686  Q14686  Q14686  Q14686  Q14686  Q14686  Q14686  Q14686  Q14686  Q14686  Q14686  Q14686  Q14686  Q14686  Q14686  Q14686  Q14686  Q14686  Q14686  Q14686  Q14686  Q14686  Q14686  Q14686  Q14686  Q14686  Q14686  Q14686  Q14686  Q14686  Q14690  Q14690  Q14690  Q14690  Q14690  Q14690  Q14690  Q14690  Q14690  Q14690  Q14690  Q14690  Q14690  Q14690  Q14690  Q14690  Q14690  Q14690  Q14690  Q14690  Q14690  Q14690  Q14690  Q14690  Q14690  Q14690  Q14690  Q14690  Q14690  Q14690  Q14690  Q14690  Q14690  Q14694  Q14694  Q14694  Q14694  Q14694  Q14694  Q14694  Q14694  Q14694  Q14694  Q14694  Q14694  Q14694  Q14694  Q14694  Q14694  Q14694  Q14694  Q14694  Q14694  Q14694  Q14694  Q14694  Q14694  Q14694  Q14694  Q14694  Q14694  Q14694  Q14694  Q14694  Q14703  Q14703  Q14703  Q14703  Q14703  Q14703  Q14703  Q14703  Q14703  Q14703  Q14703  Q14703  Q14703  Q14703  Q14703  Q14703  Q14703  Q14703  Q14703  Q14703  Q14703  Q14703  Q14703  Q14703  Q14703  Q14703  Q14703  Q14703  Q14703  Q14703  Q14703  Q14739  Q14739  Q14739  Q14739  Q14739  Q14739  Q14739  Q14739  Q14739  Q14739  Q14739  Q14739  Q14739  Q14739  Q14739  Q14739  Q14739  Q14739  Q14739  Q14739  Q14739  Q14739  Q14739  Q14739  Q14739  Q14739  Q14739  Q14739  Q14739  Q14739  Q14739  Q14764  Q14764  Q14764  Q14764  Q14764  Q14764  Q14764  Q14764  Q14764  Q14764  Q14764  Q14764  Q14764  Q14764  Q14764  Q14764  Q14764  Q14764  Q14764  Q14764  Q14764  Q14764  Q14764  Q14764  Q14764  Q14764  Q14764  Q14764  Q14764  Q14764  Q14764  Q14764  Q14764  Q14766  Q14766  Q14766  Q14766  Q14766  Q14766  Q14766  Q14766  Q14766  Q14766  Q14766  Q14766  Q14766  Q14766  Q14766  Q14766  Q14766  Q14766  Q14766  Q14766  Q14766  Q14766  Q14766  Q14766  Q14766  Q14766  Q14766  Q14766  Q14766  Q14766  Q14766  Q14790  Q14790  Q14790  Q14790  Q14790  Q14790  Q14790  Q14790  Q14790  Q14790  Q14790  Q14790  Q14790  Q14790  Q14790  Q14790  Q14790  Q14790  Q14790  Q14790  Q14790  Q14790  Q14790  Q14790  Q14790  Q14790  Q14790  Q14790  Q14790  Q14790  Q14790  Q14814  Q14814  Q14814  Q14814  Q14814  Q14814  Q14814  Q14814  Q14814  Q14814  Q14814  Q14814  Q14814  Q14814  Q14814  Q14814  Q14814  Q14814  Q14814  Q14814  Q14814  Q14814  Q14814  Q14814  Q14814  Q14814  Q14814  Q14814  Q14814  Q14814  Q14847  Q14847  Q14847  Q14847  Q14847  Q14847  Q14847  Q14847  Q14847  Q14847  Q14847  Q14847  Q14847  Q14847  Q14847  Q14847  Q14865  Q14865  Q14865  Q14865  Q14865  Q14865  Q14865  Q14865  Q14865  Q14865  Q14865  Q14865  Q14865  Q14865  Q14865  Q14865  Q14865  Q14865  Q14865  Q14865  Q14865  Q14865  Q14865  Q14865  Q14865  Q14865  Q14865  Q14865  Q14865  Q14865  Q14865  Q14865  Q14865  Q14934  Q14934  Q14934  Q14934  Q14934  Q14934  Q14934  Q14934  Q14934  Q14934  Q14934  Q14934  Q14934  Q14934  Q14934  Q14934  Q14934  Q14934  Q14934  Q14934  Q14934  Q14934  Q14934  Q14934  Q14934  Q14934  Q14934  Q14934  Q14934  Q14934  Q14934  Q14974  Q14974  Q14974  Q14974  Q14974  Q14974  Q14974  Q14974  Q14974  Q14974  Q14974  Q14974  Q14974  Q14974  Q14974  Q14974  Q14974  Q14974  Q14974  Q14974  Q14974  Q14974  Q14974  Q14974  Q14974  Q14974  Q14974  Q14974  Q14974  Q14974  Q14974  Q14974  Q14974  Q14980  Q14980  Q14980  Q14980  Q14980  Q14980  Q14980  Q14980  Q14980  Q14980  Q14980  Q14980  Q14980  Q14980  Q14980  Q14980  Q14980  Q14980  Q14980  Q14980  Q14980  Q14980  Q14980  Q14980  Q14980  Q14980  Q14980  Q14980  Q14980  Q14980  Q14980  Q14994  Q14994  Q14994  Q14994  Q14994  Q14994  Q14994  Q14994  Q14994  Q14994  Q14994  Q14994  Q14994  Q15004  Q15004  Q15004  Q15004  Q15004  Q15004  Q15004  Q15004  Q15004  Q15004  Q15005  Q15005  Q15005  Q15005  Q15005  Q15005  Q15005  Q15005  Q15005  Q15005  Q15005  Q15005  Q15005  Q15005  Q15005  Q15005  Q15005  Q15005  Q15005  Q15005  Q15005  Q15005  Q15007  Q15007  Q15007  Q15007  Q15007  Q15007  Q15007  Q15007  Q15007  Q15007  Q15007  Q15007  Q15007  Q15007  Q15007  Q15007  Q15007  Q15007  Q15007  Q15007  Q15007  Q15007  Q15007  Q15007  Q15007  Q15007  Q15007  Q15007  Q15007  Q15007  Q15007  Q15007  Q15007  Q15020  Q15020  Q15020  Q15020  Q15020  Q15020  Q15020  Q15020  Q15020  Q15020  Q15020  Q15020  Q15020  Q15020  Q15020  Q15020  Q15020  Q15020  Q15020  Q15020  Q15020  Q15020  Q15020  Q15020  Q15020  Q15020  Q15020  Q15020  Q15020  Q15020  Q15020  Q15020  Q15020  Q15024  Q15024  Q15024  Q15024  Q15024  Q15024  Q15024  Q15024  Q15024  Q15024  Q15024  Q15024  Q15024  Q15024  Q15024  Q15024  Q15024  Q15024  Q15024  Q15024  Q15024  Q15025  Q15025  Q15025  Q15025  Q15025  Q15025  Q15025  Q15025  Q15025  Q15025  Q15025  Q15025  Q15025  Q15025  Q15025  Q15025  Q15025  Q15025  Q15025  Q15025  Q15025  Q15025  Q15025  Q15025  Q15025  Q15025  Q15025  Q15025  Q15025  Q15025  Q15025  Q15025  Q15025  Q15027  Q15027  Q15027  Q15027  Q15027  Q15027  Q15027  Q15027  Q15027  Q15027  Q15027  Q15027  Q15027  Q15027  Q15027  Q15027  Q15027  Q15027  Q15027  Q15027  Q15027  Q15027  Q15027  Q15027  Q15027  Q15027  Q15027  Q15027  Q15027  Q15027  Q15027  Q15046  Q15046  Q15046  Q15046  Q15046  Q15046  Q15046  Q15046  Q15046  Q15046  Q15046  Q15046  Q15046  Q15046  Q15046  Q15046  Q15046  Q15046  Q15046  Q15046  Q15050  Q15050  Q15050  Q15050  Q15050  Q15050  Q15050  Q15050  Q15050  Q15050  Q15050  Q15054  Q15054  Q15054  Q15054  Q15054  Q15054  Q15054  Q15054  Q15054  Q15054  Q15054  Q15054  Q15054  Q15054  Q15054  Q15054  Q15054  Q15054  Q15054  Q15054  Q15054  Q15054  Q15054  Q15054  Q15054  Q15054  Q15054  Q15054  Q15054  Q15054  Q15054  Q15054  Q15054  Q15056  Q15056  Q15056  Q15056  Q15056  Q15056  Q15056  Q15056  Q15056  Q15056  Q15056  Q15056  Q15056  Q15056  Q15056  Q15056  Q15056  Q15056  Q15056  Q15078  Q15078  Q15078  Q15078  Q15078  Q15078  Q15078  Q15078  Q15078  Q15078  Q15078  Q15078  Q15078  Q15078  Q15078  Q15078  Q15078  Q15078  Q15078  Q15078  Q15078  Q15078  Q15078  Q15078  Q15078  Q15078  Q15078  Q15078  Q15078  Q15078  Q15078  Q15080  Q15080  Q15080  Q15080  Q15080  Q15080  Q15080  Q15080  Q15080  Q15080  Q15080  Q15080  Q15080  Q15080  Q15080  Q15080  Q15080  Q15080  Q15080  Q15080  Q15080  Q15080  Q15084  Q15084  Q15084  Q15084  Q15084  Q15084  Q15084  Q15084  Q15084  Q15084  Q15084  Q15084  Q15084  Q15084  Q15084  Q15084  Q15084  Q15084  Q15084  Q15084  Q15084  Q15084  Q15084  Q15084  Q15084  Q15084  Q15084  Q15084  Q15084  Q15118  Q15118  Q15118  Q15118  Q15118  Q15118  Q15118  Q15118  Q15118  Q15118  Q15118  Q15118  Q15118  Q15118  Q15118  Q15118  Q15118  Q15118  Q15118  Q15118  Q15118  Q15118  Q15118  Q15118  Q15118  Q15118  Q15118  Q15118  Q15118  Q15118  Q15118  Q15118  Q15118  Q15139  Q15139  Q15139  Q15139  Q15139  Q15139  Q15139  Q15139  Q15139  Q15139  Q15139  Q15139  Q15139  Q15139  Q15139  Q15139  Q15139  Q15139  Q15139  Q15139  Q15139  Q15139  Q15139  Q15139  Q15139  Q15139  Q15139  Q15139  Q15139  Q15139  Q15139  Q15147  Q15147  Q15147  Q15147  Q15147  Q15147  Q15147  Q15147  Q15147  Q15147  Q15147  Q15147  Q15147  Q15147  Q15147  Q15147  Q15147  Q15147  Q15147  Q15147  Q15147  Q15147  Q15147  Q15147  Q15147  Q15147  Q15147  Q15147  Q15147  Q15147  Q15147  Q15147  Q15147  Q15149  Q15149  Q15149  Q15149  Q15149  Q15149  Q15149  Q15149  Q15149  Q15149  Q15149  Q15149  Q15149  Q15149  Q15149  Q15149  Q15149  Q15149  Q15149  Q15149  Q15149  Q15149  Q15149  Q15149  Q15149  Q15149  Q15149  Q15149  Q15149  Q15149  Q15149  Q15170  Q15170  Q15170  Q15170  Q15170  Q15170  Q15170  Q15170  Q15170  Q15170  Q15170  Q15170  Q15170  Q15185  Q15185  Q15185  Q15185  Q15185  Q15185  Q15185  Q15185  Q15185  Q15208  Q15208  Q15208  Q15208  Q15208  Q15208  Q15208  Q15208  Q15208  Q15208  Q15208  Q15208  Q15208  Q15208  Q15208  Q15208  Q15208  Q15208  Q15208  Q15208  Q15208  Q15208  Q15208  Q15287  Q15287  Q15287  Q15287  Q15287  Q15287  Q15287  Q15287  Q15287  Q15287  Q15287  Q15287  Q15287  Q15287  Q15287  Q15287  Q15287  Q15287  Q15287  Q15287  Q15287  Q15287  Q15287  Q15287  Q15287  Q15287  Q15287  Q15287  Q15287  Q15287  Q15287  Q15291  Q15291  Q15291  Q15291  Q15291  Q15291  Q15291  Q15291  Q15291  Q15291  Q15291  Q15291  Q15291  Q15291  Q15291  Q15291  Q15291  Q15291  Q15291  Q15291  Q15291  Q15291  Q15291  Q15291  Q15291  Q15291  Q15291  Q15291  Q15291  Q15291  Q15291  Q15293  Q15293  Q15293  Q15293  Q15293  Q15293  Q15293  Q15293  Q15293  Q15303  Q15303  Q15303  Q15303  Q15303  Q15303  Q15303  Q15303  Q15303  Q15303  Q15303  Q15303  Q15303  Q15303  Q15303  Q15303  Q15303  Q15303  Q15303  Q15303  Q15303  Q15303  Q15303  Q15303  Q15303  Q15303  Q15303  Q15303  Q15303  Q15303  Q15303  Q15303  Q15303  Q15334  Q15334  Q15334  Q15334  Q15334  Q15334  Q15334  Q15334  Q15334  Q15334  Q15334  Q15334  Q15334  Q15334  Q15334  Q15334  Q15334  Q15334  Q15334  Q15334  Q15334  Q15334  Q15334  Q15334  Q15334  Q15334  Q15334  Q15334  Q15334  Q15334  Q15334  Q15370  Q15370  Q15370  Q15370  Q15370  Q15370  Q15370  Q15398  Q15398  Q15398  Q15398  Q15398  Q15398  Q15398  Q15398  Q15398  Q15398  Q15398  Q15398  Q15398  Q15398  Q15398  Q15398  Q15398  Q15398  Q15398  Q15398  Q15398  Q15398  Q15398  Q15398  Q15398  Q15398  Q15398  Q15398  Q15398  Q15398  Q15398  Q15424  Q15424  Q15424  Q15424  Q15424  Q15424  Q15424  Q15424  Q15424  Q15424  Q15424  Q15424  Q15424  Q15424  Q15424  Q15424  Q15424  Q15424  Q15424  Q15424  Q15424  Q15424  Q15424  Q15424  Q15424  Q15424  Q15424  Q15424  Q15424  Q15424  Q15424  Q15424  Q15424  Q15427  Q15427  Q15427  Q15427  Q15427  Q15427  Q15427  Q15427  Q15427  Q15427  Q15427  Q15427  Q15427  Q15427  Q15427  Q15427  Q15427  Q15427  Q15427  Q15427  Q15427  Q15428  Q15428  Q15428  Q15428  Q15428  Q15428  Q15428  Q15428  Q15428  Q15428  Q15428  Q15428  Q15428  Q15428  Q15428  Q15428  Q15428  Q15428  Q15428  Q15428  Q15485  Q15485  Q15485  Q15485  Q15485  Q15485  Q15485  Q15485  Q15485  Q15485  Q15485  Q15485  Q15485  Q15485  Q15485  Q15545  Q15545  Q15545  Q15545  Q15545  Q15545  Q15545  Q15545  Q15545  Q15545  Q15545  Q15545  Q15545  Q15545  Q15545  Q15545  Q15545  Q15545  Q15545  Q15554  Q15554  Q15554  Q15554  Q15554  Q15554  Q15554  Q15554  Q15554  Q15554  Q15554  Q15554  Q15554  Q15554  Q15554  Q15554  Q15554  Q15554  Q15554  Q15554  Q15554  Q15554  Q15554  Q15554  Q15554  Q15554  Q15554  Q15554  Q15554  Q15554  Q15554  Q15554  Q15554  Q15637  Q15637  Q15637  Q15637  Q15637  Q15637  Q15637  Q15637  Q15637  Q15637  Q15637  Q15637  Q15637  Q15637  Q15637  Q15637  Q15637  Q15637  Q15637  Q15637  Q15637  Q15637  Q15637  Q15637  Q15637  Q15637  Q15637  Q15637  Q15637  Q15637  Q15637  Q15648  Q15648  Q15648  Q15648  Q15648  Q15648  Q15648  Q15648  Q15648  Q15648  Q15648  Q15648  Q15648  Q15648  Q15648  Q15648  Q15648  Q15648  Q15648  Q15648  Q15648  Q15648  Q15648  Q15648  Q15648  Q15648  Q15648  Q15648  Q15648  Q15648  Q15648  Q15648  Q15648  Q15653  Q15653  Q15653  Q15653  Q15653  Q15653  Q15653  Q15653  Q15653  Q15653  Q15653  Q15653  Q15653  Q15653  Q15653  Q15654  Q15654  Q15654  Q15654  Q15654  Q15654  Q15654  Q15654  Q15654  Q15654  Q15654  Q15654  Q15654  Q15654  Q15654  Q15654  Q15654  Q15654  Q15654  Q15654  Q15654  Q15654  Q15654  Q15654  Q15654  Q15691  Q15691  Q15691  Q15691  Q15691  Q15691  Q15691  Q15691  Q15691  Q15691  Q15691  Q15717  Q15717  Q15717  Q15717  Q15717  Q15717  Q15717  Q15717  Q15717  Q15717  Q15717  Q15717  Q15717  Q15717  Q15717  Q15717  Q15717  Q15717  Q15717  Q15717  Q15717  Q15717  Q15717  Q15717  Q15717  Q15726  Q15726  Q15726  Q15726  Q15726  Q15726  Q15726  Q15726  Q15726  Q15726  Q15726  Q15726  Q15726  Q15746  Q15746  Q15746  Q15746  Q15746  Q15746  Q15746  Q15746  Q15746  Q15746  Q15746  Q15746  Q15746  Q15746  Q15746  Q15746  Q15746  Q15746  Q15746  Q15746  Q15746  Q15746  Q15746  Q15746  Q15746  Q15746  Q15746  Q15746  Q15746  Q15746  Q15746  Q15746  Q15746  Q15750  Q15750  Q15750  Q15750  Q15750  Q15750  Q15750  Q15750  Q15750  Q15750  Q15750  Q15750  Q15750  Q15750  Q15750  Q15750  Q15750  Q15750  Q15750  Q15750  Q15750  Q15750  Q15750  Q15750  Q15750  Q15750  Q15750  Q15750  Q15750  Q15750  Q15750  Q15788  Q15788  Q15788  Q15788  Q15788  Q15788  Q15788  Q15788  Q15788  Q15788  Q15788  Q15788  Q15788  Q15788  Q15788  Q15788  Q15788  Q15788  Q15788  Q15788  Q15788  Q15788  Q15788  Q15788  Q15788  Q15788  Q15788  Q15788  Q15788  Q15788  Q15788  Q15796  Q15796  Q15796  Q15796  Q15796  Q15796  Q15796  Q15796  Q15796  Q15796  Q15796  Q15796  Q15796  Q15796  Q15796  Q15796  Q15796  Q15796  Q15796  Q15796  Q15796  Q15796  Q15796  Q15796  Q15796  Q15796  Q15796  Q15796  Q15796  Q15831  Q15831  Q15831  Q15831  Q15831  Q15831  Q15831  Q15831  Q15831  Q15831  Q15831  Q15831  Q15831  Q15831  Q15831  Q15831  Q15831  Q15831  Q15831  Q15831  Q15831  Q15835  Q15835  Q15835  Q15835  Q15835  Q15835  Q15835  Q15835  Q15835  Q15835  Q15835  Q15835  Q15835  Q15835  Q15835  Q15835  Q15835  Q15835  Q15835  Q15835  Q15835  Q15835  Q15835  Q15835  Q15835  Q15835  Q15835  Q15835  Q15835  Q15835  Q15848  Q15848  Q15848  Q15848  Q15848  Q15848  Q15848  Q15907  Q15907  Q15907  Q15907  Q15907  Q15907  Q15907  Q15907  Q15907  Q15907  Q15907  Q15907  Q15907  Q15907  Q15907  Q15910  Q15910  Q15910  Q15910  Q15910  Q15910  Q15910  Q15910  Q15910  Q15910  Q15910  Q15910  Q15910  Q15910  Q15910  Q15910  Q15910  Q15910  Q15910  Q15910  Q15910  Q15910  Q15910  Q15910  Q15910  Q15910  Q15910  Q15910  Q15910  Q15910  Q15910  Q15942  Q15942  Q15942  Q15942  Q15942  Q15942  Q15942  Q15942  Q15942  Q15942  Q15942  Q15942  Q15942  Q15942  Q15942  Q15942  Q15942  Q15942  Q15942  Q15942  Q15942  Q15942  Q15942  Q15942  Q15942  Q15942  Q15942  Q15942  Q15942  Q15942  Q15942  Q15942  Q15942  Q16236  Q16236  Q16236  Q16236  Q16236  Q16236  Q16236  Q16236  Q16236  Q16236  Q16236  Q16236  Q16236  Q16236  Q16236  Q16236  Q16236  Q16236  Q16236  Q16236  Q16236  Q16236  Q16236  Q16236  Q16236  Q16236  Q16236  Q16236  Q16236  Q16236  Q16236  Q16270  Q16270  Q16270  Q16270  Q16270  Q16270  Q16270  Q16270  Q16270  Q16270  Q16270  Q16270  Q16270  Q16270  Q16270  Q16270  Q16270  Q16270  Q16270  Q16270  Q16270  Q16270  Q16401  Q16401  Q16401  Q16401  Q16401  Q16401  Q16401  Q16401  Q16401  Q16401  Q16401  Q16401  Q16401  Q16401  Q16401  Q16401  Q16401  Q16401  Q16401  Q16401  Q16401  Q16401  Q16401  Q16401  Q16401  Q16401  Q16401  Q16401  Q16401  Q16401  Q16401  Q16401  Q16401  Q16512  Q16512  Q16512  Q16512  Q16512  Q16512  Q16512  Q16512  Q16512  Q16512  Q16512  Q16512  Q16512  Q16512  Q16512  Q16512  Q16512  Q16512  Q16512  Q16512  Q16512  Q16512  Q16512  Q16512  Q16512  Q16512  Q16512  Q16512  Q16512  Q16512  Q16512  Q16512  Q16512  Q16513  Q16513  Q16513  Q16513  Q16513  Q16513  Q16513  Q16513  Q16513  Q16513  Q16513  Q16513  Q16513  Q16513  Q16513  Q16513  Q16513  Q16513  Q16513  Q16513  Q16513  Q16513  Q16513  Q16513  Q16513  Q16513  Q16513  Q16513  Q16513  Q16513  Q16513  Q16518  Q16518  Q16518  Q16518  Q16518  Q16518  Q16518  Q16518  Q16518  Q16518  Q16518  Q16518  Q16518  Q16518  Q16518  Q16518  Q16518  Q16518  Q16518  Q16518  Q16518  Q16518  Q16518  Q16518  Q16518  Q16566  Q16566  Q16566  Q16566  Q16566  Q16566  Q16566  Q16566  Q16566  Q16566  Q16566  Q16566  Q16566  Q16566  Q16566  Q16566  Q16566  Q16566  Q16566  Q16566  Q16566  Q16566  Q16566  Q16566  Q16566  Q16566  Q16566  Q16581  Q16581  Q16581  Q16581  Q16581  Q16581  Q16581  Q16581  Q16581  Q16581  Q16581  Q16581  Q16581  Q16581  Q16581  Q16581  Q16581  Q16581  Q16581  Q16581  Q16581  Q16581  Q16581  Q16581  Q16581  Q16581  Q16581  Q16581  Q16581  Q16581  Q16581  Q16581  Q16581  Q16584  Q16584  Q16584  Q16584  Q16584  Q16584  Q16584  Q16584  Q16584  Q16584  Q16584  Q16584  Q16584  Q16584  Q16584  Q16584  Q16584  Q16584  Q16584  Q16584  Q16584  Q16584  Q16584  Q16584  Q16584  Q16584  Q16584  Q16584  Q16584  Q16584  Q16584  Q16623  Q16623  Q16623  Q16623  Q16623  Q16623  Q16623  Q16623  Q16623  Q16623  Q16623  Q16623  Q16623  Q16623  Q16623  Q16623  Q16623  Q16623  Q16623  Q16623  Q16623  Q16623  Q16623  Q16625  Q16625  Q16625  Q16625  Q16625  Q16625  Q16625  Q16625  Q16625  Q16625  Q16625  Q16625  Q16625  Q16625  Q16625  Q16625  Q16625  Q16625  Q16625  Q16625  Q16625  Q16625  Q16625  Q16625  Q16625  Q16625  Q16625  Q16625  Q16625  Q16625  Q16625  Q16637  Q16637  Q16637  Q16637  Q16637  Q16637  Q16637  Q16637  Q16637  Q16637  Q16637  Q16637  Q16637  Q16637  Q16637  Q16637  Q16637  Q16637  Q16637  Q16637  Q16637  Q16637  Q16637  Q16643  Q16643  Q16643  Q16643  Q16643  Q16643  Q16643  Q16643  Q16643  Q16643  Q16643  Q16643  Q16643  Q16643  Q16643  Q16643  Q16643  Q16643  Q16643  Q16643  Q16643  Q16643  Q16643  Q16643  Q16643  Q16643  Q16643  Q16643  Q16643  Q16643  Q16643  Q16655  Q16655  Q16655  Q16655  Q16656  Q16656  Q16656  Q16656  Q16656  Q16656  Q16656  Q16656  Q16656  Q16656  Q16656  Q16656  Q16656  Q16656  Q16656  Q16656  Q16656  Q16656  Q16656  Q16656  Q16656  Q16656  Q16656  Q16656  Q16656  Q16656  Q16656  Q16656  Q16656  Q16656  Q16658  Q16658  Q16658  Q16658  Q16658  Q16658  Q16658  Q16658  Q16658  Q16658  Q16658  Q16658  Q16658  Q16658  Q16658  Q16658  Q16658  Q16658  Q16658  Q16658  Q16658  Q16658  Q16658  Q16658  Q16658  Q16658  Q16658  Q16658  Q16658  Q16658  Q16658  Q16659  Q16659  Q16659  Q16659  Q16659  Q16659  Q16659  Q16659  Q16659  Q16659  Q16659  Q16659  Q16659  Q16659  Q16659  Q16659  Q16659  Q16659  Q16659  Q16659  Q16659  Q16659  Q16659  Q16659  Q16659  Q16659  Q16659  Q16659  Q16659  Q16659  Q16659  Q16665  Q16665  Q16665  Q16665  Q16665  Q16665  Q16665  Q16665  Q16665  Q16665  Q16665  Q16665  Q16665  Q16665  Q16665  Q16665  Q16665  Q16665  Q16665  Q16665  Q16665  Q16665  Q16665  Q16665  Q16665  Q16665  Q16665  Q16665  Q16665  Q16665  Q16665  Q16666  Q16666  Q16666  Q16666  Q16666  Q16666  Q16666  Q16666  Q16666  Q16666  Q16666  Q16666  Q16666  Q16666  Q16666  Q16666  Q16666  Q16666  Q16666  Q16666  Q16666  Q16666  Q16666  Q16666  Q16666  Q16666  Q16666  Q16666  Q16666  Q16695  Q16695  Q16719  Q16719  Q16719  Q16719  Q16719  Q16719  Q16719  Q16719  Q16719  Q16719  Q16719  Q16719  Q16719  Q16719  Q16719  Q16719  Q16719  Q16719  Q16719  Q16773  Q16773  Q16773  Q16773  Q16773  Q16773  Q16773  Q16773  Q16773  Q16773  Q16773  Q16773  Q16773  Q16773  Q16773  Q16773  Q16773  Q16773  Q16773  Q16773  Q16773  Q16773  Q16773  Q16773  Q16773  Q16790  Q16790  Q16790  Q16790  Q16790  Q16790  Q16790  Q16790  Q16790  Q16790  Q16790  Q16790  Q16790  Q16790  Q16790  Q16790  Q16790  Q16790  Q16790  Q16790  Q16790  Q16790  Q16790  Q16790  Q16790  Q16790  Q16790  Q16790  Q16790  Q16790  Q16832  Q16832  Q16832  Q16832  Q16832  Q16832  Q16832  Q16832  Q16832  Q16832  Q16832  Q16832  Q16832  Q16832  Q16832  Q16832  Q16832  Q16832  Q16832  Q16832  Q16832  Q16832  Q16832  Q16832  Q16832  Q16832  Q16832  Q16832  Q16832  Q16832  Q16832  Q16832  Q16832  Q16853  Q16853  Q16853  Q16853  Q16853  Q16853  Q16853  Q16853  Q16853  Q16853  Q16853  Q16853  Q16853  Q16853  Q16853  Q16853  Q16853  Q16853  Q16853  Q16853  Q16853  Q16853  Q16853  Q16853  Q16853  Q16853  Q16853  Q16853  Q16853  Q16853  Q16853  Q16853  Q16853  Q16875  Q16875  Q16875  Q16875  Q16875  Q16875  Q16875  Q16875  Q16875  Q16875  Q16875  Q16875  Q16875  Q16875  Q16875  Q16875  Q16875  Q16875  Q16875  Q16875  Q16875  Q16875  Q16875  Q16875  Q16875  Q16875  Q16875  Q16875  Q16875  Q16875  Q16875  Q1KMD3  Q1KMD3  Q1KMD3  Q1KMD3  Q1KMD3  Q1KMD3  Q1KMD3  Q1KMD3  Q1KMD3  Q1KMD3  Q1KMD3  Q1KMD3  Q1KMD3  Q1KMD3  Q1KMD3  Q1KMD3  Q1KMD3  Q1KMD3  Q1KMD3  Q1KMD3  Q1KMD3  Q1KMD3  Q1KMD3  Q1KMD3  Q1KMD3  Q1KMD3  Q1KMD3  Q1KMD3  Q1KMD3  Q1KMD3  Q1KMD3  Q24JP5  Q24JP5  Q24JP5  Q24JP5  Q24JP5  Q24JP5  Q24JP5  Q24JP5  Q24JP5  Q24JP5  Q24JP5  Q24JP5  Q24JP5  Q24JP5  Q24JP5  Q24JP5  Q24JP5  Q24JP5  Q24JP5  Q24JP5  Q24JP5  Q24JP5  Q24JP5  Q24JP5  Q24JP5  Q24JP5  Q24JP5  Q24JP5  Q24JP5  Q24JP5  Q24JP5  Q2HXU8  Q2HXU8  Q2HXU8  Q2HXU8  Q2HXU8  Q2HXU8  Q2HXU8  Q2HXU8  Q2HXU8  Q2HXU8  Q2HXU8  Q2HXU8  Q2HXU8  Q2HXU8  Q2HXU8  Q2HXU8  Q2HXU8  Q2HXU8  Q2HXU8  Q2HXU8  Q2HXU8  Q2HXU8  Q2HXU8  Q2HXU8  Q2HXU8  Q2HXU8  Q2HXU8  Q2HXU8  Q2HXU8  Q2HXU8  Q2M2Z5  Q2M2Z5  Q2M2Z5  Q2M2Z5  Q2M2Z5  Q2M2Z5  Q2M2Z5  Q2M2Z5  Q2M2Z5  Q2M2Z5  Q2M2Z5  Q2M2Z5  Q2M2Z5  Q2M2Z5  Q2M2Z5  Q2M2Z5  Q2M2Z5  Q2M2Z5  Q2M2Z5  Q2M2Z5  Q2M2Z5  Q2M2Z5  Q2M2Z5  Q2M2Z5  Q2M2Z5  Q2M2Z5  Q2M2Z5  Q2M2Z5  Q2M2Z5  Q2M2Z5  Q2M2Z5  Q2M2Z5  Q2M2Z5  Q2NKX8  Q2NKX8  Q2NKX8  Q2NKX8  Q2NKX8  Q2NKX8  Q2NKX8  Q2NKX8  Q2NKX8  Q2NKX8  Q2NKX8  Q2NKX8  Q2NKX8  Q2NKX8  Q2NKX8  Q2NKX8  Q2NKX8  Q2NKX8  Q2NKX8  Q2NKX8  Q2NKX8  Q2NKX8  Q2NKX8  Q2NKX8  Q2NKX8  Q2NKX8  Q2NKX8  Q2NKX8  Q2NKX8  Q2NKX8  Q2NKX8  Q2NKX8  Q2NKX8  Q2PPJ7  Q2PPJ7  Q2PPJ7  Q2PPJ7  Q2PPJ7  Q2PPJ7  Q2PPJ7  Q2PPJ7  Q2PPJ7  Q2PPJ7  Q2PPJ7  Q2PPJ7  Q2PPJ7  Q2PPJ7  Q2PPJ7  Q2PPJ7  Q2PPJ7  Q2PPJ7  Q2PPJ7  Q2PPJ7  Q2PPJ7  Q2PPJ7  Q2PPJ7  Q2PPJ7  Q2PPJ7  Q2PPJ7  Q2PPJ7  Q2PPJ7  Q2PPJ7  Q2PPJ7  Q2PPJ7  Q2TAY7  Q2TAY7  Q2TAY7  Q2TAY7  Q2TAY7  Q2TAY7  Q2TAY7  Q2TAY7  Q2TAY7  Q2TAY7  Q2TAY7  Q2TAY7  Q2TAY7  Q2TAY7  Q2TAY7  Q2TAY7  Q2TAY7  Q2TAY7  Q2TAY7  Q2TAY7  Q2TAY7  Q2TAY7  Q2TAY7  Q2TAY7  Q2TAY7  Q2TAY7  Q2TAY7  Q2TAY7  Q2TAY7  Q2TAY7  Q2TAY7  Q2TAY7  Q2TAY7  Q2V2M9  Q2V2M9  Q2V2M9  Q2V2M9  Q2V2M9  Q2V2M9  Q2V2M9  Q2V2M9  Q2V2M9  Q2V2M9  Q2V2M9  Q2V2M9  Q2V2M9  Q2V2M9  Q2V2M9  Q2V2M9  Q2V2M9  Q2V2M9  Q2V2M9  Q2V2M9  Q2V2M9  Q2V2M9  Q2V2M9  Q2V2M9  Q2V2M9  Q2V2M9  Q2V2M9  Q2V2M9  Q2V2M9  Q2V2M9  Q2V2M9  Q2V2M9  Q2V2M9  Q3MHD2  Q3MHD2  Q3MHD2  Q3MHD2  Q3MHD2  Q3MHD2  Q3MHD2  Q3MHD2  Q3MHD2  Q3MHD2  Q3MHD2  Q3MHD2  Q3MHD2  Q3MHD2  Q3MHD2  Q3MHD2  Q3MHD2  Q3MHD2  Q3SXM5  Q3SXM5  Q3SXM5  Q3SXM5  Q3SXM5  Q3SXM5  Q3SXM5  Q3SXM5  Q3SXM5  Q3SXM5  Q3SXM5  Q3SXM5  Q3SXM5  Q3SXM5  Q3SXM5  Q3SXM5  Q3SXM5  Q3SXM5  Q3SXM5  Q3SXM5  Q3SXM5  Q3SXM5  Q3SXM5  Q3SXM5  Q3SXM5  Q3SXM5  Q3SXM5  Q3SXM5  Q3V6T2  Q3V6T2  Q3V6T2  Q3V6T2  Q3V6T2  Q3V6T2  Q3V6T2  Q3V6T2  Q3V6T2  Q3V6T2  Q3V6T2  Q3V6T2  Q3V6T2  Q3V6T2  Q3V6T2  Q3V6T2  Q3V6T2  Q3V6T2  Q3V6T2  Q3V6T2  Q3V6T2  Q3V6T2  Q3V6T2  Q3V6T2  Q3V6T2  Q3V6T2  Q3V6T2  Q3V6T2  Q3V6T2  Q3V6T2  Q3V6T2  Q4G163  Q4G163  Q4G163  Q4G163  Q4G163  Q4G163  Q4G163  Q4G163  Q4G163  Q4G163  Q4G163  Q4G163  Q4G163  Q4G163  Q4G163  Q4G163  Q4G163  Q4G163  Q4G163  Q4G163  Q4G163  Q4G163  Q4G163  Q4G163  Q4G163  Q4G163  Q4G163  Q4G163  Q4G163  Q4G163  Q4G163  Q4G163  Q4G163  Q53EL6  Q53EL6  Q53EL6  Q53EL6  Q53EL6  Q53EL6  Q53EL6  Q53EL6  Q53EL6  Q53EL6  Q53EL6  Q53EL6  Q53EL6  Q53EL6  Q53EL6  Q53EL6  Q53EL6  Q53EL6  Q53EL6  Q53EL6  Q53EL6  Q53EL6  Q53EL6  Q53EL6  Q53EL6  Q53EL6  Q53EL6  Q53EL6  Q53EL6  Q53EL6  Q53EL6  Q53ET0  Q53ET0  Q53ET0  Q53ET0  Q53ET0  Q53ET0  Q53ET0  Q53ET0  Q53ET0  Q53ET0  Q53ET0  Q53ET0  Q53ET0  Q53ET0  Q53ET0  Q53ET0  Q53ET0  Q53ET0  Q53ET0  Q53ET0  Q53ET0  Q53ET0  Q53ET0  Q53ET0  Q53ET0  Q53ET0  Q53ET0  Q53ET0  Q53ET0  Q53ET0  Q53ET0  Q53EZ4  Q53EZ4  Q53EZ4  Q53EZ4  Q53EZ4  Q53EZ4  Q53EZ4  Q53EZ4  Q53EZ4  Q53EZ4  Q53EZ4  Q53EZ4  Q53EZ4  Q53EZ4  Q53EZ4  Q53EZ4  Q53EZ4  Q53EZ4  Q53EZ4  Q53EZ4  Q53EZ4  Q53EZ4  Q53EZ4  Q53EZ4  Q53EZ4  Q53FA7  Q53FA7  Q53FA7  Q53FA7  Q53FA7  Q53FA7  Q53FA7  Q53FA7  Q53FA7  Q53FA7  Q53FA7  Q53FA7  Q53FA7  Q53GL7  Q53GL7  Q53GL7  Q53GL7  Q53GL7  Q53GL7  Q53GL7  Q53GL7  Q53GL7  Q53GL7  Q53GL7  Q53GL7  Q53GL7  Q53GL7  Q53GL7  Q53GL7  Q53GL7  Q53GL7  Q53GL7  Q53GL7  Q53GL7  Q53GL7  Q53GL7  Q53GL7  Q53GL7  Q53GL7  Q53GL7  Q53GL7  Q53GL7  Q53GL7  Q53GL7  Q53GL7  Q53GL7  Q53H47  Q53H47  Q53H47  Q53H47  Q53H47  Q53H47  Q53H47  Q53H47  Q53H47  Q53H47  Q53H47  Q53H47  Q53H47  Q53H47  Q53H47  Q53H47  Q53H47  Q53H47  Q53H47  Q53H47  Q53H47  Q53H47  Q53H47  Q53H47  Q53H47  Q53H47  Q53H47  Q53H47  Q53H47  Q53H47  Q53H47  Q53HL2  Q53HL2  Q53HL2  Q53HL2  Q53HL2  Q53HL2  Q53HL2  Q53HL2  Q53HL2  Q53HL2  Q53HL2  Q53HL2  Q5FBB7  Q5FBB7  Q5FBB7  Q5FBB7  Q5FBB7  Q5FBB7  Q5FBB7  Q5FBB7  Q5FBB7  Q5FBB7  Q5FBB7  Q5FBB7  Q5FBB7  Q5FBB7  Q5FBB7  Q5FBB7  Q5FBB7  Q5FBB7  Q5FBB7  Q5FBB7  Q5FBB7  Q5FBB7  Q5FBB7  Q5FBB7  Q5FBB7  Q5FBB7  Q5FBB7  Q5FBB7  Q5FBB7  Q5FBB7  Q5FBB7  Q5FYB1  Q5FYB1  Q5FYB1  Q5FYB1  Q5FYB1  Q5FYB1  Q5FYB1  Q5FYB1  Q5FYB1  Q5FYB1  Q5FYB1  Q5FYB1  Q5FYB1  Q5FYB1  Q5FYB1  Q5FYB1  Q5FYB1  Q5FYB1  Q5FYB1  Q5FYB1  Q5FYB1  Q5FYB1  Q5FYB1  Q5FYB1  Q5FYB1  Q5FYB1  Q5FYB1  Q5FYB1  Q5FYB1  Q5FYB1  Q5FYB1  Q5FYB1  Q5FYB1  Q5JRA6  Q5JRA6  Q5JRA6  Q5JRA6  Q5JRA6  Q5JRA6  Q5JRA6  Q5JRA6  Q5JRA6  Q5JRA6  Q5JRA6  Q5JRA6  Q5JRA6  Q5JRA6  Q5JRA6  Q5JRA6  Q5JRA6  Q5JRA6  Q5JRA6  Q5JRA6  Q5JRA6  Q5JRA6  Q5JRA6  Q5JRA6  Q5JRA6  Q5JRA6  Q5JRA6  Q5JRA6  Q5JRA6  Q5JRA6  Q5JRA6  Q5JVF3  Q5JVF3  Q5JVF3  Q5JVF3  Q5JVF3  Q5JVF3  Q5JVF3  Q5JVF3  Q5JVF3  Q5JVF3  Q5JVF3  Q5JVF3  Q5JVF3  Q5JVF3  Q5JVF3  Q5JVF3  Q5JVF3  Q5JVF3  Q5JVF3  Q5JVF3  Q5KSL6  Q5KSL6  Q5KSL6  Q5KSL6  Q5KSL6  Q5KSL6  Q5KSL6  Q5KSL6  Q5KSL6  Q5KSL6  Q5KSL6  Q5KSL6  Q5KSL6  Q5KSL6  Q5KSL6  Q5KSL6  Q5KSL6  Q5KSL6  Q5KSL6  Q5KSL6  Q5KSL6  Q5KSL6  Q5KSL6  Q5KSL6  Q5KSL6  Q5KSL6  Q5KSL6  Q5KSL6  Q5KSL6  Q5KSL6  Q5KSL6  Q5KSL6  Q5KSL6  Q5SQ64  Q5SQ64  Q5SQ64  Q5SQ64  Q5SQ64  Q5SQ64  Q5SQ64  Q5SQ64  Q5SQ64  Q5SQ64  Q5SQ64  Q5SQ64  Q5SQ64  Q5SQ64  Q5SQ64  Q5SQ64  Q5SQ64  Q5SQ64  Q5SQ64  Q5SQ64  Q5SQ64  Q5SQ64  Q5SQ64  Q5SQ64  Q5T1C6  Q5T1C6  Q5T1C6  Q5T1C6  Q5T1C6  Q5T1C6  Q5T1C6  Q5T1C6  Q5T1C6  Q5T1C6  Q5T1C6  Q5T1C6  Q5T1C6  Q5T1C6  Q5TA31  Q5TA31  Q5TA31  Q5TA31  Q5TA31  Q5TA31  Q5TAQ9  Q5TAQ9  Q5TAQ9  Q5TAQ9  Q5TAQ9  Q5TAQ9  Q5TAQ9  Q5TAQ9  Q5TAQ9  Q5TAQ9  Q5TAQ9  Q5TAQ9  Q5TAQ9  Q5TAQ9  Q5TAQ9  Q5TAQ9  Q5TAQ9  Q5TAQ9  Q5TAQ9  Q5TAQ9  Q5TAQ9  Q5TAQ9  Q5TAQ9  Q5TAQ9  Q5TAQ9  Q5TAQ9  Q5TAQ9  Q5TAQ9  Q5TAQ9  Q5TAQ9  Q5TAQ9  Q5TAQ9  Q5TAQ9  Q5VT25  Q5VT25  Q5VT25  Q5VT25  Q5VT25  Q5VT25  Q5VT25  Q5VT25  Q5VT25  Q5VT25  Q5VT25  Q5VT25  Q5VT25  Q5VT25  Q5VT25  Q5VT25  Q5VT25  Q5VT25  Q5VT25  Q5VT25  Q5VT25  Q5VT25  Q5VT25  Q5VT25  Q5VT25  Q5VT25  Q5VT25  Q5VT25  Q5VT25  Q5VT25  Q5VT25  Q5VTD9  Q5VTD9  Q5VTD9  Q5VTD9  Q5VTD9  Q5VTD9  Q5VTD9  Q5VTD9  Q5VTD9  Q5VTD9  Q5VTD9  Q5VTD9  Q5VTD9  Q5VTD9  Q5VTD9  Q5VTD9  Q5VTD9  Q5VTD9  Q5VTD9  Q5VTD9  Q5VTD9  Q5VTD9  Q5VTD9  Q5VTD9  Q5VTD9  Q5VTD9  Q5VTD9  Q5VTD9  Q5VTD9  Q5VTD9  Q5VTD9  Q5VTD9  Q5VTD9  Q5VWQ8  Q5VWQ8  Q5VWQ8  Q5VWQ8  Q5VWQ8  Q5VWQ8  Q5VWQ8  Q5VWQ8  Q5VWQ8  Q5VWQ8  Q5VWQ8  Q5VWQ8  Q5VWQ8  Q5VWQ8  Q5VWQ8  Q5VWQ8  Q5VWQ8  Q5VWQ8  Q5VWQ8  Q5VWQ8  Q5VWQ8  Q5VWQ8  Q5VWQ8  Q5VWQ8  Q5VWQ8  Q5VWQ8  Q5VWQ8  Q5VWQ8  Q5VWQ8  Q5VWQ8  Q5VWQ8  Q5VWZ2  Q5VWZ2  Q5VWZ2  Q5VWZ2  Q5VWZ2  Q5VWZ2  Q5VWZ2  Q5VWZ2  Q5VWZ2  Q5VWZ2  Q5VWZ2  Q5VWZ2  Q5VWZ2  Q5VWZ2  Q5VWZ2  Q5VWZ2  Q5VWZ2  Q5VWZ2  Q5VWZ2  Q5VY43  Q5VY43  Q5VY43  Q5VY43  Q5VY43  Q5VY43  Q5VY43  Q5VY43  Q5VY43  Q5VY43  Q5VY43  Q5VY43  Q5VY43  Q5VY43  Q5VY43  Q5VY43  Q5VY43  Q5VY43  Q5VY43  Q5VY43  Q5VY43  Q5VY43  Q5VY43  Q5VY43  Q5VY43  Q5VY43  Q5VY43  Q5VY43  Q5VY43  Q5VY43  Q5VY43  Q5XUX0  Q5XUX0  Q5XUX0  Q5XUX0  Q5XUX0  Q5XUX0  Q5XUX0  Q5XUX0  Q5XUX0  Q5XUX0  Q5XUX0  Q5XUX0  Q5XUX0  Q5XUX0  Q5XUX0  Q5XUX0  Q5XUX0  Q5XUX0  Q5XUX0  Q5XUX0  Q5XUX0  Q5XUX0  Q5XUX0  Q684P5  Q684P5  Q684P5  Q684P5  Q684P5  Q684P5  Q684P5  Q684P5  Q684P5  Q684P5  Q684P5  Q684P5  Q684P5  Q684P5  Q684P5  Q684P5  Q684P5  Q684P5  Q684P5  Q684P5  Q684P5  Q684P5  Q684P5  Q684P5  Q684P5  Q684P5  Q684P5  Q684P5  Q684P5  Q684P5  Q684P5  Q69YN4  Q69YN4  Q69YN4  Q69YN4  Q69YN4  Q69YN4  Q69YN4  Q69YN4  Q69YN4  Q69YN4  Q69YN4  Q69YN4  Q69YN4  Q69YN4  Q69YN4  Q69YN4  Q69YN4  Q69YN4  Q69YN4  Q69YN4  Q69YN4  Q69YN4  Q69YN4  Q69YN4  Q69YN4  Q69YN4  Q69YN4  Q69YN4  Q69YN4  Q69YN4  Q69YN4  Q69YN4  Q69YN4  Q6AI12  Q6AI12  Q6AI12  Q6AI12  Q6AI12  Q6AI12  Q6AI12  Q6AI12  Q6AI12  Q6AI12  Q6AI12  Q6AI12  Q6AI12  Q6AI12  Q6AI12  Q6AI12  Q6AI12  Q6AI12  Q6AI12  Q6AI12  Q6AI12  Q6AI12  Q6AI12  Q6AI12  Q6B0I6  Q6B0I6  Q6B0I6  Q6B0I6  Q6B0I6  Q6B0I6  Q6B0I6  Q6B0I6  Q6B0I6  Q6B0I6  Q6B0I6  Q6B0I6  Q6B0I6  Q6B0I6  Q6B0I6  Q6B0I6  Q6B0I6  Q6B0I6  Q6B0I6  Q6B0I6  Q6B0I6  Q6B0I6  Q6B0I6  Q6B0I6  Q6B0I6  Q6B0I6  Q6B0I6  Q6B0I6  Q6B0I6  Q6B0I6  Q6B0I6  Q6B0I6  Q6B0I6  Q6DN03  Q6DN03  Q6DN03  Q6DN03  Q6DN03  Q6DN03  Q6DN03  Q6DN03  Q6DN03  Q6DN03  Q6DN03  Q6DN03  Q6DN03  Q6DN03  Q6DN03  Q6DN03  Q6DN03  Q6DN03  Q6DN03  Q6DN03  Q6DN03  Q6DN72  Q6DN72  Q6DN72  Q6DN72  Q6DN72  Q6DN72  Q6DN72  Q6DN72  Q6DN72  Q6DN72  Q6DN72  Q6DN72  Q6DN72  Q6DN72  Q6DN72  Q6DN72  Q6DN72  Q6DN72  Q6DN72  Q6DN72  Q6DN72  Q6DN72  Q6DN72  Q6DN72  Q6DN72  Q6DN72  Q6DN72  Q6DN72  Q6DN72  Q6DN72  Q6DN72  Q6DN72  Q6DN72  Q6GTX8  Q6GTX8  Q6GTX8  Q6GTX8  Q6GTX8  Q6GTX8  Q6GTX8  Q6GTX8  Q6GTX8  Q6GTX8  Q6GTX8  Q6GTX8  Q6GTX8  Q6GTX8  Q6GTX8  Q6GTX8  Q6GTX8  Q6GTX8  Q6GTX8  Q6GTX8  Q6GTX8  Q6GTX8  Q6GTX8  Q6GTX8  Q6GTX8  Q6GTX8  Q6GTX8  Q6GTX8  Q6JBY9  Q6JBY9  Q6JBY9  Q6JBY9  Q6JBY9  Q6JBY9  Q6JBY9  Q6JBY9  Q6JBY9  Q6JBY9  Q6JBY9  Q6JBY9  Q6JBY9  Q6JBY9  Q6JBY9  Q6JBY9  Q6JBY9  Q6JBY9  Q6JBY9  Q6JBY9  Q6JBY9  Q6JBY9  Q6JBY9  Q6JBY9  Q6JBY9  Q6JBY9  Q6JBY9  Q6JBY9  Q6JBY9  Q6NXS1  Q6NXS1  Q6NXS1  Q6NXS1  Q6NXS1  Q6NXS1  Q6NXS1  Q6NXS1  Q6NXS1  Q6NXS1  Q6NXS1  Q6NXS1  Q6NXS1  Q6NXS1  Q6NXS1  Q6NZI2  Q6NZI2  Q6NZI2  Q6NZI2  Q6NZI2  Q6NZI2  Q6NZI2  Q6NZI2  Q6NZI2  Q6NZI2  Q6NZI2  Q6NZI2  Q6NZI2  Q6NZI2  Q6NZI2  Q6NZI2  Q6P1J9  Q6P1J9  Q6P1J9  Q6P1J9  Q6P1J9  Q6P1J9  Q6P1J9  Q6P1J9  Q6P1J9  Q6P1J9  Q6P1J9  Q6P1J9  Q6P1J9  Q6P1J9  Q6P1J9  Q6P1J9  Q6P1J9  Q6P1J9  Q6P1J9  Q6P1J9  Q6P1J9  Q6P1J9  Q6P1J9  Q6P1J9  Q6P1J9  Q6P1J9  Q6P1N0  Q6P1N0  Q6P1N0  Q6P1N0  Q6P1N0  Q6P1N0  Q6P1N0  Q6P1N0  Q6P1N0  Q6P1N0  Q6P1N0  Q6P1N0  Q6P1N0  Q6P1N0  Q6P1N0  Q6P1N0  Q6P1N0  Q6P1N0  Q6P1N0  Q6P1N0  Q6P1N0  Q6P1N0  Q6P1N0  Q6P1N0  Q6P1N0  Q6P1N0  Q6P1N0  Q6P1N0  Q6P1N0  Q6P1N0  Q6P1N0  Q6P2Q9  Q6P2Q9  Q6P2Q9  Q6P2Q9  Q6P2Q9  Q6P2Q9  Q6P2Q9  Q6P2Q9  Q6P2Q9  Q6P2Q9  Q6P2Q9  Q6P2Q9  Q6P2Q9  Q6P2Q9  Q6P2Q9  Q6P2Q9  Q6P2Q9  Q6P2Q9  Q6P2Q9  Q6P2Q9  Q6P2Q9  Q6P2Q9  Q6P2Q9  Q6P2Q9  Q6P2Q9  Q6P2Q9  Q6P2Q9  Q6P2Q9  Q6P2Q9  Q6P2Q9  Q6P2Q9  Q6P2Q9  Q6P2Q9  Q6P5Z2  Q6P5Z2  Q6P5Z2  Q6P5Z2  Q6P5Z2  Q6P5Z2  Q6P5Z2  Q6P5Z2  Q6P5Z2  Q6P5Z2  Q6P5Z2  Q6P5Z2  Q6P5Z2  Q6P5Z2  Q6P5Z2  Q6P5Z2  Q6P5Z2  Q6P5Z2  Q6P5Z2  Q6P5Z2  Q6P5Z2  Q6P5Z2  Q6P5Z2  Q6P5Z2  Q6P5Z2  Q6P5Z2  Q6P5Z2  Q6P5Z2  Q6P5Z2  Q6P5Z2  Q6P5Z2  Q6P5Z2  Q6P5Z2  Q6P988  Q6P988  Q6P988  Q6P988  Q6P988  Q6P988  Q6P988  Q6P988  Q6P988  Q6P988  Q6P988  Q6P988  Q6P988  Q6P988  Q6P988  Q6P988  Q6P988  Q6P988  Q6P988  Q6P988  Q6P988  Q6P988  Q6P988  Q6P988  Q6P988  Q6P988  Q6P988  Q6P988  Q6P988  Q6P988  Q6P988  Q6PCB0  Q6PCB0  Q6PCB0  Q6PCB0  Q6PCB0  Q6PCB0  Q6PCB0  Q6PCB0  Q6PCB0  Q6PCB0  Q6PCB0  Q6PCB0  Q6PCB0  Q6PCB0  Q6PCB0  Q6PCB0  Q6PCB0  Q6PCB0  Q6PCB0  Q6PCB0  Q6PCB0  Q6PCB0  Q6PCB0  Q6PCB0  Q6PCB0  Q6PCB0  Q6PCB0  Q6PCB0  Q6PCB0  Q6PCB0  Q6PCB0  Q6PCD5  Q6PCD5  Q6PCD5  Q6PCD5  Q6PCD5  Q6PCD5  Q6PCD5  Q6PCD5  Q6PCD5  Q6PCD5  Q6PCD5  Q6PCD5  Q6PCD5  Q6PCD5  Q6PCD5  Q6PCD5  Q6PCD5  Q6PCD5  Q6PCD5  Q6PCD5  Q6PCD5  Q6PCD5  Q6PCD5  Q6PCD5  Q6PCD5  Q6PCD5  Q6PCD5  Q6PCD5  Q6PCD5  Q6PCD5  Q6PCD5  Q6PHR2  Q6PHR2  Q6PHR2  Q6PHR2  Q6PHR2  Q6PHR2  Q6PHR2  Q6PHR2  Q6PHR2  Q6PHR2  Q6PHR2  Q6PHR2  Q6PHR2  Q6PHR2  Q6PHR2  Q6PHR2  Q6PHR2  Q6PHR2  Q6PHR2  Q6PHR2  Q6PHR2  Q6PHR2  Q6PHR2  Q6PHR2  Q6PHR2  Q6PHR2  Q6PHR2  Q6PHR2  Q6PKG0  Q6PKG0  Q6PKG0  Q6PKG0  Q6PKG0  Q6PKG0  Q6PKG0  Q6PKG0  Q6PKG0  Q6PKG0  Q6PKG0  Q6PKG0  Q6PKG0  Q6PKG0  Q6PKG0  Q6PKG0  Q6PKG0  Q6PKG0  Q6PKG0  Q6PKG0  Q6PKG0  Q6PKG0  Q6PKG0  Q6PKG0  Q6PKG0  Q6PKG0  Q6PKG0  Q6PKG0  Q6PKG0  Q6PKG0  Q6PKG0  Q6Q788  Q6Q788  Q6Q788  Q6Q788  Q6Q788  Q6Q788  Q6Q788  Q6Q788  Q6Q788  Q6Q788  Q6Q788  Q6Q788  Q6Q788  Q6Q788  Q6Q788  Q6Q788  Q6Q788  Q6Q788  Q6Q788  Q6Q788  Q6R327  Q6R327  Q6R327  Q6R327  Q6R327  Q6R327  Q6R327  Q6R327  Q6R327  Q6R327  Q6R327  Q6R327  Q6R327  Q6R327  Q6R327  Q6R327  Q6R327  Q6R327  Q6R327  Q6R327  Q6R327  Q6R327  Q6R327  Q6R327  Q6R327  Q6R327  Q6R327  Q6R327  Q6R327  Q6R327  Q6R327  Q6R327  Q6R327  Q6S5L8  Q6S5L8  Q6S5L8  Q6S5L8  Q6S5L8  Q6S5L8  Q6S5L8  Q6S5L8  Q6S5L8  Q6S5L8  Q6S5L8  Q6S5L8  Q6S5L8  Q6S5L8  Q6S5L8  Q6S5L8  Q6S5L8  Q6S5L8  Q6S5L8  Q6S5L8  Q6S5L8  Q6S5L8  Q6S5L8  Q6S5L8  Q6S5L8  Q6S5L8  Q6S5L8  Q6S5L8  Q6S5L8  Q6S5L8  Q6S5L8  Q6S5L8  Q6S5L8  Q6TGC4  Q6TGC4  Q6TGC4  Q6TGC4  Q6TGC4  Q6TGC4  Q6TGC4  Q6TGC4  Q6TGC4  Q6TGC4  Q6TGC4  Q6TGC4  Q6TGC4  Q6TGC4  Q6TGC4  Q6TGC4  Q6TGC4  Q6TGC4  Q6TGC4  Q6TGC4  Q6TGC4  Q6TGC4  Q6TGC4  Q6TGC4  Q6TGC4  Q6TGC4  Q6TGC4  Q6TGC4  Q6TGC4  Q6TGC4  Q6TGC4  Q6U736  Q6U736  Q6U736  Q6U736  Q6U736  Q6U736  Q6U736  Q6U736  Q6U736  Q6U736  Q6U736  Q6U736  Q6U736  Q6U736  Q6U736  Q6U736  Q6U736  Q6U736  Q6U736  Q6U736  Q6U736  Q6U736  Q6U736  Q6U736  Q6U736  Q6U736  Q6U736  Q6U736  Q6UUV9  Q6UUV9  Q6UUV9  Q6UUV9  Q6UUV9  Q6UUV9  Q6UUV9  Q6UUV9  Q6UUV9  Q6UUV9  Q6UUV9  Q6UUV9  Q6UUV9  Q6UUV9  Q6UUV9  Q6UUV9  Q6UUV9  Q6UUV9  Q6UUV9  Q6UUV9  Q6UUV9  Q6UUV9  Q6UUV9  Q6UUV9  Q6UUV9  Q6UUV9  Q6UUV9  Q6UUV9  Q6UUV9  Q6UUV9  Q6UUV9  Q6UWZ7  Q6UWZ7  Q6UWZ7  Q6UWZ7  Q6UWZ7  Q6UWZ7  Q6UWZ7  Q6UWZ7  Q6UWZ7  Q6UWZ7  Q6UWZ7  Q6UWZ7  Q6UWZ7  Q6UWZ7  Q6UWZ7  Q6UWZ7  Q6UWZ7  Q6UWZ7  Q6UWZ7  Q6UWZ7  Q6UWZ7  Q6UWZ7  Q6UWZ7  Q6UWZ7  Q6UWZ7  Q6UWZ7  Q6UWZ7  Q6UWZ7  Q6UWZ7  Q6UWZ7  Q6UWZ7  Q6WN34  Q6WN34  Q6WN34  Q6WN34  Q6WN34  Q6WN34  Q6WN34  Q6WN34  Q6WN34  Q6WN34  Q6WN34  Q6WN34  Q6WN34  Q6WN34  Q6WN34  Q6WN34  Q6WN34  Q6WN34  Q6WN34  Q6WN34  Q6WN34  Q6WN34  Q6WN34  Q6WN34  Q6WN34  Q6WN34  Q6WN34  Q6WN34  Q6WN34  Q6ZMU5  Q6ZMU5  Q6ZMU5  Q6ZMU5  Q6ZMU5  Q6ZMU5  Q6ZMU5  Q6ZMU5  Q6ZMU5  Q6ZMU5  Q6ZMU5  Q6ZMU5  Q6ZMU5  Q6ZMU5  Q6ZMU5  Q6ZMU5  Q6ZMU5  Q6ZMU5  Q6ZMU5  Q6ZMU5  Q6ZMU5  Q6ZMU5  Q6ZMU5  Q6ZMU5  Q6ZMU5  Q6ZMU5  Q6ZN04  Q6ZN04  Q6ZN04  Q6ZN04  Q6ZN04  Q6ZN04  Q6ZN04  Q6ZN04  Q6ZN04  Q6ZN04  Q6ZN04  Q6ZN04  Q6ZN04  Q6ZN04  Q6ZN04  Q6ZN04  Q6ZN04  Q6ZN04  Q6ZN04  Q6ZN04  Q6ZN04  Q6ZN04  Q6ZN04  Q6ZN04  Q6ZN04  Q6ZN04  Q6ZN04  Q6ZN04  Q6ZN04  Q6ZN04  Q6ZSY5  Q6ZSY5  Q6ZSY5  Q6ZSY5  Q6ZSY5  Q6ZSY5  Q6ZSY5  Q6ZSY5  Q6ZSY5  Q6ZSY5  Q6ZSY5  Q6ZSY5  Q6ZSY5  Q6ZSY5  Q6ZSY5  Q6ZSY5  Q6ZSY5  Q6ZSY5  Q6ZSY5  Q6ZSY5  Q6ZSY5  Q6ZSY5  Q6ZSY5  Q6ZSY5  Q6ZSY5  Q6ZSY5  Q6ZSY5  Q6ZSY5  Q6ZSY5  Q6ZSY5  Q6ZSY5  Q6ZUJ8  Q6ZUJ8  Q6ZUJ8  Q6ZUJ8  Q6ZUJ8  Q6ZUJ8  Q6ZUJ8  Q6ZUJ8  Q6ZUJ8  Q6ZUJ8  Q6ZUJ8  Q6ZUJ8  Q6ZUJ8  Q6ZUJ8  Q6ZUJ8  Q6ZUJ8  Q6ZUJ8  Q6ZUJ8  Q6ZUJ8  Q6ZUJ8  Q6ZUJ8  Q6ZUJ8  Q6ZUJ8  Q6ZUJ8  Q6ZUJ8  Q6ZUJ8  Q6ZUJ8  Q6ZUJ8  Q6ZUJ8  Q6ZUJ8  Q6ZUJ8  Q6ZUJ8  Q6ZUJ8  Q70E73  Q70E73  Q70E73  Q70E73  Q70E73  Q70E73  Q70E73  Q70E73  Q70E73  Q70E73  Q70E73  Q70E73  Q70E73  Q70E73  Q70E73  Q70E73  Q70E73  Q70E73  Q70E73  Q70E73  Q70E73  Q70E73  Q70E73  Q70E73  Q70E73  Q70E73  Q70E73  Q70E73  Q70E73  Q70E73  Q70E73  Q70E73  Q70E73  Q712K3  Q712K3  Q712K3  Q712K3  Q712K3  Q712K3  Q712K3  Q712K3  Q712K3  Q712K3  Q712K3  Q712K3  Q712K3  Q712K3  Q712K3  Q712K3  Q712K3  Q71F23  Q71F23  Q71F23  Q71F23  Q71F23  Q71F23  Q71F23  Q71F23  Q71F23  Q71F23  Q71F23  Q71F23  Q71F23  Q71F23  Q71F23  Q71F23  Q71F23  Q71F23  Q71F23  Q71F23  Q71F23  Q71F23  Q71F23  Q71F23  Q71F23  Q71F23  Q71F23  Q71F23  Q71F23  Q71F23  Q71F23  Q71F23  Q71F23  Q71RC2  Q71RC2  Q71RC2  Q71RC2  Q71RC2  Q71RC2  Q71RC2  Q71RC2  Q71RC2  Q71RC2  Q71RC2  Q71RC2  Q71RC2  Q71RC2  Q71RC2  Q71RC2  Q71RC2  Q71RC2  Q71RC2  Q71RC2  Q71RC2  Q71RC2  Q71RC2  Q71RC2  Q71RC2  Q71RC2  Q71RC2  Q71RC2  Q71RC2  Q71RC2  Q71RC2  Q71RC2  Q71RC2  Q7KZF4  Q7KZF4  Q7KZF4  Q7KZF4  Q7KZF4  Q7KZF4  Q7KZF4  Q7KZF4  Q7KZF4  Q7KZF4  Q7KZF4  Q7KZF4  Q7KZF4  Q7KZF4  Q7KZF4  Q7KZF4  Q7KZF4  Q7KZF4  Q7KZF4  Q7KZF4  Q7KZF4  Q7KZF4  Q7KZF4  Q7KZF4  Q7KZF4  Q7KZF4  Q7KZF4  Q7KZF4  Q7KZF4  Q7KZF4  Q7KZF4  Q7KZF4  Q7KZF4  Q7L2H7  Q7L2H7  Q7L2H7  Q7L2H7  Q7L2H7  Q7L2H7  Q7L2H7  Q7L2H7  Q7L2H7  Q7L2H7  Q7L2H7  Q7L2H7  Q7L2H7  Q7L2H7  Q7L2H7  Q7L2H7  Q7L2H7  Q7L2H7  Q7L2H7  Q7L2H7  Q7L2H7  Q7L2H7  Q7L2H7  Q7L5D6  Q7L5D6  Q7L5D6  Q7L5D6  Q7L5D6  Q7L5D6  Q7L5D6  Q7L5D6  Q7L5D6  Q7L5D6  Q7L5D6  Q7L5D6  Q7L5D6  Q7L5D6  Q7L5D6  Q7L5D6  Q7L5D6  Q7L5D6  Q7L5D6  Q7L5D6  Q7L5D6  Q7L5D6  Q7L5D6  Q7L5D6  Q7L5D6  Q7L5D6  Q7L5D6  Q7L5D6  Q7L5D6  Q7L5D6  Q7L5D6  Q7L5D6  Q7L5Y1  Q7L5Y1  Q7L5Y1  Q7L5Y1  Q7L5Y1  Q7L5Y1  Q7L5Y1  Q7L5Y1  Q7L5Y1  Q7L5Y1  Q7L5Y1  Q7L5Y1  Q7L5Y1  Q7L5Y1  Q7L5Y1  Q7L5Y1  Q7L5Y1  Q7L5Y1  Q7L804  Q7L804  Q7L804  Q7L804  Q7L804  Q7L804  Q7L804  Q7L804  Q7L804  Q7L804  Q7L804  Q7L804  Q7L804  Q7L804  Q7L804  Q7L804  Q7L804  Q7L804  Q7L804  Q7L804  Q7L804  Q7L804  Q7L804  Q7L804  Q7L804  Q7L804  Q7L804  Q7L804  Q7L804  Q7L804  Q7L804  Q7RTN6  Q7RTN6  Q7RTN6  Q7RTN6  Q7RTN6  Q7RTN6  Q7RTN6  Q7RTN6  Q7RTN6  Q7RTN6  Q7RTN6  Q7RTN6  Q7RTN6  Q7RTN6  Q7RTN6  Q7RTN6  Q7RTN6  Q7RTN6  Q7RTN6  Q7RTN6  Q7RTN6  Q7RTN6  Q7RTN6  Q7RTN6  Q7RTN6  Q7RTN6  Q7RTN6  Q7RTN6  Q7RTN6  Q7RTN6  Q7RTN6  Q7RTN6  Q7RTN6  Q7Z406  Q7Z406  Q7Z406  Q7Z406  Q7Z406  Q7Z406  Q7Z406  Q7Z406  Q7Z406  Q7Z406  Q7Z406  Q7Z406  Q7Z406  Q7Z406  Q7Z406  Q7Z406  Q7Z406  Q7Z406  Q7Z406  Q7Z406  Q7Z406  Q7Z406  Q7Z406  Q7Z406  Q7Z406  Q7Z406  Q7Z406  Q7Z406  Q7Z406  Q7Z406  Q7Z406  Q7Z406  Q7Z406  Q7Z417  Q7Z417  Q7Z417  Q7Z417  Q7Z417  Q7Z417  Q7Z417  Q7Z417  Q7Z417  Q7Z417  Q7Z417  Q7Z417  Q7Z417  Q7Z417  Q7Z417  Q7Z417  Q7Z417  Q7Z417  Q7Z417  Q7Z417  Q7Z417  Q7Z417  Q7Z417  Q7Z417  Q7Z417  Q7Z417  Q7Z417  Q7Z417  Q7Z417  Q7Z417  Q7Z417  Q7Z4W1  Q7Z4W1  Q7Z4W1  Q7Z4W1  Q7Z4W1  Q7Z4W1  Q7Z4W1  Q7Z4W1  Q7Z4W1  Q7Z4W1  Q7Z4W1  Q7Z4W1  Q7Z4W1  Q7Z4W1  Q7Z4W1  Q7Z5L9  Q7Z5L9  Q7Z5L9  Q7Z5L9  Q7Z5L9  Q7Z5L9  Q7Z5L9  Q7Z5L9  Q7Z5L9  Q7Z5L9  Q7Z5L9  Q7Z5L9  Q7Z5L9  Q7Z5L9  Q7Z5L9  Q7Z5L9  Q7Z5L9  Q7Z5L9  Q7Z5L9  Q7Z5L9  Q7Z5L9  Q7Z5L9  Q7Z5L9  Q7Z5L9  Q7Z5L9  Q7Z5L9  Q7Z5L9  Q7Z5L9  Q7Z5L9  Q7Z5L9  Q7Z5L9  Q7Z6J0  Q7Z6J0  Q7Z6J0  Q7Z6J0  Q7Z6J0  Q7Z6J0  Q7Z6J0  Q7Z6J0  Q7Z6J0  Q7Z6J0  Q7Z6J0  Q7Z6J0  Q7Z6J0  Q7Z6J0  Q7Z6J0  Q7Z6J0  Q7Z6J0  Q7Z6J0  Q7Z6J0  Q7Z6J0  Q7Z6J0  Q7Z6J0  Q7Z6J0  Q7Z6J0  Q7Z6J0  Q7Z6J0  Q7Z6J0  Q7Z6J0  Q7Z6J0  Q7Z6J0  Q7Z6J0  Q86T82  Q86T82  Q86T82  Q86T82  Q86T82  Q86T82  Q86T82  Q86T82  Q86T82  Q86T82  Q86T82  Q86T82  Q86T82  Q86T82  Q86T82  Q86T82  Q86T82  Q86T82  Q86T82  Q86T82  Q86T82  Q86T82  Q86T82  Q86T82  Q86T82  Q86T82  Q86T82  Q86T82  Q86T82  Q86T82  Q86T82  Q86U42  Q86U42  Q86U42  Q86U42  Q86U42  Q86U42  Q86U42  Q86U42  Q86U42  Q86U42  Q86U42  Q86U42  Q86U42  Q86U42  Q86U42  Q86U42  Q86U42  Q86U42  Q86U42  Q86UC2  Q86UC2  Q86UC2  Q86UC2  Q86UC2  Q86UC2  Q86UC2  Q86UC2  Q86UC2  Q86UC2  Q86UC2  Q86UC2  Q86UC2  Q86UC2  Q86UC2  Q86UC2  Q86UC2  Q86UC2  Q86UC2  Q86UC2  Q86UC2  Q86UC2  Q86UC2  Q86UC2  Q86UC2  Q86UC2  Q86UC2  Q86UC2  Q86UC2  Q86UC2  Q86UC2  Q86UC2  Q86UC2  Q86UP2  Q86UP2  Q86UP2  Q86UP2  Q86UP2  Q86UP2  Q86UP2  Q86UP2  Q86UP2  Q86UP2  Q86UP2  Q86UP2  Q86UP2  Q86UP2  Q86UP2  Q86UP2  Q86UP2  Q86UP2  Q86UP2  Q86UP2  Q86UP2  Q86UP2  Q86UP2  Q86UP2  Q86UP2  Q86UP2  Q86UP2  Q86UP2  Q86UP2  Q86UP2  Q86UP2  Q86UQ8  Q86UQ8  Q86UQ8  Q86UQ8  Q86UQ8  Q86UQ8  Q86UQ8  Q86UQ8  Q86UQ8  Q86UQ8  Q86UQ8  Q86UQ8  Q86UQ8  Q86UQ8  Q86UQ8  Q86UQ8  Q86UQ8  Q86UQ8  Q86UR1  Q86UR1  Q86UR1  Q86UR1  Q86UR1  Q86UR1  Q86UR1  Q86UR1  Q86UR1  Q86UR1  Q86UR1  Q86UR1  Q86UR1  Q86UR1  Q86UR1  Q86UR1  Q86UR1  Q86UR1  Q86UR1  Q86UR1  Q86V48  Q86V48  Q86V48  Q86V48  Q86V48  Q86V48  Q86V48  Q86V48  Q86V48  Q86V48  Q86V48  Q86V48  Q86V48  Q86V48  Q86V48  Q86V48  Q86V48  Q86V48  Q86V48  Q86V48  Q86V48  Q86V48  Q86V48  Q86V48  Q86V48  Q86V48  Q86V48  Q86V48  Q86V48  Q86V48  Q86V48  Q86V48  Q86V48  Q86V81  Q86V81  Q86V81  Q86V81  Q86V81  Q86V81  Q86V81  Q86V81  Q86V81  Q86V81  Q86V81  Q86V81  Q86V81  Q86VP1  Q86VP1  Q86VP1  Q86VP1  Q86VP1  Q86VP1  Q86VP1  Q86VP1  Q86VP1  Q86VP1  Q86VP1  Q86VP1  Q86VP1  Q86VP1  Q86VP1  Q86VP1  Q86VP1  Q86VP1  Q86VP1  Q86VP1  Q86VP1  Q86VP1  Q86VP1  Q86VP1  Q86VP1  Q86VP1  Q86VP1  Q86VP1  Q86VP1  Q86VP1  Q86VP1  Q86W56  Q86W56  Q86W56  Q86W56  Q86W56  Q86W56  Q86W56  Q86W56  Q86W56  Q86W56  Q86W56  Q86W56  Q86W56  Q86W56  Q86W56  Q86W56  Q86W56  Q86W56  Q86W56  Q86W56  Q86W56  Q86W56  Q86W56  Q86W56  Q86W56  Q86W56  Q86W56  Q86W56  Q86W56  Q86W56  Q86W56  Q86WB0  Q86WB0  Q86WB0  Q86WB0  Q86WB0  Q86WB0  Q86WB0  Q86WB0  Q86WB0  Q86WB0  Q86WB0  Q86WB0  Q86WB0  Q86WB0  Q86WB0  Q86WB0  Q86WB0  Q86WB0  Q86WB0  Q86WB0  Q86WB0  Q86WB0  Q86WB0  Q86WB0  Q86WB0  Q86WB0  Q86WB0  Q86WB0  Q86WB0  Q86WB0  Q86WB0  Q86WR7  Q86WR7  Q86WR7  Q86WR7  Q86WR7  Q86WR7  Q86WR7  Q86WR7  Q86WR7  Q86WR7  Q86WR7  Q86WR7  Q86WR7  Q86WR7  Q86WR7  Q86WR7  Q86WR7  Q86WR7  Q86WR7  Q86WR7  Q86WR7  Q86WR7  Q86WR7  Q86WR7  Q86WR7  Q86WR7  Q86WR7  Q86WR7  Q86WR7  Q86WR7  Q86WR7  Q86WV6  Q86WV6  Q86WV6  Q86WV6  Q86WV6  Q86WV6  Q86WV6  Q86WV6  Q86WV6  Q86WV6  Q86WV6  Q86WV6  Q86WV6  Q86WV6  Q86WV6  Q86WV6  Q86WV6  Q86WV6  Q86WV6  Q86WV6  Q86WV6  Q86WV6  Q86WV6  Q86WV6  Q86WV6  Q86WV6  Q86WV6  Q86WV6  Q86X55  Q86X55  Q86X55  Q86X55  Q86X55  Q86X55  Q86X55  Q86X55  Q86X55  Q86X55  Q86X55  Q86X55  Q86X55  Q86X55  Q86X55  Q86X55  Q86X55  Q86X55  Q86X55  Q86X55  Q86X55  Q86X55  Q86X55  Q86X55  Q86X55  Q86X55  Q86X55  Q86X55  Q86X55  Q86X55  Q86X55  Q86XR7  Q86XR7  Q86XR7  Q86XR7  Q86XR7  Q86XR7  Q86XR7  Q86XR7  Q86XR7  Q86XR7  Q86XR7  Q86XR7  Q86XR7  Q86XR7  Q86XR7  Q86XR7  Q86YS7  Q86YS7  Q86YS7  Q86YS7  Q86YS7  Q86YS7  Q86YS7  Q86YS7  Q86YS7  Q86YS7  Q86YS7  Q86YS7  Q86YS7  Q86YS7  Q86YS7  Q86YS7  Q86YS7  Q86YS7  Q86YS7  Q86YS7  Q86YS7  Q86YS7  Q86YS7  Q86YS7  Q86YS7  Q86YS7  Q86YS7  Q86YS7  Q86YS7  Q86YS7  Q86YS7  Q8IU85  Q8IU85  Q8IU85  Q8IU85  Q8IU85  Q8IU85  Q8IU85  Q8IU85  Q8IU85  Q8IU85  Q8IU85  Q8IU85  Q8IU85  Q8IU85  Q8IU85  Q8IU85  Q8IU85  Q8IU85  Q8IU85  Q8IU85  Q8IU85  Q8IU85  Q8IU85  Q8IU85  Q8IU85  Q8IU85  Q8IU85  Q8IU85  Q8IU85  Q8IU85  Q8IU85  Q8IU85  Q8IU85  Q8IUR7  Q8IUR7  Q8IUR7  Q8IUR7  Q8IUR7  Q8IUR7  Q8IUR7  Q8IUR7  Q8IUR7  Q8IUR7  Q8IUR7  Q8IUR7  Q8IUR7  Q8IUR7  Q8IUR7  Q8IUR7  Q8IUR7  Q8IUR7  Q8IUR7  Q8IUR7  Q8IUR7  Q8IUR7  Q8IUR7  Q8IUR7  Q8IUR7  Q8IUR7  Q8IUR7  Q8IUR7  Q8IUR7  Q8IUR7  Q8IUR7  Q8IUR7  Q8IUR7  Q8IVP5  Q8IVP5  Q8IVP5  Q8IVP5  Q8IVP5  Q8IVP5  Q8IVP5  Q8IVP5  Q8IVP5  Q8IVT2  Q8IVT2  Q8IVT2  Q8IVT2  Q8IVT2  Q8IVT2  Q8IVT2  Q8IVT2  Q8IVT2  Q8IVT2  Q8IVT2  Q8IVT2  Q8IVT2  Q8IVT2  Q8IVT2  Q8IVT2  Q8IVT2  Q8IVT2  Q8IVT2  Q8IVT2  Q8IVT2  Q8IVT2  Q8IVT2  Q8IVT2  Q8IVT2  Q8IVT2  Q8IVT2  Q8IVT2  Q8IVT2  Q8IVT2  Q8IVT2  Q8IW19  Q8IW19  Q8IW19  Q8IW19  Q8IW19  Q8IW19  Q8IW19  Q8IW19  Q8IW19  Q8IW19  Q8IW19  Q8IW19  Q8IW19  Q8IW19  Q8IW19  Q8IW19  Q8IW19  Q8IW19  Q8IW19  Q8IW19  Q8IW19  Q8IW19  Q8IW19  Q8IW19  Q8IW19  Q8IW19  Q8IW19  Q8IW19  Q8IW19  Q8IW19  Q8IW19  Q8IW41  Q8IW41  Q8IW41  Q8IW41  Q8IW41  Q8IW41  Q8IW41  Q8IW41  Q8IW41  Q8IW41  Q8IW41  Q8IW41  Q8IW41  Q8IW41  Q8IW41  Q8IW41  Q8IW41  Q8IW41  Q8IW41  Q8IW41  Q8IW41  Q8IW41  Q8IW41  Q8IW41  Q8IW41  Q8IWL1  Q8IWL1  Q8IWL1  Q8IWL1  Q8IWL1  Q8IWL1  Q8IWL1  Q8IWL1  Q8IWL1  Q8IWL1  Q8IWL1  Q8IWT6  Q8IWT6  Q8IWT6  Q8IWT6  Q8IWT6  Q8IWT6  Q8IWT6  Q8IWT6  Q8IWT6  Q8IWT6  Q8IWT6  Q8IWT6  Q8IWT6  Q8IWT6  Q8IWT6  Q8IWT6  Q8IWT6  Q8IWT6  Q8IWT6  Q8IWT6  Q8IWT6  Q8IWT6  Q8IWT6  Q8IWT6  Q8IWT6  Q8IWT6  Q8IWT6  Q8IWT6  Q8IWT6  Q8IWT6  Q8IWT6  Q8IWT6  Q8IWT6  Q8IX03  Q8IX03  Q8IX03  Q8IX03  Q8IX03  Q8IX03  Q8IX03  Q8IX03  Q8IX03  Q8IX03  Q8IX03  Q8IX03  Q8IX03  Q8IX03  Q8IX03  Q8IX03  Q8IX03  Q8IX03  Q8IX03  Q8IX03  Q8IX03  Q8IX03  Q8IX03  Q8IX03  Q8IX03  Q8IX03  Q8IX03  Q8IX03  Q8IX03  Q8IX03  Q8IX03  Q8IXJ6  Q8IXJ6  Q8IXJ6  Q8IXJ6  Q8IXJ6  Q8IXJ6  Q8IXJ6  Q8IXJ6  Q8IXJ6  Q8IXJ6  Q8IXJ6  Q8IXJ6  Q8IXJ6  Q8IXJ6  Q8IXJ6  Q8IXJ6  Q8IXJ6  Q8IXJ6  Q8IXJ6  Q8IXJ6  Q8IXJ6  Q8IXJ6  Q8IXJ6  Q8IXJ6  Q8IXJ6  Q8IXJ6  Q8IXJ6  Q8IXJ6  Q8IY84  Q8IY84  Q8IY84  Q8IY84  Q8IY84  Q8IY84  Q8IY84  Q8IY84  Q8IY84  Q8IY84  Q8IY84  Q8IY84  Q8IY84  Q8IY84  Q8IY84  Q8IY84  Q8IY84  Q8IY84  Q8IY84  Q8IY84  Q8IY84  Q8IY84  Q8IY84  Q8IY84  Q8IY84  Q8IY84  Q8IY84  Q8IY84  Q8IY84  Q8IY84  Q8IY84  Q8IYA6  Q8IYA6  Q8IYA6  Q8IYA6  Q8IYA6  Q8IYA6  Q8IYA6  Q8IYA6  Q8IYA6  Q8IYA6  Q8IYA6  Q8IYA6  Q8IYA6  Q8IYA6  Q8IYA6  Q8IYA6  Q8IYA6  Q8IYA6  Q8IYA6  Q8IYA6  Q8IYA6  Q8IYA6  Q8IYA6  Q8IYA6  Q8IYA6  Q8IYA6  Q8IYA6  Q8IYA6  Q8IYA6  Q8IYA6  Q8IYA6  Q8IYA6  Q8IYA6  Q8IYB3  Q8IYB3  Q8IYB3  Q8IYB3  Q8IYB3  Q8IYB3  Q8IYB3  Q8IYB3  Q8IYB3  Q8IYB3  Q8IYB3  Q8IYB3  Q8IYB3  Q8IYB3  Q8IYB3  Q8IYB3  Q8IYB3  Q8IYB3  Q8IYB3  Q8IYB3  Q8IYB3  Q8IYB3  Q8IYB3  Q8IYB3  Q8IYB3  Q8IYB3  Q8IYB3  Q8IYB3  Q8IYB3  Q8IYB3  Q8IYB3  Q8IZP0  Q8IZP0  Q8IZP0  Q8IZP0  Q8IZP0  Q8IZP0  Q8IZP0  Q8IZP0  Q8IZP0  Q8IZP0  Q8IZP0  Q8IZP0  Q8IZP0  Q8IZP0  Q8IZP0  Q8IZP0  Q8IZP0  Q8IZP0  Q8IZP0  Q8IZP0  Q8IZP0  Q8IZP0  Q8IZP0  Q8IZP0  Q8IZP0  Q8IZP0  Q8IZP0  Q8IZP0  Q8IZP0  Q8IZP0  Q8IZP0  Q8IZP0  Q8IZP0  Q8IZS5  Q8IZS5  Q8IZS5  Q8IZS5  Q8IZS5  Q8IZS5  Q8IZS5  Q8IZS5  Q8IZS5  Q8IZS5  Q8IZS5  Q8IZS5  Q8IZS5  Q8IZS5  Q8IZS5  Q8IZS5  Q8IZS5  Q8IZS5  Q8IZS5  Q8IZS5  Q8IZS5  Q8N122  Q8N122  Q8N122  Q8N122  Q8N122  Q8N122  Q8N122  Q8N122  Q8N122  Q8N122  Q8N122  Q8N122  Q8N122  Q8N122  Q8N122  Q8N122  Q8N122  Q8N122  Q8N122  Q8N122  Q8N122  Q8N122  Q8N122  Q8N122  Q8N122  Q8N122  Q8N122  Q8N122  Q8N122  Q8N122  Q8N122  Q8N163  Q8N163  Q8N163  Q8N163  Q8N163  Q8N163  Q8N163  Q8N163  Q8N163  Q8N163  Q8N163  Q8N163  Q8N163  Q8N163  Q8N163  Q8N163  Q8N163  Q8N163  Q8N163  Q8N163  Q8N163  Q8N163  Q8N163  Q8N163  Q8N163  Q8N163  Q8N163  Q8N163  Q8N163  Q8N163  Q8N163  Q8N163  Q8N163  Q8N1G4  Q8N1G4  Q8N1G4  Q8N1G4  Q8N1G4  Q8N1G4  Q8N1G4  Q8N1G4  Q8N1G4  Q8N1G4  Q8N1G4  Q8N1G4  Q8N1G4  Q8N1G4  Q8N1G4  Q8N1G4  Q8N1G4  Q8N1G4  Q8N1G4  Q8N1G4  Q8N1G4  Q8N1G4  Q8N1G4  Q8N1G4  Q8N1G4  Q8N1G4  Q8N1G4  Q8N1G4  Q8N1G4  Q8N1G4  Q8N1G4  Q8N1G4  Q8N1G4  Q8N264  Q8N264  Q8N264  Q8N264  Q8N264  Q8N264  Q8N264  Q8N264  Q8N264  Q8N264  Q8N264  Q8N264  Q8N264  Q8N264  Q8N264  Q8N264  Q8N264  Q8N264  Q8N264  Q8N264  Q8N264  Q8N264  Q8N264  Q8N264  Q8N264  Q8N264  Q8N264  Q8N264  Q8N264  Q8N264  Q8N264  Q8N2E6  Q8N2E6  Q8N2E6  Q8N2E6  Q8N2E6  Q8N2E6  Q8N2E6  Q8N2E6  Q8N2E6  Q8N2E6  Q8N2E6  Q8N2E6  Q8N2E6  Q8N2E6  Q8N2E6  Q8N4F0  Q8N4F0  Q8N4F0  Q8N4F0  Q8N4F0  Q8N4F0  Q8N4F0  Q8N4F0  Q8N4F0  Q8N4F0  Q8N4F0  Q8N4F0  Q8N4F0  Q8N4F0  Q8N4F0  Q8N4F0  Q8N4F0  Q8N4F0  Q8N4F0  Q8N4F0  Q8N4F0  Q8N4F0  Q8N4F0  Q8N4F0  Q8N4F0  Q8N4F0  Q8N4F0  Q8N4F0  Q8N4F0  Q8N4F0  Q8N4F0  Q8N4N8  Q8N4N8  Q8N4N8  Q8N4N8  Q8N4N8  Q8N4N8  Q8N4N8  Q8N4N8  Q8N4N8  Q8N4N8  Q8N4N8  Q8N4N8  Q8N4N8  Q8N4N8  Q8N4N8  Q8N4N8  Q8N4N8  Q8N4N8  Q8N4N8  Q8N4N8  Q8N4N8  Q8N4N8  Q8N4N8  Q8N4N8  Q8N4N8  Q8N4N8  Q8N4N8  Q8N4N8  Q8N4N8  Q8N4N8  Q8N4N8  Q8N5C8  Q8N5C8  Q8N5C8  Q8N5C8  Q8N5C8  Q8N5C8  Q8N5C8  Q8N5C8  Q8N5C8  Q8N5C8  Q8N5C8  Q8N5C8  Q8N5C8  Q8N5C8  Q8N5C8  Q8N5C8  Q8N5C8  Q8N5C8  Q8N5C8  Q8N5C8  Q8N5C8  Q8N5C8  Q8N5C8  Q8N5C8  Q8N5C8  Q8N5C8  Q8N5C8  Q8N5C8  Q8N5C8  Q8N5C8  Q8N5C8  Q8N5Z0  Q8N5Z0  Q8N5Z0  Q8N5Z0  Q8N5Z0  Q8N5Z0  Q8N5Z0  Q8N5Z0  Q8N5Z0  Q8N5Z0  Q8N5Z0  Q8N5Z0  Q8N5Z0  Q8N5Z0  Q8N5Z0  Q8N5Z0  Q8N5Z0  Q8N5Z0  Q8N5Z0  Q8N5Z0  Q8N5Z0  Q8N5Z0  Q8N5Z0  Q8N5Z0  Q8N5Z0  Q8N5Z0  Q8N5Z0  Q8N5Z0  Q8N5Z0  Q8N5Z0  Q8N5Z0  Q8N5Z0  Q8N5Z0  Q8NB16  Q8NB16  Q8NB16  Q8NB16  Q8NB16  Q8NB16  Q8NB16  Q8NB16  Q8NB16  Q8NB16  Q8NB16  Q8NB16  Q8NB16  Q8NB16  Q8NB16  Q8NB16  Q8NB16  Q8NB16  Q8NB16  Q8NB16  Q8NB16  Q8NB16  Q8NB16  Q8NB16  Q8NB16  Q8NB16  Q8NB16  Q8NBJ4  Q8NBJ4  Q8NBJ4  Q8NBJ4  Q8NBJ4  Q8NBJ4  Q8NBJ4  Q8NBJ4  Q8NBJ4  Q8NBJ4  Q8NBJ4  Q8NBJ4  Q8NBJ4  Q8NBJ4  Q8NBJ4  Q8NBJ4  Q8NBJ4  Q8NBJ4  Q8NBJ4  Q8NBP7  Q8NBP7  Q8NBP7  Q8NBP7  Q8NBP7  Q8NBP7  Q8NBP7  Q8NBP7  Q8NBP7  Q8NBP7  Q8NBP7  Q8NBP7  Q8NBP7  Q8NBP7  Q8NBP7  Q8NBP7  Q8NBP7  Q8NBP7  Q8NBP7  Q8NBP7  Q8NBP7  Q8NBP7  Q8NBP7  Q8NBP7  Q8NBP7  Q8NBP7  Q8NBP7  Q8NBP7  Q8NBP7  Q8NBP7  Q8NBP7  Q8NBX0  Q8NBX0  Q8NBX0  Q8NBX0  Q8NBX0  Q8NBX0  Q8NBX0  Q8NBX0  Q8NBX0  Q8NBX0  Q8NBX0  Q8NBX0  Q8NBX0  Q8NBX0  Q8NBX0  Q8NBX0  Q8NBX0  Q8NBX0  Q8NBX0  Q8NBX0  Q8NBX0  Q8NBX0  Q8NBX0  Q8NBX0  Q8NBX0  Q8NBX0  Q8NBX0  Q8NBX0  Q8NBX0  Q8NBX0  Q8NBX0  Q8NCD3  Q8NCD3  Q8NCD3  Q8NCD3  Q8NCD3  Q8NCD3  Q8NCD3  Q8NCD3  Q8NCD3  Q8NCD3  Q8NCD3  Q8NCD3  Q8NCD3  Q8NCD3  Q8NCD3  Q8NCD3  Q8NCD3  Q8NCD3  Q8NCD3  Q8NCD3  Q8NCD3  Q8NCD3  Q8NCD3  Q8NCD3  Q8NCD3  Q8NCD3  Q8NCD3  Q8NCD3  Q8NCD3  Q8NCD3  Q8NCD3  Q8ND76  Q8ND76  Q8ND76  Q8ND76  Q8ND76  Q8ND76  Q8ND76  Q8ND76  Q8ND76  Q8ND76  Q8ND76  Q8ND76  Q8ND76  Q8ND76  Q8ND76  Q8ND76  Q8ND76  Q8ND76  Q8NDC0  Q8NDC0  Q8NDC0  Q8NDC0  Q8NDC0  Q8NDC0  Q8NDC0  Q8NDC0  Q8NDC0  Q8NDC0  Q8NDC0  Q8NDC0  Q8NDC0  Q8NDC0  Q8NDC0  Q8NDC0  Q8NDC0  Q8NDC0  Q8NDC0  Q8NDC0  Q8NDC0  Q8NE71  Q8NE71  Q8NE71  Q8NE71  Q8NE71  Q8NE71  Q8NE71  Q8NE71  Q8NE71  Q8NE71  Q8NE71  Q8NE71  Q8NE71  Q8NE71  Q8NE71  Q8NE71  Q8NE71  Q8NE71  Q8NE71  Q8NE71  Q8NE71  Q8NE71  Q8NE71  Q8NE71  Q8NE71  Q8NE71  Q8NFW8  Q8NFW8  Q8NFW8  Q8NFW8  Q8NFW8  Q8NFW8  Q8NFW8  Q8NFW8  Q8NFW8  Q8NFW8  Q8NFW8  Q8NFW8  Q8NFW8  Q8NFW8  Q8NFW8  Q8NFW8  Q8NFW8  Q8NFW8  Q8NFW8  Q8NFW8  Q8NFW8  Q8NFW8  Q8NFW8  Q8NFW8  Q8NFW8  Q8NFW8  Q8NFW8  Q8NFW8  Q8NFW8  Q8NFW8  Q8NFW8  Q8NFW8  Q8NFW8  Q8NG66  Q8NG66  Q8NG66  Q8NG66  Q8NG66  Q8NG66  Q8NG66  Q8NG66  Q8NG66  Q8NG66  Q8NG66  Q8NG66  Q8NG66  Q8NG66  Q8NG66  Q8NG66  Q8NG66  Q8NG66  Q8NG66  Q8NG66  Q8NG66  Q8NG66  Q8NG66  Q8NG66  Q8NG66  Q8NG66  Q8NG66  Q8NG66  Q8NG66  Q8NG66  Q8NG66  Q8NHV4  Q8NHV4  Q8NHV4  Q8NHV4  Q8NHV4  Q8NHV4  Q8NHV4  Q8NHV4  Q8NHV4  Q8NHV4  Q8NHV4  Q8NHV4  Q8NHV4  Q8NHV4  Q8NHV4  Q8NHV4  Q8NHV4  Q8NHV4  Q8NHV4  Q8NHV4  Q8NHV4  Q8NHV4  Q8NHV4  Q8NHV4  Q8NHV4  Q8NHV4  Q8NHV4  Q8NHV4  Q8NHV4  Q8NHV4  Q8NHV4  Q8NHW3  Q8NHW3  Q8NHW3  Q8NHW3  Q8NHW3  Q8NHW3  Q8NHW3  Q8NHW3  Q8NHW3  Q8NHW3  Q8NHW3  Q8NHW3  Q8NHW3  Q8NHW3  Q8NHW3  Q8NHW3  Q8NHW3  Q8NHW3  Q8NHW3  Q8NHW3  Q8NHW3  Q8NHW3  Q8NHW3  Q8TAE6  Q8TAE6  Q8TAE6  Q8TAE6  Q8TAE6  Q8TAE6  Q8TAE6  Q8TAE6  Q8TAE6  Q8TAE6  Q8TAE6  Q8TAE6  Q8TAE6  Q8TAE6  Q8TAP9  Q8TAP9  Q8TAP9  Q8TAP9  Q8TAP9  Q8TAP9  Q8TAP9  Q8TAP9  Q8TAP9  Q8TAP9  Q8TAP9  Q8TAP9  Q8TAP9  Q8TAP9  Q8TAP9  Q8TAP9  Q8TAP9  Q8TB45  Q8TB45  Q8TB45  Q8TB45  Q8TB45  Q8TB45  Q8TB45  Q8TB45  Q8TB45  Q8TB45  Q8TB45  Q8TB45  Q8TB45  Q8TB45  Q8TB45  Q8TB45  Q8TB45  Q8TB45  Q8TB45  Q8TB45  Q8TB45  Q8TB45  Q8TB45  Q8TB45  Q8TB45  Q8TB45  Q8TB45  Q8TB45  Q8TB45  Q8TCT0  Q8TCT0  Q8TCT0  Q8TCT0  Q8TCT0  Q8TCT0  Q8TCT0  Q8TCT0  Q8TCT0  Q8TCT0  Q8TCT0  Q8TCT0  Q8TCT0  Q8TCT0  Q8TCT0  Q8TCT0  Q8TCT0  Q8TCT0  Q8TCT0  Q8TCT0  Q8TCT0  Q8TCT0  Q8TCT0  Q8TCT0  Q8TCT0  Q8TCT0  Q8TCT0  Q8TCT0  Q8TCT0  Q8TCT0  Q8TCT0  Q8TD08  Q8TD08  Q8TD08  Q8TD08  Q8TD08  Q8TD08  Q8TD08  Q8TD08  Q8TD08  Q8TD08  Q8TD08  Q8TD08  Q8TD08  Q8TD08  Q8TD08  Q8TD08  Q8TD08  Q8TD08  Q8TD08  Q8TD08  Q8TD08  Q8TD08  Q8TD08  Q8TD08  Q8TD08  Q8TD08  Q8TD08  Q8TD08  Q8TD08  Q8TD08  Q8TD08  Q8TD08  Q8TD08  Q8TD30  Q8TD30  Q8TD30  Q8TD30  Q8TD30  Q8TD30  Q8TD30  Q8TD30  Q8TD30  Q8TD30  Q8TD30  Q8TD30  Q8TD30  Q8TD30  Q8TD30  Q8TD30  Q8TD30  Q8TD30  Q8TD30  Q8TD30  Q8TD30  Q8TD30  Q8TD30  Q8TD30  Q8TD30  Q8TD30  Q8TD30  Q8TD30  Q8TD30  Q8TD30  Q8TD30  Q8TD30  Q8TD30  Q8TDC3  Q8TDC3  Q8TDC3  Q8TDC3  Q8TDC3  Q8TDC3  Q8TDC3  Q8TDC3  Q8TDC3  Q8TDC3  Q8TDC3  Q8TDC3  Q8TDC3  Q8TDC3  Q8TDC3  Q8TDC3  Q8TDC3  Q8TDC3  Q8TDC3  Q8TDC3  Q8TDC3  Q8TDC3  Q8TDC3  Q8TDC3  Q8TDC3  Q8TDC3  Q8TDC3  Q8TDC3  Q8TDC3  Q8TDC3  Q8TDC3  Q8TEP8  Q8TEP8  Q8TEP8  Q8TEP8  Q8TEP8  Q8TEP8  Q8TEP8  Q8TEP8  Q8TEP8  Q8TEP8  Q8TEP8  Q8TEP8  Q8TEP8  Q8TEP8  Q8TEP8  Q8TEP8  Q8TEP8  Q8TEP8  Q8TEP8  Q8TEP8  Q8TEP8  Q8TEP8  Q8TEP8  Q8TEP8  Q8TEP8  Q8TEP8  Q8TEP8  Q8TEP8  Q8TEP8  Q8TEP8  Q8TEP8  Q8TEP8  Q8TEP8  Q8TEW0  Q8TEW0  Q8TEW0  Q8TEW0  Q8TEW0  Q8TEW0  Q8TEW0  Q8TEW0  Q8TEW0  Q8TEW0  Q8TEW0  Q8TEW0  Q8TEW0  Q8TEW0  Q8TEW0  Q8TEW0  Q8TEW0  Q8TEW0  Q8TEW0  Q8TEW0  Q8TEW0  Q8TEW0  Q8TEW0  Q8TEW0  Q8TEW0  Q8TEW0  Q8TEW0  Q8TEW0  Q8TEW0  Q8TEW0  Q8TEW0  Q8TEX9  Q8TEX9  Q8TEX9  Q8TEX9  Q8TEX9  Q8TEX9  Q8TEX9  Q8TEX9  Q8TEX9  Q8TEX9  Q8TEX9  Q8TEX9  Q8TEX9  Q8TEX9  Q8TEX9  Q8TEX9  Q8TEX9  Q8TEX9  Q8TEX9  Q8TEX9  Q8TEX9  Q8TEX9  Q8TEX9  Q8TEX9  Q8TEX9  Q8TEX9  Q8TEX9  Q8TEX9  Q8TEX9  Q8TEX9  Q8TEX9  Q8TEX9  Q8TEX9  Q8TF09  Q8TF09  Q8TF09  Q8TF09  Q8TF76  Q8TF76  Q8TF76  Q8TF76  Q8TF76  Q8TF76  Q8TF76  Q8TF76  Q8TF76  Q8TF76  Q8TF76  Q8TF76  Q8TF76  Q8TF76  Q8TF76  Q8TF76  Q8TF76  Q8TF76  Q8TF76  Q8TF76  Q8TF76  Q8TF76  Q8TF76  Q8TF76  Q8TF76  Q8TF76  Q8TF76  Q8TF76  Q8TF76  Q8TF76  Q8TF76  Q8WTQ7  Q8WTQ7  Q8WTQ7  Q8WTQ7  Q8WTQ7  Q8WTQ7  Q8WTQ7  Q8WTQ7  Q8WTQ7  Q8WTQ7  Q8WTQ7  Q8WTQ7  Q8WTQ7  Q8WTQ7  Q8WTQ7  Q8WTQ7  Q8WTQ7  Q8WTQ7  Q8WTQ7  Q8WTQ7  Q8WTQ7  Q8WTQ7  Q8WTQ7  Q8WTQ7  Q8WTQ7  Q8WTQ7  Q8WTQ7  Q8WU03  Q8WU03  Q8WU03  Q8WU03  Q8WU03  Q8WU03  Q8WU03  Q8WU03  Q8WU03  Q8WU03  Q8WU03  Q8WU03  Q8WU03  Q8WU03  Q8WU03  Q8WU03  Q8WU03  Q8WUI4  Q8WUI4  Q8WUI4  Q8WUI4  Q8WUI4  Q8WUI4  Q8WUI4  Q8WUI4  Q8WUI4  Q8WUI4  Q8WUI4  Q8WUI4  Q8WUI4  Q8WUI4  Q8WUI4  Q8WUI4  Q8WUI4  Q8WUI4  Q8WUI4  Q8WUI4  Q8WUI4  Q8WUI4  Q8WUI4  Q8WUI4  Q8WUI4  Q8WUI4  Q8WUI4  Q8WUI4  Q8WUI4  Q8WUI4  Q8WUI4  Q8WUM4  Q8WUM4  Q8WUM4  Q8WUM4  Q8WUM4  Q8WUM4  Q8WUM4  Q8WUM4  Q8WUM4  Q8WUM4  Q8WUM4  Q8WUM4  Q8WUM4  Q8WUM4  Q8WUM4  Q8WUM4  Q8WUM4  Q8WUM4  Q8WUM4  Q8WUM4  Q8WUM4  Q8WUM4  Q8WUM4  Q8WUM4  Q8WUM4  Q8WUM4  Q8WUM4  Q8WUM4  Q8WUM4  Q8WUM4  Q8WUM4  Q8WUM4  Q8WUM4  Q8WV28  Q8WV28  Q8WV28  Q8WV28  Q8WV28  Q8WV28  Q8WV28  Q8WV28  Q8WV28  Q8WV28  Q8WV28  Q8WV28  Q8WV28  Q8WV28  Q8WV28  Q8WV28  Q8WV28  Q8WV28  Q8WV28  Q8WV28  Q8WV28  Q8WV28  Q8WV28  Q8WV28  Q8WV28  Q8WV28  Q8WV28  Q8WV28  Q8WV28  Q8WV28  Q8WV28  Q8WV28  Q8WV28  Q8WVM8  Q8WVM8  Q8WVM8  Q8WVM8  Q8WVM8  Q8WVM8  Q8WVM8  Q8WVM8  Q8WVM8  Q8WVM8  Q8WVM8  Q8WVM8  Q8WVM8  Q8WVM8  Q8WVM8  Q8WVM8  Q8WVM8  Q8WVM8  Q8WVM8  Q8WVM8  Q8WVM8  Q8WVM8  Q8WVM8  Q8WVM8  Q8WVM8  Q8WVM8  Q8WVM8  Q8WVM8  Q8WVM8  Q8WVM8  Q8WVM8  Q8WVM8  Q8WVM8  Q8WW12  Q8WW12  Q8WW12  Q8WW12  Q8WW12  Q8WW12  Q8WW12  Q8WW12  Q8WW12  Q8WW12  Q8WW12  Q8WW12  Q8WW12  Q8WW12  Q8WW12  Q8WW12  Q8WW12  Q8WW12  Q8WW12  Q8WW12  Q8WW12  Q8WWA1  Q8WWA1  Q8WWA1  Q8WWA1  Q8WWA1  Q8WWA1  Q8WWA1  Q8WWA1  Q8WWA1  Q8WWA1  Q8WWA1  Q8WWA1  Q8WWA1  Q8WWA1  Q8WWA1  Q8WWA1  Q8WWA1  Q8WWA1  Q8WWA1  Q8WWA1  Q8WWA1  Q8WWA1  Q8WWA1  Q8WWA1  Q8WWM7  Q8WWM7  Q8WWM7  Q8WWM7  Q8WWM7  Q8WWM7  Q8WWM7  Q8WWM7  Q8WWM7  Q8WWM7  Q8WWM7  Q8WWM7  Q8WWM7  Q8WWM7  Q8WWM7  Q8WWM7  Q8WWM7  Q8WWM7  Q8WWM7  Q8WWM7  Q8WWM7  Q8WWM7  Q8WWM7  Q8WWM7  Q8WWM7  Q8WWM7  Q8WWM7  Q8WWM7  Q8WWM7  Q8WWM7  Q8WWM7  Q8WWM7  Q8WWM7  Q8WX93  Q8WX93  Q8WX93  Q8WX93  Q8WX93  Q8WX93  Q8WX93  Q8WX93  Q8WX93  Q8WX93  Q8WX93  Q8WX93  Q8WX93  Q8WX93  Q8WX93  Q8WX93  Q8WX93  Q8WX93  Q8WX93  Q8WX93  Q8WX93  Q8WX93  Q8WX93  Q8WX93  Q8WX93  Q8WX93  Q8WX93  Q8WX93  Q8WX93  Q8WX93  Q8WX93  Q8WYJ6  Q8WYJ6  Q8WYJ6  Q8WYJ6  Q8WYJ6  Q8WYJ6  Q8WYJ6  Q8WYJ6  Q8WYJ6  Q8WYJ6  Q8WYJ6  Q8WYJ6  Q8WYJ6  Q8WYJ6  Q8WYJ6  Q8WYJ6  Q8WYK2  Q8WYK2  Q8WYK2  Q8WYK2  Q8WYK2  Q8WYK2  Q8WYK2  Q8WYK2  Q8WYL5  Q8WYL5  Q8WYL5  Q8WYL5  Q8WYL5  Q8WYL5  Q8WYL5  Q8WYL5  Q8WYL5  Q8WYL5  Q8WYL5  Q8WYL5  Q8WYL5  Q8WYL5  Q8WYL5  Q8WYL5  Q8WYL5  Q8WYL5  Q8WYL5  Q8WYL5  Q8WYL5  Q8WYL5  Q8WYL5  Q8WYL5  Q8WYL5  Q8WYL5  Q8WYL5  Q8WYL5  Q8WYL5  Q8WYL5  Q8WYL5  Q92504  Q92504  Q92504  Q92504  Q92504  Q92504  Q92504  Q92504  Q92504  Q92504  Q92504  Q92504  Q92504  Q92504  Q92504  Q92504  Q92504  Q92504  Q92504  Q92504  Q92504  Q92504  Q92504  Q92504  Q92504  Q92504  Q92504  Q92504  Q92504  Q92504  Q92504  Q92522  Q92522  Q92522  Q92522  Q92522  Q92522  Q92522  Q92522  Q92522  Q92522  Q92522  Q92522  Q92538  Q92538  Q92538  Q92538  Q92538  Q92538  Q92538  Q92538  Q92538  Q92538  Q92538  Q92538  Q92538  Q92538  Q92538  Q92538  Q92538  Q92538  Q92538  Q92538  Q92538  Q92538  Q92538  Q92538  Q92538  Q92538  Q92538  Q92538  Q92538  Q92538  Q92538  Q92538  Q92538  Q92556  Q92556  Q92556  Q92556  Q92556  Q92556  Q92556  Q92556  Q92556  Q92556  Q92556  Q92556  Q92556  Q92556  Q92556  Q92556  Q92556  Q92556  Q92556  Q92556  Q92556  Q92556  Q92556  Q92556  Q92556  Q92556  Q92556  Q92556  Q92556  Q92556  Q92556  Q92556  Q92556  Q92563  Q92563  Q92563  Q92563  Q92563  Q92563  Q92563  Q92563  Q92563  Q92563  Q92563  Q92563  Q92563  Q92563  Q92563  Q92563  Q92563  Q92563  Q92563  Q92563  Q92563  Q92563  Q92563  Q92563  Q92563  Q92563  Q92563  Q92563  Q92563  Q92563  Q92563  Q92574  Q92574  Q92574  Q92574  Q92574  Q92574  Q92574  Q92574  Q92574  Q92574  Q92574  Q92574  Q92574  Q92574  Q92574  Q92574  Q92574  Q92574  Q92574  Q92574  Q92574  Q92574  Q92574  Q92574  Q92574  Q92574  Q92574  Q92574  Q92574  Q92574  Q92574  Q92597  Q92597  Q92597  Q92597  Q92597  Q92597  Q92597  Q92597  Q92597  Q92597  Q92597  Q92597  Q92597  Q92597  Q92597  Q92597  Q92597  Q92597  Q92597  Q92597  Q92597  Q92597  Q92597  Q92600  Q92600  Q92600  Q92600  Q92600  Q92600  Q92600  Q92600  Q92600  Q92600  Q92600  Q92600  Q92600  Q92600  Q92600  Q92600  Q92600  Q92600  Q92616  Q92616  Q92616  Q92616  Q92616  Q92616  Q92616  Q92616  Q92616  Q92616  Q92616  Q92616  Q92616  Q92616  Q92616  Q92616  Q92616  Q92616  Q92616  Q92616  Q92616  Q92616  Q92616  Q92616  Q92616  Q92616  Q92616  Q92616  Q92616  Q92616  Q92616  Q92616  Q92616  Q92620  Q92620  Q92620  Q92620  Q92620  Q92620  Q92620  Q92620  Q92620  Q92620  Q92620  Q92620  Q92620  Q92620  Q92620  Q92620  Q92620  Q92620  Q92620  Q92620  Q92620  Q92620  Q92620  Q92620  Q92620  Q92620  Q92620  Q92620  Q92620  Q92620  Q92620  Q92620  Q92620  Q92625  Q92625  Q92625  Q92625  Q92625  Q92625  Q92625  Q92625  Q92625  Q92625  Q92625  Q92625  Q92625  Q92625  Q92625  Q92625  Q92625  Q92625  Q92625  Q92625  Q92625  Q92625  Q92625  Q92625  Q92625  Q92625  Q92625  Q92625  Q92625  Q92625  Q92625  Q92625  Q92625  Q92630  Q92630  Q92630  Q92630  Q92630  Q92630  Q92630  Q92630  Q92630  Q92630  Q92630  Q92630  Q92630  Q92630  Q92630  Q92630  Q92630  Q92630  Q92630  Q92630  Q92630  Q92630  Q92630  Q92630  Q92630  Q92630  Q92630  Q92630  Q92630  Q92630  Q92630  Q92673  Q92673  Q92673  Q92673  Q92673  Q92673  Q92673  Q92673  Q92673  Q92673  Q92673  Q92673  Q92673  Q92673  Q92673  Q92673  Q92673  Q92673  Q92673  Q92673  Q92673  Q92673  Q92673  Q92673  Q92673  Q92673  Q92673  Q92673  Q92673  Q92673  Q92673  Q92686  Q92686  Q92686  Q92731  Q92731  Q92731  Q92731  Q92731  Q92731  Q92731  Q92731  Q92731  Q92731  Q92731  Q92731  Q92731  Q92731  Q92731  Q92731  Q92731  Q92731  Q92731  Q92731  Q92731  Q92731  Q92731  Q92731  Q92731  Q92731  Q92731  Q92731  Q92731  Q92731  Q92734  Q92734  Q92734  Q92734  Q92734  Q92734  Q92734  Q92734  Q92734  Q92734  Q92734  Q92734  Q92734  Q92734  Q92734  Q92734  Q92734  Q92734  Q92734  Q92734  Q92734  Q92734  Q92734  Q92734  Q92734  Q92734  Q92734  Q92734  Q92734  Q92734  Q92734  Q92734  Q92734  Q92736  Q92736  Q92736  Q92736  Q92736  Q92736  Q92736  Q92736  Q92736  Q92736  Q92736  Q92736  Q92736  Q92736  Q92736  Q92736  Q92736  Q92736  Q92736  Q92736  Q92736  Q92736  Q92736  Q92736  Q92736  Q92736  Q92736  Q92736  Q92736  Q92736  Q92736  Q92783  Q92783  Q92783  Q92783  Q92783  Q92783  Q92783  Q92783  Q92783  Q92783  Q92783  Q92783  Q92783  Q92783  Q92783  Q92783  Q92783  Q92783  Q92783  Q92783  Q92783  Q92783  Q92783  Q92783  Q92783  Q92783  Q92783  Q92783  Q92783  Q92783  Q92783  Q92783  Q92783  Q92793  Q92793  Q92793  Q92793  Q92793  Q92793  Q92793  Q92793  Q92793  Q92793  Q92793  Q92793  Q92793  Q92793  Q92793  Q92793  Q92793  Q92793  Q92793  Q92793  Q92793  Q92793  Q92793  Q92793  Q92793  Q92793  Q92793  Q92793  Q92793  Q92793  Q92793  Q92794  Q92794  Q92794  Q92794  Q92794  Q92794  Q92794  Q92794  Q92794  Q92794  Q92794  Q92794  Q92794  Q92794  Q92794  Q92794  Q92794  Q92794  Q92794  Q92794  Q92794  Q92794  Q92794  Q92794  Q92794  Q92794  Q92794  Q92794  Q92794  Q92794  Q92794  Q92794  Q92794  Q92804  Q92804  Q92804  Q92804  Q92804  Q92804  Q92804  Q92804  Q92804  Q92804  Q92804  Q92804  Q92804  Q92804  Q92804  Q92804  Q92804  Q92804  Q92804  Q92804  Q92804  Q92804  Q92804  Q92804  Q92804  Q92804  Q92804  Q92804  Q92804  Q92804  Q92804  Q92804  Q92804  Q92888  Q92888  Q92888  Q92888  Q92888  Q92888  Q92888  Q92888  Q92888  Q92888  Q92888  Q92888  Q92888  Q92888  Q92888  Q92888  Q92888  Q92888  Q92888  Q92888  Q92888  Q92888  Q92888  Q92888  Q92888  Q92888  Q92888  Q92888  Q92888  Q92888  Q92888  Q92888  Q92888  Q92900  Q92900  Q92900  Q92900  Q92900  Q92900  Q92900  Q92900  Q92900  Q92900  Q92900  Q92900  Q92900  Q92900  Q92900  Q92900  Q92900  Q92900  Q92900  Q92900  Q92900  Q92900  Q92900  Q92900  Q92900  Q92900  Q92900  Q92900  Q92900  Q92900  Q92900  Q92905  Q92905  Q92905  Q92905  Q92905  Q92905  Q92905  Q92905  Q92905  Q92905  Q92905  Q92905  Q92905  Q92905  Q92905  Q92905  Q92905  Q92905  Q92905  Q92905  Q92905  Q92905  Q92918  Q92918  Q92918  Q92918  Q92918  Q92918  Q92918  Q92918  Q92918  Q92918  Q92918  Q92918  Q92918  Q92918  Q92918  Q92918  Q92918  Q92918  Q92918  Q92918  Q92918  Q92918  Q92918  Q92918  Q92918  Q92918  Q92918  Q92918  Q92918  Q92918  Q92918  Q92922  Q92922  Q92922  Q92922  Q92922  Q92922  Q92922  Q92922  Q92922  Q92922  Q92922  Q92922  Q92922  Q92922  Q92922  Q92922  Q92922  Q92922  Q92922  Q92922  Q92922  Q92922  Q92922  Q92922  Q92922  Q92922  Q92922  Q92922  Q92922  Q92922  Q92922  Q92922  Q92922  Q92934  Q92934  Q92934  Q92934  Q92934  Q92934  Q92934  Q92934  Q92934  Q92934  Q92934  Q92934  Q92934  Q92934  Q92934  Q92934  Q92934  Q92934  Q92934  Q92945  Q92945  Q92945  Q92945  Q92945  Q92945  Q92945  Q92945  Q92945  Q92945  Q92945  Q92945  Q92945  Q92945  Q92945  Q92945  Q92945  Q92945  Q92945  Q92945  Q92945  Q92945  Q92945  Q92945  Q92945  Q92974  Q92974  Q92974  Q92974  Q92974  Q92974  Q92974  Q92974  Q92974  Q92974  Q92974  Q92974  Q92974  Q92974  Q92974  Q92974  Q92974  Q92974  Q92974  Q92974  Q92974  Q92974  Q92974  Q92974  Q92974  Q92974  Q92974  Q92974  Q92974  Q92974  Q92974  Q92979  Q92979  Q92979  Q92979  Q92979  Q92979  Q92979  Q92979  Q92979  Q92979  Q92979  Q92979  Q92979  Q92979  Q92979  Q92979  Q92985  Q92985  Q92985  Q92985  Q92985  Q92985  Q92985  Q92985  Q92985  Q92985  Q92985  Q92985  Q92985  Q92985  Q92985  Q92985  Q92985  Q92985  Q92985  Q92985  Q92985  Q92985  Q92985  Q92985  Q92985  Q92985  Q92985  Q92985  Q92985  Q92985  Q92985  Q92993  Q92993  Q92993  Q92993  Q92993  Q92993  Q92993  Q92993  Q92993  Q92993  Q92993  Q92993  Q92993  Q92993  Q92993  Q92993  Q92993  Q92993  Q92993  Q92993  Q92993  Q92993  Q92993  Q92993  Q92993  Q92993  Q92993  Q92993  Q92993  Q92993  Q92993  Q92997  Q92997  Q92997  Q92997  Q92997  Q92997  Q92997  Q92997  Q92997  Q92997  Q92997  Q92997  Q92997  Q92997  Q92997  Q92997  Q92997  Q92997  Q92997  Q92997  Q92997  Q92997  Q92997  Q92997  Q92997  Q92997  Q92997  Q92997  Q92997  Q92997  Q92997  Q93009  Q93009  Q93009  Q93009  Q93009  Q93009  Q93009  Q93009  Q93009  Q93009  Q93009  Q93009  Q93009  Q93009  Q93009  Q93009  Q93009  Q93009  Q93009  Q93009  Q93009  Q93009  Q93009  Q93009  Q93009  Q93009  Q93009  Q93009  Q93009  Q93009  Q93009  Q93084  Q93084  Q93084  Q93084  Q93084  Q93084  Q93084  Q93084  Q93084  Q93084  Q93084  Q93084  Q93084  Q93084  Q93084  Q93084  Q93084  Q93084  Q93084  Q93084  Q93084  Q93084  Q93084  Q93084  Q93084  Q93084  Q93084  Q93084  Q93084  Q93084  Q93084  Q93084  Q93084  Q969H0  Q969H0  Q969H0  Q969H0  Q969H0  Q969H0  Q969H0  Q969H0  Q969H0  Q969H0  Q969H0  Q969H0  Q969H0  Q969H0  Q969H0  Q969H0  Q969H0  Q969H0  Q969H0  Q969H0  Q969H0  Q969H0  Q969H0  Q969H0  Q969H0  Q969H0  Q969H0  Q969H0  Q969H0  Q969H0  Q969H0  Q969M7  Q969M7  Q969M7  Q969M7  Q969M7  Q969M7  Q969M7  Q969Q0  Q969Q0  Q969Q0  Q969S2  Q969S2  Q969S2  Q969S2  Q969S2  Q969S2  Q969S2  Q969S2  Q969S2  Q969S2  Q969S2  Q969S2  Q969S2  Q969S2  Q969S2  Q969S2  Q969S2  Q969S2  Q969S2  Q969S2  Q969S2  Q969S2  Q969S2  Q969S2  Q969S2  Q969S2  Q969S2  Q969S2  Q969U6  Q969U6  Q969U6  Q969U6  Q969U6  Q969U6  Q969U6  Q969U6  Q969U6  Q969U6  Q969U6  Q969U6  Q969U6  Q969U6  Q969U6  Q969U6  Q969U6  Q969U6  Q969U6  Q969U6  Q969U6  Q969U6  Q969U6  Q969U6  Q969U6  Q969U6  Q969U6  Q969U6  Q969U6  Q969U6  Q969U6  Q96A00  Q96A00  Q96A00  Q96A00  Q96A00  Q96A00  Q96A00  Q96A00  Q96A00  Q96A00  Q96A00  Q96A65  Q96A65  Q96A65  Q96A65  Q96A65  Q96A65  Q96A65  Q96A65  Q96A65  Q96A65  Q96A65  Q96A65  Q96A65  Q96A65  Q96A65  Q96A65  Q96A65  Q96A65  Q96A65  Q96A65  Q96A65  Q96A65  Q96A65  Q96A65  Q96A65  Q96A65  Q96A65  Q96A65  Q96A65  Q96A65  Q96A65  Q96A65  Q96A65  Q96A72  Q96A72  Q96A72  Q96A72  Q96A72  Q96A72  Q96A72  Q96A72  Q96AD5  Q96AD5  Q96AD5  Q96AD5  Q96AD5  Q96AD5  Q96AD5  Q96AD5  Q96AD5  Q96AD5  Q96AD5  Q96AD5  Q96AD5  Q96AD5  Q96AD5  Q96AD5  Q96AD5  Q96AD5  Q96AD5  Q96AD5  Q96AD5  Q96AD5  Q96AD5  Q96AD5  Q96AD5  Q96AD5  Q96AD5  Q96AD5  Q96AD5  Q96AD5  Q96AD5  Q96AX2  Q96AX2  Q96AX2  Q96AX2  Q96AX2  Q96AX2  Q96AX2  Q96AX2  Q96AX2  Q96AX2  Q96AX2  Q96AX2  Q96AX2  Q96B36  Q96B36  Q96B36  Q96B36  Q96B36  Q96B36  Q96B36  Q96B36  Q96B36  Q96B36  Q96B36  Q96B36  Q96B36  Q96B36  Q96B36  Q96B36  Q96B36  Q96B36  Q96BD6  Q96BD6  Q96BD6  Q96BD6  Q96BD6  Q96BD6  Q96BD6  Q96BD6  Q96BD6  Q96BD6  Q96BD6  Q96BD6  Q96BR1  Q96BR1  Q96BR1  Q96BR1  Q96BR1  Q96BR1  Q96BR1  Q96BR1  Q96BR1  Q96BR1  Q96BR1  Q96BR1  Q96BR1  Q96BR1  Q96BR1  Q96BR1  Q96BR1  Q96BR1  Q96BR1  Q96BR1  Q96BR1  Q96BR1  Q96BR1  Q96BR1  Q96BR1  Q96BR1  Q96BR1  Q96BT3  Q96BT3  Q96BT3  Q96BT3  Q96BT3  Q96BT3  Q96BT3  Q96BT3  Q96BT3  Q96BT3  Q96BT3  Q96BT3  Q96BT3  Q96BT3  Q96BT3  Q96BT3  Q96BT3  Q96BT3  Q96BT3  Q96BT3  Q96BT3  Q96BT3  Q96BT3  Q96BT3  Q96BT3  Q96BT3  Q96BT3  Q96BT3  Q96BT3  Q96BT3  Q96BT3  Q96C19  Q96C19  Q96C19  Q96C19  Q96C19  Q96C19  Q96C19  Q96C19  Q96C19  Q96C19  Q96C19  Q96C19  Q96C19  Q96C19  Q96C19  Q96C36  Q96C36  Q96C36  Q96C36  Q96C36  Q96C36  Q96C36  Q96C36  Q96C36  Q96C36  Q96C36  Q96C36  Q96C36  Q96C36  Q96C36  Q96C36  Q96C36  Q96C36  Q96C36  Q96C36  Q96C36  Q96C36  Q96C36  Q96C36  Q96C36  Q96C86  Q96C86  Q96C86  Q96C86  Q96C86  Q96C86  Q96C86  Q96C86  Q96C86  Q96C86  Q96C86  Q96C86  Q96C86  Q96C86  Q96CF2  Q96CF2  Q96CF2  Q96CF2  Q96CF2  Q96CF2  Q96CF2  Q96CF2  Q96CF2  Q96CF2  Q96CF2  Q96CF2  Q96CF2  Q96CF2  Q96CF2  Q96CF2  Q96CN5  Q96CN5  Q96CN5  Q96CN5  Q96CN5  Q96CN5  Q96CN5  Q96CN5  Q96CN5  Q96CN5  Q96CN5  Q96CN5  Q96CN5  Q96CN5  Q96CN5  Q96CN5  Q96CN5  Q96CN5  Q96CN5  Q96CN5  Q96CN5  Q96CN5  Q96CN5  Q96CN5  Q96CN5  Q96CN5  Q96CN5  Q96CN5  Q96CN5  Q96CN5  Q96CN5  Q96CT7  Q96CT7  Q96CT7  Q96CT7  Q96CT7  Q96CT7  Q96CT7  Q96CT7  Q96CV9  Q96CV9  Q96CV9  Q96CV9  Q96CV9  Q96CV9  Q96CV9  Q96CV9  Q96CV9  Q96CV9  Q96CV9  Q96CV9  Q96CV9  Q96CV9  Q96CV9  Q96CV9  Q96CV9  Q96CV9  Q96CV9  Q96CV9  Q96CV9  Q96CV9  Q96CV9  Q96CV9  Q96CV9  Q96CV9  Q96CV9  Q96CV9  Q96CV9  Q96CV9  Q96CV9  Q96DA6  Q96DA6  Q96DA6  Q96DA6  Q96DA6  Q96DG6  Q96DG6  Q96DG6  Q96DG6  Q96DG6  Q96DG6  Q96DG6  Q96DG6  Q96DG6  Q96DG6  Q96DG6  Q96EB6  Q96EB6  Q96EB6  Q96EB6  Q96EB6  Q96EB6  Q96EB6  Q96EB6  Q96EB6  Q96EB6  Q96EB6  Q96EB6  Q96EB6  Q96EB6  Q96EB6  Q96EB6  Q96EB6  Q96EB6  Q96EB6  Q96EB6  Q96EB6  Q96EB6  Q96EB6  Q96EB6  Q96EB6  Q96EB6  Q96EB6  Q96EB6  Q96EB6  Q96EB6  Q96EB6  Q96EG1  Q96EG1  Q96EG1  Q96EG1  Q96EG1  Q96EG1  Q96EG1  Q96EG1  Q96EG1  Q96EG1  Q96EG1  Q96EG1  Q96EG1  Q96EG1  Q96EG1  Q96EG1  Q96EG1  Q96EG1  Q96EG1  Q96EG1  Q96EG1  Q96EG1  Q96EG1  Q96EG1  Q96EG1  Q96EG1  Q96EG1  Q96EG1  Q96EG1  Q96EG1  Q96EP5  Q96EP5  Q96EP5  Q96EP5  Q96EP5  Q96EP5  Q96EP5  Q96EP5  Q96EP5  Q96EP5  Q96EP5  Q96EP5  Q96EP5  Q96EP5  Q96EP5  Q96EP5  Q96EP5  Q96EP5  Q96EP5  Q96EP5  Q96EP5  Q96EP5  Q96EP5  Q96EP5  Q96EP5  Q96EY1  Q96EY1  Q96EY1  Q96EY1  Q96EY1  Q96EY1  Q96EY1  Q96EY1  Q96EY1  Q96EY1  Q96EY1  Q96EY1  Q96EY1  Q96EY1  Q96EY1  Q96EY1  Q96EY1  Q96EY1  Q96EY1  Q96EY1  Q96EY1  Q96EY1  Q96EY1  Q96EY1  Q96EY1  Q96EY1  Q96EY1  Q96EY1  Q96EY1  Q96EY1  Q96EY1  Q96EY1  Q96EY1  Q96EY5  Q96EY5  Q96EY5  Q96EY5  Q96EY5  Q96EY5  Q96EY5  Q96EY5  Q96EY5  Q96EY5  Q96EY5  Q96EY5  Q96EY5  Q96EY5  Q96EY5  Q96EY5  Q96EY5  Q96EY5  Q96EY5  Q96EY5  Q96EY5  Q96EY5  Q96EY5  Q96EY5  Q96EY5  Q96EY5  Q96EY5  Q96EY5  Q96G03  Q96G03  Q96G03  Q96G03  Q96G03  Q96G03  Q96G03  Q96G03  Q96G03  Q96G03  Q96G03  Q96G03  Q96G03  Q96G03  Q96G03  Q96G03  Q96G03  Q96G03  Q96G03  Q96G03  Q96G03  Q96G03  Q96G03  Q96G03  Q96G03  Q96G03  Q96G03  Q96G03  Q96G03  Q96G03  Q96G03  Q96G03  Q96G03  Q96G74  Q96G74  Q96G74  Q96G74  Q96G74  Q96G74  Q96G74  Q96G74  Q96G74  Q96G74  Q96G74  Q96G74  Q96G74  Q96G74  Q96G74  Q96G74  Q96G74  Q96G74  Q96G74  Q96G74  Q96G74  Q96G74  Q96G74  Q96G74  Q96G74  Q96G74  Q96G74  Q96G74  Q96G74  Q96G74  Q96G74  Q96GA7  Q96GA7  Q96GA7  Q96GA7  Q96GA7  Q96GA7  Q96GA7  Q96GA7  Q96GA7  Q96GA7  Q96GA7  Q96GA7  Q96GA7  Q96GA7  Q96GA7  Q96GA7  Q96GA7  Q96H79  Q96H79  Q96H79  Q96H79  Q96H79  Q96H79  Q96H79  Q96H79  Q96H79  Q96H79  Q96H79  Q96H79  Q96H79  Q96H79  Q96H79  Q96H79  Q96H79  Q96H79  Q96H79  Q96HC4  Q96HC4  Q96HC4  Q96HC4  Q96HC4  Q96HC4  Q96HC4  Q96HC4  Q96HC4  Q96HC4  Q96HC4  Q96HC4  Q96HC4  Q96HC4  Q96HC4  Q96HC4  Q96HC4  Q96HC4  Q96HC4  Q96HC4  Q96HC4  Q96HC4  Q96HC4  Q96HC4  Q96HC4  Q96HC4  Q96HC4  Q96HC4  Q96HC4  Q96HC4  Q96HC4  Q96HC4  Q96HC4  Q96HQ2  Q96HQ2  Q96HQ2  Q96HQ2  Q96HQ2  Q96HQ2  Q96HQ2  Q96HQ2  Q96HQ2  Q96I15  Q96I15  Q96I15  Q96I15  Q96I15  Q96I15  Q96I15  Q96I15  Q96I15  Q96I15  Q96I15  Q96I15  Q96I15  Q96I15  Q96I15  Q96I15  Q96I15  Q96I15  Q96I15  Q96I15  Q96I15  Q96I15  Q96I15  Q96I15  Q96I15  Q96I15  Q96J02  Q96J02  Q96J02  Q96J02  Q96J02  Q96J02  Q96J02  Q96J02  Q96J02  Q96J02  Q96J02  Q96J02  Q96J02  Q96J02  Q96J02  Q96J02  Q96J02  Q96J02  Q96J02  Q96J02  Q96J02  Q96J02  Q96J02  Q96J02  Q96J02  Q96J02  Q96J02  Q96J02  Q96J02  Q96J02  Q96J02  Q96JB2  Q96JB2  Q96JB2  Q96JB2  Q96JB2  Q96JB2  Q96JB2  Q96JB2  Q96JB2  Q96JB2  Q96JB2  Q96JB2  Q96JB2  Q96JB2  Q96JB2  Q96JB2  Q96JB2  Q96JB2  Q96JB2  Q96JB2  Q96JB2  Q96JB2  Q96JB2  Q96JB2  Q96JB2  Q96JB2  Q96JB2  Q96JB2  Q96JB2  Q96JB2  Q96JB2  Q96JB2  Q96JB2  Q96KP4  Q96KP4  Q96KP4  Q96KP4  Q96KP4  Q96KP4  Q96KP4  Q96KP4  Q96KP4  Q96KP4  Q96KP4  Q96KP4  Q96KP4  Q96KP4  Q96KP4  Q96KP4  Q96KP4  Q96KP4  Q96KP4  Q96KP4  Q96KP4  Q96KP4  Q96KP4  Q96KP4  Q96KP4  Q96KP4  Q96KP4  Q96KQ7  Q96KQ7  Q96KQ7  Q96KQ7  Q96KQ7  Q96KQ7  Q96KQ7  Q96KQ7  Q96KQ7  Q96KQ7  Q96KQ7  Q96KQ7  Q96KQ7  Q96KQ7  Q96KQ7  Q96KQ7  Q96KQ7  Q96KQ7  Q96KQ7  Q96KQ7  Q96KQ7  Q96KQ7  Q96KQ7  Q96KQ7  Q96KQ7  Q96KQ7  Q96KQ7  Q96KQ7  Q96KQ7  Q96KQ7  Q96KQ7  Q96KQ7  Q96KQ7  Q96L34  Q96L34  Q96L34  Q96L34  Q96L34  Q96L34  Q96L34  Q96L34  Q96L34  Q96L34  Q96L34  Q96L34  Q96L34  Q96L34  Q96L34  Q96L34  Q96L34  Q96L34  Q96L34  Q96L34  Q96L34  Q96L34  Q96L34  Q96L34  Q96L34  Q96L34  Q96L34  Q96L34  Q96L34  Q96L34  Q96L34  Q96L34  Q96L34  Q96LA8  Q96LA8  Q96LA8  Q96LA8  Q96LA8  Q96LA8  Q96LA8  Q96LA8  Q96LA8  Q96LA8  Q96LA8  Q96LA8  Q96LA8  Q96LA8  Q96LA8  Q96LA8  Q96LA8  Q96LA8  Q96LA8  Q96LA8  Q96LA8  Q96LA8  Q96LA8  Q96LC7  Q96LC7  Q96LC7  Q96LC7  Q96LC7  Q96LC7  Q96LC7  Q96LC7  Q96LC7  Q96LC7  Q96LC7  Q96LC7  Q96LC7  Q96LC7  Q96LC7  Q96LC7  Q96LC7  Q96LC7  Q96LC7  Q96LC7  Q96LC7  Q96LC7  Q96LC7  Q96LC7  Q96LC7  Q96LC7  Q96LC7  Q96LC7  Q96LC7  Q96LC7  Q96LC7  Q96LC7  Q96LC7  Q96P11  Q96P11  Q96P11  Q96P11  Q96P11  Q96P11  Q96P11  Q96P11  Q96P11  Q96P11  Q96P11  Q96P11  Q96P11  Q96P11  Q96P11  Q96P11  Q96P11  Q96P11  Q96P11  Q96P11  Q96P11  Q96P11  Q96P11  Q96P11  Q96P11  Q96P11  Q96P11  Q96P11  Q96P11  Q96P11  Q96P11  Q96P11  Q96P16  Q96P16  Q96P16  Q96P16  Q96P16  Q96P16  Q96P16  Q96P16  Q96P16  Q96P16  Q96P16  Q96P16  Q96P16  Q96P16  Q96P16  Q96P16  Q96P16  Q96P16  Q96P16  Q96P16  Q96P16  Q96P16  Q96P16  Q96P16  Q96P16  Q96P16  Q96P70  Q96P70  Q96P70  Q96P70  Q96P70  Q96P70  Q96P70  Q96P70  Q96P70  Q96P70  Q96P70  Q96P70  Q96P70  Q96P70  Q96P70  Q96P70  Q96P70  Q96P70  Q96P70  Q96P70  Q96P70  Q96P70  Q96P70  Q96P70  Q96P70  Q96P70  Q96P70  Q96P70  Q96P70  Q96P70  Q96P70  Q96P70  Q96P70  Q96PD5  Q96PD5  Q96PD5  Q96PD5  Q96PD5  Q96PD5  Q96PD5  Q96PD5  Q96PD5  Q96PD5  Q96PD5  Q96PD5  Q96PD5  Q96PD5  Q96PD5  Q96PD5  Q96PD5  Q96PD5  Q96PD5  Q96PD5  Q96PD5  Q96PD5  Q96PD5  Q96PD5  Q96PD5  Q96PD5  Q96PD5  Q96PD5  Q96PD5  Q96PD5  Q96PD5  Q96PU5  Q96PU5  Q96PU5  Q96PU5  Q96PU5  Q96PU5  Q96PU5  Q96PU5  Q96PU5  Q96PU5  Q96PU5  Q96PU5  Q96PU5  Q96PU5  Q96PU5  Q96PU5  Q96PU5  Q96PU5  Q96PU5  Q96PU5  Q96PU5  Q96PU5  Q96PU5  Q96PU5  Q96PU5  Q96PU5  Q96PU5  Q96PU5  Q96PU5  Q96PU5  Q96PU5  Q96PU8  Q96PU8  Q96PU8  Q96PU8  Q96PU8  Q96PU8  Q96PU8  Q96PU8  Q96PU8  Q96PU8  Q96PU8  Q96Q83  Q96Q83  Q96Q83  Q96Q83  Q96Q83  Q96Q83  Q96Q83  Q96Q83  Q96Q83  Q96Q83  Q96Q83  Q96Q83  Q96Q83  Q96Q83  Q96Q89  Q96Q89  Q96Q89  Q96Q89  Q96Q89  Q96Q89  Q96Q89  Q96Q89  Q96Q89  Q96Q89  Q96Q89  Q96Q89  Q96Q89  Q96Q89  Q96Q89  Q96Q89  Q96Q89  Q96Q89  Q96Q89  Q96Q89  Q96Q89  Q96Q89  Q96Q89  Q96Q89  Q96Q89  Q96Q89  Q96Q89  Q96Q89  Q96Q89  Q96Q89  Q96Q89  Q96Q89  Q96Q89  Q96R06  Q96R06  Q96R06  Q96R06  Q96R06  Q96R06  Q96R06  Q96R06  Q96R06  Q96R06  Q96R06  Q96R06  Q96R06  Q96R06  Q96R06  Q96R06  Q96R06  Q96R06  Q96R06  Q96R06  Q96R06  Q96R06  Q96R06  Q96R06  Q96R06  Q96R06  Q96R06  Q96R06  Q96R06  Q96R06  Q96R06  Q96RG2  Q96RG2  Q96RG2  Q96RG2  Q96RG2  Q96RG2  Q96RG2  Q96RG2  Q96RG2  Q96RG2  Q96RG2  Q96RG2  Q96RG2  Q96RG2  Q96RG2  Q96RG2  Q96RG2  Q96RG2  Q96RG2  Q96RG2  Q96RG2  Q96RG2  Q96RG2  Q96RG2  Q96RG2  Q96RG2  Q96RG2  Q96RG2  Q96RG2  Q96RG2  Q96RG2  Q96RG2  Q96RG2  Q96RI1  Q96RI1  Q96RI1  Q96RI1  Q96RI1  Q96RI1  Q96RI1  Q96RI1  Q96RI1  Q96RI1  Q96RI1  Q96RI1  Q96RI1  Q96RI1  Q96RI1  Q96RI1  Q96RI1  Q96RI1  Q96RI1  Q96RI1  Q96RI1  Q96RI1  Q96RI1  Q96RI1  Q96RI1  Q96RI1  Q96RI1  Q96RI1  Q96RI1  Q96RI1  Q96RI1  Q96RL1  Q96RL1  Q96RL1  Q96RL1  Q96RL1  Q96RL1  Q96RL1  Q96RL1  Q96RL1  Q96RL1  Q96RL1  Q96RL1  Q96RL1  Q96RL1  Q96RL1  Q96RL1  Q96RL1  Q96RL1  Q96RL1  Q96RL1  Q96RL1  Q96RL1  Q96RL1  Q96RL1  Q96RL1  Q96RL1  Q96RL1  Q96RL1  Q96RL1  Q96RL1  Q96RL1  Q96RU2  Q96RU2  Q96RU2  Q96RU2  Q96RU2  Q96RU2  Q96RU2  Q96RU2  Q96RU2  Q96RU2  Q96RU2  Q96RU2  Q96RU2  Q96RU2  Q96RU2  Q96RU2  Q96RU2  Q96RU2  Q96RU2  Q96RU2  Q96RU2  Q96RU2  Q96RU2  Q96RU2  Q96RU2  Q96RU2  Q96RU2  Q96RU2  Q96RU2  Q96RU2  Q96RU2  Q96S19  Q96S19  Q96S19  Q96S19  Q96S19  Q96S19  Q96S19  Q96S19  Q96S19  Q96SB4  Q96SB4  Q96SB4  Q96SB4  Q96SB4  Q96SB4  Q96SB4  Q96SB4  Q96SB4  Q96SB4  Q96SB4  Q96SB4  Q96SB4  Q96SB4  Q96SB4  Q96SB4  Q96SB4  Q96SB4  Q96SB4  Q96SB4  Q96SB4  Q96SB4  Q96SB4  Q96SB4  Q96SB4  Q96SB4  Q96SB4  Q96SB4  Q96SB4  Q96SB4  Q96SB4  Q96SD1  Q96SD1  Q96SD1  Q96SD1  Q96SD1  Q96SD1  Q96SD1  Q96SD1  Q96SD1  Q96SD1  Q96SD1  Q96SD1  Q96SD1  Q96SD1  Q96SD1  Q96SD1  Q96SD1  Q96SD1  Q96SD1  Q96SD1  Q96SD1  Q96SD1  Q96SD1  Q96SD1  Q96SD1  Q96SD1  Q96SD1  Q96SD1  Q96SD1  Q96SD1  Q96SD1  Q96T51  Q96T51  Q96T51  Q96T51  Q96T51  Q96T51  Q96T51  Q96T51  Q96T51  Q96T51  Q96T51  Q96T51  Q96T51  Q96T51  Q96T51  Q96T51  Q96T51  Q96T51  Q96T51  Q96T51  Q96T51  Q96T51  Q96T51  Q96T51  Q96T51  Q96T51  Q96T51  Q96T51  Q96T51  Q96T51  Q96T51  Q96T51  Q96T51  Q96T88  Q96T88  Q96T88  Q96T88  Q96T88  Q96T88  Q96T88  Q96T88  Q96T88  Q96T88  Q96T88  Q96T88  Q96T88  Q96T88  Q96T88  Q96T88  Q96T88  Q96T88  Q96T88  Q96T88  Q96T88  Q96T88  Q96T88  Q96T88  Q96T88  Q96T88  Q96T88  Q96T88  Q96T88  Q96T88  Q96T88  Q96TA1  Q96TA1  Q96TA1  Q96TA1  Q96TA1  Q96TA1  Q96TA1  Q96TA1  Q96TA1  Q96TA1  Q96TA1  Q96TA1  Q96TA1  Q96TA1  Q96TA1  Q96TA1  Q96TA1  Q96TA1  Q96TA1  Q96TA1  Q96TA1  Q96TA1  Q96TA1  Q96TA1  Q96TA1  Q96TA1  Q96TA1  Q96TA1  Q96TA1  Q96TA1  Q96TA1  Q99259  Q99259  Q99259  Q99259  Q99259  Q99259  Q99259  Q99259  Q99259  Q99259  Q99259  Q99259  Q99259  Q99259  Q99259  Q99259  Q99259  Q99259  Q99259  Q99259  Q99259  Q99259  Q99259  Q99259  Q99259  Q99259  Q99259  Q99259  Q99259  Q99259  Q99259  Q99259  Q99259  Q99426  Q99426  Q99426  Q99426  Q99426  Q99426  Q99426  Q99426  Q99426  Q99426  Q99426  Q99426  Q99426  Q99426  Q99426  Q99426  Q99426  Q99459  Q99459  Q99459  Q99459  Q99459  Q99459  Q99459  Q99459  Q99459  Q99459  Q99459  Q99459  Q99459  Q99459  Q99459  Q99459  Q99459  Q99459  Q99459  Q99459  Q99459  Q99459  Q99459  Q99459  Q99459  Q99459  Q99459  Q99459  Q99459  Q99459  Q99459  Q99490  Q99490  Q99490  Q99490  Q99490  Q99490  Q99490  Q99490  Q99490  Q99490  Q99490  Q99490  Q99490  Q99490  Q99490  Q99490  Q99490  Q99490  Q99490  Q99490  Q99490  Q99490  Q99490  Q99490  Q99490  Q99490  Q99490  Q99490  Q99490  Q99490  Q99490  Q99490  Q99490  Q99497  Q99497  Q99497  Q99497  Q99497  Q99497  Q99497  Q99497  Q99504  Q99504  Q99504  Q99504  Q99504  Q99504  Q99504  Q99504  Q99504  Q99504  Q99504  Q99504  Q99504  Q99504  Q99504  Q99504  Q99504  Q99504  Q99504  Q99504  Q99504  Q99504  Q99504  Q99504  Q99504  Q99504  Q99504  Q99504  Q99504  Q99504  Q99504  Q99536  Q99536  Q99536  Q99536  Q99536  Q99536  Q99536  Q99536  Q99536  Q99536  Q99536  Q99536  Q99536  Q99536  Q99536  Q99536  Q99536  Q99558  Q99558  Q99558  Q99558  Q99558  Q99558  Q99558  Q99558  Q99558  Q99558  Q99558  Q99558  Q99558  Q99558  Q99558  Q99558  Q99558  Q99558  Q99558  Q99558  Q99558  Q99558  Q99558  Q99558  Q99558  Q99558  Q99558  Q99558  Q99558  Q99558  Q99558  Q99558  Q99558  Q99572  Q99572  Q99572  Q99572  Q99572  Q99572  Q99572  Q99572  Q99572  Q99572  Q99572  Q99572  Q99572  Q99572  Q99572  Q99572  Q99572  Q99572  Q99572  Q99572  Q99572  Q99572  Q99572  Q99572  Q99572  Q99572  Q99572  Q99572  Q99572  Q99572  Q99572  Q99572  Q99572  Q99613  Q99613  Q99613  Q99613  Q99613  Q99613  Q99613  Q99613  Q99613  Q99613  Q99613  Q99613  Q99613  Q99613  Q99613  Q99613  Q99613  Q99613  Q99613  Q99613  Q99613  Q99613  Q99613  Q99613  Q99613  Q99613  Q99613  Q99613  Q99613  Q99613  Q99613  Q99615  Q99615  Q99615  Q99615  Q99615  Q99615  Q99615  Q99615  Q99615  Q99615  Q99615  Q99615  Q99615  Q99615  Q99615  Q99615  Q99615  Q99623  Q99623  Q99623  Q99623  Q99623  Q99623  Q99623  Q99623  Q99623  Q99623  Q99623  Q99623  Q99623  Q99623  Q99623  Q99623  Q99623  Q99623  Q99623  Q99627  Q99627  Q99627  Q99627  Q99627  Q99627  Q99627  Q99627  Q99627  Q99627  Q99638  Q99638  Q99638  Q99638  Q99638  Q99638  Q99638  Q99638  Q99638  Q99638  Q99638  Q99638  Q99638  Q99638  Q99638  Q99638  Q99638  Q99638  Q99638  Q99638  Q99638  Q99638  Q99638  Q99638  Q99638  Q99638  Q99638  Q99638  Q99638  Q99638  Q99638  Q99640  Q99640  Q99640  Q99640  Q99640  Q99640  Q99640  Q99640  Q99640  Q99640  Q99640  Q99640  Q99640  Q99640  Q99640  Q99640  Q99640  Q99640  Q99640  Q99640  Q99640  Q99640  Q99640  Q99640  Q99640  Q99640  Q99640  Q99640  Q99640  Q99640  Q99640  Q99661  Q99661  Q99661  Q99661  Q99661  Q99661  Q99661  Q99661  Q99661  Q99661  Q99661  Q99661  Q99661  Q99661  Q99661  Q99661  Q99661  Q99661  Q99661  Q99661  Q99661  Q99661  Q99661  Q99661  Q99661  Q99661  Q99661  Q99661  Q99661  Q99661  Q99665  Q99665  Q99665  Q99665  Q99665  Q99665  Q99665  Q99665  Q99665  Q99665  Q99665  Q99665  Q99665  Q99665  Q99665  Q99665  Q99665  Q99665  Q99665  Q99665  Q99665  Q99665  Q99665  Q99665  Q99665  Q99665  Q99665  Q99665  Q99665  Q99665  Q99665  Q99665  Q99665  Q99683  Q99683  Q99683  Q99683  Q99683  Q99683  Q99683  Q99683  Q99683  Q99683  Q99683  Q99683  Q99683  Q99683  Q99683  Q99683  Q99683  Q99683  Q99683  Q99683  Q99683  Q99683  Q99683  Q99683  Q99683  Q99683  Q99683  Q99683  Q99683  Q99683  Q99683  Q99704  Q99704  Q99704  Q99704  Q99704  Q99704  Q99704  Q99704  Q99704  Q99704  Q99704  Q99704  Q99704  Q99704  Q99704  Q99704  Q99704  Q99704  Q99704  Q99704  Q99704  Q99704  Q99704  Q99704  Q99704  Q99704  Q99704  Q99704  Q99704  Q99704  Q99704  Q99704  Q99704  Q99708  Q99708  Q99708  Q99708  Q99708  Q99708  Q99708  Q99708  Q99708  Q99708  Q99708  Q99708  Q99708  Q99708  Q99708  Q99708  Q99708  Q99708  Q99708  Q99708  Q99708  Q99708  Q99708  Q99708  Q99708  Q99708  Q99708  Q99708  Q99708  Q99708  Q99708  Q99759  Q99759  Q99759  Q99759  Q99759  Q99759  Q99759  Q99759  Q99759  Q99759  Q99759  Q99759  Q99759  Q99759  Q99759  Q99759  Q99759  Q99759  Q99759  Q99759  Q99759  Q99759  Q99759  Q99759  Q99759  Q99759  Q99759  Q99759  Q99759  Q99759  Q99759  Q99828  Q99828  Q99828  Q99828  Q99828  Q99828  Q99828  Q99828  Q99828  Q99828  Q99828  Q99828  Q99828  Q99828  Q99828  Q99828  Q99828  Q99828  Q99832  Q99832  Q99832  Q99832  Q99832  Q99832  Q99832  Q99832  Q99832  Q99832  Q99832  Q99832  Q99832  Q99832  Q99832  Q99832  Q99832  Q99832  Q99832  Q99832  Q99832  Q99832  Q99832  Q99832  Q99935  Q99935  Q99935  Q99935  Q99935  Q99935  Q99935  Q99935  Q99935  Q99935  Q99935  Q99935  Q99935  Q99935  Q99935  Q99935  Q99935  Q99935  Q99935  Q99935  Q99935  Q99935  Q99935  Q99935  Q99935  Q99935  Q99935  Q99935  Q99935  Q99958  Q99958  Q99958  Q99958  Q99958  Q99958  Q99958  Q99958  Q99958  Q99958  Q99958  Q99958  Q99958  Q99958  Q99958  Q99958  Q99958  Q99958  Q99958  Q99958  Q99958  Q99958  Q99958  Q99958  Q99958  Q99958  Q99958  Q99958  Q99958  Q99958  Q99958  Q99983  Q99983  Q99983  Q99983  Q99983  Q99983  Q99983  Q99983  Q99983  Q99983  Q99983  Q99983  Q99983  Q99983  Q99983  Q99983  Q99983  Q99983  Q99983  Q99983  Q99983  Q99983  Q99983  Q99983  Q99983  Q99986  Q99986  Q99986  Q99986  Q99986  Q99986  Q99986  Q99986  Q99986  Q99986  Q99986  Q99986  Q99986  Q99986  Q99986  Q99986  Q99986  Q99986  Q99986  Q99986  Q99986  Q9BPZ7  Q9BPZ7  Q9BPZ7  Q9BPZ7  Q9BPZ7  Q9BPZ7  Q9BPZ7  Q9BPZ7  Q9BPZ7  Q9BPZ7  Q9BPZ7  Q9BPZ7  Q9BPZ7  Q9BPZ7  Q9BPZ7  Q9BPZ7  Q9BPZ7  Q9BPZ7  Q9BPZ7  Q9BPZ7  Q9BPZ7  Q9BPZ7  Q9BPZ7  Q9BPZ7  Q9BPZ7  Q9BPZ7  Q9BPZ7  Q9BPZ7  Q9BPZ7  Q9BPZ7  Q9BPZ7  Q9BPZ7  Q9BPZ7  Q9BQ15  Q9BQ15  Q9BQ15  Q9BQ15  Q9BQ15  Q9BQ15  Q9BQ15  Q9BQ15  Q9BQ15  Q9BQ15  Q9BQ15  Q9BQ15  Q9BQ15  Q9BQ15  Q9BQ15  Q9BQ15  Q9BQ15  Q9BQ15  Q9BQ15  Q9BQA1  Q9BQA1  Q9BQA1  Q9BQA1  Q9BQA1  Q9BQA1  Q9BQA1  Q9BQA1  Q9BQA1  Q9BQA1  Q9BQA1  Q9BQA1  Q9BQA1  Q9BQA1  Q9BQA1  Q9BQA1  Q9BQA1  Q9BQA1  Q9BQA1  Q9BQA1  Q9BQA1  Q9BQA1  Q9BQA1  Q9BQA1  Q9BQA1  Q9BQA1  Q9BQA1  Q9BQA1  Q9BQA1  Q9BQA1  Q9BQA1  Q9BQA1  Q9BQA1  Q9BR39  Q9BR39  Q9BR39  Q9BR39  Q9BR39  Q9BR39  Q9BR39  Q9BR39  Q9BR39  Q9BR39  Q9BR39  Q9BR39  Q9BR39  Q9BR39  Q9BR39  Q9BR39  Q9BR39  Q9BR39  Q9BR39  Q9BR39  Q9BR39  Q9BR39  Q9BR39  Q9BR39  Q9BR39  Q9BR39  Q9BR39  Q9BR39  Q9BR39  Q9BR39  Q9BR39  Q9BR76  Q9BR76  Q9BR76  Q9BR76  Q9BR76  Q9BR76  Q9BR76  Q9BR76  Q9BR76  Q9BR76  Q9BR76  Q9BR76  Q9BR76  Q9BR76  Q9BR76  Q9BR76  Q9BR76  Q9BR76  Q9BR76  Q9BR76  Q9BR76  Q9BR76  Q9BR76  Q9BR76  Q9BR76  Q9BR76  Q9BR76  Q9BR76  Q9BR76  Q9BR76  Q9BR76  Q9BRK3  Q9BRK3  Q9BRK3  Q9BRK3  Q9BRK3  Q9BRK3  Q9BRK3  Q9BRK3  Q9BRK3  Q9BRK3  Q9BRK3  Q9BRK3  Q9BRK3  Q9BRK3  Q9BRK3  Q9BRK3  Q9BRK3  Q9BRK3  Q9BRK3  Q9BRK3  Q9BRK3  Q9BRK3  Q9BRK3  Q9BRK3  Q9BRK3  Q9BRK3  Q9BRK3  Q9BRK3  Q9BSJ6  Q9BSJ6  Q9BSJ6  Q9BSJ6  Q9BSJ6  Q9BSJ6  Q9BSJ6  Q9BSJ6  Q9BSJ6  Q9BSJ6  Q9BSJ6  Q9BSJ6  Q9BSJ6  Q9BSJ6  Q9BSJ6  Q9BSJ6  Q9BSJ6  Q9BSJ6  Q9BSJ6  Q9BSJ6  Q9BSJ6  Q9BSJ6  Q9BSJ6  Q9BSJ6  Q9BSJ6  Q9BSJ6  Q9BT78  Q9BT78  Q9BT78  Q9BT78  Q9BT78  Q9BT78  Q9BT78  Q9BT78  Q9BT78  Q9BT78  Q9BT78  Q9BT78  Q9BT78  Q9BT78  Q9BT78  Q9BT78  Q9BT78  Q9BT78  Q9BT78  Q9BT78  Q9BT78  Q9BT78  Q9BT78  Q9BTC0  Q9BTC0  Q9BTC0  Q9BTC0  Q9BTC0  Q9BTC0  Q9BTC0  Q9BTC0  Q9BTC0  Q9BTC0  Q9BTC0  Q9BTC0  Q9BTC0  Q9BTC0  Q9BTC0  Q9BTC0  Q9BTC0  Q9BTC0  Q9BTC0  Q9BTC0  Q9BTC0  Q9BTC0  Q9BTC0  Q9BTC0  Q9BTC0  Q9BTC0  Q9BTC0  Q9BTC0  Q9BTC0  Q9BTC0  Q9BTC0  Q9BTC0  Q9BTC0  Q9BTM9  Q9BTM9  Q9BTY2  Q9BTY2  Q9BTY2  Q9BTY2  Q9BTY2  Q9BTY2  Q9BTY2  Q9BTY2  Q9BTY2  Q9BTY2  Q9BTY2  Q9BTY2  Q9BTY2  Q9BTY2  Q9BTY2  Q9BTY2  Q9BTY2  Q9BTY2  Q9BTY2  Q9BTY2  Q9BTY2  Q9BTY2  Q9BTY2  Q9BTY2  Q9BTY2  Q9BTY2  Q9BTY2  Q9BTY2  Q9BTY7  Q9BTY7  Q9BTY7  Q9BTY7  Q9BTY7  Q9BTY7  Q9BTY7  Q9BTY7  Q9BTY7  Q9BTY7  Q9BTY7  Q9BUA3  Q9BUA3  Q9BUA3  Q9BUA3  Q9BUA3  Q9BUA3  Q9BUA3  Q9BUA3  Q9BUA3  Q9BUA3  Q9BUA3  Q9BUA3  Q9BUA3  Q9BUA3  Q9BUA3  Q9BUA3  Q9BUA3  Q9BUA3  Q9BUA3  Q9BUA3  Q9BUA3  Q9BUA3  Q9BUA3  Q9BUA3  Q9BUA3  Q9BUA3  Q9BUA3  Q9BUA3  Q9BUB5  Q9BUB5  Q9BUB5  Q9BUB5  Q9BUB5  Q9BUB5  Q9BUB5  Q9BUB5  Q9BUB5  Q9BUB5  Q9BUB5  Q9BUB5  Q9BUB5  Q9BUB5  Q9BUB5  Q9BUB5  Q9BUB5  Q9BUB5  Q9BUB5  Q9BUB5  Q9BUB5  Q9BUB5  Q9BUB5  Q9BUB5  Q9BUB5  Q9BUB5  Q9BUB5  Q9BUB5  Q9BUB5  Q9BUB5  Q9BUB5  Q9BUB5  Q9BUB5  Q9BV73  Q9BV73  Q9BV73  Q9BV73  Q9BV73  Q9BV73  Q9BV73  Q9BV73  Q9BV73  Q9BV73  Q9BV73  Q9BV73  Q9BV73  Q9BV73  Q9BV73  Q9BV73  Q9BV73  Q9BV73  Q9BV73  Q9BV73  Q9BV73  Q9BV73  Q9BV73  Q9BV73  Q9BV73  Q9BV73  Q9BV73  Q9BV73  Q9BV73  Q9BV73  Q9BV73  Q9BV86  Q9BV86  Q9BV86  Q9BV86  Q9BV86  Q9BV86  Q9BV86  Q9BV86  Q9BV86  Q9BV86  Q9BV86  Q9BV86  Q9BWF3  Q9BWF3  Q9BWF3  Q9BWF3  Q9BWF3  Q9BWF3  Q9BWF3  Q9BWF3  Q9BWF3  Q9BWF3  Q9BWF3  Q9BWF3  Q9BWF3  Q9BWF3  Q9BWF3  Q9BWF3  Q9BWF3  Q9BWF3  Q9BWF3  Q9BWF3  Q9BWF3  Q9BWF3  Q9BWF3  Q9BWF3  Q9BWF3  Q9BWT1  Q9BWT1  Q9BWT1  Q9BWT1  Q9BWT1  Q9BWT1  Q9BWT1  Q9BWT1  Q9BWT1  Q9BWT1  Q9BWT1  Q9BWT1  Q9BWT1  Q9BWT1  Q9BWT1  Q9BWT1  Q9BWT1  Q9BWT1  Q9BWT1  Q9BWT1  Q9BWT1  Q9BWT1  Q9BWT1  Q9BWT1  Q9BWT1  Q9BWT1  Q9BWT1  Q9BWT1  Q9BWT1  Q9BWT1  Q9BWT1  Q9BWT1  Q9BWT1  Q9BX63  Q9BX63  Q9BX63  Q9BX63  Q9BX63  Q9BX63  Q9BX63  Q9BX63  Q9BX63  Q9BX63  Q9BX63  Q9BX63  Q9BX63  Q9BX63  Q9BX63  Q9BX63  Q9BX63  Q9BX63  Q9BX63  Q9BX63  Q9BX63  Q9BX63  Q9BX63  Q9BX63  Q9BX63  Q9BX63  Q9BX63  Q9BX63  Q9BX63  Q9BX63  Q9BX63  Q9BX84  Q9BX84  Q9BX84  Q9BX84  Q9BX84  Q9BX84  Q9BX84  Q9BX84  Q9BX84  Q9BX84  Q9BX84  Q9BX84  Q9BX84  Q9BX84  Q9BX84  Q9BX84  Q9BX84  Q9BX84  Q9BX84  Q9BX84  Q9BX84  Q9BX84  Q9BX84  Q9BX84  Q9BX84  Q9BX84  Q9BX84  Q9BX84  Q9BX84  Q9BX84  Q9BX84  Q9BX84  Q9BX84  Q9BXA7  Q9BXA7  Q9BXA7  Q9BXA7  Q9BXA7  Q9BXA7  Q9BXA7  Q9BXA7  Q9BXA7  Q9BXA7  Q9BXA7  Q9BXA7  Q9BXA7  Q9BXA7  Q9BXA7  Q9BXA7  Q9BXA7  Q9BXA7  Q9BXA7  Q9BXA7  Q9BXA7  Q9BXA7  Q9BXA7  Q9BXA7  Q9BXA7  Q9BXA7  Q9BXA7  Q9BXM7  Q9BXM7  Q9BXM7  Q9BXM7  Q9BXM7  Q9BXM7  Q9BXM7  Q9BXM7  Q9BXM7  Q9BXM7  Q9BXM7  Q9BXM7  Q9BXM7  Q9BXM7  Q9BXM7  Q9BXM7  Q9BXM7  Q9BXM7  Q9BXM7  Q9BXM7  Q9BXM7  Q9BXM7  Q9BXM7  Q9BXM7  Q9BXM7  Q9BXM7  Q9BXM7  Q9BXM7  Q9BXM7  Q9BXM7  Q9BXS0  Q9BXS0  Q9BXS0  Q9BXS0  Q9BXS0  Q9BXS0  Q9BXS0  Q9BXS0  Q9BXS0  Q9BXS0  Q9BXS0  Q9BXS0  Q9BXS0  Q9BXS0  Q9BXS0  Q9BXS0  Q9BXS0  Q9BXS0  Q9BXS0  Q9BXS0  Q9BXS5  Q9BXS5  Q9BXS5  Q9BXS5  Q9BXS5  Q9BXS5  Q9BXS5  Q9BXS5  Q9BXS5  Q9BXS5  Q9BXS5  Q9BXS5  Q9BXS5  Q9BXS5  Q9BXS5  Q9BXS5  Q9BXS5  Q9BXS5  Q9BXS5  Q9BXS5  Q9BXS5  Q9BXS5  Q9BXS5  Q9BXS5  Q9BXS5  Q9BXS5  Q9BXS5  Q9BXS5  Q9BXS5  Q9BXS5  Q9BXS5  Q9BXS5  Q9BXS5  Q9BXS6  Q9BXS6  Q9BXS6  Q9BXS6  Q9BXS6  Q9BXS6  Q9BXS6  Q9BXS6  Q9BXS6  Q9BXS6  Q9BXS6  Q9BXS6  Q9BXS6  Q9BXS6  Q9BXS6  Q9BXS6  Q9BXS6  Q9BXS6  Q9BXS6  Q9BXS6  Q9BXS6  Q9BXS6  Q9BXS6  Q9BXS6  Q9BXS6  Q9BXS6  Q9BXS6  Q9BXS6  Q9BXS6  Q9BXS6  Q9BXS6  Q9BXW9  Q9BXW9  Q9BXW9  Q9BXW9  Q9BXW9  Q9BXW9  Q9BXW9  Q9BXW9  Q9BXW9  Q9BXW9  Q9BXW9  Q9BXW9  Q9BXW9  Q9BXW9  Q9BXW9  Q9BXW9  Q9BXW9  Q9BXW9  Q9BXW9  Q9BXW9  Q9BXW9  Q9BXW9  Q9BXW9  Q9BXW9  Q9BXW9  Q9BXW9  Q9BXW9  Q9BXW9  Q9BXW9  Q9BXW9  Q9BXW9  Q9BY32  Q9BY32  Q9BY32  Q9BY32  Q9BY32  Q9BY32  Q9BY41  Q9BY41  Q9BY41  Q9BY41  Q9BY41  Q9BY41  Q9BY41  Q9BY41  Q9BY41  Q9BY41  Q9BY41  Q9BY41  Q9BY41  Q9BY41  Q9BY41  Q9BY41  Q9BY41  Q9BY41  Q9BY41  Q9BY41  Q9BY41  Q9BY41  Q9BY44  Q9BY44  Q9BY44  Q9BY44  Q9BY44  Q9BY44  Q9BY44  Q9BY44  Q9BY44  Q9BY44  Q9BY44  Q9BY44  Q9BY44  Q9BY44  Q9BY44  Q9BY44  Q9BY44  Q9BY44  Q9BY44  Q9BY44  Q9BY44  Q9BY44  Q9BY44  Q9BY44  Q9BY44  Q9BY44  Q9BY44  Q9BY44  Q9BY44  Q9BY44  Q9BY44  Q9BY44  Q9BY44  Q9BY77  Q9BY77  Q9BY77  Q9BY77  Q9BY77  Q9BY77  Q9BY77  Q9BY77  Q9BY77  Q9BY77  Q9BY77  Q9BY77  Q9BY77  Q9BY77  Q9BY77  Q9BY77  Q9BY77  Q9BY77  Q9BY77  Q9BY77  Q9BY77  Q9BY77  Q9BY77  Q9BY77  Q9BY77  Q9BY77  Q9BY77  Q9BY84  Q9BY84  Q9BY84  Q9BY84  Q9BY84  Q9BY84  Q9BY84  Q9BY84  Q9BY84  Q9BY84  Q9BY84  Q9BY84  Q9BY84  Q9BY84  Q9BY84  Q9BY84  Q9BY84  Q9BY84  Q9BY84  Q9BY84  Q9BY84  Q9BY84  Q9BY84  Q9BY84  Q9BY84  Q9BY84  Q9BY84  Q9BY84  Q9BY84  Q9BY84  Q9BY84  Q9BYE7  Q9BYE7  Q9BYE7  Q9BYE7  Q9BYE7  Q9BYE7  Q9BYE7  Q9BYE7  Q9BYE7  Q9BYE7  Q9BYE7  Q9BYE7  Q9BYE7  Q9BYE7  Q9BYE7  Q9BYE7  Q9BYE7  Q9BYG3  Q9BYG3  Q9BYG3  Q9BYG3  Q9BYG3  Q9BYG3  Q9BYG3  Q9BYG3  Q9BYG3  Q9BYG3  Q9BYG3  Q9BYG3  Q9BYG3  Q9BYG3  Q9BYG3  Q9BYV9  Q9BYV9  Q9BYV9  Q9BYV9  Q9BYV9  Q9BYV9  Q9BYV9  Q9BYV9  Q9BYV9  Q9BYV9  Q9BYV9  Q9BYV9  Q9BYV9  Q9BYV9  Q9BYV9  Q9BYV9  Q9BYV9  Q9BYV9  Q9BYV9  Q9BYV9  Q9BYV9  Q9BYV9  Q9BYV9  Q9BYV9  Q9BYV9  Q9BYV9  Q9BYV9  Q9BYV9  Q9BYV9  Q9BYV9  Q9BYV9  Q9BZE4  Q9BZE4  Q9BZE4  Q9BZE4  Q9BZE4  Q9BZE4  Q9BZE4  Q9BZE4  Q9BZE4  Q9BZE4  Q9BZE4  Q9BZE4  Q9BZE4  Q9BZE4  Q9BZE4  Q9BZE4  Q9BZE4  Q9BZE4  Q9BZE4  Q9BZE4  Q9BZE4  Q9BZE4  Q9BZE4  Q9BZE4  Q9BZE4  Q9BZE4  Q9BZE4  Q9BZE4  Q9BZE4  Q9BZE4  Q9BZL4  Q9BZL4  Q9BZL4  Q9BZL4  Q9BZL4  Q9BZL4  Q9BZL4  Q9BZL4  Q9BZL4  Q9BZL4  Q9BZL4  Q9BZL4  Q9BZL4  Q9BZL4  Q9BZL4  Q9BZL4  Q9BZL4  Q9BZL4  Q9BZL4  Q9BZL4  Q9BZL4  Q9BZL4  Q9BZL4  Q9BZL4  Q9BZL4  Q9BZL4  Q9BZL4  Q9BZL4  Q9BZL4  Q9BZL4  Q9BZL4  Q9BZL4  Q9BZL4  Q9BZL6  Q9BZL6  Q9BZL6  Q9BZL6  Q9BZL6  Q9BZL6  Q9BZL6  Q9BZL6  Q9BZL6  Q9BZL6  Q9BZL6  Q9BZL6  Q9BZL6  Q9BZL6  Q9BZL6  Q9BZL6  Q9BZL6  Q9BZL6  Q9BZL6  Q9BZL6  Q9BZL6  Q9BZL6  Q9BZL6  Q9BZL6  Q9BZL6  Q9BZL6  Q9BZL6  Q9BZL6  Q9BZL6  Q9BZL6  Q9BZL6  Q9BZS1  Q9BZS1  Q9BZS1  Q9BZS1  Q9BZS1  Q9BZS1  Q9BZS1  Q9BZS1  Q9BZS1  Q9BZS1  Q9BZS1  Q9BZS1  Q9BZS1  Q9BZS1  Q9BZS1  Q9BZS1  Q9BZS1  Q9BZS1  Q9BZS1  Q9BZS1  Q9BZS1  Q9BZS1  Q9BZS1  Q9BZS1  Q9BZS1  Q9BZS1  Q9BZS1  Q9BZS1  Q9BZS1  Q9BZS1  Q9BZS1  Q9BZZ5  Q9BZZ5  Q9BZZ5  Q9BZZ5  Q9BZZ5  Q9BZZ5  Q9BZZ5  Q9BZZ5  Q9BZZ5  Q9BZZ5  Q9BZZ5  Q9BZZ5  Q9BZZ5  Q9BZZ5  Q9BZZ5  Q9BZZ5  Q9BZZ5  Q9BZZ5  Q9BZZ5  Q9BZZ5  Q9BZZ5  Q9BZZ5  Q9BZZ5  Q9BZZ5  Q9BZZ5  Q9BZZ5  Q9BZZ5  Q9BZZ5  Q9BZZ5  Q9GZN2  Q9GZN2  Q9GZN2  Q9GZN2  Q9GZN2  Q9GZN2  Q9GZN2  Q9GZN2  Q9GZN2  Q9GZN2  Q9GZN2  Q9GZN2  Q9GZN2  Q9GZN2  Q9GZN2  Q9GZN2  Q9GZN2  Q9GZN2  Q9GZN2  Q9GZN2  Q9GZN2  Q9GZN2  Q9GZT4  Q9GZT4  Q9GZT4  Q9GZT4  Q9GZT4  Q9GZT4  Q9GZT4  Q9GZT4  Q9GZT4  Q9GZT4  Q9GZT4  Q9GZT4  Q9GZT4  Q9GZT4  Q9GZT4  Q9GZT4  Q9GZT4  Q9GZT4  Q9GZT4  Q9GZT4  Q9GZT4  Q9GZT9  Q9GZT9  Q9GZT9  Q9GZT9  Q9GZT9  Q9GZT9  Q9GZT9  Q9GZT9  Q9GZT9  Q9GZT9  Q9GZT9  Q9GZT9  Q9GZT9  Q9GZT9  Q9GZT9  Q9GZT9  Q9GZT9  Q9GZT9  Q9GZT9  Q9GZT9  Q9GZT9  Q9GZT9  Q9GZT9  Q9GZT9  Q9GZV1  Q9GZV1  Q9GZV1  Q9GZV1  Q9GZV1  Q9GZV1  Q9GZV1  Q9GZV1  Q9GZV1  Q9GZV5  Q9GZV5  Q9GZV5  Q9GZV5  Q9GZV5  Q9GZV5  Q9GZV5  Q9GZV5  Q9GZV5  Q9GZV5  Q9GZV5  Q9GZV5  Q9GZV5  Q9GZV5  Q9GZV5  Q9GZV5  Q9GZV5  Q9GZV5  Q9GZV5  Q9GZV5  Q9GZV5  Q9GZV5  Q9GZV5  Q9GZV5  Q9GZV5  Q9GZV5  Q9GZV5  Q9GZV5  Q9GZV5  Q9GZV5  Q9GZV5  Q9GZX7  Q9GZX7  Q9GZX7  Q9GZX7  Q9GZX7  Q9GZX7  Q9GZX7  Q9GZX7  Q9GZY6  Q9GZY6  Q9GZY6  Q9GZY6  Q9GZY6  Q9GZY6  Q9GZY6  Q9GZY6  Q9GZY6  Q9GZY6  Q9GZY6  Q9GZY6  Q9GZY6  Q9GZY6  Q9GZY6  Q9GZY6  Q9GZY6  Q9GZY6  Q9GZY6  Q9GZY6  Q9GZY6  Q9GZY6  Q9GZY6  Q9GZY6  Q9GZY6  Q9GZY6  Q9H063  Q9H063  Q9H063  Q9H063  Q9H063  Q9H063  Q9H063  Q9H063  Q9H063  Q9H063  Q9H063  Q9H063  Q9H063  Q9H063  Q9H063  Q9H063  Q9H063  Q9H063  Q9H063  Q9H063  Q9H063  Q9H063  Q9H063  Q9H063  Q9H063  Q9H063  Q9H063  Q9H093  Q9H093  Q9H093  Q9H093  Q9H093  Q9H093  Q9H093  Q9H093  Q9H093  Q9H093  Q9H093  Q9H093  Q9H093  Q9H093  Q9H093  Q9H093  Q9H093  Q9H093  Q9H093  Q9H093  Q9H093  Q9H093  Q9H093  Q9H093  Q9H093  Q9H093  Q9H093  Q9H093  Q9H093  Q9H093  Q9H093  Q9H093  Q9H0H5  Q9H0H5  Q9H0H5  Q9H0H5  Q9H0H5  Q9H0H5  Q9H0H5  Q9H0H5  Q9H0H5  Q9H0H5  Q9H0H5  Q9H0H5  Q9H0H5  Q9H0H5  Q9H0H5  Q9H0H5  Q9H0H5  Q9H0H5  Q9H0H5  Q9H0H5  Q9H0H5  Q9H0H5  Q9H0H5  Q9H0H5  Q9H0H5  Q9H0H5  Q9H0H5  Q9H0H5  Q9H0H5  Q9H0H5  Q9H0H5  Q9H0K1  Q9H0K1  Q9H0K1  Q9H0K1  Q9H0K1  Q9H0K1  Q9H0K1  Q9H0K1  Q9H0K1  Q9H0K1  Q9H0K1  Q9H0K1  Q9H0K1  Q9H0K1  Q9H0K1  Q9H0K1  Q9H0K1  Q9H0K1  Q9H0K1  Q9H0K1  Q9H0K1  Q9H0K1  Q9H0K1  Q9H0K1  Q9H0K1  Q9H0K1  Q9H0K1  Q9H0K1  Q9H0K1  Q9H0K1  Q9H0K1  Q9H0K1  Q9H0K1  Q9H0N0  Q9H0N0  Q9H0N0  Q9H0N0  Q9H0N0  Q9H0N0  Q9H0N0  Q9H0N0  Q9H0N0  Q9H0N0  Q9H0N0  Q9H0N0  Q9H0N0  Q9H0N0  Q9H0N0  Q9H0N0  Q9H0N0  Q9H0N0  Q9H0N0  Q9H0N0  Q9H0N0  Q9H0N0  Q9H0S4  Q9H0S4  Q9H0S4  Q9H0S4  Q9H0S4  Q9H0S4  Q9H0S4  Q9H0S4  Q9H0S4  Q9H0S4  Q9H0S4  Q9H0S4  Q9H0S4  Q9H0S4  Q9H0S4  Q9H0S4  Q9H0S4  Q9H0S4  Q9H0S4  Q9H0S4  Q9H0S4  Q9H0S4  Q9H0S4  Q9H0S4  Q9H1A4  Q9H1A4  Q9H1A4  Q9H1A4  Q9H1A4  Q9H1A4  Q9H1A4  Q9H1A4  Q9H1A4  Q9H1A4  Q9H1A4  Q9H1A4  Q9H1A4  Q9H1A4  Q9H1A4  Q9H1A4  Q9H1A4  Q9H1A4  Q9H1A4  Q9H1A4  Q9H1A4  Q9H1A4  Q9H1A4  Q9H1A4  Q9H1A4  Q9H1A4  Q9H1A4  Q9H1A4  Q9H1A4  Q9H1A4  Q9H1A4  Q9H1D0  Q9H1D0  Q9H1D0  Q9H1D0  Q9H1D0  Q9H1D0  Q9H1D0  Q9H1D0  Q9H1D0  Q9H1D0  Q9H1D0  Q9H1D0  Q9H1D0  Q9H1D0  Q9H1D0  Q9H1D0  Q9H1D0  Q9H1D0  Q9H1D0  Q9H1D0  Q9H1D0  Q9H1D0  Q9H1D0  Q9H1D0  Q9H1D0  Q9H1D0  Q9H1D0  Q9H1D0  Q9H1D0  Q9H1D0  Q9H1D0  Q9H1D0  Q9H1D0  Q9H1Y3  Q9H1Y3  Q9H1Y3  Q9H1Y3  Q9H1Y3  Q9H1Y3  Q9H1Y3  Q9H1Y3  Q9H1Y3  Q9H1Y3  Q9H1Y3  Q9H1Y3  Q9H1Y3  Q9H1Y3  Q9H1Y3  Q9H1Y3  Q9H1Y3  Q9H1Y3  Q9H1Y3  Q9H1Y3  Q9H1Y3  Q9H1Y3  Q9H1Y3  Q9H1Y3  Q9H1Y3  Q9H1Y3  Q9H1Y3  Q9H1Y3  Q9H1Y3  Q9H1Y3  Q9H211  Q9H211  Q9H211  Q9H211  Q9H211  Q9H211  Q9H211  Q9H211  Q9H211  Q9H211  Q9H211  Q9H211  Q9H211  Q9H211  Q9H211  Q9H211  Q9H211  Q9H211  Q9H211  Q9H211  Q9H211  Q9H211  Q9H211  Q9H211  Q9H211  Q9H211  Q9H211  Q9H211  Q9H211  Q9H211  Q9H211  Q9H211  Q9H211  Q9H2D6  Q9H2D6  Q9H2D6  Q9H2D6  Q9H2D6  Q9H2D6  Q9H2D6  Q9H2D6  Q9H2D6  Q9H2D6  Q9H2D6  Q9H2D6  Q9H2D6  Q9H2D6  Q9H2D6  Q9H2D6  Q9H2D6  Q9H2D6  Q9H2D6  Q9H2D6  Q9H2D6  Q9H2D6  Q9H2D6  Q9H2D6  Q9H2D6  Q9H2D6  Q9H2D6  Q9H2D6  Q9H2D6  Q9H2D6  Q9H2D6  Q9H2D6  Q9H2D6  Q9H2G4  Q9H2G4  Q9H2G4  Q9H2G4  Q9H2G4  Q9H2G4  Q9H2G4  Q9H2G4  Q9H2G4  Q9H2G4  Q9H2G4  Q9H2G4  Q9H2G4  Q9H2G4  Q9H2G4  Q9H2G4  Q9H2G4  Q9H2G4  Q9H2G4  Q9H2G4  Q9H2G4  Q9H2G4  Q9H2G4  Q9H2G4  Q9H2G4  Q9H2G4  Q9H2G4  Q9H2G4  Q9H2G4  Q9H2G4  Q9H2G4  Q9H2K2  Q9H2K2  Q9H2K2  Q9H2K2  Q9H2K2  Q9H2K2  Q9H2K2  Q9H2K2  Q9H2K2  Q9H2K2  Q9H2K2  Q9H2K2  Q9H2K2  Q9H2K2  Q9H2K2  Q9H2K2  Q9H2K2  Q9H2K2  Q9H2K2  Q9H2K2  Q9H2K2  Q9H2K2  Q9H2K2  Q9H2K2  Q9H2K2  Q9H2K2  Q9H2K2  Q9H2K2  Q9H2K2  Q9H2K2  Q9H2K2  Q9H2K2  Q9H2K8  Q9H2K8  Q9H2K8  Q9H2K8  Q9H2K8  Q9H2K8  Q9H2K8  Q9H2K8  Q9H2K8  Q9H2K8  Q9H2K8  Q9H2K8  Q9H2K8  Q9H2K8  Q9H2K8  Q9H2K8  Q9H2K8  Q9H2K8  Q9H2K8  Q9H2K8  Q9H2K8  Q9H2K8  Q9H2K8  Q9H2K8  Q9H2K8  Q9H2K8  Q9H2K8  Q9H2K8  Q9H2K8  Q9H2K8  Q9H2W2  Q9H2W2  Q9H2W2  Q9H2W2  Q9H2W2  Q9H2W2  Q9H2W2  Q9H2W2  Q9H2W2  Q9H2W2  Q9H2W2  Q9H2W2  Q9H2W2  Q9H2W2  Q9H2W2  Q9H2W2  Q9H2W2  Q9H2W2  Q9H2W2  Q9H300  Q9H300  Q9H300  Q9H300  Q9H300  Q9H300  Q9H300  Q9H300  Q9H300  Q9H300  Q9H300  Q9H300  Q9H300  Q9H300  Q9H300  Q9H300  Q9H300  Q9H300  Q9H300  Q9H300  Q9H300  Q9H300  Q9H300  Q9H300  Q9H300  Q9H300  Q9H300  Q9H307  Q9H307  Q9H307  Q9H307  Q9H307  Q9H307  Q9H307  Q9H307  Q9H307  Q9H307  Q9H307  Q9H307  Q9H307  Q9H307  Q9H307  Q9H307  Q9H307  Q9H307  Q9H307  Q9H307  Q9H307  Q9H307  Q9H307  Q9H307  Q9H307  Q9H307  Q9H307  Q9H307  Q9H307  Q9H307  Q9H307  Q9H307  Q9H307  Q9H3K6  Q9H3K6  Q9H3K6  Q9H3Y6  Q9H3Y6  Q9H3Y6  Q9H3Y6  Q9H3Y6  Q9H3Y6  Q9H3Y6  Q9H3Y6  Q9H3Y6  Q9H3Y6  Q9H3Y6  Q9H3Y6  Q9H3Y6  Q9H3Y6  Q9H3Y6  Q9H3Y6  Q9H3Y6  Q9H3Y6  Q9H3Y6  Q9H3Y6  Q9H3Y6  Q9H3Y6  Q9H3Y6  Q9H3Y6  Q9H3Y6  Q9H3Y6  Q9H3Y6  Q9H3Y6  Q9H3Y6  Q9H3Y6  Q9H3Y6  Q9H3Y6  Q9H3Y6  Q9H3Z4  Q9H3Z4  Q9H3Z4  Q9H3Z4  Q9H3Z4  Q9H3Z4  Q9H3Z4  Q9H3Z4  Q9H3Z4  Q9H3Z4  Q9H400  Q9H400  Q9H400  Q9H400  Q9H400  Q9H400  Q9H400  Q9H400  Q9H400  Q9H400  Q9H400  Q9H400  Q9H400  Q9H400  Q9H400  Q9H400  Q9H400  Q9H400  Q9H400  Q9H400  Q9H400  Q9H400  Q9H400  Q9H425  Q9H425  Q9H425  Q9H425  Q9H425  Q9H425  Q9H425  Q9H425  Q9H425  Q9H425  Q9H425  Q9H425  Q9H425  Q9H425  Q9H425  Q9H425  Q9H425  Q9H425  Q9H425  Q9H425  Q9H425  Q9H425  Q9H425  Q9H425  Q9H425  Q9H425  Q9H425  Q9H425  Q9H425  Q9H425  Q9H425  Q9H425  Q9H425  Q9H479  Q9H479  Q9H479  Q9H479  Q9H479  Q9H479  Q9H479  Q9H479  Q9H479  Q9H479  Q9H479  Q9H479  Q9H479  Q9H479  Q9H492  Q9H492  Q9H492  Q9H492  Q9H492  Q9H4X1  Q9H4X1  Q9H4X1  Q9H4X1  Q9H4X1  Q9H4X1  Q9H4X1  Q9H4X1  Q9H4X1  Q9H4X1  Q9H4X1  Q9H4X1  Q9H4X1  Q9H4X1  Q9H4X1  Q9H4X1  Q9H4X1  Q9H4X1  Q9H4X1  Q9H4X1  Q9H5V8  Q9H5V8  Q9H5V8  Q9H5V8  Q9H5V8  Q9H5V8  Q9H5V8  Q9H5V8  Q9H5V8  Q9H5V8  Q9H5V8  Q9H5V8  Q9H5V8  Q9H5V8  Q9H5V8  Q9H5V8  Q9H5V8  Q9H5V8  Q9H5V8  Q9H5V8  Q9H5V8  Q9H5V8  Q9H5V8  Q9H5V8  Q9H5V8  Q9H5V8  Q9H5V8  Q9H5V8  Q9H5V8  Q9H5V8  Q9H5V8  Q9H5V8  Q9H5V8  Q9H706  Q9H706  Q9H706  Q9H706  Q9H706  Q9H706  Q9H706  Q9H706  Q9H706  Q9H706  Q9H706  Q9H706  Q9H706  Q9H706  Q9H706  Q9H706  Q9H706  Q9H706  Q9H706  Q9H706  Q9H706  Q9H706  Q9H706  Q9H706  Q9H706  Q9H706  Q9H706  Q9H706  Q9H706  Q9H706  Q9H706  Q9H706  Q9H706  Q9H7P6  Q9H7P6  Q9H7P6  Q9H7P6  Q9H7P6  Q9H7P6  Q9H7P6  Q9H7P6  Q9H7P6  Q9H7P6  Q9H7P6  Q9H7P6  Q9H7P6  Q9H7P6  Q9H7P6  Q9H7P6  Q9H7P6  Q9H7P6  Q9H7P6  Q9H7P6  Q9H7P6  Q9H7P6  Q9H7P6  Q9H7P6  Q9H7P6  Q9H7X0  Q9H7X0  Q9H7X0  Q9H7X0  Q9H7X0  Q9H7X0  Q9H7X0  Q9H7X0  Q9H7X0  Q9H7X0  Q9H7X0  Q9H7X0  Q9H7X0  Q9H7X0  Q9H7X0  Q9H7X0  Q9H7X0  Q9H7X0  Q9H7X0  Q9H7X0  Q9H7X0  Q9H7X0  Q9H7X0  Q9H7Z6  Q9H7Z6  Q9H7Z6  Q9H7Z6  Q9H7Z6  Q9H7Z6  Q9H7Z6  Q9H7Z6  Q9H7Z6  Q9H7Z6  Q9H7Z6  Q9H7Z6  Q9H7Z6  Q9H7Z6  Q9H7Z6  Q9H7Z6  Q9H7Z6  Q9H7Z6  Q9H7Z6  Q9H7Z6  Q9H7Z6  Q9H7Z6  Q9H7Z6  Q9H7Z6  Q9H7Z6  Q9H7Z6  Q9H7Z6  Q9H7Z6  Q9H7Z6  Q9H8M9  Q9H8M9  Q9H8M9  Q9H8M9  Q9H8M9  Q9H8M9  Q9H8M9  Q9H8M9  Q9H8M9  Q9H8M9  Q9H8M9  Q9H8M9  Q9H8M9  Q9H8M9  Q9H8M9  Q9H8S9  Q9H8S9  Q9H8S9  Q9H8S9  Q9H8S9  Q9H8S9  Q9H8S9  Q9H8S9  Q9H8S9  Q9H8S9  Q9H8S9  Q9H8V3  Q9H8V3  Q9H8V3  Q9H8V3  Q9H8V3  Q9H8V3  Q9H8V3  Q9H8V3  Q9H8V3  Q9H8V3  Q9H8V3  Q9H8V3  Q9H8V3  Q9H8V3  Q9H8V3  Q9H8V3  Q9H8V3  Q9H8V3  Q9H8V3  Q9H8V3  Q9H8V3  Q9H8V3  Q9H8V3  Q9H8V3  Q9H8V3  Q9H8V3  Q9H8V3  Q9H8V3  Q9H8V3  Q9H8V3  Q9H8V3  Q9H8V3  Q9H8V3  Q9H8Y8  Q9H8Y8  Q9H8Y8  Q9H8Y8  Q9H8Y8  Q9H8Y8  Q9H8Y8  Q9H8Y8  Q9H8Y8  Q9H8Y8  Q9H8Y8  Q9H8Y8  Q9H8Y8  Q9H8Y8  Q9H8Y8  Q9H8Y8  Q9H8Y8  Q9H8Y8  Q9H8Y8  Q9H8Y8  Q9H8Y8  Q9H8Y8  Q9H8Y8  Q9H8Y8  Q9H8Y8  Q9H8Y8  Q9H8Y8  Q9H8Y8  Q9H8Y8  Q9H8Y8  Q9H8Y8  Q9H8Y8  Q9H8Y8  Q9H9B4  Q9H9B4  Q9H9B4  Q9H9B4  Q9H9B4  Q9H9B4  Q9H9B4  Q9H9B4  Q9H9B4  Q9H9B4  Q9H9B4  Q9H9B4  Q9H9B4  Q9H9B4  Q9H9B4  Q9H9B4  Q9HAW4  Q9HAW4  Q9HAW4  Q9HAW4  Q9HAW4  Q9HAW4  Q9HAW4  Q9HAW4  Q9HAW4  Q9HAW4  Q9HAW4  Q9HAW4  Q9HAW4  Q9HAW4  Q9HAW4  Q9HAW4  Q9HAW4  Q9HAW4  Q9HAW4  Q9HAW4  Q9HAW4  Q9HAW4  Q9HAW4  Q9HAW4  Q9HAW4  Q9HAW4  Q9HAW4  Q9HAW4  Q9HAW4  Q9HAW4  Q9HAW4  Q9HAW4  Q9HAW4  Q9HB71  Q9HB71  Q9HB71  Q9HB71  Q9HB71  Q9HB71  Q9HB71  Q9HB71  Q9HB71  Q9HB71  Q9HB71  Q9HB71  Q9HB71  Q9HB75  Q9HB75  Q9HB75  Q9HB75  Q9HB75  Q9HB75  Q9HB75  Q9HB75  Q9HB75  Q9HB75  Q9HB75  Q9HB75  Q9HB75  Q9HB75  Q9HB75  Q9HB75  Q9HB75  Q9HB75  Q9HB75  Q9HB75  Q9HB75  Q9HB75  Q9HB75  Q9HB75  Q9HB75  Q9HB75  Q9HB75  Q9HB75  Q9HB75  Q9HB75  Q9HB75  Q9HB96  Q9HB96  Q9HB96  Q9HB96  Q9HB96  Q9HB96  Q9HB96  Q9HB96  Q9HB96  Q9HB96  Q9HB96  Q9HB96  Q9HB96  Q9HB96  Q9HB96  Q9HB96  Q9HB96  Q9HB96  Q9HB96  Q9HB96  Q9HB96  Q9HB96  Q9HB96  Q9HB96  Q9HB96  Q9HB96  Q9HB96  Q9HB96  Q9HB96  Q9HB96  Q9HB96  Q9HBH9  Q9HBH9  Q9HBH9  Q9HBH9  Q9HBH9  Q9HBH9  Q9HBH9  Q9HBH9  Q9HBH9  Q9HBH9  Q9HBH9  Q9HBH9  Q9HBH9  Q9HBH9  Q9HBH9  Q9HBH9  Q9HBH9  Q9HBH9  Q9HBH9  Q9HBH9  Q9HBH9  Q9HBH9  Q9HBH9  Q9HBH9  Q9HBH9  Q9HBH9  Q9HBH9  Q9HBH9  Q9HBH9  Q9HBH9  Q9HBH9  Q9HBH9  Q9HBL0  Q9HBL0  Q9HBL0  Q9HBL0  Q9HBL0  Q9HBL0  Q9HBL0  Q9HBL0  Q9HBL0  Q9HBL0  Q9HBL0  Q9HBL0  Q9HBL0  Q9HBL0  Q9HBL0  Q9HBL0  Q9HBL0  Q9HBL0  Q9HBL0  Q9HBL0  Q9HBL0  Q9HBL0  Q9HBL0  Q9HBL0  Q9HBL0  Q9HBL0  Q9HBL0  Q9HBL0  Q9HBL0  Q9HBL0  Q9HBL0  Q9HBV2  Q9HBV2  Q9HBV2  Q9HBV2  Q9HBV2  Q9HBV2  Q9HBV2  Q9HBV2  Q9HBV2  Q9HBV2  Q9HBV2  Q9HBV2  Q9HBV2  Q9HBV2  Q9HBV2  Q9HBV2  Q9HBV2  Q9HBV2  Q9HBV2  Q9HBV2  Q9HBV2  Q9HC16  Q9HC16  Q9HC16  Q9HC16  Q9HC16  Q9HC16  Q9HC16  Q9HC16  Q9HC16  Q9HC16  Q9HC16  Q9HC16  Q9HC16  Q9HC16  Q9HC16  Q9HC16  Q9HC16  Q9HC16  Q9HC16  Q9HC16  Q9HC77  Q9HC77  Q9HC77  Q9HC77  Q9HC77  Q9HC77  Q9HC77  Q9HC77  Q9HC77  Q9HC77  Q9HC77  Q9HC77  Q9HC77  Q9HC77  Q9HC77  Q9HC77  Q9HC77  Q9HC77  Q9HC77  Q9HC77  Q9HC77  Q9HC77  Q9HC77  Q9HC77  Q9HC77  Q9HC77  Q9HC77  Q9HC77  Q9HC77  Q9HC77  Q9HC77  Q9HC98  Q9HC98  Q9HC98  Q9HC98  Q9HC98  Q9HC98  Q9HC98  Q9HC98  Q9HC98  Q9HC98  Q9HC98  Q9HC98  Q9HC98  Q9HC98  Q9HC98  Q9HC98  Q9HC98  Q9HCC9  Q9HCC9  Q9HCC9  Q9HCC9  Q9HCC9  Q9HCC9  Q9HCC9  Q9HCC9  Q9HCC9  Q9HCC9  Q9HCC9  Q9HCC9  Q9HCC9  Q9HCC9  Q9HCC9  Q9HCC9  Q9HCC9  Q9HCC9  Q9HCC9  Q9HCC9  Q9HCC9  Q9HCC9  Q9HCC9  Q9HCC9  Q9HCC9  Q9HCC9  Q9HCC9  Q9HCC9  Q9HCC9  Q9HCC9  Q9HCC9  Q9HCE9  Q9HCE9  Q9HCE9  Q9HCE9  Q9HCE9  Q9HCE9  Q9HCE9  Q9HCE9  Q9HCE9  Q9HCE9  Q9HCE9  Q9HCE9  Q9HCE9  Q9HCE9  Q9HCE9  Q9HCE9  Q9HCE9  Q9HCE9  Q9HCE9  Q9HCE9  Q9HCE9  Q9HCE9  Q9HCE9  Q9HCE9  Q9HCE9  Q9HCE9  Q9HCE9  Q9HCE9  Q9HCE9  Q9HCE9  Q9HCE9  Q9HCN4  Q9HCN4  Q9HCN4  Q9HCN4  Q9HCN4  Q9HCN4  Q9HCN4  Q9HCN4  Q9HCN4  Q9HCN4  Q9HCN4  Q9HCN4  Q9HCN4  Q9HCN4  Q9HCN4  Q9HCN4  Q9HCN4  Q9HCN4  Q9HCN4  Q9HCN4  Q9HCN4  Q9HCN4  Q9HCN4  Q9HCN4  Q9HCN4  Q9HCN4  Q9HCN4  Q9HCN4  Q9HCN4  Q9HCQ7  Q9HCQ7  Q9HCQ7  Q9HCQ7  Q9HCQ7  Q9HCQ7  Q9HCQ7  Q9HCQ7  Q9HCQ7  Q9HCQ7  Q9HCQ7  Q9HCQ7  Q9HCQ7  Q9HCQ7  Q9HCQ7  Q9HCQ7  Q9HCQ7  Q9HCQ7  Q9HCX4  Q9HCX4  Q9HCX4  Q9HCX4  Q9HCX4  Q9HCX4  Q9HCX4  Q9HCX4  Q9HCX4  Q9HCX4  Q9HCX4  Q9HCX4  Q9HCX4  Q9HCX4  Q9HCX4  Q9HCX4  Q9HCX4  Q9HCX4  Q9HCX4  Q9HCX4  Q9HCX4  Q9HCX4  Q9HCX4  Q9HCX4  Q9HCX4  Q9HCX4  Q9HCX4  Q9HCX4  Q9HCX4  Q9HCX4  Q9HCX4  Q9HCX4  Q9HCX4  Q9HD40  Q9HD40  Q9HD40  Q9HD40  Q9HD40  Q9HD40  Q9HD40  Q9HD40  Q9HD40  Q9HD40  Q9HD40  Q9HD40  Q9HD40  Q9HD40  Q9HD40  Q9HD40  Q9HD40  Q9HD40  Q9HD40  Q9HD40  Q9HD40  Q9HD40  Q9HD40  Q9HD40  Q9HD40  Q9HD40  Q9HD40  Q9HD40  Q9HD40  Q9HD40  Q9HD40  Q9HD40  Q9HD40  Q9NP72  Q9NP72  Q9NP72  Q9NP72  Q9NP72  Q9NP72  Q9NP72  Q9NP72  Q9NP72  Q9NP79  Q9NP79  Q9NP79  Q9NP79  Q9NP79  Q9NP79  Q9NP79  Q9NP79  Q9NP79  Q9NP79  Q9NP79  Q9NP79  Q9NP79  Q9NP79  Q9NP79  Q9NP79  Q9NP79  Q9NPD3  Q9NPD3  Q9NPD3  Q9NPD3  Q9NPD3  Q9NPD3  Q9NPD3  Q9NPD3  Q9NPD3  Q9NPD3  Q9NPD3  Q9NQ92  Q9NQ92  Q9NQ92  Q9NQ92  Q9NQ92  Q9NQ92  Q9NQ92  Q9NQ92  Q9NQ92  Q9NQ92  Q9NQ92  Q9NQ92  Q9NQ92  Q9NQ92  Q9NQ92  Q9NQC3  Q9NQC3  Q9NQC3  Q9NQC3  Q9NQC3  Q9NQC3  Q9NQC3  Q9NQC3  Q9NQC3  Q9NQC3  Q9NQC3  Q9NQC3  Q9NQC3  Q9NQC3  Q9NQC3  Q9NQC3  Q9NQC3  Q9NQC3  Q9NQC3  Q9NQC3  Q9NQC3  Q9NQC3  Q9NQC3  Q9NQC3  Q9NQC3  Q9NQC3  Q9NQC3  Q9NQC3  Q9NQC3  Q9NQC3  Q9NQC3  Q9NQC7  Q9NQC7  Q9NQC7  Q9NQC7  Q9NQC7  Q9NQC7  Q9NQC7  Q9NQC7  Q9NQC7  Q9NQC7  Q9NQC7  Q9NQC7  Q9NQC7  Q9NQC7  Q9NQC7  Q9NQC7  Q9NQC7  Q9NQC7  Q9NQC7  Q9NQC7  Q9NQC7  Q9NQC7  Q9NQC7  Q9NQC7  Q9NQC7  Q9NQC7  Q9NQC7  Q9NQC7  Q9NQC7  Q9NQC7  Q9NQC7  Q9NQR1  Q9NQR1  Q9NQR1  Q9NQR1  Q9NQR1  Q9NQR1  Q9NQR1  Q9NQR1  Q9NQR1  Q9NQR1  Q9NQR1  Q9NQR1  Q9NQR1  Q9NQR1  Q9NQR1  Q9NQR1  Q9NQR1  Q9NQR1  Q9NQR1  Q9NQR1  Q9NQR1  Q9NQR1  Q9NQR1  Q9NQR1  Q9NQS7  Q9NQS7  Q9NQS7  Q9NQS7  Q9NQS7  Q9NQS7  Q9NQS7  Q9NQS7  Q9NQS7  Q9NQS7  Q9NQS7  Q9NQS7  Q9NQS7  Q9NQS7  Q9NQS7  Q9NQS7  Q9NQS7  Q9NQS7  Q9NQS7  Q9NQS7  Q9NQS7  Q9NQS7  Q9NQS7  Q9NQS7  Q9NQS7  Q9NQS7  Q9NQS7  Q9NQS7  Q9NQS7  Q9NQS7  Q9NQS7  Q9NQT5  Q9NQT5  Q9NQT5  Q9NQT5  Q9NQT5  Q9NQT5  Q9NQT5  Q9NQT5  Q9NQT5  Q9NQT5  Q9NQT5  Q9NQT5  Q9NQT5  Q9NQT5  Q9NQT8  Q9NQT8  Q9NQT8  Q9NQT8  Q9NQT8  Q9NQT8  Q9NQT8  Q9NQT8  Q9NQT8  Q9NQT8  Q9NQT8  Q9NQT8  Q9NQT8  Q9NQT8  Q9NQT8  Q9NQT8  Q9NQT8  Q9NQT8  Q9NQT8  Q9NQT8  Q9NQT8  Q9NQT8  Q9NQT8  Q9NQT8  Q9NQT8  Q9NQT8  Q9NQT8  Q9NQT8  Q9NQT8  Q9NQT8  Q9NQT8  Q9NQX4  Q9NQX4  Q9NQX4  Q9NQX4  Q9NQX4  Q9NQX4  Q9NQX4  Q9NQX4  Q9NQX4  Q9NQX4  Q9NQX4  Q9NQX4  Q9NQX4  Q9NQX4  Q9NQX4  Q9NQX4  Q9NQX4  Q9NQX4  Q9NQX4  Q9NQX4  Q9NQX4  Q9NQX4  Q9NQX4  Q9NQX4  Q9NQX4  Q9NQX4  Q9NQX4  Q9NQX4  Q9NQX4  Q9NQX4  Q9NQX4  Q9NQX4  Q9NQX4  Q9NR20  Q9NR20  Q9NR20  Q9NR20  Q9NR20  Q9NR20  Q9NR20  Q9NR20  Q9NR20  Q9NR20  Q9NR20  Q9NR20  Q9NR20  Q9NR20  Q9NR20  Q9NR20  Q9NR20  Q9NR20  Q9NR20  Q9NR20  Q9NR20  Q9NR20  Q9NR20  Q9NR20  Q9NR20  Q9NR20  Q9NR20  Q9NR20  Q9NR20  Q9NR20  Q9NR20  Q9NR20  Q9NR22  Q9NR22  Q9NR22  Q9NR22  Q9NR22  Q9NR22  Q9NR22  Q9NR22  Q9NR22  Q9NR22  Q9NR22  Q9NR22  Q9NR22  Q9NR22  Q9NR22  Q9NR22  Q9NR22  Q9NR22  Q9NR22  Q9NR22  Q9NR22  Q9NR22  Q9NR30  Q9NR30  Q9NR30  Q9NR30  Q9NR30  Q9NR30  Q9NR30  Q9NR30  Q9NR30  Q9NR30  Q9NR30  Q9NR30  Q9NR30  Q9NR30  Q9NR30  Q9NR30  Q9NR30  Q9NR30  Q9NR30  Q9NR30  Q9NR30  Q9NR30  Q9NR30  Q9NR30  Q9NR30  Q9NR30  Q9NR30  Q9NR30  Q9NR30  Q9NR30  Q9NR30  Q9NR50  Q9NR50  Q9NR50  Q9NR50  Q9NR50  Q9NR50  Q9NR50  Q9NR50  Q9NR50  Q9NR50  Q9NR50  Q9NR50  Q9NR50  Q9NR50  Q9NR50  Q9NR50  Q9NR50  Q9NR50  Q9NR50  Q9NR50  Q9NR50  Q9NR50  Q9NR50  Q9NR50  Q9NR50  Q9NR50  Q9NR50  Q9NR50  Q9NR50  Q9NR50  Q9NR50  Q9NR50  Q9NR50  Q9NRD5  Q9NRD5  Q9NRD5  Q9NRD5  Q9NRD5  Q9NRD5  Q9NRD5  Q9NRD5  Q9NRD5  Q9NRD5  Q9NRD5  Q9NRD5  Q9NRD5  Q9NRD5  Q9NRD5  Q9NRD5  Q9NRD5  Q9NRF9  Q9NRF9  Q9NRF9  Q9NRF9  Q9NRF9  Q9NRF9  Q9NRF9  Q9NRF9  Q9NRF9  Q9NRF9  Q9NRH2  Q9NRH2  Q9NRH2  Q9NRH2  Q9NRH2  Q9NRH2  Q9NRH2  Q9NRH2  Q9NRH2  Q9NRH2  Q9NRH2  Q9NRH2  Q9NRH2  Q9NRH2  Q9NRH2  Q9NRH2  Q9NRH2  Q9NRH2  Q9NRH2  Q9NRH2  Q9NRH2  Q9NRH2  Q9NRH2  Q9NRH2  Q9NRH2  Q9NRH2  Q9NRH2  Q9NRH2  Q9NRH2  Q9NRH2  Q9NRH2  Q9NRH2  Q9NRH2  Q9NRM7  Q9NRM7  Q9NRM7  Q9NRM7  Q9NRM7  Q9NRM7  Q9NRM7  Q9NRM7  Q9NRM7  Q9NRM7  Q9NRM7  Q9NRM7  Q9NRM7  Q9NRM7  Q9NRM7  Q9NRM7  Q9NRM7  Q9NRM7  Q9NRM7  Q9NRM7  Q9NRM7  Q9NRM7  Q9NRM7  Q9NRM7  Q9NRM7  Q9NRM7  Q9NRM7  Q9NRM7  Q9NRM7  Q9NRM7  Q9NRM7  Q9NRY4  Q9NRY4  Q9NRY4  Q9NRY4  Q9NRY4  Q9NRY4  Q9NRY4  Q9NRY4  Q9NRY4  Q9NRY4  Q9NRY4  Q9NRY4  Q9NRY4  Q9NRY4  Q9NRY4  Q9NRY4  Q9NRY4  Q9NRY4  Q9NRY4  Q9NRY4  Q9NRY4  Q9NRY4  Q9NRY4  Q9NRY4  Q9NRY4  Q9NRY4  Q9NRY4  Q9NRY4  Q9NRY4  Q9NRY4  Q9NRY4  Q9NRY4  Q9NRY4  Q9NS18  Q9NS18  Q9NS18  Q9NS18  Q9NS18  Q9NS18  Q9NS18  Q9NS18  Q9NS18  Q9NS18  Q9NS18  Q9NS18  Q9NS28  Q9NS28  Q9NS28  Q9NS28  Q9NS28  Q9NS28  Q9NS28  Q9NS28  Q9NS28  Q9NS28  Q9NS28  Q9NS28  Q9NS28  Q9NS28  Q9NS28  Q9NS28  Q9NS28  Q9NS28  Q9NS28  Q9NS56  Q9NS56  Q9NS56  Q9NS56  Q9NS56  Q9NS56  Q9NS56  Q9NS56  Q9NS56  Q9NS56  Q9NS56  Q9NS56  Q9NS56  Q9NS56  Q9NS56  Q9NS56  Q9NS56  Q9NS56  Q9NS56  Q9NS56  Q9NS56  Q9NS56  Q9NS56  Q9NS56  Q9NS56  Q9NS56  Q9NS56  Q9NS56  Q9NS56  Q9NS56  Q9NS56  Q9NS69  Q9NS69  Q9NS69  Q9NS69  Q9NS69  Q9NS69  Q9NS69  Q9NUB1  Q9NUB1  Q9NUB1  Q9NUB1  Q9NUB1  Q9NUB1  Q9NUB1  Q9NUB1  Q9NUB1  Q9NUB1  Q9NUB1  Q9NUB1  Q9NUB1  Q9NUB1  Q9NUB1  Q9NUB1  Q9NUB1  Q9NUB1  Q9NUB1  Q9NUB1  Q9NUB1  Q9NUB1  Q9NUB1  Q9NUB1  Q9NUB1  Q9NUB1  Q9NUB1  Q9NUB1  Q9NUB1  Q9NUB1  Q9NUB1  Q9NUB1  Q9NUB1  Q9NUP9  Q9NUP9  Q9NUP9  Q9NUP9  Q9NUP9  Q9NUP9  Q9NUP9  Q9NUP9  Q9NUP9  Q9NUP9  Q9NUP9  Q9NUP9  Q9NV92  Q9NV92  Q9NV92  Q9NV92  Q9NV92  Q9NV92  Q9NV92  Q9NV92  Q9NV92  Q9NV92  Q9NV92  Q9NV92  Q9NV92  Q9NV92  Q9NV92  Q9NV92  Q9NV92  Q9NV92  Q9NV92  Q9NV92  Q9NV92  Q9NV92  Q9NV92  Q9NV92  Q9NV92  Q9NV92  Q9NV92  Q9NV92  Q9NV92  Q9NVI1  Q9NVI1  Q9NVI1  Q9NVI1  Q9NVI1  Q9NVI1  Q9NVI1  Q9NVI1  Q9NVI1  Q9NVI1  Q9NVI1  Q9NVI1  Q9NVI1  Q9NVI1  Q9NVI1  Q9NVI1  Q9NVI1  Q9NVI1  Q9NVI1  Q9NVI1  Q9NVI1  Q9NVI1  Q9NVI1  Q9NVI1  Q9NVI1  Q9NVI1  Q9NVI1  Q9NVI1  Q9NVI1  Q9NVI1  Q9NVI1  Q9NVI7  Q9NVI7  Q9NVI7  Q9NVI7  Q9NVI7  Q9NVI7  Q9NVI7  Q9NVI7  Q9NVI7  Q9NVI7  Q9NVI7  Q9NVI7  Q9NVI7  Q9NVI7  Q9NVI7  Q9NVI7  Q9NVI7  Q9NVI7  Q9NVI7  Q9NVI7  Q9NVI7  Q9NVI7  Q9NVI7  Q9NVI7  Q9NVI7  Q9NVI7  Q9NVI7  Q9NVI7  Q9NVI7  Q9NVJ2  Q9NVJ2  Q9NVJ2  Q9NVJ2  Q9NVJ2  Q9NVJ2  Q9NVJ2  Q9NVJ2  Q9NVJ2  Q9NVX2  Q9NVX2  Q9NVX2  Q9NVX2  Q9NVX2  Q9NVX2  Q9NVX2  Q9NVX2  Q9NVX2  Q9NVX2  Q9NVX2  Q9NVX2  Q9NVX2  Q9NVX2  Q9NVX2  Q9NVX2  Q9NVX2  Q9NVX2  Q9NVX2  Q9NVX2  Q9NVX2  Q9NVX2  Q9NVX2  Q9NVX2  Q9NVX2  Q9NVX2  Q9NVX2  Q9NVX2  Q9NVX2  Q9NVX2  Q9NVX2  Q9NVX2  Q9NVX2  Q9NW13  Q9NW13  Q9NW13  Q9NW13  Q9NW13  Q9NW13  Q9NW13  Q9NW13  Q9NW13  Q9NW13  Q9NW13  Q9NW13  Q9NW13  Q9NW13  Q9NW13  Q9NW13  Q9NW13  Q9NW13  Q9NW13  Q9NW13  Q9NW13  Q9NW13  Q9NW13  Q9NW13  Q9NW13  Q9NW13  Q9NW13  Q9NW13  Q9NW13  Q9NW13  Q9NW13  Q9NW13  Q9NW13  Q9NWH9  Q9NWH9  Q9NWH9  Q9NWH9  Q9NWH9  Q9NWH9  Q9NWH9  Q9NWH9  Q9NWH9  Q9NWH9  Q9NWH9  Q9NWH9  Q9NWH9  Q9NWH9  Q9NWH9  Q9NWH9  Q9NWH9  Q9NWH9  Q9NWH9  Q9NWH9  Q9NWH9  Q9NWH9  Q9NWH9  Q9NWH9  Q9NWH9  Q9NWH9  Q9NWH9  Q9NWH9  Q9NWH9  Q9NWH9  Q9NWH9  Q9NWH9  Q9NWH9  Q9NWQ8  Q9NWQ8  Q9NWQ8  Q9NWQ8  Q9NWQ8  Q9NWQ8  Q9NWQ8  Q9NWQ8  Q9NWQ8  Q9NWQ8  Q9NWQ8  Q9NWQ8  Q9NWQ8  Q9NWQ8  Q9NWQ8  Q9NWQ8  Q9NWQ8  Q9NWQ8  Q9NWQ8  Q9NWQ8  Q9NWQ8  Q9NWQ8  Q9NWQ8  Q9NWQ8  Q9NWQ8  Q9NWQ8  Q9NWQ8  Q9NWQ8  Q9NWQ8  Q9NWQ8  Q9NWQ8  Q9NWQ8  Q9NWQ8  Q9NWZ3  Q9NWZ3  Q9NWZ3  Q9NWZ3  Q9NWZ3  Q9NWZ3  Q9NWZ3  Q9NWZ3  Q9NWZ3  Q9NWZ3  Q9NWZ3  Q9NWZ3  Q9NWZ3  Q9NWZ3  Q9NWZ3  Q9NWZ3  Q9NWZ3  Q9NWZ3  Q9NWZ3  Q9NWZ3  Q9NWZ3  Q9NWZ3  Q9NWZ3  Q9NWZ3  Q9NWZ3  Q9NWZ3  Q9NWZ3  Q9NWZ3  Q9NWZ3  Q9NWZ3  Q9NX09  Q9NX09  Q9NX09  Q9NX09  Q9NX09  Q9NX09  Q9NX09  Q9NX09  Q9NX09  Q9NX09  Q9NX09  Q9NX09  Q9NX09  Q9NX09  Q9NX09  Q9NX09  Q9NX09  Q9NX09  Q9NX09  Q9NX09  Q9NX09  Q9NX09  Q9NX09  Q9NX09  Q9NX09  Q9NX09  Q9NX09  Q9NX09  Q9NX09  Q9NX09  Q9NX09  Q9NX76  Q9NX76  Q9NX76  Q9NX76  Q9NX76  Q9NX76  Q9NX76  Q9NX76  Q9NX76  Q9NX76  Q9NX76  Q9NX76  Q9NX76  Q9NX76  Q9NXV6  Q9NXV6  Q9NXV6  Q9NXV6  Q9NXV6  Q9NXV6  Q9NXV6  Q9NXV6  Q9NXV6  Q9NXV6  Q9NXV6  Q9NXV6  Q9NXV6  Q9NXV6  Q9NXV6  Q9NXV6  Q9NXV6  Q9NXV6  Q9NXV6  Q9NXV6  Q9NXV6  Q9NXV6  Q9NXV6  Q9NXV6  Q9NXV6  Q9NXV6  Q9NXV6  Q9NXV6  Q9NXV6  Q9NXV6  Q9NXV6  Q9NXV6  Q9NXV6  Q9NYA1  Q9NYA1  Q9NYA1  Q9NYA1  Q9NYA1  Q9NYA1  Q9NYA1  Q9NYA1  Q9NYA1  Q9NYA1  Q9NYA1  Q9NYA1  Q9NYA1  Q9NYA1  Q9NYA1  Q9NYA1  Q9NYA1  Q9NYA1  Q9NYA1  Q9NYA1  Q9NYA1  Q9NYF0  Q9NYF0  Q9NYF0  Q9NYF0  Q9NYF0  Q9NYF0  Q9NYF0  Q9NYF0  Q9NYF0  Q9NYF0  Q9NYF0  Q9NYF0  Q9NYF0  Q9NYF0  Q9NYF0  Q9NYF0  Q9NYF0  Q9NYF0  Q9NYF0  Q9NYF0  Q9NYF0  Q9NYF0  Q9NYF0  Q9NYF0  Q9NYF0  Q9NYF0  Q9NYF0  Q9NYF0  Q9NYF0  Q9NYF0  Q9NYF0  Q9NYF8  Q9NYF8  Q9NYF8  Q9NYF8  Q9NYF8  Q9NYF8  Q9NYF8  Q9NYF8  Q9NYF8  Q9NYF8  Q9NYF8  Q9NYF8  Q9NYF8  Q9NYF8  Q9NYF8  Q9NYF8  Q9NYF8  Q9NYF8  Q9NYF8  Q9NYF8  Q9NYF8  Q9NYF8  Q9NYF8  Q9NYF8  Q9NYF8  Q9NYF8  Q9NYF8  Q9NYF8  Q9NYF8  Q9NYF8  Q9NYF8  Q9NYL2  Q9NYL2  Q9NYL2  Q9NYL2  Q9NYL2  Q9NYL2  Q9NYL2  Q9NYL2  Q9NYL2  Q9NYL2  Q9NYL2  Q9NYL2  Q9NYL2  Q9NYL2  Q9NYL2  Q9NYL2  Q9NYL2  Q9NYL2  Q9NYL2  Q9NYL2  Q9NYL2  Q9NYL2  Q9NYL2  Q9NYL2  Q9NYL2  Q9NYL2  Q9NYL2  Q9NYL2  Q9NYL2  Q9NYL2  Q9NYL2  Q9NYV4  Q9NYV4  Q9NYV4  Q9NYV4  Q9NYV4  Q9NYV4  Q9NYV4  Q9NYV4  Q9NYV4  Q9NYV4  Q9NYV4  Q9NYV4  Q9NYV4  Q9NYV4  Q9NYV4  Q9NYV4  Q9NYV4  Q9NYV4  Q9NYV4  Q9NYV4  Q9NYV4  Q9NYV4  Q9NYV4  Q9NYV4  Q9NYV4  Q9NYV4  Q9NYV4  Q9NYV4  Q9NYV4  Q9NYV4  Q9NYV4  Q9NYV4  Q9NYV4  Q9NYV6  Q9NYV6  Q9NYV6  Q9NYV6  Q9NYV6  Q9NYV6  Q9NYV6  Q9NYV6  Q9NYV6  Q9NYV6  Q9NYV6  Q9NYV6  Q9NYV6  Q9NYV6  Q9NYV6  Q9NYV6  Q9NYV6  Q9NYV6  Q9NYV6  Q9NYV6  Q9NYV6  Q9NYV6  Q9NYV6  Q9NYV6  Q9NYV6  Q9NYV6  Q9NYV6  Q9NYV6  Q9NYV6  Q9NYV6  Q9NYV6  Q9NYV6  Q9NYV6  Q9NZ53  Q9NZ53  Q9NZ53  Q9NZ53  Q9NZ53  Q9NZ53  Q9NZ53  Q9NZ53  Q9NZ53  Q9NZ53  Q9NZ53  Q9NZ53  Q9NZ53  Q9NZ53  Q9NZ53  Q9NZ53  Q9NZ53  Q9NZ53  Q9NZ53  Q9NZ53  Q9NZ53  Q9NZ53  Q9NZ53  Q9NZ53  Q9NZ53  Q9NZ53  Q9NZ53  Q9NZ53  Q9NZ53  Q9NZ53  Q9NZ53  Q9NZ53  Q9NZ53  Q9NZC7  Q9NZC7  Q9NZC7  Q9NZC7  Q9NZC7  Q9NZC7  Q9NZC7  Q9NZC7  Q9NZC7  Q9NZC7  Q9NZC7  Q9NZC7  Q9NZC7  Q9NZC7  Q9NZC7  Q9NZC7  Q9NZC7  Q9NZC7  Q9NZC7  Q9NZC7  Q9NZC7  Q9NZC7  Q9NZC7  Q9NZC7  Q9NZC7  Q9NZJ0  Q9NZJ0  Q9NZJ0  Q9NZJ0  Q9NZJ0  Q9NZJ0  Q9NZJ0  Q9NZJ0  Q9NZJ0  Q9NZJ0  Q9NZJ0  Q9NZJ0  Q9NZJ0  Q9NZJ0  Q9NZJ0  Q9NZJ0  Q9NZJ0  Q9NZJ0  Q9NZJ0  Q9NZJ0  Q9NZJ0  Q9NZJ0  Q9NZJ0  Q9NZJ0  Q9NZJ0  Q9NZJ0  Q9NZJ0  Q9NZJ0  Q9NZJ0  Q9NZJ0  Q9NZJ0  Q9NZJ0  Q9NZJ0  Q9NZJ5  Q9NZJ5  Q9NZJ5  Q9NZJ5  Q9NZJ5  Q9NZJ5  Q9NZJ5  Q9NZJ5  Q9NZJ5  Q9NZJ5  Q9NZJ5  Q9NZJ5  Q9NZJ5  Q9NZJ5  Q9NZJ5  Q9NZJ5  Q9NZJ5  Q9NZJ5  Q9NZJ5  Q9NZJ5  Q9NZJ5  Q9NZJ5  Q9NZJ5  Q9NZJ5  Q9NZJ5  Q9NZJ5  Q9NZJ5  Q9NZJ5  Q9NZJ5  Q9NZJ5  Q9NZJ5  Q9NZJ5  Q9NZJ5  Q9NZN3  Q9NZN3  Q9NZN3  Q9NZN3  Q9NZN3  Q9NZN3  Q9NZN3  Q9NZN3  Q9NZN3  Q9NZN3  Q9NZN3  Q9NZN3  Q9NZN3  Q9NZN3  Q9NZN3  Q9NZN3  Q9NZN3  Q9NZN3  Q9NZN3  Q9NZN3  Q9NZN3  Q9NZN3  Q9NZN3  Q9NZN3  Q9NZN3  Q9NZT1  Q9NZT1  Q9NZT1  Q9NZT1  Q9NZT1  Q9NZU7  Q9NZU7  Q9NZU7  Q9NZU7  Q9NZU7  Q9NZU7  Q9NZU7  Q9NZU7  Q9NZU7  Q9NZU7  Q9NZU7  Q9NZU7  Q9NZU7  Q9NZU7  Q9NZU7  Q9NZU7  Q9NZU7  Q9NZU7  Q9NZU7  Q9NZU7  Q9NZU7  Q9NZU7  Q9NZU7  Q9NZU7  Q9NZU7  Q9NZU7  Q9NZU7  Q9NZU7  Q9NZU7  Q9NZU7  Q9P013  Q9P013  Q9P013  Q9P013  Q9P013  Q9P013  Q9P013  Q9P013  Q9P013  Q9P013  Q9P013  Q9P013  Q9P013  Q9P0L2  Q9P0L2  Q9P0L2  Q9P0L2  Q9P0L2  Q9P0L2  Q9P0L2  Q9P0L2  Q9P0L2  Q9P0L2  Q9P0L2  Q9P0L2  Q9P0L2  Q9P0L2  Q9P0L2  Q9P0L2  Q9P0L2  Q9P0L2  Q9P0L2  Q9P0L2  Q9P0L2  Q9P0L2  Q9P0L2  Q9P0L2  Q9P0L2  Q9P0L2  Q9P0L2  Q9P0L2  Q9P0L2  Q9P0L2  Q9P0L2  Q9P0L2  Q9P126  Q9P126  Q9P126  Q9P126  Q9P126  Q9P126  Q9P126  Q9P126  Q9P126  Q9P126  Q9P126  Q9P126  Q9P126  Q9P126  Q9P1Z0  Q9P1Z0  Q9P1Z0  Q9P1Z0  Q9P1Z0  Q9P1Z0  Q9P1Z0  Q9P1Z0  Q9P1Z0  Q9P1Z0  Q9P1Z0  Q9P1Z0  Q9P1Z0  Q9P1Z0  Q9P1Z0  Q9P1Z0  Q9P1Z0  Q9P1Z0  Q9P1Z0  Q9P1Z0  Q9P1Z0  Q9P1Z0  Q9P1Z0  Q9P1Z0  Q9P1Z0  Q9P1Z0  Q9P1Z0  Q9P1Z0  Q9P1Z0  Q9P1Z0  Q9P1Z0  Q9P1Z0  Q9P1Z0  Q9P2R3  Q9P2R3  Q9P2R3  Q9P2R3  Q9P2R3  Q9P2R3  Q9P2R3  Q9P2R3  Q9P2R3  Q9P2R3  Q9P2R3  Q9P2R3  Q9P2R3  Q9P2R3  Q9P2R3  Q9P2R3  Q9P2R3  Q9P2R3  Q9P2R3  Q9P2R3  Q9P2R3  Q9P2R3  Q9P2R3  Q9P2R3  Q9P2R3  Q9P2R3  Q9P2R3  Q9P2R3  Q9P2R3  Q9P2R3  Q9P2R3  Q9P2R3  Q9P2R3  Q9P2Y5  Q9P2Y5  Q9P2Y5  Q9P2Y5  Q9P2Y5  Q9P2Y5  Q9P2Y5  Q9P2Y5  Q9P2Y5  Q9P2Y5  Q9P2Y5  Q9P2Y5  Q9P2Y5  Q9P2Y5  Q9P2Y5  Q9P2Y5  Q9P2Y5  Q9P2Y5  Q9P2Y5  Q9P2Y5  Q9P2Y5  Q9P2Y5  Q9P2Y5  Q9P2Y5  Q9P2Y5  Q9P2Y5  Q9P2Y5  Q9P2Y5  Q9P2Y5  Q9P2Y5  Q9P2Y5  Q9UBC9  Q9UBC9  Q9UBC9  Q9UBC9  Q9UBF6  Q9UBF6  Q9UBF6  Q9UBF6  Q9UBF6  Q9UBF6  Q9UBF6  Q9UBF6  Q9UBF6  Q9UBF6  Q9UBF8  Q9UBF8  Q9UBF8  Q9UBF8  Q9UBF8  Q9UBF8  Q9UBF8  Q9UBF8  Q9UBF8  Q9UBF8  Q9UBF8  Q9UBF8  Q9UBF8  Q9UBF8  Q9UBF8  Q9UBF8  Q9UBF8  Q9UBF8  Q9UBF8  Q9UBF8  Q9UBF8  Q9UBF8  Q9UBF8  Q9UBF8  Q9UBF8  Q9UBF8  Q9UBF8  Q9UBF8  Q9UBF8  Q9UBF8  Q9UBF8  Q9UBK5  Q9UBK5  Q9UBK5  Q9UBK5  Q9UBK5  Q9UBK5  Q9UBK5  Q9UBK5  Q9UBK5  Q9UBL3  Q9UBL3  Q9UBL3  Q9UBL3  Q9UBL3  Q9UBL3  Q9UBL3  Q9UBL3  Q9UBL3  Q9UBL3  Q9UBL3  Q9UBL3  Q9UBL3  Q9UBL3  Q9UBL3  Q9UBL3  Q9UBL3  Q9UBL3  Q9UBL3  Q9UBL3  Q9UBL3  Q9UBL3  Q9UBL3  Q9UBL3  Q9UBL3  Q9UBL3  Q9UBL3  Q9UBL3  Q9UBL3  Q9UBL3  Q9UBL3  Q9UBL3  Q9UBL3  Q9UBN4  Q9UBN4  Q9UBN4  Q9UBN4  Q9UBN4  Q9UBN4  Q9UBN4  Q9UBN4  Q9UBN4  Q9UBN4  Q9UBN4  Q9UBN4  Q9UBN4  Q9UBN4  Q9UBN4  Q9UBN4  Q9UBN4  Q9UBN4  Q9UBN4  Q9UBN4  Q9UBN4  Q9UBN4  Q9UBN4  Q9UBN4  Q9UBN4  Q9UBN4  Q9UBN4  Q9UBN4  Q9UBN4  Q9UBN4  Q9UBN4  Q9UBN4  Q9UBN4  Q9UBP6  Q9UBP6  Q9UBP6  Q9UBP6  Q9UBP6  Q9UBP6  Q9UBP6  Q9UBP6  Q9UBQ5  Q9UBQ5  Q9UBQ5  Q9UBQ5  Q9UBQ5  Q9UBQ5  Q9UBQ5  Q9UBQ5  Q9UBQ5  Q9UBR4  Q9UBR4  Q9UBR4  Q9UBR4  Q9UBR4  Q9UBR4  Q9UBR4  Q9UBR4  Q9UBR4  Q9UBR4  Q9UBR4  Q9UBR4  Q9UBR4  Q9UBR4  Q9UBR4  Q9UBR4  Q9UBR4  Q9UBR4  Q9UBR4  Q9UBR4  Q9UBR4  Q9UBR4  Q9UBR4  Q9UBR4  Q9UBR4  Q9UBR4  Q9UBR4  Q9UBR4  Q9UBR4  Q9UBR4  Q9UBW8  Q9UBW8  Q9UBW8  Q9UBW8  Q9UBW8  Q9UBW8  Q9UBW8  Q9UBW8  Q9UBW8  Q9UBW8  Q9UBW8  Q9UBW8  Q9UBW8  Q9UBW8  Q9UD71  Q9UD71  Q9UD71  Q9UD71  Q9UD71  Q9UD71  Q9UD71  Q9UD71  Q9UD71  Q9UD71  Q9UD71  Q9UD71  Q9UD71  Q9UD71  Q9UD71  Q9UD71  Q9UD71  Q9UD71  Q9UER7  Q9UER7  Q9UER7  Q9UER7  Q9UER7  Q9UER7  Q9UER7  Q9UER7  Q9UER7  Q9UER7  Q9UER7  Q9UER7  Q9UER7  Q9UER7  Q9UER7  Q9UER7  Q9UER7  Q9UER7  Q9UER7  Q9UER7  Q9UER7  Q9UER7  Q9UER7  Q9UER7  Q9UER7  Q9UER7  Q9UER7  Q9UER7  Q9UER7  Q9UER7  Q9UER7  Q9UEW8  Q9UEW8  Q9UEW8  Q9UEW8  Q9UEW8  Q9UEW8  Q9UEW8  Q9UEW8  Q9UEW8  Q9UEW8  Q9UEW8  Q9UEW8  Q9UEW8  Q9UEW8  Q9UEW8  Q9UEW8  Q9UEW8  Q9UEW8  Q9UEW8  Q9UEW8  Q9UEW8  Q9UEW8  Q9UEW8  Q9UEW8  Q9UEW8  Q9UEW8  Q9UEW8  Q9UG01  Q9UG01  Q9UG01  Q9UG01  Q9UG01  Q9UG01  Q9UG01  Q9UG01  Q9UG01  Q9UG01  Q9UG01  Q9UG01  Q9UG01  Q9UG01  Q9UG01  Q9UG01  Q9UG01  Q9UG01  Q9UG01  Q9UG01  Q9UG01  Q9UG01  Q9UG01  Q9UG01  Q9UG01  Q9UG01  Q9UG01  Q9UG01  Q9UG01  Q9UG01  Q9UG01  Q9UG01  Q9UG01  Q9UGC7  Q9UGC7  Q9UGC7  Q9UGC7  Q9UGC7  Q9UGC7  Q9UGC7  Q9UGC7  Q9UGC7  Q9UGC7  Q9UGC7  Q9UGC7  Q9UGC7  Q9UGC7  Q9UGC7  Q9UGC7  Q9UGC7  Q9UGC7  Q9UGC7  Q9UGC7  Q9UGK3  Q9UGK3  Q9UGK3  Q9UGK3  Q9UGK3  Q9UGK3  Q9UGK3  Q9UGK3  Q9UGK3  Q9UGK3  Q9UGK3  Q9UGK3  Q9UGK3  Q9UGK3  Q9UGK3  Q9UGK3  Q9UGK3  Q9UGK3  Q9UGK3  Q9UGK3  Q9UGK3  Q9UGK3  Q9UGK3  Q9UH99  Q9UH99  Q9UH99  Q9UH99  Q9UH99  Q9UH99  Q9UH99  Q9UH99  Q9UH99  Q9UH99  Q9UH99  Q9UH99  Q9UH99  Q9UH99  Q9UH99  Q9UH99  Q9UH99  Q9UH99  Q9UH99  Q9UH99  Q9UH99  Q9UH99  Q9UH99  Q9UH99  Q9UH99  Q9UH99  Q9UH99  Q9UH99  Q9UH99  Q9UH99  Q9UH99  Q9UHD1  Q9UHD1  Q9UHD1  Q9UHD1  Q9UHD1  Q9UHD1  Q9UHD1  Q9UHD1  Q9UHD1  Q9UHD1  Q9UHD1  Q9UHD1  Q9UHD1  Q9UHD1  Q9UHD1  Q9UHD1  Q9UHD1  Q9UHD1  Q9UHD1  Q9UHD1  Q9UHD1  Q9UHD1  Q9UHD2  Q9UHD2  Q9UHD2  Q9UHD2  Q9UHD2  Q9UHD2  Q9UHD2  Q9UHD2  Q9UHD2  Q9UHD2  Q9UHD2  Q9UHD2  Q9UHD2  Q9UHD2  Q9UHD2  Q9UHD2  Q9UHD2  Q9UHD2  Q9UHD2  Q9UHD2  Q9UHD2  Q9UHD2  Q9UHD2  Q9UHD2  Q9UHD2  Q9UHD2  Q9UHD2  Q9UHD2  Q9UHD2  Q9UHD2  Q9UHD2  Q9UHM6  Q9UHM6  Q9UHM6  Q9UHM6  Q9UHM6  Q9UHM6  Q9UHM6  Q9UHM6  Q9UHM6  Q9UHM6  Q9UHM6  Q9UHM6  Q9UHM6  Q9UHM6  Q9UHM6  Q9UHM6  Q9UHM6  Q9UHM6  Q9UHM6  Q9UHM6  Q9UHM6  Q9UHM6  Q9UHM6  Q9UHM6  Q9UHM6  Q9UHM6  Q9UHM6  Q9UHM6  Q9UHM6  Q9UHM6  Q9UHM6  Q9UHM6  Q9UHM6  Q9UHP3  Q9UHP3  Q9UHP3  Q9UHP3  Q9UHP3  Q9UHP3  Q9UHP3  Q9UHP3  Q9UHP3  Q9UHP3  Q9UHP3  Q9UHP3  Q9UHP3  Q9UHP3  Q9UHP3  Q9UHP3  Q9UHP3  Q9UHP3  Q9UHP3  Q9UHP3  Q9UHP3  Q9UHP3  Q9UHP3  Q9UHP3  Q9UHP3  Q9UHP3  Q9UHP3  Q9UHP3  Q9UHP3  Q9UHP3  Q9UHP3  Q9UHP3  Q9UHP3  Q9UIA9  Q9UIA9  Q9UIA9  Q9UIA9  Q9UIA9  Q9UIA9  Q9UIA9  Q9UIA9  Q9UIA9  Q9UIA9  Q9UIA9  Q9UIA9  Q9UIA9  Q9UIA9  Q9UIA9  Q9UIA9  Q9UIA9  Q9UIA9  Q9UIA9  Q9UIA9  Q9UIA9  Q9UIA9  Q9UIA9  Q9UIA9  Q9UIA9  Q9UIA9  Q9UIA9  Q9UIA9  Q9UIA9  Q9UIA9  Q9UIA9  Q9UIA9  Q9UIA9  Q9UIB8  Q9UIB8  Q9UIB8  Q9UIB8  Q9UIB8  Q9UIB8  Q9UIB8  Q9UIB8  Q9UIB8  Q9UIB8  Q9UIB8  Q9UIB8  Q9UIB8  Q9UIB8  Q9UIB8  Q9UIB8  Q9UIB8  Q9UIB8  Q9UIB8  Q9UIB8  Q9UIB8  Q9UIB8  Q9UIB8  Q9UIB8  Q9UIB8  Q9UIB8  Q9UIB8  Q9UIF9  Q9UIF9  Q9UIF9  Q9UIF9  Q9UIF9  Q9UIF9  Q9UIF9  Q9UIF9  Q9UIF9  Q9UIF9  Q9UIF9  Q9UIF9  Q9UIF9  Q9UIF9  Q9UIF9  Q9UIF9  Q9UIF9  Q9UIF9  Q9UIF9  Q9UIF9  Q9UIF9  Q9UIF9  Q9UIF9  Q9UIF9  Q9UIF9  Q9UIF9  Q9UIF9  Q9UIF9  Q9UIF9  Q9UIF9  Q9UIF9  Q9UIF9  Q9UIF9  Q9UJ70  Q9UJ70  Q9UJ70  Q9UJ70  Q9UJ70  Q9UJ70  Q9UJ70  Q9UJ70  Q9UJ70  Q9UJ70  Q9UJ70  Q9UJ70  Q9UJ70  Q9UJ70  Q9UJ70  Q9UJ70  Q9UJ70  Q9UJ70  Q9UJ70  Q9UJ70  Q9UJ70  Q9UJ70  Q9UJ70  Q9UJ70  Q9UJQ4  Q9UJQ4  Q9UJQ4  Q9UJQ4  Q9UJQ4  Q9UJQ4  Q9UJQ4  Q9UJQ4  Q9UJQ4  Q9UJQ4  Q9UJQ4  Q9UJQ4  Q9UJQ4  Q9UJQ4  Q9UJQ4  Q9UJQ4  Q9UJQ4  Q9UJQ4  Q9UJQ4  Q9UJQ4  Q9UJQ4  Q9UJQ4  Q9UJQ4  Q9UJQ4  Q9UJQ4  Q9UJQ4  Q9UJQ4  Q9UJQ4  Q9UJQ4  Q9UJQ4  Q9UJQ4  Q9UJX2  Q9UJX2  Q9UJX2  Q9UJX2  Q9UJX2  Q9UJX2  Q9UJX2  Q9UJX2  Q9UJX2  Q9UJX2  Q9UJX2  Q9UJX2  Q9UJX2  Q9UJX2  Q9UJX2  Q9UJX2  Q9UJX2  Q9UJX2  Q9UJX2  Q9UJX2  Q9UJX2  Q9UJX2  Q9UJX2  Q9UJX2  Q9UJX2  Q9UJX2  Q9UJX2  Q9UJX2  Q9UJX2  Q9UJX2  Q9UJX2  Q9UJX2  Q9UJX2  Q9UJX4  Q9UJX4  Q9UJX4  Q9UJX4  Q9UJX4  Q9UJX4  Q9UJX4  Q9UJX4  Q9UJX4  Q9UJX4  Q9UJX4  Q9UJX4  Q9UJX4  Q9UJX4  Q9UJX4  Q9UJX4  Q9UJX4  Q9UJX4  Q9UJX4  Q9UJX4  Q9UJX4  Q9UJX4  Q9UJX4  Q9UJX4  Q9UJX4  Q9UJX4  Q9UJX4  Q9UJX4  Q9UJX4  Q9UJX4  Q9UJX4  Q9UJX5  Q9UJX5  Q9UJX5  Q9UJX5  Q9UJX5  Q9UJX5  Q9UJX5  Q9UJX5  Q9UJX5  Q9UJX5  Q9UJX5  Q9UJX5  Q9UJX5  Q9UJX5  Q9UJX5  Q9UJX5  Q9UJX5  Q9UJX5  Q9UJX5  Q9UJX5  Q9UJX5  Q9UJX5  Q9UJX5  Q9UJX5  Q9UJX5  Q9UJX5  Q9UJX5  Q9UJX5  Q9UJX5  Q9UJX5  Q9UJX5  Q9UJX6  Q9UJX6  Q9UJX6  Q9UJX6  Q9UJX6  Q9UJX6  Q9UJX6  Q9UJX6  Q9UJX6  Q9UJX6  Q9UJX6  Q9UJX6  Q9UJX6  Q9UJX6  Q9UJX6  Q9UJX6  Q9UJX6  Q9UJX6  Q9UJX6  Q9UJX6  Q9UJX6  Q9UJX6  Q9UJX6  Q9UJX6  Q9UJX6  Q9UJX6  Q9UJX6  Q9UJX6  Q9UJX6  Q9UJX6  Q9UJX6  Q9UJY1  Q9UJY1  Q9UJY1  Q9UJY1  Q9UJY1  Q9UJY1  Q9UJY1  Q9UJY1  Q9UJY1  Q9UJY1  Q9UJY1  Q9UJY1  Q9UJY1  Q9UJY1  Q9UJY1  Q9UJY1  Q9UJY1  Q9UJZ1  Q9UJZ1  Q9UJZ1  Q9UJZ1  Q9UJZ1  Q9UJZ1  Q9UJZ1  Q9UJZ1  Q9UJZ1  Q9UJZ1  Q9UJZ1  Q9UJZ1  Q9UJZ1  Q9UJZ1  Q9UJZ1  Q9UJZ1  Q9UJZ1  Q9UJZ1  Q9UJZ1  Q9UJZ1  Q9UJZ1  Q9UJZ1  Q9UJZ1  Q9UJZ1  Q9UJZ1  Q9UJZ1  Q9UK32  Q9UK32  Q9UK32  Q9UK32  Q9UK32  Q9UK32  Q9UK32  Q9UK32  Q9UK32  Q9UK32  Q9UK32  Q9UK32  Q9UK32  Q9UK32  Q9UK32  Q9UK32  Q9UK32  Q9UK32  Q9UK32  Q9UK32  Q9UK32  Q9UK32  Q9UK32  Q9UK32  Q9UK32  Q9UK32  Q9UK32  Q9UK32  Q9UK32  Q9UK32  Q9UK32  Q9UK55  Q9UK55  Q9UK55  Q9UK55  Q9UK55  Q9UK55  Q9UK55  Q9UK55  Q9UK55  Q9UK55  Q9UK55  Q9UK55  Q9UK55  Q9UK55  Q9UK55  Q9UK55  Q9UK55  Q9UK55  Q9UK55  Q9UK55  Q9UK55  Q9UK55  Q9UK55  Q9UK55  Q9UK76  Q9UK76  Q9UK76  Q9UK76  Q9UK76  Q9UK76  Q9UK76  Q9UK76  Q9UK76  Q9UK76  Q9UK76  Q9UK76  Q9UK76  Q9UK76  Q9UK76  Q9UK76  Q9UKG1  Q9UKG1  Q9UKG1  Q9UKG1  Q9UKG1  Q9UKG1  Q9UKG1  Q9UKG1  Q9UKG1  Q9UKG1  Q9UKG1  Q9UKG1  Q9UKG1  Q9UKG1  Q9UKG1  Q9UKG1  Q9UKG1  Q9UKG1  Q9UKG1  Q9UKG1  Q9UKG1  Q9UKG1  Q9UKG1  Q9UKG1  Q9UKG1  Q9UKG1  Q9UKG1  Q9UKG1  Q9UKG1  Q9UKG1  Q9UKG1  Q9UKI8  Q9UKI8  Q9UKI8  Q9UKI8  Q9UKI8  Q9UKI8  Q9UKI8  Q9UKI8  Q9UKI8  Q9UKI8  Q9UKI8  Q9UKI8  Q9UKI8  Q9UKI8  Q9UKI8  Q9UKI8  Q9UKI8  Q9UKI8  Q9UKI8  Q9UKI8  Q9UKI8  Q9UKI8  Q9UKI8  Q9UKI8  Q9UKI8  Q9UKI8  Q9UKI8  Q9UKI8  Q9UKI8  Q9UKI8  Q9UKI8  Q9UKM9  Q9UKM9  Q9UKM9  Q9UKM9  Q9UKM9  Q9UKM9  Q9UKM9  Q9UKM9  Q9UKM9  Q9UKM9  Q9UKM9  Q9UKM9  Q9UKM9  Q9UKM9  Q9UKM9  Q9UKM9  Q9UKM9  Q9UKM9  Q9UKM9  Q9UKT5  Q9UKT5  Q9UKT5  Q9UKT5  Q9UKT5  Q9UKT5  Q9UKT5  Q9UKT5  Q9UKT5  Q9UKT5  Q9UKT5  Q9UKT5  Q9UKT5  Q9UKT5  Q9UKT5  Q9UKT5  Q9UKT5  Q9UKT5  Q9UKT5  Q9UKT5  Q9UKT5  Q9UKT5  Q9UKT5  Q9UKT5  Q9UKT5  Q9UKT5  Q9UKT5  Q9UKT5  Q9UKT5  Q9UKT5  Q9UKT5  Q9UKV3  Q9UKV3  Q9UKV3  Q9UKV3  Q9UKV3  Q9UKV3  Q9UKV3  Q9UKV3  Q9UKV3  Q9UKV3  Q9UKV3  Q9UKV3  Q9UKV3  Q9UKV3  Q9UKV3  Q9UKV3  Q9UKV3  Q9UKV3  Q9UKV3  Q9UKV3  Q9UKV3  Q9UKV3  Q9UKV3  Q9UKV3  Q9UKV3  Q9UKV3  Q9UKV3  Q9UKV3  Q9UKV3  Q9UKV3  Q9UKV3  Q9UKV8  Q9UKV8  Q9UKV8  Q9UKV8  Q9UKV8  Q9UKV8  Q9UKV8  Q9UKV8  Q9UKV8  Q9UKV8  Q9UKV8  Q9UKV8  Q9UKV8  Q9UKV8  Q9UKV8  Q9UKV8  Q9UKV8  Q9UKV8  Q9UKV8  Q9UKV8  Q9UKV8  Q9UKV8  Q9UKV8  Q9UKV8  Q9UKV8  Q9UKV8  Q9UKV8  Q9UKV8  Q9UKV8  Q9UKV8  Q9UKV8  Q9UKV8  Q9UKV8  Q9UL25  Q9UL25  Q9UL25  Q9UL25  Q9UL25  Q9UL25  Q9UL25  Q9UL25  Q9UL25  Q9UL25  Q9UL25  Q9UL25  Q9UL42  Q9UL42  Q9UL42  Q9UL42  Q9UL42  Q9UL42  Q9UL42  Q9UL42  Q9UL42  Q9UL42  Q9UL42  Q9UL42  Q9UL42  Q9UL42  Q9UL42  Q9UL42  Q9UL42  Q9UL42  Q9UL42  Q9UL42  Q9UL46  Q9UL46  Q9UL46  Q9UL46  Q9UL46  Q9UL46  Q9UL46  Q9UL46  Q9UL46  Q9UL54  Q9UL54  Q9UL54  Q9UL54  Q9UL54  Q9UL54  Q9UL54  Q9UL54  Q9UL54  Q9UL54  Q9UL54  Q9UL54  Q9UL54  Q9UL54  Q9UL54  Q9UL54  Q9UL54  Q9UL54  Q9UL54  Q9UL54  Q9UL54  Q9UL54  Q9UL54  Q9UL54  Q9UL54  Q9UL54  Q9UL54  Q9UL54  Q9UL54  Q9UL54  Q9UL54  Q9ULC4  Q9ULC4  Q9ULC4  Q9ULC4  Q9ULV5  Q9ULV5  Q9ULV5  Q9ULV5  Q9ULV5  Q9ULV5  Q9ULV5  Q9ULV5  Q9ULV5  Q9ULV5  Q9ULV5  Q9ULV5  Q9ULV5  Q9ULV5  Q9ULV5  Q9ULV5  Q9ULV5  Q9ULV5  Q9ULV5  Q9ULV5  Q9ULV5  Q9ULV5  Q9ULV5  Q9ULV5  Q9ULV5  Q9ULV5  Q9ULV5  Q9ULV5  Q9ULV5  Q9ULV5  Q9ULV5  Q9ULV8  Q9ULV8  Q9ULV8  Q9ULV8  Q9ULV8  Q9ULV8  Q9ULV8  Q9ULV8  Q9ULV8  Q9ULV8  Q9ULV8  Q9ULV8  Q9ULV8  Q9ULV8  Q9ULV8  Q9ULV8  Q9ULV8  Q9ULV8  Q9ULV8  Q9ULV8  Q9ULV8  Q9ULV8  Q9ULV8  Q9ULV8  Q9ULV8  Q9ULV8  Q9ULV8  Q9ULV8  Q9ULV8  Q9ULV8  Q9ULV8  Q9UM07  Q9UM07  Q9UM07  Q9UM07  Q9UM07  Q9UM07  Q9UM07  Q9UM07  Q9UM07  Q9UM07  Q9UM07  Q9UM07  Q9UM07  Q9UM07  Q9UM07  Q9UM07  Q9UM07  Q9UM07  Q9UM07  Q9UM07  Q9UM07  Q9UM07  Q9UM07  Q9UM07  Q9UM07  Q9UM07  Q9UM07  Q9UM07  Q9UM07  Q9UM07  Q9UM07  Q9UM07  Q9UM07  Q9UM11  Q9UM11  Q9UM11  Q9UM11  Q9UM11  Q9UM11  Q9UM11  Q9UM11  Q9UM11  Q9UM11  Q9UM11  Q9UM11  Q9UM11  Q9UM11  Q9UM11  Q9UM11  Q9UM11  Q9UM11  Q9UM11  Q9UM11  Q9UM11  Q9UM11  Q9UM11  Q9UM11  Q9UM11  Q9UM11  Q9UM11  Q9UM11  Q9UM11  Q9UM11  Q9UM11  Q9UM11  Q9UM11  Q9UM21  Q9UM21  Q9UM21  Q9UM21  Q9UM21  Q9UM21  Q9UM21  Q9UM21  Q9UM21  Q9UM21  Q9UM21  Q9UM21  Q9UM21  Q9UM21  Q9UM21  Q9UM21  Q9UM21  Q9UM21  Q9UM21  Q9UM21  Q9UM21  Q9UM21  Q9UM21  Q9UM21  Q9UM21  Q9UM21  Q9UM21  Q9UM21  Q9UM21  Q9UM21  Q9UM21  Q9UM73  Q9UM73  Q9UM73  Q9UM73  Q9UM73  Q9UM73  Q9UM73  Q9UM73  Q9UM73  Q9UM73  Q9UM73  Q9UM73  Q9UM73  Q9UM73  Q9UM73  Q9UM73  Q9UM73  Q9UM73  Q9UM73  Q9UM73  Q9UM73  Q9UM73  Q9UM73  Q9UM73  Q9UM73  Q9UM73  Q9UM73  Q9UM73  Q9UM73  Q9UM73  Q9UM73  Q9UM73  Q9UM73  Q9UMD9  Q9UMD9  Q9UMD9  Q9UMD9  Q9UMD9  Q9UMD9  Q9UMD9  Q9UMD9  Q9UMD9  Q9UMD9  Q9UMD9  Q9UMD9  Q9UMD9  Q9UMD9  Q9UMD9  Q9UMD9  Q9UMD9  Q9UMD9  Q9UMD9  Q9UMD9  Q9UMD9  Q9UMD9  Q9UMD9  Q9UMD9  Q9UMD9  Q9UMD9  Q9UMD9  Q9UMD9  Q9UMD9  Q9UMD9  Q9UMD9  Q9UMS4  Q9UMS4  Q9UMS4  Q9UMS4  Q9UMS4  Q9UMS4  Q9UMS4  Q9UMS4  Q9UMS4  Q9UMS4  Q9UMS4  Q9UMS4  Q9UMS4  Q9UMS4  Q9UMS4  Q9UMS4  Q9UMS4  Q9UMS4  Q9UMS4  Q9UMS4  Q9UMS4  Q9UMS4  Q9UMS4  Q9UMS4  Q9UMS4  Q9UMS4  Q9UMS4  Q9UMS4  Q9UMS4  Q9UMS4  Q9UMS4  Q9UMS4  Q9UMS4  Q9UNE7  Q9UNE7  Q9UNE7  Q9UNE7  Q9UNE7  Q9UNE7  Q9UNE7  Q9UNE7  Q9UNE7  Q9UNE7  Q9UNE7  Q9UNE7  Q9UNE7  Q9UNF1  Q9UNF1  Q9UNF1  Q9UNF1  Q9UNF1  Q9UNF1  Q9UNF1  Q9UNF1  Q9UNF1  Q9UNF1  Q9UNF1  Q9UNF1  Q9UNF1  Q9UNF1  Q9UNF1  Q9UNF1  Q9UNF1  Q9UNF1  Q9UNF1  Q9UNF1  Q9UNF1  Q9UNF1  Q9UNF1  Q9UNF1  Q9UNF1  Q9UNF1  Q9UNF1  Q9UNF1  Q9UNF1  Q9UNF1  Q9UNF1  Q9UNF1  Q9UNF1  Q9UNL4  Q9UNL4  Q9UNL4  Q9UNL4  Q9UNL4  Q9UNL4  Q9UNL4  Q9UNL4  Q9UNL4  Q9UNL4  Q9UNL4  Q9UNL4  Q9UNL4  Q9UNL4  Q9UNL4  Q9UNL4  Q9UNL4  Q9UNL4  Q9UNL4  Q9UNL4  Q9UNS2  Q9UNS2  Q9UNS2  Q9UNS2  Q9UNS2  Q9UNS2  Q9UNS2  Q9UNS2  Q9UNS2  Q9UNS2  Q9UNS2  Q9UNS2  Q9UNS2  Q9UNS2  Q9UNS2  Q9UNS2  Q9UNS2  Q9UNS2  Q9UNS2  Q9UNS2  Q9UNS2  Q9UNS2  Q9UNS2  Q9UNS2  Q9UNS2  Q9UNS2  Q9UNS2  Q9UP65  Q9UP65  Q9UP65  Q9UP65  Q9UP65  Q9UP65  Q9UP65  Q9UP65  Q9UP65  Q9UP65  Q9UP65  Q9UP65  Q9UP65  Q9UP65  Q9UP65  Q9UP65  Q9UP65  Q9UP65  Q9UP65  Q9UP65  Q9UP65  Q9UP65  Q9UP65  Q9UP65  Q9UP65  Q9UP65  Q9UP65  Q9UP65  Q9UP65  Q9UP65  Q9UP65  Q9UPN6  Q9UPN6  Q9UPN6  Q9UPN6  Q9UPN6  Q9UPN6  Q9UPN6  Q9UPN6  Q9UPN6  Q9UPN6  Q9UPN6  Q9UPN6  Q9UPN6  Q9UPN6  Q9UPN6  Q9UPN6  Q9UPN6  Q9UPN6  Q9UPN6  Q9UPN6  Q9UPN6  Q9UPN6  Q9UPN6  Q9UPN6  Q9UPN6  Q9UPN6  Q9UPN6  Q9UPN6  Q9UPN6  Q9UPN6  Q9UPN6  Q9UPN6  Q9UPN6  Q9UPP1  Q9UPP1  Q9UPP1  Q9UPP1  Q9UPP1  Q9UPP1  Q9UPP1  Q9UPP1  Q9UPP1  Q9UPP1  Q9UPP1  Q9UPP1  Q9UPP1  Q9UPP1  Q9UPP1  Q9UPP1  Q9UPP1  Q9UPP1  Q9UPP1  Q9UPP1  Q9UPP1  Q9UPP1  Q9UPP1  Q9UPP1  Q9UPP1  Q9UPP1  Q9UPP1  Q9UPP1  Q9UPP1  Q9UPP1  Q9UPP1  Q9UPT6  Q9UPT6  Q9UPT6  Q9UPT6  Q9UPT6  Q9UPT6  Q9UPT6  Q9UPT6  Q9UPT6  Q9UPT6  Q9UPT6  Q9UPT6  Q9UPT6  Q9UPT6  Q9UPT6  Q9UPT6  Q9UPT6  Q9UPT6  Q9UPT6  Q9UPT6  Q9UPT6  Q9UPT6  Q9UPT6  Q9UPT6  Q9UPT6  Q9UPT6  Q9UPT6  Q9UPT6  Q9UPT6  Q9UPT6  Q9UPT6  Q9UPV0  Q9UPV0  Q9UPV0  Q9UPV0  Q9UPV0  Q9UPV0  Q9UPV0  Q9UPV0  Q9UPV0  Q9UPV0  Q9UPV0  Q9UPV0  Q9UPV0  Q9UPV0  Q9UPV0  Q9UPV0  Q9UPV0  Q9UPV0  Q9UPV0  Q9UPV0  Q9UPV0  Q9UPV0  Q9UPV0  Q9UPV0  Q9UPV0  Q9UPV0  Q9UPV0  Q9UPV0  Q9UPV0  Q9UPV0  Q9UPV0  Q9UPY6  Q9UPY6  Q9UPY6  Q9UPY6  Q9UPY6  Q9UPY6  Q9UPY6  Q9UPY6  Q9UPY6  Q9UPY6  Q9UPY6  Q9UPY6  Q9UPY6  Q9UPY6  Q9UPY6  Q9UPY6  Q9UPY6  Q9UPY6  Q9UPY6  Q9UPY6  Q9UPY6  Q9UPY6  Q9UPY6  Q9UPY6  Q9UPY6  Q9UPY6  Q9UPY6  Q9UPY6  Q9UPY6  Q9UPY6  Q9UPY6  Q9UPY6  Q9UPY6  Q9UPZ9  Q9UPZ9  Q9UPZ9  Q9UPZ9  Q9UPZ9  Q9UPZ9  Q9UPZ9  Q9UPZ9  Q9UPZ9  Q9UPZ9  Q9UPZ9  Q9UPZ9  Q9UPZ9  Q9UPZ9  Q9UPZ9  Q9UPZ9  Q9UPZ9  Q9UPZ9  Q9UPZ9  Q9UPZ9  Q9UPZ9  Q9UPZ9  Q9UPZ9  Q9UPZ9  Q9UPZ9  Q9UPZ9  Q9UPZ9  Q9UPZ9  Q9UPZ9  Q9UPZ9  Q9UPZ9  Q9UPZ9  Q9UPZ9  Q9UQ35  Q9UQ35  Q9UQ35  Q9UQ35  Q9UQ35  Q9UQ35  Q9UQ35  Q9UQ35  Q9UQ35  Q9UQ35  Q9UQ35  Q9UQ35  Q9UQ35  Q9UQ35  Q9UQ35  Q9UQ35  Q9UQ35  Q9UQ35  Q9UQ35  Q9UQ35  Q9UQ35  Q9UQ35  Q9UQ35  Q9UQ35  Q9UQ35  Q9UQ35  Q9UQ35  Q9UQ35  Q9UQ80  Q9UQ80  Q9UQ80  Q9UQ80  Q9UQ80  Q9UQ80  Q9UQ80  Q9UQ80  Q9UQ80  Q9UQ80  Q9UQ80  Q9UQ80  Q9UQ80  Q9UQ80  Q9UQ80  Q9UQ80  Q9UQ80  Q9UQ80  Q9UQ80  Q9UQ80  Q9UQ80  Q9UQ80  Q9UQ80  Q9UQ84  Q9UQ84  Q9UQ84  Q9UQ84  Q9UQ84  Q9UQ84  Q9UQ84  Q9UQ84  Q9UQ84  Q9UQ84  Q9UQ84  Q9UQ84  Q9UQ84  Q9UQ84  Q9UQ84  Q9UQ84  Q9UQ84  Q9UQ84  Q9UQ84  Q9UQ84  Q9UQ84  Q9UQ84  Q9UQ84  Q9UQ84  Q9UQ84  Q9UQ84  Q9UQ84  Q9UQ84  Q9UQ84  Q9UQ84  Q9UQ84  Q9UQC2  Q9UQC2  Q9UQC2  Q9UQC2  Q9UQC2  Q9UQC2  Q9UQC2  Q9UQC2  Q9UQC2  Q9UQC2  Q9UQC2  Q9UQC2  Q9UQC2  Q9UQC2  Q9UQC2  Q9UQC2  Q9UQC2  Q9UQC2  Q9UQC2  Q9UQC2  Q9UQC2  Q9UQC2  Q9UQC2  Q9UQC2  Q9UQC2  Q9UQC2  Q9UQC2  Q9UQC2  Q9UQC2  Q9UQC2  Q9UQC2  Q9UQE7  Q9UQE7  Q9UQE7  Q9UQE7  Q9UQE7  Q9UQE7  Q9UQE7  Q9UQE7  Q9UQE7  Q9UQE7  Q9UQE7  Q9UQE7  Q9UQE7  Q9UQE7  Q9UQE7  Q9UQE7  Q9UQE7  Q9UQE7  Q9UQE7  Q9UQE7  Q9UQE7  Q9UQE7  Q9UQE7  Q9UQE7  Q9UQE7  Q9UQE7  Q9UQE7  Q9UQE7  Q9UQE7  Q9UQE7  Q9UQE7  Q9UQE7  Q9UQE7  Q9UQF2  Q9UQF2  Q9UQF2  Q9UQF2  Q9UQF2  Q9UQF2  Q9UQF2  Q9UQF2  Q9UQF2  Q9UQF2  Q9UQF2  Q9UQF2  Q9UQF2  Q9UQF2  Q9UQF2  Q9UQF2  Q9UQF2  Q9UQF2  Q9UQF2  Q9UQF2  Q9UQF2  Q9UQF2  Q9UQF2  Q9UQF2  Q9UQF2  Q9UQF2  Q9UQF2  Q9UQF2  Q9UQF2  Q9UQF2  Q9UQF2  Q9UQL6  Q9UQL6  Q9UQL6  Q9UQL6  Q9UQL6  Q9UQL6  Q9UQL6  Q9UQL6  Q9UQL6  Q9UQL6  Q9UQL6  Q9UQL6  Q9UQL6  Q9UQL6  Q9UQL6  Q9UQL6  Q9UQL6  Q9UQL6  Q9UQL6  Q9UQL6  Q9UQL6  Q9UQL6  Q9UQL6  Q9UQL6  Q9UQL6  Q9UQL6  Q9UQL6  Q9UQL6  Q9UQL6  Q9UQL6  Q9Y230  Q9Y230  Q9Y230  Q9Y230  Q9Y230  Q9Y230  Q9Y230  Q9Y230  Q9Y230  Q9Y230  Q9Y230  Q9Y230  Q9Y230  Q9Y230  Q9Y230  Q9Y230  Q9Y230  Q9Y230  Q9Y230  Q9Y230  Q9Y230  Q9Y230  Q9Y230  Q9Y230  Q9Y230  Q9Y230  Q9Y230  Q9Y230  Q9Y232  Q9Y232  Q9Y232  Q9Y232  Q9Y232  Q9Y232  Q9Y232  Q9Y232  Q9Y232  Q9Y232  Q9Y232  Q9Y232  Q9Y232  Q9Y232  Q9Y232  Q9Y232  Q9Y232  Q9Y232  Q9Y232  Q9Y232  Q9Y232  Q9Y232  Q9Y232  Q9Y232  Q9Y232  Q9Y232  Q9Y232  Q9Y232  Q9Y232  Q9Y232  Q9Y232  Q9Y232  Q9Y232  Q9Y233  Q9Y233  Q9Y233  Q9Y233  Q9Y233  Q9Y233  Q9Y233  Q9Y233  Q9Y233  Q9Y233  Q9Y233  Q9Y233  Q9Y233  Q9Y233  Q9Y233  Q9Y233  Q9Y233  Q9Y233  Q9Y233  Q9Y233  Q9Y233  Q9Y233  Q9Y233  Q9Y233  Q9Y233  Q9Y233  Q9Y233  Q9Y233  Q9Y233  Q9Y233  Q9Y233  Q9Y233  Q9Y233  Q9Y237  Q9Y237  Q9Y237  Q9Y237  Q9Y237  Q9Y237  Q9Y237  Q9Y241  Q9Y241  Q9Y241  Q9Y241  Q9Y241  Q9Y241  Q9Y259  Q9Y259  Q9Y259  Q9Y259  Q9Y259  Q9Y259  Q9Y259  Q9Y259  Q9Y259  Q9Y259  Q9Y259  Q9Y259  Q9Y259  Q9Y259  Q9Y259  Q9Y259  Q9Y259  Q9Y259  Q9Y259  Q9Y259  Q9Y259  Q9Y259  Q9Y259  Q9Y259  Q9Y259  Q9Y259  Q9Y259  Q9Y259  Q9Y262  Q9Y262  Q9Y262  Q9Y262  Q9Y262  Q9Y262  Q9Y262  Q9Y262  Q9Y262  Q9Y262  Q9Y262  Q9Y262  Q9Y262  Q9Y262  Q9Y262  Q9Y262  Q9Y262  Q9Y262  Q9Y262  Q9Y262  Q9Y262  Q9Y262  Q9Y262  Q9Y262  Q9Y262  Q9Y262  Q9Y262  Q9Y262  Q9Y262  Q9Y262  Q9Y262  Q9Y262  Q9Y262  Q9Y266  Q9Y266  Q9Y266  Q9Y266  Q9Y266  Q9Y266  Q9Y266  Q9Y266  Q9Y266  Q9Y266  Q9Y266  Q9Y266  Q9Y266  Q9Y266  Q9Y266  Q9Y266  Q9Y272  Q9Y272  Q9Y272  Q9Y272  Q9Y272  Q9Y272  Q9Y272  Q9Y272  Q9Y272  Q9Y272  Q9Y272  Q9Y272  Q9Y272  Q9Y272  Q9Y272  Q9Y272  Q9Y272  Q9Y272  Q9Y272  Q9Y272  Q9Y272  Q9Y272  Q9Y272  Q9Y283  Q9Y283  Q9Y283  Q9Y283  Q9Y283  Q9Y283  Q9Y283  Q9Y283  Q9Y283  Q9Y283  Q9Y283  Q9Y283  Q9Y283  Q9Y283  Q9Y283  Q9Y283  Q9Y283  Q9Y283  Q9Y283  Q9Y283  Q9Y283  Q9Y283  Q9Y283  Q9Y283  Q9Y283  Q9Y283  Q9Y283  Q9Y283  Q9Y283  Q9Y283  Q9Y283  Q9Y283  Q9Y283  Q9Y285  Q9Y285  Q9Y285  Q9Y285  Q9Y285  Q9Y285  Q9Y285  Q9Y285  Q9Y285  Q9Y285  Q9Y285  Q9Y285  Q9Y285  Q9Y285  Q9Y285  Q9Y285  Q9Y285  Q9Y285  Q9Y285  Q9Y285  Q9Y285  Q9Y285  Q9Y285  Q9Y285  Q9Y285  Q9Y285  Q9Y285  Q9Y285  Q9Y285  Q9Y285  Q9Y285  Q9Y285  Q9Y285  Q9Y294  Q9Y294  Q9Y294  Q9Y294  Q9Y294  Q9Y294  Q9Y294  Q9Y294  Q9Y294  Q9Y294  Q9Y294  Q9Y294  Q9Y294  Q9Y294  Q9Y294  Q9Y295  Q9Y295  Q9Y295  Q9Y295  Q9Y295  Q9Y295  Q9Y295  Q9Y295  Q9Y295  Q9Y295  Q9Y295  Q9Y295  Q9Y295  Q9Y2I7  Q9Y2I7  Q9Y2I7  Q9Y2I7  Q9Y2I7  Q9Y2I7  Q9Y2I7  Q9Y2I7  Q9Y2I7  Q9Y2I7  Q9Y2I7  Q9Y2I7  Q9Y2I7  Q9Y2I7  Q9Y2I7  Q9Y2I7  Q9Y2I7  Q9Y2I7  Q9Y2I7  Q9Y2I7  Q9Y2I7  Q9Y2I7  Q9Y2I7  Q9Y2I7  Q9Y2I7  Q9Y2I7  Q9Y2I7  Q9Y2I7  Q9Y2I7  Q9Y2I7  Q9Y2I7  Q9Y2K2  Q9Y2K2  Q9Y2K2  Q9Y2K2  Q9Y2K2  Q9Y2K2  Q9Y2K2  Q9Y2K2  Q9Y2K2  Q9Y2K2  Q9Y2K2  Q9Y2K2  Q9Y2K2  Q9Y2K2  Q9Y2K2  Q9Y2K2  Q9Y2K2  Q9Y2K2  Q9Y2K2  Q9Y2K2  Q9Y2K2  Q9Y2K2  Q9Y2K2  Q9Y2K2  Q9Y2K2  Q9Y2K2  Q9Y2K2  Q9Y2K2  Q9Y2K2  Q9Y2K2  Q9Y2K2  Q9Y2K2  Q9Y2K2  Q9Y2N7  Q9Y2N7  Q9Y2N7  Q9Y2N7  Q9Y2N7  Q9Y2N7  Q9Y2N7  Q9Y2N7  Q9Y2N7  Q9Y2N7  Q9Y2N7  Q9Y2N7  Q9Y2N7  Q9Y2N7  Q9Y2N7  Q9Y2N7  Q9Y2N7  Q9Y2N7  Q9Y2N7  Q9Y2N7  Q9Y2N7  Q9Y2N7  Q9Y2N7  Q9Y2N7  Q9Y2N7  Q9Y2N7  Q9Y2N7  Q9Y2N7  Q9Y2N7  Q9Y2N7  Q9Y2N7  Q9Y2N7  Q9Y2N7  Q9Y2R2  Q9Y2R2  Q9Y2R2  Q9Y2R2  Q9Y2R2  Q9Y2R2  Q9Y2R2  Q9Y2R2  Q9Y2R2  Q9Y2R2  Q9Y2R2  Q9Y2R2  Q9Y2R2  Q9Y2R2  Q9Y2R2  Q9Y2R2  Q9Y2R2  Q9Y2R2  Q9Y2R2  Q9Y2R2  Q9Y2R2  Q9Y2R2  Q9Y2R2  Q9Y2R2  Q9Y2R2  Q9Y2R2  Q9Y2R2  Q9Y2R2  Q9Y2R2  Q9Y2R2  Q9Y2R2  Q9Y2S0  Q9Y2S0  Q9Y2S0  Q9Y2S0  Q9Y2S0  Q9Y2S0  Q9Y2V2  Q9Y2V2  Q9Y2V2  Q9Y2V2  Q9Y2V2  Q9Y2V2  Q9Y2W1  Q9Y2W1  Q9Y2W1  Q9Y2W1  Q9Y2W1  Q9Y2W1  Q9Y2W1  Q9Y2W1  Q9Y2W1  Q9Y2W1  Q9Y2W1  Q9Y2W1  Q9Y2W1  Q9Y2W1  Q9Y2W1  Q9Y2W1  Q9Y2W1  Q9Y2W1  Q9Y2W1  Q9Y2W1  Q9Y2W1  Q9Y2W1  Q9Y2W1  Q9Y2W1  Q9Y2W1  Q9Y2W1  Q9Y2W1  Q9Y2W1  Q9Y2W1  Q9Y2W1  Q9Y2W7  Q9Y2W7  Q9Y2W7  Q9Y2W7  Q9Y2W7  Q9Y2W7  Q9Y2W7  Q9Y2W7  Q9Y2W7  Q9Y2W7  Q9Y2W7  Q9Y2W7  Q9Y2W7  Q9Y2W7  Q9Y2W7  Q9Y2Z0  Q9Y2Z0  Q9Y2Z0  Q9Y2Z0  Q9Y2Z0  Q9Y2Z0  Q9Y2Z0  Q9Y2Z0  Q9Y2Z0  Q9Y2Z0  Q9Y2Z0  Q9Y2Z0  Q9Y2Z0  Q9Y2Z0  Q9Y2Z0  Q9Y2Z0  Q9Y2Z0  Q9Y2Z0  Q9Y2Z0  Q9Y2Z0  Q9Y2Z0  Q9Y2Z0  Q9Y2Z0  Q9Y2Z0  Q9Y2Z0  Q9Y2Z0  Q9Y365  Q9Y365  Q9Y365  Q9Y365  Q9Y365  Q9Y365  Q9Y365  Q9Y365  Q9Y365  Q9Y365  Q9Y365  Q9Y365  Q9Y365  Q9Y365  Q9Y365  Q9Y365  Q9Y365  Q9Y365  Q9Y365  Q9Y365  Q9Y371  Q9Y371  Q9Y371  Q9Y371  Q9Y371  Q9Y371  Q9Y371  Q9Y371  Q9Y371  Q9Y371  Q9Y371  Q9Y371  Q9Y371  Q9Y371  Q9Y371  Q9Y371  Q9Y371  Q9Y371  Q9Y371  Q9Y371  Q9Y371  Q9Y371  Q9Y371  Q9Y371  Q9Y371  Q9Y371  Q9Y371  Q9Y383  Q9Y383  Q9Y383  Q9Y383  Q9Y383  Q9Y383  Q9Y383  Q9Y383  Q9Y383  Q9Y383  Q9Y383  Q9Y383  Q9Y383  Q9Y383  Q9Y383  Q9Y383  Q9Y383  Q9Y383  Q9Y383  Q9Y383  Q9Y383  Q9Y383  Q9Y383  Q9Y383  Q9Y383  Q9Y383  Q9Y383  Q9Y383  Q9Y383  Q9Y383  Q9Y383  Q9Y383  Q9Y383  Q9Y3A5  Q9Y3A5  Q9Y3A5  Q9Y3A5  Q9Y3A5  Q9Y3A5  Q9Y3A5  Q9Y3A5  Q9Y3A5  Q9Y3C5  Q9Y3C5  Q9Y3C5  Q9Y3C5  Q9Y3C5  Q9Y3C5  Q9Y3C5  Q9Y3C5  Q9Y3C5  Q9Y3D6  Q9Y3D6  Q9Y3D6  Q9Y3D6  Q9Y3D6  Q9Y3D6  Q9Y3D6  Q9Y3P8  Q9Y3P8  Q9Y3P8  Q9Y3P8  Q9Y3P8  Q9Y3P8  Q9Y3P8  Q9Y3P8  Q9Y3P8  Q9Y3P8  Q9Y3P8  Q9Y3P8  Q9Y3P8  Q9Y3P8  Q9Y3P8  Q9Y3P8  Q9Y3P8  Q9Y3S1  Q9Y3S1  Q9Y3S1  Q9Y3S1  Q9Y3S1  Q9Y3S1  Q9Y3S1  Q9Y3S1  Q9Y3S1  Q9Y3S1  Q9Y3S1  Q9Y3S1  Q9Y3S1  Q9Y3S1  Q9Y3S1  Q9Y3S1  Q9Y3S1  Q9Y3S1  Q9Y3S1  Q9Y3S1  Q9Y3S1  Q9Y3S1  Q9Y3S1  Q9Y3S1  Q9Y3S1  Q9Y3S1  Q9Y3S1  Q9Y3S1  Q9Y3S1  Q9Y3S1  Q9Y3S1  Q9Y3Z3  Q9Y3Z3  Q9Y3Z3  Q9Y3Z3  Q9Y3Z3  Q9Y3Z3  Q9Y3Z3  Q9Y3Z3  Q9Y3Z3  Q9Y3Z3  Q9Y3Z3  Q9Y3Z3  Q9Y3Z3  Q9Y3Z3  Q9Y3Z3  Q9Y3Z3  Q9Y3Z3  Q9Y3Z3  Q9Y3Z3  Q9Y3Z3  Q9Y3Z3  Q9Y3Z3  Q9Y3Z3  Q9Y3Z3  Q9Y3Z3  Q9Y3Z3  Q9Y3Z3  Q9Y3Z3  Q9Y3Z3  Q9Y3Z3  Q9Y3Z3  Q9Y3Z3  Q9Y4E8  Q9Y4E8  Q9Y4E8  Q9Y4E8  Q9Y4E8  Q9Y4E8  Q9Y4E8  Q9Y4E8  Q9Y4E8  Q9Y4E8  Q9Y4E8  Q9Y4E8  Q9Y4E8  Q9Y4E8  Q9Y4E8  Q9Y4E8  Q9Y4E8  Q9Y4E8  Q9Y4E8  Q9Y4E8  Q9Y4E8  Q9Y4E8  Q9Y4E8  Q9Y4E8  Q9Y4E8  Q9Y4E8  Q9Y4E8  Q9Y4E8  Q9Y4E8  Q9Y4E8  Q9Y4E8  Q9Y4E8  Q9Y4E8  Q9Y4P1  Q9Y4P1  Q9Y4P1  Q9Y4P1  Q9Y4P1  Q9Y4P1  Q9Y4P1  Q9Y4P1  Q9Y4P1  Q9Y4P1  Q9Y4P1  Q9Y4P1  Q9Y4P1  Q9Y4P1  Q9Y4P1  Q9Y4P1  Q9Y4P1  Q9Y4P1  Q9Y4P1  Q9Y4P1  Q9Y4P1  Q9Y4P1  Q9Y4P1  Q9Y4P1  Q9Y4P1  Q9Y4P1  Q9Y4R8  Q9Y4R8  Q9Y4R8  Q9Y4R8  Q9Y4R8  Q9Y4R8  Q9Y4R8  Q9Y4R8  Q9Y4R8  Q9Y4R8  Q9Y4R8  Q9Y4R8  Q9Y4R8  Q9Y4R8  Q9Y4R8  Q9Y4R8  Q9Y4R8  Q9Y4R8  Q9Y4R8  Q9Y4R8  Q9Y4R8  Q9Y4R8  Q9Y4R8  Q9Y4R8  Q9Y4R8  Q9Y4R8  Q9Y4R8  Q9Y4R8  Q9Y4R8  Q9Y4R8  Q9Y4R8  Q9Y4X4  Q9Y4X4  Q9Y4X4  Q9Y4X4  Q9Y4X4  Q9Y4X4  Q9Y4X4  Q9Y4X4  Q9Y4X4  Q9Y4X4  Q9Y4X4  Q9Y4X4  Q9Y4X4  Q9Y4X4  Q9Y4X4  Q9Y4X4  Q9Y4X4  Q9Y4X4  Q9Y4X4  Q9Y4X4  Q9Y4X4  Q9Y4X4  Q9Y4X4  Q9Y4X4  Q9Y4X4  Q9Y4X4  Q9Y4X4  Q9Y4X4  Q9Y4X4  Q9Y4X4  Q9Y4X4  Q9Y4X4  Q9Y4X4  Q9Y4Z0  Q9Y4Z0  Q9Y4Z0  Q9Y572  Q9Y572  Q9Y572  Q9Y572  Q9Y572  Q9Y572  Q9Y572  Q9Y572  Q9Y572  Q9Y572  Q9Y572  Q9Y572  Q9Y572  Q9Y572  Q9Y572  Q9Y572  Q9Y572  Q9Y572  Q9Y572  Q9Y572  Q9Y572  Q9Y572  Q9Y572  Q9Y572  Q9Y572  Q9Y572  Q9Y572  Q9Y572  Q9Y572  Q9Y572  Q9Y572  Q9Y5A9  Q9Y5A9  Q9Y5A9  Q9Y5A9  Q9Y5A9  Q9Y5A9  Q9Y5A9  Q9Y5A9  Q9Y5A9  Q9Y5A9  Q9Y5A9  Q9Y5A9  Q9Y5A9  Q9Y5A9  Q9Y5A9  Q9Y5A9  Q9Y5A9  Q9Y5A9  Q9Y5A9  Q9Y5A9  Q9Y5A9  Q9Y5A9  Q9Y5A9  Q9Y5A9  Q9Y5A9  Q9Y5A9  Q9Y5A9  Q9Y5A9  Q9Y5A9  Q9Y5A9  Q9Y5A9  Q9Y5A9  Q9Y5A9  Q9Y5B9  Q9Y5B9  Q9Y5B9  Q9Y5B9  Q9Y5B9  Q9Y5B9  Q9Y5B9  Q9Y5B9  Q9Y5B9  Q9Y5B9  Q9Y5B9  Q9Y5B9  Q9Y5B9  Q9Y5B9  Q9Y5B9  Q9Y5B9  Q9Y5B9  Q9Y5B9  Q9Y5B9  Q9Y5B9  Q9Y5B9  Q9Y5B9  Q9Y5B9  Q9Y5B9  Q9Y5B9  Q9Y5B9  Q9Y5B9  Q9Y5B9  Q9Y5B9  Q9Y5B9  Q9Y5B9  Q9Y5B9  Q9Y5B9  Q9Y5P4  Q9Y5P4  Q9Y5P4  Q9Y5P4  Q9Y5P4  Q9Y5P4  Q9Y5P4  Q9Y5P4  Q9Y5P4  Q9Y5P4  Q9Y5P4  Q9Y5P4  Q9Y5P4  Q9Y5P4  Q9Y5P4  Q9Y5P4  Q9Y5P4  Q9Y5P4  Q9Y5P4  Q9Y5P4  Q9Y5P4  Q9Y5P4  Q9Y5P4  Q9Y5P4  Q9Y5P4  Q9Y5P4  Q9Y5P4  Q9Y5P4  Q9Y5P4  Q9Y5P4  Q9Y5P4  Q9Y5Q6  Q9Y5Q6  Q9Y5Q6  Q9Y5Q6  Q9Y5Q6  Q9Y5Q6  Q9Y5Q6  Q9Y5Q6  Q9Y5Q6  Q9Y5Q6  Q9Y5Q6  Q9Y5Q6  Q9Y5Q6  Q9Y5Y2  Q9Y5Y2  Q9Y5Y2  Q9Y5Y2  Q9Y5Y2  Q9Y5Y2  Q9Y5Y2  Q9Y5Y2  Q9Y5Y2  Q9Y5Y2  Q9Y5Y2  Q9Y5Y2  Q9Y5Y2  Q9Y5Y2  Q9Y5Y2  Q9Y5Y2  Q9Y608  Q9Y608  Q9Y608  Q9Y608  Q9Y608  Q9Y608  Q9Y608  Q9Y608  Q9Y608  Q9Y608  Q9Y608  Q9Y608  Q9Y608  Q9Y608  Q9Y608  Q9Y608  Q9Y608  Q9Y608  Q9Y608  Q9Y608  Q9Y608  Q9Y608  Q9Y608  Q9Y608  Q9Y608  Q9Y608  Q9Y608  Q9Y608  Q9Y608  Q9Y608  Q9Y608  Q9Y657  Q9Y657  Q9Y657  Q9Y657  Q9Y657  Q9Y657  Q9Y657  Q9Y657  Q9Y657  Q9Y657  Q9Y657  Q9Y657  Q9Y657  Q9Y657  Q9Y696  Q9Y696  Q9Y696  Q9Y696  Q9Y696  Q9Y696  Q9Y696  Q9Y696  Q9Y696  Q9Y696  Q9Y696  Q9Y696  Q9Y696  Q9Y696  Q9Y696  Q9Y6E0  Q9Y6E0  Q9Y6E0  Q9Y6E0  Q9Y6E0  Q9Y6E0  Q9Y6E0  Q9Y6E0  Q9Y6E0  Q9Y6E0  Q9Y6E0  Q9Y6E0  Q9Y6E0  Q9Y6E0  Q9Y6E0  Q9Y6E0  Q9Y6E0  Q9Y6E0  Q9Y6E0  Q9Y6E0  Q9Y6E0  Q9Y6E0  Q9Y6E0  Q9Y6E0  Q9Y6E0  Q9Y6E0  Q9Y6E0  Q9Y6E0  Q9Y6E0  Q9Y6E0  Q9Y6E0  Q9Y6F6  Q9Y6F6  Q9Y6F6  Q9Y6F6  Q9Y6F6  Q9Y6F6  Q9Y6F6  Q9Y6F6  Q9Y6F6  Q9Y6F6  Q9Y6F6  Q9Y6F6  Q9Y6F6  Q9Y6F6  Q9Y6F6  Q9Y6F6  Q9Y6F6  Q9Y6F6  Q9Y6F6  Q9Y6F6  Q9Y6F6  Q9Y6F6  Q9Y6F6  Q9Y6F6  Q9Y6F6  Q9Y6F6  Q9Y6F6  Q9Y6F6  Q9Y6F6  Q9Y6F6  Q9Y6F6  Q9Y6G9  Q9Y6G9  Q9Y6G9  Q9Y6G9  Q9Y6G9  Q9Y6G9  Q9Y6G9  Q9Y6G9  Q9Y6G9  Q9Y6G9  Q9Y6G9  Q9Y6G9  Q9Y6G9  Q9Y6G9  Q9Y6G9  Q9Y6G9  Q9Y6G9  Q9Y6G9  Q9Y6G9  Q9Y6G9  Q9Y6G9  Q9Y6G9  Q9Y6G9  Q9Y6G9  Q9Y6G9  Q9Y6G9  Q9Y6G9  Q9Y6G9  Q9Y6G9  Q9Y6G9  Q9Y6G9  Q9Y6I3  Q9Y6I3  Q9Y6I3  Q9Y6I3  Q9Y6I3  Q9Y6I3  Q9Y6I3  Q9Y6I3  Q9Y6I3  Q9Y6I3  Q9Y6I3  Q9Y6I3  Q9Y6I3  Q9Y6I3  Q9Y6I3  Q9Y6I3  Q9Y6I3  Q9Y6I3  Q9Y6I3  Q9Y6I3  Q9Y6I3  Q9Y6I3  Q9Y6I3  Q9Y6I3  Q9Y6I3  Q9Y6I3  Q9Y6I3  Q9Y6I3  Q9Y6I3  Q9Y6I3  Q9Y6I3  Q9Y6K5  Q9Y6K5  Q9Y6K5  Q9Y6K5  Q9Y6K5  Q9Y6K5  Q9Y6K5  Q9Y6K5  Q9Y6K5  Q9Y6K5  Q9Y6K5  Q9Y6K5  Q9Y6K5  Q9Y6K5  Q9Y6K5  Q9Y6K5  Q9Y6K5  Q9Y6K5  Q9Y6K5  Q9Y6K5  Q9Y6K5  Q9Y6K5  Q9Y6K5  Q9Y6K5  Q9Y6K5  Q9Y6K5  Q9Y6K5  Q9Y6K5  Q9Y6K5  Q9Y6K5  Q9Y6K5  Q9Y6K5  Q9Y6K5  Q9Y6K9  Q9Y6K9  Q9Y6K9  Q9Y6K9  Q9Y6K9  Q9Y6K9  Q9Y6K9  Q9Y6K9  Q9Y6K9  Q9Y6K9  Q9Y6K9  Q9Y6K9  Q9Y6K9  Q9Y6K9  Q9Y6K9  Q9Y6K9  Q9Y6K9  Q9Y6K9  Q9Y6K9  Q9Y6K9  Q9Y6M9  Q9Y6M9  Q9Y6M9  Q9Y6M9  Q9Y6M9  Q9Y6N7  Q9Y6N7  Q9Y6N7  Q9Y6N7  Q9Y6N7  Q9Y6N7  Q9Y6N7  Q9Y6N7  Q9Y6N7  Q9Y6N7  Q9Y6N7  Q9Y6N7  Q9Y6N7  Q9Y6N7  Q9Y6N7  Q9Y6N7  Q9Y6N7  Q9Y6N7  Q9Y6N7  Q9Y6N7  Q9Y6N7  Q9Y6N7  Q9Y6N7  Q9Y6N7  Q9Y6N7  Q9Y6N7  Q9Y6N7  Q9Y6N7  Q9Y6N7  Q9Y6N7  Q9Y6N7  Q9Y6N7  Q9Y6N7  Q9Y6Q9  Q9Y6Q9  Q9Y6Q9  Q9Y6Q9  Q9Y6Q9  Q9Y6Q9  Q9Y6Q9  Q9Y6Q9  Q9Y6Q9  Q9Y6Q9  Q9Y6Q9  Q9Y6Q9  Q9Y6Q9  Q9Y6Q9  Q9Y6Q9  Q9Y6Q9  Q9Y6Q9  Q9Y6Q9  Q9Y6Q9  Q9Y6Q9  Q9Y6Q9  Q9Y6Q9  Q9Y6Q9  Q9Y6Q9  Q9Y6Q9  Q9Y6Q9  Q9Y6Q9  Q9Y6Q9  Q9Y6Q9  Q9Y6Q9  Q9Y6Q9  Q9Y6R1  Q9Y6R1  Q9Y6R1  Q9Y6R1  Q9Y6R1  Q9Y6R1  Q9Y6R1  Q9Y6R1  Q9Y6R1  Q9Y6R1  Q9Y6R1  Q9Y6R1  Q9Y6R1  Q9Y6R1  Q9Y6R1  Q9Y6R1  Q9Y6R1  Q9Y6R1  Q9Y6R1  Q9Y6R1  Q9Y6R1  Q9Y6R1  Q9Y6R1  Q9Y6R1  Q9Y6R1  Q9Y6R1  Q9Y6R1  Q9Y6R1  Q9Y6R1  Q9Y6R1  Q9Y6R1 | 18  60  78  87  102  116  148  170  210  236  276  286  289  307  309  330  423  453  521  559  595  599  600  613  626  671  729  760  788  803  807  38  51  67  78  102  127  152  216  230  259  284  318  754  799  871  936  991  996  1187  1192  1218  1242  1268  1380  1411  1503  1586  1601  1709  1790  1921  1970  1974  11  13  17  23  31  67  73  77  128  132  133  137  145  175  192  31  37  59  84  90  108  118  123  130  133  134  152  153  157  165  170  176  186  187  194  213  215  225  235  239  250  38  86  142  177  195  219  225  263  276  277  325  367  387  403  408  432  492  554  583  589  592  677  736  766  844  878  898  907  916  937  975  1009  1022  20  23  34  95  110  116  136  161  18  43  53  66  68  90  93  102  112  128  135  147  151  153  162  189  192  206  210  212  232  247  248  255  266  22  35  57  82  18  61  62  83  103  106  115  163  203  21  24  45  48  50  71  90  94  112  118  123  124  137  155  160  188  220  239  244  247  255  263  290  318  324  332  338  347  350  352  357  8  10  16  23  52  118  123  175  185  203  228  236  242  266  322  347  374  390  391  402  413  438  449  459  494  573  584  610  631  645  647  669  695  14  17  23  29  49  55  79  81  91  119  135  151  174  181  192  214  230  241  244  250  272  294  298  320  343  351  352  357  366  408  413  21  49  58  62  64  91  95  164  176  206  239  242  260  269  284  291  318  332  335  343  385  389  394  429  434  437  15  36  90  99  100  124  134  147  156  182  192  225  267  281  291  293  333  336  345  349  381  407  415  432  434  442  467  482  489  490  500  529  531  14  18  28  122  143  154  158  15  36  120  162  195  214  347  367  379  442  522  526  691  738  773  782  804  812  818  847  891  901  907  917  929  935  942  946  959  970  976  1013  1057  12  28  37  44  55  81  90  99  107  110  118  136  137  142  155  164  188  204  207  212  228  232  233  239  254  256  257  302  305  308  310  312  315  9  25  45  47  65  101  105  128  136  142  164  183  191  205  212  218  266  267  276  284  295  299  310  329  346  25  36  38  48  63  65  85  105  119  126  154  157  162  168  185  196  200  245  254  264  281  294  296  327  364  392  397  414  416  418  473  475  489  8  13  33  37  39  57  59  68  107  156  197  245  247  255  257  306  353  396  406  463  480  493  538  549  573  588  604  643  674  706  720  8  21  96  109  121  19  29  42  43  58  62  75  102  145  183  207  252  283  297  315  328  331  352  353  356  364  393  426  430  432  449  456  465  479  20  40  71  83  139  141  142  188  235  239  262  270  334  476  491  562  606  726  763  767  772  843  848  851  852  899  918  957  960  998  1022  1040  1083  31  54  70  74  135  165  241  243  266  311  320  329  345  368  377  380  386  392  396  416  438  441  462  492  521  550  575  586  612  622  689  35  39  40  44  119  126  175  179  248  257  275  330  380  412  428  450  529  533  534  538  544  552  563  572  596  653  665  693  694  703  724  16  124  181  188  211  220  234  251  336  363  368  444  469  629  702  819  884  948  1036  1084  1176  1235  1281  1335  1403  1410  1428  1518  1566  1615  1630  34  119  142  144  158  160  176  191  234  262  263  273  313  321  336  358  359  390  397  426  438  502  525  530  538  551  562  637  649  650  658  688  700  23  33  36  54  60  74  87  88  131  145  148  170  171  205  220  225  226  252  259  261  285  289  29  108  173  275  300  326  329  345  373  435  458  497  505  578  585  644  673  682  698  780  793  797  820  923  964  1026  1081  1110  1138  1160  1205  23  28  57  76  92  109  114  125  137  152  228  290  425  429  442  456  470  476  492  520  588  594  605  606  609  611  614  619  621  623  654  24  65  83  84  95  104  112  139  167  205  206  235  237  240  248  278  279  287  292  305  316  344  352  44  106  135  174  403  405  522  562  677  698  745  860  921  1048  1120  1165  1199  1215  1231  1235  1329  1448  1655  1672  1801  1831  2117  2224  2247  2347  2357  62  115  117  118  122  141  157  212  245  291  296  310  330  332  360  388  390  447  486  503  506  513  534  539  541  559  567  569  570  573  574  591  598  38  43  58  69  87  98  112  137  154  163  171  194  198  21  24  26  28  32  33  40  56  61  84  86  37  283  292  293  307  311  361  382  417  481  501  588  601  611  622  636  646  655  680  687  697  710  764  787  796  811  840  932  988  1018  1138  1139  1210  49  54  79  17  29  40  46  55  56  106  114  123  128  129  175  48  57  251  281  403  422  427  431  439  483  507  514  570  649  728  733  751  775  777  810  856  874  893  917  931  957  1007  1052  1061  1066  1107  1113  1122  36  41  91  100  113  153  247  248  263  269  337  339  340  347  377  423  433  441  455  504  528  546  584  585  605  609  612  616  630  663  700  706  740  10  11  12  13  14  16  17  20  23  25  28  29  34  36  37  41  46  49  50  53  57  77  115  122  124  153  163  168  176  188  9  10  12  18  21  39  42  75  76  82  85  90  112  165  185  188  215  222  227  233  242  254  257  264  285  286  321  328  12  15  16  69  74  87  94  103  148  149  214  234  247  273  279  288  310  321  375  411  417  418  434  439  446  477  478  513  522  566  573  578  608  77  143  170  173  181  186  191  217  247  288  299  308  312  313  315  316  317  326  339  340  349  12  75  118  121  134  206  231  274  290  309  311  334  344  456  480  496  559  619  679  731  796  797  802  824  944  957  993  1041  1045  1067  1104  88  147  193  212  214  219  236  251  284  288  291  305  307  308  315  316  326  331  333  343  357  365  366  389  407  415  444  467  468  8  15  21  44  55  62  66  75  76  113  116  121  139  201  202  235  238  242  252  295  315  319  321  362  432  496  534  544  551  595  620  10  17  40  49  64  65  80  149  179  181  183  185  186  204  225  226  247  263  267  295  300  302  325  330  342  356  365  11  30  49  93  113  121  130  150  167  201  216  37  72  240  256  278  291  296  417  431  509  572  581  604  614  643  658  692  718  763  765  789  793  794  820  877  1002  1068  1103  1116  1132  1228  51  127  131  207  233  239  256  257  267  332  372  393  402  409  411  436  463  466  471  474  476  489  498  507  550  587  600  613  634  663  679  24  55  64  82  214  246  314  319  332  337  348  364  395  403  417  472  543  562  570  575  577  582  586  595  600  602  650  674  792  871  879  29  60  15  16  36  38  61  62  113  150  151  173  176  208  277  302  341  367  10  53  54  66  67  83  89  98  104  116  123  155  186  226  245  249  266  278  283  284  314  361  369  387  388  391  198  292  304  313  401  455  509  520  521  525  526  547  552  564  578  589  610  618  714  749  776  781  782  785  789  790  795  808  877  894  921  8  17  24  26  28  44  48  49  67  92  93  94  98  116  117  127  136  141  156  160  169  188  201  241  245  293  300  363  372  397  401  407  11  12  33  37  54  57  61  65  75  84  105  154  197  272  289  311  320  338  394  405  416  447  453  457  481  491  500  520  545  549  552  10  36  38  39  61  88  133  160  183  208  275  415  479  500  580  589  596  619  756  786  808  813  840  943  954  987  1067  1071  1105  1145  1245  11  84  86  131  150  161  194  206  215  282  303  320  382  385  393  460  463  481  512  582  821  844  880  899  951  987  993  994  1023  1024  1027  16  45  98  117  151  152  154  171  172  182  199  206  212  231  232  247  249  259  283  303  307  315  336  337  344  352  365  376  398  413  414  11  13  15  91  96  132  139  144  146  161  168  214  217  232  244  246  264  271  300  314  335  344  383  391  400  401  403  408  409  438  473  12  34  60  99  156  157  226  232  254  258  284  309  312  323  11  37  45  54  103  134  157  182  183  231  238  249  260  307  310  41  43  46  57  91  132  157  166  222  225  228  281  325  328  338  339  359  542  561  579  581  589  595  611  616  669  682  757  776  782  819  25  47  63  66  79  90  91  93  107  129  145  157  162  184  185  213  229  235  255  256  281  305  328  370  389  398  407  412  431  459  460  9  37  65  95  100  102  121  125  131  153  161  170  172  173  199  209  229  238  252  253  266  284  290  329  360  454  459  471  505  559  573  29  67  72  104  121  129  211  219  224  256  289  333  358  382  389  399  439  458  462  474  479  487  529  560  565  577  601  628  635  640  650  706  717  27  33  38  41  47  57  62  95  96  123  143  196  208  257  261  278  327  350  357  41  50  86  90  105  124  165  173  179  202  204  276  278  304  313  322  331  338  340  381  389  391  401  404  423  443  462  10  11  33  56  83  129  137  169  171  174  185  230  237  254  277  287  302  315  344  352  377  383  422  453  474  477  519  581  588  595  598  11  62  74  78  93  148  161  195  255  308  365  369  391  399  433  442  451  504  516  552  562  571  594  616  684  693  747  768  805  833  871  903  1003  21  26  35  70  97  102  107  111  115  116  147  159  20  21  26  34  47  48  69  70  77  83  92  104  117  135  166  208  260  333  406  438  439  467  468  469  471  477  479  495  511  516  555  563  574  51  145  151  157  158  214  240  263  269  352  397  435  504  523  529  547  554  564  667  719  740  771  900  906  914  929  980  1040  1068  1104  1107  1122  1176  10  14  15  16  17  31  34  106  122  127  136  139  147  161  163  208  222  232  235  236  254  268  290  302  316  16  29  30  31  92  93  94  98  130  138  166  179  183  205  210  300  338  20  81  82  88  19  20  29  45  75  81  100  125  126  129  131  136  137  144  147  173  174  205  225  234  253  13  25  27  31  64  66  84  85  98  109  124  126  128  136  163  172  205  248  285  309  359  420  459  467  470  488  490  155  160  161  197  198  205  206  249  261  282  315  337  339  342  350  407  420  464  475  497  498  514  562  571  614  672  673  694  731  737  776  783  846  30  38  46  49  56  65  71  72  88  106  112  124  167  174  185  188  192  193  12  38  77  91  106  130  135  137  147  179  224  227  278  364  441  446  481  482  487  609  630  632  635  641  648  668  680  686  789  797  803  816  823  16  17  22  27  35  36  45  64  92  94  105  135  160  176  191  231  234  250  258  262  263  344  366  381  389  390  391  392  421  423  431  10  13  74  83  132  153  175  181  186  207  212  214  235  263  279  286  293  386  395  406  407  418  429  430  24  53  66  68  137  153  171  178  181  224  238  314  334  367  372  386  387  388  411  84  101  106  108  146  193  208  256  324  344  380  384  391  421  559  566  597  611  619  651  668  673  703  726  758  784  838  898  913  1061  1071  76  77  80  87  107  115  142  155  184  191  216  221  253  259  282  308  355  357  381  454  457  473  486  511  551  567  609  639  686  690  691  12  14  37  55  103  105  127  166  179  183  185  212  251  280  287  293  311  323  326  349  362  371  383  407  409  473  494  498  501  512  513  517  10  39  45  46  56  76  109  121  132  146  167  174  180  186  194  223  282  294  10  94  110  124  130  12  25  47  67  79  107  163  192  225  226  232  243  258  268  279  288  290  322  356  367  417  429  480  511  605  620  623  662  684  701  704  733  755  9  16  21  37  46  49  50  61  68  82  113  117  118  130  143  153  166  177  186  192  194  203  216  258  265  284  315  359  362  380  383  403  418  11  12  13  23  67  82  111  138  204  207  214  272  276  318  324  331  350  374  417  430  455  458  461  478  486  492  495  519  565  582  597  8  13  17  23  40  63  86  87  100  143  160  161  8  25  29  33  102  180  183  207  214  241  267  271  282  316  318  319  332  345  348  349  355  361  374  399  401  423  428  442  459  478  485  22  23  78  86  89  95  113  118  154  157  202  228  242  252  267  301  311  360  362  426  428  530  542  558  561  583  596  611  9  27  30  176  192  288  304  305  424  487  492  559  594  596  600  649  760  778  806  925  967  1050  1086  1102  1218  1226  1265  1409  1412  1459  1517  9  14  15  29  36  70  72  104  113  140  199  224  268  280  319  325  376  381  391  17  19  24  25  46  86  232  279  284  288  290  293  300  301  312  322  388  391  410  443  490  543  550  578  582  595  596  600  693  719  732  760  785  57  69  71  87  149  208  231  302  359  449  453  513  548  598  627  647  658  676  682  683  715  718  734  741  746  748  854  881  884  890  932  987  45  56  68  94  98  126  164  170  182  193  196  210  236  238  245  258  267  270  290  315  337  347  352  362  365  375  379  382  419  428  451  17  26  38  46  57  69  9  10  35  54  59  77  86  87  90  92  93  94  98  104  109  113  118  129  133  43  55  85  97  120  173  181  200  218  297  299  318  321  350  353  359  411  428  432  438  439  480  527  553  567  579  596  609  619  630  644  225  228  252  275  337  342  353  438  456  458  487  585  589  595  598  609  664  678  727  737  756  759  762  786  791  794  805  808  825  850  854  855  863  15  34  113  121  122  199  303  352  374  433  468  478  483  490  503  522  539  551  584  672  715  722  738  753  762  792  803  808  819  839  843  16  27  41  58  94  102  103  119  130  142  158  163  187  249  274  296  299  317  322  329  333  355  364  377  379  394  414  444  463  467  483  15  84  86  99  103  123  201  217  222  231  243  377  422  431  472  481  490  498  508  533  534  544  556  568  580  582  585  589  604  618  647  660  702  35  38  40  41  43  69  74  84  91  96  97  101  106  109  116  131  139  168  173  178  183  190  193  196  206  209  213  217  224  240  241  8  27  28  31  42  47  58  63  71  75  96  102  113  117  154  173  209  210  228  234  245  248  268  287  360  385  391  396  397  398  411  87  95  98  160  165  174  180  184  188  190  203  11  60  68  81  144  146  169  176  211  230  267  311  329  373  466  500  516  521  529  554  557  563  574  577  580  587  589  591  601  604  606  70  99  119  174  186  199  250  314  375  381  436  454  484  511  525  557  563  579  596  602  661  667  668  672  679  727  760  784  844  854  950  969  1046  14  41  49  54  72  82  96  112  144  152  155  158  164  194  199  43  51  84  108  113  9  58  59  213  214  24  27  29  30  35  66  79  99  103  114  124  127  132  166  189  192  198  240  277  303  304  313  342  346  357  359  9  10  19  22  101  108  109  110  116  127  131  136  141  145  167  181  193  198  205  218  223  233  246  255  286  296  378  384  407  85  109  164  215  236  267  277  313  323  325  367  371  388  394  407  413  476  499  500  503  514  516  532  550  552  562  573  579  582  635  636  11  27  66  96  117  158  168  257  264  340  402  424  429  449  452  476  482  496  529  530  534  539  544  556  584  600  605  614  628  646  655  23  32  87  106  152  172  292  333  351  387  411  424  460  597  600  617  648  649  672  682  741  757  771  782  787  1050  1059  1136  1189  1192  1196  26  34  65  72  79  85  153  12  16  39  99  117  140  150  201  225  283  288  318  361  389  484  509  537  564  569  619  682  683  697  720  733  740  788  797  824  884  946  54  65  104  153  159  165  199  202  207  213  234  247  251  253  302  344  354  411  461  464  470  474  542  615  617  620  623  700  720  785  796  22  74  88  144  171  178  190  373  404  439  507  564  566  578  629  646  665  668  697  785  793  861  866  889  908  947  1002  1012  1048  1067  1201  1231  1235  10  27  28  59  146  149  153  177  216  232  278  336  341  367  374  379  380  386  407  450  472  473  480  536  551  558  613  683  745  750  773  15  17  18  72  107  126  151  203  241  280  299  306  336  364  398  411  428  488  550  593  599  605  698  748  755  797  833  1016  1029  1035  1039  1056  1115  59  65  108  143  153  171  203  204  205  207  223  242  270  287  328  339  343  357  366  371  392  414  428  435  436  446  470  475  492  508  514  14  19  23  34  44  48  54  73  81  85  88  127  130  154  170  187  188  194  208  238  267  283  302  304  307  342  363  8  11  15  97  122  168  169  171  312  384  436  480  481  506  525  538  573  587  630  670  675  704  713  731  767  771  777  815  862  891  905  12  33  48  74  76  106  111  148  196  245  270  301  405  436  466  483  516  548  585  603  631  719  758  765  776  803  868  880  883  902  922  21  42  50  76  98  101  102  121  145  148  170  202  219  251  255  280  295  304  356  387  407  420  422  451  453  455  473  485  494  12  40  66  93  121  123  157  196  267  337  411  460  464  466  557  575  584  708  717  718  746  833  847  990  1001  1022  1058  1104  1129  1140  1187  24  41  53  87  111  18  47  49  58  69  100  195  208  213  214  215  219  220  222  223  228  235  236  238  244  245  247  248  251  259  321  359  366  325  347  358  427  489  490  563  579  581  593  643  662  673  692  703  711  858  871  952  965  1045  1051  1055  1070  1122  1126  1139  1204  1219  1223  1314  14  27  61  63  105  111  113  128  133  169  197  205  221  230  231  246  250  251  263  272  291  301  307  331  333  347  53  88  93  118  195  204  207  330  347  387  415  418  438  454  462  477  486  488  518  530  535  547  604  611  612  630  661  667  669  673  706  34  44  50  51  93  101  108  144  157  175  189  207  216  219  232  235  236  258  278  280  341  357  409  432  472  479  501  504  508  511  512  64  80  162  176  179  223  224  273  287  305  378  427  435  492  510  531  630  650  659  681  695  754  843  859  872  876  909  916  928  964  983  1013  1027  52  64  69  80  82  84  91  100  108  131  141  153  160  170  189  231  250  254  274  310  314  344  345  367  369  370  378  418  428  124  132  134  279  304  312  423  425  427  433  436  457  472  493  496  535  553  588  629  638  700  724  730  762  823  924  1072  1099  1111  1158  1187  1238  1362  41  42  44  89  114  122  127  141  162  224  244  259  262  265  266  267  270  344  346  353  389  416  421  426  432  436  437  477  480  487  494  33  86  95  147  198  204  275  279  286  359  387  408  459  534  597  599  608  625  638  640  647  655  733  757  873  891  942  985  1004  1041  1059  8  12  52  88  112  124  136  140  148  162  249  255  315  329  344  379  397  422  429  439  451  560  591  646  661  698  701  705  713  714  716  10  18  28  32  97  115  140  185  207  302  364  480  485  491  492  571  640  659  682  746  779  812  836  846  917  929  969  1037  1100  1197  1204  1209  1226  49  89  112  151  209  237  276  1062  1146  1182  1472  1606  1607  1629  1731  1738  1811  1940  1950  1957  2041  2056  2073  2103  2126  2204  2303  2347  2405  2411  2444  23  65  68  70  10  14  16  102  120  138  140  146  158  166  170  173  187  190  202  205  263  278  308  313  316  335  336  340  343  344  345  356  358  11  14  15  19  39  44  70  100  104  153  156  158  172  206  214  218  232  234  247  249  251  252  260  261  262  265  268  271  273  275  282  286  335  20  50  52  57  91  94  110  115  144  153  159  179  208  300  305  315  316  347  361  382  411  437  454  476  494  497  542  549  568  689  759  24  75  80  93  99  118  121  125  139  140  184  275  276  280  286  301  312  315  320  326  344  350  383  394  408  417  429  449  470  472  474  19  20  32  34  36  45  49  60  72  73  85  95  96  168  176  189  200  201  24  25  29  34  43  46  67  75  81  87  105  118  128  137  156  159  168  208  210  241  259  267  291  300  321  323  346  348  359  369  422  423  433  22  74  35  57  73  75  129  190  194  217  242  287  322  332  344  349  485  488  598  611  637  657  685  723  779  851  908  956  992  1124  1150  1189  1196  1223  1271  8  9  38  47  57  87  90  101  131  145  154  170  190  203  223  247  252  253  271  283  286  291  295  313  317  328  22  57  83  104  118  130  148  167  176  193  196  202  210  217  221  232  235  265  277  279  290  296  61  71  92  96  122  127  209  263  343  387  479  488  622  665  694  818  885  921  942  1005  1006  1128  1129  1132  1280  1469  1508  1521  1533  1539  1544  8  15  70  111  144  187  199  240  253  263  268  283  417  436  438  448  458  517  522  530  533  607  624  628  633  757  762  763  765  768  783  13  16  36  44  49  104  110  126  128  143  147  151  158  9  28  38  45  52  98  104  140  146  148  149  159  215  222  224  227  240  241  243  257  261  270  283  33  43  45  55  59  75  91  99  128  148  204  250  301  304  315  342  9  32  169  180  205  208  220  244  254  258  262  263  271  288  23  30  101  102  103  125  145  178  215  218  222  240  249  252  259  264  307  336  362  364  388  409  421  426  450  457  466  471  604  636  637  638  660  8  28  93  108  123  127  133  156  217  223  228  247  264  266  279  286  301  72  151  152  173  178  190  202  208  217  18  30  38  56  62  109  114  138  13  76  83  161  205  229  269  279  289  290  328  332  352  446  466  468  476  483  485  488  506  517  550  577  578  607  609  614  617  624  626  634  639  51  59  83  93  94  110  128  176  183  187  190  39  45  54  58  70  71  81  86  131  182  236  261  263  283  330  335  339  344  345  393  421  424  442  481  488  505  519  520  642  649  662  10  21  25  26  37  38  57  68  81  108  117  131  142  146  149  153  155  158  160  187  193  223  243  247  271  329  330  339  349  355  357  8  10  11  18  20  75  87  90  105  127  131  141  150  151  167  182  187  191  206  212  238  265  278  314  325  392  400  413  427  445  467  469  474  70  157  235  236  261  308  316  334  353  356  368  411  418  423  430  446  448  469  551  605  617  631  654  662  743  748  784  816  826  846  855  38  39  46  75  101  122  133  192  203  206  209  213  235  264  266  285  287  288  16  60  103  139  152  159  211  299  327  329  332  385  390  410  417  424  448  468  489  508  511  513  524  541  584  610  611  620  621  625  630  635  637  24  32  56  70  97  143  164  176  179  190  191  193  205  245  261  263  276  13  66  73  109  140  152  183  201  229  243  275  279  294  295  298  331  338  359  375  378  408  414  418  427  440  452  454  471  478  488  490  23  49  77  101  107  132  133  152  164  175  183  199  203  48  101  104  112  126  141  189  200  212  224  227  239  240  298  337  477  486  538  553  581  597  738  764  786  830  860  870  908  910  933  940  980  997  43  49  51  57  58  134  207  230  234  278  297  301  336  381  404  417  432  467  482  497  511  550  596  617  628  695  702  712  750  813  838  844  1026  61  62  99  218  304  534  681  889  900  915  997  1113  1272  1507  1664  1717  1935  2040  2098  2152  2200  2330  2340  2347  2417  2489  2591  2711  2735  2745  2886  2929  2968  20  75  82  100  168  171  184  229  304  335  451  454  649  693  758  767  786  794  809  814  847  956  1025  1129  1143  1166  1307  1331  1334  1413  1440  26  31  36  38  46  50  63  90  104  107  111  136  140  146  147  164  217  225  252  277  305  315  360  366  385  391  404  26  36  94  250  386  518  519  523  550  628  913  1074  1341  1510  1534  1537  1679  1759  1782  1848  1905  1923  1927  2181  2223  2325  2349  2369  2414  2596  2778  22  25  44  62  63  64  86  95  107  108  118  120  121  124  135  138  151  153  157  209  211  266  280  316  318  341  351  33  104  109  124  135  164  214  240  249  254  268  272  273  280  304  321  405  412  433  462  489  501  505  519  534  635  670  685  705  754  875  10  18  21  30  32  36  45  46  99  111  135  136  143  167  192  205  207  216  273  276  303  377  405  466  473  494  518  545  592  594  601  15  82  99  118  145  180  192  198  210  225  227  255  264  332  333  334  391  446  468  501  505  530  534  607  616  656  657  687  708  711  736  778  784  98  158  168  202  203  237  273  16  22  36  62  64  71  73  75  86  91  108  119  184  196  204  206  208  210  219  221  229  232  234  247  252  258  260  32  37  45  79  102  124  139  142  168  190  193  282  334  388  402  438  466  467  490  570  601  620  657  661  668  710  720  776  858  946  1020  1026  1027  35  39  61  87  101  110  118  160  167  194  218  280  309  330  342  368  373  399  402  412  466  474  530  536  562  570  572  583  587  596  625  46  68  100  104  118  122  123  134  135  146  151  170  194  202  208  213  240  277  286  346  348  362  367  389  401  432  455  464  507  542  543  551  559  107  212  264  305  420  523  783  870  969  1028  1114  1138  1141  1147  1150  1240  1262  1309  1343  1506  1520  1540  1691  1731  1782  1805  1845  1953  1976  2185  2209  34  37  60  69  79  95  98  101  103  112  115  130  138  144  159  165  166  187  193  201  216  225  229  254  303  333  349  8  19  23  39  51  82  204  214  215  219  236  272  305  308  345  346  354  362  368  388  389  399  407  409  25  26  29  49  64  72  98  126  137  160  170  181  189  204  205  220  221  234  242  39  49  63  151  187  191  199  233  256  257  310  335  406  421  436  503  506  512  572  577  582  625  742  832  833  841  845  846  848  887  903  69  182  222  228  421  628  909  1036  1078  1299  1343  1349  1354  1373  1687  2173  2247  2506  2719  2763  2781  2811  2847  3160  3241  3364  3574  3647  4152  4206  4215  4258  4531  18  41  47  48  49  79  202  225  391  402  416  457  509  543  730  738  785  817  836  868  887  891  952  968  973  983  1010  1012  1059  1106  1155  1201  1244  1259  1394  1414  1517  1576  1629  8  27  125  144  207  223  226  233  274  308  325  362  364  378  411  433  465  497  532  555  599  601  654  688  698  764  793  844  870  891  906  16  24  27  32  35  89  106  108  112  169  177  192  204  226  235  241  264  274  277  283  284  287  288  307  320  332  338  344  361  364  366  384  398  45  58  84  130  139  204  216  218  244  289  308  322  425  438  446  482  569  635  690  703  771  803  818  859  876  884  968  1016  1017  1037  1107  20  25  55  63  76  90  112  114  117  120  143  165  175  190  209  214  221  231  249  13  22  34  39  49  57  67  68  69  86  89  99  103  116  138  208  220  226  31  32  52  66  77  80  149  166  167  171  172  175  178  215  225  226  242  261  279  280  281  284  292  299  334  351  356  366  375  28  31  39  42  74  87  89  102  103  107  127  142  171  176  181  183  184  187  12  15  46  61  74  93  97  99  134  142  162  163  173  217  233  242  243  244  245  246  247  331  343  429  443  445  457  466  500  544  580  15  16  19  24  28  31  32  35  39  40  42  50  56  57  61  67  140  155  164  194  210  228  260  372  398  412  435  500  505  516  518  20  51  86  98  113  116  123  165  172  195  225  233  249  265  270  272  312  318  326  332  347  359  369  375  392  393  398  402  412  15  23  27  23  25  69  76  105  106  107  108  119  23  55  76  109  145  148  165  178  183  194  207  266  279  290  299  303  325  342  365  12  21  23  26  54  94  117  119  128  140  157  166  227  242  261  270  291  316  333  336  357  370  384  391  438  450  462  469  473  498  501  508  517  23  36  40  67  70  87  110  137  196  231  250  251  273  295  298  303  310  339  349  350  359  391  411  436  439  26  35  60  69  99  103  106  108  135  143  14  37  52  114  121  181  191  229  261  269  337  360  361  362  405  432  507  541  590  617  622  641  682  725  777  790  865  891  939  955  1012  19  229  308  372  428  543  553  564  603  714  729  806  846  849  906  934  969  1046  1258  1288  1317  1432  1445  1488  1573  1733  1743  2037  2113  2114  2125  2201  2225  60  81  90  96  123  132  133  146  156  164  184  185  192  203  207  228  267  296  318  321  340  346  19  33  59  142  163  176  199  220  239  251  276  281  11  89  92  104  110  123  148  162  171  209  46  57  62  77  87  115  136  153  155  174  175  203  256  271  305  320  321  364  390  393  399  19  38  45  48  49  51  58  87  136  178  181  184  20  24  36  66  76  77  125  134  144  162  199  202  203  207  239  246  307  318  346  358  375  385  394  397  430  442  475  491  521  539  568  576  589  18  68  79  84  94  107  148  150  152  175  188  194  195  202  207  213  223  280  292  295  353  419  422  513  516  541  566  634  644  675  681  11  25  49  70  99  107  148  156  169  182  184  187  217  220  255  329  350  354  365  385  406  411  423  430  448  11  13  23  43  63  74  93  130  146  150  183  189  190  191  197  267  351  362  374  389  393  394  395  419  438  465  476  16  32  35  83  116  128  129  131  144  198  202  213  222  268  351  354  355  358  384  403  455  460  477  514  613  632  655  741  755  779  797  16  18  21  29  41  46  67  83  96  109  159  204  212  215  221  242  246  266  283  292  302  312  341  353  371  376  383  395  413  421  21  103  110  150  162  191  192  207  220  265  266  291  302  332  333  18  30  37  44  49  51  66  78  86  88  100  106  116  126  130  131  137  183  188  189  198  207  216  218  220  221  229  232  11  20  35  57  84  88  101  111  114  116  144  148  169  170  174  183  214  223  236  259  262  278  280  282  323  377  381  397  412  417  426  22  60  69  71  77  80  89  132  145  164  260  261  307  309  316  325  337  343  354  405  8  16  49  77  122  135  145  173  182  199  206  238  266  271  274  292  305  307  309  314  323  330  339  351  365  373  389  415  427  442  468  23  98  179  192  336  452  541  550  558  640  649  650  715  763  808  892  893  976  1008  1075  1097  1234  1297  1305  1439  1454  1488  1555  1571  1586  1619  9  21  38  91  99  103  123  130  132  157  164  184  22  27  28  64  70  124  16  21  23  46  50  68  73  87  115  116  120  174  220  329  339  348  377  386  391  403  419  527  528  531  550  566  583  599  604  621  634  13  15  18  21  26  28  36  38  39  68  70  84  88  102  122  133  258  261  281  283  288  295  308  333  339  340  353  363  364  368  371  18  73  81  92  94  114  136  139  146  159  172  192  202  203  204  207  214  217  220  222  225  226  277  288  293  303  314  344  373  405  415  17  39  65  89  106  127  136  145  177  22  23  27  44  70  81  17  18  34  56  83  87  94  109  127  130  9  21  31  32  41  55  92  93  94  95  97  98  108  125  138  140  168  208  230  243  246  11  18  25  61  66  85  89  90  110  114  142  179  183  191  207  8  25  33  69  77  81  83  88  97  105  111  121  126  134  158  170  13  22  30  34  35  43  64  75  76  79  82  89  123  124  69  289  498  734  884  887  913  1067  1088  1094  1265  1370  1414  1433  1450  1619  1668  1824  1828  1832  1873  2050  2083  2121  2297  2344  2425  2529  2534  2546  2565  2728  2740  18  22  30  32  34  36  42  54  60  63  68  105  108  109  150  152  17  21  24  33  53  76  88  91  98  105  112  113  116  126  149  156  157  161  14  16  17  20  40  49  59  65  81  93  10  17  31  50  68  69  75  82  19  22  27  54  73  18  43  63  70  74  88  92  107  122  144  155  42  47  48  73  77  88  111  118  9  11  21  23  26  27  58  65  67  69  72  74  78  82  98  19  26  36  40  44  51  74  94  96  104  107  110  118  123  131  17  21  23  24  25  30  31  56  63  71  75  85  106  107  109  25  28  30  32  37  62  64  70  72  76  103  15  19  25  27  30  57  65  74  79  83  84  13  19  36  53  61  73  82  85  103  131  134  21  35  41  43  53  66  76  85  115  135  136  138  139  153  17  51  66  71  75  107  153  239  295  326  334  398  406  423  428  429  437  507  525  533  568  571  573  603  613  615  618  619  625  645  651  16  30  49  55  60  76  79  82  111  166  191  225  228  248  252  255  40  62  71  72  94  112  157  193  215  241  281  308  15  32  35  63  88  12  131  179  185  215  274  276  290  299  300  325  326  338  343  351  390  460  546  549  551  562  580  598  618  626  641  656  668  698  826  833  8  30  42  58  67  126  138  141  142  143  144  173  174  189  200  207  214  217  230  237  252  261  262  289  330  371  388  406  418  425  473  476  481  21  22  43  56  81  99  100  112  125  131  132  142  190  213  227  245  263  266  284  326  332  339  358  381  384  404  16  19  26  36  40  58  59  67  87  96  112  114  124  128  130  136  146  153  194  205  210  236  244  246  266  270  285  29  106  127  185  193  212  214  290  318  328  334  349  350  418  465  510  529  574  613  633  641  644  657  667  690  700  745  762  773  799  801  852  856  12  14  23  33  36  39  62  63  68  71  86  92  98  117  120  122  138  150  159  167  169  185  199  16  19  76  88  105  118  148  167  174  177  178  179  186  202  230  234  237  9  25  30  113  125  157  175  176  231  240  243  283  436  609  625  639  650  719  734  744  800  804  932  1065  1075  1133  1137  1256  1260  1339  1340  1489  1546  1557  1667  1775  1867  1922  2135  2154  2227  2240  11  48  58  107  143  171  18  74  101  126  178  186  196  204  235  257  280  293  297  334  346  18  28  43  66  70  72  97  105  120  132  135  137  8  14  15  18  216  217  226  244  256  294  296  297  311  328  336  366  451  459  478  494  504  541  18  30  34  42  64  83  84  135  147  191  216  217  244  286  290  296  327  343  353  359  368  408  415  465  503  504  513  514  527  576  601  17  25  34  36  44  54  79  90  10  19  65  106  132  137  179  195  273  296  315  324  326  327  378  447  454  460  473  492  497  499  526  548  554  620  644  654  687  706  750  31  54  122  133  175  210  212  238  278  302  312  327  353  381  384  387  391  392  436  439  440  466  477  501  509  516  520  607  620  644  699  10  19  28  33  49  119  15  14  15  28  30  108  110  170  173  232  234  237  274  292  294  296  297  299  354  356  358  361  22  26  30  31  38  43  10  47  84  91  102  137  154  173  178  193  236  282  301  305  309  329  338  341  395  432  433  450  463  468  512  554  559  566  573  575  578  28  52  61  76  96  98  99  111  17  19  142  153  162  172  223  227  229  239  243  263  280  318  366  367  382  406  16  17  77  87  95  124  146  147  165  173  183  197  199  214  219  237  248  257  289  292  294  327  331  332  338  18  22  25  26  33  35  57  67  107  110  134  143  148  176  192  202  209  211  212  218  233  256  258  313  345  348  349  351  356  23  45  49  97  100  106  157  171  225  244  264  266  289  296  297  299  326  336  357  364  381  386  393  403  406  407  410  435  440  464  17  25  28  29  30  45  53  54  84  103  117  119  124  136  161  195  223  232  246  258  294  312  323  346  374  378  382  402  409  421  439  17  36  39  46  100  132  160  176  218  245  272  276  281  301  309  336  346  354  356  28  45  46  143  170  173  182  189  201  229  244  273  302  304  322  20  26  50  53  56  58  75  88  103  122  123  138  143  144  146  157  165  95  223  338  391  568  671  731  851  897  1198  1370  1449  1458  1608  1792  1959  2172  2429  2870  2885  2924  2978  2990  3360  3494  3605  3608  4027  4470  4489  4517  16  44  56  71  83  125  161  189  241  266  269  305  317  320  327  340  370  394  395  403  404  410  429  440  508  512  612  673  674  682  708  10  22  27  52  99  106  127  139  145  15  20  21  24  27  40  83  84  86  103  112  183  186  187  188  193  295  302  321  365  428  15  30  33  62  63  64  145  183  194  23  42  62  64  71  72  76  121  131  145  149  156  211  221  253  255  287  288  289  315  316  341  352  355  369  371  375  377  380  399  432  13  18  33  39  47  64  75  77  88  178  233  254  324  375  394  399  410  420  425  491  508  530  531  534  541  551  557  583  584  598  608  612  620  25  51  98  125  143  192  210  266  283  284  288  292  293  321  27  42  83  112  130  133  170  175  184  201  229  234  247  254  269  274  278  281  282  292  298  319  336  340  351  365  391  409  417  421  433  446  447  22  72  196  202  250  294  310  373  387  413  451  457  573  602  609  649  653  656  760  779  783  792  977  1002  1007  1054  1073  1100  1107  1134  1151  1214  1235  14  21  30  46  101  118  131  139  146  153  190  204  235  241  277  287  304  326  371  373  396  402  419  433  474  475  477  498  552  632  672  732  773  34  67  70  88  95  116  146  153  186  191  192  218  221  231  245  90  94  95  96  99  106  116  121  127  149  166  185  215  227  240  241  260  261  303  313  314  362  366  367  371  376  378  18  19  27  58  81  88  123  127  128  132  138  147  160  178  192  214  234  241  259  260  290  304  326  355  356  429  452  469  485  493  495  497  9  43  49  50  74  135  154  155  156  158  176  187  8  20  42  46  48  49  64  98  140  144  157  182  206  217  231  235  236  239  245  253  272  298  301  314  317  320  329  391  432  508  577  54  57  124  159  162  193  221  271  282  292  305  343  346  348  370  407  528  544  546  573  582  587  596  614  623  628  656  667  679  697  750  9  67  110  115  133  162  169  37  46  85  104  110  123  173  188  194  237  239  269  308  310  317  348  363  364  379  393  411  471  495  500  536  563  569  595  596  602  667  725  778  16  22  31  32  83  99  136  138  163  170  214  227  346  349  364  365  384  457  489  490  496  522  571  591  605  627  644  653  667  692  730  11  20  45  46  18  20  21  22  24  26  50  62  69  83  93  94  112  146  8  46  60  64  65  83  94  100  153  155  156  185  191  208  231  249  284  315  322  335  340  345  406  432  481  485  591  648  687  698  720  722  731  15  36  49  50  65  75  80  104  135  136  146  174  197  204  12  58  83  16  19  29  44  64  74  17  21  23  29  37  48  83  84  123  129  132  149  154  156  160  162  165  174  182  183  189  267  292  296  318  12  18  20  35  45  69  74  86  91  114  115  117  120  123  130  131  139  142  143  73  75  83  92  94  112  153  160  163  225  237  254  325  331  350  396  16  21  51  87  98  100  102  106  109  121  159  166  179  190  191  220  254  260  298  324  354  364  384  408  428  464  485  22  37  41  46  69  74  81  102  105  119  141  181  183  185  187  191  223  225  247  258  264  326  357  362  374  383  388  415  420  428  429  11  51  85  95  100  111  120  192  206  208  218  234  251  258  260  264  270  279  295  311  326  352  359  408  459  476  521  552  563  565  628  23  44  47  49  86  93  110  127  230  231  242  272  278  286  312  347  420  27  50  51  56  59  65  70  80  100  102  112  118  124  142  147  160  165  200  205  28  58  120  170  97  139  192  202  214  319  376  436  440  445  457  550  604  613  622  623  653  749  829  949  1081  1165  1184  1204  1243  1274  1276  1284  1304  1338  1343  1354  1394  112  225  237  244  323  324  366  400  464  503  610  623  630  646  694  711  726  758  763  784  787  810  835  917  969  974  995  1001  1009  1033  1231  1297  1333  26  76  81  92  107  117  151  152  219  233  238  241  243  252  254  263  281  315  366  371  390  396  438  453  492  494  515  517  523  535  552  31  47  54  74  78  89  93  107  15  31  51  125  154  163  174  206  209  232  244  261  304  311  328  378  430  455  498  506  524  537  552  562  572  590  600  636  654  673  715  726  769  82  83  114  127  187  230  260  302  318  391  414  463  474  499  503  560  565  608  634  644  646  671  707  751  758  834  862  882  888  895  919  25  39  54  70  108  113  168  175  200  273  344  378  397  449  456  486  549  554  561  666  712  713  733  754  757  767  772  814  865  866  867  85  23  75  90  24  53  64  82  83  92  124  141  146  199  207  14  27  37  40  63  79  104  115  141  157  249  254  263  268  272  282  291  310  336  349  353  370  373  401  419  31  87  147  193  246  277  315  319  363  430  437  450  461  473  517  524  547  561  562  626  652  664  668  752  789  809  813  814  831  836  838  22  41  56  74  107  131  138  160  175  176  203  204  210  237  247  275  278  283  298  359  367  441  455  476  509  532  537  541  30  44  55  94  95  96  104  109  171  180  187  188  199  202  204  229  243  275  315  43  48  82  88  106  112  143  149  195  207  214  217  218  222  8  27  53  82  88  91  92  98  100  103  125  128  154  160  186  44  55  93  118  125  128  177  188  202  226  293  307  326  333  349  350  354  379  386  390  398  425  435  460  465  467  469  477  480  487  509  21  66  11  39  56  100  39  44  70  85  90  106  129  138  152  162  168  184  185  197  198  203  211  225  238  256  263  269  275  291  303  320  14  40  43  55  63  72  119  134  136  156  162  165  168  181  188  198  226  236  247  265  289  307  355  403  456  466  467  468  486  510  514  543  560  28  49  64  65  72  118  120  141  149  190  211  212  246  279  283  287  305  307  334  385  424  452  470  482  490  492  498  503  515  522  525  558  22  44  103  133  172  194  206  238  240  248  281  286  321  324  334  343  347  369  394  396  436  488  501  517  538  547  553  569  596  628  656  665  668  19  22  29  45  46  49  56  66  71  90  92  98  104  124  131  171  185  186  9  57  71  74  9  40  52  56  67  78  96  103  117  132  140  149  234  235  270  283  408  410  411  429  432  485  494  508  522  577  591  615  676  739  759  806  808  47  55  67  157  188  227  288  290  304  343  374  380  459  470  493  495  524  560  632  688  730  736  738  759  771  807  836  850  908  935  938  940  948  26  30  37  46  50  55  71  72  103  150  174  185  236  238  250  252  255  269  270  272  286  297  329  395  432  483  501  502  519  537  558  578  581  27  55  63  101  107  124  130  170  199  210  257  261  333  341  369  373  378  389  428  446  461  467  470  476  486  514  515  545  565  601  607  641  644  18  31  34  58  87  134  147  154  158  160  163  54  58  59  60  62  66  110  113  116  117  124  132  136  8  17  41  74  111  120  137  143  161  165  203  204  207  220  236  319  329  364  396  401  28  30  57  58  72  77  92  15  30  38  66  74  100  102  155  161  16  39  41  55  77  83  88  93  103  129  152  53  85  90  91  107  118  162  170  207  309  330  434  481  547  649  688  715  739  750  816  880  944  954  1035  1044  1085  1174  1241  1272  1350  1478  31  39  50  52  53  63  68  113  129  164  165  169  211  315  330  391  442  460  470  476  505  543  589  595  623  641  658  673  674  677  709  13  16  31  38  47  100  107  119  120  121  138  156  171  175  177  182  183  185  209  222  240  11  26  28  30  40  44  45  46  55  56  58  65  66  76  77  79  83  84  111  133  144  152  187  195  197  276  340  352  355  453  457  500  524  32  128  131  148  153  158  307  363  365  379  396  401  406  411  434  445  493  518  536  649  686  696  767  774  819  832  853  904  909  1002  1021  1026  1054  40  56  81  87  120  126  127  134  201  271  281  288  289  296  317  318  319  326  334  344  379  446  463  498  510  535  540  661  698  720  735  9  72  95  158  217  287  290  291  318  343  473  490  522  599  612  621  635  642  754  791  816  847  881  909  915  982  1040  1052  1070  1110  1282  1302  1310  12  24  70  102  148  170  200  231  243  276  324  339  343  357  410  427  449  457  538  544  546  559  560  576  607  640  716  750  838  862  937  939  1071  14  24  32  41  54  60  62  72  93  129  134  253  296  301  320  383  390  394  402  408  410  417  432  434  495  504  513  518  527  531  575  581  586  15  28  37  45  57  69  75  96  141  146  147  165  184  262  284  288  290  337  344  45  62  48  53  60  127  132  168  175  183  192  203  236  251  257  318  347  389  415  427  435  440  451  503  514  557  594  599  737  764  789  831  841  965  974  9  13  16  21  27  130  152  239  275  312  314  327  331  370  371  16  40  43  53  57  132  167  170  182  183  197  199  222  230  231  299  302  304  309  102  108  132  135  156  163  290  336  353  407  435  468  483  525  553  661  687  691  717  737  755  769  905  940  988  997  1074  1197  1268  1329  1331  1338  1370  29  59  106  116  117  129  154  182  184  193  202  211  230  250  261  304  328  372  402  450  461  482  511  517  529  549  587  596  663  664  710  11  20  23  26  45  65  71  101  109  118  140  149  182  192  221  248  254  297  318  352  369  372  376  382  387  402  407  408  410  432  439  450  459  28  72  83  103  105  112  113  120  127  163  171  179  186  207  250  274  282  292  329  339  342  374  380  393  396  399  404  423  441  453  34  47  49  54  83  108  111  149  161  186  214  220  21  72  78  91  95  107  108  128  140  143  163  176  218  256  285  295  296  303  318  341  351  360  399  434  437  462  478  492  506  519  549  565  569  43  75  78  79  138  152  170  190  249  256  268  277  13  28  36  39  45  60  64  65  67  76  85  101  103  29  35  46  48  63  64  71  91  115  202  225  22  52  57  62  75  77  126  138  151  189  206  227  242  250  255  270  28  43  47  66  106  135  150  178  185  196  8  39  63  84  24  39  49  51  59  97  99  111  115  118  119  163  169  193  200  201  223  224  226  36  45  87  123  132  186  215  229  245  252  271  18  36  40  57  62  116  168  180  200  233  260  283  297  313  324  332  341  345  347  391  406  407  420  428  445  451  473  485  488  504  524  533  541  55  66  82  83  105  187  213  274  286  360  449  527  548  571  573  623  625  656  712  717  744  769  781  795  799  884  1005  1006  1043  1046  1047  1055  1062  16  29  35  38  58  65  72  79  81  97  107  24  41  76  90  94  116  125  172  8  25  34  39  62  69  74  88  93  115  144  157  176  189  200  237  265  302  336  343  364  375  380  442  489  491  500  546  554  555  568  627  632  16  20  27  140  177  179  185  224  232  372  375  385  455  463  479  504  537  588  598  702  721  757  785  786  808  864  902  904  939  947  976  977  983  12  25  29  33  53  155  172  183  200  201  216  220  266  342  357  358  376  448  462  523  529  532  537  548  563  598  609  647  664  47  76  125  150  172  239  287  293  311  316  319  363  421  423  494  513  538  555  584  585  593  604  628  639  647  667  676  679  711  721  758  9  12  15  27  81  90  106  113  114  125  134  162  172  186  212  223  231  236  265  291  299  301  353  380  381  431  486  533  546  581  595  27  120  122  227  359  399  474  480  511  528  530  615  655  663  696  734  741  776  812  910  928  991  1002  1232  1286  1294  1301  1333  1506  1532  1553  40  60  86  105  135  138  151  158  17  79  92  104  140  228  239  263  357  363  364  657  663  665  713  835  854  866  1030  1032  1135  1187  1255  1260  1307  1465  1526  1532  1538  1570  1574  19  30  84  210  295  326  333  343  369  406  412  588  622  656  742  814  876  880  890  894  906  912  1062  1167  1304  1307  1331  1392  1524  1535  1551  41  51  75  89  16  28  41  57  71  8  24  47  91  101  103  116  121  19  41  79  81  100  103  106  116  138  178  186  191  192  219  265  292  299  302  378  380  388  393  410  416  438  448  503  28  59  178  193  223  251  282  306  309  343  382  385  395  408  427  429  435  452  475  480  511  585  607  614  616  617  622  651  660  661  691  160  163  174  201  219  242  296  300  308  322  326  328  422  424  426  430  431  494  509  529  613  647  662  703  740  753  759  782  791  851  853  36  51  54  57  65  66  67  68  74  95  103  115  157  175  177  213  214  229  232  287  330  386  388  417  424  443  445  447  449  451  452  24  50  51  62  105  116  117  161  167  205  213  216  222  49  162  169  171  36  38  64  65  80  87  98  100  139  184  202  213  214  227  247  301  341  358  395  426  449  475  484  496  497  512  516  543  566  593  610  613  617  32  34  63  64  78  89  94  134  142  143  145  149  156  167  212  238  240  299  319  326  345  366  369  392  394  28  44  46  50  67  90  46  68  113  128  227  232  235  238  255  276  282  318  341  355  369  382  388  411  428  438  443  447  452  501  502  512  558  575  633  637  730  9  14  34  77  83  101  112  139  147  155  157  193  199  230  238  251  254  258  299  307  321  363  23  41  45  48  53  68  98  105  112  113  125  126  136  161  188  191  207  259  272  339  343  370  371  375  397  424  436  438  25  38  44  104  123  147  174  187  197  239  244  251  279  375  395  397  465  501  614  628  639  712  717  729  867  868  877  931  943  962  966  15  67  70  83  122  132  159  175  187  225  232  247  252  253  256  284  383  398  410  453  488  497  498  499  537  18  57  87  98  103  104  125  127  128  133  143  144  149  153  154  155  162  163  184  189  190  205  207  215  219  221  244  267  273  282  290  308  317  14  27  52  53  61  62  67  120  131  155  187  237  396  401  412  436  450  452  486  516  551  572  706  719  730  896  897  992  1054  1112  1208  1275  1434  1442  1515  1552  1559  1598  16  40  85  113  120  121  153  208  221  254  275  276  277  281  286  296  329  340  362  381  385  400  432  489  494  511  538  541  544  613  633  637  638  38  56  144  180  267  351  373  376  418  441  445  461  491  498  510  536  551  578  628  664  684  686  695  702  740  776  792  796  797  812  849  13  18  20  39  40  85  87  100  105  106  110  129  161  218  237  239  139  201  202  236  303  333  341  347  354  367  459  461  468  473  479  504  540  572  586  638  819  843  894  919  937  969  1030  1039  1045  1065  1234  1236  1260  174  265  278  308  511  519  917  1078  1092  1199  1230  1242  1267  1297  1301  1324  1381  1476  1484  1503  1645  1707  1714  1829  1917  1936  1937  1960  1989  2015  2057  14  24  28  36  38  40  47  49  55  57  59  61  65  69  72  77  78  79  127  137  142  145  150  166  188  206  237  280  299  318  351  24  42  63  76  91  96  115  124  135  136  180  238  271  332  346  388  393  420  433  434  436  439  518  588  596  597  681  770  777  785  786  794  797  21  47  50  52  73  74  97  111  250  298  306  320  387  394  415  423  432  433  467  534  595  618  643  667  686  719  53  148  149  249  304  316  390  395  464  471  497  600  601  641  707  709  717  750  802  891  910  918  999  1045  1115  1162  1303  1423  1425  1428  1491  8  29  40  73  84  106  117  123  132  157  160  179  180  184  188  189  278  281  296  332  418  448  455  486  26  27  72  73  87  95  110  114  118  199  241  245  250  251  253  255  259  263  277  337  339  12  40  43  58  76  94  120  130  140  147  153  173  186  202  224  242  248  259  280  317  20  68  122  142  176  209  295  386  411  424  444  576  599  603  679  714  719  757  758  800  802  806  809  835  856  880  910  927  939  943  1086  1141  1151  16  21  128  132  176  189  208  228  251  255  284  292  293  296  307  311  313  316  317  325  328  342  370  376  385  397  398  427  471  487  489  32  33  34  73  91  100  118  135  160  167  177  195  200  213  228  241  265  267  282  303  309  310  325  352  353  354  395  410  435  440  445  10  31  32  33  39  42  43  46  54  74  76  112  134  141  152  161  170  172  183  186  222  223  228  230  167  220  373  407  454  599  821  845  881  961  998  1037  1202  1241  1348  1552  1612  1793  1806  2164  2253  2346  2463  2516  2652  2679  2703  2818  3026  3031  3064  3079  3089  22  42  46  69  119  127  167  180  213  219  228  242  276  120  440  453  462  583  614  668  742  775  851  919  1104  1112  1245  1295  1304  1335  1340  1344  1430  1452  1481  1560  1680  1704  1720  1999  2022  2085  2145  2172  50  135  172  192  234  480  596  606  619  652  709  939  1041  1398  1462  1490  1611  1662  1731  1745  1782  1794  1847  1876  1893  1932  2037  2108  2234  2238  2285  2302  2312  11  45  74  75  19  21  43  49  77  102  122  129  139  145  146  149  167  205  220  227  233  278  280  298  301  305  308  22  25  47  50  74  76  101  112  119  133  136  145  146  154  155  218  255  257  262  293  298  312  318  351  363  366  367  379  382  43  73  91  102  109  140  145  172  243  244  250  284  348  356  362  428  442  469  471  576  657  669  677  744  754  763  777  797  817  832  872  881  883  40  68  129  145  167  178  181  211  310  332  336  346  427  577  752  827  833  889  900  944  960  1034  1044  1066  1080  1131  1160  1193  1195  1199  1233  9  19  36  70  105  131  133  162  180  191  236  237  270  280  306  327  465  496  507  543  559  572  586  624  635  649  684  829  838  840  847  850  853  27  33  37  45  53  63  69  73  77  78  96  139  142  144  155  162  180  184  214  222  237  257  270  314  365  373  378  379  552  560  569  21  23  29  69  140  143  168  175  214  244  255  258  266  318  335  341  348  349  378  379  436  441  460  462  526  591  635  650  692  695  712  10  20  31  48  17  42  63  98  118  153  168  44  50  56  57  86  22  24  27  28  54  58  14  23  29  52  74  75  85  97  104  106  148  174  194  200  259  268  285  323  345  347  355  395  411  432  444  451  473  475  532  533  555  556  566  50  176  222  308  478  511  519  557  589  631  642  654  776  809  821  839  877  898  902  945  962  977  1045  1094  1159  1188  1231  1248  1276  1362  1373  303  368  467  739  794  923  947  1103  1133  1283  1440  1470  1577  1693  1712  1747  1935  1972  1986  2081  2316  2386  2429  2493  2530  2603  2699  2826  2833  3206  3260  40  108  140  197  203  262  285  331  371  426  428  455  474  493  520  536  537  551  653  704  712  727  823  833  868  909  924  982  983  984  1023  23  33  38  76  82  92  113  133  190  197  216  274  279  360  399  405  418  422  530  541  573  584  593  623  678  774  793  802  828  829  832  8  9  11  20  30  33  38  50  59  117  144  153  203  221  231  251  300  308  317  337  340  368  369  375  379  407  432  437  438  445  448  23  40  96  106  127  148  150  151  154  204  222  226  243  263  270  280  294  296  304  322  333  343  349  356  365  368  369  375  382  461  474  510  512  9  15  37  82  98  70  83  107  110  111  117  119  170  200  202  243  265  290  296  418  421  474  535  536  537  539  540  547  549  563  588  8  85  97  132  136  175  187  231  235  245  280  296  303  321  329  342  357  362  400  425  446  451  513  540  547  550  579  593  618  622  638  639  710  10  122  169  259  407  452  453  468  469  486  507  526  532  546  559  563  565  569  573  578  593  607  647  652  702  704  706  710  718  720  723  45  103  105  106  135  141  145  153  154  161  165  166  192  207  218  228  331  362  378  386  404  408  433  467  486  510  522  538  568  595  611  613  655  34  43  62  74  108  126  230  249  258  267  282  293  309  310  335  343  349  399  413  442  445  451  461  478  493  498  18  98  115  119  143  144  148  152  163  181  185  209  215  225  228  229  233  238  241  245  264  283  296  303  304  315  316  321  322  323  324  333  11  13  32  46  62  75  80  97  111  112  134  149  154  186  208  232  233  244  275  299  300  311  312  314  320  406  407  409  410  415  416  432  444  101  123  145  186  200  235  364  511  560  565  583  589  659  664  767  886  928  967  982  987  991  992  1012  1081  1449  1519  1570  1754  1765  1771  1795  21  36  103  109  147  171  173  178  189  191  208  215  246  277  283  288  310  315  359  370  379  381  37  55  57  67  77  97  100  127  172  182  202  205  222  243  249  269  287  333  346  362  406  420  425  434  437  519  38  42  73  119  172  187  201  213  225  227  231  256  283  366  385  392  403  501  515  523  544  545  551  552  601  607  637  643  650  674  746  9  35  53  69  95  116  168  228  256  310  342  370  375  407  413  420  10  30  35  55  70  71  84  89  95  96  124  149  153  194  200  226  243  254  281  297  301  310  312  340  381  384  412  430  480  488  497  56  79  138  160  16  17  34  35  67  91  101  102  110  199  230  242  255  265  280  290  303  352  370  380  399  401  447  459  462  467  468  502  516  525  544  547  553  11  40  78  81  167  232  278  322  335  360  366  397  466  493  517  527  533  550  554  555  588  606  627  632  652  699  708  713  722  730  734  29  129  174  176  180  188  190  193  205  240  250  258  326  357  381  417  454  464  473  478  480  486  500  530  531  536  539  542  543  551  553  42  58  63  80  88  182  186  193  228  232  242  246  248  253  255  261  278  288  289  298  300  313  319  321  330  360  374  380  383  418  434  36  37  87  110  114  116  123  126  127  273  306  314  317  319  333  335  339  409  419  431  520  571  579  637  657  675  683  713  727  732  750  23  77  98  128  134  160  202  211  215  227  264  282  283  291  303  306  20  44  62  72  85  87  89  107  134  168  181  190  197  211  212  227  247  249  280  335  338  341  368  383  384  389  391  397  399  402  406  407  408  19  22  37  63  85  123  137  138  194  197  200  201  204  207  214  218  236  243  247  248  253  254  260  263  276  278  289  293  294  300  43  44  67  72  95  96  150  186  225  234  237  242  250  290  295  332  333  359  368  372  378  403  406  417  418  419  432  475  66  112  127  144  148  149  243  249  366  413  482  488  500  504  505  535  536  539  127  136  168  179  181  191  203  204  237  238  300  307  310  314  321  379  388  396  427  437  438  445  455  456  458  460  464  465  467  475  497  43  53  65  103  118  127  140  141  146  153  179  183  199  206  208  213  224  227  269  306  353  359  377  382  383  393  418  437  456  489  497  499  530  19  24  124  369  438  559  620  622  631  632  633  638  641  642  768  15  18  39  13  30  32  90  146  151  176  190  201  206  214  217  240  265  272  274  290  291  298  300  302  304  305  309  312  313  315  326  341  11  12  13  39  52  56  72  114  115  134  140  170  174  177  184  189  198  238  240  241  250  257  24  71  90  440  610  962  966  968  1015  1016  1028  1050  1222  1233  8  12  25  30  36  46  47  51  91  107  116  131  154  155  161  169  170  174  182  186  251  274  319  320  341  343  351  373  377  393  404  10  30  47  51  161  226  246  279  352  401  409  430  505  628  664  711  717  724  747  766  771  809  810  818  856  889  931  958  1003  1017  1018  18  43  45  58  71  179  180  182  183  184  188  190  209  215  226  228  246  305  306  381  383  390  430  449  478  562  596  627  630  656  717  20  35  42  48  51  57  63  70  84  87  103  104  114  139  143  144  147  156  161  175  183  193  197  219  229  233  328  336  337  365  379  30  56  69  139  140  143  149  151  160  191  192  205  213  243  22  307  396  433  764  794  826  834  892  903  925  960  973  1011  1154  1175  1312  1370  1417  1442  1447  1490  1533  1584  1617  1646  1690  1696  1731  1736  1756  1770  1872  12  40  48  49  76  80  88  89  98  103  108  111  114  117  121  129  143  156  161  178  179  237  246  260  270  43  56  89  171  212  322  377  389  404  416  427  470  541  635  719  776  787  797  847  854  876  890  903  923  947  961  984  1016  1042  1048  1054  1080  1081  23  41  65  86  87  104  153  203  212  221  224  234  241  299  333  346  348  377  379  397  433  441  448  489  506  529  583  587  638  696  700  714  729  44  54  61  81  89  92  105  116  49  50  55  62  79  101  107  108  160  171  172  178  185  187  194  10  13  21  24  32  62  63  67  68  86  99  111  124  164  241  308  310  316  347  355  394  397  408  409  418  436  457  460  462  468  569  596  600  10  23  44  56  69  90  92  109  112  129  130  164  165  169  185  219  235  254  260  300  309  317  334  348  352  361  366  28  46  94  139  8  9  14  64  18  27  43  50  69  73  86  158  163  180  182  201  217  220  244  251  272  273  279  282  296  297  306  364  372  15  33  54  110  115  131  160  213  253  260  263  264  326  8  13  31  32  42  45  46  47  48  51  57  72  81  82  92  231  266  290  298  301  330  344  358  361  414  424  432  438  460  23  65  76  110  111  119  123  141  218  220  221  223  225  227  228  229  230  231  237  257  261  271  288  321  325  19  29  38  50  53  66  68  69  73  102  106  109  113  121  154  167  171  182  207  216  229  235  251  254  260  263  289  290  298  312  324  9  10  16  28  29  42  46  75  77  103  112  117  118  124  142  145  170  178  193  218  223  226  256  267  274  313  329  344  348  369  401  432  12  23  72  86  130  134  154  198  223  252  255  275  276  277  306  313  314  339  398  401  420  427  464  479  529  538  551  558  560  644  645  720  757  14  40  84  91  92  96  188  194  232  237  30  31  76  90  97  118  121  138  140  145  151  153  155  156  161  170  183  192  194  202  204  231  254  68  90  115  140  162  294  325  489  496  504  527  605  677  714  752  963  965  968  969  978  1015  1111  1185  1205  1207  1237  1246  1268  1279  1282  1291  1296  1322  12  19  27  34  35  92  116  119  157  158  194  204  208  212  222  240  268  323  329  372  374  391  405  407  420  429  432  444  460  491  513  537  544  13  15  28  55  70  80  104  118  146  151  187  190  201  203  205  216  222  270  285  286  295  363  365  372  393  404  102  129  149  178  214  215  11  21  112  117  194  207  218  238  252  260  288  346  380  383  434  443  566  600  726  755  787  791  795  820  914  981  1009  1026  1060  1067  1084  1101  1113  20  21  24  25  56  63  66  100  107  116  129  133  140  142  14  23  31  58  118  134  137  152  155  189  201  20  26  29  64  83  85  90  104  108  125  129  162  179  233  236  256  267  288  292  295  300  311  317  336  348  349  355  387  403  405  406  11  17  57  65  74  75  91  95  97  99  101  133  137  145  151  181  184  193  219  224  229  236  268  328  336  416  418  578  593  603  614  23  39  55  71  78  97  117  182  185  221  246  353  438  473  515  539  625  648  654  660  662  674  788  816  845  862  898  905  955  957  962  26  61  74  92  97  122  131  148  185  192  196  216  220  310  314  317  363  423  439  485  538  563  586  620  644  694  788  824  869  875  878  23  46  53  56  86  109  139  140  172  179  190  195  204  298  329  354  359  368  397  476  484  492  510  554  566  571  574  586  589  593  603  638  646  18  28  43  87  126  205  346  348  391  445  478  482  514  541  598  612  631  733  739  866  954  981  1009  1067  1080  1091  1150  1197  1233  1251  1263  32  52  70  75  88  89  109  122  269  285  298  340  364  382  415  424  438  445  447  514  547  580  583  603  682  733  792  811  816  827  872  889  893  96  145  147  220  224  233  25  28  61  87  109  128  160  166  183  194  196  198  202  231  303  304  308  326  339  341  343  371  375  380  394  399  400  402  409  411  416  39  59  93  97  143  148  167  171  174  179  190  192  196  202  203  204  209  213  219  221  226  337  343  344  354  357  407  461  462  492  520  530  12  17  32  36  43  49  52  55  69  76  78  88  91  95  96  102  103  104  129  143  151  222  225  312  314  317  336  380  384  399  427  22  34  37  46  50  59  60  129  137  173  199  225  245  275  284  293  298  323  330  331  332  380  433  456  544  560  581  630  634  635  710  737  743  27  55  74  79  98  120  130  131  157  193  383  484  494  509  525  570  611  627  652  673  679  685  690  722  736  768  783  787  790  794  922  18  30  37  55  73  98  129  139  156  226  239  250  263  339  343  348  363  414  448  473  507  521  590  610  628  631  651  721  763  788  806  843  876  20  65  73  112  210  223  236  248  301  328  342  371  399  405  426  439  449  567  571  617  632  661  663  684  699  731  759  851  855  910  947  8  42  52  72  253  262  360  381  580  584  586  595  600  616  769  837  853  958  1034  1037  1044  1059  1064  1139  1142  1151  1175  1177  1215  1241  1245  1246  1249  28  54  68  71  97  102  104  115  124  128  131  135  158  164  166  178  181  186  194  195  248  305  307  314  323  324  326  342  351  355  20  29  37  54  57  14  15  31  32  37  46  53  109  117  124  146  147  165  188  190  208  27  42  52  77  81  97  116  142  156  180  190  14  32  37  75  76  94  102  114  120  20  23  25  31  34  37  40  43  67  80  83  84  85  87  88  91  93  98  114  176  195  199  240  253  322  364  377  428  486  504  524  20  24  40  56  72  79  17  35  43  58  80  81  84  99  125  134  137  158  167  168  213  219  230  293  338  415  488  504  558  566  567  568  572  573  589  593  594  627  637  41  99  122  186  217  227  275  278  288  290  308  311  315  317  335  336  351  367  369  374  378  382  385  390  396  398  400  401  404  425  427  11  68  94  112  118  135  161  194  241  254  293  324  343  350  379  384  425  428  578  614  884  936  1169  1194  1317  1337  1454  1617  1702  1735  1737  1759  1769  24  25  49  52  59  72  78  131  138  147  151  156  176  223  238  269  291  312  321  327  333  38  43  65  72  82  113  161  223  229  234  277  283  294  301  303  318  320  341  353  358  384  507  525  571  616  642  658  693  713  742  785  8  27  36  55  83  117  122  138  141  160  165  179  212  213  217  222  269  288  306  344  345  362  8  24  27  38  81  94  184  190  209  223  227  250  251  334  345  383  403  442  478  497  499  508  510  14  15  38  44  49  90  105  123  129  131  134  155  165  171  186  190  192  226  232  242  247  248  251  263  304  327  333  336  351  353  358  359  374  19  31  48  50  79  81  99  158  159  184  187  205  207  218  221  223  232  239  268  271  275  287  337  354  37  174  188  253  290  466  524  529  728  796  838  841  859  1051  1095  1141  1158  1564  1567  1579  1655  1657  1672  1678  1710  1719  1799  1818  1845  1856  1861  30  85  95  110  114  123  131  171  193  231  237  250  266  271  272  283  287  16  33  38  56  83  102  113  127  149  150  166  173  187  206  228  248  249  274  301  303  306  308  315  331  333  341  344  346  348  383  386  399  35  43  44  46  57  101  102  104  137  162  167  215  234  240  241  260  9  18  63  64  66  85  108  165  180  183  192  193  205  225  230  248  298  53  60  68  101  122  128  129  171  212  215  216  225  250  253  257  303  309  328  340  365  385  411  419  427  430  450  482  487  515  541  574  608  670  9  11  38  49  52  60  87  89  119  132  133  202  27  35  57  62  107  141  166  179  213  219  223  230  262  264  274  276  301  332  340  349  364  365  370  375  379  383  394  400  405  412  28  35  73  74  78  140  222  249  251  276  281  284  305  309  414  617  620  621  694  736  781  803  824  855  866  877  883  991  999  1020  1023  11  19  21  26  38  53  55  62  76  80  95  8  15  20  22  131  171  237  296  376  441  452  483  486  511  520  526  589  601  604  605  606  648  704  705  767  799  833  839  844  849  854  858  19  31  76  112  116  127  129  132  150  152  173  176  188  190  35  73  74  87  122  159  190  201  212  228  331  343  358  371  403  450  466  467  479  512  534  550  597  605  606  617  670  676  755  772  797  52  54  82  84  86  90  91  100  125  126  127  129  134  140  142  162  191  193  257  275  378  385  413  431  441  455  485  495  533  550  553  23  29  43  89  93  101  105  114  120  147  151  178  197  220  236  263  301  313  344  345  355  364  373  389  392  35  39  81  82  90  105  117  129  167  23  27  49  115  173  190  232  233  43  49  57  64  68  72  81  97  100  107  109  110  114  128  129  135  137  139  170  193  196  230  280  13  18  42  47  85  107  109  121  158  187  248  265  325  353  354  468  493  497  503  532  540  570  574  631  638  692  764  816  912  998  1050  54  106  107  123  128  129  130  139  145  159  171  172  225  268  275  341  374  383  392  410  420  432  491  496  521  596  598  654  656  660  20  30  43  11  19  43  63  75  96  108  154  170  171  206  221  228  237  263  285  299  311  393  440  444  462  482  501  502  513  529  563  606  625  685  35  83  104  129  139  149  160  8  21  49  72  86  105  110  114  117  137  150  153  160  40  53  75  78  176  203  243  268  278  334  375  380  384  398  401  403  417  421  427  462  464  479  489  493  503  515  43  52  71  153  154  228  259  260  289  304  367  383  398  458  468  485  508  515  524  627  640  683  738  767  816  914  996  1043  1056  1062  1066  1082  1083  14  111  120  145  199  286  294  316  318  339  341  405  432  440  452  484  490  523  540  551  669  683  684  687  689  702  706  715  758  770  820  40  42  77  78  83  154  183  187  198  355  359  361  420  23  24  41  94  108  113  119  120  16  31  39  57  71  88  149  152  160  183  263  272  276  309  317  340  350  352  354  409  424  430  434  442  495  507  544  559  572  588  591  23  30  79  84  95  104  130  153  155  180  187  197  222  268  308  311  316  322  337  346  352  364  365  377  389  426  428  430  447  454  459  41  43  55  90  97  111  131  166  197  201  207  219  225  234  257  258  11  26  28  33  36  57  71  80  123  128  139  149  13  22  69  95  144  183  189  196  213  224  272  336  339  347  387  390  474  501  503  507  573  590  607  608  616  626  667  668  673  752  753  791  794  20  23  36  41  66  105  125  133  145  163  179  187  199  206  233  8  20  37  44  48  62  72  95  104  119  137  135  217  593  760  807  908  994  1000  1143  1157  1310  1398  1487  1496  1509  1521  1538  1558  1566  1578  1639  1699  1703  1719  1739  1788  1828  1832  1900  1995  2131  25  28  59  73  90  100  109  194  195  222  242  248  302  308  309  310  317  343  344  366  381  391  121  194  269  338  508  593  622  759  768  782  826  867  1074  1508  1555  1592  1612  1637  1647  1663  1679  1745  1759  1770  1871  1962  34  41  108  135  144  171  172  269  275  277  288  292  302  306  348  373  477  522  564  586  595  602  604  634  728  734  740  742  762  771  790  10  29  38  52  63  68  87  111  114  130  131  197  208  213  264  279  285  286  54  107  118  10  13  19  29  33  36  11  13  16  34  35  56  63  87  97  101  127  135  140  142  155  158  215  63  76  159  166  174  191  204  234  252  262  35  115  169  209  227  288  398  411  438  480  482  549  679  770  786  826  840  919  953  954  1030  1076  1152  1230  1342  1352  1359  1418  1434  1556  1603  28  30  32  34  41  43  53  55  62  79  142  204  223  239  243  251  257  281  294  299  336  346  349  365  366  407  414  438  13  18  21  25  26  33  44  64  68  74  86  120  125  128  137  153  161  172  199  214  217  218  223  228  261  267  274  283  305  308  311  357  371  21  32  33  37  51  58  77  116  131  140  141  159  167  181  240  258  262  272  282  288  300  315  337  342  353  392  409  433  464  466  475  476  500  15  48  123  124  140  217  293  329  349  363  483  517  525  570  617  690  774  799  811  864  866  881  971  974  981  989  998  1016  1061  1085  1098  1159  1182  25  33  72  80  87  100  104  111  179  237  286  298  304  320  324  338  359  368  369  370  372  387  396  406  418  9  12  14  21  28  34  39  41  43  45  46  48  52  58  102  188  192  207  209  210  211  214  217  233  252  260  273  279  286  289  294  10  18  22  24  38  49  90  100  113  117  141  165  176  214  231  238  265  57  58  74  110  135  139  170  219  225  230  240  263  265  283  286  299  301  26  54  56  66  100  115  120  123  129  135  143  164  201  252  275  288  290  298  302  307  8  13  16  25  35  63  93  103  111  129  132  139  144  169  214  248  78  96  146  211  241  319  443  532  569  586  631  872  940  1052  1113  1220  1267  1396  1407  1423  1570  1609  1719  1928  1956  1999  2089  2100  2108  2121  2197  13  51  85  88  89  97  105  117  125  128  140  150  157  190  205  253  279  288  346  435  490  500  526  628  681  760  788  798  809  818  871  62  71  79  94  116  156  175  200  205  295  298  414  426  432  602  662  713  723  742  764  793  799  822  853  865  867  887  896  899  971  1121  28  32  52  58  80  82  101  115  147  158  164  166  167  203  204  216  13  17  34  40  49  64  67  77  159  184  203  207  219  226  239  242  270  282  287  288  291  298  305  314  316  352  392  422  432  453  457  32  62  63  75  116  119  137  165  174  176  242  257  289  298  321  406  420  440  441  483  509  573  585  594  657  682  714  725  891  899  931  1006  1021  34  86  103  169  198  226  242  276  299  364  389  445  489  514  515  548  572  581  643  655  813  867  876  919  920  944  980  994  1000  1011  1037  41  61  121  126  133  134  166  217  229  245  268  273  284  292  333  334  339  352  354  356  359  361  365  404  406  409  164  219  235  256  260  270  302  328  338  388  545  562  577  578  584  620  681  682  742  767  801  809  869  878  898  911  919  936  937  972  1004  1011  1232  74  113  114  126  153  169  193  218  288  354  363  400  424  426  431  448  455  457  481  491  577  636  749  790  796  798  802  844  880  899  910  10  26  32  40  42  57  103  116  138  140  148  151  160  186  203  236  250  285  294  299  378  398  416  424  442  474  496  528  534  553  556  582  56  57  85  125  137  168  246  359  391  409  470  483  526  572  594  599  600  608  619  624  625  654  725  765  772  815  867  878  894  943  1171  22  30  33  36  78  95  130  133  143  149  153  170  209  218  223  242  271  292  315  320  326  354  355  358  380  48  221  230  252  314  330  341  366  399  493  514  547  560  562  572  580  591  625  634  647  650  654  668  705  739  742  773  774  780  804  824  98  134  183  210  227  296  339  342  406  409  418  431  491  522  570  593  660  663  720  723  752  758  790  792  817  819  858  884  887  942  973  1063  1103  37  44  64  65  70  71  74  86  93  106  118  119  196  197  220  231  272  52  83  128  131  132  134  211  252  275  283  307  308  322  12  25  31  53  80  82  83  94  105  114  172  189  190  219  234  244  37  102  135  141  150  177  220  222  230  233  255  281  336  351  364  369  378  384  387  434  443  478  489  493  513  540  556  566  578  803  806  28  31  45  67  113  136  165  193  196  203  204  211  220  242  247  270  364  383  419  435  438  444  454  471  472  539  559  573  592  622  626  18  34  40  57  77  116  126  156  162  186  192  212  235  239  261  263  266  268  271  283  287  298  317  320  342  361  367  434  435  479  485  15  36  42  50  100  103  104  106  117  151  160  178  182  186  187  188  219  229  238  257  291  294  327  373  397  416  465  470  476  490  569  8  14  15  17  20  23  75  78  79  90  101  102  146  147  148  161  207  209  247  263  277  287  296  315  349  397  431  449  451  8  12  17  23  25  45  80  87  88  118  138  143  144  152  164  178  189  190  202  214  217  238  289  302  303  318  323  325  343  358  8  14  19  34  79  162  179  242  280  281  295  35  100  106  110  128  191  201  202  204  216  250  285  298  313  347  418  442  447  505  515  560  584  608  630  635  658  664  694  783  853  871  875  884  9  12  15  19  23  31  67  112  140  151  207  253  257  261  289  290  301  306  323  334  359  395  407  412  434  438  447  448  454  474  19  33  46  76  77  80  100  114  139  166  176  223  228  18  19  21  24  26  44  47  53  63  70  97  113  8  44  48  52  81  98  130  161  177  199  214  243  265  268  272  321  339  344  346  384  445  470  474  492  505  509  514  518  530  540  588  607  628  9  31  45  73  77  90  14  26  52  55  58  66  110  120  126  127  138  153  161  171  176  184  205  32  66  129  137  148  205  294  423  446  537  540  551  684  707  727  759  765  791  847  848  863  1021  1036  1090  1121  1189  1268  1331  1352  1421  1459  1485  1487  8  44  48  52  62  91  149  174  189  190  199  214  224  226  242  252  269  277  336  365  372  395  404  420  513  532  545  555  559  574  584  31  56  92  109  130  141  201  242  261  268  302  374  379  398  411  432  458  16  28  42  45  76  139  148  164  180  189  309  326  398  459  460  481  500  519  16  35  41  71  78  94  14  21  66  76  79  81  98  108  119  121  130  154  171  175  177  182  189  191  206  207  218  219  220  253  256  41  94  174  201  234  320  397  542  580  584  602  664  680  701  793  827  879  889  943  984  1015  1035  1062  1073  1074  1178  1190  1204  1207  1230  1244  58  61  67  73  78  103  111  122  130  135  171  196  232  247  277  295  298  318  394  403  410  422  423  439  580  592  610  620  645  664  687  66  70  85  103  130  139  141  204  208  212  214  218  225  257  262  265  276  282  292  295  303  306  307  311  314  17  21  39  58  59  64  71  99  101  120  130  140  164  174  243  60  78  113  142  143  171  197  208  216  241  252  272  294  309  314  339  365  388  392  409  410  422  424  468  500  513  544  595  602  613  640  643  670  674  686  18  25  53  74  80  82  120  135  162  242  249  264  287  295  302  336  341  349  359  408  425  455  458  466  470  513  8  26  49  83  86  114  115  127  17  20  26  35  36  41  53  55  57  65  71  81  103  117  136  138  178  188  16  17  29  41  53  58  67  74  119  121  161  164  169  203  206  233  243  275  290  294  339  354  365  381  394  399  410  437  470  473  27  40  55  86  114  127  143  148  164  229  243  328  365  383  409  413  418  500  503  506  508  563  565  566  568  569  572  579  13  19  29  47  60  61  69  75  84  90  102  112  120  123  140  161  165  180  193  203  222  224  268  274  285  292  303  308  309  326  22  24  32  34  37  40  47  58  92  100  112  130  166  175  189  206  212  251  333  369  378  382  389  397  401  415  425  429  434  17  64  83  118  133  225  295  314  322  361  366  391  439  441  453  455  477  507  515  580  615  655  696  754  770  794  810  814  817  836  840  886  898  47  60  71  73  111  129  179  184  222  234  250  311  318  341  348  351  352  374  411  425  473  502  605  646  663  680  681  715  718  721  771  9  17  54  63  106  110  121  134  177  189  246  253  263  277  292  65  69  123  175  203  253  261  275  278  279  280  287  305  308  311  341  342  347  348  351  355  358  359  396  398  399  406  415  442  485  486  16  29  32  35  40  59  103  108  115  117  130  136  137  145  156  157  237  243  252  13  28  40  49  50  51  77  79  83  84  98  107  139  142  153  165  166  24  54  70  91  94  105  114  117  128  155  173  230  233  235  255  271  275  277  285  290  317  318  327  347  355  357  359  363  378  380  403  13  17  49  52  54  58  80  100  103  108  121  139  189  201  219  220  228  232  251  258  265  269  283  360  374  387  393  401  412  424  434  475  513  13  19  95  103  105  108  160  161  185  192  193  235  238  252  259  285  307  319  354  371  389  390  392  405  437  442  443  450  460  470  471  19  23  27  32  50  61  63  73  117  123  147  174  175  199  202  217  227  254  285  326  349  352  377  387  420  466  467  476  500  520  524  525  61  128  130  152  191  268  438  814  885  901  1037  1135  1188  1222  1345  1451  1508  1561  1565  1583  1830  1953  2313  2424  2530  2537  2613  2621  2643  2654  2855  12  19  22  49  90  118  145  151  154  163  169  195  230  233  239  252  286  299  340  364  379  385  392  393  404  411  422  462  499  534  549  579  581  105  189  243  268  315  329  330  393  398  402  413  441  486  513  531  604  682  721  792  807  815  853  869  920  925  1000  1043  1057  1078  1218  1231  133  135  162  175  190  195  196  225  249  259  264  294  306  353  355  366  404  415  420  427  457  464  483  521  542  600  613  632  650  669  695  96  98  105  110  123  138  139  153  213  237  247  318  321  322  344  358  455  471  472  522  528  539  549  558  621  632  663  667  672  689  718  13  30  37  44  48  53  61  84  86  89  107  115  117  119  120  121  127  163  164  165  183  185  186  190  200  205  221  235  282  300  360  439  462  71  72  99  121  122  131  139  169  173  189  196  211  271  273  276  279  297  335  11  14  15  22  34  47  49  57  94  96  118  129  293  295  302  305  309  322  323  330  333  334  344  387  414  415  38  48  161  174  217  404  430  439  473  519  537  565  596  623  660  692  729  734  749  760  762  814  871  953  996  1046  1095  1113  1198  1268  1281  1341  1344  21  35  68  112  123  155  178  208  237  310  410  414  427  430  487  515  525  618  633  687  971  972  974  975  1181  1185  1190  1193  1200  1221  1235  1266  1296  26  30  76  94  127  139  144  154  174  198  204  216  232  245  248  252  256  306  312  372  9  12  17  18  55  63  66  87  126  131  139  186  233  255  272  275  290  295  365  20  31  32  42  45  95  134  151  201  206  247  278  307  309  319  334  338  346  369  372  383  400  408  460  477  483  485  498  505  509  520  541  547  18  46  50  57  71  159  205  304  319  339  355  389  402  448  454  468  471  498  504  543  585  633  638  652  684  724  740  840  869  935  985  8  9  21  24  38  55  86  87  110  120  168  174  187  189  191  193  237  238  243  282  289  310  313  338  397  419  436  439  462  495  17  18  30  35  70  71  73  81  84  93  102  115  138  152  153  161  195  204  223  245  260  271  273  292  295  320  328  336  340  344  374  11  83  94  145  155  163  185  9  42  37  65  151  220  223  226  73  91  102  116  135  141  143  168  174  175  208  215  219  221  222  227  228  261  273  280  293  307  308  315  316  326  328  329  332  363  365  368  380  104  123  186  377  387  425  451  551  573  589  708  725  956  1050  1063  1097  1139  1320  1330  1370  1431  1448  1466  1497  1551  1566  1569  1572  1613  1631  1633  43  94  98  217  218  222  235  237  245  246  247  248  250  252  262  305  310  320  323  358  370  372  384  386  390  424  446  447  462  493  548  29  34  42  48  89  148  162  164  212  235  253  266  320  322  336  337  376  378  408  444  469  473  533  549  550  554  616  627  638  644  657  662  665  10  12  31  62  82  84  93  95  175  218  305  306  318  325  328  333  347  368  397  30  53  80  82  164  174  180  195  244  274  289  317  384  410  465  519  539  578  582  614  656  677  704  708  716  722  739  772  783  888  917  954  978  15  27  31  98  116  123  137  153  16  25  36  38  62  94  101  146  157  162  210  232  237  255  293  299  317  323  329  331  335  349  351  352  363  35  122  133  238  248  270  283  318  327  331  339  489  520  659  692  710  725  726  731  732  736  740  749  780  789  820  825  838  853  857  862  44  67  23  25  48  51  69  92  113  194  219  226  269  273  319  372  403  405  458  465  476  509  513  540  560  599  611  614  629  636  649  668  701  19  23  28  89  111  123  141  143  146  153  188  189  217  241  242  245  268  273  277  287  309  326  10  16  18  20  33  41  47  51  69  146  210  246  250  261  276  280  289  309  310  317  326  16  32  37  55  62  95  104  107  108  131  164  171  198  225  252  261  282  302  305  316  320  374  399  410  412  418  444  10  30  40  63  82  122  124  148  163  167  182  188  216  229  231  261  54  59  146  177  189  196  205  234  243  260  271  287  308  337  366  385  427  432  444  453  462  477  480  506  509  511  512  542  554  563  694  9  16  26  29  30  47  84  109  131  139  165  182  193  203  236  248  249  251  256  262  271  284  290  293  307  323  350  365  395  428  439  37  42  74  80  95  108  114  123  131  189  190  221  227  16  43  53  57  75  76  87  95  99  138  143  147  148  151  162  170  183  190  236  262  265  303  318  330  346  378  380  413  441  443  461  10  78  109  121  136  139  158  186  238  246  273  280  284  295  323  331  340  341  367  371  381  432  8  24  36  45  53  60  66  74  75  92  106  125  214  221  246  257  263  274  295  305  312  334  338  358  368  403  413  426  427  431  435  16  50  87  101  161  172  186  223  275  279  281  314  359  11  23  44  46  59  81  82  99  123  194  206  207  210  223  226  246  248  264  310  320  359  363  388  411  433  451  458  486  491  495  544  554  577  40  128  138  179  193  210  271  277  325  330  393  413  420  434  446  449  452  473  521  541  549  552  556  620  655  664  680  710  716  717  726  774  777  11  40  56  91  103  156  160  164  189  195  223  234  260  393  395  432  472  514  588  622  687  703  741  758  806  866  881  882  901  910  919  960  961  26  53  77  167  183  215  309  371  416  423  463  464  520  528  579  631  636  680  702  710  749  760  769  848  855  857  904  912  922  1017  1027  78  178  276  337  422  441  678  720  831  850  923  926  932  963  1093  1131  1153  1295  1297  1302  1367  1582  1641  1707  1777  1855  2091  2165  2413  2514  2534  10  12  24  26  29  32  36  58  63  65  104  109  112  113  120  150  176  198  205  209  213  218  249  251  267  15  22  36  57  95  98  129  151  180  231  239  255  276  284  294  295  308  322  346  359  368  462  468  484  498  506  521  522  543  544  546  591  611  25  57  146  150  159  188  275  311  394  431  531  546  549  577  602  604  631  633  669  690  702  721  775  783  817  820  837  840  1033  1051  1052  1097  1154  24  82  103  108  153  194  209  210  221  228  234  242  248  269  272  277  287  320  339  378  405  406  453  454  462  463  478  12  43  56  13  217  415  417  464  475  555  614  621  634  643  673  1033  1058  1075  1076  1171  1210  1467  1741  2057  2078  2112  2157  2306  2407  2888  3003  3041  3057  3124  31  79  84  93  94  116  117  124  129  135  136  155  157  163  186  193  219  223  225  258  271  333  352  389  392  416  431  432  442  444  445  451  452  14  22  37  79  80  85  99  109  114  157  188  195  208  234  294  302  307  314  362  426  471  501  511  592  596  606  620  621  631  674  689  748  807  19  20  22  27  58  73  83  90  109  111  127  128  140  149  165  169  182  183  194  197  224  270  277  286  289  396  398  429  449  457  462  14  22  53  54  87  119  129  134  138  157  174  207  224  229  240  241  245  246  247  280  283  287  299  305  346  347  355  373  383  385  392  403  407  42  88  106  144  166  167  179  214  257  258  260  275  289  301  313  317  320  351  436  446  478  483  491  497  517  518  520  524  534  573  599  602  610  9  35  54  84  93  96  149  174  178  186  227  245  279  285  291  306  307  443  479  488  511  516  550  551  553  557  567  19  21  28  42  84  105  109  117  129  136  149  150  156  233  346  350  355  371  377  382  12  16  18  26  35  36  55  80  83  84  90  115  124  135  155  156  182  184  194  233  240  251  257  278  280  288  292  312  329  331  337  339  353  24  29  50  77  85  102  120  138  139  173  174  278  301  356  407  427  453  454  464  471  477  478  504  505  506  509  525  568  572  574  579  11  12  19  40  41  43  50  52  55  58  64  65  74  83  85  96  119  121  125  175  194  196  198  208  225  272  12  27  56  83  106  110  112  125  138  140  160  161  175  178  183  25  146  225  378  435  579  836  854  951  1004  1100  1235  1273  1409  1689  1711  1970  2121  2148  2221  2226  2289  2303  2312  2329  2341  2352  2439  2467  2470  2491  2492  2516  22  53  55  62  119  136  139  146  11  19  28  31  46  68  86  101  146  172  182  185  194  212  249  253  270  286  295  315  16  28  48  50  76  83  93  98  101  123  132  155  175  181  182  184  193  194  195  199  231  252  258  266  274  277  280  16  68  83  106  130  139  14  15  26  37  68  76  91  92  115  120  24  34  52  75  119  125  142  184  185  187  51  54  97  130  146  14  176  377  464  468  556  1084  1096  1205  1212  1254  1415  1433  1522  1530  1573  1609  1722  1739  1782  1803  1816  1939  1970  2005  2016  2055  2072  2074  2153  2156  2184  2348  21  94  103  105  149  163  183  184  208  217  229  257  276  298  336  340  350  366  381  382  388  393  405  406  408  419  436  473  479  480  488  19  200  360  380  440  459  469  480  482  598  636  721  728  932  1004  1014  1036  1057  1104  1133  1147  1152  1170  1212  1256  1339  1345  1448  1540  1556  1593  34  85  95  97  130  165  168  17  56  110  111  135  147  168  178  183  206  278  284  313  314  370  395  410  431  434  441  454  458  474  501  508  515  573  581  584  715  731  26  34  41  42  70  73  76  90  117  118  141  150  160  161  171  182  184  192  204  207  226  245  263  43  45  55  86  95  218  245  246  258  270  272  318  328  334  371  384  399  424  434  456  460  483  8  12  16  27  30  46  61  70  74  78  88  96  219  336  373  383  396  447  454  465  466  495  574  587  655  660  682  713  719  731  750  755  13  26  31  66  90  142  10  17  19  47  50  77  86  91  102  119  126  129  133  145  147  155  163  166  180  197  19  36  60  91  115  130  135  164  172  183  195  201  219  224  264  276  303  305  313  330  345  381  26  89  265  279  28  31  38  50  74  91  114  123  167  174  190  209  215  223  236  238  241  259  322  351  357  364  384  392  450  467  473  485  486  491  499  502  504  15  142  150  183  264  271  295  333  341  397  418  433  434  436  452  544  559  564  579  622  626  644  651  675  778  791  798  917  942  977  1078  1095  1134  26  36  51  79  95  97  152  154  175  180  235  270  346  363  380  401  428  434  463  466  518  10  15  17  31  100  108  134  202  227  229  234  249  300  301  317  326  332  365  371  408  423  443  47  82  144  204  227  230  247  257  353  417  446  449  451  452  454  504  513  517  582  594  595  636  650  753  760  761  871  915  974  998  1032  16  32  73  74  96  98  103  109  135  140  169  178  188  216  226  227  243  250  258  261  272  302  303  320  321  340  343  393  414  489  536  50  67  112  148  164  169  179  196  216  219  243  248  265  292  295  339  358  379  9  26  46  66  69  102  113  126  138  164  167  182  188  197  229  233  244  259  265  398  401  451  463  478  131  145  154  163  327  392  445  452  684  709  730  731  806  867  947  1012  1037  1183  1237  1309  1382  1394  1451  1542  1584  1675  1723  1948  2074  2081  2107  2350  2407  11  18  24  45  79  84  97  107  124  135  141  147  149  158  159  166  169  170  212  243  244  252  297  333  380  385  414  426  458  465  524  534  13  86  126  163  193  202  215  234  265  266  302  303  320  330  340  341  32  36  37  41  57  68  25  29  74  86  97  103  104  108  111  124  142  8  28  29  103  105  188  237  249  256  342  356  387  403  493  494  520  554  603  617  627  645  698  704  711  719  732  769  775  864  867  886  924  937  12  13  32  46  56  61  69  91  100  110  186  233  235  244  254  261  277  24  25  26  34  36  40  48  61  80  86  87  121  146  191  245  294  301  304  349  350  351  355  356  358  16  17  21  27  28  61  65  85  251  266  269  277  282  299  319  332  348  349  89  123  139  152  168  181  31  32  170  209  220  229  286  296  309  314  342  351  393  405  466  480  530  541  566  610  640  644  655  689  748  759  801  823  878  883  892  45  59  95  115  139  220  226  229  231  278  282  374  375  382  384  388  516  621  622  627  628  630  642  653  669  675  693  760  764  796  803  10  17  24  32  36  37  38  39  50  55  58  63  83  113  114  125  129  131  132  133  136  167  242  249  301  320  330  339  343  359  444  9  17  23  28  51  59  102  104  132  141  169  170  178  212  230  254  289  290  313  319  324  337  353  357  365  366  390  397  401  438  13  22  77  81  112  128  131  41  153  172  182  215  225  242  305  314  426  482  491  534  543  560  571  643  805  842  910  920  942  1018  1152  1178  1201  1299  1302  1381  1399  1449  1457  1458  22  25  30  35  48  50  62  65  72  80  86  90  102  113  148  156  159  173  192  198  199  11  18  48  51  55  58  60  95  152  191  217  218  229  241  282  285  308  319  323  353  356  357  379  395  401  403  432  441  442  454  463  235  322  422  428  433  455  494  601  609  687  983  989  1094  1269  1274  1296  1334  1336  1341  1388  1427  1431  1437  1454  1464  1466  1482  1526  1557  1563  1774  13  21  25  29  35  55  66  78  118  119  147  168  174  203  215  219  236  237  242  261  318  338  368  369  398  9  12  14  35  61  52  53  58  62  66  98  110  114  120  123  141  142  143  159  163  173  175  176  180  185  186  187  189  192  193  194  195  196  197  198  199  21  37  51  62  75  76  79  156  181  218  274  280  318  350  359  21  24  131  137  140  147  150  154  161  170  171  187  194  265  280  284  289  295  304  306  363  366  367  368  370  371  372  373  402  405  532  8  9  34  46  95  108  128  134  149  191  245  255  258  289  303  305  314  324  325  329  337  338  339  342  32  108  49  70  85  88  106  112  161  217  242  251  262  296  17  90  98  115  124  180  226  242  247  306  317  322  329  334  36  48  86  106  160  165  174  206  213  234  242  273  296  298  351  367  373  378  385  410  413  427  435  462  474  491  505  506  521  525  541  23  90  124  126  150  161  162  167  170  189  194  210  213  214  215  216  233  243  269  317  349  353  373  380  425  456  465  537  11  27  60  69  174  191  200  239  268  311  382  384  397  403  406  409  413  420  423  438  450  463  464  466  474  494  496  499  506  528  542  546  548  60  85  58  59  63  64  66  99  130  192  194  239  244  286  337  76  112  163  195  206  313  378  489  511  652  795  1001  1121  1178  1261  1263  1424  1770  1792  2052  2148  2201  2445  2483  2546  2704  2773  2807  2954  3020  3058  68  110  120  144  150  163  177  178  185  186  188  344  371  373  376  451  454  536  541  546  549  556  568  588  591  601  607  630  652  654  655  690  691  18  20  47  65  73  80  108  128  135  147  153  154  17  40  60  74  97  136  153  159  173  190  220  222  249  263  302  325  330  359  385  386  397  400  403  411  433  460  477  539  560  567  571  38  46  73  92  104  107  108  118  167  168  175  197  206  15  54  94  98  186  187  198  204  212  218  220  253  254  276  283  304  306  325  333  336  402  425  468  511  529  568  569  590  591  619  626  647  17  28  33  34  46  49  60  105  113  131  138  154  177  185  192  201  206  218  237  244  250  254  255  262  293  302  304  317  320  14  15  69  78  95  121  122  210  219  220  222  230  233  235  246  254  267  282  307  18  29  43  54  96  131  184  192  197  201  220  261  299  300  304  368  377  378  400  408  14  43  50  75  84  112  116  163  203  209  212  220  256  259  284  285  336  341  350  355  356  358  366  370  22  29  86  111  124  136  138  182  204  225  243  254  262  365  377  397  432  446  452  467  468  481  513  528  575  588  618  633  637  691  701  718  24  54  55  62  77  87  88  105  126  134  140  149  152  169  173  179  194  219  220  234  276  305  342  384  387  406  445  461  483  488  490  497  512  20  42  85  89  143  164  189  197  433  444  449  473  497  504  580  622  663  703  727  769  789  860  897  930  989  998  1005  1012  1027  1029  1087  1089  1102  26  32  34  62  83  105  113  117  139  9  48  82  96  99  24  34  47  62  80  81  96  101  115  124  148  174  176  179  191  12  23  51  75  147  338  356  362  381  410  413  417  432  457  463  494  501  518  582  595  603  605  613  627  638  699  720  757  771  835  838  16  53  61  110  123  132  135  159  181  214  345  368  39  84  159  232  237  331  337  340  401  452  487  511  553  554  572  591  617  646  652  748  749  780  797  836  852  858  859  860  936  965  1046  51  54  110  113  226  253  288  350  391  392  470  512  525  576  626  639  644  645  646  689  739  751  769  775  778  779  787  791  794  802  811  847  869  9  85  95  106  119  123  126  137  160  175  208  215  219  244  251  276  12  14  44  55  59  61  101  29  31  61  66  124  172  231  266  272  273  49  71  87  99  107  224  229  236  254  280  307  326  331  383  387  390  412  418  431  439  466  467  487  519  526  529  565  578  592  593  595  21  34  51  52  57  212  230  242  326  329  366  444  606  652  698  706  714  745  787  810  815  909  925  953  1076  1217  1238  1267  1295  1414  1417  83  114  183  186  260  263  285  308  312  325  442  444  446  451  455  459  481  525  649  656  661  667  675  695  754  769  789  843  886  922  923  926  1016  20  56  58  60  83  130  154  157  167  172  181  216  247  274  275  281  286  296  298  313  321  427  430  435  439  446  453  463  497  534  637  8  10  32  34  48  77  81  93  97  107  128  129  130  133  138  152  173  180  185  189  190  228  242  10  31  40  42  50  72  96  125  167  182  199  217  248  316  322  330  337  349  401  415  417  423  424  430  431  432  437  438  440  447  451  452  456  58  100  101  115  145  476  556  862  1042  1057  1127  1195  1224  1228  1244  1275  1410  1441  1469  1493  1572  1633  1643  1730  1749  1839  1877  1916  1929  1991  2014  12  46  50  51  52  55  56  57  58  73  104  111  42  45  50  55  62  67  71  90  117  120  175  193  195  197  198  210  37  79  112  125  191  211  231  290  294  304  377  417  485  487  494  495  497  533  625  627  656  658  697  701  820  840  858  863  873  894  896  66  82  95  172  188  252  253  262  298  312  339  358  372  376  381  408  425  429  466  496  502  530  578  612  613  623  674  684  694  734  785  11  50  63  73  152  174  229  265  355  364  376  378  392  394  407  419  431  535  557  577  582  589  677  722  776  896  994  1075  1129  1131  1147  85  95  104  113  114  153  160  212  226  232  242  249  253  266  274  279  296  298  302  307  308  317  324  347  354  360  365  367  393  404  417  57  95  107  126  137  138  145  162  205  221  224  225  226  230  262  273  312  338  345  358  366  386  404  436  444  461  498  516  518  54  97  165  173  176  199  240  274  282  305  310  313  344  345  350  355  377  408  464  481  483  484  489  491  500  501  509  513  523  526  527  529  534  66  87  145  167  180  185  233  308  331  334  341  413  435  467  468  472  503  544  548  631  704  721  750  775  795  819  843  901  907  913  948  953  981  28  52  63  66  79  81  86  87  106  113  121  122  138  152  184  190  194  231  236  248  273  280  282  337  343  351  352  378  444  497  553  35  53  63  102  112  155  166  196  203  211  221  315  342  366  377  406  421  439  471  515  566  579  610  680  716  740  762  781  789  904  931  936  945  13  37  40  42  73  78  101  171  197  276  282  326  352  416  444  457  459  462  511  541  555  581  652  664  683  705  748  765  770  775  784  787  794  31  48  50  67  75  87  99  133  144  156  175  183  195  196  236  242  259  271  274  300  307  310  333  334  339  344  346  353  359  361  377  382  392  79  104  107  120  150  196  211  218  226  241  242  268  280  37  49  82  86  150  192  195  204  215  220  226  273  294  325  329  345  65  80  105  165  207  234  370  409  425  597  771  773  840  841  873  949  977  1052  1062  1088  1100  1126  1298  1338  1350  1359  1441  1475  1565  1654  1655  13  53  66  73  76  124  130  11  32  33  36  76  78  96  104  105  108  124  125  130  157  166  172  184  212  223  229  233  235  249  250  254  269  271  280  292  301  309  311  313  29  51  85  96  103  110  118  21  23  41  63  90  123  132  151  161  186  187  257  269  272  281  282  285  310  363  368  375  383  388  394  403  406  407  413  417  428  429  431  441  51  52  64  95  185  212  276  307  328  343  382  392  423  447  461  465  471  473  575  590  617  641  655  671  674  713  728  738  740  743  789  31  36  43  52  87  102  150  155  190  212  216  220  230  32  59  71  83  96  97  118  119  147  166  174  193  200  212  225  230  234  243  248  286  290  308  313  314  345  356  376  386  388  389  418  453  18  23  113  121  159  184  202  260  300  355  430  431  434  438  440  471  489  492  494  513  533  583  630  645  687  694  712  800  810  839  841  66  117  135  176  181  209  221  248  296  426  437  466  467  478  482  494  512  514  516  521  534  540  545  567  618  634  644  674  704  723  775  20  26  29  30  34  41  47  61  67  70  72  74  82  97  113  123  128  130  179  192  195  206  208  220  231  326  337  350  356  358  359  370  391  8  16  18  32  43  45  63  70  71  80  97  104  107  108  109  114  127  147  149  170  190  201  220  228  240  249  46  52  60  10  26  27  42  43  54  66  69  73  115  147  152  27  46  63  106  145  167  168  175  200  202  203  207  218  285  317  352  386  399  410  412  415  430  432  19  29  38  43  49  63  77  83  10  59  113  170  179  207  226  227  229  287  294  302  305  338  355  360  361  362  364  26  31  74  93  95  103  105  22  30  71  83  86  88  89  124  158  9  18  23  46  71  81  122  123  131  144  178  217  224  227  229  260  263  285  312  338  341  344  16  23  24  28  50  52  73  79  127  167  175  19  23  85  86  112  158  159  184  185  187  33  40  133  152  155  170  188  220  232  250  263  268  297  322  325  350  354  382  386  406  11  20  42  45  49  52  59  107  108  117  118  121  129  132  138  140  141  142  144  151  17  43  60  75  111  156  170  174  180  191  216  222  9  78  86  94  21  37  51  53  64  14  16  31  40  48  53  72  75  77  81  108  10  18  19  55  58  62  66  89  101  9  37  57  71  72  82  83  100  102  137  161  166  36  51  77  81  82  89  91  121  127  141  154  188  276  302  340  347  353  355  367  372  401  417  420  430  454  11  22  42  48  85  100  129  177  182  207  224  268  284  292  310  26  92  123  126  135  26  46  59  64  65  117  148  156  178  187  210  213  217  233  15  45  64  129  96  67  74  110  114  135  35  56  9  30  35  80  87  97  105  23  33  36  46  60  64  70  77  123  141  144  154  186  215  229  237  239  248  257  271  276  295  355  371  396  400  12  31  39  47  48  49  60  121  151  154  164  26  73  85  88  91  123  160  185  53  78  82  96  107  139  148  20  25  39  42  78  79  98  114  145  154  164  188  194  94  135  150  153  78  103  31  67  72  74  84  97  98  108  122  136  147  160  161  189  191  201  207  227  245  265  275  277  279  281  316  331  11  15  62  65  85  29  51  59  100  122  140  158  14  130  138  152  154  159  160  223  21  40  51  77  99  110  147  153  20  57  84  115  123  134  16  18  20  22  26  37  39  43  46  48  50  52  56  63  64  71  77  83  85  87  95  97  99  126  148  155  194  237  266  270  276  278  280  49  90  164  172  185  188  190  194  239  249  274  347  387  427  438  477  497  514  531  543  569  605  623  646  647  672  680  687  688  726  781  865  915  31  32  50  61  14  21  64  29  53  54  55  18  20  31  65  68  15  23  44  46  75  100  105  139  33  35  63  67  72  73  80  82  110  114  115  122  124  146  148  152  157  160  161  167  201  205  209  242  255  276  278  279  288  292  10  13  14  35  45  95  116  124  128  136  165  196  220  234  238  256  265  277  283  313  314  342  357  369  373  384  390  397  399  413  70  89  95  37  69  89  93  98  24  26  30  43  75  93  120  171  173  201  212  225  238  261  285  21  32  36  44  136  209  313  8  11  69  125  148  149  205  16  35  54  62  143  147  157  201  234  235  236  237  241  267  273  283  302  325  340  346  350  352  360  18  21  53  76  83  107  128  157  163  175  194  205  224  291  316  329  354  383  396  414  416  454  38  54  140  147  151  158  165  170  178  187  198  236  237  241  277  287  340  379  419  28  51  106  149  194  212  217  224  277  287  294  311  336  337  338  343  347  348  353  356  357  10  45  50  73  90  14  16  17  62  107  129  130  160  272  287  348  403  432  443  448  462  473  479  500  540  583  589  593  601  602  641  653  667  697  781  788  793  795  13  31  55  68  82  85  94  95  157  163  171  172  188  189  213  215  222  243  251  17  28  32  40  181  228  258  280  304  319  453  484  515  547  602  636  651  677  679  699  710  722  764  820  830  839  877  914  924  957  971  17  23  24  25  35  40  53  83  101  104  146  171  180  199  240  251  297  304  306  377  383  386  397  446  478  487  499  9  10  11  52  93  138  180  192  253  308  312  380  393  428  429  430  448  454  461  469  475  490  494  496  497  552  562  588  616  620  640  676  28  41  54  55  60  88  101  115  143  144  150  168  169  193  208  212  254  260  333  345  363  389  404  413  438  458  470  496  506  75  505  728  777  882  1120  1160  1288  1323  1554  1631  2041  2124  2166  2567  2654  2662  2849  2883  3083  3109  3131  3191  3253  3370  3400  3402  3560  3688  3907  3993  40  46  48  63  73  86  91  105  125  169  266  303  322  355  360  441  474  504  515  546  553  590  599  608  704  711  767  770  785  786  808  21  22  31  39  60  63  79  83  88  98  110  118  131  146  23  25  34  83  88  119  125  132  147  166  176  178  108  115  119  129  271  324  372  508  545  576  632  651  652  663  697  713  714  726  809  812  841  845  865  910  929  952  1013  1019  1023  1025  1040  95  115  118  130  131  138  139  162  163  165  168  186  187  24  47  49  55  68  104  198  211  463  812  1081  1363  1497  1534  1556  1679  1705  1746  1753  1873  2269  2652  2745  2750  2850  2890  3305  3419  3556  3579  3593  4192  4200  4213  4272  4278  38  40  43  45  65  78  101  105  108  123  133  150  162  169  186  218  239  241  245  246  253  261  278  347  385  400  402  406  426  430  436  30  45  64  71  105  116  147  175  201  202  241  244  260  261  265  268  272  288  290  315  317  322  325  328  342  344  362  363  425  426  462  27  28  33  46  61  71  85  102  104  114  120  132  147  16  48  12  17  27  31  34  43  44  48  94  115  124  128  177  195  217  11  17  31  56  74  118  130  152  193  216  262  317  336  363  454  475  478  624  641  678  706  707  727  836  840  993  1015  1166  1223  1237  1238  1245  1247  57  86  138  155  156  171  178  194  195  204  222  223  256  258  290  293  296  312  317  36  58  64  65  86  89  90  110  146  184  213  235  312  319  336  340  365  391  410  417  436  448  461  475  478  479  480  484  67  70  97  131  146  147  185  299  326  403  432  663  687  707  717  810  825  886  938  942  944  958  963  1016  1080  1135  1136  1167  1222  1249  1462  1544  1618  13  43  68  123  127  136  156  174  218  250  251  261  266  275  283  319  320  333  338  375  393  412  428  434  438  457  461  480  498  501  513  22  69  70  99  108  123  188  201  214  222  226  318  320  381  386  413  424  457  541  562  584  612  711  727  739  762  769  802  812  814  820  26  59  66  70  118  128  187  188  192  267  271  302  307  309  310  315  330  364  368  378  382  414  420  435  673  687  690  695  746  764  766  799  817  17  22  78  148  149  150  154  160  188  190  196  211  213  215  229  256  269  286  290  298  342  350  370  373  388  398  400  401  402  406  428  19  34  43  58  59  61  70  145  178  195  197  199  204  206  231  30  31  37  43  77  104  105  131  144  146  184  193  235  240  253  279  285  288  307  318  327  332  336  337  340  349  352  360  364  375  392  401  403  15  26  101  119  121  128  132  136  138  142  145  147  155  157  159  161  163  165  169  173  175  177  179  181  187  189  191  200  202  204  206  208  212  16  58  66  105  22  25  34  49  91  102  109  135  137  188  222  228  243  246  248  251  253  255  256  266  275  290  295  301  308  310  338  369  394  421  424  431  434  44  107  144  208  238  240  277  326  393  397  475  476  477  486  494  495  557  617  628  684  719  791  799  809  905  922  930  940  1014  1039  1054  1080  1103  17  117  121  143  204  212  217  218  222  229  260  292  295  313  341  354  356  366  389  421  451  465  469  497  539  571  633  637  661  679  710  19  40  69  87  111  153  154  171  189  197  202  207  209  219  225  230  239  248  252  258  259  266  274  282  286  293  295  371  416  438  443  453  510  12  37  55  93  105  107  136  180  193  261  288  303  306  311  327  335  336  349  36  94  95  156  160  238  273  287  288  317  324  338  434  510  581  617  734  764  770  815  818  841  844  863  868  869  873  897  898  921  938  29  118  230  294  329  333  351  352  411  447  449  469  471  554  569  572  585  632  652  670  673  697  762  771  772  776  782  861  864  879  882  891  919  17  24  49  62  110  132  134  135  184  238  261  334  336  337  339  344  380  381  418  501  509  518  587  618  631  636  655  47  69  111  231  233  278  291  349  379  416  543  587  700  789  918  945  1002  1027  1103  1291  1463  1686  1689  1690  1823  1954  2447  2512  2564  2720  2813  2826  2859  57  73  135  153  166  167  201  282  305  306  309  317  334  355  359  360  366  378  403  427  458  512  514  544  566  646  687  712  713  744  757  13  17  31  36  94  123  142  160  164  201  269  276  405  425  555  570  582  586  635  648  654  658  669  726  735  754  931  934  937  956  1019  1084  1091  11  15  26  69  70  78  115  118  217  224  229  251  258  263  270  298  304  305  307  321  336  341  350  430  431  446  451  44  82  93  109  193  224  256  320  378  444  19  22  52  64  70  85  201  203  205  212  216  226  229  237  277  9  80  88  104  123  136  145  149  168  15  21  23  28  32  105  24  55  57  124  135  164  212  226  322  372  489  602  626  636  717  782  797  846  864  893  939  1098  1103  1133  1154  1189  1240  1259  1264  1329  1381  1386  1409  35  52  83  84  86  87  163  202  221  222  232  239  268  335  336  377  410  480  504  514  518  521  524  540  551  564  565  577  593  603  619  17  45  51  75  112  131  169  180  203  205  238  240  261  269  316  319  335  337  338  353  354  356  370  374  377  437  457  472  535  543  9  66  67  75  77  87  132  182  185  202  225  262  286  34  45  54  85  198  261  263  267  269  284  296  377  409  416  419  450  476  626  679  737  895  1041  1097  1110  1124  1144  1271  1273  1311  1469  1475  10  16  19  39  58  83  91  126  174  212  216  241  297  323  345  370  390  433  472  479  497  559  564  577  590  657  662  8  18  21  26  45  69  94  114  120  137  31  40  49  172  197  232  274  335  353  368  382  390  411  414  429  432  475  479  486  495  541  542  545  559  565  573  578  581  595  598  602  48  90  134  137  165  180  210  212  252  268  284  431  479  494  599  623  656  746  774  776  843  904  998  1031  1039  1084  1115  1187  1195  1240  1315  1381  10  14  18  31  32  33  39  42  52  71  88  91  143  151  155  174  190  191  11  22  56  88  93  94  100  107  147  159  166  180  207  210  212  217  233  248  264  278  298  314  329  353  358  503  512  571  661  683  718  744  854  19  39  83  86  205  208  214  232  324  332  342  466  476  491  499  503  517  537  551  567  596  606  613  639  649  661  670  684  689  697  700  704  708  13  15  18  48  66  78  92  94  108  109  131  45  54  74  92  106  179  248  265  281  305  307  355  357  443  461  522  531  539  556  568  593  603  664  705  716  725  745  759  773  850  992  11  16  37  57  82  89  117  133  173  215  240  249  306  331  341  342  359  466  528  533  546  559  575  626  631  647  49  68  216  285  348  406  414  458  703  729  750  819  830  853  854  891  963  986  998  1053  1179  1244  1289  1356  1443  1450  1543  1549  1585  1612  1648  1721  1762  8  38  51  86  87  93  156  212  247  275  289  309  342  353  366  371  378  386  394  436  538  543  553  564  572  575  576  578  592  632  649  78  98  129  150  151  158  176  181  206  211  222  228  263  269  285  287  324  346  361  364  384  387  392  406  428  429  430  434  445  453  461  79  125  140  183  211  213  238  293  298  311  331  352  381  447  481  527  543  545  578  580  589  593  626  645  680  689  691  692  693  712  725  14  19  26  67  97  106  121  181  183  208  214  223  233  239  296  317  11  69  114  158  191  197  206  211  217  277  343  380  381  387  409  447  460  473  494  497  504  511  514  524  557  577  589  599  600  603  620  14  16  33  36  67  69  105  119  143  152  177  189  217  218  224  231  244  262  276  279  287  292  293  294  301  329  331  332  343  358  30  32  60  77  80  106  126  152  181  191  9  26  79  96  99  123  140  145  174  179  195  198  201  216  247  299  302  319  364  382  433  436  441  460  461  470  492  502  535  542  576  12  14  15  18  20  24  29  35  44  51  58  70  90  94  113  117  137  150  196  202  272  281  379  388  390  394  407  422  14  18  23  25  28  43  56  72  73  74  106  110  122  145  154  164  203  25  43  60  64  67  73  150  155  178  183  202  206  245  247  255  269  285  293  26  44  48  62  69  75  84  93  101  134  137  162  164  171  196  197  225  247  389  418  445  460  493  534  543  545  553  557  77  94  96  136  149  166  230  245  308  450  470  498  530  552  564  588  598  607  664  700  728  757  767  774  793  800  810  820  856  867  881  898  985  137  138  226  475  674  762  932  1135  1149  1225  1562  1669  1774  1858  2124  2215  2301  2364  2595  2645  2646  2735  2763  2946  3168  3178  3308  3416  3483  3880  3925  3997  4523  91  116  119  126  131  133  182  199  201  205  207  209  215  217  219  221  223  225  227  231  234  238  25  28  35  53  98  100  101  110  128  197  240  284  313  340  408  435  481  498  499  516  569  581  582  589  610  643  647  672  704  717  732  8  26  50  59  65  67  77  80  99  125  143  191  196  209  226  238  253  291  299  341  346  361  416  424  430  446  471  538  566  582  618  635  676  8  45  48  50  52  56  66  67  78  115  123  142  205  213  259  262  265  335  360  363  366  396  496  509  544  565  631  739  824  871  889  892  899  25  58  67  175  189  195  205  211  262  282  293  298  328  342  349  354  359  367  368  387  397  411  420  421  463  522  597  601  615  626  642  668  695  19  28  52  84  89  90  120  121  147  151  163  211  240  249  250  251  261  265  282  295  296  312  318  324  327  340  341  352  11  21  22  28  29  71  81  85  87  90  92  110  113  124  134  138  152  170  197  211  242  260  269  287  299  307  309  311  315  377  383  12  17  27  75  82  83  89  97  103  107  135  138  169  203  216  229  236  257  273  276  294  35  86  120  134  187  258  278  284  309  315  336  344  349  375  388  405  470  481  502  504  505  531  621  641  644  651  653  659  685  695  700  74  76  98  121  124  219  225  266  270  281  330  344  413  420  427  481  505  520  603  659  790  822  827  861  908  943  946  995  1068  1080  1110  85  119  132  151  155  159  165  189  233  244  272  319  322  334  339  349  431  452  469  475  496  542  564  575  617  623  642  647  665  685  721  44  62  73  83  96  100  110  112  146  159  164  195  197  208  234  241  245  259  263  266  268  280  293  312  315  335  336  348  355  378  381  394  417  33  45  48  66  115  132  144  175  176  201  280  308  324  386  418  422  429  438  443  460  491  494  499  558  562  564  629  643  674  680  734  17  21  30  32  72  128  140  141  145  159  204  209  212  214  37  45  60  75  89  132  152  175  184  261  290  298  303  363  386  390  393  394  418  432  447  448  468  489  505  509  521  532  550  580  640  10  31  41  43  46  48  55  75  89  93  130  140  142  143  183  233  244  252  258  265  273  274  277  322  12  49  51  63  64  65  104  160  193  194  205  210  212  230  231  246  247  266  268  275  283  326  338  368  378  389  394  400  408  444  491  26  28  68  88  103  105  107  109  116  140  143  146  161  166  205  214  225  66  186  205  273  285  291  295  312  318  356  360  368  371  384  432  491  492  522  543  570  626  636  686  771  835  881  901  913  968  970  972  979  991  31  39  54  76  107  121  155  165  234  235  247  250  251  257  265  287  292  295  299  307  365  367  369  457  468  482  483  29  280  333  345  356  372  439  447  482  532  533  557  674  698  765  786  830  867  876  877  894  943  1037  1067  1160  1288  1304  1320  1481  1485  1554  20  36  46  56  149  190  225  384  435  442  506  550  674  687  691  700  710  711  744  758  783  785  805  812  823  840  844  854  860  863  868  872  873  20  35  55  60  63  64  83  87  88  89  102  109  123  160  249  250  262  266  304  319  327  348  359  384  408  453  454  455  467  476  483  16  19  28  35  36  44  81  93  101  112  113  147  156  164  181  183  184  186  188  205  31  95  168  194  211  236  256  300  314  319  379  416  581  586  606  623  717  730  731  736  761  895  937  940  944  957  961  962  975  991  1045  1050  1066  9  10  66  68  90  113  116  120  135  178  199  204  218  243  249  251  260  272  276  309  29  112  113  120  139  148  154  290  297  309  331  343  344  393  409  449  539  560  575  599  611  624  670  710  720  739  772  809  834  856  859  21  33  62  118  119  124  126  127  130  152  172  187  191  216  223  236  255  266  288  297  301  348  384  392  396  483  515  530  586  592  607  16  62  64  77  90  107  138  213  223  231  247  277  283  286  463  561  587  599  667  711  748  768  782  822  860  1003  1094  1128  1130  1170  1213  26  54  72  92  114  218  225  237  306  314  317  319  328  335  339  366  376  381  412  416  447  451  467  470  480  502  508  551  566  570  594  609  644  34  56  65  136  234  246  247  261  344  372  419  495  533  562  756  758  796  824  835  841  905  917  938  956  977  1494  1536  1551  1592  2116  2203  50  52  99  104  105  119  156  160  179  186  194  209  216  220  223  227  240  285  312  313  314  315  336  340  379  383  414  419  421  433  439  13  31  41  57  64  80  82  99  103  112  117  122  125  151  152  159  174  185  229  230  257  318  322  332  338  345  347  362  376  379  380  28  38  76  79  110  133  137  139  155  156  165  249  259  262  281  333  351  382  392  402  419  422  449  491  511  528  529  10  12  13  14  16  17  18  41  47  69  122  128  144  167  190  197  200  26  35  74  83  87  129  132  133  137  139  142  144  148  149  188  211  221  222  238  248  276  287  305  329  337  341  345  365  380  389  431  14  31  44  70  132  150  151  186  210  235  282  300  322  333  404  410  460  472  489  496  548  552  562  585  604  620  623  679  720  772  776  794  803  9  15  18  37  40  49  62  72  79  88  106  108  129  206  211  248  285  316  323  330  333  341  344  355  360  369  385  392  406  421  444  70  113  137  147  208  209  218  245  277  324  395  403  480  503  532  572  580  656  693  695  713  721  750  751  755  794  836  841  843  894  896  9  13  58  87  134  137  146  151  159  167  209  215  229  241  243  289  293  300  308  317  330  390  450  469  481  484  492  493  10  23  92  93  118  119  155  157  186  192  194  196  198  206  208  212  218  222  224  230  257  263  272  278  293  295  297  310  324  332  334  16  25  30  40  80  89  93  114  120  130  148  150  185  14  17  26  33  37  41  43  49  89  102  103  126  138  164  170  200  223  280  355  364  433  437  460  466  596  600  665  681  730  756  816  38  39  47  67  99  119  170  180  208  230  250  253  256  262  280  286  290  339  354  362  388  396  21  32  52  54  143  195  218  254  275  303  322  342  346  378  430  14  26  45  50  111  132  143  188  191  192  200  244  252  257  263  266  279  318  319  325  344  345  346  353  13  14  19  27  34  39  48  64  75  96  99  121  132  157  168  189  191  196  203  212  219  230  238  241  253  254  256  264  291  318  320  341  391  12  32  39  51  87  96  127  137  175  206  212  230  237  257  289  315  319  322  332  341  342  360  456  493  527  528  531  557  584  600  608  611  614  49  225  363  421  433  497  549  644  698  824  864  875  891  1104  1281  1362  1411  1673  1695  1770  1799  1863  1878  1905  2000  2162  2376  2581  2591  2764  2812  9  62  71  98  112  133  158  174  201  208  235  239  241  273  287  288  289  306  317  322  342  346  421  426  438  453  466  499  522  556  565  17  19  52  53  111  116  127  139  150  231  241  266  270  289  291  349  401  406  419  446  449  457  458  505  522  555  558  561  576  603  618  627  675  12  71  74  100  109  184  196  251  262  291  344  354  379  389  393  407  413  415  435  447  474  490  508  518  533  545  562  572  677  741  774  776  58  100  109  124  131  158  167  208  221  230  294  298  320  324  346  364  403  406  408  18  19  20  39  40  41  54  92  97  116  165  166  180  219  220  225  226  284  288  34  66  95  96  106  114  116  130  27  59  60  75  97  109  132  142  175  178  210  238  243  262  275  276  314  367  380  391  408  451  458  463  464  498  506  520  525  600  606  10  17  38  41  46  47  88  95  102  138  153  209  215  229  261  269  276  277  296  308  326  338  368  377  384  402  427  455  499  506  507  12  15  23  30  41  48  50  53  76  89  92  105  110  167  193  219  232  244  248  252  259  269  299  303  304  309  315  16  20  56  64  129  182  186  308  324  328  338  381  391  394  450  463  476  497  526  533  565  570  594  613  621  669  692  714  762  765  771  806  849  25  33  40  48  55  56  75  78  95  115  138  145  153  168  176  188  230  234  239  278  322  335  338  339  364  365  371  382  413  420  43  46  47  62  67  107  113  125  130  148  152  157  16  18  19  32  38  41  42  43  58  65  67  72  98  105  111  114  115  126  138  147  154  79  115  176  191  267  404  424  435  437  441  476  536  580  834  1140  1213  1464  1468  1607  1616  1645  1648  1893  2183  2240  2355  2449  2460  2465  2552  2615  12  14  25  44  91  15  32  89  113  243  291  309  335  342  345  346  357  362  373  376  416  441  457  470  471  499  520  521  536  553  554  555  559  581  610  657  69  78  85  88  113  117  120  145  148  197  236  246  255  263  265  267  290  346  348  406  409  410  434  435  26  79  109  114  146  182  276  280  315  327  331  343  358  362  367  394  395  397  436  447  451  456  471  479  494  513  522  533  567  595  635  637  23  40  42  70  83  127  152  187  191  202  203  211  219  249  269  285  299  307  320  324  363  391  27  56  97  122  130  137  156  198  215  226  241  267  277  307  347  352  356  371  431  467  15  44  72  74  107  109  127  135  166  206  214  219  223  227  232  236  240  241  242  258  266  276  306  324  339  356  358  364  396  398  406  14  26  29  33  36  37  83  92  107  114  154  156  182  190  224  232  234  252  370  378  389  402  415  420  446  451  479  481  514  518  520  521  16  31  46  58  59  98  126  128  149  170  191  197  221  232  236  238  276  284  311  336  345  9  21  24  26  33  36  43  46  47  49  53  143  148  174  187  224  296  313  339  362  381  408  412  455  477  542  560  622  624  625  630  8  37  49  97  127  128  163  169  301  311  324  346  494  502  509  510  512  513  514  519  522  555  605  635  639  640  645  657  660  668  671  40  51  71  109  111  112  122  126  134  151  152  154  156  174  198  19  96  115  119  134  178  244  250  254  292  304  310  320  321  322  330  349  407  414  439  488  509  563  569  583  590  636  637  651  719  726  25  29  31  34  65  70  72  81  99  114  117  132  151  16  28  38  91  152  161  174  210  227  228  287  293  307  314  347  358  390  396  399  431  466  468  470  485  495  505  517  574  575  593  618  645  666  11  18  37  58  122  130  142  152  153  165  170  173  175  190  212  227  265  271  273  275  301  21  28  75  84  86  107  111  117  144  147  159  161  199  222  234  255  271  279  287  305  344  360  365  368  372  382  392  415  442  443  23  42  50  87  107  140  205  208  219  225  227  232  234  254  269  280  283  291  295  320  337  383  418  422  427  435  443  464  481  489  490  514  525  31  40  78  91  102  149  161  169  179  293  296  313  352  353  398  417  421  423  477  489  533  642  646  652  696  820  841  928  934  938  964  966  1058  22  24  28  33  40  118  128  165  172  243  256  275  294  305  308  322  365  378  380  388  409  413  432  441  453  465  467  477  480  501  509  8  29  49  52  55  90  114  132  149  150  162  166  179  180  259  270  287  391  430  438  467  485  556  558  704  710  712  729  744  812  813  835  878  27  36  38  56  83  91  101  114  146  161  164  175  190  192  203  207  221  240  315  319  388  408  411  416  37  108  130  133  156  168  215  225  227  242  261  449  495  513  527  561  751  770  895  907  929  978  1028  1059  1188  1195  1256  1257  1263  1308  1355  80  123  156  204  206  235  270  304  313  332  370  392  404  445  484  493  514  549  551  559  591  599  606  627  651  653  732  752  755  768  789  31  44  79  90  91  96  100  151  206  218  275  317  344  366  376  377  383  497  507  519  521  522  530  539  560  582  603  627  650  673  695  8  14  30  80  114  115  119  131  136  143  163  177  179  226  257  259  282  292  304  331  337  385  405  427  442  448  495  518  521  522  524  540  542  83  165  308  344  354  492  514  554  567  603  612  628  631  636  705  761  822  923  930  962  991  1051  1136  1215  1254  1255  1263  1273  1292  1296  1386  17  66  80  90  113  116  263  267  273  287  289  290  301  304  316  346  358  366  367  377  385  389  33  47  81  98  106  109  117  135  150  156  172  187  192  209  224  261  277  282  298  303  322  331  332  345  348  417  418  426  432  438  447  457  512  49  77  99  143  199  291  332  336  337  361  375  389  394  399  473  520  571  583  606  667  678  746  747  752  758  762  778  788  789  866  930  967  969  101  142  379  434  462  559  607  652  743  864  1120  1181  1644  1745  1848  1859  1940  1955  1980  2052  2137  2152  2194  2210  2275  2405  2428  2509  2523  2624  2633  9  12  22  31  39  59  62  90  114  124  156  173  192  228  239  242  248  249  250  268  282  285  304  101  107  161  181  189  190  204  230  231  254  257  271  307  334  337  386  412  413  426  526  532  546  579  587  623  640  643  660  676  706  712  10  15  22  30  64  79  104  113  127  177  234  249  279  295  337  341  346  354  409  412  414  421  13  20  23  28  59  70  73  77  110  111  112  114  115  117  120  122  133  179  182  217  220  221  222  227  228  236  247  40  52  58  66  68  74  76  82  84  97  106  117  121  125  127  186  193  205  228  246  256  285  290  301  333  336  337  382  384  394  404  410  469  72  73  137  170  194  195  202  309  333  372  375  378  384  395  425  426  495  503  510  563  576  584  621  624  667  685  690  734  779  803  818  11  172  199  246  262  459  464  581  799  802  910  1066  1092  1099  1104  1135  1218  1315  1349  1672  1738  1822  1904  1953  1964  1969  1970  1972  1976  1979  1996  12  30  90  134  191  232  237  245  248  252  270  273  304  313  326  340  353  383  398  399  400  405  410  431  448  449  511  515  556  586  608  661  713  29  51  62  196  279  358  367  378  382  383  397  400  432  446  466  485  544  592  598  601  635  671  727  728  753  754  821  831  833  874  893  904  911  30  82  112  118  123  131  133  159  173  175  221  231  251  259  266  296  303  336  339  351  385  402  405  37  78  82  103  113  116  118  119  124  184  208  212  225  248  265  273  303  327  331  354  363  372  17  40  63  214  233  319  327  472  643  650  655  657  662  665  699  703  736  740  762  786  799  800  803  813  815  830  947  985  1059  1073  1110  14  113  225  432  458  662  815  816  841  883  894  916  920  960  969  991  1125  1205  1281  1308  1325  1475  1501  1623  1660  1704  1797  1820  1926  1936  2031  23  136  196  248  292  376  453  485  495  516  544  598  713  763  882  988  1112  1132  1143  1154  1225  1294  1460  1471  1522  1523  1581  1594  1711  1719  1753  1766  1810  20  70  95  119  233  234  237  249  323  390  419  421  497  513  585  609  641  656  674  701  743  807  879  951  972  987  1018  1086  1108  1126  1231  49  100  105  118  125  184  188  194  233  235  236  275  291  306  335  338  352  363  382  400  405  468  486  544  549  580  602  19  33  96  111  162  179  202  205  215  218  219  235  241  250  14  36  39  54  102  118  142  159  289  306  318  383  495  514  549  653  715  735  924  943  951  962  1015  1033  1042  1076  1089  1092  1093  1178  1213  19  39  233  323  334  794  822  852  896  1019  1117  1142  1188  1192  1333  1337  1377  1465  1481  1529  1538  1552  1613  1649  1710  1719  1809  1838  1976  1997  2029  79  101  254  344  438  575  609  626  696  768  909  977  1034  1138  1259  1268  1296  1299  1300  1360  1362  1407  1454  1493  1545  1559  1573  1589  1614  1757  1789  1799  1825  14  28  35  80  81  109  112  128  170  172  188  211  215  253  265  293  319  329  349  352  353  549  556  590  612  618  619  622  637  670  744  29  49  74  75  80  99  141  153  181  372  401  417  449  483  550  552  559  565  567  670  719  730  789  792  847  853  871  894  967  976  980  19  20  32  34  69  70  80  93  101  128  130  156  159  160  167  182  239  341  348  360  361  417  425  469  477  488  506  516  529  537  559  23  66  307  327  350  404  445  459  488  499  563  577  585  595  609  636  637  642  657  668  697  713  718  728  740  782  824  829  836  864  867  873  876  19  65  88  141  183  287  294  296  329  415  438  494  519  522  539  664  694  705  768  810  1006  1236  1321  1329  1332  1415  1475  1476  1502  1674  1699  16  21  26  57  60  99  109  113  115  119  129  170  182  186  187  192  197  209  217  219  230  256  305  316  338  375  386  411  424  428  451  36  51  106  110  133  149  157  167  201  219  242  258  275  301  328  341  346  351  357  405  411  413  437  440  446  450  471  472  477  508  61  78  82  99  118  129  134  146  150  151  167  182  194  198  214  222  73  99  200  239  264  266  271  389  439  504  526  594  688  700  705  731  839  868  894  909  927  971  975  1002  1032  1051  1066  1075  1085  1089  1100  1113  1123  81  92  120  122  178  183  186  207  246  274  278  279  335  349  361  578  581  594  597  609  615  641  654  669  716  757  789  798  819  830  859  12  41  50  64  67  108  110  142  170  187  201  213  246  284  298  400  421  461  476  502  527  539  566  570  572  582  610  633  708  715  764  799  853  77  112  158  161  228  287  317  332  367  479  595  649  745  1103  1109  1130  1183  1203  1278  1280  1288  1308  1352  1500  1637  1682  1806  1856  1947  2062  2082  40  42  52  56  83  107  184  192  233  273  298  325  341  8  28  29  35  37  39  40  76  88  89  11  14  18  24  30  33  54  59  63  91  110  120  133  138  160  161  162  178  192  200  213  218  14  58  104  110  143  151  199  200  220  244  247  250  257  263  282  288  290  305  306  313  314  316  326  333  338  341  345  348  353  359  378  387  388  8  16  40  63  74  78  79  139  158  208  249  280  351  383  400  467  471  539  558  642  650  679  699  700  703  714  758  769  778  795  806  914  924  43  46  48  86  88  117  118  127  153  177  183  193  222  225  229  232  246  251  269  272  279  15  18  22  33  79  82  99  107  118  119  122  123  135  138  139  169  206  261  266  284  311  326  372  428  434  435  478  504  531  568  611  612  627  14  20  29  56  86  93  133  214  231  258  277  292  323  333  353  357  364  371  386  432  444  449  455  459  464  502  563  568  720  724  731  14  19  44  49  70  81  104  113  138  160  165  197  207  248  256  359  368  470  478  561  42  137  203  214  228  231  248  277  307  329  344  27  64  66  73  97  102  111  124  151  158  163  164  182  186  198  222  236  269  284  331  332  340  348  350  356  371  376  389  407  409  413  422  458  13  14  21  24  30  32  66  69  94  110  140  141  144  150  156  169  182  193  230  10  33  45  48  59  65  72  73  89  91  96  112  115  117  123  124  135  149  170  184  213  214  218  233  235  242  252  263  269  270  287  11  23  37  51  67  77  79  80  115  118  136  140  159  161  166  185  201  222  250  262  263  299  10  22  23  24  33  42  44  88  106  143  147  148  154  157  158  169  177  205  230  248  259  263  270  315  343  375  377  389  394  25  27  30  32  34  35  38  53  55  58  67  75  95  106  112  117  128  166  187  190  199  209  211  247  259  271  317  337  347  362  377  393  396  31  50  58  69  73  134  197  219  235  265  277  337  354  379  421  460  469  512  522  563  565  567  578  716  763  768  809  829  888  890  892  52  63  70  102  131  168  193  288  289  298  407  413  439  486  488  503  521  566  631  632  648  670  810  860  867  890  891  903  951  973  1044  1073  1088  54  323  447  476  754  782  974  981  982  1045  1099  1202  1565  2041  2169  2211  3116  3176  3250  3284  3479  3491  3565  3716  3730  3828  3900  4221  4272  4323  4670  14  26  52  53  56  57  62  63  68  72  99  107  121  24  34  39  44  64  72  82  100  124  10  11  13  32  67  91  146  155  202  222  229  252  255  307  324  350  378  380  398  403  408  416  426  21  35  39  52  67  70  74  79  80  81  83  90  94  99  103  110  112  117  118  120  121  127  137  139  141  202  251  274  276  287  297  8  25  63  71  83  85  91  108  138  140  151  158  182  188  196  203  260  270  282  312  314  315  360  379  396  426  433  440  445  479  515  43  69  136  158  159  234  247  292  315  25  27  40  67  79  179  227  302  311  344  433  444  454  464  535  550  581  630  642  679  774  817  853  905  963  975  997  1046  1129  1140  1228  1251  1263  159  184  227  242  250  255  348  392  398  403  425  429  553  630  659  714  718  720  749  751  891  902  910  914  928  948  978  985  989  1009  1040  21  22  64  94  95  108  110  8  28  30  155  202  208  221  244  273  288  306  332  353  368  376  415  439  533  629  634  648  650  673  683  689  720  746  760  767  774  787  23  24  32  86  119  143  159  163  234  247  284  322  331  332  344  350  373  384  403  427  497  504  505  550  557  569  604  617  619  832  857  860  883  25  63  90  121  141  151  155  175  182  189  202  219  226  227  230  242  277  291  307  329  360  13  18  19  20  22  53  68  125  131  153  185  222  250  272  299  327  369  417  431  451  18  43  116  152  168  189  209  230  234  242  244  273  286  295  302  14  26  34  65  76  97  113  119  159  178  200  201  205  227  228  229  230  337  338  12  23  40  49  62  64  65  113  140  161  173  194  195  225  255  269  288  293  294  338  343  380  390  402  410  412  416  422  427  431  455  456  497  14  137  182  267  268  272  290  302  315  328  342  357  360  364  374  376  396  453  458  486  494  514  515  516  519  520  535  540  554  592  610  10  14  51  151  289  296  581  599  671  673  692  693  762  784  812  823  979  1079  1090  1109  1116  1146  1194  1245  1263  1281  1289  1335  1371  1416  1451  1470  1525  45  81  108  192  243  280  308  312  314  317  319  333  334  336  346  51  71  92  101  102  115  135  142  147  156  161  189  192  215  231  249  272  308  343  383  400  424  440  441  461  9  12  16  27  40  55  140  155  156  157  165  38  41  42  48  60  88  94  99  100  111  127  135  146  158  166  175  176  197  202  221  229  232  241  242  318  27  31  40  50  65  70  72  77  78  80  87  91  116  10  22  181  245  269  299  365  438  460  469  605  609  647  725  729  741  963  983  998  999  1010  1063  1091  1154  1208  1209  1356  1388  1419  1461  1617  1689  1852  11  16  36  38  52  58  85  116  130  163  193  219  259  263  289  339  344  373  391  395  396  399  401  404  413  445  452  453  455  457  469  8  227  231  261  278  324  389  430  437  468  482  488  516  534  602  610  664  666  668  730  762  853  910  970  1092  1197  1254  1265  1275  1372  1433  21  25  47  79  82  88  103  105  110  118  139  199  214  227  233  260  269  276  293  296  306  308  317  351  353  359  397  417  433  19  59  69  142  169  193  213  216  232  240  271  283  299  307  334  378  401  404  419  421  422  13  25  26  27  30  45  49  53  55  60  78  124  168  205  247  252  334  347  372  410  420  423  476  536  548  551  552  553  554  555  113  116  146  174  198  200  232  20  25  29  40  42  52  78  115  126  149  154  158  177  190  199  40  43  44  73  76  87  106  114  212  220  228  229  271  280  363  375  405  406  433  438  474  475  533  551  596  605  610  619  639  664  690  11  13  15  44  103  116  142  143  150  152  153  169  170  182  185  186  202  205  206  218  240  258  259  267  278  281  290  303  313  481  505  508  545  33  94  103  111  136  142  153  164  168  230  253  259  294  306  310  335  347  351  356  360  363  383  387  399  400  408  414  433  448  577  597  24  25  26  27  28  29  39  90  108  115  122  129  137  142  152  161  180  236  251  253  258  260  51  66  98  105  114  148  153  165  173  193  194  196  199  233  254  262  266  267  285  306  324  350  366  367  390  403  404  434  445  452  466  486  496  69  80  81  121  126  205  297  356  372  380  392  418  495  533  537  559  561  573  575  576  578  579  584  632  659  672  713  773  791  813  826  896  916  19  25  29  78  89  132  163  166  289  297  313  349  360  366  367  370  373  375  408  416  428  434  561  590  591  615  701  714  755  833  944  23  24  41  54  121  173  201  221  223  232  238  242  266  267  269  281  307  397  410  465  468  474  488  492  511  8  10  15  16  20  27  37  43  50  58  97  115  137  189  226  263  270  337  341  343  344  345  348  352  356  456  457  15  26  31  74  78  100  109  141  180  181  196  241  246  251  266  271  281  286  291  299  302  304  308  349  357  371  373  400  415  428  449  459  470  15  19  35  55  78  97  101  160  244  255  297  300  303  307  345  371  433  493  569  570  594  597  598  602  604  633  634  635  746  765  774  59  64  80  91  95  99  109  110  121  124  132  139  162  171  179  185  186  193  200  208  225  249  259  8  25  40  45  60  69  96  101  109  113  116  159  163  179  203  206  210  277  302  310  326  341  344  358  425  445  448  458  471  505  508  18  49  63  80  88  99  103  115  139  143  163  166  170  173  175  180  192  230  238  253  262  266  270  25  38  46  55  69  73  100  119  120  255  272  274  302  304  307  312  324  341  342  345  361  426  474  487  500  553  564  594  599  601  609  20  61  84  87  36  56  61  99  102  136  138  161  169  183  196  216  228  245  257  296  303  311  318  335  349  376  384  393  408  432  457  465  485  487  36  57  64  102  105  120  127  146  156  175  187  218  234  237  274  282  325  328  329  333  350  357  366  373  409  410  428  437  439  443  481  89  103  145  183  199  247  317  328  363  386  450  462  477  487  493  511  558  564  567  569  571  591  592  595  599  627  665  684  688  700  704  14  15  31  51  65  166  186  205  274  366  380  410  415  451  484  502  509  517  581  586  587  592  641  643  653  668  692  751  761  776  786  30  110  112  113  132  145  166  180  181  211  277  312  384  401  415  418  424  438  465  480  494  506  530  546  550  568  668  680  752  88  99  60  75  115  147  167  174  186  214  221  274  311  332  341  345  361  394  408  443  456  26  55  78  143  156  157  158  173  192  201  228  231  244  250  276  284  291  303  304  323  326  337  345  382  386  28  47  53  54  67  71  102  107  139  153  162  201  237  256  287  294  304  314  319  322  330  352  360  369  389  396  397  408  428  448  19  53  65  131  173  208  215  308  311  330  380  396  433  444  451  452  463  504  513  539  674  675  676  677  683  732  737  755  758  790  793  823  841  36  57  92  98  139  150  220  234  273  301  304  324  328  338  364  376  445  449  466  482  496  497  554  581  591  594  609  620  626  644  645  646  719  8  22  26  34  52  83  84  112  153  171  172  180  192  197  211  233  269  272  273  283  299  303  362  403  431  438  478  485  487  510  512  13  22  67  128  138  168  185  188  193  224  253  260  271  279  283  312  319  323  333  375  390  393  463  507  527  543  556  597  636  655  724  73  76  78  192  196  319  338  339  392  445  578  610  615  618  620  635  648  652  669  687  729  759  770  778  783  889  922  927  946  976  988  15  36  68  73  75  79  93  99  103  111  114  115  136  153  168  177  184  193  200  207  210  211  214  221  224  226  229  239  253  256  13  30  118  193  206  212  229  256  263  271  276  295  296  317  331  341  357  376  383  424  439  458  499  504  509  540  541  547  591  619  626  647  652  14  84  137  214  278  338  386  431  485  644  759  769  774  790  791  807  810  820  835  864  870  900  913  943  945  946  960  1004  1042  1054  1081  1157  1163  52  77  108  225  291  362  465  470  713  750  776  778  844  913  927  996  1213  1247  1280  1310  1414  1417  1457  1498  1531  1640  1702  1729  1732  1809  1831  8  22  43  46  65  95  124  136  137  154  159  215  223  233  273  307  319  320  326  349  350  366  368  369  384  389  392  402  434  436  437  470  503  129  245  345  372  375  407  415  425  439  441  447  502  504  533  545  555  586  631  712  724  744  920  930  1044  1124  1141  1290  1291  1296  1337  1340  1343  1378  10  13  16  36  45  46  47  64  81  85  89  100  106  110  157  160  175  182  17  37  46  58  73  89  98  127  128  166  182  204  205  207  219  221  229  238  245  254  259  266  271  281  286  294  296  320  182  208  215  422  471  504  549  568  666  681  775  879  1019  1116  1118  1141  1179  1186  1406  1425  1540  1544  1550  1566  1596  1640  1695  1734  1787  1807  1810  11  21  36  53  71  161  171  179  204  238  251  261  288  315  321  327  334  385  407  415  418  423  424  427  438  524  529  557  577  591  611  612  659  20  22  25  46  48  49  82  87  104  106  198  206  214  220  224  232  239  281  303  305  323  341  345  374  378  389  393  394  422  424  449  49  65  95  97  141  142  173  178  195  237  300  339  342  346  348  355  366  391  398  400  401  415  419  460  464  465  504  516  546  579  628  12  16  19  21  23  40  46  96  122  133  137  144  152  162  163  215  225  243  259  263  285  325  330  338  396  24  37  58  66  151  174  198  220  252  260  264  268  290  37  44  116  128  165  183  199  239  260  266  297  304  307  334  350  351  364  378  384  391  407  503  509  520  713  761  796  804  847  905  925  933  1016  88  100  107  125  176  190  225  239  263  272  293  302  303  311  314  322  326  329  333  399  406  410  441  476  477  538  546  616  655  657  670  8  15  23  41  148  154  164  180  194  215  250  266  10  38  73  105  119  129  130  154  193  207  214  220  277  282  303  314  330  353  356  367  371  372  374  394  396  420  448  468  491  527  544  8  11  14  19  40  67  96  98  114  141  170  204  208  217  222  224  244  290  295  296  304  331  341  363  378  383  403  457  468  525  538  540  555  33  194  215  252  255  281  405  407  408  435  498  633  678  718  742  961  982  988  1096  1151  1217  1323  1326  1387  1419  1421  1465  1717  1731  1744  1847  19  24  30  45  90  132  147  154  163  197  198  205  224  234  243  244  287  339  378  387  50  71  121  212  269  270  333  365  421  435  446  457  465  477  485  489  500  595  597  715  729  828  871  907  925  939  1125  1151  1192  1208  1212  1237  1243  37  48  52  57  81  90  96  129  132  139  147  151  156  172  176  190  194  211  213  217  221  228  253  270  27  35  47  52  72  78  106  114  192  198  211  216  224  228  69  89  135  155  213  219  18  22  27  37  44  49  61  64  78  99  129  130  142  165  210  248  251  295  320  386  396  405  416  417  447  452  454  466  496  512  542  576  579  27  267  344  381  406  459  484  643  672  674  750  794  813  906  1131  1171  1212  1429  1463  1616  1620  1627  1629  1631  1671  1679  1691  1697  1703  1707  1724  9  41  43  47  86  93  96  98  105  107  124  134  136  140  145  153  157  173  185  187  206  215  219  232  233  236  243  261  286  288  289  295  305  10  35  50  53  68  92  139  150  177  183  208  289  295  300  377  499  532  534  602  618  658  679  717  722  842  851  863  874  876  949  972  15  21  23  30  33  65  74  93  111  124  148  149  155  164  185  192  196  205  214  24  30  42  44  97  154  167  219  225  234  247  260  273  358  370  420  489  493  566  605  609  705  795  802  812  826  884  908  918  920  1013  13  47  59  66  81  89  130  227  239  294  311  320  367  400  403  413  453  454  480  505  507  510  523  35  57  128  182  200  219  250  378  496  505  507  574  580  586  589  599  600  605  612  613  643  646  662  665  678  682  683  698  706  709  722  23  89  227  302  352  529  578  608  616  790  1189  1234  1301  1303  1316  1325  1387  1396  1444  1464  1507  1567  1575  1578  1602  1647  1691  1728  1766  1772  1781  1784  1801  32  38  61  66  85  115  140  149  154  160  181  191  194  202  214  216  218  221  222  225  230  271  340  351  40  67  80  107  133  141  143  200  234  250  308  311  320  355  359  379  385  393  403  404  409  412  425  426  430  431  434  435  440  447  484  505  515  33  39  65  79  88  92  95  106  109  119  121  126  128  136  140  151  154  162  166  172  179  48  62  69  82  84  98  115  119  126  141  148  205  240  252  260  275  281  283  287  293  305  332  341  372  378  381  385  395  396  408  411  412  419  30  32  43  64  66  75  78  80  82  90  92  110  113  124  129  136  137  145  148  156  171  199  239  245  246  268  271  278  15  17  19  40  50  73  82  126  127  132  135  143  151  164  171  192  193  209  211  219  220  242  288  333  351  382  393  394  398  20  23  24  28  35  41  44  72  77  130  162  182  188  192  195  25  26  46  56  88  94  104  108  127  171  175  216  224  237  293  311  31  59  70  103  105  107  111  121  172  174  178  195  212  226  240  246  255  322  328  345  357  370  389  420  465  500  28  153  160  179  203  232  238  262  292  301  309  327  337  424  468  475  537  569  594  644  653  654  700  707  721  837  841  847  849  887  928  127  236  364  475  560  665  714  819  979  1027  1173  1236  1253  1305  1329  1512  1619  1640  1713  1756  1764  1928  1934  2017  2020  2057  2079  2094  2189  2217  2233  2312  2327  12  93  152  164  165  169  205  206  226  251  268  306  307  309  328  353  400  434  445  466  467  488  524  544  548  597  603  624  632  633  717  783  866  12  51  65  72  76  98  105  114  117  138  148  149  163  164  184  185  192  193  195  221  232  265  388  395  406  417  421  425  439  448  486  12  20  29  41  42  44  46  51  92  104  129  147  148  169  179  183  208  225  226  240  250  287  311  315  341  347  351  358  409  413  416  29  45  106  115  116  125  140  156  166  171  185  198  213  221  234  266  317  353  417  427  460  507  563  573  592  616  625  647  671  707  735  22  50  55  84  100  123  134  146  147  165  198  215  217  219  227  240  256  260  277  280  305  339  349  351  361  406  449  462  52  186  191  225  228  270  323  336  391  392  443  455  464  492  497  554  577  667  672  700  716  794  795  847  849  861  876  909  920  1060  1086  16  19  32  35  59  78  80  178  197  200  212  220  225  232  258  278  322  333  338  354  21  115  352  645  667  767  795  1026  1028  1037  1042  1043  1058  1122  1138  1162  1177  1231  1234  1254  1260  1278  1280  1302  1373  1415  1421  1501  1568  1585  1625  1628  1661  8  31  37  45  76  128  132  140  162  175  205  208  223  247  258  267  270  332  350  354  357  421  431  444  449  477  489  511  513  532  554  601  609  13  18  21  38  66  78  89  91  93  125  139  200  230  266  274  276  278  336  339  341  345  395  412  415  422  441  453  506  508  554  601  26  29  47  58  59  83  93  120  128  139  161  182  193  218  228  229  230  238  242  243  272  275  282  288  297  328  343  344  11  86  92  120  145  158  167  273  275  276  277  302  312  317  345  377  419  424  440  450  461  464  473  478  519  522  530  540  543  572  574  11  27  44  48  76  77  90  106  126  138  143  152  174  183  185  188  192  200  221  228  236  291  300  307  309  310  339  360  370  377  383  9  10  23  41  45  64  70  102  110  125  132  142  169  179  187  190  202  203  220  224  237  260  324  327  328  343  345  347  361  13  32  66  94  110  113  150  189  211  214  246  255  269  296  297  301  305  306  307  314  341  377  405  418  425  430  14  57  63  74  75  198  259  282  289  299  326  383  394  395  398  399  402  403  442  491  494  496  497  498  499  506  507  515  519  524  27  102  120  174  202  230  232  233  327  362  401  435  440  462  473  547  584  588  589  598  608  624  651  652  665  695  708  725  735  742  782  52  63  72  108  142  162  174  219  233  240  250  304  312  366  421  446  477  493  523  528  572  592  640  667  718  720  721  727  731  734  757  759  772  44  139  140  152  214  218  281  304  313  436  556  569  574  597  622  651  790  822  845  853  860  933  980  984  999  1012  1066  1069  1112  1130  1154  1160  1224  8  9  19  36  71  95  106  111  126  129  138  145  154  170  198  202  203  20  29  50  53  77  94  97  107  108  111  116  120  136  139  141  145  153  170  174  183  194  204  215  222  224  246  296  349  350  359  369  384  386  73  74  75  110  115  132  137  148  221  236  288  292  318  346  356  385  388  404  431  470  490  505  523  545  546  568  569  586  631  632  660  673  700  52  126  150  189  238  254  267  300  362  372  456  474  508  527  536  538  578  600  619  628  638  642  679  681  727  755  774  781  785  816  834  884  887  10  24  30  34  62  66  69  85  100  107  126  133  152  178  182  191  198  246  251  265  293  335  337  12  33  55  57  68  75  81  86  92  113  119  127  133  134  137  162  169  183  185  190  210  211  213  225  265  292  293  304  308  309  317  319  10  19  35  97  193  200  265  280  291  329  334  340  375  377  379  405  415  422  31  48  65  76  90  162  184  190  201  223  229  232  237  238  270  288  310  329  334  335  358  369  372  388  404  415  421  422  426  429  503  16  46  47  52  54  60  61  108  122  148  154  195  212  216  219  222  245  264  315  317  321  324  326  330  335  339  341  349  369  371  377  387  412  42  131  138  184  200  220  221  260  337  364  651  652  686  809  942  955  1012  1081  1146  1178  1187  1310  1318  1329  1535  1536  1652  1684  1690  1716  1769  1797  1969  15  42  65  107  159  167  195  216  266  329  333  348  349  373  382  383  385  394  404  415  447  452  461  476  492  510  527  578  586  592  607  38  46  73  103  115  135  136  139  147  151  185  192  224  227  233  15  78  87  135  163  195  212  217  226  242  243  244  272  323  347  381  399  406  409  423  441  449  462  463  496  497  501  526  534  544  551  22  89  114  127  252  286  315  333  335  350  352  512  534  551  553  568  620  627  640  653  666  671  695  698  724  727  735  772  804  817  826  14  54  70  114  134  138  140  145  170  185  210  236  249  280  281  293  297  344  361  364  613  624  663  698  721  725  818  928  942  964  966  19  52  90  95  150  162  173  197  209  221  225  229  235  255  275  276  278  282  286  10  30  78  81  86  93  100  105  117  119  138  145  152  156  162  172  177  216  329  345  355  392  411  415  419  449  480  496  500  506  508  529  530  15  87  170  204  206  212  243  290  323  397  434  442  534  542  556  632  712  722  860  926  975  1002  1009  1058  1087  1194  1221  1295  1318  1319  1327  12  20  21  31  32  33  55  65  85  97  98  121  132  144  146  153  154  162  31  93  130  143  144  150  156  162  208  234  239  262  282  310  335  336  355  373  409  411  13  60  152  248  252  317  328  367  417  443  459  460  492  535  558  561  569  611  620  651  720  795  801  833  839  840  905  913  926  957  961  989  1034  8  18  34  78  94  111  118  142  145  183  194  234  239  25  58  69  115  124  212  229  240  254  299  313  344  345  378  467  470  491  499  501  508  510  607  617  627  641  676  708  722  744  752  761  22  26  33  53  68  71  95  108  111  112  130  142  249  268  316  333  334  335  342  375  400  592  626  632  654  655  701  790  804  863  944  53  69  74  80  86  98  118  119  127  163  198  215  240  252  264  268  273  290  297  303  358  381  409  410  411  415  417  472  479  481  486  8  11  15  18  20  27  31  34  36  38  41  50  71  93  97  99  102  190  235  243  255  311  312  347  354  358  365  382  400  416  417  9  27  53  65  75  80  102  108  127  145  162  195  213  241  243  272  275  280  305  321  322  324  326  345  349  353  355  366  66  83  91  97  106  119  135  138  144  199  271  317  324  352  381  447  461  464  487  491  501  522  526  529  549  553  556  564  575  582  595  10  14  22  26  32  34  37  41  49  64  132  156  160  169  199  213  46  81  151  301  305  307  415  417  419  428  606  612  637  652  657  661  662  693  727  734  735  778  812  832  834  881  905  965  975  981  982  8  9  10  11  64  65  90  102  122  154  159  164  169  199  206  227  242  292  294  304  326  330  331  336  337  340  343  351  355  358  359  360  364  12  15  20  21  75  89  100  116  135  172  199  223  319  337  339  367  394  396  397  410  412  447  448  458  512  523  540  550  610  633  653  655  658  13  17  40  41  49  90  92  142  143  28  41  67  69  156  178  213  228  269  278  281  335  348  376  449  451  460  466  485  544  553  589  590  593  600  602  608  614  626  644  670  39  66  70  90  102  144  149  162  164  196  206  214  224  238  239  241  242  246  263  266  305  329  330  335  343  347  349  364  378  426  467  15  21  33  43  85  92  93  126  160  206  221  226  243  247  262  271  274  306  316  348  354  373  386  438  440  16  29  38  126  130  140  141  145  176  194  201  64  84  140  150  151  158  166  174  181  193  198  199  227  301  323  327  347  355  360  361  363  387  398  514  528  553  556  586  609  612  745  748  782  108  147  151  152  255  257  261  262  264  270  287  425  429  432  441  444  448  457  538  541  584  644  654  706  733  777  841  914  949  974  1003  46  50  53  73  88  90  98  100  122  193  197  199  207  215  226  238  245  249  263  271  274  279  293  311  351  356  364  366  24  27  33  34  56  82  86  94  119  123  124  144  163  169  173  178  182  206  218  221  233  282  289  295  345  348  359  381  382  402  420  31  40  52  75  88  105  120  128  129  135  175  207  213  220  234  251  262  275  280  291  320  338  388  402  474  499  518  547  567  602  611  690  698  18  41  82  177  187  200  242  284  286  290  294  356  358  360  361  363  365  367  379  492  494  496  498  590  592  657  664  667  683  685  725  14  22  57  60  81  94  119  183  187  216  222  225  231  240  243  248  252  256  267  277  297  305  309  323  326  327  329  330  338  344  356  410  466  19  21  45  46  50  81  112  131  140  148  153  154  157  165  178  187  188  196  198  207  222  44  92  136  197  236  265  285  504  590  606  653  704  734  763  766  780  791  803  806  836  881  886  887  1082  1171  1173  1218  1231  1246  1305  1320  19  43  81  117  165  176  195  254  262  265  284  302  307  308  365  367  397  478  519  534  611  626  627  640  675  681  746  789  804  824  843  848  912  9  58  62  79  92  107  133  137  157  167  182  195  203  210  215  247  253  275  315  326  358  431  433  460  467  477  485  488  518  528  546  561  568  102  119  197  200  233  255  276  284  337  344  371  375  379  381  385  406  440  463  464  502  549  558  559  563  568  574  609  625  649  650  664  13  22  32  90  100  103  104  117  124  133  144  156  180  181  214  21  35  38  126  133  135  141  150  152  154  170  176  178  201  206  209  215  219  253  260  266  287  295  348  354  357  369  371  380  391  392  14  85  96  103  127  130  137  200  240  249  275  290  307  318  325  409  413  422  425  461  474  497  512  513  521  567  575  589  616  641  643  47  83  148  161  183  191  226  298  302  306  326  347  357  392  408  409  415  416  423  442  444  505  507  545  563  579  600  627  631  632  645  11  17  27  32  35  75  77  105  115  117  143  148  161  164  170  176  207  210  248  260  262  266  267  296  304  327  391  392  395  401  403  404  406  52  55  80  83  92  106  113  128  132  161  202  204  215  239  259  280  323  334  335  373  378  393  397  417  439  454  458  10  13  36  37  39  86  87  94  153  157  191  204  231  233  239  240  246  307  360  9  89  91  127  148  153  188  191  221  246  249  262  274  372  373  383  447  462  465  485  487  490  492  563  564  599  636  658  662  666  668  16  37  38  48  69  70  84  119  124  151  152  156  179  185  191  199  208  217  236  245  251  254  270  285  317  322  335  346  387  390  406  93  114  182  198  221  222  240  246  247  280  281  295  328  347  382  393  490  496  498  508  520  522  532  548  555  559  608  641  660  691  717  10  11  12  21  45  60  66  109  112  153  155  180  224  268  269  280  306  319  15  20  23  37  44  47  48  51  56  60  74  91  94  147  149  150  170  211  222  229  232  20  183  244  254  273  285  287  292  293  302  310  312  355  453  461  505  567  621  652  665  733  744  777  796  816  818  11  13  20  31  55  82  87  91  112  113  116  119  120  122  146  168  171  181  216  245  294  302  312  317  325  330  335  336  346  348  376  387  395  12  14  18  39  84  97  161  179  185  200  213  217  235  239  266  277  299  334  380  387  414  430  441  454  485  511  522  539  553  574  616  20  21  54  87  94  108  147  164  180  194  196  215  238  268  282  288  302  308  309  315  348  377  396  404  455  518  519  566  568  574  631  13  51  52  56  60  64  67  72  87  88  98  108  120  145  228  234  236  248  270  284  291  325  336  8  15  25  33  36  37  38  40  42  43  47  121  127  149  23  25  26  40  58  63  64  66  89  91  97  115  119  143  156  161  165  9  12  14  15  17  19  80  145  168  203  211  235  237  238  252  260  267  274  279  280  286  291  292  308  316  347  358  374  389  12  24  37  51  55  128  162  214  229  230  247  252  257  266  272  284  286  300  321  330  357  397  418  427  462  470  485  486  489  498  508  75  116  130  141  161  191  192  203  222  238  247  252  259  274  294  311  331  337  349  353  362  364  392  403  415  442  447  454  457  503  505  522  531  18  19  23  25  26  32  42  52  124  126  146  149  151  153  188  192  205  220  224  278  308  315  328  329  335  338  340  372  380  398  403  408  482  71  73  193  275  384  388  399  413  430  432  434  437  441  443  450  480  496  512  527  587  635  643  651  655  669  670  676  682  689  721  758  16  61  120  124  177  180  195  268  303  350  381  393  418  509  588  661  755  771  827  856  865  868  1009  1030  1069  1095  1159  1473  1484  1530  1591  1755  1871  94  95  100  114  123  152  201  210  248  380  428  596  598  623  665  748  781  801  881  892  932  955  1046  1103  1107  1146  1149  1214  1222  1304  1335  47  82  99  104  126  129  173  279  380  431  433  544  588  594  615  618  639  658  661  684  697  734  781  850  893  898  906  972  1006  1011  1012  1028  1065  13  37  55  73  47  50  127  171  179  185  188  192  199  220  243  251  288  337  353  389  400  415  421  430  434  438  453  458  524  568  586  627  661  678  705  23  27  53  58  104  115  127  169  187  247  248  253  260  269  291  297  333  367  369  380  399  441  445  476  490  544  545  16  20  24  90  92  96  120  122  144  145  164  171  188  213  220  250  267  42  49  82  132  139  168  197  201  203  210  211  225  239  249  266  304  317  464  507  508  550  595  644  654  751  790  797  816  907  928  940  13  31  47  85  103  108  115  169  180  188  261  289  340  348  362  366  381  416  421  423  454  536  546  582  600  618  632  637  642  718  730  770  792  12  54  65  79  81  95  124  129  133  141  144  151  196  197  209  219  225  229  243  263  269  293  327  329  330  332  335  368  374  375  421  432  439  37  78  113  120  123  125  138  172  196  213  239  252  297  298  303  316  340  347  360  373  376  384  387  430  443  463  469  504  511  528  579  580  624  14  37  38  39  44  45  46  48  53  69  77  84  87  92  93  108  119  142  147  156  168  8  14  41  50  51  52  53  54  55  65  66  67  68  69  70  72  88  99  127  137  147  186  196  212  45  56  98  133  154  168  188  211  217  248  275  276  367  426  434  459  469  475  496  500  558  589  594  598  630  657  694  736  786  810  882  892  931  9  69  99  149  350  365  378  392  399  400  415  496  532  572  573  632  641  713  719  757  798  808  855  1042  1065  1167  1200  1277  1335  1344  1352  19  34  39  44  60  96  100  122  132  142  222  230  296  309  312  357  10  15  27  28  66  104  145  151  28  41  130  136  216  219  232  265  387  464  465  515  521  576  622  688  698  706  720  754  760  794  804  883  897  912  927  929  988  993  1032  46  50  59  70  84  93  97  100  113  120  146  160  167  174  177  197  209  211  225  247  255  260  269  293  318  340  373  376  408  444  448  27  31  33  39  49  65  66  92  113  154  171  204  27  74  278  279  283  289  318  413  476  533  766  772  921  965  991  997  1069  1078  1140  1233  1318  1362  1395  1453  1515  1534  1587  1660  1761  1778  1779  1788  1839  42  56  57  80  96  109  127  135  172  177  191  196  207  235  273  336  341  342  344  350  403  416  417  451  497  498  504  510  520  580  594  603  643  46  47  49  52  96  113  134  146  150  156  157  168  169  190  222  227  229  230  236  245  249  255  260  288  298  336  351  352  373  383  388  54  102  108  146  183  208  237  263  276  303  350  394  403  410  472  525  530  561  565  575  832  840  893  1030  1039  1046  1060  1086  1116  1141  1147  43  105  114  133  178  181  193  203  233  262  263  274  296  317  319  342  344  347  354  362  367  378  384  30  31  43  48  59  76  87  102  108  127  139  173  175  178  207  221  225  240  111  189  269  309  330  337  378  438  504  684  729  731  781  871  883  979  1251  1311  1333  1361  1517  1628  1665  1677  1744  1859  1913  1988  2246  2295  2429  2445  2486  9  29  34  74  78  132  148  159  182  192  199  211  213  224  232  245  256  286  513  559  592  737  780  811  823  923  941  962  979  1058  1105  1150  1194  29  30  40  56  57  210  249  292  331  372  382  479  482  531  563  601  622  624  628  634  643  645  652  685  700  730  790  793  845  861  971  1116  1118  22  58  70  82  136  137  139  142  177  232  305  321  331  362  371  385  407  457  458  471  474  482  493  549  556  562  564  570  571  572  574  275  323  329  432  594  609  636  691  732  843  927  990  1037  1047  1049  1228  1266  1293  1306  1448  1463  1510  1544  1598  1693  1757  1847  1919  2056  2168  2175  10  36  48  8  9  12  14  15  19  34  35  39  75  96  112  129  153  157  176  264  283  286  293  333  408  444  447  448  452  469  506  510  518  8  71  72  75  81  99  100  122  132  148  150  152  153  157  166  183  193  197  204  212  213  214  215  273  275  277  297  305  345  352  365  369  376  51  195  268  307  404  437  554  654  922  1252  1295  1398  1402  1536  1754  2219  2276  2501  2543  2687  2728  2811  3028  3107  3196  3799  4007  4028  4311  4407  4550  18  49  74  91  94  129  147  156  183  191  195  200  285  316  320  355  358  369  382  396  411  423  429  436  437  439  446  453  466  481  515  516  524  27  93  114  139  318  417  471  728  803  868  889  938  945  976  980  1078  1436  1533  1599  1618  1657  1687  1754  1791  1881  1915  1923  2307  2322  2418  2421  138  186  240  283  586  670  678  1031  1100  1113  1126  1136  1247  1326  1436  1523  1539  1567  1583  1593  1594  1595  1597  1623  1634  1703  1706  1730  1745  1752  1866  1960  1982  16  18  25  36  44  51  54  55  59  61  66  76  94  97  115  128  148  157  180  201  202  226  228  231  289  330  364  375  387  438  442  539  554  48  145  159  215  240  255  266  267  325  333  349  350  356  365  374  386  463  472  512  517  545  625  630  631  691  712  746  776  788  809  833  876  899  9  92  97  152  154  215  247  409  430  436  449  499  513  599  600  694  720  812  821  851  925  956  962  968  985  1042  1052  1057  1073  1084  1092  24  58  71  95  139  148  153  177  201  227  231  232  251  252  253  254  270  276  284  296  307  320  39  98  108  200  230  299  325  326  366  368  430  444  446  447  457  500  508  540  547  554  618  630  685  697  704  728  778  803  817  818  825  19  45  50  143  200  203  211  212  246  295  301  310  319  328  330  343  395  456  508  614  689  691  723  759  760  816  822  825  830  850  876  894  1022  10  15  16  17  25  32  34  49  57  58  59  71  74  97  124  144  145  146  153  54  59  99  120  125  129  131  132  144  174  191  200  213  319  333  341  392  395  430  461  563  581  616  670  675  15  20  121  122  129  133  145  149  151  177  186  216  223  224  295  309  324  339  405  427  519  569  575  629  643  711  721  722  834  842  952  14  51  71  91  98  145  164  177  179  184  192  193  209  218  220  225  23  24  53  73  74  101  112  125  190  200  245  263  272  274  276  285  309  333  386  388  390  425  439  447  471  472  475  476  483  484  487  33  38  98  102  135  136  137  138  155  162  168  190  202  215  218  221  272  311  356  389  393  407  416  419  431  433  445  449  457  477  497  80  84  116  140  175  177  182  188  199  202  203  208  227  236  241  311  378  386  388  410  469  573  575  578  583  596  598  605  630  636  639  49  79  84  114  131  151  155  207  227  252  253  255  270  282  290  330  486  506  522  600  608  610  672  698  710  752  842  893  967  1026  1071  48  114  132  170  178  184  335  350  362  423  488  493  495  520  556  582  685  693  722  729  736  755  823  902  915  917  921  940  963  974  1005  1016  1030  18  24  92  122  128  131  132  147  148  158  159  176  182  243  349  353  398  407  426  436  438  476  478  516  556  601  625  641  665  668  678  17  24  26  31  118  124  145  32  46  79  15  28  29  36  39  67  68  93  101  102  104  115  134  140  147  162  183  185  186  187  198  227  247  250  253  255  268  323  12  20  40  68  99  101  109  111  126  144  156  165  177  200  203  214  216  229  247  258  262  275  284  319  348  411  480  486  515  530  540  12  16  18  26  85  90  99  128  130  134  136  15  26  34  86  147  159  183  219  220  226  252  253  254  314  357  410  413  420  447  452  555  601  636  638  646  683  722  729  732  753  761  766  807  40  57  69  100  104  108  115  137  11  27  47  73  87  106  111  117  122  130  134  145  158  170  185  188  191  198  199  211  223  281  288  323  344  347  391  407  411  476  489  19  23  25  56  94  116  117  148  149  155  173  203  212  88  92  116  123  146  150  155  157  168  173  183  187  202  203  211  212  221  247  47  55  62  83  126  139  158  176  189  214  244  265  12  15  17  20  21  39  43  121  136  137  144  152  172  229  257  272  279  296  315  379  385  390  421  423  466  469  474  8  10  33  100  101  107  121  125  126  129  160  171  173  183  184  230  251  279  280  288  312  315  318  345  392  400  403  429  430  468  553  11  50  56  74  76  90  146  149  169  178  183  184  192  204  205  26  33  34  43  55  63  94  102  103  154  155  174  176  220  233  247  252  261  270  278  294  298  303  304  312  24  45  56  72  101  109  114  168  170  172  197  229  272  290  13  14  16  21  87  99  118  146  150  190  191  192  204  205  214  215  9  15  66  71  115  118  227  245  256  277  280  315  335  337  356  363  399  401  429  431  447  464  470  478  492  538  559  609  620  624  648  12  79  92  93  122  155  194  207  15  17  19  73  90  118  122  129  170  171  174  206  212  218  234  262  269  272  297  308  321  346  367  370  384  415  439  473  513  526  528  39  43  46  70  96  37  67  88  92  111  146  153  161  188  208  223  16  111  265  267  275  324  365  441  442  453  454  508  525  538  540  549  550  562  569  571  585  590  605  615  634  667  668  670  675  682  693  14  16  28  69  82  85  87  90  110  145  162  188  217  218  235  237  267  278  315  330  359  364  379  392  399  411  443  499  500  508  20  28  31  50  70  107  110  112  166  193  195  204  208  264  268  291  328  335  357  364  367  372  377  381  389  23  40  44  50  54  68  82  86  136  146  159  167  169  172  195  196  197  273  283  287  336  348  353  398  418  422  426  445  446  449  453  457  472  9  18  19  21  29  32  41  45  50  57  58  61  80  87  95  98  100  125  175  178  188  192  195  208  213  230  237  246  71  104  112  113  116  130  139  152  186  210  214  224  241  261  265  306  343  367  376  377  380  381  442  447  452  480  516  528  539  541  542  560  593  54  99  144  149  259  302  306  328  337  354  363  372  375  406  431  435  436  443  450  457  458  460  477  489  496  497  500  512  527  529  556  23  27  44  46  70  71  97  99  153  224  228  249  259  285  300  303  316  8  33  78  86  87  123  128  142  196  210  218  231  239  256  257  259  263  284  289  32  52  76  85  115  137  142  145  147  148  159  162  164  195  211  214  217  260  282  298  313  318  319  332  342  354  362  372  377  379  391  567  572  15  29  31  33  58  66  68  80  108  17  20  27  62  63  66  81  97  102  109  120  129  141  142  147  169  172  210  347  362  383  390  397  402  417  421  10  18  26  40  85  136  157  162  175  188  193  208  217  242  257  275  280  287  312  315  429  471  514  567  569  601  628  679  706  747  868  42  69  71  78  83  94  150  157  212  219  237  238  276  284  285  346  356  368  501  514  516  527  531  533  536  586  598  636  663  727  769  773  816  30  32  58  73  87  113  130  168  170  194  223  229  235  249  299  313  315  324  326  341  359  370  387  390  417  439  446  58  65  119  125  143  165  211  216  232  242  246  258  271  278  350  356  391  451  472  541  582  605  606  619  641  697  830  872  895  954  991  1073  1140  15  27  34  37  98  99  149  167  218  251  253  280  354  400  406  413  464  466  473  478  494  496  504  524  533  534  543  545  553  567  661  666  710  11  52  55  97  114  154  165  168  169  188  197  209  220  234  242  259  278  282  301  308  310  338  346  8  9  14  28  42  44  85  102  220  225  244  331  339  367  375  385  391  405  433  465  469  488  491  497  506  515  549  589  592  619  630  645  652  16  20  27  28  47  56  116  126  132  147  160  166  167  215  228  242  266  277  290  292  307  309  311  315  327  350  357  360  396  408  414  415  14  17  19  22  27  37  70  93  94  99  109  115  152  153  156  158  178  189  198  208  213  273  281  285  290  298  34  78  120  127  209  285  286  301  322  347  417  456  464  503  510  523  551  600  617  624  634  722  804  826  845  859  860  899  926  943  947  971  1025  16  29  51  56  60  87  117  144  169  202  210  217  235  253  266  287  295  297  301  316  328  330  357  431  448  453  475  505  556  558  561  13  28  41  85  137  140  164  179  184  251  297  308  321  328  336  340  377  389  455  475  538  541  594  672  686  708  754  795  906  919  932  29  30  50  64  69  128  188  211  217  299  308  20  32  50  81  83  88  144  175  187  192  197  206  208  265  52  55  102  114  156  182  206  215  232  243  247  270  365  406  423  473  488  803  825  864  952  965  1180  1267  1331  1539  1558  1685  1690  1691  1712  1740  1805  12  47  79  80  84  157  222  223  243  299  338  353  430  469  470  506  533  605  662  698  734  871  883  957  982  985  990  1062  1099  1154  1174  38  55  65  70  71  76  99  104  116  214  255  280  338  390  533  560  615  629  713  738  756  771  772  777  789  832  833  839  895  996  1057  1149  1226  15  19  23  31  57  60  72  73  74  75  78  92  127  145  164  219  238  248  252  273  293  337  346  356  359  369  372  381  385  406  467  12  92  120  124  139  163  168  171  195  204  250  330  371  379  421  430  437  438  453  463  466  495  498  499  554  602  644  662  668  681  705  19  20  81  179  192  205  228  257  401  460  465  475  481  485  488  493  494  520  526  550  633  668  708  736  821  856  943  962  1053  1059  1067  36  38  57  66  101  114  149  152  182  26  37  39  92  97  130  134  222  241  249  252  264  333  334  369  396  405  406  408  413  429  430  436  438  443  448  473  519  526  536  619  15  84  100  161  184  192  197  220  268  298  310  317  320  330  364  444  459  475  518  525  529  534  538  540  562  567  603  617  626  635  655  42  52  63  80  83  96  105  123  130  137  165  235  275  283  337  352  401  476  486  508  538  545  546  553  572  573  618  620  658  676  677  684  694  20  82  102  119  165  171  172  174  201  244  265  301  350  351  354  368  392  393  437  439  529  571  625  661  682  689  700  709  751  758  769  43  82  90  119  127  138  202  238  292  321  345  355  393  404  411  424  425  468  484  485  486  500  514  594  605  613  691  715  724  727  728  8  9  12  13  56  61  65  72  73  78  82  96  135  159  164  192  207  240  241  249  255  259  287  293  383  398  414  445  521  528  549  571  578  16  17  18  24  29  31  47  50  76  91  102  123  157  158  163  173  207  32  36  57  228  253  273  283  286  293  317  327  332  334  335  339  347  390  393  410  427  460  463  549  571  595  610  625  648  653  691  761  81  175  233  248  253  370  373  388  395  401  417  420  480  491  581  584  585  610  634  648  662  703  720  752  777  784  808  882  912  919  958  981  1172  47  57  83  85  121  142  155  161  20  34  41  42  50  69  82  138  140  143  157  162  169  178  179  216  239  256  260  262  264  271  296  297  324  384  386  393  509  517  524  17  18  27  35  44  66  126  140  151  191  222  224  253  267  310  339  360  14  38  48  79  93  173  224  346  365  476  508  528  582  610  643  737  747  758  787  796  814  817  819  820  825  830  833  840  841  856  892  918  931  8  23  47  59  60  86  101  130  131  165  184  274  286  339  342  359  360  365  390  402  407  420  440  470  473  481  485  510  540  543  560  571  584  95  139  154  178  181  192  201  205  208  235  238  240  242  254  381  428  466  532  580  638  639  654  703  732  754  787  796  797  808  820  865  63  88  94  108  118  197  200  207  305  377  393  414  417  439  449  480  484  39  91  92  96  105  110  119  135  143  151  161  176  188  190  243  261  267  286  291  9  12  66  69  76  99  122  127  157  183  19  21  37  43  44  46  79  82  86  100  105  106  125  131  140  160  188  227  231  237  254  266  270  291  305  319  324  326  336  350  363  22  40  48  60  89  100  104  109  114  129  141  193  204  226  291  329  330  334  377  416  420  428  429  435  439  441  452  454  458  482  490  28  106  139  144  175  180  213  226  320  352  363  441  453  465  468  470  489  504  542  551  557  566  570  571  595  628  629  632  685  715  9  45  52  63  84  162  205  208  210  238  262  264  296  311  337  351  449  476  503  516  523  565  567  579  608  663  746  748  749  772  775  803  836  12  19  59  72  73  75  82  88  101  157  228  268  269  275  411  417  474  605  615  668  745  763  821  941  952  979  1125  1157  1227  1231  1288  14  33  48  49  50  56  72  97  106  107  123  136  144  147  153  192  200  217  226  269  281  291  302  313  316  321  416  443  446  450  455  460  466  17  135  169  207  209  242  269  311  335  377  389  392  402  413  454  459  480  509  512  533  535  539  549  555  618  627  714  738  789  836  858  43  115  122  134  145  147  169  194  200  209  218  223  225  227  237  259  282  289  311  314  345  355  390  399  403  407  450  464  482  511  535  9  44  47  48  60  80  85  87  96  99  108  129  145  147  160  171  178  180  15  16  24  27  59  85  96  164  165  169  208  221  284  291  329  333  338  388  404  417  424  507  515  525  14  19  21  25  48  55  59  62  73  74  76  79  85  92  138  166  168  176  177  178  183  184  191  193  200  226  232  233  238  8  20  30  36  42  49  55  76  125  144  152  160  174  191  243  246  255  271  332  337  359  363  370  423  429  456  464  469  472  476  483  64  74  75  110  123  129  163  175  201  221  226  233  234  239  244  246  247  265  286  323  347  348  367  390  402  13  14  58  59  61  64  75  116  125  136  150  158  181  229  239  274  284  333  365  376  388  17  21  52  69  74  84  113  116  122  128  136  140  143  161  186  200  250  269  290  319  339  356  367  379  380  389  420  431  447  449  488  491  512  49  53  76  103  109  116  129  132  134  141  144  147  156  172  176  182  195  196  200  38  47  50  57  74  98  100  129  132  135  142  161  162  176  181  185  187  204  209  217  238  244  246  250  251  264  267  273  275  279  285  286  306  39  41  65  101  102  157  162  164  179  182  185  192  251  258  262  265  306  309  310  389  392  412  517  521  523  527  555  568  592  597  598  10  32  38  66  100  102  119  150  173  183  193  199  240  243  266  267  290  291  312  321  330  356  381  391  399  407  413  415  423  431  443  17  18  24  26  27  34  35  38  40  43  56  93  111  113  121  149  256  271  303  308  309  311  334  373  377  380  402  423  11  26  34  39  45  68  88  91  97  103  106  135  137  140  144  151  164  169  177  185  188  189  195  197  217  234  15  16  18  37  58  62  79  98  105  112  172  178  212  220  237  243  297  298  311  313  340  376  389  154  186  377  431  504  523  537  609  664  811  825  875  877  952  1038  1051  1141  1165  1260  1302  1331  1528  1541  1629  1690  1708  1788  1809  1823  1827  2052  2124  2217  62  91  30  40  65  68  71  97  124  133  144  147  182  196  197  198  207  227  240  251  267  278  311  328  346  358  375  388  405  435  11  17  75  132  153  181  184  213  214  259  320  55  61  72  81  82  86  102  108  121  123  126  133  152  154  181  186  191  251  269  280  289  292  337  343  358  365  367  368  16  39  52  87  89  131  148  168  175  180  188  191  192  197  199  221  226  237  244  259  286  296  327  344  345  352  364  393  394  413  433  437  442  196  264  295  363  374  581  602  697  764  867  914  1025  1083  1187  1281  1295  1313  1397  1487  1608  1616  1618  1691  1803  1896  2132  2189  2267  2381  2390  2394  14  38  39  44  45  63  128  156  181  182  196  200  20  70  74  77  86  146  148  160  172  180  204  208  230  240  250  262  266  268  280  300  302  331  334  337  343  27  31  32  33  34  37  40  43  62  67  80  91  95  98  102  104  106  110  134  138  142  152  154  169  185  190  226  229  232  255  265  268  362  38  53  66  77  85  123  128  220  241  342  409  469  520  522  601  625  682  710  766  854  861  880  895  1007  1043  1063  1089  1095  1106  1129  1162  15  198  246  302  445  552  565  590  655  703  713  721  750  774  791  990  1034  1200  1203  1252  1306  1437  1438  1478  1497  1498  1524  1623  1697  1787  1868  1935  1986  22  28  31  71  82  121  153  156  158  166  172  178  201  213  219  238  268  279  281  290  291  304  308  336  339  346  349  73  118  123  136  161  167  187  199  225  229  230  238  245  261  283  284  301  335  365  393  401  419  432  463  465  477  495  499  510  535  17  42  48  68  74  101  116  137  176  247  307  308  319  390  398  408  468  483  517  526  28  47  71  78  82  92  102  128  158  161  178  184  192  197  205  228  230  245  254  266  281  287  289  305  322  331  339  344  347  364  377  388  401  11  15  23  57  60  62  63  72  101  106  111  148  149  154  158  163  169  170  198  215  243  251  255  260  262  285  296  305  333  363  383  88  126  132  214  375  409  422  445  449  452  505  564  612  720  726  773  784  859  877  881  898  934  973  1096  1112  1157  1175  1257  1326  1345  1407  53  111  121  126  137  176  10  13  21  26  30  43  63  83  93  138  150  190  193  199  204  215  226  246  276  328  329  351  13  28  35  45  63  72  99  122  136  191  196  217  240  249  265  283  284  285  323  353  358  360  395  404  412  459  474  503  506  517  524  526  528  32  42  44  63  100  122  126  193  204  215  216  217  220  222  243  244  254  357  366  368  370  375  386  387  405  406  409  21  40  72  99  100  114  148  236  266  309  347  362  374  406  410  412  421  452  459  479  481  490  516  563  583  585  604  638  642  653  656  11  49  57  59  69  102  105  126  135  157  166  232  233  237  243  254  280  13  63  66  77  79  85  95  142  145  187  195  197  204  268  283  9  16  124  127  159  173  219  255  262  277  288  343  350  409  430  436  442  447  476  481  499  522  546  548  589  593  603  641  710  743  805  14  24  63  122  147  159  182  183  204  258  300  312  321  393  423  468  470  472  498  526  546  548  558  562  563  567  569  571  578  623  96  143  165  176  306  325  336  338  344  350  360  398  407  417  428  453  460  476  483  486  498  499  502  519  526  532  542  544  550  602  647  650  757  30  46  75  126  145  183  197  198  212  223  236  237  241  245  259  271  344  353  362  444  456  490  529  531  559  593  641  736  771  774  849  10  13  23  25  33  57  58  59  60  67  82  107  125  130  142  162  175  184  190  241  271  274  275  279  296  304  315  319  390  400  422  62  77  111  123  138  156  179  199  203  208  230  250  255  294  295  341  349  434  437  442  457  462  464  469  470  485  489  490  506  14  29  47  53  55  61  94  101  109  110  112  117  126  128  131  136  153  162  174  196  197  221  8  22  31  32  54  66  83  84  124  131  134  203  212  220  242  243  295  302  313  321  322  12  14  34  38  39  61  75  104  125  136  153  154  166  174  242  245  247  248  274  275  289  339  368  418  35  83  133  173  189  235  248  268  349  34  41  42  51  58  60  62  65  66  69  70  87  90  93  117  173  174  181  185  202  205  220  290  295  296  307  314  322  332  376  388  41  43  66  83  85  105  169  173  23  29  36  44  51  55  59  86  92  93  99  102  106  125  135  165  167  176  181  183  190  196  198  205  217  223  8  14  31  33  73  85  89  103  111  115  119  123  125  134  137  167  174  182  185  202  205  207  209  233  235  238  244  10  15  30  74  122  123  142  148  197  217  233  263  274  331  340  373  377  386  402  416  430  462  480  523  537  540  550  551  553  573  590  606  23  112  114  118  123  135  144  151  169  185  187  234  245  259  356  359  364  420  454  486  533  540  559  563  573  590  591  592  595  611  613  90  202  257  322  339  379  445  451  509  512  515  521  545  579  652  657  659  664  670  674  688  707  743  762  775  781  782  788  792  853  856  875  893  23  28  37  52  77  86  100  117  137  179  184  189  202  207  210  213  214  215  224  229  239  246  9  14  72  109  117  118  123  132  134  136  205  229  230  247  265  271  298  300  305  315  324  344  360  424  103  192  227  254  298  307  329  339  351  699  731  736  744  748  752  835  870  893  920  922  968  984  997  1000  1151  1293  1449  1530  1681  1773  1883  22  44  57  68  72  87  152  182  184  195  208  215  234  261  315  325  326  331  358  401  486  488  546  572  596  656  696  703  723  727  729  731  732  31  37  50  84  86  91  101  120  122  128  152  165  194  200  201  228  291  294  300  319  343  353  368  369  370  377  380  382  386  392  31  42  45  47  78  79  81  93  113  121  125  150  199  211  246  265  289  316  318  372  380  386  390  391  394  411  463  467  474  485  491  503  511  44  104  140  150  186  192  250  294  344  346  405  431  468  685  686  723  826  827  869  896  964  1092  1248  1347  1487  1766  1796  1900  2062  2091  2107  2210  2274  16  17  99  114  127  137  138  139  150  182  185  186  204  303  332  337  361  363  366  369  401  441  453  498  510  558  559  561  584  628  671  100  167  187  212  253  294  352  408  454  463  486  527  540  680  830  834  836  847  849  852  853  856  857  858  865  877  935  949  974  1033  1068  1106  47  58  109  140  170  206  228  243  249  336  343  346  349  352  366  375  376  386  391  393  421  462  504  505  551  572  655  775  814  858  34  69  72  83  85  92  94  128  141  145  148  195  197  198  199  208  211  216  220  20  25  77  91  97  115  123  126  130  142  163  187  198  202  207  212  215  218  231  233  234  235  251  256  260  273  277  68  96  259  289  375  441  443  466  496  500  534  552  560  573  579  581  587  591  595  597  602  603  612  616  617  621  626  628  631  649  663  690  701  32  34  41  13  33  50  71  94  98  111  116  123  126  136  149  151  152  157  159  172  178  228  252  262  293  339  381  383  384  385  404  407  411  465  466  472  12  28  34  81  102  103  151  161  177  188  23  54  61  70  72  74  87  88  95  107  118  146  157  161  179  201  247  256  267  268  278  279  282  13  34  35  37  88  120  124  129  134  136  139  144  150  156  159  162  167  171  173  175  189  198  213  217  246  259  272  273  289  303  311  312  313  26  58  64  95  97  98  101  194  220  237  242  258  298  300  29  61  87  90  92  9  22  29  44  64  65  67  69  71  72  75  77  79  82  87  89  91  97  101  107  72  81  84  101  116  179  184  207  215  265  272  312  322  367  391  416  434  447  479  497  503  536  555  579  620  630  635  647  656  751  781  797  817  10  17  18  28  210  356  363  371  426  431  442  520  539  546  565  569  579  622  647  679  689  690  693  702  739  778  784  785  795  809  812  826  831  10  30  39  58  88  99  105  155  184  200  201  207  209  210  213  224  236  246  252  262  266  271  284  295  301  8  11  14  41  48  54  87  90  100  114  119  125  166  199  203  205  208  219  223  228  231  232  234  15  37  42  68  72  78  123  128  195  222  244  247  303  308  336  340  344  348  354  360  363  366  381  386  390  398  402  428  440  8  18  25  28  40  58  77  78  79  81  82  84  88  93  96  9  10  23  38  83  88  110  134  146  169  182  9  20  24  30  38  90  112  121  136  158  167  212  232  277  282  340  415  426  435  440  443  667  680  716  775  824  827  834  866  869  882  889  894  26  42  73  83  87  120  133  146  151  182  189  214  243  245  255  260  263  270  289  292  346  362  374  375  376  379  385  386  391  408  409  436  441  19  46  77  94  126  137  153  173  218  232  264  291  292  294  297  309  30  34  65  110  150  265  291  426  586  703  714  723  733  755  764  771  820  826  833  931  945  950  958  969  982  1067  1096  1275  1299  1300  1302  1314  1316  34  48  76  83  112  124  126  137  141  142  180  188  217  20  23  27  88  94  136  156  202  231  235  249  321  325  378  446  456  470  492  523  527  540  546  550  696  763  773  790  838  860  883  900  52  100  108  112  116  149  154  157  180  194  204  210  238  241  247  277  320  322  352  356  366  377  378  380  389  400  428  456  470  486  526  15  27  36  43  51  74  107  124  166  169  183  191  220  231  240  243  253  266  270  280  290  301  321  338  346  387  431  437  440  442  452  455  92  136  219  494  583  623  681  731  733  752  753  792  855  858  1028  1087  1089  1099  1160  1228  1231  1299  1327  1349  1365  1369  1417  1606  1619  1623  1725  9  26  56  71  106  128  130  133  142  160  167  176  202  203  210  211  262  265  266  272  274  18  28  45  60  75  93  95  137  162  167  196  284  286  290  300  306  336  341  368  372  8  12  25  93  183  192  317  471  497  551  552  615  663  717  726  727  731  765  768  772  881  892  1019  1026  1075  1081  1085  1107  1109  1231  1320  13  14  41  57  111  131  158  165  198  199  215  228  232  245  256  275  283  58  134  177  321  334  368  392  394  428  448  472  477  478  480  484  526  529  553  569  594  621  641  683  685  686  692  723  747  848  851  854  12  155  267  360  408  449  452  538  618  641  669  705  726  736  935  942  943  947  968  995  1032  1039  1042  1083  1089  1127  1129  1193  1196  1215  1219  13  30  94  116  133  135  145  146  158  160  168  175  197  221  226  228  236  237  244  260  279  285  301  312  314  320  338  351  364  17  18  22  33  37  45  56  74  84  101  112  119  139  145  151  154  163  179  39  50  66  140  162  211  213  215  217  234  267  301  307  309  359  375  394  407  453  480  516  534  540  564  571  619  626  631  717  751  765  817  818  14  27  49  63  98  111  112  147  174  178  222  225  259  260  281  301  307  315  317  319  328  331  346  349  356  372  393  401  410  415  424  438  466  17  22  23  51  76  144  151  178  188  16  51  61  117  122  185  194  195  196  197  202  206  231  236  258  281  289  8  38  61  77  78  82  96  119  161  174  193  17  24  31  42  43  61  66  75  87  97  118  136  138  158  170  13  64  226  245  270  277  488  525  583  589  603  630  635  642  666  732  738  742  749  752  775  789  823  860  983  1030  1049  1053  1084  1094  1118  45  58  62  137  237  260  293  323  332  333  342  344  352  361  362  369  392  399  439  457  467  538  543  547  552  560  634  673  704  741  839  51  93  97  100  105  116  148  152  173  184  185  188  231  232  235  241  253  278  308  320  341  364  381  384  61  72  87  97  98  106  188  202  208  218  223  230  234  235  253  255  291  300  336  357  411  426  458  481  533  787  798  852  884  909  910  11  15  54  61  93  95  104  125  136  140  142  145  183  262  207  219  239  242  250  279  322  439  519  568  578  624  663  798  910  912  1011  1041  1226  1306  1333  1336  1346  1420  1562  1570  1579  1689  1691  1765  1803  51  225  266  349  353  366  424  581  631  685  711  1014  1094  1113  1153  1157  1219  1418  1442  1446  1459  1483  1490  1550  1599  1625  1649  1662  1672  1686  1701  1721  1733  9  16  28  50  55  81  84  114  203  205  213  222  245  254  267  272  289  332  340  359  372  386  403  418  437  438  455  489  498  501  505  509  23  28  31  50  54  62  72  79  102  120  126  143  144  146  149  184  195  289  292  325  359  386  38  65  74  84  108  109  168  189  194  239  277  281  288  358  392  466  487  512  520  525  575  576  592  597  623  644  700  706  751  759  768  14  21  22  89  105  127  138  162  185  220  223  226  238  239  241  242  260  265  294  296  331  342  343  356  368  369  373  377  378  395  402  407  428  36  77  113  130  131  140  181  192  204  216  262  276  288  309  339  342  396  30  35  38  42  50  67  71  103  116  122  174  200  221  259  291  297  351  390  397  404  410  431  495  518  531  536  539  540  548  563  576  584  597  599  609  610  612  630  637  661  691  708  756  12  32  91  126  128  172  195  238  272  274  277  335  340  380  535  543  557  584  596  755  822  829  839  941  954  980  1000  1015  1018  1059  1064  22  56  110  167  229  302  362  500  636  672  677  684  716  726  769  836  843  845  849  901  925  985  1004  1072  1134  1136  1142  1153  1176  1195  1286  1383  1450  16  18  20  39  43  46  47  48  65  73  76  78  9  16  28  34  63  64  66  76  86  92  109  125  164  172  178  188  195  216  218  25  31  55  65  132  313  357  426  428  474  475  533  534  552  604  608  626  635  646  668  708  735  771  818  840  846  1006  1013  1023  1032  1035  15  45  61  70  81  91  128  16  21  32  42  44  62  111  125  175  200  203  282  285  295  297  357  362  408  409  415  435  458  486  491  500  556  578  601  605  640  654  669  676  27  44  64  65  66  85  89  115  120  142  147  183  12  15  19  37  46  69  70  83  85  115  117  118  126  137  138  146  147  152  153  162  178  182  205  220  251  271  282  321  323  87  225  238  306  333  361  374  409  476  478  489  554  559  573  577  590  732  830  860  895  965  993  999  1018  1050  1116  1155  1187  1220  1282  1284  48  68  91  101  111  125  127  129  153  168  210  273  311  331  337  352  357  369  371  391  417  450  474  492  501  564  574  584  592  14  31  42  46  80  107  150  161  163  31  79  108  109  114  123  131  133  144  161  163  165  199  221  222  223  227  248  262  264  296  312  348  381  391  395  409  426  433  434  436  465  473  14  25  41  53  95  122  126  200  209  212  217  259  280  284  288  293  305  308  320  329  346  375  459  485  512  539  578  595  632  644  712  717  745  32  93  139  209  244  274  324  353  354  378  382  389  392  393  412  421  433  465  477  479  492  507  510  515  583  725  815  878  904  912  922  998  1011  10  20  38  39  50  61  67  80  81  94  97  111  113  118  130  150  169  171  201  239  249  282  288  295  296  298  301  314  330  351  371  380  419  8  23  43  65  68  98  114  144  150  151  152  157  161  167  169  186  252  253  269  275  284  314  328  336  368  372  423  425  431  434  9  10  11  12  15  16  18  32  36  46  48  51  56  59  60  62  85  103  111  125  137  156  159  168  183  195  205  207  211  223  224  8  23  49  60  75  77  82  104  105  121  126  132  145  163  10  56  141  152  162  190  193  198  202  204  216  227  231  243  246  262  278  286  298  308  312  338  349  350  376  380  382  401  411  418  421  509  564  36  48  66  79  107  112  119  148  154  159  165  168  181  220  247  266  270  295  347  363  371  178  210  229  235  243  280  330  333  334  335  344  361  385  395  414  420  423  449  467  474  478  489  583  609  634  682  732  736  770  776  785  8  20  38  112  114  144  153  156  217  237  241  274  276  414  427  443  485  540  559  564  600  604  623  634  643  711  753  757  759  769  884  37  61  136  234  252  258  264  268  326  333  357  366  402  424  518  567  610  635  636  648  649  654  661  668  674  685  700  706  718  764  781  25  50  84  133  151  200  228  230  236  310  325  332  349  366  440  444  456  601  614  651  826  921  949  954  991  1026  1142  1165  1170  1306  1349  1427  1466  18  19  27  32  44  98  122  138  153  170  218  233  279  323  361  373  394  409  442  476  488  497  508  542  548  570  585  625  626  633  635  636  640  15  34  44  46  65  79  108  109  129  207  210  211  225  230  231  238  260  262  286  308  316  328  333  369  426  435  464  492  536  558  562  570  596  14  110  134  143  162  166  189  219  250  259  260  262  271  278  281  296  304  312  318  329  334  351  385  390  401  27  83  198  206  259  267  275  276  317  334  404  410  444  446  447  453  468  478  487  488  496  510  526  557  599  630  632  647  676  681  682  687  705  141  143  180  206  281  313  363  385  399  404  460  555  587  686  694  715  721  733  734  754  793  798  817  818  844  864  879  1002  1009  1022  1059  1061  1092  22  31  47  68  96  148  150  159  166  197  201  239  256  259  284  312  313  320  349  369  376  402  456  474  479  18  45  54  58  65  26  32  52  59  60  65  67  70  80  83  98  104  111  112  116  117  134  154  157  159  160  176  184  193  199  213  214  221  270  325  24  27  31  32  37  86  87  88  90  121  165  176  217  20  49  393  399  403  412  429  444  447  470  475  534  535  554  556  558  588  593  595  608  609  612  624  633  635  654  666  671  679  695  711  779  21  24  26  27  28  55  88  100  112  126  162  166  176  179  51  62  86  96  100  102  104  105  106  121  145  173  228  265  332  391  397  431  440  454  470  489  593  636  669  754  803  827  828  835  842  863  939  52  90  92  94  160  192  203  216  243  288  291  335  339  344  384  448  533  536  560  637  747  812  824  852  882  886  892  905  956  1045  1066  1099  1108  26  72  83  109  111  172  195  228  277  280  292  323  326  349  359  372  375  393  472  473  482  509  525  536  546  548  554  603  610  677  689  25  43  114  155  15  17  19  20  22  24  31  42  83  90  19  28  85  146  151  226  231  256  275  282  286  308  311  318  331  354  357  413  445  447  478  489  495  548  560  629  648  695  758  766  779  26  27  36  38  40  44  46  61  77  56  57  71  79  92  101  134  135  144  171  180  192  262  274  278  291  316  321  395  408  413  441  457  459  471  477  478  501  516  539  567  575  595  32  47  91  183  184  185  193  213  239  261  282  353  372  384  460  508  562  608  623  645  662  665  688  745  760  774  787  795  807  818  888  903  932  47  62  95  113  139  177  226  237  137  166  168  175  180  184  191  195  209  53  59  75  137  174  179  191  192  240  244  251  256  262  275  287  294  313  320  326  335  336  343  349  367  368  371  374  375  384  387  21  58  62  100  144  157  168  186  193  211  232  255  259  267  12  18  42  45  46  52  58  66  78  88  92  94  102  137  148  172  178  188  40  50  51  52  110  118  138  176  184  366  370  377  412  415  420  424  429  461  499  531  534  580  587  588  609  612  625  641  682  690  707  71  102  114  117  127  142  145  205  214  258  315  323  393  401  405  418  420  428  430  434  448  449  461  483  488  523  528  11  66  75  146  157  163  189  249  254  273  299  342  343  351  374  483  486  535  545  671  1097  1125  1198  1243  1305  1308  1458  1520  1521  1525  1561  1653  1673  27  28  30  53  121  147  172  181  183  187  217  247  259  272  279  297  317  321  362  371  19  24  53  79  80  88  136  152  158  180  188  222  225  235  272  289  292  293  329  330  331  373  374  20  21  30  63  84  113  132  136  153  164  208  254  282  295  296  370  381  393  455  462  540  553  557  560  564  575  585  587  616  637  640  20  41  51  55  66  110  123  125  136  137  156  163  171  180  193  200  244  250  255  261  291  314  44  93  102  110  151  206  218  243  247  266  270  315  328  347  348  383  398  413  430  499  509  510  518  524  527  632  645  666  679  680  716  18  27  31  32  34  35  37  38  44  98  115  130  156  157  183  202  210  220  227  281  290  313  332  379  384  387  391  395  398  401  404  416  439  11  186  204  255  264  357  375  438  460  461  476  479  481  505  507  514  546  550  571  582  631  648  719  765  772  835  892  912  991  1016  1030  1032  1046  40  55  57  201  260  275  293  305  339  366  376  420  480  509  512  590  621  634  645  657  706  738  833  837  878  918  919  921  937  953  1000  1026  1072  24  36  56  59  68  97  140  144  147  156  169  200  203  205  207  228  241  242  261  274  285  293  302  307  317  329  333  48  72  77  105  130  295  334  338  344  363  390  426  481  492  496  892  985  1027  1207  1214  1253  1267  1271  1281  1293  1356  1412  1505  1557  1750  1791  1854  1877  15  21  34  70  74  76  99  101  111  125  129  139  141  155  170  231  271  275  300  301  310  311  317  332  85  106  112  126  129  151  210  265  269  281  285  301  304  349  356  469  471  515  530  580  604  621  800  811  815  849  881  883  926  974  1031  21  24  27  51  52  55  61  111  121  125  137  159  223  260  262  263  267  284  304  311  314  317  342  345  382  433  483  499  510  530  576  578  588  45  53  85  92  118  120  130  147  149  179  204  208  217  235  273  288  290  299  334  378  405  421  435  487  501  558  582  586  621  642  746  57  103  122  123  127  153  161  181  212  218  243  259  268  285  336  410  488  504  505  521  562  565  610  612  622  672  680  690  698  716  736  14  28  45  96  101  128  164  205  225  251  338  377  402  413  446  474  492  493  498  499  503  507  521  597  675  714  732  738  742  769  811  9  14  24  27  28  47  57  58  66  104  122  136  157  159  175  180  181  21  29  30  78  90  116  136  142  152  156  189  207  213  229  245  275  284  287  293  300  307  332  333  335  336  345  24  25  45  83  111  220  223  251  253  258  297  318  351  378  394  436  438  460  465  516  521  528  547  555  643  645  700  712  729  730  736  10  25  28  39  61  71  80  85  91  101  106  143  153  164  182  199  277  362  368  371  380  403  409  429  14  17  18  28  31  49  61  65  70  71  79  91  92  115  119  140  21  34  55  90  98  104  137  148  170  209  215  329  352  353  380  418  430  445  491  514  543  591  597  601  636  667  673  674  681  691  693  9  15  17  27  33  72  77  88  94  97  100  103  105  134  181  183  188  190  208  226  272  293  311  444  468  528  628  652  679  705  755  8  18  21  36  43  52  63  110  170  176  177  184  185  199  206  239  244  252  253  8  29  37  39  72  75  84  109  111  112  117  129  154  156  157  166  170  192  196  199  200  219  238  253  260  274  308  320  321  329  332  27  54  96  125  202  207  343  350  567  581  582  598  601  605  641  643  645  675  676  702  703  749  777  819  873  1011  1050  1232  1325  1327  1329  34  136  139  171  180  189  209  220  242  253  290  362  371  380  417  469  478  500  561  610  617  656  727  752  760  763  775  776  796  798  828  831  834  19  34  55  92  106  143  149  153  162  195  196  217  14  20  45  76  80  118  130  138  169  171  180  209  227  229  243  246  266  323  340  345  10  51  59  102  165  169  179  221  222  29  66  111  144  172  210  260  347  350  351  356  363  367  411  422  449  452  486  493  521  603  833  923  963  1002  1014  1015  1082  1093  1210  1223  12  20  107  151  15  40  42  45  49  52  66  85  105  152  177  201  210  217  218  253  263  269  271  278  280  281  338  340  368  375  401  438  474  478  482  38  40  43  61  76  91  105  115  129  163  177  203  207  260  266  279  280  283  295  334  346  357  372  381  383  400  413  416  417  432  461  31  32  39  41  62  65  66  86  95  116  161  162  174  206  223  226  235  270  275  288  322  402  413  440  452  464  468  477  493  540  577  618  655  35  42  51  82  92  120  131  133  137  146  151  154  172  196  197  202  213  226  231  234  238  286  288  330  344  346  364  400  421  443  478  485  487  17  22  56  59  60  79  87  99  102  107  121  136  150  158  197  201  202  228  281  282  291  357  366  367  397  441  468  504  509  514  519  78  109  124  131  171  229  235  257  329  340  350  358  426  430  433  522  536  546  565  591  629  631  711  754  888  957  988  1038  1186  1314  1448  1487  1509  61  128  148  152  170  174  200  204  255  382  384  460  542  640  669  772  811  978  1009  1010  1024  1066  1079  1160  1195  1197  1202  1245  1252  1295  1306  8  18  67  89  104  148  149  205  218  220  232  253  269  275  281  283  292  297  307  313  323  324  325  333  349  372  392  397  401  414  415  475  492  93  109  122  137  149  159  163  166  191  216  236  248  295  19  23  24  25  46  58  64  70  84  101  127  162  191  194  197  206  212  247  264  303  327  350  377  407  434  509  544  546  552  554  558  562  598  13  51  52  55  57  58  118  119  123  124  125  126  145  150  168  175  180  184  205  218  11  16  31  40  55  67  70  79  86  140  217  223  231  246  257  268  273  283  300  301  318  324  329  350  359  407  415  49  64  82  84  89  90  119  129  133  144  153  156  182  203  210  216  241  275  290  291  321  337  339  353  376  377  409  413  447  452  483  13  22  89  149  292  340  345  347  400  410  418  420  433  443  473  533  605  653  662  714  719  741  749  779  786  821  824  838  863  865  956  1125  1243  39  96  104  129  134  135  189  228  232  278  373  510  548  565  589  593  603  610  619  734  751  778  791  793  802  834  874  904  947  969  1021  21  30  35  182  195  207  236  267  278  279  282  488  558  577  581  582  584  595  609  637  750  817  863  864  1001  1168  1212  1217  1224  1244  1286  25  106  171  175  201  235  261  270  337  359  455  461  544  577  643  705  822  993  1112  1123  1238  1273  1289  1325  1326  1331  1338  1380  1397  1433  1443  24  32  42  45  68  91  103  113  116  155  221  229  230  235  243  249  257  266  290  292  316  341  361  374  376  412  413  415  417  442  487  489  493  26  53  204  232  254  292  300  335  356  362  370  384  411  415  427  429  431  451  458  466  472  507  521  525  529  533  536  540  545  549  558  580  604  203  279  459  463  510  678  706  802  808  912  1021  1034  1271  1287  1562  1592  1675  1713  1831  2081  2083  2088  2230  2326  2421  2504  2554  2740  34  39  40  41  44  47  79  81  90  94  176  187  231  232  252  262  267  335  345  360  363  375  383  68  222  311  320  353  355  368  395  404  426  452  456  509  517  559  560  585  587  612  619  625  649  673  676  679  686  690  696  700  720  728  16  36  40  54  91  124  147  158  185  212  242  250  255  285  322  376  379  398  422  432  460  472  548  559  561  571  574  580  625  633  667  13  54  97  131  175  177  243  320  467  511  565  635  737  759  762  772  776  790  864  886  948  961  994  1041  1065  1077  1081  1083  1085  1089  1101  1158  1196  15  20  29  50  59  85  147  197  221  232  262  297  308  310  320  341  368  372  375  395  404  420  421  561  574  607  608  614  627  645  676  11  24  44  193  195  279  308  309  313  317  321  356  362  373  390  421  494  499  504  579  631  725  837  847  886  891  904  1015  1024  1093  26  43  106  110  114  126  156  181  203  208  220  243  252  262  270  308  310  318  339  342  358  363  383  398  410  431  437  439  14  36  45  47  49  50  53  88  129  140  146  149  182  183  201  205  214  216  256  287  304  331  337  358  361  380  393  397  481  486  498  551  580  8  24  37  39  54  58  80  101  123  152  181  187  218  269  301  325  374  389  391  424  436  443  487  563  577  587  602  605  606  650  750  766  767  9  21  23  60  72  89  104  8  11  18  50  57  82  11  26  39  40  42  75  79  86  93  115  120  130  150  164  217  231  234  252  259  268  269  327  343  349  357  362  364  376  10  21  64  77  80  81  98  149  196  198  208  217  226  238  249  255  264  273  282  303  306  348  385  414  416  422  448  458  462  504  507  515  517  34  60  97  100  136  179  231  232  255  259  260  277  281  285  298  304  13  15  18  32  33  42  66  80  92  104  110  126  177  182  183  199  202  210  232  254  257  267  270  13  38  126  173  212  280  318  325  371  425  614  634  637  661  665  678  685  714  721  725  760  766  772  804  808  809  851  888  929  937  991  1008  1043  17  23  43  57  75  83  92  99  105  109  118  157  179  184  186  193  198  201  221  232  254  255  301  307  330  332  352  416  421  435  453  460  490  16  35  47  50  59  133  142  165  166  172  175  181  183  187  189  74  78  82  89  174  198  207  223  254  271  307  314  342  47  81  274  305  445  465  505  509  550  558  560  610  697  845  978  1076  1130  1167  1204  1230  1271  1332  1379  1572  1602  1704  1712  1852  2004  2010  2027  167  397  493  545  573  672  690  696  717  746  778  808  823  846  847  920  925  975  1000  1018  1056  1057  1080  1086  1105  1111  1123  1185  1202  1216  1226  1234  1248  19  25  48  55  110  128  152  170  174  192  212  243  265  273  283  287  324  331  342  352  442  465  538  545  553  555  562  563  577  609  642  645  648  69  78  201  220  260  271  325  331  374  386  426  494  538  539  547  571  580  593  644  660  668  674  676  691  704  722  724  734  749  777  793  12  17  54  76  78  117  17  26  58  73  93  113  20  49  109  119  159  190  207  211  217  219  220  264  308  330  331  336  392  444  499  508  600  619  777  780  784  786  789  823  916  917  11  14  25  38  53  54  60  62  65  70  95  156  193  247  248  11  17  19  71  79  127  128  135  137  148  161  163  170  181  202  207  214  233  239  279  280  318  321  335  341  345  21  32  35  48  53  97  118  135  149  166  175  180  194  203  204  242  246  250  266  271  20  47  89  139  190  191  200  217  218  254  257  261  267  273  281  282  286  288  294  296  299  305  307  321  325  336  342  18  31  50  75  92  116  152  176  180  181  194  261  263  265  273  281  283  285  294  307  309  311  313  315  319  327  336  353  354  358  378  383  384  41  55  61  73  96  143  149  199  233  10  14  19  21  25  54  92  133  138  10  23  29  31  33  45  66  18  22  37  58  64  71  73  80  83  102  129  149  155  159  162  171  182  300  531  646  668  705  835  1021  1137  1179  1185  1294  1392  1397  1448  1452  1470  1608  1663  1704  1743  1798  1844  1846  1906  1909  2058  2091  2128  2129  2230  2273  10  18  23  27  33  52  84  92  93  102  161  168  192  214  237  247  278  283  295  302  368  410  433  475  482  519  538  561  601  603  614  616  68  102  153  196  229  236  242  247  261  267  302  308  432  444  510  513  586  587  621  665  673  678  680  747  775  826  862  881  916  922  937  952  961  23  34  44  48  68  107  110  121  124  138  162  163  186  199  217  252  262  298  309  316  340  357  368  371  372  383  20  22  34  71  89  114  202  209  212  245  282  317  326  379  414  434  451  501  508  525  553  637  639  655  659  671  690  775  776  780  799  38  71  92  97  98  102  106  108  109  111  114  117  118  119  121  126  135  138  140  151  177  181  232  236  240  258  293  299  306  308  331  361  387  31  47  64  54  58  66  101  103  146  170  172  176  202  206  267  268  275  310  316  320  339  346  359  360  367  388  391  402  410  413  435  482  496  507  39  54  58  63  69  88  93  127  138  139  143  146  151  158  169  196  201  209  213  215  218  223  225  244  303  340  383  426  434  464  475  526  569  113  116  117  128  165  215  237  258  281  284  365  378  455  508  511  516  577  650  821  865  878  888  932  939  967  973  976  979  1017  1026  1035  1038  1039  12  20  57  72  89  119  123  125  126  135  138  144  147  149  150  212  260  302  352  355  375  379  385  387  394  408  502  510  532  537  560  12  18  22  25  43  44  64  74  88  106  110  113  127  21  29  32  57  86  88  91  119  138  166  192  204  207  225  249  257  89  119  122  129  139  147  162  166  173  174  178  195  224  227  228  231  232  260  263  279  285  291  296  308  309  314  323  333  560  599  631  13  23  32  38  39  43  47  85  121  147  196  212  226  241  27  31  38  53  82  108  114  126  132  157  167  174  226  236  245  25  46  87  91  100  118  149  165  206  210  216  232  256  272  294  311  315  316  317  320  329  332  334  357  373  377  381  391  400  419  434  38  62  74  76  117  162  173  176  182  185  196  197  216  217  255  285  320  333  349  370  448  462  514  641  645  655  691  707  745  805  865  12  13  18  19  32  50  54  57  60  62  140  153  161  167  215  222  269  286  381  412  414  415  427  440  450  453  458  465  468  477  478  18  29  36  37  41  58  89  161  162  167  180  204  218  220  255  293  330  353  354  361  366  376  387  442  473  486  489  501  515  536  550  63  86  104  124  135  145  168  173  247  339  371  409  454  461  534  557  567  677  736  760  804  805  824  827  872  874  907  916  918  924  944  965  987  8  17  141  148  156  158  178  186  196  208  221  247  248  252  341  346  350  364  377  383  29  85  90  110  132  13  53  138  158  251  263  269  305  342  402  403  475  646  661  689  735  785  855  879  885  930  1010  1157  1160  1209  1315  1357  1358  1376  1378  1388  1527  1605  42  274  288  402  413  429  437  452  464  524  560  589  594  602  644  655  661  662  711  748  767  772  776  894  925  1009  1021  1132  1330  1359  1374  12  38  65  76  77  78  102  154  178  232  245  255  285  336  349  399  459  471  590  593  654  655  735  763  848  862  916  969  1000  1061  1064 | NHVNWR**S**SESKPE  NIRDFL**S**ELELKR  EVYDPG**S**EDPRGT  PRGTGP**S**QGPEDN  GDGEEA**S**GADGVP  EAEPLP**S**LEYWPQ  RGVTRA**S**VYMQPP  VVRKMI**S**QAQKVI  YIIVDE**S**NVKYFL  KNLRVR**S**SGGTEF  SYSFTW**S**AARTDR  TDRNVI**S**VLSGQV  NVISVL**S**GQVVEM  QELYLM**S**HSVSLK  LYLMSH**S**VSLKGI  EPIVLP**S**VVPLVP  PDPEPG**S**DILGYI  IKIRDT**S**QASAQH  VLARDS**S**DIGWVL  PLQRQL**S**VTQDDP  TLSDQD**S**HSGSSG  QDSHSG**S**SGRGPG  DSHSGS**S**GRGPGP  RRPSVA**S**SVSEEY  FEVREH**S**VPLRRR  GSPWAQ**S**RGREEA  AADSVQ**S**STRNAG  LLPDPG**S**PRLAQN  PFGIPY**S**KLSQSK  KARTGG**S**QWASSD  GGSQWA**S**SDSKRR  DRREKE**S**ELGDRP  LQVGER**S**DYAGFR  CQTLGI**S**PEEKFV  FVITTT**S**RKEITC  TLYLLQ**S**VNQLLL  YDTLVK**S**GMYEYY  AELIDN**S**LSATSR  DFESDH**S**GYVRPV  VPRSLN**S**DISYFG  ISKPAD**S**QDVHEL  NKEAIY**S**GYIRNR  EEKEKD**S**FTAVVI  LKVILH**S**SSGNKE  QVVLNE**S**NADTYA  LEASGL**S**LHYEEI  LKVKPD**S**EILVIE  CSSSGT**S**ILTGSA  TSILTG**S**AIQVQN  NQIQAF**S**PSSLSS  FSPSSL**S**SLSIAG  ENTQSI**S**VRGIKF  FTWREF**S**DFIRVQ  WPELKE**S**IPVING  KYDKDA**S**FLAGGL  INPARI**S**MKMWKL  PPTPAV**S**NVRSVA  SSPGRD**S**TEYFIV  PRLPLL**S**RTLEPY  KKKPRR**S**CTLPNY  LPDWKR**S**LPHFRN  AVCKLD**S**VNKDLN  IRKCND**S**LRHSPK  NDSLRH**S**PKVETT  PALLLL**S**LSGCFS  LLLLSL**S**GCFSIQ  SLSGCF**S**IQGPES  SIQGPE**S**VRAPEQ  RAPEQG**S**LTVQCH  LIETRG**S**EQGEKS  SEQGEK**S**DRVSIK  EKSDRV**S**IKDNQK  DPEGAA**S**TTASSP  AASTTA**S**SPTNSN  ASTTAS**S**PTNSNM  ASSPTN**S**NMAVFI  MAVFIG**S**HKRNHY  ILWLKG**S**QRVPEE  PIYMNF**S**EPLTKD  EQPLAI**S**RVTPGS  SRVTPG**S**KAALAN  IDGENT**S**NMTHLE  TLTVAR**S**EHKVWS  SEHKVW**S**PLVTEE  YKMNLA**S**EPQEVL  EVLHIG**S**AHNRSA  GSAHNR**S**AMPFTA  AMPFTA**S**PASSTT  FTASPA**S**STTARV  TASPAS**S**TTARVI  NPAGLY**S**SENISN  PAGLYS**S**ENISNF  YSSENI**S**NFNNAL  FNNALE**S**KTAASG  ESKTAA**S**GVEANS  SGVEAN**S**RPLDHA  DHAQPP**S**SLVIDK  HAQPPS**S**LVIDKE  LVIDKE**S**EVYKML  NEPPKQ**S**TSFLVL  PPKQST**S**FLVLQE  LQEILE**S**EEKGDP  GDPNKP**S**GFRSVK  KPSGFR**S**VKAPVT  VTKVAA**S**IGNAQK  VRVTME**S**ALTARD  IGPVLV**S**VNPYRD  VMISGE**S**GAGKTE  RDRLLQ**S**NPVLEA  TLRNDN**S**SRFGKY  VGGHIL**S**YLLEKS  SYLLEK**S**RVVHQN  LERNPQ**S**YLYLVK  GQCAKV**S**SINDKS  QCAKVS**S**INDKSD  FAANEE**S**NAQVTT  KGEELL**S**PLNLEQ  LAKAVY**S**RTFTWL  INRSLA**S**KDVESP  ASKDVE**S**PSWRST  EVFQHN**S**FEQFCI  KFKGII**S**ILDEEC  AGEVTY**S**VTGFLD  SKNPIM**S**QCFDRS  SQCFDR**S**ELSDKK  FDRSEL**S**DKKRPE  FLQRYK**S**LCPETW  LEVRRQ**S**LATKIQ  SAICIQ**S**WWRGTL  PALREA**S**ELLREL  QQKAVA**S**EIFKGK  VPRLFI**S**TRLGTD  LGTDEI**S**PRVLQA  VLQALG**S**EPIQYA  KGYKPR**S**RQLLLT  VSSLSD**S**LFVLHV  LTKTAL**S**ANRVNS  ININQG**S**ITFAGG  HQITDE**S**LESTRR  TDESLE**S**TRRILG  LGLAIE**S**QDAGIK  RTKNFE**S**GKAYKT  GDGGEN**S**PCNVVS  SPCNVV**S**KQPGPV  PTTGAA**S**GGYIKR  NLTQVG**S**ILGNLK  GFPGPR**S**HRDPFF  EEEEGG**S**WGRGNP  GNPRFH**S**PQHPPE  EFGFGF**S**FSPGGG  GFGFSF**S**PGGGIR  LVRDFN**S**IFSDMG  DFNSIF**S**DMGAWT  GAWTLP**S**HPPELP  ELPGPE**S**ETPGER  GQTLRD**S**MLKYPD  MLKYPD**S**HQPRIF  FGGVLE**S**DARSES  LESDAR**S**ESPQPA  SDARSE**S**PQPAPD  PAPDWG**S**QRPFHR  EDNDLD**S**QVSQEG  DLDSQV**S**QEGLGP  LQPQPK**S**YFKSIS  PKSYFK**S**ISVTKI  SYFKSI**S**VTKITK  RRTVVD**S**EGRTET  TRHEAD**S**SPRGDP  RHEADS**S**PRGDPE  PRGDPE**S**PRPPAL  ALDDAF**S**ILDLFL  TMAKAE**S**PKEHDP  FTYDYQ**S**LQIGGL  GILIVL**S**RRCRCK  EEGTFR**S**SIRRLS  LLALGD**S**GVGKTT  AQGPNG**S**SGKAFK  QGPNGS**S**GKAFKV  GQERFR**S**LTTAFF  LMFDLT**S**QQSFLN  DLTSQQ**S**FLNVRN  NVRNWM**S**QLQANA  IPYFET**S**AATGQN  TVNGGN**S**GNLDGE  TYEDDI**S**EISQKL  DDISEI**S**QKLPGE  FPVGLY**S**LESISL  GLYSLE**S**ISLAEN  YSLESI**S**LAENTQ  IITYPK**S**GTTWMI  LKEGDP**S**WIRSVP  DPSWIR**S**VPIWER  TIVGAF**S**LPDQYS  SLPDQY**S**PRLMSS  YSPRLM**S**SHLPIQ  SPRLMS**S**HLPIQI  FTKAFF**S**SKAKVI  PRDVVV**S**LYHYSK  VSLYHY**S**KIAGQL  GEVQFG**S**WFDHIK  QQDLQG**S**VERICG  GKEALG**S**VVAHST  GSVVAH**S**TFSAMK  VAHSTF**S**AMKANT  MKANTM**S**NYTLLP  YTLLPP**S**LLDHRR  HFTVAQ**S**EAFDRA  DPEEDG**S**PDPEPS  SPDPEP**S**PEPEPK  EPEPKP**S**LEPNTS  SLEPNT**S**LEREPR  REPRPN**S**SPSPSP  RPNSSP**S**PSPGQA  NSSPSP**S**PGQASE  PSPGQA**S**ETPHPR  SVPSSL**S**QSAINA  PSSLSQ**S**AINANS  SAINAN**S**HGGPAL  HGGPAL**S**LPLPLH  GPKDLR**S**AMGEGG  GLIHLY**S**ELELSA  YSELEL**S**AHNAAN  EEEDLS**S**PPGLPE  LPEPLE**S**VEAPPR  DGPREH**S**KSASLL  SSWATL**S**QGSPSY  GSPSYG**S**PEDTDS  SPEDTD**S**FWNPNA  MRVQDT**S**GTYYWH  FWKDEP**S**DEAPME  FPAQSL**S**PEPLPQ  KCFAVR**S**LGWVEM  ELAPGR**S**SVAVNN  LAPGRS**S**VAVNNC  NCIRQL**S**YHKNNL  NLHDPM**S**GGWGEG  KLVEPQ**S**QALLHA  HAQPII**S**IRVWGV  WGVGRD**S**GRERDF  AKNIAT**S**LHEICS  ESVLSS**S**SREQWT  WTPSHV**S**VAPATL  CRVRFL**S**FLAVGR  MAAGPA**S**FCCHMF  CEPNAA**S**LSEAVQ  PNAASL**S**EAVQAA  DARSQA**S**TSCLPA  VRRGVQ**S**LWGSLK  EFQRAQ**S**LLSTDR  RAQSLL**S**TDREAS  STDREA**S**IDILHS  SIDILH**S**IVKRDI  VQVKEQ**S**ILELGS  SILELG**S**LLAKTG  VRPFLN**S**ISKAKA  PFLNSI**S**KAKAAR  AARLVR**S**LLDLFL  CIEWAK**S**EKRTFL  LEARLV**S**LYFDTK  EALHLG**S**QLLREL  EVQLLE**S**KTYHAL  KTYHAL**S**NLPKAR  ARAALT**S**ARTTAN  ATLDMQ**S**GIIHAA  DWKTAY**S**YFYEAF  AFEGYD**S**IDSPKA  GYDSID**S**PKAITS  SPKAIT**S**LKYMLL  DVQALV**S**GKLALR  KCVAQA**S**KNRSLA  QASKNR**S**LADFEK  RDDPII**S**THLAKL  RVIEPF**S**RVQIEH  VQIEHI**S**SLIKLS  QIEHIS**S**LIKLSK  SSLIKL**S**KADVER  DVERKL**S**QMILDK  ETIQNM**S**KVVDSL  MSKVVD**S**LYNKAK  KMEVDY**S**ATVDQR  VIETLL**S**LEKQTR  KQTRTA**S**DMVSTS  TASDMV**S**TSRILV  SDMVST**S**RILVAV  ENIMLL**S**KRRSQL  LLSKRR**S**QLKQAV  DVKEAA**S**ILQELQ  QVETYG**S**MEKKER  IRTQII**S**KKINTK  LDQHEG**S**YLSICK  HEGSYL**S**ICKHYR  PCIQAE**S**EKWQQA  WQQALK**S**VVLYVI  PFDNEQ**S**DLVHRI  DLVHRI**S**GDKKLE  MELMRW**S**TLVEDY  MELRKG**S**LESPAT  RKGSLE**S**PATDVF  ATDVFG**S**TEEGEK  AQLLDL**S**VDESEA  DLSVDE**S**EAFLSN  ESEAFL**S**NLVVNK  NLLNDW**S**QKLNSL  WSQKLN**S**LMSLVN  KLNSLM**S**LVNKTT  HVFAVE**S**IEKKRI  VKWRGW**S**PKYNTW  PTFARR**S**NVLTGL  LTGLQD**S**STDNRA  TGLQDS**S**TDNRAK  HQYELN**S**KKHHQY  HQYQPH**S**KERAGK  PPPPGK**S**GKYYYQ  YYYQLN**S**KKHHPY  GHKEAP**S**PTCPDL  PDLGAK**S**HPPDKW  GAPGKG**S**EKGPPN  RIVIVM**S**KYMENG  QAVKIK**S**GEVAEG  AEGEAR**S**PSHKKR  GEARSP**S**HKKRAA  RREEEV**S**GVSDPQ  EEVSGV**S**DPQPQD  QPQDAG**S**RKLSPT  AGSRKL**S**PTKEAF  RNTHPP**S**HHPHPH  AVGLNL**S**HVRKRC  VRKRCL**S**ETHGER  KRLTAR**S**ISTPTC  LTARSI**S**TPTCLG  PTCLGG**S**PAAERP  EVILLD**S**DLDEPI  RCVKTR**S**EAGEPP  EAGEPP**S**SLQVKP  AGEPPS**S**LQVKPE  KPETPA**S**AAVAVA  QDEPAE**S**LSEFKP  EPAESL**S**EFKPFF  ATGADP**S**DLESGG  DPSDLE**S**GGLLHE  LHEIFT**S**PLNLLL  FAGRDA**S**RGLATF  DEYDDL**S**DLTAAQ  AQQETL**S**DWESQF  TLSDWE**S**QFTFKY  FSEEED**S**ERSSDG  RRSAAG**S**EKEEEP  VLDEDR**S**GARRLQ  GGSDEL**S**DDITQQ  ERATAI**S**LMRKFI  TPLQIK**S**VVAPEH  DAEKIR**S**LGGDVA  FEGNRY**S**RKGFLF  FKSFAM**S**AVITEG  LQGKIL**S**VDGNKI  RDLQLC**S**ETASGV  LCSETA**S**GVDVGG  GSPGGG**S**GGMSRG  ARVELH**S**TCQTIS  RTPMYG**S**QTPMYG  PMYGSG**S**RTPMYG  RTPHYG**S**QTPLHD  TPLHDG**S**RTPAQS  SRTPAQ**S**GAWDPN  DDEPTP**S**PQAYGG  YNTDQF**S**PYAAPS  APSPQG**S**YQPSPS  SYQPSP**S**PQSYHQ  YHQVAP**S**PAGYQN  NTHSPA**S**YHPTPS  SYHPTP**S**PMAYQA  PMAYQA**S**PSPSPV  QASPSP**S**PVGYSP  MTPGAP**S**PGGYNP  NPHTPG**S**GIEQNS  SGIEQN**S**SDWVTT  VTGGMC**S**VYLKDS  ATGVLL**S**IDGEDG  DAGVPE**S**GEIRTL  LLRRNY**S**REQHGV  QHGVAA**S**CLEDLR  CLEDLR**S**KACDIL  ILAIDK**S**LTPVTL  YFLCLP**S**NTKFVA  KFVALA**S**NEKWAY  KWAYNN**S**DGGTAW  GGTAWI**S**QESFDV  AWISQE**S**FDVDET  DVDETD**S**GAGLKW  QLKEDL**S**SIILLS  LKEDLS**S**IILLSE  SSIILL**S**EEDLQM  LVDAPC**S**DLAQEL  AQELRQ**S**CATVQR  REEVRQ**S**KQLLQL  ALEKEG**S**LLSKQE  KEGSLL**S**KQEESK  LSKQEE**S**KAAFGE  AVDTGI**S**RETSSD  GISRET**S**SDVALA  ISRETS**S**DVALAS  SDVALA**S**HILTAL  KQAPEL**S**LSSQDL  APELSL**S**SQDLEL  PELSLS**S**QDLELV  RLQQTQ**S**LHSLRS  QTQSLH**S**LRSISA  SLHSLR**S**ISASKA  HSLRSI**S**ASKASP  LRSISA**S**KASPPG  ISASKA**S**PPGDLQ  TPAVPV**S**APPATP  PAAAPA**S**VPAPTP  PAAAPA**S**SSDPAA  AAPASS**S**DPAAAA  PGQTPA**S**AQAPAQ  HPVILA**S**IVDSYE  LASIVD**S**YERRNE  GTVDKH**S**VEVTNC  EVTNCF**S**VPHNES  SVPHNE**S**EDEVAV  ELHKKV**S**PNELIL  HDITEH**S**VLIHEY  LIHEYY**S**REAPNP  HLTVDT**S**LQNGRM  LQNGRM**S**IKAYVS  SIKAYV**S**TLMGVP  NRVIGL**S**SDLQQV  RVIGLS**S**DLQQVG  QQVGGA**S**ARIQDA  RIQDAL**S**TVLQYA  YAEDVL**S**GKVSAD  VLSGKV**S**ADNTVG  VGRFLM**S**LVNQVP  FETMLN**S**NINDLL  LANLTQ**S**QIALNE  GFPGRR**S**VGLVKG  KGALGW**S**VSRGAN  ALGWSV**S**RGANWR  NWRWFH**S**TQWLRG  IKILMP**S**LSPTME  ILMPSL**S**PTMEEG  KEGEAV**S**AGDALC  VVTLDA**S**DDGILA  IVVEEG**S**KNIRLG  KNIRLG**S**LIGLIV  GPPPPV**S**KPSEPR  PPVSKP**S**EPRPSP  PSEPRP**S**PEPQIS  SPEPQI**S**IPVKKE  TLRFRL**S**PAARNI  NILEKH**S**LDASQG  KHSLDA**S**QGTATG  ATPTAP**S**PLQATA  QATAGP**S**YPRPVI  PVIPPV**S**TPGQPN  FTEIPA**S**NIRRVI  AKRLTE**S**KSTVPH  RLTESK**S**TVPHAY  KDDIKV**S**VNDFII  LPFIDI**S**VAVATD  IQEIAD**S**VKALSK  DSVKAL**S**KKARDG  EEYQGG**S**FSISNL  YQGGSF**S**ISNLGM  GGSFSI**S**NLGMFG  ITVTMS**S**DSRVVD  VTMSSD**S**RVVDDE  ATRFLK**S**FKANLE  RRCNSG**S**GPPPSL  GSGPPP**S**LLLLLL  ANAAPR**S**ALYSPS  PRSALY**S**PSDPLT  SALYSP**S**DPLTLL  RGAVLG**S**RSAWAV  AVLGSR**S**AWAVEF  AVEFFA**S**WCGHCI  CAEETN**S**AVCRDF  LIDALE**S**HHDTWP  IFEKGG**S**YLGREV  LLFRNG**S**VSRVPV  FRNGSV**S**RVPVLM  VPVLME**S**RSFYTA  VLMESR**S**FYTAYL  YMADLE**S**ALHYIL  VQNFLH**S**VNEWLK  WIGCQG**S**EPHFRG  FRGFPC**S**LWVLFH  EQMAAA**S**MHRVGS  VLWLWS**S**HNRVNA  RLAGAP**S**EDPQFP  KAHFSP**S**NIILDF  DFPAAG**S**AARRDV  GALELE**S**RNSTLD  KPEMMK**S**PTNTTP  AEGPEA**S**RPPKLH  LGQWHL**S**KRDTGA  VRRVGR**S**SKQLVD  VLGGGF**S**YLDISL  VGLYSL**S**FMGLLA  AEKTQK**S**VKIAPG  AVVCVE**S**EIRGDV  FEVGCY**S**QAMKMG  DNNVIE**S**KAYVGR  RNVILT**S**GCIIGA  RVTNVG**S**LLLTPQ  TPQENE**S**LFTFLG  KKCVTM**S**SAVVQL  KCVTMS**S**AVVQLY  DRNCMW**S**KKCSGV  MWSKKC**S**GVACLV  KDNPQR**S**YFLRIF  NNFVYN**S**PRGYFH  GRRQRK**S**EKRRDP  PQVNNI**S**HTKEKK  ADIGTP**S**NFQHIG  LKDRET**S**KVIYDF  PPPPPP**S**RGGPPP  PPPPHN**S**GPPPPP  APPPPP**S**RAPTAA  PPPPPP**S**RPSVAV  PPPSRP**S**VAVPPP  PPPALP**S**SAPSGP  PPALPS**S**APSGPP  LPSSAP**S**GPPPPP  PPPPPP**S**VLGVGP  PPPGLP**S**DGDHQV  KKVEQN**S**RPVSCS  QNSRPV**S**CSGRDA  SRPVSC**S**GRDALL  QGIQLK**S**VADGQE  VADGQE**S**TPPTPA  PTPAPT**S**GIVGAL  EVMQKR**S**KAIHSS  LLGNLL**S**PDNVVR  ENIPGQ**S**KITFLL  LLRRLL**S**SAFDEV  VYPALP**S**DVQTAI  LKFLFD**S**VSSQNV  FLFDSV**S**SQNVGL  LFDSVS**S**QNVGLR  QDQEHP**S**IRTLSA  CYQNDD**S**VLKSLV  DDSVLK**S**LVEIAD  EATLQL**S**LKLCGD  KLCGDT**S**LNNMQR  EDDDFD**S**NAVAGE  TEDCPK**S**LLIPYL  LVKHLH**S**IMVLKL  KTIECI**S**LIGLAV  QISYMI**S**AWARMC  RVAAAE**S**MPLLLE  IGTEPD**S**DVLSEI  PDSDVL**S**EIMHSF  LSEIMH**S**FAKCIE  YILTKV**S**DILHSI  VSDILH**S**IFSSYK  ILHSIF**S**SYKEKV  LHSIFS**S**YKEKVL  EHCSPA**S**FKYAEY  QYVCDN**S**PEVRQA  LVRVIQ**S**ADSKTK  VIQSAD**S**KTKENV  VLPHWL**S**WLPLHE  LCDLIE**S**NHPIVL  NLPKIF**S**IIAEGE  ECIAQL**S**PEQQAA  GDSDGD**S**DDEEGY  SNQNVN**S**KVNKYY  TKSERY**S**SSGSPA  RYSSSG**S**PANSFH  VLIKMA**S**QPFGRG  QQWKGA**S**NYVAKR  KYIKYN**S**NSGFVR  IKYNSN**S**GFVRDD  HFTFER**S**GHQLIV  MALFFY**S**HACNRI  CNRICE**S**MGLAPF  LAPFDL**S**PRERDA  NTKLLQ**S**AKTILR  VRTLSG**S**RPPLLR  PLLRPL**S**ENSGDE  RPLSEN**S**GDENMS  SGDENM**S**DVTFDS  SDVTFD**S**LPSSPS  FDSLPS**S**PSSATP  LHWPVF**S**DLDNMA  HRESEN**S**GDSGYP  SENSGD**S**GYPSEK  PREHGH**S**YSNRKY  WNLLNS**S**RLHLPR  EKKIGK**S**ILGKVH  EEWDQE**S**AVFHLE  GLGLMY**S**QLPHHI  HILADV**S**LKETEE  EAGDRQ**S**MILVAR  VARAFD**S**GQNLSP  EKDPQR**S**GDLYTQ  VVVGTQ**S**SGKSSV  TQSSGK**S**SVLESL  QSSGKS**S**VLESLV  KSSVLE**S**LVGRDL  NETERI**S**GNNKGV  GNNKGV**S**PEPIHL  LILRFI**S**NPNSII  FISNPN**S**IILAVT  IGVVNR**S**QLDINN  DINNKK**S**VTDSIR  LQKKYP**S**LANRNG  EPVDDK**S**ATLLQL  FGRTLE**S**VDPLGG  LFVPEV**S**FELLVK  KRLEEP**S**LRCVEL  QHCSNY**S**TQELLR  ELPSAV**S**RDKSSK  AVSRDK**S**SKVPSA  VSRDKS**S**KVPSAL  KSSKVP**S**ALAPAS  SALAPA**S**QEPSPA  EPSPAA**S**AEADGK  GKLIQD**S**RRETKN  ETKNVA**S**GGGGVG  RGMLKT**S**KAEELL  IERLIK**S**YFLIVR  RKNIQD**S**VPKAVM  VGQLYK**S**SLLDDL  GQLYKS**S**LLDDLL  DDLLTE**S**EDMAQR  KALQGA**S**QIIAEI  FKECPS**S**HPEPTR  PVTPIL**S**PSFSAQ  RMPTFP**S**TEPIYL  TEPIYL**S**LPGQSP  PFHPQG**S**LPIYRP  IYRPVV**S**TDMAKL  DKIAST**S**EFLKNG  DLEITD**S**KVSNLQ  VATVTR**S**QSLNIR  DPNGTS**S**LPTGSS  SSLPTG**S**SLLQEV  TFTCDV**S**STVEII  NQVDVG**S**YVLKVC  GEDTSR**S**STRGSL  ISSNWV**S**NYEKYY  TSSHTN**S**VPGTVT  ILHKDS**S**LGLSKE  ALELLD**S**KFADQE  VLGALL**S**VGGKRL  SMERVQ**S**FFQKNK  VIFKCL**S**TGRDRG  SENFIY**S**CAGCCV  HAQMFG**S**FKRDRA  LFLNLL**S**LMIPSG  RFSGLP**S**NDEPIL  NDEPIL**S**FSPKTY  GRIKEV**S**VFTYHK  QSLMNA**S**TDVAEC  GGAVKL**S**ISYRNG  KRKTKI**S**RKTRNP  NEMLVY**S**GYSKET  GDVLVV**S**RAALQA  DLPEQF**S**PPDVAP  ERTGLD**S**ESHYRP  TGLDSE**S**HYRPEL  APRTDW**S**LSDVDQ  RTDWSL**S**DVDQWD  LADGIK**S**FLLALP  LVTPEA**S**AEARRA  HLGRVA**S**RAPALG  RAPPPP**S**SPPPGG  APPPPS**S**PPPGGA  GGAPDG**S**EPSPDF  PKAKPA**S**TVLANG  VLANGG**S**PPSLQD  WYWGDI**S**REEVNE  FLVRDA**S**SKIQGE  LVRDAS**S**KIQGEY  DGHYGF**S**EPLTFC  EPLTFC**S**VVDLIN  RLLYPV**S**KYQQDQ  QIVKED**S**VEAVGA  QTQEKC**S**KEYLER  QRILLN**S**ERLKSR  NSERLK**S**RIAEIH  IAEIHE**S**RTKLEQ  QLRAQA**S**DNREID  IDKRMN**S**LKPDLM  QAEEML**S**GKRDGT  TFLIRE**S**SQRGCY  FLIRES**S**QRGCYA  RGCYAC**S**VVVDGD  PYNLYG**S**LKELVL  LHYQHA**S**LVQHND  QAQTAF**S**ANPANP  ANPAIL**S**EASAPI  AILSEA**S**APIPHD  RLYPEL**S**QYMGLS  SQYMGL**S**LNEEEI  ANVAVV**S**GAPLQG  QLVARP**S**SINYMV  LVARPS**S**INYMVA  IGLRLK**S**IDNGIF  QLVQAN**S**PASLVG  QANSPA**S**LVGLRF  ENCAGW**S**SDKAHK  NCAGWS**S**DKAHKV  ITMHKD**S**TGHVGF  KNGKIT**S**IVKDSS  TSIVKD**S**SAARNG  SIVKDS**S**AARNGL  VIGLKD**S**QIADIL  QIADIL**S**TSGTVV  ADILST**S**GTVVTI  IKRMAP**S**IMKSLM  APSIMK**S**LMDHTI  YMIQKK**S**REESSG  PFVEKF**S**IEIETY  TGRGPL**S**DDWART  QPPGKP**S**TEARSA  PPGPDA**S**PDASFG  QHGGAV**S**PQSLSE  GAVSPQ**S**LSEWRM  ARDSEN**S**SEEEFF  EMTKWN**S**NDFIDA  LFLILH**S**GNILDS  DVQTLS**S**AFEAVT  ALVSNL**S**PYSHDG  YSHDGD**S**LSRSQD  FDALCH**S**ANAGTG  ANAGTG**S**RGSSRR  SPEPEG**S**QNSLQA  FCPPAA**S**SEAPDG  APDGPS**S**TARLDF  GFFLFG**S**PLGLVL  DTLQTH**S**SLFLEE  LEMLVP**S**TPTSTS  VPSTPT**S**TSGAFW  AQPAAP**S**TTSEVV  TSNHRA**S**DTVVCE  IMTQPL**S**GKWIHF  TEAVVF**S**IDGSFT  RVVAWL**S**QHNFPH  KAMFLQ**S**LVQEVE  YAALGL**S**PSQTYI  AQCQFL**S**DGYVAH  DFLRKQ**S**QLLRSR  AGLDLN**S**SDNQSG  NSSDNQ**S**GGSTAS  GFYDKD**S**SGWSSS  SSFGSR**S**DSRGKS  SDRGSG**S**RGRFDD  DYDGIG**S**RGDRSG  GSRGDR**S**GFGKFE  FERGGN**S**RWCDKS  SDEDDW**S**KPLPPS  LEQELF**S**GGNTGI  ACAQTG**S**GKTAAF  EEARKF**S**YRSRVR  VVWVEE**S**DKRSFL  EESDKR**S**FLLDLL  NATGKD**S**LTLVFV  TKKGAD**S**LEDFLY  EGYACT**S**IHGDRS  SIHGDR**S**QRDREE  QFRSGK**S**PILVAT  INFDLP**S**DIEEYV  GSSRGR**S**KSSRFS  SKSSRF**S**GGFGAR  ARDYRQ**S**SGASSS  RDYRQS**S**GASSSS  RQSSGA**S**SSSFSS  SSGASS**S**SFSSSR  ASSSSF**S**SSRASS  FSSSRA**S**SSRSGG  SSRASS**S**RSGGGG  RASSSR**S**GGGGHG  YGGNYN**S**QGVDWW  LTRLAL**S**TCPAAC  QLNEDC**S**KTQPCD  ECNFGA**S**STALKG  CNFGAS**S**TALKGI  GICRAQ**S**EGRPCE  RPCEYN**S**RIYQNG  IYQNGE**S**FQPNCK  LCPQEL**S**LPNLGC  WVCDED**S**IKDPME  IAVGKG**S**SLKRLP  AVGKGS**S**LKRLPV  CIVQTT**S**WSQCSK  VQTTSW**S**QCSKTC  TSWSQC**S**KTCGTG  TCGTGI**S**TRVTND  CGQPVY**S**SLKKGK  GQPVYS**S**LKKGKK  KKGKKC**S**KTKKSP  CSKTKK**S**PEPVRF  TYAGCL**S**VKKYRP  RPKYCG**S**CVDGRC  EDGETF**S**KNVMMI  NVMMIQ**S**CKCNYN  EANLIP**S**QEPFPA  PRNPLS**S**SDAAPS  DTNGLS**S**SARPQG  GSFDDY**S**SDEDSV  VPTLPW**S**GSGLTV  TLPWSG**S**GLTVEW  LFGRDC**S**IQRRHQ  KTVGYV**S**AGTVEY  ELNFRS**S**KNVWGY  LHEFAD**S**QFGHCF  NLLETE**S**FQNNDI  DGGLLL**S**YNGNSY  HVEAGS**S**YAEMEV  LQEIMT**S**VAGRIP  LVQRYR**S**GIRGYM  QFKPDM**S**QVLDCI  GPDPSL**S**DELISI  LTQLSK**S**EHCKVA  RQILIA**S**HLPSYE  IASHLP**S**YELRHN  FQFMLP**S**SHPNRM  LRTFVQ**S**KKNILV  LFITNE**S**GYYLDI  EVTDSR**S**GNIMFH  ITFRIG**S**FGPGED  IYVAAN**S**GARIGM  DPANLD**S**EAKIIQ  MYADKE**S**RGGVLE  KKDLIK**S**MRRIDP  QEILQA**S**GELSHV  SHVHIQ**S**MLRRWF  DAGASF**S**RHFAAN  AAPYGH**S**RSSEDV  PYGHSR**S**SEDVST  YGHSRS**S**EDVSTH  RSSEDV**S**THAATK  FSLRNM**S**LCVVDG  MWHRRA**S**PEPDAA  DDAAAG**S**GGSAQW  FVPPKA**S**RPKVSI  ILETID**S**LQKHSW  DSLQKH**S**WVADIQ  CVDPGD**S**EEDTEL  LASRVA**S**CSCELL  SRVASC**S**CELLTD  VVTAPH**S**RGRDAV  LESPGG**S**GSDSNN  SPGGSG**S**DSNNTG  LFVIRQ**S**ETRPGE  QHLWFQ**S**VLDMLR  HPIPLE**S**GGSADI  PLESGG**S**ADITLR  ADITLR**S**YVRAQD  PPAAPA**S**PACWSD  ASPACW**S**DSPGQH  PACWSD**S**PGQHYF  AACPPA**S**PSDAAG  SDAAGA**S**SSSASS  AAGASS**S**SASSSS  AGASSS**S**ASSSSA  SSSSAS**S**SSAASG  SSSASS**S**SAASGP  PVEGQL**S**ARSRSN  ARSRSN**S**AERLLE  VTAFIG**S**NIVTSQ  GSNIVT**S**QTIWEG  MNCVVQ**S**TGQMQC  QCKVYD**S**LLALPQ  RALVII**S**IIVAAL  ALGVLL**S**VVGGKC  NCLEDE**S**AKAKTM  MVIVPV**S**WTAHNI  YNPLVA**S**GQKREM  KREMGA**S**LYVGWA  YVGWAA**S**GLLLLG  RTDKPY**S**AKYSAA  PYSAKY**S**AARSAA  ALNAAG**S**VHSPST  AAGSVH**S**PSTSMA  GSVHSP**S**TSMATS  VHSPST**S**MATSSQ  STSMAT**S**SQYRQL  TSMATS**S**QYRQLL  QYRQLL**S**DYGPPS  TQGTGN**S**QVPQSK  NSQVPQ**S**KYAELL  RPTYAG**S**KSAMER  TYAGSK**S**AMERLK  SEEESV**S**LTVEER  DPNSGQ**S**WYFLGR  FLGRCY**S**SIGKVQ  LGRCYS**S**IGKVQD  FISYRQ**S**IDKSEA  RQSIDK**S**EASADT  LGTLYE**S**CNQPQD  ARSKRC**S**NTSTLA  PVQQVY**S**LCLTPQ  GAITDS**S**LPTNSV  ALTRVS**S**VSQPGV  RKQLSN**S**AQGLHK  SQSSCL**S**GPNEEQ  EEQPLF**S**TGSAQY  QYHQAT**S**TGIKKA  EHLTLP**S**NSVPQG  PQGDAD**S**HLSCHT  SCHTAT**S**GGQQGI  SLVPET**S**RHTGDT  RHTGDT**S**NGCADV  ADVKGL**S**NHVHQL  IADAVS**S**PNHGDS  TDHSVA**S**SPSSAI  EQRSIN**S**VTSLNS  SLNSPH**S**GLHTVN  GLGKSQ**S**STKVDL  VSICPS**S**TEVLKA  LDLGLF**S**TKTLVE  YAQYQA**S**SFQESL  STSSEN**S**GRRRKG  NLNFLM**S**SWWPNL  LNFLMS**S**WWPNLE  EWNKLK**S**VKSPVP  ADIEKA**S**IMYPSN  ASIMYP**S**NNCDKI  RCLNPK**S**KQARLI  QVHEIQ**S**CMGRLE  ETADKQ**S**VHIVEN  ENEIQA**S**IDQIFS  SIDQIF**S**RLERLE  ERLEIL**S**SKEPPN  RLEILS**S**KEPPNK  QREELL**S**RTFTTN  TFTTND**S**DTTIPM  TIPMDE**S**LQFNSS  ESLQFN**S**SLQKVH  SLQFNS**S**LQKVHN  ANMLGL**S**NTVMRL  ALIGTG**S**SCPGAM  PGAMWL**S**TATGSR  GHRKEL**S**GVFRLC  VVVQLL**S**IRRCGH  RRFVTP**S**EPVAHS  LPRGRR**S**RRAVSV  RSRRAV**S**VPASFF  AVSVPA**S**FFRRLA  FRRLAP**S**PARPRH  KEDQEG**S**GGDYMP  SGGGQG**S**NGQGSS  NGQGSS**S**HSSGGN  PGGGHG**S**GGGQGP  TGGKGK**S**GGRFRL  PQNVSA**S**KKRHSR  ASKKRH**S**RSPFED  MMFPRV**S**PPPAPS  DSKDND**S**ESDYMF  KDNDSE**S**DYMFMA  SWSSYF**S**LPNPFR  DPKDAA**S**KPSGEG  GDGGSP**S**KPSDHE  KRPNRL**S**FITKGY  EQREAD**S**SSDYVN  DFTKRE**S**NTPAPS  PRQSAF**S**NYVNVE  PLSATG**S**NAIEEE  YVVDPF**S**ECCMDI  CMDISL**S**PSRCSE  LSPSRC**S**EPPPVA  SAFPTD**S**LERDLS  SLERDL**S**PSSAPA  SAPAVA**S**AAEPTL  RHYEGL**S**YNVDSL  LSYNVD**S**LHQKHQ  EFKVET**S**NKVLDY  VLDYDT**S**HIYTGH  IYGEEG**S**FSHGSV  RTLPFH**S**VIYHED  AVIAQI**S**SHVKAI  VIAQIS**S**HVKAID  YQTTDF**S**GIRNIS  SGIRNI**S**FMVKRI  AWVGAP**S**GSSGGI  VGAPSG**S**SGGICE  GAPSGS**S**GGICEK  GGICEK**S**KLYSDG  HVPPKV**S**HITFAH  MYARAT**S**GDKLNN  LNNNKF**S**LCSIRN  CSIRNI**S**QVLEKK  NNCFVE**S**GQPICG  KPGKQC**S**PSQGPC  EKCRDD**S**DCAREG  TALCPA**S**DPKPNF  EECTCA**S**SDGKDD  ECTCAS**S**DGKDDK  MKKMDP**S**TCASTG  DPSTCA**S**TGSVQW  TCASTG**S**VQWSRH  TGSVQW**S**RHFSGR  ITLQPG**S**PCNDFR  LKKAIF**S**PELYEN  GFIKIC**S**VHTPSS  SVHTPS**S**NPKLPP  RQRPRE**S**YQMGHM  KGKKRH**S**SSSSQS  GKKRHS**S**SSSQSS  KKRHSS**S**SSQSSE  KRHSSS**S**SQSSEI  RHSSSS**S**QSSEIS  SSSSSQ**S**SEISTK  SSSSQS**S**EISTKS  SQSSEI**S**TKSKSV  SEISTK**S**KSVDSS  ISTKSK**S**VDSSLG  KSKSVD**S**SLGGLS  SKSVDS**S**LGGLSR  SSLGGL**S**RSSTVA  LGGLSR**S**STVASL  GGLSRS**S**TVASLD  RSSTVA**S**LDTDST  ASLDTD**S**TKSSGQ  DTDSTK**S**SGQSNN  TDSTKS**S**GQSNNN  TKSSGQ**S**NNNSDT  GQSNNN**S**DTCAEF  IEKLKL**S**EGKGLE  EFIMGV**S**KYGIKV  KYGIKV**S**TSDQYD  GIKVST**S**DQYDVL  GLGAGK**S**LLALKT  LKTTDA**S**NEEYSL  ASNEEY**S**LWVYQC  WVYQCN**S**LEQAQA  AICKVL**S**TAFDSV  RNNLGN**S**SDSKNE  NNLGNS**S**DSKNED  LGNSSD**S**KNEDGS  SKNEDG**S**VFSQTE  EDGSVF**S**QTEHNI  IMAGMI**S**IISNII  GMISII**S**NIIVLG  VTDIGV**S**SIGYPM  TDIGVS**S**IGYPMS  SIGYPM**S**AASDLY  YPMSAA**S**DLYGSW  ASDLYG**S**WKFGYA  IFFGMA**S**IGLLTV  PIIGWA**S**YAPDPT  WRKNDR**S**FVSYTM  NDRSFV**S**YTMTVI  YYHVTL**S**IKHHTT  IKHHTT**S**DCTESL  TSDCTE**S**LNRDWS  SLNRDW**S**DQIDVT  IDVTKM**S**VIMICM  MFLVAW**S**PYSIVC  VAWSPY**S**IVCLWA  IVCLWA**S**FGDPKK  APLFAK**S**STFYNP  PLFAKS**S**TFYNPC  QTMPVT**S**ILPMDV  ILPMDV**S**QNPLAS  VGGAGG**S**RVSSGR  AGGSRV**S**SGRDLN  GGSRVS**S**GRDLNC  PGPQTR**S**DLLLSG  RSDLLL**S**GRDWNT  LIVGKL**S**PWIRPD  PWIRPD**S**KVEKIR  EKIRRN**S**EAAMLQ  IHTGHH**S**SMFWMR  HTGHHS**S**MFWMRV  IGADLP**S**NHVIDR  AAILPT**S**IFLTNK  KGFPVL**S**KMHQRL  TGTNHH**S**EKEFCS  SEKEFC**S**YLQYLE  QYLEYL**S**QNRPPP  YEDYLQ**S**PLQPLM  LMDNLE**S**QTYEVF  GPLVNA**S**LRAAKQ  QFEEWG**S**QVTVVS  SQVTVV**S**SDMREW  QVTVVS**S**DMREWV  KADIIV**S**ELLGSF  VSELLG**S**FADNEL  FADNEL**S**PECLDG  FLAPIS**S**SKLYNE  LAPISS**S**KLYNEV  HNFHQL**S**APQPCF  QPCFTF**S**HPNRDP  YQDITL**S**IRPETH  IRPETH**S**PGMFSW  HSPGMF**S**WFPILF  FWRCSN**S**KKVWYE  THQQVV**S**RIRAAL  PREADK**S**HPEQRE  YGFNLH**S**DKSKPG  NLHSDK**S**KPGQFI  PGQFIR**S**VDPDSP  RSVDPD**S**PAEASG  DSPAEA**S**GLRAQD  QHGDVV**S**AIRAGG  KCRVIP**S**QEHLNG  RPALVR**S**ASSDTS  TSEELN**S**QDSPPK  SPPKQD**S**TAPSST  QDSTAP**S**STSSSD  DSTAPS**S**TSSSDP  TAPSST**S**SSDPIL  APSSTS**S**SDPILD  PSSTSS**S**DPILDF  ILDFNI**S**LAMAKE  RAHQKR**S**SKRAPQ  AHQKRS**S**KRAPQM  APQMDW**S**KKNELF  RCRAVR**S**LLRSHY  PSFRQV**S**CLKELV  PEAFTT**S**VRSYLP  FTTSVR**S**YLPNTV  TDALRG**S**GAWGLL  ERAWNH**S**VREAGV  GGSASR**S**LPLPKR  RGFCVV**S**PARPAE  SLEGAL**S**GTRHSH  HHAGPP**S**TSRPPR  AGPPST**S**RPPRPW  TKHFLY**S**SGDKEQ  KEQLRP**S**FLLSSL  QLLRQH**S**SPWQVY  PPGLWG**S**RHNERR  NTKKFI**S**LGKHAK  VVELLR**S**FFYVTE  RPALLT**S**RLRFIP  PGLLGA**S**VLGLDD  LTEVIA**S**IIKPQN  VVIEQS**S**SLNEAS  VIEQSS**S**LNEASS  SSLNEA**S**SGLFDV  VRIRGK**S**YVQCQG  RTLEVQ**S**DYSSYA  RTSIRA**S**LTFNRG  LDLQVN**S**LQTVCT  VISDTA**S**LCYSIL  TASLCY**S**ILKAKN  AAGPLP**S**EAVQWL  TAQTQL**S**RKLPGT  LFLEYC**S**GGELFD  RDNLKI**S**DFGLAT  EPVDVW**S**CGIVLT  LPWDQP**S**DSCQEY  WDQPSD**S**CQEYSD  DSCQEY**S**DWKEKK  PWKKID**S**APLALL  ILVENP**S**ARITIP  VTSGGV**S**ESPSGF  GVSESP**S**GFSKHI  ESPSGF**S**KHIQSN  DFSPVN**S**ASSEEN  SPVNSA**S**SEENVK  PVNSAS**S**EENVKY  EENVKY**S**SSQPEP  ENVKYS**S**SQPEPR  EPRTGL**S**LWDTSP  LSLWDT**S**PSYIDK  LWDTSP**S**YIDKLV  KLVQGI**S**FSQPTC  DHMLLN**S**QLLGTP  LLGTPG**S**SQNPWQ  LGTPGS**S**QNPWQR  KLDADK**S**YQCLKE  GYQWKK**S**CMNQVT  MNQVTI**S**TTDRRN  LVDFRL**S**KGDGLE  KLIDIV**S**SQKIWL  LIDIVS**S**QKIWLP  KRSSVS**S**GGAGRL  GGAGRL**S**MQELRS  SMQELR**S**QDVNKQ  PTFGKL**S**INKPTS  TSERKV**S**LFGKRT  LFGKRT**S**GHGSRN  RTSGHG**S**RNSQLG  SQLGIF**S**SSEKIK  QLGIFS**S**SEKIKD  GYAHNV**S**MKSLQA  HNVSMK**S**LQAPSV  KSLQAP**S**VKDFLK  YGFLCP**S**YELPDT  HTAMKE**S**SPLFDD  TAMKES**S**PLFDDG  TIKCYE**S**FMSGAD  CYESFM**S**GADSFD  FMSGAD**S**FDEMNA  MNAELQ**S**KLKDLF  EPNRLE**S**LRKLKA  KYQAYM**S**NLESHS  YMSNLE**S**HSAILD  SNLESH**S**AILDQK  IDNQKY**S**VADIER  PKGAEN**S**KGYDFE  NAMITE**S**KRSVRT  CASELE**S**LEKHKH  HKHLLE**S**TVNQGL  TVNQGL**S**EAMNEL  VATHVG**S**VEKHLE  CMSEDL**S**ENIKEI  AALLRV**S**VLCIWM  VLCIWM**S**ALFLGV  VQQNVP**S**GTDTGD  DTGDPQ**S**KPLGDW  GTMDPE**S**SIFIED  TMDPES**S**IFIEDA  YFKEKV**S**TQNLLL  EFPRLK**S**ELEDNI  TIANVV**S**GSLSIS  ANVVSG**S**LSISSG  VVSGSL**S**ISSGIL  SGSLSI**S**SGILTL  GSLSIS**S**GILTLV  PFTEGG**S**LVLLEP  ALTGIT**S**STMDYG  LTGITS**S**TMDYGK  HDLVIK**S**LDKLKE  FLGENI**S**NFLSLA  NISNFL**S**LAGNTY  ARANLQ**S**VPHASA  QSVPHA**S**ASRPRV  VPHASA**S**RPRVTE  ERVNEP**S**ILEMSR  PSILEM**S**RGVKLT  TDVAPV**S**FFLVLD  VYLVYE**S**KHLHEG  LHEGAK**S**ETAEEL  RAITVF**S**PDGHLF  EAVKKG**S**TAVGVR  LGVEKK**S**VAKLQD  ARVECQ**S**HRLTVE  ITRYIA**S**LKQRYT  KQRYTQ**S**NGRRPF  RRPFGI**S**ALIVGF  LYQTDP**S**GTYHAW  IGRGAK**S**VREFLE  LLEVVQ**S**GGKNIE  VMRRDQ**S**LKILNP  KKTAEA**S**RWHEKW  YLLEGC**S**CERTPA  CSPHAE**S**MRKRNQ  TMVEAE**S**EYVHQL  PLRMAA**S**SKKPPI  SHDDVS**S**IFLNSE  SSIFLN**S**ETIMFL  SLEFAK**S**KLEELS  VMHDEV**S**DTENIR  LICTRS**S**GGKLHL  VVLLAP**S**RQEKAA  EKAAWM**S**DISQCV  IVFEEN**S**KVTVPH  VPHMIK**S**DARLHK  PQIRYA**S**VERLLE  TDLRFL**S**IDFLNT  YKRPFT**S**IPVRSL  HLVDGK**S**PRLCRK  ALDLTT**S**SSPTTT  DLTTSS**S**PTTTTQ  QIPLDL**S**RGLSSP  DLSRGL**S**SPEQSP  LSRGLS**S**PEQSPG  CNKLKR**S**IQKAVL  NAHCSV**S**PASAFA  ECFESL**S**AMELAE  MNYADV**S**SRANAI  TSALNR**S**AIYRLK  KTWAKV**S**KQTKAL  LQKTVS**S**EGRFKN  DTLYEL**S**LKIEPR  QCRQEL**S**PRNRER  AILTLL**S**DIASAL  LLSDIA**S**ALRYLH  VTVDYW**S**FGTLAF  QPVQWH**S**KVRQKS  SKVRQK**S**EVDIVV  NGTVKF**S**SSLPYP  GTVKFS**S**SLPYPN  YPNNLN**S**VLAERL  PVTEDE**S**LQSLKA  PATQCI**S**DGKLNE  VFLFDN**S**KITYET  TYETQI**S**PRPQPE  PRPQPE**S**VSCILQ  PQPESV**S**CILQEP  WGQVWH**S**IQTLKE  NLLRNN**S**CLSKMK  RNNSCL**S**KMKNSM  LSKMKN**S**MASMSQ  MKNSMA**S**MSQQLK  NSMASM**S**QQLKAK  LDFFKT**S**IQIDLE  IDLEKY**S**EQTEFG  TEFGIT**S**DKLLLA  IVDLQR**S**PMGRKQ  QRTEGD**S**QEMVRL  LLQAIQ**S**FEKKVR  VIYTQL**S**KTVVCK  KVEEVV**S**LMNEDE  LLKIAC**S**KVRGPV  PDSMNA**S**RLSQPG  IGKGPF**S**VVRRCI  TSSPGL**S**TEDLKR  DLKREA**S**ICHMLK  ELLETY**S**SDGMLY  ILFILL**S**GCLPFY  RQWSHI**S**ESAKDL  VLAAVS**S**HKFNSF  SSHKFN**S**FYGDPP  EELPDF**S**EDPTSS  FSEDPT**S**SGLLAA  AAERAV**S**QVLDSL  HALTDC**S**EKDLDF  KINTKS**S**PQIRNP  QIRNPP**S**DAVQRA  EVLEEI**S**CYPENN  PYLNGD**S**PESANG  REINGI**S**VANQTV  LREMRG**S**ITFKIV  TFKIVP**S**YRTQSS  PSYRTQ**S**SSCERD  YRTQSS**S**CERDSP  SSCERD**S**PSTSRQ  RDSPST**S**RQSPAN  SPANGH**S**STNNSV  HSSTNN**S**VSDLPS  STNNSV**S**DLPSTT  DIIQII**S**KDDHNW  TAGLIP**S**PELQEW  KNYYFV**S**HDQMMQ  GLNEDE**S**LQRLQK  QRLQKE**S**DILQRT  FAMFDQ**S**QIQEFK  LHDMLA**S**LGKNPT  VGIYFL**S**SCRAEE  GIYFLS**S**CRAEEG  GKDRVV**S**LSEKNF  DRVVSL**S**EKNFKQ  YYHEPV**S**SDKVTQ  YHEPVS**S**DKVTQK  GFDEEG**S**LYILKG  DPVEII**S**SKLEVQ  PVEIIS**S**KLEVQA  LIGFFK**S**EDSEYY  FFKSED**S**EYYKAF  GVAKKL**S**LKMNEV  VAFAEK**S**DPDGYE  TDNPDL**S**ILWIDP  NVTDAD**S**VWMEIP  WIEDVL**S**GKINTE  SKENCI**S**GPVKAT  VLCPSN**S**SQRVPL  LCPSNS**S**QRVPLQ  QAQKLV**S**SHKPVQ  AQKLVS**S**HKPVQN  KQLQAT**S**VPHPVS  SVPHPV**S**RPLNNT  LNNTQK**S**KQPLPS  SKQPLP**S**APENNP  PEEELA**S**KQKNEE  KQKNEE**S**KKRQWA  LAREKQ**S**KFILAL  REVEIQ**S**HLRHPN  RELQKL**S**KFDEQR  ELANAL**S**YCHSKR  ALSYCH**S**KRVIHR  ENLLLG**S**AGELKI  IADFGW**S**VHAPSS  WSVHAP**S**SRRTTL  SVHAPS**S**RRTTLC  EKVDLW**S**LGVLCY  GARDLI**S**RLLKHN  LLKHNP**S**QRPMLR  PWITAN**S**SKPSNC  WITANS**S**KPSNCQ  ANSSKP**S**NCQNKE  DVRHAK**S**GGTALH  KQNLLH**S**EKRDKK  KSPLIE**S**TANMDN  NMDNNQ**S**QKTFKN  VTTPTV**S**SGQATP  LAEITA**S**KEGQKE  RRLAST**S**DIEEKE  KENRDS**S**SLRTSS  ENRDSS**S**LRTSSS  SSSLRT**S**SSYTRR  SSLRTS**S**SYTRRK  SSVNEG**S**TYHKSC  GSTYHK**S**CSFGRR  RQDDLI**S**SSVPST  STPTVT**S**AAGLQK  QKSLLS**S**TSTTTK  GTQSST**S**NRLWAE  RLWAED**S**TEKEKD  EKTIGR**S**RSTRTR  KKESET**S**REDEYK  RPVSTS**S**STTPSS  SSSTTP**S**SSLSTM  SSTTPS**S**SLSTMS  TPSSSL**S**TMSSSL  SLSTMS**S**SLYASS  LSTMSS**S**LYASSQ  SSLYAS**S**QLNRPN  SLVGIT**S**AYSRGI  SDTEEG**S**NKKETQ  SRYETS**S**TSAGDR  EERKPY**S**SRLEKD  EVPPRL**S**HVPPPL  PPPLFP**S**APATLA  APATLA**S**RSLSHW  ATLASR**S**LSHWRP  LASRSL**S**HWRPRP  LAPLLP**S**LAPSSA  LPSLAP**S**SARQGA  PSLAPS**S**ARQGAR  VTAQQP**S**RLAGGA  FRRHFK**S**SSIQRS  RRHFKS**S**SIQRSA  RHFKSS**S**IQRSAA  SSSIQR**S**AAAAAA  QHPPAD**S**SVTMED  HPPADS**S**VTMEDM  EDMNEY**S**NIEEFA  EEFAEG**S**KINASK  GSKINA**S**KNQQDD  MFIGGL**S**WDTSKK  GLSWDT**S**KKDLTE  DLTEYL**S**RFGEVV  DPVTGR**S**RGFGFV  LFKDAA**S**VDKVLE  VFVGGL**S**PDTSEE  GLSPDT**S**EEQIKE  VKKLLE**S**RYHQIG  RYHQIG**S**GKCEIK  GYGNYN**S**AYGGDQ  GGDQNY**S**GYGGYD  QGYADY**S**GQQSTY  DYSGQQ**S**TYGKAS  STYGKA**S**RGGGNH  RRRLSD**S**SFMANL  RRLSDS**S**FMANLP  RPDSST**S**SPASPA  STSSPA**S**PAMERR  LAASFS**S**PGSSLF  SFSSPG**S**SLFSSL  PGSSLF**S**SLSSAM  LFSSLS**S**AMKQAP  QAPQAT**S**GLMEPP  MEPPGP**S**TPIVQR  DAHTDW**S**KYFHGK  NGTKVV**S**RSFKPD  AVNSLY**S**VYNFCS  DFQDIT**S**VVAMAK  TEAFID**S**KYDIRI  YMRTSI**S**GNWKAN  WKANTG**S**AMLEQV  YRLWVD**S**CSEMFG  LVVSKM**S**QLPMPG  PGGTAP**S**PLRPWA  WAPQIK**S**AKSPGQ  GPRQAQ**S**PQPQRS  SPQPQR**S**GSPSQQ  QRSGSP**S**QQRLSP  SPQQQR**S**PGSPQL  PQLSRA**S**SGSSPN  SSPNQA**S**KPGATL  VQGRST**S**QQGEES  SQSLTN**S**LSTSDT  TNSLST**S**DTSQRG  LSTSDT**S**QRGTPS  YAEFPP**S**PSNPTK  EDVDMS**S**GSSGHE  VDMSSG**S**SGHETN  DMSSGS**S**GHETNE  GSDCDD**S**GKELGM  SLMMAK**S**EHNPST  KAKGKA**S**TLATLK  YYQLLM**S**SEGHPC  EMESVT**S**EHIVKN  GKILYI**S**DQVASI  SFFCRV**S**VRKSHE  TLDTSW**S**SFINPW  PVPHSG**S**SGYGSL  LMSQTS**S**SDSNGH  KNQPTC**S**YQQISC  QISCLD**S**VIRYLE  VIRYLE**S**CNEAAT  VPALRS**S**DKRKAT  KLSIFQ**S**HCHYYL  NTSGID**S**PWKKTG  VKPRDS**S**ESTGSG  SSESTG**S**GGPVSA  SDTSQS**S**CPAVPF  LTSEMA**S**ASQPEF  EFPSRT**S**IPRQPC  FQSRSS**S**PLQLNL  LNEDLC**S**ASGSAA  LCSASG**S**AASESL  TSKYFG**S**IDSSEN  LMADAD**S**SVMMTY  KEDENG**S**PLNHRI  KLRDQP**S**LVQAIF  LHRAVA**S**CSEEAV  RAVASC**S**EEAVQV  ALVPLL**S**NVNVSD  LHHAAF**S**GHGEMV  MVKLLL**S**RGANIN  VVKLLV**S**HGAEVT  TCKDKK**S**YTPLHA  PLHAAA**S**SGMISV  LHFAAA**S**THGALC  ADVNMK**S**KDGKTP  ALHGRF**S**RSQTII  LHLAAL**S**GFSDCC  AALSGF**S**DCCRKL  CCRKLL**S**SGFDID  LFALVG**S**GASVND  LVGSGA**S**VNDLDE  LHYAAT**S**DTDGKC  YNAVHY**S**AAYGHR  LDVRNS**S**GRTPLD  IVNATD**S**KGRTPL  CLQLLL**S**HNAQVN  MLVSSA**S**AELTLQ  ALHLAC**S**KGHETS  LLGKGA**S**VLAVDE  ATMMPV**S**SSSPLS  SSSSPL**S**SLTFNA  SSSPLS**S**LTFNAI  IMRNEP**S**SYCSFN  MRNEPS**S**YCSFNN  EPSSYC**S**FNNIGG  VRRLWR**S**RAPGGE  GEGATD**S**RAHGAG  LKALTH**S**VLKKLK  LLQAVE**S**RGGTRT  QPAQPP**S**SYSLPL  PAQPPS**S**YSLPLL  QPPSSY**S**LPLLLC  WPDLRH**S**SEVKRL  PDLRHS**S**EVKRLC  RLCCCE**S**YGKINP  CNPHHL**S**RLCELE  RLCELE**S**PPPPYS  SPPPPY**S**RYPMDF  CPDAVP**S**SAETGG  PDAVPS**S**AETGGT  LAPGGL**S**DSQLLL  PGGLSD**S**QLLLEP  LEPGDR**S**HWCVVA  YCVQEP**S**LDIFYD  CLGQLN**S**DNKSQL  LNSDNK**S**QLVQKV  LVQKVR**S**KIGCGI  VWVYNR**S**SYPIFI  WVYNRS**S**YPIFIK  YPIFIK**S**ATLDNP  TLDNPD**S**RTLLVH  KVFPGF**S**IKAFDY  DYEKAY**S**LQRPND  GFTVQI**S**FVKGWG  YTRQFI**S**SCPCWL  TRQFIS**S**CPCWLE  STSAQC**S**TSDSAC  SAQCST**S**DSACRI  QCSTSD**S**ACRISP  LGRQSF**S**VKDPSP  FSVKDP**S**PLYDML  HSMDIP**S**QDQLKQ  QDQLKQ**S**AEESST  QSAEES**S**TSRKRT  AEESST**S**RKRTTE  IPTLPT**S**EHKCIH  EHKCIH**S**REDEDL  SNYTPR**S**NGSTDL  TPRSNG**S**TDLQTN  VGTAIV**S**DTTDDL  LWFLNE**S**VSEQLG  FLNESV**S**EQLGVG  ADTEQT**S**EEVGKV  EEVGKV**S**DKKVIE  TDVEVT**S**EDEWQC  ECKKFN**S**PSKRYC  DWYSDC**S**KLTHSL  THSLST**S**DITAIP  YIKKEN**S**KLFDPC  LFDPCN**S**VEFLDL  FLDLAH**S**SESQET  LDLAHS**S**ESQETI  LAHSSE**S**QETISS  ESQETI**S**SMGEQL  SQETIS**S**MGEQLD  NLLKPC**S**LCEKRP  LKKAGA**S**CPICKK  AVEEMR**S**RVVLGE  DFPGNY**S**GYDDAW  VHMDEN**S**LEFDMV  LVYNNT**S**IVQDEI  PHAAKD**S**SDPNEL  HAAKDS**S**DPNELY  KDHAKF**S**PVATAS  SPVATA**S**YRLLPD  EAAEEL**S**RCFSPG  ELSRCF**S**PGVIEV  PRLDTF**S**REIFRN  RDHYIF**S**VESTGV  YIFSVE**S**TGVLPP  PPDVLV**S**EAIKVL  NSQMNA**S**HPETNL  QGPPGY**S**GYPGPQ  YPGPQV**S**YPPPPA  PPPAGH**S**GPGPAG  PGLEYL**S**QIDQIL  KYEIKN**S**FGQRVY  RNCCGP**S**RPFTLR  ERPLRC**S**SCCCPC  RPLRCS**S**CCCPCC  EDVLKI**S**GPCVVC  GPCVVC**S**CCGDVD  VDFEIK**S**LDEQCV  CVVGKI**S**KHWTGI  DFMFFE**S**TGSQEQ  FFESTG**S**QEQKSG  TDPRPA**S**YSFCSG  PRPASY**S**FCSGKG  ASYSFC**S**GKGVGI  GIKGET**S**TATPRR  WAESLH**S**LLDDQD  KLEPCD**S**NEEKRL  DNNGIV**S**RQTKPA  TKPATK**S**FIKGCI  ESPKVC**S**DQSSGS  KVCSDQ**S**SGSGTG  SDQSSG**S**GTGKGI  TAAPRV**S**SSRRYS  NDSEQQ**S**LSSDAD  EQQSLS**S**DADTLS  TLSLTD**S**SVDGIP  LSLTDS**S**VDGIPP  RREMQE**S**VQVNGR  HHHVHH**S**TARPKE  TRRAQS**S**FAWGLE  ARSRGY**S**ESVGAA  SRGYSE**S**VGAAPN  GAAPNA**S**DGLAHS  SDGLAH**S**GKVGVA  NAKKAE**S**GKSAST  ESGKSA**S**TEVPGA  ENSRPL**S**LEHPWA  GPQLRT**S**VQPSHL  HVVPAV**S**DMELSE  RKVGGG**S**AQPCDS  SAQPCD**S**IVVAYY  LLTKKG**S**YRYYFK  PLLLLP**S**AAPDPV  TRGPGG**S**PGRRPS  PDGAPA**S**GTSEPG  APASGT**S**EPGRAR  GAGVCK**S**RPLDLV  LVFIID**S**SRSVRP  VFIIDS**S**RSVRPL  IIDSSR**S**VRPLEF  KVKTFV**S**RIIDTL  AVVNYA**S**TVKIEF  AYTDKQ**S**LKQAVG  GRITPL**S**TGTMSG  LSTGTM**S**GLAIQT  AGAREP**S**SNIPKV  GAREPS**S**NIPKVA  AARAQA**S**GIELYA  DRADMA**S**LKMMAS  SLKMMA**S**EPLEEH  GVIEKL**S**SRFQET  VIEKLS**S**RFQETF  CQHVCI**S**DGEGKH  ADKKTC**S**ALDRCA  VNDRSG**S**YHCECY  VNDRTG**S**HHCECY  ADKKTC**S**VRDKCA  DKCALG**S**HGCQHI  CQHICV**S**DGAASY  VSDGAA**S**YHCDCY  EDKKTC**S**ATEEAR  AFQDKV**S**SYLQRL  FQDKVS**S**YLQRLN  ALRLSA**S**ALFCLL  QPPPLS**S**EHKEPV  VRGARN**S**EPQDEG  ALDRPA**S**PPAPSG  ASPPAP**S**GSQQGP  PPAPSG**S**QQGPEE  LTETVR**S**QTHSLP  VRSQTH**S**LPAPES  SLPAPE**S**PEPAAP  PEASDP**S**EELEAL  ELEALA**S**LLQELR  QELRDF**S**PSSAKR  LRDFSP**S**SAKRQQ  RDFSPS**S**AKRQQE  TRVNLE**S**PGPERV  ERVWRA**S**WGEFQA  LPPPAP**S**QFQARM  QARMPD**S**GPLPET  KFGEGV**S**SPKTHL  FGEGVS**S**PKTHLG  EALAPL**S**KAYQGV  KARRPE**S**ALLGGS  SALLGG**S**EAGERL  RLADLA**S**DLLLQY  AAEERE**S**AREEEE  DPQTID**S**LIELST  DSLIEL**S**TKLHLP  PADDVV**S**IIEEVE  APTHVR**S**PQPPPP  RTLQPP**S**ALRRRH  HHALPP**S**RHYPGR  QVDSLL**S**ESQLKE  NALQKL**S**KADESA  LSKADE**S**APVANY  GLAVTI**S**RENITE  TEEEEE**S**ESEDSE  SEDSED**S**GGEEED  TYLEPY**S**EEEEGQ  EEEGQE**S**SEEGSE  ESSEEG**S**EEDVEA  AVQKAI**S**EAGIFC  VEDTNG**S**ETGFRA  PAGFRP**S**TLSQLL  QPELMP**S**QLAFRD  SRPSRI**S**LILTLW  LILTLW**S**CKMIPL  IPLPGM**S**IQVLSR  PKTWTF**S**PQVTRI  GDCFIR**S**NSASPD  IRSNSA**S**PDLGIL  LFELGI**S**YIRNST  ISYIRN**S**TGERGE  GERGEL**S**CGWVFL  EVDPSI**S**RRAHGS  RSLNRR**S**RNVLSL  RSRNVL**S**LLPETL  LIGNMC**S**IHLLIF  DRMSLQ**S**TDLISH  VMDALR**S**SWAGKE  SWAGKE**S**TLKRSE  ESTLKR**S**EKRDKE  DKEFLK**S**TFLLVY  ALQALL**S**PDGVHE  HEPFDL**S**EQTYDF  LPKTYV**S**PTHVGS  SPTHVG**S**GAYGSV  GSGAYG**S**VCSAID  AYGSVC**S**AIDKRS  SAIDKR**S**GEKVAI  VAIKKL**S**RPFQSE  LSRPFQ**S**EIFAKR  DVFTPA**S**SLRNFY  VFTPAS**S**LRNFYD  IMGMEF**S**EEKIQY  GLKYIH**S**AGVVHR  APEVIL**S**WMHYNQ  QTVDIW**S**VGCIMA  NDKAAK**S**YIQSLP  AKSYIQ**S**LPQTPR  QLFPRA**S**PQAADL  QQPFDD**S**LEHEKL  KEIVNF**S**PIARKD  PIARKD**S**RRRSGM  IIRLLF**S**KTYKLQ  YKLQER**S**DLTVKE  LNYNIV**S**GPPSHK  IVSGPP**S**HKTVVN  ECINFA**S**FNFLGL  KAAALA**S**LKKYGV  EEAIIY**S**YGFATI  GFATIA**S**AIPAYS  SAIPAY**S**KRGDIV  QKGLQA**S**RSDIKL  GLQASR**S**DIKLFK  RIFLEE**S**LSFGVL  FLEESL**S**FGVLGE  DDIDLI**S**ANMENA  MENALA**S**IGGFCC  GFCCGR**S**FVIDHQ  IDHQRL**S**GQGYCF  GQGYCF**S**ASLPPL  GYCFSA**S**LPPLLA  KALQGI**S**GLKVVG  LKVVGE**S**LSPAFH  VVGESL**S**PAFHLQ  HLQLEE**S**TGSREQ  LEESTG**S**REQDVR  DQCMNR**S**IALTQA  KCLPPP**S**IRVVVT  ELERAA**S**TIKEVA  QTTSVG**S**SCLDLW  TTSVGS**S**CLDLWR  AKVAQN**S**GLTLRR  LRGLLH**S**LQGLPA  FIILRA**S**LAQGFT  DSVLRA**S**CLLPEL  LLPELL**S**ALHRLV  LETLNG**S**QSGASK  TLNGSQ**S**GASKDL  GSQSGA**S**KDLLLL  LLLKTW**S**PPAEEL  NPDKAL**S**SLHEAA  SLHEAA**S**GLCPRP  VYTALG**S**CHRKMG  AALKEG**S**AWGPPL  PPLLEA**S**RLYQQL  TTAELE**S**LELLVE  ALNVPC**S**SKAPQF  LHCGTQ**S**QTKHIL  TKHILA**S**RCLQTG  LALLLD**S**SEPRFS  SSEPRF**S**PPPSPP  LCEELL**S**RTSSLL  YCPLWV**S**ATHLLQ  AQKVAI**S**EFSRCL  VAISEF**S**RCLELL  RAAALI**S**RGLEWV  EAQTKG**S**HEDALW  HEDALW**S**LPLYLE  LPLYLE**S**YLSWIR  YLESYL**S**WIRPSD  VGNVAD**S**TEPTKR  GVLLLL**S**SIHFQC  CRRLDR**S**AHFSTL  DRSAHF**S**TLAIKQ  LLAEAY**S**NLGNVY  AVQAYV**S**ALQYNP  DLYCVR**S**DLGNLL  NFAVAW**S**NLGCVF  LRALSL**S**PNHAVV  ALKEKG**S**VAEAED  EFAAAH**S**NLASVL  AHSNLA**S**VLQQQG  KEAIRI**S**PTFADA  TFADAY**S**NMGNTL  AFADAH**S**NLASIH  ASIHKD**S**GNIPEA  IPEAIA**S**YRTALK  EKNRLP**S**VHPHHS  SMLYPL**S**HGFRKA  PKDLKL**S**DGRLRV  LRVGYV**S**SDFGNH  FGNHPT**S**HLMQSI  VFCYAL**S**PDDGTN  NHFIDL**S**QIPCNG  ITDQET**S**PAEVAE  EVAEQY**S**EKLAYM  LKAFLD**S**LPDVKI  GDNADS**S**NTALNM  INGFSI**S**NGLATT  IIVTTR**S**QYGLPE  LKRVPN**S**VLWLLR  QNRIIF**S**PVAPKE  VWKQRI**S**SPLFNT  QLKVRV**S**QRELSR  VSQREL**S**RGVCAE  VCAERY**S**QEVFEK  VVHLPE**S**PKPGPT  DGRLIR**S**LGQASE  RSLGQA**S**EADPSG  ASEADP**S**GVASIA  DPSGVA**S**IACSSC  VASIAC**S**SCVRAV  ASIACS**S**CVRAVD  TCWGCG**S**VACTLC  CGLVDC**S**DMYEKV  TFEHLW**S**SLEPDS  FEHLWS**S**LEPDST  SSLEPD**S**TYFDLP  YFDLPQ**S**SRGNNE  VVGGTD**S**SMDVFH  VGGTDS**S**MDVFHL  AQFNLL**S**STMDQM  QFNLLS**S**TMDQMS  TMDQMS**S**RAASAS  SRAASA**S**PYTPEH  TPEHAA**S**VPTHSP  PYAQPS**S**TFDTMS  PAPVIP**S**NTDYPG  VTFQQS**S**TAKSAT  PIQIKV**S**TPPPPG  DFNEGQ**S**APASHL  YNFMCN**S**SCVGGM  KRAFKQ**S**PPAVPA  SHLQPP**S**YGPVLS  GQPPPH**S**SAATPN  QPPPHS**S**AATPNL  PANGEM**S**SSHSAQ  ANGEMS**S**SHSAQS  NGEMSS**S**HSAQSM  EMSSSH**S**AQSMVS  SAQSMV**S**GSHCTP  QSMVSG**S**HCTPPP  ADPSLV**S**FLTGLG  CIEYFT**S**QGLQSI  TSQGLQ**S**IYHLQN  KQGHDY**S**TAQQLL  AQQLLR**S**SNAATI  TISIGG**S**GELQRQ  LVRDSE**S**VAGAFA  PPLPPR**S**GSTSIS  SGSTSI**S**APTGPS  SAPTGP**S**SPLPAP  APTGPS**S**PLPAPE  TRTLAT**S**CRRLHS  KVFDQQ**S**SPMVTR  GEQELE**S**LVLKLS  SLVLKL**S**VLKDFL  LRRQRD**S**QEDWTT  KDFIFV**S**ARKREA  GTWNMG**S**VPPPKN  RPIAMQ**S**LWNIKV  EHENRI**S**HVSTSS  SHVSTS**S**VKTGIA  KGAVGV**S**FMFNGT  FMFNGT**S**FGFVNC  VNCHLT**S**GNEKTA  YRYERG**S**RDTYAW  IVTSDH**S**PVFGTF  ISKKGL**S**KTSDQA  FFIEFY**S**TCLEEY  DEAGAK**S**KAPSVS  SKAPSV**S**RGSQEP  GSQEPR**S**GSRKPA  EASCPL**S**RLFEEP  APPPKN**S**FNNPAY  VGEGSS**S**DEESGG  AIFLPP**S**LDPLPG  PLPPGP**S**PASTFL  PGPSPA**S**TFLGEV  GDDRSC**S**VLQMAK  PPRIRE**S**IQEDLA  RKEGTG**S**TATSSS  TGSTAT**S**SSSTAG  GSTATS**S**SSTAGA  STATSS**S**STAGAA  TATSSS**S**TAGAAG  GKGKGG**S**GDSAVK  KGGSGD**S**AVKQVQ  QMEMMR**S**LRHVNI  HVGWYQ**S**TYYGSF  QSTYYG**S**FVTRAL  TRALLD**S**QFSYQH  LLDSQF**S**YQHAIE  QHAIEE**S**VVLIYD  IKTAQG**S**LSLKAY  TAQGSL**S**LKAYRL  PIVIKN**S**HLINVL  WELEKK**S**AVADKH  DKHELL**S**LASSNH  ELLSLA**S**SNHLGK  LLSLAS**S**NHLGKN  DRVDEM**S**QDIVKY  TYMRNT**S**KQQQQK  ENMQRQ**S**RGEPPL  LPEEDL**S**KLFKPP  PPARMD**S**LLIAGQ  WARKLL**S**VPWLLC  GPRRYA**S**SSFKAA  PRRYAS**S**SFKAAD  RRYASS**S**FKAADL  LTLHPA**S**SSLHYS  TLHPAS**S**SLHYSL  LHPASS**S**LHYSLQ  SSSLHY**S**LQLFEG  MDRMLR**S**AMRLCL  MRLCLP**S**FDKLEL  PDAAGT**S**LYVRPV  LIGNEP**S**LGVSQP  EPSLGV**S**QPTRAL  AYFPGG**S**VTPVSL  GSVTPV**S**LLADPA  PGVVRQ**S**LLDMAQ  VREVFG**S**GTACQV  LKDHRI**S**TFKNWP  EEHKKH**S**SGCAFL  EHKKHS**S**GCAFLS  SGCAFL**S**VKKQFE  QLAQRI**S**SNIQKI  LAQRIS**S**NIQKIT  QKITQC**S**VEIQRT  LGTPQD**S**PELRQQ  YIKEFG**S**LPTTPS  SLPTTP**S**EQRQRK  VAEFTT**S**LTNFQK  VARVRA**S**SRVSGS  ARVRAS**S**RVSGSF  RASSRV**S**GSFPED  SSRVSG**S**FPEDSS  GSFPED**S**SKERNL  SFPEDS**S**KERNLV  KERNLV**S**WESQTQ  NLVSWE**S**QTQPQV  LIHERE**S**SIRQLE  IHERES**S**IRQLEA  QGDVID**S**IEANVE  QANQQL**S**RAADYQ  ADYQRK**S**RKTLCI  IGVAII**S**LIIWGL  GDAARF**S**CPPNFT  TAKPPA**S**ESPRFS  KPPASE**S**PRFSLE  SESPRF**S**LEALTG  GRHVPL**S**GSQIVK  HVPLSG**S**QIVKGK  HRYRVL**S**SCPQAG  RYRVLS**S**CPQAGE  ATLLAP**S**TEAGGG  GGLTCA**S**APQGTL  LEGPQQ**S**LSGSPL  GPQQSL**S**GSPLQP  QQSLSG**S**PLQPIP  LQPIPA**S**PPPQIP  PVTGPR**S**ALAPNL  APNLLT**S**GKKKKE  VDMALG**S**PEMDVR  LGVLFP**S**TTKKRK  LEDTVL**S**PTKKRK  EGVTVE**S**QPQVKV  EMKPLE**S**PGGTMA  PQAAPT**S**TKKKKK  ARATPG**S**TKKRKK  KKRKKQ**S**QESRMP  KKQSQE**S**RMPETV  PGPPLN**S**ESGEEA  PPLNSE**S**GEEAPT  LITLDL**S**HNGLSS  LSHNGL**S**STKLGT  SHNGLS**S**TKLGTQ  LDIFAN**S**SLKKLE  DIFANS**S**LKKLEL  LKKLEL**S**SNQIKE  KKLELS**S**NQIKEF  LELANT**S**IRNLSL  LSNSQL**S**TTSNTT  LTMLDL**S**YNNLNV  NIQHLF**S**HSLHGL  RSFTKQ**S**ISLASL  FTKQSI**S**LASLPK  QSISLA**S**LPKIDD  PKIDDF**S**FQWLKC  FVSLAH**S**PLHILN  LTKNKI**S**KIESDA  IFEIYL**S**YNKYLQ  LQLTRN**S**FALVPS  ALKNVD**S**SPSPFQ  LKNVDS**S**PSPFQP  LTILDL**S**NNNIAN  YFLKGL**S**HLHILN  HILNLE**S**NGFDEI  NQVSLK**S**LNLQKN  TNIPEL**S**SHYLCN  NIPELS**S**HYLCNT  VRLFDT**S**SCKDSA  FEGWRI**S**FYWNVS  SFYWNV**S**VHRVLG  VWEHFS**S**MEKEDQ  MEKEDQ**S**LKFCLE  IEQNLD**S**IILVFL  GIFLHK**S**ELGCDT  LGCDTG**S**TGKFEW  GKFEWG**S**KHSKEN  EWGSKH**S**KENRNF  KENRNF**S**EDVLGW  VLGWRE**S**FDLLLS  SFDLLL**S**SKNGVA  FDLLLS**S**KNGVAA  FLKTEF**S**EENLEF  EFKKIR**S**ATKLAS  SATKLA**S**RAHQIF  FEEFIC**S**EAPKEV  TLMEKD**S**YPRFLK  YPRFLK**S**PAYRDL  DLAAQA**S**AASATL  AQASAA**S**ATLSSC  AASATL**S**SCSLDE  ASATLS**S**CSLDEP  SCSKMS**S**IVDRDD  DKAKRV**S**RNKSEK  STVLQK**S**IDFLRK  KEITAQ**S**DASEIR  WKPTFL**S**NEEFTQ  AIMTDG**S**IIYVSE  GSIIYV**S**ESVTSL  IIYVSE**S**VTSLLE  LLEHLP**S**DLVDQS  HLLESD**S**LTPEYL  NFKSLN**S**VSSSAH  SLNSVS**S**SAHNGF  PNEEFT**S**RHSLEW  TYHQWN**S**RPEFIV  RSSRKS**S**HTAVSD  SSHTAV**S**DPSSTP  QRRSSF**S**SQSINS  RRSSFS**S**QSINSQ  SSQSIN**S**QSVGSS  PTNQIQ**S**GMNTGH  QQQTLQ**S**TSTQSQ  QTLQST**S**TQSQQN  QSTSTQ**S**QQNVLS  SQQNVL**S**GHSQQT  GHSQQT**S**LPSQTQ  YNTMVI**S**QPAAGS  SMVQIP**S**SMPQNS  SSMPQN**S**TQSAAV  QQFLQT**S**RLLHGN  LLHGNP**S**TQLILS  STQLIL**S**AAFPLQ  QSTFPQ**S**HHQQHQ  HHQQHQ**S**QQQQQL  DAVPIQ**S**SVVLCS  AVPIQS**S**VVLCSC  SSVVLC**S**CPSPSM  CSCPSP**S**MVRTQT  VRTQTE**S**STPPGI  RTQTES**S**TPPGIP  PGIPGG**S**RQGPAM  PRPGAG**S**LQHAQP  KILGEG**S**FSTVVL  LGEGSF**S**TVVLAR  ARELAT**S**REYAIK  RERDVM**S**RLDHPF  KLYFGL**S**YAKNGE  YIRKIG**S**FDETCT  YTAEIV**S**ALEYLH  GTAKVL**S**PESKQA  KVLSPE**S**KQARAN  GTAQYV**S**PELLTE  ELLTEK**S**ACKSSD  EKSACK**S**SDLWAL  KSACKS**S**DLWALG  AHPFFE**S**VTWENL  AYLPAM**S**EDDEDC  NYDNLL**S**QFGCMQ  FGCMQV**S**SSSSSH  GCMQVS**S**SSSSHS  CMQVSS**S**SSSHSL  MQVSSS**S**SSHSLS  YIHDLD**S**NSFELD  HDLDSN**S**FELDLQ  ELDLQF**S**EDEKRL  QLAGAG**S**MGSPGA  GAGSMG**S**PGATTG  DLEPQF**S**IITKLK  TKLKGV**S**VTQIKE  RCPEHP**S**VPLANC  GEGGTH**S**HGVKTG  RTCEIW**S**WCPVES  SWCPVE**S**GVVPSR  ESGVVP**S**RPLLAQ  KNTVTF**S**KFNFSK  FSKFNF**S**KSNALE  KFNFSK**S**NALETW  RYEPQF**S**PYCPVF  LALLGG**S**VGIRVH  DLDTGD**S**GCWPHY  GCWPHY**S**FQLQEK  FQLQEK**S**YNFRTA  PKATAN**S**VWRELA  RELALA**S**QARLAE  AECLRR**S**SAPAPT  ECLRRS**S**APAPTA  TATAAG**S**QTQTPG  PGWPCP**S**SDTHLP  GWPCPS**S**DTHLPT  LLEQEK**S**LAGWAL  LWFGGC**S**WALYLF  LVKCTI**S**ISTFLL  KCTISI**S**TFLLLC  LGAPLT**S**PQPWPG  QGEALL**S**LAMLLR  RAVLLR**S**GVLLNA  GVLLNA**S**YRSIGA  LNASYR**S**IGALNQ  TTAWVL**S**VAERQA  NATGHL**S**DTLWLI  TKEMKE**S**AARVLQ  HTRRKE**S**HAARRH  LREQVN**S**MVDISK  NSMVDI**S**KMHMIL  DLQQNL**S**SSHRAL  LQQNLS**S**SHRALE  QQNLSS**S**HRALEK  ALTELL**S**TALGPR  LTFVLS**S**TCVKEQ  ELFSEL**S**ACLYSP  LSACLY**S**PSSQKP  ACLYSP**S**SQKPAA  LTFYEP**S**ILPRLG  CQDHPR**S**LDELEQ  LGDLFA**S**FLPGIS  EQLKRI**S**KVQAKP  LLKCSQ**S**LVECAG  GLVNDE**S**PEIQAQ  LSESLH**S**LATSLP  LHSLAT**S**LPRLMN  LPRLMN**S**QDDQGK  INFVLN**S**VAHLQR  LREIVT**S**ILEEYT  ILEEYT**S**QENWYL  GLQAIT**S**GEHTCQ  TSFLAF**S**KPSPTI  PSPTIC**S**MNSNIW  FCLLLM**S**ALYPVL  DQTLLI**S**QVATST  ISQVAT**S**TMMDVC  YLVNGI**S**LNLRHL  EVMLRN**S**DANLLP  RAASFV**S**VLHALM  GHLQEQ**S**LGEEGS  NEEEEQ**S**VPPKVD  CVVVLQ**S**HKNQLL  AHQAWP**S**LVHRLT  FTPPHP**S**LHPVQL  VQLHGA**S**GQQNPY  DVPKEN**S**SGSSPL  VPKENS**S**GSSPLD  ENSSGS**S**PLDQVD  PLDQVD**S**SLPSEP  EIPTCG**S**ALNQTT  LNQTTE**S**SQSFVA  TNLHNH**S**EGEYIP  GACSAS**S**VQNGIA  NDHLQL**S**AEVVEG  AEVVEG**S**RYQESL  EAYTGL**S**PPVPSF  LSPPVP**S**FNCEVR  EFVHQN**S**QEIQRS  SQEIQR**S**SQDEMV  ERQTEH**S**PEDAAC  REKNHG**S**SPEQVV  ALEVEE**S**GSDDLL  EVEESG**S**DDLLIK  FLDPPY**S**RVITQR  EKDQSS**S**DESWET  QSSSDE**S**WETLPG  NEPELQ**S**DSSGPE  EENQEL**S**LQEGEQ  YNEVNE**S**SSDEGN  DLDVDW**S**LFDGFA  GVAEAI**S**YVDPQF  VANPPA**S**KESIDG  CCPICC**S**EYIKDD  PAVIEA**S**AAPSSE  EASAAP**S**SEPDPD  ASAAPS**S**EPDPDA  LRKVLI**S**DSLDPC  KVLISD**S**LDPCCR  VEKQNL**S**KEELIA  EGLIVR**S**ATKVTA  NTPNGN**S**LSAAEL  PNGNSL**S**AAELTC  IPQATA**S**MKDGKW  VATRMQ**S**FGMKTI  GYDPII**S**PEVSAS  IISPEV**S**ASFGVQ  SPEVSA**S**FGVQQL  HTPLLP**S**TTGLLN  LLRALQ**S**GQCAGA  DHENVI**S**CPHLGA  CPHLGA**S**TKEAQS  STKEAQ**S**RCGEEI  DMVKGK**S**LTGVVN  NAQALT**S**AFSPHT  ALTSAF**S**PHTKPW  MRAWAG**S**PKGTIQ  VITQGT**S**LKNAGN  NAGNCL**S**PAVIVG  GLLKEA**S**KQADVN  GLNVTT**S**HSPAAP  NVTTSH**S**PAAPGE  LFRTQT**S**DPAMLP  AGVRLL**S**YQTSLV  LLSYQT**S**LVSDGE  YQTSLV**S**DGETWH  WHVMGI**S**SLLPSL  HVMGIS**S**LLPSLE  ISSLLP**S**LEAWKQ  PSSTGP**S**ANKPCS  PAAATI**S**AAGPGS  SAAGPG**S**SAVPAA  AAGPGS**S**AVPAAA  AAAAVI**S**GPGGGG  QHHELT**S**LFECPV  QCRQKL**S**CCPTCR  RGALTP**S**IRNLAM  AMEKVA**S**AVLFPC  YATTGC**S**LTLHHT  CEYRPY**S**CPCPGA  CPCPGA**S**CKWQGS  SCKWQG**S**LEAVMS  SLEAVM**S**HLMHAH  LMHAHK**S**ITTLQG  DWVMMQ**S**CFGHHF  WEATPR**S**IHDGVA  AAAIMN**S**DCLVFD  KKTSVR**S**QDPGQR  LEEQNL**S**VAEGPN  ACAGPP**S**RPQRPF  AVCGFP**S**PYTCVS  SPYTCV**S**CGARYC  TEVLAH**S**GRLEKE  DLGTRI**S**RLTRRV  MISKKY**S**EFLPSM  TQVDKL**S**EDIDLL  LKSRIE**S**EVRRDL  RDSVVL**S**LLKQLQ  KILKSL**S**MELTIQ  VWKFPP**S**KDTSSL  TPMPPI**S**SVLLAF  PMPPIS**S**VLLAFS  SVLLAF**S**VLGELH  LHSKLK**S**FGQMLL  ILRPLA**S**CPSLHA  LHAVIE**S**QPNIVI  VIIRFE**S**IMTNLE  TNLEYP**S**PSEVFT  LEYPSP**S**EVFTKI  LENEKT**S**TVPLAE  SIPTNS**S**KLQQYE  YEEIIQ**S**TEEFEN  ARNLMT**S**EIHNTV  VKIIPD**S**KINVPE  LPTCRI**S**ESVKKL  AVQLFY**S**VRNIFH  ELLERL**S**SARNFS  DDEENY**S**AASKAV  ENYSAA**S**KAVRQV  LLNTAI**S**EVIGKI  DGDRLY**S**LCKTVM  QVFAPL**S**EESKNK  APLSEE**S**KNKKYQ  MMMLQA**S**LQEIGD  AAAFSS**S**EVKALI  DLDALL**S**DLETTT  DLETTT**S**HMPRSG  TSHMPR**S**GAPKER  PLTPPP**S**YGHQPQ  HQPQTG**S**GESSGA  QTGSGE**S**SGASGD  TGSGES**S**GASGDK  DKDHLY**S**TVCKPR  TVCKPR**S**PKPAAP  APPFSS**S**SGVLGT  ITDEIM**S**QFPSSK  IMSQFP**S**SKVASG  MSQFPS**S**KVASGE  EQKEDQ**S**EDKKRP  SLPSSP**S**PGLPKA  PKASAT**S**ATLELD  RLMASL**S**DFRVQN  QNHLPA**S**GPTQPP  TQPPVV**S**STNEGS  SSTNEG**S**PSPPEP  TNEGSP**S**PPEPTG  EPTGKG**S**LDTMLG  LLQSDL**S**RRGVPT  FVCGGC**S**TALGGS  TALGGS**S**FFEKDG  CYFERF**S**PRCGFC  EHFCCV**S**CGEPFG  ILDNYI**S**ALSALW  NYISAL**S**ALWHPD  ECFAPF**S**GGSFFE  APFSGG**S**FFEHEG  FHARRG**S**LCATCG  VTGRCV**S**ALGRRF  SAASSS**S**SSSAGE  AASSSS**S**SSAGEM  ASSSSS**S**SAGEMI  EMIEAP**S**QVLNFE  AIKQIE**S**ESERKA  VELRQL**S**RVNHPN  EYAEGG**S**LYNVLH  SWCLQC**S**QGVAYL  PEVFEG**S**NYSEKC  FEGSNY**S**EKCDVF  EKCDVF**S**WGIILW  CWSKDP**S**QRPSME  DPSQRP**S**MEEIVK  SATSTG**S**FMDIAS  SFMDIA**S**TNTSNK  TNTSNK**S**DTNMEQ  TIKRLE**S**KLLKNQ  LGASRG**S**SVESLP  GHRKTA**S**FGNILD  VPEIVI**S**GNGQPR  EPGQVS**S**RSSSPS  QVSSRS**S**SPSVRM  SRSSSP**S**VRMITT  SEKPTR**S**HPWTPD  PWTPDD**S**TDTNGS  STDTNG**S**DNSIPM  TNGSDN**S**IPMAYL  CPNSKE**S**MAVFEQ  KDQQNT**S**RLVQEH  DENKSL**S**TYYQQC  QLEVIR**S**QQQKRQ  AAAAEL**S**LLEKSL  LSLLEK**S**LGLSKG  EKSLGL**S**KGNKYS  SKGNKY**S**AQGERQ  QTNNGP**S**LTGLTT  KEYLLG**S**TAEEKA  TQVDGH**S**SKNDIH  QVDGHS**S**KNDIHT  LLKDLN**S**YLEDKV  EKYLNV**S**RWFCHI  GIRQHL**S**SVVFIK  IRQHLS**S**VVFIKN  NGEAIC**S**ALPTIP  ADLRYL**S**RGASGT  YLSRGA**S**GTVSSA  GASGTV**S**SARHAD  EYMPNG**S**LNELLH  SLSQSR**S**SKSAPE  QSRSSK**S**APEGGT  GQKSRA**S**IKHDIY  IKHDIY**S**YAVITW  PLQIMY**S**VSQGHR  HRARMI**S**LIESGW  MISLIE**S**GWAQNP  NPDERP**S**FLKCLI  KKTKLQ**S**VSSAIH  TKLQSV**S**SAIHLC  KLQSVS**S**AIHLCD  KKKMEL**S**LNIPVN  HGPQEE**S**CGSSQL  QEESCG**S**SQLHEN  EESCGS**S**QLHENS  SQLHEN**S**GSPETS  SGSPET**S**RSLPAP  QDNDFL**S**RKAQDC  SWDSTI**S**GSQRAA  DSTISG**S**QRAAFC  AIINPL**S**TAGNSE  LSTAGN**S**ERLQPG  AQQWIQ**S**KREDIV  EACLNQ**S**LDALLS  EDYELV**S**TKPTRT  TKPTRT**S**KVRQLL  EEPMDT**S**SVTHTE  EPMDTS**S**VTHTEH  NEEGAL**S**VLQQFK  LQQFKE**S**DLSHVQ  FKESDL**S**HVQNKS  SHVQNK**S**AFLCGV  QREKQG**S**KVQEST  GSKVQE**S**TKGPDE  GGPPPD**S**VYSGVQ  PPDSVY**S**GVQPGI  LMMDPL**S**GQNRGY  AVKLCD**S**YEIRPG  HLGVCI**S**VANNRL  NRLFVG**S**IPKNKT  NILEEF**S**KVTEGL  EYEDHK**S**AAQARR  ARRRLM**S**GKVKVW  EEILEK**S**FSEFGK  ILEKSF**S**EFGKLE  QAARQA**S**RSTAYE  ARQASR**S**TAYEDY  RGRAGY**S**QRGAPL  LGPPRG**S**RGGRGG  QQRGRG**S**RGSRGN  GRGSRG**S**RGNRGG  GYNQPD**S**KRRQTN  NQQNWG**S**QPIAQQ  QQGGDY**S**GNYGYN  SQPQTR**S**PFFQRP  RATIPN**S**SPSIRP  IPNSSP**S**IRPGAQ  PIGRPT**S**TPTPPQ  SQVPEH**S**PVVYGT  PTSSAL**S**SQPIFT  DDRCEL**S**SPREDT  DRCELS**S**PREDTI  PPTPPA**S**PPHTPV  TEEILD**S**QNLNSR  DSQNLN**S**RRSPVP  HPERDP**S**DLKKVK  EGIDAN**S**GSTDSS  IDANSG**S**TDSSGD  SGSTDS**S**GDGVTF  EGLPPI**S**DVVLDK  LFRKVR**S**ILNKLT  QLMKQV**S**GLTVDT  IDEPSF**S**VAYANM  KNHDEE**S**LECLCR  KERKTS**S**RIRFML  SRVLDP**S**KFLKIT  SGGAKA**S**ETDALR  SSLNRF**S**ALQPPA  HDKAAL**S**EEELER  EELERK**S**KSIIDE  VRVGVE**S**TLERSQ  DFIESD**S**PCSSEA  ESDSPC**S**SEALSK  DEIQMS**S**PTFLRA  LYALQA**S**IVKLDQ  ENLKGC**S**VCCKSS  CSVCCK**S**SWNQLQ  SVCCKS**S**WNQLQD  CRLAKL**S**CPALGI  CPALGI**S**KRNLYD  WRGYPD**S**ESTWEP  GYPDSE**S**TWEPRQ  LRRHHR**S**KTPRHL  PRHLDP**S**LANYLV  ELNAKR**S**HLGRIT  GCCPGA**S**LHKFAY  PIYECN**S**RCRCGY  EKIRKN**S**FVMEYV  VGEIIT**S**EEAERR  AYYGNI**S**HFVNHS  SHFVNH**S**CDPNLQ  DPVDME**S**TRMDSN  ESTRMD**S**NFGLAG  LAGLPG**S**PKKRVR  LGLCGN**S**FSGGQP  LCGNSF**S**GGQPSS  FSGGQP**S**STDAPK  SGGQPS**S**TDAPKA  NYETQD**S**HKAGPI  LQKAYE**S**KIDYDK  AFTDLN**S**INSVLG  MNSTLK**S**LHQQST  KSLHQQ**S**TQLSSS  QQSTQL**S**SSLTSV  STQLSS**S**LTSVKT  LSSSLT**S**VKTSLR  VKTSLR**S**SLNDPL  KTSLRS**S**LNDPLC  LCLVHP**S**SETCNS  CNSIRL**S**LSQLNS  IKRVLN**S**IGSDID  VLNSIG**S**DIDNVT  DILSAF**S**VYVNNT  GGLVIC**S**LLTLIV  MVGVGL**S**FLFCWI  DWEYYL**S**GKLFNK  GKLFNK**S**KMKLTF  TLHLQN**S**FNISEH  QNSFNI**S**EHLNIN  EHTGSI**S**SELESL  HTGSIS**S**ELESLK  ISSELE**S**LKVNLN  PIEQSL**S**TLYQSV  VTRILA**S**LDFAQN  FITNNT**S**SVIIEE  LQWIEF**S**ISEKVA  VDVFLC**S**YIIDPL  PAAESQ**S**SLRRQK  KREKET**S**ESRGIS  EKETSE**S**RGISRF  WLKKQK**S**YTLVVA  VKEEKP**S**VSKEEK  LKAEKA**S**QKVTKK  LDGTEY**S**CDLEKH  FYPPDP**S**QLTEDI  HGSIDL**S**EFQFAP  PKILKI**S**YKRSNF  KISYKR**S**NFYIKV  KFLTLG**S**KFRYSG  TSSKRV**S**RSLDGA  GRREVR**S**PTKAPH  NIYVRH**S**NLMLEE  ILKHQA**S**ISELKR  KRNFME**S**TPEPRP  RRITPL**S**LQTQGS  SLQTQG**S**SHETLN  LQTQGS**S**HETLNI  MNGKEI**S**PGSGPG  KEISPG**S**GPGEIR  PVTQKD**S**TSLSSE  TSLSSE**S**SSSSSE  ESSSSS**S**ESEEED  SSSSSE**S**EEEDVG  KVNGEV**S**HVDIDV  TEMVTI**S**DASQRT  VTISDA**S**QRTEIS  SQRTEI**S**TKEVPI  QTITSE**S**VSTTTT  EQHPDM**S**VTRVVV  NVSTHP**S**LSQRPG  PGGSTK**S**HPEPQT  TPKDSP**S**KSSAEA  AKTQKD**S**SNKSGA  KDSSNK**S**GAEAKT  KDSTSK**S**HPELQT  AKTQKD**S**PSKSGS  SPSKSG**S**EAQTTK  KDVPNK**S**GADGQT  QTPKDG**S**SKSGAE  KDGSSK**S**GAEDQT  KDVPNK**S**GAEKQT  GPIDGP**S**KSGAEE  IDGPSK**S**GAEEQT  GAEEQT**S**KDSPNK  VVPEQP**S**RKDHSK  DHSKPI**S**NPSDNK  KPISNP**S**DNKELP  ADKGKL**S**PHAFKT  QEEVKS**S**EPTEDV  TGPEEG**S**PPKEEK  EEKEKM**S**GSASSE  MSGSAS**S**ENREGT  EGTLSD**S**TGSEKD  LSDSTG**S**EKDDLY  DLYPNG**S**GNGSAE  NGSGNG**S**AESSHF  GNGSAE**S**SHFFAY  VLEGKR**S**KVTRRP  TRRPKA**S**DYQRLD  PAPPKR**S**FLPQVL  DTMKNP**S**IVGVLC  GVLCTD**S**QGLNLG  GCRGTL**S**DEHAGV  EHAGVI**S**VLAQQA  QAAKLT**S**DPTDIP  PVVCLE**S**DNGNIM  KQPSDV**S**SECDRE  QPSDVS**S**ECDREG  RPGAPT**S**LQTEPQ  HGGEGD**S**CPHGSP  DSCPHG**S**PQGPLA  GPFATR**S**PLFIFM  FIFMRR**S**SLLSRS  IFMRRS**S**LLSRSS  RRSSLL**S**RSSSGY  SSLLSR**S**SSGYFS  SLLSRS**S**SGYFSF  LLSRSS**S**GYFSFD  SSSGYF**S**FDTDRS  SFDTDR**S**PAPMSC  RSPAPM**S**CDKSTQ  PMSCDK**S**TQTPSP  KSTQTP**S**PPCQAF  AFNHYL**S**AMASMR  YLSAMA**S**MRQAEP  RPELQA**S**PAKPSG  GETAAD**S**MIPEEE  IGGGGG**S**GTLGSG  GLLLED**S**ARVLAP  TAAGGL**S**GGTQAL  TRAIES**S**PDKRLT  DKRLTL**S**QIYEWM  FKDKGD**S**NSSAGW  HNLSLH**S**RFMRVQ  WPGSPT**S**RSSDEL  GSPTSR**S**SDELDA  RTNSNA**S**TVSGRL  SNASTV**S**GRLSPI  SPMLYS**S**SASLSP  LYSSSA**S**LSPSVS  SLSPSV**S**KPCTVE  GGLMQR**S**SSFPYT  GLGSPT**S**SFNSTV  PTSSFN**S**TVFGPS  STVFGP**S**SLNSLR  TVFGPS**S**LNSLRQ  LTSDSL**S**HSDVMM  AQPNQG**S**LVNQNL  SRALSN**S**VSNMGL  ESSSLG**S**AKHQQQ  QSPVSQ**S**MQTLSD  SSLYST**S**ANLPVM  GHEKFP**S**DLDLDM  LDMFNG**S**LECDME  MESIIR**S**ELMDAD  LDFNFD**S**LISTQN  GNVLAT**S**LRSLRF  LATSLR**S**LRFLQI  TWKLLG**S**AICAHS  FLTLIL**S**SFLVYL  LIAATF**S**LIGVSF  FSLIGV**S**FFALPA  PAGILG**S**GLALKV  LSNPRG**S**NTKGKL  VDAIEE**S**PSKEPK  AIEESP**S**KEPKPV  YAFWQS**S**EDAGTG  TPKHKK**S**QKGSAF  KKSQKG**S**AFTFPS  SAFTFP**S**QQSPRN  TFPSQQ**S**PRNEPY  PYVARP**S**TSEIED  YPTKGT**S**SPAEAE  KEDNRY**S**DLKTII  PRGGPS**S**GKVQAT  QATPPS**S**ATTYVE  ILTLLD**S**RVSCHS  LLDSRV**S**CHSQAD  SRVSCH**S**QADLQG  RSITRD**S**DTPLSL  DSDTPL**S**LMSVNH  TPLSLM**S**VNHEEL  ELERSP**S**GFSISQ  RSPSGF**S**ISQDRD  GPNGGS**S**WMREKR  PFTPSG**S**MPLSST  SGSMPL**S**STGDGI  GSMPLS**S**TGDGIS  GDGISD**S**VWTPSN  GVYPGP**S**GEKKLK  DPGAPD**S**TRDGAL  LVLSVF**S**TIKEYE  IKEYEK**S**SEGALY  KEYEKS**S**EGALYI  ATSALR**S**LRFLQI  FTLIGV**S**FFALPA  FYATNL**S**RTDLHS  TVPMYS**S**QTQTYG  GCCPGR**S**SQKVSL  TVRRSP**S**ADQSLE  SLEDSP**S**KVPKSW  PSKVPK**S**WSFGDR  WSFGDR**S**RARQAF  RIKGAA**S**RQNSEE  DIVDDK**S**CPCEFV  TPGLKV**S**IRAVCV  VMRFLV**S**KRKFKE  MLSRIK**S**LQSRVD  PAPPYH**S**PEDSRE  PVQCPP**S**TSWQPQ  TSWQPQ**S**HPRQGH  PVGDHG**S**LVRIPP  AHERSL**S**AYGGGN  GGGNRA**S**MEFLRQ  TSISIP**S**VDHEEL  ELERSF**S**GFSISQ  FSGFSI**S**QSKENL  NLDALN**S**CYAAVA  YIAEGE**S**DTDSDL  GESDTD**S**DLCTPC  VRRLWR**S**RVVPDR  DREEGG**S**GGGGGG  EDGSLG**S**RAEPAP  GGGCGR**S**EVRPVA  GPPRPM**S**EPGAGA  PGAGAG**S**SLLDVA  GAGAGS**S**LLDVAE  PGWLPE**S**DCETVT  VTCCLF**S**ERDAAG  GAPRDA**S**DPLAGA  PAGGGR**S**REARSR  RSREAR**S**RLLLLE  KRLKER**S**LDTLLE  PLCGCH**S**FAAAAD  RLCGPE**S**PPPPYS  PLDLSD**S**TLSYTE  LSDSTL**S**YTETEA  TAPGEF**S**DASMSP  FSDASM**S**PDATKP  PDATKP**S**HWCSVA  KPSHWC**S**VAYWEH  VYDQAV**S**IFYDLP  YDLPQG**S**GFCLGQ  LNLEQR**S**ESVRRT  LEQRSE**S**VRRTRS  GFGILL**S**KEPDGV  HPIFVN**S**PTLDAP  VFDFER**S**GLQHAP  GPYDPN**S**VRISFA  PNSVRI**S**FAKGWG  SRQFIT**S**CPCWLE  LTDLQS**S**RNNVRH  SLARHH**S**PSPWIA  ARHHSP**S**PWIAWL  ETNGAT**S**TEVSLC  ATSTEV**S**LCAPPT  LKIHID**S**FQGSVT  LKLLDK**S**LHFQTD  DCALRG**S**PVYVSR  GSPVYV**S**RVVCAM  VCAMIN**S**NDDNGV  VLNGNW**S**ENYTDG  YCCGPA**S**VRAIKE  LHQDTS**S**VGNFIS  NFISTK**S**IQSDER  ARSFHG**S**QRGAEL  GAELQP**S**RPTSLS  TSLSQD**S**PRSLHT  RSLHTP**S**LRPSDV  SDVVQV**S**LKFKLL  LLALNM**S**SQFKDL  LALNMS**S**QFKDLK  DLKVNL**S**AQSLLH  HDGSPL**S**PFWQDT  TAFITL**S**PKEAKT  TYPCKI**S**YSQYSQ  PCKISY**S**QYSQYL  ISYSQY**S**QYLSTD  QYSQYL**S**TDKLIR  ALGEEK**S**SPEKIL  NKIITL**S**YPSITI  IFSNPL**S**EQVEDC  VLTVEG**S**GLFKKQ  IQANMR**S**NKFKDI  CHRLPG**S**YDSTSS  LPGSYD**S**TSSDSL  GSYDST**S**SDSLYP  SYDSTS**S**DSLYPR  DSTSSD**S**LYPRGI  AYPPVT**S**YPPLSQ  TSYPPL**S**QPDLLP  LLPIPR**S**PQPLGG  PQPLGG**S**HRTPSS  GSHRTP**S**SRRDSD  SHRTPS**S**RRDSDG  PSSRRD**S**DGANSV  DSDGAN**S**VASYEN  GANSVA**S**YENEGA  YENEGA**S**GIRGAQ  WGVWGP**S**WTRLTP  TRLTPV**S**LPPEPA  LVVLPD**S**TPATST  DSTPAT**S**TAAPSA  TSTAAP**S**APALST  PSAPAL**S**TPGIRD  TPGIRD**S**AFSMES  IRDSAF**S**MESIDD  SAFSME**S**IDDYVN  YVNVPE**S**GESAEA  VPESGE**S**AEASLD  GESAEA**S**LDGSRE  EASLDG**S**REYVNV  REYVNV**S**QELHPG  TEPAAL**S**SQEAEE  EPAALS**S**QEAEEV  KTLPLH**S**HCTEMQ  EMLTLG**S**SFCSLQ  MLTLGS**S**FCSLQG  LGSSFC**S**LQGEFC  FCQAMD**S**FTTVSH  DSFTTV**S**HVGMCE  CEETDA**S**FNVFSP  ASFNVF**S**PKFQFD  KFQFDR**S**HCQSLR  DRSHCQ**S**LRFHQN  DERDKT**S**RNMRGS  SRNMRG**S**RMNGLP  LPSPTH**S**AHCSFY  THSAHC**S**FYRTRT  GIVYAV**S**SDRFRS  DLTRSL**S**DNINLP  GESYVC**S**SDNFFK  ESYVCS**S**DNFFKK  NVNPNW**S**VNVKTS  SVNVKT**S**ANMKAP  APQSLA**S**SNSAQA  SLASSN**S**AQAREN  LVTIIR**S**GVKPRK  NKKTAH**S**FEQVLT  VMKGNP**S**ATAGPK  PGPMRR**S**KSPADS  SKSPAD**S**ANGTSS  DSANGT**S**SSQLST  SANGTS**S**SQLSTP  ANGTSS**S**QLSTPK  SKQSPI**S**TPTSPG  ANIQAV**S**LKIQTL  KIQTLK**S**NNSMAQ  TLKSNN**S**MAQAMK  EEDEEE**S**DAVVSQ  ESDAVV**S**QVLDEL  LDELGL**S**LTDELS  SLTDEL**S**NLPSTG  ELSNLP**S**TGGSLS  LPSTGG**S**LSVAAG  STGGSL**S**VAAGGK  KAEAAA**S**ALADAD  EVLAEE**S**IVCLQK  MIAEEE**S**LKERLI  KERLIK**S**ISVCQK  ELNTLC**S**ELHVEP  PHYDID**S**ASVPSL  YDIDSA**S**VPSLEE  LRETKA**S**RREEFV  RREEFV**S**IKRQII  EDAFCL**S**LENIAT  QLEMQK**S**QNEAVC  ATIMSG**S**KAKVRK  WDQCFY**S**QEQRQA  AEDYTE**S**LLQLHD  EFERKA**S**DPNRFT  TEMLYG**S**APRTPS  SNATAN**S**SIRPIF  VYHSPV**S**RLPPSG  VSRLPP**S**GSKPVA  SKPVAA**S**TCSGKK  NLELNG**S**ILSGGY  LNGSIL**S**GGYPGS  SGGYPG**S**APLQRN  RNFSIN**S**VASTYS  SINSVA**S**TYSEFA  SVASTY**S**EFAKDP  EFAKDP**S**LSDSST  AKDPSL**S**DSSTVG  DPSLSD**S**STVGLQ  GLQREL**S**KASKSD  RELSKA**S**KSDATS  LSKASK**S**DATSGI  NDPRFI**S**YCLKFA  GIGTLS**S**PLYIAW  GELQHA**S**AVLQRG  LNQMIT**S**KSNPGN  NNMACI**S**KNQGSE  LSGVIS**S**ACDKES  RGESEF**S**FEELRA  HEDLPA**S**QERSEV  ISAALV**S**PATSQS  SPATSQ**S**IAPPVP  HKVANT**S**SFHTTP  MVQATP**S**KVQPSP  PTLPDI**S**DDKDEW  QKNVRS**S**GAWGVN  IISSLS**S**AFHVFE  RTFGER**S**VSRLPS  SVSRLP**S**KPKEEV  EEFLDD**S**TVWGIR  LAPSPK**S**PGDFTS  SPGDFT**S**AAQLAS  SPIQEK**S**PKQALS  SPKQAL**S**SHMYSA  PKQALS**S**HMYSAS  LSSHMY**S**ASLLRL  ASLLRL**S**QPAAGG  EWMQMS**S**LGTVDA  LSKPVS**S**YPNTFE  TEFQLG**S**KLVYVH  MERLKP**S**MQHMFM  MFMKFY**S**AHLFQN  LIDLGQ**S**IDMKLF  TAKCET**S**GFQCVE  DCHHLP**S**LDLLRQ  AAKAGG**S**FDLRRF  FALIVF**S**CIYGEG  IYGEGY**S**NAHESK  YSNAHE**S**KQMYCV  DACRYG**S**AIGVLA  VLAFLA**S**AFFLVV  AYFPQI**S**NATDRK  IGDLLF**S**ALWTFL  VLVGAD**S**VRAAIT  RAAITF**S**FFSIFS  ITFSFF**S**IFSWGV  SFFSIF**S**WGVLAS  SWGVLA**S**LAYQRY  PNTAYA**S**YPGASV  ASYPGA**S**VDNYQQ  LKAKYP**S**LGQKPG  GQKPGG**S**DFLMKR  KNKQLP**S**AGPDKN  DLPQRK**S**SLVTSK  KSSLVT**S**KLAGGQ  VVPPNR**S**QTGWPR  PLYEKD**S**SVAARF  LYEKDS**S**VAARFQ  GYGPII**S**SLPQLL  YGPIIS**S**LPQLLS  GGLVLF**S**ANSNSS  VLFSAN**S**NSSCME  FSANSN**S**SCMELS  SANSNS**S**CMELSK  SSCMEL**S**KKIAER  RVQIQE**S**VRGKDV  FIIQTV**S**KDVNTT  VYACKT**S**CAKSII  KTSCAK**S**IIGVIP  IPYFPY**S**KQCKMR  KMRKRG**S**IVSKLL  KRGSIV**S**KLLASM  VSKLLA**S**MMCKAG  VDNLRA**S**PFLLQY  AVIVAK**S**PASAKR  VAKSPA**S**AKRAQS  SAKRAQ**S**FAERLR  VAAIHP**S**LEIPML  IIDDVD**S**FLAAAE  ATHGLL**S**SDAPRR  THGLLS**S**DAPRRI  PRRIEE**S**AIDEVV  IKTVDI**S**MILSEA  DISMIL**S**EAIRRI  RIHNGE**S**MSYLFR  HNGESM**S**YLFRNI  VFVRFN**S**SHGFPV  FVRFNS**S**HGFPVE  FPVEVD**S**DTSIFQ  EVDSDT**S**IFQLKE  CEREPQ**S**LTRVDL  LTRVDL**S**SSVLPG  TRVDLS**S**SVLPGD  RVDLSS**S**VLPGDS  SVLPGD**S**VGLAVI  VILHTD**S**RKDSPP  TDSRKD**S**PPAGSP  DSPPAG**S**PAGRSI  GSPAGR**S**IYNSFY  GRSIYN**S**FYVYCK  KLRVQC**S**TCRQAT  TLTQGP**S**CWDDVL  LIPNRM**S**GECQSP  MSGECQ**S**PHCPGT  PHCPGT**S**AEFFFK  CGAHPT**S**DKETSV  TSDKET**S**VALHLI  HLIATN**S**RNITCI  TCTDVR**S**PVLVFQ  LVFQCN**S**RHVICL  DPQLGY**S**LPCVAG  VAGCPN**S**LIKELH  YHEGEC**S**AVFEAS  SAVFEA**S**GTTTQA  ARWEAA**S**KETIKK  RVVAIK**S**IRKDKI  EIEIMS**S**LNHPHI  FFRQIV**S**AVHYCH  LQTFCG**S**PLYASP  RGPEVD**S**WALGVL  LIRQIS**S**GEYREP  REPTQP**S**DARGLI  NWGYKS**S**VCDCDA  CDALHD**S**ESPLLA  ALHDSE**S**PLLARI  MLERQR**S**LKKSKK  QRSLKK**S**KKENDF  QDAVPE**S**PSKLSS  SPSKLS**S**KRPKGI  LKKRSN**S**EHRSHS  SEHRSH**S**TGFIEG  KTQQRE**S**GYYSSP  SNDVMG**S**SIPSPS  NDVMGS**S**IPSPSP  MGSSIP**S**PSPPDP  DPARVT**S**HSLSCR  ARVTSH**S**LSCRRK  KHSSKY**S**AGTMDP  EMPTLE**S**LSEPGV  PTLESL**S**EPGVPA  VPAEGL**S**RSYSRP  RPSSVI**S**DDSVLS  SDDSVL**S**SDSFDL  SVLSSD**S**FDLLDL  RNRLAD**S**SFSLLT  NRLADS**S**FSLLTD  VPDEAE**S**AEAGAV  AAWAAE**S**GAAAGL  HRGVDA**S**GETTYK  TVGHLS**S**KPERDV  ESIFFP**S**EGSNLT  PDDYLY**S**LCNEPL  EPLIEL**S**NPGASG  KFDLKG**S**TYKRRA  TYKRRA**S**KKEKEK  QAQGAQ**S**TSDEKR  IIDILQ**S**YRFIKK  HDGDTV**S**VHRPSF  VSVHRP**S**FYAERF  VFRKNS**S**LKSSPS  KNSSLK**S**SPSKKG  GPTAAF**S**ASQIPS  SASQIP**S**EREEAQ  DLRGAR**S**YPTLED  SIATTL**S**STSLSI  IATTLS**S**TSLSIP  LSSTSL**S**IPERSP  LSIPER**S**PSETSE  RSPSET**S**EQPRYR  RRRTQS**S**GQDGRP  QVEPAC**S**VEIVVP  DAGVEA**S**PAGASA  ASPAGA**S**AAVEVE  VEVETA**S**QASDEE  APASQA**S**DEEDAP  FPTDER**S**WVYSPL  YSPLHY**S**AQAPPA  EPEPGV**S**AQPGPG  PGPGKP**S**DKRFRL  EVILVL**S**APFLRC  GASGGT**S**PSATQP  QPDDPE**S**QMACHV  QVPDVI**S**SIRQLS  QPALTS**S**RVCFPE  HEGSQK**S**QPRRRH  HVQPSD**S**EKNRTM  NFKDIS**S**CSQGIK  SPEPGL**S**QYICYV  FQCASE**S**LVDEVM  EACPMH**S**LHKLCE  SFERSN**S**LASEKD  RSNSLA**S**EKDYSP  PGTPPA**S**PPSSAW  TFSHPP**S**STKRKL  FSHPPS**S**TKRKLN  SPLLRQ**S**SSEQCS  QCSNLS**S**VRRMYK  DTASES**S**DGEGRK  STCSNE**S**LSVGGT  VTPRRI**S**WRQRIF  IFLRVA**S**PMNKSP  ASPMNK**S**PSAMQQ  YRPDMM**S**LQIQMY  IQMYQL**S**RLLHDY  LIMECE**S**FENIVE  QDELQE**S**SYSCED  LQESSY**S**CEDSET  SYSCED**S**ETLEKL  DAYGPP**S**NFLEID  FLEIDV**S**NPQTVG  IFKLKE**S**TVRRRY  TVRRRY**S**DFEWLR  DFEWLR**S**ELERES  SELERE**S**KVVVPP  DEIIDK**S**YTPSKI  KEGGAL**S**EAMSLE  ALSEAM**S**LEGDEW  RQGRIM**S**TLQGAL  GKESNM**S**TLLERA  GEKRYY**S**DPRFLN  EPLDMY**S**YLHNQG  NQGIGV**S**LAQFYI  QFQARV**S**RQTLLA  SSVPQR**S**TLAELK  ENADEA**S**TAELSK  ASTAEL**S**KPTVQP  PWNTGR**S**LEHRPR  PCKIEP**S**INHILS  QSHQQA**S**EEKKEK  TKLQIA**S**ESQKIP  CCARET**S**LAENIW  FDEFLL**S**EKKNKS  LAVLKT**S**ESITSN  TSESIT**S**NEDVSP  RAARFV**S**TPFHEI  SREATH**S**SGFSGS  REATHS**S**GFSGSS  SVASTS**S**IKCLQI  SENPTQ**S**PWCSQY  RRQLLK**S**LPELSA  LPELSA**S**AELCIE  WVAPRN**S**AELTVI  LTVIKV**S**SQPVPW  DFDHFC**S**CYQYQD  IVHGDL**S**PRCLIL  KILANC**S**SPYQVD  TERLLL**S**FNYIRT  RTVTAS**S**FPFLEQ  RILDLG**S**SKIYFL  LTRLDL**S**KNQIRS  SKNQIR**S**LYLHPS  SLYLHP**S**FGKLNS  LQGKTL**S**FFSLAA  KTLSFF**S**LAANSL  FSLAAN**S**LYSRVS  SLYSRV**S**VDWGKC  LEILDV**S**GNGWTV  DITGNF**S**NAISKS  NFSNAI**S**KSQAFS  SNAISK**S**QAFSLI  SHGFVF**S**LNSRVF  LQVLNL**S**YNLLGE  LGELYS**S**NFYGLP  IPDIFL**S**GNKLVT  LNQNRF**S**SCSGDQ  NRFSSC**S**GDQTPS  SGDQTP**S**ENPSLE  TPSENP**S**LEQLFL  NRLTVL**S**HNDLPA  YCVYPD**S**FSGVSL  VYPDSF**S**GVSLFS  DSFSGV**S**LFSLST  SGVSLF**S**LSTEGC  DAYLCF**S**SKDFTW  HLDTQY**S**DQNRFN  CLSDLN**S**ALIMVV  VVVGSL**S**QYQLMK  DKKKGR**S**PDELPS  HRNSSA**S**YGDDPT  QSLQDV**S**DEQVLV  QKESSK**S**AMMYIQ  LESLRV**S**LNNNPV  LNNNPV**S**WVQTFG  GAEGLA**S**LLDILK  EQGEED**S**YDLKGR  LNTVKD**S**KAEPHF  LIEECI**S**QIVLHK  EMKKME**S**DFEQKL  AKKEMA**S**LSAAAI  KEMASL**S**AAAITV  VPPSVP**S**RAPVPP  GGVCIS**S**PPSLPG  SPPPPL**S**GDATIP  EGVGIP**S**PSSLPG  GIPSPS**S**LPGGTA  PPPLPG**S**AGIPPP  LRRPNW**S**KLVAED  LVAEDL**S**QDCFWT  KTAQNL**S**IFLGSF  LSIFLG**S**FRMPYQ  EAVLTE**S**MIQNLI  EQLKML**S**ELKDEY  LFKLQF**S**EQVENI  AFGFNI**S**FLCKLR  KLRDTK**S**TDQKMT  AHVEKA**S**RVSAEN  QMKKQI**S**DVERDV  ETGVMD**S**LLEALQ  AGCAVT**S**LLASEL  VTSLLA**S**ELTKDD  APSIHI**S**ERLVAE  PAAPPL**S**SGGPRL  AAPPLS**S**GGPRLP  SELRDG**S**GKKVAV  AEALCR**S**IRSSKL  LCRSIR**S**SKLSEN  IRSSKL**S**ENTVIV  SVMPFI**S**AGFTRR  KLRACN**S**VFTALE  DAIEIT**S**EDRFIQ  LLDTIN**S**CIRIGK  NKAEKI**S**ECVQSD  ISECVQ**S**DTHTDN  DVKAVA**S**RATEVS  RATEVS**S**QRRHSS  SSQRRH**S**SMARIH  SQRRHS**S**MARIHS  SMARIH**S**MTIEAP  NAAQES**S**PMPVTE  LVGGLM**S**DGLRRL  KNTQMV**S**SNIITP  NTQMVS**S**NIITPI  NIITPI**S**LDDVPP  FLHCSE**S**TLRSWL  IEANYH**S**SNPYHN  SNPYHN**S**THSADV  FLCNAG**S**ELAILY  VNKFVN**S**INKPLA  EWAARI**S**EEYFSQ  ISEEYF**S**QTDEEK  FDRNTC**S**IPKSQI  TEMEGT**S**TSSIYQ  MEGTST**S**SIYQNG  EGTSTS**S**IYQNGD  EICIAA**S**KACGIT  VFHIDE**S**TRHNVL  FPRWYC**S**GSNRAY  LDDFVM**S**YLFAQW  PLAIYN**S**ISYKTF  RFIQQF**S**QCKATA  EPGSGP**S**GEEIFA  NGGIQW**S**RGKHKE  RGKHKE**S**ETLTEQ  QANQEG**S**NESRVV  SLREAL**S**FVSLID  VLENIQ**S**NCHGPI  SMDFAI**S**KLKKAG  LYVLRC**S**PKDFNK  QMETVR**S**DNIIFQ  DLIFNE**S**LGQGTF  HRNYSE**S**FFEAAS  SFFEAA**S**MMSKLS  SMMSKL**S**HKHLVL  PPFIKL**S**DPGISI  TLWEIC**S**GGDKPL  GGDKPL**S**ALDSQR  EPDFRP**S**FRAIIR  IGALGF**S**GAFEDR  VKEPGE**S**PIFWYA  PESLTE**S**KFSVAS  SKFSVA**S**DVWSFG  VASDVW**S**FGVVLY  YIEKSK**S**PPAEFM  NVNQRP**S**FRDLAL  CNRLFT**S**AEETFS  SAEETF**S**HCKSEH  TVEYMN**S**IYNPVP  DLYEPV**S**VPFSYP  SYPNGL**S**ENTSVV  MEARAL**S**AEAALA  TDVRTC**S**SSTSVI  DVRTCS**S**STSVIA  VRTCSS**S**TSVIAD  TCSSST**S**VIADLQ  DGVYFS**S**YGHYGI  DKIRTE**S**YRDFIY  CGTGIL**S**MFAAKA  VLGVDQ**S**EILYQA  KVDVII**S**EWMGYF  YFLLFE**S**MLDSVL  FESMLD**S**VLYAKN  YLAKGG**S**VYPDIC  PDICTI**S**LVAVSD  ISLVAV**S**DVNKHA  VYGFKM**S**CMKKAV  DPKTLI**S**EPCGIK  IDCHTT**S**ISDLEF  ISDLEF**S**SDFTLK  SDLEFS**S**DFTLKI  LKITRT**S**MCTAIA  HNRVVF**S**TGPQST  FSTGPQ**S**TKTHWK  LLEKPF**S**VKAGEA  KVTVHK**S**KKDPRS  SKKDPR**S**LTVTLT  LEELER**S**TLQDSD  RSTLQD**S**DEYSNP  QDSDEY**S**NPAPLP  LPLDQH**S**RKETNL  TNLDET**S**EILSIQ  ETSEIL**S**IQDNTS  SIQDNT**S**PLPAQL  QELNVY**S**EAQEPK  AQEPKE**S**PPPSKT  KESPPP**S**KTSAAA  PPPSKT**S**AAAQLD  KQDHKA**S**LDSMLG  HKASLD**S**MLGGLE  PKGHCA**S**CQKPIA  IHALGQ**S**WHPEHF  CKEEIG**S**SPFFER  KEEIGS**S**PFFERS  SPFFER**S**GLAYCP  DYHQLF**S**PRCAYC  PEHFFC**S**HCGEVF  DFLAMF**S**PKCGGC  VLENYL**S**AMDTVW  CGDCFT**S**FSTGSF  DCFTSF**S**TGSFFE  TSFSTG**S**FFELDG  ITGRCI**S**AMGYKF  FCLTQL**S**KGIFRE  DDSEVE**S**TASILA  EVESTA**S**ILASVK  TASILA**S**VKEQEA  HSHLLY**S**TIPRMQ  DPEGAM**S**VVSVET  GLPVDA**S**SVSNNY  LPVDAS**S**VSNNYI  VDASSV**S**NNYIQT  RRTGTP**S**DPRRRL  RLDAVK**S**NAAAYL  GALKNI**S**FGRDQD  GTLWNL**S**SHDSIK  TLWNLS**S**HDSIKM  EVIIPH**S**GWEREP  RHIEWE**S**VLTNTA  GCLRNV**S**SERSEA  GQKDSD**S**KLVENC  CLLRNL**S**YQVHRE  GKDEWF**S**RGKKPI  VVRIYI**S**LLKESK  ISLLKE**S**KTPAIL  YGRYIR**S**ALRQEK  RQEKAL**S**AIADLL  RVVKAA**S**GALRNL  GGQQNS**S**WNFSED  NSSWNF**S**EDTVIS  SEDTVI**S**ILNTIN  NKSGNR**S**EKEVRA  ASRSQS**S**HSYDDS  REEIQM**S**NMGSNT  SLDNNY**S**TPNERG  GVMGGQ**S**AGPQHT  CDRVAS**S**TLLDDR  AVRALK**S**LSKKYR  VLQTDR**S**DSEIIG  QTDRSD**S**EIIGYA  ENSTRQ**S**EDLGSQ  QSEDLG**S**QFTEIF  GVKLLT**S**LLKQLG  LQALTR**S**NGAIQK  LLKNNN**S**NQNFFK  EVGDEN**S**GWSAQK  NPPGAT**S**SCQKAM  GQGEIV**S**TLLPST  FSTDSL**S**NWCAAV  RVQLAT**S**IGNPPV  QCTNIL**S**QGSKIQ  THFLHN**S**ANVPFL  ALLLGI**S**IYFNDN  EKLGFI**S**KHELYS  PQPNFP**S**PEYMIF  TKAIYK**S**SEEDKK  QHQGSY**S**EGAQMN  QLTEKD**S**MIENMK  MIENMK**S**SQTSGT  GTNEQS**S**AIVSAR  LKSQLN**S**QSVEIT  NEIKAL**S**EERTAI  IKEQLD**S**SNSTIA  QLDSSN**S**TIAILQ  ELEITD**S**KKEQDD  QDQKIL**S**LKNKLK  KKKERK**S**LPEEDV  FLIKPE**S**KVAKLD  VAKLDT**S**QWPLLL  TPLACG**S**NPLKRE  INLDKP**S**NPSSHE  DKPSNP**S**SHEVVA  KPSNPS**S**HEVVAW  VEKTGH**S**GTLDPK  ATRLVK**S**QQSAGK  LVKSQQ**S**AGKEYV  EGGTQL**S**RALETL  VRTIYE**S**KMIEYD  LGIFWV**S**CEAGTY  ELRRVR**S**GVMSEK  VRSGVM**S**EKDHMV  DNHKDE**S**YLRRVV  LEKLLT**S**HKRLVM  RLVMKD**S**AVNAIC  MTTAVI**S**TCDHGI  GLGPKA**S**QKKLMI  HGKPTD**S**TPATWK  QEYVDY**S**ESAKKE  YVDYSE**S**AKKEVV  AKRKRE**S**ESESDE  RKRESE**S**ESDETP  RESESE**S**DETPPA  KKEKKK**S**KKDKKA  AKAGLE**S**GAEPGD  EPGDGD**S**DTTKKK  KNKSED**S**TKDDID  EQEPQK**S**KGKKKK  KELEEL**S**LEAQGI  NEEEFT**S**KDKKKK  ELEDKD**S**KSKKTA  EDKDSK**S**KKTAKP  KGKAQK**S**NKKWDG  SDEFLQ**S**RKGQKK  TGKKDQ**S**KQKESQ  EKKKGP**S**KATVKA  GKLLTK**S**QREARA  IPQQLE**S**KEVSES  LESKEV**S**ESMELC  SKEVSE**S**MELCAA  EEEEGE**S**EGSEGD  VSDEKD**S**GKTLDK  TLDKKP**S**KEMSSD  DTPGHE**S**FSNLRN  NLRNRG**S**SLCDIA  LRNRGS**S**LCDIAI  SINLLK**S**KKCPFI  TGDGMG**S**LIYLLV  LTQTML**S**KRLAHC  QASTLG**S**LEALLE  LEFLKT**S**EVPYAG  KDVMKA**S**VMLEHD  LGVRIF**S**AEIIYH  PQYIFN**S**RDPIVM  TPMCVP**S**KNFVDI  DIGIVT**S**IEINHK  ILVSKI**S**RQSIDA  TAAQAK**S**KQAILA  GEDVET**S**KKWAAG  GQNKQH**S**ITKNTA  IQQGRQ**S**KGLTQK  VIADYE**S**GRAIPN  IANRIK**S**KGLQKL  FKCHCM**S**ESHQRQ  CHCMSE**S**HQRQLL  RQLLLA**S**ENPQQF  QFMDYF**S**EEFRND  VYNEYI**S**HREHIH  PTFTEL**S**RENDEE  KVTFNL**S**KGACSS  LSKGAC**S**SSGATS  SKGACS**S**SGATSS  KGACSS**S**GATSSK  SSSGAT**S**SKSSTL  SSGATS**S**KSSTLG  GATSSK**S**STLGPS  ATSSKS**S**TLGPSA  SSTLGP**S**ALKTIG  ALKTIG**S**SASVKR  LKTIGS**S**ASVKRK  TIGSSA**S**VKRKES  SVKRKE**S**SQSSTQ  VKRKES**S**QSSTQS  RKESSQ**S**STQSKE  KESSQS**S**TQSKEK  SQSSTQ**S**KEKKKK  EKKKKK**S**ALDEIM  VVKMID**S**GDKLKL  NEGTLE**S**INEKTF  INEKTF**S**ATIVIE  GQRRES**S**RPVKPP  HPAPEK**S**SKVSEQ  EQLKCC**S**GILKEM  DVRLMF**S**NCYKYN  PSSSDS**S**SDSSSD  SSSDSS**S**DSSSDS  VEENKK**S**KAKEPP  TKKNNS**S**NSNVSK  KNNSSN**S**NVSKKE  EPAPMK**S**KPPPTY  IQSREP**S**LKNSNP  FETLKP**S**TLRELE  LERYVT**S**CLRKKR  VDVIAG**S**SKMKGF  GFSSSE**S**ESSSES  SSSESS**S**SDSEDS  NQHAVV**S**PPALHN  ALPQQP**S**RPSNRA  PSVKVQ**S**QPPPPL  PPPPHP**S**VQQQLQ  VIQHHH**S**PRHHKS  SPRHHK**S**DPYSTG  HKSDPY**S**TGHLRE  SPLMIH**S**PQMSQF  HSPIIR**S**EPFSPS  IRSEPF**S**PSLRPE  PPKHPE**S**IKAPVH  NMGSWA**S**LVQKHP  PSSTAK**S**SSDSFE  AKSSSD**S**FEQFRR  ATPQAQ**S**SQPQSM  VIAPLL**S**QRYRRM  VTKDGH**S**TLQMDG  WMMLVF**S**ASFVVH  MLVFSA**S**FVVHWL  CVKYIT**S**FTAAFS  SFTAAF**S**FSLETQ  TAAFSF**S**LETQLT  YGTMFP**S**GDCPSA  PSGDCP**S**AIALLA  PKNRAF**S**IRFTDT  VANTRP**S**PLTSVR  LTSVRV**S**AVLYQE  GKLYQT**S**VDFHLD  FHLDGI**S**SDECPF  HLDGIS**S**DECPFF  PLTYYH**S**ITPSSP  YHSITP**S**SPLATL  HSITPS**S**PLATLL  LQHENP**S**HFELVV  ELVVFL**S**AMQEGT  RTSYLP**S**EIMLHH  LHHCFA**S**LLTRGS  SLLTRG**S**KGEYQI  FPTPLV**S**KSPNRT  TPLVSK**S**PNRTDL  IHINGQ**S**IDNFQI  VLTANF**S**VTNLSQ  KMQNGF**S**RTLKSG  FSRTLK**S**GDGITF  PLVACS**S**CLDVSG  QPPQIE**S**FYPPLD  PPLDEP**S**IGSKNV  DEPSIG**S**KNVDLS  DPQGHP**S**TGLKTT  SLSQGV**S**VDEKLM  PKEIKV**S**KMEQKF  TSSNNN**S**MVSNTL  NNNSMV**S**NTLAKM  SPTKLP**S**INKSKD  QQQQTN**S**IRNYFQ  RNYFQP**S**TKKRER  ENQEMS**S**CKSARI  SARIET**S**CSLLEQ  RIETSC**S**LLEQTQ  EPVDTN**S**DNNLFT  TDTDLK**S**IVKNSA  KSIVKN**S**ASKSHA  AAEKLR**S**NKKREM  ETNDTF**S**DEAVPE  DEAVPE**S**SKISQE  EAVPES**S**KISQEN  RELKED**S**LWSAKE  LLTEFR**S**LVIKNS  SLVIKN**S**TSRNPS  VIKNST**S**RNPSGI  STSRNP**S**GINDDY  PHIIGG**S**DLIAHH  KSQPLK**S**SSSAEH  AEHPGA**S**KPPISS  SKPPIS**S**SSMTSR  KPPISS**S**SMTSRI  QQRVPV**S**QTPAIN  TPAINV**S**VPTTLP  VPTTLP**S**ATQVPM  QVKQYL**S**TTLANK  HANQVL**S**LPCPNQ  MPPVPG**S**SAPNSP  AMLTLN**S**NCEKEG  EQNRAE**S**ECPGMN  PGMNTH**S**RASCMQ  NTHSRA**S**CMQMDD  VIDDII**S**LESSYN  DIISLE**S**SYNEEI  IISLES**S**YNEEIL  ANTLPV**S**GNLIDL  PPGLTI**S**NSCPAN  GLTISN**S**CPANLP  GTLIPK**S**NDPDMR  GTILKA**S**VDYIRK  GLSLIP**S**TGLCSP  PVLENC**S**QDLLQH  EANQAY**S**VPTKMG  VPTKMG**S**KLEDIL  VTDPLL**S**SVSPGA  PLLSSV**S**PGASKT  SVSPGA**S**KTSSRR  PGASKT**S**SRRSSM  GASKTS**S**RRSSMS  EIEMDY**S**RNLEKL  FLAKTR**S**TKDQQF  VLNELY**S**VMKTYH  YNADSI**S**AQSKLK  DSISAQ**S**KLKEAE  EKHVRR**S**SVKKIE  KHVRRS**S**VKKIEK  YYIHDL**S**DLIDQC  DLGYHA**S**LNRALR  ELNLEQ**S**KHEGLD  QLQSRL**S**TLKIEN  SVKSTV**S**ETFMSK  TFMSKP**S**IAKRRA  CSLARR**S**STVRKQ  IPLVVE**S**CIRFIS  IFRVSG**S**QVEVND  NHLSQF**S**EENMMD  FGPSLM**S**VPEGHD  EGHDQV**S**CQAHVN  HENIFP**S**PRELEG  VYSRGG**S**MEDYCD  SFKKGA**S**LLLYQR  QRKRPE**S**GSIRKT  DSHGLS**S**SLTDSS  SPGVGA**S**CRPSSQ  GASCRP**S**SQPIMS  SISRHS**S**LKNRLD  LKNRLD**S**PQIRKT  TATAGR**S**KSFNNH  RELERQ**S**SVKHTP  LEPLKT**S**PVVAPT  AFQRSA**S**TAGDIA  AFRPVK**S**VKMAAP  GAQRWR**S**ENFERP  PVDLEG**S**GDDDSF  GSGDDD**S**FPDDEL  ELDDLY**S**GSGSGY  DDLYSG**S**GSGYFE  LYSGSG**S**GYFEQE  GYFEQE**S**GIETAM  ETAMRF**S**PDVALA  DVALAV**S**TTPAVL  PFEELP**S**ERPTLE  TLEPAT**S**PLVVTE  EVPEEP**S**QRATTV  QRATTV**S**TTMATT  ATTAAT**S**TGDPTV  VATATP**S**TPAAPP  TTPEAP**S**PPTTAA  PTPRLV**S**TATSRP  LVSTAT**S**RPRALP  PDIPER**S**TLPLGT  EPEVPV**S**GGPSGD  PVSGGP**S**GDFELP  GAAAKA**S**SPPGTL  AAAKAS**S**PPGTLP  LDNAID**S**GSSAAQ  NAIDSG**S**SAAQLP  AIDSGS**S**AAQLPQ  AQLPQK**S**ILERKE  KKKDEG**S**YTLEEP  EEPKQA**S**VTYQKP  YAMKLL**S**KFEMIK  FEMIKR**S**DSAFFW  MIKRSD**S**AFFWEE  RECDWW**S**VGVFLY  SLVGTY**S**KIMDHK  IMDHKN**S**LCFPED  RENLLL**S**DSPSCR  NLLLSD**S**PSCRET  LLSDSP**S**CRETDS  SCRETD**S**IQSRKN  ETDSIQ**S**RKNEES  TLEEHL**S**NEMQAK  LEQKCK**S**VNTRLE  EITLRK**S**VESALR  LRKSVE**S**ALRQLE  LENDVN**S**LKDQLE  NQNSQI**S**TEKVNQ  RKTQAE**S**SKQIQQ  LQSALE**S**ERRDRT  RDRTHG**S**EIINDL  LKVIQQ**S**LEQEEA  KNKIYE**S**IEEAKS  SIEEAK**S**EAMKEM  EAEKRC**S**LLDCDL  VNTLKM**S**EKQLKQ  TLTKAD**S**EQLARS  LHMELK**S**EREKLT  AQIAEE**S**QIRIEL  LQMTLD**S**KDSDIE  RLEGWL**S**LPVRNN  KILFYD**S**EQDKEQ  YANEGE**S**KKEQEF  DPFARS**S**PRTSMK  EKICTR**S**SSSPTG  KICTRS**S**SSPTGS  CTRSSS**S**PTGSDW  EISLKT**S**EGDSME  DKEIKV**S**YTVYNR  TVYNRL**S**LLLKSL  LSLLLK**S**LLAITR  TPAYRL**S**RKQGHE  FGEVQL**S**GLGEGF  RPYPSS**S**PMHPCN  TGVIYP**S**VEDSQE  TTSFST**S**PPSQLS  FSTSPP**S**QLSSSR  SPPSQL**S**SSRLSY  PPSQLS**S**SRLSYQ  PSQLSS**S**RLSYQP  LSSSRL**S**YQPAAL  HCAATP**S**SSEDTE  AATPSS**S**EDTETV  EDTETV**S**NSSEGR  NQVTLT**S**LDIPFA  MVNPPD**S**PETESP  DSPETE**S**PLQGSL  ESPLQG**S**LHSDGS  SLHSDG**S**SGGSSG  DGSSGG**S**SGNTHD  GSSGGS**S**GNTHDD  QNPPQL**S**SLSIDI  PQLSSL**S**IDIGAQ  IDIGAQ**S**MAEDLD  MAEDLD**S**LPEKLA  KDWFHG**S**CVGVEE  KPVQNG**S**QLFIKE  FIKELR**S**RTFPSA  APTFYV**S**DVENYV  VTNLEF**S**DTRMSS  SDTRMS**S**FVEPPD  YLIRPA**S**ANISLY  PASANI**S**LYERWR  LYERWR**S**ASNHSE  RRLKLG**S**LTQFPN  FKGSHK**S**GKQLPP  LNGAFR**S**WTKKQA  VNTVAS**S**DEVCDG  KKKGKK**S**RESASP  IREQTK**S**KSEAKW  EQTKSK**S**EAKWKY  KWKYKN**S**KPDSLL  EQKLEK**S**PLAGNK  DNKFSF**S**FSNKKL  KFSFSF**S**NKKLLG  NKKLLG**S**KALRPP  ALRPPT**S**PGVFGA  AYKSDD**S**SDEGSL  ARVKKE**S**GSSAAG  DACFKD**S**DYVYPS  DNPIFK**S**RSKKRK  AAAAKL**S**QQEEQK  TSTTPA**S**TTPAST  TASSQA**S**QEGSSP  AQAGRT**S**QPMAPG  RRPSAS**S**PNNNTA  ASAPPA**S**PPGSEP  PASPPG**S**EPPGPD  GGPDPQ**S**PGLDEP  PRDPGP**S**APPPRS  PGPGPR**S**EAPLPE  ELDPLF**S**WTEEPE  EECGPA**S**CPESAP  PASCPE**S**APFRLQ  PFRLQG**S**SSSHRA  GEVDVF**S**PFPAPT  TKYLDP**S**GLGVIS  SGLGVI**S**FEDFYQ  AYMGSE**S**TYSECE  FTDEDT**S**TLVHPE  PEGDAD**S**AGGSAV  PHPHGQ**S**VITVIG  EDYGEG**S**EAELSP  PAFLTP**S**PTKRLS  PTKRLS**S**KKVARY  ARYLHQ**S**GALTME  EALEDP**S**PELMEG  ENSELR**S**CTPCLK  SLTLRL**S**EEQENK  EQRRGR**S**SSMGLQ  HSRARE**S**ELEQEV  SIQGAK**S**LFSTAF  GAKSLF**S**TAFSES  LFSTAF**S**ESLAAE  SLAAEI**S**SVSRDE  LAAEIS**S**VSRDEL  AEISSV**S**RDELME  AAFHIS**S**LLEKMT  LEKMTS**S**DKDFRF  FRFMAT**S**DLMSEL  ATSDLM**S**ELQKDS  LCTNMR**S**DKEQLR  GLKTVL**S**ELPPAA  ITGQLT**S**AIAQQE  CLLPQL**S**SPRLAV  HLAAAC**S**TDLFVE  HVPNVT**S**LCLQYI  CIAALI**S**SRPDLL  HMPVLV**S**GIIFSL  VSGIIF**S**LADRSS  SLADRS**S**SSTIRM  LADRSS**S**STIRMD  PYVGEM**S**AVTLAR  LTLVAV**S**PLQLDL  ALHILA**S**FLRKNQ  LDALAQ**S**QGLSLP  LLRLLR**S**PLLPAG  DYAKLI**S**LLTAPV  RCVAAL**S**AACPQE  ARSPHS**S**TGVKVL  KVLAFL**S**LAEVGQ  QYLLLH**S**LREALG  GAAQPD**S**LKPYAE  LVLVNP**S**FLLPRL  TLAFFN**S**AVHNKP  MYSLLE**S**CLGQLD  IPEVGK**S**PIMADF  PIMADF**S**SQIRSN  FSSQIR**S**NPELAA  IQKDSA**S**APSTDS  SRARSA**S**SPRGMV  PSSSES**S**SDSDNS  GGGGGT**S**SNNSEE  PMLETA**S**KLLLSG  RLTSSV**S**CALDEA  GQSDNR**S**LAEACS  LLCLAC**S**AGYYEL  TPSPII**S**PSAMLP  ADIEAQ**S**ERTKDT  KEHRNV**S**DYTPLS  EKLREE**S**RRLALA  NGESKS**S**STSESG  GESKSS**S**TSESGD  SDESSN**S**NSSRKS  RRSKKV**S**VPSTVI  VPSTVI**S**RVIGRG  PKNRLK**S**SSANSK  PARATN**S**PKPHMV  HMVPRH**S**NQNSSG  NQNSSG**S**QVNSAG  KEHYPV**S**SPSSPS  AQPGGV**S**RNSPLD  SPNKVA**S**SSEQEA  SSSGSS**S**AHSNQQ  RPPLQQ**S**QVPPPE  SSVAVL**S**VNHIKR  LHQSDT**S**KAPGFR  NFASNI**S**GGQMYG  APSPAP**S**SVPLGS  SSVPLG**S**EKPSNV  TGTSAP**S**VIGSNL  RAAEKV**S**EARKRK  EAAALG**S**RGSCST  ALGSRG**S**CSTEVE  GSRGSC**S**TEVEKE  RGGKKK**S**TKTSRS  KKSTKT**S**RSAKAG  STKTSR**S**AKAGVI  KGVTIA**S**GGVLPN  LAKKRG**S**KGKLEA  PAKKAK**S**PSQKKP  KKAKSP**S**QKKPVS  SQKKPV**S**KKAGGK  KKGARK**S**KKKQGE  KKQGEV**S**KAASAD  EVSKAA**S**ADSTTE  KAASAD**S**TTEGTP  DGFTVL**S**TKSLFL  TVLSTK**S**LFLGQK  KLNLIH**S**EISNLA  LIHSEI**S**NLAGFE  VAGAAV**S**AGHGLP  FVIHCN**S**PVWGAD  DDKKLK**S**IAFPSI  KSIAFP**S**IGSGRN  AFPSIG**S**GRNGFP  LILKAI**S**SYFVST  ILKAIS**S**YFVSTM  ISSYFV**S**TMSSSI  YFVSTM**S**SSIKTV  FVSTMS**S**SIKTVY  VSTMSS**S**IKTVYF  YFVLFD**S**ESIGIY  VLFDSE**S**IGIYVQ  EQAEQP**S**QPSSTP  EQPSQP**S**STPGSE  QPSQPS**S**TPGSEN  PSSTPG**S**ENVLPR  VKFLQN**S**RVRQSP  NSRVRQ**S**PLATRR  DMAFQQ**S**GTAADE  LISQPY**S**PAGSRW  PYSPAG**S**RWRDYG  RMEAGL**S**ELSGSV  AGLSEL**S**GSVAQT  LSELSG**S**VAQTVT  LQTTLA**S**VQELLI  TNWILE**S**QNINEL  NINELK**S**EINSLK  LKSEIN**S**LKGLLL  RRQFPP**S**PSAPKI  QFPPSP**S**APKIPS  WQIPVK**S**PSPSSP  IPVKSP**S**PSSPAA  VKSPSP**S**SPAAVN  KSPSPS**S**PAAVNH  AAVNHH**S**SSDISP  AVNHHS**S**SDISPV  VNHHSS**S**DISPVS  HSSSDI**S**PVSNES  SDISPV**S**NESTSS  SPVSNE**S**TSSSPG  VSNEST**S**SSPGKE  NESTSS**S**PGKEGH  PGKEGH**S**PEGSTV  GHSPEG**S**TVTYHL  EEDDDV**S**HVDEED  WAKWTW**S**AVRGGA  SQAETK**S**LSFSSD  AETKSL**S**FSSDSE  LSFSSD**S**EGNFET  AGPGAK**S**QESQEA  GAKSQE**S**QEADEQ  EVVEKC**S**SKTCSK  CSSKTC**S**KPSENE  EPEHDF**S**KISIVR  SIVRPF**S**IETKDS  SIETKD**S**TDISAV  GCVTAV**S**GKALPS  GVTLEA**S**AEADLK  TPGTLS**S**DTNDSG  SSDTND**S**GVELGE  LGEESR**S**SPLKLE  GEESRS**S**PLKLEF  LGRKLG**S**TLTPKI  KDGISK**S**AGLEQP  PLSQTS**S**KPDPSQ  NSPPLS**S**EGSYHF  PTTTLT**S**SDFCSP  VNEILE**S**PKKAKS  KKHETQ**S**LALDAC  AVISQI**S**DISNRD  SQISDI**S**NRDGHA  CSNVPV**S**TINHAF  TINHAF**S**SSEAGI  KMEEDG**S**TVLGLL  SVERSL**S**DLFRRY  TKAKAE**S**AALHAG  LLGNYD**S**AMVYYQ  IMKTLE**S**FKLDST  ESFKLD**S**TPLKAA  QHDLPA**S**EGEVWS  SEGEVW**S**MPVPVE  PRKRQS**S**QYSDPK  RQSSQY**S**DPKSHG  QYSDPK**S**HGNRPS  TVRVHR**S**SAQNVH  VRVHRS**S**AQNVHN  ETNKFD**S**TGYDKD  TFFNVS**S**STLTSK  FFNVSS**S**TLTSKY  SSSTLT**S**KYRGES  SKYRGE**S**EKLVRL  EMARFY**S**PATIFI  FIDEID**S**ICSRRG  EIDSIC**S**RRGTSE  CSRRGT**S**EEHEAS  SEEHEA**S**RRVKAE  DGVGGT**S**ENDDPS  SENDDP**S**KMVMVL  IYIPLP**S**AKGREE  EELLRI**S**LRELEL  DDVDLA**S**IAENME  ENMEGY**S**GADITN  NVCRDA**S**LMAMRR  EEIRNL**S**KEEMHM  MALKKV**S**KSVSAA  LKKVSK**S**VSAADI  KVSKSV**S**AADIER  KENIKN**S**SVPRRT  ENIKNS**S**VPRRTL  LKMIQP**S**ASGSLV  MIQPSA**S**GSLVGR  QPSASG**S**LVGREN  GRENEL**S**AGLSKR  ELSAGL**S**KRKHRN  RNDHLT**S**TTSSPG  GVIVPE**S**SENKNL  VIVPES**S**ENKNLG  GGVTQE**S**FDLMIK  MIKENP**S**SQYWKE  IKENPS**S**QYWKEV  PLDNFE**S**LDNQEF  DNQEFD**S**EEETVE  DSLVED**S**EIGTCA  CAEGTV**S**SSTDAK  AEGTVS**S**STDAKP  AKKQKL**S**SDENSN  KKQKLS**S**DENSNP  LSSDEN**S**NPDLSG  NSNPDL**S**GDENDD  ENDDAV**S**IESGTN  DAVSIE**S**GTNTER  NAPGRK**S**WGKGKW  GKGKWK**S**KKCKYS  SKKCKY**S**FKCVNS  SFKCVN**S**LKEDHN  VQFNWH**S**KEGDPL  VFATVG**S**NRVTLY  TLYECH**S**QGEIRL  EIRLLQ**S**YVDADA  CAWTYD**S**NTSHPL  TYDSNT**S**HPLLAV  LLAVAG**S**RGIIRI  DPNLLL**S**VSKDHA  NLLLSV**S**KDHALR  HRDEVL**S**ADYDLL  SCGMDH**S**LKLWRI  KLWRIN**S**KRMMNA  TNRPFI**S**QKIHFP  IHFPDF**S**TRDIHR  LGDLIL**S**KSCENA  DLILSK**S**CENAIV  IDKIKP**S**ESNVTI  KIKPSE**S**NVTILG  LGRFDY**S**QCDIWY  IWYMRF**S**MDFWQK  TSFSRD**S**SILIAV  SFSRDS**S**ILIAVC  AVCDDA**S**IWRWDR  GEKPVG**S**LAGIGE  GANAKQ**S**RDCFGC  QGVGLD**S**TGYYDQ  FAGYVT**S**IAATEL  DDDYSS**S**TSLLGQ  DYSSST**S**LLGQKK  RRTMII**S**PERLDP  VNGAAA**S**QPPSKR  AASQPP**S**KRKRRW  TPKKLS**S**WDQAET  PGRAKG**S**ETPGAT  HGGATS**S**ARKNRW  TDRGGD**S**IGETPT  TPTPGA**S**KRKSRW  WDETPA**S**QMGGST  ASQMGG**S**TPVLTP  LVDVDE**S**TLSPEE  VDESTL**S**PEEQKE  EGREII**S**NLAKAA  GLATMI**S**TMRPDI  AFAVVA**S**ALGIPS  VCKSKK**S**WQARHT  ILPHLR**S**LVEIIE  TPYGIE**S**FDSVLK  LIREFQ**S**PDEEMK  GAAEII**S**RIVDDL  EQTTED**S**VMLNGF  QAADLI**S**RTAVVM  YPEVLG**S**ILGALK  IVAETC**S**PFTVLP  QNGVLK**S**LSFLFE  VHRQTA**S**AVVQHM  AVVQHM**S**LGVYGF  PNVFET**S**PHVIQA  YWKIYN**S**IYIGSQ  PNPSST**S**SPYPLP  NPSSTS**S**PYPLPE  LKGENL**S**KKAKEK  AKEKRE**S**LIKKIK  KIKDVK**S**IYLQEF  GPPDTI**S**LASERY  DTISLA**S**ERYDKD  KDDEAP**S**DGAQFP  KRRKDH**S**FLGFEW  KRWCAL**S**KTVFYY  VFYYYG**S**DKDKQQ  FAIDGY**S**VRMNNT  DCCFEI**S**APDKRI  YQFTAA**S**PKDAEE  VLQDME**S**DIIPED  DHPLPI**S**NPLTSS  ISNPLT**S**SQPIDD  SNPLTS**S**QPIDDE  PEEEED**S**APVKVE  EEQRKM**S**QDSVHH  RKMSQD**S**VHHTSG  DSVHHT**S**GDKSTD  HTSGDK**S**TDYANF  DCTGAF**S**DELSFK  AFSDEL**S**FKRGDV  DVIYIL**S**KEYNRY  EDSGTF**S**LGKMIT  VYECER**S**DVQIHV  CTGEGT**S**KKLAKH  ILKANA**S**ICFAVP  PLMPDP**S**KQPKNQ  QLNPIG**S**LQELAI  LPEYTL**S**QEGGPA  TICRLE**S**FMETGK  ETGKGA**S**KKQAKR  KFLAKF**S**NISPEN  AKFSNI**S**PENHIS  SPENHI**S**LTNVVG  TNVVGH**S**LGCTWH  LGCTWH**S**LRNSPG  WHSLRN**S**PGEKIN  INLLKR**S**LLSIPN  LKRSLL**S**IPNTDY  LDIDEL**S**ANGQYQ  QCLAEL**S**TSPITV  LAELST**S**PITVCH  CHGSGI**S**CGNAQS  SCGNAQ**S**DAAHNA  AVDFVF**S**HGLIYW  IYWSDV**S**EEAIKR  VQNVVV**S**GLLSPD  VVSGLL**S**PDGLAC  TNRIEV**S**NLDGSL  VSNLDG**S**LRKVLF  LNFIHK**S**NLDGTN  GLREIH**S**DIFSPM  TDLRRI**S**LDTPDF  VRAIRR**S**FIDGSG  RAALDG**S**DRVVLV  VVLVNT**S**LGWPNG  IGFELI**S**DMKTCI  TGVKEA**S**ALDFDV  RAFMNG**S**ALEHVV  TNLIES**S**NMLGLN  ILVFHS**S**RQSGWN  GCPAHY**S**LNADNR  LLFSQK**S**AINRMV  GFTVVV**S**SVPSQN  FTVVVS**S**VPSQNL  DLRRIE**S**SDLSGA  LRRIES**S**DLSGAN  IESSDL**S**GANRIV  TECEDH**S**DELNCP  AHVTGA**S**SSSSSS  FGYSSN**S**PSTHRS  YSYRPY**S**YRHFAP  PPTTPC**S**TDVCDS  STDVCD**S**DYAPSR  DSDYAP**S**RRMTSV  EEEGGS**S**GGAAGT  GGAAGT**S**ADGGDG  FLVRKI**S**GHDTGK  LEHIRQ**S**PFLVTL  ELFTHL**S**QRERFT  ENILLD**S**NGHVVL  LTDFGL**S**KEFVAD  KAVDWW**S**LGVLMY  ELLTGA**S**PFTVDG  VDGEKN**S**QAEISR  NSQAEI**S**RRILKS  PYPQEM**S**ALAKDL  SAMMKD**S**PFYQHY  KPLGEG**S**FSICRK  LGEGSF**S**ICRKCV  KCVHKK**S**NQAFAV  FAVKII**S**KRMEAN  KKKKHF**S**ETEASY  FSETEA**S**YIMRKL  IMRKLV**S**AVSHMH  KLVSAV**S**HMHDVG  ESCDLW**S**LGVILY  GQVPFQ**S**HDRSLT  FQSHDR**S**LTCTSA  RSLTCT**S**AVEIMK  TSTETR**S**SSSESS  RSSSSE**S**SHSSSS  SSSSES**S**HSSSSH  SSESSH**S**SSSHSH  SSHSSS**S**HSHGKT  TKTLQP**S**NPADSN  AALAFL**S**QESRTR  AFLSQE**S**RTRAGG  APVTMD**S**FFFGCE  FFGCEL**S**GHTRSF  LSGHTR**S**FTFKVE  VANLKL**S**CQPMLS  SCQPML**S**LDDFQL  VTFRLK**S**GSGPVR  FRLKSG**S**GPVRIT  HQIVTM**S**NDVSEE  TMSNDV**S**EEESEE  DVSEEE**S**EEEEED  EEEEED**S**DEEEVE  LLWILP**S**LWLLLL  KTQEHP**S**CPGPRE  PRELEA**S**KVVLLP  KVVLLP**S**CPGAPG  CPGAPG**S**PGEKGA  NCRELL**S**QGATLS  SQGATL**S**GWYHLC  QRRQDG**S**VDFFRS  SVDFFR**S**WSSYRA  DFFRSW**S**SYRAGF  FFRSWS**S**YRAGFG  GFGNQE**S**EFWLGN  LALGKF**S**EGTAGD  EGTAGD**S**LSLHSG  TAGDSL**S**LHSGRP  DSLSLH**S**GRPFTT  YDADHD**S**SNSNCA  DADHDS**S**NSNCAV  DHDSSN**S**NCAVIV  GAWWYA**S**CYRSNL  YASCYR**S**NLNGRY  NGRYAV**S**EAAAHK  YGIDWA**S**GRGVGH  PDPDWA**S**YTLGVF  GVFICL**S**CSGIHR  FICLSC**S**GIHRNI  RNIPQV**S**KVKSVR  QVSKVK**S**VRLDAW  QVEFMA**S**HGNDAA  FESKVP**S**FYYRPT  YYRPTP**S**DCQLLR  EKQEPY**S**AGYREG  DNGQFL**S**RKFVLT  TYLKDN**S**TRNIFI  DLVPKL**S**RNYLKE  GEVFIG**S**KESGYT  FIGSKE**S**GYTVLH  LHGFPP**S**TQGHHW  FACETE**S**DQREWV  HALCVC**S**RGTVII  LGEGGF**S**YVDLVE  VLMDLG**S**MNQACI  CIHVEG**S**RQALTL  RAPELF**S**VQSHCV  ELFSVQ**S**HCVIDE  ERTDVW**S**LGCVLY  VFQKGD**S**VALAVQ  AVQNQL**S**IPQSPR  QLSIPQ**S**PRHSSA  PQSPRH**S**SALRQL  QSPRHS**S**ALRQLL  LRQLLN**S**MMTVDP  HIPLLL**S**QLEALQ  HPFFLL**S**PVMGLL  PVMGLL**S**RAWSRL  TLGLKT**S**SSLPEA  LGLKTS**S**SLPEAW  GLKTSS**S**LPEAWG  GEREAT**S**VPRGQG  RAPLSP**S**LLIRTL  EGVNKF**S**YPPSHR  SALSPG**S**KPSTWV  SPGSKP**S**TWVSCP  KPSTWV**S**CPGEEE  DKRTER**S**KGARKT  ARKTSV**S**PRSSGS  TSVSPR**S**SGSDPR  SGSDPR**S**WEYRSG  RSWEYR**S**GEASEE  SWWCQP**S**DEEEGE  PCIPPP**S**AFLKAW  EEEDED**S**DSGSDE  EDEDSD**S**GSDEEE  TGVFLK**S**WVYQPG  EDSDTG**S**AEDERE  EAETSA**S**TPPASA  ASTPPA**S**AFLKAW  EDEDVD**S**EDKEDD  EDKEDD**S**EAALGE  ALGEAE**S**DPHPSH  ESDPHP**S**HPDQRA  QAQEEL**S**PCLTPA  LTQTLP**S**SSVPSS  TQTLPS**S**SVPSSP  QTLPSS**S**VPSSPV  ATPSRS**S**AAAAAA  PTGDFD**S**KPSWAD  DDKCVT**S**ELLKGI  IETRKA**S**KAVARR  WKKFGN**S**EFDPPG  VATTTV**S**DDVSMT  TVSDDV**S**MTFITS  SMTFIT**S**KEDLNC  KGQKIV**S**CRICKG  GKYVPP**S**LRDGAS  SLRDGA**S**RRGESM  ASRRGE**S**MQPNRR  IRVTNL**S**EDTRET  LFRPFG**S**ISRIYL  RPFGSI**S**RIYLAK  DKTTGQ**S**KGFAFI  KGFAFI**S**FHRRED  RAIAGV**S**GFGYDH  KPEVKI**S**EKKKIA  IDAMNP**S**SRDDFT  DAMNPS**S**RDDFTE  ITQYEK**S**LYYASF  KSLYYA**S**FLEVLV  VRDVCI**S**LEIDDL  LKKITN**S**LTVLCS  SLTVLC**S**EKQKQE  QKQEKQ**S**KAKKKK  DDFFPG**S**DRFARP  PDFRDI**S**KWNNRV  WNNRVV**S**NLLYYQ  VAAMMI**S**IVGFLS  SIVGFL**S**PFNMIL  MVVMLA**S**YFLISM  ASYFLI**S**MFGGVM  LMFIHA**S**LRLRNL  LKQMVM**S**LRVSEL  MTPADL**S**IPNVHS  IPNVHS**S**PMPATL  SLASDN**S**QRFRET  QLRFCL**S**ETSCPQ  VNTKPC**S**LPGYLP  PNTIVV**S**WTAEIG  EIGRNY**S**MAVYLV  YLVKQL**S**STVLLQ  LVKQLS**S**TVLLQR  SEIATT**S**LRVSLL  TTSLRV**S**LLCPLG  CRALTC**S**HLQCFD  LEHQVA**S**HHQSSN  IDLTID**S**SSDEEE  LTIDSS**S**DEEEEE  EEEEEP**S**AKRTCP  AKRTCP**S**LSPTSP  RTCPSL**S**PTSPLN  PSLSPT**S**PLNNKG  HQASPV**S**RTPSLP  LPAVDT**S**YINTSL  DFFPFL**S**GDNQHY  DQDLLH**S**SRFFPY  QDLLHS**S**RFFPYT  LPTTNG**S**SSGSNS  TTNGSS**S**GSNSSL  SSGSNS**S**LVSSNS  SNSSLV**S**SNSLRE  SNSLRE**S**HSHTVT  SLRESH**S**HTVTNR  TVTNRS**S**TDTASI  SSTDTA**S**IFGIIP  PTKNYL**S**YLTAPD  LTAPDY**S**AFETDI  QPIELL**S**MKRYEL  YELPAP**S**SGQKND  ELPAPS**S**GQKNDI  QECVNN**S**MAQLEH  ENLELM**S**QHGCNA  MQLTAG**S**KLREME  KLREME**S**NWVSLV  MESNWV**S**LVSKNY  NWVSLV**S**KNYEIE  STITAT**S**LGVNNS  SLGVNN**S**SHRRKN  RRKNGP**S**TLESSR  GPSTLE**S**SRFPAR  RKRGNL**S**SLEQIY  KRGNLS**S**LEQIYG  IYGLEN**S**KEYLSE  NSKEYL**S**ENEPWV  QPKQGG**S**ILLITG  GMFNTE**S**SFHMFP  NQFYRD**S**HTLHEV  IFIISD**S**LSGDNN  IISDSL**S**GDNNQR  QEECSI**S**NISFNP  LLCQGC**S**GDIRSA  CSGDIR**S**AINSLQ  IRSAIN**S**LQFSSS  NSLQFS**S**SKGENN  SLQFSS**S**KGENNL  IGGKDV**S**LFLFRA  DSPRLP**S**HLSEYE  RLPSHL**S**EYERDT  EEVVEM**S**HMPGDL  SFADIL**S**GDWNTR  GDWNTR**S**LLREYS  TRGVMH**S**NKARGY  HCQGGG**S**SFRPLH  CQGGGS**S**FRPLHK  TVPETW**S**LPLSQN  LPLSQN**S**ASELPA  SQPQPF**S**AQGDME  RKPSNA**S**EKEPTQ  TQKKKL**S**LQRSSS  KLSLQR**S**SSFKDF  LSLQRS**S**SFKDFA  FAKSKP**S**SPVVSE  AKSKPS**S**PVVSEK  NIPEDD**S**GVPTPE  PEDAGK**S**GKKLGK  KWRAVI**S**RTMNRK  DTLEEG**S**ASPTSP  PTSPDY**S**LDSPGP  KMALAF**S**EQEEHE  HELPVL**S**RQASTG  VLSRQA**S**TGSELC  RQASTG**S**ELCSPS  TGSELC**S**PSPGSG  SELCSP**S**PGSGSF  CSPSPG**S**GSFGEE  PSPGSG**S**FGEEPP  HTDFTP**S**PYDHDS  SPYDHD**S**LKLQKG  LNGKVG**S**FKFIYV  VGHARP**S**RRQSKG  RPSRRQ**S**KGKRPK  GLEEHT**S**TLLLNG  AEEGAE**S**SQEPVA  EEGAES**S**QEPVAH  PVAHTV**S**EPKVDI  VDIPRD**S**GCFEGS  SGCFEG**S**ESGRDD  CFEGSE**S**GRDDAE  EDSASA**S**LSSAAA  SASASL**S**SAAATG  ASASLS**S**AAATGT  AAATGT**S**TSTPAA  ATGTST**S**TPAAPT  VLWKIP**S**KELRVR  RLTLPH**S**IRSDSE  LPHSIR**S**DSEDIC  TKDEPN**S**TPEKTE  HGIKTV**S**QIISLQ  TVSQII**S**LQTLKK  LKKEYK**S**YEAKLR  AKLRLL**S**SFDFFL  KLRLLS**S**FDFFLT  IRRLLP**S**LIGRHF  RKKVPV**S**VNLLSK  VSVNLL**S**KNLSRE  LLSKNL**S**REINDC  GTVLNI**S**KSGSCS  SKSGSC**S**AIRIGH  AVTKGL**S**EKLPEK  AALPIF**S**SFVSNW  DEATKR**S**LLNKKK  QARKTA**S**VLSKDD  DDVAPE**S**GDTTVK  HATGKK**S**PAKSPN  AKSPNP**S**TPRGKK  RKALPA**S**ETPKAA  SETPGK**S**PEKKPK  VKEKSP**S**LGKKDA  KFFTTP**S**KSVRKA  FTTPSK**S**VRKASH  KSVRKA**S**HTPKKW  QAQHTR**S**RERADG  DRRGIT**S**ENEREV  MLERLV**S**SESKNY  LERLVS**S**ESKNYI  AKGVIP**S**SLFLQD  YLIDMA**S**RAGMHW  AGMHWL**S**TIIPTH  IFFFIV**S**NLTIDF  LVIFYL**S**FISMVI  FYLSFI**S**MVICTL  LKVFQD**S**KAWENF  YAHFLL**S**VFFVIF  VFFVIF**S**FPIASK  FSFPIA**S**KDCIPC  KDCIPC**S**ELAVIT  TVTSYL**S**LSTHAE  TSYLSL**S**THAEPY  VTAGLL**S**LLPSMP  VVILLE**S**TGLGLL  VTVAVC**S**VPLLLR  RWWTKA**S**FSVVGM  KSLTRS**S**MVKLIL  WFYVYR**S**EGMKVY  GMKVYN**S**TLTWQQ  EAYPAC**S**PGNTST  CSPGNT**S**TAEEEL  TVGMPF**S**SGADGS  FKSVLL**S**LRQGSL  GSLIEF**S**TILEGR  FELKAI**S**CLNCMA  NCMAQL**S**PTRRHV  EGPQDR**S**SQQKGR  GPQDRS**S**QQKGRL  QQKGRL**S**LQNTAE  ECFENN**S**CEIRGL  FDAQGK**S**FIKDAL  HRFGCI**S**RKCPAI  AIREMV**S**QLQREC  KEAITH**S**VQVQCE  CEQNWG**S**LCSILS  NWGSLC**S**ILSFCT  SLCSIL**S**FCTSAI  ILSFCT**S**AIQKPP  VDRTKL**S**RAHHGE  AKGERG**S**KSHPNA  GERGSK**S**HPNAHA  LGAQGP**S**GSSEWE  AQGPSG**S**SEWEDE  QGPSGS**S**EWEDEQ  SVPALW**S**EVNRYG  CLIQNG**S**FKEALN  ALKTIE**S**ANQQTD  RDLVRN**S**QDDYDE  ERKTNL**S**AVVAAQ  AVVAAQ**S**NWEKVV  LCRRSL**S**EDTDGT  GVEFKL**S**KKQLQA  EQCRKI**S**ASLQSQ  CRKISA**S**LQSQSP  ISASLQ**S**QSPEHL  MAQLKI**S**QGNISK  ISQGNI**S**KACLIL  HKPGMV**S**ALVTMY  ALVTMY**S**HEEDID  HEEDID**S**AIEVFT  KSPAHL**S**LIREAA  RKKEAI**S**DLQQLW  TLAQLI**S**AYSLVD  LSKHLP**S**SDSMSL  HLPSSD**S**MSLKVD  PSSDSM**S**LKVDVE  VEALEN**S**AGATYI  GKVTGD**S**QPKEQG  LPMRER**S**YYRGRK  GATAGA**S**SELDAS  ATAGAS**S**ELDASK  DASKTV**S**SPPTSP  ASKTVS**S**PPTSPR  VSSPPT**S**PRPGSA  TSPRPG**S**AATVSA  GSAATV**S**ASTSNI  AATVSA**S**TSNIIP  VRALPE**S**LGQHAL  GQHALR**S**AKGEEV  YVGVLG**S**KLGLQV  ELPADE**S**LPDCVF  TRPGAP**S**RRKEVD  EFFVGL**S**KRTNQR  FKDYAV**S**TVPVAD  DGLHLK**S**FCSMAG  HLKSFC**S**MAGPNL  NLIAIG**S**SESAQK  LIAIGS**S**ESAQKA  AIGSSE**S**AQKALK  KIMQQM**S**DHRYDK  PEEYPE**S**AKVYEK  HMLIPV**S**MSELEK  LIPVSM**S**ELEKVD  GLLTCC**S**VLINKK  ETPPDP**S**PPSAPA  ALRERL**S**TLPDKL  TLPDKL**S**YNIMVP  NWFAKC**S**AKQAVG  VMKNFE**S**RVEFTE  EDLQKM**S**DAAGDI  IAHKPH**S**KPKTSD  IANDVK**S**KDLLAD  LLGELD**S**KPDTVI  NGEDTT**S**SEEEKE  HKDVAS**S**EPFSGQ  ASSEPF**S**GQVNSQ  CSVNGS**S**SYHSDD  SVNGSS**S**YHSDDD  GSSSYH**S**DDDDDD  LGVGDN**S**IPTIYF  IPTIYF**S**HTVEPK  NTTLKF**S**EKKEEA  RKNSTG**S**GHSAQE  STGSGH**S**AQELPT  EYVPRK**S**ILKSRS  SILKSR**S**RENSVC  SRSREN**S**VCSDTS  SDTSES**S**AAEFDD  RRGVLR**S**ISCEEA  ATCSDT**S**ESILEE  CSDTSE**S**ILEEEP  KKLLPL**S**VTPEAF  VTPEAF**S**GTVIEK  IEKEFV**S**PSLTPP  KEFVSP**S**LTPPPA  KSPGDP**S**KDRAAK  AASPEL**S**ALEEAF  MHGKNW**S**KLCKDC  DVDIVF**S**KIKGKS  SKIKGK**S**CRTITF  KRFKDK**S**SEEAVR  RFKDKS**S**EEAVRE  GKAPII**S**GVTKAI  ISSPTV**S**RLTDTT  TTKFTG**S**HKERFD  KERFDP**S**GKGKGK  VDLVDE**S**GYVSGY  DESGYV**S**GYKHAG  IGEVVV**S**VNPYKL  DTCIVI**S**GESGAG  IVISGE**S**GAGKTE  AGKTEA**S**KYIMQY  AAITNP**S**QRAEVE  KNMLLK**S**NCVLEA  NYLLEK**S**RVIVQQ  QQPGER**S**FHSFYQ  QLLQGG**S**EQMLRS  SLHLQK**S**LSSYNY  LQKSLS**S**YNYIHV  VGAQLK**S**SINDAA  GAQLKS**S**INDAAE  ENGKVV**S**IIAELL  HTEQEA**S**YGRDAF  FLEALN**S**KLGKHA  GKHAHF**S**SRKLCA  KRLMYN**S**SNPVLK  WPEGKL**S**ITEVTK  LVDNLA**S**KEPYYV  KPNDKK**S**PQIFDD  RRYKVK**S**YIHEVA  KHVKWP**S**PPKVLR  FNRWRA**S**QLIKSI  EGNYLA**S**KPDTPQ  YMNVLF**S**CHVRKV  RKVNRF**S**KVEDRA  YNLTGL**S**VSNGKD  LTGLSV**S**NGKDQL  LIVCLF**S**KQPTHE  KQPTHE**S**RIGELV  GKKCTV**S**VETRLN  DFTKNR**S**GFILSV  YPQHAQ**S**LDGTTS  SLDGTT**S**SSIPLY  DGTTSS**S**IPLYRS  SIPLYR**S**SEEEKR  IPLYRS**S**EEEKRV  LYNPPY**S**AQSHPA  DTRKFR**S**EPRSIF  QHERPA**S**LYQSSI  PASLYQ**S**SIDRSL  QSASRT**S**PGRVDL  LTKSFT**S**SSPSSP  FTSSSP**S**SPSRAK  SELDYC**S**TYRQHL  VSPKIK**S**RSCDDL  PKVKSE**S**MGSLLC  EEDSKE**S**CPMAWG  YVPEVR**S**NGRSRI  RKDLMN**S**EVICSV  RILQYE**S**EQQHKD  RAWSQC**S**TEEVPR  MVPTRI**S**EFEKLI  CPKRRF**S**IEYLLE  RAHVEF**S**DSDQDG  IHLEGS**S**FCSESD  SDFDHF**S**FTSSES  EMDPGL**S**KLAFLV  KLAFLV**S**PVPFRR  FRRKKN**S**APKKQT  AEKKRG**S**LPDNSI  RMPRSA**S**FQDVDT  ALQDRE**S**PRSYSS  SPRSYS**S**TLTDMG  RIHSLS**S**NKPQRP  QFDQLI**S**SMSSVA  FDQLIS**S**MSSVAE  YEYRPR**S**STKSKG  SPHVVQ**S**VISLIM  QTTFEL**S**SRKKHS  RPMCMT**S**VQMSNK  LERSPY**S**NVFHVV  AGSVRC**S**PPETLA  LASTPD**S**GYSIDS  IGIPSP**S**SLFKHI  ADAGVI**S**HSASGG  CALKAM**S**AVLCCG  VSYYQL**S**EELARA  KENIEE**S**YVHLDI  NIRTVA**S**VLLRNK  LSSSST**S**SSISLG  SYLGYN**S**NARSNS  PLDKSE**S**REKIEN  VDPSQL**S**GFPLNI  HMMSLY**S**THTYSR  LSSMQQ**S**LLQIIY  VIAVTR**S**TSSTSS  TSSGSN**S**NALVPV  NALVPV**S**WKRPQL  VAVEDN**S**SEQQFG  QEDTDE**S**SEEEAA  IQEQQE**S**LVCQGI  FTFHVF**S**RLFQTI  VSFLGD**S**LQRIGT  IGTKFK**S**SLEVMM  KEAESA**S**ENEEID  QVKAFR**S**LWPSDI  SFAVIG**S**NLDMSE  DLESPK**S**LYSRES  SSAPSS**S**SMGGAC  SMGGAC**S**SFTTSS  STSVTD**S**KAMQVE  EGCGLE**S**EQSCSM  GLESEQ**S**CSMWME  DSPSNF**S**NMSTSS  DIFDAD**S**AKAPHY  TEGSRG**S**VKDRTQ  VFVGND**S**GRVKPH  PEILKK**S**LHSCSV  LKKSLH**S**CSVKGE  GNGSFS**S**PSSSHL  GTFPAV**S**ASSQLP  TLQQQI**S**SNIFPS  IFPSPN**S**VSQLQN  AGSFTG**S**TASGSS  ASGSSG**S**VDLVQQ  EAQQQL**S**SVLFSA  LSSVLF**S**APDGNE  VSPGMF**S**STEPTV  MQCELF**S**SPPAVS  PQVNLF**S**STKSMM  IAVLQG**S**SVPQDQ  STNIFL**S**QSPMNN  FFAAPN**S**ISPLQS  QEQQNQ**S**IFHQQS  MQFQSQ**S**TVSSLQ  QSQSTV**S**SLQNPG  GIQGAT**S**SPQPQA  QNSPGS**S**QQTSGM  DLLDPK**S**AAQNSK  KSAAQN**S**KPRLSF  NSKPRL**S**FSTKPT  KPRLSF**S**TKPTVL  KPTVLA**S**RVESDT  LASRVE**S**DTTINV  MKWKTV**S**TIFLVV  EQPHEI**S**QRTTIV  QKQTFI**S**QHSCVN  TFISQH**S**CVNSTE  QHSCVN**S**TELDEL  IPLGNT**S**NQISHW  NTSNQI**S**HWDLGS  SHWDLG**S**SFFFAG  HWDLGS**S**FFFAGT  IGFGNI**S**PRTEGG  FIKWNV**S**QTKIRI  TKIRII**S**TIIFIL  KHIEGW**S**ALDAIY  DYVAGG**S**DIEYLD  YFAAVL**S**MIGDWL  DWLRVI**S**KKTKEE  KFQRAT**S**IKRKLS  SIKRKL**S**AELAGN  PCRRTL**S**VNHLTS  SVNHLT**S**ERDVLP  PLLKTE**S**IYLNGL  DRLREV**S**EKLNKY  NKYNLN**S**HPPLNV  DGSKLN**S**NSGAGR  PGEDLM**S**LLDADI  ELLAVS**S**KGELYQ  KNKKPK**S**SAGISS  NKKPKS**S**AGISSM  KSSAGI**S**SMPNIT  AGAVAF**S**ISAGIP  SIASSA**S**MPYKRR  MLQTFI**S**HRCDGN  GPSTEP**S**VVESKD  DWNALK**S**MIMFGS  IDAMQG**S**EELFSV  SSSSSQ**S**QSSYII  SSQSQS**S**YIIRNP  DSDSSS**S**QSDDIE  RRSGTI**S**TSAAAA  SYLTSA**S**SLARAY  MVSIMD**S**TEAQLR  RRATLL**S**ARQGMM  FLNYAL**S**LMRSHN  ALSLMR**S**HNDEHS  FMGMVI**S**HDMLLG  GFEVKE**S**KFRREM  NKGTHT**S**LMQRLR  EREMRR**S**SGLRAG  DFRRQL**S**IDTRPF  MQPAFA**S**KITGML  DADAVF**S**AMDLAF  LYVPLY**S**SKQILK  CAVGQV**S**PYSAPW  GQVSPY**S**APWKPT  PVVLPQ**S**TLNLAK  EAKLEV**S**SSCGPQ  AKLEVS**S**SCGPQC  KLEVSS**S**CGPQCH  EAKQYL**S**YETLYA  TLYANG**S**RTETQV  VGIYIL**S**SSGDGA  GIYILS**S**SGDGAQ  GAQHRD**S**GSSGKS  QHRDSG**S**SGKSRR  HRDSGS**S**GKSRRK  SGSSGK**S**RRKRQI  QIYGYD**S**RFSIFG  GYDSRF**S**IFGKDF  LLNYPF**S**TSVKLS  NYPFST**S**VKLSTG  STSVKL**S**TGCTGT  GRGAND**S**TSAMPE  GANDST**S**AMPEQM  FMKIGV**S**PPAKQL  GGRIHF**S**GYDNDR  DAQPGA**S**GSGVYV  QPGASG**S**GVYVRM  KIIGIF**S**GHQWVD  WVDMNG**S**PQDFNV  TAADLG**S**VVRKNL  LQAPKD**S**FALKSN  DSFALK**S**NERGIG  THRFTF**S**QIFGPE  PEVGQA**S**FFNLTV  TYGVTN**S**GKTHTI  EVIWLD**S**KQIRQE  EEELST**S**LKRSVY  RSVYIE**S**RIGTST  ESRIGT**S**TSFDSG  AGLSSI**S**QCTSSS  SISQCT**S**SSQLDE  ISQCTS**S**SQLDET  SQLDET**S**HRWAQP  RFSIWI**S**FFEIYN  DLLEPP**S**QQRKRQ  PKISEL**S**LCDLAG  LCDLAG**S**ERCKDQ  AGNINT**S**LHTLGR  LVPFRD**S**KLTRVF  NVNPCA**S**TYDETL  LHVAKF**S**AIASQL  KFSAIA**S**QLVHAP  MQLGFP**S**LHSFIK  SLQVSP**S**LEKGAK  LKESLT**S**FYQEEI  AHQQSG**S**ELALRR  RLAASA**S**TQQLQE  CKAELN**S**TTEELH  LQKLGE**S**LQSAER  YARILR**S**RRSPLL  RKRNAG**S**SSDGTE  SDGTED**S**DFSTDL  TEDSDF**S**TDLEHT  LEHTDS**S**ESDGTS  HTDSSE**S**DGTSRR  SESDGT**S**RRSARV  SARVTR**S**SARLSQ  ARVTRS**S**ARLSQS  PLRQTR**S**SGSETE  EQVVDF**S**DRETKN  PTGNAP**S**SESDID  TGNAPS**S**ESDIDI  ESDIDI**S**SPNVSH  DSGSDL**S**HRPKRR  PTPGCN**S**LGHLTG  KHERHF**S**ISGCPL  ERHFSI**S**GCPLYH  PLYHNL**S**ADECKV  LRKKRN**S**GLSKEQ  KRNSGL**S**KEQKEK  LLENLT**S**EYDLDL  CLKYMK**S**QTILRR  YRKGSI**S**VFEVDG  FSKEKN**S**FLNYNV  FLNYNV**S**CILTMP  KMLIDF**S**YLLSKV  SDLGLI**S**YRSYWK  ISIKEI**S**QETAVN  AKEAKR**S**NSNKTM  EAKRSN**S**NKTMDP  NKTMDP**S**CLKWTP  EAPRKP**S**HGCRAA  DGNLCR**S**NQLLQV  HRIVIS**S**AELLQK  DALAKN**S**PGLCLK  MFKMDA**S**LTDTME  INARDW**S**RKLTQR  RIKSNT**S**KKRKVS  SKKRKV**S**LLFDHL  LEPEEL**S**EHLTYL  KSFRRI**S**FSDYQN  FRRISF**S**DYQNYL  ALCNGI**S**QWVQLM  VQLMVL**S**RPTPQL  EMTELL**S**SSRNYD  MTELLS**S**SRNYDN  TELLSS**S**RNYDNY  ALYNHI**S**ELVQLQ  APPLTP**S**KPPVVV  PDPKTI**S**KHVQRM  FEKIAA**S**FPFSFC  AASFPF**S**FCVMDK  AYFMRA**S**SIYSKL  RASSIY**S**KLGLGF  PTENNT**S**VGPVSN  PVSNLC**S**LGAKDL  IMLMGV**S**SQKISL  MLMGVS**S**QKISLR  IGSEGP**S**GPFVLS  TLYVLP**S**PTSPCP  VLPSPT**S**PCPSPV  KDSLIK**S**KEELRH  AQKKIE**S**LQLEKS  SLQLEK**S**NHVLAQ  EPGGEL**S**WEGNGP  HQAMHY**S**RILPNI  PNIWLG**S**CPRQVE  WDIVQN**S**SGCNRY  DIVQNS**S**GCNRYP  MPTPDM**S**TEGRVQ  NAGVGR**S**TAAVCG  VIAAPD**S**RRWLWS  SRRWLW**S**VLAAAL  LLTAGV**S**ALEVYT  LTCKFK**S**TSTTGG  CKFKST**S**TTGGLT  TTGGLT**S**VSWSFQ  GGLTSV**S**WSFQPE  LTSVSW**S**FQPEGA  GADTTV**S**FFHYSQ  VSFFHY**S**QGQVYL  PFKDRI**S**WAGDLD  LDKKDA**S**INIENM  GLTLLI**S**MILAVL  LYRRKN**S**KRDYTG  RDYTGC**S**TSESLS  YTGCST**S**ESLSPV  GCSTSE**S**LSPVKQ  STSESL**S**PVKQAP  KQAPRK**S**PSDTEG  APRKSP**S**DTEGLV  TEGLVK**S**LPSGSH  LVKSLP**S**GSHQGP  KSLPSG**S**HQGPVI  YAQLDH**S**GGHHSD  HSGGHH**S**DKINKS  SDKINK**S**ESVVYA  KINKSE**S**VVYADI  LNEAHK**S**LNFVST  KSLNFV**S**TLLQIT  LLQITM**S**EQLDLP  TAPGDI**S**PYTIPE  VEAIIH**S**PELIRV  IKHDYP**S**RWTAIV  IGFYLQ**S**DNSACW  YLQSDN**S**ACWLGI  KKPEER**S**PLVAAM  RFIQLL**S**DQSDQS  QLLSDQ**S**DQSVLI  GSPGNV**S**KEYNEF  YINQGV**S**HALTWK  VFEDFI**S**PTTAAQ  LLFTAC**S**KRKEVL  ALHMIG**S**LAEILL  HVFPLF**S**SELGYM  VFPLFS**S**ELGYMR  CEVKFK**S**DQNLQT  KMICEY**S**EEVTPI  GPDEEG**S**DDKAVT  TIDTLL**S**VVEDHK  FYEEIF**S**LAHSLT  IFSLAH**S**LTCQQV  LTCQQV**S**PQMWQL  DTDTLL**S**DTKYLE  YLEMIY**S**MCKKVL  TREVKT**S**ELRTMC  QVLNQV**S**GQILPA  TALEGY**S**TIIDDE  HGGYKF**S**APVVPS  SAPVVP**S**SFNFGG  APVVPS**S**FNFGGP  GEFSGI**S**DGPSVS  GISDGP**S**VSALTN  QKLKKH**S**MDFLLF  SKYITK**S**FNFYVF  KPFNRS**S**PDVKFV  VKFVCQ**S**SSIDFL  SIDFLA**S**QGFDFN  NGAGAL**S**YVSPNT  YVSPNT**S**KCPVTI  IEDLLQ**S**EENKNL  LIYQTL**S**WKYPKG  IHAIAN**S**GKLVIG  PLPADL**S**EFKEMT  DTKLMA**S**TQPFKD  DIINNT**S**LAELEK  SAEGFP**S**YDTASE  PSYDTA**S**EQLHEA  MANYLG**S**FLSPPK  YLGSFL**S**PPKIHV  IHVSAR**S**KLIEPF  DLYQLF**S**AFGNIQ  FGNIQI**S**WIDDTS  RKWTED**S**WKEADS  SWKEAD**S**KRLNPQ  NSFTAP**S**TVGKRN  VGKRNL**S**PSQEEA  KRNLSP**S**QEEAGL  GLEDGV**S**GEISDT  GVSGEI**S**DTELEQ  ELEQTD**S**CAEPLS  SPAGSI**S**KNSPAT  WQLIAW**S**VVWTLL  QPESLW**S**RFKKKC  KTKDDI**S**KNMSFL  DISKNM**S**FLKVDK  YVKALP**S**QGLSSS  LPSQGL**S**SSAVLE  PSQGLS**S**SAVLEK  EKLKEY**S**SMDAFW  KLKEYS**S**MDAFWQ  WQEGRA**S**GTVYSG  ASGTVY**S**GEEKLT  YGDFAW**S**NPLHPD  IVRIAC**S**LFNGGP  FNGGPD**S**CGCVTS  SCGCVT**S**GGTESI  TSGGTE**S**ILMACK  EIVAPQ**S**AHAAFN  AMRRAI**S**RNTAML  TAMLVC**S**TPQFPH  RVKGVT**S**ISADTH  KGVTSI**S**ADTHKY  YAPKGS**S**LVLYSD  SSLVLY**S**DKKYRN  QGGIYA**S**PTIAGS  SRPGGI**S**AACWAA  TARFLK**S**ELENIK  SVIALG**S**RDFDIY  FDIYRL**S**NLMTAK  LKDIRE**S**VTQIMK  NMVAEL**S**SVFLDS  MVAELS**S**VFLDSL  FLDSLY**S**TDTVTQ  DTVTQG**S**QMNGSP  IVARLF**S**DARRLL  LGDQEV**S**ELCGLP  TKTLLH**S**LGTLAQ  QIYQAV**S**RIVCGH  GMWEEL**S**PKIWTF  VWLINK**S**MELLDE  IFASLL**S**PVAFGF  YWFGEE**S**DEKSHP  DIRSEM**S**TIRQNL  PSSKLK**S**KTSQLS  KLCCVG**S**SLFLKN  KKDVES**S**LSSCRN  VESSLS**S**CRNSSS  SCRNSS**S**TVSYLK  NSSSTV**S**YLKKED  ISSYGI**S**ETTLEE  GVDAET**S**DGTLPA  RETDLL**S**GMDGKG  LLIARR**S**RKGFFA  LTGRNI**S**DYLVKT  VQIIAK**S**LKNKIW  GFSLGV**S**NTQALP  KHLQFI**S**GVKPVI  QQKSYV**S**STNLPV  FIGING**S**VATFVL  INDILK**S**VFLIFP  RFVSPL**S**WDLVGR  VNGAGK**S**STFKML  AFLNKN**S**ILSNIH  QLPSSL**S**SLARIF  IEDYSV**S**QTTLDQ  LCVEFA**S**VASCDA  EFASVA**S**CDAAVA  MERALN**S**YFEPPV  EPPVEE**S**ALERRP  RRPETI**S**EPKTYV  NEETTD**S**TTSKIS  TTDSTT**S**KISPSE  STTSKI**S**PSEDTQ  TSKISP**S**EDTQQE  TQQENG**S**MFSLIT  ENGSMF**S**LITWNI  LDLNNL**S**ERARGV  RARGVC**S**YLALYS  SYLALY**S**PDVIFL  VIPPYY**S**YLKKRS  SYLKKR**S**SNYEII  YLKKRS**S**NYEIIT  AIMLKK**S**RVKLKS  SRVKLK**S**QEIIPF  EIIPFP**S**TKMMRN  CVHVNV**S**GNELCL  ELCLMT**S**HLESTR  MTSHLE**S**TRGHAA  MQEAPE**S**ATVIFA  WDTQMN**S**NLGITA  GHIIPR**S**LDLLGL  DCGRFP**S**DHWGLL  SDHGDV**S**LPPEDR  DRVRAL**S**QLGSAV  ALSQLG**S**AVEVNE  PRRYFR**S**GVEIIR  RMASIY**S**EEGNIE  KHRDYK**S**AVIPEK  PDLEKP**S**LDVFPT  FPTLTV**S**SIQPSD  PTLTVS**S**IQPSDC  VSSIQP**S**DCHTTV  PPVVDR**S**LKPGAL  QFLQLA**S**ANTARG  VLIPKQ**S**AGSDYC  PKQSAG**S**DYCNTE  TQTAFL**S**SVDLHT  QTAFLS**S**VDLHTH  DLHTHC**S**YQMMLP  QMMLPE**S**VAIVCS  SVAIVC**S**PKFQET  HGLEEI**S**SCRQKG  GLEEIS**S**CRQKGF  KGFHPH**S**KDPPLF  DPPLFC**S**CSHVTV  PLFCSC**S**HVTVVD  WAVGFV**S**SMGSGN  AVGFVS**S**MGSGNP  FVSSMG**S**GNPAPG  GQEATC**S**LVLQTD  RAECCA**S**GNIDTA  NIDTAW**S**NLTHPG  CLPCKD**S**CDGVEC  ECAPDC**S**GLPARL  RLQVCG**S**DGATYR  RGHPDL**S**VMYRGR  RGRCRK**S**CEHVVC  VCPRPQ**S**CVVDQT  VVDQTG**S**AHCVVC  APCPVP**S**SPGQEL  PCPVPS**S**PGQELC  NNVTYI**S**SCHMRQ  NVTYIS**S**CHMRQA  TCFLGR**S**IGVRHA  GVRHAG**S**CAGTPE  AGGTMK**S**AEEEHY  EHYGYA**S**SNVSPA  PLPTAH**S**TLPAPC  LPSSKR**S**PSTATL  SCNSEA**S**SYESNY  EASSYE**S**NYSYPY  YSYPYA**S**PQTSPW  ACTLLG**S**PRHSPS  SWLGAR**S**SRPASP  WLGARS**S**RPASPC  TTQYTS**S**AIVAAI  DGVPVK**S**RKTTLE  KPLSPT**S**YMSPTL  LDWQLP**S**HSGPYE  IEVQPK**S**HHRAHY  ITGKTV**S**TTSHEA  KTVSTT**S**HEAILS  SHEAIL**S**NTKVLE  VHVPQP**S**GRTLSL  PSGRTL**S**LQVASN  LSLQVA**S**NPIECS  HNFLQD**S**KVIFVE  MPPDPS**S**CLVAGF  VSAPPS**S**SCPPGL  SAPPSS**S**CPPGLE  PPGLEH**S**LCPSSP  EHSLCP**S**SPSPPL  HSLCPS**S**PSPPLP  LCPSSP**S**PPLPPA  TVRRDE**S**PTAGPR  EVHEDG**S**PNLAPI  DDSVEP**S**GTKKED  PVDKKS**S**RPAGKG  EDADLC**S**ELLQES  SELLQE**S**LDALRA  PTSHKG**S**LQEVIG  ENGQVW**S**WGDGDY  ERARAL**S**ALLPCA  VARRIS**S**CLDFEQ  GQTSDI**S**SPELMG  PDLGSL**S**SPLIDT  LQSSIF**S**GGLQTS  SGGLQT**S**QIHYSY  TSQIHY**S**YNEEKD  PGGTPA**S**KSRLCS  KNFLLP**S**VQYAMF  INSQLR**S**ITHSFV  KITVQF**S**DMRTCR  GWLLDH**S**DIQVTE  MFPING**S**RFKCRN  AVFCGR**S**GKQLKR  PGMLLD**S**WSRMVK  SEPCWQ**S**SGSQGK  DSSYMP**S**LVVVSG  IQVACG**S**RDAQTL  FRLGHG**S**DVHVRK  ADSSAA**S**NKISGA  VLSAVL**S**GMGTAY  EFDRQC**S**TERRHD  VDIACG**S**GDAQTL  VKVECG**S**QFSVAL  SVALTK**S**GAVYTW  IAIATG**S**LHCVCC  RAPVHS**S**MFRFLG  TVAVIT**S**DGRMIV  NLILDE**S**HERVFS  SHERVF**S**SSQGVE  HERVFS**S**SQGVEQ  ERVFSS**S**QGVEQV  IDEETD**S**ALDLGN  EGLAQP**S**EWGLPT  TVNSEA**S**VERLQP  KEPFGG**S**SGAGSP  SPSPEA**S**ALLYQP  GAAVGL**S**ACVFCG  YEEDPA**S**QPGTSQ  PGCQQL**S**IRDFPL  LASTPY**S**LQLGRN  LNRVGV**S**YNVRHF  NVRHFI**S**AEEVKA  DTRAGL**S**SHARAH  QELLAT**S**AAEQPP  PGGPPG**S**FLTSRR  AYGDAQ**S**LTTCEV  TRKGLS**S**HARSHL  LSSHAR**S**HLRQLG  PRPGLL**S**LAKPLD  VNKAIK**S**PPGFSA  KSPPGF**S**AKGLGH  LGHPPS**S**PLLKKT  DKSPQL**S**LSPRPA  SPQLSL**S**PRPASP  ENRKGL**S**SHARSH  PNPPGP**S**PKALAK  GHSPTA**S**PPPTAR  HGELHP**S**EGPWGA  NRKGLS**S**HARSHL  MGVTEW**S**VNGSPI  EKTSHS**S**TEACCE  NRKALA**S**HARAHL  ERPLAA**S**PPGTVK  RVRPVP**S**LVPRPP  ILEMNF**S**KADPPP  TTEQRR**S**LQAFQD  DPTYIL**S**YMAPWF  IPTDII**S**DLSECL  EILQIC**S**TKGMMA  EDKMET**S**DIQIFY  PECQNL**S**ENSCPP  QNLSEN**S**CPPSEV  CPPSEV**S**DTNLYS  CGKTFV**S**LLICEH  VYEQQK**S**VFSKYF  YRVTGI**S**GATAEN  KKGTIP**S**LSIFTL  GTIPSL**S**IFTLMI  DECHNT**S**KQHPYN  VIGLTA**S**VGVGDA  ICKLCA**S**LDASVI  FFRKVE**S**RISDKF  KDLENL**S**QIQNRE  PDKDEE**S**RICKAL  NDALII**S**EHARMK  KLQELE**S**VSRDPS  QELESV**S**RDPSNE  EGNPKL**S**FLKPGI  LDAFKA**S**GDHNIL  NILIAT**S**VADEGI  EKMMND**S**ILRLQT  EKFIRD**S**QEKPKP  FKECFV**S**RPHPKP  CARQNC**S**HDWGIH  PVIKIE**S**FVVEDI  GVQTLY**S**KWKDFH  KFQHPG**S**DMRQEK  MRQEKP**S**SPSPMP  EKPSSP**S**PMPSST  PSPMPS**S**TPSPSL  MPSSTP**S**PSLNLG  QISLEG**S**VKGIQN  LSKYQA**S**TSNTVS  KYQAST**S**NTVSKL  STSNTV**S**KLLEKS  ENEIPA**S**VFVKQP  FVKQPV**S**GAVEGK  LPDENK**S**LEETLH  HTVDLS**S**DDDLPH  EEKVEE**S**RAEKIK  EKIKRS**S**LKKVDS  SLKKVD**S**LKKAFS  LGTKIV**S**VERREK  REKIKK**S**LTSNHQ  IKKSLT**S**NHQKIS  SNHQKI**S**SGKSSP  NHQKIS**S**GKSSPF  KISSGK**S**SPFKVS  ISSGKS**S**PFKVSP  KVREGE**S**HAENET  KSEDLP**S**SEQMPN  NDQEEE**S**FAEGHS  SFAEGH**S**EASLAS  SEASLA**S**ALVEGE  AAEKAT**S**RGSNSG  KATSRG**S**NSGMDS  TSRGSN**S**GMDSNI  EDEEEE**S**VALEQA  KVRYEG**S**YALTSE  RNLSKP**S**DAAKAE  HNMSKM**S**TEDPRQ  LQEIRN**S**LLPFAN  QKTNNR**S**IEAAIE  AAIEFI**S**KMSYQD  VAYHSE**S**PNSQTD  DVGRPL**S**GSGISA  GRPLSG**S**GISAFV  PPPQVR**S**VTPPPP  YVISRI**S**PVPPGA  PPPLNT**S**PMNPPN  GQRGIS**S**VPVGRQ  MELYNI**S**VPGLQT  NWPQSS**S**APAQSS  PAQSSP**S**SGHEIP  SANSQP**S**ATTVTA  LLHQNP**S**VPPYES  SRIQSY**S**PQAFKF  MLCQKE**S**NYIRLK  RAKMDK**S**MFVKIK  VVRLYY**S**FQDKDN  MGIFPE**S**LARFYI  LTCAVE**S**VHKMGF  FRWTHD**S**KYYQSG  QDSMDF**S**NEWGDP  EWGDPS**S**CRCGDR  VINWQT**S**LHIPPQ  FKTIDF**S**SDLRQQ  KTIDFS**S**DLRQQS  THPTDT**S**NFDPVD  YINSQG**S**EQQSDE  NRKPNY**S**ELQDSN  YSELQD**S**NPEFTF  ILNPTA**S**LPMLIW  PMLIWD**S**VLAPQA  QPIAWA**S**LRLQES  RVAELT**S**LSDEDS  PSPAPS**S**FSSTSV  PAPSSF**S**STSVSS  SSFSST**S**VSSLEA  SSTSVS**S**LEAEAY  KQLAQL**S**EAKDLQ  CNKEYL**S**LGALKM  LKMHIR**S**HTLPCV  TCGKAF**S**RPWLLQ  TGEKPF**S**CPHCSR  FSCPHC**S**RAFADR  RAFADR**S**NLRAHL  AHLQTH**S**DVKKYQ  RTFSRM**S**LLHKHQ  LLPLLL**S**RAQGNP  QGNPGA**S**LDGRPG  GDRVNL**S**CGGVSH  LSCGGV**S**HPIRWV  RWVWAP**S**FPACKG  PACKGL**S**KGRRPI  RPILWA**S**SSGTPT  PILWAS**S**SGTPTV  ILWASS**S**GTPTVP  FVGRLR**S**LDSGIR  RLRSLD**S**GIRRLE  RLELLL**S**AGDSGT  LLSAGD**S**GTFFCK  GRHEDE**S**RTVLHV  PGPTHG**S**VYPQLL  DLDQEP**S**LLYADL  LDHLAL**S**RPRRLS  SRPRRL**S**TADPAD  MAEGGQ**S**SIALLA  AEGGQS**S**IALLAE  EELLEE**S**PGEQPR  PRLLGH**S**LRYMLE  LELYRR**S**ADSHGH  YRRSAD**S**HGHPRE  LTRFNL**S**CHVEPW  PTNHFP**S**SEGDSS  TNHFPS**S**EGDSSK  PSSEGD**S**SKPSLM  SSEGDS**S**KPSLMS  GDSSKP**S**LMSNAW  SKPSLM**S**NAWKEM  CQQQKD**S**GGLELW  ELWHGT**S**SLDIAF  LWHGTS**S**LDIAFL  FNDTHK**S**IRKAKF  EFMERE**S**LLRRTR  SAEVTA**S**SSKHSG  AEVTAS**S**SKHSGP  EVTASS**S**KHSGPE  ASSSKH**S**GPENNQ  PENNQC**S**LHPFQI  LHPFQI**S**FRQLGW  LRDGLN**S**PNHAII  NQLVDQ**S**VPRPSC  QSVPRP**S**CVPYKY  YKYVPI**S**VLMIEA  MIEANG**S**ILYKEY  DGLKLG**S**GPSIKA  KLGSGP**S**IKALDG  KALDGR**S**QVSTPR  DGRSQV**S**TPRFGK  NRATEK**S**VKTKGP  LKQKQP**S**FSAKKM  QKQPSF**S**AKKMTE  KTVKAK**S**SVPASD  TVKAKS**S**VPASDD  KSSVPA**S**DDAYPE  NPLDFE**S**FDLPEE  IAHLPL**S**GVPLMI  SPVKMP**S**PPWESN  PSPPWE**S**NLLQSP  ESNLLQ**S**PSSILS  NLLQSP**S**SILSTL  LLQSPS**S**ILSTLD  SPSSIL**S**TLDVEL  KKRVEI**S**APSNFE  VEISAP**S**NFEHRV  QSLIEE**S**ARRPKP  DPACIT**S**IQPGAP  KTIVRG**S**KGAKDG  DEFENM**S**VTRSNS  NMSVTR**S**NSLRRD  SVTRSN**S**LRRDSP  GPGKAG**S**RGRFAG  GRFAGH**S**EAGGGS  PEKRPK**S**SREGSG  EKRPKS**S**REGSGG  SGGPQE**S**SRDKRP  ADTDHP**S**RGAQGE  VAPNGP**S**AGGLAI  GLAIPQ**S**SSSSSR  LAIPQS**S**SSSSRP  AIPQSS**S**SSSRPP  IPQSSS**S**SSRPPT  PQSSSS**S**SRPPTR  QSSSSS**S**RPPTRA  IKIGEG**S**TGIVCI  IATVRS**S**GKLVAV  AVLQAL**S**VLHAQG  IHRDIK**S**DSILLT  RDIKSD**S**ILLTHD  DGRVKL**S**DFGFCA  GFCAQV**S**KEVPRR  PEVDIW**S**LGIMVI  LHKVSP**S**LKGFLD  KAGPPA**S**IVPLMR  AQQSHG**S**SACSQP  QQSHGS**S**ACSQPH  HGSSAC**S**QPHGSV  CSQPHG**S**VTQSQG  HGSVTQ**S**QGSSSQ  VTQSQG**S**SSQSQG  TQSQGS**S**SQSQGI  QGSSSQ**S**QGISSS  SQSQGI**S**SSSTST  QSQGIS**S**SSTSTM  QGISSS**S**TSTMPN  STMPNS**S**QSSHSS  SQSSHS**S**SGTLSS  QSSHSS**S**GTLSSL  SSSGTL**S**SLETVS  SSLETV**S**TQELYS  DKYRTY**S**KKHFRI  EVGPKN**S**YIAYIE  AYIEDH**S**GNGTFV  EIALSL**S**RNKVFV  LTVDDQ**S**VYPKAL  MSKTLG**S**GACGEV  RKFAIG**S**AREADP  ITDFGH**S**KILGET  APEVLV**S**VGTAGY  RAVDCW**S**LGVILF  EHRTQV**S**LKDQIT  KFQDLL**S**EENEST  LSEENE**S**TALPQV  QVLAQP**S**TSRKRP  LAQPST**S**RKRPRE  LVFDIG**S**YTVRAG  VERDDG**S**TLMEID  ENMEAI**S**PLKNGM  MVEDWD**S**FQAILD  YKMHVK**S**EASLHP  HVKSEA**S**LHPVLM  LHPVLM**S**EAPWNT  AFANGR**S**TGLILD  TGLILD**S**GATHTT  QQGIVK**S**PLAGDF  PPYMIA**S**KEAVRE  EAVREG**S**PANWKR  LPQVTR**S**WHNYMC  IQDFQA**S**VLQVSD  ASVLQV**S**DSTYDE  VLQVSD**S**TYDEQV  EGLFDP**S**NVKGLS  SNVKGL**S**GNTMLG  NTMLGV**S**HVVTTS  SHVVTT**S**VGMCDI  RPGLYG**S**VIVAGG  GNTLIQ**S**FTDRLN  RLNREL**S**QKTPPS  SQKTPP**S**MRLKLI  TVERRF**S**SWIGGS  VERRFS**S**WIGGSI  SSWIGG**S**ILASLG  GGSILA**S**LGTFQQ  FQQMWI**S**KQEYEE  VLYFAK**S**AEITGV  EITGVR**S**ETISVP  VRSETI**S**VPQEIK  IQKHNH**S**KSTWLI  KHNHSK**S**TWLILH  FEDVGH**S**TDAREM  TDAREM**S**KTFIIG  LITTID**S**SSSWWT  ITTIDS**S**SSWWTN  TTIDSS**S**SWWTNW  TIDSSS**S**WWTNWV  WVIPAI**S**AVAVAL  EVKKPF**S**IEDVEV  TDDHVV**S**GNLVTP  AAGIVE**S**VGEGVT  VCKNPE**S**NYCLKN  HHFLGT**S**TFSQYT  LGTSTF**S**QYTVVD  AKIDAA**S**PLEKVC  LIGCGF**S**TGYGSA  FSTGYG**S**AVNVAK  AKVTPG**S**TCAVFG  LGGVGL**S**AVMGCK  DGGVDF**S**FEVIGR  LDTMMA**S**LLCCHE  HEACGT**S**VIVGVP  VGVPPA**S**QNLSIN  PASQNL**S**INPMLL  VYGGFK**S**KEGIPK  FMAKKF**S**LDALIT  GFDLLH**S**GKSIRT  GEALLL**S**RAGPAA  GPAALG**S**ASADSA  AALGSA**S**ADSAAL  GSASAD**S**AALLGW  AARRHY**S**EAVADR  DLRTRE**S**EEQKRN  PCNHVL**S**LSFPIR  NHVLSL**S**FPIRRD  IRRDDG**S**WEVIEG  GYRAQH**S**QHRTPC  RYSTDV**S**VDEVKA  EVKALA**S**LMTYKC  VPAPDM**S**TGEREM  IADTYA**S**TIGHYD  VTGKPI**S**QGGIHG  GIHGRI**S**ATGRGV  NEASYM**S**ILGMTP  GNVGLH**S**MRYLHR  CIAVGE**S**DGSIWN  VGESDG**S**IWNPDG  FKLQHG**S**ILGFPK  AKPYEG**S**ILEADC  ILIPAA**S**EKQLTK  EKQLTK**S**NAPRVK  AGGVTV**S**YFEWLK  KNLNHV**S**YGRLTF  FKYERD**S**NYHLLM  NYHLLM**S**VQESLE  LMSVQE**S**LERKFG  EFQDRI**S**GASEKD  DRISGA**S**EKDIVH  EKDIVH**S**GLAYTM  AYTMER**S**ARQIMR  DFGQET**S**YIEDNC  NQNGAI**S**LIFSLK  AISLIF**S**LKEEVG  NLTHIE**S**RPSRLK  HIESRP**S**RLKKDE  THLDKR**S**LPALTN  ATVHEL**S**RDKKKD  FANQIL**S**YGAELD  VFKTLK**S**LYKTHA  PQLEDV**S**QFLQTC  PVAGLL**S**SRDFLG  VAGLLS**S**RDFLGG  QYIRHG**S**KPMYTP  GHVPLF**S**DRSFAQ  PLFSDR**S**FAQFSQ  RSFAQF**S**QEIGLA  QEIGLA**S**LGAPDE  LCKQGD**S**IKAYGA  YGAGLL**S**SFGELQ  GAGLLS**S**FGELQY  ELQYCL**S**EKPKLL  LYYVAE**S**FNDAKE  TIPRPF**S**VRYDPY  LKILAD**S**INSEIG  LADSIN**S**EIGILC  NFEQKE**S**NGPVKV  PVKVWG**S**IKGLTE  NTAGCT**S**AGPHFN  PHFNPL**S**RKHGGP  DGVADV**S**IEDSVI  DVSIED**S**VISLSG  IEDSVI**S**LSGDHC  DSVISL**S**GDHCII  KGGNEE**S**TKTGNA  KTGNAG**S**RLACGV  IFLFLC**S**TPAWAK  TTWDYA**S**DHGEKK  SVDTEH**S**NIYLQN  HLKNLA**S**RPYTFH  RPYTFH**S**HGITYY  VTRIYH**S**HIDAPK  APKDIA**S**GLIGPL  VVDENF**S**WYLEDN  ESNRMY**S**VNGYTF  NGYTFG**S**LPGLSM  PGEWML**S**CQNLNH  VQECNK**S**SSKDNI  QECNKS**S**SKDNIR  ECNKSS**S**KDNIRG  TAPGSD**S**AVFFEQ  REYTDA**S**FTNRKE  SVPPSA**S**HVAPTE  LAKMYY**S**AVDPTK  VFDENE**S**LLLEDN  DEDFQE**S**NKMHSM  ESNKMH**S**MNGFMY  TMCKGD**S**VVWYLF  NLFPQT**S**LTLHMW  RRQSED**S**TFYLGE  GEFYIG**S**KYKKVV  YRQYTD**S**TFRVPV  WKIPER**S**GAGTED  QVKDLY**S**GLIGPL  DNIKTY**S**DHPEKV  DEEFIE**S**NKMHAI  KHRGVY**S**SDVFDI  LLRFCF**S**ATRRYY  EGKSWH**S**ETKNSL  QASLEI**S**PITFLT  DDDLTD**S**EMDVVR  DDRSYK**S**QYLNNG  EDGPTK**S**DPRCLT  CLTRYY**S**SFVNME  MERDLA**S**GLIGPL  VFDENR**S**WYLTEN  ILGCHN**S**DFRNRG  ALLKVS**S**CDKNTG  KIQNVS**S**SDLLML  SPGAID**S**NNSLSE  AIDSNN**S**LSEMTH  TSNNLI**S**TIPSDN  MPVHYD**S**QLDTTL  DSKLLE**S**GLMNSQ  LIENSP**S**VWQNIL  RQNVEG**S**YDGAYA  KHTAHF**S**KKGEEE  ACTTRI**S**PNTSQQ  VLFQDN**S**SHLPAA  SYRKKD**S**GVQESS  GTSATN**S**VTYKKV  RVATES**S**AKTPSK  DYGMSS**S**PHVLRN  LRNRAQ**S**GSVPQF  TLFLVY**S**NKCQTP  GARQKF**S**SLYISQ  ARQKFS**S**LYISQF  QFIIMY**S**LDGKKW  MPLGME**S**KAISDA  FATWSP**S**KARLHL  KYMLWL**S**ADLKFR  PLLQGK**S**LGMIFE  MIFEKR**S**TRTRLS  STRTRL**S**TETGFA  HLGVNE**S**LTDTAR  DTARVL**S**SMADAV  TARVLS**S**MADAVL  ARVYKQ**S**DLDTLA  TLAKEA**S**IPIING  PIINGL**S**DLYHPI  TLQEHY**S**SLKGLT  LQEHYS**S**LKGLTL  LKGLTL**S**WIGDGN  GNNILH**S**IMMSAA  LHSIMM**S**AAKFGM  GYEPDA**S**VTKLAE  ITDTWI**S**MGQEEE  TAKVAA**S**DWTFLH  DDEVFY**S**PRSLVF  VFYSPR**S**LVFPEA  IMAVMV**S**LLTDYS  SLLTDY**S**PQLQKP  TAEWLL**S**HTKHRP  VAIICG**S**GLGGLT  IPNFPR**S**TVPGHA  INLPGF**S**GQNPLR  DRFPAM**S**DAYDRT  MRQRAL**S**TWKQMG  VMVAGP**S**FETVAE  ADAVGM**S**TVPEVI  LRVFGF**S**LITNKV  VIMDYE**S**LEKANH  KLEQFV**S**ILMASI  VSILMA**S**IPLPDK  SPGVVI**S**DDEPGY  KALNRN**S**DRSIPM  NRNSDR**S**IPMTVD  DFIRLK**S**YCNDQS  SYCNDQ**S**TGDIKV  IGGDDL**S**TLTGKN  TMQTLL**S**LVRQYN  KMVKVA**S**LLVKRT  VKRTPR**S**VGYKPD  NHVCVI**S**ETGKAK  IKAAVP**S**IKFCLD  LDNGAK**S**VVLMSH  KSVVLM**S**HLGRPD  PMPDKY**S**LEPVAV  VAVELK**S**LLGKDV  ANPAAG**S**VILLEN  GKGKDA**S**GNKVKA  IEAFRA**S**LSKLGD  AFRASL**S**KLGDVY  TAHRAH**S**SMVGVN  AHRAHS**S**MVGVNL  FAKALE**S**PERPFL  NMEIGT**S**LFDEEG  IVKDLM**S**KAEKNG  GQATVA**S**GIPAGW  LDCGPE**S**SKKYAE  DCGPES**S**KKYAEA  EVVKAT**S**RGCITI  NTEDKV**S**HVSTGG  DKVSHV**S**TGGGAS  STGGGA**S**LELLEG  VVGGPG**S**GKGTQC  YGYTHL**S**TGDLLR  TGDLLR**S**EVSSGS  LLRSEV**S**SGSARG  LRSEVS**S**GSARGK  SEVSSG**S**ARGKKL  ARGKKL**S**EIMEKG  VAKVNT**S**KGFLID  LKRGET**S**GRVDDN  KVNAEG**S**VDSVFS  AEGSVD**S**VFSQVC  SVDSVF**S**QVCTHL  ALAALC**S**LVHSQH  LCSLVH**S**QHVFLA  APQQAR**S**LLQRVR  CVEETC**S**YEEAFE  AFEALE**S**STATDV  FEALES**S**TATDVF  HVNITR**S**GIECQL  ECQLWR**S**RYPHKP  HKPEIN**S**TTHPGA  FCRNPD**S**STTGPW  VAMTPR**S**EGSSVN  TPRSEG**S**SVNLSP  PRSEGS**S**VNLSPP  GSSVNL**S**PPLEQC  PCLAWA**S**AQAKAL  AQAKAL**S**KHQDFN  DGLDED**S**DRAIEG  EGRTAT**S**EYQTFF  PLFEKK**S**LEDKTE  ERELLE**S**YIDGRI  DAEIGM**S**PWQVML  VMLFRK**S**PQELLC  ELLCGA**S**LISDRW  CGASLI**S**DRWVLT  VRIGKH**S**RTRYER  RNIEKI**S**MLEKIY  KKPVAF**S**DYIHPV  DRETAA**S**LLQAGY  VGKGQP**S**VLQVVN  RPVCKD**S**TRIRIT  DACEGD**S**GGPFVM  GPFVMK**S**PFNNRW  YQMGIV**S**WGEGCD  FCRAGG**S**IPIPQK  QFDLEP**S**EGCFYD  YDYVKI**S**ADKKSL  ISADKK**S**LGRFCG  FCGQLG**S**PLGNPP  GKKEFM**S**QGNKML  DLDECA**S**RSKSGE  DECASR**S**KSGEED  CASRSK**S**GEEDPQ  VGGYFC**S**CRPGYE  LQEDTH**S**CQAECS  SCQAEC**S**SELYTE  CQAECS**S**ELYTEA  ELYTEA**S**GYISSL  ASGYIS**S**LEYPRS  SLEYPR**S**YPPDLR  DLRCNY**S**IRVERG  PDLDTS**S**NAVDLL  LFFTDE**S**GDSRGW  TDESGD**S**RGWKLR  GNQVLH**S**FTAVCQ  MQTRAG**S**RESEQG  RAGSRE**S**EQGVYT  KEHEAQ**S**NASLDV  EAQSNA**S**LDVFLG  HPIRRV**S**VHPDYR  LLELEN**S**VTLGPN  NRMDVF**S**QNMFCA  FCAGHP**S**LKQDAC  VATGIV**S**WGIGCS  SWGIGC**S**RGYGFY  NMIMAE**S**PGLITI  LLGYLL**S**AECTVF  RPKRYN**S**GKLEEF  CMEEKC**S**FEEARE  DGDQCE**S**NPCLNG  PCLNGG**S**CKDDIN  EQFCKN**S**ADNKVV  DNKVVC**S**CTEGYR  LAENQK**S**CEPAVP  FPCGRV**S**VSQTSK  CGRVSV**S**QTSKLT  VSVSQT**S**KLTRAE  LDNITQ**S**TQSFND  ITQSTQ**S**FNDFTR  DAFCGG**S**IVNEKW  EPLVLN**S**YVTPIC  IFLKFG**S**GYVSGW  FGSGYV**S**GWGRVF  VFHKGR**S**ALVLQY  RATCLR**S**TKFTIY  HEGGRD**S**CQGDSG  DSCQGD**S**GGPHVT  TEVEGT**S**FLTGII  FLTGII**S**WGEECA  GIYTKV**S**RYVNWI  LHLVLL**S**ASLAGL  LVLLSA**S**LAGLLL  LLLLGE**S**LFIRRE  RVTRAN**S**FLEEMK  CMEETC**S**YEEARE  REVFED**S**DKTNEF  GDQCET**S**PCQNQG  FTRKLC**S**LDNGDC  CHEEQN**S**VVCSCA  QNSVVC**S**CARGYT  LERRKR**S**VAQATS  SVAQAT**S**SSGEAP  VAQATS**S**SGEAPD  AQATSS**S**GEAPDS  SGEAPD**S**ITWKPY  CGGTIL**S**EFYILT  ERDWAE**S**TLMTQK  QKTGIV**S**GFGRTH  HEKGRQ**S**TRLKML  PYVDRN**S**CKLSSS  RNSCKL**S**SSFIIT  NSCKLS**S**SFIITQ  SCKLSS**S**FIITQN  DACQGD**S**GGPHVT  FVTGIV**S**WGEGCA  LKWIDR**S**MKTRGL  GLPKAK**S**HAPEVI  LLLFLK**S**GQGEPL  VNTQGA**S**LFSVTK  QGASLF**S**VTKKQL  AENRKS**S**IIIRMR  NYRGTM**S**KTKNGI  ITCQKW**S**STSPHR  TCQKWS**S**TSPHRP  QKWSST**S**PHRPRF  SPATHP**S**EGLEEN  NYDGKI**S**KTMSGL  KISKTM**S**GLECQA  QAWDSQ**S**PHAHGY  AHGYIP**S**KFPNKN  CTTPPP**S**SGPTYQ  EYCKIP**S**CDSSPV  KIPSCD**S**SPVSTE  IPSCDS**S**PVSTEQ  CDSSPV**S**TEQLAP  YHGDGQ**S**YRGTSS  KCQSWS**S**MTPHRH  CNLKKC**S**GTEASV  CSGTEA**S**VVAPPP  PDVETP**S**EEDCMF  QEPHRH**S**IFTPET  CGGTLI**S**PEWVLT  KSPRPS**S**YKVILG  VQEIEV**S**RLFLEP  LNGRVQ**S**TELCAG  LAGGTD**S**CQGDSG  ILQGVT**S**WGLGCA  GVYVRV**S**RFVTWI  LCVLVV**S**DSKGSN  VLVVSD**S**KGSNEL  VSDSKG**S**NELHQV  ELHQVP**S**NCDCLN  NGGTCV**S**NKYFSN  VSNKYF**S**NIHWCN  HCEIDK**S**KTCYEG  FYRGKA**S**TDTMGR  PCLPWN**S**ATVLQQ  TYHAHR**S**DALQLG  DGKKPS**S**PPEELK  RRHRGG**S**VTYVCG  TYVCGG**S**LISPCW  CGGSLI**S**PCWVIS  SPCWVI**S**ATHCFI  IVYLGR**S**RLNSNT  GRSRLN**S**NTQGEM  ILHKDY**S**ADTLAH  ALLKIR**S**KEGRCA  GRCAQP**S**RTIQTI  QTICLP**S**MYNDPQ  DPQFGT**S**CEITGF  TVVKLI**S**HRECQQ  QPHYYG**S**EVTTKM  PQWKTD**S**CQGDSG  DSCQGD**S**GGPLVC  GGPLVC**S**LQGRMT  TLTGIV**S**WGRGCA  GVYTRV**S**HFLPWI  FLPWIR**S**HTKEEN  HVHLDG**S**IKPETI  YVEVRY**S**PHLLAN  PHLLAN**S**KVEPIP  FGVKAR**S**ILCCMR  RHQPNW**S**PKVVEL  DETIPG**S**SLLPGH  ETIPGS**S**LLPGHV  YQEAVK**S**GIHRTV  HAGEVG**S**AEVVKE  FEICPW**S**SYLTGA  EICPWS**S**YLTGAW  NDQANY**S**LNTDDP  DPLIFK**S**TLDTDY  NINAAK**S**SFLPED  INAAKS**S**FLPEDE  NGPEQW**S**KLYPIA  ANGNNQ**S**PVDIKT  PVDIKT**S**ETKHDT  ETKHDT**S**LKPISV  TSLKPI**S**VSYNPA  LKPISV**S**YNPATA  IINVGH**S**FHVNFE  EDNDNR**S**VLKGGP  LKGGPF**S**DSYRLF  GGPFSD**S**YRLFQF  FHFHWG**S**TNEHGS  STNEHG**S**EHTVDG  VDGVKY**S**AELHVA  HVAHWN**S**AKYSSL  WNSAKY**S**SLAEAA  NSAKYS**S**LAEAAS  SLAEAA**S**KADGLA  FTNFDP**S**TLLPSS  PSTLLP**S**SLDFWT  STLLPS**S**LDFWTY  FWTYPG**S**LTHPPL  HPPLYE**S**VTWIIC  WIICKE**S**ISVSSE  ICKESI**S**VSSEQL  KESISV**S**SEQLAQ  ESISVS**S**EQLAQF  QLAQFR**S**LLSNVE  QFRSLL**S**NVEGDN  VIGTVT**S**GKRKVY  RKVYLL**S**LLLIGF  CVTCHG**S**PVDICT  PMCIYR**S**PEKKAT  RRVWEL**S**KANSRF  ELSKAN**S**RFATTF  YQHLAD**S**KNDNDN  NDNIFL**S**PLSIST  IFLSPL**S**ISTAFA  LSPLSI**S**TAFAMT  FKFDTI**S**EKTSDQ  TISEKT**S**DQIHFF  YRKANK**S**SKLVSA  RKANKS**S**KLVSAN  KSSKLV**S**ANRLFG  RLFGDK**S**LTFNET  KENAEQ**S**RAAINK  AINKWV**S**NKTEGR  ITDVIP**S**EAINEL  FKGLWK**S**KFSPEN  LWKSKF**S**PENTRK  YKADGE**S**CSASMM  ADGESC**S**ASMMYQ  GESCSA**S**MMYQEG  LPKPEK**S**LAKVEK  GLVDLF**S**PEKSKL  LFSPEK**S**KLPGIV  RDDLYV**S**DAFHKA  EVNEEG**S**EAAAST  GSEAAA**S**TAVVIA  VVIAGR**S**LNPNRV  CCLVPV**S**LAEDPQ  LAEFAF**S**LYRQLA  RQLAHQ**S**NSTNIF  LAHQSN**S**TNIFFS  STNIFF**S**PVSIAT  IFFSPV**S**IATAFA  TAFAML**S**LGTKAD  TLNQPD**S**QLQLTT  GNGLFL**S**EGLKLV  VKKLYH**S**EAFTVN  QHCKKL**S**SWVLLM  HCKKLS**S**WVLLMK  ENEDRR**S**ASLHLP  EDRRSA**S**LHLPKL  LHLPKL**S**ITGTYD  GTYDLK**S**VLGQLG  GITKVF**S**NGADLS  SNGADL**S**GVTEEA  EAPLKL**S**KAVHKA  IEQNTK**S**PLFMGK  RKRAPQ**S**EMAPAG  MAPAGV**S**LRATIL  LVIHNE**S**TCEQLA  PIQAKT**S**PVDEKA  RIYGMH**S**ELWGVV  HGATVL**S**PTAVFG  VFGTLA**S**LYLGAL  KDKNCT**S**RLDAHK  DAHKVL**S**ALQAVQ  AQGRAD**S**QAQLLL  QAQLLL**S**TVVGVF  PVVLPR**S**LDFTEL  GWKTGC**S**LMGASV  CSLMGA**S**VDSTLA  MGASVD**S**TLAFNT  GKMKGF**S**LLAEPQ  EFWVDN**S**TSVSVP  WVDNST**S**VSVPML  DNSTSV**S**VPMLSG  VSVPML**S**GMGTFQ  GTFQHW**S**DIQDNF  DIQDNF**S**VTQVPF  QVPFTE**S**ACLLLI  IQPHYA**S**DLDKVE  LTFQQN**S**LNWMKK  NWMKKL**S**PRTIHL  QLVLQG**S**YDLQDL  LNLQKL**S**NDRIRV  VGEVLN**S**IFFELE  EREPTE**S**TQQLNK  FAVYDQ**S**ATALHF  LPLALG**S**PMYSII  ANREFK**S**EKGRNK  IPVKQD**S**LSSQNQ  LGVLPL**S**WDIPEL  VILHSG**S**DMVQAE  YSTVGN**S**NNYLHL  YTLIGA**S**GQREVV  REVVAD**S**VWVDVK  WVDVKD**S**CVGSLV  DYAGVF**S**DAGLTF  GLTFTS**S**SGQQTA  LTFTSS**S**GQQTAQ  RRTRFI**S**LGEACK  AEENIV**S**RSEFPE  WEILAV**S**MSDKKG  VTIPPK**S**SLSVPY  TIPPKS**S**LSVPYV  QVPDTE**S**ETRILL  HLIVTP**S**GCGEQN  FVKRAP**S**TWLTAY  NLIAID**S**QVLCGA  YNVEAT**S**YALLAL  ELNLDV**S**LQLPSR  QLPSRS**S**KITHRI  ELDKAF**S**DRNTLI  LDKVSH**S**EDDCLA  YYNLEE**S**CTRFYH  LVKVQL**S**NDFDEY  IEQTIK**S**GSDEVQ  QQRTFI**S**PIKCRE  GEKPNL**S**YIIGKD  PFEPLA**S**GILLLL  LWLIAP**S**RACTCV  QTAFCN**S**DLVIRA  YTPAME**S**VCGYFH  CGYFHR**S**HNRSEE  HRSHNR**S**EEFLIA  LHITTC**S**FVAPWN  FVAPWN**S**LSLAQR  APWNSL**S**LAQRRG  TVFPCL**S**IPCKLQ  IPCKLQ**S**GTHCLW  SEKGFQ**S**RHLACL  AVALAV**S**PAAGSS  VSPAAG**S**SPGKPP  SPAAGS**S**PGKPPR  GEYNKA**S**NDMYHS  SNDMYH**S**RALQVV  KRKAFC**S**FQIYAV  CSRLLL**S**LTQESQ  LSLTQE**S**QSEEID  LTQESQ**S**EEIDCN  ALKKYN**S**QNQSNN  YNSQNQ**S**NNQFVL  ATKTVG**S**DTFYSF  GSDTFY**S**FKYEIK  GDCPVQ**S**GKTWQD  ATVGKR**S**STKFSV  TVGKRS**S**TKFSVA  RSSTKF**S**VATQTC  NNNTQH**S**SLFMLN  LTPDCK**S**LWNGDT  ARETTC**S**KESNEE  NEELTE**S**CETKKL  TKKLGQ**S**LDCNAE  QPLGMI**S**LMKRPP  KRPPGF**S**PFRSSR  FSPFRS**S**RIGEIK  KEETTV**S**PPHTSM  QDEERD**S**GKEQGH  TEHLAS**S**SEDSTT  EHLASS**S**EDSTTP  ASSSED**S**TTPSAQ  GPTPIP**S**LAKPGV  FSDFQD**S**DLIATM  SPAPIQ**S**DDDWIP  IDPNGL**S**FNPISD  LSFNPI**S**DFPDTT  PGRPWK**S**VSEINP  TTQMKE**S**YYFDLT  NADYEA**S**SSRCSS  DYEASS**S**RCSSAS  ASSSRC**S**SASPAG  SRCSSA**S**PAGDSL  ASPAGD**S**LSYYHS  PAGDSL**S**YYHSPA  YHSPAD**S**FSSMGS  SPADSF**S**SMGSPV  PADSFS**S**MGSPVN  PTVTAI**S**TSPDLQ  VTAIST**S**PDLQWL  QPALVS**S**VAPSQT  VSSVAP**S**QTRAPH  FGVPAP**S**AGAYSR  TGGRAQ**S**IGRRGK  GKVEQL**S**PEEEEK  SVEPVK**S**ISSMEL  PVKSIS**S**MELKTE  PASSRP**S**GSETAR  SSRPSG**S**ETARSV  GSETAR**S**VPDMDL  VPDMDL**S**GSFYAA  DWEPLH**S**GSLGMG  VVTCTP**S**CTAYTS  SCTAYT**S**SFVFTY  CTAYTS**S**FVFTYP  EADSFP**S**CAAAHR  AHRKGS**S**SNEPSS  HRKGSS**S**NEPSSD  SSSNEP**S**SDSLSS  NEPSSD**S**LSSPTL  YDLDYD**S**VQPYFY  SGLCSP**S**YVAVTP  VAVTPF**S**LRGDND  NDGGGG**S**FSTADQ  GGGGSF**S**TADQLE  GDMVNQ**S**FICDPD  IQDCMW**S**GFSAAA  CMWSGF**S**AAAKLV  AAAKLV**S**EKLASY  QAARKD**S**GSPNPA  RGHSVC**S**TSSLYL  SECIDP**S**VVFPYP  PYPLND**S**SSPKSC  YPLNDS**S**SPKSCA  PLNDSS**S**PKSCAS  DSSSPK**S**CASQDS  CASQDS**S**AFSPSS  QDSSAF**S**PSSDSL  SAFSPS**S**DSLLSS  FSPSSD**S**LLSSTE  SSDSLL**S**STESSP  SDSLLS**S**TESSPQ  QAPGKR**S**ESGSPS  PSAGGH**S**KPPHSP  HSKPPH**S**PLVLKR  LKRCHV**S**THQHNY  NYAAPP**S**TRKDYP  NNRKCT**S**PRSSDT  RNELKR**S**FFALRD  ATAYIL**S**VQAEEQ  EEQKLI**S**EEDLLR  AGGVGK**S**ALTIQL  DPTIED**S**YRKQVV  AGQEEY**S**AMRDQY  AINNTK**S**FEDIHQ  IKRVKD**S**DDVPMV  AARTVE**S**RQAQDL  AQDLAR**S**YGIPYI  IPYIET**S**AKTRQG  LNPPDE**S**GPGCMS  WCVEGC**S**SQHWSY  CVEGCS**S**QHWSYG  CSSQHW**S**YGLRPG  AENLID**S**FQEIVK  TTHQPR**S**PLRDLK  LKGALE**S**LIEEET  LGLLAF**S**SACYFQ  GLLAFS**S**ACYFQN  GGKRAM**S**DLELRQ  GRCFGP**S**ICCADE  EENYLP**S**PCQSGQ  LPSPCQ**S**GQKACG  GQKACG**S**GGRCAA  VCCNDE**S**CVTEPE  HRRARA**S**DRSNAT  ARASDR**S**NATQLD  RSCCSR**S**GALLLA  ALLLQA**S**MEVRGW  RGWCLE**S**SQCQDL  GWCLES**S**QCQDLT  QDLTTE**S**NLLECI  ACKPDL**S**AETPMF  RFGRRN**S**SSSGSS  FGRRNS**S**SSGSSG  GRRNSS**S**SGSSGA  RRNSSS**S**GSSGAG  NSSSSG**S**SGAGQK  SSSSGS**S**GAGQKR  QKREDV**S**AGEDCG  GGPEPR**S**DGAKPG  PREGKR**S**YSMEHF  EGKRSY**S**MEHFRW  NGAEDE**S**AEAFPL  QADLEH**S**LLVAAE  EHFRWG**S**PPKDKR  YGGFMT**S**EKSQTP  FMTSEK**S**QTPLVT  GSPWKG**S**LLLLLV  LLLLLV**S**NLLLCQ  NLLLCQ**S**VAPLPI  HYIHNL**S**SEMFSE  LSSEMF**S**EFDKRY  ITKAIN**S**CHTSSL  INSCHT**S**SLATPE  NSCHTS**S**LATPED  NQKDFL**S**LIVSIL  FLSLIV**S**ILRSWN  APEAIL**S**KAVEIE  EIYPVW**S**GLPSLQ  VWSGLP**S**LQMADE  QMADEE**S**RLSAYY  HCLRRD**S**HKIDNY  ATGSRT**S**LLLAFG  PWLQEG**S**AFPTIP  FPTIPL**S**RLFDNA  PKEQKY**S**FLQNPQ  LQNPQT**S**LCFSES  QTSLCF**S**ESIPTP  SLCFSE**S**IPTPSN  ESIPTP**S**NREETQ  EETQQK**S**NLELLR  LELLRI**S**LLLIQS  SLLLIQ**S**WLEPVQ  PVQFLR**S**VFANSL  RSVFAN**S**LVYGAS  VYGASD**S**NVYDLL  GRLEDG**S**PRTGQI  IFKQTY**S**KFDTNS  SPFLAL**S**ILVLLQ  VLLQAG**S**LHAAPF  HAAPFR**S**ALESSP  FRSALE**S**SPADPA  RSALES**S**PADPAT  ADPATL**S**EDEARL  YVQMKA**S**ELEQEQ  EQEREG**S**SLDSPR  QEREGS**S**LDSPRS  EGSSLD**S**PRSKRC  SLDSPR**S**KRCGNL  KRCGNL**S**TCMLGT  GKKRDM**S**SDLERD  KKRDMS**S**DLERDH  CQNDGR**S**CWCVGA  RFLAVQ**S**VISGRF  RGTFNF**S**QFFQQL  TPCQLQ**S**EQAFLR  QYPGSY**S**DFSTPL  GSYSDF**S**TPLAHF  ELEGMR**S**EPSKLP  GSLTAR**S**LQIPQC  TSGLLS**S**WKQARS  SWKQAR**S**QENPSP  PGPLIC**S**LESGRW  VNVTWK**S**RLEDIP  FQLHLD**S**KTFPAE  GDHFGT**S**PRTWFG  FYQVLT**S**EASQDG  CSFFTV**S**TTEPEI  CQVKVR**S**HGQDSP  VYLWKD**S**DMGSRP  KDSDMG**S**RPESMG  MGSRPE**S**MGCRKD  NGNQSL**S**SQKHWL  RILDCG**S**PDIEVH  PLVVLP**S**LTEKVS  VSTAAT**S**NFSAVR  NVAPNA**S**VLVFFH  GVFGFL**S**SGSGEV  SPAAVI**S**HERAQQ  VKQFEE**S**RGRTSS  ESRGRT**S**SKTAFY  YQALQN**S**LGGEDS  AATWYY**S**LEHSTD  ISSLKT**S**ADGAKG  GGQSAE**S**EEEELT  VMLVQG**S**WQRSLQ  QGSWQR**S**LQDTEE  QDTEEK**S**RSFSAS  TEEKSR**S**FSASQA  EKSRSF**S**ASQADP  SRSFSA**S**QADPLS  SQADPL**S**DPDQMN  NEDKRH**S**QGTFTS  SQGTFT**S**DYSKYL  TFTSDY**S**KYLDSR  YSKYLD**S**RRAQDF  AEGTFT**S**DVSSYL  TFTSDV**S**SYLEGQ  FTSDVS**S**YLEGQA  RRHADG**S**FSDEMN  HADGSF**S**DEMNTI  VLLTLL**S**VLFSQT  LLSVLF**S**QTSAWP  VLFSQT**S**AWPLYR  PLYRAP**S**ALRLGD  NEPDQV**S**LKEDID  TPYYDV**S**RNARHA  ADGVFT**S**DFSKLL  VFTSDF**S**KLLGQL  KLLGQL**S**AKKYLE  AKKYLE**S**LMGKRV  LMGKRV**S**SNISED  MGKRVS**S**NISEDP  RVSSNI**S**EDPVPV  VPVKRH**S**DAVFTD  VKKYLN**S**ILNGKR  ILNGKR**S**SEGESP  LNGKRS**S**EGESPD  RSSEGE**S**PDFPEE  FVILTL**S**NSSHCS  ILTLSN**S**SHCSPP  LTLSNS**S**HCSPPP  SNSSHC**S**PPPPLT  DAIFTN**S**YRKVLG  KVLGQL**S**ARKLLQ  LLQDIM**S**RQQGES  SRQQGE**S**NQERGA  LGRQVD**S**MWAEQK  KQMELE**S**ILVALL  NKRLGL**S**GLTLAL  GLTLAL**S**LLVCLG  LAEAYP**S**KPDNPG  DMARYY**S**ALRHYI  QRYGKR**S**SPETLI  RYGKRS**S**PETLIS  SPETLI**S**DLLMRE  DLLMRE**S**TENVPR  LALAAF**S**EASWKP  AAFSEA**S**WKPRSQ  ASWKPR**S**QQPDAP  EQQGPA**S**HHRRQL  HLVADP**S**KKQGPW  LCIVLG**S**LGCYCQ  YFNAGH**S**DVADNG  KNWKEE**S**DRKIMQ  DRKIMQ**S**QIVSFY  MQSQIV**S**FYFKLF  NFKDDQ**S**IQKSVE  DQSIQK**S**VETIKE  NVKFFN**S**NKKKRD  EKLTNY**S**VTDLNV  QVMAEL**S**PAAKTG  TGKRKR**S**QMLFRG  KCARIT**S**RIIRSS  TSRIIR**S**SEDPNE  SRIIRS**S**EDPNED  NNRENI**S**DPTSPL  NISDPT**S**PLRTRF  RFVYHL**S**DLCKKC  IVTATQ**S**NICDED  NICDED**S**ATETCY  VLTQPP**S**VSGTPG  TQPPSV**S**GTPGQR  GQRVTI**S**CSGGSS  RVTISC**S**GGSSNG  ISCSGG**S**SNGTGN  SCSGGS**S**NGTGNN  RDDKRP**S**GVPDRF  GVPDRF**S**GSKSGT  PDRFSG**S**KSGTSA  RFSGSK**S**GTSASL  GSKSGT**S**ASLAIS  KSGTSA**S**LAISGL  SASLAI**S**GLRSED  AISGLR**S**EDEAHY  AWDYRL**S**AVVFGG  AATRVH**S**QTQLVQ  QTQLVQ**S**GAEVRK  VRKPGA**S**VRVSCK  GASVRV**S**CKASGY  RVSCKA**S**GYTFID  GYTFID**S**YIHWIR  GWINPN**S**GGTNYA  TMTRDA**S**FSTAYM  TRDASF**S**TAYMDL  AYMDLR**S**LRSDDS  DLRSLR**S**DDSAVF  SLRSDD**S**AVFYCA  VFYCAK**S**DPFWSD  KSDPFW**S**DYYNFD  YYNFDY**S**YTLDVW  VVQPGR**S**LRLSCS  GRSLRL**S**CSSSGF  SLRLSC**S**SSGFIF  LRLSCS**S**SGFIFS  RLSCSS**S**GFIFSS  SSGFIF**S**SYAMYW  SGFIFS**S**YAMYWV  IIWDDG**S**DQHYAD  DQHYAD**S**VKGRFT  KGRFTI**S**RDNSKN  TISRDN**S**KNTLFL  LFLQMD**S**LRPEDT  GGHGFC**S**SASCFG  GHGFCS**S**ASCFGP  GFCSSA**S**CFGPDY  TLTCTF**S**GFSLST  CTFSGF**S**LSTSRM  FSGFSL**S**TSRMRV  GFSLST**S**RMRVSW  TSRMRV**S**WIRRPP  BDKFYW**S**TSLRTR  KFYWST**S**LRTRLS  SLRTRL**S**ISKNDS  RTRLSI**S**KNDSKN  SISKND**S**KNQVVL  CARVVN**S**VMAGYY  PGLVRP**S**QTLSLT  RPSQTL**S**LTCTVS  SLTCTV**S**GSTFSN  TCTVSG**S**TFSNDY  VSGSTF**S**NDYYTW  VFYHGT**S**DDTTPL  DTTPLR**S**RVTMLV  TMLVDT**S**KNQFSL  TSKNQF**S**LRLSSV  QFSLRL**S**SVTAAD  FSLRLS**S**VTAADT  ERTIIV**S**MWAKIS  SMWAKI**S**TQADTI  LERLFL**S**HPQTKT  FDLHPG**S**AQLRAH  QLRAHG**S**KVVAAV  VGDAVK**S**IDDIGG  DIGGAL**S**KLSELH  GALSKL**S**ELHAYI  VNFKLL**S**HCLLVT  AWDKFL**S**VVSSVL  KFLSVV**S**SVLTEK  FPFHSP**S**RLFDQF  GEHLLE**S**DLFPTS  SDLFPT**S**TSLSPF  LFPTST**S**LSPFYL  FYLRPP**S**FLRAPS  WFDTGL**S**EMRLEK  LEKDRF**S**VNLDVK  LDVKHF**S**PEELKV  DEHGFI**S**REFHRK  DPLTIT**S**SLSSDG  PLTITS**S**LSSDGV  TITSSL**S**SDGVLT  ITSSLS**S**DGVLTV  GPRKQV**S**GPERTI  RSGAQA**S**STPLSP  YIDRVR**S**LETENA  RLRITE**S**EEVVSR  ESEEVV**S**REVSGI  VVSREV**S**GIKAAY  RLQLEL**S**KVREEF  ALSTAL**S**EKRTLE  KQREFE**S**RLADAL  HEELQQ**S**RIRIDS  LRDLED**S**LARERD  ARERDT**S**RRLLAE  SPTSQR**S**RGRASS  GRASSH**S**SQTQGG  KKRKLE**S**TESRSS  ESTESR**S**SFSQHA  STESRS**S**FSQHAR  SQHART**S**GRVAVE  GAGATH**S**PPTDLV  TWGCGN**S**LRTALI  RTALIN**S**TGEEVA  LHHHHG**S**HCSSSG  HHGSHC**S**SSGDPA  GSHCSS**S**GDPAEY  KASASG**S**GAQVGG  VGGPIS**S**GSSASS  GPISSG**S**SASSVT  SSGSSA**S**SVTVTR  SGSSAS**S**VTVTRS  SVTVTR**S**YRSVGG  DNLVTR**S**YLLGNS  SYLLGN**S**SPRTQS  VLFLTG**S**QARHFW  QDEPPQ**S**PWDRVK  VDVLKD**S**GRDYVS  SGRDYV**S**QFEGSA  VSQFEG**S**ALGKQL  LLDNWD**S**VTSTFS  NWDSVT**S**TFSKLR  SVTSTF**S**KLREQL  GLRQEM**S**KDLEEV  ELQEKL**S**PLGEEM  THLAPY**S**DELRQR  KATEHL**S**TLSEKA  EHLSTL**S**EKAKPA  LLPVLE**S**FKVSFL  LESFKV**S**FLSALE  FKVSFL**S**ALEEYT  QQTEWQ**S**GQRWEL  RWVQTL**S**EQVQEE  VQEELL**S**SQVTQE  QEELLS**S**QVTQEL  ELKAYK**S**ELEEQL  ETRARL**S**KELQAA  LRVRLA**S**HLRKLR  GAERGL**S**AIRERL  RAATVG**S**LAGQPL  RMEEMG**S**RTRDRL  FQARLK**S**WFEPLV  QAAVGT**S**AAPVPS  LLLTIC**S**LEGALV  KEPCVE**S**LVSQYF  CVESLV**S**QYFQTV  LQAEAK**S**YFEKSK  ELVNFL**S**YFVELG  IVCLVL**S**VVGTAW  NTYNRV**S**EDLRSR  IDIKIR**S**CRGSCS  SCRGSC**S**RALARE  AKDLLP**S**RDRQHL  EITRGG**S**TSYGTG  TRGGST**S**YGTGSE  ESPRNP**S**SAGSWN  GSWNSG**S**SGPGST  SWNSGS**S**GPGSTG  ATWKPG**S**SGPGST  TWKPGS**S**GPGSTG  GSWNSG**S**SGTGST  GSSGTG**S**TGNQNP  GNQNPG**S**PRPGST  GQWHSE**S**GSFRPD  TTTTRR**S**CSKTVT  FVSETE**S**RGSESG  ETESRG**S**ESGIFT  ESRGSE**S**GIFTNT  NTKESS**S**HHPGIA  GKSSSY**S**KQFTSS  GDSTFE**S**KSYKMA  DHEGTH**S**TKRGHA  KRGHAK**S**RPVRDC  VLQTHP**S**GTQSGI  IKLPGS**S**KIFSVY  YCDQET**S**LGGWLL  YKRGFG**S**LNDEGE  IYYPGG**S**YDPRNN  YDPRNN**S**PYEIEN  KRMVSW**S**FHKLKT  CVFLVK**S**QGVNDN  NEEGFF**S**ARGHRP  KREEAP**S**LRPAPP  PAPPPI**S**GGGYRA  ERPIRN**S**VDELNN  NNVEAV**S**QTSSSS  EAVSQT**S**SSSFQY  AVSQTS**S**SSFQYM  VSQTSS**S**SFQYMY  SQTSSS**S**FQYMYL  NVVNEY**S**SELEKH  VVNEYS**S**ELEKHQ  IDETVN**S**NIPTNL  NLRVLR**S**ILENLR  ILENLR**S**KIQKLE  KIQKLE**S**DVSAQM  KLESDV**S**AQMEYC  RTPCTV**S**CNIPVV  CNIPVV**S**GKECEE  RKGGET**S**EMYLIQ  YLIQPD**S**SVKPYR  LIQPDS**S**VKPYRV  QNRQDG**S**VDFGRK  LGNDKI**S**QLTRMG  ANKYQI**S**VNKYRG  ALMDGA**S**QLMGEN  HNGMFF**S**TYDRDN  NDGWLT**S**DPRKQC  DPRKQC**S**KEDGGG  WMNWKG**S**WYSMRK  WKGSWY**S**MRKMSM  YSMRKM**S**MKIRPF  YALLFL**S**STCVAY  ALLFLS**S**TCVAYV  LDERFG**S**YCPTTC  GIADFL**S**TYQTKV  QVENKT**S**EVKQLI  TYNPDE**S**SKPNMI  YNPDES**S**KPNMID  DAATLK**S**RKMLEE  IMKYEA**S**ILTHDS  SILTHD**S**SIRYLQ  ILTHDS**S**IRYLQE  LQEIYN**S**NNQKIV  NKGAKQ**S**GLYFIK  YCEIDG**S**GNGWTV  QKRLDG**S**VDFKKN  EGFGHL**S**PTGTTE  EKIHLI**S**TQSAIP  HLISTQ**S**AIPYAL  DWNGRT**S**TADYAM  DFGDDP**S**DKFFTS  SDKFFT**S**HNGMQF  HNGMQF**S**TWDNDN  CAEQDG**S**GWWMNK  YQGGTY**S**KASTPN  GTYSKA**S**TPNGYD  WKTRWY**S**MKKTTM  LNAEKA**S**TNSETN  EKASTN**S**ETNRGE  ETNRGE**S**EKKRNL  RNLGEL**S**RTTSED  ELSRTT**S**EDNEVF  NQNNGT**S**SQDTAV  QNNGTS**S**QDTAVT  DTAVTD**S**KRTADP  HPADPG**S**RPHLIR  HLIRLF**S**RDAPGR  TFKDRP**S**ESDELQ  KDRPSE**S**DELQTI  QTIQED**S**AATSES  EDSAAT**S**ESLDVM  SAATSE**S**LDVMAS  SLDVMA**S**QKRPSQ  PSQRHG**S**KYLATA  KYLATA**S**TMDHAR  RGSGKD**S**HHPART  RTAHYG**S**LPQKSH  GSLPQK**S**HGRTQD  PRTPPP**S**QGKGRG  GKGRGL**S**LSRFSW  GRGLSL**S**RFSWGA  GYGGRA**S**DYKSAH  RASDYK**S**AHKGFK  DAQGTL**S**KIFKLG  DPDIPE**S**QMEEPA  SHLTFW**S**LLELRR  LDLQET**S**LAGVAN  TRSGDP**S**QPLLPQ  PLLPQH**S**SLETQL  GGTEGH**S**PSGILE  TEGHSP**S**GILEKI  AAATLM**S**ERVFRI  EGFLDC**S**LVLPPT  PPTDAP**S**EQALLS  SEQALL**S**LVPVQR  LRRRYQ**S**SPAKPD  RRRYQS**S**PAKPDS  IYFAAL**S**PAITFG  LLVVGF**S**GPLLVF  VVAFEG**S**FLVRFI  IFSFLI**S**LIFIYE  PNTALL**S**LVLMAG  DFGVPI**S**ILIMVL  TYTQKL**S**VPDGFK  PDGFKV**S**NSSARG  FKVSNS**S**ARGWVI  HPLGLR**S**EFPIWM  IWMMFA**S**ALPALL  ITTLIV**S**KPERKM  RKMVKG**S**GFHLDL  TVMGKA**S**TPGAAA  VKEQRI**S**GLLVAV  AVLVGL**S**ILMEPI  LYMGVT**S**LSGIQL  MGVTSL**S**GIQLFD  VLWVVK**S**TPASLA  VKSTPA**S**LALPFV  CFLVLT**S**LSHAFG  LVLTSL**S**HAFGQT  FGQTDM**S**RKAFVF  FVFPKE**S**DTSYVS  PKESDT**S**YVSLKA  SDTSYV**S**LKAPLT  HFYTEL**S**STRGYS  FYTELS**S**TRGYSI  SSTRGY**S**IFSYAT  RGYSIF**S**YATKRQ  EILIFW**S**KDIGYS  SKDIGY**S**FTVGGS  SFTVGG**S**EILFEV  PVHICT**S**WESASG  ICTSWE**S**ASGIVE  TSWESA**S**GIVEFW  KPRVRK**S**LKKGYT  TVGAEA**S**IILGQE  LGQEQD**S**FGGNFE  GGNFEG**S**QSLVGD  NFEGSQ**S**LVGDIG  MWDFVL**S**PDEINT  YLGGPF**S**PNVLNW  LCVLAI**S**LASMVT  LAISLA**S**MVTEDL  QGEPGP**S**GNPGKV  VGYPGP**S**GPLGAR  IKGTKG**S**PGNIKD  QPRPAF**S**AIRRNP  EPYQNH**S**GRFVCT  FTFQVL**S**QWEICL  QWEICL**S**IVSSSR  ICLSIV**S**SSRGQV  CLSIVS**S**SRGQVR  LSIVSS**S**RGQVRR  RGQVRR**S**LGFCDT  GLFQVV**S**GGMVLQ  GHIYQG**S**EADSVF  QGSEAD**S**VFSGFL  EADSVF**S**GFLIFP  MKIPWG**S**IPVLML  LGLIDI**S**QAQLSC  ISQAQL**S**CTGPPA  PGPKGE**S**GDYKAT  TQKIAF**S**ATRTIN  NNYEPR**S**GKFTCK  YFTYHA**S**SRGNLC  FTYHAS**S**RGNLCV  QATDKN**S**LLGMEG  GMEGAN**S**IFSGFL  GANSIF**S**GFLLFP  QTTSSG**S**GPFTDV  NYTDCT**S**EGRRDN  QTYPSS**S**GPVEVF  TPSQPN**S**HPIQWN  PQPSHI**S**KYILRW  RWRPKN**S**VGRWKE  GETTPF**S**PLVATS  VTEITA**S**SFVVSW  VSWVSA**S**DTVSGF  SEDGEQ**S**LILSTS  EQSLIL**S**TSQTTA  MWTPPE**S**AVTGYR  ASEYTV**S**LVAIKG  IKGNQE**S**PKATGV  APREVT**S**DSGSIV  VTSDSG**S**IVVSGL  VKDDKE**S**VPISDT  KESVPI**S**DTIIPA  EYVVSV**S**SVYEQH  YVVSVS**S**VYEQHE  GETGGN**S**PVQEFT  TEIDKP**S**QMQVTD  TDVQDN**S**ISVKWL  RYRVTY**S**SPEDGI  NLAPDS**S**SVVVSG  IKPDVR**S**YTITGL  PNSLLV**S**WQPPRA  TSEYII**S**CHPVGT  FRVPGT**S**TSATLT  WERMSE**S**GFKLLC  QCLGFG**S**GHFRCD  WVLTVL**S**LLPLLE  KWFYIA**S**AFRNEE  NEEYNK**S**VQEIQA  RENGTI**S**RYVGGQ  EKNWGL**S**VYADKP  CLRIPK**S**DVVYTD  QLWGCH**S**APHGPG  VWPQQP**S**GELFEI  TPVARC**S**VRQLKE  KLDGKF**S**VVYAKC  NAQNNG**S**NFQLEE  FQLEEI**S**RAQLVP  LVPLPP**S**TYVEFT  YVEFTV**S**GTDCVA  FCKATL**S**EKLGGA  QTQPVT**S**QPQPEG  DPDAPP**S**PPLGAP  GLPPAG**S**PPDSHV  AGSPPD**S**HVLLAA  SPSGEV**S**HPRKTR  RTVVQP**S**VGAAAG  AGLVFV**S**EAGPTG  PTGTGE**S**KCPLMV  LDAVRG**S**PAINVA  TWEPFA**S**GKTSES  FASGKT**S**ESGELH  SGKTSE**S**GELHGL  VEIDTK**S**YWKALG  WKALGI**S**PFHEHA  VFTAND**S**GPRRYT  TIAALL**S**PYSYST  ALLSPY**S**YSTTAV  LSPYSY**S**TTAVVT  KWVTFI**S**LLFLFS  SLLFLF**S**SAYSRG  LLFLFS**S**AYSRGV  LFSSAY**S**RGVFRR  RDEGKA**S**SAKQRL  DEGKAS**S**AKQRLK  QRLKCA**S**LQKFGE  WAVARL**S**QRFPKA  AEFAEV**S**KLVTDL  ICENQD**S**ISSKLK  ENQDSI**S**SKLKEC  NQDSIS**S**KLKECC  KPLLEK**S**HCIAEV  MPADLP**S**LAADFV  AADFVE**S**KDVCKN  RRHPDY**S**VVLLLR  PTLVEV**S**RNLGKV  NLGKVG**S**KCCKHP  CAEDYL**S**VVLNQL  HEKTPV**S**DRVTKC  TKCCTE**S**LVNRRP  ADICTL**S**EKERQI  LLNFTE**S**RTLHRN  NEYGIA**S**ILDSYQ  IASILD**S**YQCTAE  QCTAEI**S**LADLAT  ATYKEV**S**KMVKDA  PTGDEQ**S**SGCLEN  TGDEQS**S**GCLENQ  KKPTPA**S**IPLFQV  VPEPVT**S**CEAYEE  YDKIIP**S**CCKAEN  TKELRE**S**SLLNQH  KELRES**S**LLNQHA  ITVTKL**S**QKFTKV  DGEKIM**S**YICSQQ  IMSYIC**S**QQDTLS  SQQDTL**S**NKITEC  EKPEGL**S**PNLNRF  RDFNQF**S**SGEKNI  KNIFLA**S**FVHEYS  SFVHEY**S**RRHPQL  HPQLAV**S**VILRVA  QKYIQE**S**QALAKR  QALAKR**S**CGLFQK  ATCCQL**S**EDKLLA  VGQCCT**S**SYANRR  GQCCTS**S**YANRRP  NRRPCF**S**SLVVDE  RRPCFS**S**LVVDET  YVPPAF**S**DDKFIF  AVIADF**S**GLLEKC  EGQKLI**S**KTRAAL  LIFLTL**S**GIQGVP  IQGVPL**S**RTVRCT  VRCTCI**S**ISNQPV  CTCISI**S**NQPVNP  QPVNPR**S**LEKLEI  LEIIPA**S**QFCPRV  RCLNPE**S**KAIKNL  NLLKAV**S**KERSKR  QARSAF**S**NLFGGE  FGGEPL**S**YTRFSL  PKRCSG**S**ICYGTI  RLAGTE**S**PVREEP  DLKRKL**S**EKLDST  LSEKLD**S**TDFTGT  FREFKL**S**KVWRDQ  KIQVKD**S**AQNSVI  KVANAE**S**LNAIGV  IVNAEL**S**FFGHAH  YTPGFP**S**FNHTQF  HTQFPP**S**RSSGLP  QFPPSR**S**SGLPNI  FPPSRS**S**GLPNIP  NVKLTV**S**NVLKEI  GFQPSR**S**IIFASW  IIFASW**S**AGDFGS  SAGDFG**S**VGATEW  LEGYLS**S**LHLKAF  KAVLGT**S**NFKVSA  TSNFKV**S**ASPLLY  NFKVSA**S**PLLYTL  QFLYQD**S**NWASKV  FPFLAY**S**GIPAVS  SGIPAV**S**FCFCED  YNSQLL**S**FVRDLN  SLQWLY**S**ARGDFF  DFFRAT**S**RLTTDF  VEYHFL**S**PYVSPK  VFWGSG**S**HTLPAL  GAANAL**S**GDVWDI  VQWCAV**S**QPEATK  VRGPPV**S**CIKRDS  VVKKGG**S**FQLNEL  ELQGLK**S**CHTGLR  ARFFSA**S**CVPGAD  SQEPYF**S**YSGAFK  EPYFSY**S**GAFKCL  TVFEDL**S**DEAERD  HAVVAR**S**VNGKED  KFGKDK**S**PKFQLF  QLFGSP**S**GQKDLL  DSAIGF**S**RVPPRI  IQNLRK**S**EEEVAA  RKCNQW**S**GLSEGS  NQWSGL**S**EGSVTC  SGLSEG**S**VTCSSA  EGSVTC**S**SASTTE  GSVTCS**S**ASTTED  LAENYK**S**QQSSDP  NYKSQQ**S**SDPDPN  YKSQQS**S**DPDPNC  VRRSDT**S**LTWNSV  SVKGKK**S**CHTAVD  LFNQTG**S**CKFDEY  KFDEYF**S**QSCAPG  QSCAPG**S**DPRSNL  PGSDPR**S**NLCALC  PVTEAR**S**CHLAMA  PNHAVV**S**RMDKVE  KFGRNG**S**DCPDKF  LKKCST**S**PLLEAC  QIRQNY**S**TDVEAA  VEAAVN**S**LVNLYL  NLYLQA**S**YTYLSL  ASYTYL**S**LGFYFD  VALEGV**S**HFFREL  DLHALG**S**ARTDPH  ILALMV**S**MIGADS  VALLAL**S**SAQNLN  ALLALS**S**AQNLNE  DVSQEE**S**PSLIAG  SQEESP**S**LIAGNP  PPQGDK**S**RSPRSP  QGDKSR**S**PRSPPG  PQGDNK**S**RSSRSP  DNKSRS**S**RSPPGK  PQGDNK**S**QSARSP  GDNKSQ**S**ARSPPG  KSQSAR**S**PPGKPQ  PQGGNK**S**QGPPPP  PPPQGG**S**KSRSSR  PQGGSK**S**RSSRSP  GGSKSR**S**SRSPPG  GSKSRS**S**RSPPGK  KSRSSR**S**PPGKPQ  PPPQGG**S**KSRSAR  PQGGSK**S**RSARSP  GGSKSR**S**ARSPPG  KSRSAR**S**PPGKPQ  CFTPGE**S**QRGPRG  QAGAKP**S**GAESSK  KPSGAE**S**SKGAAF  PSGAES**S**KGAAFV  KGAAFV**S**KQEGSE  VSKQEG**S**EVVKRP  TLHTKA**S**GMALLH  EVYLDS**S**KPAVYN  LPYGPG**S**EAAAFG  EAAAFG**S**NGLGGF  GFPPLN**S**VSPSPL  YLENEP**S**GYTVRE  AFYRPN**S**DNRRQG  STNDKG**S**MAMESA  GSMAME**S**AKETRY  VCNDYA**S**GYHYGV  DKNRRK**S**CQACRL  GRGEVG**S**AGDMRA  PLMIKR**S**KKNSLA  KRSKKN**S**LALSLT  KNSLAL**S**LTADQM  EPPILY**S**EYDPTR  DPTRPF**S**EASMMG  RPFSEA**S**MMGLLT  IGLVWR**S**MEHPGK  DMLLAT**S**SRFRMM  MLLATS**S**RFRMMN  EFVCLK**S**IILLNS  GVYTFL**S**STLKSL  LSSTLK**S**LEEKDH  QLLLIL**S**HIRHMS  RLHAPT**S**RGGASV  TSRGGA**S**VEETDQ  VEETDQ**S**HLATAG  HLATAG**S**TSSHSL  ATAGST**S**SHSLQK  GSTSSH**S**LQKYYI  TLAQDN**S**RYTHFL  DDRYCE**S**IMRRRG  RRRGLT**S**PCKDIN  IHGNKR**S**IKAICE  RENLRI**S**KSSFQV  NLRISK**S**SFQVTT  LRISKS**S**FQVTTC  CKLHGG**S**PWPPCQ  LFWGVV**S**HSFPAT  WGVVSH**S**FPATLE  KAFQLW**S**NVTPLT  LTFTKV**S**EGQADI  QADIMI**S**FVRGDH  GDHRDN**S**PFDGPG  AHELGH**S**LGLSHS  GHSLGL**S**HSTDIG  SLGLSH**S**TDIGAL  GALMYP**S**YTFSGD  YPSYTF**S**GDVQLA  QAIYGR**S**QNPVQP  TPKACD**S**KLTFDA  VELNFI**S**VFWPQL  YPKDIY**S**SFGFPR  PKDIYS**S**FGFPRT  HIDAAL**S**EENTGK  YDEYKR**S**MDPGYP  YLFKNI**S**SVGPWD  LFKNIS**S**VGPWDG  YILVNV**S**FGGFLL  FLLCIF**S**VFPVFV  FPVFVA**S**CNGYFV  GLVTGW**S**LAFLAF  FGNFRF**S**SKHALT  GNFRFS**S**KHALTV  TIGIGV**S**IPPFFG  PPFFGW**S**RFIPEG  PEGLQC**S**CGPDWY  VGTKYR**S**ESYTWF  TKYRSE**S**YTWFLF  CFIVPL**S**LICFSY  LSLICF**S**YTQLLR  AAQQQE**S**ATTQKA  KAEREV**S**RMVVVM  VVVMVG**S**FCVCYV  RLVTIP**S**FFSKSA  TIPSFF**S**KSACIY  PSFFSK**S**ACIYNP  KAMTDE**S**DTCSSQ  DESDTC**S**SQKTEV  ESDTCS**S**QKTEVS  SQKTEV**S**TVSSTQ  GRHPQD**S**YEDSTQ  QDSYED**S**TQSSIF  YEDSTQ**S**SIFTYT  EDSTQS**S**IFTYTN  IFTYTN**S**NSTRGP  TYTNSN**S**TRGPFE  WVYHLT**S**VWMIFV  IFVVIA**S**VFTNGL  AETVIA**S**TISVVN  VIASTI**S**VVNQVY  LEGYTV**S**LCGITG  GITGLW**S**LAIISW  WSLAII**S**WERWMV  IVGIAF**S**WIWAAV  PPIFGW**S**RYWPHG  PHGLKT**S**CGPDVF  CGPDVF**S**GSSYPG  PDVFSG**S**SYPGVQ  DVFSGS**S**YPGVQS  SYPGVQ**S**YMIVLM  CCITPL**S**IIVLCY  AKQQKE**S**ESTQKA  QQKESE**S**TQKAEK  PAFFAK**S**ATIYNP  KKVDDG**S**ELSSAS  DDGSEL**S**SASKTE  DGSELS**S**ASKTEV  SELSSA**S**KTEVSS  ASKTEV**S**SVSSVS  ALADQE**S**CKGRCT  QCDELC**S**YYQSCC  LCSYYQ**S**CCTDYT  EQVGGP**S**LTSDLQ  GGPSLT**S**DLQAQS  SDLQAQ**S**KGNPEQ  AEEELC**S**GKPFDA  TDLKNG**S**LFAFRG  TYLFKG**S**QYWRFE  DYPRNI**S**DGFDGI  LALPAH**S**YSGRER  LPAHSY**S**GRERVY  QFQHQP**S**QEECEG  QEECEG**S**SLSAVF  EECEGS**S**LSAVFE  CEGSSL**S**AVFEHF  LFWGRT**S**AGTRQP  RQPQFI**S**RDWHGV  AGRIYI**S**GMAPRP  GMAPRP**S**LAKKQR  NRKGYR**S**QRGHSR  RSQRGH**S**RGRNQN  RGRNQN**S**RRPSRA  SRATWL**S**LFSSEE  TWLSLF**S**SEESNL  WLSLFS**S**EESNLG  LFSSEE**S**NLGANN  TCEPIQ**S**VFFFSG  QSVFFF**S**GDKYYR  DPPYPR**S**IAQYWL  VATWGI**S**GTPAPL  TPAPLD**S**VFSSSE  PLDSVF**S**SSERAH  LDSVFS**S**SERAHQ  DSVFSS**S**ERAHQV  IRKRAN**S**FLEELR  LEELRH**S**SLEREC  EELRHS**S**LERECI  DTLAFW**S**KHVDGD  LEHPCA**S**LCCGHG  CIDGIG**S**FSCDCR  DGIGSF**S**CDCRSG  FSCDCR**S**GWEGRF  FCQREV**S**FLNCSL  VGWRRC**S**CAPGYK  RMEKKR**S**HLKRDT  MTRRGD**S**PWQVVL  QVVLLD**S**KKKLAC  AVLIHP**S**WVLTAA  AHCMDE**S**KKLLVR  HPNYSK**S**TTDNDI  AQPATL**S**QTIVPI  PICLPD**S**GLAERE  TGWGYH**S**SREKEA  VPHNEC**S**EVMSNM  ECSEVM**S**NMVSEN  VMSNMV**S**ENMLCA  DACEGD**S**GGPMVA  GGPMVA**S**FHGTWF  FLVGLV**S**WGEGCG  GVYTKV**S**RYLDWI  EQKKEL**S**DIAHRI  ILAADE**S**TGSIAK  ADESTG**S**IAKRLQ  IAKRLQ**S**IGTENT  FPQVIK**S**KGGVVG  QGLDGL**S**ERCAQY  IGEHTP**S**ALAIME  VLARYA**S**ICQQNG  AVYKAL**S**DHHIYL  ACTQKF**S**HEEIAM  TGITFL**S**GGQSEE  FLSGGQ**S**EEEASI  QSEEEA**S**INLNAI  PWALTF**S**YGRALQ  GRALQA**S**ALKAWG  KRALAN**S**LACQGK  QGKYTP**S**GQAGAA  QAGAAA**S**ESLFVS  GAAASE**S**LFVSNH  VQTVKS**S**KGGPGS  YPTFNP**S**SDVAAL  PTFNPS**S**DVAALH  LIEILA**S**RTNKEI  LAKDIT**S**DTSGDF  DITSDT**S**GDFRNA  FRNALL**S**LAKGDR  LAKGDR**S**EDFGVN  NEDLAD**S**DARALY  TILTTR**S**YPQLRR  QKYTKY**S**KHDMNK  IVKCAT**S**KPAFFA  LIRIMV**S**RSEIDM  RIMVSR**S**EIDMND  QKMYGI**S**LCQAIL  LLLNQF**S**RAVADS  SRAVAD**S**WMEEVI  IAICGM**S**TWSKRS  CGMSTW**S**KRSLSQ  STWSKR**S**LSQEDA  WSKRSL**S**QEDAPQ  VAEIVP**S**FINKDT  ETINMM**S**EFVANL  ELKLTL**S**EMQPAL  VPVLKD**S**SLLFEE  PVLKDS**S**LLFEEF  LIRNRQ**S**EAADSS  QSEAAD**S**SPSELK  SEAADS**S**PSELKY  AADSSP**S**ELKYLG  LGLDTH**S**RKKRQL  KKRQLY**S**ALANKC  SFILKT**S**QCTLKE  PIRTGI**S**PLALIK  LKKLTI**S**EQNIQR  CGQPQC**S**THILQW  YLMLMR**S**PSQADI  NYLPKE**S**MLKTTL  QVPDGV**S**KVLVDH  QLQISS**S**GVIAPG  IPDFAR**S**GVQMNT  NFPVDL**S**DYPKSL  SNLYNW**S**ASYSGG  LGNNPV**S**KGLLIF  LLIFDA**S**SSWGPQ  YQADYE**S**LRFFSL  LQLQPY**S**LVTTLN  HLVSRK**S**ISAALE  NFNEKL**S**QLQTYM  LIKKLK**S**FDYHQF  KNTLEL**S**NGVIVK  NQLTLD**S**NTKYFH  HIAWTS**S**GKGSWK  QNLVYE**S**GSLNFS  SKLEIQ**S**QVDSQH  AELFNQ**S**DIVAHL  SYFSIE**S**STKGDV  LVQVHA**S**QPSSFH  VHASQP**S**SFHDFP  KWNFYY**S**PQSSPD  EKIAEL**S**ATAQEI  ATKKII**S**DYHQQF  EKFIAE**S**KRLIDL  GREENP**S**SVLAQE  TVKVSA**S**SPSLAV  VASQSD**S**KQRRLL  FPKGSV**S**NAQQPD  DLSKAV**S**LSMGLY  LKLLEE**S**IANLNR  EFPKTH**S**DVSSEQ  LYTTDQ**S**TFDILQ  FLLEGN**S**NEDCKP  NGDLVL**S**SPSNVT  LVLSSP**S**NVTLPQ  TVYCQA**S**FPGANI  IIGNKM**S**AISVHG  NKMSAI**S**VHGVST  VHGVST**S**GGQMYH  YDMNTA**S**LSQQQD  WNRCQG**S**GDDNLT  VFSNGY**S**SPSMRP  FSNGYS**S**PSMRPD  SMRPDV**S**SPPSSS  MRPDVS**S**PPSSSS  SPPSSS**S**TATTGP  VCSDEA**S**GCHYGV  GVLTCG**S**CKVFFK  QATTGV**S**QETSEN  GVSQET**S**ENPGNK  FALGWR**S**YRQSSA  KTLLLL**S**SVPKDG  TLLLLS**S**VPKDGL  PKDGLK**S**QELFDE  VKREGN**S**SQNWQR  RAVCGT**S**RQLAPA  ALGYLG**S**RQKHSL  GSRQKH**S**LPDLPY  IMQLHH**S**KHHAAY  GGHINH**S**IFWTNL  IFWTNL**S**PNGGGE  IKRDFG**S**FDKFKE  EKLTAA**S**VGVQGS  SVGVQG**S**GWGWLG  QRFAVL**S**RGVHSS  LSRGVH**S**SVASAT  SRGVHS**S**VASATS  VHSSVA**S**ATSVAT  SVASAT**S**VATKKT  VQGPPT**S**DDIFER  KYFDFL**S**SYSAVN  YFDFLS**S**YSAVNQ  DFLSSY**S**AVNQGH  IVNALK**S**QVDKLT  DKLTLT**S**RAFYNN  FWGRTL**S**AISSST  RTLSAI**S**SSTDPT  TLSAIS**S**STDPTS  LSAISS**S**TDPTSY  SSTDPT**S**YDGFGP  LLGKAL**S**GGLYPV  GGLYPV**S**AVLCDD  KPGEHG**S**TYGGNP  ELMKLP**S**DVVTAV  EDELRE**S**IEIINK  VLPGSP**S**KTRGQI  ILGPMF**S**GKSTEL  PMFSGK**S**TELMRR  AKDTRY**S**SSFCTH  KDTRYS**S**SFCTHD  DTRYSS**S**FCTHDR  LVPLAE**S**VVKLTA  GADKYH**S**VCRLCY  CYFKKA**S**GQPAGP  PDLEFD**S**LQPCFY  YFGGPD**S**TPPGED  LPTPPL**S**PSRGFA  TPPLSP**S**RGFAEH  RGFAEH**S**SEPPSW  GFAEHS**S**EPPSWV  HSSEPP**S**WVTEML  CMWSGF**S**AREKLE  KLERAV**S**EKLQHG  GPPTAG**S**TAQSPG  AGSTAQ**S**PGAGAA  PGAGAA**S**PAGRGH  VPAAPA**S**APAAGP  AGPAVA**S**GAGIAA  GDHKAL**S**TSGEDT  HKALST**S**GEDTLS  TVEKRR**S**SSNTKA  VEKRRS**S**SNTKAV  EKRRSS**S**NTKAVT  GPGRAQ**S**SELILK  PGRAQS**S**ELILKR  PSPYVE**S**EDAPPQ  PQKKIK**S**EASPRP  KIKSEA**S**PRPLKS  IPPKAK**S**LSPRNS  PKAKSL**S**PRNSDS  SLSPRN**S**DSEDSE  SPRNSD**S**EDSERR  NSDSED**S**ERRRNH  RRNDLR**S**SFLTLR  ATEYVH**S**LQAEEH  SRSGYR**S**GGGFSS  RSGGGF**S**SGSAGI  YQRRTT**S**SSTRRS  SSSTRR**S**GGGGGR  GGGGRF**S**SCGGGG  AGGGFG**S**RSLVNL  NLGGSK**S**ISISVA  GGSKSI**S**ISVARG  RGGGRG**S**GFGGGY  EIQKVK**S**REREQI  LEPYFE**S**FINNLR  DQSRLD**S**ELKNMQ  LYQAEL**S**QMQTQI  AESLYQ**S**KYEELQ  GDSVRN**S**KIEISE  NSKIEI**S**ELNRVI  VIQRLR**S**EIDNVK  NVKKQI**S**NLQQSI  ISNLQQ**S**ISDAEQ  LLEGEE**S**RMSGEC  SVSVST**S**HTTISG  GYGSGG**S**SYGSGG  YGSGGS**S**YGSGGG  GGSSYG**S**GGGSYG  GGGSYG**S**GGGGGG  GGGGRG**S**YGSGGS  SYGSGG**S**SYGSGG  SYGSGS**S**SGGYRG  YGSGSS**S**GGYRGG  GGGGGG**S**SGGRGS  RGSGGG**S**SGGSIG  GGSSGG**S**IGGRGS  GGRGSS**S**GGVKSS  TRAAFN**S**GKVDIV  YMFQYD**S**THGKFH  AEYVVE**S**TGVFTT  VIISAP**S**ADAPMF  HEKYDN**S**LKIISN  KTVDGP**S**GKLWRD  QNIIPA**S**TGAAKA  KVVKQA**S**EGPLKG  TEHQVV**S**SDFNSD  EHQVVS**S**DFNSDT  VSSDFN**S**DTHSST  FNSDTH**S**STFDAG  NSDTHS**S**TFDAGA  DNEFGY**S**NRVVDL  MEKFNA**S**IAYDRH  EVDVQG**S**KAYSRG  GTFKLN**S**NDEDIH  KLHTGR**S**RNDQVV  WMRQTC**S**TLSGLL  QTCSTL**S**GLLWEL  AQPIRW**S**HWILSH  WSHWIL**S**HAVALT  VALTRD**S**ERLLEV  NVLPLG**S**GAIAGN  GAITLN**S**MDATSE  NSMDAT**S**ERDFVA  EFLFWA**S**LCMTHL  LCMTHL**S**RMAEDL  YCTKEF**S**FVQLSD  FSFVQL**S**DAYSTG  QLSDAY**S**TGSSLM  DAYSTG**S**SLMPQK  AYSTGS**S**LMPQKK  QKKNPD**S**LELIRS  SLELIR**S**KAGRVF  TLKGLP**S**TYNKDL  EAVFEV**S**DTMSAV  EVSDTM**S**AVLQVA  VATGVI**S**TLQIHQ  NMGQAL**S**PDMLAT  RQAHEA**S**GKAVFM  VALNQL**S**LQELQT  QELQTI**S**PLFSGD  TISPLF**S**GDVICV  VWDYGH**S**VEQYGA  LGGTAR**S**SVDWQI  GGTARS**S**VDWQIR  LPPGAA**S**TQVCTG  PTNASL**S**FLQDIQ  RACHPC**S**PMCKGS  SPMCKG**S**RCWGES  CTGPKH**S**DCLACL  RYTFGA**S**CVTACP  LSTDVG**S**CTLVCP  CKKIFG**S**LAFLPE  FDGDPA**S**NTAPLQ  TGYLYI**S**AWPDSL  LQGLGI**S**WLGLRS  SWLGLR**S**LRELGS  CQPQNG**S**VTCFGP  CVARCP**S**GVKPDL  GVKPDL**S**YMPIWK  PAEQRA**S**PLTSII  RASPLT**S**IISAVV  PLTSII**S**AVVGIL  VLRENT**S**PKANKE  VMAGVG**S**PYVSRL  VGSPYV**S**RLLGIC  LGICLT**S**TVQLVT  ELVSEF**S**RMARDP  PASPLD**S**TFYRSL  DSTFYR**S**LLEDDD  RSSSTR**S**GGGDLT  EEEAPR**S**PLAPSE  AAKGLQ**S**LPTHDP  LPTHDP**S**PLQRYS  VAPLTC**S**PQPEYV  VRPQPP**S**PREGPL  HPPPAF**S**PAFDNL  ERGAPP**S**TFKGTP  GQLGWH**S**WAAGPG  WAAGPG**S**LLAWLI  AWLILA**S**AGAAPC  CCPHGS**S**GLRCTR  NLTIVK**S**GLRFVA  HFTPRL**S**RLNLSF  NALESL**S**WKTVQG  KTVQGL**S**LQELVL  LQELVL**S**GNPLHC  GNPLHC**S**CALRWL  AHMPNA**S**CGVPTL  VQVPNA**S**VDVGDD  LTELEQ**S**ATVMKS  SATVMK**S**GGLPSL  VGRAEV**S**VQVNVS  NVSFPA**S**VQLHTA  HWCIPF**S**VDGQPA  SVLNET**S**FIFTEF  NPFGQA**S**ASIMAA  FGQASA**S**IMAAFM  PIPVSF**S**PVDTNS  SPVDTN**S**TSGDPV  ETPFGV**S**VAVGLA  FACLFL**S**TLLLVL  FMTLGG**S**SLSPTE  MTLGGS**S**LSPTEG  LGGSSL**S**PTEGKG  ENPQYF**S**DACVHH  LKEASE**S**ARQDFQ  QLLAVA**S**QVAAGM  IGDFGM**S**RDIYST  QPWYQL**S**NTEAID  EPQQRH**S**IKDVHA  VLGGLI**S**GAGGGG  ILGGVI**S**AISEAA  GVISAI**S**EAAAQY  PPRTHY**S**NIEANE  NIEANE**S**EEVRQF  GDDMEV**S**ATELMN  GIDTCR**S**MVAVMD  MVAVMD**S**DTTGKL  QFDTDR**S**GTICSS  RSGTIC**S**SELPGA  SGTICS**S**ELPGAF  MIIRRY**S**DESGNM  RRYSDE**S**GNMDFD  DFDNFI**S**CLVRLD  MFRAFK**S**LDKDGT  APAPAP**S**WPLSSS  APSWPL**S**SSVPSQ  PSWPLS**S**SVPSQK  SWPLSS**S**VPSQKT  LSSSVP**S**QKTYQG  QKTYQG**S**YGFRLG  RLGFLH**S**GTAKSV  HSGTAK**S**VTCTYS  SVTCTY**S**PALNKM  VQLWVD**S**TPPPGT  MAIYKQ**S**QHMTEV  HERCSD**S**DGLAPP  RNTFRH**S**VVVPYE  EPPEVG**S**DCTTIH  YNYMCN**S**SCMGGM  NYMCNS**S**CMGGMN  IITLED**S**SGNLLG  ITLEDS**S**GNLLGR  HELPPG**S**TKRALP  ALPNNT**S**SSPQPK  LPNNTS**S**SPQPKK  GKEPGG**S**RAHSSH  GGSRAH**S**SHLKSK  GSRAHS**S**HLKSKK  HSSHLK**S**KKGQST  KSKKGQ**S**TSRHKK  KKGQST**S**RHKKLM  FCWAQY**S**SNTQQG  CWAQYS**S**NTQQGR  TQQGRT**S**IVHLFE  FGGVQV**S**PPNENV  ERYQPV**S**YKLCTR  YKLCTR**S**GNEDEF  MCGNAV**S**AGTSST  AVSAGT**S**STCGSY  VSAGTS**S**TCGSYF  TSSTCG**S**YFNPGS  SYFNPG**S**RDFPAV  FPAVPY**S**GWDFND  GKCKTG**S**GDIENY  VRDCRL**S**GLLDLA  GKDYVR**S**KIAEYM  GFRIDA**S**KHMWPG  KLHNLN**S**NWFPEG  NWFPEG**S**KPFIYQ  GGEPIK**S**SDYFGN  GEPIKS**S**DYFGNG  WNGEKM**S**YLKNWG  GWGFMP**S**DRALVF  HGAGGA**S**ILTFWD  GFTRVM**S**SYRWPR  FTRVMS**S**YRWPRY  NWYDNG**S**NQVAFG  NDDWTF**S**LTLQTG  TYCDVI**S**GDKING  GIKIYV**S**DDGKAH  DGKAHF**S**ISNSAE  KAHFSI**S**NSAEDP  HFSISN**S**AEDPFI  ERRVPF**S**LLRGPS  RLPEEW**S**QWLGGS  SQWLGG**S**SWPGYV  QWLGGS**S**WPGYVR  VAAPAY**S**RALSRQ  DEHGYI**S**RCFTRK  VDPTQV**S**SSLSPE  DPTQVS**S**SLSPEG  PTQVSS**S**LSPEGT  QVSSSL**S**PEGTLT  PKLATQ**S**NEITIP  IPVTFE**S**RAQLGG  QPTLLL**S**LLGAVG  GLAAVN**S**MPVDNR  CIIEVL**S**NALSKS  VLSNAL**S**KSSAPP  SNALSK**S**SAPPIT  NALSKS**S**APPITP  RQVLKT**S**RKDVKD  ASEAHE**S**SSRGEA  ADEPQW**S**LYPSDS  QWSLYP**S**DSQVSE  EVKTRH**S**EKSQRE  GERGED**S**SEEKHL  NERKQA**S**AIKKEE  EELVAR**S**ETHAAG  SQEKTH**S**REKSSQ  THSREK**S**SQESGE  HSREKS**S**QESGEE  EKSSQE**S**GEETGS  SGEETG**S**QENHPQ  ENHPQE**S**KGQPRS  GEEDAT**S**EVDKRR  RSSQGG**S**LPSEEK  QGGSLP**S**EEKGHP  QEESEE**S**NVSMAS  SEESNV**S**MASLGE  SNVSMA**S**LGEKRD  EKRDHH**S**THYRAS  DKRNYP**S**LELDKM  PRAYFM**S**DTREEK  QDKQYS**S**HHTAEK  WEKKPF**S**EDVNWG  QNGKWD**S**DPSGTK  KWDSDP**S**GTKTCI  LVGEFV**S**DALLVP  VAKETC**S**EKSTNL  ETCSEK**S**TNLHDY  CPLAEE**S**DNVDSA  TDYADG**S**EDKVVE  ATERTT**S**IATTTT  TTTTTE**S**VEEVVR  VVREVC**S**EQAETG  PCRAMI**S**RWYFDV  CMAVCG**S**AMSQSL  VCGSAM**S**QSLLKT  GSAMSQ**S**LLKTTQ  LPTTAA**S**TPDAVD  KHRERM**S**QVMREW  KAAQIR**S**QVMTHL  YERMNQ**S**LSLLYN  RMNQSL**S**LLYNVP  QKEQNY**S**DDVLAN  VLANMI**S**EPRISY  ISEPRI**S**YGNDAL  NDALMP**S**LTETKT  PVNGEF**S**LDDLQP  DLQPWH**S**FGADSV  HSFGAD**S**VPANTE  LTTRPG**S**GLTNIK  IKTEEI**S**EVKMDA  AEFRHD**S**GYEVHH  FAEDVG**S**NKGAII  PEERHL**S**KMQQNG  MLLLLL**S**ALAGLF  CIQANY**S**LMENGK  KLEVKF**S**WFMPSA  FSWFMP**S**APYWIL  NYALVY**S**CTCIIQ  PPETVD**S**LKNILT  LKNILT**S**NNIDVK  CTTRGV**S**SCQQCL  QQCLAV**S**PMCAWC  SIEFPV**S**EARVLE  KGSGDS**S**QVTQVS  SQVTQV**S**PQRIAL  RLRPDD**S**KNFSIQ  QMRKLT**S**NLRIGF  FVDKPV**S**PYMYIS  SPYMYI**S**PPEALE  EEVKKQ**S**VSRNRD  VKKQSV**S**RNRDAP  GWRNDA**S**HLLVFT  GSDNHY**S**ASTTMD  DNHYSA**S**TTMDYP  TTMDYP**S**LGLMTE  NLYQNY**S**ELIPGT  GVLSMD**S**SNVLQL  VLSMDS**S**NVLQLI  AYGKIR**S**KVELEV  DLPEEL**S**LSFNAT  VIPGLK**S**CMGLKI  AQAEPN**S**HRCNNG  GPGWLG**S**QCECSE  GSQCEC**S**EEDYRP  GQCVCH**S**SDFGKI  YKGEMC**S**GHGQCS  SGHGQC**S**CGDCLC  RTDTCM**S**SNGLLC  TDTCMS**S**NGLLCS  SNGLLC**S**GRGKCE  CRDEIE**S**VKELKD  ILVVLL**S**VMGAIL  LYKEAT**S**TFTNIT  ALVGLL**S**LGCVLS  SLGCVL**S**QECTKF  CTKFKV**S**SCRECI  TKFKVS**S**CRECIE  DIMDPT**S**LAETQE  GGQKQL**S**PQKVTL  YYLMDL**S**YSMLDD  LMDLSY**S**MLDDLR  LNEITE**S**GRIGFG  GRIGFG**S**FVDKTV  LKLTNN**S**NQFQTE  VGKQLI**S**GNLDAP  SAVGEL**S**EDSSNV  GELSED**S**SNVVQL  NAYNKL**S**SRVFLD  AYNKLS**S**RVFLDH  LKVTYD**S**FCSNGV  DQSRDR**S**LCHGKG  CQTQGR**S**SQELEG  QTQGRS**S**QELEGS  SQELEG**S**CRKDNN  QCLCHT**S**DVPGKL  HPGFEG**S**ACQCER  PRRVEC**S**GRGRCR  NVCECH**S**GYQLPL  PCGKYI**S**CAECLK  PFGKNC**S**AACPGL  CPGLQL**S**NNPVKG  TCKERD**S**EGCWVA  LIYVDE**S**RECVAG  KALIHL**S**DLREYR  LEKALN**S**IIDVYH  DVYHKY**S**LIKGNF  AAAKDK**S**SDKKVQ  AAKDKS**S**DKKVQT  GNEFQV**S**LSSSMS  EFQVSL**S**SSMSVS  FQVSLS**S**SMSVSE  QVSLSS**S**MSVSEL  SLSSSM**S**VSELKA  SSSMSV**S**ELKAQI  RLAVHP**S**GVALQD  DRVPLA**S**QGLGPG  QGLGPG**S**TVLLVV  KCDEPL**S**ILVRNN  RNNKGR**S**STYEVR  NNKGRS**S**TYEVRL  HLKQQV**S**GLEGVQ  YGLKPL**S**TVFMNL  GVPFFS**S**LRCMVD  LATPQP**S**EGAAPA  LGEVDT**S**LVLSSM  DTSLVL**S**SMEEAK  TSLVLS**S**MEEAKQ  YKERRE**S**IKQRLR  LRSGSA**S**PMELLS  SPMELL**S**YFKQPV  AQLNVL**S**KSSGCA  LNVLSK**S**SGCAYQ  NVLSKS**S**GCAYQD  MCNNRR**S**PTLGAS  SPTLGA**S**NRAFVR  EYEDGF**S**LPYGWT  ALARAV**S**NEIVRF  TPDQER**S**LMFMQW  GVNCET**S**CVQQPP  CIPFFR**S**CPACPG  CPACPG**S**NITIRN  QINALT**S**FVDASM  TSFVDA**S**MVYGSE  ASMVYG**S**EEPLAR  LAGDTR**S**SEMPEL  LATELK**S**LNPRWD  YLPTYR**S**YNDSVD  YRSYND**S**VDPRIA  ALNMQR**S**RDHGLP  IWMGGV**S**EPLKRK  ENEGVF**S**MQQRQA  QALAQI**S**LPRIIC  KNNIFM**S**NSYPRD  NIFMSN**S**YPRDFV  RDFVNC**S**TLPALN  FGPVAF**S**LGLLLV  VPPGED**S**KDVAAP  HRQPLT**S**SERIDK  RQPLTS**S**ERIDKQ  YILDGI**S**ALRKET  KETCNK**S**NMCESS  KSNMCE**S**SKEALA  KDGCFQ**S**GFNEET  LQNRFE**S**SEEQAR  QNRFES**S**EEQARA  ARAVQM**S**TKVLIQ  DPTTNA**S**LLTKLQ  THLILR**S**FKEFLQ  FKEFLQ**S**SLRALR  ELACIY**S**ALILHD  ANVNIG**S**LICNVG  AGGPAP**S**TAAAPA  AALGGN**S**SPSAKD  GGNSSP**S**AKDIKK  IKKILD**S**VGIEAD  RLNKVI**S**ELNGKN  GIGKLA**S**VPAGGA  GGAVAV**S**AAPGSA  DDALNA**S**FLPSES  NASFLP**S**ESGPYG  SFLPSE**S**GPYGYS  SGPYGY**S**NPKILK  PKILKQ**S**MTLNLA  LADPVG**S**LKPHLR  ERLIIQ**S**SNGHIT  RLIIQS**S**NGHITT  ALAELH**S**QNTLPS  SQNTLP**S**VTSAAQ  TLPSVT**S**AAQPVN  VAPAVA**S**VAGGSG  ASVAGG**S**GSGGFS  VAGGSG**S**GGFSAS  SGSGGF**S**ASLHSE  SGGFSA**S**LHSEPP  FSASLH**S**EPPVYA  PVYANL**S**NFNPGA  FNPGAL**S**SGGGAP  NPGALS**S**GGGAPS  SGGGAP**S**YGAAGL  RNRIAA**S**KCRKRK  TLKAQN**S**ELASTA  QNSELA**S**TANMLR  VMNHVN**S**GCQLML  SYFMLI**S**CLMFLS  SCLMFL**S**QSQGQE  LMFLSQ**S**QGQEAQ  LPQARI**S**CPEGTN  GTNAYR**S**YCYYFN  YCQNMN**S**GNLVSV  NSGNLV**S**VLTQAE  EGAFVA**S**LIKESG  ASLIKE**S**GTDDFN  NRRWHW**S**SGSLVS  RRWHWS**S**GSLVSY  WHWSSG**S**LVSYKS  SSGSLV**S**YKSWGI  SLVSYK**S**WGIGAP  WGIGAP**S**SVNPGY  GIGAPS**S**VNPGYC  NPGYCV**S**LTSSTG  YCVSLT**S**STGFQK  CVSLTS**S**TGFQKW  VIVEAL**S**KSKAEL  VEALSK**S**KAELME  AELMEI**S**EDKTKI  KTKIRR**S**PSKPLP  KIRRSP**S**KPLPEV  NDVKNR**S**VYIKGF  HKAFKG**S**IFVVFD  IFVVFD**S**IESAKK  VFDSIE**S**AKKFVE  EDAEMK**S**LEEKIG  GCLLKF**S**GDLDDQ  DLHILF**S**NHGEIK  IEDQQE**S**LNKWKS  SLNKWK**S**KGRRFK  KAAQPG**S**GKGKVQ  DKEEPA**S**KQQKTE  IFLIIT**S**AWGGSK  TSAWGG**S**KGPLDQ  LNNKNL**S**MPLLPA  DLEKIF**S**EDDDYI  YIDIVD**S**LSVSPT  DIVDSL**S**VSPTDS  VDSLSV**S**PTDSDV  SVSPTD**S**DVSAGN  PTDSDV**S**AGNILQ  QLFHGK**S**RIQRLN  IAPVGI**S**TAMGMI  TAMGMI**S**LGLKGE  THEQVH**S**ILHFKD  KDFVNA**S**SKYEIT  DFVNAS**S**KYEITT  FGYTLR**S**VNDLYI  AQIADF**S**DPAFIS  SDPAFI**S**KTNNHI  CIYFKG**S**WVNKFP  REVVKV**S**MMQTKG  EYVGGI**S**MLIVVP  VVPHKM**S**GMKTLE  VERWQK**S**MTNRTR  NYNLVE**S**LKLMGI  GNMAGI**S**DQRIAI  VGFMPL**S**TQVRFT  IYEHRT**S**CLLFMG  DRHDGT**S**NGTARL  LGTVGQ**S**PYTSAP  GQSPYT**S**APPLSH  TSAPPL**S**HTPNAD  QPIYPQ**S**QDPYSH  QSQDPY**S**HVNDPY  HVNDPY**S**LNPLHA  WPGQRQ**S**QESGLL  QRQSQE**S**GLLHTH  GLPHQL**S**GLDPRR  HGPHAL**S**SGLGDL  IKKGPV**S**LSKSNS  KGPVSL**S**KSNSNA  PVSLSK**S**NSNAVS  SLSKSN**S**NAVSAI  SNSNAV**S**AIPINK  RLSLLS**S**TSKYKV  SLLSST**S**KYKVTV  PECLNA**S**LLGGVL  VLRRAK**S**KNGGRS  SKNGGR**S**LREKLD  FLNRQH**S**DPNEQV  LLAQDR**S**PLGNSR  RSPLGN**S**RPNPIL  LEPGIQ**S**CLTHFN  THFNLI**S**HGFGSP  ISHGFG**S**PAVCAA  MDKMYL**S**NNPNSH  LSNNPN**S**HTDNNA  TDNNAK**S**SDKEEK  DNNAKS**S**DKEEKH  AAGPPP**S**EGEEST  KQPTFC**S**HCTDFI  HEFVTF**S**CPGADK  ADKGPA**S**DDPRSK  ASDDPR**S**KHKFKI  KIHTYS**S**PTFCDH  FCDHCG**S**LLYGLI  MDPNGL**S**DPYVKL  LIPDPK**S**ESKQKT  PDPKSE**S**KQKTKT  TKTIKC**S**LNPEWN  RFQLKE**S**DKDRRL  WDWDLT**S**RNDFMG  RNDFMG**S**LSFGIS  DFMGSL**S**FGISEL  SLSFGI**S**ELQKAS  SELQKA**S**VDGWFK  GWFKLL**S**QEEGEY  PVPPEG**S**EANEEL  FERAKI**S**QGTKVP  KTTNTV**S**KFDNNG  MVLGKG**S**FGKVML  FGKVML**S**ERKGTD  FLTQLH**S**CFQTMD  GLFFLQ**S**KGIIYR  DNVMLD**S**EGHIKI  YQPYGK**S**VDWWAF  EDELFQ**S**IMEHNV  NVAYPK**S**MSKEAV  AYPKSM**S**KEAVAI  RDKRDT**S**NFDKEF  GSVQAP**S**YGARPV  GAGGSG**S**RISVSR  GSGSRI**S**VSRSTS  GSRISV**S**RSTSFR  EKETMQ**S**LNDRLA  LNDRLA**S**YLDRVR  ENRRLE**S**KIREHL  PQVRDW**S**HYFKII  LQAQIA**S**SGLTVE  QAQIAS**S**GLTVEV  EVDAPK**S**QDLAKI  ELDKYW**S**QQIEES  SQQIEE**S**TTVVTT  TVVTTQ**S**AEVGAA  LEIDLD**S**MRNLKA  ILLHLE**S**ELAQTR  VDGKVV**S**ETNDTK  TNPTPG**S**LGPWKI  KRMEFT**S**SCPNVS  RMEFTS**S**CPNVSE  SSCPNV**S**ERSFDN  PNVSER**S**FDNVRS  SFDNVR**S**LKVESG  RSLKVE**S**GAWIGY  IGYEHT**S**FCGQQF  PRWDAW**S**GSNAYH  WDAWSG**S**NAYHIE  HIERLM**S**FRPICS  SFRPIC**S**ANHKES  SANHKE**S**KMTIFE  GRQWEI**S**DDYPSL  ISDDYP**S**LQAMGW  FNNEVG**S**MKIQSG  GSMKIQ**S**GAWVCY  HWREWG**S**HAQTSQ  GSHAQT**S**QIQSIR  LSSSEE**S**ITEYKQ  QDKIYP**S**FQPQPL  VMPVLK**S**PTIPFF  PPQPLW**S**VPQPKV  ECCPVC**S**QTPGGG  PAGPPG**S**QGPRGE  GLSRPF**S**AQMAGL  AGLDEK**S**GLGSQV  VGLMPG**S**VGPVGP  EVGAPG**S**KGEAGP  PLGIPG**S**SGFPGN  AKGPTG**S**PGTSGP  TGSPGT**S**GPPGSA  TSGPPG**S**AGPPGS  SPGPQG**S**TGPQGI  ERGPVG**S**SGPKGS  RPGPPG**S**IGIRGQ  IRGQPG**S**MGLPGP  LPGPKG**S**SGDPGK  PGPKGS**S**GDPGKP  DGEVGP**S**GPVGPP  KGSPGP**S**GTPGDT  LVGPPG**S**RGNPGS  LRGDPG**S**HGRVGD  PAGLPG**S**QGAPGT  PNGEQG**S**AGIPGP  PGPVGP**S**GKEGNP  PPGVRG**S**VGEAGP  MGHYDE**S**MPDPLP  VHATLK**S**LSSQIE  ATLKSL**S**SQIETM  QIETMR**S**PDGSKK  DLKLCH**S**AKQSGE  TGETCI**S**ANPSSV  ISANPS**S**VPRKTW  TWWASK**S**PDNKPV  TFLRLL**S**KEASQN  LTVIRG**S**RLFFNY  CPTICK**S**HGCTAE  EGLCCH**S**ECLGNC  ECLGNC**S**QPDDPT  SGYTMN**S**SNLLCT  GYTMNS**S**NLLCTP  CTVING**S**LIINIR  YLKIRR**S**YALVSL  NPKLCL**S**EIHKME  NELLKF**S**YIRTSF  RTYGAK**S**DIIYVQ  TDATNP**S**VPLDPI  VPLDPI**S**VSNSSS  LKWKPP**S**DPNGNI  PFESED**S**QKHNQS  SAGECC**S**CPKTDS  LKELEE**S**SFRKTF  AEDPRP**S**RKRRSL  PSRKRR**S**LGDVGN  AAFPNT**S**STSVPT  PNTSST**S**VPTSPE  KESLVI**S**GLRHFT  TPEERC**S**VAAYVS  CRLRGL**S**PGNYSV  IFVFLF**S**VVIGSI  FSVVIG**S**IYLFLR  GPLYAS**S**NPEYLS  SNPEYL**S**ASDVFP  SDVFPC**S**VYVPDE  RELGQG**S**FGMVYE  VLWEIT**S**LAEQPY  KDDLHP**S**FPEVSF  VPLDRS**S**HCQREE  CMLIGK**S**HTEDDI  RFKKPQ**S**LTKWSD  QSLTKW**S**DIWNAT  ATKYAN**S**CCQNID  FPGFHG**S**EMWNPN  NPNTDL**S**EDCLYL  GFQTGT**S**SLHVYD  FQTGTS**S**LHVYDG  FGGNPK**S**VTLFGE  AGAASV**S**LHLLSP  VSLHLL**S**PGSHSL  HLLSPG**S**HSLFTR  LSPGSH**S**LFTRAI  TRAILQ**S**GSFNAP  AILQSG**S**FNAPWA  APWAVT**S**LYEARN  AKLTGC**S**RENETE  PYGTPL**S**VNFGPT  YGAPGF**S**KDNNSI  FSKDNN**S**IITRKE  IFFPGV**S**EFGKES  SEFGKE**S**ILFHYT  EFTKKF**S**EWGNNA  YYFEHR**S**SKLPWP  KAEEIL**S**RSIVKR  EEILSR**S**IVKRWA  NETQNN**S**TSWPVF  TQNNST**S**WPVFKS  SWPVFK**S**TEQKYL  LTLNTE**S**TRIMTK  QCRFWT**S**FFPKVL  PADPAG**S**GLQRAE  RRQLRV**S**QRTDGE  QRTDGE**S**RAHLGA  QARKAP**S**GRMSIV  APSGRM**S**IVKNLQ  LQNLDP**S**HRISDR  DPSHRI**S**DRDYMG  MDFGRR**S**AEEYEY  ALLCAL**S**LALCAL  VRAATA**S**RGASQA  PEARPN**S**MVVEHP  ECSQDE**S**GAAAIF  EVQGFE**S**ATFLGY  FLGYFK**S**GLKYKK  KKGGVA**S**GFKHVV  ATEVPV**S**WESFNN  VPVSWE**S**FNNGDC  QWCGSN**S**NRYERL  LKATQV**S**KGIRDN  RARVHV**S**EEGTEP  AKLYKV**S**NGAGTM  NGAGTM**S**VSLVAD  AQGALK**S**EDCFIL  PKQTQV**S**VLPEGG  AATLHT**S**TAMAAQ  IWRIEG**S**NKVPVD  NWQGAQ**S**TQDEVA  QDEVAA**S**AILTAQ  GGTPVQ**S**RVVQGK  EPAHLM**S**LFGGKP  IYKGGT**S**REGGQT  GQTAPA**S**TRLFQV  FQVRAN**S**AGATRA  KAGALN**S**NDAFVL  FVLKTP**S**AAYLWV  VQVAEG**S**EPDGFW  KAAYRT**S**PRLKDK  PRLFAC**S**NKIGRF  VWVGKD**S**QEEEKT  KTEALT**S**AKRYIE  WDDDYW**S**VDPLDR  LTWEKV**S**SVDGVL  TWEKVS**S**VDGVLG  VDLDEM**S**FTFTEL  QKNIEI**S**VHKFFN  ISTEIN**S**ALVLKV  DYFIKL**S**PPMLLK  RRGQNR**S**ARIAKQ  FIPFMN**S**LGLVTS  PEVENL**S**KRYEEI  LMMILN**S**ASDQPS  TVNPKE**S**ILKRVK  MESMLK**S**EEERLS  LSIQNF**S**KLLNDN  VVMATY**S**RSTSQN  TYSRST**S**QNLDSG  EHRIME**S**LAWLSD  ESLAWL**S**DSPLFD  AADMYL**S**PVRSPK  AETQAT**S**AFQTQK  TQKPLK**S**TSLSLF  KPLKST**S**LSLFYK  LCERLL**S**EHPELE  DQIMMC**S**MYGICK  KEEEYD**S**IIVFYN  IIVFYN**S**VFMQRL  RSRILV**S**IGESFG  DRVLKR**S**AEGSNP  RFDIEG**S**DEADGS  SDEADG**S**KHLPGE  KHLPGE**S**KFQQKL  KQKMND**S**MDTSNK  PSPEVG**S**PLLCRP  AGPFPG**S**QTSDTL  VSAIPI**S**LDGLLF  CQGQDP**S**DEKTQD  SPPEKD**S**GLLDSV  DSGLLD**S**VLDTLL  SPLMSR**S**GCKVGD  GCKVGD**S**SGTAAA  QLLLPA**S**ESPHWS  LVPKED**S**RFSAPR  AFAPPR**S**SPCASS  DAYPLY**S**DFQPPA  EEGAEA**S**ARSPRS  PASASV**S**SASSSG  SASSSG**S**TLECIL  CKAPGA**S**GCLLPR  NYLRPD**S**EASQSP  DSEASQ**S**PQYSFE  PQYSFE**S**LPQKIC  VGVPNE**S**QALSQR  TKPDTS**S**SLLTSL  KPDTSS**S**LLTSLN  LSVVKW**S**KSLPGF  ITLIQY**S**WMSLMV  IQYSWM**S**LMVFGL  FGLGWR**S**YKHVSG  RSYKHV**S**GQMLYF  FVKLQV**S**QEEFLC  RQKGVV**S**SSQRFY  QKGVVS**S**SQRFYQ  KGVVSS**S**QRFYQL  EDEEAE**S**ATGKRA  NTFHQY**S**VKLGHP  NADKQL**S**FEEFIM  ARLTWA**S**HEKMHE  EEEKTE**S**NQEVAN  FFKNDK**S**KTWQAN  ANLRLI**S**KFDTVE  YNHIQL**S**SNLMPG  NHIQLS**S**NLMPGC  MPGCDY**S**LFKDGI  NKQQRR**S**DLDRFW  LCLIGE**S**FDDYSD  ESFDDY**S**DDVCGA  IVIGYQ**S**HADTAT  ADTATK**S**GSTTKN  AREIFD**S**RGNPTV  EVDLFT**S**KGLFRA  FRAAVP**S**GASTGI  AVPSGA**S**TGIYEA  YMGKGV**S**KAVEHI  IAPALV**S**KKLNVT  DGTENK**S**KFGANA  NAILGV**S**LAVCKA  ADLAGN**S**EVILPV  NVINGG**S**HAGNKL  GMDVAA**S**EFFRSG  ASEFFR**S**GKYDLD  YDLDFK**S**PDDPSR  KSPDDP**S**RYISPD  DPSRYI**S**PDQLAD  LADLYK**S**FIKDYP  KDYPVV**S**IEDPFD  WQKFTA**S**AGIQVV  KAVNEK**S**CNCLLL  KVNQIG**S**VTESLQ  IGSVTE**S**LQACKL  GWGVMV**S**HRSGET  VMVSHR**S**GETEDT  TGAPCR**S**ERLAKY  IEEELG**S**KAKFAG  VAELKK**S**FNRHLH  KRVYYL**S**LEFYMG  AACFLD**S**MATLGL  GNPWEK**S**RPEFML  NTMRLW**S**ARAPND  NLAENI**S**RVLYPN  IRRFKA**S**KFGSTR  KASKFG**S**TRGAGT  IEKLPW**S**KAWELT  DRLRRM**S**LIEEEG  LIEEEG**S**KRINMA  HLCIVG**S**HAVNGV  GVAKIH**S**DIVKTK  KVFKDF**S**ELEPDK  DYVKDL**S**QLTKLH  QLTKLH**S**FLGDDV  ENKLKF**S**QFLETE  KVKINP**S**SMFDVQ  VKINPS**S**MFDVQV  IIKLIT**S**VADVVN  LENYRV**S**LAEKVI  IPATDL**S**EQISTA  DLSEQI**S**TAGTEA  IDNGFF**S**PKQPDL  KCQDKV**S**QLYMNP  LKNIAA**S**GKFSSD  AASGKF**S**SDRTIK  ASGKFS**S**DRTIKE  IWNVEP**S**DLKISL  PSDLKI**S**LSNESN  DLKISL**S**NESNKV  WYREHR**S**ELNLRR  DRFNHF**S**LTLNTN  HILVDY**S**KNLVTE  LVDLAK**S**RGVEAA  VALRNR**S**NTPILV  VLDKMK**S**FCQRVR  FCQRVR**S**GDWKGY  NIGIGG**S**DLGPLM  EALKPY**S**SGGPRV  ALKPYS**S**GGPRVW  AQLNPE**S**SLFIIA  QLNPES**S**LFIIAS  SLFIIA**S**KTFTTQ  QAAKDP**S**AVAKHF  KHFVAL**S**TNTTKV  WVGGRY**S**LWSAIG  GRYSLW**S**AIGLSI  WSAIGL**S**IALHVG  NFEQLL**S**GAHWMD  QQGDME**S**NGKYIT  GKYITK**S**GTRVDH  ALMRGK**S**TEEARK  LQAAGK**S**PEDLER  GNRPTN**S**IVFTKL  IIWDIN**S**FDQWGV  EPELDG**S**AQVTSH  GSAQVT**S**HDASTN  VTSHDA**S**TNGLIN  NFEKNV**S**QAIHKY  AYRKAA**S**VIAKYP  YPHKIK**S**GAEAKK  IRQDDT**S**SSINFL  RQDDTS**S**SINFLT  QDDTSS**S**INFLTR  NFLTRV**S**GIGPSA  VSGIGP**S**AARKFV  EVKKVD**S**EYIATV  IATVCG**S**FRRGAE  FRRGAE**S**SGDMDV  RRGAES**S**GDMDVL  VLLTHP**S**FTSEST  THPSFT**S**ESTKQP  PSFTSE**S**TKQPKL  FITDTL**S**KGETKF  GVCQLP**S**KNDEKE  VLYFTG**S**DIFNKN  EPLPVD**S**EKDIFD  ENEHQL**S**LRTVSL  LSLRTV**S**LGAGAK  LATLKM**S**VQPTVS  SVQPTV**S**LGGFEI  LRLKCG**S**GPVHIS  SGPVHI**S**GQHLVA  SISGKR**S**APGGGS  SAPGGG**S**KVPQKK  KAPVKK**S**IRDTPA  AKNAQK**S**NQNGKD  NQNGKD**S**KPSSTP  GKDSKP**S**STPRSK  KDSKPS**S**TPRSKG  PSSTPR**S**KGQESF  RLPLLV**S**AGVLLV  PCRALL**S**RGPVPG  FQPPPQ**S**EQPQQP  LGNLNK**S**PAAPLS  SPAAPL**S**PASSLL  APLSPA**S**SLLAGG  PLSPAS**S**LLAGGS  SLLAGG**S**GSRPSP  LAGGSG**S**RPSPEQ  GSGSRP**S**PEQATA  LLLPRR**S**LDSPAA  PRRSLD**S**PAALAE  PERERR**S**EEPPIS  SEEPPI**S**LDLTFH  LAQQAH**S**NRKLME  GPSLTG**S**PWPGTA  TAAPAA**S**YTPTPR  AAAAVP**S**EPGDPL  VLNLLF**S**PRATKP  PRATKP**S**ALSRAV  TKPSAL**S**RAVKVF  DLAALL**S**GVRQVS  VSEDVR**S**PAGPKV  WFPRKV**S**ELDKCH  LDHPGF**S**DQVYRQ  ALLERF**S**GYREDN  PQLEDV**S**RFLKER  PVAGLL**S**ARDFLA  ARDFLA**S**LAFRVF  QYIRHA**S**SPMHSP  YIRHAS**S**PMHSPE  ASSPMH**S**PEPDCC  RTFAQF**S**QDIGLA  QDIGLA**S**LGASDE  LASLGA**S**DEEIEK  EEIEKL**S**TLYWFT  YGAGLL**S**SYGELL  ELLHCL**S**EEPEIR  QDQTYQ**S**VYFVSE  QSVYFV**S**ESFSDA  VYFVSE**S**FSDAKD  FVSESF**S**DAKDKL  AKDKLR**S**YASRIQ  KLRSYA**S**RIQRPF  RIQRPF**S**VKFDPY  PQAVRR**S**LEGVQD  HLKTKP**S**DEEMLF  NELKGT**S**KEDAMK  GKYQLQ**S**QENFEA  KDIKGV**S**EIVQNG  FTITAG**S**KVIQNE  TFKNIK**S**VTELNG  GMACAI**S**ILGKSL  ISILGK**S**LADELA  MDLQHG**S**LFLQTP  VADKDY**S**VTANSK  YSVTAN**S**KIVVVT  RQQEGE**S**RLNLVQ  PQIVKY**S**PDCIII  CIIIVV**S**NPVDIL  YVTWKL**S**GLPKHR  KHRVIG**S**GCNLDS  SGCNLD**S**ARFRYL  KLGIHP**S**SCHGWI  LGIHPS**S**CHGWIL  LGEHGD**S**SVAVWS  GEHGDS**S**VAVWSG  SSVAVW**S**GVNVAG  VNVAGV**S**LQELNP  MGTDND**S**ENWKEV  HKMVVE**S**AYEVIK  NWAIGL**S**VADLIE  VADLIE**S**MLKNLS  SMLKNL**S**RIHPVS  SRIHPV**S**TMVKGM  ENEVFL**S**LPCILN  NARGLT**S**VINQKL  VAQLKK**S**ADTLWD  LTFREK**S**RIIQEV  RFNIPP**S**TLSTIL  IPPSTL**S**TILKNK  KRAILA**S**ERKYGV  RKYGVA**S**TCRKTN  RKTNKL**S**PYDKLE  MDDFTA**S**NGWLDR  RRHGVV**S**CSGVAR  HGVVSC**S**GVARAR  TPAAPA**S**PAAVPS  SPAAVP**S**EGSGGS  AVPSEG**S**GGSTTG  SEGSGG**S**TTGWRA  REEQPP**S**VAEGYA  VAEGYA**S**QDVFSA  FSATET**S**LWYDFL  QATQRL**S**VLLCAN  CANADG**S**EKLPPL  PLVAGK**S**AKPRAG  CDYTAN**S**KGGVTT  TRMAAE**S**RRVLLL  AQSLDT**S**GLRHVQ  LEGQDP**S**GLQLGL  ITTSLK**S**EGEEEE  EEGDVD**S**DEEEEE  EEEDEE**S**SSEGLE  EEDEES**S**SEGLEA  EDEESS**S**EGLEAE  VVEAGG**S**FGAYGA  LEGGED**S**DSDSEE  EDSDSD**S**EEEDDE  DEVPVP**S**FGEAMA  VKRYLT**S**FPIDDR  EPQPGG**S**QCVEHD  ATFLNA**S**QICDGL  HLMTVR**S**SVAADV  LMTVRS**S**VAADVI  VAADVI**S**LLLNGD  TGDNNT**S**YSRWAR  DNNTSY**S**RWARLD  PLCVAV**S**AAEATV  AEATVP**S**EPIWEE  AAAAAV**S**ITYGTP  QALPVG**S**SAAVAP  ALPVGS**S**AAVAPL  PGAWDC**S**VENGGC  LQADGR**S**CTASAT  GRSCTA**S**ATQSCN  TASATQ**S**CNDLCE  NPDQPG**S**YSCMCE  DQPGSY**S**CMCETG  DCILEP**S**PCPQRC  QPLNQT**S**YLCVCA  DPNTQA**S**CECPEG  ENGGFC**S**GVCHNL  CICGPD**S**ALARHI  IGTDCD**S**GKVDGG  KVDGGD**S**GSGEPP  DGGDSG**S**GEPPPS  SGEPPP**S**PTPGST  PSPTPG**S**TLTPPA  AVGLVH**S**GLLIGI  GLLIGI**S**IASLCL  IGISIA**S**LCLVVA  YKCAAP**S**KEVVLQ  LLVLPV**S**EANFLS  RKRRAN**S**LLEETK  FTAARQ**S**TNAYPD  NEDGYM**S**CKDGKA  NINGGC**S**QICDNT  NGFVML**S**NKKDCK  KDVDEC**S**LKPSIC  YRYNLK**S**KSCEDI  YNLKSK**S**CEDIDE  EDIDEC**S**ENMCAQ  LAQDQK**S**CEVVSV  KSCEVV**S**VCLPLN  FRLPEI**S**RFSAEF  PEISRF**S**AEFDFR  DFRTYD**S**EGVILY  VILYAE**S**IDHSAW  AESIDH**S**AWLLIA  LKNEHT**S**KITTGG  VEELEH**S**ISIKIA  ELEHSI**S**IKIAKE  FPRKVE**S**ELIKPI  GSYYPG**S**GIAQFH  IDYNNV**S**SAEGWH  TLNIRP**S**TGTGVM  TVPFAV**S**LVDSTS  DSTSEK**S**QDILLS  SQDILL**S**VENTVI  QALSLC**S**DQQSHL  LKIETI**S**HEDLQR  LPDVPF**S**ATPVNA  DLDEAI**S**KHNDIR  NDIRAH**S**CPSVWK  RAHSCP**S**VWKKTK  PKRAKA**S**KKSTDH  AKASKK**S**TDHPKY  TDHPKY**S**DMIVAA  EKNRAG**S**SRQSIQ  KNRAGS**S**RQSIQK  AGSSRQ**S**IQKYIK  IQKYIK**S**HYKVGE  VGENAD**S**QIKLSI  DSQIKL**S**IKRLVT  TKGVGA**S**GSFRLA  GVGASG**S**FRLAKS  SFRLAK**S**DEPKKS  SDEPKK**S**VAFKKT  ATPKKA**S**KPKKAA  KPKKAA**S**KAPTKK  AKPVKA**S**KPKKAK  VKPKAK**S**SAKRAG  KPKAKS**S**AKRAGK  EGNTLI**S**VDYEIF  QLQGPI**S**KVRHMQ  WLETRG**S**PKSHID  TRGSPK**S**HIDKAN  FSSELC**S**PQGHGV  MAQRVK**S**DREYAG  GLLHHM**S**LQDSGG  HMSLQD**S**GGQSRA  RAISPD**S**PISQSW  SWAEIT**S**QTEGLS  HAEDLN**S**GPLSKL  GPLSKL**S**LLIRER  QLRKTY**S**EQWQQL  ELTKTH**S**QDIEKL  DIEKLK**S**QYRALA  RALARD**S**AQAKRK  QEYLEI**S**SLVQDE  EYLEIS**S**LVQDEV  FLRQYG**S**APDVPP  CVTFDE**S**LLEEGE  LQDDRH**S**TSSSEQ  DDRHST**S**SSEQER  DRHSTS**S**SEQERE  TLEILK**S**HISGIF  ILKSHI**S**GIFRPK  DFLVRE**S**QGKQEY  KQEYVL**S**VLWDGL  RHFIIQ**S**LDNLYR  EGEGFP**S**IPLLID  NFGEVF**S**GRLRAD  TLVAVK**S**CRETLP  RILKQY**S**HPNIVR  GMEYLE**S**KCCIHR  NYGRYS**S**ESDVWS  TFSLGA**S**PYPNLS  EPGQRP**S**FSTIYQ  GQRPSF**S**TIYQEL  RCVGNG**S**VEWDGP  EWDGPP**S**PHWTLY  YSDGSS**S**ILSTNN  HTNYSF**S**PWHGFT  GGRKVM**S**ISIRLK  QIVCSA**S**SVDVNF  NVQGKH**S**TSMFFR  QGKHST**S**MFFRVV  SAYLNL**S**SEQNLI  TYLGPF**S**DHQPEP  LPRLKP**S**EAGRYS  SEAGRY**S**FLARNP  PYPEVL**S**QEPFHK  HKVTVQ**S**LLTVET  AHNSVG**S**GSWAFI  NSVGSG**S**WAFIPI  VVVACM**S**IMALLL  ESYEGN**S**YTFIDP  EKEALM**S**ELKIMS  MLGPSL**S**PGQDPE  VEMRPV**S**TSSNDS  STSSND**S**FSEQDL  SSNDSF**S**EQDLDK  RDLLHF**S**SQVAQG  GMAFLA**S**KNCIHR  RDIMND**S**NYIVKG  CVYTVQ**S**DVWSYG  LLWEIF**S**LGLNPY  TFQQIC**S**FLQEQA  SSRSGG**S**GSSSSE  SGGSGS**S**SSELEE  GSGSSS**S**ELEEES  LEEESS**S**EHLTCC  AALGCL**S**LPGSRG  CLSLPG**S**RGERPH  RGERPH**S**FGSNAV  SNAVNK**S**FAKSRQ  NKSFAK**S**RQMRSV  KSRQMR**S**VDVTLM  CELSSW**S**SWTTCD  ELSSWS**S**WTTCDP  GEPCNF**S**DKEVED  NDCGDQ**S**DEANCR  QYWGIG**S**LASGIN  INLFTN**S**FEGPVL  ILKEYE**S**YSDFER  KEYESY**S**DFERNV  VTEKMA**S**KSGFSF  EKMASK**S**GFSFGF  ASKSGF**S**FGFKIP  IFELGI**S**SQSDRG  FELGIS**S**QSDRGK  LGISSQ**S**DRGKHY  RRTKRF**S**HTKSVF  VFLHAR**S**DLEVAH  RLPLEY**S**YGEYRD  IEEVYV**S**LGVSVG  LVRGGA**S**EHITTL  TDFAYS**S**TVRQNM  EFQKEV**S**SCHCAP  FQKEVS**S**CHCAPC  VPVLKG**S**RCDCIC  GLACEV**S**YRKNTP  SNWSSC**S**GRRKTR  PPQNGG**S**PCSGPA  NGGSPC**S**GPASET  EVSKVA**S**HLEVNC  TTILHL**S**ENLLYT  NLLYTF**S**LATLMP  LGTLDL**S**HNQLQS  SHNQLQ**S**LPLLGQ  LTVLDV**S**FNRLTS  SFNRLT**S**LPLGAL  PKLEKL**S**LANNNL  LLLQEN**S**LYTIPK  PKGFFG**S**HLLPFA  DVKAMT**S**NVASVQ  MTSNVA**S**VQCDNS  YSWSTA**S**LDSQMP  DSQMPS**S**LHPTQE  FTLHME**S**ITFSKT  MESITF**S**KTPKST  FSKTPK**S**TTEPTP  TPSPTT**S**EPVPEP  TTPEPT**S**EPAPSP  TSEPAP**S**PTTPEP  IPTIAT**S**PTILVS  SPTILV**S**ATSLIT  ILVSAT**S**LITPKS  SLITPK**S**TFLTTT  TTTKPV**S**LLESTK  LQGHLE**S**SRNDPF  QGHLES**S**RNDPFL  FWLLFA**S**VVLILL  KPQALD**S**GQGAAL  LLFLRG**S**LPTFRS  SLPTFR**S**SLFLWV  RGQDLL**S**TVSIRY  DLLSTV**S**IRYSGH  LLLAAG**S**LGQKPQ  RPRRPA**S**PISTIQ  RPASPI**S**TIQPKA  LLVAVG**S**ACRFLQ  GTAMAV**S**TFRKLD  AETDYQ**S**FAVLYL  ERAGQL**S**VKLYAR  VKLYAR**S**LPVSDS  ARSLPV**S**DSVLSG  SLPVSD**S**VLSGFE  VSDSVL**S**GFEQRV  HLMGKK**S**TGESSS  KKSTGE**S**SSVSER  KSTGES**S**SVSERG  STGESS**S**VSERGS  GESSSV**S**ERGSLK  SVSERG**S**LKQQLR  LGNQQP**S**WDSEDS  QQPSWD**S**EDSSNF  SWDSED**S**SNFKDV  WDSEDS**S**NFKDVG  NFKDVG**S**KGKVGR  GKVGRL**S**APGSQR  RLSAPG**S**QREGRN  GQPGNG**S**AFLLAP  LLAPNG**S**HAPDHD  GMGIVM**S**LIVLAI  TNYFIT**S**LACADL  WCEFWT**S**IDVLCV  VLCVTA**S**IETLCV  RYFAIT**S**PFKYQS  SPFKYQ**S**LLTKNK  LMVWIV**S**GLTSFL  IVSGLT**S**FLPIQM  QAYAIA**S**SIVSFY  AYAIAS**S**IVSFYV  IASSIV**S**FYVPLV  IMVFVY**S**RVFQEA  LQKIDK**S**EGRFHV  WIGYVN**S**GFNPLI  PLIYCR**S**PDFRIA  GNTGEQ**S**GYHVEQ  HQGTVP**S**DNIDSQ  PSDNID**S**QGRNCS  IVGYKD**S**PSVWAA  GYKDSP**S**VWAAVP  LVGKDR**S**SFYVNG  VGKDRS**S**FYVNGL  LGGQKC**S**VIRDSL  CSVIRD**S**LLQDGE  MDLRTK**S**TGGAPT  VEQRIR**S**FPDFPT  VVFRDI**S**PVLKDP  VLKDPA**S**FRAAIG  YIAGLD**S**RGFLFG  GFLFGP**S**LAQELG  GPTLWA**S**YSLEYG  TLWASY**S**LEYGKA  EVLECV**S**LVELTS  SLVELT**S**LKGREK  LGLCLL**S**VGVWGQ  QTPYKV**S**ISGTTV  PYKVSI**S**GTTVIL  CPQYPG**S**EILWQH  DDKNIG**S**DEDHLS  SDEDHL**S**LKEFSE  LSLKEF**S**ELEQSG  FSELEQ**S**GYYVCY  VCYPRG**S**KPEDAN  MEMDVM**S**VATIVI  LLVYYW**S**KNRKAK  IFTDVN**S**ILRYLA  DHWLEF**S**ATKLSS  FSATKL**S**SCDSFT  SATKLS**S**CDSFTS  ELNHCL**S**LRTYLV  LVGNSL**S**LADLCV  AQQAFQ**S**VGTKWD  GTKWDV**S**TTKARV  RFPPEA**S**GYLHIG  REQRID**S**KHRKNP  EEMKKG**S**QFGQSC  LNNTVL**S**KRKLTW  IAAQGS**S**RSVVNM  LKPVWY**S**PKVFIE  QYVNKN**S**KHEELM  QPYEPV**S**PYSCKE  EMPTSG**S**KEKTKV  KERPTP**S**LNNNCT  CTTSED**S**LVLYNR  QNISSN**S**SASILE  PGQPPL**S**QSSDSS  LKAQYK**S**LIGVEY  VEYKPV**S**ATGAED  SQVITK**S**EMIEYH  IEYHDI**S**GCYILR  PMFVSQ**S**ALEKEK  LWQEGH**S**AFATME  AIQGGT**S**HHLGQN  QFAYQN**S**WGLTTR  DLRDNY**S**PGWKFN  PSMGAK**S**LCIPFK  EIAQLM**S**LIINTF  IINTFY**S**NKEIFL  FLRELI**S**NSSDAL  RELISN**S**SDALDK  ELISNS**S**DALDKI  DKIRYE**S**LTDPSK  ESLTDP**S**KLDSGK  LGTIAK**S**GTKAFM  QAGADI**S**MIGQFG  EQYAWE**S**SAGGSF  QYAWES**S**AGGSFT  ESSAGG**S**FTVRTD  EIVKKH**S**QFIGYP  YGEFYK**S**LTNDWE  LAVKHF**S**VEGQLE  IRGVVD**S**EDLPLN  KFYEQF**S**KNIKLG  QNRKKL**S**ELLRYY  RYYTSA**S**GDEMVS  SGDEMV**S**LKDYCT  KDQVAN**S**AFVERL  EGKTLV**S**VTKEGL  VEKVVV**S**NRLVTS  SNRLVT**S**PCCIVT  QALRDN**S**TMGYMA  EINPDH**S**IIETLR  ADKNDK**S**VKDLVI  YETALL**S**SGFSLE  ETALLS**S**GFSLED  LLSSGF**S**LEDPQT  PTADDT**S**AAVTEE  NKTDPR**S**MNSRVF  DPRSMN**S**RVFIGN  TLVVKK**S**DVEAIF  DVEAIF**S**KYGKIV  GKIVGC**S**VHKGFA  KAGVKR**S**AAEMYG  AAEMYG**S**VTEHPS  SPSPLL**S**SSFDLD  PSPLLS**S**SFDLDY  SPLLSS**S**FDLDYD  YYDRMY**S**YPARVP  ARAVVP**S**KRQRVS  TSRRGK**S**GFNSKS  GKSGFN**S**KSGQRG  SGFNSK**S**GQRGSS  KSGQRG**S**SKSGKL  SGQRGS**S**KSGKLK  QRGSSK**S**GKLKGD  IKQKVD**S**LLENLE  KIEKEQ**S**KQAVEM  EEEQSS**S**SVKKDE  KSKENK**S**PAIKYR  NTPEPV**S**TSVSHY  PEPVST**S**VSHYGA  PVSTSV**S**HYGAEP  AEPTTV**S**PCPSSS  TVSPCP**S**SSAKGT  VSPCPS**S**SAKGTA  SPCPSS**S**AKGTAV  GTAVNF**S**SLSMTP  TAVNFS**S**LSMTPF  VNFSSL**S**MTPFGG  MTPFGG**S**SGVTPF  TPFGGS**S**GVTPFG  PFGGAS**S**SFSVVP  FGGASS**S**FSVVPS  GASSSF**S**VVPSSY  SFSVVP**S**SYPAGL  FSVVPS**S**YPAGLT  RTTEDL**S**FKKGER  DWWEAR**S**IATGKN  KNGYIP**S**NYVAPA  YVAPAD**S**IQAEEW  IFLVRE**S**ETTKGA  TTKGAY**S**LSIRDW  KGAYSL**S**IRDWDE  WEIPRE**S**LRLEVK  PLYAVV**S**EEPIYI  IVTEFM**S**KGSLLD  EFMSKG**S**LLDFLK  GRFTIK**S**DVWSFG  IKSDVW**S**FGILQT  PQGCPE**S**LHELMN  TFEYIQ**S**FLEDYF  ALGLYF**S**RDAYWE  YLKVFL**S**PTSLRE  VFLSPT**S**LREGEC  CARVYF**S**FFNTSF  FSFFNT**S**FPACSS  TSFPAC**S**SLKPRE  ADVVPA**S**GELVRR  VLNRNL**S**ISENRT  NRNLSI**S**ENRTMQ  AVLVND**S**DFQGPG  LLHFNV**S**VLPVSL  VSVLPV**S**LHLPST  VSLHLP**S**TYSLSV  PSTYSL**S**VSRRAR  ENCQAF**S**GINVQY  QYKLHS**S**GANCST  ATDQQT**S**RQAQAQ  EAGCPL**S**CAVSKR  ECGGLG**S**PTGRCE  LFSFIV**S**VLLSAF  AQAFPV**S**YSSSGA  SGARRP**S**LDSMEN  KENASP**S**ELRDLL  ELRDLL**S**EFNVLK  RGFLRE**S**RKVGPG  YLGSGG**S**RNSSSL  TMGDLI**S**FAWQIS  DVYEED**S**YVKRSQ  DSYVKR**S**QGRIPV  PVFADI**S**KDLEKM  YLDLAA**S**TPSDSL  ASTPSD**S**LIYDDG  LPRALP**S**TWIENK  AFSQAR**S**SSTGSS  GGGGQE**S**QPSPLA  NENSNN**S**QGPSQS  SQGPSQ**S**GGTGEL  SGATPT**S**KEQSGS  SKEQSG**S**STNGSN  KEQSGS**S**TNGSNG  TNGSNG**S**ESSKNR  QVQQDG**S**GQIQII  TLLPVN**S**VSAATL  ATLTPS**S**QAVTIS  QAVTIS**S**SGSQES  AVTISS**S**GSQESG  GSQESG**S**QPVTSG  VSSQAS**S**SSFFTN  SSQASS**S**SFFTNA  SQASSS**S**FFTNAN  FFTNAN**S**YSTTTT  STTTTT**S**NMGIMN  IMNFTT**S**GSSGTN  NQTSGG**S**LQAGQQ  LQAVPN**S**GPIIIR  GPNGQV**S**WQTLQL  LGQTSS**S**NTTLTP  PIASAA**S**IPAGTV  LQTINL**S**ALGTSG  LSALGT**S**GIQVHP  PFMCTW**S**YCGKRF  PKRFMR**S**DHLSKH  GPGVAL**S**VGTLPL  GAGSEG**S**GTATPS  SGSGGG**S**PTSLWG  KAEDYR**S**YRFPKL  RVAGLE**S**LGDLFP  LSTVDW**S**LILDAV  CQKMCP**S**TCGKRA  FCANIL**S**AESSDS  NILSAE**S**SDSEGF  ILSAES**S**DSEGFV  IRNGSQ**S**MYCIPC  KTKTID**S**VTSAQM  GTKGRQ**S**KGDINT  ERASCE**S**DVLHFT  DYRDLI**S**FTVYYK  HIRGAK**S**EILYIR  TNASVP**S**IPLDVL  LDVLSA**S**NSSSQL  VKWNPP**S**LPNGNL  LPNGNL**S**YYIVRW  TTMSSR**S**RNTTAA  KERTVI**S**NLRPFT  AEKLGC**S**ASNFVF  EPRPEN**S**IFLKWP  DQRECV**S**RQEYRK  ARIQAT**S**LSGNGS  SLSGNG**S**WTDPVF  VNPEYF**S**AADVYV  TVNEAA**S**MRERIE  EFLNEA**S**VMKEFN  RLLGVV**S**QGQPTL  LAPPSL**S**KMIQMA  EVSFYY**S**EENKLP  EPENME**S**VPLDPS  PLDPSA**S**SSSLPL  TPPPIS**S**YGEPCC  CLRPLP**S**QGAPSV  EGPLFS**S**PRSAVK  QTVIRT**S**PSSLVA  QKGPSP**S**FGVQPC  MIPHPQ**S**RGPFPT  PLEGDM**S**SPNSTG  EKREPE**S**VYETDC  CRWDGC**S**QEFDSQ  CHWGGC**S**RELRPF  LKTHLR**S**HTGEKP  CEHEGC**S**KAFSNA  GCSKAF**S**NASDRA  HQNRTH**S**NEKPYV  PRAPSI**S**TVEPKR  GPIREE**S**RLTVPE  GAQSSC**S**SDHSPA  DHSPAG**S**AANTDS  RVGPPV**S**LERRSS  SLERRS**S**SSSSIS  ERRSSS**S**SSISSA  YTVSRR**S**SLASPF  TVSRRS**S**LASPFP  PPENGA**S**SLPGLM  TSPTAA**S**SLDRIG  GVTRRA**S**DPAQAA  GTSMVG**S**GLNPYM  GARGPG**S**LPLGPG  CLNAHP**S**EGPPHP  PPQYLQ**S**GPYTQP  LGGSQV**S**PSRAKA  GSQVSP**S**RAKAPV  HDQRGS**S**GHTPPP  ASIAVV**S**IPRQLP  QLPGSH**S**EAGVQG  AGVQGL**S**AGDDSE  DDSELG**S**HCVAQT  GLELLA**S**GDPLPS  SGDPLP**S**ASQNAE  DPLPSA**S**QNAEMI  EMIETG**S**DCVTQA  DPPALA**S**KNAEVT  QPMNAA**S**GAAMSL  ASGAAM**S**LAGAEK  RLDYLS**S**LKVKGL  FDSLLQ**S**AKKKSI  QSAKKK**S**IRVILD  GENSWF**S**TQVDTV  IAGTNS**S**DLQQIL  DLQQIL**S**LLESNK  ILSLLE**S**NKDLLL  KDLLLT**S**SYLSDS  SSYLSD**S**GSTGEH  YLSDSG**S**TGEHTK  TGEHTK**S**LVTQYL  TGNRWC**S**WSLSQA  NRWCSW**S**LSQARL  MLWDES**S**FPDIPG  DIPGAV**S**ANMTVK  MTVKGQ**S**EDPGSL  QSEDPG**S**LLSLFR  SLFRRL**S**DQRSKE  RLSDQR**S**KERSLL  FGDVGL**S**AGLQAS  SAGLQA**S**DLPASA  ASDLPA**S**ASLPAK  ATAEEN**S**PKMRVI  RVGTRK**S**QLARIQ  ARIQTD**S**VVATLK  VATLKA**S**YPGLQF  FEIIAM**S**TTGDKI  ILDTAL**S**KIGEKS  SKIGEK**S**LFTKEL  VDLVVH**S**LKDLPT  ETLPEK**S**VVGTSS  KSVVGT**S**SLRRAA  SVVGTS**S**LRRAAQ  PHLEFR**S**IRGNLN  DEQQEF**S**AIILAT  HLEGGC**S**VPVAVH  LTGGVW**S**LDGSDS  VWSLDG**S**DSIQET  SLDGSD**S**IQETMQ  AQNLGI**S**LANLLL  LANLLL**S**KGAKNI  NANTFI**S**PQQRWR  ERIRER**S**KPVHEL  YAGPNG**S**QFGFSL  GSQFGF**S**LDFHKD  LDFHKD**S**HGRVAI  RQGLGA**S**VVSWSD  ASVVSW**S**DVIVAC  FLAQPE**S**GRRAEY  GRRAEY**S**PCRGNT  CRGNTL**S**RIYVEN  YVENDF**S**WDKRYC  YCEAGF**S**SVVTQA  PVADIF**S**SYRPGI  WHVSSQ**S**LSFDSS  SLSFDS**S**NPEYFD  RGEQMA**S**YFGHSV  APLYME**S**RADRKL  LYGRFG**S**AIAPLG  APYGGP**S**GRGQVL  LVFLGQ**S**EGLRSR  GLRSRP**S**QVLDSP  PSQVLD**S**PFPTGS  GSAFGF**S**LRGAVD  LNPAVK**S**CVLPQT  QTKTPV**S**CFNIQM  RVLLLG**S**QQAGTT  DFRDKL**S**PIVLSL  LSPIVL**S**LNVSLP  GIAMLV**S**VGNLEE  NSQNPN**S**KIVLLD  GNSFPA**S**LVVAAE  VNGLHL**S**IHLPGQ  PGQSQP**S**DLLYIL  AWFNVS**S**LPYAVP  YAVPPL**S**LPRGEA  KMLAAV**S**RVLSGA  AVSRVL**S**GASQKP  RVLSGA**S**QKPASR  ASQKPA**S**RVLVAS  SRVLVA**S**RNFAND  TFTRGL**S**VREILA  AKGKGG**S**MHMYAK  VERAAA**S**TDYYKR  AAAYCR**S**GKGPIL  EIQEVR**S**KSDPIM  QEVRSK**S**DPIMLL  KDRMVN**S**NLASVE  VNSNLA**S**VEELKE  LGYHIY**S**SDPPFE  GYHIYS**S**DPPFEV  YLVKKG**S**VFNTWK  EFYKKK**S**DNSPKG  KKKSDN**S**PKGMIP  MIPLKG**S**TLTSPC  KGSTLT**S**PCQDFG  LGALYL**S**MKDTEK  VIDWLV**S**NQSVRN  WLVSNQ**S**VRNRQE  EGLMIA**S**SLLNEG  GLMIAS**S**LLNEGY  QPAGDM**S**KSAVDG  AGDMSK**S**AVDGTA  FYYFPD**S**GFFCEE  FFCEEN**S**SDDDVI  FCEENS**S**DDDVIL  RGCVVT**S**VESNSN  VVTSVE**S**NSNGRK  TSVESN**S**NGRKSE  NSNGRK**S**EEENLF  FPCQDC**S**SKANLS  SSKANL**S**GGVWKD  YDDQLI**S**CGSVNR  QLISCG**S**VNRGTC  HTADIQ**S**EVHCIF  EVHCIF**S**PQIEEP  INSGLH**S**YMEMPL  ARQIGA**S**LNDDIL  AQSKPD**S**AEPMDR  TLLRNS**S**GCEARR  LFMGQF**S**EVLLTS  FMQVVV**S**RSGPST  VNFLLD**S**HPVSPE  GCRHFQ**S**CSQCLS  RSEECL**S**GTWTQQ  VDPVIT**S**ISPKYG  YLNSGN**S**RHISIG  GNSRHI**S**IGGKTC  TPAQTI**S**TEFAVK  RETSIF**S**YREDPI  PTKSFI**S**GGSTIT  VGKNLN**S**VSVPRM  DLLKLN**S**ELNIEW  LIAGVV**S**ISTALL  RLVSAR**S**VSPTTE  PTTEMV**S**NESVDY  HVVIGP**S**SLIVHF  GMKYLA**S**KKFVHR  TKSDVW**S**FGVLLW  KAEMRP**S**FSELVS  EMRPSF**S**ELVSRI  ELVSRI**S**AIFSTF  PYPSLL**S**SEDNAD  VRWCAT**S**DPEQHK  LCVRGT**S**ADHCVQ  DQEVGT**S**YYAVAV  VAVVRR**S**SHVTID  AVVRRS**S**HVTIDT  TLKGVK**S**CHTGIN  VESGRL**S**VMGCDV  AGETSY**S**ESLCRL  ETSYSE**S**LCRLCR  RLCRGD**S**SGEGVC  EGVCDK**S**PLERYY  ERYYDY**S**GAFRCL  VAFVKH**S**TVLENT  WGQALL**S**QDFELL  LLCRDG**S**RADVTE  EGQRLF**S**HEGSSF  DLLFKD**S**TSELVP  LRWCVL**S**TPEIQK  QCVSAK**S**PQHCME  PEDSSN**S**YYVVAV  AVVRRD**S**SHAFTL  CHAGFG**S**PAGWDV  DVLTAV**S**EFFNAS  SEFFNA**S**CVPVNN  NPKNYP**S**SLCALC  NKCVGN**S**QERYYG  NTNGHN**S**EPWAAE  WAAELR**S**EDYELL  GFKMFD**S**SNYHGQ  FKMFDS**S**NYHGQD  MSSQQC**S**GAAAPA  WGLLVL**S**WSCLQG  CLQGPC**S**VFSPVS  GPCSVF**S**PVSAME  SVFSPV**S**AMEPLG  PNQEQV**S**PLTLLK  GQTALK**S**PPGVCS  SPPGVC**S**RDPTPE  SLVAQT**S**TCPNLI  CPNLIL**S**PLSVAL  SVALAL**S**HLALGA  QVLHAG**S**GPCLPH  CLPHLL**S**RLCQDL  EDFLEQ**S**EQLFGA  FGAKPV**S**LTGKQE  KIQEFL**S**GLPEDT  RNKFDP**S**LTQRDS  SLTQRD**S**FHLDEQ  PFKNNM**S**FVVLVP  QVLANL**S**WDTLHP  DLVATL**S**QLGLQE  PDLRGI**S**EQSLVV  RGISEQ**S**LVVSGV  EQSLVV**S**GVQHQS  SGVQHQ**S**TLELSE  QSTLEL**S**EVGVEA  ATSIAM**S**RMSLSS  MSRMSL**S**SFSVNR  SRMSLS**S**FSVNRP  MSLSSF**S**VNRPFL  LPLFVG**S**VRNPNP  VRNPNP**S**APRELK  LKEQQD**S**PGNKDF  NKDFLQ**S**LKGFPR  GGVAKA**S**GGETRD  EELRPG**S**LERECK  CKEEQC**S**FEEARE  TKLFWI**S**YSDGDQ  LFWISY**S**DGDQCA  DGDQCA**S**SPCQNG  GDQCAS**S**PCQNGG  PCQNGG**S**CKDQLQ  CKDQLQ**S**YICFCL  GCEQYC**S**DHTGTK  HTGTKR**S**CRCHEG  RCHEGY**S**LLADGV  LLADGV**S**CTPTVE  LEKRNA**S**KPQGRI  NTIWVV**S**AAHCFD  LGEHDL**S**EHDGDE  HDGDEQ**S**RRVAQV  AQVIIP**S**TYVPGT  LPERTF**S**ERTLAF  LAFVRF**S**LVSGWG  VRFSLV**S**GWGQLL  QDCLQQ**S**RKVGDS  SRKVGD**S**PNITEY  MFCAGY**S**DGSKDS  AGYSDG**S**KDSCKG  SDGSKD**S**CKGDSG  DSCKGD**S**GGPHAT  YLTGIV**S**WGQGCA  GVYTRV**S**QYIEWL  LQKLMR**S**EPRPGV  WQCAPC**S**AEKLAL  CPPVSA**S**CSEVTR  PVSASC**S**EVTRSA  CSEVTR**S**AGCGCC  RCARGL**S**CRALPG  GACVQE**S**DASAPH  VQESDA**S**APHAAE  PSEEDH**S**ILWDAI  ISTYDG**S**KALHVT  LYRVVE**S**LAKAQE  KNGFYH**S**RQCETS  SRQCET**S**MDGEAG  FLWEAE**S**HAASRP  TQHLAA**S**PLCTPS  SPLCTP**S**RAAFMT  GRYPVR**S**GMASWS  VRSGMA**S**WSRTGV  VFLFTA**S**SGGLPT  FLFTAS**S**GGLPTD  LKDQGY**S**TALIGK  KWHLGM**S**CHSKTD  LGMSCH**S**KTDFCH  NYFYGI**S**LTNLRD  CKPGEG**S**VFTTGF  PLGVFF**S**LLFLAA  IIQQPM**S**YDNLTQ  PFLLVL**S**YLHVHT  VHTALF**S**SKDFAG  HTALFS**S**KDFAGK  KDFAGK**S**QHGVYG  VEEMDW**S**VGQILN  TLIYFT**S**DQGAHV  AHVEEV**S**SKGEIH  GEIHGG**S**NGIYKG  KIDEPT**S**NMDIFP  PLLEGK**S**QRSDHE  EGKSQR**S**DHEFLF  HPQNST**S**IWKAFF  NFNPVG**S**NGCFAT  VCFCFG**S**YVTHHD  PLLFDI**S**KDPRER  NPLTPA**S**EPRFYE  EVPDQF**S**WNNFLW  LQLCCP**S**TGLSCQ  CPSTGL**S**CQCDRE  YIYKRK**S**DGIYII  ENPADV**S**VISSRN  ADVSVI**S**SRNTGQ  DVSVIS**S**RNTGQR  QPLTEA**S**YVNLPT  ALCNTD**S**PLRYVD  NNKGAH**S**VGLMWW  RMRGTI**S**REHPWE  PEVADW**S**EGVQVP  EGVQVP**S**VPIQQF  FPTEDW**S**AQPATE  PATEDW**S**AAPTAQ  GARMFG**S**LLLFAL  AGVAPL**S**WDLPEP  DLPEPR**S**RASKIR  EPRSRA**S**KIRVHS  SKIRVH**S**RGNLWA  HFMGKK**S**LEPSSP  KKSLEP**S**SPSPLG  KSLEPS**S**PSPLGT  LEPSSP**S**PLGTAP  GTAPHT**S**LRDQRL  DQRLQL**S**HDLLGI  KKALGV**S**LSRPAP  ALGVSL**S**RPAPQI  KDELKK**S**LYAIFS  SLYAIF**S**QFGQIL  ILDILV**S**RSLKMR  DILVSR**S**LKMRGQ  VIFKEV**S**SATNAL  IFKEVS**S**ATNALR  ATNALR**S**MQGFPF  QYAKTD**S**DIIAKM  EKRKPK**S**QETPAT  PPAQPL**S**ENPPNH  TNELML**S**MLFNQF  PGGCQI**S**GRGARG  RPRRHP**S**VNPRSR  PSVNPR**S**RAAGSP  RSRAAG**S**PRTRGR  RTEERP**S**GSRLGD  EERPSG**S**RLGDRG  APAARG**S**RPGPAG  GTMAAG**S**ITTLPA  LPEDGG**S**GAFPPG  DGVREK**S**DPHIKL  EERGVV**S**IKGVCA  DGRLLA**S**KCVTDE  FFERLE**S**NNYNTY  NYNTYR**S**RKYTSW  RSRKYT**S**WYVALK  GQYKLG**S**KTGPGQ  LADQGQ**S**WKEEVV  ETWQEG**S**LKASCL  EGSLKA**S**CLYGQL  DLTLYQ**S**NTILRH  LRCKYI**S**LIYTNY  PFETLL**S**QNQGGK  IVGDQI**S**FADYNL  DAFPLL**S**AYVGRL  AYVGRL**S**ARPKLK  LKAFLA**S**PEYVNL  ACGLVA**S**NLNLKP  LNLGKD**S**NNLCLH  NTIVCN**S**KDGGAW  FPFQPG**S**VAEVCI  GRGALG**S**RCVQAF  RNWWVA**S**VDVVEN  VENEEA**S**ASIIVK  NEEASA**S**IIVKMT  IVKMTD**S**FTEQAD  AGGNAK**S**KSLFKN  GNAKSK**S**LFKNCD  DLMWKQ**S**IWTSTI  KQSIWT**S**TISSHL  IWTSTI**S**SHLATK  WTSTIS**S**HLATKH  VHQLCQ**S**LAGKNS  SLAGKN**S**GMPPGA  TPMNRK**S**MPEADF  MPEADF**S**SWTPLE  PEADFS**S**WTPLEF  TGKNRP**S**SGSLIQ  GKNRPS**S**GSLIQV  NRPSSG**S**LIQVVT  KAAEDR**S**KQLEDE  LEDELV**S**LQKKLK  AEADVA**S**LNRRIQ  EKAADE**S**ERGMKV  GMKVIE**S**RAQKDE  EERAEL**S**EGKCAE  AQAEKY**S**QKEDRY  EEIKVL**S**DKLKEA  AEFAER**S**VTKLEK  VTKLEK**S**IDDLED  LKYKAI**S**EELDHA  PTTWLG**S**LLLLVC  SITEEV**S**EYCSHM  EVSEYC**S**HMIGSG  LQRLID**S**QMETSC  DSQMET**S**CQITFE  VQLQEL**S**LRLKSC  KDWNIF**S**KNCNNS  SFAECS**S**QDVVTK  PKAIPS**S**DPASVS  EGTEGS**S**LLPGEQ  PRSTCQ**S**FEPPET  SPQPRP**S**VGAFNP  MEDILD**S**AMGTNW  EASGEA**S**EIPVPQ  PQGTEL**S**PSRPGG  SRPGGG**S**MQTEPA  TEPARP**S**NFLSAS  RPSNFL**S**ASSPLP  SNFLSA**S**SPLPAS  QKTDHP**S**ALLRDP  PGSPRI**S**SLRPQG  GSPRIS**S**LRPQGL  SNPSTL**S**AQPQLS  QPQLSR**S**HSSGSV  ELEGRR**S**TRDRRS  STRDRR**S**PAEPEG  PLPRFN**S**VPLTDT  TGHERQ**S**EGSFSP  ERQSEG**S**FSPQLQ  FHLLVP**S**VILVLL  YRWRRR**S**HQEPQR  EPQRAD**S**PLEQPE  LEQPEG**S**PLTQDD  TFVLTC**S**GSAPVV  VVWERM**S**QEPPQE  AQDGTF**S**SVLTLT  QDGTFS**S**VLTLTN  FCTHND**S**RGLETD  GIFEDR**S**YICKTT  YRLQVS**S**INVSVN  MPYHIR**S**ILHIPS  SAELED**S**GTYTCN  NRTLGD**S**SAGEIA  QPNIIW**S**ACRDLK  VIVVPH**S**LPFKVV  VVLTII**S**LIILIM  RWKVIE**S**VSSDGH  KVIESV**S**SDGHEY  ATAHGL**S**HSQATM  AHGLSH**S**QATMKV  SELKIM**S**HLGPHL  DKRRPP**S**AELYSN  PSAELY**S**NALPVG  GGYMDM**S**KDESVD  YADIES**S**NYMAPY  YDNYVP**S**APERTC  ATLINE**S**PVLSYM  NESPVL**S**YMDLVG  SLYTTL**S**DVWSFG  RSPLDT**S**SVLYTA  SPLDTS**S**VLYTAV  PLEGSP**S**LASSTL  GSPSLA**S**STLNEV  SPSLAS**S**TLNEVN  LNEVNT**S**STISCD  STISCD**S**PLEPQD  LLLLGG**S**AARPAP  PRARRH**S**DGTFTS  SDGTFT**S**ELSRLR  TFTSEL**S**RLREGA  GLVGKR**S**EQDAEN  EQDAEN**S**MAWTRL  MAWTRL**S**AGLLCP  AGLLCP**S**GSNMPI  LLCPSG**S**NMPILQ  PLDGTW**S**PWLPPG  PPGPMV**S**EPAGAA  QGTLAQ**S**IKGNHL  DYQEDG**S**VLLTCD  KKWNLG**S**NAKDPR  MYQCKG**S**QNKSKP  KGSQNK**S**KPLQVY  LNAATI**S**GFLFAE  LFAEIV**S**IFVLAV  REDDQY**S**HLQGNQ  WCIVLF**S**LLAWVY  MYGEIL**S**PNYPQA  YPQAYP**S**EVEKSW  PSEVEK**S**WDIEVP  HLDIEL**S**ENCAYD  ENCAYD**S**VQIISG  DSVQII**S**GDTEEG  LCGQRS**S**NNPHSP  SSNNPH**S**PIVEEF  IFKSDF**S**NEERFT  FVDVPC**S**HFCNNF  IGGYFC**S**CPPEYF  NCGVNC**S**GDVFTA  LIGEIA**S**PNYPKP  KPYPEN**S**RCEYQI  AGNCLD**S**LVFVAG  LNIETK**S**NALDII  KEDTPN**S**VWEPAK  GRVGAT**S**FYSTCQ  FYSTCQ**S**NGKWSN  DCGIPE**S**IENGKV  KVEDPE**S**TLFGSV  ESTLFG**S**VIRYTC  QRIIGG**S**DADIKN  PTMYVG**S**TSVQTS  MYVGST**S**VQTSRL  TSRLAK**S**KMLTPE  KMGPTV**S**PICLPG  ICLPGT**S**SDYNLM  CLPGTS**S**DYNLMD  GDLGLI**S**GWGRTE  GEKGMD**S**CKGDSG  DSCKGD**S**GGAFAV  RVEYAK**S**GRASCK  AKSGRA**S**CKKCSE  CKKCSE**S**IPKDSL  KGQVRL**S**KKMVDP  GFRPEY**S**ASQLKG  RPEYSA**S**QLKGFS  SQLKGF**S**LLATED  EVAKKK**S**KKEKDK  KEKDKD**S**KLEKAL  AATPPP**S**TASAPA  PPPSTA**S**APAAVN  AVNSSA**S**ADKPLS  ANIRVV**S**EDFLQD  DFLQDV**S**ASTKSL  FLAHIL**S**PWGAEV  KSGAAL**S**KKSKGQ  AAVDPD**S**GLEHSA  RYWIFR**S**WGRVGT  VGTVIG**S**NKLEQM  MPLGKL**S**KRQIQA  EVQQAV**S**QGSSDS  LLNNAD**S**VQAKVE  RGGSDD**S**SKDPID  GGSDDS**S**KDPIDV  KVVDRD**S**EEAEII  RLLWHG**S**RTTNFA  YFADMV**S**KSANYC  ADMVSK**S**ANYCHT  KHASHI**S**KLPKGK  LPKGKH**S**VKGLGK  PLGTGI**S**SGVNDT  LGTGIS**S**GVNDTS  SGVNDT**S**LLYNEY  VTVATG**S**QWFAGT  DDYIYL**S**LVGSAG  YLSLVG**S**AGCSEK  VGSAGC**S**EKHLLD  ERGAVD**S**YDVTVD  NPGFPL**S**IDAKCH  RDIQFD**S**EKGVDF  DFVLNY**S**KAMENL  FMHMFQ**S**SWNDFA  MHMFQS**S**WNDFAD  KIFVKI**S**NTISER  KISNTI**S**ERVMNH  TEMVEC**S**LERQLS  NPIFLP**S**DAKYDW  AKIWVR**S**SDFHVH  KIWVRS**S**DFHVHQ  LRTHLV**S**EVFGIA  KDLTYA**S**LCFPEA  KARGME**S**KEDIPY  GMRGRK**S**SGFPKS  SSGFPK**S**VKSREQ  FPKSVK**S**REQLSE  KSREQL**S**EYLTVV  VVIFTA**S**AQHAAV  GQYDWC**S**WIPNAP  LPDRGR**S**CWHLGA  GAVWAL**S**QFQENE  NLEAIV**S**VIAERN  LPYYYL**S**PDRIPN  GPAVAN**S**VARKHG  RGVTKR**S**LSPHRP  PQQWYL**S**GVTQRD  GHGIVV**S**ILDDGI  NYDPGA**S**FDVNDQ  DAVEAR**S**LGLNPN  GRGGLG**S**IFVWAS  SIFVWA**S**GNGGRE  CDGYTN**S**IYTLSI  NSIYTL**S**ISSATQ  YTLSIS**S**ATQFGN  RQKCTE**S**HTGTSA  GVGRKV**S**HSYGYG  GRKVSH**S**YGYGLL  QARLTL**S**YNRRGD  SPMGTR**S**TLLAAR  AFMTTH**S**WDEDPS  LEIENT**S**EANNYG  LPVPPE**S**SGCKTL  PVPPES**S**GCKTLT  CKTLTS**S**QACVVC  VCEEGF**S**LHQKSC  VLDTHY**S**TENDVE  VETIRA**S**VCAPCH  CAPCHA**S**CATCQG  SCPSHA**S**LDPVEQ  PVEQTC**S**RQSQSS  QTCSRQ**S**QSSRES  RAGLLP**S**HLPEVV  EVVAGL**S**CAFIVL  MDRGLI**S**YKGLPP  EIVDTC**S**LASPAS  DTCSLA**S**PASVCR  SLASPA**S**VCRTKH  HLHLRC**S**VDFTRR  ALGERQ**S**YKGSPM  GSPMEI**S**LPIALS  EIVIEI**S**FETSPK  FETSPK**S**SALQWL  ETSPKS**S**ALQWLT  LTPEQT**S**GKEHPY  EHPYLF**S**QCQAIH  TYTAEV**S**VPKELV  ELVALM**S**AIRDGE  PDPEDP**S**RKIYKF  VVGALE**S**RQIGPR  PRTLVW**S**EKEQVE  KEQVEK**S**AYEFSE  KSAYEF**S**ETESML  LLVLPP**S**FPYGGM  AGDKSL**S**NVIAHE  VIAHEI**S**HSWTGN  AHEISH**S**WTGNLV  WGELQN**S**VKTFGE  DPDVAY**S**SVPYEK  PDVAYS**S**VPYEKG  WKDFLY**S**YFKDKV  KEDDLN**S**FNATDL  FNAINN**S**EIRFRW  LRLCIQ**S**KWEDAI  LAAFDK**S**HDQAVR  YQEHKA**S**MHPVTA  PRLLLF**S**PSVVHL  SPWLKD**S**LSRTTN  WLKDSL**S**RTTNIQ  DGLESN**S**STQFEV  EEAELT**S**WYFVSS  MSGSPA**S**GIPVKV  GGPGFL**S**IERPDS  SIERPD**S**RPPRVG  YYYMIL**S**RGQIVF  PKRTLT**S**VSVFVD  RTLTSV**S**VFVDHH  YAAGSK**S**HKPLNM  AAGLAF**S**DGDQWT  GDQWTL**S**RKRLSC  KLGQYA**S**PTAKRC  CREPFL**S**CCQFAE  CCQFAE**S**LRKKSR  DDIPVR**S**FFPENW  WEIHGL**S**LSKTKG  TAAAAV**S**LKVVAR  PVGDAV**S**KVLQIE  PLDTLG**S**EGALSP  SPGGVA**S**LLRLPR  DNLYWG**S**VTGSQS  EMADQA**S**AWLTRQ  WLTRQG**S**FQGGFR  FQGGFR**S**TQDTVI  LNVTLS**S**TGRNGF  DLEKLT**S**LSDRYV  FDSVPT**S**RECVGF  GLVQPA**S**ATLYDY  PRVIVG**S**ARARPP  QLAAEE**S**PRPRVI  RPRVIV**S**SPWPAV  PRTPVG**S**PWPRVV  ADADPA**S**APSQGA  DPASAP**S**QGALQG  RRQDEH**S**GTRAEG  GTRAEG**S**RPGGAA  EKQEAK**S**GILEAA  YHYEPH**S**VHGVHG  PPALSG**S**PVISDI  ISLIRL**S**PHPAGP  HSSPTL**S**MISAAR  RVTPRL**S**RKRALS  LSISPL**S**DASLDL  VAYINN**S**RSSSAA  TNQNKQ**S**SESAVS  SSESAV**S**STVNPV  SESAVS**S**TVNPVA  SGLCQS**S**PGAQSS  SPGAQS**S**CSSEPS  GAQSSC**S**SEPSPL  SALAAP**S**AGGLQL  YFSSRR**S**SEASPL  NASSAD**S**YDPIST  TDASRR**S**SEASQC  YSPRPP**S**ISENVA  PRPPSI**S**ENVAME  LGFGAP**S**SLNKNN  GQYPGY**S**PQGLQA  QVHPQL**S**PSTISG  LSPSTI**S**GALNQF  ELGVPD**S**ALAGVP  GLENLG**S**CQVMRS  DHSSLF**S**GALSPS  SGALSP**S**LLHSLS  SLLHSL**S**QNSSRL  AVGDMS**S**MLTSLA  MSSMLT**S**LAEESK  KKKVEN**S**IVKCST  STRTDV**S**EKAVAS  SSDERA**S**LIKKEI  IRSLHS**S**PSLSMI  SRKRTL**S**ISPLSD  ILNNSR**S**SSSASG  SSSASG**S**YGHLSA  LSASAI**S**PALSFT  ILSRQQ**S**LGSAFG  LNPVQV**S**SGPSES  SSGPSE**S**SQNKPT  GCNKAF**S**NASDRA  KRYTDP**S**SLRKHV  PPPPRD**S**GSHSQS  GSIGDL**S**AIDETP  IGNGTQ**S**NNTCSL  CFSSRR**S**SEASQA  RRSSEA**S**QAEGRP  GRPQNV**S**VADSYD  NVSVAD**S**YDPIST  TDASRR**S**SEASQS  SSEASQ**S**DGLPSL  QSRNFH**S**SPCPPS  QWNEVS**S**GSADLS  PVAPNE**S**AGSMVN  PNESAG**S**MVNGMQ  HQLLGD**S**MQHPGA  CASFGG**S**RRQAMP  LMSGAL**S**PSIIQN  QNLSHS**S**SRLTTP  LPFPAL**S**MSTTNM  APREDA**S**PEELNR  LNRYYA**S**LRHYLN  GPDTLL**S**KTFFPD  EDRPVR**S**RSEGPD  LAAFLI**S**AALCEG  GAVLPR**S**AKELRC  QCIKTY**S**KPFHPK  ELRVIE**S**GPHCAN  EIIVKL**S**DGRELC  VPKLFT**S**QICLLL  LLAVEG**S**LHVKPP  QHINMT**S**QQCTNA  PNMTCP**S**NKTRKN  RKNCHH**S**GSQVPL  NCHHSG**S**QVPLIH  CNLTTP**S**PQNISN  PSPQNI**S**NCRYAQ  EKQIAN**S**QDGYVW  RFLCFG**S**EGGTYY  VIQEIK**S**FSQEGR  QEIKSF**S**QEGRTT  FALAIC**S**QCSDIS  AICSQC**S**DISTKQ  SQCSDI**S**TKQAAF  AAFKAV**S**EVCRIP  KKDLKE**S**MKCGMW  KQRNGW**S**HKDLLR  KDLLRL**S**HLKPSS  LSHLKP**S**SEGLAI  SHLKPS**S**EGLAIV  YKEKAL**S**VETEKL  LTNHLK**S**KEVWKA  GKMTAN**S**VLEPGN  VLEPGN**S**EVSLVC  PGNSEV**S**LVCEKL  LLAVDV**S**ASMNQR  AVDVSA**S**MNQRVL  NQRVLG**S**ILNAST  GSILNA**S**TVAAAM  TRTEKD**S**YVVAFS  SYVVAF**S**DEMVPC  QVLMAM**S**QIPAGG  AGGTDC**S**LPMIWA  IVCGMT**S**NGFTIA  VPEQRD**S**KCKVKW  DWKFLA**S**HFPNRT  VKNHWN**S**TIKRKV  GGFLSE**S**KDCKPP  PTEGQG**S**LLTNWP  LAAATT**S**KEQEPI  REDQEG**S**PPETSL  LIPAVG**S**SLSEAL  AVGSSL**S**EALDLI  EDSINN**S**LVQLQA  HTISDL**S**RSSRGE  SDLSRS**S**RGELIP  LIPISP**S**TEVGGS  GIGTPP**S**VLKRQR  SPVTEN**S**TSLSFL  VTENST**S**LSFLDS  SLSFLD**S**CNSLTP  VKTLPF**S**PSQFLN  ESPSLT**S**TPVCSQ  TSTPVC**S**QKVVVT  TPDQKY**S**MDNTPH  IKKVRK**S**LALDIV  TLPKSL**S**LPTTAP  LPTTAP**S**NSSSLT  TTAPSN**S**SSLTLS  TAPSNS**S**SLTLSG  SSSLTL**S**GIKEDN  VAQKPR**S**HFTTPA  TTPAPM**S**SAWKTV  TPAPMS**S**AWKTVA  LGRLKP**S**HTSRTL  DDSAAP**S**TLSLLG  AAPSTL**S**LLGPTF  TFPGLS**S**CSADLK  QQQEAV**S**EGSSSG  SGAPTS**S**KDNYLG  ELCKAV**S**VSMGLG  GSLLDD**S**AGKSTE  DDSAGK**S**TEDTAE  EDTAEY**S**PFKGGY  KGLEGE**S**LGCSGS  GESLGC**S**GSAAAG  SLGCSG**S**AAAGSS  GAAGPG**S**GSPSAA  AGPGSG**S**PSAAAS  PGSGSP**S**AAASSS  SPSAAA**S**SSWHTL  PSAAAS**S**SWHTLF  GLAGQE**S**DFTAPD  YPGGMV**S**RVPYPS  MGPWMD**S**YSGPYG  RRKNCP**S**CRLRKC  EEGEAS**S**TTSPTE  TQKLTV**S**HIEGYE  FAALLS**S**LNELGE  MAVIQY**S**WMGLMV  FAMGWR**S**FTNVNS  SFTNVN**S**RMLYFA  HKSRMY**S**QCVRMR  VRMRHL**S**QEFGWL  KRKNPT**S**CSRRFY  KNPTSC**S**RRFYQL  PMLGGL**S**PPGALT  QHQLPV**S**GYSTPS  LPVSGY**S**TPSPAT  SGYSTP**S**PATIET  ATIETQ**S**SSSEEI  TIETQS**S**SSEEIV  IETQSS**S**SEEIVP  ETQSSS**S**EEIVPS  SEEIVP**S**PPSPPP  FVCQDK**S**SGYHYG  GYHYGV**S**ACEGCK  KGFFRR**S**IQKNMV  VGMSKE**S**VRNDRN  VPKPEC**S**ESYTLT  KPECSE**S**YTLTPE  KYTTNN**S**SEQRVS  YTTNNS**S**EQRVSL  DLWDKF**S**ELSTKC  DKFSEL**S**TKCIIK  QDTMTF**S**DGLTLN  AETGLL**S**AICLIC  KITDLR**S**ISAKGA  TDLRSI**S**AKGAER  QEMLEN**S**EGLDTL  EGLDTL**S**GQPGGG  LAPPPG**S**CSPSLS  PPPGSC**S**PSLSPS  PGSCSP**S**LSPSSN  SCSPSL**S**PSSNRS  SPSLSP**S**SNRSSP  PSLSPS**S**NRSSPA  YIHYKL**S**QRGYEW  PAPGIF**S**SQPGHT  APGIFS**S**QPGHTP  TPHPAA**S**RDPVAR  QAGDDF**S**RRYRRD  RDFAEM**S**SQLHLT  DFAEMS**S**QLHLTP  GVMCVE**S**VNREMS  SVNREM**S**PLVDNI  VELYGP**S**MRPLFD  RPLFDF**S**WLSLKT  FDFSWL**S**LKTLLS  SLKTLL**S**LALVGA  WLNPDP**S**QKQNLL  YDGRGD**S**VVYGLR  VVYGLR**S**KSKKFR  YGLRSK**S**KKFRRP  GTPRVT**S**RSGPAP  PRVTSR**S**GPAPAR  LCGWTP**S**SGATPR  CGWTPS**S**GATPRN  LLQLLG**S**PGRRYY  PGRRYY**S**LPPHQK  QKVPLP**S**LSPTMQ  VPLPSL**S**PTMQAG  ATVGFE**S**LEECYM  NYTLDS**S**AAPTPQ  TPAATA**S**PPTPSA  SAQAPG**S**SYPPHM  AQAPGS**S**YPPHMQ  VLLPAL**S**PTMTMG  KVGEKL**S**EGDLLA  EKEADI**S**AFADYR  PLAPTP**S**APCPAT  KGRVFV**S**PLAKKL  IDSFVP**S**KVAPAP  FTDIPI**S**NIRRVI  IPHYYL**S**IDVNMG  EGRSKI**S**VNDFII  DFIIKA**S**ALACLK  KVPEAN**S**SWMDTV  VPEANS**S**WMDTVI  NHVVDV**S**VAVSTP  DVSVAV**S**TPAGLI  IANDVV**S**LATKAR  GGTFTI**S**NLGMFG  ILAIGA**S**EDKLVP  KGFDVA**S**MMSVTL  DVASMM**S**VTLSCD  MMSVTL**S**CDHRVV  RAAEDD**S**ASPPGA  AEDDSA**S**PPGAAS  VVCGDK**S**SGKHYG  VCGDKS**S**GKHYGV  TCEGCK**S**FFKRSI  SIRRNL**S**YTCRSN  LSYTCR**S**NRDCQI  RGRIPH**S**LPGAVA  PGAVAA**S**SGSPPG  GAVAAS**S**GSPPGS  VAASSG**S**PPGSAL  SGSPPG**S**ALAAVA  ALAAVA**S**GGDLFP  FPGQPV**S**ELIAQL  AARLLF**S**TVEWAR  VALLRL**S**WSELFV  LLRLSW**S**ELFVLN  GRLQVD**S**AEYGCL  PDACGL**S**DPAHVE  DPAHVE**S**LQEKAQ  VRAQYP**S**QPQRFG  LRAVPA**S**LISQLF  VPASLI**S**QLFFMR  IRDMLL**S**GSTFNW  DMLLSG**S**TFNWPY  LVVVDF**S**ATWCGP  IKPFFH**S**LSEKYS  PFFHSL**S**EKYSNV  SLSEKY**S**NVIFLE  DCQDVA**S**ECEVKC  QKVGEF**S**GANKEK  DAGLKE**S**PLQTPT  ETSDAK**S**TPTAED  GIGDTP**S**LEDEAA  VTQEPE**S**GKVVQE  DRDVDE**S**SPQDSP  ESSPQD**S**PPSKAS  PQDSPP**S**KASPAQ  SPPSKA**S**PAQDGR  AAREAT**S**IPGFPA  DFLSKV**S**TEIPAS  STEIPA**S**EPDGPS  QKEQAH**S**EEHLGR  PEARGP**S**LGEDTK  ADLPEP**S**EKQPAA  PRGKPV**S**RVPQLK  ARMVSK**S**KDGTGS  SKDGTG**S**DDKKAK  KNRPCL**S**PKHPTP  PLIQPS**S**PAVCPE  CPEPPS**S**PKYVSS  SSPKYV**S**SVTSRT  YVSSVT**S**RTGSSG  TSRTGS**S**GAKEMK  APKTPP**S**SGEPPK  PKTPPS**S**GEPPKS  PKSGDR**S**GYSSPG  SPSSAK**S**RLQTAP  DLKNVK**S**KIGSTE  YKPVDL**S**KVTSKC  DLSKVT**S**KCGSLG  HLSNVS**S**TGSIDM  SGSTAA**S**EEARSL  ASEEAR**S**LRECEL  QALLKD**S**IVQLCT  AGTRTD**S**REDEIS  SREDEI**S**PPPPNP  RRRGAI**S**AEVYTE  TEEDAA**S**YVRKVI  EKNVLF**S**HLDDNE  LDDNER**S**DIFDAM  IFDAMF**S**VSFIAG  DAMFSV**S**FIAGET  NNEWAT**S**VGEGGS  SVGEGG**S**FGELAL  WGIDRD**S**YRRILM  RRILMG**S**TLRKRK  MYEEFL**S**KVSILE  EFLSKV**S**ILESLD  KVSILE**S**LDKWER  FIILEG**S**AAVLQR  AVLQRR**S**ENEEFV  VGRLGP**S**DYFGEI  RVLGPC**S**DILKRN  TALPVN**S**PMNKGD  CIVEVI**S**DTLSKP  VISDTL**S**KPSPMP  DTLSKP**S**PMPVSQ  PSPMPV**S**QECFET  GDERIL**S**ILRHQN  HQQKKH**S**GFEDEL  GFEDEL**S**EVLENQ  EVLENQ**S**SQAELK  VLENQS**S**QAELKE  EAVEEP**S**SKDVME  AVEEPS**S**KDVMEK  MEKRED**S**KEAEKS  PEPMQE**S**KAEGNN  NTHPPA**S**LPSQKY  PPASLP**S**QKYPGP  GDSEGL**S**QGLVDR  VLNPHP**S**LGYKEI  RKGESR**S**EALAVD  SKEWED**S**KRWSKM  EDSKRW**S**KMDQLA  EEDNRD**S**SMKLSF  EDNRDS**S**MKLSFR  DSSMKL**S**FRARAY  RRGWRP**S**SREDSL  KKEEEG**S**ANRRPE  EDQELE**S**LSAIEA  QELESL**S**AIEAEL  RVQTGS**S**QPSVSP  GEPSPP**S**IHPGKS  SIHPGK**S**DLIVRV  TNKHGL**S**NSIYVF  LFLVDR**S**LYGKED  PEVTNY**S**LKGCQG  AGIMIK**S**VKRAYH  RLCLHC**S**VDQEGK  EGKSVL**S**EKFILK  CTIKDV**S**SSVYST  VSSSVY**S**TWKREN  TWKREN**S**QTKLQE  ATLTIS**S**ARVNDS  SNIRYV**S**ELHLTR  TYTFLV**S**NSDVNA  TFLVSN**S**DVNAAI  VQTLNS**S**GPPFGK  NDVGKT**S**AYFNFA  AYGLIK**S**DAAMTV  VKMLKP**S**AHLTER  EREALM**S**ELKVLS  LLHSKE**S**SCSDST  ESSCSD**S**TNEYMD  DMKPGV**S**YVVPTK  ELFSLG**S**SPYPGM  LFSLGS**S**PYPGMP  PGMPVD**S**KFYKMI  EKQISE**S**TNHIYS  SNLANC**S**PNRQKP  RINSVG**S**TASSSQ  VGSTAS**S**SQPLLV  RQMRPV**S**RVLAPH  TVIIEQ**S**WGSPKV  IEQSWG**S**PKVTKD  GVTVAK**S**IDLKDK  ATVLAR**S**IAKEGF  EGFEKI**S**KGANPV  AELKKQ**S**KPVTTP  AQVATI**S**ANGDKE  EIGNII**S**DAMKKV  FDRGYI**S**PYFINT  PYFINT**S**KGQKCE  DAYVLL**S**EKKISS  LSEKKI**S**SIQSIV  SEKKIS**S**IQSIVP  KISSIQ**S**IVPALE  VDGEAL**S**TLVLNR  QLDVTT**S**EYEKEK  ERLAKL**S**DGVAVL  LKVGGT**S**DVEVNE  CIPALD**S**LTPANE  NAGVEG**S**LIVEKI  VEKIMQ**S**SSEVGY  EKIMQS**S**SEVGYD  KIMQSS**S**EVGYDA  DAAGVA**S**LLTTAE  LEMQIN**S**NQIPGL  DACLFR**S**WAIHTG  FRCAMN**S**LPDIEE  EEVKDQ**S**RNKGSS  QSRNKG**S**SAVRVY  SRNKGS**S**AVRVYR  QRKERK**S**KSSRDA  KERKSK**S**SRDAKS  ERKSKS**S**RDAKSK  SSRDAK**S**KAKRKS  RKSCGD**S**SPDTFS  KSCGDS**S**PDTFSD  SSPDTF**S**DGLSSS  TFSDGL**S**SSTLPD  FSDGLS**S**STLPDD  SDGLSS**S**TLPDDH  TLPDDH**S**SYTVPG  LPDDHS**S**YTVPGY  ALTPAL**S**PCAVSS  LSPCAV**S**STLPDW  SPCAVS**S**TLPDWH  VEVVPD**S**TSDLYN  VVPDST**S**DLYNFQ  LYNFQV**S**PMPSTS  QVSPMP**S**TSEATT  SPMPST**S**EATTDE  MKLLEQ**S**EWQPTN  PGVQPT**S**VYGDFS  SVYGDF**S**CKEEPE  EEPEID**S**PGGDIG  GGDIGL**S**LQRVFT  DATWLD**S**LLTPVR  TPVRLP**S**IQAIPC  VFLIVL**S**VALNHL  KATPIE**S**HQVEKR  ANFLVH**S**SNNFGA  NFLVHS**S**NNFGAI  NFGAIL**S**STNVGS  FGAILS**S**TNVGSN  SSTNVG**S**NTYGKR  DTTWWQ**S**QTMLAG  AGVQYP**S**SINLTL  RLKFHT**S**RPESFA  SCENTY**S**KANRGF  AYNFDN**S**PVLQEW  GNNEAC**S**SCHCSP  CSSCHC**S**PVGSLS  LSTQCD**S**YGRCSC  CQPGFH**S**LTEAGC  PCSCDP**S**GSIDEC  SCDPSG**S**IDECNI  GFFNLE**S**SNPRGC  YSVYSI**S**STFQID  QDIAVI**S**DSYFPR  LGKQVL**S**YGQNLS  FCEMCL**S**GYRRET  PNLGPY**S**PCVLCA  CACNGH**S**ETCDPE  GTMKQQ**S**SCNPVT  TMKQQS**S**CNPVTG  HPEGSL**S**LQCKDD  KLQELE**S**LIANLG  VNNTLS**S**QISRLQ  KTANDT**S**TEAYNL  ASVAQL**S**PLDSET  EAERIA**S**AVQKNA  VQKNAT**S**TKAEAE  SVTSLL**S**IINDLL  KDEMKV**S**DLDRKV  DLDRKV**S**DLENEA  IRKTLP**S**GCFNTP  DLGTTY**S**CVGVFQ  GNRTTP**S**YVAFTD  DDAVVQ**S**DMKHWP  YKGETK**S**FYPEEV  FYPEEV**S**SMVLTK  YPEEVS**S**MVLTKM  PAYFND**S**QRQATK  GGTFDV**S**ILTIED  GIFEVK**S**TAGDTH  KHKKDI**S**ENKRAV  RAKRTL**S**SSTQAS  AKRTLS**S**STQASI  KRTLSS**S**TQASIE  SSSTQA**S**IEIDSL  ASIEID**S**LYEGID  GIDFYT**S**ITRARF  DAKLDK**S**QIHDIV  IVLVGG**S**TRIPKI  GKELNK**S**INPDEA  VQAAIL**S**GDKSEN  ILSGDK**S**ENVQDL  LDVTPL**S**LGIETA  QTFTTY**S**DNQPGV  NGILNV**S**AVDKST  VSAVDK**S**TGKENK  NDKGRL**S**KEDIER  QRDKVS**S**KNSLES  KVSSKN**S**LESYAF  SKNSLE**S**YAFNMK  ITKLYQ**S**AGGMPG  GGGAPP**S**GGASSG  PPSGGA**S**SGPTIE  PSGGAS**S**GPTIEE  EPQQEE**S**CQTAAE  EQKLKA**S**NGDTPT  ELKTDP**S**LDLHSL  EGLEEC**S**KIEVKE  SKTWLD**S**AKEIKK  HGVDYV**S**DFKLAP  VMELHK**S**YRSMTP  LHKSYR**S**MTPAQA  ILGVCS**S**GLLVYK  PKVLKI**S**YKRSSF  KISYKR**S**SFFIKI  EQEQYE**S**TIGFKL  TFFRLT**S**TDTIPK  TDTIPK**S**KFLALG  GSKFRY**S**GRTQAQ  HFERTA**S**KRASRS  GAAAVD**S**ADRSPR  GGVLDA**S**AKKTVV  EVTVPT**S**NGDQTQ  NIYIRH**S**NLMLED  IKKHHA**S**ISELKK  KHHASI**S**ELKKNF  KKNFME**S**VPEPRP  VPEPRP**S**EWDKRL  TQTVTI**S**DNANAV  AQTDDN**S**GDLDPG  TAQTIT**S**ETPSST  ITSETP**S**STTTTQ  TSETPS**S**TTTTQI  TVKGGI**S**ETRIEK  EQHPDM**S**VTKVVV  LPLLLL**S**MVAASY  LSMVAA**S**YSETVT  MVAASY**S**ETVTCE  PAVIAC**S**SPGING  AVIACS**S**PGINGF  PGNPGP**S**GSPGPK  NPGPSG**S**PGPKGQ  KGDPGK**S**PDGDSS  KSPDGD**S**SLAASE  SPDGDS**S**LAASER  DSSLAA**S**ERKALQ  KKWLTF**S**LGKQVG  CVKFQA**S**VATPRN  EPNNAG**S**DEDCVL  WNDVPC**S**TSHLAV  DVPCST**S**HLAVCE  RPGAAA**S**GERDDR  EVSDRI**S**SLGSQA  VSDRIS**S**LGSQAM  PYRGRS**S**ESSCGV  LRSQST**S**EQEKRL  GYTPDC**S**SNENLT  NENLTS**S**EEDFSS  SEEDFS**S**GQSSRV  GQSSRV**S**PSPTTY  RMFRDK**S**RSPSQN  LPYIDD**S**PSSSPH  YIDDSP**S**SSPHLS  SSPHLS**S**KGRGSR  SSKGRG**S**RDALVS  SRDALV**S**GALEST  MRKWVL**S**GILASE  SQPVLT**S**QQIETI  PRVQQW**S**HQQRVG  LFQKLA**S**QLGVYR  DPTTKN**S**LETLLY  RLKKKL**S**EQESLL  HSRNGK**S**YTFLIS  KLQTVH**S**IPLTIN  LNVIVH**S**ATGFKQ  CTLEVD**S**FGYFVN  EIELEG**S**QTLRIL  GIEVKL**S**VKFNSR  FNSREF**S**LKRMPS  SLKRMP**S**RKQTGV  VTKRER**S**KVPYIV  PITMTD**S**WSLEVM  TMTDSW**S**LEVMSQ  AIPAPD**S**KRQSIL  EGRETR**S**AKDALL  ENPDEK**S**IITYVV  AFYHYF**S**KMKVLA  KMIEKY**S**GLASDL  NILRLW**S**YLQELL  LQELLQ**S**RRQRLE  ANSLVE**S**GHPRSR  DFQAWL**S**ITQKAV  SEDMPE**S**LPEAEQ  HLEPPD**S**LEAAEA  VLSPVD**S**GNKLVA  AEGNLY**S**DKIKEK  EKAQEA**S**VLLRDN  NDKLLT**S**QDVSYD  LTSQDV**S**YDEARN  FVAELA**S**HEGWLE  LSAARS**S**DLRLQT  RKKQLE**S**SRAKLQ  RAKLQI**S**RDLEDE  RLPLAQ**S**ADYGTN  NIKQLA**S**RAQGLL  DLEQWI**S**EKELVA  EKELVA**S**SPEMGQ  DVGLDA**S**TAESFH  MARDLL**S**WMESII  ERPRDV**S**SVELLM  RPRDVS**S**VELLMK  TRSKNF**S**ACLELG  KLQQVM**S**RRKEMN  LEVCQF**S**RDASVA  HEAFEK**S**TASWAE  PLTPPF**S**ALPDPA  DPAGAP**S**RRQSRQ  APSRRQ**S**RQRPQL  QRPQLS**S**DSPSAF  PQLSSD**S**PSAFRA  LSSDSP**S**AFRASR  AFRASR**S**HSRNAT  RASRSH**S**RNATRS  SRNATR**S**HSHSHS  NATRSH**S**HSHSPR  TRSHSH**S**HSPRHS  SHSHSH**S**PRHSLR  SHSPRH**S**LRHSPG  RHSLRH**S**PGSGSC  LRHSPG**S**GSCGSS  GSGSCG**S**SSGHRP  SGSCGS**S**SGHRPC  GSCGSS**S**GHRPCA  EKEPLE**S**QYQVGP  VGPLLG**S**GGFGSV  GSGGFG**S**VYSGIR  GFGSVY**S**GIRVSD  YSGIRV**S**DNLPVA  VEKDRI**S**DWGELP  VLLKKV**S**SGFSGV  WFERPD**S**FVLILE  QEELAR**S**FFWQVL  KLIDFG**S**GALLKD  DGTRVY**S**PPEWIR  RSAAVW**S**LGILLY  FFRQRV**S**SECQHL  LCTARP**S**PTLPEQ  APVEVE**S**FLVHPG  LRDDVQ**S**INWLRD  GVQLAE**S**NRTRIT  EVEVQD**S**VPADSG  DSVPAD**S**GLYACV  SDTTYF**S**VNVSDA  VSDALP**S**SEDDDD  DDDDDS**S**SEEKET  DDDDSS**S**EEKETD  VKFKCP**S**SGTPNP  VENEYG**S**INHTYQ  KTVALG**S**NVEFMC  LHLRNV**S**FEDAGE  TCLAGN**S**IGLSHH  TGAFLI**S**CMVGSV  ISCMVG**S**VIVYKM  VHKLAK**S**IPLRRQ  VTVSAD**S**SASMNS  TVSADS**S**ASMNSG  SADSSA**S**MNSGVL  SSASMN**S**GVLLVR  AVKMLK**S**DATEKD  EYCYNP**S**HNPEEQ  NPEEQL**S**SKDLVS  PEEQLS**S**KDLVSC  RIYTHQ**S**DVWSFG  QEYLDL**S**MPLDQY  MPLDQY**S**PSFPDT  SFPDTR**S**STCSSG  FPDTRS**S**TCSSGE  CSSGED**S**VFSHEP  GEDSVF**S**HEPLPE  DFRLND**S**HKHKDK  EKDREK**S**KHSNSE  REKSKH**S**NSEHKD  KSKHSN**S**EHKDSE  TKHKDG**S**SEKHKD  KHKDGS**S**EKHKDK  EEKVRA**S**GDAKIK  EKENGF**S**SPPQIK  GKVMKL**S**PKAEEV  NIITNL**S**KCDFTQ  CDFTQM**S**QYFKAQ  EARKQM**S**KEEKLK  DIIINC**S**KDAKVP  KDAKVP**S**PPPGHK  KVTWLV**S**WTENIQ  TENIQG**S**IKYIML  YIMLNP**S**SRIKGE  IMLNPS**S**RIKGEK  YREDWK**S**KEMKVR  DFLGKD**S**IRYYNK  FRTYNA**S**ITLQQQ  IPAKIL**S**YNRANR  PKTFEK**S**MMNLQT  ARRDLK**S**AKADAK  TKKVVE**S**KKKAVQ  QIALGT**S**KLNYLD  PDTYIG**S**VELVTQ  FGQLLT**S**SNYDDD  GQLLTS**S**NYDDDE  AYDIAG**S**TKDVKV  EVCLTM**S**EKGFQQ  QISFVN**S**IATSKG  QPKSFG**S**TCQLSE  GSTCQL**S**EKFIKA  ILTEGD**S**AKTLAV  AKTLAV**S**GLGVVG  LNVREA**S**HKQIME  EFEEWK**S**STPNHK  FEEWKS**S**TPNHKK  RIQFKY**S**GPEDDA  KELILF**S**NSDNER  LILFSN**S**DNERSI  NERSIP**S**MVDGLK  VAQLAG**S**VAEMSS  GGKDSA**S**PRYIFT  PLPMLP**S**YKNFKG  PNQYVI**S**GEVAIL  EVAILN**S**TTIEIS  TSLTCN**S**MVLFDH  GMLGAE**S**AKLNNQ  EKETEK**S**DSVTDS  DTLKRK**S**PSDLWK  SESDRS**S**DESNFD  KKTAAK**S**QSSTST  TAAKSQ**S**STSTTG  KSQSST**S**TTGAKK  AVTSKK**S**KGESDD  AEQVAL**S**RTQVCG  GDAFHQ**S**DTHIFI  FIIMGA**S**GDLAKK  IVGYAR**S**RLTVAD  ADIRKQ**S**EPFFKA  DFFARN**S**YVAGQY  QYDDAA**S**YQRLNS  SYQRLN**S**HMNALH  NALHLG**S**QANRLF  TKNIHE**S**CMSQIG  IHESCM**S**QIGWNR  FGRDLQ**S**SDRLSN  GRDLQS**S**DRLSNH  QSSDRL**S**NHISSL  RLSNHI**S**SLFRED  LSNHIS**S**LFREDQ  AMEKPA**S**TNSDDV  KPASTN**S**DDVRDE  KVLKCI**S**EVQANN  PTVPRG**S**TTATFA  FFNPEE**S**ELDLTY  LDVFCG**S**QMHFVR  QMHFVR**S**DELREA  IPYIYG**S**RGPTEA  PDSPKG**S**SETETE  DSPKGS**S**ETETEP  GGKLVL**S**SLPKRL  GKLVLS**S**LPKRLC  VCGDVA**S**GYHYGV  YHYGVA**S**CEACKA  KRTIQG**S**IEYSCP  QGSIEY**S**CPASNE  EYSCPA**S**NECEIT  PVNALV**S**HLLVVE  EIVVTI**S**WAKSIP  TISWAK**S**IPGFSS  KSIPGF**S**SLSLSD  SIPGFS**S**LSLSDQ  PGFSSL**S**LSDQMS  FSSLSL**S**DQMSVL  SLSDQM**S**VLQSVW  QMSVLQ**S**VWMEVL  LGVAQR**S**LPLQDE  ALALAN**S**DSVHIE  ALANSD**S**VHIEDA  SAEEKH**S**RELEKK  LLGAGE**S**GKSTIV  AGESGK**S**TIVKQM  IHQDGY**S**LEECLE  YGNTLQ**S**ILAIVR  NIQYGD**S**ARQDDA  TMPKEM**S**DIIQRL  QRLWKD**S**GIQACF  ACFERA**S**EYQLND  EYQLND**S**AGYYLS  SAGYYL**S**DLERLV  EQDVLR**S**RVKTTG  IIETQF**S**FKDLNF  DVGGQR**S**ERKKWI  IFIAAL**S**AYDMVL  VNRMHE**S**LHLFNS  SLHLFN**S**ICNHRY  RYFATT**S**IVLFLN  IKKAHL**S**ICFPDY  DVKEIY**S**HMTCAT  LGIRRT**S**TAPAAS  RTVAIY**S**EQDTGQ  PGYGFL**S**ERADFA  VRFIGP**S**PEVVRK  TDAPIT**S**LHEAHE  GMRVVH**S**YEELEE  TRLTSD**S**VKLAKQ  TEDPAR**S**FQPDTG  IRLDNA**S**AFQGAV  ISPHYD**S**LLVKVI  TAATKM**S**RALAEF  FRDAHQ**S**LLATRV  YVAHNF**S**KLFSME  NFSKLF**S**MENWGG  VFRVFD**S**LNYLPN  GDVADP**S**RTKYSL  PSRTKY**S**LQYYMG  ACTMLV**S**SLRDRF  CTMLVS**S**LRDRFP  VDVAAD**S**MSGMTS  VAADSM**S**GMTSQP  SMSGMT**S**QPSMGA  GMTSQP**S**MGALVA  ERVFDY**S**EYWEGA  CTATMK**S**GNSDVY  LHFQAH**S**MGLGSK  LIKVTP**S**SKIVGD  MVQNGL**S**RAEAEA  AQAEEL**S**FPRSVV  ELSFPR**S**VVEFLQ  LNGQLR**S**ILVKDT  QPLCVL**S**AMKMET  METVVT**S**PMEGTV  AALGRG**S**ALGGSL  GSALGG**S**LNRTPT  GGYGPV**S**GAVSGA  PVSGAV**S**GAKPGK  KKAYEL**S**TLTGTQ  VLLLVA**S**ETGHVY  LQPMIT**S**ETGKAL  PDSPPR**S**DPTTDQ  DLTYQV**S**ESDSSG  QVSESD**S**SGETKD  TIQTAP**S**TSTTMQ  STTMQV**S**SGPSFP  TTMQVS**S**GPSFPI  QVSSGP**S**FPITNY  NYLAPV**S**ASVSPS  PVSASV**S**PSAVSS  SASVSP**S**AVSSAN  VSPSAV**S**SANGTV  SPSAVS**S**ANGTVL  NGTVLK**S**TGSGPV  VLKSTG**S**GPVSSG  LMQLPT**S**FTLMPG  QAPQQA**S**PSRDSS  SPSRDS**S**TDLTQT  LTQTSS**S**GTVTLP  PATIMT**S**SVPTTV  ATIMTS**S**VPTTVG  SGLGDG**S**LTVLNA  TVQIPV**S**AVQLHQ  IGQQAG**S**SSNLTE  QQAGSS**S**NLTELQ  QKINEV**S**SSDDKD  KINEVS**S**SDDKDA  INEVSS**S**DDKDAF  AVKCND**S**KAIVKT  TGFDCA**S**KTEIQL  EIQLVQ**S**LGVPPE  NPCKQV**S**QIKYAA  QMMTFD**S**EVELMK  RIATDD**S**KAVCRL  KAVCRL**S**VKFGAT  GATLRT**S**RLLLER  IDVVGV**S**FHVGSG  VSFHVG**S**GCTDPE  TFVQAI**S**DARCVF  GAEVGF**S**MYLLDI  GGGFPG**S**EDVKLK  LDKYFP**S**DSGVRI  KYFPSD**S**GVRIIA  GRYYVA**S**AFTLAV  LKEQTG**S**DDEDES  SDDEDE**S**SEQTFM  DDEDES**S**EQTFMY  NDGVYG**S**FNCILY  PDEKYY**S**SSIWGP  DEKYYS**S**SIWGPT  EKYYSS**S**IWGPTC  YTVAAA**S**TFNGFQ  TIYYVM**S**GPAWQL  VEEQDA**S**TLPVSC  ASTLPV**S**CAWESG  VSCAWE**S**GMKRHR  ARLVQG**S**ILKKVL  EACWDI**S**SSGVNL  ACWDIS**S**SGVNLQ  CWDISS**S**GVNLQS  SGVNLQ**S**MDSSHV  NLQSMD**S**SHVSLV  LQSMDS**S**HVSLVQ  MDSSHV**S**LVQLTL  VQLTLR**S**EGFDTY  MGVNLT**S**MSKILK  VNLTSM**S**KILKCA  PNQEKV**S**DYEMKL  IPEQEY**S**CVVKMP  CVVKMP**S**GEFARI  RICRDL**S**HIGDAV  GDAVVI**S**CAKDGV  KDGVKF**S**ASGELG  GVKFSA**S**GELGNG  NGNIKL**S**QTSNVD  IKLSQT**S**NVDKEE  TKATPL**S**STVTLS  KATPLS**S**TVTLSM  SSTVTL**S**MSADVP  TVTLSM**S**ADVPLV  PSAELK**S**ADVNVF  VSCVHS**S**VSPERA  VALKQA**S**VFSFGL  TVPEFR**S**FGDLQE  FLVDGS**S**ALGLAN  EEIAFD**S**SLVFIP  PLTTYV**S**GGVEEV  LFLFDG**S**ANLVGQ  IAVAQY**S**DDVKVE  RVDGPA**S**NLKQSG  FILAAE**S**LPKIGD  VFLLDG**S**EGVRSG  TVQQVI**S**ERVTQL  VFLIDG**S**QSAGPE  LISSGK**S**DDEVDD  LVLGGK**S**QDDVSR  EERIMN**S**FGPSAA  GKIASN**S**ATAFRV  GNVQEL**S**ELSEQV  GNPGQD**S**QERGPK  ISGPRG**S**GGAAGA  RGRRGN**S**GPPGIV  EIRFAD**S**KRKSVL  KVAVFF**S**NTPTRA  VRQLDM**S**PDPKAS  ESVDNA**S**MPPVKV  KLVDFL**S**RGMTQL  FGRLLP**S**FVSSEN  YDLTVT**S**AHDQSL  TSAHDQ**S**LVLKQN  VVCYLR**S**QVRATY  SFSTKK**S**QPPPPQ  PPQPAR**S**ASSSTI  GVAAAV**S**KTAVAP  LQVQHA**S**KQISAE  HASKQI**S**AEKQYK  KEQGFL**S**FWRGNL  FAGNLA**S**GGAAGA  GAAGAT**S**LCFVYP  IIKIFK**S**DGLRGL  YQGFNV**S**VQGIII  NVHIFV**S**WMIAQS  SWMIAQ**S**VTAVAG  AVAGLV**S**YPFDTV  RRMMMQ**S**GRKGAD  FFKGAW**S**NVLRGM  PQGIRY**S**KLSEGA  VFKNMA**S**RPYSIY  PHGVTF**S**PYEDEV  EDEVNS**S**FTSGRN  DPKFYE**S**NIMSTI  VQWHFC**S**VGTQNE  MLTSMN**S**SPRSKK  IRSFRN**S**SLNQEE  TDIIVG**S**NYSSPS  TGIRLL**S**LGAGEF  QDTGSP**S**RMRPWK  PDHNQN**S**SNDTGQ  NDTGQA**S**CPPGLY  TLSPDL**S**HTTLSL  FSQTNL**S**PELSHM  LSHMTL**S**PELSQT  TLSLDF**S**QTNLSP  FSQTNL**S**PELSQT  NLSPEL**S**QTNLSP  FGQMSL**S**PDLSQV  TLLPDL**S**QISPPP  LQEFNE**S**FPYPDL  VRTNIN**S**SRDPDN  GLSYEK**S**SEGKTY  NAVQPN**S**SYTYVW  ERSGPE**S**PGSACR  FYVAYS**S**NQINWQ  MYFNGN**S**DASTIK  NKQITA**S**SFKKSW  QGCKSL**S**SEMYVK  PYRLKS**S**MVDKIF  EKFKVE**S**EQQYFE  RTNERL**S**QELEYL  LDELQA**S**DVSVKY  EKELLH**S**QNTWLN  NKKEEV**S**RLEEQM  KANKQS**S**VLERDN  LEQLRK**S**RQHQMQ  QMQLVD**S**IVRQRD  MYRILL**S**QTTGVA  STSQTV**S**TPAPVP  QVTDLR**S**QNTKIS  DVDDLV**S**QLRQTE  ELKKTL**S**SVQNEV  NQNLIQ**S**LKEDLN  METSAQ**S**SGDHQE  NQAETK**S**KSLESQ  QYEGRI**S**RLEREL  RGIAST**S**DPPTAN  SQEAMQ**S**EGPVEH  PVFGST**S**GSVRST  VQPTQQ**S**HPQIEP  PANQEL**S**SNIVEV  EASDQV**S**DDTVEM  EVMAEE**S**TDGEVE  NQDSQD**S**IGEGVT  DQQTTT**S**SQDGQG  AADSQN**S**GEGNTG  QMTRRQ**S**VGRGLQ  STTSDA**S**EHASQS  DASEHA**S**QSVPMV  AGLEID**S**QQEEEP  DESDLP**S**TSQDPP  DPPSSS**S**VDTSSS  QLLFRA**S**PATLLL  NFDIPK**S**VRPNDE  VKTYLI**S**SIPLQG  KTYLIS**S**IPLQGA  RDYPDF**S**PSVDAE  YPDFSP**S**VDAEAI  DEKMLI**S**ILTERS  SILTER**S**NAQRQL  DLKGDL**S**GHFEHL  AKQLKK**S**MKGAGT  ILTTRT**S**RQMKDI  RQMKDI**S**QAYYTV  YTVYKK**S**LGDDIS  SLGDDI**S**SETSGD  LGDDIS**S**ETSGDF  DISSET**S**GDFRKA  DGRRDE**S**LKVDEH  EILCLR**S**FPQLKL  DEYRNI**S**QKDIVD  QKDIVD**S**IKGELS  SIKGEL**S**GHFEDL  LNRIMV**S**RSEIDL  RIMVSR**S**EIDLLD  KKHYGY**S**LYSAIK  YGYSLY**S**AIKSDT  LYSAIK**S**DTSGDY  AIKSDT**S**GDYEIT  VLLGGA**S**HASLIP  GGASHA**S**LIPETG  HAGGRR**S**GQSHEL  GRRSGQ**S**HELLRD  RRRPQP**S**KSAVIP  RPQPSK**S**AVIPDY  EEEQIH**S**TGLEYP  YPERPA**S**RANTVR  RANTVR**S**FHHEEH  ENIPGT**S**ENSAFR  PGTSEN**S**AFRFLF  RFLFNL**S**SIPENE  FLFNLS**S**IPENEV  PENEVI**S**SAELRL  ENEVIS**S**AELRLF  WETFDV**S**PAVLRW  GQHVRI**S**RSLPQG  HVRISR**S**LPQGSG  RSLPQG**S**GNWAQL  RRRAKR**S**PKHHSQ  RSPKHH**S**QRARKK  KNCRRH**S**LYVDFS  SLYVDF**S**DVGWND  LADHLN**S**TNHAIV  VQTLVN**S**VNSSIP  LVNSVN**S**SIPKAC  VNSVNS**S**IPKACC  CVPTEL**S**AISMLY  TELSAI**S**MLYLDE  FTAWCN**S**HLRKAG  LLLEVI**S**GERLAK  MRVHKI**S**NVNKAL  ALDFIA**S**KGVKLV  KGVKLV**S**IGAEEI  FAIQDI**S**VEETSA  ISVEET**S**AKEGLL  IQNFHI**S**WKDGLG  AIMTYV**S**SFYHAF  IMTYVS**S**FYHAFS  SFYHAF**S**GAQKAE  DYEKLA**S**DLLEWI  QTKLRL**S**NRPAFM  RPAFMP**S**EGRMVS  SEGRMV**S**DINNAW  YETATL**S**EIKALL  KHEAFE**S**DLAAHQ  ELDYYD**S**PSVNAR  DYYDSP**S**VNARCQ  GIHNEV**S**KIVQTY  EEIGRI**S**IEMHGT  TLEDQL**S**HLRQYE  LRQYEK**S**IVNYKP  RDAKGI**S**QEQMNE  MNEFRA**S**FNHFDR  HFDRDH**S**GTLGPE  FKACLI**S**LGYDIG  EFARIM**S**IVDPNR  AFIDFM**S**RETADT  ADQVMA**S**FKILAG  PYTGPD**S**VPGALD  GALDYM**S**FSTALY  LDYMSF**S**TALYGE  LQPGNF**S**ADEAGA  FQSVAA**S**WAHDTN  AVRTLG**S**ANLPLA  QYNALL**S**NMSRIY  KTATCW**S**LDPDLT  LTNILA**S**SRSYAM  ILASSR**S**YAMLLF  EDFTAL**S**NEAYKQ  PNLDVT**S**TMLQQG  AEEFFT**S**LELSPM  FTSLEL**S**PMPPEF  PEFWEG**S**MLEKPA  GDVLAL**S**VSTPEH  KVLQAG**S**SRPWQE  DMETTY**S**VATVCH  AGDSWR**S**MYETPS  SMYETP**S**LEQDLE  MWAQTW**S**NIYDLV  LVVPFP**S**APSMDT  PEFWNK**S**MLEKPT  EVVCHA**S**AWDFYN  TPKHLH**S**LNLLSS  LSSEGG**S**DEHDIN  IAFIPF**S**YLVDQW  WRVFDG**S**ITKENY  YIRYFV**S**FIIQFQ  KCDIYQ**S**KEAGQR  TGQPNM**S**ASAMLS  QPNMSA**S**AMLSYF  SASAML**S**YFKPLL  TPNSAR**S**EGPLPD  PWSRSL**S**ALLLLL  LLLQVS**S**WLCQEP  PGFDAE**S**YTFTVP  QRTAYF**S**LDTRFK  LVYAWD**S**TYRKFS  PPPHQA**S**VSGIQA  PHQASV**S**GIQAEL  WVIPPI**S**CPENEK  NLVQIK**S**NKDKEG  EGKVFY**S**ITGQGA  LFSHAV**S**SNGNAV  FSHAVS**S**NGNAVE  QEVFKG**S**VMEGAL  GALPGT**S**VMEVTA  IAYTIL**S**QDPELP  RNTGVI**S**VVTTGL  VVPFEV**S**LTTSTA  EKRVEV**S**EDFGVG  VGQEIT**S**YTAQEP  PDTGAI**S**TRAELD  FEHVKN**S**TYTALI  IATDNG**S**PVATGT  TLLLIL**S**DVNDNA  DLPPNT**S**PFTAEL  ELTHGA**S**ANWTIQ  NDPTQE**S**IILKPK  VTTLEV**S**VCDCEG  TAPPYD**S**LLVFDY  VFDYEG**S**GSEAAS  DYEGSG**S**EAASLS  EAASLS**S**LNSSES  SLSSLN**S**SESDKD  SLNSSE**S**DKDQDY  EENLEA**S**GDYKYS  SGDYKY**S**GRDSLI  KYSGRD**S**LIFLVD  IFLVDA**S**KAMFES  AMFESQ**S**EDELTP  LTPFDM**S**IQCIQS  SIQCIQ**S**VYISKI  IQSVYI**S**KIISSD  YISKII**S**SDRDLL  ISKIIS**S**DRDLLA  TEKDKN**S**VNFKNI  DMMGHG**S**DYSLSE  GHGSDY**S**LSEVLW  GSDYSL**S**EVLWVC  VCANLF**S**DVQFKM  DVQFKM**S**HKRIML  NPHGND**S**AKASRA  NDSAKA**S**RARTKA  PGGFDI**S**LFYRDI  FYRDII**S**IAEDED  VHFEES**S**KLEDLL  TRKRAL**S**RLKLKL  NKDIVI**S**VGIYNL  GGLLLP**S**DTKRSQ  HHYLRP**S**LFVYPE  FVYPEE**S**LVIGSS  ESLVIG**S**STLFSA  SLVIGS**S**TLFSAL  DNEGSG**S**KRPKVE  RPKVEY**S**EEELKT  ELKTHI**S**KGTLGK  DVGFTM**S**NSIPGI  GFTMSN**S**IPGIES  SIPGIE**S**PFEQAK  GTDNPL**S**GGDQYQ  IFTDLS**S**RFSKSQ  DLSSRF**S**KSQLDI  QFFLPF**S**LGKEDG  LGKEDG**S**GDRGDG  VKMVMI**S**LEGEDG  KKIERH**S**IHWPCR  CRLTIG**S**NLSIRI  TIGSNL**S**IRIAAY  RIAAYK**S**ILQERV  QGFRYG**S**DIVPFS  EQMKYK**S**EGKCFS  SEGKCF**S**VLGFCK  VLGFCK**S**SQVQRR  LGFCKS**S**QVQRRF  AAAVAL**S**SLIHAL  AAVALS**S**LIHALD  LRQYMF**S**SLKNSK  FSSLKN**S**KKYAPT  VDALID**S**MSLAKK  ALIDSM**S**LAKKDE  AEVTTK**S**QIPLSK  SVTSVG**S**VNPAEN  TPYFMK**S**IDCIRA  EEAIKF**S**EEQRFN  ITKEEA**S**GSSVTA  EEASGS**S**VTAEEA  APKDKP**S**GDTAAV  GPRGAL**S**LLLLLL  LLLAPP**S**RPAAGC  GCPAPC**S**CAGTLV  RGLTWA**S**LPTAFP  NEQALA**S**GLILIT  WYFGAY**S**IVAGVF  GKRKKG**S**TMERWG  VLHLLL**S**VPAGFL  ACLAIA**S**GIYLLA  TIKQPP**S**NPPPRP  EARKKP**S**EEEAAV  FTNRKI**S**VQRLAS  SVQRLA**S**YRRITS  SYRRIT**S**SKCPKE  YRRITS**S**KCPKEA  QKWVQD**S**MDHLDK  ALCAPA**S**ASPYSS  CAPASA**S**PYSSDT  ASASPY**S**SDTTPC  SASPYS**S**DTTPCC  KEYFYT**S**GKCSNP  YTSGKC**S**NPAVVF  WLGAAL**S**LIPLIF  PLIFLI**S**GAEAAS  SGAEAA**S**FQRNQL  NVQKFP**S**PEMIRA  QAHKEE**S**SPDYNP  AHKEES**S**PDYNPY  NPYQGV**S**VPLQQK  KENGDE**S**HLPERD  HLPERD**S**LSEEDW  PERDSL**S**EEDWMR  NFPMDM**S**DDYETQ  PMYEEN**S**RDNPFK  EQYTPQ**S**LATLES  SLATLE**S**VFQELG  VEEKIE**S**QTQEEV  QEEVRD**S**KENIEK  NDEMKR**S**GQLGIQ  LVNAAG**S**GRLQNG  FEKPLD**S**QSIYQL  KPLDSQ**S**IYQLIE  YQLIEI**S**RNLQIP  VDLDDI**S**EADLDH  FQNRML**S**KSGYPK  ALPDGL**S**VEDILN  NLLGME**S**AANQKT  AANQKT**S**YFPNPY  PYGAGR**S**RSNQLP  GAGRSR**S**NQLPKA  RVPGQG**S**SEDDLQ  VPGQGS**S**EDDLQE  EHLNQG**S**SQETDK  HLNQGS**S**QETDKL  DKLAPV**S**KRFPVG  DSADNL**S**EKLERE  RVLDKI**S**IGQLVS  WELLQA**S**AFCGLG  VRYFNS**S**AFFFSG  MGELEP**S**EGKIKH  NIIFGV**S**YDEYRY  DEYRYR**S**VIKACQ  GQRARI**S**LARAVY  EKEIFE**S**CVCKLM  YFYGTF**S**ELQNLQ  LQPDFS**S**KLMGCD  DSFDQF**S**AERRNS  LNLMTH**S**VNQGQN  TELDIY**S**RRLSQE  ETGLEI**S**EEINEE  FFDDME**S**IPAVTT  LAEVAA**S**LVVLWL  KGNSTH**S**RNNSYA  THSRNN**S**YAVIIT  HTLITV**S**KILHHK  VLQAPM**S**TLNTLK  GILNRF**S**KDIAIL  QLKQLE**S**EGRSPI  NWFLYL**S**TLRWFQ  VDSLMR**S**VSRVFK  YKNGQL**S**KVMIIE  AILENI**S**FSISPG  LLGRTG**S**GKSTLL  DGVSWD**S**ITLQQW  LARSVL**S**KAKILL  LLLDEP**S**AHLDPV  GWLSDA**S**VRHPVT  TASPSL**S**KEPQMV  QSTTGV**S**HYATDS  PIHVTE**S**SVEMTK  LAHGTL**S**VEAATV  YATMEG**S**ALGEVE  PQFAHT**S**EVEGLA  KIDHSV**S**YPPGAV  GPKVSL**S**PGPEQK  REYFTT**S**SPPATQ  FESVAP**S**QNFSDS  AVQPNE**S**TETTES  GEEVEK**S**TSVTYT  TTIYPV**S**EQPSAK  VSETDT**S**EWISST  YSSTQR**S**DQLILP  PLEEDF**S**GDFREY  DTQTSP**S**TVPTSV  ISHISD**S**EGPSST  KEEVKV**S**GTVSTN  EVGPLV**S**QTDIFE  QDSNKN**S**STAEIN  DLYYEP**S**GEGSGE  VMLPLH**S**EQNKSS  FREFED**S**TLKPNR  IDTEVP**S**EPHDSN  LPIPRK**S**ATVIPE  LSSQTP**S**SPLTIY  SPLTIY**S**GSEASG  QGAHLT**S**ILSHEE  QPDSFF**S**AGEDCV  YNVDTE**S**ALLYQG  EQLQLG**S**PNGEPC  QPGENG**S**IVTCGH  FGENFA**S**CQAGIS  SCQAGI**S**SFYTKD  GAGHFR**S**QHTTEV  GKAYIF**S**IDEKEL  VGAPMQ**S**TIREEG  AARFGE**S**IVNLGD  GLQISK**S**LSMFGQ  QISKSL**S**MFGQSI  AVGAFR**S**DSAVLL  IVDASL**S**HPESVN  VENGWP**S**VCIDLT  VLFYNM**S**LDVNRK  PPRFYF**S**SNGTSD  PRFYFS**S**NGTSDV  TGSIQV**S**SREANC  TYLAVG**S**MKTLML  CEVTDN**S**GVVQLD  VVQLDC**S**IGYIYV  LSRIDI**S**FLLDVS  SMAPNV**S**VEIMVP  MVPNSF**S**PQTDKL  ALEQQK**S**AMQTLK  NFGKME**S**GKEASV  QLEGRP**S**ILEMDE  FTIVII**S**SSLLLG  TIVIIS**S**SLLLGL  IVIISS**S**LLLGLI  NRRDSW**S**YINSKS  ANIRNM**S**VIAHVD  HVDHGK**S**TLTDSL  KSTLTD**S**LVCKAG  IKSTAI**S**LFYELS  SLFYEL**S**ENDLNF  LNFIKQ**S**KDGAGF  GHVDFS**S**EVTAAL  VVVDCV**S**GVCVQT  NVNVII**S**TYGEGE  TYGEGE**S**GPMGNI  GTVGFG**S**GLHGWA  PANGKF**S**KSATSP  FSKSAT**S**PEGKKL  ITIHLP**S**PVTAQK  PLMMYI**S**KMVPTS  SKMVPT**S**DKGRFY  AFGRVF**S**GLVSTG  VFSGLV**S**TGLKVR  LKRLAK**S**DPMVQC  QCIIEE**S**GEHIIA  CIPIKK**S**DPVVSY  SYRETV**S**EESNVL  SNVLCL**S**KSPNKH  IDKGEV**S**ARQELK  LNEIKD**S**VVAGFQ  GHVFEE**S**QVAGTP  YLPVNE**S**FGFTAD  FTADLR**S**NTGGQA  GDPFDN**S**SRPSQV  DPFDNS**S**RPSQVV  DNSSRP**S**QVVAET  SLRLQS**S**SASYGG  LRLQSS**S**ASYGGG  LQSSSA**S**YGGGFG  GGFGGG**S**CQLGGG  GGGRGV**S**TCSTRF  RGVSTC**S**TRFVSG  CSTRFV**S**GGSAGG  GYGGGV**S**CGFGGG  FGGGAG**S**GFGGGY  LNDRLA**S**YLEKVR  DWHLKQ**S**PASPER  SPERDY**S**PYYKTI  ELALRQ**S**VEADIN  LDELTL**S**KTDLEM  LEMQIE**S**LNEELA  EEMKEF**S**NQVVGQ  EWFHTK**S**AELNKE  ELNKEV**S**TNTAMI  TAMIQT**S**KTEITE  LEIELQ**S**QLSMKA  ELQSQL**S**MKAGLE  QIQGLI**S**SIEAQL  IQGLIS**S**IEAQLS  SIEAQL**S**ELRSEM  QLSELR**S**EMECQN  EIATYR**S**LLEGQD  VSPRST**S**VTTTSS  TSVTTT**S**SASVTT  SVTTTS**S**ASVTTT  ASVTTT**S**NASGRR  TTTSNA**S**GRRTSD  GLLLTA**S**LLTFWN  AQLTTE**S**MPFNVA  TPGPAN**S**GRETIY  TIYPNA**S**LLIQNV  TLQVIK**S**DLVNEE  PELPKP**S**ISSNNS  LPKPSI**S**SNNSNP  PKPSIS**S**NNSNPV  SISSNN**S**NPVEDK  RTLTLL**S**VTRNDT  EIQNPV**S**ANRSDP  PVSANR**S**DPVTLN  PDTPTI**S**PSDTYY  LSCYAA**S**NPPAQY  NPPAQY**S**WLINGT  NGTFQQ**S**TQELFI  NITVNN**S**GSYTCH  TVNNSG**S**YTCHAN  TCHANN**S**VTGCNR  IIVTEL**S**PVVAKP  KPQIKA**S**KTTVTG  VTGDKD**S**VNLTCS  SVNLTC**S**TNDTGI  TNDTGI**S**IRWFFK  WFFKNQ**S**LPSSER  KNQSLP**S**SERMKL  NQSLPS**S**ERMKLS  SERMKL**S**QGNTTL  QGNTTL**S**INPVKR  GKTGRA**S**DQRDLT  EHKPSV**S**NHTQDH  TQPTSA**S**PSLTAT  PTSASP**S**LTATEI  IYRDLI**S**HDEMFS  SHDEMF**S**DIYKIR  VEGKMV**S**RTEGNI  HHLQET**S**FTKEAY  IKDYMK**S**IKGKLE  DQDGRI**S**FDEFIK  IFHGLK**S**TDVAKT  CAIGGT**S**EQSSVG  GGTSEQ**S**SVGTQH  GTSEQS**S**VGTQHS  SVGTQH**S**YSEEEK  GTQHSY**S**EEEKYA  CKMINL**S**VPDTID  LNLALN**S**ASAIGC  LALNSA**S**AIGCHV  FADIEL**S**RNEALI  EDLMKL**S**PEELLL  NKIGNF**S**TDIKDS  STDIKD**S**KAYYHL  RVNHLY**S**DLSDAL  HLYSDL**S**DALVIF  KNQAKF**S**LVGIGG  LREAKK**S**SSISSF  REAKKS**S**SISSFK  EAKKSS**S**ISSFKD  KKSSSI**S**SFKDPK  KSSSIS**S**FKDPKI  FKDPKI**S**TSLPVL  DPKIST**S**LPVLDL  DAIQPG**S**INYDLL  NAKYAI**S**MARKIG  ERQVLL**S**EPEEAA  AGPAGN**S**VETRGE  ELLSRE**S**PSGTMK  KNRKLK**S**FNLSAL  LKSFNL**S**ALEKHG  KRPKAE**S**FFQTKA  ALDVSA**S**DDEIAR  CVLDVE**S**GNISVL  VESGNI**S**VLEGVP  GVPENV**S**PGQAFW  FCTNRR**S**ALYYVD  GKCELL**S**DDSLAV  DDSLAV**S**SPRLSP  VYLQYP**S**LIPHHQ  IPHHQC**S**QLCLYD  WYTKVT**S**VVVDVV  QLGENF**S**GIYCSL  FSGIYC**S**LLPLGC  QVGTVT**S**LTAGGS  LMVAQF**S**TPSLPP  SAGKEQ**S**VLWVSL  QSVLWV**S**LEEAEP  HGGPHS**S**FVTAWM  LVNYRG**S**TGFGQD  TGFGQD**S**ILSLPG  GQDSIL**S**LPGNVG  EEHFDA**S**HVALMG  SHGGFI**S**CHLIGQ  PVINIA**S**MLGSTD  IASMLG**S**TDIPDW  EAGFPF**S**SDCLPD  AGFPFS**S**DCLPDL  KSTHAL**S**EVEVES  NRTLSM**S**SLPGLE  LLDVGA**S**AWALER  GEFLAQ**S**EEKPHV  HVFTTV**S**QITAIE  GRKLYE**S**LLVGSL  HNMLDD**S**SDPILT  NMLDDS**S**DPILTT  RIGLFN**S**SADRVK  IGLFNS**S**ADRVKV  EFLSST**S**PLLPVD  HLGVFP**S**YYEPWG  TVMGIP**S**ISTNLS  SISTNL**S**GFGCFM  EHIADP**S**AYGIYI  LDRRFR**S**LDDSCS  FRSLDD**S**CSQLTS  SLDDSC**S**QLTSFL  SCSQLT**S**FLYSFC  LTSFLY**S**FCQQSR  YSFCQQ**S**RRQRII  NRTERL**S**DLLDWK  LGRYYM**S**ARHMAL  SVPPSP**S**LSRHSS  PSLSRH**S**SPHQSE  RASCTS**S**TSGSKR  SCTSST**S**GSKRNS  TSSTSG**S**KRNSVD  SGSKRN**S**VDTATS  DTATSS**S**LSTPSE  ATSSSL**S**TPSEPL  SSLSTP**S**EPLSPT  IHEFKK**S**AKTTLI  LWFPFN**S**MSSGVK  FPFNSM**S**SGVKKE  PFNSMS**S**GVKKEF  IIGKGR**S**YKGTVS  SYKGTV**S**ITKSGI  TVSITK**S**GIKCQP  IKCQPW**S**SMIPHE  KCQPWS**S**MIPHEH  MIPHEH**S**FLPSSY  EHSFLP**S**SYRGKD  HSFLPS**S**YRGKDL  GPWCFT**S**NPEVRY  CDIPQC**S**EVECMT  MTCNGE**S**YRGLMD  LMDHTE**S**GKICQR  PCQRWD**S**QYPHEH  NPDGSE**S**PWCFTT  IRVGYC**S**QIPNCD  IPNCDM**S**HGQDCY  NYMGNL**S**QTRSGL  NLSQTR**S**GLTCSM  FWEPDA**S**KLNENY  WDYCPI**S**RCEGDT  LDHPVI**S**CAKTKQ  NIGWMV**S**LRYRNK  KHICGG**S**LIKESW  ARQCFP**S**RDLKDY  KQVLNV**S**QLVYGP  VLDDFV**S**TIDLPN  TIPEKT**S**CSVYGW  PEKTSC**S**VYGWGY  KVTLNE**S**EICAGA  HHFYDE**S**KPFTCL  FTCLDG**S**ATIPFD  CDCKDG**S**DEPGTA  AACPNG**S**FHCTNT  GTDEYN**S**GVICEN  GRKERE**S**LQQMAE  DMDGTV**S**VTELQT  DGDGAL**S**EAEAQA  EAQALL**S**GDTQTD  TQTDAT**S**FYDRVW  IRDKYR**S**EALPTD  TDLPAP**S**APDLTE  EQPPVP**S**SPTEEE  QPPVPS**S**PTEEEE  EEEEED**S**EEAPPP  EAPPPL**S**PPQPAS  SPPQPA**S**PAEEDK  NLEQEI**S**FDFGPN  EFAYLY**S**QCYELT  KPKLGG**S**PTSLGT  LGGSPT**S**LGTWGS  SLGTWG**S**WIGPDH  PDHDKF**S**AMKYEQ  WQGPNR**S**TTVRLL  KETMVT**S**TTEPSR  TSTTEP**S**RCEYLM  LEEQIN**S**NTIPGL  EEVKDK**S**IKKGNN  YRMLPL**S**ERPSKK  PLSERP**S**KKGKKP  KQEPVE**S**SLGLSN  QEPVES**S**LGLSNG  ESSLGL**S**NGVSDL  GLSNGV**S**DLSPEY  EYAVLT**S**TIKNEV  IIVVGQ**S**HLDSNI  GQSHLD**S**NIENQE  VEVTTE**S**DEQPVS  SDEQPV**S**MSELYP  LYPLQI**S**PVSSYA  LQISPV**S**SYAESE  QISPVS**S**YAESET  VSSYAE**S**ETTDSV  ESETTD**S**VPSDEE  TTDSVP**S**DEESAE  VPSDEE**S**AEGRPH  EGKQYL**S**NMGTRG  MASFVT**S**NKPDLQ  VTIKEE**S**NPVPYN  NPVPYN**S**SWPPFQ  PVPYNS**S**WPPFQD  QDLPLS**S**SMTPAS  DLPLSS**S**MTPASS  SSMTPA**S**SSSRPD  SMTPAS**S**SSRPDR  MTPASS**S**SRPDRE  TPASSS**S**RPDRET  DRETRA**S**VIKKTS  VVGHDV**S**VSVETQ  GHDVSV**S**VETQGD  DFVNDI**S**EKEQRW  AKTIEG**S**GRTEHI  QLRNKV**S**EEHDVL  RKKEME**S**GPKASH  ESGPKA**S**HGYGGR  RDRMDK**S**AVGHEY  AEVEKH**S**SQTDAA  EVEKHS**S**QTDAAK  RDRADK**S**AVGFDY  EVEKHT**S**QKDYSR  TSQKDY**S**RGFGGR  ETEKHE**S**QRDYAK  KDRVDK**S**AVGFNE  TPIEAA**S**SGTRGL  PIEAAS**S**GTRGLK  LKAKFE**S**MAEEKR  KAVTKR**S**PEAPQP  PLPKKI**S**SEAWPP  LPKKIS**S**EAWPPV  PVGTPP**S**SESEPV  VGTPPS**S**ESEPVR  TPPSSE**S**EPVRTS  SEPVRT**S**REHPVP  VLEPED**S**SFSSAL  LEPEDS**S**FSSALA  PEDSSF**S**SALAGS  EDSSFS**S**ALAGSS  SSALAG**S**SGCPAG  SALAGS**S**GCPAGA  AVALGI**S**AVAVYD  DYQGEG**S**DELSFD  WRPWND**S**LIPWCF  QDMTTT**S**IGVEAK  FGNDIN**S**VLFTTQ  FTGPTV**S**DTLYDV  VAQNPF**S**IQVIRK  ISTRLP**S**DYIYGI  SRWNYK**S**LDVVKE  DMNEVS**S**FIQGST  KQYDVH**S**LYGYSM  HSLYGY**S**MAIATE  VFPNKR**S**FILTRS  SFILTR**S**TFAGSG  GAFYPF**S**RNHNSD  FSRNHN**S**DGYEHQ  IWYDYE**S**GAKRPW  ILGLTD**S**VTEVRV  NLGRNF**S**VQWNQI  VWRTGS**S**LSKAPE  FPRQDN**S**YSVNSA  NSYSVN**S**ARYSSM  VNSARY**S**SMGITA  NSARYS**S**MGITAD  ARIKLP**S**DPISTL  QIRRRS**S**GRVIWD  ILSDGT**S**VLHYDV  IGMMEF**S**LFGMSY  RRQDPA**S**WNETFA  NQTTLT**S**TILKRG  RGYINK**S**ETRLGS  SETRLG**S**LHVWGK  YNGNKN**S**LPFNED  EKRFVP**S**QHYVYM  VKWQDL**S**EKVVYR  YCGTLM**S**LPTKIS  SLPTKI**S**RCPHLL  MPKDGK**S**TATDIT  ANYEKT**S**GSEMAL  YEKTSG**S**EMALST  GSEMAL**S**TGDVVE  VEVVEK**S**ESGWWF  VVEKSE**S**GWWFCQ  RGWIPA**S**FLEPLD  FLEPLD**S**PDETED  VEGDEV**S**LLEGEA  VTGYFP**S**MYLQKS  SMYLQK**S**GQDVSQ  KSGQDV**S**QAQRQI  SIRNAH**S**IHQRSR  HSIHQR**S**RKRLSQ  ERQTQR**S**KPQPAV  AVPPRP**S**ADLILN  LILNRC**S**ESTKRK  LNRCSE**S**TKRKLA  CRLDID**S**PPITAR  CTIGPA**S**RSVETL  IGPASR**S**VETLKE  LKEMIK**S**GMNVAR  VARLNF**S**HGTHEY  VRTATE**S**FASDPI  ATESFA**S**DPILYR  TGLIKG**S**GTAEVE  KVVEVG**S**KIYVDD  VDDGLI**S**LQVKQK  EVENGG**S**LGSKKG  NGGSLG**S**KKGVNL  VDLPAV**S**EKDIQD  VDMVFA**S**FIRKAS  SFIRKA**S**DVHEVR  KNIKII**S**KIENHE  DEILEA**S**DGIMVA  ATQMLE**S**MIKKPR  PTRAEG**S**DVANAV  ADCIML**S**GETAKG  RLAPIT**S**DPTEAT  VGAVEA**S**FKCCSG  ASFKCC**S**GAIIVL  IIVLTK**S**GRSAHQ  LTKSGR**S**AHQVAR  TGWRPG**S**GFTNTM  EEDLGK**S**REGSRT  GKSREG**S**RTDDEV  RELREK**S**EKFAFQ  DKIRLI**S**LTDENA  IAKSGT**S**EFLNKM  AQEDGQ**S**TSELIG  FGVGFY**S**AFLVAD  DKVIVT**S**KHNNDT  TQHIWE**S**DSNEFS  HIWESD**S**NEFSVI  SDSNEF**S**VIADPR  VLKEEA**S**DYLELD  FPIYVW**S**SKTETV  KSFSKE**S**DDPMAY  GEVTFK**S**ILFVPT  ILFVPT**S**APRGLF  LFDEYG**S**KKSDYI  GVIEDH**S**NRTRLA  LLRFQS**S**HHPTDI  HPTDIT**S**LDQYVE  IYFMAG**S**SRKEAE  YFMAGS**S**RKEAES  SRKEAE**S**SPFVER  RKEAES**S**PFVERL  GVKFDE**S**EKTKES  SEKTKE**S**REAVEK  IEKAVV**S**QRLTES  SQRLTE**S**PCALVA  PCALVA**S**QYGWSG  QTGKDI**S**TNYYAS  ERMLRL**S**LNIDPD  LRVTRN**S**KINAEN  TAPAAT**S**KPGLRP  DIGNKV**S**EQLQAK  KKEAKP**S**ATGKVI  LVPVPV**S**EPVPEP  VKEEKL**S**PEPILV  ADPNLC**S**EYVKDI  TMYMTV**S**IIDRFM  TAMFIA**S**KYEEMY  HFLRRA**S**KIGEVD  MVHFPP**S**QIAAGA  TLQHYL**S**YTEESL  LSYTEE**S**LLPVMQ  KNKYAT**S**KHAKIS  SKHAKI**S**TLPQLN  TLPQLN**S**ALVQDL  GFQQIG**S**EDGEPP  LVLAVF**S**AVLGSL  FSAVLG**S**LQFGYN  QKVIEQ**S**YNETWL  QGPEGP**S**SIPPGT  GPEGPS**S**IPPGTL  TTLWAL**S**VAIFSV  LSVAIF**S**VGGMIS  SVGGMI**S**SFLIGI  VGGMIS**S**FLIGII  LAVLGG**S**LMGLAN  FLIGAY**S**GLTSGL  AYSGLT**S**GLVPMY  QVLGLE**S**LLGTAS  SLLGTA**S**LWPLLL  LPFCPE**S**PRYLYI  EGPARK**S**LKRLTG  TGWADV**S**GVLAEL  LLQLLG**S**RTHRQP  AVVLQL**S**QQLSGI  QLSQQL**S**GINAVF  NAVFYY**S**TSIFET  VFYYST**S**IFETAG  TVFTLV**S**VLLVER  ERVPAM**S**YVSIVA  PAMSYV**S**IVAIFG  IVAELF**S**QGPRPA  GFSNWT**S**NFIIGM  RTFDQI**S**AAFHRT  AFHRTP**S**LLEQEV  EQEVKP**S**TELEYL  KIKPKN**S**KQAERE  RGENLV**S**MTVEGP  RGVGGP**S**QQVMTP  AAAATA**S**IAGAPT  FIALLI**S**SLEADK  IALLIS**S**LEADKC  EKIILV**S**SANEID  KIILVS**S**ANEIDV  DSKTPV**S**TEQASR  PAKVED**S**GHYYCV  YCVVRN**S**SYCLRI  CVVRNS**S**YCLRIK  CLRIKI**S**AKFVEN  NYTCHA**S**YTYLGK  TRPVIV**S**PANETM  MEVDLG**S**QIQLIC  NVTGQL**S**DIAYWK  YWKWNG**S**VIDEDD  LGEDYY**S**VENPAN  PANKRR**S**TLITVL  NISEIE**S**RFYKHP  TVIIVC**S**VFIYKI  VLWYRD**S**CYDFLP  FLPIKA**S**DGKTYD  KTVGEG**S**TSDCDI  VGEGST**S**DCDIFV  NENVKK**S**RRLIII  ILVRET**S**GFSWLG  RETSGF**S**WLGGSS  FSWLGG**S**SEEQIA  SWLGGS**S**EEQIAM  YEKMPE**S**IKFIKQ  HGAIRW**S**GDFTQG  FTQGPQ**S**AKTRFW  MPVQRR**S**PSSKHQ  QRRSPS**S**KHQLLS  SKHQLL**S**PATKEK  SETSKP**S**MESGDG  PVGGAI**S**TAQAQA  SKSNEE**S**GDSQQP  NEESGD**S**QQPSQP  MTQIPL**S**QPIQIA  TQLPQQ**S**QANLLQ  KRIDTP**S**LEEPSD  LYGNDF**S**QTTISR  FEALNL**S**FKNMCK  ENLSSD**S**SLSSPS  SSLSSP**S**ALNSPG  RVALEK**S**FLENQK  LVATTP**S**LVTSSA  AAVTNL**S**VTGTSD  TAPPAS**S**AVTSPS  SPSLSP**S**PSASAS  SPSASA**S**TSEASS  STTQTT**S**TPLSSP  TTSTPL**S**SPLGTS  TSTPLS**S**PLGTSQ  QLPANA**S**LAAMAA  PSLMAP**S**QFAAGG  TLSGAL**S**PALMSN  LSPALM**S**NSTLAT  GSLPIT**S**LDATGN  VSAAAA**S**AGNSAP  NSAPVA**S**LHATST  ASLHAT**S**TSAESI  AESIQN**S**LFTVAS  FTVASA**S**GAASTT  SASGAA**S**TTTTAS  DEQRRR**S**GAMVKM  QEFGPI**S**YVVVMP  PAFVNY**S**TSQKIS  FVNYST**S**QKISRP  STSQKI**S**RPGDSD  PGDSDD**S**RSVNSV  DSDDSR**S**VNSVLL  DSRSVN**S**VLLFTI  ILNPIY**S**ITTDVL  AMVEFD**S**VQSAQR  AQRAKA**S**LNGADI  NGADIY**S**GCCTLK  PHGGYH**S**HYHDEG  GHRRGP**S**RYGPQY  YGPHAD**S**PVLMVY  KVKFMK**S**KPGAAM  KLNVCV**S**KQPAIM  AIMPGQ**S**YGLEDG  LEDGSC**S**YKDFSE  CSYKDF**S**ESRNNR  YKDFSE**S**RNNRFS  SRNNRF**S**TPEQAA  NRIQHP**S**NVLHFF  LGVKRP**S**SVKVFS  GVKRPS**S**VKVFSG  SSVKVF**S**GKSERS  KVFSGK**S**ERSSSG  SGKSER**S**SSGLLE  GKSERS**S**SGLLEW  GLLEWE**S**KSDALE  LEWESK**S**DALETL  LFAVFP**S**LNEEQT  VPTGLD**S**ISHDSA  DSISHD**S**ANCELP  CSKAVM**S**QALKAT  ALKATF**S**GFKKEQ  DQYEEN**S**HLTSVP  ENSHLT**S**VPHWIN  VPHWIN**S**NTLGFG  CPASTP**S**VLSSEQ  TPSVLS**S**EQEFQM  FQMFPK**S**RLSSVS  PKSRLS**S**VSVTYC  SRLSSV**S**VTYCSV  VSVTYC**S**VSQDFP  VTYCSV**S**QDFPGS  SQDFPG**S**NLNLLT  TPKDHD**S**PENGAD  GADSFE**S**SDSLLQ  ADSFES**S**DSLLQS  LLQSWN**S**QSSLLD  QSWNSQ**S**SLLDVQ  RVPSFE**S**FEDDCS  SFEDDC**S**QSLCLN  EDDCSQ**S**LCLNKP  LNKPTM**S**FKDYIQ  LAGFTG**S**GPIQLW  FLLELL**S**DKSCQS  SDKSCQ**S**FISWTG  SCQSFI**S**WTGDGW  MNYEKL**S**RGLRYY  NIIHKT**S**GKRYVY  GAGAAA**S**SAADPA  AGAAAS**S**AADPAI  AYEEYT**S**KLDALQ  GNGTDF**S**VSSSAS  DFSVSS**S**ASMDTV  SVSSSA**S**MDTVTS  MDTVTS**S**SSSSLS  VTSSSS**S**SLSVLP  TSSSSS**S**LSVLPS  KFHQRC**S**TEVPLM  IPQEEA**S**LAETAL  AETALT**S**GSSPSA  ALTSGS**S**PSAPAS  TSGSSP**S**APASDS  GPQILT**S**PSPSKS  QILTSP**S**PSKSIP  SPSPSK**S**IPIPQP  PASLPG**S**LTNVKA  VKALQK**S**PGPQRE  ERKSSS**S**SEDRNR  LLFMGY**S**TKPQLA  DYLHAK**S**IIHRDL  IHRDLK**S**NNIFLH  NPYSFQ**S**DVYAFG  TGQLPY**S**NINNRD  VGRGYL**S**PDLSKV  DLSKVR**S**NCPKAM  FPQILA**S**IELLAR  LPKIHR**S**ASEPSL  RSASEP**S**LNRAGF  SLYACA**S**PKTPIQ  GLGTWK**S**PPGQVT  EELFIV**S**KLWCTY  ACQKTL**S**DLKLDY  FFPLDE**S**GNVVPS  SGNVVP**S**DTNILD  VKAIGI**S**NFNHLQ  LIQYCQ**S**KGIVVT  IVVTAY**S**PLGSPD  AYSPLG**S**PDRPWA  AKPEDP**S**LLEDPR  LVVIPK**S**VTPERI  VFDFEL**S**SQDMTT  FDFELS**S**QDMTTL  DMTTLL**S**YNRNWR  RVCALL**S**CTSHKD  ALLSCT**S**HKDYPF  VALLVL**S**WPPPGT  PGFLGD**S**VTLPCY  MEVTHV**S**QLTWAR  WARHGE**S**GSMAVF  HQTQGP**S**YSESKR  TQGPSY**S**ESKRLE  GPSYSE**S**KRLEFV  AELRNA**S**LRMFGL  FPQGSR**S**VDIWLR  PMARCV**S**TGGRPP  AQITWH**S**DLGGMP  GGMPNT**S**QVPGFL  QVPGFL**S**GTVTVT  LWILVP**S**SQVDGK  WILVPS**S**QVDGKN  CKVEHE**S**FEKPQL  YYPPEV**S**ISGYDN  PPEVSI**S**GYDNNW  PTGYNW**S**TTMGPL  VKEGPP**S**EHSGIS  GPPSEH**S**GISRNA  SEHSGI**S**RNAIIF  GIYFYW**S**KCSREV  HCHLCP**S**STEHAS  CHLCPS**S**TEHASA  SSTEHA**S**ASANGH  TEHASA**S**ANGHVS  SANGHV**S**YSAVSR  NGHVSY**S**AVSREN  VSYSAV**S**RENSSS  AVSREN**S**SSQDPQ  VSRENS**S**SQDPQT  SRENSS**S**QDPQTE  LTAPDG**S**LCSFAT  PDGSLC**S**FATTDD  DDPCFD**S**PDLRFF  LKPEEH**S**HFPAAV  EHVRAP**S**GHHQAG  RERRRL**S**KVNEAF  TLKRCT**S**SNPNQR  LKRCTS**S**NPNQRL  RGGEHY**S**GDSDAS  EHYSGD**S**DASSPR  SGDSDA**S**SPRSNC  GDSDAS**S**PRSNCS  DASSPR**S**NCSDGM  SPRSNC**S**DGMMDY  DGMMDY**S**GPPSGA  DYSGPP**S**GARRRN  YYNEAP**S**EPRPGK  EPRPGK**S**AAVSSL  GKSAAV**S**SLDCLS  KSAAVS**S**LDCLSS  SSLDCL**S**SIVERI  SLDCLS**S**IVERIS  SIVERI**S**TESPAA  ERISTE**S**PAAPAL  LLADVP**S**ESPPRR  ADVPSE**S**PPRRQE  QEAAAP**S**EGESSG  APSEGE**S**SGDPTQ  PSEGES**S**GDPTQS  SGDPTQ**S**PDAAPQ  GCYGHP**S**STTPNL  CYGHPS**S**TTPNLD  DFYVPV**S**LCTPSR  VSLCTP**S**RAALLT  PGVLVP**S**SRGGLP  GVLVPS**S**RGGLPL  FLGIPY**S**HDQGPC  PLLANL**S**VEAQPP  FFLYYA**S**HHTHYP  THYPQF**S**GQSFAE  PQFSGQ**S**FAERSG  QSFAER**S**GRGPFG  RGPFGD**S**LMELDA  PETMRM**S**RGGCSG  MSRGGC**S**GLLRCG  VTHELA**S**SLDLLP  THELAS**S**LDLLPT  LDGFDL**S**PLLLGT  LLGTGK**S**PRQSLF  GKSPRQ**S**LFFYPS  SLFFYP**S**YPDEVR  HFFTQG**S**AHSDTT  TQGSAH**S**DTTADP  DPACHA**S**SSLTAH  PACHAS**S**SLTAHE  ACHASS**S**LTAHEP  PLLYDL**S**KDPGEN  AVTFGP**S**QVARGE  KLDKKV**S**AQEVRK  VKEGIL**S**DEIYCP  TAVLLG**S**YAVQAK  NKEVHK**S**GYLSSE  HKSGYL**S**SERLIP  KSGYLS**S**ERLIPQ  KIGFPW**S**EIRNIS  SEIRNI**S**FNDKKF  KAEREL**S**EQIQRA  AVDQIK**S**QEQLAA  PVYEPV**S**YHVQES  SYHVQE**S**LQDEGA  AEPTGY**S**AELSSE  GYSAEL**S**SEGIRD  YSAELS**S**EGIRDD  RQLLTL**S**SELSQA  QLLTLS**S**ELSQAR  TLSSEL**S**QARDEN  SKIEEP**S**VVETTH  ETTHQD**S**PLPHPE  AIVRPA**S**LQVPNV  NVLLTS**S**DSSVII  LLTSSD**S**SVIIQQ  IQQAVP**S**PTSSTV  VITQAP**S**SNRPIV  ITQAPS**S**NRPIVP  IPASIT**S**SNVHVP  PASITS**S**NVHVPA  TVKGHG**S**GLVRTQ  GLVRTQ**S**EESRPQ  RTQSEE**S**RPQSLQ  EESRPQ**S**LQQPAT  LQQPAT**S**TTETPA  RKVWVQ**S**LEKKAE  KKAEDL**S**SLNGQL  LNGQLQ**S**EVTLLR  TAMQKK**S**GYHTAD  TADKDD**S**SEDISV  ADKDDS**S**EDISVP  EDISVP**S**SPHTEA  TEAIQH**S**SVSTSN  EAIQHS**S**VSTSNG  IQHSSV**S**TSNGVS  HSSVST**S**NGVSST  STSNGV**S**STSKAE  TSNGVS**S**TSKAEA  NGVSST**S**KAEAVA  AEAVAT**S**VLTQMA  QIVMAP**S**SQSQPS  QCLKGT**S**DGPTQQ  TQQLTW**S**RESPLK  KPFLKL**S**LGLPGL  CQPGPP**S**EKAWQP  TVNVEG**S**GELFRW  LFRWNV**S**DLGGLG  CGLKNR**S**SEGPSS  GLKNRS**S**EGPSSP  SSEGPS**S**PSGKLM  PSGKLM**S**PKLYVW  CLPPRD**S**LNQSLS  RDSLNQ**S**LSQDLT  GSTLWL**S**CGVPPD  CGVPPD**S**VSRGPL  VPPDSV**S**RGPLSW  VSRGPL**S**WTHVHP  HPKGPK**S**LLSLEL  GPKSLL**S**LELKDD  RGNLTM**S**FHLEIT  LIFCLC**S**LVGILH  QYGNVL**S**LPTPTS  SLPTPT**S**GLGRAQ  LGGTAP**S**YGNPSS  PSYGNP**S**SDVQAD  SYGNPS**S**DVQADG  ADGALG**S**RSPPGV  PDSEED**S**EFYEND  QLSQDG**S**GYENPE  EDEDSF**S**NAESYE  GSAWDP**S**REATSL  EATSLG**S**QSYEDM  TSLGSQ**S**YEDMRG  HEEDAD**S**YENMDN  VLLLLL**S**ILHPSR  LSILHP**S**RPGGVP  VGGLGV**S**AGAVVP  AVPGVV**S**PEAAAK  VPGVGI**S**PEAQAA  VAAAAK**S**AAKVAA  DEGVRR**S**LSPELR  GVRRSL**S**PELREG  LREGDP**S**SSQHLP  REGDPS**S**SQHLPS  EGDPSS**S**QHLPST  SSQHLP**S**TPSSPR  HLPSTP**S**SPRVPG  LPSTPS**S**PRVPGA  RPGFGL**S**PIFPGG  VLALMI**S**MISADS  LMISMI**S**ADSHEK  FHEKHH**S**HREFPF  RECPFP**S**WRFPGL  MVLLLY**S**FSDACE  LLLYSF**S**DACEEP  HTWLPV**S**DDACYR  YCELKG**S**VAIWSG  GSVAIW**S**GKPPIC  NGKHTF**S**EVEVFE  LDAVTY**S**CDPAPG  PGPDPF**S**LIGEST  FSLIGE**S**TIYCGD  IYCGDN**S**VWSRAA  GDNSVW**S**RAAPEC  ENGKQI**S**GFGKKF  GFYLDG**S**DTIVCD  DTIVCD**S**NSTWDP  IVCDSN**S**TWDPPV  LKVLPP**S**STKPPA  KVLPPS**S**TKPPAL  TKPPAL**S**HSVSTS  PPALSH**S**VSTSST  ALSHSV**S**TSSTTK  SHSVST**S**STTKSP  HSVSTS**S**TTKSPA  TSSTTK**S**PASSAS  TTKSPA**S**SASGPR  TKSPAS**S**ASGPRP  SPASSA**S**GPRPTY  TYKPPV**S**NYPGYP  EEGILD**S**LDVWVI  GFESYG**S**SSYGGA  FESYGS**S**SYGGAG  ESYGSS**S**YGGAGG  SQAEKK**S**RARAQH  IVPCTI**S**QLLSAT  TISQLL**S**ATLVDE  IGNVEI**S**QVTIVG  VDTDDT**S**SENTVV  DTDDTS**S**ENTVVP  VAGHLR**S**FQNKKS  SFQNKK**S**LVAFKI  NAHMVL**S**KANSQP  VLSKAN**S**QPSAGR  KANSQP**S**AGRAPI  AGRAPI**S**NPGMSE  ISNPGM**S**EAGNFG  GNFGGN**S**FMPANG  NQLKHM**S**VSSIKQ  LKHMSV**S**SIKQAV  KHMSVS**S**IKQAVD  QAVDFL**S**NEGHIY  NEGHIY**S**TVDDDH  LTVVTG**S**GHASST  SPGSGS**S**TTQGQD  TEPASG**S**AATWGQ  PAHGVT**S**APDTRP  TRPAPG**S**TAPPAH  HNVTSA**S**GSASGS  SASGSA**S**GSASTL  SGSASG**S**ASTLVH  STKTDA**S**STHHSS  TKTDAS**S**THHSSV  VPPLTS**S**NHSTSP  FLSFHI**S**NLQFNS  GRYVPP**S**STDRSP  SPYEKV**S**AGNGGS  EFPGLG**S**LGTSEP  LGSLGT**S**EPLPQF  VDPALV**S**STPESG  VSSTPE**S**GVFFPS  SGVFFP**S**GPEGLD  GLDAAA**S**STAPST  LDAAAS**S**TAPSTA  ASSTAP**S**TATAAA  EGIPGG**S**PYAGWA  TGLYPA**S**TVCPTR  CPTRED**S**PPQAVE  DGKGST**S**FLETLK  LGPALP**S**SLPVPN  GPALPS**S**LPVPNS  SLPVPN**S**AYGGPD  YGGPDF**S**STFFSP  GGPDFS**S**TFFSPT  FSSTFF**S**PTGSPL  TGSPLN**S**AAYSSP  LNSAAY**S**SPKLRG  KKRLIV**S**KRAGTQ  LWRRNA**S**GDPVCN  GKKKRG**S**SLGGTG  KKKRGS**S**LGGTGA  MVVAGG**S**GSGNCG  VAGGSG**S**GNCGEV  NCGEVA**S**GLTLGP  LGPVVL**S**GPVSHL  VLSGPV**S**HLMPFP  LGSPTG**S**FPTGPM  PMPPTT**S**TTVVAP  PRRPAG**S**RLRLLL  LLLLRG**S**HAGNLT  LPLANT**S**YPWSWA  NTSYPW**S**WARVGP  TTRAGP**S**YAKLGD  FAEDDL**S**HYTRLL  RVIYIC**S**SPDAFR  LDIFGQ**S**LQGGQG  VNTIPA**S**FHDGLL  YLKIDS**S**GDRETD  DRETDF**S**LWDMDP  LNYNGT**S**QELVAV  LEKELA**S**ELWRVR  DILENE**S**ITLDWM  HGNLKS**S**NCVVDG  ELLRMA**S**PPVRGS  SPPVRG**S**QAGDVY  QAGDVY**S**FGIILQ  VEGLDL**S**PKEIIE  QPPFRP**S**LALQSH  PSLALQ**S**HLEELG  KFNREN**S**SNILDN  FNRENS**S**NILDNL  ILDNLL**S**RMEQYA  YQILPH**S**VAEQLK  VGFTAL**S**AESTPM  DAYMVV**S**GLPVRN  LLDAVR**S**FRIRHR  DTVNTA**S**RMESNG  ALKIHL**S**SETKAV  LKIHLS**S**ETKAVL  LCLVPL**S**LAQIDL  EKNGRY**S**ISRTEA  NGRYSI**S**RTEAAD  LCKAFN**S**TLPTMA  QMEKAL**S**IGFETC  PTDDDV**S**SGSSSE  TDDDVS**S**GSSSER  DDVSSG**S**SSERSS  DVSSGS**S**SERSST  VSSGSS**S**ERSSTS  SSSERS**S**TSGGYI  SERSST**S**GGYIFY  PIPDED**S**PWITDS  SPWITD**S**TDRIPA  PATTLM**S**TSATAT  TTLMST**S**ATATET  ETWDWF**S**WLFLPS  DDEDFI**S**STISTT  DEDFIS**S**TISTTP  EEETPH**S**TSTIQA  ETPHST**S**TIQATP  TIQATP**S**STTEET  TGTAAA**S**AHTSHP  PSPEDS**S**WTDFFN  DMDSSH**S**ITLQPT  LLEGYT**S**HYPHTK  TVGDSN**S**NVNRSL  SESDGH**S**HGSQEG  DGHSHG**S**QEGGAN  WLIILA**S**LLALAL  HLVNKE**S**SETPDQ  SPDALG**S**TTAVQT  SGEPLV**S**TSEPLS  TSEPLS**S**KMYTTS  SKMYTT**S**ITSDPK  YTTSIT**S**DPKADS  SDPKAD**S**TGDQTS  STGDQT**S**ALPPST  ALPPST**S**INEGSP  WTSIGA**S**TGSPLP  IGASTG**S**PLPEPT  EVSIKM**S**SVPQET  VSIKMS**S**VPQETP  ETPHAT**S**HPAVPI  GTITTN**S**PETSSR  TNSPET**S**SRTSGA  NSPETS**S**RTSGAP  ETSSRT**S**GAPVTT  PVTTAA**S**SLETSR  ASSLET**S**RGTSGP  LTMATV**S**LETSKG  ETSKGT**S**GPPVTM  VTMATD**S**LETSTG  TDSLET**S**TGTTGP  EPSSGA**S**GPQVSS  VSSVKL**S**TMMSPT  KLSTMM**S**PTTSTN  SGGDKG**S**GFPDGE  FPDGEG**S**SRRPTL  PDGEGS**S**RRPTLT  EELKSG**S**GPSLKG  EEPLVA**S**EDGAVD  DLCRLF**S**GDVVLT  LQAEGL**S**PRFHQL  DIDDLQ**S**IRALRD  GRVVNV**S**SIMSVR  RVVNVS**S**IMSVRA  NVSSIM**S**VRALKS  SVRALK**S**CSPELQ  RALKSC**S**PELQQK  LQQKFR**S**ETITEE  QKEGWP**S**SAYGVT  KEGWPS**S**AYGVTK  IGVTVL**S**RIHARK  IHARKL**S**EQRKGD  GPKATK**S**PEEGAE  FLRAAR**S**GNLDKA  KTKNGL**S**PIHMAA  LLKTGA**S**IDAVTE  GASPNV**S**NVKVET  ASPNEV**S**SDGTTP  VVTDET**S**FVLVSD  EGEELI**S**FKAERR  KAERRD**S**RDVDEE  EDSLIP**S**SPATET  ETSDNI**S**PVASPV  VDARGG**S**MRGSRH  VKPQKL**S**TPPPLA  EEEGLA**S**RIIALG  ELVVLR**S**ENGSVW  RIPLPP**S**WTDNPR  SLRLLC**S**VIGGTD  KAAQQR**S**FHFQSF  PPCAKG**S**GAEDRR  SEHLGL**S**WAELAR  RVENPN**S**LLEQSV  NSLLEQ**S**VALLNL  EGSGRQ**S**RNLKPD  LGCALS**S**PLRADQ  VTAEDS**S**LECSKA  RGPELG**S**LELVED  QEEGQR**S**EEKLPG  THSPTV**S**QVTERS  SQVTER**S**QDRLQD  VTQGPH**S**FQGTST  HSFQGT**S**TMTEGL  YEKVLV**S**VSEHTW  EQPEAE**S**SQADRD  DYFMKH**S**KDHTST  GAENQQ**S**GDAAVT  ATLAQV**S**MPAAHA  PAAHAT**S**SAPTVT  AAHATS**S**APTVTL  IQAAQP**S**VIQSPQ  QPSVIQ**S**PQVQTV  QVQTVQ**S**SCKDLK  VQTVQS**S**CKDLKR  DLKRLF**S**GTQIST  FSGTQI**S**TIAESE  ISTIAE**S**EDSQES  IAESED**S**QESVDS  SEDSQE**S**VDSVTD  SQESVD**S**VTDSQK  VDSVTD**S**QKRREI  KRREIL**S**RRPSYR  ILNDLS**S**DAPGVP  RIEEEK**S**EEETSA  KSEEET**S**APAITT  TPIYQT**S**SGQYIA  PIYQTS**S**GQYIAI  QQILVP**S**NQVVVQ  VVVQAA**S**GDVQTY  IRTAPT**S**TIAPGV  PGVVMA**S**SPALPT  KVVQLN**S**SFSLRC  FGESEV**S**WQYPMS  TVLEVS**S**ASAAHT  EGVVPA**S**YDSRQG  YALKAT**S**ELDLEM  EIKPTF**S**QLEAVN  QEIRYR**S**KLKLIR  RAKEED**S**GHYTIV  NEDAVK**S**YTFELL  LTQVPS**S**ILDLVD  VDDHHG**S**TGGQTV  ILANNV**S**NIITEI  LVIVII**S**LIVLVV  LKPTAR**S**SEKQAL  ADESTR**S**YVILSF  KKSMLD**S**EVKNLL  LLSDDN**S**EGLTLL  TLLDLL**S**FTYQVA  RDIMHD**S**NYVSKG  NYVSKG**S**TFLPVK  NLYTTL**S**DVWSYG  LLWEIF**S**LGGTPY  PGMMVD**S**TFYNKI  KPDHAT**S**EVYEIM  PSFYHL**S**EIVENL  PGQYKK**S**YEKIHL  ARMRVD**S**DNAYIG  QRLSAD**S**GYIIPL  KRNRHS**S**QTSEES  SQTSEE**S**AIETGS  SAIETG**S**SSSTFI  DDIGID**S**SDLVED  DIGIDS**S**DLVEDS  LTLLLC**S**SLEGQE  NSVDMK**S**LPDWTV  ADVSTT**S**HVKPQH  VLFYNI**S**SMKSTE  LFYNIS**S**MKSTES  EVRIYD**S**GTYKCT  IVRVNC**S**VPEEKA  EQDRVL**S**FRCQAR  CQARII**S**GIHMQT  HMQTSE**S**TKSELV  TSESTK**S**ELVTVT  TVTESF**S**TPKFHI  TPKFHI**S**PTGMIM  MAMVEH**S**GNYTCK  KPELES**S**FTHLDQ  GERLNL**S**CSIPGA  RLNLSC**S**IPGAPP  DFTKIA**S**KSDSGT  TKIASK**S**DSGTYI  DKVVKK**S**NTVQIV  IEVRCE**S**ISGTLP  SGTLPI**S**YQLLKT  YQLLKT**S**KVLENS  SHAKML**S**EVLRVK  VDEVQI**S**ILSSKV  CAVNEG**S**GPITYK  RANHAS**S**VPRSKI  ASSVPR**S**KILTVR  QMPVEM**S**RPAVPL  YTEVQV**S**SAESHK  QVSSAE**S**HKDLGK  DTETVY**S**EVRKAV  AVESRY**S**RTEGSL  ATGPPV**S**ELITKA  TKAVAA**S**KERNGL  KERNGL**S**LAALKK  DVEKNN**S**RIKLGL  IKLGLK**S**LVSKGT  GLKSLV**S**KGTLVQ  TKGTGA**S**GSFKLN  LNKKAA**S**GEAKPK  PAVVLA**S**SRGIAS  AVVLAS**S**RGIASF  SSRGIA**S**FVCEYA  FVCEYA**S**PGKATE  VLRQAD**S**QVTEVC  LTFLDD**S**ICTGTS  SICTGT**S**SGNQVN  ICTGTS**S**GNQVNL  PEPCPD**S**DFLLWI  WILAAV**S**SGLFFY  ILAAVS**S**GLFFYS  SGLFFY**S**FLLTAV  FLLTAV**S**LSKMLK  LTAVSL**S**KMLKKR  KMLKKR**S**PLTTGV  SHVDTS**S**TVSEAV  DTSSTV**S**EAVAEE  AVAEEV**S**LFSMTD  EEVSLF**S**MTDMIL  TDMILF**S**LIVGLL  KIQTLT**S**SVRESS  IQTLTS**S**VRESSF  TSSVRE**S**SFVEKM  SSVRES**S**FVEKMK  IIVFYG**S**QTGTAE  EFANRL**S**KDAHRY  YGMRGM**S**ADPEEY  DLADLS**S**LPEIDN  ETDVDL**S**GVKFAV  ATGEES**S**IRQYEL  HLELDI**S**DSKIRY  ELDISD**S**KIRYES  SKIRYE**S**GDHVAV  DLDVVM**S**LNNLDE  NNLDEE**S**NKKHPF  ELAQYA**S**EPSEQE  QYASEP**S**EQELLR  LRKMAS**S**SGEGKE  RKMASS**S**GEGKEL  GKELYL**S**WVVEAR  ILQDCP**S**LRPPID  LQARYY**S**IASSSK  RYYSIA**S**SSKVHP  YSIASS**S**KVHPNS  SKVHPN**S**VHICAV  YYGCRR**S**DEDYLY  QLNVAF**S**REQSHK  AFSREQ**S**HKVYVQ  IPVAED**S**DFPIHN  LPYGVF**S**TRGDPR  DQILDL**S**IIKHLF  FTGPVL**S**KHQDVF  NQPTLN**S**FMGLGQ  FLQNLL**S**VSQARL  QNLLSV**S**QARLRD  RKCAFI**S**QASATM  AFISQA**S**ATMHLP  DYTDFY**S**SRQHAT  YTDFYS**S**RQHATN  GYHGRA**S**SVVVSG  YHGRAS**S**VVVSGT  ASSVVV**S**GTPIRR  QMKPDD**S**KPPVYG  GEPIPI**S**KAHEHI  VLMNDW**S**ARDIQK  GPFLGK**S**FGTTVS  SFGTTV**S**PWVVPM  TFDINL**S**VNLKGE  LKGEGM**S**QAATIC  AATICK**S**NFKYMY  QQLTHH**S**VNGCNL  PGDLLA**S**GTISGP  LASGTI**S**GPEPEN  EPENFG**S**MLELSW  GSMLEL**S**WKGTKP  LILSPR**S**KESVPE  PKKKDL**S**LEEIQK  EENNNF**S**KMAEEK  VRKNKE**S**KDPADE  SESSSK**S**SQPLAS  ESSSKS**S**QPLASK  SSQPLA**S**KQEKDG  RGRPKG**S**KNKGAA  DKVQAY**S**TAGGKV  GGKVWL**S**VLFIFR  LGTAVE**S**AWGDEQ  AWGDEQ**S**AFRCNT  NVCYDK**S**FPISHV  DKSFPI**S**HVRFWV  LQIIFV**S**VPTLLY  LRTYII**S**ILFKSI  ISILFK**S**IFEVAF  WYIYGF**S**LSAVYT  IYGFSL**S**AVYTCK  QVDCFL**S**RPTEKT  IFMLVV**S**LVSLAL  LVVSLV**S**LALNII  DRVKGK**S**DPYHAT  DPYHAT**S**GALSPA  AYFNGC**S**SPTAPL  YFNGCS**S**PTAPLS  SPTAPL**S**PMSPPG  APLSPM**S**PPGYKL  TGDRNN**S**SCRNYN  GDRNNS**S**CRNYNK  NYNKQA**S**EQNWAN  IVDQRP**S**SRASSR  RASSRA**S**SRPRPD  KGSEQE**S**VKEFLA  FLKKWE**S**PAQNTA  KTLGTG**S**FGRVML  LVKLEF**S**FKDNSN  FSFKDN**S**NLYMVM  PGGEMF**S**HLRRIG  TFEYLH**S**LDLIYR  APEIIL**S**KGYNKA  IYEKIV**S**GKVRFP  GKVRFP**S**HFSSDL  RFPSHF**S**SDLKDL  FPSHFS**S**DLKDLL  KGPGDT**S**NFDDYE  SQAYSS**S**QRVSSY  SSQRVS**S**YRRTFG  PLGSPL**S**SPVFPR  LGSPLS**S**PVFPRA  PRAGFG**S**KGSSSS  GFGSKG**S**SSSVTS  FGSKGS**S**SSVTSR  GSKGSS**S**SVTSRV  SKGSSS**S**VTSRVY  SSSSVT**S**RVYQVS  SRVYQV**S**RTSGGA  LGSLRA**S**RLGTTR  GTTRTP**S**SYGAGE  TTRTPS**S**YGAGEL  GELLDF**S**LADAVN  LERRIE**S**LNEEIA  QVEMDM**S**KPDLTA  IAAKNI**S**EAEEWY  AEEWYK**S**KVSDLT  WYKSKV**S**DLTQAA  YRHQIQ**S**YTCEID  LKGTND**S**LMRQMR  LEDRFA**S**EASGYQ  RFASEA**S**GYQDNI  LLEGEE**S**RINLPI  LPIQTY**S**ALNFRE  LNFRET**S**PEQRGS  SPEQRG**S**EVHTKK  RDGEVV**S**EATQQQ  PPPAFK**S**MEVANF  PAGELG**S**IGDHER  ERAIDF**S**PYLEPL  PAPAPA**S**SGQHHD  APAPAS**S**GQHHDF  QHHDFL**S**DLFSDD  FLSDLF**S**DDYGGK  AEYGYV**S**LGRLGA  GYQAVP**S**GSSGSL  QAVPSG**S**SGSLST  AVPSGS**S**GSLSTS  PSGSSG**S**LSTSSS  GSSGSL**S**TSSSSS  SGSLST**S**SSSSPP  GSLSTS**S**SSSPPG  SLSTSS**S**SSPPGT  LSTSSS**S**SPPGTP  STSSSS**S**PPGTPS  SPPGTP**S**PADAKA  GAAPAP**S**QVKSKA  APSQVK**S**KAKKTV  KTVDKH**S**DEYKIR  NIAVRK**S**RDKAKM  KKVEQL**S**RELSTL  QLSREL**S**TLRNLF  LLEVWF**S**RQQPDA  PDANQG**S**GDLRTI  LRTIPR**S**EWDILL  LKDVQC**S**IISVTK  VQCSII**S**VTKTDK  QEAYVL**S**ESSMFV  AYVLSE**S**SMFVSK  YVLSES**S**MFVSKR  ESSMFV**S**KRRFIL  KLARDY**S**GFDSIQ  DYSGFD**S**IQSFFY  GFDSIQ**S**FFYSRK  IQSFFY**S**RKNFMK  KNFMKP**S**HQGYPH  CMGRMN**S**DCWYLY  TLDFPE**S**RVISQP  PESRVI**S**QPDQTL  TLEILM**S**ELDPAV  KDVTRE**S**GIRDLI  RDLIPG**S**VIDATM  FNPCGY**S**MNGMKS  SMNGMK**S**DGTYWT  TPEPEF**S**YVSFET  PEFSYV**S**FETNLS  SFETNL**S**QTSYDD  TNLSQT**S**YDDLIR  TLFVNQ**S**SKCRTV  LFVNQS**S**KCRTVL  CRTVLA**S**PQKIEG  KRLDCQ**S**AMFNDY  YNFVFT**S**FAKKQQ  PYMIQM**S**SKGNLP  YMIQMS**S**KGNLPS  SKGNLP**S**ILDVHV  VNVGGR**S**SVPGKM  NVGGRS**S**VPGKMK  GRKARW**S**VRPSDM  RWSVRP**S**DMAKKT  PNKTMI**S**LSIGDP  KTMISL**S**IGDPTV  MKDALD**S**GKYNGY  YNGYAP**S**IGFLSS  PSIGFL**S**SREEIA  SIGFLS**S**REEIAS  SREEIA**S**YYHCPE  KDVILT**S**GCSQAI  ILTSGC**S**QAIDLC  VPRPGF**S**LYKTLA  YKTLAE**S**MGIEVK  NLLPEK**S**WEIDLK  LIVNNP**S**NPCGSV  PSNPCG**S**VFSKRH  PCGSVF**S**KRHLQK  YGDMVF**S**DCKYEP  EPLATL**S**TDVPIL  TDVPIL**S**CGGLAK  DGLVKL**S**QRILGP  VQGALK**S**ILCRTP  FYHNTL**S**FLKSNA  TLSFLK**S**NADLCY  LRPVRP**S**GAMYLM  RLVAEQ**S**VHCLPA  MMLEAC**S**RIQEFC  LEKLRA**S**GAGKAI  AIGVLT**S**GGDAQG  KQANWL**S**VSNIIQ  GGTIIG**S**ARCKAF  GANIFR**S**EWGSLL  FRSEWG**S**LLEELV  TTARTY**S**HLNIAG  ITTTAQ**S**HQRTFV  LVSALA**S**GADWLF  RLGETR**S**RGSRLN  ETRSRG**S**RLNIII  RNGKPI**S**SSYVKD  NGKPIS**S**SYVKDL  GKPISS**S**YVKDLV  QRGGTP**S**AFDRIL  AFDRIL**S**SKMGME  FDRILS**S**KMGMEA  ACVVTL**S**GNQSVR  KPPKEK**S**NFSLAI  KEKSNF**S**LAILNV  MNAAVR**S**AVRTGI  AVRTGI**S**HGHTVY  WLGRGG**S**MLGTKR  PKGQLE**S**IVENIR  VIPATI**S**NNVPGT  VPGTDF**S**LGSDTA  VNAAME**S**CDRIKQ  CDRIKQ**S**ASGTKR  RIKQSA**S**GTKRRV  FLYNLY**S**SEGKGV  LYNLYS**S**EGKGVF  KKAVAF**S**PVTELK  LAQYRI**S**MAAYVS  HDALSG**S**GNPNPQ  GGYPGA**S**YPGAYP  VYPGPP**S**GPGAYP  GPGAYP**S**SGQPSA  PGAYPS**S**GQPSAT  PSSGQP**S**ATGAYP  GREERQ**S**VFPFES  SVFPFE**S**GKPFKI  KKLNEI**S**KLGISG  ISKLGI**S**GDIDLT  VARAGA**S**SAGLGP  ARAGAS**S**AGLGPV  CLTCAL**S**EGQPCG  YTERCG**S**GLRCQP  GLRCQP**S**PDEARP  GLCVNA**S**AVSRLR  VNASAV**S**RLRAYL  PAPGNA**S**ESEEDR  PGNASE**S**EEDRSA  ESEEDR**S**AGSVES  SAGSVE**S**PSVSST  GSVESP**S**VSSTHR  VESPSV**S**STHRVS  ESPSVS**S**THRVSD  SSTHRV**S**DPKFHP  KFHPLH**S**KIIIIK  KGHAKD**S**QRYKVD  YKVDYE**S**QSTDTQ  VDYESQ**S**TDTQNF  DTQNFS**S**ESKRET  QNFSSE**S**KRETEY  KFLNVL**S**PRGVHI  KKQCRP**S**KGRKRG  SLPEMV**S**KESERL  NGKQFC**S**TLTLNT  YLAVPT**S**KKKETE  PFVEMY**S**EIPEII  IPCRVT**S**PNITVT  HANIFY**S**VLTIDK  FKSVNT**S**VHIYDK  CSNNEE**S**FILDAD  FILDAD**S**NMGNRI  MGNRIE**S**ITQRMA  TLVVAD**S**RISGIY  TMHYSI**S**KQKMAI  TVAISS**S**TTLDCH  ILGPGS**S**TLFIER  LTVQGT**S**DKSNLE  LDSVTS**S**ESFASS  SVTSSE**S**FASSGF  SSESFA**S**SGFQED  SESFAS**S**GFQEDK  QEDKSL**S**DVEEEE  GMEFLS**S**RKCIHR  MDEDFC**S**RLREGM  NSGFTY**S**TPAFSE  SAPKFN**S**GSSDDV  PKFNSG**S**SDDVRY  LLPNAT**S**MFDDYQ  DDYQGD**S**STLLAS  DSKPKA**S**LKIDLR  LRVTSK**S**KESGLS  TSKSKE**S**GLSDVS  SDVSRP**S**FCHSSC  PSFCHS**S**CGHVSE  RKIACC**S**PPPDYN  SVFGDR**S**TGETIR  TGETIR**S**QNVMAA  NVMAAA**S**IANIVK  IANIVK**S**SLGPVG  ANIVKS**S**LGPVGL  VGDGTT**S**VVIIAA  QKIHPT**S**VISGYR  HPTSVI**S**GYRLAC  AAKTSM**S**SKIIGI  AKTSMS**S**KIIGIN  PRYPVN**S**VNILKA  LKAHGR**S**QMESML  GRSQME**S**MLISGY  MESMLI**S**GYALNC  LNCVVG**S**QGMPKR  IACLDF**S**LQKTKM  QIRQRE**S**DITKER  KRIAKA**S**GATILS  SGATIL**S**TLANLE  NTKART**S**ASIILR  KARTSA**S**IILRGA  CDEMER**S**LHDALC  VKRVLE**S**KSVVPG  RVLESK**S**VVPGGG  AVEAAL**S**IYLENY  LENYAT**S**MGSREQ  YATSMG**S**REQLAI  IAEFAR**S**LLVIPN  VNAAQD**S**TDLVAK  WIGLDL**S**NGKPRD  TIVKVK**S**LKFATE  IKLHPE**S**KDDKHG  KDDKHG**S**YEDAVH  FEQIDK**S**GSWAAI  QIDKSG**S**WAAIYQ  DIRHEA**S**DFPCRV  VSPFDH**S**RIKLHQ  NDYINA**S**LIKMEE  MEEAQR**S**YILTQG  MVWEQK**S**RGVVML  RVMEKG**S**LKCAQY  LKLTLI**S**EDIKSY  ISEDIK**S**YYTVRQ  DFGVPE**S**PASFLN  VPESPA**S**FLNFLF  LFKVRE**S**GSLSPE  KVRESG**S**LSPEHG  RESGSL**S**PEHGPV  PVVVHC**S**AGIGRS  SAGIGR**S**GTFCLA  ADQLRF**S**YLAVIE  KFIMGD**S**SVQDQW  FIMGDS**S**VQDQWK  DQWKEL**S**HEDLEP  APYGIE**S**MSQDTE  YGIESM**S**QDTEVR  QDTEVR**S**RVVGGS  PAKGEP**S**LPEKDE  DEDHAL**S**YWKPFL  RGINGV**S**PKVRKV  VKLNKA**S**INMLRI  GYPNLK**S**VNELIY  NALIAR**S**LGKYGI  LWPFKL**S**SPRGGM  WPFKLS**S**PRGGMK  HTRTIE**S**ILEPVA  PVAQQI**S**HLVIMH  DYLIDG**S**RGILSG  GSRGIL**S**GTSDLL  RVMLVN**S**MNTVKE  LLPVLI**S**AMKIFV  FVTTKN**S**KNQGIE  FTVEKM**S**AEINEI  RVLQLT**S**WDEDAW  DEDAWA**S**KDTEAM  GWLRDP**S**ASPGDA  ARGQGS**S**PVAMQK  LEAMTN**S**KQSIAK  MTNSKQ**S**IAKKID  RDDILR**S**LGEISA  EISALT**S**KLADLR  ARGEGE**S**PQARAL  EVSDVF**S**DTTTPI  KSLLDA**S**EEAIKK  LVAGAT**S**IARRAN  EAVKAA**S**DELSKT  AASDEL**S**KTISPM  ELSKTI**S**PMVMDA  QKSFLD**S**GYRILG  EARKWS**S**KPGIPA  REATKW**S**SKGNDI  RLVRGG**S**GTKRAL  KDIAKA**S**DEVTRL  ERIPTI**S**TQLKIL  TQLKIL**S**TVKATM  NISDEE**S**EQATEM  AQNLMQ**S**VKETVR  REAEAA**S**IKIRTD  YGIIMH**S**SVYSSP  GIIMHS**S**VYSSPA  MHSSVY**S**SPAAAG  HSSVYS**S**PAAAGL  LPDFDG**S**EPPGAG  EPPGAG**S**PASAPR  GAGSPA**S**APRAAA  KVLDQL**S**AGKHLQ  AGKHLQ**S**LVARGV  ARGVGG**S**LGGGAG  DDAEPL**S**KRHSDG  PLSKRH**S**DGIFTD  DGIFTD**S**YSRYRK  IFTDSY**S**RYRKQM  LIRHGE**S**AWNLEN  NLENRF**S**GWYDAD  WYDADL**S**PAGHEE  FDICFT**S**VQKRAI  VKIWRR**S**YDVPPP  PDHPFY**S**NISKDR  PFYSNI**S**KDRRYA  TEDQLP**S**CESLKD  QLPSCE**S**LKDTIA  IAAHGN**S**LRGIVK  KHLEGL**S**EEAIME  ADAIPK**S**KKVKVS  SKKVKV**S**HRSHST  VKVSHR**S**HSTEPG  KKPALV**S**IPEDVV  MHTVCL**S**KSGQVY  TVCLSK**S**GQVYSF  KSGQVY**S**FGCNDE  ALGRDT**S**VEGSEM  DTSVEG**S**EMVPGK  EKVVQV**S**AGDSHT  QVSAGD**S**HTAALT  LEPMKK**S**MVPVQV  PVVKVA**S**GNDHLV  KCVMLK**S**RGSRGH  MLKSRG**S**RGHVRF  YFTFAI**S**HEGHVY  VYGFGL**S**NYHQLG  IPQNLT**S**FKNSTK  LTSFKN**S**TKSWVG  FKNSTK**S**WVGFSG  KSWVGF**S**GGQHHT  HTVCMD**S**EGKAYS  SEGKAY**S**LGRAEY  EGAEEK**S**IPTLIS  SRLPAV**S**SVACGA  RLPAVS**S**VACGAS  SVACGA**S**VGYAVT  QDEDAW**S**PVEMMG  ENRVVL**S**VSSGGQ  RVVLSV**S**SGGQHT  VVLSVS**S**GGQHTV  RLRHVV**S**CSSQDS  SCSSQD**S**THCAEN  KEEQIH**S**VDIGND  DIGNDG**S**AFVEVL  VEVLVG**S**SAGGAG  EVLVGS**S**AGGAGE  EVLLVT**S**SFMSPS  VTSSFM**S**PSESRS  SSFMSP**S**ESRSGS  FMSPSE**S**RSGSNP  SPSESR**S**GSNPNR  RVKIVC**S**QPYSKD  VCSQPY**S**KDSPFG  DSPFGL**S**FVRFHS  SFVRFH**S**PPDKDE  VKEEDE**S**ANSLRP  EDESAN**S**LRPGAL  PGALFF**S**RINKTS  AATLQA**S**SAASSA  ASSAAS**S**ASPVSR  SSASPV**S**RAIGST  RAIGST**S**KPQESP  PAQLSP**S**VPKRPK  GVVVVL**S**GFQNPF  FQNPFR**S**ELRDKA  SEEDEA**S**HSGGSG  EDEASH**S**GGSGDE  ELEDNM**S**DRVQFV  AQEWDP**S**FEEALM  ALMDNP**S**LAFVRP  RPRWIY**S**CNEKQK  SCLAFR**S**PLSVFK  ETTRPF**S**NECLGT  VVPIDS**S**DFALDI  GVTPKQ**S**DTYFCM  DTYFCM**S**MRIPVD  DFKPRA**S**MDTVHH  MPSSTG**S**YWFCDE  DNNKDC**S**GVSLHL  KDCSGV**S**LHLTRL  VVNSDI**S**CHYKNY  HLGKVV**S**GYRVRN  IPIPVK**S**DMVMMH  KKDLGR**S**DAREGA  TLRPPE**S**RVFSLQ  LLPGQV**S**GVALDP  HVWDGN**S**FDSKFV  RSMQPG**S**DQNHFC  TGAIYV**S**DGYCNS  SDGYCN**S**RIVQFS  SRIVQF**S**PSGKFI  IVQFSP**S**GKFITQ  QWGEES**S**GSSPLP  PHDIVA**S**EDGTVY  EKLEHR**S**VKKAGI  ENKPTS**S**ELQKMQ  LIKEPG**S**GVPVVL  SRAFGD**S**EHKLET  EHKLET**S**SGRVLG  EKEDDG**S**ESEEEY  EDDGSE**S**EEEYSA  ESEEEY**S**APLPAL  QASVEA**S**GEIALC  LLNVKF**S**NCNGKR  KVQYES**S**EPADFK  MVYAVR**S**FPLSSE  RSFPLS**S**EHAKFL  QVAVKL**S**LKPTLT  PTLTEE**S**VKESAE  VFPRQF**S**KHSGHL  ELVRIR**S**DRDKNL  DRDKNL**S**LRYSVT  NLSLRY**S**VTGPGA  FIINPI**S**GQLSVT  PISGQL**S**VTKPLD  LRYRIV**S**QAPSTP  IVSQAP**S**TPSPNM  QAPSTP**S**PNMFTI  NPTYGL**S**NTATAV  NAVYRI**S**GGDPTG  IQTDPN**S**NDGLVT  QSTATV**S**VTVIDV  IRYTKL**S**DPANWL  AVLDRE**S**PNVKNN  NGIPPM**S**GTGTLQ  ETPDPN**S**INITAL  AFDLPL**S**PVTIKR  PPKSNI**S**ILRVKV  DQDYDL**S**QLQQPD  PQYPVR**S**AAPHPG  SGSTAG**S**LSSLNS  SLSSLN**S**SSSGGE  SLNSSS**S**GGEQDY  YICQYL**S**TVRERR  RAQLPE**S**APEDPD  APEDPD**S**WDSIFG  DPDSWD**S**IFGDIE  YYPALT**S**WPSLLG  GFTWAS**S**PACTEL  FLHHHP**S**SQGGGV  LHHHPS**S**QGGGVL  ILEMKT**S**EPDADE  EPDADE**S**CLNARL  RLVAYA**S**DQAHSS  ASDQAH**S**SVEKAG  EKAGLI**S**LVKMKF  GIEYAD**S**FTFNPS  KLQQTF**S**VNPIYL  HWQIPL**S**RRFRSV  LSRRFR**S**VKLWFV  LWFVIR**S**FGVKNL  LVRNDP**S**FEIPAK  AATLIL**S**QHCTSQ  HCTSQP**S**PRVGNL  RVGNLI**S**QIRGAR  TSLQSV**S**GAGDDP  PVDDCF**S**EEAPDA  ATKHKL**S**SFLFSY  LSSFLF**S**YLSVQT  FLFSYL**S**VQTKKK  KTVRSL**S**CNSVPV  RSLSCN**S**VPVSAQ  CNSVPV**S**AQKPLP  PLPTEA**S**VKNGGS  KLIKFY**S**VPSFPE  PSFPEC**S**SQCGLQ  CGPGAP**S**DAEVLH  VLHLCR**S**LEVGTV  LFYSKK**S**QRPERK  IRPGKT**S**RDFDRY  LSLQAT**S**EDEVNM  TDLEQR**S**GDITYG  TGDQFS**S**ESSLEA  DQFSSE**S**SLEAYA  TTKIKF**S**DVLHTI  TKPVEI**S**ADGLPS  YEEVPT**S**MMYSEN  PTSMMY**S**ENDISN  HYFVLT**S**SKIYYS  PKEVSS**S**TELHSN  VGDYTL**S**FWRNGK  QHCRIH**S**RQDAGT  DNLVFD**S**LYDLIT  GNSEFD**S**LVDLIS  SLVDLI**S**YYEKHP  EHLDEN**S**PLGDLL  KIALEL**S**ELVVYC  ACYRDM**S**SFPETK  YNRLQL**S**RIYPKG  GYVLQP**S**TMRDEA  FDPFDK**S**SLRGLE  LEPCAI**S**IEVLGA  PFHFQI**S**NPEFAF  PLKNNY**S**EDLELA  SLRERG**S**DASGQL  REGSFE**S**RYQQPF  FEDFRI**S**QEHLAD  LYAMRL**S**DETLID  EMKNGL**S**RDFNPT  LPTFVR**S**IPDGSE  RSIPDG**S**EKGDFI  ALDLGG**S**SFRILR  LDLGGS**S**FRILRV  QNVHME**S**EVYDTP  ENIVHG**S**GSQLFD  AFGDDG**S**LEDIRT  REIDRG**S**LNPGKQ  LFEKMV**S**GMYLGE  FNTSDV**S**AIEKNK  RLGVEP**S**DDDCVS  TIVSFR**S**ANLVAA  TVGVDG**S**LYKTHP  KTHPQY**S**RRFHKT  RRLVPD**S**DVRFLL  DVRFLL**S**ESGSGK  RFLLSE**S**GSGKGA  VVKMLP**S**FVRRTP  LLVKIR**S**GKKRTV  LFDHIV**S**CISDFL  HIVSCI**S**DFLDYM  PLGFTF**S**FPCQQT  LIVGTG**S**NACYME  RLVDEY**S**LNAGKQ  FLSQIE**S**DRLALL  QQLGLN**S**TCDDSI  NSTCDD**S**ILVKTV  TVCGVV**S**RRAAQL  KLHPHF**S**RIMHQT  SPKCNV**S**FLLSED  NVSFLL**S**EDGSGK  NGWLPI**S**AMNKVA  PCMLRN**S**DSILEA  MLRNSD**S**ILEAIQ  PKPGPR**S**GRFSCE  PRSGRF**S**CEPAGG  PAGGLT**S**LTEPPK  GNGHII**S**WTSRDG  HIISWT**S**RDGGEF  MNYDKL**S**RALRYY  FVYKFV**S**YPEVAG  PEVSVT**S**TMPNVA  APGDTV**S**GKPGTP  MRSGLY**S**TFTIQS  STFTIQ**S**LQPQPP  PAVVLP**S**AAPAGA  GAAAPP**S**GSRSTS  AAPPSG**S**RSTSPS  PPSGSR**S**TSPSPL  SRSTSP**S**PLEACL  EAPNLK**S**EELNVE  AGGHAA**S**SPEISQ  GGHAAS**S**PEISQP  ASSPEI**S**QPQKGR  ELPLSP**S**LLGGPG  PERTPG**S**GSGSGL  RTPGSG**S**GSGLQA  PGSGSG**S**GLQAPG  VLLTPS**S**LPPSIH  PSSLPP**S**IHFWST  PSIHFW**S**TLSPIA  RSPAKL**S**FQFPSS  LSFQFP**S**SGSAQV  SFQFPS**S**GSAQVH  QFPSSG**S**AQVHIP  AQVHIP**S**ISVDGL  VHIPSI**S**VDGLST  ISVDGL**S**TPVVLS  PHAKKK**S**KISASR  PEGEGR**S**KKEAKN  LTTTNS**S**EGLSMG  NSSEGL**S**MGNYIG  MGQKEY**S**IGTGST  YSIGTG**S**TKQEAK  AYLQIL**S**EETSVK  ILSEET**S**VKSDYL  EETSVK**S**DYLSSG  KSDYLS**S**GSFATT  TTCESQ**S**NSLVTS  CESQSN**S**LVTSTL  SNSLVT**S**TLASES  STLASE**S**SSEGDF  TLASES**S**SEGDFS  LASESS**S**EGDFSA  SSEGDF**S**ADTSEI  DFSADT**S**EINSNS  SEINSN**S**DSLNSS  INSNSD**S**LNSSSL  DSLNSS**S**LLMNGL  DYDPET**S**DDSLES  SDDSLE**S**SDYDPE  DDSLES**S**DYDPEN  PENSKN**S**SRSKTK  SKNSSR**S**KTKCLF  GVDYIH**S**KKLIHR  MSPEQI**S**SQDYGK  SPEQIS**S**QDYGKE  DTAFET**S**KFFTDL  LLQKLL**S**KKPEDR  EDRPNT**S**EILRTL  FLPLDF**S**TQVNSS  FSTQVN**S**SLTSPT  GSMAAP**S**LHPSLG  APSLHP**S**LGPGIG  LGPGIG**S**PGQLHS  SPGQLH**S**PISTLS  QLHSPI**S**TLSSPI  SPISTL**S**SPINGM  PPFSVI**S**SPMGPH  SPMGPH**S**MSVPTT  MGPHSM**S**VPTTPT  TPTLGF**S**TGSPQL  LGFSTG**S**PQLSSP  TGSPQL**S**SPMNPV  GSPQLS**S**PMNPVS  SPMNPV**S**SSEDIK  PMNPVS**S**SEDIKP  MNPVSS**S**EDIKPP  PSGNMA**S**FTKHIC  ICGDRS**S**GKHYGV  KHYGVY**S**CEGCKG  NENEVE**S**TSSANE  EVESTS**S**ANEDMP  NELLIA**S**FSHRSI  LLIASF**S**HRSIAV  ASFSHR**S**IAVKDG  LHVHRN**S**AHSAGV  VLFNPD**S**KGLSNP  PDSKGL**S**NPAEVE  REKVYA**S**LEAYCK  RLPALR**S**IGLKCL  TAMAEP**S**PGTLPR  RKAGVF**S**DLSNQE  GVFSDL**S**NQELKA  ELKAVH**S**FLWSKK  VHSFLW**S**KKELRL  ELRLQP**S**STTTMA  LRLQPS**S**TTTMAK  CYMRAL**S**PRPGYQ  RPGYQS**S**WASRPI  LNTTGF**S**FQDCHD  ASGQRR**S**WLIIQR  LLVDHG**S**TDAGHW  NGKFYG**S**PEELAR  GGKGHD**S**TEEPPL  EPPLFS**S**HKPRGD  PRGDFP**S**PIHVSG  PSPIHV**S**GPRLVQ  VLYGGW**S**FAFRLR  FAFRLR**S**SSGLQV  AFRLRS**S**SGLQVL  FRLRSS**S**GLQVLN  VGWGLG**S**VTHELA  LRRHFN**S**NFKGGF  LVLRTT**S**TVYNYD  NITNPW**S**PRHRVV  LEQTQY**S**WERQAA  KYLLFT**S**PQENPW  VTKYRE**S**ELCSSS  RESELC**S**SSIYHQ  ESELCS**S**SIYHQN  EDPSLA**S**RDTVIV  PEDRDC**S**MPPPFS  SMPPPF**S**YNGTYR  IPINGL**S**EFVDYE  NRRYQR**S**LPGESE  DQVTLY**S**YKVQST  YSYKVQ**S**TITSRM  SKVVNN**S**PQPQNV  AFISNF**S**MTVDGK  DGKTFR**S**SIKEKT  GKTFRS**S**IKEKTV  TAGLVR**S**SALDME  KWRKLG**S**YEHRIY  IEKIQP**S**GGTNIN  GNQDTS**S**QLKKFY  KFYNQV**S**TPLLRN  FNYPHT**S**VTDVTQ  HNYFGG**S**EIVVAG  DLQDFL**S**KDKHAD  KRRITR**S**ILQMSL  IVTPLT**S**LVIENE  QDPSCC**S**GALYYG  PSWANP**S**PTPVIS  SPTPVI**S**MLAQGS  SMLAQG**S**QVLEST  GSQVLE**S**TPPPHV  ICFNID**S**EPGKIL  LVSDPE**S**GIVVNG  LGFYFQ**S**EDIKIE  TETITL**S**HGSSTF  TLSHGS**S**TFSLSW  HGSSTF**S**LSWSDT  TFSLSW**S**DTAQVT  CWFVHN**S**GKGFID  LCMYLV**S**LLILQA  AMPALG**S**ATGRSK  ATGRSK**S**SEKRQA  DGVFIR**S**LKVNCK  AHYVVT**S**QVVNTA  PKTAFI**S**DFAVTA  YRKAAI**S**GENAGL  AGLVRA**S**GRTMEQ  LTVNPQ**S**KVTFQL  TIKKSF**S**GKKGHV  LFRPTV**S**QQQSCP  CPTCST**S**LLNGHF  KVTYDV**S**RDKICD  FGTRVQ**S**WKGSLV  VQSWKG**S**LVQASE  GSLVQA**S**EANLQA  DFVRGF**S**LDEATN  EGVTDR**S**QILKNV  NFLEVM**S**MENNGR  QLQGFY**S**QVAKPL  KQYYEG**S**EIVVAG  IADNKQ**S**SFKADV  EERANL**S**SQALQM  TPLTSM**S**IRGMAD  PTIDKP**S**EDSPPL  DKPSED**S**PPLEML  LSALQP**S**PTHSSS  IGNKAR**S**PGQHDG  FGGPVF**S**WRDQAV  KRNLVV**S**VDDGGT  HRVWKG**S**SVHQDF  PIGFEV**S**DIHPGS  GLQKDY**S**KDPWHG  MFHLDP**S**LTHTIF  YVCEGP**S**HGGLPG  GGLPGA**S**SEKNKK  IHLHAH**S**LVGKHC  TKEMDL**S**VVRLMF  TAFLPD**S**TGSFTR  RLEPVV**S**DAIYDS  SKAPNA**S**NLKIVR  EGFGDF**S**PTDVHR  NITKPA**S**VFVQLR  KSDLET**S**EPKPFL  KLMPNF**S**DSFGGG  GGGGTG**S**TGPGYS  STGPGY**S**FPHYGF  HPGTTK**S**NAGMKH  GTMDTE**S**KKDPEG  PEGCDK**S**DDKNTV  DLLEVT**S**GLISDD  VTSGLI**S**DDIINM  LDRLGN**S**VLHLAA  GHDKVL**S**ILLKHK  IHLAMM**S**NSLPCL  LAMMSN**S**LPCLLL  NAQEQK**S**GRTALH  VEHDNI**S**LAGCLL  IAAGRG**S**TRLAAL  LYDLDD**S**WENAGE  NNAFRL**S**PAPSKT  RLSPAP**S**KTLMDN  LPLSPA**S**TRQQID  SLTSGA**S**LLTLNK  GDMANN**S**VAYSGV  ALMELR**S**TDALRK  LRKIQE**S**YGDVYG  SPNEGL**S**GNPADL  SDHVKK**S**LDKDPL  KDPLLL**S**GTHVME  LPKKEK**S**VLQGKL  KAGLLM**S**AITVII  KVYTFN**S**VRKSMS  FNSVRK**S**MSTVLK  SVRKSM**S**TVLKNS  LKNSDG**S**YRIFSK  GSYRIF**S**KGASEI  KCFKIL**S**ANGEAK  RVLARS**S**PTDKHT  TDDNFT**S**IVKAVM  NVYDSI**S**KFLQFQ  KFFDID**S**GRNAPL  GKPFSC**S**ELSIEQ  FSCSEL**S**IEQWLW  IEQWLW**S**IFLGMG  LWGQLI**S**TIPTSR  ISTIPT**S**RLKFLK  ALRRQP**S**IASQHH  RQPSIA**S**QHHDVT  HDVTNI**S**TPTHIR  GLEKPE**S**RSSIHN  EKPESR**S**SIHNFM  APTKRN**S**SPPPSP  TIEMNK**S**ATSSSP  NKSATS**S**SPGSPL  KSATSS**S**PGSPLH  TSSSPG**S**PLHSLE  WLQVQE**S**VTVQEG  PYYDKN**S**PVHGYW  REGAII**S**RDSPVA  AIISRD**S**PVATNK  RLLGDP**S**RNNCSL  PSRNNC**S**LSIVDA  RNNCSL**S**IVDARR  RRRDNG**S**YFFRME  FRMERG**S**TKYSYK  RGSTKY**S**YKSPQL  TKYSYK**S**PQLSVH  YKSPQL**S**VHVTDL  TLEPGH**S**KNLTCS  SKNLTC**S**VSWACE  NLTCSV**S**WACEQG  GTPPIF**S**WLSAAP  PIFSWL**S**AAPTSL  LSAAPT**S**LGPRTT  GPRTTH**S**SVLIIT  PRTTHS**S**VLIITP  IFPGDG**S**GKQETR  THPTTG**S**ASPKHQ  PTTGSA**S**PKHQKK  PKHQKK**S**KLHGPT  HGPTET**S**SCSGAA  GPTETS**S**CSGAAP  TETSSC**S**GAAPTV  EELHYA**S**LNFHGM  FHGMNP**S**KDTSTE  NPSKDT**S**TEYSEV  AVTFAA**S**LIPQFG  PQFGLF**S**KYRTPN  YRTPNC**S**QYRLPG  FNPVCG**S**DMSTYA  VCGSDM**S**TYANEC  RYGQKE**S**SDQNFD  YGQKES**S**DQNFDY  ILIIGN**S**SVGKTS  LIIGNS**S**VGKTSF  SSVGKT**S**FLFRYA  FRYADD**S**FTPAFV  FTPAFV**S**TVGIDF  DITNEE**S**FNAVQD  NAVQDW**S**TQIKTY  TQIKTY**S**WDNAQV  EDERVV**S**SERGRQ  DERVVS**S**ERGRQL  FEFFEA**S**AKDNIN  VICEKM**S**ESLDTA  CEKMSE**S**LDTADP  KQGPQL**S**DQQVPP  NAGTGK**S**CLLHQF  KKFKDD**S**NHTIGV  IGVEFG**S**KIINVG  GQERFR**S**VTRSYY  FRSVTR**S**YYRGAA  LVYDIT**S**RETYNA  DARMLA**S**QNIVII  VTFLEA**S**RFAQEN  LMFLET**S**ALTGEN  ILNKIE**S**GELDPE  DPERMG**S**GIQYGD  AVFFLV**S**TQLFAE  DDLNYW**S**DWYDSD  WSDWYD**S**DQIKEE  GKRDAD**S**SIEKQV  KRDADS**S**IEKQVA  YGHGQI**S**HKRHKT  KRHKTD**S**FVGLMG  GKRALN**S**VAYERS  SVAYER**S**AMQNYE  ITYIGS**S**GSSPSR  IGSSGS**S**PSRTSP  SSGSSP**S**RTSPES  SRTSPE**S**LYSDNS  SPESLY**S**DNSNGS  SLYSDN**S**NGSFQS  SDNSNG**S**FQSLTQ  SNGSFQ**S**LTQGCP  TQDPAR**S**FGSIPP  SLSDDG**S**PSSSSS  DDGSPS**S**SSSSSS  DGSPSS**S**SSSSSS  GSPSSS**S**SSSSSS  PSSSSS**S**SSSSSS  SSSSSS**S**SSSSSF  SSSSSS**S**SSFYNG  SSSSSS**S**FYNGSP  SSFYNG**S**PPGSLQ  EDSSRV**S**PSKSTS  LKNENC**S**IVRINR  RFKKCL**S**VGMSRD  CLSVGM**S**RDAVRF  QCPLET**S**PTQHPT  PGPMGP**S**PPPAPV  ANHASG**S**PPATTP  LRQAPS**S**YPPTWP  PGPAHH**S**CHQSNS  MYPHGR**S**GRTVQE  LMVRFA**S**LFNVKD  LSRTTY**S**LQELGA  SAMFDF**S**EKLNSL  KAVGAF**S**ATDSFD  AFSATD**S**FDHKKF  VGLKKK**S**ADDVKK  MLDKDK**S**GFIEED  FILKGF**S**PDARDL  PDARDL**S**AKETKM  ADPAAA**S**HPLLLN  AQKNPG**S**VAENNL  AENNLC**S**QYEEKV  CIDLID**S**LRALGV  AVIGDQ**S**SGKSSV  VIGDQS**S**GKSSVL  DQSSGK**S**SVLEAL  VALPRG**S**GIVTRC  KWRGKV**S**YQDYEI  DYEIEI**S**DASEVE  IEISDA**S**EVEKEI  GEGMGI**S**HELITL  LITLEI**S**SRDVPD  ITLEIS**S**RDVPDL  QRQETI**S**LVVVPS  SLVVVP**S**NVDIAT  ATTEAL**S**MAQEVD  QDQLSL**S**EALQRE  ITHICK**S**LPLLEN  HEFHKW**S**TIIENN  LAFTDV**S**IKNFEE  LHRTAK**S**KIEDIR  EEKKKK**S**WDFGAF  DFGAFQ**S**SSATDS  FGAFQS**S**SATDSS  GAFQSS**S**ATDSSM  SSSATD**S**SMEEIF  SSATDS**S**MEEIFQ  AYHQEA**S**KRISSH  EASKRI**S**SHIPLI  ASKRIS**S**HIPLII  QDKDTY**S**WLLKER  KERSDT**S**DKRKFL  RPLRAP**S**PEPAAP  PTRAMG**S**PFPLVN  PMEHPL**S**ADIAMA  VITTLP**S**VVRLLV  LACEHR**S**PTCLRA  LRALLD**S**AAPGTL  DAVDIK**S**GRSPLI  DIKSGR**S**PLIHAV  HAVENN**S**LSMVQL  VENNSL**S**MVQLLL  VNAQMY**S**GSSALH  QMYSGS**S**ALHSAS  GSSALH**S**ASGRGL  SALHSA**S**GRGLLP  VRSGAD**S**SLKNCH  RSGADS**S**LKNCHN  PLMVAR**S**RRVIDI  KATRPA**S**TSQPDP  TRPAST**S**QPDPSP  TSQPDP**S**PDRSAN  DPSPDR**S**ANTSPE  DRSANT**S**PESSSR  ANTSPE**S**SSRLSS  ESSSRL**S**SNGLLS  SSNGLL**S**ASPSSS  NGLLSA**S**PSSSPS  LLSASP**S**SSPSQS  LSASPS**S**SPSQSP  SPSSSP**S**QSPPRD  PNFFLP**S**PSPPAF  FFLPSP**S**PPAFLP  TRWKAR**S**ALRPGA  CATRRS**S**KGPDVA  KQLYPA**S**AFPEDF  VKAKKG**S**QAFLVS  SQAFLV**S**IYNEQG  GLELGR**S**PVFLYE  FRGINL**S**DGKWHR  TKFLDR**S**DHPMID  DYCEHY**S**PDCDTA  VPDTPQ**S**QDPNPD  GKEPTP**S**KKPVEA  APMPET**S**EGAGKE  DYDYVP**S**EDYYTP  EDYYTP**S**PYDDLT  GAEIPT**S**TADTSN  TSTADT**S**NSSNPA  PYYDPT**S**SPSEIG  DPTSSP**S**EIGPGM  PVGPPG**S**GGLKGE  RRGRAG**S**DGARGM  RQGPKG**S**IGFPGF  PGPKGN**S**GGDGPA  EPGQKG**S**KGDKGE  IGQPGP**S**GADGEP  KGESGP**S**GAAGPP  DDGPKG**S**PGPVGF  EQGLPG**S**PGPDGP  IFGSLN**S**LKLEIE  PKENPG**S**WFSEFK  TFLRLL**S**ASAHQN  LRLLSA**S**AHQNVT  QDAATG**S**YDKALR  ALRFLG**S**NDEEMS  QALVRC**S**SPLPLV  ALVRCS**S**PLPLVV  AWRALA**S**GGSALD  ALASGG**S**ALDAVE  ALDAVE**S**GCAMCE  REQCDG**S**VGFGGS  SVGFGG**S**PDELGE  TLLVGE**S**ATTFAQ  ATTFAQ**S**MGFINE  FINEDL**S**TTASQA  DLSTTA**S**QALHSD  ASQALH**S**DWLARN  NVIPDP**S**KYCGPY  HIAAGT**S**TNGIKF  HGRVGD**S**PIPGAG  LMRFLP**S**YQAVEY  ACQKVI**S**RIQKHF  CANVTG**S**YGAACN  AACNKL**S**TFTQFS  STFTQF**S**FMVYNS  SFMVYN**S**EKNQPT  IKRLKN**S**DDRDSK  NSDDRD**S**KRDSLE  EITIRN**S**PYRRED  PYRRED**S**MEDRGE  RGEEDD**S**LAIKPP  EKRRHR**S**HSAEGG  EMAREH**S**RRERDR  TMREDY**S**DKVKAS  SDKVKA**S**HWSRSP  ASHWSR**S**PPRPPR  EERDLL**S**DLQDIS  SDLQDI**S**DSERKT  KTSSAE**S**SSAESG  SSAESG**S**GSEEEE  AESGSG**S**EEEEEE  EEEEEG**S**TSEESE  EEEGST**S**EESEEE  EETGSN**S**EEASEQ  QSAEEV**S**EEEMSE  VSEEEM**S**EDEERE  DRDSGE**S**EEAEEE  REIVVG**S**NMDKIY  VEHDLK**S**LMETMK  TSNLLL**S**HAGILK  LGAKEY**S**TAVDMW  PLFPGK**S**EIDQIN  KDLGTP**S**EKIWPG  RFGALL**S**DQGFDL  FPGRRI**S**AEDGLK  PTWPAK**S**EQQRVK  TTNQGA**S**AAGPGF  TEFWLI**S**APGEKT  KLHAAT**S**KNNNLA  NNLAVT**S**KFNIPD  DVLVGL**S**DELAKL  ADVLED**S**KDKVQE  KYPIKQ**S**LKNISE  QSLKNI**S**EIIAKG  IDNDLK**S**RASAYN  DLKSRA**S**AYNNLK  ERKNAG**S**LLTRSL  GSLLTR**S**LAEIVK  DDFVLD**S**EYLVTL  EMVVPR**S**SNVLSE  MVVPRS**S**NVLSED  RSSNVL**S**EDQDSY  LSEDQD**S**YLCNVT  EEMNRL**S**TDKKKQ  WLKVNF**S**EAFIAW  LRVFVE**S**VLRYGL  LYKHLD**S**SAAAII  YKHLDS**S**AAAIID  IPGLNL**S**QQEYYP  ENQEKA**S**IAGHMF  VIGGGI**S**GLSAAK  GGISGL**S**AAKLLT  LTEYGV**S**VLVLEA  NRILRL**S**KELGIE  TYKVNV**S**ERLVQY  VNINVT**S**EPHEVS  SEPHEV**S**ALWFLW  GTTRIF**S**VTNGGQ  RKFVGG**S**GQVSER  GGSGQV**S**ERIMDL  VTHVDQ**S**SDNIII  THVDQS**S**DNIIIE  DEDAPI**S**ITLDDT  DTKPDG**S**LPAIMG  YAKVLG**S**QEALHP  WCEEQY**S**GGCYTA  ETATKW**S**GYMEGA  WVQEPE**S**KDVPAV  WERNLP**S**VSGLLK  RNLPSV**S**GLLKII  LKIIGF**S**TSVTAL  IIGFST**S**VTALGF  LVKAHR**S**SVSDYV  VKAHRS**S**VSDYVN  TGKLNI**S**ADKENS  SADKEN**S**IKLTSV  NSIKLT**S**VVFILI  IGNLAL**S**DLLAGV  TANLLL**S**GATTYK  WFLREG**S**MFVALS  SMFVAL**S**ASVFSL  FVALSA**S**VFSLLA  LSASVF**S**LLAIAI  MKLHNG**S**NNFRLF  RLFLLI**S**ACWVIS  SACWVI**S**LILGGL  MGWNCI**S**ALSSCS  CISALS**S**CSTVLP  SALSSC**S**TVLPLY  LYCRIY**S**LVRTRS  SLVRTR**S**RRLTFR  TFRKNI**S**KASRSS  ISKASR**S**SEKSLA  SKASRS**S**EKSLAL  SRSSEK**S**LALLKT  TVIIVL**S**VFIACW  VLAVLN**S**GTNPII  AFIRIM**S**CCKCPS  SCCKCP**S**GDSAGK  KCPSGD**S**AGKFKR  IAGMEF**S**RSKSDN  GMEFSR**S**KSDNSS  RSKSDN**S**SHPQKD  SKSDNS**S**HPQKDE  NPETIM**S**SGNVNS  ALLKPL**S**IPNQLL  LLGPGP**S**NLPPRI  GLQMIG**S**MSKDMY  QMIGSM**S**KDMYQI  PLTLVI**S**GSGHCA  TLVISG**S**GHCALE  VLEPGD**S**FLVGAN  FLTHGE**S**STGVLQ  LTHGES**S**TGVLQP  CLLLVD**S**VASLGG  LVDSVA**S**LGGTPL  GIDILY**S**GSQKAL  DILYSG**S**QKALNA  NAPPGT**S**LISFSD  PGTSLI**S**FSDKAK  TSLISF**S**DKAKKK  AKKKMY**S**RKTKPF  RKTKPF**S**FYLDIK  HTIPVI**S**LYSLRE  PVISLY**S**LRESLA  LYSLRE**S**LALIAE  EQGLEN**S**WRQHRE  DWRDIV**S**YVIDHF  MGGLGP**S**TGKVLR  AGGGPF**S**LAGFLF  KDKDQD**S**ITGEKV  FSSSSD**S**ESEMGP  PGKNVP**S**VWRSAR  SEVSQK**S**LWNYDY  DDKPWY**S**IFPIDN  EEATSN**S**PSKESK  NSPSKE**S**KKESSL  TVYCHT**S**PFLGSL  EVPGPN**S**KRANTH  KAFPSH**S**ESSIRK  PSHSES**S**IRKRLK  KRTGMD**S**NWWVLK  SKFARG**S**RFSVAE  SSSAED**S**DFEEMG  AAGSAA**S**GNNHRD  SVTSLN**S**SATGRC  SKHKYQ**S**RESFLD  KYQSRE**S**FLDDVN  NLILAN**S**VKYNGP  LYDTNT**S**LSMSRD  DTNTSL**S**MSRDAS  QDESNM**S**VLDIPS  SVLDIP**S**ATPEKQ  VQQPQA**S**VLYEDL  YEDLLM**S**EGEDDE  LEDSNI**S**YGSYEE  TQDTSF**S**SIGGYE  SGPSVL**S**QVHLSE  SEDEED**S**EDFHSI  DSEDFH**S**IAGDSD  TPVDKT**S**NTLRVP  AVADFL**S**CLALPI  LPILFT**S**IVQHHH  FGGAAC**S**ILPSLI  ACSILP**S**LILLNM  LLNMYA**S**ILLLAT  LLLATI**S**ADRFLL  LLLTIP**S**FLYRVV  LCGVDY**S**HDKRRE  ILLRTW**S**RRATRS  SRRATR**S**TKTLKV  VVAVVA**S**FFIFWL  VTGIMM**S**FLEPSS  MSFLEP**S**SPTFLL  SFLEPS**S**PTFLLL  LLKKLD**S**LCVSFA  LDSLCV**S**FAYINC  CLLLWG**S**TELCYP  LLQGGA**S**HPETSV  ASHPET**S**VQPVLV  TLMVMV**S**KDLFGT  ACEPLV**S**MDTEDV  LHECGN**S**MQVTDD  DDALVY**S**TFLLHD  RPVGNL**S**IVRTNR  PRQGNV**S**SQAILP  RQGNVS**S**QAILPT  FRTTVF**S**EEKLTF  EEKLTF**S**LRLMEE  WNAEKR**S**PTFHLG  AEIHTG**S**HVPLRL  TPDQNA**S**PYHTIV  DGLTDA**S**SAFKVP  GLTDAS**S**AFKVPR  FHFAND**S**RNMIYI  ELNKAC**S**FSKPSN  NKACSF**S**KPSNSW  CSFSKP**S**NSWFPV  FSKPSN**S**WFPVEG  WFPVEG**S**ADICQC  GDCGTP**S**HSRRQP  CGTPSH**S**RRQPHV  RQPHVM**S**QWSRSA  HVMSQW**S**RSASRN  MSQWSR**S**ASRNRR  QWSRSA**S**RNRRHV  EQWALP**S**DTSVVL  ALPSDT**S**VVLLGV  GLAVVV**S**LTLTAV  LDLKTK**S**ENGLEF  NGLEFT**S**SGSANT  GLEFTS**S**GSANTE  EFTSSG**S**ANTETT  TTKVTG**S**LETKYR  LKLTFD**S**SFSPNT  KLTFDS**S**FSPNTG  TFDSSF**S**PNTGKK  FDIAGP**S**IRGALV  NFETAK**S**RVTQSN  KSRVTQ**S**NFAVGY  AWTAGN**S**NTRFGI  DPDACF**S**AKVNNS  SAKVNN**S**SLIGLG  AKVNNS**S**LIGLGY  GIKLTL**S**ALLDGK  TALILL**S**ILGMAC  GMACAF**S**MKNLHR  RFPVQG**S**SDSSEE  FPVQGS**S**DSSEEN  VQGSSD**S**SEENGD  EEEEET**S**NEGENN  AENTTL**S**ATTLGY  GNENEE**S**EAEVDE  QGINGT**S**TNSTEA  NGTSTN**S**TEAENG  AENGNG**S**SGGDNG  ENGNGS**S**GGDNGE  EEGEEE**S**VTGANA  RQGKGT**S**KTTTSP  TSKTTT**S**PNGGFE  QVYRTT**S**PPFGKT  AYEDEY**S**YFKGQG  GRNYEA**S**VDSLTF  VDSLTF**S**VVTGPA  VTGPAP**S**QEAGTK  QQDCTL**S**LQLTTP  RLSLEA**S**TGYQGS  STGYQG**S**SFVLGH  TGYQGS**S**FVLGHF  GFIYQG**S**AKFIKN  NAGRDC**S**RRSSPV  RDCSRR**S**SPVYVG  DCSRRS**S**PVYVGR  YVGRVV**S**GMVNCN  NYGDGV**S**PMSWIG  DGVSPM**S**WIGSVD  PMSWIG**S**VDILRR  VVTNYN**S**AHDQNS  SAHDQN**S**NLLIEY  EIQGDK**S**EMIWNF  FHCWVE**S**WMTRPD  PTPQEK**S**EGTYCC  IKEGDL**S**TKYDAP  IQQDDG**S**VHKSIN  HKSINR**S**LIVGLK  IVGLKI**S**TKSVGR  LKISTK**S**VGRDER  KYPEGS**S**EEREAF  RIRVGQ**S**MNMGSD  QSMNMG**S**DFDVFA  LCARTV**S**YNGILG  EPFSEK**S**VPLCIL  VEPVIN**S**YLLAER  KLVAEV**S**LQNPLP  LVVNFE**S**DKLKAV  WKSGGA**S**HSELIH  SGGASH**S**ELIHNL  MLATDR**S**HYAKCN  CNPYMD**S**PQSIGF  YMDSPQ**S**IGFQAT  GFQATI**S**APHMHA  KALDVG**S**GSGILT  LDVGSG**S**GILTAC  KELVDD**S**VNNVRK  DDPTLL**S**SGRVQL  DPTLLS**S**GRVQLV  DKLQDG**S**IKMKPL  VYELLD**S**PGKVLL  GKVLLQ**S**KDQITA  EGKAAI**S**NKITSC  ISNKIT**S**CIFQLL  RRIATG**S**FLKRNP  NNDPQW**S**EEQLIA  TEVDIM**S**HATQAI  FEILEK**S**WLPQNC  DVIDND**S**WRLWPS  SWRLWP**S**GDRSQQ  WPSGDR**S**QQKDKQ  QQKDKQ**S**YRDLKE  VELLLK**S**ESQCRV  LLLKSE**S**QCRVVV  VVVLMG**S**TSDLGH  VLMGST**S**DLGHCE  CELRVT**S**AHKGPD  VAVAGR**S**NGLGPV  GLGPVM**S**GNTAYP  TAYPVI**S**CPPLTP  GVQDVW**S**SLRLPS  VQDVWS**S**LRLPSG  SSLRLP**S**GLGCST  PSGLGC**S**TVLSPE  GCSTVL**S**PEGSAQ  VLSPEG**S**AQFAAQ  AQIFGL**S**NHLVWS  SNHLVW**S**KLRASI  WSKLRA**S**ILNTWI  ILNTWI**S**LKQADK  NCSPAQ**S**VLSEVP  VLSEVP**S**VPTNGM  MKRLQT**S**SVLVSG  KRLQTS**S**VLVSGL  TSSVLV**S**GLRGLG  RLAELN**S**YVPVTA  PLSAMV**S**MVTKDN  ESGDFV**S**FSEVQG  GDFVSF**S**EVQGMV  LGPYTF**S**ICDTSN  FSICDT**S**NFSDYI  CDTSNF**S**DYIRGG  KKISFK**S**LVASLA  FKSLVA**S**LAEPDF  EVMKAC**S**GKFMPI  IPFLTE**S**YSSSQD  LTESYS**S**SQDPPE  TESYSS**S**QDPPEK  LEAVQR**S**LVLQRP  PPDQLT**S**SGAPFW  TYGLTG**S**QDRAAV  PEFTPK**S**GVKIHV  NASVDD**S**RLEELK  FEKDDD**S**NFHMDF  DFIVAA**S**NLRAEN  ENYDIP**S**ADRHKS  SADRHK**S**KLIAGK  LEITML**S**QGVSML  GVSMLY**S**FFMPAA  PMTEIV**S**RVSKRK  EIVSRV**S**KRKLGR  SALLLA**S**LLLAAA  LLAAAL**S**ASAGLW  AAALSA**S**AGLWSP  ASAGLW**S**PAKEKR  RGWTLN**S**AGYLLG  AVGNHR**S**FSDKNG  GNHRSF**S**DKNGLT  DKNGLT**S**KRELRP  DDMKPG**S**FDRSIP  PGSFDR**S**IPENNI  TIIEFL**S**FLHLKE  AGNVKK**S**SGAGGG  SGAGGG**S**GSGGSG  GSGSGG**S**GSGGLI  GSGGSG**S**GGLIGL  KTKQTI**S**LFKEGK  LKGIFP**S**GLFQGD  TCNDYI**S**VFEFDI  SYIFRL**S**CTRLGQ  LYCEMG**S**TFQLCK  DPRGSG**S**LLRQGA  GAEGAP**S**PNYDDD  KVERPP**S**PFSMAP  RPPSPF**S**MAPQAS  QRVCVP**S**SASALG  SALGTA**S**KAASGS  SKAASG**S**LHKDKP  SSRLGD**S**WLPRPI  IPKVPV**S**APSSSD  VPVSAP**S**SSDPWT  PVSAPS**S**SDPWTG  VSAPSS**S**DPWTGR  SLDTSM**S**MNSSPL  TEYMTP**S**SRPLRP  EYMTPS**S**RPLRPL  NTGPEE**S**ENEDDG  ISNASS**S**FGWLSL  FPRRIN**S**ERKAGS  SERKAG**S**CQQGSG  GSCQQG**S**GPAASA  GSGPAA**S**AATASP  ASAATA**S**PQLSSE  TASPQL**S**SEIENL  AAGPGP**S**LGDEAI  IHCPPC**S**EEKLAR  YTPRCG**S**GLRCYP  IEAIQE**S**LQPSDK  QESLQP**S**DKDEGD  GDHPNN**S**FSPCSA  HPNNSF**S**PCSAHD  NSFSPC**S**AHDRRC  AKIRDR**S**TSGGKM  IRDRST**S**GGKMKV  RPVPQG**S**CQSELH  PQGSCQ**S**ELHRAL  LERLAA**S**QSRTHE  RLAASQ**S**RTHEDL  PEPDGR**S**RRGGGR  DSRGRG**S**SSGTRR  SRGRGS**S**SGTRRP  GSRGSD**S**RRPVSR  DLLSSR**S**DQNRRE  LSRTYE**S**SDRITL  PVGKGG**S**GGWKAQ  AQVVKA**S**GQNLNL  NLRVHT**S**PNAIIG  FTVRTQ**S**DAGEFQ  ISAMVN**S**LDDNGV  VLIGNW**S**GDYSRG  PSAWVG**S**VEILLS  YLRTGY**S**VPYGQC  TVTNFN**S**AHDTDT  KRPDLP**S**GFDGWQ  ATPQET**S**SGIFCC  TPQETS**S**GIFCCG  GPCSVE**S**IKNGLV  QRQDDG**S**FKIVYV  VTKAIS**S**NMREDI  YKHPEG**S**DAERKA  GQDLMV**S**VMLINH  MLINHS**S**SRRTVK  LINHSS**S**RRTVKL  KLHLYL**S**VTFYTG  AMLLNV**S**GHVKES  SGHVKE**S**GQVLAK  TVTLRQ**S**FVPVRP  LIASLD**S**PQLSQV  GDGGFF**S**DAGGDS  APTALP**S**FSTFMD  TALPSF**S**TFMDGY  QPCSSA**S**SSASST  CSSASS**S**ASSTSS  SASSSA**S**STSSSS  SASSTS**S**SSATSP  ASSTSS**S**SATSPA  TSPASA**S**FKFEDF  PVDEAL**S**SSGSDY  VDEALS**S**SGSDYY  DEALSS**S**GSDYYG  ALSSSG**S**DYYGSP  GSDYYG**S**PCSAPS  SPCSAP**S**PSTPSF  CSAPSP**S**TPSFQP  GSFGHF**S**PSQTYE  QPPAFF**S**FSPPTG  PAFFSF**S**PPTGPS  TSTKAR**S**GAPGGS  VCGDNA**S**CQHYGV  HLDSGP**S**TAKLDY  TAKLDY**S**KFQELV  YDLLSG**S**LEVIRK  PGFAEL**S**PADQDL  QDLLLE**S**AFLELF  LRLAYR**S**KPGEGK  FGDWID**S**ILAFSR  FSRSLH**S**LLVDVP  LQNRIA**S**CLKEHV  GEPQPA**S**CLSRLL  QPASCL**S**RLLGKL  LHRVEP**S**VFLPAS  SVFLPA**S**KANDVL  RWKRAG**S**YLLEEL  RRYKGG**S**PCISQP  GGSPCI**S**QPCLHN  PCLHNG**S**CQDSIW  NGSCQD**S**IWGYTC  GYTCTC**S**PGYEGS  SPGYEG**S**NCELAK  CLPGQE**S**YTCSCA  QESYTC**S**CAQGYR  ACGVLT**S**EKRAPD  QVKLTN**S**EGKDFC  LTTAKC**S**LLHRNI  TYFNRT**S**QDPLMI  AGENDL**S**LLELEW  RTRGLL**S**GWARNG  GTDLGN**S**LTTRPV  RTYCER**S**SVAAMH  TYCERS**S**VAAMHW  MHWMDG**S**VVTREH  TREHRG**S**WFLTGV  LTGVLG**S**QPVGGQ  VLVTKV**S**RYSLWF  TKVSRY**S**LWFKQI  DFHTYL**S**RCNSIK  YLSRCN**S**IKVEGG  GLNDRL**S**SCRAVH  LNDRLS**S**CRAVHL  RAVHLP**S**GGQYKI  FEKGDF**S**GQMYET  TTEDCP**S**IMEQFH  HMREIH**S**CKVLEG  IDWGAA**S**PAVQSF  PAELPA**S**VRASIE  PASVRA**S**IERKRQ  LAARPY**S**ATAAAA  GKEFMD**S**YLMNHF  KKNPHH**S**QWGDMK  LQIVKR**S**LEVWGS  LRRAVR**S**SVWKRE  RRAVRS**S**VWKRET  TLELLQ**S**TRIGMS  STRIGM**S**VNAIRK  NAIRKQ**S**TDEEVT  TDEEVT**S**LAKSLI  VTSLAK**S**LIKSWK  AKSLIK**S**WKKLLD  KLLDGP**S**TEKDLD  KEPAIT**S**QNSPEA  AITSQN**S**PEAREE  PEAREE**S**TSSGNV  AREEST**S**SGNVSN  REESTS**S**GNVSNR  TSSGNV**S**NRKDET  ARDTYV**S**SFPRAP  RDTYVS**S**FPRAPS  SFPRAP**S**TSDSVR  PRAPST**S**DSVRLK  APSTSD**S**VRLKCR  DEEELG**S**QIEEAI  YKNRVR**S**RISNLK  RVRSRI**S**NLKDAK  TAEEMA**S**DELKEM  TQVQTR**S**ADEPMT  LCLLYL**S**AGLLSR  LSAGLL**S**RLGAAF  KYGDPG**S**LFGFSL  GSLFGF**S**LAMHWQ  RTGGLY**S**CDITAR  NDADPT**S**ESKEDQ  ADPTSE**S**KEDQWM  MGVTVQ**S**QGPGGK  GRCYVL**S**QNLRIE  GHEKFG**S**CQQGVA  ETEHDE**S**LVPVPA  GLLFLT**S**VSYTDP  APRANH**S**GAVVLL  DGEGLA**S**SFGYDV  GEGLAS**S**FGYDVA  QVLKGI**S**PYFGYS  PDVAVG**S**LSDSVT  VGSLSD**S**VTIFRS  SVTIFR**S**RPVINI  TACGAP**S**GICLQV  ICLQVK**S**CFEYTA  EKERRK**S**GLSSRV  RKSGLS**S**RVQFRN  VEIQEP**S**SRRRVN  SRRRVN**S**LPEVLP  GNQDKF**S**YLPIQK  FPEKQL**S**CVANQN  LKLETT**S**NQDNLA  YLVKVE**S**KGLEKV  RSRLWN**S**TFLEEY  KTVAQY**S**GVPWWI  GPGPGQ**S**GPKPPI  PPPPQD**S**SKPVVA  PPPQDS**S**KPVVAQ  PAPGVG**S**APPASS  GSAPPA**S**SSAPPA  SAPPAS**S**SAPPAT  APPASS**S**APPATP  PATPPT**S**GAPPGS  SGAPPG**S**GPGPTP  PPPAVT**S**APPGAP  PPPTPP**S**SGVPTT  PPTPPS**S**GVPTTP  GGGPGL**S**TPGGHP  GGPGGR**S**EEKISD  EEKISD**S**EGFKAN  GFIKLE**S**RALAEI  THAAAL**S**VRNLSP  NLSPYV**S**NELLEE  LLEEAF**S**QFGPIE  VDDRGR**S**TGKGIV  GIVEFA**S**KPAARK  KAFERC**S**EGVFLL  TFEYEY**S**QRWKSL  YSQRWK**S**LDEMEK  AKDKLE**S**EMEDAY  RRQREE**S**YSRMGY  QREESY**S**RMGYMD  VPPATM**S**GSMMGS  PATMSG**S**MMGSDM  SGSMMG**S**DMRTER  NVFHAH**S**GKEGDK  GDKYKL**S**KKELKE  LLQTEL**S**GFLDAQ  RPWLHA**S**VLWAGV  LWAGVA**S**LLLPPA  FRNRNN**S**TGVAGL  LRHHLH**S**PRDHPD  DENKDV**S**TENGHH  KEDEDV**S**KEYGHL  LLPGHR**S**QDHKVG  HRHHLP**S**HRSHSH  DEDEVV**S**SEHHHH  EDEVVS**S**EHHHHI  EEEEEA**S**TEYGHQ  RHRGHG**S**EEDEDV  EEDEDV**S**DGHHHH  HHHHGP**S**HRHQGH  DDDDDV**S**IEYRHQ  EEDEDV**S**DGHHHR  PSHRHR**S**HEEDDN  NDDDDV**S**TEYGHQ  FGHYVA**S**HQPRGH  LGHQAP**S**HRQSHQ  APSHRQ**S**HQDEET  GSIKEM**S**HHPPGH  HLRKDD**S**EEEKEK  HEEDDE**S**SEQGEK  EEDDES**S**EQGEKG  KGTHHG**S**RDQEDE  EEGHGL**S**LNQEEE  EVGAPL**S**PDHSEE  ASSEEE**S**GEDTGP  GNYQPG**S**LCGYCS  VDYFSS**S**LYQALA  LAEDGY**S**GVEVRV  FGFPEG**S**VELYAE  AIAQAE**S**LRYKLL  LRFIME**S**GAKGCE  GCEVVV**S**GKLRGQ  RGQRAK**S**MKFVDG  DGLMIH**S**GDPVNY  ALRVVR**S**VRALLC  TLRAVP**S**PAAPCP  TGPALL**S**VRKFTE  IGTVGI**S**NFAQEA  GDVVYC**S**LPEVGT  EFGALE**S**VKAASE  ESVKAA**S**ELYSPL  AASELY**S**PLSGEV  ELYSPL**S**GEVTEI  PGLVNK**S**CYEDGW  LIKMTL**S**NPSELD  MTLSNP**S**ELDELM  ELDELM**S**EEAYEK  QPEDAG**S**EDELEE  GGQLNE**S**MDHGGV  CEKFEI**S**ETSVNR  FEISET**S**VNRGPE  LILEYL**S**GGELFM  FYLAEI**S**MALGHL  FGLCKE**S**IHDGTV  PEILMR**S**GHNRAV  RAVDWW**S**LGALMY  LKRNAA**S**RLGAGP  FKPLLQ**S**EEDVSQ  QSEEDV**S**QFDSKF  DVSQFD**S**KFTRQT  VDSPDD**S**TLSESA  PDDSTL**S**ESANQV  DSTLSE**S**ANQVFL  FTYVAP**S**VLESVK  APSVLE**S**VKEKFS  SVKEKF**S**FEPKIR  FWGRGA**S**ASTANP  GRGASA**S**TANPQT  EYPMET**S**GIEQMD  QMDVTM**S**GEASAP  TMSGEA**S**APLPIR  PIRQPN**S**GPYKKQ  QAFPMI**S**KRPEHL  EVIFYL**S**DREPLR  EPLRLG**S**GEYTAE  AQACRI**S**PLCHNL  TPLLDA**S**SLEYLF  PLLDAS**S**LEYLFA  PETLNK**S**IRQRNL  NKTICD**S**SVSTHD  KTICDS**S**VSTHDL  AEIFET**S**MLLISS  EMNWFH**S**NDGGNV  EEWNNF**S**YFPEIT  IKESVV**S**INKQDN  MELKLS**S**HEEALS  KLRQEG**S**EEGMYV  MYVLRW**S**CTDFDN  VTCFEK**S**EQVQGA  VQKGRY**S**LHGSDR  LHGSDR**S**FPSLGD  SLGDLM**S**HLKKQI  LKVLDP**S**HRDISL  AFFEAA**S**MMRQVS  LFMHRK**S**DVLTTP  GPFIKL**S**DPGIPI  PECVED**S**KNLSVA  CRPVTP**S**CKELAD  KSLKPE**S**GGNHIA  GMDYLG**S**RQYVHR  VKDDRD**S**PVFWYA  PECLMQ**S**KFYIAS  SKFYIA**S**DVWSFG  IASDVW**S**FGVTLH  TYCDSD**S**SPMALF  YCDSDS**S**PMALFL  KYGNDS**S**AELSEL  REEDEY**S**ELRSEL  RSELSQ**S**QHEVNE  SIPENQ**S**TMVTAD  EQLTTA**S**EHCDLA  LQTRLQ**S**VQATGP  QATGPS**S**PGRLTS  GELSTS**S**SSNDIP  LSTSSS**S**NDIPIA  SKTRSE**S**SSSDRP  TRSESS**S**SDRPVL  ETERLN**S**RIEHLK  SNAERM**S**MLVGKY  LVGKYE**S**NATALR  RLALQY**S**EQCIEA  RAAGVG**S**SPGDQS  SSPGDQ**S**GDENIT  LMKLDG**S**CGGAFA  SVQPWE**S**LSSNSH  SHTSTT**S**STASSC  HIEHLK**S**EVEEQK  QRMRSL**S**STSSGS  RMRSLS**S**TSSGSK  SLSSTS**S**GSKDKP  SSTSSG**S**KDKPGK  ECADAA**S**PALSLA  AASPAL**S**LAELRT  ELRTTC**S**ENELAA  EIRHQQ**S**AEFVND  DLKRAN**S**NLVAAY  LLEEEN**S**RPHTNE  RMRERY**S**ASKPLK  RERYSA**S**KPLKGA  GAEVQW**S**SCNIFS  AEVQWS**S**CNIFST  SSCNIF**S**TQDHAA  PGIRGI**S**EETTTG  AINVND**S**VTKSKF  NDSVTK**S**KFDNLY  LYGCRE**S**LIDGIK  CAMGHP**S**FVMSNS  HPSFVM**S**NSFTNQ  SFVMSN**S**FTNQVM  AQYLGM**S**CDGPFK  DMKVRK**S**STPEEV  MKVRKS**S**TPEEVK  AVLFCL**S**EDKKNI  TYETKE**S**KKEDLV  IFWAPE**S**APLKSK  ESAPLK**S**KMIYAS  SKMIYA**S**SKDAIK  KMIYAS**S**KDAIKK  KKGKTI**S**LTDFLA  GGTGGG**S**TYVSKP  YVSKPV**S**WADETD  VSTTWH**S**NDDDVY  APPIDR**S**ILPTAP  EPNIDR**S**RLPKSP  EFEDLD**S**LLSALS  DLDSLL**S**ALSLNE  LSLNEE**S**LGNRRI  KDRDDR**S**FGRDRN  RYDDRG**S**RDYDRG  YDRGYD**S**RIGSGR  YDSRIG**S**GRRAFG  DRRDDR**S**WSSRDD  SSRDDY**S**RDDYRR  LNLKPR**S**TPKEDD  EDDSSA**S**TSQSTR  DSSAST**S**QSTRAA  SASTSQ**S**TRAASI  RHPSWR**S**EETQER  SRTGSE**S**SQTGTS  SSQTGT**S**TTSSRN  GTSTTS**S**RNARRR  NARRRE**S**EKSLEN  SNPPAR**S**QSSDTE  EQQSPT**S**GGGKVA  GQTGNS**S**RGPGDG  RDHWKE**S**DRKDGK  KDQDSR**S**APEPKK  NPASKF**S**SASKYA  SKFSSA**S**KYAALS  GQNYPR**S**GFPLEV  GFPLEV**S**TPLGQG  SRQLRV**S**HGCVSK  VSHGCV**S**KILCRY  RYQETG**S**IRPGAI  PGAIGG**S**KPKQVT  ENPGMF**S**WEIRDK  TVPSVS**S**ISRILR  PSVSSI**S**RILRSK  RKEAEE**S**EKKAKH  EKKAKH**S**IDGILS  ILSERA**S**APQSDE  KRKQRR**S**RTTFTA  RVQVWF**S**NRRARW  LPTYQL**S**ETSYQP  YQLSET**S**YQPTSI  TSYQPT**S**IPQAVS  SIPQAV**S**DPSSTV  PQPLPP**S**TVHQST  HQSTIP**S**NPDSSS  SNPDSS**S**AYCLPS  STRHGF**S**SYTDSF  TRHGFS**S**YTDSFV  VPPSGP**S**NPMNPT  TIGNGL**S**PQVMGL  EPTTTV**S**ASCSQR  TTTVSA**S**CSQRLD  TVSASC**S**QRLDHM  SLPTSQ**S**YCPPTY  YCPPTY**S**TTGYSM  YSTTGY**S**MDPVTG  EETCAP**S**VSYFKC  TCAPSV**S**YFKCVQ  QKEVLP**S**MRKIVA  YLDRFL**S**LEPVKK  LEPVKK**S**RLQLLG  TCMFVA**S**KMKETI  CIYTDN**S**IRPEEL  FIEHFL**S**KMPEAE  TDVKFI**S**NPPSMV  FISNPP**S**MVAAGS  SMVAAG**S**VVAAVQ  QGLNLR**S**PNNFLS  SPNNFL**S**YYRLTR  RLTRFL**S**RVIKCD  IEALLE**S**SLRQAQ  EALLES**S**LRQAQQ  RFLGDL**S**HLLAII  LLKIWK**S**RSCAGI  KIWKSR**S**CAGISG  RSCAGI**S**GKSQVL  AGISGK**S**QVLFAV  LFTNYI**S**LYNTCM  VVYIAC**S**FTTVWL  TVWLIY**S**KFKATY  EILWTF**S**IYLESV  FSIYLE**S**VAILPQ  PQLFMV**S**KTGEAE  EAETIT**S**HYLFAL  GLLFPV**S**CLVLLQ  VLLQVA**S**SGNMKV  QLVFLL**S**EAHTCI  LMDDVV**S**ADNYTL  TLLLTW**S**NPYPPD  VTYLEP**S**LRIAAS  SLRIAA**S**TLKSGI  TLKSGI**S**YRARVR  CYNTTW**S**EWSPST  STKWHN**S**YREPFE  IPNPAR**S**RLVAII  EMPFQG**S**GKSAWC  FQGSGK**S**AWCPVE  WCPVEI**S**KTVLWP  KGSFCA**S**PESSRD  FCASPE**S**SRDDFQ  EPSPPA**S**PTQSPD  PAYRSF**S**NSLSQS  YRSFSN**S**LSQSPC  SNSLSQ**S**PCPREL  PVSAPT**S**GYQEFV  QGGTQA**S**AVVGLG  AGYKAF**S**SLLASS  GYKAFS**S**LLASSA  LASSAV**S**PEKCGF  CGFGAS**S**GEEGYK  PPRSPQ**S**SHLPSS  PRSPQS**S**HLPSSS  SSHLPS**S**SPEHLG  CCCGDR**S**SPPTTP  CCGDRS**S**PPTTPL  ISEKSK**S**SSSFHP  KSKSSS**S**FHPAPG  YAGPAQ**S**LGSFVH  PAQSLG**S**FVHCEP  CDEKAL**S**MCPPSP  LSMCPP**S**PLGCEL  ALAEGQ**S**CGVYTE  VCLNEK**S**YREQVK  HEEPTT**S**EMAEET  MAEETY**S**PKIFRP  PKHTRI**S**ELKAEA  RKKLTQ**S**KFVGGA  AHPRII**S**APEMRQ  PEMRQE**S**EQGPCR  RRHMEA**S**LQELKA  LQELKA**S**PRMVPR  RKQCKP**S**RGRKRG  AEQATK**S**VLFVCL  LGNICR**S**PIAEAV  VTDQNI**S**ENWRVD  ENWRVD**S**AATSGY  VDSAAT**S**GYEIGN  PDYRGQ**S**CMKRHG  RHGIPM**S**HVARQI  ILCMDE**S**NLRDLN  RDLNRK**S**NQVKTC  KIELLG**S**YDPQKQ  PYYGND**S**DFETVY  LENLVS**S**LREQCT  FTGEDC**S**QLACPS  LDCGQH**S**CPSDCN  EGEITK**S**LRRPET  TRLDAP**S**QIEVKD  VSQTDN**S**ITLEWR  PKDLQV**S**ETAETS  SETAET**S**LTLLWK  ATPYTV**S**IYGVIQ  YRTPVL**S**AEASTG  VPGSLR**S**MEIPGL  NLTLPG**S**LRAVDI  TVEYNI**S**GAERTA  ERTAHI**S**GLPPST  DFIVYL**S**GLAPSI  IRTKTI**S**ATATTE  LENLTI**S**DINPYG  SENAFD**S**FLVTVV  LVTVVD**S**GKLLDP  PQEFTL**S**GTQRKL  PDGFRL**S**WTADEG  ISKGRR**S**QTVSAI  RRSQTV**S**AIATTA  PKEVIF**S**DITENS  PTAQVE**S**FRITYV  MKGFEE**S**EPVSGS  AIATVD**S**YVISYT  VDSYVI**S**YTGEKV  TATEVQ**S**ETALLT  LNGDTT**S**GLYTIY  FDKDTD**S**AITNCA  DGGAEF**S**ARSRKR  AEFSAR**S**RKRKAN  ARDQCG**S**QPWDNN  VCADPC**S**LIPTPD  DRVYPN**S**TCKPRI  PRIIAP**S**RGSPLP  SPLPVL**S**WANREE  LQLIGI**S**SLFIAA  QLIGIS**S**LFIAAK  VTDGAC**S**GDEILT  ALKWRL**S**PLTIVS  SPLTIV**S**WLNVYM  YGILAA**S**ALYHFS  SALYHF**S**SSELMQ  ALYHFS**S**SELMQK  LYHFSS**S**ELMQKV  ELMQKV**S**GYQWCD  VIRETG**S**SKLKHF  IRETGS**S**KLKHFR  IQTHRD**S**LDLLDK  AKKAML**S**EQNRAS  RASPLP**S**GLLTPP  SKLLVD**S**NNPKIK  QPRIRR**S**GLELYA  AVVMQG**S**ARNQDD  SGRPLK**S**LKQRLK  NLHLPQ**S**LETRAE  TGKQIF**S**LIIPGH  GPYKHI**S**PGDTKV  GSSAQK**S**LSEYNN  EYNNFK**S**MVVSGA  GSKINI**S**QVIAVV  RGFVEN**S**YLAGLT  QRRLIK**S**MESVMV  LLGEIE**S**KFNQAI  FFGSAP**S**PMGGIS  SAASDA**S**GFSPGY  GGAMSP**S**YSPTSP  GGYTPQ**S**PSYSPT  PTSPNY**S**PTSPSY  PSYSPT**S**PSYSPT  YSPTSP**S**YSPTSP  PTSPSY**S**PTSPSY  PSYSPT**S**PNYSPT  PNYTPT**S**PSYSPT  PTSPSY**S**PTSPNY  PKYSPT**S**PKYSPT  LTSPAI**S**PDDSDE  DQGIYQ**S**KVRELI  KVRELI**S**DNQYRL  LEGSFG**S**KHVSPR  GIVTKC**S**LVRPKV  RPKVVR**S**VHYCPA  LVAFPS**S**SVYPTK  VAFPSS**S**VYPTKD  CNVKQM**S**KDAQPS  SKDAQP**S**FSAEDI  DAQPSF**S**AEDIAK  AKIKKF**S**KTRSKD  KFSKTR**S**KDIFDQ  FDQLAK**S**LAPSIH  AKSLAP**S**IHGHDY  LLIGDP**S**VAKSQL  PTTGRG**S**SGVGLT  LQDSLL**S**RFDLLF  DAMPLG**S**AVDILA  KKEKMV**S**AAFMKK  PVLTQE**S**ATYIAE  YIAEEY**S**RLRSQD  RLRSQD**S**MSSDTA  RSQDSM**S**SDTART  HAKARM**S**KTVDLQ  TPKTAD**S**QETKES  SQETKE**S**QKVELS  SQKVEL**S**ESRLKA  KVELSE**S**RLKAFK  REAHAQ**S**IGMNRL  MNRLTE**S**INRDSE  EIQAAL**S**KMQDDN  MVPVLL**S**LLLLLG  NQDGRY**S**LTYIYT  YIYTGL**S**KHVEDV  AFQALG**S**LNDLQF  QFFRYN**S**KDRKSQ  NSKDRK**S**QPMGLW  EDWKQD**S**QLQKAR  VEYYND**S**NGSHVL  YNDSNG**S**HVLQGR  EIENNR**S**SGAFWK  IENNRS**S**GAFWKY  RKYLKY**S**KNILDR  DRQDPP**S**VVVTSH  PSVVVT**S**HQAPGE  GNGTYQ**S**WVVVAV  QDTAPY**S**CHVQHS  SCHVQH**S**SLAQPL  CHVQHS**S**LAQPLV  HLCVLA**S**NCDEPM  RKVVGC**S**CVVVKD  KDYGKE**S**QAKDVI  ETCPCP**S**GGSCTC  PCPSGG**S**CTCADS  SCTCAD**S**CKCEGC  EGCKCT**S**CKKSCC  CTSCKK**S**CCSCCP  CKKSCC**S**CCPAEC  GTGYDL**S**ASTFSP  GYDLSA**S**TFSPDG  LSASTF**S**PDGRVF  MKAVEN**S**STAIGI  KAVENS**S**TAIGIR  VEKLVL**S**KLYEEG  KLYEEG**S**NKRLFN  LLADAR**S**LADIAR  IAREEA**S**NFRSNF  EASNFR**S**NFGYNI  HAYTLY**S**AVRPFG  VRPFGC**S**FMLGSY  CSFMLG**S**YSVNDG  FMLGSY**S**VNDGAQ  LYMIDP**S**GVSYGY  IDPSGV**S**YGYWGC  AFELEL**S**WVGELT  QEVPRG**S**EPWKQQ  LTEDGD**S**FLHLAI  EQGCLA**S**VGVLTQ  VGVLTQ**S**CTTPHL  TTPHLH**S**ILKATN  TCLHLA**S**IHGYLG  IVELLV**S**LGADVN  QNPDLV**S**LLLKCG  VTYQGY**S**PYQLTW  LTWGRP**S**TRIQQQ  KDLERD**S**LTEKEC  NGNQAR**S**EARRVG  RQTTIT**S**HFAKGP  KRRRVT**S**RERVAR  EPERAK**S**GTRTEK  DEKKHR**S**QPKDLA  NESGFE**S**YEALPQ  HKLTCF**S**VYCKHG  ELFFSG**S**AKPIYD  KALIGF**S**TSFAEY  LIGFST**S**FAEYIL  NRFTED**S**LLRHAQ  MVKFGG**S**GRSKQA  SVIPDD**S**SKPLYL  TALWED**S**SNGQMF  CEDMQL**S**YIHSKV  VIYKAP**S**ENWAME  QLEDLD**S**RVLYYS  TFNIKL**S**SPVKRP  FNIKLS**S**PVKRPR  PENTHK**S**TPASYH  ECVQVY**S**MGGPNR  SGCGGL**S**EGFHQA  PPCQGF**S**GMNRFN  PRACQL**S**VVVDDK  DDKKFV**S**NITRLS  NITRLS**S**GPFRTI  HICKDM**S**ALVAAR  IPLAPG**S**DWRDLP  VRECAR**S**QGFPDT  LAKARE**S**ASAKIK  NRHRKR**S**HSRSRS  HRKRSH**S**RSRSRD  KRSHSR**S**RSRDRK  SHSRSR**S**RDRKRR  RDRKRR**S**RSRDRR  RKRRSR**S**RDRRNR  RNRDQR**S**ASRDRR  RDQRSA**S**RDRRRR  RDRRRR**S**KPLTRG  HGGLIR**S**PRHEKK  PVPVVG**S**QMTRQA  AFLEFR**S**VDETTQ  IIFQGQ**S**LKIRRP  QPLPGM**S**ENPSVY  GMSENP**S**VYVPGV  YVPGVV**S**TVVPDS  STVVPD**S**AHKLFI  VKELLT**S**FGPLKA  FNLVKD**S**ATGLSK  DSATGL**S**KGYAFC  LLVQRA**S**VGAKNA  KNATLV**S**PPSTIN  TLVSPP**S**TINQTP  QVPGLM**S**SQVQMG  VPGLMS**S**QVQMGG  DVRDEC**S**KYGLVK  KYGLVK**S**IEIPRP  IFVEFT**S**VFDCQK  TMEPQV**S**NGPTSN  VSNGPT**S**NTSNGP  GPTSNT**S**NGPSSN  NTSNGP**S**SNNRNC  TSNGPS**S**NNRNCP  NNRNCP**S**PMQTGA  GATTDD**S**KTNLIV  TQEEFR**S**LFGSIG  FRSLFG**S**IGEIES  SIGEIE**S**CKLVRD  DKITGQ**S**LGYGFV  TKTIKV**S**YARPSS  VSYARP**S**SASIRD  ARPSSA**S**IRDANL  DANLYV**S**GLPKTM  ELEQLF**S**QYGRII  YGRIIT**S**RILVDQ  DQVTGV**S**RGVGFI  LNGQKP**S**GATEPI  KFANNP**S**QKSSQA  NNPSQK**S**SQALLS  NPSQKS**S**QALLSQ  SSQALL**S**QLYQSP  LSQLYQ**S**PNRRYP  GVKRLM**S**GPVPPS  SGPVPP**S**ACPPRF  ACPPRF**S**PITIDG  TIDGMT**S**LVGMNI  IFVYNL**S**PDSDES  YNLSPD**S**DESVLW  SPDSDE**S**VLWQLF  AAMAIA**S**LNGYRL  DRVLQV**S**FKTNKA  GSDELF**S**TCVTNG  NGPFIM**S**SNSASA  GPFIMS**S**NSASAA  MSSNSA**S**AANGND  KKFKGD**S**RSAGVP  RSAGVP**S**RVIHIR  TEGEVI**S**LGLPFG  MVNYYT**S**VTPVLR  PIYIQF**S**NHKELK  KELKTD**S**SPNQAR  ELKTDS**S**PNQARA  AVNSVQ**S**GNLALA  NLALAA**S**AAAVDA  MAMAGQ**S**PVLRII  AQHAKL**S**LDGQNI  TLRIDF**S**KLTSLN  DFSKLT**S**LNVKYN  KYNNDK**S**RDYTRP  TRPDLP**S**GDSQPS  SGDSQP**S**LDQTMA  AAAFGL**S**VPNVHG  APLAIP**S**AAAAAA  LAGAGN**S**VLLVSN  NSVLLV**S**NLNPER  ERVTPQ**S**LFILFG  QAQLAM**S**HLNGHK  PIRITL**S**KHQNVQ  TKDYGN**S**PLHRFK  LSNIPP**S**VSEEDL  NIPPSV**S**EEDLKV  DLKVLF**S**SNGGVV  LKVLFS**S**NGGVVK  ALIQMG**S**VEEAVQ  HPDAFP**S**LRALIA  LQPPPT**S**RTPFPP  PALGLR**S**SAQDPQ  ALGLRS**S**AQDPQA  ALGRAL**S**PLEEWL  SGARPL**S**HQPGPE  GEKKDV**S**GPMPDS  YGRPNV**S**AANPRG  PPNVTG**S**LHLGHA  TNAIQD**S**LTRWHR  WSCTLN**S**AISDIE  EFGVLV**S**FAYKVQ  AYKVQG**S**DSDEEV  VIHPFL**S**RSLPIV  AISIMD**S**RGALIN  EMAQAA**S**AAVTRG  AKEFGV**S**PDKISL  SGLFPL**S**ILGWPN  NQSEDL**S**VFYPGT  AHGRKM**S**KSLGNV  GRKMSK**S**LGNVID  DVIYGI**S**LQGLHN  GKGFVP**S**PTSQPG  FVPSPT**S**QPGGHE  QPGGHE**S**LVDRWI  VDRWIR**S**RLTEAV  TEAVRL**S**NQGFQA  VTTAQY**S**FWLYEL  VGLRLL**S**PFMPFV  MPQAPP**S**LCVTPY  PEPSEC**S**WKDPEA  YVQALA**S**AGVVAV  CAVALA**S**DRCSIH  LIAAQY**S**GAQVRV  AQVRVL**S**APPHFH  GFCVFE**S**NAIAYY  AIAYYV**S**NEELRG  NEELRG**S**TPEAAA  QVVQWV**S**FADSDI  WVSFAD**S**DIVPPA  DIVPPA**S**TWVFPT  KQVLEP**S**FRQAFP  PRKEKG**S**REEKQK  FAHLPK**S**TFVLDE  EFKRKY**S**NEDTLS  SNEDTL**S**VALPYF  FDKDGW**S**LWYSEY  GWSLWY**S**EYRFPE  LTQTFM**S**CNLITG  RKNAFA**S**VILFGT  LFGTNN**S**SSISGV  FGTNNS**S**SISGVW  GTNNSS**S**ISGVWV  NNSSSI**S**GVWVFR  ELAFPL**S**PDWQVD  WQVDYE**S**YTWRKL  RKLDPG**S**EETQTL  LVREYF**S**WEGAFQ  LTAIYE**S**LLSLSP  IYESLL**S**LSPDVP  ESLLSL**S**PDVPVP  PDVPVP**S**DHGGTE  DHGGTE**S**SPGWGS  SSPGWG**S**SGPWSL  GSSGPW**S**LSPSDS  SGPWSL**S**PSDSSP  PWSLSP**S**DSSPSG  SLSPSD**S**SPSGVT  LSPSDS**S**PSGVTS  PSDSSP**S**GVTSRL  SPSGVT**S**RLPGRS  SRLPGR**S**TSLVEG  ATSTTP**S**RYKTEL  RQSISF**S**GLPSGR  SFSGLP**S**GRRTSP  PGLAGP**S**LSSSSF  LAGPSL**S**SSSFSP  AGPSLS**S**SSFSPS  GPSLSS**S**SFSPSS  LSSSSF**S**PSSSPP  SSFSPS**S**SPPPPG  LSPSAF**S**AAPGTP  TPVCCP**S**CRRATP  RRATPI**S**VWGPLG  GLVRTP**S**VQSLGS  SVQSLG**S**DPDEYA  DPDEYA**S**SGSSLG  EYASSG**S**SLGGSD  GSSLGG**S**DSPVFE  VQYLTR**S**AIRRAS  AQRLPD**S**FKDSPS  PDSFKD**S**PSKGLG  SFKDSP**S**KGLGPC  WILVAF**S**FLFTVI  VITFPI**S**IWMCIK  ILPCTD**S**FIKVDM  VDMRTI**S**FDIPPQ  EILTKD**S**VTISVD  KDSVTI**S**VDGVVY  NITNAD**S**ATRLLA  NLSQIL**S**DREEIA  IAHNMQ**S**TLDDAT  AAEAEA**S**REARAK  EGEMNA**S**RALKEA  RALKEA**S**MVITES  IAAEKN**S**TIVFPL  GAYGMV**S**SAYDHV  AYGMVS**S**AYDHVR  VAIKKI**S**PFEHQT  RDILRA**S**TLEAMR  LYKLLK**S**QQLSND  LKSQQL**S**NDHICY  HRDLKP**S**NLLINT  PEIMLN**S**KGYTKS  SKGYTK**S**IDIWSV  KSIDIW**S**VGCILA  ILAEML**S**NRPIFP  ILGILG**S**PSQEDL  GILGSP**S**QEDLNC  ARNYLQ**S**LPSKTK  YLQSLP**S**KTKVAW  AKLFPK**S**DSKALD  LFPKSD**S**KALDLL  EPEAKK**S**KTAAKK  PPDQKT**S**PSGKPA  DQKTSP**S**GKPATL  ATLKIC**S**WNVDGL  LQETKC**S**ENKLPA  QELPGL**S**HQYWSA  LSHQYW**S**APSDKE  QYWSAP**S**DKEGYS  SDKEGY**S**GVGLLS  SGVGLL**S**RQCPLK  QCPLKV**S**YGIGDE  IVAEFD**S**FVLVTA  FLKGLA**S**RKPLVL  AVPLAD**S**FRHLYP  YMMNAR**S**KNVGWR  LDYFLL**S**HSLLPA  YFLLSH**S**LLPALC  LPALCD**S**KIRSKA  CDSKIR**S**KALGSD  RSKALG**S**DHCPIT  ATPPKR**S**CPSFSA  RSCPSF**S**ASSEGT  PSFSAS**S**EGTRIK  TRIKKI**S**IEGNIA  NIAAGK**S**TFVNIL  RWCNVQ**S**TQDEFE  EKPERW**S**FTFQTY  QTYACL**S**RIRAQL  IRAQLA**S**LNGKLK  VLFFER**S**VYSDRY  FERSVY**S**DRYIFA  DRYIFA**S**NLYESE  ASNLYE**S**ECMNET  NNQFGQ**S**LELDGI  LHYKHE**S**WLLHRT  FKDKYE**S**LVEKVK  LCKWFE**S**SGIHVA  ECCPTP**S**HWSATR  QNGTEP**S**SLPFLD  WDHALD**S**QEYEGL  TLSRVL**S**EPNPRP  TNANDG**S**NEGIVH  IATVQT**S**QGLADK  GEHVAP**S**EAANSL  LGSGFA**S**NREELS  PAFAHT**S**QVLVDK  GIHTGE**S**IVVAPS  AKCLGF**S**DKQIAL  HVLVLG**S**GVYRIG  ENRFKF**S**RLLDTI  LERFLS**S**AAAVSK  ECNVRV**S**RSFPFV  VKVPQF**S**FSRLAG  ELVINL**S**MRGAGG  GGRRLS**S**FVTKGY  RLAADF**S**VPLIID  SELRLD**S**VVQWME  AQLTQR**S**VHICHV  LLLTAV**S**EGRLSL  VGQHIL**S**VQQFTK  MVQKER**S**LDILKG  SFSEAT**S**SVQKGE  HGRTVH**S**LACLLT  LTQYRV**S**LRYVAP  RYVAPP**S**LRMPPT  VRAFVA**S**RGTKQE  ISVEVD**S**DPRAAY  PSERSL**S**LSLPGP  QRGYSD**S**AERAER  LRTTRM**S**WPSSFH  TRMSWP**S**SFHGTG  RMSWPS**S**FHGTGT  HGTGTG**S**GGAGGG  GAGGGS**S**RRFEAE  ENGPTP**S**PGRSPL  GRSPLD**S**QASPGL  PLDSQA**S**PGLVLH  HAGAAT**S**QRRESF  ESFLYR**S**DSDYDM  DSDYDM**S**PKTMSR  VLASLR**S**VRSNFS  TNVPVP**S**NKRSPL  TMQTYR**S**VSEMAS  LSEMSR**S**GNQVSE  QVSEYI**S**TTFLDK  KLMHSN**S**LNNSNI  DVAYHN**S**LHAADV  FLINTN**S**ELALMY  LMYNDE**S**VLENHH  DIFQNL**S**KRQRQS  ERGMEI**S**PMCDKH  NRDWYY**S**AIRQSP  ASPAQE**S**LEVMAQ  QAQSTG**S**APVAPD  APDEFS**S**REEFVV  VVAVSH**S**SPSALA  SALALQ**S**PLLPAW  TFGEDT**S**ALPAPG  KTGNSE**S**KKKPCS  KPCSET**S**QIEDTP  IEDTPS**S**KPTLLA  GHGVEG**S**DTTGSP  YQEYPN**S**QNWPED  ADLVFP**S**SATADT  NDPLKD**S**YGMSPC  ALNSPH**S**ESFVSP  HSESFV**S**PEAVAE  AKDMQP**S**MESDMA  MQPSME**S**DMALVK  KDLVLL**S**EIEVAQ  QANDII**S**STEISS  SSTEIS**S**AEKVAL  QTQKGI**S**EDSHLE  SQPSEL**S**SETSGI  TQPAKT**S**TSKAKT  AKTQPT**S**LPKQPA  LNKKPM**S**LASGLV  VASARP**S**ILPSKD  SPSKPA**S**APASRS  SAPASR**S**GSKSTQ  VASTGP**S**SRSPST  KKMTAK**S**VPADLS  SRPKST**S**TSSMKK  PKSTST**S**SMKKTT  AAGVVP**S**RVKATP  KATPMP**S**RPSTTP  PMPSRP**S**TTPFID  TTRKPE**S**NAVTKT  EAGAPT**S**ASGLNG  FTPDWE**S**REVSTG  WESREV**S**TGTTIM  VVLGAD**S**RTTTGS  SRTTTG**S**YIANRV  RIFCCR**S**GSAADT  FCCRSG**S**AADTQA  YQLGFH**S**IELNEP  LVHTAA**S**LFKEMC  EGGQVY**S**VPMGGM  GMMVRQ**S**FAIGGS  SFAIGG**S**GSSYIY  AIGGSG**S**SYIYGY  IGGSGS**S**YIYGYV  AMERDG**S**SGGVIR  MERDGS**S**GGVIRL  LAAIAE**S**GVERQV  ENTSLS**S**TTNSLM  LSSTTN**S**LMQLND  YSNDFN**S**GEANTS  SGEANT**S**DAFNWT  FNWTVD**S**ENRTNL  EGCLSP**S**CLSLLH  LSPSCL**S**LLHLQE  LQEKNW**S**ALLTAV  YLDVLF**S**TASIMH  QNPIHH**S**RFNSRT  IAVWTI**S**VGISMP  TISVGI**S**MPIPVF  FGLQDD**S**KVFKEG  KVFKEG**S**CLLADD  NFVLIG**S**FVSFFI  LIGSFV**S**FFIPLT  EATLCV**S**DLGTRA  AKLASF**S**FLPQSS  FSFLPQ**S**SLSSEK  SFLPQS**S**LSSEKL  PQSSLS**S**EKLFQR  EKLFQR**S**IHREPG  IHREPG**S**YTGRRT  GRRTMQ**S**ISNEQK  RTMQSI**S**NEQKAC  AVICKE**S**CNEDVI  TYRSAF**S**RYIQCQ  ALAYKS**S**QLQMGQ  MGQKKN**S**KQDAKT  ALGKQH**S**EEASKD  QHSEEA**S**KDNSDG  DEQPGP**S**TSQEEG  KKEKNV**S**SFQLKL  KEKNVS**S**FQLKLA  AAKAPK**S**EKEMDP  QPSAQK**S**PTSPLN  AQKSPT**S**PLNMKL  KKDEKQ**S**LISAGD  SESRKT**S**NVCIRF  CIRFEV**S**PSYVKG  RFEVSP**S**YVKGGP  MVLVPK**S**TLHNWM  FKRWVP**S**LRVICF  WDVCVT**S**YEMVIK  MVIKEK**S**VFKKFH  RIKNEK**S**KLSEIV  KTDVEK**S**LPPKKE  KIYLGL**S**KMQREW  DIDVLN**S**SGKMDK  IDVLNS**S**GKMDKM  TDEHIV**S**NSGKMV  SRVLIF**S**QMTRLL  KFIFML**S**TRAGGL  LGINLA**S**ADVVIL  VVILYD**S**DWNPQV  RLIDQQ**S**NKLAKE  VFASKE**S**ELTDED  QKMGES**S**LRNFRM  RMDIEQ**S**LYKFEG  REVEGK**S**PEEVME  EEVMEY**S**AVFWER  RIQRRI**S**IKKALD  FDWFIK**S**RTAMEF  RCNTLI**S**LIEKEN  LNLDEG**S**AAEALD  MEGNCV**S**LTRILR  SLIQFF**S**KMKKWM  QRRIPC**S**VKDLFN  GDDLVN**S**YHLLLC  QDLLNP**S**FKGLPS  TADFTA**S**EEPPCI  YFKPYI**S**KLFDRK  SSFTDN**S**KAVNKE  HFEKKR**S**FAPSTP  VITPVA**S**ATQSVS  CQHYTQ**S**TDEQPG  LHGMDM**S**VLLEQD  IVLFAY**S**SPRTFP  VLFAYS**S**PRTFPW  RSEEGL**S**RDMVKH  LAWSHD**S**ALWEAL  WEALQV**S**ANKVPT  QPLSPI**S**VHERYS  SSPTAG**S**AKRRLF  CLKLDV**S**NELRRK  FQEIMK**S**YRNQPQ  NQPQAN**S**HVYRSV  TKTPDC**S**SGPVKE  KTPDCS**S**GPVKEE  YVGRVK**S**FALKYD  GSPRRI**S**QQHSIY  SPHKNG**S**GLTPRS  SGLTPR**S**ALLYKF  KFNGSP**S**KSLKDI  RVIAID**S**DAESPA  DILIRN**S**CVKEED  DRKQLR**S**VLNNLK  ETDERD**S**TNRASF  DSTNRA**S**FKCPVC  FKCPVC**S**STFTDL  KCPVCS**S**TFTDLE  EVEEDE**S**AMPKKD  IPALKQ**S**KDHAAT  TTAGAA**S**LAGGHH  WATKGP**S**YEDLYT  EDLHRA**S**LEGKSA  ASLEGK**S**AKERPI  PIWLRE**S**TVQGAY  VQGAYG**S**EDMKEG  IHEKKT**S**SAMAGS  HEKKTS**S**AMAGSV  SSAMAG**S**VGAAAP  VTAANG**S**DSESET  AANGSD**S**ESETSE  NGSDSE**S**ETSESD  DSESET**S**ESDDDS  ESETSE**S**DDDSPP  SESDDD**S**PPRPAA  VAGRPF**S**YSEVSQ  GRPFSY**S**EVSQRP  FSYSEV**S**QRPELV  VANNGS**S**QANKLI  NEDGDL**S**KSTVLR  EAQEYG**S**FGTAEM  DDGNLL**S**IVTSGG  LLSIVT**S**GGAHGT  HGTHVA**S**IAAGHF  IGDTRL**S**TMETGT  CDLVNY**S**YGEATH  ATHWPN**S**GRICEV  GVGAYV**S**PDMMVA  VQVAAP**S**DHGVGI  PENTEN**S**EKISLQ  LALTSN**S**SWVQCP  ALTSNS**S**WVQCPS  SWVQCP**S**HLELMN  CGYDIA**S**PNAGPL  VTVCSC**S**SEVSAK  TVCSCS**S**EVSAKF  RWWASL**S**DVNIDY  QLNIHA**S**EGINRF  QTLRPV**S**AKTKPL  KTKPLG**S**RDVLPN  NKRQMG**S**GDAYPH  AYPHQY**S**LKLEKG  IRHEQI**S**DLERLK  DLPFIV**S**HRLSNT  RLSNTL**S**LDIHEN  LLGKKK**S**SNLTLP  LGKKKS**S**NLTLPP  GCYLAG**S**LTLSKT  TKTKNG**S**KDKEKD  KDKEKD**S**EKEKDL  GWTHCA**S**FTENWL  SVCNVM**S**GDQDNW  SFPGGA**S**SCKETF  FPGGAS**S**CKETFN  NLYYAE**S**DLDYGT  PDEITV**S**SDFEAR  LNVEER**S**VGPLTR  ACVALL**S**VRVYYK  PETIAG**S**DAPSLA  KFEASE**S**PCLECP  WTPPQD**S**GGREDI  REDIVY**S**VTCEQC  RTSVTV**S**DLEPHM  VSGLVT**S**RSFRTA  GLVTSR**S**FRTASV  RSFRTA**S**VSINQT  VRLEGR**S**TTSLSV  TTSLSV**S**WSIPPP  SLSVSW**S**IPPPQQ  KKGDSN**S**YNVRRT  RRTEGF**S**VTLDDL  PEDVYF**S**KSEQLK  GMLKTS**S**GKKEVP  RNILVN**S**NLVCKV  TAPEAI**S**YRKFTS  SYRKFT**S**ASDVWS  RKFTSA**S**DVWSFG  SASDVW**S**FGIVMW  TPMDCP**S**AIYQLM  LIRAPD**S**LKTLAD  IRLPST**S**GSEGVP  VPFRTV**S**EWLESI  WFHRDL**S**GLDAET  GRGVHG**S**FLARPS  SFLARP**S**RKNQGD  KNQGDF**S**LSVRVG  QGDFSL**S**VRVGDQ  HIRIQN**S**GDFYDL  KYPLNC**S**DPTSER  WYHGHM**S**GGQAET  TFLVRE**S**LSQPGD  LVRESL**S**QPGDFV  PGDFVL**S**VLSDQP  FVLSVL**S**DQPKAG  PKAGPG**S**PLRVTH  GLETFD**S**LTDLVE  TGIEEA**S**GAFVYL  LNKKQE**S**EDTAKA  FWEEFE**S**LQKQEV  ILPFDH**S**RVILQG  ILQGRD**S**NIPGSD  DSNIPG**S**DYINAN  RAYGPY**S**VTNCGE  LRTLQV**S**PLDNGD  WHYQYL**S**WPDHGV  PDHGVP**S**EPGGVL  INQRQE**S**LPHAGP  MLMENI**S**TKGLDC  MVRAQR**S**GMVQTE  KLEVLQ**S**QKGQES  SQKGQE**S**EYGNIT  NAHAKA**S**RTSSKH  AKASRT**S**SKHKED  KVKKQR**S**ADKEKS  PEHSPP**S**SPLTQP  EHSPPS**S**PLTQPP  ITYDTL**S**AQAQQD  APEQLL**S**QARDFI  INQYYS**S**IKRSGS  TYQLRE**S**ELVFGA  DFRIWN**S**QLVRYA  FSGWYM**S**TEIGTR  LDTRTT**S**SLWKDK  NVAVLH**S**YQLAKV  MVNYFL**S**PAFRYQ  PDPWKG**S**AAKGTG  ATILYG**S**ETGRAQ  LVLVVT**S**TFGNGD  AALMEM**S**GPYNSS  MSGPYN**S**SPRPEQ  SGPYNS**S**PRPEQH  RPEQHK**S**YKIRFN  FNSISC**S**DPLVSS  CSDPLV**S**SWRRKR  SDPLVS**S**WRRKRK  CVFGLG**S**RAYPHF  AARDIF**S**PKRSWK  FQATIR**S**VENLQS  VENLQS**S**KSTRAT  LVEALL**S**RVEDPP  FFLDIT**S**PPSPQL  QLLRLL**S**TLAEEP  QELEAL**S**QDPRRY  PRYYSV**S**SAPSTH  RTQEVT**S**RIRTQS  FQQFRK**S**KENNIF  ENNIFY**S**PISITS  IFYSPI**S**ITSALG  SPISIT**S**ALGMVL  TYHVDR**S**GNVHHQ  LTEFNK**S**TDAYEL  KKFYQT**S**VESVDF  YQTSVE**S**VDFANA  ANAPEE**S**RKKINS  SRKKIN**S**WVESQT  INSWVE**S**QTNEKI  PEGNIG**S**NTTLVL  NKNTYK**S**IQMMRQ  MMRQYT**S**FHFASL  TSFHFA**S**LEDVQA  YKGKDL**S**MIVLLP  KLMEWT**S**LQNMRE  RFKVEE**S**YDLKDT  NGDADL**S**GMTGSR  LSGMTG**S**RGLVLS  SRGLVL**S**GVLHKA  AVVGFG**S**SPTSTN  VVGFGS**S**PTSTNE  FGSSPT**S**TNEEFH  RQNKTN**S**ILFYGR  TERAPA**S**EEEFQF  CALLDS**S**HSELKC  ELKCDI**S**AEIQQR  ALQEQD**S**AFGAVH  DYEEMA**S**RLGRLD  QRIRTG**S**ALVQRM  RMKCYA**S**DQEVLD  RQEEPQ**S**LQAAVR  GKDAAV**S**KKASPE  KVIKME**S**EEGKEA  QPRPST**S**KAVSPP  NSNHVA**S**GAGEAE  ERVVVI**S**SSEDSD  VVVISS**S**EDSDAE  DAENSS**S**RELDDS  ELDDSS**S**ESSDLQ  LQLEGP**S**TLRVLD  NETQKI**S**QLAAVN  AAVNRE**S**KFRVVI  QPEAFF**S**IYSKAV  AFFSIY**S**KAVSLE  IYSKAV**S**LEVGLQ  FLSFLS**S**MRRPIL  EFQEAI**S**GFLAAL  YLARNM**S**ERSAMA  RNMSER**S**AMAAVL  QHVYPF**S**SLQCFA  HVYPFS**S**LQCFAS  SLQCFA**S**LQPLVQ  LALHNV**S**FMELLS  GGLKKY**S**RYLSLQ  EIPRDA**S**LMLYFR  PGTEAS**S**DQTAQG  HHFKNE**S**LGMAFL  EVAKKT**S**FKDCIP  RHIRQH**S**ALTRLR  PCYIRD**S**GVAPTD  VNKEEG**S**SGSSGR  EEGSSG**S**SGRNPQ  NKCLEL**S**LPSRAA  LELSLP**S**RAAALS  AALSFV**S**LVDGYF  RLTADS**S**HYLCHE  LTVAQR**S**QAPDGM  LEGWGR**S**FPSVRE  MRGARA**S**PRTLNL  KEITQL**S**HLGQGT  AFYETA**S**LMSQVS  ETASLM**S**QVSHTH  VAQQLA**S**ALSYLE  QLASAL**S**YLENKN  GLAEGT**S**PFIKLS  SPFIKL**S**DPGVGL  LPGGAN**S**LSTAMD  GGANSL**S**TAMDKW  GEAPLQ**S**RSPSEK  APLQSR**S**PSEKEH  EPTQRP**S**FRTILR  LTVNPD**S**PASDPT  NPDSPA**S**DPTVFH  CGPQHR**S**GWKQEI  EDQGEK**S**LQLVME  VREDGD**S**PVFWYA  HCDSSQ**S**PPTKFL  GPVAGA**S**RQENGA  RQENGA**S**VILRDI  QKSLAG**S**SGPGAS  KSLAGS**S**GPGASS  SSGPGA**S**SGTSGD  SGPGAS**S**GTSGDH  GASSGT**S**GDHGEL  LVVRIA**S**LEVENQ  LEVENQ**S**LRGVVQ  ELQQAI**S**KLEARL  LNVLEK**S**SPGHRA  NVLEKS**S**PGHRAT  PALVAK**S**SILLDV  ALVAKS**S**ILLDVK  LEACVR**S**IQLDGL  GLVWGA**S**KLVPVG  FEEHVQ**S**VDIAAF  SPAAAE**S**GAKEEL  SGAASP**S**AAEKGE  AEGEAA**S**AASSTS  EAASAA**S**STSSPK  AASAAS**S**TSSPKA  SAASST**S**SPKAED  AEAGAA**S**GEQAAA  PEKPPA**S**DETKAA  AEEAGA**S**AAACEA  AACEAP**S**AAGPGA  PAAAAA**S**SACAAP  AAAAAS**S**ACAAPS  EAQPEC**S**PEAPPA  PRAAFL**S**PLLPLL  LGFLLL**S**APHGGS  SAPHGG**S**GLHTKG  YKVIPK**S**KFVLVK  KRLAEN**S**ASSDDL  LAENSA**S**SDDLLV  AENSAS**S**DDLLVA  VAEVGI**S**DYGDKL  KLNMEL**S**EKYKLD  YKLDKE**S**YPVFYL  GEFIRA**S**GVEARQ  QGQDNL**S**SVKETQ  GQDNLS**S**VKETQK  GEDFPA**S**EMTRIA  IEKNKM**S**DGKKEE  KEELQK**S**LNILTA  LYAEVH**S**EEALFL  VFNKQK**S**HDDIVT  DRLAKG**S**ECYQKS  SECYQK**S**LSLNPF  LNPFLW**S**PFESLC  TSLQNF**S**NCLPNS  RLNLES**S**NSKYSL  NLESSN**S**KYSLNT  YSLNTD**S**SVSYID  NTDSSV**S**YIDSAV  TGTSIL**S**KQVQNK  LSPLTP**S**FGILPL  KSVFSQ**S**GNSREV  ILAQTQ**S**SGPQTS  TTPQVL**S**PTITSP  LSPTIT**S**PPNALP  NALPRR**S**SRLFTS  SSRLFT**S**DSSTTK  LFTSDS**S**TTKENS  EITKLD**S**SIISEG  ISEGKI**S**TITPQI  GYLALC**S**YNCKEA  EAINIL**S**HLPSHH  ILSHLP**S**HHYNTG  RAYFEL**S**EYMQAE  EGMEIY**S**TTLWHL  VALSVL**S**KDLTDM  TDMDKN**S**PEAWCA  AAGNCF**S**LQREHD  IMGTDE**S**QESSMT  TDESQE**S**SMTDAD  RQKLIF**S**PCSDCE  LIFSPC**S**DCEEEE  EEEEEG**S**GHSTGE  PLPPAR**S**PTEPGP  GGAEGD**S**WEEEGF  APYFLG**S**SFSPVR  AGEGRR**S**PRPDHP  TPHTPK**S**LLSKAR  TPKSLL**S**KARGID  KARGID**S**SSVKLR  ARGIDS**S**SVKLRG  SVKLRG**S**SLFMDT  FMDTEK**S**GKREFD  NPFTPD**S**LLLHSS  DSLLLH**S**SGQCRR  GEDMEA**S**DYELED  HVVRYF**S**AWAEDD  EYCNGG**S**LADAIS  GLRYIH**S**MSLVHM  PSNIFI**S**RTSIPN  IFISRT**S**IPNAAS  SIPNAA**S**EEGDED  DEDDWA**S**NKVMFK  GHVTRI**S**SPQVEE  HVTRIS**S**PQVEEG  RIPQVL**S**QEFTEL  DPERRP**S**AMALVK  SVLLSA**S**RKSAEQ  AEKFKN**S**LLQKEL  DRMATR**S**TTQSNR  TRSTTQ**S**NRTSRL  RLLFAC**S**PPPASQ  KALFGA**S**AAGGLS  SAAGGL**S**PVTNLT  QLQGLG**S**DYEQPL  LQRMGS**S**ESTDSG  QKLLGC**S**PALKRS  KRSHSD**S**LDHDIF  KPVRPV**S**RGCLHS  SRGCLH**S**HGLQEG  PARMLS**S**NERDSS  SNERDS**S**EPGNFI  PVTATL**S**DEDDGF  NEEETP**S**CMASLW  TPSCMA**S**LWTAPL  RCKLFD**S**PSLCSS  KLFDSP**S**LCSSST  DSPSLC**S**SSTRSV  PSLCSS**S**TRSVLK  CSSSTR**S**VLKRPE  ERSQEE**S**PPGSTK  EESPPG**S**TKRRKS  KSMSGA**S**PKESTN  TLHQSL**S**LASSPK  QSLSLA**S**SPKGTI  DLIGDF**S**KGYLFH  QDLKYI**S**PEIMAS  SPEIMA**S**VLNGKF  VFHCEF**S**SERGPR  FHCEFS**S**ERGPRM  FFMKCQ**S**YCEPPS  SYCEPP**S**YRPMHH  APGSAL**S**PAGVCG  PGLLLG**S**HGLLGS  SHGLLG**S**PVRAAA  VRAAAS**S**PVTTLT  ASESSL**S**SESSES  SSLSSE**S**SESSDA  SLSSES**S**ESSDAG  SSESSE**S**SDAGLC  LCMDSP**S**PMDPHM  AIRRFQ**S**MPVRLL  VRLLGH**S**PVLRNI  PDGRRK**S**EAGSGA  RKSEAG**S**GAASSS  AGSGAA**S**SSGEDK  GSGAAS**S**SGEDKE  SGAASS**S**GEDKEN  ALAEWA**S**RREAFA  AFAQRP**S**SAPDLM  PDLMCL**S**PDRKME  LALGRF**S**LTPAEG  VPPGME**S**LISAPL  GMESLI**S**APLVKT  SPSMPC**S**VIRPIL  KARVLR**S**KSLCHD  ELIGDY**S**KAFLLQ  QDLKYI**S**PETMVA  LERDAE**S**FLLKSP  ESFLLK**S**PIAPCS  SPIAPC**S**LDKRVI  IFHCEF**S**SERGPR  SWAGER**S**RRELCS  STELFS**S**TREEGS  STREEG**S**SGSGPS  TREEGS**S**GSGPSF  EEGSSG**S**GPSFRS  SSGSGP**S**FRSNQR  SGPSFR**S**NQRKML  KRCLDL**S**NLSSGE  LDLSNL**S**SGEITA  DLSNLS**S**GEITAT  ATQLTT**S**ADLDET  ETGHLD**S**SGLQEV  TGHLDS**S**GLQEVH  KRDAMC**S**SSANKE  RDAMCS**S**SANKEN  DAMCSS**S**ANKEND  NGNLVD**S**EMKYLG  DQEAKV**S**RSGLYR  EAKVSR**S**GLYRSP  VKKKYF**S**GQGKLR  CLKKTV**S**LCDITI  QMLEED**S**NQGHLI  HLIGDF**S**KVCALP  CALPTV**S**GKHQDL  TVAALL**S**GKFQGL  GALNLY**S**QEELFN  LREEDR**S**LNQYPA  ELCEPQ**S**YCPMHH  ELLRCR**S**QSKVQE  LRCRSQ**S**KVQEGE  FSPWKI**S**MFLSVR  KISMFL**S**VREDSV  LSVRED**S**VPTTAS  SVPTTA**S**FSADML  PTTASF**S**ADMLNV  NGTFAQ**S**KCPQVE  ENIFVL**S**VFCLHK  VFCLHK**S**SCTVAE  FCLHKS**S**CTVAEI  FWAITI**S**NNFDWL  VVNAII**S**MNLYSS  ISMNLY**S**SICFLM  SMNLYS**S**ICFLML  CFLMLV**S**IDRYLA  ALVKTM**S**MGRMRG  RWAKLY**S**LVIWGC  GCTLLL**S**SPMLVF  CTLLLS**S**PMLVFR  RTMKEY**S**DEGHNV  VTACVI**S**YPSLIW  CVISYP**S**LIWEVF  GFLLPL**S**VITFCT  WLPFQI**S**TFLDTL  HRLGIL**S**SCQDER  RLGILS**S**CQDERI  VITQIA**S**FMAYSN  ASFMAY**S**NSCLNP  FMAYSN**S**CLNPLV  KRFRKK**S**WEVYQG  QKGGCR**S**EPIQME  SAEVET**S**EGVDES  SEGVDE**S**EKKNSG  ESEKKN**S**GALEKE  MRMADL**S**ELLKEG  ALYFTY**S**ALEEEM  RYMGDL**S**GGQVLK  RALKLP**S**TGEGTQ  ELDQAG**S**TLARET  KGALEG**S**SCPFRT  GALEGS**S**CPFRTA  AVLRKP**S**LQFILA  GTQAEE**S**PFVGNP  DDWIVV**S**QLRITS  SQLRIT**S**LQLSDT  ITSLQL**S**DTGQYQ  GHQTFV**S**QPGYVG  GHGPQR**S**LHVPGL  PGLNKT**S**SFSCEA  GLNKTS**S**FSCEAH  NKTSSF**S**CEAHNA  AKGVTT**S**RTATIT  AWTPGL**S**GIYPLT  PEEPLT**S**QASVPP  HQLRLG**S**LHPHTP  IRVACT**S**SQGPSS  SATRNG**S**QAFVHW  AGDGPW**S**LPVPLE  QLVKEP**S**TPAFSW  PSTPAF**S**WPWWYV  RVRKSY**S**RRTTEA  TEATLN**S**LGISEE  QLNQDD**S**ILKVAV  ELEDFL**S**EAVCMK  GVCFQG**S**ERESFP  KHGDLH**S**FLLYSR  HSFLLY**S**RLGDQP  FMADIA**S**GMEYLS  SGMEYL**S**TKRFIH  VADFGL**S**KKIYNG  GLYALM**S**RCWELN  DPKDSC**S**CLTAAE  RYVLCP**S**TTPSPA  CPSTTP**S**PAQPAD  QPADRG**S**PAAPGQ  AGGDHG**S**PDSYRS  DHGSPD**S**YRSPLA  SPDSYR**S**PLASRY  YRSPLA**S**RYASPE  LASRYA**S**PEMCFV  EMCFVF**S**DRYKFR  QIQEMK**S**NLENID  IHLGAT**S**CYVGDN  KLARVI**S**RLADFA  FAKERA**S**LPTLGF  TTGTQA**S**FLQLFE  VDIEVL**S**VLASLG  VLSVLA**S**LGASVH  LASLGA**S**VHKICT  EKQQIG**S**SAMPYK  KQQIGS**S**AMPYKR  KRNPMR**S**ERCCSL  RSERCC**S**LARHLM  DPLQTA**S**VQWFER  ERTLDD**S**ANRRIC  NTLQNI**S**EGLVVY  MVKAGG**S**RQDCHE  EKIRVL**S**QQAASV  LSQQAA**S**VVKQEG  QVDAYF**S**PIHSQL  YFSPIH**S**QLDHLL  DHLLDP**S**SFTGRA  HLLDPS**S**FTGRAS  SFTGRA**S**QQVQRF  LLKPYE**S**VMKVKA  PRGARC**S**NGFPER  PPRPGP**S**RPAEKP  PRPEAK**S**AQPADG  NLAAAY**S**SILSSL  LAAAYS**S**ILSSLG  AYSSIL**S**SLGENP  TPWRAA**S**AMQFFT  GYQETI**S**DVLNDA  KDIDMF**S**MCEHHL  KQVLGL**S**KLARIV  RIVEIY**S**RRLQVQ  GVQKMN**S**KTVTST  NSKTVT**S**TMLGVF  MLLLAF**S**SWSLCS  LLLAFS**S**WSLCSD  LAFSSW**S**LCSDSE  SSWSLC**S**DSEEEM  WSLCSD**S**EEEMKA  LTNMHT**S**KISKAH  MHTSKI**S**KAHVPS  SKAHVP**S**WKMTLL  TLLNVC**S**LVNNLN  LVNNLN**S**PAEETG  TALDGF**S**LEAMLT  LHKICH**S**RAFQHW  SGVRGL**S**RLLSAR  DGNKRA**S**AKVSDS  RASAKV**S**DSISAQ  KVSDSI**S**AQYPVV  RAAFGL**S**EAGFNT  KLFPTR**S**HTVAAQ  YDTVKG**S**DWLGDQ  NYGMPF**S**RTEDGK  RAFGGQ**S**LKFGKG  ADRTGH**S**LLHTLY  SLRYDT**S**YFVEYF  LCIEDG**S**IHRIRA  YGRTYF**S**CTSAHT  TYFSCT**S**AHTSTG  CTSAHT**S**TGDGTA  GGILIN**S**QGERFM  VAKDLA**S**RDVVSR  ASRDVV**S**RSMTLE  RDVVSR**S**MTLEIR  TRLPGI**S**ETAMIF  GEAACA**S**VHGANR  GRACAL**S**IEESCR  ALSIEE**S**CRPGDK  PNAGEE**S**VMNLDK  LRFADG**S**IRTSEL  DGSIRT**S**ELRLSM  TSELRL**S**MQKSMQ  RLSMQK**S**MQNHAA  AVFRVG**S**VLQEGC  EGCGKI**S**KLYGDL  AEARKE**S**RGAHAR  IDEYDY**S**KPIQGQ  WRKHTL**S**YVDVGT  NTQAER**S**IIGMID  DKIEKP**S**LLTMMK  NFPNFL**S**ACDKKG  DKKIDF**S**EFLSLL  DFSEFL**S**LLGDIA  TDYHKQ**S**HGAAPC  GNYYVD**S**FLLGAD  DAADEL**S**VGRYAP  AEHPDF**S**PCSFQS  PDFSPC**S**FQSKAT  SPCSFQ**S**KATVFG  ATVFGA**S**WNPVHA  DGRYMR**S**WLEPTP  PTPGAL**S**FAGLPS  SFAGLP**S**SRPYGI  FAGLPS**S**RPYGIK  IKPEPL**S**ARRGDC  LDTHTL**S**LTDYAC  TDYACG**S**PPVDRE  DREKQP**S**EGAFSE  PSEGAF**S**ENNAEN  NNAENE**S**GGDKPP  NWLHAR**S**TRKKRC  HQKWKF**S**RPGIRL  SFGHPS**S**VAGEVV  SNGIKV**S**GLLVLD  LLVLDY**S**KDYNHW  HWLATK**S**LGQWLQ  NLIAEV**S**TKDVKV  EPLFGI**S**TGNLIT  ASRVEV**S**KVLILG  EFDYSG**S**QAVKAM  VKVLGT**S**VESIMA  LGTSVE**S**IMATED  EDRQLF**S**DKLNEI  QMLRRT**S**INVVRH  FALHPT**S**MEYCII  RSSALA**S**KATGYP  VVSGKT**S**ACFEPS  SACFEP**S**LDYMVT  SSRIGS**S**MKSVGE  KELSEP**S**STRIYA  ELSEPS**S**TRIYAI  AIDDNM**S**LDEIEK  CNPETV**S**TDFDEC  LYFEEL**S**LERILD  DRAEDR**S**IFSAVL  ALEFAK**S**VDYPCL  KDGRVI**S**HAISEH  RSFPFV**S**KTLGVD  LRCEMA**S**TGEVAC  FLKAML**S**TGFKIP  GQNPSL**S**SIRKLI  RRTAVD**S**GIPLLT  KSRKVD**S**KSLFHY  RKVDSK**S**LFHYRQ  ETTPLN**S**QKQLSA  VESGQI**S**NGYSAV  QISNGY**S**AVPSPG  GYSAVP**S**PGAGDD  GDDTRH**S**IPATTT  KVDFLL**S**VIGYAV  HRNGCI**S**IWRKIC  IAFYIA**S**YYNTIM  ALYYLI**S**SFTDQL  LYYLIS**S**FTDQLP  DQLPWT**S**CKNSWN  NCTNYF**S**EDNITW  ITWTLH**S**TSPAEE  WTLHST**S**PAEEFY  VLQIHR**S**KGLQDL  QDLGGI**S**WQLALC  FTVIYF**S**IWKGVK  WKGVKT**S**GKVVWV  AAQIFF**S**LGPGFG  QDALVT**S**VVNCMT  VVNCMT**S**FVSGFV  MRNEDV**S**EVAKDA  AKDAGP**S**LLFITY  IANMPA**S**TFFAII  IEAVAV**S**WFYGIT  KEMLGF**S**PGWFWR  ICWVAI**S**PLFLLF  LLFIIC**S**FLMSPP  ICSFLM**S**PPQLRL  YNYPYW**S**IILGYC  GYCIGT**S**SFICIP  RYFLLK**S**DGSFIG  PPLNNF**S**VAECQL  RTFHVD**S**PDEREE  IQMVAN**S**LKQRAP  CGSPSD**S**STTEEM  EMEVAV**S**KARAKV  AHTVTE**S**RVLQNT  ELFFHL**S**RERVFT  YGAEIV**S**ALEYLH  ALEYLH**S**RDVVYR  LCKEGI**S**DGATMK  RFPRTL**S**PEAKSL  LSPEAK**S**LLAGLL  RLGGGP**S**DAKEVM  EHRFFL**S**INWQDV  FKPQVT**S**EVDTRY  PPDRYD**S**LGLLEL  KGNKAL**S**VGNIDD  DALQCY**S**EAIKLD  HNHVLY**S**NRSAAY  VLYSNR**S**AAYAKK  DWGKGY**S**RKAAAL  LYQKLE**S**DPRTRT  RTRTLL**S**DPTYRE  QLRNKP**S**DLGTKL  RIMTTL**S**VLLGVD  LGVDLG**S**MDEEEE  YARIGN**S**YFKEEK  IHFYNK**S**LAEHRT  KDAKLY**S**NRAACY  KALDLD**S**SCKEAA  ALDLDS**S**CKEAAD  QYNRHD**S**PEDVKR  EVQQIM**S**DPAMRL  KDPQAL**S**EHLKNP  TERCIE**S**LIAVFQ  GYNYTL**S**KTEFLS  SKTEFL**S**FMNTEL  KKLDTN**S**DGQLDF  DGQLDF**S**EFLNLI  AMACHD**S**FLKAVP  PVLATE**S**DWADCK  DWADCK**S**PQPWGH  NVLQED**S**VTLTCR  TCRGTH**S**PESDSI  GTHSPE**S**DSIQWF  HSPESD**S**IQWFHN  PTHTQP**S**YRFKAN  KANNND**S**GEYTCQ  CQTGQT**S**LSDPVH  TGQTSL**S**DPVHLT  VHLTVL**S**EWLVLQ  IVLRCH**S**WKDKPL  FFQNGK**S**KKFSRS  GKSKKF**S**RSDPNF  SKKFSR**S**DPNFSI  RSDPNF**S**IPQANH  IPQANH**S**HSGDYH  QANHSH**S**GDYHCT  IGYTLY**S**SKPVTI  GYTLYS**S**KPVTIT  ITVQAP**S**SSPMGI  TVQAPS**S**SPMGII  VQAPSS**S**PMGIIV  CRKKRI**S**ANSTDP  KRISAN**S**TDPVKA  PVITEQ**S**PRRLVV  QSPHSG**S**FTITGN  RIYWMN**S**KILHIK  FANVLT**S**DNHSDY  RVKATN**S**MIDRKP  AENSLG**S**ARHAYY  LSNVQP**S**DTMVTQ  TFTCQA**S**FDPSLQ  GRLVIH**S**LDYSDQ  IHSLDY**S**DQGNYS  ELDVVE**S**RAQLLV  APEKWY**S**LGKVPG  STTLKL**S**PYVHYT  YGPGEP**S**PVSETV  KVQAVN**S**QGKGPE  IEILNS**S**AVLVKW  VPANTT**S**VILSGL  SGLRPY**S**SYHLEV  RWQPPL**S**HNGVLT  HNLTDL**S**PHLRYR  GGTMAL**S**GISDFG  ENYSVV**S**WVPKEG  EEKGGA**S**LSPQYV  YVSYNQ**S**SYTQWD  VSYNQS**S**YTQWDL  TFGEYR**S**LESDNE  EEKAFG**S**SQPSLN  DIKPLG**S**DDSLAD  PLGSDD**S**LADYGG  SFIGQY**S**GKKEKE  AGGNDS**S**GATSPI  KPPCDL**S**MQSVEV  CDLSMQ**S**VEVAGS  SVEVAG**S**GGARRS  SGGARR**S**ALLDSD  RSALLD**S**DEPLVY  QRGIQV**S**NNGPCL  NGPCLG**S**RKPDQP  QPYEWL**S**YKQVAE  KQVAEL**S**ECIGSA  LSECIG**S**ALIQKG  QGCFAY**S**MVIVPL  VNKAEL**S**LVFVDK  VMDAYG**S**ELVERG  CGVEVT**S**MKAMED  AVICFT**S**GTTGNP  THRNIV**S**DCSAFV  NIVSDC**S**AFVKAT  PDDTLI**S**FLPLAH  WLLDFA**S**KRKEAE  KEAELR**S**GIIRNN  GIIRNN**S**LWDRLI  IFHKVQ**S**SLGGRV  FHKVQS**S**LGGRVR  TGAAPV**S**ATVLTF  ENIYMR**S**EPVAQV  VFVHGE**S**LQAFLI  DVETLC**S**WAQKRG  KRGFEG**S**FEELCR  VRLGKD**S**GLKPFE  LHPELF**S**IDNGLL  LRNYFR**S**QIDDLY  KQEMLL**S**LKPSEA  LLSLKP**S**EAPELD  EGFGDW**S**QRPEQR  AQGALD**S**GEPPQC  AYEKED**S**DEVHLE  VHLEEL**S**LSKEGP  LEELSL**S**KEGPGP  LIDRTE**S**LNRSIE  TESLNR**S**IEKSNS  NRSIEK**S**NSVKKS  SIEKSN**S**VKKSQP  SNSVKK**S**QPDLPI  QPDLPI**S**KIDQWL  ASIELP**S**MAVAST  PSMAVA**S**TKSRWE  AVASTK**S**RWETGE  GEVQAQ**S**AAKTPS  SAAKTP**S**CKDIVA  IVAGDM**S**KKSLWE  GDMSKK**S**LWEQKG  WEQKGG**S**KTSSTI  KGGSKT**S**STIKST  GGSKTS**S**TIKSTP  TSSTIK**S**TPSGKR  TIKSTP**S**GKRYKF  CYHFLT**S**LLRSAM  LTSLLR**S**AMQNAR  AEAAVL**S**GPGPPL  GIPRPL**S**SAGRLS  IPRPLS**S**AGRLSQ  SSAGRL**S**QGCRGA  QGCRGA**S**TVGAAG  PETPAI**S**PSKRAR  TPAISP**S**KRARPA  LRFARL**S**EHATAP  TAPTRG**S**ARAAGY  AGYDLY**S**AYDYTI  IQIALP**S**GCYGRV  GRVAPR**S**GLAAKH  DDTERG**S**GGFGST  DYNKFM**S**VLDTNK  FVEYVR**S**LACLCL  LMMEQR**S**RDPGMV  LYFQGP**S**SNKPRV  YFQGPS**S**NKPRVI  GIVTRV**S**EVKPKM  TYQPIQ**S**PTFMPL  PLIMCP**S**QECQTN  ECQTNR**S**GGRLYL  MKMQEH**S**DQVPVG  VGNIPR**S**ITVLVE  QPGDHV**S**VTGIFL  VVQGLL**S**ETYLEA  IVKMNK**S**EDDESG  KSEDDE**S**GAGELT  YEKLAA**S**IAPEIY  VGGVDQ**S**PRGMKI  DPGVAK**S**QLLSYI  AKSQLL**S**YIDRLA  YTTGRG**S**SGVGLT  TTGRGS**S**GVGLTA  AAVLRD**S**VSGELT  VLRDSV**S**GELTLE  MEQQTI**S**IAKAGI  RYNPRR**S**LEQNIQ  LPAALL**S**RFDLLW  TYVHQH**S**RQPPSQ  RREAWA**S**KDATYT  KDATYT**S**ARTLLA  LAILRL**S**TALARL  IRLMEM**S**KDSLLG  MEMSKD**S**LLGDKG  TVRELV**S**GGRSVR  LVSGGR**S**VRFSEA  AEQRCV**S**RGFTPA  QLLLVL**S**AAGMGA  AAGMGA**S**GAPQPP  GVYGEP**S**RETPNL  LFPNFY**S**ANPLCS  SANPLC**S**PSRAAL  NPLCSP**S**RAALLT  VGGIPD**S**EQLLPE  KKAGYV**S**KIVGKW  FDEWFG**S**PNCHFG  HAPVYA**S**KPFLGT  KPFLGT**S**QRGRYG  VREIDD**S**IGKILE  TFVFFT**S**DNGAAL  NGAALI**S**APEQGG  APEQGG**S**NGPFLC  VTAGQV**S**HQLGSI  VSHQLG**S**IMDLFT  MDLFTT**S**LALAGL  AGLTPP**S**DRAIDG  FWTWTN**S**WENFRQ  CPGQNV**S**GVTTHN  GERFPL**S**FASAEY  FPLSFA**S**AEYQEA  EYQEAL**S**RITSVV  ALSRIT**S**VVQQHQ  CLTPPE**S**IPKKCL  ALQRTH**S**LLLLLL  LGLVQP**S**YGQDGM  PEETGG**S**DRYCNL  DIWNIR**S**ICSTTN  NIRSIC**S**TTNIQC  DCRDTG**S**SRAPNC  CRDTGS**S**RAPNCR  RYRAIA**S**TRRVVI  GLVACV**S**AESRAE  ACVSAE**S**RAELTS  SRAELT**S**DKDMYL  DMYLDN**S**SIEEAS  MYLDNS**S**IEEASG  SSIEEA**S**GVYPID  DDDDYA**S**ASGSGA  DDYASA**S**GSGADE  YASASG**S**GADEDV  ADEDVE**S**PELTTS  SPELTT**S**RPLPKI  PKILLT**S**AAPKVE  IPAQTK**S**PEETDK  KVHLSD**S**ERKMDP  VYTEKH**S**DSLFKR  TEKHSD**S**LFKRTE  RKKDEG**S**YDLGER  GERKPS**S**AAYQKA  KDADLW**S**SHDKML  DADLWS**S**HDKMLA  AQPLKD**S**DVEVYN  NIIKKE**S**NRQRVG  GLELIA**S**ENFASR  ASENFA**S**RAVLEA  VLEALG**S**CLNNKY  CLNNKY**S**EGYPGQ  VNVQPY**S**GSPANF  VQPYSG**S**PANFAV  TDKKKI**S**ATSIFF  KKISAT**S**IFFESM  TSIFFE**S**MPYKVN  LIIAGT**S**CYSRNL  AGTSCY**S**RNLEYA  ADMAHI**S**GLVAAG  AAGVVP**S**PFEHCH  YRKGVK**S**VDPKTG  ILYNLE**S**LINSAV  LESLIN**S**AVFPGL  ANCRAL**S**EALTEL  KIVTGG**S**DNHLIL  ILVDLR**S**KGTDGG  KVLEAC**S**IACNKN  TCPGDR**S**ALRPSG  RSALRP**S**GLRLGT  GTPALT**S**RGLLEK  LTLQIQ**S**DTGVRA  LREEVE**S**FASLFP  EVESFA**S**LFPLPG  GKRKGK**S**KKWKEI  LKFPHI**S**QCEDLR  IDRDYC**S**LCDKQP  YIQFLD**S**VAEYEV  KYLTPK**S**PVFIAQ  VGQDLV**S**QTEEKL  PCKELF**S**ACAQSV  FSACAQ**S**VHEYLR  FHEYLD**S**MFFDRF  KKRKGE**S**MALNEK  ILEKVN**S**QFVVNL  YGHIRI**S**DLGLAV  NQRYGL**S**PDYWGL  EMIEGQ**S**PFRGRK  ETEEVY**S**HKFSEE  VYSHKF**S**EEAKSI  FSEEAK**S**ICKMLL  TDDDFY**S**KFSTGS  DFYSKF**S**TGSVSI  SKFSTG**S**VSIPWQ  FSTGSV**S**IPWQNE  RQHQNN**S**KSSPSS  HQNNSK**S**SPSSKT  QNNSKS**S**PSSKTS  NSKSSP**S**SKTSFN  SKSSPS**S**KTSFNH  SPSSKT**S**FNHHIN  FNHHIN**S**NHVSSN  TEIANG**S**KDGLDS  SKDGLD**S**NPMKDY  KDYMIL**S**GPQKTA  TLLGLL**S**ALENVA  VLYLIL**S**SHQLRR  LYLILS**S**HQLRRK  QLRRKP**S**YLFIGS  SYLFIG**S**LAGADF  GADFLA**S**VVFACS  SVVFAC**S**FVNFHV  VFHGVD**S**KAVFLL  FLLKIG**S**VTMTFT  TMTFTA**S**VGSLLL  FTASVG**S**LLLTAI  CLRYPP**S**YKALLT  GIMWVL**S**ALVSYL  VLSALV**S**YLPLMG  CCPRPC**S**ELFPLI  PNDYLL**S**WLLFIA  FIAFLF**S**GIIYTY  AHQHVA**S**LSGHQD  QHVASL**S**GHQDRQ  LALMAH**S**LATTLS  SLATTL**S**DQVKKA  KAFAFC**S**MLCLIN  MLCLIN**S**MVNPVI  VIYALR**S**GEIRSS  RSGEIR**S**SAHHCL  SGEIRS**S**AHHCLA  CVRGLG**S**EAKEEA  LGLNPV**S**ASLQDQ  LNPVSA**S**LQDQHC  QDQHCE**S**LSLASN  QHCESL**S**LASNIS  ESLSLA**S**NISGLQ  SLASNI**S**GLQCNA  GLQCNA**S**VDLIGT  GTCWPR**S**PAGQLV  ECLANG**S**WAARVN  AARVNY**S**ECQEIL  LNEEKK**S**KVHYHV  YLGHCI**S**LVALLV  EVGAPW**S**GAPFQV  PFQVRR**S**IRCLRN  IHWNLI**S**AFILRN  VVQLTM**S**PEVHQS  SPEVHQ**S**NVGWCR  AIVLTY**S**TDRLRK  LRASTT**S**ETIQYR  PGEDEV**S**RVVFIY  VFIYFN**S**FLESFQ  FNSFLE**S**FQGFFV  FQGFFV**S**VFYCFL  FYCFLN**S**EVRSAI  LNSEVR**S**AIRKRW  RWQDKH**S**IRARVA  RVARAM**S**IPTSPT  AMSIPT**S**PTRVSF  TSPTRV**S**FHSIKQ  FKWDPK**S**LEIRTL  AHVLAA**S**VEQATE  DKIAKE**S**QFLKEE  ADDPCS**S**VKRGNM  AARALL**S**AVTRLL  PILYTA**S**QACLQH  IIVDPL**S**FSEERF  RLESII**S**GAALMA  AALMAD**S**SCTRDD  AGRKER**S**DALNSA  RSDALN**S**AIDKMT  AVMDHV**S**DSFLET  VANLAC**S**ISNNEE  NLACSI**S**NNEEGV  VKLVRM**S**ASQLEA  LVRMSA**S**QLEALC  LAAKPQ**S**KLAQEN  AVDDIT**S**IDDFLA  DDFLAV**S**ENHILE  EATKLL**S**NTVMPR  NEFIDA**S**RLVYDG  DVRSRT**S**VQTEDD  SFQEEK**S**KLDAEV  KIAEAG**S**RMDKLG  ADHCPD**S**ACKQDL  HQLNIC**S**KVKAEV  GGELVV**S**GVDSAM  VVSGVD**S**AMSLIQ  GVDSAM**S**LIQAAK  VQTVKA**S**YVASTK  KASYVA**S**TKYQKS  TKIKRA**S**QKKHVN  NPVQAL**S**EFKAMD  TTAPSL**S**GKGNPE  EEDVDT**S**QVLYEW  EWEQGF**S**QSFTQE  EQGFSQ**S**FTQEQV  EGMQIP**S**TQFDAA  QRLAEP**S**QMLKHA  VMVHQL**S**KKEASR  LSKKEA**S**RHAIMR  GTLHNL**S**HHREGL  LLAIFK**S**GGIPAL  LGSPVD**S**VLFYAI  AYGNQE**S**KLIILA  KLIILA**S**GGPQAL  KLLWTT**S**RVLKVL  RVLKVL**S**VCSSNK  KVLSVC**S**SNKPAI  VLSVCS**S**NKPAIV  LHLTDP**S**QRLVQN  LVQLLG**S**DDINVV  CAAGIL**S**NLTCNN  ALRHLT**S**RHQEAE  KLLHPP**S**HWPLIK  FVQLLY**S**PIENIQ  LTELLH**S**RNEGVA  AVLFRM**S**EDKPQD  LSVELT**S**SLFRTE  SVELTS**S**LFRTEP  YRQDDP**S**YRSFHS  DDPSYR**S**FHSGGY  SYRSFH**S**GGYGQD  GLPPGD**S**NQLAWF  QAHGGR**S**RAQPLT  AQPLTL**S**LGAAMT  LMLDVR**S**LGAVEP  AVEPIC**S**VNTPRE  EFLKIP**S**NFVSPE  IPSNFV**S**PEDLDI  DIPGHA**S**KDRYKT  ILPNPQ**S**RVCLGR  PMPNTV**S**DFWEMV  VWQEEV**S**LIVMLT  YQEERR**S**VKHILF  VKHILF**S**AWPDHQ  DHQTPE**S**AGPLLR  VAEVEE**S**PETAAH  PIVVHC**S**AGIGRT  EEEMPE**S**GAGSEF  PESGAG**S**EFNRKL  GVTENT**S**YYIFTQ  KVLNHF**S**IMQQRR  RGRRKA**S**ELRIHD  KKKKKG**S**DDEAFE  DEAFED**S**DDGDFE  QEVDYM**S**DGSSSS  DYMSDG**S**SSSQEE  YMSDGS**S**SSQEEP  MSDGSS**S**SQEEPE  SQEEPE**S**KAKAPQ  KGVDEQ**S**DSSEES  DEQSDS**S**EESEEE  SDSSEE**S**EEEKPP  KKRRKD**S**SEESDS  KRRKDS**S**EESDSS  SSEESD**S**SEESDI  SEESDS**S**EESDID  SDSSEE**S**DIDSEA  EESDID**S**EASSAL  DIDSEA**S**SALFMA  IDSEAS**S**ALFMAK  PSAEGG**S**TSSTLR  AEGGST**S**STLRAA  EGGSTS**S**TLRAAA  TLRAAA**S**KLEQGK  EQGKRV**S**EMPAAK  STPQPP**S**GKTTPN  TKKTGL**S**SEQTVN  KKTGLS**S**EQTVNV  CLLTGA**S**RGFGRT  LAPLLA**S**LLSPGS  LLASLL**S**PGSVLV  SLLSPG**S**VLVLSA  GSVLVL**S**ARNDEA  ELGAER**S**GLRVVR  LINNAG**S**LGDVSK  GSLGDV**S**KGFVDL  KGFVDL**S**DSTQVN  FVDLSD**S**TQVNNY  WALNLT**S**MLCLTS  SMLCLT**S**SVLKAF  MLCLTS**S**VLKAFP  LKAFPD**S**PGLNRT  RTVVNI**S**SLCALQ  TVVNIS**S**LCALQP  LVDCKV**S**AQKLLS  SAQKLL**S**LLEKDE  EKDEFK**S**GAHVDF  ALMYLG**S**LAFLGA  ARLDVA**S**EFRKKW  WNKWAL**S**RGKREL  KRELRM**S**SSYPTG  RELRMS**S**SYPTGL  ELRMSS**S**YPTGLA  QDMKGA**S**RSPEDS  MKGASR**S**PEDSSP  SRSPED**S**SPDAAR  RSPEDS**S**PDAARI  VKRYRQ**S**MNNFQG  NFQGLR**S**FGCRFG  DNVAPR**S**KISPQG  APRSKI**S**PQGYGR  GRRRRR**S**LPEAGP  PGRTLV**S**SKPQAH  GRTLVS**S**KPQAHG  NKTTMA**S**PQLMPL  VLYAVR**S**ERKLHT  LYIVSL**S**VADLIV  ILYLLM**S**KWSLGR  LLMSKW**S**LGRPLC  LCLFWL**S**MDYVAS  YVASTA**S**IFSVFI  STASIF**S**VFILCI  CIDRYR**S**VQQPLR  LGAWFL**S**FLWVIP  HFMQQT**S**VRREDK  RELINR**S**LPSFSE  INRSLP**S**FSEIKL  RSLPSF**S**EIKLRP  KKPGKE**S**PWEVLK  KDAGGG**S**VLKSPS  GGSVLK**S**PSQTPK  SVLKSP**S**QTPKEM  TPKEMK**S**PVVFSQ  KSPVVF**S**QEDDRE  QAAAEG**S**SRDYVA  AAAEGS**S**RDYVAV  YVAVNR**S**HGQLKT  HGASEI**S**EDQMLG  DQMLGD**S**QSFSRT  MLGDSQ**S**FSRTDS  GDSQSF**S**RTDSDT  SFSRTD**S**DTTTET  GKGKLR**S**GSNTGL  GKLRSG**S**NTGLDY  HSRQYV**S**GLHMNR  APDPAA**S**EPGSSG  AASEPG**S**SGADAA  APVRRQ**S**YSSTSR  RRQSYS**S**TSRGIS  QSYSST**S**RGISVT  STSRGI**S**VTKKTH  KICGDK**S**SGIHYG  KGFFRR**S**QQSNAT  FRRSQQ**S**NATYSC  QSNATY**S**CPRQKN  CLIDRT**S**RNRCQH  CLAVGM**S**RDAVKF  TPTYNI**S**ANGLTE  ELHDDL**S**NYIDGH  KADSAV**S**SFYLDI  ADSAVS**S**FYLDIQ  YLDIQP**S**PDQSGL  QPSPDQ**S**GLDING  CDYTPA**S**GFFPYC  GFFPYC**S**FTNGET  FTNGET**S**PTVSMA  ETSPTV**S**MAELEH  AQNISK**S**HLETCQ  VLLKAG**S**LEVVFI  MCRAFD**S**QNNTVY  FDGKYA**S**PDVFKS  SPDVFK**S**LGCEDF  GCEDFI**S**FVFEFG  FGKSLC**S**MHLTED  DEIALF**S**AFVLMS  LMSADR**S**WLQEKV  KLICKV**S**TLRALC  YKELFT**S**EFEPAM  NSSASL**S**PDRLNS  SPDRLN**S**PVTIPA  QPLCEY**S**TFILLF  FILLFF**S**LSGLSI  LLFFSL**S**GLSIIC  FSLSGL**S**IICAMS  PNMGLG**S**SRLQYP  NMGLGS**S**RLQYPD  TAHAAY**S**YMYAGF  YMYAGF**S**SFLILA  MYAGFS**S**FLILAT  HAAAAA**S**VASRGH  AAASVA**S**RGHPAA  PALPRL**S**DFRRRR  DFRRRR**S**FRRIAG  LVVLIC**S**IPLVVR  SLEREV**S**KNPDLQ  QAIRIA**S**VNPILD  FCRIGG**S**RRERSG  DSQRTS**S**AMSGHS  RELKEI**S**STSQTL  ELKEIS**S**TSQTLL  KEISST**S**QTLLPD  LSLPDL**S**ENGLGG  LRTLRI**S**ETSDSS  ISETSD**S**SQGQDS  SETSDS**S**QGQDSE  QGQDSE**S**VLLVDE  VDEAGG**S**GRAGPA  GPAPKG**S**SLQVTF  PAPKGS**S**LQVTFP  TGCPHR**S**GPHSAK  HRSGPH**S**AKGSLE  PHSAKG**S**LEKGSP  GSLEKG**S**PEDKEA  IRPDAP**S**RCTWQL  QLGRPA**S**ESPHHH  GRPASE**S**PHHHTA  HTAPAK**S**PKILPD  FFNAGG**S**VKDRIS  SVKDRI**S**LRMIED  TIIEPT**S**GNTGIG  VMPEKM**S**SEKVDV  MPEKMS**S**EKVDVL  TNARFD**S**PESHVG  RFDSPE**S**HVGVAW  KNEIPN**S**HILDQY  DQYRNA**S**NPLAHY  LDMLVA**S**VGTGGT  GVDPEG**S**ILAEPE  VDKWFK**S**NDEEAF  GLLCGG**S**AGSTVA  CGGSAG**S**TVAVAV  VVILPD**S**VRNYMT  YMTKFL**S**DRWMLQ  VQELGL**S**APLTVL  TLGNML**S**SLLAGK  LGNMLS**S**LLAGKV  AGKVQP**S**DQVGKV  DTLGRL**S**HILEMD  EQIQYH**S**TGKSSQ  YHSTGK**S**SQRQMV  HSTGKS**S**QRQMVF  GPNVCG**S**RYNAYC  RCMNGG**S**CSDDHC  MNGGSC**S**DDHCLC  GQPVCE**S**GCLNGG  PCFTVI**S**NQMCQG  CINTVG**S**FECKCP  VEYLYP**S**REPPRV  IDECES**S**PCINGV  LGAAWG**S**PCTLCQ  ICGKGY**S**RIKGTQ  ECKMIP**S**LCTHGK  CHNTEG**S**YRCECP  CNPGYH**S**TPDRLF  EGSYEC**S**CQPGFA  CTNTAG**S**FKCSCS  EDIDEC**S**LPNICV  CVNTPG**S**YICDCP  VGVSKA**S**CCCSLG  KASCCC**S**LGKAWG  CPAVNT**S**EYKILC  CINTAG**S**YRCDCK  CINTVG**S**FQCQCN  CLNTRG**S**YTCECN  CVNDRG**S**YHCICK  DNNECT**S**DINLCG  DINLCG**S**KGICQN  DENECL**S**AHICGG  HICGGA**S**CHNTLG  FQYEQF**S**GGCQDI  DINECG**S**AQAPCS  YDKDYL**S**GELGDN  QNGLNL**S**AKVVQG  AKVVQG**S**LDSLPQ  VQGSLD**S**LPQAVR  IHICDG**S**EEENGR  DVARIE**S**KTVIVT  QLGRWM**S**EEDFEK  MYVIPF**S**MGPLGS  SMGPLG**S**PLSKIG  PLGSPL**S**KIGIEL  GIELTD**S**PYVVAS  SPYVVA**S**MRIMTR  FVKCLH**S**VGCPLP  DRREII**S**FGSGYG  EIISFG**S**GYGGNS  SGYGGN**S**LLGKKC  FALRMA**S**RLAKEE  LAAAFP**S**ACGKTN  LAMMNP**S**LPGWKV  GVAPGT**S**VKTNPN  TNVAET**S**DGGVYW  IDEPLA**S**GVTITS  SGVTIT**S**WKNKEW  WKNKEW**S**SEDGEP  KNKEWS**S**EDGEPC  PCAHPN**S**RFCTPA  RFCTPA**S**QCPIID  IDAAWE**S**PEGVPI  VGAAMR**S**EATAAA  YLAHWL**S**MAQHPA  PGFGEN**S**RVLEWM  RIDGKA**S**TKLTPI  NMMELF**S**ISKEFW  MELFSI**S**KEFWEK  SEAEQD**S**WYQALL  YRLCLT**S**KTISFV  WMQVDD**S**VVAQNM  DEFRPR**S**KSQSSS  TATSPA**S**MVGGKP  SFRVRA**S**SDGEGT  FRVRAS**S**DGEGTM  TSPVSL**S**SSSTSG  LSSSST**S**GHGSTS  STSGHG**S**TSDCLF  LFPRRS**S**ASVSGS  PCDFRS**S**FRSVTP  NGHYIL**S**RGGNGH  AGDEAA**S**AADLDN  THSAGT**S**PTITHQ  HHRPDS**S**TLHTDD  GGGPSS**S**SSSSNA  KPPVES**S**GGKLLP  HQHLRL**S**TSSGRL  AATADD**S**SSSTSS  SSTSSD**S**LGGGYC  TAAQTN**S**RLARPT  LGDPKA**S**TLPRAR  AFHSSP**S**VRCPSQ  PSVRCP**S**QLQPAP  MSCPRQ**S**YVDTSP  SSAASA**S**PTGPQG  AELAAH**S**SLLGGP  FTRVNL**S**PNRNQS  GSGESS**S**TRRSSE  DLSAYA**S**ISFQKQ  VNDLRG**S**DSIAYD  DLRGSD**S**IAYDKG  ADLFGW**S**QLIYNH  ITTRVN**S**EQEHFL  PFGLLY**S**EVTASS  YSEVTA**S**SLVKIN  SEVTAS**S**LVKINL  AGFTLH**S**AIYAAR  PAGAAV**S**AMKCGL  CGLLPI**S**PEALSL  ISPEAL**S**LGEVAY  KNLGPK**S**KVLILR  RNHGLV**S**VGESVE  EKYKAK**S**RSPGSP  YKAKSR**S**PGSPVG  VGEGTG**S**PPKWQI  PALREK**S**KKYSDV  DVEVPA**S**VTGYSF  ASVTGY**S**FASDGD  FASDGD**S**GTCSPL  GRGDEA**S**EEGQNG  EEGQNG**S**SPKSKT  GHRTST**S**AVPNLF  KTAGPQ**S**QVLCGV  GELVTA**S**KAIIEK  REQKEK**S**PPDQPA  VPHPPP**S**TPIKLE  PTTGDD**S**DAATFK  LSPDEP**S**EALGFP  EAHRPP**S**PTEAPT  AEEAAP**S**AVEEGA  IADFMA**S**TSHAVF  DFMAST**S**HAVFPT  HAVFPT**S**SMNVSM  TSSMNV**S**MMTPIN  DLHTAD**S**LNLAKG  LMRCKI**S**SVYRLL  MRCKIS**S**VYRLLD  YGWAQL**S**DTYVTL  TGFCLH**S**AIYAAR  PATAAV**S**AMKWGL  WGLLPV**S**HNALLV  ACEIQV**S**ALSSAG  IQVSAL**S**SAGGVE  QVSALS**S**AGGVEN  RPHEVG**S**VQWAGS  FGPMQK**S**RLGEHE  VQRSMG**S**PRPKTT  ADEVEK**S**SSGMPI  DEVEKS**S**SGMPIR  QSQLLA**S**VIAEKS  SVIAEK**S**RSPSTE  TESQLM**S**KGDEDT  EDTKDD**S**EETVPN  TVPNPF**S**QLTDQE  LVSPSK**S**LEEGTK  TKKTET**S**KAATTE  TAEEIL**S**KGLSQM  ILSKGL**S**QMTTSA  LSQMTT**S**ADTDVD  KTESVT**S**GPMSPE  PSFLKK**S**KKKEKV  TQQATQ**S**YGAYPT  GYSQQS**S**QPYGQQ  QPYGQQ**S**YSGYSQ  YSGYSQ**S**TDTSGY  SQSTDT**S**GYGQSS  TSGYGQ**S**SYSSYG  YSSYGQ**S**QNTGYG  TGGYGS**S**QSSQSS  GYGSSQ**S**SQSSYG  SSQSSQ**S**SYGQQS  GQQPAP**S**STSGSY  TSGSYG**S**SSQSSS  GSYGSS**S**QSSSYG  YGSSSQ**S**SSYGQP  GSSSQS**S**SYGQPQ  SSSQSS**S**YGQPQS  SYGQPQ**S**GSYSQQ  QQNQYN**S**SSGGGG  QNQYNS**S**SGGGGG  NQYNSS**S**GGGGGG  YGQDQS**S**MSSGGG  QDQSSM**S**SGGGSG  DQSSMS**S**GGGSGG  MSSGGG**S**GGGYGN  YGNQDQ**S**GGGGSG  QSGGGG**S**GGYGQQ  GRGRGG**S**GGGGGG  GGGYNR**S**SGGYEP  DSEQDN**S**DNNTIF  ENVTIE**S**VADYFK  FDGKEF**S**GNPIKV  CENMNF**S**WRNECN  GGGPGG**S**HMGGNY  RLTMQV**S**SLQREP  LTMQVS**S**LQREPF  RIHFFL**S**KKKTDE  NRPGTV**S**SLKKNV  RPGTVS**S**LKKNVG  KNVGQF**S**GFPFEK  FPFEKG**S**VQYKKK  VLDLER**S**GVNSEL  ERSGVN**S**ELVKRI  LMHPKP**S**GKPLPK  GKPLPK**S**KKTCSK  KKERNS**S**GMARKA  PKQKAT**S**KSKKSV  QKATSK**S**KKSVKS  TSKSKK**S**VKSANV  SKKSVK**S**ANVKKA  KKNQNS**S**KKESES  IKKLLA**S**ANLEEV  RRSFHR**S**LSSSLQ  FHRSLS**S**SLQAPV  HRSLSS**S**LQAPVV  LQAPVV**S**TVGMQR  RLGTTP**S**VYGGAG  GRGIRI**S**NSRHTV  GIRISN**S**RHTVNY  HTVNYG**S**DLTGGG  VRTLEQ**S**NSKLEV  TLEQSN**S**KLEVQI  RAGRDY**S**AYYRQI  QIEELR**S**QIKDAQ  TELRRT**S**QSLEIE  LRRTSQ**S**LEIELQ  LEIELQ**S**HLSMKE  ELQSHL**S**MKESLE  HLSMKE**S**LEHTLE  ETKARY**S**SQLANL  TKARYS**S**QLANLQ  QLANLQ**S**LLSSLE  NLQSLL**S**SLEAQL  LQSLLS**S**LEAQLM  QLMQIR**S**NMERQN  TTEYQL**S**TLEERD  VDGKVV**S**SEVKEV  DGKVVS**S**EVKEVE  LNITEE**S**HVIDTG  DTGDSL**S**ISCRGQ  WVPCLV**S**IPGLNV  TLRSQS**S**VLWPDG  GDQDFL**S**NPFLVH  TLALWN**S**AAGLRR  IHEKEA**S**SPSIYS  SIYSRH**S**RQALTC  KMFAQR**S**LRRRQQ  GKNKTV**S**KLVIQN  MYKCVV**S**NKVGQD  TIESKP**S**EELLEG  WYRLNL**S**TLHDAH  ATPLAA**S**LEEVAP  EVQDRR**S**HDKHCH  DLLVNV**S**DSLEMQ  GVDLAD**S**NQKLSI  DSNQKL**S**IQRVRE  AGRYLC**S**VCNAKG  KGCVNS**S**ASVAVE  CVNSSA**S**VAVEGS  IKTGYL**S**IIMDPG  FGIHKG**S**SCDTVA  SPCAEK**S**PEQRGR  GGARRA**S**PDQEAE  ARNILL**S**ESDVVK  KVYTTQ**S**DVWSFG  IFSLGA**S**PYPGVQ  QSSEEG**S**FSQVST  PTTYKG**S**VDNQTD  SGMVLA**S**EEFEQI  SEYGEL**S**EPSEED  GELSEP**S**EEDHCS  VETRAA**S**VGLPSV  LDLPRL**S**IQKDIL  LWPNNQ**S**GSEQRV  RETDLA**S**VIYVYV  YVQDYR**S**PFIASV  PCLGSI**S**NLNVSL  PDGNRI**S**WDSKKG  AKINDE**S**YQSIMY  SHGIEL**S**VGEKLV  LYTCAA**S**SGLMTK  ISKEKQ**S**HVVSLV  KQSHVV**S**LVVYVP  PQIGEK**S**LISPVD  GEKSLI**S**PVDSYQ  PCEEWR**S**VEDFQG  GKNKTV**S**TLVIQA  IQAANV**S**ALYKCE  LNATMF**S**NSTNDI  MELKNA**S**LQDQGD  GESIEV**S**CTASGN  SITSSQ**S**SASSGF  ITSSQS**S**ASSGFV  SSQSSA**S**SGFVEE  SQSSAS**S**GFVEEK  YIVLPI**S**ETLSME  PISETL**S**MEEDSG  LSMEED**S**GLSLPT  EEDSGL**S**LPTSPV  LPTSPV**S**CMEEEE  DNTAGI**S**QYLQNS  RKSRPV**S**VKTFED  SGMVLA**S**EELKTL  SRESVA**S**EGSNQT  EGPSPT**S**EGASEA  PTSEGA**S**EANLVD  DDFERI**S**ELGAGN  KVQHRP**S**GLIMAR  VLHECN**S**PYIVGF  FYGAFY**S**DGEISI  YSDGEI**S**ICMEHM  EHMDGG**S**LDQVLK  EILGKV**S**IAVLRG  HRDVKP**S**NILVNS  SNILVN**S**RGEIKL  LCDFGV**S**GQLIDS  SFVGTR**S**YMAPER  LQGTHY**S**VQSDIW  THYSVQ**S**DIWSMG  VQSDIW**S**MGLSLV  IWSMGL**S**LVELAV  PPGRPV**S**GHGMDS  SGHGMD**S**RPAMAI  HTFIKR**S**EVEEVD  CARPLI**S**VYSEKG  PLISVY**S**EKGESS  YSEKGE**S**SGKNVT  SEKGES**S**GKNVTL  RQPYAV**S**ELAGHQ  LAGHQT**S**AESWGT  HQTSAE**S**WGTGRA  GGGTHR**S**GQGAFG  KRYAIC**S**ALAASA  CSALAA**S**ALPALV  LPALVM**S**KGHRIE  IKKVYA**S**QRMRAG  ITLLNV**S**KLNILK  FCIWTE**S**AFRKLD  TWRKAA**S**LKSNYN  KAASLK**S**NYNLPM  MINTDL**S**RILKSP  LSRILK**S**PEIQRA  AALQAK**S**DEKAAV  DQKPGT**S**GLRKRV  RVKVFQ**S**SANYAE  VKVFQS**S**ANYAEN  AENFIQ**S**IISTVE  FIQSII**S**TVEPAQ  GQNGIL**S**TPAVSC  GIKFNI**S**NGGPAP  DKIFQI**S**KTIEEY  YATMLR**S**IFDFSA  RSIFDF**S**ALKELL  LGAPAN**S**AVNCVP  LVETMK**S**GEHDFG  GFFVNP**S**DSVAVI  FVNPSD**S**VAVIAA  IAANIF**S**IPYFQQ  VRGFAR**S**MPTSGA  ARSMPT**S**GALDRV  ALDRVA**S**ATKIAL  GNLMDA**S**KLSLCG  MDASKL**S**LCGEES  ESFGTG**S**DHIREK  AVLAWL**S**ILATRK  LATRKQ**S**VEDILK  FVGKQF**S**ANDKVY  ADNFEY**S**DPVDGS  SDPVDG**S**ISRNQG  PVDGSI**S**RNQGLR  LIFTDG**S**RIVFRL  RIVFRL**S**GTGSAG  RLSGTG**S**AGATIR  IRLYID**S**YEKDVA  MLAPLI**S**IALKVS  SIALKV**S**QLQERT  PLLVVF**S**AMIFGT  HKNNDS**S**VGKSSS  DSSVGK**S**SSYPMV  SSYPMV**S**ESPEDL  CALRPQ**S**SGTVYE  TILFTV**S**IRNTLL  CDSQGE**S**CKEESP  FEMSTY**S**TNRTMI  LFAFVS**S**VARNDT  SKHPSQ**S**ALVTIV  FINATN**S**SEDYEI  WTFSRK**S**FPCEQK  GLDNGY**S**ISKFCN  LAEASA**S**QASCFS  SQASCF**S**DGYPLP  WTWKKC**S**DKSPNC  KKCSDK**S**PNCTEE  FGQWVS**S**STLNMS  SSTLNM**S**EAIKGF  FIQDNI**S**FYATIG  VQVTGS**S**DNEYFY  ATAYGI**S**KTGVSI  ISKTGV**S**IQVAVK  LKEKAD**S**SEREAL  LGACTL**S**GPIYLI  FKEHNF**S**FYPTFQ  NSSMPG**S**REVQIH  RDIMSD**S**NYVVRG  GIYTIK**S**DVWSYG  DSRKRP**S**FPNLTS  MDLGLL**S**PQAQVE  AESAGA**S**SFFPLV  ESAGAS**S**FFPLVV  VLLLAG**S**GGSGPR  LAGSGG**S**GPRGVQ  LLCACT**S**CLQANY  DGACMV**S**IFNLDG  KPFYCL**S**SEDLRN  PFYCLS**S**EDLRNT  IDLRVP**S**GHLKEP  KEPEHP**S**MWGPVE  LDMEDP**S**CEMCLS  SCEMCL**S**KDKTLQ  DLVYDL**S**TSGSGS  VYDLST**S**GSGSGL  DLSTSG**S**GSGLPL  STSGSG**S**GLPLFV  VAVKIF**S**SREERS  AVKIFS**S**REERSW  SSREER**S**WFREAE  TQLWLV**S**DYHEHG  DYHEHG**S**LFDYLN  MIKLAL**S**AASGLA  LALSAA**S**GLAHLH  AHRDLK**S**KNILVK  NMKHFD**S**FKCADI  IARRCN**S**GGVHEE  YYDLVP**S**DPSIEE  LVPSDP**S**IEEMRK  IPNWWQ**S**YEALRV  RIKKTL**S**QLSVQE  GALLGH**S**SCQNPA  ALLGHS**S**CQNPAS  SPPEEG**S**PDPDST  GSPDPD**S**TGALVE  DLYRVR**S**STSPTT  LYRVRS**S**TSPTTN  RVRSST**S**PTTNVL  TTNVLL**S**PLSVAT  VLLSPL**S**VATALS  TALSAL**S**LGAEQR  AEQRTE**S**IIHRAL  YYDLIS**S**PDIHGT  PQKNLK**S**ASRIVF  KKLRIK**S**SFVAPL  KLRIKS**S**FVAPLE  VAPLEK**S**YGTRPR  KGKLAR**S**TKEIPD  EIPDEI**S**ILLLGV  WVTKFD**S**RKTSLE  VRVPMM**S**DPKAVL  GLDSDL**S**CKIAQL  QLPLTG**S**MSIIFF  PLTGSM**S**IIFFLP  LTLIEE**S**LTSEFI  IEESLT**S**EFIHDI  VPKLKL**S**YEGEVT  EGEVTK**S**LQEMKL  QEMKLQ**S**LFDSPD  LQSLFD**S**PDFSKI  FDSPDF**S**KITGKP  GAGTTP**S**PGLQPA  GPAYGL**S**REVQQK  VKKIQA**S**TMAFKQ  KQMEQI**S**QFLQAA  RDDGLF**S**GDPNWF  NWFPKK**S**KENPRN  ENPRNF**S**DNQLQE  GTNRGA**S**QAGMTG  VFMKGL**S**KAKEGV  GVLYVG**S**KTKEGV  TDGVKH**S**MNPFCE  KEVIAV**S**CGPAQC  PQGTFA**S**QVTLEG  LGVDLT**S**KLSVIS  DLTSKL**S**VISVED  SKLSVI**S**VEDPPQ  RDMNGK**S**LDGKAI  TKPSFE**S**GRRGPP  PPPPPR**S**RGPPRG  RGGRGG**S**GGTRGP  MDDGGY**S**MNFNMS  SMNFNM**S**SSRGPL  NFNMSS**S**RGPLPV  PKRSAP**S**GPVRSS  SGPVRS**S**SGMGGR  GPVRSS**S**GMGGRA  RRDVYL**S**PRDDGY  PRDDGY**S**TKDSYS  GYSTKD**S**YSSRDY  STKDSY**S**SRDYPS  TKDSYS**S**RDYPSS  SSRDYP**S**SRDTRD  SRDYPS**S**RDTRDY  YPSRGY**S**DRDGYG  GRDRDY**S**DHPSGG  DHPSGG**S**YRDSYE  SYGNSR**S**APPTRG  PPSYGG**S**SRYDDY  PSYGGS**S**RYDDYS  RYDDYS**S**SRDGYG  YDDYSS**S**RDGYGG  YGGSRD**S**YSSSRS  GSRDSY**S**SSRSDL  SRDSYS**S**SRSDLY  SYSSSR**S**DLYSSG  YPPPRD**S**YSSSSR  PPRDSY**S**SSSRGA  DSYSSS**S**RGAPRG  GGGRGG**S**RSDRGG  GLEYAN**S**YNFAKK  HLKDEV**S**IIQSMG  IELGSD**S**SEDTVN  PWITLN**S**SIQKVN  KVNEWF**S**RSDELL  VDEYSG**S**SEKIDL  ERVHSK**S**VESNIE  VMNITN**S**GHENKT  NPNPIE**S**LEKESA  KAEPIS**S**SISNME  LTNAPG**S**FTKCSN  KEFVNP**S**LPREEK  SRFCLS**S**QFRGNE  INEVGS**S**TNEVGS  SINEIG**S**SDENIQ  PEVYKQ**S**LPGSNC  LEQPMG**S**SHASQV  DPFLIG**S**SKQMRH  MRHQSE**S**QGVGLS  NLGEAA**S**GCESET  PSNSYP**S**IISDSS  LRNPEQ**S**TSEKAV  SSEYPI**S**QNPEGL  PGVERS**S**PSKCPS  HDLTET**S**YLPRQD  GTPYLE**S**GISLFS  YLESGI**S**LFSDDP  SGISLF**S**DDPESD  VAESAQ**S**PAAAHT  YNAMEE**S**VSREKP  AMEESV**S**REKPEL  VVTDLI**S**LIRQRF  RGVAEN**S**VVISNG  ENSVVI**S**NGDINL  WANQRC**S**SPKGSA  ANQRCS**S**PKGSAR  CSSPKG**S**ARNSLV  KAKRKG**S**VSVCSK  KRKGSV**S**VCSKES  CSKESP**S**SSSESE  SKESPS**S**SSESES  KESPSS**S**SESESC  ESPSSS**S**ESESCD  PSSSSE**S**ESCDES  SSSESE**S**CDESIS  SISDGP**S**KVTLEA  GFSLTP**S**KGKTSG  PSKGKT**S**GTTSSS  SSSSDS**S**AESDDQ  SDSSAE**S**DDQCLM  RPGPGL**S**SQTAGA  AAGWRR**S**GSNGGG  GWRRSG**S**NGGGQA  APGASP**S**VSLPAS  GASPSV**S**LPASLG  SVSLPA**S**LGRGWG  GRGHPV**S**CVVNRS  NDVVKN**S**STIIQN  DVVKNS**S**TIIQNP  TPKKDY**S**LLPLLA  SYSPDV**S**DYKEGR  YAVTQE**S**KITVFW  AARHQD**S**WNGLSH  DSWNGL**S**HEAFRL  EAFRLV**S**RRDYAS  SRRDYA**S**EAIKGA  GARTTP**S**VVAFTA  FKIVRA**S**NGDAWV  AHGKLY**S**PSQIGA  GKLYSP**S**QIGAFV  KDAGQI**S**GLNVLR  AYGLDK**S**EDKVIA  GGTFDI**S**ILEIQK  GVFEVK**S**TNGDTF  KAKCEL**S**SSVQTD  KCELSS**S**VQTDIN  PYLTMD**S**SGPKHL  YLTMDS**S**GPKHLN  MQDAEV**S**KSDIGE  DAEVSK**S**DIGEVI  LFGRAP**S**KAVNPD  LDVTPL**S**LGIETL  TIPTKK**S**QVFSTA  KKSQVF**S**TAADGQ  NGIVHV**S**AKDKGT  QQIVIQ**S**SGGLSK  QIVIQS**S**GGLSKD  QSSGGL**S**KDDIEN  KLKEEI**S**KMRELL  LLARKD**S**ETGENI  NIRQAA**S**SLQQAS  SSLQQA**S**LKLFEM  AYKKMA**S**EREGSG  ASEREG**S**GSSGTG  REGSGS**S**GTGEQK  TATMAT**S**GSARKR  TMATSG**S**ARKRLL  KVEFET**S**EEVDVT  YGFEKP**S**AIQQRA  RDVIAQ**S**QSGTGK  VIAQSQ**S**GTGKTA  GKTATF**S**ISVLQC  TATFSI**S**VLQCLD  DMIRRR**S**LRTRAI  TQVVLI**S**ATLPHE  EANFTV**S**SMHGDM  ANFTVS**S**MHGDMP  PQKERE**S**IMKEFR  IMKEFR**S**GASRVL  EFRSGA**S**RVLIST  ASRVLI**S**TDVWAR  LDVPQV**S**LIINYD  IHRIGR**S**GRYGRK  DIEQYY**S**TQIDEM  EVEERR**S**WQENIS  LLKLQV**S**SQRTGL  SAAALP**S**NSFTSG  AALPSN**S**FTSGDI  YHSGPA**S**SLIEVL  EVLFGR**S**APSPAS  SAPSPA**S**EIHPLT  NTCLDT**S**QKEAVL  VLCCAP**S**NIAVDN  PARLLE**S**IQQHSL  VLARSD**S**AQIVAD  QDKREK**S**NFRNEI  AQALEA**S**CWIPLL  LPPTTV**S**HKAALA  GQLTAH**S**SVARHL  GEVRLV**S**LHIQAL  RDIAVV**S**PYNLQV  KEAVIL**S**FVRSNR  ILSFVR**S**NRKGEV  VAVICD**S**RTVNNH  NYSHEN**S**QGSSHA  TSTRTG**S**QRQEGG  AGKSLA**S**EAPSQP  LASEAP**S**QPSLNG  PSLNGG**S**PEGVES  SPEGVE**S**QDGVDH  IVEFMA**S**KKMQLE  GLRHDS**S**GEGKRR  RRFITV**S**KRAPRP  DFEALV**S**AAVKAD  QFCQLC**S**RRYCLS  GVLYAG**S**GTKNGS  RLDKKL**S**ELSNQR  RQNPCG**S**KACRRL  LFGPVD**S**EQLSRD  VDSEQL**S**RDCDAL  GRRPGT**S**PALLQG  HVDLSL**S**CTLVPR  CTLVPR**S**GEQAEG  PGGPGD**S**QGRKRR  MTDFYH**S**KRRLIF  IADVAP**S**AIREND  RENDIK**S**YFGRKV  KVAIDA**S**MSIYQF  AIDASM**S**IYQFLI  EEGETT**S**HLMGMF  KPPQLK**S**GELAKR  GELAKR**S**ERRAEA  ECKHLL**S**LMGIPY  PYLDAP**S**EAEASC  PSEAEA**S**CAALVK  IQEFHL**S**RILQEL  LCILLG**S**DYCESI  GSDYCE**S**IRGIGP  LIQKHK**S**IEEIVR  EVLDPE**S**VELKWS  SVELKW**S**EPNEEE  CGEKQF**S**EERIRS  SEERIR**S**GVKRLS  SGVKRL**S**KSRQGS  VKRLSK**S**RQGSTQ  SKSRQG**S**TQGRLD  FFKVTG**S**LSSAKR  KVTGSL**S**SAKRKE  VTGSLS**S**AKRKEP  EPEPKG**S**TKKKAK  GYISPE**S**PVVQLH  YGITII**S**GLPPEK  EKPKNL**S**CIVNEG  NSEELS**S**ILKLTW  LTWTNP**S**IKSVII  KDASTW**S**QIPPED  TASTRS**S**FTVQDL  DWSEEA**S**GITYED  TYEDRP**S**KAPSFW  RPSKAP**S**FWYKID  WYKIDP**S**HTQGYR  RGNLAE**S**KCYLIT  LKQAPP**S**KGPTVR  PNVPDP**S**KSHIAQ  GNFTDV**S**VVEIEA  FPEDLK**S**LDLFKK  INTEGH**S**SGIGGS  NTEGHS**S**GIGGSS  SSGIGG**S**SCMSSS  SGIGGS**S**CMSSSR  GSSCMS**S**SRPSIS  MSSSRP**S**ISSSDE  SSDENE**S**SQNTSS  VQVFSR**S**ESTQPL  TQPLLD**S**EERPED  YFKQNC**S**QHESSP  CSQHES**S**PDISHF  ERSKQV**S**SVNEED  RLKQQI**S**DHISQS  QISDHI**S**QSCGSG  ISQSCG**S**GQMKMF  CEGINI**S**GNFYRN  RMNTNP**S**RGPYHF  QLHQLY**S**DSFPME  HQLYSD**S**FPMELR  DWAYAA**S**KESHAT  YAASKE**S**HATLVF  EIDQQY**S**RFLQES  IKQFLQ**S**RYLEKP  RCLWEE**S**RLLQTA  LNGNNQ**S**VTRQKM  MRRSIV**S**ELAGLL  ELAGLL**S**AMEYVQ  LENWIT**S**LAESQL  ITSLAE**S**QLQTRQ  FRNLMK**S**AFVVER  VCIDKD**S**GDVAAL  EESNNG**S**LSAEFK  SNNGSL**S**AEFKHL  IDLETH**S**LPVVVI  LPVVVI**S**NICQMP  MPNAWA**S**ILWYNM  QVAEVL**S**WQFSST  VLSWQF**S**STTKRG  GPGVNY**S**GCQITW  MAGKGF**S**FWVWLD  RERAIL**S**TKPPGT  TFLLRF**S**ESSKEG  LRFSES**S**KEGGVT  WVEKDI**S**GKTQIQ  GKTQIQ**S**VEPYTK  QQLNNM**S**FAEIIM  ATNILV**S**PLVYLY  PEADPG**S**AAPYLK  AGQIAY**S**LLYSIG  AYSLLY**S**IGNGSV  YSIGNG**S**VFGKDQ  VAILVG**S**MPRREG  NVKIFK**S**QGAALD  DKYAKK**S**VKVIVV  TNCLTA**S**KSAPSI  CLTASK**S**APSIPK  ASKSAP**S**IPKENF  IPKENF**S**CLTRLD  IIWGNH**S**STQYPD  IWGNHS**S**TQYPDV  EALKDD**S**WLKGEF  IKARKL**S**SAMSAA  KARKLS**S**AMSAAK  KLSSAM**S**AAKAIC  PEGEFV**S**MGVISD  VSMGVI**S**DGNSYG  VISDGN**S**YGVPDD  PDDLLY**S**FPVVIK  LPINDF**S**REKMDL  LTEEKE**S**AFEFLS  ALARPA**S**AALRRS  SAALRR**S**FSTSAQ  ALRRSF**S**TSAQNN  RRSFST**S**AQNNAK  VAVLGA**S**GGIGQP  GIGQPL**S**LLLKNS  SLLLKN**S**PLVSRL  KNSPLV**S**RLTLYD  GVAADL**S**HIETKA  IANPVN**S**TIPITA  TIIPLI**S**QCTPKV  AKAGAG**S**ATLSMA  AGSATL**S**MAYAGA  GARFVF**S**LVDAMN  EGVVEC**S**FVKSQE  ECSFVK**S**QETECT  TECTYF**S**TPLLLG  LGIGKV**S**SFEEKM  GIGKVS**S**FEEKMI  FEEKMI**S**DAIPEL  IPELKA**S**IKKGED  LGQPHL**S**RQDLTT  TKLTPL**S**HEVISR  LSHEVI**S**RQATIN  HVAHGK**S**TVVKAI  TVVKAI**S**GVHTVR  YKLDDP**S**CPRPEC  RPECYR**S**CGSSTP  CYRSCG**S**STPDEF  YRSCGS**S**TPDEFP  KLVRHV**S**FVDCPG  LIAGNE**S**CPQPQT  CPQPQT**S**EHLAAI  IDLVKE**S**QAKEQY  APIIPI**S**AQLKYN  PPRDFT**S**EPRLIV  RLIVIR**S**FDVNKP  GGVAGG**S**ILKGVL  VRPGIV**S**KDSEGK  GIVSKD**S**EGKLMC  MCKPIF**S**KIVSLF  IFSKIV**S**LFAEHN  FTELEI**S**YFLLRR  AKVQKL**S**KNEVLM  LMVNIG**S**LSTGGR  VNIGSL**S**TGGRVS  STGGRV**S**AVKADL  GEKIAL**S**RRVEKH  LRSIAF**S**RAVFAE  VFFGLG**S**ALNWPQ  WPQALP**S**VLQIAM  QALGHI**S**GAHINP  LVGCHV**S**VLRAAF  LAVNAL**S**NSTTAG  VNALSN**S**TTAGQA  VLCIFA**S**TDERRG  PGTPAL**S**IGFSVA  ALSIGF**S**VALGHL  IHYTGC**S**MNPARS  SMNPAR**S**LAPAVV  VGAILG**S**LLYNYV  LFPPAK**S**LSERLA  PPAKSL**S**ERLAVL  QSVELH**S**PQSLPR  TVLMAC**S**GLFYSI  CSGLFY**S**IFTDQL  REEFLN**S**RMLMPQ  SAPGCE**S**RAFAPS  SLYSGL**S**TPPASY  TPPASY**S**MYSHLP  SHLPVS**S**LLFSDE  RALPCD**S**ARPVPG  PVPGEY**S**RPTLEV  CHSNIY**S**PKETIP  IPEEAR**S**DMHYSV  LKPAAP**S**ARNAPY  EEERPS**S**EDEIAL  PQSPQK**S**DCQPNS  GSPPAK**S**PTDPKA  KFIVLN**S**LNQNAK  ENLDLQ**S**PTKLSA  QSPTKL**S**ASGEDS  STIPQA**S**RLNNIV  NNIVNR**S**MTGSPR  GSPRSS**S**ESHSPL  HPPKCT**S**CGSQSP  KCTSCG**S**QSPQHA  EMGETQ**S**EYSDSS  ETQSEY**S**DSSCEN  QSEYSD**S**SCENGA  SEYSDS**S**CENGAF  HTLQTH**S**DKPYKC  CDRCQA**S**FRYKGN  YKGNLA**S**HKTVHT  KVQYRV**S**ATDLPP  ETPAQC**S**IKQERI  IKQERI**S**YTPPES  PESPVP**S**YASSTP  PVPSYA**S**STPLHV  VPSYAS**S**TPLHVP  LRMEED**S**IRLPAH  SLRPID**S**NTFEMN  RYRSPH**S**GDVLYE  KPRILF**S**PFFHPG  FFHPGN**S**IHTQPE  QRTPRP**S**VDNVHH  IELLHR**S**RSPITT  TTNHRP**S**PDPEQR  EQRPLR**S**PLDNMI  QESYPL**S**VSPMEN  NNHCPA**S**SESHPK  NHCPAS**S**ESHPKP  CPASSE**S**HPKPSS  ESHPKP**S**SPRQES  SSPRQE**S**TRVIQL  VIQLMP**S**PIMHPL  ILNPRH**S**VDFKQS  SVDFKQ**S**RLSEDG  FKQSRL**S**EDGLHR  GKPINL**S**HREDLA  HIMVSV**S**PPEEHA  YVYQLL**S**DSRYEN  RWEDKE**S**KIFRIV  MTYEKM**S**RALRHY  TPDEIM**S**GRTDRL  RLEHLE**S**QELDEQ  YFYHGL**S**WPQLSY  LSWPQL**S**YIAEDE  PHGHIT**S**LAVKRS  SLAVKR**S**HRRLGL  KLMDQA**S**RAMIEN  FNAKYV**S**LHVRKS  SLHVRK**S**NRAALH  AALHLY**S**NTLNFQ  TLNFQI**S**EVEPKY  KGNSPP**S**SGEACR  GNSPPS**S**GEACRE  VSETTE**S**TDVKDS  STDVKD**S**SEASDS  MDMADY**S**AALDPA  GNDTSP**S**EGTNLN  NLNAPN**S**LGVSAL  PNSLGV**S**ALCAIC  GKHYGA**S**SCDGCK  KHYGAS**S**CDGCKG  KGFFRR**S**VRKNHM  RKNHMY**S**CRFSRQ  MYSCRF**S**RQCVVD  NERDRI**S**TRRSSY  ISTRRS**S**YEDSSL  RSSYED**S**SLPSIN  SSYEDS**S**LPSINA  EDSSLP**S**INALLQ  LQAEVL**S**RQITSP  QITSPV**S**GINGDI  RAKKIA**S**IADVCE  IADVCE**S**MKEQLL  LGATKR**S**MVFKDV  PELAEM**S**RVSIRI  AEMSRV**S**IRILDE  PDAKGL**S**DPGKIK  RSQVQV**S**LEDYIN  NDRQYD**S**RGRFGE  LLPTLQ**S**ITWQMI  EMLLGG**S**PSDAPH  LLGGSP**S**DAPHAH  TMPTHL**S**NGQMCE  PSPPGG**S**GSEPYK  PPGGSG**S**EPYKLL  TIVKPL**S**AIPQPT  IQAAWP**S**GTECIA  KAGTKL**S**LMPWFH  LFLVRE**S**TNYPGD  DYTLCV**S**CDGKVE  RIMYHA**S**KLSIDE  YHASKL**S**IDEEVY  LVEHYT**S**DADGLC  QDEFYR**S**GWALNM  AFLAEA**S**VMTQLR  MTQLRH**S**NLVQLL  EYMAKG**S**LVDYLR  LVDYLR**S**RGRSVL  LRSRGR**S**VLGGDC  DCLLKF**S**LDVCEA  ARNVLV**S**EDNVAK  DNVAKV**S**DFGLTK  GLTKEA**S**STQDTG  LTKEAS**S**TQDTGK  KKFSTK**S**DVWSFG  TKSDVW**S**FGILLW  LLWEIY**S**FGRVPY  DAAMRP**S**FLQLRE  EYMSTG**S**DNKEEI  IKHLNV**S**DVIDIM  MENLYA**S**EEPAVY  PAVYEP**S**LMTMCQ  MTMCQD**S**NQNDER  NQNDER**S**KSLLLS  SKSLLL**S**GQEVPW  QEVPWL**S**SVRYGT  EVPWLS**S**VRYGTV  AFANHI**S**NTAKHF  GQRPQE**S**GILLNM  GRYQID**S**DVLLIP  EAGEGG**S**VLEKLE  VLEKLE**S**CGPMRE  GLDFLH**S**KKVIHH  HHDIKP**S**NIVFMS  SNIVFM**S**TKAVLV  LVDFGL**S**VQMTED  GTEIYM**S**PEVILC  ILCRGH**S**TKADIY  TKADIY**S**LGATLI  VKRYPR**S**AYPSYL  PRSAYP**S**YLYIIH  DIADDC**S**PGMREL  RELIEA**S**LERNPN  RCQSLD**S**ALLERK  ERKRLL**S**RKELEL  PENIAD**S**SCTGST  ENIADS**S**CTGSTE  DSSCTG**S**TEESEM  TGSTEE**S**EMLKRQ  IRNTNE**S**GEEVTT  LLPPLY**S**LVFIFG  LLNLAI**S**DLLFLI  LPLWAH**S**AANEWV  TFGVVT**S**VITWLV  LVAVFA**S**VPGIIF  KCQKED**S**VYVCGP  IMVICY**S**GILKTL  QEFFGL**S**NCESTS  GLSNCE**S**TSQLDQ  SNCEST**S**QLDQAT  VGEKFR**S**LFHIAL  GRGKGK**S**IGRAPE  RDSSTM**S**HTVAGG  GGSGDH**S**HQVRVK  ITHFEP**S**ISFEGL  HFEPSI**S**FEGLCN  EVRDMC**S**FDNEQL  GDPCTV**S**SQLELE  DPCTVS**S**QLELEE  YELNKD**S**ELLIHV  CPGEDK**S**IYRRGA  IECGRH**S**LPQEPV  VMPMDQ**S**SMHSDH  MPMDQS**S**MHSDHA  DQSSMH**S**DHAQTV  IPYNPS**S**HESLDQ  NPSSHE**S**LDQVGE  RESGKA**S**SSLGLQ  SGKASS**S**LGLQDF  RVIGRG**S**YAKVLL  HVFEQA**S**NHPFLV  FLVGLH**S**CFQTES  EHARFY**S**AEISLA  FYSAEI**S**LALNYL  DNVLLD**S**EGHIKL  RPGDTT**S**TFCGTP  GEDYGF**S**VDWWAL  EMMAGR**S**PFDIVG  PFDIVG**S**SDNPDQ  RIPRSL**S**VKAASV  LSVKAA**S**VLKSFL  AASVLK**S**FLNKDP  PFKPNI**S**GEFGLD  GLDNFD**S**QFTNEP  VRKIDQ**S**EFEGFE  LAQWIE**S**QPWDAI  EANNCS**S**PAGILV  ILVDAM**S**QKHLQI  IIQYQE**S**LRIQAQ  AQLAQL**S**PQERLS  LQQKQV**S**LEAWLQ  GGPPEG**S**LDVLQS  SLDVLQ**S**WCEKLA  TITDII**S**ALVTST  ISALVT**S**TFIIEK  NTRNEC**S**GEILNN  QATGTL**S**AHFRNM  AHFRNM**S**LKRIKR  DRRGAE**S**VTEEKF  FTVLFE**S**QFSVGS  LFESQF**S**VGSNEL  SQFSVG**S**NELVFQ  VVIVHG**S**QDHNAT  FKAEVQ**S**NRGLTK  QKLFNN**S**SSHLED  SHLEDY**S**GLSVSW  EDYSGL**S**VSWSQF  GLSVSW**S**QFNREN  TFLLRF**S**DSEIGG  RDFSIR**S**LADRLG  DRLGDL**S**YLIYVF  PKDEVF**S**KYYTPV  PEFVNA**S**ADAGGS  SADAGG**S**SATYMD  ADAGGS**S**ATYMDQ  YMDQAP**S**PAVCPQ  LRRPMD**S**LDSRLS  PMDSLD**S**RLSPPA  FGDELF**S**VFEGDS  WKGPPG**S**ADKAGK  GKLQSE**S**TNNGKN  SITEDL**S**LADLMP  PRVKVQ**S**VETVEG  CVDNNQ**S**VLVSAH  NQSVLV**S**AHTSAG  LVSAHT**S**AGKTVC  QRVIFT**S**PIKALS  SPIKAL**S**NQKYRE  TINPTA**S**CLVMTT  TTEILR**S**MLYRGS  IHYMRD**S**ERGVVW  QPVIIF**S**FSKKDC  VIIFSF**S**KKDCEA  NAIDCL**S**DEDKKL  TIEILF**S**EGLIKA  DFRWIS**S**GEYIQM  EYMLEK**S**FYQFQH  VIPNEE**S**VVIYYK  SNVKPN**S**GELDPL  EVLLRC**S**KESLKN  LLSAIS**S**VRLYIP  PVDNRQ**S**VLKSIQ  FEHRMY**S**HPLHND  RLGFAT**S**SDVIEM  RVACEI**S**SADELL  VACEIS**S**ADELLL  GLFNDL**S**AEQATA  QATALL**S**CFVFQE  VFQENS**S**EMPKLT  DEETYL**S**SFKPHL  EETYLS**S**FKPHLM  VDSQIA**S**DGSIPV  PREATI**S**YIKQML  LLMDID**S**YMFACV  LSLVGL**S**WMDWLK  PPEHEP**S**IPENLE  VFSFQV**S**PNMNPI  AINRNS**S**NLPLPL  CKTIVS**S**EVSGKN  KVKTKK**S**TKTINP  TKTINP**S**KYQTIR  IILHSW**S**SFPDEL  ILHSWS**S**FPDELE  KAAEIA**S**SDSANV  DSANVS**S**RGGKKF  LPKLLL**S**IKWNKL  GCLRQM**S**DEELSQ  MSDEEL**S**QYLLQL  LFWHLR**S**EVHIPA  EAYCRG**S**VGHMKV  GHMKVL**S**KQVEAL  HTCLKQ**S**AYREAL  ALSDLQ**S**PLNPCV  NPCVIL**S**ELYVEK  LATGDR**S**GLIEVV  GLIEVV**S**TSETIA  IEVVST**S**ETIADI  IEEFTL**S**CAGYCV  AGYCVA**S**YVLGIG  GIGDRH**S**DNIMVK  GLPELT**S**VKDIQY  IQYLKD**S**LALGKS  FELVSS**S**DANERK  LRELAI**S**VPTFFF  ELVRIS**S**MEGERL  VGLLGY**S**SHQGLM  TMNHVL**S**CVKKEK  QALGLL**S**VAVRSE  RYCVLA**S**LDERFD  TVGRLS**S**MNPAFV  MDMLQD**S**SLLAKR  LGQLVA**S**TGYVVE  LSESKS**S**QDSSDY  SKSSQD**S**SDYSTS  SSDYST**S**EMLVNM  RIFRDQ**S**LSHHHT  SPGRIV**S**IKLLAA  PEAPLP**S**RKAALE  DFTDYA**S**RIIHPI  LELLKD**S**SSPSLR  LLKDSS**S**PSLRSC  SSPSLR**S**CWALAQ  AEFMEH**S**DKGPLP  TAMAGE**S**YSRAYG  KILMVR**S**LVVSPH  MKNMWK**S**ARKIDA  KVLQYY**S**AATEHD  SESEAE**S**TENSPT  CRKYMK**S**GNVKDL  SLQVIT**S**KQRPRK  LREHKD**S**VMAVLE  LTGRDF**S**HDDTLD  LIKQAT**S**HENLCQ  TENSVD**S**KSIKNL  NSVDSK**S**IKNLEP  PKIIHG**S**ESMDSG  IIHGSE**S**MDSGIS  GSESMD**S**GISLDN  SMDSGI**S**LDNSYK  GISLDN**S**YKMDYP  NKNFHK**S**TGMTSR  KSTGMT**S**RSGTDV  TGMTSR**S**GTDVDA  ELMRDV**S**KEDHSK  VSKEDH**S**KRSSFV  EDHSKR**S**SFVCVL  DHSKRS**S**FVCVLL  FVCVLL**S**HGEEGI  RGDRCR**S**LTGKPK  CGIETD**S**GVDDDM  DFLYAY**S**TAPGYY  TAPGYY**S**WRNSKD  YYSWRN**S**KDGSWF  RNSKDG**S**WFIQSL  GSWFIQ**S**LCAMLK  VATEFE**S**FSFDAT  TEFESF**S**FDATFH  QIPCIV**S**MLTKEL  EILIKR**S**QQKKKT  QQKKKT**S**PLNYKE  LFVLTK**S**MLTYYE  KGFIDV**S**KIKCVE  LYIFAP**S**PQSRDL  FAPSPQ**S**RDLWVK  KFWTDG**S**YQCCRQ  KYNLFE**S**SIRKAL  LEEEDN**S**EEIVVA  NEGYIP**S**NYVTGK  YVTGKK**S**NNLDQY  CRNMNR**S**KAEQLL  FMVRDS**S**QPGLYT  PGLYTV**S**LYTKFG  KFGGEG**S**SGFRHY  FGGEGS**S**GFRHYH  IKETTT**S**PKKYYL  EKHAFG**S**IPEIIE  RLRYPV**S**VKGKNA  PTTAGF**S**YEKWEI  KWEINP**S**ELTFMR  QRQGHF**S**RDVLLS  SRDVLL**S**MCQDVC  EYLERN**S**FIHRDL  ARNCLV**S**EAGVVK  AGVVKV**S**DFGMAR  LDDQYT**S**SSGAKF  DDQYTS**S**SGAKFP  FNYSRF**S**SKSDVW  NYSRFS**S**KSDVWS  SRFSSK**S**DVWSFG  YQPKLA**S**NYVYEV  KPEGRP**S**FEDLLR  PRGIRG**S**SAARPS  QHDHFA**S**CVEDGF  NQNGEW**S**EVRSKN  EWSEVR**S**KNGQGW  GQGWVP**S**NYITPV  AAEYLL**S**SLINGS  PTVYGV**S**PIHDKW  GVWKKY**S**LTVAVK  YMATQI**S**SAMEYL  VADFGL**S**RLMTGD  SPADRP**S**FAETHQ  ETMFHD**S**SISEEV  FHDSSI**S**EEVAEE  RLPILP**S**KTRTLK  QDATEN**S**ASSLAP  ATENSA**S**SLAPGF  RKQRDK**S**PSSLLE  QRDKSP**S**SLLEDA  PTPPKR**S**SSFREM  ELTGNF**S**SVASLQ  QHADGF**S**FTPAQQ  PKCYGG**S**FAQRNL  TASDDT**S**KPFPRS  PFPRSN**S**TSSMSS  QLERTV**S**TSSQPE  RTVSTS**S**QPEENV  DMLPKK**S**EESAAP  PKKSEE**S**AAPSRE  LGAVPI**S**GKAGRP  QVPLPT**S**SISPAK  VPLPTS**S**ISPAKM  ECADLL**S**SALTEP  QELQVS**S**AAAGVP  VQQNIP**S**TLLQDH  KDNPQK**S**YFIRLY  WEQELY**S**QLVYST  YSQLVY**S**TPTPFF  KRNQRQ**S**GDRRQL  PPVGPL**S**LGLATV  QNPDIT**S**SRYRGL  NPDITS**S**RYRGLP  LPAPGP**S**PADKKR  PADKKR**S**GKKKIS  SGKKKI**S**KADIGA  ADIGAP**S**GFKHVS  SGFKHV**S**HVGWDP  LDPDLR**S**LFSRAG  DLRSLF**S**RAGISE  FSRAGI**S**EAQLTD  LTDAET**S**KLIYDF  PPPPPP**S**RGGNQL  GGNKGR**S**GPLPPV  PPATGR**S**GPLPPP  PPPPPP**S**SGNGPA  PPPPPS**S**GNGPAP  TPGAPE**S**SALQPP  PGAPES**S**ALQPPP  LQPPPQ**S**SEGLVG  QPPPQS**S**EGLVGA  HVMQKR**S**RAIHSS  GSSMEP**S**ADWLAT  LPNAPN**S**YGRRPI  QVMMMG**S**ARVAEL  LMKAFE**S**LKSFQQ  PCLTRT**S**KRPEES  EESGGR**S**RSGSIV  SGGRSR**S**GSIVEL  RSDVSS**S**ALTASV  SVKDEI**S**GELAAS  NDGTQA**S**SPISDS  ILPDEA**S**EAFRNS  EAFRNS**S**MALQQA  HLLKNM**S**HCRQPS  RQPSDS**S**VDKFVL  KGDIGQ**S**TDDDSA  EALCLL**S**TAFPVC  LSASDE**S**RKSCTV  MILTLL**S**SAWFPL  ILTLLS**S**AWFPLD  IKAALP**S**LTNPPS  SEASAA**S**RQSDTS  NYCLLD**S**DQVFIG  LPEETF**S**RFLLQL  RHQRLY**S**LLDRFR  SPSPPV**S**SHPLDG  SQCWTR**S**DSALLE  LGMSEI**S**GGQKSA  FVTHAC**S**LIYCVH  RLPLVN**S**YTRVPP  LERLLL**S**EQLSRL  VIGEFL**S**NQQPYP  WVMLSL**S**NFTQRA  MATWSL**S**CFFVSA  VVAAPG**S**PYHRLL  PQGATV**S**LWETVQ  PDGEPG**S**FVNVSC  GSFVNV**S**CPWYLP  WYLPWA**S**SVPQGH  YLPWAS**S**VPQGHV  WLQKDN**S**SLPWRD  LQKDNS**S**LPWRDL  LPWRDL**S**ECEESK  LSECEE**S**KRGERS  SKRGER**S**SPEEQL  KRGERS**S**PEEQLL  TVGYAL**S**FSALVI  GYALSF**S**ALVIAS  SALVIA**S**AILLGF  HLNLFA**S**FILRAL  FILRAL**S**VFIKDA  QWDGLL**S**YQDSLS  LLSYQD**S**LSCRLV  SYQDSL**S**CRLVFL  YTLLAF**S**VLSEQW  IFRLYV**S**IGWGVP  VICIVV**S**KLKANL  KCRLAK**S**TLTLIP  KLFTEL**S**FTSFQG  TELSFT**S**FQGLMV  QLEFRK**S**WERWRL  LHIQRD**S**SMKPLK  HIQRDS**S**MKPLKC  LKCPTS**S**LSSGAT  CPTSSL**S**SGATAG  PTSSLS**S**GATAGS  SGATAG**S**SMYTAT  GATAGS**S**MYTATC  SSLSRD**S**QGHGRD  GHGRDL**S**AAGIGL  AATQSL**S**MPASLG  LSSAST**S**SHNLQS  SSASTS**S**HNLQSI  SSHNLQ**S**IFNIGS  GPLPLS**S**QHRGDA  GDADQA**S**NILASF  ASNILA**S**FGLSAR  EEGPTL**S**YGRDGR  RHFRRD**S**FDDRGP  FDDRGP**S**LNPVLD  YDHGSR**S**QESGYY  ERCRDD**S**FFGETS  GYPHLC**S**ICDLPV  CDLPVH**S**NKEWSQ  HSNKEW**S**QHINGA  QHINGA**S**HSRRCQ  LGPPPP**S**FHLGGP  EPFGVI**S**NHLILN  PVRVHL**S**QKYKRI  GRVIHL**S**NLPHSG  HSGYSD**S**AVLKLA  LLKKDK**S**RKRSYS  DKSRKR**S**YSPDGK  DGKESP**S**DKKSKT  GSQKTE**S**STEGKE  SQKTES**S**TEGKEQ  KEQEEK**S**GEDGEK  LLESGS**S**VGDETD  NLGDVA**S**DGKKEP  EPGAES**S**ENADDP  FYCKLC**S**LFYTNE  QVPYTF**S**SKSPGN  VPYTFS**S**KSPGNG  YTFSSK**S**PGNGSL  KSPGNG**S**LREALI  DLFQDL**S**HFQETW  EAQVPD**S**DEQFVP  FVPDFH**S**ENLAFH  ENLAFH**S**PTTRIK  RTDPAL**S**CSRKPP  DPALSC**S**RKPPLP  GEQCLY**S**SAYDPP  EQCLYS**S**AYDPPR  RQIAIK**S**PAPGAL  PGALGQ**S**PLQPFP  QRNFLR**S**SGTSQP  LRSSGT**S**QPHPGH  GYLGEH**S**SVFQQP  YLGEHS**S**VFQQPL  PLDICH**S**FTSQGG  ICHSFT**S**QGGGRE  PPYPQQ**S**FKQEYH  TDFAYD**S**DVTGCA  DVTGCA**S**MYLHTE  LHTEGF**S**GPSPGD  EGFSGP**S**PGDGAM  MNYDKL**S**RSLRYY  YDKLSR**S**LRYYYE  EPEALF**S**LAFPDN  EFDRPV**S**EEDTVP  EDTVPL**S**HLDESP  LSHLDE**S**PAYLPE  ILGGRD**S**HPAAGG  PAAGGG**S**VLCFGQ  LGPEYI**S**SRMAGG  GPEYIS**S**RMAGGG  YNGWAH**S**ITQQNV  VQLKDG**S**YHEDVG  DVGYGV**S**EGLKSK  VSEGLK**S**KALSLE  LKSKAL**S**LEKARK  LKRALR**S**FGNALG  DKDYLR**S**LNKLPR  EEARYN**S**CRPNMA  QLQQVT**S**PSRPSH  TSPSRP**S**HAVIPA  PADQDC**S**SRSLSS  ADQDCS**S**RSLSSS  CSSRSL**S**SSAVES  SSRSLS**S**SAVESE  SRSLSS**S**AVESEA  KQQVRV**S**TPSAEK  VRVSTP**S**AEKSEA  TPSAEK**S**EAAPPA  APPVTH**S**TPVTVS  STPVTV**S**EPLLEK  DGVVKP**S**SRADPA  GVVKPS**S**RADPAQ  ADPAQT**S**DTLALN  QNRTPH**S**VCHQKP  QKPQAK**S**GSWDLQ  PQAKSG**S**WDLQTY  WDLQTY**S**ADQRTT  TTGNWE**S**HRKSQD  WESHRK**S**QDMKKR  LRQCLR**S**LGGYVL  ELCEFY**S**RDPDGL  KPCNRP**S**GLEPQP  LEQAII**S**QAPQVE  RMPWYH**S**SLTREE  MPWYHS**S**LTREEA  AERKLY**S**GAQTDG  VYHYLI**S**QDKAGK  KEACPN**S**SASNAS  EACPNS**S**ASNASG  CPNSSA**S**NASGAA  TLPAHP**S**TLTHPQ  RIDTLN**S**DGYTPE  EPARIT**S**PDKPRP  PMPMDT**S**VYESPY  DTSVYE**S**PYSDPE  VYESPY**S**DPEELK  GCGNFG**S**VRQGVY  REEIPV**S**NVAELL  ELLHQV**S**MGMKYL  RHYAKI**S**DFGLSK  ISDFGL**S**KALGAD  ALGADD**S**YYTARS  SYYTAR**S**AGKWPL  INFRKF**S**SRSDVW  NFRKFS**S**RSDVWS  RKFSSR**S**DVWSYG  SRSDVW**S**YGVTMW  TMWEAL**S**YGQKPY  ELYALM**S**DCWIYK  MRACYY**S**LASKVE  CYYSLA**S**KVEGPP  VEGPPG**S**TQKAEA  SSGMAD**S**ANHLPF  LVQGGM**S**DGLYLL  LGGFAL**S**VAHGRK  GGRTHA**S**PADLCH  DLCHYH**S**QESDGL  HYHSQE**S**DGLVCL  LEQAII**S**QKPQLE  WFHGKI**S**REESEQ  KISREE**S**EQIVLI  QIVLIG**S**KTNGKF  DKTGKL**S**IPEGKK  QLVEHY**S**YKADGL  RPQLPG**S**HPATWS  SHPATW**S**AGGIIS  SAGGII**S**RIKSYS  KPGHRK**S**SPAQGN  PGHRKS**S**PAQGNR  GICEAE**S**WMLVME  ELVHQV**S**MGMKYL  MKYLEE**S**NFVHRD  QHYAKI**S**DFGLSK  ISDFGL**S**KALRAD  INYYKF**S**SKSDVW  NYYKFS**S**KSDVWS  YKFSSK**S**DVWSFG  SKSDVW**S**FGVLMW  LMWEAF**S**YGQKPY  DEIPAL**S**VSRPQT  IPALSV**S**RPQTGL  RPQTGL**S**FLGPEP  DLEDLY**S**RYKKLQ  EVKRIQ**S**IPLVIG  NTAIVG**S**TTGSNY  VGSTTG**S**NYYVRI  YYVRIL**S**TIDREL  LLKPNA**S**VALHKH  VALHKH**S**NALVDV  LPPEAD**S**SIMMLT  PPEADS**S**IMMLTS  SIMMLT**S**DQKPDV  FIRVVG**S**EFVQKY  QKRLIF**S**TITSKM  IFSTIT**S**KMNLSE  TSKMNL**S**EEVDLE  ARPDKI**S**GADINS  SGADIN**S**ICQESG  NSICQE**S**GMLAVR  PSGGGG**S**GGGSGS  GGSGGG**S**GSGTPG  SGGGSG**S**GTPGPV  TPGPVG**S**PAPGHP  PGHPAV**S**SMQGKR  GHPAVS**S**MQGKRK  ANPPFK**S**TARFTL  ERLRTH**S**IESSGK  RTHSIE**S**SGKLKI  THSIES**S**GKLKIS  SGKLKI**S**PEQHWD  GRGAYG**S**VNKMVH  KMVHKP**S**GQIMAV  AVKRIR**S**TVDEKE  LDVVMR**S**SDCPYI  DVVMRS**S**DCPYIV  ICMELM**S**TSFDKF  MELMST**S**FDKFYK  FYKYVY**S**VLDDVI  HRDIKP**S**NILLDR  NILLDR**S**GNIKLC  LCDFGI**S**GQLVDS  SGQLVD**S**IAKTRD  PERIDP**S**ASRQGY  RIDPSA**S**RQGYDV  QGYDVR**S**DVWSLG  VRSDVW**S**LGITLY  PYPKWN**S**VFDQLT  GDPPQL**S**NSEERE  PPQLSN**S**EEREFS  SEEREF**S**PSFINF  EREFSP**S**FINFVN  CLTKDE**S**KRPKYK  VAGGQL**S**FKGKSL  LSFKGK**S**LKLNTA  EIEDFD**S**LEALRL  KALEKK**S**ELKRCH  LKRCHW**S**DMFTGR  IPPALI**S**LGEGLI  LVELDL**S**DNAFGP  FEALLK**S**SACFTL  EALLKS**S**ACFTLQ  TECHRK**S**SAQGKP  ECHRKS**S**AQGKPL  GDCLVR**S**KGAVAI  LKELNL**S**FCEIKR  MAKVLA**S**LSDDED  RGQGEK**S**ATPSRK  EPAPVL**S**SPPPAD  LRLGPK**S**SVLIAQ  RLGPKS**S**VLIAQQ  AQQTDT**S**DPEKVV  DPEKVV**S**AFLKVS  SAFLKV**S**SVFKDE  AFLKVS**S**VFKDEA  MQKAFN**S**SSFNSN  QKAFNS**S**SFNSNT  KAFNSS**S**FNSNTF  NSSSFN**S**NTFLTR  HMGLLK**S**EDKVKA  FVTKPN**S**ALESCS  PNSALE**S**CSFARH  SALESC**S**FARHSL  CSFARH**S**LLQTLY  FDSEER**S**SWYWGR  DSEERS**S**WYWGRL  WYWGRL**S**RQEAVA  VFLVRD**S**STSPGD  FLVRDS**S**TSPGDY  VRDSST**S**PGDYVL  PGDYVL**S**VSENSR  DYVLSV**S**ENSRVS  LSVSEN**S**RVSHYI  SENSRV**S**HYIINS  SHYIIN**S**SGPRPP  HYIINS**S**GPRPPV  RPPVPP**S**PAQPPP  QPPPGV**S**PSRLRI  PPGVSP**S**RLRIGD  GDQEFD**S**LPALLE  TLIEPV**S**RSRQGS  IEPVSR**S**RQGSGV  SRSRQG**S**GVILRQ  WWNAED**S**EGKRGM  EKYRPA**S**ASVSAL  YRPASA**S**VSALIG  PASASV**S**ALIGGN  GGNQEG**S**HPQPLG  GPYAQP**S**VNTPLP  VTKINV**S**GQWEGE  VSNGSP**S**LERMDA  AEHPKP**S**ACRNLF  RDMEEA**S**QRKWNF  QEVEKG**S**LPEFYY  KVPAQE**S**QDVSGS  QESQDV**S**GSRPAA  SQDVSG**S**RPAAPL  IGAPAN**S**EDTHLV  DPKTDP**S**DSQTGL  KTDPSD**S**QTGLAE  RPATDD**S**STQNKR  PATDDS**S**TQNKRA  RTEENV**S**DGSPNA  ENVSDG**S**PNAGSV  GSPNAG**S**VEQTPK  ARGPRC**S**QPGETC  QADPCA**S**NPCANG  YVPCSP**S**PCQNGG  LNDACI**S**NPCNEG  CINTLG**S**FECQCL  DPCHYG**S**CKDGVA  LAPCAP**S**PCRNGG  EDYESF**S**CVCPTG  DINECA**S**DPCRNG  CVDGIN**S**FTCLCP  CDVPSV**S**CEVAAQ  VDPVSR**S**PKCFNN  DVNECL**S**NPCDAR  CEPTSE**S**PFYRCL  RQCVQA**S**SQCFQS  ALASLG**S**LNIPYK  PLHAAV**S**ADAQGV  EYNLVR**S**PQLHGA  PNGYLG**S**LKPGVQ  SPPLLP**S**PFQQSP  PSPFQQ**S**PSVPLN  STVLGS**S**SGGALN  TVGGST**S**LNGQCE  GQCEWL**S**RLQSGM  YNPLRG**S**VAPGPL  LSTQAP**S**LQHGMV  MVGPLH**S**SLAASA  FLSGEP**S**QADVQP  SPALPT**S**LPSSLV  LPTSLP**S**SLVPPV  PSQHSY**S**SPVDNT  SQHSYS**S**PVDNTP  LTPSPE**S**PDQWSS  APTPAP**S**TIPGPR  APAGRP**S**ASRGHR  AGRPSA**S**RGHRKR  RGHRKR**S**RRVLYP  DAPNAA**S**LAPTPV  EPFNLT**S**EPSDYA  NLTSEP**S**DYALDL  DYALDL**S**TFLQQH  ASSQPA**S**MPQSKG  PQSKGK**S**KRKKDL  KKDLRI**S**CMSKPP  LRISCM**S**KPPAPN  PPRNLD**S**RTFITI  DDLVTI**S**ELGRGA  KVRHAQ**S**GTIMAV  IRATVN**S**QEQKRL  MELMDT**S**LDKFYR  LGEIAV**S**IVRALE  ALEHLH**S**KLSVIH  HLHSKL**S**VIHRDV  HRDVKP**S**NVLINK  MCDFGI**S**GYLVDS  KGYNVK**S**DVWSLG  VKSDVW**S**LGITMI  LRFPYE**S**WGTPFQ  QVVEEP**S**PQLPAD  LPADRF**S**PEFVDF  NPAERM**S**YLELME  QAVHLE**S**DAFLVC  CLNHAL**S**TEKEEV  LNDDTR**S**DSKFAY  DDTRSD**S**KFAYTG  RIVHIH**S**VIILRR  VIILRR**S**DKRKDR  KDRVEI**S**PEQLSA  ISPEQL**S**AASTEA  EQLSAA**S**TEAERL  VVGWYH**S**HPHITV  HITVWP**S**HVDVRT  FVGLIF**S**CFIEDK  LYTCFQ**S**IQAQKS  SIQAQK**S**SESLHG  IQAQKS**S**ESLHGP  AQKSSE**S**LHGPRD  GPRDFW**S**SSQHIS  PRDFWS**S**SQHISI  RDFWSS**S**QHISIE  SSSQHI**S**IEGQKE  GKVCLE**S**AVELPK  AYRRIH**S**LTHLDS  SLTHLD**S**VTKIHN  TKIHNG**S**VFTKNL  FTKNLC**S**QMSAVS  NLCSQM**S**AVSGPL  SQMSAV**S**GPLLQW  KLRGHV**S**HGHGRI  HLKRNQ**S**FCPTVN  KLWTLV**S**EQTRVN  IIDVVR**S**GYYKVL  VKAKFF**S**RRAEEK  AEEKIK**S**VGGACV  MVVRNC**S**SFLIKR  VVRNCS**S**FLIKRN  RNKQTY**S**TEPNNL  NLKARN**S**FRYNGL  VVIKRR**S**GQRKPA  QRKPAT**S**YVRTTI  NARATL**S**SIRHMI  ARATLS**S**IRHMIR  AAIRRA**S**AILRSQ  ASAILR**S**QKPVMV  KLFGKW**S**TDDVQI  VQINDI**S**LQDYIA  AKYLPH**S**AGRYAA  VERLTN**S**MMMHGR  VNAIIN**S**GPREDS  SGPRED**S**TRIGRA  RQAVDV**S**PLRRVN  INAAKG**S**SNSYAI  NAAKGS**S**NSYAIK  AKGSSN**S**YAIKKK  VMKAMQ**S**LKSRGY  AMQSLK**S**RGYVKE  PATLRR**S**RPETGR  RDTYRR**S**AVPPGA  AEAGAG**S**ATEFQF  ATEPEP**S**GSIANP  EIGELL**S**TTHPAN  NIKMKR**S**IEEACF  EVDLPI**S**YLTSVS  PISYLT**S**VSSLIV  KPLPSK**S**VRKESK  GREPAS**S**IHDETL  LPGGSE**S**EATASD  DYNASA**S**TISPPS  TISPPS**S**MEEDKF  SEKVSP**S**KSPSLS  AYESFL**S**ADDKAS  SPFEEK**S**GKQGSP  DTKMSI**S**EGTVSD  GTVSDK**S**ATPVDE  TDDVSP**S**LHAEVG  ETEMSP**S**KEECPR  DLSELI**S**VSQVEA  SSAHTP**S**QIASPL  EKLSPK**S**DISPLT  SPTFSD**S**TSAVKE  KTATCH**S**SSSPPI  PEDGDY**S**YEIIEK  TSPPEV**S**GYSYEK  HTLGDP**S**YSYETT  TTEKIT**S**FPESEG  TRTPQA**S**TYSYET  YTAEKK**S**PSEARQ  AEKKSP**S**EARQDV  PTSVSE**S**APSQTD  VSESAP**S**QTDSDV  IDSEDE**S**ETIPTD  TKTTKS**S**AVPPGL  GQGQPP**S**QPPQGQ  LRKLPD**S**FFKPPE  KPPEPK**S**HSRQAS  PEPKSH**S**RQASTD  TPTGVV**S**GPAATP  AQHLRQ**S**SFEIPD  WEMAKT**S**SGQRYF  EMAKTS**S**GQRYFL  PRKAML**S**QMNVTA  NVTAPT**S**PPVQQN  NMMNSA**S**GPLPDG  HKNKTT**S**WLDPRL  NQRISQ**S**APVKQP  GGVMGG**S**NSNQQQ  MRNINP**S**TANSPK  NPSTAN**S**PKCQEL  QELALR**S**QLPTLE  GTQNPV**S**SPGMSQ  RTMTTN**S**SDPFLN  TMTTNS**S**DPFLNS  SDPFLN**S**GTYHSR  NSGTYH**S**RDESTD  DSGLSM**S**SYSVPR  SGLSMS**S**YSVPRT  LSMSSY**S**VPRTPD  PDDFLN**S**VDEMDT  NQSTLP**S**QQNRFP  GEELMP**S**LQEALS  SLQEAL**S**SDILND  LQEALS**S**DILNDM  ILNDME**S**VLAATK  ARPHYG**S**VLDNER  IQMPAF**S**KIGGIL  KQQKRQ**S**GQTDPL  GVDAAN**S**AAQQYQ  ATLQRQ**S**PEHNLT  AVEMLS**S**VALINR  INRALE**S**GDVNTV  TVWKQL**S**SSVTGL  WKQLSS**S**VTGLTN  QEIQDE**S**AVLWLD  INEAVE**S**GDVGKT  QNSMQL**S**REEIQS  REEIQS**S**ISGVTA  TLQDVV**S**HSKKLT  STKFMD**S**VIFTLY  TLYNYA**S**NQREEY  LQEEIK**S**KVDQIQ  VIKMVV**S**FNRGAR  WVNQME**S**QTGEAS  VKTRLD**S**SIRNMR  VTDKFL**S**AIVSSV  LSAIVS**S**VDKIPY  AKVLKD**S**LHEKFP  FDIIDL**S**AGGQLT  IINEYL**S**QSYQKF  EVPTIE**S**LIGESS  SLIGES**S**GNLNDP  KSVKED**S**NLTLQE  CLDNLA**S**KGKVSK  EMKGKK**S**KKISLK  NVIFEI**S**PTEEVG  QNPSES**S**EPIPLE  EPEKME**S**SISSSS  SSSEEM**S**LSKCAE  SEEMSL**S**KCAEQF  RRMNEN**S**HVQVPF  VPFPPF**S**DISNPT  PPFSDI**S**NPTAHE  IVEHQY**S**HKFTVV  ISTTPD**S**RKRTRH  TATFTV**S**SMKVGE  ATFTVS**S**MKVGEK  EMVLEM**S**LEVCSC  CPDLRF**S**MALCDQ  KEHIRE**S**MKKLLG  LLGPKN**S**EGLHSA  NSEGLH**S**ARDVPV  RAMVGF**S**GVMKAL  VKRYVE**S**LWKKKS  SLWKKK**S**SGQPVT  RMNTTL**S**SLKEKV  MNTTLS**S**LKEKVN  APDLFG**S**KFFMGT  LMGVWG**S**AFSILF  VLGVSG**S**QSRGST  TTKHIV**S**NDSSDS  HIVSND**S**SDSDDE  SDSDDE**S**HEPKGT  ENEDAG**S**DYQSDN  AGSDYQ**S**DNQASW  MIMALV**S**DSALFN  GLNLNT**S**YPLSPL  YPLSPL**S**DFATQD  DFATQD**S**FDDDEL  GVDLII**S**FDFSAR  DFSARP**S**DSSPPF  ARPSDS**S**PPFKEL  KEAMVE**S**IEYRRQ  RCSVSL**S**NVEARR  QIEKNL**S**DLIDLV  LIDLVP**S**LCEDLL  LCEDLL**S**SVDQPL  CEDLLS**S**VDQPLK  YNRDGD**S**YRSPWS  DGDSYR**S**PWSNKY  SYRSPW**S**NKYDPP  EDGAMP**S**ARLRKL  YFEGGV**S**SVYLWD  FEGGVS**S**VYLWDL  KKAGDG**S**KKIKGC  IKGCWD**S**IHVVEV  VEVQEK**S**SGRTAH  EVQEKS**S**GRTAHY  AHYKLT**S**TVMLWL  WLQTNK**S**GSGTMN  QTNKSG**S**GTMNLG  TMNLGG**S**LTRQME  EKDETV**S**DCSPHI  ETVSDC**S**PHIANI  MENKIR**S**TLNEIY  IVNGLR**S**IDAIPD  QLQREL**S**QVLTQR  NNEWHE**S**KSGKKF  EWHESK**S**GKKFAT  FATCNP**S**TREQIC  VAFQRG**S**PWRRLD  RRLDAL**S**RGRLLH  TALYLG**S**LIKEAG  TVGAAI**S**SHPQIN  VGAAIS**S**HPQINK  KIAFTG**S**TEVGKL  LVKEAA**S**RSNLKR  KEAASR**S**NLKRVT  QCCTAA**S**RVFVEE  VEEQVY**S**EFVRRS  SEFVRR**S**VEYAKK  ILELIE**S**GKKEGA  KLECGG**S**AMEDKG  IKPTVF**S**EVTDNM  PILKFK**S**IEEVIK  VIKRAN**S**TDYGLT  KALKLA**S**ALESGT  LASALE**S**GTVWIN  FGGFKM**S**GNGREL  AALDSL**S**LFTSLG  SLSLFT**S**LGLSEQ  FTSLGL**S**EQKARE  RETLKN**S**ALSAQL  LKNSAL**S**AQLREA  AQQTLG**S**TIDKAT  LLYGLA**S**RLRDTR  RDTRRL**S**FLVSYI  RLSFLV**S**YIASKK  LVSYIA**S**KKIHTE  HTEPQL**S**AALEYV  ALEYVR**S**HPLDPI  TADQTL**S**LMEQLR  YKVTYA**S**DYFDQL  GHNTLP**S**PWRDRP  DRPMEE**S**LLLFEA  MRKGKF**S**EGEATL  THCLCD**S**IEHITH  IEHITH**S**LCTKEF  EFQARR**S**SYFWLC  FQARRS**S**YFWLCN  LHYAVV**S**KRKILQ  AMAVLE**S**LRVIIT  NFPAAK**S**LDIQVP  HVVKGP**S**GCVESL  PSGCVE**S**LEVTCR  AFIHWV**S**QPLMCE  VPGGFL**S**DLNLAS  SDLNLA**S**LHVVDA  AALVDC**S**VALAKP  ERLGYF**S**VDPDSH  FSVDPD**S**HQGKLV  HTTHNQ**S**RKWHRN  GIKKPR**S**QRYESL  RSQRYE**S**LKGVDP  NNAKAM**S**ARAEAI  KIPKGV**S**RKLDRL  QAAAPA**S**VPAQAP  PAVSGL**S**RQVRCF  RQVRCF**S**TSVVRP  VRCFST**S**VVRPFA  YATALY**S**AASKQN  ALYSAA**S**KQNKLE  EPKVAA**S**VLNPYV  NPYVKR**S**IKVKSL  RSIKVK**S**LNDITA  TAKERF**S**PLTTNL  AENGRL**S**NTQGVV  NTQGVV**S**AFSTMM  GVVSAF**S**TMMSVH  AFSTMM**S**VHRGEV  VPCTVT**S**ASPLEE  CTVTSA**S**PLEEAT  LEEATL**S**ELKTVL  LKTVLK**S**FLSQGQ  VLKSFL**S**QGQVLK  EAKTDP**S**ILGGMI  EKYVDM**S**VKTKIQ  IRVLTE**S**MFKHLR  TRFFGH**S**RQRARL  GNVEAQ**S**RFIFFV  ITAFLG**S**WFFFGL  LPEFHP**S**ANHTPC  NINGLT**S**AFLFSL  TSAFLF**S**LETQVT  FLLIFQ**S**ILGVII  LGVIIN**S**FMCGAI  AILAKI**S**RPKKRA  AKTITF**S**KNAVIS  SKNAVI**S**KRGGKL  VANLRK**S**LLIGSH  KSLLIG**S**HIYGKL  ENLFFI**S**PLTIYH  HVIDHN**S**PFFHMA  LDGTVE**S**TSATCQ  GTVEST**S**ATCQVR  TCQVRT**S**YVPEEV  RFAPIV**S**KTKEGK  VDFHNF**S**KTVEVE  NPNFIL**S**EVNETD  AEKIVN**S**NGELYH  IKAMNN**S**WHPECF  IEGDVV**S**ALNKAW  VNCFAC**S**TCNTKL  SLVFGI**S**YDSPDY  FGISYD**S**PDYTDE  PDYTDE**S**CTFKIS  SLRNFR**S**ILSWEL  LLYTIM**S**KPEDLK  CANTTR**S**FCDLTD  TVLEGF**S**GNTTLF  TTLFSC**S**HNFWLA  ELQFDL**S**LVIEEQ  LVIEEQ**S**EGIVKK  EIKGNM**S**GNFTYI  NTNYCV**S**VYLEHS  SVYLEH**S**DEQAVI  EQAVIK**S**PLKCTL  LPPGQE**S**ESAESA  PGQESE**S**AESAKI  ESESAE**S**AKIGGI  IALVLT**S**TIVTLK  YNYDDE**S**DSDTEA  RPLGQA**S**ATSTES  SATSTE**S**QLIDPE  QLIDPE**S**EEEPDL  PTMPKD**S**PQQLEL  QQLELL**S**GPCERR  EAPLML**S**SHLEEM  DPDNVQ**S**NHLLAS  SNHLLA**S**GEGTQP  PTFPSP**S**SEGLWS  TFPSPS**S**EGLWSE  SSEGLW**S**EDAPSD  DAPSDQ**S**DTSESD  SDQSDT**S**ESDVDL  QSDTSE**S**DVDLGD  SDVHQD**S**IVLTYL  LLQSFS**S**RLQTVA  LQTVAL**S**QQIRQS  CYGVAS**S**HLKTLL  KPSVAC**S**QLALLL  QLALLL**S**SEAHLQ  NANQAA**S**ERLAAM  GQARTS**S**SKLMAS  KLMASK**S**SATVFQ  KPMNGH**S**HSERGS  TTIDEY**S**DNNPSF  DSSGDE**S**SYSNCV  SSGDES**S**YSNCVP  GDESSY**S**NCVPID  KHRTEK**S**ESDQPV  SVIESP**S**TNRTTP  TPPLLT**S**SKAGSP  TSSKAG**S**PINLSQ  LVIKWN**S**PPYVCS  NEGAQN**S**ATFSAS  QNSATF**S**ASKLLQ  GMQSSM**S**VEEQRP  VEEQRP**S**KQLLTG  LNSPLL**S**NKTNAV  LSHDAK**S**APFLGM  APAVQR**S**APALPV  APALPV**S**EDFKSE  YPAGHG**S**ASESEH  LKQLLL**S**ENCVRD  NSKPEF**S**ISSLNG  LQKGGN**S**VTSRET  IWREAS**S**AESVSQ  HMGNNA**S**RPHSAN  KDQDRK**S**RLMGLE  GLEALK**S**HIMAAK  ANTMRT**S**LGPNGL  DGATIL**S**MMDVDH  KLMVEL**S**KSQDDE  MVELSK**S**QDDEIG  EHLDKI**S**DSVLVD  LDKISD**S**VLVDIK  AKTTLG**S**KVVNSC  GSKVVN**S**CHRQMA  IVDKDF**S**HPQMPK  HKLDVT**S**VEDYKA  RIVPRF**S**ELTAEK  GLVQEI**S**FGTTKD  IEQCKN**S**RAVTIF  IEEAKR**S**LHDALC  GGAAEI**S**CALAVS  SCALAV**S**QEADKC  VIPMAL**S**ENSGMN  MALSEN**S**GMNPIQ  GKKQQI**S**LATQMV  YLRVVR**S**LCRASG  RSLCRA**S**GSRPAW  LCRASG**S**RPAWAP  ALTAPT**S**QEQPRR  DQVTID**S**ALATQK  LATQKY**S**VAVKCA  LKKMWK**S**PNGTIR  FTPKDG**S**GVKEWE  MYNTDE**S**ISGFAH  NTDESI**S**GFAHSC  ISGFAH**S**CFQYAI  KWPLYM**S**TKNTIL  VAQVLK**S**SGGFVW  AQVLKS**S**GGFVWA  YDGDVQ**S**DILAQG  LAQGFG**S**LGLMTS  SLGLMT**S**VLVCPD  QKGRPT**S**TNPIAS  STNPIA**S**IFAWTR  CVETVE**S**GAMTKD  GCIHGL**S**NVKLNE  FLDTIK**S**NLDRAL  EFVLPT**S**QRKCKS  LRALET**S**VAADFY  VQRHPP**S**EESQAF  MHPWVT**S**KPLPEY  FIVIHR**S**TTSQTI  IHRSTT**S**QTIKVS  PGAILQ**S**FFTKMA  KMAKKK**S**LMDIPE  ESVFTV**S**LWDCDR  NVWLEF**S**IKIKDL  ALSSKA**S**AESPSS  SKASAE**S**PSSESK  ASAESP**S**SESKGK  SAESPS**S**ESKGKV  ESPSSE**S**KGKVQL  NADKLT**S**ATNPDK  NPDKEN**S**MSISIL  ENSMSI**S**ILLDNY  WHFRYE**S**LKHPKA  AYPKLF**S**SVKWGQ  YPKLFS**S**VKWGQQ  LLDCNF**S**DENVRA  AVQKLE**S**LEDDDV  LDIKSL**S**AEKYDV  AEKYDV**S**SQVISQ  EKYDVS**S**QVISQL  LPYGCI**S**TGDKIG  HWLKEK**S**PTEEKF  ILGNYK**S**FLGINK  LFVMGT**S**GKKTSP  LLIILF**S**MMLMTG  DLESTT**S**HISKRP  SEETPY**S**YPTGNH  DQWQPS**S**SRFIHQ  QWQPSS**S**RFIHQQ  SSAKTS**S**VSNPQD  AKTSSV**S**NPQDSV  VSNPQD**S**VGSPCS  SVGSPC**S**RVGEEE  PSPTVM**S**TSLGSN  MSTSLG**S**NLSELD  PADEAN**S**SPPLPG  PLPGAL**S**PLYGVP  GVPETN**S**PLGGKA  LEDVRP**S**VESLLD  LLDELE**S**SVPSPV  LDELES**S**VPSPVP  VNQGEM**S**SPQRVT  SPQRVT**S**TQQQTR  QQQTRI**S**ASSATR  TRISAS**S**ATRELD  LDELMA**S**LSDFKI  PRDGGR**S**SPGGQD  RDGGRS**S**PGGQDE  AQGKTG**S**SSPPGG  QGKTGS**S**SPPGGP  LDSMLG**S**LQSDLN  MLGSLQ**S**DLNKLG  CQEEIG**S**RNFFER  DYHNLF**S**PRCYYC  ILENYI**S**ALNTLW  RRGSLC**S**GCQKPI  PQFHVK**S**GLQIKK  DDYKVT**S**QVLGLG  ELHWRA**S**QCPHIV  DGGELF**S**RIQDRG  FTEREA**S**EIMKSI  ASEIMK**S**IGEAIQ  AIQYLH**S**INIAHR  ENLLYT**S**KRPNAI  FAKETT**S**HNSLTT  ETTSHN**S**LTTPCY  PEKYDK**S**CDMWSL  KSCDMW**S**LGVIMY  GYPPFY**S**NHGLAI  FPNPEW**S**EVSEEV  PEWSEV**S**EEVKML  QTPLHT**S**RVLKED  VKEEMT**S**ALATMR  KKIEDA**S**NPLLLK  TGTFVV**S**QPLNYR  VEPADA**S**GTEKAF  IATFTC**S**GEKEVN  AAFKIW**S**QKSGME  KIWSQK**S**GMERCR  CINNGK**S**IFEARL  RLDIDI**S**WQCLEY  YAGLAA**S**MAGEHI  IQLPGG**S**FGYTRR  YPFQIA**S**WKSAPA  QIASWK**S**APALAC  AMVFKP**S**PFTPVS  SPFTPV**S**ALLLAE  LLAEIY**S**EAGVPP  PDVAKV**S**FTGSVP  KVSFTG**S**VPTGMK  MKIMEM**S**AKGIKP  LELGGK**S**PLIIFS  SPLIIF**S**DCDMNN  IFGPVM**S**ILSFDT  PVMSIL**S**FDTEAE  INNYNV**S**PVELPF  FGGYKK**S**GFGREN  VTIEYY**S**QLKTVC  ETLVGY**S**MVGCQR  MMANRL**S**FFFDFR  FDFRGP**S**IALDTA  LDTACS**S**SLMALQ  LIGSTK**S**NMGHPE  GNVGIN**S**FGFGGS  EQGLRH**S**QDLAFL  QDLAFL**S**MLNDIA  GGMAFH**S**YFMEAI  REPKPR**S**ARWLST  SSLART**S**SAEYNV  SLARTS**S**AEYNVN  IGRLHL**S**GIDANP  IYNIDT**S**SESPDH  SRAFEV**S**ENGNLV  QGILEA**S**LEGDSG  DTMLQM**S**ILGSAK  ELPRLL**S**AACRLQ  AVENMP**S**LKMKVV  APSALG**S**ADLLVC  AWESLF**S**RVSLRL  LVGLKK**S**FYGSTL  AINCAT**S**GVVGLV  LTRGDL**S**SIRWVC  LATGKL**S**PDAIPG  ETLLIH**S**GSGGVG  STSFAN**S**RDTSFE  STSNIS**S**LEGARG  ILVETM**S**TNDTIV  TNDTIV**S**GTLPQR  QPHMVL**S**SFVLAE  VLAYTQ**S**YRAKLT  VDLIIK**S**HQGLDR  RPVLVL**S**QNTKRE  QNTKRE**S**GRKVQS  SGRKVQ**S**GNINAA  TCLGPK**S**MMKMLL  QHPAAK**S**MIEISR  KSMIEI**S**RTQDEE  VGDGTT**S**VIILAG  LAGEML**S**VAEHFL  HPTVVI**S**AYRKAL  ALDDMI**S**TLKKIS  STLKKI**S**IPVDIS  SIPVDI**S**DSDMML  PVDISD**S**DMMLNI  MLNIIN**S**SITTKA  LNIINS**S**ITTKAI  ITTKAI**S**RWSSLA  KAISRW**S**SLACNI  AISRWS**S**LACNIA  GGIIED**S**CVLRGV  RIVLLD**S**SLEYKK  IVLLDS**S**LEYKKG  EYKKGE**S**QTDIEI  ITEKGI**S**DLAQHY  CGARIV**S**RPEELR  ILLRGA**S**KEILSE  ASKEIL**S**EVERNL  VPGGGA**S**EMAVAH  HALTEK**S**KAMTGV  IQNCGA**S**TIRLLT  TIRLLT**S**LRAKHT  RIDDIV**S**GHKKKG  KKGDDQ**S**RQGGAP  RVFKKA**S**PNGKLT  FVANVQ**S**FPPAPE  PPNLPC**S**VTLQPG  KIHKRN**S**VRLVIR  TRQFLM**S**DKPLHL  PLHLEA**S**LDKEIY  YHGEPI**S**VNVHVT  VKKIKI**S**VRQYAD  DDTVAP**S**STFCKV  DTVAPS**S**TFCKVY  EDTNLA**S**STLLRE  DTNLAS**S**TLLREG  ILGIIV**S**YKVKVK  KVKLVV**S**RGGLLG  LLGDLA**S**SDVAVE  LGDLAS**S**DVAVEL  PSRRGP**S**LGASSH  GPSLGA**S**SHQHSR  PSLGAS**S**HQHSRR  ASSHQH**S**RRRQGW  IRKLQK**S**THLLIR  IRKLPF**S**RLAREI  DPPAGV**S**GAPSEN  GVSGAP**S**ENNIMV  PTVRFV**S**KMFHPN  NVYADG**S**ICLDIL  ILQNRW**S**PTYDVS  SPTYDV**S**SILTSI  PTYDVS**S**ILTSIQ  VSSILT**S**IQSLLD  ILTSIQ**S**LLDEPN  PNSPAN**S**QAAQLY  EYEKRV**S**AIVEQS  DSTLTA**S**EIRQRF  EHTYVH**S**SATIPL  HTYVHS**S**ATIPLD  FFEMLG**S**WSFGDY  EMLGSW**S**FGDYFK  GPCGPC**S**EIHYDR  KPLPKK**S**IDTGMG  GLERLV**S**VLQNKM  VLQNKM**S**NYDTDL  HEKLNA**S**RGFFAT  VDVVVQ**S**LGDAFP  QFLKTL**S**RGRRIL  IQSLGD**S**KTIPGD  YNYHLD**S**SGSYVF  NYHLDS**S**GSYVFE  MFVEEV**S**TGQECG  LVKVDD**S**SEDKTE  RRRPIM**S**NHTATH  LNFALR**S**VLGEAD  EADQKG**S**LVAPDR  TAKGAM**S**TQQIKK  DPVRVV**S**IGVPVS  SIGVPV**S**ELLDDP  ELLDDP**S**GPAGSL  PAGSLT**S**VEFCGG  THLRNS**S**HAGAFV  ALRKAE**S**LKKCLS  SLKKCL**S**VMEAKV  VILEME**S**GASAKA  EMESGA**S**AKALNE  MHSPQT**S**AMLFTV  EWVQQV**S**GLMDGK  GGGKDV**S**AQATGK  FKLCAG**S**SYRHMR  KLCAGS**S**YRHMRN  QAVMAI**S**QELNRR  LGGPTP**S**TWINQV  NQVRRR**S**SLLGSR  RSSLLG**S**RLEETL  LEETLY**S**DQELAY  KALGIL**S**NQEGWK  EGWKKE**S**QQDNGD  NGDKVM**S**KVVPDV  GPRDFV**S**VRCAKR  LHPLAG**S**PSKTKL  PLAGSP**S**KTKLTW  KLTWLL**S**IDLKGW  KGWLPK**S**IINQVL  IINQVL**S**QTQVDF  LRKRLE**S**HPASEA  ALLENF**S**SSYDYG  LLENFS**S**SYDYGE  LENFSS**S**YDYGEN  DYGENE**S**DSCCTS  GENESD**S**CCTSPP  SDSCCT**S**PPCPQD  PCPQDF**S**LNFDRA  FLPALY**S**LLFLLG  VAAVLL**S**RRTALS  SRRTAL**S**STDTFL  RRTALS**S**TDTFLL  VQWVFG**S**GLCKVA  LLLACI**S**FDRYLN  PDFIFL**S**AHHDER  LAVLLV**S**RGQRRL  RNCGRE**S**RVDVAK  RVDVAK**S**VTSGLG  VAKSVT**S**GLGYMH  GLQRQP**S**SSRRDS  LQRQPS**S**SRRDSS  QRQPSS**S**RRDSSW  SSSRRD**S**SWSETS  SSRRDS**S**WSETSE  RRDSSW**S**ETSEAS  EPRPPM**S**SHLQSP  PRPPMS**S**HLQSPP  MSSHLQ**S**PPHAPS  SPPHAP**S**SAAFGF  PPHAPS**S**AAFGFP  ICEHET**S**IDISAY  ETSIDI**S**AYIDPA  ADLFQH**S**RQQEKA  GLPGPG**S**ALKGLG  HPDLRA**S**GGSGAG  LRASGG**S**GAGKAK  AGKAKK**S**VDKNSN  KSVDKN**S**NEYRVR  NIAVRK**S**RDKAKQ  KVLELT**S**DNDRLR  KRVEQL**S**RELDTL  FRQLPE**S**SLVKAM  RQLPES**S**LVKAMG  KPYTLM**S**MVANLL  FKPFIC**S**LDLIGC  TDDFVV**S**GTCAEQ  MYGMCE**S**LWEPNM  HLFETI**S**QAMLNA  VDRDAV**S**GMGVIV  TSSPGR**S**SRRTDA  SSPGRS**S**RRTDAL  DEEMIE**S**IENLED  LRTHVD**S**HGHNVF  VFKERI**S**DMCKEN  CKENRE**S**LVVNYE  HIHVRI**S**HLPLVE  LVEELR**S**LRQLHL  NQLIRT**S**GVVTSC  TSGVVT**S**CTGVLP  LGPFCQ**S**QNQEVK  QEVKPG**S**CPECQS  AGRLPR**S**KDAILL  LADLVD**S**CKPGDE  DVKMIT**S**LSKDQQ  GEKIFA**S**IAPSIY  DPGTAK**S**QFLKYI  YIEKVS**S**RAIFTT  VQRHPV**S**REWTLE  MEQQSI**S**ISKAGI  GGRYDP**S**LTFSEN  DPSLTF**S**ENVDLT  LTEPII**S**RFDILC  HVRHHP**S**NKEEEG  KVAKMY**S**DLRKES  ESMATG**S**IPITVR  IRVMLE**S**FIDTQK  TFARYL**S**FRRDNN  INIHNL**S**AFYDSE  LSAFYD**S**ELFRMN  FRMNKF**S**HDLKRK  MIPVPM**S**IMAPAP  VLVPTV**S**MVGKHL  VFVGNI**S**EKASDM  KCGLVL**S**WKRVQG  EYKEPE**S**TLRALR  LIREYS**S**ELNAPS  SELNAP**S**QESDSH  NAPSQE**S**DSHPRK  PSQESD**S**HPRKKK  DKRDLI**S**REISKF  LISREI**S**KFRDTH  DRDRER**S**SDRNKD  RDRERS**S**DRNKDR  DRNKDR**S**RSREKS  NKDRSR**S**REKSRD  SRSREK**S**RDRERE  PKYYRG**S**ALQKRL  PTLRPI**S**SAPSVS  TLRPIS**S**APSVSS  SSAPSV**S**SASGNA  SAPSVS**S**ASGNAT  PSVSSA**S**GNATPN  NTPGDE**S**PCGIII  IIPHEN**S**PDQQQP  RPKIGL**S**LKLGAS  SLKLGA**S**NSPGQP  KKLPVD**S**VFNKFE  AYPLDW**S**IVDSIL  DWSIVD**S**ILMERR  LVDFVC**S**KVMAHS  KVMAHS**S**PQSILD  PRRYHS**S**ERGSRG  ERGSRG**S**YREHYR  YREHYR**S**RKHKRR  KHKRRR**S**RSWSSS  RRSRSW**S**SSSDRT  RSRSWS**S**SSDRTR  SRSWSS**S**SDRTRR  RSWSSS**S**DRTRRR  RRRRED**S**YHVRSR  DSYHVR**S**RSSYDD  HVRSRS**S**YDDRSS  SSYDDR**S**SDRRVY  YRRNDY**S**RDRGDA  SYRSQR**S**SRRKHR  YRSQRS**S**RRKHRR  RRRRRR**S**RTFSRS  RRSRTF**S**RSSSQH  SRTFSR**S**SSQHSS  RTFSRS**S**SQHSSR  TFSRSS**S**QHSSRR  RSSSQH**S**SRRAKS  ERYEIV**S**TLGEGT  HGHMCI**S**FELLGL  FELLGL**S**TFDFLK  NILFVN**S**DYELTY  DERSVK**S**TAVRVV  RVVDFG**S**ATFDHE  FDHEHH**S**TIVSTR  HHSTIV**S**TRHYRA  ILELGW**S**QPCDVW  LRRYLT**S**EAEEHH  ELPAPL**S**YFQNAQ  FQNAQM**S**EDNHLS  SEDNHL**S**NTVRSQ  LSNTVR**S**QNDNRE  GHPEPL**S**NGRPQG  GRPQGN**S**RQVVEQ  VVATIK**S**VSFYTR  ATIKSV**S**FYTRKD  GQRALH**S**ILNAAI  NAAIMI**S**VIVVMT  HAWLII**S**SLLLLF  AWLIIS**S**LLLLFF  LLLFFF**S**FIYLGE  GVVGMI**S**IHWKGP  AYLIMI**S**ALMALV  LILAVI**S**VYDLVA  FPALIY**S**STMVWL  PALIYS**S**TMVWLV  RRVSKN**S**KYNAES  SKYNAE**S**TERESQ  ESTERE**S**QDTVAE  NDDGGF**S**EEWEAQ  HLGPHR**S**TPESRA  HRSTPE**S**RAAVQE  AAVQEL**S**SSILAG  AVQELS**S**SILAGE  GDFIFY**S**VLVGKA  VLVGKA**S**ATASGD  KASATA**S**GDWNTT  LPALPI**S**ITFGLV  QHLIKP**S**VVFLKT  FLKTEL**S**FALVNR  KHFHGT**S**LTFSMQ  GTSLTF**S**MQDGPE  DFHRND**S**IYEELQ  KEDFPA**S**WRSEEE  FPASWR**S**EEEMAA  QHQGIL**S**RVTESV  HCQPST**S**SAFPIG  VKEIKD**S**TSQHDD  HDDDNI**S**TTSGFS  SPEAER**S**HSLSQH  SQHTAT**S**SKKPAF  FGTLSP**S**LGNSSI  QSYGVT**S**STARRI  ARRILQ**S**LEKMSS  QRESGF**S**YPNFSL  SLPITS**S**SLPTFN  PTFNFS**S**PEITTS  SSPIVK**S**TEANVL  ANVLPP**S**SIGFTF  AKTAEL**S**GSSSTL  TLEPII**S**SSAHHV  YTRPAI**S**SFSSSG  CSVCCV**S**NNAEDN  SLPSGG**S**LGLEKF  KFGVSS**S**SSGPSQ  PSQTLT**S**TGNFKF  VSSDSG**S**INPMSE  EELPKS**S**SAGFSF  SSKSTF**S**FSMTKP  AFGAQT**S**TTADQG  NSSSSS**S**TPATSA  ATTTSS**S**AGSSFV  TSSSAG**S**SFVFGT  GQQPSQ**S**AFGSGT  SAFQFG**S**STTNFN  AAFTVG**S**NGKNVF  GKNVFS**S**SGTSFS  KNVFSS**S**GTSFSG  EADRPP**S**MSSHDT  RPPSMS**S**HDTASP  SSHDTA**S**PAAPSR  ASPAAP**S**RNPCCL  CWCCCC**S**CSWNQE  CCCCSC**S**WNQERR  RRAWQA**S**RESKLQ  WQASRE**S**KLQPLP  KLQPLP**S**CEVCAT  EVCATP**S**PEEVQS  SPEEVQ**S**WAQSFD  VQSWAQ**S**FDKLMH  HSPAGR**S**VFRAFL  FLRTEY**S**EENMLF  IYEDYV**S**ILSPKE  LSPKEV**S**LDSRVR  KEVSLD**S**RVREGI  KKMQEP**S**AHTFDD  TLMHRD**S**YPRFLS  SYPRFL**S**SPTYRA  YPRFLS**S**PTYRAL  NNYGQT**S**NGVADE  NGVADE**S**PNMLVY  PIRTVK**S**FLSKIP  TVKSFL**S**KIPSVF  FLSKIP**S**VFSGSD  KIPSVF**S**GSDIVQ  PSVFSG**S**DIVQWL  GYFFPI**S**DHVLTL  ADYEAE**S**LARLQR  ERKILD**S**QERAFW  EVDIKK**S**SRMRNP  VDIKKS**S**RMRNPH  PHKTRK**S**VYGLQN  NDIRSH**S**PTHTPT  MSKVAD**S**LLSYTE  VADSLL**S**YTEQYL  PSNPWL**S**DDTTFW  FWELEA**S**KEPSQQ  EASKEP**S**QQRVKR  FLKFLE**S**EFSSEN  FLESEF**S**SENLRF  LESEFS**S**ENLRFW  PIKEVP**S**RVQEIW  LAPGAP**S**AINLDS  SAINLD**S**KSYDKT  INLDSK**S**YDKTTQ  IYKLMK**S**DSYPRF  YPRFIR**S**SAYQEL  PRFIRS**S**AYQELL  LQAKKK**S**GNSMDR  SMDRRT**S**FEKFAQ  NCLPAE**S**LPLFIV  PTSVLP**S**FYQAMA  DQRPES**S**LLNLIS  SLLNLI**S**YRAQSI  ISYRAQ**S**IHPAKD  MERFFR**S**ESRGAV  VVISQL**S**HIPEDK  QLHYKH**S**YTLPIA  TLPIAS**S**IRLQAF  LGSVPY**S**LLFRVL  CRSISV**S**EHVVRS  SEHVVR**S**RIQTSL  LQSGPE**S**SSSPGV  PPPLPR**S**NTVASF  RSNTVA**S**FSSLYQ  SVSWAD**S**AVVMEE  RRTDAY**S**RSSSVS  TDAYSR**S**SSVSSQ  RSSSVS**S**QEEKSL  LSKSSS**S**PELQTL  GTLDGE**S**AAWSAS  GESAAW**S**ASGEDS  SASGED**S**RGQPEG  SSPRSP**S**GLRPRG  PRGYTI**S**DSAPSR  GYTISD**S**APSRRG  ERDALK**S**RATASN  LPNESQ**S**FERSVQ  GEGQSN**S**ELAILS  SELAIL**S**NEHGSY  VHPPSH**S**KAPAQT  TTSFAE**S**CKPVQQ  KPVQQP**S**AFGSMK  QPSAFG**S**MKVSRD  FGSMKV**S**RDKDGS  SRDKDG**S**KVTTVV  DRPQEV**S**YTDTKV  KVIGNG**S**FGVVYQ  QAKLCD**S**GELVAI  LRYFFY**S**SGEKKD  RYFFYS**S**GEKKDE  RVARHY**S**RAKQTL  MYQLFR**S**LAYIHS  SLAYIH**S**FGICHR  KLCDFG**S**AKQLVR  RGEPNV**S**YICSRY  NVSYIC**S**RYYRAP  GATDYT**S**SIDVWS  ATDYTS**S**IDVWSA  SSIDVW**S**AGCVLA  PIFPGD**S**GVDQLV  EAIALC**S**RLLEYT  LEACAH**S**FFDELR  FTTQEL**S**SNPPLA  TTQELS**S**NPPLAT  TNATAA**S**DANTGD  EGMSNN**S**TTSISQ  SNNSTT**S**ISQARK  NSTTSI**S**QARKAV  MDRVKV**S**QAAADL  IIPVPA**S**ENPFRE  RRLSPP**S**SSAASS  RLSPPS**S**SAASSY  SSSAAS**S**YSFSDL  ASSYSF**S**DLNSTR  SFSDLN**S**TRGDAD  DDYYEE**S**YFTTRT  TYGEPE**S**AGPSRA  PESAGP**S**RAVRQS  SRAVRQ**S**VTSFPD  VRQSVT**S**FPDADA  HDDDLL**S**SSEEEC  DDDLLS**S**SEEECK  DDLLSS**S**EEECKD  PMYGRD**S**AYQSIT  RDSAYQ**S**ITHYRP  YRPVSA**S**RSSLDL  PVSASR**S**SLDLSY  VSASRS**S**LDLSYY  RSSLDL**S**YYPTSS  LSYYPT**S**SSTSFM  SYYPTS**S**STSFMS  YYPTSS**S**TSFMSS  PTSSST**S**FMSSSS  SSTSFM**S**SSSSSS  STSFMS**S**SSSSSS  TSFMSS**S**SSSSSW  SFMSSS**S**SSSSWL  FMSSSS**S**SSSWLT  MSSSSS**S**SSWLTR  SSSSSS**S**SWLTRR  SSSSSS**S**WLTRRA  MCKQDP**S**VLHTEE  LREWVE**S**MGGKVP  ATQKAK**S**EENTKE  KEEKPD**S**KKVEED  LKADEP**S**SEESDL  KADEPS**S**EESDLE  EPSSEE**S**DLEIDK  LYAKRA**S**VFVKLQ  IEINPD**S**AQPYKW  DYDEDA**S**AMLKEV  EEARRQ**S**GAQYGS  SGAQYG**S**FPGGFP  GLNEIL**S**DPEVLA  NMSKYQ**S**NPKVMN  KVMNLI**S**KLSAKF  AYKPES**S**PGSRQI  PESSPG**S**RQIQLW  GGAVPQ**S**APPVPS  SAPPVP**S**GGSHFR  PVPSGG**S**HFRFPP  HFRFPP**S**TPSEVL  FPPSTP**S**EVLSPT  TPSEVL**S**PTEDPR  PTEDPR**S**PPACSS  ACSSSS**S**SLFSAV  CSSSSS**S**LFSAVV  LGRGSV**S**DCSDGT  DCSDGT**S**ELEEPL  LLPPQL**S**PALPMT  HLAYTP**S**PTLSPM  TPSPTL**S**PMYPSG  LSPMYP**S**GGGGPS  SGGGGP**S**GSGGGS  GGGSHF**S**FSPEDM  GSHFSF**S**PEDMKR  TPPVPS**S**ASSSSS  VPSSAS**S**SSSSSS  PSSASS**S**SSSSSS  SSASSS**S**SSSSSP  ASSSSS**S**SSSPFK  SSSSSS**S**SSPFKF  SSSSSS**S**SPFKFK  SSSSSS**S**PFKFKL  VAGADK**S**GGSAGG  ADKSGG**S**AGGLAE  TPRRVS**S**DLQHAT  SETVIC**S**SRATVM  ETVICS**S**RATVML  TGPQAF**S**RVQIYH  HNPTAN**S**FRVVGR  WGLNFG**S**KEDAAQ  FAAGMA**S**ALEALE  PALPTW**S**VPNGPS  SVPNGP**S**PEEVEQ  RQQPGP**S**EHIERR  PPGLPP**S**GVPAAA  SKQEEA**S**GGPTAP  TAPKAE**S**GRSGGG  KAESGR**S**GGGGLM  KTPKDE**S**ANQEEP  ARVPAQ**S**ESVRRP  VPAQSE**S**VRRPWE  RPWEKN**S**TTLPRM  PRMKSS**S**SVTTSE  RMKSSS**S**VTTSET  SSSVTT**S**ETQPCT  TQPCTP**S**SSDYSD  QPCTPS**S**SDYSDL  PCTPSS**S**DYSDLQ  PSSSDY**S**DLQRVK  FLLLQA**S**DGIHHW  DVEIRL**S**HEHQAY  IKLGHR**S**EAKDGI  KLLQEL**S**HPNIIG  DAFGHK**S**NISLVF  GHKSNI**S**LVFDFM  VIIKDN**S**LVLTPS  SLVLTP**S**HIKAYM  DFGLAK**S**FGSPNR  PFLPGD**S**DLDQLT  QWPDMC**S**LPDYVT  DYVTFK**S**FPGIPL  PLHHIF**S**AAGDDL  LKMKYF**S**NRPGPT  PFCDEV**S**KYEKLA  ICRTKA**S**PYNRCK  YNRCKG**S**IYLVFD  DLAGLL**S**NVLVKF  LVKFTL**S**EIKRVM  FSLAKN**S**QPNRYT  AEMWTR**S**PIMQGN  HQLALI**S**QLCGSI  ISQLCG**S**ITPEVW  PAQRID**S**DDALNH  NHDFFW**S**DPMPSD  WSDPMP**S**DLKGML  DLKGML**S**THLTSM  LSTHLT**S**MFEYLA  QAEVRL**S**VPPLVE  EVMRGK**S**VILDCT  SAEMQG**S**ELQVTM  PPYQLD**S**QGRLVL  PNKGTL**S**VMEDSA  LSVMED**S**AQEIAT  EIATCN**S**RNGNPA  PEGYMT**S**RTVREA  RTVREA**S**GLLSLT  KDDRDA**S**FHCAAH  HCAAHY**S**LPEGRH  VQFWVG**S**PSTPAG  LCRGDG**S**PSPEYT  RGDGSP**S**PEYTLF  ADDVQL**S**KTLELR  LDPLEL**S**EGKVLS  SEGKVL**S**LPLNSS  LSLPLN**S**SAVVNC  SAVVNC**S**VHGLPT  GDGPML**S**LSSITF  PMLSLS**S**ITFDSN  TYVCEA**S**LPTVPV  PTVPVL**S**RTQNFT  EPKADG**S**WREGDE  EVTLIC**S**ARGHPD  WSQLGG**S**PAEPIP  RQGWVS**S**SLTLKV  QGWVSS**S**LTLKVT  LSRDGI**S**CEASNP  GISCEA**S**NPHGNK  FHFGTV**S**PQTSQA  EGAKHF**S**GLEEAV  KMIVMA**S**HMQEQE  LLRIGL**S**VSEVIE  RIGLSV**S**EVIEGY  PNLVCC**S**AKNLRD  RDIDEV**S**SLLRTS  DIDEVS**S**LLRTSI  SSLLRT**S**IMSKQY  LRTSIM**S**KQYGNE  IAQACV**S**IFPDSG  VSIFPD**S**GHFNVD  VCKILG**S**GISSSS  ILGSGI**S**SSSVLH  LGSGIS**S**SSVLHG  GSGISS**S**SVLHGM  SGISSS**S**VLHGMV  TEGDVT**S**VKDAKI  AKIAVY**S**CPFDGM  EELMNF**S**KGEENL  MLVRLN**S**KWDLRR  EMGHCD**S**VYLSEV  CDSVYL**S**EVGDTQ  KEDGAI**S**TIVLRG  TIVLRG**S**TDNLMD  LAKQIT**S**YGETCP  RALAEN**S**GVKANE  KANEVI**S**KLYAVH  GGPKPP**S**GKKDWD  TVEVRG**S**NGAFYK  KDVHED**S**LTVVFE  DIKKEI**S**EGDEVE  DEVEVY**S**RANDQE  LMILSA**S**EATVKR  SDMHLR**S**IRTKLM  TKLMLM**S**RNEEAT  AIGTHG**S**NIQQAR  FRIYGE**S**ADAVKK  QEIVDK**S**GVVRVR  RQIGSR**S**YSGRGR  IGSRSY**S**GRGRGR  RGPNYT**S**GYGTNS  SGYGTN**S**ELSNPS  GTNSEL**S**NPSETE  SELSNP**S**ETESER  NPSETE**S**ERKDEL  ERKDEL**S**DWSLAG  DELSDW**S**LAGEDD  SRHQRD**S**RRRPGG  GRGRSV**S**GGRGRG  GPRGGK**S**SISSVL  PRGGKS**S**ISSVLK  GGKSSI**S**SVLKDP  VLKDPD**S**NPYSLL  TADTDA**S**ESHHST  DTDASE**S**HHSTNR  ASESHH**S**TNRRRR  TNRRRR**S**RRRRTD  TESDTA**S**VNENGL  TVADYI**S**RAESQS  YISRAE**S**QSRQRN  SRAESQ**S**RQRNLP  KPTVFI**S**GVIARG  HQKPHA**S**MDKHPS  EKGYQT**S**SGLISS  KGYQTS**S**GLISSV  TSSGLI**S**SVSVKL  SSGLIS**S**VSVKLK  GLISSV**S**VKLKGL  PAQGDN**S**FVVMTN  GICKED**S**GCTPGK  TLFIKN**S**ISFPRF  FIKNSI**S**FPRFKV  GYVVQE**S**GQNFST  ESGQNF**S**TLAEKG  YEEKNL**S**PGFNFR  TMTTIG**S**GIGIFG  SYNQLA**S**TPIIFK  GRNVPN**S**RHKSLR  KSVVCG**S**LFHTPK  DPDMSW**S**SSLATP  TPPTLS**S**TVLIVR  SFSLCF**S**KCRTKN  HQKPFE**S**GSDKIS  AVKQAI**S**GTSPVA  IFRIRE**S**PKETFN  SCSQND**S**EEPTLS  KESYKM**S**DKLKGN  GLLGPI**S**NHSFGG  STILEE**S**GSQFEF  SIGQVD**S**SKQFEG  VHPISL**S**SSKCHD  PISLSS**S**KCHDSV  IKDFET**S**DTFFQT  SKNKLD**S**GIEPVL  SFSKVI**S**NVKDAN  SYNVVN**S**SAFSGF  SSENNH**S**IKVSPY  GKTETF**S**DVPVKT  GHGSDD**S**KNKIND  PLDLIT**S**LQNARD  LYTYGV**S**KHCIKI  ANISET**S**SNKTSS  PLEAPE**S**LMLKIS  FPLPLS**S**LFSDGG  IRKAME**S**AEQKEQ  SKSKSK**S**ERANIQ  REPLHF**S**KFLDPD  SGQRTA**S**VLWPWI  PPAPLP**S**PGTTAP  TAPRPS**S**IPAPAE  SASTFL**S**PAFPGS  SPAFPG**S**QTHSGP  ELGLVP**S**PASLWP  PPSPAP**S**STKPGP  PSPAPS**S**TKPGPE  TKPGPE**S**SVSLLQ  KPGPES**S**VSLLQG  GPESSV**S**LLQGAR  HGDIKS**S**NVLLDE  SRFAGS**S**PSQSSM  FAGSSP**S**QSSMVA  SSPSQS**S**MVARTQ  AGVALR**S**TQSTLQ  ALRSTQ**S**TLQAGL  GHSEAA**S**CIPPSP  ASCIPP**S**PQENSY  PSPQEN**S**YVSSTG  QENSYV**S**STGRAH  STGRAH**S**GAAPWQ  QPLAAP**S**GASAQA  PNQPVE**S**DESLGG  PVESDE**S**LGGLSA  LSAALR**S**WHLTPS  SWHLTP**S**CPLDPA  DTAGES**S**WGSGPG  ALGSSA**S**SSSEPP  GSSASS**S**SEPPQI  SSASSS**S**EPPQII  LQLLSS**S**SLPGLG  QLLSSS**S**LPGLGL  LFMDDD**S**YSHHSG  MDDDSY**S**HHSGLE  DPHRLN**S**HLKLGF  EPVTTH**S**FDKVWI  DKVWIC**S**HALFEI  HALFEI**S**KYVMYK  ILFATL**S**CLHIWI  CLMVLP**S**VQTIWK  VQTIWK**S**VTDVII  IAPLCT**S**VGRCFS  SVGRCF**S**SVSLQL  VGRCFS**S**VSLQLS  VVAMLL**S**LDFPGQ  TCHQVR**S**FFQRLQ  ETPVPG**S**DLQVCL  KGPTCC**S**RKMEEK  QLLQSA**S**MELKFL  FKNNYP**S**LTPQAF  EFFTDV**S**LYILGS  SLYILG**S**DINVDD  VNELFD**S**LFPVIY  NPGLPD**S**ALDINE  LIMTQV**S**KSLQVT  MTQVSK**S**LQVTRI  TDHLKF**S**KDCGRM  TRMWYC**S**YCQGLM  WREYIL**S**LEELVN  VLLGLF**S**TIHDSI  FSTIHD**S**IQYVQK  QQRQYR**S**AYYPED  EHEETL**S**SRRREL  HEETLS**S**RRRELI  LIQKLK**S**FISFYS  KLKSFI**S**FYSALP  SFISFY**S**ALPGYI  LPGYIC**S**HSPVAE  ELVERY**S**QKAARN  GPEPVV**S**QIIDKL  QLLRTM**S**MPKGRV  DDAPGN**S**QQATPK  NLGNVH**S**PLKLLT  PLKLLT**S**MAISVV  LTSMAI**S**VVCFFF  ILAKIP**S**ENNKLT  NNKLTY**S**HGNYLF  DDDFER**S**RAFNFL  FQTTYG**S**RAQTAL  LPYAMN**S**EFSSVL  AMNSEF**S**SVLAAQ  MNSEFS**S**VLAAQL  AQLKHH**S**ENKGLD  TENLVD**S**SVTFKT  ENLVDS**S**VTFKTT  VTFKTT**S**RNLARA  IIIIIV**S**IVFIYI  FIYIIV**S**PLCGGF  ELLLKR**S**QQKKKM  RGSRKG**S**IEIKKI  LLYVYA**S**NEESRS  YASNEE**S**RSQWLK  LKMDAP**S**SSTTLA  KMDAPS**S**STTLAQ  AQYDNE**S**KKNYGS  SKKNYG**S**QPPSSS  PPSSST**S**LAQYDS  SLAQYD**S**NSKKIY  AQYDSN**S**KKIYGS  RKLKSS**S**SSEDVA  KLKSSS**S**SEDVAS  HTTSKI**S**WEFPES  WEFPES**S**SSEEEE  AGNISR**S**QSEQLL  NISRSQ**S**EQLLRQ  FMVRNS**S**QVGMYT  VGMYTV**S**LFSKAV  YTVSLF**S**KAVNDK  ANKVPD**S**VSLGNG  LLKELG**S**GQFGVV  QTMMKL**S**HPKLVK  GKGLEP**S**QLLEMC  GMAFLE**S**HQFIHR  LDDQYV**S**SVGTKF  DDQYVS**S**VGTKFP  FHYFKY**S**SKSDVW  HYFKYS**S**KSDVWA  YDLYDN**S**QVVLKV  QVVLKV**S**QGHRLY  IYQIMY**S**CWHELP  ALIGGT**S**GQYYDY  DYDFPL**S**IYGQSS  LSIYGQ**S**SPNCAP  SIYGQS**S**PNCAPE  ECNCPE**S**YPSAMY  CPESYP**S**AMYCDE  DELKLK**S**VPMVPP  HNLLEN**S**KIKGRV  IKGRVF**S**KLKQLK  HNNLTE**S**VGPLPK  VGPLPK**S**LEDLQL  KITKLG**S**FEGLVN  LKEDAV**S**AAFKGL  AFKGLK**S**LEYLDL  LEYLDL**S**FNQIAR  QIARLP**S**GLPVSL  PSGLPV**S**LLTLYL  LDNNKI**S**NIPDEY  LQYLRL**S**HNELAD  HNELAD**S**GIPGNS  SGIPGN**S**FNVSSL  GNSFNV**S**SLVELD  NSFNVS**S**LVELDL  LVELDL**S**YNKLKN  EKFDIK**S**FCKILG  KILGPL**S**YSKIKH  LGPLSY**S**KIKHLR  LDGNRI**S**ETSLPP  NRISET**S**LPPDMY  KRHWTF**S**SEEQLA  RHWTFS**S**EEQLAR  RLLEFC**S**VFKPAM  KPAMPR**S**VVGTAC  RFYLNN**S**VMEYHP  VDEFNV**S**SPQFVG  DEFNVS**S**PQFVGN  YLLYTP**S**QIALTA  ALTAIL**S**SASRAG  LTAILS**S**ASRAGI  AILSSA**S**RAGITM  AGITME**S**YLSESL  TMESYL**S**ESLMLK  ESYLSE**S**LMLKEN  ENRTCL**S**QLLDIM  LLDIMK**S**MRNLVK  KYEPPR**S**EEVAVL  KLERCH**S**AELALN  DYVSKK**S**KHEEEE  YTIGTG**S**YGRCQK  QKIRRK**S**DGKILV  KELDYG**S**MTEAEK  EKQMLV**S**EVNLLR  EGGDLA**S**VITKGT  KECHRR**S**DGGHTV  GTPYYM**S**PEQMNR  EQMNRM**S**YNEKSD  MSYNEK**S**DIWSLG  EKSDIW**S**LGCLLY  PPFTAF**S**QKELAG  KDYHRP**S**VEEILE  PEKSQD**S**SPVLSE  EKSQDS**S**PVLSEL  DSSPVL**S**ELKLKE  KFLSLA**S**NPELLN  ELLNLP**S**SVIKKK  LLNLPS**S**VIKKKV  IMRSEN**S**ESQLTS  SQLTSK**S**KCKDLK  GRPQPD**S**GRRRRR  LFIGGL**S**FETTDD  FETTDD**S**LREHFE  DPQTKR**S**RGFGFV  FGFVTY**S**CVEEVD  EPKRAV**S**REDSVK  AVSRED**S**VKPGAH  VMEDRQ**S**GKKRGF  EVKKAL**S**KQEMQS  SKQEMQ**S**AGSQRG  EMQSAG**S**QRGRGG  RGRGGG**S**GNFMGR  GGGGGG**S**RGSYGG  GGGSRG**S**YGGGDG  GGGPGY**S**SRGGYG  GGPGYS**S**RGGYGG  NDFGNY**S**GQQQSN  YSGQQQ**S**NYGPMK  GPMKGG**S**FGGRSS  GSFGGR**S**SGSPYG  SFGGRS**S**GSPYGG  GGRSSG**S**PYGGGY  YGGGYG**S**GGGSGG  YGSGGG**S**GGYGSR  IKMEEE**S**GAPGVP  GAPGVP**S**GNGAPG  FDVKWQ**S**LKDLVK  MDAEGK**S**RGCAVV  EFKMEE**S**MKKAAE  EVLNKH**S**LSGRPL  LNKHSL**S**GRPLKV  MITIPP**S**ILNNPN  QAGRLG**S**TVFVAN  KLKEVF**S**MAGVVV  EDKDGK**S**RGIGTV  TVTFEQ**S**IEAVQA  EAVQAI**S**MFNGQL  NMGRFG**S**GMNMGR  RINEIL**S**NALKRG  GGGGGG**S**VPGIER  GMDRVG**S**EIERMG  VMDRMG**S**VERMGS  SVERMG**S**GIERMG  GLDHMA**S**SIERMG  LDHMAS**S**IERMGQ  TMERIG**S**GVERMG  TIERMG**S**GVERMG  IERMGL**S**MERMVP  ERMGAN**S**LERMGL  ERMGAN**S**LERMGP  GGGGGA**S**FDRAIE  RGNFGG**S**FAGSFG  GGSFAG**S**FGGAGG  KMENGK**S**KGCGVV  GVVKFE**S**PEVAER  MNGMKL**S**GREIDV  KNKGKD**S**TEMRRR  LKRRNV**S**SFPDDA  KRRNVS**S**FPDDAT  FPDDAT**S**PLQENR  QGTVNW**S**VDDIVK  IVKGIN**S**SNVENQ  VKGINS**S**NVENQL  AARKLL**S**REKQPP  LIPKFV**S**FLGRTD  LGRTDC**S**PIQFES  SPIQFE**S**AWALTN  ALTNIA**S**GTSEQT  NIASGT**S**EQTKAV  AIPAFI**S**LLASPH  FISLLA**S**PHAHIS  SPHAHI**S**EQAVWA  NIAGDG**S**VFRDLV  LAVPDM**S**SLACGY  AVPDMS**S**LACGYL  NLTWTL**S**NLCRNK  DTCWAI**S**YLTDGP  VKLLGA**S**ELPIVT  ALAVFP**S**LLTNPK  LVPFLV**S**VLSKAD  FLVSVL**S**KADFKT  AVTNYT**S**GGTVEQ  VILDAI**S**NIFQAA  GETEKL**S**IMIEEC  QNHENE**S**VYKASL  ESVYKA**S**LSLIEK  VYKASL**S**LIEKYF  LIEKYF**S**VEEEED  VVPETT**S**EGYTFQ  RSCSLL**S**TEAGAL  GPPQRL**S**FSFGDH  SCWFPP**S**HIFSVE  PPSHIF**S**VEDAST  LFAQHR**S**DLVSGR  EQGECL**S**LAVLDL  ELLKTV**S**YKACLP  KACLPP**S**LRDLIQ  GCLIRR**S**PTGTFL  FLLVGL**S**RPHSSL  LSRPHS**S**LRELLA  VAVTLT**S**CCIPRP  GHSPPT**S**SLVQPQ  SLVQPQ**S**QYQLSQ  SLMSQV**S**YRHLVL  GHLVPA**S**WKLQVV  REGADG**S**PPFIKL  REAQTL**S**LEADKW  GVTMPI**S**ALDPAK  EPVQRP**S**FRAVIR  SDYELL**S**DPTPGA  VKQLQH**S**GPDQQR  YGPGRQ**S**LRLVME  SRLLLY**S**SQICKG  VREPGQ**S**PIFWYA  FWYAPE**S**LSDNIF  LSDNIF**S**RQSDVW  RQSDVW**S**FGVVLY  FTYCDK**S**CSPSAE  YCDKSC**S**PSAEFL  SPQDRP**S**FSALGP  QDRPSF**S**ALGPQL  DMLWSG**S**RGCETH  KKFDRV**S**RLHCES  SRLHCE**S**ESFKMD  LHCESE**S**FKMDLI  FRLVIA**S**TLYEDG  PTDDRP**S**RADQFE  IEGDET**S**TEAATR  EAATRL**S**AYVSYG  RLSAYV**S**YGGLLM  HGFEVD**S**RVYLLM  APPAFE**S**FLLFEG  LGNIIK**S**QLLKDP  QTTPDY**S**PQEAFT  AITDLI**S**ELSLLE  DLISEL**S**LLEERF  NEEDEH**S**VNYKPP  KPPAQK**S**IQEIQE  LDKDDE**S**LRKYKE  LGRVAV**S**ADPNVP  GLTLVC**S**SAPGPL  LTLVCS**S**APGPLE  LTGDLE**S**FKKQSF  ESFKKQ**S**FVLKEG  EYRIKI**S**FRVNRE  VNREIV**S**GMKYIQ  TDYMVG**S**YGPRAE  GMLARG**S**YSIKSR  LARGSY**S**IKSRFT  GSYSIK**S**RFTDDD  DKTDHL**S**WEWNLT  MLQNLD**S**PFQDQL  QLHQLY**S**HSLLPV  QEAALG**S**DDSKAT  YECGRC**S**QDPESL  QQHEIE**S**RILDLR  LILKTG**S**KFTVRT  LQEGNE**S**LTVEVS  SLTVEV**S**IDRNPP  KFNILT**S**NQKTLT  TLVEQR**S**GGSGKG  EQRSGG**S**GKGSNK  GGSGKG**S**NKGPLG  EELHII**S**FTVKYT  LPVVII**S**NMNQLS  SNMNQL**S**IAWASV  PPKAPW**S**LLGPAL  LLGPAL**S**WQFSSY  LNSDQL**S**MLRNKL  RIMGFV**S**RSQERR  LLKKTM**S**GTFLLR  TFLLRF**S**ESSEGG  LLRFSE**S**SEGGIT  EGGITC**S**WVEHQD  DKVLIY**S**VQPYTK  TKEVLQ**S**LPLTEI  HRLIVV**S**NRQVDE  PEPELE**S**LELELG  LESVLE**S**TLEPVI  PTLCMV**S**QTVPEP  VDEVYV**S**RPSHFY  VYVSRP**S**HFYTDG  VPKLYR**S**VIEDVI  ENKLMQ**S**RAVDGF  AVDGFH**S**EEQQLL  QVLIPA**S**QQATAP  QVIVPD**S**KLIQHM  IQHMNA**S**NMSAAA  MNASNM**S**AAATAA  QQILTN**S**GQLLQV  IFQPQQ**S**VVLQQQ  PLPGGI**S**PQTGVI  YDKIHR**S**KNKWKF  GRDYIF**S**KAIGDA  RKASAH**S**IVECDP  QIDVYR**S**VVCPIL  EFSVKV**S**LLEIYN  TLMNAY**S**SRSHSV  YSSRSH**S**VFSVTI  GGRTRT**S**IIATIS  SIIATI**S**PASLNL  ATISPA**S**LNLEET  KNGVYI**S**EENFRV  ELDQCK**S**DLQNKT  ITSALE**S**TEEKLH  ETTKDV**S**GLHSKL  ELIKDG**S**SKQKAM  LIKDGS**S**KQKAML  LFGNLL**S**SSVSAL  ALGSLT**S**IPENVS  QSLAAE**S**KTVLQE  LSPTVV**S**ILKINS  SILKIN**S**QLKHIF  NIQKPL**S**SVQENI  IQKPLS**S**VQENIQ  ADSDGF**S**QELRNF  TKLVEE**S**VKHSDK  SEQWVS**S**LNEREQ  NLLEVV**S**QCCEAS  SQCCEA**S**SSDITE  QCCEAS**S**SDITEK  CCEASS**S**DITEKS  KSYLYP**S**TLVRTE  LMMLNC**S**ENNKEE  EHLVTK**S**RLPLRA  QLLHNL**S**PGSIDL  HNLSPG**S**IDLKDI  DFGKVI**S**AVSRLS  KVISAV**S**RLSLHS  IEKNYM**S**PLRLVL  LIKVHH**S**FLRAID  IYGEYC**S**HMEHAQ  KELLSH**S**AERPER  KISEFQ**S**SIENLQ  ISEFQS**S**IENLQV  NKDVKK**S**HGKMWS  QFEMAM**S**NIKPDK  ANANHH**S**FQMYTF  PPCKFT**S**PADLDA  LRGDPE**S**PWWEGR  LVQTRK**S**GYFPSS  KSGYFP**S**SSVKPC  SGYFPS**S**SVKPCP  GYFPSS**S**VKPCPV  TDNLLK**S**HASGTY  EAKKFD**S**LLELVE  EYYQCH**S**LKESFK  LKYPYK**S**RERSAS  SRERSA**S**RASSRS  RSASRA**S**SRSPAS  SASRAS**S**RSPASC  SPASCA**S**YNFSFL  CASYNF**S**FLSPQG  YNFSFL**S**PQGLSF  QGLSFA**S**QGPSAP  PSAPFW**S**VFTPRV  DVVRIY**S**RIGGDQ  RIGWFP**S**TYVEEE  DFDDRV**S**DEEKVR  HGDLGN**S**RFLDPR  DPRNKI**S**FKFDHL  HLRKEA**S**DPQPEE  ADGGLK**S**WRESCD  LKSWRE**S**CDSALR  WRESCD**S**ALRAYV  YVKDHY**S**NGFCTV  IIACIE**S**HQFQPK  WNGRWR**S**EWKFTI  GNVQLV**S**HKDVQD  HKDVQD**S**LTVSNE  QDSLTV**S**NEAQTA  EYQTAI**S**ENYQTM  ENYQTM**S**DTTFKA  DWNKIL**S**YKIGKE  EGAGQP**S**TSAQGQ  AGQPST**S**AQGQPA  EPTGEP**S**PKRPRG  RGRPKG**S**KNKSPS  KGSKNK**S**PSKAAQ  SKNKSP**S**KAAQKK  EETEET**S**SQESAE  GPAPEF**S**ASPPAC  APEFSA**S**PPACLY  GALEQG**S**PPDISP  GSPPDI**S**PYEVPP  PFPWMK**S**TKAHAW  LFNKYI**S**RPRRVE  QDCAVT**S**GEELLA  RLPPGL**S**ASPQPS  SASPQP**S**SVAPRR  ASPQPS**S**VAPRRP  VLVDPR**S**RRRYVR  AKCFEI**S**DADTKE  GKIVPK**S**LLLKPH  HQREKM**S**MEISIH  EISIHR**S**LAHQHV  IAPEVL**S**KKGHSF  LSKKGH**S**FEVDVW  FEVDVW**S**IGCIMY  KPPFET**S**CLKETY  INPVAA**S**LIQKML  NDEFFT**S**GYIPAR  TIPPRF**S**IAPSSL  FSIAPS**S**LDPSNR  MLQQLH**S**VNASKP  LHSVNA**S**KPSERG  VNASKP**S**ERGLVR  IPIFWV**S**KWVDYS  SKWVDY**S**DKYGLG  YQLCDN**S**VGVLFN  GVLFND**S**TRLILY  ESYLTV**S**SHPNSL  SYLTVS**S**HPNSLM  YFRNYM**S**EHLLKA  TWFRTR**S**AIILHL  AIILHL**S**NGSVQI  LHLSNG**S**VQINFF  FRTYRL**S**LLEEYG  CCKELA**S**RLRYAR  MVDKLL**S**SRSASN  VDKLLS**S**RSASNR  KLLSSR**S**ASNRLK  TGEELG**S**GQFAVV  KKCREK**S**TGLQYA  KKRRTK**S**SRRGVS  KRRTKS**S**RRGVSR  SSRRGV**S**REDIER  ITYILL**S**GASPFL  ETLANV**S**AVNYEF  FEDEYF**S**NTSALA  RSFLSR**S**NMSVAR  LSRSNM**S**VARSDD  LQHLLG**S**LSNYDV  LDVKDK**S**GEMALH  NCNLDI**S**NKYGRT  AEDLAR**S**EQHEHV  LFGHSG**S**GKTTLV  KTTLVE**S**LKCGLL  KCGLLR**S**FFRRRR  KPTVSV**S**INNLYP  THHPHC**S**ADDQST  NGVGDF**S**VWEFSG  FSVWEF**S**GNPVYF  GYDKDT**S**LLKEIR  GNDLHI**S**NKLFVL  HLQEIR**S**QIVSVC  QRLVPD**S**DVEELL  LLDSVC**S**TIENVM  TVKHYL**S**PQQLRE  QTLKET**S**LTNTMG  DVYSQA**S**LGMDIH  QEAYAS**S**CNSGTS  YASSCN**S**GTSYNS  TLDGVK**S**WLKPRL  EPFVPH**S**QAEEFY  KKEILA**S**FISGLF  ILASFI**S**GLFNFY  ADLDAK**S**GASLKL  DAKSGA**S**LKLTLL  VAGGGA**S**VVYSDT  ANYGEY**S**GAPSEQ  EYSGAP**S**EQQTYD  YAKTIL**S**LMTREK  NFLLNA**S**GSTSTP  LLNASG**S**TSTPAP  NASGST**S**TPAPSR  TSTPAP**S**RTASFS  APSRTA**S**FSESRA  TASFSE**S**RADEVA  PQDSVP**S**PRSLQG  CSRDEP**S**VAAMVY  LDNILA**S**KLYRPG  KLYRPG**S**VAYVSR  GSVAYV**S**RSGGMS  SRSGGM**S**NELNNI  ELNNII**S**RTTDGV  GDRYPG**S**TFMDHV  CATMFS**S**EVQFGH  ACANQA**S**ETAVAK  GVFVPR**S**FDELGE  PASFMT**S**ICDERG  KRLPKY**S**CQFIEM  AGKDLV**S**SLTSGL  GKDLVS**S**LTSGLL  LVSSLT**S**GLLTIG  VEKITT**S**KKPNLI  RMGEEG**S**ELPVCA  FRCCDC**S**ASLSHQ  CCDCSA**S**LSHQYY  DCSASL**S**HQYYEK  WARYGE**S**CHGCSE  YTLVEH**S**KLYCGH  EQILPD**S**PGSHLP  LPDSPG**S**HLPHTV  HTVTLV**S**IPASSH  VSIPAS**S**HGKRGL  KRGLSV**S**IDPPHG  SPDVKN**S**IHVGDR  LLIQET**S**RLLQLT  PETSPL**S**SPAYTP  ETSPLS**S**PAYTPS  SPAYTP**S**GEAGSS  PSGEAG**S**SARQKP  QKPVLR**S**CSIDRS  PVLRSC**S**IDRSPG  SLGSPA**S**QRKDLG  RKDLGR**S**ESLRVV  LRGIIK**S**MDSQYP  IIKSMD**S**QYPWSQ  DSQYPW**S**QRVSFA  PWSQRV**S**FAKDIA  FAKDIA**S**GMAYLH  GMAYLH**S**MNIIHR  IHRDLN**S**HNCLVR  QPEGLR**S**LKKPDR  EKVDVF**S**FGIVLC  TYRRGE**S**GLPAHP  SQAAKA**S**ASATVA  AAKASA**S**ATVAVN  APPAGT**S**PSPGTT  PAGTSP**S**PGTTLA  TTVPIT**S**AKAAEL  GRRAEF**S**GECSNL  EFSGEC**S**NLADRG  GFDRVR**S**IIVSAG  VRSIIV**S**AGPWVA  WVAFEQ**S**NFRGEM  PRWNTW**S**SSYRSD  RWNTWS**S**SYRSDR  WNTWSS**S**YRSDRL  WSSSYR**S**DRLMSF  RSDRLM**S**FRPIKM  AQEHKI**S**LFEGAN  QGDDAP**S**LWVYGF  LWVYGF**S**DRVGSV  FSDRVG**S**VKVSSG  VGSVKV**S**SGTWVG  GSVKVS**S**GTWVGY  FQPQMQ**S**LRRLRD  QWHLEG**S**FPVLAT  HFLYYC**S**EPTLDV  DKQVDV**S**YIAKHY  AKHYNM**S**KSKVDN  HYNMSK**S**KVDNQF  VDNQFY**S**VEVGDS  NLKPIG**S**GAQGIV  VAIKKL**S**RPFQNQ  NHKNII**S**LLNVFT  LDHERM**S**YLLYQM  GIKHLH**S**AGIIHR  SNIVVK**S**DCTLKI  ARTAGT**S**FMMTPY  ENVDIW**S**VGCIMG  PKLFPD**S**LFPADS  SLFPAD**S**EHNKLK  HNKLKA**S**QARDLL  QARDLL**S**KMLVID  DPAKRI**S**VDDALQ  YKEVMN**S**EEKTKN  VVKGQP**S**PSGAAV  KGQPSP**S**GAAVNS  SGAAVN**S**SESLPP  GAAVNS**S**ESLPPS  SESLPP**S**SSVNDI  ESLPPS**S**SVNDIS  SLPPSS**S**VNDISS  SSVNDI**S**SMSTDQ  SVNDIS**S**MSTDQT  NDISSM**S**TDQTLA  TDQTLA**S**DTDSSL  LASDTD**S**SLEASA  ASDTDS**S**LEASAG  DSSLEA**S**AGPLGC  YKDYIQ**S**ERNLEF  LFQHQN**S**SVISRL  FQHQNS**S**VISRLH  CVDANN**S**RASEIN  KKVAND**S**FLIGGL  KQFSKS**S**RAAHQD  GQPEEL**S**EAENQF  YTTCFS**S**RNFLNE  VGSGTT**S**VTSNNE  TSSEDH**S**NRNSDS  HGPLDM**S**NKMFSA  TKVEEI**S**KAGEYV  VKSKPV**S**DSSSAP  PVSDSS**S**APAFEN  KPVSAN**S**PKPACE  GQPKGV**S**SNSPKP  TQILEG**S**LGISVK  DEHCGN**S**NNKCEV  MVAIQV**S**WNIIHQ  ERQGEI**S**RIEKEH  IQERLK**S**LKKKIK  KEDYEE**S**HQRAVA  AVAAEV**S**VLENWK  LSKVQI**S**ELSFPA  ELLPES**S**GDDGQG  AARSSQ**S**PKKPFN  RSKNKN**S**LSGLSI  RTYEPS**S**ATPVTR  SSQGSP**S**VVVAPS  QWLKGQ**S**ACPACQ  SGRGWP**S**QNQELP  ELVSSS**S**SGSDSD  QKTGET**S**RALSSS  ETSRAL**S**SSKQSS  TSRALS**S**SKQSSS  SRALSS**S**KQSSSS  LSSSKQ**S**SSSRDD  SSSKQS**S**SSRDDN  SSKQSS**S**SRDDNM  SKQSSS**S**RDDNMF  GKMRYV**S**VRDFKG  PGRKGI**S**LNPEQW  LNPEQW**S**QLKEQI  TLYIAE**S**RLSWLD  IAESRL**S**WLDGSG  LSWLDG**S**GLGFSL  GSGLGF**S**LEYPTI  LEYPTI**S**LHALSR  ISLHAL**S**RDRSDC  ALSRDR**S**DCLGEH  AKFEEE**S**KEPVAD  EFRFVP**S**DKSALE  FVPSDK**S**ALEAMF  TYEEGL**S**HLTAEG  RLEGML**S**QSVSSQ  EGMLSQ**S**VSSQYN  MLSQSV**S**SQYNMA  LSQSVS**S**QYNMAG  GVRTED**S**IRDYED  GGQGQD**S**GKVVIW  VNCVRW**S**NSGMYL  STVFGS**S**GKLANV  EQWRCV**S**ILRNHS  VGKYIA**S**QADDRS  DWQLET**S**ITKPFD  VLRLSW**S**PDGHYL  KQKNGS**S**AKPSCP  GSSAKP**S**CPYCCC  CCCAVG**S**KDRSLS  KSRIHQ**S**TYGKSL  QQLDQK**S**AATREM  FNSIPL**S**GSLAGT  SIPLSG**S**LAGTML  LAGTML**S**SHSSPQ  AGTMLS**S**HSSPQL  TMLSSH**S**SPQLLP  ARPAGD**S**VNKDSM  PLAKAS**S**LSKRKL  AKASSL**S**KRKLEL  SRLMPV**S**LSVQSP  LMPVSL**S**VQSPAA  AFTLQV**S**SDPSMY  QVSSDP**S**MYIEVE  HSILAG**S**DMTVSQ  IPVMNL**S**DGKAYC  STWNLV**S**DKQDSL  VSDKQD**S**LAQCAD  CADFRS**S**LPSQDA  IIQGRT**S**NSGRQA  QGRTSN**S**GRQAAR  HGPAGT**S**VELGLQ  GLHKAL**S**TGLDYS  PPSAPR**S**VPVATT  TTPSQR**S**QLEAYS  ANMGSL**S**QTPGHK  QYVHIS**S**SPQNTG  YVHISS**S**PQNTGR  NTGRTA**S**PPAIPV  QYADSG**S**HFVPRE  TKKAES**S**RLQQAI  RYGAPS**S**ADLGLG  VPHPYE**S**RHVVVH  SPSDYS**S**RDPSGV  YSSRDP**S**GVRASV  PSGVRA**S**VMVLPN  HREASP**S**TLNDKS  GKPGHR**S**YALSPH  HRSYAL**S**PHTVIQ  PVIGYL**S**GQQQAI  LLIPVG**S**TDMEAS  STDMEA**S**GAAPAI  TTALPK**S**ENFNPE  PYFMKG**S**IIQLAN  NDLKID**S**STVERI  DLKIDS**S**TVERIE  RIEDSH**S**PGVAVI  LFDLPC**S**KLSVGD  VGDVCI**S**LTLKNL  KNLKNG**S**VKKGQP  NGINQG**S**AQMLSE  SRKLEK**S**EDEPPL  QNKDSM**S**MRSGRK  DGKAEK**S**RQTAKK  ARVEEA**S**TPKVNK  VNKQGR**S**EEISES  DSSSGL**S**QGPARP  SPQPPD**S**TPRQPE  GTGGVL**S**GPPMGP  PISVSS**S**GASGAP  GPTLAP**S**PHSLPP  SLPPAS**S**SAPAPP  PMRFPY**S**SSSSSS  RFPYSS**S**SSSSAA  SSSSSS**S**SSSASP  SSSSSS**S**SASPFP  ASQALP**S**YPHSFP  PPPTSL**S**VSNQPP  PKYTQP**S**LPSQAV  PYAMSP**S**LGSLRP  PPHSQV**S**YSQAGP  SSNSSS**S**TSQGSY  SSTSQG**S**YPCSHP  YPCSHP**S**PSQGPQ  PGYRGT**S**PPAGPG  ASGPPL**S**ATQIKQ  LDRGFN**S**CARSDL  ARPHVM**S**PGNRNH  PFPFHP**S**LGPLER  IHAASA**S**VHPLID  YRDLPA**S**LSAPMS  DLPASL**S**APMSAA  QAMHAQ**S**AELQRL  LDFLCL**S**LCRAFR  AFRDGR**S**EDFRRT  FRRTRN**S**AEAIIH  AIIHGL**S**SLTACQ  IIHGLS**S**LTACQL  RITPLE**S**ALMIWG  ALMIWG**S**IEKEHD  SHMPFK**S**KLLMII  QKDTFH**S**FFQHFS  SFFQHF**S**YNHMME  MMEKIK**S**YVNYVL  YVNYVL**S**EKSSTF  VLSEKS**S**TFLMKA  AAKVVE**S**KRTRTI  RTRTIT**S**QDKPSG  TSQDKP**S**GNDVEM  NLDTRK**S**VSDKQS  DTRKSV**S**DKQSAV  SVSDKQ**S**AVTESS  QSAVTE**S**SEGTVS  SAVTES**S**EGTVSL  TVSLLR**S**HKNLFL  HKNLFL**S**KLQHGT  RVGTPQ**S**TKKKKE  TKKKKE**S**RRATES  SRRATE**S**RIPVSK  ESRIPV**S**KSQPVT  RIPVSK**S**QPVTPE  EDKNLR**S**GVRKYG  YGEGNW**S**KILLHY  KFNNRT**S**VMLKDR  AYFVPM**S**KIADFL  LLELRV**S**YYENVI  IKAMLE**S**IGVPLE  GTDYQL**S**KEYTLD  LDVYRL**S**SVVTQH  DVYRLS**S**VVTQHD  VVTQHD**S**KKAGAE  VEHPLL**S**GLLYPG  LPALGY**S**KRVHLM  VPGLTG**S**KMSSSE  LTGSKM**S**SSEEES  TGSKMS**S**SEEESK  GSKMSS**S**EEESKI  SSSEEE**S**KIDLLD  ENNGVL**S**FIKHVL  VLFPLK**S**EFVILR  PGDLKN**S**VEVALN  ALKKLA**S**AAYPDP  AAYPDP**S**KQKPMA  KGPAKN**S**EPEEVI  PEEVIP**S**RLDIRV  KHPDAD**S**LYVEKI  EPRTVV**S**GLVQFV  KMRGVE**S**QGMLLC  GMLLCA**S**IEGINR  LDPPAG**S**APGEHV  QADFKI**S**EECIAQ  FMTKLG**S**ISCKSL  TKLGSI**S**CKSLKG  NRQKIR**S**LDVVGK  MVMEYV**S**GGELFD  GLSNMM**S**DGEFLR  GEFLRT**S**CGSPNY  LRTSCG**S**PNYAAP  PEVDIW**S**CGVILY  PEYLNR**S**VATLLM  FKQDLP**S**YLFPED  LFPEDP**S**YDANVI  KFECTE**S**EVMNSL  ESEVMN**S**LYSGDP  VMNSLY**S**GDPQDQ  SEFYLA**S**SPPSGS  EFYLAS**S**PPSGSF  SSPPSG**S**FMDDSA  GSFMDD**S**AMHIPP  PPLIAD**S**PKARCP  WHLGIR**S**QSKPYD  YLVDNR**S**YLLDFK  EVVEQR**S**GSSTPQ  VEQRSG**S**STPQRS  EQRSGS**S**TPQRSC  SSTPQR**S**CSAAGL  TPQRSC**S**AAGLHR  GLHRPR**S**SFDSTT  LHRPRS**S**FDSTTA  DSTTAE**S**HSLSGS  AESHSL**S**GSLTGS  TGSLTG**S**TLSSVS  LTGSTL**S**SVSPRL  TGSTLS**S**VSPRLG  STLSSV**S**PRLGSH  VSPRLG**S**HTMDFF  SGWEEV**S**GYDEAM  CNVRES**S**QNNWLR  SDVASA**S**SPFWME  APDESF**S**RLDAGR  VNTKVR**S**FGPLSK  RSFGPL**S**KAGFYL  TGAEPT**S**LVIAPG  LPCPPN**S**RTTSPA  YRADSD**S**ADSACT  DSDSAD**S**ACTTVP  ACTTVP**S**PPRGVI  ERRVHI**S**HLLAHT  NGVSGK**S**PLPPRY  LHSSSG**S**SLTLSW  HSSSGS**S**LTLSWA  GSSLTL**S**WAPPER  IASTVT**S**QMNSVQ  AEFETT**S**ERGSGA  TTSERG**S**GAQQLQ  AKEIDV**S**CVKIEE  EGVVTK**S**RPVMIL  ENCALD**S**FLRLND  AGMKYL**S**EMNYVH  NLVCKV**S**DFGLSR  DPTYTS**S**LGGKIP  RKFTSA**S**DVWSYG  RPYWDM**S**NQDVIN  LIRNAA**S**LKVIAS  SLKVIA**S**AQSGMS  SAQSGM**S**QPLLDR  YKESFV**S**AGFASF  VSAGFA**S**FDLVAQ  HQKKIL**S**SIQDMR  ALDMCC**S**ERLPGL  AEGLQD**S**QREMPP  PPPPPP**S**PPSDPA  PPPSPP**S**DPAQKP  PPRGAG**S**HSLTVR  RGAGSH**S**LTVRSS  HSLTVR**S**SLCLFA  SLTVRS**S**LCLFAA  CGVLWF**S**GYGHIW  GYGHIW**S**QNATNL  NATNLV**S**SLLTLL  ATNLVS**S**LLTLLK  PTAWLD**S**GTWGVP  LLLVFL**S**VGLVLV  PEDRRQ**S**VSRQPS  SVSRQP**S**FTYSEW  QPSFTY**S**EWMEEK  PEADPT**S**LTVKSM  TSLTVK**S**MGLQER  RRGSNV**S**LTLDMC  MSPREE**S**AREYLL  AREYLL**S**ASRVLQ  EYLLSA**S**RVLQAE  ILPNPH**S**RVCLTS  SRVCLT**S**PDPDDP  DPDDPL**S**SYINAN  PDDPLS**S**YINANY  TQGPIV**S**TVADFW  RLISLK**S**GTEERG  PIIVHC**S**AGIGRT  FVHHVM**S**LYEKQL  AEKFLE**S**VEGNQN  LTLLEK**S**QDNVIK  VIKVCA**S**VTFKNY  IVHLML**S**SPEQIQ  QIQKQL**S**DAISII  VLRTAH**S**LFKRYR  YRHEFK**S**NELWTE  ATIELC**S**THANDA  THANDA**S**ALRILF  LRILFS**S**LILISK  ISKLFY**S**LNFQDL  KYDLLV**S**NAIQFL  DQNTLT**S**ICEKVI  EAFEDN**S**EEYIRR  RRDLEG**S**DIDTRR  PVTGIF**S**GYVNSM  EYAKNP**S**VNWKHK  LVTSLA**S**KAQTQK  ILPDLK**S**ANVNEF  NHLQAE**S**IVVHTY  ALTLPG**S**SENEYI  MKAIMR**S**FSLLQE  AVSKNP**S**KPHFNH  HKNDIP**S**SYMALF  AFLERG**S**NTIASA  FQKLIA**S**KANDHQ  EHMPPE**S**VDQYRK  FQRLQN**S**KTTKFI  TTKFIK**S**FLVFIN  GYQTAF**S**QLAFAG  KIHLAQ**S**LHKLST  QSLHKL**S**TACPGR  CPGRVP**S**MVSTSL  SKGDDL**S**TAILKQ  AINEDN**S**VVSLSQ  EDNSVV**S**LSQPKM  NSVVSL**S**QPKMDE  AVCIVL**S**DDTCSD  LSDDTC**S**DEKIRM  RLGDVI**S**IQPCPD  VVETDP**S**PYCIVA  REDEEE**S**LNEVGY  NGPEIM**S**KLAGES  SKLAGE**S**ESNLRK  VERRIV**S**QLLTLM  ATNRPN**S**IDPALR  DLAALC**S**EAALQA  DAEVMN**S**LAVTMD  DFRWAL**S**QSNPSA  RWALSQ**S**NPSALR  LSQSNP**S**ALRETV  KFGMTP**S**KGVLFY  CQANFI**S**IKGPEL  TMWFGE**S**EANVRE  FFDELD**S**IAKARG  PLPDEK**S**RVAILK  KANLRK**S**PVAKDV  KMTNGF**S**GADLTE  IRESIE**S**EIRRER  FARRSV**S**DNDIRK  AQTLQQ**S**RGFGSF  QSRGFG**S**FRFPSG  GSFRFP**S**GNQGGA  QGGAGP**S**QGSGGG  AGPSQG**S**GGGTGG  GGGTGG**S**VYTEDN  LWDALL**S**RELFRP  DIQRAG**S**GSRRDQ  QRAGSG**S**RRDQAR  DLETRG**S**QALPLF  ALPLFI**S**CLEDTG  GQDMLA**S**FLRTNR  RQAAKL**S**KPTLEN  RPVDIG**S**GGFGDV  DVGALE**S**LRGNAD  DLAYIL**S**MEPCGH  VNFCRE**S**GLRTRT  LRTRTG**S**NIDCEK  KLRRRF**S**SLHFMV  LRRRFS**S**LHFMVE  CVVVIL**S**HGCQAS  SHGCQA**S**HLQFPG  TDGCPV**S**VEKIVN  NIFNGT**S**CPSLGG  NGTSCP**S**LGGKPK  HGFEVA**S**TSPEDE  TSPEDE**S**PGSNPE  EDESPG**S**NPEPDA  DQLDAI**S**SLPTPS  QLDAIS**S**LPTPSD  SSLPTP**S**DIFVSY  PSDIFV**S**YSTFPG  DIFVSY**S**TFPGFV  TFPGFV**S**WRDPKS  SWRDPK**S**GSWYVE  RDPKSG**S**WYVETL  FEQWAH**S**EDLQSL  HSEDLQ**S**LLLRVA  RVANAV**S**VKGIYK  NLTRRF**S**DLGFEV  LKIHEV**S**TVSHAD  HEVSTV**S**HADADC  FVCVFL**S**HGEGNH  KGDKCH**S**LVGKPK  TEVDAA**S**VYTLPA  DFLMCY**S**VAEGYY  VAEGYY**S**HRETVN  RETVNG**S**WYIQDL  MLGKYG**S**SLEFTE  LGKYGS**S**LEFTEL  DFCKDP**S**AIGKKQ  QVPCFA**S**MLTKKL  NPLLDI**S**AVVDKD  DFLDKY**S**LKPNDQ  EYHAGG**S**TQNSIK  GGSTQN**S**IKVAQW  ITGDNR**S**LIANLA  GFFLTV**S**PESVLK  LTVSPE**S**VLKVAH  KVAHHA**S**ENNRIF  IFTLNL**S**APFISQ  LSAPFI**S**QFYKES  SQFYKE**S**LMKVMP  ALPKMN**S**KRQRIV  TIMATE**S**EVTAFA  FVGGFL**S**QLVSDK  FLSQLV**S**DKPLTE  AGHYAA**S**IIIRRT  ADRLTA**S**STCGLN  QPYCIV**S**HLQDEK  ARDNPH**S**HRIQNV  AMLVER**S**ADFGRT  VCESRY**S**EIEPST  IPDPYS**S**RIQNLL  LASGNV**S**GGVCDG  DPAVCR**S**CDCDPM  DGGRCD**S**HDDPAL  TPSWTG**S**GFVRLQ  CAPLLI**S**LSTLIY  PLLISL**S**TLIYNG  SHEGAL**S**SLCEKT  HEGALS**S**LCEKTS  GQWGFP**S**CRPCVC  CHQDEY**S**QQIVCH  GHFGDP**S**RPGGRC  NPQQCP**S**PDQCHC  CHCDPS**S**GQCPCL  NFWNLT**S**GHGCQP  PCACHP**S**RARGPT  FGGRTC**S**ECQELH  NANHAL**S**GLERDR  SIRHAH**S**QSAEAE  ERRANT**S**ALAVPS  AVPSPV**S**NSASAR  PRCGGL**S**CNGAAA  EGGSIL**S**RVAETR  IAERVR**S**LADVDA  GAERAL**S**SAGERA  AERALS**S**AGERAR  RAGDRL**S**GAAARG  QVMMFG**S**TAIALE  LLKQGA**S**PNVQDT  PNVQDT**S**GTSPVH  QDTSGT**S**PVHDAA  GHTAVV**S**FLAAES  SFLAAE**S**DLHRRD  INTVDT**S**HEDMIH  TRLATC**S**SDRSVK  RLATCS**S**DRSVKI  TCSSDR**S**VKIFDV  YGNILA**S**CSYDRK  NILASC**S**YDRKVI  NGTWEK**S**HEHAGH  EHAGHD**S**SVNSVC  HAGHDS**S**VNSVCW  HDSSVN**S**VCWAPH  LILACG**S**SDGAIS  ILACGS**S**DGAISL  SSDGAI**S**LLTYTG  IGCNAV**S**WAPAVV  PAVVPG**S**LIDHPS  SLIDHP**S**GQKPNY  YIKRFA**S**GGCDNL  QKLEAH**S**DWVRDV  DVAWAP**S**IGLPTS  SIGLPT**S**TIASCS  PTSTIA**S**CSQDGR  STIASC**S**QDGRVF  WTCDDA**S**SNTWSP  TCDDAS**S**NTWSPK  ASSNTW**S**PKLLHK  DVVWHV**S**WSITAN  VWHVSW**S**ITANIL  ANILAV**S**GGDNKV  VTLWKE**S**VDGQWV  GQWVCI**S**DVNKGQ  VNKGQG**S**VSASVT  KGQGSV**S**ASVTEG  QGSVSA**S**VTEGQQ  LDLVQQ**S**CNYKQL  TLNRGI**S**EFIVMA  PYVFVR**S**KQALGR  GRACGV**S**RPVIAC  RPVIAC**S**VTIKEG  VTIKEG**S**QLKQQI  LKQQIQ**S**IQQSIE  VRGLPW**S**CSADEV  GLPWSC**S**ADEVMR  KIQNGT**S**GIRFIY  AFVELE**S**EEEVKL  EVFKSN**S**VEMDWV  GLPFGC**S**KEEIVQ  EIVQFF**S**GLEIVP  VDFQGR**S**TGEAFV  AFVQFA**S**QEIAEK  YIEIFK**S**SRAEVR  IEIFKS**S**RAEVRT  DGYGFG**S**DRFGRD  DLNYCF**S**GMSDHR  YCFSGM**S**DHRYGD  RYGDGG**S**SFQSTT  YGDGGS**S**FQSTTG  GGSSFQ**S**TTGHCV  DIYNFF**S**PLNPMR  VELFLN**S**TAGTSG  NSTAGT**S**GGAYDH  GGAYDH**S**YVELFL  VELFLN**S**TAGASG  NSTAGA**S**GGAYGS  SGGAYG**S**QMMGGM  MGGMGL**S**NQSSYG  MGLSNQ**S**SYGGPA  GLSNQS**S**YGGPAS  SYGGPA**S**QQLSGG  PASQQL**S**GGYGGG  GGYGGQ**S**SMSGYD  GYGGQS**S**MSGYDQ  GGQSSM**S**GYDQVL  QVLQEN**S**SDYQSN  AAGTEA**S**SEEVGI  AGTEAS**S**EEVGIA  AEAGPE**S**EVRTEP  AEELPG**S**HAEPPV  EADGID**S**VIVVDN  VIHKIF**S**KFGKIT  DKYMTI**S**DEWDIP  ECRDQY**S**VIFESG  DVKDPV**S**IEERAR  ETYVRW**S**PKGTYL  VQLIDF**S**PCERYL  RYLVTF**S**PLMDTQ  GFHCES**S**AHWPIF  MTLDTL**S**IYETPS  GLLDKK**S**LKISGI  KKSLKI**S**GIKDFS  SGIKDF**S**WSPGGN  IKDFSW**S**PGGNII  AWEPNG**S**KFAVLH  APRISV**S**FYHVKN  ANTIFW**S**PQGQFV  LAFVDT**S**DCTVMN  AEHYMA**S**DVEWDP  YVVTSV**S**WWSHKV  TSVSWW**S**HKVDNA  RPPTLL**S**QEQIKQ  KDLKKY**S**KIFEQK  EQKDRL**S**QSKASK  KDRLSQ**S**KASKEL  LSQSKA**S**KELVER  DTDELD**S**NVDDWE  LVAIGG**S**ENFYSV  GSENFY**S**VFEGEL  VFEGEL**S**DTIPVV  IPVVHA**S**IAGCRI  LQHIRN**S**LPDTVQ  RVEERL**S**ALGNVT  DQVLVG**S**YCVFSN  GSYCVF**S**NQGGLV  GTVNRG**S**EVIAAG  CGLDTT**S**TELSVV  TTSTEL**S**VVESVF  ELSVVE**S**VFKLNE  LNEAQP**S**TIATSM  IRLPLR**S**GLGGAP  EPGRRG**S**FVEMVD  DNLRGK**S**GQGYYV  VEMTVG**S**PPQTLN  ILVDTG**S**SNFAVG  LVDTGS**S**NFAVGA  YYQRQL**S**STYRDL  YQRQLS**S**TYRDLR  LGTDLV**S**IPHGPN  IAAITE**S**DKFFIN  KFFING**S**NWEGIL  IARPDD**S**LEPFFD  LEPFFD**S**LVKQTH  HVPNLF**S**LQLCGA  GFPLNQ**S**EVLASV  QSEVLA**S**VGGSMI  LASVGG**S**MIIGGI  IGGIDH**S**LYTGSL  HSLYTG**S**LWYTPI  EYNYDK**S**IVDSGT  DKSIVD**S**GTTNLR  FEAAVK**S**IKAASS  KSIKAA**S**STEKFP  SIKAAS**S**TEKFPD  NIFPVI**S**LYLMGE  GEVTNQ**S**FRITIL  VEDVAT**S**QDDCYK  CYKFAI**S**QSSTGT  KFAISQ**S**STGTVM  FAISQS**S**TGTVMG  RIGFAV**S**ACHVHD  IPQTDE**S**TLMTIA  YDNVAE**S**PDELSF  ESPDEL**S**FRKGDI  YQPQPD**S**VYLVPT  YLVPTP**S**KAQQGL  PHHPFP**S**PATDLY  IYQVPP**S**AGMGHD  PSMDTR**S**WEGTKP  VRGLLP**S**QYGQEV  EKGLPP**S**NHHAVY  AAPPPD**S**PPAEDV  GKRLSA**S**STGSTR  KRLSAS**S**TGSTRS  SASSTG**S**TRSSQS  TGSTRS**S**QSASSL  STRSSQ**S**ASSLEV  RLQQGV**S**ATVAHL  SAGATG**S**WRSPSE  ATGSWR**S**PSEPQE  GSWRSP**S**EPQEPL  AVAAVQ**S**AVHELL  GNAAHT**S**DRALHA  DRLVAC**S**RAVPED  NPTDKT**S**SIQSRP  SPPKFT**S**QDSPDG  ELLEKG**S**ITRQGK  ITRQGK**S**QLELQQ  RLEQEV**S**RPIDHD  FIGDTL**S**RQAKAA  KAADVR**S**QVTHYS  AALQYP**S**PSAAQD  LQYPSP**S**AAQDMV  KTRLDS**S**NLEKIY  MFDYLT**S**NGHLSE  KFWQIL**S**AVEYCH  FGNFYK**S**GEPLST  KSGEPL**S**TWCGSP  PQLDIW**S**LGVVLY  YVLVCG**S**LPFDGP  RIPFFM**S**QDCESL  PAFSAH**S**YTSNLG  PVDASC**S**GVFRPR  PRPVSP**S**SLLDTA  TQESLP**S**STGRRH  QESLPS**S**TGRRHT  HTLAEV**S**TRLSPL  EVSTRL**S**PLTAPC  CIVVSP**S**TTASPA  DSCLTF**S**ASKSPA  CLTFSA**S**KSPAGL  TFSASK**S**PAGLSG  KSPAGL**S**GTPATQ  GLLGAC**S**PVRLAS  SPVRLA**S**PFLGSQ  ASPFLG**S**QSATPV  AVLLPV**S**FQEGRR  VCQAPA**S**RASRGG  FHAPAQ**S**PGLHGG  HGGAAG**S**REGWSL  AAAPGC**S**QAPQPA  PSTLLT**S**GLPLLP  GASPVA**S**AAQLLD  EDLMPC**S**LGTFVL  LVVRGG**S**RFLATS  SRFLAT**S**IASSDD  LATSIA**S**SDDDSL  ATSIAS**S**DDDSLF  ASSDDD**S**LFIYDC  LFIYDC**S**AAEKKS  SAAEKK**S**QENKGE  APLDQG**S**GAILAS  SGAILA**S**TFSKSG  ILASTF**S**KSGSYF  ASTFSK**S**GSYFAL  TFSKSG**S**YFALTD  FALTDD**S**KRLILF  KPWQCL**S**VRTVAR  ALTFIA**S**EEKVLV  VLVADK**S**GDVYSF  KSGDVY**S**FSVLEP  GDVYSF**S**VLEPHG  WAAAPH**S**IESFCL  GHTEFV**S**RISVVP  EFVSRI**S**VVPTQP  QPGLLL**S**SSGDGT  GLLLSS**S**GDGTLR  RLWEYR**S**GRQLHC  HCCHLA**S**LQELVD  VGDQWQ**S**VPESTV  TVLKKV**S**GVLRGN  WAMLEG**S**AGADAS  SAGADA**S**FSSLYK  GADASF**S**SLYKAT  ADASFS**S**LYKATF  TFDNVT**S**YLKKKE  KKQRRR**S**PPPGPD  FRMLNS**S**FEDDPF  EDDPFF**S**ESILAH  DPFFSE**S**ILAHRE  MRQMIR**S**FSEPFG  FGRDLL**S**ISDGRG  RDLLSI**S**DGRGRA  HNDGED**S**LTHTDV  LTHTDV**S**SFQTMD  THTDVS**S**FQTMDQ  TMDQMV**S**NMRNYM  RNFGQL**S**VDPNGH  VDPNGH**S**FCSSSV  NGHSFC**S**SSVMTY  GHSFCS**S**SVMTYS  HSFCSS**S**VMTYSK  SSVMTY**S**KIGDEP  PKVFQA**S**TQTRRA  RKAMRD**S**DSGLEK  AMRDSD**S**GLEKMA  AHVIKK**S**KNKKTG  FINMNE**S**DAHAFD  FDEEWQ**S**EVLKYK  GNTRMR**S**VGHENP  GHENPG**S**RELKRR  REKPQQ**S**PAIEHG  IEHGRR**S**NVLGDK  IPEVVV**S**LAWDES  SLAWDE**S**LAPKHP  APKHPG**S**RKNMAC  TLRPYL**S**AVRATL  LCLENF**S**SQVVER  CLENFS**S**QVVERH  PEVEVR**S**SKELLL  EVEVRS**S**KELLLQ  LQPVTI**S**RNEKEK  KVLIEG**S**INSVRV  IEGSIN**S**VRVSIA  INSVRV**S**IAVKQA  VEGYDI**S**FLITNF  EIDKEI**S**EMKLSV  ISEMKL**S**VNARAR  PLLEFL**S**VKEIYN  GKLDLL**S**DTNMVD  VYKNLY**S**DDIPHA  TTRQMQ**S**TRDGRM  YECGNY**S**GAAEYL  TDRNAL**S**SLWGKL  DRNALS**S**LWGKLA  LWGKLA**S**EILMQN  ETIDNN**S**VSSPLQ  IDNNSV**S**SPLQSL  DNNSVS**S**PLQSLQ  VSSPLQ**S**LQQRTW  TWLIHW**S**LFVFFN  KVIQQE**S**YTYKDP  KLRECE**S**VLVNDF  RIHQCI**S**INMLAD  LDAKID**S**KLGHVV  MGNNAV**S**PYQQVI  VIEKTK**S**LSFRSQ  EKTKSL**S**FRSQML  KSLSFR**S**QMLAMN  KKLNQN**S**RSEAPN  LNQNSR**S**EAPNWA  SSGRSP**S**KAVAAR  AARAAG**S**TVRQRK  RQRKNA**S**CGTRSA  ASCGTR**S**AGRTTS  SAGRTT**S**AGTGGM  RFYTED**S**PGLKVG  VPVLVM**S**LLFIAS  SLLFIA**S**VFMLHI  IIKEIV**S**RNKRRY  VVRFLD**S**KHKNHY  DLDQWL**S**EDDNHV  KGVTIP**S**QRRYVY  RYVYYY**S**YLLKNH  ETIPMF**S**GGTCNP  LKVKIY**S**SNSGPT  KVKIYS**S**NSGPTR  KIYSSN**S**GPTRRE  PGPEET**S**EKVENG  EKVENG**S**LCDQEI  CDQEID**S**ICSIER  EIDSIC**S**IERADN  KANRYF**S**PNFKVK  KTVEEP**S**NPEASS  PSNPEA**S**SSTSVT  SNPEAS**S**STSVTP  NPEASS**S**TSVTPD  EASSST**S**VTPDVS  IRITLT**S**RNVKSL  TSRNVK**S**LEKVCA  TPCGEG**S**KTWDRF  RLIDLH**S**PSEIVK  IDLHSP**S**EIVKQI  IVKQIT**S**ISIEPG  KQITSI**S**IEPGVE  KTCLLI**S**YTTNKF  TTNKFP**S**EYVPTV  DRLRPL**S**YPQTDV  VFLVCF**S**VVSPSS  VCFSVV**S**PSSFEN  FSVVSP**S**SFENVK  SVVSPS**S**FENVKE  DLRDDP**S**TIEKLA  VKYVEC**S**ALTQKG  GISIYT**S**DNYTEE  YTEEMG**S**GDYDSM  GSGDYD**S**MKEPCF  FLPTIY**S**IIFLTG  YQKKLR**S**MTDKYR  KYRLHL**S**VADLLF  YTVNLY**S**SVLILA  TVNLYS**S**VLILAF  LILAFI**S**LDRYLA  IVHATN**S**QRPRKL  FIFANV**S**EADDRY  PGIVIL**S**CYCIII  CYCIII**S**KLSHSK  IIISKL**S**HSKGHQ  ISKLSH**S**KGHQKR  PYYIGI**S**IDSFIL  IGISID**S**FILLEI  TVHKWI**S**ITEALA  GAKFKT**S**AQHALT  GKRGGH**S**SVSTES  GGHSSV**S**TESESS  SSVSTE**S**ESSSFH  QKKEEE**S**AGGTKG  AGGTKG**S**SKKASA  GGTKGS**S**KKASAA  GSSKKA**S**AAQLRI  PKTCDI**S**FSDPDD  TCDISF**S**DPDDLL  DEGFYK**S**GKFVFS  SGKFVF**S**FKVGQG  PVLTIN**S**IIYGLQ  EQNVQR**S**MRGGYI  RGGYIG**S**TYFERC  FKEVLK**S**EETSKN  LKSEET**S**KNQIKV  IWHPNI**S**SVTGAI  WHPNIS**S**VTGAIC  LRTVLL**S**LQALLA  YAGAPV**S**SPEYTK  AGAPVS**S**PEYTKK  AVIVAL**S**SKSWDV  VIVALS**S**KSWDVE  VALSSK**S**WDVETA  PQFIIP**S**CIAIKE  CIAIKE**S**AKVGDQ  AEIMFE**S**FNVPGL  VLALAA**S**WTSRQV  LAASWT**S**RQVGER  TGTVID**S**GDGVTH  EGYVIG**S**CIKHIP  GIPPEQ**S**LETAKA  AVKERY**S**YVCPDL  KYDTDG**S**KWIKQY  TGINAI**S**KKEFSI  ISKKEF**S**IDVGYE  DFTQPI**S**EVVDEV  YKNIVL**S**GGSTMF  IVLSGG**S**TMFRDF  DARLKL**S**EELSGG  KLSEEL**S**GGRLKP  AVWFGG**S**MLASTP  GGSMLA**S**TPEFYQ  YEEIGP**S**ICRHNP  DDIEVE**S**DEEQPR  EQPRFQ**S**AADKRA  RDHIKD**S**FHSLRD  IKDSFH**S**LRDSVP  FHSLRD**S**VPSLQG  LRDSVP**S**LQGEKA  LQGEKA**S**RAQILD  ALEKAR**S**SAQLQT  LEKARS**S**AQLQTN  LQTNYP**S**SDNSLY  QTNYPS**S**DNSLYT  YPSSDN**S**LYTNAK  YTNAKG**S**TISAFD  AKGSTI**S**AFDGGS  SAFDGG**S**DSSSES  FDGGSD**S**SSESEP  DGGSDS**S**SESEPE  GGSDSS**S**ESEPEE  SDSSSE**S**EPEEPQ  EPEEPQ**S**RKKLRM  VKLKVD**S**FRERIT  KLLELD**S**FLKEPI  DLTQIH**S**DMNLPV  PILLTN**S**HDGLDG  PNGMLK**S**NQQLVD  GNNFGV**S**IQEETV  ELRTVE**S**EAASYL  VESEAA**S**YLDQIS  SYLDQI**S**RYYITR  TRAKLV**S**KIAKYP  DEKEYI**S**LRLIIS  SLRLII**S**ELRNQY  GKAHRL**S**AEERDQ  KVHITL**S**THECAG  HECAGL**S**ERDINL  RDINLA**S**FIEQVA  RVLVER**S**AAETVT  IMLPEK**S**QGKVLQ  TVVAVG**S**GSKGKG  VAVGSG**S**KGKGGE  GEIQPV**S**VKVGDK  AVLALL**S**LSGLEA  LALLSL**S**GLEAIQ  PKIQVY**S**RHPAEN  PAENGK**S**NFLNCY  FLNCYV**S**GFHPSD  VSGFHP**S**DIEVDL  IEKVEH**S**DLSFSK  VEHSDL**S**FSKDWS  HSDLSF**S**KDWSFY  SFSKDW**S**FYLLYY  VNHVTL**S**QPKIVK  SVVSVI**S**RFLEEY  FLEEYL**S**STPQRL  LEEYLS**S**TPQRLK  GTFPFN**S**FLSGFI  PFNSFL**S**GFISCV  FLSGFI**S**CVGSFI  FISCVG**S**FILAVC  ADFQGI**S**PERAFA  ADFLFA**S**TILHLV  ALILEP**S**LYTVKA  YDDTYP**S**VKEQKA  KTHRTD**S**EIALLE  LTVVYK**S**SIDLYF  TVVYKS**S**IDLYFY  YFYVIG**S**SYENEL  FYVIGS**S**YENELM  LNCLFD**S**LSQMLR  CLFDSL**S**QMLRKN  GGVILE**S**DPQQVV  LTEQTV**S**QVLQSA  VSQVLQ**S**AKEQIK  EQAFKR**S**RNTDEM  LRILLQ**S**KNAGAV  DYNASV**S**VPDSSG  SVSVPD**S**SGPERI  VSVPDS**S**GPERIL  GPERIL**S**ISADIE  ERILSI**S**ADIETI  PSPTAT**S**QLPLES  SQLPLE**S**DAVECL  YQHYKG**S**DFDCEL  RLLIHQ**S**LAGGII  QECCPH**S**TDRVVL  GRPMPP**S**RRDYDD  RGGRGG**S**RARNLP  DGMVGF**S**ADETWD  ADETWD**S**AIDTWS  SAIDTW**S**PSEWQM  IDTWSP**S**EWQMAY  YEPQGG**S**GYDYSY  GSGYDY**S**YAGGRG  PKDLAG**S**IIGKGG  KQIRHE**S**GASIKI  RHESGA**S**IKIDEP  DEPLEG**S**EDRIIT  QYLLQN**S**VKQYSG  EKLNLD**S**IIGRLL  LLEVQG**S**RPGKNV  RGLCLK**S**REIFLS  SREIFL**S**QPILLE  GGFPPE**S**NYLFLG  VDRGKQ**S**LETICL  GNHECA**S**INRIYG  CCHGGL**S**PDLQSM  LSPDLQ**S**MEQIRR  LCDLLW**S**DPDKDV  ENDRGV**S**FTFGAE  QLVTLF**S**APNYCG  NAGAMM**S**VDETLM  DETLMC**S**FQILKP  GKYGQF**S**GLNPGG  NPVEGF**S**AGLIDD  NGDVCI**S**ILHEPG  VETIMI**S**VISMLA  IMISVI**S**MLADPN  ADPNGD**S**PANVDA  YDEMVE**S**MKKVAG  EERNLL**S**VAYKNV  IGARRA**S**WRIISS  ASWRII**S**SIEQKE  SWRIIS**S**IEQKEE  AANTGE**S**KVFYYK  KEAAEN**S**LVAYKA  VAYKAA**S**DIAMTE  GLALNF**S**VFYYEI  YYEILN**S**PDRACR  AELDTL**S**EESYKD  DTLSEE**S**YKDSTL  EESYKD**S**TLIMQL  NLTLWT**S**DMQGDG  TARKLR**S**HRRDQK  NPFGGA**S**HAKGIV  EAKQPN**S**AIRKCV  VKVANV**S**LLALYK  VKDGKY**S**QVLANG  PFTGNV**S**IRGRIL  IRGRIL**S**GVVTKM  KRHKNM**S**VHLSPC  NMSVHL**S**PCFRDV  GECRPL**S**KTVRFN  YKGYLV**S**VDGYMN  YIDGAL**S**GHLGEV  LLNKPK**S**EMTPEE  FNTGPL**S**VLTQSV  LSVLTQ**S**VKNNTQ  WTEVPK**S**GKGKKK  GKGKKK**S**KPVNKD  NKDRYI**S**KMFLRG  MFLRGD**S**VIVVLR  IKKLIK**S**LEAARG  ARGNGT**S**MISLII  NGTSMI**S**LIIPPK  PPKDQI**S**RVAKML  DEFGTA**S**NIKSRV  TASNIK**S**RVNRLS  SRVNRL**S**VLGAIT  VLGAIT**S**VQQRLK  FKPINT**S**LYLCDN  ALTALL**S**DDSKFG  ALLSDD**S**KFGFIV  FIVIDG**S**GALFGT  HGRGGQ**S**ALRFAR  AVQLFI**S**GDKVNV  GLVLAG**S**ADFKTE  DFKTEL**S**QSDMFD  KTELSQ**S**DMFDQR  FDQRLQ**S**KVLKLV  LKLVDI**S**YGGENG  NQAIEL**S**TEVLSN  LSTEVL**S**NVKFIQ  RYFDEI**S**QDTGKY  EQEKDK**S**HFTDKE  EHELIE**S**MPLLEW  EIVTDK**S**QEGSQF  DKSQEG**S**QFVKGF  CFKCGR**S**GHWARE  RGRGMR**S**RGRGGF  GRGGFT**S**DRGFQF  RGFQFV**S**SSLPDI  GFQFVS**S**SLPDIC  FQFVSS**S**LPDICY  CYRCGE**S**GHLAKD  DEQKCY**S**CGEFGH  HVAINC**S**KTSEVN  INCSKT**S**EVNCYR  CYRCGE**S**GHLARE  CLLIVF**S**KDEFPE  DRLRPL**S**YPDTDV  VILMCF**S**VDSPDS  MCFSVD**S**PDSLEN  SVDSPD**S**LENIPE  NKKDLR**S**DEHVRT  YDYLEC**S**AKTKEG  LQKRYG**S**QNGCIN  GYVVRI**S**GGNDKQ  RVRLLL**S**KGHSCY  LLSKGH**S**CYRPRR  GERKRK**S**VRGCIV  IVDANL**S**VLNLVI  LGPKRA**S**RIRKLF  RKLFNL**S**KEDDVR  LLLIGD**S**GVGKSC  DSGVGK**S**CLLLRF  DDTYTE**S**YISTIG  YTESYI**S**TIGVDF  RFRTIT**S**SYYRGA  FRTITS**S**YYRGAH  DVTDQE**S**FNNVKQ  EIDRYA**S**ENVNKL  AKEFAD**S**LGIPFL  IPFLET**S**AKNATN  ATNVEQ**S**FMTMAA  AGGAEK**S**NVKIQS  SNVKIQ**S**TPVKQS  IMFDVT**S**RVTYKN  RKVKAK**S**IVFHRK  LQYYDI**S**AKSNYN  YDISAK**S**NYNFEK  FGMIYD**S**LDYAKK  YEKKKT**S**RKQRKE  CADATL**S**QITNNI  MHWGTD**S**RLLVSA  DSRLLV**S**ASQDGK  RLLVSA**S**QDGKLI  KLIIWD**S**YTTNKV  HAIPLR**S**SWVMTC  AIPLRS**S**WVMTCA  TCAYAP**S**GNYVAC  GLDNIC**S**IYNLKT  EGNVRV**S**RELAGH  GHTGYL**S**CCRFLD  DNQIVT**S**SGDTTC  NQIVTS**S**GDTTCA  HTGDVM**S**LSLAPD  GDVMSL**S**LAPDTR  DTRLFV**S**GACDAS  SGACDA**S**AKLWDV  TFTGHE**S**DINAIC  NAFATG**S**DDATCR  QELMTY**S**HDNIIC  IICGIT**S**VSFSKS  CGITSV**S**FSKSGR  ITSVSF**S**KSGRLL  SVSFSK**S**GRLLLA  GHDNRV**S**CLGVTD  MAVATG**S**WDSFLK  MDVDTP**S**GTNSGA  TPSGTN**S**GAGKKR  CQANQA**S**ATSEEC  NQASAT**S**EECTVA  FHFHCI**S**RWLKTR  NICVGE**S**GDRLTR  GQTPVF**S**KARYTV  ARYTVR**S**FGIRRN  LRKNNF**S**DTGNFG  GIKYDP**S**IGIYGL  LGRPGF**S**IADKKR  GAKHRI**S**KEEAMR  QRKGAG**S**VFRAHV  GKLARA**S**GNYATV  NYATVI**S**HNPETK  TRVKLP**S**GSKKVI  VKLPSG**S**KKVISS  GSKKVI**S**SANRAV  SKKVIS**S**ANRAVV  QHIGKP**S**TIRRDA  EPLGRV**S**FELFAD  ENFRAL**S**TGEKGF  GFGYKG**S**CFHRII  NGTGGK**S**IYGEKF  TGPGIL**S**MANAGP  GPNTNG**S**QFFICT  AMERFG**S**RNGKTS  SRNGKT**S**KKITIA  TLEVEP**S**DTIENV  EDGRTL**S**DYNIQK  KKRKKK**S**YTTPKK  DENGKI**S**RLRREC  LRRECP**S**DECGAG  AGVFMA**S**HFDRHY  GERESR**S**ASRSGS  RESRSA**S**RSGSAH  SRSASR**S**GSAHGS  SASRSG**S**AHGSGK  SGSAHG**S**GKSARH  RHTPAR**S**RSKEDS  TPARSR**S**KEDSRR  SRSKED**S**RRSRSK  KEDSRR**S**RSKSRS  DSRRSR**S**KSRSRS  RRSRSK**S**RSRSES  SRSKSR**S**RSESRS  SRSRSE**S**RSRSRR  RSRSRR**S**SRRHYT  SRSRRS**S**RRHYTR  RRHYTR**S**RSRSRS  SRSRSR**S**HRRSRS  SHRRSR**S**RSYSRD  RRSRSR**S**YSRDYR  SRSRSY**S**RDYRRR  DYRRRH**S**HSHSPM  RRRHSH**S**HSPMST  RHSHSH**S**PMSTRR  LGVFGL**S**LYTTER  GPIADV**S**IVYDQQ  IVYDQQ**S**RRSRGF  RIRVDF**S**ITKRPH  DDRDYY**S**RSYRGG  YRRRSP**S**PYYSRG  SPSPYY**S**RGGYRS  SRGGYR**S**RSRSRS  GGYRSR**S**RSRSYS  YRSRSR**S**RSYSPR  VGKDVS**S**LFPDVV  AIMAVN**S**FVKDCE  DQGFLD**S**LRDLIA  RDLIAD**S**NPMVVA  NAVAAL**S**EISESH  AALSEI**S**ESHPNS  LSEISE**S**HPNSNL  SESHPN**S**NLLDLN  DDREAQ**S**ICERVT  RVTPRL**S**HANSAV  ELLPKD**S**DYYNML  IMIRLA**S**QANIAQ  AIKVEQ**S**AERCVS  YPNKYE**S**IIATLC  LCENLD**S**LDEPDA  EGFHDE**S**TQVQLT  LFLKKP**S**ETQELV  SLATQD**S**DNPDLR  IYWRLL**S**TDPVTA  AKEVVL**S**EKPLIS  LICHIG**S**LASVYH  STDAGD**S**PVGTTT  QPQVIP**S**QGDLLG  VNVPQV**S**SMQMGA  NVPQVS**S**MQMGAV  QSFIPS**S**VPATFA  PATFAP**S**PTPAVV  PTPAVV**S**SGLNDL  TPAVVS**S**GLNDLF  AKGLEI**S**GTFTHR  PLMPNQ**S**IDVSLP  LNADTV**S**SKLQNN  NPNYTL**S**LKCRAP  KVIGQD**S**SEIHFK  VIGQDS**S**EIHFKV  LKKLKE**S**YCQRQG  QGVPMN**S**LRFLFE  IKNADM**S**EEMQQD  EEMQQD**S**VECATQ  VGRNFG**S**YVTHET  LNRVKV**S**QAAADL  PLLTGV**S**SSTNPF  LLTGVS**S**STNPFR  LTGVSS**S**TNPFRP  YVPRKC**S**ASNRII  PRKCSA**S**NRIIGA  GAKDHA**S**IQMNVA  IRRMGE**S**DDSILR  MGESDD**S**ILRLAK  TGDAGA**S**ATFPMQ  TFPMQC**S**ALRKNG  CKIVEM**S**TSKTGK  IVEMST**S**KTGKHG  YEDICP**S**THNMDV  IQDGYL**S**LLQDSG  LSLLQD**S**GEVRED  ILITVL**S**AMTEEA  FPDMIL**S**ASRDKT  DMILSA**S**RDKTII  RALRGH**S**HFVSDV  GHSHFV**S**DVVISS  VSDVVI**S**SDGQFA  SDVVIS**S**DGQFAL  DGQFAL**S**GSWDGT  QFALSG**S**WDGTLR  HTKDVL**S**VAFSSD  VLSVAF**S**SDNRQI  LSVAFS**S**DNRQIV  DNRQIV**S**GSRDKT  RQIVSG**S**RDKTIK  YTVQDE**S**HSEWVS  VQDESH**S**EWVSCV  SHSEWV**S**CVRFSP  VSCVRF**S**PNSSNP  VRFSPN**S**SNPIIV  RFSPNS**S**NPIIVS  SNPIIV**S**CGWDKL  LNTVTV**S**PDGSLC  TVSPDG**S**LCASGG  DGSLCA**S**GGKDGQ  INALCF**S**PNRYWL  CAATGP**S**IKIWDL  LKQEVI**S**TSSKAE  QEVIST**S**SKAEPP  EVISTS**S**KAEPPQ  EPPQCT**S**LAWSAD  CTSLAW**S**ADGQTL  VRTNRY**S**IVSSEE  NRYSIV**S**SEEDGM  RYSIVS**S**EEDGMK  GFGNGK**S**KVHTRQ  TRQQCR**S**RFVKKD  CLAFVL**S**WLFFGC  HGDLDA**S**KEGKAC  EGKACV**S**EVNSFT  CVSEVN**S**FTAAFL  TAAFLF**S**IETQTT  FMVVFQ**S**IVGCII  NETLVF**S**HNAVIA  VGNLRK**S**HLVEAH  RAQLLK**S**RITSEG  LKSRIT**S**EGEYIP  INVGFD**S**GIDRIF  DRIFLV**S**PITIVH  HEIDED**S**PLYDLS  SPLYDL**S**KQDIDN  MTTQCR**S**SYLANE  TTQCRS**S**YLANEI  YYKVDY**S**RFHKTY  PNTPLC**S**ARDLAE  EKKYIL**S**NANSFC  ILSNAN**S**FCYENE  NEVALT**S**KEEDDS  SKEEDD**S**ENGVPE  ENGVPE**S**TSTDTP  GVPEST**S**TDTPPD  DLHNQA**S**VPLEPR  FKDDYP**S**SPPKCK  HPNVYP**S**GTVCLS  SGTVCL**S**ILEEDK  AEDGCI**S**TKELGK  EVDEDG**S**GTVDFD  RCMKDD**S**KGKSEE  DDSKGK**S**EEELSD  KSEEEL**S**DLFRMF  NECKQL**S**ESQVKS  CKQLSE**S**QVKSLC  SESQVK**S**LCEKAK  EILTKE**S**NVQEVR  FRIGGK**S**PDTNYL  VDRGYY**S**VETVTL  LRGNHE**S**RQITQV  CLHGGL**S**PSIDTL  HGGLSP**S**IDTLDH  MCDLLW**S**DPDDRG  RGGWGI**S**PRGAGY  TFGQDI**S**ETFNHA  NGLTLV**S**RAHQLV  NVVTIF**S**APNYCY  DDTLKY**S**FLQFDP  PAAPAL**S**AADTKP  KPGTTG**S**GAGSGG  TGSGAG**S**GGPGGL  GPGGLT**S**AAPAGG  GVPVQG**S**KYAADR  GRRPQY**S**NPPVQG  DPPAEN**S**SAPEAE  SSSEEV**S**WISWFC  EEVSWI**S**WFCGLR  EDNPNQ**S**DLIEQA  MLPIGL**S**DIPGEA  DVYTPK**S**SRHHHT  VYTPKS**S**RHHHTD  LQLQAA**S**NFKSPV  LVCDNG**S**GLVKAG  PRAVFP**S**IVGRPR  GMGQKD**S**YVGDEA  VGDEAQ**S**KRGILT  AIQAVL**S**LYASGR  VLSLYA**S**GRTTGI  TGIVLD**S**GDGVTH  LTERGY**S**FVTTAE  EMATAA**S**SSSLEK  MATAAS**S**SSLEKS  ATAASS**S**SLEKSY  TAASSS**S**LEKSYE  SSSLEK**S**YELPDG  ETLFQP**S**FIGMES  SFIGME**S**AGIHET  HETTYN**S**IMKCDI  YANNVL**S**GGTTMY  ITALAP**S**TMKIKI  PPERKY**S**VWIGGS  SVWIGG**S**ILASLS  GGSILA**S**LSTFQQ  SILASL**S**TFQQMW  FQQMWI**S**KQEYDE  VIGHVD**S**GKSTTT  HVDSGK**S**TTTGHL  AEMGKG**S**FKYAWV  GITIDI**S**LWKFET  LWKFET**S**KYYVTI  NMITGT**S**QADCAV  EFEAGI**S**KNGQTR  GVNKMD**S**TEPPYS  STEPPY**S**QKRYEE  EIVKEV**S**TYIKKI  VAFVPI**S**GWNGDN  DNMLEP**S**ANMPWF  RKDGNA**S**GTTLLE  VTTEVK**S**VEMHHE  FNVKNV**S**VKDVRR  GNVAGD**S**KNDPPM  NHPGQI**S**AGYAPV  EKIDRR**S**GKKLED  GPKFLK**S**GDAAIV  KPMCVE**S**FSDYPP  MCVESF**S**DYPPLG  AGKVTK**S**AQKAQK  PDGQMP**S**DKTIGG  SFNTFF**S**ETGAGK  GFLVFH**S**FGGGTG  FGGGTG**S**GFTSLL  TGSGFT**S**LLMERL  LLMERL**S**VDYGKK  VDYGKK**S**KLEFSI  KSKLEF**S**IYPAPQ  YPAPQV**S**TAVVEP  VVEPYN**S**ILTTHT  HTTLEH**S**DCAFMV  LISQIV**S**SITASL  ISQIVS**S**ITASLR  VSSITA**S**LRFDGA  TYAPVI**S**AEKAYH  AYHEQL**S**VAEITN  TIKTKR**S**IQFVDW  RAVCML**S**NTTAIA  MEEGEF**S**EAREDM  EYWDYE**S**HVVEWG  LGRGKY**S**EVFEAI  IVKDPV**S**RTPALV  ALDYCH**S**MGIMHR  YNVRVA**S**RYFKGP  YQMYDY**S**LDMWSL  YSLDMW**S**LGCMLA  LGCMLA**S**MIFRKE  DILGRH**S**RKRWER  WERFVH**S**ENQHLV  ENQHLV**S**PEALDF  LRYDHQ**S**RLTARE  DQARMG**S**SSMPGG  QARMGS**S**SMPGGS  ARMGSS**S**MPGGST  SSMPGG**S**TPVSSA  GGSTPV**S**SANMMS  GSTPVS**S**ANMMSG  SSANMM**S**GISSVP  NMMSGI**S**SVPTPS  MMSGIS**S**VPTPSP  LTPEEK**S**AVTALW  TQRFFE**S**FGDLST  ESFGDL**S**TPDAVM  KVLGAF**S**DGLAHL  GTFATL**S**ELHCDK  GGASRF**S**ASSGGG  ASRFSA**S**SGGGGS  SRFSAS**S**GGGGSR  TRRDDN**S**AANNSA  LNVGVE**S**KLILKG  LEEPKY**S**SLYAQL  EEPKYS**S**LYAQLC  QPGQKQ**S**TTFRRL  DHERAK**S**LMDQYF  MCSLML**S**KELPAR  MAQGMR**S**DFFLEG  TMGRHR**S**NQLFNG  GGKFMK**S**QGLSQL  QLYHNQ**S**QGLLSQ  QSQGLL**S**QLQGQS  DMPPRF**S**KKGQLN  LNADEI**S**LRPAQS  SLRPAQ**S**FLMNKN  ITMIPP**S**AQPPRT  SKKPPP**S**KEELLK  FLPEML**S**KVIILS  SKVIIL**S**LDRSDE  ILSLDR**S**DEDKEK  EDKEKA**S**SLISLL  DKEKAS**S**LISLLK  DIPLVK**S**YLAQFA  AARAII**S**ELVSIS  LAQPLE**S**GTHFPL  TELFQQ**S**KVNMQK  SFLQYI**S**SEVNPP  SEVNPP**S**DETDSS  PSDETD**S**SSAPSK  DETDSS**S**APSKEQ  LDLRAG**S**DLKALR  AAHLGY**S**VVAINH  EKPVAV**S**ELFTTL  PIVQGK**S**RPIKIL  RLTIIV**S**DPSHCN  IIVSDP**S**HCNVLR  NVLRAT**S**SRARLY  VLRATS**S**RARLYD  AFELVY**S**PAIKDS  SPAIKD**S**TMRRYT  MRRYTI**S**SALNLM  RRYTIS**S**ALNLMQ  GKNVII**S**SAAERP  KNVIIS**S**AAERPL  GLLFGL**S**ESDAKA  LFGLSE**S**DAKAAV  DAKAAV**S**TNCRAA  TAFGII**S**TVKKPR  VKKPRP**S**EGDEDC  VEDEES**S**ESRMVV  VVTFLM**S**ALESMC  LMSALE**S**MCKELA  CKELAK**S**KAEVAC  ENKAGI**S**FIIKRP  TEPTED**S**GISLEM  NIQAGP**S**ETDDVD  QGSHHS**S**EGNEGT  HVPSET**S**EDPEVE  IEDDDY**S**PPSKRP  QLDKPA**S**GVKEEW  FAEALG**S**TEAKAV  ENIPFR**S**PSWYGI  PELLTH**S**TTEVTQ  PYPVFE**S**NPEFLY  ERIVRG**S**NKIKFV  KPELVI**S**YLPPGM  RPRSPG**S**NSKVPE  RSPGSN**S**KVPEIE  NNNPQT**S**AVRTPT  PTQTNG**S**NVPFKP  PRGREF**S**FEAWNA  PFALFE**S**FPEDFY  AIKEST**S**SKSPPR  PPRKIN**S**SPNVNT  NVNTTA**S**GVEDLN  QVNDLF**S**RKFGEA  GMPPGV**S**FKAPSY  PSYLEI**S**SMRRIL  NQLVDQ**S**ESEGPV  QESAEP**S**QLEVPA  GGVQPV**S**IQAFAS  SIQAFA**S**SSTLHG  IQAFAS**S**STLHGL  QAFASS**S**TLHGLA  GLAHIF**S**YERLSL  FSYERL**S**LKRALW  ALCFLG**S**LAVLLC  LDEVAA**S**QLTFPA  LNEFRF**S**QVSKND  FRFSQV**S**KNDLYH  DKANFR**S**FKPKPF  IRDMLL**S**CHFRGE  FRGEVC**S**AEDFKV  KCYTFN**S**GRDGRP  GETDET**S**FEAGIK  IKVQIH**S**QDEPPF  KAVTMD**S**DLDFFD  DLDFFD**S**YSITAC  DFFDSY**S**ITACRI  RYGKEL**S**MVKIPS  SMVKIP**S**KASAKY  KIPSKA**S**AKYLAK  AKKFNK**S**EQYIGE  GLFIGA**S**ILTVLE  QKEAKR**S**SADKGV  DKGVAL**S**LDDVKR  RHNPCE**S**LRGHPA  VNSEKS**S**SSERPE  NSEKSS**S**SERPEP  SEKSSS**S**ERPEPQ  EEEILG**S**DDEEQE  LGWGHF**S**TVWLCW  VRESDP**S**DPNKDM  LKWIIK**S**NYQGLP  PVRCVK**S**IIRQVL  PSGSAV**S**TAPQQK  IEENIT**S**AAPSND  ITSAAP**S**NDQDGE  DPTWIE**S**PKTNGH  IENGPF**S**LEQQLD  ESDYTY**S**SSYEQF  SDYTYS**S**SYEQFN  DYTYSS**S**YEQFNG  RHKIPE**S**QFPEFS  SQFPEF**S**TSLFSG  TSLFSG**S**LEPVAC  EPVACG**S**VLSEGS  SVLSEG**S**PLTEQE  SPSHDR**S**RTVSAS  DRSRTV**S**ASSTGD  SRTVSA**S**STGDLP  RTVSAS**S**TGDLPK  QTRQYR**S**IEVLIG  LIGAGY**S**TPADIW  YLFEPH**S**GEDYSR  PRHFAL**S**GKYSRE  ALSGKY**S**REFFNR  TKLKPW**S**LFDVLV  VPEKRA**S**AGECLR  ETARLT**S**FIGAIA  IGDLVK**S**TLGPKG  MDKILL**S**SGRDAS  DKILLS**S**GRDASL  SSGRDA**S**LMVTND  KVLVDM**S**RVQDDE  VGDGTT**S**VTVLAA  LLREAE**S**LIAKKI  AREALL**S**SAVDHG  REALLS**S**AVDHGS  SAVDHG**S**DEVKFR  IAGTTL**S**SKLLTH  AGTTLS**S**KLLTHH  VLRLKG**S**GNLEAI  IKKLGG**S**LADSYL  GGSLAD**S**YLDEGF  KIKIFG**S**RVRVDS  SRVRVD**S**TAKVAE  TGGEIA**S**TFDHPE  ELVKLG**S**CKLIEE  DKLIHF**S**GVALGE  LDEAER**S**LHDALC  AQTVKD**S**RTVYGG  VYGGGC**S**EMLMAH  EAVAME**S**YAKALR  DNAGYD**S**ADLVAQ  QLRAAH**S**EGNTTA  ILGITE**S**FQVKRQ  KRQVLL**S**AAEAAE  VRKSLN**S**IEFREC  KGPESE**S**EDHRAS  KDELLA**S**CLTFLL  NALEEW**S**IYIDRH  SDEMMK**S**YVAWDR  AHADEK**S**LGTIQQ  PRGFPP**S**ASLCLL  LGTEAQ**S**SLLKAV  AAGNRT**S**PQEGER  GSVIHF**S**HGEYFY  HWKKCD**S**WWAKDS  TLSEEM**S**QFDFST  VPRDLP**S**WMKFLH  YAKHWL**S**PLLQLA  FLLEMT**S**MSPDYP  QTADGR**S**SFDWLT  FDWLTG**S**STDPLV  FLNTTF**S**FFPPFV  SAGCLA**S**LQQPVG  ILELHY**S**QELSLL  IQNGIQ**S**FMQNYS  RLTKLQ**S**VQALTE  NRCFFL**S**KIEEKL  RKQNNF**S**LAMKLL  GSSSED**S**EKVIAG  EEAQPP**S**WSCGPA  AQPPSW**S**CGPAAG  FVARIK**S**KLDQGG  ECSPWM**S**DFKVEF  RSHFAS**S**HALICI  ALRAFR**S**DPGLLT  KLDSLL**S**DYDILS  SDYDIL**S**LSNIQQ  YDILSL**S**NIQQHS  KRDLQT**S**THVETL  ETLLTF**S**ALKRHF  KLYLTS**S**TERFSQ  SSTERF**S**QNFKVV  VDGKNE**S**EYTVKW  VVGEPD**S**RVLAHI  RMLVYK**S**EDIKNV  DIYRNT**S**WDNAGF  HYNMAK**S**YPNEEK  MLLEQF**S**FDIAEE  GLAYVG**S**PRANSH  GSPRAN**S**HGGVCP  MYPIAV**S**GDHENN  ECFQER**S**NKVCGN  NDTCCN**S**DCTLKE  KEGVQC**S**DRNSPC  ATCKGV**S**YCTGNS  YCTGNS**S**ECPPPG  REQQLE**S**CACNET  CNETDN**S**CKVCCR  VCCRDL**S**GRCVPY  LDKQYE**S**LSLFHP  LSLFHP**S**NVEMLS  TIQEDP**S**TDSHMD  EDPSTD**S**HMDEDG  KDPFPN**S**STAAKS  DPFPNS**S**TAAKSF  RSEKAA**S**FKLQRQ  AAVGFL**S**SLLRTR  AVGFLS**S**LLRTRG  RTRGCV**S**EQRLKV  QRLKVF**S**GALQEA  WFPEKP**S**KGSGYR  EKPSKG**S**GYRCIR  KMDPII**S**RVASQI  IISRVA**S**QIGLSQ  ASQIGL**S**QPQLHQ  LHQLLP**S**ELTLWV  VDPYEV**S**YRIGED  RIGEDG**S**ICVLYE  EAPLAA**S**CGLLTC  QVLLGR**S**SPSKNY  HAQAQD**S**TSDLIP  QAQDST**S**DLIPAP  IPAPPL**S**KVPLQQ  ELKEDK**S**YNVTSV  KSYNVT**S**VLFRKK  TLGNIK**S**YPGLTS  SYPGLT**S**YLVRVV  YLVRVV**S**TNYNQH  VFFKKV**S**QNREYF  RTKELT**S**ELKENF  ENFIRF**S**KSLGLP  FIRFSK**S**LGLPEN  RVGIFP**S**NYVTPR  NYVTPR**S**AFSSRC  PRSAFS**S**RCQPGG  PGGEDP**S**CYPPIQ  IHRDLK**S**SNILIL  PEVIRA**S**MFSKGS  LALPIP**S**TCPEPF  KGKFRK**S**RLKLKD  RKSLIN**S**RSSPPA  SKTWGR**S**SVVPKE  LSTPSE**S**PHFHLG  GYKQWS**S**SAPNLV  YKQWSS**S**APNLVK  VKGPRS**S**PALPGF  QHSPSQ**S**YLCIPF  EDGDGP**S**SDGIHE  DGDGPS**S**DGIHEE  EPTPVN**S**ATSTPQ  RPRRST**S**PPSRKL  RSTSPP**S**RKLFKK  LSLSSI**S**ECNSTR  SISECN**S**TRSLLR  VYEMPV**S**PVEAPP  TTPSQP**S**SHRRTP  PETLLA**S**RSPSSN  LKTPSP**S**RDPGEF  WWFVSP**S**HARSTS  SHARST**S**PANSSS  STSPAN**S**SSTETP  SPANSS**S**TETPSN  SCFASS**S**STVEER  KVVKIT**S**EIPQTE  RFNVPV**S**LESKKA  VPVSLE**S**KKAARA  AARFGI**S**SVPTKG  ARFGIS**S**VPTKGL  VPTKGL**S**SDNKPM  PTKGLS**S**DNKPMV  RFGLNV**S**SISRKS  FGLNVS**S**ISRKSE  LNVSSI**S**RKSEDD  SSISRK**S**EDDEKL  RFGIVT**S**SAGTGT  FGIVTS**S**AGTGTT  TYADYE**S**VNECME  KRMNPN**S**PSITYD  MNPNSP**S**ITYDIS  SITYDI**S**QLFDFI  DDLADL**S**CLVYRA  ISNNKI**S**TLEEGI  VAAVSF**S**AAHEGL  LQPEAC**S**AFCFST  VSRVTR**S**LDVWIG  VSRHNL**S**CSFDVV  FPVPDP**S**VAQVLV  LALVLS**S**RVNRAH  GRGRPA**S**YLWDLG  AGWNEV**S**RSEAWL  GLVVNA**S**RTVVPL  GPALAG**S**GKGFSL  ATNMLG**S**AWADCT  AELAGG**S**GVVYTW  GVVYTW**S**LEEGLS  NASNAV**S**WVSATY  GSYRVW**S**DTRDLV  ITETLV**S**LRVHTV  LGAESP**S**RMVASQ  PSRMVA**S**QAYNLT  VSTKVA**S**MAFQTQ  ARGHRS**S**ANSANS  YGAVGD**S**AYSTGH  SGEGTL**S**WPDLLS  LPAWCA**S**LAHGLS  SGWVGA**S**FPPGVS  AWLLSS**S**ASFLAS  HPSTSS**S**QLDGLS  LDGLSV**S**LGRLGT  RCEPEP**S**RLQAVF  LRPALP**S**RLARAS  SRLARA**S**RGVDLA  TFANFP**S**GSPVSA  ANFPSG**S**PVSAST  PSGSPV**S**ASTLAR  GSPVSA**S**TLARAG  DTVRCF**S**CHAAVD  RWQYGD**S**AVGRHR  GFYLEN**S**ATQSTN  ENSATQ**S**TNSGIQ  ATQSTN**S**GIQNGQ  VENYLG**S**RDHFAL  FALDRP**S**ETHADY  GQVVDI**S**DTIYPR  RNPAMY**S**EEARLK  EEARLK**S**FQNWPD  TPRELA**S**AGLYYT  PCDRAW**S**EHRRHF  RNLNIR**S**ESDAVS  LNIRSE**S**DAVSSD  SESDAV**S**SDRNFP  ESDAVS**S**DRNFPN  DRNFPN**S**TNLPRN  NLPRNP**S**MADYEA  FGTWIY**S**VNKEQL  NIHLTH**S**LEECLV  AIRMGF**S**FKDIKK  EEKIQI**S**GSNYKS  KIQISG**S**NYKSLE  SGSNYK**S**LEVLVA  DSMQDE**S**SQTSLQ  DESSQT**S**LQKEIS  SLQKEI**S**TEEQLR  PQSRPR**S**CTWPLP  EIANQP**S**EPPEVE  VHTEGR**S**EPILLP  EPILLP**S**RLPEPA  NAWGNQ**S**YAELIS  ISQAIE**S**APEKRL  KGDSNS**S**AGWKNS  NEATGK**S**SWWMLN  AASMDS**S**SKLLRG  ASMDSS**S**KLLRGR  FAKWSG**S**PCSRNR  WSGSPC**S**RNREEA  TTFRPR**S**SSNASS  TFRPRS**S**SNASSV  RSSSNA**S**SVSTRL  SNASSV**S**TRLSPL  SVSTRL**S**PLRPES  AEEIPA**S**VSSYAG  EIPASV**S**SYAGGV  GLNLTS**S**HSLLSR  NLTSSH**S**LLSRSG  HSLLSR**S**GLSGFS  LSRSGL**S**GFSLQH  SGLSGF**S**LQHPGV  GPLHTY**S**SSLFSP  LHTYSS**S**LFSPAE  GEGCFS**S**SQALEA  EGCFSS**S**QALEAL  LEAPGP**S**SLVPTL  EAPGPS**S**LVPTLS  PPTEAA**S**QDRMPQ  ALEDHF**S**SFGPIS  LEDHFS**S**FGPISE  SSFGPI**S**EVVVVK  DRETQR**S**RGFGFI  TNPEHA**S**VAMRAM  RAMNGE**S**LDGRQI  VDHAGK**S**ARGTRG  AHGRGR**S**YSRGGG  GRGRSY**S**RGGGDQ  GDQGYG**S**GRYYDS  SGRYYD**S**RPGGYG  GYGYGR**S**RDYNGR  GGYDRY**S**GGNYRD  IFIMKC**S**QCHTVE  GQAPGY**S**YTAANK  SMWGVL**S**ALGRSG  LSALGR**S**GAELCT  LCTGCG**S**RLRSPF  CGSRLR**S**PFSFVY  RLRSPF**S**FVYLPR  YLPRWF**S**SVLASC  LPRWFS**S**VLASCP  FSSVLA**S**CPKKPV  WRELPD**S**KKKIYQ  VYKEEI**S**RFKEQL  KEQLTP**S**QIMSLE  TPSQIM**S**LEKEIM  QEAKGD**S**PQEKLK  WKNLSD**S**EKELYI  YHNEMK**S**WEEQMI  AVLTQE**S**FAEHRS  SFAEHR**S**GLVPQQ  KVATLN**S**EEESDP  KAACLE**S**AQEPSG  IRPIKA**S**VITQVF  GAHLEL**S**LAKDQG  GLSIMV**S**GKLDAV  RLQTQA**S**ATVAIP  PRPDDP**S**NQIKIT  HEVLLI**S**AEQDKR  INIPPP**S**VNRTEI  IGPKGN**S**LQEILE  EIPPSD**S**ISETVI  VYAKAN**S**FTVSSV  RHLIGK**S**GANINR  VRIPPD**S**EKSNLI  PPDSEK**S**NLIRIE  PAENSN**S**ETIIIT  NCEAAR**S**RILSIQ  KGRLIR**S**IMEECG  VVIRGP**S**SDVEKA  VIRGPS**S**DVEKAK  EEKQTK**S**FTVDIR  YGGVMV**S**FPRSGT  MVSFPR**S**GTQSDK  VIGQKG**S**GIRKMM  PAPELQ**S**DIIAIT  DIRFPQ**S**GAPDPN  EEAKAP**S**RGFVVR  APWTAS**S**SEKAPD  PWTASS**S**EKAPDM  EKAPDM**S**SSEEFP  APDMSS**S**EEFPSF  EEGMPL**S**TIREVA  FDVCTV**S**RTDRET  GLDFLH**S**HRVVHR  QNILVT**S**SGQIKL  NILVTS**S**GQIKLA  GLARIY**S**FQMALT  FQMALT**S**VVVTLW  PEVLLQ**S**SYATPV  EVLLQS**S**YATPVD  TPVDLW**S**VGCIFA  KPLFRG**S**SDVDQL  PLFRGS**S**DVDQLG  PRQAFH**S**KSAQPI  QAFHSK**S**AQPIEK  NPAKRI**S**AYSALS  KRISAY**S**ALSHPY  SAYSAL**S**HPYFQD  CKENLD**S**HLPPSQ  DSHLPP**S**QNTSEL  QIGLDE**S**GGGGGS  APGELR**S**ARGPLS  SARGPL**S**SAPEIV  ARGPLS**S**APEIVH  GESDQA**S**ATSSDE  DQASAT**S**SDEVQS  QASATS**S**DEVQSP  HPPRKI**S**TEDINK  IFDKPL**S**RRLRRV  TVYKGK**S**KLTDNL  TAIREV**S**LLKDLK  IIHTEK**S**LTLVFE  GLARAK**S**IPTKTY  IPTKTY**S**NEVVTL  PDILLG**S**TDYSTQ  LGSTDY**S**TQIDMW  RPLFPG**S**TVEEQL  TWPGIL**S**NEEFKT  RAEALL**S**HAPRLD  HAPRLD**S**DGADLL  EGRNRI**S**AEDAMK  KHPFFL**S**LGERIH  KLPDTT**S**IFALKE  QLQKEA**S**LRSSSM  KEASLR**S**SSMPDS  EASLRS**S**SMPDSG  ASLRSS**S**MPDSGR  SSSMPD**S**GRPAFR  PIRRPI**S**ADSAIM  RPISAD**S**AIMNPA  FNIEMK**S**KMKAHT  NAVYHW**S**MEGESQ  KMFDRH**S**SLAGCQ  MFDRHS**S**LAGCQI  GAMQLY**S**VDRKVS  IYMNRI**S**GETIFV  RKGQVL**S**VCVEEE  TIRRFQ**S**VPAQPG  QLNKYE**S**LELCRP  SLSVED**S**LECLRA  ICVQVA**S**KYHEQL  LFESFK**S**FEGLFY  LFYFLG**S**IVNFSQ  VQKVNP**S**RLPVVI  LLDVDC**S**EDVIKN  AKIYID**S**NNNPER  NVCNEN**S**LFKSLS  ENSLFK**S**LSRYLV  SLFKSL**S**RYLVRR  DPELWG**S**VLLESN  GSVLLE**S**NPYRRP  KIVLDN**S**VFSEHR  KFDVNT**S**AVQVLI  IKADDP**S**SYMEVV  KADDPS**S**YMEVVQ  RKKARE**S**YVETEL  LLYNNV**S**NFGRLA  GARKAN**S**TRTWKE  QNHNNK**S**VNESLN  MQYASE**S**KDTELA  KLDASE**S**LRKEEE  PGAAGP**S**NVPAFL  WSPSGN**S**FHVFDQ  KHNNMA**S**FVRQLN  RKVTSV**S**TLKSED  SVSTLK**S**EDIKIR  IKIRQD**S**VTKLLT  KQECMD**S**KLLAMK  LWREVA**S**LRQKHA  MLNDSG**S**AHSMPK  SPAYSS**S**SLYAPD  PAYSSS**S**LYAPDA  PDAVAS**S**GPIISD  SSGPII**S**DITELA  TELAPA**S**PMASPG  MASPGG**S**IDERPL  ASPGRP**S**SVDTLL  SPGRPS**S**VDTLLS  PTALID**S**ILRESE  DSILRE**S**EPAPAS  TPEKCL**S**VACLDK  HLDAMD**S**NLDNLQ  GFSVDT**S**ALLDLF  VTVPDM**S**LPDLDS  SLPDLD**S**SLASIQ  LDSSLA**S**IQELLS  PPEAEN**S**SPDSGK  ENSSPD**S**GKQLVH  FLLDPG**S**VDTGSN  FELGEG**S**YFSEGD  GEGSYF**S**EGDGFA  AEDPTI**S**LLTGSE  DDFKLN**S**SIVEPK  GGLPGA**S**SEKGRK  GLPGAS**S**EKGRKT  VDLVTH**S**DPPRAH  PRAHAH**S**LVGKQC  LGICAV**S**VGPKDM  KKVMDL**S**IVRLRF  SAFLRA**S**DGSFSL  PLKPVI**S**QPIHDS  PIHDSK**S**PGASNL  SKSPGA**S**NLKISR  KRGGDV**S**DSKQFT  GGDVSD**S**KQFTYY  SLGFFP**S**SLAYSP  PSSLAY**S**PYQSGA  MAATVP**S**RDSGEE  EEAAEP**S**APSRTP  FGLAQR**S**ARALLD  VITGQT**S**VVSFLL  LDRHGD**S**AMHLAL  LRALLQ**S**GAPAVP  LAVRAR**S**PECLDL  LPSPPT**S**DSDSDS  EKDTRS**S**FRGHTP  PLDLTC**S**TKVKTL  PPLTPP**S**PAGPGL  PAGPGL**S**LGDTAL  ERLGLR**S**LVDTYR  TYRQTT**S**PSGSLL  RQTTSP**S**GSLLRS  SGSLLR**S**YELAGG  LKKRRL**S**DKGLKA  MEPGNG**S**LDLGGD  LDLGGD**S**AGRSGA  GDSAGR**S**GAGLEQ  AAGAAD**S**GPMEEE  EEEEAA**S**EDENGD  KEAAGK**S**SGPTSL  EAAGKS**S**GPTSLF  KSSGPT**S**LFAVTV  IEENKY**S**RAKSPQ  KYSRAK**S**PQPPVE  DLHFKI**S**RDRLSA  ISRDRL**S**ASSLTM  RDRLSA**S**SLTMES  DRLSAS**S**LTMESF  SSLTME**S**FAFLWA  RASYGV**S**KGKVCF  EVRIGW**S**LTTSGM  GWSLTT**S**GMLLGE  LGEEEF**S**YGYSLK  EFSYGY**S**LKGIKT  CFANFE**S**DEVELS  SDEVEL**S**YAKNGQ  GVAFKI**S**KEVLAG  EQYKEE**S**KKALPP  KKQNTG**S**KKSNKN  NTGSKK**S**NKNKSG  KSNKNK**S**GKNQFN  GGGGGG**S**GGIGYP  VFPGRG**S**YSNRGN  PGRGSY**S**NRGNYN  RGYKNQ**S**QGYNQW  WGQKPW**S**QHYHQG  DGAVTT**S**QIPASE  TSQIPA**S**EQETLV  QHIVYC**S**NDLLGD  LQEEKP**S**SSHLVS  QEEKPS**S**SHLVSR  EEKPSS**S**HLVSRP  SSSHLV**S**RPSTSS  SRPSTS**S**RRRAIS  KRHKSD**S**ISLSFD  HKSDSI**S**LSFDES  SLSFDE**S**LALCVI  ICCERS**S**SSESTG  CERSSS**S**ESTGTP  RSSSSE**S**TGTPSN  DLDAGV**S**EHSGDW  EVESLD**S**EDYSLS  EEGQEL**S**DEDDEV  VYQAGE**S**DTDSFE  GESDTD**S**FEEDPE  EEDPEI**S**LADYWK  KDKGEI**S**EKAKLE  KAKLEN**S**TQAEEG  KTIVND**S**RESCVE  VNDSRE**S**CVEEND  ITQASQ**S**QESEDY  EDYSQP**S**TSSSII  YSQPST**S**SSIIYS  SQPSTS**S**SIIYSS  QPSTSS**S**IIYSSQ  SSSIIY**S**SQEDVK  KEESVE**S**SLPLNA  KDKVNC**S**FYFKIG  RHGDRC**S**RLHNKP  HNKPTF**S**QTIALL  YRNPQN**S**SQSADG  RNPQNS**S**QSADGL  PQNSSQ**S**ADGLRC  GLRCAV**S**DVEMQE  PIHAEL**S**PVTDFR  MHLKPI**S**RELRRE  RRKKHR**S**RSRSRE  KKHRSR**S**RSRERR  HRSRSR**S**RERRSR  RSRERR**S**RSRDRG  RERRSR**S**RDRGRG  ERDRRR**S**RDRERS  ALRLLD**S**SQIVII  LRLLDS**S**QIVIIS  SQIVII**S**AAQDAS  SAAQDA**S**APPAPT  APRPTP**S**APRPAL  HQYLAE**S**SGPARG  QYLAES**S**GPARGR  KSRYET**S**LNLTTK  RFLELL**S**HSADGV  LELLSH**S**ADGVVD  QLIAKK**S**KNHIQW  HIQWLG**S**HTTVGV  TQLRLL**S**EDTDSQ  LSEDTD**S**QRLAYV  TCQDLR**S**IADPAE  LQAVDS**S**ENFQIS  SENFQI**S**LKSKQG  FQISLK**S**KQGPID  ETVGGI**S**PGKTPS  PSQEVT**S**EEENRA  ENRATD**S**ATIVSP  DSATIV**S**PPPSSP  IVSPPP**S**SPPSSL  VSPPPS**S**PPSSLT  PPSSPP**S**SLTTDP  TTDPSQ**S**LLSLEQ  PSQSLL**S**LEQEPL  EQEPLL**S**RMGSLR  LLSRMG**S**LRAPVD  VDEDRL**S**PLVAAD  HVREDF**S**GLLPEE  LPEEFI**S**LSPPHE  EEFISL**S**PPHEAL  DVEGMT**S**LKVDNL  NLTYRT**S**PDTLRR  PPDSHH**S**RRGPPP  GGYGRR**S**RSPRRR  YGRRSR**S**PRRRRR  PRRRRR**S**RSRSRS  RRSRSR**S**RSRSRS  SRSRSR**S**RSRSRS  SRSRSR**S**RSRSRY  SRSRSR**S**RYSRSK  RSRSRY**S**RSKSRS  RSRYSR**S**KSRSRT  SRSRTR**S**RSRSTS  SRTRSR**S**RSTSKS  TRSRSR**S**TSKSRS  SRSRST**S**KSRSAR  SRSTSK**S**RSARRS  STSKSR**S**ARRSKS  SRSARR**S**KSKSSS  RRSKSK**S**SSVSRS  SKSKSS**S**VSRSRS  SKSSSV**S**RSRSRS  SSSVSR**S**RSRSRS  SVSRSR**S**RSRSRS  SRSRSR**S**RSRSPP  SRSRSR**S**RSPPPV  SRSRSR**S**PPPVSK  PVSKRE**S**KSRSRS  SKRESK**S**RSRSKS  RESKSR**S**RSKSPP  SKSRSR**S**KSPPKS  SRSRSK**S**PPKSPE  SKSPPK**S**PEEEGA  RWRLVD**S**KGFDEY  LTIKTE**S**TLKTTQ  LKTTQF**S**CTLGEK  EWDGKE**S**TITRKL  GRLEAV**S**HTSDMH  EAVSHT**S**DMHRGY  HRGYAD**S**PSKAGA  YVQAFD**S**LLAGPV  ALLVTA**S**QCQQPA  PAENKL**S**DLLAPI  DLLAPI**S**EQIKEV  NHLSAV**S**ESIQAL  LSAVSE**S**IQALGW  WVKAYL**S**IWTELQ  ELSGLP**S**GPSAGS  SGPSAG**S**CPPPPP  PPPPPV**S**TISCSY  PPVSTI**S**CSYESA  VSTISC**S**YESASR  ISCSYE**S**ASRSSL  CSYESA**S**RSSLFA  YESASR**S**SLFAQI  ESASRS**S**LFAQIN  QINQGE**S**ITHALK  HALKHV**S**DDMKTH  PALKAQ**S**GPVRSG  QSGPVR**S**GPKPFS  SGPKPF**S**APKPQT  APKPQT**S**PSPKRA  KPQTSP**S**PKRATK  ENQENV**S**NLVIED  IKGKIN**S**ITVDNC  IVEIIN**S**KDVKVQ  GCHAYL**S**KNSLDC  AYLSKN**S**LDCEIV  LDCEIV**S**AKSSEM  EIVSAK**S**SEMNVL  LVAKDG**S**CLFRAV  VGQVEI**S**ALSLMY  KVLLCF**S**NGNHYD  SDSEDD**S**CKSKTA  LKNNGN**S**TSLPLS  NNGNST**S**LPLSRK  QQKRDY**S**IAAGLQ  LGKKHT**S**KNLKAP  VRQHAF**S**SHSSGS  AFSSHS**S**GSQSQK  AFPALS**S**SSVNQS  FPALSS**S**SVNQSA  PALSSS**S**VNQSAS  QSASQS**S**NPCVQR  PCVQRK**S**SHVGDR  CVQRKS**S**HVGDRK  KKLECP**S**PAEQKP  APIPVL**S**VTQTLT  LTTGPD**S**AVSQAH  AIVPPY**S**LCQTGE  PMWAPH**S**YLYPLH  IGPPTF**S**SPLVIP  PLVIPP**S**QVSESH  ESHGQL**S**YQADLE  LSKDCG**S**VSTVDE  RGEHVH**S**LPEASV  PEASVS**S**KPDEGR  EGRTEQ**S**SQTRKA  GANSVD**S**RVQRPK  ILRSGR**S**KQFYNQ  GSRKYK**S**DWGYSG  KGQPSR**S**RDEGYQ  RGDRRR**S**GMGDGH  KEHSEM**S**NNVSDP  IEMALL**S**LGYSHS  LLSLGY**S**HSSAAQ  WNPVPL**S**YVTDAP  KDMNQS**S**LAKECP  AKECPL**S**QSMISS  LSQSMI**S**SIVNST  SQSMIS**S**IVNSTY  ISSIVN**S**TYYANV  TYYANV**S**AAKCQE  DSLSEL**S**QQGANH  AQLSHG**S**QPSVRT  SHGSQP**S**VRTPLP  LVSTPI**S**PQLVNQ  RLLAQQ**S**LNQQYL  NHPPPV**S**RSMNKP  PPPVSR**S**MNKPLE  PLEQQV**S**TNTEVS  LKRAGI**S**QAVFAR  PKTASQ**S**LLVNLR  QDERER**S**LNAASA  GPAPLI**S**TPPSRP  LISTPP**S**RPPQVK  TMNINA**S**IYDEIQ  RWKEDP**S**PENRTL  AIYEQE**S**NAVHHH  RQPTVA**S**PAESDE  VASPAE**S**DEENRQ  ALGILQ**S**FIQDVG  EAIQTL**S**AQLDLP  GKLKDN**S**GLEVDV  AAQQGY**S**AYTAQP  QAYGQQ**S**YGTYGQ  QTAYAT**S**YGQPPT  TAPQAY**S**QPVQGY  VTTTQA**S**YAAQSA  ETSQPQ**S**STGGYN  TSQPQS**S**TGGYNQ  YGQSNY**S**YPQVPG  PVTAPP**S**YPPTSY  PPTSYS**S**TQPTSY  SSTQPT**S**YDQSSY  TSYDQS**S**YSQQNT  YDQSSY**S**QQNTYG  TYGQPS**S**YGQQSS  SYGQQS**S**YGQQSS  SSYGQQ**S**SYGQQP  GQQPPT**S**YPPQTG  PQTGSY**S**QAPSQY  SYSQAP**S**QYSQQS  SQYSQQ**S**SSYGQQ  QYSQQS**S**SYGQQS  SYGQQS**S**FRQDHP  RQDHPS**S**MGVYGQ  GVYGQE**S**GGFSGP  QESGGF**S**GPGENR  GPGENR**S**MSGPDN  GENRSM**S**GPDNRG  VQGLND**S**VTLDDL  KGDATV**S**YEDPPT  GKDFQG**S**KLKVSL  GSKLKV**S**LARKKP  KKPPMN**S**MRGGLP  GSRGNP**S**GGGNVQ  ASDFAF**S**PPPGGG  DPRTWL**S**FQGPPG  PGVGPG**S**EVWGIP  QGGLET**S**QPEGEA  AGVGVE**S**NSDGAS  VGVESN**S**DGASPE  EQNPEE**S**QDIKAL  LFGKVF**S**QTTICR  FEALQL**S**FKNMCK  PTLQQI**S**HIAQQL  RQKGKR**S**SSDYAQ  DFEAAG**S**PFSGGP  AAGSPF**S**GGPVSF  FSGGPV**S**FPLAPG  GTPGYG**S**PHFTAL  HFTALY**S**SVPFPE  FTALYS**S**VPFPEG  EAFPPV**S**VTTLGS  RMAEAI**S**CTLNCS  NVVFDI**S**SLMLYG  VVFDIS**S**LMLYGT  KVLDHW**S**IMTSEE  HWSIMT**S**EEEVAT  PFENLI**S**NMTFML  EQGHDQ**S**QDPKQE  HGPFPD**S**SFLTSS  GPFPDS**S**FLTSSS  ITKKED**S**THLSDS  THLSDS**S**SYNIVT  FERTQL**S**PEAKVK  NSLNLA**S**SENYKC  TPGILP**S**LPLLSS  DEQEAC**S**PQSHRV  RESVIE**S**SGAISQ  TFKNAC**S**VKIHHK  TFPSRR**S**RDRHSS  SRDRHS**S**NLNLHQ  AFTQQA**S**QTSVIF  QQASQT**S**VIFKGT  TQVHSA**S**LESYNS  HSASLE**S**YNSGPL  DLSTTS**S**MKSESS  SSMKSE**S**SSHSSW  SMKSES**S**SHSSWD  ESSSHS**S**WDSDGV  DGNCEG**S**SLVPGE  GNCEGS**S**LVPGED  ADQSLA**S**LPSGLP  LCQKTY**S**NKGTFR  ALLLSV**S**RTSGEV  RKTEYG**S**RLRIQD  SQFAIP**S**FCHFVF  ALPMPE**S**PDAANC  DYRGTA**S**TTKSGH  TASTTK**S**GHQCQP  PHSHHL**S**STDFPE  HSHHLS**S**TDFPEL  LYILVP**S**IAIPLV  RRQLMA**S**PSQDME  QLMASP**S**QDMEMP  AKLKEI**S**LSAVRF  LKEISL**S**AVRFME  PLSMIF**S**YCSHGD  EFLVMR**S**PHSDVG  VMRSPH**S**DVGSTD  DDRTVK**S**ALEPPD  KLNVKI**S**DLGLFR  YKLLGN**S**LLPIRW  IMYGKF**S**IDSDIW  GKFSID**S**DIWSYG  QPYCGY**S**NQDVVE  NSSAQT**S**GASNTT  SNTTQT**S**SLSTSP  NTTQTS**S**LSTSPV  TSSLST**S**PVSNVS  SPVSNV**S**NARYVG  MVPKPS**S**HHSGSG  KPSSHH**S**GSGSTS  YVTTAP**S**NTSMAD  TAPSNT**S**MADRAA  DRAALL**S**EGADDT  EEEEEG**S**VPETEL  KICEAV**S**LKPTAW  LKPTAW**S**LRHAVG  ALNVDD**S**RIGQTA  TKQKTN**S**PAWHDE  ELLQNG**S**RHFEDW  YVIIDL**S**GSSGEA  IIDLSG**S**SGEAPK  IDLSGS**S**GEAPKD  RQPTYC**S**HCRDFI  VGSQRF**S**VNMPHK  FCDHCG**S**LLWGLL  ESPQPA**S**GSSPSE  PQPASG**S**SPSEED  QPASGS**S**PSEEDR  ASGSSP**S**EEDRSK  PSEEDR**S**KSAPTS  EEHRAA**S**SPDGQL  EHRAAS**S**PDGQLM  KVLGKG**S**FGKVML  MFQIQR**S**RKFDEP  KFDEPR**S**RFYAAE  YAAEVT**S**ALMFLH  ELEYGP**S**VDWWAL  EDDLFE**S**ILHDDV  LYPVWL**S**KEAVSI  LSKEAV**S**ILKAFM  RLGCVA**S**QNGEDA  FLLDGS**S**SIGRSN  GLVLPF**S**GAASAQ  RAIREL**S**YKGGNT  TRPPDD**S**TSAPRD  PPDDST**S**APRDLV  LGQPLP**S**ERQEVN  VPAGET**S**VRLRGL  LLVAWR**S**VPGATG  LGPGQG**S**VLLRDL  MARTDA**S**VEQTLR  YRIIVR**S**TQGVER  PREGSA**S**VLTVRR  VTQASS**S**SVTITW  QILNAS**S**DVLRIT  LGPELS**S**YHLDGL  PAGEGP**S**AEVTAR  GQEVPG**S**PQTLPG  GVSYIF**S**LTPVLD  SYSHRP**S**PLFPLN  PQGPPG**S**ATAKGE  LPGQPG**S**PGEQGP  EDGRNG**S**PGSSGP  RNGSPG**S**SGPKGD  NGSPGS**S**GPKGDR  QGLPGP**S**GPPGLP  TAGIKA**S**ALREIV  PPGPPG**S**VGPPGA  EKGDVG**S**AGLKGD  DPGDKG**S**KGEPGD  PSGNDG**S**AGPPGP  QFIASG**S**RPLPSY  AADTAG**S**QLHAVP  SEYSEY**S**VEEYQD  AQDTQP**S**PLALLA  SKIGPP**S**PGDDEE  IPSAAT**S**SGQYVL  QNQQIF**S**VAPGSD  SSNGTV**S**SVQYQV  SNGTVS**S**VQYQVI  GGINQE**S**SQIQII  SLGLSG**S**SQTMTA  TGQAMD**S**SDNSER  GQAMDS**S**DNSERT  MDSSDN**S**ERTGER  RTGERV**S**PDINET  DLFVPT**S**SSSQLP  LQQNTN**S**LTTSSG  TNSLTT**S**SGQVHS  NSLTTS**S**GQVHSS  SGQVHS**S**DLQGNY  YIQSPV**S**EETQAQ  HLQLQE**S**QQPTSQ  IHGVQA**S**GQNISQ  AQTVTP**S**GQVTWQ  FTSTPV**S**LSTGQL  STPVSL**S**TGQLPN  PGENAD**S**PADIRI  WQLSGD**S**TLNTND  AHLRWH**S**GERPFV  FVCPEC**S**KRFMRS  KKGIHS**S**STVLAS  KGIHSS**S**TVLASV  ANIQQG**S**VSGIGT  VNTSAT**S**NQDILT  LVLCGV**S**LLLSGT  GVSLLL**S**GTVEGA  DLILIN**S**LPLVSD  NSLPLV**S**DAETSL  WKREKA**S**KINGAY  KMRQQA**S**FLPATL  GDNVNI**S**FKKVLI  VIYKNG**S**FIHSVP  NGSFIH**S**VPRHEV  GGNLFT**S**AFTRLI  TCKERC**S**GQEGCK  GQEGCK**S**YVFCLP  NHTDHF**S**VAIFTI  SGVWVC**S**VNTVAG  LNLLPK**S**QTTLNL  QPIFPS**S**EDDFYV  VEVERR**S**VQKSDQ  RRSVQK**S**DQQNIK  LTAWTL**S**DILPPQ  PENIKI**S**NITHSS  SNITHS**S**AVISWT  HSSAVI**S**WTILDG  DGYSIS**S**ITIRYK  SSNPAF**S**HELVTL  LVTLPE**S**QAPADL  LIAILG**S**AGMTCL  AFAIAN**S**TASTLS  IANSTA**S**TLSSQQ  STASTL**S**SQQLLH  RGMDYL**S**QKQFIH  SVYTTN**S**DVWSYG  KPYERP**S**FAQILV  FAQILV**S**LNRMLE  EMKATE**S**GAQSAP  TESGAQ**S**APLPME  MEGVDI**S**PKQDEG  DGTKFD**S**SLDRKD  GTKFDS**S**LDRKDK  DRKDKF**S**FDLGKG  PEYAYG**S**AGSPPK  AYGSAG**S**PPKIPP  MEKGEH**S**IVYLKP  IVYLKP**S**YAFGSV  PSYAFG**S**VGKEKF  YELHLK**S**FEKAKE  FEKAKE**S**WEMNSE  ESWEMN**S**EEKLEQ  EEKLEQ**S**TIVKER  QYKKIV**S**WLEYES  SWLEYE**S**SFSNEE  WLEYES**S**FSNEEA  EYESSF**S**NEEAQK  QALRLA**S**HLNLAM  LKLQAF**S**AAIESC  FSAAIE**S**CNKALE  KALELD**S**NNEKGL  KAKAEA**S**SGDHPT  AKAEAS**S**GDHPTD  MKEEQK**S**NTAGSQ  KSNTAG**S**QSQVET  ETPATE**S**PDTGLY  NAEDIK**S**GKLSRE  RELDFV**S**HHVRTK  LKRQEV**S**RLRMLL  RRRYLE**S**LGEEQR  KVNVPG**S**QAQLKE  ILHDIN**S**DGVLDE  LEEFLA**S**TQRKEF  TEALGR**S**QGRLEA  QKEVDT**S**EKKLLE  DGKPRT**S**PKSVKF  PRTSPK**S**VKFLFG  KNRMQL**S**GEGAKT  TREYKT**S**FHALTS  SFHALT**S**ILKAEG  GIYTGL**S**AGLLRQ  NAAQLA**S**YSQSKQ  AQLASY**S**QSKQFL  LASYSQ**S**KQFLLD  KQFLLD**S**GYFSDN  LDSGYF**S**DNILCH  LCHFCA**S**MISGLV  FCASMI**S**GLVTTA  LVTTAA**S**MPVDIA  RYEGFF**S**LWKGFT  GGKYVD**S**EGHLYT  EPEGTH**S**FDGIWK  DGIWKA**S**FTTFTV  WFYRLL**S**ALFGIP  IYFAIL**S**FLHIWA  VVPCIK**S**FLIEIQ  IEIQCI**S**RVYSIY  CISRVY**S**IYVHTV  AVGKIF**S**NVRINL  RCQAQV**S**RRISFS  SRRISF**S**ASHRLY  RISFSA**S**HRLYSK  ASHRLY**S**KFLSDE  LYSKFL**S**DEENLK  YFADVV**S**TTENVA  LQSQPV**S**NNEEMA  SVGDGL**S**TSAVGC  GDGLST**S**AVGCAS  HEASRA**S**QLVDVE  VEKEYR**S**VLDDLT  SPQAAT**S**RDINRK  IELDHA**S**LEEDAE  GPSSLG**S**MLMPVQ  KKARVL**S**KKEERL  DSEGEE**S**EYFPTE  EDDSEE**S**DAEFDE  ILHETG**S**YTHKKE  ILITSY**S**YIRLMQ  LMQDDI**S**RYDWHY  ITMGGY**S**NASPVQ  YQNFVD**S**KEVYRI  GEMQIF**S**GLIALR  KMIVVE**S**LLKIWH  VLLFSQ**S**RQMLDI  GTTTIA**S**RQPLIT  DPDWNP**S**TDTQAR  KDDPHM**S**SNVTSN  MSSNVT**S**NDRLGE  GECSNS**S**GTGKTS  DEKLGL**S**YKRERP  SKTKHH**S**VAEEET  QDSENK**S**EAKEQS  EKLFKK**S**VGVHSV  KSVGVH**S**VMKHDA  APAGKK**S**RFGKKR  EDADSS**S**GPLASS  SSGPLA**S**SSLLAK  ERLESE**S**GHLQEA  MAKGEA**S**EKIIIN  RHETYR**S**TLRTLP  DGGGVG**S**SGSSGG  GGGVGS**S**GSSGGG  GVGSSG**S**SGGGGC  VGSSGS**S**GGGGCE  ALDIFE**S**PDGGGS  AGHGAG**S**GGCRGW  LFEDPY**S**SRAARV  FEDPYS**S**RAARVV  RVVAFA**S**LFFILV  LFFILV**S**ITTFCL  RVGNIT**S**VHFRRE  VGLSGL**S**SKAARD  GLSGLS**S**KAARDV  GHTLRA**S**TNEFLL  RIGARP**S**DPRGND  NFGMYY**S**LAMAKQ  RPAQLE**S**PMYCKS  CKSEET**S**PRDSTC  ETSPRD**S**TCSDTS  PRDSTC**S**DTSPPA  STCSDT**S**PPAREE  ERKRAD**S**KQNGDA  DANAVL**S**DEEGAG  LTQPLA**S**SPTPEE  TQPLAS**S**PTPEER  RRALRR**S**TTRDRN  AACFLL**S**TGDYAC  YACADG**S**VRKGTF  DLPLQH**S**PEAACP  AEPAQA**S**GPYVEI  EGRSAG**S**IPGERS  SIPGER**S**TDTTKT  PGTVRI**S**LVTKDP  PDRCIH**S**FQNLGI  DLEQAI**S**QRIQTN  VTVRDP**S**GRPLRL  RLPPVL**S**HPIFDN  CRVNRN**S**GSCLGG  VNRNSG**S**CLGGDE  GWEARG**S**FSQADV  EARGSF**S**QADVHR  PPYADP**S**LQAPVR  QAPVRV**S**MQLRRP  KSIMKK**S**PFSGPT  MKKSPF**S**GPTDPR  RRIAVP**S**RSSASV  IAVPSR**S**SASVPK  AVPSRS**S**ASVPKP  QPYPFT**S**SLSTIN  PYPFTS**S**LSTINY  PFTSSL**S**TINYDE  PTMVFP**S**GQISQA  FPSGQI**S**QASALA  GQISQA**S**ALAPAP  AGEGTL**S**EALLQL  GALLGN**S**TDPAVF  LASVDN**S**EFQQLL  SGDEDF**S**SIADMD  IADMDF**S**ALLSQI  ELTALE**S**LIEMGF  LGREPT**S**SEQGGL  GREPTS**S**EQGGLE  QGGLEG**S**GSAAGE  GLEGSG**S**AAGEGK  EGKPAL**S**EEERQE  RQGQEL**S**AARQRL  AKKYGG**S**VGSQPP  YGGSVG**S**QPPPVA  PVPSSP**S**QEPPTK  RLPDGT**S**LTQTFR  DPVQLL**S**GFPRRA  ELGLVP**S**AVLIVA  TAPVVF**S**TPQATQ  TQMNTP**S**QPRQHF  RQHFYP**S**RAQPPS  HVYPAG**S**QVMMIP  ITEEIM**S**GARTAS  SESQPS**S**PSPTPS  SQPSSP**S**PTPSPS  SPSPTP**S**PSPVLE  SPTPSP**S**PVLEPG  PNLAVL**S**IPGDTM  MTTIQM**S**VEESTP  EPEVES**S**PELAPP  LLNGAP**S**PPAVDL  PPAVDL**S**PVSEPE  VDLSPV**S**EPEEQA  TAPSAT**S**PAQEEE  EAGEAE**S**EKGGEE  FQFIFA**S**MQKPEG  LGRTTL**S**TRGPPR  EKAWKP**S**SKRTAA  DIARRR**S**LGNIKF  PPGPPI**S**RGLPLV  LSWGKG**S**SGGSGA  KPSDAA**S**EAARPA  STLNRF**S**ALQQAV  RRVVQR**S**SLSRER  CVQELA**S**PSLLFI  QELASP**S**LLFIFV  LCAGHL**S**TAQYYQ  WIEANL**S**EQQIVS  SEQQIV**S**NTLVRA  FLRIGL**S**NFDCGS  SNFDCG**S**CQSCQG  DCGSCQ**S**CQGEAV  VKEYVE**S**ENGQMY  MYPPWD**S**TFDAHI  KNVDLI**S**ETTVEL  TTVELY**S**LAERCR  RYFLEM**S**DTKDMN  PQPTFC**S**VCHEFV  IAKCTG**S**AINSRE  TGSAIN**S**RETMFH  KVYNYK**S**PTFCEH  ALAMIE**S**TQQARC  EIGLPC**S**IKNEAR  REPQGI**S**WESPLD  LNKERP**S**LQIKLK  KMLGKG**S**FGKVFL  VEKRVL**S**LAWEHP  LMYHIQ**S**CHKFDL  CHKFDL**S**RATFYA  GLQFLH**S**KGIVYR  GQKYNH**S**VDWWSF  HSVDWW**S**FGVLLY  EMLIGQ**S**PFHGQD  EEELFH**S**IRMDNP  FRPKVK**S**PFDCSN  KSPFDC**S**NFDKEF  AEPQPP**S**GGLTDE  TDEAAL**S**CCSDAD  AALSCC**S**DADPST  CSDADP**S**TKDFLL  VKDPKK**S**LDFYTR  FPIMKF**S**LYFLAY  KIAWAL**S**RKATLE  EDDETQ**S**YHNGNS  SYHNGN**S**DPRGFG  AVPDVY**S**ACKRFE  YKCEGR**S**AGSIPG  SIPGEH**S**TDNNRT  NNRTYP**S**IQIMNY  ALPPVV**S**NPIYDN  VNKNCG**S**VRGGDE  EAKGIF**S**QADVHR  DQEVSE**S**MDFRYL  PTLASQ**S**AGITVN  RPGLLG**S**IGEGRY  KEPNLF**S**HDAVVR  MPTGVS**S**QAESYY  AESYYP**S**PGPISS  APLPSS**S**WSSVAH  PSSSWS**S**VAHPTP  GNTNPL**S**SFSTRT  NPLSSF**S**TRTLPS  IVGMEA**S**SMPSAD  EASSMP**S**ADLYGI  ADLYGI**S**DPNMLS  NMLSNC**S**VNMMTT  QLHQMS**S**SSMSAG  LHQMSS**S**SMSAGA  MSSSSM**S**AGANSN  TVFVSQ**S**DAFEGS  SDAFEG**S**DFSCAD  FSCADN**S**MINESG  NSMINE**S**GPSNST  INESGP**S**NSTNPN  HGFVQD**S**QYSGIG  VQDSQY**S**GIGSMQ  QYSGIG**S**MQNEQL  VKYVVP**S**FSAGGL  DLKSVQ**S**LATGPA  ALPALV**S**CGSSLQ  ALVSCG**S**SLQGRC  APACLF**S**AHHNRP  CPDCVA**S**PLGTRV  DAAVAA**S**FSPRSV  AVAASF**S**PRSVSI  SIEYVH**S**FHTGAF  LTVQPA**S**VTDDPS  TRLARL**S**ATEPEL  FPLLVS**S**SFSRVD  QVELVR**S**LNYLLY  NFSLGD**S**GQPVQR  NFYLHP**S**GLVPEG  RPLPKD**S**SKLRPV  VGPTNV**S**LTVTNM  PGATVA**S**VPLSLQ  EDPVVL**S**ISPNCG  PVVLSI**S**PNCGYI  WVAGNL**S**ARGDGA  VTVGGE**S**CQHEFR  NLNDLA**S**LDQTAG  AIDGLD**S**TTCVHG  TCVHGA**S**FSDSED  ERVVTH**S**DRVIGK  IQCAIK**S**LSRITE  TVKDLI**S**FGLQVA  GLQVAR**S**MEYLAE  LDREYY**S**VQQHRH  PEYCPD**S**LYQVMQ  PEQPQF**S**PMPGNV  YASAML**S**VFNSDD  MLSVFN**S**DDYSPA  FNSDDY**S**PAVQEN  IPALRR**S**SSFLCT  PALRRS**S**SFLCTE  ALRRSS**S**FLCTES  SFLCTE**S**CNSKYQ  CTESCN**S**KYQCET  CETGEN**S**KGNVQD  NAFIVW**S**RDQRRK  NPRMRN**S**EISKQL  MRNSEI**S**KQLGYQ  MLPKNC**S**LLPADP  LPADPA**S**VLCSEV  PASVLC**S**EVQLDN  CTKATH**S**RMEHQL  PPINAA**S**SPQQRD  PINAAS**S**PQQRDR  HQCYWK**S**GEPQSD  SDDIEA**S**RMKRAA  TNEFCA**S**CPTFLR  LCDPHP**S**KKGASS  PSKKGA**S**SAYLEN  SKKGAS**S**AYLENS  SAYLEN**S**KGAPNN  KGAPNN**S**CSEIKM  REREDY**S**PLIRVI  IGRVFS**S**AEALVQ  AEALVQ**S**FRKVKQ  TKEELK**S**LQAKDE  AAMEED**S**EASSSR  EEDSEA**S**SSRIGD  DSEASS**S**RIGDSS  SSRIGD**S**SQGDNN  LGPDDV**S**VDIDAI  VYTRLL**S**NEKIET  NALVYL**S**PNVECD  TYHNVY**S**RDPNYL  ENRNLH**S**PEYLEM  LFCKAM**S**KLPLAA  KLIRLW**S**KYNADQ  ITYKVI**S**NEFNSR  ISNEFN**S**RNLVND  NRIRMY**S**ERRITV  RITVLY**S**LVQGQQ  VDEGGV**S**KEFFQL  HPVLYQ**S**LKDLLE  MITFQI**S**QTDLFG  EFVNLY**S**DYILNK  HMVTNE**S**PLKYLF  LLPEYS**S**KEKLKE  RVQAMK**S**PDHNGE  MRLRRL**S**TKYRTE  LTLKTP**S**QDSDYI  KTPSQD**S**DYINAN  DHDVPS**S**FDSILD  VPSSFD**S**ILDMIS  SILDMI**S**LMRKYQ  PICIHC**S**AGCGRT  TENMVS**S**IEPEKQ  EPEKQD**S**PPPKPP  KPPRTR**S**CLVEGD  HAIKIK**S**ASPCIA  CIADKI**S**KPQELS  LNVGDT**S**QNSCVD  NSCVDC**S**VTQSNK  DCSVTQ**S**NKVSVT  PEESQN**S**DTPPRP  KGHVTW**S**FHGPEN  IPIPDL**S**EGNSSD  QTRKTV**S**LTPSPT  LFRTPL**S**FTNPLH  PLHSDD**S**DSDERN  DSDERN**S**DGAVTQ  AATSTE**S**ISTRKV  RKVLPM**S**IARHNI  IAGTTH**S**GAEKDV  EKDVDV**S**EDSPPP  PERTPE**S**FVLASE  ESFVLA**S**EHNTPV  HNTPVR**S**EWSELQ  PVRSEW**S**ELQSQE  EWSELQ**S**QERSEQ  LQSQER**S**EQKKSE  PYTEAA**S**LSTGST  TEAASL**S**TGSTVT  ASLSTG**S**TVTIKG  TEMKEE**S**DIVFHF  RRVVMN**S**REYGAW  WKQQVE**S**KNMPFQ  GQEFEL**S**ISVLPD  EFELSI**S**VLPDKY  VMVNGQ**S**SYTFDH  MVNGQS**S**YTFDHR  QVWRDI**S**LTKFNV  VFHYFE**S**NSEPTT  EPTTWA**S**IIRHGD  IQKIVD**S**HKVKHV  RLSHLR**S**EEVHWL  VDMGVS**S**VREKYE  CLEIRR**S**YWEMRG  KFFEIL**S**PVYRFD  KCALGS**S**WIISVE  GPEEGI**S**YLTDKG  VQTIQY**S**NSEDKD  TIQYSN**S**EDKDRK  RLVNGT**S**QSFIIR  VNGTSQ**S**FIIRPQ  HQGIYM**S**PENPAL  TCKNCT**S**DSVREK  YSLDLA**S**LILYAY  LYAYQL**S**TALAYL  ALAYLE**S**KRFVHR  RNVLVS**S**NDCVKL  LGDFGL**S**RYMEDS  KWMAPE**S**INFRRF  RRFTSA**S**DVWMFG  CWAYDP**S**RRPRFT  ATVSWD**S**GGSDEA  EAPPKP**S**RPGYPS  GYPSPR**S**SEGFYP  TNHYQV**S**GYPGSH  ITAMAG**S**IYPGQA  LLDQTD**S**WNHRPQ  IDREDG**S**LQGPIG  AQKLLN**S**DLGELI  LRIAFN**S**YELGSL  NSYELG**S**LQAEDE  KMKEAL**S**TERGKT  MYPEWK**S**TFDAHI  AAEEPV**S**EVTVGV  EVTVGV**S**VLAERC  QAKVLM**S**VQYFLE  CKQSMR**S**EDEAKF  GQPTFC**S**VCKDFV  TGTAAN**S**RDTIFQ  KVHNYM**S**PTFCDH  FCDHCG**S**LLWGLV  RRSDSA**S**SEPVGI  EDMQDN**S**GTYGKI  GKIWEG**S**SKCNIN  KIWEGS**S**KCNINN  KVLGKG**S**FGKVLL  GLQFLH**S**KGIIYR  GLKYTF**S**VDWWSF  FSVDWW**S**FGVLLY  EMLIGQ**S**PFHGDD  EDELFE**S**IRVDTP  RWITKE**S**KDILEK  FRPKVK**S**PRDYSN  KSPRDY**S**NFDQEF  KARLSY**S**DKNLID  HDSIQI**S**WKAPRG  LLVTPT**S**GGKTNQ  WHLNAF**S**TKDEVI  SRNLRE**S**GVELFA  DREIKA**S**AHAITG  VDGTVS**S**TVLKNL  VLKNLM**S**LTEYQI  YDVTEN**S**MRVKWD  LDDGSE**S**EVVTAV  TIVPTT**S**VTSVFQ  GDETTS**S**LRVKWD  TDGEGV**S**VSAPGK  GKTLPS**S**GPQNLR  ITWDPP**S**SPVKGY  TWDPPS**S**PVKGYR  LITNLL**S**GMDYNV  DRQKQE**S**TVGGGT  DSEYKI**S**VYTKLQ  QEIEGP**S**VSIMEK  NFNKII**S**FLYSTV  DYSELV**S**IGSKPS  LVEKDF**S**SVEGVS  PSDYTI**S**FLFRIL  RKIFYG**S**FHKLHI  GLRDDE**S**CPDLPH  CPDLPH**S**CSCSET  IPGGVG**S**PGRDGS  SPGRDG**S**PGQRGL  VPGITG**S**MGPQGA  ERGDLQ**S**QAMVRS  IPSHSS**S**IRTVQG  PGPAGP**S**GESRPG  PGYCDP**S**SCSAYG  AAVILE**S**IFLKRS  LTVHKL**S**YYEYDF  ERGRRG**S**KKGSID  PRRGEE**S**SEMEQI  RRGEES**S**EMEQIS  SEMEQI**S**IIERFP  GQYLCC**S**QTAKNA  PAAAPV**S**TSELKK  YFILEE**S**NLPWWR  VTEAED**S**IEMYEW  SKHMTR**S**QAEQLL  GFIVRD**S**SKAGKY  VCSTPQ**S**QYYLAE  AEKHLF**S**TIPELI  NYHQHN**S**AGLISR  NSAGLI**S**RLKYPV  RLKYPV**S**QQNKNA  QNKNAP**S**TAGLGY  AGLGYG**S**WEIDPK  KMIKEG**S**MSEDEF  QGVVKV**S**DFGLSR  VSDFGL**S**RYVLDD  LDDEYT**S**SVGSKF  KFPVRW**S**PPEVLM  PEVLMY**S**KFSSKS  LMYSKF**S**SKSDIW  MYSKFS**S**KSDIWA  SKFSSK**S**DIWAFG  LMWEIY**S**LGKMPY  VYTIMY**S**CWHEKA  TFKILL**S**NILDVM  YNEPHE**S**RTNSDI  GLNGCD**S**PDPDAD  DIDLMI**S**RQRLCA  PVSIPV**S**SHNSLV  VSIPVS**S**HNSLVY  HNSLVY**S**NPVSSL  LPLAHP**S**LQRNSM  PSLQRN**S**MSPGVT  MGGDLT**S**GAGTSA  TSGAGT**S**AGNGYG  YGNPRN**S**PGLLVS  SPGLLV**S**PGNLNK  VLIPPG**S**KNTMPS  SKNTMP**S**VSEDVD  NQRINN**S**QSAQSL  RINNSQ**S**AQSLAT  TYGTEY**S**LSSADL  SALHLG**S**VTGWQQ  LHNMPP**S**ALSQLG  MPPSAL**S**QLGACT  SNLSLP**S**TQSLNI  SLPSTQ**S**LNIKSE  QSLNIK**S**EPVSPP  DRTTTP**S**RYPQHT  DSLSSC**S**SSYDGS  SLSSCS**S**SYDGSD  LSSCSS**S**YDGSDR  SSSYDG**S**DREDHR  HRNEFH**S**PIGLTR  IGLTRP**S**PDERES  DERESP**S**VKRMRL  DPTGTK**S**CFETKE  DKKQCK**S**RFVTPF  LVGEFV**S**DVLLVP  QGMTLY**S**YGMLLP  QTKIIG**S**VSKEEE  KIIGSV**S**KEEEEE  DYDVYK**S**EFPTEA  NPTEPG**S**DGTMSD  GSDGTM**S**DKEITH  DVKAVC**S**QEAMTG  RWYFDL**S**KGKCVR  NRNNFE**S**EDYCMA  DVYFET**S**ADDNEH  LEKEAA**S**EKQQLV  YLAALQ**S**DPPRPH  KAAQMK**S**QVMTHL  EERRNQ**S**LSLLYK  RRNQSL**S**LLYKVP  MDQFTA**S**ISETPV  QFTASI**S**ETPVDV  PVDVRV**S**SEESEE  RVSSEE**S**EEIPPF  HPMKKG**S**GVGEQD  EEKVIN**S**KNKVDE  LEEERE**S**VGPLRE  PLREDF**S**LSSSAL  REDFSL**S**SSALIG  EDFSLS**S**SALIGL  DFSLSS**S**ALIGLL  ATVIVI**S**LVMLRK  RQYGTI**S**HGIVEV  EANADT**S**VEEESF  TSVEEE**S**FGPQPI  FGPQPI**S**RLEQCG  INIKGI**S**EAKADK  EFHQRR**S**EIIQIT  IQITTG**S**KELDKL  GGIETG**S**ITEMFG  AERYGL**S**GSDVLD  RYGLSG**S**DVLDNV  QLLYQA**S**AMMVES  SAMMVE**S**RYALLI  ALLIVD**S**ATALYR  LYRTDY**S**GRGELS  SGRGEL**S**ARQMHL  NIIAHA**S**TTRLYL  ICKIYD**S**PCLPEA  VVEVRG**S**NGAFYK  DEVEVY**S**RANEKE  TIERLR**S**VNPNKP  KAVGAF**S**VTYDPE  IDMHFR**S**LRTKLS  SLRTKL**S**LIMRNE  MRNEEA**S**KQLESS  ASKQLE**S**SRQLAS  SSRQLA**S**RFHEQF  AVKKAR**S**FLEFAE  SLPSNN**S**RVGPNA  QRVLVA**S**SVVAGE  RVLVAS**S**VVAGES  SVVAGE**S**QKPELK  FVGTKD**S**IANATV  LRQIGA**S**SRPPPN  RTDKEK**S**YVTDDG  QGMGRG**S**RPYRNR  YTSGTN**S**EASNAS  GTNSEA**S**NASETE  NASETE**S**DHRDEL  DHRDEL**S**DWSLAP  DELSDW**S**LAPTEE  TEEERE**S**FLRRGD  KGNDDH**S**RTDNRP  GRTTDG**S**LQIRVD  DCNNER**S**VHTKTL  KTLQNT**S**SEGSRL  TLQNTS**S**EGSRLR  NTSSEG**S**RLRTGK  KKEKPD**S**VDGQQP  LLAGLF**S**LSQAQY  AGLFSL**S**QAQYED  WFHYLR**S**QQSTYY  YLRSQQ**S**TYYDPY  PAYTYG**S**PSPPDP  YTYGSP**S**PPDPRD  YLPFVP**S**RMKYVY  QNNQIT**S**IQEGVF  HGNQIT**S**DKVGRK  VGRKVF**S**KLRHLE  PGPLPR**S**LRELHL  LDHNQI**S**RVPNNA  EIQEVG**S**SMRGLR  IQEVGS**S**MRGLRS  SMRGLR**S**LILLDL  LILLDL**S**YNHLRK  VPDGLP**S**ALEQLY  VYTVPD**S**YFRGAP  LLYVRL**S**HNSLTN  VRLSHN**S**LTNNGL  TNNGLA**S**NTFNSS  ASNTFN**S**SSLLEL  SNTFNS**S**SLLELD  NTFNSS**S**LLELDL  LLELDL**S**YNQLQK  NRINEF**S**ISSFCT  INEFSI**S**SFCTVV  NEFSIS**S**FCTVVD  VDVVNF**S**KLQVLR  GNEIKR**S**AMPADA  GQFKDI**S**LSDYKG  FKDISL**S**DYKGKY  TEIIAF**S**DRAEEF  CQVIGA**S**VDSHFC  IGASVD**S**HFCHLA  MNIPLV**S**DPKRTI  KADEGI**S**FRGLFI  DLPVGR**S**VDETLR  AGWKPG**S**DTIKPD  KPDVQK**S**KEYFSK  SAKQRG**S**KGGHGA  EKGAHP**S**GGADDV  GGGGGK**S**SSSSSA  AAAAAS**S**SASCSR  AASSSA**S**CSRRLG  VAAAAF**S**GWCVHH  VQQVRR**S**HQDFSR  RSHQDF**S**RQREEL  TFGTFE**S**ILRSSQ  ESILRS**S**QHKQDL  AVKQGE**S**EVSRIS  QGESEV**S**RISEVL  SEVSRI**S**EVLQKL  EILKDL**S**DGIHVV  LTELTK**S**INDNIA  VKEIQT**S**AKSREW  IQTSAK**S**REWDME  TLQTME**S**DIYTEV  RSEESV**S**RLPEEI  ELRQLK**S**DSHGPK  SARQTE**S**LESLLS  QTESLE**S**LLSKSQ  ESLLSK**S**QEHEQR  RLEGLG**S**SEADQD  LEGLGS**S**EADQDG  DQDGLA**S**TVRSLG  VEELKR**S**VGELPS  LPSTVE**S**LQKVQE  FLDRLS**S**LDNLKA  LDNLKA**S**VSQVEA  NENNLE**S**AKGLLD  DPAARM**S**RSSGRS  AARMSR**S**SGRSGS  ARMSRS**S**GRSGSM  SRSSGR**S**GSMDPS  SSGRSG**S**MDPSGA  SGSMDP**S**GAHPSV  PSGAHP**S**VRQTPS  SVRQTP**S**RQPPLP  PPLPHR**S**RGGGGG  RGGGGG**S**RGGARA  RGGARA**S**PATQPP  PPLLPP**S**ATGPDA  TPLLPP**S**ATASVK  PPSATA**S**VKMEPE  LMAEKD**S**LDPSFT  KDSLDP**S**FTHAMQ  KIQKGD**S**KKDDEE  NYLDLF**S**HKNMKL  ETGAKI**S**VLGKGS  SVLGKG**S**MRDKAK  EQFLEL**S**YLNGVP  NGVPEP**S**RGRGVP  DTYAEQ**S**YEGYEG  GYEGYY**S**QSQGDS  EGYYSQ**S**QGDSEY  SQSQGD**S**EYYDYG  HGEVQD**S**YEAYGQ  WNGTRP**S**LKAPPA  LVVDFL**S**YKLSQK  FLSYKL**S**QKGYSW  LSQKGY**S**WSQFSD  QKGYSW**S**QFSDVE  YSWSQF**S**DVEENR  APEGTE**S**EMETPS  AINGNP**S**WHLADS  NGATGH**S**SSLDAR  GATGHS**S**SLDARE  ATGHSS**S**LDAREV  RYRRAF**S**DLTSQL  AFSDLT**S**QLHITP  PGTAYQ**S**FEQVVN  RIVAFF**S**FGGALC  GALCVE**S**VDKEMQ  EMQVLV**S**RIAAWM  NNAAAE**S**RKGQER  AGLGAG**S**GGATRP  ATEKEA**S**ARREIG  GAVIGG**S**AGASPP  GGSAGA**S**PPSTLT  AGASPP**S**TLTPDS  STLTPD**S**RRVARP  LELVGE**S**GNNTST  ESGNNT**S**TDGSLP  DELYRQ**S**LEIISR  QSLEII**S**RYLREQ  TKPMGR**S**GATSRK  GRSGAT**S**RKALET  NEDDVK**S**LSRVMI  DDVKSL**S**RVMIHV  VMIHVF**S**DGVTNW  RIVTLI**S**FGAFVA  KTINQE**S**CIEPLA  IEPLAE**S**ITDVLV  TQDEII**S**KTKQVI  LKNEHN**S**ILQSLL  HNSILQ**S**LLETLK  LKKDDE**S**NLVEEK  NLVEEK**S**NMIRKS  SNMIRK**S**LEMLEL  MLELGL**S**EAQVMM  QVMMAL**S**NHLNAV  HLNAVE**S**EKQKLR  QQKLQK**S**EQSVAQ  LQKSEQ**S**VAQLEE  KYDDDI**S**PSEDKD  DDDISP**S**EDKDTD  EDKDTD**S**TKEPLD  GIQQQH**S**SAAAAA  IQQQHS**S**AAAAAQ  LVIQYA**S**QGRYEV  EDLEKT**S**GHDHPD  TKNNLA**S**CYLKQG  HEREFG**S**VDDENK  KQKDGT**S**FGEYGG  KACKVD**S**PTVTTT  EEAAMR**S**RKQGLD  DVVKYE**S**GPDGGE  DGGEEV**S**MSVEWN  GEEVSM**S**VEWNGG  EWNGGV**S**GRASFC  GVSGRA**S**FCGKRQ  KRKSWM**S**KVFSGK  EFPPHH**S**QSTFRK  PPHHSQ**S**TFRKTS  EKLGDG**S**FGVVRR  GEWDAP**S**GKTVSV  LKPDVL**S**QPEAMD  FLLGTL**S**RYAVQV  GMGYLE**S**KRFIHR  LKTRTF**S**HASDTW  TSVAGL**S**AQDISQ  HTGHGD**S**DPRHCW  DPPDLL**S**VELSTS  DQDPLS**S**DFKRLG  LWLAKP**S**ARVPGT  TKASRG**S**GAEVTL  LRPCAP**S**LAQLAM  LAMDAC**S**LLDETP  DETPPQ**S**PTRALP  GVPAGP**S**QGQTNY  GGKPPS**S**AQTAEI  AGSPAP**S**PSPGGD  RPHVQL**S**PAPPGE  PGEEET**S**QWPGPA  QWPGPA**S**PPRVPP  QGSRTP**S**PLVPPG  PLVPPG**S**SPLPPR  PPRLSS**S**PGKTMP  TMPTTQ**S**FASDPK  DGKKVS**S**THYYLL  LLPERP**S**YLERYQ  FLREAQ**S**PEEPTP  LLLPPP**S**TPAPAA  LQCHGW**S**VQRAAQ  PTCYCN**S**SFQLQA  TCYCNS**S**FQLQAD  STITPT**S**TRQTTA  RQPRVR**S**HACEND  EEDPKD**S**RRGRLE  FWTEYR**S**GSVYRL  ESNATC**S**ARTCPP  NDCEDN**S**DEENCE  LACRPP**S**HPCANN  HTCQIQ**S**YCAKHL  NYNRTV**S**CACPHL  LAVDWV**S**RNLFWT  RCNLDG**S**GLEVID  VNNGDC**S**QLCLPT  GSIKRG**S**KDNATD  LKSIHL**S**DERNLN  DTLYWT**S**YTTSTI  IMRAAL**S**GANVLT  DDCGDG**S**DEIPCN  CSATDC**S**SYFRLG  SATDCS**S**YFRLGV  HCNKFC**S**EAQFEC  DDCGDG**S**DEAAHC  HINECL**S**RKLSGC  TDWGDH**S**LIGRIG  RIGMDG**S**SRSVIV  SNLCLL**S**PGGGHK  IHVCLP**S**QFKCTN  NDCVDG**S**DEPANC  DDNEIR**S**LFPGHP  WHTGTI**S**YRSLPP  NRKTLI**S**GMIDEP  SRHSLA**S**TDEKRE  RVEFPR**S**GRGTGR  GRYGPP**S**RRSENR  GPPSRR**S**ENRVVV  ENRVVV**S**GLPPSG  VSGLPP**S**GSWQDL  GLPPSG**S**WQDLKD  DNTKFR**S**HEGETA  KVDGPR**S**PSYGRS  DGPRSP**S**YGRSRS  SPSYGR**S**RSRSRS  SYGRSR**S**RSRSRS  GRSRSR**S**RSRSRS  SRSRSR**S**RSRSNS  SRSRSR**S**RSNSRS  SRSRSR**S**NSRSRS  SRSRSN**S**RSRSYS  SRSNSR**S**RSYSPR  SNSRSR**S**YSPRRS  SRSRSY**S**PRRSRG  SYSPRR**S**RGSPRY  PRRSRG**S**PRYSPR  RGSPRY**S**PRHSRS  PVQNAP**S**ETSEEE  NAPSET**S**EEEPKR  EEEPKR**S**PAQQES  SKEVAE**S**NSCKFP  TAKGKE**S**GSSGPN  KGKESG**S**SGPNKF  GKESGS**S**GPNKFI  NKFILI**S**CGGAPT  RPQTQT**S**YDAKRT  SSDGLG**S**RSIKQE  SERPPY**S**YMAMIQ  KPGWKN**S**IRHNLS  FWTIHP**S**ANRYLT  LPEHLE**S**QQKRPN  AASLMS**S**ELARHS  GIAPLS**S**AGPGKE  RPIKVE**S**PPLEEW  PSFKEE**S**SHSWED  SFKEES**S**HSWEDS  TPRPKK**S**YSGLRS  SEGPST**S**RWAAEL  LPFPAD**S**SDPASQ  PFPADS**S**DPASQL  DPASQL**S**YSQEVG  ETLPIS**S**TPSKSV  FSPVQT**S**QGASDP  QTSQGA**S**DPLPDP  SAPPLE**S**PQRLLS  DVPKPG**S**PEPQVS  GLAANR**S**LTEGLV  TMNDSL**S**KILLDI  AALGVQ**S**INWQTA  HHTDKF**S**SQELIL  MNKGLG**S**NERLEF  RLEFIV**S**TGPYPS  STGPYP**S**ESAMTK  GPYPSE**S**AMTKAV  KAVFPL**S**NGSSGG  FPLSNG**S**SGGWSA  GNTLTI**S**ISSPAS  SQGGIS**S**VKLGTF  PWLNVD**S**VFMGNH  ILSICL**S**ILDRSL  LSILDR**S**LNFRRD  AATDVA**S**RNDPKY  LSAMIN**S**NDDNGV  VLAGNW**S**GTYTGG  PRSWNG**S**VEILKN  RSLGIP**S**RVITNF  VITNFN**S**AHDTDR  EGWFVR**S**DLGPSY  RSDLGP**S**YGGWQV  ATPQER**S**QGVFQC  WKNSVN**S**HTIGRY  TIGRYI**S**TKAVGS  STKAVG**S**NARMDV  YKYPEG**S**DQERQV  PFAATS**S**MGLETE  HEVWKD**S**ATMSLD  YEKYLK**S**DNMIRI  CKVPDE**S**EVVVER  NVQMLF**S**NPLDEP  VLMVEG**S**GLLLGN  QLLADF**S**CNKFPA  GPEALS**S**LLLLLL  DRTIPD**S**DISASS  IPDSDI**S**ASSSWS  DSDISA**S**SSWSDS  DISASS**S**WSDSTA  SSSWSD**S**TAARHS  RHSRLE**S**SDGDGA  HSRLES**S**DGDGAW  AWCPAG**S**VFPKEE  GLGKEF**S**RSYRLR  SYRLRY**S**RDGRRW  WGQEVI**S**GNEDPE  GQTMYL**S**EAVYLN  AVYLND**S**TYDGHT  YDYVGW**S**NHSFSS  VGWSNH**S**FSSGYV  SNHSFS**S**GYVEME  PRARAV**S**VPLGGR  GPWLLF**S**EISFIS  LLFSEI**S**FISDVV  SEISFI**S**DVVNNS  PPPTNF**S**SLELEP  RGNPPH**S**APCVPN  GSALLL**S**NPAYRL  TNTQAY**S**GDYMEP  PPPPQN**S**VPHYAE  HLCEVD**S**PQDLVS  AQGPTI**S**YPMLLH  GKFTTA**S**DVWAFG  GRQVYL**S**RPPACP  LMLRCW**S**RESEQR  RCWSRE**S**EQRPPF  EQRPPF**S**QLHRFL  PPVNAI**S**TTLLRD  GAEGKF**S**AGADIR  ADIRGF**S**APRTFG  LDKVVN**S**DPVEEA  RFAQRV**S**DQPLES  SDQPLE**S**RRLCNK  CNKPIQ**S**LPNMDS  SLPNMD**S**IFSEAL  FLYLLQ**S**GQARAL  RKANKW**S**TPSGAS  ASWKTA**S**ARPVSS  ASARPV**S**SVGVVG  PVIAVD**S**DKNQLA  ANKMIT**S**VLEKEA  VLEKEA**S**KMQQSG  ASKMQQ**S**GHPWSG  QSGHPW**S**GPKPRL  PKPRLT**S**SVKELG  KPRLTS**S**VKELGG  AVFEEM**S**LKKQVF  QVFAEL**S**AVCKPE  FLCTNT**S**ALDVDE  DVDEIA**S**STDRPH  VDEIAS**S**TDRPHL  ATVMNL**S**KKIKKI  MGPFRV**S**DLAGLD  KPDPWL**S**KFLSRY  WLSKFL**S**RYRKTH  IEPRTI**S**QDEILE  LERCLY**S**LINEAF  GEGIAA**S**PEHIDV  GPMFYA**S**TVGLPT  IPQLEP**S**DYLKKL  ALLCAP**S**LLVALD  VALDIC**S**KNPCHN  RGDVFP**S**YTCTCL  NGNIAN**S**QIAASS  NSQIAA**S**SVRVTF  SQIAAS**S**VRVTFL  VNAWTP**S**SNDDNP  NAWTPS**S**NDDNPW  VVTQGA**S**RLASHE  GASRLA**S**HEYLKA  AFKVAY**S**LNGHEF  VRLYPT**S**CHTACT  LGLKNN**S**IPDKQI  DKQITA**S**SSYKTW  KQITAS**S**SYKTWG  QITASS**S**YKTWGL  WGLHLF**S**WNPSYA  LFSWNP**S**YARLDK  NAWVAG**S**YGNDQW  LQVDLG**S**SKEVTG  QVDLGS**S**KEVTGI  GARNFG**S**VQFVAS  SVQFVA**S**YKVAYS  SYKVAY**S**NDSANW  VAYSND**S**ANWTEY  QDPRTG**S**SKIFPG  DPRTGS**S**KIFPGN  GNWDNH**S**HKKNLF  QKQPLT**S**PGSVSP  VSPSRD**S**SVPGSP  SPSRDS**S**VPGSPS  SVPGSP**S**SIVAKM  VPGSPS**S**IVAKMD  EPHFTY**S**LLEHVE  HVELPR**S**RERSLS  PRSRER**S**LSPKST  SRERSL**S**PKSTSP  RSLSPK**S**TSPPPS  LSPKST**S**PPPSPE  RSPGII**S**QASAPR  GIISQA**S**APRTTG  TGTPRT**S**LPHFHH  FHHPET**S**RPDSNI  ETSRPD**S**NIYKKP  IYKQRE**S**VGGSPQ  EDLIIE**S**SKFPAA  YWPCPP**S**LAVVET  WRKRKA**S**RRGAEE  RQREEL**S**KVTSNL  KEEMEK**S**LPIRRK  IRRKTR**S**LPDRTP  HQGTSK**S**SSLPAY  YGRTTL**S**RLQSTE  LQSTEF**S**PSGSET  STEFSP**S**GSETGS  EFSPSG**S**ETGSPG  SGSETG**S**PGLQNG  LSAEDF**S**RVFAMS  SRVFAM**S**PEEFGK  AALLLG**S**ACCGSA  GSACCG**S**AQLLFN  LFNKTK**S**VEFTFC  DGALNK**S**TVPTDF  TVPTDF**S**SAKIEV  VPTDFS**S**AKIEVS  SAKIEV**S**QLLKGD  LLKGDA**S**LKMDKS  SLKMDK**S**DAVSHT  DKSDAV**S**HTGNYT  LKYRVV**S**WFSPNE  RVVSWF**S**PNENIL  KTLKYR**S**GGMDEK  FVPGEY**S**LKNATG  LGLIVT**S**TGILIL  LHYYVF**S**TAIGLT  TAIGLT**S**FVIAIL  LAVVGL**S**LCIAAC  HGPLLI**S**GLSILA  LLISGL**S**ILALAQ  YMKFVA**S**NQKTIQ  GIIFKN**S**KTGKVD  SEFEKL**S**DFFKTH  FGGQLL**S**FDIGDQ  VFEIPL**S**NVSQCT  FAQNVL**S**KADVIQ  QMFFVI**S**LDPPIK  FLILLF**S**KDEDIS  SKDEDI**S**LTLNMN  TKNMSG**S**LYEMVS  SLYEMV**S**RVMKAL  GNFQGH**S**GAQCIT  AQCITC**S**YKASSG  CSYKAS**S**GLLYPL  IRFDEI**S**FVNFAR  GTTTTR**S**FDFEIE  TQYTFS**S**IEREEY  EENAND**S**SDDSGE  GEETDE**S**FNPGEE  DSNASA**S**SSSNEG  NASASS**S**SNEGDS  ASASSS**S**NEGDSD  MAKDRK**S**RKKPVE  EGGRGE**S**SKRDKS  VKMEKK**S**TPSRGS  EKKSTP**S**RGSSSK  RGSSSK**S**SSRQLS  SSSKSS**S**RQLSES  SRQLSE**S**FKSKEF  KKKRRR**S**EDSEEE  EEEELA**S**TPPSSE  ASTPPS**S**EDSASG  DSYRSM**S**ESYTLE  YRSMSE**S**YTLEGN  YVACRK**S**VPTVSK  TRILKC**S**EQSLIE  LKCSEQ**S**LIEFFN  GNFPMI**S**DDLVNS  SDDLVN**S**YHLLLC  DFHAKD**S**KPSSDP  KDSKPS**S**DPPCII  IIEKLC**S**LHDGLV  PGNFGE**S**FKAINK  YEEYVL**S**VGNLDE  RYIKEN**S**PCVTPV  PCVTPV**S**TATHSL  TATHSL**S**RLHTML  QPDEDF**S**NCAKEI  YYKVLE**S**VIEQEQ  LGDMDL**S**GILEQD  LAWKPE**S**PLWEKI  TGGLGR**S**ITSPTT  ALAGSL**S**SQQVTG  QQKQGQ**S**VTSSSN  QSVTSS**S**NRPRKT  CAKLDI**S**DELRKK  VTKEDK**S**FQNIMR  KRRNSG**S**SDSRSH  NSGSSD**S**RSHQNS  RLTGAN**S**DMEEEE  KNETML**S**PREKIF  FYYFSN**S**PSKRLR  ILLEDG**S**ESPAKR  RITGYK**S**HAKEIL  EVPQPL**S**WYPEEL  AWHTNL**S**RKILRK  FHQFLV**S**ETESGN  LVSETE**S**GNISRQ  TESGNI**S**RQEAVS  SRQEAV**S**MIPPLL  MCAAPG**S**KTTQLI  QAKRLS**S**PCIMVV  VVNHDA**S**SIPRLQ  LCDVPC**S**GDGTMR  GGRMVY**S**TCSLNP  MVYSTC**S**LNPIED  DEAVIA**S**LLEKSE  ASLLEK**S**EGALEL  LELADV**S**NELPGL  AVLVKK**S**SMPWNK  SAETRE**S**TQLSPA  GKPTDP**S**KLESPS  SKLESP**S**FTGTGD  DLENNG**S**KKDGVC  FYALDP**S**FPRMNL  RQLYMV**S**KELRNV  NVLLNN**S**EKMKVI  LYPFIN**S**RIITVS  SRIITV**S**MEDVKI  PFFRKL**S**SETYSQ  LSSETY**S**QAKDLA  LKYEPD**S**ANPDAL  GWRGKA**S**IRTFVP  VILTNE**S**AASTGQ  HALEWP**S**LTAQWL  PEGKDF**S**IHRLVL  VLGTHT**S**DEQNHL  NHLVIA**S**VQLPND  DAQFDA**S**HYDSEK  DASHYD**S**EKGEFG  EFGGFG**S**VSGKIE  GGFGSV**S**GKIEIE  IATKTP**S**SDVLVF  DYTKHP**S**KPDPSG  PSKPDP**S**GECNPD  LSGHLL**S**ASDDHT  GHLLSA**S**DDHTIC  ICLWDI**S**AVPKEG  AVVEDV**S**WHLLHE  WHLLHE**S**LFGSVA  HESLFG**S**VADDQK  MIWDTR**S**NNTSKP  TRSNNT**S**KPSHSV  NNTSKP**S**HSVDAH  TSKPSH**S**VDAHTA  AEVNCL**S**FNPYSE  FILATG**S**ADKTVA  LKLKLH**S**FESHKD  KLHSFE**S**HKDEIF  NETILA**S**SGTDRR  ETILAS**S**GTDRRL  LNVWDL**S**KIGEEQ  KIGEEQ**S**PEDAED  GHTAKI**S**DFSWNP  AKISDF**S**WNPNEP  WVICSV**S**EDNIMQ  DEDPEG**S**VDPEGQ  TEDHLE**S**LICKVG  GEKSAC**S**LESNLE  SACSLE**S**NLEGLA  DLPNYK**S**KILRLL  IRQLKE**S**LKANNY  YLVRFL**S**DLVNCH  HVIAAP**S**MVAMFE  YVYAFL**S**SLPWVG  VYAFLS**S**LPWVGK  IFANTE**S**YLKRRQ  PPHTED**S**VYPMPR  VMPGSH**S**VERFVI  LHCIIK**S**HWKERK  CKLQPG**S**LPQVLA  RFINWF**S**HHLSNF  WFSHHL**S**NFQFRW  NFQFRW**S**WEDWSD  DWSDCL**S**QDPESP  LSQDPE**S**PKPKFV  EKCMRL**S**YHQRIL  YKYGDE**S**SNSLPG  GDESSN**S**LPGHSV  NSLPGH**S**VALCLA  LHLAAK**S**FSHSFS  AKSFSH**S**FSALAK  SFSHSF**S**ALAKFH  IFSSEL**S**RDFTRL  VWEILH**S**TIRKMN  RQHKRR**S**DDDDRS  SDDDDR**S**SDRKDG  CETDGT**S**VLTPWY  IPFAKC**S**RVVSRS  KCSRVV**S**RSPPPR  PPPRLP**S**QSLRPM  PRLPSQ**S**LRPMPQ  LRATGC**S**QLGLYP  DLRRRQ**S**IINELK  KKAQWG**S**SGAASE  KAQWGS**S**GAASEP  GSSGAA**S**EPVVLG  EGCGFP**S**TNEYPD  QEEACA**S**RQLHSL  ASRQLH**S**LASFST  QLHSLA**S**FSTVTA  HSLASF**S**TVTARR  EFSQSN**S**ATSSPA  TSSPAP**S**GSAAAN  AAAGLP**S**ASAAAV  DFMSNL**S**LLEESE  FPQAPG**S**VAAAVA  PPPGPL**S**QHPPVP  AGQPRK**S**SSSRRN  ITKAIE**S**SAEKRL  EKRLTL**S**QIYEWM  ASMDNN**S**KFAKSR  AGDSPG**S**QFSKWP  FSKWPA**S**PGSHSN  ASPGSH**S**NDDFDN  STLPSL**S**EISNPE  LLSSPT**S**LTVSTQ  PTSLTV**S**TQSSPG  LTVSTQ**S**SPGTMM  TVSTQS**S**PGTMMQ  TSLNSP**S**PNYQKY  YGQSSM**S**PLPQMP  TLQDNK**S**SYGGMS  LQDNKS**S**YGGMSQ  LKELLT**S**DSPPHN  GVAQPN**S**RVLGQN  GPNSVM**S**TYGSQA  VMSTYG**S**QASHNK  KMMNPS**S**HTHPGH  GHAQQT**S**AVNGRP  STMPHT**S**GMNRLT  ALGGYS**S**VSSCNG  NVLPNQ**S**FPHSVK  QTGIQA**S**EDVKEI  YRLLKI**S**IENEQL  EQLVIG**S**YSQPSD  LVIGSY**S**QPSDSW  GSYSQP**S**DSWDKD  YSQPSD**S**WDKDYD  WDKDYD**S**FVLPLL  ILFRLD**S**QNAQGY  WIFIAW**S**PDHSHV  AWSPDH**S**HVRQKM  TVKEDV**S**LHGYKK  YKKYLL**S**QSSPAP  KYLLSQ**S**SPAPLT  YLLSQS**S**PAPLTA  GVAFPI**S**REAFQA  KRIPKD**S**ARYHFF  FFLYKH**S**HEGDYL  EGDYLE**S**IVFIYS  SIVFIY**S**MPGYTC  MPGYTC**S**IRERML  RERMLY**S**SCKSRL  ERMLYS**S**CKSRLL  LYSSCK**S**RLLEIV  QHAHKQ**S**FAKPKG  FESDLH**S**LLQLDA  MRAANR**S**HSAGRT  AANRSH**S**AGRTPG  GRTPGK**S**SSKVQT  RTPGKS**S**SKVQTT  TPGKSS**S**KVQTTP  ENQPEN**S**QTPTKK  QKATPG**S**SRKTCR  LNLVDW**S**SGNVLA  NLVDWS**S**GNVLAV  AVALDN**S**VYLWSA  NSVYLW**S**ASSGDI  VYLWSA**S**SGDILQ  QPGEYI**S**SVAWIK  PGEYIS**S**VAWIKE  YLAVGT**S**SAEVQL  LAVGTS**S**AEVQLW  RLRNMT**S**HSARVG  RNMTSH**S**ARVGSL  ARVGSL**S**WNSYIL  NSYILS**S**GSRSGH  DGRHLA**S**GGNDNL  LVNVWP**S**APGEGG  AWCPWQ**S**NVLATG  ATGGGT**S**DRHIRI  RIWNVC**S**GACLSA  CSGACL**S**AVDAHS  SAVDAH**S**QVCSIL  VCSILW**S**PHYKEL  ELKGHT**S**RVLSLT  KASAAK**S**SLIHQG  AEEELR**S**KSKICA  EELRSK**S**KICANV  KRPVCG**S**NGKTYL  DACLTG**S**KIQVDY  HCKEKK**S**VSPSAS  KEKKSV**S**PSASPV  KKSVSP**S**ASPVVC  SVSPSA**S**PVVCYQ  PVVCYQ**S**NRDELR  IPDGWF**S**KGSNYS  GWFSKG**S**NYSEIL  SKGSNY**S**EILDKY  NFDNGD**S**RLDSSE  DSRLDS**S**EFLKFV  DALIEL**S**DENADW  NADWKL**S**FQEFLK  LKCLNP**S**FNPPEK  QPALMF**S**PTQPGR  NNEEIV**S**DPIYIE  FTKQPE**S**MNVTRN  DKGLTV**S**KGVQIN  NIKAIP**S**PPTEVS  SPPTEV**S**IRNSTA  EVSIRN**S**TAHSIL  PGFDGY**S**PFRNCS  SPFRNC**S**IQVKEA  QALANY**S**IGVSCM  NYSIGV**S**CMNEIG  MNEIGW**S**AVSPWI  IGWSAV**S**PWILAS  TTEGAP**S**VAPLNV  WQSAGI**S**KELLEE  WVDYAP**S**STPAPG  VDYAPS**S**TPAPGN  GLILYI**S**LAIRKR  AFTEED**S**ELVVNY  LHSLGV**S**EELQNK  TMKLDN**S**SQREIE  EIEEFL**S**EAACMK  HTYLLY**S**RLETGP  KWIAIE**S**LADRVY  LYEIMY**S**CWRTDP  TQLLES**S**EGLAQG  LNIDPD**S**IIASCT  TPRAAI**S**VVTAEV  LVRNGV**S**WSHSSM  RNGVSW**S**HSSMLP  GVSWSH**S**SMLPLG  SMLPLG**S**SLPDEL  LLFADD**S**SEGSEV  EMLTKK**S**TLRDQI  LRDQIN**S**DHRTRA  DRYMEV**S**GNLRDL  EELNAI**S**GPNEFA  EICVPM**S**VEFEEL  KARENP**S**EEAQNL  YINLKA**S**EKLDYI  DYITYL**S**IFDQLF  GWPKET**S**SALTHA  WPKETS**S**ALTHAG  GAHLDL**S**AFSSWE  LDLSAF**S**SWEELA  DLSAFS**S**WEELAS  SWEELA**S**LGLDRL  GLDRLK**S**ALLALG  RAQRLF**S**TKGKSL  FSTKGK**S**LESLDT  KGKSLE**S**LDTSLF  LESLDT**S**LFAKNP  FAKNPK**S**KGTKRD  EEEEQI**S**ESESED  EEQISE**S**ESEDEE  QISESE**S**EDEENE  QIEDAV**S**LWAKLK  LKLQKA**S**ERWQPD  EEEYED**S**SGNVVN  EEYEDS**S**GNVVNK  EDSQPE**S**QVLEDD  EAKEQL**S**AQELME  GGCSLA**S**TPATTL  LHLLQL**S**GQRSLV  LVQDSL**S**TNSSDL  SPDAFR**S**TPFIVP  STPISQ**S**TPVFPP  PVFPPG**S**LPIPSQ  KDGDMH**S**SSLTVE  PKMESL**S**SHRIDE  KMESLS**S**HRIDED  MSPVLN**S**KFVPAE  PETPCE**S**QGEELK  SLTETQ**S**QGLCLQ  VDVKEP**S**PRVDVS  KCSDSQ**S**WEDIAP  EPVEQD**S**SQPSLP  NSLTED**S**KMANAK  ANAKQL**S**SDAEAQ  NAKQLS**S**DAEAQK  SAHASQ**S**FCESSS  STPIGI**S**NYPEST  KTMSVL**S**CICEAR  NLLHFP**S**SQGEEE  IQTMEC**S**LRVPET  ECETEV**S**PSQTGG  DLGDIS**S**FSSKAS  RTSSGT**S**LSAMHS  DGLDAS**S**PGNSFV  ASSPGN**S**FVGLRV  SEDEYF**S**AGVVKG  VMAKHS**S**VYPTQE  AVQNMV**S**HTERAL  RALKAV**S**DWIDEQ  DEQEKG**S**SEQAES  KYEILQ**S**VDDAAI  LAGETL**S**VNDPPD  RANGLK**S**CVIVIR  GEEKSP**S**KKKKKI  APIFTM**S**VEVDGN  VEVDGN**S**FEASGP  EAVSTP**S**AAFPSD  ISETGG**S**HDKRFV  AGAGYG**S**YGYGGN  SATAGY**S**QFYSNG  GYSQFY**S**NGGHSG  GHSGNA**S**GGGGGG  GGGGGG**S**SGYGSY  GGGGGS**S**GYGSYY  GGQQKP**S**YGSGYQ  HQGQQQ**S**YNQSPY  YNHGQG**S**YSYSNS  HGQGSY**S**YSNSYN  SDYNYE**S**KFNYSG  KFNYSG**S**GGRSGG  GGNSYG**S**GGASYN  GGYGGG**S**GGGSSY  GGSGGG**S**SYQGKQ  GKQGGY**S**QSNYNS  SQSNYN**S**PGSGQN  NYNSPG**S**GQNYSG  GSGQNY**S**GPPSSY  NYSGPP**S**SYQSSQ  YSGPPS**S**YQSSQG  LLQPGF**S**KTLLGT  EAKYLC**S**ACRNVL  CGHRYC**S**FCLASI  CSFCLA**S**ILSSGP  CLASIL**S**SGPQNC  LASILS**S**GPQNCA  IYEEGI**S**ILESSS  GISILE**S**SSAFPD  ISILES**S**SAFPDN  SILESS**S**AFPDNA  ARREVE**S**LPAVCP  LPAVCP**S**DGCTWK  TLKEYE**S**CHEGRC  HECPER**S**LSCRHC  HLAMLL**S**SVLEAK  LAMLLS**S**VLEAKP  PLLGDQ**S**HAGSEL  DQSHAG**S**ELLQRC  MTAEAC**S**RQHRLD  KIEALS**S**KVQQLE  VQQLER**S**IGLKDL  VLEMEA**S**TYDGVF  VFIWKI**S**DFARKR  SPAFYT**S**RYGYKM  GRGTHL**S**LFFVVM  FRPDVT**S**SSFQRP  RPDVTS**S**SFQRPV  PDVTSS**S**FQRPVN  NDMNIA**S**GCPLFC  PLFCPV**S**KMEAKN  KMEAKN**S**YVRDDA  PEAAPA**S**AASAPG  APASAA**S**APGPAP  GPAPPV**S**APAALP  APAALP**S**STAAEN  PAALPS**S**TAAENK  AAENKA**S**PAGTAG  RGAAPV**S**AGGAAP  PPEGAI**S**NGVYVL  GVYVLP**S**AANGDV  DVKPVV**S**STPLVD  VKPVVS**S**TPLVDF  RAGFEA**S**DPRIIR  RIIRLI**S**LAAQKF  AAQKFI**S**DIANDA  KMKGTA**S**GSSRSK  KGTASG**S**SRSKSK  GTASGS**S**RSKSKD  ASGSSR**S**KSKDRK  GSSRSK**S**KDRKYT  DLTPAL**S**EYGINV  APPPPG**S**RPADLE  TCEIPE**S**KYSPLG  LSPSPA**S**SISSRS  IYDDVD**S**ELNEAA  LGSPLT**S**PGGSPG  GLGHSL**S**PRQSPC  TDENWL**S**PRPASG  VPSPGH**S**PRGSVT  DTWLNA**S**VHGGSG  ASVHGG**S**GLGPAV  IDDGLG**S**QYPLKK  PIFRTS**S**LPPLDW  PSGKVL**S**LQIASI  LSLQIA**S**IPVECS  PHIEKY**S**INSCSV  EMVVTG**S**NFLPES  DLSSVP**S**LPVPHP  AQTQRP**S**SDSGCS  QTQRPS**S**DSGCSH  SSDSGC**S**HDSVLS  YASMVT**S**SHLPQL  TGQRSL**S**SPVADQ  SHPLAS**S**PLSGPP  LASSPL**S**GPPSPQ  PLSGPP**S**PQLQPM  PYQSPS**S**GTASSP  PSSGTA**S**SPSPAT  SSGTAS**S**PSPATR  MHSGQH**S**TQAQST  GLSAPS**S**LICHSL  IIGRDM**S**QISVSQ  MSQISV**S**QGAGVS  PLPSPE**S**LDLGRS  AAANSG**S**SLPLFD  AANSGS**S**LPLFDC  FTIDHQ**S**CSRVHA  IDHQSC**S**RVHAAL  FLIDLN**S**THGTFL  QQIPID**S**TVSFGA  PIDSTV**S**FGASTR  TVSFGA**S**TRAYTL  KPQTLP**S**AVKGDE  AHNKRI**S**TLTIEE  KRKRKN**S**RVTFSE  NSRVTF**S**EDDEII  PEDVDP**S**VGRFRN  RVEGPG**S**LGLEES  SLGLEE**S**GSRRMQ  GLEESG**S**RRMQNF  MQNFAF**S**GGLYGG  GLPPTH**S**EAGSQP  THSEAG**S**QPHGIH  LTPVVP**S**AVNMNP  LLWIGY**S**QGTTHV  IFGPSH**S**SSANAV  FGPSHS**S**SANAVQ  SANAVQ**S**ICNALG  RWKHQV**S**DNKDSF  KDSFYV**S**LYPDFS  SLYPDF**S**SLSRAI  TENTQV**S**SIIEKW  SIIEKW**S**MERLQA  APPKPD**S**GLLDGF  DAVHVV**S**VAVQQF  FPQMTV**S**SLQCNR  PQMTVS**S**LQCNRH  FDLDVI**S**LKEEGL  GTWDPA**S**GLNMTE  YVLFKK**S**DKPLYG  FMTLGI**S**ILYRKP  SFLNPL**S**PDIWMY  LAYLGV**S**CVLFVI  HPCNPD**S**DVVENN  NFTLLN**S**FWFGVG  ALMQQG**S**ELMPKA  MESPID**S**ADDLAK  KMWAFM**S**SRRQSV  QSVLVK**S**NEEGIQ  YAFLME**S**TTIEFV  VGTPMG**S**PYRDKI  GCPEEE**S**KEASAL  AAGLVL**S**VFVAVG  AQLEKR**S**FCSAMV  EKRSFC**S**AMVEEL  DQQQYQ**S**ALFWAD  DKVASL**S**REEPQD  AAHALR**S**RKLDKL  SGFKDP**S**SDWEMS  GFKDPS**S**DWEMSQ  SSDWEM**S**QSSIKS  DWEMSQ**S**SIKSSI  WEMSQS**S**IKSSIC  SQSSIK**S**SICLLR  RTLATY**S**YKEALK  AFDLLT**S**HHMLTA  EKELLE**S**LPLSKL  LESLPL**S**KLCNEE  KKYNKP**S**ETVIPE  ETVIPE**S**VDGLQE  NLDVVV**S**LAERHY  MCYKLT**S**VVMEKD  KDPFHA**S**CLPVHI  NELFYL**S**HKLVDL  LVDLYP**S**NPVSWF  YPSNPV**S**WFAVGC  HSFAVE**S**EHDQAM  YGLTNN**S**KLAERF  LAERFF**S**QALSIA  FFSQAL**S**IAPEDP  QNASTY**S**AIGYIH  RRDDTF**S**VTMLGH  EMYIGD**S**EAYIGA  LTPLET**S**RKTPDS  SRKTPD**S**RPSLEE  EIEMNE**S**DMMLET  VRGVDL**S**GNDFKG  QKLEHL**S**VSHNNL  LEHLSV**S**HNNLTT  LHGELS**S**LPSLRA  IVARAN**S**LKNSGV  FKLDDL**S**VLDLSH  LNLSHN**S**IDTIPN  STQRTQ**S**NLPTSL  TSLEGL**S**NLADVD  LADVDL**S**CNDLTR  CLYTLP**S**LRRLNL  VETLNL**S**RNQLTS  SRNQLT**S**LPSAIC  QLTSLP**S**AICKLS  NKKQEE**S**ADARAP  LDKKAC**S**AIHAVN  EEMGDE**S**EEFLQV  VFDNDI**S**YIEGGT  GAQATL**S**STTKAR  ALGGEP**S**EIKKHV  QINYKL**S**VEHKQR  RMRLLQ**S**LLDTRC  YILDCW**S**DVFIWL  HATVSR**S**LEGTEA  LQSPGL**S**GKVKRD  WLTFTF**S**LQKKFE  LKVPFE**S**EDNQGI  NTMFDT**S**YSKQVI  MFDTSY**S**KQVINE  TRLFRC**S**NEKGYF  WVGTQT**S**QVEIKL  CPICME**S**FTEEQL  LEKLLA**S**SINGVR  KITRIT**S**LTQLTD  IDTAGL**S**EAVGLL  PRQFCR**S**CGLVLC  EVEKSN**S**QVVEEQ  QVVEEQ**S**YLLNIA  AEVQAV**S**RCDYFL  TVNVED**S**WAMEAT  AMEATA**S**AASTSV  ATASAA**S**TSVTFR  ASAAST**S**VTFREM  FREMDM**S**PEEVVA  PEEVVA**S**PRASPA  VASPRA**S**PAKQRG  KMGAKG**S**TPGMFN  MFNLPV**S**LYVTSQ  VSLYVT**S**QGEVLV  EIRRSP**S**GIDSFV  SPSGID**S**FVLSFL  LIGVTD**S**YDNSLK  TDSYDN**S**LKVYTL  CVACHR**S**QLSKPW  CHRSQL**S**KPWGIT  GITALP**S**GQFVVT  FTVDRG**S**GVVKYS  SGVVKY**S**CLCSAV  HLEGGF**S**IGSVGP  QLGRQI**S**HFFSEN  QISHFF**S**ENEDFR  DLIVAD**S**SRKEIL  PKGGGY**S**VLIREG  HCIKIY**S**YHLRRY  NSEDEI**S**NLVKLD  SPASVG**S**DTLSDL  LSDLGI**S**SLQDGL  AVKCMR**S**IRRWSY  AFMGPP**S**QAMWAL  LGDKIA**S**SIVAQT  GDKIAS**S**IVAQTA  IPTLPW**S**GSGLRV  QAEVPG**S**PIFVMR  QYGNAI**S**LFGRDC  KMVGYV**S**AGTVEY  PIDFED**S**AHVPCP  QELNFR**S**NKNVWG  QFGHCF**S**WGENRE  DPSVMR**S**PSAGKL  SVMRSP**S**AGKLIQ  TLTAVE**S**GCIHYV  MQLDNP**S**KVQQAE  AELHTG**S**LPRIQS  SLPRIQ**S**TALRGE  LQDIMT**S**VSGRIP  PPNVEK**S**IKKEMA  VLCQFP**S**QQIANI  ATLNRK**S**EREVFF  LVQRYR**S**GIRGHM  IEESVR**S**MVMRYG  LFLTNE**S**GYYLDI  YKEVTD**S**RTAQIM  KRFQAQ**S**LGTTYI  DRESRG**S**VLEPEG  QEKGVI**S**DILDWK  LEQYAA**S**YSGLMR  QYAASY**S**GLMRIE  EIHRKL**S**EATRSS  LSEATR**S**SLRELQ  SEATRS**S**LRELQN  PDAIPE**S**GVEPPA  KNYKGN**S**IKESIR  GNSIKE**S**IRRGHD  LDCGDL**S**NALKCY  NALKCY**S**RARDYC  ARDYCT**S**AKHVIN  LNVIKV**S**VYLQNW  VYLQNW**S**HVLSYV  NWSHVL**S**YVSKAE  HVLSYV**S**KAESTP  YVSKAE**S**TPEIAE  QRGERD**S**QTQAIL  DFPELL**S**PSNVAI  LQRNVI**S**SSSFKL  QRNVIS**S**SSFKLF  RNVISS**S**SFKLFL  NVISSS**S**FKLFLE  IFKFYE**S**KYASCL  YESKYA**S**CLKMLD  ALIQYF**S**PYVSAD  YFSPYV**S**ADMHRM  ILEGLI**S**ARVDSH  ISARVD**S**HSKILY  ARVDSH**S**KILYAR  RDVDQR**S**TTFEKS  STTFEK**S**LLMGKE  LREMDC**S**VLKRLM  GGGAGG**S**GSHGTL  GTLGLP**S**GGKCLL  RPFLAH**S**AGYILG  AGYILG**S**VNVRCN  RRRAKG**S**VSLEQI  RAKGSV**S**LEQILP  VRARLR**S**GLYSAV  LRSGLY**S**AVIVYD  IVYDER**S**PRAESL  RSPRAE**S**LREDST  ESLRED**S**TVSLVV  REDSTV**S**LVVQAL  GGYERF**S**SEYPEF  GYERFS**S**EYPEFC  EYPEFC**S**KTKALA  PPPVPP**S**ATEPLD  LDLGCS**S**CGTPLH  TALLNV**S**SDCPNH  ALLNVS**S**DCPNHF  HKADIS**S**WFMEAI  QRRSII**S**PNFSFM  IISPNF**S**FMGQLL  QLLQFE**S**QVLATS  SQVLAT**S**CAAEAA  CAAEAA**S**PSGPLR  AEAASP**S**GPLRER  TPATPT**S**QFVFSF  VSVGVH**S**APSSLP  GVHSAP**S**SLPYLH  VHSAPS**S**LPYLHS  TMIGAG**S**KDAGTL  GTLNHG**S**KPLPPN  KERPEI**S**LPSDFE  PEISLP**S**DFEHTI  ARLLQT**S**NITKSE  VLEFYN**S**KKTSNS  YNSKKT**S**NSQKYM  SKKTSN**S**QKYMSF  SAEDYN**S**SNALNV  AEDYNS**S**NALNVK  LNVKAV**S**ETPAVP  KKKPKM**S**DEEILE  ILEKLR**S**IVSVGD  KLRSIV**S**VGDPKK  KIGQGA**S**GTVYTA  IVNYLD**S**YLVGDE  EYLAGG**S**LTDVVT  ALEFLH**S**NQVIHR  IHRDIK**S**DNILLG  LLGMDG**S**VKLTDF  QITPEQ**S**KRSTMV  PEQSKR**S**TMVGTP  PKVDIW**S**LGIMAI  QNPEKL**S**AIFRDF  DVEKRG**S**AKELLQ  KIAKPL**S**SLTPLI  IAKPLS**S**LTPLIA  FLVLLH**S**VSSSLS  VLLHSV**S**SSLSSS  LLHSVS**S**SLSSSE  LHSVSS**S**LSSSEL  SVSSSL**S**SSELTE  VSSSLS**S**SELTEL  SSSLSS**S**ELTELK  KLERVQ**S**GLDLFS  SGLDLF**S**MLLEQN  LRELLA**S**LRRHDL  ARQLKV**S**DTKIDS  SDTKID**S**IEDRYP  TERVRE**S**LRIWKN  LVGALR**S**CQMNLV  RDLQNR**S**GAMSPM  GAMSPM**S**WNSDAS  SPMSWN**S**DASTSE  RIKIPN**S**GAVDWT  VDWTVH**S**GPQLLF  DRITVR**S**DEEMKA  EMKAML**S**YYYSTV  MLSYYY**S**TVMEQQ  NTRAGP**S**QHSSPA  AGPSQH**S**SPAVSD  GPSQHS**S**PAVSDS  HSSPAV**S**DSLPSN  SPAVSD**S**LPSNSL  VSDSLP**S**NSLKKS  DSLPSN**S**LKKSSA  SNSLKK**S**SAELKK  NSLKKS**S**AELKKI  KAYHVP**S**GKILAV  LQKQIM**S**ELEILY  ILYKCD**S**SYIIGF  LYKCDS**S**YIIGFY  FVENRI**S**ICTEFM  EFMDGG**S**LDVYRK  GLTYLW**S**LKILHR  HRDVKP**S**NMLVNT  LCDFGV**S**TQLVNS  MAPERI**S**GEQYGI  EQYGIH**S**DVWSLG  IHSDVW**S**LGISFM  VWSLGI**S**FMELAL  IQKNQG**S**LMPLQL  CIVDED**S**PVLPVG  LPVGEF**S**EPFVHF  GNAAVV**S**MWVCRA  EDGEDG**S**AEPPGP  PAHTAA**S**VAAKNL  ALLKAR**S**FDVTFD  GAYGVV**S**SARRRL  PYGEFK**S**VYVVLD  LHQIIH**S**SQPLTL  HQIIHS**S**QPLTLE  HRDLKP**S**NLLVNE  ARGLCT**S**PAEHQY  APELML**S**LHEYTQ  MVLGTP**S**PAVIQA  VRAYIQ**S**LPPRQP  ADRQAL**S**LLGRML  MLRFEP**S**ARISAA  QIRFQP**S**LQPVAS  SLQPVA**S**EPGCPD  QPPPPV**S**EPAPPK  KKDGAI**S**DNTKAA  LLKSLR**S**RLRDGP  RLRDGP**S**APLEAP  ERGAGA**S**GGPSTD  GASGGP**S**TDPLAG  LAGLVL**S**DNDRSL  AAPALT**S**VPAPAP  TPVQPT**S**PPPGPV  TGPQPQ**S**AGSTSG  QPQSAG**S**TSGPVP  PGLPGS**S**TPGVLP  TQQLSK**S**QVEDPL  GQADSA**S**LSASLL  SASLSA**S**LLADWL  NPADIE**S**LQREIQ  REIQMD**S**PMLLAD  QPPAPK**S**KLKKLS  SKLKKL**S**EDSLTK  KKLSED**S**LTKQPE  EKLGEG**S**YGSVFK  GEGSYG**S**VFKAIH  KAIHKE**S**GQVVAI  KQVPVE**S**DLQEII  EIIKEI**S**IMQQCD  IMQQCD**S**PYVVKY  VVKYYG**S**YFKNTD  EYCGAG**S**VSDIIR  CGAGSV**S**DIIRLR  IATILK**S**TLKGLE  CVADIW**S**LGITSI  WSLGIT**S**IEMAEG  RKPELW**S**DDFTDF  KNAKPV**S**ILRDLI  EEEEEN**S**DEDELD  DEDELD**S**HTMVKT  HTMVKT**S**VESVGT  VKTSVE**S**VGTMRA  GTMRAT**S**TMSEGA  RATSTM**S**EGAQTM  TMIEHN**S**TMLESD  NSTMLE**S**DLGTMV  GTMVIN**S**EDEEEE  MKRNAT**S**PQVQRP  PQVQRP**S**FMDYFD  QDFKNK**S**HENCNQ  HEPFPM**S**KNVFPD  DFLKNL**S**LEELQM  KLQLKN**S**PPYILD  FKIYID**S**LMKKSK  RMYEEQ**S**QDRRNL  RNLTKL**S**LIFSHM  HEVHQI**S**SGLEAM  EVHQIS**S**GLEAMA  EAMALK**S**TIDLTC  LFQPWG**S**ILRNWN  ARLQKY**S**TKPGSY  QALIDG**S**REGFYL  GHLMCT**S**CLTAWQ  LTAWQE**S**DGQGCP  QNSPVT**S**PGSSPL  LQIPHL**S**LPPVPP  PTGSPK**S**SPCMVR  PPDNRL**S**RHIHHV  HIHHVE**S**VPSRDP  EGAKVF**S**NGHLGS  TGPLAN**S**LSEKTR  PLANSL**S**EKTRDP  DEYKIP**S**SHPVSL  HPVSLN**S**QPSHCH  NGTHGP**S**SEKKSN  GTHGPS**S**EKKSNI  PSSEKK**S**NIPDLS  NPKHGS**S**LNRTPS  HSLIEH**S**KPPGSS  HSKPPG**S**SSRPSS  KPPGSS**S**RPSSGQ  DYDQLP**S**CSDGSQ  DQLPSC**S**DGSQAP  APGSVT**S**RLGSVF  VTSRLG**S**VFPFLL  QLADAL**S**QFHAAV  FLAMGK**S**KAALPD  FKKVLK**S**NPSENE  VLKSNP**S**ENEEKE  EEKEAQ**S**QLIKSD  QSQLIK**S**DEMQRL  EMQRLR**S**QALNAF  ALNAFG**S**GDYTAA  EPRKAI**S**DLKAAS  SDLKAA**S**KLKNDN  EAFYKI**S**TLYYQL  LGDHEL**S**LSEVRE  DHELSL**S**EVRECL  RYTDAT**S**KYESVM  ATSKYE**S**VMKTEP  VMKTEP**S**IAEYTV  AEYTVR**S**KERICH  RICHCF**S**KDEKPV  EAIRVC**S**EVLQME  QRLLKQ**S**QKRDYY  AAKEVL**S**DPEMRK  DPLDAE**S**QQGGGG  GNPFHR**S**WNSWQG  FHRSWN**S**WQGFNP  QGFNPF**S**SGGPFR  GFNPFS**S**GGPFRF  VYIGRL**S**YNVREK  DIQRFF**S**GYGRLL  SGGGGY**S**SRRTSG  GGGGYS**S**RRTSGR  LIVENL**S**SRCSWQ  IVENLS**S**RCSWQD  GVIEFR**S**YSDMKR  IEFRSY**S**DMKRAL  EDKPRT**S**HRRSYS  SHRRSY**S**GSRSRS  RRSYSG**S**RSRSRS  SYSGSR**S**RSRSRR  SGSRSR**S**RSRRRS  SRRRSR**S**RSRRSS  RRSRSR**S**RRSSRS  SRSRRS**S**RSRSRS  SRSRSR**S**ISKSRS  SRSISK**S**RSRSRS  SISKSR**S**RSRSRS  SRSRSR**S**KGRSRS  PKSDRG**S**HSHSRS  SHSHSR**S**RSKDEY  KDEYEK**S**RSRSRS  SRSRSR**S**RSPKEN  GDIKSK**S**RSRSQS  IKSKSR**S**RSQSRS  SKSRSR**S**QSRSNS  PLPVPP**S**KARSVS  PPKRAT**S**RSRSRS  SRSRSR**S**KSRSRS  SRSRSK**S**RSRSRS  GITLRG**S**AEIVAE  IVAEFF**S**FGINSI  FSFGIN**S**ILYQRG  QRGIYP**S**ETFTRV  DWLYKC**S**VQKLVV  KLVVVI**S**NIESGE  VISNIE**S**GEVLER  KTAKDD**S**APREKS  SAPREK**S**QKAIQD  IQDEIR**S**VIRQIT  LPLLEV**S**CSFDLL  LLEVSC**S**FDLLIY  EEVRLR**S**FTTTIH  ASAAAA**S**AASGSP  AAASAA**S**GSPGPG  PGPGEG**S**AGGEKR  AGGEKR**S**TAPSAA  KRSTAP**S**AAASAS  APSAAA**S**ASASAA  SAAASA**S**ASAAAS  SASAAA**S**SPAGGG  CLHSAC**S**ACLGPA  APAAAN**S**SGDGGA  PAAANS**S**GDGGAA  CKQQCF**S**KDIVEN  NYFMRD**S**GSKAAT  DNAPAT**S**YCVECS  SYCVEC**S**EPLCET  STGPAK**S**RDGERT  LVLFCE**S**CDTLTC  TKEVRS**S**IRQVSD  ASWALE**S**DNNTAL  NTALLL**S**KKLIYF  RAPGPL**S**KQGSGS  PLSKQG**S**GSSQPM  GDDPYS**S**AEPHVS  SAEPHV**S**GVKRSR  PRLASP**S**GSTSSG  SPSGST**S**SGLEVV  VPGEEW**S**CSLCHV  LKEEDG**S**LSLDGA  ATDSTF**S**LDQPGG  KLSPPY**S**SPQEFA  VEPPPM**S**LPGAGL  RFYGKN**S**SYVHGG  FYGKNS**S**YVHGGL  VHGGLD**S**NGKPAD  IHRKVM**S**QNFTNC  QVMGLL**S**NNNQAL  VLAPEG**S**VANKFY  EVVPDD**S**GTFYDQ  YDQAVV**S**NDMEEH  PEQEPV**S**EIQEEK  PEDAQK**S**SSPAPA  EDLRTF**S**WASVTS  RTFSWA**S**VTSKNL  SWASVT**S**KNLPPS  SKNLPP**S**GAVPVT  VVKVPA**S**QPRPES  SQPRPE**S**KPESQI  PESKPE**S**QIPPQR  MVRHPD**S**HQLFIG  PHEVDK**S**ELKDFF  LKDFFQ**S**YGNVVE  FVVFDD**S**EPVQKV  PVQKVL**S**NRPIMF  VCGDKV**S**GYHYGL  GLLTCE**S**CKGFFK  HYTCTE**S**QSCKID  TCTESQ**S**CKIDKT  DYVLPP**S**LHGPEP  PGRAIK**S**EYPEPY  GYPEPF**S**GGPNVP  LQEPTK**S**RPDQPA  ADQTFI**S**IVDWAR  LLQNCW**S**ELLVFD  QHGKEG**S**ILLVTG  VATQAG**S**LLHSLV  AGSLLH**S**LVLRAQ  KFIILF**S**LDLKFL  VEVRAL**S**MQAKEY  GSPARL**S**LRQTGS  SPGMIY**S**TRYGSP  YRNLGK**S**GLRVSC  KSGLRV**S**CLGLGT  KKGWRR**S**SLVITT  ETERGL**S**RKHIIE  IEGLKA**S**LERLQL  AMYWGT**S**RWSSME  WGTSRW**S**SMEIME  GTSRWS**S**MEIMEA  EIMEAY**S**VARQFN  VGAMTW**S**PLACGI  LACGIV**S**GKYDSG  VSGKYD**S**GIPPYS  SGIPPY**S**RASLKG  PPYSRA**S**LKGYQW  LKDKIL**S**EEGRRQ  LRNEGV**S**SVLLGA  RNEGVS**S**VLLGAS  SVLLGA**S**NADQLM  QVLPKL**S**SSIIHE  VLPKLS**S**SIIHEI  LPKLSS**S**IIHEID  IIHEID**S**ILGNKP  QEIPDL**S**SNVATS  EIPDLS**S**NVATSF  SSNVAT**S**FTWGWD  TWGWDS**S**KTSELL  KTSELL**S**GMGVSA  LSGMGV**S**ALEKEE  EKEEPD**S**ENIPQE  NLGHPE**S**PPRKRL  RLKSKG**S**DKDFVI  ENFPGV**S**WDSLPD  PGVSWD**S**LPDELL  PELLKV**S**GVCKRW  RWYRLA**S**DESLWQ  VTGRLL**S**QGVIAF  AFRCPR**S**FMDQPL  VQHMDL**S**NSVIEV  HMDLSN**S**VIEVST  NSVIEV**S**TLHGIL  TLHGIL**S**QCSKLQ  SKLQNL**S**LEGLRL  LEGLRL**S**DPIVNT  NTLAKN**S**NLVRLN  LVRLNL**S**GCSGFS  LNLSGC**S**GFSEFA  ALQTLL**S**SCSRLD  LQTLLS**S**CSRLDE  TLLSSC**S**RLDELN  LDELNL**S**WCFDFT  ITQLNL**S**GYRKNL  LVHLDL**S**DSVMLK  HLDLSD**S**VMLKND  NYLQHL**S**LSRCYD  HLQINC**S**HFTTIA  SSYPMA**S**LYVGDL  MLYEKF**S**PAGPVL  PAGPVL**S**IRVCRD  DMITRR**S**LGYAYV  PIRIMW**S**QRDPSL  DPSLRK**S**GVGNVF  AFGNIL**S**CKVVCD  VCDENG**S**KGYAFV  FVGRFK**S**RKEREA  EEVDDE**S**LKELFS  SLKELF**S**QFGKTL  RDPNGK**S**KGFGFV  KGFGFV**S**YEKHED  MNGKEI**S**GKIIFV  LKQERI**S**RYQGVN  KLRKEF**S**PFGSIT  EFSPFG**S**ITSAKV  PFGSIT**S**AKVMLE  MLEDGR**S**KGFGFV  FGFVCF**S**SPEEAT  GFVCFS**S**PEEATK  NGRIVG**S**KPLYVA  PSAIRQ**S**GPRPTL  QQGLTD**S**CQSGGV  APYKYA**S**SVRSPH  PYKYAS**S**VRSPHP  YASSVR**S**PHPAIQ  QEPLTA**S**MLAAAP  LIQTMH**S**NLAGKI  LLEIDN**S**ELLHML  LLHMLE**S**PESLRS  MLESPE**S**LRSKVD  SPESLR**S**KVDEAV  LDRHSD**S**KQGKYL  CARQEK**S**SSGLNH  FNEDTR**S**LEISQS  IATQLI**S**KYPASL  LPNCEL**S**PLLMIL  TFRGIS**S**EQIQAE  TLALTT**S**IVPGTV  KDKEEL**S**FSEVEE  RCLLGL**S**EQLLNN  ICKSLA**S**FIKKPF  FNDYPD**S**SVSDAN  ANEPGE**S**QSTIGA  LAEEYL**S**KQDLLF  QDTKGD**S**SRLLKA  IQEDWK**S**LLTDCF  NSSASQ**S**TDLCDF  LEILSK**S**PDSYQK  VLEAVG**S**CLGEVG  QHSKDA**S**YTKALK  LQPFRT**S**RKKFLE  NLWIPL**S**ENHDIW  SWRNLL**S**THVQGF  SCLRHF**S**QTSRST  CCLDKK**S**QRTMLA  KEETGI**S**LQDLLL  EEMCKR**S**LESVYS  NYDGES**S**DELRNG  PEVARR**S**RITKNV  KNVPKQ**S**SQLDED  VPLSQR**S**GVLEWC  MEVQKK**S**FEEKYE  LAGCPD**S**FLHHPY  VNDMNA**S**LESLYS  SLYSAC**S**MQSDTV  ASGPPR**S**IQPQVS  RQRVQR**S**QPVHIL  DQQFRT**S**SLPAIP  PVLTPG**S**LPPSQA  QDVKVF**S**EDGTSK  QLLVYK**S**HCVDDN  HCVDDN**S**WTLVEH  LVVQVE**S**TMASES  VESTMA**S**ESKFLF  STMASE**S**KFLFRK  VTWCQQ**S**NGSQTQ  LQNFLN**S**SSCPEI  QNFLNS**S**SCPEIQ  NFLNSS**S**CPEIQG  KELGKK**S**WKKLYV  YVCLRR**S**GLYCST  RSGLYC**S**TKGTSK  LADLED**S**NIFSLI  EDSNIF**S**LIAGRK  ALLSPF**S**TPVRSV  FSTPVR**S**VSENSL  LVAMDF**S**GQTGRV  NPAEAQ**S**AALEEG  HAWRKR**S**TRMNIL  WFHGRI**S**REESHR  LFLLRD**S**QSNPKA  DGQTFF**S**LDDGNT  DGNTKF**S**DLIQLV  YVYFEN**S**SSNPYL  YFENSS**S**NPYLIR  YRRRDI**S**STLIAL  RRRDIS**S**TLIALA  HRELFL**S**RQLESL  LSRQLE**S**LPATHI  HIRGKC**S**VTLLNE  ETESLK**S**YLERED  EDFFFY**S**LVYDPQ  RALDCS**S**SVRQPS  QPSLHM**S**AAAASR  KNIYDI**S**KAISAL  DISKAI**S**ALVPQG  DEMEEW**S**ASEANL  MEEWSA**S**EANLFE  KAAEAE**S**KLKQVY  SCYTTQ**S**YQWYSW  QSYQWY**S**WGPPNM  QCRLCA**S**CWTYWK  RPGPNR**S**NMSPHG  PNRSNM**S**PHGLPA  HGLPAR**S**SGSPKF  GLPARS**S**GSPKFA  PYLPIN**S**AAIKAE  LPEASQ**S**PLVLKQ  KPDPVK**S**VSSVLS  PVKSVS**S**VLSSLT  SVSSVL**S**SLTPAK  PVINNG**S**PTILGK  KRLLMP**S**RGLANH  ARHMGP**S**RNLLLN  LLLNGK**S**YPTKVR  RKLLSS**S**ETKRAA  QQGQMA**S**GDSNLN  MGLRDR**S**FISEQM  RDRSFI**S**EQMYEH  VMYCVL**S**ELEKTF  EKTFGW**S**HLEALF  PQEALS**S**SPRCEP  PGFSSE**S**CEQLAL  DTEVLE**S**NGMIDA  DAARTY**S**TAPGEK  KEKYQE**S**PEGRDK  EEPQEA**S**SSLARC  ARCGSV**S**CLSAET  EPEKEL**S**LLPGEG  GEGEEG**S**DDCSEM  EGSDDC**S**EMCDGE  RQEASS**S**LARRGS  SLARRG**S**VSSELE  ARRGSV**S**SELENH  SEELAS**S**LLYDNV  VPGAEQ**S**AYENEK  PEARTE**S**DQACGT  VDIANN**S**TLGKPK  KRGHGW**S**RMRMRR  MRRQEN**S**QQNDNS  DGQVVS**S**EKKANV  VNLKDL**S**KIRGRK  GTRFTQ**S**DRAAQK  AQKRVR**S**RASRKH  KKRILK**S**QNNSSV  MKESPG**S**QQCCQE  LKVYCC**S**ESSFFA  VYCCSE**S**SFFAKI  AFCDAQ**S**TQEIHE  IIVRIG**S**GFDNID  DNIDIK**S**AGDLGI  CNVPAA**S**VEETAD  VEETAD**S**TLCHIL  EGTRVQ**S**VEQIRE  QIREVA**S**GAARIR  FYDPYL**S**DGVERA  LGLQRV**S**TLQDLL  QDLLFH**S**DCVTLH  ALDVHE**S**EPFSFS  HESEPF**S**FSQGPL  PHAAWY**S**EQASIE  WYSEQA**S**IEMREE  TGRIPD**S**LKNCVN  AATHWA**S**MDPAVV  VEGIVP**S**AMSLSH  IVPSAM**S**LSHGLP  PSAMSL**S**HGLPPV  KLQYYY**S**SSEDED  LQYYYS**S**SEDEDS  QYYYSS**S**EDEDSD  GRCAPA**S**SSVPAE  RCAPAS**S**SVPAEA  CAPASS**S**VPAEAE  LAGEGI**S**VNTGPK  RLIKKL**S**MTCRSH  LSMTCR**S**HLDEEE  DLQEKI**S**GKMTLK  KQVFEI**S**SGEGFL  QVFEIS**S**GEGFLD  IDKEQK**S**IVIMVH  KFCKVK**S**SVIGAS  FCKVKS**S**VIGASS  SSVIGA**S**SQFTRN  SVIGAS**S**QFTRNA  EVLVLT**S**VRNSAT  LTSVRN**S**ATCHSE  VGDGTV**S**WGLEDD  YENRIY**S**LKIECG  NMNGVN**S**SNGVVD  MNGVNS**S**NGVVDP  VDPRAI**S**VLAKWQ  LAKWQN**S**YSIKVV  KWQNSY**S**IKVVLQ  LRRLMM**S**KENMKL  LLFLLF**S**LGWVQP  ANPPNI**S**SLSPRQ  NPPNIS**S**LSPRQL  FPCAEV**S**GLSTER  QKNVKL**S**TEQLRC  CLAHRL**S**EPPEDL  LNPDAF**S**GPQACT  ACTRFF**S**RITKAN  CWGVRG**S**LLSEAD  VRGSLL**S**EADVRA  LLPRLV**S**CPGPLD  PPYGPP**S**TWSVST  PSTWSV**S**TMDALR  GQPIIR**S**IPQGIV  AAWRQR**S**SRDPSW  AWRQRS**S**RDPSWR  AREIDE**S**LIFYKK  PQGYPE**S**VIQHLG  YLFLKM**S**PEDIRK  RKWNVT**S**LETLKA  NKGHEM**S**PQAPRR  YPGYLC**S**LSPEEL  LSPEEL**S**SVPPSS  LSSVPP**S**SIWAVR  SSVPPS**S**IWAVRP  FQNMNG**S**EYFVKI  YFVKIQ**S**FLGGAP  EDLKAL**S**QQNVSM  LSQQNV**S**MDLATF  YLVLDL**S**MQEALS  SMQEAL**S**GTPCLL  DEGQDM**S**QVSGKE  QVSGKE**S**PPVSDT  PIPEDL**S**TTSGGQ  EDLSTT**S**GGQQSS  TSGGQQ**S**SKSDRV  SGGQQS**S**KSDRVV  LRMLDA**S**GEKMNG  GEKMNG**S**HRDQGS  HRDQGS**S**ALSGVG  LMVHKR**S**HTGERP  CNQCGA**S**FTQKGN  CGYCGR**S**YKQRSS  SYKQRS**S**LEEHKE  CHNYLE**S**MGLPGT  KIGSER**S**LVLDRL  VLDRLA**S**NVAKRK  NVAKRK**S**SMPQKF  VAKRKS**S**MPQKFL  DTPYDS**S**ASYEKE  ENEMMK**S**HVMDQA  NYLGAE**S**LRPLVQ  QTPPGG**S**EVVPVI  NHSAQD**S**AVENLL  ENLLLL**S**KAKLVP  KAKLVP**S**EREASP  DSTDTE**S**NNEEQR  HARNGL**S**LKEEHR  LRVVST**S**GEQMKV  NMCGYH**S**QDRYEF  QDRYEF**S**SHITRG  DRYEFS**S**HITRGE  SRIHLV**S**EPSITH  HLVSEP**S**ITHFLQ  THFLQV**S**WEKTLE  WEKTLE**S**GFVITL  TLTDGH**S**AWTGTV  AWTGTV**S**ESEISQ  TGTVSE**S**EISQEA  VSESEI**S**QEADDM  LRKALL**S**GAGPAD  VYTFNF**S**KESCYF  FNFSKE**S**CYFFFE  KNLKDV**S**FRLGSF  VSFRLG**S**FNLEKV  RFEKCV**S**AKEALE  KKTKIR**S**LHNKLL  GETAIC**S**EMTADR  DPVYDE**S**TDEESE  ENQTDL**S**GLASAA  DLSGLA**S**AAVSKD  LASAAV**S**KDDSII  KDDSII**S**SLDVTD  VTDIAP**S**RKRRQR  LQEKEN**S**RPDSSL  ENSRPD**S**SLPETS  NSRPDS**S**LPETSK  SSLPET**S**KKEHIS  SKKEHI**S**AENMSL  GLLGAG**S**PLPSWP  AGSPLP**S**WPLPNI  QDDLPI**S**LKKVLQ  KKVLQT**S**LPEPLR  VRGYAG**S**WVSICT  GHTCAL**S**WRESVH  ALSWRE**S**VHTQKP  QLVTGT**S**FSGPTV  IQNSIC**S**PDFSGG  ICSPDF**S**GGVNMD  NMDHST**S**ILGVAS  TCIMEA**S**TDFLPG  LPGLNF**S**NCSRRA  LNFSNC**S**RRALEK  VDPCCD**S**LTCQLR  PGAQCA**S**DGPCCQ  NCQLRP**S**GWQCRP  EFCPGD**S**SQCPPD  MHGRCA**S**YAQQCQ  YAQQCQ**S**LWGPGA  SCGRNP**S**GSYVSC  PSGSYV**S**CTPRDA  TQPLLG**S**IRDLLW  GTELNC**S**WVHLDL  VHLDLG**S**DVAQPL  GHGVCD**S**NRHCYC  TQLKAT**S**SLTTGL  LVMLGA**S**YWYRAR  RGTKQA**S**ALSFPA  KQASAL**S**FPAPPS  SFPAPP**S**RPLPPD  ADPVVR**S**PKSQGP  PPLVVP**S**RPAPPP  KFWEVI**S**DEHGID  EHGIDP**S**GNYVGD  GNYVGD**S**DLQLER  LQLERI**S**VYYNEA  VYYNEA**S**SHKYVP  YYNEAS**S**HKYVPR  EPGTMD**S**VRSGAF  TMDSVR**S**GAFGHL  NFIFGQ**S**GAGNNW  GAELVD**S**VLDVVR  GFQLTH**S**LGGGTG  LGGGTG**S**GMGTLL  MGTLLI**S**KVREEY  RIMNTF**S**VVPSPK  VPSPKV**S**DTVVEP  PYNATL**S**IHQLVE  DLNHLV**S**ATMSGV  LVSATM**S**GVTTSL  MSGVTT**S**LRFPGQ  PLTARG**S**QQYRAL  VFRGRM**S**MKEVDE  QMLAIQ**S**KNSSYF  AIQSKN**S**SYFVEW  IQSKNS**S**YFVEWI  PRGLKM**S**STFIGN  RGLKMS**S**TFIGNS  STFIGN**S**TAIQEL  ELFKRI**S**EQFTAM  EFTEAE**S**NMNDLV  NMNDLV**S**EYQQYQ  ATLVLT**S**DQSSPE  VLTSDQ**S**SPEIDE  LTSDQS**S**PEIDED  PNPHLK**S**TLAMSP  KSTLAM**S**PRQRKK  PEGAAE**S**TETQES  STETQE**S**RPPGIP  PDTEVE**S**RLGTSG  ESRLGT**S**GTAKKT  KTHERG**S**KEPSTK  RGSKEP**S**TKEPST  PSTKEP**S**THIPPL  GWEKRM**S**RSSGRV  EKRMSR**S**SGRVYY  KRMSRS**S**GRVYYF  NHITNA**S**QWERPS  SQWERP**S**GNSSSG  ERPSGN**S**SSGGKN  RPSGNS**S**SGGKNG  PSGNSS**S**GGKNGQ  PARVRC**S**HLLVKH  HLLVKH**S**QSRRPS  LVKHSQ**S**RRPSSW  QSRRPS**S**WRQEKI  YIQKIK**S**GEEDFE  GEEDFE**S**LASQFS  SLASQF**S**DCSSAK  SQFSDC**S**SAKARG  QFSDCS**S**AKARGD  GDLGAF**S**RGQMQK  KPFEDA**S**FALRTG  LRTGEM**S**GPVFTD  GPVFTD**S**GIHIIL  QHIMKS**S**PLMFVN  RIAATP**S**CHLLHK  EWPVVM**S**RFLSQL  HMGYLQ**S**APLQLM  LPAQPA**S**TFFSSF  AALKME**S**MEIIEE  ENLSSN**S**DGISPK  PKRRRL**S**SSLNPS  RRRLSS**S**LNPSKR  SSSLNP**S**KRAPKQ  AESLQI**S**LEYSGL  VVITWM**S**LDFYTK  IYMQVN**S**SFEDHI  IKHILE**S**LDSEDG  FNMQLL**S**SSVGIE  CLGSLL**S**HVIVAL  LNTRYK**S**SQKSTD  YKSSQK**S**TDWSGV  AEKCPH**S**KSNRNK  NRNKVD**S**MVSTVD  DTLAVA**S**FRSKAY  AVASFR**S**KAYTRA  LEMTQN**S**YRAKEP  WMMTAV**S**KSSYPM  SSTLSM**S**THFKML  LRKDAE**S**RRRELH  TFPDPT**S**WYSSRS  RSAYCR**S**TAVMSM  RSTAVM**S**MVGYIL  QREPLM**S**VLKTFL  VTGLPL**S**IEGHVH  GSGHQP**S**QSRAIP  GHQPSQ**S**RAIPTR  TRTVAI**S**DAAQLP  PGGTLF**S**TTPGGT  GTLIED**S**KVEVNN  VIKMKS**S**DFLESA  GGFGKV**S**LCFHRT  IEEGKY**S**LVMEYM  EMSTPL**S**VKGRII  LIMCIK**S**GNRPDV  FRPFYL**S**QLEESV  SLKKEY**S**NENAVV  PSSRSN**S**ATEQPG  ATEQPG**S**LHSSQG  QPGSLH**S**SQGLGM  PGSLHS**S**QGLGMG  MGPVEE**S**WFAPSL  ESWFAP**S**LEHPQE  QEENEP**S**LQSKLQ  NEPSLQ**S**KLQDEA  ERRRRV**S**HDPFAQ  KGTAYS**S**AASHGN  QPSGLT**S**QPQVLY  QNNGLY**S**SHGFGT  NNGLYS**S**HGFGTR  IPSHMP**S**LHNIPV  TPTMPF**S**SLPPTD  PTMPFS**S**LPPTDE  KYTIYN**S**TGIQIG  MEIGGT**S**SSLLDS  EIGGTS**S**SLLDST  IGGTSS**S**LLDSTN  SSSLLD**S**TNTNFK  IFDNTT**S**LTDKHL  KLGFTQ**S**QIDEID  QALHQC**S**RIDLLS  EMTKYH**S**DDYIKF  YIKFLR**S**IRPDNM  IRPDNM**S**EYSKQM  DNMSEY**S**KQMQRF  FEFCQL**S**TGGSVA  QLSTGG**S**VASAVK  TGGSVA**S**AVKLNK  LHHAKK**S**EASGFC  AKKSEA**S**GFCYVN  DRVMTV**S**FHKYGE  DGIDDE**S**YEAIFK  IFKPVM**S**KVMEMF  MEMFQP**S**AVVLQC  VVLQCG**S**DSLSGD  LQCGSD**S**LSGDRL  CGSDSL**S**GDRLGC  CVEFVK**S**FNLPML  DFKLHI**S**PSNMTN  KLHISP**S**NMTNQN  DPDKRI**S**ICSSDK  KRISIC**S**SDKRIA  RISICS**S**DKRIAC  EGGRKN**S**SNFKKA  GGRKNS**S**NFKKAK  IGKGAF**S**VVRRCV  IVRLHD**S**ISEEGF  VAREYY**S**EADASH  YSEADA**S**HCIQQI  ENLLLA**S**KCKGAA  GTPGYL**S**PEVLRK  PWVCQR**S**TVASMM  QRSTVA**S**MMHRQE  LATRNF**S**VGRQTT  TAPATM**S**TAASGT  TMSTAA**S**GTTMGL  LVEQAK**S**LLNKKA  VKPQTN**S**TKNSAA  TNSTKN**S**AAATSP  NSAAAT**S**PKGTLP  VDGIKE**S**SDSANT  DGIKES**S**DSANTT  IKESSD**S**ANTTIE  GPLPCP**S**PAPFSP  SPLPAP**S**PRISDI  APSPRI**S**DILNSV  ISDILN**S**VRRGSG  EAEGPL**S**AGPPPC  GPPPCL**S**PALLGP  SPSPRI**S**DILNSV  PEAEGP**S**PVGPPP  GPPPCP**S**PTIPGP  GPLPTP**S**RKQEII  CDPGLT**S**FEPEAL  NLLAKN**S**KPIHTT  QGRPRT**S**QSEETR  RPRTSQ**S**EETRVW  PDVYET**S**DLPEDD  DAEELT**S**TSVEHI  EELTST**S**VEHIIV  TKGLDF**S**DRIGKT  KRTGYE**S**GEYEML  KTTVKE**S**ATEEKL  KQQLVA**S**HLEKLL  LEATKN**S**KGGSGG  KNSKGG**S**GGKTTG  TGTPPD**S**SLVTYE  GTPPDS**S**LVTYEL  VTYELH**S**RPEQDK  PEQDKF**S**QAAKVA  DAQNPL**S**AGLQGA  LLQAKV**S**ALDLAV  VEARLQ**S**VLGKVN  IAKHKA**S**VEDADT  EDADTQ**S**KVHQLY  ETIQRW**S**PIASTL  RWSPIA**S**TLPELV  QQMIAN**S**LKDNTT  VEGNFA**S**IDERMK  LVAQVN**S**CQYPGL  ATRHGP**S**QDGDNT  RCALNK**S**RDFRLI  KIYEVC**S**NGPAPT  GPAPTD**S**QPPEDY  QPPEDY**S**FGAGEE  LQRMLP**S**LSLTED  PPAPDP**S**PLAPPP  GFRELL**S**EVLEPG  PGPLPA**S**LPPAGE  LPDLLI**S**PHMLPL  PRALTI**S**NPHGCR  GCRLFY**S**QLEATQ  SPEDIP**S**DKQRFY  QCKVFW**S**GPCASA  WSGPCA**S**AHDSCP  CASAHD**S**CPNPIQ  VKTKLF**S**LEHFLN  LLLEMF**S**GELSWS  LHHIWQ**S**QQRLQP  RVGYWL**S**EKKIKK  VVQLNL**S**RPIEEQ  EADQND**S**QSLELV  DQNDSQ**S**LELVHR  RTLLDR**S**KSYELI  LLDRSK**S**YELIRK  EDDRIC**S**PPFMEL  PFMELT**S**LCGDDT  VAHGTN**S**HEMAIV  VFVVGE**S**YTVVQR  TVVQRP**S**LKNFSA  PSLKNF**S**AGTSDR  NFSAGT**S**DRESIF  GTSDRE**S**IFFNSH  ESIFFN**S**HNVSKP  FNSHNV**S**KPESSS  NVSKPE**S**SSVLTE  VSKPES**S**SVLTEL  SKPESS**S**VLTELD  GVFERP**S**DEVIRE  EVIREL**S**RALRQA  RQALGV**S**LFGIDI  PGYEGV**S**EFFTDL  TVLQGQ**S**TAMAAT  VALLRH**S**KLLAEP  VGERTC**S**ASPGCC  ERTCSA**S**PGCCGS  SPGCCG**S**MMGQDA  GCNAGV**S**PSFQQH  NAGVSP**S**FQQHCV  QQHCVA**S**LATKAS  PAPTQL**S**QDQLEA  AEEKAR**S**QRSRQT  KARSQR**S**RQTSLV  QRSRQT**S**LVSSRR  RQTSLV**S**SRREPP  QTSLVS**S**RREPPP  GRKKKM**S**NALAIQ  LAIQVD**S**EGKIKY  IARQGQ**S**KDKVIY  KDKVIY**S**KYTDLV  RVALEK**S**VSQKVA  ALEKSV**S**QKVAAA  YIRYTP**S**QQGVAF  QGVAFN**S**GAKQRV  IPRGPP**S**PPAPVM  PAPVMH**S**PSRKMT  PVMHSP**S**RKMTVK  KIPPCI**S**NWKNAK  QHDRNL**S**RAAPDK  AAPDKR**S**KLQRNE  NENRDI**S**EVIALG  VPNPRT**S**NEVQYD  QRLFNQ**S**KGMDSG  QSKGMD**S**GFAGGE  GKDMAQ**S**IYRPSK  QSIYRP**S**KNLDKD  VPDKEF**S**GSDRRQ  DKEFSG**S**DRRQRG  AKQHGG**S**KRPSDS  GGSKRP**S**DSSRPK  SKRPSD**S**SRPKEH  KRPSDS**S**RPKEHE  EKGAGK**S**EFRNQK  PENQDE**S**ELLTVP  WKEPAF**S**KEDNPR  RGLLEE**S**SFATLF  GLLEES**S**FATLFP  LDLIEG**S**MTVCTT  IKLLAR**S**VSFEQA  LLARSV**S**FEQAVR  DIIKIG**S**LVRNKE  LIGPKG**S**TLKALE  VQGNTV**S**AIGPFS  SAIGPF**S**GLKEVR  PIYNIK**S**LMIKRE  RELAKD**S**ELRSQS  KDSELR**S**QSWERF  SELRSQ**S**WERFLP  PPPQPE**S**QIDKEL  IDKELA**S**GEYFLK  KQAEAI**S**KRQEER  VKPKEA**S**TETKID  TKIDVA**S**IKEKVK  KSSSCE**S**LGSQPA  AAARPP**S**VDSLSS  RPPSVD**S**LSSAST  PSVDSL**S**SASTSH  SASTSH**S**ENSVHT  TSHSEN**S**VHTKSA  VHTKSA**S**VVSSDS  KSASVV**S**SDSIST  SASVVS**S**DSISTS  SVVSSD**S**ISTSAD  SDSIST**S**ADNFSP  KIGGAS**S**RGENSY  SSRGEN**S**YGLETV  EGRTRR**S**IFENLM  KYAFPV**S**NNLPLF  QGIPNE**S**WRITKI  GVSGKR**S**KEDEKY  MDSNAQ**S**HKIFIF  KGGGYE**S**EDAYQN  IHVMRE**S**LRKLKE  EETHWL**S**NLESTH  IADKVE**S**GKTSVV  VESGKT**S**VVVHCS  VEKEWL**S**FGHRFQ  HADADR**S**PVFLQF  RTVSLW**S**YINSQL  PLYGSY**S**NHVLYP  REISNR**S**TSSSER  ISNRST**S**SSERAS  SNRSTS**S**SERASS  SSSERA**S**SPAQCV  HTGGET**S**ACKPSS  AGQMPH**S**HQYSDR  RRQPNI**S**DQQVSA  APLRKL**S**VDLIKT  QGQGDD**S**SHKKER  GQGDDS**S**HKKERK  DRYEID**S**LIGKGS  SLIGKG**S**FGQVVK  LCNPKR**S**AIKIVD  IVDFGS**S**CQLGQR  IYQYIQ**S**RFYRSP  LAIDMW**S**LGCILV  NTSNSV**S**TSPAME  SPAMEQ**S**QSSGTT  QSSGTT**S**STSSSS  SSGTTS**S**TSSSSG  GTTSST**S**SSSGGS  TTSSTS**S**SSGGSS  TSSTSS**S**SGGSSG  SSSGGS**S**GTSNSG  GGSSGT**S**NSGRAR  MDCETH**S**PQVRQQ  HHHHGN**S**SHHHHH  RPRVYN**S**PTNSSS  YNSPTN**S**SSTQDS  NSPTNS**S**STQDSM  SSSTQD**S**MEVGHS  SHHSMT**S**LSSSTT  SMTSLS**S**STTSSS  TTSSST**S**SSSTGN  SSTSSS**S**TGNQGN  LGVGKT**S**IIKRYV  YVHQLF**S**QHYRAT  KVLNWD**S**RTLVRL  FVVFDI**S**RSSTFE  VFDISR**S**STFEAV  FDISRS**S**TFEAVL  AVLKWK**S**DLDSKV  WKSDLD**S**KVHLPN  VHLPNG**S**PIPAVL  CDQNKD**S**SQSPSQ  DQNKDS**S**QSPSQV  NKDSSQ**S**PSQVDQ  DSSQSP**S**QVDQFC  AGWFET**S**AKDNIN  ILVNHQ**S**FPNEEN  GAPSPS**S**FTTGHL  TFLVRK**S**NTRQCQ  PEASGP**S**FVSSHY  GPSFVS**S**HYILES  GVSLEG**S**ELMFPD  KELEAI**S**HLGIEF  REKFKR**S**FKVRVS  SFKVRV**S**TETSSP  RVSTET**S**SPLSPP  LLRRES**S**VGYRVP  PAGSGP**S**LPPMPS  SLPPMP**S**LQEVDC  VDCGSP**S**SSEEEG  DCGSPS**S**SEEEGV  CGSPSS**S**EEEGVP  EEGVPG**S**RGSPAT  RRPLLR**S**MSAAFC  GIRQAL**S**RARAML  RARAML**S**AELGPE  EKSLHC**S**VLKPLR  SHLSLP**S**PVELEQ  LRTYSP**S**AQVKRL  LEAEYM**S**ELLEPS  SELLEP**S**LLTGEG  YYLTSL**S**ASLALL  ASLALL**S**GLGQAH  VAYQDP**S**SGCTSK  AYQDPS**S**GCTSKT  AVPPEA**S**IATLNQ  VTEEEG**S**GQSEAR  GQSEAR**S**RGEEQG  PQAETG**S**GTESDS  TGSGTE**S**DSDESV  SGTESD**S**DESVPE  ESDSDE**S**VPELEE  IDEEPV**S**KAKQSR  VSKAKQ**S**RSEKKA  KAKQSR**S**EKKARK  KARKAM**S**KLGLRQ  RVTIRK**S**KNILFV  KPDVYK**S**PASDTY  VYKSPA**S**DTYIVF  AKIEDL**S**QQAQLA  VQGEAV**S**NIQENT  KPKVIR**S**DGAPAE  EGKRNR**S**DTEQEG  QEGKYY**S**EEAEVD  EERRIQ**S**CVHFMT  KCLEFK**S**KHEEID  EEIDLV**S**LEEFYK  EAPPDI**S**KAEVTM  KYRECL**S**NKEKIL  VKKEYL**S**SLQPRL  KKEYLS**S**LQPRLN  ACDKTL**S**VAIEGS  SVAIEG**S**VDEAKA  FKPPED**S**QDDESD  QDDESD**S**DAEEEQ  LKRHPL**S**VMLDLK  LKCKDD**S**VLHLTF  ELITPI**S**AGDLLS  SAGDLL**S**PDSVLS  DLLSPD**S**VLSCLY  VGILTL**S**DYVLEL  TVIADH**S**LSASHM  IADHSL**S**ASHMET  DHSLSA**S**HMETTM  LKTRVQ**S**RLALHK  LHKQFA**S**LEHGIV  GIVPVT**S**DCQYLF  FPAKVV**S**RLVKWV  VLNPGY**S**SIPPVF  LNPGYS**S**IPPVFQ  KGEKTN**S**NDDNIR  LCGPWP**S**HQLLTN  TESHDD**S**VEGPKE  RLFRGP**S**RMKPFK  TCLCPA**S**GAKPKL  GAKPKL**S**GFKGGG  QLNVGG**S**LYYTTV  MLKAMF**S**GRMEVL  LVNMCQ**S**ALQDKK  LQDKKD**S**YQPVCN  NIPIIT**S**LKEEER  EERLIE**S**STKPVV  ERLIES**S**TKPVVK  KLLYNR**S**NNKYSY  RSNNKY**S**YTSNSD  NKYSYT**S**NSDDHL  YSYTSN**S**DDHLLK  ELFDKL**S**LRFNGR  DEICCW**S**FYGQGR  AEVCCT**S**IVYATE  PRVPDN**S**LLEATS  SLLEAT**S**RSRSQA  LEATSR**S**RSQASP  ATSRSR**S**QASPSE  IHVKRY**S**TYDDRQ  GLWDFK**S**RTDEEL  RTDEEL**S**FRAGDV  ERETVE**S**EPWFFG  WFFGCI**S**RSEAVR  FGCISR**S**EAVRRL  AFLIRV**S**EKPSAD  RVSEKP**S**ADYVLS  SADYVL**S**VRDTQA  HLNEAV**S**FLSLPE  EAVSFL**S**LPELVN  NYHRAQ**S**LSHGLR  HRAQSL**S**HGLRLA  LCRKLG**S**GYFGEV  VAIKVI**S**RDNLLH  HQQMLQ**S**EIQAMK  ALYAVV**S**VGDPVY  ELMAKG**S**LLELLR  LELLRD**S**DEKVLP  EKVLPV**S**ELLDIA  GMCYLE**S**QNYIHR  KEDVYL**S**HDHNIP  TAPEAL**S**RGHYST  LSRGHY**S**TKSDVW  GHYSTK**S**DVWSFG  TKSDVW**S**FGILLH  LLHEMF**S**RGQVPY  VPYPGM**S**NHEAFL  PLECPP**S**VHKLML  ALRERL**S**SFTSYE  LRERLS**S**FTSYEN  GGRAQR**S**GLLEVL  KVLVNL**S**EDALVL  DALVLS**S**EEGAAA  GAQPPD**S**PAGVRT  QVPESI**S**NQKRGV  KMPILI**S**KIFKGL  PYVKKG**S**PVSEIG  KKGSPV**S**EIGWET  ETPPPE**S**PRLGGS  SPRLGG**S**TSDPPS  RLGGST**S**DPPSSQ  TSDPPS**S**QSFSFH  DPPSSQ**S**FSFHRD  MCYVTR**S**MALADP  RQLEIH**S**PDAKHT  HTVILR**S**KDSATA  ILRSKD**S**ATAQAW  TAQAWF**S**AIHSNV  WFSAIH**S**NVNDLL  KTGIAG**S**REIRHL  EKVPGE**S**KKQWKP  ATRLVH**S**GPGKGS  LFRAET**S**RDLSHW  ETSRDL**S**HWTRSI  LSHWTR**S**IVQGCH  VQGCHN**S**AELIAE  ELIAEI**S**TACTYK  HYENGF**S**ITTEPQ  PKTIIQ**S**PYEKLK  YEKLKM**S**SDDGIR  EKLKMS**S**DDGIRM  IQLDLH**S**CPKPIV  IVFIIH**S**FLSAKI  KIKRTP**S**KKGKPA  GKPAEV**S**VKIPEK  VEFMST**S**AVASRS  QRQKNL**S**WLEEKE  KEKEVV**S**ALRYFK  PRIQHS**S**ALSSCY  YSRVYQ**S**LANLIR  ANLIRW**S**DQVMLE  MLEGVN**S**EDKEMV  PPKKRQ**S**APSPTR  KRQSAP**S**PTRVAV  SRATSG**S**SLPVGI  PRLSPC**S**SIGKLS  RLSPCS**S**IGKLSK  FLQQDL**S**NADQIP  LSPLPE**S**LGESGS  PESLGE**S**GSPFLG  SLGESG**S**PFLGPP  GSGCRV**S**YERHPS  SQYDNI**S**GEDLQS  DFTAPE**S**TGDPEK  GVPGKD**S**RDGSER  KDSRDG**S**ERAPKS  SERAPK**S**PDALES  PDVRGG**S**GDILLV  LLRCAT**S**SQPLAA  TLHDFH**S**HEIAEQ  KLNNFN**S**YLAILS  SYLAIL**S**ALDSAP  ILSALD**S**APIRRL  CTLIDS**S**SSFRAY  LIDSSS**S**FRAYRA  EALWEL**S**LKIKPR  NFFWDP**S**TSRRFS  FWDPST**S**RRFSPP  STSRRF**S**PPSSSL  FSPPSS**S**LQPGKM  LQPGKM**S**DVSPVV  ELVRTD**S**PNFLCS  LCSVLP**S**HWRCNK  GNDENY**S**AELRNA  AELRNA**S**AVMKNQ  SKPSLF**S**DRLSDL  GRIPHP**S**MRVGVP  SLNSAP**S**PFNPQG  PRQAQS**S**PPWSYD  YDQSYP**S**YLSQMT  SYPSYL**S**QMTSPS  HSTTPL**S**STRGTG  KSQAGA**S**ELGPFS  DPRQFP**S**ISSLTE  RQFPSI**S**SLTESR  LTESRF**S**NPRMHY  PVTSGM**S**LGMSAT  GMSLGM**S**ATTHYH  YPGSSQ**S**QSGPFQ  GPFQTS**S**TPYLYY  YGTSSG**S**YQFPMV  VPGGDR**S**PSRMLP  GGDRSP**S**RMLPPC  PPCTTT**S**NGSTLL  TTTSNG**S**TLLNPN  DADGSH**S**SSPTVL  SPTVLN**S**SGRMDE  RMSVGL**S**LLLPLS  SVVMAQ**S**HWPSEP  ENQLEA**S**MHSVLS  LEASMH**S**VLSDLH  SMHSVL**S**DLHEAV  TDLIAS**S**GDIIKV  LHWDSQ**S**HTLEGL  KGVHYI**S**VSATRL  SHIPQT**S**SVFSIE  HIPQTS**S**VFSIEV  VYPEDH**S**ELQSVR  DHSELQ**S**VRTASP  DPGEVV**S**SACAAD  PGEVVS**S**ACAADE  ENGALL**S**WKLGCS  CSLNQN**S**VPDIHG  AREGAM**S**AQLGYP  IQPTRV**S**EAGTTV  TKKPRV**S**TPKPAT  PKPATP**S**TDSTTT  RVTTKV**S**ITRLET  RIRTTT**S**GVPRGG  SWVQFN**S**NSQLMY  VQFNSN**S**QLMYGL  PDFKAT**S**ITVTGS  SITVTG**S**GSCRHL  TVTGSG**S**CRHLQF  PPRRVP**S**EAPPTE  DRDPEK**S**SEDDVY  DSKPPP**S**SSMPLI  SKPPPS**S**SMPLIL  PEYPNQ**S**VPETTP  PMEGKG**S**RPKNMT  GHVGFD**S**LPDQLV  DQLVNK**S**VSQGFC  LVNKSV**S**QGFCFN  ETGLGK**S**TLMDTL  PGVQLQ**S**NTYDLQ  TYDLQE**S**NVRLKL  LKLTIV**S**TVGFGD  QINKED**S**YKPIVE  LHTYHD**S**RIHVCL  IAPTGH**S**LKSLDL  TGHSLK**S**LDLVTM  TMKKLD**S**KVNIIP  AKADAI**S**KSELTK  ADAISK**S**ELTKFK  FKIKIT**S**ELVSNG  ITSELV**S**NGVQIY  FPTDDE**S**VAEING  PFAVIG**S**TEELKI  KDTDPD**S**KPFSLQ  PDSKPF**S**LQETYE  LEDKKK**S**LDDEVN  AAELLQ**S**QGSQAG  LLQSQG**S**QAGGSQ  GSQAGG**S**QTLKRD  LYDVMK**S**KKHRTW  EAAKEE**S**QQMVLD  PESVLL**S**AVSGED  VLLSAV**S**GEDTQD  VKFLWE**S**YRQCLD  DLLRNN**S**RVERLY  QRHHNQ**S**TAINLN  NLNNPE**S**QSMHLE  NNPESQ**S**MHLETR  QLDSAI**S**MELWQE  DIHGLF**S**LSKKPP  VSQIYQ**S**IEFSRL  RTLSFG**S**DLNYAT  IGPHLQ**S**MPSEQI  NQLTAM**S**SVLAKA  TAYLKN**S**RKEHQR  EHKNRM**S**RMLEDR  LKAARQ**S**VYEEKL  RWGDRD**S**EGTWRK  KGPEAD**S**EWRRGP  GRDEDR**S**HRRDEE  PEEDRF**S**RRGADD  LDEDRG**S**WRTADE  ADDERS**S**WRNADD  EKAREE**S**WGPPRE  WGPPRE**S**RPSEER  RGPAEE**S**SSWRDS  GPAEES**S**SWRDSS  SSWRDS**S**RRDDRD  SEREEV**S**SWRRAD  GEKEKA**S**WRAEKD  LRGCGA**S**LRLELL  NFNKVL**S**SLVTLN  RIKSFD**S**LGSQSL  TNEDEL**S**FSKGDV  EDELSF**S**KGDVIH  RTGWFP**S**NYVREV  TTAINK**S**YYNVVL  LRPLQT**S**EKLSSA  LSSANI**S**YLMGNL  QQMLVQ**S**LEECTK  YCANHP**S**AVNVLT  METKGA**S**SPGILV  VLTTGL**S**KPFMRL  AAFKNL**S**AQCQEV  GNVTYM**S**QVLIQC  LIQCAG**S**EEKNER  LLMLSA**S**PRMSGF  RNAFEI**S**GSMIER  AFEISG**S**MIERIL  IERILV**S**CNNQQD  PTIKPH**S**VPSHTL  PSHTLP**S**HPVTPS  HPVTPS**S**KHADSK  HTLPHP**S**HHGTPH  KTPKPW**S**LSCLRP  PKPWSL**S**CLRPAP  TRQTLN**S**TWQGTD  ADDDQP**S**LDSLGR  DQPSLD**S**LGRRSS  LSRLEP**S**DLSEDS  HSYRMG**S**TSRKSC  KARNKK**S**GELVAV  KVFNTT**S**YLRPRE  VEETGG**S**RQKVLV  LVMEYC**S**SGSLLS  VMEYCS**S**GSLLSV  SSGSLL**S**VLESPE  LLSVLE**S**PENAFG  VGEEGQ**S**IYKLTD  VTVDLW**S**IGVTLY  YHAATG**S**LPFIPF  LSLGLQ**S**QLVPIL  VHVFSL**S**QAVLHH  AVHKQT**S**VAPRHQ  VLEPSV**S**AQHIAH  IAHTTA**S**SPLTLF  AHTTAS**S**PLTLFS  SPLTLF**S**TAIPKG  AAAELR**S**RLRTLA  TLAEVL**S**RCSQNI  ITETQE**S**LSSLNR  ETQESL**S**SLNREL  TQESLS**S**LNRELV  NRELVK**S**RDQVHE  QVHEDR**S**IQQIQC  YKQFKK**S**RMRPGL  LDKVNF**S**HLAKRL  VQKYQA**S**LVTHGK  LRLVGC**S**VAACNT  KLLEEL**S**HQLLQD  PIAPYP**S**PTRKDL  GMKLLA**S**DLLDNN  SYQGKK**S**IPHITS  SIPHIT**S**DRLLIK  IINDDQ**S**LYADVY  TYLQKP**S**QGMTAA  VVPEPG**S**SLLTSF  VPEPGS**S**LLTSFE  GSSLLT**S**FEKWHE  EAADTK**S**CCDYSL  KSCCDY**S**LHVDIT  LHVDIT**S**WYDGVR  QDKGVN**S**FQVYMA  KDVYQM**S**DSQLYE  VYQMSD**S**QLYEAF  PEGHAL**S**RPEELE  YITKVM**S**KSAADI  TKVMSK**S**AADIIA  GEPIAA**S**LGTDGT  DGTHYW**S**KNWAKA  AAAFVT**S**PPLSPD  DLQVTG**S**GHCPYS  SGHCPY**S**TAQKAV  QFVAVT**S**TNAAKI  GRIAVG**S**DADVVI  TAKSHK**S**AVEYNI  GMECHG**S**PLVVIS  SPLVVI**S**QGKIVF  FGLQGV**S**RGMYDG  YATPAP**S**AKSSPS  PAPSAK**S**SPSKHQ  APSAKS**S**PSKHQP  SAKSSP**S**KHQPPP  LHQSNF**S**LSGAQI  QSNFSL**S**GAQIDD  NVPAIM**S**SLWKKL  RPSGQI**S**DPSRSY  PSEIHM**S**EEAIQD  KNNKNI**S**QSISSQ  SSQPME**S**NPSIVL  EKNSLS**S**NVPSES  PITSFV**S**LGCEAN  SQFCEN**S**NDTVKL  KINFHG**S**KSSDSS  DNSCLQ**S**EILPVS  LPVSVE**S**SHLNVS  EIHLGD**S**LSSTKQ  LGDSLS**S**TKQPSN  SSTKQP**S**NDSASV  HSLPPE**S**VCSSVG  ELTSAV**S**SINGEN  TFKSED**S**AVNNTQ  AVNQAV**S**PNFSQG  PNFSQG**S**AIIIAS  QNGNNF**S**TPPRQV  FPVPPK**S**QKAQGL  VPFPEE**S**IVPAAK  KSESAI**S**RHTTIR  KKQGTS**S**NNKNVL  QDIQRH**S**SVSRLA  DIQRHS**S**VSRLAD  SRLADS**S**DLPVPR  VPRTPG**S**GAGEKH  IIKAPS**S**RRFSED  PSSRRF**S**EDSSTS  RNSRPS**S**KNLTNS  LLGPGN**S**LQLWDT  PEMLRN**S**TDTTPL  GPGTPE**S**TTVEPA  EPAARR**S**TGLDAG  ANMGNL**S**TDSAAM  GNLSTD**S**AAMEIQ  AAMEAL**S**TEPSAT  ALSTEP**S**ATEALS  SATEAL**S**MEPTTK  GLFIPF**S**VSSVTH  FIPFSV**S**SVTHKG  IPFSVS**S**VTHKGI  GIPMAA**S**NLSVNY  MAASNL**S**VNYPVG  GAPDHI**S**VKQCLL  VLAVRL**S**RKGHMY  YPVRNY**S**PTEMVC  TEMVCI**S**SLLPDG  EMVCIS**S**LLPDGG  DGGEGP**S**ATANGG  TANGGL**S**KAKSPG  GLSKAK**S**PGLTPE  DFVNDV**S**EKEQRW  AKTVQG**S**GHQEHI  ETGPKA**S**HGYGGK  QDRMDK**S**AVGHEY  VGHEYQ**S**KLSKHC  EYQSKL**S**KHCSQV  HCSQVD**S**VRGFGG  MDRVDQ**S**AVGFEY  KTEKHA**S**QKDYSS  SQKDYS**S**GFGGKY  ADRVDK**S**AVGFDY  KTEKHE**S**QRDYSK  ESQRDY**S**KGFGGK  KDKVDK**S**AVGFEY  KTEKHE**S**QKDYVK  KLQLHE**S**QKDYKT  GKFGVQ**S**ERQDSA  QSERQD**S**AAVGFD  KLAKHE**S**QQDYSK  ESQQDY**S**KGFGGK  RMDKNA**S**TFEDVT  EDVTQV**S**SAYQKT  DVTQVS**S**AYQKTV  PVEAVT**S**KTSNIR  AVTSKT**S**NIRANF  TEERLP**S**SPVYED  EERLPS**S**PVYEDA  VYEDAA**S**FKAELS  SFKAEL**S**YRGPVS  SYRGPV**S**GTEPEP  EPEPVY**S**MEAADY  ADYREA**S**SQQGLA  AGDDEI**S**FDPDDI  VCFYSN**S**FNPGKN  ITSILL**S**GRIGPN  RLKHMK**S**DEIHWL  PEDFME**S**LKEDRT  NALDKK**S**NFELLE  GIRQLT**S**QDAKPT  GAPQAL**S**IKTSSL  ALSIKT**S**SLAEAE  LSIKTS**S**LAEAEN  QGEHQG**S**LIIHPR  DGEKRN**S**LPQIPM  NLEARR**S**HLSESC  RSHLSE**S**CSIESD  ESCSIE**S**DIYAEI  NKEKFM**S**EAVIMK  LERNKN**S**LKVLTL  LGDFGL**S**RYIEDE  EDYYKA**S**VTRLPI  RRFTTA**S**DVWMFA  CWDYDP**S**DRPRFT  FTELVC**S**LSDVYQ  PEGLCA**S**SPTLTS  EGLCAS**S**PTLTSP  SSPTLT**S**PMEYPS  SPMEYP**S**PVNSLH  YPSPVN**S**LHTPPL  NVFKRH**S**MREEDF  EDFIQP**S**SREEAQ  DFIQPS**S**REEAQQ  PRLGAQ**S**IQPTAN  VDDLLP**S**LPSSSR  QQNAVT**S**LSEECK  NAVTSL**S**EECKRQ  MKLENV**S**VALEFL  TLILHY**S**ISMPMW  GDANKV**S**ARGPGL  LEDKGD**S**TFRCTY  GAPITR**S**PFPVHV  GYAIPR**S**PFEVQV  VGTLGF**S**IEGPSQ  DEDIRD**S**PFIAHI  KHTIII**S**WGGVNV  HIKVDP**S**HDASKV  DGSCAV**S**YLPTEP  TFTVDC**S**EAGEAE  KCLVTV**S**IGGHGL  PHEEEP**S**EVPQLR  GNHIPG**S**PLQFYV  YVDAIN**S**RHVSAY  DKHIPG**S**PFTAKI  DDSMRT**S**QLNVGT  LSQLTA**S**IRAPSG  VWGKGL**S**EGHTFQ  EGRMKE**S**ITRRRQ  SIATIG**S**TCDLNL  TERTEI**S**KTRGGE  EVRVEE**S**TQVGGD  GEDSAY**S**VRFVPQ  VPVASL**S**DDARRL  KVNQPA**S**FAVQLN  GDPGLV**S**AYGPGL  GGTTGV**S**SEFIVN  SSSRGS**S**YSSIPK  SIPKFS**S**DASKVV  DFLPSR**S**VLSVCF  PSRSVL**S**VCFPGC  PGCLLT**S**GEAEQQ  AEQQRK**S**KEIDKC  EIDKCL**S**REKTYV  LLGAGE**S**GKSTFL  AGESGK**S**TFLKQM  FRPTIY**S**NVIKGM  IPWGDN**S**NQQHGD  HGDKMM**S**FDTRAP  RALWAD**S**GIQNAY  EFQLGE**S**VKYFLD  EPDYIP**S**QQDILL  DVGGQR**S**ERKRWF  WFECFD**S**VTSILF  CFDSVT**S**ILFLVS  SILFLV**S**SSEFDQ  ILFLVS**S**SEFDQV  LFLVSS**S**EFDQVL  TNRLTE**S**LNIFET  VNNRVF**S**NVSIIL  RVFSNV**S**IILFLN  EKVQIV**S**IKDYFL  CINKYG**S**PYTKNS  SPYTKN**S**GFATCV  KDVNEC**S**QENGGC  PGSFHC**S**CHSGFE  HSGFEL**S**SDGRTC  SGFELS**S**DGRTCQ  IDECAD**S**EACGEA  DEGFAY**S**SQEKAC  EGFAYS**S**QEKACR  EQVCVN**S**PGSYTC  CVNSPG**S**YTCHCD  RGGLKL**S**QDMDTC  GAQGSR**S**EGHIPD  PFSVAK**S**VKSLYL  VAKSVK**S**LYLGRM  AGGHQD**S**TWIVLA  GVGRVT**S**SGPVIN  VGRVTS**S**GPVINH  GMWQTI**S**VEELAR  GSFYPG**S**GFAFYS  SGFAFY**S**LDYMRT  LDVGTE**S**TWEVEV  LRAVPL**S**VALVDY  ALVDYH**S**TKKLKK  EHVVTV**S**LRDGEA  DGTRGQ**S**EVSAAQ  RGQSEV**S**AAQLQE  LERHLR**S**PVLTFA  PDVPVT**S**APVTAF  EAAYKH**S**DITAHS  SDITAH**S**CPPVEP  SKTSNN**S**TMQVSF  NSTMQV**S**FVCQRC  FVCQRC**S**QPLKLD  PLKLDT**S**FKILDR  QEEETN**S**GEEPFI  PRQDGV**S**RRFIPP  TLIGEA**S**DGGTME  GTMENL**S**RRLKVT  DLFDIM**S**GQTDVD  QMNEDD**S**EQLQME  QYQREY**S**EFKRQQ  LDDELK**S**VENQMR  TFHIWH**S**GQFGTI  RLGRLP**S**VPVEWN  VPYGNH**S**YLESLT  NHSYLE**S**LTDKSK  ESLTDK**S**KELPLY  ELPLYC**S**GGLRFF  IEDTGG**S**GGSYSI  TGGSGG**S**YSIKTQ  GSGGSY**S**IKTQFN  IKTQFN**S**EEQWTK  SPPRHQ**S**RCDGDA  RCDGDA**S**PPSPAR  GDASPP**S**PARWSL  PSPARW**S**LGRKRR  AERRPE**S**FTTPEG  EGPKPR**S**RCSDWA  KPRSRC**S**DWASAV  RCSDWA**S**AVEEDE  FGRERK**S**SSGSSD  GRERKS**S**SGSSDS  RERKSS**S**GSSDSK  RKSSSG**S**SDSKES  KSSSGS**S**DSKESM  SSGSSD**S**KESMST  SSDSKE**S**MSTVPA  DSKESM**S**TVPADF  DFETDE**S**VLMRRQ  NKFKKY**S**RRSWDQ  KKYSRR**S**WDQQIK  HPVDLE**S**AESSSE  DLESAE**S**SSEPQT  LESAES**S**SEPQTS  ESAESS**S**EPQTSS  SSEPQT**S**SQDDFD  SEPQTS**S**QDDFDV  DDFDVY**S**GTPTKV  KVRHMD**S**QVEDEF  KRKKSK**S**RSRSHE  ERKRSK**S**KERKRS  SKERKR**S**RDRERK  DRERKK**S**KSRERK  ERKKSK**S**RERKRS  SRERKR**S**RSKERR  ERKRSR**S**KERRRS  SKERRR**S**RSRSRD  ERRRSR**S**RSRDRR  FRGRYR**S**PYSGPK  SGPKFN**S**AIRGKI  KIGLPH**S**IKLSRR  PHSIKL**S**RRRSRS  KLSRRR**S**RSKSPF  SRRRSR**S**KSPFRK  RDVRMI**S**DRNSRR  DRNSRR**S**KGIAYV  VEFVDV**S**SVPLAI  PIIVQA**S**QAEKNR  NNLQKG**S**AGPMRL  MRLYVG**S**LHFNIT  IQLMMD**S**ETGRSK  DSETGR**S**KGYGFI  FITFSD**S**ECAKKA  TERTDA**S**SASSFL  TDASSA**S**SFLDSD  DASSAS**S**FLDSDE  QQALQM**S**GSLAFG  ALQMSG**S**LAFGAV  GAVAEF**S**FVIDLQ  DLQTRL**S**QQTEAS  SQQTEA**S**ALAAAA  IYVDKN**S**AQGNVY  PMQETA**S**SHEQPA  MQETAS**S**HEQPAS  VYQVPP**S**VQRSIG  YVYEYP**S**RYQKDV  VYDIPP**S**SAKGPV  YDIPPS**S**AKGPVF  AKGPVF**S**VPVGEI  PPQLGQ**S**VGSQND  EPPAET**S**EKANPQ  NRLSFS**S**TGSTRS  SFSSTG**S**TRSNMS  STGSTR**S**NMSTSS  SNMSTS**S**TSSKES  ESSLSA**S**PAQDKR  ALEMGV**S**SLMALV  LEMGVS**S**LMALVT  LQRVED**S**HQILSQ  QILSQT**S**HDLNEC  HDLNEC**S**WSLNIL  FRPGPG**S**LHLKNG  PESIMN**S**TEYPHG  EYPHGG**S**QGQLLH  CSSSDG**S**ERSWMD  SDGSER**S**WMDDYD  LEHHQL**S**QFQLLE  PVENDI**S**KWKPSQ  ISKWKP**S**QSLPTT  ALFSCV**S**SAQPPR  RNKVMN**S**SNQLCE  AALHYP**S**TTALQE  HQVTDL**S**RNAQLF  LLPRGT**S**SFRRFT  AIYTFE**S**LVKILA  WNWLDF**S**VIIMAY  VGALIQ**S**VKKLAD  LTVFCL**S**VFALIG  VDTVSR**S**SLEMSP  RSSLEM**S**PLAPVN  QPSPGT**S**APGHAL  LMELGL**S**RMSNLS  LGLSRM**S**NLSVLR  WDCMEV**S**GQSLCL  EEDEEN**S**LGTEEE  PEAPPD**S**RTWSQV  RTWSQV**S**ATASSE  VSATAS**S**EAEASA  GETPED**S**CSEGST  IFMILL**S**SGALAF  RPLRAL**S**RFEGMR  IFWLIF**S**IMGVNL  LVMFIY**S**IFGMAN  LPNSNG**S**RGDCGS  DFADAL**S**EPLRIA  RKHEEV**S**AMVIQR  LIAYVM**S**ENFSRP  RPLGPP**S**SSSISS  PSSSSI**S**STSFPP  SSSSIS**S**TSFPPS  SSISST**S**FPPSYD  STSFPP**S**YDSVTR  FPPSYD**S**VTRATS  VRGSDY**S**HSEDLA  ADILLK**S**GECAGQ  MEAPGH**S**RQLLLQ  LDHDMV**S**PAVFRL  AVLAAA**S**YLQIPD  IQACYP**S**PVGPPP  AAALCA**S**ERRCSP  ASERRC**S**PLCGLD  LCGLDL**S**KKSPPG  LDLSKK**S**PPGSAA  KKSPPG**S**AAPERP  LPPRPD**S**PPSAGP  RPDSPP**S**AGPAAY  EEAAPP**S**DPFRGG  FRGGSG**S**PGPEPP  GRPDGP**S**LLYRWM  HEPGLG**S**YGDELG  RERGSP**S**ERCEER  PPRYPG**S**LDGPGA  DGDDYK**S**SSEETG  GDDYKS**S**SEETGS  DDYKSS**S**EETGSS  SSEETG**S**SEDPSP  GSSEDP**S**PPGGHL  AYGEPE**S**FGDNLY  CGKGFP**S**SEQLNA  GKGFPS**S**EQLNAH  RPYRCA**S**CDKSYK  CASCDK**S**YKDPAT  MTRHMR**S**HLGLKP  EHMRIH**S**GEKPYE  QQRNLI**S**HMKMHA  LTAEQL**S**LKQQDK  KAAEVL**S**QGAHLA  GNFPRL**S**YPTFFP  PDDFLT**S**DEEVDS  DSVLFG**S**LRGHVV  RAGPSY**S**MPVHAA  NTITNF**S**EKDRPE  GLSKDA**S**RCSEQP  SEQPSI**S**DIKEKS  KSKFRM**S**ELSSSR  RMSELS**S**SRPKRR  MSELSS**S**RPKRRK  AVQYIE**S**SDSEEI  YIESSD**S**EEIETS  RAKAGS**S**KVIEDV  FACALT**S**SVPTTK  ACAVEG**S**KKTDVE  LIICPL**S**VLSNWI  YGTKGD**S**PLHSIR  GTPIQN**S**LKDLWS  SLKDLW**S**LLSFLK  DLWSLL**S**FLKLKP  GLRRLQ**S**LIKNIT  IQHITL**S**DEERKI  LLTNAV**S**SNGPSG  LTNAVS**S**NGPSGN  KMKLIL**S**SGSDEE  MKLILS**S**GSDEEC  EELARD**S**EKKSDM  SDMEWT**S**SSKINA  MEWTSS**S**KINALM  EIPLKA**S**GFVFTR  QKKRVE**S**IQCFQN  QNTEAG**S**PTIMLL  PTIMLL**S**LKAGGV  VAWVNK**S**RTRFRI  WKRNFR**S**ALNRKE  IYEFVN**S**GVGDFS  SGVGDF**S**QPDTSP  FSQPDT**S**PDTNGG  DTNGGG**S**TSDTQE  NGGGST**S**DTQEDI  PDPGPP**S**LAVAPE  CPQPLR**S**PSLDNP  QPLRSP**S**LDNPTP  VFQQTI**S**CPEGLR  GLRLVG**S**EVGDRT  LPDPGM**S**LTDRGV  TDRGVM**S**YVRHVL  YVRHVL**S**CLGGGL  HTYWAV**S**EELLPN  EELLPN**S**GHGPDG  ITFTEG**S**GRSPRY  TEGSGR**S**PRYALW  WFCVGE**S**WPQDQP  ARVGGA**S**SLENTV  SNSHPL**S**LTSDQY  HPLSLT**S**DQYKAY  RRARFV**S**KKGNCN  LLIFTM**S**FLCSWL  TMSFLC**S**WLLFAM  HGDLAP**S**EGTAEP  AEPCVT**S**IHSFSS  CVTSIH**S**FSSAFL  TSIHSF**S**SAFLFS  SIHSFS**S**AFLFSI  SSAFLF**S**IEVQVT  AETLIF**S**KHAVIA  VGDLRK**S**MIISAT  RKSMII**S**ATIHMQ  VVRKTT**S**PEGEVV  NGVGGN**S**IFLVAP  HVIDAN**S**PLYDLA  LYDLAP**S**DLHHHQ  TTQART**S**YLADEI  EEDGRY**S**VDYSKF  RYSVDY**S**KFGNTI  QLDEDH**S**LLEALT  EALTLA**S**ARGPLR  GPLRKR**S**VPMAKA  AVLWQD**S**FSPHLK  MPVVLT**S**GTGSQA  AAGTHS**S**PVPGSI  LSPRSE**S**GGLGVS  SSSPGD**S**CLRKGG  GPNQNG**S**EGLAQL  GLAQLT**S**TNGAKP  WGVYPA**S**LFQQQA  NNNLAS**S**SFYGNN  SFYGNN**S**LNSNSQ  NSLNSN**S**QSSSLF  LNSNSQ**S**SSLFSQ  QSSSLF**S**QGSAQP  SLFSQG**S**AQPANT  GTAVAN**S**NTGSGS  ANSNTG**S**GSRRDS  SFYNGL**S**FSSSPG  GLSFSS**S**PGPVGM  SQTPPP**S**LSSHGS  GSGRYI**S**AAPGAE  AKYRSA**S**SASSLF  KYRSAS**S**ASSLFS  SASSAS**S**LFSPSS  PSSTLF**S**SSRLRY  STLFSS**S**RLRYGM  VMPSGR**S**RLLEDF  ALEFIP**S**DQQNEM  ECVQPQ**S**LQFIID  GRPEDK**S**KIVAEI  GNVLVL**S**QHKFAS  MNDGPH**S**ALYTMM  NFGTLL**S**SQKEAE  NAAAQG**S**PEATLR  FLDDLL**S**RHVIRA  LVDSCK**S**TVVWML  HMGMTA**S**YTSNLA  ALQLLD**S**VRPEAQ  HSKAAG**S**SCHITQ  SKAAGS**S**CHITQL  HLEEAA**S**SLKHLD  GVSLLL**S**VLRDPA  PALQKS**S**KAWYLL  AYLSLP**S**NNLSHS  LPSNNL**S**HSLWEQ  MGSDIL**S**TQKAAV  KAAVET**S**FLDYGE  SEVLSC**S**EKLVCH  IAPSTN**S**SPVLKT  TQALQA**S**LNHKTP  TKLGGL**S**CCTTQL  VPGSEP**S**KTQGQK  GRQKLA**S**APLRLN  RRPQRA**S**DQARPG  DNWRKM**S**FEILRG  AFLVTE**S**VSITCR  DQLQGL**S**LQEMPG  RLALIP**S**GVTVCV  IASLEK**S**VLGCWK  GPAQEA**S**RLQELL  SFRFLL**S**YSIIKE  IKEYGA**S**PVLSQG  LLFGCS**S**AALAVR  TEQSSE**S**LRCNVE  YHRLDV**S**LPFVSR  SVIVPE**S**DEEGHS  TVEAKQ**S**EAEVVT  GVILER**S**QPPGED  QESQAG**S**DTDVEE  TTTERD**S**DTDVEE  ALVRAH**S**EKDQPP  QPPFGD**S**DDSVEA  LERSQA**S**TTVDIN  IKKHQV**S**VEGTNQ  VADVRK**S**QLPAEG  GLEAVQ**S**MEDEPT  LRESED**S**ETQPFD  KNGESA**S**PERDRE  APVPSG**S**QSGGRG  KIRTRK**S**SRMTPF  EPHPST**S**TAQPVT  VTPKPT**S**QATRSR  SRTNRS**S**VKTPEP  VTSEPT**S**QATRGR  APELRP**S**TSTDRP  ELQPST**S**TDQPVT  VTPEPT**S**QATRGR  RGRKNR**S**SVKTPE  GRKNRS**S**VKTPET  APELQP**S**TSRNQL  VTPEPT**S**RATRCR  TTDQPI**S**PEPITQ  EPITQP**S**CIKRQR  EPKSQA**S**RNQRWG  AVRAAE**S**LTAIPE  QPKASQ**S**RKRSLA  KAGDIL**S**GDQDKE  QKRRKP**S**VPCPEP  TESLSS**S**NSDDNK  VTSTPI**S**KPPPPL  PAAPTT**S**SMGSSI  AAPTTS**S**MGSSIR  TTSSMG**S**SIRHSP  PLSSGI**S**TPVTNV  CGVRKR**S**YSAGNA  SSCEAS**S**ELRENG  VVYHRG**S**RSCKDA  YHRGSR**S**CKDAAV  ELQAAG**S**RKKVDK  AQPLVF**S**KVVEAV  DRCAGR**S**VEMCDK  GSNTEE**S**VNDLTL  VDVTVC**S**PKECAS  VNTEAV**S**QVEAAV  TADQDT**S**TDLEQV  CTNTCL**S**TLDKQT  GVGTLL**S**GHSGFD  DDPVGE**S**LENPQP  INSVMK**S**ASTEEL  TQEGTL**S**PVNLTD  ACTNNE**S**TLKSIM  KDGNKD**S**NGAKKN  SSDESS**S**SESDDE  RERYEL**S**EKMLSA  DPKALT**S**KDMRFC  HEWFRV**S**SQKSAI  DVNAKA**S**QAGQTA  DKNTLG**S**DLPRIK  LFDYII**S**QDRLSE  ISQDRL**S**EEETRV  VFRQIV**S**AVAYVH  AVAYVH**S**QGYAHR  ELIQGK**S**YLGSEA  GKSYLG**S**EADVWS  SEADVW**S**MGILLY  DVPKWL**S**PSSILL  PKWLSP**S**SILLLQ  KWLSPS**S**ILLLQQ  YPVEWQ**S**KNPFIH  DCVTEL**S**VHHRNN  TMEDLI**S**LWQYDH  PVRLRL**S**SFSCGQ  LRLSSF**S**CGQASA  PFTDIK**S**NNWSLE  LEDVTA**S**DKNYVA  WCEDDL**S**TGAATP  TKYWTE**S**NGVESK  ESNGVE**S**KSLTPA  NRYTTP**S**KARNQC  IKIPVN**S**TGTDKL  ITVLTR**S**KRKGSA  RSKRKG**S**ARDGPR  LLNEIM**S**ILPKKH  LKCQTQ**S**DFGKVT  LGGGGG**S**GVGDGG  PVRGPP**S**PRPAGP  LGREPK**S**FLCRLC  EDPELD**S**DKDETG  EFYNIA**S**LVRLVK  DNENRT**S**QGPVKH  ELTQMV**S**TMSDGW  QMVSTM**S**DGWKFE  KFEQLI**S**IGSSYN  QLISIG**S**SYNYGN  LISIGS**S**YNYGNE  EFLCVV**S**RELNNS  SRELNN**S**TNGIVI  GIVIEP**S**EKAKIL  EIENFK**S**YKGRQI  IIGPNG**S**GKSNLM  PNGSGK**S**NLMDAI  VLGEKT**S**NLRVKT  RVIVGG**S**SEYKIN  VQLHEY**S**EELEKL  FQGAVE**S**IAMKNP  ALFEEI**S**RSGELA  EIKEKD**S**ELNQKR  KAKENT**S**HKIKKL  LEAAKK**S**LQNAQK  RLKEEA**S**KRAATL  IDRQES**S**RQQRKA  KRLYPG**S**VYGRLI  DAIIVD**S**EKTGRD  QKSGVI**S**GGASDL  QMRLKY**S**QSDLEQ  LNLQEK**S**KLESEL  KLEQKR**S**DRHNLL  DIKLPL**S**KGTMDD  GTMDDI**S**QEEGSS  SSQGED**S**VSGSQR  KLNEQQ**S**VLQRIA  AMEKLE**S**VRDKFQ  DKFQET**S**DEFEAA  FNACFE**S**VATNID  EIYKAL**S**RNSSAQ  KALSRN**S**SAQAFL  ALSRNS**S**AQAFLG  NYIKEQ**S**TCNFQA  QGDCVI**S**KVLTFD  YTSLCS**S**TMEDSE  EDDDTK**S**DSILED  QQQSHP**S**GSLAPP  GWNQLP**S**GALQPP  PPPAQG**S**LGTMTA  LRPPGP**S**PHMAQQ  QMMPDV**S**IQQTNM  FSGHGM**S**FNAPFS  LLQSDI**S**AGHFGV  QQPPPP**S**QPQSQQ  KMVYQE**S**PQNPSS  SGSEAP**S**VPGGPN  QQPPVN**S**LPSSHG  VNSLPS**S**HGHHFP  SNSRRS**S**PGSSRK  RSSPGS**S**RKTTPS  PRNVLV**S**PTPLAN  KKDGQP**S**DPNKLP  ENKNLV**S**PAMREA  EDLKKA**S**VIPTLQ  PTLQDL**S**SSKEPS  SLNLPH**S**NELCSS  TTSANT**S**AALPTH  GQSAAQ**S**NARPQF  QNIKFS**S**APVPPN  VPPNAL**S**SSPAPN  SSGNRR**S**PVSSSK  CKKVTG**S**LEKGEE  LVSKET**S**TTALQA  VNAAIV**S**GQSSEP  PTVASE**S**VENGHR  EILSVE**S**LCEGMR  ELELVI**S**LPNGLQ  GNGGVV**S**LSVGHS  TRVVHL**S**LRPIFL  LLSLRT**S**IIEAQY  VPKHEL**S**TEYIPD  KERMLL**S**FKLSSD  KEPAGH**S**QKKGKA  HRVLCL**S**QSEGRV  SDKFVT**S**TSDHFV  KLEVHV**S**LHQDLV  QVGQGV**S**LTLKTT  IKKHTL**S**IGDMVT  LNTHSV**S**PMEKIK  GKIGTV**S**IFHMSD  HMSDSY**S**ETPLED  DNVLTL**S**LRSSRT  LTLSLR**S**SRTNPE  TLSLRS**S**RTNPET  SHVSQH**S**PSKKAL  VSQHSP**S**KKALYN  GKPDVL**S**ASLEGQ  EEVEMP**S**KEKQQP  PKKAGL**S**EEDDSL  WNVGLD**S**LTPALP  LAESSD**S**EEDEKP  QATIKK**S**KKEREL  KAEKEL**S**RIEEAL  FDRLVL**S**SPNSSI  ALECLP**S**KEHVDV  IFENTL**S**TYPKRT  KRTDVW**S**VYIDMT  ERVIHL**S**LAPKRM  YIFGDF**S**PDEFNQ  FVTPRS**S**VELPPY  VELPPY**S**GTVLCG  TPSYSI**S**STLNPQ  PSYSIS**S**TLNPQA  GITKEA**S**YGSIDC  KEASYG**S**IDCQYP  LALDGS**S**NVEAEV  KDGGDD**S**ISTEAL  GGDDSI**S**TEALVN  NSAVPN**S**VSAEDA  TPRTCN**S**PQNSTD  CNSPQN**S**TDSVSD  GADFGQ**S**CFPAEA  AGRDTL**S**RTAGAQ  GQILES**S**GEGTAT  DPTKPE**S**ASPPAD  PADGTG**S**ASGTLP  ASLFHD**S**KPSSSS  FHDSKP**S**SSSPVA  HDSKPS**S**SSPVAY  KKLLSP**S**NEKLTI  NEKLTI**S**NGPKNH  GPRNKT**S**VTRQAD  FGGHIR**S**VVYQQS  SVVYQQ**S**SKESAT  VVYQQS**S**KESATL  QQSSKE**S**ATLQPF  LQLDIQ**S**DKIRTV  KQEVEI**S**RRVTLE  VYHHGN**S**ATGGHY  DRLEKK**S**FEKAPC  LKVEFS**S**TVVEYE  ARNSFI**S**SALKSS  RNSFIS**S**ALKSSE  SSALKS**S**EVDNWR  PSSDYP**S**DFEVIQ  SLKYAE**S**DPTVPC  CNETRW**S**QKWQSS  HATGRH**S**SRRLLR  NIARFS**S**RGMTTW  GAGVRG**S**GVKGGC  SGTSVA**S**PVVAGA  KQALIA**S**ARRLPG  SYKPQA**S**LSPSYI  NIEVAF**S**YSSVLW  EVAFSY**S**SVLWPW  SVLWPW**S**GYLAIS  SGYLAI**S**ISVTKK  YLAISI**S**VTKKAA  MYQHLR**S**MGYFVE  DVDNGL**S**LVIFSD  SDWYNT**S**VMRKVK  HDMYYA**S**GCSIAK  YYASGC**S**IAKFPE  IVLYGD**S**NCLDDS  SNCLDD**S**HRQKDC  ALLQYT**S**YGVTPP  PPSGAG**S**VTPERM  VLPNFR**S**NRPQVR  PQVRPL**S**PGESGA  PLSPGE**S**GAWDIP  RGRWPG**S**SLYYEV  GRWPGS**S**LYYEVE  EILSHD**S**TSQLYT  LSHDST**S**QLYTVK  RKGGST**S**SSPSRR  KGGSTS**S**SPSRRR  RRRGSR**S**RSRSRS  PGRPPK**S**ARRSAS  RRSASA**S**HQADIK  LKPFGN**S**ISRYNG  PFGNSI**S**RYNGEP  QEKFSL**S**QESSYI  FSLSQE**S**SYIATQ  SLSQES**S**YIATQY  YIATQY**S**LRPRRE  KLKEID**S**KEEKYV  CKQKDP**S**LLNFPP  VFCVVL**S**VYLYMR  VYLYMR**S**LKAPRN  NDLSPA**S**SGNAVY  DLSPAS**S**GNAVYD  QDRAVP**S**LAMILV  AMILVN**S**FQLLYV  WVPFIY**S**FQAFYL  QAFYLV**S**HPNEVS  VSWPMA**S**LIIVLK  IFRGAN**S**QKNAFR  AFRKNP**S**DPKLAH  LKTIHT**S**TGKNLL  GKNLLV**S**GWWGFV  IMALAW**S**LPCGFN  HVLDQN**S**NVSRVE  TVANPV**S**RDAQGL  VVKGEK**S**FFLQPG  QDVYVL**S**EQQGLL  EDEEKV**S**HQAGDH  VRAVIG**S**TYMLTQ  EKDTAK**S**LQPLAP  NKTRVV**S**YRVPHN  FGPELV**S**LGPEEQ  EQFTVL**S**LSAGRP  ETAKLF**S**VPDFVG  ACKAIA**S**RVRGAV  VRGAVA**S**VTFDDF  DDFHKN**S**ARIIRT  VFGFET**S**EAKGPD  QNGLVV**S**SVDVQS  NGLVVS**S**VDVQSV  SSVDVQ**S**VEPVDQ  RDALQR**S**VQLAIE  IEITTN**S**QEAAAK  QKILDQ**S**EAEKAR  LELEAL**S**MAVEST  LSMAVE**S**TGTAKA  AKAEAE**S**RAEAAR  RIEGEG**S**VLQAKL  QLELEV**S**KAQQLA  QVKLLQ**S**LGLKST  QSLGLK**S**TLITDG  TLITDG**S**TPINLF  LGRRVA**S**GPSPGE  RVASGP**S**PGEGIS  SPGEGI**S**PQSAQA  EGISPQ**S**AQAPQA  WAGLLA**S**SAHGRL  NARYSR**S**SAAAGA  ERTRRT**S**KPGGAA  GRQVVR**S**KVPQET  WSKAPG**S**QRCTKP  LPQQIH**S**QVTPLS  QVTPLS**S**QSVVIH  TPLSSQ**S**VVIHHG  EQSTEG**S**FPLRYV  NGGQCS**S**RDKCQC  IPVHGA**S**VPKLYQ  KHPPEA**S**VQIHQV  EAQPGQ**S**QVSYQG  PGQSQV**S**YQGLPV  TIHSTY**S**HQQVIP  PDPTFS**S**CVPDPP  QCMHPL**S**VHLTKQ  KQLCCC**S**VGKAWG  TQPVAK**S**THPPPL  PEKEIP**S**LDQEKT  DECLNP**S**TCPDEQ  QQGFSI**S**ADGRTC  DENQEY**S**PMTGQC  MTGQCR**S**RTSTDL  QCRSRT**S**TDLDVD  PAGESS**S**EAGGEN  DECQDP**S**SCIDGQ  ECQDPS**S**CIDGQC  PMVLDA**S**EKRCIR  ELNNRM**S**LCKNAK  LPGYVP**S**DKPNYC  IGEQLD**S**EDLASL  DSEDLA**S**LKFLSL  ASLKFL**S**LDYIPQ  KRMLEE**S**NLSFLK  LEESNL**S**FLKELL  PGRAQI**S**AYRVML  VMLYQI**S**EEVSRS  QISEEV**S**RSELRS  SEEVSR**S**ELRSFK  SRSELR**S**FKFLLQ  LLQEEI**S**KCKLDD  CAQINK**S**LLKIIN  NDYEEF**S**KERSSS  EFSKER**S**SSLEGS  FSKERS**S**SLEGSP  SSSLEG**S**PDEFSN  GSPDEF**S**NGEELC  CGVMTI**S**DSPREQ  SPREQD**S**ESQTLD  REQDSE**S**QTLDKV  KVYQMK**S**KPRGYC  KVPKLH**S**IRDRNG  YQLMDH**S**NMDCFI  FICCIL**S**HGDKGI  PIYELT**S**QFTGLK  IPVETD**S**EEQPYL  YLEMDL**S**SPQTRY  TVNNCV**S**YRNPAE  GTWYIQ**S**LCQSLR  IQSLCQ**S**LRERCP  EVNYEV**S**NKDDKK  KKAYEL**S**VLCDCE  LIIFNH**S**NKLFQY  EPDGED**S**LEQSPL  EDSLEQ**S**PLLEDK  LFRRYG**S**TVPAPN  PVTVPV**S**NQSSLQ  QSSLQF**S**NPSGSL  GSLVTP**S**LVTSSL  LPQRPA**S**AGAMLG  ANGACP**S**PVGNGY  PVANGN**S**LNKVIP  PPPPTH**S**TQLGAP  DLRVIT**S**QAGKGL  AQRLGV**S**QSTHSL  QGLPFS**S**MPTAYN  TDYQLT**S**AELSSL  TSAELS**S**LPAFSS  SSLPAF**S**SPGGLS  SSPGGL**S**LGNVTA  QPPQQQ**S**HLVPVS  SHLVPV**S**LSNLIP  LVPVSL**S**NLIPGS  TTHPHI**S**IKSEPV  PHISIK**S**EPVSPS  SEPVSP**S**RERSPA  SPSRER**S**PAPPPP  EPGDGL**S**SPAGGS  PGDGLS**S**PAGGSY  SSPAGG**S**YETGDR  EPEAEG**S**AVKRMR  AHYPKQ**S**FTMVAD  LRLKQQ**S**ELQSQV  QQSELQ**S**QVRYKE  NKGKGF**S**VVADTP  KTQDQI**S**NIKYHE  HEEFEK**S**RMGPSG  KSRMGP**S**GGEGME  EPERRD**S**QDGSSY  RDSQDG**S**SYRRPL  DSQDGS**S**YRRPLE  PHHIPT**S**APVYQQ  QQPVAQ**S**YGGYKE  EPAAPV**S**IQRSAP  PVSIQR**S**APGGGG  RAVYDY**S**AADEDE  ADEDEV**S**FQDGDT  RQLLSS**S**KLYFLP  DEVIAV**S**EKVIVK  IQDKPS**S**ILTDQF  TLIENE**S**ICDEFA  CPQRRD**S**FSGVKD  QRRDSF**S**GVKDSN  FSGVKD**S**NNNSDG  GNPGST**S**AATCTR  ENKTKV**S**GTKRIK  EGYQEF**S**AKPLAS  TDQGSN**S**EKVAEE  SEPQEA**S**FPSFPT  GNPGIM**S**PLAKKK  KLLSQV**S**GASLSS  VSGASL**S**SSYPYG  RDDLCS**S**LSQTHH  SSPHLH**S**LYRHTE  YRESEN**S**SFPSHR  HLQDKK**S**AAAEAP  DQPTDL**S**LPKNPH  VLGLAH**S**TTGPQE  PKKYPE**S**LSRSGK  PESLSR**S**GKPHHV  ILHRKM**S**PQNIGA  KKARAV**S**PLDPSK  KASEQE**S**EGSKAA  GHSGGG**S**EGHKLP  HKLPLS**S**PIFPGL  PGLYSG**S**LCNSGL  SGSLCN**S**GLNSRL  RLPAGY**S**HSLQYL  KNQTVL**S**PLMQPL  QPLAFH**S**LVMQRG  PPRPAP**S**PGTWES  ESQPAR**S**VRLGGP  PSIRIT**S**ISPTPE  IRITSI**S**PTPEPP  GSSSLS**S**WSFFSD  SSWSFF**S**DASDEA  SFFSDA**S**DEAALY  ELNEAA**S**RFGLGS  GRGPED**S**WLLLSA  KRRYSS**S**GTPSSA  SSSGTP**S**SASPAL  SSGTPS**S**ASPALS  KTRRTS**S**EQAVAL  RSEEPA**S**CNGKLP  PLGAEE**S**VAPPGG  SVPIEC**S**QRSAQE  IECSQR**S**AQELPQ  VEAYSP**S**ACSVRG  YSPSAC**S**VRGGEE  ELVLTG**S**NFLPDS  SNFLPD**S**KVVFIE  TVNRLQ**S**NEVTLT  LTVPEY**S**NKRVSR  QVYFYV**S**NGRRKR  GTDMDF**S**PPRPPY  QTGPPP**S**YRPGLR  LPRPFP**S**DPYGGR  YGGRGS**S**FSLGLP  PPPLPA**S**PPLEGP  GPFPSQ**S**DVHPLP  APEQEK**S**RGGYSS  ILEKTV**S**PDRLEL  TFLVEL**S**RVLANP  LANPGN**S**QVARVA  GLQIKN**S**LTSKDP  IKNSLT**S**KDPDIK  ETYRPS**S**ASQCVA  YRPSSA**S**QCVAGI  NVTNPN**S**TEHMKE  EQLQDK**S**NEILTA  MRKEEP**S**NNVKLA  TNALLN**S**LEFTKA  ANFDKE**S**ERHFIM  NLVKIM**S**LYYQYM  QGIEFW**S**NVCDEE  DLAIEA**S**EAAEQG  LEGPEP**S**QLKPLV  ELMKDP**S**VVVRDT  CLIEGL**S**AEPRVA  VCWAFS**S**LAEAAY  TYCLSS**S**FELIVQ  QNNLRS**S**AYESLM  MEIVKN**S**AKDCYP  QVLQME**S**HIQSTS  MESHIQ**S**TSDRIQ  SHIQST**S**DRIQFN  QFNDLQ**S**LLCATL  SDVVMA**S**LLRMFQ  DALMAV**S**TLVEVL  NENVHR**S**VKPQIL  VKPQIL**S**VFGDIA  LNELRE**S**CLEAYT  RVEFIL**S**FIDHIA  LTEGRR**S**KTNKAK  NRKHPS**S**PECLVS  STMSSK**S**PRDWEQ  QKAPVP**S**TCSSTF  PVPSTC**S**STFPEE  QLADER**S**NRDELE  LRDKNE**S**LTMRLH  RKINQL**S**EENGDL  KLREFA**S**HLQQLQ  QLEKEL**S**AALQDK  SSISNL**S**QAKEEL  AEEREA**S**LRERDA  REKAEL**S**RKVEEL  LKAETR**S**LVEQHK  KEKEHA**S**GSGAQS  SGSGAQ**S**EAAGRT  ALRAEV**S**KLEQQC  ELGHSQ**S**ALASAQ  TKVQDH**S**KAEDEW  LQAETA**S**NSARAA  AETASN**S**ARAAER  RAAERS**S**ALREEV  EKQRVA**S**ENLRQE  LQLEHT**S**TQALVS  VQREAQ**S**TARELE  LQEQLR**S**LEQLQK  LTAQVR**S**LEAQVA  SGRKTR**S**ARRRTT  ATSSTQ**S**LARLGS  ESRPSL**S**LGTITD  PKKLGN**S**LLRRGA  ASPNTR**S**GTRRSP  FFRRTV**S**KSIGPT  RRTVSK**S**IGPTCP  TCPFAG**S**CEVSKT  AGSCEV**S**KTQRRH  RKDMIL**S**AEALAL  QTPVQL**S**KEQEEL  DLPVFR**S**LPIEDQ  PIEDQI**S**LLKGAA  EDGARV**S**PTVGFQ  AAMALF**S**PDRPGV  MALTLQ**S**YIKGQQ  LLAELR**S**INEAYG  QHIQGL**S**AMMPLL  VRTKAD**S**VPGTYR  PRKVLG**S**STSATN  RKVLGS**S**TSATNS  STSATN**S**TSVSSR  SATNST**S**VSSRKA  TNSTSV**S**SRKAEN  NSTSVS**S**RKAENK  RLSPKD**S**EKENQI  IPEEAG**S**SGLGKA  PEEAGS**S**GLGKAK  AVQGGR**S**GGSGGC  GGRSGG**S**GGCSGA  GGSGGC**S**GAGGAS  SGAGGA**S**NCGTGS  SNCGTG**S**GRSGLL  GTGSGR**S**GLLDKW  IDKWDG**S**AVKNSL  GSAVKN**S**LDDSAK  KNSLDD**S**AKKVLL  LTICTI**S**CFFAIV  MHPFPE**S**KPVLAL  LALCVI**S**YFVMMG  ILTIYT**S**YKEKSI  TSYKEK**S**IFLVAH  DDIWQL**S**SSLKRF  DIWQLS**S**SLKRFD  IWQLSS**S**LKRFDD  LKLTFI**S**GRTKQQ  EAEFTK**S**IAKFFD  AKFFDH**S**GTLVMD  AYEPEI**S**RLHDSL  ISRLHD**S**LAIERK  PKKVRL**S**ETDFKV  VTGLRE**S**EEKLKQ  KQVQQP**S**VAQLRS  SVAQLR**S**TMVDPA  QAQNEL**S**AWKFTP  WKFTPD**S**QTGKKL  YSEELK**S**SQDELN  SEELKS**S**QDELND  EVEGMQ**S**TILVLQ  QYQQQQ**S**QASAPS  QQQSQA**S**APSTSR  SQASAP**S**TSRTTA  TSRTTA**S**EPVEQS  SEPVEQ**S**EATSKD  GPSNGS**S**SRQRTS  SSRQRT**S**GSGFHR  RQRTSG**S**GFHREG  TEDDFP**S**SPGNGN  EDDFPS**S**PGNGNK  PGNGNK**S**SNSSEE  GNGNKS**S**NSSEER  GNKSSN**S**SEERTG  RTGRGG**S**GYVNQL  GYVNQL**S**AGYESV  LSAGYE**S**VDSPTG  GYESVD**S**PTGSEN  VDSPTG**S**ENSLTH  PTGSEN**S**LTHQSN  NSLTHQ**S**NDTDSS  SNDTDS**S**HDPQEE  GNRTVG**S**RHVQNG  VQNGLD**S**SVNVQG  QNGLDS**S**VNVQGS  ATAAET**S**ASEPEA  SEPEAE**S**KAGPKA  TRRKVL**S**RAVAAA  QQEEGV**S**ESDGDE  DEYAMA**S**SAESSP  MASSAE**S**SPGEYE  ASSAES**S**PGEYEW  MARQKM**S**EIFPLT  WLHDEI**S**MAQDGL  GLEKVR**S**VFERAL  RLEKVH**S**LFRRQL  EDPIPE**S**VIQNYN  DLWIRY**S**QYLDRQ  WTVALW**S**RYLLAM  VDHQVI**S**VTFEKA  EERFNE**S**GDPSCV  NESGDP**S**CVIMQN  RAVQCT**S**DYPEHV  MERTEG**S**LEDWDI  DEEEQP**S**KRRRVE  RRRVEN**S**IPAAGE  VDVEPP**S**KQKEKA  PKVLHD**S**SKDSIT  KVLHDS**S**KDSITV  HDSSKD**S**ITVFVS  VSNLPY**S**MQEPDT  EFKEEK**S**ALQALE  LEMDRK**S**VEGRPM  GRPMFV**S**PCVDKS  FKVFRY**S**TSLEKH  KHKLFI**S**GLPFSC  KGRTQL**S**LLPRAL  RALQRP**S**AAAPQA  VETDVV**S**NTSGSA  DVVSNT**S**GSARVK  VSNTSG**S**ARVKLG  EFFVDC**S**ASATPE  FVDCSA**S**ATPEFE  RIFNNK**S**SVDLKT  IFNNKS**S**VDLKTL  LKTLCI**S**PREHCW  NLFDAI**S**IAVKAA  LEDEEG**S**KDIELS  SKDIEL**S**DDPYDC  YDCIRL**S**VENVPC  LQEEAC**S**LASLLV  EACSLA**S**LLVSVT  LASLLV**S**VTSKGV  LLVSVT**S**KGVVTC  RKVGKG**S**LDPESI  GSLDPE**S**IFEMME  GKVLHA**S**LQSVVH  LHASLQ**S**VVHKEE  VVHKEE**S**LGPKRQ  IYDPGG**S**VPSGEA  PGGSVP**S**GEASAA  VPSGEA**S**AAFERL  RLVKEN**S**RLKEKM  LPPPSP**S**LGSFDP  PSPSLG**S**FDPLAE  DSNVTA**S**PTAPAC  TAPACP**S**DKPAPV  PVQKPP**S**SGTSSE  VQKPPS**S**GTSSEF  PPSSGT**S**SEFEVV  PSSGTS**S**EFEVVT  TPEEQN**S**PESSSH  EQNSPE**S**SSHANA  QNSPES**S**SHANAM  RLETTL**S**VCAEEP  KNEQRT**S**ILQTLC  GNKEGA**S**GRPGSP  ASGRPG**S**PKMEGT  AGQQQA**S**VTAGKV  MLEQQR**S**ELLEVN  WDQHFR**S**MKQQYE  KLLLAK**S**KIEMEE  ALEEAL**S**IQTPPS  SIQTPP**S**SPPTAF  IQTPPS**S**PPTAFG  DFQRER**S**DRERMN  QAQVTL**S**NAQLKA  KRKAKA**S**GERYHV  HGFEDW**S**QIRYPP  VRNPNQ**S**SQVMDP  RNPNQS**S**QVMDPP  RPTEPE**S**PKNDRE  EECLKD**S**PRFRAS  SPRFRA**S**IELVEA  LVEAEV**S**ELETRL  RHYLAA**S**RAFVVG  LEKFTV**S**LNHKLD  LNHKLD**S**HAELLD  FWRGAE**S**LEAALT  EELSRL**S**QYRKEL  HQLVLN**S**AREKRD  GEEPEP**S**LREGPG  HLFKRA**S**NAFKTW  RWFTIQ**S**NQLVYQ  VKLCPD**S**ERRFCF  FCFEVV**S**TSKSCL  LLQLWV**S**AVQSSI  WVSAVQ**S**SIASAF  SIASAF**S**QARLDD  QARLDD**S**PRGPGQ  GHLAIG**S**AATLGS  PAPEWA**S**INLGVT  TLCIQC**S**GIHRSL  CSGIHR**S**LGVHFS  SLGVHF**S**KVRSLT  HFSKVR**S**LTLDSW  RSLTLD**S**WEPELV  KPGPSC**S**RQEKEA  SKPEPP**S**EDLGSL  PSEDLG**S**LHPGAL  DIFRDF**S**LMASDD  DFSLMA**S**DDPEKL  DDPEKL**S**RRSHDL  EVKVDG**S**EPKLSK  GSEPKL**S**KNELKR  AKQKEL**S**EKQLSQ  LSEKQL**S**QATAAA  VGPEEE**S**VDPNQY  QYYKIR**S**QAIHQL  KFHVDI**S**LTDFIQ  DFIQKY**S**HLQPGD  IHAKRA**S**GGKLIF  LQVMAN**S**RNYKSE  NSRNYK**S**EEEFIH  TKKGEL**S**IIPYEI  YEITLL**S**PCLHML  QKFIIR**S**KIITYI  IITYIR**S**FLDELG  ITEKMV**S**GMVKHI  VKHITG**S**YKVTYH  DHPQIM**S**PLAKWH  LAKWHR**S**KEGLTE  AMFLTD**S**NNIKEV  LGNLLA**S**DRNPPT  LVWDEV**S**GQWRRR  HKMQLP**S**AAGLHP  HPTGHQ**S**KEELGR  MQVAKV**S**TASVGR  AKVSTA**S**VGRFQE  EKVPRG**S**GKKRKF  LLRVMN**S**KKPQLD  AKRRKM**S**QKGKRK  RKGGPP**S**QGGKRK  LGGKMN**S**GPPGLG  VTYKWL**S**YTLGVH  HVTYLV**S**GSLIQN  TYLVSG**S**LIQNGH  LIQNGH**S**CHKVAV  KLAVTA**S**IHVYSI  ASIHVY**S**IQKAML  KAMLKD**S**GPLFNT  DYDILK**S**NLQNCS  PAESSS**S**SKKFEQ  SKKFEQ**S**HLHMSS  QSHLHM**S**SETQAN  SHLHMS**S**ETQANN  GHGPPA**S**KQVSQQ  PASKQV**S**QQPKGI  IMGMFA**S**KAAAKT  KEVTNA**S**AAGNKA  GKGNMM**S**NFFGKA  VEQPTV**S**VTEPKL  PAGLKK**S**SKKAEP  KLPESD**S**SEDEVF  LPESDS**S**EDEVFP  DEVFPD**S**PGAYEA  GAYEAE**S**PSPPPP  YEAESP**S**PPPPPS  SPPPPP**S**PPLEPV  TEPEPP**S**VKSSSG  PSVKSS**S**GENKRK  RKRVLK**S**KTYLDG  TEKVYE**S**ESCTDS  KVYESE**S**CTDSEE  SESCTD**S**EEELNM  ELNMKT**S**SVHRPP  KANRQV**S**ITGFFQ  YDDRAY**S**SFGGGR  DDRAYS**S**FGGGRG  FGGGRG**S**RGSAGG  GRGSRG**S**AGGHGS  SAGGHG**S**RSQKEL  GGHGSR**S**QKELPT  AIFKDL**S**IRSVRL  KDLSIR**S**VRLVRD  EFDEVD**S**LKEALT  ALLGDR**S**LRVDIA  DDRGMG**S**SRESRG  DRGMGS**S**RESRGG  MGSSRE**S**RGGWDS  SRGGWD**S**RDDFNS  SRDDFN**S**GFRDDF  LGGRGG**S**RPGDRR  TGPPMG**S**RFRDGP  GPPLRG**S**NMDFRE  QVANPN**S**AIFGGA  VLSLSP**S**YRKATL  YTAVQN**S**KNAKDK  KNLKRH**S**IISVLP  KRHSII**S**VLPWKR  KRIVAV**S**AKKKNS  SAKKKN**S**KKVQPN  KKVQPN**S**SYQNNI  KVQPNS**S**YQNNIT  NENLKK**S**LSCANL  NLKKSL**S**CANLST  LSCANL**S**TFAQPP  PPAPPA**S**QLSGSQ  PPASQL**S**GSQTGG  ASQLSG**S**QTGGSS  SQTGGS**S**SVKKAP  QTGGSS**S**VKKAPH  PHPAVT**S**AGTPKR  IVQAST**S**ELLRCL  YRLKHL**S**PTDPVL  LRSVDR**S**LLLQGW  LCRDVI**S**SEVGSD  CRDVIS**S**EVGSDH  ISSEVG**S**DHELQA  LTCLYL**S**YSYMGN  CLYLSY**S**YMGNEI  YMGNEI**S**YPLKPF  KPFLVE**S**CKEAFW  FWDRCL**S**VINLMS  SVINLM**S**SKMLQI  VINLMS**S**KMLQIN  YFTQVF**S**DLKNES  QQLRAE**S**DFEQLP  PDDVAI**S**ANIADI  EKRGFT**S**HFVFVI  VKTKGG**S**KYLIYR  QFHALQ**S**KLEERF  ERFGPD**S**KSSALA  FGPDSK**S**SALACT  GPDSKS**S**ALACTL  LNAYMK**S**LLSLPV  YMKSLL**S**LPVWVL  RIFFYQ**S**PYDSEQ  YQSPYD**S**EQVPQA  RTRKVK**S**VSPQGN  RKVKSV**S**PQGNSV  VSPQGN**S**VDRMAA  FDFTGN**S**KLELNF  DVIFLL**S**RINKDW  TGIFPL**S**FVKILK  YYEDTI**S**TIKDIA  AVEEDL**S**STPLLK  VEEDLS**S**TPLLKD  DLVRLL**S**DEDVAL  LVLGLV**S**CTFFLA  AVNGLY**S**SSDDVI  VNGLYS**S**SDDVIE  NGLYSS**S**DDVIEL  VIELTP**S**NFNREV  NREVIQ**S**DSLWLV  EVIQSD**S**LWLVEF  DADKHH**S**LGGQYG  TIKIFG**S**NKNRPE  DRLGGR**S**GGYSSG  GRSGGY**S**SGKQGR  RSGGYS**S**GKQGRS  SGKQGR**S**DSSSKK  QGRSDS**S**SKKDVI  GRSDSS**S**KKDVIE  IELTDD**S**FDKNVL  DKNVLD**S**EDVWMV  EWAAAA**S**EVKEQT  VNQVLA**S**RYGIRG  IFQKGE**S**PVDYDG  DGGRTR**S**DIVSRA  TRSDIV**S**RALDLF  RALDLF**S**DNAPPP  GAAGRN**S**YLEVLL  TEAGAQ**S**ELETAL  FALLKG**S**FSEQGI  LLKGSF**S**EQGINE  EFLREL**S**FGRGST  LSFGRG**S**TAPVGG  LRAAGF**S**RSFSSD  AAGFSR**S**FSSDSG  FSRSFS**S**DSGSSP  RSFSSD**S**GSSPAS  FSSDSG**S**SPASER  SSDSGS**S**PASERG  SGSSPA**S**ERGVPG  DFYARF**S**PSPLSM  YARFSP**S**PLSMKQ  FSPSPL**S**MKQFLD  QFLDFG**S**VNACEK  NACEKT**S**FMFLRQ  NIMKEI**S**LLPDNL  NLLRTP**S**VQLVQS  SVQLVQ**S**WYIQSL  QSWYIQ**S**LQELLD  LDFKDK**S**AEDAKA  VIEYKE**S**FGVDPV  LDRFYM**S**RISIRM  FYMSRI**S**IRMLLN  MLLNQH**S**LLFGGK  GGKGKG**S**PSHRKH  KGKGSP**S**HRKHIG  DLYYIN**S**PELELE  EELNAK**S**PGQPIQ  QVVYVP**S**HLYHMV  DLTVKM**S**DRGGGV  LFNYMY**S**TAPRPR  RPRVET**S**RAVPLA  GYGLPI**S**RLYAQY  GDLKLY**S**LEGYGT  IYIKAL**S**TDSIER  KALSTD**S**IERLPV  AALVPG**S**GPGPAP  APVGGI**S**FHLQIG  HLQIGL**S**REPVLL  LLLQDS**S**GDYSLA  DSSGDY**S**LAHVRE  LIEVVL**S**ASATFE  KIPNNC**S**GVRRRR  STIRTS**S**AELSTS  EPLLQK**S**PSESFI  LDKILM**S**KVKVPH  HTFVIH**S**YTRPTV  LSPGAE**S**DVVMEE  NDSERN**S**GLMDDM  AECQND**S**GEMQDP  HTKRKS**S**TVMKEG  FQNDTG**S**RYYKEI  YKEIPL**S**EILSLE  NVVNPS**S**PSPNNS  NNSVLT**S**GVGADV  HRDISV**S**ISVSNC  DISVSI**S**VSNCQI  SVSISV**S**NCQIQE  QENVDI**S**TVYQIF  ENVLLA**S**ADPFPQ  NKGYNR**S**LDMWSV  RSLDMW**S**VGVIIY  NPWKEI**S**HEAIDL  KMRKRY**S**VDKTLS  THLINP**S**ASHSDT  LINPSA**S**HSDTPE  NPSASH**S**DTPETE  FFLTWR**S**EGKEGQ  GQVLEC**S**LINSIR  LINSIR**S**GAIPKD  RIVCVC**S**GTDLVN  WVEGLR**S**IIHNFR  GKIPVR**S**ITRTFA  KELGLP**S**GKNDEI  KKKGLI**S**SDGFCR  KKGLIS**S**DGFCRY  FCRYLM**S**DENAPV  EYPVIL**S**FENHCS  SFENHC**S**KYQQYK  LKQALE**S**HPLEPG  MMEAGE**S**ASPANI  EAGESA**S**PANILE  NEEEIE**S**ADQEEE  KFGNEL**S**ADDLGH  NIHPYL**S**TMINYA  KGGRVD**S**SNYMPQ  GGRVDS**S**NYMPQI  AGCQMV**S**LNYQTP  KFEYNG**S**CGYLLK  EGNKPL**S**LPTIFC  AMGIET**S**DIADVP  DIADVP**S**DTSKND  NVTPQS**S**SELRPT  VTPQSS**S**ELRPTT  TTAALA**S**GVEAKK  KHAKEH**S**TMQKLH  QYDKEK**S**THEKIL  IRDLHL**S**QQCELL  LSHDRE**S**KEMRAH  KISMEN**S**KAISQD  VMRAMA**S**LRARGL  CDNFTS**S**WRDGRL  ERRFPS**S**FEEIEI  EADKNR**S**KGIYQS  RLRSLE**S**LHSFVA  EVGFDW**S**DRNTNM  VGPAQP**S**HWKVLS  HWKVLS**S**SGSEAA  WKVLSS**S**GSEAAV  DVQLIR**S**WSLATF  REYGSC**S**HHYQQL  AAPTLR**S**ELELTL  LQQLRQ**S**SEAEIQ  LRKASD**S**ELERQK  ERAEQE**S**ARQLQL  TLQQEQ**S**VLDQLR  CFEGLR**S**LVPAAE  EAGQKL**S**IYNALK  DPYTGQ**S**VSLFQA  GGIVDP**S**KSHRVP  TTVKDL**S**ELGSVR  RTLLQG**S**GCLAGI  LHEQLL**S**AEKAVT  DIPGGG**S**HGGSTM  LWEVMQ**S**DLIPEE  YLYGTG**S**VAGVYL  LHDRLL**S**AERAVT  EILTDP**S**DDTKGF  KTSSKS**S**VRKRRV  EITISS**S**DGVVKS  YGRRYA**S**GSSASL  NEEEPQ**S**RPRPMR  RRGLRW**S**TLPKSS  RSSFLR**S**SCLSSC  SSFLRS**S**CLSSCL  LRSSCL**S**SCLRCS  RSSCLS**S**CLRCSS  SSCLRC**S**SRRTPS  SCLRCS**S**RRTPSA  SSRRTP**S**AGLSRK  TPSAGL**S**RKDLFE  HKLEEG**S**FKERLA  KERLAR**S**RPQFRG  IHGRNL**S**NEEMIQ  EFCVED**S**KDVNVN  NVNFEK**S**KLTFSC  KSKLTF**S**CLGGSD  FSCLGG**S**DNFKHL  CIDPND**S**KHKRTD  HKRTDR**S**ILCCLR  CLRKGE**S**GQSWPR  AKLNWL**S**VDFNNW  SNFDRF**S**EMMNNM  TGSTPC**S**SMSNHT  GSTPCS**S**MSNHTK  TPCSSM**S**NHTKER  TLENFY**S**NLIAQH  EKRLRR**S**AHARKE  GLEDFE**S**LKVIGR  ILVEAD**S**LWVVKM  VVKMFY**S**FQDKLN  TVLAID**S**IHQLGF  DNLLLD**S**KGHVKL  KGHVKL**S**DFGLCT  YRNLNH**S**LPSDFT  LNHSLP**S**DFTFQN  KLCDWW**S**LGVIMY  GYPPFC**S**ETPQET  PPEVPI**S**EKAKDL  GVEEIK**S**NSFFEG  EEIKSN**S**FFEGVD  ERPAAI**S**IEIKSI  ISIEIK**S**IDDTSN  KSIDDT**S**NFDEFP  FDEFPE**S**DILKPT  KPTVAT**S**NHPETD  KENNKK**S**STRAPS  TKRKDR**S**DEKSKD  DRSDEK**S**KDRSKD  KGATKE**S**SEKDRG  KTRKRR**S**ASSGSS  KRRSAS**S**GSSSTR  ASSGSS**S**TRSRSS  SSTRSR**S**SSTSSS  STRSRS**S**STSSSG  TRSRSS**S**TSSSGS  SRSSST**S**SSGSST  SSGSST**S**TGSSSG  STSTGS**S**SGSSSS  SSSGSS**S**SSASSR  SSSSSA**S**SRSGSS  SRSGSS**S**TSRSSS  SGSSST**S**RSSSSS  TSRSSS**S**SSSSGS  SRSSSS**S**SSSGSP  SSSSSS**S**SGSPSP  SSSSSS**S**GSPSPS  SGSPSP**S**RRRHDN  HDNRRR**S**RSKSKP  NRRRSR**S**KSKPPK  RRSRSK**S**KPPKRD  RMHPHL**S**KGYAYV  PPPRRF**S**PPRRML  PRMRRR**S**RSPRRR  MRRRSR**S**PRRRSP  SPVRRR**S**RSPGRR  GRRRHR**S**RSSSNS  NLELLE**S**FGQNYP  GTLDCI**S**MALTCT  GIAKII**S**AHIHPV  HIHPVC**S**LCWSRD  DGHKLV**S**ASTDNI  HKLVSA**S**TDNIVS  STDNIV**S**QWDVLS  QRFRFP**S**PILKVQ  PVMLTL**S**DSKHVV  MLTLSD**S**KHVVLP  LPVDDD**S**DLNVVA  DLNVVA**S**FDRRGE  LVLKTD**S**QDLVAS  SQDLVA**S**FRVTTG  RVTTGT**S**NTTAIK  NTTAIK**S**IEFARK  WKKCCF**S**GDGEYI  EYIVAG**S**ARQHAL  LYIWEK**S**IGNLVK  VRPIIA**S**ISSGVV  PIIASI**S**SGVVSI  IIASIS**S**GVVSIW  IEDEDK**S**EPEQTG  EEVDVT**S**VDPIAA  DEELED**S**KALLYL  PDAVQT**S**LMDEGA  LMDEGA**S**SEKKRQ  SEKKRQ**S**SADGSQ  QSSADG**S**QPPKKK  VKGDGK**S**KKKQAG  GLPLEG**S**AKGKVQ  RVVRPD**S**ELGERP  FLGKED**S**KTFDQL  DKDDKI**S**WEEYKQ  PAEFHD**S**SDHHTF  AEFHDS**S**DHHTFK  YIADMF**S**HEENGP  EPDWVL**S**EREQFN  RHLVYE**S**DKNKDE  WNMFVG**S**QATNYG  TVQPSD**S**QSVCAG  QPSDSQ**S**VCAGTE  NKLSSL**S**DLEQQY  GNLEIT**S**IEHNRD  DLSFLR**S**VREVTG  SNLTLV**S**TNGSSG  CYGPYV**S**DCCHRE  NFVVDS**S**SCVRAC  VRACPS**S**KMEVEE  GTGSLM**S**AQTVDS  VLYSGL**S**LLILKQ  KQQGIT**S**LQFQSL  QSLKEI**S**AGNIYI  IYITDN**S**NLCYYH  GRICIE**S**CNLYDG  REFENG**S**ICVECD  DNCTKC**S**HFKDGP  GCNGPT**S**HDCIYY  YPWTGH**S**TLPQHA  VYVRRK**S**IKKKRA  EALIMA**S**MDHPHL  HKDNIG**S**QLLLNW  RNVLVK**S**PNHVKI  HQSDVW**S**YGVTIW  WMIDAD**S**RPKFKE  ELAAEF**S**RMARDP  DRMKLP**S**PNDSKF  RIDSNR**S**EIGHSP  SSTQRY**S**ADPTVF  VFAPER**S**PRGELD  LKNNIL**S**MPEKAK  HSLPPR**S**TLQHPD  DYLQEY**S**TKYFYK  LGTEGS**S**VFFLDV  PGEVLR**S**VPDDYR  VIWNQA**S**QCVDHI  GNQQLE**S**LCWGRD  CWGRDS**S**TVVSSH  SSTVVS**S**HSDGSY  DFFTVH**S**TRPEDE  YLAPLH**S**SAITCS  SSAITC**S**AHVASV  CSAHVA**S**VPAKLW  QSPQPV**S**SALSWP  PVSSAL**S**WPITGG  PVEQAV**S**VAIIDL  FDYQRK**S**PVLARC  VKSLKK**S**LRQSFR  RRIEPR**S**ADDSLS  PRSADD**S**LSGVVR  SADDSL**S**GVVRCL  MWAGTN**S**GSVFAY  AGTNSG**S**VFAYAL  GDVHVF**S**VPGLRP  RPQVHY**S**CIRKED  IRKEDI**S**GIASCV  DISGIA**S**CVFTRH  QGFYLI**S**PSEFER  ITEPLC**S**LDINWP  QANGTP**S**ILLAPQ  ILLAPQ**S**LDGSPD  PQSLDG**S**PDPAHS  PPEAAL**S**PMSIDS  VKDFLG**S**SEESEK  FTDAKE**S**STVFEL  TDAKES**S**TVFELK  GECGFT**S**QTARPQ  LCIEPF**S**SPPELP  CIEPFS**S**PPELPD  VMKPQD**S**GSSANE  KPQDSG**S**SANEQA  SSSHFA**S**RHRKDI  KIAHRK**S**LSQKEN  AHRKSL**S**QKENRH  SVRITR**S**KAKDQM  QPVMPT**S**LRMTRS  SLRMTR**S**ATQAAK  QVPRTV**S**STTARK  PEGKVP**S**KGRPAK  EENTLN**S**QTNATS  NPDGVL**S**KMENLP  KIKGKN**S**FAPKDF  TPMTPR**S**ANAFLT  KTEVDE**S**QATKEI  QKCKTY**S**TKTIQQ  KTIQQD**S**NKLPCP  PIKEVP**S**LERNEG  FRNILQ**S**ETEKLT  LIKLEE**S**GWQVNN  FSGLSV**S**SEGPSQ  VSSEGP**S**QRLGTP  SVNKAV**S**QSRNEM  NKAVSQ**S**RNEMGI  GPQNTK**S**EHVKKT  KKTLFL**S**IPESRS  SIPESR**S**SIEDAQ  LKVDCL**S**SERMSL  NKKEGI**S**DVVEGM  LNSSIT**S**QDVLMS  QDVLMS**S**PEKNTA  PEKNTA**S**QNSILE  EGETKI**S**QSELFD  AALSSA**S**SETGTR  ALSSAS**S**ETGTRR  TGTRRL**S**DLRVID  EGNKKT**S**KRSSKG  QEDVET**S**LENLQD  AEIDNG**S**VADCVE  ADNLQE**S**LSDSRE  QESLSD**S**RELVEG  EPVKEE**S**SELEQP  FAQDTS**S**VGPDRK  TAHAQS**S**KADSLL  PAVEQS**S**AASELA  SELAEA**S**SEELAE  ELAEAS**S**EELAEA  APTEAP**S**PEARDS  SPEARD**S**KEDGRK  PAPKES**S**TSEGAD  ADQKMS**S**PEDDSD  EEKGRS**S**CGRNFW  DLKNLF**S**KYGKVV  TSDKRD**S**DGKKEK  DGKKEK**S**SNSDRS  GKKEKS**S**NSDRST  PGPSER**S**RATKSG  RATKSG**S**RGTERT  TVVMDK**S**KGVPVI  EKRSVV**S**FDKVKE  PRKSRD**S**ESHSRV  KSRDSE**S**HSRVRE  RREDDR**S**WQGTAD  GGERSM**S**GHSGPG  RSMSGH**S**GPGHMM  FAPGGA**S**RGHPIP  GLDEKV**S**EPLLWE  GFVEFL**S**EEDADY  IRVNKA**S**AHNKNL  LLYDTF**S**AFGVIL  DPDTGN**S**KGYAFI  AFINFA**S**FDASDA  FASFDA**S**DAAIEA  NRPITV**S**YAFKKD  YAFKKD**S**KGERHG  KGERHG**S**AAERLL  AAQNPL**S**QADRPH  DAPPPP**S**APNPVV  APNPVV**S**SLGSGL  PNPVVS**S**LGSGLP  VVSSLG**S**GLPPPG  GMPPPG**S**FPPPVP  AGHGPP**S**AGTPGA  HPGHGH**S**HPHPFP  MPHPGM**S**QMQLAH  HAGPPG**S**GGQPPP  RGPPFG**S**PMGHPG  PGGKTG**S**GGVASS  GSGGVA**S**SSESNR  SGGVAS**S**SESNRD  GGVASS**S**ESNRDR  VASSSE**S**NRDRRE  MKNHLG**S**YECKLC  LHNNEG**S**YLAHTQ  VTKQRD**S**EMGQQS  SEMGQQ**S**LLFQID  PRHRFM**S**AYEQRI  IAFKVP**S**REIDKA  KPPAPP**S**LPAGPP  RPPLPE**S**LPPPPP  PTGPAP**S**GPPGPP  VVHPPA**S**GVHPPA  GVHPPT**S**GVHPPA  GVHPPP**S**AGVHPQ  PPGVHP**S**APGVHP  PPGVHP**S**NPGVHP  LRPPLP**S**EGPGNI  AATLLL**S**FLGMAW  MVGLEG**S**DKLTIL  DRGHFL**S**GWHTIY  QRRVDG**S**VDFYRD  YKQGFG**S**RLGEFW  LTAQGT**S**ELRVDL  QFAKYR**S**FKVADE  GAFVEG**S**AGDSLT  EGSAGD**S**LTFHNN  TFHNNQ**S**FSTKDQ  HNNQSF**S**TKDQDN  YKNCHV**S**NLNGRY  LRGTHG**S**FANGIN  NGINWK**S**GKGYNY  GKGYNY**S**YKVSEM  APHELE**S**QFILRL  LPPEYA**S**TVRRAV  VRRAVQ**S**GHVNLK  DRVPLA**S**KLVDLP  LPCVME**S**LKTIDK  ICQMLV**S**TVDGDL  VEEPVA**S**TDPKAS  STDPKA**S**KKKDKD  KKKYIE**S**PDVEKE  TDAEAV**S**TRWEII  NQGLDI**S**SPGMSG  QGLDIS**S**PGMSGH  ISSPGM**S**GHRQGH  EIFNDL**S**SSSEDE  IFNDLS**S**SSEDED  FNDLSS**S**SEDEDE  NDLSSS**S**EDEDET  REKEQL**S**SLQEEL  EKEQLS**S**LQEELE  GTAGPA**S**GPGVVR  VRDPAA**S**QPRKRP  GEGARR**S**DTMAGG  MAGGGG**S**SDGSGR  AAGRRA**S**RSSGRA  GRRASR**S**SGRARR  RRASRS**S**GRARRG  LRAFRG**S**RYGDFR  GKEHTV**S**RLLRVM  GENLDC**S**FDMEAE  ELTPLE**S**AINVLE  TEAVVE**S**SRKLVK  EAVVES**S**RKLVKE  ILKKHM**S**KDPTTQ  PVIQNF**S**YETFQQ  MLRFLE**S**HLDDAE  AKKALK**S**ESAASS  KSESAA**S**STGKED  SESAAS**S**TGKEDK  AAFKTL**S**GAQDSE  LSGAQD**S**EAAFAK  RKDENE**S**SAPADG  ADGEGG**S**ELQPKN  NKRMTI**S**RLVLEE  LVLEED**S**QSTEPS  LEEDSQ**S**TEPSAG  SQSTEP**S**AGLNSS  SAGLNS**S**QEAASA  SSQEAA**S**APPSKP  AASAPP**S**KPTVLN  PKGKWN**S**SNGVEE  KGKWNS**S**NGVEEK  KWTVEE**S**EWVKAG  TPLDFP**S**KKRKRS  PPATRV**S**DKVMIP  MIRGKG**S**VKEGKV  ILRPWQ**S**SETRSI  LRPWQS**S**ETRSIT  QSSETR**S**ITNTTV  GAGHIA**S**DCKFQR  RPGDPQ**S**AQDKAR  MDKEYL**S**LMAELG  EAPVPA**S**VGSTSG  ATTPLA**S**APRPAA  NNPPPP**S**LMSTTQ  PPPSLM**S**TTQSRP  LMSTTQ**S**RPPWMN  WMNSGP**S**ESRPYH  NSGPSE**S**RPYHGM  PGGGPH**S**FPHPLP  MDQYLG**S**TPVGSG  GSTPVG**S**GVYRLH  PPPPPP**S**GQPPPP  QPPPPP**S**GPLPPW  PPPPPP**S**SSMASS  PPPPPS**S**SMASST  PPPPSS**S**MASSTP  PSSSMA**S**STPLPW  SSSMAS**S**TPLPWQ  TTTTTT**S**AGTGSI  TSAGTG**S**IPPWQQ  QAAAAA**S**PGAPQM  PPPPPG**S**AGMMYA  PPPMDP**S**NFVTMM  QGETEE**S**EKLSKM  EESEKL**S**KMSSLL  EKRVVM**S**SGGHQH  KNFDEF**S**KHLKGL  KWTPSF**S**SITSAN  SITSAN**S**VDLPAC  LFNMSM**S**IKDRHE  EDFSKV**S**QNPILT  PLERQN**S**SSGSPR  ERQNSS**S**GSPRME  KTKKKK**S**SRLPPE  TKKKKS**S**RLPPEK  VPHPQP**S**IQRMVR  DVTDIL**S**DIAEEA  PLRDSS**S**SGHSQS  QSTLFD**S**DVFQTN  KEGNGT**S**NSTLSG  MVGKPS**S**HSQYTS  TSSGSV**S**SSGSKS  SSSSSA**S**TSGKMK  TSGKMK**S**SKSEGS  SSQSKN**S**SQSGGK  KPNISP**S**HSRPPG  SSSGMK**S**SSGLGS  QKTPPS**S**NSCTAS  SSGSSM**S**SSQNQH  SQNQHG**S**SKGKSP  MGVSTN**S**SSHPMS  VSTSGS**S**VDSSKK  LQKPGE**S**SGEGLR  RGSPSH**S**KSPAYT  ESESGS**S**IAEKSY  DRDKKK**S**HSIKPE  ELGPGL**S**WAPLVF  DFLLGF**S**AGTEYM  AILGET**S**TVEKLY  EKEEEE**S**EEDWKL  EPTCGR**S**PLHLAV  GRTPLG**S**AMLRPN  EGEDEK**S**GPCSSS  EKSGPC**S**SSSDSD  SGPCSS**S**SDSDSG  CSSSSD**S**DSGDEG  SSSDSD**S**GDEGDE  DDIVVH**S**SRSQTR  DIVVHS**S**RSQTRL  VVHSSR**S**QTRLPP  LPPTPA**S**KPLPDD  NFCPLP**S**EQCYQA  GPAWVG**S**HGVLQH  GGLRPG**S**LDAEID  AEIDLL**S**STLAEL  EIDLLS**S**TLAELN  GGRGHA**S**RRPDRQ  PAYRTG**S**LKPNPA  LKPNPA**S**PLPASP  ASPLPA**S**PYGGPT  GGPTPA**S**YTTAST  ASYTTA**S**TPAGPA  PPRRGA**S**QASGPL  RGASQA**S**GPLPGP  WGPGYR**S**QREPGP  EEAAGV**S**GPAGRG  GPQVPL**S**QPPEDE  DMNHPP**S**GEYFGQ  VGCFVC**S**TCRAQL  EKCATC**S**QPILDR  FTVDAT**S**QIHCIE  KFAPRC**S**VCGGAI  IVALDR**S**FHIGCY  ECGLLL**S**SEGECQ  CGLLLS**S**EGECQG  ILCKAC**S**AWRIQE  VNVYST**S**VTSDNL  YSTSVT**S**DNLSRH  VTSDNL**S**RHDMLA  LAWINE**S**LQLNLT  KIEQLC**S**GAAYCQ  DMLFPG**S**IALKKV  ETAVAP**S**LVAPAL  PKKPLT**S**SSAAPQ  KKPLTS**S**SAAPQR  KPLTSS**S**AAPQRP  APQRPI**S**TQRTAA  TQDELR**S**LFSSIG  ELRSLF**S**SIGEVE  LRSLFS**S**IGEVES  SIGEVE**S**AKLIRD  DKVAGH**S**LGYGFV  NGLRLQ**S**KTIKVS  SKTIKV**S**YARPSS  VSYARP**S**SEVIKD  SYARPS**S**EVIKDA  DANLYI**S**GLPRTM  DVEDMF**S**RFGRII  FGRIIN**S**RVLVDQ  DQTTGL**S**RGVAFI  IRFDKR**S**EAEEAI  AEEAIT**S**FNGHKP  GHKPPG**S**SEPITV  HKPPGS**S**EPITVK  KNVALL**S**QLYHSP  LSQLYH**S**PARRFG  AQRFRF**S**PMGVDH  MGVDHM**S**GLSGVN  DHMSGL**S**GVNVPG  NVPGNA**S**SGWCIF  VPGNAS**S**GWCIFI  DKILQV**S**FKTNKS  PLEKVA**S**VGNSRP  VASVGN**S**RPTGQQ  TGQQLE**S**LGLLAP  LAPGEQ**S**LPCTER  AATARL**S**RRGTSL  LSRRGT**S**LSPPPE  RRGTSL**S**PPPESS  LSPPPE**S**SGSPQQ  SPPPES**S**GSPQQP  PPESSG**S**PQQPGL  PQQPGL**S**APHSRQ  GLSAPH**S**RQIPAP  PNYNWN**S**FGLRFG  VKLVAS**S**HISKTS  SLSVDP**S**RVDSMP  GQMGRF**S**CKITGR  SGKASM**S**AELSIQ  ETKATN**S**DVRKEV  KSKNCS**S**PQRGGS  PGLGVL**S**PSGEER  KFRCEV**S**GIPKPE  VRRQEG**S**IEVYED  VYEDAG**S**HYLCLL  TVHEKK**S**SRKSEY  KKSSRK**S**EYLLPV  TMTVQV**S**GNPPPE  TQPWFI**S**KPRSVT  FISKPR**S**VTASLG  GQSVLI**S**CAIAGD  LAKKGT**S**KTPVPE  ATPDFR**S**VLGGKK  PAENGS**S**SAETLN  AENGSS**S**AETLNA  NAKAVE**S**SKPLSN  ENLKSA**S**KEELKK  TDNEKR**S**ESQGTA  QEGSLC**S**VSIEKA  KSRRPK**S**SLPPVL  SRRPKS**S**LPPVLG  LSWYGS**S**YDGGSA  ATCRST**S**FNVQDL  SEPSQE**S**ELTTVG  NTEQKV**S**DFYDIE  RLENAG**S**LKVLFG  EAFDEI**S**DDAKDF  DQSIRE**S**RHFQID  RRSLLQ**S**EQQPSW  QSEQQP**S**WTDDLP  GSASNR**S**YSADGK  ASNRSY**S**ADGKGT  SHPPED**S**WLKFRS  SWLKFR**S**ENNCFL  FVAQRL**S**AELLLG  FDVVER**S**FLESID  ALAEKA**S**LQSQLP  TLEREI**S**GGAMAV  RALLCK**S**TVDGLQ  DELFRL**S**QLGLDA  TDIDLL**S**AAKSKP  LLSAAK**S**KPIIAE  GFLVLM**S**EGLYKA  RVKRIH**S**DTFASG  HSDTFA**S**GGERAR  YPLGEM**S**QPTPSP  GRVYPV**S**VPYSSA  PVSVPY**S**SAQSTS  VSVPYS**S**AQSTSK  PYSSAQ**S**TSKTSV  SSAQST**S**KTSVTL  QSTSKT**S**VTLSLV  LSLVMP**S**QGQMVN  PTLTLQ**S**TNTHTQ  TNTHTQ**S**SSSSSD  NTHTQS**S**SSSSDG  HTQSSS**S**SSDGGL  QSSSSS**S**DGGLFR  RSRPAH**S**LPPGED  SGLGDS**S**SDPANP  MQCFTV**S**QPKSIQ  TVSQPK**S**IQEDGE  AITGVE**S**FMTKQD  IISIDT**S**SLRAAG  MTRGTA**S**SPSYRF  IPRVNP**S**VNPSIS  SDLHSS**S**HSNSSN  HSNSSN**S**QGSFGC  SQSSNP**S**LNLNNS  MEGTGI**S**LAQFMS  SLAQFM**S**PRRQVT  PNISTL**S**SPVGMT  NNNRSY**S**NIPVTS  IQSDNS**S**SDGKPL  DGKPLD**S**GLLHNN  LSCTGT**S**NSASAN  CTGTSN**S**ASANSS  GTSNSA**S**ANSSGG  QVQGNS**S**IKLELD  DEKDLR**S**TPNLSL  SEILPA**S**LQSATA  SEDQCI**S**SQLDEL  GGLDVL**S**ERFPPQ  VGINMR**S**GMQQQI  AANPEA**S**LANRNS  SLSPGS**S**MVPMPI  PIPPPQ**S**SLLQQT  QQTPPA**S**GYQSPD  LYCNQL**S**STDLLK  PQAQQK**S**LLQQLL  LLGWKK**S**AGGSGG  KKSAGG**S**GGAGGG  CEKAVK**S**LVKKLK  KCVTIP**S**TCSEIW  TIPSTC**S**EIWGLS  SEIWGL**S**TPNTID  DTTGLY**S**FSEQTR  TGLYSF**S**EQTRSL  FSEQTR**S**LDGRLQ  DGRLQV**S**HRKGLP  RWPDLH**S**HHELKA  LDDYTH**S**IPENTN  AGIEPQ**S**NYIPET  PPPGYI**S**EDGETS  SEDGET**S**DQQLNQ  LSPVNH**S**LDLQPV  LQPVTY**S**EPAFWC  EPAFWC**S**IAYYEL  GETFHA**S**QPSLTV  FHASQP**S**LTVDGF  DGFTDP**S**NSERFC  FTDPSN**S**ERFCLG  FCLGLL**S**NVNRNA  VFAECL**S**DSAIFV  AECLSD**S**AIFVQS  SAIFVQ**S**PNCNQR  AALLAQ**S**VNQGFE  MCTIRM**S**FVKGWG  RRQTVT**S**TPCWIE  TEGELM**S**VGMDTF  DLLGEG**S**YGKVKE  VKEVLD**S**ETLCRR  MQEMLD**S**VPEKRF  GLEYLH**S**QGIVHK  GGTLKI**S**DLGVAE  DDTCRT**S**QGSPAF  CRTSQG**S**PAFQPP  NGLDTF**S**GFKVDI  FKVDIW**S**AGVTLY  ENIGKG**S**YAIPGD  DCGPPL**S**DLLKGM  EPAKRF**S**IRQIRQ  RQIRQH**S**WFRKKH  TKDRWR**S**MTVVPY  VPEEEA**S**HNGQRR  TEAAQL**S**TKSRAE  AQLSTK**S**RAEGRA  PARKAC**S**ASSKIR  RKACSA**S**SKIRRL  KACSAS**S**KIRRLS  ETVVAN**S**AFIAAR  RGSFDG**S**SSQPSR  GSFDGS**S**SQPSRD  SFDGSS**S**QPSRDK  GSSSQP**S**RDKKYL  LKLPPL**S**KCESLR  PLSKCE**S**LRDSLS  CESLRD**S**LSLEFE  SLRDSL**S**LEFESV  LSLEFE**S**VCLEQP  FQQFLQ**S**AEKHLP  QAKLFC**S**FLDEGI  FQEYLG**S**LYFLRF  GGFGEV**S**ACQMKA  ILMKVH**S**RFIVSL  HSRFIV**S**LAYAFE  DGNVRI**S**DLGLAV  ELLDGQ**S**KTKGYA  GEEYDF**S**VDYFAL  LKHRII**S**EPVKYP  KYPDKF**S**QASKDF  DKFSQA**S**KDFCEA  PPFIPD**S**KTVYAK  ELNVWR**S**DGQMPD  DDMKGI**S**GGSSSS  KGISGG**S**SSSSKS  GISGGS**S**SSSKSG  ISGGSS**S**SSKSGM  SGGSSS**S**SKSGMC  GGSSSS**S**KSGMCL  GAYVYR**S**AFSVGL  VYRSAF**S**VGLETY  QNHYDG**S**TGKFHC  MKDVKV**S**LFKKDK  NNVDQA**S**GSVLLH  VDQASG**S**VLLHLE  ADNDND**S**TFTGFL  VVLIGD**S**GVGKSN  DSGVGK**S**NLLSRF  GKSNLL**S**RFTRNE  NEFNLE**S**KSTIGV  FNLESK**S**TIGVEF  VEFATR**S**IQVDGK  RYRAIT**S**AYYRGA  LRDHAD**S**NIVIML  MLVGNK**S**DLRHLR  AEKNNL**S**FIETSA  LSFIET**S**ALDSTN  ETSALD**S**TNVEEA  EIYRIV**S**QKQIAD  RAAHDE**S**PGNNVV  NNVVDI**S**VPPTTD  RADEVK**S**MFSSNR  EVKSMF**S**SNRQKI  VKSMFS**S**NRQKIL  PVHILT**S**VSSLRG  ILTSVS**S**LRGTRE  RECSVT**S**DLDFPT  TLNAVA**S**VPIMYS  PIMYSW**S**PLQQNF  HRDDKE**S**RPPRKF  PPRKFP**S**DKIFEA  KIFEAI**S**SMFPDK  IFEAIS**S**MFPDKG  DGPNAK**S**VQREQS  REQSLH**S**FHTLFC  RLPNNS**S**RPSTPT  TINVLE**S**KDTDSD  EKKDET**S**SSSEAN  KKDETS**S**SSEANS  PENVEW**S**GAEASM  WSGAEA**S**MFRVLI  EFRVKE**S**SIIAPA  FRVKES**S**IIAPAP  RQPCDS**S**CPCVIA  KFCQCS**S**ECQNRF  AADHWD**S**KNVSCK  VSCKNC**S**IQRGSK  CSIQRG**S**KKHLLL  HLLLAP**S**DVAGWG  QKNEFI**S**EYCGEI  YDKYMC**S**FLFNLN  IRFANH**S**VNPNCY  RPSPAI**S**VSVSAP  SPAISV**S**VSAPAF  AISVSV**S**APAFYA  PFRPGD**S**EPPPAP  PPPIEE**S**FPPAPL  EEEIFP**S**PPPPPE  QPREKV**S**SIDLEI  PREKVS**S**IDLEID  IDLEID**S**LSSLLD  LEIDSL**S**SLLDDM  EIDSLS**S**LLDDMT  PFKARV**S**SGYVPP  FKARVS**S**GYVPPP  PVATPF**S**SKSSTK  TPFSSK**S**STKPAA  PFSSKS**S**TKPAAG  PLPPWK**S**PSSSQP  PWKSPS**S**SQPLPQ  WKSPSS**S**QPLPQV  VPAPAQ**S**QTQFHV  VQLHVQ**S**QTQPVS  PRGPPA**S**SPAPAP  RGPPAS**S**PAPAPK  APAPKF**S**PVTPKF  KFTPVA**S**KFSPGA  PVASKF**S**PGAPGG  APGGSG**S**QPNQKL  GHPEAL**S**AGTGSP  GSPQPP**S**FTYAQQ  RPLEGT**S**FIVDQA  QYAPRC**S**VCSEPI  PRCSVC**S**EPIMPE  DCGKPL**S**IEADDN  DIDLGV**S**REVFDF  PAQHIQ**S**ETSGSA  SGSANY**S**QVAHIP  VAHIPK**S**DALYFD  VDDNEV**S**SATFQS  SSATFQ**S**LVPDIP  IPGHIE**S**PVFIAT  ATNQAQ**S**PETSVA  AQSPET**S**VAQVAP  DNYHFY**S**SIPSME  LNAFED**S**FSSILS  SFSSIL**S**TEDPNQ  AFIAEP**S**ISNSMP  PSPATL**S**HSLSEL  TLSHSL**S**ELLNGP  NQNHPE**S**TAEFND  DSDSGI**S**LNTSPS  GISLNT**S**PSVASP  TSPSVA**S**PEHSVE  VASPEH**S**VESSSY  PEHSVE**S**SSYGDT  EVEELD**S**APGSVK  LDSAPG**S**VKQNGP  PKTPVH**S**SGDMVQ  KTPVHS**S**GDMVQP  DMVQPL**S**PSQGQS  SPSQGQ**S**THVHDA  EKELPV**S**PGHRKT  TKDKHS**S**RLEAHL  EDGKPY**S**PSEYSL  VFLVPK**S**KKPDVK  LLLLPL**S**SSSSSD  LLLPLS**S**SSSSDT  LLPLSS**S**SSSDTC  LPLSSS**S**SSDTCG  PLSSSS**S**SDTCGP  LSSSSS**S**DTCGPC  GPCEPA**S**CPPLPP  GMECVK**S**RKRRKG  AGGPGV**S**GVCVCK  GVCVCK**S**RYPVCG  RYPVCG**S**DGTTYP  DGTTYP**S**GCQLRA  CQLRAA**S**QRAESR  ASQRAE**S**RGEKAI  KAITQV**S**KGTCEQ  TCEQGP**S**IVTPPK  GAQVYL**S**CEVIGI  TGWVLV**S**PLSKED  EYECHA**S**NSQGQA  ECHASN**S**QGQASA  NSQGQA**S**ASAKIT  QGQASA**S**AKITVV  RLGPLF**S**LLNENH  KTTLCV**S**ILERLL  LIHPDD**S**VKILTL  VKILTL**S**QIGRIV  GRIVEN**S**DAVTEI  AKAAIK**S**LSRISL  KSLSRI**S**LTQAGL  LEALFE**S**NLLDDL  LLDDLK**S**VMKTND  ELIIEI**S**SVSPES  LIIEIS**S**VSPESL  IEISSV**S**PESLNY  SSVSPE**S**LNYCTT  CIEMVT**S**LAYTHH  GVIDQI**S**NIIVGA  IIVGAD**S**DPFSSF  ADSDPF**S**SFYLPG  DSDPFS**S**FYLPGF  NLAVMD**S**PQQICE  VFEMIE**S**QDPTMI  TVGILG**S**NVEGKQ  MRIGHQ**S**KNAPVE  RCLDAI**S**SLLYLP  CLDAIS**S**LLYLPP  TESWFS**S**LSRDPL  ELFRGI**S**SQPFPE  LFRGIS**S**QPFPEL  QKLMFN**S**PGFVEY  EYVVDR**S**VEHDKA  VEHDKA**S**KDAKYE  VKALAN**S**KTIAEI  RLRTYL**S**EGPYYV  YYVKPV**S**TTAVEG  TTDLGR**S**LGPVEL  ELLLRG**S**SRRLDL  LLLRGS**S**RRLDLL  AGGPTC**S**ATNLSR  CSATNL**S**RVAGLE  PDDTQG**S**PDLGAV  ELAAAS**S**AAFSTR  GPGTPD**S**RPPFLS  RGLYSR**S**GSLSGR  SLSGRS**S**LKAEAE  ENTSEV**S**TVLKLD  PNAWDQ**S**FTLELE  RQKKIF**S**KQQGKA  TGTGTF**S**PGASPG  TFSPGA**S**PGSEAR  LNLGTD**S**DSSPQK  LGTDSD**S**SPQKSS  SRDPPS**S**PSSLSS  DPPSSP**S**SLSSPI  PPSSPS**S**LSSPIQ  SSPSSL**S**SPIQES  SPSSLS**S**PIQEST  SSPIQE**S**TAPELP  FGKVLL**S**EFRPSG  ARDEVE**S**LMCEKR  ILAAVT**S**AGHPFL  IHSDVF**S**EPRAIF  GYGDRT**S**TFCGTP  EVLTDT**S**YTRAVD  EMLVGE**S**PFPGDD  EEEVFD**S**IVNDEV  PFVPTL**S**GRTDVS  GEAPTL**S**PPRDAR  TELQGD**S**RSLPFS  SRSLPF**S**ENVSAV  PFSENV**S**AVQKLD  VTTDKK**S**LAYVDN  DNILKK**S**NKKLEE  NNDPRC**S**TSNNRL  NMIQMY**S**NGSSKD  QMYSNG**S**SKDRKL  PKNHPK**S**RIIIEE  IIIEEL**S**LVAASP  PRQSMI**S**TQNQYS  ENVPGR**S**KATSVA  VALPGW**S**PSETRS  SPSETR**S**SFMSRT  PSETRS**S**FMSRTS  TRSSFM**S**RTSKSK  SFMSRT**S**KSKSGS  MSRTSK**S**KSGSSR  TVVGQT**S**WKPISN  KPISNQ**S**WDQKFT  TLELDR**S**RELEIS  SRELEI**S**VYWRDW  APPASD**S**TVTKLD  LGEIDE**S**SELRVL  GEIDES**S**ELRVLD  IQNDRN**S**ILPKSQ  ARDEVD**S**LMCEKR  IFETVN**S**VRHPFL  IHTDVF**S**EPRAVF  EVLTET**S**YTRAVD  RGREDV**S**NFDDEF  ETVEEL**S**SPLTAH  TVEELS**S**PLTAHV  PLWLTG**S**LLRCGP  GLFEVG**S**EPFYHL  IFSRFF**S**YFRGVE  DLCNYV**S**VNGATA  CFGKNF**S**IAYNIV  DKEDPI**S**KSEIVV  EDPISK**S**EIVVQF  VVQFPC**S**DRFKPS  SDRFKP**S**YVHSFG  KPSYVH**S**FGLTPN  NLFKFL**S**SWSLWG  LFKFLS**S**WSLWGA  KFLSSW**S**LWGANY  YMDCFE**S**NETMGV  NNKYRT**S**PFNLFH  ATAILC**S**DETIWL  EPEVLF**S**GPRQAF  VWQEPD**S**YPSEPI  EPDSYP**S**EPIFVS  SEPIFV**S**HPDALE  DDGVVL**S**VVVSPG  VLSVVV**S**PGAGQK  LNAKDL**S**EVARAE  LKVTVP**S**CSASSC  VTVPSC**S**ASSCSS  CSASSC**S**SVTASA  SASSCS**S**VTASAA  CSSVTA**S**AAPGTA  AAPGTA**S**LVPDYW  DYWIDG**S**NRDALS  SNRDAL**S**DFFEVE  DFFEVE**S**ELGRGA  LGRGAT**S**IVYRCK  GVLLRL**S**HPNIIK  ETPTEI**S**LVLELV  VEKGYY**S**ERDAAD  IADFGL**S**KIVEHQ  PEVDMW**S**VGIITY  CEYYFI**S**PWWDEV  PWWDEV**S**LNAKDL  KAVVAS**S**RLGSAS  ASSRLG**S**ASSSHG  SRLGSA**S**SSHGSI  RLGSAS**S**SHGSIQ  LGSASS**S**HGSIQE  ASSSHG**S**IQESHK  HGSIQE**S**HKASRD  QESHKA**S**RDPSPI  PREGQG**S**SAVGFE  REGQGS**S**AVGFEV  NSTDLL**S**QPWNEP  EPPVIL**S**MVILSL  LSMVIL**S**LTFLLG  DLLCCL**S**LPFSLA  CLSLPF**S**LAHLAL  LCKLIP**S**IIVLNM  VLNMFA**S**VFLLTA  NVGMAC**S**ICGCIW  YKFGLS**S**SLDYPD  KFGLSS**S**LDYPDF  DPLENR**S**LENIVQ  QRPSAD**S**LPRGSA  DSLPRG**S**ARLTSQ  GSARLT**S**QNLYSN  KPADVV**S**PKIPSG  VSPKIP**S**GFPIED  IEDHET**S**PLDNSD  TSPLDN**S**DAFLST  NSDAFL**S**THLKLF  HLKLFP**S**ASSNSF  LFPSAS**S**NSFYES  PSASSN**S**FYESEL  SNSFYE**S**ELPQGF  VGFLLP**S**VIMIAC  IMIACY**S**FIVFRM  RGRFAK**S**QSKTFR  RFAKSQ**S**KTFRVA  HIFGVL**S**LLTDPE  LGKTLM**S**WDHVCI  ALASAN**S**CFNPFL  RKKARQ**S**IQGILE  ILEAAF**S**EELTRS  RSTHCP**S**NNVISE  LKSPLG**S**WNGSGS  LGSWNG**S**GSGGGG  GGRPEG**S**PKAAGY  LFDYEP**S**GQDELA  SRDAAI**S**GDEGWW  QVGIFP**S**NYVSRG  FPSNYV**S**RGGGPP  ISVTAE**S**VRQEAR  IHRDLK**S**NNILLL  LLQPIE**S**DDMEHK  PEVIKA**S**TFSKGS  IKASTF**S**KGSDVW  STFSKG**S**DVWSFG  KGSDVW**S**FGVLLW  LTLPIP**S**TCPEPF  RRPDFA**S**ILQQLE  AAREQR**S**QAEQLR  DGGERI**S**MPLDFK  CWAWGP**S**SPKPGE  WAWGPS**S**PKPGEA  ATWYLD**S**DDSSPL  YLDSDD**S**SPLGSP  LDSDDS**S**PLGSPS  DSSPLG**S**PSTPPA  SPLGSP**S**TPPALN  VPAERG**S**SSGTPK  PAERGS**S**SGTPKL  AERGSS**S**GTPKLI  SPPPGT**S**RSAPGT  PPLGLI**S**RPRPSP  RPSPLR**S**RIDPWS  EVKRKH**S**AILASP  HSAILA**S**PNPDEK  ELEELM**S**DIKKTA  TANKVR**S**KLKSIE  VRSKLK**S**IEQSIE  LKSIEQ**S**IEQEEG  EEGLNR**S**SADLRI  EGLNRS**S**ADLRIR  IRKTQH**S**TLSRKF  TQHSTL**S**RKFVEV  KFVEVM**S**EYNATQ  EYNATQ**S**DYRERC  TGRTTT**S**EELEDM  LEDMLE**S**GNPAIF  NPAIFA**S**GIIMDS  SGIIMD**S**SISKQA  GIIMDS**S**ISKQAL  ISKQAL**S**EIETRH  EIETRH**S**EIIKLE  IIKLEN**S**IRELHD  MAMLVE**S**QGEMID  YVERAV**S**DTKKAV  KAVKYQ**S**KARRKK  SSRPLE**S**PPPYRP  PNHYAP**S**NDIYGG  HVRPML**S**QPAYSF  LSQPAY**S**FYPEDE  HFYKWT**S**PPGVIR  GVIRIL**S**MLIIVM  DRGYGT**S**LLGGSV  TSLLGG**S**VGYPYG  GYPYGG**S**GFGSYG  GGSGFG**S**YGSGYG  GFGSYG**S**GYGYGY  LVIFVT**S**VIRSEM  VTSVIR**S**EMSRTR  LSVIIV**S**AILGIM  VNPTAQ**S**SGSLYG  TAQSSG**S**LYGSQI  SGSLYG**S**QIYALC  MDRYDK**S**NILWDK  EWVKNV**S**AGTQDV  GTQDVP**S**PPSDYV  DSPMAY**S**SNGKVN  RFYPES**S**YKSTPV  PESSYK**S**TPVPEV  QELPLT**S**PVDDFR  EYPPIT**S**DQQRQL  GLQEYK**S**LQSELD  EYKSLQ**S**ELDEIN  EINKEL**S**RLDKEL  DDYREE**S**EEYMAA  HCKQLK**S**KLSHIK  QLKSKL**S**HIKKMV  VPEQED**S**VLFRRG  YDKAVA**S**FKHALK  GDICET**S**GKPKTT  PAKKNK**S**QKKNTA  KKNTAA**S**LQQWKV  KVGDKC**S**AIWSED  KCSAIW**S**EDGCIY  YPATIA**S**IDFKRE  REEQNL**S**DLLSPI  NLSDLL**S**PICEVA  QENENE**S**QVSTDE  ENESQV**S**TDESEN  QVSTDE**S**ENSRSP  TDESEN**S**RSPGNK  ESENSR**S**PGNKSD  RSPGNK**S**DNIKPK  KSAPWN**S**FLPPPP  PPPHLL**S**CWLPPF  WLPPFP**S**GPPIIP  PPICPD**S**LDDADA  DADALG**S**MLISWY  LGSMLI**S**WYMSGY  LISWYM**S**GYHTGY  EVIREE**S**AADWAL  YTYEDG**S**DDLKLA  DLKLAA**S**GEGGLQ  GGLQEL**S**GHFENQ  VMYGFC**S**VKDSQA  FCSVKD**S**QAALPK  RKCACA**S**HVAKVA  DVIVNA**S**SVEDID  VIVNAS**S**VEDIDA  RRLKEQ**S**IFGDHR  ETHMKK**S**ESEVEE  HMKKSE**S**EVEEAA  QQERVA**S**ASAGSC  ERVASA**S**AGSCDV  ASASAG**S**CDVPSP  GSCDVP**S**PFNHRP  PGSHLD**S**HRRMAP  TRSPSD**S**STASTP  RSPSDS**S**TASTPV  SDSSTA**S**TPVAEQ  ALDEVT**S**SQPPPL  DLMFME**S**AEQAVL  VPPAAT**S**LIDLWP  GNGEGA**S**TLQGEP  RAPTPP**S**GTEVTL  EEVEGE**S**LAAPQT  QTPTLP**S**ALEELE  KEGTQA**S**EGYFSQ  ASEGYF**S**QSQEEE  EGYFSQ**S**QEEEFA  EEEFAQ**S**EELCAK  KKGHGH**S**YTTAEE  RALMDK**S**LHVGTQ  GFDHRD**S**KVSLQE  HRDSKV**S**LQEKNC  ATYTEH**S**MLSADE  DTSYDD**S**DILNST  DSDILN**S**TAADEV  RPHVFE**S**NPSIRK  VFESNP**S**IRKRQQ  AIVLCI**S**PSKPNP  VLCISP**S**KPNPVF  VVRKYK**S**MILEDL  ILEDLE**S**ALAEHA  APQEVN**S**ELPPLT  IDGIPV**S**VDKMTQ  PEMLKY**S**TGRGKP  PGWGKE**S**CKPIWW  PWANVR**S**DVRTEE  EQKQRV**S**WTQALR  QATATH**S**IAHLVP  IAHLVP**S**QTVVQT  TVVQTF**S**NPDGTV  NPDGTV**S**LIQVGT  ATLADA**S**ELPTTV  VAQVNY**S**AVADGE  IQTTQA**S**EATQAV  ATQAVA**S**LAEAAV  EAAVAA**S**QEMQQG  VTMALN**S**EAAAHA  GGQIVL**S**GETAAA  LVQIPV**S**MYQTVV  YQTVVT**S**LAQGNG  PVTTRI**S**DSAVTM  TTRISD**S**AVTMDG  GFKVNA**S**ASSLKK  PPDEAG**S**AAVCLR  AAVCLR**S**HLGRYL  HDDGRW**S**LQSEAH  GRWSLQ**S**EAHRRY  GTEDRL**S**CFAQTV  CFAQTV**S**PAEKWS  PQVNIY**S**VTRKRY  KRYAHL**S**ARPADE  VPWGVD**S**LITLAF  FQDQRY**S**VQTADH  YTLEFR**S**GKVAFR  GRYLAP**S**GPSGTL  LAPSGP**S**GTLKAG  ANERNV**S**TRQGMD  RQGMDL**S**ANQDEE  ATGGVQ**S**TASSKN  GVQSTA**S**SKNASC  VQSTAS**S**KNASCY  ASSKNA**S**CYFDIE  RITLRA**S**NGKFVT  NGKFVT**S**KKNGQL  NGQLAA**S**VETAGD  VETAGD**S**ELFLMK  TLDANR**S**SYDVFQ  LDANRS**S**YDVFQL  AYNIKD**S**TGKYWT  KYWTVG**S**DSAVTS  WTVGSD**S**AVTSSG  SDSAVT**S**SGDTPV  AGVLKA**S**AETVDP  ILGPSG**S**QLTDDV  SLTELN**S**VYIVQE  GLKYIH**S**ANVLHR  IMDPHY**S**HKGHLS  VTKWYR**S**PRLLLS  MQLILE**S**IPVVHE  LSHPYM**S**IYSFPM  PMDEPI**S**SHPFHI  YHDCQF**S**EHDWPV  LDPRAL**S**DVTDEE  CNYKTR**S**SSYLDN  NLVWRE**S**EVNHYY  KLIIDL**S**NWKEQS  EQSKEK**S**DKKGKS  SDKKGK**S**KCERNG  IALEEA**S**QQLAGK  DLNSSV**S**QLELKS  SQLELK**S**LISKSV  ELKSLI**S**KSVSQE  KSLISK**S**VSQEKQ  LISKSV**S**QEKQEK  LEALYQ**S**SWDSQF  EALYQS**S**WDSQFV  YQSSWD**S**QFVSGG  WDSQFV**S**GGEDCF  KENTYT**S**YLDKFF  EGFLNN**S**GEFLFN  GIPQFH**S**PVGSPL  FHSPVG**S**PLKSIQ  QATLTP**S**AMKSSP  TPSAMK**S**SPQIPH  NDKKKI**S**SERRKE  DKKKIS**S**ERRKEK  AARSRR**S**KESEVF  PLPHNV**S**SHLDKA  VMRLTI**S**YLRVRK  EQNTQR**S**FFLRMK  RTMNIK**S**ATWKVL  HVYDTN**S**NQPQCG  EELLGR**S**IYEYYH  VLKPVE**S**SDMKMT  LFTKVE**S**EDTSSL  AGDTII**S**LDFGSN  ISLDFG**S**NDTETD  NINLAM**S**PLPTAE  LEPNPE**S**LELSFT  DQTPSP**S**DGSTRQ  DGSTRQ**S**SPEPNS  PEPNSP**S**EYCFYV  RSFDQL**S**PLESSS  LSPLES**S**SASPES  SPLESS**S**ASPESA  SSASPE**S**ASPQST  IKILIA**S**PSPTHI  ILIASP**S**PTHIHK  IHKETT**S**ATSSPY  TQSRTA**S**PNRAGK  RSPNVL**S**VALSQR  AATTSL**S**WKRVKG  VKGCKS**S**EQNGME  TIILIP**S**DLACRL  CRLLGQ**S**MDESGL  MVKSLL**S**NDLKLN  ATSPAP**S**TSSTVK  SPAPST**S**STVKTE  PAPSTS**S**TVKTEG  AQKRKK**S**TKEKAG  PKGSKV**S**EEQTQP  STAMGR**S**PSPKTS  SAPPNS**S**STENPK  APPNSS**S**TENPKT  VIVKVL**S**TTKPFE  LEVNEE**S**TVSEAG  ILHKQA**S**GNIVYG  LISEMH**S**FIQIKK  RNNDPK**S**MKLPQE  RQLPYP**S**EASTTF  PYPSEA**S**TTFPES  STTFPE**S**HLRTPQ  PPTTPS**S**SFFTKK  QILKEG**S**HFPGPF  SIGPAE**S**HPHTPQ  PPSTPS**S**SFLTTK  KSEDTI**S**KMNDFM  PGPFMT**S**IGPAES  TPQMPP**S**TPSSSF  PPSTPS**S**SFLTTL  TEPEEV**S**IEDSAQ  PKGLIR**S**ASVTPK  KINQLC**S**QTKGSF  NTGELR**S**VIHSHI  DLRFQS**S**AVMALQ  LQEACE**S**YLVGLF  LPPVDL**S**LVNKDE  IYFLGN**S**LGLQPK  WITGDE**S**IVGLMK  LHLLML**S**FFKPTP  EAKAFP**S**DHYAIE  DHYAIE**S**QLQLHG  GLNIEE**S**MRMIKP  IEKEGD**S**IAVILF  IAVILF**S**GVHFYT  DFACWC**S**YKYLNA  WFGHEL**S**TRFKMD  VCGFRI**S**NPPILL  PILLVC**S**LHASLE  VCSLHA**S**LEIFKQ  KALRKK**S**VLLTGY  VNIITP**S**HVEERG  QLTITF**S**VPNKDV  PVPLYN**S**FHDVYK  FTNLLT**S**ILDSAE  EFVKLA**S**EHDVVN  AFQHAV**S**GDFMLN  LTKILA**S**FFGELL  GRPVFV**S**LKPGPI  QNGELG**S**SSNWQL  NGELGS**S**SNWQLD  GELGSS**S**NWQLDP  LAGKFT**S**RTKALV  PLGKVF**S**REELEL  ELELVA**S**LCQQHD  DGHQHI**S**IASLPG  QHISIA**S**LPGMWE  RTLTIG**S**AGKTFS  SAGKTF**S**ATGWKV  RTVHQN**S**VFHCPT  FHCPTQ**S**QAAVAE  QAAVAE**S**FEREQL  LLFRQP**S**SYFVQF  LFRQPS**S**YFVQFP  RDHMIR**S**LQSVGL  MIRSLQ**S**VGLKPI  PIIPQG**S**YFLITD  FLITDI**S**DFKRKM  LVAIPV**S**IFYSVP  PVSIFY**S**VPHQKH  TVQLLL**S**LLLLVP  PRMQED**S**PLGGGS  SPLGGG**S**SGEDDP  PLGGGS**S**GEDDPL  GEEDLP**S**EEDSPR  LPSEED**S**PREEDP  PEVKPK**S**EEEGSL  KSEEEG**S**LKLEDL  KEGDDQ**S**HWRYGG  PPWPRV**S**PACAGR  CAGRFQ**S**PVDIRP  LRNNGH**S**VQLTLP  AAGRPG**S**EHTVEG  IHVVHL**S**TAFARV  EGPEEN**S**AYEQLL  AYEQLL**S**RLEEIA  EIAEEG**S**ETQVPG  VPGLDI**S**ALLPSD  ISALLP**S**DFSRYF  LLPSDF**S**RYFQYE  YFQYEG**S**LTTPPC  NQTVML**S**AKQLHT  KQLHTL**S**DTLWGP  LWGPGD**S**RLQLNF  GRVIEA**S**FPAGVD  FPAGVD**S**SPRAAE  PAGVDS**S**PRAAEP  EPVQLN**S**CLAAGD  LLFAVT**S**VAFLVQ  GTKGGV**S**YRPAEV  LLLPIL**S**SAKAQV  TASSQW**S**ESTAAK  KYGRLD**S**EEGDGA  DGTRWI**S**WRNRHG  IPVTDH**S**MNVCMR  FVLPGG**S**IIYLND  IIYLND**S**VYDGAV  VQCYFR**S**EASEWE  YFRSEA**S**EWEPNA  LDDVNP**S**ARFVTV  SEALPT**S**PMAPTT  MLKVDD**S**NTRILI  KMLEKA**S**RRMLDD  DDEMTV**S**LSLPSD  LSLPSD**S**SMFNNN  SLPSDS**S**MFNNNR  NRSSSP**S**EQGSNS  EEESGC**S**GVVKPV  VKPVQP**S**GPEGVP  TGGNTY**S**VPAVTM  RHEPPN**S**SSSDVR  HEPPNS**S**SSDVRT  EPPNSS**S**SDVRTV  PPNSSS**S**DVRTVS  SDVRTV**S**YTNLKF  IADFGM**S**RNLYSG  MSRNLY**S**GDYYRI  LPIRWM**S**WESILL  RWMSWE**S**ILLGKF  CQEQPY**S**QLSDEQ  QPYSQL**S**DEQVIE  PAICPD**S**VYKLML  DTKNRP**S**FQEIHL  GDGGEP**S**QLPHCP  WTHPGQ**S**QLFADL  AAQARP**S**DNCVFS  SDNCVF**S**VELQLP  QPQPNV**S**ELVVGP  GPLPHP**S**YMRDVT  APRGLQ**S**GDRATW  GLYYNI**S**GAGFFL  QGRYYD**S**LAQLEA  GTGGSW**S**LKSPVP  GSWSLK**S**PVPPGP  PQGPRF**S**VQGSRV  RFSVQG**S**RVASSL  SSLWTF**S**FGLGAF  RLVYEI**S**LQEALA  AIYGGN**S**PAAMTT  PLRRHH**S**DLYSHY  HHSDLY**S**HYFGGL  LVVRSM**S**TLLNYD  DTVFHP**S**GAIEIR  YATGYI**S**SAFLFG  ATGYIS**S**AFLFGA  PMAVPW**S**PEHQLQ  AAFLVG**S**ATPRYL  RYLYLA**S**NHSNKW  YLASNH**S**NKWGHP  YRIQML**S**FAGEPL  PLPQNS**S**MARGFS  SMARGF**S**WERYQL  KEEEPS**S**SSVFNQ  EEEPSS**S**SVFNQN  EEPSSS**S**VFNQND  EDPSFY**S**ADSIYF  PLELTQ**S**RVQKIW  PVDHRP**S**LPRSCG  RPSLPR**S**CGPKLT  GPKLTN**S**PTVIVM  RGKTYI**S**KKLTRY  EAVKQY**S**SYNFFR  AVKQYS**S**YNFFRP  ALRDVK**S**YLAKEG  KAFFIE**S**VCDDPT  IMEVKI**S**SPDYKD  MEVKIS**S**PDYKDC  DYKDCN**S**AEAMDD  DFMKRI**S**CYEASY  ISCYEA**S**YQPLDP  KCDRDL**S**LIKVID  VQDHIQ**S**RIVYYL  GRIGGD**S**GLSSRG  GGDSGL**S**SRGKKF  GDSGLS**S**RGKKFA  KFASAL**S**KFVEEQ  DLRVWT**S**QLKSTI  WTSQLK**S**TIQTAE  RYPTGE**S**YQDLVQ  AYFLDK**S**AEEMPY  YGCRVE**S**IYLNVE  IYLNVE**S**VCTHRE  KKPRIN**S**FEEHVA  FEEHVA**S**TSAALP  EHVAST**S**AALPSC  GQNMKG**S**RSSADS  NMKGSR**S**SADSSR  KVTELR**S**ELQRRG  QRRGLD**S**RGLKVD  PRPVAA**S**GGGPGG  AAEPDA**S**EKPAEA  AEATAG**S**GGVNGG  GDETPG**S**EVPGDK  QGDDQD**S**EKSKPA  DQDSEK**S**KPAGSD  KSKPAG**S**DGERRG  REEAYH**S**RSKSPL  NLDTYT**S**DLHFQV  DLHFQV**S**KDRYGG  GGQPLF**S**EKFPTL  KFPTLW**S**GARSTY  LWSGAR**S**TYGVTK  EGCTEV**S**LLRVGW  LLRVGW**S**VDFSRP  GWSVDF**S**RPQLGE  LGEDEF**S**YGFDGR  TEEVEL**S**FSKNGE  GVAFWI**S**KDSLAD  FWISKD**S**LADRAL  MVGLPG**S**GKTQWA  PEMDPK**S**RDLLVQ  KLVQIA**S**RTKRNF  QCNVYN**S**GQRRKL  LLFKTF**S**RKVVVV  EMKANF**S**LPEKCD  RKLLPP**S**EKRTNR  RNRQNR**S**RGQGYV  YNRDWQ**S**YYYHHP  YPPANS**S**LSSRSE  ANSSLS**S**RSETFL  SSLSSR**S**ETFLLL  VELELP**S**HWFSQA  LPSHWF**S**QASTTR  LDRFKG**S**RHHTTL  GLTEPD**S**SPLELS  LTEPDS**S**PLELSE  VWEILV**S**ERDIRA  EHVGCE**S**ANTQVL  DWLLDV**S**HLVAPH  REPGVT**S**IEVRSP  TSIEVR**S**PLSDSI  EVRSPL**S**DSILGE  RSPLSD**S**ILGEQA  VTDDKV**S**VLELRV  QPVMGI**S**LTLSRG  GISLTL**S**RGTAHP  ATCWAQ**S**ALPAPK  SLWLSF**S**DHTVAP  QLGVVV**S**GAGAEG  HRVPLA**S**GTAWLG  LGLPPA**S**TPAPAL  PAPALP**S**SPAWSP  PSSPAW**S**PPATEA  SATDPT**S**PQPHNW  PPKGEG**S**CPCESG  GSCPCE**S**GGGGEA  PPGGTT**S**SSSTLA  PAPPAQ**S**PEEPVG  GAPAVQ**S**ILVAGE  TLTFQD**S**AGARNN  RGHPAP**S**PIWRHA  MMFLQI**S**NDINSD  ISNDIN**S**DSEKLS  NDINSD**S**EKLSQL  SDSEKL**S**QLQKTI  QQQDNL**S**QQLGNS  SQQLGN**S**NNLSME  GNSNNL**S**MEEEFL  EEEFLK**S**QISSVL  FLKSQI**S**SVLKRQ  LKSQIS**S**VLKRQE  ELIIHT**S**DHRCNP  WQWYQN**S**CYYFTT  EKTWAN**S**RKDCID  DCIDKN**S**TLVKID  TLVKID**S**LEEKDF  EKDFLM**S**QPLLMF  QPLLMF**S**FFWLGL  FFWLGL**S**WDSSGR  LGLSWD**S**SGRSWF  GLSWDS**S**GRSWFW  WDSSGR**S**WFWEDG  WFWEDG**S**VPSPSL  EDGSVP**S**PSLFST  GSVPSP**S**LFSTKE  PSPSLF**S**TKELDQ  LDQING**S**KGCAYF  KGNIYI**S**RCSAEI  IYISRC**S**AEIFWI  SAVPLS**S**PDYYER  QHGLRD**S**EKKRLD  IKKMLC**S**KDSLGL  SIPDPH**S**HRQTAQ  SSNVTD**S**CVVQTS  SCVVQT**S**NDTQCL  NIDGKA**S**LQIGEK  HCLEIG**S**NTRHGK  NTRHGK**S**NLSEGK  LSEGKK**S**AELNSP  KSAELN**S**PLRERL  TDLKCD**S**SSGSEG  DLKCDS**S**SGSEGE  VEEKRA**S**PPVSPI  VSEYCE**S**ENKWSQ  WSQEKH**S**PWEGVS  AHREPK**S**QKPFRK  ESWSTS**S**DLTISI  DLTISI**S**EDDLIL  QERVAL**S**TEKNCI  TLSSPD**S**EKESST  PGQTPD**S**DVPRAQ  EECGRR**S**AIHSSE  RSAIHS**S**ESSCSL  SSESSC**S**LPSILN  TREQEV**S**SGCGDK  REQEVS**S**GCGDKS  SGCGDK**S**KKENVA  KTDASV**S**HLSGLN  SEASFS**S**SEGSPL  SEGSPL**S**RHENKK  NALWDE**S**DDSNSE  ESDDSN**S**EIEAAL  PEAEAL**S**PEQAAH  FTDVCN**S**GLLLYR  QIIAFL**S**GMFDAS  NWQQLS**S**FRGQEF  DFACQG**S**LLGTLK  DVQKKK**S**SNPEAR  IYRKFV**S**LDHIKE  LNLGTF**S**AQDGNE  TLVFSQ**S**RQILNI  TQLQLQ**S**LHAAQR  QPSPLL**S**THHTQE  TQEEDI**S**SKMASV  ISSKMA**S**VVIDDL  GEKQDL**S**SIKVNV  EKQDLS**S**IKVNVT  DGKGTG**S**ADSIAT  GTGSAD**S**IATLPK  LPKGFG**S**VEELCT  SLGMEK**S**FATKNE  QEDPLE**S**FNYVLS  SFNYVL**S**KSTKAD  NPWPII**S**ITNESQ  NAESNV**S**IIEIAD  KLEEEP**S**ASSPQY  EEEPSA**S**SPQYAC  EEPSAS**S**PQYACD  NLFLED**S**ADNRQN  CLSWEF**S**EKDDEP  SFKDTS**S**INPFNT  TSLFQF**S**SVKQFD  FSSQIP**S**SVNKSM  LSSENK**S**SWLMTS  SSWLMT**S**KPSALA  FFETNY**S**QIYFIF  LKGNNK**S**QREELD  FRWHYQ**S**IGSTLK  MVIQAA**S**LEWKNK  DNENIY**S**TKIPYM  EQDKSH**S**NSSTLS  GFSETD**S**KEASSE  DSKEAS**S**ESSGHK  EPMRFR**S**ATTSGA  NAPAAG**S**GHLTVG  IPEPLC**S**DSSQGQ  EPLCSD**S**SQGQKA  CRERQK**S**ESTNSD  CLTDDC**S**IIAGGS  TGWHPD**S**AAVLWR  PLRMFA**S**WLFKAA  VLQLLV**S**YWEKLQ  LPSAEY**S**SVETDK  VLLHPV**S**TAVLEE  CCVCGS**S**TYTQQS  NAHVEG**S**ELSFEV  VEGSEL**S**FEVFRS  SPVGSL**S**DVRVIV  PSFLIS**S**WHRDTF  ENIGHT**S**PECLLP  ELKNLD**S**RQCRET  GLQRNG**S**TGQTAP  TRMPSD**S**DDSLTK  PSDSDD**S**LTKKLR  SGKLLP**S**LVCATC  LIPLYQ**S**FYEERA  SIEIES**S**DVIRLI  QYLKEN**S**LHRALA  SLNTVD**S**IESFVA  TVDSIE**S**FVADIN  VLQAIQ**S**LKLPDK  ELGAAR**S**LLRQTD  ENLLAR**S**YFDPRE  EAYPDG**S**SKEKRR  AYPDGS**S**KEKRRA  ALAGEV**S**VVPPSR  VSVVPP**S**RLMALL  IKFGQK**S**HVECAR  VECARF**S**PDGQYL  QYLVTG**S**VDGFIE  VLCMCF**S**RDTEML  RFERAH**S**KGVTCL  LSFSKD**S**SQILSA  SFSKDS**S**QILSAS  SQILSA**S**FDQTIR  KEFRGH**S**SFVNEA  EFRGHS**S**FVNEAT  DGHYII**S**ASSDGT  HYIISA**S**SDGTVK  YIISAS**S**DGTVKI  MKTTEC**S**NTFKSL  CSNTFK**S**LGSTAG  TFKSLG**S**TAGTDI  TDITVN**S**VILLPK  QGQIVR**S**FSSGKR  QIVRSF**S**SGKREG  IVRSFS**S**GKREGG  FVLYCF**S**TVTGKL  NLIATY**S**EDGLLK  LRRALF**S**LKQIFQ  RGVKPW**S**NIMEIL  RDRRRA**S**VCSSGG  SVQSIK**S**TLSAPT  SIKSTL**S**APTSPC  SNFGNN**S**YHSSRP  HSSRPS**S**GSSVPT  VPTTPT**S**SVSPPQ  ARLERS**S**PSGLLT  LERSSP**S**GLLTSS  SGLLTS**S**FRQHQE  KELRSR**S**VSRGRA  LRSRSV**S**RGRADL  SHSPSS**S**DSQEAL  LTVSAS**S**PGTPHH  PHHPQA**S**AGDPEP  AGQDIA**S**AHEGAE  CSASSV**S**SSSSTL  EAEAVA**S**LATRIS  STLQAN**S**QTQDES  CLDNRG**S**VKAFAE  CREFLW**S**KLEPIK  PIKVDT**S**RLEHLF  SSISEL**S**ARLHLW  DTVHKQ**S**LLHHVC  ENFPDS**S**DLYSEI  SGKFSG**S**SPAPPS  GKFSGS**S**PAPPSQ  SSPAPP**S**QPQGLS  LGVRTR**S**RASRGS  RTRSRA**S**RGSTSS  SRASRG**S**TSSWTM  SATQVP**S**QRVVPR  PPGEYF**S**VGSQVS  EYFSVG**S**QVSCRT  SVGSQV**S**CRTCQE  VAFDYQ**S**KMLALK  LALKCP**S**SSGKPN  ALKCPS**S**SGKPNH  LKCPSS**S**GKPNHA  INLQYV**S**EVEIIN  TPPPLA**S**LNVSKL  LASLNV**S**KLASKA  NVSKLA**S**KARTEK  EKEEKL**S**QAYAIS  SQAYAI**S**AGVSLE  AISAGV**S**LEGQQL  CKGKEG**S**ALSHVR  KEGSAL**S**HVRKIV  HFRDVE**S**QKILQR  QKILQR**S**QAQQPQ  YREIAR**S**CNCYME  WYTARK**S**ITVICD  VICDFY**S**LIRLHF  FIPRLG**S**RADLIK  GRWAVV**S**GATDGI  YAEELA**S**RGLNII  LNIILI**S**RNEEKL  IIVADF**S**SGREIY  IVADFS**S**GREIYL  QYFTQL**S**EDKLWD  VNIAAA**S**LMVHVV  GAIVTI**S**SGSCCK  AIVTIS**S**GSCCKP  VTISSG**S**CCKPTP  PQLAAF**S**ASKAYL  LAAFSA**S**KAYLDH  AYLDHF**S**RALQYE  LQYEYA**S**KGIFVQ  KGIFVQ**S**LIPFYV  PFYVAT**S**MTAPSN  TSMTAP**S**NFLHRC  NFLHRC**S**WLVPSP  CSWLVP**S**PKVYAH  YAHHAV**S**TLGISK  VSTLGI**S**KRTTGY  RTTGYW**S**HSIQFL  TGYWSH**S**IQFLFA  ANILNR**S**LRKEAL  MEVTDM**S**QEDIEP  DERDEH**S**ETIIEL  ETIIEL**S**EERDGL  EMAQKQ**S**MDESLH  LEMENQ**S**LTKTVE  KENQRL**S**KKVEIL  ETLREN**S**ERQIKI  HLNQTV**S**SLRQRS  ALEQEN**S**ELEREN  LKKTLD**S**FKNLTF  QKTLEN**S**NKKIQQ  IGIYKE**S**CVRLKE  EERMVQ**S**SPPISG  ENSTLN**S**QSTSLM  STLNSQ**S**TSLMNQ  LENENE**S**VIKERE  EYESLI**S**KHGTLK  KHGTLK**S**AHKNLE  MRKLIK**S**KKDINR  TLTPTR**S**DSSEGF  TTAINF**S**TVNSSA  NFSTVN**S**SAGFRS  SSAGFR**S**KQLVNN  TSFEDI**S**PQGVSD  SGRTST**S**NSNNNA  AWSSSG**S**SPIQYL  ESNKLT**S**VQIKSS  SCGLAR**S**VSGKTP  IKLVKE**S**SLSRQS  TLPRAS**S**VISTAE  RASSVI**S**TAEGTT  DKDERI**S**CLEAYV  AYVTLT**S**KSSRFT  TEILKM**S**QRHSGQ  AGNGAD**S**PPIVNS  RDFCST**S**SFQDSG  RRRLNV**S**FALLKG  LKGDFE**S**QNSSLE  NSSLES**S**ISQVIN  SRANNF**S**PLVTST  TSTIDD**S**KDDCSL  FEVECI**S**PIQGNN  GNNFKD**S**ITHDFS  LLGSSV**S**GTTCGT  NIRFNA**S**QILSPS  SQILSP**S**PEVRGS  SPEVRG**S**ISTPED  ISTPED**S**GFNSLS  GRSRRL**S**TLREQS  QIVHPD**S**EKRAAA  KRAAAA**S**AISEGQ  AAASAI**S**EGQLSS  ISEGQL**S**SDESGD  SEGQLS**S**DESGDL  QLSSDE**S**GDLTFS  FSLKNL**S**KTPALQ  TAESLC**S**VWKVSR  CSVWKV**S**RNWREI  TQLKTD**S**EGAVLN  LQLLNR**S**ALRSVQ  QARIPG**S**QREQGS  VLTPLA**S**SSVTHL  LTPLAS**S**SVTHLS  KKRGLC**S**RTACGF  ADPDNL**S**DSLFSG  PDNLSD**S**LFSGDE  LSDSLF**S**GDEENA  INGNWI**S**ASSINE  GNWISA**S**SINEAR  NWISAS**S**INEARI  SVSDSG**S**DALRSG  GSDALR**S**GLTVPT  RLLDRR**S**RSGKGR  LDRRSR**S**GKGRGL  NLGEMK**S**GVPVLA  VPVLAV**S**LALEGK  ALEGKA**S**HREMTS  SHREMT**S**KLLSDL  MTSKLL**S**DLCGTV  LCGTVM**S**TTDVEK  TTDVEK**S**FDKLLK  CNTYID**S**YKGTVD  KATVLL**S**MSKGGK  TVLLSM**S**KGGKRK  SGGGQQ**S**VNHLVK  LKEYLL**S**GDISEA  LLSGDI**S**EAEHCL  IIMVLE**S**TGESTF  LESTGE**S**TFKMIL  ILDLLK**S**LWKSST  LKSLWK**S**STITVD  KSLWKS**S**TITVDQ  NLDVPH**S**YSVLER  DVPHSY**S**VLERFV  LRDLCP**S**RGRKRF  VMMDIG**S**TRLQAQ  LAYTRS**S**HYGGSL  HSPLDS**S**RSTRHH  PLDSSR**S**TRHHGL  LSPPPE**S**SWRRTM  SPPPES**S**WRRTMA  NRTSSD**S**ALHTSV  DSALHT**S**VMNPSP  PGPTPP**S**ILPSRR  DAKKLS**S**SSSRPR  EETAYP**S**LSGGNS  GLHSPL**S**HPSLQS  SPLSHP**S**LQSSLS  HPSLQS**S**LSNPNL  SLQSSL**S**NPNLQA  NPNLQA**S**LSSPQP  QPQLQG**S**HSHPSL  TSLGHP**S**LSAPAL  LSAPAL**S**SSSSSS  APALSS**S**SSSSST  PALSSS**S**SSSSTS  PVLGAP**S**YPASTP  APSYPA**S**TPGASP  QFSPTM**S**PTLSSI  TMSPTL**S**SITQGV  MSPTLS**S**ITQGVP  QPHTPK**S**LQQPGL  LPSQSC**S**VQSSGG  SGHGQQ**S**YHRPMS  LDPPGF**S**EGPGFL  DSSPGF**S**KEIAAA  TKDLIK**S**KWGSKP  IKSKWG**S**KPSNSK  KWGSKP**S**NSKSET  GSKPSN**S**KSETTL  KPSNSK**S**ETTLEK  IAHLKT**S**VDEITS  SVDEIT**S**GKGKLT  QLKARY**S**TTTLLE  QVLKAL**S**EEKDVL  VLKQQL**S**AATSRI  QLSAAT**S**RIAELE  RIAELE**S**KTNTLR  TNTLRL**S**QTVAPN  APNCFN**S**SINNIH  PNCFNS**S**INNIHE  TETAAH**S**LPQQTK  QTKKPE**S**EGYLQE  YNDLLA**S**AKKDLE  QTITQL**S**FELSEF  QLSFEL**S**EFRRKY  LNQLLY**S**QRRADV  EEEKKR**S**EELLSQ  RSEELL**S**QVQFLY  VQFLYT**S**LLKQQE  QITQLE**S**LKQLHE  KEVAKP**S**PGEGEV  LLKVAA**S**ALNRAD  DPPPGA**S**NILGLE  ILGLEA**S**GHVAEL  LIHAGL**S**GVGTAA  PLVTAG**S**QKKLQM  YKKEDF**S**EATLKF  LDCIGG**S**YWEKNV  INGPLF**S**KLLFKR  LLFKRG**S**LITSLL  RGSLIT**S**LLRSRD  ITSLLR**S**RDNKYK  QILPHF**S**TEGPQR  FENRRR**S**GGGPVL  GGGPVL**S**WQRLGC  QALLRA**S**GLPVQP  PCCALA**S**PRPDRA  LEGTLV**S**LARVPQ  VVGDGA**S**VDLLLL  LENERR**S**GGGPLE  EHRLQG**S**ELSLVP  ELAENT**S**GGDHPS  SGGDHP**S**TQGPRA  GTVTMG**S**GEEPGQ  GEEPGQ**S**GASLRT  PGQSGA**S**LRTGPM  PGQSGT**S**LRTGPM  GQAEQV**S**SMPMGS  QAEQVS**S**MPMGSL  EHEGLV**S**LRPVGL  EQEGPM**S**LGPVGS  SLGPVG**S**AGPVET  AGPVET**S**KGLLGQ  VEIAMD**S**PEQEGL  AEEFLR**S**LLGSIS  SLLGSI**S**CHVLCL  CLEHPG**S**ARFLLG  AQLVVH**S**AFEQDV  ERCHGV**S**VALRGD  AGPWDQ**S**LAFPLA  AFPLAA**S**GPTLAG  LDAARS**S**IRVVRV  AHGFNR**S**FCGRNA  YFARRA**S**LSVQDR  SVQDRY**S**PPNADG  ASPDDP**S**GLPGRS  CLPGTC**S**CLRHGE  ENYDDN**S**CLRDIG  CLRDIG**S**GGKYAE  NVLCRC**S**DHCRNR  GEVLGF**S**EVQRRI  LQTKSD**S**NYIIAI  GRFLNH**S**CEPNLL  IPVRID**S**MVPKLA  ELSYDY**S**GRYLNL  YLNLTV**S**EDKERL  CYCGAK**S**CTAFLP  AFLPFD**S**SLYCPV  FLPFDS**S**LYCPVE  YCPVEK**S**NISCGN  VEKSNI**S**CGNEKE  GNEKEP**S**MCGSAP  EPSMCG**S**APSVFP  MCGSAP**S**VFPSCK  APSVFP**S**CKRLTL  FCKGDE**S**LEDEER  LEDEER**S**GRPSEV  ERSGRP**S**EVDNDQ  ELNVNH**S**TVVRHL  NRRFEV**S**SSLILR  RRFEVS**S**SLILRN  MVTIWW**S**AAGLIH  AGLIHY**S**FLNPGE  LPHPPY**S**PDLLPT  FQEFVE**S**QSTDFY  EFVESQ**S**TDFYAT  GINQLI**S**RWQKCV  APRKGS**S**RVAKTN  RVAKTN**S**LRRRKL  RRRKLA**S**FLKDFD  RIKQIE**S**DRQNLL  VKRCPP**S**KKRTQS  SKKRTQ**S**IQGKGK  KGKGKR**S**SRANTV  VGRLEV**S**MVKPTP  LTPRFD**S**RVFKTP  ERIYNI**S**GNGSPL  SDLQRH**S**IAQLDP  GNIKKL**S**NRLAQI  ERCLKK**S**FQDSLE  EIGKRR**S**FIAAPC  ALENEK**S**KVKEAQ  LKGKLT**S**QQTVEP  QNQEIC**S**SGMDPN  DPNSDD**S**SRNLFV  PNSDDS**S**RNLFVK  LPGQGE**S**FQIEDQ  RTVSVR**S**SLKKHC  SICQFD**S**LDDFET  LDDFET**S**HLAGKS  SHLAGK**S**FEFERV  DILESK**S**EQTKSK  KSEQTK**S**KQRDTQ  KANRRK**S**KRMSKY  KYKENK**S**ENKKTV  KMHKSV**S**SNDAYN  PFRQKV**S**NDSNRE  QKVSND**S**NREENN  NNESEV**S**LCESSG  EVSLCE**S**SGSGDD  VSLCES**S**GSGDDS  LCESSG**S**GDDSDD  YIQNPT**S**NSDRPV  QNPTSN**S**DRPVTR  EKETEG**S**KPTKTP  KDITNV**S**LYPVVK  PKKNKA**S**PAVALP  KEPTLA**S**KLRRGD  KRALEV**S**PAKEAI  YVREFV**S**RFPDCR  HTLTGF**S**LVSLLS  TGFSLV**S**LLSFGY  SLVSLL**S**FGYLSW  LSFGYL**S**WDWAKP  EAGEQP**S**AAPPQP  DVGYHG**S**DIETPT  QPICTP**S**RSQLLT  ICTPSR**S**QLLTGR  HTGLQH**S**IIRPQQ  LQEAGY**S**THMVGK  FDTFLG**S**LTGNVD  NVAWGL**S**GQYSTM  GLSGQY**S**TMLYAQ  LYAQRA**S**HILASH  ASHILA**S**HSPQRP  HILASH**S**PQRPLF  VHTPLQ**S**PREYLY  YGFYNN**S**VIIFSS  NSVIIF**S**SDNGGQ  SVIIFS**S**DNGGQT  NGGQTF**S**GGSNWP  GLGFVH**S**PLLKRK  KRKQRT**S**RALMHI  LAGGTT**S**AADGLD  DVWPAI**S**EGRASP  ISEGRA**S**PRTEIL  NHAQHG**S**LEGGFG  NLERMA**S**VRQAVW  VWLFNI**S**ADPYER  AWGPWA**S**DEEEEE  EEGRAR**S**FSRGRR  GRARSF**S**RGRRKK  KICKLR**S**FFRKLN  STGRRF**S**EHKLCA  ESDSVF**S**ENTEDL  HHSHAN**S**QANHAQ  NKTSNS**S**QVSNEQ  SNSSQV**S**NEQDKI  LKTKFG**S**TADALV  DTMDLE**S**SSSEEE  MDLESS**S**SEEEKE  DLESSS**S**EEEKED  QSATDY**S**DPDNVD  GMTVHS**S**VHSNNL  QNQPRF**S**SPDEID  SEKIRL**S**EGEAKE  GGPAFL**S**KVEEDD  AINAKR**S**KEKNPG  MSSKLK**S**AQQESL  DKVFRA**S**ESQILS  SESQIL**S**IAEKML  IGDTHA**S**EVSQKP  AILLIY**S**FMFYLT  VTEQQI**S**EKLKTI  VISMNA**S**EFSEVQ  MNASEF**S**EVQIAL  IKSFEK**S**QKDLEV  NLLECE**S**ESEGQN  LECESE**S**EGQNKG  RTQTAI**S**VVEEDL  LPHPRW**S**AEASGK  SPSDPG**S**GTATMM  NSSSRG**S**SPTRVL  PFRPLG**S**LGPREY  VYEAID**S**RDGASC  DSRDGA**S**CAELVS  SCAELV**S**FKHPHV  PRLQMA**S**PEEKCQ  QTVIVQ**S**FLRAFQ  LVKKGK**S**KVGDML  AAELLM**S**CFRVCA  CFRVCA**S**DTRAGI  RAGIED**S**KKWGML  LIRAID**S**SNLKDD  IRAIDS**S**NLKDDY  NLKDDY**S**TAQRVT  RKAMFD**S**DFKQAE  QAEEYL**S**FAFEHC  FEHCHR**S**SQKNKR  EHCHRS**S**QKNKRM  EVTRAV**S**EGNLLL  LKTHQL**S**LDAFLV  HVKGYI**S**HQHQKL  HQKLVV**S**KQNPFP  PLLSEA**S**PEPIPE  PCPEAT**S**ESATEL  PEPATE**S**APEPTP  RTPMSW**S**RIKKIL  QATVAE**S**SCRNLC  ATVAES**S**CRNLCH  MHCWYS**S**YSHRTQ  EVCKVK**S**HRLCAL  CHESCG**S**YQRLQD  RCLWCN**S**TVHDDC  DCRRRF**S**KECCFR  FRSHRS**S**VIPPTA  IPPTAL**S**DPKGDG  GQLVVS**S**DFWNLD  FWNLDW**S**SACSCP  DWSSAC**S**CPLLIF  IFINSK**S**GDHQGI  GAFWNK**S**KSPLDI  FWNKSK**S**PLDILN  LMYDRL**S**VLIDVL  EEAAAT**S**AEKSAT  GTLSSI**S**SLKSED  GLDAKI**S**LDFNTR  KELLQR**S**YRKLEE  CDGETI**S**LPNLQG  VVLNIT**S**YAGGIN  IFYGQD**S**GNEMGA  AVDVTF**S**LKGLYD  KEFKKL**S**EIDWMN  VPEEKS**S**DTDSRS  KSSDTD**S**RSLRLK  EERKPK**S**GQSVQS  SGQSVQ**S**FIGNLW  VELPCP**S**PPTLHG  HGDEHL**S**WFCSPA  HLSWFC**S**PAAGSF  CSPAAG**S**FTTLVA  GKPGRE**S**RLRLLG  RLLGNY**S**LWLEGS  SLWLEG**S**KEEDAG  VLVLKG**S**QLSARA  LKGSQL**S**ARAADG  ARAADG**S**PCNVLL  CNVLLC**S**VVPSRR  LCSVVP**S**RRMDSV  PSRRMD**S**VTWQEG  VRGRVQ**S**FWGSEA  VQSFWG**S**EAALLL  CPGEGL**S**EPRSRR  GLSEPR**S**RRPRII  THNKGV**S**FSLAAS  NKGVSF**S**LAASID  SFSLAA**S**IDASPA  AASIDA**S**PALCAP  PALCAP**S**TGWDMP  VVILAL**S**IVLWRQ  APGRDA**S**IPQFKP  GRRLPG**S**EPRPEL  PRPELR**S**FSSEEV  VILKDC**S**VPNPSW  CSVPNP**S**WNKDLR  KKCEDG**S**WKRLPS  SWKRLP**S**YKRTPT  MKEEQM**S**QAQLFT  AQLFTR**S**FDDGLG  RPIPLC**S**VVMINS  SVVMIN**S**QLDKVE  GRKFFV**S**CNVQSV  VSCNVQ**S**VDEKTL  DEKTLY**S**EATSLF  LYSEAT**S**LFIKLN  PGRPPL**S**RRLLAL  ARDGPA**S**EAALQL  GKENKG**S**VEIMRK  LHGQAE**S**AAAVWK  PTERFR**S**LLQAVS  SLLQAV**S**ELEKKH  TDLANG**S**LSSSPE  NGSLSS**S**PEEMSG  SSPEEM**S**GAEEGR  EGRETS**S**GIEVEA  GIEVEA**S**DLSLSL  ASDLSL**S**LTGDDG  GGPNRT**S**TESRGT  NRTSTE**S**RGTDTE  SGEDKD**S**DSMEDT  NRVHDR**S**EEEEEE  ANRDQD**S**SDDERA  NRDQDS**S**DDERAL  LEDWVS**S**ETSALP  RERELG**S**SARFVY  RGTWLA**S**GSDDLK  AKFLPN**S**GDSTLA  LPNSGD**S**TLAMCA  LALEPD**S**PCTFLS  RQDRPA**S**KLVVTK  HLVNSE**S**KANITC  ITCLVY**S**HDGTEL  GTELLA**S**YNDEDI  DIYLFN**S**SHSDGA  IYLFNS**S**HSDGAQ  NFYGPK**S**EFVVSG  KSEFVV**S**GSDCGH  EFVVSG**S**DCGHIF  FLWEKS**S**CQIIQF  LPVLAT**S**GLDHDV  APTAEA**S**TELTGL  QTDLFD**S**HMLWFL  DADSDE**S**PSSSDT  SDESPS**S**SDTSDE  TNGQCF**S**VETLLD  PECDWW**S**LGVCMY  KKHPFF**S**GIDWDN  DDCLKN**S**ETMPPP  VGFTYT**S**SCVLSD  QEKLEL**S**RKLQES  DGPLTA**S**KDLEIK  ALAAEA**S**KDRKLR  LKQKQI**S**YSPGVC  QKQISY**S**PGVCSI  EKTRRE**S**QSEREE  TLYENL**S**IHNQQL  LADKKE**S**VAHWEA  LNKVKA**S**NIITEC  QPSVVI**S**QVIDMR  VTASQL**S**ASNNKC  NKFRDR**S**VYVPKE  YLLCFN**S**IGIYTD  YNAPYL**S**VYSENA  GSVSIP**S**ITKSRP  IPSITK**S**RPEPGR  RPEPGR**S**MSASSG  EPGRSM**S**ASSGLS  GRSMSA**S**SGLSAR  PSEGSL**S**SGGMDQ  GGMDQG**S**DAPARD  DFDGED**S**DSPRHS  SDSPRH**S**TASNSS  STASNS**S**NLSSPP  NSSNLS**S**PPSPAS  KSLSLE**S**TDRGSW  RSFLVK**S**KKAHTY  PRDQAP**S**NSPVLS  DQAPSN**S**PVLSTL  SNSPVL**S**TLFPNQ  EGPIVL**S**RPQDGD  RPQDGD**S**PLSDSP  DGDSPL**S**DSPPFY  DSPLSD**S**PPFYKP  PPFYKP**S**FSWDTL  FYKPSF**S**WDTLAT  SYRQAP**S**TMQSAF  SAFLEH**S**VSLYGS  FLEHSV**S**LYGSPL  SVSLYG**S**PLVPST  GSPLVP**S**TEPALD  EPALDF**S**LRYSPG  DFSLRY**S**PGMDAY  KCNKVF**S**TPHGLE  EVHVRR**S**HSGTRP  HVRRSH**S**GTRPFA  TFGHAV**S**LEQHTH  QHTHVH**S**QERSFE  VHSQER**S**FECRMC  GKAFKR**S**STLSTH  KAFKRS**S**TLSTHL  KRSSTL**S**THLLIH  THLLIH**S**DTRPYP  KRFHQK**S**DMKKHT  VCGKAF**S**QSSNLI  GKAFSQ**S**SNLITH  KAFSQS**S**NLITHS  SNLITH**S**RKHTGF  TGFKPF**S**CELCTK  GGSARK**S**TGRSSY  QRSRSR**S**RTRPAR  PQERPG**S**RRSLPG  RPGSRR**S**LPGSLS  SPSMEP**S**AATPFR  SIKRTK**S**QPKLDR  SHESLL**S**PSSAVE  VEALDL**S**MEEEVV  FEVTTS**S**GSKCFS  SGSKCF**S**CRSAAE  HPNKDN**S**RRVEHI  KKKERN**S**YLGLVS  SYLGLV**S**LPAASV  VSLPAA**S**VAGRQF  TKEEMA**S**ALVHIL  LKEVFA**S**WRQECS  LCPAIM**S**PSLFNL  PAIMSP**S**LFNLLQ  SNPETL**S**NTAGFE  DLGREL**S**SLHSLL  ALSTPG**S**GQLPGT  SGSSSI**S**AGLQKM  FFVTRS**S**GVQPSP  SSGVQP**S**PARSSS  AAGLPL**S**PRGLGD  GLGDSG**S**EGHSSL  LSSHSN**S**EELAAA  AAAKLG**S**FSTAAE  AKLGSF**S**TAAEEL  LQYPRP**S**SGTLAS  RLRQQS**S**SSKGDS  LQRCIV**S**PAGRHS  SPAGRH**S**ASLIFL  AGRHSA**S**LIFLHG  LIFLHG**S**GDSGQG  LHGSGD**S**GQGLRM  PTAPPR**S**YTPMKG  PMKGGI**S**NVWFDR  CPEHLE**S**IDVMCQ  IDEEVK**S**GIKKNR  ILIGGF**S**MGGCMA  AGVFAL**S**SFLNKA  GVFALS**S**FLNKAS  SFLNKA**S**AVYQAL  YQALQK**S**NGVLPE  DELVLH**S**WAEETN  WAEETN**S**MLKSLG  TNSMLK**S**LGVTTK  VTTKFH**S**FPNVYH  NVYHEL**S**KTELDI  AGTLNP**S**DPNTCS  SDPNTC**S**FWESFT  TTTTKE**S**HSRPFS  TTKESH**S**RPFSLL  CHGFYE**S**RGFCVP  CSCGNN**S**SCDPKS  GVCSCP**S**GLQPPN  GPSCDV**S**CSQGTS  SCSQGT**S**GFFCPS  FCPSTH**S**CQNGGV  FQTPQG**S**CSCPPG  WMGTIC**S**LPCPEG  FHGPNC**S**QECRCH  DGFYGL**S**CQAPCT  TCDREH**S**LSCHPM  GVCQAT**S**GLCQCA  PGTWGF**S**CNASCQ  GFSCNA**S**CQCAHE  GARCHL**S**CPEGLW  PGFRGP**S**CQRSCQ  GPSCQR**S**CQPGRY  TFGANC**S**QPCQCG  HLAVAY**S**SGRLDG  SGRLDG**S**EYVMPD  MPDVPP**S**YSHYYS  PSYHTL**S**QCSPNP  PLDRGS**S**RLDRSY  YNKGLI**S**EEELGA  LGASVA**S**LSSENP  ASVASL**S**SENPYA  YDSPKN**S**HIPGHY  LCGVGP**S**RGCRRR  EERIEA**S**AGVGGG  GLCAGP**S**PPPPRC  PPPPRC**S**LLELPP  LVEIFA**S**LPGTDL  PGTDLP**S**LAQVCT  LEITGV**S**CRDVYA  VKKDEF**S**TKCNQT  TDHHRM**S**GGRQEE  RIYLPP**S**RPDDLI  FKGTYG**S**HGLEIV  LEIVML**S**FHGRRA  RNFNEL**S**RIVLEV  RQGPRE**S**QPSPAQ  PRESQP**S**PAQPRA  PRAEAP**S**KGPDGT  VLPVGV**S**SRNEDY  LPVGVS**S**RNEDYP  AGHGFT**S**PERTPG  VWLELK**S**FSLYSR  LELKSF**S**LYSRVQ  KSFSLY**S**RVQATF  RNADAP**S**PQAFDE  QELANS**S**DATLPD  PPTMKS**S**EFFEML  TPTSLG**S**SICEEE  LGNLIL**S**VKCEEA  LRVILR**S**KLKTVH  GLSKLP**S**VPQIAK  ASQMIV**S**YDEHEV  VPDMIA**S**NFLHAY  HGGFLE**S**FKRAIR  RAIRVR**S**HSMETM  IRVRSH**S**METMVG  PRLHTG**S**EGQGDS  SEGQGD**S**RARCDS  SRARCD**S**TSSTPK  RCDSTS**S**TPKTPD  TPDGGH**S**SQEIKS  PDGGHS**S**QEIKSE  SSQEIK**S**ETSSNP  ETSSNP**S**SPEICP  TSSNPS**S**PEICPN  SSSSTS**S**VSSTAG  STSSVS**S**TAGEGE  EGDSGG**S**QPSTTS  SGGSQP**S**TTSPFK  QEVFVY**S**PSPSSE  VYSPSP**S**SESPSL  YSPSPS**S**ESPSLG  ATPIIM**S**RSPTDA  SPTDAK**S**RNSPRS  DAKSRN**S**PRSNLK  FRFDKL**S**HASSGA  HPSAEQ**S**SHIDVV  VFDRLG**S**LEYDEN  ISPDRN**S**VPQEGQ  DGYEQI**S**SDEDGI  VPLLYF**S**CPYKTT  GRQNEK**S**GYQKLL  HLAEKT**S**SLPNHS  ENEVEA**S**MDMDLL  DMDLLE**S**SNISEG  SEETDH**S**DLLGTL  QLCDLA**S**PTALLI  LLDALA**S**HKACKL  IALILP**S**SSEGSI  LILPSS**S**EGSISE  LEQLSN**S**LPNKEL  NKELMT**S**ICDCLL  RVVSTF**S**KDTGEL  TGELAS**S**FLEFMR  LKQLLQ**S**KEESPE  KLVLEH**S**KDDDNL  VLSAPE**S**LQNLFN  SDFDLH**S**ELERSF  LERSFL**S**EPSSPG  SFLSEP**S**SPGRTK  HETFIT**S**SGKSEY  QRKQNT**S**RPPSMH  SSRGGF**S**GNRGGR  TRGSSW**S**AQNTPR  STGYRP**S**PRDRAS  SPRDRA**S**RGRGGL  RGGLGP**S**WASANS  LGPSWA**S**ANSGSG  KFVSGG**S**GRGRHV  VQKLVE**S**GVDVNS  SGVDVN**S**QNEVNG  NHGQVV**S**YLLKSG  VSYLLK**S**GADKEI  MPVQLT**S**RREIRK  PQLKKE**S**ELPFVP  TPTAED**S**AQMQNG  MQNGGP**S**TPPASP  PSTPPA**S**PPADGS  SPPADG**S**PPLLPP  FPRDHT**S**LALVQN  VQNGDV**S**APSAIL  GDVSAP**S**AILRTP  ILRTPE**S**TKPGPV  VCQPPV**S**QSRSLF  QPPVSQ**S**RSLFSS  PVSQSR**S**LFSSVP  QSRSLF**S**SVPSKP  SRSLFS**S**VPSKPP  LFSSVP**S**KPPMSL  PSKPPM**S**LEPQNG  VRIQNP**S**LRENDF  ELVLMI**S**ENNFLF  LFRNAA**S**TLTERP  YIAYME**S**QGAHRA  ETYDNI**S**EILIAT  PLQQVA**S**GRAGVF  YRHLAN**S**KKYQTP  KNRIYN**S**PIYGAD  IYGADI**S**GSLFDE  GADISG**S**LFDENT  EDMDLY**S**INYLHL  RELFPG**S**SRGCGA  HKVALI**S**PTVLKE  DYGKMA**S**QCSCGE  KMASQC**S**CGEARV  EARVTF**S**MDAFVR  EPRVPA**S**QELSTQ  PASQEL**S**TQKEVQ  GLRQLP**S**HWARHS  SHWARH**S**PWPMAA  WPMAAR**S**GTRCHT  CHTLVC**S**SLPRRS  HTLVCS**S**LPRRSA  SSLPRR**S**AVSGTA  PRRSAV**S**GTATQP  RAAAVH**S**SKKPSS  AAAVHS**S**KKPSST  HSSKKP**S**STPSST  SSKKPS**S**TPSSTP  KPSSTP**S**STPGPS  PSSTPS**S**TPGPSA  SSTPGP**S**AQIIHP  AQIIHP**S**NGRRGR  GTTCTA**S**GPEPEP  DKPVPL**S**PGLQHP  QHPVKA**S**GCSWAP  GKKRKR**S**RKESYS  SRKESY**S**IYVYKV  AMGIMN**S**FLNDIF  RIAGEA**S**RLAHYN  AHYNKR**S**TITSRR  KRSTIT**S**RRSRRP  TITSRR**S**RRPCAC  ACCCPA**S**WPSTPC  CPASWP**S**TPCPRA  PRAPRR**S**PSTPAP  APRRSP**S**TPAPSE  PSTPAP**S**ESLPGP  TPAPSE**S**LPGPGA  PGPGAR**S**LPPSLP  ARSLPP**S**LPPRVA  VAGCFV**S**KGSFQG  CFVSKG**S**FQGHLT  QGHLTT**S**VKESFL  TTSVKE**S**FLCCQS  SFLCCQ**S**QLMFLA  QLMFLA**S**RLVNFR  WKNTPL**S**QVKFYR  GKFLHF**S**KENQTL  KENQTL**S**MGAATV  QSRGQY**S**CSGQVM  RGQYSC**S**GQVMYI  QTFTQT**S**ETAMVQ  FPPPVL**S**AIPSPE  VLSAIP**S**PEPREG  PEPREG**S**LVTLRC  KLHPLR**S**ALRLLF  ALRLLF**S**FHKDGH  RVQAPV**S**RPVLTL  SPPILY**S**FYLDEK  KIVGNH**S**APCGGT  PCGGTT**S**LLFPVK  QDAGNY**S**CEAENS  SCEAEN**S**VSRERS  EAENSV**S**RERSEP  SVSRER**S**EPKKLS  SEPKKL**S**LKGSQV  VLFTPA**S**NWLVPW  LLVYVR**S**WRKAGP  KAGPLP**S**QIPPTA  DEGVVY**S**VVHRTS  SVVHRT**S**KRSEAR  HRTSKR**S**EARSAE  KRSEAR**S**AEFTVG  TVGRKD**S**SIICAE  VGRKDS**S**IICAEV  VRCLQP**S**EVSSTE  LQPSEV**S**STEVNM  QPSEVS**S**TEVNMR  TEVNMR**S**RTLQEP  EDLPRP**S**ISAEPG  LPRPSI**S**AEPGTV  TVIPLG**S**HVTFVC  FRLERE**S**RSTYND  LERESR**S**TYNDTE  NDTEDV**S**QASPSE  EDVSQA**S**PSESEA  VSQASP**S**ESEARF  QASPSE**S**EARFRI  ARFRID**S**VSEGNA  FRIDSV**S**EGNAGP  YKPPKW**S**EQSDYL  PKWSEQ**S**DYLELL  LLVKET**S**GGPDSP  TSGGPD**S**PDTEPG  PDTEPG**S**SAGPTQ  DTEPGS**S**AGPTQR  GPTQRP**S**DNSHNE  QRPSDN**S**HNEHAP  NEHAPA**S**QGLKAE  YILIGV**S**VVFLFC  KQGPPR**S**KDEEQK  DRETDT**S**ALAAGS  SALAAG**S**SQEVTY  ALAAGS**S**QEVTYA  RTARAV**S**PQSTKP  RAVSPQ**S**TKPMAE  TKPMAE**S**ITYAAV  NANVDN**S**ASPSVA  NVDNSA**S**PSVAQL  DNSASP**S**VAQLAG  AKETPA**S**KPTRRK  RRKPPC**S**LPLFPP  KSPPNA**S**HPPKFK  PKFKVK**S**SPLIEK  SPPSTP**S**SPGVRS  PPSTPS**S**PGVRSR  SSPGVR**S**RPSEAE  GVRSRP**S**EAEEVP  AEEVPV**S**FDQPPE  DQPPEG**S**HLPCYN  KVRTRG**S**IKRRPP  IKRRPP**S**RRFRRS  DFRAVE**S**SQQNGA  FRAVES**S**QQNGAK  GDEVLP**S**KSKAPG  EVLPSK**S**KAPGSP  APGSPL**S**SEGAAG  PGSPLS**S**EGAAGE  KPPLRR**S**PSRTEK  PESPQT**S**GPEAEN  VGPEHD**S**QETKKL  VKETPH**S**PPGGVK  VLEPGC**S**PQTGPA  PAQLET**S**SEVQSE  AQLETS**S**EVQSEP  TSSEVQ**S**EPAVPK  ILKNKT**S**TTSSMV  NKTSTT**S**SMVASA  KTSTTS**S**MVASAE  TSSMVA**S**AEQPRR  AEQPRR**S**VDEELS  SVDEEL**S**KKSQKW  EELSKK**S**QKWDEI  MKIDEP**S**PPYHSM  PSPPYH**S**MMGDDE  EEDSDL**S**PEEREK  LARQLI**S**KDLHDD  ETADGE**S**MNTEES  SMNTEE**S**NQGSTP  EESNQG**S**TPSDQQ  NQGSTP**S**DQQQNK  AEAPEP**S**SAGAQA  EAPEPS**S**AGAQAA  SEELIK**S**DQVNGV  NGVLVL**S**LLDKII  MEGAVQ**S**IQGELS  SIQGEL**S**KLGKAH  KAHATT**S**NTVSKL  TTSNTV**S**KLLEKV  VKTVRG**S**LERQAG  KLSISK**S**LKESEA  SKSLKE**S**EALPEK  EEVIEE**S**RAERIK  AERIKR**S**GLRRVD  DFKKAF**S**KEKMEK  REKLKT**S**RDKLRK  HVVYAR**S**KTAVYK  VIFGEF**S**WPKNVK  EYYTLD**S**ILFLLN  LNNVHL**S**HPVYVR  YLNGEA**S**TSASID  NGEAST**S**ASIDRS  EASTSA**S**IDRSAP  SASIDR**S**APLEIG  EIGLQR**S**TQVKRA  QTEQIR**S**LSEAMS  EQIRSL**S**EAMSVE  SLSEAM**S**VEKIAA  IMAKKR**S**TIKTDL  TALKQR**S**FVDAEV  VTRDIV**S**RERVWR  RTTILQ**S**TGKNFS  STGKNF**S**KNIFAI  IFAILQ**S**VKAREE  HGMTLK**S**VTEGAS  SVTEGA**S**ARKTQT  PVPRPV**S**QARPPP  PNQKKG**S**RTPIII  IPAATT**S**LITMLN  DLKFVP**S**DEKKKQ  PGGTAI**S**VTVPYR  WLLPDG**S**PVDIFA  VTVLEL**S**YHKRHL  GLLVDL**S**PDGLMI  YQTAIE**S**ARQAGD  ARQAGD**S**AKMRRY  LENLLA**S**IRKGNA  IGKGPA**S**TPTYSP  VTLEGP**S**ATAPAS  SATAPA**S**SPGLAK  PLAQLQ**S**RQRDYK  HFRVAK**S**FDAVLE  AVLEAL**S**RGEPVD  GEPVDL**S**CLPPPP  DPPSPP**S**QPPTPA  TPATAP**S**TTEVPP  TKPTQQ**S**LVGVLE  QPKAPP**S**RTPQSG  RTPQSG**S**APTAKA  EPMLEA**S**RNGLPV  RPGPGL**S**QEAARR  EMCLNH**S**NQFTQL  FEQRTF**S**VIKIFP  KIFPDL**S**SNDMLL  IFPDLS**S**NDMLLF  AQKDKT**S**VIKNTD  VIKNTD**S**PEFKEQ  KLCINR**S**HRGFRR  PAPARE**S**GNRSAR  RESGNR**S**ARPLHS  SARPLH**S**LSVLAF  RPLHSL**S**VLAFDQ  QDIMQR**S**QWQRAQ  RLGNDG**S**RDAAKE  HITGAI**S**FVNEIP  FTLPMM**S**TLYRLA  PISHRH**S**VKSQEP  KRYLFR**S**FKATKF  TKLVVD**S**HVQYRL  QFEGRH**S**KGVAKT  TILQHL**S**EAWRCW  TVHWLE**S**RRFSPI  CNVMLE**S**RFEKMY  DMNHTN**S**YGIIRG  TVQWEN**S**FVSVYS  LRVDDE**S**MQRFHN  ILMASG**S**TTFTKI  IKIGLN**S**KMPSRF  GGLGML**S**MGHVLI  ASGFEE**S**MKWKKL  TIHPRK**S**YKMNSS  SYKWNV**S**RPSLLA  LAYNLH**S**AYGNWF  GLQLYS**S**EPTEPY  PTEPYL**S**SQNYGE  DWLKTI**S**SYTAFS  SSYTAF**S**RLILIL  ILGMEI**S**APSQQR  MEISAP**S**QQRQQI  GDEIIT**S**TTSNYE  WRVRAI**S**AANLHL  NHIYVS**S**DDIKET  NESPQL**S**PQDVTT  TIIITC**S**FTPGSC  AYKLTP**S**GYEWGR  HEVHRP**S**HFLNFA  QEGEVY**S**ADREDL  PRQPGP**S**QWPPED  PAEPVA**S**GPRPWA  QQMLRD**S**QLKVAL  LLRMKI**S**SLEASG  LRMKIS**S**LEASGS  ISSLEA**S**GSPEPG  NVVKLL**S**SRRTQD  VVKLLS**S**RRTQDR  AQLQES**S**QKLDLL  PAHPLR**S**RVTREL  PGYPQP**S**GTPVKP  PAAALA**S**SPSEGW  AAALAS**S**PSEGWL  ALASSP**S**EGWLRT  GRGELA**S**EVLAVL  GQVAEQ**S**WDQTFV  NACHQL**S**LSLVPQ  RQERIF**S**KRRGQD  QDFLRA**S**QMNLGM  NLLPPC**S**SPSTIS  LLPPCS**S**PSTISP  TTLREA**S**DPATPS  LPQEPT**S**EETPRT  RTRRGP**S**PPASPT  GPSPPA**S**PTRKPP  KKQEVL**S**RDEIES  SRDEIE**S**LYCEKR  GHPFLL**S**LLACFQ  LACFQT**S**SHACFV  ACFQTS**S**HACFVT  GFGDRT**S**TFCGTP  PYPGFL**S**VQGLEF  TPPAPH**S**LLTARQ  RVLLLL**S**LLHCAG  AGQPVE**S**FPLDFT  VEGNMD**S**FMAQVK  FMAQVK**S**LAQSLY  VKSLAQ**S**LYPCSA  HLLLNT**S**VTCNDG  VTCNDG**S**PAGYYL  GYYLKE**S**RGSRRW  LKESRG**S**RRWLLF  NRENCD**S**RYDTMR  TMRRLM**S**SRDWPR  MRRLMS**S**RDWPRT  TGTGIL**S**SQPEEN  GTGILS**S**QPEENP  VFIPYC**S**SDVWSG  FIPYCS**S**DVWSGA  DVWSGA**S**SKSEKN  VWSGAS**S**KSEKNE  SGASSK**S**EKNEYA  LLGRGL**S**GAKVLL  LLLAGS**S**AGGTGV  VRGLAD**S**GWFLDN  FAPACL**S**HEIIIR  HEIIIR**S**HWTDVQ  VQVKGT**S**LPRALH  LHCWDR**S**LHDSHK  DRSLHD**S**HKASKT  HDSHKA**S**KTPLKG  PVHLVD**S**CPWPHC  WPHCNP**S**CPTVRD  PQGLEP**S**ELLGML  ALGLAL**S**LRLALA  RLALAR**S**GAERGP  ERGPPA**S**APRGDL  LMFLLD**S**SASVSH  MFLLDS**S**ASVSHY  LLDSSA**S**VSHYEF  DSSASV**S**HYEFSR  VSHYEF**S**RVREFV  FPFGQH**S**SGEAAQ  QDAVRA**S**AQRMGD  QLFAEA**S**GARPGV  WVTDGG**S**SDPVGP  VTDGGS**S**DPVGPP  VTVFIV**S**TGRGNF  GNFLEL**S**AAASAP  ELSAAA**S**APAEKH  VQELRG**S**ILDAMR  HATEIT**S**SGFRLA  ATEITS**S**GFRLAW  PLLTAD**S**GYYVLE  VLELVP**S**AQPGAA  VALVPE**S**NVRLLR  EAGPGA**S**GPESGA  GASGPE**S**GAGPAP  PERIVI**S**HARPRS  SHARPR**S**LRVSWA  PRSLRV**S**WAPALG  WAPALG**S**AAALGY  VTAAFR**S**GRESAL  FRSGRE**S**ALSAKA  GRESAL**S**AKACTP  PAGMAS**S**QGGPAL  VPADVV**S**SQGVPS  EQHRQG**S**DGNHTI  NHTIPA**S**SLHSMT  HTIPAS**S**LHSMTN  SMTNFI**S**GLQRLH  LEFLRP**S**SSNHSV  RTRRRV**S**ASRRAR  RARAGG**S**QRTDSA  GSQRTD**S**ARLRAP  DAYFQV**S**RTQPDL  PATTYD**S**ETRNPV  ELQVSS**S**SDSDSD  DSDSDS**S**AEYGGV  VDQAEE**S**GAVILE  TLPKQP**S**PQKSEP  FGYRCI**S**TWLKGQ  LRALDT**S**EQERMK  LQQPRG**S**QAWVLS  VLSCSP**S**SQGQHK  AYCDAL**S**CLVISQ  RGLAFS**S**YLRGLL  AGLANG**S**ILVYDV  YDVRNT**S**SHVQEL  CPLVSL**S**YMPRAA  GTLEDA**S**FWEQKM  EQKMDF**S**HWPHVL  FQTENS**S**RHCLVT  SVLMEM**S**YRLDDT  KNAIFQ**S**PENDGN  WDAASG**S**LLQDLQ  LTERLG**S**GTYATV  KCVAKK**S**LNKASV  KSLNKA**S**VENLLT  KDFQWD**S**DNIYLI  CAGGDL**S**RFIHTR  FMQQLA**S**ALQFLH  LHERNI**S**HLDLKP  PQNILL**S**SLEKPH  QNILLS**S**LEKPHL  GFAQHM**S**PWDEKH  ARVDLW**S**MGVILY  GQPPFA**S**RSFSEL  PPFASR**S**FSELEE  FASRSF**S**ELEEKI  LEEKIR**S**NRVIEL  PLRPLL**S**RDCRDL  LLERDP**S**RRISFQ  DPSRRI**S**FQDFFA  DLEHMP**S**GESLGR  HMPSGE**S**LGRATA  DSAAAL**S**LYCKAL  KVGQYV**S**RAEELK  ELKAIV**S**SSNQAL  KAIVSS**S**NQALLR  LLRQGT**S**ARDLLR  LDLYQH**S**LGELLL  QVKMRE**S**RWEADT  LDKEGL**S**ESVRSS  GGEPDG**S**ARRPRP  GEIAHK**S**VQPQSH  KSVQPQ**S**HKPQPT  ESPKTK**S**DESGEE  KTKSDE**S**GEEKNG  PREKLA**S**RPTRPP  HDQDET**S**SVKSDG  AGGARA**S**FRGRGR  YYFDNV**S**STELYS  YFDNVS**S**TELYSV  PITLIA**S**FHRVQA  ALTTDI**S**LIFAAL  FAALKD**S**KVVEIV  PPIVDY**S**QTDFSQ  YSQTDF**S**QLLNCP  SLPDLD**S**ENWIEV  PKKSEE**S**RFSHLT  RTGNHT**S**RAKMSA  TSRAKM**S**AELAKV  KFEPEY**S**QIKQEV  KKVNMI**S**REQFDT  TPQLKD**S**SQTSRF  PQLKDS**S**QTSRFY  HRPRTA**S**ISSSPS  PRTASI**S**SSPSEG  GTPTVG**S**YGCTPQ  PKFQHP**S**HELLKE  RLGIGQ**S**QEMNTL  TLFRFW**S**FFLRDH  CPSQSS**S**RPAAMI  EDAKWT**S**QHSNTQ  WALALL**S**AFSATQ  ALLSAF**S**ATQARK  GFWDYF**S**QTSGDK  DYFSQT**S**GDKGRV  PATLKD**S**LEQDLN  EKLRPL**S**GSEAPR  LRPLSG**S**EAPRLP  LLQGLQ**S**RVVHHT  FHPYAE**S**LVSGIG  YAESLV**S**GIGRHV  VQELHR**S**VAPHAP  APHAPA**S**PARLSR  ASPARL**S**RCVQVL  RCVQVL**S**RKLTLK  QLREEL**S**RAFAGT  PDPQML**S**EEVRQR  PPPPGH**S**AFAPEF  EFQQTD**S**GKVLSK  DSGKVL**S**KLQARL  WEDITH**S**LHDQGH  VRGRND**S**GEENVP  YLIQDS**S**ILQKVL  FIEALL**S**VDPGRF  GMKPER**S**LQNNGL  LFIGTL**S**CHPHGV  DKNKTI**S**SEALDI  QMKPAL**S**HLGDKG  STLSLN**S**ESTSSR  LSLNSE**S**TSSRHN  SRHNSE**S**ESVPSS  ESESVP**S**SMFILE  SESVPS**S**MFILED  FGSSST**S**TFFLDI  KGGKLS**S**ESKTSN  RTLTEP**S**VDFNHS  TLQLET**S**FMGNKH  DTGSTP**S**IGENDL  TTSGIS**S**MSSSPS  GISSMS**S**SPSRET  MDTDCG**S**MSTVVS  SMSTVV**S**TKTIKT  PQSNHL**S**LSKSNS  SNHLSL**S**KSNSVS  LPRRAQ**S**LKAPSI  KANSFE**S**RLTPSR  VRSMVS**S**ATYGGS  SATYGG**S**DDYIGL  SIHSDA**S**LFLEST  PLEVVP**S**KFSGIS  GVSQEG**S**ASSTKS  LVINLS**S**SVSTKC  NLSSSV**S**TKCHET  EVSHLL**S**HCTFRL  RERGQD**S**LAGLVL  LHRAKY**S**RFRNES  SRFRNE**S**ITSLDE  TSLDEG**S**SGGSVG  EDATLP**S**QESPTP  SRDPGS**S**GPSSPE  GSSGPS**S**PETSLS  ETSLSR**S**GTAPPP  TALTPD**S**CPLPGP  GEPTLR**S**RQDRHF  CVEVLQ**S**MRSLDF  VLQSMR**S**LDFGMR  VTREAI**S**RLCEAV  PPVKFL**S**TVLGKS  KSNLQF**S**GMNIKL  NIKLTI**S**TCSLTL  LTISTC**S**LTLMNL  MAQDVI**S**TIGQAF  QYLKNP**S**LNTSCE  NPSLNT**S**CESEEV  LNTSCE**S**EEVHID  PGNSKC**S**SVYENC  ENCLEQ**S**RAIGNV  HPRGVQ**S**QRDTSL  QSQRDT**S**LLKHTC  LQSTPG**S**AGNQRS  SAQPLG**S**PWHCGK  TAQPAS**S**HSLPHI  QPASSH**S**LPHIKQ  CYHGKL**S**RKAAES  VRESAT**S**PGQYVL  RYHMDN**S**LPIISS  PIISSG**S**EVSLKQ  RAMSFQ**S**IIHLSL  QSIIHL**S**LDSPVH  IHLSLD**S**PVHAVC  EICLDL**S**GCAPQK  VANTVI**S**EKEDAT  TIWWPL**S**DPTYAT  ATVKMT**S**PSPSVD  VKMTSP**S**PSVDAD  MTSPSP**S**VDADKV  LTGIEV**S**LEVDIY  NGQVEM**S**SDKQAK  LNVQGP**S**CILKKY  WPQKDN**S**STFELV  EAIAFP**S**AEFSGL  EFSGLI**S**YSVSLV  SGLISY**S**VSLVEE  LISYSV**S**LVEESQ  DTVTKL**S**EKSNSQ  TKLSEK**S**NSQVAS  LSEKSN**S**QVASVY  SNSQVA**S**VYEDPN  PMKYSL**S**PGIGYM  EDHKVA**S**MDSIGN  KVASMD**S**IGNLMV  IGNLMV**S**PPVKVQ  GRVLIG**S**SFYPSA  AEGRAM**S**KTLRDF  FLLLLA**S**PSACYK  LLLASP**S**ACYKLF  QLLADE**S**LKKQNE  KLTNIP**S**DQQPKR  DGDPFA**S**KLSWEA  PFASKL**S**WEADLV  TIIGIL**S**TFGNGY  GYVLYM**S**SRRKKK  YVLYMS**S**RRKKKL  VCDLGI**S**VVGKPF  KPFTII**S**CFCHRW  FFFGCG**S**LITMTA  ITMTAV**S**LDRYLK  LKICYL**S**YGVWLK  AIWAYA**S**FWTTMP  PEPFGT**S**CTLDWW  WWLAQA**S**VGGQVF  TAVIVF**S**YVKIIA  IIAKVK**S**SSKEVA  IAKVKS**S**SKEVAH  AKVKSS**S**KEVAHF  EVAHFD**S**RIHSSH  FDSRIH**S**SHVLEM  DSRIHS**S**HVLEMK  IPYAVV**S**VWSAFG  AVVSVW**S**AFGRPD  AFGRPD**S**IPIQLS  SIPIQL**S**VVPTLL  PTLLAK**S**AAMYNP  KATKKK**S**LEGFRL  VTTVRK**S**SAVLEI  TTVRKS**S**AVLEIH  NNPRKF**S**EKIALH  FQTPFQ**S**SGLDTS  SSGLDT**S**RTTRHH  PHRRPL**S**VDKHGR  SPPADT**S**WRRTNS  DSALHQ**S**TMTPTQ  TPTQPE**S**FSSGSQ  PTFPAL**S**SSSSTG  FPALSS**S**SSTGNL  PALSSS**S**STGNLA  ALSSSS**S**TGNLAA  GMSTPG**S**SPQHRP  HRPAGV**S**PLSLST  VSPLSL**S**TEARRQ  VAMDAL**S**LEQQLP  PPPPPA**S**QQPPPP  LAVTVP**S**SLPQSP  PSSLPQ**S**PPENPG  MGIDIA**S**APALQQ  LQQYRT**S**AGSPAN  ANQSPT**S**PVSNQG  SPTSPV**S**NQGFSP  GFSPGS**S**PQHTST  SSPQHT**S**TLGSVF  NAISSS**S**LYSPGS  SSSSLY**S**PGSTLN  GSTLNY**S**QAAMMG  MMGLTG**S**HGSLPD  LTGSHG**S**LPDSQQ  TGESPP**S**LSKELT  ESPPSL**S**KELTSS  STSAVL**S**GFVLGA  QHLNTD**S**DTEGFL  KGEAKN**S**ITDSQM  KNSITD**S**QMDDVE  LFSFYN**S**SGEVNE  FSFYNS**S**GEVNEQ  ALKKIL**S**NVKKNV  YKFRRH**S**DQIMTF  NLQEHF**S**NQDLVF  FLLLTP**S**IITESC  PSIITE**S**CSTHRL  THRLEH**S**LYKPQK  VANLGM**S**EQLGYK  LGYKTV**S**GSCMST  YKTVSG**S**CMSTGF  VSGSCM**S**TGFSRA  CMSTGF**S**RAVQTH  AVQTHS**S**KFFEED  INEMYA**S**LQEELK  LQEELK**S**ICKKVE  CKKVED**S**EQAVDK  RTFFPN**S**EFLHSC  LHSCVM**S**LKNRHV  LKNRHV**S**KSSCNY  NRHVSK**S**SCNYNH  RHVSKS**S**CNYNHH  PEASPA**S**TPQIIK  RWQFKR**S**RLLDTQ  DTQDKR**S**KADTGS  KADTGS**S**NQDKAS  SNQDKA**S**KMSSPE  PEVRVL**S**SLLGLA  EVRVLS**S**LLGLAL  LWFPLD**S**HARARP  FHGKRY**S**PGESWH  RYSPGE**S**WHPYLE  CLRCTC**S**EGAHVS  SEGAHV**S**CYRLHC  VEPHTP**S**GLRAPP  LRAPPK**S**CQHNGT  QHGEIF**S**AHELFP  AHELFP**S**RLPNQC  NQCVLC**S**CTEGQI  PLPLPD**S**CCQACK  ACKDEA**S**EQSDEE  QSDEED**S**VQSLHG  EEDSVQ**S**LHGVRH  HPQDPC**S**SDAGRK  PQDPCS**S**DAGRKR  PAPTGL**S**APLSFI  GLSAPL**S**FIPRHF  RPKGAG**S**TTVKIV  HGGKTY**S**HGEVWH  KADPGH**S**EISSTR  PGHSEI**S**STRCPK  GHSEIS**S**TRCPKA  RVLVHT**S**VSPSPD  LVHTSV**S**PSPDNL  HTSVSP**S**PDNLRR  ALEHEA**S**DLVEIY  LLHQEL**S**CPLCLQ  TAECGH**S**FCRACL  TRPQAL**S**TNLQLA  EHLDPL**S**IYCEQD  VCGVCA**S**LGSHRG  VCASLG**S**HRGHRL  CMRKEK**S**VAVLEH  LAALEG**S**LDREAE  LRRELG**S**LNSYLE  ELGSLN**S**YLEQLR  KYCLVT**S**RLQKIL  QKILAE**S**PPPARL  IQLPII**S**DDFKFQ  ELTFDP**S**SAHPSL  LTFDPS**S**AHPSLV  PSSAHP**S**LVVSSS  HPSLVV**S**SSGRRV  PSLVVS**S**SGRRVE  SLVVSS**S**GRRVEC  GRRVEC**S**EQKAPP  VAHQQL**S**EGEHYW  RLHAVP**S**QGLWLL  EPRALR**S**PERRPT  RIGLYL**S**FGDGVL  FGDGVL**S**FYDASD  LSFYDA**S**DADALV  DLERNG**S**GGGGGG  GASLYD**S**EPRKKS  SEPRKK**S**VNMTEC  ECVPVP**S**SEHVAE  CVPVPS**S**EHVAEI  TYIVTP**S**RDKEPV  HHGSGG**S**GPGSLW  PGRKPF**S**SYRNDS  SYRNDS**S**SSLGSA  GSASTD**S**YFGGGT  PPSPAL**S**FAHNGN  WAQFER**S**PGGGPA  PAAPVS**S**SCSSSA  AAPVSS**S**CSSSAS  VSSSCS**S**SASSSA  SSSCSS**S**ASSSAS  CSSSAS**S**SASSSS  SSSASS**S**ASSSSV  TSCPRL**S**PPLHMA  LQPSDT**S**GSSSSS  SDTSGS**S**SSSSSS  TSGSSS**S**SSSSSS  SGSSSS**S**SSSSSS  GSSSSS**S**SSSSSS  SSSSSS**S**SSSSSS  SSSSSS**S**SSGLRR  SSSSSS**S**SGLRRK  GLRRKG**S**RDCSVC  KGSRDC**S**VCFESE  CSVCFE**S**EVIAAL  AGEPRT**S**VEAAVA  EACPEP**S**PLCPVP  YLVPTF**S**LPPAPG  VRVLNR**S**FEKAVH  ARYVPR**S**PPWAGA  LGPGQA**S**ASSPDD  PGQASA**S**SPDDGG  GQASAS**S**PDDGGR  EARQLK**S**CMKPVR  HPDVQE**S**VGPLVA  GDVPRS**S**PPVAFT  SPRDQA**S**GPDASE  ASGPDA**S**EGATGP  EATWGV**S**SENGGG  GGLEAV**S**GSEELL  PERALN**S**ALAEEI  EGEEGL**S**VTPSSP  GLSVTP**S**SPEGDS  LSVTPS**S**PEGDSP  GDSPKE**S**PPEILS  ILSGAR**S**VVATMG  LPWAEG**S**GCDGPV  GMGKDT**S**SLHMNR  MGKDTS**S**LHMNRV  IAGVTE**S**LGEAGT  SGKEPA**S**PVLLQG  QNPTLL**S**PLGAEV  VARPHV**S**SQDEKD  EKDAGP**S**LEPPKK  LEPPKK**S**PTLAVP  VPVALN**S**GVSLLV  RLGPEA**S**FSAEDL  DLSLFL**S**TRCVVV  CVVVLL**S**AELVQH  LCGVRD**S**EEFLDF  AVKKAI**S**EDSGCD  EDEKVV**S**YSKQQN  NLPTVT**S**PGNLMV  SPEDSP**S**VRMEAK  ENEYTI**S**VKAPNL  VKAPNL**S**SGNVSL  VSLKIY**S**GDLVVC  DKLLTE**S**LKNNIP  KNNIPA**S**GLHLFG  GALQAY**S**VANKHG  ADAVYE**S**MAHLST  DEDLYE**S**MAAFVP  IPGDGF**S**RATKDS  KFLEGN**S**MGMTNL  DDDEAF**S**VDLASR  FSVDLA**S**RPPVPV  PGNFYV**S**SESIRK  RDRPQS**S**IYDPFA  LNQKKR**S**ESFRFQ  QREKQK**S**GKQTDL  GDWKTD**S**TSSTAS  WKTDST**S**STASST  KTDSTS**S**TASSTS  STASST**S**NRSSTR  STSNRS**S**TRSLLS  NRSSTR**S**LLSVSS  VPEVTR**S**RSPGPP  EVTRSR**S**PGPPQV  DGTPTM**S**LERPPR  LTQSLD**S**DKPMEP  LKGLSS**S**SNRIAK  KGLSSS**S**NRIAKP  PSHASY**S**LDDVTA  SHSSIT**S**AASSMD  ITSAAS**S**MDSLDI  VHMSDD**S**SKTMMV  DNLMDK**S**HCGYSL  GYSLDW**S**LVETVS  KGKAKV**S**RDLVCF  HSNQSD**S**GVSDTQ  PAGHVR**S**QSIVSS  RSQSIV**S**SVFSEA  SKARME**S**MNRPYT  VTPYTA**S**QPSPPL  PPPPLP**S**QSAPSA  TIPAPT**S**TKTVAP  QPAFPA**S**YIPPSP  TLPKQQ**S**FCAKPP  CAKPPP**S**PLSPVP  PLSPVP**S**VVKQIA  FPPPPP**S**PVPAPP  TPQRNS**S**IKSSSG  NSSIKS**S**SGAEHP  KRPSVD**S**LVSKFT  PPAESG**S**PSKETL  SVVEFP**S**PPSDSD  EFPSPP**S**DSDFPP  PQPQQW**S**KMSVKK  RPKRND**S**TRLTQA  VPQVPT**S**PKSSLS  SPKSSL**S**VQPGFL  YGGSHI**S**GYATLR  AQQQMT**S**SQKALM  QQQMTS**S**QKALML  LMLELK**S**LQEEPV  ITLVDE**S**DLYNWE  PIDYPY**S**PPTFRF  NGDVCI**S**ILHPPV  PVDDPQ**S**GELPSE  QSGELP**S**ERWNPT  VRTILL**S**VISLLN  ILLSVI**S**LLNEPN  NEPNTF**S**PANVDA  PANVDA**S**VMFRKW  FRKWRD**S**KGKDKE  IIRKQV**S**ATKAEA  IKTKVP**S**NDNSSD  VPSNDN**S**SDLLYD  PSNDNS**S**DLLYDD  SEGARR**S**KNTLER  TLERTH**S**MKDKAG  FDFPDN**S**DVSSIG  PDNSDV**S**SIGRLG  FDPPLH**S**TAIYAD  SKHCGL**S**LSSTPP  CGLSLS**S**TPPGKE  GKEAKR**S**SDTSGN  KEAKRS**S**DTSGNE  KRSSDT**S**GNEASE  TSGNEA**S**EIESVK  EASEIE**S**VKISAK  RKLRPI**S**DDSESI  RPISDD**S**ESIEES  ISDDSE**S**IEESDT  SESIEE**S**DTRRKV  TRRKVK**S**AEKIST  VIRTTA**S**SELSEK  TASSEL**S**EKPAES  PAESVT**S**KKTGPL  PLSAQP**S**VEKENL  ENLAIE**S**QSKTQK  QKKGKI**S**HDKRKK  HDKRKK**S**RSKAIG  KRKKSR**S**KAIGSD  PEGMKT**S**DIKELN  IKMLKE**S**QMLTNL  ELKERK**S**SLRNAA  LKERKS**S**LRNAAY  NAAYFL**S**NLKQLY  QLYQDY**S**DVQAQE  VKETYD**S**SSLPAL  ETYDSS**S**LPALLF  EYEVMY**S**SSCETT  YEVMYS**S**SCETTR  EVMYSS**S**CETTRN  YDVSGE**S**NSAVST  ESNSAV**S**TEDLKE  QLEFCF**S**RENLSK  FSRENL**S**KDLYLI  LISQMD**S**DQFIPI  VKGLFK**S**ENCPKV  CEFAHN**S**NWYITF  GYRLMD**S**SIYSHP  MDSSIY**S**HPIQTQ  NPHQQY**S**VYSIVP  APFPNG**S**FVNGFN  GFNSPG**S**YKTNAA  FRSSGG**S**EHSTEG  SGGSEH**S**TEGSVS  GQLNRY**S**SRNFPA  YLQKET**S**TLQVEQ  HPSTAE**S**KAPTPK  FPPLPG**S**SSRMPG  VLENRM**S**DVVKGV  NEELTI**S**CPVPAD  QLNMST**S**SPCAAE  LNMSTS**S**PCAAEL  KDLIED**S**SVQKDG  DLIEDS**S**VQKDGL  IPVSPP**S**TTKPSR  KPPKEP**S**SVLVQP  PPKEPS**S**VLVQPL  NGAPEN**S**VEKPHE  KPEARA**S**KDYSGF  EQRRQF**S**HRAIPQ  ERQINL**S**NIRAGN  GENIAE**S**LVAEGL  PEQNRL**S**ECEEQA  PRHFVD**S**HHQKPV  RREADG**S**ETPEPF  AKFFTE**S**RLLQRD  VQIILE**S**CHNQNI  ARCVDW**S**IAVYTR  IVVKLN**S**GDYKTI  KTIHLS**S**IRPPRL  IAEALV**S**KGLATV  DDDQRS**S**HYDELL  HRVADI**S**GDTQKA  LQRAGR**S**EAVVEY  VVEYVF**S**GSRLKL  EYVFSG**S**RLKLYL  QEGEPF**S**EEATLF  VEVEVE**S**MDKAGN  IDGANL**S**VLLVEH  LVEHAL**S**KVHFTA  FTAERS**S**YYKSLL  RSSYYK**S**LLSAEE  LEEKER**S**ASYKPV  EKERSA**S**YKPVFV  HPPVEG**S**YAPRRG  RVEKVE**S**PAKIHV  NREVLP**S**TRLGTL  TRLGTL**S**PAFSTR  TLSPAF**S**TRVLPA  RTDAVD**S**VVRDIQ  LNVEHL**S**AGCPHV  YLNAQE**S**AKSARL  AQESAK**S**ARLNLW  PAFIDI**S**EEDQAA  LRAYLK**S**KGAEIS  SKGAEI**S**EENSEG  EISEEN**S**EGGLHV  DDKDVE**S**VMNSVV  VESVMN**S**VVSLLL  VMNSVV**S**LLLILE  QEALIE**S**LCEKLV  REGERP**S**LRLQLL  LRLQLL**S**NLFHGM  RYTVYC**S**LIKVAA  LIKVAA**S**CGAIQY  QVRKWI**S**DWNLTT  LVDCKK**S**DAASKV  KKSDAA**S**KVMVEL  MVELLG**S**YTEDNA  YTEDNA**S**QARVDA  LLTIFV**S**AKLASY  VSAKLA**S**YVKFYQ  NKDFID**S**LGLLHE  VENKEI**S**FDTMQQ  QRKVVV**S**HSTHRT  KVVVSH**S**THRTFG  AMAEQE**S**ARNGGR  EGKLRA**S**VEKGDY  LFFRYM**S**QSKHTE  FRYMSQ**S**KHTEAR  ARELMY**S**GALLFF  GALLFF**S**HGQQNS  SHGQQN**S**AADLSM  NSAADL**S**MLVLES  SMLVLE**S**LEKAEV  NLAKVF**S**LMDPNS  SLMDPN**S**PERVTF  ERVTFV**S**RALKWS  SRALKW**S**SGGSGK  RALKWS**S**GGSGKL  KWSSGG**S**GKLGHP  EQNYCE**S**RYHFLH  RYHFLH**S**ADGEGC  NMLVEY**S**TSRGFR  LVEYST**S**RGFRSE  TSRGFR**S**EVDMFV  LCLKNK**S**SASVVF  CLKNKS**S**ASVVFT  KNKSSA**S**VVFTTY  YTQKHP**S**IEDGPP  CEQYQP**S**LRRDPM  VPPKQT**S**SYGGLL  PPKQTS**S**YGGLLG  LGNLLT**S**LMGSSE  LTSLMG**S**SEQEDG  TSLMGS**S**EQEDGE  QEDGEE**S**PSDGSP  DGEESP**S**DGSPIE  GRISRL**S**VRDVRF  DVRFPT**S**LGGHGA  HTDPDY**S**AAYVVI  FYRQLT**S**DGQLRW  YPAYTT**S**CAWLGY  CAWLGY**S**DDTLKQ  EAVEWM**S**KLAKFK  WIEEPT**S**PDDILG  LGHATI**S**KALVPL  QFLQID**S**CRLGSV  DSCRLG**S**VNENLS  SVNENL**S**VLLMAK  IIFDYI**S**VSASLE  FDYISV**S**ASLENR  YISVSA**S**LENRVC  VMIQRA**S**YMPPKD  PKDPGY**S**TEMKEE  TEMKEE**S**VKKHQY  LKPKGK**S**GTNDTY  LGKEKY**S**TSVAEK  VWKEEA**S**FELPGL  GLLIQG**S**PEKYIL  LIVMHR**S**LVGLDK  MKDKTR**S**PFAKLK  GTFSDT**S**SAIIPS  SSAIIP**S**THMPDA  DANSEF**S**SGEIQM  LGPQRL**S**SAHSMS  SSAHSM**S**DLSGSH  HSMSDL**S**GSHMSS  LSGSHM**S**SEKLKA  SGSHMS**S**EKLKAG  ESGSLK**S**PHRRTL  KMNQPD**S**IVDEGE  FTNVTA**S**LPQKFA  NPFEES**S**ETWDSS  SSETWD**S**SMNLFS  SETWDS**S**MNLFSK  EKREKV**S**LFERVT  VTGKKD**S**RRSDKL  KKDSRR**S**DKLNNG  SPCDLK**S**PNAFSE  DYFDYE**S**TNPFTA  TAKFRA**S**NIMPSS  SNIMPS**S**SFHMSP  NIMPSS**S**FHMSPT  SSSFHM**S**PTSNED  FHMSPT**S**NEDLRK  RVPYEP**S**RKAGKF  RIRRWV**S**EKFIVE  RKTNDA**S**SESIAS  KTNDAS**S**ESIASF  SSESIA**S**FSKQEV  ESIASF**S**KQEVMS  SKQEVM**S**SFLPEG  KQEVMS**S**FLPEGG  INLEAC**S**NEMVTF  QGELHV**S**KLFNHP  ELWVVT**S**FMAYGS  SFMAYG**S**AKDLIC  MGYVHR**S**VKASHI  DGKVYL**S**GLRSNL  YLSGLR**S**NLSMIS  GLRSNL**S**MISHGQ  SNLSMI**S**HGQRQR  KVLPWL**S**PEVLQQ  AKSDIY**S**VGITAC  ELTMSP**S**RSVANS  TMSPSR**S**VANSGL  SRSVAN**S**GLSDSL  VANSGL**S**DSLTTS  NSGLSD**S**LTTSTP  SDSLTT**S**TPRPSN  TSTPRP**S**NGDSPS  RPSNGD**S**PSHPYH  SNGDSP**S**HPYHRT  PYHRTF**S**PHFHHF  NPDARP**S**ASTLLN  DARPSA**S**TLLNHS  STLLNH**S**FFKQIK  QIKRRA**S**EALPEL  SQSQDH**S**GIFGLV  AGGGPG**S**GTSPQV  LRERYY**S**GLIYTY  GLIYTY**S**GLFCVV  TEGAYR**S**MLQDRE  ILCTGE**S**GAGKTE  YLAHVA**S**SPKGRK  LAHVAS**S**PKGRKE  VKNDNS**S**RFGKFI  TNGPSS**S**PGQERE  SHEEII**S**MLRMVS  VGLEQV**S**SLGDGP  GLEQVS**S**LGDGPP  RLMATL**S**NTNPSF  VTDIIV**S**FQAAAR  ELELVV**S**ELEARV  GEEEEC**S**RQMQTE  LLEDQN**S**KLSKER  RRLDGE**S**SELQEQ  AQEDLE**S**ERVART  LEDTLD**S**TNAQQE  AQQELR**S**KREQEV  AELENV**S**GALNEA  ALNEAE**S**KTIRLS  LSKELS**S**TEAQLH  ELEALL**S**SKDDVG  LEALLS**S**KDDVGK  LKAQMA**S**AGQGKE  VEETRT**S**REEIFS  SREEIF**S**QNRESE  QEELAA**S**DRARRQ  EEEQSN**S**ELLNDR  SAERSF**S**AKAESG  LEEGVA**S**DEEAEE  QPQHHH**S**HHHPHH  YYFYNH**S**HNHHHH  QHGAEG**S**PKAQPK  AGEREI**S**LKNLSS  NFIQKN**S**MDKKNG  DKKNGK**S**YENKSG  GVVTNN**S**GYITNG  DGSGSE**S**GYTTPK  TFKPDY**S**EQKGNR  HASAVA**S**KEDSWT  VASKED**S**WTLFKP  VFPVDN**S**SAKIVP  FPVDNS**S**AKIVPK  NKTIQN**S**SVSPTS  SPTSSS**S**SSSSTG  PTSSSS**S**SSSTGE  SSSSSS**S**STGETQ  GETQTQ**S**SSRLSQ  LSQVPM**S**ALKSVT  VTSANF**S**NGPVLA  NTLTPI**S**SGTDSV  ISSGTD**S**VLQDMS  QDMSLT**S**AAVEQI  SLFIYP**S**NMQTML  AQVDLP**S**QTDQQN  QNQWGL**S**FINEPS  TVTGKS**S**EHKVME  SPQVLG**S**ILKSGT  LKSGTT**S**ESGALS  SESGAL**S**LEPSHI  LQKADT**S**SQGALV  ARVVAV**S**RTQADL  TQADLD**S**LVRECP  TERALG**S**VGPVDL  KEAFDR**S**FEVNLR  RAVIQV**S**QIVARG  GAIVNV**S**SQCSQR  AIVNVS**S**QCSQRA  NVSSQC**S**QRAVTN  RAVTNH**S**VYCSTK  NHSVYC**S**TKGALD  PTVVMT**S**MGQATW  MGQATW**S**DPHKAK  AILFLL**S**DRSGMT  FLLSDR**S**GMTTGS  SGMTTG**S**TLPVEG  AASRRQ**S**CYLCDL  PPGAAA**S**AAAKPP  AKPPPL**S**AKDILL  LGSDFG**S**SRPAAS  LVPNGF**S**KLEEPP  VPLMNG**S**ATPLPT  GGRAAA**S**LAAVSG  ASLAAV**S**GTAAAS  AAASLG**S**AQPTDL  KRPASV**S**SSAAVE  RPASVS**S**SAAVEH  PASVSS**S**AAVEHE  HRGPAD**S**LSTAAG  HSGPFE**S**KFKKEP  ANGANG**S**KAVART  EAQPWL**S**TSTEGL  PTSSFV**S**PPPPTA  PPPPTA**S**PHSNRT  PTASPH**S**NRTTPP  AAQNGQ**S**PMAALI  AGGSHA**S**KDANQV  DANQVH**S**TTRRNS  NSPPSP**S**SMNQRR  SPPSPS**S**MNQRRL  PASLPD**S**SLATSA  ASLPDS**S**LATSAP  DSSLAT**S**APLCCT  QCPSVP**S**HKFCFP  KFCFPC**S**RQSIKQ  IKQQGA**S**GEVYCP  GEVYCP**S**GEKCPL  LERLDA**S**AKVLPC  PGPGGG**S**GTNCTN  SSKDLQ**S**SQGGQQ  PRVQSW**S**PPVRGI  IGIFPI**S**YVEFNS  SSAAAQ**S**STAPKH  TMANKS**S**QASQNR  SPPVLI**S**SSNPTA  PVLISS**S**NPTAAA  SELSGL**S**CSAPSQ  LSGLSC**S**APSQVH  RAVTNA**S**QAKVPM  VTMVSP**S**TAGGPA  GNGVAG**S**PSVVPA  GVAGSP**S**VVPAAV  AAHIQT**S**PQAKVL  AGLSPA**S**VGLSHH  VGLSHH**S**LASPQP  APLMPG**S**ATHTAA  ISISRA**S**APLACA  AAAPLT**S**PSITSA  TSPSIT**S**ASLEAE  LPTSPD**S**ASSACG  SPDSAS**S**ACGNSS  GLLKLL**S**GASTKR  KLLSGA**S**TKRKPR  KRKPRV**S**PPASPT  GHGRAG**S**CPVDGD  KASSLD**S**AVPIAP  PPRQAC**S**SLGPVL  GPVLNE**S**RPVVCE  GPIRIR**S**MQTGIT  PRIFQL**S**HNIKNV  PSGAKQ**S**RLMLTL  PAAMKP**S**QGSGSF  TSQKET**S**RQLSYS  ETSRQL**S**YSDNQA  SRQLSY**S**DNQASA  YSDNQA**S**AKRGSL  GNPGRG**S**IKTVAG  IARTIP**S**LTSTST  KRKRMI**S**TGSELN  KAMTDP**S**RKYLTS  SREKQL**S**LKQSEE  AGSKEH**S**SGGTNL  GSKEHS**S**GGTNLD  DRTNVS**S**QTPSAK  VSSQTP**S**AKRSLG  QQLQGF**S**NLGNTC  ILQSLF**S**LQSFAN  SLFSLQ**S**FANDLL  SAHMAI**S**RPLKAS  ASQMVN**S**CITSPS  KRSVAL**S**QRLCEM  KSELEN**S**GFDRMS  ISKRDA**S**PSLSHE  DASPSL**S**HEDDDK  QQALAQ**S**LQEQEA  YNDLEV**S**KIQEAA  QSDRDR**S**GYIFFY  LETEKN**S**QSLSTE  TEKNSQ**S**LSTEVG  AAGGRG**S**GPGRRR  YGNGLE**S**EELEPE  PGPGPG**S**GAPGSQ  GSGAPG**S**QEEEEE  EKQMNM**S**PPPGNA  AGPVIM**S**IEEKME  MEADAR**S**IYVGNV  HFHGCG**S**VNRVTI  ILCDKF**S**GHPKGF  FAYIEF**S**DKESVR  EFSDKE**S**VRTSLA  KESVRT**S**LALDES  SLALDE**S**LFRGRQ  TNRPGI**S**TTDRGF  RTTNYN**S**SRSRFY  TTNYNS**S**RSRFYS  NYNSSR**S**RFYSGF  SRSRFY**S**GFNSRP  FYSGFN**S**RPRGRV  KPAKAA**S**LARNLA  GGAAGR**S**QEPEVP  GLCGAG**S**EPSIAP  GAGSEP**S**IAPTSC  PSIAPT**S**CAGNLP  CAGNLP**S**RPPPLL  RPPPLL**S**PLLASR  LSPLLA**S**RNPCPW  WHYLHL**S**GSHNTL  YLHLSG**S**HNTLAP  LHRKRG**S**QPPDMA  QPPDMA**S**ALTDRT  ALTDRT**S**RAPSTY  RTSRAP**S**TYTYTS  STYTYT**S**RPRALP  ALPCQR**S**RYRDSL  RSRYRD**S**LTQPDE  LLGRPD**S**LELQRQ  GKTIEQ**S**LLEVME  LANLRA**S**QREYEE  YEELRN**S**ERAEVQ  HKHNET**S**QKIAAR  LADLLP**S**VFGSLR  LPSVFG**S**LRDSGY  FGSLRD**S**GYFYDP  EKTMEY**S**MVGRTV  GEDTHQ**S**PEPEDE  PGAMTE**S**LEASEF  TESLEA**S**EFLEQS  SEFLEQ**S**MSQTRE  FLEQSM**S**QTRELL  TTYDRR**S**SQERKF  TYDRRS**S**QERKFM  FIVLIP**S**IVITVI  PRDFKL**S**DALAVE  KPGQKK**S**KNGSDD  QETKQE**S**GSGKKK  TKQESG**S**GKKKAS  SGKKKA**S**SKKQKT  MDNADS**S**PVVDKR  FKDFLL**S**LKTMMF  DALKKS**S**KGELTT  QNKIHV**S**YQETQQ  ILRDAV**S**NTTNQL  TTNQLE**S**KQSAEL  EVQSLH**S**KLTDTL  LTDTLV**S**KQQLEQ  LMQLME**S**EQKRVN  ERDRLT**S**KEEELK  QNKALK**S**EVQKLQ  KLQTLV**S**EQPNKD  QQLSIT**S**KVQELQ  KEASSA**S**QFEELE  ENKLFK**S**QIEQLK  ELLKVI**S**EREKEI  EREKEI**S**GLWNEL  DKVNKT**S**KERQQQ  PKVSVP**S**NLSYGE  ELERLR**S**ENKDIE  KAEMER**S**TYVTEV  SLELIQ**S**KIVKAA  VSPETE**S**SEKETM  SPETES**S**EKETMS  KETMSV**S**LNQTVT  CWHTLK**S**LNGYKN  NGYKNL**S**SGAETR  GYKNLS**S**GAETRE  TREGLR**S**SSPVDL  REGLRS**S**SPVDLP  EGLRSS**S**PVDLPL  GQRKLL**S**LQLLLC  LLCACT**S**VTDLTY  ATAPHR**S**LLAIHL  LHLVPA**S**SAAMKA  HLVPAS**S**AAMKAT  PQGHAP**S**AEDPTG  TGTWTV**S**GPCKDH  HPHPFL**S**QSNPPT  HPFLSQ**S**NPPTRI  NPPTRI**S**SALPLK  PPTRIS**S**ALPLKT  LPLKTD**S**ALEQTP  RALHLF**S**GVPAPP  RFQEAL**S**DFWLAL  VLHNVA**S**AQCQLG  LWTEAA**S**SLREAM  WTEAAS**S**LREAMS  SLREAM**S**KWPEGS  SKWPEG**S**LNGLDS  SLNGLD**S**ALDQVQ  KAKVVA**S**AIPDDQ  ANHDAR**S**LIMDSP  RSLIMD**S**PRAGTH  GADRCT**S**TAYQEQ  GKQAPL**S**PGLPAM  GAGAGG**S**EPLVTV  RRGADL**S**SLRALL  RGADLS**S**LRALLG  AQLGQL**S**YLAPGE  PIPEEE**S**LQRAWQ  QVVAQH**S**YSAQGP  VAQHSY**S**AQGPED  YKETAS**S**RHLRFK  AEGSNS**S**MLAEIE  NLTKKI**S**SELEML  IEDGIS**S**TLPSKE  ISSTLP**S**KESRRK  ELKKMK**S**KNNDLQ  LQDNYL**S**EQNKNK  EDAFLS**S**KGRHER  NENYSL**S**NRQVSS  MGVSTD**S**GTQETK  DRFVPG**S**SQSEGK  RFVPGS**S**QSEGKK  KAWKGT**S**KPGTES  KASDTS**S**ETVFGK  VTQAAN**S**GCSKAI  AANSGC**S**KAIGAL  AIGALA**S**SRRSSS  YPYSCR**S**QENILQ  NILQGF**S**TSHKEG  RCRVIK**S**SGREKP  ENESVK**S**VRASTN  PNKVTS**S**ITIYPS  SITIYP**S**DSSSPR  GLAELT**S**VSNHVS  SVSNHV**S**SPFELS  VSNHVS**S**PFELSI  IRWKSH**S**APSEVG  PSEVGF**S**DARHVT  VRNAWK**S**RRDLKS  AYTQRS**S**TDFSEL  RSSTDF**S**ELEQPR  GDAPEP**S**SRRTQS  GEDCTL**S**VYRQLH  ADKMDM**S**LDDIIK  IIKLNR**S**QRGGRG  GRGRAG**S**QGGRGG  NRPAPY**S**RPKQLP  QHDLFD**S**GFGGGA  GGKLLV**S**NLDFGV  NLDFGV**S**DADIQE  AVHYDR**S**GRSLGT  YDRSGR**S**LGTADV  NIQLVT**S**QIDAQR  QRRPAQ**S**VNRGGM  RGAGRN**S**KQQLSA  NSKQQL**S**AEELDA  FQNVAK**S**YLPNAH  IFKVGW**S**TARDYY  YYTFLW**S**PMPEHY  GEIRGA**S**TPFQFR  FQFRAS**S**PVEELL  LTEVTQ**S**LKMENE  RFSDAT**S**KAHQLE  LEEDIV**S**VTHKAI  KETELD**S**LKDKLK  ENTKLM**S**EVQTLK  LDGNKE**S**VITHFK  TFLLTT**S**SKEDTC  FLLTTS**S**KEDTCF  FLAKEL**S**DAVNVR  KQINKL**S**DQSANN  NKLSDQ**S**ANNNNV  QKVNDA**S**VNTDPA  NTDPAT**S**ASTVDV  DPATSA**S**TVDVKP  TVDVKP**S**PSAAEA  DVKPSP**S**AAEADF  KMEGQN**S**QSPQCF  QCFKTC**S**EQNGYV  GYVLTL**S**NAQPVL  YGNPYA**S**QETRDG  EDNVVC**S**QPARNF  TAPDPP**S**QHLRGH  TGFCFD**S**SFDVHK  PPNYDQ**S**KFEEHV  FEEHVE**S**HWKVCP  KVCPMC**S**EQFPPD  WGAATT**S**PAASDA  TTSPAA**S**DARSFP  DARSFP**S**RQRRVL  QFRVPP**S**SPACVP  AGQHRG**S**ATSLVF  HRGSAT**S**LVFKQK  KTAESE**S**LDSKEN  NNTRIE**S**MMSSVQ  RIESMM**S**SVQKDN  IESMMS**S**VQKDNF  EKLENV**S**QLSLDK  KSPTEK**S**TQYLNQ  PGEDCA**S**CQQDEI  PLSDVG**S**EDVGTG  KNSCQD**S**EADEET  DEQEDG**S**SSQTAN  EQEDGS**S**SQTANK  QEDGSS**S**QTANKP  QTANKP**S**RFQARD  QFEGGE**S**RTGMND  NVECRN**S**KQHGKK  AQHLYQ**S**ILPDMV  NHSITM**S**QEQIAS  SQEQIA**S**LLANAF  KMKSEY**S**SYPDIN  MKSEYS**S**YPDINF  VTFTRQ**S**LEDFPE  TGTEQY**S**EYTGYA  TYRWSR**S**HEDGSE  VSSENL**S**AVATGN  EECRNC**S**TPGPDI  VDAKDT**S**ATSQSV  PQAEQP**S**LESTSK  PSLEST**S**KEAFFS  SKEAFF**S**RVETFS  SRVETF**S**SLKWAG  GKPFEL**S**PLVCAK  CDMLKC**S**SCQAFL  DMLKCS**S**CQAFLC  QAFLCA**S**LQPAFD  FWPDSP**S**PDRFGM  LDLQLP**S**LRPEDL  LTEDKI**S**LLLHLL  TTIKLG**S**DIQVHV  VTACIL**S**VCGWAC  CSSSLE**S**MQLSLI  LESMQL**S**LITCSQ  LSLITC**S**QCMRKV  FQQIES**S**MTDLDA  MTDLDA**S**FGLTSS  SFGLTS**S**PIPGLE  TRSWDS**S**SPVDRP  TRPVTR**S**MGTGDT  ARLCSS**S**SSDTSS  RLCSSS**S**SDTSSR  LCSSSS**S**DTSSRS  SSSDTS**S**RSFFDP  SDTSSR**S**FFDPTS  LAHKQS**S**QPAETD  QPAETD**S**MSLSEK  AETDSM**S**LSEKSR  MSLSEK**S**RKVFRI  PVTHRK**S**DASDMN  HRKSDA**S**DMNSDT  DASDMN**S**DTSPSC  DMNSDT**S**PSCRLR  NSDTSP**S**CRLRAF  CRLRAF**S**RGGSLE  AFSRGG**S**LESRSS  RGGSLE**S**RSSSSR  GSLESR**S**SSSRSR  LESRSS**S**SRSRSF  RSSSSR**S**RSFTLD  FTLDDE**S**LKYLTH  FEETID**S**LDEDFE  CCLCSP**S**LEESTS  SPSLEE**S**TSSPSE  SLEEST**S**SPSEPE  ESTSSP**S**EPEDVI  HPRLLR**S**VPTPLV  AARGPR**S**GDPGPG  DPGPGP**S**HPAQPK  KAPRFP**S**NIIVTN  GAPGGG**S**SPERVA  APGGGS**S**PERVAR  LANGFP**S**AHEALK  AHEALK**S**APSSFA  LKSAPS**S**FAPAGK  FAPAGK**S**LCFRPG  STRARQ**S**FPGPRQ  DWRRAD**S**LPRPQG  QFAGRG**S**SEEARR  FAGRGS**S**EEARRE  HSSLHP**S**IPCPRG  AALVLL**S**ACLVTL  LVLHLA**S**LQLGLL  LLNGVC**S**LAEELR  ELRHIH**S**RYRGSY  HSRYRG**S**YWRTVR  GALLLL**S**IYFYYS  SIYFYY**S**LPNAVG  LALLGL**S**QALNIL  LAPAEI**S**AVCEKG  AHGLAW**S**YYIGYL  LLRGAV**S**QRLYIL  GVPDNL**S**MADPNI  IKDRVY**S**NSIYEL  DRVYSN**S**IYELLE  QTLFAM**S**QYSQAG  FAMSQY**S**QAGFSR  YSQAGF**S**REDRLE  LADAPE**S**QNNCRL  QEPADD**S**SFSLSQ  EPADDS**S**FSLSQE  ADDSSF**S**LSQEVL  DSSFSL**S**QEVLRH  EEVTVG**S**LKTSAV  VGSLKT**S**AVPSTS  KTSAVP**S**TSTMSQ  SAVPST**S**TMSQEP  EPELLI**S**GMEKPL  VRAGPD**S**AGIALY  VCVFKC**S**VSRETE  SRETEC**S**RVGKQS  SRVGKQ**S**FIITLG  ITLGCN**S**VLIQFA  TPNDFC**S**FYNILK  GHTLER**S**VFSERT  LERSVF**S**ERTEES  SERTEE**S**SAVQYF  CGSGIL**S**FFAAQA  NERMLE**S**YLHAKK  NFWYQP**S**FHGVDL  FHGVDL**S**ALRGAA  RILMAK**S**VKYTVN  KFHMLH**S**GLVHGL  IANKRQ**S**YDISIV  QVDQTG**S**KSSNLL  QTGSKS**S**NLLDLK  PSPPPG**S**HYTSPS  PGSHYT**S**PSENMW  NMWNTG**S**TYNLSS  TAYDLS**S**VIASGS  LSSVIA**S**GSSVGH  VIASGS**S**VGHNNL  IVNHTH**S**RMGSIM  THSRMG**S**IMSTGI  RMGSIM**S**TGIVQG  GIVQGS**S**GAQGSG  SGGGST**S**AHYAVN  AHYAVN**S**QFTMGG  PAISMA**S**PMSIPT  GKSKIN**S**CPLSLS  INSCPL**S**LSWGKR  SWGKRH**S**VDTSPG  RHSVDT**S**PGYHES  SPGYHE**S**DSKKSE  GYHESD**S**KKSEDL  ESDSKK**S**EDLSLC  KKSEDL**S**LCNVAE  CNVAEH**S**NTTEGP  KQEGAQ**S**VEEMFE  DDAVNG**S**AWTILL  NFQFYT**S**LMNSVN  YTSLMN**S**VNRQHK  RQHKYN**S**VIPMRP  NALEEE**S**RGFPTQ  ERIFQE**S**VYKTQQ  TDVYLK**S**LNPQWN  LDHDTY**S**ANDAIG  KFFCTT**S**IPKCYR  SPSKSY**S**RQSSSS  SYSRQS**S**SSDTDL  SRQSSS**S**DTDLSL  HAVVGY**S**ESTSIC  VVGYSE**S**TSICEE  GYSEST**S**ICEEVC  EEVCIL**S**ASGTAA  KTPNDG**S**YEQHIS  SYEQHI**S**HMQKKI  INPPEI**S**EEIIGS  PEPRQR**S**RLLRSQ  RSRLLR**S**QSESSD  LRSQSE**S**SDEVTE  RSQSES**S**DEVTEL  AMEDVH**S**LLTDVP  EIQMFT**S**VRVIRL  VRVIRL**S**SLNLTN  RVIRLS**S**LNLTNQ  HVNFTV**S**LPEDEL  KTPVEK**S**LQRAST  FPLELC**S**DSLPSH  LELCSD**S**LPSHPF  VKMTPL**S**FIPGAK  FIRETT**S**LREEGG  QCLINV**S**GDAVVF  VVFVRE**S**DLEVVS  SDLEVV**S**SQQPTT  DLEVVS**S**QQPTTN  ARENGE**S**SSSWKK  RENGES**S**SSWKKQ  ENGESS**S**SWKKQA  NGESSS**S**WKKQAE  ALKGKE**S**SIENEI  LKGKES**S**IENEIA  LEDIYE**S**PNHLYL  LVMQLV**S**GGELFD  YTEKDA**S**TLIRQV  ENLLYY**S**QDEESK  YSQDEE**S**KIMISD  ESKIMI**S**DFGLSK  ISDFGL**S**KMEGKG  LAQKPY**S**KAVDCW  KAVDCW**S**IGVIAY  FYDEND**S**KLFEQI  AEYEFD**S**PYWDDI  NKNIHE**S**VSAQIR  NIHESV**S**AQIRKN  RKNFAK**S**KWRQAF  RKLHLG**S**SLDSSN  LGSSLD**S**SNASVS  GSSLDS**S**NASVSS  SSNASV**S**SSLSLA  SNASVS**S**SLSLAS  SVSSSL**S**LASQKD  SSLSLA**S**QKDCLA  KDCLAP**S**TLCSFI  APSTLC**S**FISSSS  TLCSFI**S**SSSGVS  LCSFIS**S**SSGVSG  CSFISS**S**SGVSGV  SSSSGV**S**GVGAER  ETPIRM**S**VLSEVT  IRMSVL**S**EVTASS  LSEVTA**S**SRHYVD  SEVTAS**S**RHYVDR  LLQQET**S**STELKT  CAVVLG**S**LAMGTE  TENNVK**S**LLDCHI  LLQGLL**S**PDLKFI  LRTIFT**S**PVTPEE  YICQIF**S**HCCKGP  IAHLLT**S**LSYKVR  FENPQV**S**MTLVNV  ELQRIA**S**ITDHLI  YFKYPS**S**VSAITD  KYPSSV**S**AITDIK  AFKLYA**S**LGANDE  RIVTGL**S**ESSVKV  VTGLSE**S**SVKVRL  TGLSES**S**VKVRLA  AVRCLH**S**LSRSVQ  RCLHSL**S**RSVQQL  EILVVA**S**SMLCNL  ILVVAS**S**MLCNLL  NLLLEF**S**PSKEPI  DILRSL**S**TEQLFR  FRLLSD**S**DLNVLM  LLRNLL**S**TRPHID  HIDKIM**S**THGKQI  KYYMGH**S**HVKLQL  WNEEEG**S**QERQDK  DILHKL**S**QSPDSN  LHKLSQ**S**PDSNLC  LSQSPD**S**NLCDKA  PPQDYE**S**DDDSYE  YESDDD**S**YEVLDL  NRVFGH**S**SGPMVE  RVFGHS**S**GPMVEK  PMVEKY**S**VATQIV  LLLQIA**S**HSGYVQ  LQIASH**S**GYVQID  KQNIVI**S**SGFVGG  QNIVIS**S**GFVGGF  VLDGDT**S**YTYHLV  CMGPEA**S**GWGQDE  VQRQGV**S**YSVHAY  RQGVSY**S**VHAYTG  QAVRKS**S**TVATLQ  TPGPPR**S**TPLEEN  NKGAPH**S**SPARGT  GTTPGA**S**QAPKAF  AVPTWA**S**VQVVDD  VVDDPG**S**LASVES  DPGSLA**S**VESPGT  PLLTKL**S**LITAPR  RERGRP**S**LYVQRD  LHVGRA**S**TPDWVS  AFGKPS**S**LSTAEA  GKPSSL**S**TAEAKA  EAKAAT**S**PKATMS  SPKATM**S**PRHLSE  STKQEA**S**KPPRGC  LQKSQS**S**DLLERE  LERERE**S**VLRREQ  DQNSRS**S**SQASGI  QNSRSS**S**QASGIT  RSSSQA**S**GITGSY  GITGSY**S**VSESPF  TGSYSV**S**ESPFFS  SESPFF**S**PIHLHS  SPIHLH**S**NVAWTV  VEDPVD**S**APPGQR  YAGINP**S**DGINSE  MAERWE**S**RIYASE  ITDKRV**S**RRHAIL  NPCFYQ**S**SEKSQL  YQSSEK**S**QLLPLK  NPGDSF**S**LLVDKY  YIFRIL**S**IPSEVE  HETTGA**S**QLEGST  ASQLEG**S**TEIAKT  QMTPTN**S**VSFLGE  TPTNSV**S**FLGENR  MLAEHL**S**DQNLSV  LSVPAI**S**GGNVIQ  GNVIQG**S**GKEEIC  EICKDK**S**QLNTTQ  GRRQLI**S**SGSSEN  RRQLIS**S**GSSENT  QLISSG**S**SENTSA  LISSGS**S**ENTSAE  GSSENT**S**AEQDTG  NTDQEE**S**TISSKE  QEESTI**S**SKEMPQ  EELGKV**S**KHKIAT  SCSENC**S**SAQGDS  CSENCS**S**AQGDSL  SSAQGD**S**LQDESQ  QDESQG**S**HSESSS  QGSHSE**S**SSNPSN  SHSESS**S**NPSNPE  HAKATD**S**VLQGSE  NKVKRT**S**CMYGAN  ECPYGP**S**CYRKNP  EYDLND**S**FLDDEE  KAIKET**S**ILEEYS  SILEEY**S**INWTQK  KLGAGI**S**GPVRVC  RVCVKK**S**TQERFA  IEVFAN**S**VQFPHE  VQFPHE**S**SPRARL  QFPHES**S**PRARLL  FTEKQA**S**QVTKQI  LLFKDN**S**LDAPVK  RHQKEK**S**GIIPTS  PYTYNK**S**CDLWSL  KSCDLW**S**LGVIIY  GYPPFY**S**KHHSRT  FYSKHH**S**RTIPKD  RKIMTG**S**FEFPEE  FPEEEW**S**QISEMA  EEWSQI**S**EMAKDV  DHPWLN**S**TEALDN  LDNVLP**S**AQLMMD  IQDLKV**S**LKPLHS  SLKPLH**S**VNNPIL  GTKPKD**S**VYIHDH  ENGAED**S**NVALEK  LRDTLQ**S**FSWNGR  DTLQSF**S**WNGRGF  LILMAA**S**GAACEV  KDVCVG**S**PGIPGT  IPGTPG**S**HGLPGR  QTRGAL**S**LQGSIM  ALSLQG**S**IMTVGE  VGEKVF**S**SNGQSI  GEKVFS**S**NGQSIT  FSSNGQ**S**ITFDAI  ENEAIA**S**FVKKYN  GLTEGP**S**PGDFRY  PGDFRY**S**DGTPVN  KWVTKD**S**CNDSFR  EPTYPN**S**TILPTP  LIFLAC**S**NFWFKF  FKFPRT**S**SKLEHF  KFPRTS**S**KLEHFV  KLEHFV**S**ILLKCF  LLKCFD**S**PWTTRA  WTTRAL**S**ETVVEE  ETVVEE**S**DPKPAF  FSKMNG**S**MDKKSS  GSMDKK**S**STVSED  SMDKKS**S**TVSEDV  QGIVDR**S**ETGVLD  CTVDIE**S**LTGYRT  LFKILA**S**FYISLV  LASFYI**S**LVIFYG  WWMLRR**S**LKKYSF  KKYSFE**S**IREESS  ESIREE**S**SYSDIP  SIREES**S**YSDIPD  REESSY**S**DIPDVK  QYDPLY**S**KRFAVF  VFLSEV**S**ENKLRQ  IPLWIY**S**LKTLEE  HLTGNL**S**AENNRY  KVLRLK**S**NLSKLP  RLKSNL**S**KLPQVV  KLIVLN**S**LKKMAN  LERIPH**S**IFSLHN  IPHSIF**S**LHNLQE  GNNVLQ**S**LPSRVG  VLQSLP**S**RVGELT  CPLLKR**S**GLVVEE  VAQEAL**S**AQKEIY  SQVSLV**S**GSSSSS  LVSGSS**S**SSKYDP  VSGSSS**S**SKYDPE  FRTDRG**S**HSDLWS  TDRGSH**S**DLWSSS  SHSDLW**S**SSSSLE  HSDLWS**S**SSSLES  DLWSSS**S**SLESSS  SSLESS**S**FPLPKQ  SSQTDI**S**GSFGIN  LHSQLK**S**LSSSMQ  LKSLSS**S**MQSLSS  LSSSMQ**S**LSSGSS  SGSSPG**S**LTSSRG  SPGSLT**S**SRGSLV  LTSSRG**S**LVASSL  ASSLDS**S**TSASFT  TSLSPR**S**SLSSPS  SPRSSL**S**SPSPPC  ELSLGN**S**AQERYR  VQRLGA**S**EAAAFD  AFDSDE**S**EAVGAT  VAVLPC**S**ESTTCL  NEVFWV**S**MSYPAL  CRSGER**S**TRWYNL  TQTLED**S**WRYEET  RRVGTP**S**QGPFLR  NRSDSD**S**STLSKK  VRMKRP**S**SVKSLR  ATRTWH**S**QLTQEI  FLRNLF**S**QTLSLG  LFSQTL**S**LGSQKE  QTLSLG**S**QKERLL  VARYMQ**S**ERCRRV  LVGAGI**S**TSAGIP  GAGIST**S**AGIPDF  GIPDFR**S**PSTGLY  PDFRSP**S**TGLYDN  EAIFEI**S**YFKKHP  HGTFYT**S**HCVSAS  YTSHCV**S**ASCRHE  SHCVSA**S**CRHEYP  RHEYPL**S**WMKEKI  MKEKIF**S**EVTPKC  KCEDCQ**S**LVKPDI  IVFFGE**S**LPARFF  LPARFF**S**CMQSDF  FFSCMQ**S**DFLKVD  LLVMGT**S**LQVQPF  QVQPFA**S**LISKAP  PFASLI**S**KAPLST  ISKAPL**S**TPRLLI  KEKAGQ**S**DPFLGM  GGMDFD**S**KKAYRD  VRREHA**S**IDAQSG  ASIDAQ**S**GAGVPN  AGVPNP**S**TSASPK  VPNPST**S**ASPKKS  RWDRRD**S**VESGCQ  RRDSVE**S**GCQTES  SGCQTE**S**SKEGEE  GCQTES**S**KEGEEG  KLTQDM**S**QDEKVV  IRGEIG**S**GNFSQV  IGSGNF**S**QVKLGI  VKLGIH**S**LTKEKV  KTQRLL**S**REISSM  LLSREI**S**SMEKLH  LSREIS**S**MEKLHH  EVVETL**S**KLHLVM  ELFGKI**S**TEGKLS  STEGKL**S**EPESKL  KLSEPE**S**KLIFSQ  ESKLIF**S**QIVSAV  IFSQIV**S**AVKHMH  ENVFYT**S**NTCVKV  VGDFGF**S**TVSKKG  FGFSTV**S**KKGEML  LNTFCG**S**PPYAAP  VAKLKK**S**ILEGTY  ILEGTY**S**VPPHVS  SVPPHV**S**EPCHRL  LDPKHL**S**ETSTLK  KHLSET**S**TLKEEE  EENEVK**S**TLEHLG  QGRDAR**S**SITGVY  GRDARS**S**ITGVYR  RKKALE**S**VPVMML  RDLKKG**S**RVYRGI  AKGKLK**S**QNTKPY  TKPYLK**S**KNNCQN  NQPPSK**S**TIRPKN  KPKRSI**S**IKLQPR  PPNTAG**S**QKPKLE  LGKRLT**S**ECVSSN  SKPSSK**S**FQQCEA  QQCEAG**S**STTGEL  QCEAGS**S**TTGELS  STTGEL**S**RKPVGS  IHVENE**S**LDNFLK  PKLYTR**S**KPKTDS  SKPKTD**S**YNQTKN  YNQTKN**S**LVPKQA  GKSSVN**S**AVLKDR  FVGETQ**S**RTFPVK  VKSQQL**S**RGADLA  RPGVKP**S**RTVPSH  PSRTVP**S**HFIRTL  TLSKVQ**S**SKKPVV  NETKIR**S**YPVTEQ  KPRTYP**S**LLQGEY  TVGRFN**S**AIPSTP  IRPNGT**S**GNKHNN  LEEWQK**S**KGKTYK  IKEMNI**S**FWKSIE  KAQLEL**S**SKINNT  ILNILS**S**IPEAEK  KAKLLA**S**KGTFDV  LNILQD**S**NRTTEG  TTEGIT**S**DSLVAE  ITPVRR**S**SRIERA  RIERAV**S**RYPEML  EQDNRF**S**NKQKKL  EKKVDM**S**KVNLEV  EVKNPD**S**KMMQIN  SPRRRK**S**RSPSPR  SPRRRS**S**PVRRER  KRSHSR**S**PRHRTK  QEATST**S**DILKVP  SRSRSK**S**RSRTRS  SRSKSR**S**RTRSRS  SRSRTR**S**RSPSHT  TRSRSP**S**HTRPRR  RSSASL**S**GSSSSS  SASLSG**S**SSSSSS  SLSGSS**S**SSSSSR  LSGSSS**S**SSSSRS  GSSSSS**S**SSRSRS  SSSSSS**S**RSRSPP  SSSSSR**S**RSPPKK  KPPKRT**S**SPPRKT  RRQNQQ**S**SSDSGS  QNQQSS**S**DSGSSS  QQSSSD**S**GSSSSS  SSSDSG**S**SSSSED  PPPRRR**S**PSPRRY  PRRRSP**S**PRRYSP  RRSPSL**S**SKHRKG  SKHRKG**S**SPSRST  RKGSSP**S**RSTREA  QPNKRH**S**PSPRPR  NKRHSP**S**PRPRAP  RPIRRV**S**RTPEPK  LEEEIP**S**GKRALI  KRALIE**S**YQNLTR  KAYTTQ**S**LASVAY  TTQSLA**S**VAYQIN  LLDIQA**S**QLRRME  SSINHI**S**QTVDIH  TTNKNT**S**RTHKII  PTQKPP**S**PPMSGR  PPSPPM**S**GRGTLG  PNDYMT**S**PARLGS  SPARLG**S**QHSPGR  RLGSQH**S**PGRTAS  SPGRTA**S**LNQRPR  QRPRTH**S**GSSGGS  RTHSGS**S**GGSGSR  SSGGSG**S**RENSGS  SGSREN**S**GSSSIG  ENSGSS**S**IGIPIA  IAVPTP**S**PPTIGP  IGPENI**S**VPPPSG  APLLPV**S**TVIAAP  VIAAPG**S**APGSQY  PGSAPG**S**QYGTMT  QISRHN**S**TTSSTS  RHNSTT**S**STSSGG  HNSTTS**S**TSSGGY  STTSST**S**SGGYRR  TTSSTS**S**GGYRRT  GYRRTP**S**VTAQFS  SVTAQF**S**AQPHVN  NGGPLY**S**QNSISI  IPMFDD**S**PPPPPP  DKDDEL**S**FMEGAI  QTKQKK**S**KSAEFL  KQKKSK**S**AEFLMV  NPAFNM**S**SPDLSA  PAFNMS**S**PDLSAC  MSSPDL**S**ACQTAE  KFRLPA**S**AEPKGN  QCATEV**S**REDDDR  TKLFDE**S**RQGEPQ  GEPQDE**S**GREETL  REETLN**S**EAPGSS  NSEAPG**S**SNKSHE  SEAPGS**S**NKSHEI  PGSSNK**S**HEIHKE  EIHKEA**S**EATTAH  LEEFQR**S**QKTIIL  TIILLG**S**SPLEQE  IILLGS**S**PLEQEI  LEQEIR**S**TSLHCM  QEIRST**S**LHCMED  CMEDEM**S**HPWILL  VTAVIR**S**RRYYRE  KIEGSK**S**LAQSWR  CWIDPL**S**MGPQKA  VKKLCT**S**LRRNAK  IFVYDC**S**NAGLIV  QMPLPP**S**MKNCIQ  PADLFT**S**CLTTPI  CMQKCV**S**LVPGVT  ILAVDS**S**CQADLV  IWQNFD**S**ARWCGV  AHEKLY**S**LLSDPI  MLAQLV**S**DGSPMV  ATTEGG**S**LTPVRD  AVATAR**S**LNKSLQ  GNLSTS**S**SASSTL  STSSSA**S**STLGSP  NEEHIL**S**FETIDK  DKMRRA**S**SYSSLN  NSLIGV**S**FNSVYT  IGVSFN**S**VYTQIW  AMKVLN**S**IAYKAT  GGSPPA**S**STSSSS  ASSTSS**S**SLTNDV  SSTSSS**S**LTNDVA  LNGQDC**S**LLLTAT  ADSCVT**S**LSCDSH  SCVTSL**S**CDSHRS  AWVVKA**S**LQKRPD  GHIVSV**S**VNGDVR  DPRMPE**S**VNVLQI  QRVGAI**S**CLAFHP  PHLAVG**S**NDYYIS  PGGRNF**S**GTASTS  PVATEL**S**QNARHL  EVFFQL**S**VVKGRL  VKVQTL**S**NQPLLK  FPQKPL**S**LFQTSH  SHTLHL**S**HLNRFP  RLDQGR**S**DDYDSK  RSLLVP**S**DFLSVH  FLSVHL**S**WLSAFP  VHLSWL**S**AFPLSQ  PSRIQV**S**SEKEAA  EPITAD**S**DPAYSS  DSDPAY**S**SKVLLL  SDPAYS**S**KVLLLS  LVGGEW**S**PSLDGL  GGEWSP**S**LDGLDP  QTGIDL**S**GCTKWW  EQAADT**S**RRNAET  PGCVNL**S**LHGIVE  RPKERI**S**FEVMVL  EGPATE**S**EAPLKE  LLPKPL**S**SGGEEE  LPKPLS**S**GGEEEE  KPRGEA**S**EDLCEM  EDSEVR**S**VASNQS  SVASNQ**S**EMEFSS  QAKQLV**S**RVVTQN  LPPPGK**S**TKPGAA  EHKALV**S**HNGSLI  RAEQQD**S**GRLYLE  ELKLEE**S**HNRFSA  ESHNRF**S**ATEVTN  VVEKAD**S**WVEKEE  AAAVSE**S**WPELEL  LHYLEV**S**GCGSLR  EVSGCG**S**LRAPGP  GLPQLH**S**LVLRRN  ALGPGL**S**PELGPL  LRVLDL**S**GNALEA  GLPQLQ**S**LNLSGN  LQSLNL**S**GNRLRE  CAPRLQ**S**LNLTGN  TGNCLD**S**FPAELF  GALPLL**S**ELAAAD  NCLREL**S**PDIAHL  DIAHLA**S**LKTLDL  LKTLDL**S**NNQLSE  LSNNQL**S**EIPAEL  RLEKMV**S**GCQTRS  SGCQTR**S**ILEYLR  KGRAEG**S**EKEESR  LRVLHV**S**ENPVPL  PLTVRV**S**PEVRDV  LKRFLT**S**QTKLHE  KQKKRQ**S**VSGLHR  KKRQSV**S**GLHRYL  ADGDVI**S**FPPITN  FPPITN**S**EKTKVK  KVKKTT**S**DLFLEV  LFLEVT**S**ATSLQI  EVTSAT**S**LQICKD  ENKEEG**S**LSDTEA  TEADAV**S**GQLPDP  AGKDGP**S**LLVVEQ  VVDLEG**S**LKVVYP  LKVVYP**S**KADLAT  MTANHE**S**YLLMAS  MEDWVK**S**IRRVIW  DCGEKP**S**FDSNTD  EKPSFD**S**NTDVHT  KYEDFL**S**CAKLLS  LAKQVK**S**LPVVNY  FLDEVQ**S**YSGVNK  SGVNKM**S**VQNLAT  KDAELQ**S**KPQDGV  KPQDGV**S**NNNEIQ  NTKDSP**S**RQCSWD  SPSRQC**S**WDKSES  QCSWDK**S**ESPQRS  SWDKSE**S**PQRSSM  SESPQR**S**SMNNGS  TNSPKN**S**VHKLDV  TNGSFS**S**SNAEGL  LQARRS**S**SLKVSG  QARRSS**S**LKVSGT  TNVRNM**S**WLPNGY  NLDDKQ**S**IDSATW  SATWST**S**SCEISL  ATWSTS**S**CEISLP  TSSCEI**S**LPENSN  ISLPEN**S**NSCRSS  SNSCRS**S**TTTCPE  HPRDYE**S**KSDHRS  RSSRAT**S**SSDNSE  ALHSLV**S**SLKQEM  LHSLVS**S**LKQEMT  QKIEYE**S**RIKSLE  GCRPWG**S**LLGLLG  GLLGLV**S**AAAAAW  AAWDLA**S**LRCTLG  TKPLVL**S**LHGWTG  WTGTGK**S**YVSSLL  TGKSYV**S**SLLAHY  GKSYVS**S**LLAHYL  FQGGLR**S**PRVHHF  PRVHHF**S**PVLHFP  LHFPHP**S**HIERYK  YKKDLK**S**WVQGNL  LTACGR**S**LFLFDE  LRPFLG**S**SWVVYG  RPFLGS**S**WVVYGT  RAPPRR**S**GALPPA  LPVVGA**S**TPGTVV  LNKAAL**S**YVSEIG  AALSYV**S**EIGKAP  TRVTQS**S**IRTPVV  IRTPVV**S**ISACSL  TPVVSI**S**ACSLFS  SACSLF**S**GHANEF  ANEFDG**S**NSTSHA  EFDGSN**S**TSHALL  DGSNST**S**HALLVL  HIKAVL**S**NKLCLS  SNKLCL**S**ISNLVQ  KLCLSI**S**NLVQGV  NPVGPE**S**QIRYSM  ESQIRY**S**MVSVPT  IRYSMV**S**VPTVTS  SVPTVT**S**DYISLE  VTSDYI**S**LEVNAV  HVGTEG**S**MATVGL  MATVGL**S**QQLFDS  SQQLFD**S**ALLLLQ  ITGQLR**S**DDNLLN  DNLLNT**S**ALGRLI  EVLATA**S**NSAFQS  SNSAFQ**S**LFSLDV  AFQSLF**S**LDVVVN  NLRLQL**S**VSKVKL  RLQLSV**S**KVKLQG  KLQGTT**S**VLGDVQ  VQLTVA**S**SNVGFI  QLTVAS**S**NVGFID  PESPCL**S**PLKPLK  LNPALD**S**AEHPMP  MPPPPL**S**PLALAP  PLALAP**S**SAIRDQ  QKNQTA**S**GDSLDV  QTASGD**S**LDVRVP  LDVRVP**S**KPCLMK  YRRHLD**S**SKISVL  DIITVP**S**DNVVMV  VVMVHE**S**KQKVDL  AFDDKA**S**NELVYQ  AQPLVE**S**IFRKGM  AYGQTG**S**GKTYTM  TMGGDF**S**GTAQDC  GTAQDC**S**KGIYAL  LVEIGN**S**CRTSRQ  GNSCRT**S**RQTPVN  TPVNAH**S**SRSHAV  NAHSSR**S**HAVFQI  ADTTKA**S**RKRQLE  GAEINK**S**LLALKE  HTPFRA**S**KLTLVL  SFIGQN**S**STCMIA  FIGQNS**S**TCMIAT  CMIATI**S**PGMTSC  APRMLK**S**HIGNSE  IGNSEM**S**LQRDEF  KIPYVQ**S**EEQKEI  TISGKG**S**SQWLEN  DFCIAR**S**LSILEQ  CIARSL**S**ILEQKI  ACCRAL**S**QESSKY  GIHSPS**S**YHPGDG  MNEQNR**S**AATPPS  QPPQQP**S**SMQTGM  PPPPPP**S**YMHIPR  MHIPRY**S**TNPITV  PSNLYG**S**PGSIYI  YHSPPP**S**QCPSPF  PPSQCP**S**PFSSPQ  CPSPFS**S**PQHQVQ  FMPPSP**S**TTPPHP  QKQGSH**S**VAYLPY  LPYTAS**S**LSKGSM  PISNQP**S**PWNQHS  ATTPPS**S**SPSRGI  TTPPSS**S**PSRGIS  SPSRGI**S**SQPKPP  PSRGIS**S**QPKPPF  QPKPPF**S**VNPVYI  GPSCTP**S**PSPRVI  SCTPSP**S**PRVIPN  HKYQRS**S**SSGSDD  YQRSSS**S**GSDDYA  ELERLK**S**EVNGME  RRLRRV**S**CTTAIP  EMTRLR**S**MNRQLQ  EVDLLQ**S**RGNFDP  VVPPKP**S**KKDSSD  KPSKKD**S**SDPCTI  PSKKDS**S**DPCTIE  RKARRI**S**VTSKVQ  RFITAA**S**AARNPS  SAARNP**S**PIRTMT  TMTDIL**S**RGPKSM  LSRGPK**S**MISLAG  GPKSMI**S**LAGGLP  KRALQY**S**PSAGIP  ALQYSP**S**AGIPEL  TIHYPP**S**QGQMDL  MDLCVT**S**GSQQGL  LCVTSG**S**QQGLCK  LDEPAY**S**GTLQSL  YSGTLQ**S**LHPLGC  NIINVA**S**DESGIV  NVASDE**S**GIVPDS  SGIVPD**S**LRDILS  SLRDIL**S**RWKPED  NNPTGN**S**LTSERK  TGNSLT**S**ERKKEI  RVPTFL**S**MDVDGR  RVIRAD**S**FSKIIS  IRADSF**S**KIISSG  SFSKII**S**SGLRIG  FSKIIS**S**GLRIGF  VSTLHP**S**TFNQLM  FNQLMI**S**QLLHEW  RVIDFY**S**NQKDAI  NAFYVD**S**SAPSPY  AFYVDS**S**APSPYL  VDSSAP**S**PYLRAS  SPYLRA**S**FSSASP  YLRASF**S**SASPEQ  LRASFS**S**ASPEQM  ASFSSA**S**PEQMDV  QDQGKR**S**VPSEKL  GKRSVP**S**EKLTTA  GEIEKF**S**NRSNIC  EKFSNR**S**NICRFL  CRFLTA**S**QDKILF  DVNRKL**S**DVWKEL  DVWKEL**S**LLLQVE  MPVSPI**S**QGASWA  PISQGA**S**WAQEDQ  NEKIEA**S**LRRLEI  IKKEQL**S**GSPWIL  KEQLSG**S**PWILLR  LRENEV**S**TLYKGE  KKLQAG**S**IAIVRQ  TMKKFE**S**PNILRI  VTPPQF**S**IVMEYC  LYRLHH**S**EAPELH  LHGKIR**S**SNFLVT  HGKIRS**S**NFLVTQ  KTDRVK**S**TAYLSP  KSTAYL**S**PQELED  YQYDVK**S**EIYSFG  VKSEIY**S**FGIVLW  PFQGCN**S**EKIRKL  LGEDCP**S**ELREII  CRAHDP**S**VRPSVD  DPSVRP**S**VDEILK  LGNGRR**S**MKSPPL  GRRSMK**S**PPLVLA  FNYWIA**S**SRSVDL  NYWIAS**S**RSVDLQ  WIASSR**S**VDLQTR  QLDKIQ**S**SHNFQL  LDKIQS**S**HNFQLE  HNFQLE**S**VNKLYQ  NLERKF**S**YDLSQC  KFSYDL**S**QCINQM  VASRDL**S**ENNDQR  QQLQAL**S**EPQPRL  GNVLGN**S**KSQTPA  VLGNSK**S**QTPAPS  SQTPAP**S**SEVVLD  QTPAPS**S**EVVLDS  SEVVLD**S**KRQVEK  QVQAAL**S**VSQENP  DENEAE**S**ETDKQA  TVSSRR**S**WWPLPL  KEETHL**S**QSERTA  ETHLSQ**S**ERTARR  GFLVKM**S**GDLLEL  YIEEDS**S**VFAQSI  SSVFAQ**S**IPWNLE  VYLLDT**S**IQSDHR  LDTSIQ**S**DHREIE  RFHRQA**S**KCDSHG  GVAKGA**S**MRSLRV  KGASMR**S**LRVLNC  QGKGTV**S**GTLIGL  LEFIRK**S**QLVQPV  EDIIGA**S**SDCSTC  DIIGAS**S**DCSTCF  TCFVSQ**S**GTSQAA  VAALPP**S**THGAGW  FCRTVW**S**AHSGPT  TVWSAH**S**GPTRMA  PDEELL**S**CSSFSR  EELLSC**S**SFSRSG  LSCSSF**S**RSGKRR  CSSFSR**S**GKRRGE  HVLTGC**S**SHWEVE  VLTGCS**S**HWEVED  EASIHA**S**CCHAPG  WTLTGC**S**ALPGTS  NTCVVR**S**RDVSTT  VRSRDV**S**TTGSTS  DVSTTG**S**TSEGAV  STTGST**S**EGAVTA  LVVFGA**S**GFTGQF  QVDPER**S**SRLPWA  VDPERS**S**RLPWAV  WAVAGR**S**REKLQR  LGRPTL**S**SEVGII  GRPTLS**S**EVGIII  DIANPA**S**LDEMAK  CIENGA**S**CIDISG  ASCIDI**S**GEPQFL  GVYIIG**S**SGFDSI  VYIIGS**S**GFDSIP  GSSGFD**S**IPADLG  TLTAVE**S**FLTIHS  SFLTIH**S**GPEGLS  SGPEGL**S**IHDGTW  HDGTWK**S**AIYGFG  YGFGDQ**S**NLRKLR  RKLRNV**S**NLKPVP  KRRWPI**S**YCRELK  RELKGY**S**IPFMGS  SIPFMG**S**DVSVVR  FMGSDV**S**VVRRTQ  YENLEE**S**PVQYAA  TVGGIT**S**VIKLMF  KFPWFF**S**FGYFSK  FSFGYF**S**KQGPTQ  KQIDAA**S**FTLTFF  FFGQGY**S**QGTGTD  AAMTLL**S**DASHLP  TLLSDA**S**HLPKAG  TPGAAF**S**KTKLID  ADRTDG**S**VQAAAW  TVLGAD**S**KSGEVD  RVTPLP**S**LASPAV  GYCSRI**S**RKSPGD  WDPLHP**S**STDMAL  DPLHPS**S**TDMALV  LSLQET**S**SSSFLS  SSSSFL**S**SQPFED  SSSFLS**S**QPFEDD  LLSTKP**S**SIISTK  LSTKPS**S**IISTKT  IMQNWN**S**RRRHRY  ENFIPC**S**EPVKGT  KNVLDV**S**CRKTGL  ERKVTP**S**KYSSLI  LIYFDS**S**ATYNLD  RLSLPS**S**KAKAKS  SKAKAK**S**LSEAFE  AKAKSL**S**EAFENL  ENLGKR**S**LEAGRC  CLPKSD**S**SSSLPK  PKSDSS**S**SLPKTN  KTNPTH**S**ATRPQQ  LHVQGN**S**SGIFRK  SGIFRK**S**VSPSKT  RKSVSP**S**KTLSVP  PLCIGV**S**TDKASM  GFQKLP**S**SPLGCR  TAIEAP**S**STCVAR  LSEPQG**S**GRQGNS  GDQGSS**S**QPNSEE  TTSCCV**S**SSPKLR  TSCCVS**S**SPKLRR  SCCVSS**S**PKLRRN  LRRNAH**S**RLESYR  CNLQHI**S**DRENID  NMEFNP**S**DHPRAS  SDHPRA**S**TIFLSK  TIFLDD**S**TVSQPN  LDDSTV**S**QPNLKY  ENLHPL**S**KSEVPP  LHPLSK**S**EVPPDY  FVRTLF**S**AAQLTA  GAILLA**S**KVWDDQ  FNINVP**S**SVYAKY  NINVPS**S**VYAKYY  YYFDLR**S**LAEANN  HKLEAI**S**RLCEDK  YKDLRR**S**ARKRSA  DALPEH**S**PAKTSA  HSPAKT**S**AVSNTK  AKTSAV**S**NTKPGQ  PQGWPG**S**NPWNNP  NPWNNP**S**APSSVP  NNPSAP**S**SVPSGL  NPSAPS**S**VPSGLP  APSSVP**S**GLPPSA  PSGLPP**S**ATPSTV  PPSATP**S**TVPFGP  PTGMYP**S**VPPTGP  PAPFPP**S**GPSCPP  FPPSGP**S**CPPPGG  PLGPWG**S**MSSGPW  GPWGSM**S**SGPWAP  PWGSMS**S**GPWAPG  PNMPYP**S**PGPYPA  YPAPTG**S**YPTPGL  GLYPTP**S**NPFQVP  NPFQVP**S**GPSGAP  QVPSGP**S**GAPPMP  WIGDGE**S**TSPSDK  KGKEEK**S**KGKAKP  EEEGGE**S**KADDPY  DPYAHL**S**KKEKKK  YERQVA**S**LKAANA  AAENDF**S**VSQAEM  ENDFSV**S**QAEMSS  VSQAEM**S**SRQAML  SQAEMS**S**RQAMLE  AMLENA**S**DIKLEK  IKLEKF**S**ISAHGK  LEKFSI**S**AHGKEL  IANRAL**S**IPPNID  RPTQKF**S**GGWRMR  GWRMRV**S**LARALF  KTLLIV**S**HDQGFL  LKAGGK**S**TKQAEK  PDPPPL**S**PPVLGL  FGIDMD**S**RICIVG  PNGVGK**S**TLLLLL  GRFGLE**S**HAHTIQ  IQICKL**S**GGQKAR  NNLDIE**S**IDALGE  GAVIVV**S**HDARLI  WVVEEQ**S**VSQIDG  VEEQSV**S**QIDGDF  EKGAAT**S**VSNPRG  GAATSV**S**NPRGRP  NPRGRP**S**RGRPPK  PKLQRN**S**RGGQGR  ILARGG**S**KGIPLK  LRAALD**S**GAFQSV  DSGAFQ**S**VWVSTD  FQSVWV**S**TDHDEI  AQVHRR**S**SEVSKD  QVHRRS**S**EVSKDS  RRSSEV**S**KDSSTS  SEVSKD**S**STSLDA  EVSKDS**S**TSLDAI  SKDSST**S**LDAIIE  GNIQAT**S**PCLHPT  REEGYD**S**VFSVVR  GYDSVF**S**VVRRHQ  RHQFRW**S**EIQKGV  ELYENG**S**FYFAKR  EMRAEH**S**VDIDVD  NGHIYV**S**GDQKEI  DQKEII**S**YDVKDA  KDAIGI**S**LLKKSG  ISLLKK**S**GIEVRL  IEVRLI**S**ERACSK  ISERAC**S**KQTLSS  CSKQTL**S**SLKLDC  SKQTLS**S**LKLDCK  DCKMEV**S**VSDKLA  KMEVSV**S**DKLAVV  YLGNEV**S**DEECLK  LKRVGL**S**GAPADA  APADAC**S**TAQKAV  EAAKCV**S**GSTAIS  AKCVSG**S**TAISTY  SGSTAI**S**TYPKTL  QKLGSG**S**FGTVYL  LEAQLL**S**KLDHPA  IVKFHA**S**FVEQDN  LHRDLK**S**KNVFLK  IGDFGV**S**RLLMGS  SRLLMG**S**CDLATT  GTPHYM**S**PEALKH  QGYDTK**S**DIWSLA  TKSDIW**S**LACILY  NHAFAG**S**NFLSIV  AGSNFL**S**IVLKIV  LNAIME**S**MLNKNP  NPSLRP**S**AIEILK  NLMCRY**S**EMTLED  QTLRAL**S**EVQKMT  RMQELR**S**RNFQQL  RNFQQL**S**VDVLHE  EGRLSC**S**PQDEDE  QGREEE**S**DEPTLE  LENLPE**S**QPIPSM  DLHELE**S**IVEDAT  YADAFD**S**YCEESD  EIRNEG**S**QPAYRT  RTNQQD**S**DIEALA  NVLGCT**S**LDTKTI  TMAEDM**S**PGPPIF  MKRMRE**S**AMQKLG  KVVPQA**S**DCFEVD  IKIWDA**S**SMTLVD  KIWDAS**S**MTLVDK  FLVTAS**S**SGDKIV  TCVNLN**S**TSMYLV  TSMYLV**S**GGLNNT  NIWDLK**S**KRVHRS  GEIILH**S**VTTNLS  GHGSNQ**S**VRHLKY  KKSLLG**S**VSDNGI  TLWDVN**S**QSPYHN  WDVNSQ**S**PYHNFD  ASGICF**S**PVNELL  ILYDTS**S**KKLVKT  TLAIGS**S**RGKIYQ  DLRMLK**S**PVKTIS  SPVKTI**S**AHKTSV  CIAFQY**S**TVLTKS  STVLTK**S**SLNKGC  TVLTKS**S**LNKGCS  SLNKGC**S**NKPTTV  REAPAT**S**IATVLP  KAGLPR**S**INTDTL  GKNQDF**S**SFDDTG  FDDTGK**S**SLGDMF  PVTSST**S**VLHSSP  NLNTSP**S**SNQTRN  LNTSPS**S**NQTRNS  TAGVAS**S**LSEKIA  GVASSL**S**EKIADS  SEKIAD**S**IGNNRQ  QLNEMH**S**LLERYS  MGAELP**S**SPLAIE  LPPGSL**S**STPLST  PPGSLS**S**TPLSTP  LSSTPL**S**TPCSSV  PLSTPC**S**SVPSSP  PCSSVP**S**SPSFCA  SVPSSP**S**FCAPSP  PSFCAP**S**PGTGGG  AGGGGG**S**SQAGGA  GGGGGS**S**QAGGAP  GAPGPP**S**GGPGAV  GAVGGT**S**GKPALE  EDLYWM**S**GYQHHL  VEALIG**S**GHHGAH  RLEERF**S**DDQLVS  SDDQLV**S**MSVREL  DQLVSM**S**VRELNR  RQLRGF**S**KEEVIR  NRGYAQ**S**CRFKRV  QRHILE**S**EKCQLQ  EKCQLQ**S**QVEQLK  GRGGPG**S**AGGAGF  GFPREP**S**PPQAGP  SVATGS**S**ETAGGA  ETAGGA**S**GGGARV  ARVFFQ**S**PRGGAG  RGGAGG**S**PGSSSG  AGGSPG**S**SSGSGS  GGSPGS**S**SGSGSS  GSPGSS**S**GSGSSR  PGSSSG**S**GSSRED  SSSGSG**S**SREDSA  SSGSGS**S**REDSAP  GSSRED**S**APVATA  DLLDAD**S**DEERAS  SDEERA**S**KLQEAL  FIKELL**S**RIRGMR  GGGGWG**S**GSSFRG  GGWGSG**S**SFRGTP  GWGSGS**S**FRGTPG  GGPRPP**S**PRDGYG  PPYGPR**S**RPYGSS  RSRPYG**S**SHSPRH  SRPYGS**S**HSPRHG  PYGSSH**S**PRHGGS  PGGYPG**S**YSRSPA  GYPGSY**S**RSPAGS  SRSPAG**S**QQQFGY  QTHPQG**S**PRTSTP  QGSPRT**S**TPFGSG  ENYFKP**S**MLEDPW  AGLEPV**S**VVDISQ  VSVVDI**S**QQYSNT  DISQQY**S**NTQTFT  EGGSTG**S**AGSDSS  STGSAG**S**DSSTSG  GSAGSD**S**STSGSG  SAGSDS**S**TSGSGG  GSDSST**S**GSGGAQ  DSSTSG**S**GGAQQR  IEHKEA**S**DRETAI  LYEKLM**S**PENTLL  ERTFMA**S**EFLDWL  GIIQHV**S**NKHPFV  KHPFVD**S**NLLYQF  ELLNEK**S**PSSQET  LNEKSP**S**SQETHD  NEKSPS**S**QETHDS  FCLRKQ**S**HDNRKS  DNRKST**S**FMSVSP  FMSVSP**S**KEIKIV  KEIKIV**S**AVRRSS  VSAVRR**S**SMSSCG  SAVRRS**S**MSSCGS  SMSSCG**S**SGYFSS  GSSGYF**S**SSPTLS  SSGYFS**S**SPTLSS  VLCNPK**S**VLKRPV  LKRPVT**S**EELLTP  GFVVRG**S**KPCHIQ  IQAVDP**S**GPAAAA  VCQFVV**S**VNGLNV  VDYRTV**S**NLILTG  AAEPLQ**S**VLWVKQ  QQRCAV**S**LEPARA  LLRWWR**S**PGPGAG  PGADAC**S**VPVSEI  ACSVPV**S**EIIAVE  MLEKLT**S**RPKHLL  PLFTLA**S**ITTDII  IGRTQR**S**AGVDQN  RAVLVP**S**SLRIGI  AVLVPS**S**LRIGII  TDCVCY**S**TVGTSD  YSTVGT**S**DAETSA  TSDAET**S**ALHIVV  HIVVGD**S**LAMDVS  SLAMDV**S**SVHHNS  STLLRY**S**VSLLGY  LLRYSV**S**LLGYGF  GDIIKD**S**EKKRWL  GLKTFL**S**HHCYEG  CYEGTV**S**FLPAQH  CFVCRQ**S**KQQLEE  INATNM**S**CACRRS  HLGDGS**S**DLILIR  ILIRKC**S**RFNFLR  KKFQFT**S**KHMEDE  HMEDED**S**DLKEGG  RFGHIC**S**SHPSCC  FGHICS**S**HPSCCC  ICSSHP**S**CCCTVS  CTVSNS**S**WNCDGE  DGEVLH**S**PAIEVR  DHPNII**S**LLDVIR  QDVHVR**S**IFYQLL  ATRFLH**S**GHVVHR  HRDQKP**S**NVLLDA  DFGLAR**S**LGDLPE  APEVLL**S**SHRYTL  PEVLLS**S**HRYTLG  LGVDMW**S**LGCILG  PLFPGT**S**TLHQLE  ETIPPP**S**EEDLLA  DLLALG**S**GCRASV  GSGCRA**S**VLHQLG  VLHQLG**S**RPRQTL  LLPPDT**S**PEALDL  APDKRL**S**ATQALQ  QRFHCP**S**DEWARE  HEGVQL**S**VPEYRS  SVPEYR**S**RVYQMI  ILECGG**S**SGTSRE  GGSSGT**S**REKGPE  KGPEGV**S**PSQAHL  PEGVSP**S**QAHLHK  PRPRPQ**S**SPGHDP  DPAEHE**S**PRAAKN  NVPRQN**S**APLLQT  APPLTL**S**LVKPSG  LSLVKP**S**GRGAAP  GRGAAP**S**LTSQAA  AAPSLT**S**QAAAQV  PGRRMF**S**TSALQG  RRMFST**S**ALQGAQ  ALLGGY**S**QAYGTV  YGTVCH**S**ALGHLP  CGPRTP**S**SWGRSQ  GPRTPS**S**WGRSQS  PSSWGR**S**QSSAAA  SWGRSQ**S**SAAAEA  WGRSQS**S**AAAEAS  SAAAEA**S**AVLKVR  KVRPER**S**RRERIL  RILTLE**S**MNPQVK  YPNLLD**S**PSFPED  NLLDSP**S**FPEDAK  QACGGN**S**LGSYSA  GGNSLG**S**YSASQG  NSLGSY**S**ASQGVN  LGSYSA**S**QGVNCI  YLTTGA**S**DGISTI  GASDGI**S**TILKIL  VSGGGK**S**RTGVMI  PQYPLY**S**AVISEL  LYSAVI**S**ELDAIQ  PTGQVQ**S**RKCIED  YQDNVY**S**PDCRFH  PDCRFH**S**FKKVLY  EMGPEY**S**SNVELA  MGPEYS**S**NVELAS  SNVELA**S**FHSTSK  ELASFH**S**TSKGYM  ASFHST**S**KGYMGE  QLVKLL**S**VRLCPP  RLCPPV**S**GQAAMD  PVAGEE**S**FEQFSR  ESFEQF**S**REKESV  FSREKE**S**VLGNLA  ICVVPG**S**GFGQRE  VNREKL**S**ESVLMK  REKLSE**S**VLMKVE  LETSCG**S**PHYACP  EPEKRL**S**LEQIQK  PRKRVD**S**PMLSRH  VDSPML**S**RHGKRR  RRPERK**S**MEVLSI  DAGGGG**S**PVPTRR  AQHSQR**S**RSVSGA  HSQRSR**S**VSGAST  QRSRSV**S**GASTGL  RSVSGA**S**TGLSSS  GASTGL**S**SSPLSS  STGLSS**S**PLSSPR  PLSSPR**S**PVFSFS  KTQTLP**S**RGPRGG  EQPPPP**S**ARSTPL  PGSPRS**S**GGTPLH  LHTPRA**S**PTGTPG  SLTPES**S**PELAKR  LSIPSL**S**HSVLSQ  SVLSQT**S**FRAEYK  RAEYKA**S**GGPSVF  KASGGP**S**VFQKPV  FQVDIS**S**SEGPEP  QVDISS**S**EGPEPS  SEGPEP**S**PRRDGS  SPRRDG**S**GGGGIY  GGGGIY**S**VTFTLI  STHDQP**S**VQALAD  DPELSS**S**PRRGPP  FGYFIR**S**PEKREP  QSQAQL**S**EGSITL  DSMLRI**S**TIASAI  RISTIA**S**AIAEAS  PNDLEK**S**NGSNAL  LEKSNG**S**NALDME  LKKTEV**S**RYESAL  FRTINS**S**NSVTNR  NDEKAT**S**ISTPSD  VSEISN**S**EKHVTF  EHGGRG**S**EDEQES  SFRPST**S**PLSHSS  ALESFG**S**AAQQQQ  RGKGTL**S**SIIQNN  QVGSAT**S**HPVSCQ  LTQPSL**S**AAPFAQ  KPFPVP**S**VGTNCG  WDSGVT**S**GLGSVR  GEKVDL**S**TYRCLV  VLFIPS**S**PGVFRC  VFRCTF**S**VASWPC  CTFSVA**S**WPCSTD  SPKKQI**S**SSDILD  VDIEVD**S**PNPTPV  LAKVAS**S**RKQHLP  KVPEQG**S**HFSVDP  SGSKPL**S**PGPCLD  FRDCIS**S**REFLQP  QPSSKA**S**LESTSD  PLDQLA**S**EEPWTV  GCVAPE**S**KLQILV  HGIQKV**S**ITFLPR  VNLRNN**S**FITHSL  GDGTSA**S**STGTQS  DGTSAS**S**TGTQSP  SSTGTQ**S**PEIFGS  LGTNNV**S**AFQPYQ  QPYQAT**S**EIEVTP  PALIGL**S**TSVSDS  KDENYR**S**LPRDTS  RDTSNW**S**NQFQRD  GTEEDN**S**RVEPVG  KNNYYS**S**RFSPDS  PHSAHP**S**GKPPSA  EVPLND**S**GSAGLG  PLNDSG**S**AGLGVS  LGIFVK**S**IINGGA  METLRR**S**MSTEGN  SGKYQL**S**PTVNMP  DQSSSS**S**HDDVGF  WAKAAI**S**DSADCS  SRDVGP**S**LGLKKS  KSSSLE**S**LQTAVA  RAAIDK**S**YDKPAV  EEDTEE**S**SRSGRE  KIKIQE**S**FTSEEE  MYGGVS**S**YEGSMA  VSSYEG**S**MALNAR  PVDSNR**S**TPSNHD  SNRSTP**S**NHDRIQ  QEERES**S**QQAQRQ  QAQRQY**S**SLPRQS  MKKQPP**S**EGPSNY  RQDVPP**S**PSQVAR  LCDLLA**S**AADPQI  QRESLK**S**LILTAL  EHCVSL**S**LAQLSA  LSLAQL**S**ATIFRK  LQLLQH**S**THSPHS  LQHSTH**S**PHSPER  TLGEVG**S**PGLLFY  FLVKVK**S**KALLKN  LVLAVL**S**DGAGDH  LQPHIS**S**YSREVM  PHISSY**S**REVMPL  QPVQIQ**S**LETLGV  LRRCTY**S**LFAALS  SLFAAL**S**GLMGEG  TTLMLL**S**LRSTEG  MLLSLR**S**TEGIVP  LLFDDE**S**DGEEEE  VEEEDD**S**EISGYS  EDDSEI**S**GYSVEN  AAVGEI**S**VNTSVA  FLPYME**S**VFEEVF  LHKACQ**S**CPSEPN  LTGVLR**S**CGTLTL  AAAGGD**S**FAPFFA  QGLGAA**S**AQFVSR  ASAQFV**S**RLLPVL  LLPVLL**S**TAQEAD  ARLLMA**S**PTRKPE  TIGRLF**S**FLYQSS  FSFLYQ**S**SPDQVI  SFLYQS**S**PDQVID  ELLRIC**S**LILADN  FQAALG**S**LPVDKA  TLKRIQ**S**HKGVIG  RTTLDN**S**TTVQYA  LTMKAK**S**TVRDID  TFLRIR**S**KKHEIM  RRFFNS**S**GSSDAS  FNSSGS**S**DASIGD  RPPQKC**S**TPCGPL  SASLFS**S**LASPCP  ASPCPG**S**PTPRDS  SPTPRD**S**VISIGT  PRDSVI**S**IGTSAC  VISIGT**S**ACLVAA  ACLVAA**S**AVPSGL  RASLPC**S**QEEATG  TRASLR**S**VLFGLM  LFGLMN**S**GTPEDS  EGPGLS**S**TGKRRA  LERTRS**S**RKSKHQ  ETSLLH**S**HRFKKG  KTRASF**S**FHKKKI  KIVTDV**S**EVCSIY  ATSLSG**S**LLSECS  SLLSEC**S**NRPVMN  PVMNRT**S**GAPSSW  RTSGAP**S**SWHSSS  APSSWH**S**SSMYLL  LNTLSI**S**NKKASD  ISNKKA**S**DAEKVY  PDLVNG**S**HQKTFE  VHCVQG**S**YPPLLL  YNSTKG**S**ANDRPD  SLATAK**S**ILHQLT  VLLKKT**S**LKKLHY  KSSTIP**S**CGLQVS  VVFCDV**S**MDEDLF  LQARKP**S**DCDSKE  KPSDCD**S**KELQRR  ELRQKL**S**LNFHSL  LSLNFH**S**LCEQQP  EGPTKD**S**ALQGLV  LVATCA**S**APAPGN  NPQPFL**S**QAVATK  FKDFVT**S**AFYDKF  FEMQPV**S**DKYFTE  EILEKV**S**SPFIVS  ILEKVS**S**PFIVSL  SSPFIV**S**LAYAFE  LAYAFE**S**KTHLCL  HLCLVM**S**LMNGGD  TRGLDM**S**RVIFYS  SRVIFY**S**AQIACG  LGNCRL**S**DLGLAV  ILMEKV**S**YSYPVD  MEKVSY**S**YPVDWF  WFAMGC**S**IYEMVA  DYKEKV**S**KEDLKQ  PEQRLG**S**REKSDD  LGSREK**S**DDPRKH  PFVPDP**S**VVYAKD  AEIDDF**S**EVRGVE  GCEEGN**S**SKSGVC  CEEGNS**S**KSGVCL  LQILYK**S**LEKSIP  YKSLEK**S**IPESIK  EKSIPE**S**IKVYGA  KLEEVL**S**YSNVIS  EEVLSY**S**NVISWE  SYSNVI**S**WEQTLQ  IRKVAT**S**KSVQVD  KVATSK**S**VQVDYM  PKKHKT**S**SNDKME  KKHKTS**S**NDKMEL  NKEGNF**S**NMFLDA  NMFLDA**S**HAGLVN  FGKNER**S**LKYIER  PEGQLV**S**WIVMEQ  WIVMEQ**S**CELRMG  HLEKYL**S**QKEIPF  ADNNEK**S**LQALNN  GLQQQR**S**VEPMRL  VEPMRL**S**MDTPMP  KRSAVA**S**SVVKQK  TRSMLS**S**FLPPVP  FLPPVP**S**LPSDPP  RYKPKK**S**LERRKN  AETLGD**S**SPSSSS  GDSSPS**S**SSTPAS  SSPSSS**S**TPASGC  TPASGC**S**SPNDSE  PASGCS**S**PNDSEH  PNPILG**S**EALLGQ  LRLQET**S**VAPFAL  FALPTV**S**LLPAIT  APARAD**S**DRRTHP  AGGTLP**S**RLQPIL  LLDPSG**S**HAPLLT  GRLPRG**S**TGDTVL  SQARVL**S**SSETPA  QARVLS**S**SETPAR  HAGRIQ**S**IWSRLQ  YGTNPL**S**RLKLDN  ARWAAG**S**VTDLAF  LAFKVA**S**RELKNG  DEVGAG**S**GEGFNV  PIAREF**S**PDLVLV  PDLVLV**S**AGFDAA  LGGYHV**S**AKCFGY  LASCPD**S**WVPRVP  AVTALA**S**LSVGIL  LAEDRP**S**EQLVEE  VQLKKT**S**EVDLAK  IQQTYP**S**GGEEQA  RAAEEL**S**KLRRAA  EPKFPF**S**ENQICL  DAFDKG**S**LFGGSV  GSLFGG**S**VKLALA  VKLALA**S**LGYEKS  IKETVL**S**ALSREP  EPTVDI**S**PDTVGT  DTVGTL**S**LIMLAQ  NAEYHQ**S**ILAKQQ  LIKTVA**S**RYDEYV  KATLVK**S**TPVNVP  PVNVPI**S**QKFTDL  EKMVPV**S**VQQSLA  PVSVQQ**S**LAAYNQ  ADLVNR**S**IAQMRE  GDTVPQ**S**ILTKSR  QSILTK**S**RSVIEQ  ILTKSR**S**VIEQGG  REILDE**S**LRLLDE  LNAAIP**S**ANPAKT  AKTMQG**S**EVVNVL  VNFDMT**S**KFLTAL  INEEAL**S**VTELDR  TTKVQE**S**LKKQEG  LKNIQV**S**HQEFSK  VSHQEF**S**KMKQSN  FSKMKQ**S**NNEANL  SIAREP**S**APSIPT  TPAYQS**S**PAGGHA  PANRAP**S**ATAPSP  PSQTPG**S**APPPQA  KITVPA**S**QKLRQL  PRRDYA**S**ESPADE  DEEEQW**S**DDFDSD  ENPDEH**S**DSEMYV  PDEHSD**S**EMYVMP  EENADD**S**YEPPPV  EYIDNR**S**SQRHSP  RSSQRH**S**PPFSKT  RHSPPF**S**KTLPSK  TLPSKP**S**WPSEKA  SKPSWP**S**EKARLT  EKARLT**S**TLPALT  IHPTES**S**SPPPEK  HPTESS**S**PPPEKA  APMVNR**S**TKPNSS  NSSTPA**S**PPGTAS  SPPGTA**S**GRNSGA  TASGRN**S**GAWETK  PPPAAP**S**PLPRAG  KTTPVA**S**QQNASS  SQQNAS**S**VCEEKP  RQEAVQ**S**PVFPPA  VDGPLP**S**FSSNST  GPLPSF**S**SNSTIS  PLPSFS**S**NSTISE  PSFSSN**S**TISEQE  SSNSTI**S**EQEAGV  RSNKDG**S**FLIRKS  SFLIRK**S**SGHDSK  FLIRKS**S**GHDSKQ  GEEYFG**S**VAEIIR  IRNHQH**S**PLVLID  PLVLID**S**QNNTKD  VPHIKN**S**TGEPVW  LHLLLH**S**DRDPIP  RNQLYE**S**YYLNFI  YYLNFI**S**AISRSK  NFISAI**S**RSKLED  ISAISR**S**KLEDIA  NAALAA**S**AVTQVA  QNKELV**S**YRAINR  MDTIVD**S**LFCFFV  VPIIRC**S**RGTAAE  LRDARN**S**LFTGDT  LGAGQF**S**FQRPLL  RVNLEE**S**SGVENS  VNLEES**S**GVENSP  SSGVEN**S**PAGARP  KRKNKK**S**YDLTPV  FPEVAE**S**VQQELE  VQQELE**S**YRAQED  EVKRLK**S**IMGLEG  EDEGAI**S**MLSDNT  GAISML**S**DNTAKL  NTAKLT**S**AVSSLP  KLTSAV**S**SLPELL  YEEKIM**S**KTTLDK  SLLDII**S**DPDAGT  FLIYYI**S**TQQAPS  STQQAP**S**EADLEQ  AFTKMA**S**APASYG  APASYG**S**TTTKPM  RVMNTG**S**QFVMEG  MLRGND**S**SVPRNK  LRGNDS**S**VPRNKN  HILYGC**S**ELFNAT  DEKPEK**S**QRAGAA  VKTKTV**S**SSNGGE  KTKTVS**S**SNGGES  TKTVSS**S**NGGESS  SSNGGE**S**SSRSAE  SNGGES**S**SRSAEK  NGGESS**S**RSAEKR  GESSSR**S**AEKRSA  RSAEKR**S**AEEEAA  TKPTKI**S**KFGFAI  FGFAIG**S**QTTKKA  QTTKKA**S**AISIKL  KKASAI**S**IKLGSS  ISIKLG**S**SKPKET  SIKLGS**S**KPKETV  LAPKTL**S**VAAAFN  FNEDED**S**EPEEMP  GRDTPT**S**AGPNSF  TSAGPN**S**FNKGKH  KGKHGF**S**DNQKLW  WERNIK**S**HLGNVH  ETSASS**S**QPQDNS  SQPQDN**S**QVHRET  GKAGLF**S**QEQYER  QYERNK**S**SSSSSS  YERNKS**S**SSSSSS  ERNKSS**S**SSSSSS  RNKSSS**S**SSSSSS  NKSSSS**S**SSSSSS  KSSSSS**S**SSSSSS  SSSSSS**S**SSSSSE  SSSSSS**S**SSSSES  SSSSSS**S**SSSESN  SSSSSS**S**SSESND  SSSSSS**S**SESNDE  SSSSSS**S**ESNDED  SSSSSE**S**NDEDQQ  TGKHRR**S**LGAGYP  YPHGNG**S**PGPGHG  PGEVVP**S**GESGLR  GLRRRG**S**DPASGE  SGEVEA**S**QLRRLN  YADWFM**S**LGVGLL  GLLTFA**S**LETVGI  LVYRIH**S**VLQGFI  PGPLAT**S**AAPPGP  GPPAAA**S**PCLGPV  GAAAIG**S**ARGQST  LTAVVG**S**TCDVKV  IFKTLS**S**KFELAV  AVHRKA**S**EPAGGP  TMVFKP**S**DVMLVH  KDKFTD**S**AIAMNS  SAIAMN**S**KVNGEH  DLESDM**S**NGWDPN  TYDSSL**S**SYTVPL  YDSSLS**S**YTVPLE  RGGVRC**S**SSRGGR  KTLSSP**S**NRPSGE  RPSGET**S**VPPPPA  AAPAPI**S**ASCPEP  PEPPIG**S**AVPTSS  SAVPTS**S**ASIPVT  GSISPA**S**PKISLA  PASPKI**S**LAPTDV  FKLQPS**S**SPENSL  VDGLLT**S**EPMGSP  TSEPMG**S**PVSSKT  MGSPVS**S**KTESVS  PPPPCP**S**QTGSPP  AEQVKK**S**TLNPNA  RTHSTP**S**IPVLTA  PYPVSN**S**VPGQQG  AATPYS**S**YIPYNP  PMAHYP**S**QPVFAP  ATTPTG**S**QPQSQH  SQHAAP**S**PVQHQA  SLPPGP**S**AQSPQS  GTSSHE**S**FYDSLS  EISQIF**S**TSPASL  RPQDNR**S**TPVQPL  RGAKTP**S**TNVKPK  CLATNP**S**GSDTTS  VFIEGA**S**STDSDS  ESLAFK**S**RAGAMP  AQKKTT**S**VSLTIG  VSLTIG**S**SSPKTG  SLTIGS**S**SPKTGV  AVIQPL**S**VPVQQV  LQKKPR**S**TAEPEE  DYGSAT**S**TAQLVV  PPILET**S**SLELAS  PILETS**S**LELASK  KANSNK**S**LPTPAV  TPAVLL**S**PTKEPP  ASPEPM**S**ALASRS  SALASR**S**APAMQS  TPASSP**S**SSSLPS  WGSSSP**S**PPPPPP  PPPPVF**S**PTAAFP  TPAAFL**S**ALLPSQ  KDGKQI**S**PKSDHY  SLHTTA**S**TLDDDG  LPTPDL**S**WQLDGK  IIEPVT**S**RDAGIY  NESLTH**S**TDRVSM  WHQQSQ**S**TKPKKV  PKKVRP**S**ASRYAA  SRYAAL**S**DQGLDI  NQLHRK**S**VKKGFD  LMVAGE**S**GLGKST  ESGLGK**S**TLINSL  KSTLIN**S**LFLTNL  RQVPEA**S**ARLTQT  TPGFGD**S**VDCSDC  GDSVDC**S**DCWLPV  QYLRDE**S**GLNRKN  RKNIQD**S**RVHCCL  CCLYFI**S**PFGRGL  DAEMKE**S**IPFAVV  PFAVVG**S**CEVVRD  RARCLQ**S**LARPGA  RDRASR**S**KLSRQS  ASRSKL**S**RQSATE  QAQMQQ**S**QAQGEQ  GQIPDP**S**VTTGSL  PSVTTG**S**LPGLGP  PLTGLP**S**SALTVE  LTGLPS**S**ALTVEE  RPQPVK**S**ELDEEE  EFLQRE**S**ERLELM  CIVRTD**S**VKTPES  SVKTPE**S**EGNPLL  SELEAG**S**EEDRKL  NLSLSE**S**FFMVKG  LLGVDF**S**SKESKS  SSKESK**S**CTIGMV  YESCIS**S**EQSCIN  CISSEQ**S**CINEWN  AMQDLE**S**TRPDSP  LRSIMM**S**QDLENV  KAKRNH**S**KCLVHC  WRQQTD**S**SLQQPV  RQQTDS**S**LQQPVD  CCFRRL**S**DPLLPS  SDPLLP**S**PEDETG  KKLEFG**S**PKGRSG  ENLNNN**S**KRSCPN  FLPHIT**S**SPVAHL  AHLASR**S**RVPEKP  VPEKPA**S**GPTEPP  FLPPAG**S**RRADTS  PKVLPK**S**LLLKNS  SLLLKN**S**HCDKNP  DLRLLF**S**NESEKP  EKPTTN**S**YLMQHQ  AGSDEK**S**EAAPAS  EGGSLK**S**PPPFFY  DHTSSF**S**KDFLKT  YTPTSS**S**MSSNLT  PTSSSM**S**SNLTRS  LGSLTF**S**TEDLSS  FSTEDL**S**SEADPS  AATSKP**S**GKPAPE  EDFHGH**S**HRHSHE  GHSHRH**S**HEDFHH  DFHHGH**S**HAHGHG  HGHTHE**S**IWHGHT  DHDHGH**S**HEDLHH  DLHHGH**S**HGYSHE  GHSHGY**S**HESLYH  HGYSHE**S**LYHRGH  GHDHEH**S**HGGYGE  HGGYGE**S**GAPGIK  GATVLI**S**AAPFFV  FLIPVE**S**NSPRHR  NSPRHR**S**LLQILL  LLQILL**S**FASGGL  ILLSFA**S**GGLLGD  HALEPH**S**HHTLEQ  QPGHGH**S**HSGQGP  GHGHSH**S**GQGPIL  VGLWVL**S**GIVAFL  KGGHGH**S**HGHGHA  GHGHAH**S**HTRGSH  HSHTRG**S**HGHGRQ  HGRQER**S**TKEKQS  QKRRGG**S**TVPKDG  GLDLRV**S**GYLNLA  GLAIGA**S**FRGGRG  FAILVQ**S**GCSKKQ  LVQSGC**S**KKQAMR  EGGAVG**S**EIAGGA  ELLREA**S**PLQSLL  EASPLQ**S**LLEVLG  VTKAGG**S**AALSPS  GGSAAL**S**PSKKRK  SAALSP**S**KKRKNS  SKKRKN**S**KKKNQP  NQPGKY**S**QLVVET  LGERNG**S**SLAKIY  GERNGS**S**LAKIYT  RTYLKY**S**IKALVQ  GTGANG**S**FKLNRK  APGAAG**S**RRADKK  QKPEQR**S**HKKGAG  KKAAKP**S**VPKVPK  KRNARW**S**THTPLD  FLEVIR**S**EDTTGP  TPISSA**S**SEAASA  PISSAS**S**EAASAV  ASSEAA**S**AVVSPS  SAVVSP**S**TDSGLE  PGSPGY**S**TATEPG  LFRFLI**S**LTNPHD  LNLYAA**S**LRVCFL  QLWRIP**S**FVTELY  NIDLLE**S**FVSTFS  SFVSTF**S**FQGLRL  LRVPTA**S**YDLDLF  RKCAMI**S**AHYGLS  TALSSE**S**IENLPS  SIENLP**S**VFGSNP  QREETP**S**NRGEST  GESTVL**S**FVSWLT  LMKALV**S**VTPDEE  LLLMKP**S**VLSRVS  LDRGYT**S**DSEVYT  NSKPGP**S**RPGPSP  LLKCVE**S**LSFIVR  NRFKKK**S**KEGSML  AASIYS**S**WAEEQR  QKIEAD**S**RTLWAH  DALEWE**S**CFNKVL  YMHAGS**S**DLLSEA  PPPEIP**S**ELGACD  ESPRAA**S**SSSPGS  SPRAAS**S**SSPGSP  PGSPVA**S**SPSRLS  ALIEAT**S**PVPLLA  EVCDGW**S**LANHEY  ALQHAD**S**SNFYIT  LQHADS**S**NFYITE  ILRLTT**S**PAQNAQ  ERIQSS**S**MDAKLE  ALKDLA**S**LSRDVT  INLDGI**S**LLTQMV  LTQMVE**S**GTERYQ  MDHGIV**S**WDTFSV  VSWDTF**S**VAFIKK  ASFVNK**S**AIDISI  KSAIDI**S**ILQRSL  SLAILE**S**MVLNSH  IPHLQG**S**DQEIQT  AQKQLR**S**IILTHV  IAFDAE**S**EPNNSS  ESEPNN**S**SGSMEK  SEPNNS**S**GSMEKR  PNNSSG**S**MEKRKS  SMEKRK**S**MYTRDY  RIVLEN**S**SREDKH  ECPFGR**S**SIELTK  CPFGRS**S**IELTKM  FFTHDR**S**FEEFFC  ALTTKP**S**SLDQFK  LTTKPS**S**LDQFKS  SLDQFK**S**KLQNLS  SKLQNL**S**YTEILK  ILKIRQ**S**ERMNQE  FWYCRL**S**PNHKVL  YGDLEE**S**PQGEVP  GEVPHD**S**LQDKLP  VLELAF**S**ILYDSN  EDEQWL**S**SISQYS  DEQWLS**S**ISQYSG  QWLSSI**S**QYSGKI  SSISQY**S**GKIKHW  CQKVKC**S**RHKVCI  QRAMCI**S**RKKLEH  LHGNKD**S**ICKPCH  HMAQLA**S**VCGSDG  LASVCG**S**DGHTYS  SDGHTY**S**SVCKLE  DGHTYS**S**VCKLEQ  EQQACL**S**SKQLAV  QQACLS**S**KQLAVR  TEQAAT**S**TADGKP  QLLHEN**S**KQNGSA  NSKQNG**S**ASSVAG  KQNGSA**S**SVAGPA  QNGSAS**S**VAGPAS  SVAGPA**S**GLDKSL  DKSLGA**S**CKDSIG  GASCKD**S**IGWMFS  SIGWMF**S**KLDTSA  FSKLDT**S**ADLFLD  IRPFFN**S**CDTYKD  YKDGRV**S**TAEWCF  PGIFIP**S**CDEDGY  KMQCDQ**S**SGDCWC  MQCDQS**S**GDCWCV  GTRTHG**S**PDCDDI  DDIVGF**S**GDFGSG  FSGDFG**S**GVGWED  YYLETS**S**QPALHI  VIRLQP**S**WKHKLS  SWKHKL**S**QAPLLP  LPMIPQ**S**GKQHLL  LVHLHA**S**VYALFH  FLRSHY**S**MKENLE  PELVTG**S**KDHELD  IECAKI**S**LDPTEA  SYEDGY**S**VSHQIS  YADTQN**S**YGCATS  ATLWSP**S**MVCGMT  LGTPAT**S**PPPAPL  PAPLCH**S**DDYVHI  DDYVHI**S**LPQATV  LASEED**S**IEKDKE  THSAAS**S**SQGASV  SSSQGA**S**VNPEPL  IDLPCG**S**ADESPA  CGSADE**S**PAGDRE  DRECQT**S**LETSIF  LLLSQV**S**QKLSNS  KLSNSE**S**VQQQME  ELEKNR**S**HVLQQT  SSARGS**S**GSRGGG  RGGGGS**S**SSSSEL  SSSSEL**S**TPEKPP  QRAGPF**S**SRWETT  SLPSSK**S**FLGMKA  TSSLSE**S**LKTELG  LDGPHP**S**PPTPDS  SPPTPD**S**VGQLHI  IETLHG**S**VHVTLC  QQDGAA**S**FPAGYM  AGYMYP**S**MDQLAE  QQFGLK**S**IIGMGT  WMDWAA**S**KISGWT  WAASKI**S**GWTQAL  LPDMVV**S**HLFGKE  GKEEMQ**S**NVEVVH  FINAYN**S**RRDLEI  LLVVGD**S**SPAVDA  LVVGDS**S**PAVDAV  AVVECN**S**KLDPTK  GGLPQI**S**QPAKLA  GMGYMP**S**ASMTRL  GYMPSA**S**MTRLMR  SLDGTR**S**RSHTSE  DGTRSR**S**HTSEGT  RSRSHT**S**EGTRSR  EGTRSR**S**HTSEGT  TSEGTR**S**RSHTSE  RSRSHT**S**EGAHLD  LDITPN**S**GAAGNS  SGAAGN**S**AGPKSM  QWINEL**S**SPETRE  WINELS**S**PETREN  NALLEL**S**KKRESV  LSKKRE**S**VPDLAP  APMLWH**S**FGTIAA  IVNIYP**S**INPPTL  TLTAHQ**S**NRVCNA  LLQCVA**S**HPETRS  SHPETR**S**AFLAAH  PFLHTV**S**KTRPFE  EYLRLT**S**LGVIGA  CLRIME**S**GSELSK  RIMESG**S**ELSKTV  ESGSEL**S**KTVATF  QTYERF**S**HVAMIL  KMVLQL**S**KEPSAR  QLSKEP**S**ARLLKH  RCYLRL**S**DNPRAR  PSKSSG**S**AALLAL  YLSAIL**S**LEPNQN  LPTIQK**S**LLRSPE  LAGHLK**S**NSPRLM  NLARQC**S**DSSAME  DSSAME**S**LTKHLF  HVVSGP**S**SQVLNG  AFSLKT**S**TSAVRH  KLSVAD**S**QAEAKL  SLVAVQ**S**GLWPAL  SMNAMG**S**LSVLSP  NAMGSL**S**VLSPDR  DKSIIQ**S**AQQDSI  ILAKNP**S**GLTQYI  IPVLVD**S**FLPLLK  LSAPAF**S**LVFPFL  SQYLDS**S**QVKPLF  NAPNDA**S**YDAVRQ  AKHLDK**S**DPKVKP  VQESVA**S**CLPPLV  WRTKAG**S**VELLGA  RAFQDR**S**TDTRKM  TPGLKA**S**LLDPVP  PEVRTV**S**AKALGA  EIVATA**S**KVDIAP  MTTETA**S**EDDNFG  VWKIVV**S**NTPRTL  GVCIGL**S**EIMKST  RLIGNE**S**KGEHVP  LVIRLT**S**ADALRP  IRKNIV**S**LLLSML  EDNTRI**S**SAGCLG  MVRHGR**S**LALSVA  DTSEDA**S**IHRLEG  GGLICK**S**KSAASE  KSKSAA**S**EQHVFK  GEDKKK**S**KVSSYK  KKSKVS**S**YKDWEE  EEFWER**S**RQRERE  HGVYAS**S**KEEKDW  DWKKEK**S**RDRDYD  SRHSSR**S**ERDGGS  GGSERS**S**RRNEPE  RRNEPE**S**PRHRPK  KDAATP**S**RSTWEE  AATPSR**S**TWEEED  EDSGYG**S**SRRSQW  RRSQWE**S**PSPTPS  YRDSER**S**HRLSTR  TRDRDR**S**VRGKYS  DRRHLG**S**TPRLSR  DHMKRK**S**EASSEF  VVGETG**S**GKTTQL  RRVAAM**S**VAKRVS  SLQVHL**S**GAPGDI  AVLPIY**S**QLPSDL  TNIAET**S**LTVDGI  IMFVID**S**GYCKLK  EDNMLN**S**MYQLWI  NTGGLT**S**TGRLMV  SKMLIV**S**CDMGCS  LIVSML**S**VPAIFY  MVQQRM**S**LASCGT  CHLHPT**S**SLFGMG  LGPMFY**S**VKQAGK  QEQEKR**S**PLGSVR  LSGKRL**S**SGFGGG  SGKRLS**S**GFGGGG  GGGGGG**S**GGGGGG  GGGGLG**S**SSHPLS  GGGLGS**S**SHPLSS  AHPNLL**S**CNTKKH  YQTEMG**S**ALHEAA  TVRELP**S**QKSQQI  LISSMD**S**ISQKSQ  NAISCH**S**LDSMAS  MASGRS**S**DQDSTN  TKDHRR**S**SSSRSQ  HRRSSS**S**RSQDSA  QSHPDG**S**PQQGAC  PSALDQ**S**KRVGYL  AEEGDR**S**GARSRA  ELKLSR**S**LSKSDS  KLSRSL**S**KSDSDL  SLSKSD**S**DLLTCS  SDLLTC**S**PTEDAT  EDATMG**S**RSESLS  ATMGSR**S**ESLSNC  ESLSNC**S**IGKKRL  GEGIDF**S**QERQKI  LRTLEQ**S**VGEWLE  DVHFLG**S**NVMEEQ  LQDYVH**S**FLSSGY  YVHSFL**S**SGYSSI  QKPPRF**S**QLRCQD  QTSSPL**S**QNDSCT  CAKMRK**S**TEHMKK  DAQSHA**S**VSWVVD  QSHASV**S**WVVDPK  TGRGGD**S**AVRQLQ  LPPLRA**S**NAAAAA  AHTIGG**S**KHTMND  DHLHVG**S**HAHGQI  IHRRQG**S**STSLKS  HRRQGS**S**TSLKSM  RQGSST**S**LKSMEG  SSTSLK**S**MEGMGK  EHHEIF**S**YPEIYF  KVIGKG**S**FGQVVK  MTFELL**S**MNLYEL  NKFQGF**S**LPLVRK  VRKFAH**S**ILQCLD  LKQQGR**S**GIKVID  KVIDFG**S**SCYEHQ  VYTYIQ**S**RFYRAP  MPIDMW**S**LGCILA  RAKNFV**S**SKGYPR  AKNFVS**S**KGYPRY  CTVTTL**S**DGSVVL  TTLSDG**S**VVLNGG  VLNGGR**S**RRGKLR  LRGPPE**S**REWGNA  PTGEKT**S**VKRITE  VKRITE**S**TGAITS  STGAIT**S**ISKLPP  GAITSI**S**KLPPPS  SKLPPP**S**SSASKL  KLPPPS**S**SASKLR  LPPPSS**S**ASKLRT  PPSSSA**S**KLRTNL  HEPSGY**S**TVFRST  GSEQQS**S**VQLWVS  SVQLWV**S**FGRKPM  ATLING**S**MNEENM  TEPGEK**S**TVFTIF  NKENVH**S**WLILQV  YKLWSP**S**DERGNE  DFGFKM**S**EDLSLE  RGYRKI**S**GDTCSG  LAFEPL**S**QLLYWV  KWPNGI**S**VDDQWI  FRASKY**S**GSQMEI  LPKANN**S**RSCRCP  RCPEDV**S**SSVLPS  PEDVSS**S**VLPSGD  TDCQDG**S**DEDPVN  RDCSDG**S**DEQHCE  QQCLFH**S**MVCDGI  IQCRDG**S**DEDAAF  RDCADG**S**DEEACP  ANVTAA**S**TPTQLG  ANCPTH**S**TLTCMS  LDCSDE**S**DEKACS  YRVVGE**S**IWKTLE  IVEYSR**S**GSKMWA  PDIHID**S**YGENYL  VRPPAP**S**LKAKAI  VPYQGP**S**SDYVVV  EKHFNE**S**RGYEIH  HRRLQS**S**FTAFAN  FTAFAN**S**HYSSRL  CTENAC**S**KPDDDI  AAKIQA**S**FRGHMA  ARKKIK**S**GERGRK  DIKNSP**S**SLNSPS  IKNSPS**S**LNSPSS  SPSSLN**S**PSSYNC  SSLNSP**S**SYNCSQ  SLNSPS**S**YNCSQS  PSSYNC**S**QSILPL  GSIYIP**S**SYVDSH  SIYIPS**S**YVDSHH  PSSYVD**S**HHEYPA  PGRQTT**S**PNVLWP  VVHRQL**S**HLYAEP  PWCEAR**S**LEHTLP  TLKRKV**S**GNRCAS  HFCAVC**S**DYASGY  VCSDYA**S**GYHYGV  KAFFKR**S**IQGHND  LLLDAL**S**PEQLVL  PPHVLI**S**RPSAPF  VLISRP**S**APFTEA  APFTEA**S**MMMSLT  QVRLLE**S**CWMEVL  AMILLN**S**SMYPLV  VWVIAK**S**GISSQQ  IAKSGI**S**SQQQSM  AKSGIS**S**QQQSMR  ISSQQQ**S**MRLANL  SHVRHA**S**NKGMEH  LRGCKS**S**ITGSEC  KSSITG**S**ECSPAE  CSPAED**S**KSKEGS  NGQLDL**S**GKLIIK  LITIFD**S**SDLSFA  ITIFDS**S**DLSFAI  FDSSDL**S**FAIQCS  SFAIQC**S**RILKLT  QPRPLE**S**SQVKYL  PRPLES**S**QVKYLR  VNRLLD**S**LEPPGE  PGEPGP**S**TNIPEN  DGREEK**S**ASDSSG  REEKSA**S**DSSGKQ  EKSASD**S**SGKQST  KSASDS**S**GKQSTQ  DSSGKQ**S**TQVMAA  VMAASM**S**AFDPLK  INKNVM**S**AFGLTD  LTDDQV**S**GPPSAP  QVSGPP**S**APAEDR  APAEDR**S**GTPDSI  TPDSIA**S**SSSAAH  PDSIAS**S**SSAAHP  DSIASS**S**SAAHPP  SIASSS**S**AAHPPG  QYGIQY**S**ASYSQQ  GIQYSA**S**YSQQTG  QYSASY**S**QQTGPQ  YGQQPT**S**QAPAPA  APAPAF**S**GQPQQL  YTVAPA**S**QPGMAP  QPGMAP**S**QPGAYQ  PRPGFT**S**LPGSTM  FTSLPG**S**TMTPPP  TMTPPP**S**GPNPYA  RLCFLE**S**TSNSKN  ERYLHL**S**YGNGSL  YEGGAV**S**VHARSL  TTGKYL**S**LMEDKN  HMDDGI**S**LSRSQH  RGLDAL**S**KKAKAS  CAQFSG**S**LDWLIS  LVNHVS**S**MRPNIF  PCLVEF**S**KLPEQE  DITMWL**S**KRLPQF  SFGSQN**S**NTDIMF  RTKPDY**S**TSHSAR  DYSTSH**S**ARLTED  YYQVEP**S**TKLFPA  LPGIGL**S**TSLRPR  AMFDHL**S**YLLENS  AGCGLQ**S**CQMLVS  DLRAAA**S**LDTAAL  GTEHHA**S**LIDSLL  MESNYV**S**MMEKQS  NKYAEH**S**HDKWSM  RRISQT**S**QVSVDA  FGNDAT**S**IVNCLH  LLPMLS**S**LFEHIG  RERAAL**S**LPTNVE  LAGLMQ**S**CSVLDL  DMLVES**S**NNVEMI  KLKDLT**S**SDTFKE  FFRIIC**S**LLLGGS  GLSDLM**S**NPVPMP  VTSLDS**S**SHRIIA  DVEKAT**S**EMNTAE  PKDCLR**S**IMRRVN  LLGACV**S**NCGKIF  CSRDFA**S**EVSNVL  DFASEV**S**NVLNKG  PQLSLI**S**AMIKNL  TFPAIG**S**QAAEQA  AEQAKA**S**PALVAK  AKAIEL**S**LKEQRQ  KEQRQQ**S**TTLSTL  QQSTTL**S**TLYPST  LSTLYP**S**TSSLLT  KKTVQF**S**DDVQVE  LLQMLQ**S**TDPSDD  LQSTDP**S**DDQPDL  DIDRKH**S**ELSELN  RKHSEL**S**ELNVKV  KVMEAL**S**LYTKLM  NEDPMY**S**MYAKLQ  QPYYMQ**S**SGVSGS  YAGPPP**S**GAYLVA  AGNAQM**S**HLQSYS  SHLQSY**S**LPPEQL  LPPEQL**S**SLSQAV  PPEQLS**S**LSQAVV  EQLSSL**S**QAVVPP  QAVVPP**S**ANPALP  ANPALP**S**QQTQAA  YPNTMV**S**SVQGNT  SQAPVY**S**PPPAAT  PNYNLT**S**STLPQP  NYNLTS**S**TLPQPG  LPQPGG**S**QQPPQP  GFSAND**S**TDFGSL  GNVSAS**S**PVQQGL  NSANMA**S**LSAMGK  LPKQAA**S**TSGPTP  TDIKNT**S**VTNVPN  SSRQII**S**HWKNCT  QNATSL**S**NPNPID  NSFNPM**S**LGNVQL  NQFPSS**S**GAMSVG  TAAGMP**S**LQHTTP  AAPTQP**S**TPVSSS  TPVQPP**S**VATPQS  VATPQS**S**QQQPTP  NRVPTP**S**SVASAE  TPSSVA**S**AETNSQ  SQSTSP**S**QPRKKI  YISYLD**S**IHFFRP  TEDRLT**S**AKELPY  TNKNKS**S**ISRANK  NVSNDL**S**QKLYAT  DPDPLL**S**CDLMDG  LRRSKW**S**TLCMLV  GLDDEG**S**SQGEPQ  CRNANC**S**LPSCQK  RNVPQQ**S**LPSPTS  PQPSPV**S**MSPAGF  SPAGFP**S**VARTQP  MKQQIG**S**PGQPNP  PQQHML**S**GQPQAS  LNTPSR**S**ALSSEL  PSRSAL**S**SELSLV  SALFGG**S**AASGFH  ESCESL**S**CLPPVS  GNSGHP**S**CLKFSP  NMLFCD**S**CDRGFH  EVDGNV**S**TIYCQN  DFSYLL**S**KREGQA  REGQAG**S**PEKPLS  KRKHHN**S**SVVTET  LRCQSS**S**KRKSKD  EEEDEE**S**DDADDT  PILKPV**S**LLRKRD  KRDVKN**S**PLEPDT  EEDAAS**S**EVPAAS  DDGHLE**S**TKKKEL  SLTQEE**S**SEHEGA  GSLSAP**S**MQNMET  MDVPSV**S**DHSQQV  ENYENP**S**SYDSTM  ICGNSS**S**QSSCSY  CSYGGL**S**SSSSLT  SYGGLS**S**SSSLTQ  YGGLSS**S**SSLTQS  GLSSSS**S**LTQSSC  SMMQQS**S**VQPAAN  ANCSIK**S**PQSCVV  QQQPPL**S**QCSMNN  PPLSQC**S**MNNSFT  GSTGNI**S**IYERIP  FGAGSY**S**QPSATF  QPSATF**S**LAKLQQ  KSAPLP**S**AAAHQQ  QMGMMG**S**QAYTQQ  MMYTGP**S**HHSYMN  SGGEQQ**S**YSTYGN  GEQQSY**S**TYGNPG  TYGNPG**S**QGYGQA  QASQSY**S**GYGQTT  YGQTTD**S**SYGQNY  SYGQNY**S**GYSSYG  QNYSGY**S**SYGQSQ  NYSGYS**S**YGQSQS  YSSYGQ**S**QSGYSQ  SYGQSQ**S**GYSQSY  QSGYSQ**S**YGGYEN  YENQKQ**S**SYSQQP  QQQNME**S**SGSQGG  NMESSG**S**QGGRAP  DYGQQD**S**YDQQSG  YDQHQG**S**YDEQSN  YSQNQQ**S**YHSQRE  SQRENY**S**HHTQDD  NRGYGG**S**QGGGRG  RGPMTG**S**SGGDRG  GPMTGS**S**GGDRGG  PRTDAD**S**ESDNSD  TDADSE**S**DNSDNN  DSESDN**S**DNNTIF  KGEATV**S**FDDPPS  FMRGGG**S**GGGRRG  WVCPNP**S**CGNMNF  NFARRN**S**CNQCNE  EPRPED**S**RPSGGD  SSGGGY**S**GDRSGG  GYSGDR**S**GGGYGG  GDRGGG**S**GYGGDR  GYGGDR**S**GGGYGG  QNSQFQ**S**LEQVKR  VQEVVQ**S**QQVAVG  QLEDFR**S**KRLMGM  STDEEK**S**AAVVNA  KSGDKK**S**GRNFFR  VMGNRR**S**DEPAKT  KTKKGL**S**SILDAA  TKKGLS**S**ILDAAR  QDTPGV**S**LHPLSL  HPLSLD**S**PDREPG  PLELGD**S**SPQGPM  LELGDS**S**PQGPMS  SPQGPM**S**LESLAP  SLAPPE**S**TDEGAE  EGAETE**S**PEPGDE  EGEPGR**S**GLELEP  LQNIFP**S**LDELIE  ELIEVH**S**LFLDRL  SWFQKI**S**SRFCSR  ISSRFC**S**RQSFAL  FVQEAE**S**RPRCRR  QRRLDL**S**HLRQSS  LSHLRQ**S**SDPMLS  SHLRQS**S**DPMLSE  LLLKSH**S**RTLTPT  PVLRLT**S**AMTREV  LVAQTV**S**ERKNWC  RPKPRP**S**PSSTRE  EPLLSS**S**ENGNGG  RTERIL**S**DLLPFC  ALRKVL**S**LKQLLF  IQENLL**S**LEETMK  RLRPLL**S**QLGGNS  VEAYGP**S**SQTLTF  NGAVDD**S**VAKTSQ  DSVAKT**S**QLLAEL  NGRGNT**S**GSHIVN  RGNTSG**S**HIVNHL  CRQPCA**S**QSSLKD  WLVKIP**S**EQEQLR  AIELRS**S**VGAPVE  FVWKST**S**FDRMQS  SFDRMQ**S**ALKTFA  VDETSV**S**GYIYHK  VLQRPL**S**LIQGPP  TGKTVT**S**ATIVYH  DETGEL**S**SADEKR  ETGELS**S**ADEKRY  KAGLSQ**S**LFERLV  RMHPAL**S**AFPSNI  PYEGQR**S**YLVQYM  VQYMQF**S**GSLHTK  KDFIIL**S**CVRANE  ESLMQF**S**KPRKLV  EAIIPG**S**VYDRSS  SVYDRS**S**QGRPSS  SQGRPS**S**MYFQTH  MISAGP**S**HVAAMN  FGLPGP**S**QTNLPN  LPNSQA**S**QDVASQ  ASQDVA**S**QPFSQG  QGYISM**S**QPSQMS  MSQPGL**S**QPELSQ  PELSQD**S**YLGDEF  NMQEAQ**S**IDEIYK  FKYCKI**S**ALALLK  MVMHAR**S**GGNLEV  TMIIMD**S**FALPVE  AIGWYH**S**HPGYGC  GYGCWL**S**GIDVST  LSGIDV**S**TQMLNQ  DPTRTI**S**AGKVNL  PPDEGP**S**EYQTIP  YYALEV**S**YFKSSL  EVSYFK**S**SLDRKL  VSYFKS**S**LDRKLL  YWVNTL**S**SSSLLT  WVNTLS**S**SSLLTN  VNTLSS**S**SLLTNA  NTLSSS**S**LLTNAD  GQVFDL**S**EKLEQS  SEKLEQ**S**EAQLGR  AQLGRG**S**FMLGLE  ETHDRK**S**EDKLAK  AKATRD**S**CKTTIE  AIHGLM**S**QVIKDK  KARDKV**S**GDLVAL  EFCGAG**S**LQDIYQ  IYQVTG**S**LSELQI  ELCDIW**S**LGITAI  LFLMTK**S**GYQPPR  NPGKGP**S**IGDIED  RSTHRS**S**SLGIPD  STHRSS**S**LGIPDA  PPRDLR**S**SSPRKQ  RDLRSS**S**PRKQLS  VLVRCA**S**GPPPNS  RPGPPP**S**TSSPHL  GPPPST**S**SPHLTA  PPPSTS**S**PHLTAH  TAHSEP**S**LWNPPS  CPLRIH**S**TAAWTH  AAWTHP**S**TKDQHL  LEMLFP**S**RTTWVY  RTTWVY**S**INNVLM  INNVLM**S**LSGKTP  VAEGAS**S**GGPFLC  CGALET**S**VVLLQW  PGRPGK**S**VLFHTV  VRFGAL**S**CWLGEM  CWLGEM**S**TEHRGP  MVLMDG**S**VKLVTP  QVWALG**S**DQLLQE  SPRLEC**S**GTISPH  NLLLPG**S**SNSPAS  LLLPGS**S**NSPASA  NSPASA**S**RVAGIT  AVGATG**S**GIAAAA  ATKFWE**S**PETVSQ  ESPETV**S**QLDSVR  FDLQNP**S**RMDRNV  TFTDEK**S**KASHHI  DEKSKA**S**HHIYPY  HIYPYS**S**SQDDEE  IYPYSS**S**QDDEEW  YDTWVH**S**NDVDAE  ENRKPV**S**FRQRIS  SFRQRI**S**TKNEEP  NEEPVR**S**PERRDR  RRDRKA**S**ANARKR  ARKRKH**S**PSPPPP  KRKHSP**S**PPPPTP  TESRKK**S**GKKGQA  VNLKKD**S**ENTPVK  IIPSYA**S**WFDYNC  PQEYLT**S**TACRRN  KKTLAK**S**KGASAG  YQPVPF**S**QSGNPV  PVPFSQ**S**GNPVMS  AALEEF**S**RVREEV  PTYGLE**S**SCIAGT  TYGLES**S**CIAGTG  GENEKN**S**EKEQDS  SEKEQD**S**EVSEDT  EQDSEV**S**EDTKSE  VSEDTK**S**EEKETE  TCKERE**S**DTGKKK  AAAALA**S**AATKAK  EERKIK**S**LVALLV  QIPGPG**S**MMPGQH  IPEFEP**S**EQEDSS  PSEQED**S**SSAERG  SEQEDS**S**SAERGL  EQEDSS**S**AERGLG  ERGLGP**S**PAGDGP  PAGDGP**S**GSGKHH  GDGPSG**S**GKHHRQ  GLLWDA**S**HQQEQP  QQEQPT**S**SSHHGG  QEQPTS**S**SHHGGA  EQPTSS**S**HHGGAG  GAVEIR**S**RHSSYP  EIRSRH**S**SYPAGT  SPFRGR**S**RSAPPN  SDEFVD**S**FKKGLP  ATQMRQ**S**SSWTRV  TQMRQS**S**SWTRVF  QMRQSS**S**WTRVFQ  WTRVFQ**S**WWDRNL  GGPGGG**S**AGGPSQ  GSAGGP**S**QPPGGG  ATTVNN**S**TPDFGF  DGDQPE**S**KKLASQ  ESKKLA**S**QGDSIS  LASQGD**S**ISSQLG  SQGDSI**S**SQLGPI  QGDSIS**S**QLGPIH  HPPPRT**S**MTEEYR  NKIQQD**S**GCKVQI  GGLPER**S**VSLTGA  LTGAPE**S**VQKAKM  MLDDIV**S**RGRGGP  DRNEYG**S**RIGGGI  VPVPRH**S**VGVVIG  GVVIGR**S**GEMIKK  INDLLQ**S**LRSGPP  LLQSLR**S**GPPGPP  GGEMTF**S**IPTHKC  GAFVEI**S**RQLPPN  PAPHDP**S**KAAAAA  AWAAYY**S**HYYQQP  PPPTGQ**S**DYTKAW  PGAPPG**S**QPDYSA  GSQPDY**S**AAWAEY  RARIDR**S**RELASK  RSRELA**S**KTREKE  TIRERP**S**SAIYPS  IRERPS**S**AIYPSD  AIYPSD**S**FRQSLL  SDSFRQ**S**LLGSRR  RGRSSL**S**LAKSVS  SLSLAK**S**VSTTNI  SLAKSV**S**TTNIAG  LSQSTD**S**LNMRNR  MRNRTL**S**VESLID  KDFAAD**S**WSLAVD  WSLAVD**S**SFLQQH  SLAVDS**S**FLQQHK  IHTRFL**S**QLLERR  QALCPG**S**TRNFVI  LGDLLI**S**QFSGPS  QMCKTY**S**EFCSRH  SRILQH**S**HGIEEE  LVKELL**S**NVDEGI  PTLDKP**S**VVSLQN  IRVIQQ**S**VRTCPS  SVRTCP**S**REDFPL  AEEDGG**S**GMALPT  PRGLFR**S**ESLESP  ARTFNG**S**IELCRA  CRADSD**S**SQRDRN  RADSDS**S**QRDRNG  RATEAG**S**LEARLR  EARLRE**S**EQARAL  GSEEEG**S**SRLSPP  FKPRER**S**GGEQAQ  VVLEGA**S**LETVKV  NCDKHK**S**ILLKNG  PDITHQ**S**LLMLMD  LLMLMD**S**PLNRAG  QLLHKL**S**VRAADG  VIKNPV**S**DHFPVG  CMKVGT**S**FSIPVV  KVGTSF**S**IPVVSD  FSIPVV**S**DVRELV  VRELVP**S**SDPIVF  RELVPS**S**DPIVFV  FAHGKV**S**VEYTEK  YTEKMV**S**ISNYPL  EKMVSI**S**NYPLSA  ISNYPL**S**AALTCA  WLLGEI**S**SGCYEG  LLGEIS**S**GCYEGL  FARKDL**S**EADARI  RGRWPP**S**SRGGGP  GRWPPS**S**RGGGPP  FRCALR**S**TRRFVM  VMLRDN**S**GDPADP  HKVYAL**S**RELCWR  LQAVQQ**S**CLADHL  DHLLTA**S**WGADPV  AVETTP**S**PGPQPA  EAAAPE**S**PHQAEP  QAEPYL**S**PSPSAC  EPYLSP**S**PSACTA  YLSPSP**S**ACTAVQ  TAVQEP**S**PGALDV  KVVGHP**S**CTFLYG  QQVAFP**S**PAELPD  VGGPPG**S**ASPSTP  GPPGSA**S**PSTPAC  PGSASP**S**TPACLL  ARQRRG**S**PRYTIY  GFGQDL**S**AGRPKE  GRPKEK**S**LVLVKL  TQREGV**S**SLDSSS  QREGVS**S**LDSSSL  GVSSLD**S**SSLSLC  VSSLDS**S**SLSLCL  SLSLCL**S**SANSLY  LSLCLS**S**ANSLYD  CLSSAN**S**LYDDIE  PLAEIL**S**VKDISG  LSVKDI**S**GRKLFY  EREVPA**S**AQASGK  PASAQA**S**GKTLPI  EPDQPL**S**SSSCLQ  PDQPLS**S**SSCLQP  DQPLSS**S**SCLQPN  QPLSSS**S**CLQPNH  RKVEVV**S**PATPVP  PATPVP**S**ETAPAS  SETAPA**S**VFPQNG  PGRKRK**S**NCLGTD  DEDSQD**S**SDGIPS  APRMTG**S**LVSDRS  MTGSLV**S**DRSHDD  SLVSDR**S**HDDIVT  CLKYGR**S**LKCLQR  DGRKNK**S**YSQNLC  HIVGYF**S**KEKEST  KLLIEF**S**YELSKV  EFSYEL**S**KVEGKT  TPEKPL**S**DLGLLS  GLLSYR**S**YWSQTI  SYRSYW**S**QTILEI  ILMGLK**S**ESGERP  MGLKSE**S**GERPQI  ITINEI**S**EITSIK  EISEIT**S**IKKEDV  KKEDVI**S**TLQYLN  QYILTL**S**EDIVDG  RLLRID**S**KCLHFT  VVSWLV**S**AEGSHP  LVSAEG**S**HPDPAP  GDSRPP**S**FHPHAG  ETDSLV**S**AQRERP  EPGGYD**S**SSTLMS  GGYDSS**S**TLMSSE  SSTLMS**S**ELETTS  SELETT**S**FFDSDE  DEDDST**S**RFSSST  DSTSRF**S**SSTEQS  STSRFS**S**STEQSS  SSSTEQ**S**SASRLM  RRKQKV**S**RIERSS  ERSSSF**S**SITDST  FSSITD**S**TMSLNI  INFENM**S**NDDAVR  FPAYGM**S**PSLSTI  SLSTIT**S**TSSSIT  STITST**S**SSITSS  FHLSIH**S**DMAAIV  EARKYA**S**NLLKAG  SQHSEG**S**RSSGSN  HSEGSR**S**SGSNRS  GSRSSG**S**NRSGSD  GSNRSG**S**DRRKEK  DPKAGD**S**KSGGSG  KAGDSK**S**GGSGSE  GGSGSE**S**DHTTRS  RSGPAA**S**EHSHRS  SEHSHR**S**HHSLAS  SHRSHH**S**LASSLR  NGNVAL**S**DGHNTA  FTVERF**S**RLSESV  FSRLSE**S**VLSPPC  DRPHQK**S**VGFFLQ  SDSTSW**S**CHAQAV  DDEKSF**S**RRISHL  SFSRRI**S**HLFFHK  HGVAWD**S**KKHTGY  ATCYMN**S**LLQTLF  PTEGDD**S**SKSVPL  TEGDDS**S**KSVPLA  GDDSSK**S**VPLALQ  FYELQH**S**DKPVGT  TKKLTK**S**FGWETL  GWETLD**S**FMQHDV  FRGKMV**S**YIQCKE  FDDDVV**S**RCTKEE  GHDDDL**S**VRHCTN  LVYIRE**S**KLSEVL  FKVLKN**S**SLAEFV  LAEFVQ**S**LSQTMG  EFVQSL**S**QTMGFP  DPELAA**S**GATLPK  YDPKTR**S**LNYCGH  HIYTPI**S**CKIRDL  IQDYDV**S**LDKALD  LLQFFK**S**QGYRDG  DFENRR**S**FKCIWL  CLSPAT**S**RTFRIE  VMKRIQ**S**LLDIQE  PQPGNM**S**HPRPWL  PSEEGK**S**LWELVL  QERNAE**S**AIEALK  MGKVIR**S**DRKGVQ  RLIEIK**S**TTLRVD  TLRVDQ**S**ILTGES  SILTGE**S**VSVTKH  KNAIVR**S**LPSVET  CTSVIC**S**DKTGTL  LTTNQM**S**VCRMFV  CALCND**S**ALDYNE  EFTLEF**S**RDRKSM  FSRDRK**S**MSVYCT  RDRKSM**S**VYCTPT  VKGAPE**S**VIERCS  RDWGSG**S**DTLRCL  MELDDC**S**KFVQYE  VEPAHK**S**RIVENL  IVENLQ**S**FNEITA  IGIAMG**S**GTAVAK  GTAVAK**S**AAEMVL  AAEMVL**S**DDNFAS  EGRAIY**S**NMKQFI  MEKLPR**S**PREALI  PTTMAL**S**VLVTIE  MCNALN**S**VSENQS  NALNSV**S**ENQSLL  SVSENQ**S**LLRMPP  LVAVAM**S**MALHFL  FQVTPL**S**GRQWVV  VVVLQI**S**LPVILL  GLLRTV**S**QAWSRQ  RQPLTT**S**WTPDHT  RNEPEV**S**AGNRVE  RRRTGG**S**LRGNPS  SLRGNP**S**SSQVDE  SVDEDS**S**GNQEEQ  EEMDQE**S**DDFDQS  SDDFDQ**S**DDSSRE  FDQSDD**S**SREDEH  DQSDDS**S**REDEHT  NSVTNS**S**SIVDLP  SVTNSS**S**IVDLPV  LPVHQL**S**SPFYTK  PVHQLS**S**PFYTKT  RKLDHG**S**EVRSFS  SEVRSF**S**LGKKPC  WLKMFQ**S**WSGPEK  KPGFIH**S**PWKSAY  IHSPWK**S**AYIRQH  NRIVSG**S**DDNTLK  NTLKVW**S**AVTGKC  HTGGVW**S**SQMRDN  RDNIII**S**GSTDRT  NIIISG**S**TDRTLK  HEKRVV**S**GSRDAT  KRVVSG**S**RDATLR  DGRRVV**S**GAYDFM  DGIHVV**S**GSLDTS  VSGNAD**S**TVKIWD  GPNKHQ**S**AVTCLQ  NFVITS**S**DDGTVK  NLVTLE**S**GGSGGV  TLESGG**S**GGVVWR  VWRIRA**S**NTKLVC  DDGLKG**S**RTAATA  RTAATA**S**DSTRRV  AATASD**S**TRRVSV  DSTRRV**S**VRDKLL  TGEICL**S**LLREHS  SLLREH**S**IDGTGW  VVWGLN**S**LFTDLL  YKKGKD**S**LYAQGR  RYDRKQ**S**GYGGQT  VEPNCR**S**KRMLAI  KFHHLV**S**PFVGQQ  VVKTGG**S**SKKLQP  VKTGGS**S**KKLQPA  KKLQPA**S**LQSLWL  QPASLQ**S**LWLQDT  EMGPPG**S**SPTPEP  MGPPGS**S**PTPEPP  KQVGEP**S**GQKTLD  QKTLDG**S**SRSAEL  KTLDGS**S**RSAELV  LDGSSR**S**AELVPQ  PQGEDD**S**EYLERD  GRWLRV**S**FGLFGS  SFGLFG**S**VWVNDF  VWVNDF**S**RAKKAN  GDWRDP**S**PRLVLH  FYNCQL**S**WSSSPV  NCQLSW**S**SSPVVT  CQLSWS**S**SPVVTP  QLSWSS**S**PVVTPT  PTCDIL**S**EKFHRG  LDQRYF**S**GLGNII  AGIHPL**S**LGSVLS  HPLSLG**S**VLSASR  SLGSVL**S**ASRREV  GSVLSA**S**RREVLV  DHVVEF**S**TAWLQG  QCQPQL**S**EEPEQC  TPLLPD**S**LVYQIF  VYQIFL**S**LGPADV  RQWQAV**S**RDEFLW  RHPAAM**S**WYEEFQ  DQVLHL**S**FSHSGY  VLHLSF**S**HSGYQF  SGYQFA**S**CSKDCT  YQFASC**S**KDCTVK  SNDLTI**S**LLHSAD  WSYTQF**S**QFNKDD  DSLLLA**S**GVFLGP  FLGPHN**S**SSGEIA  AVISLD**S**FALLSR  CWLTET**S**LISGNL  TETSLI**S**GNLHRI  RIGDIT**S**CSVLWL  GDITSC**S**VLWLNN  AFQDVE**S**ENVNVV  IQNLNA**S**TVRTVM  VMVADC**S**RFDSPD  DCSRFD**S**PDLLLE  AGDPAT**S**PCRIFD  RIFDLG**S**DNEEVV  RAQPQL**S**ERMLET  SATGAK**S**KYLIFT  IIGMGL**S**PDNRYL  FIFLDV**S**RDFVAS  SRDFVA**S**GAEDRH  HEDVVN**S**VVFSPQ  ELLLTA**S**DDATIK  TIKAWR**S**PRTMRV  LGKRVL**S**KLQSPS  VLSKLQ**S**PSRARG  SKLQSP**S**RARGPG  ARGPGG**S**PGGLQK  ELLELE**S**EEERSR  ESEEER**S**RKIQGL  IQGLLK**S**CGKPVE  PGLRQP**S**PSHDGS  LRQPSP**S**HDGSLS  SPSHDG**S**LSPLQD  SHDGSL**S**PLQDRA  KYRSTV**S**KSKDPS  PSGLLI**S**VIRTLS  IRTLST**S**DDVEDR  TERITN**S**RNKIKQ  LEQCMA**S**KHYLSA  ATDMLV**S**AVESLE  LRLELH**S**KKMNLH  KEKGKI**S**SLVKDA  EKGKIS**S**LVKDAS  SLVKDA**S**VPLIDV  HYSTAG**S**SSVREI  YSTAGS**S**SVREIN  STAGSS**S**VREINL  KQIVKR**S**TTQVAD  AVAAAH**S**VVLGYL  KNTRTA**S**EPSAQL  RTASEP**S**AQLSYA  AQLSYA**S**TGREFA  LFKFES**S**SHAISM  SSSHAI**S**MSAYLR  EGVTKT**S**DPLKIL  HDLSAY**S**DQFLNM  EEKLVI**S**ASWAKD  KLVISA**S**WAKDDD  AKDDDI**S**RLLKSL  AAFGKE**S**EVLIGN  SLEWLA**S**RTKSAF  RTKSAF**S**NLSTSQ  SAFSNL**S**TSQMLS  TDLPPV**S**EQIMQT  QIMQTL**S**ELAKSF  LSELAK**S**FQDMAD  IVANVE**S**MDYDPL  LRYANN**S**NYKNDV  EAYVHK**S**VMEELK  KRIIDD**S**EITKED  IGDEHI**S**FTTSKI  HISFTT**S**KIGSLI  TTSKIG**S**LIDVNQ  LIDVNQ**S**KDPEGL  LKCLVF**S**LIGLHF  EKTWNI**S**FAGCGF  YYVGVA**S**CLREHA  THIYGA**S**AGALTA  AKFIEV**S**KEARKR  LGPLHP**S**FNLVKI  KVLPAD**S**HEHASG  DSHEHA**S**GRLGIS  SGRLGI**S**LTRVSD  ISLTRV**S**DGENVI  GENVII**S**HFNSKD  IISHFN**S**KDELIQ  IQANVC**S**GFIPVY  CGLIPP**S**LQGVRY  YVDGGI**S**DNLPLY  KNTITV**S**PFSGES  ITVSPF**S**GESDIC  SPFSGE**S**DICPQD  DICPQD**S**STNIHE  ICPQDS**S**TNIHEL  LRVTNT**S**IQFNLR  RNLYRL**S**KALFPP  KDQAVE**S**AQAEDY  AQAEDY**S**QLPGED  DLLTTL**S**NMLPVR  YTLPLE**S**ALSFTI  PLESAL**S**FTIRLL  LGRHLP**S**RLPEQV  RVQSLP**S**VPLSCA  LPSVPL**S**CAAYRE  APADPA**S**PQHQLA  GPAPLL**S**TPAPEA  GEAPER**S**PPCSPS  ERSPPC**S**PSYDLT  SPPCSP**S**YDLTGK  KDGAFL**S**GTFIAT  GQERFR**S**VTHAYY  YDITNK**S**SFDNIR  DITNKS**S**FDNIRA  GNKADM**S**SERVIR  NKADMS**S**ERVIRS  SERVIR**S**EDGETL  VPFLET**S**AKTGMN  HQADEP**S**FQIRDY  IRDYVE**S**QKKRSS  PAPQPP**S**PTPSPP  PPSPTP**S**PPRPTL  PTETET**S**GEQLGI  GEQLGI**S**DNGGLF  LPPFCE**S**DPESTD  CESDPE**S**TDDGSL  ESTDDG**S**LSEETP  TDDGSL**S**EETPAG  AGPPTC**S**VPPASA  CSVPPA**S**ALPTQQ  TQQYAK**S**LPVSVP  AKSLPV**S**VPVWGF  KRTEAR**S**SDEENG  RTEARS**S**DEENGP  EENGPP**S**SPDLDR  ENGPPS**S**PDLDRI  LDRIAA**S**MRALVL  RPRLNT**S**DFQKLK  LDMPPV**S**YDVQLL  DVQLLH**S**WNNNDR  WNNNDR**S**LNVFVK  RHPVAQ**S**TDAIRG  ADAPLH**S**VGYTTL  VGNNHE**S**WGWDLG  DGKNQP**S**KTYPAF  TFIVPD**S**FLVALD  MDDGTL**S**FIVDGQ  KLYPVV**S**AVWGHC  MDLCRR**S**VRLALG  TLPLPA**S**LKAYLL  TMDYKE**S**CPSVSI  YKESCP**S**VSIPSS  ESCPSV**S**IPSSDE  PSVSIP**S**SDEHRE  SVSIPS**S**DEHREK  VYKVLV**S**VGRSEW  LVSVGR**S**EWFVFR  AFLQMD**S**PKHQSD  EDEDER**S**SQKLHS  DEDERS**S**QKLHST  QKLHST**S**QNINLG  NINLGP**S**GNPHAK  KVIGKG**S**FGKVLL  LVGLHY**S**FQTTEK  HLQRER**S**FPEHRA  YAAEIA**S**ALGYLH  ALGYLH**S**IKIVYR  ENILLD**S**VGHVVL  KEGIAI**S**DTTTTF  ILHKPL**S**LRPGVS  SLRPGV**S**LTAWSI  VSLTAW**S**ILEELL  NHPFFE**S**LSWADL  PFFESL**S**WADLVQ  YSVCVS**S**DYSIVN  CVSSDY**S**IVNASV  YSIVNA**S**VLEADD  ADHNPD**S**DSTPRT  HNPDSD**S**TPRTLL  TPRRPR**S**ARAGAR  LLTAPE**S**SILMPE  LTAPES**S**ILMPES  SILMPE**S**VVKPVP  PQAVQP**S**RQESSC  QPSRQE**S**SCGSLE  PSRQES**S**CGSLEL  QESSCG**S**LELQLP  KQRLRL**S**VFQQGV  GVDQGL**S**LSQEPQ  DQGLSL**S**QEPQGN  QGNADA**S**SLTRSL  GNADAS**S**LTRSLN  RDLRDT**S**LAPPNI  SQPMVG**S**PNVYHS  AGRKTQ**S**SGPGLQ  GRKTQS**S**GPGLQK  PGLQKN**S**PGKPAQ  FALGFL**S**TSSGVS  GFLSTS**S**GVSGED  STSSGV**S**GEDEVE  EEGVSV**S**EMEATG  SGDEDA**S**GRAASP  RAASPE**S**ASSTPE  SPESAS**S**TPESLQ  PGAAVL**S**SEPAEP  GAAVLS**S**EPAEPL  HYVKLF**S**FYAKMP  LIPCAY**S**GNSVFP  ELATKL**S**RRLQME  AAEALG**S**ADCELS  SADCEL**S**AKLLRR  GIGEPQ**S**PSRRVF  GEPQSP**S**RRVFNP  TEFKEF**S**RKQIKD  VDEDFD**S**KLSFRE  DFDSKL**S**FREFLL  GELQED**S**GLCVLA  CVLARL**S**EIDVSS  LSEIDV**S**SEGVKG  SEIDVS**S**EGVKGA  GVKGAK**S**FFEAKV  VQAINV**S**SRFEEE  QAINVS**S**RFEEEI  TAAGIL**S**AHKIIA  AHKIIA**S**SPEMNL  HKIIAS**S**PEMNLP  MNLPTV**S**ALRKMG  GVNLTR**S**NKETVK  KETVKH**S**DVLFLA  ARHIVV**S**CAAGVT  AAGVTI**S**SVEKKL  AGVTIS**S**VEKKLM  LLEQLM**S**SVGFCT  LEQLMS**S**VGFCTE  DAVTGL**S**GSGPAY  VTGLSG**S**GPAYAF  AKMLLD**S**EQHPCQ  LKDNVC**S**PGGATI  ALHFLE**S**GGFRSL  ESGGFR**S**LLINAV  INAVEA**S**CIRTRE  RTRELQ**S**MADQEK  ADQEKI**S**PAALKK  DRVKLE**S**PTVSTL  LESPTV**S**TLTPSS  VSTLTP**S**SPGKLL  STLTPS**S**PGKLLT  GKLLTR**S**LALGGK  EEAHAA**S**TEEKEA  PVRLPF**S**GFRLQK  QKVLRE**S**ARDKII  GKVNEA**S**GDGDGE  AQLLTG**S**PELQLQ  ELQLQF**S**NDIYST  FSNDIY**S**TYHLFP  TLPHLE**S**QSLSIQ  PHLESQ**S**LSIQWV  LESQSL**S**IQWVYN  FENPDP**S**DGFVLI  HRRGIR**S**LRDLTP  YLHYLP**S**YYHLHV  GFEAPG**S**GVERAH  FFKGGG**S**SKSRAA  FKGGGS**S**KSRAAP  GGGSSK**S**RAAPSP  KSRAAP**S**PQEALV  QIDGTL**S**TIEFQR  REALEN**S**HTNTEV  AAKAMK**S**VHENMD  DIAQEI**S**EAFSQR  EISEAF**S**QRVGFG  RLPNVP**S**SSLPAQ  LPNVPS**S**SLPAQP  PNVPSS**S**LPAQPN  NRKPGM**S**STARRS  RKPGMS**S**TARRSR  RRSRAA**S**SQRAEE  RSRAAS**S**QRAEEE  EFRRSY**S**RLCRES  SRLCRE**S**GAEPQE  CTELVL**S**DCMLSE  LSDCML**S**EEGATL  LLQQNK**S**IQSLTL  QNKSIQ**S**LTLEWN  EQAMGH**S**QDRLTT  ARTHVL**S**KEVQHL  HLREEK**S**KQFLDL  EEMAKS**S**RASAAR  AKSSRA**S**AARVGQ  EAALAL**S**EQKAQD  AEQEQL**S**LSQRQA  QEQLSL**S**QRQAKE  EAAERE**S**KLLRDL  KLLRDL**S**AANEKN  TRQEMT**S**MSAELK  QEMTSM**S**AELKMR  RQSLED**S**ESLRIK  SLEDSE**S**LRIKEV  TRHLEE**S**EKAMQE  LEAARL**S**LEEELS  SLEEEL**S**RVKAAA  VKAAAL**S**ERGQAE  ELIKAK**S**QARLEE  EAQTRV**S**QLGLQV  ELQQEL**S**LKDQER  QLKVMA**S**DHREAL  ALLDRE**S**ENASLR  RESENA**S**LREKLR  EEAQRA**S**FLQNAV  QGENTK**S**AAARAR  LLEEED**S**KLKGGK  APRVAT**S**SKVTRA  PRVATS**S**KVTRAQ  TAEKAK**S**HLEVPL  DAIAVL**S**VAEEAA  NPNMRL**S**QLKQLL  KKEWLR**S**PDNPMN  LTEKED**S**PSESTG  EKEDSP**S**ESTGNG  EDSPSE**S**TGNGPP  GRFEEL**S**AWTEKQ  QFFEIQ**S**KEAKER  GKLKGK**S**ERSSED  GKSERS**S**EDPTDD  EDPTDD**S**RLPRAE  LQLKLN**S**SGSSED  QLKLNS**S**GSSEDS  LNSSGS**S**EDSFVE  GPTRTV**S**TGTALS  STGTAL**S**KYRSRS  SKYRSR**S**ADGAKN  HEELTV**S**QLLLCL  EAKERV**S**DFEKKT  DFEKKT**S**NRSEIE  KKTSNR**S**EIETQT  GPETVG**S**EVEALN  LNLQVT**S**LFKELQ  EAHTKL**S**KAELMK  KNSAIP**S**ELNEKQ  LELQVE**S**MLSEIK  QVESML**S**EIKMEQ  KTEDEK**S**KLTVLQ  ELTRKE**S**EKVDRA  AEKALA**S**KQLQMD  QMEVYC**S**DFHAER  EDGGRQ**S**LMEMQS  RHGART**S**DSDQQA  GARTSD**S**DQQAYL  VKQVFQ**S**LPKSAF  FQSLPK**S**AFSGGY  LPKSAF**S**GGYYRG  ALILGV**S**PTANKG  HPDKGG**S**PYIAAK  KAYVTK**S**PVDAGK  YIADMI**S**GNGYTT  QEPWDP**S**GDWSIF  DPSGDW**S**IFPEWL  KIDREI**S**AILKYL  HLMMKY**S**EFRAGV  EFRAGV**S**VYGIVK  YGIVKD**S**EDIYNL  IPLKDV**S**LLTQKL  YQIKTF**S**GQTHGF  RKREDC**S**PADKPY  QPGGSP**S**AAGADR  PGLQGP**S**REPPLA  LTGAGV**S**VSCGIP  GAGVSV**S**CGIPDF  GIPDFR**S**RDGIYA  PGQFQP**S**LCHKFI  IIQCHG**S**FATASC  LLIVIG**S**SLKVRP  LIVIGS**S**LKVRPV  PVALIP**S**SIPHEV  VALIPS**S**IPHEVP  CNPVKL**S**EITEKP  KELAYL**S**ELPPTP  LHVSED**S**SSPERT  VSEDSS**S**PERTSP  RTSPPD**S**SVIVTL  TSPPDS**S**VIVTLL  LDQAAK**S**NDDLDV  NDDLDV**S**ESKGCM  DLDVSE**S**KGCMEE  PQEVQT**S**RNVESI  TSRNVE**S**IAEQME  DLKNVG**S**STGEKN  EKNERT**S**VAGTVR  VAKEQI**S**RRLDGN  SEDDVL**S**SSSCGS  EDDVLS**S**SSCGSN  DVLSSS**S**CGSNSD  SSCGSN**S**DSGTCQ  DSGTCQ**S**PSLEEP  EPMEDE**S**EIEEFY  VLLAGV**S**FSGFLY  LAGVSF**S**GFLYPL  LVDFCI**S**GKTRGQ  NLDKMA**S**EGMRFV  DFHAAA**S**TCSPSR  AAASTC**S**PSRASL  ASTCSP**S**RASLLT  CSPSRA**S**LLTGRL  RNFAVT**S**VGGLPL  HLGHHG**S**YHPNFR  YFGIPY**S**HDMGCT  PQGDGP**S**RNLQRD  EQPVNL**S**SLAQKY  QPVNLS**S**LAQKYA  QFIQRA**S**TSGRPF  IQRAST**S**GRPFLL  AAPRGR**S**LYGAGL  GLWEMD**S**LVGQIK  KCELAG**S**VGPFTG  QTRQGG**S**PAKQTT  VPVNVT**S**TALLSV  TSTALL**S**VLDIFP  VALAQA**S**LPQGRR  FDGVDV**S**EVLFGR  EVLFGR**S**QPGHRV  VLFHPN**S**GAAGEF  ARACDG**S**TGPELQ  IANDNI**S**SADYTQ  ANDNIS**S**ADYTQD  DYTQDP**S**VTPCCN  VGGLDW**S**TTQETL  TQETLR**S**YFSQYG  TLRSYF**S**QYGEVV  DKTTNQ**S**RGFGFV  VGTVLA**S**RPHTLD  WQKGPR**S**DNSKSN  GPRSDN**S**KSNKIF  RSDNSK**S**NKIFVG  TFEDEQ**S**VDQAVN  RAEPRD**S**KSQAPG  EPRDSK**S**QAPGQP  PGQPGA**S**QWGSRV  GASQWG**S**RVVPNA  PPPPFT**S**YIVSTP  FTSYIV**S**TPPGGF  GAPPQF**S**FGYGPP  AFPPPP**S**QAAPDM  QAAPDM**S**KPPTAQ  GYGQDL**S**GFGQGF  GFGQGF**S**DPSQQP  QGFSDP**S**QQPPSY  PSQQPP**S**YGGPSV  PSYGGP**S**VPGSGG  GPSVPG**S**GGPPAG  GPPAGG**S**GFGRGQ  PRLPAI**S**GRGARP  VVGAWL**S**RKLSVP  WLSRKL**S**VPAFAS  SVPAFA**S**SLTSCG  FASSLT**S**CGPRAL  TLRPGV**S**LTGTKH  PFICTA**S**FHTSAP  TASFHT**S**APLAKE  KAKEKF**S**QLAEAY  EAYEVL**S**DEVKRK  QYDAYG**S**AGFDPG  GFDPGA**S**GSQHSY  DPGASG**S**QHSYWK  ASGSQH**S**YWKGGP  IFGEFS**S**SSFGDF  FGEFSS**S**SFGDFQ  GEFSSS**S**FGDFQT  GPFVMR**S**TCRRCG  RCGGRG**S**IIISPC  RGSIII**S**PCVVCR  TFRVQK**S**PVFRRD  DGADIH**S**DLFISI  HSDLFI**S**IAQALL  GIPRIN**S**YGYGDH  VPKRLT**S**RQQSLI  LTSRQQ**S**LILSYA  QQSLIL**S**YAEDET  NGVTLT**S**SGGSTM  GVTLTS**S**GGSTMD  LTSSGG**S**TMDSSA  GGSTMD**S**SAGSKA  MDSSAG**S**KARREA  DEEGFL**S**KLKKMF  PVPGTD**S**APLAGL  LAGLAW**S**SASAPP  AGLAWS**S**ASAPPP  LAWSSA**S**APPPRG  PPPRGF**S**AISCTV  RGFSAI**S**CTVEGA  VEGAPA**S**FGKSFA  PASFGK**S**FAQKSG  KSFAQK**S**GYFLCL  GYFLCL**S**SLGSLE  YFLCLS**S**LGSLEN  CLSSLG**S**LENPQE  QIVVDK**S**PLPLGF  PLPLGF**S**PVCDPM  VCDPMD**S**KASVSK  PMDSKA**S**VSKKKR  DSKASV**S**KKKRMC  VFDVRL**S**GKTKTV  LSLDAA**S**QPSKGG  DAASQP**S**KGGLLE  LLERTA**S**RLGSRA  TASRLG**S**RASTLR  RLGSRA**S**TLRRND  SIYEAS**S**LYGISA  SSLYGI**S**AMDGVP  PRFEGK**S**CSPLAF  CSPLAF**S**AFGDLT  GDLTIK**S**LADIEE  AMGPGI**S**RMNDLT  QKGIVI**S**FDARAH  DARAHP**S**SGGSSR  ARAHPS**S**GGSSRR  HPSSGG**S**SRRFAR  AATTFI**S**QGIPVY  IPVYLF**S**DITPTP  FVPFTV**S**HLKLCA  NGAQII**S**PHDKGI  PQAWDD**S**LIDSSP  DDSLID**S**SPLLHN  LHNPSA**S**INNDYF  KYCFHR**S**VNRETK  VHGVGH**S**FVQSAF  GHSFVQ**S**AFKAFD  KGVLTL**S**FALADK  GEWRVF**S**GNELGA  EKNQDR**S**ALKDTY  KDTYML**S**STVSSK  DTYMLS**S**TVSSKI  MLSSTV**S**SKILRA  LSSTVS**S**KILRAI  LDKDGV**S**AAVISA  VSAAVI**S**AELASF  ISAELA**S**FLATKN  YHITKA**S**YFICHD  CGKFEI**S**AIRDLT  TTGYDD**S**QPDKKA  KAVLPT**S**KSSQMI  VLPTSK**S**SQMITF  LPTSKS**S**QMITFT  VATMRT**S**GTEPKI  ELNELV**S**AIEEHF  GDRDRD**S**GVVGAR  PRPQQA**S**PPPCGG  GVGGCC**S**GPGHSK  CSGPGH**S**KRRRQA  KNADYF**S**NYVTED  VEVYQY**S**TGTSAV  QYSTGT**S**AVEPIN  DEPIRV**S**YHRNIH  RNIHYN**S**VVNPNK  VGLGLP**S**FKPGFA  PGFAEQ**S**LMKNAI  KNAIKT**S**EESWIE  IKTSEE**S**WIEQQM  EQVARE**S**YLQWLR  SQPRKA**S**ATCSSA  KASATC**S**SATAAA  ASATCS**S**ATAAAS  ATAAAS**S**GLEEWT  GLEEWT**S**RSPRQR  RSPRQR**S**SASSPE  SPRQRS**S**ASSPEH  RQRSSA**S**SPEHPE  LGMKPP**S**PGTVLA  ALAKPP**S**PCAPGT  PCAPGT**S**SQFSAG  CAPGTS**S**QFSAGA  GTSSQF**S**AGADRA  ATSPLV**S**LYPALE  ALIQQM**S**PSAFGL  IQQMSP**S**AFGLND  QQEYLD**S**MKKNKV  VTPLLE**S**WALSQV  LESWAL**S**QVAGMP  CENVQP**S**GSFKIR  NVQPSG**S**FKIRGI  CRHLVC**S**SGGNAG  RHLVCS**S**GGNAGI  TIVLPE**S**TSLQVV  VLPEST**S**LQVVQR  IWKGHA**S**LVQELK  TLPDIT**S**VAKSLG  ITSVAK**S**LGAKTV  QVCKIH**S**EVVEDT  EDTEAV**S**AVQQLL  ALAAIY**S**GLLRRL  EGCLPP**S**LTSVVV  LPPSLT**S**VVVIVC  GGNNIN**S**RELQAL  AEPTVC**S**FLTKVL  RGHVEL**S**EARLRD  VGGGGT**S**AWRVVA  WRVVAV**S**SVRLCA  RVVAVS**S**VRLCAR  PNRDCW**S**TCTLSH  WSTCTL**S**HDIHTP  NMQVLK**S**HGLFGL  KFHVCK**S**FVKGEC  LQTCKR**S**HQLIHA  QLIHAA**S**LKLLQD  QGLNIP**S**VVNFQI  VNFQII**S**TYKHMK  LENTDN**S**SPSTEH  ENTDNS**S**PSTEHS  TDNSSP**S**TEHSQG  SPSTEH**S**QGLEKQ  EAGPLA**S**VPAQSA  ASVPAQ**S**AKKPCP  MPLTIS**S**LKDGGK  IGDVVL**S**IDGINA  IKGCTG**S**LNMTLQ  MTLQRA**S**AAPKPE  ITSPAV**S**KVTSTN  PFGSVS**S**PKVTSI  SSPKVT**S**IPSPSS  KVTSIP**S**PSSAFT  TSIPSP**S**SAFTPA  SIPSPS**S**AFTPAH  AHATTS**S**HASPSP  TTSSHA**S**PSPVAA  SSHASP**S**PVAAVT  QSPSAL**S**AGKTAV  RQPTVT**S**VCSETS  TVTSVC**S**ETSQEL  SVCSET**S**QELAEG  PTHSDA**S**KKRLIE  GTTQSR**S**FRILAQ  TEHLKE**S**EADNTK  NNSQEP**S**PQLASS  PSPQLA**S**SVASTR  SPQLAS**S**VASTRS  MPESLD**S**PTSGRP  GRPGVT**S**LTAAAA  AFKPVG**S**TGVIKS  GVIKSP**S**WQRPNQ  PNQGVP**S**TGRISN  PSTGRI**S**NSATYS  TGRISN**S**ATYSGS  SVAPAN**S**ALGQTQ  DTCFVC**S**VCCESL  CSVCCE**S**LEGQTF  AVEELV**S**GVRQAA  FAEQFR**S**YSESEK  EQFRSY**S**ESEKQW  FRSYSE**S**EKQWKA  RDPPDG**S**GRLDQL  RLDQLL**S**LSMVWA  DQLLSL**S**MVWANH  HLFLGC**S**YNKDLL  PQFTTR**S**ELMKKH  APAPAA**S**QPSGCG  PAASQP**S**GCGKHN  GCGKHN**S**PERKVY  EAWGNP**S**SPYSAG  AWGNPS**S**PYSAGR  NPSSPY**S**AGRKAK  INAARE**S**LAKMIG  QDIIFT**S**GGTESN  TSGGTE**S**NNLVIH  NNLVIH**S**VVKHFH  FHANQT**S**KGHTGG  HTGGHH**S**PVKGAK  KPHFIT**S**SVEHDS  PHFITS**S**VEHDSI  SSVEHD**S**IRLPLE  VTFVPV**S**KVSGQA  VPVSKV**S**GQAEVD  MPVPEI**S**QRIKAL  KRIHLN**S**QFPGTQ  PNTCNF**S**IRGPRL  CRVLMA**S**VGAACH  VGAACH**S**DHGDQP  DHGDQP**S**PVLLSY  PSPVLL**S**YGVPFD  RNALRL**S**VGRSTT  RLSVGR**S**TTRAEV  SGSQLG**S**MGSLTM  GSLTMK**S**QLQITV  LQITVI**S**AKLKEN  KNWFGP**S**PYVEVT  LHFRVW**S**HQTLKS  ETIGDL**S**ICLDGL  NGETTC**S**ENGVSL  CSENGV**S**LCLPRL  ECNSAI**S**AHCNLC  LPGLSD**S**PISASR  DSPISA**S**RVAGFT  SQNDDG**S**RSKDET  KDETRV**S**TNGSDD  SGNNSP**S**LSNGGF  SRPPRP**S**RPPPPT  SVNGSP**S**ATSESD  PSATSE**S**DGSSTG  DGSSTG**S**LPPTNT  IIPLTI**S**GGSGPR  LTISGG**S**GPRPLN  QDLFAT**S**QSKEFD  QWEDPR**S**QGQLNE  DPRTGK**S**ALDNGP  SFQQIM**S**FSPQDL  QQIMSF**S**PQDLRR  EWFFLL**S**HEVLNP  LQINPA**S**YINPDH  GLKDLE**S**IDPEFY  DLEMYF**S**VDKEIL  VAEWRL**S**RGVEEQ  ENWLPR**S**HTCFNR  TDRQTD**S**VLELKA  DLCSLT**S**QSLPIE  CSLTSQ**S**LPIELT  LPIELT**S**VVPEST  TSVVPE**S**TEDILL  LLKGFT**S**LGMEEE  ILNDVN**S**ALQHLE  ALQHLE**S**LQKQYL  INTKLN**S**PTLSVN  PTLSVN**S**DGFIPM  DCITYI**S**SHPNFK  CITYIS**S**HPNFKD  TLQTLT**S**QLLKRD  LLKRDP**S**SVPNAD  LKRDPS**S**VPNADN  ELLLGP**S**IACTVA  TVAELT**S**QNNRDH  HCALVR**S**GCAFMV  MEQIAQ**S**LKDEQK  KVPSEA**S**FSDVHL  PSEASF**S**DVHLEE  EEGESN**S**LTKSGS  SNSLTK**S**GSTESL  SLTKSG**S**TESLNP  KSGSTE**S**LNPRPQ  LSQEAL**S**ACIQSL  LLGASE**S**ISKNKT  FTIKEI**S**LDLKKT  RFFRLN**S**NNALIE  ALKTMA**S**QGGPKY  VTLRSM**S**LYLSNK  SMSLYL**S**NKDTEF  QIIACP**S**MEQLSL  KWVAIQ**S**VSAWPE  VAIQSV**S**AWPEKR  VKQLGG**S**VELVDI  QKLPDG**S**EIPLPP  LLGRLG**S**DPQKKT  LEDGWD**S**EPFTLV  KLYGRG**S**TDDKGP  LEGMEE**S**GSEGLD  GMEESG**S**EGLDEL  VDYVCI**S**DNYWLG  FIEVEC**S**NKDLHS  SNKDLH**S**GVYGGS  SGVYGG**S**VHEAMT  LILLMG**S**LVDKRG  AQILLH**S**HKKDIL  HRWRYP**S**LSLHGI  WRYPSL**S**LHGIEG  GIEGAF**S**GSGAKT  EGAFSG**S**GAKTVI  KVVGKF**S**IRLVPN  VGEQVT**S**YLTKKF  KFAELR**S**PNEFKV  GGKPWV**S**DFSHPH  PWVSDF**S**HPHYLA  LTREGG**S**IPVTLT  MLLPVG**S**ADDGAH  ADDGAH**S**QNEKLN  PKATPD**S**LEPAGP  LEPAGP**S**SPASVT  TKSFPS**S**PSKGGS  SPSKGG**S**CPSRAK  AGKSPP**S**VQSLAM  GAAAAG**S**EPPPAT  IQHFRM**S**DDVHSL  MSDDVH**S**LGKVTS  KRRKLN**S**GGGLSE  LSEELG**S**ARRSGE  LGSARR**S**GEVTLT  TKGDPG**S**LEEWET  VVGDDF**S**LYYDSY  LYYDSY**S**VDERVD  KKWRKD**S**PWVKPS  SPWVKP**S**RKRRKR  MEVPLG**S**LELPSE  KCMATE**S**VDGELS  RETMRP**S**SRVALM  HCGEDA**S**EAQEVT  ADTSQP**S**ARMRGH  LADTID**S**SGPSLT  ADTIDS**S**GPSLTL  PNGGCL**S**AVGLPL  ALVIQE**S**ERRKKL  AAAQKG**S**VEICHV  WASFTG**S**AAIAEV  CVLLFL**S**RGANPE  DLTPER**S**DVWFAL  EDYKYI**S**ENCETS  CLCGQL**S**IRCWYD  YVGELI**S**DAEADV  PRIAFF**S**SRDIRT  PGNDRN**S**DTHGTL  LGSGRS**S**DKGPSW  DKGPSW**S**SRSLGA  PSWSSR**S**LGARCR  KTQLNP**S**SLQKLF  TQLNPS**S**LQKLFR  VFDYLV**S**HGRMKE  KFRQIV**S**AVHYCH  LDTFCG**S**PPYAAP  ILYTLV**S**GSLPFD  YTLVSG**S**LPFDGH  RVPFYM**S**TDCESI  IKESLT**S**QKYNEV  TNGTSS**S**KGTSHS  SKGTSH**S**KGQRSS  KGQRSS**S**STYHRQ  SCSTAG**S**GSRGLP  STAGSG**S**RGLPPS  RGLPPS**S**PMVSSA  SSPMVS**S**AHNPNK  PERRKD**S**TSTPNN  RRKDST**S**TPNNLP  PNNLPP**S**MMTRRN  PGAERP**S**LLPNGK  PNGKEN**S**SGTPRV  NGKENS**S**GTPRVP  PRVPPA**S**PSSHSL  VPPASP**S**SHSLAP  HSLAPP**S**GERSRL  RGSTIR**S**TFHGGQ  LLRFPW**S**VKLTSS  WSVKLT**S**SRPPEA  GGPEPL**S**HFEVEV  KKRKLE**S**GGGGEG  LYYECY**S**DVSVHE  ECYSDV**S**VHEEMI  AGTGIL**S**IFCAQA  VYAVEA**S**AIWQQA  QVDAIV**S**EWMGYG  YGLLHE**S**MLSSVL  LHESML**S**SVLHAR  HESMLS**S**VLHART  GLLLPA**S**AELFIA  LFIAPI**S**DQMLEW  WRLGFW**S**QVKQHY  HYGVDM**S**CLEGFA  RCLMGH**S**EIVVQG  IVVQGL**S**GEDVLA  FAQLEL**S**RAGLEQ  GGRFRC**S**CYGSAP  RCSCYG**S**APMHGF  TFPGGE**S**EKPLVL  EKPLVL**S**TSPFHP  PLVLST**S**PFHPAT  EQDTDV**S**GEITLL  EITLLP**S**RDNPRR  LLPLLL**S**SLLGGS  LPLLLS**S**LLGGSQ  SSLLGG**S**QAMDGR  WIRVQE**S**VMVPEG  CISVPC**S**FSYPRQ  SVPCSF**S**YPRQDW  SREVEM**S**TRGRFQ  PAKGNC**S**LVIRDA  TCHVDF**S**RKGVSA  FSRKGV**S**AQRTVR  PRDLVI**S**ISRDNT  AENRLG**S**QQRALD  QRALDL**S**VQYPPE  NLGNGT**S**LPVLEG  PVLEGQ**S**LCLVCV  VCVTHS**S**PPARLS  SPPARL**S**WTQRGQ  LSPSQP**S**DPGVLE  ARHPLG**S**QHVSLS  GLHCSC**S**SQASPA  SCSSQA**S**PAPSLR  LLEGNS**S**QDSFEV  GNSSQD**S**FEVTPS  SFEVTP**S**SAGPWA  GPWANS**S**LSLHGG  LHGGLS**S**GLRLRC  LISTAF**S**NGAFLG  TPRPRF**S**RHSTIL  PRFSRH**S**TILDYI  QKATPN**S**PRTPLP  LPPGAP**S**PESKKN  KQYQLP**S**FPEPKS  FPEPKS**S**TQAPES  VLAGVE**S**RQGSIK  VESRQG**S**IKGLVY  IKGLVY**S**SNFQNV  KGLVYS**S**NFQNVK  CETQRY**S**AVLDAV  LDAVIA**S**AGLLRA  KVHRGV**S**RNEDLL  DLLEVG**S**RPGPAS  SRPGPA**S**QLPRFV  NTLKTC**S**DDVVDY  FKRQGF**S**YQGRAS  SYQGRA**S**SLDDLR  YQGRAS**S**LDDLRA  ILQDRA**S**CLPAML  LDPPPG**S**HVIDAC  APGNKT**S**HLAALL  DAKRLA**S**MATLLA  LARAGV**S**CCELAE  EDFLAV**S**PSDPRY  FLAVSP**S**DPRYHE  YILLDP**S**CSGSGM  LLDPSC**S**GSGMPS  DPSCSG**S**GMPSRQ  SGSGMP**S**RQLEEP  PGAGTP**S**PVRLHA  HALTFP**S**LQRLVY  LQRLVY**S**TCSLCQ  LVYSTC**S**LCQEEN  WPHRGL**S**TFPGAE  EHCLRA**S**PETTLS  SPETTL**S**SGFFVA  PETTLS**S**GFFVAV  ALEKKL**S**ELSNSQ  KKLSEL**S**NSQQSV  LSELSN**S**QQSVQT  LSNSQQ**S**VQTLSL  QSVQTL**S**LWLIHH  IHHRKH**S**RPIVTV  NDVIQN**S**KRKGPE  EAFKHV**S**SETDES  AFKHVS**S**ETDESC  SSETDE**S**CKKHLG  HLGRVL**S**IWEERS  SIWEER**S**VYENDV  DENENC**S**SLGSPS  ENENCS**S**LGSPSE  NCSSLG**S**PSEPPQ  SSLGSP**S**EPPQTL  DLENAA**S**GDAAVH  VHQRIA**S**LPVEVQ  VEVQEV**S**LLDKIT  KITDKE**S**GERLSK  ESGERL**S**KMVEDA  RKLARV**S**LVRKEL  VRKELR**S**RIQSLP  LRSRIQ**S**LPDLSR  QSLPDL**S**RLPNVT  LPNVTG**S**HMHLPF  TLTGIL**S**PVQEVR  AIRQLA**S**VILKQY  PNGLRE**S**ISKVRS  ISKVRS**S**VAYAVS  YGIRTR**S**RAVEIF  FPKHMV**S**SMQQIL  PKHMVS**S**MQQILP  WNTLTE**S**AAFYVR  VEDPVD**S**DGEVLG  HALLEN**S**KFKSTV  TDFQNE**S**AAALAA  CMLALG**S**VKAIIT  KAIITD**S**VKNGRI  RALWAA**S**RFTVAM  RFTVAM**S**PELIQQ  FLQATV**S**GLHETQ  CDQLKV**S**ESTHVL  DPEFTA**S**MESKIC  AIFLKY**S**NDPVVA  NDPVVA**S**LAQDIF  DIFKEL**S**QIEACQ  CLRAYV**S**VTLEQV  TLSVMQ**S**LIMVFA  LLEFLC**S**LPGPTG  VMAEWT**S**RQHLFY  QYEGKV**S**SVALCK  YEGKVS**S**VALCKL  EGIRTR**S**KSAKNP  LIINEL**S**NVMEAN  ATPAEW**S**QDDSND  EWSQDD**S**NDMWED  LAGQLL**S**DILATS  PCYIMF**S**GHLNDN  LGLLLW**S**DPGTAS  LPLLMD**S**VIQALA  KTRHTA**S**AWLMSA  ASAWLM**S**APNSGP  LMSAPN**S**GPHNRL  LDPCPL**S**PELLGL  VLAPDG**S**TVAVEP  INLPLD**S**MAAPWE  PDVRAT**S**SPGLRD  CPDVQA**S**LPDAKA  PDAKAK**S**PPTMVD  PPTMVD**S**LLAVTL  LTFLRG**S**QTQSHP  GCWDQL**S**APRTFT  LLDPKA**S**LLTMAF  ILGDYL**S**RTPEPR  TPEPRP**S**LSHLLS  EPRPSL**S**HLLSQY  SLSHLL**S**QYYGAG  RDPGFR**S**NFRRQN  NGAALT**S**ASILAQ  AALTSA**S**ILAQQV  LQLQCM**S**QEQLAQ  CAANMR**S**MQRYHQ  WGDIGY**S**FVVGSD  YSFVVG**S**DGYVYE  HTLGHN**S**RGFGVA  VRDTLP**S**CAVRAG  KPRPAR**S**VSKRSR  RPARSV**S**KRSRRE  RSVSKR**S**RREPPP  EPVYGL**S**EDEGES  LRVKVV**S**GIDLAK  KDIFGA**S**DPYVKL  YFRVNP**S**NHRLLF  FLLRPR**S**HKSRVK  RPRSHK**S**RVKGFL  GQDEEN**S**DQRDDM  GWEVVD**S**NDSASQ  DSNDSA**S**QHQEEL  AHRRFR**S**RRHISE  LPPPPA**S**PGSRTS  TSPQEL**S**EELSRR  LQITPD**S**NGEQFS  NGEQFS**S**LIQREP  IQREPS**S**RLRSCS  PSSRLR**S**CSVTDA  GEEPTP**S**VAYVHT  TTPGLP**S**GWEERK  SPTVTL**S**APLEGA  AVKDTL**S**NPQSPQ  FPVHMR**S**KTSLNP  HMRSKT**S**LNPNDL  GPAVPY**S**REFKQK  EWFFLL**S**KEMFNP  YGLFEY**S**ATDNYT  CNEDHL**S**YFTFIG  DMESVD**S**EYYNSL  DLKPNG**S**EIMVTN  QFVTGT**S**RVPMNG  FAELYG**S**NGPQLF  TIEQWG**S**PEKLPR  NDKKLM**S**SLPNFC  DKKLMS**S**LPNFCG  LLDEEI**S**RVRKDM  NDTLNG**S**TEKRSA  GSTEKR**S**AELPDA  MVRGKG**S**MRDKKK  AAEGED**S**LKKMQL  RDANIK**S**PALAFS  SPALAF**S**LAATAQ  TLAPAT**S**ILEYPI  EYPIEP**S**GVLGAV  WAAPVK**S**QAIAQP  PATTAK**S**HLHQKP  NKEHHL**S**DREFVF  EGVYEI**S**LSPTGV  VYEISL**S**PTGVSR  LSPTGV**S**RVCLYP  ELPYTY**S**RITMEP  TGHTFN**S**LLCNLY  YRNEKD**S**VDWHSD  DSVDWH**S**DDEPSL  HSDDEP**S**LGRCPI  RCPIIA**S**LSFGAT  PIIASL**S**FGATRT  VPKEYH**S**REPRVN  PNTEAN**S**FESKDY  EANSFE**S**KDYLQV  CILGRL**S**EKSSGQ  QMAQKF**S**FSKVFG  TYGLTN**S**GKTYTF  LNVLFD**S**LQERLY  EYLRLS**S**EQEKEE  EKEEIA**S**KSALLR  VTVHND**S**DDTLYG  YGSLTN**S**LNISEF  TNSLNI**S**EFEESI  ANSIKF**S**VWVSFF  ILQIED**S**EMSRVI  TGNINT**S**LLTLGK  LKNSEK**S**KFQQHV  LNVLKF**S**AIAQKV  PDTLNS**S**QEKLFG  NDKADT**S**SLIINN  VPKDSK**S**KICSER  TEDQKK**S**EEVRPN  ELHVQK**S**KNQEQE  EKIMKL**S**NEIETA  KVECSH**S**AKLEQD  KEELSA**S**SARTQN  KKKNQC**S**QELDMK  LKALIS**S**NVQKDN  RIISET**S**KIETQI  NLKFPI**S**DDRNSS  ISDDRN**S**SVKKEQ  SDDRNS**S**VKKEQK  KTYSLR**S**QASIIG  GDFLQH**S**PSILQS  DQKMKE**S**DHQIIK  KLSLSL**S**PSPQTG  KRSPAC**S**SLTPSL  NKRTDL**S**SEHFSH  KRTDLS**S**EHFSHS  LSSEHF**S**HSSKWL  TMAETN**S**ISLNGP  CSKESL**S**SRTEAV  SKESLS**S**RTEAVR  SNAFLP**S**SVLWLS  DQALVS**S**VEDILS  LGSDTE**S**WMSPLA  EKGVNT**S**VMLENL  PDLTAL**S**RHDLED  HPETQD**S**STQTDT  PETQDS**S**TQTDTS  ARNVMQ**S**WVLISK  EDKTTV**S**QESRRA  LEQDLA**S**MREFRG  TALLSR**S**RQLTEK  RVLEQV**S**AQLEEC  ILANMD**S**QLKELQ  EKTRQY**S**QKLGLL  LTEQLQ**S**LTLFLQ  RVASMV**S**LQPAET  MTTELQ**S**LCSLLQ  ELQSLC**S**LLQESK  CSLLQE**S**KEEAIR  LEQIDK**S**GELISL  LAGQLD**S**NCQPMA  EENLRR**S**DKELEK  IYKTLL**S**IPEVVR  QTTAEP**S**RSFSSA  SRRNGL**S**RLCQSR  QSRTAL**S**EDRWSS  LSEDRW**S**SYCLSS  SEDRWS**S**YCLSSL  SSYCLS**S**LAAQNI  PEHTDP**S**EPRGSV  PSEPRG**S**VSCCSL  LLRGLS**S**GWSSPL  GEKIPV**S**VWMKRM  TVTSCD**S**LFAHLH  ITDLIP**S**VQLPPS  VSGYRA**S**VWVFCT  LIPGFY**S**YMDLAY  QDLLGE**S**RSEPVD  PAEDGG**S**DAGMCG  HCPCYG**S**EWGLWW  SQDLAP**S**PSGMAG  GCTGSS**S**ACYALA  QEVDVN**S**FSWNLK  DQTDQT**S**SNCSCA  ELRETP**S**SLAVGS  LRETPS**S**LAVGSD  SSLAVG**S**DPDVGS  SLQEQG**S**CVLDDR  LEVCLV**S**SEHYAA  EVCLVS**S**EHYAAS  SEHYAA**S**DRESPG  IQEGAY**S**GSCYHR  ACEGEY**S**QKYSTM  LEIAIL**S**RVEHAN  KLIDFG**S**AAYLER  VSKELM**S**LVSGLL  ENPIQI**S**PHCSCT  QISPHC**S**CTPSGF  HCSCTP**S**GFFMEM  FFMEMM**S**MKPAKG  LEVEPY**S**QYSNVQ  EPYSQY**S**NVQFPQ  QVQPQI**S**SSSYYS  VQPQIS**S**SSYYSN  QPQISS**S**SYYSNL  PQISSS**S**YYSNLG  SSSSYY**S**NLGFYP  QPEEWY**S**PGIYEL  KPRMGA**S**AGRIKG  VCGDRA**S**GYHYNA  KGFFRR**S**ITKNAV  TEIQCK**S**KRLRKN  QTVNED**S**EGRDLR  DLRQVT**S**TTKSCR  VTSTTK**S**CREKTE  LHFIMD**S**YNKQRM  ILKEEF**S**AEENFL  IALLKG**S**AVEAMF  EAMFLR**S**AEIFNK  FNKKLP**S**GHSDLL  KLPSGH**S**DLLEER  EERIRN**S**GISDEY  IRNSGI**S**DEYITP  YITPMF**S**FYKSIG  MFSFYK**S**IGELKM  TAIVIL**S**PDRQYI  HAEMLM**S**WRVNDH  KKVKEV**S**ESRNLE  ALALKM**S**EQEARE  IAESLN**S**CRPSDA  LNSCRP**S**DASATR  PLATGP**S**SQSHQE  WQLVPP**S**LFKGSH  PSLFKG**S**HISQGN  FKGSHI**S**QGNEAE  TEEEPV**S**GSSGSW  SGSWDQ**S**SQPVFE  LKAVQG**S**GDTSRH  VLPRPP**S**LIQNEC  RASDWH**S**KTKDFQ  TKDFQE**S**SIKSLK  SVPASQ**S**VAALTS  ALTSKR**S**LVLMPE  LVLMPE**S**SAEEIT  VLMPES**S**AEEITV  ETQLSS**S**ETFDLE  DLEREV**S**PGSRDI  REVSPG**S**RDILDG  EKEVAI**S**TFSSSN  VAISTF**S**SSNQVS  AISTFS**S**SNQVSC  GTAAQT**S**LDIDKN  GSGRAC**S**TVEGKW  DADIKS**S**ETGAFR  MEEAGC**S**REMQSS  SREMQS**S**FTRRDL  NESPVK**S**FVSISE  VTVQPG**S**RTRTKA  AADGHG**S**SCQMLL  ADGHGS**S**CQMLLN  PSEVEG**S**AANKEV  FSAVIQ**S**LFQLPE  FRRLVL**S**YSLPQN  VLENCR**S**HTEKRN  FALMMG**S**NRKFVD  EQQQDV**S**EFTHKL  DRYMYR**S**KELIRN  TKPASE**S**CPPESD  ESCPPE**S**DTHMTL  MTLPLS**S**VHCSVS  SVHCSV**S**DQTSKE  SVSDQT**S**KESTST  DQTSKE**S**TSTESS  ESTSTE**S**SSQDVE  STSTES**S**SQDVES  PLTSSR**S**SMEMPS  SSMEMP**S**QPAPRT  CLQRWR**S**EIEQDI  EEVERD**S**YGGLRN  TESDQM**S**EVEALS  KIPQME**S**STNSSS  GVRCLS**S**EHAVIV  ETPTSH**S**DPRLQH  FADKNL**S**YDERSI  RRGVKE**S**VIALYR  LNAKAA**S**LFETND  PTIRPN**S**PYDLCS  SPYDLC**S**RFAAVM  FAAVME**S**IQGVST  RVLEVA**S**GSGQHA  LEVASG**S**GQHAAH  LAEWQP**S**DVDQRC  DQRCLD**S**IAATTQ  GGILPQ**S**LDLLLC  INMAHV**S**PLRCTE  AINGKI**S**PQSNVD  GKISPQ**S**NVDFDL  EDLGKA**S**GLLLER  DKAQRK**S**ETQHRG  RGSAPH**S**ESDLPE  SAPHSE**S**DLPEQE  LGWGHF**S**TVWLSW  FSTVWL**S**WDIQGK  EIRLLK**S**VRNSDP  LKSVRN**S**DPNDPN  PENILL**S**VNEQYI  ATEWQR**S**GAPPPS  APPPSG**S**AVSTAP  PSGSAV**S**TAPQPK  KPADKM**S**KNKKKK  QEKLEE**S**STIGQD  EKLEES**S**TIGQDQ  INYTQN**S**NNETLR  NLNQES**S**FLSSQN  SSQNGD**S**STSQET  SQNGDS**S**TSQETD  NGDSST**S**QETDSC  TSQETD**S**CTPITS  DTMVCQ**S**SSTVGQ  TMVCQS**S**STVGQS  SSTVGQ**S**FSEQHI  TVGQSF**S**EQHISQ  FSEQHI**S**QLQESI  ISQLQE**S**IRAEIP  LDNKGK**S**TAGNFL  QTRQYR**S**LEVLIG  LEVLIG**S**GYNTPA  TPADIW**S**TACMAF  VEKYEW**S**QEEAAG  AEYPTI**S**IDRFDR  WKKRII**S**IEIETP  SLVDEA**S**GEKEEI  RVKDIQ**S**VYLDTT  SREECL**S**GVLELV  VLELVR**S**WITRSP  RSWITR**S**PYHVVW  YLFTNL**S**EELGVQ  EEYFQW**S**KLPCGI  MWFGER**S**RKTNVI  IVRTGE**S**SYRACF  SYRACF**S**FHSSYS  ACFSFH**S**SYSEIK  EIKDFL**S**YLCPVN  LCRSSQ**S**TEPKYK  AECMQS**S**RFTNFV  EESNSE**S**EEEVGI  LQGDLG**S**VLHLQK  VAGGSQ**S**PKLFSD  PKLFSD**S**DGESTH  SDSDGE**S**THISSQ  ESTHIS**S**QNSSQS  ISSQNS**S**QSTHIT  SQNSSQ**S**THITEQ  DTVLLS**S**QERNSG  SSQERN**S**GDITSL  CPKDTY**S**DLKSRD  DVTIVP**S**TGEPTT  EPTTLS**S**ETHIPE  HIPEEK**S**LLNLST  SDFEVP**S**TPEAEL  FEIVDR**S**QLPGPG  GPGDLR**S**ATRPRA  RAAEGW**S**APILTL  TGNLSA**S**CGSALR  LSASCG**S**ALRAAA  GLGGGD**S**GDGTAR  GTARAA**S**KCQMME  MHMMKL**S**IKVLLQ  IKVLLQ**S**ALSLGR  ALSLGR**S**LDADHA  GLKVKK**S**FIGQNK  DNKHLL**S**EFYEPE  KGEDLD**S**QVGVID  VGVIDF**S**LYLKDV  GLEKTN**S**KLQEEL  ATDRIC**S**LQEEQQ  GLDEMY**S**DVWKQL  AQNAES**S**LQQKNE  KNEAIT**S**FEGKTN  EERLQH**S**ERARQG  ALQLQL**S**QLHEQC  QLHEQC**S**SLEKEL  LHEQCS**S**LEKELK  LEKELK**S**EKEQRQ  QHEKDT**S**SLLRME  HEKDTS**S**LLRMEL  EMGLHL**S**QSKLKM  GLHLSQ**S**KLKMED  EKEFSI**S**RRKHHC  IFCNTC**S**SNELAL  FCNTCS**S**NELALP  NELALP**S**YPKPVR  PVRVCD**S**CHTLLL  HTVDSL**S**RLTKVE  SLVLPH**S**TKERDS  GCCLGQ**S**ESDKSS  AAAETD**S**RPADED  VTRKAP**S**RDEPCS  SRDEPC**S**STSRPA  RDEPCS**S**TSRPAL  EPCSST**S**RPALEE  GVVQMN**S**RDVRAR  WYDAEI**S**RKRETR  VVLGDD**S**LNDCRI  RRKSGP**S**CKHCKD  CLDPPL**S**SVPSED  LDPPLS**S**VPSEDE  PLSSVP**S**EDEWYC  ECRNDA**S**EVVLAG  MASATS**S**SQRDWG  ASATSS**S**QRDWGK  RFRVQV**S**ESGVHR  RVQVSE**S**GVHRPH  EAKDWR**S**GKPVRV  WPEKGK**S**GFLVWR  EREKEN**S**KREEEE  GPSRAG**S**PRRTSK  LTAQQS**S**LIREDK  LIREDK**S**NAKLWN  WNEVLA**S**LKDRPA  DRPASG**S**PFQLFL  KDCLDR**S**FRAQVF  FRAQVF**S**CPACRY  RYDLGR**S**YAMQVN  GVALFN**S**MRHEIE  FQHQED**S**KKWRNR  KWRNRF**S**LVPHNY  PRAVIN**S**AGYKIL  GYKILT**S**VDQYLE  LELIGN**S**LPGTTA  NGIPED**S**KVEGPA  NEVQIL**S**NLVMEE  RFEEVL**S**KVQQVQ  SKEHLA**S**KIRAFI  VQPYIP**S**ILEALM  ALMVPT**S**QGFTEV  EYMEKL**S**RLAYHP  HPLKMQ**S**CYEKME  CYEKME**S**LRLDGL  QQRFDV**S**STSVFK  QRFDVS**S**TSVFKQ  KEELCK**S**IQRVLE  KKYDYD**S**SSVRKR  KYDYDS**S**SVRKRF  YDYDSS**S**VRKRFF  EALLQI**S**IPFLLK  LAPTCK**S**ELPRFQ  DWGEEY**S**NSGGGG  GGSPSP**S**TPESAT  PESATL**S**EKRRRA  KAAPEA**S**SPPASP  LGPPKP**S**DQETGE  ETGEQV**S**SPSSHP  EQVSSP**S**SHPALH  QVSSPS**S**HPALHT  ASSTPS**S**SATSSN  SSTPSS**S**ATSSNA  PSSSAT**S**SNAGAD  SSSATS**S**NAGADP  FLQRTN**S**LEEKSR  NSLEEK**S**RLVSAF  EKSRLV**S**AFKERQ  AFKERQ**S**SKNLLS  FKERQS**S**KNLLSC  SSKNLL**S**CENSDR  LLSCEN**S**DRDARF  RTETDF**S**NLFARD  RKTFDR**S**TKVLDF  GFNLEL**S**DHPESL  LSDHPE**S**LEQILV  RFFNQL**S**TGLDII  AGEWLT**S**TANTNM  REIVGW**S**SKDGDG  EIVGWS**S**KDGDGI  DGDGIF**S**PGGAIS  SPGGAI**S**NMYSIM  AISNMY**S**IMAARY  KLVLFT**S**EQSHYS  SEQSHY**S**IKKAGA  GGGLLM**S**RKHRHK  GIERAN**S**VTWNPH  GVLLQC**S**AILVKE  DKQYDV**S**YDTGDK  FWYIPQ**S**LRGVPD  LRGVPD**S**PQRREK  KALMME**S**GTTMVG  FFRMVI**S**NPAATQ  NPAATQ**S**DIDFLI  TVTVFI**S**SSLNTF  VTVFIS**S**SLNTFR  TVFISS**S**LNTFRS  SLNTFR**S**EKRYSR  RSEKRY**S**RSLTIA  EKRYSR**S**LTIAEF  LELLVG**S**PASCME  LVGSPA**S**CMELEL  EDALLG**S**YPVDDG  IHVIDH**S**GARLGE  GEYEDV**S**RVEKYT  RQDTVR**S**FLKRSK  EEKAQA**S**SIPVGS  EKAQAS**S**IPVGSR  SSIPVG**S**RCEVRA  VRAAGQ**S**PRRGTV  LGKNDG**S**VNGKRY  YGKNQW**S**RIASLL  QWSRIA**S**LLHRKS  YEWLDP**S**IKKTEW  LGFYDT**S**EENYQA  LDGELR**S**EKEGRD  LKRKKE**S**DLPSAI  SAILQT**S**GVSEFT  LQTSGV**S**EFTKKR  EFTKKR**S**KLVLPA  VKVGQA**S**EIARQT  RQTAEE**S**GITNSA  ESGITN**S**ASSTLL  GITNSA**S**STLLSE  ITNSAS**S**TLLSEY  ASSTLL**S**EYNVTN  YNVTNN**S**VALRTP  NTPLHE**S**DFSGVT  LHESDF**S**GVTPQR  TPNTVL**S**TPFRTP  EGLTPR**S**GTTPKP  DGMADY**S**DPSYVK  ADYSDP**S**YVKQME  KDLPRP**S**EVNETI  LTDLQK**S**EELIKK  HHPYEP**S**GNKKGK  GFGTNN**S**EHITYL  NPYEKF**S**KEELKK  VVKQGM**S**HGELSS  MSHGEL**S**SEAYNQ  KKDRIE**S**LEKRLE  LKKHED**S**AIPRRL  ALWIST**S**SAGAGG  PHPGTG**S**RRLKVA  SAGTGA**S**VSAAAT  AAGGGG**S**TASTSG  GSTAST**S**GGVGAG  PLPGPP**S**LSSGSG  GPPSLS**S**GSGSRE  GAELRA**S**PKAVIN  PKAVIN**S**QEWTLS  SQEWTL**S**RSIPEL  VLGDAR**S**GKSSLI  DARSGK**S**SLIHRF  AVIFVF**S**LEDENS  NSFQAV**S**RLHGQL  LLAACK**S**LPSSPS  ACKSLP**S**SPSHSA  CKSLPS**S**PSHSAA  HTSDYS**S**SLPSSP  AAVAGL**S**TPGSLH  AAKRRT**S**LFANRR  SDSEKR**S**LDSRGE  KKYVTL**S**SNGFLL  INDYIH**S**THGKEM  AFGPSA**S**INGLVK  ATTPMP**S**PSPSPS  PSPSPS**S**LQPPPD  NLARAL**S**TDCTPS  FEFLIV**S**STGQTW  ESQILA**S**LQCCES  LQCCES**S**KVKLRT  PNPTWA**S**LNLGAL  NLGTHL**S**RVRSLD  SITATP**S**PRRRSS  KDPVQC**S**RDVVIC  VICPDA**S**LEDAKK  LGAQNL**S**ESAAVK  AQNLSE**S**AAVKEI  HEIGFG**S**KVTTHP  GGHYTY**S**ENRVEK  DGLILT**S**RGPGTS  SRGPGT**S**FEFALA  KAKMQE**S**GEQTIS  VSNPDV**S**DQKPET  DQKPET**S**SLASNL  QKPETS**S**LASNLP  ASNLPM**S**EEIMTC  SSNDYT**S**QMYSAK  PYAHIL**S**VPVSET  GLIQTP**S**PSQHSV  IQTPSP**S**QHSVLT  PSPSQH**S**VLTCTT  LTTSQP**S**PAHYSY  PSPAHY**S**YPIQAS  YPIQAS**S**TNASLI  ASLIST**S**STIANI  SLISTS**S**TIANIP  ACYPSS**S**FGVTGQ  AATTYQ**S**EKPSVM  AAQRLS**S**GDPSTS  LSSGDP**S**TSPSLS  SGDPST**S**PSLSQT  DPSTSP**S**LSQTTP  LSQTTP**S**KDTDDQ  RKADAT**S**SQDSEL  KADATS**S**QDSELE  HSLLTG**S**YAQKYG  QDLSNY**S**FSTDGF  LSNYSF**S**TDGFSG  TDGFSG**S**GGSGSH  PIENIY**S**ATKIGK  TKIGKE**S**CFERIV  CFERIV**S**RFGKKV  ATGEDA**S**SPPPKT  TGEDAS**S**PPPKTE  PKTEAA**S**DPQHPA  PQHPAA**S**EGAAAA  AAAAAA**S**PPLLRC  DKVKLQ**S**RPAAPP  AVGEGV**S**DRKAGD  VMVLNR**S**GMWQEE  EEVTVP**S**VQTFLI  NLQPGH**S**VLVHMA  TVFGTA**S**ASKHEA  FGTASA**S**KHEALK  DEIKKI**S**PKGVDI  MDPLGG**S**DTAKGY  TWWNQF**S**VTALQL  GEVELV**S**GVVARL  IKPHID**S**VWPFEK  CPGAPG**S**AVGQQK  LGKKQS**S**VYKLEA  LEAVEK**S**PVFCGK  AGPAAI**S**IIAQAE  ENSQEF**S**PTFSER  RTPEQE**S**CTIPVQ  RPALPR**S**ELHKLI  ALQGSV**S**SGQAHS  TWAARG**S**RSREPS  ELLEGG**S**LGQLVK  GLEYLH**S**RRILHG  LLSSDG**S**HAALCD  KVDVWS**S**CCMMLH  LCLKIA**S**EPPPVR  EPIHRV**S**AAELGG  TLSKEE**S**GMWEPL  EPLPLS**S**LEPAPA  PARNPS**S**PERKAT  SLSQPF**S**LEEQEQ  EQEQIL**S**CLSIDS  DSEKNP**S**KASQSS  KNPSKA**S**QSSRDT  PSKASQ**S**SRDTLS  SKASQS**S**RDTLSS  SSRDTL**S**SGVHSW  LSSGVH**S**WSSQAE  GVHSWS**S**QAEARS  QAEARS**S**SWNMVL  AEARSS**S**WNMVLA  RPTDTP**S**YFNGVK  IATGIS**S**QIPAAA  DMEVPD**S**GIDLQC  TLAPDG**S**FAWSWR  PACCSC**S**DVFQYE  KVTRIQ**S**MNYGTI  VCFALV**S**DKLYQR  RKEPVI**S**SVHTKV  KEPVIS**S**VHTKVK  VKKLVH**S**VFDTAD  FPLQGN**S**FFVMTN  TRRTLC**S**SDRGCK  RRTLCS**S**DRGCKK  QKTCEV**S**AWCPIE  RPALLN**S**AENFTV  HCRPKY**S**FRRLDD  DKTTNV**S**LYPGYN  LVVYIG**S**TLSYFG  YIGSTL**S**YFGLAA  FLIDTY**S**SNCCRS  LIDTYS**S**NCCRSH  SSNCCR**S**HIYPWC  YRKKCE**S**IVEPKP  PTLKYV**S**FVDESH  VSFVDE**S**HIRMVN  QQLLGR**S**LQDVKG  MDFTDL**S**RLPLAL  KEATPR**S**RDSPVW  TPRSRD**S**PVWCQC  VWCQCG**S**CLPSQL  CGSCLP**S**QLPESH  GACITT**S**ELFRKL  LALDVD**S**TNSRLR  DVDSTN**S**RLRHCA  ATWRFG**S**QDMADF  DFAILP**S**CCRWRI  RKEFPK**S**EGQYSG  AYGKAK**S**IVDKEG  NNAKAL**S**TLRQKI  YNRDFE**S**HITSYK  EKNEED**S**EGSSDE  EEDSEG**S**SDEDED  EDEDGV**S**AATFLK  TFLKKK**S**EAPSGE  KKSEAP**S**GESRKF  EAPSGE**S**RKFLKK  EDWDTG**S**TSSDSD  DTGSTS**S**DSDSEE  GSTSSD**S**DSEEEE  TSSDSD**S**EEEEGK  KQTALA**S**RFLKKA  KFNIIA**S**LYDYNP  ENILEE**S**ENLHNA  QNTDPH**S**QEYVEH  PEGSSK**S**EQDQAE  YHHALH**S**RWYQAR  ALLDIQ**S**SGRAKE  LLDIQS**S**GRAKEL  QGLLLR**S**LQERNQ  YMAAHE**S**DARRRM  LLGPPE**S**MREHVV  DWKTCH**S**FIINEK  RKIQEE**S**LRTYLF  TYLFTY**S**SVYDSI  YLFTYS**S**VYDSIS  ISMETL**S**DMFELD  DLPTVH**S**IISKMI  LAEKLG**S**LVENNE  MCPKNA**S**YYGNRA  LGDAQQ**S**VRLDDS  SVRLDD**S**FVRGHL  EGKCHL**S**LGNAMA  AMAACR**S**FQRALE  RYPEAQ**S**VASDIL  EAQSVA**S**DILRMD  DILRMD**S**TNADAL  NRGTVN**S**KLRKLD  QLELKK**S**KRKDYY  GVDKNA**S**EDEIKK  HHPDRH**S**GASAEV  DRHSGA**S**AEVQKE  EAFTIL**S**DPKKKT  KKTRYD**S**GQDLDE  GGPGGF**S**FEASGP  GFSFEA**S**GPGNFF  AYGVRE**S**VFTVEG  ARPRKI**S**SPTGSK  RPRKIS**S**PTGSKD  ISSPTG**S**KDLQMV  LQMVNI**S**LRVLSR  ISLRVL**S**RPNAQE  NAQELP**S**MYQRLG  EERVLP**S**IVNEVL  VNEVLK**S**VVAKFN  VAKFNA**S**QLITQR  TQRAQV**S**LLIRRE  ERAKDF**S**LILDDV  VAITEL**S**FSREYT  ITELSF**S**REYTAA  MLGEAL**S**KNPGYI  RAAQNI**S**KTIATS  SKTIAT**S**QNRIYL  LNLQDE**S**FTRGSD  ESFTRG**S**DSLIKG  VAVMAE**S**AFSFKK  MAESAF**S**FKKLLD  IPPAIK**S**ANSELG  AIKSAN**S**ELGGIW  ELGGIW**S**VGQRIW  INAHQW**S**ETVQPI  RAFALV**S**QAYTSI  VSQAYT**S**IIADDF  QGWQAD**S**TTRMVL  NKFIPL**S**EPAPVP  LGKAVH**S**LSRIGD  KAVHSL**S**RIGDEL  PLEDGL**S**LRTVNS  SLRTVN**S**SRSAYA  LRTVNS**S**RSAYAC  TVNSSR**S**AYACFL  CKILMK**S**FLSVFR  LMKSFL**S**VFRSLA  FLSVFR**S**LAMLEK  VEKCCI**S**LNGRSS  ISLNGR**S**SRLVVQ  SLNGRS**S**RLVVQL  RKTHNL**S**FQDCES  SFQDCE**S**LQAVFD  AVFDPA**S**CPHMLR  EAVLPF**S**PALAEV  HEEEAD**S**TAKAMV  EFRGLL**S**FAESAN  LLSFAE**S**ANLNLS  SANLNL**S**IHFDAP  IFTIKD**S**LLDGHF  FVLATL**S**DTDSHS  TLSDTD**S**HSQDLG  PQLQAH**S**TPHPDD  ANDDID**S**YMIAME  TIGNEG**S**RVLPSI  GSRVLP**S**ISLSPG  RVLPSI**S**LSPGPQ  GPQPPK**S**PGPHSE  EDEAEP**S**TVPGTP  PPKKFR**S**LFFGSI  GTPPPL**S**GTPIPV  HAEPGF**S**LKRPRG  KRPRGL**S**RSLPPP  PPPAKG**S**IPISRL  SFRGEA**S**ETLQSP  SPGYDP**S**RPESFF  DPSRPE**S**FFQQSF  ESFFQQ**S**FQRLSR  QSFQRL**S**RLGHGS  EVFKVR**S**KEDGRL  LYAVKR**S**MSPFRG  TELCGP**S**LQQHCE  CEAWGA**S**LPEAQV  ALAHLH**S**QGLVHL  TAADVF**S**LGLTIL  EFTAGL**S**SELRSV  FTAGLS**S**ELRSVL  LSSELR**S**VLVMML  MAAEAL**S**RGWALW  PATPPG**S**PPCSLL  PGSPPC**S**LLLDSS  LLDSSL**S**SNWDDD  LDSSLS**S**NWDDDS  SNWDDD**S**LGPSLS  DDSLGP**S**LSPEAV  SLGPSL**S**PEAVLA  LARTVG**S**TSTPRS  RTVGST**S**TPRSRC  STSTPR**S**RCTPRD  PRGSFP**S**FEPRNL  EPRNLL**S**LFEDTL  SNGLIH**S**ANVRTV  IPAPKE**S**LRSRST  LPAAAN**S**RKQFSV  NSRKQF**S**VPPAPT  MEEQVH**S**IRGSSS  HSIRGS**S**SANPVN  EKKAQN**S**EMRMKR  AQEYDS**S**FPNWEF  AFDETA**S**NEVVYR  AYGQTG**S**GKTHTM  TMGGDL**S**GKAQNA  QEHLVN**S**ADDVIK  KMIDMG**S**ACRTSG  GQTFAN**S**NSSRSH  FANSNS**S**RSHACF  NSNSSR**S**HACFQI  RMHGKF**S**LVDLAG  RGADTS**S**ADRQTR  HTPFRE**S**KLTQVL  TQVLRD**S**FIGENS  SFIGEN**S**RTCMIA  CMIATI**S**PGISSC  TISPGI**S**SCEYTL  ISPGIS**S**CEYTLN  SPHSGP**S**GEQLIQ  KEEEEL**S**SQMSSF  EEEELS**S**QMSSFN  ELSSQM**S**SFNEAM  FVNKAE**S**ALAQQA  QLEEQA**S**RQISSK  HTFRGC**S**LAFMFI  HVILLG**S**TVNITC  TVNITC**S**LKPRQG  QGCFHY**S**RRNKLI  NFHHGH**S**LNSQVT  EYTLQL**S**GPKNLT  KVTAVN**S**LGSSSS  AVNSLG**S**SSSLPS  NSLGSS**S**SLPSTF  IKFQKA**S**VSRCTL  RLRYRP**S**NSRLWN  RYRPSN**S**RLWNMV  YEFQIS**S**KLHLYK  WSDWSE**S**LRAQTP  KRHIDY**S**RQQISL  WKNLSV**S**EARGKI  PPRKDP**S**AVQEYV  PLNWLR**S**RPYNVS  IRVYAL**S**GDQGGC  SSILGN**S**KHKAPL  KHKAPL**S**GPHINA  YWKERD**S**NSQPQL  KERDSN**S**QPQLCE  EIPYRV**S**QNSHPI  TAAGES**S**HGNERE  LRPQWC**S**REIPDP  EKDMMH**S**ASSPPP  DMMHSA**S**SPPPPR  MMHSAS**S**PPPPRA  LYKVLE**S**RGSDPK  VLESRG**S**DPKPEN  HDGYLP**S**NIDDLP  SVFPSS**S**LHPLTF  DEGITF**S**VPPFAP  VPPFAP**S**GFCTIP  SFWNVE**S**AAAPGI  GCPAAT**S**SSSATR  CPAATS**S**SSATRG  AATSSS**S**ATRGRG  ATRGRG**S**SVGGGS  SSVGGG**S**RRTTVA  YVINEA**S**QGQLVV  IAVVEM**S**DAFRQP  KVYCCD**S**SFMKGL  KVAQAS**S**SQYFRE  VAQASS**S**QYFRES  SQYFRE**S**ILNDIR  NFTDTE**S**RDHGAS  SRDHGA**S**WFKKAF  NLEKLQ**S**YWEVGF  IHEWNF**S**ASSVRG  VRGVSI**S**KFEERC  EEGDCE**S**DLLEYD  QYLGSF**S**ENGFIK  VPGGSL**S**ALLRSK  SGVLKI**S**DFGTSK  DEFLKV**S**SKKKKT  KTQPKL**S**ALSAGS  VEDTSS**S**SEYGSV  FDSHGI**S**QVQVVL  WMFALD**S**IIRKAV  SGVSTL**S**STVSHD  TLSSTV**S**HDSQSA  KHLKLK**S**QPIEIP  GPLFLQ**S**QRFGTK  AVLYPA**S**PHGVAR  FFDHKG**S**SSGGGR  FDHKGS**S**SGGGRG  DHKGSS**S**GGGRGS  SGGGRG**S**SRRLDC  RLAECV**S**VAPVTV  LDTAQR**S**HLLAAD  LAADAP**S**SAAWVQ  AADAPS**S**AAWVQT  NAFPKG**S**WTLAPT  DNPPKL**S**ALEMLE  LEMLEN**S**LYSPTW  LENSLY**S**PTWEGS  SPTWEG**S**QFWVTV  LTVGAQ**S**QILEPL  ILEPLL**S**WPYTLL  RDKVMF**S**FEAGRR  AGRRCP**S**GPGTFT  DVLRAD**S**HEGEVA  AEGKLP**S**PPGPQE  PQELLD**S**PPALYA  YAEPLD**S**LRIAPC  PCPSQD**S**LYSDPL  SQDSLY**S**DPLDST  YSDPLD**S**TSAQAG  AVPPPR**S**TKPLLA  TGSGIK**S**HNSALY  GIKSHN**S**ALYSQV  HNSALY**S**QVQKSG  YSQVQK**S**GASGSW  KSGASG**S**WDCGLS  SWDCGL**S**RVGTDK  PNSADT**S**SDFKDL  EENKKL**S**EQLQQK  SPITAF**S**FSGVNR  NEMRKV**S**KSSTHP  MRKVSK**S**STHPQH  AKAHGT**S**SYTPDK  LGVQEE**S**ETQGPM  SLRFSD**S**TSKTPP  VFGATS**S**IKSGLD  RLEKTR**S**KSEDSA  ALFTHH**S**LGSEVN  THHSLG**S**EVNKII  KIIIQS**S**NKQILI  LINKNI**S**ESLGEQ  KKTEEE**S**EHEVSC  ESEHEV**S**CPQASF  PMDNQF**S**MNGDCV  QEKSQG**S**ETSKNK  SQGSET**S**KNKFRQ  TIPKGF**S**SSRKAS  PKGFSS**S**RKASDG  SSSRKA**S**DGNCTL  CTLPKD**S**PGEPCS  SPGEPC**S**QECIIL  IKIQTR**S**DHGGCE  GGCELA**S**VLQLNP  QKGEKS**S**NEERKM  THEEYE**S**CLADSF  GDERET**S**LQNFPH  KKLASC**S**RHRFRY  WEVGFP**S**TQTCME  GHSNRQ**S**DVRIKF  RSSSMK**S**LRILLL  LRILLL**S**QDRNHN  NSSSPH**S**GVSRQV  QVRIKA**S**QSAGDI  RIKASQ**S**AGDINT  RHLSVS**S**QNPGRS  HIARQG**S**YTSINS  SYTSIN**S**EGEFIP  EFIPET**S**EQCMLD  CMLDPL**S**SAENSL  LSSAEN**S**LSGSCQ  SAENSL**S**GSCQSL  ENSLSG**S**CQSLDR  LDRSAD**S**PSFRKS  DNRQEY**S**DRETQL  PRRYHV**S**VHHKDY  VHHKDY**S**DGRRTF  LFTLVP**S**SRSLST  LVPSSR**S**LSTNGE  DSENAL**S**VQERNV  RNVPTK**S**PSAPIN  TGRELA**S**KQVQFD  VQFDPD**S**PETSKE  PDSPET**S**KEVSAL  ETSKEV**S**ALECEI  EYMPGG**S**VKDQLK  YGALTE**S**VTRKYT  GMSYLH**S**NMIVHR  LGDFGA**S**KRLQTI  GTPYWM**S**PEVISG  GSGSRL**S**KELLAE  LPQEQR**S**VESSLR  EQRSVE**S**SLRAQV  QRSVES**S**LRAQVP  PFEQIL**S**LPELKA  CRVFST**S**PAKDSL  TSPAKD**S**LSFEDF  PAKDSL**S**FEDFLD  DFLDLL**S**VFSDTA  DLLSVF**S**DTATPD  ATPDIK**S**HYAFRI  LNREDL**S**RLVNCL  GEDTRL**S**ASEMKQ  DTRLSA**S**EMKQLI  DNILEE**S**DIDRDG  DGTINL**S**EFQHVI  EFQHVI**S**RSPDFA  QHVISR**S**PDFASS  LKEGTD**S**SQGIPQ  KEGTDS**S**QGIPQL  GIPQLV**S**NISACQ  QLVSNI**S**ACQVIA  RGKATI**S**NDGATI  LVDIAK**S**QDAEVG  VGDGTT**S**VTLLAA  CAMTAL**S**SKLISQ  AMTALS**S**KLISQQ  LSSKLI**S**QQKAFF  GGALED**S**QLVAGV  AFKKTF**S**YAGFEM  LEKIHH**S**GAKVVL  GAKVVL**S**KLPIGD  MMACGG**S**IQTSVN  GGSIQT**S**VNALSA  TSVNAL**S**ADVLGR  MEETER**S**LHDAIM  RAIKND**S**VVAGGG  AIEMEL**S**KYLRDY  KYLRDY**S**RTIPGK  NALTAA**S**EAACLI  AACLIV**S**VDETIK  TIKNPR**S**TVDAPT  GLLALI**S**CFTPSE  ISCFTP**S**ESQRFS  CFTPSE**S**QRFSRR  SESQRF**S**RRPYLP  PRWVPP**S**PPPPYD  PPPPYD**S**RLNSPL  YDSRLN**S**PLSLPF  RLNSPL**S**LPFVPG  PGRVPP**S**SFSRFS  GRVPPS**S**FSRFSQ  VPPSSF**S**RFSQAV  SSFSRF**S**QAVILS  SQAVIL**S**QLFPLE  QLFPLE**S**IRQPRL  AIYLPI**S**NPEPQI  TATATT**S**TSTKPT  TATTST**S**TKPTMT  KPTMTI**S**SSTVPI  PTMTIS**S**STVPIS  TMTISS**S**TVPISS  SSTVPI**S**STPEPA  STVPIS**S**TPEPAT  TPEPAT**S**ISAATP  EPATSI**S**AATPAA  AATPAA**S**TENTTQ  TVQVTT**S**NQTILS  SNQTIL**S**SPAFKS  NQTILS**S**PAFKSF  SSPAFK**S**FWQKLF  QARYSV**S**DPNALG  GVVPYL**S**EQNYYR  YYRAAG**S**YGGMAS  SYGGMA**S**PMGVYS  SPMGVY**S**GHPEQY  GHPEQY**S**AGMGRS  SAGMGR**S**YAPYHH  LVKPPY**S**YIALIT  SIRHNL**S**LNECFV  KKPGKG**S**YWTLDP  WTLDPD**S**YNMFEN  NMFENG**S**FLRRRR  FKKKDV**S**KEKEER  EPPPAA**S**KGAPAT  LQGSPR**S**AASTPA  SPRSAA**S**TPAGSP  AGSPDG**S**LPEHHA  NGLPGF**S**VENIMT  AGGYQC**S**MRAMSL  CSMRAM**S**LYTGAE  ALDEAL**S**DHPSGP  ALSDHP**S**GPTSPL  GPTSPL**S**ALNLAA  AAAQAA**S**WYLNHS  SWYLNH**S**GDLNHL  VREMFN**S**HRLGIE  RLGIEN**S**TLGESQ  NSTLGE**S**QVSGNA  LGESQV**S**GNASCQ  QVSGNA**S**CQLPYR  CQLPYR**S**TPPLYR  YTLGCV**S**ECFCPT  CPTNFP**S**SMYCDN  PTNFPS**S**MYCDNR  EAVTAN**S**FINATH  LKEINL**S**HNKIKS  SHNKIK**S**QKIDYG  PFPLPK**S**LERLLL  LGYNEI**S**KLQTNA  YNYLHD**S**LLKDKI  MQLNLC**S**NRLESM  CSNRLE**S**MPPGLP  MPPGLP**S**SLMYLS  PPGLPS**S**LMYLSL  SSLMYL**S**LENNSI  LSLENN**S**ISSIPE  LENNSI**S**SIPEKY  ENNSIS**S**IPEKYF  LHTLRM**S**HNKLQD  PNIVEL**S**VGHNKL  LTVMCP**S**IDPLHY  KLKEPI**S**SYIFFC  LKEPIS**S**YIFFCF  YYGEQR**S**TNGQTI  PDDDDE**S**EDHDDP  PDNAHE**S**PEQEGA  AQAGRQ**S**SAKRHL  QAGRQS**S**AKRHLA  YLADMN**S**SESVGS  LADMNS**S**ESVGSD  DMNSSE**S**VGSDAP  SSESVG**S**DAPCVV  VVKVEP**S**DNGPLF  VPKYWG**S**GLHDKN  HDKNGK**S**YRFMIM  IMDRFG**S**DLQKIY  ANAKRF**S**RKTVLQ  KTVLQL**S**LRILDI  HGDIKA**S**NLLLNY  GTIEFT**S**IDAHNG  HNGVAP**S**RRGDLE  PKYVRD**S**KIRYRE  YRENIA**S**LMDKCF  GLKAIG**S**KDDGKL  KKEIEE**S**KEPGVE  VEDTEW**S**NTQTEE  EAIQTR**S**RTRKRV  LAHIRQ**S**HVTSDD  RQSHVT**S**DDTGMC  MPGDSG**S**EIQGSN  GYVYAQ**S**VDITSS  QSVDIT**S**SWDFGI  FGIRRR**S**NTAQRL  QWKERN**S**KQSAQE  ERNSKQ**S**AQELKS  SAQELK**S**LFEKKS  SLFEKK**S**LKEKPP  KEKPPI**S**GKQSIL  PISGKQ**S**ILSVRL  GKQSIL**S**VRLEQC  NPFNEY**S**KFDGKG  YLPLHS**S**QDRLLP  TVVTMA**S**ARVQDL  DFPPLD**S**NEPIHK  ALVEKY**S**SPGLTS  NAAHGF**S**LIQVDN  KGSQKV**S**GPQYRL  VAVDLD**S**TLESQS  CLVREN**S**SRADGV  GVFEED**S**QIDIAT  TVQDML**S**SHHYKS  VQDMLS**S**HHYKSF  YKSFKV**S**MIHRLR  VTNQKA**S**TKFWIK  IKQKPI**S**IDSDLL  DLAEEK**S**PSHAIF  AEEKSP**S**HAIFKL  VNYILE**S**RASTAR  ILESRA**S**TARADY  NRRTSF**S**FQKEKK  VADKTG**S**INISVW  TGSINI**S**VWDDVG  LTKGYA**S**VFKGCL  EFCMVY**S**EVPNFS  SEVPNF**S**EPNPEY  EPNPEY**S**TQQAPN  KAVQND**S**NPSASQ  QNDSNP**S**ASQPTT  DSNPSA**S**QPTTGP  QPTTGP**S**AASPAS  TGPSAA**S**PASENQ  SAASPA**S**ENQNGN  QNGNGL**S**APPGPG  HPPHTP**S**HPPSTR  TPSHPP**S**TRITRS  STRITR**S**QPNHTP  AGPPGP**S**SNPVSN  GPPGPS**S**NPVSNG  PSSNPV**S**NGKETR  EAARYR**S**DGALLL  ALLLGA**S**SLSGRC  LGASSL**S**GRCWAG  GRCWAG**S**LWLFKD  PNEGFC**S**AGVQTE  RGILVA**S**DSGAVE  ILVASD**S**GAVELW  EHDDIV**S**TVSVLS  DIVSTV**S**VLSSGT  STVSVL**S**SGTQAV  SGTQAV**S**GSKDIC  AQQVVL**S**SYRAHA  QQVVLS**S**YRAHAA  VTCVAA**S**PHKDSV  ASPHKD**S**VFLSCS  KDSVFL**S**CSEDNR  SVFLSC**S**EDNRIL  RCPKPA**S**QIGCSA  ASQIGC**S**APGYLP  PGYLPT**S**LAWHPQ  DENGTV**S**LVDTKS  SLVDTK**S**TSCVLS  VDTKST**S**CVLSSA  STSCVL**S**SAVHSQ  TSCVLS**S**AVHSQC  VTGLVF**S**PHSVPF  LVFSPH**S**VPFLAS  SVPFLA**S**LSEDCS  PFLASL**S**EDCSLA  SLSEDC**S**LAVLDS  SLAVLD**S**SLSELF  LAVLDS**S**LSELFR  VRDATW**S**PLNHSL  KGQGEY**S**GSWNFG  QGEYSG**S**WNFGFE  TFEGYW**S**QGKRHG  GIRQSS**S**SGAKYE  IRQSSS**S**GAKYEG  MAVVVR**S**PLRTSL  RSPLRT**S**LSSLRS  PLRTSL**S**SLRSEH  GTVAPD**S**PASPAS  APDSPA**S**PASDGP  SPASPA**S**DGPALP  DGPALP**S**PAIPRG  RVSFLK**S**DLSSGA  DLSSGA**S**DAASTA  GASDAA**S**TASLGE  DAASTA**S**LGEAAE  RSGFGV**S**ERSSGL  FGVSER**S**SGLRYE  GVSERS**S**GLRYEG  KAEIAA**S**RTSHAK  IAASRT**S**HAKAKA  LAANQE**S**NIARTL  AWNGEP**S**GEGSRS  EPSGEG**S**RSVTPS  SGEGSR**S**VTPSEG  SRSVTP**S**EGAGRR  QAPPAP**S**REPEVA  LYQGYH**S**YAVRTT  EPEVSG**S**ESAPSS  GSESAP**S**SPATAP  SESAPS**S**PATAPL  RKVVRQ**S**KFRHVF  YEDIRV**S**RVTWDS  SRVTWD**S**TFCAVN  FLVLPL**S**KTGRID  NDEVIA**S**GSEDCT  EVIASG**S**EDCTVM  PENGLT**S**PLTEPV  ARNVLL**S**AGCDNV  ELYRLD**S**LHPDLI  DLIYNV**S**WNHNGS  NGSLFC**S**ACKDKS  SACKDK**S**VRIIDP  VFTTGF**S**RMSERQ  TGFSRM**S**ERQLAL  ALQELD**S**SNGALL  LQELDS**S**NGALLP  VCGKGD**S**SIRYFE  CGKGDS**S**IRYFEI  FLNTFT**S**KEPQRG  PQRGMG**S**MPKRGL  KRGLEV**S**KCEIAR  MTVPRK**S**DLFQDD  EAEEWV**S**GRDADP  ADPILI**S**LREAYV  REAYVP**S**KQRDLK  QRDLKI**S**RRNVLS  SRRNVL**S**DSRPAM  RNVLSD**S**RPAMAP  PAMAPG**S**SHLGAP  HLGAPA**S**TTTAAD  DATPSG**S**LARAGE  KLVLLQ**S**SAVLLH  LVLLQS**S**AVLLHS  SAVLLH**S**GSSVPA  VLLHSG**S**SVPAAA  LLHSGS**S**VPAAAG  VPAAAG**S**SVVSES  PAAAGS**S**VVSESA  AGSSVV**S**ESAVSW  SSVVSE**S**AVSWEA  VSESAV**S**WEAGAR  AVLRCQ**S**PRMVWT  RLLDLY**S**AGEQRV  RGRLEL**S**ASAFDD  RLELSA**S**AFDDGN  FDDGNF**S**LLIRAV  YCHLYE**S**LAVRLE  FERGDF**S**LRIEPL  ADEGTY**S**CHLHHH  EPPPRG**S**PGNGSS  GSPGNG**S**SHSGAP  SPGNGS**S**HSGAPG  GNGSSH**S**GAPGPD  NVIVPE**S**RAHFFQ  RGGYEY**S**DQKSGK  EYSDQK**S**GKSKGK  DQKSGK**S**KGKDVN  DQMLYR**S**EDIQLD  RAELAH**S**PLPAKY  WQNMGT**S**VRRRSL  QEQLED**S**KELQPV  ELQPVV**S**HQETSV  VSHQET**S**VGALGS  SVGALG**S**LCRQFQ  NLRAGP**S**WKRLET  LQAAAR**S**AKSALG  AARSAK**S**ALGAVS  SALGAV**S**QRIQES  SQRIQE**S**CQSGTK  IQESCQ**S**GTKWLV  SGSPTH**S**LSQKST  SPTHSL**S**QKSTRL  HSLSQK**S**TRLSGA  QKSTRL**S**GAAPAH  GAAPAH**S**AADPWE  KEHHRL**S**VRMGSH  LSVRMG**S**HAHPLR  AHPLRR**S**RREAAF  REAAFR**S**PYSSTE  AFRSPY**S**STEPLC  FRSPYS**S**TEPLCS  STEPLC**S**PSESDS  EPLCSP**S**ESDSDL  QHLQKL**S**QELDEA  ERKQAL**S**DRQGFI  LAQLMN**S**SGSHKD  AQLMNS**S**GSHKDL  LMNSSG**S**HKDLAG  EKAIQL**S**GAEQLE  MVNENV**S**LVISRQ  NVSLVI**S**RQLLTD  LPNLPD**S**TAKEIY  IQPRVI**S**FEEQVA  FEEQVA**S**IRQHLA  IRQHLA**S**IYEKEE  AYINRA**S**LLQNES  SLLQNE**S**TNEQLQ  QRYNEL**S**YKTIVH  KTIVHE**S**ERLEAL  HCTILA**S**AGQQRS  SAGQQR**S**RMLATL  ATTADG**S**SILDRA  TTADGS**S**ILDRAV  IEHNLL**S**ASKLYN  HNLLSA**S**KLYNNI  KAEKIA**S**QMITEG  WDKQIQ**S**LCFQVN  NLLEKI**S**QTAPEW  HDDTSD**S**DSDGLT  PLKGIQ**S**RLRKKR  EKAANP**S**GKKKLK  TMKFLS**S**GKEQKP  CESSTP**S**WASDHN  EKTAAP**S**PSLLYK  TKEDRR**S**EEKAAA  TPSSGA**S**AARQAG  AMSAAP**S**QPNSQI  SPPVSD**S**EEQQES  RAVPEK**S**TAPLLD  PKKQKL**S**ASVKKE  KQKLSA**S**VKKEDL  LSPCPA**S**CGSGVV  NISTSE**S**RSPPEG  DTTLFL**S**RLSTIW  SLYSYF**S**SRGRFG  LYLIPL**S**AQDPVP  STTPPG**S**PPPPPP  ASTAAS**S**TASSAS  KKSFDP**S**AREPPG  SPPPKS**S**LPKAEL  FQQEQQ**S**ADKPAS  EKPPAG**S**EQDGWK  TCPGFA**S**QDKALG  DPRNLH**S**AGRSSS  PEENIA**S**NDGPRG  QKGPIP**S**LFSGQH  PPPYGD**S**RGPSPS  GDSRGP**S**PSYLGG  PPSALS**S**SAPGQG  ERGRNW**S**RERDWD  RKDRSK**S**KESARD  LFIQGD**S**VRPGIL  QLQDQD**S**VLFIST  PPCPAH**S**ATRFDP  FDPTWE**S**LDARQL  IHWGVF**S**VPSFGS  GVFSVP**S**FGSEWF  SVPSFG**S**EWFWWY  KDNYPP**S**FKYEDF  ADIFQA**S**GAKYIV  KYIVLT**S**KHHEGF  GFTLWG**S**EYSWNW  LWGSEY**S**WNWNAI  RFGLYY**S**LFEWFH  LFLEDE**S**SSFHKR  FLEDES**S**SFHKRQ  LEDESS**S**FHKRQF  KRQFPV**S**KTLPEL  QPEVLW**S**DGDGGA  PDQYWN**S**TGFLAW  AWLYNE**S**PVRGTV  DRWGAG**S**ICKHGG  GGFYTC**S**DRYNPG  RREAGI**S**DYLTIE  QLVETV**S**CGGNLL  TLDGTI**S**VVFEER  RLRQMG**S**WLKVNG  ETHTWR**S**QNDTVT  PDVWYT**S**KPKEKL  FLKWPT**S**GQLFLG  QPLNWI**S**LEQNGI  GAGAGA**S**GGPEAS  SGGPEA**S**PEAEVV  MELAPA**S**APARDA  AALANL**S**REPAPC  AAEPAD**S**GLERLV  YLAPLL**S**NLSQRP  PLLSNL**S**QRPAAR  LTQYPD**S**SVRRGG  TQYPDS**S**VRRGGV  AGPEDF**S**EEEMER  ILRELH**S**WEPEPD  TPPPRP**S**PLEAGS  SPLEAG**S**DGCEEP  EPKQQV**S**WEQEFL  QEFLVG**S**SPGGSG  EFLVGS**S**PGGSGR  GSSPGG**S**GRALCM  AEIRAP**S**ADTARS  SADTAR**S**HILEQH  PHTLDL**S**PSEKSN  TLDLSP**S**EKSNIL  LSPSEK**S**NILEAW  NILEAW**S**EGVALL  QPSPPN**S**DSGQDA  SPPNSD**S**GQDAHP  IVVLLD**S**EDNPSL  DSEDNP**S**LPKRSR  PSLPKR**S**RPRGLR  EPPSPD**S**PTETFA  RHFTDG**S**FPAGFV  FVLQLF**S**HTQLRG  QLRGPD**S**KDSPKD  GPDSKD**S**PKDREV  EEPPAV**S**LLQDWS  SLLQDW**S**RHPQGT  VGAGDT**S**DWPTVL  DWPTVL**S**ESSTTV  PTVLSE**S**STTVAG  TVLSES**S**TTVAGK  PIEMGS**S**EPLPIA  RGRATD**S**LPGKFE  DMYKLT**S**ELLGEG  EKQAGH**S**RSRVFR  QAGHSR**S**RVFREV  EKLQGG**S**ILAHIQ  FNEREA**S**RVVRDV  HTKDKV**S**LCHLGW  LCHLGW**S**AMAPSG  WSAMAP**S**GLTAAP  LTAAPT**S**LGSSDP  APTSLG**S**SDPPTS  PTSLGS**S**DPPTSA  SSDPPT**S**ASQVAG  DPPTSA**S**QVAGTT  ENILCE**S**PEKVSP  ESPEKV**S**PVKICD  CDFDLG**S**GMKLNN  GMKLNN**S**CTPITT  LTTPCG**S**AEYMAP  KRCDLW**S**LGVVLY  VLYIML**S**GYPPFV  QNKLFE**S**IQEGKY  KDWAHI**S**SEAKDL  DWAHIS**S**EAKDLI  EAKDLI**S**KLLVRD  DAKQRL**S**AAQVLQ  QVLQRN**S**STMDLT  VLQRNS**S**TMDLTL  ALNRQL**S**QHEENE  LADGLC**S**MKLSPP  LCSMKL**S**PPCKSR  LSPPCK**S**RLARRR  HFLEMK**S**ATDRDL  KEAHER**S**QELIQL  SALLTQ**S**QKQNED  DNIAQG**S**GHENSL  SLELDS**S**IFSQFD  QSIAEL**S**SSENTL  AAAVKL**S**ALNEAL  EIQKKL**S**ESRHQQ  QLQGLS**S**AKELLE  KWEKER**S**WHQQEL  ERTQAE**S**ALCQMQ  LVAQDD**S**QRLVEQ  IRQQEL**S**ALRQDM  QLASLY**S**ALQQAL  DTEAEK**S**QVHTEL  DLQRQL**S**QNQEEK  KQNSLE**S**ELMELH  KNEEVE**S**ERERAQ  ELEKCR**S**VLEHLP  QELQAQ**S**SQIHDL  QIHDLE**S**HSTVLA  HDLESH**S**TVLARE  LEQIKL**S**LRERGR  KEQSLQ**S**QLDEAQ  GDLRAE**S**REQEKA  PHSHKT**S**PMEEQS  TKASVS**S**LQEVAM  DGMEKQ**S**WRQRLE  TRSAQT**S**RELAGL  LAGLHH**S**LSHSLL  HHSLSH**S**LLAVAQ  DEKQFY**S**KAKTYW  GGYGHI**S**SIDINS  GYGHIS**S**IDINSS  SSIDIN**S**SRKFLQ  SIDINS**S**RKFLQR  PNKTGT**S**CALDCG  FTPEPD**S**YDVIWI  LRRCKG**S**LRPNGI  ILDDVD**S**SVCRDL  LDDVDS**S**VCRDLD  VRRIIC**S**AGLSLL  ICSAGL**S**LLAEER  TEQEIR**S**LFEQYG  NINVEA**S**KNKSKT  EASKNK**S**KTSTKL  KNKSKT**S**TKLHVG  LHVGNI**S**PTCTNK  RMHVQL**S**TSRLRT  HVQLST**S**RLRTAP  PGMGDQ**S**GCYRCG  GKEGHW**S**KECPID  ECPIDR**S**GRVADL  RTPYTM**S**YGDSLY  TMSYGD**S**LYYNNA  RCRAAR**S**YEAVAA  VAAAAA**S**VYNYAE  YAEQTL**S**QLPQVQ  QNTAMA**S**HLTSTS  MASHLT**S**TSLDPY  SHLTST**S**LDPYDR  RHLLPT**S**GAAATA  AAVTAA**S**TSYYGR  VTAAST**S**YYGRDR  YGYGHE**S**ELSQAS  GHESEL**S**QASAAA  SELSQA**S**AAARNS  SAAARN**S**LYDMAR  RYVKLI**S**METSSS  LISMET**S**SSSDDS  ISMETS**S**SSDDSC  SMETSS**S**SDDSCD  METSSS**S**DDSCDS  SSSSDD**S**CDSFAS  SDDSCD**S**FASDNF  SCDSFA**S**DNFANT  EGCRTR**S**QCRHSG  RSQCRH**S**GPLRVA  MKFPAR**S**TRGATN  TNKKAE**S**RQPSEN  AESRQP**S**ENSVTD  RQPSEN**S**VTDSNS  ENSVTD**S**NSDSED  SVTDSN**S**DSEDES  TDSNSD**S**EDESGM  SDSEDE**S**GMNFLE  MLAKLM**S**ELESFP  LMSELE**S**FPGSFR  LESFPG**S**FRGRHP  RHPLPG**S**DSQSRR  PLPGSD**S**QSRRPR  TFPGVA**S**RRNPER  PLTRSR**S**RILGSL  RSRILG**S**LDALPM  EDDLPR**S**RRSRSS  LPRSRR**S**RSSVTL  SRRSRS**S**VTLPHI  ELENVC**S**NSREKI  EKIYNR**S**LGSTCH  YNRSLG**S**TCHQCR  VHAYLK**S**LKQEFE  ILRGLN**S**KQHCLL  PTGSGK**S**LALLCS  ALAWQQ**S**LSGKPA  PADEGV**S**EKAEVQ  KAEVQL**S**CCCACH  ERNGTS**S**TCQDSP  SSTCQD**S**PEKTTL  CSRCCC**S**TKQGNS  KDHTGK**S**KIPKIY  DIEELV**S**LGKKLK  RESASY**S**VTEVQL  SACKIW**S**GNEMLL  REVPVI**S**ASTQIM  VPVISA**S**TQIMLK  NPAVAF**S**DINGKV  PMKSFS**S**ELGVTF  VGALLL**S**VCQTVS  LKERWL**S**TGLWHN  VCRGKV**S**EGLDFS  RFRNNP**S**RYISGL  RYISGL**S**KWVRQQ  FESALE**S**LAEFSK  QKVLNV**S**IKDRTN  SWSSFN**S**LGQYFT  KTEKME**S**KTVLPF  NLTVNT**S**FGSCPQ  LTRKNH**S**EHPLCS  SEHPLC**S**EEALDP  DPDIEL**S**LVSEED  TEAEDE**S**IYFTPE  NRLANN**S**DCILAK  RLQSQK**S**WIKGVF  KSHSSH**S**LRKIWT  TLNSMH**S**HFILSD  GPNVIL**S**VWETVK  ALEQAM**S**DALVMD  KHQRHS**S**GNRNES  AESTLH**S**QFIRTA  HKSRKK**S**KEQNVS  AHEAKE**S**HMVDDA  YELRNW**S**NSTCLK  CLKLAV**S**GGLRPF  GLRPFV**S**HTCTQM  WLKIII**S**IILPPT  MSHVPQ**S**QDFQFM  DQNASS**S**KESASV  MAIVLL**S**FGVARK  GEIDVC**S**SQPSCP  VSFIKD**S**LLSLDS  IKDSLL**S**LDSQVG  CKKLPH**S**WSNVIC  LLEITN**S**KREATN  PEPMTM**S**SPLSQA  EPMTMS**S**PLSQAK  WQTCLP**S**TCDSDS  QKQAQD**S**SLSDNS  KQAQDS**S**LSDNST  WLQPNT**S**FWINPL  ISEEEY**S**KNWFTV  IGVDKI**S**ASLKSP  KAMRVV**S**TWSEDD  FLIYCH**S**ANQWLT  ENLTDP**S**VIKPEV  LKRNDY**S**PERINS  INLGEG**S**YAKVKS  SYAKVK**S**AYSERL  KVKSAY**S**ERLKFN  AMLNHC**S**IIKTYE  YEIFET**S**HGKVYI  KKFHQL**S**LAIKYC  DFNIKL**S**DFSFSK  IKLSDF**S**FSKRCL  LSDFSF**S**KRCLRD  RCLRDD**S**GRMALS  SGRMAL**S**KTFCGS  SKTFCG**S**PAYAAP  KVYDIW**S**LGVILY  YIMVCG**S**MPYDDS  SMPYDD**S**NIKKML  RVNFPR**S**KHLTGE  HIDEIL**S**HCWMQP  QPKARG**S**PSVAIN  KARGSP**S**VAINKE  INKEGE**S**SRGTEP  NKEGES**S**RGTEPL  WTPEPG**S**DKKSAT  PGSDKK**S**ATKLEP  GTAMQM**S**RQSEIL  MQMSRQ**S**EILGFP  EILGFP**S**KPSTME  GFPSKP**S**TMETEE  LRFFRQ**S**VAGLAA  EEKQAE**S**RRAVSA  ESRRAV**S**ACQEIQ  AIFTQK**S**KPGPDP  EYLIGQ**S**IGKGCS  SIGKGC**S**AAVYEA  NLEVTK**S**TGLLPG  GRGPGT**S**APGEGQ  KMMWNI**S**AGSSSE  NISAGS**S**SEAILN  ISAGSS**S**EAILNT  AILNTM**S**QELVPA  QELVPA**S**RVALAG  AVTYRK**S**KRGPKQ  VLRAFT**S**SVPLLP  LRAFTS**S**VPLLPG  YPDVLP**S**RLHPEG  LCVNTP**S**PRLAAM  AHRDLK**S**DNILVE  CCLADE**S**IGLQLP  GLQLPF**S**SWYVDR  LMAPEV**S**TARPGP  RAVIDY**S**KADAWA  GKAHLE**S**RSYQEA  AHLESR**S**YQEAQL  LPALPE**S**VPPDVR  LLQREA**S**KRPSAR  EASKRP**S**ARVAAN  ANVLHL**S**LWGEHI  GWLLQQ**S**AATLLA  GGREPR**S**EDPTPA  VLAALL**S**VVAVVS  SVVAVV**S**CLYLGV  RIAALE**S**AKGAPS  SAKGAP**S**IHLLPD  RLLAQK**S**YEHMAK  IAREAP**S**ECNCPA  RGRRGE**S**GPPGQP  INHGFL**S**ADQQLI  PPGQKG**S**IGAPGI  EKGDPG**S**SAAGIK  KGDPGS**S**AAGIKG  KGEPGE**S**GRPGQK  KGKQGE**S**GTRGPK  TRGPKG**S**KGDRGE  RGEKGD**S**GAQGPR  PKGEQG**S**PGIPGM  EQGLKG**S**KGDMGD  KGEKGD**S**GMPGPQ  PGPQGP**S**IIGPPG  RGDVDM**S**EVEHFM  EEEGML**S**PILAHG  LYLVAT**S**KKNACV  KKNACV**S**LVFSFL  CVSLVF**S**FLYKVV  KVVQVF**S**EYFKEL  KELEEE**S**IRDNFV  YPQTTD**S**KILQEY  TVTNAV**S**WRSEGI  NAVSWR**S**EGIKYR  FLDVIE**S**VNLLVS  SVNLLV**S**ANGNVL  NGNVLR**S**EIVGSI  RSEIVG**S**IKMRVF  KMRVFL**S**GMPELR  NTGRGK**S**KSVELE  GRGKSK**S**VELEDV  HQCVRL**S**RFENDR  ENDRTI**S**FIPPDG  GEFELM**S**YRLNTH  PLIWIE**S**VIEKHS  SVIEKH**S**HSRIEY  IEKHSH**S**RIEYMI  SQFKRR**S**TANNVE  VPNDAD**S**PKFKTT  FKTTVG**S**VKWVPE  KWVPEN**S**EIVWSI  NSEIVW**S**IKSFPG  IVWSIK**S**FPGGKE  AHFGLP**S**VEAEDK  EGKPPI**S**VKFEIP  IPYFTT**S**GIQVRY  LKIIEK**S**GYQALP  SLEELD**S**LKYSDL  LDSLKY**S**DLQNLA  LQNLAK**S**LGLRAN  NENQDE**S**QTSASS  QDESQT**S**ASSCDE  ESQTSA**S**SCDETE  SQTSAS**S**CDETEI  ETEIQI**S**NQEEAE  VRVDPD**S**QQNHSE  DSQQNH**S**EIKISN  HSEIKI**S**NPTEFQ  EAENAV**S**SGNRDS  AENAVS**S**GNRDSK  SSGNRD**S**KVPSEG  RDSKVP**S**EGKKSL  PSEGKK**S**LYTDES  SLYTDE**S**SKPGKN  LYTDES**S**KPGKNK  HFKEME**S**IDQYIE  HFEEHN**S**MNELKQ  GRLSVA**S**TPISQR  PISQRR**S**QGRSCG  RRSQGR**S**CGPASQ  RSCGPA**S**QSTLGL  CGPASQ**S**TLGLKG  KTGVRF**S**AATKDN  DNEHKR**S**LTKTPA  KTPARK**S**AHVTVS  KTITGN**S**AAVITP  VFDLKA**S**LSRPLN  LKPWGQ**S**KENNYL  TLRRHP**S**YPKIIE  LQDEEA**S**MGASYS  SMGASY**S**KSLIKL  QHDIIT**S**LPEILG  ENTASV**S**EHKVFD  LRNKIR**S**GCIQEQ  LLQSTF**S**VHYLVL  AQSLLH**S**LDQSII  LHSLDQ**S**IISFGS  LDQSII**S**FGSLLY  LVVLNP**S**AMMMNA  VIRKQL**S**STVFKY  QCTQVT**S**LLQLVH  PVTSQE**S**GQKLVS  SGQKLV**S**PLCLAP  PGEKLE**S**MSAKER  ERSFMC**S**LIFLTL  HTVTAI**S**AKIRKK  KQKTDG**S**KTSSSD  DGSKTS**S**SDTLSE  ECDPTP**S**HRGQLN  LDIEVF**S**ILHCGL  FLLEDL**S**QKLESM  ALHVLS**S**RLKQGE  PLEELL**S**QSVHYL  NKEKIA**S**LARQFL  SGDKEK**S**NISNDQ  PGTAAD**S**QQIHEE  HREDVL**S**LLETFQ  HHLCGH**S**KIHQDT  SQNSQE**S**TADESE  GEPDEI**S**IQKCQE  AGFEDK**S**AYALCT  LCTFAL**S**TGDPSQ  LSTGDP**S**QPVRLF  LFRGRT**S**GRIVAP  AEKNAV**S**HRFRAL  PEEPAD**S**GQSLVP  PADSGQ**S**LVPVYI  VPVYIY**S**PEYVSM  YSPEYV**S**MCDSLA  YVSMCD**S**LAKIPK  RASMVH**S**LIEAYA  VKPKVA**S**MEEMAT  QHLQKV**S**QEGDDD  DDDHPD**S**IEYGLG  KVAINW**S**GGWHHA  AKKDEA**S**GFCYLN  GVEDAF**S**FTSKVM  DAFSFT**S**KVMTVS  SKVMTV**S**LHKFSP  VSLHKF**S**PGFFPG  PGTGDV**S**DVGLGK  GKGRYY**S**VNVPIQ  YYQICE**S**VLKEVY  AGDPMC**S**FNMTPV  ILGKTL**S**SEIPDH  LGKTLS**S**EIPDHE  VLEITP**S**CRPDRN  LLTVRG**S**EGLYMV  PPHFTE**S**TVFPRE  TVFPRE**S**GKNCKV  CKVCIF**S**KDGTLF  EKVNII**S**VTNKGL  NKGLLH**S**FDLLKA  WQPYTT**S**KDGTAG  TGTCLK**S**FIQKKM  NWCPSW**S**EDETLC  AVYVPG**S**KGAPSF  GSKGAP**S**FVRLYQ  AALANK**S**FFKADK  AVLVIA**S**TDVDKT  VDKTGA**S**YYGEQT  IATNGE**S**AVVQLP  YDVVWN**S**SSTEFC  DVVWNS**S**STEFCA  VVWNSS**S**TEFCAV  RNAAYY**S**PHGHIL  KNYKLI**S**KPVASD  ISKPVA**S**DSTYFA  KPVASD**S**TYFAWC  IWHYTG**S**ILHKYD  HKYDVP**S**NAELWQ  AELWQV**S**WQPFLD  NKPITN**S**KLHEEE  QNMKPQ**S**GNDKPL  AKQEAR**S**DKSPDL  EARSDK**S**PDLAPT  PTPAPQ**S**TPRNTV  TPRNTV**S**QSISGD  RNTVSQ**S**ISGDPE  TVSQSI**S**GDPEID  GVGGVR**S**RVGIQQ  IQQGLL**S**QSTRTA  QGLLSQ**S**TRTATF  RQKIGL**S**DARLKL  AREMLN**S**RKQQTT  DAREKI**S**LKRSSP  KISLKR**S**SPAAFI  DDDGIA**S**VPTKQM  QMKFAA**S**GGFLHH  HHMAGL**S**SSKLSM  HMAGLS**S**SKLSMS  MAGLSS**S**KLSMSK  LSSSKL**S**MSKALP  SSKLSM**S**KALPLT  TAPALP**S**SIRTKA  APALPS**S**IRTKAL  KALTNM**S**RTLVNK  NGNVIT**S**DQPILL  PILLRL**S**DSPSMK  LLRLSD**S**PSMKKE  RLSDSP**S**MKKESE  PSMKKE**S**ELPRRV  RVNSAS**S**SNPPAE  VNSASS**S**NPPAEV  LKALFK**S**SGASVT  KALFKS**S**GASVTT  FKSSGA**S**VTTQPT  LVALLE**S**GTEKVL  FVEYNT**S**HILEAI  TELIQH**S**AKHKVD  QDVASL**S**SDCFLT  DVASLS**S**DCFLTV  LGKLEK**S**FNSVHL  LVPTCI**S**QPCLPV  IEKAKA**S**NGCVLV  MKRMDM**S**LDEAYR  GASGPK**S**KLKLLH  CADSAT**S**EAAGQR  HPASVP**S**VPSVQP  PSLLED**S**PLVQAL  FSLDIK**S**VSYSAS  IKSVSY**S**ASMAAS  SVSYSA**S**MAASLH  ASLHGF**S**SSEDAL  SPVQEL**S**EQTPET  EQTPET**S**PDKEEA  TARPSD**S**QSKRLH  RPSDSQ**S**KRLHSV  LHSVRT**S**SSGTAQ  EDNYHT**S**FLFGLS  WYFATE**S**SHFYSA  ASYSAY**S**CSQLPT  YSAYSC**S**QLPTCG  RRRQKP**S**DRADSR  FGESIM**S**ENRSRE  IMSENR**S**REELGK  GKVGSQ**S**SFSGSM  GSQSSF**S**GSMEII  AVVTAG**S**VGAAKT  EGPAPL**S**ETGAPG  TGAPGC**S**GSRPPE  APGCSG**S**RPPELE  ELEPER**S**LGRFRG  EEEEDM**S**HFSLRL  EDMSHF**S**LRLEGG  ERLINL**S**ELTPYI  TPYILC**S**ICKGYL  LHTFCK**S**CIVRHF  VRHFYY**S**NRCPKC  VPQPVP**S**SKGRSK  PQPVPS**S**KGRSKK  PSSKGR**S**KKVLES  SKKVLE**S**VFRIPP  PPELDM**S**LLLEFI  KKFVRV**S**GEATIG  PAGPIL**S**LNPQED  DETQIF**S**YFSQFG  QIFSYF**S**QFGTVT  VTRFRL**S**RSKRTG  RFRLSR**S**KRTGNS  SKRTGN**S**KGYAFV  AFVEFE**S**EDVAKI  IPFKQP**S**YPSVKR  KQPSYP**S**VKRYNR  IDYDFP**S**LILQKT  ILQKTE**S**ISKTNR  QKTESI**S**KTNRQT  KTNRQT**S**TKGQVL  VFKQPI**S**CVKEEI  TQTPTH**S**RKKRRR  VDEKPD**S**PMYVYE  PMYVYE**S**TVHCTN  MHNLED**S**CFSFLQ  LEDSCF**S**FLQTQL  HEDCEN**S**AGEEED  EEETMD**S**ETAKMA  PTDTKE**S**SEKDAL  STSGFA**S**TFREDN  TFREDN**S**SNSLKP  ARGQIK**S**EPPSEE  EENEEE**S**ITLCLS  SPSCLR**S**LFSITK  LFSITK**S**VELSGL  SNFTMG**S**PLRGPG  GELDRR**S**VIFSSS  SVIFSS**S**ACDQVS  SACDQV**S**TSVHSY  VSTSVH**S**YSGVSS  AGQSLP**S**SQAYSH  PSSQAY**S**HGGLMA  RMRPNT**S**CPVPIK  RTRTSS**S**CSSYSY  LPLCEF**S**SSPCSQ  LCEFSS**S**PCSQGA  QSYGTN**S**SDESGS  TNSSDE**S**GSFSEA  SEADSE**S**CPVQDR  KMHKLT**S**EQLEFI  DNFSCL**S**QEVCRD  DLPTAS**S**INPAPL  TDPGTF**S**ERGPPL  KITVVP**S**AKDFID  FIDLTL**S**KTQRKT  NYHDRL**S**QILTDF  LMKYGD**S**LYRCKQ  IKRQKQ**S**LEYLEQ  QVRQHL**S**RLPTID  YPNVGK**S**SFINKV  PNVGKS**S**FINKVT  YAFTTK**S**LFVGHM  LYVMDL**S**EQCGHG  KRIAEL**S**EDDQKI  IFTDLQ**S**EGFPVI  FPVIET**S**TLTEEG  RMETEE**S**RKKRER  WDLMNL**S**EKHDKI  AAGEYD**S**VSESED  GEYDSV**S**ESEDEE  YDSVSE**S**EDEEML  KLKILE**S**KEKNTQ  LEKEMR**S**LGVDMD  AVQARR**S**RSITRK  QARRSR**S**ITRKRK  KRKRED**S**APPSSV  EDSAPP**S**SVARSG  DSAPPS**S**VARSGS  PSSVAR**S**GSCSRT  SVARSG**S**CSRTPR  ARSGSC**S**RTPRDV  RTPRDV**S**GLRDVK  KPKHLL**S**GKRKAG  ARAVLD**S**TNADGI  PLHVAA**S**CGYLDI  NIAAVN**S**DGDLPL  PLDLAE**S**DAMEGL  ADEEVL**S**LLEELA  RNQKEA**S**QSRGQE  QEPQAP**S**SSKHRR  PQAPSS**S**KHRRSS  SKHRRS**S**VCRLSS  SVCRLS**S**REKISL  ISLQDL**S**KERRPG  RTLNGV**S**SPPHPS  PHPSPK**S**PVQLEE  LEEAPF**S**RRFGLL  LLKTGS**S**GALGPP  LQRSAS**S**SWLEGT  SWLEGT**S**TQAKEL  RITPTP**S**PKLPEP  PKLPEP**S**VLSEVT  PEPSVL**S**EVTKPP  PPCLEN**S**SPPSRI  PCLENS**S**PPSRIP  ENSSPP**S**RIPEPE  PNVPTA**S**TAPPAD  TAPPAD**S**RDRRRS  SRDRRR**S**YQMPVR  PVRDEE**S**ESQRKA  RDEESE**S**QRKARS  SQRKAR**S**RLMRQS  VPGVEN**S**DSPAQR  AEPADR**S**QESSTL  ADRSQE**S**STLEGG  EELKAL**S**DLRADN  GGLELQ**S**PPPLLP  APGSGV**S**FHIQIG  VKQLAC**S**IVDQKF  LVEVVL**S**ASATFE  HALTVH**S**YRAPAF  HKRCAF**S**IPNNCS  ARKRRL**S**STSLAS  RKRRLS**S**TSLASG  SVRLGT**S**ESLPCT  CTAEEL**S**RSTTEL  LPRRPP**S**SSSSSS  PRRPPS**S**SSSSSA  PSSSSS**S**SASSYT  SSSSAS**S**YTGRPI  LDKMLL**S**KVKVPH  HTFLIH**S**YTRPTV  SALMDE**S**EDSGVI  SGVIPG**S**HSENAL  ENALHA**S**EEEEGE  YKEIPL**S**EILTVE  ESAQNF**S**LVPPGT  GTPGGP**S**GQGAEA  APHRQA**S**LSISVS  HRQASL**S**ISVSNS  PDEVLG**S**GQFGVV  FPTKQE**S**QLRNEV  LEMILS**S**EKGRLP  RSLDMW**S**VGVIMY  AFMYPA**S**PWSHIS  YPASPW**S**HISAGA  EHPLPG**S**GLPTDR  PRPGKP**S**APSLAL  GKPSAP**S**LALGPS  GPSPGA**S**PSWRAA  SPGASP**S**WRAAPK  RAAPKA**S**DLLGAR  RGGAHA**S**SSSLNP  GGAHAS**S**SSLNPM  GAHASS**S**SLNPMP  AHASSS**S**LNPMPP  LNPMPP**S**QLQLPT  LVMVAP**S**GARLGP  HFMHQL**S**TVDAHA  QVHPLE**S**PAMISL  ESPAMI**S**LTPPTT  TATGVF**S**LKARPG  ASLEWV**S**REPALL  CTFPNP**S**APRKDS  RKDSTL**S**AVPQSS  SAVPQS**S**YPLLAN  QREMVQ**S**LEQQLV  ALTKAS**S**VASSDK  KASSVA**S**SDKGSC  ASSVAS**S**DKGSCC  ASSDKG**S**CCIVAA  PVVPAW**S**GPREAP  PREAPD**S**LFAVRR  RRHLWG**S**HGNSTF  WGSHGN**S**TFPEFL  AIRHNL**S**LHKCFV  CFVRVE**S**EKGAVW  KRSQRP**S**RCSNPT  FPELAD**S**AINAQL  CEDEDV**S**IRRQAI  LLQTDD**S**AEFNLV  VNNALL**S**IFKMDA  TLGGLF**S**QILQGE  RAIKFL**S**TKLKTL  ELILTE**S**KKVLED  LFMKIL**S**GLKSLQ  ILSGLK**S**LQTVSG  KSLQTV**S**GRQQLV  EQTFNP**S**DPDCVD  QAVPLF**S**KNVHST  FSKNVH**S**TRFVTY  KLLAEM**S**SFCGDM  LLAEMS**S**FCGDME  EPKLQF**S**YVECLL  VECLLY**S**FHQLGR  LFHIPP**S**YKSTVT  IPPSYK**S**TVTLSW  KSTVTL**S**WKPVQK  IGQKRA**S**EDTTSG  ASEDTT**S**GSPPKK  EDTTSG**S**PPKKSS  GSPPKK**S**SAGPKR  SPPKKS**S**AGPKRD  QIYNPP**S**GKYSSN  PPSGKY**S**SNLGNF  PSGKYS**S**NLGNFN  RGAFRG**S**RGGRGW  EDEGLL**S**LAGKRK  GNLPKE**S**VKILRD  RYNAYP**S**EQEKLS  SEQEKL**S**LSGQTN  QEKLSL**S**GQTNLS  SGQTNL**S**VLQICN  PNQFTI**S**RRGGKA  RRGGKA**S**DVALPR  VALPRG**S**SPSVLA  ALPRGS**S**PSVLAV  PRGSSP**S**VLAVSV  PSVLAV**S**VPAPTN  APTNVL**S**LSVCSM  TNVLSL**S**VCSMPL  LSLSVC**S**MPLHSG  CSMPLH**S**GQGEKP  PRGELE**S**PKPLVT  PLVTPG**S**TLTLLT  TRAEAG**S**PTGGLF  QDKEDF**S**SFQLLV  DKEDFS**S**FQLLVE  QKQQDP**S**LPLLHT  CAQYCI**S**FADVEK  HINIRD**S**IHLTPV  LTPVLT**S**SILNQL  TPVLTS**S**ILNQLT  LFQKTG**S**FKIRGA  ALNAVR**S**LVPDAL  KAVVTH**S**SGNHGQ  AVVTHS**S**GNHGQA  IQAYGA**S**IVYCEP  IVYCEP**S**DESREN  CEPSDE**S**RENVAK  VKALKP**S**VKVYAA  VYAAEP**S**NADDCY  ADDCYQ**S**KLKGKL  IADGVK**S**SIGLNT  ADGVKS**S**IGLNTW  GVAAVL**S**QHFQTV  QHFQTV**S**PEVKNI  NICIVL**S**GGNVDL  GNVDLT**S**SITWVK  NVDLTS**S**ITWVKQ  GGPGGP**S**PSERDR  PGGPSP**S**ERDRQY  ENLLRC**S**RCRSSF  RCSRCR**S**SFYCCK  CSRCRS**S**FYCCKE  KLVCQG**S**EGALGH  VGPHQH**S**GPAPPA  ARRDNA**S**GDAAKG  DPAAAA**S**PCRAAA  AAGGQG**S**AVAAEA  EEPPAR**S**SLFQEK  EPPARS**S**LFQEKA  ANLYPP**S**NTPGDA  TPGDAL**S**PGGGLR  TDGQLV**S**QKSDSS  QLVSQK**S**DSSKDI  VSQKSD**S**SKDIRG  SQKSDS**S**KDIRGD  TIGLLM**S**SMDDLI  IGLLMS**S**MDDLIR  CNGKLG**S**YKINGR  DWDAKV**S**GGILRI  RLLFFW**S**DRRNPH  VELNKP**S**DSVGKD  DGTMED**S**EAVQRA  KHHGAQ**S**AALQKV  RDALAA**S**HEPPPE  FLADGG**S**ADTCDQ  TALHRA**S**LEGHME  VVKLLQ**S**HGADTN  VRDKLL**S**TPLHVA  IVEHFL**S**LGLEIN  LEGPND**S**GRETPQ  LEALFN**S**VMNPKP  VMNPKP**S**SWRKKI  MNPKPS**S**WRKKIL  KKILPE**S**FFKEPD  FFKEPD**S**GSHSRQ  KEPDSG**S**HSRQSS  PDSGSH**S**RQSSTD  GSHSRQ**S**STDSSG  SHSRQS**S**TDSSGG  RQSSTD**S**SGGHPG  QSSTDS**S**GGHPGP  GAQHVR**S**HSSPAS  HVRSHS**S**PASLQL  SHSSPA**S**LQLGTG  AHLRQQ**S**YDVTDE  NLHPAV**S**STPVPQ  LHPAVS**S**TPVPQR  TPVPQR**S**MAVSQP  QRSMAV**S**QPNLVM  QQQMAP**S**TLSQQN  MAPSTL**S**QQNHPT  PPAGLM**S**MPNALT  MTPDMR**S**ITNNSS  RSITNN**S**SDPFLN  SITNNS**S**DPFLNG  NGGPYH**S**REQSTD  REQSTD**S**GLGLGC  LGLGCY**S**VPTTPE  TPEDFL**S**NVDEMD  DLGTLE**S**EDLIPL  LFNDVE**S**ALNKSE  RRDSAT**S**FSLDFG  DSATSF**S**LDFGYL  LFLRYI**S**DWDLDP  RVTWFT**S**WSPCYD  TWFTSW**S**PCYDCA  RGNPNL**S**LRIFTA  EGLHEN**S**VRLSRQ  ENSVRL**S**RQLRRI  LLGVAA**S**LCVRCS  SLCVRC**S**RPGAKR  RPGAKR**S**EKIYQQ  KIYQQR**S**LREDQQ  LREDQQ**S**FTGSRT  QQSFTG**S**RTYSLV  TGSRTY**S**LVGQAW  LLQFYP**S**LEDPAS  SLEDPA**S**SRYQNF  LEDPAS**S**RYQNFS  SRYQNF**S**KGSRHG  QNFSKG**S**RHGSEE  KGSRHG**S**EEAYID  YNWGRF**S**KPPEDD  EDDDAN**S**YENVLI  LCRGDL**S**LSLALK  RGDLSL**S**LALKTG  LKTGPT**S**GLCPSA  TSGLCP**S**ASPEED  GLCPSA**S**PEEDEE  PEEDEE**S**EDYQNS  SEDYQN**S**ASIHQW  DYQNSA**S**IHQWRE  IHQWRE**S**RKVMGQ  QLQREA**S**PGPVGS  SPGPVG**S**PDEEDG  KLLENS**S**FEAINS  SFEAIN**S**QLTVET  IIGRIE**S**YSCKMA  GRIESY**S**CKMAGD  LSPSRL**S**KSQGGE  EEEGPL**S**DKCSRK  PLSDKC**S**RKTLFY  IATLNE**S**FRPDYD  RPDYDF**S**TARSHE  DFSTAR**S**HEFSRE  ARSHEF**S**REPSLS  EFSREP**S**LSWVVN  SREPSL**S**WVVNAV  VNAVNC**S**LFSAVR  VNCSLF**S**AVREDF  AECDIY**S**YNPDLD  YNPDLD**S**DPFGED  PFGEDG**S**LWSFNY  EDGSLW**S**FNYFFY  KRIVFF**S**CRSISG  VFFSCR**S**ISGSTY  FSCRSI**S**GSTYTP  CRSISG**S**TYTPSE  EEVEEE**S**RSGGSG  VEEESR**S**GGSGAE  ESRSGG**S**GAEETS  SGAEET**S**TMEEDR  LVFARR**S**GPTPSA  RSGPTP**S**AAELAR  AEGLIK**S**PKPLMK  KKARES**S**GRLVAI  HEVFEN**S**SKIVIV  EVFENS**S**KIVIVM  DLYDYI**S**ERQQLS  SERQQL**S**EREARH  IADFGL**S**NLYHQG  GSPLYA**S**PEIVNG  TGPEVD**S**WSLGVL  ILVKQI**S**NGAYRE  REPPKP**S**DACGLI  SDSARA**S**MADWLR  DWLRRS**S**RPLLEN  GLERQH**S**LKKSRK  QHSLKK**S**RKENDM  ENDMAQ**S**LHSDTA  AHRPGK**S**NLKLPK  ILKKKV**S**ASAEGV  EDPPEL**S**PIPASP  ESGYYS**S**PEPSES  AGDVFV**S**GDPKEQ  APTTFG**S**LDELAP  RPLARA**S**RPSGAV  ARASRP**S**GAVSED  SEDSIL**S**SESFDQ  EDSILS**S**ESFDQL  SILSSE**S**FDQLDL  PLRGCV**S**VDNLTG  PSEGPG**S**CLRRWR  LGDSCF**S**LTDCQE  RRVEIL**S**EGNEVQ  MLMCDT**S**GSIQLS  MCDTSG**S**IQLSEE  SGSIQL**S**EEQKSA  LSEEQK**S**ALAFLN  NRGQPS**S**SNAGNK  AGNKRL**S**TIDESG  TIDESG**S**ILSDIS  ESLDWD**S**SLVKTF  KREKRR**S**TSRQFV  EKRRST**S**RQFVDG  GPIEAV**S**TIETVP  VPYWTR**S**RRKTGT  QPWNSD**S**TLNSRQ  MLADFV**S**QTSPMI  DFVSQT**S**PMIPSI  TSPMIP**S**IVVHCV  DIHAIC**S**LLKDFL  ITDEDN**S**IAAMYQ  LQRVAQ**S**PHTKMD  VVERLL**S**LPLEYW  LPLEYW**S**QFMMVE  LHVIEN**S**NAFSTP  ENSNAF**S**TPQTPD  TPDIKV**S**LLGPVT  QLLKTP**S**SSSLSQ  LLKTPS**S**SSLSQR  LKTPSS**S**SLSQRV  PSSSSL**S**QRVRST  NTPRFG**S**KSKSAT  PRFGSK**S**KSATNL  QVMETK**S**MLYLVT  PQLDIW**S**MGVVLY  MLVLDP**S**KRLTIA  ESLQNK**S**YNHFAA  LVERLK**S**HRSSFP  LPVTMH**S**PNMRLL  GCQSLP**S**NMMETS  SNMMET**S**IDEGLE  SMNDSP**S**LDSVDS  DSPSLD**S**VDSEYD  SLDSVD**S**EYDMGS  SEYDMG**S**VQRDLN  MLANQP**S**PRMTSP  HNRSPV**S**FREGRR  CPQEEV**S**QQQESV  VSQQQE**S**VSTLPA  QQQESV**S**TLPASV  VSTLPA**S**VHPQLS  SVHPQL**S**PRQSLE  QLSPRQ**S**LETQYL  HRLQKP**S**LLSKAQ  CKEPPR**S**LEQQLQ  MQIAES**S**YPQPSQ  QETPPP**S**QQAPPF  SLTQPL**S**PVLEPS  SPVLEP**S**SEQMQY  PVLEPS**S**EQMQYS  SEQMQY**S**PFLSQY  QYSPFL**S**QYQEMQ  QTCELP**S**AASPAP  ELPSAA**S**PAPDYP  VDGAQQ**S**DLTGPD  GLQEAP**S**SYDPLA  VFLGEQ**S**VAKTSL  QSVAKT**S**LITRFR  TRFRYD**S**FDNTYQ  IGIDFL**S**KTMYLE  GQERLR**S**LIPRYI  PRYIRD**S**AAAVVV  DITNVN**S**FQQTTK  VRTERG**S**DVIITL  ADKRQV**S**VEEGER  ALPGME**S**TQDGSR  ESTQDG**S**REDMSD  GSREDM**S**DIKLEK  PQEQTV**S**EGGCSC  VSEGGC**S**CYSPMS  GGCSCY**S**PMSSST  SCYSPM**S**SSTLPQ  CYSPMS**S**STLPQK  YSPMSS**S**TLPQKP  PQKPPY**S**FIDCSV  YSFIDC**S**VNIGLN  GLNLFP**S**LITFCN  LITFCN**S**SLLPVS  APEEHD**S**PTEASQ  DSPTEA**S**QPIVEE  GLAETG**S**GKTGAF  ELAFQI**S**EQFEAL  QFEALG**S**SIGVQS  FEALGS**S**IGVQSA  SSIGVQ**S**AVIVGG  IVGGID**S**MSQSLA  GGIDSM**S**QSLALA  IDSMSQ**S**LALAKK  RKTFLF**S**ATMTKK  PVKCAV**S**SKYQTV  VKCAVS**S**KYQTVE  YYIFIP**S**KFKDTY  NELAGN**S**FMIFCS  SFMIFC**S**TCNNTQ  PLHGQM**S**QSKRLG  HGQMSQ**S**KRLGSL  QSKRLG**S**LNKFKA  FKAKAR**S**ILLATD  LATDVA**S**RGLDIP  FDIPTH**S**KDYIHR  TARAGR**S**GKAITF  GEKKKR**S**REDAGD  GNMVIW**S**KGSKSQ  GLLFER**S**ASSHEV  TPLVCK**S**GSLFGS  FLNTDP**S**IVMTYD  PQNVAT**S**SSLTAH  LTAHLR**S**LSKGDS  SSIHSQ**S**RSTSSP  SSPSLH**S**RSPSIS  SNMAAL**S**RAHSPA  PKKARP**S**ETGSDD  NRSLCL**S**PSEASQ  LSPSEA**S**QMKDED  MKDEDF**S**QNLSLD  DFSQNL**S**LDSSTL  NLSLDS**S**TLLFTH  GFMHHP**S**FFTSEP  PGICER**S**RLVVLS  LVSDES**S**QYLTRI  RFSFRH**S**TSVSSL  SFRHST**S**VSSLAE  CREQPA**S**DWPEAV  IGRQDL**S**KQACEG  NLPKGK**S**VLSSDV  KGKSVL**S**SDVPSG  LKIAPA**S**QIDSAW  YCTDRE**S**YSLAAG  EIELPC**S**EDLNLE  AMVMAG**S**GNLKVL  ELLIDL**S**KGTQHL  ILDLFS**S**VLYECV  LEESQL**S**MLACFL  DVAPRL**S**PVRVWP  LHPMGL**S**LPKEKG  LILCLW**S**KFCRWF  WFQRRE**S**WAQSRD  RESWAQ**S**RDEQNL  QKRIWE**S**PLLLAA  VFEPMT**S**ELYEGQ  LLARRA**S**VSARAT  ARRASV**S**ARATGT  GTAFRR**S**PCNLIY  FGEHPL**S**FAACVN  FAACVN**S**EEIVRL  DIRAQD**S**LGNTVL  MYNLLL**S**YDRHGD  TYGPLT**S**TLYDLT  DLTEID**S**SGDEQS  LTEIDS**S**GDEQSL  SSGDEQ**S**LLELII  PVKELV**S**LKWKRY  RTNNRT**S**PRDNTL  MVMRLI**S**ASGEVV  MRLISA**S**GEVVPM  VILGFA**S**AFYIIF  YPMALF**S**TFELFL  DLPFMY**S**ITYAAF  RCLWPR**S**GICGRE  AFHTRG**S**EDLDKD  EDLDKD**S**VEKLEL  LSLPMP**S**VSRSTS  MPSVSR**S**TSRSSA  SVSRST**S**RSSANW  SRSTSR**S**SANWER  RSTSRS**S**ANWERL  APAGTL**S**PAPLFS  SPAPLF**S**PGTYER  LALLLG**S**IGLLGV  LLLVNI**S**LSDLLV  LVNISL**S**DLLVSL  LSDLLV**S**LFGVTF  VTFTFV**S**CLRNGW  CVWDGF**S**GSLFGI  WDGFSG**S**LFGIVS  SLFGIV**S**IATLTV  ARVINF**S**WAWRAI  TYIWLY**S**LAWAGA  CTVDWK**S**KDANDS  SKDAND**S**SFVLFL  KDANDS**S**FVLFLF  YGHILY**S**IRMLRC  LVTPTI**S**IVSYLF  PTISIV**S**YLFAKS  SYLFAK**S**NTVYNP  IRKFRR**S**LLQLLC  DLPAAG**S**EMQIRP  IRPIVM**S**QKDGDR  KKVTFN**S**SSIIFI  KVTFNS**S**SIIFII  VTFNSS**S**IIFIIT  IIFIIT**S**DESLSV  IITSDE**S**LSVDDS  TSDESL**S**VDDSDK  SLSVDD**S**DKTNGS  SDKTNG**S**KVDVIQ  LACRTP**S**PARPAL  ALRAPA**S**ATSGSR  APASAT**S**GSRKRA  ASATSG**S**RKRARP  LSVDEV**S**SPSTPE  SVDEVS**S**PSTPEA  DEVSSP**S**TPEAPD  DIPACP**S**PGQKIK  QPPHLT**S**AQDQDT  QDQDTI**S**ELASCL  TISELA**S**CLQRAR  AQDAGE**S**CTPEAE  LAEMFR**S**MDTIVG  GMLHNR**S**ETPTFA  KTVYPA**S**YRFRQE  KDGTRR**S**DYQLTI  APQLTA**S**RLLQRR  HKAFLA**S**LSPAMV  AFLASL**S**PAMVVP  RARNLI**S**PRMEKA  RMEKAL**S**QLALRS  SQLALR**S**AAPSSP  LRSAAP**S**SPGSPR  RSAAPS**S**PGSPRP  APSSPG**S**PRPALP  ATPPAA**S**PSALKG  LARVLR**S**VFVSER  LRSVFV**S**ERKPAL  ERKPAL**S**MEVACA  CARMVG**S**CCTIMS  SCCTIM**S**PGEMEK  KHLLLL**S**ELLPDW  LLPDWL**S**LHRIRT  QELRSP**S**GAEVPY  PTAAPR**S**RSRELE  PTSSPD**S**ATPDDT  DDTSNS**S**SVDWDT  EGPRAD**S**SQRAPS  SSQRAP**S**LLTRSP  SSMTPH**S**GPRSTT  DTSRAS**S**TQQEIS  NTPRAS**S**PSRSTQ  PRASSP**S**RSTQLD  RENSRT**S**CAQRDN  RDNPRT**S**CAQRDN  RASRTS**S**PNRATR  RDNPRA**S**SPSRTI  DNPRAS**S**PSRTIQ  QENPRT**S**CARRDN  QQNIPR**S**SSTQQD  QNIPRS**S**STQQDN  NPGTSS**S**QCCTQK  NNPRNS**S**PHRTNK  HDPPQS**S**FGPTQY  PFLPDT**S**DAEHQC  PSPSPG**S**SGGSRG  QLLRRQ**S**SPAPSR  SREYKE**S**WGQPEA  FNPFLL**S**LGVLRW  EPGEPP**S**PSLTTT  VTKLSD**S**NKENAL  ERRGPP**S**DGHEAL  QGEAPQ**S**ALRSQE  IPPGYI**S**QEACER  VLSEQY**S**QKCLEI  GNGCGR**S**NERSSC  AKTRRL**S**SSESPQ  KTRRLS**S**SESPQR  ECPGWD**S**TIESGY  APPPTE**S**LEALPT  PEASGG**S**LEIDFQ  DFQVVQ**S**SSFGGE  FQVVQS**S**SFGGEG  QVVQSS**S**FGGEGA  GALETC**S**AVGWAP  DEDERE**S**MRSSRR  ERESMR**S**SRRRRR  RESMRS**S**RRRRRR  RKVKRE**S**RERNAE  QDLRHI**S**MGYKMK  EFQRNR**S**GRLVSH  RSGRLV**S**HSTPIR  HGNQDA**S**HSFFSW  NQDASH**S**FFSWFS  ASHSFF**S**WFSNHS  SFFSWF**S**NHSLPE  YLRERG**S**RIKRKK  DIFSEI**S**DIDETI  IHDIKI**S**DFMETT  TTDNNE**S**ADDHET  TTDNNE**S**ADDNNE  HGNNQD**S**SDSDNE  GNNQDS**S**DSDNEA  NQDSSD**S**DNEADE  EGDNEG**S**DDDGNE  DVIEII**S**DESVEE  GEDSDD**S**DLEDVL  PLHNAC**S**FGHAEV  LDLADP**S**AKAVLT  LLESAR**S**GNEEKM  ASDGRK**S**TPLHLA  PLHNAC**S**YGHYEV  NRVEVC**S**LLLSYG  RIKKHL**S**LEMVNF  TPLHVA**S**EKAHND  TCRLLL**S**YGCDPN  CDPNII**S**LQGFTA  LLQEGI**S**LGNSEA  DIEGRQ**S**TPLHFA  AGYNRV**S**VVEYLL  DTQGRH**S**TPLHLA  ALSSGP**S**SPSSLS  GPSSPS**S**LSAASS  SSPSSL**S**AASSLD  LDNLSG**S**FSELSS  NLSGSF**S**ELSSVV  GSFSEL**S**SVVSSS  SFSELS**S**VVSSSG  ELSSVV**S**SSGTEG  LSSVVS**S**SGTEGA  SSVVSS**S**GTEGAS  GTEGAS**S**LEKKEV  VPGVDF**S**ITQFVR  GVERLI**S**GQQGLN  YLTLNT**S**GSGTIL  VEEEMQ**S**TVREHR  RMLFHG**S**PFVNAI  AENSSK**S**NQYVYG  RVTLGK**S**FLQFSA  ATNAHT**S**EVVAIK  IKKMSY**S**GKQTHE  YCLGSA**S**DLLEVH  GLAYLH**S**HALIHR  ADFGSA**S**MASPAN  GKVDIW**S**LGITCI  FNMNAM**S**ALYHIA  DSPTLQ**S**NEWTDS  SNEWTD**S**FRRFVD  DSEHGT**S**LNREMD  LNREMD**S**LGSNHS  EMDSLG**S**NHSIPS  SLGSNH**S**IPSMSV  SNHSIP**S**MSVSTG  QSSSVN**S**MQEVMD  EVMDES**S**SELVMM  VMDESS**S**ELVMMH  MMHDDE**S**TINSSS  ESTINS**S**SSVVHK  TINSSS**S**VVHKKD  EPRPTQ**S**VQSQAL  ELREQM**S**GYKRMR  ETHANN**S**SIELEK  THANNS**S**IELEKL  LTTFLE**S**QKKQYK  EMNEDH**S**TPKKEK  LIRHDE**S**TRELEY  AEQYEQ**S**INEMMA  LLNAYQ**S**KIKMQT  ALQKER**S**ERIKNL  ALLPPP**S**PAAALL  APPPPA**S**LGSPAP  PPASLG**S**PAPPKG  KGAAAP**S**ASQRRK  AAAPSA**S**QRRKRT  QRRKRT**S**FSAEQL  RKRTSF**S**AEQLQL  LTLLPE**S**RIQVWF  QNRRAK**S**RRQSGK  AKSRRQ**S**GKSFQP  RRQSGK**S**FQPLAR  GVGGGI**S**DSSSQG  GGGISD**S**SSQGQN  GGISDS**S**SQGQNF  GISDSS**S**QGQNFE  QNFETC**S**PLSEDI  ETCSPL**S**EDIGSK  LSEDIG**S**KLDSWE  IGSKLD**S**WEEHIF  GQAWGA**S**VGGRSC  ASVGGR**S**CEELTA  GEAYKR**S**ALIPPV  ETVFYP**S**PYPIRS  SPYPIR**S**LIKPLF  TGCAFG**S**AAIWQY  AIWQYE**S**LKSRVQ  QYESLK**S**RVQSYF  LKSRVQ**S**YFDGIK  KADWLD**S**IRPQKE  KWWNNL**S**DGQRTV  CLWRVP**S**LQRTMI  MIRYFT**S**NPASKV  FTSNPA**S**KVLCSP  ASKVLC**S**PMLLST  CSPMLL**S**TFSHFS  MLLSTF**S**HFSLFH  STFSHF**S**LFHMAA  NMYVLW**S**FSSSIV  YVLWSF**S**SSIVNI  VLWSFS**S**SIVNIL  LWSFSS**S**IVNILG  FMAVYL**S**AGVISN  LSAGVI**S**NFVSYV  VISNFV**S**YVGKVA  TGRYGP**S**LGASGA  GPSLGA**S**GAIMTV  RRGFSD**S**GGGPPA  RRTRRE**S**RQESDP  QKLIEE**S**QRKMNA  ARPRRQ**S**MKEKEH  ETEVRE**S**EKQQDS  PDKECK**S**LSPGKE  KECKSL**S**PGKENV  EKEEKE**S**EPQPEP  SQPVLQ**S**QPPSQP  LQSQPP**S**QPEDLS  KDFPVE**S**VKLTEV  LTVHPE**S**KSKTKT  SKTKTR**S**RSRGRA  RNKTSK**S**RSRSSS  SRSRSS**S**SSSSSS  SRSSSS**S**SSSSSS  SSSSSS**S**TSSSSG  SSSTSS**S**SGSSSS  SSSSGS**S**SSSGSS  SSGSSS**S**SGSSSS  SSSGSS**S**SRSSSS  SSGSSS**S**RSSSSS  SSSSSS**S**TSGSSS  SSSTSG**S**SSRDSS  SSTSGS**S**SRDSSS  GSSSRD**S**SSSTSS  DSSSST**S**SSSESR  SSSTSS**S**SESRSR  TSSSSE**S**RSRSRG  DRKHRR**S**VDRKRR  TSGLER**S**HKSSKG  RSDRKR**S**ISESSR  SRSGKR**S**SRSERD  TTLNRC**S**CSFRVL  LNRCSC**S**FRVLVV  FRVLVV**S**AKFEGK  RRLAFL**S**FFWDKI  DHGTPG**S**LDPNTD  LPAEPC**S**PFPQLF  RCGGEL**S**VRRGDR  IFARRL**S**GQPSAG  RLSGQP**S**AGLVPI  THVAKA**S**PETLSD  ASPETL**S**DQPWYF  DQPWYF**S**GVSRTQ  WYFSGV**S**RTQAQQ  AQQLLL**S**PPNEPG  AFLIRP**S**ESSLGG  LIRPSE**S**SLGGYS  IRPSES**S**LGGYSL  SSLGGY**S**LSVRAQ  LGGYSL**S**VRAQAK  VCHYRV**S**MAADGS  SMAADG**S**LYLQKG  VWERPH**S**EFALGR  EGLWLG**S**LPVAIK  AIKVIK**S**ANMKLT  RLHAVC**S**GGEPVY  QVAEGM**S**YLEEQR  LKDDIY**S**PSSSSK  DDIYSP**S**SSSKIP  DIYSPS**S**SSKIPV  IYSPSS**S**SKIPVK  ANYRVF**S**QKSDVW  RVFSQK**S**DVWSFG  QKSDVW**S**FGVLLH  MLECWR**S**SPEERP  LECWRS**S**PEERPS  SPEERP**S**FATLRE  QRSLST**S**GESLYH  LDKNAT**S**DDIKKS  SDDIKK**S**YRKLAL  IYDKYG**S**LGLYVA  NTYFVL**S**SWWAKA  TYFVLS**S**WWAKAL  ETEFYV**S**PEDLEA  LEAQLQ**S**DEREAT  IVIQPA**S**ATETTQ  TQLTAD**S**HPSYHT  CCALLL**S**LWALCT  RARLQG**S**ATAAEA  ATAAEA**S**LLRRTH  RRTHLC**S**LSKSDT  THLCSL**S**KSDTRL  LCSLSK**S**DTRLHE  LHRGPR**S**SRALRP  HRGPRS**S**RALRPA  RALRPA**S**MDLLRP  PHWLEV**S**RDITGP  GPQAAP**S**AFPHQE  GLEATY**S**NVGLAA  AALPGV**S**LAASPV  GVSLAA**S**PVVAEY  RKGTHR**S**PQEPQQ  QVDVLY**S**RVCKPK  RALDVD**S**GPLENV  LENVYE**S**IRELGD  GDPAGR**S**STCGAG  DPAGRS**S**TCGAGT  AGTPPA**S**SCPSLG  GTPPAS**S**CPSLGR  PASSCP**S**LGRGWR  AIAASR**S**AVMSGN  KRFTYF**S**SLSPMA  RFTYFS**S**LSPMAR  TYFSSL**S**PMARKI  PRDPGD**S**EELTRF  TWQDEH**S**APFSWE  EHSAPF**S**WETKSQ  FSWETK**S**QMEFSI  KSQMEF**S**ISALSI  QMEFSI**S**ALSIQE  FSISAL**S**IQEPSN  LSIQEP**S**NGTAAS  SNGTAA**S**EPRPLS  SEPRPL**S**KASQGS  RPLSKA**S**QGSQAL  SKASQG**S**QALKSS  GSQALK**S**SQGSRS  LKSSQG**S**RSSSLD  SSQGSR**S**SSLDAL  QGSRSS**S**LDALGP  RKEEEA**S**FWKINA  KINAER**S**RGEGPE  FQSLTP**S**QIKSME  TPSQIK**S**MEKGEK  AKVERP**S**TLRQEQ  RPLPNV**S**TERERP  QPVQAF**S**SALHEA  PVQAFS**S**ALHEAA  LEGKLP**S**PDVRQD  GEDTLF**S**EPKFAQ  PKFAQV**S**SSNVVL  KFAQVS**S**SNVVLK  FAQVSS**S**NVVLKT  PGAGCI**S**EGRAYD  FEGEVA**S**LEALRS  SLEALR**S**TGLVRV  EHLKMK**S**LSSQAS  LKMKSL**S**SQASKL  KMKSLS**S**QASKLG  SLSSQA**S**KLGEQM  EARELW**S**RLQVKI  LHGDLW**S**GNVAED  IIYDPA**S**FYGHSE  ASFYGH**S**EFELAI  FGGFPR**S**FFTAYH  FGREYR**S**PSLGTM  REYRSP**S**LGTMRR  IRDQHP**S**KIPVII  PDHVNM**S**ELVKII  LLVNQH**S**MVSVST  NQHSMV**S**VSTPIA  HSMVSV**S**TPIADI  PPAAQG**S**PAAAAA  AAPALD**S**AAAEDL  AAAEDL**S**DALCEF  VLADFA**S**PFHERH  ERMKRR**S**SASVSD  RMKRRS**S**ASVSDS  KRRSSA**S**VSDSSG  RSSASV**S**DSSGFS  SASVSD**S**SGFSDS  ASVSDS**S**GFSDSE  SDSSGF**S**DSESAD  SSGFSD**S**ESADSL  GFSDSE**S**ADSLYR  DSESAD**S**LYRNSF  DSLYRN**S**FSFSDE  LYRNSF**S**FSDEKL  RNSFSF**S**DEKLNS  SDEKLN**S**PTDSTP  LNSPTD**S**TPALLS  STPALL**S**ATVTPQ  TMLSIK**S**GERIVF  RIVFTF**S**CQSPEN  FTFSCQ**S**PENHFV  KNIDCM**S**GPCPFG  QLQPST**S**LLPTLN  RIGTFC**S**NGTVSR  CSNGTV**S**RIKMQE  FHPRNV**S**GFSIAN  FSIANR**S**SIKRLC  HLRASV**S**FLNFNL  FLNFNL**S**NCERKE  AGNFNL**S**LQGCDQ  CDQDAQ**S**PGILRL  PRPVKQ**S**RKFVPG  SNLTLT**S**GSKHKI  NVEKTI**S**CTDHRY  YSLQVP**S**DILHLP  VELHDF**S**WKLLVP  EKPCNT**S**FSYLVA  QDLYFG**S**FCPGGS  SFCPGG**S**IKQIQV  RQGLTV**S**FIPYFK  VTPDTK**S**KVYLRT  SVSWNI**S**VPRDQV  RAEEIF**S**LDEDVL  DVLPKP**S**FHHHSF  PSFHHH**S**FWVNIS  SNCSPT**S**GKQLDL  QLDLLF**S**VTLTPR  HLLQDS**S**GSFLQP  SPPTIC**S**RAPTAK  EEPPPR**S**PPESES  PNNGDV**S**SKDTDI  APSLGC**S**LKDVKW  LKDVKW**S**SVAVPL  KDVKWS**S**VAVPLD  PLDLLV**S**TYRLPQ  RTNESI**S**LPFQCK  WNEECK**S**PKKGRC  PKKGRC**S**GHNHVP  HNHVPN**S**LSYARD  ILPYQD**S**GDSGSD  DSGDSG**S**DYLFPE  PEASEE**S**AGIPGK  PPVPPK**S**EAVREE  PPVPPR**S**AKPLST  AKPLST**S**PSIPPR  QQTRSP**S**PTLSYY  SPSPTL**S**YYSSGL  SGLHNI**S**VTKTDT  SAEAVS**S**RLSWPN  DFLLDP**S**RSYSYP  GCELLA**S**PTSPVT  PVTAEF**S**SSVSGC  VTAEFS**S**SVSGCP  EFSSSV**S**GCPKSA  PKSASY**S**LESTDV  VEEKVA**S**ETSPLP  GMQDIF**S**ASYPFS  SASYPF**S**SPLHLQ  ASYPFS**S**PLHLQL  LQLAPR**S**CGDGSP  QPPADL**S**GLSIEE  ADLSGL**S**IEEVSK  LRFIGL**S**EDVISF  LSEDVI**S**FFVTEK  CFCVRR**S**RDPPPP  QRGTDQ**S**TMPEVK  PEVKDL**S**EALPET  GVGVVA**S**RNRAPT  KDGLFK**S**KVTRYL  YLCFTR**S**FSKENS  SFSKEN**S**HLGNVL  KFIPRD**S**TEAAIC  FIGELN**S**MGIWYR  VPRNHD**S**SQPTTP  PRNHDS**S**QPTTPS  SQPTTP**S**QSSAAS  PTTPSQ**S**SAASTP  TTPSQS**S**AASTPA  SQSSAA**S**TPAPNL  NLPRHI**S**LTLPAT  TFRGRN**S**TRTDYE  DYEYQH**S**NLYAIS  SNLYAI**S**AMDGVP  GVPFMI**S**EKFSCV  MISEKF**S**CVPESM  FSCVPE**S**MQPFDL  LGITIK**S**LAEIEK  EKEYEY**S**FRTEQS  SFRTEQ**S**AAARLP  TEVVPS**S**ALSEVS  VPSSAL**S**EVSLRL  SALSEV**S**LRLLCH  PIEYPD**S**WYRDIT  WYRDIT**S**NKKFFS  SNKKFF**S**LAATYR  DGDILA**S**NFSVDT  ILASNF**S**VDTQVA  QVAYIL**S**LGVVKE  RKHGIG**S**LLLESL  GSLLLE**S**LKDHIS  SLKDHI**S**TTAQDH  YLPYYY**S**IRGVLK  YIQHLG**S**ALASLS  LGSALA**S**LSPCSI  SALASL**S**PCSIPH  ASLSPC**S**IPHRVY  VYRQAH**S**LLCSFL  AHSLLC**S**FLPWSG  CSFLPW**S**GISSKS  LPWSGI**S**SKSGIE  PWSGIS**S**KSGIEY  SGISSK**S**GIEYSR  AVAAGT**S**GVAGEG  AEGTAP**S**PGRVSP  PSPGRV**S**PPTPAR  LCRRPD**S**TWHSAE  PDSTWH**S**AEVIQS  SAEVIQ**S**RVNDQE  DAVQKN**S**EKYLSE  NSEKYL**S**ELAEQP  IDAWYF**S**PFPEDY  YMKYEK**S**YRFHLG  KEIYRK**S**NISVYE  YRKSNI**S**VYEVDG  HIVGYF**S**KEKESP  FSKEKE**S**PDGNNV  KFLIAF**S**YELSKL  AFSYEL**S**KLESTV  ELSKLE**S**TVGSPE  LESTVG**S**PEKPLS  SPEKPL**S**DLGKLS  SDLGKL**S**YRSYWS  GKLSYR**S**YWSWVL  SYRSYW**S**WVLLEI  DFRGTL**S**IKDLSQ  LSIKDL**S**QMTSIT  DLSQMT**S**ITQNDI  TQNDII**S**TLQSLN  IISTLQ**S**LNMVKY  VEEHLK**S**AQYKKP  PPITVD**S**VCLKWA  RLPLSH**S**PEHVEM  VEMALL**S**NILAAY  NILAAY**S**FVSENP  AAYSFV**S**ENPERA  AALYFV**S**GVCIGL  ALVIRI**S**CHTDCR  FLQDRE**S**SSDSSD  LQDRES**S**SDSSDS  QDRESS**S**DSSDSE  RESSSD**S**SDSEDG  ESSSDS**S**DSEDGS  SSDSSD**S**EDGSED  SDSEDG**S**EDTVSD  GSEDTV**S**DLSVRR  DTVSDL**S**VRRHRR  FLFSSR**S**SKTFKP  LFSSRS**S**KTFKPK  KNIPEG**S**HQYELL  AEATLG**S**GNLRQA  EFCTEA**S**CPVMSA  ASCPVM**S**AGPRYE  KKPIKC**S**APKYID  DETLFP**S**KIGVPF  FPKNFM**S**VAKTIL  YHQHFD**S**VMQLQE  EAHLNT**S**FKHFIF  ENSVLT**S**TTGRTS  TSLADS**S**IFDSKV  DSSIFD**S**KVTEIS  SKVTEI**S**KENLLI  ENLLIG**S**TSYVEE  ESCPGK**S**DEKLIK  GFVKME**S**VEEFEG  EFEGLD**S**PEFENV  VTDFQD**S**VFNDLY  PVVLNC**S**QKGEPL  GEPLPF**S**CRPLYC  IRKDFN**S**KVTHLV  KFRVAV**S**LGTPIM  FQDCIL**S**FLGFSD  LSFLGF**S**DEEKTN  QEWFWG**S**IQMDAR  HSLSIG**S**LLDISN  SNTPES**S**INYGDT  YGDTPK**S**CTKSSK  KSCTKS**S**KSSTPV  TKSSKS**S**TPVPSK  CPANLL**S**SHRSLV  QRVETI**S**LGEHPC  VIGTFR**S**PHGQTR  LSFQMT**S**DELPKE  DMDSTL**S**RASRAI  STLSRA**S**RAIKKT  RAIKKT**S**KKVTRA  GSVEGR**S**PSSNDK  EGRSPS**S**NDKHVM  SRLSST**S**SLAGIP  SLAGIP**S**PSLVSL  PSPSLV**S**LPSFFE  LRVQEN**S**PGHRAG  FFDFIV**S**INGSRL  VKMLIY**S**SKTLEL  LELRET**S**VTPSNL  ETSVTP**S**NLWGGQ  HVLEVE**S**NSPAAL  AGLRPH**S**DYIIGA  DTVMNE**S**EDLFSL  ESEDLF**S**LIETHE  VIITPN**S**AWGGEG  AWGGEG**S**LGCGIG  EEGKKI**S**LPGQMA  SSVNPP**S**LSPPGT  VNPPSL**S**PPGTTG  TTGIEQ**S**LTGLSI  QSLTGL**S**ISSTPP  TGLSIS**S**TPPAVS  TPPAVS**S**VLSTGV  PPQVNQ**S**LTSVPP  VNQSLT**S**VPPMNP  GLPPLP**S**MPPRNL  APLPLP**S**EFLPSF  FPLVPE**S**SSAASS  PLVPES**S**SAASSG  LVPESS**S**AASSGE  ESSSAA**S**SGELLS  SSGELL**S**SLPPTS  SGELLS**S**LPPTSN  SSLPPT**S**NAPSDP  AKADAA**S**SLTVDV  KADAAS**S**LTVDVT  GDSTPV**S**EKPVSA  VSEKPV**S**AAVDAN  EPRWDQ**S**TFIGRA  TNEQLE**S**ARKIVH  AKYIYD**S**AFHPDT  ILIGRM**S**AQVPMN  WQWINQ**S**FNAVVN  VNYTNR**S**GDAPLT  LGTAYV**S**ATTGAV  ALTKHV**S**PLIGRF  GNRLGE**S**ANAAKQ  ITQVVV**S**RILMAA  KRFPWM**S**APIQVG  ALFPQK**S**SMSVTS  LFPQKS**S**MSVTSL  PQKSSM**S**VTSLEA  SSMSVT**S**LEAELQ  QAKIQE**S**HPELRR  ADSPSD**S**GQGSYE  SDSGQG**S**YETIGP  RKVLQD**S**DSETED  YKTVAD**S**DESYME  FTTDRK**S**SKKHIH  HSFEEG**S**ELSKGT  ALKQLH**S**ETQRLI  PSPGDS**S**VLQQES  KKLDGA**S**HTKPGE  KENNDG**S**SEIGKA  KAVGFL**S**VPKSLS  KSLSSD**S**TLLLFK  LFKDSS**S**KMGYFP  NSGKQP**S**KLDEDD  DEDDSC**S**LLTKES  LLTKES**S**HNSSFE  FRASLV**S**SASKSS  SSASKS**S**GKLSEP  GKLSEP**S**LPIEDS  KHLPRK**S**DKKENM  ELLNLC**S**GKFTSQ  CSGKFT**S**QDASTP  DASTPA**S**SELNKQ  KQEKES**S**MGDPME  EALALC**S**GSFPTD  SGSDVG**S**EDEYDG  SDEELQ**S**QIKKIH  LSDHNP**S**APRNSR  AEAAKE**S**SKSQVK  EAAKES**S**KSQVKK  AKESSK**S**QVKKRG  GPSFMT**S**PSPKHL  SFMTSP**S**PKHLKT  ALTAEK**S**KIETEI  NKMQQK**S**QKKAEL  GYTVKI**S**NYGWDQ  NYGWDQ**S**DKFVKI  VHFTER**S**FDLLVK  KNLNGK**S**YSMIVN  LNGKSY**S**MIVNNL  NLLKPI**S**VEGSSK  PISVEG**S**SKKVKT  ISVEGS**S**KKVKTD  KEKEKP**S**YDTETD  DTETDP**S**EGLMNV  NKAWVE**S**REKQAK  AAAGDA**S**EDSDAG  GDASED**S**DAGSRA  EDSDAG**S**RALPFL  LAQLPQ**S**LSCLRS  SLSCLR**S**LVLKGG  LDLSFN**S**LETLPA  LGALLL**S**HNCLSE  LQRLDL**S**QNLLDT  ASNRLQ**S**LPASLA  LQSLPA**S**LAGLRS  RLLVLH**S**NLLASV  PRLFLT**S**DLDSFP  LTSDLD**S**FPVTPQ  PHDALL**S**HVLELQ  CQVPHF**S**WFLVVS  VVSRPV**S**NACLVP  EGTLLC**S**SGHPGV  EEPRRV**S**MQVVRM  LLCLSQ**S**GPPSFL  SQSGPP**S**FLQPVT  VQLPLP**S**GITGLS  SGITGL**S**LDRSRL  GLSLDR**S**RLHLLY  ICFVFY**S**HLKNVK  LPRLRG**S**EGPRRG  RRGAGL**S**LAPLNL  TGFLTQ**S**NLLSVA  IRHMLF**S**WAERQA  VQALEQ**S**DRQDVA  RRKYQD**S**IRRMGL  PALPGS**S**APQPPE  VLRALG**S**RGWEPF  CQRNLM**S**LLMAVR  LMAVRP**S**LPESGL  RPSLPE**S**GLLSVL  PESGLL**S**VLQIAQ  DLGVGT**S**MEGASP  TSMEGA**S**PLSERC  EGASPL**S**ERCQRQ  GGRRLK**S**PQAPDP  EEENRD**S**QQPGKR  GKRRKD**S**EEEAAS  SEEEAA**S**PEGKRV  ERPEHK**S**LESLAD  EHKSLE**S**LADGGS  SLADGG**S**ASPIKD  AKGLAE**S**LELPKA  QLLHEC**S**PSQMDL  LHECSP**S**QMDLLC  TWLLAL**S**PDLSLS  ALSPDL**S**LSNATV  ATVLTR**S**LFLGRI  RILSLT**S**SASRLL  ILSLTS**S**ASRLLT  SLTSSA**S**RLLTTA  LTTALT**S**FCAKYT  YTYPVC**S**ALLDPV  CLVKME**S**LEPDAQ  TFLVLQ**S**LLERQV  MTPEKF**S**VLMEKL  GLAATT**S**MAYAKL  TTFLRK**S**LKAALK  LQGFHR**S**FKGQNP  PFELAF**S**LDQPDH  QPDHGD**S**DFGLQC  DFGLQC**S**ARPDMP  RPDMPA**S**QPIDIP  RGRATD**S**FSGRFE  CINLIT**S**QEYAVK  QPGHIR**S**RVFREV  EKMRGG**S**ILSHIH  RGGSIL**S**HIHKRR  FNELEA**S**VVVQDV  VVQDVA**S**ALDFLH  EHPNQV**S**PVKICD  CDFDLG**S**GIKLNG  KLNGDC**S**PISTPE  GDCSPI**S**TPELLT  LLTPCG**S**AEYMAP  EVVEAF**S**EEASIY  AFSEEA**S**IYDKRC  KRCDLW**S**LGVILY  ILYILL**S**GYPPFV  FVGRCG**S**DCGWDR  QNMLFE**S**IQEGKY  KDWAHI**S**CAAKDL  AAKDLI**S**KLLVRD  MVLQRN**S**CAKDLT  VLVRAT**S**RCLQLS  SRCLQL**S**PPSQSK  LQLSPP**S**QSKLAQ  LSPPSQ**S**KLAQRR  QRRQRA**S**LSSAPV  QRASLS**S**APVVLV  ALEKIC**S**ICKAMD  YSNISA**S**ADQALD  MQPVYT**S**GIYNIP  NGGALA**S**ERETDI  PMRPSY**S**AQEGLA  FRSQSF**S**EAEPQL  LVASRP**S**PQPLAE  AAPLHK**S**QSVPGA  PLHKSQ**S**VPGAWP  SQPLSG**S**SRQSHP  QPLSGS**S**RQSHPL  SVPPGR**S**YSPYDY  SPKEAT**S**DPSRTP  EATSDP**S**RTPEEE  PRSYVE**S**VARTAV  PILSAD**S**TSVGSF  LSADST**S**VGSFPS  FPSGES**S**DQGPRT  FSSSPE**S**QARAQF  GTGFHG**S**TVSSPQ  FHGSTV**S**SPQSSA  ASPGSP**S**LGRHLG  VAYGGY**S**TPEDRR  SGYQAP**S**TPSFPV  YYPGLS**S**PATSPS  LSSPAT**S**PSPDSA  VSSPVA**S**GMSSPS  NVLFVN**S**VDMESL  TGPQAI**S**KATSET  AISKAT**S**ETLAAD  AIVNFV**S**KVMLNA  PRGTGC**S**AGLLMT  LLAGLQ**S**ARGTNV  ETENND**S**ETAENY  PETEDV**S**NRNVVK  GCPGGE**S**KCVVRV  GWGKPI**S**ESLESV  GKPISE**S**LESVRL  ISESLE**S**VRLACI  LACIHT**S**PLNRFK  LRQDQQ**S**IILVND  IILVND**S**AILEVR  LEVRKE**S**HPLAFE  KFTVYT**S**SELQMR  FTVYTS**S**ELQMRR  ELQMRR**S**SLPATD  LQMRRS**S**LPATDA  STPEVQ**S**EQSSVR  EVQSEQ**S**SVRYKD  VQSEQS**S**VRYKDS  SVRYKD**S**TSLDQL  RYKDST**S**LDQLPT  MYRDTF**S**YNFYNR  YNRPIL**S**RRNTVW  VKTKGP**S**RPPLDA  FRGQVY**S**ELKYHP  RFFHWF**S**KWRKLH  EVTWYI**S**WSPCTK  TWYISW**S**PCTKCT  YQEALR**S**LCQKRD  EFQHCW**S**KFVYSQ  WSKFVY**S**QRELFE  GEILRH**S**MDPPTF  RVTCFT**S**WSPCFS  TCFTSW**S**PCFSCA  SWSPCF**S**CAQEMA  EMAKFI**S**KNKHVS  SKNKHV**S**LCIFTA  EAGAKI**S**IMTYSE  ISIMTY**S**EFKHCW  DGLDEH**S**QDLSGR  EHSQDL**S**GRLRAI  FLMPTS**S**ELNSGQ  TSSELN**S**GQNFLT  QWMTNP**S**RAGVIL  KPQSDK**S**ETHTAF  KLLTMV**S**GQCTLP  CTLPGL**S**LLPDDQ  QKHDDS**S**EVANIE  RKSLSP**S**GLKIQT  FENKVT**S**NNKENV  TMKESE**S**SLDVSL  MKESES**S**LDVSLQ  KGHRMS**S**TPVKAV  QLHSLS**S**ADELRE  DVTIKP**S**TEDRER  DRERGI**S**SREDSP  RERGIS**S**REDSPQ  ISSRED**S**PQVCDD  SDKDYS**S**DESIME  DYSSDE**S**IMESIK  DESIME**S**IKHKVS  PKPKSD**S**HLGNEL  ELKLNI**S**QDQPPG  KETKWS**S**THSRLR  THSRLR**S**QIQMLV  DKLANT**S**VRFQNS  SVRFQN**S**QISSGT  QNSQIS**S**GTQVEK  GNPPRR**S**KSAPPR  PPRRSK**S**APPRDL  EVLHFS**S**GQIEKH  QETKYR**S**GRIRVK  HMPHGG**S**SNNLCH  MPHGGS**S**NNLCHT  TLSFRC**S**LADFQI  IGRGQF**S**EVYKAT  IIKYLD**S**FIEDNE  ADAGDL**S**QMIKYF  YFVQLC**S**AVEHMH  AVEHMH**S**RRVMHR  GLGRFF**S**SETTAA  LGRFFS**S**ETTAAH  GTPYYM**S**PERIHE  NGYNFK**S**DIWSLG  FKSDIW**S**LGCLLY  EMAALQ**S**PFYGDK  DKMNLF**S**LCQKIE  LPGEHY**S**EKLREL  KLRELV**S**MCICPD  LVSQFR**S**CQDNVL  AKELTR**S**LEDVRG  FELSYV**S**AMVPVK  PPEGPL**S**AKAKDP  DAELAC**S**MQYDDQ  PPIACQ**S**PAHRPG  GRPRLR**S**GSDEEE  PRLRSG**S**DEEERV  PFGWAG**S**TWADPQ  GGAAGI**S**LPASEK  EGTDGA**S**LAGTSS  ASLAGT**S**SCSCLD  SLAGTS**S**CSCLDS  AGTSSC**S**CLDSRL  SCSCLD**S**RLHLDG  NPKSPT**S**LDSAVA  SPTSLD**S**AVATQE  GGPHKL**S**TGATNC  SCVCCG**S**CGDSRE  GGVIGA**S**YAAGLA  PPSEDA**S**NGREPK  LPHTSG**S**QVDTAS  PQALSG**S**SSSTAG  ALSGSS**S**STAGSC  LSGSSS**S**TAGSCS  STAGSC**S**SDKMGP  RSRFHG**S**HDLIHR  LQTNYA**S**DLRSIL  CGKIFC**S**RCSSHS  IFCSRC**S**SHSAPL  SRCSSH**S**APLPRY  SGAGGT**S**LEGERG  IYENVE**S**ELRFFT  YPAVFG**S**VLYTFT  LFQLLV**S**LPLCLA  MLALLV**S**VSAEGY  LFQFVN**S**YLSLFY  FVNSYL**S**LFYIGF  EGGGGG**S**GGGGRR  LRLKKV**S**FAERGA  ALLEEG**S**PTMVEK  AEGAPG**S**PEREPP  PGSNSD**S**TRRQRR  PPEEEH**S**PQLTQA  TQAELE**S**CMKKYE  EARAEG**S**GLDPAT  GLDPAT**S**SEKASA  LDPATS**S**EKASAK  TSSEKA**S**AKAKGS  RPKRPG**S**LLAPNN  LSSGAT**S**SLAAAG  SFKFLK**S**PETRRD  PETRRD**S**ERSHSP  RRDSER**S**HSPPKA  GTPSSG**S**SRVQRS  SSRVQR**S**GPVDEA  ALRTRR**S**RSPAPP  RTRRSR**S**PAPPPP  PLPGDA**S**FYSLPP  GDASFY**S**LPPPPL  LETPAP**S**PSPSPS  APSPSP**S**PSPQAV  AAELQA**S**GGPRHP  VLGMAG**S**GKTTFV  NGGIVT**S**LNLFAT  EKAQNM**S**KYVLID  IEVFTW**S**ASGTII  VFTWSA**S**GTIITE  ITEALA**S**SFPTVV  TEALAS**S**FPTVVI  IYVMDT**S**RSTNPV  VMDTSR**S**TNPVTF  NPVTFM**S**NMLYAC  NMLYAC**S**ILYKTK  TDIIDH**S**FAVEWM  QETTYV**S**NLTRSM  VSNLTR**S**MSLVLD  NLTRSM**S**LVLDEF  VLDEFY**S**SLRVVG  LDEFYS**S**LRVVGV  LRVVGV**S**AVLGTG  LFVQVT**S**AAEEYE  YERLKK**S**LANAES  SLANAE**S**QQQREQ  LRKDMG**S**VALDAG  AGTAKD**S**LSPVLH  TAKDSL**S**PVLHPS  SPVLHP**S**DLILTR  EDEEAD**S**DTDDID  HRVTEE**S**HEEPAF  QNFMQE**S**MAQYWK  LLTLAT**S**SLLTSN  LTLATS**S**LLTSNI  TSSLLT**S**NIFCAD  ADELVI**S**NLHSKE  VISNLH**S**KENYDK  ENYDKY**S**EPRGYP  YPKGER**S**LNFEEL  KNVIKM**S**TPAVNK  VNKMPH**S**FANLPL  NVQEER**S**AGATAN  ANLPLR**S**GRNMEV  GRNMEV**S**LVRRVP  RTTTAK**S**VCRMLS  SVCRML**S**DLCQGS  SDLCQG**S**MHSPCA  CQGSMH**S**PCANDL  ANDLFY**S**MTCQHQ  NPDQKQ**S**RRLLFK  FNEKGT**S**LTPEEE  EERFLD**S**AEYGNI  RKMLEE**S**KTLNFN  GQRLTL**S**PLEQEL  EDGTRF**S**HDITPI  EKQRKD**S**FSHSRS  QRKDSF**S**HSRSRM  KDSFSH**S**RSRMNA  SFSHSR**S**RMNAYK  AAYLSL**S**SEDPVL  NDYRKL**S**MQCKDF  VNFQVW**S**DHHRPS  SDHHRP**S**LSRIKL  HHRPSL**S**RIKLAI  LAVFGV**S**IGLPFL  YWIAPC**S**KLGRTL  FVAHAV**S**FTIFLG  LLVVNA**S**DRFEGV  VLGMIW**S**ECKEIW  LDFGML**S**IFVASF  DTLHNV**S**LPPEVA  RDKWWP**S**DPQIIS  SDPQII**S**EGLYAI  ILPANE**S**FGPLQI  FGPLQI**S**LGRTVK  FTTVEE**S**FKTLFW  FKTLFW**S**IFGLSE  WSIFGL**S**EVISVV  LVPSPK**S**FYYLIM  EMGMLN**S**KFKKTR  QAGMRN**S**ENLTAN  EIKQDI**S**SLRYEL  IKQDIS**S**LRYELL  AGERLV**S**PAYVRQ  GCEARR**S**HEHLIR  ENGWDE**S**TLELFL  ELAIMD**S**NNFLGN  IHGIGR**S**GDISAV  QPKAAG**S**SLLNKI  PKAAGS**S**LLNKIT  PMATGM**S**LTLCFL  PRIDQK**S**CFKSMI  QKSCFK**S**MITAGF  CILCIH**S**TTSCFA  CIHSTT**S**CFAPRV  NAYGVQ**S**SKCMHL  AYGVQS**S**KCMHLI  IDAFVQ**S**LDKNFM  IAGFND**S**FIQEIS  SFIQEI**S**KMYPGR  MYPGRA**S**ASPSLD  PGRASA**S**PSLDVL  RASASP**S**LDVLIT  VLITLL**S**LGSNGY  TLLSLG**S**NGYKKL  ERKEMF**S**YLSNQI  EMFSYL**S**NQIKKL  NQIKKL**S**EAYNER  TPHNPI**S**LAMTLK  AVTQLG**S**MLFTRQ  LFTRQV**S**GARVVP  RVVPLG**S**MQTVSG  GSMQTV**S**GYTFRG  TFRGFM**S**HTNNYP  AYLNAA**S**AIGMKM  AVRKER**S**KESDDN  ILIIGE**S**GVGKSS  ESGVGK**S**SLLLRF  SGVGKS**S**LLLRFT  FKVKTI**S**VDGNKA  FRTLTP**S**YYRGAQ  KFARKH**S**MLFIEA  MLFIEA**S**AKTCDG  TPGLWE**S**ENQNKG  NKGVKL**S**HREEGQ  LPAQFK**S**IQHHLR  TGMKID**S**KTPECR  ECRKFL**S**KLMDQL  HKNMIK**S**FYTASL  KSFYTA**S**LLIDVI  EDAGAA**S**LPTQPT  TQPTQP**S**SSSTYD  QPTQPS**S**SSTYDP  PTQPSS**S**STYDPS  TQPSSS**S**TYDPSN  SSTYDP**S**NMPSGN  DPSNMP**S**GNYTGI  PAEVPH**S**TGVASN  HSTGVA**S**NTIQPT  ALFNTI**S**QGDVRL  YCKYAG**S**ALQYED  LQYEDV**S**TAVQNL  AGLELL**S**DQGYRV  FAQADG**S**AYIEQG  PHEIRG**S**RARALP  LVNCQY**S**SATFST  VNCQYS**S**ATFSTG  YSSATF**S**TGERKR  PHGDRK**S**CEMGLQ  TQLHPR**S**QIDIYV  DFVCAC**S**AGFVDG  TALADL**S**HVEEAA  LALLPA**S**GQIALL  QGAAEP**S**RGPPLP  RGPPLP**S**ARGAPP  ARGAPP**S**PEAGFA  FATADH**S**SQERET  ATADHS**S**QERETE  LARGTQ**S**IPNDSP  QSIPND**S**PARGEG  RGEGTH**S**EEEGFA  AMDEED**S**DGELNT  LNTWEL**S**EGTNCP  FNEDWD**S**ELKADQ  ADDIQE**S**ISQELK  DIQESI**S**QELKPW  DMIYDP**S**WHHPPP  PLIPYY**S**KMVFET  SPLVSS**S**DSPPRP  KPAAGL**S**AAPVPT  GQEDFP**S**VLLETA  SPLSAA**S**FKEHEY  TLQENV**S**EASKEV  EASKEV**S**EKAKTL  LLGDPT**S**ENKTDE  LVAAQD**S**ETDYVT  MDLVQT**S**EVMQES  SEVMQE**S**LYPAAQ  CPSFEE**S**EATPSP  VPSAGA**S**VIQPSS  ASVIQP**S**SSPLEA  SSPLEA**S**SVNYES  EEAMSV**S**LKKVSG  QPVPDH**S**ELVEDS  SELVED**S**SPDSEP  EDSSPD**S**EPVDLF  EPVDLF**S**DDSIPD  DLFSDD**S**IPDVPQ  ESLTET**S**FESMIE  ENKEKL**S**ALPPEG  DEVSTL**S**KKEKIP  RETETF**S**DSSPIE  AEKKLP**S**DTEKED  SLFLLL**S**LTVFSI  IALALL**S**VTISFR  LLSVTI**S**FRIYKG  ESEVAI**S**EELVQK  VQKYSN**S**ALGHVN  VDDLVD**S**LKFAVL  LKVPKG**S**IGQYIQ  DRSVGH**S**RIPSAK  GHSRIP**S**AKGKKN  VKVQLR**S**GEEKFP  EINSRV**S**LKVGET  VLPGKE**S**LGYFVG  SFACVE**S**TILLHI  PKLAFM**S**RGVGDK  VGDKGS**S**SHNKPK  GDKGSS**S**HNKPKA  KPKATG**S**TSDPGN  KATGST**S**DPGNRN  PGNRNR**S**ELFYTL  FYTLNG**S**SVDSQP  YTLNGS**S**VDSQPQ  VDSQPQ**S**KSKNTW  KSLTEI**S**TDFDRS  TDFDRS**S**PPLQPP  IGHSPL**S**LSAQSV  TAPVQE**S**PPLAMP  AMPPGN**S**HGLEVG  LFVKLK**S**CRPDSR  KSCRPD**S**RFASLQ  PDSRFA**S**LQPVSN  ASLQPV**S**NQIERC  QIERCN**S**LAFGGY  NDVEYY**S**ETQELL  EKVEAA**S**GFTSEE  PLLKIR**S**AGQKVQ  EWSFIN**S**NLKFAE  HKYNPV**S**LPKDLP  AAGGKM**S**KPCAVE  NVFTGQ**S**KIYSYM  GQSKIY**S**YMSPNK  KIYSYM**S**PNKCSG  MSPNKC**S**GMRFPL  PLQEEN**S**VTHHEV  AGNAVR**S**AMKSEE  VRSAMK**S**EEQKIK  PFPNQK**S**EAAEPP  PPKTPP**S**SCDSTN  PKTPPS**S**CDSTNA  PPSSCD**S**TNAAIA  FYPVRR**S**SRKSKA  YPVRRS**S**RKSKAE  RRSSRK**S**KAELQS  SKAELQ**S**EERKRI  IDELIE**S**GKEEGM  IATKQF**S**RGDFVV  LYAQDP**S**TGCYMY  YYFQYL**S**KTYCVD  GRLINH**S**KCGNCQ  HLILIA**S**RDIAAG  YDYGDR**S**KASIEA  GDRSKA**S**IEAHPW  LMPKTP**S**QKNRRK  RKKRRI**S**YVQDEN  PIRRRL**S**RRKSRS  SRSSQL**S**SRRLRS  RSSQLS**S**RRLRSK  RLRSKD**S**VEKLAT  HVTQLM**S**TEPLPR  LSPTPA**S**ATAPTS  SATAPT**S**QGIPTS  PTSDEE**S**TPKKSK  ESTPKK**S**KARILE  KARILE**S**ITVSSL  LESITV**S**SLMATP  ESITVS**S**LMATPQ  GVGTGR**S**ASKLRI  GTGRSA**S**KLRIAQ  ILPDNF**S**TPTGSR  TGSRTD**S**QSVRHS  SVVRRA**S**RRLAKK  GRIICH**S**YLERLL  DTEIAN**S**TPNPKP  SSPETP**S**AGQQEA  SYKQAV**S**ELDEEQ  RSKTPS**S**PCPASK  RMDPKC**S**FVEKER  KEAAGA**S**KALNVT  VTVDVQ**S**PACTSY  ARGTPL**S**QAIIHQ  EDIFKK**S**KPRYHK  QGARVP**S**SLAYSL  GARVPS**S**LAYSLK  ASVAAE**S**LAGSRA  AESLAG**S**RARAAR  AVERPL**S**LNARAC  LNARAC**S**RVRVVC  RHKEPG**S**GSGGGV  KEPGSG**S**GGGVYW  GVYWVD**S**QQKRYV  GIVTAK**S**GDIFKV  KVDVGG**S**EPASLS  GGSEPA**S**LSYLSF  SEPASL**S**YLSFEG  ASLSYL**S**FEGATK  EMVCID**S**CGRANG  ACEHMT**S**DQRKQI  MSEGNK**S**RTVAAT  TNMNEE**S**SRSHAV  TLYDVK**S**GTSGEK  DVKSGT**S**GEKVGK  EKVGKL**S**LVDLAG  GSNINK**S**LTTLGL  DSLGGN**S**KTAMVA  GISLQS**S**GIKVGD  TFVNGS**S**VSSPIQ  MKNENS**S**EQLDVD  DVDGDS**S**SEVSSE  HEEEKR**S**ALERQR  FSFHSP**S**AQQRLR  DEQENH**S**LIGVAN  APEVDT**S**SSSVSK  EVDTSS**S**SVSKEP  CPVEVI**S**AKDVPT  VKSVQE**S**GTLPLM  EVKAEA**S**WDSAVH  VTFEIV**S**NIPEDA  NVENPA**S**ADSEAY  NPASAD**S**EAYIEK  IEKYLR**S**VLAVEN  VSQTTV**S**RGIAPA  GPPSPL**S**EASSGY  ASSGYF**S**HSVSTA  VSTATL**S**DALGPG  GGQALA**S**DSEEAD  QALASD**S**EEADEV  GLLVRP**S**RVRRAT  SATNLA**S**LTAALA  GTELDY**S**VNPESL  GKYTEI**S**FDEQNQ  FYQLCA**S**AQQSEF  NERSSV**S**EDDSHL  SVSEDD**S**HLKVFC  ELLGLE**S**GRVAQW  NQALQF**S**GKQHTF  VEILRA**S**KFHLCA  GSKFRS**S**LYLLME  IRISAQ**S**YPSRWT  KQELSF**S**DKKEVC  RMLLEK**S**FELKTQ  VEKHVQ**S**QKREMR  TKQLLE**S**YDIEDV  ATRVLE**S**HFQSQK  LESHFQ**S**QKDCYE  DFKQQI**S**ELEKQK  DANMLK**S**LMNSTI  EDFEML**S**FWLSNT  MLSFWL**S**NTCHFL  NCLKQY**S**GEEEFM  LNNFDL**S**EYRQIL  EYRQIL**S**DVAIRI  DGYTMT**S**VLQQLS  LRKDMC**S**CRKGMQ  DKNLQN**S**LAKETL  VKKTTD**S**DAKEIY  ERCTSL**S**AVQIIK  IIKILN**S**YTPIDD  EKRVTP**S**FVRKVQ  LNSRED**S**SQLMLD  TFPFTP**S**PHALEM  MIQIPS**S**FKLGFL  ASELKA**S**EIPFHP  EIPFHP**S**IKTQDP  PKAEEK**S**PKKQKV  LFKNQL**S**PYEQSE  LSPYEQ**S**EILGYA  TAPEKF**S**KTSFDD  EKFSKT**S**FDDEHG  ETIGKG**S**FGQVAK  NNFQGF**S**LSIVRR  FQGFSL**S**IVRRFT  VRRFTL**S**VLKCLQ  KCLQML**S**VEKIIH  YQKGQA**S**VKVIDF  VIDFGS**S**CYEHQK  VYTYIQ**S**RFYRSP  QSRFYR**S**PEVILG  VAIDMW**S**LGCITA  GFIQTA**S**RRQTFF  RQTFFD**S**KGFPKN  KKRYPD**S**KDLTMV  LKTYDT**S**FLDFLR  CLVWEP**S**LRMTPD  HAWIHQ**S**RNLKPQ  PQTLRK**S**NSFFPS  VQGCHH**S**SRKADE  QGCHHS**S**RKADEI  TEKTKD**S**PTKHVQ  VDAPKK**S**EAAVGA  AVGAEV**S**MTSPGQ  AEVSMT**S**PGQSKN  MTSPGQ**S**KNFSLK  GQSKNF**S**LKNTNV  AENAAE**S**TEVNSP  ESTEVN**S**PPSQPP  EVNSPP**S**QPPQPV  QCVHHV**S**TQPSCP  HVSTQP**S**CPGRGK  PGRGKM**S**KLLNPE  NPEEMT**S**RDYYFD  RDYYFD**S**YAHFGI  TLTYRN**S**MYHNKH  VVLDVG**S**GTGILS  SGTGIL**S**MFAAKA  VFGIEC**S**SISDYS  FGIECS**S**ISDYSE  IECSSI**S**DYSEKI  SSISDY**S**EKIIKA  KVDIII**S**EWMGYC  YCLFYE**S**MLNTVI  VKTEEL**S**FTSAFC  EELSFT**S**AFCLQI  HKKMGF**S**TAPDAP  EIYGTI**S**MKPNAK  GQLCET**S**VSNDYK  KKEKPK**S**DKTEEI  KKKAEP**S**EVDMNS  DMNSPK**S**KKAKKK  KKKEEP**S**QNDISP  IEKKVV**S**SKTKKV  EKKVVS**S**KTKKVT  NPSEAA**S**EESNSE  QKEGAF**S**NFPISE  FSNFPI**S**EETIKL  GTGKTF**S**FAIPLI  ELANQV**S**KDFSDI  QVSKDF**S**DITKKL  DITKKL**S**VACFYG  QVEEIL**S**VAYKKD  AKKYMK**S**TYEQVD  IKQDAQ**S**LHGDIP  KGFRNG**S**FGVLVA  VDLVIQ**S**SPPKDV  PPKDVE**S**YIHRSG  ESYIHR**S**GRTGRA  TEIIKA**S**SKDAIR  EIIKAS**S**KDAIRL  VPPTAI**S**HFKQSA  ISHFKQ**S**AEKLIE  HISGAT**S**VDQRSL  TMILQC**S**IEMPNI  QEKWHD**S**RRWQLS  SRRWQL**S**VATEQP  RGQREG**S**RGPRGQ  GPRGQR**S**GGGNKS  GNKSNR**S**QNKGQK  MAVGGG**S**RMTDLT  RMTDLT**S**SIPKPL  MTDLTS**S**IPKPLL  DMGTAD**S**LRYIYP  TDVLVL**S**CDLITD  FRAYDA**S**LAMLMR  MRKGQD**S**IEPVPG  DFIGVD**S**TGKRLL  ELVIKG**S**ILQKHP  FLMENG**S**ITSIRS  ENGSIT**S**IRSELI  SITSIR**S**ELIPYL  LVRKQF**S**SASSQQ  VRKQFS**S**ASSQQG  KQFSSA**S**SQQGQE  QFSSAS**S**QQGQEE  KKKELK**S**LDIYSF  KSLDIY**S**FIKEAN  DRWEDL**S**RSQVRC  WEDLSR**S**QVRCYV  QVPKLL**S**ALCPEE  EEPPVH**S**SAQIVS  EPPVHS**S**AQIVSK  HLVGVD**S**LIGPET  TQIGEK**S**SIKRSV  QIGEKS**S**IKRSVI  KSSIKR**S**VIGSSC  KRSVIG**S**SCLIKD  RSVIGS**S**CLIKDR  NCLLMN**S**VTVEEG  VTVEEG**S**NIQGSV  GSNIQG**S**VICNNA  KDCLIG**S**GQRIEA  QNLIGI**S**IGGGAQ  TGVNGR**S**IKGKTK  DPKQGM**S**LDIVLK  RLVENM**S**SGTADA  LVENMS**S**GTADAL  ADALGL**S**RAILCN  RAFYEL**S**QTHRAF  AFGDVF**S**VIGVRE  EPQPAA**S**EAFVKF  FADAHR**S**IEKFGI  VKFEYL**S**YCLKVK  MDDEEY**S**CIALGE  EPLYRV**S**TGNYEY  EARARF**S**QMRKDV  QLQRLV**S**TMSKYY  RLVSTM**S**KYYNDC  TAAGEP**S**RDTRGA  PDGVNI**S**KEARSA  ISKEAR**S**AISRAA  EARSAI**S**RAASVF  AISRAA**S**VFVLYA  FVLYAT**S**CANNFA  RKTLNA**S**DVLSAM  NASDVL**S**AMEEME  KGKKEA**S**EQKKKD  KDKKTD**S**EEQDKS  SEEQDK**S**RDEDND  GKKLTT**S**CGSLAY  PAVDIW**S**LGVILF  FQEAND**S**ETLTMI  DPKRRA**S**LEEIEN  VSYKNL**S**EEEHNS  SEEEHN**S**IIQRMV  IQTRSA**S**PSNIKA  HATVPQ**S**PARAAD  PARAAD**S**VLNGHR  VLNGHR**S**KGLCDS  SKGLCD**S**AKKDDL  STVPPA**S**LKPTAS  IFEEGE**S**DDEFDM  LKMNIA**S**PGTVHK  RYHRRK**S**QGRGSS  KSQGRG**S**SCSSSE  GRGSSC**S**SSETSD  RGSSCS**S**SETSDD  ETSDDD**S**ESRRRL  DSGFTY**S**WHRRDS  SEGPPG**S**EGDGGG  GDGGGQ**S**KPSNAS  GGVDKA**S**PSENNA  VDKASP**S**ENNAGG  AGGGSP**S**SGSGGN  GGGSPS**S**GSGGNP  GSPSSG**S**GGNPTN  RRCAGP**S**NSMQLA  NSMQLA**S**RSAGEL  GSQLHG**S**TKYIID  WKMCIS**S**TGNAGQ  GGIKFF**S**DHMADT  EGLLCA**S**SPASCC  FPATTY**S**GNSRQR  LKQPSK**S**SVQGLP  LPFANE**S**GTSAAA  ALKQTG**S**RSIEAA  KQTGSR**S**IEAALE  PVTRRP**S**FEGTGD  TPYEGP**S**FGADGP  PKGYGA**S**VEAAGA  GYGVQR**S**PSFQSK  GVQRSP**S**FQSKTP  RSPSFQ**S**KTPPET  HVLGSR**S**QVFASD  RSQVFA**S**DSPPQS  TLARRD**S**LQKPGL  EQYDLD**S**LCAGME  CAGMEQ**S**LRAGPN  PEGGDK**S**RKSAKG  VPVRKN**S**RDEEKR  RESRIK**S**YSPYAF  PGGDMM**S**LLIRME  FRWTHN**S**KYYQKG  KYYQKG**S**HVRQDS  QDSMEP**S**DLWDDV  PAQVKL**S**PEARDL  ITKLCC**S**ADHRLG  SAIDFS**S**DIRKQP  SHPMDT**S**NFDPVD  SPWNDA**S**EGSTKA  NDASEG**S**TKAWDT  FRCPKP**S**GAEASQ  PSGAEA**S**QAESSD  ISVVGL**S**GTEKEK  FHLDHT**S**VLSTSD  TFQPHR**S**TALQPY  LLGIDV**S**RGMNRN  LQVVET**S**ARSNVN  SRKMQA**S**PEYQDY  DEIDHL**S**CIKAKK  ELLLEY**S**ELFYEL  GKMYEL**S**LRPIEG  NSKESL**S**YVVESI  LSYVVE**S**IEKSRE  IEKSRE**S**TLGRRD  SGETLH**S**LIQQGQ  QGQQIA**S**KLQCVF  RNLNLV**S**STASIK  KKRIEL**S**VLSYHS  VLSYHS**S**FSIRKS  SYHSSF**S**IRKSRL  SFSIRK**S**RLVHGY  VLDNDL**S**REQLTE  FTSIPC**S**QPQHKL  NSNLQD**S**EEDIEP  EDTSLP**S**LSKDHS  GQRKSV**S**SSPWLP  NGSGNG**S**DSEMDT  SGNGSD**S**EMDTSS  SEMDTS**S**LERGRK  RKVSIV**S**KPVLYR  SYRTSF**S**VGSDDE  KEEDQA**S**QGYKGD  EGIYRV**S**GNKSEM  SHLNKV**S**HNNKVN  PPGARP**S**SPSAVA  GTRLVW**S**RSGSAG  RLVWSR**S**GSAGWL  VWSRSG**S**AGWLDR  AAAAAA**S**GMESNT  AASGME**S**NTSSSL  GMESNT**S**SSLENL  MESNTS**S**SLENLA  ESNTSS**S**LENLAT  QIQETI**S**DNCVVI  NCVVIF**S**KTSCSY  VIFSKT**S**CSYCTM  FSKTSC**S**YCTMAK  TTLLFF**S**QINMCE  QINMCE**S**KEKTFF  FKLIHG**S**GKEETS  SGKEET**S**KEAKIR  FHEDTR**S**SRSGHL  HEDTRS**S**RSGHLA  DTRSSR**S**GHLAKE  AKETRV**S**PEEAVK  AVKWGE**S**FDKLLS  SFDKLL**S**HRDGLE  FLKTEF**S**EENIEF  CEDFKK**S**KGPQQI  KEVITN**S**ITQPTL  TQPTLH**S**FDAAQS  SFDAAQ**S**RVYQLM  QLMEQD**S**YTRFLK  YTRFLK**S**DIYLDL  TNLRRR**S**RSFTCN  LRRRSR**S**FTCNEF  PPPAPA**S**EGRRRS  SEGRRR**S**RRVRLR  CRELAA**S**APARPA  RPAPAS**S**EIMASA  RCVQEW**S**KNKAEC  LFGAHG**S**LVNIVQ  FIHEFI**S**FARSPF  TPGPSY**S**SSEQVH  GPSYSS**S**EQVHVT  NNDSDD**S**SDNCVI  NDSDDS**S**DNCVIV  SDVSRC**S**SPHSVL  DVSRCS**S**PHSVLG  NKGHCD**S**STRIKS  RSSDSR**S**QSRSGH  SRSQSR**S**GHDQKN  GKKRMK**S**KRSRSR  SRSRES**S**RPRGRR  RRDKKR**S**RTRDSS  LSSEST**S**RSRSRS  WEYTYY**S**RNKDRD  HYSRQS**S**SPEFRV  ERHRSR**S**LSSNRS  PSREFA**S**KAKDSH  GNYKNE**S**DTFSDS  SDTFSD**S**RSSDRE  EESTFV**S**DLENQP  DLENQP**S**NIVSLQ  SLQTEP**S**RQLPSP  LPSPRT**S**LMSVCL  PRTSLM**S**VCLGRD  GAGEPQ**S**PDELLP  ELDETL**S**ERLWGL  FPERVR**S**AAGATF  GATFDL**S**LFVAQK  QKMYRF**S**RAALWI  LWIGTT**S**FMILVL  GPNTGL**S**GGMPGA  VGRLLG**S**LRGLSG  GSLRGL**S**GQPARP  RPPCGV**S**APRRAA  RAASGP**S**GSAPAV  ASGPSG**S**APAVAA  GSYPAL**S**AQAARE  GGQLNV**S**VNCLDQ  VRKSPE**S**VALIWE  AIYMPV**S**PLAVAA  VIFAGF**S**AESLAG  AGFSAE**S**LAGRIN  PVCAPE**S**MGSEDM  APESMG**S**EDMLFM  LFMLYT**S**GSTGMP  MLYTSG**S**TGMPKG  LCNGAT**S**VLFEST  TSVLFE**S**TPVYPN  VKKYDR**S**SLRTLG  KKYDRS**S**LRTLGS  SLRTLG**S**VGEPIN  HRVVGD**S**RCTLVD  CIAPRP**S**EEGAEI  LMDEKG**S**VVEGSN  GSVVEG**S**NVSGAL  SGALCI**S**QAWPGM  DDVINI**S**GHRLGT  HPAVPE**S**AVIGYP  FIVVKD**S**AGDSDV  KDSAGD**S**DVVVQE  RLPKTR**S**GKVMRR  LRKIIT**S**EAQELG  TTLEDP**S**IIAEIL  IIAEIL**S**VYQKCK  LEKLQR**S**GEVPPQ  LQRVLQ**S**EFCNAV  YETVDI**S**SSPEVR  ETVDIS**S**SPEVRA  TVDISS**S**PEVRAN  VAAFAA**S**EGHSHP  AASEGH**S**HPRVVE  GGKEQN**S**PIYISR  NSPIYI**S**RIIPGG  RGDQLL**S**VNGVSV  LSVNGV**S**VEGEHH  VLEEME**S**RFEKMR  SQRVCA**S**GPSMLN  VCASGP**S**MLNSAR  GPSMLN**S**ARGAPE  ATNAEV**S**AAAAGA  AAGATG**S**EELPPG  GPAATT**S**STGVAV  PAATTS**S**TGVAVG  AEHGED**S**LSRKPD  HGEDSL**S**RKPDPE  LNEEDN**S**ESSAIE  EEDNSE**S**SAIEQP  EDNSES**S**AIEQPP  IEQPPT**S**NPAPQI  QIVQAA**S**SAPALE  IVQAAS**S**APALET  PALETD**S**SPPPYS  ALETDS**S**PPPYSS  SSPPPY**S**SITVEV  SPPPYS**S**ITVEVP  VEVPTT**S**DTEVYG  PVPPPY**S**VATSLP  PYSVAT**S**LPTYDE  AAAAET**S**QRIQEE  PPRDDF**S**DADQLR  WLGFCL**S**FCITNT  ICGFGL**S**LIKWIL  ILIVRF**S**DYFTGY  LKVRNM**S**ESMAAA  VRNMSE**S**MAAAHR  LQKEIA**S**EIIGLL  VLSSKG**S**RKSVLE  GIIAFF**S**ALDKQH  DSNNNL**S**PFSIAL  LDLLKT**S**VVKSFK  PHRSYV**S**TMILEV  VKNSVH**S**WDHVTQ  TIETSP**S**LSRMPN  APLVLQ**S**CSSKVT  LVLQSC**S**SKVTEA  EAFDYL**S**FLPLQT  KVLGSL**S**SSQCSQ  LSSSQC**S**QSLSVS  VHVDVH**S**HYNSVA  VHSHYN**S**VANETF  CLEIMD**S**LRRCLS  SADFSQ**S**TSIGIK  SHQESL**S**VLRSSN  KETGHV**S**GPDGQN  PTSVEE**S**GKKEKG  IRQFQR**S**LLNLLS  TVLTSL**S**KLLEPS  SKLLEP**S**SPQFVQ  KICKEN**S**REDALF  ILLRDL**S**QDIHGH  VSQETL**S**EEASSQ  VQTALP**S**GSCVDT  LQVCQS**S**GGIPKN  SYVQNK**S**KSLNYT  MQHMKL**S**TSRDFK  HMKLST**S**RDFKIK  APKDKW**S**NFDPTG  ARELEH**S**RYAKDA  LQLEQQ**S**KLKMRL  MRLEAL**S**LLHTLV  TLVWAW**S**LCRAGA  QTQERL**S**GSASPE  QERLSG**S**ASPEQV  RLSGSA**S**PEQVPA  AVEQLK**S**EQIRAQ  ERRKTL**S**EETRQH  LRKQEE**S**VQKQEA  RQTVLE**S**IRTAGT  LAVGVY**S**AKNATL  ARLGKP**S**LVRETS  SLVRET**S**RITVLE  RHPIQV**S**RRLLSR  VSRRLL**S**RPQDAL  LEGVVL**S**PSLEAR  GVVLSP**S**LEARVR  NTKKNR**S**LYRNIL  KKLALH**S**GMDYAI  FDWANT**S**RRGLLL  RATEKI**S**EDLRAT  YRTGQH**S**NKFMLV  FMLVLA**S**NQPEQF  DYGRKC**S**EVARLT  RLTEGM**S**GREIAQ  IAQLAV**S**WQATAY  QATAYA**S**EDGVLT  LLDWFR**S**LFWKEE  LVGLQY**S**GKTTFV  FVNVIA**S**GQFSED  IASGQF**S**EDMIPT  GQPRFR**S**MWERYC  REKIEA**S**RNELHN  IEKMNL**S**AIQDRE  REICCY**S**ISCKEK  ICCYSI**S**CKEKDN  GGQLLG**S**PFDVPV  LGKTLE**S**QAVETE  AVTRCT**S**SLEGHS  VTRCTS**S**LEGHSE  SSLEGH**S**EAVISV  VISVAF**S**PTGKYL  TGKYLA**S**GSGDTT  KYLASG**S**GDTTVR  VRFWDL**S**TETPHF  HRHWVL**S**ISWSPD  HWVLSI**S**WSPDGR  VLSISW**S**PDGRKL  RTLAGH**S**KWITGL  ECRYVA**S**SSKDGS  CRYVAS**S**SKDGSV  RYVASS**S**KDGSVR  SSSKDG**S**VRIWDT  LTGHTQ**S**VTCLRW  GDGLLY**S**ASQDRT  GLLYSA**S**QDRTIK  VNTMAL**S**TDYALR  FEPAEA**S**VNPQDL  ERLVSG**S**DDFTLF  INQVLF**S**PDSRIV  RIVASA**S**FDKSIK  SASFDK**S**IKLWDG  TGKYLA**S**LRGHVA  IAWSAD**S**RLLVSG  RLLVSG**S**SDSTLK  LLVSGS**S**DSTLKV  VSGSSD**S**TLKVWD  VYAVDW**S**PDGQRV  DGQRVA**S**GGKDKC  VGRLPP**S**ARSEQL  QLEELF**S**QVGPVK  VVTEKG**S**KACRGF  FGYVTF**S**MLEDVQ  KGKNEN**S**ECPKKE  LIIRNL**S**FKCSED  NLSFKC**S**EDDLKT  KYKDTQ**S**VSAIGE  AIGEEK**S**HESKHQ  EEKSHE**S**KHQESV  ESKHQE**S**VKKKGR  EEENIE**S**KVTKPV  RPAPAK**S**SDHSEE  AKSSDH**S**EEDSDL  DHSEED**S**DLEESD  DSDLEE**S**DSIDDG  GEELAQ**S**DTSTEE  LAQSDT**S**TEEQED  DKAVQV**S**NKKKRK  KKRKLP**S**DVNEGK  RNLSFD**S**EEEELG  HPDTEH**S**KGCAFA  KAAEGV**S**AADMAK  DQNIFV**S**RTRLCL  LLLSAT**S**GEKGVR  GNMKGQ**S**LGYAFA  RPIVEF**S**LEDRRK  ELRIQR**S**LQKMRS  HHTEEQ**S**KVPPEQ  QKRKAG**S**TSWTGF  QEKQQL**S**SEQVSR  LSSEQV**S**RKKAKG  QKLLGP**S**KGAPLA  RVIDLK**S**ELKRRN  HEADEL**S**GDASVE  EEENVH**S**KELLSA  DGTQEV**S**KPLPSE  AEDDNI**S**VTIQAE  NVKITD**S**EASKPK  EKEARE**S**SKKAES  ASGQAK**S**SSKESK  SGQAKS**S**SKESKD  TSSTSG**S**SGSSTK  SGSSGS**S**TKNIWV  TKNIWV**S**GLSSNT  IWVSGL**S**SNTKAA  WVSGLS**S**NTKAAD  KYGKVL**S**AKVVTN  VVTNAR**S**PGAKCY  YGIVTM**S**SSTEVS  KVKGDP**S**KKEMKK  KENDEK**S**SSRSSG  NDEKSS**S**RSSGDK  KNTSDR**S**SKTQAS  KEEKRS**S**EKSEKK  KRSSEK**S**EKKESK  KSEKKE**S**KDTKKI  EKIHGR**S**KEKERA  EQEKRN**S**LKRPRD  ARREDP**S**FERYPK  EAGPNP**S**RPTSWK  VERPER**S**GREVSG  REVSGH**S**VRGAPP  APPGNR**S**SASGYG  GMITQH**S**SNASPI  NRIVQI**S**GNSMPR  AGSLLG**S**GQMQIT  QITLWG**S**LAAVAI  FLIFLC**S**SCDREK  LIFLCS**S**CDREKK  KKPRQH**S**GDHENL  NLMNVP**S**DKEMFS  SDKEMF**S**RSVTSL  ATDAPA**S**SEQNGA  TDAPAS**S**EQNGAL  TNGDIL**S**EDSTLT  DILSED**S**TLTCMQ  YEEVQT**S**ASDLLD  EVQTSA**S**DLLDSQ  ASDLLD**S**QDSTGK  KPKCHQ**S**RELPRI  TMLTAR**S**VDGDQG  YEVLKD**S**SSQENM  VLKDSS**S**QENMVE  HLEKGH**S**GKAKST  NKKCRQ**S**VNVESI  ESILGN**S**CDPEEE  GGEAEE**S**ATDTTS  SATDTT**S**ETNKRF  ETNKRF**S**SLSYKS  TNKRFS**S**LSYKSR  KRFSSL**S**YKSREE  SSLSYK**S**REEDPT  LTEEEI**S**AMYSSV  GQLVNK**S**GQSLTV  QGDPQR**S**PSSCND  FEKTPN**S**TLPPAG  PPAGRP**S**EEPEPD  KENDYE**S**ISDLQQ  NKPITP**S**TYVRCL  GLIRKL**S**DFIDPQ  VAIKKP**S**GDDRYN  LLQTGK**S**PTSELL  TGKSPT**S**ELLFDW  EFFAPA**S**LLLPDA  TANTLP**S**KEAITV  VQNLEQ**S**YMPPDS  SYMPPD**S**SSPENK  YMPPDS**S**SPENKS  MPPDSS**S**PENKSL  SSPENK**S**LEVSDT  NKSLEV**S**DTRFHS  SDTRFH**S**FSFYEL  TRFHSF**S**FYELKN  FDERPI**S**VGGNKM  VELLGF**S**SDGDDL  ELLGFS**S**DGDDLC  VYMPNG**S**LLDRLS  SLLDRL**S**CLDGTP  DGTPPL**S**WHMRCK  IHRDIK**S**ANILLD  AFTAKI**S**DFGLAR  FGLARA**S**EKFAQT  GEITPK**S**DIYSFG  PKSDIY**S**FGVVLL  KMNDAD**S**TSVEAM  NDADST**S**VEAMYS  SVEAMY**S**VASQCL  AMYSVA**S**QCLHEK  SLWDRF**S**SSSTSS  LWDRFS**S**SSTSSS  WDRFSS**S**STSSSP  DRFSSS**S**TSSSPS  SSSSTS**S**SPSSLP  SSSTSS**S**PSSLPR  STSSSP**S**SLPRTP  PDRPPR**S**AWGSAT  PRSAWG**S**ATREEG  EEGFDR**S**TSLESS  GFDRST**S**LESSDC  RSTSLE**S**SDCESL  ESSDCE**S**LDSSNS  DCESLD**S**SNSGFG  CESLDS**S**NSGFGP  SLDSSN**S**GFGPEE  PDFELL**S**DPEDEH  MQLLQE**S**LAQARL  AQARLG**S**RRPARL  MPSQLV**S**QVGKEL  LLRLAY**S**EPCGLR  CVEQGK**S**CHSVGQ  QGKSCH**S**VGQLAL  QLALDP**S**LVPTFQ  LVLRLD**S**RLWPKI  IQGLFS**S**ANSPFL  PFLPGF**S**QSLTLS  LPGFSQ**S**LTLSTG  SQSLTL**S**TGFRVI  IKKKLY**S**SEQLLI  KKKLYS**S**EQLLIE  ENGAVY**S**PTTEED  PARGPR**S**GLAAYF  GLQLLL**S**LLAFIC  ICEEVV**S**QCTLCG  YFFEFV**S**CSAFLL  FEFVSC**S**AFLLSL  CSAFLL**S**LLILIV  DTTKVK**S**SDFYIT  TTKVKS**S**DFYITL  CVFLLA**S**IIFVST  ASIIFV**S**THDRTS  STHDRT**S**AEIAAI  VFGFIA**S**FMFLLD  YEKRQE**S**QLRKPE  EVSEYL**S**QNPRVA  GGAASA**S**TDEAAD  EGVEEP**S**KKRVIE  IEGKNS**S**AVEQDH  QDHAKT**S**AKTERA  KSESGN**S**ARSSGI  SGNSAR**S**SGISSQ  RSSGIS**S**QNSSTS  ISSQNS**S**TSDGDR  SQNSST**S**DGDRSV  VSSQSS**S**SVSSQV  QVTTAG**S**GKASEA  AGSGKA**S**EAEAPD  DKHGSA**S**FVSLLK  GSASFV**S**LLKSSV  MTQSTD**S**RQQSGS  SALEGS**S**ASASQS  SASQSS**S**EIEVPL  LLGSSG**S**SEVELP  ELPLLS**S**KPSSET  LSSKPS**S**ETASSG  SVAKNS**S**SSGTSL  SLLTPK**S**SSSTNT  LLTPKS**S**SSTNTS  LLASKS**S**SQTSGS  KSSSQT**S**GSLVSK  SSQTSG**S**LVSKST  QLASKS**S**SQTSTS  STSQLP**S**KSTSQS  KSTSQS**S**ESSVKF  SQSSES**S**VKFSCK  CELRCK**S**VYLGTG  VLLDEE**S**RPVNLP  ALQLFR**S**HVQPLL  LAEAEI**S**FTLMLT  ARELVR**S**EELGRW  DALVVM**S**GDGLMH  IQKPLC**S**LPAGSG  CSLPAG**S**GNALAA  GNALAA**S**LNHYAG  LCRRLL**S**PMNLLS  SPMNLL**S**LHTASG  LSLHTA**S**GLRLFS  SGLRLF**S**VLSLAW  RLFSVL**S**LAWGFI  ADVDLE**S**EKYRRL  PVGRVG**S**KTPASP  LEEPVP**S**HWTVVP  VLALLH**S**HLGSEM  LHSHLG**S**EMFAAP  YVRAGV**S**RAMLLR  DGELMV**S**EAVQGQ  NYFWMV**S**GCVEPP  CVEPPP**S**WKPQQM  LSNSSN**S**VFSECL  SDGCPK**S**ADLIGL  HCEDQA**S**GAVCRS  SGAVCR**S**LSTPQF  STPQFN**S**LDVIAD  HAVAVQ**S**PMFLLC  SSLWSA**S**HPSSSK  WSASHP**S**SSKKMD  SASHPS**S**SKKMDG  ASHPSS**S**KKMDGY  MDGYIL**S**LVQKKT  TNKPRT**S**VNADPT  RAPGGV**S**QGNSVN  SVNLKN**S**KQACLP  LNNGTF**S**PPKQWS  SPPKQW**S**KESKAE  KQWSKE**S**KAEQAE  AASDLQ**S**KHLPKT  QEHARC**S**AIGTGE  AIGTGE**S**PKESAQ  GESPKE**S**AQLSGA  GASPKE**S**PSRGPA  GVVKNS**S**LKHRGP  EKTRAG**S**KKCRFP  SSKGRK**S**GGGPEA  YRRWKS**S**AEISYE  YSAECE**S**LFHSTV  CESLFH**S**TVVDTS  GEFVGE**S**TTTSDS  STTTSD**S**EESGGL  SGGLIW**S**QFVQTL  GRSNSR**S**HSSRSK  KSRSQS**S**SRSRSR  KRYSSR**S**RSRTYS  RRGRSR**S**RSPKRR  GRSRSR**S**PKRRSV  SPRSSS**S**RSSSPY  SSPYSK**S**PVSKRR  YSKSPV**S**KRRGSQ  DIWPGL**S**AYDNSP  PIATPP**S**QSSSCS  PPSQSS**S**CSDAPM  SPERSG**S**GSVGNG  ERSGSG**S**VGNGSS  YRQFRK**S**VLADQG  KSFATA**S**HRNTEE  KYKSKV**S**LKGNRE  KEEDKN**S**ERITVK  RIKMIA**S**DSHRPE  PVPLDD**S**NRPASL  DSNRPA**S**LTKDRL  LPQASK**S**TSESFI  SKSTSE**S**FIQHIV  KEQYFK**S**AAMTLN  LNERFT**S**YQKATE  KATEEH**S**TRQKSP  SKERGD**S**KGSRES  DHSRSS**S**SSASPS  SSSSSA**S**PSSPSS  SSSASP**S**SPSSRE  SREEKE**S**KKEREE  FKKSGS**S**PKWTHD  YRAKWI**S**QDKEVA  EILSVL**S**HRNIIQ  IHRDLK**S**RNVVIA  PSSCPR**S**FAELLH  DAKKRP**S**FKQIIS  SFKQII**S**ILESMS  SILESM**S**NDTSLP  SMSNDT**S**LPDKCN  QKLTEQ**S**NTPLLP  NTPLLP**S**FEIGAW  LVRKGD**S**SAEMSV  EMSVYA**S**LFKENN  HIIHFK**S**AIEKLT  PPLIKD**S**GGEPEE  CTVTYE**S**DVRTPK  PGSRSD**S**SADCQW  HFDGQD**S**YAAAVR  PVNQSR**S**SSPTQY  VNQSRS**S**SPTQYG  GLTKNF**S**SLHLNS  LTKNFS**S**LHLNSR  SSLHLN**S**RDSGFS  RDSGFS**S**GNTDTS  GNTDTS**S**ERGRYS  SERGRY**S**DRSRNK  NKYGRG**S**ISLNSS  GRYSGK**S**QHSTPS  SQHSTP**S**RGRYPG  GKFYRV**S**QSALNP  EEDSKV**S**EGGWTK  KKPHRP**S**PAKTNK  GTLQPS**S**GGGSSN  RHKSKH**S**KDMGLV  SDSDTF**S**DDMAFK  QTEKEK**S**QEVSSK  DRISGS**S**KRSNEE  HKDRSK**S**HRKRET  SPHRKW**S**DSSKQD  HRKWSD**S**SKQDDS  SSKQDD**S**PSGASY  PYSRRR**S**SSYERS  YSGRSP**S**PYGRRR  PYGRRR**S**SSPFLS  SRSPLP**S**RKSMKS  PAYSRH**S**SSHSKK  SVEAKD**S**GLESKK  KDSGLE**S**KKLPRS  SVKLEK**S**APDTEL  VSSQAN**S**QPPVQV  SVKTQV**S**VTAAIP  PKETLP**S**KPVKKE  LMGLLE**S**GLVHFS  PAIDVW**S**CGCILG  AQLELI**S**RLCGSP  ISRLCG**S**PCPAVW  EFSFIP**S**AALDLL  LKDVEL**S**KMAPPD  QLLNIH**S**NPEMQQ  ALTEAT**S**QQQDSE  TSQQQD**S**ETMAPE  ALLQLL**S**QPEAEP  GPETGF**S**AIDTDE  LKAEGS**S**NSVVHA  GGPTQS**S**AYGKLY  GDAAAS**S**SAVKKL  DAAASS**S**AVKKLG  VKKLGA**S**RTGISN  ASRTGI**S**NMRALE  ENDFFN**S**PPRKTV  LLEFRS**S**IMYLTK  LPWLNR**S**QTVVEE  FLGNLV**S**AQTVFL  CLSMIA**S**HFVPPR  EGDVDV**S**DSDDED  FPFVRK**S**ERTLEC  HNLLRI**S**VYFPTL  TCGGTD**S**TEGLFN  LMSLVL**S**YMKDVC  LLPTHA**S**CHVQFF  FMFYLC**S**FKLGFA  KKLQDP**S**NPAIIR  AGNYIG**S**FLARAK  YLNNQD**S**GTKAFC  RHKQLL**S**GNLKEG  GLQYLQ**S**LNFERI  FERIVM**S**QLNPLK  LKICLP**S**VVNFFA  MLPVIR**S**TAGGDS  STAGGD**S**VQICTN  PCVLKR**S**KKFIDP  QVWEDM**S**AEELQE  VIGITP**S**SFDTHF  IGITPS**S**FDTHFR  FDTHFR**S**PSSSVG  THFRSP**S**SSVGSP  HFRSPS**S**SVGSPP  PSSSVG**S**PPVLYM  RLPPLL**S**PLLLLL  GACVAG**S**DEPGPE  GPEGLT**S**TSLLDL  EGLTST**S**LLDLLL  LDSEEP**S**ETMGLG  GLGAPG**S**GFPSEE  EEELND**S**SLDLGP  EELNDS**S**LDLGPT  LTEKAG**S**IEDTSQ  PQVRDF**S**LTSSSQ  RDFSLT**S**SSQTPG  DFSLTS**S**SQTPGA  KSRHED**S**GDQASS  DSGDQA**S**SGVEVE  SGDQAS**S**GVEVES  GVEVES**S**MGPSLL  TPGDQD**S**TSQEAE  GDQDST**S**QEAEAT  EAPQEA**S**EEATAG  EVPALP**S**FPQTTA  PQTTAP**S**GAEHPD  DEDPLG**S**RTSASS  GSRTSA**S**SPLAPG  QAAEAQ**S**RIPWDS  VLPRHG**S**GHHGAW  HGAWHI**S**LSKPSE  PTQDVL**S**MLGDIR  SCQARA**S**QVRSDY  PKLKHV**S**HGEELR  PTLDVA**S**DSQSEM  VASDSQ**S**EMQEKH  MQEKHP**S**LNGGGA  KRDPED**S**DVFEED  GLDDTD**S**EDELPP  RQRYDG**S**TTAMEI  VVTGAN**S**GIGFET  GFETAK**S**FALHGA  RNMARA**S**EAVSRI  RASEAV**S**RILEEW  DLALLR**S**VQHFAE  TFALPW**S**LTKDGL  QDVLCR**S**APARVI  ARVIVV**S**SESHRF  RVIVVS**S**ESHRFT  IVVSSE**S**HRFTDI  FTDIND**S**LGKLDF  LGKLDF**S**RLSPTK  LDFSRL**S**PTKNDY  MLAYNR**S**KLCNIL  LCNILF**S**NELHRR  ELHRRL**S**PRGVTS  SPRGVT**S**NAVHPG  PGNMMY**S**NIHRSW  YSNIHR**S**WWVYTL  ARPFTK**S**MQQGAA  CCRCMP**S**PEAQSE  PSPEAQ**S**EETART  RTLWAL**S**ERLIQE  SQYPLQ**S**LLTGYQ  RLYNTE**S**QSFRKK  SDKQTP**S**KPKKKQ  PKKKQN**S**KGLAPS  RQEPIA**S**KSFLYP  SFLYPG**S**STRKLG  TRKLGY**S**SLILDS  RKLGYS**S**LILDST  FNGHQN**S**TFYVKS  DDQFLV**S**GSSDEA  PGGDKL**S**TVGWAS  STVGWA**S**QKKKES  CNPSNS**S**PSSAAC  PSNSSP**S**SAACAP  SNSSPS**S**AACAPS  SAACAP**S**CAGDLP  SNTPTF**S**IKTSPA  SPAKAR**S**PINRRG  NRRGSV**S**SVSPKP  RRGSVS**S**VSPKPP  SPKPPS**S**FKMSIR  WVTRTP**S**SSPPIT  SETKIM**S**PRKALI  VKRRLD**S**SCLESV  GNQEDL**S**KDSLGP  PISPYA**S**ESCGTL  SPYASE**S**CGTLPL  RPCGEG**S**EMVGKE  RKAENP**S**PRSPSS  PSPRSP**S**SQTPNS  SPRSPS**S**QTPNSR  SSQTPN**S**RRQSGK  VTITPS**S**MRKICT  GSLVSS**S**LSKPEV  LVSSSL**S**KPEVFG  VPFTVE**S**LLESSY  LTTYGL**S**AYSGKV  RAGFIE**S**TFKPNE  DIVIKV**S**VADWKV  DDTSYT**S**NDDVLE  RGATEN**S**VYLGMY  GQLYLQ**S**SVRISE  QSSVRI**S**EKFPSS  LSNDKF**S**HEEYSN  HRQRKE**S**ETQCQT  KNSGYI**S**RYLTDF  TDWPLS**S**PSPMDA  SPMDAP**S**VKIRRM  IEIIAP**S**PQRSRS  SPQRSR**S**FSVGIS  SCDQTS**S**SESQFS  CDQTSS**S**ESQFSP  HEDISE**S**VDAAYN  LCPSEA**S**PYVRSR  ASPYVR**S**RERTSS  SGCDNA**S**SKEEPK  GCDNAS**S**KEEPKT  TAFKPT**S**SKSSSE  PRPTTL**S**LDLTKN  EKLQPS**S**PKVYLY  IHGNSY**S**HKVDIF  HKVDIF**S**LGLILF  ELLYPF**S**TQMERV  MVQDML**S**PSPMER  QDMLSP**S**PMERPE  TVLRQR**S**RSLSSS  EVFQTV**S**EGLKKL  LKKLYK**S**KLLPLE  RFHEFH**S**PALEDA  LLVGQY**S**TGKTTF  PEPTTD**S**FIAVMQ  PNPVLE**S**ISVIDT  PVLESI**S**VIDTPG  DTPGIL**S**GEKQRI  GEKQRI**S**RGYDFA  AHKLDI**S**DEFSEV  DISDEF**S**EVIKAL  YGALMW**S**LGKIVN  IRVYIG**S**FWSHPL  YIGSFW**S**HPLLIP  LFRDIQ**S**LPRNAA  VHAYII**S**SLKKEM  HAYIIS**S**LKKEMP  LKKEMP**S**VFGKDN  EREHQI**S**PGDFPN  LQAQDF**S**KFQPLK  KFQPLK**S**KLLEVV  LVRQEE**S**QRPIQM  EIFYTL**S**PVDGKI  KKEMVR**S**KLPNSV  RSKLPN**S**VLGKIW  QYKKAF**S**AVDTDG  ATGKNL**S**EAQLRK  QLRKLI**S**EVDSDG  LISEVD**S**DGDGEI  DGDGEI**S**FQEFLT  RVLGLG**S**RREPRS  SRREPR**S**LPAGGP  PPPGHA**S**AGPAAM  AGPAAM**S**SHIAKS  GPAAMS**S**HIAKSE  SSHIAK**S**ESKTSL  HIAKSE**S**KTSLLK  KSESKT**S**LLKAAA  AAAAAA**S**GGSRAP  AAASGG**S**RAPRHG  RDPGLP**S**RRLPGS  SRRLPG**S**CPATPQ  CPATPQ**S**SGDPSS  PATPQS**S**GDPSSR  QSSGDP**S**SRRPLC  SSGDPS**S**RRPLCR  EEGARG**S**QRVLPQ  ALPAAA**S**RPSPSS  AAASRP**S**PSSPLP  ASRPSP**S**SPLPPA  SRPSPS**S**PLPPAR  GEERGL**S**PALGLR  ALGLRG**S**LRARGR  ARGRGD**S**VPAAAS  SVPAAA**S**EADPFL  RLRPML**S**SAFGQD  LRPMLS**S**AFGQDR  AFGQDR**S**LRPEEI  MELIEL**S**QQINMN  DGEIST**S**ELREAM  KGEGDL**S**QLSKQY  GDLSQL**S**KQYSSR  QLSKQY**S**SRDLPS  LSKQYS**S**RDLPSH  SSRDLP**S**HTKIKY  TREHTT**S**SSVSKK  REHTTS**S**SVSKKP  EHTTSS**S**VSKKPR  TTSSSV**S**KKPRLD  EDFEEE**S**DDDDTA  RMENIL**S**GNPLLN  LNLTGP**S**QPQANF  VNDTLR**S**EFHKKF  DTENHT**S**VDGYTE  CRNSIT**S**ATDEQP  CQRSRP**S**SDLNNS  SSDLNN**S**TLQSPA  NNSTLQ**S**PAHLKV  HLKVQR**S**ISANQK  SDHAGP**S**IPPAVS  KRPQAN**S**VESEQK  QANSVE**S**EQKEEW  TTVGSK**S**EMTASP  KSEMTA**S**PLVGPE  MSVSSI**S**SAGSSV  SVSSIS**S**AGSSVA  RPRHQK**S**MSTSGH  RHQKSM**S**TSGHPI  QKSMST**S**GHPIKV  QRVPAA**S**PSAHSI  ASPSAH**S**ISTATP  PSAHSI**S**TATPDR  TRFPRG**S**SSRSTF  RFPRGS**S**SRSTFH  RGSSSR**S**TFHGEQ  QLRERR**S**VAYNGP  YNGPPA**S**PSHETG  GPPASP**S**HETGAF  TSTGII**S**KITSKF  FVRRDP**S**EGEASG  PSEGEA**S**GRTDTS  RTDTSR**S**TSGEPK  KEEGKD**S**KPRSLR  SMKTTS**S**MDPNDM  KRISGT**S**IAFKNI  RKPALI**S**VGSASS  ALISVG**S**ASSSWW  ISVGSA**S**SSWWRV  SVGSAS**S**SWWRVM  VGSASS**S**WWRVMA  VALGIW**S**VMQRNY  QYVVKQ**S**ELKGTF  FKGHKC**S**PCDTNW  WRYYGD**S**CYGFFR  NLTWEE**S**KQYCTD  IRWVGL**S**RQKSNE  GLSRQK**S**NEVWKW  WKWEDG**S**VISENM  EDGSVI**S**ENMFEF  RSVLAA**S**SPFFRE  REALLT**S**APLPLP  TTTAAS**S**SSSSSS  SSSSSS**S**SSSSAS  SSSSSS**S**ASSSSS  SSSSSA**S**SSSSSS  SSSASS**S**SSSSSS  SSASSS**S**SSSSSS  SASSSS**S**SSSSSP  ASPPAS**S**PPRVLE  VLNFIY**S**ARLALP  GRRLGI**S**RLQGLG  APDLQC**S**LPRRPL  GASTRG**S**TGLGAG  RHSNVH**S**WRRKYP  RAFHGI**S**PGLLAS  SPGLLA**S**EKTPNG  RPYKTY**S**QGAPEA  APEAPL**S**PTLNTP  PVAMPA**S**PPPGPP  EPGPPP**S**VITFAH  VHGGSS**S**GGGGSG  GAGRGP**S**QLQAPP  GEEMEE**S**EEDEEE  LWRPYY**S**YKPKRK  HGGGSH**S**SRAGRR  TPVIAY**S**KGSAGT  EMQVSS**S**SGEAGG  MQVSSS**S**GEAGGG  GEAGGG**S**TAAEEA  TAAEEA**S**ETASLQ  EPPVVA**S**GGSYVY  AYPYNF**S**NLAALP  SSESFI**S**RLLAIV  FVLAAR**S**DSWSLA  LAARSD**S**WSLANL  ARSDSW**S**LANLSS  CEKGVM**S**LVNVRN  CAEIIA**S**HWDDLR  LRKEDF**S**SMSAQL  LYKMIK**S**KTEYPL  YLIEMD**S**QLPGKL  VDMVDK**S**GWSLLH  VDKSGW**S**LLHKGI  LVALYS**S**KKHSAD  YSSKKH**S**ADVMSE  HSADVM**S**EMAQIA  GNEYVF**S**QLLQCK  RLIQRG**S**HTDAPD  LPKEAA**S**LTSLAD  EAASLT**S**LADSVH  NHPDVV**S**VILEQK  QRQDSK**S**ALFLLE  ACFLIR**S**GCDVNS  PIHVAI**S**SQHGVI  IIQLLV**S**HPDIHL  TFKNNK**S**AEAILK  HVAVQN**S**DIESVL  QNSDIE**S**VLFLIS  SVLFLI**S**VHANVN  SRVQDA**S**KLTPLH  DLPTIC**S**VLLENG  KPDADG**S**TVLLLA  CRAIVR**S**GARLGV  RLLDML**S**KEPPWC  PPWCDG**S**YCYECT  AALPPG**S**AARALH  FTLHLC**S**TEKIYK  YKEFYR**S**EVIKNS  PDRLDT**S**VSCFVV  RLDTSV**S**CFVVKI  FEHKGY**S**NAQKTI  NSYDVF**S**LLRLHR  EKLRLT**S**TSNELK  ALQDKG**S**AFSAEH  DKGSAF**S**AEHLKL  LQLQKE**S**LNELRK  RCRQLL**S**ELSYIY  QLLSEL**S**YIYPID  GVKLPN**S**EDFQAK  QAKDDG**S**IAVALG  YTAHLV**S**MISFFL  HLVSMI**S**FFLQVP  IHKGSR**S**TIKDNI  CDRHHT**S**SAIPVP  DRHHTS**S**AIPVPK  PVPKRQ**S**SIFGGA  HRKRAS**S**ENERLQ  PPPSYN**S**ALAQPV  PVTTVP**S**MGETER  TERKIT**S**LSSSLD  RKITSL**S**SSLDTS  SSSLDT**S**LDFSKE  NGTLLP**S**EQAGSA  EQAGSA**S**VQLPGE  GEFEEF**S**RRIYAL  LNENVS**S**FRRPRR  QQVKQP**S**QPPPQE  TKEPCH**S**KVPQPG  KVPEPG**S**IKVPDQ  LPEPCP**S**TVTPGP  ETCALA**S**HSGSSG  CALASH**S**GSSGSK  LASHSG**S**SGSKSG  ASHSGS**S**GSKSGG  HSGSSG**S**KSGGDK  GSSGSK**S**GGDKMF  GGDKMF**S**LKKWNA  NAVAMW**S**WDVECD  WGECNH**S**FHNCCM  FHNCCM**S**LWVKQN  PTSEPT**S**GPPGNN  PGNNGG**S**LLSVIT  NGDGVD**S**EIRCLD  DISMAI**S**YLYNSK  ISYLYN**S**KEPGVQ  SSDMHI**S**TQRHSR  ISTQRH**S**RGTKLR  RKRELP**S**LSPAPD  KRTHQR**S**KSDATA  KSDATA**S**ISLSSN  TASISL**S**SNLKRT  DEELSS**S**TESIDN  LSSSTE**S**IDNSFS  IDNSFS**S**PVRLAP  EREFIK**S**LMAIGK  KTQRLI**S**ELSLLN  RLISEL**S**LLNHKL  ENFDTT**S**VPARIP  HEQRAG**S**FSTVPN  QRAGSF**S**TVPNYD  PEVHTN**S**CDNISQ  SQFSVD**S**ITSQES  SITSQE**S**KEPVFI  RRIREG**S**PYGHLP  PNWRLL**S**VIVKCG  KKQSQL**S**LLDYFL  TTEAFL**S**AQRNFV  DFGFIL**S**SSPRNL  EIMQQG**S**QLPCFH  LPCFHG**S**STIRNL  KERFHM**S**MTEEQL  TTPGER**S**SLPAFY  TPGERS**S**LPAFYP  AFYPGT**S**GSCSGC  YPGTSG**S**CSGCGS  GTSGSC**S**GCGSLS  SCSGCG**S**LSLPLL  SGCGSL**S**LPLLAG  AADAVA**S**LLIVGA  CARPRR**S**PAQEDG  APTVEP**S**SGEAEG  PTVEPS**S**GEAEGG  ANLVDV**S**GGLETE  GLETES**S**NGKDTL  EGAGDT**S**EVMDTQ  MDTQAG**S**VDEENG  TFGIDT**S**SCLPFM  FGIDTS**S**CLPFMT  PFMTNY**S**FHCNVC  LKEMCL**S**ALANLT  ANLTWQ**S**RTQDEH  HPKTMF**S**KDKDII  LLDQDL**S**NIGPAY  YDNQKQ**S**SAVSTS  KQSSAV**S**TSGNLN  GGIAAG**S**SGKGRG  TTKKAR**S**DPLFSA  RSDPLF**S**AQRLPP  APQLKI**S**DDRLTV  VGEKGY**S**MVRASH  YSMVRA**S**HGVRKG  AARLGW**S**QPLGNL  LGYDKF**S**YSWRSK  YDKFSY**S**WRSKKG  GTKFHQ**S**IGKHYS  SIGKHY**S**SGYGQG  IGKHYS**S**GYGQGD  DTETAK**S**LPDTYK  ALIKFK**S**YLYFEE  LKQTPH**S**EIIFYK  VYFPAI**S**LYKSCT  YKSCTV**S**INFGPC  LTYRPM**S**DMGWGA  ESELSP**S**EKAYLN  EKGDYA**S**VKKSLE  LIELLL**S**FNVYVG  NCVECV**S**SSDVDS  CVECVS**S**SDVDSL  VECVSS**S**DVDSLR  VDSLRH**S**RSRLNI  SLIALS**S**EDPFLT  VENEFK**S**EYEELS  LLDQTR**S**SRELEI  SLIEEQ**S**GNDLAR  YLIAPK**S**PLGLFI  FICHTA**S**YLTFLF  FLLLLA**S**QHIDRS  VAFVKY**S**ALNPRE  LGPLQI**S**LGRMLL  KQNNAF**S**TLFETL  GTYNVI**S**LVVLLN  IAMMNN**S**YQLIAD  RTKLWM**S**YFEEGG  PFNVIP**S**PKSLWY  VIPSPK**S**LWYLIK  MRRKPE**S**FGTIGR  LKQDIS**S**FRFEVL  LRGSKL**S**TIQSAN  SKESSN**S**ADSDEK  SDSEGN**S**KDKKKN  DKKKNF**S**LFDLTT  TLIHPR**S**AAIASE  SERHNI**S**NGSALV  RNIQLE**S**RGLASR  LSIPGL**S**EQCVLV  RVCPFK**S**EKVVVE  PEEMDW**S**ELYPEF  PLTQNQ**S**HDDPKD  GLLVEL**S**PLFPDT  EIRVKV**S**DYVQDR  NIACLR**S**NAMKHL  HKWRII**S**PTLLAE  VPLEDL**S**EDPVVG  VGHLGT**S**TEEGKK  ITGFED**S**VRKFIC  EMLGDL**S**DSQLKV  LGDLSD**S**QLKVWM  QLKVWM**S**KYGWSA  MSKYGW**S**ADESGQ  GWSADE**S**GQIFIC  GQIFIC**S**QEESIK  ICSQEE**S**IKPKNI  EKIDFD**S**VSSIMA  LDRHWH**S**KCLKCS  SKCLKC**S**DCHTPL  CFSRGE**S**VYCKDD  FYLMED**S**RLVCKA  QLETLK**S**AYNTSP  KSAYNT**S**PKPARH  HVREQL**S**SETGLD  VREQLS**S**ETGLDM  SRGGSK**S**DKDSVQ  SKSDKD**S**VQEGQD  VQEGQD**S**DAEVSF  DSDAEV**S**FPDEPS  SFPDEP**S**LAEMGP  ANGLYG**S**LGEPTQ  QALGRP**S**GALGNF  GALGNF**S**LEHGGL  RELRPG**S**PYGVPP  PYGVPP**S**PAAPQS  SPAAPQ**S**LPGPQP  GPQPLL**S**SLVYPD  PQPLLS**S**LVYPDT  LVYPDT**S**LGLVPS  SLGLVP**S**GAPGGP  LAGNGP**S**SDLSTG  AGNGPS**S**DLSTGS  GPSSDL**S**TGSSGG  SDLSTG**S**SGGYPD  DLSTGS**S**GGYPDF  YPDFPA**S**PASWLD  FPASPA**S**WLDEVD  FLLLAK**S**AKGAAL  VRELAE**S**DFASTF  AESDFA**S**TFRLLT  NKLRHL**S**VVTLAA  ADVLRG**S**LDQRNQ  RLEVDY**S**IGRDIQ  IQRQDL**S**AIARTL  GCEVVL**S**GIEEQV  GIEEQV**S**RANQHK  LKQQIE**S**EVANLK  AAAAAT**S**QDPEQH  TNQRQP**S**KKASKG  QPSKKA**S**KGKGLR  GKGLRG**S**AKIWSK  RKKIQF**S**VPAPPS  SVPAPP**S**QLDPRQ  AMLFRL**S**EHSSPE  FRLSEH**S**SPEEEA  RLSEHS**S**PEEEAS  SPEEEA**S**PHQRAS  SPHQRA**S**GEGHHL  EGHHLK**S**KRPNPC  CAYTPP**S**LKAVQR  VQRIAE**S**HLQSIS  AESHLQ**S**ISNLNE  SHLQSI**S**NLNENQ  LNENQA**S**EEEDEL  EEEEED**S**QAEVLK  LKVIRQ**S**AGQKTT  PPPLDE**S**ERDGGS  SERDGG**S**EDQVED  VEDPAL**S**EPGEEP  PGAEAP**S**SSEPHG  PHGARG**S**SSSGGK  HGARGS**S**SSGGKK  GARGSS**S**SGGKKC  EFCNIL**S**RVLSRA  VLSRAR**S**RPAKLY  TVLKAH**S**AKKKLN  NAENTA**S**QSPRTR  SPRTRG**S**RRQIQR  RLRENR**S**LAMSRL  NRSLAM**S**RLDEVI  RLDEVI**S**KYAMLQ  ADTPEA**S**LDSGEG  PEASLD**S**GEGPSG  DSGEGP**S**GMASQG  GPSGMA**S**QGCPSA  ASQGCP**S**ASRAET  EEEATD**S**EEEEDL  DKSPMS**S**LQISNE  GRIVSP**S**LLSEEP  VSPSLL**S**EEPLAP  LPLDTP**S**SVETDI  SVETDI**S**SSRKQS  VETDIS**S**SRKQSE  NGAGMV**S**STSFNG  GMVSST**S**FNGGVS  PHNWGD**S**GPPCKK  EKKQTG**S**GPLGNS  LAPVAD**S**STRVDS  TRVDSP**S**HGLVTS  PSPARL**S**QTPHSQ  LQEVIG**S**GATAVV  LEKCQT**S**MDELLK  KEIQAM**S**QCSHPN  QAMSQC**S**HPNVVT  VVTYYT**S**FVVKDE  LVMKLL**S**GGSMLD  KLLSGG**S**MLDIIK  LLGEDG**S**VQIADF  IADFGV**S**AFLATG  FKADMW**S**FGITAI  SFRKLL**S**LCLQKD  CLQKDP**S**KRPTAA  DEMDEK**S**EEGKAA  EGKAAF**S**QEKSRR  AFSQEK**S**RRVKEE  NPEIAV**S**ASTIPE  EIAVSA**S**TIPEQI  IPEQIQ**S**LSVHDS  EQIQSL**S**VHDSQG  SLSVHD**S**QGPPNA  EDYREA**S**SCAVNL  DYREAS**S**CAVNLV  VLRLRN**S**RKELND  DTADGV**S**QELFSA  VSQELF**S**AGLVDG  LTFKLA**S**GCDGSE  ASGCDG**S**EIPDEV  HLRTLL**S**PQDGAA  MKYGRK**S**YMVKGM  VKGMAF**S**PDSTKI  TKTNKS**S**TIYGTE  TESYVV**S**LTTNCS  SLTTNC**S**GKGILS  DEGSGE**S**QGKLVN  FTTAVS**S**PGGQSV  SSPGGQ**S**VVLGSY  NWIPRR**S**IWEEAK  AWKRDG**S**RLCVGT  VIVKNL**S**SGTRVV  IVKNLS**S**GTRVVL  TRVVLK**S**HYGYEV  YLVAHT**S**ETLLLG  YNIGTV**S**HESRVD  GTVSHE**S**RVDWLE  MQWVPG**S**DVLVAQ  VAQNRN**S**LCVWYN  EIADQV**S**REYGGE  AYLWAK**S**LGGEAA  HAADNC**S**FEFAFE  EAHDPD**S**VAEVLV  KEAGLW**S**DALRIC  YLKVRD**S**GNSGLA  VRDSGN**S**GLAEKC  HLIREG**S**SAQALA  CENLVK**S**SEANSP  ENLVKS**S**EANSPA  KSSEAN**S**PAHEEF  TVAARL**S**VSLLRH  DWVLTV**S**MDQRLE  RGAYEA**S**LVAAST  PARRPL**S**SGSPPL  ARRPLS**S**GSPPLE  RPLSSG**S**PPLEEL  LERQAG**S**EAHLKV  ILLLVP**S**EETDEN  EAMLFT**S**EIFDMY  TLEYFP**S**ELGGLR  GGLRHA**S**ASIGGS  LRHASA**S**IGGSEA  SASIGG**S**EAYRHM  QGRVHT**S**TMTVAI  IDTKRA**S**GAGGQH  HVNTTD**S**AVRIVH  LPTGVV**S**ECQQER  ECQQER**S**QLKNKE  LRAKLY**S**MHLEEE  RKIQIG**S**KGRSEK  IGSKGR**S**EKIRTY  LDELVQ**S**LKEYAD  EYADYE**S**LVEIIS  PKGVLP**S**HYYESF  PSHYYE**S**FLEKKG  TIYFYN**S**NRDFQH  DEIPWG**S**SRDPGT  EIPWGS**S**RDPGTH  DPGTHF**S**LILRDQ  GHLYMM**S**EVLAKE  RALETP**S**CFLKVS  SCFLKV**S**RLEAQL  NLLLRP**S**GDGADG  DGADGV**S**VTTRQM  DVEQPF**S**CTSLDA  QPFSCT**S**LDAVVN  VVNYFV**S**HTKKAL  NVWVAP**S**APGPGP  GGPKPL**S**PASSQD  KPLSPA**S**SQDKLP  PLSPAS**S**QDKLPP  ENQDVA**S**SSWPVI  NQDVAS**S**SWPVIL  QDVASS**S**WPVILK  GRKLPV**S**SAQPLF  RKLPVS**S**AQPLFP  GDDDGS**S**SSGGSS  DDDGSS**S**SGGSSV  GSSVAG**S**QSTLFK  PQLGPS**S**DAHTSY  SWFPPR**S**SLEELH  TGGSES**S**RASGLV  DFLGSS**S**GYSSED  SSSGYS**S**EDDYVG  DQQSSS**S**RLRSAV  AVSRAG**S**LLWMVA  VLTRRF**S**SLKTFL  WWAAKD**S**RRPDEG  VMSRVH**S**LERRLE  ALAAEF**S**SNWQKE  LAAEFS**S**NWQKEA  AEHQQD**S**EDLFKK  KKIVRA**S**QESEAR  RIQQLK**S**EWQSMT  VRDDVE**S**QFPAWI  QFPAWI**S**QFLARG  QALQRY**S**EDRIGL  ADYALE**S**GGASVI  LESGGA**S**VISTRC  GGASVI**S**TRCSET  VISTRC**S**ETYETK  TKTALL**S**LFGIPL  IPLWYH**S**QSPRVI  LWYHSQ**S**PRVILQ  FAVVRL**S**ARIRPT  KALSPN**S**TISSAP  SPNSTI**S**SAPKDF  FDPETN**S**DDACTY  DALKGW**S**CCKRRT  RRTTDF**S**DFLSIV  DFSDFL**S**IVGCTK  TKGRHN**S**EKPPEP  EAIKRP**S**PDEPMT  NLELKI**S**ASLKQA  ELKISA**S**LKQALD  LDKLKL**S**SGNEEN  DKLKLS**S**GNEENK  EIKIGT**S**CKNGGC  CKNGGC**S**KTYQGL  TYQGLE**S**LEEVCV  EVCVYH**S**GVPIFH  EGMKYW**S**CCRRKT  CCRRKT**S**DFNTFL  GGEVTI**S**VYAKNS  SVYAKN**S**LPELSR  NSLPEL**S**RVEANS  SRVEAN**S**TLLNVH  VIDVKR**S**YVTMTA  EPMQWA**S**LELPAA  KVFNNI**S**FLRPVD  EFCPCG**S**LYTVLE  TVLEEP**S**NAYGLP  AYGLPE**S**EFLIVL  IGEDGQ**S**VYKLTD  ATVDLW**S**IGVTFY  YHAATG**S**LPFRPF  IITGKP**S**GAISGV  KPSGAI**S**GVQKAE  SGDMPV**S**CSLSRG  PVSCSL**S**RGLQVL  MVIHVF**S**LQQMTA  HKIYIH**S**YNTATI  KQTKII**S**SNQELI  QTKIIS**S**NQELIY  NPIFVV**S**REPLNT  LIYEKI**S**LPKVHP  DLDGDA**S**MAKAIT  YACRIA**S**TLLLYQ  AELGEI**S**DIHTKL  TKLLRL**S**SSQGTI  KLLRLS**S**SQGTIE  QGTIET**S**LQDIDS  SLQDID**S**RLSPGG  DIDSRL**S**PGGSLA  LRKQLL**S**LTNQCF  DIEEEV**S**KYQEYT  QKMFTA**S**SGIKHT  MTPIYP**S**SNTLVE  TPIYPS**S**NTLVEM  ILERFG**S**LTMDGG  SPTQEP**S**CMATPA  ATPAPP**S**WWDSSQ  PPSWWD**S**SQSSIS  PSWWDS**S**QSSISS  WWDSSQ**S**SISSLG  WDSSQS**S**ISSLGR  SSQSSI**S**SLGRLP  SQSSIS**S**LGRLPS  SLGRLP**S**ISPTAP  IYTFCR**S**RSLRTP  IINLAV**S**DFLMSF  APVFFT**S**SLYKQW  GALFGI**S**SMITLT  ALFGIS**S**MITLTA  ATFGVA**S**KRRAAF  LYALAW**S**LPPFFG  PPFFGW**S**AYVPEG  PEGLLT**S**CSWDYM  CSWDYM**S**FTPAVR  CKGNGE**S**LWQRQR  QRQRLQ**S**ECKMAK  LSWAPY**S**AVALVA  VLTPYM**S**SVPAVI  GVSRRH**S**RPYPSY  HSRPYP**S**YRSTHR  PYPSYR**S**THRSTL  YRSTHR**S**TLTSHT  HRSTLT**S**HTSNLS  TLTSHT**S**NLSWIS  SHTSNL**S**WISIRR  SNLSWI**S**IRRRQE  ESLGSE**S**EVGWTH  QQANGR**S**LYGQGL  QNVLQQ**S**AAQKHQ  FSAVIQ**S**LFNLLE  LNYKPP**S**NAQDLP  LKDAFK**S**NDSQQQ  SQQQDV**S**EFTHKL  SENSGK**S**GQEHWF  VLTFEL**S**RFEFNQ  RLERYL**S**YGSGPK  YALEFA**S**SKPVCT  ALEFAS**S**KPVCTS  DDIDAS**S**PPSGSI  DASSPP**S**GSIPSQ  SSPPSG**S**IPSQTL  SELPST**S**PSSVAA  LPSTSP**S**SVAAIS  SVAAIS**S**RSVIHK  ITEEEL**S**VLESCL  ELSVLE**S**CLHRWR  DLQESI**S**RIHRTI  TIELMY**S**DKSMIQ  DIAVTK**S**SWEELV  GGYRNA**S**AYCLMY  QEKLLA**S**QKLRES  QIITKA**S**HEHEDK  HEHEDK**S**PETVLQ  FGDRNL**S**FDERCH  ENFQRE**S**YIDSLL  NNKELL**S**KGLYRG  MRNRWC**S**YLGQEM  PKLLDC**S**MEIKSF  EPPKLP**S**YSTHEL  PKLPSY**S**THELCE  FARIML**S**LSRTPA  LVEFTN**S**PDCLSK  LLLERG**S**SSYSQL  LERGSS**S**YSQLLA  NLLKQA**S**GKNLNL  SAFLDS**S**TLQLFF  YHSIPP**S**FSPLVL  IASVRR**S**LFNNAE  ERAKFL**S**HLVDGV  LLARLK**S**NYQLGE  ANFTVT**S**LQHWEF  WEFAPN**S**VHYLLS  ITSRLE**S**VHIILR  ELLQSA**S**ASPMDI  VIGGRV**S**FASTDE  GRVSFA**S**TDEQDA  DETMVL**S**VFIGKI  QLLNDL**S**IGYSSV  RKLVKL**S**AVQFML  MLNNHT**S**EHFSFL  LGINNQ**S**NLTDMR  AVAQMF**S**TNSFNE  AFNAKT**S**FMMLFE  LKLKGI**S**ICFSML  GISICF**S**MLKAAL  LLSIPH**S**DLLDYP  VIMYIL**S**SISEGL  IMYILS**S**ISEGLT  YILSSI**S**EGLTAL  VCTGCC**S**CLDHIV  FKQLSR**S**TKKRTT  DCRNQW**S**MSRPLL  RNSIVN**S**QPPEKQ  RREVND**S**MKNSTY  EAAGKD**S**EIFTVN  NGILGE**S**VTFPVN  KIIAWT**S**KTSVAY  AWTSKT**S**VAYVTP  YVTPGD**S**ETAPVV  NYNLVI**S**DLRMED  KPKITQ**S**LMASVN  TQSLMA**S**VNSTCN  LMASVN**S**TCNVTL  NVTLTC**S**VEKEEK  NVTYNW**S**PLGEEG  TAQNPV**S**NNSDSI  NPVSNN**S**DSISAR  VSNNSD**S**ISARQL  NNSDSI**S**ARQLCA  HHTGLL**S**VLAMFF  LLVLIL**S**SVFLFR  LVLILS**S**VFLFRL  RIFPEG**S**CLNTFT  KNPYAA**S**KKTIYT  YTYIMA**S**RNTQPA  NTQPAE**S**RIYDEI  YDEILQ**S**KVLPSK  QSKVLP**S**KEEPVN  PVNTVY**S**EVQFAD  DKMGKA**S**TQDSKP  KASTQD**S**KPPGTS  FPQQGK**S**LNGDVN  TSGILN**S**APHSSS  NSAPHS**S**STSHLH  PSANPG**S**NLKDPP  NGILGG**S**RQPSSP  EDTPIL**S**EDSLEP  KLPLED**S**PVISAL  EDSPVI**S**ALDCPS  SALDCP**S**LNNATA  DSQTST**S**IFASPT  FDLNNG**S**DAEQEE  QLHPAT**S**PAVSPT  AVLPAV**S**LEVPLT  LTASVT**S**PKASPV  VTSPKA**S**PVTSPA  LLCQGD**S**LGEVQD  VHELNG**S**TLIINE  AKRTGR**S**EVEMEG  QPRHLK**S**PVRGQD  PVRGQD**S**EQPQAQ  FLEQEG**S**PLSLGQ  QHDLSQ**S**AFLSWL  SQSAFL**S**WLSQTQ  QTQSHS**S**LLSSSV  VLTPDS**S**PGKLDP  SPQQLA**S**SKPMNR  RRGRPP**S**KFFKQM  EGIMSW**S**PKEKTY  AYCEHL**S**DSQEDI  KRKSGY**S**LNFSEG  SKRRRL**S**MRNHHS  LRGGYT**S**SEEFAA  TFNEDD**S**EVGKAG  EGGGTR**S**EVLLVS  SEVLLV**S**EDGKIL  AEADGL**S**TNHWLI  PLVPLR**S**LGLSLS  LRSLGL**S**LSGGDQ  SLGLSL**S**GGDQED  DRFPYL**S**ESYLIT  FPYLSE**S**YLITTD  TTDAAG**S**IATATP  GGVVLI**S**GTGSNC  LISGTG**S**NCRLIN  LINPDG**S**ESGCGG  NPDGSE**S**GCGGWG  MMGDEG**S**AYWIAH  VKIVFD**S**IDNLEA  QQGDPL**S**RYIFRK  PILCVG**S**VWKSWE  VGSVWK**S**WELLKE  QAQNFF**S**SFTLMK  AQNFFS**S**FTLMKL  LMKLRH**S**SALGGA  MKLRHS**S**ALGGAS  SALGGA**S**LGARHI  LLPMDY**S**ANAIAF  AEFFSI**S**EFLEHK  VLIMND**S**EGPVPS  SEGPVP**S**EDFSGA  LSHQPT**S**PGSKDC  QPTSPG**S**KDCHRE  EKPDAE**S**VVYLKT  PVPGAN**S**IPWVLE  TLGSHM**S**QQVSAA  HMSQQV**S**AAVALL  LSQKAG**S**QGLSLD  AGSQGL**S**LDALKQ  PHANIP**S**ATSSLS  NIPSAT**S**SLSPGL  LFQSPF**S**TVALDT  TVALDT**S**KKGKGK  DPIDEP**S**LSLDSK  IDEPSL**S**LDSKPV  PGPSPE**S**EGGPTL  VGPNYN**S**PRAGGF  VLSCQS**S**LKMHYR  ICGRAF**S**TKGNLK  VHRTNT**S**IKTQHS  QAESIK**S**KSPDAG  AGSKAE**S**SENSRT  AESSEN**S**RTEMEG  GTFVGP**S**TLSPGM  CGKNFS**S**ASALQI  KNFSSA**S**ALQIHE  HGANNN**S**ARRGRK  VWNQYT**S**MLNGGL  ADVEKP**S**ATDGVP  AVAPVL**S**INSDFS  PVLSIN**S**DFSDLR  SINSDF**S**DLREIK  ERGLLH**S**SKWSAE  RGLLHS**S**KWSAEL  LHSSKW**S**AELAFS  SAELAF**S**LPALPL  FLHGCN**S**KKAYFL  YFLYMY**S**RYLSGE  MYSRYL**S**GEKKKD  DDETVD**S**LGPLEK  ELRVEL**S**KKHQAR  EMLKFL**S**LPDTWM  LIDVGF**S**KSSYIV  DVGFSK**S**SYIVSQ  VGFSKS**S**YIVSQI  KSSYIV**S**QIAVAY  DIDKAL**S**IFNELR  ENMDTF**S**NLLYVR  NLLYVR**S**MKSELS  YVRSMK**S**ELSYLA  SMKSEL**S**YLAHNL  VIGNYY**S**LRSQHE  NYYSLR**S**QHEKAA  MEMKNT**S**AAIQAY  QLRPND**S**RMLVAL  HEQLTE**S**EQAAQC  YIQDIY**S**CGEIVE  VEHLEE**S**TAFRYL  KLWDEA**S**TCAQKC  PFFLPA**S**LSANNT  FLPASL**S**ANNTPT  TPTRRV**S**PLNLSS  VLLNEM**S**RTGEGA  TGEGAV**S**LMERRR  YKLIEE**S**CPQLAN  CPQLAN**S**VQIRIK  FDDLSD**S**FSGTEP  DLSDSF**S**GTEPEV  PEVHKT**S**VVGLFL  LAYSKL**S**FSQVFK  YSKLSF**S**QVFKLY  ADMELT**S**RDEGER  EEEVSC**S**GPLSQK  SCSGPL**S**QKQAEF  QAEFFL**S**QQASLL  KALTPA**S**LQKELN  VFSSTH**S**LLHYFD  ILTGAE**S**KSNGEE  TGAESK**S**NGEEGY  EEGYGR**S**LRYAAL  IRIAQE**S**NDHVCL  GLPYLA**S**LGIQSL  MDALKD**S**DLLHWK  SELIDI**S**IAQKTA  WRLYGR**S**TMALQQ  GCFAAA**S**EVLKHL  ERFPPN**S**QHAQLW  QAQNQM**S**EAHKLL  NTEMVI**S**VLLSVA  VISVLL**S**VAELYW  RLQYLA**S**ETVLNL  IPEQAL**S**LLHMAI  LHQELP**S**HGVPLI  SFHRVW**S**FPPNEN  KPESLH**S**FSVEAP  MEVTVE**S**SVLTSF  EVTVES**S**VLTSFY  ESSVLT**S**FYNAED  KNYSNT**S**KIFSEE  IFSEEN**S**DEIIKL  ILVLGG**S**SGFIEL  LALCLS**S**DLKSLS  SDLKSL**S**VVTEVS  ETNLLY**S**FLPEVT  RKFTHI**S**ALLQYI  LQYINL**S**LTCMCE  ILMQMD**S**RLTKFV  LKKLGQ**S**IESSYS  LQVIDS**S**MKNFKA  EDDDLV**S**PPNTEG  YDFLQN**S**SHLKES  DFLQNS**S**HLKESP  PYYPRK**S**LHFVKR  LYRDTR**S**EDSTRR  DTRSED**S**TRRLFK  HTDISQ**S**VSNGLI  DISQSV**S**NGLIAI  IAIKFG**S**FTYATT  LVQLPL**S**LVYNSE  VYNSED**S**AEYQFT  QFTGTY**S**TRLDEQ  RLDEQC**S**AIPTRT  HWRLLE**S**MKAQYV  VSCVLS**S**NLRHVR  AEGDSD**S**RPGQEL  VAWNTV**S**TGLVPP  LVSSRT**S**GAVPPK  EFWNAI**S**QCENSA  ISQCEN**S**ADEPQC  LDPYLR**S**LELLEK  RGVLFF**S**TPRTFQ  LEGELD**S**RYARRR  PLCAGC**S**SDKQQC  QVLHRL**S**LLERVS  FYRIYA**S**LRIEEL  RQQLLV**S**LKAALE  IITLYI**S**AIKALR  LRVLDP**S**MVILEV  AGLTGD**S**DGTGDL  QDSEDD**S**GEPEDW  DADPGK**S**SSKRRS  ADPGKS**S**SKRRSS  SSSKRR**S**SDIISL  SSKRRS**S**DIISLL  RSSDII**S**LLVSIY  IISLLV**S**IYGSKD  FINEYR**S**LLADRL  VYAVIL**S**SEFWPP  YFQDQA**S**WTLEEL  EPPGTF**S**VIEEER  NMVLID**S**DDESDS  SDDESD**S**GMASQA  SDSGMA**S**QADQKE  TNLESL**S**LDRIYN  DQQLVY**S**AGVYRL  DGQMPF**S**CHYPSR  FSCHYP**S**RLRRDP  RDPFRD**S**PLSSRL  FRDSPL**S**SRLLDD  RDSPLS**S**RLLDDG  PDDLTA**S**WPDWAL  WALPRL**S**SAWPGT  ALPRLS**S**AWPGTL  WPGTLR**S**GMVPRG  VCVNVH**S**FKPEEL  DGYVEV**S**GKHEEK  QEGGIV**S**KNFTKK  PVTVFA**S**LSPEGL  TVFASL**S**PEGLLI  PQVPPY**S**TFGESS  YSTFGE**S**SFNNEL  STFGES**S**FNNELP  RGSLLA**S**GRAPRR  RAPRRA**S**SGLPRN  APRRAS**S**GLPRNT  RIRYVQ**S**LKEIVI  INVPEQ**S**AVTLDN  MDPYKA**S**YGVEDP  AQTTMR**S**ELGKLS  SELGKL**S**LDKVFR  VFRERE**S**LNASIV  RESLNA**S**IVDAIN  PPRVKE**S**MQMQVE  RATVLE**S**EGTRES  SEGTRE**S**AINVAE  QAQILA**S**EAEKAE  QAAGEA**S**AVLAKA  NGDAAA**S**LTVAEQ  VAEQYV**S**AFSKLA  QYVSAF**S**KLAKDS  SKLAKD**S**NTILLP  NTILLP**S**NPGDVT  NPGDVT**S**MVAQAM  GTPDSL**S**SGSSRD  TPDSLS**S**GSSRDV  DSLSSG**S**SRDVQG  SLSSGS**S**RDVQGT  VQGTDA**S**LDEELD  FSGGGA**S**SGEVNG  SGGGAS**S**GEVNGL  EEGEAD**S**CHDEGV  KVLGQG**S**FGKVFL  KVLKKA**S**LKVRDR  LTDFGL**S**KESVDQ  FGLSKE**S**VDQEKK  VNRRGH**S**QSADWW  RRGHSQ**S**ADWWSY  QSADWW**S**YGVLMF  GMPQFL**S**AEAQSL  PANRLG**S**EGVEEI  PPFKPA**S**GKPDDT  SPGLPA**S**ANAHQL  FSFVAT**S**IAEEYK  EDIGVG**S**YSVCKR  IGVGSY**S**VCKRCI  VKIIDK**S**KRDPSE  KSKRDP**S**EEIEIL  LKQKCF**S**EREASD  FSEREA**S**DILYVI  DILYVI**S**KTVDYL  HRDLKP**S**NILYMD  ILYMDE**S**ASADSI  IGNGKF**S**LSGGNW  NGKFSL**S**GGNWDN  PKRNDV**S**HVVKGA  AMVATY**S**ALTHKT  LEPVAA**S**SLAQRR  EPVAAS**S**LAQRRS  SLAQRR**S**MKKRTS  VPSLLL**S**VLLAQV  VPGLAP**S**PQSPET  LAPSPQ**S**PETPAP  APQNQT**S**RVVQAP  ASEEKA**S**EEEKAW  KAWLMA**S**RQQLAK  QLAKET**S**NFGFSL  TSNFGF**S**LLRKIS  SLLRKI**S**MRHDGN  DGNMVF**S**PFGMSL  FSPFGM**S**LAMTGL  KPGLLP**S**LFKGLR  GLRETL**S**RNLELG  LGLTQG**S**FAFIHK  ETFFNL**S**KRYFDT  MNFRNA**S**QAKRLM  GAGKFA**S**TFDKNF  GIRRIF**S**PFADLS  SPFADL**S**ELSATG  ADLSEL**S**ATGRNL  GRNLQV**S**RVLQRT  AVAGIL**S**EITAYS  SEITAY**S**MPPVIK  MIYEET**S**GMLLFL  KGVDPN**S**RNSSRV  DPNSRN**S**SRVLRP  PNSRNS**S**RVLRPP  RPPGGG**S**NFSLGF  GGGSNF**S**LGFDEP  RKNKMA**S**NIFGTP  PEENQA**S**WAKSAG  QASWAK**S**AGAKSS  KSAGAK**S**SGGRED  SAGAKS**S**GGREDL  GREDLE**S**SGLQRR  RNSSEA**S**SGDFLD  NSSEAS**S**GDFLDL  DTDLPG**S**LGQSEE  PGSLGQ**S**EEKPVP  APAPVP**S**RRNPPG  DSPQTR**S**LLGVFE  EDATAI**S**NYMNQL  DAQNEL**S**AATHLT  STLQQF**S**KVIDEL  VIDELS**S**CHAVLS  SCHAVL**S**TQLADA  EVFQIA**S**NDHDAA  AAINRY**S**RLSKKR  TEDVYT**S**RKKQHQ  YMQAQI**S**FFKMGS  SFFKMG**S**ENLNEQ  MDIDNC**S**VMAVDC  SFDGKK**S**SILQAE  FDGKKS**S**ILQAES  SKQIYL**S**ENPEET  RPAAGQ**S**RPPTAR  RTSSSG**S**LGSEST  AALSLD**S**LVAPDT  SGGSTK**S**ETEDSI  GSMEVK**S**DDHPDV  IFRMTE**S**HLLVTC  GFVLRT**S**SGRSES  SSGRSE**S**NLSSVC  SESNLS**S**VCYIFE  ELDRRA**S**EKQKEI  KDLEEQ**S**RLIAAS  SRLIAA**S**SRPNQA  RLIAAS**S**RPNQAS  RPNQAS**S**EGQFVV  FVVLSS**S**QSEESD  VLSSSQ**S**EESDLG  VQSSSG**S**LEGPPS  SLEGPP**S**WSQLST  EGPPSW**S**QLSTSP  TSPTPG**S**AAAARS  SAAAAR**S**LLNHTP  RFTGVA**S**GSTGST  ASGSTG**S**TGSCSV  SVGAKA**S**TNNESS  STNNES**S**NHSFGS  NESSNH**S**FGSLGS  SNHSFG**S**LGSLSD  SFGSLG**S**LSDKES  GSLGSL**S**DKESET  ENQNES**S**QGKSIG  HSHSTP**S**SSVRPN  HSTPSS**S**VRPNSP  SSVRPN**S**PSPTAL  VRPNSP**S**PTALAF  VQPKQL**S**FKIIQT  KLAALE**S**NKIQDL  RLNKCI**S**MSKKLL  LSSREK**S**MQDRLR  TVRHGA**S**FTEQWT  INNEDN**S**QFKDHP  LGRGGF**S**EVYKAF  KLYDYF**S**LDTDTF  DGMDLT**S**QGAGTY  KEPPKI**S**NKVDVW  PFGHNQ**S**QQDILQ  VKPVVS**S**EAKAFI  MAGLTA**S**PTPPSS  SLKLQA**S**NVTNKN  NKNDPK**S**INSRVF  DPKSIN**S**RVFIGN  TALVKK**S**DVETIF  DVETIF**S**KYGRVA  GRVAGC**S**VHKGYA  YAFVQY**S**NERHAR  AASAIY**S**GYIFDY  VKLFAR**S**TAVTTS  STAVTT**S**SAKIKL  TAVTTS**S**AKIKLK  AKIKLK**S**SELQAI  KIKLKS**S**ELQAIK  ELTQIK**S**NIDALL  NIDALL**S**RLEQIA  GGGGGG**S**GGGGSG  GSGGGG**S**GGGGGG  GGGGGG**S**SRPPAP  GGGGGS**S**RPPAPQ  AGSEPR**S**GTNSPP  LEAAIL**S**GWKTFW  WKTFWQ**S**VSKERV  TFWQSV**S**KERVAR  VQLYIL**S**FLSPHD  YILSFL**S**PHDLCQ  DLCQLG**S**TNHYWN  LLRDLP**S**WSSVDW  RDLPSW**S**SVDWKS  DLPSWS**S**VDWKSL  SSVDWK**S**LPDLEI  ILKKPI**S**EVTDGA  PYTRRA**S**KSSRPM  TRRASK**S**SRPMYG  RRASKS**S**RPMYGA  MYGAVT**S**FLHSLI  VTSFLH**S**LIIQNE  LEELNT**S**LVLSLM  NTSLVL**S**LMSSEE  LVLSLM**S**SEELCP  VLSLMS**S**EELCPT  QIDGIG**S**GVNFQL  NILILY**S**TTRKER  AREEHT**S**AVNKMF  AVNKMF**S**RHNEGD  QQGSRY**S**VIPQIQ  EWQDEF**S**HIMAMT  TDPAFG**S**SGRPLL  DPAFGS**S**GRPLLV  RPLLVL**S**CISQGD  LVLSCI**S**QGDVKR  RGVEYG**S**GRGHLG  AVGTDP**S**TSRKMA  AKSGQK**S**ALVKRL  AAFQPN**S**QIGEEM  LELSRH**S**PRKSSS  HSPRKS**S**SISEEK  GGRFTR**S**QEEARK  QEEARK**S**HLARQQ  SHTLLP**S**HRLKQS  DSSSSR**S**SSSSSS  SSSSRS**S**SSSSSS  RSRSPD**S**SGSRSH  SPDSSG**S**RSHSPL  SGSRSH**S**PLRSKQ  SESRSR**S**RSRSRS  SRSRSR**S**RSRSAS  SRSRSR**S**RSASSN  TETKDP**S**SGQEVA  ETKDPS**S**GQEVAT  RTSTSS**S**SVQARR  TSTSSS**S**VQARRL  ATQPQT**S**ETQTSH  EVTMDT**S**ENRPEN  ESSLPK**S**FKRKIS  TTESLK**S**LIPDIK  PPRGKI**S**NIVHIS  WIDKIK**S**HCFVTY  WLPLTD**S**QIVQKE  RDTKRH**S**RSRSRS  TKRHSR**S**RSRSTP  RHSRSR**S**RSTPVR  RPDFGT**S**GRTIKL  VSIKWV**S**CVSLQA  KWVSCV**S**LQALHD  VMRHLP**S**MRYTPV  YTPVGR**S**FFTASE  TASEGC**S**NPLGGG  HQSVRP**S**LWKMML  MLNIDV**S**ATAFYK  EVLDFK**S**IEEQQK  QKPLTD**S**QRVKFT  VTRRPA**S**HQTFPL  LTDNQT**S**TMIRAT  IRATAR**S**APDRQE  DRQEEI**S**KLMRSA  RVLQPP**S**ILYGGR  TEVHLK**S**FTEQLR  EQLRKI**S**RDAGMP  YAQGAD**S**VEPMFR  TTPQTL**S**NLCLKI  GDGKKP**S**IAAVVG  IAAVVG**S**MDAHPN  LIQFYK**S**TRFKPT  NERVGK**S**GNIPAG  FDFYLC**S**HAGIQG  AGIQGT**S**RPSHYH  QGTSRP**S**HYHVLW  WDDNRF**S**SDELQI  DDNRFS**S**DELQIL  YVRCTR**S**VSIPAP  RCTRSV**S**IPAPAY  HDSAEG**S**HTSGQS  AEGSHT**S**GQSNGR  SHTSGQ**S**NGRDHQ  AAGRAY**S**FKVVLL  GCVGKT**S**LVLRYC  ITTLQA**S**FLTKKL  PIYYRD**S**NGAILV  DITDED**S**FQKVKN  EKERHV**S**IQEAES  SIQEAE**S**YAESVG  AESYAE**S**VGAKHY  AKHYHT**S**AKQNKG  RAKGNG**S**SQPGTA  AKGNGS**S**QPGTAR  EPQAQT**S**GGGCCS  DWCRIM**S**VDEQKS  SVDEQK**S**LMVTGI  LQETLK**S**LGRYRL  LEDTDV**S**AIPSEV  DVSAIP**S**EVQGKG  KEGQTV**S**GMFRAL  LGQEGV**S**PATVPC  ATVPCI**S**PELLAH  RKLRVF**S**GSAVPA  LRVFSG**S**AVPAPE  PAPEEE**S**FEVWLE  KRWLAE**S**LRGPAL  VQADNP**S**ISVEEC  ADNPSI**S**VEECLE  FKQVFG**S**LESRRT  VFGSLE**S**RRTAQV  EEGEKV**S**AYVLRL  DQGPPP**S**FLELMK  EEEEEA**S**FENESI  ASFENE**S**IEEPEE  PCGVRL**S**GEARKQ  QLLQED**S**LNVADL  NVADLT**S**LRAPLD  GNEKVL**S**LLALVK  AFQTTI**S**KYFSER  TISKYF**S**ERGDAV  DAVAKA**S**KETHVM  ELYHII**S**SNLEKI  LYHIIS**S**NLEKIV  DPEKLF**S**DLREIG  SYSGKQ**S**NEKWQD  MEYCLG**S**ASDLLE  GLAYLH**S**HNMIHR  KLGDFG**S**ASIMAP  GKVDVW**S**LGITCI  FRNFVD**S**CLQKIP  RAGTLT**S**LESSHS  TLTSLE**S**SHSVPS  LTSLES**S**HSVPSM  SSHSVP**S**MSISAS  MSISAS**S**QSSSVN  ASSQSS**S**VNSLAD  GEHTVT**S**HSSIIH  IHRLPG**S**DNLYDD  AAPAPT**S**TTSSAR  APTSTT**S**SARRRA  QEHEQD**S**ALREQL  ALREQL**S**GYKRMR  GEREEH**S**ARLQRE  ELQENP**S**TPKREK  SPQKHG**S**LVDEEV  PGDGCP**S**PDIPPE  FAVGSS**S**GLLPLL  LVGLGA**S**YLLLCT  TALHLP**S**SLFLLL  ALHLPS**S**LFLLLA  RVRRGI**S**RLWLRV  RVLLRL**S**PMAFRA  QLGPPA**S**RQPLPG  TLAGRR**S**RTRQSR  DEKENV**S**NCIQLK  CIQLKT**S**VIKGIK  AIKFVL**S**GANIMC  VGVMKM**S**AEDIEK  PTEPGP**S**PVPAFL  DHLIRW**S**PSGTSF  LIRWSP**S**GTSFLV  WSPSGT**S**FLVSDQ  GTSFLV**S**DQSRFA  FLVSDQ**S**RFAKEV  PQYFKH**S**NMASFV  GFRKVV**S**IEQGGL  VEFQHP**S**FVRGRE  LRGVQE**S**TEARLR  VVTLRQ**S**HGQQHR  PLQAGP**S**NAGGKR  GGKRKL**S**LMLDEG  LMLDEG**S**SCPTPA  MLDEGS**S**CPTPAK  ETNLGL**S**PHRARG  ARGPII**S**DIPEDS  SDIPED**S**PSPEGT  IPEDSP**S**PEGTRL  PEGTRL**S**PSSDGR  GTRLSP**S**SDGRRE  TRLSPS**S**DGRREK  ILEGKG**S**FSPEGP  EGKGSF**S**PEGPRN  GPLGLE**S**GDRSPE  GDRSPE**S**LLPPML  LDVLGP**S**LQGREW  AVKGLN**S**PSPGKD  GALTIY**S**TPESRT  IYSTPE**S**RTASYL  PESRTA**S**YLGPEA  CVDPRL**S**VSPPSL  DPRLSV**S**PPSLRD  LSVSPP**S**LRDLLP  LREVAH**S**RRAAGG  PGGPGG**S**GDFLLI  ANLEAK**S**RQVAAL  PPRGRR**S**ANDELF  ELFRAG**S**RLRRQL  KLAIIF**S**HMHAEL  HTFWRE**S**CGARCV  PWAEFE**S**LLGTCH  TIDLTC**S**GHVSIF  TCSGHV**S**IFEFDV  CRDKPG**S**YIFRPS  SYIFRP**S**CTRLGQ  WAIGYV**S**SDGSIL  AIGYVS**S**DGSILQ  YVSSDG**S**ILQTIP  PANKPL**S**QVLLEG  QQRIHV**S**EEQLQL  LYWAMD**S**TFELCK  CKICAE**S**NKDVKI  CGHLLC**S**CCLAAW  LAAWQH**S**DSQTCP  AWQHSD**S**QTCPFC  KGWEAV**S**IYQFHG  QATAED**S**GNSSDQ  AEDSGN**S**SDQEGR  EDSGNS**S**DQEGRE  LGQVPL**S**APPLPP  RLLKGN**S**PPAALG  TQLDIC**S**SAPEDC  QLDICS**S**APEDCT  APEDCT**S**FSINAS  EDCTSF**S**INASPG  PPAKKK**S**TGSSTW  KKKSTG**S**STWPLD  KKSTGS**S**TWPLDP  MKVASG**S**TGDQKV  DQKVQI**S**YYGPKT  LTGVEI**S**LCADIT  DRDNLE**S**SAMDCE  RDNLES**S**AMDCED  DDEVLD**S**EDLQDM  VLHVAR**S**EMDKVR  TRGKLS**S**KCSVVL  KLSSKC**S**VVLGPK  LGPKWP**S**HYLMVP  LITLTI**S**LLDTSN  ISLLDT**S**NLELPE  AVVFQD**S**VVFRVA  NEDFLK**S**VTTLAM  PQTGGI**S**GLDSFG  FGNLEV**S**PPVTVR  CYPSND**S**RQMHQA  ALQDFL**S**AQQVQA  APVKLY**S**DWLSVG  LYSDWL**S**VGHVDE  HVDEFL**S**FVPAPD  FRLLLA**S**PRSCYK  TLREHN**S**FVERCI  FKLKEF**S**KAEAFF  LEEKVC**S**LLEPLG  VRRKPF**S**FKWWNM  RTLTPA**S**SPVSSP  SPVSSP**S**KHGDRF  GDRFIP**S**RAGANW  KAKDAT**S**DNGKDG  KDGLAY**S**ALLKNE  DRRLQP**S**TPEKKG  KGLFTY**S**LSTKRS  LFTYSL**S**TKRSSP  SLSTKR**S**SPDDGN  DDGNDV**S**PYSLSP  VSPYSL**S**PVSNKS  YSLSPV**S**NKSQKL  KPTRKI**S**KIPFKV  LNLVDW**S**SLNVLS  NLVDWS**S**LNVLSV  SSLNVL**S**VGLGTC  TCVYLW**S**ACTSQV  TRLCDL**S**VEGDSV  LSVEGD**S**VTSVGW  EGDSVT**S**VGWSER  VTSVGW**S**ERGNLV  NAEQLS**S**GSRDRM  EQLSSG**S**RDRMIL  DHQLLA**S**GGNDNK  LVWNHS**S**LSPVQQ  WNHSSL**S**PVQQYT  VKAIAW**S**PHQHGL  QCIDTG**S**QVCNLA  VSTHGY**S**QNQILV  AKLTGH**S**YRVLYL  VFSKTR**S**TKVKWE  TKVKWE**S**VSVLNL  VKWESV**S**VLNLFT  ALAFIT**S**FLTLSW  TSFLTL**S**WYTTWQ  IAEHRI**S**QRSSEL  HRISQR**S**SELNTI  RISQRS**S**ELNTIV  GAETNG**S**KDALNK  DALNKF**S**DNTLKL  LLKELT**S**KKSLQV  ELTSKK**S**LQVPSI  KSLQVP**S**IYYHLP  LLKNEG**S**LQPAVQ  NGRTGV**S**IVMGIP  VKREVK**S**YLIETL  LIETLH**S**LIDNLY  NLEKEF**S**KEISSG  EFSKEI**S**SGLVEV  FSKEIS**S**GLVEVI  KETFGD**S**KERVRW  NFALQL**S**SEEWMI  FALQLS**S**EEWMIL  WMILEF**S**QLGFIG  RIRFRP**S**LFQHVG  QHVGLH**S**SLSGKI  HVGLHS**S**LSGKIQ  NPPAEV**S**TSLKVY  KPVNVE**S**YLFHSG  EVLPFK**S**EGLEIS  EGMVDP**S**LNPISA  PSLNPI**S**AFRLSV  ISAFRL**S**VIQNSA  LSVIQN**S**AVWAIL  LLPPSS**S**ELKAGR  GPAPGV**S**WTAGSP  AEARTL**S**RVLKGG  RVLKGG**S**VRKLRR  LLQFNL**S**ELFSWW  GHSSLE**S**PTNMPS  SPTNMP**S**PSPDYF  DSFPFL**S**HRSRYG  LNTSAD**S**KHTILS  LSPWMR**S**SSEHCT  HCTLAV**S**VHRHLQ  HRHLQP**S**GRYIAQ  FALKNC**S**EGTSPG  NCSEGT**S**PGSKMA  EGTSPG**S**KMALQS  DHALLL**S**TTDVPA  ESATVT**S**ATFPAP  PAPIKS**S**PCELRM  VLRGNV**S**LVLVEN  AAYEGL**S**LWQWMV  VAFDNI**S**ISLDCY  FDNISI**S**LDCYLT  YQNSNL**S**VEVGSE  KNTMMR**S**HGVSVL  GWNDNT**S**LLWAGK  DGEDGV**S**FISPLG  KHYLNC**S**HCEVDE  PLSLIL**S**VVTSAL  VRCIGV**S**LQSLPR  SKTDTW**S**FGVLLW  PPLPTT**S**SGKAAK  SQSNPP**S**ELHKVH  WNPTYG**S**WFTEKP  QSLTHG**S**SGYINS  PRKEFA**S**SSTRGR  IRVRLQ**S**ASPSTR  LQSASP**S**TRWTEL  LLKGSR**S**ASVSPT  SRSASV**S**PTRNSS  KIVTAS**S**QSVSGT  ASSQSV**S**GTYDAT  NAYSAG**S**VFGVPN  ASIAAT**S**FSEDTL  IAATSF**S**EDTLKK  AWCPCG**S**CCSWWK  DKIGLH**S**DSQEEL  EAGPPG**S**GEKGER  SVGPKG**S**SGSPGP  RGPPGP**S**GDPGKP  PGKIVT**S**EGSSML  GALGIP**S**GPSEGG  PPGSIS**S**SGQEIQ  PGSISS**S**GQEIQQ  ISEYMQ**S**DSIRSY  LASHVV**S**YLRTSG  YGVSLF**S**SSISSE  PGLPGT**S**YEELLS  PGNVWS**S**ISVEDL  NVWSSI**S**VEDLSS  ISVEDL**S**SYLHTA  AAENSD**S**FRSELI  FRSELI**S**YLTSPD  SRGSSS**S**SHSSSV  SVRRGS**S**YSSSMS  SLICSI**S**NEVPEH  PEHPCV**S**PVSNHV  IRPKPP**S**ATSIPA  DAVMLH**S**FTLRQQ  TTRQEL**S**HALYQH  VPQAVP**S**SQPSVV  PQAVPS**S**QPSVVG  VKPEEL**S**KYRQVA  SHVGLH**S**ASIPGI  VGLHSA**S**IPGILA  ALDLCP**S**DTNKIL  VVFDKS**S**EQILAT  HTKKVT**S**VVFHPS  SVVFHP**S**QDLVFS  SQDLVF**S**ASPDAT  DLVFSA**S**PDATIR  ATIRIW**S**VPNASC  WSVPNA**S**CVQVVR  VVRAHE**S**AVTGLS  SAVTGL**S**LHATGD  TGDYLL**S**SSDDQY  GDYLLS**S**SDDQYW  DYLLSS**S**DDQYWA  DQYWAF**S**DIQTGR  KVTDET**S**GCSLTC  GTGTMD**S**QIKIWD  ANFPGH**S**GPITSI  HSGPIT**S**IAFSEN  ITSIAF**S**ENGYYL  ATAADD**S**SVKLWD  TAADDS**S**VKLWDL  LHFTEH**S**GLTTGV  HAKFIA**S**TGMDRS  LELDGQ**S**VKAHFF  CQLEME**S**YDEAIA  NLQRAY**S**LAKEQR  FGDDIP**S**ALRIAK  KKKRWN**S**IEERRI  RRIHQE**S**ELHSYL  QESELH**S**YLSRLI  ELHSYL**S**RLIAAE  EGDEDD**S**HVRAQQ  DMDELF**S**QVDEKR  YLCGKI**S**FELMRE  EPCITP**S**GITYDR  VIDAFI**S**ENGWVE  RFQAEA**S**EKDSSS  EASEKD**S**SSMMQT  ASEKDS**S**SMMQTL  SEKDSS**S**MMQTLL  PETPKA**S**KALEVS  SEDVKV**S**KASGVS  SKASGV**S**KATEVS  SKATEV**S**KTPEAR  APATQA**S**STTQLT  LAAENK**S**LAADTK  TETKKV**S**HVADTK  ESAAAQ**S**QENQDT  GEEDGS**S**DQSQAS  DGSSDQ**S**QASGTT  SDQSQA**S**GTTGGR  TGGRRV**S**KALMAS  SKALMA**S**MARRAS  ALLSLR**S**PKARRG  RAAKLQ**S**SQEPEA  KIPIKR**S**DMLKDI  IERAGY**S**LEKVFG  HLYILL**S**TLEPTD  LLMVLL**S**IIFMNG  RPGIHH**S**LFGDVK  YARVPN**S**NPPEYE  MGIGLG**S**ENAAGP  KAKAQE**S**GSASTG  KAQESG**S**ASTGAS  SASTGA**S**TSTNNS  STGAST**S**TNNSAS  STSTNN**S**ASASAS  NNSASA**S**ASTSGG  ASTSGS**S**GACGFS  LEHYLD**S**IENLPF  LATEYM**S**SARSLS  ATEYMS**S**ARSLSS  YMSSAR**S**LSSEEK  SSARSL**S**SEEKLA  SARSLS**S**EEKLAL  KEKQIE**S**SDYDSS  EKQIES**S**DYDSSS  ESSDYD**S**SSSKGK  SSDYDS**S**SSKGKK  SDYDSS**S**SKGKKK  DYDSSS**S**KGKKKG  KAARAR**S**KGKNSD  RSKGKN**S**DEEAPK  LKLVRT**S**PEYGMP  PEYGMP**S**VTFGSV  PSVTFG**S**VHPSDV  FGSVHP**S**DVLDMP  CLCHQV**S**YGEMIG  CDNPDC**S**IEWFHF  LEQFVN**S**VRQLSA  NSVRQL**S**AQGQMT  CELINK**S**GELLAK  LLAKNL**S**HLDTVL  LDVQEH**S**LGVLAV  VLFVKF**S**MPSVPD  VKFSMP**S**VPDFET  DFETLF**S**QVQLFI  QVQLFI**S**TCNGEH  NTNQLT**S**IHADLC  TPAMAV**S**HIMLES  SHIMLE**S**YKKYIL  KKYILV**S**LILLGK  QLPKYT**S**QIVGRF  RFIKPL**S**NAYHEL  ELAQVY**S**TNNPSE  YSTNNP**S**ELRNLV  NLVNKH**S**ETFTRD  LVKQCL**S**SLYKKN  VKQCLS**S**LYKKNI  KTFLTL**S**LQDMAS  SLQDMA**S**RVQLSG  ASRVQL**S**GPQEAE  DGEIFA**S**INQKDG  QKDGMV**S**FHDNPE  PQFVQK**S**MGSQED  GSQEDD**S**GNKPSS  VVAVLG**S**GGGLRA  ACLGVL**S**EMKEQG  TYLAGV**S**GSTWAI  LAGVSG**S**TWAISS  GSTWAI**S**SLYTND  STWAIS**S**LYTNDG  EWDLAK**S**LQKTIQ  TIQAAR**S**ENYSLT  ARSENY**S**LTDFWA  WAYMVI**S**KQTREL  TRELPE**S**HLSNMK  LPESHL**S**NMKKPV  DNDLQP**S**WQEARA  PHHAGF**S**ALGAFV  ALGAFV**S**ITHFGS  SITHFG**S**KFKKGR  LRGLWG**S**ALGNTE  AVANAK**S**IGHLIF  LLRLQE**S**SQGEHP  LRLQES**S**QGEHPP  ENWTRT**S**LEKQEQ  DPERKG**S**LSNLMD  ERKGSL**S**NLMDFV  KTGICA**S**KWEWGT  IRDKIM**S**SRKHLH  RDKIMS**S**RKHLHL  EVHLIL**S**FDFSAG  ILSFDF**S**AGDPFE  AELDLW**S**KAPASC  WSKAPA**S**CYILKG  GDIEAW**S**DTYDTF  FNSELY**S**LNDYKP  DYKPPI**S**KAKMTQ  VFAPRF**S**NNIIST  VTPVLA**S**TTTAMS  SQLSHV**S**ESVNNS  QEGTFG**S**EHSASP  GSEHSA**S**PSQGSS  EHSASP**S**QGSSQQ  VAVRSR**S**RTHSRS  SRSRSR**S**PRKRRS  RKRRSR**S**RSGSRK  RRSRSR**S**GSRKRK  HRKRSR**S**RSRERK  ERKRKS**S**RSYSSE  IRSKTL**S**VCSTTL  RALQKL**S**SGSYKI  WETVKS**S**EPVKET  PAVPTV**S**LVPPAF  PPAFPV**S**MPVPPP  PPVVPT**S**LVQPSL  TSLVQP**S**LSMTPE  LVIPGG**S**VASNLA  ASNLAT**S**ALPAGN  LIDHQI**S**SGENTR  SGENTR**S**VIPNDI  VQRPNV**S**SNSEIL  PNVSSN**S**EILGVR  SNVSSS**S**GIIAAQ  IQPPSV**S**NSSGLL  PPSVSN**S**SGLLGV  RGIPPP**S**VLDSAL  ERGRFR**S**GNYRFD  YEKLTS**S**NEINKE  GRAKMA**S**VPVYCL  EVLHGP**S**IMKKRR  MKKRRG**S**SKGHDT  FVRELR**S**RTFDSS  RSRTFD**S**SDEVIL  SRTFDS**S**DEVILK  VEHYVG**S**DKEIDV  KVLNVI**S**LEFSDT  VISLEF**S**DTRLSN  LMSVRD**S**YTDFHI  GGNFLH**S**LNIEMQ  LTRPAH**S**TSVSMS  RKGKES**S**ALGPAG  NLMDTY**S**HQALKT  GACLND**S**DDDSPD  NDSDDD**S**PDLDLD  DLDGNE**S**PLALLM  PLALLM**S**NGSTKR  STKRVK**S**LSKSRR  LDLLKA**S**RQVGGP  LTEAPA**S**PSTQEA  SSPATS**S**LQAWWT  GGQDRS**S**GSSSSG  QDRSSG**S**SSSGLG  SGLGTV**S**NSPASQ  EEEENA**S**LDEQDS  PKKKKN**S**DDAPWS  EGTRVA**S**IETGLA  QPRPQD**S**NLSLTV  QLVTSS**S**PLPPPE  LTQRRP**S**VGSQSN  YQDDYC**S**GSVMSE  VMSERV**S**GLAGSI  VSGLAG**S**IYREFE  VEHIER**S**KMQQVG  GNSQTE**S**SLPGRR  RKERPT**S**LNVFPL  GDHWHL**S**DLGQLQ  SAAATP**S**TTGTKS  KSNTPT**S**SVPSAA  SNTPTS**S**VPSAAV  PTSSVP**S**AAVTPL  ELKRVK**S**EAIIAR  TEMIRA**S**REHPSV  TIWQFF**S**RLFSSS  FFSRLF**S**SSSSPP  FSRLFS**S**SSSPPP  RLFSSS**S**SPPPAK  AKRPYP**S**VNIHYK  PTTAGF**S**QRRNHA  DDDCTS**S**ARREQK  GDGEPK**S**AHTSPE  SSIPAA**S**DSDYPP  VPRSNC**S**SRGDTP  PRSNCS**S**RGDTPV  SIKLKD**S**VLSLVH  GNGVVI**S**IPLTET  VYGDDS**S**DRAASS  SSDRAA**S**SFIPYC  SFIPYC**S**MAQAQL  AVKFFV**S**VPGNVL  RNVLVL**S**GGEGYI  DETYIP**S**EQEILE  RAKLST**S**GAIKKK  QSVSLG**S**SVESGR  LGSSVE**S**GRQLGE  TKGLLG**S**IYEDKT  QSVHSS**S**EPLRNL  EESLRT**S**QPEEKK  EEKKDV**S**LDSDAA  KADPTG**S**EPAKAS  DAGEEG**S**RREEAA  KDDSQS**S**QDELQS  SQDELQ**S**KQSKGL  KGKEQH**S**QAEELG  VSPEVR**S**TEPVAP  HQQKEQ**S**LSSLRE  LREELE**S**QQKAER  HRVHQK**S**YHVAGY  HTHLLQ**S**NQQLRE  EGVALR**S**AKEFLV  LVQQTR**S**MRRRQT  ESSESF**S**PPHREW  SLRQIS**S**QLSSVL  DSLNPQ**S**PPPLLA  ARFSAL**S**SATPTS  RFSALS**S**ATPTST  SSATPT**S**TQWAWD  TQWAWD**S**GQGPRL  SPTPLE**S**RLGYMS  LRLLQH**S**HSQVPE  PRLPLF**S**STPKPK  KPKATL**S**LLQLGL  LPEGIT**S**ELECVT  LECVTN**S**TLAAII  AIIRQL**S**SLSKHA  RQLSSL**S**KHAEDI  FYIRAN**S**LQDRID  STVEEV**S**LQDINM  MKKAFK**S**STVQDQ  QDQQVV**S**KNSIPN  QVVSKN**S**IPNPVA  KFYTDP**S**YFFDLW  RPDNRL**S**QSVYHG  SVYHGA**S**SEGSLS  VYHGAS**S**EGSLSP  SSEGSL**S**PDTRSH  DTRSHA**S**DVTDYS  SDVTDY**S**YPATPN  PATPNH**S**LHPQPV  PQPVTP**S**YAAGDV  HEYRPP**S**ASARHM  YRPPSA**S**ARHMAL  PQAPEG**S**QASAPM  IEYYNP**S**GPPPPP  AQTAFV**S**PLQMPM  QPPFPA**S**ASSTHA  PFPASA**S**STHAAP  GPPGPG**S**SLSSSP  PPGPGS**S**LSSSPM  GPGSSL**S**SSPMHG  GSSLSS**S**PMHGPP  PISDAR**S**DLLAAI  RIAVEY**S**DSDDDS  AVEYSD**S**DDDSEF  SDSDDD**S**EFDEND  LGRSIE**S**GELIAI  NLREVK**S**LKKLNH  PLFPGA**S**EIDTIF  EGYQLS**S**AMNFRW  TLIPNA**S**SEAVQL  VGHPLG**S**TTQNLQ  TQNLQD**S**EKPQKG  PHTRIS**S**RQHQAS  PYKAEV**S**RTDHPS  SRTDHP**S**HLQEDK  LQEDKP**S**PLLFPS  HNKHPQ**S**KITAGL  WGLISR**S**TKDSDD  SRSTKD**S**DDWADL  LDDLDF**S**PSLSRI  DLDFSP**S**LSRIDL  DFSPSL**S**RIDLKN  TLCRFE**S**VLDLKP  VLDLKP**S**EPVGTG  PVGTGN**S**APTQTS  SAPTQT**S**YQRRDT  IRNGIL**S**NPGKEF  PPNPWS**S**SGLSGK  WSSSGL**S**GKSSGT  GLSGKS**S**GTMSVI  KSSGTM**S**VISKVN  GTMSVI**S**KVNSVG  VISKVN**S**VGSSST  NSVGSS**S**TSSSGL  SSSTSS**S**GLTGNY  TGNYVP**S**FLKKEI  APIPDP**S**PGYSSL  FHTQPR**S**TPGLIP  GRRSES**S**SPRRER  DTSRSR**S**RSAAAK  ISSSPT**S**KNRSHG  PTSKNR**S**HGRAKR  GHSRSR**S**PQWRRS  RTPARR**S**GRSRSR  GRSRSR**S**LVRRGR  RSRSGS**S**QPKAKS  SQPKAK**S**RTPPRR  RQSPSR**S**SSPQPK  PCPQEK**S**KDSLVQ  SCPGSL**S**LCAGVK  TSNFES**S**PEVEER  SLTLDQ**S**QSQASL  RSESDS**S**PDSKAK  DSKSRL**S**PRRSRS  RRGSRS**S**PEPKTK  SRSASS**S**PETRSR  PARQES**S**RTSSRR  IRRRSA**S**GSSSDR  RRSASG**S**SSDRSR  GSSSDR**S**RSATPP  PAANLA**S**RIPAAS  AANYPS**S**SRTPQA  SQSRMT**S**ERAPSP  APGVPH**S**DVGEPP  SSSSSS**S**SSGSSS  RRRETP**S**PRPMRH  ANRVLR**S**LVEASS  RSLVEA**S**SSGVSV  SLVEAS**S**SGVSVL  LVEASS**S**GVSVLS  ASSSGV**S**VLSLCE  SGVSVL**S**LCEKGD  GIAFPT**S**ISVNNC  AFPTSI**S**VNNCVC  NCVCHF**S**PLKSDQ  HFSPLK**S**DQDYIL  WNKVAH**S**FNCTPI  PIEGML**S**HQLKQH  AVDVLV**S**SGEGKA  VDVLVS**S**GEGKAK  IYKRDP**S**KQYGLK  GLKMKT**S**RAFFSE  TSRAFF**S**EVERRF  GPMRIT**S**GPFEPD  EPDLYK**S**EMEVQD  LKALLQ**S**SASRKT  LLQSSA**S**RKTQKK  KKKKKA**S**KTAENA  TAENAT**S**GETLEE  FVNMLL**S**HGIKPI  RYMCIL**S**GCDYLS  VDPETL**S**YAGQYV  GQYVDD**S**IALQIA  MPAHSR**S**HSWDDK  AHSRSH**S**WDDKTC  QKSANV**S**SIWHRN  QLKENP**S**TVGVER  GVERVI**S**TKGLNL  PRSAEL**S**EDDLLS  SSEGNK**S**LSFSEV  NKSLSF**S**EVFVPD  RSRFFC**S**SDSTDC  DSTDCV**S**NKVSIQ  TDVARN**S**SDDIPN  DVARNS**S**DDIPNN  VFTDEE**S**YSFESS  TDEESY**S**FESSKF  RSCFSW**S**GGLGDF  GGLGDF**S**RTPSPS  SRTPSP**S**PSTALQ  LPENNM**S**DVSQLK  LREEAC**S**SQSQES  EACSSQ**S**QESGEF  SSQSQE**S**GEFSLQ  GEFSLQ**S**SNASKL  LQSSNA**S**KLSQCS  SKLSQC**S**SKDSDS  QCSSKD**S**DSEESD  SQSDQT**S**KLRLSH  LRLSHF**S**KKDTPL  TGWLRK**S**PPEKKL  RWFILR**S**GRMSGD  LRSGRM**S**GDPDVL  YYKNDH**S**KKPLRI  VFDIKT**S**ERTFYL  FNQAEE**S**TDSLRN  SPAELS**S**SSQHLL  LLRERK**S**SAPSHS  HMQPTL**S**TSAPQE  ARSASF**S**QGTRAS  HCVNGI**S**GQVHGF  QVHGFY**S**LPKPSR  YSLPKP**S**RHNTEF  KGSLTG**S**ETDNED  VPATPL**S**AYQIPR  QQRPPI**S**ENSRSV  PPISEN**S**RSVAAT  LPAMDN**S**RLHRAS  GESAGR**S**AESMSD  MSDGVG**S**FLPGKM  PMNPGS**S**TLLAME  ERAGDN**S**QSVYIP  KSPITK**S**WSRANH  NHTFNS**S**SSQYCR  TFNSSS**S**QYCRPI  RPISTQ**S**ITSTDS  STQSIT**S**TDSGDS  STDSGD**S**EENYVP  FQPSSP**S**PHRKPS  HRKPST**S**SVTSDE  WTDVRQ**S**SEPSKG  IIQGFR**S**YRDQTI  VLSDEF**S**HLRPEQ  IDKEEV**S**LRRVIG  LESAGF**S**RSNPYY  DERKEE**S**ISLMKE  RKEESI**S**LMKETE  AKLDEL**S**AKRETS  DELAGN**S**EQRKRL  KKDELQ**S**ERNYLW  ILNGID**S**INKVLD  FYHIVD**S**DEVSTK  KTLICR**S**MEVSTQ  SRDSIL**S**EMKMLK  FMPKQR**S**LQSLEA  KQRSLQ**S**LEASLH  SLHAME**S**TRESLK  MESTRE**S**LKAELG  DLLSQL**S**LEDQKR  VLTATT**S**ELEAIN  SEDLDN**S**IDKTEA  KIRELG**S**LPQEAF  EKYQTL**S**LKQLFR  DQFVNF**S**EQKEKL  QVSKNF**S**EVFQKL  KGDVEG**S**QSQDEG  GEGSGE**S**ERGSGS  GESERG**S**GSQSSV  SERGSG**S**QSSVPS  RGSGSQ**S**SVPSVD  SQSSVP**S**VDQFTG  GVGIRV**S**FTGKQG  QHRKAV**S**DMIMEL  KFRNKV**S**HIDVIT  LGGGAA**S**PPAASP  ASPPAA**S**PFLGLH  LGLHIA**S**PPNFRL  FEDEDL**S**EITDEC  TDECGI**S**LQCKDT  GGGGAG**S**RLQAEM  GQSQGQ**S**QGPGSG  RVSRSS**S**PLKTGE  DELPPQ**S**GPAPTT  TTDRGT**S**TDSPCR  PGGRGH**S**HRDRIH  DAAEPT**S**AFLPPT  PTESRM**S**VSSDPD  ESRMSV**S**SDPDPA  DPAAYP**S**TAGRPH  GFDCLS**S**PERAEP  PPRASL**S**SDTSAL  SLSSDT**S**ALSYDS  SDTSAL**S**YDSVKY  AQLELV**S**LRPCFG  PCFGDY**S**DESDSA  DNCASV**S**SPYESA  NCASVS**S**PYESAI  AALAKN**S**DWVDQF  RVKFLG**S**VQVPYH  VHFNPP**S**SCVLEI  HFNPPS**S**CVLEIS  SCVLEI**S**VRGVKI  GVKADD**S**QEAKGN  FQLKNI**S**FCGYHP  ACHVFV**S**EDSTKA  NESDGM**S**GREPSL  EILPRT**S**LHSIPV  LPRAMP**S**SMGGGG  LQEFLL**S**KSKEPT  EFLLSK**S**KEPTPG  VAERRS**S**PLLRRK  GAGPGA**S**SVCNSA  AGPGAS**S**VCNSAP  ASSVCN**S**APGSGP  CNSAPG**S**GPSSPN  PGSGPS**S**PNSSHS  RALPLD**S**SPNQFS  SSPNQF**S**LYTSPS  PSLPNI**S**LGLQAT  NSHLTA**S**PKLSTQ  GKFMST**S**SIPGCL  PRHRPL**S**RTQSSP  LSRTQS**S**PLPQSP  SSPLPQ**S**PQALQQ  REGSTE**S**ESTQED  GYKKLF**S**DAQPLQ  QETGLL**S**KCERIR  GHHAEE**S**TAMGFC  GFCFFN**S**VAITAK  AFYNDP**S**VLYISL  PSVLYI**S**LHRYDN  GNFFPG**S**GAPEEV  TAICDA**S**EACVSA  CVSALL**S**VELQPL  SAMALL**S**VGAEQA  ERIGAH**S**HIRGLG  LEPRQA**S**QGMVGQ  FTAIAG**S**EIFSLE  AGSEIF**S**LEMSKT  IFSLEM**S**KTEALT  TQAFRR**S**IGVRIK  PATGTG**S**KVGKLT  GTKMIE**S**LTKDKV  KATGKI**S**KLGRSF  ISKLGR**S**FTRARD  DYDAMG**S**QTKFVQ  EVVHTV**S**LHEIDV  EIDVIN**S**RTQGFL  GFLALF**S**GDTGEI  DTGEIK**S**EVREQI  HMLDIE**S**FSFLNR  LDIESF**S**FLNRAL  LNRALE**S**DMAPVL  TRIRGT**S**YQSPHG  RGTSYQ**S**PHGIPI  DRLLIV**S**TTPYSE  VSTTPY**S**EKDTKQ  EEDVEM**S**EDAYTV  RIGLET**S**LRYAIQ  QLITAA**S**LVCRKR  DIKRVY**S**LFLDES  SLFLDE**S**RSTQYM  FLDESR**S**TQYMKE  RSAWGK**S**RKKNWQ  LKRNNV**S**APDGPS  DGPSDP**S**ISVSSE  PSDPSI**S**VSSEQS  DPSISV**S**SEQSGA  PSISVS**S**EQSGAQ  SVSSEQ**S**GAQQPP  RWKGYD**S**EDDTWE  TRTNRT**S**PNNARK  RKQISR**S**TNSNFS  STNSNF**S**KTSPKA  SNFSKT**S**PKALVI  NTAPSL**S**SRKNMD  TAPSLS**S**RKNMDL  KILVPK**S**PVKSRT  PKSPVK**S**RTAVDG  AVDGFQ**S**ESPEKL  DGFQSE**S**PEKLDP  ERARMG**S**RPRIHP  VNGKGT**S**PFMDAL  TTNIQT**S**VTGVTA  DKRLRF**S**VRQTES  SVRQTE**S**AYRYRD  FTHILL**S**TKSSEN  ILLSTK**S**SENNSL  EVQSAL**S**TAAADD  SKLVLL**S**AVGSVF  LLSAVG**S**VFCCGL  YTTFGQ**S**PDGCST  QSPDGC**S**TVMFPK  KIMGGA**S**ANEMLL  PVVLEE**S**KALVRC  LKKIWG**S**AQGMDS  RIEERK**S**QHLTGL  KVKAYL**S**LHPQVL  DEFVSE**S**VSAETV  FVSESV**S**AETVEK  KRKNNK**S**EDESAP  NKSEDE**S**APKEVS  VVYELN**S**YIEQRL  LLYELS**S**IIKIAT  LGECNN**S**LCIFTP  TQGTTV**S**AYVAKS  RGTGLE**S**GTRIQS  SGTRIQ**S**VLCLPI  KEAFCL**S**HQEVAT  NIVAID**S**LLEHIM  KNKELY**S**DLFDIG  TKEIRF**S**IEKGIA  LCMPIV**S**RGSVIG  QMVNKI**S**GSAFSK  VNKISG**S**AFSKTD  YHRIRH**S**ECIYRV  VTMEKL**S**YHSICT  YHSICT**S**EEWQGL  VYMVHR**S**CGTSCF  HRGFSN**S**YLQKFD  PLAALY**S**TSTMEQ  MEQHHF**S**QTVSIL  EGHNIF**S**TLSSSE  NIFSTL**S**SSEYEQ  IFSTLS**S**SEYEQV  LNLNNQ**S**HRDRVI  ACRDNL**S**QWEKVI  ETATWI**S**SPSVAQ  TATWIS**S**PSVAQK  PKGKSG**S**GKAGKG  GGAASG**S**DSADKK  AASGSD**S**ADKKAQ  AMEKLK**S**GMRFNE  EVAAQY**S**EDKARQ  GWMTRG**S**MVGPFQ  AFALPV**S**GMDKPV  STDTGV**S**LPSYEE  TGVSLP**S**YEEDQG  YEEDQG**S**KLIRKA  GLYKLK**S**RGNTKM  RGNTKM**S**IHLIHM  TVGMGY**S**MYREFW  ATAVAG**S**GAVGGC  KDGLQQ**S**KCPDTT  PKRRRA**S**SLSRDA  KRRRAS**S**LSRDAE  RRASSL**S**RDAERR  LRVYPV**S**GGLSNL  PVSGGL**S**NLLFRC  NLLFRC**S**LPDHLP  LPDHLP**S**VGEEPR  ILQGVD**S**LVLESV  DSLVLE**S**VMFAIL  AILAER**S**LGPQLY  LEQYIP**S**RPLKTQ  LREPVL**S**AAIATK  NLLEMY**S**LKDEMG  LRKLLE**S**TPSPVV  LLESTP**S**PVVFCH  GNILLL**S**EPENAD  EPENAD**S**LMLVDF  LVDFEY**S**SYNYRG  VDFEYS**S**YNYRGF  KKGETL**S**QEEQRK  DLLVEV**S**RYALAS  SRYALA**S**HFFWGL  FFWGLW**S**ILQASM  WSILQA**S**MSTIEF  ILQASM**S**TIEFGY  YLDYAQ**S**RFQFYF  PADDYE**S**EAAYDP  DPYAYP**S**DYDMHT  YFHKTV**S**DLIDQK  VYELQA**S**RVSSDV  LQASRV**S**SDVIDQ  QASRVS**S**DVIDQK  QDIYEN**S**WTKLTE  KVSGGP**S**LEQRFE  FIYQFQ**S**FSQYRC  YQFQSF**S**QYRCKT  CKTAKK**S**EEEIDF  EIDFLR**S**NPKIWN  KIWNVH**S**VLNVLH  HSLVDK**S**NINRQL  QLEVYT**S**GGDPES  SGGDPE**S**VAGEYG  GEYGRH**S**LYKMLG  KMLGYF**S**LVGLLR  GLLRLH**S**LLGDYY  IELNKK**S**MYSRVP  NKKSMY**S**RVPECQ  YIQRTK**S**MFQRTT  PMRIDE**S**IHLQLR  VYEELF**S**YSCPKF  EELFSY**S**CPKFLS  SCPKFL**S**PVVPNY  QQLKVF**S**DEVQQQ  QQQAQL**S**TIRSFL  QLSTIR**S**FLKLYT  KNLVWT**S**GISALD  VWTSGI**S**ALDGEF  LDGEFQ**S**ASEVDF  GEFQSA**S**EVDFYI  LVNTFF**S**FLRRKT  LITQTF**S**HHNQLA  LAKEAK**S**ETSGPQ  EAKSET**S**GPQIKE  AQLKNG**S**LDSPGK  RWTQTL**S**ELDLAV  EVKVEE**S**SWLIED  VKVEES**S**WLIEDG  NKMEWW**S**RLVSSD  WWSRLV**S**SDPEIN  WSRLVS**S**DPEINT  PENSKL**S**DLDSET  KLSDLD**S**ETRSMV  LDSETR**S**MVEKMM  YDQRQK**S**MGLPTS  SMGLPT**S**DEQKKQ  IKKMCP**S**DSELSI  KMCPSD**S**ELSIPA  PSDSEL**S**IPAKNC  RMVILG**S**SKVGKT  MVILGS**S**KVGKTA  GKTAIV**S**RFLTGR  FHRKFY**S**IRGEVY  LDILDT**S**GNHPFP  PAMRRL**S**ILTGDV  VFILVF**S**LDNRDS  SLDNRD**S**FEEVQR  QILDTK**S**CLKNKT  CAYFEI**S**AKKNSS  ISAKKN**S**SLDQMF  SAKKNS**S**LDQMFR  AMAKLP**S**EMSPDL  KLPSEM**S**PDLHRK  DLHRKV**S**VQYCDV  KLLRAG**S**GGGGGD  PFARRP**S**VHSDLM  RRPSVH**S**DLMYIR  YIREKA**S**AGSQAK  EKASAG**S**QAKDKE  LLFAGS**S**LASQVH  RLIVGN**S**ALKDKE  LTTRHR**S**PKCLAL  LLIKHD**S**NIGIPD  DAAPTE**S**LLNWQD  LLERNK**S**GTIPSD  PSVKDD**S**DLEGRT  DLEGRT**S**FMWAAG  ALSGHV**S**TVKLLL  VDQDGH**S**LLHWAA  QQKGRR**S**PDSCRP  STQDVP**S**RQSRAP  DVPSRQ**S**RAPSKQ  PRDSRG**S**PGGSLG  RGSPGG**S**LGGALQ  KEQHVS**S**DLQGTN  DLQGTN**S**RRPNET  NEGSDG**S**RHPGVP  RHPGVP**S**VEKSRG  VPSVEK**S**RGETAG  DEKGED**S**RRAAAS  SRRAAA**S**LPPHDS  SLPPHD**S**HWKPSR  LRGGRC**S**PAGSSR  RCSPAG**S**SRPGSA  CSPAGS**S**RPGSAR  YSHLPQ**S**TEELRS  GPLSGQ**S**VNIDLL  IQRAWR**S**YQLRKH  QLRKHL**S**HLRHMK  SPSKGT**S**GTKSTK  KQIYGC**S**HEGKIH  IHLLEN**S**GRSKNF  LRRLEA**S**DGGLDS  SDGGLD**S**AELAAE  VVGAVK**S**LQALGE  IEAELR**S**TKHWEL  EIAREG**S**HEARVF  EARVFR**S**IPPEGL  PEGLAQ**S**ELMRLP  ELMRLP**S**GKVGFS  SGKVGF**S**KAMSNK  GFSKAM**S**NKWIRV  WIRVDK**S**AADGPR  LGEKER**S**ELRKRK  YWVSKG**S**AFSTSI  GSAFST**S**ISKQET  AFSTSI**S**KQETEL  KQETEL**S**PEMISS  LSPEMI**S**SGSWRD  EMISSG**S**WRDRPF  HGVLPD**S**GHLHPL  PLLKVR**S**QFRQIF  TDNFIE**S**SFWNFD  DNFIES**S**FWNFDA  RVKRTH**S**QGGYGS  SQGGYG**S**QGYKYN  LRTHTT**S**ASARAL  THTTSA**S**ARALYR  TPVKYF**S**IDRVFR  NPYTEP**S**MEVFSY  PSMEVF**S**YHQGLK  WVEVGN**S**GVFRPE  GLPENV**S**VIAWGL  VIAWGL**S**LERPTM  LQMVYD**S**PLCRLD  VVLDNP**S**PFYNPF  ECIEDL**S**EDLEWK  KIIYVG**S**AESEEY  YVGSAE**S**EEYDQV  YDQVLD**S**VLVGPV  PVKPDF**S**KLQRNI  QRNILA**S**NPRVTR  KLEDAE**S**SNPNLQ  LEDAES**S**NPNLQS  SNPNLQ**S**LLSTDA  NLQSLL**S**TDALPS  STDALP**S**ASKGWS  DALPSA**S**KGWSTS  SASKGW**S**TSENSL  SKGWST**S**ENSLNV  GFVGFP**S**VGKSTL  FPSVGK**S**TLLSNL  GKSTLL**S**NLAGVY  NLAGVY**S**EVAAYE  FGIRLN**S**KPPNIG  TATCPQ**S**ELDAET  DAETVK**S**ILAEYK  ADVTLR**S**DATADD  NKIDQI**S**IEELDI  PHCVPI**S**AHHRWN  QLPDYT**S**PVVLPY  PVVLPY**S**RTTVED  ALVWGL**S**VKHNPQ  PFKSAY**S**SFVNLF  TSPQLP**S**RTQSVR  DDLAWQ**S**LIHPDS  SPARNR**S**ASITNL  LQSTEF**S**ETPSPD  EGHSEP**S**WFKDIK  SVSSFQ**S**TVDSDS  FQSTVD**S**DSAASI  QKEYLI**S**DTGGQQ  TGGQQL**S**ISDAFI  GQQLSI**S**DAFIKE  AMERLL**S**ANHNHM  AHKKMS**S**CIKNPK  IKLRGG**S**DYELAR  FAPVPE**S**LLPLPV  EKGMRC**S**TRDYFA  SIQVLP**S**HELVST  RIQPKN**S**DPFAHS  LSDAVW**S**TKVDCL  SSSAQS**S**NAPSAC  PSYQCP**S**MFCDTP  VALSNE**S**WSMSFA  YNQMVA**S**FSYSPI  SSQSST**S**STHLQL  LDTASS**S**EDVFDG  EGLPTN**S**TSDSRP  SDSRPK**S**SSPIRL  IRSLSH**S**SPWQAR  LRTSIH**S**DSHFLS  SDSHFL**S**SHLIID  VGRDDT**S**NELVVG  LKTWCG**S**PPYAAP  DEGEEP**S**PEALVR  PLGRRA**S**DGGANI  DQEAVQ**S**STYKDS  FSDGAA**S**IQAFKA  RLRIQP**S**SPPPNH  FRQPSN**S**PPPMSS  SPPPMS**S**AMIQPH  QGLPSR**S**AIFQQQ  MQQPAQ**S**QQVTIQ  GRGISI**S**PSAGQM  PLSKQL**S**ADSAEA  LNVNRF**S**PANYDQ  QSRGSP**S**SYSPST  SRGSPS**S**YSPSTG  LLSPRH**S**LTGHSD  HSLTGH**S**DIRLPP  AGSLAP**S**LGGQSM  SYHHHT**S**PQHLLQ  CVSQAS**S**PTPPHG  FQDSKS**S**STLTKG  QDSKSS**S**TLTKGC  GPGDPE**S**LLGTVS  SLLGTV**S**HAQELG  QPTAAF**S**KNKVPS  SKNKVP**S**REPVIG  GNCMDR**S**SPGQAV  VAGKAL**S**SARMSD  QSSLMG**S**QQFQDG  NEECGA**S**LGGHEH  HEHPDL**S**DGSQHL  GSQHLN**S**SCYPST  ITDILL**S**YKHPEV  ELRKEK**S**RDAARS  SRDAAR**S**RRSQET  PFARGV**S**AHLDKA  AHLDKA**S**IMRLTI  GDMAYL**S**ENVSKH  LELIGH**S**IFDFIH  TPQQTL**S**RRKVEA  FSLRMK**S**TLTSRG  MKSTLT**S**RGRTLN  WKVLNC**S**GHMRAY  PAGSPD**S**EPPLQC  GRGAFL**S**RHSLDM  AEVAGY**S**PDDLIG  DDLIGC**S**AYEYIH  YIHALD**S**DAVSKS  LDSDAV**S**KSIHTL  TQATVV**S**GGRGPQ  GGRGPQ**S**ESIVCV  CVHFLI**S**QVEETG  ETGVVL**S**LEQTEQ  ATRHPQ**S**PLSADL  VHRLFT**S**GKDTEA  PPALEP**S**LLPRWG  LLPRWG**S**DPRLSC  PRLSCS**S**PSRGDP  LSCSSP**S**RGDPSA  RGDPSA**S**SPMAGA  GDPSAS**S**PMAGAR  RTLAQS**S**EDEDEG  FLLFPL**S**LSFLLT  PLGLGP**S**LLSPYS  LGPSLL**S**PYSDED  SLLSPY**S**DEDTTQ  ILPYDY**S**RVELSL  ELSLIT**S**DEDSSY  DHDVPS**S**IDPILE  CYQEDD**S**VPICIH  ENFSVF**S**LIREMR  MRTQRP**S**LVQTQE  LQADSY**S**PNLPKS  SPNLPK**S**TTKAAK  VLHPAK**S**STSFDF  FLELNY**S**FDKNAD  LLFEGC**S**NSKPVN  KVMHVS**S**AELNYS  VENPYF**S**SWPPSG  ENPYFS**S**WPPSGT  PPSGTS**S**KMSLDL  SLLPTS**S**TSLFSY  LFSYYN**S**HDSLSL  NSPTNI**S**SLLNQE  VPKSLS**S**AVKVKI  LEWGGT**S**EPKKFD  PKKFDD**S**VILRPS  SVILRP**S**KSVKLR  ILRPSK**S**VKLRSP  ELHQDR**S**SPPPPL  PERTLE**S**FFLADE  QSIETY**S**TSYPDT  IETYST**S**YPDTME  TMENST**S**SKQTLK  GKSFTR**S**KSLKIL  PAESVQ**S**NNSSSF  GFANRF**S**KPKGPR  ELERKI**S**GLKTSM  ISGLKT**S**MAEGER  DHTLGN**S**LRYMIM  YTTTHP**S**ESKINL  TTHPSE**S**KINLRI  LDKFEA**S**IKDYKD  PPTHQA**S**VGLLDT  LLDTPR**S**RERSPS  SATVRA**S**QGPVYK  CKCFCR**S**KGHGFI  DIFLHI**S**DVEGEY  VTYKMC**S**IPPKNE  SRSRSA**S**RSRSRS  RRLSSR**S**RSRSYS  GYGNYR**S**NWQNYR  NYRQAY**S**PRRGRS  RSRRSS**S**SRSSSN  SKDSRP**S**QAAGDN  AKEQTF**S**GGTSQD  TFSGGT**S**QDTKAS  SQDTKA**S**ESSKPW  DTKASE**S**SKPWPD  TKASES**S**KPWPDA  VVVRRR**S**PRPSPV  FDHGSG**S**LSPSKK  TGSTYG**S**SQKEES  GSTYGS**S**QKEESA  SSQKEE**S**AASGGA  GKMKSD**S**FAPKTD  AKGRKE**S**EFDDEP  ESFPER**S**KKEDRG  EDRGKR**S**EGGHRG  CRDLVH**S**NKKEQE  HIQSAQ**S**QRSPSE  RRARDR**S**RSSSSS  RDRSRS**S**SSSSQS  RSSSSS**S**QSSHSY  SSSSSQ**S**SHSYKA  SSQSSH**S**YKAEEY  KDFVGP**S**ERGGGR  RFMFRK**S**STSPKW  FMFRKS**S**TSPKWA  KEVTKA**S**DGSLLG  TKASDG**S**LLGDLG  LGHTPL**S**KKEGIK  WQRPRL**S**RQALMR  LVKWIL**S**STAPQG  VKWILS**S**TAPQGS  STAPQG**S**DSSDSE  APQGSD**S**SDSELE  GSDSSD**S**ELELST  DSELEL**S**TVRHQP  TKKELQ**S**LYRGFK  DFVVGL**S**ILLRGT  MLAIMK**S**IYDMMG  KDENIM**S**SMQLFE  DENIMS**S**MQLFEN  AAGTAT**S**QRFFQS  SQRFFQ**S**FSDALI  RFFQSF**S**DALIDE  VADAKK**S**LELNPN  ELNPNN**S**TAMLRK  GLQLLT**S**SDPPAL  LQLLTS**S**DPPALD  DPPALD**S**QSAGIT  PALDSQ**S**AGITGA  GADANF**S**VWIKRC  QEAQNG**S**ESEVWT  AQNGSE**S**EVWTHQ  EVWTHQ**S**KIKYDW  DWYQTE**S**QVVITL  DVNVEF**S**EKELSA  FSEKEL**S**ALVKLP  ALVKLP**S**GEDYNL  PIIPEQ**S**TFKVLS  STFKVL**S**TKIEIK  VKNLYP**S**SSPYTR  KNLYPS**S**SPYTRN  LFQQIY**S**DGSDEV  QIYSDG**S**DEVKRA  NKSFME**S**GGTVLS  SGGTVL**S**TNWSDV  VLSTNW**S**DVGKRK  PVLGRE**S**VQVPDD  DDQDFR**S**FRSECE  DFRSFR**S**ECEAEV  GWNLTY**S**RAGVSV  YSRAGV**S**VWVQAV  YRKKWD**S**NVIETF  ADVGYY**S**WRCPKP  DVITLR**S**WLPMGA  YIIMNY**S**VKHPKY  DLVRAV**S**IQTGYL  TGYLIQ**S**TGPKSC  QSTGPK**S**CVITYL  QVDPKG**S**LPKWVV  KWVVNK**S**SQFLAP  WVVNKS**S**QFLAPK  WLHPEQ**S**PLPSLA  EQSPLP**S**LALSEL  LPSLAL**S**ELSVQH  LENIDE**S**AVAESR  ESAVAE**S**REERMG  DAGTFL**S**RAVQFT  HLENLL**S**KAECTK  LDRKAP**S**RINNPE  RELIQT**S**ALNFLT  AAETRN**S**SEQELR  AETRNS**S**EQELRI  ELRITQ**S**EFDRQA  LLLEGI**S**STHAHH  LLEGIS**S**THAHHL  LQKQLG**S**FPSNYL  QLGSFP**S**NYLSNN  FPSNYL**S**NNNQTS  SNNNQT**S**VTPVPS  SVTPVP**S**VLPNAI  LPNAIG**S**SAMAST  PNAIGS**S**AMASTS  GSSAMA**S**TSGLVI  SAMAST**S**GLVITS  SGLVIT**S**PSNLSD  LVITSP**S**NLSDLK  TSPSNL**S**DLKECS  SDLKEC**S**GSRKAR  LKECSG**S**RKARVL  DYDAAN**S**TELSLL  ANSTEL**S**LLADEV  EVITVF**S**VVGMDS  SVVGMD**S**DWLMGE  DQLMGT**S**RDGDTT  RQRIKF**S**DDRVCK  CPHDVL**S**GTRMDL  ADYEIA**S**KEQDFF  AMDHLQ**S**FIADCD  ETQEEI**S**AEVAAK  EGNVEE**S**QKVMDE  EEVYRN**S**MPASSF  RNSMPA**S**SFQQQK  NSMPAS**S**FQQQKL  RVCEVC**S**AYLGLH  REKLRR**S**RSHSKN  KLRRSR**S**HSKNPK  RRSRSH**S**KNPKRS  NPKRSR**S**REHRRH  EHRRHR**S**RSMSRE  RRHRSR**S**MSRERK  HRSRSM**S**RERKRR  RKRRTR**S**KSREKR  HRHRSR**S**SSRSRS  HRSRSS**S**RSRSRS  SRSSSR**S**RSRSHQ  SSSRSR**S**RSHQRS  SRSRSR**S**HQRSRH  SRSHQR**S**RHSSRD  HSSRDR**S**RERSKR  RSKRRS**S**KERFRD  SCDRDR**S**SRDRSP  CDRDRS**S**RDRSPR  RSSRDR**S**PRDRDR  ESANGR**S**EDRRSS  RSEDRR**S**SEEREA  SEDRRS**S**EEREAG  KVVGWR**S**GVEKDL  EVLQTH**S**VFVNVS  SVFVNV**S**KGQVAK  KKEDLI**S**AFGTDD  KGEVQV**S**DKERHT  MKDIHY**S**VKTNKS  SVKTNK**S**TKQQAL  LIKVIE**S**EDYGQQ  ETKGKG**S**LEVLNL  CLKSPT**S**DDISLL  PTSDDI**S**LLHESQ  ISLLHE**S**QSDRAS  LLHESQ**S**DRASFG  SQSDRA**S**FGEGTE  VYHPTP**S**QTRLAT  DPGRDG**S**EKKIRE  DDWLMR**S**FTCPSC  RSFTCP**S**CMEPVD  VLNELV**S**VEDLLK  FEKKFQ**S**EKAAGS  SEKAAG**S**VSKSTQ  KAAGSV**S**KSTQFE  AGSVSK**S**TQFEYA  AWCLVR**S**KYNDDI  ELLPKG**S**KEEQRD  CLWTLT**S**AAMSRG  LTSAAM**S**RGDNCT  LALGIP**S**ITQAWG  TLLFLI**S**LAAHLS  SLAAHL**S**QWTRGR  QWTRGR**S**RSHPGQ  TRGRSR**S**HPGQGR  HPGQGR**S**GESVEE  QGRSGE**S**VEEVPL  LQTGRL**S**QDPEPD  EVMCYT**S**LQLRPP  GTPVKY**S**EVVLDS  SEVVLD**S**EPKSQA  LDSEPK**S**QASGPE  EPKSQA**S**GPEPEL  EPELYA**S**VCAQTR  TRRARA**S**FPDQAY  KPKVLR**S**WCRQIL  VKIVAK**S**IRDRVA  LADAAP**S**PAQCVC  LPQSLP**S**LGAYQQ  FPDPAM**S**FAPVLP  PPPQYF**S**PAVILP  PAATPG**S**QILLGH  LPQSCE**S**YGGSDV  RSTRAR**S**RQERAS  SRQERA**S**RPRLTI  GEESRQ**S**QANAPV  YKDQLS**S**KEQPSF  SSKEQP**S**FLASQQ  TMPEPA**S**GTASQA  PASGTA**S**QAGGPG  TSELET**S**QPLAET  VPTSSA**S**AGTPVE  QPLVEK**S**ELAPTR  RQVASD**S**HVVPSV  SAEPPP**S**DMGTVG  DEWTLA**S**PHSLRY  VGEPVS**S**DSGDEG  EPVSSD**S**GDEGPR  MKVPTI**S**VTSFHS  PTISVT**S**FHSQSS  SVRASL**S**SDICSG  ERVTYK**S**SSKPRA  SSRSST**S**SLAPGP  SRSSTS**S**LAPGPE  PAPGPL**S**TTVIPG  TACCGH**S**TQPRGG  ADSEQP**S**KRPRCD  RPRCDD**S**PRTPSN  DSPRTP**S**NTPSAE  TPSNTP**S**AEADWS  SAEADW**S**PGLELH  GPEQVC**S**FLRRGG  LPCLDE**S**RFENLG  FENLGV**S**SLGERK  ENLGVS**S**LGERKK  ERKKLL**S**YIQRLV  YVFPGA**S**HNRFEH  HNRFEH**S**LGVGYL  QPELQI**S**ERDVLC  LGHGPF**S**HMFDGR  WTHEQG**S**VMMFEH  FEHLIN**S**NGIKPV  IVGPLE**S**PVEDSL  ESPVED**S**LWPYKG  GRPENK**S**FLYEIV  FLYEIV**S**NKRNGI  MFHTRN**S**LHRRAY  GKKYRI**S**TAIDDM  FLEILY**S**TDPKLK  KREDYE**S**LPKEVA  LPKEVA**S**AKPKVL  NPIDHV**S**FYCKTA  ITKNQV**S**QLLPEK  KKVDRK**S**LYAARQ  KKEWND**S**TSVQNP  EWNDST**S**VQNPTR  TRLREA**S**KSRVQL  LREASK**S**RVQLFK  PGPIDN**S**GLLKDG  GWNKLV**S**WYTLME  VVTRRF**S**KADTID  PLNKPD**S**TIQDAG  GPSTPK**S**PGASNF  PGASNF**S**TLPKIS  STLPKI**S**PSSLSN  ISPSSL**S**NNYNNM  NRNVKN**S**NYCLPS  SNYCLP**S**YTAYKN  NTCFMN**S**AIQCLS  SAIQCL**S**NTPPLT  HLKRND**S**IIVDIF  FHGLFK**S**TLVCPE  DLCTAL**S**ALSGIP  TALSAL**S**GIPADK  YTHHTG**S**SLFGQP  THHTGS**S**LFGQPF  CRYVKI**S**TETEET  DEPDDE**S**SQDQEL  QDQELP**S**ENENSQ  PSENEN**S**QSEDSV  ENENSQ**S**EDSVGG  LRLDER**S**FLALDW  DFEKHE**S**VEYKPP  KKLDLW**S**LPPVLV  INDLDM**S**EFLINP  YNLIAV**S**NHYGGM  SSVSTA**S**EDQIVS  SEDQIV**S**KAAYVL  QRQDTF**S**GTGFFP  RETKGA**S**AATGIP  TGIPLE**S**DEDSND  EDFPET**S**EPVWIL  ILGRKY**S**IFTEKD  EKDEIL**S**DVASRL  ILSDVA**S**RLWFTY  GGTGPT**S**DTGWGC  RKRQPD**S**YFSVLN  QPDSYF**S**VLNAFI  FIDRKD**S**YYSIHQ  RKDSYY**S**IHQIAQ  GVGEGK**S**IGQWYG  AVFDTW**S**SLAVHI  VFDTWS**S**LAVHIA  RRLCRT**S**VPCAGA  TAFPAD**S**DRHCNG  EVTNRP**S**PWRPLV  CFMMPQ**S**LGVIGG  IGGKPN**S**AHYFIG  CFIPDE**S**FHCQHP  HPPCRM**S**IAELDP  IAELDP**S**IAVGFF  QQVKKL**S**LLGGAL  LVELQP**S**HLACPD  PDVLNL**S**LDSSDV  LNLSLD**S**SDVERL  NLSLDS**S**DVERLE  LERFFD**S**EDEDFE  EAIHAL**S**SSEDGG  IHALSS**S**EDGGHI  IFCTLE**S**LKRYLG  CLASRL**S**PAWLEL  LEELWA**S**FFLEGP  EGAAGP**S**FRLMKM  LQAVVD**S**LQGGLD  LQGGLD**S**SVSFVS  GLDSSV**S**FVSQVL  AALTQG**S**YLHQRV  LGPEVL**S**RLLGNL  TTPMLQ**S**LLGHLA  GHLAMD**S**QRRPLL  EPELRD**S**RDELLA  IVAEVV**S**ARIHPE  YEEDEL**S**LELLAL  PAGDGA**S**EAGTSL  FVPYDM**S**GDRELK  GDRELK**S**SKAPAY  VEALTT**S**EDIERW  TATREV**S**VELAKV  RTPQPG**S**PSPNTP  PQPGSP**S**PNTPCL  AVSQPG**S**AVASDW  PGSAVA**S**DWRVVV  VEERIR**S**KTQRLS  GPAGSP**S**RFNSVA  GLLSAV**S**SVLLSL  LLSAVS**S**VLLSLP  VSSVLL**S**LPAARL  ELLEAR**S**WLADVA  KTELLE**S**EQGSPN  PPEDSL**S**VDHFQT  INKART**S**PTAVSS  TSPTAV**S**SSPVSM  SPTAVS**S**SPVSMT  VSSSPV**S**MTASAS  PVSMTA**S**ASSPSS  SMTASA**S**SPSSTS  MTASAS**S**PSSTST  ASASSP**S**STSTSS  SSPSST**S**TSSSSS  SSTSTS**S**SSSSRL  STSTSS**S**SSSRLA  TSTSSS**S**SSRLAS  TSSSSS**S**RLASSP  SSRLAS**S**PTVITS  VITSVS**S**ASSSST  SVSSAS**S**SSTVLT  SSASSS**S**TVLTPG  PGPLVA**S**ASGVGG  SPMNLQ**S**NKLSHV  LQSNKL**S**HVHRIP  MDPRGL**S**PRQSKS  GLSPRQ**S**KSDSDD  RQSKSD**S**DDDDLP  SVNETG**S**TALSIA  KFPCSI**S**PFSIES  SPFSIE**S**TRRQRR  TRRQRR**S**ESPDSR  RQRRSE**S**PDSRKR  NKVYTK**S**SHLKAH  TWKFAR**S**DELTRH  DCDRSF**S**RSDHLA  YNGHLV**S**CDNWMN  REVICT**S**RDGDKF  ECYIRG**S**TIKYLR  AVKIVN**S**KAISRE  VNSKAI**S**REVKAM  EVKAMA**S**LDNEFV  KFMENG**S**LSGLLQ  MENGSL**S**GLLQSQ  HRDLKP**S**NVLLDP  STFQGG**S**QSGTGS  FQGGSQ**S**GTGSGE  SQSGTG**S**GEPGGT  RKASTA**S**DVYSFG  TASDVY**S**FGILMW  LMQLCW**S**SEPKDR  MQLCWS**S**EPKDRP  EPKDRP**S**FQECLP  FLSQLR**S**SNRRFS  SSNRRF**S**IPESGQ  RFSIPE**S**GQGGTE  TIENQH**S**RNDVMV  RNDVMV**S**EWLNKL  NLEEPP**S**SVPKKC  LEEPPS**S**VPKKCP  VPKKCP**S**LTKRSR  AWTAGT**S**SDSMAQ  AGTSSD**S**MAQPPQ  PQTPET**S**TFRNQM  FRNQMP**S**PTSTGT  QMPSPT**S**TGTPSP  RQGMNW**S**CRTPEP  TWGLAP**S**GKGRGL  HPPPVG**S**QEGPKD  KDPEAW**S**RPQGWY  DFEPYL**S**PQARPN  YTAMSD**S**YLPSYY  SDSYLP**S**YYSPSI  PSYYSP**S**IGFSYS  SIGFSY**S**LGEAAW  AMPYLT**S**YGQLSN  TSYGQL**S**NGEPHF  GFNFFP**S**GIDFSA  SAWGNN**S**SQGQST  AWGNNS**S**QGQSTQ  NSSQGQ**S**TQSSGY  QGQSTQ**S**SGYSSN  QSSGYS**S**NYAYAP  NYAYAP**S**SLGGAM  AMIDGQ**S**AFANET  AALKLG**S**TEVASN  GSTEVA**S**NVPKVV  VPKVVG**S**AVGSGS  VGSAVG**S**GSITSN  SAVGSG**S**ITSNIV  GSGSIT**S**NIVASN  TSNIVA**S**NSLPPA  NIVASN**S**LPPATI  SWADIA**S**KPAKQQ  GQPTQG**S**PQPVGQ  PQPAQL**S**VQQQAA  GSGSTP**S**EPHPVL  EDDIHR**S**IKYNIW  KYNIWC**S**TEHGNK  LFSVNG**S**GHFCGV  GVAEMK**S**AVDYNT  NKPVTN**S**RDTQEV  RQEEEE**S**VKKERQ  IREKNE**S**NKSSFD  KNESNK**S**SFDKMI  NESNKS**S**FDKMIE  IEAIKE**S**KNGKKI  FDKIDI**S**AVVAYT  DEKVRH**S**KLAESV  LAGADP**S**TVEMCY  NYNLKF**S**VVSDKN  MGIRFK**S**YCSNLV  RFKSYC**S**NLVRTL  GSLVIN**S**KNQYKL  KKGMVF**S**INLGFS  DLLGRG**S**RAALLT  IQKARK**S**NVSYKN  ARKSNV**S**YKNPSL  VSYKNP**S**LMPKEP  NFYCPG**S**ALGRNE  GIVKQD**S**LVINLN  CLLQPT**S**SALVNA  IVYKDY**S**KKVTMI  NAIPVA**S**LDPIKE  IKEWLN**S**CDLKYT  EPEGEG**S**DAEEGD  DAEEGD**S**ESEIED  DSDEDY**S**SEAEES  SSEAEE**S**DYSKES  AEESDY**S**KESLGS  SDYSKE**S**LGSEEE  EQSRSM**S**RKRKAS  RKASVH**S**SGRGSN  RGSNRG**S**RHSSAP  NRGSRH**S**SAPPKK  RGSRHS**S**APPKKK  SWNSSG**S**EEDPET  EDPETE**S**GPPVER  ALSYYK**S**EDETEY  RGSICL**S**KAVITP  ECRFDI**S**VNDSVW  EQHKTE**S**GYGSES  TESGYG**S**ESSLRR  SGYGSE**S**SLRRHG  GYGSES**S**LRRHGS  RHGSMV**S**LVSGAS  SMVSLV**S**GASGYS  SGASGY**S**ATSTSS  SGYSAT**S**TSSFKK  YSATST**S**SFKKGH  SATSTS**S**FKKGHS  DFPTTR**S**DGDFLH  GILATL**S**HCIELM  TELKKK**S**HFGGPD  RLHWPT**S**LPSGDA  WPTSLP**S**GDAFSS  QKPYSR**S**SSMSSI  SRSSSM**S**SIDLVS  SSIDLV**S**ASDDVH  IDLVSA**S**DDVHRF  DDVHRF**S**SQVEEM  QNHMTY**S**LQDVGG  KRVWPA**S**QRDVLY  RDVLYL**S**VIRKIP  WIVCNF**S**VDHDSA  FSVDHD**S**APLNNR  ICQTLV**S**PPEGNQ  FTLFLF**S**VLFAIS  SVLFAI**S**EVRSKE  AISEVR**S**KESVRL  EVRSKE**S**VRLCGL  VIYICA**S**SRWRRH  IYICAS**S**RWRRHQ  QAETGN**S**FQLPHK  PHKREF**S**EENPAQ  LPKVDA**S**GEDRLW  TEELWK**S**KKHSVM  WKSKKH**S**VMSRQD  KKHSVM**S**RQDLQT  CCTDGC**S**MTDLSA  HIILVL**S**GKGGVG  KGGVGK**S**TISTEL  VGKSTI**S**TELALA  VDLCGP**S**IPRMLG  FLDREQ**S**ISLMSV  DREQSI**S**LMSVGF  QSISLM**S**VGFLLE  LIKQFV**S**DVAWGE  DTPPGT**S**DEHMAT  TTPQAV**S**VGDVRR  GIVENM**S**GFTCPH  HCTECT**S**VFSRGG  ECTSVF**S**RGGGEE  GVPFLG**S**VPLDPA  IQEFPG**S**PAFAAL  AFAALT**S**IAQKIL  RYSHRS**S**HHRPYL  MFKDRS**S**RLSSLN  DRSSRL**S**SLNHSY  SLNHSY**S**HSHGMK  GMKKRS**S**DSHKDL  SHKDLL**S**GLYFDQ  YSSLRH**S**KPTSAY  RHSKPT**S**AYYTRQ  AYYTRQ**S**SSLYSD  YYTRQS**S**SLYSDP  QSSSLY**S**DPLATY  ASPTAN**S**GLLRSA  RTPSEC**S**YYSSRI  SECSYY**S**SRISSA  ECSYYS**S**RISSAR  YYSSRI**S**SARSSP  YSSRIS**S**ARSSPG  SRGRRE**S**VVSAAD  RRESVV**S**AADYFS  RRGSVV**S**EVDDIS  SEVDDI**S**IPDLSS  SIPDLS**S**LDEKSD  SSLDEK**S**DKQYAE  ENYTRP**S**SRNSAS  NYTRPS**S**RNSASA  SSRNSA**S**ATTPLS  TPLSGN**S**SRRGSG  GSGDTS**S**LIDPDT  AAQVLE**S**AGEGPL  EERQKC**S**RNDGTV  DANRQI**S**EYKFKL  KTPGQR**S**RADAGH  AGHAGV**S**ANMMKK  MMKKRT**S**HKKHRS  SHKKHR**S**SVGPSK  HKKHRS**S**VGPSKP  RSSVGP**S**KPVSQP  GPSKPV**S**QPRRNI  QVPVNP**S**LYLIKY  PDRVAT**S**RISDAH  FETEDG**S**KDEWRG  LRIMPD**S**NDSPPA  PGEVVD**S**LVGKQV  YAKEDG**S**KRTGMV  QVEAKP**S**VYFIKF  LFVKAG**S**DGESIG  AGSDGE**S**IGNCPF  IGNCPF**S**QRLFMI  LKGVVF**S**VTTVDL  PFITFN**S**EVKTDV  PKYLKL**S**PKHPES  SPKHPE**S**NTAGMD  DIFAKF**S**AYIKNS  SAYIKN**S**RPEANE  LDEYLN**S**PLPDEI  DEIDEN**S**MEDIKF  MEDIKF**S**TRKFLD  YLTNAY**S**RDEFTN  FTNTCP**S**DKEVEI  EVEIAY**S**DVAKRL  LPHPGG**S**TNLKAD  EKIGKG**S**FGEVFK  QEITVL**S**QCDSPY  VLSQCD**S**PYVTKY  VTKYYG**S**YLKDTK  EYLGGG**S**ALDLLE  GLDYLH**S**EKKIHR  AANVLL**S**EHGEVK  PEVIKQ**S**AYDSKA  KQSAYD**S**KADIWS  SKADIW**S**LGITAI  RGEPPH**S**ELHPMK  TLEGNY**S**KPLKEF  CLNKEP**S**FRPTAK  RNAKKT**S**YLTELI  RWKAEQ**S**HDDSSS  EQSHDD**S**SSEDSD  QSHDDS**S**SEDSDA  SHDDSS**S**EDSDAE  DSSSED**S**DAETDG  ETDGQA**S**GGSDSG  GQASGG**S**DSGDWI  ASGGSD**S**GDWIFT  NGALQP**S**DLDRNK  IPKRPF**S**QCLSTI  PFSQCL**S**TIISPL  CLSTII**S**PLFAEL  AELKEK**S**QACGGN  CGGNLG**S**IEELRG  EACPGI**S**DTMVAQ  QRLQRY**S**LSGGGT  PGTRGH**S**QQEAAM  EPQAAQ**S**PAGQGP  PPAAGV**S**CSPTPT  AAGVSC**S**PTPTIV  HRHLKV**S**TASLTS  EEAKLV**S**ERFLTR  TRRGRK**S**RSSPGD  GRKSRS**S**PGDSPS  SPGDSP**S**AVSPNL  DSPSAV**S**PNLSPS  PSASPT**S**SRSNSL  SASPTS**S**RSNSLT  GDEADV**S**SPHPGE  DEADVS**S**PHPGEP  PPPVEK**S**KEIAIE  LAPVTN**S**SGKMAL  GSPLPR**S**PTQDAA  GVGPPA**S**QGRGPA  MGPEAG**S**KAELPP  RGLSWD**S**GPEEPG  LKLPDL**S**EAAEQE  GLPSEL**S**PAIEEE  NVFVQL**S**LAFRND  NQNSSR**S**CGPSED  SRSCGP**S**EDGVPR  VPRTAR**S**MSLTLG  QTPSSS**S**IPSLPA  SPNGKG**S**LPVTSA  SCLEEL**S**QETKAR  EKGPRS**S**KLEELV  LGRSTC**S**AAQRDS  RVGSFG**S**SPPGLS  VGSFGS**S**PPGLSS  SSPPGL**S**STYTGG  SPPGLS**S**TYTGGP  LGNEIA**S**GNGGAA  DGQNLW**S**CILSEV  LWSCIL**S**EVSTRS  CILSEV**S**TRSRSK  SEVSTR**S**RSKLPA  VSTRSR**S**KLPAGK  FSLDAV**S**LKDTLV  MLVVDM**S**KPWTAL  PWTALD**S**LQKWAS  SLQKWA**S**VVREHV  QRRNTA**S**QEDKDD  QEDKDD**S**VVLPLG  HFDFIQ**S**HIRKFC  AALIYT**S**VKENKN  FLMKLQ**S**LLAKQP  PRTPNR**S**VSSNVA  TPNRSV**S**SNVASV  PNRSVS**S**NVASVS  SPIPAG**S**KKIDPN  MKAGAT**S**EGVLAN  LANFFN**S**LLSKKT  FFNSLL**S**KKTGSP  LSKKTG**S**PGGPGV  PGGPGV**S**GGSPAG  PGVSGG**S**PAGGAG  GGAGGG**S**SGLPPS  GAGGGS**S**GLPPST  NIVHNY**S**EAEIKV  KVREAT**S**NDPWGP  NDPWGP**S**SSLMSE  DPWGPS**S**SLMSEI  PSSSLM**S**EIADLT  AFSEIM**S**MIWKRL  YLIKTG**S**ERVSQQ  AQTATA**S**SAAVGS  QTATAS**S**AAVGSG  SSAAVG**S**GPPPEA  EQAWPQ**S**SGEEEL  EADQPP**S**CGPEDD  QLQLAL**S**LSREEH  QLALSL**S**REEHDK  TGGKEE**S**SLMDLA  PTAAPT**S**DPWGGP  AAPAGP**S**VDPWGG  TPDPWG**S**SDGGVP  PDPWGS**S**DGGVPV  DGGVPV**S**GPSASD  VSGPSA**S**DPWTPA  TPAPAF**S**DPWGGS  GSPAKP**S**TNGTTA  PGAFDM**S**GVRGSL  TRKTPE**S**FLGPNA  ALVDLD**S**LVSRPG  DLDSLV**S**RPGPTP  PPGAKA**S**NPFLPG  GPATGP**S**VTNPFQ  LNQLRL**S**PVPPVP  APPTYI**S**PLGGGP  TVKGGS**S**GRGTAL  FLDCFK**S**YVDQRA  LSEMRA**S**LESWWQ  LTFPEQ**S**VPGALQ  LQFRLT**S**VDLEDW  EDWMDV**S**LVPAFN  PKPQVY**S**TLLNSG  YSTLLN**S**GCQGGE  CKKDAF**S**LAEGLR  MGDPVQ**S**WKGPGL  TPENSK**S**LNAVYP  GMALDL**S**QIPTKE  NCVHKA**S**RVSKGG  RVSKGG**S**FGRGTD  LQFQLV**S**TALKSW  AVGQLS**S**GTKPNP  PNPQVY**S**RLLTSG  YWTVNY**S**TEDPAM  LSRDGT**S**VQPWDV  DLDKFI**S**EFLQPN  KVVKGG**S**SAKGTA  VVKGGS**S**AKGTAL  DLVVFL**S**CFSQFT  VFLSCF**S**QFTEQG  ENPRVL**S**FSLTSQ  PRVLSF**S**LTSQTM  SGSRPS**S**QVYVDL  YVDLIH**S**YSNAGE  DLIHSY**S**NAGEYS  SNAGEY**S**TCFTEL  RPTKLK**S**LIRLVK  ISKGRG**S**LPPQHG  EQGGKD**S**QFNMAE  NRHLWK**S**QLCEMV  CEMVQP**S**GGPAAD  MAEDKA**S**VKAQVT  VKAQVT**S**LLGELQ  LGELQE**S**QSRLEA  ELQESQ**S**RLEAAT  GRARAA**S**EQARQL  QARQLE**S**EREALQ  ALQQQH**S**VQVDQL  LRMQGQ**S**VEAALR  MERQAA**S**EEKRKL  YDNHIK**S**SVVGSE  DNHIKS**S**VVGSER  KSSVVG**S**ERKRGM  QLQREY**S**KLKASC  YSKLKA**S**CQESAR  KASCQE**S**ARIEDM  KRHVEV**S**QAPLPP  APAYLS**S**PLALPS  SPLALP**S**QRRSPP  ALRHLE**S**WCVQRD  PYIFPD**S**PGGTSY  DSPGGT**S**YERYDC  LDDWHP**S**EKAMYP  KKLRRE**S**WEREVK  PFLVMI**S**LLSLSP  SDNDDN**S**LGYTGS  IVHGRK**S**RPDEGV  YLGEAV**S**HNASLE  MVGERE**S**EVAELT  TVLERP**S**FVKRPS  SFVKRP**S**NLAVTV  DGELPK**S**RYEIRD  VGKAEA**S**ATLTVQ  YQPPQS**S**SRFSVS  QPPQSS**S**RFSVSQ  DGTFVL**S**CVATGS  YGISDP**S**QISDPV  QDVLPT**S**QGVDHK  PTVLSS**S**SIEVHW  RTPAKN**S**VVIPDL  PQGVTV**S**KNDGNG  SVEVAA**S**TGAGSG  AHGNPV**S**PEDQVS  SPEDQV**S**LAQQIS  KRNGLT**S**TYAGIR  SNLTTY**S**RPADCI  NSSDRG**S**STSGSQ  DRGSST**S**GSQGHK  NISVDE**S**YDQEMP  HTYGYI**S**GPLVSD  LEQTPA**S**SVGDLE  EQTPAS**S**VGDLES  MINGWG**S**ASEEDN  NGWGSA**S**EEDNIS  NISSGR**S**SVSSSD  SSDRKG**S**SYKGRE  RDPSSS**S**SMSSRG  RRREQE**S**KYIEEL  ITRHDL**S**GKVVNI  TNSLRS**S**MRPGFE  SSVGGM**S**MSPNQG  QGLQMP**S**SRAYGL  STTGQM**S**GARYGG  ARYGGS**S**NIASLT  PGMQSP**S**SYQNNN  NYGLNM**S**SPPHGS  FSSSSL**S**ALQAIS  MNITQP**S**KVSNQD  SMCQSN**S**RDHLSD  NSRDHL**S**DKESKE  KESKES**S**VEGAEN  TNSPLD**S**SCKESS  SSVSVT**S**PSGVSS  SPSGVS**S**STSGGV  PSGVSS**S**TSGGVS  ATGKDT**S**SITSCG  LDRDDP**S**DALSKE  GVDNKM**S**QCTSST  MSQCTS**S**TIPSSS  TSSTIP**S**SSQEKD  NPRMMD**S**QENYGS  DWGLPN**S**KAGRME  ASNQLG**S**WPDGML  LSMEQV**S**HGTQNR  FHLQGQ**S**PSFNSM  AFGRVS**S**PPNAMM  AASIYQ**S**SEMKGW  GNLARN**S**SFSQQQ  VLDRGA**S**FLKHVC  GVHVPK**S**YRRRRR  RISENY**S**DKSDIE  IENADE**S**SSSILK  ENADES**S**SSILKP  NADESS**S**SILKPL  LGEEDD**S**PAPPQL  PHVATL**S**LHSLFE  LDREAS**S**LPQLVE  DIGKTV**S**SASRMF  TNPDNG**S**PAMTHR  THRNLT**S**SSLNDI  PRDAEA**S**NVLVGE  PKGKAK**S**YHEIGR  AIATLM**S**DEVFHD  PPKSLP**S**SDKRKN  KAPFFA**S**DFYDAL  LNIQAL**S**AILFIY  TEEGFS**S**LISFIF  GFSSLI**S**FIFIYD  EYLPTM**S**STDMYH  YLPTMS**S**TDMYHN  DFAIIL**S**ILIFCV  SEFKPT**S**PNRGWF  VAATVI**S**IAHIDS  KMETET**S**APGEQP  LYMGVA**S**LNGVQF  LLWILK**S**TVAAII  FSQHDL**S**FLDDVI  QPFLSD**S**KPSDRE  LSDSKP**S**DRERSP |
